# Lack of host phylogenetic structure in the gut bacterial communities of New Zealand cicadas and their interspecific hybrids

**DOI:** 10.1038/s41598-022-24723-3

**Published:** 2022-11-29

**Authors:** Diler Haji, Jason Vailionis, Mark Stukel, Eric Gordon, Emily Moriarty Lemmon, Alan R. Lemmon, Chris Simon

**Affiliations:** 1 Department of Ecology and Evolutionary Biology, University of Connecticut, Storrs, CT USA; 2 Department of Biological Science, Florida State University, Tallahassee, FL USA; 3 Department of Scientific Computing, Florida State University, Tallahassee, FL USA; 4 Present Address: Department of Integrative Biology, University of California, Berkeley, CA USA

**Keywords:** Phylogenetics, Microbial ecology, Metagenomics, Symbiosis

## Abstract

Host-microbe interactions are intimately linked to eukaryotic evolution, particularly in sap-sucking insects that often rely on obligate microbial symbionts for nutrient provisioning. Cicadas (Cicadidae: Auchenorrhyncha) specialize on xylem fluid and derive many essential amino acids and vitamins from intracellular bacteria or fungi (*Hodgkinia*, *Sulcia*, and *Ophiocordyceps*) that are propagated via transmission from mothers to offspring. Despite the beneficial role of these non-gut symbionts in nutrient provisioning, the role of beneficial microbiota within the gut remains unclear. Here, we investigate the relative abundance and impact of host phylogeny and ecology on gut microbial diversity in cicadas using 16S ribosomal RNA gene amplicon sequencing data from 197 wild-collected cicadas and new mitochondrial genomes across 38 New Zealand cicada species, including natural hybrids between one pair of two species. We find low abundance and a lack of phylogenetic structure and hybrid effects but a significant role of elevation in explaining variation in gut microbiota.

## Introduction

The study of patterns of associations between microbes and their hosts have provided many evolutionary insights^1^, from the endosymbiotic origins of the eukaryotes^2^ to the microbial basis of adaptations that are not directly encoded by the eukaryotic genome itself^3,4^. The reliance of hosts on functions provided by their microbes can lead to selection for specificity in host-microbe relationships when microbes are inherited through vertical transmission from mothers to offspring^5^ or selected by hosts from the environment every generation^6^. Often, such microbes are required for host development and are spatially segregated into specific cells and tissues within the body of a host individual^7–12^.

Cicadas (Hemiptera: Auchenorrhyncha: Cicadidae) represent a well-studied example of obligate and specific associations between animals and microbes. They obtain essential amino acids and vitamins absent in their diets from two obligate symbionts, “*Candidatus* Sulcia muelleri” (hereafter *Sulcia*) and “*Candidatus* Hodgkinia cicadicola” (hereafter *Hodgkinia*). These microbes have largely non-overlapping metabolic functions required by their hosts for development and are housed in bacteriomes outside of the gut but within the cicada abdomen^12–16^. As previously predicted by early microscopy^17,18^, recent molecular work shows that the loss of *Hodgkinia* is coincident with replacements by yeast-like *Ophiocordyceps* fungi^19^.

Although these obligate symbionts—*Sulcia, Hodgkinia*, and *Ophiocordyceps*—are important in supplying nutrients to cicada hosts, they are not known to reside in the cicada gut. Comparatively little is known about cicada gut-associated microbes^20–22^ despite the increasingly recognized nutritional role of animal gut microbiota^8,23–25^. Previous studies on cicada gut-associated microbiota have focused on *Meimuna mongolica*^20,21^ and *Platypleura kaempferi*^21^. As opposed to the specificity of obligate symbionts, gut microbiota may be facultative or transient with little specificity and involve complex microbial communities with varying functions across fluctuating ecological conditions^26^, but may nonetheless be essential for host development^27–30^.

Variation in microbial communities among animal hosts may be explained by many factors, including the host phylogeny itself^25,31,32^, particularly when microbes are inherited. However, many ecological correlates have also been shown to impact host-associated microbial communities, particularly the conditions of the gut niche which experiences continuous input of environmental microbes^33–35^. It remains unclear, however, to what extent microbial community assembly within many host species is influenced by fluctuating ecological conditions as compared to host evolution. Indeed, transient microbes may not have functions relevant to host fitness^36,37^. Yet these microbes may be a source of adaptive functions of novel microbial metabolism as hosts diversify^38^. Broader surveys are needed to enhance our understanding of the causes and consequences of variation in host-associated gut microbiota and whether they play important roles in organisms with other symbiotic associations such as cicadas.

In this study, we generate 16S rRNA gene amplicons of microbial communities and newly assembled mitochondrial genomes across 38 New Zealand (NZ) cicada species and hybrids between one pair of two species to understand the relative contributions of host phylogeny and ecology to variation in gut microbiota. These 38 species comprise three genera and represent a radiation of species from a single colonizing ancestor which now occupy a wide variety of habitats and elevations. We show that phylogenetic distances among cicada hosts are poor predictors of gut microbial communities compared to elevational differences, suggesting little host specificity and a role for ecological heterogeneity. Further supporting this lack of host specificity, we found no evidence of significant differences in gut microbial communities between interspecific hybrids and their parental species. These results point to the stochasticity of bacterial communities across an island landscape and may be related to the fact that most essential nutrients are supplied by specialized bacteriome-associated symbionts.

## Methods

### Sample collection and processing

#### Collection and outgroup choice

WE collected New Zealand cicadas in 95% ETOH from 1995 to 2018 and stored them at − 80 °C until processing (see Table S1 for specimen details). Based on previous work^39^, we included several *Caledopsalta* (Cicadettini, Cicadettinae) cicada specimens from New Caledonia as an outgroup comparison to the New Zealand cicada clade comprising *Kikihia, Maoricicada*, and *Rhodopsalta.* We also included several *Magicicada* (Lamotialnini, Cicadettinae) from North America. Although we focus on the aforementioned groups, our dataset also includes, for comparative purposes, *Neotibicen* (North American, Cryptotympanini, Cicadinae) and *Platypedia* (North America, Tibicininae) specimens but these are not discussed as part of our main findings. In summary, we caught cicadas by net or lured them using manually produced mating clicks, taking advantage of the attraction that male cicadas have for wing flicking sounds and movements associated with conspecific females^40,41^. We identified each specimen to species in the field using a combination of song, morphology, and knowledge of their evolutionary history and distribution^42–44^. One hybrid-descended lineage, *K.* “muta × tuta”, was sampled. This lineage possesses *K. muta* nuclear DNA, song, and morphology, but *K.* “tuta” mtDNA as a result of hybridization and introgression of the mitochondrion^44^. The identity of *K.* “muta × tuta” samples were inferred based on the above criteria and the geographic distribution of the *K. muta* and *K.* “muta × tuta” lineages. For specimens sampled from localities known to have both *K. muta* and *K.* “muta × tuta” lineages, we sequenced the mitochondrial COI gene and treated 100% matches to *K.* “tuta” sequences in GenBank as confirmation of *K.* “muta × tuta” identity.

#### Dissection

WE sterilized specimens by submerging them in 2% bleach, letting them sit for 1–2 min and then washing them in both 50–70% alcohol and sterile water. We then dissected specimens using small scissors, forceps, and pins to access gut tissue ventrally. The complex structure of the cicada gut required prolonged (15–30 min) and relatively tedious dissection compared to insects with relatively simpler guts (Fig. S1). We either extracted both gut and reproductive tissue or only gut tissue depending on the specific dataset produced (dataset specifications provided below). Tissue samples were either directly placed into Powersoil bead tubes or stored in sterile cryotubes and kept frozen until DNA extraction. All dissection equipment was sterilized with 10% bleach and then treated with UV light in a crosslinker for at least one minute prior to dissection. We carried out dissections over the course of several months, with 2–15 dissections on any given day.

We binned processed samples into three sample batches based on the timing and methodological differences in processing, the workers who processed them, and sampling design: B1, B2, B3 (Table S1). Dataset-specific variations are described as follows:

- **B1 dataset**: Combined gut and male reproductive tissue from *Kikihia muta* and *Kikihia* “tuta” representing nine parental populations and six previously identified hybrid populations extracted using a Powersoil DNA Isolation kit (MO BIO Laboratories) under the standard protocol. The entire purification process was performed using the Powersoil DNA Isolation protocol (not DNeasy).
- **B2 dataset**: Gut tissue from a broad sampling of New Zealand cicada species mostly within *Kikihia* extracted by mechanical lysing within Powersoil bead tubes containing Powersoil lysis buffer and subsequent DNeasy 96-well plate extraction under standard protocols (beginning after Proteinase-K treatment and incubation).
- **B3 dataset**: Gut tissue from a broad sampling of New Zealand cicada species including outgroup species from New Caledonia and various North American cicadas extracted by mechanical lysing within Powersoil bead tubes containing Powersoil lysis buffer and subsequent DNeasy 96-well plate extraction under standard protocols (beginning after Proteinase-K treatment and incubation).

### Amplicon sequencing of 16S V4 rRNA

We amplified the V4 region of bacterial 16S rRNA using universal barcoded primers 515F (5′-GTGCCAGCMGCCGCGGTAA-3′) and 806R (5′-GGACTACHVGGGTWTCTAAT-3′) with attached Illumina-compatible adapters and indices (Microbial Analysis, Resources, and Services Facility, University of Connecticut) under the following PCR conditions: 95 °C for 2 min, 35 cycles of 95 °C for 30 s, 55 °C for 1 min, 72 °C for 1 min, and then a final extension at 72 °C for 5 min. All libraries were quantified with QIAxcel and manually inspected for proper marker alignment. We normalized libraries by pooling to the lowest concentration for each of the sample sets. Samples that could not be quantified due to low concentration were pooled using the maximum product available. Pooled samples were cleaned and size-selected using a bead-based approach. Final 16S amplicon libraries were sequenced paired end on Illumina MiSeq. Dataset-specific variations are described as follows:

- **B1 dataset:** Amplified with V4 16S rRNA primers as above using EmeraldAmp GT PCR Master Mix (TAKARA BIO).
- **B2 dataset:** Amplified in a separate facility (Microbial Analysis, Resources, and Services Facility, University of Connecticut) with V4 16S rRNA primers as above using GoTaq DNA Polymerase (PROMEGA).
- **B3 dataset:** First amplified with primers 27F (5′-AGAGTTTGATCMTGGCTCAG-3′) and 1492R (5′-GGTTACCTTGTTACGACTT-3′) using EmeraldAmp GT PCR Master Mix (TAKARA BIO) to minimize the amplification of non-bacterial taxa under the following cycling conditions: 94 °C for 5 min, then 5 cycles of 94 °C for 45 s, 56 °C for 45 s, 72 °C for 1.5 min, and then a final extension at 72 °C for 10 min. The product was used as a template for amplification with V4 16S rRNA primers as in dataset B1.

### Negative controls

We took various controls when processing the B2 and B3 datasets, most of which were taken as part of the B3 dataset. Six PCR controls across the two datasets were taken (two controls in the B2 dataset and four controls in B3 dataset) during library amplification using V4 16S rRNA primers (Fig. S2, PCR and control). The remaining controls are associated with the B3 dataset and are as follows: Six dissection reagent controls of fluid used prior to dissection (Fig. S2, dissection), 10 controls of surface contents of forceps after transferring tissue from dissection plates to extraction tubes and sterilizing (Fig. S2, transfer), five surface sterilization controls of the fluid used to wash specimens prior to dissection (Fig. S2, wash), and six extraction kit controls from both the DNeasy and powersoil kits (Fig. S2, dneasy and powersoil).

### 16S rRNA amplicon data processing

We denoised and merged reverse and forward reads in QIIME 2^45^ using the DADA2 pipeline^46^ separately for each dataset, with the exception of the B3 dataset in which we only considered forward reads due to high error rates in the reverse reads. We aligned the denoised sequences in MAFFT^47^, filtered the alignments, and constructed a midpoint-rooted phylogeny using the “align-to-tree-mafft-fasttree” pipeline in QIIME 2. We classified amplicon sequence variants (ASVs) with the QIIME 2 “classify-sklearn” plug-in after training a classifier with the “fit-classifier-naive-bayes” plug-in on sequences of the V4 region of 16S rRNA extracted from SILVA 99% OTUs (Release 132) database. The resulting feature tables, phylogenies, classifications, and sample metadata were primarily processed using the R package *phyloseq*^48^. We removed ASVs classified to mitochondria, chloroplast, cicada bacterial endosymbiont (*Hodgkinia* and *Sulcia*), eukaryote, and archaea. To minimize noise introduced by sequencing errors, unclassified taxa, and low sequencing coverage, we removed ASVs that could not be classified at the Kingdom, Phylum, or Class taxonomic levels and ASVs with a total abundance across all samples within a dataset of less than three. We included only samples with a total abundance greater than 100. The remaining ASVs per dataset were further collapsed using the “tip_glom” function in *phyloseq* to cluster ASVs based on cophenetic distances with a tree height of 0.03. We then used the R package *decontam*^49^ to identify putative contaminants. However, we were unsuccessful in identifying contaminants with our post-PCR DNA concentration data using the frequency-based filters under reasonable thresholds due to large inter-sample variability in the presence and abundance of different ASVs. Instead, we relied on control samples in the B2 and B3 datasets. We used the prevalence-based filter with a threshold of 0.3 to remove ASVs that were enriched in the controls and then we subsequently removed all ASVs in all datasets that were classified to these putative contaminant genera (Fig. S2).

### Quantitative PCR (qPCR) of 16S rRNA amplicons

To estimate how initial copy numbers of target molecules may differ across our samples, we submitted a subset of samples for qPCR using universal V4 region primers and a CFX Opus 384 Real-Time PCR System. We sought to assess whether quantification of samples after PCR but before pooling would be predictive of the number of target molecules initially present. Thus we analyzed DNA extracts of 24 samples including those of six negative controls and other samples with a range of expected amplicon copy numbers based upon initial quantification as well as sequencing results (Fig. S3 and Table S4). Samples were quantified in triplicate and average initial amplicon copy number estimated based upon correlation to curve calculated with standard.

### Cicada mitochondrial genome phylogeny

We assembled host mitochondrial genomes using off-target capture data from an anchored hybrid enrichment dataset of worldwide cicada lineages^50–52^. We first deduplicated merged and unmerged reads using the function clumpify in BBMap^53^, trimmed adapters and reads with Q < 20 using Trimmomatic^54^, and then assembled reads using both merged and unmerged data in SPAdes v. 3.12.0^55,56^. We extracted mitochondrial contigs from the resulting assembly using tblastn with a published partial *K. muta* reference mitochondrial genome—Genbank MG737737^57^—used BWA v. 0.7.5a^58^ in a second processing step, and then reassembled the resulting reads with SPAdes 3.12.0. The final sets of mitochondrial contigs for each sample were aligned to various published and nearly complete mitochondrial genomes^57^ using the MAFFT v. 7 E-INS-i algorithm^59^, combined into single mitochondrial genome sequences, and manually edited to exclude misassembled regions in Geneious v. 10.1.3^60^. We used these mitochondrial sequences as sample-specific baits in MITObim v. 1.9.1^61^ to assemble improved, higher-quality mitochondrial genomes that were aligned with the MAFFT v. 7 E-INS-i algorithm and manually edited in Geneious v. 10.1.3 for a final mitochondrial genome alignment. We designed a partitioning scheme that included combined 1st and 2nd codon positions and the 3rd codon position for each protein-coding gene, partitions for each of the 12S rRNA and 16S rRNA loci, and a single partition for all tRNAs. We ran a maximum likelihood analysis with this partitioning scheme in RAxML v.8^62^ on the CIPRES web server^63^ to produce a final phylogeny.

## Results

### Patterns of bacterial communities of New Zealand cicadas suggest a low abundance and highly variable microbiota not present in eggs

**Figure 1:**
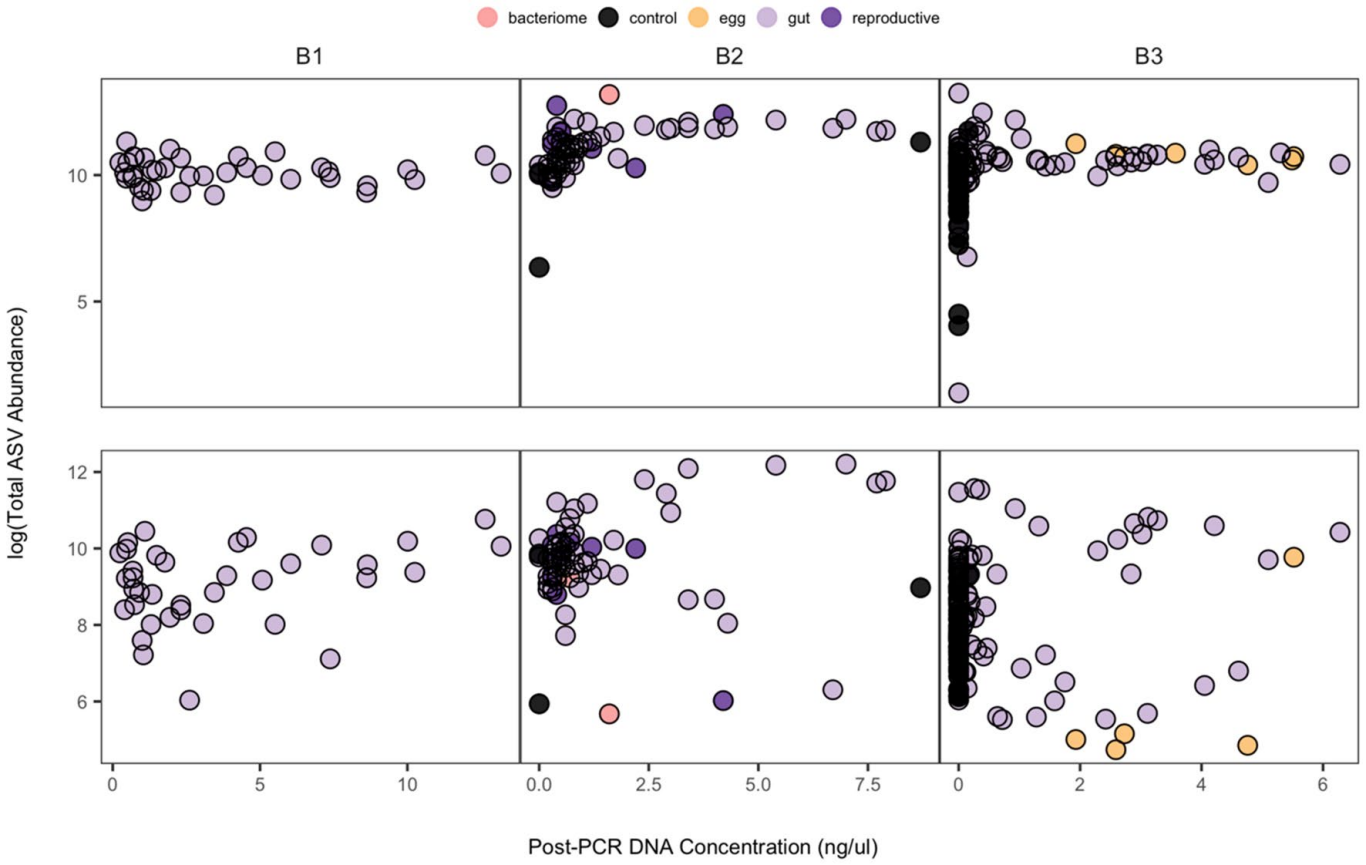
The relationship between DNA concentrations of the amplicon libraries and logged total abundance of sequenced ASVs per sample. The top three panels represent unfiltered data corresponding to each dataset and the bottom three panels represent filtered bacteria-only data corresponding to each dataset. Colors correspond to tissue type.

Prior to filtering each dataset, some samples contained low total ASV abundances (Fig. 1, top), suggesting that cicada gut tissues sometimes contain low amounts of bacterial cells or that the dissection procedure failed to isolate many microbial cells. Our qPCR data of 16S rRNA amplicons from a subset of our samples corroborate these patterns (some of which were quantified as possessing fewer than 100 initial copies of the 16S ribosomal rRNA) and show that the absolute quantity of 16S rRNA amplicons inferred from qPCR is positively correlated with the DNA concentration data we collected post-PCR for a subset of samples (Fig. S3, R^2^ = 0.5375). Our results also showed that controls were consistently quantified as having very few initial target molecule copies (fewer than 0.5 in all cases). Herein, we use our DNA concentration data to inform our filtering procedure.

**Figure 2:**
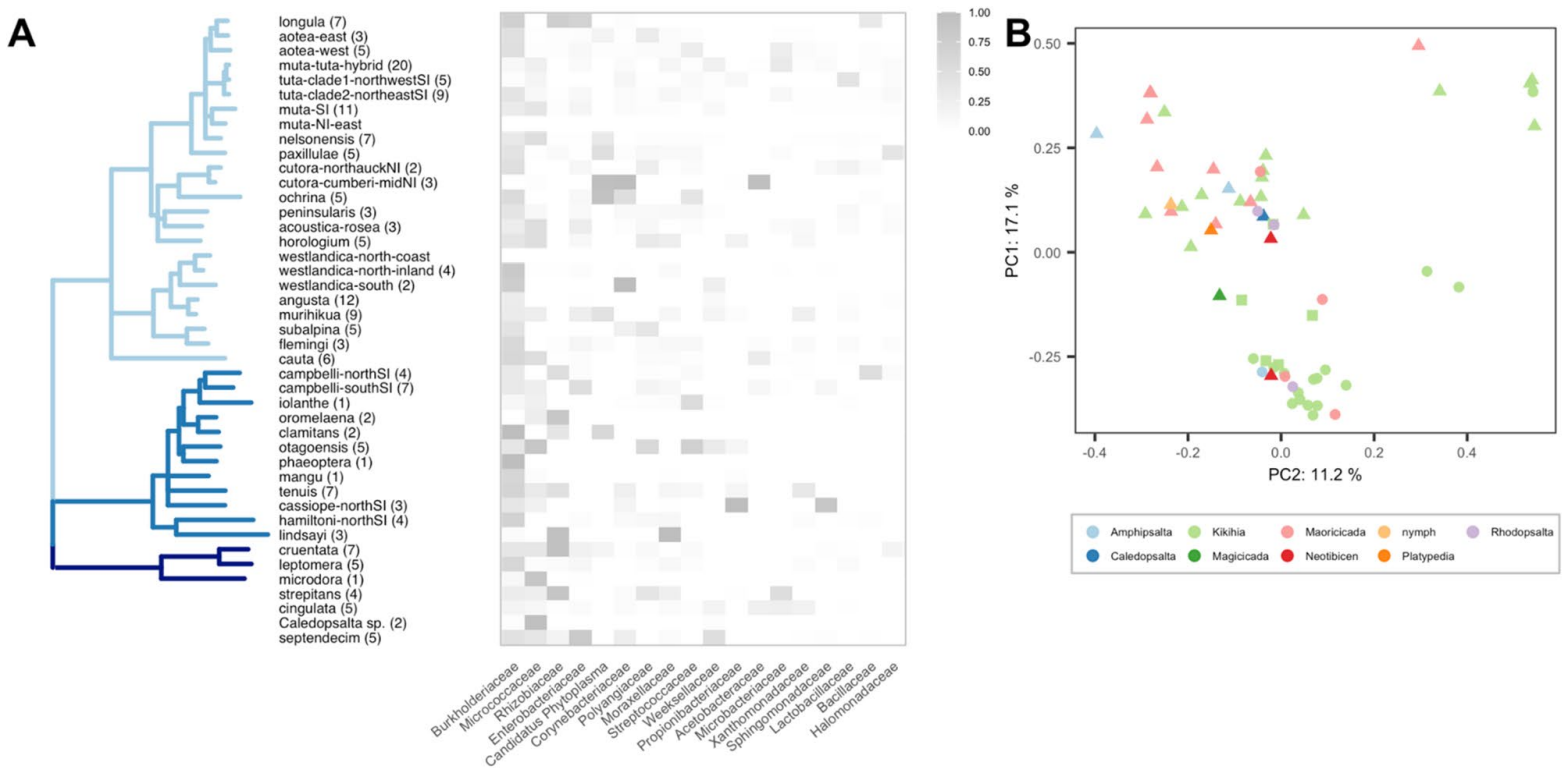
Distribution of major taxonomic groups using 16S V4 rRNA classification of amplicon sequence variants (ASVs) across all datasets. (**A**) Maximum likelihood phylogeny using nearly complete mitochondrial genomes of the sampled host species. Branches are collapsed at a bootstrap threshold of 90; bootstrap supports for all nodes are shown in Fig. S8. Tip labels contain the number of individuals sampled in parentheses and match specific epithets in Table S1. Branch colors represent different genera. Species not used to construct the phylogeny are added below the tree (*Amphipsalta strepitans*, *Amphipsalta cingulata*, *Caledopsalta* sp., *Magicicada septendecim*). (Right) Heatmap of relative abundances of major bacterial families averaged across individuals and datasets per species. (**B**) Ordination using weighted UniFrac distances of filtered bacteria-only samples using relative abundances of major bacterial families averaged across individuals within species. Colors correspond to host genera. Shapes correspond to datasets (square = B1, circle = B2, triangle = B3). The axis labels report the percent variation explained by the corresponding principal components.

After filtering samples (see “16S rRNA amplicon data processing” in “Methods”), we identified 18 major bacterial families with high relative abundance and prevalence across samples and datasets, with Burkholderiaceae, Micrococcaceae, Rhizobiaceae (non-*Hodgkinia*), and Enterobacteriaceae among the top four groups in terms of relative abundance (Fig. 2A heatmap, Figs. S4, S5). We could not detect a qualitative structuring of these major bacterial families among clades defined by the host mitochondrial genome phylogeny. We used principal coordinates analysis (PCoA) ordinations of Bray distances derived from relative abundances of ASVs belonging to these major bacterial groups and averaged across samples within species (Fig. 2B) to show that variation in bacterial communities surveyed in this study are primarily explained by differences in datasets (permanova, R^2^ = 0.17, p < 0.01**) rather than differences in genera or species sampled (permanova, p > 0.05). In addition, we could not find significant contributions of host genera or species in structuring communities within datasets using either weighted and unweighted UniFrac distances of unmerged samples (see Fig. S6). Although specimens used in this study were collected over many years, we do not find a qualitative effect of collection date in structuring communities (see Fig. S7) and the effect of collection year was insignificant in explaining microbial community differences when considering each dataset separately (permanova, p > 0.05). After filtering, we were left with one nymphal sample with a similar microbial composition as adult samples (see Fig. 2B), but we are unable to make conclusions about the nymphal microbiome with these data alone.

### Mitochondrial genome phylogeny improves resolution of relationships among New Zealand cicadas

Our host phylogeny is based on a whole mitogenome data, nearly seven-fold increase in alignment length (~ 14,000 bp) from previous phylogenies^64,65^, which were based on approximately 2000 bp of sequence data. Our phylogeny (Fig. 2A, Fig. S8) recovers *Rhodopsalta microdora* as sister to *R. leptomera* + *R. cruentata* with 100% bootstrap support, confirming results with fewer genes^66^, and groups *Rhodopsalta* with *Maoricicada* with 100% bootstrap support. The *Maoricicada* taxon sampling is reduced compared to the previous *Maoricicada* mitochondrial tree, but despite the missing taxa the mitogenome phylogeny is congruent with the previous tree^64^. For the *Kikihia* taxa, the mitogenome phylogeny recovers the same major well-supported clades previously identified^65^.

### Elevational differences explain bacterial community variation better than host phylogeny

We used various statistical tests to assess the effect of host phylogeny on bacterial community variation and found no evidence of host phylogenetic structure. Mantel tests between unweighted and weighted UniFrac distances among gut microbial communities and cophenetic distances among host mitochondrial genomes were insignificant based on permutation tests for both B2 and B3 datasets (p > 0.05). Tests of phylogenetic signal (Pagel’s lambda and K statistic) using the first axis positions of either unweighted or weighted UniFrac PCoA ordinations as phylogenetically distributed traits were insignificant for each dataset as well (p > 0.05). In addition, ordinations of both weighted and unweighted UniFrac distances did not produce clustering that corresponded to host taxonomy at either the genus or species levels (Fig. 2B and Fig. S6).

**Figure 3:**
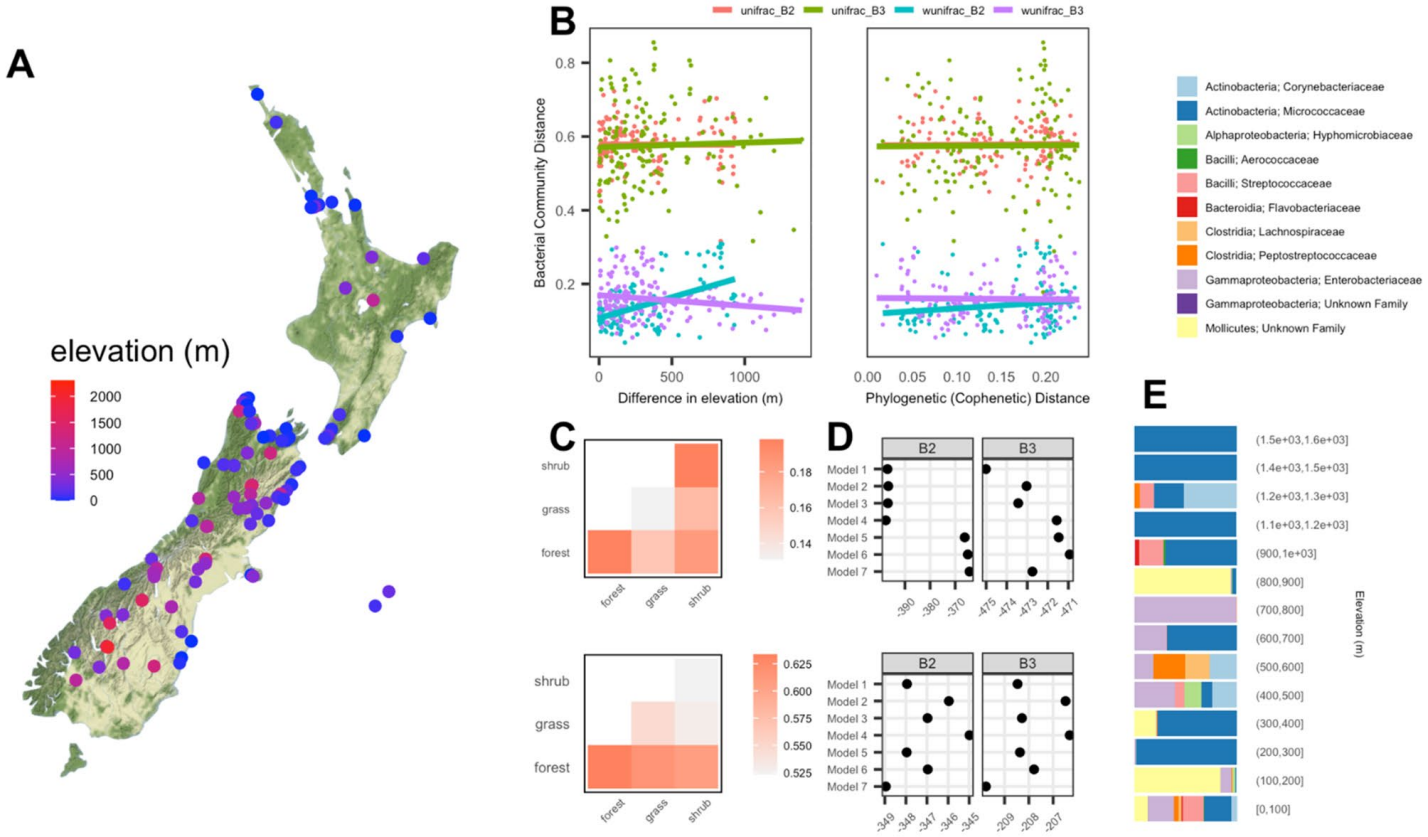
Correlates of bacterial community differences in New Zealand cicadas using filtered 16S V4 rRNA ASVs from the B2 and B3 datasets. (**A**) Sampled sites with labels representing state and locality codes found in Table S1. The map was created using the R package ggmap (version 3.0.1). (**B**) Pairwise bacterial community differences using both unweighted (unifrac) and weighted (wunifrac) UniFrac distances with increasing differences in elevation (left) and increasing cophenetic distances calculated using the host mitochondrial phylogeny (right) per dataset. Pairwise comparisons between samples representing different host genera were excluded. (**C**) Average pairwise bacterial community differences among *Kikihia* samples in the B2 dataset using weighted (top) and unweighted (bottom) UniFrac distances for comparisons between samples of the same and different habitats. (**D**) AIC values across beta regression models using weighted (top) and unweighted (bottom) UniFrac distances as response variables. Model formulas, including explanatory variables, associated with each model are provided in Table S2. (**E**) Relative abundance of differentially abundant family-level ASVs across samples binned into increasing elevation categories. Family-level bacterial ASV classifications (above) were found to be significantly differentially abundant between high and low elevation samples based on analysis with DESeq. Note that the group “Mollicutes; Unknown Family” (yellow) is represented entirely by ASVs belonging to the plant pathogen *Candidatus Phytoplasma*. multivariate analysis (Table S3).

We were able to collect specimens from a wide range of elevations throughout New Zealand (Fig. 3A), allowing us to isolate the effects of phylogenetic and elevational relationships among specimens. We did not find a positive correlation between phylogenetic distance and pairwise bacterial community dissimilarity using either weighted or unweighted UniFrac distances in either the B2 or B3 datasets (Fig. 3B, right), in accordance with the lack of host phylogenetic structure among the most abundant and prevalent bacterial families (Fig. 2). However, we found a strong positive correlation between differences in elevation and weighted UniFrac pairwise distances in the B2 dataset, suggesting elevational differences may play an important role in structuring New Zealand cicada microbial communities (Fig. 3B, left). In addition to elevation, we examined the effect of habitat type (forest, grassland, or shrub habitats) specifically in *Kikihia* species because these species occupy a broad range of habitat types. Our collections and observations of these species allow us to group them into discrete habitat types (Fig. S9), however we did not find significant differences between samples of the same habitat type and samples from different habitat types (Fig. 3C).

In addition, we compared multiple Beta regression models in which cophenetic distances between host lineages were used in combination with other covariates or excluded. Both the best and worst fitting models (AIC) included the effect of cophenetic distances, suggesting that host phylogeny had negligible explanatory power compared to the ecological covariates that were also included in these models (Fig. 3D and Table S2). We do not find similarly strong patterns in the B3 dataset, but this dataset was enriched for low-abundance bacteria resulting from nested PCR amplification (see “Amplicon sequencing of 16S V4 rRNA” in “Methods”) which likely erased signals of a positive relationship between elevation and weighted bacterial community distances given methodological effects on relative abundances. However, we find that in both the B2 and B3 datasets, the best fitting Beta regression models of weighted UniFrac distances always included elevation as an explanatory variable (Fig. 3D, top) and that elevation was significant in all models using the B2 dataset despite having small effect sizes (Table S2).

### Microbial communities in hybrids between *Kikihia muta* and *K.* “tuta” resemble those of their parental species

**Figure 4:**
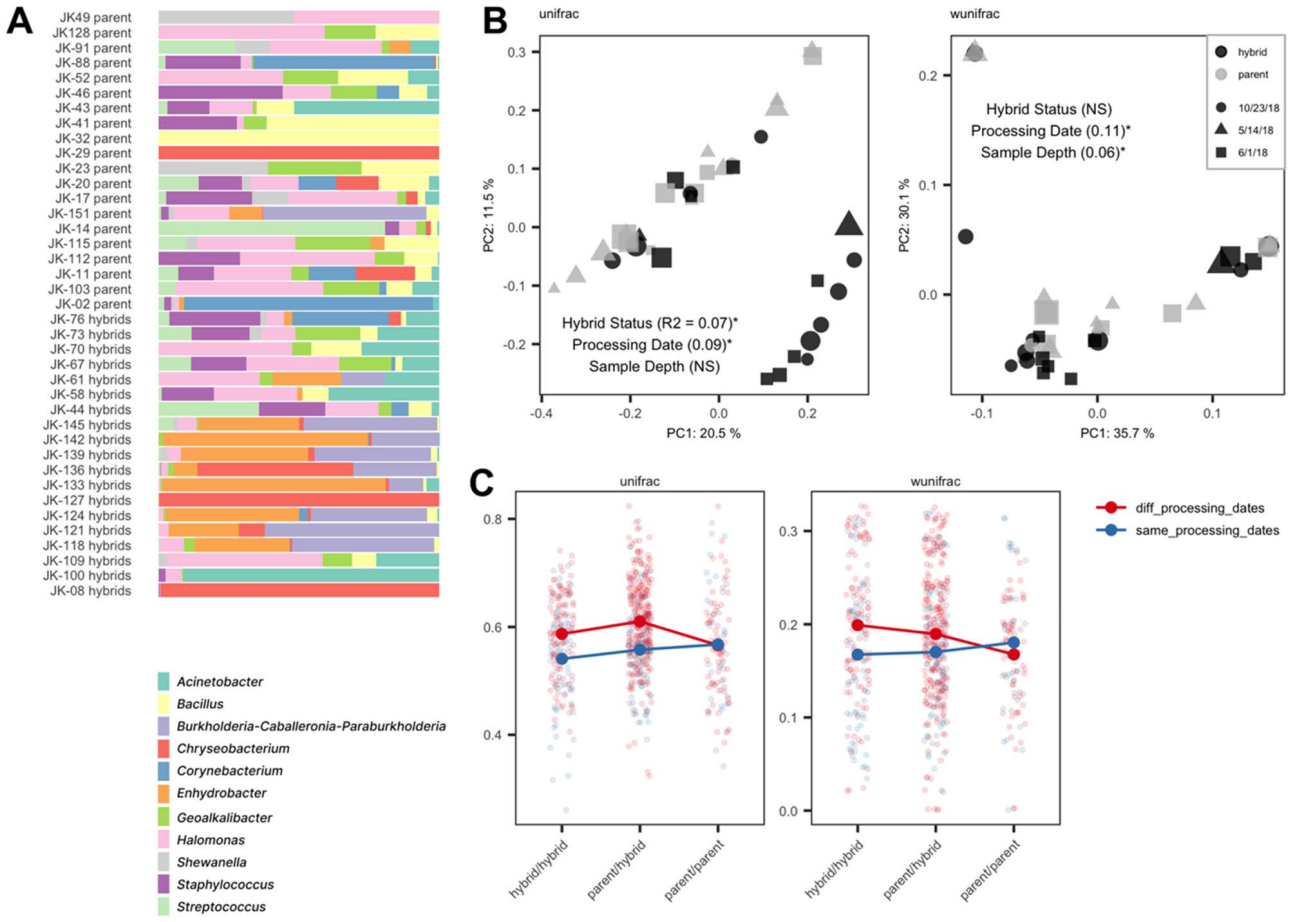
Bacterial community differences among hybrid and parental *Kikihia muta* and *Kikihia tuta* using filtered 16S V4 rRNA ASVs from the B1 dataset. (**A**) Relative abundance of the most abundant Genus-level bacterial ASVs across hybrids and parental samples. (**B**) PCoA ordination of samples from dataset B1 using unweighted UniFrac (left) distances and weighted UniFrac distances (right). Grey samples represent hybrid cicadas and black samples represent parental samples of either *Kikihia muta* or *Kikihia tuta*. Shapes represent different times of dissection and sample processing and their size represents relative sample depth (i.e., total ASV abundance). R-squared values from permanova analyses of the effect of hybrid status, processing date, and sample depth on each respective distance measure are reported in parentheses when explanatory variables were significant at a 0.05 significance level. “NS” stands for not-significant (i.e., p > 0.05) (**C**) Unweighted (left) and weighted (right) pairwise bacterial community distances for hybrid/hybrid, hybrid/parent, and parent/parent sample comparisons. Colors represent comparisons in which samples were processed either within the same processing date or within different processing dates. Lines connect mean values for each category of comparisons.

Specimens with evidence of introgression between *K*. *muta* and *K.* “tuta”, which we refer to as hybrids, in the B1 dataset did not show qualitative differences in the relative abundance of bacterial ASVs compared to parental species (Fig. 4A). Ordination of gut microbial communities using unweighted and weighted UniFrac distances showed that hybrids cluster with parental species (Fig. 4B) with significant effects of processing date (i.e., time at which samples were purified for DNA) and sample depth (i.e., total abundance of all ASVs). The processing date explained 9% and 11% of variation in unweighted and weighted UniFrac distances (permanova, p < 0.05***), respectively. However, hybrid status did not significantly explain variation in weighted UniFrac distances (permanova, p > 0.05) and explained less variation than processing date in unweighted UniFrac distances (permanova, R^2^ = 7%, p < 0.05***).

Pairwise unweighted and weighted UniFrac distances were elevated in comparisons of samples that included a hybrid specimen (Fig. 4C, hybrid/hybrid and hybrid/parent pairwise comparisons). Although this pattern may suggest that hybrids have a divergent and more variable microbiome compared to parents, these results include the effects of processing date. Considering only comparisons of samples processed on the same day (Fig. 4C, same_processing_dates), comparisons between hybrid and parental samples have indistinguishable differences in their bacterial communities. We found that the mean and variance of bacterial community distances are indistinguishable between comparisons that included a hybrid specimen and comparisons that included only parental specimens (Kolmogorov–Smirnov Test, p > 0.05; *F* test, p > 0.05).

## Discussion

### Lack of stable microbial communities in New Zealand cicadas

Studies of plant sucking bugs with specialized bacteriome-dwelling endosymbionts have with few exceptions^21,67^ not focused on the microbiota outside the bacteriome. Our dataset suggests that adult cicadas have low abundance transient microbial communities in their guts as shown in some other insects^36,37^. For example, in caterpillars^37^ the structural simplicity of the gut niche in combination with the rapid pace at which ingested material moves it is suggested to contribute to the lack of resident microbes (i.e., bacteria that are able to replicate and establish at a higher rate than they are lost due to death or excretion). However, the cicada gut niche is structurally more complex and larger in relative volume and especially surface area than that of most other insects, and includes many separate functional compartments^20,68^ that may be amenable to hosting resident microbes. The paucity of abundant and consistent microbial residents in this study may be explained by similar factors as in caterpillars, including the physiological pace of food processing driven by plant transpiration^69^ and osmotic pressure gradients via the cicada filter chamber^70^. These facets of cicada physiology are likely not amenable to microbial colonization as the quick flow of nutritionally poor xylem fluid through the gut may create a harsh environment in which resident bacteria might not be able to establish and proliferate. In addition to being rapidly transported through the cicada alimentary canal, xylem fluid is primarily composed of water with major solutes that include potassium, sodium, calcium, magnesium, chloride, and phosphate^70^. This is analogous to the rapid movement of food in the highly alkaline gut environments of caterpillars, which have been reported to lack appreciable evidence of a resident and functional gut microbiota^37^. We assume that relatively few microbes other than those adapted to living in xylem vessels (i.e., plant pathogens) can live in this cicada gut environment. Second, the presence of obligate bacterial and fungal symbionts outside of the gut circumvents any obvious need for the functional dependence of adult cicadas on microbe-derived essential nutrients in the gut, reducing the likelihood of selection for life-history or physiological traits that maintain either vertical inheritance or faithful horizontal acquisition of gut microbiota.

While the patterns shown in this study suggest the lack of a consistent resident gut microbiota, low-abundance microbial communities are difficult to detect. We were unable to detect differences in total bacterial abundance that correlated with host phylogeny or other abiotic correlates we considered. The lack of a consistent gut microbiota across host taxa is not completely unexpected because it is uncertain what role gut microbial colonization may have in supplementing an already complete cicada diet or providing other transient functions (e.g., immunity to pathogens) as cicadas navigate fluctuating environments. We could not conclude this without looking. Any questions regarding a possible functional role of what we found to be low abundance bacteria would require ecological experiments and tests of fitness. Given the long life of most cicada nymphs^16^ compared to mammals, within which gut microbiota are structured by host phylogeny, and the much shorter span of the adult stage^71,72^, it is unlikely that transient microbes are able to establish within adult cicada guts. A greater sampling of nymphal gut microbial diversity and abundance is needed to understand the contribution of environmentally acquired microbiota in the much longer-lived nymphal stage, as well as the microbes that are transmitted to the adult stage from the nymphal stage. Previous work has shown that *Magicicada* nymphs had gut microbial communities that differed from both the adults and the soil in which they were found^67^. While the lack of microbial diversity in eggs dissected from the abdomens of female cicadas in this study suggests a lack of vertical transmission of non-endosymbiotic bacteria, both the overwhelming relative abundance of cicada endosymbiont cells and the lack of other bacterial cells in these samples may prevent detection of low-abundance microbes.

### New Zealand cicada mitochondrial phylogeny compared to previous studies with much less data

An added feature of our study is a new phylogeny of the host NZ cicada species using nearly complete mitochondrial genomic data (~ 14 kb). Support among the NZ cicada species is improved by the inclusion of most mitochondrial genes. Relationships within the *Maorocicada* are better supported but otherwise compatible with that of previous studies based on 2274 bp of mtDNA with slightly larger taxon sampling^64,73^. Relationships involving *M. campbelli* (Fig. 2A, campbelli-northSI and campbelli-southSI) and *M. iolanthe* (Fig. 2A, iolanthe) remain inconsistent with species trees generated from nuclear loci that group these taxa with the other lower elevation species, *M. hamiltoni* (Fig. 2A, hamiltoni-northSI) and *M. lindsayi* (Fig. 2A, lindsayi), in agreement with similarities in male genitalia, mating songs, and habitat specialization^64,73^. Low support at the base of the *Maoricicada* alpine clade suggests rapid diversification during a period of intense mountain building^64^. Relationships among *Kikihia* match those of previous mitochondrial phylogenies, including a lack of resolution among some clades despite seven times more data. This is not surprising given evidence for rapid radiation and extensive hybridization (including mtDNA capture) in this group^44,74^. Bacterial communities in other cicada genera—*Magicicada*, *Caledopsalta*, *Neotibicen*, and *Platypedia*—cluster haphazardly among the NZ cicadas (Fig. 2).

### Lack of host specificity in New Zealand cicada microbial communities

Our analysis suggested no host phylogenetic structure among bacterial communities in New Zealand cicadas, even when accounting for variation explained by ecological differences, though several other studies have shown a significant effect of host phylogeny in structuring microbial communities in, for example, corbiculate bees, mammals, tropical birds, ants, deer mice, fruit flies, mosquitoes, and wasps^31,32,34,75,76^. Small phylogenetic distances among host taxa and the suggested lack of functional importance of gut microbial communities given the presence of specialized endosymbionts likely contributed to these results.

In addition, we could not find significant differences in gut bacterial composition between hybrids and their parental species, reinforcing our conclusions that cicadas lack resident gut microbiota that are structured by host phylogeny. Indeed, if host adaptations that maintain assembly of bacterial communities have evolved, we would expect that these mechanisms in hybrids would deteriorate when genomic variants that have undergone divergent ecological selection for gut microbial community assembly^77^ are combined into different genetic backgrounds. Improper regulation of gut bacteria in hybrid hosts may be one source of hybrid lethality, however support for this hypothesis is uncertain^78,79^. We found little evidence that host genetics influenced microbial community assembly in cicadas, yet other hybrid crosses have shown a host genetic effect in mammals^77,80^.

Although gut microbial communities in New Zealand cicadas do not seem to be host specific, the composition of these communities may be highly influenced by elevational differences among host individuals. Elevational diversity is a prominent feature of the New Zealand landscape, with dramatically varying ecosystems along elevational gradients. Understanding the many ecological factors contributing to variation in the microbiome is an important next step for understanding host-associated microbial diversity. While our data suggest an association with elevation, we lack sufficient power to identify specific bacterial taxa enriched at high or low elevations. In addition, future studies with broader within-species sampling are needed to investigate the causes of intraspecific variation in the microbiome. Although we do not have sufficient intraspecific sampling of elevationally diverse species in this study, some *Kikihia,* in particular, occupy broad elevational ranges and may be good candidates for additional sampling. Nonetheless, elevation is the best predictor of microbial community variation in our analyses.

### Phytoplasma plant pathogens in New Zealand cicadas

We report the first sequence-based identification, to our knowledge, of the plant pathogen *Candidatus* Phytoplasma in cicadas. Phytoplasmas (Phylum Tenericutes: Class Mollicutes) comprise a group of prokaryotic relatives to mycoplasmas and spiroplasmas and are transmitted among plant hosts by insect vectors through phloem. These insect vectors primarily represent the phloem-feeding members of the hemipteran groups Cicadellidae, Fulgoromorpha, and Psyllidae, but may be transmitted by the families Pentatomidae and Tingidae as well^81^. Despite interactions with both plant host and insect vector, phytoplasma strains do not appear to have insect-vector specificity and many different vectors may transmit a single phytoplasma strain or vice versa^81^. This pattern is recapitulated in other insect-vectored plant pathogens, particularly *Xylella* pathogens which are found in xylem^82^. All cicadas are xylem feeders so far as is known^83^ and *Xylella* transmission has been reported in the apache cicada *Diceroprocta apache* into California grapevine^84^ and by various Brazilian cicadas into coffee^85^. Rather than *Xylella*, we found that phytoplasmas were in high abundance across six specimens representing *K. paxillulae, K.* “murihikua”, *K.* “aotea”, *K. muta*, *K.* “nelsonensis”, and *K. ochrina* (see Fig. 2 heatmap, “*Candidatus* Phytoplasma”). We did not detect phytoplasmas in any New Zealand cicada genus other than *Kikihia*, which tend to occupy lower elevation habitats characterized by grasses and shrubs. Phytoplasmas comprise a large majority of the total rRNA abundance when present in these samples. The high abundance of 16S rRNA amplicons that were classified to phytoplasmas after our filtering procedure suggests that cicadas may be able to vector these phloem-specific plant pathogens despite being xylem feeders. While this is unlikely and requires further investigation, a similar situation has been found in some phloem-feeding hemipterans which acquire and transmit xylem-specific Xylella pathogens^86^.

## Conclusion

In summary, we find that gut microbial communities in NZ cicadas are low abundance and not strongly structured by host phylogeny and that hybrid cicadas resemble parental species, suggesting a lack of host specificity. Instead, we find that elevation differences among individuals explain variation in gut microbiota better than phylogenetic relatedness among hosts. Our new and nearly complete mitochondrial genomes improved phylogenetic relationships among NZ cicadas, providing a valuable resource for investigations of the evolutionary history of this group and our discovery of agriculturally-important phytoplasma plant pathogens in *Kikihia* cicadas is the first in this Hemipteran subgroup.

## Data Availability:

The datasets generated and/or analyzed during the current study are available in the GenBank repository (Accession: PRJNA879614, https://www.ncbi.nlm.nih.gov/bioproject/PRJNA879614).

## Supplementary Information

Supplementary Information 1.

Supplementary Information 2.

Supplementary Information 3.

Supplementary Information 4.

Supplementary Information 5.
