# Supplementary material for: Lack of host phylogenetic structure in the gut bacterial communities of New Zealand cicadas and their interspecific hybrids
Source: Sci Rep. 2022 Nov 29;12:20559. doi: 10.1038/s41598-022-24723-3 (PMC9709078; doi:10.1038/s41598-022-24723-3)
Supplement: Supplementary file 1 — Supplementary Information 1. [file 41598_2022_24723_MOESM1_ESM.docx]

>2045e96ad2e2cf90078d900f67bb6f8f

TACGTGAGAGACTAGTGTTATTCATCTTAATTGGGTTTAAAGGGTACCTAGACAGTCAATATAACTTCTATAATGCTAATACTTGACTAGAGTTTTAAGTAAGAGGGAAGTACTTAAGGAGTAAGAGATGAAATATCTGTGATACCAAAGGGACTCCGTAAAGGCGAAGGCATCCCTTTATCTAAAAACTAACGTTGAAGGACGAAGGCTTAGATAACAAATAGG

>c31219c21d19f783a7cc6b509a9e0fc4

AACAGAGGATACAAGCGTTATCCGGATTTATTGGGTTTAAAGGGTGCGTAGGTGGTTTTTTAAGTCAGTAGTGAAATCTTAAAGCTTAACTTTAAAAGTGCTATTGATACTGATAAACTAGAGTGAGGTTGGAGTAACTGGAATGTGTGGTGGAGCGGTGAAATGCATAGAGATCACACAGAACACCAATCGCGAAGGCATGTTACTAAACATAGACTGACACTGAGGCACGAAAGCATGGGTAGCAAACAGG

>5673ae66819e3bdbbc09461e4959159e

TACGGAGGGTGCAAGCGTTAATCGGAATTACTGGGCGTAAAGCGCACGCAGGCGGTCTGTTAAGTCAGATGTGAAATCCCCGGGCTTAACCTGGGAACTGCATTTGAAACTGGCAGGCTTGAGTCTCGTAGAGGGGGGTAGAATTCCAGGTGTAGCGGTGAAATGCGTAGAGATCTGGAGGAATACCGGTGGCGAAGGCGGCCCCCTGGACGAAGACTGACGCTCAGGTGCGAAAGCGTGGGGAGCAAACAGG

>3902f671cea4650ba271a2cbc90c7eb8

TACGTAGGGCGCAAGCGTTATCCGGAATTATTGGGCGTAAAGAGCTCGTAGGCGGTTTGTCGCGTCTGCTGTGAAAGTCCGGGGCTCAACTCCGGTTCTGCAGTGGGTACGGGCAGGCTTGAGTGATGTAGGGGAGACTGGAATTCCTGGTGTAGCGGTGAAATGCGCAGATATCAGGAGGAACACCGATGGCGAAGGCAGGTCTCTGGGCATTAACTGACGCTGAGGAGCGAAAGCATGGGGAGCGAACAGG

>9dcd05e2f900431a5c267a4b853bc2ae

TACGGAGGGTGCAAGCGTTATCCGGATTTATTGGGTTTAAAGGGTCCGTAGGCTGATGTGTAAGTCAGTGGTGAAATCTCACAGCTTAACTGTGAAACTGCCATTGATACTGCATGTCTTGAGTGTTGTTGAAGTAGCTGGAATAAGTAGTGTAGCGGTGAAATGCATAGATATTACTTAGAACACCAATTGCGAAGGCAGGTTACTAAGCAACAACTGACGCTGATGGACGAAAGCGTGGGGAGCGAACAGG

>c1cf0c4ee7d879c86ca0523506557623

TACGTAGGGCGCAAGCGTTATCCGGAATTATTGGGCGTAAAGAGCTCGTAGGCGGTTTGTCGCGTCTGCTGTGAAAGACCGGGGCTCAACTCCGGTTCTGCAGTGGGTACGGGCAGACTGGAGTGATGTAGGGGAGACTGGAATTCCTGGTGTAGCGGTGAAATGCGCAGATATCAGGAGGAACACCGATGGCGAAGGCAGGTCTCTGGGCATTAACTGACGCTGAGGAGCGAAAGCATGGGGAGCGAACAGG

>3cd9ce63f826137d18ca1a11155a21ad

TACGTAGGGTGCGAGCGTTGTCCGGAATTATTGGGCGTAAAGAGCTCGTAGGCGGTCTGTCGCGTCTGCTGTGAAAACCCGAGGCTCAACCTCGGGCCTGCAGTGGGTACGGGCAGACTAGAGTGCGGTAGGGGAGAATGGAATTCCTGGTGTAGCGGTGGAATGCGCAGATATCAGGAGGAACACCGATGGCGAAGGCAGTTCTCTGGGCCGTAACTGACGCTGAGGAGCGAAAGCGTGGGGAGCGAACAGG

>0606870e7caf9d39f42f23dff84c6190

TACGGAGGGTGCGAGCGTTAATCGGAATTACTGGGCGTAAAGCGCGCGTAGGCGGCTTGATAAGCCGGTTGTGAAAGCCCCGGGCTCAACCTGGGAACGGCATCCGGAACTGTCAGGCTAGAGTGCAGGAGAGGAAGGTAGAATTCCCGGTGTAGCGGTGAAATGCGTAGAGATCGGGAGGAATACCAGTGGCGAAGGCGGCCTTCTGGACTGACACTGACGCTGAGGTGCGAAAGCGTGGGTAGCAAACAGG

>9fe4c3dfc721f87da8ff9661f9600667

TACGTAGGGCGCAAGCGTTATCCGGAATTATTGGGCGTAAAGAGCTCGTAGGCGGTTTGTCGCGTCTGCTGTGAAATCCGGAGGCTCAACCTCCGGCCTGCAGTGGGTACGGGCAGACTAGAGTGCGGTAGGGGAGATTGGAATTCCTGGTGTAGCGGTGGAATGCGCAGATATCAGGAGGAACACCGATGGCGAAGGCAGATCTCTGGGCCGTAACTGACGCTGAGGAGCGAAAGGGTGGGGAGCAAACAGG

>17f42ee9caaaa916caa2e2f582808a9b

TACGTAGGGTGCGAGCGTTAATCGGAATTACTGGGCGTAAAGCGTGCGCAGGCGGTTTGTTAAGACAGATGTGAAATCCCCGGGCTCAACCTGGGAACTGCATTTGTGACTGGCAGGCTAGAGTATGGCAGAGGGGGGTAGAATTCCACGTGTAGCAGTGAAATGCGTAGAGATGTGGAGGAATACCGATGGCGAAGGCAGCCCCCTGGGCCAATACTGACGCTCATGCACGAAAGCGTGGGGAGCAAACAGG

>6376ea6dab7d0fde3cd66f53b57e1484

TACAGAGGGTGCAAGCGTTAATCGGAATTACTGGGCGTAAAGCGCGCGTAGGTGGTTCGTTAAGTTGGATGTGAAATCCCCGGGCTCAACCTGGGAACTGCATTCAAAACTGACGAGCTAGAGTATGGTAGAGGGTGGTGGAATTTCCTGTGTAGCGGTGAAATGCGTAGATATAGGAAGGAACACCAGTGGCGAAGGCGACCACCTGGACTGATACTGACACTGAGGTGCGAAAGCGTGGGGAGCAAACAGG

>465d5de7fc208880ff9aeac9fae9e22e

TACGTAAGAGACTAGTGTTATTCATCTTAATTAGGTTTAAAGGGTACCTAGACGGTCAATATAGCTTCTAGAATGTTAGTACTTGACTAGAGTTTGATATAAGAGGGCAGTACTTGAGGAGGAGAGATGAAATTCTATTATACCAAAGGGACTCGGTAAAGGCGAAGGCAGCCCTCTATGTAAAAACTGACGTTGAAGGACGAAGGCACAGAGCACAAACAGG

>e5c19d7800b18015f3a917fc015fc42f

TACAGAGGGTGCGAGCGTTAATCGGAATTACTGGGCGTAAAGCGAGTGTAGGTGGCTCATTAAGTCACATGTGAAATCCCCGGGCTTAACCTGGGAACTGCATGTGATACTGGTGGTGCTAGAATATGTGAGAGGGAAGTAGAATTCCAGGTGTAGCGGTGAAATGCGTAGAGATCTGGAGGAATACCGATGGCGAAGGCAGCTTCCTGGCATAATATTGACACTGAGATTCGAAAGCGTGGGTAGCAAACAGG

>d56218023269c95d871ef294d8a2fff9

TACGTAGGGTGCAAGCGTTGTCCGGAATTACTGGGCGTAAAGAGCTCGTAGGTGGTTTGTCGCGTCGTCTGTGAAATCCCGGGGCTTAACTCCGGGTCTGCAGGCGATACGGGCATAACTAGAGTGCTGTAGGGGAGACTGGAATTCCTGGTGTAGCGGTGAAATGCGCAGATATCAGGAGGAACACCGATGGCGAAGGCAGGTCTCTGGGCAGTAACTGACGCTGAGGAGCGAAAGCATGGGGAGCAAACAGG

>681c1740d5917dc84caacdd55ece7ca2

TACAGAGGGTGCAAGCGTTAATCGGAATTACTGGGCGTAAAGCGCGCGTAGGTGGTTCGTTAAGTTGGATGTGAAAGCCCCGGGCTCAACCTGGGAACTGCATCCAAAACTGGCGAGCTAGAGTATGGTAGAGGGTGGTGGAATTTCCTGTGTAGCGGTGAAATGCGTAGATATAGGAAGGAACACCAGTGGCGAAGGCGACCACCTGGACTGATACTGACACTGAGGTGCGAAAGCGTGGGGAGCAAACAGG

>9d11a49f7b76486ab9585dc1418c6d20

TACGTAGGGTGCAAGCGTTAATCGGAATTACTGGGCGTAAAGCGTGCGCAGGCGGTTATGCAAGACAGAGGGGAAATCCCCGGGCTCAACCTGGGAACTGCCTTTGTGACTGCATGGCTAGAGTACGGTAGAGGGGGATGGAATTCCGCGTGTAGCAGTGAAATGCGTAGATATGCGGAGGAACACCGATGGCGAAGGCAATCCCCTGGACCTGTACTGACGCTCATGCACGAAAGCGTGGGGAGCAAACAGG

>aa1087482a5bd53ec4580a739d282d60

TACGGAGGGTCCGAGCGTTAATCGGAATTACTGGGCGTAAAGCGTGCGCAGGCGGTTTGTTAAGCGAGATGTGAAAGCCCTGGGCTCAACCTAGGAATAGCATTTCGAACTGGCGAACTAGAGTCTTGTAGAGGGGGGTAGAATTCCAGGTGTAGCGGTGAAATGCGTAGAGATCTGGAGGAATACCGGTGGCGAAGGCGGCCCCCTGGACAAAGACTGACGCTCATGCACGAAAGCGTGGGGAGCAAACAGG

>a2f988ce2503c28864c1ee290da7cce7

TACGAAGGGGGCTAGCGTTGCTCGGAATCACTGGGCGTAAAGGGCGCGTAGGCGGCCATTCAAGTCGGGGGTGAAAGCCTGTGGCTCAACCACAGAATTGCCTTCGATACTGTTTGGCTTGAGTCTGGTAGAGGTTGGTGGAACTGCGAGTGTAGAGGTGAAATTCGTAGATATTCGCAAGAACACCGGTGGCGAAGGCGGCCAACTGGACCAGTACTGACGCTGAGGCGCGAAAGCGTGGGGAGCAAACAGG

>bdf8a26094624622d68509a87fa75ba7

TACGTAGGTGGCAAGCGTTATCCGGAATTATTGGGCGTAAAGCGCGCGCAGGTGGTTTCTTAAGTCTGATGTGAAAGCCCACGGCTCAACCGTGGAGGGTCATTGGAAACTGGGAGACTTGAGTGCAGAAGAGGAAAGTGGAATTCCATGTGTAGCGGTGAAATGCGTAGAGATATGGAGGAACACCAGTGGCGAAGGCGACTTTCTGGTCTGTAACTGACACTGAGGCGCGAAAGCGTGGGGAGCAAACAGG

>bcc6da5f621cbdfc1f0c9becbac6cdff

TACGTAGGGCGCAAGCGTTATCCGGAATTATTGGGCGTAAAGAGCTCGTAGGCGGTTTGTCGCGTCTGCTGTGAAAGACCGGGGCTCAACTCCGGTTCTGCAGTGGGTACGGGCAGACTAGAGTGCAGTAGGGGAGACTGGAATTCCTGGTGTAGCGGTGAAATGCGCAGATATCAGGAGGAACACCGATGGCGAAGGCAGGTCTCTGGGCTGTAACTGACGCTGAGGAGCGAAAGCATGGGGAGCGAACAGG

>65d43491988bfe557da4d86a5ba25dae

TACGTAGGTGGCAAGCGTTATCCGGAATTATTGGGCGTAAAGCGCGCGTAGGCGGTTTTTTAAGTCTGATGTGAAAGCCCACGGCTCAACCGTGGAGGGTCATTGGAAACTGGAAAACTTGAGTGCAGAAGAGGAAAGTGGAATTCCATGTGTAGCGGTGAAATGCGCAGAGATATGGAGGAACACCAGTGGCGAAGGCGACTTTCTGGTCTGTAACTGACGCTGATGTGCGAAAGCGTGGGGATCAAACAGG

>1fbc230d68b6bacb0a1d23a81e315294

CAAATTTATGTCTTCAGAAAGACAAAATATTTTTCCAGGGTCCATTTCAACCATGTCGGTCATTTTCAACTAGCAGTGCCTTGTAGATATTTGTGAAAGATTTTGAATGTGTCTTGTCCATAGAGTTCAGTTTGTTCATTAATGATTTTTTTATGTAGTTTGTAACGTAATATATTTAGGCTTTCAAGTAAATTGATTTTGTTTCCTTTTTCCGGTTGTG

>410a1a55ad49a0b1059d6edefd74087e

TACGAAGGGGGCTAGCGTTGCTCGGAATTACTGGGCGTAAAGGGAGCGTAGGCGGACATTTAAGTCAGGGGTGAAATCCCGGGGCTCAACCTCGGAATTGCCTTTGATACTGGGTGTCTTGAGTATGAGAGAGGTATGTGGAACTCCGAGTGTAGAGGTGAAATTCGTAGATATTCGGAAGAACACCAGTGGCGAAGGCGACATACTGGCTCATTACTGACGCTGAGGCTCGAAAGCGTGGGGAGCAAACAGG

>d20279309173cf4f1bd242adeb32b8e2

TACAGAGGGTGCAAGCGTTAATCGGAATTACTGGGCGTAAAGCGCGCGTAGGTGGTTCGTTAAGTTGGATGTGAAAGCCCCGGGCTCAACCTGGGAACTGCATCCAAAACTGGCGAGCTAGAGTATGGTAGAGGGTGGTGGAATTTCCCGTGTAGCGGTGAAATGCGTAGATATAGGAAGGAACACCAGTGGCGAAGGCGACCACCTGGACTGATACAGACATTGAGGTGCGAAAGCGTGGGGAGCAAACAGG

>fccbdff8fac6bb4a77fa3e3d96e7ec2a

TACGGAGGGTGCAAGCGTTATCCGGATTTATTGGGTTTAAAGGGTCCGTAGGCGGATCTGTAAGTCAGTGGTGAAATCTCACAGCTTAACTGTGAAACTGCCATTGATACTGCAGGTCTTGAGTGTTGTTGAAGTAGCTGGAATAAGTAGTGTAGCGGTGAAATGCATAGATATTACTTAGAACACCAATTGCGAAGGCAGGTTACTAAGCAACAACTGACGCTGATGGACGAAAGCGTGGGGAGCGAACAGG

>aa9b3a1418d146c262ec63305292065a

TACGTAGGGTGCGAGCGTTGTCCGGAATTACTGGGCGTAAAGGGCTCGTAGGTGGTTTGTCGCGTCGTCTGTGAAATTCCGGGGCTTAACTCCGGGCGTGCAGGCGATACGGGCATAACTTGAGTACTGTAGGGGTAACTGGAATTCCTGGTGTAGCGGTGAAATGCGCAGATATCAGGAGGAACACCGATGGCGAAGGCAGGTTACTGGGCAGTTACTGACGCTGAGGAGCGAAAGCATGGGTAGCGAACAGG

>ddaf21e19046f783553c274bb76d6293

TACGAAGGGGGCTAGCGTTGCTCGGAATTACTGGGCGTAAAGGGAGCGTAGGCGGACATTTAAGTCAGGGGTGAAATCCCGGGGCTCAACCTCGGAATTGCCTTTGATACTGGGTGTCTTGAGTATGAGAGAGGTGTGTGGAACTCCGAGTGTAGAGGTGAAATTCGTAGATATTCGGAAGAACACCAGTGGCGAAGGCGACACACTGGCTCATCACTGACGCTGAGGCTCGAAAGCGTGGGGAGCAAACAGG

>ed79d5727c8dac7ed36c4ce71063fa6b

AACAGAGGATACAAGCGTTATCCGGATTTATTGGGTTTAAAGGGTGCGTAGGTGGTTTTTTAAGTCAGTAGTGAAATCTTAAAGCTTAACTTTAAAAGTGCTATTGATACTGATAAACTAGAGTGAGGTTGGAGTAACTGGAATGTGTGGTGGAGCGGTGAAATGCATAGAGATCACACAGAACACCAATCGCGAAGGCATGTTACTAAACATAGACTGACACTGAGGTACGAAAGCATGGGTAGCAAACAGG

>fccd274e718e26b32c71f188d97d9e59

TACGGAGGGGGCTAGCGTTGTTCGGAATTACTGGGCGTAAAGCGCACGTAGGCGGACTGGAAAGTTGGGGGTGAAATCCCGGGGCTCAACCTCGGAACTGCCTTCAAAACTATCAGTCTGGAGTTCGAGAGAGGTGAGTGGAATTCCGAGTGTAGAGGTGAAATTCGTAGATATTCGGAGGAACACCAGTGGCGAAGGCGGCTCACTGGCTCGATACTGACGCTGAGGTGCGAAAGCGTGGGGAGCAAACAGG

>d588304bb15a0e51e74d0faea125edce

TACGGAGGGTGCAAGCGTTGTTCGGAATTATTGGGCGTAAAGCGCGTGCAGGCGGCTGTTCAAGTCCGATGTGAAAGCCCGGGGCTCAACCCCGGAAGTGCATTGGAAACTGGACAGCTTGAGTACGGGAGAGGGAGGTAGAATTCCGAGTGTAGGGGTGAAATCCGTAGATATTCGGAGGAATACCGGTGGCGAAGGCGGCCTCCTGGACCGATACTGACGCTGAGACGCGAAAGCGTGGGGAGCAAACAGG

>820f6693f569e339f183638cd73a7fe6

TACGTAGGGTGCGAGCGTTGTCCGGAATTACTGGGCGTAAAGAGCTCGTAGGCGGTTTGTCACGTCGTCTGTGAAATCCTAGGGCTTAACCCTGGACGTGCAGGCGATACGGGCTGACTTGAGTACTACAGGGGAGACTGGAATTTCTGGTGTAGCGGTGGAATGCACAGATATCAGGAAGAACACCGATGGCGAAGGCAGGTCTCTGGGTAGTAACTGACGCTGAGGAGCGAAAGCATGGGTAGCGAACAGG

>aaf720cb05ddb528efa9bacbd90f7c58

TACGGAGGGAGCTAGCGTTGTTCGGAATTACTGGGCGTAAAGCGCACGTAGGCGGCTTTGTAAGTTAGAGGTGAAAGCCTGGAGCTCAACTCCAGAATTGCCTTTAAGACTGCATCGCTCGAATCCAGGAGAGGTGAGTGGAATTCCGAGTGTAGAGGTGAAATTCGTAGATATTCGGAAGAACACCAGTGGCGAAGGCGGCTCACTGGACTGGTATTGACGCTGAGGTGCGAAAGCGTGGGGAGCAAACAGG

>06f825b512d903b9230e1a55d87359ee

TACGTAGGTCCCGAGCGTTGTCCGGATTTATTGGGCGTAAAGCGAGCGCAGGCGGTTAGATAAGTCTGAAGTTAAAGGCTGTGGCTTAACCATAGTACGCTTTGGAAACTGTTTAACTTGAGTGCAAGAGGGGAGAGTGGAATTCCATGTGTAGCGGTGAAATGCGTAGATATATGGAGGAACACCGGTGGCGAAAGCGGCTCTCTGGCTTGTAACTGACGCTGAGGCTCGAAAGCGTGGGGAGCAAACAGG

>e2bd3a9338e344ff658675239fdea3ff

TACGTAGGGTGCGAGCGTTGTCCGGAATTACTGGGCGTAAAGAGCTCGTAGGTGGTTTGTCGCGTCGTCTGTGAAATTCCGGGGCTTAACTCCGGGCGTGCAGGCGATACGGGCATAACTTGAGTGCTGTAGGGGAGACTGGAATTCCTGGTGTAGCGGTGAAATGCGCAGATATCAGGAGGAACACCGATGGCGAAGGCAGGTCTCTGGGCAGTTACTGACGCTGAGGAGCGAAAGCATGGGTAGCGAACAGG

>0e2e91fba1ca9ed7de2d11ea4786c914

TACAGAGGGTGCGAGCGTTAATCGGATTTACTGGGCGTAAAGCGTGCGTAGGCGGCTAATTGAGTCGGATGTGAAATCCCCGAGCTTAACTTGGGAATTGCATTCGATACTGGTTAGCTAGAGTGTGGGAGAGGATGGTAGAATTCCAGGTGTAGCGGTGAAATGCGTAGAGATCTGGAGGAATACCGATGGCGAAGGCAGCCATCTGGCCTAACACTGACGCTGAGGTACGAAAGCATGGGGAGCAAACAGG

>9e701e82d552ee66d9007fa3883b6560

GACAGAGGATGCAAGCGTTATCCGGAATGATTGGGCGTAAAGCGTCTGTAGGTGGCTTTTTAAGTCCGCCGTCAAATCCCAGGGCTCAACCCTGGACAGGCGGTGGAAACTACCAAGCTGGAGTACGGTAGGGGCAGAGGGAATTTCCGGTGGAGCGGTGAAATGCGTAGAGATCGGAAAGAACACCAACGGCGAAAGCACTCTGCTGGGCCGACACTGACACTGAGAGACGAAAGCTAGGGGAGCGAATGGG

>c94a1bbbc3d8dc87a75398feb6c75704

CACAGTACGTCATCCATCGATGGGCTTCAATCTGTGACCACCTCTCATCTACAGCCTACCTAAGTGTTCTCCCCATCTATAGCCTGTATAATACATTAAATACCTTGCATATATAACATACATATTCGTACATTGTATTATAATTCATTATAAGGAATAATAATAATGCATATTTA

>e8386d3a307c208c4b9f0a756259cd6b

TACAGAGGGTGCGAGCGTTAATCGGATTTACTGGGCGTAAAGCGTGCGTAGGCGGCTTCTTAAGTCGGATGTGAAATCCCTGAGCTTAACTTAGGAATTGCATTCGATACTGGGAAGCTAGAGTATGGGAGAGGATGGTAGAATTCCAGGTGTAGCGGTGAAATGCGTAGAGATCTGGAGGAATACCGATGGCGAAGGCAGCCATCTGGCCTAATACTGACGCTGAGGTACGAAAGCATGGGGAGCAAACAGG

>116cdb5a4a431cf5aa2b1949dcf57159

TACGAAGGGTGCAAGCGTTAATCGGAATTACTGGGCGTAAAGCGCGCGTAGGTGGTTTGATAAGTTGGATGTGAAAGCCCCGGGCTCAACCTGGGAATTGCATCCAAAACTGTCTGACTAGAGTATGGCAGAGGGTGGTGGAATTTCCTGTGTAGCGGTGAAATGCGTAGATATAGGAAGGAACACCAGTGGCGAAGGCGACCACCTGGGCTAATACTGACACTGAGGTGCGAAAGCGTGGGGAGCAAACAGG

>d829bee4984f82ffc2453212157caf96

TACGAAGGGGGCTAGCGTTGCTCGGAATCACTGGGCGTAAAGGGTGCGTAGGCGGGTCTTTAAGTCAGGGGTGAAATCCTGGAGCTCAACTCCAGAACTGCCTTTGATACTGAAGATCTTGAGTTCGGGAGAGGTGAGTGGAACTGCGAGTGTAGAGGTGAAATTCGTAGATATTCGCAAGAACACCAGTGGCGAAGGCGGCTCACTGGCCCGATACTGACGCTGAGGCACGAAAGCGTGGGGAGCAAACAGG

>5a0ae6a10829f1b6d01debcce81fa0c6

TACGAAGGGGGCTAGCGTTGCTCGGAATCACTGGGCGTAAAGGGCGCGTAGGCGGACTTTTAAGTCGGGGGTGAAAGCCCAGGGCTCAACCCTGGAATTGCCTTCGATACTGAGAGTCTTGAGTTCGGAAGAGGTTGGTGGAACTGCGAGTGTAGAGGTGAAATTCGTAGATATTCGCAAGAACACCAGTGGCGAAGGCGGCCAACTGGTCCGATACTGACGCTGAGGCGCGAAAGCGTGGGGAGCAAACAGG

>dfa71f3681c7d9119bae9c51e2edee4c

ACATTCTTTGTACTTCATTAGTTATTTAATTTACACCACTTGTCACCCCCTTACATTGTACAAACGAGTTTGCTGGCACATTATGTAAAATACACGTTCCTGTTACAGCTTATCCCTGACCTGCTAAAACATGAAAGCAATATTTCTATGACCACAAAAACTATGGACCAAGAAGCTA

>ce6280b5620c33233ef1ac71b6777acb

CACGATTAACCCAAGTCAATAGAAGCCGGCGTAAAGAGTGTTTTAGATCACCCCCTCCCCAATAAAGCTAAAACTCACCTGAGTTGTAAAAAACTCCAGTTGACACAAAATAGACTACGAAAGTGGCTTTAACATATCTGAACACACAATAGCTAAGACCCAAACTGGGATTA

>68e66f60d2c3fc5abd282de594c3f16a

CACAGTACGTCATCCATCGATGGGCTTCAATCTGTGACCACCTCTCATCTACAGCCTAGTTAAGTGTTCTCCCCATCTATAGCCTGTATAATACATTAAATACCTTGCATATATAACATACATATTCGTACATTGTATTATAATTCATTATAAGGAATAATAATAATGCATATTTA

>144668ec621c7036d7503ccda344e48c

TACGGAGGGTGCAAGCGTTAATCGGAATTACTGGGCGTAAAGCGCACGCAGGCGGTTTGTTAAGTCGGATGTGAAATCCCCGGGCTCAACCTGGGAACTGCATTCGAAACTGGCAAGCTTGAGTCTTGTAGAGGGGGGTAGAATTCCAGGTGTAGCGGTGAAATGCGTAGAGATCTGGAGGAATACCGGTGGCGAAGGCGGCCCCCTGGACAAAGACTGACGCTCAGGTGCGAAAGCGTGGGGAGCAAACAGG

>3ebe761bfb1238c87195d431f41bf976

TACAGAGGGTGCGAGCGTTAATCGGATTTACTGGGCGTAAAGCGTGCGTAGGCGGCTTTTTAAGTCGGATGTGAAATCCCTGAGCTTAACTTAGGAATTGCATTCGATACTGGGAAGCTAGAGTATGGGAGAGGATGGTAGAATTCCAGGTGTAGCGGTGAAATGCGTAGAGATCTGGAGGAATACCGATGGCGAAGGCAGCCATCTGGCCTAATACTGACGCTGAGGTACGAAAGCATGGGGAGCAAACAGG

>b5c8ed4496d79b916859402cdf11f0a7

TACGGAGGGGGCTAGCGTTGTTCGGAATTACTGGGCGTAAAGCGCACGTAGGCGGCTTTGTAAGTTAGAGGTGAAAGCCTGGAGCTCAACTCCAGAACTGCCTTTAAGACTGCATCGCTTGAATCCAGGAGAGGTGAGTGGAATTCCGAGTGTAGAGGTGAAATTCGTAGATATTCGGAAGAACACCAGTGGCGAAGGCGGCTCACTGGACTGGTATTGACGCTGAGGTGCGAAAGCGTGGGGAGCAAACAGG

>10afda2baef44de4c584a6641de399b1

TACGGAGGGGGCTAGCGTTGTTCGGAATTACTGGGCGTAAAGCGCACGTAGGCGGCTTTGTAAGTCAGAGGTGAAAGCCTGGAGCTCAACTCCAGAACTGCCTTTGAGACTGCATCGCTTGAATCCAGGAGAGGTCAGTGGAATTCCGAGTGTAGAGGTGAAATTCGTAGATATTCGGAAGAACACCAGTGGCGAAGGCGGCTGACTGGACTGGTATTGACGCTGAGGTGCGAAAGCGTGGGGAGCAAACAGG

>0c579d21280801f02a641e1608606927

TACGTAGGGTGCGAGCGTTATCCGGAATTATTGGGCGTAAAGAGCTCGTAGGCGGTTTGTCGCGTCTGTCGTGAAAGTCCGGGGCTTAACCCCGGATCTGCGGTGGGTACGGGCAGACTAGAGTGCAGTAGGGGAGACTGGAATTCCTGGTGTAGCGGTGGAATGCGCAGATATCAGGAGGAACACCGATGGCGAAGGCAGGTCTCTGGGCTGTAACTGACGCTGAGGAGCGAAAGCATGGGGAGCGAACAGG

>d7e2e2fb85fd90f929aa59891717271d

TACGGAGGGTGCGAGCGTTAATCGGAATTACTGGGCGTAAAGCGCGCGTAGGCGGCTTGATAAGCCGGTTGTGAAAGCCCCGGGCTCAACCTGGGAACGGCATCCGGAACTGTCAGGCTAGAGTGCAGGAGAGGAAGGTAGAATTCCCGGTGTAGCGGTGAAATGCGTAGAGATCGGGAGGAATACCAGTGGCGAAGACGGCCTTCTGGACTGACACTGACGCTGAGGTGCGAAAGCGTGGGTAGCAAACAGC

>3d5baff45729bf2214074c2e9f77ad10

TACAGAGGGTGCAAGCGTTAATCGGATTTACTGGGCGTAAAGCGCGCGTAGGCGGCTAATTAAGTCAAATGTGAAATCCCCGAGCTTAACTTGGGAATTGCATTCGATACTGGTTAGCTAGAGTGTGGGAGAGGATGGTAGAATTCCAGGTGTAGCGGTGAAATGCGTAGAGATCTGGAGGAATACCGATGGCGAAGGCAGCCATCTGGCCTAACACTGACGCTGAGGTGCGAAAGCATGGGGAGCAAACAGG

>807c7c80fbfbb05418264cb86c7b6427

TACGGAGGGGGCTAGCGTTGTTCGGAATTACTGGGCGTAAAGCGCACGTAGGCGGACAAGACAGTCAGAGGTGAAATCCCGGGGCTCAACCCCGGAACGGCCTTTGAAACTCCTTGTCTTGAGGTCGAGAGAGGTGAGTGGAATTCCGAGTGTAGAGGTGAAATTCGTAGATATTCGGAGGAACACCAGTGGCGAAGGCGGCTCACTGGCTCGATACTGACGCTGAGGTGCGAAAGCGTGGGGAGCAAACAGG

>dcba105f35d8ebc9e22269c7491ad3a7

TACGAAGGGTGCAAGCGTTACTCGGAATTACTGGGCGTAAAGCGTGCGTAGGTGGTCGTTTAAGTCCGTTGTGAAAGCCCTGGGCTCAACCTGGGAACTGCAGTGGATACTGGGCGACTAGAGTGTGGTAGAGGGTAGCGGAATTCCTGGTGTAGCAGTGAAATGCGTAGAGATCAGGAGGAACATCCATGGCGAAGGCAGCTACCTGGACCAACACTGACACTGAGGCACGAAAGCGTGGGGAGCAAACAGG

>a537d8bab85c83b0e74c73c55790324b

TACGAAGGGGGCTAGCGTTGCTCGGAATTACTGGGCGTAAAGGGAGCGTAGGCGGACATTTAAGTCAGGGGTGAAATCCCGGGGCTCAACCTCGGAATTGCCTTTGATACTGGGTGTCTTGAGTATGAGAGAGGTGTGTGGAACTCCGAGTGTAGAGGTGAAATTCGTAGATATTCGGAAGAACACCAGTGGCGAAGGCGACACACTGGCTCATTACTGACGCTGAGGCTCGAAAGCGTGGGGAGCAAACAGG

>60828e70a7f4f35c0badbbf562c86621

TACGTAGGTGGCAAGCGTTGTCCGGAATTATTGGGCGTAAAGCGCGCGCAGGCGGTCTCTTAAGTCTGATGTGAAAGCCCCCGGCTCAACCGGGGAGGGTCATTGGAAACTGGGAGACTTGAGTACAGAAGAGGAGAGTGGAATTCCACGTGTAGCGGTGAAATGCGTAGAGATGTGGAGGAACACCAGTGGCGAAGGCGACTCTCTGGTCTGTAACTGACGCTGAGGCGCGAAAGCGTGGGGAGCAAACAGG

>ef96546161304fc5ff8ca3fcc65a826a

TACACTTATCTCTTTAGAAAGACAAAATATTTCTCCAGGATGCAGTTCAACCATGTCGGTCATTTTCAACTGGCAGTGCCTTGTAGATAGTTGTGAAAGAATGTGAATGTGTCTTGTCCATAGAGTTCAGTTTGTTCAGTAATGATTTTGTTATGTAGTTTGTAACGTAATGTATTTAGACTGTCAAGGAAATTCATTTTATTTCCTTTTTTGTCGTTTGTG

>394eda29c886632f514dd94b58381186

TACGGAGGGTGCGAGCGTTAATCGGAATAACTGGGCGTAAAGGGCACGCAGGCGGTGACTTAAGTGAGGTGTGAAAGCCCCGGGCTTAACCTGGGAATTGCATTTCATACTGGGTCGCTAGAGTACTTTAGGGAGGGGTAGAATTCCACGTGTAGCGGTGAAATGCGTAGAGATGTGGAGGAATACCGAAGGCGAAGGCAGCCCCTTGGGAATGTACTGACGCTCATGTGCGAAAGCGTGGGGAGCAAACAGG

>9207bf5e78e77c8ecb9d079b8ce7dc8b

TACGTAGGGTGCGAGCGTTGTCCGGAATTACTGGGCGTAAAGAGCTCGTAGGTGGTTTGTCGCGTCGTCTGTGAAATCCCGGGGCTTAACTTCGGGCGTGCAGGCGATACGGGCATAACTAGAGTGCTGTAGGGGAGACTGGAATTCCTGGTGTAGCGGTGAAATGCGCAGATATCAGGAGGAACACCGATGGCGAAGGCAGGTCTCTGGGCAGTTACTGACGCTGAGGAGCGAAAGCATGGGTAGCGAACAGG

>c41e38ec0abb37da79acd32fd3cd7c66

TACGTAGGGTGCGAGCGTTGTCCGGATTTACTGGGCGTAAAGAGCTCGTAGGTGGCTTGTCGCGTCGTCTGTGAAAGTCTGGGGCTTAACTCCGGGTGTGCAGGCGATACGGGCTGGCTTGAGTGCTGTAGGGGAGACTGGAATTCCTGGTGTAGCGGTGGAATGCGCAGATATCAGGAGGAACACCGATGGCGAAGGCAGGTCTCTGGGCAGTCACTGACGCTGAGGAGCGAGAGCATGGGTAGCGAACAGG

>604166449f6224b1baa3c192504c94b4

CACACTTATGTCTTCAGAAAGACAAAATATTTCCCCATGATGCATTTCAACCATGTCGGTTATTTTCAACTGGCAGTGCCTTGTTGATATTTGTGAAAGATTTTGAATATGTCTTGTCCATAGAGTTGAGTTTGTTCATTAATGATTTTGTTATGTAGTTTGTAACGTAATACATTTAGGCTTTCAAGTAAATTCATTTTGTTTATTTTTTTGCCGGTTGTG

>5fe8a79baa0e9601bc73ff3938dbf497

TACGGAGGGAGCTAGCGTTGTTCGGAATTACTGGGCGTAAAGCGCACGTAGGCGGCGATTTAAGTCAGAGGTGAAAGCCCGGGGCTCAACCCCGGAACTGCCTTTGAGACTGGATTGCTAGAATCTTGGAGAGGCGAGTGGAATTCCGAGTGTAGAGGTGAAATTCGTAGATATTCGGAAGAACACCAGTGGCGAAGGCGGCTCGCTGGACAAGTATTGACGCTGAGGTGCGAAAGCGTGGGGAGCAAACAGG

>5baf6c8c6c5e4668663a09bc7f34981e

TACGGAGGGTGCGAGCGTTAATCGGAATTACTGGGCGTAAAGCGCGCGTAGGCGGCTTGATAAGCCGGTTGTGAAAGCCCCAGGCTCAACCTGGGAACGGCATCCGGAACTGTCAGGCTAGAGTGCAGGAGAGGAAGGTAGAATTCCCGGTGTAGCGGTGAAATGCGTAGAGATCGGGAGGAATACCAGTGGCGAAGGCGGCCTTCTGGACTGACACTGACGCTGAGGTGCGAAAGCGTGGGTAGCAAACAGG

>146aa4974f1075d717ba466857a63498

TTCTTACTGCAAACGTATTGTCACACACATATGTTTTGTAGTAATTATTGTGAGCAGCATCCTCTATGTTATAAATGTAATAAGGAAATCTTAAAACAATGATAATAATCTGTTTATAGTAATTAATCTTCATCATCTTCGCCATTCCAGGATTAGAAACCCGAGTAGTCCGG

>2740cf2417c92847cc298cbd71dd1fcd

TACGTAGGTGGCAAGCGTTGTCCGGAATTATTGGGCGTAAAGCGCGCGCAGGCGGATAGGTCAGTCTGTCTTAAAAGTTCGGGGCTTAACCCCGTGATGGGATGGAAACTGCCAATCTAGAGTATCGGAGAGGAAAGTGGAATTCCTAGTGTAGCGGTGAAATGCGTAGATATTAGGAAGAACACCAGTGGCGAAGGCGACTTTCTGGACGAAAACTGACGCTGAGGCGCGAAAGCCAGGGGAGCGAACGGG

>60803a35b6be5e2be55881846942896b

TACGTGAGAGACTAGTGTTATTCATCTTAATTGGGTTTAAAGGGTACCTAGACAGTCAATATATCTTCTAGAATGCTAATACTTGACTAGAGTTTTAAGGAAGAGGGAAGTACTTAAGGAGTAAGAGATGAAATATCTGTGATACCAAAGGGACTCTGTAAAGGCGAAGGCATTCCTTTATCTAAAAACTAACGTTGAAGGACGAAGGCTTAGATAACAAATAGG

>a40c6cc9186e4d6cd58f6eee99ec8611

TACGTAGGTGGCAAGCGTTGTCCGGAATTATTGGGCGTAAAGCGCGCGCAGGCGGTCTTTTAAGTCTGATGTGAAAGCCCCCGGCTCAACCGGGGAGGGTCATTGGAAACTGGGAGACTTGAGTACAGAAGAGGAGAGTGGAATTCCACGTGTAGCGGTGAAATGCGTAGATATGTGGAGGAACACCAGTGGCGAAGGCGACTCTCTGGTCTGTAACTGACGCTGAGGCGCGAAAGCGTGGGGAGCAAACAGG

>a0f16b7e9d429146ba60084db814176f

TACGTAGGTGGCAAGCGTTGTCCGGAATTATTGGGCGTAAAGCGCGCGCAGGCGGTCTTTTAAGTCTGATGTGAAAGCCCACGGCTCAACCGTGGAGGGTCATTGGAAACTGGAAGACTTGAGTACAGAAGAGAAGAGTGGAATTCCACGTGTAGCGGTGAAATGCGTAGATATGTGGAGGAACACCAGTGGCGAAGGCGACTCTCTGGTCTGTAACTGACGCTGAGGCGCGAAAGCGTGGGGAGCAAACAGG

>68c860ba582d3bd877e86f2335c58844

CACACTTATGTCTTCAGAAAGACAAAATATTTATCCAGGATGCATTTCAACCATGTCGGTCATTTTCAACTGGCAGTGCCTTGTAGATATTTGTGAACGATTTTGAATGTGTCTTGTCAATAGAGTTCAGTTTGTTCATTAATGATTTTGTTATGTAGTTTGTAACGTAATATATTTAGGCTATCAAGTAAATTCATTTTGTTTCCTTTTTGCCGCTTGTG

>886813d6c66ef41ef648c9b92ffde619

TACGTAGGGTGCGAGCGTTGTCCGGAATTACTGGGCGTAAAGAGCTCGTAGGTGGTTTGTCGCGTCGTTTGTGTAAGCCCGCAGCTTAACTGCGGGACTGCAGGCGATACGGGCATAACTTGAGTGCTGTAGGGGAGACTGGAATTCCTGGTGTAGCGGTGGAATGCGCAGATATCAGGAGGAACACCGATGGCGAAGGCAGGTCTCTGGGCAGTAACTGACGCTGAGGAGCGAAAGCATGGGTAGCGAACAGG

>5a7b179b1b45f0fe2282f260bf073f60

TACGTAGGGTGCGAGCGTTGTCCGGATTTATTGGGCGTAAAGGGCTCGTAGGTGGTTGATCGCGTCGGAAGTGTAATCTTGGGGCTTAACCCTGAGCGTGCTTTCGATACGGGTTGACTTGAGGAAGGTAGGGGAGAATGGAATTCCTGGTGGAGCGGTGGAATGCGCAGATATCAGGAGGAACACCAGTGGCGAAGGCGGTTCTCTGGGCCTTTCCTGACGCTGAGGAGCGAAAGCGTGGGGAGCGAACAGG

>56df37e07aea5ad9be2f654378a5ccc2

TACGTAGGTGGCAAGCGTTTTCCGGAATTATTGGGCGTAAAGCGCGCGCAGGCGGTCTCTTAAGTCTGATGTGAAAGCCCCCGGCTCAACCGGGGAGGGTCATTGGAAACTGGGAGACTTGAGTACAGAAGAGGAGAGTGGAATTCCACGTGTAGCGGTGAAATGCGTAGATATGTGGAGGAACACCAGTGGCGAAGGCGACTCTCTGGTCTGTAACTGACGCTGAGGCGCGAAAGCGTGAGGAGCAAACAGG

>985c62c37efedae38c6a8f81b5b7246c

CACACTTATGTCTTCAGAAATACAAACCATTTCTCCAGGATGCATTTCAACCATGTCGGTCATTTTCAACTGTCAGTGCCTTGTAGATATTTGTGGAAGATTTTGAATGTGTCTTGTCCATGGAGTTCAGTTTGTTCATTAATGATTTTGTTGTGTAGTTTGTAACGTAATATATTTAGGGTTTTAAGTAAATTCATTTTATTTCCTTTTTGCCTGTTGTA

>2542d180b48084a6fca1019781927566

TACGGAGGATCCAAGCGTTATCCGGATTTATTGGGTTTAAAGGGTCCGTAGGCGGCCTATTAAGTCAGTGGTGAAAGACGGCAGCTTAACTGTCGCAGTGCCTTTGATACTGATAGGCTTGAATCTATTTGAAGTGGGCGGAATAAGACAAGTAGCGGTGAAATGCATAGATATGTCTTAGAACTCCGATTGCGAAGGCAGCTCACTAAGTTAGTATTGACGCTGATGGACGAAAGCGTGGGGATCAAACAGG

>097c9225e333f794d3f45e8335461c1f

CTCTTACTGCAAACGTATTGTCACACACATATGTTTTGTAGTAATTATTGTGAGCAGCATCCTCTATGTTATAAAGTTAATAAGGAAATCTTAAAACAATGATAATAATCTGTTTATAGTAATTAATCTTCATCATCTTCGCCATTCCAGGATTAGAAACCCGAGTAGTCCGG

>e452d6f927f3beebf9486846554b4497

TACGGAGGGTGCGAGCGTTAATCGGAATTACTGGGCGTAAAGCGCGCGTAGGCGGCTTGATAAGCCGGTTGTGAAAGCCCCGGGCTCAACCTGGGAACGGCATCCGGAACTGTCAGGCTAGAGTGCAGGAGAGGAAGGTAGAATTCCCGGTGTAGCGGTGAAATGCGTAGAGATCGGTAGGAATACCAGTGGCGAAGGCGGCCTTCTGGACTGACACTGACGCTGAGGTGCGAAAGCGTGGGTAGCAAACAGG

>b7ae2d92c6199a9e58c97d0e75865d36

TACGTAGGGCGCAAGCGTTGTCCGGAATTATTGGGCGTAAAGAGCTCGTAGGCGGTTTGTCGCGTCTGCTGTGAAAGCCCGGGGCTTAACCCCGGGTGTGCAGTGGGTACGGGCAGACTAGAGTGCAGTAGGGGAGACTGGAATTCCTGGTGTAGCGGTGGAATGCGCAGATATCAGGAGGAACACCGATGGCGAAGGCAGGTCTCTGGGCTGTTACTGACGCTGAGGAGCGAAAGCATGGGGAGCGAACAGG

>f13472c21bf87849221e9f3a2f2ae5e0

TACGGAGGGTGCAAGCGTTACCCGGAATCACTGGGCGTAAAGGGCGTGTAGGCGGACGCTTAAGTCTGACTTTAAAGACTGCGGCTCAACCGCAGGACTGGGTTGGAGACTGGGCGTCTGGACCTCTGGAGAGGGAACCGGAATTCCTGGTGTAGCGGTGGAATGCGTAGATACCAGGAGGAACACCGATGGCGAAGGCAGGTTCCTGGACAGAAGGTGACGCTGAGGCGCGAAAGTGTGGGGAGCGAACCGG

>7862a3f7fef22be627908a45556f56f8

TACGTAGGTGGCAAGCGTTGTCCGGAATTATTGGGCGTAAAGCGCGCGCAGGCGGTCTTTTAAGTCTGATGTGAAAGCCCTCGGCTCAACCGGGGAGGGTCATTGGAAACTGGGAGACTTGAGTACAGAAGAGGAGAGTGGAATTCCACGTGTAGCGGTGAAATGCGTAGATATGTGGAGGAACACCAGTGGCGAAGGCGACTCTCTGGTCTGTAACTGACGCTGAGGCGCGAAAGCGTGGGGAGCAAACAGG

>8df2b2e6c1cb64bd18b81d5bae7f0898

TACGTAGGGTGCAAGCGTTAATCGGAATTACTGGGCGTAAAGCGTGCGCAGGCGGTTTTGTAAGTCTGTCGTGAAAGCCCCGGGCTCAACCTGGGAATTGCGATGGAGACTGCAAGGCTTGAATCTGGCAGAGGGGGGTAGAATTCCACGTGTAGCAGTGAAATGCGTAGAGATGTGGAGGAACACCGATGGCGAAGGCAGCCCCCTGGGTCAAGATTGACGCTCATGCACGAAAGCGTGGGGAGCAAACAGG

>6b1110c61a09521ccc9a0c9a803492f5

CATTGAACTATCGTGAGAACGGCACGCCGCCAAAGGGAATTATATTATAGTAAATATTGGCGTAAATAAACATTTTATTAATAGTTGTAATATATGAAAATGTGCAGAAGATAAAGACGATGAAAACGGTGTTTAACAATCGCTTATCGAGACAATATTTAATTAATTAGGAATTAAAATATGTGTATTATTTGTATTTAACAAGGAATAATCTTGAAAATAAATACAGTGGTAAACGGAACTAATAGAGTCACGCAAGTGAACTTAGAACAATTTTAAGAAC

>a72dafec771261af801964e35e5229a6

TACGTAGGGTGCGAGCGTTAATCGGAATTACTGGGCGTAAAGCGTGCGCAGGCGGTTTTGTAAGACAGAGGGGAAATCCCCGGGCTCAACCTGGGAACTGCCTTTGTGACTGCAAGGCTAGAGTACGGCAGAGGGGGATGGAATTCCGCGTGTAGCAGTGAAATGCGTAGATATGCGGAGGAACACCGATGGCGAAGGCAATCCCCTGGGTCTGTACTGACGCTCATGCACGAAAGCGTGGGGAGCAAACAGG

>8400dd8baa70ab70f07c34b0d0024559

TACGAAGGGGGCTAGCGTTGCTCGGAATCACTGGGCGTAAAGGGTGCGTAGGCGGGTCTTTAAGTCAGGGGTGAAATCCTGGAGCTCAACTCCAGAACTGCCTTTGATACTGAGGATCTTGAGTCCGGAAGAGGTGAGTGGAACTGCGAGTGTAGAGGTGAAATTCGTAGATATTCGCAAGAACACCAGTGGCGAAGGCGGCTCACTGGTCCGGTACTGACGCTGAGGCACGAAAGCGTGGGGAGCAAACAGG

>fef3cc3b04dd00134dd9debf9de2c674

TCGAACCCAAGCCACGTGGTGTAGCGAACCAACACACTACACACCACGAGTACTCCCCCCCCCCTTCCAATCAACAAAAAATCACAATACGATTACAGGAAATTGTTCAGAATGTAGCGCCAAATGAAATTACAAAGAGGAGGATTTTGAATATTGCAGAAGGATCGTGCAAAACAAACGTTTGCTAGCAGCCTTCCTCACGTGTATACTGCACTGATGCTTCGAGAAATAGTCTGCAGAACTAT

>701415696ad5f15da397c612e03bd2ea

CACACTTATGTCTTCAGAAATACAAACCATTTCTCCAGGATGCATTTCAAACATGTCGGTCATTTTCAACTGTCAGTGCCTTGTAGATATTTGTGGAAGATTTTGAATGTGTCTTGTCCATGGAGTTCAGTTTGTTCATTAATGATTTTGTTGTGTAGTTTGTAACGTAATATATTTAGGGTTTTAAGTAAATTCATTTTATTTCCTTTTTGCCTGTTGTA

>3c0a6d8306cd7370af30c05627913780

AACAGAGGATACAAGCGTTATCCGGATTTATTGGGTTTAAAGGGTGCGCAGGTGGTTTTTTAAGTCAGTAGTGAAATCTTAAAGCTTAACTTTAAAAGTGCTATTGATACTGATAAACTAGAGTGAGGTTGGAGTAACTGGAATGTGTGGTGGAGCGGTGAAATGCATAGAGATCACACAGAACACCAATCGCGAAGGCATGTTACTAAACATAGACTGACACTGAGGTACGAAAGCATGGGTAGCAAACAGG

>7ba04a00b7566eb1959c8f459fbc8322

TACGTAGGTCCCGAGCGTTGTCCGGATTTATTGGGCGTAAAGCGAGCGCAGGCGGTTAGATAAGTCTGAAGTTAAAGGCAGTGGCTCAACCATTGTAGGCTTTGGAAACTGTTTAACTTGAGTGCAGAAGGGGAGAGTGGAATTCCATGTGTAGCGGTGAAATGCGTAGATATATGGAGGAACACCGGTGGCGAAAGCGGCTCTCTGGTCTGTAACTGACGCTGAGGCTCGAAAGCGTGGGGAGCGAACAGG

>c22b16cc6108c04f29fea3b6d4c81571

TACAGAGGGTGCAAGCGTTAATCGGATTTACTGGGCGTAAAGCGCGCGTAGGTGGCCAATTAAGTCAAATGTGAAATCCCCGAGCTTAACTTGGGAATTGCATTCGATACTGGTTGGCTAGAGTATGGGAGAGGATGGTAGAATTCCAGGTGTAGCGGTGAAATGCGTAGAGATCTGGAGGAATACCGATGGCGAAGGCAGCCATCTGGCCTAATACTGACACTGAGGTGCGAAAGCATGGGGAGCAAACAGG

>90b18d5a29d812561b456aa7f9876232

TTCTTACTGCAAACGTATTGTCACACACATATGTTTTGTAGTAATTATTGTGAGCAGCATCCTCTATGTTATAAATGTAATAAGGAAATCTTAAAACAATGATAATAATCTGTTTATAGTAATTAATCTTCATCATCTTCGCCATTCCAGGATTAGAAACCCCAGTAGTCCGG

>dbed58e8215ab3e25941e1df25be40ba

TTCTTACTGCAAACGTATTGTCACACACATATGTTTTGTAGTAATTATTGTGAGCAGCATCCTCTATGTTATAAATGTAATAAGGAAATCTTAAAACAATGATAATAATCTGTTTATAGTAATTAATCTTCATCATCTTCGCCATTCCAGGATTAGAAACCCTAGTAGTCCGG

>8d68c0332cfaf5b0eb23d0ab375e41d9

CTCTTACTGCAAACGTATTGTCACACACATATGTTTTGTAGTAATTATTGTGAGCAGCATCCTCTATGTTATAAAGTTAATAAGGAAATCTTAAAACAATGATAATAATCTGTTTATAGTAATTAATCTTCATCATCTTCGCCATTCCAGGATTAGATACCCCAGTAGTCCGG

>85c44c83eddc5d3028261a1000b7d0e1

TACGTAGGTGGCAAGCGTTGTCCGGAATTATTGGGCGTAAAGCGCGCGCAGGTGGTTTAATAAGTCTGATGTGAAAGCCCACGGCTCAACCGTGGAGGGTCATTGGAAACTGTTAAACTTGAGTGCAGGAGAGAAAAGTGGAATTCCTAGTGTAGCGGTGAAATGCGTAGAGATTAGGAGGAACACCAGTGGCGAAGGCGGCTTTTTGGCCTGTAACTGACACTGAGGCGCGAAAGCGTGGGGAGCAAACAGG

>28330edc6de99db0c03ee5275d032287

TACGGAGGGTGCAAGCATTGTTCGGAATTATTGGGCGTAAAGCGCGTGCAGGCGGCTGTTCAAGTCCGATGTGAAAGCCCGGGGCTCAACCCCGGAAGTGCATTGGAAACTGGACAGCTTGAGTACGGAAGAGGGAGGTAGAATTCCGAGTGTAGGGGTGAAATCCGTAGATATTCGGAGGAATACCGGTGGCGAAGGCGGCCTCCTGGACCGATACTGACGCTGAGACGCGAAAGCGTGGGGAGCAAACAGG

>6c9e867446471a7175ee09c7bfeef890

CACAGTACGTCATCCATCGATGGGCTTCAATCTGTGACCACCTCTCATCTACAGCCTAGTTAAGTGTTCTCCCCATCTATAGCCTGTATAATACATTAAATACCTTGCATATACAACATACATATTCGTACATTGTATTATAATTCATTATAAGGAATAATAATAATGCATATTTA

>43c951b8ebd7cc900898c8fa6445dee3

GACAGAGGGTGCAAACGTTGTTCGGAATTACTGGGCGTAAAGCGTGTGTAGGCGGCCATGTAAGTTGGATGTGAAAGCCCCGGGCTCAACCCGGGAAGTGCATTCAAAACTGCGTGGCTTGAGTACTGGAGAGGTTGGTAGAATTCTCGGTGTAGAGGTGAAATTCGTAGATATCGAGAGGAATACCGGTGGCGAAGGCGGCCAACTGGACAGATACTGACGCTGAGACACGAAAGCGTGGGGAGCAAACAGG

>e9d22068e812ec6f689184b26624717e

CAAATTTACGTCTTCAGAAAGACAAAATATTTTTCCAGGGTACATTTCAACCATGTCGGTCATTTTCAACTAGCAGTGCCTTGTAGATATTTGTGAAAGATTTTGAATGTGTCTTGTCCATAGAGTTCAGTTGTTCATTAATGATTTTTTTATGTAGTTCGTAACGTAATATATTTAGGCTTTCAAGTAAATTGATTTTGTTTCCTTTTTCCGGTTGCG

>821403c34165d7b42378e24adb047f71

CACACTTATGTCTTCAGAAAGACAAAATATTTCTCCAGGATGCATTTCGACCATGTCGGTCATTGTCAACTGGCAGTGCATTGTAGATATCTGTGAAAGATTTTGAATGTGTCTTGTCCATAGAGTTCAGTTTGTTCATTAATAATTTTTTATGTAGTTTTTAAAGTAATATATTTAGACTTTCAAGTAAATTCGTTTTGTTTCCTTTTTTGCCGGTTGTG

>2d318b3e49d3861aadbaf7558de826d0

CATTGAACTATCGTGAGAAAGTCAAACCGCCAAAGGGAATTATATTATAGTAAATATTGGCGTAAATAAACATTTTATTAATAGTTGTAATATATGAAAATGTGCAGAAGATAAAGACGATTAAAACGGTGTTTAACAATCGCTTATCGAGACAATATTTAATTAATTAGGAATTAAAATATGTGTATTATTTGTATTTAACAAGGAATAAACTTGAAAATAAATACAGTGGTGAACGGAACTAATAGAGTCACGCAAGTGAACTTAGAACATTTTAATAAC

>ac007328bce609a715815c6a7fbb9e95

TACGAGGAAGACTAGTGTTATTCATCTTTATTAGGTTTAAAGGGTACCTAGACGGCATATCAAGCCCCAAAAGGGAACAGATATACTAGAGTTTTATGTGAGAGGAATATATTAGTACTATTGGTGTAGAGATGAAATTCTTTGATACTAATAGGACGGATAACAGCAAAAGCAAACCTTTATGTAAAAACTGACGTTGAGGGACGAAGGCTTGGGTCGCGAATAGG

>e7e5ba6e4b07e96f52be0b8ceaf7844d

TACGAGGGGAGCGAGTGTTGTTCGGTTTTATTGGGCGTAAAGGGTGTTCAGGTTGTTCAATAAGTTGATCTCTAAATCTTGGAACTCAACCCCATTCAAGGGGTCAATACTGTTGGACTTGAGCTTACAAGGGACGAATGGAATTCCAAGTGTAGAGGTGAAATTCGTTGATATTTGGGGGAACACCGGAGGCGAAAGCGATTCGTCATACTAAGCTGACACTGAAGACACGAAAGCATGGGGAGCAAACAGG

>367e53ef4d44c3e18044e4a99c6be547

CCGCCGCGGTAATACGATTTCTTTAATTTAAATATTTAAGTTTCAGTTAATATAACAATAATATAAAATATCTAAAATTTTGGTGAAATATATTTTATCTTTAAAAATTAATTTTATGTCTGAAAAATTTTTGTATAAACTAGGATTAGAAACCCCAGTAGTCCGGCTGACTGACTTAACGTCCA

>922d33a2963d3e87a3d4df6b82371235

TACGTAGGTGGCAAGCGTTATCCGGAATTATTGGGCGTAAAGCGCGCGTAGGCGGTTTCTTAAGTCTGATGTGAAAGCCCACGGCTCAACCGTGGAGGGTCATTGGAAACTGGGAAACTTGAGTGCAGAAGAGGAAAGTGGAATTCCATGTGTAGCGGTGAAATGCGCAGAGATATGGAGGAACACCAGTGGCGAAGGCGACTTTCTGGTCTGTAACTGACGCTGATGTGCGAAAGCGTGGGGATCAAACAGG

>41e6c0b55654af1d5cd503359fb77d18

TACGAAGGGGGCTAGCGTTGCTCGGAATTACTGGGCGTAAAGGGAGCGTAGGCGGACATTTAAGTCAGGGGTGAAATCCCAGAGCTCAACTCTGGAACTGCCTTTGATACTGGGTGTCTTGAGTGTGATAGAGGTATGTGGAACTCCGAGTGTAGAGGTGAAATTCGTAGATATTCGGAAGAACACCAGTGGCGAAGGCGACATACTGGATCATTACTGACGCTGAGGCTCGAAAGCGTGGGGAGCAAACAGG

>2f49affea1aa1db3f453ce4b3066a63f

TACGTAGGGTACTAGCGTTGTCCGGAATTATTGGGCGTAAAGAGCTCGTAGGTGGTTTGTCGCGTCTGCTGTGGAAACGTGCCGCTTAACGGTGCGCGTGCAGTGGGTACGGGCGGACTAGAGTGCAGTAGGGGAGTCTGGAATTCCTGGTGTAGCGGTGAAATGCGCAGATATCAGGAGGAACACCGGTGGCGAAGGCGGGACTCTGGGCTGTTACTGACGCTGAGGAGCGAAAGCATGGGGAGCGAACAGG

>f04323f393f38c95b7ac34efa5912801

TTCTTACTGCAAACGTATTGTCACACACATATGTTTTGTAGTAATTATTGTGAGCAGCATCCTCTATGTTATAAATGTAATAAGGAAATCTTAAAACAATGATAATAATCTGTTTATAGTAATTAATCTTCATCATCTTCGCCATTCCAGGATTAGATACCCCTGTAGTCCGG

>bf17611a292130e24329ccc421a261cb

TACGTAGGTCCCGAGCGTTGTCCGGATTTATTGGGCGTAAAGCGAGCGCAGGCGGTTAGATAAGTCTGAAGTTAAAGGGTGTGGCTTAACCATAGTACGCTTTGGAAACTGTTTAACTTGAGTGCAAGAGGGGAGAGTGGAATTCCATGTGTAGCGGTGAAATGCGTAGATATATGGAGGAACACCGGTGGCGAAAGCGGCTCTCTGGCTTGTAACTGACGCTGAGGCTCGAAAGCGTGGGGAGCAAACAGG

>eba44921be3517ff5f8ce91bc770fe01

CTCGTATCATACTACACGCTACACACTGCTTTGCTCTCCTTAACACCAAACAAGATACGAGTATATAAAGTTTTACACCAATATAAATCAATATTACACTACAGACCAACGATACGGGAGAGAGCTAAACATTTCTCGTTGTTCATACGAGAGAGGGCGTCAAATAGTTAACACAGTCTCAAACTAAACATACATTTTACCGCATGTTATTTTTCTTTCCTTTATGCCGTGAGGTTATCTCACTTATCTGTAGGTGGTAAATCTAATGATATTGTCGCATTTATTAGTTAATTAATTAGTTTATT

>8c5f7c86116d413272990a0306ee9ccb

TTCTTACTGCAAACGTATTGTCACACACATATGTTTTGTAGTAATTATTGTGAGCAGCATCCTCTATGTTATAAATGTAATAAGGAAATCTTAAAACAATGATAATAATCTGTTTATAGTAATTAATCTTCATCATCTTCGCCATTCCAGGATTAGATACCCTAGTAGTCCGG

>6f3f68e5c8e2a11b388ddbbea9fa182d

TACGAAGGGTGCAAGCGTTAATCGGAATTACTGGGCGTAAAGCGCGCGTAGGTGGTTCGTTAAGTTGGATGTGAAAGCCCCGGGCTCAACCTGGGAACTGCATCCAAAACTGGCGAGCTAGAGTATGGCAGAGGGTGGTGGAATTTCCTGTGTAGCGGTGAAATGCGTAGATATAGGAAGGAACACCAGTGGCGAAGGCGACCACCTGGGCTAATACTGACACTGAGGTGCGAAAGCGTGGGGAGCAAACAGG

>fd496fd32dc8c08ade2e8b6c9d8ee13d

TACGTAGGTCCCGAGCGTTGTCCGGATTTATTGGGCGTAAAGCGAGCGCAGGCGGTTTGATAAGTCTGAAGTTAAAGGCTGTGGCTCAACCATAGTTCGCTTTGGAAACTGTCAAACTTGAGTGCAGAAGGGGAGAGTGGAATTCCATGTGTAGCGGTGAAATGCGTAGATATATGGAGGAACACCGGTGGCGAAAGCGGCTCTCTGGTCTGTAACTGACGCTGAGGCTCGAAAGCGTGGGGAGCGAACAGG

>b5e6d073ba80b055fa84cb7c17917c16

TACGGAGGGAGCTAGCGTTGTTCGGAATTACTGGGCGTAAAGCGCACGTAGGCGGCGATTTAAGTCAGAGGTGAAAGCCCGGGGCTCAACCCCGGAACTGCCTTTGAGACTGGATTGCTTGAATCCTGGAGAGGTGAGTGGAATTCCGAGTGTAGAGGTGAAATTCGTAGATATTCGGAAGAACACCAGTGGCGAAGGCGGCTCACTGGACAGGTATTGACGCTGAGGTGCGAAAGCGTGGGGAGCAAACAGG

>22cdb8de4aea60f0713d7a1f35c86e29

TACGGAGGGAGCTAGCGTTGTTCGGAATTACTGGGCGTAAAGCGCACGTAGGCGGCTTTGTAAGTTAGAGGTGAAAGCCTGGAGCTCAACTCCAGAATTGCCTTTAAGACTGCATCGCTCGAATCCGGGAGAGGTGAGTGGAATTCCGAGTGTAGAGGTGAAATTCGTAGATATTCGGAAGAACACCAGTGGCGAAGGCGGCTCACTGGACCGGTATTGACGCTGAGGTGCGAAAGCGTGGGGAGCAAACAGG

>77a920bd965da12d31f93c1adf2c5ea1

TACGTAGGGTGCGAGCGTTGTCCGGAATTACTGGGCGTAAAGAGCTCGTAGGTGGTTTGTTGCGTCGTCTGTGAAATTCCGGGGCTTAACTTCGGGGTGGCAGGCGATACGGGCATAACTAGAGTGCTGTAGGGGAGACTGGAATTCCTGGTGTAGCGGTGGAATGCGCAGATATCAGGAGGAACACCGATGGCGAAGGCAGGTCTCTGGGCAGTAACTGACGCTGAGGAGCGAAAGCATGGGGAGCGAACAGG

>a563624b2dda7623750bde3a4863a4f5

AAAAAAAGCTGTCTACTACGAATTTGTATGAACATCAAATTTGTGTCACTAACGAAAACAAATTCATTCGTAAAACATACCCGGCACTATCAAGAAAGGGTCGATGTTGAAATCCAATGGATGGTGGATCAAGGGGTTATCAAACGATCCAACAGTGATTTTCTTAACCCGGTGGTAACCGTTAAGGAATATAAGGCTGTGCCTCGATATGCACAACTCAAAAATAGTATCGTGTTGGGCTCCGAACGCTGAGTCAGTTTTTATTAAGTGTCAGGGTGTCCGCTACATGTCCCG

>22b3c70cdfb1714224e1bf8a6314bee9

TACGTATGGAGCGAGCGTTGTCCGGAATTATTGGGCGTAAAGGGTACGCAGGCGGTTTAATAAGTCGAATGTTAAAGATCGGGGCTCAACCCCGTAAAGCATTGGAAACTGATAAACTTGAGTAGTGGAGAGGAAAGTGGAATTCCTAGTGTAGTGGTGAAATACGTAGATATTAGGAGGAATACCAGTAGCGAAGGCGACTTTCTGGACACAAACTGACGCTGAGGTACGAAAGCGTGGGGAGCAAACAGG

>a50512643b1f30896e862955c91a08cb

TACGGAGGGGGCTAGCGTTGTTCGGAATTACTGGGCGTAAAGCGCACGTAGGCGGATCAGAAAGTTGGGGGTGAAATCCCGGGGCTCAACCCCGGAACTGCCTTCAAAACTATTGGTCTGGAGTTCGAGAGAGGTGAGTGGAATTCCGAGTGTAGAGGTGAAATTCGTAGATATTCGGAGGAACACCAGTGGCGAAGGCGGCTCACTGGCTCGATACTGACGCTGAGGTGCGAAAGCGTGGGGAGCAAACAGG

>901c738324d8fc7c901555c77695ec61

TACGTAGGGTGCGAGCGTTAATCGGAATTACTGGGCGTAAAGCGTGCGCAGGCGGTTGTGTAAGACAGGCGTGAAATCCCCGGGCTCAACCTGGGAATGGCGCTTGTGACTGCACGGCTGGAGTGCGGCAGAGGGGGATGGAATTCCGCGTGTAGCAGTGAAATGCGTAGATATGCGGAGGAACACCGATGGCGAAGGCAATCCCCTGGGCCTGCACTGACGCTCATGCACGAAAGCGTGGGGAGCAAACAGG

>12760674f167960a4a43f35f016d9a96

CCGCCGCGGTAATACGATTTCTTTAATTTAAATATTTAAGTTTCAGTTAATATAATAATAATATAAAATATCTAAAATTTTGGTGAAATATATTTTATCTTTAAAAATTAATTTTATGTCTGAAAAATTTTTGTATAAACTAGGATTAGAAACCCCAGTAGTCCGGCTGACTGACTTGCGTCAAA

>0e3b6ceee02f82f5f529f14d9feafed1

TACGAGGGGAGCGAGTGTTGTTCGGTTTTATTGGGCGTAAAGGGTATGTAGGCGGTTTTGTAAGTCAACACTTAAATCTTGAGACTTAATCTCATTACAGGGGTTGATACTGCATAACTACGAACTTAATAAGGATGAACACAATTCCAAGTGTAGAGGTGAAATTCGTTGATATTTGGAGGAGTACCAAAGGCGAAGGCAGTTCATTGGGTTAAGTTGACGCTGAGGTACGAAAGCGTGGGGAGCAAACAGG

>34a58b1e5c8204bd085356fb94aca6a7

AACAGGGGATACCAGCGTTATCCGGATTTATTGGGTTTAAAGGGTGCGTAGGTTGTTTTTTAAGTCAGTAGTGAAATCTTAAAGCTTAACTTTAAAAGTGCTATTGAGACTGATAAACTAGAGTGAGGTTGGAGTAACTGGAATGTGTGGTGGAGCGGTGAAATGCATAGAGATCACACAGAACACCAATAGCGCAAGCAGGTTACTAAACCTAGACTGACACTGAGGCACGAAAGCATGGGTAGCAAACAGG

>5cec8695a5f52db36057462f8fe08270

CCGCCGCGGTAATACGATTTCTTTAATTTAAATATTTAAGTTTCAGTTAATATAACAATAATATAAAATATCTAAAATTTTGGTGAAATATATTTTATCTTTAAAAATTAATTTTATGTCTGAAAAATTTTTGTATAAACTAGGATTAGAAACCCTAGTAGTCCGGCTGACTGACTTGCGTCAAA

>51ab822467f70275f0771ff0fe493d9d

TACGTAGGGGGCAAGCGTTGTCCAGAATTATTGGGCGTAAAGCGCGCGCAGGCGGCTTACTAAGTCTGGTGTGAAAGCCCACGGCTCAACCGTGGAGGGCCATTGGAAACTGGTAAGCTTGAGTGCAGGAGAGGAGAGCGGAATTCCCGGTGTAGCGGTGAAATGCGTAGATATCGGGAGGAACACCCGTGGCGAAGGCGGCTCTCTGGCCTGTAACTGACGCTGAGGCGCGAAAGCGTGGGGAGCAAACAGG

>6202e2ba7f6fb0aa30f0778636db5f62

CATTGAACTATCGTGAGAAAGTCAAACCGCCAAAGGGAATTATATTATAGTAAATATTGGCGTAAATAAACATTTTATTAATAGTTGTAATATATGAAAATGTGCAGAAGATAAAGACGATTAAAACGGTGTTTAACAATCGCTTATCGAGACACTATTAAAGTAATTAGGAATTAAAATATGTTTATTATGTGTATTATTCAAGGAATAAACTTGAAAATAAATACAGTGTTAAACGGAACTAATAGAGTCACGCAAGTGAACTTAGAACATTTTAATAAC

>945184b6386c192c0066e0a98a154780

TACGGAGGGTGCAAGCGTTAATCGGAATTACTGGGCGTAAAGCGCACGCAGGCGGTCTGTCAAGTCGGATGTGAAATCCCCGGGCTCAACCTGGGAACTGCATTCGAAACTGGCAGGCTAGAGTCTTGTAGAGGGGGGTAGAATTCCAGGTGTAGCGGTGAAATGCGTAGAGATCTGGAGGAATACCGGTGGCGAAGGCGGCCCCCTGGACAAAGACTGACGCTCAGGTGCGAAAGCGTGGGGAGCAAACAGG

>aafb6ca6537c21f170ece1de474769ab

TACGTAGGTGGCAAGCGTTATCCGGAATTATTGGGCGTAAAGCGCGCGTAGGTGGTTTTTTAAGTCTGATGTGAAAGCCCACGGCTCAACCGTGGAGGGTCATTGGAAACTGGAAAACTTGAGTGCAGAAGAGGAAAGTGGAATTCCATGTGTAGCGGTGAAATGCGCAGAGATATGGAGGAACACCAGTGGCGAAGGCGACTTTCTGGTCTGTAACTGACGCTGATGTGCGAAAGCGTGGGGATCAAACAGG

>32b65de73d46ada99ba69a3ebd820844

CCGCCGCGGTAATACGATTTCTTTAATTTAAATATTTAAGTTTCAGTTAATATAACAATAATATAAAATATCTAAAATTTTGGTGAAATATATTTTATCTTTAAAAATTAATTTTATGTCTGAAAAATTTTTGTATAAACTAGGATTAGATACCCTAGTAGTCCGGCTGACTGACTTAACGTCCA

>cfac06aff518f81ba29c1d4660ef6379

CACACTTATGTCTTCAGAAAGACAAAATATTTCCCCATGATGCATTTCAACCATGTCGGTTATTTTCAACTGGCAGTGCCTTGTTGATATTTGTGAAAGATTTTGAATATGTCTTGTCTATAGAGTTGAGTTTGTTCATTAATGATTTTGTTATGTAGTTTGTAACGTAATACATTTAGGCTTTCAAGTAAATTCATTTTGTTTAATTTTTTGCCGGTTGTG

>be80fe6e84c2bae9737bd5ee5b9ca751

TACGTAGGGCGCAAGCGTTATCCGGAATTATTGGGCGTAAAGAGCTCGTAGGCGGTTTGTCGCGTCTGCCGTGAAAGTCCGGGGCTTAACCCCGGATCTGCGGTGGGTACGGGCAGACTAGAGTGCAGTAGGGGAGACTGGAATTCCTGGTGTAGCGGTGAAATGCGCAGATATCAGGAGGAACACCGATGGCGAAGGCAGGTCTCTGGGCTGTAACTGACGCTGAGGAGCGAAAGCATGGGGAGCGAACAGG

>49dafa7aab4a538e6d8e91455bb6d243

CACACTTATGTCTTTAGAAAGACAACACGTTTTTCCAGAATGCATTTCAACCTTCTCGGCCATTTTCAACTGGCAGTGTCTTTTCAATGTGTTTCTGCAAGATAAACGTCTTGAAAATTAAACTATCCGATTCTTTTTCACGAACACGGTCATGTGAACATAACAAATCTGTAATCAGCCTCCAAAT

>4490a1dff5ed7f1a014487420c58ef58

TACGTAGGGCGCGAGCGTTATCCGGAATTATTGGGCGTAAAGAGCTCGTAGGCGGTTTGTCACGTCTGCTGTGAAAGCCCGGGGCTTAACCCCGGGTGTGCAGTGGGTACGGGCAGACTGGAGTGCAGTAGGGGAGACTGGAATTCCTGGTGTAGCGGTGAAATGCGCAGATATCAGGAGGAACACCGATGGCGAAGGCAGGTCTCTGGGCTGTTACTGACGCTGAGGAGCGAAAGCATGGGGAGCGAACAGG

>a7e8c9fd3f736301eab2d73d022a48ad

CCGCCGCGGTAATACGATTTCTTTAATTTAAATATTTAAGTTTCAGTTAATATAACAATAATATAAAATATCTAAAATTTTGGTGAAATATATTTTATCTTTAAAAATTAATTTTATGTCTGAAAAATTTTTGTATAAACTAGGATTAGAAACCCGAGTAGTCCGGCTGACTGACTTAACGTCCA

>9cb7ffa2588fff8cffc97481ee396c02

CCGCCGCGGTAATACGATTTCTTTAATTTAAATATTTAAGTTTCAGTTAATATAACAATAATATAAAATATCTAAAATTTTGGTGAAATATATTTTATCTTTAAAAATTAATTTTATGTCTGAAAAATTTTTGTATAAACTAGGATTAGAAACCCTAGTAGTCCGGCTGACTGACTTAACGTCCA

>7911816f5e81f650f769aba0d5c708cb

GACAGAGGATGCAAGCGTTATCCGGAATGATTGGGCGTAAAGCGTCTGTAGGTGGCTTTTCAAGTCCGCCGTCAAATCCCAGGGCTCAACCCTGGACAGGCGGTGGAAACTACCAAGCTGGAGTACGGTAGGGGCAGAGGGAATTTCCGGTGGAGCGGTGAAATGCATTGAGATCGGAAAGAACACCAACGGCGAAAGCACTCTGCTGGGCCGACACTGACACTGAGAGACGAAAGCTAGGGGAGCAAATGGG

>2aee1c97df88875ec4e56cee5e981021

CCGCCGCGGTAATACGATTTCTTTAATTTAAATATTTAAGTTTCAGTTAATATAACAATAATATAAAATATCTAAAATTTTGGTGAAATATATTTTATCTTTAAAAATTAATTTTATGTCTGAAAAATTTTTGTATAAACTAGGATTAGAAACCCGAGTAGTCCGGCTGACTGACTCTACGACCA

>06e1674aa5f80ede79b5b7f1504a8afd

TACGAAGGGGGCTAGCGTTGTTCGGATTTACTGGGCGTAAAGCGCACGTAGGCGGGCTAATAAGTCAGGGGTGAAATCCCGGGGCTCAACCCCGGAACTGCCTTTGATACTGTTAGTCTTGAATATGGTAGAGGTGAGTGGAATTCCGAGTGTAGAGGTGAAATTCGTAGATATTCGGAGGAACACCAGTGGCGAAGGCGGCTCACTGGACCATTACTGACGCTGAGGTGCGAAAGCGTGGGGAGCAAACAGG

>82dec3eeac5d034d118bba43fddd01fe

TACGGAGGGAGCTAGCGTTGTTCGGAATTACTGGGCGTAAAGCGCACGTAGGCGGCCATTCAAGTCAGAGGTGAAAGCCCGGGGCTCAACCCCGGAACTGCCTTTGAAACTAGATGGCTTGAATCTTGGAGAGGCGAGTGGAATTCCGAGTGTAGAGGTGAAATTCGTAGATATTCGGAAGAACACCAGTGGCGAAGGCGACTCGCTGGACAAGTATTGACGCTGAGGTGCGAAAGCGTGGGGAGCAAACAGG

>7c6c449702794385ac4f7f39b63a6d05

CACACTTATGTCTTCAGAAAGACAAAATATTTCCCCATGATGCATTTCAACCATGTCGGTCATTTTCAACTCGCAGTGCCTTGTTGATATTTGTGAAAGATTTTGAATATGTCTTGTCCATAGAGTTGAGTTTGTTCATTAATGATTTTGTTATGTAGTTTGTAACGTAATACATTTAGGCTTTCAAGTAAATTCATTTTGTTTACTTTTTTGCCGGTTGTG

>ddfd49f939f92958b1ec816741055348

TACGTAGGGTCCAAGCGTTAATCGGAATTACTGGGCGTAAAGCGTGCGCAGGCGGTTGTGCAAGACCGATGTGAAATCCCCGAGCTTAACTTGGGAATTGCATTGGTGACTGCACGGCTAGAGTGTGTCAGAGGGGGGTAGAATTCCACGTGTAGCAGTGAAATGCGTAGAGATGTGGAGGAATACCGATGGCGAAGGCAGCCCCCTGGGATAACACTGACGCTCATGCACGAAAGCGTGGGGAGCAAACAGG

>a1d83433e07f97fd446f7ff512e44fc6

TACGTAGGGTGCAAGCGTTAATCGGAATTACTGGGCGTAAAGCGTGCGCAGGCGGTTTTGTAAGACAGTGGTGAAATCCCCGGGCTCAACCTGGGAACTGCCATTGTGACTGCAAGGCTAGAGTGCGGCAGAGGGGGATGGAATTCCGCGTGTAGCAGTGAAATGCGTAGATATGCGGAGGAACACCGATGGCGAAGGCAATCCCCTGGGCCTGCACTGACGCTCATGCACGAAAGCGTGGGGAGCAAACAGG

>1d54ce9c901f70456cde6f4e95343df2

CCGCCGCGGTAATACGATTTCTTTAATTTAAATATTTAAGTTTCAGTTAATATAATAATAATATAAAATATCTAAAATTTTGGTGAAATATATTTTATCTTTAAAAATTAATTTTATGTCTGAAAAATTTTTGTATAAACTAGGATTAGAAACCCCTGTAGTCCGGCTGACTGACTTGCGTCAAA

>aad59f2fc230eec626de61094fa01e39

CATTGAACTATCGTGAGAAAGTCAAACCGCCAAAGGGAATTATATTATAGTAAATATTGGCGTAAATAAACATTTTATTAATAGTTGTAATATATGAAAATGTGCAAAAGATAGAGACGATTAAAACGGTGTTTAACAATCGCTTATCGAGACAATATTTAATTAATTAGGAATTAAAATATGTGTATTATTTGTATTTAACAAGGAATAAACGTGAAAATAAATACAGTGGTGAACGGAACTAATAGAGTCACGCAAGTGAACTTAGAACAGTTTTAAGAAC

>b1497adc59b7b0dedd4c9836720e64eb

TACGAAGGGTGCGAGCGTTAATCGGAATTACTGGGCGTAAAGCGCGCGTAGGTGGTTTGTTAAGTTGGAAGTGAAAGCCCCGGGCTCAACCTGGGAATTGCTTTCAAAACTGACAGGCTAGAGTACGGTAGAAGGTAGTGGAATTTCCTGTGTAGCGGTGAAATGCGTAGATATAGGAAGGAACATCAGTGGCGAAGGCGACTACCTGGACTGATACTGACACTGAGGTGCGAAAGCGTGGGGAGCAAACAAG

>56e932dd341109fb7128fb6b9b1c1f8a

GCCGCCGCGGTAAGTGCTCAGAAAACATCAATCTTTCACTTCGTTCTCACTTTCTCTTTTTACTGCCTCCTCTTCTGACCCTTTTCCACCATACCCCTTCTTCTCCTTTTCGTCACCCCTTTCGGTCTATTTGTAGCATAATATATTAGAAACCCGAGTAGTCCGGCTGACTGACTTAACGTCCAT

>01f3a7682563f79b10f6ce3f5cfe5718

CCGCCGCGGTAATACGATTTCTTTAATTTAAATATTTAAGTTTCAGTTAATATAATAATAATATAAAATATCTAAAATTTTGGTGAAATATATTTTATCTTTAAAAATTAATTTTATGTCTGAAAAATTTTTGTATAAACTAGGATTAGAAACCCTAGTAGTCCGGCTGACTGACTTGCGTCAAA

>966b5161c75d8b2d6a16e0662b45ddff

TACGTGAGAGACTAGTGTTATTCATCTTAACTGGGTTTAAAGGGTACCTAGACAGTCAATATAACTTCTATAATGCTAATACTTGACTAGAGTTTTAAGTAAGAGGGAAGTACTTAAGGAGTAAGAGATGAAATATCTGTGATACCAAAGGGACTCCGTAAAGGCGAAGGCATCCCTTTATCTAAAAACTAACGTTGAAGGACGAAGGCTTAGATAACAAATAGG

>f0dbfbc9bb9c17a0dd24728e4fa860b1

TACGTAGGTGGCAAGCGTTGTCCGGAATTATTGGACGTAAAGCGCGCGCAGGCGGTCTCTTAAGTCTGATGTGAAAGCCCCCGGCTCAACCGGGGAGGGTCATTGGAAACTGGGAGACTTGAGTACAGAAGAGGAGAGTGGAATTCCACGTGTAGCGGTGAAATGCGTAGATATGTGGAGGAACACCAGTGGCGAAGGCGACTCTCTGATCTGTAACTGACGCTGAGGCGCGAAAGCGTGGGGAGCAAACAGG

>5c60f84b3d868e8544e0b7805e20ae77

TACAGAGGGTGCGAGCGTTAATCGGATTTACTGGGCGTAAAGCGTGCGTAGGCGGCCAATTAAGTCAAATGTGAAATCCCCGAGCTTAACTTGGGAATTGCATTCGATACTGGTTGGCTAGAGTATGGGAGAGGATGGTAGAATTCCAGGTGTAGCGGTGAAATGCGTAGAGATCTGGAGGAATACCGATGGCGAAGGCAGCCATCTGGCCTAATACTGACGCTGAGGTACGAAAGCATGGGGAGCAAACAGG

>9d5a2ce98b74f5213467f9ac19096252

CCGCCGCGGTAATACGATTTCTTTAATTTAAATATTTAAGTTTCAGTTAATATAATAATAATATAAAATATCTAAAATTTTGGTGAAATATATTTTATCTTTAAAAATTAATTTTATGTCTGAAAAATTTTTGTATAAACTAGGATTAGAAACCCGAGTAGTCCGGCTGACTGACTTGCGTCAAA

>f0ca9eda7919577348bff8b8a1b45c3e

CCGCCGCGGTAATACGATTTCTTTAATTTAAATATTTAAGTTTCAGTTAATATAACAATAATATAAAATATCTAAAATTTTGGTGAAATATATTTTATCTTTAAAAATTAATTTTATGTCTGAAAAATTTTTGTATAAACTAGGATTAGAAACCCTAGTAGTCCGGCTGACTGACTGAGACTTAA

>7026285bdd096c9455c3e501e4346dfb

CCGCCGCGGTAATACGATTTCTTTAATTTAAATATTTAAGTTTCAGTTAATATAACAATAATATAAAATATCTAAAATTTTGGTGAAATATATTTTATCTTTAAAAATTAATTTTATGTCTGAAAAATTTTTGTATAAACTAGGATTAGAAACCCCTGTAGTCCGGCTGACTGACTGAGACTTAA

>a43ad7a83aaafe86f998c7a6374bc892

TACGGAGGGTGCAAGCGTTGTCCGGATTTATTGGGTTTAAAGGGTGCGTAGGTGGGATCATAAGTCTGGTTTGAAAGCTGGTGGCTCAACCATCAGATGTGGCTGGAAACTGTGGTTCTTGAATGGCGTAGCGGTAGCCGGAATGGGTCATGTAGCGGTGAAATGCATAGATATGACCCGGAACACCGATTGCGAAGGCAGGCTACTGGGCGTCGATTGACACTGAGGCACGAGAGCATGGGTAGCAAACAGG

>bf69d21a91b8377ff9c37a8ed36c70aa

CCGCCGCGGTAATACGATTTCTTTAATTTAAATATTTAAGTTTCAGTTAATATAACAATAATATAAAATATCTAAAATTTTGGTGAAATATATTTTATCTTTAAAAATTAATTTTATGTCTGAAAAATTTTTGTATAAACTAGGATTAGATACCCCAGTAGTCCGGCTGACTGACTGAGACTTAA

>1bc0322855afa3337d7cc668a965cef2

TACGGAGGGTGCAAGCGTTAATCGGAATTACTGGGCGTAAAGCGCGCGTAGGCGGTTATTTAAGTCAGATGTGAAATCCCCGGGCTTAACCTGGGAATTGCATTTGAGACTGGATGGCTAGAGTATGGTAGAGGTGAGTGGAATTTCAGGTGTAGCGGTGAAATGCGTAGATATCTGAAGGAACATCAGTGGCGAAGGCGACTCACTGGGCCATTACTGACGCTGAGGTGCGAAAGCGTGGGTAGCAAACAGG

>115fcebb776eda0401839622d1eca5b8

TACGAAGGGGGCTAGCGTTGCTCGGAATCACTGGGCGTAAAGGGCGCGTAGGCGGTCTTTTAAGTCGGGGGTGAAAGCCTGTGGCTCAACCACAGAATTGCCTTCGATACTGGGAGACTTGAGACCGGAAGAGGACAGCGGAACTGCGAGTGTAGAGGTGAAATTCGTAGATATTCGCAAGAACACCAGTGGCGAAGGCGGCTGTCTGGTCCGGTTCTGACGCTGAGGCGCGAAAGCGTGGGGAGCAAACAGG

>40b403a1df8ff3c99ff92545e81b3919

CCGCCGCGGTAATACGATTTCTTTAATTTAAATATTTAAGTTTCAGTTAATATAATAATAATATAAAATATCTAAAATTTTGGTGAAATATATTTTATCTTTAAAAATTAATTTTATGTCTGAAAAATTTTTGTATAAACTAGGATTAGAAACCCCGGTAGTCCGGCTGACTGACTTGCGTCAAA

>dc1d0dc8db4e7c2314ef9d14052de96d

GACAGAGGGTGCAAACGTTGTTCGGAATTACTGGGCGTAAAGCGTGTGTAGGCGGTCTTGTAAGTCGGATGTGAAAGCCCCGGGCTCAACCCGGGAAGTGCACTCGATACTGCGAGACTTGAGTATCGGAGAGGTTGGTGGAATTCTCGGTGTAGAGGTGAAATTCGTAGATATCGAGAGGAACACCGGTGGCGAAAGCGGCCAACTGGACGAATACTGACGCTGAGACACGAAAGCGTGGGGAGCAAACAGG

>6aae9a17fbd9c7a4f55a65ef18d63320

TACGAAGGGGGCTAGCGTTGCTCGGAATCACTGGGCGTAAAGGGCGCGTAGGCGGCCATTCAAGTCGGGGGTGAAAGCCTGTGGCTCAACCACAGAATTGCCTTCGATACTGTTTGGCTTGAGTATGGTAGAGGTCGGTGGAACTGCGAGTGTAGAGGTGAAATTCGTAGATATTCGCAAGAACACCAGTGGCGAAGGCGGCCGACTGGACCATTACTGACGCTGAGGCGCGAAAGCGTGGGGAGCAAACAGG

>4f6f12c1983a7c7a339ba8b21c544a84

TACGTAGGGTGCGAGCGTTAATCGGAATTACTGGGCGTAAAGCGTGCGCAGGCGGTTTTGTAAGACAGGCGTGAAATCCCCGGGCTTAACCTGGGAATGGCGCTTGTGACTGCAAGGCTAGAGTGCGTCAGAGGGGGGTAGAATTCCACGTGTAGCAGTGAAATGCGTAGAGATGTGGAGGAATACCGATGGCGAAGGCAGCCCCCTGGGACGAGACTGACGCTCATGCACGAAAGCGTGGGGAGCAAACAGG

>1727bd8544639e521c5efff9f8c225a6

TACAGAGGGTGCAAGCGTTGTTCGGAATTACTGGGCGTAAAGCGTGCGTAGTCGGTATTGAGAGTCACGGGTGAAATCCCAGGGCTTAACCCTGGAACTGCCTGTGAGACCTCAGTACTAGAGTGTGAGAGGGGATAGTGGAATACCCAGTGTAGCGGTGAAATGCGTAGAGATTGGGTGGAACACCGGTGGCGAAGGCGGCTATCTGGCTCACAACTGACGATCAGGCACGAAAGCGTGGGGAGCAAACAGG

>2987717251dc6b65e72066c24484bec4

CCGCCGCGGTAATACGATTTCTTTAATTTAAATATTTAAGTTTCAGTTAATATAACAATAATATAAAATATCTAAAATTTTGGTGAAATATATTTTATCTTTAAAAATTAATTTTATGTCTGAAAAATTTTTGTATAAACTAGGATTAGATACCCGAGTAGTCCGGCTGACTGACTTAACGTCCA

>e4e28a40134239b4ec2f4d9bb83bf037

TTCTTACTGCAAAATATTGTCACACACATATGTTTTGTAGTAATTATTGTGAGCAGCATCCTCTATGTTATAAATGTCATAAGGAAATCTTAAAACAATGATAATAATCTGTTTATAGTAATTATTCTTCATCATCTTCGCTATTCCTGGATTAGAAACCCTTGTAGTCCGGC

>63d29d8c99c33d97ca9d500371d221c9

TACGGAGGGTCCGAGCGTTAATCGGAATTACTGGGCGTAAAGCGTGCGCAGGCGGTTTGTTAAGCGAGATGTGAAAGCCCTGGGCTCAACCTAGGAATAGCATTTCGAACTGGCGAACTAGAGTCTTGTAGAGGGGGGTAGAATTCCAGGTGTAGCGGTGAAATGCGTAGAGATCTGGAGGAATACCGGTGGCGAAGGCGGCCCCCTGGACGAAGACTGACGCTCATGCACGAAAGCGTGGGGAGCAAACAGG

>ca75a3930be5f6d0e8a5a7e9b2f31e09

CCGAGATCTACACTGCGTCACTATGGTAATTGTGTGCCAGCCGCCGCGGTAACAGCCCAACGCCCTGGAAACACTACCACGACCGGCTGGAACGATACGAGTGTCATTTTCCTATTCTGTTTTAATATGGCAGTTTACCTATCGATTAGAAACCCCGGTAGTCCGGCTGACTGACTTGCGTCAAATCTCGTATGCCGTCTTCTGCTTGAAAAAAAAAAAAAAAAA

>a7da93a9744a7863292bcf97d47b6a10

TACGTAGGGTGCGAGCGTTGTCCGGAATTATTGGGCGTAAAGAGCTTGTAGGCGGTTTGTCGCGTCTGCTGTGAAAATCCGGGGCTCAACCCCGGACTTGCAGTGGGTACGGGCAGACTAGAGTGTGGTAGGGGAGACTGGAATTCCTGGTGTAGCGGTGAAATGCGCAGATATCAGGAGGAACACCGATGGCGAAGGCAGGTCTCTGGGCCACTACTGACGCTGAGAAGCGAAAGCATGGGGAGCGAACAGG

>6f470466d3eca81e322a799e116a719f

TACGTATGTCACAAGCGTTATCCGGATTTATTGGGCGTAAAGCGCGTCTAGGCGGTTAATTAAGTCTGATGTGAAAATGCGGAGCTCAACTCCGTATTGCGTTGGAAACTGGTTAACTAGAGTACTGGAGAGGTAAGCGGAACTACAAGTGTAGAGGTGAAATTCGTAGATATTTGTAGGAATGCCGATGGGGAAGCCAGCTTACTGGACAGATACTGACGCTAAAGCGCGAAAGCGTGGGTAGCAAACAGG

>ac02d7e5a954e30c3cdd73a625ec45b6

CATTGAACTATCGTGAGAAAGTCAAGCCGCCAAAGGGAATTATATTATAGTAAATATTGGCGTAAATAAACATTTTATTAATAGTTGTAATATATGAAAATGTGCAGAAGATAAAGACGATTAAAACGGTGTTTAACAATCGCTTATCGAGACAATATTTAATTAATTAGTAATTAAAATATGTTTATTATGTGTATTATTCAAGGAATAGACTTGACAATAAATACAGTGGTAAACGGAACTAATAGAGTCACGCAAGTGAACTTAGAACATTTTTAATAAC

>4473ac847f597164696bc96da74ca753

TACGAAGGGGGCTAGCGTTGCTCGGAATCACTGGGCGTAAAGGGCGCGTAGGCGGACTCTTAAGTCGGGGGTGAAAGCCCAGGGCTCAACCCTGGAATTGCCTTCGATACTGAGAGTCTTGAGTTCGGAAGAGGTTGGTGGAACTGCGAGTGTAGAGGTGAAATTCGTAGATATTCGCAAGAACACCAGTGGCGAAGGCGGCCAACTGGTCCGATACTGACGCTGAGGCGCGAAAGCGTGGGGAGCAAACAGG

>a04c5d991aa692295a0b91c8f258c984

GGTATTGCAGCGACGCGATAGCGTTTTCCATTTACCGAAGTCGGAATATCTGGGGAAAGCGAAATATCTCTCAGACGCGTCTCCCAGCGAGATTTACAGGCGGCAGCAGTCTTTTCAATAATTCATATAAAGTGAATTTTATTTCCATTAACCGGATAAAAAAACGTTGAACACGAGTGAAGTCGAATAACCGTGG

>28e260728976404c3e3e1ef2357c68a6

GACAGAGGATGCAAGCGTTATCCGGAATGATTGGGCGTAAAGCGTCTGTAGGTGGCTTTTCAAGTCCGCCGTCAAATTCCAGGGCTCAACCCTGGACAGGCGGTAGAAACTACCAAGCTGGAGTACGGTAGGGGCAGAGGGAATTTCCGGTGGAGCGGTGAAATGCGTTGAGATCGGAAAGAACACCAACGGCGAAAGCACTCTGCTGGGCCGACACTGACACTGAGAGACGAAAGCTAGGGGAGCAAATGGG

>a581b2d4607e97c5afb8a61b67a6ecd9

TACGAAGGGGGCTAGCGTTGTTCGGATTTACTGGGCGTAAAGCGCGCGTAGGCGGATATTTAAGTCAGAGGTGAAATCCCAGGGCTCAACCCTGGAACTGCCTTTGATACTGGGTATCTAGAGTATGGAAGAGGTGAGTGGAATTCCGAGTGTAGAGGTGAAATTCGTAGATATTCGGAGGAACACCAGTGGCGAAGGCGGCTCACTGGTCCATTACTGACGCTGAGGTGCGAAAGCGTGGGGAGCAAACAGG

>7a53ea3b23204b31fa844fc7a65a152e

CCGCCGCGGTAATACGATTTCTTTAATTTAAATATTTAAGTTTCAGTTAATATAACAATAATATAAAATATCTAAAATTTTGGTGAAATATATTTTATCTTTAAAAATTAATTTTATGTCTGAAAAATTTTTGTATAAACTAGGATTAGAAACCCCTGTAGTCCGGCTGACTGACTCTACGACCA

>2f77ac274cc33232a9055b49e8b99708

TACGGAGGGGACTAGCGTTGTTCGGAATTACTGGGCGTAAAGCGCACGTAGGCGGACTGGAAAGTTGGGGGTGAAATCCCGGGGCTCAACCTCGGAACTGCCTTCAAAACTATCAGTCTGGAGTTCGAGAGAAGTGAGTGGAATTCCGAGTGTAGAAGTGAAATTCGTAGATATTCGGAGGAACACCAGTGGCAAAGGCGGCTCACTGGCTCGATACTGACGCTGAGGTGCGAAAGCGTGGGGAGCAAACAGG

>d6efd2da2728fd74ded268122ee05036

TACGGAGGGAGCTAGCGTTATTCGGAATTACTGGGCGTAAAGCGCACGTAGGCGGCTTTGTAAGTTAGAGGTGAAAGCCTGGAGCTCAACTCCAGAATTGCCTTTAAGACTGCATCGCTTGAATCCAGGAGAGGTGAGTGGAATTCCGAGTGTAGAGGTGAAATTCGTAGATATTCGGAAGAACACCAGTGGCGAAGGCGGCTCACTGGACTGGTATTGACGCTGAGGTGCGAAAGCGTGGGGAGCAAACAGG

>b21d5535b0132f715924ffb0a14044cc

TACGTAGGGTGCGAGCGTTAATCGGAATTACTGGGCGTAAAGCGTGCGCAGGCGGTTATGTAAGACAGATGTGAAATCCCCGGGCTCAACCTGGGAACTGCATTAGTGACTGCATAGCTGGAATACGGCAGAGGGGGATGGAATTCCGCGTGTAGCAGTGAAATGCGTAGATATGCGGAGGGACACCGATGGCGTAGGCAATCCCCTGGGCCTGTACTGACGCTCATGCACGAAAGCGTGGGGAGCAAACAGG

>cb89ac968f0ea4fa3b48de62ae889cf6

TACGTAGGTGGCAAGCGTTGTCCGGAATTATTGGGCGTAAAGCGCGCGCAGGCGGTCTCTTAAGTCTGATGTGAAAGCCCCCGGCTCAACCGGGGAGGGTCATTGGAAACTGGGAGACTTGAGTACAGAAGAGGAGAGTGGAATTCCACGTGTAGCGGTGAAATGCGTAGATATGTGGAGGAACACCAGTGGCGAAGGCGACTCTCTGGTCTGTAACTGACGCTGAGGCGCGAAAGCGTGGGGAGCAAACAGG

>7629f0928583d9f14def2b39ac1121b7

TACGAGGGGAGCGAGTGTTGTTCGGTTTTATTGGGCGTAAAGGGTACGTAGGCGGTTTTGTAAGTCAACACTTAAATCTTGAGACTTAATCTCATTACAGGTGTTGATACTGCATAACTATGAACTTAATAGGGGTGAACACAATTCCAAGTGTAGAGGTGAAATTCGTTGATATTTGGAGGAGTACCAAAGGCGAAGGCAGTTCATTGGGTTAAGTTGACGCTGAGGTACGAAAGCGTGGGGAGCAAACAGG

>7d78ed99b08bd1723065fdd795d17e9c

TACGTAGGGTGCGAGCGTTGTCCGGAATTACTGGGCGTAAAGGGCTCGTAGGTGGTTTGTCGCGTCGTCTGTGAAATTCTGGGGCTTAACTCCGGGCGTGCAGGCGATACGGGCATAACTTGAGTGCTGTAGGGGTAACTGGAATTCCTGGTGTAGCGGTGAAATGCGCAGATATCAGGAGGAACACCGATGGCGAAGGCAGGTTACTGGGCAGTTACTGACGCTGAGGAGCGAAAGCATGGGTAGCGAACAGG

>dc40901d2a75ad6678f667d9e7827119

CCGCCGCGGTAATACGATTTCTTTAATTTAAATATTTAAGTTTCAGTTAATATAATAATAATATAAAATATCTAAAATTTTGGTGAAATATATTTTATCTTTAAAAATTAATTTTATGTCTGAAAAATTTTTGTATAAACTAGGATTAGATACCCCGGTAGTCCGGCTGACTGACTTGCGTCAAA

>92946b8c74cdc5b9e9166c281f22a13a

TTCCTCTTTTTTCAACGAAGTTCCGCAATTCATTTCTATTTGCTGCCATTCATTTGCCTATTTCAATAGATGCCTCTAGTTCTGAACGCTGATACTATACTATCGTTCCATGCGATTCTAGGTCTTCCTCTACAGTTTTTTTCTTTTTCCTGGTTTCCCATAGCTTTTTGACCAGTCTCTCTGTGCATGCTT

>7d0fd17647460e5425eb0ce0aa995036

TACGGAGGGAGCTAGCGTTGTTCGGAATTACTGGGCGTAAAGCGCACGTAGGCGGCTTTGTAAGTCAGAGGTGAAAGCCTGGAGCTCAACTCCAGAACTGCCTTTGAGACTGCATCGCTTGAATCCAGGAGAGGTGAGTGGAATTCCGAGTGTAGAGGTGAAATTCGTAGATATTCGGAAGAACACCAGTGGCGAAGGCGGCTCACTGGACTGGTATTGACGCTGAAGTGCGAAAGCGTGGGGAGCAAACAGG

>a3ca6538736db7607c2de7e43d7496f9

CATTTTACCATAGTGTCTGCCTCCACGGGCACGTTTATGGCTTTGCCGGTTATCTTTTAAGACTGTTTCTATTCTGAGACGACGGATCTGCATACGTGGAAATCCGGATGAAATCAGCCTCTCAGTGATACGATCGAGCGGCAGATGGGTCGGCTCTTCCGGATACTTATAACTATTACTTTGGCTCGGCGGAGGCACTTTACCCTCATTAAGCAAGCGGCGGTATGTGGAGCACA

>86ac24ef0e869ee1d5436e231f5bcca7

TACGGAGGGTGCAAGCGTTGTTCGGAATTATTGGGCGTAAAGCGCGTGCAGGCGGCTGTTCAAGTCCGATGTGAAAGCCCGGGGCTCAACCCCGGAAGTGCATTGGAAACTGGACAGCTTGAGTACGGGAGAGGGAGGTAGAATTCCGAGTGTAGGGGTGAAATCCGTAGATATTTGGAGGAATACCGGTGGCGAAGGCGGCCTCCTGGACCGATACTGACGCTGAGACGCGAAAGCGTGGGGAGCAAACAGG

>2f9103a7492f2978e9c17a2c45995b2e

TACGAAGGGGGCTAGCGTTGCTCGGAATTACTGGGCGTAAAGGGCGCGTAGGCGGTCATTTTAGTCAGGCGTGAAATTCCCGGGCTTAACCTGGGGACTGCGCTTGATACTGGGTGACTTGAGGGTGGAAGAGGGTCGTGGAATTCCCAGTGTAGAGGTGAAATTCGTAGATATTGGGAAGAACACCGGTGGCGAAGGCGGCGACCTGGTCCATTACTGACGCTGAGGCGCGACAGCGTGGGGAGCAAACAGG

>e502ebea2a54a6f839d310d6d57e7d47

TACGTATGTCACGAGCGTTATCCGGATTTATTGGGCGTAAAGCGCGTCTAGGTGGTTATGTAAGTCTGATGTGAAAATGCAGGGCTCAACTCTGTATTGCGTTGGAAACTGTATAACTAGAGTACTGGAGAGGTAAGCGGAACTACAAGTGTAGAGGTGAAATTCGTAGATATTTGTAGGAATGCCGATGGGGAAGCCAGCTTACTGGACAGATACTGACGCTAAAGCGCGAAAGCGTGGGTAGCAAACAGG

>0b9d81bf74392ad0ae77b55a24d4445e

TACGTAAAAGACTAGTGTTAGTCATCTTTATTAGGTTTAAAGGGTACCTAGACGGTAAATTAAACTCTAAATGAGTACTTTTTTACTAGAGTTTTATAAGAGAAGGAAGAATTTCTGGAGTAGCGATAAAATACTCTTATACCAGAAGGACTGGTAACAGCGAAGGCATCCTTCTAAGTAAAAACTGACGTTGAGGGACGAAGGCTTGGGTAGCAATAAGG

>2fb70975701695c4e584c8a0c9965d54

CACACATATGTCTTTAGAATGACAAAACATTTTTCCATAATGCATTTCAACCATGTCGGTCATTTTCAAATGGCAGTGCATTGTAGATAGTTGGGGTAGATTTTGATTGTGTCTTGTCCATAGAGTTCAGTTTGTTTATTAATGATTTTGTTATGTAGTTTGTAACGTAGTACGAGTATATTTAGATTTTCGAGTAAATTCATTCTTTTATGATAAAATTCAATGTATTTCATTTTTTGCCGGTTGTG

>62d599597a31cc0a682c5031b93d84a4

TACGAGGGGAGCGAGTGTTGTTCGGTTTTATTGGGCGTAAAGGGCACTTAGGCTGTTTTACAAGTTAACAGTTAAATCTTGGAACTCAATTCCATGCCAATTGTTAATACTGTTTTACTAAGAATTTAGTGGAGATGATCATAATTCCAAGTGTAGAGGTGAAATTCGTTGATATTTGGAGGAGTACCAAAGGCGAAGGCAGATCATCATGTTAAGTTGACGCTAAAAGTGCGAAAGCGTGGGGAGCAAACAGG

>04b788106dacc021b0ea1f32f3b7513f

CATTGAACTATCGTGAGAAAGTCAAACCGCCAAAGGGAATTATATTATAGTAAATATTGGCGTAAATAAACATTTTATTAATAGTTGTAATATATGAAAATGTGCAGAAGATAGAGACGATTAAAACGGTGTTTAACAATCGCTTATCGAGACAATATTTAATTAATTAGGAATTAAAATATGTGTATTATTTGTATTTAACAAGGAATAAACGTGAAAATAAATACAGTGGTAAACGGAACTAATAGAGTCACGCAAGTGAACTTAGAACAATTTTAAGAAC

>bc3cacfb5767a65d7f54284deedd9e95

TACGGAGGGGGCTAGCGTTGTTCGGAATTACTGGGCGTAAAGCGCACGTAGGCGGCTTTGTAAGTTAGAGGTGAAAGCCTGGAGCTCAACTCCAGAATTGCCTTTAAGACTGCATCGCTTGAATCCAGGAGAGGTGAGTGGAATTCCGAGTGTAGAGGTGAAATTCGTAGATATTCGGAAGAACACCAGTGGCGAAGGCGGCTCACTGGACTGGTATTGACGCTGAGGTGCGAAAGCGTGGGGAGCAAACAGG

>4e84f05b8251d7ed308a00ab22fa0724

TACGGAGGGTGCAAGCGTTGTTCGGAATTATTGGGCGTAAAGCGCGTGCAGGCGGCTGTTCAAGTCCGATGTGAAAGCCCGGGGCTCAACCCCGGAAGTGCATTGGAAACTGGACAGCTTGAGTACGGGAGAGGGAGGTAGAATTCCGAGTGTAGGGGTGAAATCCGTAGATATTCGGAGAAATACCGGTGGCGAAGGCGGCCTCCTGGACCGATACTGACGCTGAAACGCGAAAGCGTGGGGAGCAAACAGG

>cbe9e5f34c7b6768d85759c1248cfddd

TACGAAGGGGGCTAGCGTTGTTCGGAATTACTGGGCGTAAAGCGCACGTAGGCGGATATTTAAGTCAGGGGTGAAATCCCGCAGCTCAACTGCGGAACTGCCTTTGATACTGGGTATCTTGAGTATGGAAGAGGTAAGTGGAATTGCGAGTGTAGAGGTGAAATTCGTAGATATTCGCAGGAACACCAGTGGCGAAGGCGGCTTACTGGTCCATTACTGACGCTGAGGTGCGAAAGCGTGGGGAGCAAACAGG

>93fec914068220be020251cc9c36396b

TACGTATGTCGCGAGCGTTATCCGGAATTATTGGGCATAAAGGGCATCTAGGCGGCCTAACAAGTCAGGGGTGAAAACCTGCGGCTCAACCGCAGGCCTGCCTTTGAAACTGTAAGGCTGGAGTACCGGAGAGGTGGACGGAACTGCACGAGTAGAGGTGAAATTCGTAGATATGTGCAGGAATGCCGATGATGAAGATAGTCCACTGGACGGAAACTGACGCTGAAGTGCGAAAGCCGGGGGAGCGAACAGG

>8743ff835d67f75f7e1534ad77a4a02a

TACGTAGGGTGCGAGCGTTAATCGGAATTACTGGGCGTAAAGCGTGCGCAGGCGGTTATGTAAGACAGATGTGAAATCCCCGGGCTCAACCTGGGAACTGCATTAGTGACTGCATAGCTGGAATACGGCAGAGGGGGATGGAATTCCGCGTGTAGCAGTGAAATGCGTAGATATGCGGAGGAACACCGATGGCGAAGGCAATCCCCTGGGCCTGTACTGACGCTCATGCACGAAAGCGTGGGGAGCAAACAGG

>9bd0501d2a9bdd871282c1aa312a5b74

GCCGCCGCGGTAAGTGCTCAGAAAACATCAATCTTTCACTTCGTTCTCACTTTCTCTTTTTACTGCCTCCTCTTCTGACCCTTTTCCACCATACCCCTTCTTCTCCTTTTCGTCACCCCTTTCGGTCTATTTGTAGCATAATATATTAGATACCCCTGTAGTCCGGCTGACTGACTTAACGTCCAT

>a689bb9a3a1a27abe1333699f6d4aebc

TACGGAGGATCCAAGCGTTATCCGGAATCATTGGGTTTAAAGGGTCCGTAGGCGGCCTTGTAAGTCAGTGGTGAAATCTCCCCGCTCAACGGGGAAACTGCCATTGATACTGCAGGGCTTGAATTATGGTGAAGTAACTAGAATATGTAGTGTAGCGGTGAAATGCTTAGAGATTACATGGAATACCAATTGCGAAGGCAGGTTACTAACCATATATTGACGCTGATGGACGAAAGCGTGGGGAGCGAACAGG

>c53e7420108f44aca5e7076ac700dfec

TACGAAGGGTGCAAGCGTTGTTCGGAATTATTGGGCGTAAAGCGCGTGCAGGCGGCTGTTCAAGTCCGATGTGAAAGCCCGGGGCTCAACCCCGGAAGTGCATTGGAAACTGGACAGCTTGAGTACGGGAGAGGGAGGTAGAATTCCGAGTGTAGGGGTGAAATCCGTAGATATTCGGAGGAATACCGGTGGCGAAGGCGGCCTCCTGGACCGATACTGACGCTGAGACGCGAAAGCGTGGGGAGCAAACAGG

>6a42f6b31b1958a4fd7c015ad7affbf4

TACGTAGGGGGCAAGCGTTGTCCGGAATTATTGGGCGTAAAGCGCGCGCAGGCGGTCATTTAAGTCTGGTGTTTAATCCCGGGGCTCAACCCCGGATCGCACTGGAAACTGGGTGACTTGAGTGCAGAAGAGGAGAGTGGAATTCCACGTGTAGCGGTGAAATGCGTAGATATGTGGAGGAACACCAGTGGCGAAGGCGACTCTCTGGGCTGTAACTGACGCTGAGGCGCGAAAGCGTGGGGAGCAAACAGG

>0071d9b8eee5e0d14c9cf04604a7711a

CCGCCGCGGTAATACGATTTCTTTAATTTAAATAGTTAAGTTTCAGTTAATATAATAGTAATATAAAATATCTATAATTTTGGTGAAATATATTTTATCTTGAAAAATTAATTTTATGTCTGAAAAATTTTTGTTTAAACTAGGATTAGAAACCCCAGTAGTCCGGCTGACTGACTCGCGATATA

>ff48ea2b9d30c737b53aeb9841179d57

TACGTATGGGGCGAGCGTTGTCCGGAATTATTGGGCGTAAAGGGTACGTAGGCGGCCTTTTAAGTCAGGTGTGAAAGCGTGAGGCTTAACCTCATTAAGCACTTGAAACTGGAAGGCTTGAGTGAAGGAGAGGAAAGTGGAATTCCTAGTGTAGCGGTGAAATGCGTAGATATTAGGAGGAATACCGGTGGCGAAGGCGACTTTCTGGACTTTTACTGACGCTCAGGTACGAAAGCGTGGGGAGCAAACAGG

>1e43f75985b946a268b2fdc23cc35925

GCCGCCGCGGTAAGTGCTCAGAAAACATCAATCTTTCACTTCGTTCTCACTTTCTCTTTTTACTGCCTCCTCTTCTGACCCTTTTCCACCATACCCCTTCTTCTCCTTTTCGTCACCCCTTTCGGTCTATTTGTAGCATAATATATTAGATACCCTAGTAGTCCGGCTGACTGACTTAACGTCCAT

>b0427d6905d31250f86cd04748e78ac9

AGATCTACACGATAGCGTTATGGTAATTGTGTGCCAGCCGCCGCGGTAACACACGTATGTCTTTAGAAAGACAACACGTTTTTGCAGAATGCATTTCAACCTTCTCGGTCATTTTCAACTGGCAGTGACTTCCTGAAAATGACCATTAGAAACCCGAGTAGTCCGGCTGACTGACTACTGTGTAATCTCGTATGCCGTCTTCTGCTTGAAAAAAAAAAAAAA

>b45ed3e05b49d86d5db595b1255d4c53

TCGAACCCAAGCCACGTGGTGTAACGAACCAACACACTACACACCACGAGTACTCCCCCCCCCCTTCCAATCAACAAAAAATCACAATACGATTACAGGAAATTGTTCAGAATGTAGCGCCAAATGAAATTACAAAGAGGAGGATTTTGAATATTGCAGAAGGATCGTATTGGTGCAAAACAAACGTTTGCTAGCAGCCTTCCTCACGTGTATACTGCACTGATGCTTCGAGAAATAGTCTGCAGAACTAT

>cdf2b7893a7c66be2475defd929d7c02

TACGGAGGGGGCTAGCGTTGTTCGGAATTACTGGGCGTAAAGCGCACGTAGGCGGATCAGTCAGTCAGGGGTGAAATCCCGGGGCTCAACCCCGGAACTGCCTTTGATACTGCTGGTCTTGAGGTCGAGAGAGGTGAGTGGAATTCCGAGTGTAGAGGTGAAATTCGTAGATATTCGGAGGAACACCAGTGGCGAAGGCGGCTCACTGGCTCGATACTGACGCTGAGGTGCGAAAGCGTGGGGAGCAAACAGG

>62ca4b170b3674c7de46826de4d55aa8

TTCTTACTGCAAACGTATTGTCAAACACATATGTTTTGTAGTAATTATTGTGAGCAGCATCCTCTATGTTATAAATGTAATAAGGAAATCTTAAAACAATGATAATAATCTGTTTATAGTAATTAATCTTCATCATCTTCGCTATTCCAGGATTAGAAACCCCAGTAGTCCGG

>9c029f349d00d4a0139761afd64c55d1

TTCTTACTGCAAACGTATTGTCACACACATGTGTTTTGTAGTAATTTTTGTGAGCAGCATCCTCTATGTTATAAATGTAATAAGGAAATCTTAAAACAATGATAATAATCTGTTTATAGTAATTAATCTTCATCATCTTCGCTATTCCAGGATTAGAAACCCGAGTAGTCCGG

>c2d5b1fe7e78d3d6f19b77c071c5c0f8

TACGAAGGGTGCAAGCGTTACTCGGAATTACTGGGCGTAAAGCGTGCGTAGGTGGTGGTTTAAGTCTGTTGTGAAAGCCCTGGGCTCAACCTGGGAATTGCAGTGGATACTGGGTCACTAGAGTGTGGTAGAGGGTAGCGGAATTCCCGGTGTAGCAGTGAAATGCGTAGAGATCGGGAGGAACATCCGTGGCGAAGGCGGCTACCTGGACCAACACTGACACTGAGGCACGAAAGCGTGGGGAGCAAACAGG

>3cac8e98742ad2b953e0a7e62e7020c4

TACGATTAACCCAAACTAATTATCTTCGGCGTAAAACGTGTCAACTATAAATAAATAAATAGAATTAAAATCCAACTTATATGTGAAAATTCATTGTTAGGACCTAAACTCAATAACGAAAGTAATTCTAGTCATTTATAATACACGACAGCTAAGACCCAAACTGGGATTAG

>b4b925835dca6941390f98c2af7c7d70

TACGAAGGGTGCAAGCGTTAATCGGAATTACTGGGCGTAAAGCACGCGTAGGTGGTTTGTTAAGTTGGATGTGAAAGCCCCGGGCTCAACCTGGGAACTGCATCCAAAACTGGCAAGCTAGAGTATGGCAGAGGGTGGTGGAATTTCCTGTGTAGCGGTGAAATGCGTAGATATAGGAAGGAACACCAGTGGCGAAGGCGACCACCTGGGCTAATACTGACACTGAGGTGCGAAAGCGTGGGGAGCAAACAGG

>9067fca59d6b4741ae6709ea44cd5fda

CATTGAACTATCGTGAGAAAGTCAAGCCGCCAAAGGGAATTATATTATAGTAAATATTGGCGTAAATAAACATTTTATTAATAGTTGTAATATATGAAAATGTGCAGAAGATAAAGACGATTAAAACGGTGTTTAACAATCGCTTATCGAGACAATATTTAATTAATTAGTAATAAAAATATGTTTATCATGTGTATTATTCAAGGAATAGACTTGACAATAAATACAGTGGTAAACGGAACTAATAGAGTCACGCAAGTGAACTTAGAACATTTTTAATAAC

>41b11d50e98c1a315d61f5b7a585d156

TACGTAGGGTGCGAGCGTTAATCGGAATTACTGGGCGTAAAGCGTGCGCAGGCGGTTATGTAAGACAGATGGGAAATCCCCGGGCTCAACCTGGGAACTGCATTTGTGACTGCATGGCTAGAGTACGGTAGAGGGGGATGGAATTCCGCGTGTAGCAGTGAAATGCGTAGATATGCGGAGGAACACCGATGGCGAAGGCAATCCCCTGGACCTGTACTGACGCTCATGCACGAAAGCGTGGGGAGCAAACAGG

>4846f2a7c7565bec29924451d1994b81

TACGGAGGGTGCGAGCGTTAATCGGAATTACTGGGCGTAAAGCGCGCGTAGGCGGCTTGATAAGCCGGTTGTGAAAGCCCCGAGCTCAACCTGGGAACGGCATCCGGAACTGTCAGGCTAGAGTGCAGGAGAGGAAGGTAGAATTCCCGGTGTAGCGGTGAAATGCGTAGAGATCGGGAGGAATACCAGTGGCGAAGGCGGCCTTCTGGACTGACACTGACGCTGAGGTGCGAAAGCGTGGGTAGCAAACAGG

>c61e678c4585c76f06da173838515021

TACGTAGGGTGCGAGCGTTAATCAGAATTACTGGGCGTAAAGCGTGCGCAGGCGGTTTTGTAAGACAGAGGTGAAATCCCCGGGCTCAACCTGGGAACTGCCTTTGTGACTGCAAGGCTAGAGTACGGCAGAGGGGGATGGAATTCCGCGTGTAGCAGTGAAATGCGTAGATATGCGGAGGAACACCGATGGCGAAGGCAATCCCCTGGGCCTGTACTGACGCTCATGCACGAAAGCGTGGGGAGCAAACAGG

>e056c59afbcce6fab9f7550ff6213aeb

TACGGAGGGGGCTAGCGTTGTTCGGAATTACTGGGCGTAAAGCGCACGTAGGCGGACTGGAAAGTTGGGGGTGAAATCCCGGGGCTCAACCTCGGAACTGCCTTCAAAACTATCAGTCTGGAGTTCGAGAGAGGTAAGTGGAATTCCGAGTGTAGAGGTGAAATTCGTAGATATTCGGAGGAACACCAGTGGCGAAGGCGGCTCACTGGCTCGATACTGACGCTGAGGTGCGAAAGCGTGGGGAGCAAACAGG

>eef6d3c42ab7df7398b35f58e10d7f8d

TACGTAGGGTGCGAGCGTTAATCGGAATTACTGGGCGTAAAGCGTGCGCAGGCGGTTTGTTAAGACAGATGTGAAATCCCCGGGCTTAACCTGGGAACTGCATTTGTGACTGGCAAGCTAGAGTATGGCAGAGGGGGGTAGAATTCCACGTGTAGCAGTGAAATGCGTAGAGATGTGGAGGAATACCGATGGCGAAGGCAGCCCCCTGGGCCAATACTGACGCTCATGCACGAAAGCGTGGGGAGCAAACAGG

>d7926eba30ca0f01bac6fc5d090e9ab9

TACGGAGGATGCGAGCGTTATCCGGAATCATTGGGTTTAAAGGGTCCGTAGGCGGGCTAATAAGTCAGAGGTGAAAGCGCTCAGCTCAACTGAGCAACTGCCTTTGAAACTGTTAGTCTTGAATGGTTGTGAAGTAGTTGGAATGTGTAGTGTAGCGGTGAAATGCTTAGATATTACACAGAACACCGATAGCGAAGGCATATTACTAACAATTAATTGACGCTGATGGACGAAAGCGTGGGGAGCGAACAGG

>3fd71acc3bc9455281006f5fc35c3f7b

TACGGAGGGTGCAAGCATTGTTCGGAATTATTGGGCGTAAAGCGCGTGCAGGCGGCTGTTCAAGTCCGATGTGAAAGCCCGGGGCTCAACCCCGGAAGTGCATTGGAAACTGGACAGCTTGAGTACGGAAGAGGGAGGTAGAATTCCGAGTGTAGGGGTGAAATCCGTAGATATTCGGAGGAATACCGGTGGCGAAGGCGGCCTCCTGGACCGATACCGACGCTGAGACGCGAAAGCGTGGGGAGCAAACAGG

>0ed3a6836e138f14044f6950ad7280d2

TACGTAGGGCGCGAGCGTTGTCCGGAATTATTGGGCGTAAAGAGCTCGTAGGCGGCTGGTCGCGTCTGTCGTGAAATCCTCTGGCTTAACTGGGGGCTTGCGGTGGGTACGGGCCGGCTTGAGTGCGGTAGGGGAGACTGGAACTCCTGGTGTAGCGGTGGAATGCGCAGATATCAGGAAGAACACCGGTGGCGAAGGCGGGTCTCTGGGCCGTTACTGACGCTGAGGAGCGAAAGCGTGGGGAGCGAACAGG

>2f3f456b876776fd76e8eff1c212f0db

TACGGAAGGTCCGGGCGTTATCCGGATTTATTGGGTTTAAAGGGAGCGCAGACGGTCTTTTAAGCGTGCTGTGAAATATTGCGGCTCAACCGTAAGATTGCAGCGCGAACTGGTAGACTTGAGTTCGCTGGAGGTACGCGGAATTCGTGGTGTAGCGGTGAAATGCTTAGATATCACGAAGAACTCCGATTGCGAAGGCAGCGTACCGTGGCGTTACTGACGTTCATGCTCGAAAGCGCGGGTATCGAACAGG

>7b11c44eef75fb0ebb0680d6a08cccad

TACGTAGGGTGCAAGCGTTATCCGGAATTATTGGGCGTAAAGAGCTCGTAGGCGGTTTGTCGCGTCTGCTGTGAAATCCCGAGGCTCAACCTCGGGCCTGCAGTGGGTACGGGCAGACTAGAGTGCGGTAGGGGAGATTGGAATTCCTGGTGTAGCGGTGGAATGCGCAGATATCAGGAGGAACACCGATGGCGAAGGCAGATCTCTGGGCCGTAACTGACGCTGAGGAGCGAAAGGGTGGGGAGCAAACAGG

>729908645785ff8a0409ea99258f95bf

TACGTAGGTGGCAAGCGTTATCCGGAATTATTGGGCGTAAAGCGCGCGCAGGTGGTTTCTTAAGTCTGATGTGAAAGCCCACGGCTCAACCGTGGAGGGTCATTGGAAACTGGGAGACTTGAGTGCAGAAGAGGAAAGTGGAATTCCATGTGTAGCGGTGAAATGCGTAGAGATATGGAGGAACACCAGTGGCGAAGGCGACTTTCTGGTCTGCAACTGACACTGAGGCGCGAAAGCGTGGGGAGCAAACAGG

>8307dc29315d2433794a7fc239cf3cfa

TACGGAGGGTGCGAGCGTTAATCGGAATTACTGGGCGTAAAGCGCGCGTAGGCGGCTTGATAAGCCGGTTGTGAAAGCCCCGGGCTCAACCTGGGAACGGCATCCGGAACTGTCAGGCTAGAGTGCAGGAGAGGAAGGTAGAATTCCCGGTGTAGCGGAGAAATGCGTAGAGATCGGGAGGAATACCAGTGGCGAAGGCGGCCTTCTGGACTGACACTGACGCTGAGGTGCGAAAGCGTGGGTAGCAAACAGG

>e6b39f795c5239b7bf061b93e2bb6ce3

CACACTTATGTCTTCAGAAAGACAAAATATTTATCCAGGATGCATTTCAACCATGTCGGTCATTTTTAACTGGCAGTGCCTTGTAGATATTTGTGAACGATTTTGAATGTGTCTTGTCAATAGAGTTCAGTTTGTTCATTAATGATTTTGTTATGTAGTTTGTAACGTAATATATTTAGGCTATCAAGTAAATTCATTTTGTTTCCTTTTTGCCGGTTGTG

>732b7603b170da1718e9b5af0ae47e7a

AGGCTTCTATTGCCCATCATCGCGTCGTGCCGCCGCAGCGGACTTATCTGGAGGTCACCTCTATTGAGATCCACGGAGCTCTTGACGGCGTCCTCTTTGTGTCAGCTTATAAGCCCTTCTTAGAAAGAACTTCTGGTCTGCCACTTGGAGACCGTCTTCGATGCACATCGTAGGGTGATCATGGCCGGCGACCTGA

>87461b5f2af4769574e5c3af8b7a3d03

TACGGAGGATGCGAGCGTTATTCGGAATCATTGGGTTTAAAGGGTCTGTAGGCGGGCTATTAAGTCAGGGGTGAAAGGTTTCAGCTTAACTGAGAAATTGCCTTTGATACTGGTAGTCTTGAATATCTGTGAAGTTCTTGGAATGTGTAGTGTAGCGGTGAAATGCTTAGATATTACACAGAACACCGATTGCGGAGGCAGGGGACTAACAGACGATTGACGCTGAGAGACGAAAGCGTGGGGAGCGAACAGG

>5737e058385a6a34a3bcfdec9f73df47

TACGGAGGGTGCGAGCGTTATTCGGAATTACTGGGCGTAAAGCGCACGCAGGCGGTTGCCCAAGTCAGATGTGAAAGCCCCGGGCTTAACCTGGGAACTGCATTTGAAACTGGGCGACTAGAGTATGAAAGAGGAAAGCGGAATTTCCAGTGTAGCAGTGAAATGCGTAGATATTGGAAGGAACACCGATGGCGAAGGCAGCTTTCTGGGTCGATACTGACGCTCATGTGCGAAAGCGTGGGGAGCAAACAGG

>956ecd93aea363dd7f1b86fadc65dfcc

CCGCCGCGGTAATACGATTTCTTTAATTTAAATATTTAAGTTTCAGTTAATATAACAATAATATAAAATATCTAAAATTTTGGTGAAATATATTTTATCTTTAAAAATTAATTTTATGTCTGAAAAATTTTTGTATAAACTAGGATTAGAAACCCCAGTAGTCCGGCTGACTGACTTGCGTCAAA

>58a9a501230a08491f94325868b26299

TACGAAGGGTGCAAGCGTTAATCGGAATTACTGGGCGTAAAGCGCGCGTAGGTGGTTTGTTAAGTTGGATGTGAAAGCCCCGGGCTCAACCTGGGAACTGCATCCAAAACTGGCAAGCTAGAGTATGGCAGAGGGTGGTGGAATTTCCTGTGTAGCGGTGAAATGCGTAGATATAGGAAGGAACACCAGTGGCGAAGGCGACCACCTGGGCTAATACTGACACTGAGGTGCGAAAGCGTGGGGAGCAAACAGG

>ed1375bdaaea965f8dd917230d269be0

TACGGAGGGGGCTAGCGTTGTTCGGAATTACTGGGCGTAAAGCGCACGTAGGCGGACTGGAAAGTTGGGGGTGAAATCCCGGGGCTCAACCTCGGAACTACCTTCAAAACTATCAGTCTGGAGTTCGAGAGAGGTGAGTGGAATTCCGAGTATAGAGGTGAAATTCGTAGATATTCGGAGGAACACCAGTGGCGAAGGCGGCTCACTGGCTCGATACTGACGCTGAGGTGCGAAAGCGTGGGGAGCAAACAGG

>f1860fe716257bd5fd5c4c6a16cf3b95

TACGTAGGGTGCGAGCGTTAATCGGAATTACTGGGCGTAAAGCGGGCGCAGACGGTTACTTAAGCAGGATGTGAAATCCCCGGGCTCAACCTGGGAACTGCGTTCTGAACTGGGTGACTAGAGTGTGTCAGAGGGAGGTAGAATTCCACGTGTAGCAGTGAAATGCGTAGAGATGTGGAGGAATACCGATGGCGAAGGCAGCCTCCTGGGATAACACTGACGTTCATGCCCGAAAGCGTGGGTAGCAAACAGG

>6af118db0608fbf49242f0355e1fd56d

TACGAGGGGAGCGAGTGTTGTTCAGTTTTATTGGGCGTAAAGGGTGTTCAGGTGGCTAAGCAAGTTAACAACAAAATCTTGAGGCTCAACCTCATAACGTTCGGTTAATACTGCTTGGCTAGAGCTTGGATAGGAACAAACGGAATTCCAAGTGTAGAGGTGAAATTCGTTGATACTTGGAGGAACACCAGAGGCGAAGGCGGTTTGTTATGCCAAGCTGACACTGAAGACACGAAAGCATGGGGAGCAAACAGG

>e573f4124ee82ff94d3215f1876cb988

GCCGCCGCGGTAATACAAGTAAATCTTTTTTTAAAAAATTAAAAATAATAAAAAAATTTTTGTATTTTAAAAATTTATAAAGGTGAAATTTATAATTTTAAAAAGATTAATTTGATTTTATAAAAATTTAATTTTTAAACTAGGATTAGAAACCCTAGTAGTCCGGCTGACTGACTCGCGATATAT

>92f1720367db58c68a96eceb9feb416a

TACGAAGGGGGCTAGCGTTGCTCGGAATCACTGGGCGTAAAGGGCGCGTAGGCGGCCGATTAAGTCGGGGGTGAAAGCCTGTGGCTCAACCACAGAATTGCCTTCGATACTGGTTGGCTTGAGACCGGAAGAGGACAGCGGAACTGCGAGTGTAGAGGTGAAATTCGTAGATATTCGCAAGAACACCAGTGGCGAAGGCGGCTGTCTGGTCCGGTTCTGACGCTGAGGCGCGAAAGCGTGGGGAGCAAACAGG

>01c2b37d190d266468669acd2603ba22

TACGGAGGGTGCAAGCGTTGTTCGGAATTATTGGGCGTAAAGCGCGTGCAGGCGGCTGTTCAAGTCTGATGTGAAAGCCCGGGGCTCAACCCCGGAAGTGCATTGGAAACTGGACAGCTTGAGTACGGGAGAGGGAGGTAGAATTCCGAGTGTAGGGGTGAAATCCGTAGATATTCGGAGGAATACCGGTGGCGAAGGCGGCCTCCTGGACCGATACTGACGCTGAGACGCGAAAGCGTGGGGAGCAAACAGG

>dee0d5ea04ef493783599ca61807d792

ACTCTTACTGCAAACGTATTGTCACACACATGTTTTGTAGTAATTATTGTGAGCAGCATCCTCTATGTTATAAATTTAATAAGGAAATCTTAAAACAATGATAATAATCTGTTTATAGTAATTAATCTTCTTCGCCATTCCAGGATTAGAAACCCGAGTAGTCCGGCTGACTGA

>bd0fd792491b3f8a561a79da2beaeb45

TACGGAGGGGGCTAGCGTTGTTCGGAATCACTGGGCGTAAAGCGCACGTAGGCGGCGATCTAAGTCAGAGGTGAAAGCCCGGGGCTCAACCCCGGAATTGCCTTTGAGACTGGATTGCTTGAACGTCGGAGAGGTGGGTGGAATTCCGAGTGTAGAGGTGAAATTCGTAGATATTCGGAAGAACACCAGTGGCGAAGGCGGCCCACTGGACGACTGTTGACGCTGAGGTGCGAAAGCGTGGGGAGCAAACAGG

>f792998b877a4fe4b284db74afaf14de

TACGTATGTCGCGAGCGTTATCCGGAATTATTGGGCATAAAGGGCATCTAGGTGGCCTAACAAGTCAGGGGTGAAAACCTGCGGCTCAACCGCAGGCTTGCCTTTGAAACTGTAAGGCTGGAGTATCGGAGAGGTGGACGGAACTGCACGAGTAGAGGTGAAATTCGTAGATATGTGCAGGAATGCCGATGATGAAGATAGTTCACTGGACGATAACTGACGCTGAAGTGCGAAAGCCGGGGGAGCAAACAGG

>35e6d0a378f763e3902d599c2d573475

CATTGAACTATCGTGAGAAAGTCAAGCCGCCAAAGGGAATTATACTATAGTAAATATTGGCGTAAATAAACATTTTATTAATAGTTGTAATATATGAAAATGTGCAGAGGATAAAGACGATTAAAACGGTGTTTAACAATCGCTTATCGAGACAATATTTAATTAATTAGTAATTAAAATATGTTTATTATGTGTATTATTCAAGGAATAGACTTGACAATAAATACAGTGGTAAACGGAACTAATAGAGTCACGCAAGTGAACTTAGAACATTTTTAATAAC

>8feff4fb993943279e1768300901c845

TACGTAGGGTGCAAGCGTTGTCCGGAATTATTGGGCGTAAAGAGCTCGTAGGCGGTTTGTCGCGTCTGCTGTGAAAACCCGAGGCTCAACCTCGGGCCTGCAGTGGGTACGGGCAGACTAGAGTGCAGTAGGGGAGATTGGAATTCCTGGTGTAGCGGTGGAATGCGCAGATATCAGGAGGAACACCAATGGCGAAGGCAGATCTCTGGGCTGTAACTGACGCTGAGGAGCGAAAGCATGGGGAGCGAACAGG

>f2c611d7fe31ef93b37b4e6a764017fb

GACGGGGGGGGCAAGTGTTCTTCGGAATGACTGGGCGTAAAGGGCACGTAGGCGGTGAATCGGGTTGAAAGTGAAAGTCGCAAAAAACTGGCGGAATGCTCTCGAAACCAATTCACTTGAGTGAGACAGAGGAGAGTGGAATTTCGTGTGTAGGGGTGAAATCCGGAGATCTACGAAGGAACGCCAAAAGCGAAGGCAGCTCTCTGGGTCCCTACCGACGCTGGGGTGCGAAAGCATGGGGAGCGAACGGG

>0dbf680e00aa85eb226b129f0adeff7e

TACGTAGGGTGCGAGCGTTGTCCGGAATTATTGGGCGTAAAGAGCTTGTAGGCGGTTTGTCGCGTCTGCTGTGAAAGACCGGGGCTTAACTCCGGTTCTGCAGTGGGTACGGGCAGACTAGAGTGTGGTAGGGGAGACTGGAATTCCTGGTGTAGCGGTGAAATGCGCAGATATCAGGAGGAACACCGATGGCGAAGGCAGGTCTCTGGGCCATTACTGACGCTGAGAAGCGAAAGCATGGGGAGCGAACAGG

>58d311836793b18400e882b9ecc57579

TACGGAGGGTGCAAGCGTTAATCGGAATTACTGGGCGTAAAGCGCACGCAGGCGGTCTGTCAAGTCGGATGTGAAATCCCCGGGCTCAACCTGGGAACTGCATTCGAAACTGGCAGACTAGAGTCTTGTAGAGGGGGGTAGAATTCCAGGTGTAGCGGTGAAATGCGTAGAGATCTGGAGGAATACCGGTGGCGAAGGCGGCCCCCTGGACAAAGACTGACGCTCAGGTGCGAAAGCGTGGGGAGCAAACAGG

>4cc817431b3c62a5fc9a845599d17ac5

TACGAAGGGTGCAAGCGTTAATCGGAATTACTGGGCGTAAAGCGCGCGTAGGTGGTTTTGTAAGTTGGAGGTGAAATCCCCGGGCTCAACCTGGGAACTGCCTCCAAAACTGCATGACTAGAGTACGGTAGAGGGTGGTGGAATTTCCTGTGTAGCGGTGAAATGCGTAGATATAGGAAGGAACACCAGTGGCGAAGGCGACCACCTGGACTGATACTGACACTGAGGTGCGAAAGCGTGGGGAGCAAACAGG

>7aaa1c634a0d6d8deea816c16fdaf092

AAATGGCTACTTTCACGGGTGCAGTACGGGCTAACTGTGTGTTAAGGTTTCATGACACAAACTCTGCAACAACAGTTCAGCATAATTTTTGCACCGAGTGTGGTAAAGATCCTCTTACTAGACCTACAATTTACACTTGACCTCAGAACTTCGTTGAGAGTGGTTGTTCGGTTCAGC

>923f521b9cf313f1f95c9367e09bbc1c

TACGTAGGTGGCAAGCGTTGTCCGGAATTATTGGGCGTAAAGCGCGCGCAGGCGGATTGGTCAGTCTGTCTTAAAAGTTCGGGGCTTAACCCCGTGATGGGATGGAAACTGCCAATCTAGAGTATCGGAGAGGAAAGTGGAATTCCTAGTGTAGCGGTGAAATGCGTAGATATTAGGAAGAACACCAGTGGCGAAGGCGACTTTCTGGACGAAAACTGACGCTGAGGCGCGAAAGCCAGGGGAGCGAACGGG

>827992b22906f7a8b7f7a87580b21cfa

GACAGAGGATGCAAGCGTTATCCGGAATGATTGGGCGTAAAGCGTCTGTAGGTGGCTTTTCAAGTCCGCCGTCAAATCCCAGGGCTCAACCCTGGACAGGCGGTGGAAACTACCAAGCTGGAGTACGGTAGGGGCAGAGGGAATTTCCGGTGGAGCGGTGAAATGCGTAGAGATCGGAAAGAACACCAACGGCGAAAGCACTCTGCTGGGCCGACACTGACACTGAGAGACGAAAGCTAGGGGAGCAAATGGG

>9538dde07e438fbc3295e5bb7e27fb99

CATTGAACTATCGTGAGAAAGTCACGCCGCCAAAGGGAATTATATAATAGTAAATATTAGCGTAAATAAACATTTTATTAATAGTTGTAATATATGATAATGTGCAAAAGATAGAGACGATTAAAACGGTGTTTAACAATCGCTTATCGAGACACTATTAAAGTAATTAGGAATTAAAATATGTTTATTATGTGTATTATTCAAGGAATAAACTTGAAAATAAATACAGTGTTAAACGGAACTAATAGAGTCACGCAAGTGAACTTAGAACATTTTAATAAC

>273c6f86e31372c1292901e1935d0c07

CACACTTATGTCTTCAGAAAGACAAAATATTTCTCCAGGATGCATTTCAACTATGTCGGTCATTTTCAACTGGCAGTGCCTTGTAGATATTTGTGAAAGATTTTGAGTGTGTCTTGTCCATAGAGTTGAGTTTGTTCATTAATAATTGTGTTATGTAGTTGTACGTAATACATTCAGGCTTTCAAGTAAATTCATTTTGTTTACTTCTTTGCCGGTTGTG

>78f1224136a594084b29c27221ef08b9

TACGTAGGGGGCAAGCGTTATCCGGATTCATTGGGCGTAAAGCGCTCGTAGGCGGTCTGTTAGGTCGGGAGTTAAATCCGGGGGCTCAACCCCCGCTCGCTCTCGATACCGGCAGACTTGAGTTTGGTAGGGGAAGGTGGAATTCCTAGTGTAGCGGTGGAATGCGCAGATATTAGGAAGAACACCAGTGGCGAAGGCGGCCTTCTGGGCCATAACTGACGCTGAGGAGCGAAAGCTAGGGGAGCAAACAGG

>54a43d2113c13203e813d22b17f3f370

TACGGAGGATGCGAGCGTTATCCGGATTTATTGGGTTTAAAGGGTGCGTAGGCGGTTTTTTAAGTTAGTGGTTAAACGTCGGGGCTTCACCTTGATTTGCCATTAATACTGTAAGACTCGAGTTCAGACGAGGTAGGCGGAATAAGTTAAGTAGCGGTGAAATGCTTAGATATAACTTAGAACTCCGATAGCGAAGGCAGCTTACCAGACTGCGACTGACGCTGAAGCACGAGAGCGTGGGTAGCGAACAGG

>173dc4050d5f308554c0d0868b7d4953

TACGGAGGGTGCAAGCGTTGTTCGGAATTATTGGGCGTAAAGCGCGTGCAGGCGGCTGTTCAAGTCCGATGTGAAAGCCCGGGACTCAACCCCGGAAGTGCATTGGAAACTGGACAGCTTGAATACGGGAGAGGGAGGTAGAATTCCGAGTGTAGGGGTGAAATCCGTAGATATTCGGAGGAATACCGGTGGCGAAGGCGGCCTCCTGGACCGATACTGACGCTGAGACGCGAAAGCGTGGGGAGCAAACAGG

>814d36cce47414becceb7665900831e6

TACGTATGTCGCGAGCGTTATCCGGAATTATTGGGCATAAAGGGCATCTAGGCGGCCTTTCAAGTCAGGGGTGAAAACCTGCGGCTCAACCGCAGGCCTGCCTTTGAAACTGATAGGCTGGAGTACCGGAGAGGTGGACGGAACTGCACGAGTAGAGGTGAAATTCGTAGATATGTGCAGGAATGCCGATGATGAAGATAGTTCACTGGACGGTAACTGACGCTGAAGTGCGAAAGCCGGGGGAGCGAACAGG

>2bd9309f2f97cae51d18d06ea1ca519a

TACGTAGGTGGCAAGCGTTGTCCGGATTTATTGGGCGTAAAGCGAGCGCAGGCGGTTCCTTAAGTCTGATGTGAAAGCCCCCGGCTCAACCGGGGAGGGTCATTGGAAACTGGGGAACTTGAGTGCAGAAGAGGAGAGTGGAATTCCATGTGTAGCGGTGAAATGCGTAGATATATGGAGGAACACCAGTGGCGAAGGCGACTCTCTGGTCTGTAACTGACGCTGAGGCTCGAAAGCGTGGGTAGCAAACAGG

>2084f436bb855d8d888265dcd2fda277

TACGTAGGGTGCAAGCGTTAATCGGAATTACTGGGCGTAAAGCGTGCGCAGGCGGTTTTGTAAGACAGTGGTGAAATCCCCGGGCTCAACCTGGGAACTGCCATTGTGACTGTAAGGCTAGAGTGCGGCAGAGGGGGATGGAATTCCGCGTGTAGCAGTGAAATGCGTAGATATGCGGAGGAACACCGATGGCGAAGGCAATCCCCTGGGCCTGCACTGACGCTCATGCACGAAAGCGTGGGGAGCAAACAGG

>bc56a7361c9a3b49f1f1c51874321e12

TACGTAGGGCGCAAGCGTTGTCCGGAATTATTGGGCGTAAAGAGCTCGTAGGCGGTTTGTCGCGTCTGCTGTGAAAGCCCGGGGCTTAACCCCGGGTGTGCAGTGGGTACGGGCAGACTTGAGTGCAGTAGGGGAGACTGGAATTCCTGGTGTAGCGGTGAAATGCGCAGATATCAGGAGGAACACCGATGGCGAAGGCAGGTCTCTGGGCTGTTACTGACGCTGAGGAGCGAAAGCATGGGGAGCGAACAGG

>2de541767a82bc14e1e864f280f8216d

TTCTTACTGCAAACGTATTGTCACACACATGTTTTGTAGTAATTATTGTGAGCAGCATCCTCTATGTTATAAATGTAATAAGGAAATCTTAAAACAATGATAATAATCTGTTTATAGTAATTAATCTTCATCATCTTCGCCATTCCAGGATTAGAAACCCCTGTAGTCCGGCT

>30fce74018285af76fc89ebf67d1ae64

ATTTTCTTTATAGAGACGTTCATTTAGTTTTATCGTGTGGCCACGTACTCCCATTCGTTGACGAACCAGATAGATCGAAAACGTACATCGGGAGTCACTATGCTAGTGAAAGTAACTGATTTCAACTAAGGTTCTACAAACAAATAATAAAATTGCATTAATTAGATACCCCT

>6606f680e9a7a36d254524c2b17cd969

TACGTAGGGTGCAAGCGTTGTCCGGAATTATTGGGCGTAAAGAGCTCGTAGGCGGTCTGTCGCGTCGGCTGTGAAAACCCGAGGCTCAACCTCGGGCCTGCAGTCGATACGGGCAAACTAGAGTGTTGCAGGGGAGACTGGAATTCCTGGTGTAGCGGTGAAATGCGCAGATATCAGGAGGAACACCGGTGGCGAAGGCGGGTCTCTGGGCAACAACTGACGCTGAGGAGCGAAAGCGTGGGGAGCGAACAGG

>e03bc28d05bf9b737d4ea6bf0b5eedb8

TACGGAGGGTGCGAGCGTTAATCGGAATTACTGGGCGTAAAGCGCGCGTAGGCGGCTTGATAAGCCGGTTGTGAAAGCCCCGGGCTCAACCTGGGAACGGCATCCGGAACTGTCAGGCTAGAGTGCAGGAGAGGAAGGTAGAATTCCCGGTGTAGCGGTGAAATGCGTAGAGATCGGGAGGAATACCAGTGGCGAAGGCGGCCTTCTGGACTGACACTGACGCTGAGGCGCGAAAGCGTGGGTAGCAAACAGG

>70c3ca05e9fa73277618890c44dda7d6

GCTCGATGTCGGAGCCGATAACAGGATTATCCGGCTTCACCTATGTGCCTCTCACTGCTTTTGTATATTATCCCTTCCGTAGTAAACCTTACTGAGATAATATTATACAGCGGCGAGTGGTACAAAGTAGCTGCTTCGGAAGAAAAGAAAAACATTCTTACTGGTAGACTATACTAAAGAAAAATATTCATGAGAAGCGTAGCATTGTGTAGAGCTGAAACATGAAAGTAGGAATGTCAGATTATCTGAAGAATTTCGAATATGATATTGGAGAACAATGCAGAATACATGTTGGACAGACGTG

>7ad6416c369e26f09e5a945e288ea934

CATTGAACTATCGTGAGAAAGTCAAACCGCCAAAGGGCTATAAAAAAGGGTTATAAAGGGTTATAGTAAATATTAACGTAAATAAACATTTTATTAATAGTTGTAATATATGATAATGTGCAAAAGATAGAGACGATTAAAACGGTGTTTAACAATCGCTTATCGAGACACTATTAAAGTAATTAGGAATTAAAATATGTTTATTATGTGTATTATTCAAGGAATAAACTTGAAAATAAATACAGTGTTAAACGGAACTAATAGAGTCACGCAAGTGAACTTAGAACATTTTAATAAC

>dac0f04ba8c53c4b0b20316b3db7c6a1

TACGTAGGTGGCAAGCGTTATCCGGAATTATTGGGCGTAAAGCGCGCGTAGGCGGTTTTTTAAGTCTGATGTGAAAGCCCACGGCTCAACCGTGGAGGGTCATTGGAAACTGGAAAACTTGAGTGCAGAAGAGGAAAGTGGAATTCCATGTGTAGCGGTGAAATGCGCAGAGATATGGAGGAACACCAGTGGCGAAGGCGACTTTCTGGTCTGTAACTGACGCTGATGCGCGAAAGCGTGGGGATCAAACAGG

>1368aca6d12ec4a9bbf16a2ff67104e1

TACGTAGGGCGCAAGCGTTGTCCGGAATTATTGGGCGTAAAGAGCTCGTAGGCGGTTTGTCGCGTCTGCTGTGAAAGCCCGGGGCTCAACCCCGGGTCTGCAGTGGGTACGGGCAGACTAGAGTGCAGTAGGGGAGACTGGAATTCCTGGTGTAGCGGTGAAATGCGCAGATATCAGGAGGAACACCGATGGCGAAGGCAGGTCTCTGGGCTGTTACTGACGCTGAGGAGCGAAAGCATGGGGAGCGAACAGG

>ba62734792851a13bc5c7d92e179b00f

CATTGAACTATCGTGAAAAAGTCAAACCGCCAAAGGGAATTATATTATAGTAAATATTGGCGTAAATAAACATTTTATTAATAGTTGTAATATATGATAATGTGCAGAAGATAAAGACGATTAAAACAGTGTTTAATAATCGCTTATCGAGACACTATTAAAGTAATACGGAATTAAAATATGTTTATTATGTGTATTAAACAAGGGATAAACTTGAAAATAAATACAGTGGTAAACGGAACTAATAGAGTCACGCAAGTGAACTTAGAACATTTTTAATAAC

>25272650f673d0d96fa1fee3db0dcee8

TTCTTACTGCAAACGTATTGTCACACACATATGTTTTGTAGTAATTATTGTGAGCAGCATCCTCTATGTCATAAATGTAATAAGGAAATCTTAAAACAATGATAATAATCTGTTTATAGTAATTAATCTTTATCATCTTCGCCATTCCAGGATTAGAAACCCTAGTAGTCCGG

>f7a52ddf9dbbe8fe6d3afea2447b99aa

GACGAACAGTGCGGGTGTTACTCGGATTTATTGGGCGTAAAGGGTATGTAGGTGGCCATTTAAACTTTGAACGAAATACTAGGGTAATAACCTTAGAAGGTGTTCTTTAAAATTATTTGGCTTTGAGTTAATGAGAGGTTAGAAGAAATTCTAGTGGTAGATATTAAATCCTTCGATATTAGAATGAATACCAAGGGCGAAGGCATCTTTCTGGCATTAACTGACGCTGATATACGTAAGTGTAGGGAGCGAATCGG

>6400be98be706e381faa4df752eeba93

TACGTAGGGCGCAAGCGTTGTCCGGAATTATTGGGCGTAAAGAGCTTGTAGGTGGCTTGTCGCGTCTGCCGTGAAAACCCGAGGCTCAACCTCGGGCGTGCGGTGGGTACGGGCAGGCTAGAGTGTGGTAGGGGAGACTGGAACTCCTGGTGTAGCGGTGATATGCGCAGATATCAGGAAGAACACCGATGGCGAAGGCAGGTCTCTGGGCCATTACTGACACTGAGAAGCGAAAGCATGGGGAGCGAACAGG

>2d03d27411a5fda13c4f35261f9f174f

CCGCCGCGGTAATACGATTTCTTTAATTTAAATATTTAAGTTTCAGTTAATATAACAATAATATAAAATATCTAAAATTTTGGTGAAATATATTTTATCTTTAAAAATTAATTTTATGTCTGAAAAATTTTTGTATAAACTAGGATTAGATACCCGAGTAGTCCGGCTGACTGACTGAGACTTAA

>b316e238f824ade50a3c069fa87d21b2

TACGTAGGTGGCAAGCGTTGTCCAGAATTATTGGGCGTAAAGCGCGCGCAGGCGGTCTCTTAAGTCTGATGTGAAAGCCCCCGGCTCAACCGGGGAGGGTCATTGGAAACTGGGAGACTTGAGTACAGAAGAGGAGAGTGGAATTCCACGTGTAGCGGTAAAATGCGTAGATATGTGGAGGAACACCAGTGGCGAAGGCGACTCTCTGGTCTGTAACTGACGCTGAGGCGCGAAAGCGTGGGGAGCAAACAGG

>015eee080b971f4284683cc08e976fea

TACGTAGGTGGCAAGCGTTGTCCGGAATTATTGGGCGTAAAGGGCGTGTAGGTGGATTCTTAAGTCGTGTGTCTAAGTGCGGTGCTCAACACCGTATGGGCGCAGGAAACTGGGAATCTTGAGTGTAGGAGAGGAAAGTGGAATTCCCAGTGTAGCGGTGAAATGCGTAGATATTGGGAGGAATACCAGTGGCGAAGGCGACTTTCTGGACTGTGTCTGACACTGAGGCGCGAAAGCCAGGGGAGCGAACGGG

>162dcb3929e23b071e65fda88174be74

TACGTAGGGTGCGAGCGTTGTCCGGAATTACTGGGCGTAAAGAGCTCGTAGGTGGTTTGTCGCGTCGTTTGTGGAATACCGCAGCTTAACTGCGGGGTTGCAGGCGATACGGGCATAACTTGAGTGCTGTAGGGGAGACTGGAATTCCTGGTGTAGCGGTGGAATGCGCAGATATCAGGAGGAACACCGATGGCGAAGGCAGGTCTCTGGGCAGTAACTGACGCTGAGGAGCGAAAGCATGGGGAGCGAACAGG

>31f8990842e5ca7f845c65400a9baa24

TACCAGCACCCCGAGTGGTCGGGACGATTATTGGGCCTAAAGCATCCGTAGCCGGTTCTGCAAGTCTTCCGTTAAATCCAGCCGCTCAACGGATGGGCCGCGGAGGATACTATAGGACTAGGAGGCGGGAGAGGCAAGCGGTACTCAGTGGGTAGGGGTAAAATCCTTTGATCCATTGAAGACCACCAGTGGCGAAGGCGGCTTGCCAGAACGCGCTCGACGGTGAGGGATGAAAGCTGGGGGAGCAAACCGG

>d4f6defc65534cfe1b42bc3d5a23a1da

TTCTTACTGCAAACGTATTGTCACACACATGTGTTTTGTAGTAATTTTTGTGAGCAGCATCCTCTATGTTATAAATGTAATAAGGAAATCTTAAAACAATGATAATAATCTGTTTATAGTAATTAATCTTCATCATCTTCGCTATTCCAGGATTAGATACCCGAGTAGTCCGG

>a0901407705992dc213ac509ede97d47

TACGGAGGGTGCAAGCGTTAATCGGAATTACTGGGCGTAAAGCGCACGCAGGCGGTCTGTCAAGTCGGATGTGAAATCCCCGGGCTCAACCTGGGAACTGCATCCGAAACTGGCAGGCTTGAGTCTCGTAGAGGGGGGTAGAATTCCAGGTGTAGCGGTGAAATGCGTAGAGATCTGGAGGAATACCGGTGGCGAAGGCGGCCCCCTGGACGAAGACTGACGCTCAGGTGCGAAAGCGTGGGGAGCAAACAGG

>f67106d07b885d4d811daca7688633ed

TACGGAAGGTCCGGGCGTTATCCGGATTTATTGGGTTTAAAGGGAGCGTAGGCCGTGGATTAAGCGTGTTGTGAAATGCAGGTGCTCAACGTCTGCACTGCAGCGCGAACTGGTCCACTTGAGTGTGCGCAACGCAGGCGGAATTCGTCGTGTAGCGGTGAAATGCTTAGATATGACGAAGAACTCCGATTGCGAAGGCAGCTTGCGGGAGCACAACTGACGCTGAAGCTCGAAAGTGCGGGTATCGAACAGG

>d15bc449222795a9ff230013aa633686

TACAGAGGGTGCGAGCGTTAATCGGATTTACTGGGCGTAAAGCGTGCGTAGGCGGCTTTTTAAGTCGGATGTGAAATCCCCGAGCTTAACTTGGGAATTGCATTCGATACTGGGAAGCTAGAGTATGGGAGAGGATGGTAGAATTCCAGGTGTAGCGGTGAAATGCGTAGAGATCTGGAGGAATACCGATGGCGAAGGCAGCCATCTGGCCTAATACTGACGCTGAGGTACGAAAGCATGGGGAGCAAACAGG

>79c5fb2d82862a1f28ef7fa0497415ab

TTCTGACTGCAAACGTATTGTCACACACATATGTTTTGTAGTAATTATTGTGAGCAGCATCCTCTATGTTATAAATGTAATAAGGAAATCTTAAAACAATGATAATAATCTGTTTATAGTAATTAATCTTCATCATCTTCGCCATTCCAGGATTAGAAACCCTTGTAGTCCGG

>029fbe1ecd8a63470b3caf53f1af615d

TACGTAGGTCCCGAGCGTTGTCCGGATTTATTGGGCGTAAAGCGAGCGCAGGCGGTTAGATAAGTCTGAAGTTAAAGGGTGTGGCTTAACCATAGTACGCTTTGGAAACTGTTTAACTTGAGTGCAGAAGGGGAGAGTGGAATTCCATGTGTAGCGGTGAAATGCGTAGATATATGGAGGAACACCGGTGGCGAAAGCGGCTCTCTGGTCTGTAACTGACGCTGAGGCTCGAAAGCGTGGGGAGCGAACAGG

>edfcadddeda0e2a18488c28d0000b50d

TACGTAGGTGGCAAGCGTTGTCCGGAATTATTGGGCGTAAAGGGCGTGTAGGTGGATCTTTAAGTCGTGTGTCTAAGTGCGGTGCTCAACGCCGTATGGGCGCAGGAAACTGGGGATCTTGAGTGCAGGAGAGGAAAGTGGAATTCCCAGTGTAGCGGTGAAATGCGTAGATATTGGGAGGAACACCAGTGGCGAAGGCGACTTTCTGGACTGTGTCTGACACTGAGGCGCGAAAGCCAGGGGAGCGAACGGG

>5346ea829c6831c580ea689dea68dfae

TACGGAGGGTGCAAGCGTTGTTCGGAATTATTGGGCGTAAAGCGCGTGCAGGCGGCTGTTCAAGTCCGATGTGAAAGCCCGGGGCTCAACCCCGGAAGTGCATTGGAAACTGGACAGCTTGAGTACGGGAGAGGGAGGTAGAATTCCGAGTATAGGGGTGAAATCCGTAGATATTCGGAGGAATACCGGTGGCGAAGGCGGCCTCCTGGACCGATACTGACGCTGAGACGCGAAAGCGTGGGGAGCAAACAGG

>685a1812a6e46f72d7cf1f4a060b8e96

TACGTAGGGTGCGAGCGTTGTCCGGAATTACTGGGCGTAAAGAGCTCGTAGGTGGTTTGTCGCGTCGTTTGTGTAAGCCCGCAGCTTAACTGCGGGACTGCAGGCGATACGGGCATAACTTGAGTGCTGTAGGGGAGACTGGAATTCCTGGTGTAGCGGTGGAATGCGCAGATATCAGGAGGAACACCGATGGCGAAGGCAGGTCTCTGGGCAGTAACTGACGCTGAGGAGCGAAAGCATGGGGAGCGAACAGG

>f30afbee7698d335ae83b7a8e06e98de

CCGCCGCGGTAATACGATTTCTTTAATTTAAATATTTAAGTTTCAGTTAATATAACAATAATATAAAATATCTAAAATTTTGGTGAAATATATTTTATCTTTAAAAATTAATTTTATGTCTGAAAAATTTTTGTATAAACTAGGATTAGAAACCCTGGTAGTCCGGCTGACTGACTTGCGTCAAA

>d246c1d97375468760c6d624a11e2cfb

CCGCCGCGGTAATACGATTTCTTTAATTTAAATAGTTAAGTTTCAGTTAATATAATAGTAATATAAAATATCTATAATTTTGGTGAAATATATTTTATCTTGAAAAATTAATTTTATGTCTGAAAAATTTTTGTTTAAACTAGGATTAGATACCCGAGTAGTCCGGCTGACTGACTCGCGATATA

>fae6d0c5cd2a53341a4ec984541b1541

TACGTATGTCACGAGCGTTATCCGGATTTATTGGGCGTAAAGCGCGTCTAGGTGGTTATGTAAGTCTGATGTGAAAATGCAGGGCTCAACTCTGTATTGCGTTGGAAACTGTATAACTAGAGTACTGGAGAGGTAAGCGGAACTACAAGTGTAGAGGTGAAATTCGTAGATATTTGTAGGAATGCCGATGGGGAAGCCAGCTTACTGGACAGATACTGACGCTGAAGCGCGAAAGCGTGGGTAGCAAACAGG

>d23879268372baf125c87b2d92bedd8f

CATTGAACTATCGTGAGAAAGTCAAACCGCCAAAGGGAATTATATTATAGTAAATATTGGCGTAAATAAACATTTTATTAATAGTTGTAATATATGAAAATGTGCAGAAGATAGAGACGATTAAAACGGTGTTTAACAATCGCTTATCGAGACAATATTTAATTAATTAGTAATTAAAATATGTTTATTACGTGTATTATTCAAGGAATAGACTTGACAATAAATACAGTGGTAAACGGAACTAATAGAGTCACGCAAGTGAACTTAGAACAGTTTTAAGAAC

>0920dcf0f62fb2b3ab9e32f1c4edec37

TACGTAGGGTGCAAGCGTTAATCGGAATTACTGGGCGTAAAGCGTGCGCAGGCGGTTATGCAAGACAGAGGTGAAATCCCCGGGCTCAACCTGGGAACTGCCTTTGTGACTGCATGGCTAGAGTACGGTAGAGGGGGATGGAATTCCGCGTGTAGCAGTGAAATGCGTAGATATGCGGAGGAACACCGATGGCGAAGGCAATCCCCTGGACCTGTACTGACGCTCATGCACGAAAGCGTGGGGAGCAAACAGG

>1bc14b7a0febeb970d0584a038d1ece8

TACGTAGGTGGCAAGCGTTGTCCGGAATTATTGGGCGTAAAGGGCGTGTAGGTGGATTCTTAAGTCGTGTGTCTAAGTGCGGTGCTCAACACCGTATGGGCGCAGGAAACTGGGAATCTTGAGTGCAGGAGAGGAAAGTGGAATTCCCAGTGTAGCGGTGAAATGCGTAGATATTGGGAGGAACACCAGTGGCGAAGGCGACTTTCTGGACTGTGTCTGACACTGAGGCGCGAAAGCCAGGGGAGCGAACGGG

>d82fb3fd0f591f414b5ae023381ed9bb

TACGGAGGGTGCGAGCGTTAATCGGAATTACTGGGCGTAAAGCGCGCGTAGGCGGCTTGATAAGCCGGTTGTGAAAGCCCCGGGCTCAACCTGGGAACGGCATCCGGAACTGTCAGGCTAGAGTGCAGGAGAGGAAGGTAGAATTCCCGGTGTAGCGGTGAAATGCGTAGAGATCGGGAGGAATACCAGTGGAGAAGGCGGCCTTCTGGACTGACACTGACGCTGAGGTGCGAAAGCGTGGGTAGCAAACAGG

>c714ed959d8f9e86eac94b0a972c0ed6

TTCTTACTGCAAACGTATTGTCACACACATGTGTTTTGTAGTAATTATTGTGAGCAGCATCCTCTATGTTATAAATGTAATAAGGAAATCTTAAAACAATGATAATAATCTGTTTATAGTAATTAATCTTCATCATCTTCGCCATTCCAGGATTAGATACCCTAGTAGTCCGG

>f42309a74e8f72143785a7207584b6f7

TACGTAAGGACCGAGCGTTGTCCGGAATCATTGGGCGTAAAGGGTACGTAGGCGGTTAGAAAAGTTAGAAGTGAAAGGCTATAGCTCAACTATAGTAAGCTTTTAAAACTGTTTAACTTGAGAGATGGAAGGGAAAGTGGAATTCCTAGTGTAGCGGTGAAATGCGCAGATATTAGGAGGAATACCGGTGGCGAAGGCGACTTTCTGGCCATTATCTGACGCTGAGGTACGAAAGCGTGGGTAGCAAACAGG

>853db0ade7009a2872dc479f2465aba1

TACGAAGGGGGCTAGCGTTGCTCGGAATCACTGGGCGTAAAGGGCGCGTAGGCGGCTTTTTAAGTCGGGGGTGAAAGCCTGTGGCTCAACCACAGAATTGCCTTCGATACTGGAAAGCTTGAGACCGGAAGAGGACAGCGGAACTGCGAGTGTAGAGGTGAAATTCGTAGATATTCGCAAGAACACCAGTGGCGAAGGCGGCTGTCTGGTCCGGTTCTGACGCTGAGGCGCGAAAGCGTGGGGAGCAAACAGG

>23c6c8ff495ba110d6bbcf5aaabdd41a

GCCGCCGCGGTAAGTGCTCAGAGAACATCAATCTTTCACTTCGTTCTCACTTTCTCTTTTTACTGCCTCCTCTTCTGACCCTTTTCCACCATACCCCTTCTTCTCCTTTTCGTCATCTCTTTCGGTCTATTTGTAGCATAATATATTAGATACCCTTGTAGTCCGGCTGACTGACTGAGACTTAAT

>ce0d877c8ac4bc448fcd008ceb717e9b

CATTGAACTATCGTGAGAAAGTCAAACCGCCAAAGGGAATTATATTATAGTAAATATTGGCGTAAATAAACATTTTATTAATAGTTGTAATATATGAAAATGTGCAGAAGATAAAGACGATTAAAACGGTGTTTAACAATCGCTTATCGAGACAATATTTAATTAATTAGGAATTAAAATATGTGTATTATTTGTATTTAACAAGGAATAAACGTGAAAATAAATACAGTGGTAAACGGAACTAATAGAGTCACGCAAGTGAACGTAGAACATTTTTAATAACATTAGAACTAATGGAAA

>fce627c0abc22e5cff75b9a8b9429da9

CATTGAACTATCGTGAGAAAGTCAAACCGCCAAAGGGAATTATATTATAGTAAATATTGGCGTAAATAAACATTTTATTAATAGTTGTAATATATGAAAATGTGCAGAAGATAGAGACGATTAAAACGGTGTTTAACAATCGCTTATCGAGACAATATTTAATTAATTAGGAATTAAAATATGTGTATTATTTGTATTTAACAAAGAATAAACTTGAAAATAAATACAGTGGTAAACGGAACTAATAGAGTCACGCAAGTGAACTTAGAACAATTTTAAGAAC

>357bfe394c102cb7b0129e31c6d1b1e7

GCCGCCGCGGTAATACAAGTAAATCTTTTTTTAAAAAATTAAAAATAATAAAAAAATTTTTGTATTTTAAAAATTTATAAAGGTGAAATTTATAATTTTAAAAAGATTAATTTGATTTTATAAAAATTTAATTTTTAAACTAGGATTAGAAACCCTTGTAGTCCGGCTGACTGACTCGCGATATAT

>2e0c24f8279ab7459bda6aed21917872

GCCGCCGCGGTAAGTGCTCAGAGAACATCAATCTTTCACTTCGTTCTCACTTTCTCTTTTTACTGCCTCCTCTTCTGACCCTTTTCCACCATACCCCTTCTTCTCCTTTTCGTCATCTCTTTCGGTCTATTTGTAGCATAATATATTAGAAACCCCAGTAGTCCGGCTGACTGACTGAGACTTAAT

>f3484d87806dcf8dcb259c949a33f174

TACGAAGGGTGCAAGCGTTAATCGGAATTACTGGGCGTAAAGCGCGCGTAGGTGGTTCGTTAAGTTAGATGTGAAAGCCCCGGGCTCAACCTGGGAACTGCATCCAAAACTGGCGAGCTAGAGTATGGCAGAGGGTGGTGGAATTTCCTGTGTAGCGGTGAAATGCGTAGATATAGGAAGGAACACCAGTGGCGAAGGCGACCACCTGGGCTAATACTGACACTGAGGTGCGAAAGCGTGGGGAGCAAACAGG

>4f6dd0a5111c6487ad5ca440178570de

CGCCGCGGTAATACAATTTTTTCAATTTAATATGTAAGTTTCAGTTAATTGATTATTTTTAATAATTTATATCAATTTTGGTGAAATAATATTTATTAATATAAATAATTTAAATGTGTCTGAGAAACTATAAATTAAACTAGGATTAGAAACCCGTGTAGTCCGGCTGACTGACTCTACGACC

>de56ba5808191bf7ab9551871830fa1f

TACGTGAGAGACTAGTGTTATTCATCTTAATTGGGTTTAAAGGGTACCTAGACAGTCAATATAACTTCTATAATGCTAATACTTGACCAGAGTTTTAAGTAAGAGGGAAGTACTTAAGGAGTAAGAGATGAAATATCTGTGATACCAAAGGGACTCCGTAAAGGCGAAGGCATCCCTTTATCTAAAAACTAACGTTGAAGGACGAAGGCTTAGATAACAAATAGG

>09f9294499192d4eecf29e85501cc425

TACGGAGGGTGCAAGCGTTATCCGGATTTATTGGGTTTAAAGGGTCCGTAGGTGGGCTGATAAGTCAGCGGTGAAATCCTGCAGCTTAACTGTAGAACTGCCGTTGATACTGTTAGTCTTGAGTGTATTTGAAGTGGCTGGAATAAGTAGTGTAGCGGTGAAATGCATAGATATTACTTAGAACACCAATTGCGAAGGCAGGTCACTAAGATACAACTGACGCTGAGGGACGAAAGCGTGGGGAGCGAACAGG

>b9dcb33216458d58a1966a7646d0dda8

ATTTGTTTTCTTAGTACAGACAGACGTACGTAGTGCAGTTGATATTGGAAGTGCATTACTATGCATTTTTTATCTTTTCATTGGTTCGTTAACTATGAATCGGCGAAACCCGTCGGTCATTTCCTACTATACAATCCATTTTTATGTTACACACAGTATTTGATTTGTGGCGAGTAATTTGTCACTCTTTGTTCGTTGTCCGGCTGT

>b7f5af401f678ffbe9f7299cfa59e3bf

TACGAAGGGGGCTAGCGTTGTTCGGATTTACTGGGCGTAAAGCGCACGTAGGCGGTTTGTTAAGTCAGGGGTGAAATCCCGGAGCTCAACTCCGGAACTGCCTTTGATACTGGCAAGCTAGAGTCCGGAAGAGGTAAGTGGAACTCCTAGTGTAGAGGTGGAATTCGTAGATATTAGGAAGAACACCAGTGGCGAAGGCGGCTTACTGGTCCGGAACTGACGCTGAGGTGCGAAAGCGTGGGGAGCAAACAGG

>184f9a852daf0096b9b6e4053033ad99

CCGCCGCGGTAATACGATTTCTTTAATTTAAATATTTAAGTTTCAGTTAATATAACAATAATATAAAATATCTATAATTTTGGTGAAATATATTTTATCTTTAAAAATTAATTTTATGTCTGAAAAATTTTTGTATAAACTAGGATTAGAAACCCGAGTAGTCCGGCTGACTGACTGAGACTTAA

>7c693d2c9bd099a1489c916ff5eab38e

TACGTAGGGTGCGAGCGTTGTCCGGAATTACTGGGCGTAAAGAGCTCGTAGGCGGTTTGTCGCGTCGTCTGTGAAATCCTACAGCTTAACTGTGGGCGTGCAGGCGATACGGGCAGACTTGAGTACTACAGGGGAGACTGGAATTCCTGGTGTAGCGGTGAAATGCGCAGATATCAGGAGGAACACCGGTGGCGAAGGCGGGTCTCTGGGTAGTAACTGACGCTGAGGAGCGAAAGCATGGGTAGCGAACAGG

>7fb08fa9b7b2eef6a3db57d0de769227

TACGTAGGGGGCTAGCGTTGTCCGGAATCACTGGGCGTAAAGGGTTCGCAGGCGGAAATGCAAGTCAGATGTAAAAGGCAGTAGCTTAACTACTGTAAGCATTTGAAACTGCATATCTTGAGAAGAGTAGAGGTAAGTGGAATTTTTAGTGTAGCGGTGAAATGCGTAGATATTAAAAAGAATACCGGTGGCGAAGGCGACTTACTGGGCTCATTCTGACGCTGAGGAACGAAAGCGTGGGTAGCAAACAGG

>fc92775f178c2e8ccf5fb3442d32a246

CATTGAACTATCGTGAGAATGGCACGCCGCCAAAGGGAATTATATTATAGTAAATATTGGCGTAAATAAACATTTTATTAATAGTTGTAATATATGAAAATGTGCAGAAGATAGAGACGATGAAAACGGTGTTTAACAATCGCTTATCGAGACAATATTTAATTAATTAGGAAATAAAATAAGTGTATTATTTGTATTTAACAAGAAATAAACGTGAAAATAAATACAGTGGTAAACGGAACTAATAGAGTCACGCAAGTGAACGTAGAACATTTTTAATAAC

>126ba5d922b6650c28122fcd93ca85cf

TACGGAGGGTGCAAGCGTTATCCGGATTTATTGGGTTTAAAGGGTCCGTAGGCGGATCTGTAAGTCAGTGGTGAAATCTCGCAGCTTAACTGCGAAACTGCCATTGATACTGCAGGTCTTGAGTGTTGTTGAAGTAGCTGGAATAAGTAGTGTAGCGGTGAAATGCATAGATATTACTTAGAACACCAATTGCGAAGGCAGGTTACTAAGCAACAACTGACGCTGATGGACGAAAGCGTGGGGAGCGAACAGG

>1efe887de1bdd596c9461281fe80d17f

TACGAAGGGGGCTAGCGTTGCTCGGAATTACTGGGCGTAAAGGGCGCGTAGGCGGCGGCCCAAGTCAGGCGTGAAATTCCTGGGCTCAACCTGGGGACTGCGCTTGATACTGGGTTGCTTGAGGATGGAAGAGGCTCGTGGAATTCCCAGTGTAGAGGTGAAATTCGTAGATATTGGGAAGAACACCGGTGGCGAAGGCGGCGAGCTGGTCCATTACTGACGCTGAGGCGCGACAGCGTGGGGAGCAAACAGG

>fee5bfae6c902b64bb55bf94af8c8f72

TACGTAGGGCGCGAGCGTTGTCCGGAATTATTGGGCGTAAAGAGCTTGTAGGCGGCTTGTTGCGCCTGCTGTGAAAACTTGGGGCTTAACCCTGAGCGTGCAGTGGGTACGGGCAGGCTAGAGTGTGGTAGGGGTGATTGGAACTCCAGGTGTAGCGGTGGAATGCGCAGATATCTGGAAGAACACCGATGGCGAAGGCAGGTCACTGGGCCATTACTGACGCTGAGAAGCGAAAGCGTGGGGAGCGAACAGG

>7af8eb0a3bc0cf4a6050f6ae57f05ca8

TACGGAGGGTGCGAGTGTTAATCGGAATTACTGGGCGTAAAGCGCGCGTAGGCGGCTTGATAAGCCGGTTGTGAAAGCCCCGGGCTCAACCTGGGAACGGCATCCGGAACTGTCAGGCTAGAGTGCAGGAGAGGAAGGTAGAATTCCCGGTGTAGCGGTGAAATGCGTAGAGATCGGGAGGAATACCAGTGGCGAAGACGGCCTTCTGGACTGACACTGACGCTGAGGTGCGAAAGCGTGGGTAGCAAACAGG

>e0b19e8fbe6d66f27f37565419254f93

TAATTTATTTATTTCTTTACTTAGGGATTACACGTCCAGCATCTGGAATTATAGACACGAAAACGGCGAGTAGCTGGCATACTTCGTGCACGGATCGTGTTAAGCTTAAGACTGACAAACAAACTATAGAAATGATCATTAAACATTAGAAACCCCAGTAGTCCGGCTGACTGACT

>6e2b13d81452bee95443fab306241bc6

CACACTTATGTCTTTAGAAATACAAACCATTTCTCCAGGATGCATTTCAACCATGTCGGTCATTTTCAACTGGCAGTGCCTTGTAGATATTTGTGAAATATTTTGAATGTGTCTTGTCCATAGACAGTTTGTAAATTAATGATTTTGTTATGTAGTTTGTAACGTAATATATTTAGGCTTTTAAGTAAATTCATTTTATTTTCTTTTTTCCGGTCGTG

>f2dcc03dea2f55efd8f2b13e4820a9bc

TACGGAGGATGCGAGCGTTATCCGGATTTATTGGGTTTAAAGGGTGCGTAGGCGGGCAGATAAGTCAGCGGTAAAATTGAGAGGCTCAACCTCTTACCGCCGTTGAAACTGTCAGTCTTGAGTGGGCGAGAAGTATGCGGAATGCGTGGTGTAGCGGTGAAATGCATAGATATCACGCAGAACTCCGATTGCGAAGGCAGCATACCGGCGCCCGACTGACGCTGAAGCACGAAAGCGTGGGTATCGAACAGG

>ed88e6a4322eb1a05cbbadd5b6f565f6

CTATGGTAATTGTGTGCCAGCCGCCGCGGTAATAGTGATGTTTTGGCTGACTTTATCTGCTGCTTTGTTACAGTCAAGCTTAATAAAACCACCATAAACACACACATTAAGTAAGATGCTAACTCAAGTCACACACAACAATACATTAGAAACCCCAGTAGTCCGGCTGACTGACTTAACGTCCATCTCGTATGCCGTCTTCTGC

>837369d11dd2db2474feb75c4ad8048c

CACGATTAACCCAAGTCAATAGAAGCCGGCGTAAAGAGTGTCTTAGATCACCCCCTCCCCAATAAAGCTAAAACTCACCTGAGTTGTAAAAAACTCCAGTTGACACAAAATAGGCTACGAAAGTGGCTTTAACATATCTGAACACACAATAGCTAAGACCCAAACTGGGATTA

>180001bc11090727de7ab462cf67c27b

GCTCGATGTCGGAGCCGATAACAGGATTATCCGGCTTCACCTGTGTGCCTCTCACTGCTTTTGTATATTATCCCTTCCGTAGTAAACCTTACTGAGATAATATTATACAGCGGCGAGTGGTACAAAGTAGCTGCTTCGGAAGAAAAGAAAAACATTCTTACTGGTAGACTATACTAAAGAAAAATATTCATGAGAAGCGTAGCATTGTGTAGAGCTGAAACATGAAAGTAGGAATGTCAGATTATCTGAAGAATTTCGAATATGATATTGGAGAACAATGCAGAATACATGTTGGACAGACGTG

>895ceb83b02c3c744fc0669719a77c16

GACGAACCGTGCGAACGTTATTCGGAATCACTGGGCTTAAAGAGCGCGTAGGCGGATTGCTACGTCGGTGTCTGAAATCCCCCGGCTCAACCGGGGAAGTGGATCCGATACGGGCAGTCTTGAGGGACGTAGGGGGGGCTGGAACTTCCGGTGGAGCGGTGAAATGCGTTGAGATCGGAAGGAACGCCCGTGGCGAAAGCGAGCCCCTGGACGTCTACTGACGCTGAGGCGCGAAAGCTAGGGGAGCGAACGGG

>2721fd42d5373667f111c60faaf21eac

TACGTAGGGCGCGAGCGTTGTCCGGAATTATTGGGCGTAAAGAGCTTGTAGGCGGTTTGTCGCGTCTGCTGTGAAAGGCCGGGGCTTAACTCCGTGTATTGCAGTGGGTACGGGCAGACTAGAGTGCAGTAGGGGAGACTGGAATTCCTGGTGTAGCGGTGGAATGCGCAGATATCAGGAGGAACACCGATGGCGAAGGCAGGTCTCTGGGCTGTAACTGACGCTGAGAAGCGAAAGCATGGGGAGCGAACAGG

>43df41213733cc1695586369fcb3caa9

CATTGAACTATCGTGAGAAAGTCAAACCGCCAAAGGGAATTATATAATAGTAAATATTAGCGTAAATAAACATTTTATTAATAGTTGTAATATATGAAAATGTGCAGAAGATAAAGACGATTAAAACGGTGTTTAACAATCGCTTATCGAGACAATATTTAATTAATTAGGAATTAAAATATGTGTATTATTTGTATTTAACAAGGAATAAACTTGAAAATAAATACAGTGTTAAACGGAACTAATAGAGTCACGCAAGTGAACTTAGAACATTTTAATAAC

>8a9535e2c1533c81065d0b6bca4b4646

CATTGAACTATCGTGAGAAAGTCAAACCGCCAAAGGGAATTATATAATAGTAAATATTAGCGTAAATAAACATTTTATTAATAGTTGTAATATATGAAAATGTGCAGAAGATAAAGACGATTAAAACGGTGTTTAACAATCGCTTATCGAGACACTATTTAATTAATTAGGAATTAAAATATGTGTATTATTTGTATTTAACAAGGAATAAACTTGAAAATAAATACAGTGGTGAACGGAACTAATAGAGTCACGCAAGTGAACTTAGAACAATTTTAAGAAC

>f3c795f0cd315cbfe63c8a0d71b00c51

CCGCCGCGGTAATACGATTTCTTTAATTTAAATATTTAAGTTTCAGTTAATATAACAATAATATAAAATATCTATAATTTTGGTGAAATATATTTTATCTTTAAAAATTAATTTTATGTCTGAAAAATTTTTGTATAAACTAGGATTAGAAACCCTAGTAGTCCGGCTGACTGACTGAGACTTAA

>f0140aec257756213c446d6d7c286873

TACGTAGGTGGCGAGCGTTGTCCGGAATCATTGGGCGTAAAGGGAGCGCAGGCGGGCATGTAAGTCTTTCTTAAAAGTGCGGGGCTCAACCCCGTGATGGGAAAGAAACTATGTGTCTTGAGTACAGGAGAGGAAAGCGGAATTCCCAGTGTAGCGGTGAAATGCGTAGATATTGGGAGGAACACCAGTGGCGAAGGCGGCTTTCTGGACTGCAACTGACGCTGAGGCTCGAAAGCCAGGGGAGCGAACGGG

>0717fcc2431a114b3c74c470a09c52de

TACGAAGGGGGCTAGCGTTGCTCGGAATCACTGGGCGTAAAGGGCGCGTAGGCGGCCATTCAAGTCGGGGGTGAAAGCCTGTGGCTCAACCACAGAATTGCCTTCGATACTGTTTGGCTTGAGTCTGGTAGAGGTTGGTGGAACTGCGAGTGTAGAGGTGAAATTCGTAGATATTCGCAAGAACACCGGTGGCGAAGGCGGCCAACTGGACCAGTACTGACGCTGAGGCGCGAAAGCGTGGGGGGCAAACAGG

>37e035a88185ca6ab6aae45f3a5ab278

CCGCCGCGGTAATACGATTTCTTTAATTTAAATATTTAAGTTTCAGTTAATATAACAATAATATAAAATATCTAAAATTTTGGTGAAATATATTTTATCTTTAAAAATTAATTTTATGTCTGAAAAATTTTTGTATAAACTAGGATTAGATACCCTTGTAGTCCGGCTGACTGACTCTACGACCA

>b1937d4985b612a6e9c822da87bdf76e

TACGTAGGGTGCAAGCGTTGTCCGGAATTATTGGGCGTAAAGAGCTCGTAGGCGGTTTGTCGCGTCTGCTGTGAAATCCCGAGGCTCAACCTCGGGTCTGCAGTGGGTACGGGCAGACTAGAGTGCGGTAGGGGAGATTGGAATTCCTGGTGTAGCGGTGGAATGCGCAGATATCAGGAGGAACACCGATGGCGAAGGCAGATCTCTGGGCCGTAACTGACGCTGAGGAGCGAAAGCATGGGGAGCGAACAGG

>b88830496eb7d9ce1ffb3628ce9b3bed

TACGGAGGGGGCTAGCGTTGTTCGGAATTACTGGGCGTAAAGCGCACGTAGGCGGACTAGAAAGTTGGGGGTGAAATCCCGGGGCTCAACCTCGGAACTGCCTTCAAAACTATCAGTCTGGAGTTCGAGAGAGGTGAGTGGAATTCCGAGTGTAGAGGTGAAATTCGTAGATATTCGGAGGAACACCAGTGGCGAAGGCGGCTCACTGGCTCGATACTGACGCTGAGGTGCGAAAGCGTGGGGAGCAAACAGG

>9355696f0bdfd9750e5391941ed08b68

TACGAAGGGGGCTAGCGTTGTTCGGAATCACTGGGCGTAAAGCGCACGTAGGCGGATCTTTAAGTCAGAGGTGAAATCCCAAGGCTCAACCTTGGAACTGCCTTTGATACTGGGGATCTTGAGTCCGGGAGAGGTGAGTGGAACTGCGAGTGTAGAGGTGAAATTCGTAGATATTCGCAAGAACACCAGTGGCGAAGGCGGCTCACTGGCCCGGTACTGACGCTGAGGTGCGAAAGCGTGGGGAGCAAACAGG

>e821202cde86e63a7561b773eb42cd7b

TTCTTACTGCAAACGTATTGTCACACACATGTTTTGTAGTAATTATTGTGAGCAGCATCCTCTATGTTATAAATGTAATAAGGAAATCTTAAAACAATGATAATAATCTGTTTATAGTAATTAATCTTCATCATCTTCGCCATTCCAGGATTAGAAACCCTGGTAGTCCGGCT

>e8a7b99d544d4334ced3d3d4e6900de0

TACGTAGGTGGCAAGCGTTGTCCGGAATTATTGGGCGTAAAGGGCGTGTAGGTGGATTCTTAAGTCGTGTGTCTAAGTGCGGTGCTCAACACCGTATGGGCGCAGGAAACTGGGAATCTTGAGTGCAGGAGAGGAAAGTAGAATTCCCAGTGTAGCGGTGAAATGCGTAGATATTGGGAGAAACACCAGTGGCGAAGGCGACTTTCTGGACTGTGTCTGACACTGAGGCGCGAAAGCCAGGGGAGCGAACGGG

>7b05e54017a28ac048ba285f4546ec73

CACGCGTGATATGGTAATTGTGTGCCAGCCGCCGCGGTAATACTACACAAGGCACCGAGGACGAAGACGAGGAGGATATGAAAGTTGAATAAAGGAAACGAAGACACGGCAGGACGACGTTGAGTGTATCACGTGACAGAAGTTATTAGAAACCCCTGTAGTCCGGCTGACTGACTTAACGTCCATCTCGTATGCCGTCTTCTGCTTGAAAAA

>06c6bccd9698ea80cd8568bdbba0efb2

GTATGGTAATTGTGTGCCAGCCGCCGCGGTAATAGTGATGTTTTGGCTGACTTTATCTGCTGCTTTGTTACAGTCAAGCTTAATAAAACCACCATAAACACACACATTAAGTAAGATGCTAACTCAAGTCACACACAACAATACATTAGAAACCCCAGTAGTCCGGCTGACTGACTCTACGACCATCTCGTATGCCGTCTTCTGC

>59c511c68563d2270dd4325b6d66d11a

TACGGAAGGTCCGGGCGTTATCCGGATTTATTGGGTTTAAAGGGAGCGTAGGCCGTGGATTAAGCGTGTTGTGAAATGCAGGTGCTCAACGTCTGCACTGCAGCGCGAACTGGTTCACTTGAGTGTGCGCAACGCAGGCGGAATTCGTCGTGTAGCGGTGAAATGCTTAGATATGACGAAGAACTCCGATTGCGAAGGCAGCTTGCGGGAGCACAACTGACGCTGAAGCTCGAAAGTGCGGGTATCGAACAGG

>534ae1292f8ab63bdd6f97344ef4656d

TACGAAGGGGGCTAGCGTTGCTCGGAATCACTGGGCGTAAAGGGTGCGTAGGCGGGTTTTTAAGTCAGAGGTGAAATCCTGGAGCTCAACTCCAGAACTGCCTTTGATACTGAAAGTCTTGAGTATGGGAGAGGTGAGTGGAACTGCGAGTGTAGAGGTGAAATTCGTAGATATTCGCAAGAACACCAGTGGCGAAGGCGGCTCACTGGCCCATTACTGACGCTGAGGCACGAAAGCGTGGGGAGCAAACAGG

>e3ba80d2874f0a557f1523d37bf8130b

CATTGAACTATCGTGAGAACAGCACGCCGCCAAAGGGAATTATATTATAGTAAATATTGGCGTAAATAAACATTTTATTAATAGTTGTAATATATGAAAATGTGCAGAAGATAAAGACGATGAAAACGGTGTTTAACAATCGCTTATCGAGACAATATTTAATTAATTAGGAATTAAAATATGTGTATTATTTGTATTTAACAAGGAATAATCTTGAAAATAAATACAGTGGTAAACGGAACTAATAGAGTCACGCAAGTGAACTTAGAACAATTTTAAGAAC

>214f20d6092a780cbacafcbc25f67176

TACGGAGGGGGCTAGCGTTGTTCGGAATTACTGGGCGTAAAGCGCACGTAGGTGGACTGGAAAGTTGGGGGTGAAATCCCGGGGCTCAACCTCGGAACTGCCTTCAAAACTATCAGTCTGGAGTTCGAGAGAGGTGAGTGGAATTCCGAGTGTAGAGGTGAAATTCGTAGATATTCGGAGGAACACCAGTGGCGAAGGCGGCTCACTGGCTCGATACTGACGCTGAGGTGCGAAAGCGTGGGGAGCAAACAGG

>61449a347bfde2e502c47d60c439cef9

GCCGCGGTAATATGGTAAAACAAATCTCTTTGGAAAGGACCTCACACGCTAGTGGAAAGCGCCTAGACTGCACGCCAGCCGGCTGGCGCATATAATAATATGTAATCTCATTATAAAAATTAAAAAATATATTGCAAAAATTATATTAGAAACCCGAGTAGTCCGGCTGACTGACTTAACGTC

>55d1ca6c99f7db903653974224cdabb0

CATTGAACTATCGTGAGAACGGCACGCCGCCAAAGGGAATTATATTATAGTAAATATTGGCGTAAATAAACATTTTATTAATAGTTGTAATATATGAAAATGTGCAGAAGATAAAGACGATGAAAACGGTGTTTAACAATCGCTTATCGAGACAATATTTAATTAATTAGGAATTAAAATATGTGTATTATTTGTATTTAACAAGGAATAATCTTGAAAATAAATACAGTGGTAAACGGAACTAATAGAGTCACGCAAGTAAACTTAGAACAATTTTAAGAAC

>be58bdf5b7e55e13a17fc6daaf228a92

CACACTTATGTCTTCAGAAAGACAAAATATTTCCCCATGATGCATTTCAACCATGTCGGTTATTTTCAACTGGCAGTGCCTTGTAGATATTTGTGAAAGATTTTGAATGCGTCTTGTCCATAGAGTTGAGTTTGTTCATTAATGATTTTGTTATGTAGTTTGTAACGTAATACATTTAGGCTTTCAAGTAAATTCATTTTGTTTAATTTTTTGCCGGTTGTG

>0fc6b2e82df1810fe8922b3ca6ddc081

CAAATTTATGTCTTCAGAAAGACAAAATATTTTTCCAGGGTCCATTTCAACCATGTCGGTCATTTTCAACTAGCAGTGCCTTGTAGATATTTGTGAAAGATTTTGAATGTGTCTTGTCCATAGAGTTCAGTTTGTTCATTAATGATTTTTTTATGTAGTTTGTAACATAATATATTTAGGCTTTCAAGTAAATTGATTTTGTTTCCTTTTTCCGGTTGTG

>83d43568dd12fc5450e4f6596e918cb9

CATTGAACTATCGTGAGAATGGCACGCCGCCAAAGGGAATTATATTATAGTAAATATTGGCGTAAATAAACATTTTATTAATAGTTGTAATATATGAAAATGTGCAAAAGATAGAGACGATTAAAACGGTGTTTAACAATCGCTTATCGAGACAATATTTAATTAATTAGGAATTAAAATATGTGTATTATTTGTATTTAACAAGGAATAAACGTGAAAATAATTACAGTGGTAAACGGAACTAATAGAGTCACGCAAGTGAACTTAGAACAATTTTAAGAAC

>9e3e61ab8f8501e49dfd6f9a0af91d14

TACGTAAGGACCGAGCGTTGTCCGGAATCATTGGGCGTAAAGGGTACGTAGGCGGCTAGAAAAGTTAGAAGTCAAAGGCTATAGCTCAACTATAGTAAGCTTCTAAAACTATTTAGCTTGAGAGATGGAAGGGAAAGTGGAATTCCTAGTGTAGCGGTGGAATGCGCAGATATTAGGAGGAATACCGGTGGCGAAGGCGACTTTCTGGCCATTTTCTGACGCTGAGGTACGAAAGCGTGGGTAGCAAACAGG

>d32e579b3ae7b2aae8d5bf9f027c29af

TACGTAGGGTGCGAGCGTTAATCGGAATTACTGGGCGTAAAGCGTGCGCAGGCGGTTATGTAAGACAGATGTGAAATCCCCGGGCTCAACCTGGGAACTGCATTTGTGACTGCATGGCTAGAGTACGGTAGAGGGGGATGGAATTCCGCGTGTAGCAGTGAAATGCGTAGATATGCGGAGGAACACCGATGGCGAAGGCAATCCCCTGGACCTGTACTGACGCTCATGCACGAAAGCGTGGGGAGCAAACAGG

>cc02d8e02665dcb672be926e61f5d0ec

TACGTAGGGTGCGAGCGTTGTCCGGAATTACTGGGCGTAAAGAGCTCGTAGGTGGTTTGTCGCGTCGTTTGTGGAATACCGCAGCTTAACTGCGGGGTTGCAGGCGATACGGGCATAACTTGAGTGCTGTAGGGGAGACTGGAATTCCTGGTGTAGCGGTGGAATGCGCAGATATCAGGAGGAACACCGATGGCGAAGGCAGGTCTCTGGGCAGTAACTGACGCTGAGGAGCGAAAGCATGGGTAGCGAACAGG

>34244f46ca2dbc7e4f45efefd6ee8dce

TACGGAGGATCCGAGCGTTATCCGGATTTATTGGGTTTAAAGGGTGCGTAGGCGGCACTTTAAGTCAGGGGTGAAAGACGGCAGCTCAACTGTCGCAGTGCCCTTGATACTGAAGTGCTTGAATGCGGTTGAAGACGGCGGAATGAGACAAGTAGCGGTGAAATGCATAGATATGTCTCAGAACACCGATTGCGAAGGCAGCTGTCTAAGCCGTTATTGACGCTGATGCACGAAAGCGTGGGGATCGAACAGG

>4b7ab4c4043601d889df52b02f9b2fa0

CTATGGTAATTGTGTGCCAGCCGCCGCGGTAATAGTGATGTTTTGGCTGACTTTATCTGCTGCTTTGTTACAGTCAAGCTTAATAAAACCACCATAAACACACACATTAAGTAAGATGCTAACTCAAGTCACACACAACAATACATTAGATACCCTTGTAGTCCGGCTGACTGACTTAACGTCCATCTCGTATGCCGTCTTCTGC

>907aa67614fb431673dcd7b913a839c5

TACAGAGGGTGCAAGCGTTAATCAGAATTACTGGGCGTAAAGCGCGCGTAGGCGGCTAGGTAAGATGGGTGTGAAATCCCCGGACTCAACCTGGGAACTGCATCCATAACTGCCTGGCTAGAGTACAGTAGAGGGTGGTGGAATTTCCTGTGTAGCGGTGAAATGCGTAGATATAGAAAGGAACACCAGTGGCGAAGGCGACCACCTGGACTGATACTGACGCTGAGGTGCGAAAGCGTGGGGAGCAAACAGG

>8f7c737007cfbed8b5ea17503e45422c

TACGTAGGGTGCGAGCGTTGTCCGGAATTACTGGGCGTAAAGAGCTCGTAGGCGGTTTGTCGCGTCGTCTGTGAAATTCTGCAACTCAATTGCAGGCGTGCAGGCGATACGGGCAGACTTGAGTACTACAGGGGAGACTGGAATTCCTGGTGTAGCGGTGAAATGCGCAGATATCAGGAGGAACACCGGTGGCGAAGGCGGGTCTCTGGGTAGTAACTGACGCTGAGGAGCGAAAGCGTGGGTAGCGAACAGG

>04c326eb626c415548956b31ad96939f

TACGTAGGGTGCGAGCGTTGTCCGGAATTATTGGGCGTAAAGAGCTTGTAGGCGGTTTGTCGCGTCTGCTGTGAAAACTCGGGGCTTAACCCCGAGCCTGCAGTGGGTACGGGCAGACTAGAGTGTGGTAGGGGAGACTGGAATTCCTGGTGTAGCGGTGGAATGCGCAGATATCAGGAGGAACACCGATGGCGAAGGCAGGTCTCTGGGCCATAACTGACGCTGAGAAGCGAAAGCGTGGGGAGCGAACAGG

>e389818573ef2bcd5950f80a5cf60535

TGCCAGCCGCCGCGGTAAGGAAAATATATAAACAGAAATACTAAAATGATCATGTTTAAGAGTAAGTTAATAAGTATTAATAAGTTAACCTGTATTAACTTACAGAAGTGAGGCATTAGTTATGACGCCCAGTAATGAACACCTATTAGATACCCGAGTAGTCCGGCTGACTGACTTGCGTCAAATCTCGT

>df8456a1abbfb4c8a2c450b44378d4cb

TACGTAGGGCGCGAGCGTTGTCCGGAATTATTGGGCGTAAAGGGCTTGTAGGCGGTTGGTCGCGTCTGCCGTGAAATCCTCTGGCTTAACTGGGGGCGTGCGGTGGGTACGGGCTGACTTGAGTGCGGTAGGGGAGACTGGAACTCCTGGTGTAGCGGTGGAATGCGCAGATATCAGGAAGAACACCGGTGGCGAAGGCGGGTCTCTGGGCCGTTACTGACGCTGAGGAGCGAAAGCGTGGGGAGCGAACAGG

>dcf04ca109f9b5f324e7495410313405

CTATGGTAATTGTGTGCCAGCCGCCGCGGTAATAGTGATGTTTTGGCTGACTTTATCTGCTGCTTTGTTACAGTCAAGCTTAATAAAACCACCATAAACACACACATTAAGTAAGATGCTAACTCAAGTCACACACAACAATACATTAGAAACCCTGGTAGTCCGGCTGACTGACTTAACGTCCATCTCGTATGCCGTCTTCTGC

>b03d2e5ac94c7a0d115e5c542e9315e8

TACGTAGGTGACAAGCGTTGTCCGGATTTATTGGGCGTAAAGGGAGCGCAGGCGGTCTGTTTAGTCTAATGTGAAAGCCCACGGCTTAACCGTGGAACGGCATTGGAAACTGACAGACTTGAATGTAGAAGAGGAAAATGGAATTCCAAGTGTAGCGGTGGAATGCGTAGATATTTGGAGGAACACCAGTGGCGAAGGCGATTTTCTGGTCTAACATTGACGCTGAGGCTCGAAAGCGTGGGGAGCGAACAGG

>969a0174367db1fdf96dcc65cb846ba5

CACACTTATGTCTCCAGAAAGACAAAATATTTATCCAGGATGCATTTCAACCATGTCGGTCATTTTCAACTGGCAGTGCCTTGTAGATATTTGTGAACGATTTTGAATGTGTCTTGTCCATAGAGTTCAGTTTGTTCATTAATGATTTTGTTATGTAGTTTGTAACGTAATATATTTAGGCTATCAAGTAAATTCATTTTGTTTCCTTTTCGCCGGTTGTG

>e6dae8dc2537455f21eb32a6ee3cea7f

GCCGCCGCGGTAAGTGCTCAGAGAACATCAATCTTTCACTTCGTTCTCACTTTCTCTTTTTACTGCCTCCTCTTCTGACCCTTTTCCACCATACCCCTTCTTCTCCTTTTCGTCATCTCTTTCGGTCTATTTGTAGCATAATATATTAGAAACCCGGGTAGTCCGGCTGACTGACTGAGACTTAAT

>dc1af5b08fecce4be1a45f50ed11afc2

TACGGAGGGTGCAAGCGTTACCCGGAATCACTGGGCGTAAAGGGCGTGTAGGCGGATATTTAAGTCTGGTTTTAAAGACCGAGGCTCAACCTCGGGAGTGGACTGGATACTGGATGTCTTGACCTCTGGAGAGGTAACTGGAATTCCTGGTGTAGCGGTGGAATGCGTAGATACCAGGAGGAACACCAATGGCGAAGGCAAGTTACTGGACAGAAGGTGACGCTGAGGCGCGAAAGTGTGGGGAGCAAACCGG

>e44d163e9ce8b2021b49f3633afc74ff

TACGGAGGGGGCTAGCGTTGTTCGGAATTACTGGGCGTAAAGCGCACGTAGGCGGATTGGAAAGTTGGGGGTGAAATCCCGGGGCTCAACCCCGGAACTGCCTTCAAAACTCCCAGTCTTGAGGTCGAGAGAGGTGAGTGGAATTCCGAGTGTAGAGGTGAAATTCGTAGATATTCGGAGGAACACCAGTGGCGAAGGCGGCTCACTGGCTCGATACTGACGCTGAGGTGCGAAAGCGTGGGGAGCAAACAGG

>710265966728bcda02d087db3abd5ed8

ACATCTACACACGCGTGATATGGTAATTGTGTGCCAGCCGCCGCGGTAAGGAACAAAGAAAACTGTGAATGAACAAGTTATGTAGAAATACACGCGGTAGCCGAGTGCGCGCTGGAGAGCTTTACGACCAGAACCCCTGATGGCATTAGAAACCCGAGTAGTCCGGCTGACTGACTTAACGTCCATCTCGTATGCCGTCTTCTGCTTGAAAAAAAAAAAAAA

>e78ecb138a8bee2a728274b849db12c1

TACGGAGGGTGCGAGCGTTAATCGGAATAACTGGGCGTAAAGGGCACGCAGGCGGCTATTTAAGTGAGGTGTGAAATCCCCGGGCTTAACCTGGGAATTGCATTTCAGACTGGGTAGCTAGAGTACTTTAGGGAGGGGTAGAATTCCACGTGTAGCGGTGAAATGCGTAGAGATGTGGAGGAATACCGAAGGCGAAGGCAGCCCCTTGGGAATGTACTGACGCTCATGTGCGAAAGCGTGGGGAGCAAACAGG

>0074eedb126413b872c0a337dcd5b912

TTCTTACTGCAAACGTATTGTCACACACATATGTTTTGTAGTAATTATTGTGAGCAGCATCCTCTATGTTATAAATGTAATAAGGAAATCTTAATGATAATAATCTGTTTATAGTAATTAATCTTCATCATCTTCGCCATTCCAGGATTAGAAACCCGAGTAGTCCGGCTGAC

>062db844caf59b575cbcedf3400614ff

CATTGAACTATCGTGAGAAAGTCAAACCGCCAAAGGGAATTATATTATAGTAAATATTGGCGTAAATAAACATTTTATTAATAGTTGTAATATATGATAATGTGCAAAAGATAGAGACGATTAAAACGGTGTTTAATAATCGCTTATCGAGACACTATTAAAGTAATTAGGAATTAAAATATGTTTATTATGTGTATTATTCAAGGAATAAACTTGAAAATAAATACAGTGTTAAACGGAACTAATAGAGTCACGCAAGTGAACTTAGAACATTTTAATAAC

>d68062e7adff0d894287f76670e7f51e

TACGTAGGGTGCGAGCGTTGTCCGGAATTATTGGGCGTAAAGAGCTTGTAGGCGGTTTGTCGCGTCTGCTGTGAAAATTCGGGGCTCAACCCCGGACTTGCAGTGGGTACGGGCAGACTAGAGTGTGGTAGGGGAGACTGGAATTCCTGGTGTAGCGGTGAAATGCGCAGATATCAGGAGGAACACCGATGGCGAAGGCAGGTCTCTGGGCCACTACTGACGCTGAGAAGCGAAAGCATGGGGAGCGAACAGG

>98a6d1bc72d98035a11d9700f7277f65

TACGTAGGTGGCAAGCGTTATCCGGAATTATTGGGCGTAAAGCGCACGTAGGCGGTTTTTTAAGTCTGATGTGAAAGCCCACGGCTCAACCGTGGAGGGTCATTGGAAACTGGAAAACTTGAGTGCAGAAGAGGAAAGTGGAATTCCATGTGTAGCGGTGAAATGCGCAGAGATATGGAGGAACACCAGTGGCGAAGGCGACTTTCTGGTCTGTAACTGACGCTGATGTGCGAAAGCGTGGGGATCAAACAGG

>a1ed615a4ed0b08c534fcc504a4608b2

TACGTAGGGCGCAAGCGTTGTCCGGAATTATTGGGCGTAAAGAGCTCGTAGGCGGTTTGTCGCGTCTGGTGTGAAAACTCGAGGCTCAACCTCGAGCTTGCATCGGGTACGGGCAGACTAGAGTGCGGTAGGGGAGACTGGAATTCCTGGTGTAGCGGTGGAATGCGCAGATATCAGGAGGAACACCGATGGCGAAGGCAGGTCTCTGGGCCGCAACTGACGCTGAGGAGCGAAAGCATGGGGAGCGAACAGG

>fd94bf5a8e2a81f72fcd840bbb9f6f64

TACGTAGGGTGCGAGCGTTGTCCGGAATTATTGGGCGTAAAGAGCTTGTAGGCGGTTTGTCGCGTCTGCTGTGAAAGACCGGGGCTTAACTCCGGTTCTGCAGTGGGTACGGGCAGACTAGAGTATGGTAGGGGAGACTGGAATTCCTGGTGTAGCGGTGAAATGCGCAGATATCAGGAGGAACACCGATGGCGAAGGCAGGTCTCTGGGCCATTACTGACGCTGAGAAGCGAAAGCATGGGGAGCGAACAGG

>604e51688f77dbab757f5d9253d64b29

TAATTTATTTATTTCTTTACTTAGGGATTACACGTCCAGCATCTGGAATTATAGACACGAAAACGGCGAGTAGCTGGCATACTTCGTGCACGGATCGTGTTAAGCTTAAGACTGACAAACAAACTATAGAAATGATCATTAAACATTAGATACCCCAGTAGTCCGGCTGACTGACT

>e5b87e1925bea86ca8b66c9fd52114ed

TACAGAGGCCCCAAGCGTTGTTCGGATTTACTGGGCGTAAAGGGTGTGTAGGGGGTCGTGTAAGTTTGACGTGAAATCCCGTTGCTCAACAACGGAACTGCGTCGAATACTGCTCGGCTGGAGGTTCGGAGATGAGGGCGGAATTCTCGGTGTAGCGGTGAAATGCGTAGATATCGAGAGGAACGCCGATGGCGAAAGCAGCCCTCAAGACGAAATCTGACCCTGAAACACGAAGGCCAGGGGAGCAAACGGG

>8e9dd1ce14e4bcf856499ea42516118e

CATGGATGATACAGGAGCTCATTGGGCTGAGACCCAGACAGCGCACACACGATTCGGGTGCTAGACACCCCGCACAGCATCAGTCAGTCAGTAATATTGGTTATCGTAGGGCTATTGATCTACTAAATAACTGCGACAAAACTATTTACCCATCTATTCACTTTCTACTAAAGATTTCTGTCACCTTGTCATATTCTGTTCCAACTGCTGATAGAACATTATCATTGCTCAGAACACTTACAACTTAG

>e9fc9bcab719b759892b46f7f729bfef

TACGGAGGGGGCTAGCGTTGTTCGGAATTACTGGGCGTAAAGCGCACGTAGGCGGACTGGAAAGTTGGGGGTGAAATCCCGGGGCTCAACCTCGGAACTGCCTTCAAAACTATCAGTCTGGAGTTCGAGAGAGGTGAGTGGAATTCCGAGTGTAGAGGTGAAATTCGTAGATATTCGGAGGAACACCAGTGGCGAAGGCGACTCACTGGCTCGATACTGACGCTGAGGTGCGAAAGCGTGGGGAGCAAACAGG

>2e33dac18ad52f6982b9601afed83858

GTATGGTAATTGTGTGCCAGCCGCCGCGGTAATAGTGATGTTTTGGCTGACTTTATCTGCTGCTTTGTTACAGTCAAGCTTAATAAAACCACCATAAACACACACATTAAGTAAGATGCTAACTCAAGTCACACACAACAATACATTAGAAACCCCAGTAGTCCGGCTGACTGACTTAACGTCCATCTCGTATGCCGTCTTCTGC

>4626652d75470cf25ec54760c98f4403

TACAGAGGGTGCAAGCGTTAATCGGAATTACTGGGCGTAAAGCGCGTGTAGGTGGTTCGTTAAGTTGGATGTGAAAGCCCCGGGCTCAACCTGGGAACTGCATCCAAAACTGGCGAGCTAGAGTATGGTAGAGGGTGGTGGAATTTCCTGTGTAGCGGTGAAATGCGTAGATATAGGAAGGAACACCAGTGGCGAAGGCGACCACCTGGACTGATACTGACACTGAGGTGCGAAAGCGTGGGGAGCAAACAGG

>48b62684cb6f0fcbed918e49c59ec58e

CATTGAACTATCGTGAGAAAGTCACGCCGCCAAAGGGAATTATATAATAGTAAATATTAGCGTAAATAAACATTTTATTAATAGTTGTAATATATGATAATGTGCAAAAGATAGAGACGATTAAAACGGTGTTTAACAATCGCTTATCGAGACACTATTAAAGTAATTAGGAATTAAAATATGTTTATTATGTGTATTATTCAAGGAATAAACTTGAAAATAAATACAGTGTTAAACGGAACTAATAGAGTCACGCAAGTGAACTTAAAACATTTTAATAAC

>6a4c0e5943a7eb8c9f0b5b5e69171828

TACGTAGGGTGCGAGCGTTGTCCGGAATTACTGGGCGTAAAGAGCTCGTAGGTGGTTTGTCGCGTCGTCTGTGAAATTCCGGGGCTTAACTTCGGGCGTGCAGGCGATACGGGCATAACTTGAGTGCTGTAGGGGAGACTGGAATTCCTGGTGTAGCGGTGAAATGCGCAGATATCAGGAGGAACACCGATGGCGAAGGCAGGTCTCTGGGCAGTTACTGACGCTGAGGAGCGAAAGCATGGGTAGCAAACAGG

>4a0b292ba716582f9af46694458c0b9b

TACAGAGGATGCAAGCGTTATCCGGAATGATTGGGCGTAAAGCGTCTGTAGGTGGCTTTTTAAGTCCGCCGTCAAATCCCAGGGCTCAACCCTGGACAGGCGGTGGAAACTACCAAGCTGGAGTACGGTAGGGGCAGAGGGAATTTCCGGTGGAGCGGTGAAATGCGTAGAGATCGGAAAGAACACCAACGGCGAAAGCACTCTGCTGGGCCGACACTGACACTGAGAGACGAAAGCTAGGGGAGCGAATGGG

>a71e39410bdca160bf261526199c659f

CCGCCGCGGTAATACGATTTCTTTAATTTAAATAGTTAAGTTTCAGTTAATATAACAATAATATAAAATATCTATAATTTTGGTGAAATATATTTTATCTTGAAAAATTAATTTTATGTCTGAAAAATTTTTATTTAAACTAGGATTAGAAACCCCAGTAGTCCGGCTGACTGACTCTATCGTGA

>62b7733e19e1a6768ca9ec2827363f82

GAGATCTACACTGCGTCACTATGGTAATTGTGTGCCAGCCGCCGCGGTAATACTGACGTAAACACTTTGAAACCCTAACGCGTATACTAGTTGTCTCAACACATGTGAATGAACTTCTCGCTGTCTAATATAACTCTTGCGAAGATTAGAAACCCTTGTAGTCCGGCTGACTGACTGAGACTTAATCTCGTATGCCGTCTTCTGCTTGAAAAAAAAAAAAATA

>146186e7792beb9c5ef3b3f875662620

CACACTTATGTCTTTAGAATGACAAAACATTTTTCCATAATGCATTTCAACCATGTCGGTCATTTTCAAATGGCAGTGCCTTGTAGATAGTTGGGGTAGATTTTGATTGTGTCTTGTCCATAGAGTTCAGTTTGTTTATTAATGATTTTGTTATGTAGTTTGTAGCGTAGTGTATTTAGATTTTCGAGTAAATTCATTCTTTTATGATAAAATTCAATGTATTTCCTTTTTTGCCGGTTGTG

>f56db2fce56a335343f6fc679bcc9151

TACGGAAGATGCGAGCGTTATCCGGATTTATTGGGTTTAAAGGGAGCGCAGACGGGAGGTCAAGTCAGTTGTGAAAGCTCACGGCTCAACCGTGGAACTGCAGTTGAAACTGGCCTTCTTGAGTGCGGCACAGGCAGGCGGAATTCGTGGTGTAGCGGTGAAATGCTTAGATATCACGAAGAACTCCGATCGCGAAGGCAGCTTGCCGGACCGTAACTGACGTTCATGCTCGAAAGTGCGGGTATCAAACAGG

>e31678836ca29c0ebde1bd1003bcdca6

TACGTAGGGCGCGAGCGTTGTCCGGAATTATTGGGCGTAAAGAGCTCGTAGGCGGCTGGTCGCGTCTGTCGTGAAATCCTCTGGCTTAACTGGGGGCGTGCGGTGGGTACGGGCCGGCTTGAGTGCGGTAGGGGAGGCTGGAATTCCTGGTGTAGCGGTGGAATGCGCAGATATCAGGAGGAACACCGGTGGCGAAGGCGGGTCTCTGGGCCGTGTACTGACGCTGAGGAGCGAAAGCGTGGGGAGCGAACAGG

>610a4d77ea9acfd0aeed31ef1eabb9f4

TACGGAGGGTCCGAGCGTTAATCGGAATTACTGGGCGTAAAGCGTGCGCAGGCGGTTTGTTAAGCGAGATGTGAAAGCCCTGGGCTCAACCTAGGAATAGTATTTCGAACTGGCGAACTAGAGTCTTGTAGAGGGGGGTAGAATTCCAGGTGTAGCGGTGAAATGCGTAGAGATCTGGAGGAATACCGGTGGCGAAGGCGGCCCCCTGGACAAAGACTGACGCTCATGCACGAAAGCGTGGGGAGCAAACAGG

>96924d233782df39847a45f5d73bf98f

TACGTAGGGTGCAAGCGTTAATCGGAATTACTGGGCGTAAAGCGTGCGCAGGCGGTTATGCAAGACAGAGGGGAAATCCCCGGGCTCAACCTGGGAACTGCCTTTGTGACTGCATGGCTAGAGTACGGTAGAGGGGGATGGAATTCCGCGTGTAGCAGTGAAATGCGTAGATATGCGGAGGAACACCGATGGCGAAGGCAATCCCCTGGACCTGTACTGACGCTCAAGCACGAAAGCGTGGGGAGCAAACAGG

>89a0a58e5f22d2fc6ba3566fbfb26a77

GCCTGTTATGACCCGACACGCTTGCTAAATACAACGGTATCTTCACTCTGTTTTGTAGTAACCCAATACATGTATATATATGTATGTGTGTGTGTGTGTGTGCATGCGCGCGCGCGCACTTATGCACCTAGATTTCCCGACGATGAAGTCTAGTTTTATGGAAGATACAATGGAGATGGAGAAGAAGATTA

>c6f5ceaa7871f6f7612a9fa40623bebc

TAGTATGTATGGTAATTGTGTGCCAGCCGCCGCGGTAATGAGTGTGATATAATTTTACAATACACTATAACATTAGATACCACAAGCCACACACAGAAGTCGGGGGCGCAAACACAATCATATAATTTTACCATACGCTATAACATTAGAAACCCTAGTAGTCCGGCTGACTGACTCTACGACCATCTCGTATGCCGTCTTCTGCTTGAAA

>3a3f626d6c24b02d34ff6c102d61f321

GCCGCCGCGGTAATACAAGTAAATCTTTTTTTAAAAAATTAAAAATAATAAAAAAATTTTTGTATTTTAAAAATTTATAAAGGTGAAATTTATAATTTTAAAAAGATTAATTTGATTTTATAAAAATTTAATTTTTAAACTAGGATTAGATACCCGGGTAGTCCGGCTGACTGACTCGCGATATAT

>e5084584f5af583447aa94ffc29a7c9a

ATATTTTCGATTATCATTAAACCTAATCGTCGGAGTAGAATAACGCGGGATGCACTGTCTCGAGCTCAGTTTACGTACCGTACTTACGTTTTCAACTGGAATTTTATCTCTGTTTCATTGGCCTTATATGTATGTAGTCCGATTAGTCCAATAAGTGCTTAACTGTTAGTAAAATGTAAAGGCAAATAAATAAGCCTAGAGCAAAACAGAAACTGGTGCC

>29fbc7e5021229480248fa7466a499a2

TACGTAGGTGGCGAGCGTTGTCCGGATTTATTGGGCGTAAAGGGAGTGTAGGCGGTCTTTTAAGTCTGATGTGAAAGCCCACGGCTCAACCGTGGAGGGTCATTGGAAACTGGGAGACTTGAGTGCAGAAGAGGAGAGCGGAATTCCATGTGTAGCGGTGAAATGCGTAGATATATGGAGGAACACCAGTGGCGAAGGCGGCTCTCTGGTCTGTAACTGACGCTGAGGCTCGAAAGCGTGGGGAGCAAACAGG

>e2f4cd7b2677e79d936664244c943b7e

CATTGAACTATCGTGAGAAAGTCAAACCGCCAAAGGGAATTATATTATAGTAAATATTGGCGTAAATAAACATTTTATTAATAGTTGTAATATATGAAAATGTGCAGAAAATAAAGACGATTAAAACGGTGTTTAAATATCGCTTATCGAGACAATATTTAATTAATTAGGAATTAAAATATGTGTATTATTTGTATTTAACAAGGAATAAACTTGAAAATAAATACAGTGGTGAACGGAACTAATAGAGTCACGCAAGTGAACTTAGAACAATTTTAAGAAC

>a972133c1851d5aabf8204d48f2d32cb

TACAGAGGGTGCGAGCGTTAATCGGAATTACTGGGCGTAAAGCGAGTGTAGGTGGCTCATTAAGTCACATGTGAAATCGCCGGGCTTAACCTGGGAACTGCATGTGATACTGGTGGTGCTAGAATATGTGAGAGGGAAGTAGAATTCCAGGTGTAGCGGTGAAATGCGTAGAGATCTGGAGGAATACCGATGGCGAAGGCAGCTTCCTGGCATAATATTGACACTGAGATTCGAAAGCGTGGGTAGCAAACAGG

>8dd6a39f4ec303a01bbb7fe2d41f81e9

TTCTTACTGCAAACGTATTGTCACACACATATGTTTTGTAGTAATTATTGTGAGCAGCATCCTCTATGTTATAAATGTAATAAGGAAATCTCAAAACAATGATAATAATCTGTTTATAGTAATTAATCTTCATCATCTTCACCATTCCAGGATTAGATACCCTAGTAGTCCGG

>947361b860f6efd4d153b0c00de2e8f2

TACGGAGGGGGCTAGCGTTGTTCGGAATTACTGGGCGTAAAGCGCACGTAGGCGGACTGGAAAGTTGGGGGTGAAATCCCGGGGCTCAACCTCGGAACTGCCTTCAAAACTATCAGTCTGGAGTTCGAGAGAGGTGAGTGGAATTCCGAGTGTAGAGGTGAAATTCGTAGATATTCGGAGGAACACCAGTGGCGAAGGCGGCTCACTGGCTCGATACTGATGCTGAGGTGCGAAAGCGTGGGGAGCAAACAGG

>810b2f918e619779585d280c08ddb1c9

TACGTAGGTGGCAAGCGTTGTCCGGAATTATTGGGCGTAAAGCGCGCGCAGGCGGTCTTTTAAGTCTGATGTGAAAGCCCCCGGCTCAACCGGGGAGGGTCATTGGAAACTGGGAGACTTGAGTACAGAAGAGGAGAGTGGAATTCCACGTGTAGCGGTGAAATGCGTAGATATGTGGAGGAACACCAGTGGCGAAGGCGACTCTCTGGTCTGTAACTGACGCTGAGGCGCGAAAGCGTGGGAAGCAAACAGG

>1e8863549e1b50b8eec22ceae706d20d

AGATCTACACCGATCTACTATGGTAATTGTGTGCCAGCCGCCGCGGTAAGGAACAAAGAAAACTGTGAATGAACAAGTTATGTAGAAATACACGCGGTAGCCGAGTGCGCGCTGGAGAGCTTTACGACCAGAACCCCTGATGGCATTAGAAACCCCAGTAGTCCGGCTGACTGACTTAACGTCCATCTCGTATGCCGTCTTCTGCTTGAAAAAAAAAAAAAA

>6f6f77efda61edf3a9f145b5edd14c21

TTCTTACTGCAAAATATTGTCACACACATATGTTTTGTAGTAATTATTGTGAGCAGCATCCTCTATGTTATAAATGTCATAAGGAAATCTTAAAACAATGATAATAATCTGTTTATAGTAATTATTCTTCATCATCTTCGCTATTCCTGGATTAGAAACCCGAGTAGTCCGGC

>7d217addfd80cb5393d81dae5f160367

TACGTAGGGTGCGAGCGTTAATCGGAATTACTGGGCGTAAAGCGTGCGCAGGCGGTTGTGCAAGACAGATGTGAAATCCCCGGGCTTAACCTGGGAACTGCATTTGTGACTGCACGGCTAGAGTGCGGCAGAGGGGAGTGGAATTCCGCGTGTAGCAGTGAAATGCGTAGATATGCGGAGGAACACCGATGGCGAAGGCAGCTCCCTGGGCCTGCACTGACGCTCATGCACGAAAGCGTGGGGAGCAAACAGG

>43e0d08ba757a8cc44b870e9cfac1c11

CACCGATCTACTATGGTAATTGTGTGCCAGCCGCCGCGGTAAAGTAAACACAATATCTGGCAGGACTGCCACTCGTTACTTGATTCAGCACTCTCCTTATATAGTTTAGTTTAGATTCGTCTGTTTTACAGATTGACTCACTTGATTAGAAACCCTAGTAGTCCGGCTGACTGACTCTACGACCATCTCGTATGCCGTCTTCTGCTTGAAAAAAA

>3f445b8a9804ab5ca0ac04149a3ec36e

GATCTACTATGGTAATTGTGTGCCAGCCGCCGCGGTAAAAACCCATACGATGCTCAACAAAGTAAAGAATAGTACCGGTACTTGCTGAAGAACTGCAGATGTCCCCAGACTCTGCAGGGTAACGTTAGGCCACCCCCACTAAATATTAGAAACCCCGGTAGTCCGGCTGACTGACTTAACGTCCATCTCGTATGCCGTCTTCTGCTTGAAA

>9bbe8dafed150aa646185fbe3e0247f6

CCGCCGCGGTAATACGATTTCTTTAATTTAAATATTTAAGTTTCAGTTAATAAAACAATAATATAAAATATCTATAATTTTGGTGAAATATATTTTATTTTTAAAAATTAATTTTATGTCTGAAAAATTTTTGTATAAACTAGGATTAGAAACCCCAGTAGTCCGGCTGACTGACTGAGACTTAA

>24e4a3997e50367598b3152939b4fb75

TACGTATGTCACGAGCGTTATCCGGATTTATTGGGCGTAAAGCGCGTCTAGGTGGTTATATAAGTCTGATGTGAAAATGCAGGGCTCAACTCTGTATTGCGTTGGAAACTGTATAACTAGAGTACTGGAGAGGTAAGCGGAACTACAAGTGTAGAGGTGAAATTCGTAGATATTTGTAGGAATGCCGATGGGGAAGCCAGCTTACTGGACAGATACTGACGCTAAAGCGCGAAAGCGTGGGTAGCAAACAGG

>e73600ba189bb11421a313e70155442f

CATTGAACTATCGTGAGAAAGTCAAACCGCCAAAGGGAATTATATAATAGTAAATATTAGCGTAAATAAACATTTTATTAATAGTTGTAATATATGAAAATGTGCAAAAGATAGAGACGATTAAAACGGTGTTTAACAATCGCTTATCGAGACACTATTAAAGTAATTAGGAATTAAAATATGTTTATTATGTGTATTATTCAAGGAATAAACTTGAAAATAAATACAGTGTTAAACGGAACTAATAGAGTCACGCAAGTGAACTTAGAACATTTTAATAAC

>c40531cdcd9e334d7139a2f977786fdc

CATTGAACTATCGTGAGAAAGCCAAACCGCCAAAGGGAATTATATTATAGTAAATATTGGCGTAAATAAACATTTTATTAATAGTTGTAATATATGAAAATGTGCAGAAGATAAAGACGATTAAAACGGTGTTTAACAATCGCTTATCGAGACAATATTTAATTAATTAGGAATTAAAATATGTGTATTATTTGTATTTAACAAGGAATAAACGTGAAAATAAATACAGTGGTAAACGGAACTAATAGAGTCACGCAAGTGAACGTAGAACATTTTTAATAAC

>4dec9fc8d46193294c61f7b3d1af6dd0

TACGTAGGTCCCGAGCGTTGTCCGGATTTATTGGGCGTAAAGCGAGCGCAGGGGGTTAGATAAGTCTGAAGTTAAAGGCTGTGGCTTAACCATAGTACGCTTTGGAAACTGTTTAACTTGAGTGCAAGAGGGGAGAGTGGAATTCCATGTGTAGCGGTGAAATGCGTAGATATATGGAGGAACACCGGTGGCGAAAGCGGCTCTCTGGCTTGTAACTGACGCTGAGGCTCGAAAGCGTGGGGAGCAAACAGG

>c4f7f7d843eca4862537b5052067f2bb

TACGAAGGGGGCTAGCGTTGCTCGGAATCACTGGGCGTAAAGGGCGCGTAGGCGGCCTTGTAAGTTGGGGGTGAAAGCCCGTGGCTCAACCACGGAATTGCCTTCGATACTGCTTGGCTTGAGTGTGGTAGAGGTTGGTGGAACTGCGAGTGTAGAGGTGAAATTCGTAGATATTCGCAAGAACACCGGTGGCGAAGGCGGCCAACTGGACCATCACTGACGCTGAGGCGCGAAAGCGTGGGGAGCAAACAGG

>16fc3e9e8f1ef8d39263273da78476f7

TTCTGACTGCAAACGTATTGTCACACACATATGTTTTGTAGTAATTATTGTGAGCAGCATCCTCTATGTTATAAATGTAATAAGGAAATCTTAAAACAATGATAATAATCTGTTTATAGTAATTAATCTTCATCATCTTCGCCATTCCAGGATTAGAAACCCGGGTAGTCCGG

>85a80e036fe0460409c983f1a325fc00

TCAAACGAAAGCACAAAAAAAGGTAGCCAAGCTAATCCACCCCGATCACCAGCAATAACATCCACACATACGTTTTACCCAAGAATGGTTAACATACATAACAACATAACATTCACTAACGATCAGAAACAACTTTTAAACAAAGGGATAAATCACAATCTACACTACACGCAGAACAATAACAACATCAAGAAACGG

>c0696f3ea461c9d227737482b0c1a8b0

TACGTAGGGCGCAAGCGTTATCCGGAATTATTGGGCGTAAAGAGCTCGTAGGCGGTTTGTCGCGTCTGCTGTGAAAGTCCGGGGCTCAACTCCGGTTCTGCAGTGGGTACGGGCAGGCTAGAGTGATGTAGGGGAGACTGGAATTCCTGGTGTAGCGGTGAAATGCGCAGATATCAGGAGGAACACCGATGGCGAAGGCAGGTCTCTGGGCATTAACTGACGCTGAGGAGCGAAAGCATGGGGAGCGAACAGG

>1063e92b4acc17f22a826d2c79da73b9

TACGTAGGGCGCGAGCGTTGTCCGGAATTATTGGGCGTAAAGGGCTCGTAGGCGGCTTGTCGCGTCTGCTGTGAAAATGCGGGGCTTAACTCCGTACGTGCAGTGGGTACGGGCAGGCTAGAGTGCGGTAGGGGTGACTGGAATTCCTGGTGTAGCGGTGGAATGCGCAGATATCAGGAGGAACACCGATGGCGAAGGCAGGTCACTGGGCTGTTACTGACGCTGAGGAGCGAAAGCGTGGGGAGCGAACAGG

>8e075e5a3b1084bd1f0e4fed245aed10

CATTGAACTATCGTGAGAAAGTCAAACCGCCAAAGGGAATTATATTATAGTAAATATTAGAGTAAATAAACATTTTATTAATAGTTGTAATATATGAAAATGTGCAGAAGATAGAGACGATTAAAACGGTGTTTAACAATCGCTTATCGAGACAATATTTAATTAATTAGGAATTAAAATATGTGTATTATTTGTATTTAACAAGGAATAAACGTGAAAATAAATACAGTGGTAAACGGAACTAATAGAGTCACGCAAGTGAACTTAGAACAATTTTAAGAAC

>04ecfad5772d2e09a84a0f5ef460536c

TACGAAGGGGGCTAGCGTTGCTCGGAATCACTGGGCGTAAAGGGCGCGTAGGCGGCGTTTTAAGTCGGGGGTGAAAGCCTGTGGCTCAACCACAGAATGGCCTTCGATACTGGGACGCTTGAGTATGGTAGAGGTTGGTGGAACTGCGAGTGTAGAGGTGAAATTCGTAGATATTCGCAAGAACACCGGTGGCGAAGGCGGCCAACTGGACCATTACTGACGCTGAGGCGCGAAAGCGTGGGGAGCAAACAGG

>5dd96305408bd66fa4454484c8d3172d

TACGGAGGGTGCAAGCGTTGTTCGGAATTATTGGGCGTAAAGCGCGTGCAGGCGGCTGTTCAAGTCCGATGTGAAAGCCCGGGGCTCAACCCCAGAAGTGCATTGGAAACTGGACAGCTTGAGTACGGGAGAGGGAGGTAGAATTCCGAGTGTAGGGGTGAAATCCGTAGATATTCGGAGGAATACCGGTGGCGAAGGCGGCCTCCTGGACCGATACTGACGCTGAGACGCGAAAGCGTGGGGAGCAAACAGG

>d90581f2ec69e041afdc4a85e6e2e019

TACGTAGGGCGCAAGCGTTGTCCGGAATTATTGGGCGTAAAGAGCTCGTAGGCGGCTTGTCGCGTCGGATGTGAAAGCCCGGGGCTTAACCCCGGGTCTGCATTCGATACGGGCAGGCTAGAGTTCGGTAGGGGAGATCGGAATTCCTGGTGTAGCGGTGAAATGCGCAGATATCAGGAGGAACACCGGTGGCGAAGGCGGATCTCTGGGCCGATACTGACGCTGAGGAGCGAAAGCGTGGGGAGCGAACAGG

>0e2d370f860f8262fe2d1fd778dd030f

TACGTAGGGTGCGAGCGTTGTCCGGAATTACTGGGCGTAAAGAGCTCGTAGGTGGTTTGTCGCGTCGTCTGTGAAATTCCGGGGCTTAACTCCGGGCGTGCAGGCGATACGGGCATAACTTGAGTACTGTAGGGGAGACTGGAATTCCTGGTGTAGCGGTGAAATGCGCAGATATCAGGAGGAACACCGGTGGCGAAGGCGGGTCTCTGGGCAGTAACTGACGCTGAGGAGCGAAAGCATGGGGAGCGAACAGG

>9ed2c152392c6f3bce82d27e6fa038ac

GCCGCCGCGGTAAGTGCTCAGAAAACATCAATCTTTCACTTCGTTCTCACTTTCTCTTTTTACTGCCTCCTCTTCTGACCCTTTTCCACCATACCCCTTCTTCTCCTTTTCGTCACCCCTTTCGGTCTATTTGTAGCATAATATATTAGAAACCCTAGTAGTCCGGCTGACTGACTTGCGTCAAAT

>1f0019d84564bd3488dcf8fe4333ce92

TACAAGTAAGACTAGTGTTATTCATCTTTATTAGGTTTAAAGGGTACCTAGACAGTATATCTAGCCTCAAAAGGGAACAGATTTACTAGAGTTTTATGTGAGAGGAAAATATTAGAACCATTGGAGTAGTGATAGAATATTTTGATACTAATGGGACGGATAACGGCGAAGGCAAACCTCTATGTAATAACTGACGTTGAGGGACGAAGGCTTGGGGAGCGAATAGG

>2da8ad9975124bee965f13916a5d6657

CACACTTATGTCTTCAGAAATACAAACCATTTCTCCAGGATGCATTTCAAACATGTCGGTCATTTTCAACTGTCAGTGCCTTGTAGATATTTGTGGAAGATTTTGAATGTGTCTTGTCCATAGACAGTTTGTAAATTAATGATTTTGTTATGTAGTTTGTAACGTAATATATTTAGGCTTTTAAGTAAATTCATTTTATCTTATTTTTTCCGGTCGTG

>a33a75b67c18816b646d880bb420d860

TACGTAGGTGGCAAGCGTTATCCGGAATTATTGGGCGTAAAGCGCGCGTAGGCGGTTTTTTAAGTCTGATGTGAAAGCCCACGGCTCAACCGTGGAGGGTCATTGGAAACTGGAAAACTTGAGTGCAGAAGAGGAAAGTGGAATTCCATGTGTAGCGGTGAAATGCGTAGAGATATGGAGGAACACCAGTGGCGAAGGCGACTTTCTGGTCTGTAACTGACGCTGATGTGCGAAAGCGTGGGGGTCAAACAGG

>fdf7a93fb7059b18b8a2011df50a9562

CCGCCGCGGTAATACGATTTCTTTAATTTAAATATTTAAGTTTCAGTTAATAAAACAATAATATAAAATATCTATAATTTTGGTGAAATATATTTTATTTTTAAAAATTAATTTTATGTCTGAAAAATTTTTGTATAAACTAGGATTAGAAACCCTAGTAGTCCGGCTGACTGACTACTGTGTAA

>cc76632b2535760cb32dbf5f6cc2b04e

TACGAAGGGTGCGAGCGTTAATCGGAATTACTGGGCGTAAAGCGCGCGTAGGTGGTTTGTTAAGTTGGAAGTGAAAGCCCCGGGCTCAACCTGGGAATTGCTTTCAAAACTGACAGGCTAGAGTACGGTAGAGGGTAGTGGAATTTCCTATGTAGCGGTGAAATGCGTAGATATAGGAAGGAACATCAGTGGCGAAGGCGACTACCTAGACTGATACTGACACTGAGGTGCGAAAGCGTGGGGAGCAAACAGG

>4e6bbcb4038123fe8cdc0c4a3a9a1f37

CCGCCGCGGTAATACGATTTCTTTAATTTAAATATTTAAGTTTCAGTTAATATAACAATAATATAAAATATCTAAAATTTTGGTGAAATATATTTTATCTTTAAAAATTAATTTTATGTCTGAAAAATTTTTGTATAAACTAGGATTAGATACCCTGGTAGTCCGGCTGACTGACTCTACGACCA

>27e024a4ebe7e69987948a71139275bd

TTCTTACTGCAAACGTATTGTCACACACATGTTTTGTAGTAATTATTGTGAGCAGCATCCTCTATGTTATAAATGTAATAAGGAAATCTTAAAACAATGATAATAATCTGTTTATAGTAATTAATCTTCATCATCTTCGCCATTCCAGGATTAGATACCCCTGTAGTCCGGCT

>9802230c7a12e24d962db1545438a923

CACACTTATGTCTTCAGAAGTACAAACCATTTCTCCAGGATGCATTTCAACCATGTCGGTCATTTTCAACTGGTAGTGCCTTGTAGATATTTGTGAAAGATTTTGAATGTGTCTTGTCCATAGAGTTCAGTTTGTAAATTAATGATTTTGTTATGTAGTTTGTAACGTAATATACAGTATTTAGGCTTTTAAGTAAATTAATTTTATTTCCTTTTTTTCGGTTGTG

>c69a9aa53498590fd9a56eeb3fc75bab

CACACTTATGTCTCCAGAAAGACAAAATATTTATCCAGGATGCATTTCAACCATGTCGGTCATTTTCAACTGGCAGTGCCTTGTAGATATTTGTGAACGATTTTGAATGTGTCTTGTCCATAGAGATCAGTTTGTTCATTAATGATTTTGTTATGTAGTTTGTAACGTAATATATTTAGGCTATCAAGTAAATTCATTTTGTTTCCTTTTTTCCGGTTGTG

>7f85ac088481e3811666998c6ba22231

TACGTATGTCACAAGCGTTATCCGGATTTATTGGGCGTAAAGCGCGTCTAGGTGGTTATATAAGTCTGATGTGAAAATGCAGGGCTCAACTCTGTATTGCGTTGGAAACTGTGTAACTAGAGTACTGGAGAGGTAAGCGGAACTACAAGTGTAGAGGTGAAATTCGTAGATATTTGTAGGAATGCCGATGGGGAAGCCAGCTTACTGGACAGATACTGACGCTGAAGCGCGAAAGCGTGGGTAGCAAACAGG

>75a3e20e916ee5ec7dceae34a1bc0209

TACGTAGGGCGCGAGCGTTGTCCGGAATTATTGGGCGTAAAGAGCTCGTAGGCGGCTGGTCGCGTCTGTCGTGAAATCCTCTGGCTTAACTGGGGGCTTGCGGTGGGTACGGGCCGGCTTGAGTGCGGTAGGGGAGGCTGGAACTCCTGGTGTAGCGGTGGAATGCGCAGATATCAGGAAGAACACCGGTGGCGAAGGCGGGTCTCTGGGCCGTTACTGACGCTGAGGAGCGAAAGCGTGGGGAGCGAACAGG

>c25c212ecf21fc7bf5c28563e4f75444

TACGGAGGGAGCTAGCGTTGTTCGGAATTACTGGGCGTAAAGCGCACGTAGGCGGCTTTGTAAGTTAGAGGTGAAAGCCTGGAGCTCAACTCCAGAATTGCCTTTAAGACTGCATCGCTCGAATACAGGAGAGGTGAGTGGAATTCCGAGTGTAGAGGTGAAATTCGTAGATATTCGGAAGAACACCAGTGGCGAAGGCGGCTCACTGGACTGGTATTGACGCTGAGGTGCGAAAGCGTGGGGAGCAAACAGG

>565b59725452b37f481c73bbb7e4ff26

TACGGAGGGTGCGAGCGTTAATCGGAATTACTGGGCGTAAAGCGCGCGTAGGCGGCGTGATAAGCCGGTTGTGAAAGCCCCGGGCTCAACCTGGGAACGGCATCCGGAACTGTCAGGCTAGAGTGCAGGAGAGGAAGGTAGAATTCCCGGTGTAGCGGTGAAATGCGTAGAGATCGGGAGGAATACCAGTGGCGAAGGCGGCCTTCTGGACTGACACTGACGCTGAGGTGCGAAAGCGTGGGTAGCAAACAGG

>7a1f55fe0ae75add4938a1f92fc101ac

TACGTAGGTGGCAAGCGTTATCCGGAATTATTGGGCGTAAAGCGCGCGTAGGCGGTTTTTTAAGTCTGATGTGAAAGCCCACGGCTCAACCGTGGAGGGTCATTGGAAACTGAAAAACTTGAGTGCAGAAGAGGAAAGTGGAATTCCATGTGTAGCGGTGAAATGCGCAGATATATGGAGGAACACCAGTGGCGAAGGCGACTTTCTGGTCTGTAACTGACGCTGATGTGCGAAAGCGTGGGGATCAAACAGG

>f62148561c91f95decfc8489ae413d3d

TACAGAGGGTGCAAGCGTTAATCGGAATTACTGGGCGTAAAGCGCGCGTAGGTGGTTTGTTAAGTTGGATGTGAAAGCCCCGGGCTCAACCTGGGAACTGCATCCAAAACTGGCAAGCTAGAGTACGGTAGAGGGTGGTGGAATTTCCTGTGTAGCGGTGAAATGCGTAGATATAGGAAGGAACACCAGTGGCGAAGGCGACCGCCTGGACTGATACTGACACTGAGGTGCGAAAGCGTGGGGAGCAAACAGG

>d34c2ce628a1f43d762cefdb24b05821

TACGTAGGGTGCGAGCGTTGTCCGGAATTACTGGGCGTAAAGAGCTCGTAGGTGGTTTGTCGCGTCGTCTGTGAAATTCCGGGGCTTAACTTCGGGCGTGCAGGCGATACGGGCATAACTTGAGTGCTGTAGGGGAGACTGGAATTCCTGGTGTAGCGGTGGAATGCGCAGATATCAGGAGGAACACCGATGGCGAAGGCAGGTCTCAGGGCAGTAACTGACGCTGAGGAGCGAAAGCATGGGGAGCGAACAGG

>7e33f84c71f9c8e99b72be958a32f78c

TACCAGCACCTCGAGTGGTCAGGACGTTTATTGGGCCTAAAGCATCCGTAGCCGGCTCTGCAAGTCTTCGGTCAAATCCACCTGCTCAACAGATGGGCTGCTGGAGATACTACAGAGCTAGGGAGTGGGAGAGGCAGACGGTATTCAGTGGGTAGGGGTAAAATCCTCTGATCCATTGAGGACCACCAGTGGCGAAGGCGGTCTGCCAGAACACGTTCGACGGTGAGGGATGAAAGCTGGGGGAGCAAACCGG

>3e1501828c1b85dfe918938ec7af8567

TACGAGGGGAGCGAGTGTTGTTCGGTTTTATTGGGCGTAAAGGGTACGTAGGCGGTTTTGTAAGTCAACATTTAAATCTTGAGACTTAATCTCATTACAGGTGTTGATACTGCATAACTATGAACTTAATAGGGATGAACACAATTCCAAGTGTAGAGGTGAAATTCGTTGATATTTGGAGGAGTACCAAAGGCGAAGGCAGTTCATTGGGTTAAGTTGACGCTGAGGTACGAAAGCGTGGGGAGCAAACAGG

>41da1f67db882e54aa2020701805031e

TACGAAGGGGGCTAGCGTTGCTCGGAATCACTGGGCGTAAAGGGTGCGTAGGCGGGTTTTTAAGTCAGAGGTGAAATCCTGGAGCTCAACTCCAGAACTGCCTTTGATACTGGGAATCTTGAGTATGGAAGAGGTGAGTGGAACTGCGAGTGTAGAGGTGAAATTCGTAGATATTCGCAAGAACACCAGTGGCGAAGGCGGCTCACTGGTCCATAACTGACGCTGAGGCACGAAAGCGTGGGGAGCAAACAGG

>dee632b7432fc9dd0f5077f1f879363f

TACGTAGGGTGCGAGCGTTGTCCGGAATTACTGGGCGTAAAGAGCTCGTAGGTGGTTTGTCGCGTCGTGTGTGAAATTCCGGGGCTTAACTTCGGGGTTGCATACGATACGGGCATAACTTGAGTGCTGTAGGGGAGACTGGAATTCCTGGTGTAGCGGTGAAATGCGCAGATATCAGGAGGAACACCGATGGCGAAGGCAGGTCTCTGGGCAGTAACTGACGCTGAGGAGCGAAAGCATGGGGAGCGAACAGG

>dc5265416587dc62aeb31f7f553b257c

CTGCGTCACTATGGTAATTGTGTGCCAGCCGCCGCGGTAACACACATTGTTATTGTTGTTGTGATGTTTATATATGTGTAGGTTTTAATTTATGTTTATTTTTTGGCCTTTGGAAATGTTTTTAATTAGTTTGAGGTTGTTGGGATTAGAAACCCGAGTAGTCCGGCTGACTGACTTAACGTCCATCTCGTATGCCGTCTTCTGCTTGAAAAA

>a1da1fc93a3f33d0b5bfbb5650157cf3

TACGTAGGTGGCAAGCGTTATCCGGAATTATTGGGCGTAAAGCGCGCGTAGGCGGTTTTTTAAGTCTGATGTGAAAGCCCACGGCTCAACCGTGTAGGGTCATTGGAAACTGGAAAACTTGAGTGCAGAAGAGGAAAGTGGAATTCCATGTGTAGCGGTGAAATGCGCAGAGATATGGAGGAACACCAGTGGCGAAGGCGACTTTCTGGTCTGTAACTGACGCTGATGTGCGAAAGCGTGGGGATCAAACAGG

>41f3f39a0335a7392afa80327caf0f77

TACGTAAGGACCGAGCGTTGTCCGGAATCATTGGGCGTAAAGGGTACGTAGGCGGCTAGAAAAGTTAGAAGTCAAAGGCTATAGCTCAACTATAGTAAGCTTCTAAAACTATTTAGCTTGAGAAATGGAAGGGAAAGTGGAATTCCTAGTGTAGCGGTGGAATGCGCAGATATTAGGAAGAATACCGGTGGCGAAGGCGACTTTCTGGCCATTATCTGACGCTGAGGTACGAAAGCGTGGGTAGCAAACAGG

>c255314cae48ee26907b6f0300ca73ca

TACGGAAGGTCCGGGCGTTATCCGGATTTATTGGGTTTAAAGGGAGCGTAGGCCGTATTTTAAGCGTGCCGTGAAATGCCGTGGCTCAACCATGGCACTGCGGCGCGAACTGGATTACTTGAGTGCGCGGAAGGTAGGCGGAATTCGTGGTGTAGCGGTGAAATGCTTAGATATCACGAAGAACTCCGATTGCGAAGGCAGCTTACCGCAGCGTAACTGACGCTGAAGCACGAAGGTGCGGGTATCGAACAGG

>b0c94ca146677930438a5130d29dc9ff

GATCTACTATGGTAATTGTGTGCCAGCCGCCGCGGTAATGAGTGTGATATAATTTTACAATACACTATAACATTAGATACCACAAGCCACACACAGAAGTCGGGGGCGCAAACACAATCATATAATTTTACCATACGCTATAACATTAGAAACCCGAGTAGTCCGGCTGACTGACTTAACGTCCATCTCGTATGCCGTCTTCTGCTTGAAA

>466510aa4213ebb5cc61138b10842987

TATTAGTGTTCCACTATTCTATATATCACTAAAAGTATTTTGCCACTCTTCATTAACATGTTTCTATGTTTAACCAATACAAAATTTGTTGCAATCATTGCCTTTAATTTTATAGATGCCATGTTTAGTGTATCATTGCAATTGTATATATTGTTTATATTTATATATAATCATATACAAACATATAATAGAATCATTTGACCCCAAAGGAATATGG

>f8b63f43327963bd5f791f6038621d1e

ATATGGTAATTGTGTGCCAGCCGCCGCGGTAATAGTGATGTTTTGGCTGACTTTATCTGCTGCTTTGTTACAGTCAAGCTTAATAAAACCACCATAAACACACACATTAAGTAAGATGCTAACTCAAGTCACACACAACAATACATTAGAAACCCGTGTAGTCCGGCTGACTGACTTAACGTCCATCTCGTATGCCGTCTTCTGC

>6414af71861e11af2a331d1aef1b144f

GTAATTGTGTGCCAGCCGCCGCGGTAATACTGTATCCAAGCATATAGACATCAAATTGATTGACAAATCTATCATATTGTCAAAGTATATTAGGTATAAAATCATATAACTCGTATCAGCTTCGGAAAAATAGTCAGACATTTAATTAGATACCCTAGTAGTCCGGCTGACTGACTGAGACTTAATCTCGTATGCCGTCT

>1b51357852b0b814b4d9918c6f0ab762

TACGTAGGTCCCGAGCGTTGTCCGGATTTATTGGGCGTAAAGCGAGCGCAGGCGGTTAGATAAGTCTGAAGTTAAAGGCTGTGGCTTAACCATAGTATGCTTTGGAAACTGTTTAACTTGAGTGCAGAAGGGGAGAGTGGAATTCCATGTGTAGCGGTGAAATGCGTAGATATATGGAGGAACACCGGTGGCGAAAGCGGCTCTCTGGTCTGTAACTGACGCTGAGGCTCGAAAGCGTGGGGAGCAAACAGG

>fd65ee1057b5bfffe8a8c7f630e57fef

CATTGAACTATCGTGAGAAAGTCAAGCCGCCAAAGGGAATTATATAATAGTAAATATTAGCGTAAATAAACATTTTATTAATAGTTGTAATATATGATAATGTGCAAAAGATAGAGACGATTAAAACGGTGTTTAACAATCGCTTATCGAGACACTATTAAAGTAATTAGGAATTAAAATATGTTTATTATGTGTATTATTCAAGGAATAAACTTGAAAATAAATACAGTGTTAAACGGAACTAATAGAGTCACGCAAGTGAACTTAGAACATTTTAATAAC

>89d81e7e65cc84f32033ddd2b170dccb

CACGCGTGATATGGTAATTGTGTGCCAGCAGCCGCGGTAATACTACACAAGGCACCGAGGACGAAGACGAGGAGGATATGAAAGTTGAATAAAGGAAACGAAGACACGGCAGGACGACGTTGAGTGTATCACGTGACAGAAGTTATTAGAAACCCCTGTAGTCCGGCTGACTGACTTAACGTCCATCTCGTATGCCGTCTTCTGCTTGAAAAA

>50d4dc82ae2bcb70031b64b0ff56ef0f

CATTGAACTATCGTGAGAAAGTCAAACCGCCAAAGGGAATTATATTATAGTAAATATTAACGTAAATAAACATTTTATTAATAGTTGTAATATATGATAATGTGCAAAAGATAGAGACGATTAAAACGGTGTTTAACAATCGCTTATCGAGACACTATTAAAGTAATTAGGAATTAAAATATGTTTATTATGTGTATTATTCAAGGAATAAACTTGAAAATAAATACAGTGTTAAACGGAACTAATAGAGTCACGCAAGTGAACTTAGAACATTTTAATAAC

>b6997dde67a9edc81f394ceea2d01409

GCCGCCGCGGTAAGTGCTCAGAAAACATCAATCTTTCACTTCGTTCTCACTTTCTCTTTTTACTGCCTCCTCTTCTGACCCTTTTCCACCATACCCCTTCTTCTCCTTTTCGTCACCCCTTTCGGTCTATTTGTAGCATAATATATTAGAAACCCCTGTAGTCCGGCTGACTGACTGAGACTTAAT

>1d4b4053c1e2019d392854d3ee82c363

CCGCCGCGGTAATACGATTTCTTTAATTTAAATATTTAAGTTTCAGTTAATATAATAATAATATAAAATGTCTATAATTTTGGTGAAATATATTTTATCTTTAAAAATTAATTTTATGTCTGAAAAATTTTTGTATAAACTAGGATTAGAAACCCTTGTAGTCCGGCTGACTGACTTGCGTCAAA

>dd8752d4075126510ca67a9a26782910

TACGTGAGAGACTAGTGTTATTCATCTTAATTGGGCTTAAAGGGTACCTAGACAGTCAATATAACTTCTATAATGCTAATACTTGACTAGAGTTTTAAGTAAGAGGGAAGTACTTAAGGAGTAAGAGATGAAATATCTGTGATACCAAAGGGACTCCGTAAAGGCGAAGGCATCCCTTTATCTAAAAACTAACGTTGAAGGACGAAGGCTTAGATAACAAATAGG

>7e05d89be13c66e8f27a25fbf1208fa4

TACGTAGGTGGCAAGCGTTGTCCGGAATTATTGGGCGTAAAGTGCGCGCAGGCGGTCTTTTAAGTCTGATGTGAAAGCCCCCGGCTCAACCGGGGAGGGTCATTGGAAACTGGGAGACTTGAGTACAGAAGAGGAGAGTGGAATTCCACGTGTAGCGGTGAAATGCGTAGATATGTGGAGGAACACCAGTGGCGAAGGCGACTCTCTGGTCTGTAACTGACGCTGAGGCGCGAAAGCGTGGGGAGCAAACAGG

>cbef40f459197faa8de7bba6a4764a34

TACGGAAGGTCCAGGCGTTATCCGGATTTATTGGGTTTAAAGGGAGTGTAGGCGGTTTGTTAAGCGTGTTGTGAAATTTAGGTGCTCAACATTTAACTTGCAGCGCGAACTGTCAGACTTGAGTACACGCAACGTATGCGGAATTCATGGTGTAGCGGTGAAATGCTTAGATATCATGAAGAACTCCGATTGCGAAGGCAGCATACGGGAGTGTAACTGACGCTTAAGCTCGAAGGTGCGGGTATCGAACAGG

>e97bbb90270d4d7c850d7b41986f2bb1

TACGGAGGGAGCTAGCGTTATTCGGAATTACTGGGCGTAAAGCGCACGTAGGCGGCTTTGTAAGTTAGAGGTGAAAGCCCGGAGCTCAACTCCGGAATTGCCTTTAAGACTGCATCGCTCGAATCCAGGAGAGGTGAGTGGAATTCCGAGTGTAGAGGTGAAATTCGTAGATATTCGGAAGAACACCAGTGGCGAAGGCGGCTCACTGGACTGGTATTGACGCTGAGGTGCGAAAGCGTGGGGAGCAAACAGG

>f8bcddaea88eb790743420346bb1bc80

TACGTAGGGCGCGAGCGTTGTCCGGAATTATTGGGCGTAAAGAGCTTGTAGGCGGTTGGTCGCGTCTGCTGTGAAAGGCCGGGGCTTAACTCCGGTTTTGCAGTGGGTACGGGCTAACTAGAGTGCAGTAGGGGAGACTGGAATTCCTGGTGTAGCGGTGGAATGCACAGATATCAGGAGGAACACCGATGGCGAAGGCAGGTCTCTGGGCTGTAACTGACGCTGAGAAGCGAAAGCATGGGGAGCGAACAGG

>a894dd1ea1b8f48947a9e9ff688f06cc

TACGGAGGGTGCAAGCGTTATCCGGATTTATTGGGTTTAAAGGGTCCGTAGGCGGACTCGTAAGTCAGTGGTGAAATCTCATAGCTTAACTATGAAACTGCCATTGATACTGCGGGTCTTGAGTAAGGTAGAGGTAGCTGGAATAAGTAGTGTAGCGGTGAAATGCATAGATATTACTTAGAACACCAATTGCGAAGGCAGGTTACCATGTCTTAACTGACGCTGATGGACGAAAGCGTGGGGAGCGAACAGG

>751f320499e9b53c56f40330f77ecc2d

TACGGAGGGTGCAAGCGTTAATCGGAATTACTGGGCGTAAAGCGCACGCAGGCGGTTGATTAAGTTAGATGTGAAATCCCCGGGCTTAACCTGGGAATGGCATCTAAGACTGGTCAGCTAGAGTCTTGTAGAGGGGGGTAGAATTCCATGTGTAGCGGTGAAATGCGTAGAGATGTGGAGGAATACCGGTGGCGAAGGCGGCCCCCTGGACAAAGACTGACGCTCAGGTGCGAAAGCATGGGGAGCAAACAGG

>8b78c8758a8347430f4ac9dfc6ceb58e

TACGTAGGGTGCAAGCGTTAATCGGAATTACTGGGCGTAAAGCGTGCGCAGGCGGTTGTGGAAGTCAGATGTGAAATCCCCGGGCTCAACCTGGGAATTGCATTTGAGACTGCACGGCTAGAGTGTGTCAGAGGGGGGTAGAATTCCACGTGTAGCAGTGAAATGCGTAGATATGTGGAGGAATACCGATGGCGAAGGCAGCCCCCTGGGATAACACTGACGCTCATGCACGAAAGCGTGGGGAGCAAACAGG

>3959dadb949dc75fe090a8582324520e

TACGAAGGGTGCAAGCGTTAATCGGAATTACTGGGCGTAAAGCGCGCGTAGGTGGCTTGATAAGTTGGATGTGAAATCCCCGGGCTCAACCTGGGAACTGCATCCAAAACTGTCTGGCTAGAGTGCGGTAGAGGGTAGTGGAATTTCCAGTGTAGCGGTGAAATGCGTAGATATTGGAAGGAACACCAGTGGCGAAAGCGACTACCTGGACTGACACTGACACTGAGGTGCGAAAGCGTGGGGAGCAAACAGG

>c31e26bdb7606350be8d78e93490be7d

TACGGAGGGTGCGAGCGTTAATCGGAATTACTGGGCGTAAAGCGCGCGTAGGCGGCTTGATAAGCCGGTTGTGAAAGCCCCGGGTTCAACCCGGGAACGGCATCCGGAACTGTCAGGCTAGAGTGCAGGAGAGGAAGGTAGAATTCCCGGTGTAGCGGTGAAATGCGTAGAGATCGGGAGGAATACCAGTGGCGAAGGCGGCCTTCTGGACTGACACTGACGCTGAGGTGCGAAAGCGTGGGTAGCAAACAGG

>df9a03a7b0a0b0c251578ccd3351ff2a

GCCGCCGCGGTAATACAAAATTTTCAGTTAAATAAATTAAGCTTACAGTTAATAAATATTTTTAATAAATTATTTAAATTTTAGTGAAATATATTTATATAAATTTAAATTTACATATCTGAGAAACTATTAAATTAAACTAGGATTAGAAACCCCTGTAGTCCGGCTGACTGACTTGCGTCAAAT

>028aab1022506da2818f79628e34e42b

AACAGAGGATACAAGCGTTATCCGGATTTATTGGGTTTAAAGGGTGCGTAGGTGGTTTTTTAAGTCAGTAGTGAAATCTTAAAGCTTAACTTTAAAAGTGCTATTGATACTGATAAACTAGAGTGAGGTTGGAGTATCTGGAATGTGTGGTGGAGCGGTGAAATGCATAGAGATCACACAGAACACCAATCGCGAAGGCATGTTACTAAACATAGACTGACACTGAGGCACGAAAGCATGGGTAGCAAACAGG

>56d5e0bff844ead5dc7fd49dcfb0c411

CCGCCGCGGTAATACGATTTCTTTAATTTAAATATTTAAGTTTCAGTTAATAAAACAATAATATAAAATATCTATAATTTTGGTGAAATATATTTTATTTTTAAAAATTAATTTTATGTCTGAAAAATTTTTGTATAAACTAGGATTAGAAACCCCAGTAGTCCGGCTGACTGACTACTGTGTAA

>17adfd78f834a58bade2f8e4befcdb88

GCCGCCGCGGTAAGTGCTCAGAGAACATCAATCTTTCACTTCGTTCTCACTTTCTCTTTTTACTGCCTCCTCTTCTGACCCTTTCCACCATACCCCTGCTTCTCCTTTTCGTCATCCCTTTTCGGTCTATTTGTAGCATAATATATTAGATACCCTTGTAGTCCGGCTGACTGACTTAACGTCCAT

>b2b073f218beadc8ac47899ead3e756c

TACAGAGGCCCCAAGCGTTGTTCGGATTTACTGGGCGTAAAGGGTGTGTAGGGGGTTGGGTAAGTTTGACGTGAAATCCCGTTGCTCAACAACGGAACTGCGTCGAATACTGCTCGGCTGGAGGTTCGGAGATGAGGGCGGAATTCTCGGTGTAGCGGTGAAATGCGTAGATATCGAGAGGAACGCCGATGGCGAAAGCAGCCCTCAAGACGAAATCTGACCCTGAAACACGAAGGCCAGGGGAGCAAACGGG

>0fbbfcd1e0e4d919dcdff7c2bee23f8a

CGCGTACCAGCACCTGTAGCTGTGGCGACTATATTACGGCCCACCAACGCGTCCTCACACCCCACAACGTGTTACGTCCACCATAAGCGAAGAGCAATGTCCGCCAAACTTTGTTAATAATATAGAATTGCCGAAAACATCGTGCGTGAAATCCCATTATCCGAACCCTTATT

>86d6db0b6e15510fa5d27ceae8fd5e0d

TACAGAGGGTGCAAGCGTTAATCGGAATTACTGGGCGTAAAGCGCGCGTAGGTGGTTTGTTAAGTTGGATGTGAAATCCCCGGGCTCAACCTGGGAACTGCATCCAAAACTGGCAAGCTAGAGTAGGGCAGAGGGTGGTGGAATTTCCTGTGTAGCGGTGAAATGCGTAGATATAGGAAGGAACACCAGTGGCGAAGGCGACCACCTGGGCTCATACTGACACTGAGGTGCGAAAGCGTGGGGAGCAAACAGG

>5079182f1a0d70b86d0f1846c4b0260f

TACGGAGGGTCCGAGCGTTAATCGGAATTACTGGGCGTAAAGCGTGCGCAGACGGTTTGTTAAGCGAGATGTGAAAGCCCTGGGCTCAACCTAGGAATAGCATTTCGAACTGGCGAACTAGAGTCTTGTAGAGGGGGGTAGAATTCCAGGTGTAGCGGTGAAATGCGTAGAGATCTGGAGGAATACCGGTGGCGAAGGCGGCCCCCTGGACAAAGACTGACGCTCATGCACGAAAGCGTGGGGAGCAAACAGG

>d8b5ff27bbbbfeb8ec63428858c6ab79

TACGAAGGGTGCAAGCGTTACTCGGAATTACTGGGCGTAAAGCGTGCGTAGGTGGTGGTTTAAGTCTGTTGTGAAAGCCCTGGGCTCAACCTGGGAATTGCAGTGGATACTGGGTCACTAGAGTGTGGTAGAGGGTAGCGGAATTCCCGGTGTAGCAGTGAAATGCGTAGAGATCGGGAGGAACATCCGTGGCGAAGGCGGCTACCTGGACCAACACTGACACTGAAGCACGAAAGCGTGGGGAGCAAACAGG

>d6083db79a57ad9b168d1643197bb17e

CGCCGCGGTAATACAATTTTTTCAATTTAATATGTAAGTTTCAGTTAATTGATTATTTTTAATAATTTATATCAATTTTGGTGAAATAATATTTATTAATATAAATAATTTAAATGTGTCTGAGAAACTATAAATTAAACTAGGATTAGAAACCCCGGTAGTCCGGCTGACTGACTTGCGTCAA

>f2f6952030b919897e6baaae00a5786f

GACGGGGGGGGCAAGTGTTCTTCGGAATGACTGGGCGTAAAGGGCACGTAGGCGGTGAATCGGGTGGAAAGTTCAAGTCGCCAAAAAGTGGCGGAATGCTCTCGAAACCAATTCACTTGAGTGAGACAGAGGAGAGTGGAATTTCGTGTGTAGGGGTGAAATCCGTAGATCTACGAAGGAAGGCCAAAAGCGAAGGCAGCTCTCTGGGTCTCTACCGACGCTGGGGTGCGAAAGCATGGGGAGCGAACAGG

>63de2ce8c53a93ffb6c59dac90c36bdc

TAGTATGTATGGTAATTGTGTGCCAGCCGCCGCGGTAATGAGTGTGATATAATTTTACAATACACTATAACATTAGATACCACAAGCCACACACAGAAGTCGGGGGCGCAAACACAATCATATAATTTTACCATACGCTATAACATTAGAAACCCGAGTAGTCCGGCTGACTGACTTAACGTCCATCTCGTATGCCGTCTTCTGCTTGAAA

>ca6939a543403a640bc148dafaa34f5a

GTATGGTAATTGTGTGCCAGCCGCCGCGGTAATAGTGATGTTTTGGCTGACTTTATCTGCTGCTTTGTTACAGTCAAGCTTAATAAAACCACCATAAACACACACATTAAGTAAGATGCTAACTCAAGTCACACACAACAATACATTAGATACCCGAGTAGTCCGGCTGACTGACTTAACGTCCATCTCGTATGCCGTCTTCTGC

>bdbaaf5282c2be17511a4dd3218e6dd9

CATTGAACTATCGTGAGAAAGTCAAACCGCCAAAGGGAATTATATTATAGTAAATATTGGCGTAAATAAACATTTTATTAATAGTTGTAATATATGAAAATGTGCAGAAGATAAAGACGATTAAAACGGTGTTTAACAATCGCTTATCGAGACAATATTTAATTAATTAGGAATTAAAATATGTGTATTATGTGTATTAAACAAGGGATAAACTTGAAAATAAATACAGTGGTAAACGGAACTAATAGAGTCACGCAAGTGAACGTAGAACATTTTTAATAAC

>9d238be62c6f3180505133b577437b5d

GCGTCACTATGGTAATTGTGTGCCAGCCGCCGCGGTAATGAGTGTGATATAATTTTACAATACACTATAACATTAGATACCACAAGCCACACACAGAAGTCGGGGGCGCAAACACAATCATATAATTTTACCATACGCTATAACATTAGAAACCCGTGTAGTCCGGCTGACTGACTTGCGTCAAATCTCGTATGCCGTCTTCTGCTTGAAA

>9e3243e54b3584fefcaf5a943a45b80b

CCGCCGCGGTAAGTGCTCAGAGAACATCAATCTTTCACTTCGTTCTCACTTTCTCTTTTTACTGCCTCGTCTTCTGACCCTTTTCCACCATACCCCTTCTTCTCCTTTTCGTCATCCTTTTTCGGCCTATTTGTAGCATAATATATTAGAAACCCCGGTAGTCCGGCTGACTGACTTGCGTCAAA

>e2ae077d9b5cc408fc8ff004ca301e74

TTCTGACTGCAAACGTATTGTCACACACATATGTTTTGTAGTAATTATTGTGAGCAGCATCCTCTATGTTATAAATGTAATAAGGAAATCTTAAAACAATGATAATAATCTGTTTATAGTAATTAATCTTCATCATCTTCGCCATTCCAGGATTAGAAACCCCGGTAGTCCGG

>fa3be2840777c910e6ffcce73ddc962d

TACAGAGGGTGCGAGCGTTAATCGGATTTACTGGGCGTAAAGCGCGCGTAGGTGGTTAATTAAGTCAAATGTGAAATCCCCGAGCTTAACTTGGGAATTGCATTCGATACTGGTTAGCTAGAGTATGGGAGAGGATGGTAGAATTCCAGGTGTAGCGGTGAAATGCGTAGAGATCTGGAGGAATACCGATGGCGAAGGCAGCCATCTGGCCTAATACTGACACTGAGGTGCGAAAGCATGGGGAGCAAACAGG

>b867bdfb2b54821bffa49aabc613eff5

CCGCGGTAACATTGTTTCTCGACCCTGCCGACGTTGGTCTCGGTTGAATATAACCGGACTAAAATCTAAATATGCCCTTACGTAGCTCGTTACTGTGTCAGATTACAGCACAAGTTCGGGCCCACTTACCGCGGCGGCTGACACATTAGAAACCCCAGTAGTCCGGCTGACTGACTTAACGT

>0c7b6fca84c10d3c273e9c92542568dc

CATTGAGCTATCGTGAGAAAGTCAAGCCGCCAAAGGGAATTATATTATAGTAAATATTGGCGTAAATAAACATTTTATTAATAGTTGTAATATATGAAAATGTGCAGAAGATAGAGACGATTAAAACGGTGTTTAACAATCGCTTATCGAGACAATATTTAATTAATTAGTAATTAAAATATGTTTATTATGTGTATTATTCAAGGAATAGACTTGACAATAAATACAGTGGTAAACGGAACTAATAGAGTCACGCAAGTGAACTTAGAACATTTTTAATAAC

>4ee98d73789553e0934f4b3f2f4fdb7e

TACGTAGGTGGCAAGCGTTGTCCGGAATTATTGGGCGTAAAGCGCGCGCAGGCGGTCTTTTAAGTCTGACGTGAAAGCCCCCGGCTCAACCGGGGAGGGTCATTGGAAACTGGGAGACTTGAGTACAGAAGAGGAGAGTGGAATTCCACGTGTAGCGGTGAAATGCGTAGATATGTGGAGGAACACCAGTGGCGAAGGCGACTCTCTGGTCTGTAACTGACGCTGAGGCGCGAAAGCGTGGGGAGCAAACAGG

>2ae5603293f1067eb773701eb6fb6b30

CCGCCGCGGTAATACGATTTCTTTAATTTAAATATTTAAGTTTCAGTTAATATAACAATAATATAAAATATCTAAAATTTTGGTGAAATATATTTTATCTTTAAAAATTAATTTTATGTCTGAAAAATTTTTGTATAAACTAGGATTAGATACCCTAGTAGTCCGGCTGACTGACTCTACGACCA

>17789b897abe5eb8d710dbf134e051a5

TACGTAGGGGGCGAGCGTTGTCCGGAATCACTGGGCGTAAAGAGCGTGTAGGCGGCCCGGTAAGTCTGCTGTGAAAACCCGGGGCTCAACCCCGGGCGTGCAGTGGAAACTGCCGGGCTAGAGGGCGGCAGAGGCGAGTGGAATTCCCGGTGTAGCGGTGAAATGCGCAGATATCGGGAGGAACACCAGTAGCGAAGGCGGCTCGCTGGGCCGTCCCTGACGCTGAGACGCGAAAGCTAGGGGAGCGAACAGG

>b0ba550020361212ab847a06ef70cd5b

TACGAAGGGGGCTAGCGTTGCTCGGAATCACTGGGCGTAAAGGGCGCGTAGGCGGCCATTCAAGTCGGGGGTGAAAGCCTGTGGCTCAACCACAGAATTGCCTTCGATACTGTTTGGCTTGAGTCTGGTAGATGTTGGTGGAACTGCTAGTGTAGAGGTGAAATTCGTAGATATTCGCAAGAACACCGGTGGCGAAGGCGGCCAACTGGACCAGTACTGACGCTGAGGCGCGAAAGCGTGGGGAGCAAACAGG

>3b874d78d6d75379a0094dc4f75c035d

TATGTAGGTGGCAAGCGTTGTCCGGAATTATTGGGCGTAAAGCGCGCGCAGGCGGTCTTTTAAGTCTGATGTGAAAGCCCCCGGCTCAACCGGGGAGGGTCATTGGAAACTGGGAGATTTGAGTACAGAAGAGGAGAGTGGAATTCCACGTGTAGCGGTGAAATGCGTAGATATGTGGAGGAACACCAGTGGCGAAGGCGACTTTCTGGTCTGTAACTGACGCTGAGGTGCGAAAGCGTGGGGAGCAAACAGG

>ed9b8f487299175dcc9046f932fb3aff

TACGTAGGGTGCAAGCGTTAATCGGAATTACTGGGCGTAAAGCGTGCGCAGGCAGTTATGCAAGACAGAGGTGAAATCCCCGGGCTCAACCTGGGAACTGCCTTTGTGACTGCATGGCTAGAGTACGGTAGAGGGGGATGGAATTCCGCGTGTAGCAGTGAAATGCGTAGATATGCGGAGGAACACCGATGGCGAAGGCAATCCCCTGGACCTGTACTGACGCTCATGCACGAAAGCGTGGGGAGCAAACAGG

>02cbe4fe2d07be07a1d265aa81f2f8f6

TCGAACCCAAGCCACGTGGTGTAGCGAACCAACACACTACACACCACGAGTACTCCCCCCCTTCCAATCAACAAAAAATCACAATACGATTACAGGAAATTGTTCAGAATGTAGCGCCAAATTAAATTACAAAGAGGAGGATTTTGAATATTGCAGAAGAATCGTATTGGTGCAAAACAAACGTTTGCTAGCAGCCTTCCTCACGTGTATACTGCACTGATGCTTCGAGAAATAGTCTGCAGAACTAT

>fc571fa1d217cf94eaae1f9c421bd989

AGCCATGTAGGGCTTTTAAATAAAAAACCACAACACGCTTTGTGGCGGCCGCTGACGTTCACTAAGTGCACTATTGTTTATTTAGAGGCAAAAAACTGCTGTGGTTGTTTACAGCGTGATGCATCGAACCTTAGTCAATAATGCATCTGTAGGCACAAGATCGATAAGTGACA

>cd28e997667886159259b77e1a172c3c

CACCGATCTACTATGGTAATTGTGTGCCAGCCGCCGCGGTAAAGTAAACACAATATCTGGCAGGACTGCCACTCGTTACTTGATTCAGCACTCTCCTTATATAGTTTAGTTTAGATTCGTCTGTTTTACAGATTGACTCACTTGATTAGAAACCCCAGTAGTCCGGCTGACTGACTTAACGTCCATCTCGTATGCCGTCTTCTGCTTGAAAAAAA

>d77686cda10c2849bcf82aad2f67948b

TACGAAGGGTGCAAGCGTTAATCGGAATTACTGGGCGTAAAGCGCGCGTAGGTGGTTTGATAAGTTGGATGTGAAAGCCCCGGGCTCAACCTGGGAATTGCATCCATAACTGTCTGACTAGAGTATGGCAGAGGGTGGTGGAATTTCCTGTGTAGCGGTGAAATGCGTAGATATAGGAAGGAACACCAGTGGCGAAGGCGACCACCTGGGCTAATACTGACACTGAGGTGCGAAAGCGTGGGGAGCAAACAGG

>87bbbcbdc650f0e9e4f29d4321cde287

GACAGAGGGTGCAAACGTTGTTCGGAATTACTGGGCGTAAAGCGTGTGTAGGCGGTCTTGTAAGTCGGATGTGAAAGCCCCGGGCTCAACCCGGGAAGTGCACTCGATACTGCGAGACTTGAGTATCGGAGAGGTTGGTGGAATTCTCGGTGTAGAGGTGAAATTCGTAGATATCGAGGGGAACACCGGTGGCGAAAGCGGCCAACTGGACGAATACTGACGCTGAGACACGAAAGCGTGGGGAGCAAACAGG

>de72622233e8b592b10620706e965d4d

GTATGGTAATTGTGTGCCAGCCGCCGCGGTAATAGTGATGTTTTGGCTGACTTTATCTGCTGCTTTGTTACAGTCAAGCTTAATAAAACCACCATAAACACACACATTAAGTAAGATGCTAACTCAAGTCACACACAACAATACATTAGATACCCGTGTAGTCCGGCTGACTGACTCTACGACCATCTCGTATGCCGTCTTCTGC

>1c4ff75fd7b0f67d3c916b2b37426126

CACGATTAACCCAAGTCAATAGAAGCCGGCGTAAAGAGTGTTTTAGATCACCCCCTCCCCAATAAAGCTAAAACTCACCTGAGTTGTAAAAAACTCCAGTTGACACAAAATAGACTACGAAAGTGGCTTTAACGTATCTGAACACACAATAGCTAAGACCCAAACTGGGATTA

>20c04628001f703ed3fde277fe3d42a7

GCAGCCGCGGTAAGTGCTCAAAGAACATCAATCTTTCACTTCGTTCTCACTTTCTCTTTTTACTGCCTCCTCTTCTGAACCTTTCCACCATACCCCTTCCTCTCCTTTTCTTCATCCCTTTTCGGTCTATTTGTAGCATAATATATTAGAAACCCCGGTAGTCCGGCTGACTGACTTGCGTCAAAT

>63464d37480177dc9d4ff07ea357295a

ATGACAAAATCATTGAACATGTTCAACATATTATTCAAAGTGACAGAAGAAAATCTAACAACCTCTGATGCGTTTCTCTTGTATATGTATATTAGCCAGATCTTCAGATTCTAATGGTCTTTACTCAACAGATAAAAACTACATCAAACTATAATTGTAGTTACAATATTTACAAAGTAAGTTGTAAATCAATTTTTAAATACTTAAAATTGTAACTAGCAGAAAAATGGAGAAACTTATAACCAACCCTAATAATTAGCAAACGATATTCCTATATAGCTATA

>3fb11383d86dfad2dfd111339161741f

TACGGAGGGTGCAAGCGTTATCCGGATTTATTGGGTTTAAAGGGTCCGTAGGCGGATCTGTAAGTCAGTGGTGAAATCTCATAGCTTAACTATGAAACTGCCATTGATACTGCAGGTCTTGAGTGAGATTGAGGTAGCTGGAATAAGTAGTGTAGCGGTGAAATGCATAGATATTACTTAGAACACCAATTGCGAAGGCAGGTTACCAAGTCTTAACTGACGCTGATGGACGAAAGCGTGGGGAGCGAACAGG

>ad5e81c085edc51f625eb37b86d79aae

ATGACAAAATCATTGAACATGTTCAACATATTATTCAAAGTGACAGAAGAAAATCTAACAACCTCTGATGCGTTTCTCTTGTATATGTATATTAGCCAGATCTTCAGATTCTAATGGTCTTTACTCAACAGATAAAAACTACATCAAACTATAATTGTAGTTACAATATTTACAAAGTAAGTTGTAAATCAATTTTTAAATACTTAAAATTGTAACTAGCAGAAAAATGGAGAAACTTATAACCAACCCTAATAATTAGCAAACAATATTCCTATATAGCTATA

>2a04f6e1a4800d292044386c97f59d1c

CATTGAACTATCGTGAGAACGGCACGCCGCCAAAGGGAATTATATTATAGTAAATATTGGCGTAAATAAACATTTTATTAATAGTTGTAATATATGAAAATGTGCAGAAGATAAAGACGATGAAAACGGTGTTTAACAATCGCTTATCGAGACAATATTTAATTAATTAGGAATTAAAATATGTGTATTATTTGTATTTAACAAGGAATAATCTTGAGAATAAATACAGTGGTAAACGGAACTAATAGAGTCACGCAAGTGAACTTAGAACAATTGTAAGAAC

>7ccf78e87e31646ca07831039eff1cfb

TTCTTACTGCAAACGTATTGTCACACACATGTTTTGTAGTAATTATTGTGAGCAGCATCCTCTATGTTATAAATGTAATAAGGAAATCTTAAAACAATGATAATAATCTGTTTATAGTAATTAATCTTCATCATCTTCGCCATTCCAGGATTAGATACCCGGGTAGTCCGGCT

>e194c4fb3e1695ff337d391543d41946

CGCCGCGGTAAAATAATTTCTTTAATTTAAATAGTTAAGTTTCACTTAATGTAATAATAATATAAAATAACTATAATTTTGGTCAAATATTTTTTATTAGAAAAAAATTAATTTTATTTCTGAAAGCATTTTGTGTAAACTAGGATTAGATACCCTTGTAGTCCGGCTGACTGACTTAACGTCC

>8f803bee68447aba5130336832a85c60

CATTGAACTATCGTGAGAAAGTCAAACCGCCAAAGGGAATTATATTATAGTAAATATTGGCGTAAATAAACATTTTATTAATAGTTGTAATATATGATAATGTGCAAAAGATAGAGACGATTAAAACGGTGTTTAACAATCGCTTATCGAGACAATATTTAATTAATTAGGAAATAAAATATATTTATTATTGGTATTAAACAAGGGATAATCTTGAAAATAAATACAGTGGTAAACGGAACTAATAGAGTCACGCAAGTGAACTTAGAACATTTTTAATAAC

>9bb14d66aa439e9c13202bd3662ce57b

GCGTCACTATGGTAATTGTGTGCCAGCCGCCGCGGTAATGAGTGTGATATAATTTTACAATACACTATAACATTAGATACCACAAGCCACACACAGAAGTCGGGGGCGCAAACACAATCATATAATTTTACCATACGCTATAACATTAGATACCCCGGTAGTCCGGCTGACTGACTTGCGTCAAATCTCGTATGCCGTCTTCTGCTTGAAA

>6baed13a8f4e9135bb51c57708acc93f

TACGTAGGGTGCGAGCGTTAATCGGAATTACAGGGCGTAAAGCGTGCGCAGGCGGTTTGTTAAGACAGATGTGAAATCCCCGGGCTCAGCCTGGGAACTGCATTTGTGACTGGCAGGCTAGAGTATGGCAGAGGGGGGTAGAATTCCACGTGTAGCAGTGAAATGCGTAGAGATGTGGAGGAATACCGATGGCGAAGGCAGCCCCCTGGGCCAATACTGACGCTCATGCACGAAAGCGTGGGGAGCAAACAGG

>9bb276530d60c2d320b7fd8bc8cf8613

TACGTAGGGTGCGAGCGTTAATCGGAATTACTGGGCGTAAAGCGTGCGCAGGCGGTGATGTAAGACAGATGTGAAATCCCCGGGCTCAACCTGGGAACTGCATTTGTGACTGCATCGCTGGAGTGCGGCAGAGGGGGATGGAATTCCGCGTGTAGCAGTGAAATGCGTAGATATGCGGAGGAACACCGATGGCGAAGGCAATCCCCTGGGCCTGCACTGACGCTCATGCACGAAAGCGTGGGGAGCAAACAGG

>0e2ab3feec497be058299ccba476ef24

CACACTTATGTCTTCAGAAATACAAACCATTTCTCCAGGATGCATTTCAAACATGTCGGTCATTTTCAACTGTCAGTGCCTTGTAGATATTTGTGGAAGATTTTGAATGTGTCTTGTCCATAGACAGTTTGTAAATTAATGATTTTGTTATGTAGTTTGTAACGTAATATATTTAGGCTTTTAAGTAAATTCATTTTATTTTATTTTTTCCGGTCGTG

>cce4293136c7241b9a3f27932987179e

TACGTAGGTGGCAAGCGTTGTCCGGAATTATTGGGCGTAAAGCGCGCGCAGGCGGTCTTTTAAGTCTGATGTGAAAGCCCCTGGCTCAACCGGGGAGGGTCATTGGAAACTGGGAGACTTGAGTACAGAAGAGGAGAGTGGAATTCCACGTGTAGCGGTGAAATGCGTAGATATGTGGAGGAACACCAGTGGCGAAGGCGACTCTCTGGTCTGTAACTGACGCTGAGGCGCGAAAGCGTGGGGAGCAAACAGG

>a1dc570a4262b29bd038e43096c11d95

GATCTACTATGGTAATTGTGTGCCAGCCGCCGCGGTAAAAACCCATACGATGCTCAACAAAGTAAAGAATAGTACCGGTACTTGCTGAAGAACTGCAGATGTCCCCAGACTCTGCAGGGTAACGTTAGGCCACCCCCACTAAATATTAGATACCCGAGTAGTCCGGCTGACTGACTTAACGTCCATCTCGTATGCCGTCTTCTGCTTGAAA

>dc298a119c31a54d5792a8ae5d3681e4

TACGAAGGGGGCTAGCGTTGTTCGGATTTACTGAGCGTAAAGCGCACGTAGGCGGGCTAATAAGTCAGGGGTGAAATCCCGGAGCTCAACCCCGGAACTGCCTTTGATACTGTTAGCCTTGAGTATGGTAGAGGTGAGTGGAATTCCGAGTGTAGAGGTGAAATTCGTAGATATTCGGAGGAACACCAGTGGCGAAGGCGGCTCACTGGACCATTACTGACGCTGAGGTGCGAAAGCGTGGGGAGCAAACAGG

>d2fce88068a0e56c69f57c78b82bb694

CACACTTATGTCTTCAGAAAGACAAAATATTTCCCCATGATGCATTTCAACCATGTCGGTCATTTTCAACTGGCAGTGCCTTGTTGATATTTGTGAAAGATTTTGAATATGTCTTGTCCATAGAGTTGAGTTTGTTCATTAATGATTTTGTTATGTAGTTTGTAACGTAATACATTTAGGCTTTCAAGTAAATTCATTTTGTTTACTTTTTTGCCGGTTGTG

>3cf62705e2069925425c64b43223f31e

TACGGAGGGTGCAAGCGTTATCCGGATTCACTGGGTTTAAAGGGTGCGTAGGTGGGCTTGTAAGTCAGTGGTGAAATCTCCGTGCTTAACATGGAAACTGCCATTGATACTATAGGTCTTGAATTTTCCGGAGGTTAGCGGAATATGTCATGTAGCGGTGAAATGCTTAGATATGACATAGAACACCAATTGCGAAGGCAGCTAACTACAGGGACATTGACACTGATGCACGAAAGCGTGAGGATCAAACAGG

>9f298142518816b0c54e327bca7fd762

TACGAAGGGTGCAAGCGTTGCTCGGAATTATTGGGCGTAAAGGGTAGGTAGGTGGTTACGTATGTCTGGGGTGAAATCCCTGAGCTCAACTCAGGAAGTGCCTTGGAAACGGCGTAACTAGAGTGCTAGAGAGGTTCGTAGAATTCCCAGTGTAGCGGTGAAATGCGTAGAGATTGGGAGGAATACCAGAGGCGAAGGCGGCGAACTGGATAGCAACTGACACTAAACTACGAAAGCGTGGGGAGCAAACAGG

>11397adbcc3ae7ea2d586775e298a19d

TACGGAGGGAGCTAGCGTTGTTCGGAATTACTGGGCGTAAAGCGCACGTAGGCGGCTTTGTAAGTCAGAGGTGAAAGCCTGGAGCTCAACTCCAGAACTGCCTTTGAGACTGCATCGCTTGAATCCAGGAGAGGTGAGTGGAATTCCGAGTGTAGAGGTGAAATTCGTAGATATTCGGAAGAACACCAGTGGCGAAGGCGGCTCACTGGACTGGTATTGACGCTGAGGTGCGAAAGCGTGGGGAGCAAACAGG

>beae9d228d51980ddcc01cb4118aec8b

CATTGAACTATCGTGAGAAAGTCAAACCGCCAAAGGGAATTATATTATAGTAAATATTGGCGTAAATAAACATTTTATTAATAGTTGTAATATATGAAAATGTGCAGAAGATAAAGACGATTAAAACGGTGTTTAACAATCGCTTATCGAGACACTATTAAAGTAATACGGAATTAAAATATGTTTATTATGTGTATTAAACAAGGGATAAACTTGAAAATAAATACAGTGGTAAACGGAACTAATAGAGTCACGCAAGTGAACTTAGAACATTTTTAATAAC

>7ff346973a282aa55de296afdb5d74af

TACGTAGGGCGCAAGCGTTATCCGGAATTATTGGGCGTAAAGAGCTCGTAGGCGGTTTGTCGCGTCTGCCGTGAAAGTCCGGGGCTCAACTCCGGATCTGCGGTGGGTACGGGCAGACTAGAGTGATGTAGGGGAGACTGGAATTCCTGGTGTAGCGGTGAAATGCGCAGATATCAGGAGGAACACCGATGGCGAAGGCAGGTCTCTGGGCATTAACTGACGCTGAGGAGCGAAAGCATGGGGAGCGAACAGG

>9e1b1776cd5973bca311645149a85b87

TACGTAGGTGGCAAGCGTTATCCGGAATTATTGGGCGTAAAGCGCGCGTAGGCGGTTTCTTAAGTCTGATGTGAAAGCCCACGGCTCAACCGTGGAGGGTCATTGGAAACTGGGAAACTTGAGTGCAGGAGAGGAAAGTGGAATTCCATGTGTAGCGGTGAAATGCGCAGAGATATGGAGGAACACCAGTGGCGAAGGCGACTTTCTGGTCTGCAACTGACGCTGATGTGCGAAAGCGTGGGGATCAAACAGG

>0b65a254095e0b913622c9f3f3cffa77

ATATGGTAATTGTGTGCCAGCCGCCGCGGTAATAGTGATGTTTTGGCTGACTTTATCTGCTGCTTTGTTACAGTCAAGCTTAATAAAACCACCATAAACACACACATTAAGTAAGATGCTAACTCAAGTCACACACAACAATACATTAGAAACCCCTGTAGTCCGGCTGACTGACTTGCGTCAAATCTCGTATGCCGTCTTCTGC

>cd9401a6bce4a63af516d06d2a843f9d

TACGTAGGTGGCAAGCGTTGTCCGGAATTATTGGGCGTAAAGCGCGCGCAGGCGGATCAGTCAGTCTGTCTTAAAAGTTCGGGGCTTAACCCCGTGATGGGATGGAAACTGCTGATCTAGAGTATCGGAGAGGAAAGTGGAATTCCTAGTGTAGCGGTGAAATGCGTAGATATTAGGAAGAACACCAGTGGCGAAGGCGACTTTCTGGACGAAAACTGACGCTGAGGCGCGAAAGCCAGGGGAGCGAACGGG

>9d9b0d36c5a09b4e2e47cead02b088d1

TTGTGTGCCAGCCGCCGCGGTAACCGCTGTGTTCAGTGTGATATACTTTTTACAATACACAATAACAGATGCCACAAGCCATACACACACACACATACATAGAAAGTCGTAGAACGCAAACACAGTCATATAATTTTACCGTACATTAGAAACCCCTGTAGTCCGGCTGACTGACTTGCGTCAAATCTCGTATGCC

>2393ace9b1cc38db9d01fbe354f1a381

TACGGAGGGGGCGAGCGTTATTCGGAATTATTGGGCGTAAAGGGCGCGTAGGCGGCCCGGTAAGTCAAAGGTGAAATCCCTCGGCTCAACTGAGGAACTGCCTTTGAAACTGTCGGGCTTGAGGCCGGGAGAGGGTAGTGGAATTCCCAGTGTAGCGGTGAAATGCGTAGATATTGGGAGGAACACCAGTGGCGAAGGCGGCTACCTGGACCGGTTCTGACGCTGAGGCGCGAAAGCGTGGGTAGCAAACAGG

>848d74306b3228ab32c2d91ade582c2b

CATTGAACTATCGTGAGAAAGTCAAACCGCCAAAGGGAATTATATTATAGTAAATATTGGCGTAAATAAACATTTTATTAATAGTTGTAATATATGAAAATGTGCAAAAGATAGAGACGATTAAAACGGTGTTTAACAATCGCTTATCGAGACAATATTTAATTAATTAGGAAATAAAATATATTTATTATTTGTATTTAACAAGGAATAAACGTGAAAATAAATACAGTGATAAACGGAACTAATAGAGTCACGCAAGTGAACTTAGAACAATTTTAAGAAC

>32dbc894defe0ac4e0baed9f8514584f

TACGTAGGTGGCAAGCGTTGTCCGGAATTATTGGGCGTAAAGGGCGTGTAGGTGGATTCTTAAGTCGTGTGTTTAAGTGCGGTGCTCAACACCGTATGGGCGCAGGAAACTGGGAATCTTGAGTGCAGGAGAGGAAAGTGGAATTCCCAGTGTAGCGGTGAAATGCGTAGATATTGGGAGGAACACCAGTGGCGAAGGCGACTTTCTGGACTGTGTCTGACACTGAGGCGCGAAAGCCAGGGGAGCGAACGGG

>f32490b8845851fe9be12251653f85f3

TACGTAGGGTGCAAGCGTTGTCCGGAATTATTGGGCGTAAAGAGCTCGTAGGCGGTTTGTCGCGTCTGCTGTGAAATCTGGGGGCTCAACCCCCAGCCTGCAGTGGGTACGGGCAGACTAGAGTGCGGTAGGGGAGATTGGAATTCCTGGTGTAGCGGTGGAATGCGCAGATATCAGGAGGAACACCGATGGCGAAGGCAGATCTCTGGGCCGTTACTGACGCTGAGGAGCGAAAGCATGGGGAGCGAACAGG

>e0cf9a8c30c0d50842d5d117372e5bda

AACAGAGGATACAAGCGTTATCCGGATTTATTGGGTTTAAAGGGTGCGTAGGTTGTTTTTTAAGTCAGTAGTGAAATCTTAAAGCTTAACTTTAAAAGTGCTATTGATACTGATAAACTAGAGTGAGGTTGGAGTAACTGGAATGTGTGGTGGAGCGGTGAAATGCATAGAGATCACACAGAACACCGATCGCGAAAGCAGATTACTAAACCTAGACTGACACTGAGGCACGAAAGCATGGGTAGCAAACAGG

>54085c3cb5c6bd0f50fa818ff400b887

CATTGAACTATCGTGAGAATGGCACGCCGCCAAAGGGAATTATATTATAGTAAATATTGGCGTAAATAAACATTTTATTAATAGTTGTAATATATGAAAATGTGCAGAAGATAAAGACGATTAAAACGGTGTTTAACAATCGCTTATCGAGACAATATTTAATTAATTAGGAAATAAAATCAGTGTATTATTTGTATTTAACAAGGAATAAACGTGAAAATAAATACAGTGGTAAACGGAACTAATAGAGTCACGCAAGTGAACTTAGAACAATTTTAAGAACATTAGAACTAATGGAAA

>a60f0b4bc20e5a162b45b3d2ed7a0d6c

TACGAAGGGGGCTAGCGTTGCTCGGAATTACTGGGCGTAAAGGGAGCGTAGGCGGACTGTTAAGTTAGAGGTGAAAGCCCAGGGCTCAACCTTGGAATTGCCTTTGATACTGGCAGTCTTGAGTACGGAAGAGGTATGTGGAACTCCGAGTGTAGAGGTGAAATTCGTAGATATTCGGAAGAACACCAGTGGCGAAGGCGACATACTGGTCCGTTACTGACGCTGAGGCTCGAAAGCGTGGGGAGCAAACAGG

>47464f7e413b2cdf641574f20b15de85

TACGTAGGGTGCGAGCGTTGTCCGGAATTACTGGGCGTAAAGAGCTTGTAGGCGGCTTGTCGCGTCGATCGTGAAAACTTGGGGCTCAACCCCAAGCTTGCGGTCGATACGGGCAGGCTTGAGTACTTCAGGGGAGACTGGAATTCCTGGTGTAGCGGTGAAATGCGCAGATATCAGGAGGAACACCGGTGGCGAAGGCGGGTCTCTGGGAAGTAACTGACGCTGAGAAGCGAAAGCGTGGGTAGCGAACAGG

>0dea5f0ff03f1943100550c7fc0c6cfb

TACGTGAGAGACTAGTGTTATTCATCTTAATTGGGTTTAAAGGGTACCTAGACAGTCAATATAACTTCTAGAATGCTAATACTTGACTAGAGTTTTAAGTAAGAGGGAAGTACTTAAGGAGTAAGAGATGAAATATCTGTGATACCAAAGGGACTCCGTAAAGGCGAAGGCATCCCTTTATCTAAAAACTAACGTTGAAGGACGAAGGCTTAGATAACAAATAGG

>e6dea759bfdcb42032cc1bab6293e4a5

TACGTGAGAGACTAGTGTTATTCATCTTAATTGGGTTTAAAGGGTACCTAGACAGTCAATATAACTTCTATAATGCTAATACTTGACTAGAGTTTTAAGTAAGAGGGTAGTACTTAAGGAGTAAGAGATGAAATATCTGTGATACCAAAGGGACTCCGTAAAGGCGAAGGCATCCCTTTATCTAAAAACTAACGTTGAAGGACGAAGGCTTAGATAACAAATAGG

>d916beb8812b7a0fb34ea7f569deb8b1

CATTGAACTATCGTGAGAACGGCACGCCGCCAAAGGGAATTATATTATAGTAAATATTGGCGTAAATAAACATTTTATTAATAGTTGTAATATATAAAAATGTGCAGAAGATAAAGACGATGAAAACGGTGTTTAACAATCGCTTATCGAGACAATATTTAATTAATTAGGAATTAAAATATGTGTATTATTTGTATTTAACAAGGAATAATCTTGAAAATAAATACAGTGGTAAACGGAACTAATAGAGTCACGCAAGTGAACTTAGAACAATTTTAAGAAC

>9d3b77775d4a589d67b1fccffef238f5

AACAGAGGATACAAGCGTTATCCGGATTTATTGGGTTTAAAGGGCGCGTAGGTGGTTTTTTAAGTCAGTAGTGAAATCTTAAAGCTTAACTTTAAAAGTGCTATTGATACTGATAAACTAGAGTGAGGTTGGAGTAACTGGAATGTGTGGTGGAGCGGTGAAATGCATAGAGATCACACAGAACACCAATCGCGAAGGCATGTTACTAAACATAGACTGACACTGAGGCACGAAAGCATGGGTAGCAAACAGG

>04182ac4701cef02edd0ed5ae4cf403a

CCGCCGCGGTAATACGATTTCTTTAATTTAAATAGTTAAGTTTCAGTTAATATAACAATAATATAAAATATCTATAATTTTGGTGAAATATATTTTATCTTGAAAAATTAATTTTATGTCTGAAAAATTTTTATTTAAACTAGGATTAGAAACCCTTGTAGTCCGGCTGACTGACTCTATCGTGA

>1b8c624667a8843ce11eb1fb18cd9b78

TACGTAGGTGGCAAGCGTTGTCCGGAATTATTGGGCGTAAAGGGCGTGTAGGTGGATTCCTAAGTCGTGTGTCTAAGTGCGGTGCTCAACACCGTATGGGCGCAGGAAACTGGGAATCTTGAGTGCAGGAGAGGAAAGTGGAATTCCCAGTGTAGCGGTGAAATGCGTAGATATTGGGAGGAACACCAGTGGCGAAGGCGACTTTCTGGACTGTGTCTGACACTGAGGCGCGAAAGCCAGGGGAGCGAACGGG

>8674dcf0cfe35939037fa9a5b986866f

TACGTATGTCACAAGCGTTATCCGGATTTATTGGGCGTAAAGCGCGTCTAGGTGGTTATGTAAGTCTGATGTGAAAATGCAGGGCTCAACTCTGTATTGCGTTGGAAACTGCATGACTAGAGTACTGGAGAGGTAAGCGGAACTACAAGTGTAGAGGTGAAATTCGTAGATATTTGTAGGAATGCCGATGGGGAAGCCAGCTTACTGGACAGATACTGACGCTAAAGCGCGAAAGCGTGGGTAGCAAACAGG

>2b48539e2f50427a717d14f39ae05e5a

CATTGAACTATCGTGAGAAAGTCAAACCGCCAAAGGGAATTAGATTATAGTAAATATTGGCGTAAATAAACATTTTATTAATAGTTGTAATATATGAAAATGTGCAGAAGATAAAGACGATGAAAACGGTGTTTAACAATCGCTTATCGAGACAATATTTAATTAATTAGGAATTAAAATATGTGTATTATTTGTATTTAACAAGGAATAAACGTGAAAATAAATACAGTGGTAAACGGAACTAATAGAGTCACGCAAGTGAACGTAGAACATTTTTAATAAC

>58ab4257cbdb7c2021a954410d08ff84

TACGTAGGTGGCAAGCGTTGTCCGGATTTACTGGGCGTAAAGAGCGCGCAGGCGGTCGAGTAAGTCGAATGTGAAAGCCCCCGGCTTAACTGGGGAGGGTCATTCGATACTGTTCGACTTGAAGGCAGGAGAGGGCAGCAGAATTCCCGGTGTAGTGGTGAAATGCGTAGATATCGGGAGGAATACCAGTGGCGAAGGCGGCTGCCTGGCCTGTTCTTGACGCTGAGGCGCGAAAGCTGGGGGAGCAAACGGG

>9a92a0854df9a0e400e4c6e7006028b1

TACGAAGGGTGCAAGCGTTAATCGGAATTACTGGGCGTAAAGCGCGCGTAGGTGGTTCGTTAAGTTGGATGTGAAAGCCCCGGGCTCAACCTGGGAACTGCATCCAAAACTGGCGAGCTAGAGTATGGTAGAGGGTGGTGGAATTTCCTGTGTAGCGGTGAAATGCGTAGATATAGGAAGGAACACCAGTGGCGAAGGCGACCACCTGGGCTAATATTGACACTGAGGTGCGAAAGCGTGGGGAGCAAACAGG

>4466b22ec8b9ccfc7c145c892e67375f

CACCGATCTACTATGGTAATTGTGTGCCAGCCGCCGCGGTAAAGTAAACACAATATCTGGCAGGACTGCCACTCGTTACTTGATTCAGCACTCTCCTTATATAGTTTAGTTTAGATTCGTCTGTTTTACAGATTGACTCACTTGATTAGAAACCCGTGTAGTCCGGCTGACTGACTTAACGTCCATCTCGTATGCCGTCTTCTGCTTGAAAAAAA

>d090a9e25bd8d67082e07744d6eed107

AGTACCCTCAAGCGCACAGTCGCAAACTCCGCGGTCGTGAAAAGCGAACAATAATATCGACACGGCGTCGTTTATTCACGATCCGAAATCACACAAATAACCGAGTATTTTGCAGAGGTATAGTGCCAAGTAAGTAGTAGAGGTATTGTGCAGTGACCGCGTACTTGCGGAATTAATACGTTAATAAAG

>92d79de98aac2f495dd40bd0f89b2d36

CCGCCGCGGTAAGTGCTCAGAGAACATCAATCTTTCGCTTCGTTCTCACTTTCTCTTTTTACTGCCTCCTCTTCTGACCCTTTTCCACCATACCCCTTCTTCTCCTTTTCGTCATCCCTTTTCGGTCTATTTGTAGCATAATATATTAGAAACCCCTGTAGTCCGGCTGACTGACTTAACGTCCA

>efbe1f58b1e2984ddc53a64f047d94ff

TACGTAGGGTGCAAGCGTTAATCGGAATTACTGGGCGTAAAGCGTGCGCAGGCGGTTTTGTAAGTCTGATGTGAAATCCCCGGGCTCAACCTGGGAATTGCATTGGAGACTGCAAGGCTAGAATCTGGCAGAGGGGGGTAGAATTCCACGTGTAGCAGTGAAATGCGTAGATATGTGGAGGAACACCGATGGCGAAGGCAGCCCCCTGGGTCAAGATTGACGCTCATGCACGAAAGCGTGGGGAGCAAACAGG

>dc8395e8d78b7b9792b0033b6f27989f

CCGCCGCGGTAAGTGCTCAGAGAACATCAATCTTTCACTTCGTTCTCACTTTCTCTTTTTACTGCCTCCTCTTCTGACCCTTTTCCACCATACCCCTTCTTCTCCTTTTCGTCATCCCTTTTCGGTCTATTTGTAGCATAATATATTAGAAACCCCTGTAGTCCGGCTGACTGACTTGCGTCAAA

>69179c4a6011d0c9d30036cf18626520

TACAGAGGGTGCGAGCGTTAATCGGATTTACTGGGCGTAAAGCGTGCGTAGGCGGCTGATTAAGTCGGATGTGAAATCCCTGAGCTTAACTTAGGAATTGCATTCGATACTGGTCAGCTAGAGTATGGGAGAGGATGGTAGAATTCCAGGTGTAGCGGTGAAATGCGTAGAGATCTGGAGGAATACCGATGGCGAAGGCAGCCATCTGGCCTAATACTGACGCTGAGGTACGAAAGCATGGGGAGCAAACAGG

>b595f96f96fe7a346c01c042c2f6276d

TACGGAAGGTCCGGGCGTTATCCGGATTTATTGGGTTTAAAGGGAGCGTAGGCCGTGGATTAAGCGTGTTGTGAAATGTAGACGCTCAACGTCTGACTTGCAGCGCGAACTGGTCCACTTGAGTGTGCACAACGCAGGCGGAATTCGTCGTGTAGCGGTGAAATGCTTAGATATGACGAAGAACCCCGATTGCGAAGGCAGCTTGCGGGAGCACGACTGACGCTGAAGCTCGAAAGTGCGGGTATCGAACAGG

>b0a611aa28ac434a1cf5855141d464a2

TACGAAGGGGGCTAGCGTTGCTCGGAATTACTGGGCGTAAAGGGAGCGTAGGCGGACATTTAAGTCAGGGGTGAAATCCCGGGGCTCAACCTCGGAATTGCCTTTGATACTGGGTGTCTTGAGTATGAGAGAGGTATGTGGAACTCCGAGTGTAGAGGTGAAATTCGTAGATATTCGGAAGAACACCAGTGGCGAAGGCGACACACTGGCTCATCACTGACGCTGAGGCTCGAAAGCGTGGGGAGCAAACAGG

>4cfc7774316e88c062bfcf114e449d11

CCGTTGTCGATAGCAGTGAAAGAATGTAAACAGGATTATCGCTGGTAATTACCTGTACACTTGGCTATGGTATGACAATATTTCATGTAGTTGTACAGTATACTGTCGGTATAACGGACCGACGAACGACCTGCTCCATGCTGCGCCGTGCCGTGTCGTGTAATTAGAAACCC

>ce54fd570e2891bb1c24b6b985436151

TACGGAGGATGCGAGCGTTATCCGGAATCATTGGGTTTAAAGGGTCCGTAGGCGGGCTGATAAGTCAGAGGTGAAAGCGCTTAGCTCAACTAAGCAACTGCCTTTGAAACTGTCAGTCTTGAATGATTGTGAAGTAGTTGGAATGTGTAGTGTAGCGGTGAAATGCTTAGATATTACACAGAACACCGATAGCGAAGGCATATTACTAACAATTTATTGACGCTGATGGACGAAAGCGTGGGGAGCGAACAGG

>3ef44db1ee077cdee8c4917f9fb9a2ba

CATTTTACCATAGTGTCTGCCTCCACGGGCACGTTTATGGCTTTGCCGGTTATCTTTTAAGACTGTTTCTATTCTGAGACGACGGATCTGCATACGTGGAAATCCGGATGAAATCAGCCTCTCAGTGATACGATCGAGCGGCAGATGGGTCGGATCTCCCGGATACTTATAACTATTACTTTGGCTCGGGGGAGGCACTTTACCCTCATTAAGCAAGCGGCGGTATGTGGAGCACA

>ecc423b8fefd03a92aa1a97fc74a654c

TACGTAGGGTGCAAGCGTTAATCGGAATTACTGGGCGTAAAGCGTGCGCAGGCGGTTCGGAAAGAAAGATGTGAAATCCCAGAGCTTAACTTTGGAACTGCATTTTTAACTACCGGGCTAGAGTGTGTCAGAGGGAGGTGGAATTCCGCGTGTAGCAGTGAAATGCGTAGATATGCGGAGGAACACCGATGGCGAAGGCAGCCTCCTGGGATAACACTGACGCTCATGCACGAAAGCGTGGGGAGCAAACAGG

>19297cd60467685f9d2dc44e5064e8d5

TACGTAGGGTGCGAGCGTTGTCCGGAATTACTGGGCGTAAAGAGCTCGTAGGTGGTTTGTCGCGTCGTCTGTGAAATTCCGGGGCTTAACTCCGGGCGTGCAGGCGATACGGGCATAACTTGAGTGCTGTAGGGGAGACTGGAATTCCTGGTGTAGCGGTGAAATGCGCAGATATCAGGAGGAACACCGATGGCGAAGGCAGGTCTCTGGGCAGTTACTGACGCTGAGGAGCGAAAGCGTGGGGAGCGAACAGG

>e60f41f3d716cc3b8316a61509696136

TCGAACCCAAGCCACGTGGTGTAGCGAACCAACACACTACACACCACGAGTACTCCCCCCCCCTTCCAATCAACAAAAAATCACAATACGATTACAGGAAATTGTTCAGAATGTAGCGCCAAATGAAATTACAAAGAGGAGGATTTTGAATATTGCAGAAGGATCGTGCAAAACAAACGTTTGCTAGCAGCCTTCCTCACGTGTATACTGCACTGATGCTTCGAGAAATAGTCTGCAGAACTAT

>d83f60183d81253a505beaeef3cd168f

TACGTAGGGCGCGAGCGTTGTCCGGAATTATTGGGCGTAAAGAGCTTGTAGGCGGTTGGTCGCGTCTGCTGTGAAAGGCTGGGGCTTAACCCTGGTTTTGCAGTGGGTACGGGCTAACTAGAGTGCAGTAGGGGAGACTGGAATTCCTGGTGTAGCGGTGGAATGCGCAGATATCAGGAGGAACACCGATGGCGAAGGCAGGTCTCTGGGCTGTAACTGACGCTGAGAAGCGAAAGCATGGGGAGCGAACAGG

>b484195da5a669012008758c2f88fb90

TACGTAGGTGGCAAGCGTTGTCCGGAATTATTGGGCGTAAAGCGCGCGCAGGCGGCTTCCTAAGTCCATCTTAAAAGTGCGGGGCTTAACCCCGTGATGGGATGGAAACTGGGAAGCTGGAGTATCGGAGAGGAAAGTGGAATTCCTAGTGTAGCGGTGAAATGCGTAGAGATTAGGAAGAACACCGGTGGCGAAGGCGACTTTCTGGACGAAAACTGACGCTGAGGCGCGAAAGCGTGGGGAGCAAACAGG

>f0c5948956661168a06d9199ebce6d97

GCCGCCGCGGTAAGTGCTCAGAGAACATCAATCTTTCACTTCGTTCTCACTTTCTCTTTTTACTGCCTCCTCTTCTGACCCTTTCCACCATACCCCTGCTTCTCCTTTTCGTCATCCCTTTTCGGTCTATTTGTAGCATAATATATTAGATACCCTAGTAGTCCGGCTGACTGACTCTACGACCAT

>818434292143daca42a3168f3728da82

TACGTAGGTGGCAAGCGTTGTCCGGAATTATTGGGCGTAAAGCGCGCGCAGGCGGCTTCCCAAGTCCCTCTTAAAAGTGCGGGGCTTAACCCCGTGATGGGAAGGAAACTGGGAAGCTGGAGTATCGGAGAGGAAAGTGGAATTCCTAGTGTAGCGGTGAAATGCGTAGAGATTAGGAAGAACACCGGTGGCGAAGGCGACTTTCTGGACGAAAACTGACGCTGAGGCGCGAAAGCGTGGGGAGCAAACAGG

>4199a9d1e3ae3c6051624042525eff53

TATCCCCACGCGATGACGCAACTTTTTCCAGCTCGTTACCATGTTACGCTAAAGCCTACAATTTCCATCGTTGTAGCAATATTATGTCGTGAGTTAATATCACCACAGAGTGAATGAAAATTCAAACCAGAGACTGTATATAGGATAGCAATTGTTATCCAGTTACTAACTTACGCTTA

>9ac3bb1d7dedb08a012692a6f536b5af

TACGGAGGGAGCTAGCGTTATTCGGAATTACTGGGCGTAAAGCGCACGTAGGCGGCTTTGTAAGTAAGAGGTGAAAGCCCAGAGCTCAACTCTGGAATTGCCTTTTAGACTGCATCGCTTGAATCATGGAGAGGTCAGTGGAATTCCGAGTGTAGAGGTGAAATTCGTAGATATTCGGAAGAACACCAGTGGCGAAGGCGGCTGACTGGACATGTATTGACGCTGAGGTGCGAAAGCGTGGGGAGCAAACAGG

>e9d1b0f78e7779404faeb2553e7b363d

CCGCCGCGGTAAGTGCTCAGAGAACATCAATCTTTCGCTTCGTTCTCACTTTCTCTTTTTACTGCCTCCTCTTCTGACCCTTTTCCACCATACCCCTTCTTCTCCTTTTCGTCATCCCTTTTCGGTCTATTTGTAGCATAATATATTAGATACCCGAGTAGTCCGGCTGACTGACTTAACGTCCA

>6e05347007600c55ebcaf7c87cd20c8a

CATTGAGCTATCGTGAGAAAGTCAAGCCGCCAAAGGGAATTATATTATAGTAAATATTGGCGTAAATAAACATTTTATTAATAGTTGTAATATATGAAAATGTGCAGAAGATAAAGACGATTAGAACGGTGTGTAACAATCGCTTATCGAGACAATATTTAATTAATTAGTAATTAAAATATGTTTATTATGTGTATTATTCAAGGAATAGACTTGACAATAAATACAGTGGTAAACGGAACTAATAGAGTCACGCAAGTGAACTTAGAACATTTTTAATAAC

>5126bf96248d0acd00414de9bbddae18

TACGGAGGGTGCGAGCGTTAATCGGAATTACTGGGCGTAAAGCGCGCGTAGGCGGCTTGATAAGCCGGTTGTGAAAGCCCCGGGCTCAACCTGGGAACGGCATCCGGAACTGTCAGGCTAGAGTGCAGGAGAGGAAGGTAAAATTCCCGGTGTAGCGGTGAAATGCGTAGAGATCGGGAGGAATACCAGTGGCGAAGGCGGCCTTCTGGACTGACACTGACGCTGAGGTGCGAAAGCGTGGGTAGCAAACAGG

>4c72d551faabfeee82ae3dbcb5fc16af

TACGTAGGGTGCGAGCGTTAATCGGAATTACTGGGCGTAAAGCGTGCGCAGGCGGTTTTGTAAGACAGAGGGGAAATCCCCGGGCTCAACCTGGGAACTGCCTTTGTGACTGCAAGGCTAGAGTACGGCAGAGGGGGATGGAATTCCGCGTGTAGCAGTGAAATGCGTAGATATGCGGAGGAACACCGATGGCGAAGGCAATCCCCTGGGCCTGTACTGACGCTCATGCACGAAAGCGTGGGGAGCAAACAGG

>5dad507afd6cecb7185e8d8fe5fe4b4a

TACGGAAGGTCCAGGTGTTATCCGGATTTATTGGGTTTAAAGGGAGCGTAGGCCGCAGGTTAAGTGTGTTGTGAAAAGCAGTCGCCCAACGTCTGCCTTGCAGCGCAAACTGTCCTGCTTGAGTGCGCACAACGCAGGCGGAATTCGTCGTGTAGCGGTGAAATGCTTAGATATGACGAAGAACTCCGATTGCGAAGGCAGCTTGCGGGAGCGCAACTGACGCTGAAGCTCGAAAGTGCGGGTATCGAACAGG

>47adb64b99b84d903582fe4681df6652

AATTGTGTGCCAGCCGCCGCGGTAACACAATAGCACACAAAAACAGATTGGGCATCACACTGCGAAGAGAGAAGGAAGGGAATGCGTACTGGCAACCTTGCACTATTATTTGTATATCACTGGCTGTGACTGAAACTGCTTGCCATTAGATACCCGAGTAGTCCGGCTGACTGACTTAACGTCCATCTCGTATGCCGT

>80bc1b80f519a29c99b1ba7d61b8c8b5

CATTGAACTATCGTGAGAAAGTCAAACCACCAAAGGGAATTATATTATAGTAAATATTGGCGTAAATAAACATTTTATTAATAGTTGTAATATATGAAAATGTGCAGAATATAAAGACGATTAAAACGGTGTTTAACAATCGCTTATCGAGACAATATTTAATTAATTAGGAATTAAAATATGTGTATTATTTGTATTTAACAAGGAATAAACTTGAAAATAAATACAGTGGTGAACGGAACTAATAGAGTCACGCAAGTGAACTTAGAACAATTTTAAGAAC

>f330e765325ac47997066c79007f76b7

TACGTAGGGCGCAAGCGTTGTCCGGAATTATTGGGCGTAAAGAGCTTGTAGGTGGCTTGTCGCGTCTGCCGTGAAAACCCGAGGCTCAACCTCGGGCGTGCGGTGGGTACGGGCAGGCTAGAGTGTGGTAGGGGAGACTGGAACTCCTGGTGTAGCGGTGAAATGCGCAGATATCAGGAAGAACACCGATGGCGAAGGCAGGTCTCTGGGCCATTACTGACACTGAGAAGCGAAAGCATGGGTAGCGAACAGG

>68b097f6ca9b2923ef62ef4c1c70c7f6

GTAATTGTGTGCCAGCCGCCGCGGTAATACTGTATCCAAGCGTATAGACATCAAATTGATTGACAAATCTATCATATTGTCAAAGTATAGTAGGTATAAAATCATATAACTCGTATCACCTTCGGAAAAATAGTCAGACATTTAATTAGAAACCCGTGTAGTCCGGCTGACTGACTCTACGACCATCTCGTATGCCGTCT

>c06ccd1f7d57566ef669942328b1a946

TACAGAGGGTGCAAGCGTTAATCGGAATTACTGGGCGTAAAGCGCGCGTAGGTGGTTTGTTAAGTTGGATGTGAAAGCCCCGGGCTCAACCTGGGAACTGCATCCAAAACTGGCAAGCTAGAGTACGGTAGAGGGTGGTGGAATTTCCTGTGTAGCGGTGAAATGCGTAGATATAGGAAGGAACACCAGTGGCGAAGGCGACCACCTGGACTGATACTGACACTGAGGTGCGAAAGCGTGGGGAGCAAACAGG

>aabc2e854cb9f141633d1c47e8bcd2b5

TACGGAGGATGCGAGCGTTATCCGGATTTATTGGGTTTAAAGGGAGCGCAGACGGGATGTTAAGTCAGCTGTGAAAGTTCGGGGCTCAACCTTGAAATTGCAGTTGAAACTGTCGTTCTTGAGTGAGGTTGCGGTATGCGGAATTCGTGGTGTAGCGGTGAAATGCTTAGATATCACGAAGAACCCCGATTGCGAAGGCAGCATACCAACCCTTTACTGACGTTCATGCTCGAAAGTGCGGGTATCAAACAGG

>1354d8eddb9222983aca5a2fd2c6c7a5

TACGTAGGGTACGAGCGTTGTCCGGAATTATTGGGCGTAAAGAGCTCGTAGGTGGTTGGTCACGTCTGCTGTGGAAACGCAACGCTTAACGTTGCGCGTGCAGTGGGTACGGGCTGACTAGAGTGCAGTAGGGGAGTCTGGAATTCCTGGTGTAGCGGTGAAATGCGCAGATATCAGGAGGAACACCGGTGGCGAAGGCGGGACTCTGGGCTGTAACTGACACTGAGGAGCGAAAGCATGGGGAGCGAACAGG

>ebbb10981a3a293d3f4de12dda1b5afc

GTATGGTAATTGTGTGCCAGCAGCCGCGGTAATAGTGATGTTTTGGCTGACTTTATCTGCTGCTTTGTTACAGTCAAGCTTAATAAAACCACCATAAACACACACATTAAGTAAGATGCTAACTCAAGTCACACACAACAATACATTAGAAACCCCAGTAGTCCGGCTGACTGACTTAACGTCCATCTCGTATGCCGTCTTCTGC

>002cf1635c9bf31e15d56dd9c4b36a61

GCCGCCGCGGTAAGTGCTCAGAAAACATCAATCTTTCACTTCGTTCTCACTTTCTCTTTTTACTGCCTCCTCTTCTGACCCTTTTCCACCATACCCCTTCTTCTCCTTTTCGTCACCCCTTTCGGTCTATTTGTAGCATAATATATTAGATACCCGGGTAGTCCGGCTGACTGACTTAACGTCCAT

>4bdef84a0be0aa781e0b0721471e0504

CATTGAACTATCGTGAGAACAGCACGCCGCCAAAGGGAATTATATTATAGTAAATATTAGCGTAAATAAACATTTTATTAATAGTTGTAATATATGAAAATGTGCAGAAGATAGAGACGATGAAAACAGTGTTTAACAATCGCTTATCGAGACAATATTTAATTAATTAGGAATTAAAATATGTGTATTATTTGTATTTAACAAGGAATAATCTTGAAAATAAATACAGTGGTAAACGGAACTAATAGAGTCACGCAAGTGAACTTAGAACAATTTTAAGAAC

>2a391dcb404bfce52d9b99b178537607

GCCGCCGCGGTAAGTGCTCAGAGAACATCAATCTTTCACTTCGTTCTCACTTTCTCTTTTTACTGCCTCCTCTTCTGACCCTTTCCACCATACCCCTGCTTCTCCTTTTCGTCATCCCTTTTCGGTCTATTTGTAGCATAATATATTAGAAACCCTAGTAGTCCGGCTGACTGACTACTGTGTAAT

>b332b9a4d638a1f226e57c94a40f1450

TACGTAGGGGGCGAGCGTTATCCGGAATTATTGGGCGTAAAGAGTGCGTAGGTGGCATCTTAAGCGCAGGGTTTAAGGCAATGGCTCAACCATTGTTCGCCTTGCGAACTGGGGTGCTTGAGTGCAGGAGGGGAAAGTGGAATTCCTAGTGTAGCGGTGAAATGCGTAGATATTAGGAGGAACACCAGTGGCGAAGGCGACTTTCTGGACTGTTACTGACACTGAGGCACGAAAGCGTGGGGAGCAAACAGG

>0e354184678b0001165081f5c149654c

TTCCTCTTTTTTCAACGAAGTTCCGCAATTCATTTCTATTTGCTGCCATTCATTTGCCTATTTCAATAGATGCCTCTAGTTCGGAACGCTGATACTATACTATCGTTCCATGCGATTCTAGGTCTTCCTCTACAGTTTTTTTCTTTTTCCTGGTTTCCCATAGCTTTTTGACCAGTCTCTCTGTGCATGCTT

>6e5c5e8ab4e07833bd4a8ec94f14dcb9

TACGTAGGGGGCAAGCGTTATCCGGATTCATTGGGCGTAAAGCGCTCGTAGGCGGTCTGTTAGGTCGGGAGTTAAATCCGGAGGCTCAACCTCCGTTCGCTCCCGATACCGGCAGACTTGAGTTTGGTAGGGGAAGGTGGAATTCCTAGTGTAGCGGTGGAATGCGCAGATATTAGGAAGAACACCAGTGGCGAAGGCGGCCTTCTGGGCCATAACTGACGCTGAGGAGCGAAAGCTAGGGGAGCAAACAGG

>46edd9a681a1c394007859666f76d477

TACGGAGGGTGCAAGCGTTAATCGGAATTACTGGGCGTAAAGCGTGCGTAGGCGGTTCGTTAAGTCTGTTGTGAAAGCCCCGGGCTCAACCTGGGAATGGCAATGGATACTGGCGAGCTAGAGTGTGTCAGAGGATGGTGGAATTCCCGGTGTAGCGGTGAAATGCGTAGAGATCGGGAGGAACATCAGTGGCGAAGGCGGCCATCTGGGACAACACTGACGCTGAGGCACGAAAGCGTGGGGAGCAAACAGG

>dab3be67af28fccd7212a2ce2ee514c7

TACGTAGAAGACTAGTGTTAGTCATCTTTATTAGGTTTAAAGGGTACCTAGACGGTAAATAAAACTTGAATAGAGTACTTATTTACTAGAGTTTTATAAGAGAAGGAAGAACTTCTGGAGTAGTGATAGAATACGTTGATACCAGAGGGACTGGTCACGGCGAAGGCATCCTTCTATGTAAAAACTGACGTTGAGGGACGAAGGCTTGGGGATCAATCAGG

>730f71ab7b06a0ef53c8aa79db82f37c

TACGTAGGTGGCAAGCGTTGTCCGGAATTATTGGGCGTAAAGCGCGCGCCGGCGGTCTTTTAAGTCTGATGTGAAAGCCCCCGGCTCAACCGGGGAGGGTCATTGGAAACTGGGAGACTTGAGTACAGAAGAGGAGAGTGGAATTCCACGTGTAGCGGTGAAATGCGTAGATATGTGGAGGAACACCAGTGGCGAAGGCGACTCTCTGGTCTGTAACTGACGCTGAGGCGCGAAAGCGTGGGGAGCAAACAGG

>779080eec63d2579feb8b5620ae36c70

TACGAAGGGGGCTAGCGTTGCTCGGAATCACTGGGCGTAAAGGGCGCGTAGGCGGACTTTTAAGTCGGGGGTGAAAGCCCAGGGCTCAACCCTGGAATTGCCTTCGATACTGAGAGTCTTGAGTTCGGAAGAGGTTGGTGGAACTGCGAGTGTAGAGGTGAAATTCGTAGATATTCGCAAGAACACTAGTGGCGAAGGCGGCCAACTGGTCCGATACTGACGCTGAGGCGCGAAAGCGTGGGGAGCAAACAGG

>ff6e7f8bb549dd47275e303dbebb1413

TACAGAGGGTGCGAGCGTTGTTCGGAATTACTGGGCGTAAAGCGCGCGCAGGCGGTCCTTTAAGTCTACCCGTGAAATGCCGGAGCTCAACTCCGTGCACGCCGGGGGATACTGGAGGACTGGAGACGAGTAGAGGCAAGCGGAATTCCGGGTGTAGCGGTGGAATGCGTAGATATCCGGAAGAACACCGGAGGCGAAGGCGGCTTGCTGGGCTTGGTCTGACGCTGAGGCGCGAAAGCGTGGGGAGCGAACAGG

>377bf1b86dd15d576b1b9edd617843ce

TACGGAAGGTCCGGGCGTTATCCGGATTTATTGGGTTTAAAGGGAGCGTAGGCCGCCCCTTAAGCGTGTTGTGAAATGCGGGTGCTCAACATCCGACTTGCAGCGCGAACTGGGGGGCTTGAGTGCGCCGAAAGTAGGCGGAATTCGTGGTGTAGCGGTGAAATGCTTAGATATCACGAAGAACTCCGATTGCGAAGGCAGCTTACTGTAGCGCAACTGACGCTGATGCTCGAAAGCGTGGGTATCGAACAGG

>df69480b570421674819d9bf0acd2634

TACGTAGGGTGCGAGCGTTGTCCGGAATTACTGGGCGTAAAGAGCTCGTAGGTGGTTTGTCGCGTTGTTCGTGAAATCTCACGGCTTAACTGTGAGCGTGCGGGCGATACGGGCAGACTTGAGTACTGCAGGGGAGACTGGAATTCCTGGTGTAGCGGTGGAATGCGCAGATATCAGGAGGAACACCGGTGGCGAAGGCGGGTCTCTGGGCAGTAACTGACGCTGAGGAGCGAAAGCGTGGGGAGCGAACAGG

>10559e3647ae9bf0798642e54fc1703b

CCGCCGCGGTAAGTGCTCAAAGAACATCAATCTTTCACTTCGTTCTCACTTTCTCTTTTTACTGCCTCCTCTTCTGACCCTTTTCCACCATACCCCTTCTTCTCCTTTTCGTCATCCCTTTTCGGTCTATTTGTAGCATAATATATTAGAAACCCGAGTAGTCCGGCTGACTGACTTGCGTCAAA

>70ede84843f07cd5312e3f1a1e032d64

CCGCCGCGGTAAGTGCTCAAAGAACATCAATCTTTCACTTCGTTCTCACTTTCTCTTTTTACTGCCTCCTCTTCTGACCCTTTTCCACCATACCCCTTCTTCTCCTTTTCGTCATCCCTTTTCGGTCTATTTGTAGCATAATATATTAGAAACCCTGGTAGTCCGGCTGACTGACTTGCGTCAAA

>12ba4640fe403f2ce4da0d118299dd73

TACGGAGGGTGCGAGCGTTAATCGGAATTACTGGGCGTAAAGCGTACGCAGGCGGTCTGTTAAGTCAGATGTGAAATCCCCGGGCTCAACCTGGGAACTGCATTTGAAACTGGCAGGCTAGAGTCTAGTAGAGGGGGGTAGAATTCCAGGTGTAGCGGTGAAATGCGTAGAGATCTGGAGGAATACCGGTGGCGAAGGCGGCCCCCTGGACGAAGACTGACGCTCAGGTACGAAAGCGTGGGGAGCAAACAGG

>db40912a1211af5210dbc0dc5e6df9ec

AATGTATTGAATGAAGAATTGTCACAGTTAATAATCCCCCATGATAGTCGGCGACTTAAACAACCTGCACAACCCAATGTAGGACTGTAGTTCCCTTAATGCAAAAGATAGAATTATTGAGGAGCTAGTGGGTGCATTTCATTTAACTATTACCAACAAAAGGGATTAGAAAC

>e78d28b0c959b6e035caad98e3288205

TACAGAGGATGCAAGCGTTATCCGGAATGATTGGGCGTAAAGCGTCTGTAGGTGGCTTTTTAAGTCCGCCGTCAAATCCCAGGGCTCAACCCTGGACAGGCGGTGGAAACTACCAAGCTGGAGTACGGTAGGGGCAGAGGGAATTTCCGGTGGAGCGGTGAAATGCGTAGAGATCGGAAAGAACACCAACGGCGAAAGCACTCTGCTGGGCCGACACTGACACTGAGAGACGAAAGCTAGGGGAGCAAATGGG

>ac60a88cb0d31f5aeaccbc0cb1de0056

GCCGCGGTAATTAGAAACATCAGTATAGGCCGCCGAGTTGCTGACTCCCAGACATTGTACCTAGCGTGCGTCATACAGGTTCAATGATGGTTTCCATGAGTCCACATACGCTCCTGCACAAGCTCTACGCTCTCAATCGCGGTAATTAGAAACCCCTGTAGTCCGGCTGACTGACTTAACGTC

>2a93c128bebeb49dfd75221634f2b78a

GTAATTGTGTGCCAGCCGCCGCGGTAAGGTTTCTTGTATTCTTGTTAACTACTGAACATCCCCCGCCCTCACAACAATGGTGACATTAAGTGTTCGGATTACCTTTCCTTCTATTCCACTTTGAAAGAAGACATGCTGTGTATCATTAGAAACCCCAGTAGTCCGGCTGACTGACTTAACGTCCATCTCGTATGCCGTCT

>29c6d4e5a7916c805fd88cf76a78a25d

ATTGTGTGCCAGCCGCCGCGGTAATCAAACGAAAGCACAACAGAAAGATAGCCCAACTAATACCACTCCGATCACCAGCAATAACACCCACACATACGTTTTACCCATGAATAGTTAACCTCACCAACATAACATTCACTAACAATTAGAAACCCTAGTAGTCCGGCTGACTGACTTAACGTCCATCTCGTATGCCG

>7d135dfbf857c62673695ef24332b100

TACGTAGGGCGCGAGCGTTGTCCGGAATTATTGGGCGTAAAGAGCTTGTAGGCGGTTTGTCGCGTCTGCTGTGAAAGGCCGGAGCTTAACTCCGTGTATTGCAGTGGGTACGGGCAGACTAGAGTGCAGTAGGGGAGACTGGAATTCCTGGTGTAGCGGTGGAATGCGCAGATATCAGGAGGAACACCGATGGCGAAGGCAGGTCTCTGGGCTGTAACTGACGCTGAGAAGCGAAAGCATGGGGAGCGAACAGG

>6b34496ffed5d3ec3059bfe9d66997a4

TACGTAGGGTGCGAGCGTTAATCGGAATTACTGGGCGTAAAGCGTGCGCAGGCGGTTTGTTAAGACAGATGTGAAATCCCCGGGCTCAACCTGGGAACTGCATTTGTGACTGGCAGGCTAGAGTATGGCAGAGGGGGGTAGAATTCCACGTGTAGCAGTGAAATGCGTAGAGATGTGGAGGAATACCGATGGCGAAGGCAGCACCCTGGGCCAATACTGACGCTCATGCACGAAAGCGTGGGGAGCAAACAGG

>c0bc46cdfe8a98aa9680c373c4ba759f

CCAATGTATATGAACATAAAATATGTGTTACAGATGAAAAAAAATTCATACGTAAAACATACCCAACCCCGTTACATTACCAGGAACGGGTTGACACGGAAATTCAACAAATTTTAGAACAAGGTATCATAGAAAGATCCAGCAGTAACTTCTTGAATCACGTGGTCATTGTAAAGAAAACAATAATGATATTCGCTT

>ab7da83f9af71e40ddbeaff9054616a6

GGTTATTTTGATTTTGTGTGCGATCAGCTGTTCGTTTGTTGTAAAGACTGACTAAATGTTGTTCTGTGTTTTTGTAGGCCATATTGTGTGGAATGTGTTTTTTGTAAAATATGTGTTCAAAATATCGATGTTGTGAAAAACATTACTCGTGCTCATGTAATTTATTTCGCTGAACCCGCAATAGAGTAGCGAAACGCTACATTACTGTACTGAAAAAACAAGAAATTTTCGTTAGTTTGAG

>f7500dd3d8693f4087bc435ba9610a8e

CATTGAACTATCGTGAGAAAGTCAAACCGCCAAAGGGAATTATATTATAGTAAATATTGGCGTAAATAAACATTTTATTAATAGTTGTAATATATGAAAATGTGCAGAAGATAAAGACGATTAAAACGGTGTTTAACAATCGCTTATCGAGACAATATTTAATTAATTAGGAATTAAAATATGTGTATTATTTGTATTTAACAAGGAATAAACTTGAAAATAAATACAGTGTTAAACGGAACTAATAGAGTCACGCAAGTGAACTTAGAACATTTTAATAAC

>a9d4df91df1592ec7fa799c2c7ec9c44

TATTAGTGTTCCACTATTCTATATATCACTAAAAGTATTTTGCCACTCTTCATTAACATGTTTCTATGTTTGACCAATATAAAATTTGTTGCAATCATTGCCTTTAATTTTATAGATGCCATGTTTAGTGTATCATTGCAATTGTATATATTGTTTATATTTATATATAATCATATACAAACATATAATAGAATCATTTGACCCCAAAGGAATATGG

>54ff95ca73c3449b6cb046fba25572f8

TCCAATCTAATATTGCAGGAAACAAGCAATTACTCAGAGGACAACAAAATGCAGGTTGCGGAAATTGGATTCTATCAGGCTTTTGAAAAAGAAAGCCTATGGACGGCTGCGAATAAGGCTATTACCAAATATGCAGTCCCGAACCGAGGCGGCAGCAATTAGAAACCCCAGTA

>c495614b6767589987787cf62f1eb8ed

TACGAAGGGGGCTAGCGTTGCTCGGAATCACTGGGCGTAAAGGGCGCGTAGGCGGCCGATCAAGTCAGAGGTGAAAGCCCAGGGCTCAACCCTGGAATTGCCTTTGATACTGTTCGGCTCGAGACCGGAAGAGGTTAGTGGAACTGCGAGTGTAGAGGTGAAATTCGTAGATATTCGCAAGAACACCAGTGGCGAAGGCGGCTAACTGGTCCGGTTCTGACGCTGAGGCGCGAAAGCGTGGGGAGCAAACAGG

>04ec24b9d8d43d2ad4caad2293187a68

TACAGAGGGTGCAAGCGTTAATCGGAATTACTGGGCGTAAAGCGCGCGTAGGTGGTTCGTTAAGTTGGATGTGAAAGCCCCGGGCTCAACCTGGGAACTGCATCCAAAATTGGCGAGCTAGAGTATGGTAGAGGGTGGTGGAATTTCCTGTGTAGCGGTGAAATGCGTAGATATAGGAAGGAACACCAGTGGCGAAGGCGACCACCTGGACTGATACTGACACTGAGGTGCGAAAGCGTGGGGAGCAAACAGG

>3770abc7f233bbde7c0a771b64b7c75f

TACGTAGGGTGCGAGCGTTATCCGGAATTATTGGGCGTAAAGAGCTCGTAGGCGGTTTGTCGCGTCTGCCGTGAAAGTCCGGGGCTTAACTCCGGATCTGCGGTGGGTACGGGCAGACTAGAGTGCAGTAGGGGAGACTGGAATTCCTGGTGTAGCGGTGGAATGCGCAGATATCAGGAGGAACACCGATGGCGAAGGCAGGTCTCTGGGCTGTAACTGACGCTGAGGAGCGAAAGCATGGGGAGCGAACAGG

>23137dcb67bc19e124027f802aeca9a0

TACGGAGGATCCGAGCGTTATCCGGATTTATTGGGTTTAAAGGGTGCGTAGGCGGCATTTTAAGTCAGGGGTGAAATACAACGGCTCAACCGTTGCAGTGCCTTTGATACTGATATGCTTGAATGTGGTTGAAGAAGGCGGAATGAGACAAGTAGCGGTGAAATGCATAGATATGTCTCAGAACCCCGATTGCGAAGGCAGCTTTCTAAGCCATGATTGACGCTGATGCACGAAAGCGTGGGGATCGAACAGG

>30ad487e1e86be71afce4e03fd7553b5

TACGGAGGGTGCAAGCGTTAATCGGAATTACTGGGCGTAAAGCGCACGCAGGCGGCTTTTTAAGTCGGATGTGAAAGCCCCGGGCTCAACCTGGGAATTGCATCTGATACTGGGAAGCTAGAGTATGTGAGAGGGGGGTAGAATTCCAAGTGTAGCGGTGAAATGCGTAGAGATTTGGAGGAATACCAGTGGCGAAGGCGGCCCCCTGGCACAATACTGACGCTCAGGTGCGAAAGCGTGGGGAGCAAACAGG

>679ed482b0d7e4c3815adf517dd9f653

AAATGGCTACTTTCACGAGTGCAGAACGGGCTAACTGTGTGTTAAGGTTTCATGACACAAACTCTGCAACAACAGTTCAGCATAATTTTTGCACCGAGTGTGGTAAAGATCCTCTTACTAGACCTACAATTTACACTTGACCTCAGAACTTCGTTGAGAGTGGTTGTTCGGTTCAGC

>538366072ad100079c8eb083d277afc3

TACGAAGGGGGCTAGCGTTGTTCGGAATTACTGGGCGTAAAGCGAGCGTAGGTTGTGCCGCAAGTCAGGGGTGAAATCCCAGAGCTTAACTCTGGAACTGCCTTTGAAACTGCGGTGCTAGAATCTCAGAGGGGGTAGCGGAATTCCAAATGTAGGGGTGAAATCCGTAGATATTTGGAGGAACACCGGTGGCGAAGGCGGCTACCTGGATGAGTATTGACACTGAGGCTCGAAAGCGTGGGGATCAAACAGG

>28b02dc4fbd41d857ac49164e69550c5

CATTGAACTATCGTGAGAATGGCACGCCGCCAAAGGGAATTATATTATAGTAAATATTGGCGTAAATAAACATTTTATTAATAGTTGTAATATATGAAAATGTGCAGAAGATAGAGACGATTAAAACGGTGTTTAACAATCGCTTATCGAGACAATATTTAATTAATTAGGAATTAAAATATGTGTATTATTTGTATTTAACAAGGAATAAACGTGAAAGTAAATACAGTGGTGAACGGAACTAATAGAGTCACGCAAGTGAACTTAGAACAATTTTAAGAAC

>8a764c265972e8a53eb2561a58576e8d

AACAGAGGATACAAGCGTTATCCGGATTTATTGGGTTTAAAGGGTGCGTAGGTGGTTTTTTAAGTCAGTAGTGAAATCTTAAAGCTTAACTTTAAAAGTGCTATTGATACTGATAAACTAGAGTGAGGTTGGAGTAACTGGAATGTGTGGTGGAGCGGTGAAATGCATAGAGATCACACAGAACACCGATCGCGAAAGCAGATTACTAAACCTAGACTGACACTGAGGCACGAAAGCATGGGTAGCAAACAGG

>75e7f70d4d2bfddbac83e2b118062207

TACGGAGGGGGCTAGCGTTGTTCGGAATTACTGGGCGTAAAGCGCACGTAGGCGGATCGGAAAGTTGGGGGTGAAATCCCGGGGCTCAACCTCGGAACTGCCTTCAAAACTATCGGTCTTGAGTTCGAGAGAGGTGAGTGGAATTCCGAGTGTAGAGGTGAAATTCGTAGATATTCGGAGGAACACCAGTGGCGAAGGCGGCTCACTGGCTCGATACTGACGCTGAGGTGCGAAAGCGTGGGGAGCAAACAGG

>18fcc616a8927df65ec59717ef3ecc13

TACGGAGGGTGCAAGCGTTGTTCGGAATTATTGGGCGTAAAGCGCGTGCAGGCGGCTGTTCAAGTCCGATGTGAAAGCCCGGGGCTCAACCCCGGAAGTGCATTGGAAACTGGACAGCTTGAGTACGGGAGAGGGAGGCAGAATTCCGAGTGTAGGGGTGAAATCCGTAGATATTCGGAGGAATACCGGTGGCGAAGGCGGCCTCCTGGACCGATACTGACGCTGAGACGCGAAAGCGTGGGGAGCAAACAGG

>16763f54ebca109b358f66c9d3f7c377

TACACTGCGTCACTATGGTAATTGTGTGCCAGCAGCCGCGGTAAGAAGAGGAAATCTTCAAACATATGTTTCTCAGGAAGATGGCTTGCTTGTAAAATAGGAGAGGGCTTACCACTGAAGAGGTCGTTGACGAAAACGTTTCGAATTAGAAACCCCGGTAGTCCGGCTGACTGACTTGCGTCAAATCTCGTATGCCGTCTTCTGCTTGAAAAAAAAA

>c7ca630b8f24261de913a98f1f79e1a0

AAAAACTTCCACCTATGACAAAAATGTCAGTAAAACCGAAATTAAGGTGAGTTCATGCGACTCAGAATTTACCATTTCATAAAATAAGCCGCTGGGAAATCTGCTGTGAACTTGATACTATTAAACAAATACCGGCAACAGACTTGCTGTAACAAACGTATCACTTATTAGAA

>0fe33d5563c2d170da882ae1f98e7bb8

CCGCCGCGGTAATACGATTTCTTTAATTTAAATATTTAAGTTTCAGTTAATATAACAATAATATAAAATATCTAAAATTTTGGTGAAATATATTTTATCTTTAAAAATTAATTTTATGTCTGAAAAATTTTTGTATAAACTAGGATTAGATACCCCGGTAGTCCGGCTGACTGACTCTACGACCA

>52c2a5d32f96347976af71f685f90c09

GTAATTGTGTGCCAGCCGCCGCGGTAATACTGTATCCAAGCGTATAGACATCAAATTGATTGACAAATCTATCATATTGTCAAAGTATAGTAGGTATAAAATCATATAACTCGTATCACCTTCGGAAAAATAGTCAGACATTTAATTAGATACCCGTGTAGTCCGGCTGACTGACTTGCGTCAAATCTCGTATGCCGTCT

>94841eb1abc47baffbb689fff0eb9bf7

TACGTATGTCGCGAGCGTTATCCGGAATTATTGGGCATAAAGGGCATCTAGGCGGCCTTTCAAGTCAGGGGTGAAAACCTGCGGCTCAACCGCAGGCCTGCCTTTGAAACTGATAGGCTGGAGTACCGGAGAGGTGGACGGAACTGCACGAGTAGAGGTGAAATTCGTAGATATGTGCAGGAATGCCGATGATGAAGATAGTTCACAGGACGGTAACTGACGCTGAAGTGCGAAAGCCGGGGGAGCGAACAGG

>b210c32db542f4ab29440a54be0295ef

CATTGAGCTATCGTGAGAACGGCACGCCGCCAAAGGGAATTATATTATAGTAAATATTGGCGTAAATAAACATTTTATTAATAGTTGTAATATATGAAAATGTGCAGAAGATAAAGACGATGAAAACGGTGTTTAACAATCGCTTATCGAGACAATATTTAATTAATTAGGAA

>88861753d7404b48123942884ab38652

CATTGAACTATCGTGAGAAAGTCAAACCGCCAAAGGGAATTATATTATAGTAATTATTAGCGTTAATAAACATTTTATTAATAGTTGTAATATATGATAACGTGTAAAAGATAAAGACGATTCAAACAGTGGTTAATAATCGCGTATCGAGACACTATTTAATTAATTAGGAATTAAAATATGTTTATTATTTGTATTAAACAAGGGATAGACTTGAAAATAAATACAGTGTTAAACGGAACTAATAGAGTCACGCAAGTGAACTTAGAACATTTTTAATAAC

>ab275f6c36f126e34fae41949ca62170

CTGCGTCACTATGGTAATTGTGTGCCAGCAGCCGCGGTAACCTCCACTCCATAACGCAATCTTTTTATCAGTCTCGTCACTATCTCACGAAATCGCGTGCAATTCTCGTCGTCGAAACAATATAACTTGGATACATTGAAAACCATTAGAAACCCTAGTAGTCCGGCTGACTGACTTGCGTCAAATCTCGTATGCCGTCTTCTGCTTGAAAAA

>b6825773b8b549f2ab996dcd37e1bedd

TACGTATGTCGCAAGCGTTATCCGGAATTATTGGGCATAAAGGGCATCTAGGCGGCCAGGCAAGTCTGGGGTGAAAACTTGCGGCTCAACCGCAAGCCTGCCCTGGAAACTGCCTGGCTAGAGTGCTGGAGAGGTGGACGGAACTGCACGAGTAGAGGTGAAATTCGTAGATATGTGCAGGAATGCCGATGATGAAGATAGTTCACTGGACGGCAACTGACGCTGAAGTGCGAAAGCTGGGGGAGCGAACAGG

>4ef40ec631485eb074db32aed115a06f

TACGTAGGGCGCAAGCGTTATCCGGATTTATTGGGCGTAAAGAGCTCGTAGGCGGTTTGTCGCGTCTGCCGTGAAAGTCCGAGGCTCAACCTCGGATCTGCGGTGGGTACGGGCAGACTAGAGTGATGTAGGGGAGACTGGAATTCCTGGTGTAGCGGTGAAATGCGCAGATATCAGGAGGAACACCGATGGCGAAGGCAGGTCTCTGGGCATCTACTGACGCTGAGGAGCGAAAGCATGGGGAGCGAACAGG

>1a4149271dd161efcb232040a7557166

TACGTAGGGCGCGAGCGTTGTCCGGAATTATTGGGCGTAAAGGGCTTGTAGGCGGCTGGTCGCGTCTGCCGTGAAATCCTCTGGCTCAGCTGGGGGCGTGCGGTGGGTACGGGCTGGCTTGAGTGCGGTAGGGGAGGCTGGAACTCCTGGTGTAGCGGTGGAATGCGCAGATATCAGGAGGAACACCGGTGGCGAAGGCGGGTCTCTGGGCCGTTACTGACGCTGAGGAGCGAAAGCGTGGGGAGCGAACAGG

>ad14ae1c180c8422aa5d0de04c066ebe

CCAATGTATATGAACATAAAATATGTGTTACAGATGAAAAAAAATGCATACGTAAAACATACCCAATCCCGTTACATTACCAGGAACGGGTTGACACGGAAATTCAACGAATTTTAGAACAAGGTATCATAGAAAGATCCAGCAGTAACTTCTTGAATCCCGTGGTCATTGTAAAGAAAACAATAATGATATTCGCTT

>9cebfa8af90c0c9a0da688b24c60feb3

TAGTATGTATGGTAATTGTGTGCCAGCAGCCGCGGTAATGAGTGTGATATAATTTTACAATACACTATAACATTAGATACCACAAGCCACACACAGAAGTCGGGGGCGCAAACACAATCATATAATTTTACCATACGCTATAACATTAGAAACCCTAGTAGTCCGGCTGACTGACTCTACGACCATCTCGTATGCCGTCTTCTGCTTGAAA

>3e3c0e6499b429e4089a61c441ea7387

TACGTAAGAGACTAGCGTTATTCATCTTAATTAGGTTTAAAGGGTACTCAGACGGTCAATATAACAAAAAAATGTTATTACTTGACTAGAGTTATATATAAGAAGGTAGTACTTTAGGAGGAGAGATTATATTCAATGATACCGAAAGGACTCGGTAAAGGCGCAGGCAACCTTTTATGTAATAACTGACGTTGAAGGACGAAGGCATGGATCACGAACAGG

>06df978d30cdedfde52905d7734cb0d3

TACGGAGGATGCGAGCGTTATCCGGATTTACTGGGTTTAAAGGGTGCGTAGGCGGAAGATTAAGTCAGCGGTAAAAAAGCGGGGCTCAACCCCGTCGAGCCGTTGAAACTGGTTTTCTAGAGTGGGTGAGAAGTACGCGGAATGCGTGGTGTAGCGGTGAAATGCATAGATATCACGCAGAACTCCGATTGCGAAGGCAGCGTACCGGCGCCCGACTGACGCTGAAGCACGAAAGCGTGGGTATCGAACAGG

>e27c014a11fa55a0a9edf311c6c74a07

AATTGTGTGCCAGCCGCCGCGGTAACATTGAACTATCGTGAGAATGGCACGCCGCCAAAGGGAATTATATTATAGTAAATATTAGCGTAAATAAACATTTTATTAATAGTTGTAATATATGAAAATGTGCAGAAGATAGAGACGATTAGAAACCCTTGTAGTCCGGCTGACTGACTTAACGTCCATCTCGTATGCCGT

>e4422ae6a8bdecad84d32a8b2e232c1d

CACACGCGTGATATGGTAATTGTGTGCCAGCAGCCGCGGTAAAGTAAACACAATATCTGGCAGGACTGCCACTCGTTACTTGATTCAGCACTCTCCTTATATAGTTTAGTTTAGATTCGTCTGTTTTACAGATTGACTCACTTGATTAGAAACCCCAGTAGTCCGGCTGACTGACTTAACGTCCATCTCGTATGCCGTCTTCTGCTTGAAAAAAA

>6fdce5a1459b63bd2b6c53ff5ed249af

CACGCGTGATATGGTAATTGTGTGCCAGCCGCCGCGGTAACACACATTGTTATTGTTGTTGTGATGTTTATATATGTGTAGGTTTTAATTTATGTTTATTTTTTGGCCTTTGGAAATGTTTTTAATTAGTTTGAGGTTGTTGGGATTAGAAACCCCGGTAGTCCGGCTGACTGACTTAACGTCCATCTCGTATGCCGTCTTCTGCTTGAAAAA

>aae3b661c8f24dfdca5df170fa139b94

GCCGCCGCGGTAAGTGCTCAGAGAACATCAATCTTTCACTTCGTTCTCACTTTCTCTTTTTACTGCCTCCTCTTCTGACCCTTTCCACCATACCCCTGCTTCTCCTTTTCGTCATCCCTTTTCGGTCTATTTGTAGCATAATATATTAGAAACCCTAGTAGTCCGGCTGACTGACTGAGACTTAAT

>698d81bb69da8029c15325c912cf4cb4

GTATGGTAATTGTGTGCCAGCCGCCGCGGTAATAGTGATGTTTTGGCTGACTTTATCTGCTGCTTTGTTACAGTCAAGCTTAATAAAACCACCATAAACACACACATTAAGTAAGATGCTAACTCAAGTCACACACAACAATACATTAGAAACCCTGGTAGTCCGGCTGACTGACTCTACGACCATCTCGTATGCCGTCTTCTGC

>325c0e5d26531e42956a36e9169fd635

GACAGAGGGTGCAAACGTTGTTCGGAATTACTGGGCGTAAAGCGTGTGTAGGCGGTGAGGTAAGTCGGATGTGAAAGCCCAGGGCTCAACCCTGGAAGTGCACTCGATACTGCTTCGCTTGAGTACTGGAGAGGTTGGTGGAATTCTCGGTGTAGAGGTGAAATTCGTAGATATCGAGAGGAACATCTGTGGCGAAGGCGGCCAACTGGACAGATACTGACGCTGAGACACGAAAGTGCGGGGAGCAAACAGG

>e36ef41408f4589f6a5e1c2f51e0c6c3

TACGGAGGGTGCGAGCGTTAATCGGAATTACTGGGCGTAAAGCGCGCGTAGGCGGCTTGATAAGCCGGTTGTGAGAGCCCCGGGCTCAACCTGGGAACGGCATCCGGAACTGTCAGGCTAGAGTGCAGGAGAGGAAGGTAGAATTCCCGGTGTAGCGGTGAAATGCGTAGAGATCGGGAGGAATACCAGTGGCGAAGGCGGCCTTCTGGACTGACACTGACGCTGAGGTGCGAAAGCGTGGGTAGCAAACAGG

>ed47dfb9dd37e678dc7a23c1bd1a56c1

TACGTAGGGTGCGAGCGTTAATCGGAATTACTGGGCGTAAAGCGTGCGCAGGCGGTTGTGTAAGACAGGCGTGAAATCCCCGGGCTCAACCTGGGAATGGGGCTTGTGACTGCACGGCTGGAGTGCGGCAGAGGGGGATGGAATTCCGCGTGTAGCAGTGAAATGCGTAGATATGCGGAGGAACACCGATGGCGAAGGCAATCCCCTGGGCCTGCACTGACGCTCATGCACGAAAGCGTGGGGAGCAAACAGG

>fbc11377615606d77a26963029f0af35

TTCTTACTGCAAACGTATTGTCACACACATGTTTTGTAGTAATTATTGTGAGCAGCATCCTCTATGTTATAAATGTAATAAGGAAATCTTAAAACAATGATAATAATCTGTTTATAGTAATTAATCTTCATCATCTTCGCCATTCCAGGATTAGATACCCCGGTAGTCCGGCT

>9cfbdb16e1a5b9d3862e3901a97e4f61

AAATGGCTACTTTCACGGGTGCAGTACGGGCTAACTGTGTGTTAAGGTCTCATGACACAAACTCTGCAACAACAGTTCAGCATAATTTTTGCACCGAGTGTGGTAAAGATCCTCTTACTAGACCTACAATTTACACTTGACCTCAGAACTTCGTTGAGAGTGGTTGTTCGGTTCAGC

>a727d6ea618966f9dba1292093324e65

CCGTTGTCGATTGCAGTGAAAGAATGTAAACAGGATTATCGCTGGTAATTACCTGTACACTTGGCTATGGTATGACAATATTTCATGTAGTTGTACAGTATACTGTCGGTATAACGGACCGACGAGCGACCTGCTCCATGCTGCGCCGTGCCGTGTCGTGTAATTAGAAACCC

>a33b29fec8bf83f6b46f6c23602dd83e

CCGCCGCGGTAATACGATTTCTTTAATTTAAATATTTAAGTTTCAGTTAATAAAACAATAATATAAAATATCTATAATTTTGGTGAAATATATTTTATTTTTAAAAATTAATTTTATGTCTGAAAAATTTTTGTATAAACTAGGATTAGATACCCGTGTAGTCCGGCTGACTGACTGAGACTTAA

>431add7da20321d6e15dace7524ba3b2

TACGTAAGGACCGAGCGTTGTCCGGAATCATTGGGCGTAAAGGGTACGTAGGCGGTTAAAAAAGTTAGAAGTGAAAGGCTATAGCTCAACTATAGTAAGCTTTTAAAACTGTTTAACTTGAGAGATGGAAGGGAAAGTGGAATTCCTAGTGTAGCGGTGAAATGCGCAGATATTAGGAGGAATACCGGTGGCGAAGGCGACTTTCTGGCCATTATCTGACGCTGAGGTACGAAAGCGTGGGTAGCAAACAGG

>d3e62e2ba3ac9a85a44f236500a04d27

TACGTAGGGTGCGAGCGTTGTCCGGAATTATTGGGCGTAAAGAGCTTGTAGGCGGTTTGTCGCGTCTGCTGTGAAAATTCAGGGCTTAACCCTGGACGTGCAGTGGGTACGGGCAGACTAGAGTGTGGTAGGGGAGACTGGAATTCCTGGTGTAGCGGTGGAATGCGCAGATATCAGGAGGAACACCGATGGCGAAGGCAGGTCTCTGGGCCATTACTGACGCTGAGAAGCGAAAGCATGGGGAGCGAACAGG

>ba63d9bbf74ad6417d998e68e875485e

CACACTTATGTCTTTAGAAACACAAAACATTTCTCCAGAATGCATTTCAACCATGTCGGTCATTTTCAACTAGCAGTGCCTAGATAGTTGTGGAAGATTTTGAATATGTCTTGTCCAAAGAGTTCAGTTTGTTCATTAATGATTTTGTTATGTAGCCTATTATGTTTAGACTTTCAAGTAAATTC

>374b724806294b861c4ed5cfb74e2f31

TACGAAGGGGGCTAGCGTTGCTCGGAATCACTGGGCGTAAAGGGCGCGTAGGCGGCCATTCAAGTCGGGGGTGAAAGCCTGTGGCTCAACCACAGAATTGCCTTCGATACTGTTTGGCTTGAGTTTGGTAGAGGTTGGTGGAACTGCGAGTGTAGAGGTGAAATTCGTAGATATTCGCAAGAACACCAGTGGCGAAGGCGGCCAACTGGACCAACACTGACGCTGAGGCGCGAAAGCGTGGGGAGCAAACAGG

>cb0ac164712deb1fa251a08b0b2c96d3

TACGGAGGGTCCGAGCGTTATCCGGAATCATTGGGTTTAAAGGGTCCGTAGGCGGCTTTATAAGTCAGTGGTGAAATCCGGCAGCTCAACTGTCGAACTGCCATTGATACTGTAGGGCTTGAATTATTGTGAAGTAACTAGAATATGTAGTGTAGCGGTGAAATGCTTAGATATTACATGGAATACCAATTGCGAAGGCAGGTTACTAACAATTGATTGACGCTGATGGACGAAAGCGTGGGGAGCGAACAGG

>67a5c29ddb5d1eeda2b8780bb4694235

TACGTAGGGGGCAAGCGTTATCCGGATTTACTGGGTGTAAAGGGAGCGCAGACGGCAATGCAAGTCTGAAGTGAAAGGCGTGGGCTCAACCCATGAACTGCTTTGGAAACTGTATAGCTTGAGTGTCGGAGGGGTAAGCGGAATTCCTAGTGTAGCGGTGAAATGCGTAGATATTAGGAGGAACACCGGAGGCGAAGGCGGCTTACTGGACGACAACTGACGTTGAGGCTCGAAGGCGTGGGGAGCAAACAGG

>2bbb28b70c3a3757e48663f4d0003ecc

TACGGAGGGAGCTAGCGTTGTTCGGAATTACTGGGCGTAAAGCGCACGTAGGCGGCTATTCAAGTCAGAGGTGAAAGCCCGGGGCTCAACCCCGGAACTGCCTTTGAAACTAGATAGCTTGAATCCAGGAGAGGTGAGTGGAATTCCGAGTGTAGAGGTGAAATTCGTAGATATTCGGAAGAACACCAGTGGCGAAGGCGGCTCACTGGACTGGTATTGACGCTGAGGTGCGAAAGCGTGGGGAGCAAACAGG

>47c369f949de40a5db3532220542a0a1

TACGGAGGGTGCAAGCGTTGTTCGGAATTATTGGGCGTAAAGCGCGTGCAGGCGGCTGTTCAAGTCCGATGTGAAAGCCCGGGGCTCAACCCCGGAAGTGCATTGGAAACTGGACAGCTTGAGTACGGGAGAGGGAGGTAGAATTCCGAGTGTAGGGGTGAAATCCGTAGATATTCGGAGGAATACCGGTGGCGAAGGCGGCCTCCTGGACCGATACTGACGCTGAGACGTGAAAGCGTGGGGAGCAAACAGG

>1e104b993416995498346089f57fd3bf

TACGTAGGGTGCGAGCGTTAATCGGAATTACTGGGCGTAAAGCGTGCGCAGGCGGTTTGGCAAGTCAGATGTGAAATCCCCGAGCTCAACTTGGGAACTGCGTTTGAAACTGCCAGACTAGAATATGTCAGAGGGGGGTAGAATTCCACGTGTAGCAGTGAAATGCGTAGAGATGTGGAGGAATACCAATGGCGAAGGCAGCCCCCTGGGATAATATTGACGCTCATGCACGAAAGCGTGGGGAGCAAACAGG

>e2c3426bc84fd0c2b7f64a4ad2f29b58

AACAGAGGATACAAGCGTTATCCGGATTTATTGGGTTTAAAGGGTGCGTAGGTGGTTTTTTAAGTCAGTAGTGAAATCTTAAAGCTTAACTTTAAAAGTGCTATTGATACTGATAAACTAGAGTGAGGTTGGAGTAACTGGAATGTGTGGTGGAGCGGTGAAATGCATAGAGATCACACAGAACACCAATCGCGAAGGCATGTTACTAAACATAGACTGACATTGAGGCACGAAAGCACGGGTAGCAAACAGG

>6add585ceb4b4d22dc542a521200cdf1

TACGAAGGGGGCTAGCGTTGCTCGGAATCACTGGGCGTAAAGGGCGCGTAGGCGGCGTTTTAAGTCGGGGGTGAAAGCCTGTGGCTCAACCACAGAATTGCCTTCGATACTGGGACGCTTGAGTCTGGTAGAGGTTGGTGGAACTGCGAGTGTAGAGGTGAAATTCGTAGATATTCGCAAGAACACCGGTGGCGAAGGCGGCCAACTGGACCAGTACTGACGCTGAGGCGCGAAAGCGTGGGGAGCAAACAGG

>8cea79d5c6b8ef42f25e65b177f67d74

GCCGCCGCGGTAAGTGCTCAGAGAACATCAATCTTTCACTTCGTTCTCACTTTCTCTTTTTACTGCCTCCTCTTCTGACCCTTTCCACCATACCCCTGCTTCTCCTTTTCGTCATCCCTTTTCGGTCTATTTGTAGCATAATATATTAGAAACCCCGGTAGTCCGGCTGACTGACTCTACGACCAT

>40016f47c525598d361577c93057edaf

GACGGGGGATGCAAGTGTTATCCGGAATCACTGGGCGTAAAGCGTCTGCAGGTGGTTTCCTAAGTCCACTGTTAAACCTTGAGGCTCAACCTCAAATCTGCATTGGAAACTAGGAGACTTGAGTATAGTAGGGGTAGAGGGAATTTCCAGTGGAGCGGTGAAATGCGTAGATATTGGAAAGAACACCGATGGCGAAGGCACTCTACTGGGCTATTACTGACACTCAGAGACGAAAGCTAGGGGAGCAAATGGG

>ce8362a05fdbfb38fa0988439b94b5e5

GTAATTGTGTGCCAGCCGCCGCGGTAATACTGTATCCAAGCATATAGACATCAAATTGATTGACAAATCTATCATATTGTCAAAGTATAGTAGGTATAAAATCATATAACTCGTATCAGCTTCGGAAAAATAGTCAGACATTTAATTAGAAACCCCTGTAGTCCGGCTGACTGACTTAACGTCCATCTCGTATGCCGTCT

>f9f3a83cc1792a24cf5b3f7349760042

CATTGAACAATCGTGAGAAAGTCACGCCGCCAAAGGGAATTATATAATAGTAAATATTAGCGTAAATAAACATTTTATTAATAGTTGTAATATATGATAATGTGCAAAAGATAGAGACGATTAAAACGGTGTTTAACAATCGCTTATCGAGACACTATTAAAGTAATTAGGAATTAAAATATGTTTATTATGTGTATTATTCAAGGAATAAACTTGAAAATAAATACAGTGTTAAACGGAACTAATAGAGTCACGCAAGTGAACTTAGAACATTTTAATAAC

>738f003227e018683046142adddfc0aa

TTCTTACTGCAAAATATTGTCACACACATATGTTTTGTAGTAATTATTGTGAGCAGCATCCTCTATGTTATAAATGCCATAAGGAAATCTTAAAACAATGATAATAATCTGTTTATAGTAATTATTCTTCATCATCTTCGCTATTCCTGGATTAGAAACCCCTGTAGTCCGGC

>f4e78e2e397dc8bd3ca2f87c245a8773

TACGTATGTCACAAGCGTTATCCGGATTTATTGGGCGTAAAGCGCGTCTAGGTGGTTATGTAAGTCTGATGTGAAAATGCAGGGCTCAACTCTGTATTGCGTTGGAAACTGTGTAACTAGAGTACTGGAGAGGTAAGCGGAACTACAAGTGTAGAGGTGAAATTCGTAGATATTTGTAGGAATGCCGATGGGGAAGCCAGCTTACTGGACAGATACTGACGCTGAAGCGCGAAAGCGTGGGTAGCAAACAGG

>39fc135c2cc21e227e3b0d31b5a72eef

CCGCCGCGGTAAGTGCTCAGAGAACATCAATCTTTCGCTTCGTTCTCACTTTCTCTTTTTACTGCCTCCTCTTCTGACCCTTTTCCACCATACCCCTTCTTCTCCTTTTCGTCATCCCTTTTCGGTCTATTTGTAGCATAATATATTAGATACCCTGGTAGTCCGGCTGACTGACTTAACGTCCA

>40786ecf8338731efe349d38d1431a20

AACAGAGGATACAAGCGTTATCCGGATTTATTGGGTTTAAAGGGTGCGTAGGTGGTTTTTTAAGTCAGTAGTGAAATCTTAAAGCTTAACTTTAAAAGTGCTATTGATACTGATAAACTAGAGTGAGGTTGGAGTAACTGGAATGTGTGGTGGAGCGGTGAAATGCATAGAGATCACACAGAACACCAATCGCAAAGGCATGTTACTAAACATAGACTGACACTGAGGCACGAAAGCATGGGTAGCAAACAGG

>da63f3f4e4c20cf97baef9e525079ed3

TAGTATGTATGGTAATTGTGTGCCAGCCGCCGCGGTAATGAGTGTGATATAATTTTACAATACACTATAACATTAGATACCACAAGCCACACACAGAAGTCGGGGGCGCAAACACAATCATATAATTTTACCATACGCTATAACATTAGATACCCCGGTAGTCCGGCTGACTGACTTAACGTCCATCTCGTATGCCGTCTTCTGCTTGAAA

>68262884f1021c896f8e1bf7348d773c

TACGGAGGATCCAAGCGTTATCCGGAATCATTGGGTTTAAAGGGTCCGTAGGCGGTTTAATAAGTCAGTGGTGAAAGCCCATCGCTCAACGGTGGAACGGCCATTGATACTGTTAAACTTGAATTATTAGGAAGTAACTAGAATATGTAGTGTAGCGGTGAAATGCTTAGAGATTACATGGAATACCAATTGCGAAGGCAGGTTACTACTAATGGATTGACGCTGATGGACGAAAGCGTGGGTAGCGAACAGG

>f7ef16c8a6ac4fcc8afca7b8a60d48ca

TACGGAAGGTCCGGGCGTTATCCGGATTTATTGGGTTTAAAGGGAGCGTAGGCCGGTGATTAAGCGTGTTGTGAAATGTAGACGCTCAACGTCTGACTTGCAGCGCGAACTGGTCGCCTTGAGTGCGCAGGAAGTTGGCGGAATTCGTGGTGTAGCGGTGAAATGCTTAGATATCACGAAGAACTCCGATTGCGAAGGCAGCTGACTGTAGCGTAACTGACGCTGAAGCTCGAAAGTGTGGGTATCGAACAGG

>511bca3d23292383d80675d057c027a3

CATTGAACTATCGTGAGAAAGTCAAACCGCCAAAGGGAATTATATTATAGTAAATATTGGCGTAAATAAACATTTTATTAATAGTTGTAATATATGAAAATGTGCAGAAGATAAAGACGATTAAAACGGTGTTTAACAATCGCTTATCGAGACAATATTTAATTAATTAGGAATTAAAATATGTGTATTATTTGTATTTAACAAGGAATAAACTTGAAAATAAATACAGTGGTAAACGGAACTAATAGAGTCACGCAAGTGAACGTAGAACATTTTTAATAAC

>ab6ed56eb14b7f11af61772a418f9556

CGCGAATCGATTCGACACGGAAAACGCGTAAATATTAGCAACGACACGTTCGAGTAATGATGCTTCGGTTCGTTTGGATTTGAAATACTCAGCCACCCTTTAAAAAATTATATTATCTGTCTACATATGTCAGATGAAAAAAAACTTAAAAGTATTTGAAAGGAAGATAATTA

>75791bf8a774ded855fd8ead2e6105b1

CATTGAACTATCGTGAGAATGGCACGCCGCCAAAGGGAATTATATTATAGTAAATATTAGCGTAAATAAACATGTTATTAATAGTTGTAATATATGAAAATGTGCAGAAGATAGAGACGATTAAAACAGTGTTTAACAATCGCTTATCGAGACAATATTTAATTAATTAGGAATTAAAATATGTGTATTATTTGTATTTAACAAGGAATAAACGTGAAAATAAATACAGTGGTAAACGGAACTAATAGAGTCACGCAAGTGAACTTAGAACAATTTTAAGAACACTAGAACTAATGGAAA

>4e0763702795e6afb59e7d22281a0099

TACGTAGGTGGCAAGCGTTATCCGGAATTATTGGGCGTAAAGCGCGCGTAGGCGGTTTTTTAAGTCTGATGTGAAAGCCCACGGCTCAACCGTGGAGGGTCATTGGAAACTGAAAAACTTGAGTGCAGAAGAGGAAAGTGGAATTCCATGTGTAGCGGTGAAATGCGCAGAGATATGGAGGAACACCAGTGGCGAAGGCGACTTTCTGGTCTGTAACTGACGCTGATGTGCGAAAGCGTGGGGATCAAACAGG

>c8f94a4eab8129bde6b26db3083e48b5

TAGTATGTATGGTAATTGTGTGCCAGCCGCCGCGGTAATGAGTGTGATATAATTTTACAATACACTATAACATTAGATACCACAAGCCACACACAGAAGTCGGGGGCGCAAACACAATCATATAATTTTACCATACGCTATAACATTAGAAACCCCTGTAGTCCGGCTGACTGACTCTACGACCATCTCGTATGCCGTCTTCTGCTTGAAA

>5b16ef448b903be87c41c1d1821c765f

CACACTTATGTCTTTAGAAAGGCAAAACGTTTTTGCTAAATTCATTTAAACTTTCTAGGTCATTTTGAACTGGCAGTGCCTTGTAGATAGTTTTGGAAGATTTTGAATGTTGCTTGTCCATAGAGATCAGTTTGTTCATTAATGATTTTGTTGTGTAGTTTGTAACGTAATATATTTAAACTTTCAAGAAGACTAATTTTATTTACGTTTTTGCCTGTTGTA

>f818e66ed1979726fe827feb517a5a31

TACGGAGGGTCCGAGCGTTAATCGGAATTACTGGGCGTAAAGCGTGCGCAGGCGGTTTGTTAAGCGAGATGTGAAAGCCCTGGGCTCAACCTAGGAATAGCATTTCGAACTGGCGAACTAGAGTCTTGTAGAGGGGGGTAGAATTCCAAGTGTAGCGGTGAAATGCGTAGAGATCTGGAGGAATACCGGTGGCGAAGGCGGCCCCCTGGACAAAGACTGACGCTCATGCACGAAAGCGTGGGGAGCAAACAGG

>7665993ad96ae8bd43bb0c6e4ec4502b

TTCTTACTGCAAACGTATTGTCACACACATATGTTTTGTAGTAATTATTGTGAGCAGCATCCTCTATGTTATAAATGTAATAAGGAAATCTTAATGATAATAATCTGTTTATAGTAATTAATCTTCATCATCTTCGCCATTCCAGGATTAGATACCCCTGTAGTCCGGCTGAC

>1110b00319d07d3afcd3a94fe2d51c42

TACGTAGGTGGCAAGCGTTGTCCGGAATTATTGGGCGTAAAGCGCGCGCAGGCGGCCTCTTAAGTCTGATGTGAAAGCCCCCGGCTCAACCGGGGAGGGCCATTGGAAACTGGGAGGCTTGAGTATAGGAGAGAAGAGTGGAATTCCACGTGTAGCGGTGAAATGCGTAGAGATGTGGAGGAACACCAGTGGCGAAGGCGACTCTTTGGCCTATAACTGACGCTGAGGCGCGAAAGTGTGGGGAGCAAACAGG

>26f173c04574871aba8876838e2d9e61

TACGGAGGGAGCTAGCGTTGTTCGGAATTACTGGGCGTAAAGCGCGCGTAGGCGGTTACTCAAGTCAGAGGTGAAAGCCCGGGGCTCAACCCCGGAACTGCCTTTGAAACTAGGTGACTAGAATCTTGGAGAGGTCAGTGGAATTCCGAGTGTAGAGGTGAAATTCGTAGATATTCGGAAGAACACCAGTGGCGAAGGCGACTGACTGGACAAGTATTGACGCTGAGGTGCGAAAGCGTGGGGAGCAAACAGG

>dfcfc4140419717168404a5adca319a8

TACGTATGGTGCAAGCGTTATCCGGATTTACTGGGTGTAAAGGGAGCGTAGACGGAGAAGCAAGTCTGGAGTGAAAGCCCGGGGCTCAACCCCGGGACTGCTTTGGAAACTGTTTATCTAGAGTGCCGGAGAGGTAAGCGGAATTCCTAGTGTAGCGGTGAAATGCGTAGATATTAGGAGGAACACCAGTGGCGAAGGCGGCTTACTGGACGGTAACTGACGTTGAGGCTCGAAAGCGTGGGGAGCAAACAGG

>df02ecd857b270cb1c282703650907d6

CACCATTATTAAAATATTTTTAAATTTATAGACTTTATGATTTTATATTTCTGTTGTAATTTCCGTATTAATAAAAATATTTAAATTTGTTCTCTTTTACAACATATTATACACGTGTATAAAATTAATAATTAATTATCAGTTTCAATTAGTGTAATTATTTAGATGTTCAA

>0ff52ecd45be79c478d82cfd5bd2e9cd

TACAGAGGGTGCGAGCGTTAATCGGATTTACTGGGCGTAAAGCGTGCGTAGGCGGCTTTTTAAGTCGGATGTGAAATCCCTGAGCTTAACTTAGGAATTGCATTCGATACTGGAAAGCTAGAGTATGGGAGAGGATGGTAGAATTCCAGGTGTAGCGGTGAAATGCGTAGAGATCTGGAGGAATACCGATGGCGAAGGCAGCCATCTGGCCTAATACTGACGCTGAGGTACGAAAGCATGGGGAGCAAACAGG

>4a3e948d85d5201615c41f6748e47a51

GCCGCCGCGGTAAGTGCTCAGAAAACATCAATCTTTCACTTCGTTCTCACTTTCTCTTTTTACTGCCTCCTCTTCTGACCCTTTTCCACCATACCCCTTCTTCTCCTTTTCGTCACCCCTTTCGGTCTATTTGTAGCATAATATATTAGAAACCCGTGTAGTCCGGCTGACTGACTTAACGTCCAT

>f3ac0741950df4896fc7f1fb5169f82f

TTCTTACTGCAAACGTATTGTCACACACATGTGTTTTGTAGTAATTATTGTGAGCAGCATCCTCTATGTTATAAATGTAATAAGGAAATCTTAAAACAATGATAATAATCTGTTTATAGTAATTAATCTTCATCATCTTCGCCATTCCAGGATTAGAAACCCTAGTAGTCCGG

>d86f5036e548ddf4ca8fd965f05943ee

GGTATTGCAGCGACGCGATAGCGTTTTCCATTTACCGAAGTCGGAATATCTGGGGAAAGCGAAATATCTCTCAGACGCGTCTCCCAGCGAGATTTACAGGCGGCAGCAATCTTTTCAATAATTCATATAAAGTGAATTTTATTTCCATTAACCGGATAAAAAAACGTTGAACACGAGTGAAGTCGAATAACCGAGG

>6a01069f1ae10b08563e1d703dddb588

TTCTTACTGCAAACGTATTGTCACACACATATGTTTTGTAGTAATTATTGTGAGCAGCATCCTCTATGTCATAAATGTAATAAGGAAATCTTAAAACAATGATAATAATCTGTTTATAGTAATTAATCTTTATCATCTTCGCCATTCCAGGATTAGATACCCCGGTAGTCCGG

>794b95faf321cb7a172a3349d3a15464

TTAAACGAAAGCACAACAAAAAGATAGCCAAACTAATACCACCCCAATCACCAGCAATAACACCCACACATACGTTTTACCCAAGAATAGTCAACCTAACCAACGTAACATTCACTAACGAACAGAAACAACTTTTAAACAAAGGGGTAAACCACAACCTACACTACACACAGAATAATAACACTATCAAGAACATGGT

>51a014156b3a7d273878c862e9caaf38

TACGAAGGGTGCGAGCGTTAATCGGAATTACTGGGCGTAAAGCGCGCGTAGGTGGTTTGTTAAGTTGGAAGTGAAAGCCCCGGGCTCAACCTGGGAATTGCTTTCAAAACTGACAGGCTAGAGTACGGTAGAGGGTAGTGGAATTTCCTGTGTAGCGGTGAAATGCGTAGATATAGGAAGGAACATCAGTGGCGAAGGCGACTACCTGGACTGATACTGACACTGAGGTGCGAAAGCGTGGGGAGCAAACAGG

>33579a77351ec8c2bfb2710fd668b4a0

AGCCGCCGCGGTAACACTTTGGTAACGTATTGATTATGTTAGAGTTTGTGCGATACAGCAAGCCTTAAGAATAACGCAGATGCTTTTGTTCCCGCGCTCCAGTTCAAAGGTGCTACCACGCTGATGTTATTTCATTTCGACTTGATTAGATACCCCAGTAGTCCGGCTGACTGACTTAACGTCCATC

>785d67b0c0232c96a21b7bf1c46f0a79

TACGGAAGGTCCAGGCGTTATCCGGATTTATTGGGTTTAAAGGGAGCGTAGGCTGGAGATTAAGTGTGTTGTGAAATGTAGACGCTCAACGTCTGACTTGCAGCGCATACTGGTTTCCTTGAGTACGCACAACGTTGGCGGAATTCGTCGTGTAGCGGTGAAATGCTTAGATATGACGAAGAACTCCGATTGCGAAGGCAGCTGACGGGAGCGCAACTGACGCTTAAGCTCGAAGGTGCGGGTATCAAACAGG

>54409f477e5967dc3c2d64fd3e06def6

GGTAATTGTGTGCCAGCCGCCGCGGTAATTGACTGGGTGAGACCCACCGCAGTTGCAGCATGTTGCAGAGGTCTCCGGTGCGACCGGATAGTAGCCTCGGTGCTCTCCTAACGTCTCTGTGGAAAACGGCCAACGTCGTGATGGATTAGAAACCCCTGTAGTCCGGCTGACTGACTGAGACTTAATCTCGTATGCCGTCTT

>5648dccee530d68ceb3e4d7d22cf8756

TACAGAGGGTGCAAGCGTTAATCGGAATTACTGGGCGTAAAGCGCGCGTAGGTGGTTTGTTAAGTTGGATGTGAAATCCCCGGGCTCAACCTGGGAACTGCATTCAAAACTGACTGACTAGAGTATGGTAGAGGGTGGTGGAATTTCCTGTGTAGCGGTGAAATGCGTAGATATAGGAAGGAACACCAGTGGCGAAGGCGACCACCTGGACTAATACTGACACTGAGGTGCGAAAGCGTGGGGAGCAAACAGG

>f8dfff4af8c51cf71f3dd51f7735e3c8

GTATGGTAATTGTGTGCCAGCCGCCGCGGTAATAGTGATGTTTTGGCTGACTTTATCTGCTGCTTTGTTACAGTCAAGCTTAATAAAACCACCATAAACACACACATTAAGTAAGATGCTAACTCAAGTCACACACAACAATACATTAGAAACCCGTGTAGTCCGGCTGACTGACTTAACGTCCATCTCGTATGCCGTCTTCTGC

>6dd800e7beb1451c6a68b25e0fce6906

TACAGAGGGTGCGAGCGTTAATCGGATTTACTGGGCGTAAAGCGTGTGTAGGCGGCTTTTTAAGTCGGATGTGAAATCCCCGAGCTTAACTTGGGAATTGCATTCGATACTGGGAAGCTAGAGTATGGGAGAGGATGGTAGAATTCCAGGTGTAGCGGTGAAATGCGTAGAGATCTGGAGGAATACCGATGGCGAAGGCAGCCATCTGGCCTAATACTGACGCTGAGGTACGAAAGCATGGGGAGCAAACAGG

>7a621e5cf70d9efff12c13e0c1a6cf4a

TACGGAGGGTGCAAGCGTTACCCGGAATCACTGGGCGTAAAGGGCGTGTAGGCGGGAACTTAAGTCTGGTTTTAAAGACCGGGGCTCAACCTCGGGAGTGGACTGGATACTGGGTTTCTTGATCTCTGGAGAGGTAACCGGAATTCCTGGTGTAGCGGTGGAATGCGTAGATACCAGGAGGAACACCAATGGCGAAGGCAGGTTACTGGACAGAAGATGACGCTGAGGCGCGAAAGTGTGGGGAGCGAACCGG

>70792b6ac0d2c723cec8c12dc6576345

TACGTAGGGAGCAAGCGTTATCCGGATTTATTGGGTGTAAAGGGTGCGTAGACGGGAAGACAAGTTGGTTGTGAAATCCCTCGGCTTAACTGAGGAACTGCAACCAAAACTATCTTTCTTGAGTGCTGGAGAGGAAAGTGGAATTCCTAGTGTAGCGGTGAAATGCGTAGATATTAGGAGGAACACCAGTGGCGAAGGCGACTTTCTGGACAGTAACTGACGTTGAGGCACGAAAGTGTGGGGAGCAAACAGG

>775a3e8fda569f4f615202216a322657

CACACTTTTGTCTAAAGGAAATACAAACCATTTCTCCAGGATGCATTTCAACCATGTCGGTCATTTTCAACTGGCAGTGCCTTGTAGATATTTGTGAAAGATTTTGAATGTGTCTTGTCCATAGAGTTCAGTTTGTAAATTAATGATTTTGTTATGTAGTTTGTAACGTAATATATTTAGGCTTTTAAGTAAATTCATTTTATTTCCTTTTTTCTGGTTGTG

>d90df1e94e242c142303eeddf2180b73

TACGGAGGGAGCTAGCGTTGTTCGGAATTACTGGGCGTAAAGCGCACGTAGGCGGCTATTCAAGTCAGAGGTGAAAGCCCGGGTCTCAACCCCGGAACTGCCTTTGAAACTAGATAGCTTGAATCCAGGAGAGGTGAGTGGAATTCCGAGTGTAGAGGTGAAATTCGTAGATATTCGGAAGAACACCAGTGGCGAAGGCGGCTCACTGGACTGGTATTGACGCTGAGGTGCGAAAGCGTGGGGAGCAAACAGG

>d1227f8c4518c979a83c19ff5d94a4c8

CCGCCGCGGTAATACGATTTCTTTAATTTAAATAGTTAAGTTTCAGTTAATATAACAATAATATAAAATATCTATAATTTTGGTGAAATATATTTTATCTTGAAAAATTAATTTTATGTCTGAAAAATTTTTATTTAAACTAGGATTAGATACCCGTGTAGTCCGGCTGACTGACTCTATCGTGA

>f6e85fd21f5f073974fea0cf395612c8

TACGTAGGGTCCAAGCGTTAATCGGAATTACTGGGCGTAAAGCGTGCGCAGGCGGTTGTGCAAGACCGATGTGAAATCCCCGAGCTTAACCTGGGAATTGCATTGGTGACTGCACGGCTAGAGTGTGTCAGAGGGGGGTAGAATTCCACGTGTAGCAGTGAAATGCGTAGAGATGTGGAGGAATACCGATGGCGAAGGCAGCCCCCTGGGATAACACTGACGCTCATGCACGAAAGCGTGGGGAGCAAACAGG

>951abe2e2b6aa89969bbbd31a3485e53

CATTGAACTATCGTGAGAATGGCACGCCGCCAAAGGGAATTATATTATAGTAAATATTAGCGTAAATAAACATTTTATTAATAGTTGTAATATATGAAAATGTGCAGAAGATAGAGACGATTAAAACAGTGTTTAACAATCGCTTATCGAGACAATATTTAATTAATTAGGAAATAAAATAAGTGTATTATTTGTATTTAACGAGGAATAAACGTGAAAATAAATACAGTGGTAAACGGAACTAATAGAGTCACGCAAGTGAACTTAGAACATTTTTAATAAC

>d7348b86110af99c20176a0c0aa0697a

GACGTAGGATGCGAGCGTTGTCCGGATTTATTGGGCGTAAAGAGTTCGTAGGTGGTTTGTTAAGTTTGGTGTTAAAGATCGGGGCTCAACCCTGGGACTGCACTGAATACTGGCAGACTCGAGTGTGGTAGAGGCTAGTGGAATTCCCAGTGTAGCGGTGAAATGCGTAGATATTGGGAAGAACACCGGTGGCGTAGGCGACTAGCTGGGCCATAACTGACGCTGAGGAACGAAAGCCAGGGGAGCGAATGGG

>1b9175ea4e4774adc7da0c0e202e61d7

TACGTAAGGACCGAGCGTTGTCCGGAATCATTGGGCGTAAAGGGTACGTAGGCGGGTTTTTAAGTTAGAAGTCAAAGGCTATAGCTCAACTATAGTAAGCTTCTAAAACTGGGAACCTTGAGTAATGGAAGGGAAAGTGGAATTCCTAGTGTAGCGGTGGAATGCGCAGATATTAGGAGGAATACCGGTGGCGAAGGCGACTTTCTGGCCATTAACTGACGCTGAGGTACGAAAGCGTGGGTAGCAAACAGG

>770a2040ea0bf46ee7c3a4ae665d86e1

CGCCGCGGTAATTAATTTTATTGGGTTTGGTATTGGAAACGTGCAAGGATCGTTTCAGGAACCGATTCTCGCTGTCGTCTTTTCTTCTTCACGTTGTGAATTAGTTCTTCTTTAGTTGTAACCTGAAATTAGTTTTCCACACAGATTAGAAACCCCTGTAGTCCGGCTGACTGACTTGCGTCAA

>600aa8f6d3b7c7fb96265a2d87f52fbb

TACGGAGGATCCGAGCGTTATCCGGATTTATTGGGTTTAAAGGGTGCGTAGGCGGCCTATTAAGTCAGGGGTGAAATACGGTGGCTCAACCATCGCAGTGCTTTTGATACTGATGGGCTTGAATCCATTTGAAGTGGGCGGAATAAGACAAGTAGCGGTGAAATGCATAGATATGTCTTAGAACTCCGATTGCGAAGGCAGCTCACTAAGCTGGTATTGACGCTGATGCACGAAAGCGTGGGGATCGAACAGG

>b3d0710439d0837fa3a24ac97e0d797b

CATTGAACTATCGTGAGAAAGTCAAACCGCCAAAGGGAATTATATTATAGTAAATATTAGCGTAAATAAACATTTTATTAATAGTTGTAATATATGATAATGTGCAGAAGATAAAGACGATTAAAACGGTGTTTAACAATCGCTTATCGAGACAATATTTAATTAATTAGGAATTAAAATATATTTTATTATTTGTATTAAACAAGGGATAAACTTGAAAATAAATACAGTGGTAAACGGAACTAATAGAGTCACGCAAGTGAACTTAGAACATTTTAATAAC

>889f1bc31887efea2354ba099342a203

TACGTAGGGCGCAAGCGTTGTCCGGAATTATTGGGCGTAAAGAGCTCGTAGGCGGCTTGTCACGTCGGTTGTGAAAGCCCGGGGCTTAACCCCGGGTCTGCAGTCGATACGGGCAGGCTAGAGTTCGGTAGGGGAGATCGGAATTCCTGGTGTAGCGGTGAAATGCGCAGATATCAGGAGGAACACCGGTGGCGAAGGCGGATCTCTGGGCCGATACTGACGCTGAGGAGCGAAAGCGTGGGGAGCGAACAGG

>28163366802c945634e88adc721ea087

ACGAACCCAAGCCACGTGGTGTAGCGAACCAACACACTACACACCACGAGTACTCCCCCCCCCTTCCAATCAACAAAAAATCACCATACGATTACAGGAAATTGTTCAGAATGTAGCGCCAAATGAAATTACAAAGAGGAGGATTTTGAATATTGCAGAAGGATCGTATTGGTGCAAAACAAACGTTTGCTAGCAGCCTTCCTCACGTGTATACTGCACTGATGCTTCGAGAAATAGTCTGCAGAACTAT

>d8267365cb27b0f67dace5ca5408ebf8

CCGCCGCGGTAATACGATTTCTTTAATTTAAATATTTAAGTTTCAGTTAATATAACAATAATATAAAATATCTAAAATTTTGGTGAAATATATTTTATCTTTAAAAATTAATTTTATGTCTGAAAAATTTTTGTATAAACTAGGATTAGAAACCCGTGTAGTCCGGCTGACTGACTGAGACTTAA

>b27b9fd33fba649558c0b40a2ec64104

ATCTACACCTAGTATGTATGGTAATTGTGTGCCAGCCGCCGCGGTAAGTTAGTATACTAATGAAAATTTCCGCCAGCACATACATACGGAATTTTGAGCAAGTCCTCCATATGAAAATGAAGAAGTTCAGATAGAGTTAGTGACATTAGAAACCCCAGTAGTCCGGCTGACTGACTTAACGTCCATCTCGTATGCCGTCTTCTGCTTGAAAAAAAAAAAA

>abed5d6df643b81799d793e1b9f0de43

CATTGAACTATTGTGAGAACGGCACGCCGCCAAAGGGAATTATATTATAGTAAATATTAGCGTAAATAAACATGTTATTAATAGTTGTAATATATGAAAATGTGCAGAAGATAGAGACGATGAAAACGGTGTTTAACAATCGCTTATCGAGACAATATTTAATTAATTAGGAAATAAAATAAGTGTATTATTTGTATTTAACAAGAATTAAACGTGAAAATAAATACAGTGGGAAACGGAACTAATAGAGTCACGCAAGTGAACTTAGAACAATTTTAAGAACATTAGAACTAGTGGAAA

>81b14a06fe51fed6aaa311ff4e370125

TACGGAAGGTCCAGGCGTTATCCGGATTTATTGGGTTTAAAGGGAGTGTAGGCGGTCTGTTAAGCGTGTTGTGAAATTTAGGTGCTCAACATCTACCTTGCAGCGCGAACTGGCGGACTTGAGTGCACGCAACGTATGCGGAATTCATGGTGTAGCGGTGAAATGCTTAGATATCATGACGAACTCCGATTGCGAAGGCAGCGTACGGGAGTGTTACTGACGCTTAAGCTCGAAGGTGCGGGTATCGAACAGG

>e1ed2377ab464966d63a95a6ff8bde64

TACGGAAGGTCCGGGCGTTATCCGGATTTATTGGGTTTAAAGGGAGCGTAGGCCGGAGATTAAGCGTGTTGTGAAATGTAGACGCTCAACGTCTGCACTGCAGCGCGAACTGGTTTCCTTGAGTACGCATAAAGTGGGCGGAATTCGTGGTGTAGCGGTGAAATGCTTAGATATCACGAAGAACTCCGATTGCGAAGGCAGCTCACTGGGGCGCAACTGACGCTGAAGCTCGAAAGCGCGGGTATCGAACAGG

>2a6e139a799a8f59d777f6a5a6d8cc87

CCGCCGCGGTAAGTGCTCAAAGAACATCAATCTTTCACTTCGTTCTCACTTTCTCTTTTTACTGCCTCCTCTTCTGACCCTTTTCCACCATACCCCTTCCTCTCCTTTTCGTCATCCCTTTTCGGTCCATTTGCACCATAATATATTAGATACCCCGGTAGTCCGGCTGACTGACTCTACGACCA

>7ec431d6ffa3c3c88ab96ee181098173

GACGTAGGTGGCAAGCGTTACTCGGAATTACTAGGCGTAAAGCGCGCGTAGGCGGAATGTTAAGTCTGTTGTGTAATCTCTGGGCTCAACCCAGAAACTGCAACGGAAACTGGCGTTCTTGAGTGGGGCAGAGGAGATCGGAATTCCTAGTGTAGCAGTGAAATGCGTAGATATTAGGAGGAACACCGGTGGCGAAGGCGGATCTCTGGGCCTTTACTGACGCTCAAGTGCGAAAGCTAGGGGAGCAAACGGG

>be637003056d804c246ac272311b0d8e

CATTGAACTATCGTGAGAAAGTCAAACCGCCAAAGGGAATTATATTATAGTAAATATTGGCGTAAATAAACATTTTATTAATAGTTGTAATATATGAAAATGTGCAGAAGATAAAGACGATTAAAACGGTGTTTAACAATCGCTTATCGAGACAATATTTAATTAATTAGGAATTAAAATATATTTATTATTGGTATTAAACAAGGGATAAACTTGAAAATAAATACAGTGGTGAACGGAACTAATAGAGTCACGCAAGTGAACTTAGAACATTTTTAATAAC

>a52970705dfd5642085748f4646b3b18

GACGGGGGGGGCAAGTGTTCTTCGGAATGACTGGGCGTAAAGGGCACGTAGGCGGTGAATCGGGTTGAAAGTGAAAGTCGCCAAAAACTGGCGGAATGCTCTCGAAACCAATTCACTTGAGTGAGACAGAGGAGAGTGGAATTTCGTGTGTAGGGGTGAAATCCGGAGATCTACGAAGGAACGCCAAAAGCGAAGGCAGCTCTCTGGGTCCCTACCGACGCTGGGGTGCGAAAGCATGGGGAGCGAACAGG

>195ed9f12150fb68c2b8c83ef9eab20b

CACACTTATGTCTTCAGAAATACAAACCATTTCTCCAGGATGCATTTCAACCATGTCGGTCATTTTCAACTGTCAGTGCCTTGTAGATATTTGTGGAAGATTTTGAATGTGTCTTGTCCATGGAGTTCAGTTTGTTCATTAATGATTTTGTTGTGTAGTTTGTAACGTAATATATTTAGGGTTTTAAGTAAATTCATTTTATTTCCTTTGTGCCTGTTGTA

>6b3181ab556d3a132f531d0ed53bfbf8

CACCGATCTACTATGGTAATTGTGTGCCAGCAGCCGCGGTAAAGTAAACACAATATCTGGCAGGACTGCCACTCGTTACTTGATTCAGCACTCTCCTTATATAGTTTAGTTTAGATTCGTCTGTTTTACAGATTGACTCACTTGATTAGAAACCCCAGTAGTCCGGCTGACTGACTTAACGTCCATCTCGTATGCCGTCTTCTGCTTGAAAAAAA

>188d8b265b94c03ead936c3731b731c5

CACACTTATGTCTTCAGAAAGACAAAATATTTCTCCAGGATGCATTTCAACCTTGTCGGTCATTTTCAACTGGCAGTGCCTTGTGAAAGATTTGTGAAAGATTTTGAATGTGTATTGTCTATAGAGTTCAGTTTGTTCGTTAATGATCTTGTTACGTAGTTTGTAACGTAATATATTTAGGCTTTCAAGTAAATTCATTTTGTTTCCTATTTTGTTGGTTTTG

>409692e7997dd6c5eafcdc16e46806a3

TACGTAGGGTGCGAGCGTTAATCGGAATTACTGGACGTAAAGCGTGCGCAGGCGGTTATGTAAGACAGATGTGAAATCCCCGGGCTCAACCTGGGAACTGCATTAGTGACTGCATAGCTGGAGTACGGCAGAGGGGGATGGAATTCCGCGTGTAGCAGTGAAATGCGTAGATATGCGGAGGAACACCGATGGCGAAGGCAATCCCCTGGGCCTGTACTGACGCTCATGCACGAAAGCATGGGGAGCAAACAGG

>c0e995afeb5ae6c970a5e27244f76c8f

TACGTAGGTGGCAAGCGTTGTCCGGAATTATTGGGCGTAAAGCGCGCGCAGGCGGTCCTTTAAGTCTGATGTGAAAGCCCACGGCTCAACCGTGGAGGGTCATTGGAAACTGGGGGACTTGAGTGCAGGAGAGAAAAGTGGAATTCCACGTGTAGCGGTGAAATGCGTAGAGATGTGGAGGAACACCAGTGGCGAAGGCGACTTTTTGGCCTGTAACTGACGCTGAGGCGCGAAAGCGTGGGGAGCAAACAGG

>d42f97e4a6f95b799b346d1171fc2266

TACGTAGGGTGCGAGCGTTGTCCGGAATTACTGGGCGAAAAGAGCTCGTAGGTGGTTTGTCGCGTCGTCTGTGAAATTCCGGGGCTTAACTCCGGGCGTGCAGGCGATACGGGCATAACTTGAGTGCTGTAGGGGAGACTGGAATTCCTGGTGTAGCGGTGAAATGCGCAGATATCAGGAGGAACACCGATGGCGAAGGCAGGTCTCTGGGCAGTTACTGACGCTGAGGAGCGAAAGCATGGGTAGCGAACAGG

>7b6805fa54360eaa665b751f05b1cd75

TACGAAGGGGGCTAGCGTTGCTCGGAATTACTGGGCGTAAAGGGAGCGTAGGCGGACATTTAAGTCAGGGGTGAAATCCCGGGGCTCAACCTCGGAATTGCCTTTGATACTGGGTGTCTTGAGTATGAGAGAGGTGTGTGGAACTCCGAGTGTAGAGGTGAAATTCGTAGATATTCGGAAGAACACCAGTGGCTAAGGCGACACACTGGCTCATCACTGACGCTGAGGCTCGAAAGCGTGGGGAGCAAACAGG

>44bf9ea0e54b100f113ea6c7088ca27c

TGTGCCAGCAGCCGCGGTAAGCGTATTTCTAAATTTTACCTAAAATAGTGAACTGTAGGTCGTGTTTTAATTACAAAATCAGTTCTTTATTTGAGTCGTATTTACTCTTTTCTTTTTGTTAGAACTCGTCCTGTACCGTCAATCATTAGAAACCCGTGTAGTCCGGCTGACTGACTGAGACTTAATCTCGTAT

>9c4cbe504b82481eb23fa8c10d3bd353

TACGGAGGGGGCTAGCGTTGTTCGGAATTACTGGGCATAAAGCGCACGTAGGCGGACTGAAAAGTTGGGGGTGAAATCCCGGGGCTCAACCTCGGAACTGCCTTCAAAACTATCAGTCTGGAGTTCGAGAGAGGTGAGTGGAATTCCGAGTGTAAAGGTGAAATTCGTAGATATTCGGAGGAACACCAGTGGCGAAGGCGGCTCACTGGCTCGATACTGACGCTGAGGTGCGAAAGCGTGGGGAGCAAACAGG

>5bac75a444201fd2a3f12fd55ea2b644

TACGTAGGGTGCGAGCGTTGTCCGGAATTACTGGGCGTAAAGAGCTCGTAGGCGGTTTGTCGCGTCGTCTGTGAAATTCTGCAACTCAATTGCAGGCGTGCAGGCGATACGGGCAGACTTGAGTACTACAGGGGAGACTGGAATTCCTGGTGTAGCGGTGAAATGCGCAGATATCAGGAGGGACACCGGTGGCGAAGGCGGGTCTCTGGGTAGTAACTGACGCTGAGGAGCGAAAGCGTGGGTAGCGAACAGG

>662d853272e6601f43867adc2c95b0a5

TACGTAGGGTGCGAGCGTTGTCCGGAATTATTGGGCGTAAAGAGCTTGTAGGCGGTTTGTCACGTCTGCTGTGAAAATCCAGGGCTTAACCCTGGACGTGCAGTGGGTACGGGCAGACTAGAGTGTGGTAGGGGAGACTGGAATTCCTGGTGTAGCGGTGGAATGCGCAGATATCAGGAGGAACACCGATGGCGAAGGCAGGTCTCTGGGCCATTACTGACGCTGAGAAGCGAAAGCATGGGGAGCGAACAGG

>5d5db5a4b1cb701462bfd0989f5a3495

TACGTAGGGTGCGAGCGTTAATCGGAATTACTGGGCGTAAAGCGTGCGCAGGCGGTCTTGTAAGACAGAGGTGAAATCCCTGGGCTCAACCTAGGAATGGCCTTTGTGACTGCAAGGCTGGAGTGCGGCAGAGGGGGATGGAATTCCGCGTGTAGCAGTGAAATGCGTAGATATGCGGAGGAACACCGATGGCGAAGGCAGTCCCCTGGGCCTGCACTGACGCTCATGCACGAAAGCGTGGGGAGCAAACAGG

>f48cbba2fe7a2cba3df1c5bcffc1fa83

TACGTAGGGTGCAAGCGTTAATCGGAATTACTGGGCGTAAAGCGTGCGCAGGCGGTTTTGTAAGTCTGACGTGAAATCCCCGGGCTCAACCTGGGAATTGCGTTGGAGACTGCAAGGCTGGAGTCTGGCAGAGGGGGGTAGAATTCCACGTGTAGCAGTGAAATGCGTAGAGATGTGGAGGAACACCGATGGCGAAGGCAGCCCCCTGGGTCAAGACTGACGCTCATGCACGAAAGCGTGGGGAGCAAACAGG

>ea511b2afcf4ae0db15964799622bbda

TTCTTACTGCAAACGTATTGTCACACACATGTGTTTTGTAGTAATTATTGTGAGCAGCATCCTCTATGTTATAAATGTAATAAGGAAATCTTAAAACAATGATAATAATCTGTTTATAGTAATTAATCTTCATCATCTTCGCCATTCCAGGATTAGAAACCCCGGTAGTCCGG

>720ced0908670804bbdd97c7b023ba6d

GCCGCGGTAATTAGAAACATCAGTATAGGCCGCCGAGTTGCTGACTCCCAGACATTGTACCTAGCGTGCGTCATACAGGTTCAATGATGGTTTCCATGAGTCCACATACGCTCCTGCACAAGCTCTACGCTCTCAATCGCGGTAATTAGAAACCCCAGTAGTCCGGCTGACTGACTTAACGTC

>d601f9044fe6d26fcda66f11774c4302

GGACTGTATGTTGATTACAGAAAAAAAGTTTATGGAAAATGTTGATATTGTGTATGTTTTGTAATTTTGTTTGGGGATTATTTATCTATATGGTTGACCGTTTAATTCAATAAAAAGACTGTTGGATTTTGAAATAATACAAAGAATGTTTGGTATTATATTTAAACAGAATAATACAAAGATAAAGTTCTATTGAAAGGATATTTCTATTTTATAAGTTGTTTTATCTTATGAATAAATGATAGTATAAATTTCTTATTTACTTTAA

>ca529b13e80095fb80571ccb65e1e9c5

TACGAAGGGGGCTAGCGTTGCTCGGATTTACTGGGCGTAAAGGGCGCGTAGGCGGATAACCAAGTTGGGGGTGAAATCCCGGGGCTCAACCTCGGAACTGCCTCCAAAACTGGTGATCTTGAGTATGAGAGAGGTGTGTGGAACTCCGAGTGTAGAGGTGAAATTCGTAGATATTCGGAAGAACACCAGTGGCGAAGGCGACACACTGGCTCATTACTGACGCTGAGGCGCGAAAGCGTGGGGAGCAAACAGG

>9ac80fd69dc98dd15b882cbaf0451d0b

TACGGAGGGTGCGAGCGTTAATCGGAATTATTGGGCGTAAAGCGCGCGTAGGCGGCTTGATAAGCCGGTTGTGAAAGCCCCGGGCTCAACCTGGGAACGGCATCCGGAACTGTCAGGCTAGAGTGCAGGAGAGGAAGGTAGAATTCCCGGTGTAGCGGTGAAATGCGTAGAGATCGGGAGGAATACCAGTGGCGAAGGCGGCCTTCTGGACTGACACTGACGCTGAGGTGCGAAAGCGTGGGTAGCAAACAGG

>263e41ea2c25dc87bd2ff5910cf30d40

TACGTATGGTGCAAGCGTTATCCGGATTTACTGGGTGTAAAGGGAGCGCAGGCGGTGCGGCAAGTCTGATGTGAAAGCCCGGGGCTCAACCCCGGTACTGCATTGGAAACTGTCGTACTAGAGTGTCGGAGGGGTAAGCGGAATTCCTAGTGTAGCGGTGAAATGCGTAGATATTAGGAGGAACACCAGTGGCGAAGGCGGCTTACTGGACGATAACTGACGCTGAGGCTCGAAAGCGTGGGGAGCAAACAGG

>2cd7ff1790d44cf8ac26c6585567ac32

CATTGAACTATCGTGAGAAAGTCAAACCGCCAAAGGGAATTATATTATAGTAAATATTGGCGTAAATAAACATTTTATTAATAGTTGTAATATATGAAAATGTGCAGAAGATAAAGACGATTAAAACGGTGTTTAACAATCGCTTATCGAGACAATATTTAATTAATTAGGAATTAAAATATGTGTATTATTTGTATTTAACAAGGAATACACTTGAAAATAAATACAGTGTTAAACGGAACTAATAGAGTCACGCAAGTGAACTTAGAACATTTTTAATAAC

>0c11526dcc7b80980d4996816efb55e3

TACGAAGGGGGCTAGCGTTGTTCGGATTTACTGGGCGTAAAGCGCGCGTAGGCGGATAAATTAGTTAGAGGTGAAATCCCAGGGCTCAACCCTGGAACTGCCTTTAATACTGTTTATCTAGAGTATGAAAGAGGTGAGTGGAATTCCGAGTGTAGAGGTGAAATTCGTAGATATTCGGAGGAACATCAGTGGCGAAGGCGGCTCACTGGTTCATTACTGACGCTGAGGTGCGAAAGCGTGGGGAGCAAACAGG

>86f5d4fe7f3b412fa35950b701ad9efd

CATTTTACCATAGTGTCTGCCTCCACGGGCACGTTTATGGCTTTGCCGGTTATCTTTTAAGACTGTTTCTATTCTGAGACGACGGATCTGCATACGTGGGAATCCAGATGAAATCAGTCTCTCAGTGATACGATCGAGCGGCAGATGGGTCGGATCTTCCGGATACTTATAACTATTAATTTGGCTCGGGGGAGGCACTTTACCCTCATTAAGCAAGCGGCGGTATGTGGAGCACA

>56dbdce55042104250b95a52c2b34096

TACGAAGGGTGCAAGCGTTATTCGGATTTATTGGGCGTAAAGGGTGTGTAGGCGGTACACTAAGTCTATTGTTAAATGCTCCGGCCTAACCGGAGACATGCGGTGGAAACTGGTGTGCTGGAGGATGGAAGAGAGAAGTGGAATTCTCGGAGTAGCGGTAAAATGCGTAGATCTCGAGAGGAACACCAATGGCGAAGGCAGCTTCTTGGTCCATACCTGACGCTGAAACACGAAAGCGTGGGGAGCAAACAGG

>4c907ee2fbaf23ad1b5f0d226997b3d9

TACAGAGGGTGCAAGCGTTAATCGGAATTATTGGGCGTAAAGCGCGCGTAGGTGGTTCGTTAAGTTGGATGTGAAAGCCCCGGGCTCAACCTGGGAACTGCATCCAAAACTGGCGAGCTAGAGTATGGTAGAGGGTGGTGGAATTTCCTGTGTAGCGGTGAAATGCGTAGATATAGGAAGGAACACCAGTGGCGAAGGCGACCACCTGGACTGATACTGACACTGAGGTGCGAAAGCGTGGGGAGCAAACAGG

>dd36571922ab7a138ee06438ab9c7891

TACGTAGGGTGCGAGCGTTAATCGGAATTACTGGGCGTAAAGCGGGCGCAGACGGTTACTTAAGCGGGATGTGAAATCCCCGGGCTCAACCCGGGAACTGCGTTCCGAACTGGGTGGCTAGAGTGTGTCAGAGGGGGGTAGAATTCCACGTGTAGCAGTGAAATGCGTAGAGATGTGGAGGAATACCGATGGCGAAGGCAGCCCCCTGGGATAACACTGACGTTCATGCCCGAAAGCGTGGGTAGCAAACAGG

>8662e86f01ced8208d2f4ee4b3d5f432

TACGGAAGGTCCGGGCGTTATCCGGATTTATTGGGTTTAAAGGGAGCGTAGGCTGGAGATTAAGTGTGTTGTGAAATGTAGACGCTCAACGTCTGAATTGCAGCGCATACTGGTTTCCTTGAGTACGCACAACGTTGGCGGAATTCGTCGTGTAGCGGTGAAATGCTTAGATATGACGAAGAACTCCGATTGCGAAGGCAGCTGACGGGAGCGCAACTGACGCTTAAGCTCGAAGGTGCGGGTATCAAACAGG

>15e1468b770babb65d201bd49a1750c7

AAAAAAAAGCTGTCTACTACGAATTTGTATGAACATCAAATTTGTGTCACTAACGAAAACAAATTCATTCGTAAAACATACCCGGCACTATCAAGAAAGGGTCGATGTTGAAATCCAATGGATGGTGGATCAAGGGGTTATCAAACGATCCAACAGTGATTTTCTTAACCCGGTGGTAACCGTTAAGGAATATAAGGCTGTGCCTCGATATGCACAACTCAAAAATAGTATCGTGTTGGGCTCCGAACGCTGAGTCAGTTTTTATTAAGTGTCAGGGTGTCCGCTACATGTCCCG

>a304db38556bd6c6144e6788e66b684d

CATTGAACTATCGTGAGAAAGTCAAACCGCCAAAGGGAATTATATTATAGTAAATATTGGCGTAAATAAACATTTTATTAATAGTTGTAATATATGAAAATGTGCAGAAGATAAAGACGATGAAAACGGTGTTTAACAATCGCTTATCGAGACAATATTTAATTAATTAGGAATTAAAATATGTGTATTATTTGTATTTAACAAGGAATAAACGTGAAAATAAATACAGTGGTAAACGAAACTAATAGAGTCACGCAAGTGAACGTAGAACATTTTTAATAAC

>170b20159dc451839513af9464e8164e

TATATACACATAGGCTTACAAAACTCCATATAACACTGGTAATTCTTTTGAACTAAAAGAGAAAGAACATGTTTCCGTTGGTTGAAAAAAACTTTCAAAAATTGCTTACCCTATAAACCCGTTAGATAAATTAAAAGTAAAAATAAAAATAAAAATATGTAGGAGTTGTAACTATACAATATTATAACTAATTAAGCAGATTCGGTAAATATCCTACAA

>b703f8afc6ddb07155bff186d8b219e1

TACGAAGGGGGCTAGCGTTGTTCGGATTTACTGGGCGTAAAGCGCGCGTAGGCGGATATTTAAGTCAGAGGTGAAATCCCAGGGCTCAACCCTGGAACTGCCTTTGATACTGGGTATCTAGAGTATGGAAGAGGTGAGTGGAATTCCGAGTGTAGAGGTGAAATTCGTAGATATTCGGAGGAACACCAGTGGCGAAGGCGGCTCACTGGTCCATTACTGACGCTGAGGAGCGAAAGCGTGGGGAGCAAACAGG

>edc9e5c16e40aff1eadce6597940f08f

TACGTAGGTCCCGAGCGTTATCCGGATTTATTGGGCGTAAAGCGAGCGCAGGCGGTTAGATAAGTCTGAAGTTAAAGGCTGTGGCTTAACCATAGTACGCTTTGGAAACTGTTTAACTTGAGTGCAGAAGGGGAGAGTGGAATTCCATGTGTAGCGGTGAAATGCGTAGATATATGGAGGAACACCGGTGGCGAAAGCGGCTCTCTGGTCTGTAACTGACGCTGAGGCTCGAAAGCGTGGGGAGCAAACAGG

>7d51287c6979ad225200bd7288e63b2c

TACGTAGGGAGCAAGCGTTATCCGGATTTATTGGGTGTAAAGGGTACGTAGGCGGCCTCATAAGTCTGTGGTTTAAGTCCGAAGCTTAACTTCGGTTCGCCACAGAAACTGTTTGGCTTGAGTATGGTAGAGGCAAGTGGAATTTCTAGTGTAGCGGTTAAATGCGTAGATATTAGAAGGAACACCAGTGGCGAAGGCGACTTGCTGGGCCATTACTGACGCTGAGGTACGAAAGCGTGGGGAGCAAATAGG

>a91f663adac0dc192ca12e548628e376

CATTGAACTATCGTGAGAAAGTCAAACCGCCAAAGGGAATTATATTATAGTAAATATTGGCGTAAATAAACATTTTATTAATAGTTGTAATATATGAAAATGTGCAGAAGATAGAGACGATTAAAACGGTGTTTAACAATCGCTTATCGAGACAATATTTAATTAATTAGGAAATAAAATATATTTATTATTTGTATTTAACAAGGAATAAACGTGAAAATAAATACAGTGGTGAACGGAACTAATAGAGTCACGCAAGTGAACTTAGAACAGTTTTAAGAAC

>97b11147f4ad97661f06274bb7c0496b

AACGTGGGAGGCAAGCGTTATTGATCATAAATGGGTCTAAAGGGTCCGTAAAATGGTAAATTAAGCGTAATGAAATTCGAACTGATTTACTAGAGTCTTATAGAGGAACAGAGGAATTGCAAGAGTAAGGATGAAATCTATTGATACTTGTAGGACCGCCAAAGGCGAAGGCATCGTTCTAGGTATAGACTGACGTTGAGGGACGTAGGCGTTGGGAGCAAAAAGG

>2b869347e522205f1c4a919196e6b45b

TACGTATGTCGCGAGCGTTATCCGGAATTATTGGGCATAAAGGGCATCTAGGCGGATATACAAGTCAGGGGTGAAAACTTAGGGCTCAACTCAAAGCTTGCCTTTGAAACTGTATATCTAGAGTGCTGGAGAGGTGGACGGAACTACACGAGTAGAGGTGAAATTCGTAGATATGTGTAGGAATGCCGATGATGAAGATAGTCCACTGGACAGCAACTGACGCTGAAGTGCGAAAGCTAGGGGAGCAAACAGG

>db5f439e3cf3518a5888e515c6fda879

GCAGCCGCGGTAAGTGCTCAGAGAACATCAATCTTTCACTTCGTTCTCACTTTCTCTTTTTACTGCCTCCTCTTCTGACCCTTTCCACCATACCCCTGCTTCTCCTTTTCGTCATCCCTTTTCGGTCTATTTGTAGCATAATATATTAGAAACCCGGGTAGTCCGGCTGACTGACTCTACGACCAT

>f1074ede5d356ad4a93ff7b67f3b98a6

TACGGAGGGTGCAAGCGTTAATCGGAATTACTGGGCGTAAAGCGCACGCAGGCGGTTGATTAAGTTAGATGTGAAATCCCCGGGCTTAACCTGGGAATGGCATCTAAGACTAGTCAGCTAGAGTCTTGTAGAGGGGGGTAGAATTCCATGTGTAGCGGTGAAATGCGTAGAGATGTGGAGGAATACCGGTGGCGAAGGCGGCCCCCTGGACAAAGACTGACGCTCAGGTGCGAAAGCGTGGGGAGCAAACGGG

>96b786989b4e37b05e30da9d379ec057

GACGTAGGAGGCGAGCGTTATCCGGATTCACTGGGCGTAAAGCGCATGTAGGCGGATGTCTAAGTCGTGTGTTAAAGCCCCTGGCTCAACTGGGGGAGGTCATGCGAGACTGGATGACTTGAGGGCAACAGAGGCACGTGGAACTCCCGGTGTAGCGGTGGAATGCGTAGAGATCGGGAAGAACACCCGTGGCGAAGGCGGCGTGCTGGGTTGCAACTGACGCTGAGATGCGAAAGTGTGGGGAGCAAACGGG

>f992dfdf7e8ebde2cd041f7e4100beff

TACGTAGGGGGCAAGCGTTATCCGGATTTACTGGGTGTAAAGGGAGCGTAGACGGAATGGCAAGTCTGAAGTGAAATGCCCGGGCTCAACCCGGGAACTGCTTTGGAAACTGTCGATCTGGAGTGTTGGAGAGGTAAGTGGAATTCCTGGTGTAGCGGTGAAATGCGTAGAGATCAGGAAGAACACCGGAGGCGAAGGCGGCTTACTGGACAAAGACTGACGTTGAGGCTCGAAAGCGTGGGAAGCAAACAGG

>2367bf61effefd58d727c21ea74e4f90

GCCGCCGCGGTAAGTGCTCAGAAAACATCAATCTTTCACTTCGTTCTCACTTTCTCTTTTTACTGCCTCCTCTTCTGACCCTTTTCCACCATACCCCTTCTTCTCCTTTTCGTCACCCCTTTCGGTCTATTTGTAGCATAATATATTAGAAACCCTGGTAGTCCGGCTGACTGACTGAGACTTAAT

>61d4b24d82ada122dc5de49ecf9c05c9

TATGGAGGGGGCTAGCGTTGTTCGGAATTACTGGGCGTAAAGCGCACGTAGGCGGACTGGAAAGTTGGGGGTGAAATCCCGGGGCTCAACCTCGGAACTGCCTTCAAAACTATCAGTCTGGAGTTCGAGAGAGGTGAGTGGAATTCCGAGTGTAGAGGTGAAATTCGTAGATATTCGGAGGAACACCAGTGGCGAAGGCGGCTCACTGGCTCGATACTGACGCTGAGGTGCGAAAGCGTGGGGAGCAAACAGG

>48a5efa5308d095c5240dd2cea1580c3

TACGTAGGTGGCAAGCGTTGTCCGGAATTATTGGGCGTAAAGCGCGCGCAGGCGGTCTTTTAAGTCTGATGTGAAAGCCCCCGGCTCAACCGGGGAGGGTCATTGGAAACTGGGAGACTTGAGTACAGAAGAGGAGAGTGGAATTCCACGTGTAGCGGTGAAATGCGTAGATATGTGAAGGAACACCAGTGGCGAAGGCGACTCTCTGGTCTGTAACTGACGCTGAGGCGCGAAAGCGTGGGGAGCAAACAGG

>c6c3ab4e828fb40d6e05967b7aac9338

TACGTAGGGGGCAAGCGTTATCCGGATTTACTGGGTGTAAAGGGAGCGTAGACGGTGTGGCAAGTCTGATGTGAAAGGCATGGGCTCAACCTGTGGACTGCATTGGAAACTGTCATACTTGAGTGCCGGAGGGGTAAGCGGAATTCCTAGTGTAGCGGTGAAATGCGTAGATATTAGGAGGAACACCAGTGGCGAAGGCGGCTTACTGGACGGTAACTGACGTTGAGGCTCGAAAGCGTGGGGAGCAAACAGG

>9a7b41ae8b757eb42354bc5f7bbdf0cd

TACGGAGGGTGCGAGCGTTAATCGGAATAACTGGGCGTAAAGGGCACGCAGGCGGTGATTTAAGTGAGGTGTGAAAGCCTCGGGCTTAACCTGGGAATTGCATTTCATACTGGGTCGCTAGAGTACTTTAGGGAGGGGTAGAATTCCACGTGTAGCGGTGAAATGCGTAGAGATGTGGAGGAATACCGAAGGCGAAGGCAGCCCCTTGGGAATGTACTGACGCTCATGTGCGAAAGCGTGGGGAGCAAACAGG

>8e9e41ad7e5a60d18c633485baa62678

TACGTAGGCGGCAAGCGTTGTCCGGAATTATTGGGCGTAAAGGGAGCGCAGGTGGGACGGTAAGTCCGTCTTAAAAGGCAGGGGCTCAGCCCCTGTAAGGGATGGAAACTATCGATCTTGAGTGCCGGAGAGGAAAGCGGAATTCCCAGTGTAGCGGTGAAATGCGTAGATATTGGGAAGAACACCAGTGGCGAAGGCGGCTTTCTGGACGGCAACTGACACTGAGGCTCGAAAGCCAGGGGAGCGAACGGG

>c7791e8423d7e57aec539d8b8fc19e6b

ATATGGTAATTGTGTGCCAGCCGCCGCGGTAATAGTGATGTTTTGGCTGACTTTATCTGCTGCTTTGTTACAGTCAAGCTTAATAAAACCACCATAAACACACACATTAAGTAAGATGCTAACTCAAGTCACACACAACAATACATTAGATACCCTAGTAGTCCGGCTGACTGACTTGCGTCAAATCTCGTATGCCGTCTTCTGC

>035c280d40578777f6faec3db9cba293

CCGCCGCGGTAAGTGCTCAAAGAACATCAATCTTTCACTTCGTTCTCACTTTCTCTTTTTACTGCCTCCTCTTCTGACCCTTTTCCACCATACCCCTTCCTCTCCTTTTCGTCATCCCTTTTCGGTCCATTTGCACCATAATATATTAGATACCCCAGTAGTCCGGCTGACTGACTTAACGTCCA

>cb0db18277323b1f0db8a57fef080696

TACGTAGATGGCAAGCGTTGTCCGGATTTATTGGGCGTAAAGCGAGCGCAGGCGGTTCTTTAAGTCTGATGTGAAAGCCCCTGGCTCAACCAGGGAGGGTCATTGGAAACTGGAGAACTTGAGTGCAGAAGAGGAAAGTGGAATTCCATGTGTAGCGGTGAAATGCGTAGATATATGGAGGAACACCAGTGGCGAAGGCGACTTTCTGGTCTGTAACTGACGCTGAGGCTCGAAAGCATGGGGAGCAAACAGG

>4707f6452fc568a0968e19fe2e5983f8

TACGTAGGGCGCGAGCGTTGTCCGGAATCATTGGGCGTAAAGCGCGTGTAGGCGGCTGAATAAGTCTGCTGTGAAAGTCCAGGGCTCAACCCTGAAATGTCGGCGGATACTGCTCAGCTGGAGTACGGGAGGGGCGAGTGGAATTCCTGGTGTAGCGGTGGAATGCGCAGATATCAGGAGGAACACCTATGGCGAAGGCAGCTCGCTGGAACGTTACTGACGCTGAGACGCGAAAGCGTGGGGAGCAAACAGG

>11143268b00d03d94483fac2c68118d7

TACGGAGGGTGCAAGCGTTAATCGGAATTACTGGGCGTAAAGCGCACGCAGGCGGTTTGTTAAGTCAGATGTGAAATCCCCGGGCTCAACCTGGGAACTGCATTTGAAACTGGCAAGCTAGAGTCTCGCAGAGGGGGGTAGAATTCCAGGTGTAGCGGTGAAATGCGTAGAGATCTGGAGGAATACCGGTGGCGAAGGCGGCCCCCTGGACGAAGACTGACGCTCAGGTGCGAAAGCGTGGGGAGCAAACAGG

>5f1e76ccef126e46611c91bcea04545a

AATTGTGTGCCAGCCGCCGCGGTAACACAATAGCACACAAAAACAGATTGGGCATCACACTGCGAAGAGAGAAGGAAGGGAATGCGTACTGGCAACCTTGCACTATTATTTGTATATCACTGGCTGTGACTGAAACTGCTTGCCATTAGAAACCCTTGTAGTCCGGCTGACTGACTGAGACTTAATCTCGTATGCCGT

>927061e59791923b7dd1fdfb957701d4

GACGTAGGGCGCGAGCGTTGTCCGGATTTATTGGGCGTAAAGAGCTCGTAGGCGGCTTGTTGCGTCGGCTGTGAAATCCCGTGGCTTAACTGCGGGCTTGCAGCTGATACGGGCGGGCTAGAGTTCGGTAGGGGAGACTGGAATTCCTGGTGTAGCGGTGAAATGCGCAGATATCAGGAGGAACACCGGTGGCGAAGGCGGGTCTCTGGGCCGATACTGACGCTGAGGAGCGAAAGCGTGGGGAGCGAACAGG

>24c2d510527caa4c1e1bab094a6298d9

TACAAGGAAGACTAGTGTTATTCATCTTTATTAGGTTTAAAGGGTACCTAGACGGCAAATTAAGCCCCAAAAGGGTACTGATATGCTAGAGTTTAATGGGAGAGGTAAATATTAGGGCTATTGGTGTAGTGATGAAATACTTTTATACTAATGGAACGGGTAACGGCGAAGGCAACCCTCTATGTATAAACTGACGTTGAGGGACGAAGGCTTGGGGAGCGAATAGG

>e722b905945568a30015955073e2e7ef

GACGAACCGTGCGAACGTTATTCGGAATCACTGGGCTTAAAGCGCGTGTAGGCGGATGGGGACGTCGGTCGCTGAAATCCCCCGGCTCAACCGGGGAACTGGCCCCGAAACGCCCCGTCTGGAGGAACGTAGGGGGATCTGGAACTTCCGGTGGAGCGGTGAAATGCGTTGAGATCGGAAGGAACGCCCGTGGCGAAAGCGAGATCCTGGACGTTTTCTGACGCTGAGACGCGAAAGCTAGGGTAGCGAACGGG

>e9ade5ee5e0b1d2081c32b459950d257

AGTAATACGTGCGTCTTATCTGGCATACCCGGACCAGATAAGTAACAAGGGCACACACAATACTACATACGCAACACTCTCCCCCCACCGCACGGCTTGCCCCCGAGCGTGGGTAAACAGGTTGGCTAGTATCTCTCTGGCTGCGTTGGAATGTGAATTCGATCACGGACAGAGGGCGCAACAGGCCTTGTCTAATGCGTGACGCAAGAAGAAGAAGATATTTTTAACAGTAATGGAATATTTGACTTGGCATTTGAATTGCGGTCGTTAGACATCACTGATTTTAA

>77ca36a2a24b0a3415aad9647292913a

TACGTAGGTGGCAAGCGTTGTCCGGAATTACTGGGTGTAAAGGGAGCGTAGGCGGGAGTGCAAGTTGAATGTGAAATCTATCGGCTCAACCGGTAGCTGCGTTCAAAACTGCACTTCTTGAGTGAAGTAGAGGCAGGCGGAATTCCTAGTGTAGCGGTGAAATGCGTAAATATTAGGAGGAACACCAGTGGCGAAGGCGGCCTGCTGGGCTTTAACTGACGCTGAGGCTCGAAAGCGTGGGTAGCAAACAGG

>aee3d7022cfa7f88d4b8ac729dca70e1

TACGGAAGATGCGAGCGTTATCCGGATTTATTGGGTTTAAAGGGTGCGCAGGCGGCGGGCCGAGTCAGCGGTAAAATAGAGGGGCTCAACCCCTTCGAGCCGTTGATACTGGCCTGCTAGAGTGGGCGAGAAGTACGCGGAATGCGTGGTGTAGCGGTGAAATGCATAGATATCACGCAGAACTCCGATTGCGAAGGCAGCGTACCGGCGCCCTACTGACGCTGAGGCACGAAAGCGCGGGTATCGAACAGG

>c37b1d04f9dd96522eb74ba88260c663

CACACTTATGACTTCAGAACAACAAAATATTTCTCCAGAATGCATTTCAACCTTGTCGGTCATTTTCAACTGGCAGTGCCTTGTAGATATTTGTGGAAGATTTTGAATTTGTCTTGTCAATAGAGTTCAGTTTGTTTATTAATCAATTTTGTTATGTAGTTTGTAACGTAATATATTTAGGCTTTCAAGTAATTTCATTTTGTTTCCTTTTTTGCCGGTTGTG

>2c3da64d93afe60fa430dba13fbde7be

TACGGAAGGTCCGAGCGTTATCCGGATTTATTGGGTTTAAAGGGAGCGTAGGCGGCGCGGAAAGTCAGCTGTGAAATCCGTCGGCTCAACCGTCGGCTTGCAGTTGAAACTGCCATGCTTGAGCGCACGCAGGGATGCCGGAATTCATGGTGTAGCGGTGAAATGCTTAGATATCATGAAGAACTCCGATCGCGAAGGCAGGTGTCCGGAGTGTGTCTGACGCTGAGGCTCGAAAGTGCGGGTATCAAACAGG

>8710c26879102dd3b934a8d4a5a676ae

CATTGAACTATCGTGAGAAAGTCAAACCGCCAAAGGGAATTATATTATAGTAAATATTGGCGTAAATAAACATTTTATTAATAGTTGTAATATATGAAAATGTGCAGAAGATAAAGACGATTAAAACGGTGTTTAACAATCGCTTATCGAGACACTATTAAAGTAATACGGAATTAAAATATGTTTATTATGGGTATTAAACAAGGGATAAACTTGAAAATAAATACAGTGGTAAACGGAACTAATAGAGTCACGCAAGTGAACTTAGAACATTTTTAATAAC

>312a2e5803a3e8126d0c4be250640826

TACGGAGGGTGCAAGCGTTGTTCGGAATTATTGGGCGTAAAGCGCGTGCAGGCGGCTGTTCAAGTCCGATGTGAAAGCCCGGGGCTCAACCCCGGAAGTGCATTGGAAACTGGACAGCTTGAGTACGGGAGAGGGAGGTAGAATTCCGAGTGTAGGGGTGAAATCCGTAGATATTCGGAGGAATACCGGTGGCGAAGGCGACCTCCTGGACCGATACTGACGCTGAGACGCGAAAGCGTGGGGAGCAAACAGG

>94ee2bf87f995c21f2cb02bd26b567e9

ATATGGTAATTGTGTGCCAGCCGCCGCGGTAACACAATATCATTTCTTTCCATGAGAAATTGTCTGTTGGTTATTCGTTTCCATTTAAAGCCGAGTTTCTTCAGTATTCTTGATAAGGTTGTTGTACAGTCACTCAAACTTCAGATTAGAAACCCCAGTAGTCCGGCTGACTGACTTAACGTCCATCTCGTATGCCGTCTTCTGC

>e3f65db4b29d94d57ce5db0d42a80213

TACAGAGGGTACAAGCGTTAATCGGAATTACTGGGCGTAAAGCGCGCGTAGGCGGCTAGGTAAGATGGGTGTGAAATCCCCGGGCTCAACCTGGGAACTGCATCCATAACTGCCTGGCTAGAGTACAGTAGAGGGTGGTGGAATTTCCTGTGTAGCGGTGAAATGCGTAGATATAGGAAGGAACACCAGTGGCGAAGGCGACCACCTGGACTGATACTGACGCTGAGGTGCGAAAGCGTGGGGAGCAAACAGG

>bb16c5ca3c011ba635c093c02269887f

GACGAAGGGGGCTAGCGTTGTTCGGAATTACTGGGCGTAAAGCGAGTGTAGGCGGTTGCCCAAGTCAGGTGTGAAAGCCTTGAGCTCAACTCAAGAAATGCACTTGGTACTGGGTGGCTAGAGGACCGGAGAGGATAGTGGAATTCCCAGTGTAGTGGTGAAATACGTAGAGATTGGGAAGAACACCAGTGGCGAAGGCGGCTATCTGGACGGTTACTGACGCTAAGACTCGAAAGCGTGGGGAGCAAACAGG

>07537d1f555e6d17ebdbfd084169c485

TACAGAGGGTGCGAGCGTTAATCGGGATTACTGGGCGTAAAGCGAGTGTAGGTGGCTCATTAAGTCACATGTGAAATCCCCGGGCTTAACCTGGGAACTGCATGTGATACTGGTGATGCTAGAATATGTAAGAGGGAAGTAGAATTCCAGGTGTAGCGGTGAAATGCGTAGAGATCTGGGGGAATACCGATGGCGAAGGCAGCTTCCTGGCATAATATTGACACTGAGATTCGAAAGCGTGGGTAGCAAACAGG

>306c0e59c852bcefbd80fe8585a989fb

CATTGAACTATCGTGAGAAAGTCAAGCCGCCAAAGGGCGGTAAATATTGGCGTAAATAAACATTTTATTAATAGTTGTAATATATGAAAATGTGCAGAAGATAAAGACGATTAAAACGGTGTTTAACAATCGCTTATCGAGACAATATTTAATTAATTAGTAATTAAAATATGTTTATTATGTGTATTATTCAAGGAATAGACTTGACAATAAATACAGTGGTAAACGGAACTAATAGAGTCACGCAAGTGAACTTAGAACATTTTTAATAAC

>cd1c64924b4669c5b30f4904ab02400c

TACGGAGGGTGCAAGCGTTGTTCGGAATTATTGGGCGTAAAGCGCGTGCAGGCGGCTGTTCAAGTCCGATGTGAAAGCCCGGGGCTCAACCCCGGAAGTGCATTGGAAACTGGACAGCTTGAGTACGGGAGAGGGAGGTAGAATTCCGAGTGTAGGGGTGAAATCCGTAGATATTCGGAGGAATACCGGTGGCGAAGGCGGTCTCCTGGACCGATACTGACGCTGAGACGCGAAAGTGTGGGGAGCAAACAGG

>d114fb4c335125128be28401522dd41a

TACGTAGGTCCCGAGCGTTGTCCGGATTTATTGGGCGTAAAGCGAGCGCAGGTGGTTTATTAAGTCTGGTGTAAAAGGCAGTGGCTCAACCATTGTATGCATTGGAAACTGGTAGACTTGAGTGCAGGAGAGGAGAGTGGAATTCCATGTGTAGCGGTGAAATGCGTAGATATATGGAGGAACACCGGTGGCGAAAGCGGCTCTCTGGCCTGTAACTGACACTGAGGCTCGAAAGCGTGGGGAGCAAACAGG

>0e4d46f3be740136aebfd6690a977258

CATTGAACTATCGTGAGAAAGTCAAACCGCCAAAGGGAATTATATTATAGTAAATATTAGCGTTAATAAACATTTTATTAATAGTTGTAATATATGATAATGTGCAGAAGATAAAGACGATTAAAACAGTGTTTAATAATCGCTTATCGAGACACTATTAAAGTAATACGGAATTAAAATATGTTTATTATGTGTATTAAACAAGGGATAAACTTGAAAATAAATACAGTGGTAAACGGAACTAATAGAGTCACGCAAGTGAACTTAGAACATTTTTAATAAC

>d06e555096e39ca54e989c7585044648

TACGGAGGGTGCGAGCGTTAATCGGAATTACTGGGCGTAAAGCGCGCGTAGGCGGCTTGATAAGCCGGTTGTGAAAGCCCCGGGCTCAACCTGGGAACGGCATCCGGAACTGTCAGGCTAGAGTGCAGGAGAGGAAGGTAGAATTCCCGGTGTAGCGGTGAAATGCGTAGAGATCGGGAGGAATACCAGTGGCGAAGGCGGCCTTCAGGACTGACACTGACGCTGAGGTGCGAAAGCGTGGGTAGCAAACAGG

>7008df4a1b297dfc30f88c015f9234e5

CCGCCGCGGTAAGTGCTCAGAGAACATCAATCTTTCACTTCGTTCTCACTTTCTCTTTTTACTGCCTCGTCTTCTGACCCTTTTCCACCATACCCCTTCTTCTCCTTTTCGTCATCCTTTTTCGGTCTATTTGTAGCATAATATATTAGATACCCCTGTAGTCCGGCTGACTGACTTAACGTCCA

>072f40a3b6a718e0333d16c0bbe3d386

TATGGTAATTGTGTGCCAGCCGCCGCGGTAAGAGAAAAACTATCGCCAACAATTAGCGCATTAAGTAGTAGTAACGGCGACGGTAACAGCTGTACGCTACTGATAAGCCAGCAGCCGACGCTACTTCTCTTCTGTCTACGATTTATTAGAAACCCTAGTAGTCCGGCTGACTGACTTAACGTCCATCTCGTATGCCGTCTTCTG

>3f2df031aadf64258d2296fd95d11d4a

CACCGATCTACTATGGTAATTGTGTGCCAGCCGCCGCGGTAAAGTAAACACAATATCTGGCAGGACTGCCACTCGTTACTTGATTCAGCACTCTCCTTATATAGTTTAGTTTAGATTCGTCTGTTTTACAGATTGACTCACTTGATTAGATACCCCTGTAGTCCGGCTGACTGACTTAACGTCCATCTCGTATGCCGTCTTCTGCTTGAAAAAAA

>5ab4d61dfea593ddcc6305887cb75fc7

CCCGCGTGATATGGTAATTGTGTGCCAGCCGCCGCGGTAACCTCCACTCCATAACGCAATCTTTTTATCAGTCTCGTCACTATCTCACGAAATCGCGTGCAATTCTCGTCGTCGAAACAATATAACTTGGATACATTGAAAACCATTAGAAACCCTAGTAGTCCGGCTGACTGACTTAACGTCCATCTCGTATGCCGTCTTCTGCTTGAAAAA

>6e89c7fc82acf5cd5a5860e8e52d6c5d

CACGCGTGATATGGTAATTGTGTGCCAGCCGCCGCGGTAACACACATTGTTATTGTTGTTGTGATGTTTATATATGTGTAGGTTTTAATTTATGTTTATTTTTTGGCCTTTGGAAATGTTTTTAATTAGTTTGAGGTTGTTGGGATTAGAAACCCGTGTAGTCCGGCTGACTGACTTAACGTCCATCTCGTATGCCGTCTTCTGCTTGAAAAA

>c7a60fb7af5ef6e03f5376a0ce798edd

TCCAATCTCTTCAACGCCAGCTAAATAAACACAAACGAGAAACAGAATTACGAGAGTGATTGCAACTTTGGTGCAACCGGCTGCGACTTGGTAGGTATAGAAACAGCATGGCTGTTACCAATAAAATGAGAAAAGGAAAGTTATGACCTCGCCGGAGAAAATTCAAACCAACACAAAAACAAATCCAAACGTTACAGAGGCCCTAAAACCACGGCCTATTATTCTGAGTAATGTAAATAACTACTCCGAATTTTAAGCAGGT

>c5fdda1fb74750610018ec0fd27756aa

TACGGAGGGGGCTAGCGTTGTTCGGAATTACTGGGCGTAAAGCGCACGTAGGCGGACTGGAAAGTTGGGGGTGAAATCCCGGGGCTTAACCTCGGAACTGCCTTCAAAACTATCAGTCTGGAGTTCGAGAGAGGTGAGTGGAATTCCGAGTGTAGAGGTGAAATTCGTAGATATTCGGAGGAACACCAGTGGCGAAGGCGGCTCACTGGCTCGATACTGACGCTGAGGTGCGAAAGCGTGGGGAGCAAACAGG

>c27e6b83b3f87bbe9123ed6079e402b2

TACGGAGGGTCCGAGCGTTAATCGGAATTACTGGGCGTAAAGCGTGCGCAGGCGGTTTGTTAAGCAAGATGTGAAAGCCCTGGGCTCAACCTAGGAATAGCATTTCGAACTGGCGAACTAGAGTCTTGTAGAGGGGGGTAGAATTCCAGGTGTAGCGGTGAAATGCGTAGAGATCTGGAGGAATACCGGTGGCGAAGGCGGCCCCCTGGACAAAGACTGACGCTCATGCACGAAAGCGTGGGGAGCAAACAGG

>7d5c88f73ba121d2422f80673ffde7bc

CATTGAACTATCGTGAGAACGGCACGCCGCCAAAGGGAATTATATTATAGTAAATATTAGCGTAAATAAACATTTTATTAATAGTTGTAATATATGAAAATGTGCAGAAAATAGAGACGATTAAAACGGTGTTTAACAATCGCTTATCGAGACAATATTTAATTAATTAGGAAATAAAATAAGTGTATTATTTGTATTTAACGAGGAATAAACGTGAAAATAAATACAGTGGTAAACGGAACTAATAGAGTCACGCAAGTGAACTTAGAACAATTTTAAGAACATTAGAACTAATGGAAA

>db7936edf9809e7467285d4150bf467c

CCGCTACTGTGGGAGAGCTCCAAAAACAGGCGTATTTGGGGACGCAAGACCCTGCCGTGTGACTTGAAACAGTAGTCTTGTGAGGGATGTAGTGGCGATTTAAAAGTGAAAGGGAGAAGTAACGGGTTATTCGAAACTCAACGAAAATAAGCGCTGGCAAAGTCCTGTACGCGATCGCCAAATTTCACTGAACTTTATAAAACACTCTAACGTTAC

>cd49953b9030d5e4d59672db29c7d4db

TTGACACTTTATCTCGCGCTCGTTCCGTTACTTAACGCCGCATTAAAAACCTACCTTTTCATTGAATTTACCTTATTTTTTCTTTAATAAAACTAAACTATCGTTTCCTATCGGTAAGTAAAACTTAACTGTTTTATAAAATTCTTTAGCTGCGTGTAGCTATAATTACCTATACACTTATCTAAACATTTATTAACGATTGTCGACATATTCCATAAAG

>ce2565c7dae637b3d3b127c6b90b4ba4

TACGGAGGGTCCGAGCGTTAATCGGAATTACTGGGCGTAAAGCGTGCGCAGGCGGTTTGTTAAGCGAGATGTGAAAGCCCTGGGCTCAACCTAGGAATAGCATTTCGAACTGGCGAACTAGAGTCTTGTAGAGGGGGGTAGAATTCCAGGTGTAGCGGTGAAATGCGTAGAGATCTGGAGGAATACCGGTGGCGAAGGTGGCCCCCTGGACAAAGACTGACGCTCATGCACGAAAGCGTGGGGAGCAAACAGG

>2e3ef71f41cae91fc5bf076309fe3762

ACATTCTTTGTACTTCATTAGTTATTTAATTTACACCACTTGTCACCCCCTTATATTGTACAAACGAGTTTGCTGGCACATTATGTGAAATACACGTTCCTGTTACAGCTTATCCCTGACCTGCTAAAACATGAACAGCAATATTTCTATGACCACAAAAACTATGGACCAAGAAGCTA

>c757ad670c5d796cb866c941c62c4e33

TACGGAGGATCCAAGCGTTATCCGGAATCATTGGGTTTAAAGGGTCCGTAGGCGGTTTAGTAAGTCAGTGGTGAAAGCCCATCGCTCAACGGTGGAACGGCCATTGATACTGCTAGACTTGAATTATTAGGAAGTAACTAGAATATGTAGTGTAGCGGTGAAATGCTTAGAGATTACATGGAATACCAATTGCGAAGGCAGGTTACTACTAATGGATTGACGCTGATGGACGAAAGCGTGGGTAGCGAACAGG

>c3e473ec18f6f3c6b732af83d989af94

TACGGAGGGGGCTAGCGTTGTTCGGAATTACTGGGCGTAAAGCGCACGTAGGCGGACTGGAAAGTTGGGGGTGAAATCCCGGGGCTCAACCTCGGAACTGCCTTCAAAACTATCAGTCTGGAGTTCGAGAGAGGTGAGTGGAATTCCGAGTGTAGAGGTGAAATTCGTAGATATTCGGAGGAACACCAGTGGCGAAGGCGGCTCACTGGCTCGATACTGACGCTGAGGTGCGAAAGCGTGAGGAGCAAACAGG

>6db2c97532abe971a75024dfeaf40e9d

TACAGAGGGTGCAAGCGTTAATCGGAATTACTGGGCGTAAAGCGAGCGTAGGTGGCTTGATAAGTCAGATGTGAAATCCCCGGGCTTAACCTGGGAACTGCATCTGATACTGTTAGGCTAGAATAGGTGAGAGGAAGGTAGAATTCCAGGTGTAGCGGTGAAATGCGTAGAGATCTGGAGGAATACCGATGGCGAAGGCAGCCTTCTGGCATCATATTGACACTGAGGTTCGAAAGCGTGGGTAGCAAACAGG

>c47375ae4f9fc628ee1a74895f8f1b3f

TACGTATGGTGCAAGCGTTATCCGGATTTACTGGGTGTAAAGGGAGCGTAGACGGATAGGCAAGTCTGGAGTGAAAGCCCGGGGCTCAACCCCGGGACTGCTTTGGAAACTGTTTATCTAGAGTGCTGGAGAGGTAAGCGGAATTCCTAGTGTAGCGGTGAAATGCGTAGATATTAGGAGGAACACCAGTGGCGAAGGCGGCTTACTGGACAGTAACTGACGTTGAGGCTCGAAAGCGTGGGGAGCAAACAGG

>a4031d45ca4c8af1d9d1cca34e9bc1e8

CATTTTACCATAGTGTCTGCCTCCACGGGCACGTTTATGGCTTTGCCGGTTATCTTTTAAGACTGTTTCTATTCTGAGACGACGGATCTGCATACGTGGGAATCCGGATGAAATCAGTCTCTCAGTGATACGATCGAGCGGCAGATGGGTCGGATCTTCCGGATACTTATAACTATTACTTTGGCTCGGCGGAGGCACTTTACCCTCATTAAGCAAGCGGCGGTATGTGGAGCACA

>cc1c987a3331d24e2567bd207b053438

TACAGAGGGTGCAAGCGTTAATCGGAATTACTGGGCGTAAAGCGCGCGTAGGCGGTTGTATAAGTTGGAGGTGAAATCCCCGGGCTCAACCTGGGAATTGCCTTCAAAACTGTACGGCTAGAGTGTGGGAGAGGAAGGTAGAATTCCAGGTGTAGCGGTGAAATGCGTAGATATCTGGAGGAATACCGATGGCGAAGGCAGCCTTCTGGCCTAACACTGACGCTGAGGTGCGAAAGCATGGGGAGCAAACAGG

>9a7c9894ffe03444593d4fb2d99e43d0

TACGAAGGGGGCTAGCGTTGCTCGGAATCACTGGGCGTAAAGGGCGCGTAGGCGGCGTTTTAAGTCGGGGGTGAAAGCCTGTGGCTCAACCACAGAATTGCCTTCGATACCGGGACGCTTGAGTCTGGTAGAGGTTGGTGGAACTGCGAGTGTAGAGGTGAAATTCGTAGATATTCGCAAGAACACCGGTGGCGAAGGCGGCCAACTGGACCAGTACTGACGCTGAGGCGCGAAAGCGTGGGGAGCAAACAGG

>c949c9593fb101d45db1c115cf1083dd

TACAGAGGGTGCAAGCGTTATCCGGAATCACTGGGTTTAAAGGGTGCGTAGGAGGGTAGATAAGTCAGAGGTGAAAGGTAGTCGCTTAACGATTAAATTGCCTTTGATACTGTTTATCTTGAATCAAGATGAGGTTGGCGGAATGAGACATGTAGCGGTGAAATGCATAGATATGTCTTAGAACACCGATTGCGAAGGCAGCTGACTGGACTTCGATTGACGCTGAGGCACGAAAGCGTGGGGATCAAACAGG

>867ed10d71e6ce90c0bfc24c4ec393b9

TACGGGGGTGGCAAGCGTTGTTCGGAATTACTGGGCGTAAAGGGCGCGTAGGCGGCCGGGGAGGTCGCATGTGAAAGCCGGGGGCTTAACCTCCGAAGGGCATGCGAAACCGCCCGGCTTGAGTGTCGGAGGGGAAGGCGGAATTCCGGGTGTAGCGGTGAAATGCGTAGATATCCGGAGGAACACCGGTGGCGAAGGCGGCCTTCTGGACGACGACTGACGCTGAGGCGCGAAAGCCAGGGGAGCAAACGGG

>541bd6e1797d0ede75da2443e3b78faf

TACGTAGGGCGCAAGCGTTGTCCGGAATTATTGGGCGTAAAGAGCTCGTAGGCGGTTTGTCGCGTCTGCTGTGAAAGCCCGGGGCTTAACTCCGGGTGTGCAGTGGGTACGGGCAGACTAGAGTGCAGTAGGGGAGACTGGAATTCCTGGTGTAGCGGTGAAATGCGCAGATATCAGGAGGAACACCGATGGCGAAGGCAGGTCTCTGGGCTGTTACTGACGCTGAGGAGCGAAAGCATGGGGAGCGAACAAG

>c4ac97285eefe4d6925afba62d8b845a

TACGTAGGGTGCGAGCGTTGTCCGGAATTACTGGGCGTAAAGGGCTCGTAGGTGGTTTGTCGCGTCGTCTGTGAAATTCCGGGGCTTAACTCCGGGCGTGCAGGCGATACGGGCATAACTTGAGTACTGTAGGGGTAACTGGAATTCCTGGTGTAGCGGTGAAATGCGCAGATATTAGGAGGAACACCGATGGCGAAGGCAGGTTACTGGGCAGTTACTGACGCTGAGGAGCGAAAGCATGGGTAGCGAACAGG

>9ee9d7f1e7a96963f1ee2668f858fced

TACGTATGGACCGAGCGTTGTCCGGAATCATTGGGCGTAAAGGGTACGTAGGCGGCCTAGTAAGTTAGAAGTGAAATAATATAGCTCAACTATATAAAGCTTTTAAAACTGTTAGGCTTGAGAGATGAAAGGGAAAGTGGAATTCCTAGTGTAGCGGTGAAATGCGCAGATATTAGGAAGAATACCGGTGGCGAAGGCGACTTTCTGGTCATCATCTGACGCTGAGGTACGAAAGCGTGGGTAGCAAACAGG

>4ad2ce56725cc253a45c5004838e684e

TACGAAGGGGGCTAGCGTTGTTCGGATTTACTGGGCGTAAAGCGCACGTAGGCGGATTTTTAAGTCAGGGGTGAAATCCCGGCGCTCAACCCCGGAACTGCCTTTGATACTGGAAGTCTTGAGTGTGGAAGAGGTGAGTGGAATTCCGAGTGTAGAGGTGAAATTCGTAGATATTCGGAGGAACACCAGTGGCGAAGGCGGCTCACTGGTCCATTACTGACGCTGAGGTGCGAAAGCGTGGGGAGCAAACAGG

>5820d21ef8a28acc6fbc3a3710071f67

GACCAGATGAATTCTTGACACATTATTAAAAAGTTTTAATAAAATATGGTTCTCTGGCATCTTTCCAACAGCAACGAATATTCCCATTCTTAAAGAAAACAAAAACCTTTCATTATTATGTAGTTATCGTCCTATCTCACTAACGTCAGTTCTAAGCAAAGTATTTGAGAAGATTATCAATAGTAGACTGCAGTGGGAACTGGAGAAAAGGAAGCTCCTATCTCCTGCCCAGTGTGGTTTCCGGTGTTCGAGATCCACTCTTGACCAGATTTTGTACCTTCATGACAATATTATCGTGTGTTTC

>d3b52387360ceed8f554547f02b16f85

GACGTAGGGCGCGAGCGTTGTCCGGATTTATTGGGCGTAAAGAGCTCGTAGGCGGCTTGTCGCGTCGACTGTGAAAACCCGCAGCTCAACTGCGGGCCTGCAGTCGATACGGGCAGGCTAGAGTTCGGTAGGGGAGACTGGAATTCCTGGTGTAGCGGTGAAATGCGCAGATATCAGGAGGAACACCGGTGGCGAAGGCGGGTCTCTGGGCCGATACTGACGCTGAGGAGCGAAAGCGTGGGGAGCGAACAGG

>3bac14d2805bf995afe9d316a5777b1e

TACGTAGGTGGCAAGCGTTGTCCGGATTTATTGGGCGTAAAGCGAGCGCAGGCGGAAGAATAAGTCTGATGTGAAAGCCCTCGGCTTAACCGAGGAACTGCATCGGAAACTGTTTTTCTTGAGTGCAGAAGAGGAGAGTGGAACTCCATGTGTAGCGGTGGAATGCGTAGATATATGGAAGAACACCAGTGGCGAAGGCGGCTCTCTGGTCTGCAACTGACGCTGAGGCTCGAAAGCATGGGTAGCGAACAGG

>63d713076c75fedf9431592437dcf475

GCCGCGGTAATTAGAAACATCAGTATAGGCCGCCGAGTTGCTGACTCCCAGACATTGTACCTAGCGTGCGTCATACAGGTTCAATGGTGGTTTCCATGAGTCCCCATGCGCTCCTGCACAAGCTCTACGCTCTCAATCGCGGTAATTAGATACCCGAGTAGTCCGGCTGACTGACTTGCGTCA

>f4c4bf63d2f22896f482b7da319faaa1

CCGCCGCGGTAATACGATTTCTTTAATTTAAATATTTAAGTTTCAGTTAATATAATAATAATATAAAATGTCTATAATTTTGGTGAAATATATTTTATCTTTAAAAATTAATTTTATGTCTGAAAAATTTTTGTATAAACTAGGATTAGATACCCCGGTAGTCCGGCTGACTGACTTGCGTCAAA

>b383a0f98e44fe8a7452ebf944105c48

TACGTAGGTGGCAAGCGTTGTCCGGAATTATTGGGCGTAAAGGGCTCGCAGGCGGTTTCTTAAGTCTGATGTGAAAGCCCCCGGCTCAACCGGGGAGGGTCATTGGAAACTGGGAAACTTGAGTGCAGAAGAGGAGAGTGGAATTCCACGTGTAGCGGTGAAATGCGTAGAGATGTGGAGGAACACCAGTGGCGAAGGCGACTCTCTGGTCTGTAACTGACGCTGAGGAGCGAAAGCGTGGGGAGCGAACAGG

>248e927163446189d429f4d36dc3c4c0

TATGGTAATTGTGTGCCAGCCGCCGCGGTAAGAGAAAAACTATCGCCAACAATTAGCGCATTAAGTAGTAGTAACGGCGAAGGTAACAGCTGTACGCTACTGATAAGCCAGCAGCCGACGCTACTTCTCTTCTGTCTACGATTTATTAGAAACCCCAGTAGTCCGGCTGACTGACTTAACGTCCATCTCGTATGCCGTCTTCTG

>99212b0bc2136b555aa8a942694b8b34

CGGTAAAAGTTTAAACCGACAATGATAAGATACGTAACTGTATCAAACGTTGACAATAATCCAAGTGACAGATAATCAAACAAGTGTAATACACGACAAAAGTATACATACATATTTATTTATGATAATGTATGCAATGCAATAATTAGAAACCCGAGTAGTCCGGCTGACTGACTCTA

>a7c367beef72574e1d66781545a0674a

TACGTAGGTGGCGAGCGTTGTCCGGAATCATTGGGCGTAAAGGGAGCGCAGGCGGGCATGTAAGTCTTTCTTAAAAGTGCGGGGCTCAACCCCGTGATGGGAAAGAAACTACATGTCTTGAGTACAGGAGAGGAAAGCGGAATTCCCAGTGTAGCGGTGAAATGCGTAGATATTGGGAGGAACACCAGTGGCGAAGGCGGCTTTCTGGACTGCAACTGACGCTGAGGCTCGAAAGCCAGGGGAGCGAACGGG

>db347806b21e364fd3f99100b060e56a

CATTGAACTATCGTGAGAAAGTCAAACCGCCAAAGGGAATTATATTATAGTAAATATTGGCGTAAATAAACATTTTATTAATAGTTGTAATATATGAAAATGTGCAGAAGATAGAGACGATTAAAACGGTGTTTAACAATCGCTTATCGAGACAATATTTAATTAATTAGAAA

>cd93017c92a76a9ce9abbee5f54f88bb

TACGGAGGATCCAAGCGTTATCCGGAATCATTGGGTTTAAAGGGTCCGTAGGCGGTTTAGTAAGTCAGTGGTGAAAGCCCATCGCTCAACGGTGGAACGGCCATTGATACTGCTAGACTTGAATTATTAGGAAGTAACTAGAATATGTAGTGTAGCGGTGAAATGCTTAGAGATTACATGGAATACCAATTGCGAAGGCAGGTTACTACTAATTGATTGACGCTGATGGACGAAAGCGTGGGTAGCGAACAGG

>9d3d5706f15af689ab054af07b4a76a2

TAATTGTGTGCCAGCAGCCGCGGTAATCCATTATTTCTGGTAGCCCAGAGTATGGACGATCTAGACAACAAGTGCAGGAAAGCATATGAGGAATGCGGAATACTAGAATTGCAAATAAATACCAAGAAAACAAAATATCAGTGGATTAGAAACCCCTGTAGTCCGGCTGACTGACTTAACGTCCATCTCGTATGCCGTC

>a381c1e1b0a4e76331d2b444223b6fc0

CGCGAATCGATTCGACACGGAAAACGCGTAAATATTAGCAACGACACGTTCGAGTAATGGTGCTTCGGTTCGTTTGGATTTGAAATACTCAGCCACCCTTTAAAAAATTATATTATCTGTCTACATATGTCAGATGAAAAAAAAATTTAAAAGTAGTTGAAAGGAAGATAATT

>2ddc0ac7b80e23d88d0c9a83c4578939

ATTTGTTTTCTTTGTACAGACAGACGTACGTGGTGCAGTTGATATTGGAAGTGCATTACTATGCATTTTTTATCTTTTCATTTGGTACGAATTACACAGAGGTCGTTAACTATGAATCGGCGAAACCCTTCGGTCATTTCCTACTATACAATCCATTTTTATGTTACACAGAGTATTTGATTTGTGGCGAGTAATTTGTCACTCTTTGTTCGCTGTCCGGCTGT

>21669341313babc4db269f444f905f78

TACGGAGGGGGCTAGCGTTGTTCGGAATTACTGGGCGTAAAGCGCACGTAGGCGGACTGGAAAGTTGGGGGTGAAATCCCGGGGCTCAACCTCGGAACTGCCTTCAAAACTATCAGTCTGGAGTTCGAGAGAGGTGAGTGGAATTCCGAGTGTAGAGGTGAAATTCGTAGATATTCGGAGAAACACCAGTGGCGAAGGCGGCTCACTGGCTCGATACTGACGCTGAGGTGCGAAAGCGTGGGGAGCAAACAGG

>e2319b6281e012202c31ea8de07a8205

TACGGAGGGGGCTAGCGTTGTTCGGAATTACTGGGCGTAAAGCGCACGTAGGCGGCTTTGTAAGTTAGGGGTGAAAGCCTGGAGCTCAACTCCAGAATTGCCTTTAAGACTGCATCGCTTGAATCCAGGAGAGGTGAGTGGAATTCCGAGTGTAGAGGTGAAATTCGTAGATATTCGGAAGAACACCAGTGGCGAAGGCGGCTCACTGGACTGGTATTGACGCTGAGGTGCGAAAGCGTGGGGAGCAAACAGG

>c340c99eb9c9f3f007a4ccc370b6ee9b

CCGCCGCGGTAAGTGCTCAGAGAACATCAATCTTTCACTTCGTTCTCACTTTCTCTTTTTACTGCCTCGTCTTCTGACCCTTTTCCACCATACCCCTTCTTCTCCTTTTCGTCATCCTTTTTCGGTCTATTTGTAGCATAATATATTAGAAACCCTGGTAGTCCGGCTGACTGACTTAACGTCCA

>a27c6e0a8a8b3ed58a66160a57674ded

CATTGAACTATCGTGAGAATGGCACGCCGCCAAAGGGAATTATATTATAGTAAATATTGGCGTAAATAAACATTTTATTAATAGTTGTAATATATGAAAATGTGCAGAAGATAGAGACGATGAAAACGGTGTTTAACAATCGCTTATCGAGACAATATTTAATTAATTAGAAA

>8254c31585eec5a802e2754cae34361e

CGGTAAAAGTTTAAACCGACAATGATAAGATACGTAACTGTATCAAACGTTGACAATAATCCAAGTGACAGATAATCAAACAAGTGTAATACACGACAAAAGTATACATACATATTTATTTATGATAATGTATGCAATGCAATAATTAGAAACCCCGGTAGTCCGGCTGACTGACTTAA

>4e558ff23ba35a3371f74bf616fd05b9

TACGTAGGTGGCAAGCGTTGTCCGGAATTATTGGGCGTAAAGCGCGCGCAGGCGGTCTTTTAAGTCTGATGTGAAAGCCCCCGGCTCAACCGGGGAGGGTCATTGGAAACTGGGAGACTTGAGTACAGAAGAGGAGAGTGGAATTCCACGTGTAGCGGTGAAATGCGTAGATATGTGGAAGAACACCAGTGGCGAAGGCGACTCTCTGGTCTGTAACTGACGCTGAGGCGCGAAAGCGTGGGGAGCAAACAGG

>821b51e82c4e1e6a91a0df1c30c19cd8

TACAGAGGGTGCGAGCGTTAATCGGAATTACTGGGCGTAAAGCGAGTGTAGGTGGCTCATTAAGTCACATGTGAAATCCCCGGGCTTAACCTGGGAACTGCATGTGATACTGGTGGTGCGAGAATATGTGAGAGGGAAGTAGAATTCCAGGTGTAGCGGTGAAATGCGTAGAGATCTGGAGGAATACCGATGGCGAAGGCAGCTTCCTGGCATAATATTGACACTGAGATTCGAAAGCGTGGGTAGCAAACAGG

>a460e1641b9befd1fd2ed03419eabff4

TACGAGGAAGACAAGTGTTATTCATCTTTATTAGGTTTAAAGGGTACCTAGACAGTATATTTAGCCTTAATTGGTACTAATTTACTAGAGTTTCGTAGAAAAGTAATCTGTAGAACTATCGGAGGAGAGATGTAATTTTGTAATACCGGTGGTACTGTTACGTGAAAACAATTCTTTATATTTAACTGACGTTGAGGGACGAAGGCTTGGGTAACGAATAGG

>1f11bff452e26637389de3486e564f6f

TACGGAGGATGCGAGCGTTATCCGGATTTATTGGGTTTAAAGGGTGCGCAGGCGGGACAGCAAGTCAGCGGTCAAATTGCGGGGCTCAACCCCGTCCAGCCGTTGAAACTGCGGTTCTTGAGTGCGCGAGAAGTATGCGGAATGCGTGGTGTAGCGGTGAAATGCATAGATATCACGCAGAACTCCGATTGCGAAGGCAGCATACCGGCGCGCAACTGACGCTCATGCACGAAAGCGTGGGGAGCAAACAGG

>d51c1cd7ce9a1f0ab0cfa5a9dc665fd0

CACACTTATGTCTTCAGAAAGACAAAATATTTCTCCAGGATGCATTTCAACTATGTCGGTCATTTTCAACTGGCAGTGCCTTGTAGATATTTGTGAAAGATTTTGAGTGTGTCTTGTCCATAGAGTTGAGTTTGTTCATTAATAATTTTGTTATGTAGTTTGTAACGTAATACATTTAGGCTTTTAAGTAAATTCATTTTGTTTAGTTCTTTGCCGGTTGTG

>0a21726cd4952a96635576553da67787

TACGGAAGGTCCAGGCGTTATCCGGATTTATTGGGTTTAAAGGGAGCGTAGGCGGATTATTAAGTCAGTGGTGAAAGACGGTGGCTCAACCATCGTTAGCCATTGAAACTGGTAGTCTTGAGTGCAGACAGGGATGCTGGAACTCGTGGTGTAGCGGTGAAATGCTTAGATATCACGATGAACTCCGATCGCGAAGGCAGGTGTCCGGGCTGCAACTGACGCTGAGGCTCGAAAGTGTGGGTATCAAACAGG

>67cfd0d3e21fd35c590648ba9d5d6b09

GGTTATGTTCGTCAACACCGGGCAAACCCGGTCATATAATTTTACCATACACTATAACACGTTGAGGTCGAGATTGCAGTGCAAGGTGAGGTTATGTTATCACATACTTTACAATGAAGTCTGGTCATATAATTGTGGCCGACAACGAAGAAACCGATCGAGATGGCAGTGGAGTCATAATTTTACCGGCAGTGGAAACGTGTGGTGAGGTTATGTTCATCAAC

>d83c5d727ff188025f2f46d856148190

TGAAACAAATCGGTTTTGTCAGACCGGACCGGTTTTACCGCACTACAAGGATTCTGGTAAACACTGTTTACGGACTGTTACAGAATCTTCACCGAGTTAATATTAAACCATTTATAGTCATTCTTCTTGACAATAGTTTTCAGAAAGTATCACGTTGTGTGTAAGTAAATATAAAATATTTTGAAACACGAGATAGTGTATTATGAACTACAGAGCTATTCCAGTTATCCGGATT

>1fa721bca8e6709e3a6e1a81c6a4a9a6

CACGATAGCGTTATGGTAATTGTGTGCCAGCCGCCGCGGTAAAGTAAACACAATATCTGGCAGGACTGCCACTCGTTACTTGATTCAGCACTCTCCTTATATAGTTTAGTTTAGATTCGTCTGTTTTACAGATTGACTCACTTGATTAGAAACCCTTGTAGTCCGGCTGACTGACTACTGTGTAATCTCGTATGCCGTCTTCTGCTTGAAAAAAA

>f699290b3632d3cf7a97e1ee23587084

TACAGAGGGTGCAAGCGTTAATCGGAATTACTGGGCGTAAAGCGCGCGTAGGTGGTTCGTTAAGTTGGATGTGAAAGCCCCGGGCTCAACCTGGGAACTGCATTCAAAACTGGCGAGCTAGAGTATGGTAGAGGGTGGTGGAATTTCCTGTGTAGCGGTGAAATGCGTAGATATAGGAAGGAACACCAGTGGCGAAGGCGACCACCTGGACTGATACTGACACTGAGGTGCGAAAGCGTGGGGAGCAAACAGG

>a83d9a056592f67f3bb5715a2081b45c

CATTGAACTATCGTGAGAAAGTCAAGCCGCCAAAGGGAATTATATTATAGTAAATATTAGCGTTAATAAACATTTTATTAATAGTTGTAATATATGATAATGTGCAGAAGATAAAGACGATTAAAACAGTGTTTAATAATCGCTTATCGAGACACTATTAAAGTAATACGGAATTAAAATATGTTTATTATGTGTATTAAACAAGGGATGAACTTGAAAATCAATACAGTGTTAAACGAAACTAATAGAGTCACGCAAGTGAACTTAGAACATTTTTAATAAC

>8612a638846acf832935cb932a1f0398

TACGGAGGGTGCAAGCGTTATCCGGATTTATTGGGTTTAAAGGGTCCGTAGGCGGACTTATAAGTCAGTGGTGAAAGCCTGTCGCTTAACGATAGAACTGCCATTGATACTGTAAGTCTTGAGTATATTTGAGGTAGCTGGAATAAGTAGTGTAGCGGTGAAATGCATAGATATTACTTAGAACACCAATTGCGAAGGCAGGTTACCAAGATATAACTGACGCTGAGGGACGAAAGCGTGGGGAGCGAACAGG

>8461eeb2fa3303459af1758c7e3b6ed3

TACGTAGGGCGCAAGCGTTGTCCGGAATTATCGGGCGTAAAGAGCTCGTAGGCGGTTTGTCGCGTCTGGTGTGAAAACTCGAGGCTCAACCTCGAGCTTGCATCGGGTACGGGCAGACTAGAGTGCGGTAGGGGAGACTGGAATTCCTGGTGTAGCGGTGGAATGCGCAGATATCAGGAGGAACACCGATGGCGAAGGCAGGTCTCTGGGCCGCAACTGACGCTGAGGAGCGAAAGCATGGGGAGCGAACAGG

>a2bf6823d0b7569faa0320954d19147e

AAGCAGCATATGGTAATTGTGTGCCAGCAGCCGCGGTAACAGACTCTACAGCTTCTCTGGTGAGTCGTTCTACATCGTCAAACTTGAAAGAAACATTTCCCTGGCGACTTTCCCTTTCACTTGAGCCCATATTAGCTCGATAGGATTAGATACCCTGGTAGTCCGGCTGACTGACTGAGACTTAATCTCGTATGCCGTCTTCTGCTTGAAAA

>db6a6439a49db4020eff203d55eba02a

TACGAAGGGTGCAAGCGTTAATCGGAATTACTGGGCGTAAAGCGCGCGTAGGTGGTTTGATAAGTTGGATGTGAAAGCCCCGGGCTCAACCTGGGAATTGCATCCATAACTGTCTGACTAGAGTATGGCAGAGGGTGGTGGAATTTCCTGTGTAGCGGTGAAATGTGTAGATATAGGAAGGAACACCAGTGGCGAAGGCGACCACCTGGGCTAATACTGACACCGAGGTGCGAAAGCGTGGGGAGCAAACAGG

>ed22dc2adede5ab5b66c7527782c345e

TACGTAGAAGACAAGTGTTATTCATCTTTAACCGGTTTAAAGGGTACCTAGACGGAAAATCAAGCCATAGTAGGGACTAATTTTCTAGAGTTTTATGTGTGAATAGTCGAATTATCTGAGTAGCAATAATATGCGATGATACAGGTAAGACGGATGGCGGCGAAGGCAATATTCTATGTAGAAACTGACGTTGAGGGACGAAGCCATGGGGAGCGAGAAGG

>850cd943df43949eb6f9b3d608b92b87

TACGTAGGTGGCGAGCGTTATCCGGAATCATTGGGCGTAAAGGGTGCGTAGGCGGCGAGTTAAGTCTGAGGTAAAAGGTTGCAGCTCAACTGTAACAAGCCTTGGAAACTGACTGGCTAGAGTGCAGGAGAGGGCAGTGGAATTCCATGTGTAGCGGTAAAATGCGTAGATATATGGAGGAACACCAGTGGCGAAGGCGGCTGTCTGGCCTGTAACTGACGCTGAGGCACGAAAGCGTGGGGAGCAAATAGG

>03372358b8b6ef450923ce1f7b34505e

TACGGAGGATGCGAGCGTTATCCGGATTTATTGGGTTTAAAGGGTGCGTAGGCGGAGCGTCAAGTCAGCGGTAAAAATTCGGGGCTCAACCCCGTCGTGCCGTTGAAACTGACGCCCTTGAGTGAGCGAGAAGTAAGCGGAATGCGTGGTGTAGCGGTGAAATGCATAGATATCACGCAGAACGCCGATTGCGAAGGCAGCTTACCGGCGCTCGACTGACGCTGAGGCACGAAAGTGCGGGTATCGAACAGG

>4aeb615b1fe1b07ac50b5840a2bc021a

CCGCCGCGGTAAGTGCTCAAAGAACATCAATCTTTCACTTCGTTCTCACTTTCTCTTTTTACTGCCTCCTCTTCTGACCCTTTTCCACCATACCCCTTCTTCTCCTTTTCGTCATCCCTTTTCGGTCTATTTGTAGCATAATATATTAGATACCCCTGTAGTCCGGCTGACTGACTTGCGTCAAA

>1f639abfb02c42ff219c3a734f90ae44

TGAAAATATCCAACGCACACATGGGCGCATGCGCATGTTTACGAACAGCGGGCCTCTAAGCGGTCAGTTGCCTTTCGCGGACTACAGCTGGCGACCCTTGTCGCTCTCTTTTAATCTTTATTCTCTTTCGCCCTCCCGTTTCCGGATTCCCTTGTCCTCGTCCGGTTCGTACCGTCTGCTCCCGTTTCTACTGTGCCGAGTATATGTTCGGTCATTTCTTACTTGGCGATTCATAGAACGCTTTAACAACTCTCACAGACTGATCGTATGTCTTGAATAAATTTGTG

>69bb24d015f345a05fdeea8400ee5bac

TACGAGGGGAGCGAGTGTTGTTCAGTTTTATTGGGCGTAAAGGGTATGTAGGCGGTTTTGTAAGTCAACACTTAAATCTTGAGACTTAATCTCATTATAGTGTTGATACTGCATAACTATGAACTTAATAGGGGTGAACACAATTCCAAGTGTAGAGGTGAAATTCGTTGATATTTGGAGGAGTACCAAAGGCGAAGGCAGTTCATTGGGTTAAGTTGACGCTGAGGTACGAAAGCGTGGGGAGCAAACAGG

>f8aeec3c1ff0f4e7e1c829e4db0618d9

TACAGAGGGTGCAAGCGTTAATCGGAATTACTGGGCGTAAAGCGCGCGTAGGTGGTTTGTTAAGTTGGATGTGAAATCCCCGGGCTCAACCTGGGAACTGCATTCAAAACTGACTGACTAGAGTATGGTAGAGGGTGGTGGAATTTCCTGTGTAGCGGTGAAATGCGTAGATATAGGAAGGAACACCAGTGGCGAAGGCGACCACCTGGACTGATACTGACACTGAGGTGCGAAAGCGTGGGGAGCAAACAGG

>6de6cae480657d65433bbaee62118c87

TACGGAAGGTCCGGGCGTTATCCGGATTTATTGGGTTTAAAGGGAGCGTAGGCGGGATGCCAAGTCAGCCGTGAAATGCCGTGGCCCAACCATGGCCCTGCGGTTGAAACTGTCGTCCTTGAGTGCGCGCAGGGGTGCCGGAATTCATGGTGTAGCGGTGAAATGCTTAGATATCATGAAGAACTCCGACCGCGAAGGCAGGTGCCCGGAGCGCAACTGACGCTGAGGCTCGAAGGTGCGGGTATCGAACAGG

>96efd726d3de34255db2e77805345706

TACGAAGGGTGCAAGCGTTACTCGGAATTACTGGGCGTAAAGCGTGCGTAGGTGGTGGTTTAAGTCAGATGTGAAAGCCCTGGGCTCAACCTGGGAACTGCATTTGATACTGGATCACTAGAGTGCGGTAGAGGATAGTGGAATTCCCGGTGTAGCAGTGAAATGCGTAGAGATCGGGAGGAACACCAGTGGCGAAGGCGACTATCTGGACCAGCACTGACACTGAGGCACGAAAGCGTGGGGAGCAAACAGG

>4cb57a4533e560227d4daacea1e6f09a

TACGAAGGGGGCTAGCGTTGTTCGGAATTACTGGGCGTAAAGGGCGCGTAGGCGGCCCGCTCAGTCAGAAGTGAAAGCCCCGGGCTCAACCTGGGAATTGCTTTTGATACTGGCGGGCTTGAGTTCCGGAGAGGATGGTGGAATTCCCAGTGTAGAGGTGAAATTCGTAGATATTGGGAAGAACACCGGTGGCGAAGGCGGCCATCTGGACGGACACTGACGCTGAGGCGCGAAAGCGTGGGGAGCAAACAGG

>11f0558dca37009cbf1a6083b0b658e5

TACGGAGGGTGCAAGCGTTAATCGGAATTACTGGGCGTAAAGCGCACGCAGGCGGTTGATTAAGTTAGATGTGAAATCCCCGGGCTTAACCTGGGAATGGCATCTAAGACTGGTCAGCTAGAGTCTTGTAGAGGGGGGTAGAATTCCATGTGTAGCGGTGAAATGCGTAGAGATGTGGAGGAATACCGGTGGCGAAGGCGGCCCCCTGGACAAAGACTGACGCTCAGGTGCGAAAGCGTGGGGAGCAAACAGG

>e56b91bc38d06da3a0be8988f2d389dc

TACGAAGGGTGCAAGCGTTGTTCGGATTTATTGGGCGTAAAGCGCGCGCAGGCGGACCTGTAAGTCAGATGTGAAATCTCGGGGCTCAACTCCGAAACTGCGTCTGAAACTACAGGTCTAGAATCTCGGAGAGGGATGGGGTATTTCACATGTAGGGGTAAAATCCGTAGATATGTGAAGGAACACCAGAGGCGAAGGCGCCATCCTGGACGAGTATTGACGCTGAGGCGCGAAAGCGTGGGGATCAAACAGG

>cc761daf51f27c423da57f3f1f0ff5cc

TACGGAGGGTGCAAGCGTTAATCGGAATTACTGGGCGTAAAGCGCACGCAGGCGGTCTGTTAAGTCAGATGTGAAATCCCCGGGCTTAACCTGGGAACTGCATTTGAAACTGGCAGGCTTGAGTCTTGTAGAGGGGGGTAGAATTCCAGGTGTAGCGGTGAAATGCGTAGAGATCTGGAGGAATACCGGTGGCGAAGGCGGCCCCCTGGACAAAGACTGACGCTCAGGTGCGAAAGCGTGGGGAGCAAACAGG

>1b33e015891dae7fb724eee39590d3d9

AATTGTGTGCCAGCCGCCGCGGTAACATTGAACTATCGTGAGAAAGTCAAACCGCCAAAGGGAATTATATTATAGTAAATATTGGCGTAAATAAACATTTTATTAATAGTTGTAATATATGAAAATGTGCAAAAGATAGAGACGATTAGATACCCCTGTAGTCCGGCTGACTGACTTAACGTCCATCTCGTATGCCGT

>47912b9ee803503acda1516a6e5fa10a

CATTGAACTATCGTGAGAAAGTCAAGCCGCCAAAGGGAATTATATTATAGTAAATATTGGCGTAAATAAACATTTTATTATTAGTTGTAATATATGAAAATGTGCAGAAGATAAAGACGATTAAAACGGTGTTTAACAATCGCTTATCGAGACATTGTTTTTAATTAATTAGTAATAAAAATATGTTTATTATGTGTATTATTCAAGGAATAGACTTGACAATAAATACAGTGGTAAACGGAACTAATAGAGTCACGCAAGTGAACTTAGAACATTTTTAATAAC

>d6680d977f3a40058a3d5ea477ea7fbc

TACGGAGGGGGCTAGCGTTGTTCGGAATTACTGGGCGTAAAGCGCACGTAGGCGGCTTTGTAAGTTAGAGGTGAAAGCCTGGAGCTCAACTCCAGAATTGCCTTTAAGACTGCATCGCTTGAACGTCGGAGAGGTGAGTGGAATTCCGAGTGTAGAGGTGAAATTCGTAGATATTCGGAAGAACACCAGTGGCGAAGGCGGCTCACTGGACGACTGTTGACGCTGAGGTGCGAAAGCGTGGGGAGCAAACAGG

>d3f15f26540c2d1c8bf24edecabf0bb6

TACGTAGGGCGCAAGCGTTGTCCGGAATTATTGGGCGTAAAGAGCTTGTAGGTGGCTTGTCGCGTCTGCCGTGAAAACCCGAGGCTCAACCTCGGGCGTGCGGTGGGTACGGGCAGGCTAGAGTGTGGTAGGGGAGACTGGAACTCCTGGTGTAGCGGTGAAATGCGCAGATATCAGGAAGAACACCGATGGCGAAGGCAGGTCTCTGGGCCATTACTGACACTGAGAAGCGAAAGCATGGGGAGCGAACAGG

>2141d93bbcf8de0a4d263185f95375a2

TACGTAGGTGGCAAGCGTTGTCCGGATTTATTGGGCGTAAAGGGAACGCAGGCGGTCTTTTAAGTCTGATGTGAAAGCCTTCGGCTTAACCGGAGATGTGCATTGGAAACTGGAAGACTTGAGTGCAGAAGAGGAGAGTGGAACTCCATGTGTAGCGGTGAAATGCGTAGATATATGGAAGAACACCAGTGGCGAAAGCGGCTCTCTGGTCTGTAACTGACGCTGAGGTTCGAAAGCGTGGGTAGCGAACAGG

>56b1aad1c3b1a60de2e507ebfb7e684d

TACGTAGGGTGCGAGCGTTGTCCGGATTTATTGGGCGTAAAGGGCTCGTAGGTGGTTGATCGCGTCGGAAGTGTAATCTTGGGGCTTAACCCTGAGCGTGCTTTCGATACGGGTTGACTTGAGGAAGGTAGGGGAGAATGGAATTCCTGGTGGAGCGGTGGAATGCGCAGATATCAGGAGGAACACCAGTGGCGAAGGCGGTTCTGTGGGCCTTTCCTGACGCTGAGGAGCGAAAGCGTGGGGAGCGAACAGG

>571c6729feb1b5d88483f4357eef77e9

GCCGCCGCGGTAAGTGCTCAGAGAACATCAATCTTTCACTTCGTTCTCACTTTCTCTTTTTACTGCCTCCTCTTCTGACCCTTTCCACCATACCCCTGCTTCTCCTTTTCGTCATCCCTTTTCGGTCTATTTGTAGCATAATATATTAGAAACCCTTGTAGTCCGGCTGACTGACTCTACGACCAT

>50a50ee3d4ca18a21a32bb96857ab253

TACGGAGGGTGCAAGCGTTGTTCGGAATTATTGGGCGTAAAGCGCGTGCAGGCGGCTGTTCAAGTCCGATGTGAAAGCCCGGGGCTCAACCCCGGAAGTGCATTGGAAACTGGACAGCTTGAGTACGGGAGAGGGAGGTAGAATTCCGAGTGTAGGGGTGAAATCCGTAGATATTCGGAGGAATACCGGTGGCGAAGGCGGCCTCCTGGACCGATACTGACGCTGAGACGCGAAAGCGTGGGAAGCAAACAGG

>10afe7c34ee7c9c720131abef4765346

CATTGAACTATCGTGAGAAAGTCAAGCCGCCAAAGGGAATTATATTATAGTAAATATTGGCGTAAATAAACATTTTATTAATAGTTGTAATATATGAAAATGTGCAGAAGATATAGACGATTAAAACGGTGTTTAACAATCGCTTATCGAGACAATATTTAATTAATTAGTAATTAAAATATGTTTATTATGTGTATTATTCAAGGAATAGACTTGACAATAAATACAGTGGTAAACGGAACTAATAGAGTCACGTAAGTGAACTTAGAACATTTTTAATAAC

>efbe2be07787c4384193c4ae0d411d16

CCGCCGCGGTAATACGATTTCTTTAATTTAAATATTTAAGTTTCAGTTAATATAACAATAATATAAAATATCTAAAATTTTGGTGAAATATATTTTATCTTTAAAAATTAATTTTATGTCTGAAAAATTTTTGTATAAACTAGGATTAGATACCCTAGTAGTCCGGCTGACTGACTACTGTGTAA

>ea403646ed22d679fa4586263d8fc32f

TACAGAGGGTGCGAGCGTTAATCGGATTTACTGGGCGTAAAGCGTGCGTAGGCGGCTTATTAAGTCGGATGTGAAATCCCCGAGCTTAACTTGGGAATTGCATTCGATACTGGTGAGCTAGAGTATGGGAGAGGATGGTAGAATTCCAGGTGTAGCGGTGAAATGCGTAGAGATCTGGAGGAATACCGATGGCGAAGGCAGCCATCTGGCCTAATACTGACGCTGAGGTACGAAAGCATGGGGAGCAAACAGG

>c365c6df832ceaee41b4ed82e51d902f

GTAATTGTGTGCCAGCCGCCGCGGTAAGGTTTCTTGTATTCTTGTTAACTACTGAACATCCCCCGCCCTCACAACAATGGTGACATTAAGTGTTCGGATTACCTTTCCTTCTATTCCACTTTGAAAGAAGACATGCTGTGTATCATTAGATACCCTGGTAGTCCGGCTGACTGACTTAACGTCCATCTCGTATGCCGTCT

>2fc77f7324ff3f20c372b59b56363404

TACGTACAAGACTAGTGTTATTCATCTTTAATAGGTTTAAAGGGTACCTAGACGGTAAGTAAAGCCTTAATCGGGACTAATTTACTAGAGTTACTTGCGAAGGAGCACTAAAGTACTAGTGGTGTAGAGATAAAATTCTATGATACCTTTTTTCGAAAGAGAAATAATGGCACAAGTATAGGCGAAAGCATCTCCTTATGTGATAACTGACGTTGAGGGACGAAGGCTTTGCGCAGCGAACAGG

>b0b8c849db125ff6c952286ea30a48f8

TACGGAGGGGGCTAGCGTTGTTCGGAATTACTGGGCGTAAAGCGCACGTAGGCGGATCAGAAAGTTGGGGGTGAAATCCCGGGGCTCAACCCCGGAACTGCCTTCAAAACTATTGGTCTGGAGTTCGAGAGAGGTGAGTGGAATTCCGAGTGTAGAGGTGAAATTCGTAGATATTCGGAGGAACACCAGTGGCGAAGGCGGCTCACTGGCTCGGTACTGACGCTGAGGTGCGAAAGCGTGGGGAGCAAACAGG

>df74f9dd527db2dc07920e568494b347

GTAATTGTGTGCCAGCCGCCGCGGTAATACTGTATCCAAGCGTATAGACATCAAATTGATTGACAAATCTATCATATTGTCAAAGTATAGTAGGTATAAAATCATATAACTCGTATCACCTTCGGAAAAATAGTCAGACATTTAATTAGAAACCCCAGTAGTCCGGCTGACTGACTTGCGTCAAATCTCGTATGCCGTCT

>c179ba48c0c4b9136966079078fd3ff6

TACAGAGGTCCCAACCGTTGTTCGGATTCACTGGGCGTAAAGGGTGCGTAGGTGGTTGGGTAAGTTTGAGGTGAAATCTCCGAGCTTAACTCGGAAAAGGCCTTGAATACTATCTGGCTTGAGGGTTGGAGGGGAGACTGGAATTCTCTGTGTAGCAGTGAAATGCGTAGATATCGAGAGGAACACCAGTGGCGAAGGCGAGTCTCTGGACAACACCTGACACTGAGGCACGAAAGCTAGGGGAGCAAACGGG

>b01f65c3da02d1690cbbb45a210380d5

TACGGAGGGTGCAAGCGTTATCCGGATTCACTGGGTTTAAAGGGTGCGTAGGCGGGTAGGTAAGTCAGAGGTGAAATCCTGGAGCTTAACTCCAGAACTGCCTTTGATACTATCTATCTTGAATATGGTGGAGGTAAGCGGAATATGTCATGTAGCGGTGAAATGCATAGATATGACATAGAACACCTATTGCGAAGGCAGCTTACTACGCCTATATTGACGCTGAGGCACGAAAGCGTGGGGATCAAACAGG

>5174f84a22f06b5330afeded33bb3d53

CACACTTCTGTCTTTAGAAAGACAAAACATGTTTCCAAGATGAATTTCAACCATGTCGGTCATTTTCAACTGGTAGTGCCTTGTAAATAGTTGTGAAACATTTTGAATGTGTCTTCTCCATAGAGTTCAGTTTGTTCATTAATGATTTTGTTATGTACTTTGTAACTTAGTATATTTAGACTTTCAAGTAAATTCATTTTATTTCCTTTTTTGCCGGTTGTC

>202ccb47d7106c5067a177dee8a9cc1a

TACAGAGGGTGCAAGCGTTAATCGGATTTACTGGGCGTAAAGGGCGCGTAGGTGGTTATTTAAGTGAGATGTGAAAGCCCCGGGCTCAACCTGGGAACTGCATCTTATACTGGGTAACTGGAGTGCAGTAGAGGGTAGTGGAATTTCCGGTGTAGCGGTGAAATGCGTAGATATCGGAAGGAACACCAGTGGCGAAGGCGGCTACCTGGACTGAGACTGACACTGAGGCGCGAAAGCGTGGGGAGCAAACAGG

>3714db21d59bf50cff3ddd13ce2ebd2c

TACGTAGGGCGCGAGCGTTGTCCGGAATTATTGGGCGTGAAGGGCTTGTAGGCGGCCGGTCGCGTCCGCCGTGAAATTCTCCGGCTCAACCGGGGGCTTGCGGTGGGTACGGGCCGGCTGGAGTGCGGTAGGGGAGGCTGGAACTCCTGGTGTAGCGGTGGAATGCGCAGATATCAGGAGGAACACCGGTGGCGAAGGCGGGTCTCTGGGCCGTTACTGACGCTGAGGAGCGAAAGCGTGGGGAGCGAACAGG

>c4d8b2bf99cdc93ce91924fe32ec9081

TACGGAGGGTGCAAGCGTTGTTCGGAATTATTGGGCGTAAAGCGCGTGCAGGCGGCTGTTCAAGTCCGATGTGAAAGCCCGAGGCTCAACCCCGGAAGTGCATTGGAAACTGGACAGCTTGAGTACGAGAGAGGGAGGTAGAATTCCGAGTGTAGGGGTGAAATCCGTAGATATTCGGAGGAATACCGGTGGCGAAGGCGGCCTCCTGGACCGATACTGACGCTGAGACGCGAAAGCGTGGGGAGCAAACAGG

>163f94c3a35b73a62562ce71439af321

TACGGAGGGTGCAAGCGTTGTTCGGAATTATTGGGCGTAAAGCGCGTGCAGGCGGCTGTTCAAGTCCGATGTGAAAGCCCGGGGCTCAACCCCGGAAGTGCATTGGAAACTGGACAGCTTGAGTACGGGAGAGGGAGGTAGAATTCCGAGTGTAGGGGTGAAATCCGTAGATATTCGGAGGAATACCGGTGGCGAAGGCGGCCTCCTGGACCGATACTGACACTGAGACGCGAAAGCGTGGGGAGCAAACAGG

>144a330066f43e4888807d3d33dc68f5

CACACTTATGTCTTCAGAAAGACAAAATGTTTCTCCAGAATGCATTTAAACCGTCTCGGTCATTTTCAGCTGGCAGTGCCTTGTAGATAATTGTGGAAGATTTTGAATGTTACTTGTCCATAGAGATCAGTTTGTTCATTAATGAGTTTGTTGTGTAGTTTGTAAATACAAACACAAGAACAAAAGAATTCTAAATTAACAAACGCAGTACAAATCGCATACAATATACCTTCAAACCAACCACC

>43961ec2faa5fa54221dfefb616f81ac

GGTATTGCAGCGACGCGATAGCGTTTTCCATTTACCGAAGTCGGAATATCTGGGGAAAGCGAAATATCTCTCAGACGCGTCTCCCAGCGAGATTTACAGGCGGCAGCAGTCTTTTCAATAATTCATATAAAGTGAATTTTATTTCCATTAACCGGATAAAAAAACGTTGAACACGAGTGAAGTCGAATAACTGTGG

>69a662cf572bf84c150e70493004248a

CATTGAACTATCGTGAGAATGGCACGCCGCCAAAGGGAATTATATTATAGTAAATATTGGCGTAAATAAACATTTTATTAATAGTTGTAATATATGAAAATGTGCAGAAGATAGAGACGATTAAAACGGTGTTTAACAATCGCTTATCGAGACAATATTTAATTAATTAGGAAATAAAATAAGTGTATTATTTGTATTTAACAAGAAATAAACGTGAAAATAAATACAGTGGTAAACGGAACTAATAGAGTCACGCAAGTGAACGTAGAACATTTTTAATAAC

>1bc599cac5647c5c30496e93ca551557

TACAGAGGGTGCAAGCGTTAATCGGAATTACTGGGCGTAAAGCGCGCGTAGGTGGTTCGTTAAGTTGGATGTGAAATCCCCGGGCTCAACCTGGGAACTGCATCCAAAACTGGCGAGCTAGAGTATGGTAGAGGGTGGTGGAATTTCCTGTGTAGCGGTGAAATGCGTAGATATAGGAAGGAACACCAGTGGCGAAGGCGACCACCTGGACTGATACTGACACTGAGGTGCGAAAGCGTGGGGAGCAAACAGG

>8904ae440972752641283831b7091a60

CCGCCGCGGTAAGTGCTCAGAGAACATCAATCTTTCGCTTCGTTCTCACTTTCTCTTTTTACTGCCTCCTCTTCTGACCCTTTTCCACCATACCCCTTCTTCTCCTTTTCGTCATCCCTTTTCGGTCTATTTGTAGCATAATATATTAGAAACCCGGGTAGTCCGGCTGACTGACTTAACGTCCA

>16d183895bad13327024246c004b102b

CACACTTATGTCTTCAGAAAGACAAAATATTTATCCAGGATGCATTTCAACCATGTCGGTCATTTTCAACTGGCAGTGCCTTGTAGACATTTGTGAAAGATTTTGAATGTGTCTTATCCATAGAGTTCAGTTTGTTCATTAATGATTTTGTTATGTAGTTTGTAACGTAATATATTTAGCCTATCAAGTAAATTCATTTTGTTTCCTTTTTGCCGGTTGTG

>a79d3662f7d61e9795ff25e7a5ec0d3e

GACGGGGGGGGCAAGTGTTATTCGGAATGACTGGGCGTAAAGGGCACGTAGGCAGTGAATCAGGTTAAAAGTCAAAGACGCCAGCTCAACTGGCGGAATGCTTTCAAAACCAATTCACTCGAGTGGGATAGAGGGGAGTGGAATTTCGTGTGTAGGGATAAAATCCGGAGATATACGAAGGAACGCCGAAGTAGCGAAGGCAGCTTTCTGGGTCTCTACTGACGCTAGGGTGCGAAAGCTTGGGGAGCAAACAGG

>f1e68550ad2b375c14dcca8e9e027f15

TACGGAGGTGGCAAGCGTTACTCGGATTGATTGGGTGTAAAGGGCGTGTAGGTGGTGGATTAAGTCGAATGTGAAATCCCTTGGCTCAACCAAGGAACTGCATTCGATACTGATTTGCTTGAGTGATAGAGAGGTAAGCGGAATTCCCGGTGTAACAGTGAAATGTGTAGATATCGGGAGGAACACCAGTGGCGAAGGCGGCTTACTGGCTATCTACTGACACTGAAACGCGAGAGCTAGGGGAGCAAACAGG

>afe248d353e544f2b70a66bf58beed76

TACGTAGGGTGCGAGCGTTAATCGGAATTACTGGGCGTAAAGCGTGCGCAGGCGGCTTTGTAAGACAGGTGTGAAATCCCCGGGCTTAACCTGGGAACTGCGCTTGTGACTGCAAGGCTTGAGTGTGGCAGAGGGGGGTGGAATTCCACGTGTAGCAGTGAAATGCGTAGAGATGTGGAGGAACACCGATGGCGAAGGCAGCCCCCTGGGTCAACACTGACGCTCATGCACGAAAGCGTGGGTAGCAAACAGG

>b57f4db3ac9eee7609f7f17ac7a4a29e

AAAATCAATTTAATGTTTTTTTTAATCCACAATATCACTTTAATCTTGATACATATTTAGTCTAAATGCTACGTTGACTACATATTTTAATTTTATAGATGCTTTCTGTGGAAAATTCTTTTACACTACAATTTACATATACAGATGCTTTATTGGGATTGATTTATTTCATGTAAACTACATATACTTCTGTATGTATAATAGGATAGGAATGGGCGAAAAAGAATGGGAGAAGGAATGTGT

>1c0d9672c5480661271d7672e33313a7

GTAGCTACTCAGAGCAATCCCACTTTACTACGAACACAATACACTATGGCCATAATTTTAAGGATTACAACATCTTGCTTAACGACTGCAACGGGGTTTAGTGTTTCCACAGCATCGTAAAGTCAAAGTTTCGAGAGGAATAATTAAAACGGCTTTACGTGAAAATTGTTTCTCTCTCGC

>39edc07f93d37fd13fbe8371c0914159

TACGAAGGGGGCTAGCGTTGCTCGGAATCACTGGGCGTAAAGGGTGCGTAGGCGGGTCTTTAAGTCAGGGGGGAAATCCTGGAGCTCAACTCCAGAACTGCCTTTGATACTGAAGATCTTGAGTATGGGAGAGGTGAGTGGAACTGCGAGTGTAGAGGTGAAATTCGTAGATATTCGCAAGAACACCAGTGGCGAAGGCGGCTCACTGGCCCATAACTGACGCTGAGGCACGAAAGCGTGGGGAGCAAACAGG

>a4b9fc120965f328d4713f34c7bd8f64

CACCGATCTACTATGGTAATTGTGTGCCAGCCGCCGCGGTAAAGTAAACACAATATCTGGCAGGACTGCCACTCGTTACTTGATTCAGCACTCTCCTTATATAGTTTAGTTTAGATTCGTCTGTTTTACAGATTGACTCACTTGATTAGAAACCCTGGTAGTCCGGCTGACTGACTTAACGTCCATCTCGTATGCCGTCTTCTGCTTGAAAAAAA

>e6b14c8384f8ab960db2fb299308b24f

TACGTAGAAGACTAGTGTTATTCATCTTTAGTAGGTTTAAAGGGTACCTAGACGGTATAATTAGTCTTTAATAGGAAACGGTTATACTAGAGTTATATATGAGGAGGTGAGTATTTGTGGAGTAGAGTTGAAATTCTTTGATACCATGAGGACTGGTAAAGGCGAAAGCAACCTTTTATCTAATAACTGACGTTGAGGGACGAAGGCTCGGGTCGCGAACAGG

>c5c1cb3d6d12e018c7426e1612c12595

CATTGAACTATCGTGAGAAAGTCAAACCGCCAAAGGGAATTATATTATAGTAAATATTGGCGTAAATAAACATTTTATTAATAGTTGTAATATATGAAAATGTGCAGAAGATAACGACGATTAAAACGGTGTTTAACAATCGCTTATCGAGACAATATTTAATTAATTAGGAATTAAAATATGTGTATTATTTGTATTATTCAAGGAATAAACTTGAAAATAAATACAGTGTTAAACGGAACTAATAGAGTCACGCAAGTGAACTTAGAACATTTTAATAAC

>dd88cd7041055eed0fb45a9ebe75cd74

TACGTAGGGGGCGAGCGTTGTCCGGAATCATTGGGCGTAAAGGGTTCGTAGGCGGACTGATAAGTCAGATGTGAAAAGATGTGGCTTAACCATGTTAAGCATTTGAAACTGTCAGTCTTGAGTGAAGGAGAGGTAAGTGGAATTCCTAGTGTAGCGGTGAAATGCGTAGATATTAGGAGGAATACCAGTAGCGAAGGCGACTTACTGGCCTTTAACTGACGCTGAGGAACGAAAGCGTGGGTAGCAAACAGG

>23891dfc4fac0293ce56091622e6896e

TACGAAGGGGGCTAGCGTTGCTCGGAATCACTGGGCGTAAAGGGCGCGTAGGCGGACTTTTAAGTCGGGGGTGAAAGCCCAGGGCTCGACCCTGGAATTGCCTTCGATACTGAGAGTCTTGAGTTCGGAAGAGGTTGGTGGAACTGCGAGTGTAGAGGTGAAATTCGTAGATATTCGCAAGAACACCAGTGGCGAAGGCGGCCAACTGGTCCGATACTGACGCTGAGGCGCGAAAGCGTGGGGAGCAAACAGG

>284b2c19fb2d22a33f9827d741445927

TACGGAGGGTCCGAGCGTTAATCGGAATTACTGGGCGTAAAGCGTGCGCAGGCGGTTTGTTAAGCGAGATGTGAAAGCCCTGGGCTCAACCTAGGAATAGCATTTCGAACTGGCGAACTAGAGTTTTGTAGAGGGGGGTAGAATTCCAGGTGTAGCGGTGAAATGCGTAGAGATCTGGAGGAATACCGGTGGCGAAGGCGGCTCCCTGGACAAAGACTGACGCTCATGCACGAAAGCGTGGGGAGCAAACAGG

>800c10d8c3784d5613b1c362eb9e251c

TACAGAGGGTGCGAGCGTTAATCGGATTTACTGGGCGTAAAGCGTGCGTAGGCGGCTTTTTAAGTCGGATGTGAAATCCCTGAGCTTAACTTAGGAATTGCATTCGATACTGGGAAGCTAGAGTATGGGAGAGGATGGTAGAATTCCAGGTGTAGCGGTGAAATACGTAGAGATCTGGAGGAATACCGATGGCGAAGGCAGCCATCTGGCCTAATACTGACGCTGAGGTACGAAAGCATGGGGAGCAAACAGG

>a4437f7303bfcc14aaa749766a3c2614

CATTGAACTATCGTGAGAAAGTCAAACCGCCAAAGGGAATTATATTATAGTAAATATTGGCGTAAATAAACATTTTATTAATAGTTGTAATATATGAAAATGTGCAGAAGACAAAGACGATTAAAACGGTGTTTAACAATCGCTTATCGAGACAATATTTAATTAATTAGGAATTAAAATATGTGTATTATTTGTATTTAACAAGGAATAAACTTGAAAATAAATACAGTGATAAACGGAACTAATAGAGTCACGCAAGTGAACTTAGAACATTTTTAATAAC

>8a6bc6067958bd68205ff2a83ba26476

CGCGAATCGATTCGACACGGAAAACGCGTAAATATTAGCAACGACACGTTCGAGTAATGGTGCTTCGGTTCGTTTGGATTTGAAATACTCAGCCACCCTTTAAAAAATTATATTATCTGTCTACATATGTCAGATGAAAAAAAAACTTAAAAGTAGTTGAAAGGAAGATAATT

>4f91bc708731545e9b076ed6494df2ad

CATTGAACTATCGTGAGAAAGTCAAACCGCCAAAGGGAATTATATTATAGTAAATATTGGCGTAAATAAACATTTTATTAATAGTGGTAATATATGATAATGTGCAGAAGGTAAAGACGATTAAAACGGTGTTTAACAATCGCTTATCGAGACACTATTAAAGTAATTAGGAATTAAAATATGTTTATTATGTGTATTATTCAAGGAATAAACTTGAAAATAAATACAGTGTTAAACGGAACTAATAGAGTCACGCAAGTGAACTTAGAACATTTTAATAAC

>ea60638f9057ef506db0120b45f7cd16

TACGTAAAAGACTAGTGTTAGTCATCTTTATTAGGTTTAAAGGGTACCTAGACGGTAAATTAAACTCTAAATGAGTATTTTTTTACTAGAGTTTTATAAAAGAAGGAAGAATTTCTGGAGTAGTGATAGAATACTTTAATACCAGAAGGACTGTTAACGGCGAAGGCATCCTTCTATGTAAAAACTGACGTTGAGGGACGAAGGCTTGGGTAGCAATAAGG

>13b059c31d945d6e700a689d1df3e034

TACGTAAGGACCGAGCGTTGTCCGGAATCATTGGGCGTAAAGGGTACGTAGGCGGTTATCTAAGTTAGAAGTCAAAGGCTATAGCTCAACTATAGTAAGCTTTCAAAACTGGATAACTTGAGAGATGGAAGGGAAAGTGGAATTCCTAGTGTAGCGGTGAAATGCGCAGATATTAGGAGGAATACCGGTGGCGAAGGCGACTTTCTGGCCATTATCTGACGCTGAGGTACGAAAGCGTGGGTAGCAAACAGG

>d65247bbe630690279883ec59e369218

TAAAGCCGAAGACGATTTCCGCAACTGAAGTCGAAACGTCCGGACTTGGTAAACAATGAACTTCAGCTCGAGAGCCCGGAAATTTTCACAACAAACTCACATAGTCTGTTTCACACAAACAAACACTGTCCACCTAATTGATACATTAGAAACCCCGGTAGTCCGGCTGACTGACT

>7803741fb67ea920be19ec58328ae7bb

GACAAGGGAGACGAGTGTTATTCATCTTTAACAGGTATATAGGGTACCTAGACGGTGTACAAAGGCTTAAATAAGTACCTGTTACACTTGAGTTTGATATGTGAGAGGAATATGTCAGAATTATTGGTGGAAAGATGAAATTTGCTAATACTAATAGGACTGATAACGGCGAAGGCAAACCTCTATGTACATGTATTTTTACATTGTTAACGAGTTGTATCGTTAACAATAACAAACGAAAAATACTATAACTGACGTTGAGGGACGAAGGCTCAGAGAGTGAAGAGG

>a7ad29436b897b9b8891b540fe68e1b0

TACGTAGGGTACAAGCGTTGTCCGGAATTATTGGGCGTAAAGAGCTCGTAGGTGGTCTGTCACGTCTGCTGTGGAAACGCAAGGCTCAACCTTGCGCGTGCAGTGGGTACGGGCGGACTAGAGTGCAGTAGGGGAGTCTGGAATTCCTGGTGTAGCGGTGAAATGCGCAGATATCAGGAGGAACACCGGTGGCGAAGGCGGGACTCTGGGCTGTAACTGACGCTGAGGAGCGAAAGCATGGGGAGCGAACAGG

>41ae6f1318836d20280526e5c708cfb9

CATTGAACTATCGTGAGAAAGTCAAACCGCCAAAGGGAATTATATTATAGTAAATATTGGCGTAAATAAACATTTTATTAATAGTTGTAATATATGAAAATGTGCAGAAGATAAAGACGATTAAAACGGTGTTTAACAATCGCTTATCGAGACAATATTTAATTAATTAGGAATTAAAATATGTGTATTATTTGTATTTAACAAGGAATAAACTTGAAAATAAATACGGTGGTAAACGGAACTAATAGAGTCACGCAAGTGAACGTAGAACATTTTTAATAAC

>4b906a5272686664a1dee5ff784896e2

TACGGAGGGTGCAAGCGTTAATCGGAATTACTGGGCGTAAAGCGCGCGTAGGCGGTTATTTAAGTCAGATGTGAAATCCCCGGGCTTAACCTGGGAATTGCATTTGAGACTGGATGGCTAGAGTATGGTAGAGATGAGTGGAATTTCAGGTGTAGCGGTGAAATGCGTAGATATCTGAAGGAACATCAGTGGCGAAGGCGACTCACTGGGCCATTACTGACGCTGAGGTGCGAAAGCGTGGGTAGCAAACAGG

>0a034e716339144b03715d35c017e6d1

TACGTAGGGTGCGAGCGTTAATCGGAATTACTGGGCGTAAAGCGTGCGCAGGCGGTTTGTTAAGACAGATGTGAAATCCCCGGGCTCAACCTGGGAACTGCATTTGTGACTGGCAGGCTAGAGTATGGCAGAGGGGGGTAGAATTCCACGTGTAGCAGTGAAATGCGTAGAGATGTGGAGGAATACTGATGGCGAAGGCAGCCCCCTGGGCCAATACTGACGCTCATACACGAAAGCGTGGGGAGCAAACAGG

>d79c5c836ba3e693ee6e33745ea40788

TACGTAGGGGGCAAGCGTTGTCCGGAATTATTGGGCGTAAAGCGCGCGCAGGCGGCTTACTAAGTCTGGTGTGAAAGCCCACGGCTCAACCGTGGAGGGCCATTGGAAACTGGTAAGCTTGAGTGCAGGAGAGGAGAGCGGAATTCCCGGTGTAGCGGTGAAATGCGTAGATATCGGGAGGAACACTCGTGGCGAAGGCGGCTCTCTGGCCTGTAACTGACGCTGAGGCGCGAAAGCGTGGGGAGCAAACAGG

>cf3299475a5258cb33f61e88aa643410

CATTTTACCATAGTGTCTGCCTCCACGGGCACGTTTATGGCTTTGCCGGTTATCTTTTAAGACTGTTTCTATTCTGAGACGACGGATCTGCATACGTGGAAATCCGGATGAAATCAGCCTCTCAGTGATACGATCGAGCGGCAGATGGGTTGGATCTTCCGGATACTTATAACTATTACTTTGGCTCGGCGGAGGCACTTTACCCTCATTAAGCAAGCGGCGGTATGTGGAGCACA

>aecbcf202302e7fb4f8352106d3c8e01

CATTGAACTATCGTGAGAGAGTCAAGCCGCCAAAGGGAATTATATTATAGTAAATATTGGCGTAAATAAACATTTTATTAATAGTTGTAATATATGAAAATGTGCAGAAGATAGAGACGATTAAAACGGTGTTTAACAATCGCTTATCGAGACAATATTTAATTAATTAGTAATTAAAATATGTTTATTATGTGTATTATTCAAGGAATAGACTTGACAATAAATACAGTGATAAACGGAACTAATAGAGTCACGCAAGTGAACGTAGAACATTTTTAATAAC

>fdbe52a323266f5930a5693e8309c281

TACGGAGGGTGCAAACGTTGTTCGGAATTATTGGGCGTAAAGCGCGTGCAGGCGGCTGTTCAAGTCCGATGTGAAAGCCCGGGGCTCAACCCCGGAAGTGCATTGGAAACTGGACAGCTTGAGTACGGGAGAGGGAGGTAGAATTCCGAGTGTAGGGGTGAAATCCGTAGATATTCGGAGGAATACCGGTGGCGAAGGCGGCCTCCTGGACCGATACTGACGCTGAGACGCGAAAGCGTGGAGAGCAAACAGG

>d451cdef9c41a4aa590f365ebc165044

TACGTAGGGCGCGAGCGTTGTCCGGAATTATTGGGCGTAAAGAGCTTGTAGGCGGTTTGTCGCGTCTGCTGTGAAAGCCCGGGGCTTAACCCCGGGTTTGCAGTGGGTACGGGCAGACTAGAGTGCAGTAGGGGAGACTGGAATTCCTGGTGTAGCGGTGAAATGCGCAGATATCAGGAGGAACACCAATGGCGAAGGCAGGTCTCTGGGCTGTAACTGACGCTGAGAAGCGAAAGCATGGGGAGCGAACAGG

>732e55c9c16f6196f62d6f67234c2633

CATTGAACTATCGTGAGAAAGTCAAACCGCCAAAGGGAATTATATTATAGTAAATATTAGCGTTAATAAACATTTTATTAATAGTAGTAATATATGATAATGTGCAGAAGATAAAGACGATTAAAACAGTGTTTAATAATCGTTTATCGAGACACTATTAAAGTAACACGGAATTAAAATATGTTTATTATGTGTGTTAAACAAGGGATGAACTTGAAAATCAATACAGTGTTAAACGGAACTAATAGAGCCACGCAAGTGAACTTAGAACATTTTTAATAAC

>df8739f6c87f0eed0a6e10285911d397

TGTGTATGCTATTATAATTTAATCCAAATATACAAGTGTGAAATTTTCCACTCCTAAATTAAATTAATATTCAGTAAATATTATAGCAAACAACCTTATTGTTATTAACACCTTATTATACACCCCTAATACGTCAACAATAAGATGACTAAACTCATGATTAGAAACCCGAG

>d32ef65e78c31325bde831171c7247f1

GAGAAAAATGATCTCACGGTGGGACCGCATTGCAACATCAATCCCGCGGAATTCGGGCCGGCCCTTCCATTATCTCCCCACCCACTTCGTCTGGCGTCCAAATTCCTTAACGTAAATATAAACGAAAGGTAATTAACAATTGTCTGGTTGTTAGTTCCTGGACATCTGCTACACGCACACGTGTTTGTAGACAAGACTCTTTGACGGACGTTGACATGCTCGGCTA

>10c537129e69bcd1429de8a1f905477a

TACGGAGGGTGCAAGCGTTATCCGGATTTATTGGGTTTAAAGGGTCCGTAGGCGGACTTGTAAGTCAGTGGTGAAATCTCATAGCTTAACTATGAAACTGCCATTGATACTGCAGGTCTTGAGTAAATTTGAAGTGGCTGGAATAAGTAGTGTAGCGGTGAAATGCATAGATATTACTTAGAACACCAATTGCGAAGGCAGGTCACTAAGATTTAACTGACGCTGATGGACGAAAGCGTGGGGAGCGAACAGG

>12994e2c41e2d0461ace0d3eadc7872e

TACAAGTAAGACTAGTGTTATTCATCTTAATTAGGTTTAAAGGGTACCTAGACAGTATTTCTAGCCTCCAAAGGGAACAGATTTACTAGAGTTTTATGTGAGAGGAAAATATTAGAACCATTGGAGTAGTGATAAAATGTTTTGATACTAATGGGACGGATAACGGCGAAGGCAAACCTCTATGTAATAACTGACGTTGAGGGACGAAGGCTTGGGTAGCGAATAGG

>4ddb5728ebcdfb72a577df600bec99b7

CACACTTATGTCTTTAGAAAGACAACACGTTTTTGCAGAATGCATTTCAACCTTCTCGGTCATTTTCAACTGGCAGTGCCTTGTAGATAATTGTGGAAGATTTTGAATGTTTCTTGTCCATAGAGTTCAGTTTGTTCATTAAAGATTTTGTTATGTAGTTTGTAACGTAGTATATTAAGACTTTCAAGTAAATTTATTTTATTTCCTTTTTTGCCGGTTGTG

>3ed48d72c0be47de93df60ec693e0f6a

ACCAAGATGTGTGGATGCAGGATACCGTTGCCGCCCCAAGCCCGGTAGAGGAGGATTGGTGAGGCTAGCAACTGGTCAACCGTGAAAAGACTTTCTGCTTAAGGGACCAACGAATTGCCTCCAGGATTAGAAAGGATTGCCTCTAGGATTAGAACGGATTGCCTCCAGGATTA

>6d0e334a7a63c1f4955aca71e450afe4

TACGGAGGGTGCAAGCGTTATCCGGATTCACTGGATTTAAAGGGTGCGTAGGCGGGTATTTAAGTCAGTGGTGAAATCCTAGAGCTTAACTCTAGAACTGCCATTGATACTATTTATCTTGAATATTGTGGAGGTAAGCGGAATATGTCATGTAGCAGTGAAATGCTTAGATATGACATAGAACACCTATTGCGAAGGCAGCTTACTACGCATATATTGACGCTGAGGCACGAAAGCGTGGGGATCAAACAGG

>f3b2a6ea2be00e253c738c4be93d46da

TACGTGAGAGACTAGTGTTATTCATCTTAATTGGGTTTAAAGGGTACCTAGACAGTCAATATATCTTCTAGAATGCTAATACTTGACTAGAGTTTTAAGGAAGAGGGAAGTACTTAAGGTGTAAGAGATGAAATATCTGTGATACCAAAGGGACTCTGTAAAGGCGAAGGCATTCCTTTATCTAAAAACTAACGTTGAAGGACGAAGGCTTAGATAACAAATAGG

>ecfd528aeb87b7f822cf42a2c3b7b2db

CACGCGTGATATGGTAATTGTGTGCCAGCCGCCGCGGTAACACACATTGTTATTGTTGTTGTGATGTTTATATATGTGTAGGTTTTAATTTATGTTTATTTTTTGGCCTTTGGAAATGTTTTTAATTAGTTTGAGGTTGTTGGGATTAGATACCCTAGTAGTCCGGCTGACTGACTTAACGTCCATCTCGTATGCCGTCTTCTGCTTGAAAAA

>b5790c383f7c9b4ef6aa6fdd806e83f2

TACGTAGGGTGCGAGCGTTATCCGGAATTATTGGGCGTAAAGAGCTCGTAGGCGGTTTGTCGCGTCTGTCGTGAAAGTCCGGGGCTTAACCCCGGATCTGCGGTGGGTACGGGCAGACTAGAGTGCAGTAGGGGAGACTGGAATTCCTGGTGTAGCGGTGGAATGCGCAGATATCAGGAGGAACACCGATGGCGAAGGCAGGTCTCTGGGCTGTAACTGACGCTGAGAAGCGAAAGCATGGGGAGCGAACAGG

>2b27e7aea7dbc220865e426c166d7846

CCGCCGCGGTAATACGATTTCTTTAATTTAAATATTTAAGTTTCAGTTAATATAACAATAATATAAAATATCTAAAATTTTGGTGAAATATATTTTATCTTTAAAAATTAATTTTATGTCTGAAAAATTTTTGTATAAACTAGGATTAGATACCCGGGTAGTCCGGCTGACTGACTCTACGACCA

>65a079291a0118d19bcc0d55175256b8

CCGCCGCGGTAATACGATTTCTTTAATTTAAATATTTAAGTTTCAGTTAATAAAACAATAATATAAAATATCTATAATTTTGGTGAAATATATTTTATTTTTAAAAATTAATTTTATGTCTGAAAAATTTTTGTATAAACTAGGATTAGATACCCTAGTAGTCCGGCTGACTGACTACTGTGTAA

>56cd04f81cbad05f32f213f8574fddef

CCGCCGCGGTAATACGATTTCTTTAATTTAAATATTTAAGTTTCAGTTAATAAAACAATAATATAAAATATCTATAATTTTGGTGAAATATATTTTATTTTTAAAAATTAATTTTATGTCTGAAAAATTTTTGTATAAACTAGGATTAGAAACCCGAGTAGTCCGGCTGACTGACTACTGTGTAA

>ef7726634aab97e046c01c65a91023b3

TACAGTAGCTTCAACACCAGCTAAAATAAAACTCATGTTACATAATTACTGATTGTGTTGTTATTTTGTTAGCCAATTGACGTTTTACGCCTATTATATTTAGATTATAACGTCAATAACAGGTACATAATTTTATTAAAAAAAAAAAGAAATTGAATCCCGTAAAACTCTGTCATCTTATTAACACAGAAACAAACTTTGTCAAAGGAATCGTCACCGTCATGTCTTCTTCGCCATATAAATTCACACAG

>b78bfc947143435063575106052035fc

TACGTAGGTGGCAAGCGTTGTCCGGAATTATTGGGCGTAAAGCGCGCGCAGGCGGTTCCTTAGGTCTGATGTGAAAGCCCACGGCTCAACCGTGGAGGGTCATTGGAAACTGGGGGACTTGAGTGCAGAAGAGGAGAGCGGAATTCCACGTGTAGCGGTGAAATGCGTAGAGATGTGGAGGAACACCAGTGGCGAAGGCGGCTCTCTGGTCTGTAACTGACGCTGAGGCGCGAAAGCGTGGGGAGCAAACAGG

>7a25be448b096a402c8b0fad3542403b

CATTGAACTATCGTGAGAACGGCACGCCGCCAAAGGGAATTATATTATAGTAAATATTGGCGTAAATAAACATTTTATTAATAGTTGTAATATATGAAAATGTGCAGAAGATAGAGACGATGAAAACAGTGTTTAACAATCGCTTATCGAGACAATATTTAATTAATTAGGAAATAAAATAAGTGTATTATTTGTATTTAACAAGGAATAATCTTGAAAATAAATACAGTGGTAAACGGAACTAATAGAGTCACGCAAGTGAACTTAGAACAATTTTAAGAAC

>ca9841cf3858340eeee6838ef001c61c

AGCTGAGACTGAAGAGCAGTCTAACACGTCTCATTATGTAAACACCCACTCAGTAGGCGGAGCCAATTTTACCCTGTATATGTTAGCATGAAAATAATGAAATCAGCCAAACGATACTGCAAAAAATAAAATAAATTAAATTGAAATTGAAATAATATCCAACTAATAAATTA

>ed8cb8a9584eb80d67e25758211c8d5d

GGACCGCATGTTAGTTAAAGAAAATGTAGATATTGTATATATTTTGTAATTTTGTTTTGTAGATTATTTATCCATATGCTTGTCCGTTTTAATGCAATAAAAAGACTGTGGATTTTTTGAAATAATACAAAGAATGTTTTGGTATTATAGTTAAACAGAATAATACAAAGATAAAGTTTATATTGAAAGGATATTTCTATTTTTATAAGCTGTTTTATCTTATGAATAAATGATAGTATGAATTTCTTATTTACTTTAA

>71b2752260c132e4f01646d908f64c75

CATTGAACTATCGTGAGAAAGTCAAACCGCCAAAGGGAATTATATTATAGTAAATATTGGCGTAAATAAACATTTTATTAATAGTTGTAATATATGAAAATGTGCAAAAGATAGAGACGATTAAAACGGTGTTTAACAATCGCTTATCGAGACAATATTTAATTAATTAGGAATTAAAATATGTGTATTATTTGTATTTAACAAGGAATAAACGTGAAAATAAATACAGTGGTGAACGGAACTAATAGAGTCACGCAAGTGAACTTAGAACAATTTTAAGAAC

>b688e82616fe00a9e0d1a20e084361fc

CATTGAACTATCGTGAGAGAGTCAAGCCGCCAAAGGGAATTATATTATAGTAAATATTGGCGTAAATAAACATTTTATTAATAGTTGTAATATATGAAAATGTGCAGAAGATAGAGACGATTAAAACGGTGTTTAACAATCGCTTATCGAGACAATATTTAATTAATTAGTAATTAAAATATGTTTATTATGTGTATTATTCAAGGAATAGACTTGACAATAAATACAGTGGTAAACGGAACTAATAGAGTCACGCAAGTGAACTTAGAACAATTTTAAGAAC

>1598c9a9a9b635ad065b888c308f979e

AGATCTACACAAGCAGCATATGGTAATTGTGTGCCAGCCGCCGCGGTAAGGAACAAAGAAAACTGTGAATGAACAAGTTATGTAGAAATACACGCGGTAGCCGAGTGCGCGCTGGAGAGCTTTACGACCAGAACCCCTGATGGCATTAGAAACCCCTGTAGTCCGGCTGACTGACTTAACGTCCATCTCGTATGCCGTCTTCTGCTTGAAAAAAAAAAAAAA

>a7317e6f4b2b73452671cd063941d538

TACGAAGGGGGCTAGCGTTGCTCGGAATTACTGGGCGTAAAGGGCGCGTAGGCGGATCGTTAAGTCAGGGGTGAAATCCCGGGGCTCAACCTCGGGACTGCCCTTGATACTGGCGATCTTGAGTATGAGAGAGATATGTGGAACTCCGAGTGTAGAGGTGAAATTCGTAGATATTCGGAAGAACACCAGTGGCGAAGGCGACATACTGGCTCATTACTGACGCTGAGGCGCGAAAGCGTGGGGAGCAAACAGG

>e33dfe5a96b466dd3655bdb759074d45

CCGCCGCGGTAATACGATTTCTTTAATTTAAATAGTTAAGTTTCAGTTAATATAACAATAATATAAAATATCTATAATTTTGGTGAAATATATTTTATCTTGAAAAATTAATTTTATGTCTGAAAAATTTTTATTTAAACTAGGATTAGATACCCTGGTAGTCCGGCTGACTGACTCTATCGTGA

>9a7c67d04b04ae8a75c38846873262d1

CCCAATCCAATATTGCAGGAAACAAGCAATTACTCAGAGGACAACAAAATGCAGGTTGCGGAAATTGGATTCTATCAGGCTTTTGAAAAAGAAAGCCTATGGACGGCTGCGAATAAGGCTATTACCAAATATGCAGTCCCGAACCGAGGCGGCAGCAATTAGAAACCCCTGTA

>aa5be0a92ef5245ac8d44d61f96c3e6b

CATTGAACTATCGTGAGAAAGTCAAGCCGCCAAAGGGAATTATATTATAGTAAATATTGGCGTAAATAAACATTTTATTAATAGTTGTAATATATGATAATGTGCAGAAGATAAAGACGATTAAAACGGTGTTTAACAATCGCTTATCGAGACAATATTTAATTAATTAGTAATTAAAATATGTTTATTATGTGTATTATTCAAGGAATAGACTTGACAATAAATACAGTGGTAAACGGAACTAATAGAGTCACGTAAGTGAACTTAGAACATTTTTAATAAC

>15f0ea1139299335a5aea77dd413b4da

TACGTAGGGGGCAAGCGTTGTCCGGAATCACTGGGCGTAAAGCGCGCGCAGGCGGCTATCTACGTCCGGGGTGAAAGCCCAGAGCTCAACTCTGGGATTGCCTTGGATACGGGATGGCTTGAGGATCGGAGAGGCAAGGGGAATTCCACGTGTAGCGGTGAAATGCGTAGAGATGTGGAGGAACACCTGTGGCGAAGGCGCCTTGCTGGCCGATTTCTGACGCTGAGGCGCGAAAGCGTGGGGAGCAAACAGG

>d1bcb676aff7a1d85092bfb173feb6d1

CATTGAACTATCGTGAGAAAGTCAAACCGCCAAAGGGAATTATATTATAGTAAATATTGGCGTAAATAAACATTTTATTAATAGTTGTAATATATGAAAATGTGCAGAAGATAAAGACGATTAAAACGGTGTTTAACAATCGCTTATCGAGACAATATTTAATTAATTAGGAAATAAAATATATTTATTATTTGTATTTAACAAGGAATAAACGTGAAAATAAATACAGTGGTAAACGGAACTAATAGAGTCACGCAAGTGAACTTAGAACAATTTTAAGAAC

>b5e419d34582d0e3575e3fe726745401

CCTAGTATGTATGGTAATTGTGTGCCAGCCGCCGCGGTAACCTCCACTCCATAACGCAATCTTTTTACCAGTCTCGTCATTATCTCACGAAATCACGTGCAATTCTCGTCGTCGAAACAATATAACTTAAATACATTGAAAACCATTAGAAACCCTTGTAGTCCGGCTGACTGACTTAACGTCCATCTCGTATGCCGTCTTCTGCTTGAAAAA

>530345bd3954a795d3c5b40d56b581c4

TACGGAGGGTGCAAGCGTTAATCGGAATTACTGGGCGTAAAGCGCACGCAAGCGGTTGATTAAGTTAGATGTGAAATCCCCGGGCTTAACCTGGGAATGACATCTAAGACTGGTCAGCTAGAGTCTTGTAGAGGGGGGTAGAATTCCATGTGTAGCGGTGAAATGCGTAGAGATGTGGAGGAATACCGGTGGCGAAGGCGGCCCCCTGGACAAAGACTGACGCTCAGGTGCGAAAGCGTGGGGAGCAAACAGG

>5fe8e445b0f6c9284ac9a94338cc715c

TACGTAGGGTGCGAGCGTTGTCCGGAATTATTGGGCGTAAAGAGCTTGTAGGCGGTTTGTCGCGTCTGCTGTGAAAATCTGGGGCTTAACCCTGGACGTGCAGTGGGTACGGGCAGACTAGAGTGTGGTAGGGGAGACTGGAATTCCTGGTGTAGCGGTGGAATGCGCAGATATCAGGAGGAACACCGATGGCGAAGGCAGGTCTCTGGGCCATTACTGACGCTGAGAAGCGAAAGCATGGGGAGCGAACAGG

>defecb8b26642820c138b00076ed0e00

CATTGAACTATCGTGAGAAAGTCAAACCGCCAAAGGGAATTATATTATAGTAAATATTGGCGTAAATAAACATTTTATTAATAGTTGTAATATATGAAAATGTGCAGAAAATAAAGACGATTAAAACGGTGTTTAAAAATCGCTTATCGAGACAATATTTAATTAATTAGGAATTAAAATATGTGTATTATTTGTATTTAACAAGGAATAAACGTGAAAATCAATACAGTGTTAAACGGAACTAATAGAGTCACGCAAGTGAACTTAGAACATTTTTAATAAC

>4e156e3c0101ddfe47691cac65ccb0a1

GGTAATTGTGTGCCAGCCGCCGCGGTAATTGACTGGATGAGACCCACCGCAGTTGCAGCATGTTGCAGAGGTCTCCGGTGCGACCAGATAGTAGCCTCGGTGCTCTCCTAACGTCTCTGTGGAAAACGGCCAACGTCGTCATGGATTAGAAACCCTGGTAGTCCGGCTGACTGACTCTACGACCATCTCGTATGCCGTCTT

>457ebcf22f795ae91136b7cfb889a0af

TACGAAGGGGGCTAGCGTTGCTCGGAATTACTGGGCGTAAAGGGAGCGTAGGCGGACATTTAAGTCAGGGGTGAAATCCCGGGGCTCAACCTCGGAATTGCCTTTGATACTGGGTGTCTTGAGTATGAGAGAGGTGTGTGGAACTCCGAGTGTAGAGGTGAAATTCGTAGATATTCGGAAGAACACCAGTGGCGAAGGCGACACACTGGCTCATTACTGACGCTGAGGCTAGAAAGCGTGGGGAGCAAACAGG

>836d43a2ff8c6ae378553d85bfb5f4e4

TACGAAGGGGGCTAGCGTTGTTCGGAATCACTGGGCGTAAAGCGCACGTAGGCGGACCATTAAGTCAGGGGTGAAAGCCTGGAGCTCAACTCCAGAACTGCCTTTGATACTGATGGTCTCGAGTTCGGAAGAGGTTGGTGGAACTGCGAGTGTAGAGGTGAAATTCGTAGATATTCGCAAGAACACCAGTGGCGAAGGCGGCCAACTGGTCCGATACTGACGCTGAGGTGCGAAAGCGTGGGGAGCAAACAGG

>01344f29aa14cf1d706092a4e7721018

TACGTAGGGTGCGAGCGTTAATCGGAATTACTGGGCGTAAAGCGAGCGCAGACGGTTTATTAAGCAAGATGTGAAATCCCCGAGCTTAACTTGGGAACTGCGTTTTGAACTGGTAAGCTAGAGTATGTCAGAGGGGGGTAGAATTCCACGTGTAGCAGTGAAATGCGTAGAGATGTGGAGGAATACCGATGGCGAAGGCAGCCCCCTGGGATAATACTGACGTTCATGCTCGAAAGCGTGGGTAGCAAACAGG

>c15d9047c056d8f0f906d4d66dfd3dd2

CATTGAACTATCGTGAGAACGGCACGCCGCCAAAGGGAATTATATTATAGTAAATATTAGCGTAAATAAACATTTTATTAATAGTTGTAATATATGAAAATGTGCAGAAGATAGAGACGATTAAAACAGTGTTTAACAATCGCTTATCGAGACAATATTTAATTAATTAGGAAATAAAATCAGTGTATTATTTGTATTTAACAAGGAATAAGCGTGAAAATAAATACAGTGGTAAACGGAACTAATAGAGTCACGCAAGTGAACTTAGAACAATTTTAAGAACACTAGAACTAATGGAAA

>1f8ec6dc41af134c1e8327e5ccba045d

GGTAATTGTGTGCCAGCCGCCGCGGTAATTGACTGGATGAGACCCACCGCAGTTGCAGCATGTTGCAGAGGTCTCCGGTGCGACCAGATAGTAGCCTCGGTGCTCTCCTAACGTCTCTGTGGAAAACGGCCAACGTCGTCATGGATTAGAAACCCTAGTAGTCCGGCTGACTGACTGAGACTTAATCTCGTATGCCGTCTT

>09ba0dcc9d08747a09b3a4ec08c8d529

TACGGAGGGGGCAAGCGTTATCCGGAATTATTGGGCGTAAAGGGTACGTAGGTGGCCTTTTAAGCGTAGGGTATAAGGCAATGGCTCAACCATTGTTCGCCCTATGAACTGGAAGGCTTGAGTGCAGGAGAGGAAAGCGGAATTCCTAGTGTAGCGGTGGAATGCATAGATATTAGGAGGAACATCAGCGGCGAAGGCGGCTTTCTGGACTGCAACTGACACTGAGGTACGAAAGCGTGGGGAGCAAACAGG

>fd698004117bd303c5c396670d15df2a

CTCGAGAACTGGTGATCCGATTTTTATAGGGTTTTCTTTAAACAATACATGTAAAGAAGACGGCATGGTTCATCACATGTCTCACAAGTATTCTTACAATTTATGAATAACAAATATTTTTATAGTTAAACTCAATTAACCCTTCAAATGAATACATGAGAAAATATTTAAATACTCGTCAATAAGTGTCCTAAAGATTAATAACAAACAAATAATAATTGTCTAAAACGTCTCCTGACAAATAACACTATTATGCAAAACCATTATTCAGTAGTA

>10cd48e59d95073ad2a1a623d55f23a0

AACTGCAGTAAGTTTTAGTGACTGCACAACGAGTGGAACACTGAATCCTGAAACCTTACTTAAATTCCGCTTTTTAACCGCCCTGGTCTCCGATCATTATAAAAGCTCTGAGATGGTTAAAACTTATTATTCAATTGTTTTTAATTAACAGAAGCATAAATTAGTATAGTTAAAGAAAATTATGTAATAATGTAATAAGTCAATAATGATGTAATAGTAAATCAAAATGAGAAAAAACTGTGATGT

>9794ee3c0e2d061610c6692b51473b11

CATTGAACTATCGTGAGAACAGCACGCCGCCAAAGAGAATTATATTATAGTAAATATTAGCGTAAATAAACATTTTATTAATAGTTGTAATATATGAAAATGTGCAGAAGATAGAGACGATTAAATCAGTGTTTAACAATCGCTTATCGAGACAATAGTTAATTAATTAGGAAATAAAATAAGTGTATTATTTGTATTTAACAAGGAATAAGCGTGAAAATAAATACAGTGGTAAACGGAACTAATAGAGTCACGCAAGTGAACTTAGAACAATTTTAAGAACACTAGAACTAATGGAAA

>f2aacebd8720eb26ca30323824efca25

ACACAAGAGAGAGGTTTTTAGAATGGACGCTATAGAAGTGGTTCTCAAAAGACTCATGGATTTTAAACAAGAGGAACCTAACTAAAAATATAGTTAGCTTCTGGAGCGCTAGAGTAAAATTTAGATTTACTTTCCTAAAACAAAATGGAAAGCGTGTCCTTAGCGTAAGAAACTTCCCTAAAAGCAACTAGAGCAATAGTAGGCTTACGTAATTTTATGAAGAAGAGGGTGGAAAGCAACTGC

>c67ba62f34a05261ea7a810ff643a52c

TACGTAAGGACCGAGCGTTGTCCGGAATCATTGGGCGTAAAGGGTACGTAGGCGGGTCATTAAGTTAGAAGTCAAAGGCTATAGCTCAACTATAGTAAGCTTCTAAAACTGGAGACCTTGAGTAATGGAAGGGAAAGTGGAATTCCTAGTGTAGCGGTGGAATGCGCAGATATTAGGAGGAATACCGGTGGCGAAGGCGACTTTCTGGCCATTAACTGACGCTGAGGTACGAAAGCGTGGGTAGCAAACAGG

>987869ff61a2f9aa291a016392a0b72d

TACGTAGGGACCAAGCGTTGTTCGGATTTACTGGGCGTAAAGGGCGCGTAGGCGGCTTGACAAGTCACTTGTGAAATCTCCGGGCTTAACTCGGAACGGCCAAGTGAAACTGTCATGCTAGAGTGCAGAAGGGGCAATCGGAATTCTTGGTGTAGCGGTGAAATGCGTAGATATCAAGAGGAACACCTGAGGTGAAGACGGGTTGCTGGGCTGACACTGACGCTGAGGCGCGAAAGCCAGGGGAGCAAACGGG

>337b14e1938677e488fda429b20b7e54

TACGGGGGGGGCAAGCGTTGTTCGGAATTACTGGGCGTAAAGGGCTCGTAGGCGGCCAACTAAGTCAGACGTGAAATCCCCAGGCTCAACCTGGGAACTGCGTCTGATACTGGATGGCTTGAATCCGGGAGAGGGATGCAGAATTCCAGGTGTAGCGGTGAAATGCGTAGATATCTGGAGGAATACCGGTGGCGAAGGCGGCATCCTGGACCGGCATTGACGCTGAGGAGCGAAAGCCAGGGGAGCAAACGGG

>83dfd321cf090563a809da72b5f198fa

TACGGAGGGTGCAAGCGTTATCCGGATTCACTGGGTTTAAAGGGTGCGTAGGCGGGTATTTAAGTCAGTGGTGAAATCCTAGAGCTTAACTCTAGAACTGCCATTGATACTATTTATCTTGAATATTGTGGAGGTAAGCGGAATATGTCATGTAGCGGTGAAATGCTTAGATATGACATAGAACACCTATTGCGAAGGCAGCTTACTACGCATATATTGACGCTGAAGCACGAAAGCGTGGGGATCAAACAGG

>be22632d5e3f6175707a3213d7a90ad2

TACGTAGGTGGCAAGCGTTGTCCGGAATTATTGGGCGTAAAGCGCGCGCAGGCGGCTTCTTAAGTCCATCTTAAAAGTGCGGGGCTTAACCCCGTGATGGGATGGAAACTGAGAGGCTGGAGTATCGGAGAGGAAAGTGGAATTCCTAGTGTAGCGGTGAAATGCGTAGAGATTAGGAAGAACACCGGTGGCGAAGGCGACTTTCTGGACGACAACTGACGCTGAGGCGCGAAAGCGTGGGGAGCAAACAGG

>fec8716195af98907bac8206e598cefa

TACGTAGGGTGCGAGCGTTGTCCGGAATTATTGGGCGTAAAGGGCTCGTAGGCGGTTTGTTGCGTCGGGAGTGAAAACTCAGGGCTTAACCCTGAGCCTGCTTCCGATACGGGCAGACTAGAGGTATGCAGGGGAGAACGGAATTCCTGGTGTAGCGGTGAAATGCGCAGATATCAGGAGGAACACCGGTGGCGAAGGCGGTTCTCTGGGCATTACCTGACGCTGAGGAGCGAAAGTGTGGGGAGCGAACAGG

>75f8d18fdbf63974ca6712a322105f9a

GAAACTGCCACTGCCTGGTATAGATAATAAACCAGTTTTAAAAAGCTGTAAAGTATTATAGTAATCAAGTACGGAAAAACACATTATCTAGATAAAAAAAAACTCTTCTAAACAAAATGTATATTTCCCTAAACTGTATATAAACTATATAAATGGTAATAAATTAAATAATACTGC

>d9df0b8acb82c53ec3fc58bd9c440992

GGTAATTGTGTGCCAGCCGCCGCGGTAATTGACTGGATGAGACCCACCGCAGTTGCAGCATGTTGCAGAGGTCTCCGGTGCGACCAGATAGTAGCCTCGGTGCTCTCCTAACGTCTCTGTGGAAAACGGCCAACGTCGTCATGGATTAGAAACCCCTGTAGTCCGGCTGACTGACTTAACGTCCATCTCGTATGCCGTCTT

>6778bcd2106d48f692f61a6de709786a

TTCTTACTGCAAACGTATTGTCACACACATGTGTTTTGTAGTAATTATTGTGAGCAGCATCCTCTATGTTATAAATGTAATAAGGAAATCTTAAAACAATGATAATAATCTGTTTATAGTAATTAATCTTCATCATCTTCGCCATTCCAGGATTAGATACCCTGGTAGTCCGG

>983bcc091cd03e5a7f535525b252e40b

TACAGAGGGTGCAAGCGTTAATCGGATTTACTGGGCGTAAAGCGCGCGTAGGTGGCCAATTAAGTCAAATGTGAAATCCCCGAGCTTAACTTGGGAATTGCATTCGATGCTGGTTGGCTAGAGTATGGGAGAGGATGGTAGAATTCCAGGTGTAGCGGTGAAATGCGTAGAGATCTGGAGGAATACCGATGGCGAAGGCAGCCATCTGGCCTAATACTGACACTGAGGTGCGAAAGCATGGGGAGCAAACAGG

>8cfd01ba0f379d69bfc0ddc960267d10

TACGGAGGGTGCAAGCGTTGTTCGGAATTATTGGGCATAAAGCGCGTGCAGGCGGCTGTTCAAGTCCGATGTGAAAGCCCGGGGCTCAACCCCGGAAGTGCATTGGAAACTGGACAGCTTGAGTACGGGAGAGGGAGGTAGAATTCCGAGTGTAGGGGTGAAATCCGTAGATATTCGGAGGAATACCGGTGGCGAAGGCGGCCTCCTGGACCGATACTGACGCTGAGACGCGAAAACGTGGGGAGCAAACAGG

>ffdd70aecdbcbad8283159affda91091

TACGTAGGTCCCGAGCGTTGTCCGGATTTATTGGGCGTAAAGCGAGCGCAGGCGGTTAGATAAGTCTGAAGGTAAAGGGTGTGGCTTAACCATAGTATGCTTTGGAAACTGTTTAACTTGAGTGCAGAAGGGGAGAGTGGAATTCCATGTGTAGCGGTGAAATGCGTAGATATATGGAGGAACACCGGTGGCGAAAGCGGCTCTCTGGTCTGTAACTGACGCTGAGGCTCGAAAGCGTGGGGAGCAAACAGG

>81c04870ea988a0e4636f3bc73b88712

CATTGAACTATCGTGAGAAAGTCAAACCGCCAAAGGGAATTATATTATAGTAAATATTAGCGTAAATAAACATTTTATTAATAGTTGTAATATATGATAATGTGCAGAAGATAAAGACGATTAAAACGGTGTTTAACAATCGCTTATCGAGACAATATTTAATTAATTAGGAATTAAAATATGTGTATTATTTGTATTTAACAAGGAATAATCTTGAAAATAAATACAGTGGTAAACGGAACTAATAGAGTCACGCAAGTGAACTTAGAACAATTTTAAGAAC

>04036f1908151f27e15d60c5d09e1302

CATTGAACTATCGTGAGAAAGTCACGCCGCCAAAGGGAATTGTATTAGAGTAAATATTGGCGTAAATAAACATTTTATTAATAGTTGTAATATATGAAAATGTGCAGAAAATAAAGACGATTAAAACGGTGTTTAAATATCGCTTATCAAGACAATATTTAATTAATTAGGAATTAAAATATGTGTATTATTTGTATTTAACAAGGAATAAACTTGAAAATAAATACAGTGGTGAACGGAACTAATAGAGTCACGCAAGTGAACTTAGAACAATTTTAAGAAC

>6b08596cd7fa0727c16c28d4e0aacdbd

TACGTAGGGAGCGAGCGTTGTCCGGAATTACTGGGTGTAAAGGGAGCGTAGGCGGGACGGCAAGTCAGATGTGAAATATACGTGCTCAACATGTAGACTGCATTTGAAACTGTCGTTCTTGAGTGAGGTAGAGGTAAGCGGAATTCCTGGTGTAGCGGTGAAATGCGTAGAGATCAGGAGGAACATCGGTGGCGAAGGCGGCTTACTGGGCCTTTACTGACGCTGAGGCTCGAAAGCGTGGGGAGCAAACAGG

>c0c67bd4538e36a2bc8df6af01acfc1e

TACGGAGGGGGCTAGCGTTGTTCGGAATTACTGGGCGTAAAGCGCACGTAGGCGGACTGGAAAGTTGGGGGTGAAATCCCGGGGCTCAACCTCGGAACTGCCTTCAAAACTATCAGTCTGGAGTTCGAGAGAGGTGAGTGGAATTCCGAGTGTAGAGGTGAAATTCGTAGATATTCGGAGGAACACCAGTGGCGAAGGCGGCTCACTAGCTCGATACTGACGCTGAGGTGCGAAAGCGTGGGGAGCAAACAGG

>1445453bbfb71357b988db22f8fa58e6

TACAGAGGGTGCGAGCGTTAATCGGAATTACTGGGCGTAAAGCGAGTGTAGGTGGCTCATTAAGTCACATGTGAAATCCCCGGGCTTAACCTGGGAACTGCATGTGATACTGGTGGTGCTAGAATATGTGAGAGGGAAGTAGAATTCCAGGTGTAGCGGTGAAATGCGTAGAGATATGGAGGAATACCGATGGCGAAGGCAGCTTCCTGGCATAATATTGACACTGAGATTCAAAAGCGTGGGTAGCAAACAGG

>f01ecd63aa4d62af5fb7b6cdd96372c8

TACGTAGGGGGCAAGCGTTATCCGGATTTACTGGGTGTAAAGGGAGCGTAGACGGCGGCACAAGTCTGGAGTGAAAGGCGGGGGCCCAACCCCCGGACTGCTTTGGAAACTGCGCGGCTGGAGTGCAGGAGAGGCAAGTGGAATTCCTAGTGTAGCGGTGAAATGCGTAGATATTAGGAGGAACACCAGTGGCGAAGGCGGCTTGCTGGACTGTAACTGACGTTGAGGCTCGAAAGCGTGGGGAGCAAACAGG

>f10dae870d41b4ba56eacbae4e53b1d1

CCGCCGCGGTAATACGATTTCTTTAATTTAAATATTTAAGTTTCAGTTAATAAAACAATAATATAAAATATCTATAATTTTGGTGAAATATATTTTATTTTTAAAAATTAATTTTATGTCTGAAAAATTTTTGTATAAACTAGGATTAGATACCCGTGTAGTCCGGCTGACTGACTACTGTGTAA

>2eeedead0f29786a7ec9cf13de5f5144

TACGAAGGGTGCAAGCGTTAATCGGAATTACTGGGCGTAAAGCGCGCGTAGGTGGTTGGGTAAGTTGAATGTGAAAGCCCCGGGCTCAACCTGGGAACTGCATCCAAAACTGCCCGACTAGAGTACGGTAGAGGGTGGTGGAATTTCCTGTGTAGCGGTGAAATGCGTAGATATAGGAAGGAACACCAGTGGCGAAGGCGACCACCTGGACTGATACTGACACTGAGGTGCGAAAGCGTGGGGAGCAAACAGG

>75096407cd1f61de1d6165e893f4d084

CATTGAACTATCGTGAGAAAGTCAAACCGCCAAAGTGAATTATATTATAGTAAATATTGGCGTAAATAAACATTTTATTAATAGTTGTAATATATGAAAATGTGCAGAAGATAAAGACGATTAAGACGGTGTTTAACAATCGCTTATCGAGACACTATTAAAGTAATTAGGAATTAAAATATGTTTATTATGTGTATTATTCAAGGAATAAACTTGAAAATAAATACAGTGTTAAACGGAACTAATAGAGTCACGCAAGTGAACTTAGAACATTTTAATAAC

>1c3d611ab1acf7f2858b497dcc2744ba

TTATCAGGGTTATTTTATCTCCCTTCTTGAATAGATCATTATTGAAGGGATCGTTGTGCTAGTTTTCCAGTGTCTTGGGACTTTGAATAGTTCCAATGACCTTTTCTGAATGACTTTGAATAGCTCCTTTAGCTCATCACATAATGTCGTTCCACCGTATTTGAGCCGCTCATTC

>ec3421cdbbaf79e9c283595549308a36

TACGGAGGGTGCAAGCGTTATCCGGATTCACTGGGTTTAAAGGGTGCGTAGGCGGGTAGGGAAGTCAAAGGTGAAATCCTGGAGCTTAACTCCAGAACTGCCTTTGATACTATCTATCTTGAATATGGTGGAGGTAAGCGAAATATGTCATGTAACGGTGAAATGCATAGATATGACATAGAACACCTATTGCGAAGGCAGCTTACTACGCCTATATTGACGCTGAGGCACGAAAGCGTGGGGATCAAACAGG

>f99ef204e999a05fa9fd70503c167928

TACGTAGAAGACTAGTGTTAATCATCTTTATTAGGTTTAAAGGGTACCTAGACGGTAAATTAAACTCTAAATGAGTACTTGTTTACTAGAGTTTTATGAAAGGAGGAAGAATTTCTGGAGTAGTGATTTAATATGAATAATCTCAGAGAGACTGGTAACGGCGAAGGCATCCTTCTATGTAAAAACTGACGTTGAGGGACGAAGGCTTGGGTAACGAGAAGG

>fbbafdf5df5e4b6c7144d317ebca3b19

CTCATTAGAACCGATCTGACTACCGCTATAACTCACTATAACTGATCTGACAACCGCGATAACTCATTAGAACCGATTTGACAACCGCTATAACTCACTATAACTGATCTGACAACCGCAATAACTCATTAGAACCGATCTGACAACCGCTATAACTTATTAGAACTGATATGACAACAGCTATAACTCACTATAACTGAACTGACAACCTCTATAACTC

>d6717900fa829f70a8c76b1c4456654b

GCAGCCAACCTCAGCCACTACACTATACAAACGTATTTACTGCGGACGATAATCTAGTTTCTCAGTTGTGCACGTTTGTCGTGTTACTGCAATTGTCATGTACCGAAGCGTGCGTTATCTCAGTCTCTATTAAGTCTTTCTTTCTGATAACATTTCTTCAGGTCTTCAAACTTTACTTAAATTTCGGTTTCTTTCTCCCATTTTCTTCTTACG

>51ff82a42dac98af81f3ea1fd5b4586a

CATTGAACTATCGTGAGAAAGTCAAACCGCCAAAGGGAATTATATTATAGTAAATATTGGCGTAAATAAACATTTTATTAATAGTTGTAATATATGAAAATGTGCAGAAGATAAAGATGATTAAAACGGTGTTTAACAATCGCTTATCGAGACAATATTTAATTAATACGGAATTAAAATATGTTTATTATGTGTATTAAACAAGGGATGAACTTGAAAATCAATACAGTGTTAAACGGAACTAATAGAGTCACGCAAGTGAACTTAGAACATTTTTAATAAC

>d6d470466c1723cae9276ef06f00f3f0

TACGTAAAAGACTAGTGTTAGTCATCTTTATTAGGTTTAAAGGGTACCTAGACGGTAAATTAAACTCTAAATGAGTATTTTTTTACTAGAGTTTTATAAAAGAAGGAAGAATTTCTGGAGTAGTGATAGAATACTTTAATACCAGAAGGACTGGTAACGGCGAAGGCATCCTTCTATGTAAAAACTGACGTTGAGGGACGAAGGCTTGGGTAGCAATAAGG

>3c0f7e7a8518453e007613864a743ace

TACGTAGGGGGCAAGCGTTGTCCGGAATCACTGGGCGTAAAGCGCGCGCAGGCGGTATCTTGCGTCTGGGGTGAAAGTCCAGAGCTCAACTCTGGGATTGCCTTGGAAACGGGGATACTTGAGCGTCGGAGAGGCAAGGGGAATTCCACGTGTAGCGGTGAAATGCGTAGAGATGTGGAGGAACACCAGTGGCGAAGGCGCCTTGCTGGCCGATTGCTGACGCTGAGGCACGAAAGCGTGGGGAGCAAACAGG

>fb97da1ed2100f745b5122ff69800d31

TACGAAGGGGGCTAGCGTTGCTCGGAATCACTGGGCGTAAAGGGCGCGTAGGCGGCCGATTAAGTCGGGGGTGAAAGCCTGTGGCTCAACCACAGAATTGCCTTCGATACTGGTTGGCTTGAGACCGGAAGAGGACAGCGGAACTGCGAGTGTAGAGGTGAAATTCGTAGATATTCGCAAGAACACCAGTGGCGAAGGCGGCTGTCTGGTCCGGTTCTGACGCTGAGGCACGAAAGCGTGGGGAGCAAACAGG

>7937ff23ca81ebbb9270fdf81a9bbcdf

CATTGAACTATCGTGAGAGAGTCAAGCCGCCAAAGGGAATTATATTATAGTAAATATTGGCGTAAATAAACATTTTATTAATAGTTGTAATATATGAAAATGTGCAGAAGATAAAGACGATGAAAACGGTGTTTAACAATCGCTTATCGAGACAATATTTAATTAATTAGTAATTAAAATATGTTTATTATGTGTATTATTCAAGGAATAGACTTGACAATAAATACAGTGGTAAACGGAACTAATAGAGTCACGCAAGTGAACTTAGAACATTTTTAATAAC

>4ed0abce9662a5fe27b3ceb5bf6ec61f

TACGGAGGGGGCTAGCGTTGTTCGGAATTACTGGGCGTAAAGCGCACGTAGGCGGCTTGGTAAGTTAGAGGTGAAAGCCTGGAGCTCAACTCCAGAATTGCCTTTAAGACTGCCGAGCTAGCAGGTGAGAGAGGTGAGTGGAATTCCGAGTGTAGAGGTGAAATTCGTAGATATTCGGAAGAACACCAGTGGCGAAGGCGGCTCACTGGCTCATACTGGACGCTGAGGTGCGAAAGCGTGGGGAGCAAACAGG

>73f531ddc7ef7e759ba1ca07514a38c4

TACGGAGGGAGCTAGCGTTGTTCGGAATTACTGGGCGTAAAGCGCACGTAGGCGGCTTTGTAAGTTAGAGGTGAAAGCCTGGAGCTCAACTCCAGAATTGCCTTTAAGACTGCATCGCTTGAATCCGGGAGAGGTGAGTGGAATTCCGAGTGTAGAGGTGAAATTCGTAGATATTCGGAAGAACACCAGTGGCGAAGGCGGCTCACTGGACCGGTATTGACGCTGAGGTGCGAAAGCGTGGGGAGCAAACAGG

>e8d76524ca9c559b3bd5684b3c6fb923

TACGTAGGGGGCAAGCGTTATCCGGATTTACTGGGTGTAAAGGGAGCGCAGACGGCCAAGCAAGTCTGAAGTGAAATGCATGGGCTCAACCCATGAATTGCTTTGGAAACTGTTAGGCTTGAGTGTCGGAGGGGTAAGCGGAATTCCTAGTGTAGCGGTGAAATGCGTAGATATTAGGAGGAACACCGGAGGCGAAGGCGGCTTACTGGACGACAACTGACGTTGAGGCTCGAAGGCGTGGGGAGCAAACAGG

>aa0df0b066bbe1038a844179c7e82e1a

TACGGAGGGTGCAAGCGTTACTCGGAATCACTGGGCGTAAAGGACGCGTAGGCGGATTATCAAGTCTCTTGTGAAATCTAACGGCTTAACCGTTAAACTGCTTGGGAAACTGATAATCTAGAGTAAGGGAGAGGCAGATGGAATTCTTGGTGTAGGGGTAAAATCCGTAGATATCAAGAAGAATACCTATTGCGAAGGCGATCTGCTGGAACTTAACTGACGCTAATGCGTGAAAGCGTGGGGAGCAAACAGG

>ffe5ae8cfd4fb1ede5e7e85d7bf817bb

TACGGAGGATGCGAGCGTTATCCGGATTTATTGGGTTTAAAGGGTGCGTAGGCGGAAGGTCAAGTCAGCGGTAAAATGTCGGGGCTCAACCCCGGCCGGCCGTTGAAACTGGTCATCTGGAGTAGGAGAGAAGTATGCGGAATCCGTGGTGTAGCAGTGAAATGCTTAGATATCACGAAGAACTCCGATTGCGAAGGCAGCGTACCGTGGCGTTACTGACGTTCATGCTCGAAAGCGCGGGTATCGAACAGG

>ea427875c613794826105b0c09874750

GACGTAGGGCGCGAGCGTTGTCCGGAATTATTGGGCGTAAAGAGCTCGTAGGCGGCTTGTTGCGTCGGCTGTGAAAACCCATGGCTTAACTGTGGGCTTGCAGCCGATACGGGCAGGCTAGAATTCGGTAGGGGAGACTGGAATTCCTGGTGTAGCGGTGGAATGCGCAGATATCAGGAGGAACACCGGTGGCGAAGGCGGGTCTCTGGGCCGATATTGACGCTGAGGAGCGAAAGCGTGGGGAGCGAACAGG

>4d0e7834692b9f7441515e7a553ddb2a

ACCTAGTATGTATGGTAATTGTGTGCCAGCCGCCGCGGTAAAGGCTTCTATTGCCCATCATCGCGTCGTGCCGCCGCAGCGGACTTATCTGGAGGTCACCTCTATTGAGATCCACGGAGCTCTTGACGGCGTCCTCTTTGTGTCATTAGAAACCCGTGTAGTCCGGCTGACTGACTTGCGTCAAATCTCGTATGCCGTCTTCTGCTTGAAAAAA

>f9463f68a88924b7f62ff633ca227c74

CATTGAACTATCGTGAGAATGGCACGCCGCCAAAGGGAATTATATTATAGTAAATATTAGCGTAAATAAACATTTTATTAATAGTTGTAATATATGAAAATGTGCAGAAGATAGAGACGATTAAAACAGTGTTTAACAATCGCTTATCGAGACAATATTTAATTAATTAGGAAATAAAATAAGTGTATTATTTGAATTTAACAAGAAATAAACGTGAAAATAAATACAGTGGGAAAAGGAACTAATAGAGTCACGCAAGTGAACTTAGAACATTTTAAGAACATTAGAACTAATGGAAA

>62727dc7f1b9c5878de3da94b2977f05

TACGAAGGGGGCTAGCGTTGTTCGGATTTACTGGGCGTAAAGCGCACGTAGGCGGACTTTTAAGTCAGGGGTGAAATCCCGGGGCTCAACCCCGGAACTGCCTTTGATACTGGAAGTCTTGAGTATGGTAGAGGTGAGTGGAATTCCGAGTGTAGAGGTGAAATTCGTAGATATTCGGAGGAACACCAGTGGCGAAGGCGGCTCACTGGACCATTACTGACGCTGAGGTGCGAAAGCGTGGGGAGCAAACAGG

>922de7f1e9f2387fb1e8cec86789920a

TACAGGGGGTGCGAGCGTTAATCGGAATTACTGGGCGTAAAGCGAGTGTAGGTGGCTCATTAAGTCACATGTGAAATCCCCGGGCTTAACCTGGGAACTGCATGTGATAGTGGTGGTGCTAGAATATGTGAGAGGGAAGTAGAATTCCAGGTGTAGCGGTGAAATGCGTAGAGATCTGGAGGAATACCGATGGCGAAGGCAGCTTCCTGGCATAATATTGACACTGAGATTCGAAAGCGTGGGTAGCAAACAGG

>c438306a422e303f2fd1e7941fa04619

CATTGAACTATCGTGAGAAAGTCAAACCGCCAAAGGGAATAATATTATAGTAAATATTGGCGTAAATAAACATTTTATTAATAGTTGTAATATATGAAAATGTGCAAAAGATAGAGACGATTAAAACGGTGTTTAACAATCGCTTATCGAGACACTATTAAAGTAATTAGGAATTAAAATATGTTTATTATGTGTATTATTCAAGGAATAAACTTGAAAATAAATACAGTGTTAAACGGAACTAATAGAGTCACGCAAGTGAACTTAGAACATTTTAATAAC

>4c161c32c40b1e411f6422cc9525847f

CATTGAACTATCGTGAGAAAGTCACGCCGCCAAAGGGAATTATATAATAGTAAATATTAGCGTAAATAAACATTTTATTAATAGTTGTAATATATGATAATGTGCAAAAGATAGAGACGATTAAAACGGTGTTTAACAATCGCTTATCGAGACACTATTAAAGTAATTAGGAATTAAAATATGTTTATTATGTGTATTATTCAAGGAATAAACTTGGAAATAAATACAGTGTTAAACGGAACTAATAGAGTCACGCAAGTGAACTTAAAACATTTTAATAAC

>c3e8240963f778387ed0cf390283dce6

CACACTTATGTCTTCAGAAAGACAAAATATTTCTCCAGGATGCATTTCGACCATGTCGGTCATTGTCAACTGGCAGTGCATTGTAGATATCTGTGAAAGATTTTGAATGCGTTTTGTTCATAGAGTTCAGTTTGTTCATTAATAATTTTTTTATGTAGTTTTTAAAGTAATATATTTATACTTTCAAGTAAATTCGTTTTGTTTCCTTTTTTGCCGGTTGTG

>82dece6e35540738ba450a0c3a90b5a0

TACGGAGGGTGCAAGCGTTAATCGGAATTACTGGGCGTAAAGCGCACGCAGGCGGTTTGTTAAGTCAGATGTGAAATCCCCGGGCTCAACCTGGGAACTGCATTTGAAACTGGCAAGCTAGAGTCTCGTAGAGGGGGGTAGAATTCCAGGTGTAGCGGTGAAATGCGTAGAGATCTGGAGGAATACCGGTGGCGAAGGCGGCCCCCTGGACGAAGACTGACGCTCAGGTGCGAAAGCGTGGGGAGCAAACAGG

>578c493ebd3fdb0b96ec0c11d19db362

TTAAACGAAAGCACAACAAAAAGATAGCCAAACTAATACCACCCCAATCACCAGCAATAATACCCACACATACGTTTTACCCAAGAATAGTTAACCTAACCAACGTAACATTCACTAACGAACAGAAACAACTTTTAAACAAAGGGATAAACCACAACCTACACTACACACAGAATAATAACACCATCAAGAACATGGT

>8b6d6427845f51ed80bc1c0257a086c6

TACGTAGGTCCCGAGCGTTATCCGGATTTATTGGGCGTAAAGCGAGCGCAGGCGGTTAGATAAGTCTGAAGGTAAAGGGTGTGGCTTAACCATAGTACGCTTTGGAAACTGTTTAACTTGAGTGCAGAAGGGGAGAGTGGAATTCCATGTGTAGCGGTGAAATGCGTAGATATATGGAGGAACACCGGTGGCGAAAGCGGCTCTCTGGTCTGTAACTGACGCTGAGGCTCGAAAGCGTGGGGAGCAAACAGG

>ef082017c0bfefb9dcf9ac41e878ea1c

TACGGAGGGTGCGAGCGTTGTCCGGATTTATTGGGTTTAAAGGGTGCGTAGGCGGCCGTTTAAGTCTGGGGTGAAAGCCCGCTGCTCAACAGCGGAACTGCCCTGGATACTGGATGGCTTGAGTACAGACGAGGTTGGCGGAATGGACAGAGTAGCGGTGAAATGCATAGATACTGTCCAGAACCCCGATTGCGAAGGCAGCTGACTAGGCTGTTACTGACGCTGAGGCACGAAAGCGTGGGGAGCGAACAGG

>ce8f5812cbcea834ef4b3cbaab501fa3

CATTGAACTATCGTGAGACAGTCAAGCCGCCAAAGGGAATTATATTATAGTAAATATTGGCGTAAATAAACATTTTATTAATAGTTGTAATATATGAAAATGTGCAGAAGATAAAGACGATTAGAACGGTGTTTAACAATCGCTTATCGAGACAATATGTAATTAATTAGTAATTAAAATATGTTTATTATGTGTATTATTCAAGGAATAGACTTGACAATAAATACAGTGGTAAACGGAACTAATAGAGTCACGCAAGTGAACTTAGAACATTTTTAATAAC

>7f862b96f58f9f2a310e849bd3a65069

TACGGAAGGTCCGGGCGTTATCCGGATTTATTGGGTTTAAAGGGAGCGTAGGCCGGGGATTAAGTGTGTTGTGAAATGTAGGCGCCCAACGTCTGACTTGCAGCGCATACTGGTTCCCTTGAGTACGCGCAACGCCGGCGGAATTCGTCGTGTAGCGGTGAAATGCTTAGATATGACGAAGAACCCCGATTGCGAAGGCAGCCGGCGGGAGCGCAACTGACGCTGAAGCTCGAAGGTGCGGGTATCGAACAGG

>7c5e6d0670382f362fe3948d9496c9c6

CGGTAAAAACACTTACCAAAGCACGAAACGTTTTCAGTTACAACTGACTATCTCCAGCGTTTCGTATCTCCAGAGACGTTGAAGATAGTCACTTGTGACTGAAAACTTTCGTCCATTGGTATATATGTCTACCAATGTATACAAATTAGAAACCCTTGTAGTCCGGCTGACTGACTGAG

>7876a5906e5be58d068237cd0b6ebb3c

TACAGAGGGTGCAAGCGTTAATCGGAATTACTGGGCGTAAAGCGTGCGTAGACGGTTACATAAGTCGGGTGTGAAAGCCCCGGGCTCAACCTGGGAATTGCATTCGAGACTGCGTAGCTAGGGTGCGGAAGAGGGAAGCGGAATTTCCGGTGTAGCGGTGAAATGCGTAGATATCGGAAGGAACACCAGTGGCGAAAGCGGCTTCCTGGTCCAGCACCGACGTTCAGGCACGAAAGCGTGGGGAGCAAACAGG

>fc56631cace5adf7cb79e21eda65aefb

TACGTAGGTGGCAAGCGTTATCCGGAATTATTGGGCGTAAAGCGCGCGTAGGCGGTTTTTTAAGTCTGATGTGAAAGCCCACGGCTCAACCGTGGAGGGTCATTGGAAACTGGAAAACTTGAGTGCAGAAGAGGAAAGTGGAATTCCATGTGTAGCGGTGAAATGCGCAGAGATATGGAGGAACACCAGTAGCGAAGGCGACTTTCTGGTCTGTAACTGACGCTGATGTGCGAAAGCGTGGGGATCAAACAGG

>51bab8ceb8cf115ecbe1c6cc85ec827b

TTATCAGGGTTTTTATTTTTGTCTCCCTTCTTGAATATAGGGATCGTCGTGCTAGTTTTCCAATCTCCTGGGACTTCTCCCTTATCCCGAATGACTTTGAACAGCTCCGTTAGCTCATCACATAATTTTGTACCACCGTATTTGAGCAGCTTATTTGTGAGACTATCAGGGCCGGGAGCTTTCCTATTTTTTAAACATCCGATCGCCTTTCCTACGTCTTCTCTCTGTATTTCCAGAGTGTTACTATTCTTATTAGTTTGCTCCCTTGCTTCCTTTGT

>67cf545e267d5bca442227b27fb1e2a3

TACAGAGGGTGCGAGCGTTAATCGGATTTACTGGGCGTAAAGCGTGCGTAGGCGGCTTTTTAAGTCGGATGTGAAATCCCCGAGCTTAACTTGGGAATTGCATTCGATACTGAGAAGCTAGAGTATGGGAGAGGATGGTAGAATTCCAGGTGTAGCGGTGAAATGCGTAGAGATCTGGAGGAATACCGATGGCGAAGGCAGCCATCTGGCCTAATACTGACGCTGAGGTACGAAAGCATGGGGAGCAAACAGG

>24567b9f51b6c8efd4ff96666a16482c

AATTGTGTGCCAGCCGCCGCGGTAACACAATAGCACACAAAAACAGATTGGGCATCACACTGCGAAGAGAGAAGGAAGGGAATGCGTACTGGCAACCTTGCACTATTATTTGTATATCACTGGCTGTGACTGAAACTGCTTGCCATTAGATACCCTTGTAGTCCGGCTGACTGACTTAACGTCCATCTCGTATGCCGT

>d719a09f0d72a573ea949ca34d377806

CATTTTACCATAGTGTCTGCCTCCACGGGCACGTTTATGGCTTTGCCGGTTATCTTTTAAGACTGTTTCTATTCTGAGACGACGGATCGCATACGTGGAAATCCGGATGAAATCAGTCTCTCAGTGATACGATCGAGCGGCAGATGGGTCGGATCTCCCGGATACTTATAACCTATTACTTTGGCTCGGCGGAGGCACTTTACCCTCATTAAGCAAGCGGCGGTATGTGGAGCACA

>ee98ed201702826bcb0dbbd56d7f2c5a

TACGTAGGGCGCGAGCGTTGTCCGGATTTATTGGGCGTAAAGAGCTCGTAGGTGGTTCGTCGCGTCTGTCGTGAAAGCCAGCAGCTTAACTGTTGGTCTGCGGTGGGTACGGGCGGGCTTGAGTGCGGTAGGGGAGATTGGAATTCCTGGTGTAGCGGTGGAATGCGCAGATATCAGGAGGAACACCGATGGCGAAGGCAGGTCTCTGGGCCGTTACTGACGCTGAGGAGCGAAAGCGTGGGGAGCGAACAGG

>1ed1341ab34ad70cb21751966bb563d4

TACGTAGGGTGCGAGCGTTGTCCGGAATTACTGGGCGTAAAGAGCTCGTAGGTGGTTTGTCGCGTCGTCTGTGAAATACCAATGCTTAACGTTGGTCGTGCAGGCGATACGGGCATTACTTGAGTGCTGTAGGGGTAACTGGAATTCCTGGTGTAGCGGTGAAATGCGCAGATATCAGGAGGAACACCGATGGCGAAGGCAGGTTACTGGGCAGTTGCTGACGCTGAGGAGCGAAAGCATGGGTAGCGAACAGG

>a84955e11368b0c4240c012a0c1ec4ba

CATTTTACCATAGTGTCTGCCTCCACGGGCACGTTTATGGCTTTGCCGGTTATCTTTTAAGACTGTTTCTATTCTGAGACGACGGATCTGCATACGTGGAAATCCGGATGAAATCAGCCTCTCAGTGATACGATCGAGCGGCAGATGGGTCGGATCTTCCGGATACTTATAACTATTACTTTGGCTCGGCGGAGGCACTTTACCCTCATTAAGCAAGCGGCGGTATGTGGAGCACA

>58494b5523d2b3c645926107d3cbf2ee

TACGAAGGTGGCTAGCGTTGTTCGGATTTATTGGGCGTAAAGGGTCCGCAGGCGGTTTGGAAAGTCAGATGTGAAATCCCACAGCTTAACTGTGGACACGCATTTGAAACTTCCAGACTAGAGTGTGAGAGGGGTGAATGGAATTTCCAGTGTAGGGGTGAAATCCGTAGATATTGGAAAGAACACCGGCCGCGAAGGCGATTCACTGGCTCATTACTGACGCTCAGGGACGAAAGCCAGGGGAGCAAACGGG

>99d07f7a502971ec8ee06b92d41f359a

TACGTATGTCACAAGCGTTATCCGGATTTATTGGGCGTAAAGCGCGTCTAGGTGGTTATGTAAGTCTGATGTGAAAATGCAGGGCTCAACTCTGTATTGCGTTGGAAACTGCATGACTAGAGTACTGGAGAGGTAAGCGGAACTACAAGTGTAGAGGTGAAATTCGTAGATATCTGTAGGAATGCCGATGGGGAAGCCAGCTTACTGGACAGATACTGACGCTAAAGCGCGAAAGCGTGGGTAGCAAACAGG

>bb84371b5a3def52df2ed8654ac8af58

TGTTCGTCCTACTACCATTCTGGTGTCACGTTATTTCATTTTTAAATAAATTCGGTGCTGCCTGCGCAGTAATATAATACGTATTGAAACAGCAACTTGTCAACAGTGATTAGATTCTTGCAGCACTTAATACACGTTAGTTTTAACTACGTCATGTGTGTCACGGATTAGAA

>aad1ab59a6783bd3e869d7f1bbab0a39

CATTGAACTATCGTGAGAATGGCACGCCGCCAAAGGGAATTATATTATAGTAAATATTAGCGTAAATAAACATTTTATTAATAGTTGTAATATATGAAAATGTGCAGAAGATAGAGACGATTAAAACAGTGTTTAACAATCGCTTATCGAGACAATATTTAATTAATTAGGAAGTAAAATATGTGTATTATTTGTATTTAACAAGGAATAAACGTGAAAATAAATACAGTGGTAAACGGAACTAATAGAGTCACGCAAGTGAACTTAGAACATTTTTAATAAC

>bb2c1673521af5cb0a4aedd6f7bb97c7

CATTGAACTATCGTGAGAAAGTCAAACCGCCAAAGGGAATTATATTATAGTAAATATTGGCGTAAATAAACATTTTATTAATAGTTGTAATATATGAAAATGTGCAGAAGATAAAGACGATTAAAACGGTGTTTAACAATCGCTTATCGAGACAATATTTAATTAATTAGGAATTAAAATATATTTATTATTGGTATTAAACAAGGGATAATCTTGAAAATAAATACAGTGGTAAACGGAACTAATAGAGTCACGCAAGTGAACGTAGAACATTTTTAATAAC

>10018212066cc356c7862b31b27142fe

TACGTAGGGGGCAAGCGTTGTCCGGATTTATTGGGCGTAAAGAGCGTGTAGGCGGCCAGGTAGGTCTGCTGTGAAAACTCGAGGCTTAACCTCGAGAGGTCGGCGGAAACCATCTGGCTAGAGTCCGGAAGAGGAGAATGGAATTCCCGGTGTAGCGGTGAAATGCGCAGATATCGGGAAGAACACCCGTGGCGAAGGCGGTTCTCTGGGACGGTACTGACGCTGAGACGCGAAAGCGTGGGGAGCGAACAGG

>d20b513165153f0e9599ac9e1c017b33

CGGTAAAAACACTTACCAAAGCACGAAACGTTTTCAGTTACAACTGACTATCTCCAGCGTTTCGTATCTCCAGAGACGTTGAAGATAGTCACTTGTGACTGAAAACTTTCGTCCATTGGTATATATGTCTACCAATGTATACAAATTAGAAACCCGAGTAGTCCGGCTGACTGACTTAA

>a92e3ddb5961c0608c0be06ee9a7d78c

ATGGTAATTGTGTGCCAGCCGCCGCGGTAAATTTTTGTCCCAGAAATCGGTGCCAGCATGGAGTAAATGTTAATATCAATGGAACACCCATACCAGTATTAGACACTGTACATTTTTTGGGAGATATCTTCGATAATAAGTTAAATTAGAAACCCTGGTAGTCCGGCTGACTGACTTAACGTCCATCTCGTATGCCGTCTTCT

>0ee6d5a7ad8c057ce547db045f4abbc1

TACGTAGGGCGCGAGCGTTATCCGGAATTATTGGGCGTAAAGAGCTCGTAGGCGGTTTGCCGCGTCTGCTGTGAAAGCCCGGGGCTTAACTCCGGGTTTGCAGTGGGTACGGGCAGACTGGAGTGCAGTAGGGGAGACTGGAATTCCTGGTGTAGCGGTGAAATGCGCAGATATCAGGAGGAACACCGATGGCGAAGGCAGGTCTCTGGGCTGTTACTGACGCTGAGGAGCGAAAGCATGGGGAGCGAACAGG

>8e2d4869b8621f73089c72904677b34d

TACGGGGGGGGCAAGTGTTCTTCGGAATGACTAGGCGTAAAGGGCACTACGGCAGTGAATCGGGTTGCAAGTTCAAGTCGCCAAAAACCGGCGGAATGCTCTCGAAACCAATTCACTTGAGTGAGACAGAGGAGAGTTTCATTTCGTGTGTAGGGGTTAAATCCGTAGATCTACGAAGGAAGGCCAAAAGCGAAGGCAGCTCTTTGGGTCCCTACTGACGCTGAGGTGCGAAAGCATGGGGAGCAAACTGG

>453cf07d3e96e304f23fbde7b355fe70

TACGTAGGGTGCAAGCGTTGTCCGGAATTATTGGGCGTAAAGAGCTCGTAGGCGGTTTGTCGCGTCTGCTGTGAAATCTGGGGGCTCAACCCCCAGCCTGCAGTGGGTACGGGCAGACTAGAGTGCGGTAGGGGAGATTGGAATTCCTGGTGTAGCGGTGGAATGCGCAGATATCAGGAGGAACACCGATGGCGAAGGCAGATCTCTGGGCCGTAACTGACGCTGAGGAGCGAAAGCATGGGGAGCGAACAGG

>93b3f7d15eaba71f3f624d0a52fb88c8

TACGTAGGGTGCAAGCGTTAATCGGAATTACTGGGCGTAAAGCGTGCGCGGGCGGTTATGCAAGACAGAGGTGAAATCCCCGGGCTCAACCTGGGAACTGCCTTTGTGACTGCATGGCTAGAGTACGGTAGAGGGGGATGGAATTCCGCGTGTAGCAGTGAAATGCGTAGATATGCGGAGGAACACCGATGGCGAAGGCAATCCCCTGGACCTGTACTGACGCTCATGCACGAAAGCGTGGGGAGCAAACAGG

>dab5a0ce7a10cf0a3c5d8822db2cd31c

CCGTTGTCGATTGCAGTGAAAGAATGTAAACAGGATTATCGCTGGTAATTACCTGTACACTTGGCTATGGTATGACAATATTTCATGTAGTTGTACAGTATACTGTCGGTATAACGGACCGACGAGCGACCTGCTCCATGCTGCGCCGTGCCGTGTCGTGTAATTAGATACCC

>ab8ca974db3b12ffabc0d73e66aba87c

TACGTAGGGTGCAAGCGTTAATCGGAATTACTGGGCGTAAAGCGTGCGCAGGCGGTTGTGTAAGTCAGATGTGAAATCCCCGGGCTCAACTTGGGAATTGCATTTGAGACTGCACGGCTAGAGTGTGTCAGAGGGGGGTAGAATTCCACGTGTAGCAGTGAAATGCGTAGATATGTGGAGGAATACCGATGGCGAAGGCAGCCCCCTGGGATAACACTGACGCTCATGCACGAAAGCGTGGGGAGCAAACAGG

>9f68e6aad35ab42586377a992b1dbd66

TACGTAGGGCGCAAGCGTTGTCCGGAATTATTGGGCGTAAAGAGCTCGTAGGCGGTTTGTCGCGTCTGCTGTGAAAGCCCGGGGCTTAACCCCGGGTGTGCAGTGGGTACGGGCAGACTTGAGTGCAGTAGGGGAGACTGGAACTCCTGGTGTAGCGGTGAAATGCGCAGATATCAGGAAGAACACCGATGGCGAAGGCAGGTCTCTGGGCTGTTACTGACGCTGAGGAGCGAAAGCATGGGGAGCGAACAGG

>8028a14ea865ee0033489eb980af7fdf

TACGAGTGCCCCAAGCGTTATCCGGAATTATTGGGCGTAAAGGGTGAGTAGGTGGTTATATTAGTCTTGTGTTAAAGCCTTCGGCTCAACCGAAGATCTGCATAGGAAACGGTATAACTAGAGTATGTGAGAGGTGTGTAGAACTCATGGAGTAGGGGTGAAATCCGTTGATATCATGGGGAATACCAAAAGCGTAGGCAGCACACTGGCACATTACTGACACTGAATCACGAAAGCGTGGGTAGCGAATGGG

>004c5cd94b8bb26093c03102b35d331d

CCCAGGAGCCACAAGTGGGAGCCACTTTTATTGGGCCTAAAGCGTTCGTAGCCGGTCGAGTAAATTTCCTGTGAAATCGTTGGGCTCAACCTAACGACGTGCAGGAAACACTGCTAGACTAGGGACCGGGAGGCGTCAGAGGTATTCCTGAGGGAGCGGTAAAATGTTATAATCTTAGGAGGACCCACTGTGGCGAAGGCGTCTGACGAGAACGGATCCGACGGTGAGGAACGAAAGCTAGGGGAGCAAACCGG

>006bbfed0b71de5d170ca5cdb45c5159

TACGGAGGGGGCTAGCGTTGTTCGGAATTACTGGGCGTAAAGCGCACGTAGGCGGACTGGAAAGTTGGGGGTGAAATCCCGGGGCTCAACCTCGGAACTGCCTTCAAAACTATCAGTCTGGAGTTCGAGAGAGGTGAGTGGAATTCCGAGTGTAGAGGTGAAATTCGTAGATATTCGGAGGAACACCAGTGGCGAAGGCAGCTCACTGGCTCGATACTGACGCTGAGGTGCGAAAGCGTGGGGAGCAAACAGG

>39dcb4557eeaf1f20d44a26a72de9410

TACGGAGGGTGCAAGCGTTGTCCGGATTTATTGGGTTTAAAGGGTGCGTAGGTGGTATTGTAAGTCTGGTTTGAAAGCTGGTCGCTTAACGATCAGATGTGGCTGGAAACTGCGGTACTTGAATGGATTGGCGGTAGCCGGAATGGGTCATGTAGCGGTGAAATGCATAGATATGACCCGGAACACCGATTGCGAAGGCAGGCTACTACGATTTGATTGACACTGAGGCACGAGAGCATGGGTAGCGAACAGG

>426a58d7fde254d8ff01fac25e36ff0e

TACGGAAGGTCCAGGCGTTATCCGGATTTATTGGGTTTAAAGGGAGCGTAGGCGGATTGTTAAGTCAGCGGTTAAAGGGTGTGGCTCAACCATACATTGCCGTTGAAACTGGCGATCTTGAGTGCAGACAGGGATGCCGGAATTCGTGGTGTAGCGGTGAAATGCTTAGATATCACGAAGAACTCCGATCGCGAAGGCAGGTGTCCGGGCTGCAACTGACGCTGAGGCTCGAAAGTGTGGGTATCAAACAGG

>6a90057b6e7a186c4d4e8974cd089f3f

TACGTAGGGGGCAAGCGTTATCCGGATTTACTGGGTGTAAAGGGAGCGTAGACGGCGAAGCAAGTCTGAAGTGAAAACCCAGGGCTCAACCCTGGGACTGCTTTGGAAACTGTTTTGCTAGAGTGCTGGAGAGGTAAGTGGAATTCCTAGTGTAGCGGTGAAATGCGTAGATATTAGGAGGAACACCAGTGGCGAAGGCGGCTTACTGGACAGTAACTGACGTTGAGGCTCGAAAGCGTGGGGAGCAAACAGG

>d8f82b1c484a3a64d5ec42e3c981fdf3

AAACCCATACGATGCTCAACAAAGTAAAGAATAGTACCGGTACTTGCTGAAGAACTGCAGATGTCCCCAGACTCTGCAGGTTAACGTTAGGCCACCCCCACTAAATATTAAAAACCAAGGTAATGTAAATACTTTGAAAATAAAATAGTAAAATAAGTAGTAACCTAGTCTTAAGAGAGAATCTCACAGACACTACCGGACTGTTTCAACTGTGAAAACGCTTTCTGAAATTTAATAAAAGTACAACTGGCTACCGAGGTTAATGACCTTGACTATATAATTG

>79bf3fc109209d28c3080a9db34a4ffc

TACGTAGGTGGCAAGCGTTGTCCGGAATTATTGGGCGTAAAGCGCGCGCAGGCGGTCTCTTAAGTCTGATGTGAAAGCCCCCGGCTCAACCGGGGAGGGTCATTGGAAACTGGGAGACTTGAGTACAAAAGAGGAGAGTGGAATTCCACGTGTAGCAGTGAAATGCGTAGAGATGTGGAGGAACACCAGTGGCGAAGGCGACTCTCTGGTCTGTAACTGACGCTGAGGCGCGAAAGCGTGGGGAGCAAACAGG

>5260f5955522cc8aed295a212e7d0524

CCTAGTATGTATGGTAATTGTGTGCCAGCCGCCGCGGTAATACTACACAAGGCACCGAGGACGAAGACGAGGAGGATATGAAAGTTGAATAAAGGAAACGAAGACACGGCAGGACGACGTTGAGTGTATCACGTGACAGAAGTTATTAGATACCCCTGTAGTCCGGCTGACTGACTCTACGACCATCTCGTATGCCGTCTTCTGCTTGAAAAA

>655942a5371537769b907ce2f8386dfa

CATTGAACTATCGTGAGAAAGTCAAACCGCCAAAGGGAATTATATTATAGTAAATATTGGCGTAAATAAACATTTTATTAATAGTTGTAATATATGAAAATGTGCAGAAGATAAAGACGATTAAAACGGTGTTTAACAATCGCTTATCGAGACAATATTTAATTAATTAGGAATTAAAATATGTGTATTATTTGTATTTAACAAAGAATAAACTTGAAAATAAATACAGTGGTAAACGGAACTAATAGAGTCACGCAAGTGAACTTAGAACAATTTTAATAAC

>22f69abd7a3ab4d62177d0def48f5c4c

TCAAACGAAAGCACAACAAAAAGATAGCCAAACTAATACCACCCCGATCACCAGCAATAACATCCACACATACGTTTTACCCAAGAATGGTTAACCTCACCAACATAACATTCACTAACGATCAGAAACAACTTTTAAACAAAGGGATAAATCACAACCTACACTACACGCAGAACAACATCAAGAAACAGGG

>8f48089d125ee77e9d7c0a8be4f275da

TACGTAGGGTCCGAGCGTTGTCCGGAATTACTGAGCGTAAAGAGCTCGTAGGTGGTTTGTCGCGTTGTTCGTGAAAACTCACAGCTTAACTGTGGGCGTGCGGGCGATACGGGCAGACTAGAGTACTGCAGGGGAGACTGGAATTCCTGGTGTAGCGGTGGAATGCGCAGATATCAGGAGAAACACCGGTGGCGAAGGCGGGTCTCTGGGCAGTAACTGACGCTGAGGAGCGAAAGCGTGGGTAGCGAACAGG

>a5beb8a4ae45a1ad4c956d40bf186dab

TACGGAGGATCCAAGCGTTATCCGGAATCATTGGGTTTAAAGGGTCCGTAGGCGGCCTTGTAAGTCAGTGGTGAAATCTCCCCGCTCAACGGGGAAACTGCCATTGATACTGCAGGGCTTGAATTATGGTGAAGTAACTAGAATATGTAGTGTAGCGGTGAAATGCTTAGAGATTACATGGAATACCAATTGCGAAGGCAGGTTACTAACCATATATTGACGCTGATGGACGAAAGAGTGGGGAGCGAACAGG

>8f0fb68673a8b3c26f3fd210e29b7916

TACGTAGGTGGCAAGCGTTGTCCGGATTTACTGGGCGTAAAGGATGCGTAGGCGGACATTTAAGTCAGATGTGAAATACCCGAGCTTAACTTGGGTGCTGCATTTGAAACTGGGTGTCTAGAGTGCAGGAGAGGTAAGTGGAATTCCTAGTGTAGCGGTGAAATGCGTAGAGATTAGGAAGAACACCAGTGGCGAAGGCGACTTACTGGACTGTAACTGACGCTGAGGCATGAAAGCGTGGGGAGCAAACAGG

>b52f26f4deed65b73682f945acfa354a

CATTGAACTATCGTGAGAAAGTCAAACCGCCAAAGGGAATTATATTATAGTAAATATTGGCGTAAATAAACATTTTATTAATAGTTGTAATATATGAAAATGTGCAGAAAATAAAGACGATTAAAACAGTGTTTAATAATCGCTTATCGAGACACTATTAAAGTAATACGGAATTAAAATATGTTTATTATGTGTATTAAACAAGAGATGAACTTGAAAATCAATACAGTGTTAAACGGAACTAATAGAGTCACGCAAGTGAACTTAGAACATTTTTAATAAC

>3bae00d865b43e800a6f297f958ac2de

TACGTAGGTGGCAAGCGTTGTCCGGAATTATTGGGCGTAAAGCGCGCGCAGGCGGATCGGTCAGTCTGTCTTAAAAGTTCGGGGCTTAACCCCGTGATGGGATGGAAACTGCCAATCTAGAGTATCGGAGAGGAAAGTGGAATTCCTAGTGTAGCGGTGAAATGCGTAGATATTAGGAAGAACACCAGTGGCGAAGGCGACTTTCTGGACGAAAACTGACGCTGAGGCGCGAAAGCCAGGGGAGCGAACGGG

>ddded45dca25d23ad7edf81c19c89ed7

GACCAGATGATTTCCACATTCATATGTTGCAACATACTGCATCTCATATTCTTGACACATTATTAATAAAATATGATTTTCTAGCATCTTTCCAACAGCGTGGAAAACAGCAACGATTATTCCAATTCTTAAAGAAAACAAAAACCCTTCATTATTAGATACCCTAGTAGTCC

>0091e4f000f82262aa13a990fa9b575b

CAGGCAACTAAGTCTATACGTGAATTGATCCGAAATAAAAGTATTTGTCAGAAGACAAAGAACAGCATATTCCACAACAAAATTGATAACAGTGATATAAGATATGGCCTTTTACGAATATTCTAAGGAGTGAAATTCAAACAATGAGATTACACTACATGTGAGAATGTCTACACGTTGCGAGGATAAAATAATAAAAAAGGTCGTATGGAGAAGGGCAGTAACATGCAG

>cb0d6cfd5930fd0266965c09471be3db

CTCGAGAACGGATGATCCGATTTTTATAGGGTTTTCTTTAAACAATACACGTAAAGAGGACGGCATGGTTCATCACATGTCTCACAAGTATTCTTACAATTTATGAATAACAAATATTTTTATAGTTAAACTCAATTAACCCTTAAAATAATACAGGAGAAAATATTTAAATAATCGTCAATAAGTGTCCTAATGATTAATAACAAACAAATAATAATTGTCTAAAACGTCTCCTGACAAATAACGCTATTATGCAAAACCATTATTCAGTAGTA

>555fa87ffd81dfdce9180672eddb68dd

GCAGCCATCCTCAGCCACTACACTATACAAACGTATTTACTGCGGACGATAATCTAGTTTCTCAGTTGTGCACGTTTGTCGTGTTACTGCAGTTACCATGTACCGAAGCGTGCGTTATCTCAGTCTCTATTAAGTCTTTCTTTCTGATATAATTTCTTCAGGTCTTCAAACTTTACTTAAATTTCGGTTTCTTTCTCCCATTTTCTTCTTACG

>460dd2dc0c85defde9408f8130feee82

CATTGAACTATCGTGAGAATGGCACGCCGCCAAAGGGAATTATATTATAGTAAATATTGGCGTAAATAAACATTTTATTAATAGTTGTAATATATGAAAATGTGCAGAAGATAGAGACGATTAAACCAGTGTTTAACAATCGCTTATCGAGACAATATTTAATTAATTAGGAAATAAAATAAGTGTATTATTTGTATTTAACAAGGAATAAACGTGAAAATAAATACAGTGGTAAACGGAACTAATAGAGTCACGCAAGTGAACGTAGAACATTTTTAATAACATTAGAACTAATAGAAA

>197857668aa86f0ce540bee63ab290ba

CCGCCGCGGTAAGTGCTCAAAGAACATCAATCTTTCACTTCGTTCTCACTTTCTCTTTTTACTGCCTCCTCTTCTGACCCTTTTCCACCATACCCCTTCCTCTCCTTTTCGTCATCCCTTTTCGGTCCATTTGCACCATAATATATTAGAAACCCGTGTAGTCCGGCTGACTGACTACTGTGTAA

>024611f1b5d2dbe9a6c9fd359d8051f9

CAGGCAACTAAGTCTATACGTGAATTGACCCGAAATAAAAGTATTTGTCAGAAGACAAAGAACAGCATATTCCACAACAAAATTGATAACAGTGATATAAGATATGGCCTTTTACGATTATTCTAAGGAGTGAAATTCAAACAATGAGATTACACTACATGTGAGAATGTCTACACGTTGCGAGGATAAAATAATAAAAAAGGTCGTATGGAGAAGGGCAGTAACATGCAG

>6a9b5423c4280b8e814d955cdfeed013

TACGGAGGATGCGAGCGTTATCCGGATTTATTGGGTTTAAAGGGTGCGTAGGCGGAAGGTCAAGTCAGCGGTAAAATGTCGGGGCTCAACCCCGGCCGGCCGTTGAAACTGGTCATCTGGAGTAGGAGAGAAGTATGCGGAATTCGTGGTGTAGCGGTGAAATGCATAGATATCACGAAGAACTCCGATTGCGAAGGCAGCATACCGGCTCCTTACTGACGCTGAGGCACGAAAGCGTGGGGATCAAACAGG

>17d341e98f23f4d6ad78d6e7be53c0e9

TACGTAGGGCGCAAGCGTTGTCCGGAATTATTGGGCGTAAAGGGCTCGTAGGCGGCTTGTCACGTCGGTTGTGAAAGCCCGGGGCTTAACCCCGGGTCTGCAGTCGATACGGGCAGGCTAGAGTGTGGTAGGGGAGATCGGAATTCCTGGTGTAGCGGTGAAATGCGCAGATATCAGGAGGAACACCGGTGGCGAAGGCGGATCTCTGGGCCATTACTGACGCTGAGGAGCGAAAGCGTGGGGAGCGAACAGG

>76620522fb7dfde9d5cb7e25cddf6aac

TACGGAGGATCCAAGCGTTATCCGGAATCATTGGGTTTAAAGGGTCCGTAGGCGGTTTAGTAAGTCAGTGGTGAAAGCCCATCGCTCAACGGTGGAACGGCCATTGATACTGCTAAACTTGAATTATTAGGAAGTAACTAGAATATGTAGTGTAGCAGTGAAATGCTTAGAGATTACATGGAATACCAATTGCGAAGGCAGGTTACTACTAATGGATTGACGCTGATGGACGAAAGCGTGGGTAGCGAACAGG

>639f903a8a0c64e4a8dc0baf77bff85c

TACGGAAGGTGCAAGCGTTAACCGGATTTATTGGGCGTAAAGAGTGCGTAGGCTGGATAATAAGTCAGATGTGAAATTTCGATACTCAATATCGAAGCTGCATTTGAAACTGTTTTTCTAGAGGATAGGTGGAGAAAGCGGAATTCCGTATGTAGCGGTGGAATGCGTAGATATACGGAGGAACACCCGTGGCGAAAGCGGCTTTCTAACTTATTCCTGACGCTGAGGCACGAAAGCGTGGGGAGCAAACAGG

>4e610fd61d60fc782d4a7f8a30637a4e

TACGTAGGTGGCAAGCGTTGTCCGGAATTATTGGGCGTAAAGGGCGTGTAGGTGGATTCTTAAGTCGTGTGTCTAAGTGCGGTGCTCAACACCGTATGGGCGCAGGAAACTGGGAATCTTGAGTGCAGGAGAGGAAAGTGGAATTCCCAGTGTAGCGGTGAAATGCGTAAATATTGGGAGGAACACCAGTGGCGAAGGCGACTTTCTGGACTGTGTCTGACACTGAGGCGCGAAAGCCAGGGGAGCGAACGGG

>c0d5395792eadbf5f62e8ffb14fa0262

GACGGGGGGGGCAAGTGTTCTTCGGAATGACTGGGCGTAAAGGGCACGTAGGCGGTGAATCGGGTTGAAAGTGAAAGTCGCCAAAAAGTGGCGGAATGCTCTCGAAACCAATTCACTTGAGTGAGACAGAGGAGAGTGGAATTTCGTGTGTAGGGGTGAAATCCGTAGATCTACGAAGGAACGCCAAAAGCGAAGGCAGCTCTCTGGGTCCCTACCGACGCTGGGGTGCGAAAGCATGGGGAGCGAACAGG

>8a3a8c5a18cf4bc29134b753897be392

TACGAAGGGGGCTAGCGTTGTTCGGAATTACTGGGCGTAAAGCGCACGTAGGCGGATTTGTAAGTCAGGGGTGAAATCCCGGGGCTCAACCTCGGAACTGCCTTTGATACTGCAAGTCTTGAGTCCGATAGAGGTGAGTGGAATTCCTAGTGTAGAGGTGAAATTCGTAGATATTAGGAAGAACACCAGTGGCGAAGGCGGCTCACTGGATCGGTACTGACGCTGAGGTGCGAAAGCGTGGGGAGCAAACAGG

>90afabd8d6f7f09b5c7adae992a1ffd5

CATTGAACTATCGTGAGAAAGTCAAACCGCCAAAGGGAATTATATTATAGTAAATATTAGCGTAAATAACCATTTTATTAATAGTTGTAATATATGATAATGTGCAAAAGATAGAGACGATTAAAACGGTGTTTAACAATCGCTTATCGAGACACTATTAAAGTAATTAGGAATTAAAATATGTTTATTATGTGTATTATTCAAGGAATAAACTTGAAAATAAATACAGTGTTAAACGGAACTAATAGAGTCACGCAAGTGAACTTAGAACATTTTAATAAC

>727606aed808856fc790f3a61a70cff9

AGCAGCATATGGTAATTGTGTGCCAGCAGCCGCGGTAATGAGTGTGATATAATTTTACAATACACTATAACATTAGATACCACAAGCCACACACAGAAGTCGGGGGCGCAAACACAATCATATAATTTTACCATACGCTATAACATTAGATACCCTGGTAGTCCGGCTGACTGACTTAACGTCCATCTCGTATGCCGTCTTCTGCTTGAAA

>dce85f8b1f9fd4f8e9ddc32993a1682b

GTGCCAGCCGCCGCGGTAACCGGACCTGTGTAGTTTGTTGTCGGCTTTGGCGTGTTAGTACTTCGTCTGTGCTCGCGTCCACCAGTGCACGATTCCCATAGAGTACGTACGCAGACTTTCACTCCCTAGAGCAACGGCCGCCTTATTAGATACCCTGGTAGTCCGGCTGACTGACTTGCGTCAAATCTCGTA

>0dd27c8fcba7c4c39afdf4eb910041b8

TCAAGTAGGCTTTGAATAACGCGCATCAATTTGCCGCAATAACGCTCGTTGTCATCGACCAGTTATTGGGACGCGACAATAACAGGCGGACATTTCACGGAGAGTTAGACCTGCTGATTGTTGGACTCAGCGCCCCGGGACGTCATTAATACCGCGATACTGTTCACGGGCGTGCTTTCACACTTATACGCGTACACATCGTGTAGCGCTACATTATCACAGCCAGTCCGACGTCGTAGATTCTATTAGAAATAATTTTAGTAACCGCCTGTTGTTAACCTAAACGTATTCGTTGTATTTT

>d46e2205f0c6ecf67b51f83d111c509c

TACGGAGGGTGCAAGCGTTAATCGGAATTACTGGGCGTAAAGCGCACGCAGGCGGTTTGTTAAGTCAGATGTGAAATCCCCGGGCTCAACCTGGGAACTGCATCTGATACTGGCAAGCTTGAGTCTCGTAGAGGGGGGTAGAATTCCAGGTGTAGCGGTGAAATGCGTAGAGATCTGGAGGAATACCGGTGGCGAAGGCGGCCCCCTGGACGAAGACTGACGCTCAGGTGCGAAAGCGTGGGGAGCAAACAGG

>b32648a253a3bb21bbe3bcc1db3f7caf

TACGTAGGGTGCGAGCGTTGTCCGGAATTACTGGGCGTAAAGAGCTCGTAGGCGGTTTGTCGCGTCGTCTGTGAAATTCTGCAGCTTAACTGTAGGCGTGCAGGCGATACGGGCAGACTTGAGTACTACAGGGGAGACTGGAATTCCTGGTGTAGCGGTGAAATGCGCAGATATCAGGAGGAACACCGGTGGCGAAGGCGGGTCTCTGGGTAGTAACTGACGCTGAGGAGCGAAAGCGTGGGTAGCGAACAGG

>d101c6da77626bb8d2c7658f047ae274

TACGGAGGATGCGAGCGTTATCCGGATTTATTGGGTTTAAAGGGTGCGTAGGCGGCGTGTCAAGTCAGCGGTCAAATCGCGGGGCTCAACACCGTGCCGCCGTTGAAACTGACAGGCTTGAGTGGGCGAGAAGTGCGCGGAATGCGTGGTGTAGCGGTGAAATGCATAGATATCACGCAGAACCCCGATTGCGAAGGCAGCGCACCGGCGCCCTACTGACGCTGAGGCACGAAAGTGCGGGGATCAAACAGG

>d6f755d0dc0492d4a62ee3748fad3ffe

TACGTATGTCGCAAGCGTTATCCGGAATTATTGGGCATAAAGGGCATCTAGGCGGCCAGGCAAGTCTGGGGTGAAAACCTGCGGCTCAACCGCAGGCCTGCCCTGGAAACTGCGTGGCTAGAGTGCTGGAGAGGTGGACGGAACTGCACGAGTAGAGGTGAAATTCGTAGATATGTGCAGGAATGCCGATGATGAAGATAGTTCACTGGACGGCAACTGACGCTGAAGTGCGAAAGCCGGGGGAGCGAACAGG

>ea93341e08300bd78c3ea01820b82240

TACGAAGGGGGCTAGCGTTGCTCGGAATCACTGGGCGTAAAGGGCGCGTAGGCGGCTGATTTAGTCGAGGGTGAAAGCCCGTGGCTCAACCACGGAATGGCCTTCGATACTGATTGGCTTGAGACCGGAAGAGGACAGCGGAACTGCGAGTGTAGAGGTGAAATTCGTAGATATTCGCAAGAACACCAGTGGCGAAGGCGGCTGTCTGGTCCGGTTCTGACGCTGAGGCGCGAAAGCGTGGGGAGCAAACAGG

>e1c7d97fe13e9127d225d76a1feb8c78

TACGAAGGGGGCTAGCGTTGTTCGGAATTACTGGGCGTAAAGCGCACGTAGGCGGATATTTAAGTCAGGGGTGAAATCCCAGAGCTCAACTCTGGAACTGCCTTTGATACTGGGTATCTTGAGTATGGAAGAGGTAAGTGGAATTGCGAGTGTAGAGGTGAAATTCGTAGATATTCGCAGGAACACCAGTGGCGAAGGCGGCTTACTGGTCCATTACTGACGCTGAGGTGCGAAAGCGTGGGGAGCAAACAGG

>11ac1400309829ee5a48419e57eec62e

TACGGAGGGTGCGAGCGTTGTCCGGAATCACTGGGCGTAAAGGGCGCGTAGGTGGCTGGAGCAGTCAACGGTGAAAGCTTGGGGCTCAACCCCGAGTCGGCCGTCGATACTCTTCGGCTCGAGCACTGTAGAGGCAGGTGGAATTCCGGGTGTAGCGGTGGAATGCGTAGAGATCCGGAAGAACACCGGTGGCGAAGGCGGCCTGCTGGGCAGTCGCTGACACTGAGGCGCGACAGCGTGGGGAGCAAACAGG

>84e5843387709d458fc6ece6da9b68c3

TCTAGTGTATGGTAATTGTGTGCCAGCCGCCGCGGTAATGAGTGTGATATAATTTTACAATACACTATAACATTAGATACCACAAGCCACACACAGAAGTCGGGGGCGCAAACACAATCATATAATTTTACCATACACTATAACATTAGAAACCCTAGTAGTCCGGCTGACTGACTTAACGTCCATCTCGTATGCCGTCTTCTGCTTGAAA

>c1908a2545058f32b44aa7377b01d54f

TGATGAATAGAATTTCAGTCAAATGCGTCTCAGTAACGTGGCCGAACCGCAAAAACCTACTAATGCCGTCAACAAAGCTTACCTCGACAATCACACGCCTATACGCGGTAAAGACAATTAAGGTTTTGGAAAACGACAACTCGTCGACGTAGCCGAACCTCTGCAGGACACTGATTGTGTAAACCTTAAGTATCTCGAAGCGCTGGTTGTACGCCCGCTAAGTGG

>b022b5312f30be684bdd2cc66030dea5

TACGTAGGTGGCAAGCGTTGTCCGGAATTATTGGGCGTAAAGCGCGCGCAGGCGGCCTATCCAGTCTGTCTTAAAAGTTCGGGGCTCAACCCCGTGATGGGATGGAAACTAGTAGGCTAGAGTATCGGAGAGGAAAGCGGAATTCCTAGTGTAGCGGTGAAATGCGTAGATATTAGGAAGAACACCAGTGGCGAAGGCGGCTTTCTGGACGAAAACTGACGCTGAGGCGCGAAAGCCAGGGGAGCGAACGGG

>fb648692eb272195ec4e6e92ef4d53a2

CATTGAACTATCGTGAGAGAGTCAAGCCGCCAAAGGGAATTATATTATAGTAAATATTAGCGTAAATAAACATTTTATTAATAGTTGTAATATATGAAAATGTGCAGAAGATAAAGACGATTAAAACGGTGTTTAACAATCGCTTATCGAGACAATATTTAATTAATTAGATA

>d9658c4713fddabad7d4647224b77c61

TACAGAGGGTGCAAGCGTTAATCGGAATTACTGGGCGTAAAGCGCGCGTAGGTGGTTTGTTAAGTTGGATGTGAAAGCCCCGGGCTCAACCTGGGAACTGCATCCAAAACTGGCAAGCTAGAGTACGGTAGAGGGTGGTGGAATTTCCTGTGTAGCGGTGAAATGCGTAGATATAGGAAGGAACACCAGTGGCGAAGGCAACCACCTGGACTGATACTGACACTGAGGTGCGAAAGCGTGGGGAGCAAACAGG

>8f789cf55997693b99bcb9b31c6d474f

ACATTCATTGTACTTCATTAGTTATTTAATTTACACCACTTGTCACCCCCTTATATTGTACAAATGAGTTTGCCGGCACATTATGTGAAATACACGTTCCTGTTACAGCTTATCCCTGACCTGCTAAAACATGAACGGCAATATTTCTATGACCACAAAAACTATGGACCAAGAAGCTA

>455219eeb206cd432bf64f51159e0b4c

TACGTAGGGTGCAAGCGTTGTCCGGATTTACTGGGCGTAAAGAGCTCGTAGGTGGTGTGTCGCGTCGTCTGTGAAATTCCGGGGCTTAACTCCGGGCGTGCAGGCGATACGGGCACGACTAGAGTGCTGTAGGGGTAACTGGAATTCCTGGTGTAGCGGTGAAATGCGCAGATATCAGGAGGAACACCGATGGCGAAGGCAGGTTACTGGGCAGTTACTGACGCTGAGGAGCGAAAGCATGGGGAGCGAACAGG

>54e6cd3e29bea5a444549455f8a6333a

CCGATCATGGTGAGGCACATCCCTAACAACGGCCGTAGTGATTACGCTCTGCAACCAATGACACAATCATTTGTTCACAAGTCTCTCACTCTATTTCTGTCCATATTCCGCTGCGCGTAATTCTCATCGTCGTGACAATGTCTTCTGTGGTGAAGTTATTCCACCTCCTCAAAAGAACTGGTTCTACATTTCCAATTCCTACATTCTTTATTTGTTTTATCGATTTTTTATATTAATCTCTACGATATTATCAATACATAAGTGGCAAAATACGTTTTAACCG

>c9f4dd74569207b9809278d8839bb40a

AAAAAAAGCTGTCTACTACGAATTTGTATGAACATCAAATTTGTGTCACTAACGAAAACAAATTCATTCGTAAAACATACCCGGCACTATCAAGAAAGGGTCGATGTTGAAATCCAATGGATGGTGGATCAAGGGGTTATCAAACGATCCAACGGTGATTTTCTTAACCCGGTGGTAACCGTTAAGGAATATAAGGCTGTGCCTCGATATGCACAACTCAAAAATAGTATCGTGTTGGGCTCCGAACGCCGAGTCAGTTTTTATTAAGTGTCAGGGTGTCCGCTACATGTCCCG

>f7468538e3346d687ca69b16628b6f3a

TACGGAGGGTGCAAGCGTTAATCGGAATTATTGGGCGTAAAGGGCGCGTAGGCGGATAGACAAGTCTGGTGTGAAATACCGTGGCTCAACCACGGTGCTGCATCGGAAACTGTATATCTTGAGGGTAGGCGGAGAAAACGGAATTCCAAGTGTAGCGGTGAAATGCGTAGATATTTGGAAGAACACCGGTGGCGAAAGCGGTTTTCTAGCTTATTCCTGACGCTGAGGCGCGAGAGCAAGGGGAGCAAACAGG

>e5595bdd74b2c5cc2ec5ffff6b04e4b8

TACGGAGGGGGCTAGCGTTGTTCGGAATTACTGGGCGTAAAGCGCACGTAGGCGGACTGGAAAGTTGGGGGTGAAATCCCGGGGCTCAACCTCGGAACTGCCTTCAAAACTATCAGTCTGGAGTTCGAGAGAGGTGAGTGGAATTCCGAGTGTAGAGGTGAAATTCGTAGATATTCGGAGGAACACCAGTGGCGAAGGCGGCTCACTGGCTCGATACTGACGCTGAGGTGCGAAAGCGTGGGAAGCAAACAGG

>b2aa4aa436bb9fb3b1a7572ddf7389eb

CACACTTATGTATTTAGAAAGACAAAATATTCCTCCATGATGCATTTCAACCATGTCGGTTATTTTTAACTGGCAATGCCTTGTAGATATTTTTGAAAGATTTTGAATGTGTCTTGTCCATACAGTTCAGTTTTATTTAGGCTTTCAATTAAATTCCTTTTTGCTTTTTTGCCGGTTGTG

>7e7deb01f0491d33ecb87427fcbeb1ee

TACGGAGGGTGCAAGCGTTATCCGGATTCACTGGGTTTAAAGGGTGCGTAGGCGGGCAGGTAAGTCAGTGGTGAAATCCTAGAGCTTAACTCTAGAACTGCCATTGATACTATCTGTCTTGAATATTGTGGAGGTAAGCGGAATATGTCATGTAGCGGTGAAATGCTTAGATATGACATAGAACACCTATTGCGAAGGCAGCTTACTACGCATATATTGACGCTGAGGCACGAAAGCGTGGGGATCAAACAGG

>1b0c35b6d03e585b89a63d355386793f

TACGGAGGCTCCAAGCGTTAATCGGAATTACTGGGCGTAAAGCGTGCGCAGGCGGTTTGTTAAGCGAGATGTGAAAGCCCTGGGCTCAACCTAGGAATAGCATTTCGAACTGGCGAACTAGAGTCTTGTAGAGGGGGGTAGAATTCCAGGTGTAGCGGTGAAATGCGTAGAGATCTGGAGGAATACCGGTGGCGAAGGCGGCCCCCTGGACAAAGACTGACGCTCATGCACGAAAGCGTGGGGAGCAAACAGG

>97354d5d1bcbea0b68a0712eacecad0a

TTCTTACTGCAAACGTATTGTCACACACATATGTTTTGTAGTAATTATTGTGAGCAGCATCCTCTATGTTATAAATGTAATAAGGAAAGCTTAAAACAATGATAATAATCTGTTTATAGTAATTAATCTTCATCATCTTCGCCATTCCAGGATTAGAAACCCGAGTAGTCCGG

>0b5b7c76ac5b705424003f48b6385075

CACGCTTATGTCTTTAGAAAGACAAAACATTTCTCCAGAATGCATTCCAACCTTGTCGGTCATTTTCAACTGGCAGTGTCTTGTAGATATTTGTGAAAGATTTTGAATGTGTCTTGTCCATAGAGTTCAGTTTGTTCGTTAATGATTTTGTTGTGTAGTTTGTAACGAAATATATTTAAACTTTCAAGAAGATTCAGTTTATTTCGTTTTTTCCGGTTGTG

>e18bc409ac37213beb4c848cfccbaa06

GGTAAGCAACTACAGCACAATAAACTGTCTACATTTAATAAACAAACTTATTGAATGTTGCAATGAGTACACAAAACTTAGTGTTTAGAAGAAAAAAGGATATTCACAATTTTTTCTGAAATATATAAAAACTCAACAGCTCAAATTAGAAACCCCAGTAGTCCGGCTGACTGACTTA

>ecfc5812c430a69c403672d746f03ccb

TACGTAGGGTGCGAGCGTTGTCCGGAATTATTGGGCGTAAAGAGCTTGTAGGCGGTTTGTCGCGTCTGCTGTGAAAGCCCGGGGCTTAACTCCGGGTCTGCAGTGGGTACGGGCAGACTAGAGTGTGGTAGGGGAGACTGGAATTCCTGGTGTAGCGGTGAAATGCGCAGATATCAGGAGGAACACCGATGGCGAAGGCAGGTCTCTGGGCCATTACTGACGCTGAGAAGCGAAAGCATGGGGAGCGAACAGG

>6cdd150edd0fdf0ca08703ac8a5c2c8d

TCGTTCTGGTGTTCTCTTCGTCGACGTCGAACGTGAGCAGATTCTTCATGGTGCTAGTGACACCGAGTCGTCTCGTGCGCGCTACTTCCACGCCGGCCAACTCGTACCGTATCTCTTCGAACAAAAAAGCTACGGCATTGTTGTCGAGACACAGATCCGTGTCCACTTTTGATGTTCCGTCTCGCTTAGTGAGTCTCCCTTCGACGACACGCTTTCGCACGGAAGTGTGTACAGGTCTTGCTGTTGAACGGCTATTCGTATCTCGTCGTTGTAAC

>cb1a1832de69f78aa14c50812b6bbc99

TACGGAGGGTGCAAGCGTTATCCGGATTTATTGGGTTTAAAGGGTCCGTAGGCGGACTTATAAGTCAGTGGTGAAAGCCTGTCGCTTAACGATAGAACTGCCATTGATACTGTAAGTCTTGAGTATATTTGAGGTAGCTGGAATAAGTAGTGTAGCGGTGAAATGCATAGATATTACTTAGAACACCAATTGCGAAGGCAGGTTACCAAGATATAACTGACGCTGAGGGACGAAAGCGTGGGTAGCGAACAGG

>a1034b5bec71d26dee3d5154ce7ca3c5

CACACTTCAGAAAGACAATATATTTCTCCAGGATGCATTTCAACCATGTCGGTCATTTTCAACTGGCAGTTCCTTCTAGATATTTGTGAAAGATTTTGAATGTGTCTTGTCCATAGAGTTGAGTTTGTTTATTAATGATTTTGTTATGTAGTTTGTAATGTAATATATTTAGGCTTTCAAGTAAATTCATTTTGTTTCCTTTTTTGCCGGTTGTG

>cf8b7bdf2ef04acee6e879cd85fdd9cf

TACAAGTAAGACTAGTGTTATTCATCTTTATTAGGTTTAAAGGGTACCTAGACAGTATTTCTAGCCTCAAAAGGGAACAGACTTACTAGAGTTTTATGTGAGAGGAAAATATTAGAACCATTGGAGTAGAGATAAAATATTTTGATACTAATGGGACGGATAACGGCGAAGGCAAACCTCTATGTAATAACTGACGTTGAGGGACGAAGGCTTGGGGAGCGAATAGG

>8a7d7de4627eb82702031c6644543c8a

TACGGAGGGTGCAAGCGTTGTCCGGAATCATTGGGCGTAAAGAGTTCGTAGGTGGTTTGTTAAGTTTGGTGTTAAATGCAGGGGCTCAACTCCTGTTCAGCATCGGATACTGGCAGACTTGAATGCGGTAGAGGTAAAGGGAATTCCTGGTGTAGCGGTGAAATGCGTAGATATCAGGAGGAACATCGGTGGCGTAAGCGCTTTACTAGGCCGTAATTGACACTGAGGAACGAAAGCCAGGGTAGCAAATGGG

>98676d575c32a9b511c4a86fd25bed83

CACACTTATGTCTTTAGAAAGGCAAAACGTTTTTGTAAAATTCATTTCAACTTTCTCGGTCATTTTCAACTGGCAGTGCCTTGTAGATAGTTTTGGAAGATTTTGAATGTTGCTTGTCCATAGAGATCAGTTTGTTCATTAATGATTTTGTTGTGTAGTTTGTAACATAATATATTTAAACTTTCAAGAAGATTAATTTTATTTCCTTTTTTGCCGTTGTA

>0973bf2cfcef7c81f368cccad2f1d4f7

GACGTAGGGGGCGAGCGTTGTCCGGAGTTACTGGGCGTAAAGGGCCTGTAGGCGGTCGGGCAGGTTCCGGCTGACAGCCCGCGGCTTCACTGCGGGAGAAGCAGGAAGACGGTCTGACTTGAGGGCCACAGAGGGACAGGGAATTCCCGGTGGAGCGGTGAAATGCGTAGAGATCGGGAAGAACACCGAAGGCGAAGGCACTGTCCTGGGTGGTACCTGACGCTGAGAGGCGAAAGCTAGGGGAGCGAACGGG

>a967000ec3e5ba2293cf1b8cfac5c0b7

TACGAAGGGGGCTAGCGTTGCTCGGAATTACTGGGCGTAAAGGGCGCGTAGGCGGCCATCTTAGTCAGACGTGAAATTCCTGGGCTCAACCTGGGGGCTGCGTTTGAGACGGGGTGGCTTGAGGATGGAAGAGGCTCGTGGAATTCCCAGTGTAGAGGTGAAATTCGTAGATATTGGGAAGAACACCGGTGGCGAAGGCGGCGAGCTGGTCCATTACTGACGCTGAGGCGCGACAGCGTGGGGAGCAAACAGG

>ec67cfb7840563f4af01be2b6b0c2fbb

AGCAGAGGATACAAGCGTCATCCGGATTTATTGGGTTTAAAGGGTGCGTAGGTGGTTTTTTAAGTCAGTAGTGAAATCTTAAAGCTTAACTTTAAAAGTGCTATTGATACTGATAAACTAGAGTGAGGTTGGAGTAACTGGAATGTGTGGTGGAGCGGTGAAATGCATAGAGATCACACAGAACACCAATCGCGAAGGCATGTTACTAAACATAGACTGACACTGAGGCACGAAAGCATGGGTAGCAAACAGG

>b532c63e6831fee25fab35743e613786

TATGATATATTTAAAACTTTAGTAAATCATATAAACCAGGGAAAATAGTCAATAGTAAAACTTATTTCAAATTTAAATAAAAACAAAACTTGAGTAATCAGTTTCAGGAAAAAATTAATAAACTTGAATAAAAAGGGAACAGCAATAATCTGGAAACCATCTGGTTTAACAAAAGCCGTATATGACACAAGTCTCATTACAGCATTATATTATTCAAACAGCAGCATATTACAGAAATTATAAGTATTCTCCTGGAACACAGTACACATCATTTCA

>0deb7b08e26a29f86d531be6e558a831

ACATTCATTGTACTTCATTAGTTATTTAATTTACACCACTTGTCACCCCCTTATATTGTACAAACGAGTTTGCTGGCACATTATGTGAAATACACGTTCCTGTTACAGCTTATCCCTGACCTGCTAAAACATGAACGGCAATATTTCTATGACCACAAAAACTATGGACCAAGAAGCTA

>864267f12bedace19272af56224498c7

TACGTAGGGTGCGAGCGTTAATCGGAATTATTGGGCGTAAAGCGAGTGTAGACGGTTATTTAAGCCAGATGTGAAATACCCGAGCCTAACTTGGGAGGTGCATATGGAACTGGGTAGCTAGAGTGTGTCAGAGGGAGGTAGAACTCCACGTGTAGCAGTGAAATGCGTAGAGATGTGGAAGAATACCGATGGCGAAGGCAGCCTCCTGGGATAACACTGACGTTGAGGCTCGAAAGCGTGGGGAGCAAACAGG

>813434cebae171e302567765c864c7ff

TACGTAGGTGGCAAGCGTTGTCCGGATTTATTGGGCGTAAAGCGAGCGCAGGCGGAAGAATAAGTCTGATGTGAAAGCCCTCGGCTTAACCGAGGAACTGCATCGGAAACTGTTTTTCTTGAGTGCAGAAGAGGAGAGTGGAACTCCATGTGTAGCGGTGGAATGCGTAGATATATGGAAGAACACCAGTGGCGAAGGCGACTCTCTGGTCTGCAACTGACGCTGAGGCTCGAAAGCATGGGTAGCGAACAGG

>488185b68bb0754ddb67dd3f496b53f2

CATTGAACTATCGTGAAAAAATCAAGCCGCCAAAGGGAATTATATTATAGTAAATATTGGCGTAAATAAACATTTTATTAATAGTTGTAATATATGAAAATGTGCAGAAGATAAAGACGATTAAAACGGTGTTTAACAATCGCTTATCGAGACAATATTTAATTAATTAGTAATTAAAATATGTTTATTATGTGTATTATTCAAGGAATAGACTTGACAATAAATACAGTGGTAAACGGAACTAATAGAGTCACGCAAGTGAACTTAGAACATTTGTAATAAC

>06d27076bf071952a4208cdbdd42ec9b

CTATGGTAATTGTGTGCCAGCCGCCGCGGTAAGTATCTGCAATGTGTGTTGCAGTAGTATCAGGCGCAGTGTCCACGCGACATGACGTTACGTGATTGTTAATCGCGTGACTAAGAGTTAATGACTTACTGACATTGTCCTGTCATTAGATACCCTTGTAGTCCGGCTGACTGACTTAACGTCCATCTCGTATGCCGTCTTCTGC

>a8420220d4097ae1b3c50143350397cb

CTCACTCATGTCTTTAGAAAGACAAAACATTTTTTCAGGATGCATTTCATTCATGTCGGTCATTTTCAACTGGCAGTGTCTTGTAGGATAGTTGTGAAAGATTTTGAATGTGTCTTGTTGATAGAGTTCAGTTTGTTCATTTAGACTTTCAAGCAAATTCATTTTATTTCCTTTTTTGCCGGTTGTG

>150b036956831ab6d0cc5d26ce1b276f

AATTGTGTGCCAGCCGCCGCGGTAACACAATAGCACACAAAAACAGATTGGGCATCACACTGCGAAGAGAGAAGGAAGGGAATGCGTACCGGCAACCTTGCACTATTATTTGTATATCACTGGCTGTGACTGAAACTGCTTGCCATTAGAAACCCCTGTAGTCCGGCTGACTGACTTGCGTCAAATCTCGTATGCCGT

>7bef673b88b3ee538417349a2d9f0c78

CATTGAACTATCGTGAGAAAGTCAAACCGCCAAAGGGAATTATATTATAGTAAATATTGGCGTAAATAAACATTTTATTAATAGTTGTAATATATGAAAATGTGCAGAAGATAAAGACGATTAAAACGGTGTTTAACAATCGCTTATCGAGACAATATTTAATTAATTAGGAATTAAAAAATATTTATTATTTGTATTTAACAAGGAATAAACTTGAAAATAAATACAGTGGTAAACGGAACTAATAGAGTCACGCAAGTGATCTTAGAACATTTTTAATAAC

>17b2bc1f09d2ef33dde06ed43812f4e9

TACGGAGGGGGCTAGCGTTGTTCGGAATCACTGGGCGTAAAGCGCACGTAGGCGGCGATCTAAGTCAGAGGTGAAAGCCCGGGGCTCAACCCCGGAATTGCCTTTGAGACTGGATTGCTTGAACGTCGGAGAGGTGGGTGGAATTCCGAGTGTAGAGGTGAAATTCGTAGATATTCGGAAGAACACCAGTTGCGAAGGCGGCCCACTGGACGACTGTTGACGCTGAGGTGCGAAAGCGTGGGGAGCAAACAGG

>6446eabedd72a822ff6a888afc61e887

CATTGAACTATCGTGAGAAAGTCAAACCGCCAAAGGGAATTATATTATAGTAAATATTGGCGTAAATAAACATTTTATTAATAGTTGTAATATATGATAATGTGCAGAAGATAAAGACGATTAAAACAGTGTTTAATAATCGCTTATCGAGACACTATTAAAGTAATACGGAATTAAAATATGTTTATTATGTGTATTAAACAAGGGATAAACTTGAAAATAAATACAGTGGTAAACGGAACTAATAGAATCACGCAAGTGAACTTAGAACATTTTTAATAAC

>52d1042d86d9df309ce326d83d3a2c83

CACACTTATGTCTTCAGAAATACAAACCATTTCTCCAGGATGCATTTCAACCATGTCGGTCATTTTCAACTGTCAGTGCCTTGTAGATATTTGTGGAAGATTTTGAATGTGTCTTGTCCATGGAGTTCAGTTTGTTCATTAATGATTTTGTTGTGTAGTTTGTAACGTAATATATTTAGGGTTTTAAGTAAATTCATTTTATGTCCTTTGTGCCTGTTGTA

>d48ae428373e0905b0ef588ba040932b

CAGGCAACTAAGTCTATACGTGAATTGACCCGAAATAAAAGTATTTGTCAGAAGACAAAGAACAGCATATTCCACTACAAAATTGATAACAGTGATATAAGATATGGCCTTTTACGAATATTCGAAGGAGTGAAATTCAAACAATGAGATTACACTACATGTGAGAATGTCTACACGTTGCGAGGATAAACTAATAAAAAAGGTCGTATGGAGAAGGGCAGTAACATGCAG

>c9a54aebf5dfcbd9adca048d100b3c16

TACGTAGGGGTCGAGCGTTGTCCGGAGTTACTGGGCGTAAAGCGCGTGCAGGTGGCTCATTACGCCCGGCGTGAAAGCCCCCGGCTCAACCGGGGAGGGTCGTCGGGGACGGATGGGCTTGAGGGTCGCAGGGGCTGGTGGAATTCCCGGTGTAGTGGTGAAATGCGTAGAGATCGGGAGGAACACCCGTGGCGAAGGCGGCCAGCTGGGCGACACCTGACACTGAGACGCGAAGGCGTGGGGAGCGAACGGG

>e8bf6301a54414495183cbfe7825ad04

TACGTAGGGTGCGAGCGTTAATCGGAATTACTGGGCGTAAAGCGTGCGCAGGCGGTTTCGTAAGCTGGAGGTGAAATCCCCGGGCTTAACCTGGGAATGGCCTTCAGGACTGCGAGGCTAGAGTGCGGCAGAGGGAGGTGGAATTCCACGTGTAGCAGTGAAATGCGTAGAGATGTGGAGGAACACCGATGGCGAAGGCAGCCTCCTGGGCCAGTACTGACGCTCATGCACGAAAGCGTGGGGAGCAAACAGG

>517f4714b21a0a716e7ca12d7527b4da

CATTGAACTATCGTGAGAATGACACGCCGCCAAAGAGAATTATATTATAGTAAATATTAGCGTAAATAAACATTTTATTAATAGTTGTAATATATGAAAATGTGCAGAAGATAAAGACGATGAAAACGGTGTTTAACAATCGCTTATCGAGACAATATTTAATTAATTAGGAATTAAAATATGTGTATTATTTGTATTTAACAAGGAATAATCTTGAAAATAAATACAGTGGTAAACGGAACTAATAGAGTCACGCAAGTGAACTTAGAACAATTTTAAGAAC

>aad5ad95b49aa024ba9bbcbbcc03d1aa

GTCCTCACGTCTGCGGCAGCGAGCCCTTGGCTACGCCACGATTTTATCGCGGCGAGAGTAGGTTCGTGCTGGCGTGAGCGGTAAGCCGCGGCCGCTGATTGGAGCGTGCACTGCACCTTCTAGCTGGCGACAACGCCGCAGGTCGCGCAAGGAGACACTTGTACATCCTTGCGAGAGCTCGAGCGCGGCTGACTTCTAGCCGACGCGGCTTTGCAGTATCCTGCGTAGTGCGGAAGATTTTTTAAGGTCATGGATGGCCCTTATATACGG

>a1aa0b690d2d90898e02d27bd442a0dc

TGTGCCAGCCGCCGCGGTAAGCGTATTTCTAAATTTTACCTAAAATAGTGAACTGTAGGTCGTGTTTTAATTACAAAATCAGTTCTTTATTTGAGTCGTATTTACTCTTTTCTTTTTGTTAGAACTCGTCCTGTACCGTCAATCATTAGATACCCCAGTAGTCCGGCTGACTGACTTAACGTCCATCTCGTAT

>75e566ca0d3111bb149bc4df7b928325

TACGTAGGTGGCAAGCGTTGTCCGGATTTATTGGGCGTAAAGGGAGCGCAGGTGGCTTCTTAAGTCTGATGTGAAAGCCCACGGCTTAACCGTGGAGGGTCATTGGAAACTGGGAAACTTGAGTACAGAAGAGGAATGTGGAACTCCATGTGTAGCGGTGGAATGCGTAGATATATGGAAGAACACCAGTGGCGAAGGCGACATTCTGGTCTGTTACTGACACTGAGGCTCGAAAGCGTGGGGAGCAAACAGG

>f29cd02e53571db81491449978191c3e

CATTGAACTATCGTGAGAATGGCACGCCGCCAAAGGGAATTATATTATAGTAAATATTAGCGTAAATAAACATTTTATTAATAGTTGTAATATATGATAATGTGCAGAAGATTAAGACGATTAAAACGGTGTTTAACAATCGCTTATCGAGACACTATTAAAGTAATTAGGAATTAAAATATGTTTATTATGTGTATTAAACATAGACTTGAAAATAAATACAGTGTTAAACGGAACTAATAGAGTCACGCAAGTGAACTTAGAACATTTTAATAAC

>187537a60ac8f41ca0f505544134d3fb

GGAAACGGACAGCAAGTTTTCTTTACTGGAAACAGTGGCCTACTTAGTTAAAATGCTACCTTCTTCCTATTATTGTTTGTGATTTAGGGGCCTAGTCAGAAAACTTAAAACCTAGAGTTAATATATCTCAAGGTAATAAAATACAAAATAGTAATGAATGAAGCAGCAATAACATACGTCAATTAGCATGTGCGTTCAGATGTAACGTCTTGAGCTGTTACTGTGTTTCGTTAAACACATTTCAGACTGTAGAGACGAATTTTACAGTGCCATTAAAACTAC

>c49f2b759e636d140adddc4aca22bf6b

CATTGAACTATCGTGAGAACGGCATGCCGCCAAAGGAAATTATATTATAGTAAATATTAGCGTAAATAAACATTTTATTAATAGTTGTAATATATGAAAATGTGCAGAAGATAGAGACGATGAAAACGGTGTTTAACAATCGCTTATCGAGACAATATTTAATTAATTAGGAATTAAAATATGTGTATTATTTGTATTTAACAAGGAATAATCTTGAAAATAAATACAGTGGTAAACGGAACTAATAGAGTCACGCAAGTGAACTTAGAACAATTTTAAGAAC

>59e7289380be2a5909212804646e6eb6

TACGTAGGTGGCAAGCGTTGTCCGGAATTATTGGGCGTAAAGCGCGCGCAGGCGGCTTCTTAAGTCTGATGTGAAATCTCGGGGCTCAACCCCGAGCGGCCATTGGAAACTGGGGAGCTTGAGTGCAGAAGAGGAGAGTGGAATTCCACGTGTAGCGGTGAAATGCGTAGAGATGTGGAGGAACACCAGTGGCGAAGGCGACTCTCTGGTCTGTAACTGACGCTGAGGCGCGAAAGCGTGGGGAGCAAACAGG

>edbbf799f4cd5930f5d4ce0a6f40386c

TGATTCTCGGTACGACGTTTCCACCTCCAAACGCCGACGATACTCGACACTCCGGGTCAATTTTTTCACCCCTTTGTGCTTTTACTCAGCGGTTGAGTTTTCGTTGGCTCTTTAAAAAAATATAACACGCTTAGTTCACACGTTTCAAAGACACGTCTTACCTTGACGACGTACAACATGTAACCAGAAAAGTCTCAAACGGTGTGTCGTAAACTTGCGTTGTCGGAATTTGTCACTGACGATGCCGCATATCTTAGAATGGGCGACCCCTATC

>9bef50a428d844532fddc31ff78c460b

TACAGAGGGTGCAAGCGTTAATCGGAATTACTGGGCGTAAAGCGCGCGTAGGCGGTTCGTTAAGTTGGATGTGAAATCCCCGGGCTCAACCTGGGAACTGCATCCAAAACTGGCGAGCTAGAGTGTGGTAGAGGGTGGTGGAATTTCCTGTGTAGCGGTGAAATGCGTAGATATAGGAAGGAACACCAGTGGCGAAGGCGACCACCTGGACCAACGCTGACGCTGAGGTGCGAAAGCGTGGGGAGCAAACAGG

>022f868b978f1d2316cf5e567072f41d

TACGGAGGGTGCAAGCGTTGTTCGGAATTATTGGGCGTAAAGCGCGTGCAGGCGGCTGTTCAAGTCCGATGTGAAAGCCCGGGGCTCAACCCCGGAAGTGCATTGGAAACTGGACAGCTTGAGTACGGGAGAGGGTGGTAGAATTCCGAGTGTAGGGGTGAAATCCGTAGATATTCGGAGGAATACCGGTGGCGAAGGCGGCCTCCTGGACCGATACTGACGCTGAGACGCGAAAGCGTGGGGAGCAAATAGG

>ba3ca2d4f0a69c23e1a4b1e56d469d73

GCCGCCGCGGTAATACGTCGGTCAAGGTGTAGCCCAGGGTACCTAAAATGATTCCACGGCGGAATCGTGTTACGGCCTATGGTTTAGTTGGCGAGGGAGCTTCATGCACGTGGAGAAGACCACGGTTGTACACTTGGTCGCTTTATTAGAAACCCTAGTAGTCCGGCTGACTGACTTAACGTCCAT

>00652925514157370f4adae8a6dcdaac

CATTGAACTATCGTGAGAAAGTCAAGCCGCCAAAGGGAATTATATTATAGTAAATATTGGCGTAAATAAACATTTTATTAATAGTTGTAATATATGAAAATGTGCAGAAGATAAAGACGATTAAAACGGTGTTTAACAATCGCTTATCGAGACAATATTTAATTAATTAGTAATTAAAATATGTTTATTAGGTGTATTATTCAAGGAATAGACTTGACAATAAATACAGTGGTAAACGGAACTAATAGAGTCACGCAAGTGAACTTAGAATATTTTTAATAAC

>f40ce11c5992f49fcabe5635f12352f7

TGCCAGCCGCCGCGGTAATTAGGTATAGGCCGCCGAGTTGCTGACTCCCAGACATTGTACCTAGCGTGCGTCATACAGGTTCAATGGTGGTTTCCATGAGTCCACATACGCTCCTGCACAAGCTCTACGGTCTCAATCGCGGTAATTAGAAACCCTAGTAGTCCGGCTGACTGACTTAACGTCCATCTCGT

>e0fada5fd884e14bf866c99a1f6805b6

TACGTAGGGGGCTAGCGTTGTCCGGATTTATTGGGCGTAAAGAGCTCGTAGGTGGTTCGGTAAGTCGGATGTGAAATCTCCAGGCTCAACCTGGAGGGGTCATTCGATACTGCCGTGACTAGAGTTCGGTAGAGGAGTGTGGAATTCCTGGTGGAGCGGTGAAATGCGCAGAGATCAGGAGGAACACCCGTAGCGAAGGCGGCACTCTGGGCCGATACTGACACTGAGGAGCGAAAGCGTGGGGAGCGAACAGG

>de96e1a951b0fa81ff1f225777ee2d0b

CATTGAACTATCGTGAGAAAGTCAAACCGCCAAAGGGAATTATATTATAGTAAATATTGGCGTAAATAAACATTTTATTAATAGTTGTAATATATGAAAATGTGCAGAAGATAAAGACGATTAAAACGGTGTTTAACAATCGCTTATCGAGACAATATTTAATTAATTAGGAATTAAAATATATTTATTATTGGTATTAAACAAGGGATAATCTTGAAAATAAATACAGTGGTGAACGGAACTAATAGAGTCACGCAAGTGAACTTAGAACAATTTTAAGAAC

>27f084489f023ae01b1ccbff1f5ce0d3

TTATCAGGGTTATTTTTGTCTCCCTTCTTGAATATAGGGATCGTCGTGCTAGTTTTCCAATCTCCTGGGACTTCTCCCTTATCCCGAATGACTTTGAACAGCTCCGTTAGCTCATCACATAATTTCGTACCACCGTATTTGAGCAGCTTATTCGTGAGACTATCAGGGCCGGGAGCTTTCCTATTTTTTAAACATCTGATCGCCTTTCCTACGTCTTCTCTCTGTATTTCCAGAGTGTTACTATTCTTATTAGTTTGCTCCCTTGCTTCCTTTGT

>8043ce781f9ae5b8ba578d45e629b985

TACGGAGGGTGCAAGCGTTACCCGGAATCACTGGGCGTAAAGGGCGTGTAGGCGGATCGTTAAGTCTGGTTTTAAAGACCGTGGCTCAACCACGGGAGTGGACTGGATACTGGCAATCTTGACCTCTGGAGAGGTAACTGGAATTCCTGGTGTAGCGGTGGAATGCGTAGATACCAGGAGGAACACCAATGGCGAAGGCAAGTTACTGGACAGAAGGTGACGCTGAGGCGCGAAAGTGTGGGGAGCGAACCGG

>a61b210e9b598bc114b16f7ba914a6b1

TACGGAGGGGGCTAGCGTTGTTCGGAATTACTGGGCGTAAAGCGCACGTAGGCGGACTGGAAAGTTGGGGGTGAAATCCCGGGGCTCAACCTCGGAACTGCCTTCAAAACTATCAGTCTGGAGTTCGAGAGAGGTGAGTGGAATTCCGAGTGTAGAGGTGAAATTCGTAGATATTCGGAGGAACACCAGTGGCGAAGGCGGCTCACTGGCTCGATACTAACGCTGAGGTGCGAAAGCGTGGGGAGCAAACAGG

>2669a82b1647a9d5f49c2e6d923fc5d5

TACGGAAGGTTCTGGCGTTATCCGGATTTATTGGGTTTAAAGGGAGCGTAGGCTGTTTTTTAAGCGTGTTGTGAAATGTACCGGCTCAACCGGTGATGTGCAGCGCGAACTGGAAGACTTGAGTGTGTTGTAAGTAGGCGGAATTCGTGGTGTAGCGGTGAAATGCTTAGATATCACGAGGAACTCCGATTGCGTAGGCAGCTTACTGTCTCACTACTGACGCTGATGCTCGAAAGCGCGGGTATCGAACAGG

>c92ac62278a485f343ab1c271bb2d30c

TACGTAGGTGGCAAGCGTTGTCCGGAATGATTGGGCGTAAAGGGCGCGCAGGCGGCTGTGTAAGTCTGTCCAGAAAGTGCGGGGCTAAACCCCGTGAGAGGATGGAAACTGGACAGCTGAGAGTGTCGGAGAGGAAAGCGGAATTCCTAGTGTAGCGGTGAAATGCGTAGATATTAGGAGGAACACCGGTGGCGAAAGCGGCTTTCTGGACGACAACTGACGCTGAGGCGCGAAAGCCAGGGGAGCAAACGGG

>646a045e916fc7747ccc949751fa2bd1

GCCGCCGCGGTAATCAACTTAGTCTAATATCACGCCATCTGGTGGCCAGTACTTCACACATACTATAATACTATTTTAACCGTGTATAATTGGATCAAACCCAGACTATGTTGCCTGTAACAGTGTTAGTACGCCGAGGGATAAATTAGATACCCGGGTAGTCCGGCTGACTGACTTAACGTCCAT

>17bd74132ce954f6ef7aebe3e5ba9151

TACAGAGGGTGCGAGCGTTAATCGGAATTACTGGGCGTAAAGCGAGTGTAGGTGGCTCATTAAGTCACATGTGAAATCCCCGGGCTTAACCTGGGAACTGCATGTGATACTGGTGGTGCTAGAATATGTGAGAGGGAAGTAGAATTCCAGGTGTAGCGGTGAAATGCGTAGAGATCTGGAGGAATACCGATGGCGAAGGCAGCTTCCTGGCACAATATTGACACTGAGATTCGAAAGCGTGGGTAGCAAACAGG

>e732689f00d992f86c50ad8420b4e328

CACAAGCAGCATATGGTAATTGTGTGCCAGCCGCCGCGGTAAAGTAAACACAATATCTGGCAGGACTGCCACTCGTTACTTGATTCAGCACTCTCCTTATATAGTTTAGTTTAGATTCGTCTGTTTTACAGATTGACTCACTTGATTAGAAACCCTAGTAGTCCGGCTGACTGACTTAACGTCCATCTCGTATGCCGTCTTCTGCTTGAAAAAAA

>55badc154ba1997ea91db947c85d5f73

TACGTAGGGTGCAAGCGTTGTCCGGAATTATTGGGCGTAAAGAGCTCGTAGGCGGTTTGTCGCGTCTGCTGTGAAATTCCGAGGCTCAACCTCGGGCGTGCAGTGGGTACGGGCAGACTTGAGTGCGGTAGGGGAGATTGGAATTCCTGGTGTAGCGGTGGAATGCGCAGATATCAGGAGGAACACCGATGGCGAAGGCAGATCTCTGGGCCGTTACTGACGCTGAGGAGCGAAAGCATGGGGAGCGAACAGG

>c9b9072640557c6b152257c819021c14

TACGTAGGGTGCGAGCGTTAATCGGAATTACTGGGCGTAAAGCGTGCGCAGGCGGTGATGTAAGACAGATGTGAAATCCCCGGGCTCAACCTGGGAACTGCATTTGTGACTGCATCGCTGGAGTGCGGCAGAGGGGGATGGAATTCCGCGTGTAGCAGTGAAATGCGTAGATATGCGGAGGAACATCGATGGCGAAGGCAATCCCCTGGGCCTGCACTGACGCTCATGCACGAAAGCGTGGGGAGCAAACAGG

>c15ba7772639f42c56f35e97e5e8d48a

TACGTATGTCGCGAGCGTTATCCGGAATTATTGGGCATAAAGGGCATCTAGGCGGCACGACAAGTCAGGGGTGAAAACTTGCGGCTCAACTGCAAGCTTGCCTTTGAAACTGTAGTGCTAGAGTATTGGAAAGGTGGGCGGAACTACACGAGTAGAGGTGAAATTCGTAGATATGTGTAGGAATGCCGATGATGAAGATAGCTCACTGGACGATAACTGACGCTGAAGTGCGAAAGCTAGGGGAGCGAACAGG

>cdf14d2fed157f8032715a22d3bf4573

TACGGAAGGTCCGGGCGTTATCCGGATTTATTGGGTTTAAAGGGAGTGTAGGCGGTCTGTTAAGCGTGTTGTGAAATTTAGGTGCTCAACATTTAACTTGCAGCGCGAACTGTCAGACTTGAGTACACGCAGCGCAGGCGGAATTCATGGTGTAGCGGTGAAATGCTTAGATATCATGAGGAACTCCGATCGCGAAGGCAGCCTGCGGGAGTGTTACTGACGCTTAAGCTCGAAGGTGCGGGTATCGAACAGG

>7bb630155c5be81bc152e1aa4781638b

TACAGAGGGTGCAAGCGTTAATCGGAATTACTGGGCGTAAAGCGCGCGTAGGTGGTTTGTTAAGTTGGATGTGAAAGCCCCGGGCTCAACCTGGGAACTGCATCCAAAACTGACAAGCTAGAGTACGGTAGAGGGTGGTAGAATTTCCTGTGTAGCGGTGAAATGCGTAGATATAGGAAGGAACACCAGTGACGAAGGCGACCACCTGGACTGATACTGACACTGAGGTGCGAAAGCGTGGGGAGCAAACAGG

>de9f116a4174da584d2fb2f122cdf2e2

TACGGAGGGGGCTAGCGTTGTTCGGAATTACTGGGCGTAAAGCGCACGTAGGCGGACTGGAAAGTTGGGGGTGAAATCCCGGAGCTCAACCTCGGAACTGCCTTCAAAACTATCAGTCTGGAGTTCGAGAGAGGTGAGTGGAATTCCGAGTGTAGAGGTGAAATTCGTAGATATTCGGAGGAACACCAGTGGCGAAGGCGGCTCACTGGCTCGATACTGACGCTGAGGTGCGAAAGCGTGGGGAGCAAACAGG

>823e4ec7477b999e35e9c38863bb44cb

CATTGAACAATCGTGAGGAAGTCAAACCGCCAAAGGGAATTATATTATAGTAAATATTGGCGTAAATAAACATTTTATTAATAGTTGTAATATATGAAAATGTGCAGAAGATAAAGACGATTAAAACGGTGTTTAACAATCGCTTATCGAGACAATATTTAATTAATTAGGAATTAAAATATGTGTATTATTTGTATTTAACAAAGAATAAACTTGAAAATAAATACAGTGGTAAACGGAACTAATAGAGTCACGCAAGTGAACTTAGAACAATTTTAAGAAC

>137e1760c442b7c8661ba69cf7cf0a42

TACGGAGGGAGCTAGCGTTATTCGGAATTACTGGGCGTAAAGCGCACGTAGGCGGCTTTGTAAGTAAGAGGTGAAAGCCTGGAGCTCAACTCCAGAATTGCCTTTTAGACTGCATCGCTTGAATCCAGGAGAGGTGAGTGGAATTCCGAGTGTAGAGGTGAAATTCGTAGATATTCGGAAGAACACCAGTGGCGAAGGCGGCTCACTGGACTGGTATTGACGCTGAGGTGCGAAAGCGTGGGGAGCAAACAGG

>3586eb9c072fd9355276e197efaa28b7

CACAAGTAAGACAAGTGTTATTCATCATTATTAGGTTTAAAGCGTACCTAGACGGCATTTTACACAGTAATGTTAATTTTTTGCTAGAGTTTAATAGGGGAGGATAATATTAGGACTACTGGTGTAGAGATGAAATTCTTTGATACTAGTAGAATGTGTAACGACGAAGGTAACCCTCTATCTATAAACTGACGTTGAGGAACGAAGGCTTGGGGAGCGAATAGG

>deaa5c4ba882229db00862782a6dc052

CCATATGTCTGGTTCATACGCCGCTAATCCAACACCTGCCTAAACAGTCACCACTTCCATTTAATCTATTACTGAATGTGACTGACTTCCGGTAATAGATTTCTATAGTTCTAAATTACTGGTTTAATATCTAATCTAATATGATGCGAGTTAACAATGATAGAAAACAGTTGTGAAATAATCTACTGACTG

>9908fffab7ed4f3bec44cda2f5084d49

TACGTAGGTGGCAAGCGTTGTCCGGATTTATTGGGCGTAAAGCGAGCGCAGGCGGTTTCTTAAGTCTGATGTGAAAGCCCCCGGCTCAACCGGGGAGGGTCATTGGAAACTGGGAGACTTGAGTGCAGAAGAGGAGAGTGGAATTCCATGTGTAGCGGTGAAATGCGTAGATATATGGAGGAACACCAGTGGCGAAGGCGGCTCTCTGGTCTGTAACTGACGCTGAGGCTCGAAAGCGTGGGGAGCAAACAGG

>c4db9ae54cc7a544b18bfd99e26df6e3

TACGGAGGGTGCAAGCGTTATCCGGATTTACTGGGTTTAAAGGGTGCGCAGGCGGATATGTAAGTCAGTGGTGAAATCTTTGGGCTTAACCCGAAAATTGCCATTGATACTATATATCTTGAATGCTGTGGAGGTAAGCGGAATATGTCATGTAGCGGTGAAATGCTTAGAGATGACATAGAACACCTATTGCGAAGGCAGCTTACTACGCAGATATTGACGCTCATGCACGAAAGCGTGGGGATCAAACAGG

>f0e0a5b77c5a8d4405fcc1b5fd67c165

TACAGAGACCTCAAGCGTTATCCGGATTCATTGGGCGTAAAGCGTCCGCAGGTGGTTTTCTAAGTTGGGAGTCAAATCTTTGGGCTTAACCTAAAGACTGCTCTCAATACTGGGAAACTTGAGACTGGGAGAGGCACACGGAACTGTTGGTGTAGTAGTAAAATGCGTTGATATCAACAGGAACACCAAAGGCGAAAGCAGTGTGCTGGAACAGTTCTGACACTCATGGACGAAAGCGTGGGGAGCGAATGGG

>fd3e907759e6ec1831fd216eacdacf4d

TACGGAGGGAGCTAGCGTTGTTCGGAATTACTGGGCGTAAAGCGCGCGTAGGCGGCTTGCCAAGTCAGGGGTGAAATCCCGGGGCTCAACCCCGGAACTGCCCTTGAAACTAGCAGGCTAGAATCTTGGAGAGGTCAGTGGAATTCCGAGTGTAGAGGTGAAATTCGTAGATATTCGGAAGAACACCAGTGGCGAAGGCGACTGACTGGACAAGTATTGACGCTGAGGTGCGAAAGCGTGGGGAGCAAACAGG

>70dbac76545ca7f0d29e40f47caf5706

TACGGAAGGTCCGGGCGTTATCCGGAATTATTGGGTTTAAAGGGAGCGCAGGCGGGAGTATAAGTCAGCTGTTAAATATCAGAGCCCAACTCTGTTATGCAGTTGAAACTATATTTCTTGAGTACGCACAGGGATGGCGGAATTCAGGGTGTAGCGGTGAAATGCTTAGATATCCTGAAGAACTCCGATCGCGAAGGCAGCCATCCGGAGCGTAACTGACGCTGAGGCTCGAAGGTGCGGGTATCGAACAGG

>1d7c764816c1b27fae786ac175a5ef9e

TACGGAGGGTGCAAGCGTTATCCGGATTTATTGGGTTTAAAGGGTCCGTAGGCGGGCTGATAAGTCAGTGGTGAAATCCTACAGCTTAACTGTAGAACTGCCATTGATACTGTTAGTCTTGAGTATATTTGAAGTAGCTGGAATAAGTAGTGTAGCGGTGAAATGCATAGATATTACTTAGAACACCAATTGCGAAGGCAGGTTACTAAGATATAACTGACGCTGAGGGACGAAAGCGTGGGGAGCGAACAGG

>153a1c462f6c9cc86cb69c231e968272

TACGGGGGGTGCGAGCGTTAATCGGAATAACTGGGCGTAAAGGGCACGCAGGCGGTGACTTAAGTGAGATGTGAAAGCCCCGGGCTTAACCTGGGAATTGCATTTCATACTGGGTCGCTAGAGTACTTTAGGGAGGGGTAGAATTCCACGTGTAGCGGTGAAATGCGTAGAGATGTGGAGGAATACCGAAGGCGAAGGCAGCCCCTTGGGAATGTACTGACGCTCATGTGCGAAAGCGTGGGGAGCAAACAGG

>5b2157227cde890e5b14c3fcdd1e3be2

GGTAATTGTGTGCCAGCCGCCGCGGTAATTGACTGGATGAGACCCACCGCAGTTGCAGCATGTTGCAGAGGTCTCCGGTGCGACCAGATAGTAGCCTCGGTGCTCTCCTAACGTCTCTGTGGAAAACGGCCAACGTCGTCATGGATTAGAAACCCCTGTAGTCCGGCTGACTGACTACTGTGTAATCTCGTATGCCGTCTT

>bf40e2d9abeb50c2b66bdef56d4cab8b

CCGCCGCGGTAATACGATTTCTTTAATTTAAATATTTAAGTTTCAGTTAATATAATAATAATATAAAATGTCTATAATTTTGGTGAAATATATTTTATCTTTAAAAATTAATTTTATGTCTGAAAAATTTTTGTATAAACTAGGATTAGATACCCGTGTAGTCCGGCTGACTGACTTGCGTCAAA

>643e79984435c2dfbb0f075b4bc0aadb

TACGTAGGGTGCGAGCGTTGTCCGGAATTACTGGGCGTAAAGAGCTCGTAGGTGGTTTGTCGCGTCGTTTGTGTAATACCGCAGCTTAACTGCGGGGTTGCAGGCGATACGGGCATAACTTGAGTGCTGTAGGGGAGACTGGAATTCCTGGTGTAGCGGTGGAATGCGCAGATATCAGGAGGAACACCGATGGCGAAGGCAGGTCTCTGGGCAGTAACAGACGCTGAGGAGCGAAAGCATGGGTAGCGAACAGG

>2973f5d53af12b2c9171483b6f242fd5

GACGTAGGGCGCGAGCGTTGTCCGGATTTATTGGGCGTAAAGAGCTCGTAGGCGGCTTGTCGCGTCGTGTGTGAAATCTCAGGGCTCAACTCTGACATTGCATTCGATACGGGCAGGCTAGAGTTCGGTAGGGGAGACTGGAATTCCTGGTGTAGCGGTGAAATGCGCAGATATCAGGAGGAACACCGGTGGCGAAGGCGGGTCTCTGGGCCGATACTGACGCTGAGGAGCGAAAGCGTGGGGAGCGAACAGG

>5a694d737402f7e578563ca1ed03c865

CACAAGTGAGACTAGTGTTATTCATCTTTATTAGGTTTAAAGGGTACCTAGACAGTATTTCCAGCCCCTAAAGGGTACAGATTTACTAGAGTTTTATACGGGAGGTAAATATTAGGACCATTGGTGTAGAGATGAAATTCTTTGATACTAATGGGATGTGTAACGGCGAAGGCAACCCTCTATGTAAAAACTGACGTTAAGGGACGAAGGCTTGGGGAGCGAATAGG

>1d21588c5cccf32224a37563606a8172

TACGTAGGGTGCAAGCGTTAATCGGAATTACTGGGCGTAAAGCGTGCGCAGGCGGTTTTGTAAGACAGTGGTGAAATCCCCGGGCTCAACCTGGGAACTGCCATTGTGACTGCAAGGCTAGAGTGCGGCAGAGGGGGATGGAATTCCGCGTGTAGCAGTGAAATGCGTAGATATGCAGAGGAACACCGATGGCGAAGGCAATCCCCTGGGCCTGCACTGACGCTCATGCACGAAAGCGTGGGGAGCAAACAGG

>da75b296ba8c702ddbea082b6b284ed2

GGACTGTATGTTGATTACAGAAAAAAAGTTTATGGAAAATGTTGATATTGTGTATGTTTTGTAATTTTGTTTGGGGATTATTTATCTATATGGTTGACCGTTTAATTCAATAAAAAGACTGTTGGATTTTGAAATAATACAAAGAATGTTTGGTATTATATTTAAACAGAATAATACAAAGATAAAGTTCATATTGAAAGGATATTTCTATTTTATAAGTTGTTTTATCTTATGAATAAATGATAGTATAAATTTCTTATTTACTTTAA

>185ad80cccc75330cb9062609ca111d2

CACAAGCAGCATATGGTAATTGTGTGCCAGCCGCCGCGGTAAAGTAAACACAATATCTGGCAGGACTGCCACTCGTTACTTGATTCAGCACTCTCCTTATATAGTTTAGTTTAGATTCGTCTGTTTTACAGATTGACTCACTTGATTAGAAACCCTAGTAGTCCGGCTGACTGACTGAGACTTAATCTCGTATGCCGTCTTCTGCTTGAAAAAAA

>399ff0f6ac8e78263b86e927255e7ad4

AAATGGCTACTTTCACGGGTGCAGTACGGGCTATCTGTGTGTTAAGGTTTCATGACACAAACTCTGCAACAACAGTTCAGCATAATTTTTGCACCGAGTGTGGTAAAGATCCTCTTACTAGACCTACAATTTACACTTGACCTCAGAACTTCGTTGAGAGTGGTTGTTCGGTTCAGC

>8febf59c6eb58b7b36f1786f625c8d5a

TACGGAGGATGCGAGCGTTATCCGGATTTATTGGGTTTAAAGGGTGCGCAGGCGGGCGGTCAAGCCGGCGGTCAAATCGAGGGGCCCAACCCCTTTCCGCCGCCGGAACTGGCCGCCTAGAGTGGGCGAGAAGTAAGCGGAATGCGTGGTGTAGCGGTGAAATGCATAGATATCACGCAGAACGCCGATTGCGAAGGCAGCTTACCGGCGCCCTACTGACGCTCAGGCACGAAAGCGTGGGGATCGAACAGG

>8d47bac6fd2fec3f9a25215c67b49b00

TACGGAGGGGGCTAGCGTTGTTCGGAATTACTGGGCGTAAAGCGCACGTAGGCGGACCAGAAAGTTGGGGGTGAAATCCCGGGGCTCAACCCCGGAACTGCCTTCAAAACTATTGGTCTGGAGTTCGAGAGAGGTGAGTGGAATACCGAGTGTAGAGGTGAAATTCGTAGATATTCGGTGGAACACCAGTGGCGAAGGCGGCTCACTGGCTCGATACTGACGCTGAGGTGCGAAAGCGTGGGGAGCAAACAGG

>0fe496ef5b223cce55881b2a62e310d5

CATTGAACTATCGTGAGAAAGTCAAACCGCCAAAGGGAATTATATTATAGTAAATATTGGCGTAAATAAACATTTTATTAATAGTTGTAATATATGAAAATGTGCAGAAGATAAAGACGATTAAAACGGTGTTTAACAATCGCTTATCGAGACAATATTTAATTAATTAGGAATTAAAATATGTGTATTATTTGTATTTAACAAGGAATAAACGTGAAAATCAATACAGTGTTAAACGGAACTAATAGAGTCACGCAAGTGAACTTAGAACATTTTTAATAAC

>3934416faa180e9c5663dc96e2186feb

CATTGAACTATCGTGAGAAAGTCAAACCGCCAAAGGGAATTATATTATAGTAAATATTGGCGTAAATAAACATTTTATTAATAGTTGTAATATATGAAAATGTGCAGAAGATAAAGACGATTAAAACGGTGTTTAACAATCGCTTATCGAGACAATATTTAATTAATTAGGAAGTAAAATATGTGTATTATTTGTATTTAACAAGGAATAAACTTGAAAATAAATACAGTGGTGAACGGAACTAATAGAGTCACGCAAGTGAACTTAGAACAATTTTAAGAAC

>594061ed4e2ea378773c474cd4803c29

TACGTAGGGCGCAAGCGTTGTCCGGAATTATTGGGCGTAAAGAGCTCGTAGGCGGTCTGTCGCGTCTGGTGTGAAATCCCATGGCTCAACTGTGGGCTTGCATCGGGTACGGGCAGACTAGAGTGCTGTAGGGGAGACTGGAATTCCTGGTGTAGCGGTGGAATGCGCAGATATCAGGAGGAACACCGATGGCGAAGGCAGGTCTCTGGGCAGTTACTGACGCTGAGGAGCGAAAGCATGGGGAGCGAACAGG

>7e2f08b0e09fd9702c6234502a1a9b01

AGTCAGTCAAAGGTGGCATCTTCTCCACCGAGCAAACCCACATCACAGACTTCAATCCACGTCCATCCGCATCTAACACTTTACAGGAATCGTAGAGTACTGCCACAAACTCACCATGAAATTCTCGGATTTCCCAGCCCTGCTACCGTTGTAGCCTCGCCACCATCTCAATCGGGTTACAACAAATAGTAAAAGGTACGGGAAAGACCACGACGGGCGCTTCCATGCTAGCTTCTGCACCCAAATCCAAGCAGCCTGAAACCCGCG

>20c4b658af7b1cc36ab8c21d60ad2e92

GTAATTGTGTGCCAGCAGCCGCGGTAACGCACTTATGTATTTAGAAAGACAAAACATTTCACCAGAATGCATTTCAGCCATGGCTTGTTGGACTATGTTCCTTCATCTCTTCTTGTATGTCACTTGGTCTTCCATTTAGCAATAATTAGAAACCCGGGTAGTCCGGCTGACTGACTTAACGTCCATCTCGTATGCCGTCT

>7b049019be4ce8a517528f9bc02e5f14

AAATGGCTACTTTCACGGGTGCAGAACGGGCTAACTGTGTGTTAAGGTTTCATGACACAAACTCTGCAACAACAGTTCAGCATAATTTTTGCACCGAGTGTGGTAAAGATCCTCTTACTAGACCTACAATTTACACTTGACCTCAGAACTTCGTTGAGAGTGGTTGTTCGATTCAGC

>fa5097768529b1682253ec991f42b0ae

CATTGAACTATCGTGGTGTTTGTCATTTAGATTTGCTTCACCATTTAGATTTGACTACTTAATTACGTACACATACGTAGATGTATATATATATATATATATATATATATATATATATTTCATATAATATAACTCACTGTTTTTCTTTGTTATATAATATATAATATATATATTTTTTAATAATTACAGACAAAGATGTAAATGTTTTTAATTTTATTTAAAGTACTTTTTCGATATCATA

>949b3558d97db5697089fa8958f35fdb

CGATCGCAAGTCTTCGCCAAGTAGTTGTCCACCGACAAGTTCCAATTGTGACGGCCGTGAAAAGATTAGCCAAATGATGCTGTTGTTAATTTAATACCTGTGGAAAAGTTCTAATACTTTGCTGGTGTGCGGAATGTGGCCGGGCAGTGCGAACTGCATTCGGTGTTGGATTTCGTGGACAGGTGGTCGCCACCTGCTCGGCCACTGACGTGATGCATGGCAACAACGTAGGCCTACGTAACGTTCTGTACGGAGTATGAGGCTGAGCCACAGTAGCCGTCTTG

>6312e18774568dc33b4821a40305812d

GTCATTTGTCCGAGCCCCAGTCATTCTCTGTCCTGTACCACAGTACTTGGCCGGATCATCAATCCGTATACCAAAGCCTGTAGCCGTGGTTTCTATATACGGTATCTTCATAGCCTCCAATCTAGTAGCACCTTCGTGACTACCTACTGTCTTCACGCCTAGCCAAATTTCCTGCCCACCTCC

>55cd773a68624a711191b1b665d6535e

TACGGAGGGTGCAAGCGTTATCCGGATTTATTGGGTTTAAAGGGTCCGTAGGCGGACCTGTAAGTCAGTGGTGAAATCTCATAGCTTAACTATGAAACTGCCATTGATACTGCAGGTCTTGAGTAAATTTGAAGTGGCTGGAATAAGTAGTGTAGCGGTGAAATGCATAGATATTACTTAGAACACCAATTGCGAAGGCAGGTCACTAAGATTTAACTGACGCTGATGGACGAAAGCGTGGGTAGCGAACAGG

>35084611a5a0ff44337c0183b285e2f9

TACGTAGGGTGCGAGCGTTGTCCGGAATTACTGGGCGTAAAGAGCTCGTAGGCGGTTTGTCGCGTCGTTCGTGAAATCTTGATGCTTAACATCAAGCGTGCGGGCGATACGGGCAGACTTGAGTACTACAGGGGAGACTGGAATTCCTGGTGTAGCGGTGAAATGCGCAGATATCAGGAGGAACACCGGTGGCGAAGGCGGGTCTCTGGGTAGTAACTGACGCTGAGGAGCGAAAGCATGGGTAGCGAACAGG

>9719509b51cac45b9fed783b4e33e464

AACAGAGGATACAAGCGTTATCCGGATTTATTGGGTTTAAAGGGTGCGTAGGTGGTTTTTTAAGTCAGTAGTGAAATCTTAAAGCTTAACTTTAAAAGTGCTATTGATACTGATAAACTAGAGTGAGGTTGGAGTAACTGGAATGTGTGGTGGAGCGGTGAAATGCATAGAGATGACACAGAACACCAATCGCGAAGGCATGTTACTAAACATAGGCTGACACTGAGGCACGAAAGCATGGGTAGCAAACAGG

>d66072bb4e903a9c1a95728d29427410

TACGTAGGGTGCAAGCGTTGTCCGGAATTATTGGGCGTAAAGAGCTCGTAGGCGGTTTGTCGCGTCTGCTGTGAAATCCCGAGGCTCAACCTCGGGCTTGCAGTGGGTACGGGCAGACTAGAGTGCGGTAGGGGAGATTGGAATTCCTGGTGTAGCGGTGGAATGCGCAGATATCAGGAGGAACACCGATGGCGAAGGCAGATCTCTGGGCCGTAACTGACGCTGAGGAGCGAAAGCGTGGGGAGCGAACAGG

>aa986ee5ca8fefd2ce80ec3319222a5d

TACGTAGGGGGCTAGCGTTGTCCGGAATCATTGGGCGTAAAGCGCGTGTAGGCGGCCCGGTAAGTCCGCTGTGAAAGTCGGGGGCTCAACCCTCGGATGCCGGTGGATACTGTCGGGCTAGAGTGCGGAAGAGGCGAGTGGAATTCCTGGTGTAGCGGTGAAATGCGCAGATATCAGGAGGAACACCAATTGCGAAGGCAGCTCGCTGGGACGTGACTGACGCTGAGACGCGAAAGCGTGGGGAGCAAACAGG

>50381276e4c242b4739b4a2cd9b5c3d7

GCCGCCGCGGTAATCAACTTAGTCTAATATCACGCCATATGGTGGCCAGTACATCACACATACTATAATACTATTTTAACCGTGTATAATTGGATCAAACCCAGACTATGTTGCCTGTAACAGTGTTAGTACGCCGAGGGATAAATTAGAAACCCCGGTAGTCCGGCTGACTGACTGAGACTTAAT

>496ecde24f9ab698992413d3d4f04b5f

TACGAAGGGTGCAAGCGTTACTCGGAATTACTGGGCGTAAAGCGTGCGTAGGTGGTTGTTTAAGTCTGTTGTGAAAGCCCTGGGCTCAACCTGGGAATTGCAGTGGATACTGGGCGACTAGAGTGTGGTAGAGGGTAGTGGAATTCCCGGTGTAGCAGTGAAATGCGTAGAGATCGGGAGGAACATCCATGGCGAAGGCAGCTACCTGGACCAACACTGACACTGAGGCACGAAAGCGTGGGGAGCAAACAGG

>a446cdf0527dac843d99a0b0e4c6e235

TACGTAGGGTGCAAGCGTTGTCCGGATTTATTGGGCGTAAAGGGCTCGTAGGCGGTTTGTTGCGTCTGGTGTGAAAGCTTACTGCTTAACGGTAGGTTGCGCTGGATACGGGCAGGCTTGAGTGCAGTAGGGGAGACTGGAATTCTCGGTGTAACGGTGGAATGTGTAGATATCGGGAAGAACACCTATGGCGAAGGCAGGTTTCTGGGCTGTTACTGACGCTGAGGAGCGAAAGCGTGGGGAGCGAACAGG

>72fb43efcd7d4b858596793363bdce59

TACAGAGGGTGCAAGCGTTAATCGGAATTACTGGGCGTAAAGCGCGCGTAGGTGGTTTGTTAAGTTGAATGTGAAATCCCCGGGCTCAACCTGGGAACTGCATCCAAAACTGGCAAGCTAGAGTAGGGCAGAGGGTGGTGGAATTTCCTGTGTAGCGGTGAAATGCGTAGATATAGGAAGGAACACCAGTGGCGAAGGCGACCACCTGGGCTCATACTGACACTAAGGTGCGAAAGCGTGGGGAGCAAACAGG

>106f34a47e6278d0fc28ef5fbd99dbc8

TACGAAGGGGGCTAGCGTTACTCGGAATTACTGGGCGTAAAGGGCGCGTAGGCGGCGTTCCAAGTTAGGCGTGAAAGTCCTGGGCTCAACCTGGGAACTGCGCTTAAGACTGGAGTGCTAGAGGATGGAAGAGGGTTGTGGAATTCCCAGTGTAGAGGTGAAATTCGTAGATATTGGGAAGAACACCGGTGGCGAAGGCGGCAACCTGGTCCATTACTGACGCTGAGGCGCGATAGCGTGGGGAGCAAACAGG

>d9100189502bb0a31832957fb967b51c

CACACTTATGTCTTTAGCAACACAAAACATTTCTCCAGAATGCATTTCAACCATGTCGGTCATTTTCAACTAGCAGTGCCTAGATAGTTGTGGAAGATTTTGAATATGTCTTGTCCAAAGAGTTCAGTTTGTTCATTAATGATTTTGTTATGTATTATGTTTAGACCTTCAAGTAAATTC

>c667664df1fb6f8705f9a8a861370a72

TACGTAGGTGGCAAGCGTTGTCCGGATTTATTGGGCGTAAAGCGAGCGCAGGCGGAATGATAAGTCTGATGTGAAAGCCCACGGCTCAACCGTGGAACTGCATCGGAAACTGTCATTCTTGAGTGCAGAAGAGGAGAGTGGAACTCCATGTGTAGCGGTGGAATGCGTAGATATATGGAAGAACACCAGTGGCGAAGGCGGCTCTCTGGTCTGCAACTGACGCTGAGGCTCGAAAGCATGGGTAGCGAACAGG

>7596cc6b6da76e4d6cdce8774317371a

CATTGAACTATCGTGAGAATGGCACGCCGCCAAAGGGAATTATATTATAGTAAATATTAGCGTAAATAAACATTTTATTAATAGTTGTAATATATGAAAATGTGCAGAAGATAGAGACGATTAAAACAGTGTTTAACAATCGCTTATCGAGACAATATTTAATTAATTAGGAATTAAAATATGTGTATTATTTGTATTTAACAAGGAATAATCTTGAAAATAAATACAATGGTAAACGGAACTAATAGAGTCACGCAAGTGAACTTAGAACAATTTTAAGAAC

>5fc4803eca1f459694e8aef3c1575e9f

CACAGAGGATACAAGCGTTATCCTGATTTATTTTTTTTCTCTTGTTTTTCTTTTTGTTTTTTATTCTTTTTTTCACTTTTAAAGCTTAACTTTAAAAGTGCTATTGATACTGATAAACTAGAGTGAGGTTGGAGTAACTGGAATGTGTGGTGGAGCGGTGAAATGCATAGAGATCACACAGAACACCAATCGCGAAGGCATGTTACTAAACATAGACTGACACTGAGGCACGAAAGCATGGGTAGCAAACAGG

>6ddb70c7e7274ae91d17c3d901adfde7

TACGTAGGGGGCAAGCGTTGTCCGGAATCATTGGGCGTAAAGCGCGTGTAGGCGGCCTGGTAAGTCCGTTCTGAAAGCCTGGGGCTCAACCCCAGGAGGCGGATGGATACTGCAAGGCTCGAGTACGGAAGAGGCGAGTGGAATTCCCGGTGTAGCGGTGAAATGCGCAGATATCGGGAGGAACACCAATGGCGAAGGCAGCTCGCTGGGACGTTACTGACGCTGAGACGCGAAAGCGTGGGGAGCAAACAGG

>396cee86b047db511fc1380ae1320d83

GACAGAGGTGGCAAGCGTTGTTCGGATTCATTGGGTGTAAAGGGTCCGTAGGTGGCCTAGTAAGTTCGGCGTGAAATCCCAGAGCTCAACTCTGGAACTGCGTTGAAAACTACTAGGCTTGAGTGCCGGAGAGGTTAGGGGAATTCCAGGTGTAAGGGTGAAATCTGTAGATATCTGGAGGAACACCAGTGGCGAAGGCGCCTAACTGGCCGGTTACTGACACTGAGGGACGAAAGCAAGGGGAGCAAACAGG

>e335f74033bc634af43ee6baa84fa247

TACGTAGGGGGCTAGCGTTATCCGGAATTACTGGGCGTAAAGGGTGCGTAGGTGGTTTCTTAAGTCAGAGGTGAAAGGCTACGGCTCAACCGTAGTAAGCCTTTGAAACTGGGAAACTTGAGTGCAGGAGAGGAGAGTGGAATTCCTAGTGTAGCGGTGAAATGCGTAGATATTAGGAGGAACACCAGTTGCGAAGGCGGCTCTCTGGACTGTAACTGACACTGAGGCACGAAAGCGTGGGGAGCAAACAGG

>146195f72933e590d98b89c38d6eda33

TACGTAGGGTGCAAGCGTTGTCCGGAATTATTGGGCGTAAAGAGCTCGTAGGCGGTTTGTCGCGTCGGCTGTGAAAGCCCGGAGCTCAACTCCGGGTCTGCAGTCGATACGGGCAGACTTGAGTGTTGCAGGGGAGACTGGAATTCCTGGTGTAGCGGTGAAATGCGCAGATATCAGGAGGAACACCGGTGGCGAAGGCGGGTCTCTGGGCAACAACTGACGCTGAGGAGCGAAAGCGTGGGGAGCGAACAGG

>3c00a9519b0f13d14a7f5eb3d0afa740

TCAAACGAAAGCACAACAAAAAGATAGCCAAACTAATACCACCCCGATCACCAGCAATAACACCCACACATACGTTTTACCCAAGAATAGTTAACCTCACCAACATAACATTCACTAACTATCAGAAACACCTTTTAAACAAAGGGATAAATCACAACCTACACTACACGCAGAACAATAACACCATCAAGAACATTGT

>8ec4e2c46151f19f0f845e4e311c8522

AGTAATACCTGCGTCTTATCTGGCATACCCGGACCAGATAAGTAACAAGGGCACACACAATACTACATACGCAACACTCTTCCCCACCGCACGGCTTGCCCCCGAGCGTGGGTAAACAGGTTGGCTAGTATCTCTCTGGCTGCGTTGGAATGTGAATTCGATCACGGACAGAGGGCGCAACAGGCCTTGTCTAATGCGTGACGCAAGAAGAAGAAGATATTTTTAACAGTAATGGAATATTTGACTTGGCATTTGAATTGCGGTCGTTAGACATCACTGATTTTAA

>0f6b6c001d7d046fa22c6d994f7bb4a6

TACGTAGGGTGCGAGCGTTGTCCGGAATTATTGGGCGTAAAGGGCTCGTAGGCGGTTTGTCGCGTCGGGAGTGAAAACATCGGGCTTAACTCGGTGCTTGCTTTCGATACGGGCAGACTTGAGGCATGCAGGGGAGAACGGAATTCCTGGTGTAGCGGTGAAATGCGCAGATATCAGGAGGAACACCGGTGGCGAAGGCGGTTCTCTGGGCATGTTCTGACGCTGAGGAGCGAAAGTGTGGGGAGCGAACAGG

>8055a304b37b3ba61aec8cf458ec4dfd

TACGTAGGGTGCGAGCGTTGTCCGGAATTATTGGGCGTAAAGAGCTTGTAGGCGGTTTGTCGCGTCTGCCGTGAAAATCCGGGGCTCAACTCCGGACTTGTGGTGGGTACGGGCAGACTAGAGTGTGGTAGGGGAGACTGGAATTCCTGGTGTAGCGGTGAAATGCGCAGATATCAGGAGGAACACCGATGGCGAAGGCAGGTCTCTGGGCCACTACTGACGCTGAGAAGCGAAAGCATGGGGAGCGAACAGG

>0c24902044a01da0859d5bcebe331b67

CCAATGTGTATGAACATAAAATATGTGTTTCAGATGAAAAAAAATGCATACGTAAAACATACCCAATCCCGTTACATTACCAGGAACGGGTAGACACGGAAATTCAACGAATGTTAGAACAAGGGGTCATAGAAATATCCAGCAGTAACTTCTTGAATCCCGTGGTCATTGTAAAGAAAAAAAATAATGATATTCGCTTATGTTTGGAC

>3c4277a0ffb8696c71e0614b7c21b4bc

CGCGAATCGATTCGACACGGAAAACGCGTAAATATTAGGAACGACACATTCGAGTAATGGTGGTTCGGTTCGTTTGGATATGAAATACTCAGCCACCCTTTAAAAAATTATATTATCTGTCTAAATATTTCATATAAAAAAAAACTTAAAAGTATTTGAAAGGAAGATGATTA

>94bd99f42c1196e3faed9aa96c51ac9d

CTGAAGAAGCCGACTAAATTGGTGAAATATATAAAAATCAGTCTTTCTGCAAAACGAAGATTGTGTTTTCGGGGAAGAGAGAGTGAGTGATTATTTAATTAAGGGGAGGGCGGCGTTATATGGGGGAATGGTATTGTAAACACGTTTATTAGAAACCCCTGTAGTCCGGCTGA

>193142b69690618887ca50c94adf20bd

GGCCAGATGAATTCCACATTTATATGGAACACACTCCATCTCATATTCTTGACACATTATTAAAAAGTTTTAATAAAATATGGTTCTCTGGCATCTTTCCAACAGTGTGGAAAACGGCAACGATTATTCCCATTCTTAAAGAAAACAAAAACCCTTCACTATTAGAAACCCCA

>574a6d8a4a9e23f799340bb050914c84

TACGTAGGGGGCAAGCGTTGTCCGGATTTATTGGGCGTAAAGAGCTCGTAGGTGGTTTGGTAAGTCGGATGTGAAATCTCCAGGCTCAACCTGGAGGGGTCATTCGATACTGCCATGACTAGAGGTCGGTAGGGGAGTGTGGAATTCCTGGTGGAGCGGTGAAATGCGCAGATATCAGGAGGAACACCCGTAGCGAAGGCGGCACTCTGGGCCGATTCTGACACTGAGGAGCGAAAGCGTGGGGAGCGAACAGG

>9f3ad2bed3fe8b3fe9347c132d369139

CATTGAACTATCGTGAGAAAGTCAAACCGCCAAAGGGAATTATATTATAGTAAATATTGGCGTAAATAAACATTTTATTAATAGTTGTAATATATGAAAATGTGCAGAAGATAAAGACGATTAAAACGGTGTTTAACAATCGCTTATCGAGACAATATTTAATTAATTAGGAATTAAAATATGTGTATTATTTGTATTTAACAAGGAATAAACGTGAAAATAAATACAGTGGTAAACGGAACTAATAGAGTCACGGAAGTGAACGTAGAACATTTTTAATAAC

>24eed4490ca0fe7edf3bac39b4b52187

CATTGAACTATCGTGAGAGAGTCAAGCCGCCAAAGGGAATTATATTATAGTAAATATTAGCGTAAATAAACATTTTATTAATAGTTGTGATATATGATAATGTGCAGAAGATAAAGACGATTAAAACGGTGTTTAACAATCGCTTATCGAGACAATATTTAATTAATTAGGAATTAAAATATATTTTATTATTTGTATTAAACAAGGGATAAACTTGAAAATAAATACAGTGGTAAACGGAACTAATAGAGTCACGCAAGTGAACTTAGAACATTTTAATAAC

>4608fae4bbc9964cdd17af8782f2155e

TACGTAGGGTGCAAGCGTTAATCGGAATTACTGGGCGTAAAGCGTGCGCAGGCGGTTTTGTAAGTCTGTCGTGAAATCCCCGGGCTCAACCTGGGAATTGCGATGGAGACTGCAAGGCTAGAATCTGGCAGAGGGGGGTAGAATTCCACGTGTAGCAGTGAAATGCGTAGAGATGTGGAGGAACACCGATGGCGAAGGCAGCCCCCTGGGTCAAGATTGACGCTCATGCACGAAAGCGTGGGGAGCAAACAGG

>958eb51cf0a66587bbc59e2dec305846

TACGGAGGGTGCAAGCGTTATCCGGATTTATTGGGTTTAAAGGGTCCGTAGGCGGATCTGTAAGTCAGTGGTGAAATCTCGCAGCTTAACTGCGAAACTGCCATTGATACTGCAGGTCTTGAGTGTTGTTGAAGTAGCTGGAATAAGTAGTGTAGCAGTGAAATGCATAGATATTACTTAGAACACCAATTGCGAAGGCAGGTTACTAAGCAACAACTGACGCTGATGGACGAAAGCGTGGGGAGCGAACAGG

>e3144c1e6da75deb6fdcb383b634abbb

TACGGAGGGTGCGAGCGTTAATCGGAATTACTGGGCGTAAAGAGTGCGTAGGTGGTTTTTTAAGTTATCTGTGAAATCCCTGGGCTTAACCTGGGGCGGTCAGATAAGACTGGAAGACTCGAGTATGGGAGGGGGTAGTGGAATTTCCGGTGTAGCGGTGAAATGCGTAGAGATCGGAAGGAACACCAGTGGCGAAGGCGGCTACCTGGCCTAATACTGACACTGAGGCACGAAAGCGTGGGGAGCAAACAGG

>2d59c68f7318eba1e6f113cad7d2105e

GTCCTCACGTCTACGGCAGCGATCCCTTGGCTACGCCACGAATTTATCGAGGTTCATGCTGGCGTGAGCGGTAAGCCGCGGCCGCTGATTGGAGCGTGCACCGTACCTGCTACCTGCCGACAACGCTGCAGCTTTCGTAAGGAGACGCTTGTACAACCTTGTGAGAGCTCGAGCTCGGCCTGGCTTCTACCCGACGCGGCTTTGCTAAGTCGACAATTTATAAGGCCTCA

>63e18afd03b92a2092fabc44bacb4995

AGCCGCCGCGGTAACACAATATCATTTCTTTCCATGAGAAATTGTCTGTAATTTATTAATTTCTATTTAAAGCTGAGTTTCTTCAGTATTCTTGATAATGTTGTTGTACTTACATTAAATAAATCACAATCACTCAAACTTCTGATTAGATACCCTAGTAGTCCGGCTGACTGACTTGCGTCAAATC

>d805249ba47eb179735f08303d59e549

CACGCAGAAGACTAGTGTTATTCATCTTTATTAGGTATAAAGGGTACCTAGACGGATTATTAAGCCAAATAAAGGGACTAATAGTCTAGAGTTTTATGAAAAAATATCGAATTACTGGAGGAAAGTCAAAATTTTGTAATACCAGTAAGACGGGTTACCACGAAGGTCTTATTTTATGTAAAAACTGACGTTGAGGAACGAAGCCTGGGGTAGCAATAAGG

>d8ddd8cd84818ef6661d3ab6f39fd5b6

CGATCAACTTACCCAACTTGCGTGTCACAGCCGGGCTGACGAGAGTCGGAAACAGTGACCTAGACGGGCTGACACCATCTCGGCGTTAGAACTACAATGATTTGTGTCACGTTTAATGAGGTGAGCACTTATACCGTTACCACGTCATAGAACCTACATGAACATTAAGTGACATAATCCGTTTTACTATGTACTGCAGTGTGCTCTGTCAGGCTAACTCTCCTTACGTTTATA

>62e0c02635d94436a619655f31ccd060

CACACTTATGTCTTTAGAAAGACAAAGCGTTTTTCCAGAATGCATTTCAACATTATCGGTCATTTTCAACTGGCAGTGCCTTGTAGATAGTTGAGTACGATTTTGAATGTTTCTTGTCCATCAGTTTGTTCATTAATGATTTTGTTGTATAGTTTGTAACGTAATATATTTAAACTTTCAAGAAGATTCATTTTATTTCCTTTTTTGCCTGTTGTA

>2f248d93f9aa5ded399aa4eb52375a0c

TACGGAGGGTGCAAGCGTTAATCGGAATTACTGGGCGTAAAGCGCACGCAGGCGGTCTGTTAAGTCAGATGTGAAATCCCCGGGCTTAACATGGGAACTGCATTTGAAACTGGCAGGCTTGAGTCTCGTAGAGGGGGGTAGAATTCCAGGTGTAGCGGTGAAATGCGTAGAGATCTGGAGGAATACCGGTGGCGAAGGCGGCCCCCTGGACGAAGACTGACGCTCAGGTGCGAAAGCGTGGGGAGCAAACAGG

>19b9a1c6a02ba216a4dd34ba86f13d42

GGTAATTGTGTGCCAGCAGCCGCGGTAATTGACTGGGTGAGACCCACCGCAGTTGCAGCATGTTGCAGAGGTCTCCGGTGCGACCGGATAGTAGCCTCGGTGCTCTCCTAACGTCTCTGTGGAAAACGGCCAACGTCGTGATGGATTAGAAACCCGGGTAGTCCGGCTGACTGACTGAGACTTAATCTCGTATGCCGTCTT

>1c6ba5e2b9b56b9b1df0278a351fd706

TTTTGGTAATTGTGTGCCAGCAGCCGCGGTAATAGTGATGTTTTGGCTGACTTTATCTGCTGCTTTGTTACAGTCAAGCTTAATAAAACCACCATAAACACACACATTAAGTAAGATGCTAACTCAAGTCACACACAACAATACATTAGAAACCCCTGTAGTCCGGCTGACTGACTCTACGACCATCTCGTATGCCGTCTTCTGC

>9c88be019b837821016e27303f375448

ATTCTTCTTCTATAAGACTCGTAAATACCTTTCCATTGTCCACTATTCTATTACCGTATGTAGCGCAATGAGTGTGTGATAATTTGGCAACGCTGCAATGACTTCGACCAATGCTTTCAAAGGCATCGGCGACGGGAACCCCACCTCCCAATACCGTCCAGCGTTCAATTCTCGAAAACAGATTTGATACAG

>60b0af62b05bff04ae3da3b160dec070

TACGTAGGGAGCGAGCGTTATCCGGATTCATTGGGCGTAAAGAGCGCGTAGGCGGCCTCTCAAGCGAGATCTCTAATCCGAGGGCTCAACCCCCGGCCGGATCCCGAACTGGGAGGCTCGAGTTCGGTAGAGGCAGGCGGAATTCCCGGTGTAGCGGTGGAATGCGCAGATATCGGGAAGAACACCGATGGCGAAGGCAGCCTGCTGGGCCGCAACTGACGCTGAGGCGCGAAAGCTAGGGGAGCGAACAGG

>34bf23d4f32959c54da54f889279b4f3

TACGTAGGTGGCAAGCGTTGTCCGGATTTATTGGGTTTAAAGGGTGCGTAGGCGGTTCCATAAGTCAGTGGTGAAATACAATAACTTAACTATTGAGGTGCCATTGATACTGCGGAACTTGAGTACAGACGAGGTAGGCGGAATTGACGGTGTAGCGGTGAAATGCTTAGATATCGTCAAGAACACCGATAGCGAAGGCAGCTTACTAGACTGTAACTGACGCTGAGGCACGAAAGTGTGGGGATCAAACAGG

>ebed4857cae9baedc7c41618448b8e63

TACGAAGGGGACGAGCGTTATTCGGAATGATTAGGCGTAAAGAGTTTGTAGGTGGTATAATTGATTCTTGGTGAAATTTTAAGTTTTTATAGTTTAAAAAGGCCTTTAATATCAATTTACTTGAGTATAGCAGAGAACAGCATAATTTTATATTTAGGAGTAAAATCCAACGAAATATAAAGGAATGCCAATGGCGGTAGCAGCTGTTTGGGCTATTTACTGACACTGAGAAACGAAAGCGTAGGGATCAAACAGG

>443a1b6e9bb8b8434623dbabf8c5a719

TACGAAGGGGGCAAGCGTTGTTCGGAATTACTGGGCGTAAAGGGCGCGTAGGCGGCTTATCAAGTCAGGCGTGAAATTCCCGGGCTCAACCTGGGGGCTGCGCTTGATACTGATGAGCTTGAATGCGGGAGAGGATAGTGGAATTCCCAGTGTAGAGGTGAAATTCGTAGATATTGGGAAGAACACCGGTGGCGAAGGCGGCTATCTGGCCCGTAATTGACGCTGAGGCGCGAAAGCGTGGGGAGCAAACAGG

>124d9ddfa90efc88afac999b9be51c76

CCGTTGTCGATTGCAGTGAAAGAATGTAAACAGGATTATCGCTGGTAATTACCTGTACACTTGGCTATGGTATGACAATATTTCATGTAGTTGTACAGTATACTGTCGGTATAACGGACCGACGAACGACCTGCTCCATGCTGCGCCGTGCCGTGTCGTGTAATTAGAAACCC

>2a78665a021e2c9cba5d1535d014f8e9

TACGTAGGGTGCGAGCGTTGTCCGGAATTATTGGGCGTAAAGGGCTCGTAGGCGGTTTGTCACGTCGGGAGTGAAAACTCAGGGCTTAACCCTGAGCCTGCTTCCGATACGGGCAGACTAGAGGTATGCAGGGGAGAACGGAATTCCTGGTGTAGCGGTGAAATGCGCAGATATCAGGAGGAACACCGGTGGCGAAGGCGGTTCTCTGGGCATTACCTGACGCTGAGGAGCGAAAGTGTGGGGAGCGAACAGG

>b5c518e124ff24160333000f6150b458

TACGGAGGGGGCTAGCGTTGTTCGGAATTACTGGGCGTAAAGCGCACGTAGGCGGACTGGAAAGTCAGAGGTGAAATCCCAGGGCTCAACCTTGGAACTGCCTTTGAAACTATCAGTCTGGAGTTCGAGAGAGGTGAGTGGAATTCCGAGTGTAGAGGTGAAATTCGTAGATATTCGGAGGAACACCAGTGGCGAAGGCGGCTCACTGGCTCGATACTGACGCTGAGGTGCGAAAGCGTGAGGAGCAAACAGG

>60ba99895405eb1d039ef1a859850637

TACGGAGGGGGCTAGCGTTGTTCGGAATTACTGGGCGTAAAGCGCACGTAGGCGGACCAGAAAGTTGGGGGTGAAATCCCGGGGCTCAACCCCGGAACTGCCTTCAAAACTATTGGTCTGGAGTTCGAGAGAGGTGAGTGGAATACCGAGTGTAGAGGTGAAATTCGTAGATATTCGGTGGAACACCAGTGGCGAAGGCGGCTCACTTGCTCGATACTGACGCTGAGGTGCGAAAGCGTGGGGAGCAAACAGG

>aedaa93585cdd5ef4f2c4340e9c961dd

CGCGTGATATGGTAATTGTGTGCCAGCCGCCGCGGTAATGAGTGTGATATAATTTTACAATACACTATAACATTAGATACCACAAGCCACACACAGAAGTCGGGGGCGCAAACACAATCATATAATTTTACCATACGCTATAACATTAGATACCCCGGTAGTCCGGCTGACTGACTTGCGTCAAATCTCGTATGCCGTCTTCTGCTTGAAA

>6c1070719a6331f88bba022f1e7b25b3

CACACTTATGTCTTTAGAAAAAAAAACATTTTTCCAGTATGCATTTCAACCATGTCGGTCATTTTCAACTGGCAGCGTCTTGTAGTTGCGAAAGATTTTGAATGCGTCTTGTCCATAGAGTTCAGTTTGTTCATTAATGATTTTGTTATGTAGCTTGTAACGTAGTATATTTCGATTTTCAAGTAAATTTATTGTCTCTCCTTTTTTGCCGGTTGTG

>dfba4ba8b14e37237bb7cc827c8143da

GGTTATGTTCGTCAACACCGGGCAAACCCGGTCATATAATTTTACCATACACTATAACACGTTGAGGTCGAGATTGCAGTGCAAGGTGAGGTTATGTTATCACATACTTTACAATGAAGTCTGGTCATATAATTGTGGCCGACAACGAAGAAACCGATCGAGATGGCAGTGGAGTCATATTTTTACCGGCAGTGGAAACGTGTGGTGAGGTTATGTTCATCAAC

>6093be3b18de97528e4735cc1736d928

TTCCTCTTTTTTCAACGAAGTTCCGCAATTCATTTCTATTTGTTGCCATTCATTTGCCTATTTCAATAGATGCCTCTAGTTCGGAACGCTGATACTATACTATCGTTCCATGCGATTCTAGGTCTTCCTCTACAGTTTTTTTCTTTTTCCTGGTTTCCCATATCTTTTTGACCAGTCTCTCTGTGCATGCTT

>d7d7a4ccff78c351297e9de2fc3f5052

CACGCGTGATATGGTAATTGTGTGCCAGCCGCCGCGGTAACACACATTGTTATTGTTGTTGTGATGTTTATATATGTGTAGGTTTTAATTTATGTTTATTTTTTGGCCTTTGGAAATGTTTTTAATTAGTTTGAGGTTGTTGGGATTAGATACCCGGGTAGTCCGGCTGACTGACTTAACGTCCATCTCGTATGCCGTCTTCTGCTTGAAAAA

>e10bffe84e708db77e0898bb3fcb6894

AACGCACGCACACAAACACACACGCATACACATACATTTAATAACATTTACAGCTGTGTATTCAAATAATTGCGAAACGGCCACTACAATCCATCAGGATTTGAATAAATACATTTAATCCTGTAATTTGAGTAACCTCTGTGCATTAGAAACCCCAGTAGTCCGGCTGACTG

>64c3b2c7f51754ca08ae156f8c9477b4

AATCGTTCATCTGCAGCAAAATGAGTACACCTATCGGATCGGAAAGGTGAGGAATGAATCCTGTAGGCCTGTATACTGCAAGGACGTTGATAACACCGATCATACAGTTTTCAAGTGCCAAATATGGGAAAGCGGACGTACTACATTAGAAACCCGAGTAGTCCGGCTGACTGAC

>bb1751cc4bb865d040f110183e885cb7

TGTAACGCGATACCCTCATCATACCCGTACGGTCCAAGTTTTCAGGTAACGTTATTATGATATTTAAATACGGCGAAAGTTCCAAATATTAGTGCTATTCTGTTATATTATCATCAGTAAGGTTCGCAATTCATTGACTTCTGTAACCGTTTTCCTGTCACAATAAGCAAATAAGAAAACCTTTGAATCCTCAAATTTCATGCACAGA

>5568a86f78c5d3e5157ff815580ba08f

CATTGAACTATCGTGAGAAAGTCAAACCGCCAAAGGGAATTATATTATAGTAAATATTGGCGTAAATAAACATTTTATTAATAGTTGTAATATATGAAAATGTGCAGAAGATAAAGACGATTAAAACGGTGTTTAACAATCGCTTATCGAGACAATATTTAATTAATTAGGAATTAAAATATGTGTATTATGTGTGTTAAACAAGGGATAAACTTGAAAATAAATACAGTGGTAAACGGAACTAATAGAGTCACGCAAGTGAACGTAGAACATTTTTAATAAC

>d15d0d5175f2af28085890d2bd59e73d

CATTGAACTATCGTGAGAAAGTCAAACCGCCAAAGGGAATTATATTATAGTAAATATTGGCGTAAATAAACATTTTATTAATAGTTGTAATATATGAAAATGTGCAGAAGATAAAGACGATTAAAACGGTGTTTAACAATCGCTTATCGAGACAATATTTAATTAATTAGGAATTCAAATATATTTTATTATTTGTATTAAACAAGGGATAAACTTGAAAATAAATACAGTGGTAAACGGAACTAATAGAGTCACGCAAGTGAACTTAGAACATTTTAATAAC

>86f769db3b94b30b473a9075e49d3281

TACGGAGGGTCCGAGCGTTAATCGGAATTACTGGGCATAAAGCGTGCGCAGGCGGTTTGTTAAGCGAGATGTGAAAGCCCTGGGCTCAACCTAGGAATAGCATTTCGAACTGGCGAACTAGAGTCTTGTAGAGGGGGGTAGAATTCCAGGTGTAGCGGTGAAATGCGTAGAGATCTGGAGGAATACCGGTGGCGAAGGCGGCCCCCTGGACAAAGACTGACGCTCATGCACGAAAGCGTGGGGAGCAAACAGG

>3192052e5cb493959af1c73c19674205

TACGGAGGATGCGAGCGTTATCCGGATTTATTGGGTTTAAAGGGTGCGTAGGCGGAGTGTCAAGTCAGCGGTAAAAATTCGGGGCTCAACCCCGTCGTGCCGTTGAAACTGACGCCCTTGAGTGAGCGAGAAGTAAGCGGAATGCGTGGTGTAGCGGTGAAATGCATGGATATCACGCAGAACGCCGATTGCGAAGGCAGCTTACTGGCGCTCGACTGACGCTGAGGCACGAAAGTGCGGGTATCGAACAGG

>f36a2bbc4fe860d9ce66de81af6a856f

TACTTATTTCTCAATCTTTATCCTTAATTATTTTTCTTAAATATCTCTTATTCTTTTTTTCTCTTCTTCTTTTAAAGTCCGGGGCTCAACTCCGGTTCTGCAGTGGGTACGGGCAGGCTTGAGTGATGTAGGGGAGACTGGAATTCCTGGTGTAGCGGTGAAATGCGCAGATATCAGGAGGAACACCGATGGCGAAGGCAGGTCTCTGGGCATTAACTGACGCTGAGGAGCGAAAGCATGGGGAGCGAACAGG

>b425228e0bf7bd5f37ccdf721064928a

ATGACAAAATCATTGAACATGTTCGACATATTATTCAAAGTGACAGAAGAAAATCTAACAACCTCTGATGCGTTTCTCTTGTATATGTATATTAGCCAGATCTTCAGATTCTAATGGTCTTTACTCAACAGATAAAAACTACATCAAACTATAATTGTAGTTACAATATTTACAAAGTAAGTTGTAAATCAATTTTTAAATACTTAAAATTGTAACTAGCAGAAAAATGGAGAAACTTATAACCAACCCTAATAATTAGCAAACAATATTCCTATATAGCTATA

>3a2c7f6c8f274f5a5b0b9059ec6d2743

CATTGAACTATCGTGAGAAAGTCAAACCGCCAAAGGGAATTATATTATAGTAAATATTGGCGTAAATAAACATTTTATTAATAGTCGTAATATATGAAAATGTGCAAAAGATAGAGACGATTAAAACGGTGTTTAACAATCGCTTATCGAGACACTATTAAAGTAATTAGGAATTAAAATATGTTTATTATGTGTATTATTCAAGGAATAAACTTGAAAATAAATACAGTGTTAAACGGAACTAATAGAGTCACGCAAGTGAACTTAGAACATTTTTAATAAC

>b129eb31db1084e80d9530c459baff96

GACGGGGGGGGCAAGTGTTCTTCGGAATGACTGGGCGTAAAGGGCACGTAGGCGGTGAATCGGGTTGAAAGTGAAAGTCGCCAAAAAGTGGCGGAATGCTCTCGAAACCAATTCACTTGAGTGAGACAGAGGAGAGTGGAATTTCGTGTGTAGGGGTTAAATCCGTAAATATACGAAGGAACGCCAAAAGCGAAGGCAGCTCTCTGGGTCCCTACCGACGCTGGGGTGCGAAAGCATGGGGAGCGAACAGG

>a19b1866d04b3c38aa8f3e906a2ef3e0

AAATGGCCACTTTCACGGGTGCAGTACGGGCTAACTGTGTGTTAAGGTTTCATGACACAAACTCTGCAACAACAGTTCAGCATAATTTTTGCACCGAGTGTGGTAAAGATCCTCTTACTAGACCTACAATTTACACTTGACCTCAGAACTTCGTTGAGAGTGGTTGTTCGGTTCAGC

>97991ce469a1cc109b514c89920eaa50

TACGGAGGGAGCTAGCGTTGTTCGGAATTACTGGGCGTAAAGCGCACGTAGGCGGCTTTGTAAGTTAGAGGTGAAAGCCTGGAGCTCAACTCCAGAACTGCCTTTAAGACTGCATCGCTTGAATCCAGGAGAGGTGAGTGGAATTCCGAGTGTAGAGGTGAAATTCGTAGATATTCGGAAGAACACCAGTGGCGAAGGCGGCTCACTGGACTGGTATTGACGCTGAGGTGCGAAAGCGTGGGGAGCAAACAGG

>e6f35e978bd7ee5baf1f0e2b955965f6

TACGTAGGGTGCAAGCGTTGTCCGGAATTATTGGGCGTAAAGAGCTCGTAGGCGGTTTGTCGCGTCTGCTGTGAAAACGCGAGGCTCAACCTCGCGCCTGCAGTGGGTACGGGCAGACTAGAGTGTGGTAGGGGAGATTGGAATTCCTGGTGTAGCGGTGGAATGCGCAGATATCAGGAGGAACACCGATGGCGAAGGCAGATCTCTGGGCCATTACTGACGCTGAGGAGCGAAAGCGTGGGGAGCGAACAGG

>ad9018f80e00f2ca4b48067195ac310d

TACGAAGGGGGCTAGCGTTGCTCGGAATTACTGGGCGTAAAGGGCGCGTAGGCGGACATTTAAGTCAGGGGTGAAATCCCAGAGCTCAACTCTGGAACTGCCTTTGATACTGGGTGTCTTGAGTGTGAGAGAGGTATGTGGAACTCCGAGTGTAGAGGTGAAATTCGTAGATATTCGGAAGAACACCAGTGGCGAAGGCGACATACTGGCTCATTACTGACGCTGAGGCGCGAAAGCGTGGGGAGCAAACAGG

>56c72f1c07fd6d56eb7eceaaf11772c4

CATTGAACTATCGTGAGAAAGTCAAACCGCCAAAGGGAATTATATTATAGTAAATATTAGCGTAAATAAACATTTTATTAATAGTTGTAATATATGATAATGTGCAGAAGATAAAGACGATTAAAACGGTGTTTAACAATGGCTTATCGAGACAATATTTAATTAATTAGGAATTAAAATATATTTTATTATTTGTATTAAACAAGGGATAAACTTGAAAATAAATACAGTGGTAAACGGAACTAATAGAGTCACGCAAGTGAACTTAGAACATTTTAATAAC

>eb1a52ddd9b9b7e63fefbf857f999f80

GCTCGATGTCGGAGCCGATAACAGGATTATCCGGCTTCACCTGTGTGCCTCTCACTGCTTTTGTATATTATCCCTTCCGTAGTAGACCTTACTGAGATAATATTATACAGCGGCGAGTGGTACAAAGTAGCTGCTTCGGAAGAAAAGAAAAACATTCTTACTGGTAGACTATACTAAAGAAAAATATTCATGAGAAGCGTAGCATTGTGTAGAGCTGAAACATGAAAGTAGGAATGTCAGATTATCTGAAGAATTTCGAATATGATATTGGAGAACAATGCAGAATACATGTTGGACAGACGTG

>8ca538ef33c35ef1823f310c022bae59

TGTAATGTCCAAAGAACCGTTAAAACTAATTTGACACCCCAGTGTAGTCTTTATTGTAACAAAAGCATCGTATTAGGAACTAAACAAAAATGTTTCTATTCCTTGTTTCACAAAATAATCGCCACCTGGCGTGTGTTGTCACGTAAATACATAACACGAACGATTAACACTTTACGATTATAACGCCATAAAAATGTTCAGAGGGTCGTTAACGGGAGAAGGTTGACAGCAGAGAGGGGCGTAATATAAATTGTCGTAAAAAAAACGAATAAATGAGAATAATATCCAAAGATCTGT

>50f5a1e6251ce3547ca41ee8e4e7593c

TACGGAGGGTGCAAGCGTTGTTCGGAATTATTGGGCGTAAAGCGCGTGCAGGCGGCTGTTCAAGTCCGATGTGAAAGCCCGGGGCTCAACCCCGGAAGTGCATTGGAAACTGGACAGCTTGAGTACGGGAGAGGGAGGTAGAATTCCGAGTGTAGGGGTGAAATCCGTAGATATTCGGAGGAATACCGGTGGCGAAAGCGGCCTCCTGGACCGATACTGACGCTGAGACGCGAAAGCGTGGGGAGCAAACAGG

>61ed6a6000a76fa8258665f512a93e5f

TTCTACATCAGTTGCAATTGGTTTATACGAAACTGTTTTGTTTTTCCAGTATGCCGAAAGTGCATTCAACAGTGCGACGTGCAACGGAAAGCCTGTAATTAAATATGCGTTTAGGAAACCCAGTTGTCTTCTCGCGTACGGTCACATTAAATTGAAACGAAGCGGGAAGGCCTCATCGCCAACTAAATAAAATGGGAATATTTCATTTGTTCCAGGAATTTCTTGAGG

>0fd3239ba6bdd2dc98fb1e5363ceff01

TTCACTGTTGTCGAGAAGTTGTAACATCTCAACGTTTCGGTGTAAGTGGAGCCTTTCCTCATGTTTCCTCGCAAATCGCTGGATTTCTTCAGTAACCATAGGTATTTTGAAATCCTTGTGGATATCCTCATTTCGAATATACCATGGTGAATTAACAATACTTCGCAATACATTATTCTGAAAACGCTGAATCACTAGAATATTACCTTTCTTGGTGCAACTCCATAGCACTCCGAACGTCCAAACTGGCTGTAACACTTGCTTATAT

>93d7d75a2eec680e10ed7a200588e4fb

CGCGGTAAGTGAAACAGTTTATTGTCTATAACGTTTAAACGGTTGGTCGGATTTTACATTTCTTTACATGTTTTGTACGACTTGATTAAGAGAATGTACCTGCTTAATTGCATTCTTATCCATTAAGTAGTTAAAGAGAAACCCATTAGAAACCCCTGTAGTCCGGCTGACTGACTTAACG

>27d4129916a7ad936238bccf39e87939

GGTAATTGTGTGCCAGCCGCCGCGGTAATTGACTGGATGAGACCCACCGCAGTTGCAGCATGTTGCAGAGGTCTCCGGTGCGACCAGATAGTAGCCTCGGTGCTCTCCTAACGTCTCTGTGGAAAACGGCCAACGTCGTCATGGATTAGAAACCCCAGTAGTCCGGCTGACTGACTTAACGTCCATATCGTATGCCGTCTT

>caf09429103d652236b0d75b7ee2f027

TGTGCCAGCCGCCGCGGTAAGCGTATTTCTAAATTTTACCTAAAATAGTGAACTGTAGGTCGTGTTTTAATTACAAAATCAGTTCTTTATTTGAGTCGTATTTACTCTTTTCTTTTTGTTAGAACTCGTCCTGTACCGTCAATCATTAGAAACCCCAGTAGTCCGGCTGACTGACTGAGACTTAATCTCGTAT

>d17fb1649c579bc67de85c71ea8a4a98

TACAGAGGGTGCAAGCGTTAATCGGAATTACTGGGCGTAAAGCGCGCGTAGGTGGCTAAGTAAGATGGGTGTGAAATCCCCGGGCTCAACCTGGGAACTGCATCCATAACTGCTTGGCTAGAGTACGGTAGAGGGTAGTGGAATTTCCTGTGTAGCGGTGAAATGCGTAGATATAGGAAGGAACACCAGTGGCGAAGGCGACTACCTGGACTGATACTGACACTGAGGTGCGAAAGCGTGGGGAGCAAACAGG

>a0da905b7974c508f32fe0aeabf7cd0c

TACGTAGGGTGCAAGCGTTGTCCGGAATTACTGGGCGTAAAGAGCTCGTAGGCGGTTTGTCGCGTCGTCTGTGAAATTCTGCAGCTTAACTGCAGGCGTGCAGGCGATACGGGCAGACTTGAGTACTACAGGGGAGACTGGAATTCCTGGTGTAGCGGTGAAATGCGCAGATATCAGGAGGAACACCGGTGGCGAAGGCGGGTCTCTGGGTAGTAACTGACGCTGAGGAGCGAAAGCGTGGGTAGCGAACAGG

>a189985b18cf7dbbb78b9c8aaafe4e47

GGACTGTATGTTGGTTACAGAAAAAAAGTTTATGGAAAATGTTGATATTGTGTATGTTTTGTAATTTTGTATGGGGATTATTTATCTATATGGTTGACTGTTTTAATTCAATAAAAAGACTGTGGATTTTGAAATAATACAAAGAATGTTTGGTATTATATTTAAACAGAATAATACAAAGATAAAGTTATATTGAAAGGATATTTCTATTTTTATAAGCTGTTTTATCTTATGAATAAATGATAGTATGAATTTCTTATTTACTTTAA

>d6e4bfd0f238fc87a3d62f525a7780f4

TATTGTGTCATTTATTGTAATGGTATCGCTTATACATGCAACGAGTGACCACGTAAATCCCTTCTACTTTTATTATTAGGTTTGCGTATTGTATCTTTCCCCTAATTAGTGTTTGTTAATATAAACATATTTTTTTAAAGTACACACACATCCTCACAATTTTAAAAATTACTACAGACGTTACAAAGGTTAAATCTGACATAATCCGAAGGTTTCAGATTAAAACTCGGATGCCACACAAACACACACACACATACATCAACCACACAAACACACACACACAGCACACATC

>907a6f4815121fe0996c3a7113c7c6be

CGCACTTATGTCTTTAGAAATACAAAACATTTATCCAGAATGCATTTCAACCTTCTCAAAGTCATTTTCATCTGGCAGTGCCTTGTAGATAGTTGTGGAAGATTTTGAATGTGTCTTGTCCATAAAGTTCAGTTTGTTCATTAATGTTTTTGTTATGTAGTTTGTAACGTAGTATATTTAGACTTTCAAGTAAATTCATTTTATTTCCTTTTTTGCCGGTTGTG

>94929634ca6022d5994d4ad1c49ae7f0

GGTAACTACTGAAAACTTCAGACATCGTCTGGGACGCCTCACGTGGGCAGCAGTCTTGCGCACATAGCCTGCAGCCAGTGCTTTGCTACCCCATGTCCTGTGATCAGTTCTACAACATGTTTTAGCTGTCGCCGGTTCAACTTAATTAGATACCCGGGTAGTCCGGCTGACTGACTTG

>dfd42bc974d222dd5ad90c5cb821ea9f

TACGTAGGGTGCGAGCGTTGTCCGGAATTATTGGGCGTAAAGAGCTCGTAGGCGGCTTGTCGCGTCGGATGTGAAAGCCCGGGGCTTAACTCCGGGTCTGCATTCGATACGGGCAGGCTAGAGTTCGGTAGGGGAGATCGGAATTCCTGGTGTAGCGGTGAAATGCGCAGATATCAGGAGGAACACCGGTGGCGAAGGCGGATCTCTGGGCCGATACTGACGCTGAGGAGCGAAAGCGTGGGGAGCGAACAGG

>1e69db06d96a1bc65a08d4a44130cd27

TACGGAGGGTGCAAGCGTTATCCTGATTTATTTTTTTTCTCTTGTCTTTCTTTTTATTTTTTATTCTTTTTTTCACTTTCACAGCTTAACTGTGAAACTGCCATTGATACTGCATGTCTTGAGTGTTGTTGAAGTAGCTGGAATAAGTAGTGTAGCGGTGAAATGCATAGATATTACTTAGAACACCAATTGCGAAGGCAGGTTACTAAGCAACAACTGACGCTGATGGACGAAAGCGTGGGGAGCGAACAGG

>9caccad382e142edfd408be3b061ad4c

TACGGAGGATCCAAGCGTTATCCGGAATCATTGGGTTTAAAGGGTCCGTAGGCGGTTTAGTAAGTCAGTGGTGAAAGCCCATCACTCAACGGTGGAACGGCCATTGATACTGCTAGACTTGAATTATTAGGAAGTAACTAGAATATGTAGTGTAGCGGTAAAATGCTTAGAGATTACATGGAATACCAATTGCGAAGGCAGGTTACTACTAATTGATTGACGCTGATGGACGAAAGCGTGGGTAGCGAACAGG

>d632de5c3fc21bbd74ec98ccfcd20ed9

TACGGAAGGTCCGGGCGTTATCCGGATTTATTGGGTTTAAAGGGAGCGTAGGCCGCGCCTTAAGCGTGTTGTGAAATCCGGGTGCTCAACATCCGGCTTGCAGCGCGAACTGGGGCGCTTGAGTGCGCAGAAAGTAGGCGGAATTCGTGGTGTAGCGGTGAAATGCTTAGATATCACGAGGAACTCCGATTGCGAAGGCAGCCTACTGTAGCGCTACTGACGCTGATGCTCGAAAGCGTGGGTATCGAACAGG

>e31300b5d144d1a83b2db48098112496

CGGTAACTACTGTGTGGTATATCCTGAGAATGTTTATACCAAAGAGAATGTTAAAGAGAGAAAGAGCTGACAGGAGATGTAGAGGACGGCCATGAACCCGCTGGCAAGATGACATCAGAAAGGACGCCGTGGAGTTACTGGGAGATTAGAAACCCCTGTAGTCCGGCTGACTGACTTGC

>dd146bc47c0b0ae0a523f33248cc93ba

CTCGTATCATACTACACGCTACACACTGCTTTGCTCTCCTTAACACCAAACAAGATATATAAAGTTTTACACCAATATAAATCAATATTACACTACAGACCAACGATACGGGGGAGAGCTAAACATTTCTCGTTGTTCATACGAGAGAGGGCGTCAAATAGTTAACACAGCCTCAAACTAAACATACATTTTACCGCATGTTATTTTTCATTCCTTTATGCCGTGAGATTATCTCACTTATCTGTAGGTGGTAAATCTAATGATATTATCGCATTTATTAGTTAATTAATTAGTTTATT

>d0be4912ca1ce4c17a0c7630dd47d818

TACGAAGGGTGCAAGCGTTGCTCGGAATTATTGGGCGTAAAGGGTAGGTAGGTGGTTACGTATGTCTGAGGTGAAAGCCCTAAGCTTAACTTAGGAAGTGCCTTGGAAACGGCGTAACTAGAGTACTGGAGAGGTTCGTAGAGTTCCCGGTGTAGCGGTGAAATGCGTAGAGATCGGGAGGAATACCAGAGGCGAAGGCGACGAACTGGACAGTAACTGACACTAAACTACGAAAGCGTGGGGAGCAAACAGG

>54c7b75b91a26fa1e4b993850ede571d

CAGACCGTTAGGAGGGTGTGTGAGACACAACGTGGGCTGGACAAAATACATATAGTAAACAGTTCTCTAACGCTCAGTTCACTGATAATCTGACACACAGCGGCGTTAATTAAACGTCACTTAAATTCATAAGATTTAAGAGATCGTAGACCTTTTTAACTGTGATTGCAGTGCATATTTAATTGGGAATA

>a0b72f40b46d8f6fc5b052dc4ce3eb2f

TACGTAGGTGGCAAGCGTTGTCCGGAATTATTGGGCGTAAAGCGCGCGCAGGCGGTCTTTTAAGTCTGATGTGAAAGCCCCCGGCTCAACCGGGGAGGGTCATTGGAAACTGGGAGACTTGAGTACAGAAGAGGAGAGTGGAATTCCACGTGTAGCGGTGAAATGCGTAGATATGTGGAGGAACACCAGTGGCGAAGGCGATTCTCTGGTCTGTAACTGACGCTGAGGCGCGAAAGCGTGGGGAGCAAACAGG

>87220bfae0f0511ebec7b77c671bd8da

TCGAACCCAAGCCACGTGGTGTAGCGAACCAACACACTACACACCACGAGTACTCCCCCCCCCTTCCAATCAACAAAAAATCACAATACGATTACAGGAAATTGTTCAGAATGTAGCGCCAAATGAAATTACAAAGAGGAGGATTTTGAATATTGCAGAAGGATCGTATTGGTGCAAAACAAACGTTTGCTAGCAGCCTTCCTCACGTGTATACTGCACTGATGCTTCGAGAAATAGTCTGCAGAACTAT

>cbf9c4405d12544c6a993fa43cfdda3f

GGTAATTGTGTGCCAGCCGCCGCGGTAATTGACTGGGTGAGACCCACCGCAGTTGCAGCATGTTGCAGAGGTCTCCGGTGCGACCGGATAGTAGCCTCGGTGCTCTCCTAACGTCTCTGTGGAAAACGGCCAACGTCGTGATGGATTAGAAACCCTTGTAGTCCGGCTGACTGACTTAACGTCCATCTCGTATGCCGTCTT

>7e8ab35113ac5c81c1e6bee769158bd2

CATTTTACCATAGTGTCTGCCTCCACGGGCACGTTTATGGCTTTGCCGGTTATCTTTTAAGACTGTTTCTATTCTGAGACGACGGATCTGCATACGTGGAAATCCGGATGAAATCAGCCTCTCAGTGATACGATCGAGCGGCAGGTGGGTCGGATCTCCCGGATACTTATAACAATTACTTTGGCTCAGGGGAGGCACTTTACCCTCATTAAGCAAGCGGCGGTATGTGGAGCACA

>c4ba3ee4039b7fea6811b1d71ff3a20c

AATTCCTGGAATAACAACCACAGCATGTTACAAGAGAAGGAGCAGATAAACGCAACTGTGACAACAACAGCAGAACAGCATTTTACGAGATAAGGAGCCAACAAACACAACATAAATACAACTTGTCAACTCAGCGGAGATAAAAAAGAGATAGACACCTGCAAACTACGCGATACCGCATAACACAGCCAGCTTCTTGTTATTGCTACATTCGTGGAAAAGAGAAG

>9801bb0ac5d94b989547476cc4216fec

TTAAACGAAAGCACAACAAAAAGATAGCCAAACTAATACCACCTCAATCACCAGCAATAACACCCACACATACGTTTTACCCAAGAATAGTTAACCTAACCAACGTAACATTCACTAACGAACAGAAACAACTTTTAAACAAAGGGATAAACCACAACCTACACTACACACAGAATAATAACACCATCAAGAACATGGT

>55c86b4e94b6493cebceebe0b0178063

GGCCAGATGAATTCCACATTTATATGGAACACACTCCATCTCATATTCTTGACACATTATTAAAAGTTTTAATAAAATATGGTTCTCTGGCATCTTTCCAACAGTGTGGAAAACGGCAACGATTATTCCCATTCTTAAAGAAAACAAAAACCCTTCACTATTAGAAACCCTGG

>3111eace3eddfe8b0074d83ae6ecc2ac

TCAAACGAAAGCACAACAAAAAGATAGCCAAACTAATACCACCCCGATCATCAGCAATAACACCCACACATACGTTTTACCCAAGAATAGTTAACCTCACCAACATAAAATTCACTAACTATCAGAAACACCTTTTAAACAAAGGGATAAATCACAACCTACACTACATGCAGAACAATAACACCATCAAGAACATTGT

>c1d61dd295ffe1b34fb74c35806b379a

TACGAAGGGGGCTAGCGTTGCTTGGAATCACTGGGCGTAAAGGGTGCGTAGGCGGGTCTTTAAGTCAGGGGTGAAATCCTGGAGCTCAACTCCAGAACTGCCTTTGATACTGAGGATCTTGAGTCCGGAAGAGGTGAGTGGAACTGCGAGTGTAGAGGTGAAATTCGTAGATATTCGCAAGAACACCAGTGGCGAAGGCGGCTCACTGGTCCGGTACTGACGCTGAGGCACGAAAGCGTGGGGAGCAAACAGG

>4157e688914e446b15f49fc8a46b0101

TACGGAGGGTGCAAGCGTTGTTCGGAATTATTGGGCGTAAAGCGCATGCAGGCGGCTGTTCAAGTCCGATGTGAAAGCCCGGGGCTCAACCCCGGAAGTGCATTGGAAACTGGACAGCTTGAGTACGGGAGAGGGAGGTAGAATTCCGAGTGTAGAGGTGAAATCCGTAGATATTCGGAGGAATACCGGTGGCGAAGGCGGCCTCCTGGACCGATACTGACACTGAGACGCGAAAGCGTGGGGAGCAAACAGG

>f4fb1b63d2a09aae5595c103fc7c2fba

TACGGAGGATGCGAGCGTTATCCGGATTTATTGGGCTTAAAGGGTGCGCAGGCGGGCTGTCAAGTCAGCGGTAAAATTGAGAGGCTCAACCTCTTCGAGCCGTTGAAACTGGCGGTCTTGAGTGAGCGAGAAGTACGCGGAATGCGTGGTGTAGCGGTGAAATGCATAGATATCACGCAGAACTCCGATTGCGAAGGCAGCGTACCGGCGCTCAACTGACGCTCATGCACGAAAGCGTGGGTATCGAACAGG

>5748a87fc2d4295ae3995e780a810196

TACGTAGGGTGCGAGCGTTGTCCGGAATTATTGGGCGTAAAGAGCTCGTAGGCGGTTTGTCGCGTCGACTGTGAAAACCTGGGGCTCAACTCTGGGCTTGCAGTCGATACGGGCAGACTTGAGTTCGGTAGGGGAGACTGGAATTCCTGGTGTAGCGGTGAAATGCGCAGATATCAGGAGGAACACCGGTGGCGAAGGCGGGTCTCTGGGCCGATACTGACGCTGAGGAGCGAAAGCGTGGGGAGCGAACAGG

>f053dc400e83a3abaaa1aeef9dc9fe84

AAATGGCTACTTTCACGGGTGCAGAACGGGCTAACTGTGTGTTAAGGTTTCATGACACAAACTCTGCAACAACAGTTCAGCATAATTTTTGCACCGAGTGTGGTAAAGATCCTCTTACTAGACCTAAAATTTACACTTGACCTCAGAACTTCGTTGAGAGTGGTTGTTCGGTTCAGC

>c2a787cf867ebfdce216002bcc8a94e1

TACGAAGGGGGCTAGCGTTGTTCGGAATTACTGGGCGTAAAGCGCACGTAGGCGGATATTTAAGTCAGGGGTGAAATCCCAGAGCTCAACTCTGGAACTGCCTTTGATACTGGGTATCTTGAGTATGGAAGAGGTAAGTGGAATTGCGAGTGTAGAGGTGAAATTCGTAGATATTCGCAGGAACACCAGTGGCGAAGGCGGCTTACTGGTCCATAACTGACGCTGAGGTGCGAAAGCGTGGGGAGCAAACAGG

>c51318150f399c6a44d58cf9b6d85d3d

TACGTAGGGTGCGAGCGTTGTCCGGAATTATTGGGCGTAAAGAGCTTGTAGGCGGTTTGTCGCGTCTGCCGTGAAAGCCCAGGGCTTAACCCTGGGTCTGCGGTGGGTACGGGCAGACTAGAGTGTGGTAGGGGAGACTGGAATTCCTGGTGTAGCGGTGAAATGCGCAGATATCAGGAGGAACACCGATGGCGAAGGCAGGTCTCTGGGCCACTACTGACGCTGAGAAGCGAAAGCATGGGGAGCAAACAGG

>ca6a921f746e33e53d78968f74a9ebf6

TACGTAGGGGGCAAGCGTTGTCCGGATTTATTGGGCGTAAAGAGCGTGTAGGCGGCCTGACAGGTCTGCTGTGAAAACTCGAGGCTCAACCTCGAGACGTCGGCGGAAACCGTCAGGCTAGAGTCCGGAAGAGGAGAGTGGAATTCCTGGTGTAGCGGTGAAATGCGCAGATATCAGGAAGAACACCTATGGCGAAGGCAGCTCTCTGGGACGGTACTGACGCTGAGACGCGAAAGCGTGGGGAGCGAACAGG

>c077ffec3c3ed58fcc51ebe96a4537be

TACAGAGGGTGCGAGCGTTAATCGGATTTACTGGGCGTAAAGCGTGCGTGGCGGCTGATTAAGACGGATGTGAAATCCCTGAGCTTAACTTAGGAATTGCATTCGATACTGGTCAGCTAGAGTATGGGAGAGGATGGTAGAATTCCAGGTGTAGCGGTGAAATGCGTAGAGATCTGGAGGAATACCGATGGCGAAGGCAGCCATCTGGCCTAATACTGACGCTGAGGTACGAAAGCATGGGGAGCAAACAGG

>76ae0421984b4b7f739287a592744da7

TACGTAGGGCGCAAGCGTTGTCCGGAATTATTGGGCGTAAAGAGCTCGTAGGCGGTTTGTCGCGTCTGCTGTGAAAGCCCGGGGCTTAACTCCGGGTGTGCAGTGGGTACGGGCAGACTAGAGTGCAGTAGGGGAGACTGGAATTCCTGGTGTAGCGGTGGAATGCGCAGATATCAGGAGGAACACCGATGGCGAAGGCAGGTCTCTGGGCTGTAACTGACGCTGAGAAGCGAAAGCATGGGGAGCGAACAGG

>7b1753c439f93fc3ab4f3de846ce2627

CACACTTATGTCTCCAGAAAGACAAAATATTTATCCAGGATGCATTTCAACCATGTCGGTCATTTTCAACTGGCAGTGCCTTGTAGATATTTGTGAACGATTTTGAATGTGTCTTGTCCATAGAGATCAGTTTGTTCATTAATGATTTTGTTATGTAGTTTGTAACGTAATATATTTAGGCTATCAAGTAAATTCATTTTGTTTCCTTTTTGCCGGTTGTG

>90df9593d04804f88a64314325258335

TACGGAGGGGGCTAGCGTTATTCGGAATTACTGGGCGTAAAGCGTACGTAGGCGGTGGTTCAAGTCAGAGGTGAAAGCCCGGAGCTCAACTCCGGAACTGCCTTTGAAACTAGATCGCTAGAACATCGGAGAGGTAAGTGGAATTCCGAGTGTAGAGGTGAAATTCGTAGATATTCGGAAGAACACCAGTGGCGAAGGCGGCTTACTGGACGATTGTTGACGCTGAGGTACGAAAGCGTGGGGAGCAAACAGG

>fc8e8c559d886c895bcbe2dc9365b4dc

TACAGAGGGTGCAAGCGTTAATCGGAATTACTGGGCGTAAAGCGCGCGTAGGTGGTTCGTTAAGTTGGATGTGAAAGCCCCGGGCTCAACCTGGGAACTGCATCCAAAACTGGCGAGCTAGAGTATGGTAGAGGGTGGTGGAATTTCTTGTGTAGCGGTGAAATGCGTAGATATAGGAAGGAACACCAGTGGCGAAGGCGACCACCTGGACTGATACTGACACTGAGGTGCGAAAGCGTGGGGAGCAAACAGG

>cf79208f07450e069e76190a78947fae

TACGGAGGGGGCTAGCGTTGTTCGGAATTACTGGGCGTAAAGCGCACGTAGGCGGACTGGAAAGTTGGGGGTGAAATCCCGGGGCTCAACCTCGGAACTGCTTTCAAAACTATCAGTCTGGAGTTCGAGAGAGGTGAGTGGAATTCCGAGTGTAGAGGTGAAATTCGTAGATATTCGGAGGAACACCAGTGGCGAAGGCGGCTCACTGGCTCGATACTGACGCTGAGGTGCGAAAGCGTGGGGAGCAAACAGG

>123850bcc6f0048d3f0ef23f5e1d6537

TACTTATTTCTCAATCTTTATCCTTAATTATTTTTCTTAAATATCTCTTATTCTTTTTTTCTCTTCTTCTTTTAAATCCGGAGGCTCAACCTCCGGCCTGCAGTGGGTACGGGCAGACTAGAGTGCGGTAGGGGAGATTGGAATTCCTGGTGTAGCGGTGGAATGCGCAGATATCAGGAGGAACACCGATGGCGAAGGCAGATCTCTGGGCCGTAACTGACGCTGAGGAGCGAAAGGGTGGGGAGCAAACAGG

>3b79391c02e904bf7d16e1143a0d1fbb

GAAATGTCCCAACATTCTTTACTTTAGACATGACCCTCGTATGAAGCTGTCTGTCAATTCACATAAGTATCCGAACATGTGTTAGAAACAACACTGTTTTTAGGTTTAACATGTTTTATTTTTCTTAATACCAAATGTTTTGCCCATTCTATAATAAAGTATCCTCAGTGGCTCAAGCCACTGAACAGTTTGTAGGATGGACGAAATGTCTAGTATTAAGAAAAACAAAATTTGTGTAAACCAACCAACACACAGCTACTGT

>df7db10977ff3e4b750f58b67a91e77f

ATGATTGTCCTGTGACCTGCTTGACCTACTTGGATAAAAATACTTAACAAAGCACAAAATGTTCTCAGTCAAAAGCTATGCTGAAGATAGTCAGTTTTGACTGAAAACATTTTGTCCTTTGGTAAGTATTTTTACCATAGTGCATAAATTAGAAACCCTGGTAGTCCGGCTGA

>f02d83647849766acc6b2ebb19a7b045

TACGAAGGGTGCAAGCGTTATCCGGATTCATTGGGTTTAAAGGGTGCGTAGGCGGGGCGTTAAGTCAGTGGTGAAATCCTGCAGCTCAACTGTAGACTTGCCATTGATACTGACGCTCTTGAGTGCGCTTGAAGTAGGCGGAATGTGCCGTGTAGCGGTGAAATGCTTAGATATGGCACAGAACACCAATTGCGAAGGCAGCTTACTAAGGCGATACTGACGCTGAGGCACGAAAGCGTGGGGATCGAACAGG

>29ebe17e0237b3883999c5b5ecff6713

TACGGAGGGTGCAAGCGTTAATCGGAATTACTGGGCGTAAAGCGCGCGTAGGCGGTATGTTAAGTCGGATGTGAAGTCCCTGGGCTCAACCTAGGCATTGCATCCGATACTGGCACACTAGAGTGTGGGAGAGGAAGGTAGAATTCCAGGTGTAGCGGTGAAATGCGTAGAGATCTGGAGGAATACCGATGGCGAAGGCAGCCTTCTGGCCTAACACTGACGCTGAGGTGCGAAAGCATGGGGAGCAAACAGG

>dc0448788e6e7b308569ff07e68ca29f

TACAGAGGGTGCGAGCGTTAATCGGAATTACTGGGCGTAAAGCGAGTGTAGGTGGCTCATTAAGTCACATGTGAAATCCCCGGGCTTAACCTGGGAACTGCATGTGATACTGGTGGTGCTAGAATATGTGAGAGGGAAGTAGAATTCCAGGTGTAGCGGTGAAATGCGTAGAGATCTGGAGGAATACCGATGGCGAAGGCAGCTTCCTGGCATAATATTGTCACTGAGATTCGAAAGCGTGGGTAGCAAACAGG

>fee4f42710f43c7afac843df35f38f2b

CATTGAACTATCGTGAGAAAGTCAAACCGCCAAAGGGAATTATATTATAGTAAATATTGGCGTAAATAAACATTTTATTAATAGTTGTAATATATGAAAATGTGCAGAAGACAAAGACGATTAAAACGGTGTTTAACAATCGCTTATCGAGACAATATTTAATTAATTAGGAATTAAAATATATTTATTATTGGTATTAAACAAGGGATAAACTTGAAAATAAATACAGTGGTAAACGGAACTAATAGAGTCACGCAAGTGAACTTAGAACATTTTTAATAAC

>69ac4f3cd27033224f2be7279ddcfd5a

CGCCGCGGTAATACAATTTTTTCAATTTAATATGTAAGTTTCAGTTAATTGATTATTTTTAATAATTTATATCAATTTTGGTGAAATAATATTTATTAATATAAATAATTTAAATGTGTCTGAGAAACTATAAATTAAACTAGGATTAGATACCCTTGTAGTCCGGCTGACTGACTTAACGTCC

>0bb7b185b0821b4585371cd1dfbf945e

CCGCGGTAACTGGAGATAATGCTAACTGTAGATGGCAGAGAAAGAGATTAGAGTAATCCTTAAGAAGAACTAAAGAGGATCTTCTCTTTATCACAAAGATCATAATTGCACAATCAATGGACAATATGGGTTTAAAAGGAAGCAATTAGAAACCCTTGTAGTCCGGCTGACTGACTTGCGTC

>185b22d14643b9835109108cdc6f4112

TATGGTAATTGTGTGCCAGCCGCCGCGGTAAGAGAAAAACTATCGCCAACAATTAGCGCATTAAGTAGTAGTAGCGGCGACGGTAACAGCTGTACGCTACTGATAAGCCAGCAGCCGACGCTACTTCTCTTCTGTCTACGATTTATTAGAAACCCCGGTAGTCCGGCTGACTGACTTGCGTCAAATCTCGTATGCCGTCTTCTG

>fad58a373d619455fdd11784cb24d8b7

GCTCGATGTCGGAGCCGATAACAGGATTATCCGGCTTCACCTGTGTGCCTCTCACTGCTTTTGTATATTATCCCTTCCGTAGTAAACCTTACTGAGATAATATTATACAGCAACGAGTGGTACAAAGTAGCTGCTTCGGAAGAAAAGAAAAACATTCTTACTGGTAGACTATACTAAAGAAAAATATTCATGAGAAGCGTAGCATTGTGTAGAGCTGAAACATGAAAGTAGGAATGTCAGATTATCTGAAGAATTTCGAATATGATATTGGAGAACAATGCAGAATACATGTTGGACAGACGTG

>a53b02d4fa1759b8b9bee6c5ac28fbbf

ATAGCGTTATGGTAATTGTGTGCCAGCAGCCGCGGTAATGAGTGTGATATAATTTTACAATACACTATAACATTAGATACCACAAGCCACACACAGAAGTCGGGGGCGCAAACACAATCATATAATTTTACCATACGCTATAACATTAGAAACCCCGGTAGTCCGGCTGACTGACTACTGTGTAATCTCGTATGCCGTCTTCTGCTTGAAA

>f9b72a31bc0101ad3f11df858e448d2f

TACGTAGGTGACAAGCGTTGTCCGGATTTACTGGGCGTAAAGAGCGCGCAGGCGGTCGATCAAGTCGAGTGTGAAAGCCCCCGGCTCAACTGGGGAGGGTCATTCGATACTGATCGACTCGAAGGCAGGAGAGGGTAGTGGAATTCCCGGTGTAGTGGTGAAATGCGTAAATATCGGGAGGAACACCAGTGGCGAAGGCGACTACCTGGCCTGTTCTTGACGCTGAGGCGCGAAAGCTAGGGGAGCAAACGGG

>1bdd416369e5fe3712baf774b620cdef

TACGGAGGGTGCAAGCGTTAATCGGAATTACTGGGCGTAAAGCGCACGCAGGCGGTCTGTCAAGTCGGATGTGAAATCCCCGGGCTCAACCTGGGAACTGCATTCGAAACTGGCAGGCTAGAGTCTTGTAGAGGGGGGTAGAATTCCAGGTGTAGCGGTGAAATGCGTAGAGATCTGGAGGAATACCGGTGGCGAAGGCGGCCCCCTGGAGAAAGACTGACGCTCAGGTGCGAAAGCGTGGGGAGCAAACAGG

>5bfa23ffdd4cca740990e5b5ee2a4cc8

GACATGATAGACTAGTGTTATTCATCTTTAATGGGTTTAAAGGGTACCTAGACGGAAGTTCAAGCCCGCATAGGGGACGGAATTTCTAGAGTTTTATGTGAGAAGGGGAGTACCTCTGGCGGAGAGATGAAATTCGTTGATACCTTTGGGACTGGTAAAGGCGAAGGCAACCTTCTAAGTAGAAACTGACGTTGAGGGACGAAGGCTTTGGTCACGAACAGG

>2c4971fe51e86843d7097a6fce579e25

GAAATGTCCCAACGGTCTTTACTTTAGACATGGCCCTTGTATGAAGCTGTCTGTCAATTCATATAAGTATCCGAACATGTGTTAGAAACAACACTGTTTTTAGGTTTACATGTTTTATTTTTCTTAATACCAAATGTTTTGCCCATTCTATAATAAAGTATCCTCAGTGGCTCAAGCCACTGAACATTTTGTAGGATGGGCAAAATGTCTAGTATTAAGAAAAACAAAATTTGTGTAAACCAACCAACACACAGCTACTGT

>b2aeb631e7d84934c46491460266f3e2

TACGTAGGGGGCGAGCGTTGTCCGAAGTTACTGGGCGTAAAGAGCGCGTAGGCGGGTTCTTAAGTGAGGGGTGAAAGTCCGAGGCTCAACCTCGGAACTGCCTTTCATACTGGGAACCTTGAGTGCGGGAGAGGCGAGTGGAATGGCCGGTGTAGCGGTGAAATGCGTAGATATCGGTCGGAACACCCATGGCGAAGGCAGCTCGCTGGCCTGTAACTGACGCTGAGGCGCGAAAGCGTGGGGAGCAAACAGG

>84886b7fa934d4a0016eac3235363ffa

TACGGAGGGTGCAAGCGTTAATCTGATTTACTTTTTTTCTCTCGCCTTCCTTTTTTCTTTTTATTCTTTTTTTCACTTCCCGGGCTTAACCTGGGAACTGCATTTGAAACTGGCAGGCTTGAGTCTCGTAGAGGGGGGTAGAATTCCAGGTGTAGCGGTGAAATGCGTAGAGATCTGGAGGAATACCGGTGGCGAAGGCGGCCCCCTGGACGAAGACTGACGCTCAGGTGCGAAAGCGTGGGGAGCAAACAGG

>2020b6bb0268d0a28f6cc282b171c759

TGAAATTGAAAACAACAAAAGCGTTAAACTTTTTTATAACGTTTTATGGTGTTTCCATACAATGCAGTGCAGACTTTTCCCCCGTTATTTACTAAAACAATCGCCTCTCTTCATTACCTGCTTTGACTTCCAGAGAAGCACTAAGGGATTAGAAACCCCAGTAGTCCGGCTGA

>fe0b88cf174330e6d5d031d0be234859

CATTGAACTATCGTGAGAAAGTCAAGCCGCCAAAGGGAATTATATTATAGTAAATATTGGCGTAAATAAACATTTTATTAATAGTTGTAATATATGAAAATGTGCAGAAGATATAGACGATTAAAACGGTGTTTAACAATCGCTTATCGAGACAATATTTAATTAATTAGTAATTAAAATATGTTTATTATGTGTATTATTCAAGGAATAGACTTGACAATAAATACAGTGGTAAACGGAACTAATAGAGTCACGCAAGTGAACTTAGAACATTTTTAATAAC

>f12041e094b5b103f404ed619583bfb0

CACACTTATGTCTTTAGAAACACAAAACATTTCTCCAGAATGCATTTCAACCATATCGGTCATTTTCAACTAGCAGTGCCTAGATAGTTGTGGAAGATTTTGAATATGTCTTGTCCATAGAGTTCAGTTTGTTCATTATTGATTTTGTTATGTATTATGTTTCGACTTTCAAGTAAATTC

>e1340ae28d8fe01fd799bf74902e288a

TATTAGTGTTCCACTATTCTATATATCACTAAAAGTATTTTGCCGCTCTTCATTAACATGTTTCTATATTTGACCAATATAAAATTTGTTGCAATCATTGCCTTTAATTTTAGAGATGCCATGTTTAGTGTATCATTGCAATTGTATATATTGTTTATATTTATATATAATCATATACAAACATATAATAGAATCATTTGACCCCAAAGGAATATGG

>78c4926c525615cd3018bc8589c67d90

TACGGAGGGAGCTAGCGTTGTTCGGAATTACTGGGCGTAAAGCGCACGTAGGCGGCTTTTCAAGTCAGGGGTGAAATCCCGGGGCTCAACCCCGGAACTGCCCTTGAAACTGGAAGGCTAGAATCCTGGAGAGGCGAGTGGAATTCCGAGTGTAGAGGTGAAATTCGTAGATATTCGGAAGAACACCAGTGGCGAAGGCGACTCGCTGGACAGGTATTGACGCTGAGGTGCGAAAGCGTGGGGAGCAAACAGG

>381c42c17c409862dddef8e488abd5d2

CACACGTATGTCTTTGGAAAGACAACACGTTTTTGCAGAATGCATTTCAACCTTCTCGGTCATTTTCAACTGGCAGTGCCTTGTAGATAATTGTGGAAGATTTTGAATGTTTCTTGTCCATAGAGTTCAGTTTGTTCATTAATGATTTTGTTGTGTAGTTTTTAACGTAATATATTTAAACTTTCAAAAGAAGATTCGTTTTATTTCCTTTTTTGCCTGTTGTA

>68cc6fa0ccaee8a782bfa53317908bec

TACGTAGGGTGCAAGCGTTGTCCGGAATTATTGGGCGTAAAGAGCTCGTAGGCGGTTGGTCGCGTCGGCTGTGAAAACCCGGAGCTCAACTCCGGGCCTGCAGTCGATACGGGCCGACTTGAGTGTTGCAGGGGAGACTGGAATTCCTGGTGTAGCGGTGAAATGCGCAGATATCAGGAGGAACACCGGTGGCGAAGGCGGGTCTATGGGCAACAACTGACGCTGAGGAGCGAAAGCGTGGGGAGCGAACAGG

>229ae1eb7937d254af4045bedc4f298f

TTCCGGCTGCAATAGCGTATAGTAATGTTGTTGGATCTGTGTGCCACGCTGTCCATGGGACGCCCTGCCGGCGGCGGTACGTGGACGTTTACACCCGAAGCACGCCGCGGTGTCTCCGAGTGTTGAGGTAGGCTCCGGTACGTTTACTTTGAACAGATTAGAGAGCTCAAATCAGGTCGTATTTAGGCCTGAATCCTGTTTGCGTGGAATACGATCTCGGTTTTGTTTTGTTGGGTTTTTTTTGGA

>92f5b8179cb20917b7f6b8815709d42a

GGACTGTATGTTGGTTACAGAAAAAAAGTTTATGGAAAATGTTGATATTGTGTATGTTTTGTAATTTTGTGTGGGGATTATTTATCTATATGGTTGACCGTTTTAATTCAATAAAAAGACTGTGGATTTTGAAATAATACAAAGAATGTTTGGTATTATATTTAAACAGAATAATACAAAGATAAAGTTCATATTGAAAGGATATATTTCTATTTTTATAAGCTGTTTTATCTTATGAATAAATGATAGTATGAATTTCTTATTTACTTTAA

>172199480656b6cd7cef26f91aea0cd8

TACGGAGGGTGCAAGCGTTAATCTGATTTACTTTTTTTCTCTCGCCTTCCTTTTTGCTTTTTATTCTTTTTTTCACTTCCCGGGCTTAACCTGGGAACTGCATTTGAAACTGGCAGGCTTGAGTCTCGTAGAGGGGGGTAGAATTCCAGGTGTAGCGGTGAAATGCGTAGAGATCTGGAGGAATACCGGTGGCGAAGGCGGCCCCCTGGACGAAGACTGACGCTCAGGTGCGAAAGCGTGGGGAGCAAACAGG

>9b4411329c4c0f52a5b91b88caab2ad5

CGCCGCGTCGAGGAGAGGACTTGCGTCACCCCGGTTGTCATTCATCAAGGCGTGCTCCGCATCATCGCCGACCACTCCGGTTTGTCAAACCGGTAGCGTCGAGAAGACGGCAGTCATCGGCACTGCCGAAACGTCTGATTCGTTTGAAACGACTGGGTCGCGGTAGAATAGCCCGGAAGAATTTAGTGATATTGTAATTGACTCCGGCCGTGAAAGCCTATGTGTTTT

>9c637cefe71e306475d089d480bf4a59

TACGTAGAAGACAAGTGTTATTCATCTTTAACAGGTTTAAAGGGTACCTAGACTGGAAATCAAGCCGTAGAAGGAACTAATTTTCTAGAGTTTTATAAGGGAGAATCGAACGATCTGATGAGCGATATAATGCTTTGATACAGATAGGACGGATAGCAGCGAAAGCGTTTTTCTATGTAATAACTGACGTTGAGGAACGAAGCCTTGGGTAGCAATAAGG

>dab7c82e81717f4835a02765ddbd8fab

CACGTAGGGTGCGAGCGTTGTCCGGAATTATTGGGCGTAAAGAGCTCGTAGGCGGTGTGTCACGTCGGCCGTGAAAACCTGCAGCTTAACTGTGGGCGTGCGGTCGATACGGGCATCACTGGAGTTCGGCAGGGGAGACTGGAATTCCTGGTGTAGCGGTGAAATGCGCAGATATCAGGAGGAGCACCGGTGGCGAAGGCGGGTCTCTGGGCCGATACTGACGCTGAGGAGCGAAAGCGTGGGGAGCGAACAGG

>87213336a3a8fb6c289cc9ab03d03245

GTGCCAGCCGCCGCGGTAAACCAAGATGTGTGGATGCAGGATACCGTTGCCGCCCCAAGCCCGGTAGAGGAGGATTGGTGAGGCTAGCAACTGGTCAACCGTGAAAAGACTTTATGCTTAAGGGACCAACGAATTGCCTCCAGGATTAGATACCCTTGTAGTCCGGCTGACTGACTTAACGTCCATCTCGTA

>1704c0d42b0d3d802e89050bc7ff129a

GACCAGATGATTTCCACATTCATATGTTGCAGCATACTGCATCACATACTCTTGACACATTATTAATAATGATTTTCTGGCATCTTTCCAACAGCGTGGAAAACAGCAACGATTATTCCCATTCTTAAAGAGAACAAAAACCCTTCATTATTAGATACCCTGGTAGTCCGGCT

>2e508b688f2d707335e70aa9eacc9042

TACGTAGGGCGCGAGCGTTGTCCGGAATTATTGGGCGTAAAGAGCTTGTAGGCGGTTTGTCGCGTCTGCTGTGAAAGGCCGGGGCTTAACTCCGTGTATTGCAGTGGGTACGGGCAGACTAGAGTGCAGTAGGGGAGACTGGAGTTCCTGGTGTAGCGGTGGAATGCGCAGATATCAGGAGGAACACCGATGGCGAAGGCAGGTCTCTGGGCTGTAACTGACGCTGAGAAGCGAAAGCATGGGGAGCGAACAGG

>aeea908759b486535bd509bf693481bf

GACGGGGGGGGCAAGTGTTCTTCGGAATGACTGGGCGTAAAGGGCACGTAGGCGGTGAATCGGGTTGAAAGTGAAAGTCGCCAAAAAGGGGCGGAATGCTCTCGAAACCAATTCACTTGAGTGAGACAGAGGAGAGTGGAATTTCGTGTGTAGGGGTGAAATCCGTAGATCTACGAAGGAACGCCAAAAGCGAAGGCAGCTCTCTGGGTCCCTACCGACGCTGGGGTGCGAAAGCATGGGGAGCGAACAGG

>55554b6e69f91db4a60a2dd56e1d2573

CCGAATCCAATATTGTAGGAAACAAGCAATTACTCAGGTGACAACAAAATGCAGGTTGGGGAAATTGGATTCTATCAGGCTTTTGAAAAAGAAAGCCTATGGACGGCTGGGAATAAGGCTATTACCAAATATGCAGTCCCGAACCGAGGCGGCAGCAATTAGAAACCCTGGTA

>a9b264a5065bf1ca90fc08547706b48c

TAATTGTGTGCCAGCAGCCGCGGTAAAACAGCCGGTTGAACCATCGTTAAAATCGCCTATGCTGTTTTTTTGACCTAAATGCTAGGTCCAAGGCTGTTTTGGGGACAAGCCCCCTTCAGCCCAATGGGGTTGGTTGGTGAATGGATTAGAAACCCCTGTAGTCCGGCTGACTGACTTGCGTCAAATCTCGTATGCCGTC

>dd348be0141270fea7bf7ff4fa4f6713

GGTAATTGTGTGCCAGCCGCCGCGGTAATTGACTGGATGAGACCCACCGCAGTTGCAGCATGTTGCAGAGGTCTCCGGTGCGACCAGATAGTAGCCTCGGTGCTCTCCTAACGTCTCTGTGGAAAACGGCCAACGTCGTCATGGATTAGAAACCCGGGTAGTCCGGCTGACTGACTACTGTGTAATCTCGTATGCCGTCTT

>0dc8009686a8b884053c62162814d531

CCGTTGTCGATTGCAGTGAAAGAATGTAAACAGGATTATCGCTGGTAATTACCTGTACACTTGGCTATGGTATGACAATATTTCATGTAGTTGTACAGTATACTGTCGGTATAACGGACCGACGAACGACCTGCTCCATGCTGCGCCGTGCCGTGTCGTGTAATTAGATACCC

>7ab35d4b4751d6858b89cb4df4379db7

TACGTAGGATCCAAGCGCTATCCGGAATTACTGGGCGTAAAGCGTGCGCAGACGGTTAAGTAGGTCTTATGCGAAATCCGGTGGCTCAACCACCTGGACTGTATAGGAAACCCCTTGACTTGAGGTAGGTAGAGGTACATGGAATTTCTGGTGTAGGAGTGACATCCGTAGATATCAGAAGGAACACCAATGGCGAAGGCAGTGTACTGGGCCTTACCTGACGTTCAGGCACGAAAGCGTGGGTAGAAAACAGG

>87d1101e0bc651e8f204055a8bf8c62d

TACGGAGGGGGCTAGCGTTGTTCGGAATTACTGGGCGTAAAGCGCACGTAGGCGGTTTTTCAAGTCAGAGGTGAAAGCCCGGGGCTCAACTCCGGAATTGCCTTTGAAACTGGGAGACTTGAACACGGGAGAGGTGAGTGGAATTCCGAGTGTAGAGGTGAAATTCGTAGATATTCGGAGGAACACCAGTGGCGAAGGCGGCTCACTGGACCGTAGTTGACGCTGAGGTGCGAAAGCGTGGGGAGCAAACAGG

>275fd29c87501b014955cecb4a5a7355

TACGGAGGATGCGAGCGTTATCCGGATTTATTGGGTTTAAAGGGTGCGCAGGCGGCGGATCAAGTCAGCGGTCAAATTGCGGGGCTCAACCCCGTACTGCCGTTGAAACTGGTCCGCTTGAGTTGGAGAGAGGCAGGCGGAATGCGCGGTGTAGCGGTGAAATGCATAGATATCGCGCAGAACTCCGATTGCGAAGGCAGCCTGCCGGCTCCATACTGACGCTGAGGCACGAAAGCGTGGGGATCGAACAGG

>b83de4a6fc3377ae24261881a4bf2fb9

TACGTAGGTCCCGAGCGTTGTCCGGATTTATTGGGCGTAAAGCGAGCGCAGGTGGTTTATTAAGTCTGGTGTAAAAGGCAGTGGCTCAACCATTGTATGCACTGGAAACTGGTAGACTTGAGTGCAGGAGAGGAGAGTGAAATTCCATGTGTAGCGGTGAAATGCGTAGATATATGGAGGAACACCGGTGGCGAAAGCGGCTCTCTGGCCTGTAACTGACACTGAGGCTCGAAAGCGTGGGGAGCAAACAGG

>8c23b2fa1097d6c80817bd9d1bba7553

CTTTTGTTGTAGCGGTGTCCTTGCCGACTTGAGCTGCGCTTTGTAATGGGACTAAATGTGTTGTGTTGGTATCAGTTTTGTACCAAACAAAGGCACAATGATGAACATTCGGGGAAAATCTACAAATATACAAACATAAAACACAAAACGTATTGATACCAGAACAAATCCAGACC

>aed20045b55af9c7297006e269502585

TTCTTACTGCAAACGTATTGTCACACACATATGTTTTGTAGTAATTATTGTGAGCAGCATCCTCTATGTTGTAAATGTAATAAGGAAAGCTTAAAACAATGATAATAATCTGTTTATAGTAATTAATCTTCATCATCTTCGCCATTCCAGGATTAGATACCCGTGTAGTCCGG

>d38d0bf01ac5b1592486b5f4a839ef9c

TACGGAGAGGGCTAGCGTTATTCGGAATTATTGGGCGTAAAGGGCGCATAGGCTGGTTAGTAAGTTAAAAGTGAAATCCCGAGGCTTAACCTTGGAACTGCTTTTAAAACTGCTAACCTAGAGATTGAAAGAGGATAGAGGAATTCCTAGTGTAGAGGTGAAATTCGTAAATATTAGGAGGAACACCAGTGGCGAAGGCGTCTATCTGGTTCAAATCTGACGCTGAGGCGCGAAGGCGTGGGGAGCAAACAGG

>b417d53604b81b3c17146214a98807bf

CATTGAACTATCGTGAGAATGGCACGCCGCCAAAGGGAATTATATTATAGTAAATATTGGCGTAAATAAACATTTTATTAATAGTTGTAATATATGAAAATGTGCAAAAGATAGAGACGATTAAAACGGTGTTTAACAATCGCTTATCGAGACAATATTTAATTAATTAGGAAATAAAATCAGTGTATTATTTGTATTTAACAAGGAATAAACGTGAAAATAAATACAGTGGTAAACGGAACTAATAGAGTCACGCAAGTGAACTTAGAACAATTTTAAGAAC

>aeeada11dd354b75f335e7bb9032fcab

TGAAGTCATTCAGAGTCTTCGCGCGAGCTGCCGGGTAATTAACTTTAATTAAGACAGTTGTGTATACATATACTATACACGACGAAGACGGCATTGTACCGACTGCGCCAGTATTTATGTGTACTGCTCTCCGCATACTGCTTGGTTTCCCCCTTTGTCACTTCCCTATATAAATCATCATCATA

>04228b730cd94d675de7d2d9559d8130

CGCGGTAAGTGAAACAGTTTATTGTCTATAACGTTTAAACGGTTGGTCGGATTTTACATTTCTTTACATGTTTTGTACGACTTGATTAAGAGAATGTACCTGCTTAATTGCATTCTTATCCATTAAGTAGTTAAAGAGAAACCCATTAGATACCCGAGTAGTCCGGCTGACTGACTCTACG

>4f078b6dbcc3d5423a5cb2003e577957

TACGTAGGGTGCGAGCGTTGTCCGGAATTATTGGGCGTAAAGAGCTTGTAGGCGGTTTGTCGCGTCTGCTGTGAAAATCCGGGGCTCAACCCCGGACTTGCAGTGGGTACGGGCAGGCTAGAGTGTGGTAGGGGAGACTGGAATTCCTGGTGTAGCGGTGAAATGCGCAGATATCAGGAGGAACACCGATGGCGAAGGCAGGTCTCTGGGCCACTACTGACGCTGAGAAGCGAAAGCATGGGGAGCGAACAGG

>dfa466a1b85a97043c137d51f338fc99

CCGAATCCAATATTGTAGGAAACAAGCAATTACTCAGGTGACAACAAAATGCAGGTTGCGGAAATTGGATTCTATCAGGCTTTTGAAAAAGAAAGCCTATGGACGGCTGGGAATAAGGCTATTACCAAATATGCAGTCCCGAACCGAGGCGGCAGCAATTAGATACCCGAGTA

>c4c97224bbfb4532b90197342caea27a

TACGTAGGGTGCAAGCGTTGTCCGGATTTACTGGGCGTAAAGAGCTCGTAGGTGGTGTGTCGCGTCGTCTGTGAAATTCTGGGGCTTAACTCCGGGCGTGCAGGCGATACGGGCACGACTAGAGTGCTGTAGGGGTAACTGGAATTCCTGGTGTAGCGGTGAAATGCGCAGATATCAGGAGGAACACCGATGGCGAAGGCAGGTTACTGGGCAGTTACTGACGCTGAGGAGCGAAAGCATGGGGAGCGAACAGG

>3a910ddaf3d810289241c7e4f0ddcc01

TACGAACTGCGCGAACGTTATTCGGAATCACTGGGCTTAAAGGGTGCGTAGGCGGCGATACAAGTCAGATGTGAAAGCCAACAGCTCAACTGTTGAATTGCGTTTGAAACTGTATTGCTTGAGTGTGACAGGGGTATTCGGAACTTCCAGTGGAGCGGTGAAATGTGTTGATATTGGAAGGAACACCGGTGGCGAAAGCGGAATACTGGGTCATTGCTGACGCTGAGGCACGAAAGCCAGGGTAGCGAACGGG

>adbda4915419b8f4cd4a001a04cdfc02

TACGAAGGGGGCTAGCGTTGCTCGGAATCACTGGGCGTAAAGGGTGCGTAGGCGGGTTTTTAAGTCAGAGGTGAAATCCTGGAGCTCAACTCCAGAACTGCCTTTGATACTGAAAGTCTTGAGTATGGGAGAGGTGAGTGGAACTGCGAGTGTAGAGGTGAAATTCGTAGATATTCGCAAGAACACCAGTGGCGAAGGCGGCTCACTGGCCCATAACTGACGCTGAGGCACGAAAGCGTGGGGAGCAAACAGG

>4111460021ea102b956cb6a82fd0652a

TTTCGAGAAGAGATACGGTTACGATTAAGGGAGTTCGGGAGATAATAAGGACTTCAATCCATAAGCTGACCAACGACATAATAACAAAACAGCTGTTCTGGTGTGAACATGTGCAACCGATGAGCAGAGAACGATTATCTGCACTAGGCACCAAGAGGAAGATGGAAACAAGGGAGACTCTGGAGTAGTTGAAAGAATGGAACTATCACAGAAATGGGAGAAAGAGGATTAAAAGAAGCCCGGTGGATGGATAGGTATAGCTGTATAGCTGGAG

>fed9de4b808b1718d9bc0520ba7fe5bd

TACAAGTAAGACTAGTGTTATTCATCTTAATTAGGTTTAAAGGGTACCTAGACAGTATTTCTAGCCTCCAAAGGGAACAGATTTACTAGAGTTTTATGTGAGAGGAAAATATTAGAACCATTGGAGTAGTGATAAAATGTTTTGATACTAATGGGACGGATAACGGCGAAGGCAAACCTCTATGTAATAACTGACGTTGAGGGACGAAGGCTTGGGTAGCGGATAGG

>e9a094aaa4c9dc3128b74402cce12d56

TACGAAGGGGGCTAGCGTTGCTCGGAATCACTGGGCGTAAAGGGTGCGTAGGCGGGGTTTTAAGTCAGGGGTGAAATCCTGGAGCTCAACTCCAGAACTGCCTTTGATACTGAAGATCTTGAGTCCGGGAGAGGTGAGTGGAACTGCGAGTGTAGAGGTGAAATTCGTAGATATTCGCAAGAACACCAGTGGCGAAGGCGGCTCACTGGCCCGGTACTGACGCTGAGGCACGAAAGCGTGGGGAGCAAACAGG

>72863bd03ba6e221cf3d2c55cbe003df

GCGTCGACGAAGAAATTGTCATGGCCATACAAAGTCACTACGCATGGAACAAACGTCGTGGTATAAATAGACAGTGCCTTTGATAACGAGTGTAAGTTGATGAGGAAAGATAACAACTTCTATAAGCGTACACAAAGAGAGACGGACACCGGAGCAGCGTTATCTCAACAGATAAGAATGTAAGTGAGCGACAGCGACAACCTTTTACAGATAAGGAACGACTTTCAGCTCAATGTCATGAGAAAGCTGTACCTGTACTACAATAAAACATACGCGCATAACA

>519ab5377b5f1f1b51dca6eb35cb9fe3

CATTGAACTATCGTGAGAACAGCACGCCGCCAAAGGGAATTATATTATAGTAAATATTAGCGTAAATAAACATTTTATTAATAGTTGTAATATATGAAAATGTGCAGAAGATAGAGACGATTAAACCAGTATTTAACAATCGCTTATCGAGACAATATTTAATTAATTAGGAAATAAAATAAGTGTATTATTTGTATTTAACGAGGAATAAACGTGAAAATAAATACAGTGGTAAACGGAACTAATAGAGTCACGCAAGTGAACGTAGAACATTTTTAATAAC

>f91bf2b2dd398e738e6661f830d137fd

TACGTAGAAGACAAGTGTTATTCATCTTTAACAGGTTTAAAGGGTACCTAGACGGGAAATCAAGCCAGTGTAAAACAGAGGTACTAATTTCCTAGAGTTTTATATGTCAAGTTTGAACTATCTGAGGAGAGATATAATGCAGAGATACAGATAGGACGGATCGCAGCGTAGGCGTACTTGTGTGTAAAAACTGACGTTGAGGGACGAAGCCCGGGGGAGCGATAAGG

>fc21fe7f3dd3b74e196bc4d66364e4df

TACAAGGAAGACTAGTGTTATTCATCTTAATTAGGTTTAAAGGGTACCTAGACAGTATTTCTAGCCTCAAAAGGGAACAGACTTACTAGAGTTTTATGGGAGAGGAAAATATTAGAACCATTGGAGTAGAGATAAAATGTTTTGATACTAATGGGACGGATAGCGGCGAAGGCAAACCTCTATGTAATAACTGACGTTGAGGGACGAAGGCTTGGGGAGCGAATAGG

>58eb1cb7caf7c6a369cc4557ba01bfa3

TCAAACGAAAGCACAACAAAAAGATAGCCAAACTAATACCACCCCGATCACCAGCAATAATATCCACACATACGTTTTACCCCAGAATGGTTAACCTCACCAACATAACATTCACTAACGATCAGAAACAACTTTTAAACAAAGGGATAAATCACAACCTACACTACACGCAGAACAATAACACCATCAAGAAACAGGG

>e9dd85eda1dd9d7eee1a78b467b3ed05

CATTGAACTATCGTGAGAATGGCACGCCGCCAAAGGGAATTATATTATAGTAAATATTGGCGTAAATAAACATTTTATTAATAGTTGTAATATATGATAATGTGCGGAAGATTAAGACGATTAAAACGGTGTTTAACAATCGCTTATCGAGACACTATTAAAGTAATTAGGAATTAAAATATGTGCAGTATGTGTATTAAACAAGGAATAGATTTGAAAATAAATACAGTGTTAAACGGAACTAATAGAGTCACGCAAGTGAACTTAGAACATTTTTAATAAC

>1fa19a198819b145e52bb266e4e7840b

TACGGAGGGGGCTAGCGTTGTTCGGAATTACTGGGCGTAAAGCGCACGTAGGCGGCTTTGTAAGTCAGGGGTGAAAGCCTGGAGCTCAACTCCAGAACTGCCTTTGAGACTGCATCGCTTGAATCCGGGAGAGGTAAGTGGAATTCCGAGTGTAGAGGTGAAATTCGTAGATATTCGGAAGAACACCAGTGGCGAAGGCGGCTTACTGGACCGGGATTGACGCTGAGGTGCGAAAGCGTGGGGAGCAAACAGG

>18a6944b1854f36ded3503485a88f1e1

TACAGAGGGTGCAAGCGTTAATCGGAATTACTGGGCGTAAAGCGCGCGTAGGCGGCTAGGTAAGATGGGTGTGAAATCCCCGGGCTCAACCTGGGAACTGCATCCATAACTGCCTGGCTAGAGTACAGTAGAGGGTGGTGGAATTTCCTGTGTAGCGGTGAAATGCGTAGATATAGGAAGGAACACCAGTGGCGAAGGCGACCACCTGGACTGATACTGACGCTGAGGTGCGAAAGCGTGGGGAGCAAACAGG

>277621ee76bb8a052742a0a96fbc196c

TACGTAGGGAGCGAGCGTTATCCGGAATTATTGGGCGTAAAGGGTGCGTAGATGGTATATTAAGTCTTTTGTAAAAATGCTAGGCTCAACCTAGTAGGGCAAAAGATACTAATGAACTAGAGTATGACAGGGGCAAGTGGAACTACATGTGTAGCGGTAAAATGCGTAAATATATGTAAGAACACCGGTGGCGAAGGCGGCTTGCTGGGTCGATACTGACATTGAGGCACGAAAGCGTGGGGAGCAAACAGG

>96f24de310f1ffc9280db41bf424af77

TACGGAGGGTCCGAGCGTTAATCGGAATTACTGGGCGTAAAGCGTGCGCAGGCGGTTTGTTAAGCGAGATGTGAAAGCCCTGGGCTCAACCTAGGAATAGCATTTCGAACTGGCGAACTAGAGTCTTGTAGAGGGGGGTAGAATTCCAGGTGTAGCGGTGAAATGCGTAGAGATCTGGAGGAATACCGGTGGCGAAGGCGGCCTCCTGGACAAAGACTGACGCTCATGCACGAAAGCGTGGGGAGCAAACAGG

>a149a9cd7a3d79c9f9c4139a406fce84

CATTGAACTATCGTGAGAAAGTCAAACCGCCAAAGGGAATTATATTATAGTAAATATTGGCGTAAATAAACATTTTATTAATAGTTGTAATATATGAAAATGTGCAGAAGATAAAGACGATTAAAACGGTGTTTAACAATCGCTTATCGAGACACTATTAAAGTAATTAGGAATTAAAATATGTTTATTATGTGTATTATTCAAGGAATAAACTTGAAAATAAATACAGTGTTAAACGGAACTAATAGAGTCACGCAAGTGAACTTAGAACATTTTTAATAAC

>7b1bcc3b0474df155947b5b4c455e60f

CCGCCGCGGTAAGTGCTCAAAGAACATCAATCTTTCACTTCGTTCTCACTTTCTCTTTTTACTGCCTCCTCTTCTGACCCTTTTCCACCATACCCCTTCCTCTCCTTTTCGTCATCCCTTTTCGGTCCATTTGCACCATAATATATTAGATACCCCAGTAGTCCGGCTGACTGACTTGCGTCAAA

>f5137df359eeb2c3c5547f69c8dba4a6

TACGTAGGTGGCAAGCGTTGTCCGGAATTATTGGGCGTAAAGCGCGCGCAGGCGGTCTCTTAAGTCTGATGTGAAAGCCCCCGGCTCAACCGGGGAGGGTCATTGGAAACTGGGAGACTTGAGTACAGAAGAGGAGAGTGGAATTCCACGTGTAGCGGTGAAATGCGTAGATATGTGGAGGAACACCAGTGGCGAAGGCGGCTCTCTGGTCTGTAGCTGACGCTGAGGCGCGAAAGCATGGGGAGCAAACAGG

>e079de18e6a1c0e118dd5be770f64d23

GCGTCACTATGGTAATTGTGTGCCAGCCGCCGCGGTAATGAGTGTGATATAATTTTACAATACACTATAACATTAGATACCACAAGCCACACACAGAAGTCGGGGGCGCAAACACAATCATATAATTTTACCATACGCTATAACATTAGAAACCCCGGTAGTCCGGCTGACTGACTTAACGTCCATCTCGTATGCCGTCTTCTGCTTGAAA

>8f3f9bc9fa2485146065c71ba28172bc

TACGTGAGAGACTAGTGTTATTCATCTTAATTGGGTTTAAATTTTATTTTTCCATTCTATATAACTTTTATAATGCTAATACTTGACTAGAGTTTTAAGTAAGAGGGAAGTACTTAAGGAGTAAGAGATGAAATATCTGTGATACCAAAGGGACTCCGTAAAGGCGAAGGCATCCCTTTATCTAAAAACTAACGTTGAAGGACGAAGGCTTAGATAACAAATAGG

>222b1a92aa22e4643dc45c0275b2dc1d

TCAAACGAAAGTACAACAAAAAATATAGTCAAACTAATACCACCCCGATCACCTGCAATAACACCCACACATACGTTTTACCCAAGAATAGTTAACCTCACCTACATAACAGTCACTAACGATCAGAAACAACTTTTAAAGAAAGGGATAAGTCACAACCTACACTACACGCAGAACAATAACACCATCAAGAACACTAT

>0cdf8af09d7d87e06f0938d31fe056ea

CTTAAAAAAATAATCCTTTTGTTGGGTCATTCTTCCATAACATATCTTCTCCTAAATGATTTCATTTTGCTCTCGGTGTGCCGTATAACTTAGAGAATATGTGGGGAGAGAGAGGGAGAGGGAGAAAGAGGGGAAGAGGGAAGTCGCAAATATTTGTCCAAACAATAAGTCTACTACGCTTGTGAAGTAGGATCTTTAAACTCAATCTGTTA

>b090c91991a307cd63bc4b92027f280d

TATGGTAATTGTGTGCCAGCAGCCGCGGTAAGAGAAAAACTATCGCCAACAATTAGCGCATTAAGTAGTAGTAACGGCGAAGGTAACAGCTGTACGCTACTGATAAGCCAGCAGCCGACGCTACTTCTCTTCTGTCTACGATTTATTAGAAACCCTGGTAGTCCGGCTGACTGACTTAACGTCCATCTCGTATGCCGTCTTCTG

>b04697499f2136757a9be58f422a41d8

TGAAATTTCGGAAAACAACAAAAGTGTTAAACTTTTTTTATAACGTTTTATGGTGTCTCCATACAATTCAGTGCAGACTTTTCCCCCGTTATTTACTAAAAGAATTGTCCCTCTTCATTAGCTGTCTGGTCTCCGCCTGCTTTGACTTCCAGAGAAGCACTAAGGGATTAGAA

>bf965766c9cf5efb5559f5f79c8ded80

TACGTAGGGTGCGAGCGTTGTCCGGAATTACTGGGCGTAAAGAGCTCGTAGGCGGTTTGTCGCGTCGTTCGTGAAAACTTGGGGCTTAACTCCAAGCGTGCGGGCGATACGGGCAGACTTGAGTACTACAGGGGAGACTGGAATTCCTGGTGTAGCGGTGAAATGCGCAGATATCAGGAGGAACACCGGTGGCGAAGGCGGGTCTCTGGGTAGTAACTGACGCTGAGGAGCGAAAGCGTGGGTAGCGAACAGG

>4da5327fec081b0dc3364b0f2d3f9fb4

AAATGGCTACTTTCACGGGTGCAGAACGGGCTAACTGTGTGTTAAGGTTTCATGACACAAACTCTGCAACAACAGTTCAGCATAATTTTTGCACCGAGTGTGGTAAAGATCCTCTTACTAGACCTAAAATTTACACTTGACCTCAGAACTTCGTTGAGAGTGGTTGTTCGATTCAGC

>4f58476407fc1e45695d402eb122ee75

AACGGAGGGTGCAAGCGTTATCCGGATTTATTGGGTTTAAAGGGTCCGTAGGCTGATGTGTAAGTCAGTGGTGAAATCTCACAGCTTAACTGTGAAACTGCCATTGATACTGATAAACTAGAGTGAGGTTGGAGTAACTGGAATGTGTGGTGGAGCGGTGAAATGCATAGAGATCACACAGAACACCAATCGCGAAGGCATGTTACTAAACATAGACTGACACTGAGGCACGAAAGCATGGGTAGCAAACAGG

>52de2693c0c79d4df79fb6f83279b3dc

AACAGAGGATACAAGCGTTATCTTGATTTTTTTTTTTTCCCTTTTTCTTCTTTTTTTTTGTAAGTTTTTCTTTTCTTCTTAAAGCTTAATTGTAAAAGTGCTATTGATACTGATAAACTAGAGTGAGGTTGGAGTAACTGGAATGTGTGGTGGAGCGGTGAAATGCATAGAGATCACACAGAACACCAATCGCGAAGGCATGTTACTAAACATAGACTGACACTGAGGCACGAAAGCATGGGTAGCAAACAGG

>391eae28df8772c9dea40f7997961935

TACGAAGGGGGCTAGCGTTGCTCGGAATCACTGGGCGTAAAGGGTGCGTAGGCGGGTCTTTAAGTCAGGGGGGAAATCCTGGAGCTCAACTCCAGAACTGCCTTTGATACTGAGGATCTTGAGTCCGGAAGAGGTGAGTGGAACTGCGAGTGTAGAGGTGAAATTCGTAGATATTCGCAAGAACACCAGTGGCGAAGGCGGCTCACTGGTCCGGTACTGACGCTGAGGCACGAAAGCGTGGGGAGCAAACAGG

>b2b2f15bba0fc1530aadaed8b2108177

TAAATTACGTTCCATACATTGTTACTGTTGTTATCTACTAAGTCCTACCAGAACACACAGTTTGCTCGTAAGTTTCCGTGATCTCACTGCACATATATTGCAGACAACAAAGTAAAGCTTCTGCGGAATAAGTGTGTCGGTTTTTCATTGTTTCTTTGAGACGCGTTTTAAAACCGTACATAAACTAGTTAATTATTAGTAGATTGTTATGGAAAAAGTTAGTACAATTAGTCGCATGGCACCA

>86bf6ba2593a73582d40e0f60762cff8

TACGGAGGGTGCGAGCGTTAATCGGAATTACTGGGCGTAAAGAGTGCGTAGGTGGTTTTTTAAGTTATCTGTGAAATCCCTGGGCTTAACCTGGGGCGGTCAGATAAGACTGGAAGACTCGAGTATGGGAGAGGGTAGTGGAATTTCCGGTGTAGCGGTGAAATGCGTAGAGATCGGAAGGAACACCAGTGGCGAAGGCGGCTACCTGGCCTAATACTGACACTGAGGCACGAAAGCGTGGGGAGCAAACAGG

>562e39c196168023c4a82410464c3dc3

TACGTAGGGGGCAAGCGTTATCCGGATTTACTGGGTGTAAAGGGAGCGGAGACGGTAATGCAAGTCTGGAGTGAAAACCCGGGGCTCAACCCCGGGACTGCTTTGGAAACTGTATAACTGGAGTGCCGGAGAGGTAAGCGGAATTCCTAGTGTAGCGGTGAAATGCGTAGATATTAGGAGGAACACCAGTGGCGAAGGCGGCTTACTGGACGGTAACTGACGTTGAGGCTCGAAAGCGTGGGGAGCGAACAGG

>44b2e42929961628c958eeb578777d0f

TACGTAGGGCGCAAGCGTTATCCTGATTTATTTTTTTTCTCTCGCTTTTCTTTTTGTTTTTTCTTCTTTTTTTCACTTCCGGGGCTCAACTCCGGTTCTGCAGTGGGTACGGGCAGGCTTGAGTGATGTAGGGGAGACTGGAATTCCTGGTGTAGCGGTGAAATGCGCAGATATCAGGAGGAACACCGATGGCGAAGGCAGGTCTCTGGGCATTAACTGACGCTGAGGAGCGAAAGCATGGGGAGCGAACAGG

>0a83be255a37e5c7d5de2f0308382293

TACGTAGGGCGCAAGCGTTATCCGGAATTATTGGGCGTAAATTTCTTTTTTTCGTGTTGTCGCGTCTTCTGTGAAAGTCCGGGGCTCAACTCCGGTTCTGCAGTGGGTACGGGCAGGCTTGAGTGATGTAGGGGAGACTGGAATTCCTGGTGTAGCGGTGAAATGCGCAGATATCAGGAGGAACACCGATGGCGAAGGCAGGTCTCTGGGCATTAACTGACGCTGAGGAGCGAAAGCATGGGGAGCGAACAGG

>2eeb3e652497dd74a24523e5bbcd84bb

GATCTACTATGGTAATTGTGTGCCAGCCGCCGCGGTAATGAGTGTGATATAATTTTACAATACACTATAACATTAGATACCACAAGCCACACACAGAAGTCGGGGGCGCAAACACAATCATATAATTTTACCATACGCTATAACATTAGATACCCTGGTAGTCCGGCTGACTGACTCTACGACCATCTCGTATGCCGTCTTCTGCTTGAAA

>cca5bae97dd3ceb6947022a02fbe297a

TGTGTGCCAGCAGCCGCGGTAACACAATAGCACACAAAAACAGATTGGCCATCACACTGCGGGAAAGAGAGAAGGAAGGGAATACATACTGGGTACCTTGCACTATTATTTGTATATCACTGGCTGTGACCGAAACTGCTTGCCATTAGATACCCTTGTAGTCCGGCTGACTGACTCTACGACCATCTCGTATGC

>f5c08c7f3a8166207f5411521e4cfe39

CGCAATAAATAAGGCCTTAACAAAGACGCCGACTTTGCGGAGGAAAGTGCAGCGCGTTTTGTGGTTTGCTAAGTTTGAATCTGCGGCCCTTGTCCAACGTGAGTATCGTCGGGTGTTCAATGAAGATCCACCAAACAACACGAACATTTATCTTTGTGATAAACAGCTTAAGGTGGCCGGAAGCCCATATGACAGAAGCGTTCTGGTAGTCATCTGTTAGCGACGAACCAGTTGAAGAC

>c847ff74d3642b425ea24f37186670e4

CATTGAACTATCGTGAGAAAGTCAAACCGCCAAAGGGAATTATATTATCGTAAATATTGGCGTAAATAAACATTTTATTAATAGTTGTAATATATGAAAATGTGCAGAAGATAAAGACGATTAAAACGGTGTTTAACAATCGCTTATCGAGACAATATTTAATTAATTAGGAATTAAAATATGTGTATTATTTGTATTTAACAAGGAATAAACTTGAAAATAAATACAGTGGTGAACGGAACTAATAGAGTCACGCAAGTGAACTTAGAACATTTTAATAAC

>51ff72c031b482c46933a3226572db43

CTGCTCTCAGATTTTATATACTGTGACTGTTGGGTAGTATCAACTTTACTTCTAGAACTCCTTGCCTTTCATAGTAGCAATTACTTTCTCGATCTTTCCACCAATCTTCTCGAACATATTGATTAGCATCTTCCCATCTTTTTCTTTAATGACAAGAATTTGCATTAGTTTTGTAGCTCAAACTTCCTTTCTTATATACTGGCCTTGAATCTG

>8860aa0c2bc8cb6f34f79e5acf51adc0

CACACTTATGTCTTCAGAAAGACAAAATATTTATCCAGGATGCATTTCAACCATGTCGGTCATTTTCAACTGGCATTGCCTTGTAGATATTCGTGAACGATTTTGAATGTGTCTCGTCAATAGAGTTCAGTTTGTTCATTAATGATTTTGTTATGTAGTTTGTAACGTAATATATTTAGGCTATCAAGTAAATTCATTTTGTTTCCTTTTTGCCGCCTGTG

>8d703908c09c7cb0da2a4398dd74f9f8

TACGTAGGGGGCTAGCGTTATCCGGATTTACTGGGCGTAAAGGGTGCGTAGGTGGTTTCTTAAGTCAGGAGTGAAAGGCTACGGCTCAACCGTAGTAAGCTCTTGAAACTGGGAAACTTGAGTGCAGGAGAGGAAAGTGGAATTCCTAGTGTAGCGGTGAAATGCGTAGATATTAGGAGGAACACCAGTAGCGAAGGCGGCTTTCTGGACTGTAACTGACACTGAGGCACGAAAGCGTGGGGAGCAAACAGG

>66e048f71fb63f55f37a325716ab3b00

TACGAAGGGGGCTAGCGTTACTCGGAATTACTGGGCGTAAAGGGCGCGTAGGCGGCGTTTCAAGTTGGACGTGAAATTCCTGGGCTCAACCTGGGGACTGCGTTCAAGACTGTGATGCTAGAGGATGGAAGAGGGTCGTGGAATTCCCAGTGTAGAGGTGAAATTCGTAGATATTGGGAAGAACACCGGTGGCGAAGGCGGCGACCTGGTCCATTACTGACGCTGAGGCGCGACAGCGTGGGGAGCAAACAGG

>23baf50b5def740fbe457bbc16cdfed1

TACGGTGGGAGCTAGTGTTATTGTGATGACTGGGCGTAGGGTACGTAGGTGGTGAAGGAGAATTTGAATAAAATCCTGGGGAATCCCCCTTGTCCGTTCAATTTATAAATTCACTAGAATCTGAGAGGGACGCTGGTATGTTTATGGAGAGGTTAAATATGAGATATAAATAGGACCGACAGTGGTGAAAGCAAGTTTCTTGTCCAGTATTGAGCTAAGGTACGAAGGCAGGGGAGCAAAAGGG

>f216b614d579c750b276f4a62739b5fc

TACGTAGGGCGCGAGCGTTGTCCGGAATTATTGGGCGTAAAGGGCTTGTAGGCGGTTGGTTGCGTCTGCCGTGAAATTCTCTGGCTTAACTGGGGGCGTGCGGTGGGTACGGGCTGACTTGAGTGCGGTAGGGGAGACTGGAACTCCTGGTGTAGCGGTGGAATGCGCAGATATCAGGAAGAACACCGGTGGCGAAGGCGGGTCTCTGGGCCGTTACTGACGCTGAGGAGCGAAAGCGTGGGGAGCGAACAGG

>ba304b6f0c4b6002b3191e123d1daf7b

TACAGAGGTCTCAAGCGTTGTTCGGAATCACTGGGCGTAAAGGGTGCGTAGGCGGCGCGGAAAGTCAGGGGTGAAATCCCGGAGCTCAACTCCGGAACTGCCTTTGATACTCCCGCGCTTGAGTGCTGGAGAGGAGTCCGGAATTCACGGTGGAGCAGTGAAATGCGTGGATATCGTGAGGAACACTAGTGGCGAAGGCGGGACTCTGGACAGCTACTGACGCTGAGGCACGAAGGCCAGGGGAGCAAACGGG

>8b48687bbc8102872caa6b3bd1a611fa

TACGAAGGGGGCTAGCGTTGCTCGGAATCACTGGGCGTAAAGGGTGCGTAGGCGGGGCTTTAAGTCAGGGGGGAAATCCTGGAGCTCAACTCCAGAACTGCCTTTGATACTGAGGATCTTGAGTCCGGAAGAGGTGAGTGGAACTGCGAGTGTAGAGGTGAAATTCGTAGATATTCGCAAGAACACCAGTGGCGAAGGCGGCTCACTGGTCCGGTACTGACGCTGAGGCACGAAAGCGTGGGGAGCAAACAGG

>0dc31aef8aecd9dab97222c5ce967686

GGACCGCATGTTAGTTAAAGAAAATGTAGATATTGTATATATTTTGTAATTTTGTTTTGTAGATTATTTATCCATATGGTTGTCCGTTTTAATGCAATAAAAAGACTGTGGATTTCTTGAAATAATACAAAGAATGTTTTGGTATTATAGTTAAACAGAATAATACAAAGATAAAGTTTATATTGAAAGGATATTTCTATTTTTATAAGCTGTTTTATCTTATGAATAAATGATAGTATGAATTTCTTATTTACTTTAA

>3763058f4fa951f03cbe5ff2d24ad565

CATTGTACTATCGTGAGAAAGTCAAACCGCCAAAGGGAATTATATTATAGTAAATATTGGCGTAAATAAACATTTTATTAATAGTTGTAATATATGAAAATGTGCAGAAGATAAAGACGATTAAAACGGTGTTTAACAATCGCTTATCGAGACACTATTAAAGTAATTAGGAATTAAAATATGTTTATTATGTGTATTATTCAAGGAATAAACTTGAATAATAAATACAGTGTTAAACGGAACTAATAGAGTCACGCAAGTGAACTTAGAACATTTTTAACAAC

>583d97b06fa0b6b27c277d1c9dedf44f

TGTGTTAGGTCTGAAGCCGGTGACAGACAAAGTAGCGTGTTTTTATCTATTTATACCGTCTGTTGTCCAAGATAACGCCGCCGTTCCCGCAACCCTGCGTCTGTACTTATCCTCGGTCTTCGTTTTCCCACAGTGCTCACTCATAATACGAATTTTGTTACACAGACTGCCTGCGCCTCGCGATTAATATAATCCTTTCAGTTATAACGTTAACTAGGCATTAAGATGCTGTCCAGGCAAGCCGCAGTTCTTTGTACTGAATGAGTGATGAAACAGTAAATCATTTGACC

>d50990124d8a3fb95912a52ea6acc8bd

CACGTAGGGGGCAAGCGTTGTCCGGAATTATTGGGCGTAAAGGGCGCGCAGGCGGCCTTGTAAGTCTGTCGTTTAAACTCGGAGCTCAACTTCGAGTCGCGATGGAAACTGCAAAGCTTGAGTGCAGAAGAGGAAAGCGGAATTCCACGTGTAGCGGTGAAATGCGTAGAGATGTGGAGGAACACCAGTGGCGAAGGCGACTTTCTGGGCTGTAACTGACGCTGAGGCGCGAAAGCGTGGGGAGCAAACAGG

>19506dda46c7f451541d32c82dbe4a3b

CATGGATGATACAGGAGCTCATTGGGCTGAGACCCAGACAGCGCACACACGATTCGGGTGCTAGACACCCCGCACAGCATCAGTCAGTCAGTAATATTGGTTATCGTAGGGCTATTGATCTACTAAATAACTGCGACAAAACTATTTACCCATCTATTCACTTTCTACTAAAGATTGCTGTCACCTTGTCATATTCTGTTCCAACTGCTGATAGAACATTATCATTGCTCAGATCACTTACAACTTAG

>0ca8ac4e6d4efcc3649a2cf1f76a50f3

TACGTGAGAGACTAGTGTTATTCTTCTTAATTTTTTTTCTCTTGTCTCTCTCTTTTCCCTATATTTTTTTTCTTGCTTATACTTGACTAGAGTTTTAAGTAAGAGGGAAGTACTTAAGGAGTAAGAGATGAAATATCTGTGATACCAAAGGGACTCCGTAAAGGCGAAGGCATCCCTTTATCTAAAAACTAACGTTGAAGGACGAAGGCTTAGATAACAAATAGG

>fd9d6b3b6e91b427f130a704a3919d2e

TACGGAGGGGGCTAGCGTTGTTCGGAATTACTGGGCGTAAAGCGCACGTAGGCGGACCAGAAAGTTGGGGGTGAAATCCCGGGGCTCAACCTCGGAACTGCCTTCAAAACTATTGGTCTTGAGTTCGAGAGAGGTGAGTGGAATTCCGAGTGTAGAGGTGAAATTCGTAGATATTCGGAGGAACACCAGTGGCGAAGGCGGCTCACTGGCTCGATACTGACGCTGAGGTGCGAAAGCGTGGGGAGCAAACAGG

>7ddc5733045569f7ddf62c0f68e2e0ff

CACACTTATGTCTTCCGAAATACAAACCATTTCTCCAGGATGCATTTCAACCATGTCGGTCATTTTCAACTGTCAGTGCCTTGTAGATATTTGTGGAAGATTTTGAATGTGTCTTGTCCATGGAGTTCAGTTTGTTCATTAATGATTTTGTTGTGTAGTTTGTAACGTAATATATTTAGGGTTTTAAGTAAATTCATTTTATTTCCTTTTTGCCTGTTGTA

>666130c9464a8490f4e0e448c55eb75b

TACGTAGGGTGCGAGCGTTAATCGGAATTACTGGGCGTAAAGCGTGCGCAGGCGGTCTTGTAAGACAGGTGTGAAATCCCCGGGCTTAACCTGGGAATTGCATTTGTGACTGCAAGGCTGGAGTGCGGCAGAGGGGGATGGAATTCCGCGTGTAGCAGTGAAATGCGTAGATATGCGGAGGAACACCGATGGCGAAGGCAATCCCCTGGGCCTGCACTGACGCTCATGCACGAAAGCGTGGGGAGCAAACAGG

>7026af09173a18ebb44c2be7d5aab071

TACGTAGGTGGCAAGCGTTGTCCGGAATTATTGGGCGTAAAGGGCGTGTAGGTGGATTCTTAAGTCGTGTGTCTAAGTGCGGTGCTCAACACCGTATGGGCGCAGGAAACTGGGAATCTTGAGTGTAGGAGAGGAAAGTGGAATTCCCAGTGTAGCGGTGAAATGCGTAGATATTGGGAGGAACACCAGTGGCGAAGGCGACTTTCTGGACTGTGTCTGACACTGAGGCGCGAAAGCCAGGGGAGCGAACGGG

>87af7c8fae89e78ec4c03848162c2851

TAAATATATAGTTGTATAACTTTAAAATATACTTTTTTTATGTCGGAATAATCGAAGAACGTGATGCCTAAGGGACGGAGAGCGCGAGTTGTGAGAGAGAAACGGCTAGCGGGGACCGAAACAAAAAGCAGATTGTACATTTGAATGGGAAAGGAGGGGGAATATTGAGAGGAGAGAAGGAATAGTCAGACGTATAGGAGCATA

>11d91c9f9f3a844ec15126dcea3f6320

TACGGAGGATCCAAGCGTTATCCGGAATCATTGGGTTTAAAGGGTCCGTAGGCGGTCAGATAAGTCAGTGGTGAAAGCCCATCGCTCAACGGTGGAACGGCCATTGATACTGTCTGACTTGAATTATTAGGAAGTAACTAGAATATGTAGTGTAGCGGTGAAATGCTTAGAGATTACATGGAATACCAATTGCGAAGGCAGGTTACTACTAATATATTGACGCTGATGGGCGAAAGCGTGGGTAGCGAACAGG

>499ee9b7a947ddf3d3441a4b49f0d887

GGACCGCATGTTAGTTAAAGAAAATGTAGATATTGTATATATTTTGTAATTTTGTTTTGTAGATTATTTATCCATATGGTTGTCCGTTTTAATGCAATAAAAAGACTGTGGATTTTTTGAAATAATACAAAGAATGTTTTGGTATTATATTTAAACAGAATAATACAAAGATAAAGTTTATATTGAAAGGATATTTCTATTTTTATAAGTTGTCTTATCTTATGAATAAATGATAGTATGAATTTCGTATTTACTTTAA

>ab3385faf556f7226dbb96444d224bcf

CACACTTATGTCTTCAGAAAGACAAAATGTTTCTCCAGAATGCATTTAAACCGTCTCGGTCATTTTCAGCTGGCAGTGCCTTGTAGATAATTGTGGAAGATTTTGAATGTTACTTGTCCATAGAGATCAGTTTGTTCATTAATGAGTTTGTTGTGTAGTTTGTAAATACAAACACAAGAACAAAAGAATTCTAAATTAACAAACGCAGTACAGATCGCATACAATATACCTTCAAACCAACCACC

>ea518d14b1ffe4d6a0323cc50b9422a3

TACGTAGGGGGCGAGCGTTGTCCGGAATCACTGGGCGTAAAGAGCGTGTAGGCGGCCCGGTAAGTCTGCTGTGAAAACCCGGGGCTCAACCCCGGGCGTGCAGTGGAAACTGCCGGGCTAGAGGGCGGCAGAGGCGAGTGGAATTCCCGGTGTAGCGGTGAAATGCGCAGATATCGGGAGGAACACCAGTAGCGAAGGCGGCTCGCTGGGCCGCCCCTGACGCTGAGACGCGAAAGCTAGGGGAGCGAACAGG

>7ce0cc8adb123cfd96881aa1f08cb56a

TACGTAGGTGGCAAGCGTTGTCCGGAATTATTGGGCGTAAAGCGCGCGCAGGCGGTCTTTTAAGTCTGATGTGAAAGCCCCCGGCTCAACCGGGGAGGGTCATTGGAAACTGGGAGACTTGAGTACAGAAGAGGAGAGTGGAATTCCACGTGTAGCGGTGAAATGCGTAGAGATGTGGAGGAACACCAGTGGCGAAGGCGACTCTCTGGTCTGTAACTGACGCTGAGGCGCGAAAGCGTGGGGAGCAAACAGG

>755dad9e7be24267e9132d56531a1b57

TACGGAGGGTGCGAGCGTTAATCGGAATTACTGGGCGTAAAGGGTGCGTAGGTGGTTAGATAAGTTAGCTGTGAAATTCCTGGGCTTAACCTGGGCTGGTCAGCTAATACTGTTTAACTTGAGTATAAGAGAGGGTAGTGGAATTTCCAGTGTAGCGGTGAAATGCGTAGAGATTGGAAGGAATACCAGTGGCGAAGGCGGCTACCTGGCTTAATACTGACACTGAGGCACGAAAGCGTGGGGAGCAAACAGG

>8e7e7d8f61f075bac312ea998cad777f

GGTGACAACGAAGAAAATGATCTACATGGCAGTGGAGAAATTGAGGAGTGTCATATAATTTTATCATTCACTATGTAACATTAGATGCCATTTGCCTCCGTGGCGAGGAGGTTAGGTTCGTCATATACTTTTACTATACATTATAACATTAGAAACCCCAGTAGTCCGGCTGA

>3b0301ca780f4e93e4dad00190683b05

CATTTTACCATAGTGTCTGCCTCCACGGGCACGTTTATGGCTTTGCCAGTTATCTTTTAAGACTGTTTCTATTCTGAGACGACGGATCTGCATACGTGGAAATCCGGATGAAATCAGCCTCTCAGTGATACGATCGAGCGGCAGATGGGTCGGCTCTTCCGGATACTTATAACTATTACTTTGGCTCGGCGGAGGCACTTTACCCTCATTAAGCAAGCGGCGGTATGTGGAGCACA

>3e3da8cc750c82d35e57b3011577bfc9

CTAGATACTTGACTCTCCATTTGACACAACAAGCACCTAAGGCAACAAAATTAAAATTAAATATCTAAGTCGGTAATAAGTCGAATTATATCGTTCGCTGCAGAGACGGGAATTAAGGCAGAAAAAAACAAAACTAACAACAGAGGTCAGACATGAAGGTATCAAAAATAAATATGCGGAAAGGCCGTACGGCATCGGGAAAGGAAAGAAAATACCAGAGTAACATGAAAAGTGGAAAAC

>27d5adde9e355487f712a810780ef794

TACGGAGGGTGCAAGCGTTATCCGGATTTATTGGGTTTAAAGGGTCCGTAGGCGGATCTGTAAGTCAGTGGTGAAATCTCACAGCTTAACTGTGAAACTGCCATTGATACTGCAGGTCTTGAGTGTTGTTGAAGTAGCTGGAATAAGTAGTGTAGCGGTGAAATGCATAGGTATTACTTAGAACACCAATTGCGAAGGCAGGTTACTAAGCAACAACTGACGCTGATGGACGAAAGCGTGGGGAGCGAACAGG

>5405b06c31242b072918eb4cb0a8a7dc

TACGGAGGGTCCGAGCGTTAATCGGAATTACTGGGCGTAAAGCGTGCGCAGGCGGTTTGTTAAGCGAGATGTGAAAGCCCTGGGCTCAACCTAGGAATAGCATTTCGAACTGGCGAACTAGAGTCTTGTAGAGGGGGGTAGAATTCCAGGTGTAGCGGTGAAATGCGTAGAGATCTGGAGGAATACCGGTGGCGAAGGCGGCCCCTTGGATAAAGACTGACGCTCATGCACGAAAGCGTGGGGAGCAAACAGG

>c8a43a055a287585d77f609c2eee8bf4

CCAATGTATATGAACATAAAATATGTGTTGCAGATGAAAAAAAATTCATACGTAAAACATACCCAATCCCATTACATTACCAGGAACGGGTTGACACGGAAATTCAACAAATGTTGGATCAAGGGGTCATAGAAAGATCCAGCAGTAACTTCTTGAATCCTGTAGTCATTGTAAAGAAAAAAAATAATGATATTCGCTTATGTCTAGAC

>1878ba52599b9f103a4f07e3b58e1f62

TACGAAGGGGGCTAGCGTTGCTCGGAATTACTGGGCGTAAAGGGCGCGTAGGCGGACAGTTAAGTTGGGGGTGAAAGCCCGGGGCTCAACCTCGGAATTGCCTTCAATACTGGCTGTCTTGAGTACGGGAGAGGTGAGTGGAACTCCGAGTGTAGAGGTGAAATTCGTAGATATTCGGAAGAACACCAGTGGCGAAGGCGACTCACTGGCCCGTTACTGACGTTGAGGCGCGAAAGCGTGGGGAGCAAACAGG

>8900e97ab218451c72ed4ca9c56b3d6e

TACAGAGGGTGCAAGCGTTAATCGGATTTGCTGGGCGTAAAGCGCGCGTAGGTGGCCAATTAAGTCAAATGTGAAATCCCCGAGCTTAACTTGGGAATTGCATTCGATACTGGTTGGCTAGAGTATGGGAGAGGATGGTAGAATTCCAGGTGTAGCGGTGAAATGTGTAGAGATCTGGAGGAATACCGATGGCGAAGGCAGCCATCTGGCCCAATACTGACACTGAGGTGCGAAAGCATGGGGAGCAAACAGG

>9b953cc0d3d5898cd10f42c157400bee

TACTTATTTTTCAATCTTTAATCTTAATTACTTTTCTTAAATCTCACTCATTCTTTCTTTTAATTCATATTTTAAATCCCCGGGCTTAACCTGGGAACTGCATTTGAAACTGGCAGGCTTGAGTCTCGTAGAGGGGGGTAGAATTCCAGGTGTAGCGGTGAAATGCGTAGAGATCTGGAGGAATACCGGTGGCGAAGGCGGCCCCCTGGACGAAGACTGACGCTCAGGTGCGAAAGCGTGGGGAGCAAACAGG

>fab142bb9658237beace44c17161df24

TACGGAGGGTGCAAGCGTTAATTTGATTTTTTTTTCTTCCTTCTCCCTCCTTCTTTCTTGTAAGTTTTCTTTTTCTTCCCCGGGCTTAATTTGGGAACTGCATTTGAAACTGGCAGGCTTGAGTCTCGTAGAGGGGGGTAGAATTCCAGGTGTAGCGGTGAAATGCGTAGAGATCTGGAGGAATACCGGTGGCGAAGGCGGCCCCCTGGACGAAGACTGACGCTCAGGTGCGAAAGCGTGGGGAGCAAACAGG

>9e045bdd7325c8ff7732c56d2423380a

TGTTCGTCCTACTACCATTCTGGTGTCACGTTATTTCATTTTTAAATAAATTCGGTGCTGCCTGCGCAGTAATATAATACGTATTGAAACAGCAACTTGTCAACAGTGATTAGATTCTTGCAGCACTTAATACACGTTAGTTTTAACTACGTCATGTGTGTCACGGATTAGAT

>b8a58b8ced331b6fe1ce4bf0fb7d6a1b

CGGGTGTAGTCGTACGTTACTGTAATTACCGTACTTCCTGACTATGCATGTGCATTCCTATGCGTCCTCGCTAACTAACTTTCCCGCCCGTCTCACAGTTTCACTTTATGTATCCACACGCATTATTTCCCTACCAACAAACTGCAGAAGAATTAATCTACCGGTGCGGTCCAGAGGTATTAACAACGTACATAATTTGCTGCAGAGTTCACTTATTTCATTTGGGAAAGAGTAACAAGGACTTTGAAAAGCTAGATCAATCAATGCTGCAGTTAAGACAGATTGGTGAGAAGGCATTAGAAT

>de5f46bdead7fe8f158847ec95aa25d4

AGATGATTGGAATTTCAGTCAAATGCGTCTCACTAACGTGGCCGAACCGCAAAAACCTACTGACGTTGTCAACAAAGCGTACCTCGTCAATCACAGGCCTATACGCGGTAAAGACGAATGGGGTTTTGAAAAACGGCGACTCGTCGACGTAGCCGAACCTTTGCAGGACACCGACTGTGTAAACCTTAAGTATGTCGAAACGCTGGTTGTACGCCCGCTAAGCGG

>c003e5e804916a713efa066d1a1be0d6

TACGTAAGGTGCGAGCGTTGTCCGGATTTATTGGGCGTAAAGGGCTCGTAGGTGGTTGACCGCGTCGGAAGTGTAATCTTGGGGCTTAACCCTGAGCGTGCTTTCGATACGGGTTGACTTGAGGAAGGTAGGGGAGAATGGAATTCCTGGTGGAGCGGTGGAATGCGCAGATATCAGGAGGAACACCAGTGGCGAAGGCGGTTCTCTGGGCCTTTCCTGACGCTGAGGAGCGAAAGCGTGGGGAGCGAACAGG

>8e91141a4215ddd9eda34c455192ba57

TACCAGCACCTCGAGTGGTCAGGACGTTTATTGGGCCTAAAGCATCCGTAGCCGGCTCTGCAAGTCTTCGGTCAAATCCACCTGCTCAACAGATGGGCTGCTGGAGATACTACAGAGCTAGGGAGTGGGAGAGGCAGACGGTATTCAGTGGGTAGGGGTAAAATCCTCTGATCCATTGAGGACTACCAGTGGCGAAGGCGGTCTGCCAGAACATGTTCGACGGTGAGGGATGAAAGCTGGGGGAGCAAACCGG

>7eb7da9bb105781f9c5502de21d786b8

TACGTAGGGTGCAAGCGTTAATCGGAATTACTGGGCGTAAAGCGTGCGCAGGCGGTTCGGAAAGAAAGATGTGAAATCCCAGGGCTCAACCTTGGAACTGCATTTTTAACTACCGGACTAGAGTATGTCAGAGGGAGGTGGAATTCCACGTGTAGCAGTGAAATGCGTAGATATGTGGAGGAACACCGATGGCGAAGGCAGCCTCCTGGGATAATACTGACGCTGAGGAGCGAAAGCATGGGGAGCGAACAGG

>7ee68f0d40595f6c482b6bf7ba4f9fd4

TACGTGAGAGACTAGTGTTATTCTTCTTAATTTTTTTTCTCTTGTCTCTCTCTTTTCCCTTTATTTTTTTTCTTGCTTATACTTGACTAGAGTTTTAAGTAAGAGGGAAGTACTTAAGGAGTAAGAGATGAAATATCTGTGATACCAAAGGGACTCCGTAAAGGCGAAGGCATCCCTTTATCTAAAAACTAACGTTGAAGGACGAAGGCTTAGATAACAAATAGG

>10534496225de098d3d09861b9fb22a9

TACGTAGGGCGCAAGCGTTGTCCGGAATTATTGGGCGTAAAGAGCTCGTAGGCGGCTTGTCACGTCGGATGTGAAAGCCCGGGGCTTAACCCCGGGTCTGCATTCGATACGGGCTAGCTAGGGTGTGGTAGGGGAGATCGGAATTCCTGGTGTAGCGGTGAAATGCGCAGATATCAGGAGGAACACCGGTGGCGAAGGCGGGACTCTGGGCTGTAACTGACACTGAGGAGCGAAAGCATGGGGAGCGAACAGG

>3976ccaed252009a1221c6ad872552f3

TCCTTCTTTCTCCCTCGTTCTCCGGCCTTCTTGGGCGTCCCGAGCTCGTCGGCGGTTTGTCGCGTCTGCTGTGCAAGTCCGGGGCTCAACTCCGGTTCTGCAGTGGGTACGGGCAGGCTTGAGTGATGTAGGGGAGACTGGAATTCCTGGTGTAGCGGTGAAATGCGCAGATATCAGGAGGAACACCGATGGCGAAGGCAGGTCTCTGGGCATTAACTGACGCTGAGGAGCGAAAGCATGGGGAGCGAACAGG

>bafc44ca7b359c7fa4c049da838f023e

TACGAGAGCCTCGAGCGTTATCCGGAATTATTGGGCGTAAAGGGTGCGTAGGTTGTTCTATTAGTCTTTTGTCAAAGCCCCGAGCTTAACTTGGGAATTGCGAAAGAAACGGTAGAACTTGAAAGTGCGAGGGGTATACGGAACTCATGGTGTAGGGGTGAAATCCGTTGATATCATGGGGAACACCAAAAGCGAAGGCAGTATACTGGCGCATATTTGACACTGAAGCACGAAAGCGTGGGTAGCGAATGGG

>7b5d1c332d144b4f3e17d09bfbdd2a71

TACGTAGGGCGCAAGCGTTGTCCGGAATTATTGGGCGTAAAGAGCTCGTAGGCGGTTTGTCGCGTCTGCTGTGAAAGACCGGGGCTCAACTCCGGTTCTGCAGTGGGTACGGGCAGACTAGAGTGCAGTAGGGGAGACTGGAATTCCTGGTGTAGCGGTGAAATGCGCAGATATCAGGAGGAACACCGATGGCGAAGGCAGGTCTCTGGGCTGTAACTGACGCTGAGGAGCGAAAGCATGGGGAGCGAACAGG

>26bc2aea7d5e9d3f6d294c0c9a9f6643

TACGTAGGGGTCGAGCGTTGTCCGGAGTTACTGGGCGTAAAGCGCGTGCAGGTGGCTCATTACGCCCGGCGTGAAAGCCCCCGGCTCAACCGGGGAGGGTCGTCGGGGACGGGTGAGCTTGAGGGTCGCAGGGGCTGGTAGAATTCCCGGTGTAGTGGTGAAATGCGTAGAGATCGGGAGGAATACCCGTGGCGAAGGCGGCCAGCTGGGCGACACCTGACACTGAGACGCGAAGGCGTGGGGAGCGAACGGG

>78df0e2af0cea56a50ec2bdac80c7d78

AATTGTGTGCCAGCAGCCGCGGTAACACAATAGCACACAAAAACAGATTGGGCATCACACTGCGAAGAGAGAAGGAAGGGAATGCGTACTGGCAACCTTGCACTATTATTTGTATATCACTGGCTGTGACTGAAACTGCTTGCCATTAGATACCCTAGTAGTCCGGCTGACTGACTGAGACTTAATCTCGTATGCCGT

>fa7cd0fd2fc6980c93ca06462b5fa817

TACAGAGACTGCAAGCGTTATTCGGATTCACTGGGCGTAAAGGGTGCGCAGGTGGCCTTGTGTGTCAGATGTGAAAGCCTGGAGCTTAACTCCAGAATTGCGTCTGAAACTACAGGGCTAGAGCATTGGAGAGGGTAGCGGAGTTCATGGTGTAGCAGTGAAATGCGTAGATATCATGAGGAACACCAGAGGCGAAGGCGGCTACCTGGACAATAGCTGACGCTCAGGCACGAAAGCGTGGGGAGCAAAAGGG

>0274e932c8f1da4dd172ee9389787bd5

TACGGAGGGTGCAAGCGTTGTTCGGAATTATTGGGCGTAAAGCGCATGCAGGCGGCTGTTCAAGTCCGATGTGAAAGCCCGGGGCTCAACCCCGGAAGTGCATTGGAGACTGGACAGCTTGAGTACGGGAGAGGGAGGCAGAATTCCGAGTGTGGGGGTGAAATCCGTAGATATTCGGAGGAATACCGGTGGCGAAGGCGGCCTCCTGGACCGATACTGACGCTGAGACGCGAAAGCATGGAGAGCAAACAGG

>00733853826dc6fe64ea2d61e8ab7226

TACGGAGGGTGCAAGCGTTAATCGGAATTACTGGGCGTAAAGCGCGCGTAGGCGGCTTGATAAGCCGGTTGTGAAAGCCCCGGGCTCAACCTGGGAACGGCATCCGGAACTGTCAGGCTAGAGTGCAGGAGAGGAAGGTAGAATTCCCGGTGTAGCGGTGAAATGCGTAGAGATCGGGAGGAATACCAGTGGCGAAGGCGGCCTTCTGGACTGACACTGACGCTGAGGTGCGAAAGCGTGGGTAGCAAACAGG

>b7711dabbb340b3f0961a20c018a3350

CCCCTCTTATACAATCTTTATCCTTATTTATTTTTTTTAAATTTTTCTTATTTTTTTTTTTACTTCCGTATTTAAATCTTAAAGCTTAACTTTAAAAGTGCTATTGATACTGATAAACTAGAGTGAGGTTGGAGTAACTGGAATGTGTGGTGGAGCGGTGAAATGCATAGAGATCACACAGAACACCAATCGCGAAGGCATGTTACTAAACATAGACTGACACTGAGGCACGAAAGCATGGGTAGCAAACAGG

>702ca77b6d0c5529a3830968c471a09f

CATTGAACTATCGTGAGAATGGCACGCCGCCAAAGGGAATTATATTATAGTAAATATTAGCGTAAATAAACATTTTATTAATAGTTGTAATATATGAAATATTGTGTACGGATGGTAAAAGTATATGATCATGTTACCCAACACAATTGTTGTGTCGATATCCATTAGATACC

>44517f96320c2d128504923f0015108e

TACGTAGGGCGCAAGCGTTATCCGGAATTATTGGGCGTAAAGAGCTCGTAGGCGGTTTGTCGCGTCTGCTGTGAAAGACCGGGGCTCAACTCCGGTTCTGCAGTGGGTACGGGCAGACTGGAGTGATGTAGGGGAGACTGGAATTCCTGGTGTAGCGGTGAAATGCGCAGACATCAGGAGGAACACCGATGGCGAAGGCAGGTCTCTGGGCATTAACTGACGCTGAGGAGCGAAAGCATGGGGAGCGAACAGG

>e6d261741257b7208437f001f3f88714

TAACGACTAATTTAATATTTTAAATACATTATCTTTTTTGTTAGCGACAGGTTTCCAATTATTCGTAGAATGCAAACAATCCCGCTTCAACAACGATGTTAAGTGAAAAGAGGTTCAGATTGTCATCTGAGCGCTGTAAAGAGTCGCGACGCGCTTTCGATTCCCGTTCGAGACATATTCGTCCTCTTTAAACGTAACAGTGATCGAACGTATGCGATAAAAACTGAAATAACGGATGCCAGCCATCGTCGAATTAATTTTTAAAGCGACGTTTCGTCTAT

>052ec41c51362f32cb3eb74ab5d7fd9d

CCTTCCTAGACAAAATTGAAATTTAATATAATATAAAACCATTCTTTTAACAATACAGAGAATATTCTTCCTATAAAATAAATTTGTCACGCTCTATTTGAGAAAGAAATACTTGTGTGTTAAAACAATGGATCGGAAGATTGTTGAACTCTCAAGAAATACTAAAGGAGCAACAACTGTTTCCACGTATTCGATGCACCTGCAGAAAATTAGCAA

>763045c9257760179c93010e4c00038e

AAAAAAAGCTGTCTACTACGAATTTGTATGAACATCAAATTTGTGTCACTAACGAAAACAAATTCATTCGTAAAACATACCCGGCACTATCAAGAAAGGGTCGATGTTGAAATCCAATGGATGGTGGATCAAGGGGTTATCAAACGATCCAACAGTGATTTTCTTAACCCGGTGGTAACCGTTAAGGAATATAAGGCTGTGCCTCGATATGCGCAACTCAAAAATAGTATCGTGTTGGGCTCCGAACGCTGAGTCAGTTTTTATTAAGTGTCAGGGTGTCCGCTACATGTCCCG

>531dfdcd0ed5f638ab1b553e806beecb

TGCCCGTAGGTTCCTTCGCTTCCGTACCGACGTCGTAACGACACCTACAATTGACAATTTATAAATTAAGTTAATTTATAACAGACAAAATACAAATTTTATACTTGTTTCCATGTTTGACTGCTTTTTTATTAATTTTTTTTACATTTTTTATCCATATAACATATGATATGTAGAACGCAGACTGAGTTCCACGTGGTTGTGTGTGCTGCAATCTGTGTGTTGTT

>fd03778b29f999201d5dd368e811fccb

GCAGACTAGTGAACCAATCAGCGTTCAGCAGGTTCTATGGAGTGGGGGACATGTGACTGTACACAACATTTCCTGTTGACACATGCAACTGTACTAATTAGCACACACGTTATCTGCTTCCACGAGAGGTGACTGACCTGGCCTGCCGCGTTACTCAGAACTCACAGATAAGAAATCTCAGAACATGTATGCTGCTCAGACCGGCTGCGTTGTACGATAAACTATCACATATTCTACATTACTAAGACCGGTGGTGAGGTTATGTTCATCATATACTTTTACAATACACTATAAC

>7de1a5f7faaacfdf4f957bfe234bff00

GACAGAGGGTGCAAACGTTGTTCGGAATTACTGGGCGTAAAGCGTGTGTAGGCGGCCATGTAAGTTGGATGTGAAAGCCCCGGGCTCAACCCGGGAAGTGCATTCAAAACTGCGTGGCTTGAGCACTGGAGAGGTTGGTAGAATTCTCGGTGTAGAAGTGAAATTCGTAGATATCGAGAGGAATACCGGTGGCGAAGGCGGCCAACTGGACAGATACTGACGCTGAGACACGGAAGCGTGGGGAGCAAACAGG

>c95698e82b3cb5df3f3ca456773bb5dc

CATTGAACTATCGTGAGAAAGTCAAACCGCCAAAGGGAATTATATTATAGTAAATATTAGCGTAAATAAACATTTTATTAATAGTTGTAATATATGAAAATGTGCAAAAGATAGAGACGATTAAAACGGTGTTTAACAATCGCTTATCGAGACACTATTAAAGTAATTAGGAATTAAAATATGTTTATTATGTGTATTATTCAAGGAATAGACTTGACAATAAATACAGTGTTAAACGGAACTAATAGAGTCACGCAAGTGAACGTAGAACATTTTTAATAAC

>e4f709064ed6eb3061fef9beca5df2e4

TACGGAGGGTGCAAGCGTTATCCGGATTTATTGGGTTTAAAGGGTCCGTAGGCTGATGTGTAAGTCAGTGGTGAAATCTCACAGCTTAACTGTGAAACTGCCATTGATACTGCATGTCTTGAGTGTTGTTGAAGTAGCTGGAATAAGTAGTGTAGCGGTGAAATGCATAGATATGTATTAGAACTCCGATTGCGAAGGCAGCTCACTAAGTTAGTATTGACGCTGATGGACGAAAGCGTGGGGATCAAACAGG

>7b75d608a7b8e8fb015dfd08d4892c9f

CACAGAGGATACAAGCGTTATCCTGATTTATTTTTTTTCTCTTGTTTTTCTTTTTTTTTTTTATTCTTTTTTTCACTTTTAAAGCTTAACTTTAAAAGTGCTATTGATACTGATAAACTAGAGTGAGGTTGGAGTAACTGGAATGTGTGGTGGAGCGGTGAAATGCATAGAGATCACACAGAACACCAATCGCGAAGGCATGTTACTAAACATAGACTGACACTGAGGCACGAAAGCATGGGTAGCAAACAGG

>eed8f1918faf9d824ff325dbe62c49ab

TACGAAGGGTGCAAGCGTTACTCGGAATTACTGGGCGTAAAGCGTGCGTAGGTGGTGGTTTAAGTCTGTTGTGAAAGCCCTGGGCTCAACCTGGGAATTGCAGTGGATACTGGGTCACTAGAGTGTGGTAGAGGGTAGCGGAGTTCCCGGTGTAGCAGTGAAATGCGTAGAGATCGGGAGGAACATCCGTGGCGAAGGCGGCTACCTGGACCAACACTGACACTGAGGCACGAAAGCGTGGGGAGCAAACAGG

>25b60da8f1af462e61067fc3545e8efb

TACGTATGGTGCAAGCGTTATCCGGATTTACTGGGTGTAAAGGGAGCGTAGACGGATAGGCAAGTCTGGAGTGAAAGCCCGGGGCTCAACCCCGGGACTGCTTTGGAAACTGTTTATCTAGAGTGCTGGAGAGGTAAGTGGAATTCCTAGTGTAGCGGTGAAATGCGTAGATATTAGGAGGAACACCAGTGGCGAAGGCGGCTTACTGGACAGTAACTGACGTTGAGGCTCGAAAGCGTGGGGAGCAAACAGG

>781c0d5b35318a48c6dc7311173f34f8

ATATTTTCGATTATCATTAAACCCAATAGTCGGAGTAGGATAACGCGTGATGCACTGTCTCGAGCTCAGTTTACGTACCGTACTTACGTTTTCAACTGGAATTTTATCTCTGTTTCATTGGCCTTATATGTATGTGGTCCGATTAGTCCAATAATTGCTTAACTGTTAGTAAAATGTAAAGGCAAATAAATAAGCATAGAGCAAAACAGAAACGGGTGCC

>47dc7e3b779745b76af7d543ef509179

CAACTTGTATATTTATTAAATTGAAATATTTTAAGCTTATGTCTGTATAATATTATTAAAAGTAATGTATAATATTGTAACTTTGAAAAAAGTTAATAAAAATATTGTATATTACATAGTCTATGTAAAAGAATATAGTCTATATTTTAAAGGTAAAGCATTTTCTAAATTAG

>821748663c14247994e3e400068269b5

TACGGAGGATCCAAGCGTTATCCGGAATCATTGGGTTTAAAGGGTCCGTAGGCGGTCAGATAAGTCAGTGGTGAAAGCCCATCGCTCAACGGTGGAATGGCCATTGATACTGTCTGACTTGAATTATTAGGAAGTAACTAGAATATGTAGTGTAGCGGTGAAATGCTTAGAGATTACATGGAATACCAATTGCGAAGGCAGGTTACTACTAATGGATTGACGCTGATGGACGAAAGCGTGGGTAGCGAACAGG

>e438e6fd403f3329acfa4152ac2ad355

ATTCTTCTTCTATAAGACTCGTAAATACCTTTTCATTGTCCACTATTCTATTACCGTATGTAGCGCAATGAGCGTGTGATAATTTGACAACGCTGCAATGACTTCGACCAATGCTTTTAAAGCCATCGGCGACGGGAAGTCCACCTCCCAATAACGTCCAGCGTTCAATTCTCGAAAACAGATTTGATATAG

>aa73d2c485650f5c8e5ba227daccb6f1

TACGAGGAAGACTAGTGTTATTCATCTTTATTAGGTTTAAAGGGTACCTAGACGGCATATCAAGCCCCAAAAGGGAACAGATATACTAGAGTTTTATGTGAGAGGAATATATTAGTACTATTGGTGTAGAGATGAAATTCTTTGATACTAATAGGACGGATAAGAGCAAAAGCAAACCTTTATGTAAAAACTGACGTTGAGGGACGAAGGCTTGGGTCGCGAATAGG

>e8b1a4a7f8e68e8eb8e5fb92cda00928

GACAGAGGGTGCAAACGTTGTTCGGAATTACTGGGCGTAAAGCGCGTGTAGGCGGTCTTGTAAGTCGGATGTGAAAGCCCCGGGCTCAACCCGGGAAGTGCACTCGATACTGCGAGACTTGAGTATCGGAGAGGTTGGTGGAATTCTCGGTGTAGAGGTGAAATTCGCAGATATCGAGAGGAACACCGGTGGCGAAAGCGGCCAACTGGACGAATACTGACGCTGAGACACGAAAGCGTGGGGAGCAAACAGG

>05877a04d1ae6324e898024da2fcf549

TACGTAGGGTGCAAGCGTTATCCGGAATTACTGGGCGTAAAGCGTCTGTAGGTGGTCTGTTAAGTTAAATGTTAAATCTATGGGCTCAACTCATAGGCTGCATTTAATACTGGCAGACTAGAAGATGGAAGAGGTAAGCGGAATTAGTAGTGGAGCGGTGAAATGCGTAGATATTACTAAGAACACCAATGGCGAAGGCAGCTTACTGGGCCATTCTTGACACTGAGAGACGAAAGCGTGGGGAGCGAACGGA

>7a806fea5aa994dfb55d62b78b6f6817

TACGGAGGATCCAAGCGTTATCCGGAATCATTGGGTTTAAAGGGTCCGTAGGTGGTTTAATAAGTCAGTGGTGAAATCTGGTCGCTCAACAATCAAACGGCCATTGATACTGTTAGACTTGAATTATTTGGAAGTAACTAGAATATGTAGTGTAGCGGTGAAATGCTTAGATATTACATGGAATACCAATTGCGAAGGCAGGTTACTACAAGTGGATTGACGCTGATGGACGAAAGCGTGGGGAGCGAACAGG

>d82322bdabca0b497dfbb3702b38e5e1

CCACAAGAAACGCTTTTCATTTTAATTGGACGTGTATGATCCACGTGTCATCAATGGCTACTAACATAGTTAACATTGTTTGTTACTCAAATTGAGGGATAGGTGGAAAGAATTGTTAAAAACATGAGGATAAACAGATGAACGAGGCACTGACGTAGGGGATAACTAGAAATTATTTTTTGCTCACTAATTCCTTTCTCGCTTGTTGCATACCAAAACAGCACTACTTCTTATAAAACTGATTAAAGGCATCACAGCATAGGTTAGCGTCTGGCAGTATTAGTCAGAATTAAACGAATGCTG

>aa1364df685911526dd52edf98af8aaf

TACGAAGGGTGCGAGCGTTAATCGGAATTACTGGGCGTAAAGCGCGCGTAGGTGGTTTGTTAAGTTGGAAGTGAAAGCCCCGGGCTCAACCTGGGAATTGCTTTCAAAACTGACAGGCTAGAGTACGGTAGAGGGTAGTGGAATTTCCTGTGTAGCGGTGAAATGCGTAGATATAGGAAGGAACATCAGTGGCGAAGGCGACTACCTGGACTGATACTGACACTAAGGTGCGAAAGCGTGGGGAGCAAACAGG

>9786924c4c66c920c7442ce735a32621

TACGTAGGTGGCAAGCGTTGTCCGGATTTATTGGGCGTAAAGCGAGCGCAGGCGGTTTCTTAAGTCTGATGTGAAAGCCTTCGGCTCAACCGAAGAAGTGCATCGGAAACTGGGAAACTTGAGTGCAGAAGAGGACAGTGGAACTCCATGTGTAGCGGTGAAATGCGTAGATATATGGAAGAACACCAGTGGCGAAGGCGGCTGCCTGGTCTGTAACTGACGCTGAGGCTCGAAAGCATGGGTAGCAAGCAGG

>9c15ad7015086d8cb403fe21c745aeb5

CAAATTTATGTCTTCAGAAAGACAAAATATTTTTCCAGGGTCCATTTCAACCACGTCGGTCATTTTCAACTAGCAGTGCCTTGTAGATATTTGTGAAAGATTTTGAATGTGTCTTGTCCATAGAGTTCAGTTTGTTCATTAATGATTTTTTTATGTAGTTTGTAACGTAATATATTTAGGCTTTCAAGTAAATTGATTTTGTTTCCTTTTTCCGGTTGTG

>f2b1ed9e1cd02bf78c3475ff73803ecd

CCCCTCTTCTCCCCTCGTTCTCCGGCTTTCTTGGGTTTCCCGGGTGCGTCGGTGGTTTTTTCCGTCCGTCGTGCAATCTTAAAGCTTAACTTTAAAAGTGCTATTGATACTGATAAACTAGAGTGAGGTTGGAGTAACTGGAATGTGTGGTGGAGCGGTGAAATGCATAGAGATCACACAGAACACCAATCGCGAAGGCATGTTACTAAACATAGACTGACACTGAGGCACGAAAGCATGGGTAGCAAACAGG

>8fb8136baae6d6775b67a3792f4f35a8

GACGTAGGATGCAAGCGTTGTCCGGATTTATTGGGCGTAAAGAGTTCGTAGGCGGCAAATCATGTCTAATGTTAAATCATAGAGCTTAACTCTATTCCTGCATTGGAAACTGGTTTGCTAGAGCAAGGTAGGGGTCAGAAGAATTCCTGGTGTAGCGGTGAAATGCGTAGATATCAGGAGGAACACCGGTGGCGAAAGCGTCTGACTGGGCCTTTGCTGACGCTGAGGAACGAAAGCTAGGGGAGCGAAAGGG

>3ae8d6aa5ef1218e8b89421a0f5a94fc

ATGACAAAATCATTGAACATGTTCAACATATTATTCAAAGTGACAGAAGAAAATCTAACAACCTCTGATGCGTTTCTCTTGTATATGTATATTAGCCAGATCTTCAGATTCTAATGGTCTTTACTCAACAGATAAAAACTACATCAAACTATAATTGTAGTTACAATATTTACAATGTAAGTTGTAAATCAATTTTTAAATACTTAAAATTGTAACTAGCAGAAAAATGGAGAAACTTATAACCAACCCTAATAATTAGCAAACAATATTCCTACATAGCTATA

>5166ae2c0767d287fc13b852ccf07def

TACGGAGGGGGCTAGCGTTGTTCGGAATTACTGGGCGTAAAGCGCACGTAGGCGGACTGGAAAGTTGGGGGTGAAATCCCGGGGCTCAACCTCGGAATTGCCTTCAAAACTATCAGTCTGGAGTTCGAGAGAGGTGAGTGGAATTCCGAGTGTAGAGGTGAAATTCGTAGATATTCGGAGGAACACCAGTGGCGAAGGCGTCTCACTGGCTCGATACTGACGCTGAGGTGCGAAAGCGTGGGGAGCAAACAGG

>87fe0d730ebe9c0ed0d1a1ff2b96fa17

TACAGAGGGTGCAAGCGTTAATCGGAATTACTGGGCGTAAAGCGCGCGTAGGTGGTTCGTTAAGTTGGATGTGAAAGCCCCGGGCTCAACCTGGGAACTGCATCCAAAACTGGCGAGCTAGAGTATGGTAGAGGGTGGTGGAATTTCCTGTGTAGCGGTGAAATGCGTAGATATAGGAAGGAACACCAGTGGCGAAGGCGGCCCCCTGGACGAAGACTGACGCTCAGGTGCGAAAGCGTGGGGGGCAAACAGG

>11b51f2746108e0498a6b529e1a0498c

TACGGAGGGTGCAAGCGTTAATCGGAATTACTGGGCGTAAATTTCATTTTTTCGTGCTGTTAAGTCATATGTGAAATCCCCGGGCTTAACCTGGGAACTGCATTTGAAACTGGCAGGCTTGAGTCTCGTAGAGGGGGGTAGAATTCCAGGTGTAGCGGTGAAATGCGTAGAGATCTGGAGGAATACCGGTGGCGAAGGCGGCCCCCTGGACGAAGACTGACGCTCAGGTGCGAAAGCGTGGGGAGCAAACAGG

>d8304285f886b70234bc5f2b014e68a6

CTTAAAACAATAATCCTTTTGTTGGGCCATTCTTCCATAACATATCTTCTCCTAAATGATTTCATTTTGCTCTCGGTGTGCCGTATAACTTAGAGAATATGTGGGGAGAGAGAGTGAGAGGGAGAAAGAGGGGAAGAGGGAAGTCGCAAATATTTGTTCAAACAATAAGTCTACTATGCTTGTAAAGAAGGATCTTTAAACTCAATCTGTTA

>b6f13359b672099d072a194f203aa329

GACCAGATGATTTCCACACTCATATGTTGCAACATGCCCCATCTCAATACTCTTCGCACATTACTAAAAATGTTTAATAAAATATGGTTTTCTGGCATCTTTCAAACAGCATAGAAAACAGTAACGATTATTCCCATTCTTGAAGAAAAACCGCCTTCATTATTAGATACCCG

>a0d7d3791ecdf9e729bb7e965f46949f

TGAAACAAATCGGTTTTGTCAGACCGGACCGGTTTTACCGCACTACAAGGATTCTGGTAAACACTGTTTACGGACTGTTACAGAATCTTCACCGAGTTAATATTAAACCATTTATAGTCATTCTTCTTGACAATAGTTTTCAGAAAGTATCACGTTGTGTGTAAGTAAATATAAAATATTTTGAAACACGAGATAGTGTATTATGAACTACAGAGCTATTACAGTTATCCGGATT

>8572b51354eb37ebdf73436e139543af

TACGTAGGGTGCGAGCGTTGTCCGGATTTATTGGGCGTAAAGGGCTCGTAGGTGGTTGATCGCGTCGGAAGTGTAATCTTGGGGCTTAACCCTGAGCGTGCTTTCGATACGGGTTGACTTGAGGAAGGTAGGGGAGAATGGAATTCCTGGTGGAGCGGTGGAATGCGCAGATATCAGGAGGAACACCAGTGGCGAAGGCGGGTCTCTGGGCCTTTCCTGACGCTGAGGAGCGAAAGCGTGGGGAGCGAACAGG

>9f21e33d214dc108db8ae749418d93aa
[truncated: 25,243 more chars]
