# Supplementary material for: Lack of host phylogenetic structure in the gut bacterial communities of New Zealand cicadas and their interspecific hybrids
Source: Sci Rep. 2022 Nov 29;12:20559. doi: 10.1038/s41598-022-24723-3 (PMC9709078; doi:10.1038/s41598-022-24723-3)
Supplement: Supplementary file 2 — Supplementary Information 2. [file 41598_2022_24723_MOESM2_ESM.docx]

>c31219c21d19f783a7cc6b509a9e0fc4

AACAGAGGATACAAGCGTTATCCGGATTTATTGGGTTTAAAGGGTGCGTAGGTGGTTTTTTAAGTCAGTAGTGAAATCTTAAAGCTTAACTTTAAAAGTGCTATTGATACTGATAAACTAGAGTGAGGTTGGAGTAACTGGAATGTGTGGTGGAGCGGTGAAATGCATAGAGATCACACAGAACACCAATCGCGAAGGCATGTTACTAAACATAGACTGACACTGAGGCACGAAAGCATGGGTAGCAAACAGG

>2045e96ad2e2cf90078d900f67bb6f8f

TACGTGAGAGACTAGTGTTATTCATCTTAATTGGGTTTAAAGGGTACCTAGACAGTCAATATAACTTCTATAATGCTAATACTTGACTAGAGTTTTAAGTAAGAGGGAAGTACTTAAGGAGTAAGAGATGAAATATCTGTGATACCAAAGGGACTCCGTAAAGGCGAAGGCATCCCTTTATCTAAAAACTAACGTTGAAGGACGAAGGCTTAGATAACAAATAGG

>0dea5f0ff03f1943100550c7fc0c6cfb

TACGTGAGAGACTAGTGTTATTCATCTTAATTGGGTTTAAAGGGTACCTAGACAGTCAATATAACTTCTAGAATGCTAATACTTGACTAGAGTTTTAAGTAAGAGGGAAGTACTTAAGGAGTAAGAGATGAAATATCTGTGATACCAAAGGGACTCCGTAAAGGCGAAGGCATCCCTTTATCTAAAAACTAACGTTGAAGGACGAAGGCTTAGATAACAAATAGG

>c0696f3ea461c9d227737482b0c1a8b0

TACGTAGGGCGCAAGCGTTATCCGGAATTATTGGGCGTAAAGAGCTCGTAGGCGGTTTGTCGCGTCTGCTGTGAAAGTCCGGGGCTCAACTCCGGTTCTGCAGTGGGTACGGGCAGGCTAGAGTGATGTAGGGGAGACTGGAATTCCTGGTGTAGCGGTGAAATGCGCAGATATCAGGAGGAACACCGATGGCGAAGGCAGGTCTCTGGGCATTAACTGACGCTGAGGAGCGAAAGCATGGGGAGCGAACAGG

>5673ae66819e3bdbbc09461e4959159e

TACGGAGGGTGCAAGCGTTAATCGGAATTACTGGGCGTAAAGCGCACGCAGGCGGTCTGTTAAGTCAGATGTGAAATCCCCGGGCTTAACCTGGGAACTGCATTTGAAACTGGCAGGCTTGAGTCTCGTAGAGGGGGGTAGAATTCCAGGTGTAGCGGTGAAATGCGTAGAGATCTGGAGGAATACCGGTGGCGAAGGCGGCCCCCTGGACGAAGACTGACGCTCAGGTGCGAAAGCGTGGGGAGCAAACAGG

>3902f671cea4650ba271a2cbc90c7eb8

TACGTAGGGCGCAAGCGTTATCCGGAATTATTGGGCGTAAAGAGCTCGTAGGCGGTTTGTCGCGTCTGCTGTGAAAGTCCGGGGCTCAACTCCGGTTCTGCAGTGGGTACGGGCAGGCTTGAGTGATGTAGGGGAGACTGGAATTCCTGGTGTAGCGGTGAAATGCGCAGATATCAGGAGGAACACCGATGGCGAAGGCAGGTCTCTGGGCATTAACTGACGCTGAGGAGCGAAAGCATGGGGAGCGAACAGG

>17f42ee9caaaa916caa2e2f582808a9b

TACGTAGGGTGCGAGCGTTAATCGGAATTACTGGGCGTAAAGCGTGCGCAGGCGGTTTGTTAAGACAGATGTGAAATCCCCGGGCTCAACCTGGGAACTGCATTTGTGACTGGCAGGCTAGAGTATGGCAGAGGGGGGTAGAATTCCACGTGTAGCAGTGAAATGCGTAGAGATGTGGAGGAATACCGATGGCGAAGGCAGCCCCCTGGGCCAATACTGACGCTCATGCACGAAAGCGTGGGGAGCAAACAGG

>76addc8524e10c6faff0161e36a1987a

CACGATTAACCCAAGTCAATAGAAGCCGGCGTAAAGAGTGTTTTAGATCACCCCCTCCCCAATAAAGCTAAAACTCACCTGAGTTGTAAAAAACTCCAGTTGACACAAAATAGACTACGAAAGTGGCTTTAACATATCTGAACACACAATAGCTAAGACCCAAACTGGG

>a41fd0f01d1eb3400f0fee992d87a837

TACGTAGGGCGCAAGCGTTATCCGGAATTATTGGGCGTAAAGAGCTCGTAGGCGGTTTGTCGCGTCTGCCGTGAAAGTCCGGGGCTTAACCCTGGTTCTGCGGTGGGTACGGGCAGACTGGAGTGCAGTAGGGGAGACTGGAATTCCTGGTGTAGCGGTGAAATGCGCAGATATCAGGAGGAACACCGATGGCGAAGGCAGGTCTCTGGGCTGTTACTGACGCTGAGGAGCGAAAGCATGGGGAGCGAACAGG

>079ced3edc02fb1d1b9cb47b15e46156

TACGTGAGAGACTAGTGTTATTCATCTTAATTGGGTTTAAAGGGTACCTAGACAGTCAATATAACTTCTATAATGTTAATACTTGACTAGAGTTTTAAGTAAGAGGGAAGTACTTAAGGAGTAAGAGATGAAATATCTGTGATACCAAAGGGACTCCGTAAAGGCGAAGGCATCCCTTTATCTAAAAACTAACGTTGAAGGACGAAGGCTTAGATAACAAATAGG

>0295d43a9b08998f52cad6706b9446cf

TACATAGGGGGCAAGCGTTATCCGGATTTATTGGGCGTAAAGGGTGCGTAGGCGGTTAAGTAAGTCTGTGGTTTAAACGCAATGCTCAACATTGTGACGCTATAGAAACTGTTTGACTAGAGTTGGATAGAGGCAAGTGGAATTCCATGTGTAGCGGTAAAATGCGTAAATATATGGAGGAACACCAGTAGCGAAGGCGGCTTGCTAGGTCTTAACTGACGCTGAGGCACGAAAGCGTGGGGAGCAAACAGG

>113e5cb2dbb0b2086e230fc9d845310f

TACAGAGGGTGCAAGCGTTAATCGGAATTACTGGGCGTAAAGCGCGCGTAGGTGGTTCGTTAAGTTGGATGTGAAAGCCCCGGGCTCAACCTGGGAACTGCATTCAAAACTGACGAGCTAGAGTATGGTAGAGGGTGGTGGAATTTCCTGTGTAGCGGTGAAATGCGTAGATATAGGAAGGAACACCAGTGGCGAAGGCGACCACCTGGACTGATACTGACACTGAGGTGCGAAAGCGTGGGGAGCAAACAGG

>c1cf0c4ee7d879c86ca0523506557623

TACGTAGGGCGCAAGCGTTATCCGGAATTATTGGGCGTAAAGAGCTCGTAGGCGGTTTGTCGCGTCTGCTGTGAAAGACCGGGGCTCAACTCCGGTTCTGCAGTGGGTACGGGCAGACTGGAGTGATGTAGGGGAGACTGGAATTCCTGGTGTAGCGGTGAAATGCGCAGATATCAGGAGGAACACCGATGGCGAAGGCAGGTCTCTGGGCATTAACTGACGCTGAGGAGCGAAAGCATGGGGAGCGAACAGG

>e5a339fe9c47193e5f06c2e6adb46d44

TACGTGAGAGACTAGTGTTATTCATCTTAATTGGGTTTAAAGGGTACCTAGGCAGTCAATATAACTTCTATAATGCTAATACTTGACTAGAGTTTTAAGTAAGAGGGAAGTACTTAAGGAGTAAGAGATGAAATATCAGTGATACCAAAGGGACTCCGTAAAGGCGAAGGCATCCCTTTATTTAAAAACTAACGTTGAAGGACGAAGGCTTAGATAACAAATAGG

>59c3b4a20fffe281a3902b09838c1e2a

TACGTGAGAGACTAGTGTTATTCATCTTAAATGGGTTTAAAGGGTACCTAGACAGTCAATATAACTTCTATAATGCTAATACTTGACTAGAGTTTTAAGTAAGAGGGAAGTACTTAAGGAGTAAGAGATGAAATATCTGTGATACCAAAGGGACTCCGTAAAGGCTAATGCATCCCTTTATCTAAAAACTAACGTTGAAGGACGAAGGCTTAGATAACAAATAGG

>0920dcf0f62fb2b3ab9e32f1c4edec37

TACGTAGGGTGCAAGCGTTAATCGGAATTACTGGGCGTAAAGCGTGCGCAGGCGGTTATGCAAGACAGAGGTGAAATCCCCGGGCTCAACCTGGGAACTGCCTTTGTGACTGCATGGCTAGAGTACGGTAGAGGGGGATGGAATTCCGCGTGTAGCAGTGAAATGCGTAGATATGCGGAGGAACACCGATGGCGAAGGCAATCCCCTGGACCTGTACTGACGCTCATGCACGAAAGCGTGGGGAGCAAACAGG

>b3d091a44dde5d01438e3ab4028e087c

TACGAAGGGGGCGAGCGTTGTTCGGAATAACTGGGCGTAAAGGGCACGTAGGCGGGTTATTAAGTCAGTGGTGAAATCCCAAGGCTCAACCTTGGAACTGCCTCTGATACTGGTTACCTAGAGTTCAGGAGAGGTGAGTGGAATTCCGAGTGTAGAGGTGAAATTCGTAGATATTCGGAGGAACACCGGTGGCGAAGGCGGCTCACTGGACTGATACTGACGCTGAGGTGCGAAAGCGTGGGGAGCAAACAGG

>901c738324d8fc7c901555c77695ec61

TACGTAGGGTGCGAGCGTTAATCGGAATTACTGGGCGTAAAGCGTGCGCAGGCGGTTGTGTAAGACAGGCGTGAAATCCCCGGGCTCAACCTGGGAATGGCGCTTGTGACTGCACGGCTGGAGTGCGGCAGAGGGGGATGGAATTCCGCGTGTAGCAGTGAAATGCGTAGATATGCGGAGGAACACCGATGGCGAAGGCAATCCCCTGGGCCTGCACTGACGCTCATGCACGAAAGCGTGGGGAGCAAACAGG

>9fb877df89cff6487d4371a1444880f8

TACGGAGGGTGCAAGCGTTATCCGGATTTATTGGGTTTAAAGGGTCCGTAGGCGGATTTGTAAGTCAGTGGTGAAATCTCACAGCTTAACTGTGAAACTGCCATTGATACTGCAAGTCTTGAGTGTTGTTGAAGTAGCTGGAATAAGTAGTGTAGCGGTGAAATGCATAGATATTACTTAGAACACCAATTGCGAAGGCAGGTTACTAAGCAACAACTGACGCTGATGGACGAAAGCGTGGGGAGCGAACAGG

>8400dd8baa70ab70f07c34b0d0024559

TACGAAGGGGGCTAGCGTTGCTCGGAATCACTGGGCGTAAAGGGTGCGTAGGCGGGTCTTTAAGTCAGGGGTGAAATCCTGGAGCTCAACTCCAGAACTGCCTTTGATACTGAGGATCTTGAGTCCGGAAGAGGTGAGTGGAACTGCGAGTGTAGAGGTGAAATTCGTAGATATTCGCAAGAACACCAGTGGCGAAGGCGGCTCACTGGTCCGGTACTGACGCTGAGGCACGAAAGCGTGGGGAGCAAACAGG

>1b51357852b0b814b4d9918c6f0ab762

TACGTAGGTCCCGAGCGTTGTCCGGATTTATTGGGCGTAAAGCGAGCGCAGGCGGTTAGATAAGTCTGAAGTTAAAGGCTGTGGCTTAACCATAGTATGCTTTGGAAACTGTTTAACTTGAGTGCAGAAGGGGAGAGTGGAATTCCATGTGTAGCGGTGAAATGCGTAGATATATGGAGGAACACCGGTGGCGAAAGCGGCTCTCTGGTCTGTAACTGACGCTGAGGCTCGAAAGCGTGGGGAGCAAACAGG

>d829bee4984f82ffc2453212157caf96

TACGAAGGGGGCTAGCGTTGCTCGGAATCACTGGGCGTAAAGGGTGCGTAGGCGGGTCTTTAAGTCAGGGGTGAAATCCTGGAGCTCAACTCCAGAACTGCCTTTGATACTGAAGATCTTGAGTTCGGGAGAGGTGAGTGGAACTGCGAGTGTAGAGGTGAAATTCGTAGATATTCGCAAGAACACCAGTGGCGAAGGCGGCTCACTGGCCCGATACTGACGCTGAGGCACGAAAGCGTGGGGAGCAAACAGG

>3d5baff45729bf2214074c2e9f77ad10

TACAGAGGGTGCAAGCGTTAATCGGATTTACTGGGCGTAAAGCGCGCGTAGGCGGCTAATTAAGTCAAATGTGAAATCCCCGAGCTTAACTTGGGAATTGCATTCGATACTGGTTAGCTAGAGTGTGGGAGAGGATGGTAGAATTCCAGGTGTAGCGGTGAAATGCGTAGAGATCTGGAGGAATACCGATGGCGAAGGCAGCCATCTGGCCTAACACTGACGCTGAGGTGCGAAAGCATGGGGAGCAAACAGG

>58f903ade24191a738f4f5765b728439

TACATAGGGGGCAAGCGTTATCCGGAATTATTGGGCGTAAAGGGTGCGTAGGCGGTTAAATAAGTTTATGGTCTAAGTGCAATGCTTAACGTTGTGATGCTATAAAAACTGTTTAGCTAGAGTTGGATAGAGGCAAGTGGAATTCCATGTGTAGTGGTAAAATGCGTAAATATATGGAGGAACACCAGAAGCGAAGGCGGCTTGCTGGGTCTTAACTGACGCTGAGGCACGAAAGCGTGGGGAGCAAACAGG

>80f80f471bb8ba856f86aea5dd91997d

TACATAGGGGGCGAGCGTTATCCGGAATTATTGGGCGTAAAGGGTGCGTAGGCGGTTAAATAAGTTTATGGTCTAAGTGCAATGCTTAACGTTGTGATGCTATAAAAACTGTTTAGCTAGAGTTGGATAGAGGCAAGTGGAATTCCATGTGTAGTGGTAAAATGCGTAAATATATGGAGGAACACCAGAAGCGAAGGCGGCTTGCTGGGTCTTAACTGACGCTGAGGCACGAAAGCGTGGGGAGCAAACAGG

>945184b6386c192c0066e0a98a154780

TACGGAGGGTGCAAGCGTTAATCGGAATTACTGGGCGTAAAGCGCACGCAGGCGGTCTGTCAAGTCGGATGTGAAATCCCCGGGCTCAACCTGGGAACTGCATTCGAAACTGGCAGGCTAGAGTCTTGTAGAGGGGGGTAGAATTCCAGGTGTAGCGGTGAAATGCGTAGAGATCTGGAGGAATACCGGTGGCGAAGGCGGCCCCCTGGACAAAGACTGACGCTCAGGTGCGAAAGCGTGGGGAGCAAACAGG

>7b11c44eef75fb0ebb0680d6a08cccad

TACGTAGGGTGCAAGCGTTATCCGGAATTATTGGGCGTAAAGAGCTCGTAGGCGGTTTGTCGCGTCTGCTGTGAAATCCCGAGGCTCAACCTCGGGCCTGCAGTGGGTACGGGCAGACTAGAGTGCGGTAGGGGAGATTGGAATTCCTGGTGTAGCGGTGGAATGCGCAGATATCAGGAGGAACACCGATGGCGAAGGCAGATCTCTGGGCCGTAACTGACGCTGAGGAGCGAAAGGGTGGGGAGCAAACAGG

>b2db4c40be62db703722123b19b2222e

TACGTAGGGCGCGAGCGTTATCCGGAATTATTGGGCGTAAAGAGCTCGTAGGCGGTTTGTCGCGTCTGCTGTGAAAGACCGGGGCTCAACTCCGGTTCTGCAGTGGGTACGGGCAGGCTGGAGTGCAGTAGGGGAGACTGGAATTCCTGGTGTAGCGGTGAAATGCGCAGATATCAGGAGGAACACCGATGGCGAAGGCAGGTCTCTGGGCTGTTACTGACGCTGAGGAGCGAAAGCATGGGGAGCGAACAGG

>06f825b512d903b9230e1a55d87359ee

TACGTAGGTCCCGAGCGTTGTCCGGATTTATTGGGCGTAAAGCGAGCGCAGGCGGTTAGATAAGTCTGAAGTTAAAGGCTGTGGCTTAACCATAGTACGCTTTGGAAACTGTTTAACTTGAGTGCAAGAGGGGAGAGTGGAATTCCATGTGTAGCGGTGAAATGCGTAGATATATGGAGGAACACCGGTGGCGAAAGCGGCTCTCTGGCTTGTAACTGACGCTGAGGCTCGAAAGCGTGGGGAGCAAACAGG

>43c951b8ebd7cc900898c8fa6445dee3

GACAGAGGGTGCAAACGTTGTTCGGAATTACTGGGCGTAAAGCGTGTGTAGGCGGCCATGTAAGTTGGATGTGAAAGCCCCGGGCTCAACCCGGGAAGTGCATTCAAAACTGCGTGGCTTGAGTACTGGAGAGGTTGGTAGAATTCTCGGTGTAGAGGTGAAATTCGTAGATATCGAGAGGAATACCGGTGGCGAAGGCGGCCAACTGGACAGATACTGACGCTGAGACACGAAAGCGTGGGGAGCAAACAGG

>4a0b292ba716582f9af46694458c0b9b

TACAGAGGATGCAAGCGTTATCCGGAATGATTGGGCGTAAAGCGTCTGTAGGTGGCTTTTTAAGTCCGCCGTCAAATCCCAGGGCTCAACCCTGGACAGGCGGTGGAAACTACCAAGCTGGAGTACGGTAGGGGCAGAGGGAATTTCCGGTGGAGCGGTGAAATGCGTAGAGATCGGAAAGAACACCAACGGCGAAAGCACTCTGCTGGGCCGACACTGACACTGAGAGACGAAAGCTAGGGGAGCGAATGGG

>dc1d0dc8db4e7c2314ef9d14052de96d

GACAGAGGGTGCAAACGTTGTTCGGAATTACTGGGCGTAAAGCGTGTGTAGGCGGTCTTGTAAGTCGGATGTGAAAGCCCCGGGCTCAACCCGGGAAGTGCACTCGATACTGCGAGACTTGAGTATCGGAGAGGTTGGTGGAATTCTCGGTGTAGAGGTGAAATTCGTAGATATCGAGAGGAACACCGGTGGCGAAAGCGGCCAACTGGACGAATACTGACGCTGAGACACGAAAGCGTGGGGAGCAAACAGG

>2721fd42d5373667f111c60faaf21eac

TACGTAGGGCGCGAGCGTTGTCCGGAATTATTGGGCGTAAAGAGCTTGTAGGCGGTTTGTCGCGTCTGCTGTGAAAGGCCGGGGCTTAACTCCGTGTATTGCAGTGGGTACGGGCAGACTAGAGTGCAGTAGGGGAGACTGGAATTCCTGGTGTAGCGGTGGAATGCGCAGATATCAGGAGGAACACCGATGGCGAAGGCAGGTCTCTGGGCTGTAACTGACGCTGAGAAGCGAAAGCATGGGGAGCGAACAGG

>3a7bcfd3dd1c23c2efae4b6b3fcd36a3

TACGAAGGGGGCTAGCGTTGCTCGGAATCACTGGGCGTAAAGGGTGCGTAGGCGGGTTTTTAAGTCAGGGGTGAAATCCTGGAGCTCAACTCCAGAACTGCCTTTGATACTGAAGATCTTGAGTCCGGGAGAGGTGAGTGGAACTGCGAGTGTAGAGGTGAAATTCGTAGATATTCGCAAGAACACCAGTGGCGAAGGCGGCTCACTGGCCCGGTACTGACGCTGAGGCACGAAAGCGTGGGGAGCAAACAGG

>565b59725452b37f481c73bbb7e4ff26

TACGGAGGGTGCGAGCGTTAATCGGAATTACTGGGCGTAAAGCGCGCGTAGGCGGCGTGATAAGCCGGTTGTGAAAGCCCCGGGCTCAACCTGGGAACGGCATCCGGAACTGTCAGGCTAGAGTGCAGGAGAGGAAGGTAGAATTCCCGGTGTAGCGGTGAAATGCGTAGAGATCGGGAGGAATACCAGTGGCGAAGGCGGCCTTCTGGACTGACACTGACGCTGAGGTGCGAAAGCGTGGGTAGCAAACAGG

>d32e579b3ae7b2aae8d5bf9f027c29af

TACGTAGGGTGCGAGCGTTAATCGGAATTACTGGGCGTAAAGCGTGCGCAGGCGGTTATGTAAGACAGATGTGAAATCCCCGGGCTCAACCTGGGAACTGCATTTGTGACTGCATGGCTAGAGTACGGTAGAGGGGGATGGAATTCCGCGTGTAGCAGTGAAATGCGTAGATATGCGGAGGAACACCGATGGCGAAGGCAATCCCCTGGACCTGTACTGACGCTCATGCACGAAAGCGTGGGGAGCAAACAGG

>eb8c47f2846f38f78ce7cd8a719a6b69

TACATAGGGGGCAAGCGTTATCCGGATTTATTGGGCGTAAAGGGTGCGTAGGCGGTTAAATAAGTCTGTGGTTTAAACGCAATGCTCAACATTGTGACGCTATAGAAACTGTTTGACTAGAGTTGGATAGAGGCAAGCGGAATTCCATGTGTAGCGGTAAAATGCGTAAAGATATGGAGGAACACCAGTAGCGAAGGCGGCTTGCTGGGTCTTAACTGACGCTGAGGCACGAAAGCGTGGGGAGCAAACAGG

>ecc423b8fefd03a92aa1a97fc74a654c

TACGTAGGGTGCAAGCGTTAATCGGAATTACTGGGCGTAAAGCGTGCGCAGGCGGTTCGGAAAGAAAGATGTGAAATCCCAGAGCTTAACTTTGGAACTGCATTTTTAACTACCGGGCTAGAGTGTGTCAGAGGGAGGTGGAATTCCGCGTGTAGCAGTGAAATGCGTAGATATGCGGAGGAACACCGATGGCGAAGGCAGCCTCCTGGGATAACACTGACGCTCATGCACGAAAGCGTGGGGAGCAAACAGG

>65d43491988bfe557da4d86a5ba25dae

TACGTAGGTGGCAAGCGTTATCCGGAATTATTGGGCGTAAAGCGCGCGTAGGCGGTTTTTTAAGTCTGATGTGAAAGCCCACGGCTCAACCGTGGAGGGTCATTGGAAACTGGAAAACTTGAGTGCAGAAGAGGAAAGTGGAATTCCATGTGTAGCGGTGAAATGCGCAGAGATATGGAGGAACACCAGTGGCGAAGGCGACTTTCTGGTCTGTAACTGACGCTGATGTGCGAAAGCGTGGGGATCAAACAGG

>8012da7c810e58147175f741af2a78d2

TACGTAGGGTGCAAGCGTTAATCGGAATTACTGGGCGTAAAGCGTGCGCAGGCGGTTATGCAAGACAGAGGTGAAATCCCCGGGCTCAACCTGGGAACTGCCTTTGTGACTGCATAGCTAGAGTACGGTAGAGGGGGATGGAATTCCGCGTGTAGCAGTGAAATGCGTAGATATGCGGAGGAACACCGATGGCGAAGGCAATCCCCTGGACCTGTACTGACGCTCATGCACGAAAGCGTGGGGAGCAAACAGG

>2bd9309f2f97cae51d18d06ea1ca519a

TACGTAGGTGGCAAGCGTTGTCCGGATTTATTGGGCGTAAAGCGAGCGCAGGCGGTTCCTTAAGTCTGATGTGAAAGCCCCCGGCTCAACCGGGGAGGGTCATTGGAAACTGGGGAACTTGAGTGCAGAAGAGGAGAGTGGAATTCCATGTGTAGCGGTGAAATGCGTAGATATATGGAGGAACACCAGTGGCGAAGGCGACTCTCTGGTCTGTAACTGACGCTGAGGCTCGAAAGCGTGGGTAGCAAACAGG

>3020e9870fc0422d68a4aecea103b122

GACGGGGGGGGCAAGTGTTCTTCGGAATGACTGGGCGTAAAGGGCACGTAGGCGGTGAATCGGGTTGAAAGTGAAAGTCGCCAAAAAGTGGCGGAATGCTCTCGAAACCAATTCACTTGAGTGAGACAGAGGAGAGTGGAATTTCGTGTGTAGGGGTGAAATCTTGAAATATACGAAGGAACGCCAAAAGCGAAGGCAGCTCTCTGGGTCCCTACCGACGCTGGGGTGCGAAAGCATGGGGAGCGAACAGG

>d46e2205f0c6ecf67b51f83d111c509c

TACGGAGGGTGCAAGCGTTAATCGGAATTACTGGGCGTAAAGCGCACGCAGGCGGTTTGTTAAGTCAGATGTGAAATCCCCGGGCTCAACCTGGGAACTGCATCTGATACTGGCAAGCTTGAGTCTCGTAGAGGGGGGTAGAATTCCAGGTGTAGCGGTGAAATGCGTAGAGATCTGGAGGAATACCGGTGGCGAAGGCGGCCCCCTGGACGAAGACTGACGCTCAGGTGCGAAAGCGTGGGGAGCAAACAGG

>04ecfad5772d2e09a84a0f5ef460536c

TACGAAGGGGGCTAGCGTTGCTCGGAATCACTGGGCGTAAAGGGCGCGTAGGCGGCGTTTTAAGTCGGGGGTGAAAGCCTGTGGCTCAACCACAGAATGGCCTTCGATACTGGGACGCTTGAGTATGGTAGAGGTTGGTGGAACTGCGAGTGTAGAGGTGAAATTCGTAGATATTCGCAAGAACACCGGTGGCGAAGGCGGCCAACTGGACCATTACTGACGCTGAGGCGCGAAAGCGTGGGGAGCAAACAGG

>5648dccee530d68ceb3e4d7d22cf8756

TACAGAGGGTGCAAGCGTTAATCGGAATTACTGGGCGTAAAGCGCGCGTAGGTGGTTTGTTAAGTTGGATGTGAAATCCCCGGGCTCAACCTGGGAACTGCATTCAAAACTGACTGACTAGAGTATGGTAGAGGGTGGTGGAATTTCCTGTGTAGCGGTGAAATGCGTAGATATAGGAAGGAACACCAGTGGCGAAGGCGACCACCTGGACTAATACTGACACTGAGGTGCGAAAGCGTGGGGAGCAAACAGG

>9662c72ee218edd84b8e39b2ec7ea972

ACATTCTTTGTACTTCACTAGTTATTTAATTTACACCACTTGTCACCCCCTTATATTGTACAAACGAGTTTGCTGGCACATTATGTGAAATACACGTTCCTGTTACAGCTTATCCCTGACCTGCTAAAACATGAAAGCAATATTTCTATGACCACAAAAACTATGGACCAAGAAGCTA

>0c579d21280801f02a641e1608606927

TACGTAGGGTGCGAGCGTTATCCGGAATTATTGGGCGTAAAGAGCTCGTAGGCGGTTTGTCGCGTCTGTCGTGAAAGTCCGGGGCTTAACCCCGGATCTGCGGTGGGTACGGGCAGACTAGAGTGCAGTAGGGGAGACTGGAATTCCTGGTGTAGCGGTGGAATGCGCAGATATCAGGAGGAACACCGATGGCGAAGGCAGGTCTCTGGGCTGTAACTGACGCTGAGGAGCGAAAGCATGGGGAGCGAACAGG

>9eab7a1249300ee6e9757c224201f94d

TACGAAGGGTGCAAGCGTTACTCGGAATTACTGGGCGTAAAGCGTGCGTAGGTGGTTATTTAAGTCCGTTGTGAAAGCCCTGGGCTCAACCTGGGAACTGCAGTGGATACTGGATGACTAGAATGTGGTAGAGGGTAGCGGAATTCCTGGTGTAGCAGTGAAATGCGTAGAGATCAGGAGGAACATCCATGGCGAAGGCAGCTACCTGGACCAACATTGACACTGAGGCACGAAAGCGTGGGGAGCAAACAGG

>394eda29c886632f514dd94b58381186

TACGGAGGGTGCGAGCGTTAATCGGAATAACTGGGCGTAAAGGGCACGCAGGCGGTGACTTAAGTGAGGTGTGAAAGCCCCGGGCTTAACCTGGGAATTGCATTTCATACTGGGTCGCTAGAGTACTTTAGGGAGGGGTAGAATTCCACGTGTAGCGGTGAAATGCGTAGAGATGTGGAGGAATACCGAAGGCGAAGGCAGCCCCTTGGGAATGTACTGACGCTCATGTGCGAAAGCGTGGGGAGCAAACAGG

>c4f7f7d843eca4862537b5052067f2bb

TACGAAGGGGGCTAGCGTTGCTCGGAATCACTGGGCGTAAAGGGCGCGTAGGCGGCCTTGTAAGTTGGGGGTGAAAGCCCGTGGCTCAACCACGGAATTGCCTTCGATACTGCTTGGCTTGAGTGTGGTAGAGGTTGGTGGAACTGCGAGTGTAGAGGTGAAATTCGTAGATATTCGCAAGAACACCGGTGGCGAAGGCGGCCAACTGGACCATCACTGACGCTGAGGCGCGAAAGCGTGGGGAGCAAACAGG

>1727bd8544639e521c5efff9f8c225a6

TACAGAGGGTGCAAGCGTTGTTCGGAATTACTGGGCGTAAAGCGTGCGTAGTCGGTATTGAGAGTCACGGGTGAAATCCCAGGGCTTAACCCTGGAACTGCCTGTGAGACCTCAGTACTAGAGTGTGAGAGGGGATAGTGGAATACCCAGTGTAGCGGTGAAATGCGTAGAGATTGGGTGGAACACCGGTGGCGAAGGCGGCTATCTGGCTCACAACTGACGATCAGGCACGAAAGCGTGGGGAGCAAACAGG

>e61c89f9d67cbc839830f5027f78caf6

CACACTTATATCTTCAGAAAGACAAAATATTTCTCCAGGATGCATTTCAACAATGTCGGTCATTTTCAACTGGCAGTGCCTTGTAGATATTTGTGAAAGATTTTGAATGTGTCCTGTCCATAGAGTTCATTAATGATTTTGTTATGTATATTGTAACGTAATATATTTAGGCTTTGAAGTAAAATCATTTTGTTTCCTTTTTTGCCGGTTGTG

>0ed3a6836e138f14044f6950ad7280d2

TACGTAGGGCGCGAGCGTTGTCCGGAATTATTGGGCGTAAAGAGCTCGTAGGCGGCTGGTCGCGTCTGTCGTGAAATCCTCTGGCTTAACTGGGGGCTTGCGGTGGGTACGGGCCGGCTTGAGTGCGGTAGGGGAGACTGGAACTCCTGGTGTAGCGGTGGAATGCGCAGATATCAGGAAGAACACCGGTGGCGAAGGCGGGTCTCTGGGCCGTTACTGACGCTGAGGAGCGAAAGCGTGGGGAGCGAACAGG

>48f35d215d0a20baa99b5f686b24fec0

CACACTTATGTTTTCAGAAAGACAAAATATTTCTCCAGGATGCATTTCAACCATGTCGCTCATTTTCAACTGGCAGTGCCTTGTAGTTATTTGTGAAAGATTATGAATGTGTCTTGTCCATAGGATTCAGTTCATTAACGATTTTGTTATGTAGTTTGTAACGTAATATATTTAGGTTTTCAAGTCAATTCATTTGTTTCCTTTTTTTCCGGTTGTG

>b2bac62667dd5b7b46224f0fdf1b2265

TACGAAGGGGGCTAGCGTTGCTCGGAATTACTGGGCGTAAAGGGCGCGTAGGCGGACAGTTAAGTTGGGGGTGAAAGCCCGGGGCTCAACCTCGGAAATGCCTTCAATACTGGCTGTCTTGAGTACGGGAGAGGTGAGTGGAACTCCGAGTGTAGAGGTGAAATTCGTAGATATTCGGAAGAACACCAGTGGCGAAGGCGACTCACTGGCCCGTTACTGACGCTGAGGCGCGAAAGCGTGGGGAGCAAACAGG

>c8be90660f94638cffbfdf7e5a6dcfa8

TACGGAGGGGGCTAGCGTTGTTCGGAATTACTGGGCGTAAAGCGCGCGTAGGCGGACGGTCAAGTTGGGGGTGAAAGCCCGGGGCTCAACCCCGGAACTGCCTTCAAAACTGATCGTCTGGAGACCGGGAGAGGTGAGTGGAATTCCCAGTGTAGAGGTGAAATTCGTAGATATTGGGAAGAACACCAGTGGCGAAGGCGGCTCACTGGACCGGATCTGACGCTGAGGTGCGAAAGCGTGGGGAGCGAACAGG

>29fbc7e5021229480248fa7466a499a2

TACGTAGGTGGCGAGCGTTGTCCGGATTTATTGGGCGTAAAGGGAGTGTAGGCGGTCTTTTAAGTCTGATGTGAAAGCCCACGGCTCAACCGTGGAGGGTCATTGGAAACTGGGAGACTTGAGTGCAGAAGAGGAGAGCGGAATTCCATGTGTAGCGGTGAAATGCGTAGATATATGGAGGAACACCAGTGGCGAAGGCGGCTCTCTGGTCTGTAACTGACGCTGAGGCTCGAAAGCGTGGGGAGCAAACAGG

>8f6ddfbe4a2848010ea6decabc798d82

CGGTTTACCAAGTATATGGCAACTTTATGCGTTCATTTTGGGTGTTGAAACTTAAAAATCTCATCTTTTTTTTTATCGTTAAGGAATTCAGGATCCAGCGAGTAGCTGTCCTGTTCACAAGTAGGATAGCGTGGTTAATGTTACAGCGTTATATCTGGACTACCTGTAATTTCTTCATGATGAGACCCAAGGTTCCAACTGCGCGTTATGAAAACCAAACTAGTTTGACACTA

>cc761daf51f27c423da57f3f1f0ff5cc

TACGGAGGGTGCAAGCGTTAATCGGAATTACTGGGCGTAAAGCGCACGCAGGCGGTCTGTTAAGTCAGATGTGAAATCCCCGGGCTTAACCTGGGAACTGCATTTGAAACTGGCAGGCTTGAGTCTTGTAGAGGGGGGTAGAATTCCAGGTGTAGCGGTGAAATGCGTAGAGATCTGGAGGAATACCGGTGGCGAAGGCGGCCCCCTGGACAAAGACTGACGCTCAGGTGCGAAAGCGTGGGGAGCAAACAGG

>9fe4c3dfc721f87da8ff9661f9600667

TACGTAGGGCGCAAGCGTTATCCGGAATTATTGGGCGTAAAGAGCTCGTAGGCGGTTTGTCGCGTCTGCTGTGAAATCCGGAGGCTCAACCTCCGGCCTGCAGTGGGTACGGGCAGACTAGAGTGCGGTAGGGGAGATTGGAATTCCTGGTGTAGCGGTGGAATGCGCAGATATCAGGAGGAACACCGATGGCGAAGGCAGATCTCTGGGCCGTAACTGACGCTGAGGAGCGAAAGGGTGGGGAGCAAACAGG

>df8456a1abbfb4c8a2c450b44378d4cb

TACGTAGGGCGCGAGCGTTGTCCGGAATTATTGGGCGTAAAGGGCTTGTAGGCGGTTGGTCGCGTCTGCCGTGAAATCCTCTGGCTTAACTGGGGGCGTGCGGTGGGTACGGGCTGACTTGAGTGCGGTAGGGGAGACTGGAACTCCTGGTGTAGCGGTGGAATGCGCAGATATCAGGAAGAACACCGGTGGCGAAGGCGGGTCTCTGGGCCGTTACTGACGCTGAGGAGCGAAAGCGTGGGGAGCGAACAGG

>5f6f7a6cbde0e54a42a1b6f69ed11225

CACACTTATGTCTTCAGAAAGACAAAATATTTCTCCAGGATGCATTTCAACCATGTCGGTCATTTTCAACTGGCAGTGCCTTGTAGATATTTGTGAAAAATTTTGAATGTGTCTTGTCCATAGAGTTGAGTTTGTTCATTAATGATTTTGTTATGTAGTTTGTAACGTAATACATTCAGGCTTTCAAGTAAATTCATTTTGTTTACTTTTTTGCCGGTTGTG

>f0bc65c62a99017dcd039d76236bd653

TACGAAGGGGGCTAGCGTTGCTCGGAATTACTGGGCGTAAAGGGAGCGTAGGCGGACTGTTTAGTCAGAGGTGAAAGCCCAGGGCTCAACCTTGGAATTGCCTTTGATACTGGCAGTCTTGAGTATGGGAGAGGTGAGTGGAACTCCGAGTGTAGAGGTGAAATTCGTAGATATTCGGAAGAACACCAGTGGCGAAGGCGACTCACTGGCCCATTACTGACGCTGAGGCTCGAAAGCGTGGGGAGCAAACAGG

>63267202e7278b39a1075b144dfbd938

CACACTTATGTTTTCAGAAAGACAAAATATTTCTCCAGGATGCATTTCAACCATGTCGCTCATTTTCAACTGACAGTGCCTTGTAGTTATTTGTGAAAGATTATGAATGTGTCTTGTCCATAGGATTCAGTTCATTAACGATTTTGTTATGTAGTTTGTAACGTAATATATTTAGGTTTTCAAGTCAATTCATTTGTTTCCTTTTTTTCCGGTTGTG

>87461b5f2af4769574e5c3af8b7a3d03

TACGGAGGATGCGAGCGTTATTCGGAATCATTGGGTTTAAAGGGTCTGTAGGCGGGCTATTAAGTCAGGGGTGAAAGGTTTCAGCTTAACTGAGAAATTGCCTTTGATACTGGTAGTCTTGAATATCTGTGAAGTTCTTGGAATGTGTAGTGTAGCGGTGAAATGCTTAGATATTACACAGAACACCGATTGCGGAGGCAGGGGACTAACAGACGATTGACGCTGAGAGACGAAAGCGTGGGGAGCGAACAGG

>d15bc449222795a9ff230013aa633686

TACAGAGGGTGCGAGCGTTAATCGGATTTACTGGGCGTAAAGCGTGCGTAGGCGGCTTTTTAAGTCGGATGTGAAATCCCCGAGCTTAACTTGGGAATTGCATTCGATACTGGGAAGCTAGAGTATGGGAGAGGATGGTAGAATTCCAGGTGTAGCGGTGAAATGCGTAGAGATCTGGAGGAATACCGATGGCGAAGGCAGCCATCTGGCCTAATACTGACGCTGAGGTACGAAAGCATGGGGAGCAAACAGG

>5bf78c5bc37644dab35424c9e54acf06

CACACTTATGTCTTCAGAAAGACAAAATATTTCTCCAGGATGCATTTCAACCATGTCGGTCATTTTCAACTGGCAGTGCCTTGTAGATATTTGTGAAAGATTTTGAATGTGTCTTGTCCATAGATTTGAGTTTGTTCATTAATGATTTTGTTATGTAGTTTGTAACGTAGTATATTTAGACTTTCAAGTAAATTCATTTTGTTTACTTTTTTGCCGGTTGTG

>2f17f16d02d8431a44ef5355a2160b62

TACCGGCAGCTCAAGTGATGACCGATATTATTGGGCCTAAAGCGTCCGTAGCCGGCCACGAAGGTTCATCGGGAAATCCGCCAGCTCAACTGGCGGGCGTCCGGTGAAAACCACGTGGCTTGGGACCGGAAGGCTCGAGGGGTACGTCCGGGGTAGGAGTGAAATCCCGTAATCCTGGACGGACCACCGATGGCGAAAGCACCTCGAGAAGACGGATCCGACGGTGAGGGACGAAAGCTAGGGTCTCGAACCGG

>30ad487e1e86be71afce4e03fd7553b5

TACGGAGGGTGCAAGCGTTAATCGGAATTACTGGGCGTAAAGCGCACGCAGGCGGCTTTTTAAGTCGGATGTGAAAGCCCCGGGCTCAACCTGGGAATTGCATCTGATACTGGGAAGCTAGAGTATGTGAGAGGGGGGTAGAATTCCAAGTGTAGCGGTGAAATGCGTAGAGATTTGGAGGAATACCAGTGGCGAAGGCGGCCCCCTGGCACAATACTGACGCTCAGGTGCGAAAGCGTGGGGAGCAAACAGG

>dd36571922ab7a138ee06438ab9c7891

TACGTAGGGTGCGAGCGTTAATCGGAATTACTGGGCGTAAAGCGGGCGCAGACGGTTACTTAAGCGGGATGTGAAATCCCCGGGCTCAACCCGGGAACTGCGTTCCGAACTGGGTGGCTAGAGTGTGTCAGAGGGGGGTAGAATTCCACGTGTAGCAGTGAAATGCGTAGAGATGTGGAGGAATACCGATGGCGAAGGCAGCCCCCTGGGATAACACTGACGTTCATGCCCGAAAGCGTGGGTAGCAAACAGG

>62727dc7f1b9c5878de3da94b2977f05

TACGAAGGGGGCTAGCGTTGTTCGGATTTACTGGGCGTAAAGCGCACGTAGGCGGACTTTTAAGTCAGGGGTGAAATCCCGGGGCTCAACCCCGGAACTGCCTTTGATACTGGAAGTCTTGAGTATGGTAGAGGTGAGTGGAATTCCGAGTGTAGAGGTGAAATTCGTAGATATTCGGAGGAACACCAGTGGCGAAGGCGGCTCACTGGACCATTACTGACGCTGAGGTGCGAAAGCGTGGGGAGCAAACAGG

>09a05e40df02f01459a639c05f5939a7

TACGGAGGGGGTTAGCGTTGTTCGGAATTACTGGGCGTAAAGCGCGCGTAGGCGGATTGGAAAGTTGGGGGTGAAATCCCGGGGCTCAACCCCGGAACTGCCTTCAAAACTCCCAGTCTAGAGTTCGAGAGAGGTGAGTGGAACTCCGAGTGTAGAGGTGAAATTCGTAGATATTCGGAAGAACACCAGTGGCGAAGGCGGCTCACTGGCTCGATACTGACGCTGAGGTGCGAAAGTGTGGGGAGCAAACAGG

>e96a18c7e3d537fc5fcb8f8ed70414c9

TACGAAGGGGGCTAGCGTTGCTCGGAATCACTGGGCGTAAAGGGTGCGTAGGCGGGTCTTTAAGTCAGGGGTGAAATCCTGGAGCTCAACTCCAGAACTGCCTTTGATACTGAAGATCTTGAGTATGGGAGAGGTGAGTGGAACTGCGAGTGTAGAGGTGAAATTCGTAGATATTCGCAAGAACACCAGTGGCGAAGGCGGCTCACTGGCCCATAACTGACGCTGAGGCACGAAAGCGTGGGGAGCAAACAGG

>e6cad09c4fd44cdb1b919a77f3d92429

TACGGAGGGTGCAAGCGTTATCCGGATTCACTGGGTTTAAAGGGTGCGTAGGCGGGTATGTAAGTCAGTGGTGAAATACCGGAGCTTAACTTCGGAACTGCCATTGATACTATATATCTTGAATATTGTGGAGGTAAGCGGAATATGTCATGTAGCGGTGAAATGCTTAGAGATGACATAGAACACCGATTGCGAAGGCAGCTTGCTACGCAAATATTGACGCTGAGGCACGAAAGCGTGGGGATCAAACAGG

>820f6693f569e339f183638cd73a7fe6

TACGTAGGGTGCGAGCGTTGTCCGGAATTACTGGGCGTAAAGAGCTCGTAGGCGGTTTGTCACGTCGTCTGTGAAATCCTAGGGCTTAACCCTGGACGTGCAGGCGATACGGGCTGACTTGAGTACTACAGGGGAGACTGGAATTTCTGGTGTAGCGGTGGAATGCACAGATATCAGGAAGAACACCGATGGCGAAGGCAGGTCTCTGGGTAGTAACTGACGCTGAGGAGCGAAAGCATGGGTAGCGAACAGG

>e27b9e73955f9ee43166443c005e4186

TACGTAGGTGGCAAGCGTTATCCGGAATTATTGGGCGTAAAGCGCGCGTAGGCGGTCTCTTAAGTCTGATGTGAAAGCCCCCGGCTCAACCGGGGAGGGTCATTGGAAACTGGGAGACTTGAGTGCAGAAGAGGAGAGTGGAATTCCATGTGTAGCGGTGAAATGCGCAGAGATATGGAGGAACACCAGTGGCGAAGGCGGCTCTCTGGTCTGTAACTGACGCTGAGGTGCGAAAGCGTGGGGATCAAACAGG

>07da22cab5c55a4badb462bcbbded642

TACGAAGGGGGCTAGCGTTGTTCGGATTTACTGGGCGTAAAGCGCACGTAGGCGGACTATTAAGTCAGGGGTGAAATCCCGGGGCTCAACCCCGGAACTGCCTTTGATACTGGTAGTCTTGAGTTCGAGAGAGGTGAGTGGAATTCCGAGTGTAGAGGTGAAATTCGTAGATATTCGGAGGAACACCAGTGGCGAAGGCGGCTCACTGGCTCGATACTGACGCTGAGGTGCGAAAGCGTGGGGAGCAAACAGG

>e5c19d7800b18015f3a917fc015fc42f

TACAGAGGGTGCGAGCGTTAATCGGAATTACTGGGCGTAAAGCGAGTGTAGGTGGCTCATTAAGTCACATGTGAAATCCCCGGGCTTAACCTGGGAACTGCATGTGATACTGGTGGTGCTAGAATATGTGAGAGGGAAGTAGAATTCCAGGTGTAGCGGTGAAATGCGTAGAGATCTGGAGGAATACCGATGGCGAAGGCAGCTTCCTGGCATAATATTGACACTGAGATTCGAAAGCGTGGGTAGCAAACAGG

>edc9e5c16e40aff1eadce6597940f08f

TACGTAGGTCCCGAGCGTTATCCGGATTTATTGGGCGTAAAGCGAGCGCAGGCGGTTAGATAAGTCTGAAGTTAAAGGCTGTGGCTTAACCATAGTACGCTTTGGAAACTGTTTAACTTGAGTGCAGAAGGGGAGAGTGGAATTCCATGTGTAGCGGTGAAATGCGTAGATATATGGAGGAACACCGGTGGCGAAAGCGGCTCTCTGGTCTGTAACTGACGCTGAGGCTCGAAAGCGTGGGGAGCAAACAGG

>e7e1373564db3d14aa3c8cb4371d2f41

TACGTATGTCGCAAGCGTTATCCGGAATTATTGGGCTTAAAGGGCATCTAGGCGGTATAACAAGTTGAAGGTGAAAAGCTGTAGCTCAACTATAGTCTTGCCTACAAAACTGTAATACTAGAGTACTGGAAAGGTGGGTGGAACTACACGAGTAGAGGTGAAATTCGTAGATATGTGTAGGAATGCCGATGATGAAGATAACTCACTGGACAGAAACTGACGCTGAAGTGCGAAAGCTAGGGGAGCAAACAGG

>63afe8e6aac58bf0d670a82ca5bc574c

TACGGAGGGTGCAAGCGTTAATCGGAATTACTGGGCGTAAAGCGCACGCAGGCGGTTTGTTAAGTCAGATGTGAAATCCCCGAGCTTAACTTGGGAACTGCATTTGAAACTGGCAAGCTAGAGTCTTGTAGAGGGGGGTAGAATTCCAGGTGTAGCGGTGAAATGCGTAGAGATCTGGAGGAATACCGGTGGCGAAGGCGGCCCCCTGGACAAAGACTGACGCTCAGGTGCGAAAGCGTGGGGAGCAAACAGG

>1ad03cb4c01f859bead2f9746936c0ad

CACGATTAACCCAAGTCAATAGAAGCCGGCGTAAAGAGTGTTTTAGATCACCCCCTCCCCAATAAAGCTAAAACTCACCTGAGTTGTAAAAAGCTCCAGTTGACACAAAATAGACTACGAAAGTGGCTTTAACATATCTGAACACACAATAGCTAAGACCCAAACTGGG

>90c1e87f5bf030c3368c2da35a3870a8

TACGGAGGGTGCAAGCGTTATCCGGATTTATTGGGTTTAAAGGGTCCGTAGGCGGATTTGTAAGTCAGTGGTGAAATCTCACAGCTTAACTGTGAAACTGCCATTGATACTGCAAGTCTTGAGTGTTGTTGAAGTAGCTGGAATAAGTAGTGTAGCAGTGAAATGCATAGATATTACTTAGAACACCAATTGCGAAGGCAGGTTACTAAGCAACAACTGACGCTGATGGACGAAAGCGTGGGGAGCGAACAGG

>2727f819185a7e8993f13df355a399fa

TACGGAGGGAGCTAGCGTTATTCGGAATTACTGGGCGTAAAGCGCACGTAGGCGGCTTTGTAAGTTAGAGGTGAAAGCCTGGAGCTCAACTCCAGAATTGCCTTTGATACTGCATGGCTTGAATCCAGGAGAGGTGAGTGGAATTCCGAGTGTAGAGGTGAAATTCGTAGATATTCGGAAGAACACCAGTGGCGAAGGCGGCTCACTGGACTGGTATTGACGCTGAGGTGCGAAAGCGTGGGGAGCAAACAGG

>465d5de7fc208880ff9aeac9fae9e22e

TACGTAAGAGACTAGTGTTATTCATCTTAATTAGGTTTAAAGGGTACCTAGACGGTCAATATAGCTTCTAGAATGTTAGTACTTGACTAGAGTTTGATATAAGAGGGCAGTACTTGAGGAGGAGAGATGAAATTCTATTATACCAAAGGGACTCGGTAAAGGCGAAGGCAGCCCTCTATGTAAAAACTGACGTTGAAGGACGAAGGCACAGAGCACAAACAGG

>fd98346394a5c79e554003012cb33826

TACGTAGGGTGCAAGCGTTATCCGGAATTATTGGGCGTAAAGAGCTCGTAGGCGGTTTGTCGCGTCTGTCGTGAAAGTCCGGGGCTCAACTCCGGATCTGCGGTGGGTACGGGCAGACTAGAGTGATGTAGGGGAGACTGGAATTCCTGGTGTAGCGGTGGAATGCGCAGATATCAGGAGGAACACCGATGGCGAAGGCAGGTCTCTGGGCATTAACTGACGCTGAGGAGCGAAAGCATGGGGAGCGAACAGG

>2e6230cd9900830571d33690cdb2d930

CACACTTATGTCTTCAGAAAGACAAAATATTTCTCCAGGATGCATTTCAACCATGTCGGTCATTTTCAACTGGCAGTGCCTTGTAGATATTTGTGAAAGATTTTGAATGTGTCTTGTCCATAGAGTTTAGTTGGTTCATTAATGATTTTGTTATGTAGTTTGTAACGTAATACATTCAGGCTTTCAAGTAAATTCATTTTGTTTACTTTTTTGCCGGTTGTG

>c41e38ec0abb37da79acd32fd3cd7c66

TACGTAGGGTGCGAGCGTTGTCCGGATTTACTGGGCGTAAAGAGCTCGTAGGTGGCTTGTCGCGTCGTCTGTGAAAGTCTGGGGCTTAACTCCGGGTGTGCAGGCGATACGGGCTGGCTTGAGTGCTGTAGGGGAGACTGGAATTCCTGGTGTAGCGGTGGAATGCGCAGATATCAGGAGGAACACCGATGGCGAAGGCAGGTCTCTGGGCAGTCACTGACGCTGAGGAGCGAGAGCATGGGTAGCGAACAGG

>ba945255fb4aff154706e5a5214f4910

TACGAAGGGGGCTAGCGTTGCTCGGAATCACTGGGCGTAAAGGGCGCGTAGGCGGCGTTTTAAGTCGGGGGTGAAAGCCTGTGGCTCAACCACAGAATGGCCTTCGATACTGGGACGCTTGAGTCTGGTAGAGGTTGGTGGAACTGCGAGTGTAGAGGTGAAATTCGTAGATATTCGCAAGAACACCGGTGGCGAAGGCGGCCAACTGGACCAGTACTGACGCTGAGGCGCGAAAGCGTGGGGAGCAAACAGG

>d6efd2da2728fd74ded268122ee05036

TACGGAGGGAGCTAGCGTTATTCGGAATTACTGGGCGTAAAGCGCACGTAGGCGGCTTTGTAAGTTAGAGGTGAAAGCCTGGAGCTCAACTCCAGAATTGCCTTTAAGACTGCATCGCTTGAATCCAGGAGAGGTGAGTGGAATTCCGAGTGTAGAGGTGAAATTCGTAGATATTCGGAAGAACACCAGTGGCGAAGGCGGCTCACTGGACTGGTATTGACGCTGAGGTGCGAAAGCGTGGGGAGCAAACAGG

>fc90e2c2753274c2740093c8dabc8751

CACACTTATGTCTTCAGAAAGACAAAATATTTCCCCATGATGCATTTCAACCATGTCGGTCATTTTCAACTGGCAGTGCCTTGTTGATATTTGTGAAAGATTTTGAATATGTCTTGTCCATAGAGTTGAGTTTGTTCATTAATGATTTTGTTATGTAGTTTGTAACGTAATACATTTAGGCTTTCAAGTAAATTCATTTTGTTTATTTTTTTGCCGGTTGTG

>ef242413607a8842e9b8e53240c7db07

GACGGGGGGGGCAAGTGTTCTTCGGAATGACTAGGCGTAAAGGGCACGTAGGCGGTGAATCGGGTTGAAAGTGAAAGTCGCCAAAAACTGGCGGAATGCTCTCGAAACCAATTCACTTGAGTGAGACAGAGGAGAGTGGAATTTCGTGTGTAGGGGTGAAATCCGGAGATCTACGAAGGAACGCCAAAAGCGAAGGCAGCTCTCTGGGTCCCTACCGACGCTGGGGTGCGAAAGCATGGGGAGCGAACGGG

>c939ee112ba982665e012437dae05c1e

TACGGAGGGTGCAAGCGTTAATCGGAATTACTGGGCGTAAAGCGCACGCAGGCGGTCTGTCAAGTCGGATGTGAAATCCCCGGGCTCAACCTGGGAACTGCATTCGAAACTGGCAGGCTGGAGTCTTGTAGAGGGGGGTAGAATTCCAGGTGTAGCGGTGAAATGCGTAGAGATCTGGAGGAATACCGGTGGCGAAGGCGGCCCCCTGGACAAAGACTGACGCTCAGGTGCGAAAGCGTGGGGAGCAAACAGG

>e07bdd14b954490ad4c99811ccc66581

TTACTTTAAAGAAACCAAGTAACTATATTTTTATAAGAATCTGCTATTAGCCACACCACACAACCCCCAACCTGACAGACCACAAATTCTGAATTCGGGCTGTTCATACCCTACAGAGACCCTTCTGTACTTCCATCTAGTCGAGAAGTTCCTGCCTGGTCGCAGAAGACCAACAGCTCCAGTAGTCCAACCGTTCCTCTACCTACACAGAAGACGCACTCCCTGCTGCT

>8df2b2e6c1cb64bd18b81d5bae7f0898

TACGTAGGGTGCAAGCGTTAATCGGAATTACTGGGCGTAAAGCGTGCGCAGGCGGTTTTGTAAGTCTGTCGTGAAAGCCCCGGGCTCAACCTGGGAATTGCGATGGAGACTGCAAGGCTTGAATCTGGCAGAGGGGGGTAGAATTCCACGTGTAGCAGTGAAATGCGTAGAGATGTGGAGGAACACCGATGGCGAAGGCAGCCCCCTGGGTCAAGATTGACGCTCATGCACGAAAGCGTGGGGAGCAAACAGG

>5d0c6af420698f1cdff612c8f7960627

TACGGAGGGGGCTAGCGTTGTTCGGAATTACTGGGCGTAAAGCGCACGTAGGCGGCTTTGTAAGTCAGGGGTGAAAGCCTGGAGCTCAACTCCAGAACTGCCTTTGAGACTGCATCGCTTGAATCCGGGAGAGGTAAGTGGAATTCCGAGTGTAGAGGTGAAATTCGTAGATATTCGGAAGAACACCAGTGGCGAAGGCGGCTTACTGGACCGGAATTGACGCTGAGGTGCGAAAGCGTGGGGAGCAAACAGG

>4cb41777a79a421ce0ea64d1d234279c

TACGTAGGTGGCAAGCGTTGTCCGGAATTATTGGGCGTAAAGCGCGCGCAGGCGGTCTCTTAAGTCCATCTTAGAAGTGCGGGGCTTAACCCCGTGAGGGGATGGAAACTGGGAGACTGGAGTATCGGAGAGGAAAGTGGAATTCCTAGTGTAGCGGTGAAATGCGTAGATATTAGGAAGAACACCGGTGGCGAAGGCGACTTTCTGGACGAAAACTGACGCTGAGGCGCGAAAGCGTGGGGAGCAAACAGG

>9e99abc7db78507408b50434015d4d61

CAAATTTATGTCTTCAGAAAGACAAAATATTTTTCCAGGGTCCATTCCAACCATGTCGGTCATTTTCAACTAGCAGTGCCTTGTAGATATTTGTGAAAGATTTTGAATGTGTCTTGTCCATAGAGTTCAGTTTGTTCATTAATGATTTTGTTATGTAGTTTGTAACGTAATATATTTAGGCTTTCAAGTAAATTGATTTTGTTTCCTTTTTCCGGTTGTG

>9cbdce1222c53f34addcd0b9044c299e

TACCGGCAGCCCGAGTGATGGCCGATCTTATTGGGCCTAAAGCGTCCGTAGCTGGCCGCGCAAGTCCATCGGGAAATCCACCTGCTCAACAGGTGGGCGCCCGGTGGAAACTGTGCGGCTTGGGACCGGAAGGCGCGACGGGTACGTCCGGGGTAGGAGTGAAATCCCGTAATCCTGGACGGACCGCCGATGGCGAAAGCACGTCGCGAGAACGGATCCGACAGTGAGGGACGAAAGCCAGGGTCTCGAACCGG

>0413593eee733b15e989cb0727027ba8

CACACTTATTTCTTCAGAAAGACAAAATATTTCTCCAGGATGCATTTCAACAATGTCGGTCATTTTCAACTGGCAGTGCCTTGTAGATATTTGTGAAAGATTTTGAATGTGTCCTGTCCATAGAGTTCATTAATGATTTTGTTATGTATATTGTAACGTAATATATTTAGGCTTTGAAGTAAAATCATTTTGTTTCCTTTTTTGCCGGTTGTG

>8ff16b044329f30bd360e6fee91fd205

TACGTAGGGGGCAAGCGTTGTCCGGAATTATTGGGCGTAAAGCGCTCGCAGGCGGTCTTTTAAGTCTGATGTGAAAGCCCACGGCTTAACCGTGGAGGGTCATTGGAAACTGGAGGACTTGAGTGCAGAAGAGGAGAGTGGAATTCCACGTGTAGCGGTGAAATGCGTAGAGATGTGGAGGAACACCAGTGGCGAAGGCGACTCTCTGGTCTGTAACTGACGCTGAGGAGCGAAAGCGTGGGGAGCGAACAGG

>f52cc1224cac807cd42d9a901ed53c9b

GACGGGGGGGGCAAGTGTTCTTCGGAATGACTGGGCGTAAAGGGCACGTAGGCGGTGAATCGGGTTGAAAGTGAAAGTCGCCAAAAACTGGTGGAATGCTCTCGAAACCAATTCACTTGAGTGAGACAGAGGAGAGTGGAATTTCGTGTGTAGGGGTGAAATCCGGAGATCTACGAAGGAACGCCAAAAGCGAAGGCAGCTCTCTGGGTCCCTACCGACGCTGGAGTGCGAAAGCATGGGGAGCGAACGGG

>556d30f1f5ebd1b695754cc7ccd58eeb

CACGATTAACCCAAGTCAATAGAAGCCGGCGTAAAGAGTGTTTTAGATCACCCCCTCCCCAATAAAGCTGAAACTCACCTGAGTTGTAAAAAACTCCAGTTGACACAAAATAGACTACGAAAGTGGCTTTAACATATCTGAACACACAATAGCTAAGACCCAAACTGGG

>6fd05e11fe57bb5ba9fd3b0fdd8b80f1

TACGTAGGGTGCAAGCGTTAATCGGAATTACTGGGCGTAAAGCGTGCGCAGGCGGTTTTGTAAGTCTGTTGTGAAATCCCCGGGCTCAACCTGGGAATGGCAATGGAGACTGCAAGGCTAGAGTTTGGCAGAGGGGGGTAGAATTCCACGTGTAGCAGTGAAATGCGTAGATATGTGGAGGAACACCGATGGCGAAGGCAGCCCCCTGGGTCAAAACTGACGCTCATGCACGAAAGCGTGGGGAGCAAACAGG

>aa73d2c485650f5c8e5ba227daccb6f1

TACGAGGAAGACTAGTGTTATTCATCTTTATTAGGTTTAAAGGGTACCTAGACGGCATATCAAGCCCCAAAAGGGAACAGATATACTAGAGTTTTATGTGAGAGGAATATATTAGTACTATTGGTGTAGAGATGAAATTCTTTGATACTAATAGGACGGATAAGAGCAAAAGCAAACCTTTATGTAAAAACTGACGTTGAGGGACGAAGGCTTGGGTCGCGAATAGG

>aa9b3a1418d146c262ec63305292065a

TACGTAGGGTGCGAGCGTTGTCCGGAATTACTGGGCGTAAAGGGCTCGTAGGTGGTTTGTCGCGTCGTCTGTGAAATTCCGGGGCTTAACTCCGGGCGTGCAGGCGATACGGGCATAACTTGAGTACTGTAGGGGTAACTGGAATTCCTGGTGTAGCGGTGAAATGCGCAGATATCAGGAGGAACACCGATGGCGAAGGCAGGTTACTGGGCAGTTACTGACGCTGAGGAGCGAAAGCATGGGTAGCGAACAGG

>6cc2e3c1e8d19522873a8978620ce410

TACCGGCAGTCCGAGTGATGGCCGATATTATTGGGCCTAAAGCGTCCGTAGCTTGCTGTGTAAGTCCATTGGGAAATCGACCAGCTCAACTGGTCGGCGTCCGGTGGAAACTACACAGCTTGGGGCCGAGAGACTCAACGGGTACGTCCGGGGTAGGAGTGAAATCCTGTAATCCTGGACGGACCACCAATGGGGAAACCACGTTGAGAGACCGGACCCGACAGTGAGGGACGAAAGCCAGGGTCTCGAACCGG

>c0d5395792eadbf5f62e8ffb14fa0262

GACGGGGGGGGCAAGTGTTCTTCGGAATGACTGGGCGTAAAGGGCACGTAGGCGGTGAATCGGGTTGAAAGTGAAAGTCGCCAAAAAGTGGCGGAATGCTCTCGAAACCAATTCACTTGAGTGAGACAGAGGAGAGTGGAATTTCGTGTGTAGGGGTGAAATCCGTAGATCTACGAAGGAACGCCAAAAGCGAAGGCAGCTCTCTGGGTCCCTACCGACGCTGGGGTGCGAAAGCATGGGGAGCGAACAGG

>9ac3bb1d7dedb08a012692a6f536b5af

TACGGAGGGAGCTAGCGTTATTCGGAATTACTGGGCGTAAAGCGCACGTAGGCGGCTTTGTAAGTAAGAGGTGAAAGCCCAGAGCTCAACTCTGGAATTGCCTTTTAGACTGCATCGCTTGAATCATGGAGAGGTCAGTGGAATTCCGAGTGTAGAGGTGAAATTCGTAGATATTCGGAAGAACACCAGTGGCGAAGGCGGCTGACTGGACATGTATTGACGCTGAGGTGCGAAAGCGTGGGGAGCAAACAGG

>69fc8436786b625796174cc7a17c9372

TACGTAGGGTGCAAGCGTTGTCCGGAATTACTGGGCGTAAAGAGCTCGTAGGTGGTTTGTCGCGTCGTCTGTGAAAGCCCGGGGCTTAACTCCGGGTCTGCAGGCGATACGGGCATAACTAGAGTGCTGTAGGGGAGACTGGAATTCCTGGTGTAGCGGTGAAATGCGCAGATATCAGGAGGAACACCGATGGCGAAGGCAGGTCTCTGGGCAGTAACTGACGCTGAGGAGCGAAAGCATGGGGAGCGAACAGG

>d7926eba30ca0f01bac6fc5d090e9ab9

TACGGAGGATGCGAGCGTTATCCGGAATCATTGGGTTTAAAGGGTCCGTAGGCGGGCTAATAAGTCAGAGGTGAAAGCGCTCAGCTCAACTGAGCAACTGCCTTTGAAACTGTTAGTCTTGAATGGTTGTGAAGTAGTTGGAATGTGTAGTGTAGCGGTGAAATGCTTAGATATTACACAGAACACCGATAGCGAAGGCATATTACTAACAATTAATTGACGCTGATGGACGAAAGCGTGGGGAGCGAACAGG

>0d8e4d5659848091e6cc768f7edc853b

TACGGAGGGTGCAAGCGTTATCCGGATTTATTGGGTTTAAAGGGTCCGTAGGCGGATTTGTAAGTCAGTGGTGAAATCTCACAGCTTAACTGTGAAACTGCCATTGATACTGCAAGTCTTGAGTGTTGTTGAAGTAGCTGGAATAAGTAGTGTAGTGGTGAAATGCATAGATATTACTTAGAACACCAATTGCGAAGGCAGGTTACTAAGCAACAACTGACGCTGATGGACGAAAGCGTGGGGAGCGAACAGG

>581a55014641e1cd55cdc272c0365a28

TACGTAGGTGGCAAGCGTTATCCGGATTTATTGGGCGTAAAGAGAGTGCAGGCGGTTTTCTAAGTCTGATGTGAAAGCCTTCGGCTTAACCGGAGAAGTGCATCGGAAACTGGATAACTTGAGTGCAGAAGAGGGTAGTGGAACTCCATGTGTAGCGGTGGAATGCGTAGATATATGGAAGAACACCAGTGGCGAAGGCGGCTACCTGGTCTGCAACTGACGCTGAGACTCGAAAGCATGGGTAGCGAACAGG

>78500c826f22cac8943b1e6a24736ddf

TACGTAGGTGGCAAGCGTTGTCCGGATTTATTGGGCGTAAAGCGAGCGCAGGCGGAAGAATAAGTCTGATGTGAAAGCCCTCGGCTTAACCGAGGAACTGCATCGGAAACTGTTTTTCTTGAGTGCAGAAGAGGAGAGTGGAACTCCATGTGTAGCGGTGGAATGCGTAGATATATGGAAGAACACCAGTGGCGAAGGCGGCTCTCTGGTCTGCAACTGACGCTGAGGCTCGAAAGCATGGGTAGTGAACAGG

>4d72007c70f4abc0bf9e8fe8b826d07d

TACGGAGGGTGCAAGCGTTAATCGGAATTACTGGGCGTAAAGCGCACGCAGGCGGTCTGTCAAGTCGGATGTGAAATCCCCGGGCTCAACCTGGGAACTGCATCCGAAACTGGCAGGCTAGAGTCTTGTAGAGGGGGGTAGAATTCCAGGTGTAGCGGTGAAATGCGTAGAGATCTGGAGGAATACCGGTGGCGAAGGCGGCCCCCTGGACAAAGACTGACGCTCAGGTGCGAAAGCGTGGGGAGCAAACAGG

>c1d9eada909d2d5dbe078f25f1038508

TACGTAGGGTGCGAGCGTTAATCGGAATTACTGGGCGTAAAGCGTGCGCAGGCGGCTTTGCAAGACAGAGGTGAAATCCCCGGGCTCAACCTGGGAACTGCCTTTGTGACTGCAAGGCTAGAGTACGGCAGAGGGGGATGGAATTCCGCGTGTAGCAGTGAAATGCGTAGATATGCGGAGGAACACCGATGGCGAAGGCAATCCCCTGGGCCTGTACTGACGCTCATGCACGAAAGCGTGGGGAGCAAACAGG

>7433f49009bcca4a3ca6cfc7db672d08

TACGAAGGGGGCTAGCGTTGTTCGGAATTACTGGGCGTAAAGCGCACGTAGGCGGATTGCTAAGTCAGGGGTGAAATCCCGGGGCTCAACCTCGGAACTGCCTTTGATACTGACAGTCTTGAGTCCGGAAGAGGTGAGTGGAATTCCTAGTGTAGAGGTGAAATTCGTAGATATTAGGAAGAACACCAGTGGCGAAGGCGGCTCACTGGTCCGGTACTGACGCTGAGGTGCGAAAGCGTGGGGAGCAAACAGG

>3bac14d2805bf995afe9d316a5777b1e

TACGTAGGTGGCAAGCGTTGTCCGGATTTATTGGGCGTAAAGCGAGCGCAGGCGGAAGAATAAGTCTGATGTGAAAGCCCTCGGCTTAACCGAGGAACTGCATCGGAAACTGTTTTTCTTGAGTGCAGAAGAGGAGAGTGGAACTCCATGTGTAGCGGTGGAATGCGTAGATATATGGAAGAACACCAGTGGCGAAGGCGGCTCTCTGGTCTGCAACTGACGCTGAGGCTCGAAAGCATGGGTAGCGAACAGG

>d83f60183d81253a505beaeef3cd168f

TACGTAGGGCGCGAGCGTTGTCCGGAATTATTGGGCGTAAAGAGCTTGTAGGCGGTTGGTCGCGTCTGCTGTGAAAGGCTGGGGCTTAACCCTGGTTTTGCAGTGGGTACGGGCTAACTAGAGTGCAGTAGGGGAGACTGGAATTCCTGGTGTAGCGGTGGAATGCGCAGATATCAGGAGGAACACCGATGGCGAAGGCAGGTCTCTGGGCTGTAACTGACGCTGAGAAGCGAAAGCATGGGGAGCGAACAGG

>0562e97bf8665edd5ae85899297a44a8

TACGGAGGGCGCGAGCGTTACCCGGATTTACTGGGCGTAAAGGGCGTGTAGGCGGCCTGGGGCGTCCCATGTGAAAGGCCACGGCTCAACCGTGGAGGAGCGTGGGATACGCTCAGGCTAGAGGGTGGGAGAGGGTGGTGGAATTCCCGGAGTAGCGGTGAAATGCGCAGATACCGGGAGGAACGCCGATGGCGAAGGCAGCCACCTGGTCCACTTCTGACGCTGAGGCGCGAAAGCGTGGGGAGCAAACCGG

>18cd7ec9a8b5fcf931acafe4e7bd413a

TACGAAGGGTGCAAGCGTTACTCGGAATTACTGGGCGTAAAGCGTGCGTAGGTGGTTCGTTAAGTCTGATGTGAAAGCCCTGGGCTCAACCTGGGAATTGCATTGGATACTGGCGAGCTAGAGTGCGGTAGAGGGTAGTGGAATTCCCGGTGTAGCAGTGAAATGCGTAGAGATCGGGAGGAACATCCGTGGCGAAGGCGACTGCCTGGACCAGCACTGACACTGAGGCACGAAAGCGTGGGGAGCAAACAGG

>6b8673c5c5f48a7effc1e60e8faf1e0a

TACGGAGGGTGCAAGCGTTATCCGGATTTATTGGGTTTAAAGGGTCCGTAGGCGGACCTGTAAGTCAGTGGTGAAATCTCATAGCTTAACTATGAAACTGCCATTGATACTGCAGGTCTTGAGTGAATTTGAGGTAGCTGGAATAAGTAGTGTAGCGGTGAAATGCATAGATATTACTTAGAACACCAATTGCGAAGGCAGGTTACCAAGATTCAACTGACGCTGAGGGACGAAAGCGTGGGGAGCGAACAGG

>b306b305fd1e6de902363b591fd28fac

TACGTAGGTGGCAAGCGTTGTCCGGAATTATTGGGCGTAAAGCGCGCGCAGGCGGTCCTTTAAGTCTGATGTGAAAGCCCACGGCTCAACCGTGGAGGGTCATTGGAAACTGGGGGACTTGAGTGCAGAAGAGGAAAGTGGAATTCCAAGTGTAGCGGTGAAATGCGTAGAGATTTGGAGGAACACCAGTGGCGAAGGCGACTTTCTGGTCTGTAACTGACGCTGAGGCGCGAAAGCGTGGGGAGCAAACAGG

>e5b87e1925bea86ca8b66c9fd52114ed

TACAGAGGCCCCAAGCGTTGTTCGGATTTACTGGGCGTAAAGGGTGTGTAGGGGGTCGTGTAAGTTTGACGTGAAATCCCGTTGCTCAACAACGGAACTGCGTCGAATACTGCTCGGCTGGAGGTTCGGAGATGAGGGCGGAATTCTCGGTGTAGCGGTGAAATGCGTAGATATCGAGAGGAACGCCGATGGCGAAAGCAGCCCTCAAGACGAAATCTGACCCTGAAACACGAAGGCCAGGGGAGCAAACGGG

>dc9e840c3957d353c56ad240a9df198a

GACAGAGGGTGCAAACGTTGTTCGGAATTACTGGGCGTAAAGCGTGTGTAGGCGGCTATGTAAGTCGGATGTGAAAGCCCTGGGCTCAACCCAGGAAGTGCACTCGATACTGCGTAGCTTGAGTCTCGGAGAGGTTGGTGGAATTCTCGGTGTAGAGGTGAAATTCGTAGATATCGAGAGGAACACCGGTGGCGAAGGCGGCCAACTGGACGAAGACTGACGCTGAGACACGAAAGCGTGGGGAGCAAACAGG

>b02386c452fc40916a7c6c959a4885ac

TACGGAGGGAGCTAGCGTTGTTCGGAATTACTGGGCGTAAAGCGTACGTAGGCGGTTTAATAAGTCAGGGGTGAAAGCCCAGAGCTCAACTCTGGAACTGCCTTTGAGACTGTTAGACTAGAACATAGAAGAGGTAAGTGGAATTCCGAGTGTAGAGGTGAAATTCGTAGATATTCGGAAGAACACCAGTGGCGAAGGCGACTTACTGGTCTATAGTTGACGCTGAGGTACGAAAGCGTGGGTAGCAAACAGG

>f7447c8e079023f4f2579d8580575dd3

TACGTAGGTGGCAAGCGTTGTCCGGATTTATTGGGCGTAAAGCGAGTGCAGGCGGCTCGATAAGTCTGATGTGAAAGCCTTCGGCTCAACCGGAGAATTGCATCAGAAACTGTCGAGCTTGAGTACAGAAGAGGAGAGTGGAACTCCATGTGTAGCGGTGAAATGCGTAGATATATGGAAGAACACCGGTGGCGAAGGCGGCTCTCTGGTCTGTTACTGACGCTGAGGCTCGAAAGCATGGGTAGCGAACAGG

>d51712f032fbe4f91fabb00955ed20cf

CACACTAATCTCTTCAGAAAGACAAAATATTTCTCCAGAATGCATTTCAACCATGACGGTCATTTTTAACTGGCAGTGCCTTATAGATATTCGTGAAAGGTTTTGAACGTGTCTTGTCCATAGAGTTCAGTTTGTTCAATAATGATTTTTTTATGTAATTTTTAACGTAGTATATTTAGGCTTTCAAGTAAATTCATTTTGTTTCCTTTTTTGCCGGCTGTG

>f3db0aee0c55abffe6dbd7cd17123f0e

TACCGGCAGCTCGAGTGATGTCCAATATTATTGGGCCTAAAGCGTCCGTAGCTGGCCGCGCAAGTCCGTCGGGAAATCCACCTGCCCAACAGGTGGGCGTCCGGCGGAAACTGTGTGGCTTGGAACCGGAAGGCTCAGAGGGTACGTCCGGGGTAGGAGTGAAATCCCGTAATCCCGGACGGACCACCGATGGCGAAAGCACTCTGAGAAGACGGCTTCGACAGTGAGGGACGAAAGCTAGGGTCTCAAACCGG

>821403c34165d7b42378e24adb047f71

CACACTTATGTCTTCAGAAAGACAAAATATTTCTCCAGGATGCATTTCGACCATGTCGGTCATTGTCAACTGGCAGTGCATTGTAGATATCTGTGAAAGATTTTGAATGTGTCTTGTCCATAGAGTTCAGTTTGTTCATTAATAATTTTTTATGTAGTTTTTAAAGTAATATATTTAGACTTTCAAGTAAATTCGTTTTGTTTCCTTTTTTGCCGGTTGTG

>61100359491dce0c2cbb71beb7216d9e

TACCGGCAGCACGAGTGATGGCCGATATTATTGGGCCTAAAGCGTCCGTAGCTTGCTGTGTAAGTCCATTGGGAAATCGACGCGCTCAACGCGTCGACGTCCGGTGGAAACTACACGGCTTGGGGCCGAGAGACTCGACGGGTACGTCCGGGGTAGGAGTGAAATCCTGTAATCCTGGACGGACCACCAATGGGGAAACCACGTCGAGAGACCGGACCCGACAGTGAGGGACGAAAGCCAGGGTCTCGAACCGG

>11397adbcc3ae7ea2d586775e298a19d

TACGGAGGGAGCTAGCGTTGTTCGGAATTACTGGGCGTAAAGCGCACGTAGGCGGCTTTGTAAGTCAGAGGTGAAAGCCTGGAGCTCAACTCCAGAACTGCCTTTGAGACTGCATCGCTTGAATCCAGGAGAGGTGAGTGGAATTCCGAGTGTAGAGGTGAAATTCGTAGATATTCGGAAGAACACCAGTGGCGAAGGCGGCTCACTGGACTGGTATTGACGCTGAGGTGCGAAAGCGTGGGGAGCAAACAGG

>912f795ef3617aeb65ff9ad69b9567a8

TACGAAGGGTGCAAGCGTTACTCGGAATTACTGGGCGTAAAGCGTGCGTAGGTGGTCGTTTAAGTCCGTTGTGAAAGCCCTGGGCTCAACCTGGGAACTGCAGTGGATACTGGACGACTAGAGTGTGGTAGAGGGTAGCGGAATTCCTGGTGTAGCAGTGAAATGCGTAGAGATCAGGAGGAACATCCATGGCGAAGGCAGCTACCTGGACCAACACTGACACTGAGGCACGAAAGCGTGGGGAGCAAACAGG

>4490a1dff5ed7f1a014487420c58ef58

TACGTAGGGCGCGAGCGTTATCCGGAATTATTGGGCGTAAAGAGCTCGTAGGCGGTTTGTCACGTCTGCTGTGAAAGCCCGGGGCTTAACCCCGGGTGTGCAGTGGGTACGGGCAGACTGGAGTGCAGTAGGGGAGACTGGAATTCCTGGTGTAGCGGTGAAATGCGCAGATATCAGGAGGAACACCGATGGCGAAGGCAGGTCTCTGGGCTGTTACTGACGCTGAGGAGCGAAAGCATGGGGAGCGAACAGG

>6e0b74c4ffc471afcc1671629302f955

TACGTAGGGTGCAAGCGTTGTCCGGAATTATTGGGCGTAAAGAGCTCGTAGGCGGTTTGTTGCGTCGGCTGTGAAAACCTGGAGCTCAACTCCAGGCCTGCAGTCGATACGAGCAGACTAGAGTGTTGCAGGGGAGACTGGAATTCCTGGTGTAGCGGTGAAATGCGCAGATATCAGGAGGAACACCGGTGGCGAAGGCGGGTCTCTGGGCAGCAACTGACGCTGAGGAGCGAAAGCGTGGGGAGCGAACAGG

>c333727b7091148aea9ec776e98843b0

CACGATTAGCCCAAGTCAATAGAAGCCGGCGTAAAGAGTGTTTTAGATCACCCCCTCCCCAATAAAGCTAAAACTCACCTGAGTTGTAAAAAACTCCAGTTGACACAAAATAGACTACGAAAGTGGCTTTAACATATCTGAACACACAATAGCTAAGACCCAAACTGGG

>8d935db4a4135e3280b62a19a1466f2d

TACGGAGGGTGCAAGCGTTATCCGGATTTATTGGGTTTAAAGGGTCCATAGGCGGATTTGTAAGTCAGTGGTGAAATCTCACAGCTTAACTGTGAAACTGCCATTGATACTGCAAGTCTTGAGTGTTGTTGAAGTAGCTGGAATAAGTAGTGTAGCGGTGAAATGCATAGATATTACTTAGAACACCAATTGCGAAGGCAGGTTACTAAGCAACAACTGACGCTGATGGACGAAAGCGTGGGGAGCGAACAGG

>cd9401a6bce4a63af516d06d2a843f9d

TACGTAGGTGGCAAGCGTTGTCCGGAATTATTGGGCGTAAAGCGCGCGCAGGCGGATCAGTCAGTCTGTCTTAAAAGTTCGGGGCTTAACCCCGTGATGGGATGGAAACTGCTGATCTAGAGTATCGGAGAGGAAAGTGGAATTCCTAGTGTAGCGGTGAAATGCGTAGATATTAGGAAGAACACCAGTGGCGAAGGCGACTTTCTGGACGAAAACTGACGCTGAGGCGCGAAAGCCAGGGGAGCGAACGGG

>a4cbe987942964b584e95e4efab6a176

TACGTAGGTGGCAAGCGTTGTCCGGAATTATTGGGCGTAAAGGGCTCGCAGGCGGTTCCTTAAGTCTGATGTGAAAGCCCCCGGCTCAACCGGGGAGGGTCATTGGAAACTGGGGAACTTGAGTGCAGAAGAGGAGAGTGGAATTCCACGTGTAGCGGTGAAATGCGTAGAGATGTGGAGGAACACCAGTGGCGAAGGCGACTCTCTGGTCTGTAACTGACGCTGAGGAGCGAAAGCGTGGGGAGCGAACAGG

>68887a1da7baf770336fe3d8c28e716c

TACGTAGGGTGCGAGCGTTAATCGGAATTACTGGGCGTAAAGCGTGCGCAGGAGGTTTGTTAAGACAGATGTGAAATCCCCGGGCTCAACCTGGGAACTGCATTTGTGACTGGCAGGCTAGAGTATGGCAGAGGGGGGTAGAATTCCACGTGTAGCAGTGAAATGCGTAGAGATGTGGAGGAATACCGATGGCGAAGGCAGCCCCCTGGGCCAATACTGACGCTCATGCACGAAAGCGTGGGGAGCAAACAGG

>9e701e82d552ee66d9007fa3883b6560

GACAGAGGATGCAAGCGTTATCCGGAATGATTGGGCGTAAAGCGTCTGTAGGTGGCTTTTTAAGTCCGCCGTCAAATCCCAGGGCTCAACCCTGGACAGGCGGTGGAAACTACCAAGCTGGAGTACGGTAGGGGCAGAGGGAATTTCCGGTGGAGCGGTGAAATGCGTAGAGATCGGAAAGAACACCAACGGCGAAAGCACTCTGCTGGGCCGACACTGACACTGAGAGACGAAAGCTAGGGGAGCGAATGGG

>09de3dde6c465b74bcb8e6f8a478e4e0

TACGTAGGGTGCGAGCGTTGTCCGGAATTACTGGGCGTAAAGAGTTCGTAGGCGGTTTGTCGCGTCGTTTGTGAAAACCCGGGGCTCAACTTCGGGCTTGCAGGCGATACGGGCAGACTTGAGTGTTTCAGGGGAGACTGGAATTCCTGGTGTAGCGGTGAAATGCGCAGATATCAGGAGGAACACCGGTGGCGAAGGCGGGTCTCTGGGAAACAACTGACGCTGAGGAACGAAAGCGTGGGTAGCAAACAGG

>569653b1659271a290facfcedd0de061

TACGTAGGTGGCAAGCGTTGTCCGGATTTATTGGGCGTAAAGCGAGCGCAGGCGGTTTCTTAAGTCTGATGTGAAAGCCCCCGGCTCAACCGGGGAGGGTCATTGGAAACTGGGAGACTTGAGTGCAGAAGAGGAGAGTGGAATTCCATGTGTAGCGGTGAAATGCGTAGATATATGGAGGAACACCAGTGGCGAAGGCGGCTCTCTGGTCTGTAACTGACGCTGAGGCTCGAAAGCGTGGGGAGCGAACAGG

>22d93a5440a1c97f06db10312de65173

TACGGAGGGTGCGAGCGTTAATCGGAATAACTGGGCGTAAAGGGCACGCAGGCGGACTTTTAAGTGAGGTGTGAAATCCCCGGGCTTAACCTGGGAATTGCATTTCAGACTGGGAGTCTAGAGTACTTTAGGGAGGGGTAGAATTCCACGTGTAGCGGTGAAATGCGTAGAGATGTGGAGGAATACCGAAGGCGAAGGCAGCCCCTTGGGAATGTACTGACGCTCATGTGCGAAAGCGTGGGGAGCAAACAGG

>abc36f66e460e9c38dc935725f284c9f

TACCGGCAGTCCGAGTGATGGCCGATATTATTGGGCCTAAAGCGTCCGTAGCTGGCTGGACAAGTCCGTTGGGAAATCTGCCCGCTTAACGGGCAGGCGTCCAGCGGAAACTGTTCAGCTTGGGACCGGAAGACCTGAGGGGTACGTCTGGGGTAGGAGTGAAATCCCGTAATCCTGGACGGACCGCCGGTGGCGAAAGCGCCTCAGGAGGACGGATCCGACAGTGAGGGACGAAAGCTAGGGTCTCGAACCGG

>10afda2baef44de4c584a6641de399b1

TACGGAGGGGGCTAGCGTTGTTCGGAATTACTGGGCGTAAAGCGCACGTAGGCGGCTTTGTAAGTCAGAGGTGAAAGCCTGGAGCTCAACTCCAGAACTGCCTTTGAGACTGCATCGCTTGAATCCAGGAGAGGTCAGTGGAATTCCGAGTGTAGAGGTGAAATTCGTAGATATTCGGAAGAACACCAGTGGCGAAGGCGGCTGACTGGACTGGTATTGACGCTGAGGTGCGAAAGCGTGGGGAGCAAACAGG

>567f18673293d0688d5f8f36d9cf3bc3

CACGATTAACCCAAGTCAATAGAAGCCGGCGTAAAGAGTGTTTTAGATCACCCCCTCCCCAATAAAGCTAAAACTCACTTGAGTTGTAAAAAACTCCAGTTGACACAAAATAGACTACGAAAGTGGCTTTAACATATCTGAACACACAATAGCTAAGACCCAAACTGGG

>e9fbe7be1dbade27399de38f098e90de

TCGAACCCAAGCCACGTGGTGTAGCGAACCAACACACTACACACCACGAGTACTCCCCCCCCCCTTCCAATCAACAAAAAATCACAATACGATTACAGGAAATTGTTCAGAATGTAGCGCCAAATGAAATTACAAAGAGGAGGATTTTGAATATTGCAGAAGGATCGTATTGGTGCAAAACAAACGTTTGCTAGCAGCCTTCCTCACGTGTATACTGCACTGATGCTTCGAGAAATAGTCTGCAGAACTAT

>8320a83941fdf8ff8c354dfb223130dd

CACACAGAAAGACAAAATATTTCTCCAGGATGCATTTCAACCATGTCGGTCATTTTCAACTGGCAGTGCCTTGTAGATATTTGTGAAAGATTTTGAATGTGTCTTGTCCATAGATTTGAGTTTGTTCATTAATGATTTTTTTATGTAGTTTGTAACGTAGTATATTTAGACTTTCAAGTAAATTCATTTTCTTTACTTTTTTGCCGGTTGTG

>606ac3a3639b3a9f2dad54fa84b8be11

CACACTTACCTATGTCTTTAGAAAGACAAAACATTTTTCCAGAATGCATTTCAACCATGTCGGTCATTTTCAACTGGCAGTGCCTTGTAGATAGTTGGGGAAGATTTTGAATGTGTTTTGTCCATAGAGTTCAGTTTGTTCATTAATGATTTTGTTATGTAGTTTGTAACGTACTATATTTAGACTTTCAAGTAAATTCATTTTTATTTCCTATTTTTGCCGGTTGTG

>e4ef1c7b616eb842c0ff7a327e83bd76

TACGGAGGATGCGAGCGTTATCCGGATTTATTGGGTTTAAAGGGTGCGTAGGTGGTTAATTAAGTCAGCGGTGAAAGTTTGTGGCTCAACCATAAAATTGCCGTTGAAACTGGTTGACTTGAGTATATTTGAGGTAGGCGGAATGCGTGGTGTAGCGGTGAAATGCATAGATATCACGCAGAACTCCGATTGCGAAGGCAGCTTACTAAACTATAACTGACACTGAAGCACGAAAGCGTGGGGATCAAACAGG

>6b6b180315def5d9a9ab20b8e1afd836

TACGGAGGGTGCAAGCGTTATCCGGATTTATTGGGTTTAAAGGGTCCGTAGGCGGATTTGTAAGTCAGTGGTGAAATCTCACAGCTTAACTGTGAAACTGCCATTGATACTGCAAGTCTTGAGTGTTGTTGAAGTAGCTGGAATAAGTAGTGTAGCGGTGAAATGCATAGATATTACTTAGAACACCAATTGCGAAGGCAGGTTACTAAGCAACAACTGACGCTGATGGACGAAAGTGTGGGGAGCGAACAGG

>3928d5bdcbbf94eca7b83e13879738f6

AACAGAGGATACAAGCGTTATCCGGATTTATTGGGTTTAAAGGGTGCGTAGGTGGTTTTTTAAGTCAGTAGTGAAATCTTAAGGCTTAACTTTAAAAGTGCTATTGATACTGATAAACTAGAGTGAGGTTGGAGTAACTGGAATGTGTGGTGGAGCGGTGAAATGCATAGAGATCACACAGAACACCAATCGCGAAGGCATGTTACTAAACATAGACTGACACTGAGGCACGAAAGCATGGGTAGCAAACAGG

>43fddf1528d4a98928fd8c3a8ac23bfd

TACAGAGGGTGCAAGCGTTAATCGGAATTACTGGGCGTAAAGCGCGCGTAGGTGGTTTGTTAAGTTGAATGTGAAATCCCCGGGCTCAACCTGGGAACTGCATCCAAAACTGGCAAGCTAGAGTATGGTAGAGGGTGGTGGAATTTCCTGTGTAGCGGTGAAATGCGTAGATATAGGAAGGAACACCAGTGGCGAAGGCGACCACCTGGACTGATACTGACACTGAGGTGCGAAAGCGTGGGGAGCAAACAGG

>4a0df1b8dce61f225bd6afe9addd0e37

TACCGGCAGCCCAAGTGATGGCCGATCTTATTGGGCCTAAAGCGTCCGTAGCTGGCCGCGCAAGTCCATCGGGAAATCCACCTGCTCAACAGGTGGGCGCCCGGTAGAAACTGCGTGGCTTGGGACCGGAAGGCGCGACGGGTACGTCCGGGGTAGGAGTGAAATCCCGTAATCCTGGACGGACCGCCGATGGCGAAAGCACGTCGCGAGAACGGATCCGACAGTGAGGGACGAAAGCCAGGGTCTCGAACCGG

>aac108ccc77b37bdebe1d9e6ede0abff

TACAGAGGATGCAAGCGTTATCCGGAATGATTGGGCGTAAAGCGTCTGTAGGTGGCTTTTTAAGTCCGCCGTCAAATCCCAGGGCTCAACCCTGGACAGGCGGTGGAAACTACCAAGCTGGAGTACGGTAGGGGCAGAGGGAATTTCCGGTGGAGCGGTGAAATGCGTAGAGATCGGAAAGAACACCAACGGCGAAAGCACTCTGCTGGGCCGACACTGACACTGAGAGGCGAAAGCTAGGGGAGCGAATGGG

>0e2e91fba1ca9ed7de2d11ea4786c914

TACAGAGGGTGCGAGCGTTAATCGGATTTACTGGGCGTAAAGCGTGCGTAGGCGGCTAATTGAGTCGGATGTGAAATCCCCGAGCTTAACTTGGGAATTGCATTCGATACTGGTTAGCTAGAGTGTGGGAGAGGATGGTAGAATTCCAGGTGTAGCGGTGAAATGCGTAGAGATCTGGAGGAATACCGATGGCGAAGGCAGCCATCTGGCCTAACACTGACGCTGAGGTACGAAAGCATGGGGAGCAAACAGG

>1fbc230d68b6bacb0a1d23a81e315294

CAAATTTATGTCTTCAGAAAGACAAAATATTTTTCCAGGGTCCATTTCAACCATGTCGGTCATTTTCAACTAGCAGTGCCTTGTAGATATTTGTGAAAGATTTTGAATGTGTCTTGTCCATAGAGTTCAGTTTGTTCATTAATGATTTTTTTATGTAGTTTGTAACGTAATATATTTAGGCTTTCAAGTAAATTGATTTTGTTTCCTTTTTCCGGTTGTG

>08bdc6c81f6b9daab3d132b54e1d45bc

TACGTAGGGTGCAAGCGTTGTCCGGAATTACTGGGCGTAAAGAGTTCGTAGGCGGTTTGTCGCGTCGTTTGTGAAAACCAGCAGCTCAACTGCTGGCTTGCAGGCGATACGGGCAGACTTGAGTACTGCAGGGGAGACTGGAATTCCTGGTGTAGCGGTGAAATGCGCAGATATCAGGAGGAACACCGGTGGCGAAGGCGGGTCTCTGGGCAGTAACTGACGCTGAGGAACGAAAGCGTGGGTAGCGAACAGG

>949f1b41b0c85763b471cbc4bbffa1ba

AACTCACACAACTAAACAGTTTTAACATTTCCCACCGAGTTTTATATTTTTTCATATTTCGTCGCTTGGCTGGCGCCTACATTGATCCGACTCCGAGAACGAGCTCCAGATGAGCTGTCATCAAAATTCTTAGAGATTCGCAGTCAAGGGTATAACATCCATATCAGGAATGTTTCTTACATATTCACATGGCGGCCAGAAATTCGTCATAACAGTAACTGGATTA

>ddfd49f939f92958b1ec816741055348

TACGTAGGGTCCAAGCGTTAATCGGAATTACTGGGCGTAAAGCGTGCGCAGGCGGTTGTGCAAGACCGATGTGAAATCCCCGAGCTTAACTTGGGAATTGCATTGGTGACTGCACGGCTAGAGTGTGTCAGAGGGGGGTAGAATTCCACGTGTAGCAGTGAAATGCGTAGAGATGTGGAGGAATACCGATGGCGAAGGCAGCCCCCTGGGATAACACTGACGCTCATGCACGAAAGCGTGGGGAGCAAACAGG

>2a97a19bc75e9e74e1b632bbe54486c1

CACGATTAACCCAAGTCAATAGAAGCCGGCGTAAGGAGTGTTTTAGATCACCCCCTCCCCAATAAAGCTAAAACTCACCTGAGTTGTAAAAAACTCCAGTTGACACAAAATAGACTACGAAAGTGGCTTTAACATATCTGAACACACAATAGCTAAGACCCAAACTGGG

>50b0253f8ee42e209eda46c466bb93dd

TACGGAGGGTGCAAGCGTTATCCGGATTTATTGGGTTTAAAGGGTCTGTAGGCGGATTTGTAAGTCAGTGGTGAAATCTCACAGCTTAACTGTGAAACTGCCATTGATACTGCAAGTCTTGAGTGTTGTTGAAGTAGCTGGAATAAGTAGTGTAGCGGTGAAATGCATAGATATTACTTAGAACACCAATTGCGAAGGCAGGTTACTAAGCAACAACTGACGCTGATGGACGAAAGCGTGGGGAGCGAACAGG

>61ed6a6000a76fa8258665f512a93e5f

TTCTACATCAGTTGCAATTGGTTTATACGAAACTGTTTTGTTTTTCCAGTATGCCGAAAGTGCATTCAACAGTGCGACGTGCAACGGAAAGCCTGTAATTAAATATGCGTTTAGGAAACCCAGTTGTCTTCTCGCGTACGGTCACATTAAATTGAAACGAAGCGGGAAGGCCTCATCGCCAACTAAATAAAATGGGAATATTTCATTTGTTCCAGGAATTTCTTGAGG

>204fd607ccb445bf0a5524be28c9df8f

CACACTTATGTCTTCAGAAAGACAAAATATTTCTCCAGGATGCATTTCAACCATGTTGGTCATTTTCAACTGGCAGTGCCTTGTAGATGTTTGTAAAAGATTTTGAATGTGTCTTGTCCATAGAGTTCAGTTTGTTGAAGCAAACTGAAATATGTTTGTTCAAGCATACATTGGCTTTCAAGTAAATTCATTTTGTGTCCTTTTTTGCCGGTTGTG

>9908fffab7ed4f3bec44cda2f5084d49

TACGTAGGTGGCAAGCGTTGTCCGGATTTATTGGGCGTAAAGCGAGCGCAGGCGGTTTCTTAAGTCTGATGTGAAAGCCCCCGGCTCAACCGGGGAGGGTCATTGGAAACTGGGAGACTTGAGTGCAGAAGAGGAGAGTGGAATTCCATGTGTAGCGGTGAAATGCGTAGATATATGGAGGAACACCAGTGGCGAAGGCGGCTCTCTGGTCTGTAACTGACGCTGAGGCTCGAAAGCGTGGGGAGCAAACAGG

>d9b23082985e1674fadf7650b58a5011

TACGGAGGGTGCAAGCGTTAATCGGAATTACTGGGCGTAAAGCGCACGTAGGTGGTTTGTTAAGCTAGCCGTGAAATCCCCGGGCTCAACCTGGGCACTGCGGTTAGAACTGGCAAGCTAGAGTAGGGTAGAGGGGTGTGGAATTCCAGGTGTAGCGGTGAAATGCGTAGATATCTGGAGGAACATCAGTGGCGAAGGCGACACCCTGGACTCATACTGACACTGAGGTGCGAAAGCGTGGGGAGCAAACAGG

>f44c3e866050273e250d38b88dcd86fd

CACGATTAACCCAAGTCAATAGAAGCCGGCGTAAAGAGTGTTTTAGATCACCCCCTCCCCAATAAAGCTAAAACTCACCTGAGTTGTAAAAAACTCCAGTTGACACAAAATAGACTACGAAAGTGGCCTTAACATATCTGAACACACAATAGCTAAGACCCAAACTGGG

>f93c192b3f8184ac914b43d868ecb3b1

TACGTAGGTGGCGAGCGTTGTCCGGAATTATTGGGCGTAAAGCGCGCGTAGGCGGGATAGTCAGTCTGTTGTAAAAGTGCGGGGCTCAACCCCGTAAGGTCAATGGAAACTGCTATTCTTGAGTGCAGGAGAGGAAAGTGGAATTCCCAGTGTAGCGGTGAAATGCGTAGATATTGGGAGGAACACCAGTGGCGAAGGCGACTTTCTGGACTGTGTCTGACGCTGAGGCGCGAAAGCCAGGGGAGCGAACGGG

>7c7a5ce62147e420298cbb52ec8c6a3a

CAAATTTATGTCTTCAGAAAGACAAAATATTTTTCCAGGGTCCATTTCAACCATGTCGGTCATTTTCAACTAGCAGTGCCTTGTAGATATTTGTGAAAGATTTTGAATGTGTCTTGTCCATAGAGTTCAGTTTGTTCATTAATGATTCTTTATGTAGTTTGTAACGTAATATATTTAGGCTTTCAAGTAAATTGATTTTGTTTCCTTTTTCCGGTTGTG

>72788fb7edfb529829d812a5f2921c62

TACGTAGGGTGCGAGCGTTAATCGGAATTACTGGGCGTAAAGCGTGCACAGGCGGTTTGTTAAGACAGATGTGAAATCCCCGGGCTCAACCTGGGAACTGCATTTGTGACTGGCAGGCTAGAGTATGGCAGAGGGGGGTAGAATTCCACGTGTAGCAGTGAAATGCGTAGAGATGTGGAGGAATACCGATGGCGAAGGCAGCCCCCTGGGCCAATACTGACGCTCATGCACGAAAGCGTGGGGAGCAAACAGG

>a537d8bab85c83b0e74c73c55790324b

TACGAAGGGGGCTAGCGTTGCTCGGAATTACTGGGCGTAAAGGGAGCGTAGGCGGACATTTAAGTCAGGGGTGAAATCCCGGGGCTCAACCTCGGAATTGCCTTTGATACTGGGTGTCTTGAGTATGAGAGAGGTGTGTGGAACTCCGAGTGTAGAGGTGAAATTCGTAGATATTCGGAAGAACACCAGTGGCGAAGGCGACACACTGGCTCATTACTGACGCTGAGGCTCGAAAGCGTGGGGAGCAAACAGG

>df63f2b021e452bc6ca64e9bfb70b862

TACGTAGGGTGCAGGCGTTAATCGGAATTACTGGGCGTAAAGCGTGCGCAGGCGGTTTGTTAAGACAGGTGTGAAATCCCCGGGCTTAACCTGGGAATTGCGCTTGTAACTGGCAGACTGGAGTGTGGCAGAGGGGGGTGGAATTCCAAGTGTAGCAGTGAAATGCGTAGATATTTGGAGGAACACCGATGGCGAAGGCAGCCCCCTGGGCTAACACTGACGCTCATGCACGAAAGCGTGGGGAGCAAACAGG

>c22b16cc6108c04f29fea3b6d4c81571

TACAGAGGGTGCAAGCGTTAATCGGATTTACTGGGCGTAAAGCGCGCGTAGGTGGCCAATTAAGTCAAATGTGAAATCCCCGAGCTTAACTTGGGAATTGCATTCGATACTGGTTGGCTAGAGTATGGGAGAGGATGGTAGAATTCCAGGTGTAGCGGTGAAATGCGTAGAGATCTGGAGGAATACCGATGGCGAAGGCAGCCATCTGGCCTAATACTGACACTGAGGTGCGAAAGCATGGGGAGCAAACAGG

>e33c5576113465ae725d3993fc43738e

TACGAAGGGTGCAAGCGTTACTCGGAATTACTGGGCGTAAAGCGTGCGTAGGTGGTTCGTTAAGTCTGATGTGAAAGCCCTGGGCTCAACCTGGGAATTGCATTGGATACTGGCGAGCTAGAGTGCGGTAGAGGATGGCGGAATTCCCGGTGTAGCAGTGAAATGCGTAGAGATCGGGAGGAACATCTGTGGCGAAGGCGGCCATCTGGACCAGCACTGACACTGAGGCACGAAAGCGTGGGGAGCAAACAGG

>b6e4e0d69a380553624ff40d56da2543

GACGGGGGGGGCAAGTGTTCTTCGGAATGACTGGGCGTAAAGGGCACGTAGGCGGTGAATCGGGTTGAAAGTGAAAGTCGCCAAAAACTGGCGGAATGCTCTCGAAACCAATTCACTTGAGTGAGACAGAGGAGAGTGGAATTTCGTGTGTAGGGGTGAAATCCATGAATCTACGAAGGAACGCCAAAAGCGAAGGCAGCTCTCTGGGTCCCTACCGACGCTGGGGTGCGAAAGCATGGGGAGCGAACAGG

>6c743f540ed34be99f959fbc3ffba98c

TCGAACCCAAGCCACGTGGTGTAGCGAACCAACACACTACACACCACGAGTACTCCCCCCCCCTTCCAATCAACAAAAAATCACAATACGATTACAGGAAATTGTTCAGAATGTAGCGCCAAATAAAATTACAAAGAGGAGGATTTTGAATATTGCAGAAGGATCGTATTGGTGCAAAACAAACGTTTGCTAGCAGCCTTCCTCACGTGTATACTGCACTGATGCTTCGAGAAATAGTCTGCAGAACTAT

>cf09df93f4d77a305cdaf5389da98f00

TACGGAGGGTGCAAGCGTTAATCGGAATTACTGGGCGTAAAGCGTGCGCAGGCGGCTGATTAAGTCGGATGTGAAAGCCCCGGGCTCAACCTGGGAATGGCATTCGATACTGGTCAGCTAGAGTCTGGTAGAGGTAAGCGGAATTCCGGGTGTAGCGGTGAAATGCGTAGATATCCGGAGGAACATCAGTGGCGAAGGCGGCTTACTGGACCAAGACTGACGCTCAGGCACGAAAGCGTGGGTAGCAAACAGG

>ea403646ed22d679fa4586263d8fc32f

TACAGAGGGTGCGAGCGTTAATCGGATTTACTGGGCGTAAAGCGTGCGTAGGCGGCTTATTAAGTCGGATGTGAAATCCCCGAGCTTAACTTGGGAATTGCATTCGATACTGGTGAGCTAGAGTATGGGAGAGGATGGTAGAATTCCAGGTGTAGCGGTGAAATGCGTAGAGATCTGGAGGAATACCGATGGCGAAGGCAGCCATCTGGCCTAATACTGACGCTGAGGTACGAAAGCATGGGGAGCAAACAGG

>7f85ac088481e3811666998c6ba22231

TACGTATGTCACAAGCGTTATCCGGATTTATTGGGCGTAAAGCGCGTCTAGGTGGTTATATAAGTCTGATGTGAAAATGCAGGGCTCAACTCTGTATTGCGTTGGAAACTGTGTAACTAGAGTACTGGAGAGGTAAGCGGAACTACAAGTGTAGAGGTGAAATTCGTAGATATTTGTAGGAATGCCGATGGGGAAGCCAGCTTACTGGACAGATACTGACGCTGAAGCGCGAAAGCGTGGGTAGCAAACAGG

>f17d959760b1fa8a8c0ac74b621bdbb4

GACGGGGGGGGCAAGTGTTCTTCGGAATGACTGGGCGTAAAGGGCACGTAGGCGGTGAATCGGGTTGAAAGTTAAAGTCGCCAAAAACTGGTGGAATGCTCTCGAAACCAATTCACTTGAGTGAGACAGAGGAGAGTGGAATTTCGTGTGTAGGGGTGAAATCCGCAGATCTACGAAGGAACGCCAAAAGCGAAGGCAGCTCTCTGGGTCCCTACCGACGCTGGAGTGCGAAAGCATGGGGAGCGAACGGG

>b6ec86c622c28f5a4d60b9c1cc117fd4

TATGGAGGGTGCAAGCGTTATCCGGATTTATTGGGTTTAAAGGGTCCGTAGGCGGATTTGTAAGTCAGTGGTGAAATCTCACAGCTTAACTGTGAAACTGCCATTGATACTGCAAGTCTTGAGTGTTGTTGAAGTAGCTGGAATAAGTAGTGTAGCGGTGAAATGCATAGATATTACTTAGAACACCAATTGCGAAGGCAGGTTACTAAGCAACAACTGACGCTGATGGACGAAAGCGTGGGGAGCGAACAGG

>6aae9a17fbd9c7a4f55a65ef18d63320

TACGAAGGGGGCTAGCGTTGCTCGGAATCACTGGGCGTAAAGGGCGCGTAGGCGGCCATTCAAGTCGGGGGTGAAAGCCTGTGGCTCAACCACAGAATTGCCTTCGATACTGTTTGGCTTGAGTATGGTAGAGGTCGGTGGAACTGCGAGTGTAGAGGTGAAATTCGTAGATATTCGCAAGAACACCAGTGGCGAAGGCGGCCGACTGGACCATTACTGACGCTGAGGCGCGAAAGCGTGGGGAGCAAACAGG

>d66a98986a0a80c97580a2a98d8ddc72

TACAGAGGGTGCAAGCGTTAATCGGATTTACTGGGCGTAAAGCGCGCGTAGGCGGCTAATTAAGTCAAATGTGAAATCCCCGAGCTTAACTTGGGAATTGCATTCGATACTGGTTAGCTAGAGTGTGGGAGAGGATGGTAGAATTCCAGGTGTAGCGGTGAAATGCGTAGAGATCTGGAGGAATACCGACGGCGAAGGCAGCCATCTGGCCTAACACTGACGCTGAGGTGCGAAAGCATGGGGAGCAAACAGG

>47f2563806791ddc9283a33644e7c658

GACGGGGGGGGCAAGTGTTCTTCGGAATGACTGGGCGTAAAGGGCACGTAGGCGGTGAATCGGGTTGAAAGTGAAAGTCGCCAAAAACTGGTGGAATGCTCTCGAAACCAATTCACTTGAGTGAGACAGAGGAGAGTGGAATTTCGTGTGTAGGGGTGAAATCCGGAGATCTACGAAGGAACGCCAAAAGCGAAGGCAGCTCTCTGGGTCCCTACCGACGCTGGGGTGCGAAAGCATGGGGAGCGAACGGG

>7876a5906e5be58d068237cd0b6ebb3c

TACAGAGGGTGCAAGCGTTAATCGGAATTACTGGGCGTAAAGCGTGCGTAGACGGTTACATAAGTCGGGTGTGAAAGCCCCGGGCTCAACCTGGGAATTGCATTCGAGACTGCGTAGCTAGGGTGCGGAAGAGGGAAGCGGAATTTCCGGTGTAGCGGTGAAATGCGTAGATATCGGAAGGAACACCAGTGGCGAAAGCGGCTTCCTGGTCCAGCACCGACGTTCAGGCACGAAAGCGTGGGGAGCAAACAGG

>20b3df08cd5fac2be26a2928155bab69

TACGTAGGGTGCAAGCGTTAATCGGAATTACTGGGCGTAAAGCGTGCGCAGGCGGTTCGGAAAGAAAGATGTGAAATCCCAGAGCTTAACTTTGGAACTGCATTTTTAACTACCGAGCTAGAGTGTGTCAGAGGGAGGTGGAATTCCGCGTGTAGCAGTGAAATGCGTAGATATGCGGAGGAACACCGATGGCGAAGGCAGCCTCCTGGGATAACACTGACGCTCATGCACGAAAGCGTGGGGAGCAAACAGG

>a6fc343dd640208ff0816cdabec0426c

TACGTATGTCGCGAGCGTTATCCGGAATTATTGGGCATAAAGGGCATCTAGGCGGCCTGACAAGTCAGGGGTGAAAACCTGCGGCTCAACCGCAGGCCTGCCTTTGAAACTGTGAGGCTGGAGTACCGGAGAGGTGGACGGAACTGCACGAGTAGAGGTGAAATTCGTAGATATGTGCAGGAATGCCGATGATGAAGATAGTTCACTGGACGGTAACTGACGCTGAAGTGCGAAAGCCGGGGGAGCAAACAGG

>8a6bca303920d729b5eba71e92098603

CACACTTATGTCTTCAGAAAGACAAAATATTTCTTCAGGATGCATTTCAACCATGTCGGTCATTTTCAACTGGCAGTGCCTTGTAGATATTTGTGAAAGATTTTGAATGTGTCTTCTCCATAGAGTTGAGTTTGTTCATTAATGATTTTGTTATGTAGTTTGTAACGTAATACATTCAGGCTTTCAAGTAAATTCATTTTGTTTACTTTTTTGCCGGTTGTG

>b3f2495b892ba8079ef230f3be00f6e0

CACGATTAACCCAAGTCAATAGAAGCCGGCGTAAAGAGTGTTTTAGATCACCCCCTCCCCAATAAAACTAAAACTCACCTGAGTTGTAAAAAACTCCAGTTGACACAAAATAGACTACGAAAGTGGCTTTAACATATCTGAACACACAATAGCTAAGACCCAAACTGGG

>20f63518d323b502ff47b07259bf7df4

GACAGAGGATGCAAGCGTTATCCGGAATGATTGGGCGTAAAGCGTCTGTAGGTGGCTTTTGAAGTCCGCCGTCAAATCCCAGGGCTCAACCCTGGACAGGCGGTGGAAACTGCCAAGCTGGAGTACGGTAGGGGCAGACGGAATTTCCGGTGGAGCGGTGAAATGCGTAGAGATCGGAAAGAACACCAACGGCGAAAGCACTCTGCTGGGCCGACACTGACACTGAGAGACGAAAGCTAGGGGAGCAAATGGG

>ff9d93d7b7e46787568f2d241caeaf3b

TACGAAGGGTGCAAGCGTTAATCGGAATTACTGGGCGTAAAGCGCGCGTAGGTGGTTCAGCAAGTTGGATGTGAAATCCCCGGGCTCAACCTGGGAACTGCATCCAAAACTACTGAGCTAGAGTACGGTAGAGGGTGGTGGAATTTCCTGTGTAGCGGTGAAATGCGTAGATATAGGAAGGAACACCAGTGGCGAAGGCGACCACCTGGACTGATACTGACACTGAGGTGCGAAAGCGTGGGGAGCAAACAGG

>d5687886c2c8e1177ae6549940c4c0e6

TACGGAGGGTGCAAGCGTTAATCGGAATTACTGGGCGTAAAGCGCACGCAGGCGGTCTGTTAAGTCAGATGTGAAATCCCCGGGCTTAACCTGGGAACTGCATTTGAAACTGGCAGGCTTGAGTCTTGTAGAGGGGGGTAGAATTCCAGGTGTAGCGGTGAAATGCGTAGAGATCTGGAGGAATACCGGTGGCGAAGGCGGCCCCCTGGACAAAGACTGACGCTCAGGTGCGAAAGCGTGGGGAGTAAACAGG

>286fb1435fc041189701a06d7b162bbb

TACCGGCAGCCCGAGTGATGGCCGATCTTATTGGGCCTAAAGCGTCCGTAGCTGGCCGCACAAGTCCATCGGAAAATCCACCCGCCCAACGGGTGGGCGTCCGGTGGAAACTGTGTGGCTTGGGACCGGAAGGCGCGACGGGTACGTCCGGGGTAGGAGTGAAATCCCGTAATCCTGGACGGACCGCCGATGGCGAAAGCACGTCGCGAGAACGGATCCGACAGTGAGGGACGAAAGCCAGGGTCTCGAACCGG

>6c1c6bc409623ce2ec693adc7c13672e

CACGATTAACCCAAGTCGATAGAAGCCGGCGTAAAGAGTGTTTTAGATCACCCCCTCCCCAATAAAGCTAAAACTCACCTGAGTTGTAAAAAACTCCAGTTGACACAAAATAGACTACGAAAGTGGCTTTAACATATCTGAACACACAATAGCTAAGACCCAAACTGGG

>f4cba96059e73ce414425143975e3108

CACACTTATGTCTTTAGAAAGGCAAAACGTTTTTGCAAAATTCATTTCAACCATCTCGGTCATTTTCAACTGGCAGTGCCTTGTAGATAGTTTTGGAAGATTTTGAATGTTGCTTGTCCATAGAGATCAGTTTGTTCATTAATGATTTTGTTGTGTAGTTTGTAACGTAATATTTTTAAACTTTCAAGAAGATTAATTTTATTTCCTTTTTTGTCTGTTGTA

>d7348b86110af99c20176a0c0aa0697a

GACGTAGGATGCGAGCGTTGTCCGGATTTATTGGGCGTAAAGAGTTCGTAGGTGGTTTGTTAAGTTTGGTGTTAAAGATCGGGGCTCAACCCTGGGACTGCACTGAATACTGGCAGACTCGAGTGTGGTAGAGGCTAGTGGAATTCCCAGTGTAGCGGTGAAATGCGTAGATATTGGGAAGAACACCGGTGGCGTAGGCGACTAGCTGGGCCATAACTGACGCTGAGGAACGAAAGCCAGGGGAGCGAATGGG

>e1af6bc045f2e53c1f6a87b9dc1c6da0

CGGCGTGACAATAGTCAAACATATCCCACACAGAGGCAAAATCATGAACGTTTCGTAAAATCTACATATATGCTAACGCAGAACACAAAACCTGTTGATATATGAACAAATCCAAATCAATACAAACACGAGACAACTTTTAAAACACCAAACATTGAAAACATTCAGAAATCAAAAATTCTCGCTGAACATATTCAAACAATGATACCAACACAAGTCACTTAACCCC

>b1a2eb74f17971864ed1ed964da108e5

TACGTATGTTGCGAGCGTTATCCGGAATTATTGGGCTTAAAGGGCATCTAGGCGGTAAGACAAGTTGAAGGTGAAAACCTGTGGCTCAACCATAGGCTTGCCTACAAAACTGTTGAACTAGAGTACTGGAAAGGTGGGTGGAACTACACGAGTAGAGGTGAAATTCGTAGATATGTGTAGGAATGCCGATGATGAAGATAACTCACTGGACAGAAACTGACGCTGAAGTGCGAAAGCTAGGGGAGCAAACAGG

>153a1c462f6c9cc86cb69c231e968272

TACGGGGGGTGCGAGCGTTAATCGGAATAACTGGGCGTAAAGGGCACGCAGGCGGTGACTTAAGTGAGATGTGAAAGCCCCGGGCTTAACCTGGGAATTGCATTTCATACTGGGTCGCTAGAGTACTTTAGGGAGGGGTAGAATTCCACGTGTAGCGGTGAAATGCGTAGAGATGTGGAGGAATACCGAAGGCGAAGGCAGCCCCTTGGGAATGTACTGACGCTCATGTGCGAAAGCGTGGGGAGCAAACAGG

>14fd1c34c1dc8d9e7a969e455e5250b1

TACGAAGGGGGCTAGCGTTGCTCGGAATTACTGGGCGTAAAGGGCGCGTAGGCGGACAGTTAAGTTGGGGGTGAAAGCCCGGGGCTCAACCTCGGAATTGCCTTCAATACTGGCTGTCTTGAGTACGGGAGAGGTGAGTGGAACTCCGAGTGTAGAGGTGAAATTCGTAGATATTCGGAAGAACACCAGTGGCGAAGGCGACTCACTGGCCCGTTACTGACGCTGAGGCGCGAAAGCGTGGGGAGCAAACAGG

>99bccd58aaeb00f98e22add40be6e36b

TACGTAGGGGGCTAGCGTTATCCGGATTTACTGGGCGTAAAGGGTGCGTAGGCGGTCTTTCAAGTCAGGAGTGAAAGGCTACGGCTCAACCGTAGTAAGCTCTTGAAACTGGGAGACTTGAGTGCAGGAGAGGAGAGTGGAATTCCTAGTGTAGCGGTGAAATGCGTAGATATTAGGAGGAACACCAGTTGCGAAGGCGGCTCTCTGGACTGTAACTGACGCTGAGGCACGAAAGCGTGGGGAGCAAACAGG

>ec790b9277154b5cb0984af539a155eb

TACGGAGGGTGCAAGCGTTAATCGGAATTACTGGGCGTAAAGCGCACGCAGGCGGTCTGTTAAGTCAGATGTGAAATCCCCGGGCTTAACCTGGGAACTGCATTTGAAACTGGCAGGCTTGAGTCTTGTAGAGGGGGGTAGAATTCCAGGTGTAGCGGTGAAATGCGTAGAGATCTGGAGGAATACCGGTGGCGAAGGCGGCCCCCTGGACAAAGACTGACGCTCAGGTGCGAAAGCGTGGGGGGCAAACAGG

>e3f1db0317972bcbd0d65ce63718ab41

TACAGAGGGTGCAAGCGTTATCCGGATTTATTGGGTTTAAAGGGTCCGTAGGCGGATTTGTAAGTCAGTGGTGAAATCTCACAGCTTAACTGTGAAACTGCCATTGATACTGCAAGTCTTGAGTGTTGTTGAAGTAGCTGGAATAAGTAGTGTAGCGGTGAAATGCATAGATATTACTTAGAACACCAATTGCGAAGGCAGGTTACTAAGCAACAACTGACGCTGATGGACGAAAGCGTGGGGAGCGAACAGG

>a8e53bd66879cd51ecb4e7daf552accb

TACGTAGGGCGCAAGCGTTATCCGGAATTATTGGGCGTAAAGAGCTTGTAGGCGGTTCGTCGCGTCTGGTGTGAAAGCCCATCGCTTAACGGTGGGTCTGCGCCGGGTACGGGCGGGCTAGAGTGCAGTAGGGGAGACTGGAATTCCCGGTGTAACGGTGGAATGTGTAGATATCGGGAAGAACACCAATGGCGAAGGCAGGTCTCTGGGCTGTTACTGACGCTGAGAAGCGAAAGCGTGGGGAGCGAACAGG

>b7ae2d92c6199a9e58c97d0e75865d36

TACGTAGGGCGCAAGCGTTGTCCGGAATTATTGGGCGTAAAGAGCTCGTAGGCGGTTTGTCGCGTCTGCTGTGAAAGCCCGGGGCTTAACCCCGGGTGTGCAGTGGGTACGGGCAGACTAGAGTGCAGTAGGGGAGACTGGAATTCCTGGTGTAGCGGTGGAATGCGCAGATATCAGGAGGAACACCGATGGCGAAGGCAGGTCTCTGGGCTGTTACTGACGCTGAGGAGCGAAAGCATGGGGAGCGAACAGG

>25796cdd0224a9f6ae7f8c9c7e66d7a2

TACAGAGGGTGCAAGCGTTAATCGGATTTACTGGGCGTAAAGCGCGCGTAGGCGGCTAATTAAGTCAAATGTGAAATCCCCGAGCTTAACTTGGGAATTGCATTCGATACTGGTTAGCTAGAGTGTGGGAGAGGATGGTAGAATTCCAGGTGTAGCGGTGAAATGCGTAGAGACCTGGAGGAATACCGATGGCGAAGGCAGCCATCTGGCCTAACACTGACGCTGAGGTGCGAAAGCATGGGGAGCAAACAGG

>15ac7e3d6852c34b4175574e8c3f7cdd

TACGGAGGGTGCAAGCGTTATCCGGATTTATTGGGTTTAAAGGGTCCGTAGGCGGATTTGTAAGTCAGTGGTGAAATCTCACAGCTTAACTGTGAAACTGCCATTGATACTGCAAGTCTTGAGTGTTGTTGAAGTAGCTGGAATAAGTAGTGTAGCGGTGAAATGCATAGATATTACTTAGAACACCAATTGCGAAGGCAGGTTACTAAGCAACAACTGACACTGATGGACGAAAGCGTGGGGAGCGAACAGG

>c94a1bbbc3d8dc87a75398feb6c75704

CACAGTACGTCATCCATCGATGGGCTTCAATCTGTGACCACCTCTCATCTACAGCCTACCTAAGTGTTCTCCCCATCTATAGCCTGTATAATACATTAAATACCTTGCATATATAACATACATATTCGTACATTGTATTATAATTCATTATAAGGAATAATAATAATGCATATTTA

>c36c7aa5e6c47fc763828a8add383940

TACGGAGGGTGCGAGCGTTAATCGGAATAACTGGGCGTAAAGGGCACGCAGGCGGTGACTTAAGTGAGGTGTGAAAGCCCCGGGCTTAACCTGGGAATTGCATTTCATACTGGGTCGCTAGAGTACTTTAGGGAGGGGTAGAATTCCACGTGTAGCGGTGAAATGCGTAGAGATGTGGAGGAATACCGAAGGCGAAGGCAGCCCCTTGGGAAAGTACTGACGCTCATGTGCGAAAGCGTGGGGAGCAAACAGG

>36ce67b3fecce277df99829617975495

TACGTAGGGCGCGAGCGTTGTCCGGAATTATTGGGCGTAAAGAGCTTGTAGGCGGTTTGTCGCGTCTGCTGTGAAAGGCCGGGGCTTAACCCCGTGTATTGCAGTGGGTACGGGCAGACTAGAGTGCAGTAGGGGAGACTGGAATTCCTGGTGTAGCGGTGGAATGCGCAGATATCAGGAGGAACACCGATGGCGAAGGCAGGTCTCTGGGCTGTAACTGACGCTGAGAAGCGAAAGCATGGGGAGCGAACAGG

>bdbb8308b169d82019f1c62368008afe

TACGTAGGGTGCGAGCGTTAATCGGAATTACTGGGCGTAAAGCGTGCGCAGGCGGTTTGTTAAGACAGATGTGAAATCCCCGGGCTCAACCTGGGAACTGCATTTGTGACTGGCAGGCTAGAGTATGGCAGAGGGGGGTAGAATTCCACGTGTAGCAGTGAAATGCGTAGAGATGTGGAGGAATACCGATGGCGAAGGCAGCCCCCTGGACCAATACTGACGCTCATGCACGAAAGCGTGGGGAGCAAACAGG

>82dec3eeac5d034d118bba43fddd01fe

TACGGAGGGAGCTAGCGTTGTTCGGAATTACTGGGCGTAAAGCGCACGTAGGCGGCCATTCAAGTCAGAGGTGAAAGCCCGGGGCTCAACCCCGGAACTGCCTTTGAAACTAGATGGCTTGAATCTTGGAGAGGCGAGTGGAATTCCGAGTGTAGAGGTGAAATTCGTAGATATTCGGAAGAACACCAGTGGCGAAGGCGACTCGCTGGACAAGTATTGACGCTGAGGTGCGAAAGCGTGGGGAGCAAACAGG

>52d998a538496179968dec55950d1516

TACGTAGGGTGCGAGCGTTAATCGGAATTACTGGGCGTAAAGCGAGCGCAGACGGTTATTTAAGCAGGATGTGAAATCCCCGGGCTTAACCTGGGAACTGCGTTCTGAACTGGATAGCTAGAGTGTGTCAGAGGGGGGTAGAATTCCACGTGTAGCAGTGAAATGCGTAGAGATGTGGAGGAATACCGATGGCGAAGGCAGCCCCCTGGGATAACACTGACGTTCATGCTCGAAAGCGTGGGTAGCAAACAGG

>dcba105f35d8ebc9e22269c7491ad3a7

TACGAAGGGTGCAAGCGTTACTCGGAATTACTGGGCGTAAAGCGTGCGTAGGTGGTCGTTTAAGTCCGTTGTGAAAGCCCTGGGCTCAACCTGGGAACTGCAGTGGATACTGGGCGACTAGAGTGTGGTAGAGGGTAGCGGAATTCCTGGTGTAGCAGTGAAATGCGTAGAGATCAGGAGGAACATCCATGGCGAAGGCAGCTACCTGGACCAACACTGACACTGAGGCACGAAAGCGTGGGGAGCAAACAGG

>1637345f45b90ef2aba65723fddc6151

TACGTAGGTCCCGAGCGTTGTCCGGATTTATTGGGCGTAAAGCGAGCGCAGGCGGTTAGATAAGTCTGAAGTTAAAGGCTGTGGCTTAACCATAGTACGCTTTGGAAACTGTTTAACTTGAGTGCAGAAGGGGAGAGTGGAATTCCATGTGTAGCGGTGAAATGCGTAGATATATGGAGGAACACCGGTGGCGAAAGCGGCTCTCTGGTCTGTAACTGACGCTGAGGCTCGAAAGCGTGGGGAGCGAACAGG

>1b9175ea4e4774adc7da0c0e202e61d7

TACGTAAGGACCGAGCGTTGTCCGGAATCATTGGGCGTAAAGGGTACGTAGGCGGGTTTTTAAGTTAGAAGTCAAAGGCTATAGCTCAACTATAGTAAGCTTCTAAAACTGGGAACCTTGAGTAATGGAAGGGAAAGTGGAATTCCTAGTGTAGCGGTGGAATGCGCAGATATTAGGAGGAATACCGGTGGCGAAGGCGACTTTCTGGCCATTAACTGACGCTGAGGTACGAAAGCGTGGGTAGCAAACAGG

>3483d4bf5a02282e81d30bb2c4a052be

CACGATTAACCCAAGTCAATAGAAACCGGCGTAAAGAGTGTTTTAGATCAATTCCCCTCAATAAAGCTAAAATTCACCTGAGTTGTAAAAAACTCCAGTTGATACAAAATAAACTACGAAAGTGGCTTTAACGCATCTGAACACACAATAGCTAAGACCCAAACTGGG

>b92728ceb7d87e55750c27bf28743466

CACGATTAACCCAAGTCAATAGAAGCCGGCGTAAAGAGTGTTTTAGATCACCCCCTCCCCAATAAAGCTAAAACTCACCTGAGTTATAAAAAACTCCAGTTGACACAAAATAGACTACGAAAGTGGCTTTAACATATCTGAACACACAATAGCTAAGACCCAAACTGGG

>45f3cbee30d6d8db06d424a7e53c5683

TACGTAGGGTGCGAGCGTTGTCCGGATTTACTGGGCGTAAAGAGCTCGTAGGTGGCTTGTCGCGTCGTCTGTGAAAGTCTGGGGCTTAACTCCGGGTGTGCAGGCGATACGGGCTGGCTTGAGTGCTGTAGGGGAGACTGGAATTCCTGGTGTAGCGGTGGAATGCGCAGATATCAGGAGGAACACCGATGGCGAAGGCAGGTCTCTGGGCAGTCACTGACGCTGAGGAGGGAGAGCATGGGTAGCGAACAGG

>7389b6c11a50061a457df7ef95f06d22

CACGTAGGGTGCGAGCGTTGTCCGGAATTATTGGGCGTAAAGAGCTCGTAGGCGGTGTGTCGCGTCGGCCGTGAAAACTTGGGGCTTAACTCTGAGCGTGCGGTCGATACGGGCATCACTTGAGTTCGGCAGGGGAGACTGGAATTCCTGGTGTAGCGGTGAAATGCGCAGATATCAGGAGGAACACCGGTGGCGAAGGCGGGTCTCTGGGCCGATACTGACGCTGAGGAGCGAAAGCGTGGGGAGCGAACAGG

>13a6b08f7caf6964a9fcb73999d152eb

GACAGAGGATGCAAGCGTTATCCGGAATGATTGGGCGTAAAGCGTCTGTAGGTGGCTTTTTAAGTTCGCCGTCAAATCCCAGGGCTCAACCCTGGACAGGTGGTGAAAACTACTAAGCTAGAGTACGGTAGGGGCAGAGGGAATTTCCGGTGGAGCGATGAAATGCGTAGAGATCGGAAGGAACACCAACGGCGAAAGCACTCTGCTGGGCCGACACTGACACTGAGAGACGAAAGCTAGGGGAGCGAATGGG

>5fbdb23e3bf239332ba46d09017dadc3

TACGGAGGGTGCAAGCGTTAATCGGAATTACTGGGCGTAAAGCGCACGCAGGCGGTCTGTCAAGTCGGATGTGAAATCCCCGAGCTTAACTTGGGAATTGCATTCGATACTGGTTAGCTAGAGTGTGGGAGAGGATGGTAGAATTCCAGGTGTAGCGGTGAAATGCGTAGAGATCTGGAGGAATACCGATGGCGAAGGCAGCCATCTGGCCTAACACTGACGCTGAGGTGCGAAAGCATGGGGAGCAAACAGG

>a404eb6d55dd50dc739fb1daf6d6841a

TACGTAGGTGGCAAGCGTTGTCCGGAATTATTGGGCGTAAAGCGCGCGCAGGCGGTCACTTAAGTCCATCTTAGAAGTGCGGGGCTTAACCCCGTGAGGGGATGGAAACTGGGAGACTGGAGTATCGGAGAGGAAAGTGGAATTCCTAGTGTAGCGGTGAAATGCGTAGATATTAGGAAGAACACCGGTGGCGAAGGCGACTTTCTGGACGAAAACTGACGCTGAGGCGCGAAAGCGTGGGGAGCAAACAGG

>2c660360fc6cfdfacc4541f63e180b9d

CACACTTATGTCTTTAGAAATACAAACCATTTCTCCAGGATGCATTTCAACCATGTCGGTAATTTTCAACTGGCAGTGCCTTGTAGATATTTGTGAAAGATTTTGAACGTGTCTTGTCCATAGAGTTCAGTTTGTAAATTAATAATTTTGTTATGTAGTTTGTAACGTAATATATTTAGGCTTTTAAGTAAATTCATTTTATTTCCTTTTTTCCGGTTGTG

>7d0fd17647460e5425eb0ce0aa995036

TACGGAGGGAGCTAGCGTTGTTCGGAATTACTGGGCGTAAAGCGCACGTAGGCGGCTTTGTAAGTCAGAGGTGAAAGCCTGGAGCTCAACTCCAGAACTGCCTTTGAGACTGCATCGCTTGAATCCAGGAGAGGTGAGTGGAATTCCGAGTGTAGAGGTGAAATTCGTAGATATTCGGAAGAACACCAGTGGCGAAGGCGGCTCACTGGACTGGTATTGACGCTGAAGTGCGAAAGCGTGGGGAGCAAACAGG

>78db7fa6b16b73b101fbb8b282b5330e

TACGAAGGGTGCAAGCGTTAATCGGAATTACTGGGCGTAAAGCGCGCGTAGGTGGTTCGTTAAGTTGGATGTGAAAGCCCCGGGCTCAACCTGGGAACTGCATCCAAAACTGGCGAGCTAGAGTACGGTAGAGGGTGGTGGAATTTCCTGTGTAGCGGTGAAATGCGTAGATATAGGAAGGAACACCAGTGGCGAAGGCGACCACCTGGACTGATACTGACACTGAGGTGCGAAAGCGTGGGGAGCAAACAGG

>4e0763702795e6afb59e7d22281a0099

TACGTAGGTGGCAAGCGTTATCCGGAATTATTGGGCGTAAAGCGCGCGTAGGCGGTTTTTTAAGTCTGATGTGAAAGCCCACGGCTCAACCGTGGAGGGTCATTGGAAACTGAAAAACTTGAGTGCAGAAGAGGAAAGTGGAATTCCATGTGTAGCGGTGAAATGCGCAGAGATATGGAGGAACACCAGTGGCGAAGGCGACTTTCTGGTCTGTAACTGACGCTGATGTGCGAAAGCGTGGGGATCAAACAGG

>fc21fe7f3dd3b74e196bc4d66364e4df

TACAAGGAAGACTAGTGTTATTCATCTTAATTAGGTTTAAAGGGTACCTAGACAGTATTTCTAGCCTCAAAAGGGAACAGACTTACTAGAGTTTTATGGGAGAGGAAAATATTAGAACCATTGGAGTAGAGATAAAATGTTTTGATACTAATGGGACGGATAGCGGCGAAGGCAAACCTCTATGTAATAACTGACGTTGAGGGACGAAGGCTTGGGGAGCGAATAGG

>3ebe761bfb1238c87195d431f41bf976

TACAGAGGGTGCGAGCGTTAATCGGATTTACTGGGCGTAAAGCGTGCGTAGGCGGCTTTTTAAGTCGGATGTGAAATCCCTGAGCTTAACTTAGGAATTGCATTCGATACTGGGAAGCTAGAGTATGGGAGAGGATGGTAGAATTCCAGGTGTAGCGGTGAAATGCGTAGAGATCTGGAGGAATACCGATGGCGAAGGCAGCCATCTGGCCTAATACTGACGCTGAGGTACGAAAGCATGGGGAGCAAACAGG

>9580aa8a52ec18a4e92e701cdb595faa

TACGTAGGTGGCGAGCGTTATCCGGATTTACTGGGCGTAAAGGGAGCGTAGGCGGATGATTAAGTGGGATGTGAAATACCCGGGCTCAACTTGGGTGCTGCATTCCAAACTGGTTATCTAGAGTGCAGGAGAGGAGAGTGGAATTCCTAGTGTAGCGGTGAAATGCGTAGAGATTAGGAAGAACACCAGTGGCGAAGGCGACTCTCTGGACTGTAACTGACGCTGAGGCTCGAAAGCGTGGGGAGCAAACAGG

>0e8efbac1ecb94ddcef73744fc125a27

GACGGGGGGGGCAAGTGTTCTTCGGAATGACTGGGCGTAAAGGGCACGTAGGCGGTGAATCGGGTTGAAAGTGAAAGTCGCCAAAAACTGGCGGAATGCTCTCGAAACCAATTCACTTGAGTGAGACAGAGGAGAGTGGAATTTCGTGTGTAGGGGTGAAATCCGGAGATCTACGAAGGAACGCCAAAAGCGAAGGCAGCTCTCTGGGTCCCTACCGACGCTGGGGTGCGAAAGCATGGGGAGCGAACGGG

>9a1467bcd36b7367898c768ee05b1910

TACGGAGGGGGTTAGCGTTGTTCGGAATTACTGGGCGTAAAGCGCGCGTAGGCGGACGGTCAAGTTGGGGGTGAAAGCCCGGGGCTCAACCCCGGAACTGCCTTCAAAACTGATCGTCTGGAGACCGGGAGAGGTGAGTGGAATTCCCAGTGTAGAGGTGAAATTCGTAGATATTGGGAAGAACACCAGTGGCGAAGGCGGCTCACTGGACCGGATCTGACGCTGAGGTGCGAAAGCGTGGGGAGCGAACAGG

>67ca78638e95eca768c0c1da3c96761a

TACGAAGGGGGCTAGCGTTGCTCGGAATCACTGGGCGTAAAGGGTGCGTAGGCGGGTTTCTAAGTCAGAGGTGAAAGCCTGGAGCTCAACTCCAGAACTGCCTTTGATACTGGAAGTCTTGAGTATGGCAGAGGTGAGTGGAACTGCGAGTGTAGAGGTGAAATTCGTAGATATTCGCAAGAACACCAGTGGCGAAGGCGGCTCACTGGGCCATTACTGACGCTGAGGCACGAAAGCGTGGGGAGCAAACAGG

>cf0df8e07f56582c371749a44a13792a

TACGGAGGGTGCAAGCGTTAATCGGAATTACTGGGCGTAAAGCGCACGCAGGCGGTCTGTCAAGTCGGATGTGAAATCCCCGGGCTCAACCTGGGAACTGCATTCGAAACTGGCAGGCTAGAGTCTTGTAGAGGGGGGTAGAATTCCAGGTGTAGCGGTGAAATGCGTAGAGATCTGGAGGAATACCGGTGGCGAAGGCGGCCCCCTGGACAAAGACTGACGCTCAGGTACGAAAGCGTGGGGAGCAAACAGG

>06ca8a0f60b4b28d67a9be2613fb7b07

TACGTAGGGTGCGAGCGTTGTCCGGAATTACTGGGCGTAAAGAGCTCGTAGGCGGTTTGTCGCGTCGTCTGTGAAATTCTGCAACTCAATTGTAGGCGTGCAGGCGATACGGGCAGACTTGAGTACTACAGGGGAGACTGGAATTCCTGGTGTAGCGGTGAAATGCGCAGATATCAGGAGGAACACCGGTGGCGAAGGCGGGTCTCTGGGTAGTAACTGACGCTGAGGAGCGAAAGCGTGGGTAGCGAACAGG

>b9dd5628d9f3a26537de68a4f1014d4d

TACGGAGGGTGCAAGCGTTAATCGGAATTACTGGGCGTAAAGCGCACGCAGGCGGTCTGTCAAGTCGGATGTGAAATCCCCGGGCTCAACCTGGGAACTGCATTCGAAACTGGCAGGCTAGAGTCTTGTAGAGGGGGGTAGAGTTCCAGGTGTAGCGGTGAAATGCGTAGAGATCTGGAGGAATACCGGTGGCGAAGGCGGCCCCCTGGACAAAGACTGACGCTCAGGTGCGAAAGCGTGGGGAGCAAACAGG

>bcba47620c299fd34809f143e3d26dff

TACGGAGGGAGCTAGCGTTGTTCGGAATTACTGGGCGTAAAGCGCACGTAGGCGGCTTTGTAAGTTAGAGGTGAAAGCCTGGAGCTCAACTCCAGAATTGCCTTTAAGACTGCATCGCTTGAATCCAGGAGAGGTGAGTGGAATTCCGAGTGTAGAGGTGAAATTCGTAGATATTCGGAAGAACACCAGTGGCGAAGGCGGCTCACTGGACTGGTATTGACGCTGAGGTGCGAAAGCGTGGGGAGCAAACAGG

>0806f3413df1dd5f4006cfe45adefccb

TACAGAGGATGCAAGCGTTATCCGGAATTATTGGGCGTAAAGCGTCTGTAGGTGGCTTTTCAAGTCCCCCGTTAAATCCCAGGGCTCAATCCTGGACAGGCGGTGGAAACTACTAAGCTAGAGTATGGTAGGGGCAGAGGGAATTTCCGGTGGAGCGGTGAAATGCGCTGAGATCGGAAAGAACACCAATGGCGAAAGCACTCTGCTGGGCCGACACTGACACTGAGAGACGAGAGCTAGGGGAGCAAATGGG

>6f7cad115c22f23d0908045c73092561

TACCGGCAGTCCAAGTGATGGCCGATATTATTGGGCCTAAAGCGTCCGTAGCTTGCTGTGTAAGTCCATTGGGAAATCGACCAGCTCAACTGGTCGGCGTCCGGTGGAAACTACACAGCTTGGGGCCGAGAGACTCAACGGGTACGTCCGGGGTAGGAGTGAAATCCTGTAATCCTGGACGGACCACCAATGGGGAAACCACGTTGAGAGACCGGACCCGACAGTGAGGGACGAAAGCCAGGGTCTCGAACCGG

>22b3c70cdfb1714224e1bf8a6314bee9

TACGTATGGAGCGAGCGTTGTCCGGAATTATTGGGCGTAAAGGGTACGCAGGCGGTTTAATAAGTCGAATGTTAAAGATCGGGGCTCAACCCCGTAAAGCATTGGAAACTGATAAACTTGAGTAGTGGAGAGGAAAGTGGAATTCCTAGTGTAGTGGTGAAATACGTAGATATTAGGAGGAATACCAGTAGCGAAGGCGACTTTCTGGACACAAACTGACGCTGAGGTACGAAAGCGTGGGGAGCAAACAGG

>26b4c7425c99bd6528739b1b4da8f833

TACGGAGGGTGCAAGCGTTAATCGGAATTACTGGGCGTAAAGCGCACGCAGGCGGTCTGTTAAGTCAGATGTGAAATCCCCGGGCTTAACCTGGGAACTGCATTTGAAACTGGCAGGCTTGAGTCTTGTAGAGGGGGGTAGAATTCCAGGTGTAGCGGTGAAATGCGTAGAGATCTGGAGGAATACCGGTGGCGAAGGCGGCCCCCTGGACAAAGACTGACGCCCAGGTGCGAAAGCGTGGGGAGCAAACAGG

>f039d097982dd35113275a61be95dbc5

TACGGAGGGTGCAAGCATTATCCGGATTTATTGGGTTTAAAGGGTCCGTAGGCGGATTTGTAAGTCAGTGGTGAAATCTCACAGCTTAACTGTGAAACTGCCATTGATACTGCAAGTCTTGAGTGTTGTTGAAGTAGCTGGAATAAGTAGTGTAGCGGTGAAATGCATAGATATTACTTAGAACACCAATTGCGAAGGCAGGTTACTAAGCAACAACTGACGCTGATGGACGAAAGCGTGGGGAGCGAACAGG

>1b19ddc52f14d0b9a5430a8f2efeb1a8

TACGTAGGGTGCAAGCGTTGTCCGGAATTATTGGGCGTAAAGAGCTCGTAGGCGGTTCGTCGCGTCGGCTGTGAAAACCTGAGGCTCAACCTCAGGCCTGCAGTCGATACGGGCGGACTTGAGTACTGCAGGGGAGACTGGAATTCCTGGTGTAGCGGTGAAATGCGCAGATATCAGGAGGAACACCGGTGGCGAAGGCGGGTCTCTGGGCAGTAACTGACGCTGAGGAGCGAAAGCGTGGGGAGCGAACAGG

>1b3a9dbef5073d8b83e169bdd738f1a3

TACGAAGGGGGCTAGCGTTGTTCGGATTTACTGGGCGTAAAGGGCGCGTAGGCGGACCTGTAAGTCAGGGGTGAAATCCCAAGGCTCAACCTTGGAACTGCCTTTGATACTGTGGGTCTTGAGTCCGGGAGAGGTGAGTGGAACTGCGAGTGTAGAGGTGAAATTCGTAGATATTCGCAAGAACACCAGTGGCGAAGGCGGCTCACTGGCCCGGAACTGACGCTGAGGCGCGAAAGCGTGGGGAGCAAACAGG

>b9a53273b7fe88c2a54712da1b268cad

TACGTAGGGTGCGAGCGTTAATCGGAATTACTGGGCGTAAAGCGTGCGCAGGCGGTTTGTTAAGACAGATGTGAAATCCCCGGGCTCAACCTGGGAACTGCATTTGTGACTGGCAGGCTAGAGTATGGCAGAGGGGGGTAGAATTCCACGTGTAGCAGTGAAATGCGTAGAGATGTGGAGGAATACCGATGGCGAAGGCATCCCCCTGGGCCAATACTGACGCTCATGCACGAAAGCGTGGGGAGCAAACAGG

>950a0f48967b4ba4c946ec28b8d32bf8

TACAGAGGGTGCGAGCGTTGTCCGGAATCACTGGGCGTAAAGGGCGCGTAGGTGGTCGGGTGCGCGTGCCGTGAAAGCCCGGGGCTCAACCCCGGGTCGGCGGTGCGAACGGCTCGACTGGAGCATGCGAGAGGCAGGCGGAATTCCGGGTGTAGCGGTGGAATGCGTAGAGATCCGGAAGAACACCGGGGGCGAAGGCGGCCTGCTGGCGCAGTGGCTGACACTGAGGCGCGACAGCGTGGGGAGCAAACAGG

>0a8648fcadc21513b0887bba5abed9a6

TACCGGCAGTCCGAGTGATGGCCGATATTATTGGGCCTAAAGCGTCCGTAGCCGGCCGGACAAGTCCGTTGGGAAATCGACGCGCTCAACGCGTCGGCGTCCAGCGGAAACTGTCCGGCTTGGGGCCGGAAGACCTGAGGGGTACGTCCGGGGTAGGAGTGAAATCCTGTAATCCTGGACGGACCACCAATGGGGAAACCACCTCAGGAAGACGGACCCGACGGTGAGGGACGAAAGCTAGGGTCTCGAACCGG

>a09f9686e65622421296cf7ea7e92ae8

TACGGAGGGTGCGAGCGTTGTCCGGAATCACTGGGCGTAAAGGGCGCGTAGGCGGTCTGCTAAGCGTGTGGTGAAAGCTCGGGGCTCAACCCCGAGTCGGCCATGCGAACTGGTGGACTAGAGCACTGTAGAGGCAGGTGGAATTCCGGGTGTAGCGGTGGAATGCGTAGAGATCCGGAAGAACACCGGTGGCGAAGGCGGCCTGCTGGGCAGTAGCTGACGCTGAGGCGCGATAGCGTGGGGAGCAAACAGG

>fdba13e6336743e284d41229d28dfa90

TACGTGAGAGACTAGTGTTATTCATCTTAATTGGGTTTAAAGGGTACCTAGGCAGTCAATATAACTTCTAGAATGCTAATACTTGACTAGAGTTTTAAGTAAGAGGGAAGTACTTAAGGAGTAAGAGATGAAATATCTGTGATACCAAAGGGACTCCGTAAAGGCGAAGGCATCCCTTTATCTAAAAACTAACGTTGAAGGACGAAGGCTTAGATAACAAATAGG

>3d3f89fadf28973358458a50d436e91e

TACGAAGGGGGCTAGCGTTGCTCGGAATCACTGGGCGTAAAGGGCGCGTAGGCGGCGTTTTAAGTTGGGGGTGAAAGCCTGTGGCTCAACCACAGAATTGCCTTCGATACTGGGACGCTTGAGTGTGGTAGAGGTTGGTGGAACTGCGAGTGTAGAGGTGAAATTCGTAGATATTCGCAAGAACACCGGTGGCGAAGGCGGCCAACTGGACCATCACTGACGCTGAGGCGCGAAAGCGTGGGGAGCAAACAGG

>43904939ba355dce37c93c12823b71e2

TACGTAGGGTGCGAGCGTTGTCCGGAATTACTGGGCGTAAAGAGCTCGTAGGTGGTTTGTCGCGTCGTTTGTGTAAGTCCACAGCTTAACTGTGGGACTGCAGGCGATACGGGCATAACTTGAGTGCTGTAGGGGAGACTGGAATTCCTGGTGTAGCGGTGAAATGCGCAGATATCAGGAGGAACACCGATGGCGAAGGCAGGTCTCTGGGCAGTAACTGACGCTGAGGAGCGAAAGCATGGGTAGCGAACAGG

>5402ba93ede19b701749226bacf6c0a7

TACGTAGGTGGCAAGCGTTGTCCGGAATTATTGGGCGTAAAGCGCGCGCAGGCGGTCTCTTAAGTCTGATGTGAAAGCCCACGGCTCAACCGTGGAGGGTCATTGGAAACTGGGAGACTTGAGTACAGAAGAGGAGAGTGGAATTCCACGTGTAGCGGTGAAATGCGTAGATATGTGGAGGAACACCAGTGGCGAAGGCGACTCTCTGGTCTGTAACTGACGCTGAGGCGCGAAAGCGTGGGGAGCAAACAGG

>c408aa26ec7afaa88df03c32f4d8c5ce

TACAAGGAAGACTAGTGTTATTCATCTTTATTAGGTTTAAAGGGTACCTAGACGGTATTTCTAGCCCCCAAAGGGTACAGAATTACTAGAGTTTTATGGAAGAGGCAAATATTAGGACCATTGGTGTAGAGATAAAATTTTTTGATACTAATGGGACGGATAACAGCGAAGGCAACCCTCTATGTATAAACTGACGTTGAGGGACGAAGGCTTGGGGAGCGAATAGG

>73373d2d94551b3c53c3e01b6a02aeb3

TACGTAGGGCGCAAGCGTTATCCGGAATTATTGGGCGTAAAGAGCTCGTAGGCGGTTTGTCGCGTCTGCTGTGAAATCCCGAGGCTCAACCTCGGGTCTGCAGTGGGTACGGGCAGACTAGAGTGCGGTAGGGGAGATTGGAATTCCTGGTGTAGCGGTGGAATGCGCAGATATCAGGAGGAACACCGATGGCGAAGGCAGATCTCTGGGCCGTAACTGACGCTGAGGAGCGAAAGGGTGGGGAGCAAACAGG

>634f593c575b88c86631fd2d621beec1

TACGTAGGTGGCAAGCGTTGTCCGGATTTATTGGGCGTAAAGCGAGTGCAGGCGGCTCGATAAGTCTGATGTGAAAGCCTTCGGCTCAACCGGAGAATTGCATCAGAAACTGTCGAGCTTGAGTACAGAAGAGGAGAGTGGAACTCCATGTGTAGCGGTGAAATGCGTAGATATATGGAAGAACACCGTTGGCGAAGGCGGCTCTCTGGTCTGTTACTGACGCTGAGGCTCGAAAGCATGGGTAGCGAACAGG

>f849febf52b4ae538554b6e65d7fc47c

TACGTAGGTGGCAAGCGTTATCCGGAATTATTGGGCGTAAAGCGCGCGTAGGCGGTTTTTTAAGTCTGATGTGAAAGCCCACGGCTCAACCGTGGAGGGTCATTGGAAACTGGAAAACTTGAGTGCAGAAGAGGAAAGTGGAATTCCATGTGTAGCGGTGAAATGCGCAGAGATATGGAGGAACATCAGTGGCGAAGGCGACTTTCTGGTCTGTAACTGACGCTGATGTGCGAAAGCGTGGGGATCAAACAGG

>0a32c15f9e935a3e4fb3bac4081a2f70

TACAGAGGGTGCAAGCGTTAATCGGATTTACTGGGCGTAAAGCGCGCGTAGGCGGCTAATTAAGTCAAATGTGAAATCCCCGAGCTTAACCTGGGAATTGCATTCGATACTGGTTAGCTAGAGTGTGGGAGAGGATGGTAGAATTCCAGGTGTAGCGGTGAAATGCGTAGAGATCTGGAGGAATACCGATGGCGAAGGCAGCCATCTGGCCTAACACTGACGCTGAGGTGCGAAAGCATGGGGAGCAAACAGG

>aa8f31dd9ded91eab0004b4a3888a385

CACGATTAACCCAAGTCAATAGAAGCCGGCGTAAAGAGTGTTTTAGATCACCCCCTCCCCAATAAAGCTAAAACTCACCTGAGTTGTAAAAAACTCCAGTTGACACAAAATAGACTACGAAAGTGGCTTTAACATATCTGAACACACAATAGCTAAGACCCAAACCGGG

>2a526245ed96af69b39c2ee0a9fd5bd7

TACCGGCAGCACGAGTGATGACCGATATTATTGGGCCTAAAGCGTCCGTAGCTGGCCAAGCAAGTCCATTGGGAAATCGACGCGCTCAACGCGTCGGCGTCCGGTGGAAACTGTTTGGCTTGGGGCCAGAAGACCTGAGGGGTACGTCCGGGGTAGGAGTGAAATCCTGTAATCCTGGACGGACCACCAATGGGGAAACCACCTCAGGAGGATGGACCCGACAGTGAGGGACGAAAGCTAGGGTCTCGAACCGG

>f84482a8e96df1f86aa9eb0b29b9d60f

CACACTTATGTCTTCAGAAAGACAAAATATTTCTCCAGGATCCATTTCATCCATGTCGGTCATTTTCAACTGGCAGAGCATTGTTGATATTTGTAAAAGACTTTGAATGTGTCTTGTCCATAGAGTTGAGTTTGTTCATTAATGATTTTGTTATGTAGTTTGTAACGTAATACATTTAGGCTTTCAAGTAAATTCATTTTCGTTGTG

>685a1812a6e46f72d7cf1f4a060b8e96

TACGTAGGGTGCGAGCGTTGTCCGGAATTACTGGGCGTAAAGAGCTCGTAGGTGGTTTGTCGCGTCGTTTGTGTAAGCCCGCAGCTTAACTGCGGGACTGCAGGCGATACGGGCATAACTTGAGTGCTGTAGGGGAGACTGGAATTCCTGGTGTAGCGGTGGAATGCGCAGATATCAGGAGGAACACCGATGGCGAAGGCAGGTCTCTGGGCAGTAACTGACGCTGAGGAGCGAAAGCATGGGGAGCGAACAGG

>c45486e332113011f595d0edd05e6fc1

TACAGAGGCCCCAAGCGTTGTTCGGATTTACTGGGCGTAAAGGGTGTGTAGGGGGTTGGGTAAGTTTGACGTGAAATCCCGTTGCTCAACAACGGAACTGCGTCGAATACTGCTCAGCTGGAGGTTCGGAGATGAGGGCGGAATTCTCGGTGTAGCGGTGAAATGCGTAGATATCGAGAGGAACGCCGATGGCGAAAGCAGCCCTCAAGACGAAATCTGACCCTGAAACACGAAGGCCAGGGGAGCAAACGGG

>0e64455de1c13f4c6d78bf483729e5fd

TACCGGCAGCCCGAGTGATGGCCGATATTATTGGGCCTAAAGCGTCCGTAGCTTGCTGTGTAAGTCCATTGGGAAATCGACCAGCTCAACTGGTCGGCGTCCGGTGGAAACTACACAGCTTGGGGCCGAGAGACTCAACGGGTACGTCCGGGGTAGGAGTGAAATCCTGTAATCCTAGACGGACCACCAATGGGGAAACCACGTTGAGAGACCGGACCCGACAGTGAGGGACGAAAGCCAGGGTCTCGAACCGG

>c4688dff464241e2b0f20f861209b62f

TACGGAGGGTGCAAGCATTAATCGGATTTATTGGGCGTAAAGGGCGCGTAGGCGGGAAGGAAAGTCAGATGTGAAATCCCGGGGCTCAACCCCGGAACAGCATTTGAAACTCCCTTTCTTGAGGGTAGACGGAGAAAATGGAATTCCACAAGTAGCGGTGAAATGCGTAGATATGTGGAAGAACACCAGTGGCGAAGGCGATTTTCTAGTTTATACCTGACGCTGAGGCGCGAAAGCAAGGGGATCAAACAGG

>64df79355e8ac5b4034ed0314aac59ea

CAACCCACTTTTTTCCTGGGCATAATTTCCGTCGTTTCTGCTGAAATATTTGAAACTGTGTTTCTACTTATCTGCAGGGCATCGGCGGCTCGCTGCATGGAATTTTGAACACGGAGTGGACCGTTGTTAATTTGTTCCTGTTCGAAATAATTACGCAGTCTATTAACAAATGTTCTCGCTTGATATTTTAATGTCAATCCACGACCAAATTTTCTAACTCTCCCTTGATCCCACGAC

>4358c342efe8e91d593b3dfb0d0e07b7

CACACTTATGTCTTCAAGAAGACAAAATATTTCTCCAGGATGCATTTCAACCATGTCGGTCATTTTCAACTGGCAGTGCCTTGTAGATATTTGTGAAAGATTTTGAATGTGTCTTGTCCATAGAGTTGAGTTTGTTCATTAATGATTTTGTTATGTAGTTTGTAACGTAATATATTTAGGCTTTCAAGTAAATTCATTTTGTTTCCTTTTTTGCCGGTTGTG

>09f9294499192d4eecf29e85501cc425

TACGGAGGGTGCAAGCGTTATCCGGATTTATTGGGTTTAAAGGGTCCGTAGGTGGGCTGATAAGTCAGCGGTGAAATCCTGCAGCTTAACTGTAGAACTGCCGTTGATACTGTTAGTCTTGAGTGTATTTGAAGTGGCTGGAATAAGTAGTGTAGCGGTGAAATGCATAGATATTACTTAGAACACCAATTGCGAAGGCAGGTCACTAAGATACAACTGACGCTGAGGGACGAAAGCGTGGGGAGCGAACAGG

>118ef820045be87c07304c7712ec3888

TACAGAGGGTGCGAGCGTTAATCGGATTTACTGGGCGTGAAGCGTGCGTAGGCGGCTTTTTAAGTCGGATGTGAAATCCCCGAGCTTAACTTGGGAATTGCATTCGATACTGGGAAGCTAGAGTATGGGAGAGGATGGTAGAATTCCAGGTGTAGCGGTGAAATGCGTAGAGATCTGGAGGAATACCGATGGCGAAGGCAGCCATCTGGCCTAATACTGACGCTGAGGTACGAAAGCATGGGGAGCAAACAGG

>a198143da18897a72b770275c2b613b5

GACAGAGGATGCAAGCGTTATCCGGAATGATTGGGCGTAAAGCGTCTGTAGGTGGCTTTTTAAGTTCGCCGTCAAATCCCAGGGCTCAACCCTGGACAGGTGGTGAAAACTACTAAGCTAGAGTACGGTAGGGGCAGAGGGAATTTCCGGTGGAGCGATGAAATGCGTAGAGATCGGAAGGAACGCCAACGGCGAAAGCACTCTGCTGGGCCGACACTGACACTGAGAGACGAAAGCTAGGGGAGCGAATGGG

>6add585ceb4b4d22dc542a521200cdf1

TACGAAGGGGGCTAGCGTTGCTCGGAATCACTGGGCGTAAAGGGCGCGTAGGCGGCGTTTTAAGTCGGGGGTGAAAGCCTGTGGCTCAACCACAGAATTGCCTTCGATACTGGGACGCTTGAGTCTGGTAGAGGTTGGTGGAACTGCGAGTGTAGAGGTGAAATTCGTAGATATTCGCAAGAACACCGGTGGCGAAGGCGGCCAACTGGACCAGTACTGACGCTGAGGCGCGAAAGCGTGGGGAGCAAACAGG

>b5160161214aa7ee577b8c869ad1c4fe

TACCGGCAGTCCAAGTGATGGCCGATATTATTGGGCCTAAAGCGTCCGTAGCCTGCTGTGTAAGTCCGTTGGGAAATCGACGCGCTCAACGCGTCGGCGTCCAGCGGAAACTACACGGCTTGGGGCCGAGAGACTTGACGGGTACGTCCGGGGTAGGAGTGAAATCCTGTAATCCTGGACGGACCACCAATGGCGAAACCACCTCAGGAAGACGGACCCGACGGTGAGGGACGAAAGCTAGGGTCTCGAACCGG

>61d96b86c674ec6276984c9be7a81efc

TACGGAGGGAGCTAGCGTTGTTCGGAATTACTGGGCGTAAAGCGCACGTAGGCGGTTACTCAAGTCAGAGGTGAAAGCCCGGGGCTCAACCCCGGAACTGCCTTTGAAACTAGGTAACTAGAATCCTGGAGAGGTGAGTGGAATTCCGAGTGTAGAGGTGAAATTCGTAGATATTCGGAAGAACACCAGTGGCGAAGGCGGCTCACTGGACAGGTATTGACGCTGAGGTGCGAAAGCGTGGGGAGCAAACAGG

>f0e3e0ac9b4fa0c81f85f0c755c725ce

TACGGAGGGTGCAAGCGTTAATCGGAATTGCTGGGCGTAAAGCGCACGCAGGCGGTCTGTCAAGTCGGATGTGAAATCCCCGGGCTCAACCTGGGAACTGCATTCGAAACTGGCAGGCTAGAGTCTTGTAGAGGGGGGTAGAATTCCAGGTGTAGCGGTGAAATGCGTAGAGATCTGGAGGAATACCGGTGGCGAAGGCGGCCCCCTGGACAAAGACTGACGCTCAGGTGCGAAAGCGTGGGGAGCAAACAGG

>eef6d3c42ab7df7398b35f58e10d7f8d

TACGTAGGGTGCGAGCGTTAATCGGAATTACTGGGCGTAAAGCGTGCGCAGGCGGTTTGTTAAGACAGATGTGAAATCCCCGGGCTTAACCTGGGAACTGCATTTGTGACTGGCAAGCTAGAGTATGGCAGAGGGGGGTAGAATTCCACGTGTAGCAGTGAAATGCGTAGAGATGTGGAGGAATACCGATGGCGAAGGCAGCCCCCTGGGCCAATACTGACGCTCATGCACGAAAGCGTGGGGAGCAAACAGG

>c3d4b58fe1f20b718bffce1c0f193134

TACGGAGGGGGCTAGCGTTGTTCGGAATTACTGGGCGTAAAGCGCACGTAGGCGGACCGGAAAGTCAGAGGTGAAATCCCAGGGCTCAACCTTGGAACTGCCTTTGAAACTATCGGTCTGGAGTTCGAGAGAGGTGAGTGGAATTCCGAGTGTAGAGGTGAAATTCGTAGATATTCGGAGGAACACCAGTGGCGAAGGCGGCTCACTGGCTCGATACTGACGCTGAGGTGCGAAAGCGTGGGGAGCAAACAGG

>68aeba2ef529bd52c4b8d6de05cb8749

CACACTTATGTCTTTAGAAAGACAAAACATTTCTCCAGAATGCATTTCACCCGTGTCGGTCACTTTCAACTGGCAGTAACTTGTTGATATTTGTGTCTTGCCCATAGAGTTCAGTTTGTTCATTAATTATTTTGTTATGTAATTTGTAACGTAATATATTTAGGCTTTCAAGTAAATTCATTTTACTTCCTTTTTGCCGGTTGAG

>7ebaf73a5b34a0493795bd3b6c630c20

GACGAAGGATGCGAGCGTTATCCGGATTCACTGGGTTTAAAGGGTGCGTAGGCGGTTTAGTAAGTTTCAAGTTAAAGACTGGTGCTTAACATCAGGACTGCTTGAAAAACTGCTAGACTTGAGATAGGTCGGCGTTGCTGGAATATGGCAAGTAGAGGTGAAATTCATAGATATGCCATAGAACACCGATAGCGAAGGCAGGCGACGAGGCCTTATCTGACGCTGAGGCACGAAAGCGTGGGGATCAAACAGG

>e9777cd0ab748da065533780f908fd5d

TACGTAGGGTGCGAGCGTTAATCGGAATTACTGGGCGTAAAGCGTGCGCAGGCGGTTTGTTAAGACAGATGTGAAATCCCCGGGCTCAACCTGGGAACTGCATTTGTGACTGGCAGGCTAGAGTATGGCAGAGGGGGGTAGAATTCCACGTGTAGCAGTGAAATGCGTAGAGATGTGGAGGAATACCGATGGCGAAGGCAGCCCCCTGGGCCAATACTGACGCTCATGCACGAAAGCGTGAGGAGCAAACAGG

>827992b22906f7a8b7f7a87580b21cfa

GACAGAGGATGCAAGCGTTATCCGGAATGATTGGGCGTAAAGCGTCTGTAGGTGGCTTTTCAAGTCCGCCGTCAAATCCCAGGGCTCAACCCTGGACAGGCGGTGGAAACTACCAAGCTGGAGTACGGTAGGGGCAGAGGGAATTTCCGGTGGAGCGGTGAAATGCGTAGAGATCGGAAAGAACACCAACGGCGAAAGCACTCTGCTGGGCCGACACTGACACTGAGAGACGAAAGCTAGGGGAGCAAATGGG

>b834275a404d714243171b36f858f7a6

TACAGAGGGTGCAAGCGTTAATCGGATTTACTGGGCGTAAAGCGCACGTAGGCGGCTAATTAAGTCAAATGTGAAATCCCCGAGCTTAACTTGGGAATTGCATTCGATACTGGTTAGCTAGAGTGTGGGAGAGGATGGTAGAATTCCAGGTGTAGCGGTGAAATGCGTAGAGATCTGGAGGAATACCGATGGCGAAGGCAGCCATCTGGCCTAACACTGACGCTGAGGTGCGAAAGCATGGGGAGCAAACAGG

>23bd044024010c3ba28e2542b98a22e1

TACGTAGGGCGCAAGCGTTATCCGGAATTATTGGGCGTAAAGAGCTTGTAGGCGGTTCGTCGCGTCTGGTGTGAAAGCCCATCGCTTAACGGTGGGTTTGCGCCGGGTACGGGCGGGCTAGAGTGCAGTAGGGGAGACTGGAATTCTCGGTGTAACGGTGGAATGTGTAGATATCGGGAAGAACACCAATGGCGAAGGCAGGTCTCTGGGCTGTTACTGACGCTGAGAAGCGAAAGCGTGGGGAGCGAACAGG

>7911816f5e81f650f769aba0d5c708cb

GACAGAGGATGCAAGCGTTATCCGGAATGATTGGGCGTAAAGCGTCTGTAGGTGGCTTTTCAAGTCCGCCGTCAAATCCCAGGGCTCAACCCTGGACAGGCGGTGGAAACTACCAAGCTGGAGTACGGTAGGGGCAGAGGGAATTTCCGGTGGAGCGGTGAAATGCATTGAGATCGGAAAGAACACCAACGGCGAAAGCACTCTGCTGGGCCGACACTGACACTGAGAGACGAAAGCTAGGGGAGCAAATGGG

>8ae518dbb29595b3f79214be0b589066

TACGTAGGTGGCAAGCGTTGTCCGGATTTATTGGGCGTAAAGCGCGCGCAGGCGGTCTTTTAAGTCTGATGTGAAAGCCCCCGGCTTAACCGGGGAGGGTCATTGGAAACTGGAAGACTGGAGTGCAGAAGAGGAGAGTGGAATTCCACGTGTAGCGGTGAAATGCGTAGATATGTGGAGGAACACCAGTGGCGAAGGCGACTCTCTGGTCTGTAACTGACGCTGAGGCGCGAAAGCGTGGGGAGCAAACAGG

>0dbf680e00aa85eb226b129f0adeff7e

TACGTAGGGTGCGAGCGTTGTCCGGAATTATTGGGCGTAAAGAGCTTGTAGGCGGTTTGTCGCGTCTGCTGTGAAAGACCGGGGCTTAACTCCGGTTCTGCAGTGGGTACGGGCAGACTAGAGTGTGGTAGGGGAGACTGGAATTCCTGGTGTAGCGGTGAAATGCGCAGATATCAGGAGGAACACCGATGGCGAAGGCAGGTCTCTGGGCCATTACTGACGCTGAGAAGCGAAAGCATGGGGAGCGAACAGG

>a94755da5b51afc8620a1f8630c4b6dd

TTCTTACTGCAAACGTATTGTCATACACATAGTGTTTTTGTAGTAATTATTGTGAGCAGCATCCTCTATGTTATAAATGTAATAAGGAAATCTTAAAATAATAATAATAATCTGTTTAAAGTAATTAATCGTTATCATCTTCGCCATTCCAGGATTAGAAACC

>2f1df086d2e5f688e86db361a188ac0a

TACGTAGGGTGCGAGCGTTAATCGGAATTACTGGGCGTAAAGCGTGCGCAGGCGGTTTGTTAAGACAGATGTGAAATCCCCGGGCTCAACCTGGGAACTGCATTTGTGACTGGCAGGCTAGAGTATGGCAGAGGGGGGTAGAATTCCACGTGTAACAGTGAAATGCGTAGAGATGTGGAGGAATACCGATGGCGAAGGCAGCCCCCTGGGCCAATACTGACGCTCATGCACGAAAGCGTGGGGAGCAAACAGG

>74eaab08ab138d53259ec0dce5b772fd

TACCGGCAGCCCGAGTAATGGCCACTCTTATTGGGCCTAAAGCGTCCGTAGCTGGCCGCGCAAGTCCATCGGGAAATCTACCTGCTCAACAGGTGGGCGCCCGGTGGAAACTGCGCGGCTTGGGACCGGAAGGCGCGACGGGTACGTCCGGGGTAGGAGTGAAATCCCGTAATCCTGGACGGACCGCCGATGGCGAAAGCACGTCGCGAGAACGGATCCGACAGTGAGGGACGAAAGCCAGGGTCTCGAACCGG

>21a30b278fd1629a1a90bee5cfed82d6

TACGGAGGGAGCTAGCGTTGTTCGGAATTACTGGGCGTAAAGAGTACGTAGGCGGCTATTCAAGTCAGAGGTGAAAGCCCGGGGCTCAACCCCGGAACTGCCTTTGAAACTAGGTAGCTAGAATCTTGGAGAGGTCAGTGGAATTCCGAGTGTAGAGGTGAAATTCGTAGATATTCGGAAGAACACCAGTGGCGAAGGCGACTGACTGGACAAGTATTGACGCTGAGGTACGAAAGCGTGGGGAGCAAACAGG

>6c06b6134e297bd5a9d4b8776b81c480

TACGGAGGGTGCAAGCGTTATCCGGATTTATTGGGTTTAAAGGGTCCGTAGGCGGACCCGTAAGTCAGTGGTGAAATCTCATAGCTTAACTATGAAACTGCCATTGATACTGCGGGTCTTGAGTAAATTTGAAGTGGCTGGAATAAGTAGTGTAGCGGTGAAATGCATAGATATTACTTAGAACACCAATTGCGAAGGCAGGTCACTAAGATTTAACTGACGCTGATGGACGAAAGCGTGGGTAGCGAACAGG

>094b49ca9853e2d508b35771e7471699

TACGTAGGGTGCGAGCGTTAATCGGAATTACTGGGCGTAAAGCGTGCGCAGGCGGTTTGTTAAGACAGATGTGAAATCCCCGGGCTCAACCTGGGAACTGCATTTGTGACTGGCAGGCTAGAGTATAGCAGAGGGGGGTAGAATTCCACGTGTAGCAGTGAAATGCGTAGAGATGTGGAGGAATACCGATGGCGAAGGCAGCCCCCTGGGCCAATACTGACGCTCATGCACGAAAGCGTGGGGAGCAAACAGG

>63e0a2e10a98e5d726a385ec387cd00f

TACCGGCAGCTCAAGTGATGTCCCATATTATTGGGCCTAAAGCGTCCGTAGCTGGCCAACCAAGTCCATCGGGAAATCCACCCGCCCAACGGGTGGGCGTCCGGTGGAAACTGGCTGGCTTGGAACCGGAAGGCTCAGAGAGTACGTCCGGGGTAGGAGTGAAATCCCGTAATCCCGGACGGACTACCGATGGCGAAAGCACTCTGAGAAGACGGCTTCGACAGTGAGGGACGAAAGCTAGGGTCTCAAACCGG

>e60f41f3d716cc3b8316a61509696136

TCGAACCCAAGCCACGTGGTGTAGCGAACCAACACACTACACACCACGAGTACTCCCCCCCCCTTCCAATCAACAAAAAATCACAATACGATTACAGGAAATTGTTCAGAATGTAGCGCCAAATGAAATTACAAAGAGGAGGATTTTGAATATTGCAGAAGGATCGTGCAAAACAAACGTTTGCTAGCAGCCTTCCTCACGTGTATACTGCACTGATGCTTCGAGAAATAGTCTGCAGAACTAT

>4cbfff144d4e7a4e0f4619ed505be070

TACGGAGGGTGCAAGCGTTAATCGGAATTACTGGGCGTAAAGCGCACGCAGGCGGTCTGTCAAGTCGGATGTGAAATCCCCGGGCTCAACCTGGGAACTGCATTCGAAACTGGCAGGCTTGAGTCTTGTAGAGGGGGGTAGAATTCCAGGTGTAGCGGTGAAATGCGTAGAGATCTGGAGGAATACCGGTGGCGAAGGCGGCCCCCTGGACAAAGACTGACGCTCAGGTGCGAAAGCGTGGGGAGCAAACAGG

>4436dafaa8f16319ae0cfb6b1447c775

TACGTAGGGTCCGAGCGTTATCCGGAATCATTGGGCGTAAAGAGCTCGTAGGCGGTTCAGCAAGTCGGCTGTGAAAGTCCGGGGCTCAACCCCGGAATTGCAGTCGATACTGCTGTGACTCGAGTCCGGTAGAGGAGAATGGAATTCCCGGTGTAGCGGTGAAATGCGCAGATATCGGGAGGAACACCAGTAGCGAAGGCGGTTCTCTGGGCCGGCACTGACGCTGAGGAGCGAAAGCGTGGGGAGCAAACAGG

>5d40c206a20a097fee7f53042ded8267

TACGAAGGGTGCAAGCGTTAATCGGAATTACTGGGCGTAAAGCGCGCGTAGGTGGCTTGATAAGTCGGATGTGAAATCCCCGGGCTCAACCTGGGAACTGCATCCAAAACTGTCTGGCTAGAGTGTGGTAGAGGGTAGTGGAATTTCCAGTGTAGCGGTGAAATGCGTAGATATTGGAAGGAACACCAGTGGCGAAGGCGACTACCAGGACTAACACTGACACTGAGGTGCGAAAGCGTGGGGAGCAAACAGG

>99deb3c5ecb022ec05609ebd1112a557

TACGGAGGATCCGAGCGTTATCCGGATTTATTGGGTTTAAAGGGAGCGTAGATGGATGTTTAAGTCAGTTGTGAAAGTTTGCGGCTCAACCGTAAAATTGCAGTTGATACTGGATATCTTGAGTGCAGTTGAGGCAGGCGGAATTCGTGGTGTAGCGGTGAAATGCTTAGATATCACGAAGAACTCCGATTGCGAAGGCAGCCTGCTAAGCTGCAACTGACATTGAGGCTCGAAAGTGTGGGTATCAAACAGG

>2a683351471a18461d48b9eef67e007f

TACGTAGGGTGCGAGCGTTAATCGGAATTACTGGGCGTAAAGCGTGCGCAGGCGGTTTGTTAAGACAGATGTGAAATCCCCGGGCTCAACCTGGGAACTGCATTTGTGACTGGTAGGCTAGAGTATGGCAGAGGGGGGTAGAATTCCACGTGTAGCAGTGAAATGCGTAGAGATGTGGAGGAATACCGATGGCGAAGGCAGCCCCCTGGGCCAATACTGACGCTCATGCACGAAAGCGTGGGGAGCAAACAGG

>2bbb28b70c3a3757e48663f4d0003ecc

TACGGAGGGAGCTAGCGTTGTTCGGAATTACTGGGCGTAAAGCGCACGTAGGCGGCTATTCAAGTCAGAGGTGAAAGCCCGGGGCTCAACCCCGGAACTGCCTTTGAAACTAGATAGCTTGAATCCAGGAGAGGTGAGTGGAATTCCGAGTGTAGAGGTGAAATTCGTAGATATTCGGAAGAACACCAGTGGCGAAGGCGGCTCACTGGACTGGTATTGACGCTGAGGTGCGAAAGCGTGGGGAGCAAACAGG

>8d0b7d5f836c15da6ee88aa14db85409

GACAGAGGATGCAAGCGTTATCCGGAATGATTGGGCGTAAAGCGTCTGTAGGTGGCTTTTTAAGTTCGCCGTCAAATCCCAGGGCTCAACCCTGGACAGGTGGTGAAAACTACTAAGCTAGAGTACGGTAGGGGCATAGGGAATTTCCGGTGGAGCGATGAAATGCGTAGAGATCGGAAGGAACACCAACGGCGAAAGCACTCTGCTGGGCCGACACTGACACTGAGAGACGAAAGCTAGGGGAGCGAATGGG

>544fd51f6a3b8ccee94195380cab02ef

TACGTAGGGTGCGAGCGTTAATCGGAATTACTGGGCGTAAAGCGTGTGCAGGCGGTTTGTTAAGACAGATGTGAAATCCCCGGGCTCAACCTGGGAACTGCATTTGTGACTGGCAGGCTAGAGTATGGCAGAGGGGGGTAGAATTCCACGTGTAGCAGTGAAATGCGTAGAGATGTGGAGGAATACCGATGGCGAAGGCAGCCCCCTGGGCCAATACTGACGCTCATGCACGAAAGCGTGGGGAGCAAACAGG

>26005dfb7ad2e8cdbb139846af329663

TACGTAGGTGGCAAGCGTTGTCCGGAATTATTGGGCGTAAAGCGCGCGCAGGCGGTCCTTTAAGTCTGATGTGAAAGCCCACGGCTCAACCGTGGAGGGTCATTGGAAACTGGGGGACTTGAGTACAGAAGAGAAGAGTGGAATTCCACGTGTAGCGGTGAAATGCGTAGAGATGTGGAGGAACACCAGTGGCGAAGGCGACTCTTTGGTCTGTAACTGACGCTGAGGCGCGAAAGCGTGGGGAGCAAACAGG

>284db2278b83f80c91619212fcf2c01c

TACGTAGGGTGCGAGCGTTAATCGGAATTACTGGGCGTAAAGCGTGCGCAGGCGGTTTGTTAAGACAGATGTGAAATCCCCGGGCTCAACCTGGGAACTGCATTTGTGACTGGCAGGCTAGAGTATGGCAGAGGGGGGTAGAATTCCACGTGTAGCAGTGAAATGCGTAGAGATGTGGAGGAATACCGATGGCGAAGGAAGCCCCCTGGGCCAATACTGACGCTCATGCACGAAAGCGTGGGGAGCAAACAGG

>985c62c37efedae38c6a8f81b5b7246c

CACACTTATGTCTTCAGAAATACAAACCATTTCTCCAGGATGCATTTCAACCATGTCGGTCATTTTCAACTGTCAGTGCCTTGTAGATATTTGTGGAAGATTTTGAATGTGTCTTGTCCATGGAGTTCAGTTTGTTCATTAATGATTTTGTTGTGTAGTTTGTAACGTAATATATTTAGGGTTTTAAGTAAATTCATTTTATTTCCTTTTTGCCTGTTGTA

>7b569cca079c2d5abadf4dc9629b2468

TACGTAGGTGGCAAGCGTTGTCCGGATTTACTGGGTGTAAAGGGTGCGCAGGCGGACCTTTAAGTAGAAAGTGAAAGGTTGGAGCTCAACTCCGACACTGCTTCCTATACTGGGGGTCTTGAGTTTCGGAGGGGGAAGCGGAACGACACGTGTAGCGGTGAAATGCGTTGATATGTGTCGGAACACCAATGGCGAAAGCAGCTTCCTGGACGAATACTGACGCTCAGGCACGAAAGCCAAGGTAGCAAACAGG

>c756ca6ccdcca2136a5e44d55982aca1

TACGGAGGGAGCTAGCGTTGTTCGGAATTACTGGGCGTAAAGCGCACGTAGGCGGCTTTGTAAGTTAGAGGTGAAAGCCCGGGGCTCAACCCCGGAATTGCCTTTGATACTGCATGGCTTGAATCCAGGAGAGGTGAGTGGAATTCCGAGTGTAGAGGTGAAATTCGTAGATATTCGGAAGAACACCAGTGGCGAAGGCGGCTCACTGGACTGGTATTGACGCTGAGGTGCGAAAGCGTGGGGAGCAAACAGG

>84c59907c413d30913b4476f773ceb6c

CTTTTTACAGAGATAAGGTCTCAAGTCTGGGAATGTGGCTATTATAAATGAGATTTTATGAATTTCTTAAAAAGTTCAAAATTAATATTAGTGAACTTTGTAAACGTCTTAAATATATTCTGGCATATAGAAACTAGTTGTAAACACTTTCGTCAATGCGAAGAAAGTAAC

>c06ccd1f7d57566ef669942328b1a946

TACAGAGGGTGCAAGCGTTAATCGGAATTACTGGGCGTAAAGCGCGCGTAGGTGGTTTGTTAAGTTGGATGTGAAAGCCCCGGGCTCAACCTGGGAACTGCATCCAAAACTGGCAAGCTAGAGTACGGTAGAGGGTGGTGGAATTTCCTGTGTAGCGGTGAAATGCGTAGATATAGGAAGGAACACCAGTGGCGAAGGCGACCACCTGGACTGATACTGACACTGAGGTGCGAAAGCGTGGGGAGCAAACAGG

>c8594093300e667cfc4a667ca15b2470

AGCTATTGCATGCTATGCCTTCAAGCCACACCTCCGCGCCCGTCGGAGTGTGGAGCGTGAGGTTTTTCGTTACTAAATTTCTGGATTCGGTCCCCGCGCTCAAGGCCCGCGATAGAAACTATCTAGCAATAGCTTAAAACTTACAATGTTGTAAATTATGCATGTTATGTATATGTTGTATGTTATTATTATGCATAAAGTTAAAATAAACGATTCTGTTGGATAATTCACT

>fe5b867a508c1fe71eac03b2d7d41488

TACGTAGGGTGCGAGCGTTGTCCGGAATTACTGGGCGTAAAGAGCTCGTAGGTGGTTTGTCGCGTCGTCTGTGAAATTCCGGGGCTTAACTCCGGGCGTGCAGGCGATACGGGCATAACTTGAGTACTGTAGGGGAGACTGGAATTCCTGGTGTAGCGGTGAAATGCGCAGATATCAGGAGGAACACCGATGGCGAAGGCAGGTCTCTGGGCAGTTACTGACGCTGAGGAGCGAAAGCATGGGTAGCGAACAGG

>1b2c0563638ede81b37fd9e544beddcc

AACAGAGGATACAAGCGTTATCCGGATTTATTGGGTTTAAAGGGTGCGTAGGTGGTTTTTTAAGTCAGTAGTGAAATCTTAAAGCTTAACTTTAAAGGTGCTATTGATACTGATAAACTAGAGTGAGGTTGGAGTAACTGGAATGTGTGGTGGAGCGGTGAAATGCATAGAGATCACACAGAACACCAATCGCGAAGGCATGTTACTAAACATAGACTGACACTGAGGCACGAAAGCATGGGTAGCAAACAGG

>183b4a36051380683ff23640aeae4c1f

TACGTAGGTGGCAAGCGTTGTCCGGATTTACTGGGCGTAAAGGGAGCGTAGGCGGATACTTAAGTGGGATGTGAAATACCCGGGCTCAACCCGGGTGCTGCATTCCAAACTGGGTATCTAGAGTGTGGGAGAGGAAAGTGGAATTCCTAGTGTAGCGGTGAAATGCGTAGAGATTAGGAAGAACACCAGTGGCGAAGGCGACTTTCTGGACCATAACTGACGCTGAGGCTCGAAAGCGTGGGGAGCAAACAGG

>d23b4024f3fadcb333e775ed8e959f63

TTAGCCGGATGGTTTCCGTTGCAGTTGCAACACTTTGCGGCGGACCTTGCGGTTAACAAATTGGTATTCGGGTCGGGCAGTGATGATGATATTCAGCCGGTTCGCACGACCGGTTGTTAAATTTTTCTCAAGTTCACCGAACCGGTAGTTACATCTTCTATATACAT

>ad9018f80e00f2ca4b48067195ac310d

TACGAAGGGGGCTAGCGTTGCTCGGAATTACTGGGCGTAAAGGGCGCGTAGGCGGACATTTAAGTCAGGGGTGAAATCCCAGAGCTCAACTCTGGAACTGCCTTTGATACTGGGTGTCTTGAGTGTGAGAGAGGTATGTGGAACTCCGAGTGTAGAGGTGAAATTCGTAGATATTCGGAAGAACACCAGTGGCGAAGGCGACATACTGGCTCATTACTGACGCTGAGGCGCGAAAGCGTGGGGAGCAAACAGG

>5e5f7b9a531dc1357012a51dc743d86d

TACCGGCAGCCCGAGTGATGGCCGATCTTATTGGGCCTAAAGCGTCCGTAGCTGGCCGCGCAAGTCCATCGGGAAATCCACCCGCTCAACCGGTGGACGTCCGGCGGAAACTGTTCAGCTTGGGGCCGGGAGACTCAGCGGGTACGTCTGGGGTAGGAGTGAAATCCCGTAATCCTAGACGGACCACCGATGGCGAAAGCACGCTGAGAGACCGGACCCGACAGTGAGGGACGAAAGCTGGGGTCTCGAACCGG

>e5e36a76164ca8707ce914cbdacdf2ba

TACGGAGGGGGTTAGCGTTGTTCGGAATTACTGGGCGTAAAGCGCGCGTAGGCGGATTGGAAAGTTGGGGGTGAAATCCCGGGGCTCAACCCCGGAACTGCCTTCAAAACTCCCAGTCTAGAGTTCGAGAGAGGTGAGCGGAACTCCGAGTGTAGAGGTGAAATTCGTAGATATTCGGAAGAACACCAGTGGCGAAGGCGGCTCACTGGCTCGATACTGACGCTGAGGTGCGAAAGTGTGGGGAGCAAACAGG

>05ebe8e85db18c7be6e1cb4845aea55f

TACGTAGGGTGCGAGCGTTGTCCGGAATTATTGGGCGTAAAGAGCTTGTAGGCGGTCTGTCGCGTCTGCTGTGAAAGACCGGGGCTTAACTCCGGTTCTGCAGTGGGTACGGGCAGACTAGAGTGTGGTAGGGGAGACTGGAATTCCTGGTGTAGCGGTGAAATGCGCAGATATCAGGAGGAACACCGATGGCGAAGGCAGGTCTCTGGGCCATTACTGACGCTGAGAAGCGAAAGCATGGGGAGCGAACAGG

>49c05a35c7e5491fa54ae401b756a857

TACGGAGGGAGCTAGCGTTGTTCGGAATTACTGGGCGTAAGGCGCACGTAGGCGGCTTTGTAAGTCAGAGGTGAAAGCCTGGAGCTCAACTCCAGAACTGCCTTTGAGACTGCATCGCTTGAATCCGGGAGAGGTGAGTGGAATTCCGAGTGTAGAGGTGAAATTCGTAGATATTCGGAAGAACACCAGTGGCGAAGGCGGCTCACTGGACCGGTATTGACGCTGAGGTGCGAAAGCGTGGGGAGCAAACAGG

>bffd79a65e1cd0c98d984f7df8f1ed09

TACGTAGGTGGCAAGCGTTGTCCGGAATTATTGGGCGTAAAGGGCGCGCAGGCGGTTCGGTAAGTCTGTCTTAAAAGTGCGGGGCTTAACCCCGTGAGGGGACGGAAACTGTCGAACTTGAGTGTCGGAGAGGAAAGCGGAATTCCTAGTGTAGCGGTGAAATGCGTAGATATTAGGAGGAACACCGGTGGCGAAAGCGGCTTTCTGGACGACAACTGACGCTGAGGCGCGAAAGCGTGGGGAGCAAACAGG

>2eac9795499a82803c375130b81f9b43

TACAAGTAAGACTAGTGTTATTCATCTTTATTAGGTTTAAAGGGTACCTAGACAGTATTTCTAGCCCCCAAAGGGAACAGATTTACTAGAGTTTTATGTGAGAGGAAAATATTAGAACCATTGGAGTAGAGATAAAATATTTTGATACTAATGGGACGGATAACGGCGAAGGCAACCCTCTATGTAATAACTGACGTTGAGGGACGAAGGCTTGGGGAGCAAATAGG

>85c44c83eddc5d3028261a1000b7d0e1

TACGTAGGTGGCAAGCGTTGTCCGGAATTATTGGGCGTAAAGCGCGCGCAGGTGGTTTAATAAGTCTGATGTGAAAGCCCACGGCTCAACCGTGGAGGGTCATTGGAAACTGTTAAACTTGAGTGCAGGAGAGAAAAGTGGAATTCCTAGTGTAGCGGTGAAATGCGTAGAGATTAGGAGGAACACCAGTGGCGAAGGCGGCTTTTTGGCCTGTAACTGACACTGAGGCGCGAAAGCGTGGGGAGCAAACAGG

>735cb9abbbc8ff2b5b354be608a3a869

TACGTAGGTCCCGAGCGTTGTCCGGATTTATTGGGCGTAAAGCGAGCGCAGGCGGTTTAATAAGTCTGAAGTTAAAGGCAGTGGCTTAACCATTGTTCGCTTTGGAAACTGTTAAACTTGAGTGCAGAAGGGGAGAGTGGAATTCCATGTGTAGCGGTGAAATGCGTAGATATATGGAGGAACACCGGTGGCGAAAGCGGCTCTCTGGTCTGTAACTGACGCTGAGGCTCGAAAGCGTGGGGAGCAAACAGG

>cdd6b958e7d71deef2b107edb5981bb4

TACGTAGGGTGCGAGCGTTAATCGGAATTACTGGGCGTAAAGCGTGCGCAGGCGGTTTGTTAAGACAGATGTGAAATCCCCGGGCTCAACCTGGGAACTGAATTTGTGACTGGCAGGCTAGAGTATGGCAGAGGGGGGTAGAATTCCACGTGTAGCAGTGAAATGCGTAGAGATGTGGAGGAATACCGATGGCGAAGGCAGCCCCCTGGGCCAATACTGACGCTCATGCACGAAAGCGTGGGGAGCAAACAGG

>c5e48d33cc9cb2091f9867c5937315dd

TACGTAGGGCGCGAGCGTTGTCCGGATTTATTGGGCGTAAAGAGCTCGTAGGTGGTTCGTCGCGTCTGTCGTGAAAGCCAGCAGCTTAACTGTTGGTCTGCGGTGGGTACGGGCGGGCTTGAGTGCGGTAGGGGTGACTGGAATTCCTGGTGTAGCGGTGGAATGCGCAGATATCAGGAGGAACACCGATGGCGAAGGCAGGTCACTGGGCCGTTACTGACACTGAGGAGCGAAAGCGTGGGGAGCGAACAGG

>6b34496ffed5d3ec3059bfe9d66997a4

TACGTAGGGTGCGAGCGTTAATCGGAATTACTGGGCGTAAAGCGTGCGCAGGCGGTTTGTTAAGACAGATGTGAAATCCCCGGGCTCAACCTGGGAACTGCATTTGTGACTGGCAGGCTAGAGTATGGCAGAGGGGGGTAGAATTCCACGTGTAGCAGTGAAATGCGTAGAGATGTGGAGGAATACCGATGGCGAAGGCAGCACCCTGGGCCAATACTGACGCTCATGCACGAAAGCGTGGGGAGCAAACAGG

>d537ae76faee48a20bd981a978a5c055

GGTTATGTTCGTCAACACCGGGCAAACCCGGTCATATAATTTTACCATACACTATAACACGTTGTGGTCGAGATTGCAGTGCGTGGTGAGGTTATGTTCATCATATACTTTACAATGAAGTCTGGTCATATAATTGTGGCCGACAACGAAGAAACCGATCGAGATGGCAGTGGAGTCATAATTTTACCGGCAGTGGAAACGTGTGGTGAGGTTATGTTCATCAAC

>4698c8c2a39cee7ceca684e06aca2e5e

TACGTAGGGTGCGAGCGTTAATCGGAATTACTGGGCGTAAAGCGTGCGCAGGCGGTTTGTTAAGACAGATGTGAAATCCCCGGGCTCAACCTGGGAACTGCATTTGTGACTGGCAGGCTAGAGTATGGCAGAGGGGGGTAGAATTCCACGTGTAGCAGTGAAATGCGTAGAGATGTGGAAGAATACCGATGGCGAAGGCAGCCCCCTGGGCCAATACTGACGCTCATGCACGAAAGCGTGGGGAGCAAACAGG

>122fe5fb1af16f9c98b455c1139199db

TACGTAGGGTGCGAGCGTTAATCGGAATTACTGGGCGTAAAGCGTGCGCAGGCGGTTTGTTAAGACAGATGTGAAATCCCCGGGCTCAACCTGGGAACTGCATTTGTGACTGGCAGGCTAGAGTATGACAGAGGGGGGTAGAATTCCACGTGTAGCAGTGAAATGCGTAGAGATGTGGAGGAATACCGATGGCGAAGGCAGCCCCCTGGGCCAATACTGACGCTCATGCACGAAAGCGTGGGGAGCAAACAGG

>93cb9694e3d71c3af5d12f3a955e20a7

TACGTAGGGTGCGAGCGTTAATCGGAATTACTGGGCGTAAAGCGTGCGCAGGCGGTTTGTTAAGACAGATGTGAAATCCCCGGGCTCAACCTGGGAACTGCATTTGTGACTGGCAGGCTAGAGTATGGCAGAGGGGGGTAGAATTCCACGTGTAGCAGTGAAATGCGTAGAGATGTGGAGGAATACCGATGGCGAAGGCAGCCCCCTGGGACAATACTGACGCTCATGCACGAAAGCGTGGGGAGCAAACAGG

>fccd274e718e26b32c71f188d97d9e59

TACGGAGGGGGCTAGCGTTGTTCGGAATTACTGGGCGTAAAGCGCACGTAGGCGGACTGGAAAGTTGGGGGTGAAATCCCGGGGCTCAACCTCGGAACTGCCTTCAAAACTATCAGTCTGGAGTTCGAGAGAGGTGAGTGGAATTCCGAGTGTAGAGGTGAAATTCGTAGATATTCGGAGGAACACCAGTGGCGAAGGCGGCTCACTGGCTCGATACTGACGCTGAGGTGCGAAAGCGTGGGGAGCAAACAGG

>d676e025fb7484f8c8f79dc642805d70

TACGGAGGGTGCAAGCGTTAATCGGAATTACTGGGCGTAAAGCGCACGCAGGCGGTCTGTTAAGTCAGATGTGAAATCCCCGGGCTCAACCTGGGAACTGCATCTGATACTGGCAGGCTTGAGTCTCGTAGAGGGGGGTAGAATTCCAGGTGTAGCGGTGAAATGCGTAGAGATCTGGAGGAATACCGGTGGCGAAGGCGGCCCCCTGGACGAAGACTGACGCTCAGGTGCGAAAGCGTGGGGAGCAAACAGG

>fd496fd32dc8c08ade2e8b6c9d8ee13d

TACGTAGGTCCCGAGCGTTGTCCGGATTTATTGGGCGTAAAGCGAGCGCAGGCGGTTTGATAAGTCTGAAGTTAAAGGCTGTGGCTCAACCATAGTTCGCTTTGGAAACTGTCAAACTTGAGTGCAGAAGGGGAGAGTGGAATTCCATGTGTAGCGGTGAAATGCGTAGATATATGGAGGAACACCGGTGGCGAAAGCGGCTCTCTGGTCTGTAACTGACGCTGAGGCTCGAAAGCGTGGGGAGCGAACAGG

>9e7424827a933dba788faeebb8325fa3

TTCTTACTGCAAACGTATTGTCATACACATAGTGTTTTTGTAGTAATTATTGTGAGCAGCATCCTCTATGTTATAAATGTAATAAGGAAATCTTAAAATAATAATAATAATCTGTTACATTGTTGTTGTCAAAAAGTAATTAATCGTTATCATCTTCGCCATTCCAGG

>1f94abc9b56f4292c21891573f77389c

CACGATTAACCCAAGTCAATAGAAGCCGGCGTAAAGAGTGTTTTAGATCACCCCCTCCCCAATAAGGCTAAAACTCACCTGAGTTGTAAAAAACTCCAGTTGACACAAAATAGACTACGAAAGTGGCTTTAACATATCTGAACACACAATAGCTAAGACCCAAACTGGG

>01b516cc5155ce07499eac04de5f44fb

CACGATTAACCCAAGTCAATAGAAGCCGGCGTAAAGAGTGTTTTAGATCACCCCCTCCCCAATAAAGCTAGAACTCACCTGAGTTGTAAAAAACTCCAGTTGACACAAAATAGACTACGAAAGTGGCTTTAACATATCTGAACACACAATAGCTAAGACCCAAACTGGG

>2e7c0d1c259d412d94d3605d3236e6b7

CACGATTAACCCAAGTCAATAGAAGCCGGCGTAAAGAGTGTTTTAGATCACCCCCTCCCCAATAAAGCTAAAACTCACCTGAGTCGTAAAAAACTCCAGTTGACACAAAATAGACTACGAAAGTGGCTTTAACATATCTGAACACACAATAGCTAAGACCCAAACTGGG

>8e493df449e84acb4e24f5811ff3599b

TACGTAGGTGGCGAGCGTTATCCGGAATTATTGGGCGTAAAGGGTGCGTAGGCGGCCTGTTAAGTAAGTGGTTAAATTGTTGGGCTCAACCCAATCCAGCCACTTAAACTGGCAGGCTAGAGTATTGGAGAGGCAAGTGGAATTCCATGTGTAGCGGTAAAATGCGTAGATATATGGAGGAACACCAGTGGCGAAGGCGGCTTGCTAGCCAAAGACTGACGCTCATGCACGAAAGCGTGGGGAGCAAATAGG

>a4da7eaffcd6ae3122ca4e695f478cb9

CGGTTTACCAAGTATATGGCAACTTTATGCGTTCATTTTGGGTGTTGAAACTTAAAAATCTCATCTTTTTTTTATCGTTAAGGAATTCAGGATCCAGCGAGTAGCTGTCCTGTTCACAAGTAGGATAGCGTGGTTAATGTTACAGCGTTATATCTGGACTACCTGTAATTTCTTCATGATGAGACCCAAGGTTCCAACTGCACGTTATGAAAACCAAACTAGTTTGACACTA

>2db83ab6ebac297ad85c15cf5734b87c

TACGTAGGGTGCGAGCGTTAATCGGAATTACTGGGCGTAAAGCGTGCGCAGGCGGTTTGTTAAGACAGATGTGAAATCCCCGGGCTCAACCTGGGAACTGCATTTGTGACTGGCAGGCTAGAGTATGGCAGAGGGGGGTAGAATTCCACGTGTAGCAGTGAAATGCGTAGAGATGTGGAGGAATACCGATGGCGAAGGCAGCCCACTGGGCCAATACTGACGCTCATGCACGAAAGCGTGGGGAGCAAACAGG

>40c00caf4aba9abc8cf5384e6e8e438c

TACGGAGGGTGCAAGCGTTAATCGGAATTACTGGGCGTAAAGCGCACGCAGGCGGTTTGTTAAGTCAGATGTGAAATCCCCGGGCTCAACCTGGGAACTGCATTTGAAACTGGCAAGCTTGAGTCTCGTAGAGGGGGGTAGAATTCCAGGTGTAGCGGTGAAATGCGTAGAGATCTGGAGGAATACCGGTGGCGAAGGCGGCCCCCTGGACGAAGACTGACGCTCAGGTGCGAAAGCGTGGGGAGCAAACAGG

>8d1af5a64100fefd616373bd51f61399

TACCGGCAGTCCGAGTGATGGCCGATATTATTGGGCCTAAAGCGTCCGTAGCTTGCTGTGTAAGTCCATTGGGAAATCGACCAGCTCAACTGGTCGGCGTCCGGTGGAAACTACACAGCTTGGGGCCGAGAGACTCAACGGGTACGTCCGGGGTAGGAGTGAAATCCTGTAATCCTAGACGGACCACCAATGGGGAAACCACGTTGAGAGACCGGACCCGACAGTGAGGGACGAAAGCCAGGGTCTCGAACCGG

>7b0141e5fa45c338cf14a1fbfb971d5d

GACAGAGGATGCAAGCGTTATCCGGAATGATTGGGCGTAAAGCGTCTGTAGGTGGCTTTTTAAGTTCGCCGTCAAATCCCAGGGCTCAACCCTGGACAGGTGGTGAAAACTACTGAGCTAGAGTACGGTAGGGGCAGAGGGAATTTCCGGTGGAGCGATGAAATGCGTAGAGATCGGAAGGAACACCAACGGCGAAAGCACTCTGCTGGGCCGACACTGACACTGAGAGACGAAAGCTAGGGGAGCGAATGGG

>a6299b4b8aac994612e4b2235fc9eabc

AACGTAGGGTGCAAGCGTTGTCCGGAATTACTGGGTGTAAAGGGAGCGCAGGCGGGAAGACAAGTTGGAAGTGAAAACCATGGGCTCAACCCATGAATTGCTTTCAAAACTGTTTTTCTTGAGTAGTGCAGAGGTAGATGGAATTCCCGGTGTAGCGGTGGAATGCGTAGATATCGGGAGGAACACCAGTGGCGAAGGCGGCCTACTGGGCACCAACTGACGCTGAGGCTCGAAAGTGTGGGTAGCAAACAGG

>e4a75c15c515de0d447a10e77195d980

TACAGAGGGTGCAAGCGTTAATCGGATTTACTGGGCGTAAAGCGCGCGTAGGCGGCTAATTAAGTCAAACGTGAAATCCCCGAGCTTAACTTGGGAATTGCATTCGATACTGGTTAGCTAGAGTGTGGGAGAGGATGGTAGAATTCCAGGTGTAGCGGTGAAATGCGTAGAGATCTGGAGGAATACCGATGGCGAAGGCAGCCATCTGGCCTAACACTGACGCTGAGGTGCGAAAGCATGGGGAGCAAACAGG

>680677097dde70225c394039d913c036

TACCGGCAGTTCGAGTGATGGCCGCTCTTATTGGGCCTAAAGCGTTCGTAGCCGGCCACGCAAGTTCGTCGGGAAATCTGCCCGCTTAACGGGCAGGCGTCCGGCGAAAACTGCGTGGCTTGGGACCGGGAGACTCGAGAAGTACGTTCGGGGTAGGAGTGAAATCCCATAATCCCGAACGGACTACCGATGGCGAAAGCATCTCGAGAGCACGGATCCGACGGTGAGGAACGAAAGCTGGGGTCTCGAACCGG

>15609c3a10ffc772203bdd01e9bdb9cb

CACGATTAACCCAAGTCAATAGAAACCGGCATAAAGAGTGTTTTAGATCAATTCCCCTCAATAAAGCTAAAATTCACGTGAGTTGTAAAAAACTCCAGTTGATACAAAATAAACTACGAAAGTGGCTTTAATGCATCTGAACACAGAATAACTAAGACCCAAACTGGG

>a81474388f8811070e09917a19ab6ca1

CACGATTAACCCAAGCCAATAGAAGCCGGCGTAAAGAGTGTTTTAGATCACCCCCTCCCCAATAAAGCTAAAACTCACCTGAGTTGTAAAAAACTCCAGTTGACACAAAATAGACTACGAAAGTGGCTTTAACATATCTGAACACACAATAGCTAAGACCCAAACTGGG

>58bd181f757283f5a4ceebdf90ff6243

TACGTATGGTGCAAGCGTTATCCGGATTTACTGGGTGTAAAGGGAGCGTAGACGGATAGGCAAGTCTGGAGTGAAAACCCAGGGCTCAACCCTGGGACTGCTTTGGAAACTGCAGATCTGGAGTGCCGGAGAGGTAAGCGGAATTCCTAGTGTAGCGGTGAAATGCGTAGATATTAGGAGGAACACCAGTGGCGAAGGCGGCTTACTGGACGGTGACTGACGTTGAGGCTCGAAAGCGTGGGGAGCAAACAGG

>282dd7867741c58a5b4084ad8dcf416e

TACGTAGGGCGCAAGCGTTATCCGGAATTATTGGGCGTAAAGAGCTTGTAGGCGGTTCGTCGCGTCTGGTGTGAAAGCCCATCGCTTAACGGTGGGTTTGCGCCGGGTACGGGCGGGCTAGAGTGCAGTAGGGGAGACTGGAATTCCCGGTGTAACGGTGGAATGTGTAGATATCGGGAAGAACACCAATGGCGAAGGCAGGTCTCTGGGCTGTTACTGACGCTGAGAAGCGAAAGCGTGGGGAGCGAACAGG

>7d78ed99b08bd1723065fdd795d17e9c

TACGTAGGGTGCGAGCGTTGTCCGGAATTACTGGGCGTAAAGGGCTCGTAGGTGGTTTGTCGCGTCGTCTGTGAAATTCTGGGGCTTAACTCCGGGCGTGCAGGCGATACGGGCATAACTTGAGTGCTGTAGGGGTAACTGGAATTCCTGGTGTAGCGGTGAAATGCGCAGATATCAGGAGGAACACCGATGGCGAAGGCAGGTTACTGGGCAGTTACTGACGCTGAGGAGCGAAAGCATGGGTAGCGAACAGG

>a7b1a0322e651913c385a6eb371b8006

TACGTAGGTGGCAAGCGTTGTCCGGATTTATTGGGCGTAAGGCGCGCGCAGGCGGTCTTTTAAGTCTGATGTGAAAGCCCCCGGCTTAACCGGGGAGGGTCATTGGAAACTGGAAGACTGGAGTGCAGAAGAGGAGAGTGGAATTCCACGTGTAGCGGTGAAATGCGTAGATATGTGGAGGAACACCAGTGGCGAAGGCGACTCTCTGGTCTGTAACTGACGCTGAGGCGCGAAAGCGCGGGGAGCAAACAGG

>c4f72d9b7cb0d5c8d2dd2cc94be1ddc0

TACCGGCAGCACGAGTGATGGCCGATCTTATTGGGCCTAAAGCGTCCGTAGCTTGCTGTGTAAGTCCATTGGGAAATCGACCAGCCCAACTGGTCGGCGTCCGGTGGAAACTACACAGCTTGGGGCCGAGAGACTCAACGGGTACGTCCGGGGTAGGGGTGAAATCCTGTAATCCTGGACGGACCACCAATGGGGAAAGCACTCTGAGAAGACGGCTTCGACAGTGAGGGACGAAAGCTAGGGTCTCAAACCGG

>b27abbca50a4139a3d4f66390b178ffa

TACGAAGGGAGCAAGCGTTGTTCGGATTTATTGGGCGTAAAGGGTGTGTAGGCGGTTTGATAAGTTAGATGTGAAAGCCCAGGGCTTAACCCTGGAATTGCATTTAAGACTGTTGAACTTGAAGTTTGGAGAGGTTAGCAGAATTCCCAGTGTAGAGGTGAAATTCGTAGATATTGGGAAGAATACCAATGGCGTAGGCAGCTAACTGGCCAAAACTTGACGCTGAGGCACGAAAGCGTGGGGAGCAAACAGG

>1f8daf471249c3ffe82189e03e02bc19

TACCGGCAGCCCGAGTGATGGCCGATTTTATTGGGCCTAAAGCGTTCGTAGCTTGCCGAGCAAGTCCGTCGGGAAATCCACGCGCTCAACGTGTGGACGTCCGGCGGAAACTGTTCGGCTTGGGACCGGAAGACCCAAGGGGTACGTTCGGGGTAGGAGTGAAATCCCGTAATCCCGGACGGACCGCCGATGGCGAAAGCACCTTGGGAAGACGGACCCGACGGTGAGGGACGAAAGCTGGGGTCTCGAACCGG

>47c9fdd2c3f36cc353473699d91fc113

CACGATTAACCCAAGTCAATAGAAGCCGGCGTAAAGAGCGTTTTAGATCACCCCCTCCCCAATAAAGCTAAAACTCACCTGAGTTGTAAAAAACTCCAGTTGACACAAAATAGACTACGAAAGTGGCTTTAACATATCTGAACACACAATAGCTAAGACCCAAACTGGG

>5489893e48cdd25076bb07a064bf899e

GCACGTTGTATTCTACGTTCACGCAGAACGCGGTAATTCGCTGCAACTTTGGTGCGCGGTGAATATGAGCACACCTGTATTATTCTCTACGCAGAACAGAAATTTGTCCTGCCTTAGGAAGTTTTTGTTACATTTTGAGTCTTTTTTACTTCCACTTCGTAAGCCAGCAATTATTACCTG

>c77662cb165c394af052f39209451d74

TACGTAGGTGGCAAGCGTTATCCGGATTTATTGGGCGTAAAGAGAGTGCAGGCGGTTTTCTAAGTCTGATGTGAAAGCCTTCGGCTTAACCGGAGAAGTGCATCGGAAACTGGATAACTTGAGTGCAGAAGAGGGTAGTGGAACTCCATGTGTAGCGGTGGAATGCGTAGATATATGGAAGAACACCAGTGGCGAAGGCGGCTACCTGGTCTGCAACTGACGCTGAGACTTGAAAGCATGGGTAGCGAACAGG

>7a9313b572df3efbbf07843dd102896c

TACGGAGGGTGCAAGCGTTAATCGGAATTACTGGGCGTAAAGGGCGCGTAGGCGGCTTGCCAAGCCGGATGTGAAAGCCCCGGGCTCAACCCGGGAACGGCATTCGGAACTGGCAGGCTAGAGTGCAGGAGAGGAAGGTGGAATTCCCGGTGTAGCGGTGAAATGCGTAGAGATCGGGAGGAATACCAGTGGCGAAGGCGGCCTTCTGGACTGACACTGACGCTGAGGCGCGAAAGCGTGGGTAGCAAACAGG

>de71d6183a932eafd5efa4020c6334e5

TACGTAGGGTGCGAGCGTTAATCGGAATTACTGGGCGTAAAGCGTGAGCAGGCGGTTTGTTAAGACAGATGTGAAATCCCCGGGCTCAACCTGGGAACTGCATTTGTGAGTGGCAGGCTAGAGTATGGCAGAGGGGGGTAGAATTCCACGTGTAGCAGTGAAATGCGTAGAGATGTGGAGGAATACCGATGGCGAAGGCAGCCCCCTGGGCCAATACTGACGCTCATGCACGAAAGCGTGGGGAGCAAACAGG

>78d7ce8a354181655b491280d6268247

CACGATTAACCCAAGTCAATAGAAGCCGGCGTAAAGAGTGTTTTAGATCACCCCCTCCCCAATAAAGCTAAAACTCACCTGAGTTGTAAAAAACTCCAGCTGACACAAAATAGACTACGAAAGTGGCTTTAACATATCTGAACACACAATAGCTAAGACCCAAACTGGG

>dfa71f3681c7d9119bae9c51e2edee4c

ACATTCTTTGTACTTCATTAGTTATTTAATTTACACCACTTGTCACCCCCTTACATTGTACAAACGAGTTTGCTGGCACATTATGTAAAATACACGTTCCTGTTACAGCTTATCCCTGACCTGCTAAAACATGAAAGCAATATTTCTATGACCACAAAAACTATGGACCAAGAAGCTA

>10b890326e2b0b15509aefb95d0d6b79

TACGGAGGATCCGAGCGTTATCCGGATTTATTGGGTTTAAAGGGTGCGTAGGCGGCCTATTAAGTCAGGGGTGAAATACGGTGGCTCAACCATCGCAGTGCCTTTGATACTGATGGGCTTGAATCCATTTGAAGTGGGCGGAATAAGACAAGTAGCGGTGAAATGCATAGATATGTCTTAGAACTCCGATTGCGAAGGCAGCTCACTAAGCTGGTATTGACGCTGATGCACGAAAGCGTGGGGATCGAACAGG

>b618b5371494839510369f90cfab5560

CACGATTAACCCAAGTCAATAGAAGCCGGCGTAAAGAGTGTTTTAGATCACTCCCTCCCCAATAAAGCTAAAACTCACCTGAGTTGTAAAAAACTCCAGTTGACACAAAATAGACTACGAAAGTGGCTTTAACATATCTGAACACACAATAGCTAAGACCCAAACTGGG

>2b60de7615b8e3463c6759765e0162df

TACCGGCAGCACGAGTGATGGCCGATCTTATTGGGCCTAAAGCGTCCGTAGCCGGCCAGACAAGTCCGTTGGGAAATCGACGTGCCTAACACGTCGGCGTCCAGCGGAAACTGTCTGGCTTGGGGCCGGAAGACTCGAGGGGTACGTCCGGGGTAGGAGTGAAATCCCGTAATCCTGGACGGACCGCCGGTGGCGAAAGCGCCTCGAGAGGACGGACCCGACGGTGAGGGACGAAAGCTAGGGTCTCGAACCGG

>d76d59ec71de0e3b22da0c9cd564d41a

TACGTAGGGGGCAAGCGTTATCCGGATTTACTGGGTGTAAAGGGAGCGTAGACGGCAAGGCAAGTCTGATGTGAAAACCCAGGGCTTAACCCTGGGACTGCATTGGAAACTGTCTGGCTCGAGTGCCGGAGAGGTAAGCGGAATTCCTAGTGTAGCGGTGAAATGCGTAGATATTAGGAAGAACACCAGTGGCGAAGGCGGCTTACTGGACGGTAACTGACGTTGAGGCTCGAAAGCGTGGGGAGCAAACAGG

>33295f1846cff2bf9c88e15798fbc91d

TACGTAGGTGGCAAGCGTTGTCCGGAATTATTGGGCGTAAAGCGCGCGCAGGCGGTTCCTTAAGTCTGATGTGAAAGCCCACGGCTCAACCGTGGAGGGTCATTGGAAACTGGGGGACTTGAGTGCAGAAGAGGAGAGCGGAATTCCACGTGTAGCGGTGAAATGCGTAGAGATGTGGAGGAACACCAGTGGCGAAGGCGGCTCTCTGGTCTGTAACTGACGCTGAGGCGCGAAAGCGTGGGGAGCAAACAGG

>5b671d25b014ef713e6d58470d06ed93

CACACTTATGTCTTCAGAAAGACAAAATATGATGCATTTCAACCATGTCGGTCATTTTCAACTGGCAGTGCCTTGTTGATATTTGTGAAAGATTTTGAATATGTCTTGCCCATAGAGTTGAGTTTGTTCATTAATGATTTTGTTATGTAGTTTGTAACGTAATACATTTAGGCTTTCAAGTAAATTCATTTTGTTTACTTTTTTGCCGGTTGTG

>87842e5852abdeb1992907f232d162f8

CACGATTAACCCAAGTCAATAGAAGCCGGCGTAAAGAGTGTTTTAGATCACCCCCTCCCCAATAAAGCTAAAACTCACCTGAGTTGTAAAAAACTCCAGTTGACACAAAATAGACTACGAAAGTGGCTTTAACATATCTGAACACACGATAGCTAAGACCCAAACTGGG

>9abfd6203f74386fb73e16165e71db92

CACGATTAACCCAAGTCAATAGAAGCCGGCGTAAAGAGTGTTTTAGATCACCCCCTCCCCAATAAAGCTAAAACTCACCTGAGTTGTAAAAAACTCCGGTTGACACAAAATAGACTACGAAAGTGGCTTTAACATATCTGAACACACAATAGCTAAGACCCAAACTAGG

>d96eb020405d71b65b6e2970c61e39ed

TACGTAGGTGGCAAGCGTTGTCCGGGTTTATTGGGCGTAAAGCGAGCGCAGGCGGTTTCTTAAGTCTGATGTGAAAGCCCCCGGCTCAACCGGGGAGGGTCATTGGAAACTGGGAGACTTGAGTGCAGAAGAGGAGAGTGGAATTCCATGTGTAGCGGTGAAATGCGTAGATATATGGAGGAACACCAGTGGCGAAGGCGGCTCTCTGGTCTGTAACTGACGCTGAGGCTCGAAAGCGTGGGGAGCAAACAGG

>708e50f6b07a7fcee8183886a5f169b1

TACGAAGGGTGCAAGCGTTACTCGGAATTACTGGGCGTAAAGCGTGCGTAGGCGGTTCGTTAAGTCCGATGTGAAAGCCCTGGGCTCAACCTGGGAACTGCATTGGAAACTGGCGGGCTGGAGTGCGGTAGAGGGTGGCGGAATTCCCGGTGTAGCAGTGAAATGCGTAGATATCGGGAGGAACACCCGTGGCGAAGGCGGCCACCTGGACCAGCACTGACGCTGAGGCACGAAAGCGTGGGGAGCAAACAGG

>68a6e4195cb3db7a2125090e6c03b731

CACACTTATGTCTTTAGAAAGACAAAACGTTTTTCCAGAATGCATTTCACCCTTCTCGGTCATTTTCAACTGACAGTGCCTTGTAGATAGTTGTGGAAGATTTTGAGTGTTTCTTGTCCATAGAGATCAGTTTGTTCATTAATGATTTTGTTGTGTAATATGTAACGCAATATATATAAACTTTCAAGAACATTAATTTTATTTCCTTTTTTGCCAGTTGTA

>932867ef9d55adcc79f5713a2cae51fe

TACGTGAGAGACTAGTGTTATTCATCTTAATTGGGTTTAAAGGGTACCTAGACAGTCAATATAACTTCTAGAATGCTAATACTTGACTAGAGTTCTAAGTAAGAGGGAAGTACTTAAGGAGTAAGAGATGAAATATCTGTGATACCAAAGGGACTCCGTAAAGGCGAAGGCATCCCTTTATCTAAAAACTAACGTTGAAGGACGAAGGCTTAGATAACAAATAGG

>ef96546161304fc5ff8ca3fcc65a826a

TACACTTATCTCTTTAGAAAGACAAAATATTTCTCCAGGATGCAGTTCAACCATGTCGGTCATTTTCAACTGGCAGTGCCTTGTAGATAGTTGTGAAAGAATGTGAATGTGTCTTGTCCATAGAGTTCAGTTTGTTCAGTAATGATTTTGTTATGTAGTTTGTAACGTAATGTATTTAGACTGTCAAGGAAATTCATTTTATTTCCTTTTTTGTCGTTTGTG

>855a5c83cc07f349b77b865579d7438a

TACGTAGGGTGCGAGCGTTAATCGGAATTACTGGGCGTAAAGCATGCGCAGGCGGTTTGTTAAGACAGATGTGAAATCCCCGGGCTCAACCTGGGAACTGCATTTGTGACTGGCAGGCTAGAGTATGGCAGAGGGGGGTAGAATTCCACGTGTAGCAGTGAAATGCGTAGAGATGTGGAGGAATACCGATGGCGAAGGCAGCCCCCTGGGCCAATACTGACGCTCATGCACGAAAGCGTGGGGAGCAAACAGG

>5af360fd49b6b745c0e4e81b285b3170

CACGATTAACCCAAGTCAATAGAAGCCGGCGTAAAGAGTGTTTTAGATCACCCCCTCCCCAATAAAGCTAAAACTCACCTGAGTTGTAAAAAACTCCAGTTGGCACAAAATAGACTACGAAAGTGGCTTTAACATATCTGAACACACAATAGCTAAGACTCAAACTGGG

>828462a817540042b378fac232a00f64

TACGGAGGGTGCAAGCGTTAATCGGAATTACTGGGCGTAAAGCGCACGCAGGCGGTCTGTTAAGTCAGATGTGAAATCCCCGGGCTTAACCTGGGAACTGCATTTGAAACTGGCAGGCTTGAGTCTTGTAGAGGGGGGTAGAATTCCAGGTGTAGCGGTGAAATGCGTAGAGATCTGGAGGAATACTGGTGGCGAAGGCGGCCCCCTGGACAAAGACTGACGCTCAGGTGCGAAAGCGTGGGGAGCAAACAGG

>8396d8db5407e887d45d7d75d7083a35

TACCGGCAGCCCGAGTGATGGCCGATCTTATTGGGCCTAAAGCGTCCGTAGCTGGCCGCGCAAGTCCATCGGGAAATCCACCTGCTCAACAGGTGGGCGCCCGGTGGAAACTGCGCGGCTTGGGACCGGAAGGCGCGACGGGTACGTCCGGGGTAGGAGTGAAATCCCGTAATCCTGGACGGACCGCCGATGGCGAAAGCACGTCGCGAGAACGGATCCGACAGTGAGGGACGAAAGCCAGGGTCTCGAACCGG

>568ecb7c820cd4dceeb2f40f19c3b2ed

TACGTGAGAGACTAGTGTTATTCATCTTAATTGGGTTTAAAGGGTACCTAGACAGTCAATATAACTTCTATAATGCTAATACTTGACTAGAGTTCTAAGTAAGAGGGAAGTACTTAAGGAGTAAGAGATGAAATATCTGTGATACCAAAGGGACTCCGTAAAGGCGAAGGCATCCCTTTATCTAAAAACTAACGTTGAAGGACGAAGGCTTAGATAACAAATAGG

>25d64737365ec1cf7ca519f6eb8052e8

TACGTAGGTGGCAAGCGTTGTCCGGATTTACTGGGCGTAAAGGGAGCGTAGGCGGATTTTTAAGTGGGATGTGAAATACCCGGGCTCAACCTGGGTGCTGCATTCCAAACTGGAAATCTAGAGTGCAGGAGGGGAAAGCGGAATTCCTAGTGTAGCGGTGAAATGCGTAGAGATTAGGAAGAACACCAGTGGCGAAGGCGACTTTCTGGACTGTAACTGACGCTGAGGCTCGAAAGCGTGGGGAGCAAACAGG

>49be5e7b2ba8725be76bb5405160dc81

TACGGAGGGTGCAAGCGTTAATCGGAATTACTGGGCGTAAAGCGCACGCAGGCGGTCTGTCAAGTCGGATGTGAAATCCCCGGGCTCAACCTGGGAACTGCATTCGAAACTGGCAGGCTAGAGTCTTGTAGAGGGGGGTAGAATTCCAGGTGTAGCGGTGAAATGCGTAGATATAGGAAGGAACACCAGTGGCGAAGGCGACCACCTGGACTGATACTGACACTGAGGTGCGAAAGCGTGGGGAGCAAACAGG

>d49783e7800974b5f2bcce554e26072e

TACGTAGGGGGCAAGCGTTATCCGGATTTACTGGGTGTAAAGGGAGCGTAGACGGAAGAGCAAGTCTGATGTGAAAGGCTGGGGCTTAACCCCAGGACTGCATTGGAAACTGTTTTTCTAGAGTGCCGGAGAGGTAAGCGGAATTCCTAGTGTAGCGGTGAAATGCGTAGATATTAGGAGGAACACCAGTGGCGAAGGCGGCTTACTGGACGGTAACTGACGTTGAGGCTCGAAAGCGTGGGGAGCAAACAGG

>e4973ec3ed58cf02bc50f785a7c3a6ec

TACGGAGGGTGCAAGCGTTATCTGGATTTATTGGGTTTAAAGGGTCCGTAGGCGGATTTGTAAGTCAGTGGTGAAATCTCACAGCTTAACTGTGAAACTGCCATTGATACTGCAAGTCTTGAGTGTTGTTGAAGTAGCTGGAATAAGTAGTGTAGCGGTGAAATGCATAGATATTACTTAGAACACCAATTGCGAAGGCAGGTTACTAAGCAACAACTGACGCTGATGGACGAAAGCGTGGGGAGCGAACAGG

>9a014920654b9c61d8a589a6dfd69d61

TACGTAGGGTGCGAGCGTTAATCGGAATTACTGGGCGTAAAGCGTGCGCAGGCGGTTTGTTAAGACAGATGTGAAATCCCCGGGCTCAACCTAGGAACTGCATTTGTGACTGGCAGGCTAGAGTATGGCAGAGGGGGGTAGAATTCCACGTGTAGCAGTGAAATGCGTAGAGATGTGGAGGAATACCGATGGCGAAGGCAGCCCCCTGGGCCAATACTGACGCTCATGCACGAAAGCGTGGGGAGCAAACAGG

>702a36a26a2717cbe3ba34fad4776a2c

TACGGAGGGTGCAAGCGTTGTCCGGATTTATTGGGTTTAAAGGGTGCGTAGGCGGCTTGTTAAGTCCGGGGTGAAAGCCCACAGCTCAACTGTGGAACTGCCCTGGATACTGGCAAGCTTGAGTCCAGACGAGGTTGGCGGAATGGATGCTGTAGCGGTGAAATGCATAGATAGCATCCAGAACCCCGATTGCGTAGGCAGCTGACTAGGCTGGTACTGACGCTGAGGCACGAAAGCGTGGGGAGCGAACAGG

>5198cab8583f3910ef121b408bc722b1

TACCGGCAGCCCGAGTGATGGCCGATCTTATTGGGCCTAAAGCGTCCGCAGCTGGCCGCGCAAGTCCATCGGGAAATCCACCTGCTCAACAGGTGGGCGCCCGGTGGAAACTGTGCGGCTTGGGACCGGAAGGCGCGACGGGTACGTCCGGGGTAGGAGTGAAATCCCGTAATCCTGGACGGACCGCCGATGGTGAAAGCACGTCGCGAGAACGGATCCGACAGTGAGGGACGAAAGCCAGGGTCTCGAACCGG

>be066e42c37b89db6680195009e0f55b

TACGTAGGGTGCAAGCGTTAATCGGAATTACTGGGCGTAAAGCGTGCGCAGGCGGTTATGCAAGACAGAGGTGAAATCCCCGGGCTCAACCTGGGAACTGCCTTTGTGACTGCATAGCTAGAGTGCGGCAGAGGGGGATGGAATTCCGCGTGTAGCAGTGAAATGCGTAGATATGCGGAGGAACACCGATGGCGAAGGCAATCCCCTGGGCCTGCACTGACGCTCATGCACGAAAGCGTGGGGAGCAAACAGG

>25a20215f664c7be802b40c5d8dbae40

TACGTAGGGTGCGAGCGTTAATCGGAATTACTGGGCGTAAAGCGTGCGCAGGCGGTTTGTTAAGACAGATGTGAAATCCCCGGGCTCAACCTGGGAACTGCATTTGTGACTGGCAGACTAGAGTATGGCAGAGGGGGGTAGAATTCCACGTGTAGCAGTGAAATGCGTAGAGATGTGGAGGAATACCGATGGCGAAGGCAGCCCCCTGGGCCAATACTGACGCTCATGCACGAAAGCGTGGGGAGCAAACAGG

>691452da30ea5319dc4252de6739a467

TACGGAGGGTGCAAGCGTTAATCGGAATTACTGGGCGTAAAGCGTGCGCAGGCGGCTGATTAAGTCGGATGTGAAAGCCCCGGGCTCAACCTGGGAATGGCATTCGATACTGGTCAGCTAGAGTCTGGTAGAGGTAAGCGGAATTCCGGGTGTAGCGGTGAAATGCGTAGATATCCGGAGGAACATCAGTGGCGAAGGCGGCTTACTGGACCAAGCCTGACGCTCAGGCACGAAAGCGTGGGTAGCAAACAGG

>d4e418bf80be1142fbe9cc43c65e14ce

TACGAAGGGGGCTAGCGTTGTTCGGAATCACTGGGCGTAAAGCGCACGTAGGCGGATTATTAAGTCAGGGGTGAAAGCCTGGAGCTCAACTCCAGAACTGCCTTTGATACTGATAGTCTCGAGTTCGGAAGAGGTTGGTGGAACTGCGAGTGTAGAGGTGAAATTCGTAGATATTCGCAAGAACACCAGTGGCGAAGGCGGCCAACTGGTCCGATACTGACGCTGAGGTGCGAAAGCGTGGGGAGCAAACAGG

>4ab45fe41f079cb2862bbc6d50ddec58

TACGGAGGGTGCAAGCGTTAATCGGAGTTACTGGGCGTAAAGCGCACGCAGGCGGTCTGTCAAGTCGGATGTGAAATCCCCGGGCTCAACCTGGGAACTGCATTCGAAACTGGCAGGCTAGAGTCTTGTAGAGGGGGGTAGAATTCCAGGTGTAGCGGTGAAATGCGTAGAGATCTGGAGGAATACCGGTGGCGAAGGCGGCCCCCTGGACAAAGACTGACGCTCAGGTGCGAAAGCGTGGGGAGCAAACAGG

>bb2792e4d5d8c4bc95a5bbff91e63df9

TACGTAGGGTGCGAGCGTTAATCGGAATTACTGGGCGTAAAGCGTGCGCAGGCGGTTTGTTAAGACAGATGTGAAATCCCCGGGCTCAACCTGGGAACTGCATTTGTGACTGGCAGGCTAGAGTATGGCAGAGGGGGGTAGAATTCCACGTGTAGCAGTGAAATGCGTAGAGATGTGGAGGAATACCGATGGCGAAGGCAGCCCCCTGGGCCAATACTGACGATCATGCACGAAAGCGTGGGGAGCAAACAGG

>3a54d4e72af90b9c2fd50d7d1bb0336b

CACGTAGGCACCAAGCGTTATCCGGATTTATTGGGCGTAAAGAGCTCGTAGGCGGTTCGGTAAGTCGGGTGTGAAAACTCTGGGCTCAACCCAGAGAGGCCACCCGATACTGCTGTGACTAGAGTGCGGTAGGGGAGCGGGGAATTCCTGGTGTAGCGGTGAAATGCGCAGATATCAGGAGGAACACCAGCGGCGAAGGCGCCGCTCTGGGCCGTAACTGACGCTGAGGAGCGAAAGCATGGGTAGCAAACAGG

>049e82de8b6d828c7d4b129c3569f196

TACCGGCAGCACGAGTGATGGCCGCTATTATTGGGCCTAAAGCGTTCGTAGCCGGCCACGCAAGTTCGTCGGGAAATCTGCCCGCTTAACGGGCAGGCGTCCGGCGAAAACTGCGTGGCTTGGGACCGGGAGACTCGAGAAGTACGTTCGGGGTAGGAGTGAAATCCCATAATCCCGAACGGACTACCGATGGCGAAAGCATCTCGAGAGCACGGATCCGACGGTGAGGAACGAAAGCTGGGGTCTCGAACCGG

>9bd861d552480ace28069ea90b1a236e

TACGGAGGGTGCAAGCATTAATCGGATTTATTGGGCGTAAAGGGCGCGTAGGCGGGAAGGAAAGTCAGATGTGAAATCCCGGGGCTCAACCCCGGAACAGCATTTGAAACTCCCTTTCTTGAGGGTAGACGGAGAAAATGGAATTCCACAAGTAGCGGTGAAATGCGTAGATATGTGGAAGAACACCAGTGGCGAAGGCGATTTTCTAGTTTACACCTGACGCTGAGGCGCGAAAGCAAGGGGATCAAACAGG

>e306df68659fcc7e67dfcf44b6fbf9b4

TACGTAGGGTGCGAGCGTTAATCGGAATTACTGGGCGTAAAGCGTGCGCAGGCGGTTTGTTAAGACAGATGTGAAATCCCCGGGCTTAACCTGGGAACTGCATTTGTGACTGGCAGGCTAGAGTATGGCAGAGGGGGGTAGAATTCCACGTGTAGCAGTGAAATGCGTAGAGATGTGGAGGAATACCGATGGCGAAGGCAGCCCCCTGGGCCAATACTGACGCTCATGCACGAAAGCGTGGGGAGCAAACAGG

>e8386d3a307c208c4b9f0a756259cd6b

TACAGAGGGTGCGAGCGTTAATCGGATTTACTGGGCGTAAAGCGTGCGTAGGCGGCTTCTTAAGTCGGATGTGAAATCCCTGAGCTTAACTTAGGAATTGCATTCGATACTGGGAAGCTAGAGTATGGGAGAGGATGGTAGAATTCCAGGTGTAGCGGTGAAATGCGTAGAGATCTGGAGGAATACCGATGGCGAAGGCAGCCATCTGGCCTAATACTGACGCTGAGGTACGAAAGCATGGGGAGCAAACAGG

>eaf6f4575767bb32d96eb671b0ccf020

TACCGGCAGCCCAAGTGATGGCCGATCTTATTGGGCCTAAAGCGTCCGTAGCTGGCCGCGCAAGTCCATCGGGAAATCCACCTGCTCAACAGGTGGGCGCCCGGTAGAAACTGCGTGGCTTGGGACCGGAAGGCGCGACGGGTACGTCCGGGGTAGGAGTGAAACCCCGTAATCCTGGACGGACCGCCGATGGCGAAAGCACGTCGCGAGAACGGATCCGACAGTGAGGGACGAAAGCCAGGGTCTCGAACCGG

>3c67f6fdc0b3ca938d1d6565a1f2eace

TACGGAGGGGGCTAGCGTTGTTCGGAATTACTGGGCGTAAAGCGCGCGTAGGCGGACGGTCAAGTTGGGGGTGAAAACCCGGGGCTCAACCCCGGAACTGCCTTCAAAACTGATCGTCTGGAGACCGGGAGAGGTGAGTGGAATTCCCAGTGTAGAGGTGAAATTCGTAGATATTGGGAAGAACACCAGTGGCGAAGGCGGCTCACTGGACCGGATCTGACGCTGAGGTGCGAAAGCGTGGGGAGCGAACAGG

>859249ba848569f52d70d3ca73f17d3f

TACGTAGGGTGCGAGCGTTAATCGGAATTACTGGGCGTAAAGCGTGCGCAGGCGGTTTGTTAAGACAGATGTGAAATCCCCGGGCTCAACCTGGGAACTGCATTTGTGACTGGCAGGCTAGAGTATGGCAGAGGGGGGTAGAATTCCACGTGTAGCAGTGAAATGCGTAGAGATGTGGAGGAATACCGATGGCGAAGACAGCCCCCTGGGCCAATACTGACGCTCATGCACGAAAGCGTGGGGAGCAAACAGG

>20e1c97367648842695dbc4aa0f577c2

TACGTAGGGGGCGAGCGTTGTCCGAAGTTACTGGGCGTAAAGCGCGCGTAGGCGGTTTCTTAAGTTCGGGGTGAAAGGTTCAACGCTCAACGTGAACAGTGCCTTGAATACTGGGAGACTTGAGTTCGGGAGAGGGCAGTGGAATTCCTGGTGTAGCGGTGAAATGCGTAGATATCAGGAGGAACACCGATGGCGAAGGCAGCTGCCTGGCCTAGAACTGACGCTGAGGTGCGAAAGCGTGGGGAGCGAACAGG

>9bb151b98abbeec1dd64ec8f4be6a87a

TACGGAGGGTGCAAGCGTTGTCCGGAATCACTGGGTGTAAAGGGTGTGCAGGCGGGGCAACAAGTCGGATGTGAAACCCCATGGCTCAACCATGGAGGTGCATTCGAAACTGTTGCTCTTGAGTCCCGGAGAGGCTGTCGGAATTCGTGGTGTAGCGGTGAAATGCGTAGATATCACGAGGAACACCAGAGGCGAAAGCGGGCAGCTGGACGGGTACTGACGCTCAGGCACGAAAGCGTGGGGAGCAAACAGG

>07e46b397c80a250bbf58073b466c48b

TACGTAGGGTGCGAGCGTTAATCGGAATTACTGGGCGTAAAGCGTGCGCAGGCGGTTTGTTAAGACAGATGTGAAATCCCCGGGCTCAACCTGGGAACTGCATTTGTGACTGGCAGGCTAGAGTATGGCAGAGGGGGGTAGAATTCCACGTGTAGCAATGAAATGCGTAGAGATGTGGAGGAATACCGATGGCGAAGGCAGCCCCCTGGGCCAATACTGACGCTCATGCACGAAAGCGTGGGGAGCAAACAGG

>e93c7a7636ec55204a7d4da06464c8ae

AACAGAGGATACAAGCGTTATCCGGATTTATTGGGTTTAAAGGGTGCGTAGGTGGTTTTTTAAGTCAGTAGTGAAATCCTAAAGCTTAACTTTAAAAGTGCTATTGATACTGATAAACTAGAGTGAGGTTGGAGTAACTGGAATGTGTGGTGGAGCGGTGAAATGCATAGAGATCACACAGAACACCAATCGCGAAGGCATGTTACTAAACATAGACTGACACTGAGGCACGAAAGCATGGGTAGCAAACAGG

>6a7a6fed3a850c6bab631c438c0556c4

TACGTAGGGCGCAAGCGTTATCCGGATTTATTGGGCGTAAAGAGCTCGTAGGCGGTTTGTCGCGTCTGCCGTGAAAGTCCGAGGCTCAACCTCGGATCTGCGGTGGGTACGGGCAGACTAGAGTGATGTAGGGGAGACTGGAATTCCTGGTGTAGCGGTGAAATGCGCAGATATCAGGAGGAACACCGATGGCGAAGGCAGGTCTCTGGGCATTTACTGACGCTGAGGAGCGAAAGCATGGGGAGCGAACAGG

>55fcef7016326a10d2bc44d69cf8ea38

TACGTAGGGTGCGAGCGTTAATCGGAATTACTGGGCGTAAAGCGTGCGCAGGCGGTTATGTAAGACAGAGGTGAAATCCCCGGGCTCAACCTGGGAACGGCCTTTGTGACTGCATAGCTAGAGTACGGTAGAGGGGGATGGAATTCCGCGTGTAGCAGTGAAATGCGTAGATATGCGGAGGAACACCGATGGCGAAGGCAATCCCCTGGACCTGTACTGACGCTCATGCACGAAAGCGTGGGGAGCAAACAGG

>aa7ab24e6ac9867bf57821561a394b99

TACAGAGGGTGCAAGCGTTAATCGGATTTACTGGGCGTAAAGCGCGCGTAGGCGGCTAATTAAGTCAAATGTGAAATCCCCGAGCTTAACTTGGGAATTGCATTCGATACTGGTTAGCTAGAGTGTGGGAGAGGATGGTAGAATTCCAGGTGTAGCGGTGAAATGCGTAGAGATCTGGCGGAATACCGATGGCGAAGGCAGCCATCTGGCCTAACACTGACGCTGAGGTGCGAAAGCATGGGGAGCAAACAGG

>9e8813539e1d5f51be859381aa075d57

GTTGAAGCCTATAAATCCGAATCAGTTAGTTTTGACGTGAAAGAAAGGAATGGATCGGTGAATCGAATGGGCACGTTTCTCACCACCACCACGTTACCGTTATGCAACGTCCAGCGACTGTGAAATTCGCGTTCGGTTTCGCGCGCGCGCACTCGCATACAGATGAGAG

>be48b3d9fd736892065ceca8f8e3ceb8

TACGGAGGGTGCAAGCGTTAATCGGAATTACTGGGCGTAAAGCGCACGCAGGCGGTTTGTTAAGTCAGATGTGAAATCCCCGAGCTTAACTTGGGAACTGCATTTGAAACTGGCAAGCTAGAGTCTTGTAGAGGGGGGTAGAATTCCAGGTGTAGCGGTGAAATGCGTAGAGATCTGGAGGAATACCGGTGGTGAAGGCGGCCCCCTGGACAAAGACTGACGCTCAGGTGCGAAAGCGTGGGGAGCAAACAGG

>7bdf3bd4cc555b679f9557ce3ebb677e

TACGGAGGGTGCAAGCGTTATCCGGATTTATTGGGTTTAAAGGGTCCGTAGGCGGATTTGTAAGTCAGTGGTGAAATCTCACAGCTTAACTGTGAAACTGCCATTGATACTGCAAGTCTTGAGTGTTGTTGAAGTAGCTGGAATAAGTAGTGTAGCGGTGAAATGCATAGATATTACTTAGAACACCAATTGCGAAGGCAGGTTACTAAGCAACAACTGACGCTGATGGACGAAAGCGTGGGGAGTGAACAGG

>6925396593d201c5774816196a8be7d0

TACGGAGGATGCGAGCGTTATCCGGATTTATTGGGTTTAAAGGGTGCGTAGGTGGTTAATTAAGTCAGCGGTGAAAGTTTGTGGCTCAACCATAAAATTGCCGTTGAAACTGGTTGACTTGAGTATATTTGAGGTAGGCGGAATGCGTGGTGTAGCGGTGAAATGCATAGATATCACGCAGAACTCCGATTGCGAAGGCAGCTTACTAAACTATAACTGACATTGAAGCACGAAAGCGTGGGGATCAAACAGG

>6ec53fc9b856399ccc61c18231a15772

TACGGAGGGTGCAAGCGTTAATCGGAATTACTGGGCGTAAGGCGCACGCAGGCGGTCTGTTAAGTCAGATGTGAAATCCCCGGGCTTAACCTGGGAACTGCATTTGAAACTGGCAGGCTTGAGTCTTGTAGAGGGGGGTAGAATTCCAGGTGTAGCGGTGAAATGCGTAGAGATCTGGAGGAATACCGGTGGCGAAGGCGGCCCCCTGGACAAAGACTGACGCTCAGGTGCGAAAGCGTGGGGAGCAAACAGG

>f930b8c59c9b0889efd77801734b6e14

TACAGAGGGTGCAAGCGTTAATCGGATTTACTGGGCGTAAAGCGCGCGTAGGCGGCTAATTAAGTCAAATGTGAAATCCCCGAGCTTAACTTGGGAATTGCATTCGATACTGGTTAGCTAGAGTGTGGGAGAGGATGGTAGAATTCCAGGTGCAGCGGTGAAATGCGTAGAGATCTGGAGGAATACCGATGGCGAAGACAGCCATCTGGCCTAACACTGACGCTGAGGTGCGAAAGCATGGGGAGCAAACAGG

>488bf501d0d09be35f4ea7e683216493

CACACTTATGTCTTTAGAAAGACAACACGTTTTTGCAGAATGCATTTCAACCATGTCGGTCAATTTCAACTGGCAGTGCCTTGTAGATAATTGTGGAAGATTTTGAATGTTTCTTGTCCATAGAGTTCAGTTTGTTCATTAACGATTTTTTTTTGTAGTTTGTAACGTAATATATTTAAACTTTCAAGAAAATTCATTTTACTTCCTTTTTTGCCTGTTATG

>3723afc1064d9251b40817b082239812

TACCGGCAGCTCAAGTGATGTCCCATATTATTGGGCCTAAAGCGTCCGTAGCTGGCCGACCAAGTCTATCGGGAAATCCACCTGCCCAACAGGTGGGCGTCCGGTAGAAACTGGCCGGCTTGGAACCGGAAGGCTCAGAGAGTACGTCCGGGGTAGGAGTGAAACCCCGTAATCCTGGACGGACCGCCGATGGCGAAAGCACGTCGCGAGAACGGATCCGACAGTGAGGGACGAAAGCCAGGGTCTCGAACCGG

>bd961e41d54ab0aad9b3826dd3778fce

TACAGAGGGTGCAAGCGTTAATCGGATTTACTGGGCGTAAAGCGCGCGTAGGCGGCTAATTAAGTCAAATGTGAAATCCCCGAGCTTAACTTGGGAATTGCATTCGATACTGGTTAGCTAGAGTGTGGGAGAGGATGGTAGAATTCCAGGTGTAGCGGTGAAATGCGTAGAGATCTGGAGGAATACCGATGGCGAAGGCAGCCATCTGGCCTAACACTGACGCTGAGGTGCGAAGGCATGGGGAGCAAACAGG

>4dd77f96055ce85443636bfa808d7acf

CACACTTATGTCTTTAGAAAGGCAACACGTTTTTGCAGAATGCATTTCAACCATGTCATTTTCAATTGGCAGTTCCTTGTAGATAATTGTGGAAGATTTTGAATGTTTCTTGTCCATAGAGTTCAGTTTGTTCATTAACGATTTTTTTGTGTAGTTTGTAACGTAATATATTTAAACTTTCAAGAAAATTCATTTTATTTCCTTTTTTGCCTGTTATG

>861c43480c852e1733dce289b09a66e5

TACGTAGGGTGCGAGCGTTAATCGGAATTATTGGGCGTAAAGCGAGTGCAGACGGTTGTTTAAGCCAGATGTGAAATCCCCGAGCTTAACTTGGGACGTGCATTTGGAACTGGATAACTAGAGTGTGTCAGAGGGAGGTAGAATTCCACATGTAGCGGTGGAATGCGTAGAGATGTGGAGGAATACCGATGGCGAAGGCAGCCTCCTGGGATAACACTGACGTTGAGGCTCGAAAGCGTGGGGAGCAAACAGG

>4597947d0d73c0ff4e7b194eb2550cce

TACCGGCAGCACGAGTGATGGCCGCTATTATTGGGCCTAAAGCGTCCGTAGCTGGCCAGACAAGTCCGTTGGGAAATCGGCGTGCCTAACACGTCGGCGTCCGGCGGAAACTGTCTGGCTTGGAGCCGGAAGACCCGAGGGGTACGTCCGGGGTAGGAGTGAAATCCTGTAATCCTGGACGGACCACCGATGGCGAAAGCACCTCGGGAAGACGGACTCGACAGTGAGGGACGAAAGCCAGGGTCTCGAACCGG

>edd096f19296dca68ac45dd3ccfbb938

CACGATTAACCCAAGTCAATAGGAGCCGGCGTAAAGAGTGTTTTAGATCACCCCCTCCCCAATAAAGCTAAAACTCACCTGAGTTGTAAAAAACTCCAGTTGACACAAAATAGACTACGAAAGTGGCTTTAACATATCTGAACACACAATAGCTAAGACCCAAACTGGG

>94816ae4d281db2303f7b0a86e9ea6e7

TACAGAGGGTGCAAGCGTTATTCGGAATTACTGGGCGTAAAGCGCGCGTAGGCGGACTGTTAAGTCGGTTGTGAAAGCCCTGGGCTCAACCTAGGAATTGCATCCGATACTGGCAATCTAGAGTTTAGGAGAGGGAAGTGGAATTCCTGGTGTAGCAGTGAAATGCGTAGATATCAGGAGGAACATCAGTGGCGAAGGCGACTTCCTGGCCTAAAACTGACGCTGAGGTGCGAAAGCTTGGGTAGCGAACGGG

>195a54476a1802d75e0e773601fe964b

TACGTAGGGTGCGAGCGTTGTCCGGAATTACTGGGCGTAAAGAGCTCGTAGGTGGTTTGTCGCGTCGTCTGTGAAATTCCGGGGCTTAACTTCGGGCGTGCAGGCGATACGGGCATAACTTGAGTGCTGTAGGGGAGACTGGAATTCCTGGTGTAGCGGTGGAATGCGCAGATATCAGGAGGAACACCGATGGCGAAGGCAGGTCTCTGGGCAGTAACTGACGCTGAGGAGCGAAAGCATGGGGAGCGAACAGG

>e78d28b0c959b6e035caad98e3288205

TACAGAGGATGCAAGCGTTATCCGGAATGATTGGGCGTAAAGCGTCTGTAGGTGGCTTTTTAAGTCCGCCGTCAAATCCCAGGGCTCAACCCTGGACAGGCGGTGGAAACTACCAAGCTGGAGTACGGTAGGGGCAGAGGGAATTTCCGGTGGAGCGGTGAAATGCGTAGAGATCGGAAAGAACACCAACGGCGAAAGCACTCTGCTGGGCCGACACTGACACTGAGAGACGAAAGCTAGGGGAGCAAATGGG

>7eedbf3323e05d0d0ad28deb4018f534

TACGTAGGGTGCGAGCGTTAATCGGAATTACTGGGCGTAAAGCGTGCGCAGGCGATTTGTTAAGACAGATGTGAAATCCCCGGGCTCAACCTGGGAACTGCATTTGTGACTGGCAGGCTAGAGTATGGCAGAGGGGGGTAGAATTCCACGTGTAGCAGTGAAATGCGTAGAGATGTGGAGGAATACCGATGGCGAAGGCAGCCCCCTGGGCCAATACTGACGCTCATGCACGAAAGCGTGGGGAGCAAACAGG

>ec1efd53bae78ac3b1f2c2985a6761ed

TACGAAGGGGGCTAGCGTTGCTCGGAATCACTGGGCGTAAAGGGCGCGTAGGCGGCGTTTTAAGTCGGGGGTGAAAGCCTGTGGCTCAACCACAGAATGGCCTTCGATACTGGGACGCTTGAGTATGGTAGAGGTTGGTGGAACTGCGAGTGTAGAGGTGAAATTCGTAGATATTCGCAAGAACACCGGTGGCGAAGGCGGCCAACTGGACCATCACTGACGCTGAGGCGCGAAAGCGTGGGGAGCAAACAGG

>8eeb8de3096e0ae8633bd4ba7f6418be

TACGGAGGGAGCTAGCGTTATTCGGAATTACTGGGCGTAAAGCGCACGTAGGCGGCTTTGTAAGTTAGAGGTGAAAGCCTGGAGCTCAACTCCAGAATTGCCTTTGATACTGCATGGCTTGAATCCAGGAGAGGTGAGTGGAATTCCGAGTGTAGAGGTGAAATTCGTAGATATTCGGAAGAACACTAGTGGCGAAGGCGGCTCACTGGACTGGTATTGACGCTGAGGTGCGAAAGCGTGGGGAGCAAACAGG

>a6d775b50af6eb4e34a64ef626da922e

TACGGAGGGTGCAAGCGTTAATCGGAATTACTGGGCGTAAAGCGCACGCAGGTGGTTTGTTAAGTCAGATGTGAAATCCCCGGGCTCAACCTGGGAACTGCATCTGATACTGGCAAGCTTGAGTCTCGTAGAGGGGGGTAGAATTCCAGGTGTAGCGGTGAAATGCGTAGAGATCTGGAGGAATACCGGTGGCGAAGGCGGCCCCCTGGACAAAGACTGACGCTCAGGTGCGAAAGCGTGGGGAGCAAACAGG

>2131c402ca6e57739d05e9caca1f1afb

CACGATTAACCCAAGTCAATAGAAGCCGGCGTAAAGAGTGTTTTAGATCACCCCCTCCCCAATAAAGCTAAAACTCACCTGAGTTGTAAAAAACCCCAGTTGACACAAAATAGACTACGAAAGTGGCTTTAACATATCTGAACACACAATAGCTAAGACCCAAACTGGG

>637b9b3f4d1cbb1a10c07817619cdf69

TACAGAGGGTGCAAGCGTTAATCGGAATTACTGGGCGTAAAGCGCGCGTAGGTGGTTAGTTAAGTTGGATGTGAAATCCCCGGGCTCAACCTGGGAACTGCATTCAAAACTGACTGACTAGAGTATGGTAGAGGGTGGTGGAATTTCCTGTGTAGCGGTGAAATGCGTAGATATAGGAAGGAACACCAGTGGCGAAGGCGACCACCTGGACTGATACTGACACTGAGGTGCGAAAGCGTGGGGAGCAAACAGG

>8465e7c09b8f09ff5814887cb4891a5f

TACGTAGGGTGCGAGCGTTAATCGGAATTACTGGACGTAAAGCGTGCGCAGGCGGTTTGTTAAGACAGATGTGAAATCCCCGGGCTCAACCTGGGAACTGCATTTGTGACTGGCAGGCTAGAGTATGGCAGAGGGGGGTAGAATTCCACGTGTAGCAGTGAAATGCGTAGAGATGTGGAGGAATACCGATGGCGAAGGCAGCCCCCTGGGCCAATACTGACGCTCATGCACGAAAGCGTGGGGAGCAAACAGG

>060cf91cfb472b04d82e15a19461c523

TACGTAGGGAGCGAGCGTTGTCCGGAATTACTGGGTGTAAAGGGAGCGTAGGCGGGGCGGCAAGTCAGGTGTGAAATTTATGGGCTTAACCCATAACCTGCATTTGAAACTGTCGCTCTTGAGTGAAGAAGAGGTTGGCGGAATTCCTAGTGTAGCGGTGAAATGCGTAGATATTAGGAGGAACACCAGTGGCGAAGGCGGCCAACTGGGCTTTTACTGACGCTGAGGCTCGAAAGCGTGGGGAGCAAACAGG

>c6ee43a6e4f937caa902186cf80864c6

TACAGAGGGTGCAAGCGTTAATCGGATTTACTGGGCGTAAAGCGCGCGTAGGTGGTTAATTAAGTCAAATGTGAAATCCCCGAGCTTAACTTGGGAATTGCATTCGATACTGGTTAGCTAGAGTATGGGAGAGGATGGTAGAATTCCAGGTGTAGCGGTGAAATGCGTAGAGATCTGGAGGAATACCGATGGCGAAGGCAGCCATCTGGCCTAATACTGACACTGAGGTGCGAAAGCATGGGGAGCAAACAGG

>83168dc906296ebbcd2b54b9dfaf71f5

TACGTAGGTGGCAAGCGTTGTCCGGAATTATTGGGCGTAAAGCGCGCGCAGGCGGTCCTTTAAGTCTGATGTGAAATCTTGCGGCTCAACTGCAAGCGGTCATTGGAAACTGGGGGACTTGAGTGCAGAAGAGGAAAGTGGAATTCCACGTGTAGCGGTGAAATGCGTAGAGATGTGGAGGAACACCAGTGGCGAAGGCGGCTTTCTGGTCTGTAACTGACGCTGAGGCGCGAAAGCGTGGGGAGCAAACAGG

>0eedb25a12c4cc261190b69f3f82cc60

TACGGAGGGGGCTAGCGTTGTTCGGAATTACTGGGCGTAAAGCGCGCGTAGGCGGCTTTGTAAGTTAGAGGTGAAAGCCCGGAGCTCAACTCCGGAACTGCCTTTAAGACTGCATCGCTAGAATTGTGGAGAGGTAAGTGGAATTCCGAGTGTAGAGGTGAAATTCGTAGATATTCGGAAGAACACCAGTGGCGAAGGCGACTTACTGGACACATATTGACGCTGAGGTGCGAAAGCGTGGGGAGCAAACAGG

>9860355e5ddf8b627774ecd467b33158

TACCGGCAGCCCGAGTGATGGCCGATCTTATTGGGCCTAAAGCGTCCGTAGCTGGCCGCACAAGTCCATCGGAAAATCCACCCGCTCAACGGGTGGGCGTCCGGTGGAAACTGTGTGGCTTGGGACCGGAAGGCGCGACGGGTACGTCCGGGGTAGGAGTGAAATCCCGTAATCCTGGACGGACCGCCGATGGCGAAAGCACGTCGCGAGAACGGATCCGACAGTGAGGGACGAAAGCTAGGGTCTCGAACCGG

>a0901407705992dc213ac509ede97d47

TACGGAGGGTGCAAGCGTTAATCGGAATTACTGGGCGTAAAGCGCACGCAGGCGGTCTGTCAAGTCGGATGTGAAATCCCCGGGCTCAACCTGGGAACTGCATCCGAAACTGGCAGGCTTGAGTCTCGTAGAGGGGGGTAGAATTCCAGGTGTAGCGGTGAAATGCGTAGAGATCTGGAGGAATACCGGTGGCGAAGGCGGCCCCCTGGACGAAGACTGACGCTCAGGTGCGAAAGCGTGGGGAGCAAACAGG

>b3d2cc3b9f0184830bec2ef73bcc6166

TACGTAGGTCCCAAGCGTTGTCCGGATTTATTGGGCGTAAAGCGAGCGCAGGTGGTTTCTTAAGTCTGATGTAAAAGGCAGTGGCTCAACCATTGTGTGCATTGGAAACTGGGAGACTTGAGTGCAGGAGAGGAGAGTGGAATTCCATGTGTAGCGGTGAAATGCGTAGATATATGGAGGAACACCGGAGGCGAAAGCGGCTCTCTGGCCTGTAACTGACACTGAGGCTCGAAAGCGTGGGGAGCAAACAGG

>0dc06389b421bb35b0938295146ad022

TACGTAGGTCCCGAGCGTTGTCCGGATTTATTGGGCGTAAAGCGAGCGCAGGCGGTTTAATAAGTCTGAAGTTAAAGGCAGTGGCTTAACCATTGTTCGCTTTGGAAACTGTTAAACTTGAGTGCAGAAGGGGAGAGTGGAATTCCATGTGTAGCGGTGAAATGCGTAGATATATGGAGGAACACCGGTGGCGAAAGCGGCCCTCTGGGCTGTAACTGACGCTGAGGCTCGAAAGCGTGGGGAGCAAACAGG

>70aee6e45235e5c70d2d0b0716af2d6d

GACGAAGGATGCGAGCGTTATCCGGATTCACTGGGTTTAAAGGGTGCGTAGGCGGTTTAGTAAGTTTCAAGTTAAAGACTGGTGCTTAACATCGGGACTGCTTGAAAAACTGCTAGACTTGAGATAGGTCGGCGTTGCTGGAATATGGCAAGTAGAGGTGAAATTCATAGATATGCCATAGAACACCGATAGCGAAGGCAGGCGACGAGGCCTTATCTGACGCTGAGGCACGAAAGCGTGGGGATCAAACAGG

>626d0fc6d20bce69c8a578f3f47d8bca

TCATTATGTAATATATTCAATGTTTCTTTGTTTTTTACTCCTGTACTACAATAGCCCACCCAAGCTTTGTATAATTCTTCGTGCATTGTTTATTTTATCCTTTGTGCATGAGTTTTTGCTTTGAAACCCATCATCTGTTTTCTTCAGGTCTACACAGATTAGA

>dd8f8b15d408d307197613b7efe3a247

CGTATTTCTTGGATGTGCATTATTTCCAGTTTTCAATTATAAAAAATATCTTAAAAACGTAAGAAAATAATTGAATTTGTATTGACGTTTTCTACTCAATTGGTACGTGGATATGTTTTATAAGTGGTCTGAATATAAGAAATATACGAAATACTCTTAAAATGTACTTAGGTATGATTGTATATTTTCAAATAATTTTAGTTTATCGTAATTATTTTTCTTATTATTACGCGGTGTTGC

>2ccced5997303ac396011c1efeeb74f8

CACGATTAACCCAAGTCAATAGAAGCCGGCGTAAAGAGTGTTTTAGATCACCCCCTCCCCAATAAAGCTAAAACTCACCTGAGTTGTAAAAAACTCCAGTTGACACAAAATGGACTACGAAAGTGGCTTTAACATATCTGAACACACAATAGCTAAGACCCAAACTGGG

>9827e3a2e67651bf4e33ba5a53378023

TACGTAGGGTGCGAGCGTTAATCGGAATTACTGGGCGTAAAGCGTGCGCAGGCGGTTTGTTAAGACAGATGTGAAATCCCCGGGCTCAACCTGGGAACTGCATTTGTGACTGGCAGGCTAGAGTATGGCAGAGGGGGGTAGAATTCCACGTGTAGCAGTGAAATGCGTAGAGATGTGGAGAAATACCGATGGCGAAGGCAGCCCCCTGGGCCAATACTGACGCTCATGCACGAAAGCGTGGGGAGCAAACAGG

>5524b77cd1ed0f26a0ed27e1f13db874

TACAGAGGGTGCAAGCGTTAATCGGATTTACTGGGCGTAAAGCGCGCGTAGGCGGCTAATTAAGTCGAATGTGAAATCCCCGAGCTTAACTTGGGAATTGCATTCGATACTGGTTAGCTAGAGTGTGGGAGAGGATGGTAGAATTCCAGGTGTAGCGGTGAAATGCGTAGAGATCTGGAGGAATACCGATGGCGAAGGCAGCCATCTGGCCTAACACTGACGCTGAGGTGCGAAAGCATGGGGAGCAAACAGG

>34a02aebf919468f6c81e6fa9ff92b9b

TACGGAGGGTGCAAGCGTTAATCGGAATTACTGGGCGTAAAGCGCACGCAGGCGGTTTGTTAAGTCAGATGTGAAATCCCCGGGCTCAACCTGGGGACTGCATTTGAAACTGGCAAGCTTGAGTCTCGTAGAGGGGGGTAGAATTCCAGGTGTAGCGGTGAAATGCGTGGAGATCTGGAGGAATACCGGTGGCGAAGGCGGCCCCCTGGACGAAGACTGACGCTCAGGTGCGAAAGCGTGGGGAGCAAACAGG

>09156ad15628688c48c6be96dcc59e05

TACGTAGGGTGCAAGCGTTAATCGGAATTACTGGGCGTAAAGCGTGCGCAGGCGGTTATGCAAGACAGAGGTGAAATCCCCGGGCTCAACCTGGGAACTGCCTTTGTGACTGCATGGCTAGAGTACGGTAGAGGGGGATGGAATTCCGCGTGTAGCAGTGAAATGCGTAGATATGCGGAGGAACACCGATGGTGAAGGCAATCCCCTGGACCTGTACTGACGCTCATGCACGAAAGCGTGGGGAGCAAACAGG

>e5de49bb019e1e347817dfae5c1d04d2

TACGTAGGTGGCAAGCGTTGTCCGGATTTATTGGGCGTAAAGCGAGCGCGGGCGGAAGAATAAGTCTGATGTGAAAGCCCTCGGCTTAACCGAGGAACTGCATCGGAAACTGTTTTTCTTGAGTGCAGAAGAGGAGAGTGGAACTCCATGTGTAGCGGTGGAATGCGTAGATATATGGAAGAACACCAGTGGCGAAGGCGGCTCTCTGGTCTGCAACTGACGCTGAGGCTCGAAAGCATGGGTAGTGAACAGG

>13fdab233df0e2f9cffec6574d8b8345

TACGTAGGTGGCGAGCGTTGTCCGGAATTACTGGGCGTAAAGCGCGCGTAGGCGGAGCTATCAGTCTGCCGTGAAAAACTACTGCTTAACAGTAGGAGGCCGGTGGAAACAGTAGCTCTTGAGTGCCGGAGAGGGAAGCGGAATTCCCAGTGTAGCGGTGAAATGCGTAGATATTGGGAGGAACACCAGTGGCGAAGGCGGCTTTCTGGACGGTGTCTGACGCTGAGGCGCGAAAGCCAGGGGAGCGAACGGG

>117956a36f81de70d0e276f6486047a2

TACGTAGGGTGCGAGCGTTAATCGGAATTACTTGGCGTAAAGCGTGCGCAGGCGGTTTGTTAAGACAGATGTGAAATCCCCGGGCTCAACCTGGGAACTGCATTTGTGACTGGCAGGCTAGAGTATGGCAGAGGGGGGTAGAATTCCACGTGTAGCAGTGAAATGCGTAGAGATGTGGAGGAATACCGATGGCGAAGGCAGCCCCCTGGGCCAATACTGACGCTCATGCACGAAAGCGTGGGGAGCAAACAGG

>5627e0a45fcd726123a95ae9f63b632b

TACCGGCAGCTCAAGTGATGACCGATATTACTGGGCCTAAAGCGTCCGTAGCCGGCCACGAAGGTTCATCGGGAAATCCGCCAGCTCAACTGGCGGGCGTCCGGTGAAAACCACGTGGCTTGGGACCGGAAGGCTCGAGGGGTACGTCCGGGGTAGGAGTGAAATCCCGTAATCCTGGACGGACCACCGATGGCGAAAGCACCTCGAGAAGACGGATCCGACGGTGAGGGACGAAAGCTAGGGTCTCGAACCGG

>1f642e94d3f784c6cfb0d196ed233c0b

CACGATTAACCCAAGTCAATAGAAGCCGGCGTAAAGAGTGTTTTAGATCACCCCCTCCCCAATAAAGCTAAAACTCACCTGAGTTGTAAAAAACTCCAGTTGACACAAAATAGACTACGAAAGTGGCTTTAGCATATCTGAACACACAATAGCTAAGACCCAAACTGGG

>1e58954aea83de19c0ff35b693e9cc29

TACGGAGGGTGCAAGCGTTAATCGGAATTACTGGGCGTAAAGCGCACGCAGGCGGTCTGTCAAGTCGGATGTGAAATCCCCGGGCTCAACCTGGGAACTGCATTCGAAACTGGCAGGCTAGAGTCTTGTAGAGGGGGGTAGAATTCCAGGTGTAGCGGTGAAATGCGTAGAGATCTGGAGGAATACCGGTGGCGAAGGCGGCCCCCTGGACAAAGACTGACGCTCAGGTGCGAAAGCATGGGGAGCAAACAGG

>8889305f80d845b27a6abc0e1d94bd7b

CACGATTAACCCAAGTCAATAGAAGCCGGCGTAAAGAGTGTTTTAGATCACCCCCTCCCCAATAAAGCTAAAACTCACCTGAGTTGTAAAAAATTCCAGTTGACACAAAATAGACTACGAAAGTGGCTTTAACATATCTGAACACACAATAGCTAAGACCCAAACTGGG

>3cc1864704c18fe2ddc46e3b4646be34

AACAGAGGATACAAGCGTTATCCGGATTTATTGGGTTTAAAGGGTGCGTAGGTGGTTTTTTAAGTCAGTAGTGAAATCTTAAAGCTTAACTTTAAAAGTGCTATTGATACTGATAAACTAGAGTGAGGTTGGAGTAACTGGAATGTGTGGTGGAGCGGTGAAATGCATAGAGATCACACAGAACACCAATCGTGAAGGCATGTTACTAAACATAGACTGACACTGAGGCACGAAAGCATGGGTAGCAAACAGG

>e96f48e017fae8d57062ead3e77aa004

CAACAAGGGGAACCTAACAATGAGCCGCCTCGCAATCAATCAATCCTCGCCACGCCACATCAACGCCATTCGTGTAGCACAATTTTTCTTCGCCTATCTACACACACGCACGCACACACACTTACACAATTTATAGGTACTTGTATAAATCTTTACTACGTTCGCGTTATGAATAATACGTATATTATACAACTGATGATTTGTAACCTTGTAGCAGTTTCAGGGCATTTATATGG

>22e94e81679617e7891af5470015b27c

TACAGAGGGTGCAAGCGTTAATCGGATTTACTGGGCGTAAAGCGCGCGTAGGCGGCTAATTAAGTCAAATGTGAAATCCCCGAGCTTAACTTGGGAATTGCATTCGATACTGGTTAGCTAGAGTGTGGGAGAGGATGGTAGAATTCCAGGTGTAGCGGTGAAATGCGTAGAGATCTGGAGGAACACCGATGGCGAAGGCAGCCATCTGGCCTAACACTGACGCTGAGGTGCGAAAGCATGGGGAGCAAACAGG

>cead08419f57661c2d1d0e75983847dc

CACACTTATATCTTCAGAAAGACAAAATATTTCTCCAGGATGCATTTCAACAATGTCGGTCATTTTCAACTGGCAGTGCCTTGTAGATATTTGTGAAAGATTTTGAATGTGTCCTGTCCATAGAGTTCATTAATGATTTTGTTATGTATATTTTAACGTAATATATTTAGGCTTTGAAGTAAAATCATTTTGTTTCCTTTTTTGCCGGTTGTG

>b95c77e4909caec983e5863d415fb97e

TACGTAGGGTGCAAGCGTTAATCGGAATTACTGGGCGTAAAGCGTGCGCAGGCGGTTATGCAAGACAGAGGTGAAATCCCCGGGCTCAACCTGGGAACTGCCTTTGTGACTGCATGGCTAGAGTACGGTAGAGGGGGATGGAATTCTGCGTGTAGCAGTGAAATGCGTAGATATGCGGAGGAACACCGATGGCGAAGGCAATCCCCTGGACCTGTACTGACGCTCATGCACGAAAGCGTGGGGAGCAAACAGG

>c3b52a0d6c29d3e644875bcfced38c90

TACGGAGGGTGCAAGCGTTAATCGGAATTACTGGGCGTAAAGCGCACGCAGGCGGTCTGTCAAGTCGGACGTGAAATCCCCGGGCTCAACCTGGGAACTGCATTCGAAACTGGCAGGCTAGAGTCTTGTAGAGGGGGGTAGAATTCCAGGTGTAGCGGTGAAATGCGTAGAGATCTGGAGGAATACCGGTGGCGAAGGCGGCCCCCTGGACAAAGACTGACGCTCAGGTGCGAAAGCGTGGGGAGCAAACAGG

>41f3f39a0335a7392afa80327caf0f77

TACGTAAGGACCGAGCGTTGTCCGGAATCATTGGGCGTAAAGGGTACGTAGGCGGCTAGAAAAGTTAGAAGTCAAAGGCTATAGCTCAACTATAGTAAGCTTCTAAAACTATTTAGCTTGAGAAATGGAAGGGAAAGTGGAATTCCTAGTGTAGCGGTGGAATGCGCAGATATTAGGAAGAATACCGGTGGCGAAGGCGACTTTCTGGCCATTATCTGACGCTGAGGTACGAAAGCGTGGGTAGCAAACAGG

>f179e3fe0763c3197267f73c17792834

TACAGAGGCCTCAAGCGTTGTTCGGAATCACTGGGCGTAAAGGGTGCGTAGGCGGCGCGGAAAGTCAGAGGTGAAATCCCGGAGCTCAACTCCGGAACTGCCTCCGATACTCCCGCGCTCGAGGACTGGAGAGGAGTCTGGAATTCACGGTGGAGCAGTGAAATGCGTGGATATCGTGAGGAACACTAGTGGCGAAGGCGAGACTCTGGACAGTACCTGACGCTGAGGCACGAAGGCCAGGGGAGCGAACGGG

>868e0165fe32a8925b7f20afa5e1dbfc

TACGTAGGGTGCAAGCGTTAATCGGAATTACTGGGCGTAAAGCGTGCGCAGGCGGTTATATAAGACAGATGTGAAATCCCCGGGCTCAACCTGGGAACTGCATTTGTGACTGTATAGCTAGAGTACGGCAGAGGGGGATGGAATTCCGCGTGTAGCAGTGAAATGCGTAGATATGCGGAGGAACACCGATGGCGAAGGCAATCCCCTGGGCCTGTACTGACGCTCATGCACGAAAGCGTGGGGAGCAAACAGG

>989bd0d39b46393d43ef36a4a3328b89

TACGGAGGGGGCTAGCGTTGTTCGGAATTACTGGGCGTAAAGCGTACGTAGGCGGCTTTGTAAGTTAGAGGTGAAAGCCTGGAGCTCAACTCCAGAACTGCCTTTAAGACTGCATCGCTTGAATCCAGGAGAGGTGAGTGGAATTCCGAGTGTAGAGGTGAAATTCGTAGATATTCGGAAGAACACCAGTGGCGAAGGCGGCTCACTGGACTGGTATTGACGCTGAGGTACGAAAGCGTGGGGAGCAAACAGG

>886813d6c66ef41ef648c9b92ffde619

TACGTAGGGTGCGAGCGTTGTCCGGAATTACTGGGCGTAAAGAGCTCGTAGGTGGTTTGTCGCGTCGTTTGTGTAAGCCCGCAGCTTAACTGCGGGACTGCAGGCGATACGGGCATAACTTGAGTGCTGTAGGGGAGACTGGAATTCCTGGTGTAGCGGTGGAATGCGCAGATATCAGGAGGAACACCGATGGCGAAGGCAGGTCTCTGGGCAGTAACTGACGCTGAGGAGCGAAAGCATGGGTAGCGAACAGG

>f1fdaade3f3d12be1223453ae532227c

TACGAAGGGGGCTAGCGTTGCTCGGAATTACTGGGCGTAAAGGGAGCGTAGGCGGATCGTTTAGTCAGGGGTGAAATCCCGGGGCTCAACCTCGGAACTGCCTTTGATACTGGCGATCTTGAGTTTGGGAGAGGTGTGTGGAACTCCGAGTGTAGAGGTGAAATTCGTAGATATTCGGAAGAACACCAGTGGCGAAGGCGACACACTGGCCCAATACTGACGCTGAGGCTCGAAAGCGTGGGGAGCAAACAGG

>d509a55b710fe965b01dabeffd3f5868

TACGTAGGGTGCGAGCGTTAATCGGAATTACTGGGCGTAAAGCGTGCGCAGGCGGTTTGTTAAGACAGATGTGAAATCCCCGGGCTCAACCTGGGAACTGCATTTGTGACTGGCAGGCTAGAGTATGGCAGAGGGGGGTAGAATTCCACGTGTAGCAGTGAAATGCGTAGAGATGTGGAGGAATACCGATAGCGAAGGCAGCCCCCTGGGCCAATACTGACGCTCATGCACGAAAGCGTGGGGAGCAAACAGG

>4e62bb0e72c9a8c85aa560265ca35a39

CACACTTATGTCTTCAGAAAGACAAAATATTTCCCCATGATGCATTTCAACCATGTCGGTCATTTTCAACTGGCAGTGCCTTGTTGATATTTGTGAAATATTTTGAATATGTCTTGTCCATAGAGTTGAGTTTGTTCATTAATGATTTTGTTATGTAGTTTGTAACGTAATACATTTAGGCTTTCAAGTAAATTCATTTTGTTTACTTTTTTGCCGGTTGTG

>2f898a8cd83fb030c7582c0ae6ea135d

TACGGAGGGTGCAAGCGTTAATCGGAATCACTGGGCGTAAAGCGCGCGTAGGCGGCTTGGTAAGTTGAATGTGAAAGCCCCGGGCTTAACCTGGGAACTGCATCCAAAACTGCCAGGCTAGAGTACGGTAGAGGGAGGTGGAATTTCCGGTGTAGCGGTGAAATGCGTAGAGATCGGAAGGAACATCAGTGGCGAAGGCGGCCTCCTGGACCGATACTGACGCTGAGGTGCGAAAGCGTGGGGAGCAAACAGG

>a07a037c7b642a63bacaedc346d014d9

TACGTAGGGGGCGAGCGTTGTTCGGAATTACTGGGCGTAAAGAGCGCGTAGGCGGTCAAATAAGTCAGGTGTGAAATCCCTCAGCTTAACTGAGGTTTGGCGCTTGAAACTGTCTGACTTGAGTTTAGGAGAGGAGAGTGGAATTCCCAGTGTAGCGGTGAAATGCGTAGATATTGGGAGGAACACCTGTGGCGAAGGCGGCTCTCTGGACTAATACTGACGCTGAGGCGCGAAAGCTAGGGGAGCAAACTGGG

>73db7cbe707961864af8be2212905a5d

TACCGGCAGTCCAAGTGATGGCCGATATTATTGGGCCTAAAGCGTCCGTAGCCTGCTGTGTAAGTCCGTTGGGAAATCGACGCGCTCAACGCGTCGGCGTCCAGCGGAAACTACACGGCTTGGGGCCGAGAGACTTGACGGGTACGTCCGGGGTAGGAGTGAAATCCTGTAATCCTGGACGGACCACCAATGGCGAAAGCACGTCAAGAGACCGGACCCGACGGTGAGGGACGAAAGCTAGGGTCTCGAACCGG

>7b4fe34a097e3008c36e5fa6689d8665

TACGGAGGGTGCAAGCGTTATCCGGATTCACTGGGTTTAAAGGGTGCGTAGGCGGGTTAGTAAGTCAGTGGTGAAATCCCCGAGCTTAACTTGGGAACTGCCGTTGATACTATTAGTCTTGAATATCGTGGAGGTAAGCGGAATATGTCATGTAGCGGTGAAATGCTTAGATATGACATAGAACACCAATTGCGAAGGCAGCTTGCTACACGATTATTGACGCTGAGGCACGAAAGCGTGGGGATCAAACAGG

>b19cf87656725313652f3b05d12eda27

TACGTAGGGTGCGAGCGTTAATCGGAATTACTGGGCGTAAAGAGTGCGCAGGCGGTTTGTTAAGACAGATGTGAAATCCCCGGGCTCAACCTGGGAACTGCATTTGTGACTGGCAGGCTAGAGTATGGCAGAGGGGGGTAGAATTCCACGTGTAGCAGTGAAATGCGTAGAGATGTGGAGGAATACCGATGGCGAAGGCAGCCCCCTGGGCCAATACTGACGCTCATGCACGAAAGCGTGGGGAGCAAACAGG

>f920d7df1d5cb79ee4badb645279b2a2

CACGATTAACCCAAGTCAATAGAAGCTGGCGTAAAGAGTGTTTTAGATCACCCCCTCCCCAATAAAGCTAAAACTCACCTGAGTTGTAAAAAACTCCAGTTGACACAAAATAGACTACGAAAGTGGCTTTAACATATCTGAACACACAATAGCTAAGACCCAAACTGGG

>ae441d62a6d4edc321b736bcc6101974

TACGGAGGGTGCAAGCGTTAATCGGAATTACTGGGCGTAAAGCGCACGCAGGCGGTCTGTCAAGTCGGATGTGAAATCCCCGGGCTCAACCTGGGAACTGCATTCGAAACTGGCAGGCTAGAGTCTTGTAGAGGGGGGTAGAATTCCAGGTGTAGCGGTGAAATGCGTAGAGATCTGGAGGAATACCGGTGGCGAAGGCGGCCCCCTGGACAGAGACTGACGCTCAGGTGCGAAAGCGTGGGGAGCAAACAGG

>6846a40f60860f1a1646fa067f1a3611

GACAGAGGATGCAAGCGTTATCCGGAATGATTGGGCGTAAAGAGTCTGTAGGTGGCTTTTTAAGTTCGCCGTCAAATCCCAGGGCTCAACCCTGGACAGGTGGTGAAAACTACTAAGCTAGAGTACGGTAGGGGCAGAGGGAATTTCCGGTGGAGCGATGAAATGCGTAGAGATCGGAAGGAACACCAACGGCGAAAGCACTCTGCTGGGCCGACACTGACACTGAGAGACGAAAGCTAGGGGAGCGAATGGG

>cea51a3090f2be455fe65db824f81b61

TACGGAGGGTGCAAGCGTTATCCGGATTTATTGGGTTTAAAGGGTCCGTAGGCGGATTTGTAAGTCAGTGGTGAAATCTCACAGCTTAACTGTGAAACTGCCATTGATACTGCAAGTCTTGAGTGTTGTTGAAGTAGCTGGAATAAGTAGTGTAGCGGTGAAATGCATAGATATTACTTAGAACACCAATTGCGAAGGCAGGTTACTAAGCAACAACTGATGCTGATGGACGAAAGCGTGGGGAGCGAACAGG

>97025232bf50b1d9e31461fed54c9db4

TACGTAGGGTGCAAGCGTTAATCGGAATTACTGGGCGTAAAGCGTGCGCAGGCGGTTCGGCAAGAAGGATGTGAAATCCCGGGGCTTAACCTCGGAACTGCATTCTTAACTGCCGGGCTAGAGTATGTCAGAGGGGGGTAGAATTCCACGTGTAGCAGTGAAATGCGTAGAGATGTGGAGGAATACCGATGGCGAAGGCAGCCCCCTGGGATAATACTGACGCTCATGCACGAAAGCGTGGGGAGCAAACAGG

>065e94e5b2735ca615634b690dbbf342

CACACTTATATCTTCAGAAAGACAAAATATTTCTCCAGGATGCATTTCAACAATGTCGGTCATTTTCAACTGGCAGTGCCTTGTAGATATTTGTGAAAGATTTTGAATGTGTCCTGTCCATAGAGTTCATTAATGATTTTGTCATGTATATTTTAACGTAATATATTTAGGCTTTGAAGTAAAATCATTTTGTTTCCTTTTTTGCCGGTTGTG

>90f6a82a72817d315e023a63631fb313

TACGTAGGTGGCAAGCGTTATCCGGAATTATTGGGCGTAAAGCGCGCGTAGGCGGTTTTTTAAGTCTGATGTGAAAGCCCACGGCTCAACCGTGGAGGGTCATTGGAAACTGGAAAACTTGAGTGCAGAAGTGGAAAGTGGAATTCCATGTGTAGCGGTGAAATGCGCAGAGATATGGAGGAACACCAGTGGCGAAGGCGACTTTCTGGTCTGTAACTGACACTGAGGCGCGAAAGCGTGGGGAGCAAACAGG

>784b37b1cdbee55717570bb61b756dc9

TACGTAGGGTGCGAGCGTTAATCGGAATTACTGGGCGTAAAGCGTGCGCAGGCGGTTTGTTAAGACAGATGTGAAATCCCCGGGCTCAACCTGGGAACTGCATTTGTGACTGGCAGGCTAGAGTATGGCAGAGGGGGGTAGAATTCCACGTGTAGCAGTGAAATGCGTAGAGATGTGGAGGAATAACGATGGCGAAGGCAGCCCCCTGGGCCAATACTGACGCTCATGCACGAAAGCGTGGGGAGCAAACAGG

>84725f8c3ad8d05195755d7c8f358335

TACGTAGGTGGCAAGCGTCATCCGGAATTATTGGGCGTAAAGCGCGCGCAGGTGGTTTCTTAAGTCTGATGTGAAAGCCCACGGCTCAACCGTGGAGGGTCATTGGAAACTGGGAGACTTGAGTGCAGAAGAGGAAAGTGGAATTCCATGTGTAGCGGTGAAATGCGTAGAGATATGGAGGAACACCAGTGGCGAAGGCGACTTTCTGGTCTGTAACTGACACTGAGGCGCGAAAGCGTGGGGAGCAAACAGG

>14a27a4907c04e375eca4c9dab622a4f

TTACTTTAAAGAAACCAAGTAACTATATTTTTATAAGAATCTGCTATTAGCCACACCACACAAAACCCCCAACCTGACAGACCACAAATTCTGAATTCGGGCTGTTCATACCCTACAGAGACCCTTCTGTACTTCCATCTAGTCGAGAAGTTCCTGCCTGGTCGCAGAAGACCAACAGCTCCAGTAGACCAACCGTTCCTCTACCTACACAGAAGACGCACTCCCTGCTGCT

>1130d7f266d2ad6443dcc1342053418e

TACAGAGGATGCAAGCGTTATCCGGAATGATTGGGCGTAAAGCGTCTGTAGGTGGCTTCTTAAGTCCGCCGTCAAATCCCAGGGCTCAACCCTGGACAGGCGGTGGAAACTACCAAGCTGGAGTACGGTAGGGGCAGAGGGAATTTCCGGTGGAGCGGTGAAATGCGTAGAGATCGGAAAGAACACCAACGGCGAAAGCACTCTGCTGGGCCGACACTGACACTGAGAGACGAAAGCTAGGGGAGCGAATGGG

>3a02ac7fa74088ef2cdfaf94759ac613

CACGATTAACCCAAGTCAATAGAAGCCGGTGTAAAGAGTGTTTTAGATCACCCCCTCCCCAATAAAGCTAAAACTCACCTGAGTTGTAAAAAACTCCAGTTGACACAAAATAGACTACGAAAGTGGCTTTAACATATCTGAACACACAATAGCTAAGACCCAAACTGGG

>6b19048f1ada1800bd7326953fc3c974

CACGATTAACCCAAGTCAATAGAAGCCGGCGTAAAGAGTGTTTTAGATCACCCCCTCCCCAATAAAGCTAAAACTCACCTGAGTTGTAGAAAACTCCAGTTGACACAAAATAGACTACGAAAGTGGCTTTAACATATCTGAACACACAATAGCTAAGACCCAAACTGGG

>93e1b326393f0ab2a89e589b7e6eb95f

CACGATTAACCCAAGTCAATAGAAGCCGGCGTAAAGAGTGTTTTGGATCACCCCCTCCCCAATAAAGCTAAAACCCACCTGAGTTGTAAAAAACTCCAGTTGACACAAAATAGACTACGAAAGTGGCTTTAACATATCTGAACACACAATAGCTAAGACCCAAACTGGG

>a1aac52828ca5430cf451c9d9f351160

TACGTAGGGTGCGAGCGTTAATCGGAATTACTGGGCGTAAAGCGTGCGCAGGCGGTTTGTTAAGACAGATGTGAAATCCCCGGGCTCAACCTGGGAACTGCATTTGTGACTGGCAGGCTAGAGTATGGCAGAGGGGGGTAGAATTCCACGTGTAGCAGTGAAATGCGTAGAGATGTGGAGGAATACCGATGGCGAAGGCAGCCCCATGGGCCAATACTGACGCTCATGCACGAAAGCGTGGGGAGCAAACAGG

>8ef752e645507636c4fc4b8c3b77f7a1

TACGGAGGATCCAAGCGTTATCCGGAATCATTGGGTTTAAAGGGTCCGTAGGCGGTTTAGTAAGTCAGTGGTGAAAGCCCATCGCTCAACGGTGGAACGGCCATTGATACTGCTGAACTTGAATTATTAGGAAGTAACTAGAATATGTAGTGTAGCGGTGAAATGCTTAGAGATTACATGGAATACCAATTGCGAAGGCAGGTTACTACTAATTGATTGACGCTGATGGACGAAAGCGTGGGTAGCGAACAGG

>d6c6048d165a11a47c4c89fef20660a0

TACGTAGGGTGCGAGCGTTAATCGGAATTACTGGGCGTAAAGCGTGCGCAGGCGGTTTTGTAAGTCGGATGTGAAATCCCCGGGCTCAACCTGGGAACTGCGTTCGAAACTGCAAGGCTAGAGTGTGTCAGAGGGGGGTAGAATTCCACGTGTAGCAGTGAAATGCGTAGAGATGTGGAGGAATACCAATGGCGAAGGCAGCCCCCTGGGATAACACTGACGCTCATGCACGAAAGCGTGGGGAGCAAACAGG

>9551f0823feaf3cbb93297a008ab536e

CACGATTAACCCAAGTCAATAGAAGCCGGCGTAAAGAGTGTTTTAGATCACCCCCTCCCCAATAAAGCTAAAACTCACCTGAGTTGTAAAAAACTCCAATTGACACAAAATAGACTACGAAAGTGGCTTTAACATATCTGAACACACAATAGCTAAGACCCAACCTGGG

>f2524e2e7f220bbc1c0986dd1eaf0ac4

TACGTAGGGTGCGAGCGTTAATCGGAATTACTGGGCGTAAAGCGTGCGCAGGCGGTTTGTTAAGACAGATGTGAAATCCCCGGGCTCAACCTGGGAACTGCATTTGTGACTGGCAGGCTAGAGTATGGCAGAGGGGGGTAGAATTCCACGTGTAGCAGTGAAATGCGTAGAGATGTGGAGGAATACAGATGGCGAAGGCAGCCCCCTGGGCCAATACTGACGCTCATGCACGAAAGCGTGGGGAGCAAACAGG

>f8333a94c407e1e7c7ba2df21cd05190

TACAGAGGGTGCAAGCGTTAATCGGATTTACTGGGCGTAAAGCGCGCGTAGGCGGCTAATTAAGTCAAATGTGAAATCCCCGAGCTTAACTTGGGAATCGCATTCGATACTGGTTAGCTAGAGTGTGGGAGAGGATGGTAGAATTCCAGGTGTAGCGGTGAAATGCGTAGAGATCTGGAGGAATACCGATGGCGAAGGCAGCCATCTGGCCTAACACTGACGCTGAGGTGCGAAAGCATGGGGAGCAAACAGG

>60b66034cbd11cd714a5a4c91fc31b54

CACGATTAACCCAAGTCAATAGAAGCCGGCGTAAAGAGTGTTTTAGATCACCCCCTCCCCAATAAAGCTAAAACTCACCTGAGTTGTAAAAAACTCCAGTTGACACAAAATAGACTACGAAAGTGGCTTTAACATATCTGAACACACAATAGCTAAGGCCCAAACTGGG

>75e02f20de6779fec32d96241f8426a5

TACCGGCAGCTCAAGTGATGTCCCATATTATTGGGCCTAAAGCGTTCGTAGCTGGCCACGCAAGTCCATCGGGAAATCCACCTGCTCAACAGGTGGGCGTCCGGTAGAAACTGGCCGGCTTGGAACCGGAAGGCTCAGAGAGTACGTCCGGGGTAGGAGTGAAATCCCGTAATCCCGGACGGACTACCGATGGCGAAAGCACTCTGAGAAGACGGCTTCGACAGTGAGGGACGAAAGCTCGGGTCTCAAACCGG

>b38965931588b5ebbac1fa824fca48fd

GACAGAGGGTGCAAACGTTGTTCGGAATTACTGGGCGTAAAGCGCGTGTAGGCGGCTCTGCAAGTCGGATGTGAAAGCCCTCGGCTCAACCGGGGAAGTGCACCCGAAACTGCAGAGCTAGAGTCTCGGAGAGGATCGTGGAATTCTCGGTGTAGAGGTGAAATTCGTAGATATCGAGAGGAACACCGGTGGCGAAGGCGGCGATCTGGACGATGACTGACGCTGAGACGCGAAAGCGTGGGGAGCAAACAGG

>27d91bcebdaae9680f3345b0285e3338

TACGTAGGTGGCAAGCGTTGTCCGGAATTATTGGGCGTAAAGGGCTCGCAGGCGGTTCCTTAAGTCTGATGTGAAAGCCCCCGGCTCAACCGGGGAGGGTCATTGGAAACTGGGGAACTTGAGTGCAGAAGAGGAGAGTGGAATTCCACGTGTAGCGGTGAAATGCGTAGAGATGTGGAGGAACACCAGTGGCGAAGGCGACTCTCTGGTCTGTAACTGACGCTGATGTGCGAAAGCGTGGGGATCAAACAGG

>2f23ba04e29d4f4fe40a157cc74d49b8

TACGAAGGGGGCTAGCGTTGCTCGGAATGACTGGGCGTAAAGGGCGCGTAGGCGGAATGCTTTGTCGGGCGTGAAATTCCAGGGCTTAACCTTGGGACTGCGTTCGAGACGGGTATTCTAGAGTGGAGAAGAGGGTCGTGGAATTCCCAGTGTAGAGGTGAAATTCGTAGATATTGGGAAGAACACCGGTGGCGAAGGCGGCGACCTGGTCTTTTACTGACGCTGAGGCGCGAAAGCGTGGGGAGCAAACAGG

>894aa8ec28e33419cb036523838ea468

CACGATTAACCCAAGTCAATAGAAGCCGGCGTAAAGAGTGTTTTAGATCACCCCCTCCCCAATAAAGCTAAAACTCACCTGAGTTGTAAAAAACTCCAGTTGACACAAAATAGACTACGAAAGTGGCTTTAACATATCTGAACACACAATAGCTAGGACCCAAACTGGG

>be29dd9311afc94b495302160c027d7e

CACGATTAACCCAAGTCAATAGAAGCCGGCGTAAAGAGTGTTTTAGATCACCCCCTCCCCAATAAAGCTAAAACTCACCTGAGTTGTAAAAAACTCCAGTTGACACAAAATAGACTACGAAAGTGGCTTTAACGTATCTGAACACACAATAGCTAAGACCCAAACTGGG

>45e3696d78d57fc35094a6cf67e6414b

TACGTAGGGGGCAAGCGTTGTCCGGAATTATTGGGCGTAAAGCGCGCGCAGGCGGCTTTTTAAGTCCGGTGTCACAGCCCAAGGCTCAACCTTGGGTCGCACTGGAAACTGGAGAGCTTGAGTACAGAAGAGGAAAGTGGAATTCCACGTGTAGCGGTGAAATGCGTAGAGATGTGGAGGAACACCAGTGGCGAAGGCGACTTTCTGGGCTGTAACTGACGCTGAGGCGCGAAAGCGTGGGGAGCAAACAGG

>14e9fe3f0d807f9d9535c1e539983df8

TACGTAGGGTGCGAGCGTTGTCCGGAATTATTGGGCGTAAAGAGCTCGTAGGCGGTCTGTCGCGTCGTTCGTGAAAACTTGGGGCTTAACCCTGAGCTTGCGGTCGATACGGGCATGACTTGAGTTCGGCAGGGGAGACTGGAATTCCTGGTGTAGCGGTGAAATGCGCAGATATCAGGAGGAACACCGGTGGCGAAGGCGGGTCTCTGGGCCGATACTGACGCTGAGGAGCGAAAGCGTGGGGAGCGAACAGG

>34bf6e1e3c280cc30498f20930a44f50

TACGTAGGGTGCGAGCGTTAATCGGAATTACTGGGCGTAAAGCGTGCGCAGGCGGTTTGTTAAGACAGATGTGAAATCCCCGGGCTCAACCTGGGAACTGCATTTGTGACTGGCAGGCTAGAGTATGGCAGAGGGGGGTAGAATTCCACGTGTAGCAGTGAAATGCGTAGATATGTGGAGGAATACCGATGGCGAAGGCAGCCCCCTGGGCCAATACTGACGCTCATGCACGAAAGCGTGGGGAGCAAACAGG

>a7a3260b46c83e5d5e4c864f8f3d7fa1

GACGGGGGGGGCAAGTGTTCTTCGGAATGACTGGGCGTAAAGGGCACGTAGGCGGTGAATCGGGTTGAAAGTGAAAGTCGCCAAAAAGTGGCGGAATGCTCTCGAAACCAATTCACTTGAGTGAGACAGAGGAGAGTGGAATTTCGTGTGTAGGGGTGAAATCCGGAGATCTACGAAGGAAGGCCAAAAGCGAAGGCAGCTCTCTGGGTCCCTACCGACGCTGGTGTGCGAAAGCATGGGGAGCGAACGGG

>d8db2ce12888cc65faaead9b7feffc27

TACGGAGGGGGCTAGCGTTGTTCGGAATTACTGGGCGTAAAGCGCGCGTAGGCGGACGGTCAAGTTGGGGGTGAAAGCCCGGGGCTCAACCCCGGAACTGCCTCCAAAACTGGGTGCCTAGAGTCCGAGAGAGGTGAGTGGAATTCCCAGTGTAGAGGTGAAATTCGTAGATATTGGGAAGAACACCAGTGGCGAAGGCGGCTCACTGGCTCGGTACTGACGCTGAGGTGCGACAGCGTGGGGAGCAAACAGG

>0df6c802966e8670279671824da4f10a

TACGTAGGTGGCAAGCGTTGTCCGGATTTATTGGGCGTAAAGCGAGTGCAGGCGGTTCAATAAGTCTGATGTGAAAGCCTTCGGCTCAACCGGAGAATTGCATCAGAAACTGTTGAACTTGAGTGCAGAAGAGGAGAGTGGAACTCCATGTGTAGCGGTGGAATGCGTAGATATATGGAAGAACACCAGTGGCGAAGGCGGCTCTCTGGTCTGCAACTGACGCTGAGGCTCGAAAGCATGGGTAGCGAACAGG

>4bb684e7960675e87f5a42973202df18

TACGGAGGGTGCAAGCGTTATCCGGATTTATTGGGTTTAAAGGGTCCGTAGGCGGATCTGTAAGTCAGTGGTGAAATCTCACAGCTTAACTGTGAAACTGCCATTGATACTGCAGGTCTTGAGTAAATTTGAAGTGGCTGGAATAAGTAGTGTAGCGGTGAAATGCATAGATATTACTTAGAACACCAATTGCGAAGGCAGGTCACTAAGATTTAACTGACGCTGATGGACGAAAGCGTGGGGAGCGAACAGG

>b3ed5c4e35fbe43dc754942f498f0fc7

CACGATTAACCCAAGTCAATAGAAGCCGGCGTAAAGAGTGTTTTAGATCACCCCCTCCCCAATAAAGCTAAAACTCACCTGAGTTGTAAAAAACTCCAGTTGACACAAAATAGACTACGAAAGTGGCCTTAGCATATCTGAACACACAATAGCTAAGACCCAAACTGGG

>b473cb52c909c60b97aa7d08fb1750e1

TACAGAGGGTGCAAGCGTTAATCGGATTTACTGGGCGTGAAGCGCGCGTAGGCGGCTAATTAAGTCAAATGTGAAATCCCCGAGCTTAACTTGGGAATTGCATTCGATACTGGTTAGCTAGAGTGTGGGAGAGGATGGTAGAATTCCAGGTGTAGCGGTGAAATGCGTAGAGATCTGGAGGAATACCGATGGCGAAGGCAGCCATCTGGCCTAACACTGACGCTGAGGTGCGAAAGCATGGGGAGCAAACAGG

>11e814ca47e4bf644524e88af8efd78c

TACAGAGGGTGCAAGCGTTAATCGGATTTACTGGGCGTAAAGCGCGCGTAGGCGGCTAATTAAGTCAAATGTGAAATCCCCGAGCTTAACTTGGGAATTGCATTTGATACTGGTTAGCTAGAGTGTGGGAGAGGATGGTAGAATTCCAGGTGTAGCGGTGAAATGCGTAGAGATCTGGAGGAATACCGATGGCGAAGGCAGCCATCTGGCCTAACACTGACGCTGAGGTGCGAAAGCATGGGGAGCAAACAGG

>9af3467db68cf6063627304cecd46a65

TACAGAGGGTGCAAGCGTTAATCGGAATTACTGGGCGTAAAGCGCGCGTAGGTGGTTCGTTAAGTTGGATGTGAAATCCCCGGGCTCAACCTGGGAACTGCATTCAAAACTGTCGAGCTAGAGTATGGTAGAGGGTGGTGGAATTTCCTGTGTAGCGGTGAAATGCGTAGATATAGGAAGGAACACCAGTGGCGAAGGCGACCACCTGGACTGATACTGACACTGAGGTGCGAAAGCGTGGGGAGCAAACAGG

>2199f68727a4e8df7171a6d2a5ac007f

TACGTAGGGTGCAAGCGTTAATCGGAATTACTAGGCGTAAAGCGTGCGCAGGCGGTTATGCAAGACAGAGGTGAAATCCCCGGGCTCAACCTGGGAACTGCCTTTGTGACTGCATGGCTAGAGTACGGTAGAGGGGGATGGAATTCCGCGTGTAGCAGTGAAATGCGTAGATATGCGGAGGAACGCCGATGGCGAAGGCAATCCCCTGGACCTGTACTGACGCTCATGCACGAAAGCGTGGGGAGCAAACAGG

>c9f3278443d8ca1fe052cd9ec91428f2

TACGGAGGATCCGAGCGTTATCCGGAATTATTGGGTTTAAAGGGTTCGTAGGCGGTTGAGTAAGTCAGTGGTGAAATCTTATAGCTTAACTATAAAATTGCCGTTGATACTGCTTGACTTGAATAGTATGGAAGTAATTAGAATATGTAGTGTAGCGGTGAAATGCTTAGATATTACATGGAATACCAATTGCGAAGGCAGATTACTACGTACTTATTGACGCTGATGAACGAAAGCGTGGGTAGCGAACAGG

>9b0a9653aac25d447f72248ea160eae4

TACCGGCAGCTCAAGTGATGACCGATATTATTGGGCCTAAAGCGTCCGTAGCCGGCCACGAAGGTTCATCGGGAAATCCGCCAGCTCAACTGGCGGGCATCCGGTGAAAACCACGTGGCTTGGGGCCGGAAGGCTCGAGGGGTACGTCCGGGGTAGGAGTGAAATCCCGTAATCCTGGACGGACCACCGATGGCGAAAGCACCTCGAGAAGACGTATCCGACGGTGAGGGACGAAAGCTAGGGTCTCGAACCGG

>7ad450db5fcb3ac8bcc87bbc82f4d841

TACAGAGGGTGCAAGCGTTAATCGGATTTACTGGGCGTAAAGCGCGCGTAGGCGGCTAATTAAGTCAAATGTGAAATCCCCGAGCTTAACTTGGGAATTGCATTCGATACTGGTTAGCTAGAGTGTGGGAGAGGATGGTAGAATTCCAGGTGTAGCGGTGAAATGCGTAGAGATCTGGAGGAATACCGATGGCGAAGGCAGCCATCTGGCCTAACACCGACGCTGAGGTGCGAAAGCATGGGGAGCAAACAGG

>8bd5272165e3399235baeed4408adf01

AACAGAGGATACAAGCGTTATCCGGATTTATTGGGTTTAAAGGGTGCGTAGGTGGTTTTTTAAGTCAGTAGTGAAATCTTAAAGCTTAACTTTAAAAGTGCTATTGATACTGATAAACTAGAGTGAGGTTGGAGTAACTGGAATGTGTGGTGGAGCGGTGAAATGCATAGAGATCACACAGAGCACCAATCGCGAAGGCATGTTACTAAACATAGACTGACACTGAGGCACGAAAGCATGGGTAGCAAACAGG

>5778c68f5094344a7aa5270d8e5ed926

TACGTAGGGTGCGAGCGTTAATCGGAATTACTGGGCGTAAAGCGTGCGCAGGCGGTTTGTTAAGACAGATGTGAAATCCCCGGGCTCAAACTGGGAACTGCATTTGTGACTGGCAGGCTAGAGTATGGCAGAGGGGGGTAGAATTCCACGTGTAGCAGTGAAATGCGTAGAGATGTGGAGGAATACCGATGGCGAAGGCAGCCCCCTGGGCCAATACTGACGCTCATGCACGAAAGCGTGGGGAGCAAACAGG

>ac08f23b84caefbf70a137c95c79be63

TACGTAGAAGACTAGTGTTATTCATCTTTAATGGGTTTAAAGGGTACCTAGACGGAAAATTAAGCCTTAATGGGAACTAATTTTCTAGAGTTTTATGTGAGAATATACGAATTACCAGAGGAGAGTCAAAATTCTATTATACTGGTAGGACGGGTAACAGCAAAGGCAGTATTTTATGTAAAAACTGACGTTGAGGGACGAAGCCTTGGGTAGCGATAAGG

>07e4e78d4dfce5aaadac2571dc84697b

TACGTAGGGTGCGAGCGTTAATCGGAATTACTGGGCGTAAAGCGTGCGCAGGCGGTTTGTTAAGACAGATGTGAAATCCCCGGGCTCAACCTGGGAACTGCATTTGTGACTGGCAGGCTAGAGTATGGCAGAGGGGGGTAGAATTCCACGTGTAGCAGTGAAATGCGTAGAGATGTGGAGGAATACCGATGGAGAAGGCAGCCCCCTGGGCCAATACTGACGCTCATGCACGAAAGCGTGGGGAGCAAACAGG

>507f0b79db3ee4df00b44e755285e80d

TACCGGCAGCCCGAGTGATGGCCGATCTTATTGGGCCTAAAGCGTCCGTAGCTGGCCGCGCAAGTCCATCGGGAAATCCACCTGCTCAACAGGTGGGCGCCCGGTGGAAACTGTGTGGCTTGGGACCGGAAGGCGCGACGGGTACGTCCGGGGTAGGAGTGAAACCCCGTAATCCTGGACGGACCGCCGATGGCGAAAGCACGTCGCGAGAACGGATCCGACAGTGAGGGACGAAAGCCAGGGTCTCGAACCGG

>906d326b2aaaf28dea94a28bbcea68a4

TACGAGGGGAGCGAGTGTTGTTCGGTTTTATTGGGCGTAAAGGGTACGTAGGCGGTTTTGTTAGTCAACAATTAAATCTTGGAACTTAATTCCATAGCAGTTGTTGATACTGCATAACTACGAACTTAATAGGGGTGGACACAATTCCAAGTGTAGAGGTGAAATTCGTTGATATTTGGAGGAGTACCAAAGGCGAAGGCAGTTCATTGGGTTAAGTTGACGCTGAGGTACGAAAGCGTGGGGAGCAAACAGG

>4a9564b5801a990a81cef5a3a230f399

CACGATTAACCCAAGTCAATAGAAGCCGGCGTAAAGAGTGTTTTAGATCACCCCCTCCCCAATAAAGCTAAAACTCACCCGAGTTGTAAAAAACTCCAGTTGACACAAAATAGACTACGAAAGTGGCTTTAACATATCTGAACACACAATAGCTAAGACCCAAACTGGG

>ebdd09eeb268370c3804dc87427ef51b

TACGTAGGGCGCGAGCGTTGTCCGGAATTATTGGGCGTAAAGAGCTTGTAGGCGGTTTGTTGCGTCTGCTGTGAAAGACCGGGGCTTAACCCCGGTATTGCAGTGGGTACGGGCAGACTAGAGTGCAGTAGGGGAGACTGGAATTCCTGGTGTAGCGGTGAAATGCGCAGATATCAGGAGGAACACCGATGGCGAAGGCAGGTCTCTGGGCTGTAACTGACGCTGAGAAGCGAAAGCATGGGGAGCGAACAGG

>1417711691ef00de91f8e2735c9c32cc

CACCACTAATGTCTTTAGAAAGACAAAACATTTCTCCAGGATGCATTTCAACCATGTCTGTCATTTTCAACTGGCAGTGCCTTGTAGATATTTTTGAAAGATTTTGAATGTGCCTTGTCTACAGAGTTCAGTTTGTTCGTAAATGATTTTGTTATATTTAGGCTTTCAATTAAATTCAATTTATTTCCTTTTTGCCGGTTGTG

>0db8d222600bcc3b9d4a7c27c743de20

CACACTTATGTCTTCAGAAAGACAAAATATTTATCCAGGATGCATTTCAACCATGTCGGTCATTTTCAACTGGCAGTGCCTTGTATCTATTTTCTTGTAAAGATTTTGAATGTGTCTTGTCCATAGAGTTCAGTTTGTTCATTAATGATTTTGTTATGTAGTTTGTAACGTAATATGTTTAGGCTATCAAGTAAATTAATTTTGTTTCCTTTTTGCCGGTTGTG

>1a250513c6b709db8f97c1070630290d

CACACTTATGTCTTCAGAGAGACAAAATATTTATCCAGGATGCGTTTCAACCATGTCGGTCATTTTCAACTGGCAGTGCCTTGTAGATATTTGTGAAAGATTTTGAATGTGTCTTGTCCATAGAGCCAGTTTGTTCATTAATGATTTTGTTATGTAGTTTTTAACGTAATATATTTAGGCTTTCAAGTAAATTCATTTTGTTTCCTTTTCGCCGGTTGTG

>f8e966fd6a44aecf3991a7dd167fca57

GACGTAGGGCGCGAGCGTTGTCCGGATTTATTGGGCGTAAAGAGCTCGTAGGCGGCTTGTCGCGTCGACCGTGAAAACTTGGGGCTCAACCCCAAGCCTGCGGTCGATACGGGCAGGCTAGAGTTCGGTAGGGGAGACTGGAATTCCTGGTGTAGCGGTGAAATGCGCAGATATCAGGAGGAACACCGGTGGCGAAGGCGGGTCTCTGGGCCGATACTGACGCTGAGGAGCGAAAGCGTGGGGAGCGAACAGG

>ded7073a8f24f54ca86dc5901839c29d

TACCGGCAGCTCAAGTGATGTCCCATATTATTGGGCCTAAAGCGTCCGTAGCTGGCCGCGCAAGTCCGTCGGGAAATCCACTCGCTTAACGAGTGGGCGTCCGGCGGAAACTGTGCGGCTTGGGACCGGAAGATCCAAGGGGTACGTCCGGGGTAGGAGTGAAATCCCGTAATCCTGGACGGACCACCGATGGCGAAAGCACCTTGGAAGGACGGATCCGACAGTGAGGGACGAAAGCTAGGGTCTCGAACCGG

>0197c1f2f56861cc0de116b23070baea

CACAGATGATACAGGTGCTCATTGGGCTGAGACCCAGATAGCGCAAACACGATTCGGGTGCTAGAAATCCCGCACAGCATCAGTCAGTCAGTAATATTGGTTATCGGAGCGCTGTTCCAGCTTTTATTGATCTACTAAATAACTGCGACAAAATTATTTACCCATGTATTCACTTTCTACTTAAGATTGCTGTCACCTTGCCATATTCTGTTGCAACTGCAGATAGAACATTTTTATTGCCCAGAAGACTTAAAACTTAG

>603f035e259d00b1bc162778dd027a6f

TACGTGAGAGACTAGTGTTATTCATCTTAATTGGGCTTAAAGGGTACCTAGACAGTCAATATAACTTCTAGAATGCTAATACTTGACTAGAGTTTTAAGTAAGAGGGAAGTACTTAAGGAGTAAGAGATGAAATATCTGTGATACCAAAGGGACTCCGTAAAGGCGAAGGCATCCCTTTATCTAAAAACTAACGTTGAAGGACGAAGGCTTAGATAACAAATAGG

>0822682f56582eb9d57d5b9456dacee5

AACGTAGGAGACTAGCGTTATCCGGATTTATTGGGCGTAAAGCGCGTGTAGGCGGTGCCTTAAGTTGGACGTAAAATCTCCTGGCCTAACTGGGAGGGGCCGTTCAATACTGGGGCACTTGAGGGCAGAAGAGGGAAGTGGAATTCCCGGTGTAGCGGTGGAATGCGTAGATATCGGGAGGAACACCTGTGGCGAAGGCGGCTTCCTGGGATGCCCCTGACGCTGAGACGCGAAAGCTAGGGGAGCAAACTGGG

>ded4f2ba6e4bf454d51caaf818c9eada

TACGTGAGAGACTAGTGTTATTCATCTTAATTGGGTTTAAAGGGTACCTAGACAGTCAATATAACTTCTATAATGCTAATACTTGACTAGAGTTTTAAGTAAGAGGGAAGTACTTAAGGAGTAAGAGATGAAATATCTGTGATACCAAAGGGACTCCGTAAAGGCGAAGGCATCCCTCTATCTAAAAACTAACGTTGAAGGACGAAGGCTTAGATAACAAATAGG

>e5415ff42bc7bb84391d637502fbf600

TACGTAGGGTGCGAGCGTTAATCGGAATTACTGGGCGTAAAGCGTGCGCAGGCGGTTTGTTAAGACAGATGTGAAATCCCCGGGCTCAACCTGGGAACTGCATTTGTGACTGGCAGGCTAGAGTATGGCAGAGGGGGGTAGAATTCCACGTGTAGCAGTGAAATGCGTAGAGATGTGGAGGAATACCGATGGCGAAGGCAGCCCCCTGGGCCAATACTGAAGCTCATGCACGAAAGCGTGGGGAGCAAACAGG

>fcd277a64a475e30fdbbe161d7878f39

CACGATTAACCCAAGTCAATAGAAGCCGGCGTAAAGAGTGTTTTAGATCACCCCCTCCCCAATAAAGCTAAAACTCACCTGAGTTGTAAAAAACTCCAGTTGTCACAAAATAGACTACGAAAGTGGCTTTAACATATCTGAACACACAATAGCTAAGACCCAAACTGGG

>107273e73071282e4d0f4a0b46548da5

TACGGAGGGTGCAAGCGTTAATCGGAATTACTGGGCGTAAAGCGCACGCAGGCGGTTGGATAAGTTAGATGTGAAAGCCCCGGGCTCAACCTGGGAATTGCATTTAAAACTGTCCAGCTAGAGTCTTGTAGAGGGGGGTAGAATTCCAGGTGTAGCGGTGAAATGCGTAGAGATCTGGAGGAATACCGGTGGCGAAGGCGGCCCCCTGGACAAAGACTGACGCTCAGGTGCGAAAGCGTGGGGAGCAAACAGG

>d504e53ccce7a7a7a428bc855ad3380b

TACGGAGGGTGCGAGCGTTACCCGGATTTACTGGGTGTAAAGGGCGTGTAGGCGGTCTCTCAAGTCCGATGCTAAAGACCGAAGCTCAACTTCGGGGGTGCGTTGGATACTGTGAGGCTAGACGGTCGGAGAGGGTAGCGGAATTTCCGGAGTAGCGGTGAAATGCGCAGATACCGGAAGGAACGCCAATAGCGAAAGCAGCTACCTGGACGATTTGTGACGCTGAGGCGCGAAAGCGTGGGGAGCAAACCGG

>33cbebf2cef9f0510d373de954f1caa2

CATTGAACTATCGTGAGAAAGTCAAACCGCCAAAGGGAATTATATTATAGTAAATATTGACGTAAATAAACATTTTATTAATAGTTGTAATATATGAAAATGTGCAGAAGATAGAGACGATTAAAACGGTGTTTAACAATCGCTTATCGAGACAATATTTAATTA

>7901295a7aacf812117647165eaa72ca

TACGGAGGGTGCAAGCGTTAATCGGAATTACTGGGCGTAAAGCGTGCGCAGGCGGCTGATTAAGTCGGATGTGAAAGCCCCGGGCTCAACCTGGGAATGGCATTCGATACTGGTCAGCTAGAGTCTGGTAGAGGTAAGCGGAATTCCGGGTGTAGCGGTGAAATGCGTAGATATCCGGAGGAACGTCAGTGGCGAAGGCGGCTTACTGGACCAAGACTGACGCTCAGGCACGAAAGCGTGGGTAGCAAACAGG

>466c28e50b16a322098b62b4e6f4e8f3

TACGGAGGGTGCAAGCGTTATCCGGATTTATTGGGTTTAAAGGGTCCGTAGGCGGATTTGTAAGTCAGTGGTGAAATCTCACAGCTTAACTGTGAAACTGCCATTGATACTGCAAGTCTTGAGTGTTGTTGAAGTAGCTGGAATAAGTAGTGTAGCGGTGAAATGCATAGATATTACTTAGAACACCAATTGCGAAGGCAGGTTACTAAGCAACAACTGACGCTGATGGACGAAAGCGTGGGGAGCAAACAGG

>a2c5520edb0b1fd1db48f2cccdbee7ff

TACGAGGGGAGCGAGTGTTGTACGGTTTTATTGGGCGTAAAGGGCTATTAGGTGGCTCAATAAGTCGATAGTTAAATCTTGGAACTTAATTCCATCTATGTTATCGATACTATTGAACTAGAGTATGTAAAAGGGACGAACGGAATTCCAAGTGTAGAGGTGAAATTCGTTGATACTTGGAGGAACACCAGAGGCGAAAGCGGTTCGTCATAACAAAACTGACACTGAAATAGCGAAAGCATGGGGAGCAAACAGG

>c6ab19a121431399ff1d082d05e8a98d

TACGTAGGGTGCGAGCGTTAATCGGAATTACTGGGCGTAAAGCGTGCGCAGGCGGTTTGTTAAGACAGATGTGAAATCCCCGGGCTCAACCTGGGAACTGCATTTGTGACTGGCAGGCTAGAGTATGGCAGAGGGGGGTAGAATTCCACATGTAGCAGTGAAATGCGTAGAGATGTGGAGGAATACCGATGGCGAAGGCAGCCCCCTGGGCCAATACTGACGCTCATGCACGAAAGCGTGGGGAGCAAACAGG

>4c53f2820b75cbdc94802d859ae02e8c

TACCGGCAGCACGAGTGATGACCGATATTATTGGGCCTAAAGCGTCCGTAGCTGGCCAAGCAAGTCCATTGGGAAATCGACGTGCTCAACGCGTCGGCGTCCGGTGGAAACTGTTTGGCTTGGGGCCAGAAGACCTGAGGGGTACGTCCGGGGTAGGAGTGAAATCCTGTAATCCTGGACGGACCACCAATGGGGAAACCACGTTGAGAGACCGGACCCGACAGTGAGGGACGAAAGCCAGGGTCTCGAACCGG

>d27b7a212ee343d62cb0529c2ee484c6

TACCGGCAGCCCGAGTGATGGCCGATCTTATTGGGCCTAAAGCGTCCGTAGCTGGCCGCGCAAGTCCATCGGGAAATCCACCTGCTCAACAGGTGGGCGCCCGGTGGAAGCTGCGCGGCTTGGGACCGGAAGGCGCGACGGGTACGTCCGGGGTAGGAGTGAAACCCCGTAATCCTGGACGGACCGCCGATGGCGAAAGCACGTCGCGAGAACGGATCCGACAGTGAGGGACGAAAGCCAGGGTCTCGAACCGG

>5f8a834c95ce47f155d23258facd12f2

CACGATTAACCCAAGTCAATAGAAGCCGGCGTAAAGAGTGTTTTAGATCACCCCCTCCCCAATAAAGCTAAAACTCACCTGAGTTGTAAAAAACTCCGGTTGACACAAAATAGACTACTAAAGTGGCTTTAACATATCTGAACACACAATAGCTAAGACCCAAACTGGG

>8629fc9bdeba614e3960f48a05c2e942

CACCGGCAGCCCGAGTGATGGCCGATATTATTGGGCCTAAAGCGTCCGTAGCTGGCCGGGGAAGTCCGTCGGGAAATCCACTCGCTCAACGGGTGGGCGTCCGGTGGAAACTGTGTGGCTTGGGACCGGAAGGCGCGACGGGTACGTCCGGGGTAGGAGTGAAATCCCGTAATCCTGGACGGACCGCCGATGGCGAAAGCACGTCGCGAGAACGGATCCGACAGTGAGGGACGAAAGCCAGGGTCTCGAACCGG

>71260dcf4bbaab6f686913d3cfac35e6

CACACTTATGTCTTCAAGAAGACAAAATATTTCTCCAGGATGCATTTCAACCATGTCGGTCATTTTCAACTGGCAGTGCCTTGTAGATATTTGTGAAAGATTTTGAATGTGTCTTGTCCATAGAGTTGAGTTTGTTCATTAATGATTTTGTTATGTAGTTTGTAACGTAATATATTTAGGCTTTCTAGTAAATTCATTTTGTTTCCTTTTTTGCCGGTTGTG

>8137e743465bee06b3274e9a822b872e

ACATTCTTTGTACTTCATTAGTTATTTAATTTACACCACTTGTCACCCCCTTATATTGTACAAACGAGTTTTTCTGGCACATTATGTGAAATACACGTTCCTGTTACAGCTTATCCTTGACCTGCTAAAACATGAACAGCAATATTTCTATGACCACAAAAACTATGGACCAAGAAGCTG

>edd9b0a12d59a5a75356978861d80cc8

CACACTTATGTCTTCAGAAAGACAAAATATTTCTCCAGGATGCATTTCAACCATGTCGGTCATTTTCAACTGGCAGTGCCTTGTAGATATTTGTGAAAGATTTTGAATGTGTCTTGTCCATAGAGTTGAGTTTGTTCATTAATGATTTTGTTATGTAGTTTGTAACGTAGTATATTTAGACTTTCAAGTAAATTCATTTTGTTTACTTTTCTGCCGGTTGTG

>40dd14df02c6278521b868bb5c22ba68

TACAGAGGGTGCAAGCGTTAATCGGATTTACTGGGCGTGAAGCGCGCGTAGGCGGCTAATTAAGTCAAATGTGAAATCCCCGAGCTTAACTTGGGAATTGCATTCGATACTGGTTAGCTAGAGTGTGGGAGAGGATGGTAGAATTCCAAGTGTAGCGGTGAAATGCGTAGAGATCTGGAGGAATACCGATGGCGAAGGCAGCCATCTGGCCTAACACTGACGCTGAGGTGCGAAAGCATGGGGAGCAAACAGG

>642a39373d892379a93af5a4fc6885a7

TACGTAGGGTGCGAGCGTTAATCGGAATTACTGGGCGTAAAGCGTGCGCAGGCGGTTTGTTAAGACAGATGTGAAATCCCCGGGCTCAACCTGGGAACTGCATTTGTGACTGGCAGGCTAGAGTATGGCAGAGGGGGGTAGAATTCCACGTGTAGCAGTGAAATGCGTAGAGATGTGGAGGAATACCGATGGCGAAGGCAGCCCCCTGGGCCAATACTGACGCTCATGCACGAAAGTGTGGGGAGCAAACAGG

>242710e9bedc08f80b5c785a36beacb5

TACATAGGGTGCAAGCGTTAATCGGATTTACTGGGCGTAAAGCGCGCGTAGGCGGCTAATTAAGTCAGATGTGAAATCCCCGGGCTTAACCTGGGAACTGCATTTGAAACTGGCAGGCTTGAGTCTTGTAGAGGGGGGTAGAATTCCAGGTGTAGCGGTGAAATGCGTAGAGATCTGGAGGAATACCGGTGGCGAAGGCGGCCCCCTGGACAAAGACTGACGCTCAGGTGCGAAAGCGTGGGGAGCAAACAGG

>8004f4f6805dd164fc549ba6c5f80eab

TACCGGCAGCACGAGTGATGGCCGATATTATTGGGCCTAAAGCGTCCGTAGCTTGCTGTGCAAGTCCATTGGGAAATCCACGCGCTCAACGCGTGGGCGCCCGGTGGAAACTGCACGGCTTGGGGCCGAGAGACTCGACGGGTACGTCCGGGGTAGGAGTGAAATCCTGTAATCCTGGACGGACCACCAATGGGGAAACCACGTCGAGAGACCGGACCCGACAGTGAGGGACGAAAGCCAGGGTCTCGAACCGG

>113e3920b984872b2dbfca83b5f3d64a

TACGGAGGGTGCAAGTGTTATCCGGATTTATTGGGTTTAAAGGGTCCGTAGGCGGATTTGTAAGTCAGTGGTGAAATCTCACAGCTTAACTGTGAAACTGCCATTGATACTGCAAGTCTTGAGTGTTGTTGAAGTAGCTGGAATAAGTAGTGTAGCGGTGAAATGCATAGATATTACTTAGAACACCAATTGCGAAGGCAGGTTACTAAGCAACAACTGACGCTGATGGACGAAAGCGTGGGGAGCGAACAGG

>3609fe015806d2490a4ee5dc152246f2

TACGTAGGGTGCAAGCGTTAATCGGAATTACTGGGCGTAAAGCGTGCGTAGACGGTTATCTAAGTCAGATGTGAAATCCCCGGGCTTAACCTGGGAACTGCATTTGAGACTGTATAGCTAGAGTACGGTAGAGGGGGATGGAATTCCGCGTGTAGCAGTGAAATGCGTAGATATGCGGAGGAACACCGATGGCGAAGGCAATCCCCTGGGCCTGCACTGACGCTCATGCACGAAAGCGTGGGGAGCAAACAGG

>63edbedfbbf47cb16a8e0feeedbc63ab

TACCGGCAGCCCGAGTGATGGCCGATCTTATTGGGCCTAAAGCGTCCGTAGCCGGCCACACAAGTCCATCGGGAAATCCGCCAGCCTAACTGGCGGGCGTCCGGTGGAAACTGTGTGGCTCGGGATCGGAAGACCCAACGGGTACGTCTTGGGTAGGAGTGAAATCCCGTAATCCTGGACGGACCACCGATAGCGAAAGCACGTTGGGAAGACGAATCCGACGGTGAGGGACGAAAGCCAGGGTCTCGAACCGG

>99116e19c5b63af17887c6fba47998fb

TACAGAGGGTGCAAGCGTTAATCGGATTTACTGGGCGTAAAGCGCGCGTAGGCGGCTAATTAAGTCAAATGTGAAATCCCCGAGCTTAACTTGGGAATTGCATTCGATACTGGTTAGCTAGAGTGTGGGAGAGGATGGTAGAATTCCAGGTGTAGCGGTGAAATGCGTGGAGATCTGGAGGAATACCGATGGCGAAGGCAGCCATCTGGCCTAACACTGACGCTGAGGTGCGAAAGCATGGGGAGCAAACAGG

>5e72ba94b3cf6dc49f8de1e2f73355b9

TACGTAGGTGGCAAGCGTTGTCCGGATTTATTGGGCGTAAAGCGCGCGCAGGCGGCTATGTAAGTCTGGTGTTAAAGCCCGGGGCTCAACCCCGGTTCGCATCGGAAACTGTGTAGCTTGAGTGCAGAAGAGGAAAGCGGTATTCCACGTGTAGCGGTGAAATGCGTAGAGATGTGGAGGAACACCAGTGGCGAAGGCGGCTTTCTGGTCTGTAACTGACGCTGAGGCGCGAAAGCGTGGGGAGCAAACAGG

>f67950b9e629d9830ce36442aeaf5782

TACGTAGAAGACAAGTGTTATTCATCTTTAACAGGTTTAAAGGGTACCTAGACGGGAAATCAAGCCTTAGTTGGGACTAATTTCCTAGAGTTTTATGTGTGAAGTATCGAATTACCTGAGAAGCAATAATATGCTGTGATACAGGTAGGACGGGTAGCAGCGAAGGCAATCTTCTATGTAAAAACTGACGTTGAGGGACGAAGCCTTGGGGAGCGAGAAGG

>b2035485a0b41d043d6ff9827beeb3ba

TACAGAGGGTGCAAGCGTTAATCGGATTTACTGGGCGTAAAGCGCGCGTAGGCGGCTAATTAAGTCAAATGTGAAATCCCCGAGCTTAACTTGGGAACTGCATTTGAAACTGGCAGGCTTGAGTCTTGTAGAGGGGGGTAGAATTCCAGGTGTAGCGGTGAAATGCGTAGAGATCTGGAGGAATACCGGTGGCGAAGGCGGCCCCCTGGACAAAGACTGACGCTCAGGTGCGAAAGCGTGGGGAGCAAACAGG

>9cf5cd8a39bf6a1ecd8fbe97b97c50af

CACGATTAATCCAAGTCAATAGAAGCCGGCGTAAAGAGTGTTTTAGATCACCCCCTCCCCAATAAAGCTAAAACTCACCTGAGTTGTAAAAAACTCCAGTTGACACAAAATAGACTACGAAAGTGGCTTTAACATATCTGAACACACAATAGCTAAGACCCAAACTGGG

>aef33a1267fec79905cf8f2d0eb78e4a

TACGTAGGGTGCAAGCGTTGTCCGGAATTATTGGGCGTAAAGAGCTCGTAGGCGGTCTGTCGCGTCGGCTGTGAAAACCCGGAGCTCAACTCCGGGCCTGCAGTCGATACGGGCAAACTGGAGTACTGCAGGGGAGACTGGAATTCCTGGTGTAGCGGTGAAATGCGCAGATATCAGGAGGAACACCGGTGGCGAAGGCGGGTCTCTGGGCAGTAACTGACGCTGAGGAGCGAAAGCGTGGGGAGCGAACAGG

>f4801b7a68515d9005fa572ee6afdf41

TACGTAGGGTCCAAGCGTTAATCGGAATTACTGGGCGTAAAGCGTGCGCAGGCGGTTGTGCAAGACCGATGTGAAATCCCCGGGCTTAACCTGGGAATTGCATTGGTGACTGCACGGCTAGAGTGTGTCAGAGGGGGGTAGAATTCCACGTGTAGCAGTGAAATGCGTAGAGATGTGGAGGAATACCGATGGCGAAGGCAGCCCCCTGGGATAACACTGACGCTCATGCACGAAAGCGTGGGGAGCAAACAGG

>afb01eb68e7ca28d495fe00994677e25

AACAGAGGATACAAGCGTTATCCGGATTTATTGGGTTTAAAGGGTGCGTAGGTGGTTTTTTAAGTCAGTAGTGAAATCTTAAAGCTTAACTTTAAAAGTGCTATTGATACTGATAAACTAGAGTGAGGTTGGAGTAACTGGAATGTGTGGTGGAGCGGTGAAATGCATAGAGATCACACAGAACACCAATCGCGGAGGCATGTTACTAAACATAGACTGACACTGAGGCACGAAAGCATGGGTAGCAAACAGG

>9b9791bec30e50a5d87c5f2fa109e6b9

TACCGGCAGCTCAAGTGATGTCCCATATTATTGGGCCTAAAGCGTCCGTAGCTGGCCGACCAAGTCTATCGGGAAATCCACCTGCCCAACAGGTGGGCGTCCGGCGGAAACTGTGTGGCTTGGAACCGGAAGGCTCAGAGAGTACGTCCGGGGTAGGAGTGAAATCCCGTAATCCCGGACGGACTACCGATGGCGAAAGCACTCTGAGAAGACGGCTTCGACAGTGAGGGACGAAAGCTCGGGTCTCAAACCGG

>992774c7b2ff93b59cbd57ce1e1ca51a

TACGTAGGGTGCGAGCGTTAATCGGAATTACTGGGCGTAAAGCGTGCGCAGGCGGTTTGTTAAGACAGATGTGAAATCCCCGGGCTCAACCTGGGAACTGCATTTGTAACTGGCAGGCTAGAGTATGGCAGAGGGGGGTAGAATTCCACGTGTAGCAGTGAAATGCGTAGAGATGTGGAGGAATACCGATGGCGAAGGCAGCCCCCTGGGCCAATACTGACGCTCATGCACGAAAGCGTGGGGAGCAAACAGG

>53ceab520f8f0418f1252931d1883350

TACGGAGGGGGCTAGCGTTGTTCGGAATTACTGGGCGTAAAGCGCACGTAGGCGGCTTTGTAAGTCAGGGGTGAAAGCCTGGAGCTCAACTCCAGAACTGCCTTTGAGACTGCATCGCTTGAATCCGGGAGAGGTAAGTGGAATTCCGAGTGTAGAGGTGAAATTCGTAGATATTCGGAAGAACACCAGTGGCGAAGGCGGCTTACTGGACCGGAATTGACGCTGAGGTGCAAAAGCGTGGGGAGCAAACAGG

>2c167bfc640abc3337203990d7f17561

TACGTAGGTGGCAAGCGTTATCCGGAATTATTGGGCGTAAAGCGCGCGTAGGCGGTTTTTTAAGTCTGATGTGAAAGCCCCCGGCTCAACCGGGGAGGGTCATTGGAAACTGGGGAACTTGAGTGCAGAAGAGGAGAGTGGAATTCCACGTGTAGCGGTGAAATGCGTAGAGATGTGGAGGAACACCAGTGGCGAAGGCGACTCTCTGGTCTGTAACTGACGCTGAGGAGCGAAAGCGTGGGGAGCGAACAGG

>40fb4a8e2e83aa75e2f8884b42dd36c0

TACGAAGGGGGCTAGCGTTGCTCGGAATCACTGGGCGTAAAGGGCGCGTAGGCGGCGTTTTAAGTCGGGGGTGAAAGCCTGTGGCTCAACCACAGAATGGCCTTCGATACTGGGACGCTTGAGTATGGTAGAGGTTGGTGAAACTGCGAGTGTAGAGGTGAAATTCGTAGATATTCGCAAGAACACCGGTGGCGAAGGCGGCCAACTGGACCATTACTGACGCTGAGGCGCGAAAGCGTGGGGAGCAAACAGG

>fa297012bf2c56cfaa0302489af48511

CACGATTAACCCAAGTCAATAGAAGCCGGCGTAAAGAGTGTTTTAGATCACCCCCTCCCCAATAAAGCTAAAACTCACCTGAGTTGTAAAAAACTCCAGTTGACACAAAATAGACTACGAAAGTGGCTTTAACACATCTGAACACACAATAGCTAAGACCCAAACTGGG

>a0f786897b3ccdf3b0c946cb27cd1312

TACAGAGGGTGCAAGCGTTAATCGGATTTACTGGGCGTAAAGCGCGCGTAGGCGGCTAATTAAGTCAAATGTGAAATCCCCGAGCTTAACTTGGGAATTGCATTCGATACTGGTTAGCTAGAGTGTGGGAGAGGATGGTAGAATTCCAGGTGTAGCGGTGAAATGCGTAGAGATCTGGAGGAATACCGGTGGCGAAGGCAGCCATCTGGCCTAACACTGACGCTGTGGTGCGAAAGCATGGGGAGCAAACAGG

>548cce36fc72bca2cd92b6a4e961a8c8

TACGTAGGGTGCGAGCGTTAATCGGAATTACTGGGCGTAAAGCGTGCGCAGGCGGTTTGTTAAGACAGATGTGAAATCCCCGGGCTCAACCTGGGAACTGCATTTGTGACTGGCAGGCTAGAGTATGGCAGAGGGGGGTAGAATTCCACGTGTAGCAGTGAAATGGGTAGAGATGTGGAGGAATACCGATGGCGAAGGCAGCCCCCTGGGCCAATACTGACGCTCATGCACGAAAGCGTGGGGAGCAAACAGG

>b3dbcf84ea2bf25089adcd8907359a6e

TACCGGCAGCCCGAGTGATGGCCGATCTTATTGGGCCTAAAGCGTCCGTAGCTGGCCGCGCAAGTCCGTCGGGAAATCCACCTGCCCAACAGGTGGGCGTCCGGCGGAAACTGTGTGGCTTGGAACCGGAAGGCTCAGAGAGTACGTCCGGGGTAGGAGTGAAATCCCGTAATCCTGGACGGACTACCGATGGCGAAAGCACCTCGAGAGCACGGATCCGACAGTGAGGAACGAAAGCTGGGGTCTCGAACCGG

>f7fcadf84287b3280620e50e430388bd

AACAGAGGATACAAGCGTTATCCGGATTTATTGGGTTTAAAGGGTGCGTAGGTGGTTTTTTAAGTCAGTAGTGAAATCTTAAAGCTTAACTTTAAAAGTGCTATTGGTACTGATAAACTAGAGTGAGGTTGGAGTAACTGGAATGTGTGGTGGAGCGGTGAAATGCATAGAGATCACACAGAACACCAATCGCGAAGGCATGTTACTAAACATAGACTGACACTGAGGCACGAAAGCATGGGTAGCAAACAGG

>ef6add0fe0befe583244a2c55d3d9887

CCAAGCGTAAGCACAACAAAAAGATAGCCAAACTAATACCACCCCGATCACCAGCAATTACGCCTACACACACTTTTTACCCAAGAATAGTTAACCTCACCGACATTACATTCACTAACGATCAGGAACATCTTTTAAACAAAGGGATAAATCACAACCTACACTACACGCAGAACAGTAACACCATAAAGAACATTAT

>be3c22899447565f854a0120983a747a

TACGTAGGTGGCAAGCGTTGTCCGGATTTACTGGGCGTAAAGGATGTGTAGGCGGATATTTAAGTGAGATGTGAAAGCCCCGGGCTCAACCTGGGGACTGCATTTCAAACTGGGTATCTAGAGTGCAGGAGAGGAAAGCGGAATTCCTAGTGTAGCGGTGAAATGCGTAGAGATTAGGAAGAACATCAGTGGCGAAGGCGGCTTTCTGGACTGTAACTGACGCTGAGGCATGAAAGCGTGGGGAGCAAACAGG

>f13ce8c0d067b7c101b7741c9ea31184

TACGGAGGGGGCTAGCGTTGTTCGGAATTACTGGGCGTAAAGCGCGCGTAGGCGGACGGTCAAGTTGGGGGTGAAAGCCCGGGGCTCAACCCCGGAACTGCCTTCAAAACTGATCGTCTGGAGACCGGGAGAGGTGAGTGGAATTCCCAGTGTAGAGGTGAAATTCGTAGATATTGGGAAGGACACCAGTGGCGAAGGCGGCTCACTGGACCGGATCTGACGCTGAGGTGCGAAAGCGTGGGGAGCGAACAGG

>9b53067e23a3f7417c2354a1e00a7b95

TACGAAGGGGGCTAGCGTTGCTCGGAATCACTGGGCGTAAAGGGTGCGTAGGCGGGTCTTTAAGTCAGAGGTGAAATCCTGGAGCTCAACTCCAGAACTGCCTTTGATACTGAAGATCTTGAGTTCGGGAGAGGTGAGTGGAACTGCGAGTGTAGAGGTGAAATTCGTAGATATTCGCAAGAACACCAGTGGCGAAGGCGGCTCACTGGCCCGATACTGACGCTGAGGCACGAAAGCGTGGGGAGCAAACAGG

>47b098c7edc1e61aef6d211df51b8d96

TACGTATGGTGCAAGCGTTATCCGGATTTACTGGGTGTAAAGGGAGCGTAGACGGAGAAGCAAGTCTGGAGTGAAAGCCCGGGGCTCAACCCCGGGACTGCTTTGGAAACTGTTTTTCTTGAGTGCCGGAGAGGTAAGCGGAATTCCTAGTGTAGCGGTGAAATGCGTAGATATTAGGAGGAACACCAGTGGCGAAGGCGGCTTACTGGACGGTAACTGACGTTGAGGCTCGAAAGCGTGGGGAGCAAACAGG

>c4c75ac680ac9ab592169bb075150a96

TACGGAGGGTGCAAGCGTTAATCGGAATTACTGGGCGTAAAGCGCACGCAGGCGGTCTGTCAAGTCGGATGTGAAATCCCCGGGCTCAACCTGGGAACTGCATTCGAAACTGGCAGGCTAGAGTCTTGTAGAGGGGGGTAGAATTCCAGGTGTGGCGGTGAAATGCGTAGAGATCTGGAGGAATACCGGTGGCGAAGGCGGCCCCCTGGACAAAGACTGACGCTCAGGTGCGAAAGCGTGGGGAGCAAACAGG

>6861f0cc9faaac2442e0b52e1eea1b59

TACCGGCAGTCCGAGTGATGGCCGATATTATTGGGCCTAAAGCGTCCGTAGCTTGCTGTGTAAGTCCATTGGGAAATCGACCAGCTCAACTGGTCGGCGTCCGGTGGAAACTACACAGCTTGGGGCCGAGAGACTCAACGGGTACGTCCGGGGTAGGAGTGAAATCCTGTAATCCTGGACGGACCACCAATGGGGAAACCACGTTGAGAGACCGGACCCGACAGTGAGGGACGAAAGCTAGGGTCTCGAACCGG

>ae950fb34d2cd243ccaa605e9fb37705

CGCACTTATGTCTTCAGAAAGACAAAATATTTATCCAGGATGCATTTCAACCATGTCGGTCATTTTCAACTGGCACTGCCTTGTAGATATTTGTGAAAGATTTTGAATGTGTCTTGTCCATAGAGTTCAGTTTGTTCATTAATGATTTTGTTATGTAGTTTGTAACGTAATATATTTAGGCTATCAAGTAAATTCATTTTGTTTCCTTTTTGCCGGTTGTG

>b30c5b09be4d9fea735fd3e899c376cf

TACAGAGGGCTCAAGCGTTAATCGGAATCACTGGGCTTAAAGCGAGCGTAGGCGGATCGGTAGGCACGTTGTGAAAGCCCACGGCTCAACCGTGGAATTGCAGCGTGAACCCCCGATCTTGAGGCGAGTAGAGGTAGCCAGAACGATAGGTGGAGCGGTGAAATGCGTAGAGATCTATCGGAATGTCGAAGGTGAAGACAGGCTACTGGGCTCGAACTGACGCTGAGGCTCGAAAGCGTGGGGATCAAACGGG

>3fe98fde3a891d7d986d62b2beae4628

TACGTATGTCGCGAGCGTTATCCGGAATTATTGGGCATAAAGGGCATCTAGGCGGCCGGGTAAGTCCGGGGTGAAAACTGCTGGCTCAACCAGCAGCCTGCCTTGGAAACTACCCGGCTTGAGTGCTGGAGAGGTGGACGGAACTGCACGAGTAGAGGTGAAATTCGTAGATATGTGCAGGAATGCCGATGATGAAGATAGTTCACTGGACGGCAACTGACGCTGAAGTGCGAAAGCTAGGGGAGCGAACAGG

>89df928867dc6988322a4fde456f6dea

TACGTAGGTGGCAAGCGTTGTCCGGAATTATTGGGCGTAAAGCGCGCGCAGGCCGATAGGTCAGTCTGTCTTAAAAGTTCGGGGCTTAACCCCGTGATGGGATGGAAACTGCCAATCTAGAGTATCGGAGAGGAAAGTGGAATTCCTAGTGTAGCGGTGAAATGCGTAGATATTAGGAAGAACACCAGTGGCGAAGGCGACTTTCTGGACGAAAACTGACGCTGAGGCGCGAAAGCCAGGGGAGCGAACGGG

>03efcbe8b4ed51eceea269d588eb55c7

TACGTAGGTCCCGAGCGTTATCCGGATTTATTGGGCGTAAAGCGAGCGCAGGCGGTTAGATAAGTCTGAAGTTAAAGGCTGTGGCTTAACCATAGTACGCTTTGGAAACTGTTTAACTTGAGTGCAGAAGGGGAGAGTGGAATTCCATGTGTAGCGGTGAAATGCGTAGATATATGGAGGAACACCGGTGGCGAAAGCGGCTCTCTGGTCTGTAACTGACGCTCATGCACGAAAGCGTGGGGAGCAAACAGG

>e77c68a347f34cf3f3ec5f350d91765e

AACGTAGGGGGCGAGCGTTATCCGGAATTACTGGGCGTAAAGCGCGTGTAGGCGGGCTGTTAAGTCGGTTGTGAAAATCCGGGGCTCAACTTCGGACATGCAGCCGATACTGACAGTCTTGAGGGCAGGAGAGGGAAGCGGAATTCCCGGTGTAGCGGTGAAATGCGTAGATATCGGGAGGAACACCAGTGGCGAAGGCGGCTTTCTGGACTGTACCTGACGCTGAGACGCGAAAGCGTGGGGAGCAAACAGG

>0c19a526bb0a576320e6ff9acab87c6d

TACGTAGGTGGCAAGCGTTGTCCGGATTTATTGGGCGTAAAGCGCGCGCAGGCGGTCTTTTAAGTCTGATGTGAAAGCCCCCGGCTTAACCGGGGAGGGTCATTGGAAACTGGAAGACTGGAGTGCAGAAGAGGAGAGTGGAATTCCACGTGTAGCGGTGAAATGCGTAGATATGTGGAGGAACACCAGTGGCGAAGGCGACCCTCTGGTCTGTAACTGACGCTGAGGCGCGAAAGCGTGGGGAGCAAACAGG

>1d5740c2be1a11ab963bc88b66efcb54

TACGTAGGTGGCAAGCGTTATCCGGATTTATTGGGCGTAAAGAGAGTGCAGGCGGTTTTCTAAGTCTGATGTGAAAGCCTTCGGCTTAACCGGAGAAGTGCATCGGAAACTGGATAACTTGAGTGCAGAAGAGGGTAGTGGAACTCCATGTGTAGCGGTGGAATGCGTAGATATATGGAAGAACACCAGTGGCGAAGGCGGCTCTCTGGTCTGTAACTGACGCTGAGGCTCGAAAGCGTGGGGAGCAAACAGG

>40ce08919318a67ffd545a218579c466

TACGTAGGGTGCGAGCGTTAATCGGAATTACTGGGCGTAAAGCGTGCGCAGGCGGTTTGTTAAGACAGATGTGAAATCCCCGGGCTCAACCTGGGAACTGCATTTGTGACTGGCAGGCTAGAGTATGGCAGAGGGGGGTAGAATTCCACGTGTAGCAGTGAAATGCGTAGAGATGTGGAGGAATACCGATGGCAAAGGCAGCCCCCTGGGCCAATACTGACGCTCATGCACGAAAGCGTGGGGAGCAAACAGG

>9c5c2ec8398d8ff5f5b2e0aab15d8cbe

TACAGAGGATGCAAGCGTTATCCGGAATGATTGGGCGTAAAGCGTCTGTAGGTGGCTTTTTAAGTCCGCCGTCAAATCCCAGGGCTCAACCCTGGACAGGCGGTGGAAACTACCAAGCTTGAGTACGGTAGGGGCAGAGGGAATTTCCGGTGGAGCGGTGAAATGCGTAGAGATCGGAAAGAACACCAACGGCGAAAGCACTCTGCTGGGCCGACACTGACACTGAGAGACGAAAGCTAGGGGAGCAAATGGG

>713d3c6eb212365260dbcf4f1ecdf380

TACGTAGGGGGCGAGCGTTGTCCGGAATTATTGGGCGTAAAGCGCGCGCAGGCGGTCCCTTAAGTCTGATGTGAAAGCCCACGGCTCAACCGTGGAGGGTCATTGGAAACTGGGGGACTTGAGTGCAGGAGAGGAGAGCGGAATTCCACGTGTAGCGGTGAAATGCGTAGAGATGTGGAGGAACACCAGTGGCGAAGGCGGCTCTCTGGCCTGCAACTGACGCTGAGGCGCGAAAGCGTGGGGAGCAAACAGG

>ca228736334356da84bc09143c3fff05

TACGTAGGTGGCAAGCGTTGTCCGGATTTACTGGGCGTAAAGGATGCGTAGGCGGATGTTTAAGTGAGATGTGAAATACCCGGGCTCAACTTGGGTGCTGCATTTCAAACTGGACATCTAGAGTGCGGGAGAGGAAAGCGGAATTCCTAGTGTAGCGGTGAAATGCGTAGAGATTAGGAAGAACACCAGTGGCGAAGGCGGCTTTCTGGACCGTAACTGACGCTGAGGCATGAAAGCGTGGGGAGCAAACAGG

>1d08082f76b8ae2f259592a9dbfecf21

CACCGGCAGCCCAAGTGATGGCCGATATTATTGGGCCTAAAGCGTCCGTAGCTGGCCGCACAAGTCCGTCGGGAAATCCACTCGCTTAACGGGTGGGCGTCCGGCGGAAACTGTGCGGCTTGGGACAGGAAGGCTCGAGGGGTACGTTCGGGGTAGGAGTGAAATCCCGTAATCCTGAACGGACCGCCGATGGCGAAAGCACCTCGAGAGGACTGATCCGACAGTGAGGGACGAAAGCTGGGGTCTCGAACCGG

>28ae12b08e0693443053339cb3189ccb

TACCGGCAGCTCAAGTGATGTCCCATATTATTGGGCCTAAAGCGTCCGTAGCTGGCCGACCAAGTCTATCGGGAAATCCACCTGCCCAACAGGTGGGCGTCCGGTAGAAACTGGCCGGCTTGGAACCGGAAGGCTCAGAGAGTACGTCCGGGGTAGGAGTGAAATCCCGTAATCCCGGACGGACTACCGATGGCGAAAGCACTCTGAGAAGACGGCTTCGACAGTGAGGGACGAAAGCTCGGGTCTCAAACCGG

>f2b5b2610927ac305c849af058198fa0

TACGGAGGGGGCTAGCGTTGTTCGGAATTACTGGGCGTAAAGCGCACGTAGGCGGGTATCCAAGTTGGGGGTGAAATCCCGGGGCTCAACCCCGGAACTGCCTCCAAAACTGGGTGCCTAGAGTCCGAGAGAGGTGAGTGGAATTCCCAGTGTAGAGGTGAAATTCGTAGATATTGGGAAGAACACCAGTGGCGAAGGCGGCTCACTGGCTCGGTACTGACGCTGAGGTGCGACAGCGTGGGGAGCAAACAGG

>3dd014905df96254258e05d21a80a3f4

TCAAGTAGGCTTTGAATAACGCGCATCAATTTGCCGCAATAACGCTCGTTGTCATCGGCCAGTTATCGGGACGCGACAATAACAGCAGGACATTTCACGGAGAGTTAGACCTGCTGATTGTTGGACTCAGCGCCCCGGGACTGTAATCCGTTGTGTTCTCTGTTCACGGGCGCGCTTTCACACATCGCGTAGCGCTACATTATCACAGTCAGTCCGACGTCTTAGATGGT

>93d9848a4e09b94ab72cbc84a01cafc2

TACCGGCAGCTCGAGTGATGTCCAATATTATTGGGCCTAAAGCGTCCGTAGCTGGCCGCGCAAGTCCATCGGGAAATCCACCTGCCCAACAGGTGGGCGTCCGGCGGAAACTGTGTGGCTTGGAACCGGAAGGCTCAGAGGGTACGTCCGGGGTAGGAGTGAAATCCCGTAATCCTGGACGGACCGCCGGTGGCGAAAGCGCCTCAGGAGGACGGACTCGACAGTGAGGGACGAAAGCCAGGGTCTCGAACCGG

>e0449413df6c170c81c535eb162ddd09

GACGTAGGATGCAAGCGTTGTCCGGATTTATTGGGCGTAAAGAGTTCGTAGGCGGTTTGTTAAGTCTGATGTTAAAGATCGGGGCCCAACCCTGGGAGTGCATTGGATACTGGCAGACTGGAGTGCGGTAGAGGCTAGTGGAATTCCCAGTGTAGCGGTGAAATGCGTAGATATTGGGAAGAACACCAGTGGCGTAGGCGACTAGCTGGGCCGTAACTGACGCTGAGGAACGAAAGCCAGGGGAGCGAATGGG

>ce68aedcc3f10ce053f74298a1639e51

CACGATTAACCCAAGTCAATAGAAGCCGGCGTAAAGAGTGTTTTAGATCACCCCCTCCCCAATAAAGCTAAAACTCACCTGAGTTGTAAAAAACTCCAGTTGACACAAAATAGACTACGAAAGTGGCTTTAACATATCCGAACACACAATAGCTAAGACCCAAACTGGG

>bd48aa56dc6b971b45ec7ac8e8a559ea

TACGTGAGAGACTAGTGTTATTCATCTTAATTGGGTTTAAAGGGTACCTAGACAGTCAATATAACTTCTATAACGTTAATACTTGACTAGAGTTTTAAGTAAGAGGGAAGTACTTAAGGAGTAAGAGATGAAATATCTGTGATACCAAAGGGACTCCGTAAAGGCGAAGGCATCCCTTTATCTAAAAACTAACGTTGAAGGACGAAGGCTTAGATAACAAATAGG

>7b60a64f22de86011c32950d81a57bb4

TACGTAGGGTGCGAGCGTTAATCGGAATTACTGGGCGTAAAGCGTGCGCAGGCGGTTTGTTAAGACAGATGTGAAATCCCCGGGCTGAACCTGGGAACTGCATTTGTGACTGGCAGGCTAGAGTATGGCAGAGGGGGGTAGAATTCCACGTGTAGCAGTGAAATGCGTAGAGATGTGGAGGAATACCGATGGCGAAGGCAGCCCCCTGGGCCAATACTGACGCTCATGCACGAAAGCGTGGGGAGCAAACAGG

>18ec4bf39c039aa44177877452b0b6c0

TACGTAGGGTGCGAGCGTTAATCGGAATTACTGGGCGTAAAGCGTGCGCAGGCGGTTTGTTAAGACAGATGTGAAATCCCCGGGCTCAACCTGGGAACTGCATTTGTGACTGGCAGGCTAGAGTATGGCAGAGGGGGGTAGAATTCCACGTGTAGCAGTGAAATGCATAGAGATGTGGAGGAATACCGATGGCGAAGGCAGCCCCCTGGGCCAATACTGACGCTCATGCACGAAAGCGTGGGGAGCAAACAGG

>55d9508e499d07e966fe1717bd80c566

TACGAAGGGGGCTAGCGTTGCTCGGAATTACTGGGCGTAAAGGGCGCGTAGGCGGACAGTTAAGTCGGGGGTGAAAGCCCGGGGCTCAACCTCGGAATTGCCTTCGATACTGGCTGTCTTGAGTACGGGAGAGGTGTGTGGAACTCCGAGTGTAGAGGTGAAATTCGTAGATATTCGGAAGAACACCAGTGGCGAAGGCGACACACTGGCCCGTTACTGACGCTGAGGCGCGAAAGCGTGGGGAGCAAACAGG

>d8d7cfff55d5789c478e164722a3c8d8

TACGTAGGTGGCAAGCGTTATCCGGAATTATTGGGCGTAAAGCGCGCGTAGGCGGTTTTTTAAGTCTGATGTGAAAGCCCACGGCTCAACCGTGGAGGGTCATTGGAAACTGGAAAACTTGAGTGCAGAAGAGGAAAGTGGAATTCCATGTGTAGCGGTGAAATGCGCAGAGATATGTAGGAACACCAGTGGCGAAGGCGACTTTCTGGTCTGTAACTGACGCTGATGTGCGAAAGCGTGGGGATCAAACAGG

>bd6ac76ba66d8fd69d058864460107d7

TACGTAGGGTGCGAGCGTTAATCGGAATTACTGGGCGTAAAGCGTGCGCAGGCGGTTTGTTAAGACAGATGTGAAATCCCCGGGCTCAACCTGGGAACTGCATTTGTGACTGGCAGGCTAGAGTATGGCAGAGGGGGGTAGAATTCCACGTGTAGCAGTGAAATGCGTAGAGATGTGGAGGAATAGCGATGGCGAAGGCAGCCCCCTGGGCCAATACTGACGCTCATGCACGAAAGCGTGGGGAGCAAACAGG

>3ba3b2be653f1c13e8c23dc7fdaf3bb6

CACGTAAGGGGCAAGCGTTGTTCGGAATTATTGGGCGTAAAGGGTGCGCAGGCGGAAACATAAGCCTGATGTAAAAGGCTCCAGCTTAACTGGAAGAATGCATTGGGAACTGTGTAACTAGAGTATAAGAGGGGGAGTTGGAATTCCAGGTGTAGGGGTGAAATCTGTAGATATCTGGAAGAACACCGGTGGCGAAGGCGAACTCCTGGCTATATACTGACGCTGAGGCACGAAAGCGTGGGGAGCGAACAGG

>46b01ca688373bf6f87ab8b97bdc193d

TACGGAGGGTGCAAGCGTTAATCGGAATTACTGGGCGTAAAGCGCACGCAGGCGGTCTGTCGAGTCGGATGTGAAATCCCCGGGCTCAACCTGGGAACTGCATTCGAAACTGGCAGGCTAGAGTCTTGTAGAGGGGGGTAGAATTCCAGGTGTAGCGGTGAAATGCGTAGAGATCTGGAGGAATACCGGTGGCGAAGGCGGCCCCCTGGACAAAGACTGACGCTCAGGTGCGAAAGCGTAGGGAGCAAACAGG

>419fd81c2cdac1a5d38831e40efdc3b0

TACGGAGGGTGCGAGCGTTAATCGGAATTACTGGGCGTAAAGGGCGCGTAGGCGGTCTGTTAAGCCAGATGTGAAAGCCCCGGGCTTAACCTGGGAACAGCATTTGGAACTGGCAGACTTGAGTGCAGGAGAGGAAGGTAGAATTCCAGGTGTAGCGGTGAAATGCGTAGAGATCTGGAGGAATACCAGTGGCGAAGGCGGCCTTCTGGACTGACACTGACGCTGAGGCGCGAAAGCGTGGGTAGCAAACAGG

>a08a9907898b3516944e8e06530dbb54

CACGATTAACCCAAGTCAATAGAAGCCGGCGTAAAGAGTGTTTTAGATCACCCCCTCCCCAATAAAGCTAAAACTCACCTGAGTTGTAAAAACCTCCAGTTGACACAAAATAGACTACGAAAGTGGCTTTAACATATCTGAACACACAATAGCTAAGACCCAAACTGGG

>9ca2a08407f6ec7d3decf5949c9e277f

TACGTAGGATGCAAGCGTTAATCGGAATTACTGGGCGTAAAGCGTGCGTAGGCGGTCCTGTAAGACCGATGTGAAATCCCCGGGCTTAACCTGGGAACTGCATTGGTGACTGCAGGGCTTGAGTGTGTCAGAGGGAGGTGGAATTCCGCGTGTAGCAGTGAAATGCGTAGAGATGCGGAGGAACACCGATGGCGAAGGCAGCCTCCTGGGATAACACTGACGCTCAGGCACGAAAGCGTGGGGAGCAAACAGG

>9802230c7a12e24d962db1545438a923

CACACTTATGTCTTCAGAAGTACAAACCATTTCTCCAGGATGCATTTCAACCATGTCGGTCATTTTCAACTGGTAGTGCCTTGTAGATATTTGTGAAAGATTTTGAATGTGTCTTGTCCATAGAGTTCAGTTTGTAAATTAATGATTTTGTTATGTAGTTTGTAACGTAATATACAGTATTTAGGCTTTTAAGTAAATTAATTTTATTTCCTTTTTTTCGGTTGTG

>467536ab6fa753b7668c3763f56b8165

CACGATTAACCCAAGTCAATAGAAGCCGGCATAAAGAGTGCTTTAGATCACCCCCTCCCCAATAAAGCTAAAACTCACCTGAGTTGTAAAAAACTCCAGTTGACACAAAATAGACTACGAAAGTGGCTTTAACATATCTGAACACACAATAGCTAAGACCCAAACTGGG

>cb5212f47a63c14d5d69aaa3375ae689

TACGTAGGTGGCAAGCGTTATCCGGATTTATTGGGCGTAAAGCGAGCGCAGGCGGTCTTTTAAGTCTGATGTGAAAGCCCCCGGCTTAACCGGGGAGGGTCATTGGAAACTGGGAGACTTGAGTGCAGAAGAGGAAAGCGGAATTCCATGTGTAGCGGTGAAATGCGTAGATATATGGAGGAACACCAGTGGCGAAGGCGGCTTTCTGGTCTGTAACTGACGCTGAGGCTCGAAAGCGTGGGGAGCAAACAGG

>a23715ec32b49109cb36a91dbdcf6a10

CACGATTAACCCAAGTCAATAGAAGCCGGCGTAAAGAGTGTTTTAGATCACCCCCTCCCCAATAAAGCTAAAACTCACCTGAGTTGTAAAAAACTCCAGTTGACACAAAATAGACTACGAAAGTGGCTTTAACATATCTGAACACACAATAGTTAAGACCCAAACTGGG

>65641452be75528754b5ae258ba661c1

TACGTAGGTGGCAAGCGTTGTCCGGATTTATTGGGCGTAAAGGGAGCGCAGGTGGTTTCTTAAGTCTGATGTGAAAGCCCACGGCTTAACCGTGGAGGGTCATTGGAAACTGGGAAACTTGAGTACAGAAGAGGAATGTGGAACTCCATGTGTAGCGGTGGAATGCGTAGATATATGGAAGAACACCAGTGGCGAAGGCGACATTCTGGTCTGTTACTGACACTGAGGCTCGAAAGCGTGGGGAGCAAACAGG

>48c6b10991383c82df6f797dc51e2bf4

TACGTAGGGGGCTAGCGTTATCCGGATTTACTGGGCGTAAAGGGTGCGTAGGCGGTCTTTCAAGTCAGGAGTGAAAGGCTACGGCTCAACCGTAGTAAGCTCTTGAAACTGGGAGACTTGAGTGCAGGAGAGGAGAGTGGAATTCCTAGTGTAGCGGTGAAATGCGTAGAGATTAGGAAGAACACCAGTGGCGGAGGCGACTCTCTGGACTGTAACTGACGCTGAGGCTCGAAAACGTGGGGAGCAAACAGG

>2c8b634438b3c756d4a3a56a313cec1b

TACGGAGGGGGCTAGCGTTGTTCGGAATTACTGGGCGTAAAGCGCGCGTAGGCGGACGGTCAAGTTGGGGGTGAAAGCCCGGGGCTCAACCCCGGAACTGCCTTCAAAACTGATCGTCTGGAGACCGGGAGAGGTGAGTGGAATTCCCAGTGTAGAGATGAAATTCGTAGATATTGGGAAGAACACCAGTGGCGAAGGCGGCTCACTGGACCGGATCTGACGCTGAGGTGCGAAAGCGTGGGGAGCGAACAGG

>13c32fe4d0072f2a2c508f759f1678d1

GACGGGGGGGCAAGTGTTCTTCGGAATGACTAGGCGTAAAGGGCACGTAGGTGGTGAATCGGGTTGAAAGTGAAAGTCGCCAAAAAACTGGCGGAATGCTCTCGAAACCAATTCACTTGAGTGAGACAGAGGAGAGTGGGATTTCATGTGTAGGGGTGAAATCCGGAGATCTACGAAGGAACTCTAGGTCCCTACAGACGCTGGGGTGCGAAAGCGTAGGGAGCGAACGGG

>842295f7abb7f824bbda9817d47f340c

TACAGAGGGTGCAAGCGTTAATCGGATTTACTGGGCGTAAAGCGCGCGTAGGCGGCTAATTAAGTCAAATGTGAAATCCCCGAGCTTAACTTGGGAATTGCATTCGATACTGGTTAGCTAGAGTGTGGGAGAGGATGGTAGAATTCCAGGTGTAGCGGTGAAATGCGTAGAGATCTGGAGGAATACCGATGGCGAAGGCAGCCATCTGGCCTAACACTGACGCTGAGGCGCGAAAGCATGGGGAGCAAACAGG

>c4e31869b36209375b754236c1074e37

GACGGGGGATGCAAGTGTTATCCGGAATAATTGGGCGTAAAGCGTCTGTAGGTGGTTTACCAAGTCTGTTGTTAAAACTCAAGGCTTAACCTTGATCAGGCAACAGAAACTAGTAGGCTTGAGTACGGTAGGGGCAGAGGGAATTCTCGGTGTAGTGGTGAAATGCGTAGATATCGAGAAGAACACCAATAGCGAAAGCACTCTGCTGGGCCGAAACTGACATTGAGAGACGAAAGCTAGGGGAGCGAAAGGG

>829e78d9d9c082584f20767aee41ed9b

TACGTAGGGTGCGAGCGTTAATCGGAATTACTGGGCGTAAAGCGTGCGCAGGCGGTGATGTAAGACCGATGTGAAACCCCCGGGCTTAACCTGGGAACTGCATTGGTGACTGCATCGCTGGAGTATGGCAGAGGGGGGTGGAATTCCACGTGTAGCAGTGAAATGCGTAGAGATGTGGAGGAACACCGATGGCGAAGGCAGCCCCCTGGGCCAATACTGACGCTCATGCACGAAAGCGTGAGGAGCAAACAGG

>b3adb5d66e7ddfe402f761dd1509ea37

CGTGGATGATACAGGAGCTCATTGGGCTGAGACCCAGACAGCGCACACACGATTCGGGTGCTAGACACCCCGCACAGCATCAGTCAGTCAGTAATATTGGTTATCGTAGGGCTATTGAGCTACTAAATAACTGCGACAAAACTATTTACCCATCTATTCACTTTCTACTAAAGATTGCTGTCACCTTGTCATATTCTGTTCCAACTGCTGATAGAACATTATCATTGCTCAGAAGACTTACAACTTAG

>63552fafe148f6b32524121ee98b37c0

CACGATTAACCCAAGTCAATAGAAGCCGGCGTAAAGAGTGTTTTAGATCACCCCCTCCCCAATAAAGCTAAAACTCACCTGAGTTGTAAAGAACTCCAGTTGACACAAAATAGACTACGAAAGTGGCTTTAACATATCTGAACACACAATAGCTAAGACCCAAACTGGG

>8ebe4fcb35d74ca7d717b60ebac2799d

TACCGGCAGCCCGAGTGATGGCCGATCTTATTGGGCCTAAAGCGTCCGTTGCTGGCCGCACAAGTCCATCGGGAAATCCACCTGCTCAACAGGTGGGCGTCCGGTGGAAACTGTGTGGCTTGGGACCGGAAGGCGCGACGGGTACGTCTGGGGCAGGAGTGAAATCCCGTAATCCCGGACGGACTACCGATGGCGAAAGCACTCTGAGAAGACGGCTTCGACAGTGAGGGACGAAAGCTCGGGTCTCAAACCGG

>896cb5e30a4bb55376012d230a115d41

TACCGGCAGTCCAAGTGATGGCCGATATTATTGGGCCTAAAGCGTCCGTAGCCTGCTGTGTAAGTCCATTGGGAAATCGACCAGCTCAACTGGTCGGCGTCCGGTGGAAACTACACAGCTTGGGGCCGAGAGACTCAACGGGTACGTCCGGGGTAGGAGTGAAATCCTGTAATCCTGGACGGACCACCAATGGGGAAACCACGTTGAGAGACCGGACCCGACAGTGAGGGACGAAAGCCAGGGTCTCGAACCGG

>87ddd7ea5d5bdd511f895aa6b6ee3c45

CACACTTATGTCTTCAGAAAGACAAAATATTTATCCAGCATGCATTTCAACCATGTCGGTCATTTTCAACTGGCAGTGCCTTGTAGATATTTGTGAAAGATTTTGAATGTGTCTTGTCCATAGAGTTCAGTTTGTTCATTAATGATTTTGTTATGCAGTTTGTAACGTAATATATTTAGGCTTTCAAGTAAATTCATTTTGTTTCCTTTTTGCCGGTTGTG

>5ee8b50788ab51000bda4a507756d4d5

TACCGGCAGTCCGAGTGATGGCCGATATTATTGGGCCTAAAGCGTCCGTAGCTTGCTGTGTAAGTCCATTGGGAAATCGACGCGCTCAACGCGTCGGCGTCCAGCGGAAACTACACGGCTTGGGGCCGAGAGACTTGACGGGTACGTCCGGGGTAGGAGTGAAATCCTGTAATCCTGGACGGACCACCAATGGGGAAACCACGTCGAGAGACCGGACCCGACAGTGAGGGACGAAAGCCAGGGTCTCGAACCGG

>af74bfa9bd8e384d61e79209d847baba

CACGATTAACCCAGGTCAATAGAAGCCGGCGTAAAGAGTGTTTTAGATCACCCCCTCCCCAATAAAGCTAAAACTCACCTGAGTTGTAAAAAACTCCAGTTGACACAAAATAGACTACGAAAGTGGCTTTAACATATCTGAACACACAATAGCTAAGACCCAAACTGGG

>3f803c6a182a7484832b04d0550ce269

TACGTAGGGTGCAAGCGTTGTCCGGAATTATTGGGCGTAAAGAGCTCGTAGGCGGTGTGTCGCGTCTGCTGTGAAAACTCAGGGCTCAACTCTGAGCTTGCAGTGGGTACGGGCACACTTGAGTGCGGTAGGGGAGACTGGAATTCCTGGTGTAGCGGTGAAATGCGCAGATATCAGGAGGAACACCGGTGGCGAAGGCGGGTCTCTGGGCCGTAACTGACGCTGAGGAGCGAAAGCATGGGGAGCGAACAGG

>b945269c5c7aff55597c3ed78ab7c986

GACGGGGGGGGCAAGTGTTCTTCGGAATGACTGGGCGTAAAGGGCACGTAGGCGGTGAATCGGGTTGAAAGTGAAAGTCGCCAAAAACTGGCGGAATGCTCTCGAAACCAATTCACTTGAGTGAGACAGAGGAGAGTGGAATTTCGTGTGTAGGGGTGAAATCTCAAGATCTACGAAGGAACGCCAAAAGCGAAGGCAGCTCTCTGGGTCCCTACCGACGCTGGGGTGCGAAAGCATGGGGAGCGAACGGG

>6a28c660ffdc398d4f7c539581ccbfcb

TACGAAGGGGGCTAGCGTTGCTCGGAATCACTGGGCGTAAAGGGCGCGTAGGCGGCGTTTTAAGTTGGGGGTGAAAGCCTGTGGCTCAACCACAGAATTGCCTTCGATACTGGGACGCTTGAGTGTAGTAGAGGTTGGTGGAACTGCGAGTGTAGAGGTGAAATTCGTAGATATTCGCAAGAACACCGGTGGCGAAGGCGGCCAACTGGACCATCACTGACGCTGAGGCGCGAAAGCGTGGGGAGCAAACAGG

>3770c35ec27e9c36ad01b719884eddd6

TACGTAGGGTGCGAGCGTTAATCGGAATTACTGGGCGTAAAGCGTGCGCAGGCGGTTTGTTAAGACAGATGTGAAATACCCGGGCTCAACCTGGGAACTGCATTTGTGACTGGCAGGCTAGAGTATGGCAGAGGGGGGTAGAATTCCACGTGTAGCAGTGAAATGCGTAGAGATGTGGAGGAATACCGATGGCGAAGGCAGCCCCCTGGGCCAATACTGACGCTCATGCACGAAAGCGTGGGGAGCAAACAGG

>40a57ccb173a1855384febea16dd8565

CACGACTAACCCAAGTCAATAGAAGCCGGCGTAAAGAGTGTTTTAGATCACCCCCTCCCCAATAAAGCTAAAACTCACCTGAGTTGTAAAAAACTCCAGTTGACACAAAATAGACTACGGAAGTGGCTTTAACATATCTGAACACACAATAGCTAAGACCCAAACTGGG

>4d312fba58214eaac2d3d5cd342fec2b

TACGGAGGGGGCTAGCGTTGTTCGGAATTACTGGGCGTAAAGCGCGCGTAGGCGGACGGTCAAGTTGGGGGTGAAAGCCCGGGGCTCAACCCCGGAACTGCCTTCAAAACTGATCGTCTGGAGACCGGGAGAGGTGAGTGGAATTCCCAGTGTAGAGGTGAAATTCGTAGATATTGGGAAGAACGCCAGTGGCGAAGGCAGCTCACTGGACCGGATCTGACGCTGAGGTGCGAAAGCGTGGGGAGCGAACAGG

>5836b62e3dea218ed2d7d28ecc599049

TACGTAGGGTGCGAGCGTTAATCGGAATTACTGGGCGTAAAGCGTGCGCAGGCGGTTTGTTAAGACAGATGTGAAATCCCCGGGCTCAACCTGGGAACTGCATTTGTGACTGGCAGGCTAGAGTATGGCAGAGGGGGGTAGAATTCCACGTGTAGCAGTGAAATGCGTAGAGATGTGGAGGAATACCGATGGCTAAGGCAGCCCCCTGGGCCAATACTGACGCTCATGCACGAAAGCGTGGGGAGCAAACAGG

>ac68c7c702432c26bc13677cb4c1ea8f

TACGTAGGTGGCAAGCGTTGTCCGGATTTATTGGGTTTAAAGGGTGCGTAGGCGGCCCTGTAAGTCAGTGCTGAAATATCCCAGCTTAACTGGGAGGGTGGCATTGATACTGCGGGGCTAGAGTACAGATGAGGTAGGCGGAATTGACGGTGTAGCGGTGAAATGCTTAGATATCGTCAAGAACACCTATAGCGAAGGCAGCTTACTAGGCTGTAACTGACGCTGAGGCACGAAAGTGTGGGGATCAAACAGG

>bb4c369edc0bdf7dc090f236a120b510

TACGTAGGGTGCGAGCGTTAATCGGAATTACTGGGCGTAAAGCGTGCGCAGGCGGTTTGTTAAGACAGATGTGAAATCCCCGGGATCAACCTGGGAACTGCATTTGTGACTGGCAGGCTAGAGTATGGCAGAGGGGGGTAGAATTCCACGTGTAGCAGTGAAATGCGTAGAGATGTGGAGGAATACCGATGGCGAAGGCAGCCCCCTGGGCCAATACTGACGCTCATGCACGAAAGCGTGGGGAGCAAACAGG

>c03f41fb299b6e9e89e42f80c3f0de38

TACGTAGGGTGCGAGCGTTAATCGGAATTACTGGGCGTAAAGCGTGCGCAGGCGGTTTGTTAAGACAGATGTGAAATCCCCGGGCTCAACCTGGGAACTGCATTTGTGACTGGCAGGCTAGAGTATGGCAGAGGGGGGTAGAATTCCACGTGTAGCAGTGAAATGCGTAGAGATGTGGAGGAATACCGATGGCGAAGGCAGCCCCCTGGGCCAATACTTACGCTCATGCACGAAAGCGTGGGGAGCAAACAGG

>e51ef7768516798733afd6733ca61f10

TACGTAGGGTGCGAGCGTTAATCGGAATTACTGGGCGTAAAGCGTGCGCAGGCGGTTTGTTAAGACAGATGTGAAATCCCCGAGCTCAACCTGGGAACTGCATTTGTGACTGGCAGGCTAGAGTATGGCAGAGGGGGGTAGAATTCCACGTGTAGCAGTGAAATGCGTAGAGATGTGGAGGAATACCGATGGCGAAGGCAGCCCCCTGGGCCAATACTGACGCTCATGCACGAAAGCGTGGGGAGCAAACAGG

>1131b4f90b77582528be93616756a214

TACGTAGGTGGCGAGCGTTATCCGGATTTACTGGGCGTAAAGGGAGCGTAGGCGGATGATTAAGTGGGATGTGAAGTACCCGGGCTCAACTTGGGTGCTGCATTCCAAACTGGTTATCTAGAGTGCAGGAGAGGAGAGTGGAATTCCTAGTGTAGCGGTGAAATGCGTAGAGATTAGGAAGAACACCAGTGGCGAAGGCGACTCTCTGGACTGTAACTGACGCTGAGGCTCGAAAGCGTGGGGAGCAAACAGG

>8af229135ed0a3ad983dc171338050ea

TACGTAGGTGGCAAGCGTTGTCCGGAACTATTGGGCGTAAAGGGCTCGCAGGCGGTTCCTTAAGTCTGATGTGAAAGCCCCCGGCTCAACCGGGGAGGGTCATTGGAAACTGGGGAACTTGAGTGCAGAAGAGGAGAGTGGAATTCCACGTGTAGCGGTGAAATGCGTAGAGATGTGGAGGAACACCAGTGGCGAAGGCGGCTACCTGGTCTGCAACTGACGCTGAGACTCGAAAGCATGGGTAGCGAACAGG

>2525972adb7a36b6f6d26d6426e8ba4d

TACGTAGGGTGCGAGCGTTAATCGGAATTACTGGGCGTAAAGCGTGCGCAGGCGGTTTGTTAAGACAGATGTGAAATCCCCGGGCTCAACCTGGGAACTGCATTTGTGACTGGCAGGCTAGAGTATGGCAGAGGGGGGTAGAATTCCACGTGTAGCAGTGAAATGCGTAGAGATATGGAGGAATACCGATGGCGAAGGCAGCCCCCTGGGCCAATACTGACGCTCATGCACGAAAGCGTGGGGAGCAAACAGG

>9c0462a7ac161bf95840b7a06a139621

TACGTAGGGTGCGAGCGTTAATCGGAATTACTGGGCGTAAAGCGTGCGCAGGCGGTTTGTTAAGACAGATGTGAAATCCCCGGGCTCAACCTGGGAACTGCATTTGTGACTGGCAGGCTAGAGTATGGCAGAGGGGGGTAGAATTCCACGTGTAGCAGTGAAATGCGTAGAGATGTGGAGGAATACCGATGGCGAAGGCAGCCACCTGGGCCAATACTGACGCTCATGCACGAAAGCGTGGGGAGCAAACAGG

>0d968a78a341bb4c75b647b59c7b55f5

ACATTCTTTGTACTTCATTAGTTATTTAATTTACACCACTTGTCACCCCCTTATATTGTACAAATGAGTTTGCTGGCACATTATGTGAAATACACGTTCCTGTTACAGCTTATCCCTGACCTGCTAAAACATGAACAGCAATATTTCTATGACCACAAAAACTATGGACCAAGAAGCTA

>c72c89c5ffa1f52b22749382b4163136

TACGTAGGGGGCGAGCGTTGCTCGGAATTACTGGGCGTAAAGGGTGTGCAGGCGGCCGATTAAGTCAACGATGAAATCCCGAAGCTCAACTTCGGAATGGTCTTTGATACTGATCGGCTTGAGGCTGGTTGAGGAGAGCGGAATTCCCGGTGGAGCGGTGAAATGCGTAGATATCGGGAGGAACACCAGAGGCGAAGGCGGTTCTCTAAACTTGTCCTGACGCTGAGACACGAAAGCTAGGGGAGCAAACTGGG

>feb6973df5dafb6a8e5cfcf18c2b249e

TACGGAGGGGGCTAGCGTTGTTCGGAATTACTGGGCGTAAAGCGCGCGTAGGCGGACGGTCAAGTTGGGGGTGAAAGCCCGGGGCTCAACCCCGGAACTGCCTTCAAAACTGATCGTCTGGAGACCGGGAGAGGTGAGTGGAATTCCCAGTGTAGAGGTGAAATTCGTAGATATTGGGAAGAACACCAGTGGCGGAGGCGGCTCACTGGACCGGATCTGACGCTGAGGTGCGAAAGCGTGGGGAGCGAACAGG

>62b8bcd9584e4c51af7416e8d84ddb2c

TACGTGAGAGACTAGTGTTATTCATCTTAATTGGGTCTAAAGGGTACCTAGACAGTCAATATAACTTCTAGAATGCTAATACTTGACTAGAGTTTTAAGTAAGAGGGAAGTACTTAAGGAGTAAGAGATGAAATATCTGTGATACCAAAGGGACTCCGTAAAGGCGAAGGCATCCCTTTATCTAAAAACTAACGTTGAAGGACGAAGGCTTAGATAACAAATAGG

>54ef7398d56cff3f50bd8f891af574f4

TACGTAGGGCGCGAGCGTTATCCGGAATTATTGGGCGTAAAGAGCTTGTAGGCGGTTTGCCGCGTCTGCTGTGAAAGCCCGGGGCTTAACTCCGGGTGTGCAGTGGGTACGGGCAGGCTAGAGTGCAGTAGGGGAGACTGGAATTCCTGGTGTAGCGGTGAAATGCGCAGATATCAGGAGGAACACCGATGGCGAAGGCAGGTCTCTGGGCTGTTACTGACGCTGAGAAGCGAAAGCATGGGTAGCGAACAGG

>c01378622ab9c1262d2b606ab5044bbd

CACACTTATGCCTTCAGAAAAACAAAATATTTATCCAGGATGCGTTTCAACCATGTCGGTCATTTTCAACTGGCAGTGCCTTGTAGATATTTGTGAAAGATTTTGAATGTGTCTTGTCCATAGAGTTCAGTTTGTTCATTAATGATTTTGTTATGTAGTTTGTAACGTAATATATTTAGGCTTTCAAGTAAATTCATTTTGTTTCCTTTTTGCCGGTTGTG

>f82ddb7154a89e23f7a80874f961bc88

TACGAAGGGGGCTAGCGTTGCTCGGAATCACTGGGCGTAAAGGGTGCGTAGGTGGATCTTTAAGTCAGGGGTGAAATCCTGGAGCTCAACTCCAGAACTGCCTTTGATACTGAGGATCTTGAGTTCGGGAGAGGTAAGTGGAACTGCGAGTGTAGAGGTGAAATTCGTAGATATTCGCAAGAACACCAGTGGCGAAGGCGGCTTACTGGCCCGATACTGACACTGAGGCACGAAAGCGTGGGGAGCAAACAGG

>34a14f58174fc2df4c56573770afd77d

TACGGAGGGTGCAAGCGTTATCCGGATTCACTGGGTTTAAAGGGTGCGTAGGCGGGTTAGTAAGTCAGTGGTGAAATCCCCGAGCTTAACTTGGGAACTGCCGTTGATACTATTAGTCTTGAATATCGTGGAGGTAAGCGGAATATGTCATGTAGCGGTGAAATGCTTAGATATGACATAGAACACCAATTGCGAAGGCAGTCTACTAAGCCGTGATTGACGCTGAGGCACGAAAGCGTGGGGAGCGAACAGG

>70863ce7cf9256c48acf086fa72d9567

TACCGGCAGCTCAAGTGATGGCCACTCTTATTGGGCCTAAAGCGTCCGTAGCTGGCCGCGCAAGTCCGTCGGGAAATCCACTGGCTCAACCAGTGGGCGTCCGGCGGAAACTGTGCGGCTTGGGACCGGAAGGCTCGAGAGGTACGTCCGGGGTAGGAGTGAAATCCCGTAATCCCGAACGGACCACCGATGGCGAAAGCATCTCGAGAAAACGGATCCGACAGTGAGGGACGAAAGCTAGGGTCTCGAACCGG

>1c069a9d0b88eca6af758e45a5bddd7f

CACACTTATGTCTTTAGAAAGACAAAACGTTTTTCCAGAATGCATTTCACCCTTCTCGGTCATTTTCAACTGACAGTGCCTTGTAGATAGTTGTGGAAGATTTTGAGTGTTTCTTGTCCATAGAGATCAGTTTGTTCATTAATGATTTTGTTGTGTAATATGTAACGCAATATATTTAAACTTTCAAGAACATTAATTTTATTTCCTTTTTTGCCAGTTGTA

>869c89488b5cce0c993034c95ced9200

TACGTAGGTGGCAAGCGTTGTCCGGAATTATTGGGCGTAAAGCGCGCGCAGGCGGCTTCTTAAGTCTGATGTGAAAGCCCCCAGCTCAACTGGGGAGGGTCATTGGAAACTGGGAAGCTTGAGTACAGAAGAGGAGAGTAGAATTCCACGTGTAGCGGTGAAATGCGTAGAGATGTGGAGGAATACCAGTGGCGAAGGCGGCTCTCTGGTCTGTAACTGACGCTGAGGCGCGAAAGCGTGGGGAGCAAACAGG

>85d20f47c9e005fc5fc77b034540ede3

CACACTTATGTCTTTAGAAAGACAAAACATTTTTCCAGAATGCATTTCAACCTTCTCGGTCATTTTCAACTGGCAGTGCCTTGTAGATATTTGTGGAAAATTTTGAATGTTTCTTGTCCATAGAGTTCAGTTTGTTCATTGATGGTTTTGTTGTGTAGTTTGTAACGTAATATATTTAAACTTTCAAGTAGATTCATTTTATTTCCTTTTTTGCCGATTGTG

>fa7dc0ba31a62ddd4d8eb47ddaf71289

TACGTAGGGTGCGAGCGTTAATCGGAATTACTGGGCGTAAAGCGTGCGCAGGCGGTTTGTTAAGACAGATGTGAAATCCCCGGGCTCAACCTGGGAACTGCATTTGTGACTGGCAGGCTAGAGTATGGCAGAGGGGGGTAGAATTCCACGTGTAGCAGTGAAATGCGTAGAGATGTGGAGGAATACCGATGGTGAAGGCAGCCCCCTGGGCCAATACTGACGCTCATGCACGAAAGCGTGGGGAGCAAACAGG

>d50be1e7e90ce37a603c9621950168c0

TACGTAGGGTGCGAGCGTTAATCGGAATTACTGGGCGTAAAGCGTGCGCAGGCGGTTTGTTAAGACAGATGTGAAATCCCCGGGCTCAACCTGGGAACTGCATTTGTGACTGGCAGGCTAGAGTATGGCAGAGGGGGGTAGAATTCCACGTGTAGCAGTGAAATGCGTAGAGATGTGGAGGAATACCGATGGCGAGGGCAGCCCCCTGGGCCAATACTGACGCTCATGCACGAAAGCGTGGGGAGCAAACAGG

>06399f0f2bbd0bd1d64c9a55a0a68330

TACGTAGGGTGCGAGCGTTAATCGGAATTACTGGGCGTAAAGCGTCCGTAGGCGGTTTGTTAAGACAGATGTGAAATCCCCGGGCTCAACCTGGGAACTGCATTTGTGACTGGCAGGCTAGAGTATGGCAGAGGGGGGTAGAATTCCACGTGTAGCAGTGAAATGCGTAGAGATGTGGAGGAATACCGATGGCGAAGGCAGCCCCCTGGGCCAATACTGACGCTCATGCACGAAAGCGTGGGGAGCAAACAGG

>cca713f9f233a8d5829e96ec58b0d86b

TACCGGCAGCCCGAGTGATGGCCGATCTTATTGGGCCTAAAGCGTCCGTAGCTGGCCGCACAAGTCCATCGGAAAATCCACCCGCTCAACGGGTGGGCGTCCGGTGGAAACTGTGTGGCTTGGGACCGGAAGGCGCGACGGGTACGTCCGGGGTAGGAGTGAAATCCCGTAATCCTGGACGGACCGCCGATGGCGAAAGCACGTCGCGAGAACGGATCCGACAGTGAGGGACGAAAGCCAGGGTCTCGAACCGG

>64cae9f9c8f27a8ee4e7aafc9a43a29b

TACGTAGGGTGCGAGCGTTAATCGGAATTACTGGGCGTAAAGCGTGCGCAGGTGGTTTGTTAAGACAGATGTGAAATCCCCGGGCTCAACCTGGGAACTGCATTTGTGACTGGCAGGCTAGAGTATGGCAGAGGGGGGTAGAATTCCACGTGTAGCAGTGAAATGCGTAGAGATGTGGAGGAATACCGATGGCGAAGGCAGCCCCCTGGGCCAATACTGACGCTCATGCACGAAAGCGTGGGGAGCAAACAGG

>a11c3dd493ba887dbdbdd08649cc1956

TACATAGGTGGCAAGCGTTATCCGGATTTATTGGGCGTAAAGCGTGCGTAGACGGTTCTGCAAGTCTGAGGTTAAATCTAGGAGCTCAACTCCTATCCGCCTTAGAAACTACAGATCTAGAGTATAGAAGAGGTTAGTGGAATTTCACAAGTAGCGGTGGAATGCGTTGATATGTGAAGGAACACCAGTGGCGAAGGCGGCTAACTGGTCTATTACTGACGTTGTGGCACGAAAGCGTGGGGAGCAAATAGG

>6716a86b7f9615f416edf59fc37b9c83

AACAGAGGATACAAGCGTTATCCGGATTTATTGGGTTTAAAGGGTGCGTAGGTGGTTTTTTAAGTCAGTAGTGAAATCTTAAAGCTTAATTTTAAAAGTGCTATTGATACTGATAAACTAGAGTGAGGTTGGAGTAACTGGAATGTGTGGTGGAGCGGTGAAATGCATAGAGATCACACAGAACACCAATCGCGAAGGCATGTTACTAAACATAGACTGACACTGAGGCACGAAAGCATGGGTAGCAAACAGG

>9c62d20e89c8c199417763d39a254a8e

CACGATTAACCCAAGTCAATAGAAGCCGGCGTAAAAAGTGTTTTAGATCACCCCCTCCCCAATAAAGCTAAAACTCACCTGAGTTGTAAAAAACTCCAGTTGACACAAAATAGACTACGAAAGTGGCTTTAACATATCTGAACACACAATAGCTAAGACCCAAACTGGG

>864267f12bedace19272af56224498c7

TACGTAGGGTGCGAGCGTTAATCGGAATTATTGGGCGTAAAGCGAGTGTAGACGGTTATTTAAGCCAGATGTGAAATACCCGAGCCTAACTTGGGAGGTGCATATGGAACTGGGTAGCTAGAGTGTGTCAGAGGGAGGTAGAACTCCACGTGTAGCAGTGAAATGCGTAGAGATGTGGAAGAATACCGATGGCGAAGGCAGCCTCCTGGGATAACACTGACGTTGAGGCTCGAAAGCGTGGGGAGCAAACAGG

>a1d83433e07f97fd446f7ff512e44fc6

TACGTAGGGTGCAAGCGTTAATCGGAATTACTGGGCGTAAAGCGTGCGCAGGCGGTTTTGTAAGACAGTGGTGAAATCCCCGGGCTCAACCTGGGAACTGCCATTGTGACTGCAAGGCTAGAGTGCGGCAGAGGGGGATGGAATTCCGCGTGTAGCAGTGAAATGCGTAGATATGCGGAGGAACACCGATGGCGAAGGCAATCCCCTGGGCCTGCACTGACGCTCATGCACGAAAGCGTGGGGAGCAAACAGG

>8e89b6b7f9810e8e6f3760168c3b6e88

TACCGGCAGTCCAAGTGATGGCCGATATTATTGGGCCTAAAGCGTCCGTAGCTTGCTGTGTAAGTCCGTTGGGAAATCGACCAGCTCAACTGGTCGGCGTCCGGTGGAAACTACACAGCTTGGGGCCGAGAGACTCAACGGGTACGTCCGGGGTAGGAGTGAAATCCCGTAATCCCGGACGGACTACCGATGGCGAAAGCACTCTGAGAAGACGGCTTCGACAGTGAGGGACGAAAGCTCGGGTCTCAAACCGG

>3360e4c34e83e563b772ba9e462a4802

TACGGAGGGGGTTAGCGTTGTTCGGAATTACTGGGCGTAAAGCGCGCGTAGGCGGCTTGGTCAGTCGGATGTGAAAGCCCCGGGCTCAACCTGGGAATTGCATTCGATACTGCCTGGCTAGAGTCTGGTAGAGGGTAGCGGAATTCCCGGTGTAGCGGTGAAATGCGTAGATATCGGGAGGAACATCAGTGGCGAAGGCGGCTGCCTGGACCAAGACTGACGCTGAGGTGCGAAAGCGTGGGGAGCAAACAGG

>a52970705dfd5642085748f4646b3b18

GACGGGGGGGGCAAGTGTTCTTCGGAATGACTGGGCGTAAAGGGCACGTAGGCGGTGAATCGGGTTGAAAGTGAAAGTCGCCAAAAACTGGCGGAATGCTCTCGAAACCAATTCACTTGAGTGAGACAGAGGAGAGTGGAATTTCGTGTGTAGGGGTGAAATCCGGAGATCTACGAAGGAACGCCAAAAGCGAAGGCAGCTCTCTGGGTCCCTACCGACGCTGGGGTGCGAAAGCATGGGGAGCGAACAGG

>b35cc2450b0da51b1410fd3636f3e0ac

TACCGGCAGTCCGAGTGATGGCCGATCTTATTGGGCCTAAAGCGTCCGTAGCTGGCCGCGCAAGTCCATCGGAAAATCCACCTGCTCAACAGGTGGGCGCCCGGTGGAAACTGCGCGGCTTGGGGCCGGAAGACCTGAGGGGTACGTCCGGGGTAGGAGTGAAATCCTGTAATCCTGGACGGACCACCAATGGGGAAACCACCTCAGGAAGACGGACCCGACGGTGAGGGACGAAAGCTAGGGTCTCGAACCGG

>50c1adc394d69456bab0180bd2d25d57

TACGGAGGGTGCAAGCGTTATCCGGATTCACTGGGTTTAAAGGGTGCGTAGGCGGGTATTTAAGTCAGTGGTGAAATCCTAGAGCTTAACTCTAGAACTGCCATTGATACTATTTATCTTGAATATTGTGGAGGTAAGCGGAATATGTCATGTAGCGGTGAAATGCTTAGATATGACATAGAACACCTATTGCGAAGGCAGCTTACTACGCATATATTGACGCTGAGGCACGAAAGCGTGGGGATCAAACAGG

>e11283c3a9a58e67a76bb0077377c7ce

GACGGGGGGGGCAAGTGTTCTTCGGAATGACTGGGCGTAAAGGGCACGTAGGCGGTGAATCGGGTTGAAAGTGAAAGTCGCCAAAAACTGGCGGAATGCTCTCGAAACCAATTCACTTGAGTGAGACAGAGGAGAGTGGAATTTCGTGTGTAGGGGTGAAATCCGGAGATCTACGAAGGAACGCCAAAAGCGAAGGCAGCTCTCTGGGTCCCTACCGACGCTGGAGTGCGAAAGCATGGGGAGCGAACGGG

>6399da4ad90d4cdb4a5eb37b37b96bbf

TACGTAGGTGGCAAGCGTTATCCGGATTTATTGGGCGTAAAGAGGGTGCAGGCGGTTTTCTAAGTCTGATGTGAAAGCCTTCGGCTTAACCGGAGAAGTGCATCGGAAACTGGATAACTTGAGTGCAGAAGAGGGTAGTGGAACTCCATGTGTAGCGGTGGAATGCGTAGATATATGGAAGGACACCAGTGGCGAAGGCGGCTACCTGGTCTGCAACTGACGCTGAGACTCGAAAGCATGGGTAGCGAACAGG

>4cd0c00fb5acc2d118e5d50d631c54b7

CACACTTATGTCTTCAGAAAGACAAAATATTTATCCAGGATGCATTTCAACCATGTCGGTCATTTTCAACTGGCAGTGCCTTGTAGATATTTGTGAACGATTTTGAATGTGTCTTGTCAATAGAGTTCAGTTTGTTCATTAATGATTTTGTTATGTAGTTTGTAACGTAATATATTTAGGCTATCAAGTAAATTCATTTTGTTTCCTTTTTGCCGGTTGTG

>f51b153bbfe8b3d7058a9cc6187a21db

CATTTTACCATAGTGTCTGCCTCCACGGGCACGTTTATGGCTTTGCCGGTTATCTTTTAAGACTGTTTCTATTCTGAGACGACGGATCGCATACGTGGAAATCCGGATGAAATCAGTCTCTCAGTGATACGATCGAGCGGCAGATGGGTCGGATCTTCCGGATACTTATAACTATTACTTTGGCTCGGGGGAGGCACTTTACACTCATTAAGCAAGCGGCGGTATGTAGAGCACA

>6b3e766b913b1574c4565efe2c483ebd

TACGTAGGGCGCAAGCGTTATCCGGAATTATTGGGCGTAAAGAGCTCGTAGGCGGTTTGTCGCGTCTGCTGTGAAAGTCCGGGGCTCAACTCCGGTTCTGCAGTGGGTACGGGCAGGCTTGAGTGATGTAGGGGAGACTGGAATTCCTGGTGTAGCGGTGAAATGCGCAGATATTAGGAGGAACACCGATGGCGAAGGCAGGTCTCTGGGCATTAACTGACGCTGAGGAGCGAAAGCATGGGGAGCGAACAGG

>163fcd6e4b231a0878777dcd81dca2f7

TACGTAGGGTGCAAGCGTTAATCGGAATTACTGGGCGTAAAGCGTGCGCAGGCGGTTATATAAGACAGATGTGAAATCCCCGGGCTCAACCTGGGAACTGCATTTGTGACTGTATAGCTAGAGTACGGCAGAGGGGGATGGAATTCCGCGTGTAGCAGTGAAATGCGTAGATATGCGGAGGAACACCGATGGCGAAGGCAATCCCCTGGGCCTGTACTGACGCTCATGCACGAAAGCGTGGAGAGCAAACAGG

>56d4288c21f1a6a3f2adc9d4df692fbb

TACGTAGGTGGCAAGCGTTGTCCGGATTTATTGGGCGTAAAGCGCGCGCAGGCGGTCTTTTAAGTCTGATGTGAAAGCCCCCGGCTCAACCGGGGAGGGTCATTGGAAACTGGGAGACTTGAGTGCAGAAGAGGAGAGTGGAATTCCATGTGTAGCGGTGAAATGCGTAGATATATGGAGGAACACCAGTGGCGAAGGCGGCTCTCTGGTCTGTAACTGACGCTGAGGCTCGAAAGCGTGGGGAGCAAACAGG

>f7791f535524377ad962dbd8d36d488a

TACGGAGGGTGCAAGCGTTATCCGGATTTATTGGGTTTAAAGGGTCCGTAGGCGGATTTGTAAGTCAGTGGTGAAATCTCACAGCTTAACTGTGAAACTGCCATTGATACTGCAAGTCTTGAGTGTTGTTGAAGTAGCTGGAATAAGTAGTGTAGCGGTGAAATGCATAGATATTACTTAGAACACCAATTGCGAAGGCAGGTTACTAAGCAACAACTGACGCTGATGGATGAAAGCGTGGGGAGCGAACAGG

>09a8bf3a775bad7f7dea015f44b2b76c

CACACTTATGTCTTCAAGAAGACAAAATATTTCTCCAGGATGCATTTCAACCATGTTGGTCATTTTCAACTGGCAGTGCCTTGTAGATATTTGTGAAAGATTTTGAATGTGTCTTGTCCATAGAGTTGAGTTTGTTCATTAATGATTTTGTTATGTAGTTTGTAACGTAATATATTTAGGCTTTCTAGTAAATTCATTTTGTTTCCTTTTTTGCCGGTTGTG

>fd62f10ca3883f5401d5669bb280f5b5

TTGTGTTCTTAAGAAATAAAACCAATTTTATTTTATTTACAAAGTAGAATCCTTTGTGATGTAGTCCTATTAAGTACTTTTAATTAATTAAAATGTTCTCTCAACAAAACAACTATTAGTATTTTTTTTATATCTGTAATAATAAAAGCATTATAAAGCATTCTTTGAGTTAGATTTATTAACCCTAATAGTACAGTAGAACTT

>7a9c05163dd0f18c1fccfa5661b1a015

CATTGAACTATCGTGAGAAAGTCACGCCGCCAAAGGGAATTATATTATAGTAATTATTGGCGTTAATAAACATTTTAGTAATAGTTGTAATATATCATATTGTGTGAAAGATAAAGACGATTAAAACAGTGTTTAACAATCGCTTATCGAGACACTATTAAAGTAGTTAGGAATTAAAATATGTTTATTATTTGTATTTAACAAGGAATAGTCTTGAAAATAATTACAGTATTAAACGGAACTAATAGAGTCACGCAAGTGAA

>e89d80b639fa0276cc709cf314eecbf0

TACCGGCAGCTCAAGTGATGTCCCATATTATTGGGCCTAAAGCGTCCGTAGCTGGCCGCACAAGTCCGTCGGGAAATCCACCTGCCCAACAGGTGGGCGTCCGGCGGAAACTGTGTGGCTTGGAACCGGAAGGCTCAGAGAGTACGTCCGGGGTAGGAGTGAAATCCCGTAATCCCGGACGGACTACCGATGGCGAAAGCACTCTGAGAAGACGGCTTCGACAGTGAGGGACGAAAGCTCGGGTCTCAAACCGG

>309c9a4548be614cd5a7ccdc869357c6

CCAAGGCATCATATGAAATTTGTTACATTCTTGGTAAGCATATGAAACCATTTACCGACGCAGAAATTGTAAAAGAATGCTTTATTAGTGGGGCTAATGCACTTTTTGATAAATTCAGCAACAAAAAACAAATATTGTCTGATATAAAAAAACTACAGCTCTCCGATTCAACCTGTATAAGGCGTATTGAAGACATTTCAAAATTCATCTACGATAACTT

>1a2618ce2ec57c2341f3fc45d0463c92

CACGATTAACCCAAGTCAATAGAAGCCGGCGTAAAGAGTGTTTTAGATCACCCCCTCCCCAATAAAGCTAAAACTCACCTGAGTTGTAAAAAACTCCAGTTGACACAAAATATACTACGAAAGTGGCCTTAACATATCTGAACACACAATAGCTAAGACCCAAACTGGG

>ca1ca2fdf94d331798552a7653fbffc8

CCGTCACTTAAAAACTCGCCATTGGATAATTGTCTGTTATCCATGTTACTCGATAATACGTTTTACAAGTTGTCTTTCTTAACGAATTTCGAAACGGCATTTTTCTGCTGTCTCAGTTTTCAGTTTCTTATCGTCTTGATGATTTGGTAGTCTTTTTGTACTATTATTTAAAATTTTTAAATAACAGATATTACGAACCTTCTAAGAAGTGAGTATAAACTAG

>3ef3255ad19423fae3b82a18bc810a0f

TACGTAGGGTGCGAGCGTTAATCGGAATTAATGGGCGTAAAGCGTGCGCAGGCGGTTTGTTAAGACAGATGTGAAATCCCCGGGCTCAACCTGGGAACTGCATTTGTGACTGGCAGGCTAGAGTATGGCAGAGGGGGGTAGAATTCCACGTGTAGCAGTGAAATGCGTAGAGATGTGGAGGAATACCGATGGCGAAGGCAGCCCCCTGGGCCAATACTGACGCTCATGCACGAAAGCGTGGGGAGCAAACAGG

>5dc2d4b50568d4d4a87a923f8433fcac

CACGATTAACCCAAGTCAATAGAAGCCGGCGTAAAGAGTGTTTTAGATCACCCCCTCCCCAATAAAGCTAAAACTCACCTGAGTCGTAAAAAACTCCAGTTGACACAAAATAGACTACGAAAGTGGCTTTAACATATCTGAACACGCAATAGCTAAGACCCAAACTGGG

>fcee56301081e95273d7535de880814b

TACGGAGGGTGCAAGCGTTATCCGGATTTATTGGGTTTAAAGGGTCCGTAGGCGGATTTGTAAGTCAGTGGTGAAATCTCACAGCTTAACTGTGAAACTGCCATTGATACTGCAAGTCTTGAGTGTTGTTGAAGTAGCTGGAATAAGTAGTGTAGCGGTGAAATGCATAGATATTACTTAGAACACCAATTGCGAAGGCAGGTTACTAAGCAACAACTGACGCTGATGGACGAAAGCGTGGGAAGCGAACAGG

>6f9e425f4e1ad0cdd8f6d9dd136ccc7d

TACGTAGGTGGCAAGCGTTGTCCGGATTTACTGGGCGTAAAGGATGCGTAGGCGGATGTTTAAGTGAGATGTGAAATACCCGGGCTCAACTTGGGTGCTGCATTTCAAACTGGACATCTAGAGTGCGGGAGAGGAAAGCGGAATTCCTAGTGTAGCGGTGGAATGCGTAGAGATTAGGAAGAACACCAGTGGCGAAGGCGGCTTTCTGGACCGTAACTGACGCTGAGGCATGAAAGCGTGGGGAGCAAACAGG

>f3fd9ccfce49167eacbaca1b8dbaeefe

CACACTTATGTCTTCAGAAAGACAAAATATTTCTCCAGGATGCATTTCAACCATGTCGGTCATTTTCAACTGGCAGTGCCTTGTAGATATTTGTGAAAGATTTTGAATGCGTCTTGTCCATAGAGTTGAGTTTGTTCATTAATGATTTTGTTATGTAGTTTGTAACGTAATATATTTAGGCTTTCAAGTAAATTCATTTTGTTTCCTTTTTTGCCGGTTGTG

>92ea8b30523b2f5e9c63ab905ba92a5b

CACGATTAACCCAAGTCAATAGAAGCCGGCGTAAAGAGTGTTTTAGATCACCCCCTCCCCAATAAAGCTAAAACTCACCTGAGTTGTAAAAAACTCCAGTTGACACAAAATAGACCACGAAAGTGGCTTTAACATATCTGAACACACAATAGCTAAGACCCAAACTGGG

>5c7eb6c1f90b62936db774b666f9667a

CACGATTAACCCAAGTCAATAGAAGCCGGCGTAAAGAGTGTTTTAGATCACCCCCTCCCCAATAAAGCTAAAACTCACCTGAGTTGTAAAAAACTCCAGTTGACACAAAATAGACTACGGAAGTGGCTTTAACATATCTGAACACACAATAGCTAAGACCCAAACTGGG

>721f82b2610dd132af131e2cd50c93bd

CATTTTACCATAGTGTCTGCCTCCACGGGCACGTTTATGGCTTTGCCGGTTATCTTTTAAGACTGTTTCTATTCTGAGACGACGGATCTGCATACGTGGAAATCCGGATGAAATCAGTCTCTCAGTGATACGATCGAGCGGCAGATGGGTCGGATCTTCCGGATACTTATAACTATTACTTTGGCTCGGCGGAGGCACTTTACCCTCATTAAGCAAGCGGCGGTATGTGGAGCACA

>7766061f86c36a201439a1df4e15a4e0

TACGTAGGGTGCAAGCGTTGTCCGGAATTATTGGGCGTAAAGAGCTCGTAGGCGGTTTGTCGCGTCTGCTGTGAAATCCCGAGGCTCAACCTCGGGCTTGCAGTGGGTACGGGCAGACTGGAGTGCGGTAGGGGAGATTGGAATTCCTGGTGTAGCGGTGGAATGCGCAGATATCAGGAGGAACACCGATGGCGAAGGCAGATCTCTGGGCCGTAACTGACGCTGAGGAGCGAAAGCGTGGGGAGCGAACAGG

>c2ab3103e0d998b8480c39984b4ddc83

TACGTAGGTCCCGAGCGTTGTCCGGATTTATTGGGCGTAAAGCGAGCGCAGGCGGTTTAATAAGTCTGAAGTTAAAGGCAGTGGCTTAACCATTGTTCGCTTTGGAAACTGTTAAACTTGAGTGCAGAAGGGGAGAGTGGAATTCCATGTGTAGCGGTGAAATGCGTAGATATATGGAGGAACACCGGTGGCGAAAGCGGTTCTCTGGTCTGTAACTGACGCTGAGGCTCGAAAGCGTGGGGAGCAAACAGG

>f91349ae28de0a066c87851436afd9a9

TACGTAGGTGGCAAGCGTTGTCCGGAATTACTGGGCGTAAAGGGTGCGTAGGCGGTCCTTTAAGTGGGATGTGAAATCCCTGGGCTTAACCCAGGAACTGCATTCCAAACTGGAGGACTAGAGTGCAGGAGAGGAAAGCGGTATTCCCAGTGTAGCGGTGAAATGCGTAGAGATTGGGAGGAACACCAGTAGCGAAGGCGGCTTTCTGGAATGTAACTGACGCTGAGGCACGAAAGCGTGGGGAGCAAACAGG

>9017410f61d53efa4c30e399f33e2804

TACCGGCAGCACGAGTGATGGCCGATCTTATTGGGCCTAAAGCGTCCGTAGCCGGCCAGACAAGTCCGTTGGGAAATCGACGTGCCTAACACGTCGGCGTCCAGCGGAAACTGTCTGGCTTGGGGCCGGAAGACTCGAGGGGTACGTCTGGGGTAGGAGTGAAATCCCGTAATCCTGGACGGACCGCCGGTGGCGAAAGCGCCTCAGGAGAACGGATCCGACAGTGAGGGACGAAAGCTAGGGTCTCGAACCGG

>256c536268ab77767583b8cd1b4cd24b

TACGTAGGGTGCGAGCGTTAATCGGAATTACTGGGCGTAAAGCGTGCGTAGGCGGTTGTGTAAGACAGGCGTGAAATCCCCGGGCTCAACCTGGGAATGGCGCTTGTGACTGCACGGCTGGAGTGCGGCAGAGGGGGATGGAATTCCGCGTGTAGCAGTGAAATGCGTAGATATGCGGAGGAACACCGATGGCGAAGGCAATCCCCTGGGCCTGCACTGACGCTCATGCACGAAAGCGTGGGGAGCAAACAGG

>82d30ee39cd361275fb98a9c2d682d0f

TACCGGCAGCTCAAGTGATGTCCCATATTATTGGGCCTAAAGCGTCCGTAGCTTGCTGTGTAAGTCCATTGGGAAATCGACCAGCTCAACTGATGGGCGTCCGGCGGAAACTACAAGGCTTGGGACCGGAAGGCTCGAGGGGTACGTCCGGGGTAGGAGTGAAATCCTGTAATCCTGGACGGACCACCAATGGGGAAACCACGTTGAGAGACCGGACCCGACAGTGAGGGACGAAAGCCAGGGTCTCGAACCGG

>a2045f5e10b399cdf754e263c2d6a374

TACGTAGGTGGCAAGCGTTGTCCGGATTTATTGGGCGTAAAGCGCGCGCAGGCGGTCTTTTAAGTCTGATGTGAAAGCCCCCGGCTTAACCGGGGAGGGTCATTGGAAACTGGAAGACTGGAGTGCAGAAGAGGAGAGTGGAATTCCACGTGTAGCGGTGAAATGCGTAGAGATGTGGAGGAACACCAGTGGCGAAGGCGACTCTCTGGTCTGTAACTGACGCTGAGGAGCGAAAGCGTGGGGAGCGAACAGG

>bb9d718b5cef478746344f408d7a9bc4

TACCGGCAGCCCGAGTGATGGCCGATCTTATTGGGCCTAAAGCGTCCGTAGCTGGCTGCGCAAGTCCGTCGGGAAATCCACTCGCCCAACGAGTGGGCGTCCGACGGAAACTGCACAGCTTGGGACCGGAAGGCTCGAAGGGTACGTTCGGGGTAGGAGTGAAATCCCATAATCCCGCACGGACCACCGATGGCGAAAGCACTTCGAGAAAACGGATCCGACAGTGAGGGACGAAAGCCAGGGTCTCGAACCGG

>abbd59db2f1cf478ac1b5455d913b13a

TACGTAGGGTGCGAGCGTTAATCGGAATTACTGGGCGTAAAGCGTGCGCAGGCGGTTTGTTAAGACAGATGTGAAATCCCCAGGCTCAACCTGGGAACTGCATTTGTGACTGGCAGGCTAGAGTATGGCAGAGGGGGGTAGAATTCCACGTGTAGCAGTGAAATGCGTAGAGATGTGGAGGAATACCGATGGCGAAGGCAGCCCCCTGGGCCAATACTGACGCTCATGCACGAAAGCGTGGGGAGCAAACAGG

>a68ad5c005e494057a9253b0c58e17e8

TACCGGCAGCCCGAGTGATGGCCGATATTATTGGGCCTAAAGCGTCCGTAGCTGGCTGGACAAGTCCGTTGGGAAATCTGCCCGCTTAACGGGCAGGCGTCCAGCGGAAACTGTTCAGCTTGGGACCGGAAGACCTGAGGGGTACGTCTGGGGTAGGAGTGAAATCCTGTAATCCTGGACGGACCACCGGTGGCGAAAGCGCCTCAGAAAAACGGACCCGACGGTGAGGGACGAAAGCCAGGGTCTCGAACCGG

>1bc599cac5647c5c30496e93ca551557

TACAGAGGGTGCAAGCGTTAATCGGAATTACTGGGCGTAAAGCGCGCGTAGGTGGTTCGTTAAGTTGGATGTGAAATCCCCGGGCTCAACCTGGGAACTGCATCCAAAACTGGCGAGCTAGAGTATGGTAGAGGGTGGTGGAATTTCCTGTGTAGCGGTGAAATGCGTAGATATAGGAAGGAACACCAGTGGCGAAGGCGACCACCTGGACTGATACTGACACTGAGGTGCGAAAGCGTGGGGAGCAAACAGG

>6e2b13d81452bee95443fab306241bc6

CACACTTATGTCTTTAGAAATACAAACCATTTCTCCAGGATGCATTTCAACCATGTCGGTCATTTTCAACTGGCAGTGCCTTGTAGATATTTGTGAAATATTTTGAATGTGTCTTGTCCATAGACAGTTTGTAAATTAATGATTTTGTTATGTAGTTTGTAACGTAATATATTTAGGCTTTTAAGTAAATTCATTTTATTTTCTTTTTTCCGGTCGTG

>7d217addfd80cb5393d81dae5f160367

TACGTAGGGTGCGAGCGTTAATCGGAATTACTGGGCGTAAAGCGTGCGCAGGCGGTTGTGCAAGACAGATGTGAAATCCCCGGGCTTAACCTGGGAACTGCATTTGTGACTGCACGGCTAGAGTGCGGCAGAGGGGAGTGGAATTCCGCGTGTAGCAGTGAAATGCGTAGATATGCGGAGGAACACCGATGGCGAAGGCAGCTCCCTGGGCCTGCACTGACGCTCATGCACGAAAGCGTGGGGAGCAAACAGG

>d7f11ab217a6deb82abe01ebbe117262

TACGGAGGGTGCAAGCGTTAATCGGGATTACTGGGCGTAAAGCGCACGCAGGCGGTCTGTCAAGTCGGATGTGAAATCCCCGGGCTCAACCTGGGAACTGCATTCGAAACTGGCAGGCTTGAGTCTTGTAGAGGGGGGGAGAATTCCAGGCGTAGCGGTGAAATGCGTAGAGATCTGGAGGAATACCGGTGGCGAAGGCGGCCCCCTGGACGAAGACTGACGCTCAGGTGCGAAAGCGTGGGGAGCAAACAGG

>77d2e830cddecd52d2d9f6ddf5192bac

TACGGAGGGTGCAAGCGTTATCCGGATTTATTGGGTTTAAAGGGTCCGTAGGCGGATTTGTAAGTCAGTGGTGAAATCTCACAGCTTAACTGTGAAACTGCCATTGATACTGCAAGTCTTGAGTGTTGTTGAAGTAGCTGGAATAAGTAGTGTAGCGGTGAAATGCATAGATATTACTTAGAACACCAATTGCAAAGGCAGGTTACTAAGCAACAACTGACGCTGATGGACGAAAGCGTGGGGAGCGAACAGG

>7bdc5fd87b237cc177cebf309afaab0b

AACGTAGGATCCTAGCGTTATCCGAATTTACTGGGCGTAAAGCGCGTGCAGGTGGTTTGGTAAGTTGGATGTGAAAGCTCCTGGCTCAACTGGGAGAGGCCGTTCAAAACTACCAGACTTGAGGGCGGTAGAGGAAGGTGGAATTCCCGGTGTAGTGGTGAAATGCGTAGATATCGGGAGGAACACCAGTGGCGAAAGCGGCCTTCTGGGCCGTCCCTGACACTCAGACGCGAAAGCTAGGGGAGCAAACTGGG

>ea93341e08300bd78c3ea01820b82240

TACGAAGGGGGCTAGCGTTGCTCGGAATCACTGGGCGTAAAGGGCGCGTAGGCGGCTGATTTAGTCGAGGGTGAAAGCCCGTGGCTCAACCACGGAATGGCCTTCGATACTGATTGGCTTGAGACCGGAAGAGGACAGCGGAACTGCGAGTGTAGAGGTGAAATTCGTAGATATTCGCAAGAACACCAGTGGCGAAGGCGGCTGTCTGGTCCGGTTCTGACGCTGAGGCGCGAAAGCGTGGGGAGCAAACAGG

>3fc981f4ea94dc108e13afee1657fdbf

GACGGGGGGGGCAAGTGTTCTTCGGAATGACTGGGCGTAAAGGGCACGTAGGCGGTGAATCGGGTTGAAAGTGAAAGTCGCCAAAAACTGGTGGAATGCTCTCGAAACCAATTCACTTGAGTGAGACAGAGGAGAGTGGAATTTCGTGTGTAGGGGTGAAATCCGCAGATATACGAAGGAACGCCAAAAGCGAAGGCAGCTCTCTGGGTCCCTACCGACGCTGGAGTGCGAAAGCATGGGGAGCGAACGGG

>342cfa81bf1359e42bdabe38d0cd1b81

TACGTAGGGTGCGAGCGTTAATCGGAATTACTGGGCGTAAAGCGTGCGCAGGCGGTTTGTTAAGACAGATGTGAAATCCCCGGGCTCAACCTGGGAGCTGCATTTGTGACTGGCAGGCTAGAGTATGGCAGAGGGGGGTAGAATTCCACGTGTAGCAGTGAAATGCGTAGAGATGTGGAGGAATACCGATGGCGAAGGCAGCCCCCTGGGCCAATACTGACGCTCATGCACGAAAGCGTGGGGAGCAAACAGG

>b4c50694fc9283e355eafc08e1d944bc

TACCGGCAGTCCAAGTGATGGCCGATATTATTGGGCCTAAAGCGTCCGTAGCTTGCTGTGTAAGTCCATTGGGAAATCGACCAGCTCAACTGGTCGGCGTCCGGTGGAAACTACACAGCTTGGAACCGGAAGGCTCAGAGAGTACGTCCGGGGTAGGAGTGAAATCCTGTAATCCTGGACGGACCACCAATGGGGAAACCACGTTGAGAGACCGGACCCGACAGTGAGGGACGAAAGCCAGGGTCTCGAACCGG

>8b0da92658dce667ebb2cc167f7df2c2

CACGATTAACCCAAGTCAATAGAAGCCGGCGTAAAGAGTGTTTTAGATCACCCCCTCCCCAATAAAGCTAAAACTCACCTGAGTTGTAAAAAACTCCAGTTGACACAAGATAGACTACGAAAGTGGCTTTAACATATCTGAACACACAATAGCTAAGACCCAAACTGGG

>1773184384024225f000f046c107fab5

TACGTAGGTGGCAAGCGTTGTCCGGATTTACTGGGTGTAAAGGGCGTGCAGCCGGGAGTGCAAGTCAGATGTGAAATGCCGAGGCTCAACCTCGGAGCTGCATTTGAAACTGTACTTCTTGAGTACTGGAGAGGCAGACGGAATTCCTAGTGTAGCGGTGAAATGCGTAGATATTAGGAGGAACACCAGTGGCGAAGGCGGTCTGCTGGACAGCAACTGACGGTGAGGCGCGAAAGCGTGGGGAGCAAACAGG

>d550c6c79cb3fc039c34e49ffc2bac14

TACGTAGGGTGCGAGCGTTAATCGGAATTACTGGGCGTAAAGCGTGCGCAGGCGGTTTGTTAAGACAGATGTGAAATCCCCGGGCTCAACCTGGGAACTGCATTTGTGACTGGCAGGCTAGAGTATGGCAGAGGGGGGTAGAATTCCGCGTGTAGCAGTGAAATGCGTAGAGATGTGGAGGAATACCGATGGCGAAGGCAGCCCCCTGGGCCAATACTGACGCTCATGCACGAAAGCGTGGGGAGCAAACAGG

>b1f87a2c98e1345ff4bbaf4aeaa026c8

TACGGAGGGTGCAAGCGTTAATCGGAATTACTGGGCGTAAAGCGCACGCAGGCGGTCTGTCAAGTCGGATGTGAAATCCCCGGGCTCAACCTGGGAACTGCATTCGAAACTGGCAGGCTAGAGTCTTGTAGAGGGGGGTAGAATTCCAGGTGTAGCGGTGAAATGCATAGAGATCTGGAGGAATACCGGTGGCGAAGGCGGCCCCCTGGACAAAGACTGACGCTCAGGTGCGAAAGCGTGGGGAGCAAACAGG

>8620cab22732f48f9e6d80bd74f3a21b

TACGTAGGGTGCAAGCGTTAATCGGAATTACTGGGCGTAAAGCGTGCGCAGGCGGTTATGCAAGACAGAGGTGAAATCCCCGGGCTCAACCTGGGAACTGCCTTTGTGACTGCATGGCTAGAGTACGGTAGAGGGGGATGGAATTCCGCGTGTAGCAGTGAAATGCATAGATATGCGGAGGAACACCGATGGCGAAGGCAATCCCCTGGACCTGTACTGACGCTCATGCACGAAAGCGTGGGGAGCAAACAGG

>38ff6dfe64cb8fb1ff1fb5dd5a752f3b

TACCGGCAGTCCGAGTGATGGCCGATATTATTGGGCCTAAAGCGTCCGTAGCTTGCTGTGTAAGTCCATTGGGAAATCGACCAGCTCAACTGGTCGGCGTCCGGTGGAAACTACACAGCTTGGGGCCGAGAGACTCAACGGGTACGTCCGGGGTAGGAGTGAAATCCTGTAATCCTGGACGGACCACCAATGGGGAAACCACCTCAGGAAGACGGACCCGACGGTGAGGGACGAAAGCTAGGGTCTCGAACCGG

>f354a1944034596c55d90602b2f17824

TACGTAGGGTGCGAGCGTTAATCGGAATTACTGGGCGTAAAGCGTGCGCAGGCGGTTTGTTAAGACAGATGTGAAATCCCCGGGCTCAACCTGGGAACTGCATTTGTGACTGGCAGGCTAGAGTATGGCAGAGGGGGGTAGAATTCCACGTGTAGCAGTGAAATGCGTAGAGATGTGGAGGAATACCGATGGCGAAGGCAGCCCCCTAGGCCAATACTGACGCTCATGCACGAAAGCGTGGGGAGCAAACAGG

>3d43dfa8f0ad516a210c3bb37f6d3f89

TACGAAGGGAGCTAGCGTTGTTCGGAATCACTGGGCGTAAAGCGCACGTAGGCGGATATGTCAGTCAGGGGTGAAATCCCGGAGCTCAACTTCGGAACTGCCTTTGATACAGCATATCTCGAGTCCGAGAGAGGTGAGTGGAATTCCTAGTGTAGAGGTGAAATTCGTAGATATTAGGAAGAACACCGGTGGCGAAGGCGGCTCACTGGCTCGGTACTGACGCTGAGGTGCGAAAGCGTGGGGAGCAAACAGG

>d44ee653ff8df06e5d8e1457d647d73f

TACCGGCAGCACGAGTGATGGCCGATCTTATTGGGCCTAAAGCGTCCGTAGCCGGCCAGGCAAGTTCGTCGGGAAATCCACCCGCTCAACGGGTGGGCGTCCGGCGAAAACTGTCTGGCTAGGGACCGGAAGACCCGAGGGGTACGTCCGGGGTAGGAGTGAAATCCTGTAATCCTGGACGGACCACCAATGGGGAAACCACGTTGAGAGACCGGACCCGACAGTGAGGGACGAAAGCCAGGGTCTCGAACCGG

>dfa6db19c843a4419a5ad0e081a52cdf

TACGGAGGGGGCTAGCGTTGTTCGGAATTACTGGGCGTAAAGCGCGCGTAGGCGGACGGTCAAGTTGGGGGTGAAAGCCCGGGGCTCAACCCCGGAACTGCCTTCAAAGCTGATCGTCTGGAGACCGGGAGAGGTGAGTGGAATTCCCAGTGTAGAGGTGAAATTCGTAGATATTGGGAAGAACACCAGTGGCGAAGGCGGCTCACTGGACCGGATCTGACGCTGAGGTGCGAAAGCGTGGGGAGCGAACAGG

>103bf6704d4b6be23872c027f58ef027

TACCGGCAGCCCGAGTGATGGCCGATCTTATTGGGCCTAAAGCGTCCGTTGCTGGCCGCACAAGTCCATCGGGAAATCCACCTGCTCAACAGGTGGGCGTCCGGTGGAAACTGTGTGGCTTGGGACCGGAAGGCGCGACGGGTACGTCTGGGGTAGGAGTGAAATCCCGTAATCCCGGACGGACTACCGATGGCGAAAGCACTCTGAGAAGACGGCTTCGACAGTGAGGGACGAAAGCTCGGGTCTCAAACCGG

>e82c23e7822595ce7ed6978baeb2f433

TACGTAGGTGGCAAGCGTTGTCCGGATTAATTGGGCGTAAAGCGAGCGCAGGCGGAGGAATAAGTCTGATGTGAAAGCCCTCGGCTTAACCGAGGAACTGCATCGGAAACTGTTTTTCTTGAGTGCAGAAGAGGAGAGTGGAACTCCATGTGTAGCGGTGGAATGCGTAGATATATGGAAGAACACCAGTGGCGAAGGCGGCTCTCTGGTCTGCAACTGACGCTGAGGCTCGAAAGCATGGGTAGTGAACAGG

>ba1e3f0141c4d62b33ce0f4aab67e09e

TACGTAGGTGGCGAGCGTTATCCGGATTTACTGGGCGTAAAGGATGCGTAGGTGGAATTTTAAGTGGGATGTGAAATACCCGGGCTCAACCTGGGAACTGCATTCCAAACTGGAATTCTAGAGTGCAGGAGAGGAAAGCGGAATTCCTAGTGTAGCGGTGAAATGCGTAGAGATTAGGAAGAACACCAGTGGCGAAGGCGGCTTTCTGGACTGTAACTGACACTGAGGCATGAAAGCGTGGGGAGCAAACAGG

>76883280dd9d3b5d03b60bc29c3b3012

GACAGAGGGTGCAAACGTTGTTCGGAATTACTGGGCGTAAAGCGTGTGTAGGCGGTCTTGTAAGTCGGATGTGAAAGCCCCGGGCTCAACCCGGGAAGTGCACTCGATACTGCGAGGCTTGAGTATCGGAGAGGTTGGTGGAATTCTCGGTGTAGAGGTGAAATTCGTAGATATCGAGAGGAACACCGGTGGCGAAAGCGGCCAACTGGACGAATACTGACGCTGAGACACGAAAGCGTGGGGAGCAAACAGG

>4045eda1d78ea6f9a8892b8ee1f4cd83

TACGTAGGGGGCAAGCGTTGTCCGGAATTATTGGGCGTAAAGCGCGCGCAGGCGGTCTTTTAAGTTTGGTGTTTAAGCCCGGGGCTCAACCCCGGTTCGCACTGAAAACTGGGAGACTTGAGTGCAGGAGAGGAAAGCGGAATTCCACGTGTAGCGGTAAAATGCGTAGAGATGTGGAGGAACACCAGTGGCGAAGGCGGCTTTCTGGACTGTAACTGACGCTGAGGCGCGAAAGCGTGGGGAGCAAACAGG

>4fa9bcfcd994a5fbb979dc149167a62c

TACGAGTGCCCCAAGCGTTATCCGGAATTATTGGGCGTAAAGGGTGCGTAGGTGGTGGTATTAGTCTTGTGTTAAACCCTTCGGCTTAACCGAAGAATCGCATAAGAAACGGTACCACTAGAGGATGTGAGAGGTGTGTAGAACTCATGGAGTAGGGGTGAAATCCGTTGATATCATGGGGAATACCAAAAGCGTAGGCAGCACACTGGCACATTTCTGACACTGAAGCACGAAAGCGTGGGTAGCGAATGGG

>6179458227379ce58644b8bf0905a69c

TACCGGCAGCTCGAGTGATGTCCAATATTATTGGGCCTAAAGCGTCCGTAGCTGGCCGCGCAAGTCCGTCGGGAAATCCACCCGCTTAACGGGTGGGCGTCCGGCGGAAACTGCACGGCTTGGGACAGGAAGGCTCGAGGGGTACGTTCGGGGTAGGAGTGAAACCCCGTAATCCTGAACGGACCGCCGATGGCGAAAGCACCTCGAGAGGACTGATCCGACAGTGAGGGACGAAAGCTGGGGTCTCGAACCGG

>fc06d4e8328b8122820d9161dd533f1f

TACGTAGGGTGCGAGCGTTAATCGGAATTACTGGGCGTAAAGCGTGCGCAGGCGGTTTGTTAAGACAGATGTGAAATCCCCGGGCTCAACCTGGGAACTGCATTTGTGACTGGCAGGCTAGAGTATGGCAGAGGGGGGTAGAATTCCACGTGTAGCAGTGAAATGCGTAGAGACGTGGAGGAATACCGATGGCGAAGGCAGCCCCCTGGGCCAATACTGACGCTCATGCACGAAAGCGTGGGGAGCAAACAGG

>83acded3f866709326169cd939774980

TACGTGAGAGACTAGTGTTATTCATCTTAATTGGGTTTAAAGGGTACCTAGACAGTCAATATAACTTCTATAATGTTAATACTTGACTAGGGTTTTAAGTAAGAGGGAAGTACTTAAGGAGTAAGAGATGAAATATCTGTGATACCAAAGGGACTCCGTAAAGGCGAAGGCATCCCTTTATCTAAAAACTAACGTTGAAGGACGAAGGCTTAGATAACAAATAGG

>d4532c8a4879d75e07bac2d76ff581b1

TACGTAGGGCGCAAGCGTTATCCGGAATTATTGGGCGTAAAGAACTCGTAGGCGGTTTGTCGCGTCTGCTGTGAAAGTCCGGGGCTCAACTCCGGTTCTGCAGTGGGTACGGGCAGGCTAGAGTGATGTAGGGGAGACTGGAATTCCTGGTGTAGCGGTGAAATGCGCAGATATCAGGAGGAACACCGATGGCGAAGGCAGGTCTCTGGGCATTAACTGACGCTGAGGAGCGAAAGCATGGGGAGCGAACAGG

>1a79c9d5c7162ac016d6f5f38bce40a8

TACGTAGGGTGCGAGCGTTAATCGGAATTACTGGGCGTAAAGCGTGCGCAGGCGGTTTGTTAAGACAGATGTGAAATCCCCGGGCTCAACCTGGGAACTGCATTTGTGACTGGCAGGCTAGAGTATGGCAGAGGGGGGTAGAATTCCACGTGTAGCAGTGAAATGCGTAGAGATGTGGAGGACTACCGATGGCGAAGGCAGCCCCCTGGGCCAATACTGACGCTCATGCACGAAAGCGTGGGGAGCAAACAGG

>eca77da0f127beac8f7a32c49bfc1874

AATTACAGTAAGCATGAATAAAGTATAGCATAAATAAAGCTTGACTAAAATTACAGTAAACATGACTTCGATAAGAAAAATTGAACACAATAATTAAGTTAGTTTAGAAAATATTTCCTTTATTAACATAGCTATAAATAAAATGAATACAAACAAAATTATTGCAATTGAGATGATCTATATGAGAATGAACTGTGGCGTTTCAAGAACTGATTAACAGAGAAATAACAAGTAGATTAGGG

>7c779e3fc2e9293849057f743730427a

TACGTAGGGTGCGAGCGTTAATCGGAATTACTGGGCGTAAAGCGTGCGCAGACGGTTTGTTAAGACAGATGTGAAATCCCCGGGCTCAACCTGGGAACTGCATTTGTGACTGGCAGGCTAGAGTATGGCAGAGGGGGGTAGAATTCCACGTGTAGCAGTGAAATGCGTAGAGATGTGGAGGAATACCGATGGCGAAGGCAGCCCCCTGGGCCAATACTGACGCTCATGCACGAAAGCGTGGGGAGCAAACAGG

>dd8e605d1e6df075c329344b58b24052

TACAGAGGGTGCAAGCGTTAATCGGATTTACTGGGCGTAAAGCGCGCGTAGGCGGCTAATTAAGTCAAATGTGAAATCCCCGAGCTTAACTTGGGAATTGCATTCGATACTGGTTAGCTAGAGTGTGGGAGAGGATGGTAGAATTCCAGGTGTAGCGGTGAAATGCGTAGAGATCTGGAGGAATACCGTTGGCGAAGGCAGCCATCTGGCCTAACACTGACGCTGAGGTGCGAAAGCATGGGGAGCAAACAGG

>2da1d3937e16973815093774f592b6fa

TACGGAGGGGGCTAGCGTTGTTCGGAATTACTGGGCGTAAAGCGCGCGTAGGCGGACGGTCAAGTTGGGGGTGAAAGCCCGGGGCTCAACCCCGGAACTGCCTTCAAAACTGATCGCCTGGAGACCGGGAGAGGTGAGTGGAATTCCCAGTGTAGAGGTGAAATTCGTAGATATTGGGAAGAACACCAGTGGCGAAGGCGGCTCACTGGACCGGATCTGACGCTGAGGTGCGAAAGCGTGGGGAGCGAACAGG

>f73fb18550903c97f784b2f7c8d5ad7a

TACGTAGGTGGCAAGCGTTATCCGGAATTATTGGGCGTAAAGCGCGCGTAGGCGGTTTTTTAAGTCTGATGTGAAAGCCCACGGCTCAACCGTGGAGGGTCATTGGAAACTGGAAAACTTGAGTGCAGAAGTGGAAAGTGGAATTCCATGTGTAGCGGTGAAATGCGCAGAGATATGGAGGAACACCAGTGGCGAAGGCGACTTTCTGGTCTGTAACTGACGCTGATGTGCGAAAGCGTGGGGATCAAACAGG

>3ea7934a4dc28ef4b7d987dce71fe5fe

CACCGGCAGCCCAAGTGATGGCCGATATTATTGGGCCTAAAGCGTCCGTAGCTGGCCGCGCAAGTTCGTCGGGAAATCCACCCGCTCAACGGGCAGGCGTCCAGCGGAAACTGTTCAGCTTGGGACCGGAAGACCTGAGGGGTACGTCCGGGGTAGGAGTGAAATCCCGTAATCCTGGACGGACCGCCGATGGCGAAAGCACGTCGCGAGAACGGATCCGACAGTGAGGGACGAAAGCCAGGGTCTCGAACCGG

>9785bd93104d73d8cb6c865a0d5f195f

TACGTAGGTGGCAAGCGTTGTCCGGAATTATTGGGCGTAAAGGGCTCGCAGGCGGTTCCTTAAGTCTGATGTGAAAGCCCCCGGCTCAACCGGGGAGGGTCATTGGAAACTGGGGAACTTGAGTGCAGAAGAGGAGAGTGGAATTCCACGTGTAGCGGTGAAATGCGTAGAGATGTGGAGGAACACCAGTGGCGAAGGCGGCTCTCTGGTCTGTAACTGACGCTGAGGAGCGAAAGCGTGGGGAGCGAACAGG

>f11328554007fb5775dc5d9fc8a6eb0e

TACGTATGGAGCAAGCGTTATCCGGATTTACTGGGTGTAAAGGGAGCGCAGGCGGGTGATCAAGTCAGCTGTGAAAACTACGGGCTTAACCCGTAGACTGCAGTTGAAACTGTTCATCTTGAGTGAAGTAGAGGTTGGCGGAATTCCGAGTGTAGCGGTGAAATGCGTAGATATTCGGAGGAACACCGGTGGCGAAGGCGGCCAACTGGGCTTTAACTGACGCTGAGGCTCGAAAGTGTGGGGAGCAAACAGG

>817f61547be84b9b85a2f23841b0558e

CACGTAAGGGGCAAGCGTTGTTCGGAATTATTGGGCGTAAAGGGTGCGCAGGCGGATACATAAGCCTGATGTGAAAGGCTCCAGCTTAACTGGAAGAATGCATTGGGAACTGTGTAACTAGAGTATAAGAGGGGGAGTTGGAATTCCAGGTGTAGGGGTGAAATCTGTAGATATCTGGAAGAACACCGGTGGCGAAGGCGAACTCCTGGCTATATACTGACGCTGAGGCACGAAAGCGTGGGGAGCGAACAGG

>368ffb6ec0761046c90e31b71208b7a9

TACGTAGGTGGCAAGCGTTGTCCGGAATTATTGGGCGTAAAGGGCTCGCAGGCGGTTCCTTAAGTCTGATGTGAAAGCCCCCGGCTCAACCGGGGAGGGTCATTGGAAACTGGGGAACTTGAGTGCAGAAGAGGAGAGTGGAATTCCATGTGTAGCGGTGAAATGCGTAGATATATGGGGGAACACCAGTGGCGAAGGCGGCTCTCTGGTCTGTAACTGACGCTGAGGCTCGAAAGCGTGGGGAGCAAACAGG

>b03bc1b561548d597bdabacf3b844b96

TACGGAGGGTGCAAGCGTTATCCGGATTTATTGGGTTTAAAGGGTCCGTAGGCGGATTTGTAAGTCAGTGGTGAAATCTCACAGCTTAACTGTGAAACTGCCATTGATACTGCAAGTCTTGAGTGTTGTTGAAGTAGCTGGAATAAGTAGTGTAGCGGTGAAATGCATAGATATTACTTAGAACACCAATTGCGAAGGCAGGTTACTAAGCAACAACTGACGCTGATGGACAAAAGCGTGGGGAGCGAACAGG

>a1137ec4f8dde8e35b6612e57e0df437

TACGTAGGGGGCGAGCGTTGTTCGGAATTACTGGGCGTAAAGAGCGCGTAGGCGGTCAAATAAGTCAGGTGTGAAATCCCTCAGCTTAACTGAGGTTTGGCGCTTGAAACTGTCTGACTTGAGTTTAGGGGAGGAGAGTGGAATTCCCAGTGTAGCGGTGAAATGCGTAGATATTGGGAGGAACACCTGTGGCGAAGGCGGCTCTCTGGACTAATACTGACGCTGAGGCGCGAAAGCTAGGGGAGCAAACTGGG

>a276272e6500c60bd3f8f58279c5ec64

TACGTAGGGAGCAAGCGTTGTTCGGAATTATTGGGCGTAAAGAGCGCGTAGGCGGTTCGGTAAGTCTGACGTGAAATCCCGGAGCTTAACTCCGGAACTGCGTTGGATACTGCCGAGCTTGAGTGTGGGAGAGGAGAGTGGAATTCCCAGTGTAGAGGTGAAATTCGTAGATATTGGGAGGAACACCCGTGGCGAAGGCGGCTCTCTGGACCACAACTGACGCTGAGGCGCGAAAGCTAGGGTAGCAAACTGGG

>80eb821df416687a4bf047d6fd0cddff

TACGTAGGGTGCAAGCGTTAATCGGAATTACTGGGCGTAAAGCGTGCGCAGGCGGTTATGCAAGACAGAGGTGAAATCCCCGGGCTCAACCTGGGAACTGCCTTTGTGACTGCATGGCTAGAGTACGGTAGAGGGGGATGGAATTCCGCGTGTAGCAGTGAAATGCGTAGATATGCGGAGGAATACCGATGGCGAAGGCAATCCCCTGGACCTGTACTGACGCTCATGCACGAAAGCGTGGGGAGCAAACAGG

>b66af2a823b63ab71dc1b397fd284f60

TACGGAGGATCCAAGCGTTATCCGGAATCATTGGGTTTAAAGGGTCCGTAGGCGGGCTTATAAGTCAGTGGTGAAATCTCCCAGCTCAACTGGGAAACTGCCATTGATACTGTAGGTCTTGAATTATTAGGAAGTAACTAGAATATGTAGTGTAGCGGTGAAATGCTTAGATATTACATGGAATACCAATTGCGAAGGCAGGTTACTACTAATATATTGACGCTGATGGACGAAAGCGTGGGTAGCGAACAGG

>ebc69674616616a5b96975d87f05cd87

CACGATTAACCCAAGTCAATAGAAGCCGGCGTAAAGAGTGTTTTAGATCACCCCCTCCCCAATAAAGCTAAAACTCACCTGAGTTGTAAAAAACTCCAGTTGACGCAAAATAGACTACGAAAGTGGCTTTAACATATCTGAACACGCAATAGCTAAGACCCAAACTGGG

>8060ce3a59e88c613cbfa576db0de96e

TACCGGCAGCCCGAGTGATGGCCGATCTTATTGGGCCTAAAGCGTCCGTAGCTGGCCGCACAAGTCCACCGGGAAATCCACCTGCCCAACAGGTGGGCGTCCGGCGGAAACTGTGTGGCTTGGAACCGGAAGGCGCGACGGGTACGTCCGGGGTAGGAGTGAAATCCCGTAATCCTGGACGGACCGCCGATGGCGAAAGCACGTCGCGAGAACGGATCCGACAGTGAGGGACGAAAGCCAGGGTCTCGAACCGG

>4a42ded1ae61d878277efa96d2060076

CATACTTATGTCTTTAGACAGACAAAGCATTTTTCCAGAATCCATTTCAACCTTGTCGGTCATTTTCAACTGGCAGTGCCTTGTAGATAGTTGCGGAAGATTATAAATGTGACTTGTCCATAGAGATCAGTTTGTTCGTTAATGATTTGGTTGTGTAGTTTGTAACGTAATATATTTAAACTTTCAAGAAGATTCATTTTATTTCCTTTTTTGCCGGTTGTG

>57c2dccb6adba677d7c6bfe138d887fd

TACGGAGGGAGCTAGCGTTGTTCGGAATTACTGGGCGTAAAGCGCACGTAGGCGGCTTTGTAAGTCAGGGGTGAAAGCCTGGAGCTCAACTCCAGAACTGCCTTTGAGACTGCATCGCTTGAATCCGGGAGAGGTGAGTGGAATTCCGAGTGTAGAGGTGAAATTCGTAGATATTCGGAAGAACACCAGTGGCGAAGGCGGCTCACTGGACCGGTATTGACGCTGAGGTGCGAAAGCGTGGGGAGCAAACAGG

>629823d2e49381c30a917604fea81181

CACCACTCATGTCTTTAGAAAGACAAAACATTTCTCCAGGATGCATTTCAACCGTGTCGGTCATTTTCAACTGGCAGTGCCTTGTAGATATTTGTGAAAGATTTTGAACGTGTCTTGTCCATAGAGTTCAGTTTGTTCATTAATGATTTTGTTATGTATTTTGTAACGTAATATATTTAGGCTTTCAAGTACAGTAAAGTCATTTTATTTCCTTTTCTGCCGGTTGCG

>006a7b3285373af2810b693202e3d911

TACAGAGGGTGCAAGCGTTAATCGGATTTACTGGGCGTAAAGCGCGCGTAGGCGGCTAATTAAGTCAAATGTGAAATCCCCGAGCTTAACTTGGGAATTGCATTCGATACTGGTTGGCTAGAGTGTGGGAGAGGATGGTAGAATTCCAGGTGTAGCGGTGAAATGCGTAGAGATCTGGAGGAATACCGATGGCGAAGGCAGCCATCTGGCCTAACACTGACGCTGAGGTGCGAAAGCATGGGGAGCAAACAGG

>cb1c9c4d48627f59971a394a8c0c93b2

AACGTAGGGGGCGAGCGTTGTCCGGAATCACTGGGCGTAAAGGGCGAGTAGGCGGCTGCCGAAGTCGAAAGTGAAAACCCAGGGCTCAACCTTGGGCCTGCTTTCGAAACCAGGCAGCTTGAGTGCAGGAGAGGTAAGTGGAATTCCCGGTGTAGCGGTGAAATGCGTAGATATCGGGAGGAACACCAGTGGCGAAAGCGGCTTACTGGCCTGTAACTGACGCTGAGGCGCGAAAGCGTGGGGAGCAAACAGG

>e5491253316a4c90a1f8390c385869c3

TACGGAGGGGGTTAGCGTTGTTCGGAATTACTGGGCGTAAAGCGCGCGTAGGCGGATTGGAAAGTTGGGGGTGAAATCCCAGGGCTCAACCCCGGAACTGCCTTCAAAACTGATCGTCTGGAGACCGGGAGAGGTGAGTGGAATTCCCAGTGTAGAGGTGAAATTCGTAGATATTCGGAAGAACACCAGTGGCGAAGGCGGCTCACTGGCTCGATACTGACGCTGAGGTGCGAAAGTGTGGGGAGCAAACAGG

>8a9ab2227458786b58a7597599c71495

TACGTAGGGGGCTAGCGTTATCCGGATTTACTGGGCGTAAAGGGTGCGTAGGTGGTTTCTTAAGTCAGGAGTGAAAGGCTACGGCTTAACCGTAGTAAGCTCTTGAAACTGGGAAACTTGAGTGCAGGAGAGGAAAGTGGAATTCCTAGTGCAGCGGTGAAATGCGTAGATATTAGGAGGAACACCAGTAGCGAAGGCGGCTTTCTGGACTGTAACTGACACTGAGGCACGAAAGCGTGGGGAGCAAACAGG

>a699597e0c7b45ba77f7ab9ba21d818e

TACGTAGGGCGCAAGCGTTGTCCGGAATTATTGGGCGTAAAGAGCTTGTAGGTGGCTTGTCGCGTCTGCCGTGAAAACCCGAGGCTCAACCTCGGGCGTGCGGTGGGTACGGGCAGGCTAGAGTGTGGTAGGGGAGACTGGAACTCCTGGTGTAGCGGTGAAATGCGCAGATATCAGGAAGAACACCGATGGCGAAGGCAGGTTTCTGGGCCATTACTGACACTGAGAAGCGAAAGCATGGGTAGCGAACAGG

>6ea51322e836ea6007a00d44cfa04f12

CACACTTATGTCTTCAGAAAGACAAAATATTTCTCCAGGATGCATTTCAACCATGTCGGTCATTTTCAACTGGCAGTGCCTTGTAGATGTTTGTGAAAGATTTTGAATGTGTCTTGCCCATAGAGTTCAGTTTGTTCATTAATGATTTTGTTATGTAGTTTGTAACGTAATATATTTAGGCTTTCAAGTAAATTCATTTTGTTTCCTTTTTTGCCGGTTGTG

>8a1b15f0d18fdf839ceffb52c0ca3ca4

CACGATTAACCCAAGTCAATAGAAGCCGGCGTAAAGAGTGTTTTAGATCACTCCCTCCCCAATAAGGCTAAAACTCACCTGAGTTGTAAAAAACTCCAGTTGACACAAAATAGACTACGAAAGTGGCTTTAACATATCTTAACACACAATAGCTGAGACCCAAACTGGG

>8a43a3be6865c25838141e52f1e4e0fe

TACGAAGGGGGCTAGCGTTGCTCGGAATGACTGGGCGTAAAGGGCGCGTAGGCGGTTGTTACAGTCAGATGTGAAATCCCCGGGCTTAACCTGGGAACTGCATTTGATACGTAACGACTAGAGTTCGAGAGAGGGTTGTGGAATTCCCAGTGTAGAGGTGAAATTCGTAGATATTGGGAAGAACACCGGTGGCGAAGGCGGCAACCTGGCTCGATACTGACGCTGAGGCGCGAAAGCGTGGGGAGCAAACAGG

>367f29caf4dc201175a66d1369d5e2d3

TACATAGGGGGCAAGCGTTATCCGGAATTATTGGGCGTAAAGGGTGCGTAGGCGGTTAAATAAGTTTATGGTCTAAGTGCAATGCTCAACATTGTGATGCTATAAAAACTGTTTAACTAGAGTTGGATAGAGGCAAGTGGAATTCCATGTGTAGTGGTAAAATGCGTAAATATATGGAGGAACACCAGTAGCGAAGGCGGCTTGCTGGGTCTTAACTGACGCTGAGGCACGAAAGCGTGGGGAGCAAACAGG

>bc3cacfb5767a65d7f54284deedd9e95

TACGGAGGGGGCTAGCGTTGTTCGGAATTACTGGGCGTAAAGCGCACGTAGGCGGCTTTGTAAGTTAGAGGTGAAAGCCTGGAGCTCAACTCCAGAATTGCCTTTAAGACTGCATCGCTTGAATCCAGGAGAGGTGAGTGGAATTCCGAGTGTAGAGGTGAAATTCGTAGATATTCGGAAGAACACCAGTGGCGAAGGCGGCTCACTGGACTGGTATTGACGCTGAGGTGCGAAAGCGTGGGGAGCAAACAGG

>9760a27409350f77d0b29b6d06eb325c

TACGTAGGGTGCGAGCGTTAATCGGAATTACTGGGCGTAAAGCGTGCGCAGGCGGTTGTGTAAGACAGGCGTGAAATCCCCGGGCTCAACCTGGGAATGGCGCTTGTGACTGCACGGCTGGAGTGCGGCAGAGGGGGATGGAATTCCGCGTGTAGCAGTGAAATGCGTAGATATGCGGAGGAACACCGATGGCGAAGGCAATCCCCTGGGCCTGCACTGACGCTTATGCACGAAAGCGTGGGGAGCAAACAGG

>7fe55e0a70cb9bee8227eeac6da8e56b

TACGAAGGGGGCTAGCGTTGCTCGGAATCACTGGGCGTAAAGGGCGCGTAGGCGGACTCTTAAGTCGGGGGTGAAAGCCCAGGGCTCAACCCTGGAATTGCCTTGGATACTGAGAGTCTTGAGTTCGGAAGAGGTTGGTGGAACTGCGAGTGTAGAGGTGAAATTCGTAGATATTCGCAAGAACACCAGTGGCGAAGGCGGCCAACTGGTCCGACACTGACGCTGAGGCGCGAAAGCGTGGGGAGCAAACAGG

>9b1109dcd8cdeb0926a372e9d201ffeb

TACCGGCAGCCCGAGTGATGGCCGCTATTATTGGGCCTAAAGCGTCCGTAGCTGGCCAGACAAGTCCGTTGGGAAATCGGCGTGCCTAACACGTCGGCGTCCGGCGGAAACTGTCTGGCTTGGAGCCGGAAGACCCGAGGGGTACGTCCGGGGTAGGAGTGAAATCCTGTAATCCTGGACGGACCACCGATGGCGAAAGCACCTCGGGAAGACGGACTCGACAGTGAGGGACGAAAGCTGGGGTCTCGAACCGG

>a0d52fc02ab404977c723e5cf17ff8eb

TACCGGCAGTCCAAGTGATGGCCGATCTTATTGGGCCTAAAGCGTCCGTAGCTTGCTGTGTAAGTCCATTGGGAAATCGACCAGCTCAACTGGTCGGCGCCCGGTGGAAACTACACAGCTTGGGGCCGAGAGACTCAACGGGTACGTCCGGGGTAGGAGTGAAATCCTGTAATCCTGGACGGACCACCAATGGGGAAACCACGTTGAGAGACCGGACCCGACAGTGAGGGACGAAAGCTCGGGTCTCAAACCGG

>685272b5e881d03d7ca3afdc2ac43542

TACGTAGGGTGCGAGCGTTAATCGGAATTACTGGGCGTAAAGCGTGCGCAGGCGGTTTGTTAAGACAGATGTGAAATCCCCGGGCTCAACCTGGGAACTGCATTTGTGACTGGCAGGCTAGAGTATGGCAGAGGGGGGTAGAATTCTACGTGTAGCAGTGAAATGCGTAGAGATGTGGATGAATACCGATGGCGAAGGCAGCCCCCTGGGCCAATACTGACGCTCATGCACGAAAGCGTGGGGAGCAAACAGG

>5000c50b1cf6af4e14a82273c3821a23

TACGTATGGAGCAAGCGTTATCCGGATTTACTGGGTGTAAAGGGAGTGTAGGTGGCCAGGCAAGTCAGAAGTGAAAGCCCGGGGCTCAACCCCGGGACTGCTTTTGAAACTGCAGGGCTAGAGTGCAGGAGGGGCAAGTGGAATTCCTAGTGTAGCGGTGAGATGCGTAGATATTAGGAGGAACACCAGTGGCGAAGGCGGCTTGCTGGACTGTAACTGACACTGAGGCTCGAAAGCGTGGGGAGCAAACAGG

>6c52571e2e388d847accd578bf775bdf

TACGTGAGAGACTAGTGTTATTCATCCTAATTGGGTTTAAAGGGTACCTAGACAGTCAATATAACTTCTATAATGCTAATACTTGACTAGAGTTTTAAGTAAGAGGGAAGTACTTAAGGAGTAAGAGATGAAATATCTGTGATACCAAAGGGACTCCGTAAAGGCGAAGGCATCCCTTTATCTAAAAACTAACGTTGAAGGACGAAGGCTTAGATAACAAATAGG

>0c12917b23686afb4300aa94a0613910

CATTGAACTATCGTGAGAAAGTCAAACCGCCAAAGGGAATTATATTATAGTAAATATTGGCGTAAATAAACATTGTATTAATAGTTGTAATATATGAAAATGTGCAGAAGATAAAGACGATTAAAACGGTGTTTAACAATCGCTTATCGAGACAATATTTAATTA

>f4fef81cfb712bf529b4b177d026d127

GACGGGGGGGGCAAGTGTTCTTCGGAATGACTGGGCGTAAAGGGCACGTAGGCGGTGAATCGGGTTGAAAGTCAAAGTCGCCAAAAACTGGCGGAATGCTCTCGAAACCAATTCACTTGAGTGAGACAGAGGAGAGTGGAATTTCGTGTGTAGGGGTGAAATCCGTAAATCTACGAAGGAACGCCAAAAGCGAAGGCAGCTCTCTGGGTCCCTACCGACGCTGGGGTGCGAAAGCATGGGGAGCGAACGGG

>2d00e465ad18c4731ecf9573550ac73f

TACGGAGGGAGCTAGCGTTGTTCGGAATTACTGGGCGTAAAGCGCACGTAGGCGGTTACTCAAGTCAGAGGTGAAAGCCCGGGGCTCAACCCCGGAACTGCCTTTGAAACTAGGTGACTGGAATCTTGGAGAGGCGAGTGGAATTCCGAGTGTAGAGGTGAAATTCGTAGATATTCGGAAGAACACCAGTGGCGAAGGCGACTCGCTGGACAAGTATTGACGCTGAGGTGCGAAAGCGTGGGGAGCAAACAGG

>7c5ab074a911dfece6e105bafbd752f9

TACCGGCAGTCCGAGTGATTGCCGATATTATTGGGCCTAAAGCGTCCGTAGCTGGCTGGACAAGTCCGTTGGGAAATCTGCCCGCTTAACGGGCAGGCGTCCAGCGGAAACTGTTCAGCTTGGGACCGGAAGACCTGAGGGGTACGTCTGGGGTAGGAGTGAAATCCCGTAATCCTGGACGGACCGCCGATGGCGAAAGCACGTCGCGAGAACGGATCCGACAGTGAGGGACGAAAGCCAGGGCCTCGAACCGG

>dc8956a28613ef5e6f1bd4baac508d8a

CACGATTAACCCAAGTCAATAGAAGCCGGCGTAAAGAGTGTTTTAGATCACCCCCTCCCCAATAAAGCTAAAATTCACCTGAGTTGTAAAAAACTCCAGTTGACACAAAATAGACTACGAAAGTGGCTTTAACATATCTGAACACACAATAGCTAAGACCCAAACTGGG

>636f36ba451124f365c7ee621232f0be

TACGTAGGGTGCAAGCGTTAATCGGAATTACTGGGCGTAAAGCGTGCGCAGGCGGTTATGCAAGACAGAGGTGAAATCCCCGGGCTCAACCTGGGAACTGCCTTTGTGACTGCATGGCTAGAGTACGATAGAGGGGGATGGAATTCCGCGTGTAGCAGTGAAATGCGTAGATATGCGGAGGAACACCGATGGCGAAGGCAATCCCCTGGACCTGTACTGACGCTCATGCACGAAAGCGTGGGGAGCAAACAGG

>a44185b32b0f2211bd7c462d0a51e2c2

CACGATTAACCCAGGTCAATAGAAGCCGGCGTAAAGAGTGTTTTAGATCACCCCCTCCCCAATAAAGCTAAAACTCACCTGAGTTGTAAAAAACTCCAGTTGACACAAAATAGACTACGAAAGTGGCTTTAACAAATCTGAACACACAATAGCTAAGACCCAAACTGGG

>191a7e8b75fa6f7eae047480b0fb00a0

CGATTCTGATGTCATAGAAACTCGTCTACAAATGCCCTGAAGACAACAATAAATTTTTTCGAAACGTGTGCGAAAATTCCTTAAATTGTTTCGTTAATTGCCATAACCTTGATAAGCGTTTCTGCATTTATCATTTCACGTTCGATACTAAAATTAGTCTTTAGTACTGTTTATTATTGATTTTTTTTTTTACACATTTTATGACCAC

>0fc1b4b38c76ff1d5614d73496426a3e

TACGTAGGGTGCGAGCGTTGTCCGGATTTACTGGGCGTAAAGGGCTCGTAGGTGGTGTGTTGCGTCGTCTGTGTAATCCAGGGGCTTAACTTTTGGTTGGCAGGCGATACGGGCATTGCTTGAGTGCTGTAGGGGAGACTGGAATTCCTGGTGTAGCGGTGAAATGCGCAGATATCAGGAGGAACACCGATGGCGAAGGCAGGTCTCTGGGCAGTTACTGACGCTGAGGAGCGAGAGCATGGGTAGCGAACAGG

>01c50bfc7a1d4dce14e9f790fbb94fba

TACCGGCAGTCCAAGTGATGGCCGATATTATTGGGCCTAAAGCGTCCGTAGCTTGCTGTGTAAGTCCATTGGGAAATCGACGCGCTCAACGCGTCGACGTCCGGTGGAAACTACACGGCTTGGGGCCGAGAGACTCGACGGGTACGTCCGGGGTAGGAGTGAAATCCTGTAATCCTGGACGGACCACCAATGGGGAAACCACGTCGAGAGACCGGACCCGACAGTGAGGGACGAAAGCCAGGGTCTCGAACCGG

>578c493ebd3fdb0b96ec0c11d19db362

TTAAACGAAAGCACAACAAAAAGATAGCCAAACTAATACCACCCCAATCACCAGCAATAATACCCACACATACGTTTTACCCAAGAATAGTTAACCTAACCAACGTAACATTCACTAACGAACAGAAACAACTTTTAAACAAAGGGATAAACCACAACCTACACTACACACAGAATAATAACACCATCAAGAACATGGT

>a4b812739599e62138a4139a73025693

TACGTAGGTGGCAAGCGTTGTCCGGATTTATTGGGCGTAAAGCGCGCGCAGGCGGTCTTTTAAGTCTGATGTGAAAGCCCCCGGCTTAACCGGGGAGGGTCATTGGAAACTGGAAGACTGGAGCGCAGAAGAGGAGAGTGGAACTCCACGTGTAGCGGTGAAATGCGTAGAGATGTGGAGGAACACCAGTGGCGAAGGCGACTCTCTGGTCTGTAACTGACGCTGAGGAGCGAAAGCGTGGGGAGCGAACAGG

>684a42d9432bd05b91b8fb53d75706ca

TACGTAGGTGGCGAGCGTTATCCGGATTTACTGGGCGTAAAGGGAGCGTAGGCGGATGATTAAGTGGGATGTGAAATACCCGGGCTCAACTTGGGTGCTGCATTCCAAACTGGTTATCTAGGGTGCAGGAGAGGAGAGTGGAATTCCTAGTGTAGTGGTGAAATGCGTAGAGATTAGGAAGAACACCAGTGGCGAAGGCGACTCTCTGAACTGTAACTGACGCTGAGGCTCGAAAGCGTGGGGAGCAAACAGG

>a9a7004d015a0f988266a82c0c15a853

TACGTAGGTGGCAAGCGTTATCCGGATTTATTGGGCGTAAAGAGAGTGCAGGCGGTTTTCTAAGTCTGATGTGAAAGCCTTCGGCTTAACCGGAGAAGTGCATCGGAAACTGGATAACTTGAGTGCAGAAGAGGGTAGTGGAACTCCATGTGTAGCGGTGGAATGCGTAGATATATGGAAGAACACCAGTGGCGAAGGCGACTTTCTGGTCTGTAACTGACGCTGATGTGCGAAAGCGTGGGGATCAAACAGG

>dec7393cb02127d40c654340757f6afe

CACGATTAACCCAAGTAAATAGAAGCCGGCGTAAAGAGTGTTTTAGATCACCCCCTCCCCAATAAAGCTAAAACTCACCTGAGTTGTAAAAAACTCCAGTTGACACAAAATAGACTACGAAAGTGGCTTTAACATATCTGAACACACAATAGCTAAGACCCAAACTGGG

>a6def887bac9fa20f66ba28077dbb75d

TACGTAGGTGGCAAGCGTTGTCCGGAATTATTGGGCGTAAAGCGCGCGCAGGCGGTTCCTTAAGTCTGATGTGAAAGCCCACGGCTCAACCGTGGAGGGTCATTGGAAACTGGGGAACTTGAGTGCAGAAGAGAAGAGCGGAATTCCACGTGTAGCGGTGAAATGCGTAGAGATGTGGAGGAACACCAGTGGCGAAGGCGGCTCTTTGGTCTGTAACTGACGCTGAGGCGCGAAAGCGTGGGGAGCAAACAGG

>2fc1b7579c8889d7b6660b8e68ea9277

CACGATTAACCCAAGTCAATAGAAGCCGGCGTAAAGAGTGTTTTAGATCACCCCCTTCCCAATAAAGCTAAACTCACCTGAGTTGTAAAAAACTCCAGTTGACACAAAATAGACTACGAAAGTGGCTTTAACATATCTGAACACACAATAGCTAAGACCCAAACTGGG

>6e3083a22a3cc975f36eba77be1afd89

CACGATTAACCCAAGTCAATAGAAGCCGGCGTAAAGAGTGTTTTAGATCACCCCCTCCCCAATAAAGCTAAAACTCACCTGAGTTGTAAAAAACTCCAGTTGACACAAAATAGACTACGAAAGTGGCTTTAACATGTCTGAACACACAATAGCTAAGACCCAAACTGGG

>64ac880ca5dd603e484da45fb4a93dc8

CATGGATGATACAGGAGCTCATTGGGCTGAGACCCAGACAGCGCACACACGATTCGGGTGCTAGACACCCCGCACAGCATCAGTCAGTCAGTAATATTGGTTATCGTAGGGCTATTGATCTACTAAATAACTGCGACAAAACTATTTACCCATCTATTCACTTTCTACTAAAGATTGCTGTCACCTTGTCATATTCTGTTCCAACTGCTGATAGAACATTATCATTGCTCAGAAGACTTACAACTTAG

>0703af4c34ef8d269ad11aca5a72ff41

TACGTAGGGGGCAAGCGTTATCCGGATTTACTGGGTGTAAAGGGAGCGTAGACGGCGAAGCAAGTCTGGTGTGAAAACCCGGGGCTCAACCCCGGGCCTGCATTGGAAACTGTTTTGCTTGAGTGCCGGAGAGGTAAGCGGAATTCCTAGTGTAGCGGTGAAATGCGTAGATATTAGGAGGAACACCAGTGGCGAAGGCGGCTTACTGGACTGTAACTGACGTTGAGGCTCGAAAGCGTGGGGAGCAAACAGG

>4c57d4ca76e7954b09b3d93dcc0865fb

TACGTAGGGTGCGAGCGTTGTCCGGAATTATTGGGCGTAAAGAGCTTGTAGGCGGTTTGTCGCGTCTGCCGTGAAAACCTAGGGCTTAACTCTGGGCGTGCGGTGGGTACGGGCAGGCTAGAGTGTGGTAGGGGAGACTGGAATTCCTGGTGTAGCGGTGAAATGCGCAGATATCAGGAGGAACACCGATGGCGAAGGCAGGTCTCTGGGCCATAACTGACGCTGAGAAGCGAAAGCATGGGGAGCGAACAGG

>2d10d3bfdbfd9ecc4bdc0b127b9d27dd

TACCGGCAGCTCAAGTGATGTCCCATATTATTGGGCCTAAAGCGTCCGTAGCTGGCCGACCAAGTCTATCGGGAAATCCACCTGCCCAACAGGTGGGCGTCCGGTAGAAACTGGCCGGCTTGGAACCGGAAGGCTCAGAGAGTACGTCCGGGGTAGGAGTGAAATCCTGTAATCCTAGACGGACCACCAATGGGGAAACCACGTCAAGAGACCGGACCCGACAGTGAGGGACGAAAGCCAGGGTCTCGAACCGG

>210bd5d7bc6bb2f821d494f43eab4ca2

TACGTAGGTGGCAAGCGTTGTCCGGAATTATTGGGCGTAAAGCGCGCGCAGGCGGTCTCTTAAGTCCATCTTAGAAGTGCGGGGCTTAACCCCGTGAGGGGATGGAAACTGGGAGACTGGAGTATCGGAGAGGAAAGTGGAATTCCTAGTGTAGCGGTGAAATGCGTAGATATTAGGAAGAACACCGGTGACGAAGGCGACTTTCTGGACGAAAACTGACGCTGAGGCGCGAAAGCGTGGGGAGCAAACAGG

>78b22e16bbafa8a3e7e80e3dcc9bd7f2

TACCGGCAGCTCGAGTGATGACCGATCTTATTGGGCCTAAAGCGTCCGTAGCTGGCCGCGCAAGTCCATCGGGAAATCCACCTGCTCAACAGGTGGGCGCCCGGTAGAAACTGCGTGGCTTGGGACCGGAAGGCGCGACGGGTACGTCCAGGGTAGGAGTGAAATCCCGTAATCCTGGACGGACCGCCGGTGGCGAAAGCGCCTCGGGAAGACGGATCCGACGGTGAGGGACGAAAGCTTGGGTCACGAACCGG

>fb8f2c3d9a31337cb9e7ba97e675aa28

TACGTAGGGTGCGAGCGTTAATCGGAATTACTGGGCGTAAAGCGTGCGCAGGCGGTTTGTTAAGACAGATGTGAAATCCCCGGGCTCAACCTGGGAACTGCATTTATGACTGGCAGGCTAGAGTATGGCAGAGGGGGGTAGAATTCCACGTGTAGCAGTGAAATGCGTAGAGATGTGGAGGAATACCGATGGCGAAGGCAGCCCCCTGGGCCAATACTGACGCTCATGCACGAAAGCGTGGGGAGCAAACAGG

>a2f9720063c0ca45aecd583c2fe2adac

TACCGGCAGCTCGAGTGATGGCCGATCTTATTGGGCCTAAAGCGTCCGTAGCTGGCCGAACAAGTCCATCGGGAAATCCATCCGCTCAACGGATGGGCGTCCAGCGGAAACTGTTCGGCTTGGGGCCGGAAGACCTGAGGGGTACGTCCGGGGTAGGAGTGAAATCCCGTAATCCCGAACGGACCACCGATGGCGAAAGCACCTCGAGAAGACGGCTCCGACAGTGAGGGACGAAAGCTAGGGTCTCGAACCGG

>801cc09fd1b2467a8d28af3f5abf9998

TACCGGCAGCTCAAGTGATGTCCCATATTATTGGGCCTAAAGCGTCCGTAGCTGGCCAACCAAGTCTATCGGGAAATCCACCCGCCCAACGGGTGGGCGTCCGGTAGAAACTGGCTGGCTTGGAACCGGAAGGCTCAGAGAGTACGTCCGGGGTAGGAGTGAAATCCCGTAATCCCGGACGGACTACCGATGGCGAAAGCACTCTGAGAAGACGGCTTCGACAGTGAGGGACGAAAGCTCGGGTCTCAAACCGG

>12eb04d3618652efc4b95ea622813e88

CAAATTTATGTCTTCAGAAAGACAAAATATTTTTCTAGGGTCCATTTCAACCATGTCGGTCATTTTCAACTAGCAGTGCCTTGTAGATATTTGTGAAAGATTTTGAATGTGTCTTGTCCATAGAGTTCAGTTTGTTCATTAATGATTTTGTTATGTAGTTTGTGACGTAATATATTTAGGCTTTCAAGTAAATTGATTTTGTTTCCTTTTTCCGTTTGTG

>6a9efbd74c28e4c03f1a50c9485c86aa

AATCCTTGTGCCGTGAACCGAAATAGTTCCGGAAACGAGGTTCTATTCCAAACTACGAAATGAAGAAGAGGAAGAGGAAGAAGAAGAAATCTGCGAGATCACTATGTGCTTCACACGACGTGATGATCGTATTGCATGCGTTTAGCGAGCAACACTATGACCACGCATGCTAAAGCTAAAGGGATAATTATTTT

>73eb8fa8648db35bd2f286bbbe6791da

TACGAAGGGGGCTAGCGTTGTTCGGAATCACTGGGCGTAAAGGGTGCGTAGGCGGGCATTTAAGTCAGGGGTGAAATCCCGAGGCTCAACCTCGGAACTGCCCTTGATACTGGATGTCTTGAGTCCGGAAGAGGTGAGTGGAACTGCGAGTGTAGAGGTGAAATTCGTAGATATTCGCAAGAACACCGGTGGCGAAGGCGGCTCACTGGTCCGGAACTGACGCTGAGGCACGACAGCGTGGGGAGCAAACAGG

>bc56a7361c9a3b49f1f1c51874321e12

TACGTAGGGCGCAAGCGTTGTCCGGAATTATTGGGCGTAAAGAGCTCGTAGGCGGTTTGTCGCGTCTGCTGTGAAAGCCCGGGGCTTAACCCCGGGTGTGCAGTGGGTACGGGCAGACTTGAGTGCAGTAGGGGAGACTGGAATTCCTGGTGTAGCGGTGAAATGCGCAGATATCAGGAGGAACACCGATGGCGAAGGCAGGTCTCTGGGCTGTTACTGACGCTGAGGAGCGAAAGCATGGGGAGCGAACAGG

>3039a2b3e40ab419660200c71468c1ab

CACCGGCAGCCCAAGTGATGGCCGATATTATTGGGCCTAAAGCGTCCGTAGCTGGCCGCACAAGTCCGTCGGGAAATCCACTCGCTTAACGAGTGGGCGTCCGGCGGAAACTGTGCGGCTTGGGACCGGAAGATCCAAGGGGTACGTCCGGGGTAGGAGTGAAATCCCGTAATCCTGGACGGACCGCCGGTGGCGAAAGCGCCTCGAGAGGACGGACCCGACGGTGAGGGACGAAAGCTAGGGTCTCGAACCGG

>3251125cebcbd205c98531c7143c09f6

TACGTAGGTGGCAAGCGTTGTCCGGAATTATTGGGCGTAAAGCGCGCGCAGGCGGCACCTTAAGTCTGATGTGAAATCTTGCGGCTCAACCGCAAGCGGTCATTGGAAACTGGGGCGCTTGAGTGCAGAAGAGGAGAGCAGAATTCCACGTGTAGCGGTGAAATGCGTAGAGATGTGGAGGAATACCGGTAGCGAAGGCGGCTCTCTGGTCTGTTACTGACGCTGAGGTGCGAAAGCGTGGGGAGCGAACAGG

>37eb45558c1b50dd0340359c5151b0fc

TACAGAGGGTGCAAGCGTTAATCGGATTTACTGGGCGTAAAGCGCGCGTAGGCGGCTAATTAAGTCAAATGTGAAATCCCCGAGCTTAACTTGGGAATTGCATTCGATACTGGTTAGCTAGAGTGTGGGAGAGGATGGTGGAATTCCAGGTGTAGCGGTGAAATGCGTAGAGATCTGGAGGAATACCGATGGCGAAGGCAGCCATCTGGCCTAACACTGACGCTGAGGTGCGAAAGCATGGGGAGCAGACAGG

>5ca69e987482826ec25fd58696ceadd8

TACCGGCAGTCCGAGTGATGGCCGATATTATTGGGCCTAAAGCGTCCGTAGCCTGCTGTGTAAGTCCATTGGGAAATCGACCAGCTCAACTGGTCGGCGTCCGGTGGAAACTACACAGCTTGGGGCCGAGAGACTCAACGGGTACGTCCGGGGTAGGAGTGAAATCCTGTAATCCTGGACGGACCACCAATGGGGAAACCACGTTGAGAGACCGGACCCGACAGTGAGGGACGAAAGCCAGGGTCTCGAACCGG

>1b831f4878d96360a84536ea3892e678

TACCGGCAGTCCGAGTGATGGCCGATCTTATTGGGCCTAAAGCGTCCGTAGCTGGCTAGACAAGTCCGTTGGGAAATCTGTCCGCTTAACGGGCAGGCGTCCAGCGGAAACTGTGTAGCTTGGGACCGGAAGACCTGAGGGGTACGTCTGGGGTAGGAGTGAAATCCCGTAATCCTGGACGGACCACCGGTGGCGAAAGCGCCTCGGGAAGACGGATCCGACGGTGAGGGACGAAAGCTTGGGTCACGAACCGG

>7ebbb1e6f79a2910951c191a4bf1df66

TACGTAGGGTGCGAGCGTTAATCGGAATTACTGGGCGTAAAGCGTGCGCAGGCGGTTGTGTAAGACAGGTGTGAAATCCCCGGGCTTAACCTGGGAACTGCGCTTGTGACTGCACGGCTAGAGTATGGCAGAGGGGGGTGGAATTCCACGTGTAGCAGTGAAATGCGTAGAGATGTGGAGGAACACCGATGGCGAAGGCAGCCCCCTGGGCCAATACTGACGCTCATGCACGAAAGCGTGGGGAGCAAACAGG

>5a7b179b1b45f0fe2282f260bf073f60

TACGTAGGGTGCGAGCGTTGTCCGGATTTATTGGGCGTAAAGGGCTCGTAGGTGGTTGATCGCGTCGGAAGTGTAATCTTGGGGCTTAACCCTGAGCGTGCTTTCGATACGGGTTGACTTGAGGAAGGTAGGGGAGAATGGAATTCCTGGTGGAGCGGTGGAATGCGCAGATATCAGGAGGAACACCAGTGGCGAAGGCGGTTCTCTGGGCCTTTCCTGACGCTGAGGAGCGAAAGCGTGGGGAGCGAACAGG

>10002ab838f2095c6c33ddf6818bfbb9

TACGTAGGGTGCGAGCGTTAATCGGAATTACTGGGCGTAAAGCGTGCGCAGGCGGTTTGTTAAGACAGATGTGAAATCCCCGGGCTCAACCTGGGAACTGCATTTGTGACTGGCAGGCTAGAGTATGGTAGAGGGGGGTAGAATTCCACGTGTAGCAGTGAAATGCGTAGAGATGTGGAGGAATACCGATGGCGAAGGCAGCCCCCTGGGCCAATACTGACGCTCATGCACGAAAGCGTGGGGAGCAAACAGG

>556bdecf95e6dc0e758c6951d2f9ed19

TACGTAGGGTGCGAGCGTTAATCGGAATTACTGGGCGTAAAGCGTGCGCAGGCGGTTTGTTAAGACAGATGTAAAATCCCCGGGCTCAACCTGGGAACTGCATTTGTGACTGGCAGGCTAGAGTATGGCAGAGGGGGGTAGAATTCCACGTGTAGCAGTGAAATGCGTAGAGATGTGGAGGAATACCGATGGCGAAGGCAGCCCCCTGGGCCAATACTGACGCTCATGCACGAAAGCGTGGGGAGCAAACAGG

>6f728b0fa7a093b4bdff1fabdd579fe8

TACCGGCAGCACGAGTGATGGCCGATCTTATTGGGCCTAAAGCGTCCGTAGCCGGCCGAGCAAGTTCGTCGGGAAATCCGCCAGCCCAACTGGCGGGCGTCCGGTGGAAACTGCACGGCTCGGGACCGGAAGACCCGAAGGGTACGTCTTGGGTAGGAGTGAAATCCCGTAATCCTGGACGGACCACCGATGGCGAAAGCACTTCGGGAAGACGGATCCGACGGTGAGGGACGAAAGCCAGGGTCTCGAACCGG

>a9c40b8fe5d98b2dcce842bd1a01c95f

TACGTAGGTGGCAAGCGTTGTCCGGATTTATTGGGCGTAAAGCGAGCGCAGGCGGTCTTTTAAGTCTGATGTGAAAGCCCCCGGCTTAACCGGGGAGGGTCATTGGAAACTGGGAGACTTGAGTGCAGAAGAGGAAAGCGGAATTCCATGTGTAGCGATGAAATGCGTAGATATATGGAGGAACACCAGTGGCGAAGGCGGCTTTCTGGTCTGTAACTGACGCTGAGGCTCGAAAGCGTGGGGAGCAAACAGT

>367537fe69982b2d80f856a4d9e02f8f

TACGTAGGTGGCAAGCGTTATCCGGAATTATTGGGCGTAAAGCGCGCGCAGGTGGTTTCTTAAGTCTGATGTGAAAGCCACGGCTCAACCGTGGAGGGTCATTGGAAACTGGGAGACTTGAGTGCAGAAGAGGAAAGTGGAATTCCATGTGTAGCGGTGAAATGCGTAGAGATATGGAGGAACACCAGTGGCGAAGGCGACTTTCTGGTCTGTAACTGACACTGAGGCGCGAAAGCGTGGGGAGCAAACAGG

>7e14404fb846fbd4f3bf632d83e812d0

CACACTTATGTCTTTAGAAAGACAAAACTTTTTTCCAGAATGCATTTCAACCATGTCGGTCATTTTCAACTGACAGTGCCTTGTAGACAGTTGTGGAAGATTTTGAATGTGTCTTGTCCATAGAGTTCAGTTTGTTCATTAATGATTTTGTTATGTAGTCTGTAACGTAGTATGTTTAGACTTTCAAGTAAATTCATTTTATTTCTTTTTTGTCAGTTGTA

>9dce3c93948777131431ce20b70240b2

CACGATTAACCCAAGCCAATAGAAGCCGGCGTAAAGAGTGTTTTAGATCACCCCCTCCCCAATAAAGCTAAAACTCACCTGAGTTGTGAAAAACTCCAGTTGACACAAAATAGACTACGAAAGTGGCTTTAACATATCTGAACACACAATAGCTAAGACCCAAACTGGG

>a2135d1620f0fc101db4630a3d9280a6

TACCGGCAGCTCGAGTGATGGCCGATCTTATTGGGCCTAAAGCGTCCGTAGCTTGCTGTGTAAGTCCATTGGGAAATCGACGCGCTCAACGCGTCGACGTCCGGTGGAAACTACACGGCTTGGGGCCGAGAGACTCGACGGGTACGTCCGGGGTAGGAGTGAAATCCCGTAATCCTGGACGGACCACCAATGGGGAAACCACGTCGAGAGACCGGACCCGACAGTGAGGGACGAAAGCCAGGGTCTCGAACCGG

>4f2cec56018d70fe8eb6b66f32e1f11b

TACGGAGGGTGCAAGCGTTATCCGGATTTATTGGGTTTAAAGGGTCCGTAGGCAGATTTGTAAGTCAGTGGTGAAATCTCACAGCTTAACTGTGAAACTGCCATTGATACTGCAAGTCTTGAGTGTTGTTGAAGTAGCTGGAATAAGTAGTGTAGCGGTGAAATGCATAGATATTACTTAGAACACCAATTGCGAAGGCAGGTTACTAAGCAACAACTGACGCTGATGGACGAAAGCGTGGGGAGCGAACAGG

>a8c7ad753953cd583a16e62b8cc4ac57

TACGGAGGGTGCAAGCGTTATCCGGATTCACTGGGTTTAAAGGGTGCGTAGGCGGGTAGGTAAGTCAGTGGTGAAATCTCGGAGCTTAACTCCGAAACTGCCATTGATACTATCTATCTTGAATATTGTTGAGGTTTGCGGAATATGTCATGTAGCGGTGAAATGCATAGATATGACATAGAACACCTATTGCGAAGGCAGCAGGCTAAACATATATTGACGCTGAGGCACGAAAGCGTGGGGATCAAACAGG

>a4ec09c37d2e8adbe679fedd0184808f

TACCGGCAGCTCAAGTGATGTCCCATATTATTGGGCCTAAAGCGTCCGTAGCTGGCCAACCAAGTCCATCGGGAAATCCACCCGCCCAACGGGTGGGCGTCCGGTGGAAACTGGCTGGCTTGGAACCGGAAGGCTCAGAGAGTACGTCCGGGGTAGGAGTGAGATCCCGTAATCCCGGACGGACTACCGATGGCGAAAGCACTCTGAGAAGACGGCTTCGACAGTGAGGGACGAAAGCTAGGGTCTCAAACCGG

>9c42ceba122d822eafeecf3d5f8bcbf3

TACGTAGGGTGCGAGCGTTAATCGGAATTACTGGGCGTAAAACGTGCGCAGGCGGTTTGTTAAGACAGATGTGAAATCCCCGGGCTCAACCTGGGAACTGCATTTGTGACTGGCAGGCTAGAGTATGGCAGAGGGGGGTAGAATTCCACGTGTAGCAGTGAAATGCGTAGAGATGTGGAGGAATACCGATGGCGAAGGCAGCCCCCTGGGCCAATACTGACGCTCATGCACGAAAGCGTGGGGAGCAAACAGG

>9bb276530d60c2d320b7fd8bc8cf8613

TACGTAGGGTGCGAGCGTTAATCGGAATTACTGGGCGTAAAGCGTGCGCAGGCGGTGATGTAAGACAGATGTGAAATCCCCGGGCTCAACCTGGGAACTGCATTTGTGACTGCATCGCTGGAGTGCGGCAGAGGGGGATGGAATTCCGCGTGTAGCAGTGAAATGCGTAGATATGCGGAGGAACACCGATGGCGAAGGCAATCCCCTGGGCCTGCACTGACGCTCATGCACGAAAGCGTGGGGAGCAAACAGG

>fa75e4a8d77483c82a344c520c19f153

TACGTAGGTGACAAGCGTTGTCCGGATTTATTGGGCGTAAAGCGAGCGCAGGCGGTCCGGTAAGTCTGATGTGAAAGCCCACGGCTCAACCGTGGAACGGCATTGGAAACTGGCGGACTTGAATGTAGCAGAGGAAAGTGGAATTCCATGTGTAGCGGTGGAATGCGTAGATATATGGAGGAACACCAGTGGCGAAAGCGACTTTCTGGGCTATGATTGACGCTGAGGCTCGAAAGCGTGGGGAGCGAACAGG

>26c9a9f47420d79637cc5cf386e54667

CACATGTCTTACGATTGTTTTAAATACTTTAACCTTACTTCGTGTGTTGAGGTGAGGTAACGTAGCACCTGCTTTGCTATTACTTGACGGTTGTCGATTTAGATGGTCTATCCATTCCTTTCTAAATTCCTTTATTTTGTTACTTAGATAATAGATTCACAATTCATCTCTTATTTTCTCATTTTCATTGTCTCCTGAAAAATTT

>c3dc283bb4863be2eed27332044390ac

TACAGAGGGTGCAAGCGTTAATCGGATTTACTGGGCGTAAAGCGCGCGTAGGCGGCTAATTAAGTCAAATGTGAAATCCCCGAGCTTAACTTGGGTATTGCATTCGATACTGGTTAGCTAGAGTGTGGGAGAGGATGGTAGAATTCCAGGTGTAGCGGTGAAATGCGTAGAGATCTGGAGGAATACCGATGGCGAAGGCAGCCATCTGGCCTAACACTGACGCTGAGGTGCGAAAGCATGGGGAGCAAACAGG

>2d18587c9ac09f6093b613d6f49c139e

TACGTAGGGTGCAAGCGTTAATCGGAATTACTGGGCGTAAAGCGTGCGCAGGCGGTTATGCAAGACAGAGGTGAAGTCCCCGGGCTCAACCTGGGAACTGCCTTTGTGACTGCATGGCTAGAGTACGGTAGAGGGGGATGGAATTCCGCGTGTAGCAGTGAAATGCGTAGATATGCGGAGGAACACCGATGGCGAAGGCAATCCCCTGGACCTGTACTGACGCTCATGCACGAAAGCGTGGGGAGCAAACAGG

>720ca43425bd4323fb341f3b58faafd1

CACGATTAACCCAAGTCAATAGAAGCCGGCGTAAAGAGTGTTTTAGATCACCCCCTCCCCAATAAAGCTAAAACTCACCTGAGTTGTAAAAAACTCCAGTTGACACAAAATAGACTACGAAAGTGGCTTTAACATATCTGGACACACAATAGCTAAGACCCAAACTGGG

>b075ffa5f46e41ba6886029b04d77987

TACGTAGGGTGCGAGCGTTAATCGGAATTACTGGGCGTAAAGCGTGCGCAGGCGGTTTGTTAAGACAGATGTGAAATCCCCGGGCTCAACCTGGGAACTGCATTTGTGACTGGCAGGCTAGAGTATGGCAGAGGGGGGTAGAATTCCACGTGTAGCAGTGAAATGCGTAAAGATGTGGAGGAATACCGATGGCGAAGGCAGCCCCCTGGGCCAATACTGACGCTCATGCACGAAAGCGTGGGGAGCAAACAGG

>2d9b102c7fcde2240e3e24bc15289c5a

TACCGGCAGCTCAAGTGATGTCCCATATTATTGGGCCTAAAGCGTCCGTAGCTGGCCGCACAAGTCCGTCGGGAAATCCACCTGCCCAACAGGTGGGCGTCCGGCGGAAACTGTGTGGCTTGGAACCGGAAGGCTCAGAGAGTACGTCCGGCGTAGGAGTGAAATCCCGTAATCCCGGACGGACTACCGATGGCGAAAGCACTCTGAGAAGACGGCTTCGACAGTGAGGGACGAAAGCCAGGGTCTCGAACCGG

>490a8050e50f8090ac35f2f56579facd

TACCGGCAGCACGAGTGATGACCGATATTATTGGGCCTAAAGCGTCCGTAGCTGGCCAAGCAAGTCCATTGGGAAATCGACGTGCTCAACGCGTCGGCGTCCGGTGGAAACTGTTTGGCTTGGGGCCAGAAGACCTGAGGGGTACGTCCGGGGTAGGAGTGAAATCCTGTAATCCTGGACGGACCACCAATGGGGAAACCACCTCAGGAAGACGGACCCGACGGTGAGGGACGAAAGCTAGGGTCTCGAACCGG

>e5cac2513ac4fd8da8f0aac8fe809b6e

CGTTTACTTGATGACGCAATCACTTTCATCAGTTTCGTCATTATCTCAGCAGCGTGACAATACAATTGAGGCCGACACAGGGAAACAAGTAAATGGATAGACAAATAAACTCGGAAAAAAGATATTGTTTACAGAATTAAGAAAATTTAATTCAAGCCGGCAGGAATTTTGGGTAGGCGAGAAAATAGACGCTTACTAAGTTTTCTAG

>85da8003768c119882171c7ea9200ff5

TACCGGCAGCTCAAGTGATGTCCCATATTATTGGGCCTAAAGCGTCCGTAGCTGGCTGGACAAGTCCGTTGGGAAATCGGCGTGCCTAACGCGTCGGCGTCCGGCGGAAACTACACAGCTTGGGGCCGAGAGACTCAACGGGTACGTCCGGGGTAGGAGTGAAATCCCGTAATCCTGGACGGACCGCCGGTGGCGAAAGCGCCTCGAGAGGACGGACCCGACGGTGAGGGACGAAAGCTAGGGTCTCGAACCGG

>7d174a43769a935bb68c2a3fe99d713c

TACGGAGGGTGCAAGCGTTAATCGGAATTACTGGGCGTAAAGCGTGCGCAGGCGGCTGATTAAGTCGGATGTGAGAGCCCCGGGCTCAACCTGGGAATGGCATTCGATACTGGTCAGCTAGAGTCTGGTAGAGGTAAGCGGAATTCCGGGTGTAGCGGTGAAATGCGTAGATATCCGGAGGAACATCAGTGGCGAAGGCGGCTTACTGGACCAAGACTGACGCTCAGGCACGAAAGCGTGGGTAGCAAACAGG

>7320ecb6ee6a12e65d6ffd5d30386f4c

TACCGGCAGTCCGAGTGATGGCCGATATTATTGGGCCTAAAGCGTCCGTAGCTTGCTGTGTAAGTCCATTGGGAAATCGACGCGCTCAACGCGTCGACGTCCGGTGGAAACTACACGGCTTGGGGCCGAGAGACTCGACGGGTACGTCCGGGGTAGGAGTGAAATCCTGTAATCCTGGACGGACCACCAATGGGGAAACCACGTTGAGAGACCGGACCCGACAGTGAGGGACGAAAGCCAGGGTCTCGAACCGG

>649f2efd13df2dff8125deb6f1a44f58

TACGGAGGGTGCGAGCGTTAATCGGAATTACTGGGCGTAAAGCGCGCGTAGGTGGCTTGATAAGCCGGTTGTGAAAGCCCCGGGCTCAACCTGGGAACGGCATCCGGAACTGTCAAGCTAGAGTGCAGGAGAGGAAGGTAGAATTCCCGGTGTAGCGGTGAAATGCGTAGAGATCGGGAGGAATACCAGTGGCGAAGGCGGCCTTCTGGACTGACACTGACACTGAGGTGCGAAAGCGTGGGTAGCAAACAGG

>1fee76576d77f7da4af5cbff889ad0f3

TACAGAGACTGCAAGCGTTATTCGGATTCACTGGGCGTAAAGGGTGCGCAGGCGGCTGGGTGTGTCAGATGTGAAATCCCGAGGCTTAACCTCGGAACTGCGTCTGAAACTACTCGGCTAGAGTATTGGAGAGGGTAACGGAATTCACGGTGTAGCAGTGAAATGCGTAGATATCGTGAGGAACACCAGAGGCGAAGGCGGTTACCTGGACAATTACTGACGCTCAGGCACGAAAGCATGGGGAGCAAAAGGG

>77f22c27b06cf72e8ab048cf20f8725b

TACGTAGGGTGCGAGCGTTAATCGGAATTACTGGGCGTAAAGCGTGCGCAGGCGGTTTGTTAAGACAGATGTGAAATCCACGGGCTCAACCTGGGAACTGCATTTGTGACTGGCAGGCTAGAGTATGGCAGAGGGGGGTAGAATTCCACGTGTAGCAGTGAAATGCGTAGAGATGTGGAGGAATACCGATGGCGAAGGCAGCCCCCTGGGCCAATACTGACGCTCATGCACGAAAGCGTGGGGAGCAAACAGG

>9215bccf440af12ae35dfd490cd7492e

TACGTAGGGTGCGAGCGTTAATCGGAATTACTGGGCGTAAAGCGTGCGCAGGCGGTTTGTTAAGACAGATGTGAAATCCCCGGGCTCAACCTGGGAACTGCATTTGTGACTGGCATGCTAGAGTATGGCAGAGGGGGGTAGAATTCCACGTGTAGCAGTGAAATGCGTAGAGATGTGGAGGAATACCGATGGCGAAGGCAGCCCCCTGGGCCAATACTGACGCTCATGCACGAAAGCGTGGGGAGCAAACAGG

>9d12cc39081e2b6de9e8f3b15fa6e451

TACCGGCAGTTCGAGTGATGGCCGATATTATTGGGCCTAAAGCGTCCGTAGCTGGCCAAGCAAGTCCATTGGGAAATCGACGCGCTCAACGCGTCGGCGTCCGGTGGAAACTGTTTGGCTTGGGGCCAGAAGACCTGAGGGGTACGTCCGGGGTAGGAGTGAAATCCTGTAATCCTGGACGGACCACCAATGGGGAAACCACGTCGAGAGACCGGACCCGACAGTGAGGGACGAAAGCCAGGGTCTCGAACCGG

>06433221f946e2bc929aabc23f37a146

TACCGGCAGCCCGAGTGATGGCCGATCTTATTGGGCCTAAAGCGTCCGTAGCTGGCCGCGCAAGTCTATCGGGAAATCCACCTGCCCAACAGGTGGGCGTCCGGTAGAAACTGGCCGGCTTGGAACCGGAAGGCTCAGAGAGTACGTCCGGGGTAGGAGTGAAATCCCGTAATCCCGGACGGACTACCGATGGCGAAAGCACTCTGAGAAGACGGCTTCGACAGTGAGGGACGAAAGCTCGGGTCTCAAACCGG

>c890b5bfec4e8d6d1795c4d1ccd09dc8

TACGGAGGGTGCAAGCGTTAATCGGAATTACTGGGCGTAAAGCGCACGCAGGCGGTCTGTTAAGTCAGATGTGGAATCCCCGGGCTTAACCTGGGAACTGCATTTGAAACTGGCAGGCTTGAGTCTTGTAGAGGGGGGTAGAATTCCAGGTGTAGCGGTGAAATGCGTAGAGATCTGGAGGAATACCGGTGGCGAAGGCGGCCCCCTGGACAAAGACTGACGCTCAGGTGCGAAAGCGTGGGGAGCAAACAGG

>c1b2a338f06bf5cfdc8fb86b8a9b65f3

CACGATTAACCCAAGTCAATAGAAGCCGACGTAAAGAGTGTTTTAGATCACCCCCTCCCCAATAAAGCTAAAACTCACCTGAGTTGTAAAAAACTCCAGTTGACACAAAATAGACTACGAAAGTGGCTTTAACATACCTGAACACACAATAGCTAAGACCCAAACTGGG

>237895c53a300fc4963d8591650ca3f9

GACGAAGGGGGCAAGCGTTGCTCGGAATCACTGGGCTTAAAGAGGGCGTAGGCGGGCCGGCCAGTCGGATGTGAAATCCCTTGGCTCAACTGAGGAATGGCATTCGATACTGCCGGACTTGAGGTGCTCAGGGGCGAGTGGAACTCTCGGTGGAGCGGTGAAATGCGTAGATGTCGAGAGGAACGCCAATGGTGAAGACAGCTCGCTGGGGGCATCCTGACGCTGAGGTCCGAAAGCTAGGGGAGCAAACTGGG

>48901746cf70ed14cf5cde8060edf41e

CACAAGTGAGACTAGTGTTATTCATCTTTATTAGGTTTAAAGGGTACCTAGACGGTATTTTAGGCCCCAAAAGGGTACAAATATACTAGAGTTTTGTAGGAGAGGTAAATATTAGGACCATTGGTGTAGAGATGAAATTCTTTGATACTAATGGGATGTATAATGGCGAAGGCAATCCTCTATAGATAAACTGACGTTGAGGGACGAAGGCTTGGGGAGCGAATAGG

>e46ded7869f162d72dfd6c9f3f9c268b

TACGTAGGGTGCGAGCGTTAATCGGAATTACTGGGCGTAAAGCGTGCGCAGGCGGTTTGTTAAGACAGATGTGAAATCCCCGGGCTCAACCTGGGAACTGCATTTGTGACTGGCAGGCTAGAGTATGGCAGAGGGGGGTAGAATTCCACGTGTAGCAGTGAAATGCGTAGAGATGTGGAGGAATACCGATGGCGAAGGCAGCCCCCTGAGCCAATACTGACGCTCATGCACGAAAGCGTGGGGAGCAAACAGG

>978106c16170224a78409c09e28451d4

TACGGGGGGTGCAAGCGTTAATCGGAATTACTGGGCGTAAAGCGCACGCAGGCGGTTGATTAAGTTAGATGTGAAATCCCCGGGCTTAACCTGGGAATGGCATCTAAAACTGGTCAGCTAGAGTCTTGTAGAGGGGGGTAGAATTCCATGTGTAGCGGTGAAATGCGTAGAGACGTGGAGGAATACCGGTGGCGAAGGCGGCCCCCTGGACAAAGACTGACGCTCAGGTGCGAAAGCGTGGGGAGCAAACAGG

>098df0f7790f0eca5d02e9f40682c194

TACGTAGGGTACGAGCGTTAATCGGAATTACTGGGCGTAAAGCGTGCGCAGGCGGTTTGTTAAGACAGATGTGAAATCCCCGGGCTCAACCTGGGAACTGCATTTGTGACTGGCAGGCTAGAGTATGGCAGAGGGGGGTAGAATTCCACGTGTAGCAGTGAAATGCGTAGAGATGTGGAGGAATACCGATGGCGAAGGCAGCCACCTGGGCCAATACTGACGCTCATGCACGAAAGCGTGGGGAGCAAACAAG

>b7a920c00b6e363db57b7cb9d46b5c04

TACCGGCAGCCCGAGTGATGGCCGATCTTATTGGGCCTAAAGCGTCCGTAGCTGGCCGCGCAAGTCCATCGGGAAATCCACCTGCTCAACAGGTGGGCGCCCGGTGGAAACTGTGCGGCTTGGGACCGGAAGGCGCGACGGGTACGTCCGGGGTAGGAGTGAAATCCCGTAATCCCGAACGGACCACCGATGGCGAAAGCACCTCGAGAAGACGGCTCCGACAGTGAGGGACGAAAGCTAGGGTCTCGAACCGG

>be3e6779f010ef871b66c77b3d3cf4cf

TACCGGCAGTCCGAGTGATGGCCGCTGTTATTGGGCCTAAAGCGTCCGTAGCTTGCCGTGCAAGTCTATCGGGAAATCGACGTGCTCAACGCGTCGGCTTCCGGTGGAAACTGCACGGCTTGGGGCCGGAAGACTCAGCGGGTACGTCCGGGGTAGGAGTGAAATCCTGTAATCCTGGACGGACCACCAATGGCGAAAGCACGCTGAGAAGCCGGACCCGACAGTGAGGGACGAAAGCCAGGGTCTCGAACCGG

>ce5627b8a95ae09494c2b57e11dfe66c

TACGTAGGGTGCGAGCGTTAATCGGAATTACTGGGTGTAAAGCGTGCGCAGGCGGTTTGTTAAGACAGATGTGAAATCCCCGGGCTCAACCTGGGAACTGCATTTGTGACTGGCAGGCTAGAGTATGGCAGAGGGGGGTAGAATTCCACGTGTAGCAGTGAAATGCGTAGAGATGTGGAGGAATACCGATGGCGAAGGCAGCCCCCTGGGCCAATACTGACGCTCATGCACGAAAGCGTGGGGAGCAAACAGG

>10edbf6c4e33a9170c6c02537ac94b70

TACGTAGGGTGCGAGCGTTAATCGGAATTACTGGGCGTAAAGCGTGCGCAGGCGGTTTGTTAAGACAGATGTGAAATCCCTGGGCTCAACCTGGGAACTGCATTTGTGACTGGCAGGCTAGAGTATGGCAGAGGGGGGTAGAATTCCACGTGTAGCAGTGAAATGCGTAGAGATGTGGAGGAATACCGATGGCGAAGGCAGCCCCCTGGGCCAATACTGACGCTCATGCACGAAAGCGTGGGGAGCAAACAGG

>5bccbebfa648aacb95a9407ca0a902f7

TACGTAGGGTGCAAGCGTTAATCGGAATTACTGGGCGTAAAGCGTGCGCAGGCGGTTATGCAAGACAGATGTGAAATCCCCGGGCTCAACCTGGGAACTGCATTTGTGACTGCATAGCTAGAGTGCGGCAGAGGGGGATGGAATTCCGCGTGTAGCAGTGAAATGCGTAGATATGCGGAGGAACACCGATGGCGAAGGCAATCCCCTGGGCCTGCACTGACGCTCATGCACGAAAGCGTGGGGAGCAAACAGG

>f84326c9790515a0835ca7ff8a8c7d54

TACAGAGGATGCAAGCGTTATCCGGAATTATTGGGCGTAAAGCGTCTGTAGGTGGCTTTTTAAGTCCGCCGTCAAATCCCAGGGCTCAACCCTGGACAGGCGGTGGAAACTACCAAGCTGGAGTACGGTAGGGGCAGAGGGAATTTCCGGTGGAGCGGTGAAATGCGTAGAGATCGGAAAGAACACCAACGGCGAAAGCACTCTGCTGGGCCGACACTGACACTGAGAGACGAAAGCTAGGGTAGCGAATGGG

>30b88c302be4fc9994137808dc71e784

TACGTAGGGTGCGAGCGTTAATCGGAATTACTGGGCGTAAAGCGTGCGCAGGCGGTTTGTTAAGACAGATGTGAAATCCCCGGGCTCAACCTGGGAACTGCATTTGTGACTGGCAGGCTAGAGTATGGCAGAGGGGGGTAGAATTCCACGTGTAGCAGTGAAATGCGTAGAGATGTGGAGGAATACCGATGGCGAAGGCAGCCCCCTGGGCCAATACTGACGCTCATGCACAAAAGCGTGGGGAGCAAACAGG

>2f3da68606254b35e1d68615524c6815

TACGGAGGATGCGAGCGTTATTCGGAATCATTGGGTTTAAAGGGTCTGTAGGCGGGCTATTAAGTCAGGGGTGAAAGGTTTCAGCTTAACTGAGAAATTGCCTTTGATACTGGTAGTCTTGAATATCTGTGAAGTTCTTGGAATGTGTAGTGTAGCGGTGAAATGCTTAGATATTACACAGAACACCGATTGCGAAGGCAGGGGACTAACAGACAATTGACGCTGAGAGACGAAAGCGTGGGGAGCGAACAGG

>9beaf174891c162d4ecbfd976e382479

TACGTAGGTGGCAAGCGTTGTCCGGATTTATTGGGCGTAAAGCGCGCGCAGGCGGTCTTTTGAGTCTGATGTGAAAGCCCCCGGCTTAACCGGGGAGGGTCATTGGAAACTGGAAGACTGGAGTGCAGAAGAGGAGAGTGGAATTCCACGTGTAGCGGTGAAATGCGTAGATATGTGGAGGAACACCAGTGGCGAAGGCGACTCTCTGGTCTGTAACTGACGCTGAGGCGCGAAAGCGTGGGGAGCAAACAGG

>a6e5a33676a59047b0186661dc31ac1c

TACGGAGGGGGCTAGCGTTGTTCGGAATTACTGGGCGTAAAGCGTACGTAGGCGGCTTTGTAAGTTAGAGGTGAAAGCCTGGAGCTCAACTCCAGAACTGCCTTTAAGACTGCATCGCTTGAATCCAGGAGAGGTGAGTGGAATTCCGAGTGTAGAGGTGAAATTCGCAGATATTCGGAAGAACACCAGTGGCGAAGGCGGCTCACTGGACTGGTATTGACGCTGAGGTACGAAAGCGTGGGGAGCAAACAGG

>16850c9a0e88f0bd71ed5c1372149fc7

TACCGGCAGCTCAAGTGATGTCCCATATTATTGGGCCTAAAGCGTCCGTAGCTGGCCGACCAAGTCTCCCGGGAAATCCACCCGCCCAACGGGTGGGCGTCCGGTAGAAACTGGCCGGCTTGGAACCGGAAGGCTCAGAGAGTACGTCCGGGGTAGGAGTGAAATCCCGTAATCCCGGACGGACTACCGATGGCGAAAGCACTCTGAGAAGACGGCTTCGACAGTGAGGGACGAAAGCTCGGGTCTCAAACCGG

>f86a1eefe102f6d38a0fc8bbfc4be7ec

GACAGAGGATGCAAGCGTTATCCGGAATGATTGGGCGTAAAGCGTCTGTAGGTGGCTTTTTAAGTTCGCCGTCAAATCCCAGGGCTCAACCCTGGACAGGTGGTGAAAACTACTAAGCTAGAGTACGGTAGGGGCAGAGGGAATTTCCGGTGGAGCGATGAAACGCGTAGAGATCGGAAGGAACACCAACGGCGAAAGCACTCTGCTGGGCCGACACTGACACTGAGAGACGAAAGCTGGGCGAGCGAATGGG

>554ada7f937e4e1856ff35177e33d13b

TACGTAGGTGGCAAGCGTTATCCGGATTTATTGGGCGTAAAGAGAGTGCAGGCGGTTTTCTAAGTCTGATGCGAAAGCCTTCGGCTTAACCGGAGAAGTGCATCGGAAACTGGATAACTTGAGTGCAGAAGAGGGTAGTGGAACTCCATGTGTAGCGGTGGAATGCGTAGATATATGGAAGAACACCAGTGGCGAAGGCGGCTACCTGGTCTGCAACTGACGCTGAGACTCGAAAGCATGGGTAGCGAACAGG

>08b0d8f19c96940b2344eddf694f0b73

TACGGAGGGTGCAAGCGTTATCCGGATTTATTGGGTTTAAAGGGTCCGTAGGTGGATTTGTAAGTCAGTGGTGAAATCTCACAGCTTAACTGTGAAACTGCCATTGATACTGCAAGTCTTGAGTGTTGTTGAAGTAGCTGGAATAAGTAGTGTAGCGGTGAAATGCATAGATATTACTTAGAACACCAATTGCGAAGGCAGGTTACTAAGCAACAACTGACGCTGATGGACGAAAGCGTGGGGAGCGAACAGG

>fe048fa7c6bb3c9638c9dbea1f487a97

TACGGAGAGTGCAAGCGTTATCCGGATTTATTGGGTTTAAAGGGTCCGTAGGCGGATTTGTAAGTCAGTGGTGAAATCTCACAGCTTAACTGTGAAACTGCCATTGATACTGCAAGTCTTGAGTGTTGTTGAAGTAGCTGGAATAAGTAGTGTAGCGGTGAAATGCATAGATATTACTTAGAACACCAATTGCGAAGGCAGGTTACTAAGCAACAACTGACGCTGATGGACGAAAGCGTGGGGAGCGAACAGG

>478fd3c49029a0dda20ce4bd1ca6ebed

TACCGGCAGCTCGAGTGATGTCCAATATTATTGGGCCTAAAGCGTCCGTAGCTGGCCGCGCAAGTCCATCGGGAAATCCACCTGCTCAACAGGTGGGCGCCCGGTGGAAACTGTGCGGCTTGGGACCGGAAGGCGCGACGGGTACGTCCGGGGTAGGAGTGAAACCCCGTAATCCTGGACGGACCGCCGATGGCGAAAGCACGTCGCGAGAACGGATCCGACAGTGAGGGACGAAAGCCAGGGTCTCGAACCGG

>002aeaa1ea2be03deeca8e6e1cdd68e1

CACACTTATGTCTTTAGAAAGACAAAACAGAATGCACTTCAACTATGTCGGTTATTTTTAACTGGCAGTGCCTAGTAGATAGTTGTGGAAGATTTTGAATGTGCCTTGTCCATAGAGTTCAGTTTGTTCATTAATGATTTTTTCATGTAGTTTGTAACGTAGTATATTTAGACTTTCAAGTAGATTCATTTTATTTCCTGATTTTTCTGTTGTG

>7249b66c172869d4d31b6944a778468b

AACAGAGGATACAAGCGTTATCCGGATTTATTAGGTTTAAAGGGTGCGTAGGTGGTTTTTTAAGTCAGTAGTGAAATCTTAAAGCTTAACTTTAAAAGTGCTATTGATACTGATAAACTAGAGTGAGGTTGGAGTAACTGGAATGTGTGGTGGAGCGGTGAAATGCATAGAGATCACACAGAACACCAATCGCGAAGGCATGTTACTAAACATAGACTGACACTGAGGCACGAAAGCATGGGTAGCAAACAGG

>df0944a492b635af5f06543af365d927

TACCGGCAGCCCGAGTGATGGCCGATCTTATTGGGCCTAAAGCGTCCGTAGCTGGCCGCGCAAGTCCGTCGGGAAATCCACCTGCTCAACAGGTGGGCGCCCGGTGGAAACTGTGCGGCTTGGGACCGGAAGGCGCGACGGGTACGTCCGGGGTAGGAGTGAAATCCCGTAATCCTGGACGGACCGCCGATGGCGAAAGCACGTCGCGAGAACGGATCCGACAGTGAGGGACGAAAGCCAGGGTCTCGAACCGG

>c35f330d97720fe3b62821f80cbc0c1a

TACGTAGGGTGCGAGCGTTAATCGGAATTACTGGGCGTAAATCGTGCGCAGGCGGTTTGTTAAGACAGATGTGAAATCCCCGGGCTCAACCTGGGAACTGCATTTGTGACTGGCAGGCTAGAGTATGGCAGAGGGGGGTAGAATTCCACGTGTAGCAGTGAAATGCGTAGAGATGTGGAGGAATACCGATGGCGAAGGCAGCCCCCTGGGCCAATACTGACGCTCATGCACGAAAGCGTGGGGAGCAAACAGG

>0d2a1629b88f2727f0818a85e03f8363

TACGGAGGGTGCAAGCGTTATCCAGATTTATTGGGTTTAAAGGGTCCGTAGGCGGATTTGTAAGTCAGTGGTGAAATCTCACAGCTTAACTGTGAAACTGCCATTGATACTGCAAGTCTTGAGTGTTGTTGAAGTAGCTGGAATAAGTAGTGTAGCGGTGAAATGCATAGATATTACTTAGAACACCAATTGCGAAGGCAGGTTACTAAGCAACAACTGACGCTGATGGACGAAAGCGTGGGGAGCGAACAGG

>f2fd591cc93edaa3475e22c9eebda096

TACCGGCAGCCCAAGTGATGGCCGATCTTATTGGGCCTAAAGCGTCCGTAGCTGGCCGCGCAAGTCCATCGGGAAATCCACCTGCTCAACAGGTGGGCGCCCGGTGGAAACTGCGCGGCTTGGGACCGGAAGGCGCGACGGGTACGTCCGGGGTAGGAGTGAAATCCCGTAATCCTGGACGGACCGCCGATGGCGAAAGCACGTCGCGAGAACGGATCCGACAGTGAGGGACGAAAGCCAGGGTCTCGAACCGG

>63d5eb4084678830c41e6f217df71582

TACGTAGGGTGCGAGCGTTAATCGGAATTACTGGGCGTAAAGCGTGCGCATGCGGTTTGTTAAGACAGATGTGAAATCCCCGGGCTCAACCTGGGAACTGCATTTGTGACTGGCAGGCTAGAGTATGGCAGAGGGGGGTAGAATTCCACGTGTAGCAGTGAAATGCGTAGAGATGTGGAGGAATACCGATGGCGAAGGCAGCCCCCTGGGCCAATACTGACGCTCATGCACGAAAGCGTGGGGAGCAAACAGG

>8a6ff0c88772f33477cc3c1e68cc67ff

GACGTAGGGGGCGAGCGTTGTTCGGATTTACTGGGCGTAAAGCGCGCGTAGGCGGCCAGATAAGTCGGGTGTGAAAGCCCTCGGCTCAACCGAGGTTCCGCATCCGATACTGCTTGGCTGGAGTCTAGGAGAGGAGAGCGGAATTCCCAGTGTAGCGGTGAAATGCGTAGATATTGGGAGGAACACCCGTGGCGAAGGCGGCTCTCTGGACTAGTACTGACGCTGAGGCGCGAAAGCTAGGGGAGCGAACGGG

>69490aabfdd1391405bea26712922121

TACGATTTCTTTAATTTAAATAGTTAAGTTTCAGTTAATGTATTTGTAATTTTAAAAAGTTATAATTTTGGTGAAATATATTTTGTCTTAAAAAATTAATTTTATATCTGAAAAATTTTTGTTCAAACTAGGATTAGAAACCCTAGTAGTCCGGCTGACTGAC

>77effe1a43455583b9f1b9398462058b

TACGTAGGTGGCAAGCGTTGTCCGGAATTATTGGGCGTAAAGCGCGCGCAGGCGGTCCTTTAAGTCTGATGTGAAAGCCCACGGCTCAACCGTGGAGGGTCATTGGAAACTGGGGGACTTGAGTGCAGAAGAGAAGAGTGGAATTCCACGTGTAGCGATGAAATGCGTAGAGATGTGGAGGAACACCAGTGGCGAAGGCGACTCTTTGGTCTGTAACTGACGCTGAGGCGCGAAAGCGTGGGGAGCAAACAGG

>9c00dbc749dbbca1fbf929e987c05ab9

TACGTAGGGTACGAGCGTTAATCGGAATTACTGGGCGTAAAGCGTGCGCAGGCGGTTTGTTAAGACAGATGTGAAATCCCCGGGCTCAACCTGGGAACTGCATTTGTGACTGGCAGGCTAGAGTATGGCAGAGGGGGGTAGAATTCCACGTGTAGCAGTGAAATGCGTAGAGATGTGGAGGAATACCGATGGCGAAGGCAGCCCCCTGGGCCAATACTGACGCTCATGCACGAAAGCGTGGGGAGCAAACAAG

>e1b159f3817a00949f42f1f2b16376dd

CACACCTATGTCTTTAGAAAGACAAAACGTTTTTCCAGAATGCATTTTCAACCATGTCGGTCATTTTCAACTTGGCAGTGCCTTGTAGATAGTTGTGGAAGATTTTGAATGTGTATTATAACTTATGGTTAAAATAGTATTTTTGTTCCGCGTTAATAAGCCACGACTTTACTACGCGTCTGTAAGTACGAAAAAGTAGTTCACAATATGAAACACGATCAGA

>323edc6997767fde654a195551ffbf19

TACGGAGGGTGCAAGCGTTATCCGGATTTATTGGGTTTAAAGGGTCCGTAGGCGGATTTGTAAGTCAGTGGTGAAATCTCACAGCTTAACTGTGAAACTGCCATTGATACTGCAAGTCTTGAGTGTTGTTGAAGTAGCTGGAATAAGTAGTGTAGCGGTGAAATGCATAGATATTACTTAGAACACCAATTGTGAAGGCAGGTTACTAAGCAACAACTGACGCTGATGGACGAAAGCGTGGGGAGCGAACAGG

>269d5170a78b5f9419b6c8bd08bde6cc

TACAGAGGGTGCAAGCGTTAATCGGATTTACTGGGCGTAAAGCGCGCGTAGGCGGCTAATTAAGTCAAATGTGAAATCCCCGAGCTTAACTTGGGAATTGCATTCGATACTGGTTAGCTAGAGTGTGGGAGAGGATGGTAGAATTCCAGGTGTAGCGGTGAAATGCGTAGAGATCTGGAGGAGTACCGATGGCGAAGGCAGCCATCTGGCCTAACACTGACGCTGAGGTGCGAAAGCATGGGGAGCAAACAGG

>8fd74e7714f2d3ed501ccb6132f03e94

TACGTAGGGTGCGAGCGTTAATCGGAATTACTGGGGGTAAAGCGTGCGCAGGCGGTTTGTTAAGACAGATGTGAAATCCCCGGGCTCAACCTGGGAACTGCATTTGTGACTGGCAGGCTAGAGTATGGCAGAGGGGGGTAGAATTCCACGTGTAGCAGTGAAATGCGTAGAGATGTGGAGGAATACCGATGGCGAAGGCAGCCCCCTGGGCCAATACTGACGCTCATGCACGAAAGCGTGGGGAGCAAACAGG

>e475a89f103bb9173f175d09f8f7dde8

TACCGGCAGCCCGAGTGATGGCCGATCTTATTGGGCCTAAAGCGTCCGTAGCTGGCCGCGCAAGTCCATCGGGAAATCCACCCGCTCAACAGGTGGGCGCCCGGTGGAAACTGTGCGGCTTGGGACCGGAAGGCGCGACGGGTACGTCCGGGGTAGGAGTGAAATCCCGTAATCCTGGACGGACCGCCGATGGCGAAAGCACGTCGCGAGAACGGATCCGACAGTGAGGGACGAAAGCCAGGGTCTCGAACCGG

>b78c3d2665eb911d2e3598a1359a0faf

TACGAAGGGGGCTAGCGTTGTTCGGATTTACTGGGCGTAAAGCGCACGTAGGCGGACTTTTAAGTCAGGGGTGAAATCCCGGGGCTCAACCCCGGAACTGCCTTTGATACTGGAAGTCTTGAGTATGGTAGAGGTGAGTGGAATTCCGAGTGTAGAGGTGGAATTCGTAGATATTCGGAGGAACACCAGTGGCGAAGGCGGCTCACTGGACCATTACTGACGCTGAGGTGCGAAAGCGTGGGGAGCAAACAGG

>6ebf58558480ab207634ac79110ca1e3

TACAGAGGGTGCAAGCGTTAATCGGATTTACTGGGCGTAAAGCGCGCGTAGGCGGCTAATTAAGTCAAATGTGAAATCCCCGAGCTTAACTTGGGAATTGCATTCGATACTGGTTAGCTAGAGTGTGGGAGAGGACGGTAGAATTCCAGGTGTAGCGGTGAAATGCGTAGAGATCTGGAGGAATACCGATGGCGAAGGCAGCCATCTGGCCTAACACTGACGCTGAGGTGCGAAAGCATGGGGAGCAAACAGG

>263d85878dfcbb64bbe2bb6ec678f194

TACCGGCAGCCCAAGTGATGGCCGATCTTATTGGGCCTAAAGCGTCCGTAGCTGGCCGCGCAAGTCCATCGGGAAATCCACCTGCTCAACAGGTGGGCGCCCGGTGGAAACTGTGCGGCTTGGGACCGGAAGGCGCGACGGGTACGTCCGGGGTAGGAGTGAAATCCCGTAATCCTGGACGGACCGCCGATGGCGAAAGCACGTCGCGAGAACGGATCCGACAGTGAGGGACGAAAGCTAGGGTCTCGAACCGG

>30ff803630af3feafb89d33f0ba7945c

TACGTAGGGTGCGAGCGTTGTCCGGAATTATTGGGCGTAAAGAGCTTGTAGGCGGTTTGTCGCGTCTGCTGTGAAAATCCGAGGCTCAACCTCGGACTTGCAGTGGGTACGGGCGAACTAGAGTGTGGTAGGGGAGACTGGAATTCCTGGTGTAGCGGTGAAATGCGCAGATATCAGGAGGAACACCGATGGCGAAGGCAGGTCTCTGGGCCACTACTGACGCTGAGAAGCGAAAGCGTGGGGAGCGAACAGG

>167757e407e7f0f3d1d021a68dfbd267

TACGTAGGGTGCGAGCGTTAATCGGAATTACTGGGCGTAAAGCGTGCGCAGGCGGTTTGTTAAGACAGATGTGAAATCCCCGGGCTCAACCTGGGAACTGCATTTGTGACTGGCAGGCTAGAGTATGGCAGAGGGGGGTAGAATTCCACGTGTAGCAGTGAAATGCCTAGAGATGTGGAGGAATACCGATGGCGAAGGCAGCCCCCTGGGCCAATACTGACGCTCATGCACGAAAGCGTGGGGAGCAAACAGG

>bc26085a25ec107c12ec96c062e0a88d

TACGTAGGGTGCGAGCGTTAATCGGAATTATTGGGCGTAAAGCGTGCGCAGGCGGTTTGTTAAGACAGATGTGAAATCCCCGGGCTCAACCTGGGAACTGCATTTGTGACTGGCAGGCTAGAGTATGGCAGAGGGGGGTAGAATTCCACGTGTAGCAGTGAAATGCGTAGAGATGTGGAGGAATACCGATGGCGAAGGCAGCCCCCTGGGCCAATACTGACGCTCATGCACGAAAGCGTGGGGAGCAAACAGG

>d892d40bc79d0c356bd2886ce8d120ee

TACGTAGGGCGCAAGCGTTGTCCGGAATTATTGGGCGTAAAGAGCTCGTAGGCGGTTTGTCGCGTCTGCTGTGAAAACCTAAGGCTCAACCTTGGGCTTGCAGTGGGTACGGGCAGACTAGAGTGCGGTAGGGGTGACTGGAATTCCTGGTGTAGCGGTGGAATGCGCAGATATCAGGAGGAACACCGATGGCGAAGGCAGGTCACTGGGCCGCAACTGACGCTGAGGAGCGAAAGCATGGGGAGCGAACAGG

>0904b22f7fd5cd91d26907bf646f6d42

TACGGAGGGTGCAAGCGTTATCCGGATTTATTGGGTTTAAAGGGTCCGTAGGCGGATTTGTAAGTCAGTGGTGAAATCTCACAGCTTAACTGTGAAACTGCCATTGATACTGCAAGTCTTGAGTGTTGTTGAAGTAGCTGGAATAAGTAGTGTAGCGGTGAAATGCATAGATATTACTTAGAACACCAATTGCGAAGGCAGGTTACTAAGCAACAACTGACGCTGATGGACGAAAGCGTGGAGAGCGAACAGG

>518c2bd074c6a339e4ce0fc763a68c94

TACGTAGGGTGCGAGCGTTAATCGGAATTACTGGGCGTAAAGCGTGCGAAGGCGGTTTGTTAAGACAGATGTGAAATCCCCGGGCTCAACCTGGGAACTGCATTTGTGACTGGCAGGCTAGAGTATGGCAGAGGGGGGTAGAATTCCACGTGTAGCAGTGAAATGCGTAGAGATGTGGAGGAATACCGATGGCGAAGGCAGCCCCCTGGGCCAATACTGACGCTCATGCACGAAAGCGTGGGGAGCAAACAGG

>3bb0bf5132b0f988573de9bd6059b22c

TACGTAGGGTGCAAGCGTTAATCGGAATTACTGGGCGTAAAGCGTGCGCAGGCGGTTATGTAAGACAGAGGTGAAATCCCCGGGCTCAACCTGGGAACTGCCTTTGTGACTGCATAGCTAGAGTACGGTAGAGGGGGATGGAATTCCGCGTGTAGCAGTGAAATGCGTAGATATGCGGAGGAACACCGATGGCGAAGGCAATCCCCTGGACCTGTACTGACGCTCATGCACGAAAGCGTGGGGAGCAAACAGG

>741c1fb7c2f12d19d00f6755c4e6ff41

TACGTAGGGTGCGAGCGTTAATCGGAATTACTGGGCGTAAAGCGTGCGCAGGCGGTTTGTTAAGACAGATGTGAAATCCCCGGGCTCAACCTGGGAACTGCATTTGTGACTGGCAGGCTAGAGTATGGCAGAGGGGGGTATAATTCCACGTGTAGCAGTGAAATGCGTAGAGATGTGGAGGAATACCGATGGCGAAGGCAGCCCCCTGGGCCAATACTGACGCTCATGCACGAAAGCGTGGGGAGCAAACAGG

>cf381b7b7ad3c8de3c5909dab3f68543

TACGTAGGGTGCGAGCGTTAATCGGAATTACTGGGCGTAAAGCGTGCGCAGGCGGTGATGTAAGACCGATGTGAAATCCCCGGGCTTAACCTGGGAACTGCATTGGTGACTGCATCGCTGGAGTATGGCAGAGGGGGGTGGAATTCCACGTGTAGCAGTGAAATGCGTAGAGATGTGGAGGAACACCGATGGCGAAGGCAGCCCCCTGGGCCAATACTGACGCTCATGCACGAAAGCGTGGGGAGCAAACAGG

>e021a65bee2dc454517c1ce70d6ed556

TACCGGCAGCACGAGTGATGGCCGATCTTATTGGGCCTAAAGCGTCCGTAGCCGGCCGAGCAAGTTCGTCGGGAAATCCACCAGCTCAACTGGTGGGCGTCCGGCGAAAACTGTTCGGCTTGGGGCCGGAAGACCCAAGGGGTACGTCCGGGGTAGGAGTGAAATCCCGTAATCCTGAACGGACCGCCGATGGCGAAAGCACCTCGAGAGGACTGATCCGACAGTGAGGGACGAAAGCTGGGGTCTCGAACCGG

>68262884f1021c896f8e1bf7348d773c

TACGGAGGATCCAAGCGTTATCCGGAATCATTGGGTTTAAAGGGTCCGTAGGCGGTTTAATAAGTCAGTGGTGAAAGCCCATCGCTCAACGGTGGAACGGCCATTGATACTGTTAAACTTGAATTATTAGGAAGTAACTAGAATATGTAGTGTAGCGGTGAAATGCTTAGAGATTACATGGAATACCAATTGCGAAGGCAGGTTACTACTAATGGATTGACGCTGATGGACGAAAGCGTGGGTAGCGAACAGG

>827f33fa58c72cb44bf8dd3fc55eb316

TACGTAGGGTGCGAGCGTTAATCGGAATTACTGGGCGTAAAGCGTGCGCAGGCGGTTTGTTAAGACAGATGTGAAATCCCCGGGATCAACCTGGGAACTGCATTTGTGACTGGCAGGCTAGAGTATGGCAGAGGGGGGTAGAATTCCACGTGTAGCAGTGAAATGCGTAGAGATGTGGAGGAATACCGATGGCGAAGGCAGCCCCCTGGGCCAATACTGACGCTCATACACGAAAGCGTGGGGAGCAAACAGG

>56c362ab608e81ed51ef02073b71bd2b

TACGGAGGGGATTAGCGTTGTTCGGAATTACTGGGCGTAAAGCGCGCGTAGGCGGATTGGAAAGTTGGGGGTGAAAGCCCGGGGCTCAACCCCGGAACTGCCTTCAAAACTGATCGTCTGGAGACCGGGAGAGGTGAGTGGAATTCCCAGTGTAGAGGTGAAATTCGTAGATATTGGGAAGAACACCAGTGGCGAAGGCGGCTCACTGGACCGGATCTGACGCTGAGGTGCGAAAGCGTGGGGAGCGAACAGG

>aa8b0d23cf530d322dabb8175614d5bd

TACGTAGGGTGCGAGCGTTAATCGGAATTACTGGGCGTAAAGGGTGCGCAGGCGGTTTGTTAAGACAGATGTGAAATCCCCGGGCTCAACCTGGGAACTGCATTTGTGACTGGCAGGCTAGAGTATGGCAGAGGGGGGTAGAATTCCACGTGTAGCAGTGAAATGCGTAGAGATGTGGAGGAATACCGATGGCGAAGGCAGCCCCCTGGGCCAATACTGACGCTCATGCACGAAAGCGTGGGGAGCAAACAGG

>979f72163b50e092c8dfcb454f4c9d1a

AACAGAGGATACAAGCGTTATCCGGATTTATTGGGTTTAAAGGGTGCGTAGGTGGTTTTTTAAGTCAGTAGTGAAATCTTAAAGCTTAACTTTAAAAGTGCTATTGATACTGATAAACTAGAGTGAGGTTGGAGTAACTGGAATGTATGGTGGAGCGGTGAAATGCATAGAGATCACACAGAACACCAATCGCGAAGGCATGTTACTAAACATAGACTGACACTGAGGCACGAAAGCATGGGTAGCAAACAGG

>bf39d305f06c34b6c999707f16519430

GACGAAGGTGGCGAGCGTTGTTCGGAATCACTGGGCTTAAAGCGCACGTAGGCGGCCTGTCAAGCGCCGTGTGAAATCCCTCGGCGTAACCGAGGAATGGCTTGGCGAACTGGCAGGCTTGAGGCAGGTATGGGCGAGTGGAACTCTTGGTGGAGCGGTGAAATGCGTAGATATCAAAAGGAACGCCGGCGGTGAAGACGACTCGCTGGGCCTGTCCTGACGCTGAGGTGCGAAAGCCAGGGGAGCAAACTGGG

>740c97487cb2bdcc8f9d9957a8e504bd

TACCGGCAGTCCGAGTGATGGCCGATATTATTGGGCCTAAAGCGTCCGTAGCTGGCCGCGCAAGTCCATCGGGAAATCCACCTGCTCAACTGGTCGGCGCCCGGTGGAAACTACACAGCTTGGGGCCGAGAGACTCAACGGGTACGTCCGGGGTAGGAGTGAAATCCTGTAATCCTGGACGGACCACCAATGGGGAAACCACGTTGAGAGACCGGACCCGACAGTGAGGGACGAAAGCCAGGGTCTCGAACCGG

>8f737567a197057c3451c9372329bb72

TACGTAGGGTGCGAGCGTTAATCGGAATTACTGGGCGTAAAGCGTGCGCAGGCGGTTTTGTAAGACAGGCGTGAAATCCCCGGGCTTAACCTGGGAATTGCGCTTGTGACTGCAAGGCTAGAGTGCGTCAGAGGGGGGTAGAATTCCACGTGTAGCAGTGAAATGCGTAGAGATGTGGAGGAATACCGATGGCGAAGGCAGCCCCCTGGGACGTGACTGACGCTCATGCACGAAAGCGTGGGGAGCAAACAGG

>e898620b82d3ae5814a1888c8888639a

TACAGAGGGTGCAAGCGTTAATCGGAATTACTGGGCGTAAAGCGCGCGTAGGTGGTTTGTTAAGTTGGATGTGAAAGCCCCGGGCTCAACCTGGGAACTGCATCCAAAACTGGCAAGCTAGAGTACAGTAGAGGGTGGTGGAATTTCCTGTGTAGCGGTGAAATGCGTAGATATAGGAAGGAACACCAGTGGCGAAGGCGACCACCTGGACTGATACTGACACTGAGGTGCGAAAGCGTGGGGAGCAAACAGG

>66ebc24b5bc4ed843fcb5c3751826511

TACGGAGGGTGCAAGCGTTAATCGGAATTACTGGGCGTAAAGCGTGCGCAGGCGGCTGATTAGGTCGGATGTGAAAGCCCCGGGCTCAACCTGGGAATGGCATTCGATACTGGTCAGCTAGAGTCTGGTAGAGGTAAGCGGAATTCCGGGTGTAGCGGTGAAATGCGTAGATATCCGGAGGAACATCAGTGGCGAAGGCGGCTTACTGGACCAAGACTGACGCTCAGGCACGAAAGCGTGGGTAGCAAACAGG

>11d4bd4cb51e000251ff58f3646d32be

CACACTTATCTCTTTAGAAAGACAAAACATTTCTCCAGAATGCATTTCAACCTTGTCGGTCATTTTCAACTGGCAGTGCCTTGTAGATACTTGTGGAAGATTTAACATGTGTCTTGTCAATAGAGTTCAGTTTGTTCATTAATGATTTTGTTGTGTAGTTTGTAACGTAGTATATTTAAACTTTCAAGTAGATTCATTTTATTTCCTTTTTTTCCGGTTGTG

>adfc0b0ff1398d6fbce1d01a2caede48

TACGGAGGGTGCAAGCGTTAATCGGAATTACTGGGCGTAAAGCGCACGCAGGCGGTTTGTTAAGTCAGATGTGAAATCCCCGGGCTCAACCTGGGAACTGCATCTGATACTGGGAAGCTTGAGTCTCGTAGAGGGGGGTAGAATTCCAGGTGTAGCGGTGAAATGCGTAGAGATCTGGAGGAATACCGGTGGCGAAGGCGGCCCCCTGGACGAAGACTGACGCTCAGGTGCGAAAGCGTGGGGAGCAAACAGG

>5ce595b1571bdb3fb613b825b9f5a52e

TACGGAGGGTGCAAGCGTTAATCGGAATTACTGGGCGTAAAGCGCGCGCAGGCGGTCTTTTAAGTCTGATGTGAAAGCCCCCGGCTTAACCGGGGAGGGTCATTGGAAACTGGAAGACTGGAGTGCAGAAGAGGAGAGTGGAATTCCACGTGTAGCGGTGAAATGCGTAGAGATGTGGAGGAACACCAGTGGCGAAGGCGACTCTCTGGTCTGTAACTGACGCTGAGGAGCGAAAGCGTGGGGAGCGAACAGG

>a65f5f9976951759604beb9352891e0f

TACGTAGGGTGCAAGCGTTAATCGGAATTACTGGGCGTAAAGCGTGCGCAGGCGGTTATATAAGTCAGATGTGAAATCCCCGGGCTCAACCTGGGAACTGCATTTGAGACTGTATAGCTAGAGTACGGTAGAGGGGGATGGAATTCCGCGTGTAGCAGTGAAATGCGTAGATATGCGGAGGAACACCGATGTCGAAGGCAATCCCCTGGACCTGTACTGACGCTCATGCACGAAAGCGTGGGGAGCAAACAGG

>297127495c321ba9b57a7a339389212d

CGTATTTCTTGGATGTGCATTACTTCCGGGTTTGAATTATAAAAAATATCTTCAAAACGTAAGAAAATAATTGAGTTTGTATTGACGGTTTCCACTCAATTGGTACGTGGACATGTTTTATAAGGGGTCCGAATATAAGAAATATACGAAATACTCTTGAAATGTACTTACGTATGATTCTATATTTTCAAATAATTTTAGTTTATCGTAATTATTTTTCGTATTCTTACGTGGAGTTGT

>d386c4be8a37ccb34ea514af42a69a31

TACGTAGGGTGCGAGCGTTAATCGGAATTACTGAGCGTAAAGCGTGCGCAGGCGGTTTGTTAAGACAGATGTGAAATCCCCGGGCTCAACCTGGGAACTGCATTTGTGACTGGCAGGCTAGAGTATGGCAGAGGGGGGTAGAATTCCACGTGTAGCAGTGAAATGCGTAGAGATGTGGAGGAATACCGATGGCGAAGGCAGCCCCCTGGGCCAATACTGACGCTCATGCACGAAAGCGTGGGGAGCAAACAGG

>2ef43569fa81843da25798540a221bea

TACGTAGGGTGCGAGCGTTAATCGGAATTACTGGGCGTAAAGCGTGCGCAGGCGGTTTGTTAAGACAGATGTGAAATCCCCGGGCTCAACCTGGGAACTGCATTTGTGACTGGCAGGCTAGAGTATGGCAGAGGGGGGTAGAATTCCACGTGTAGCAGTGAAATGCGTAGAGATGTGGAGGAATACCGATGGCGAAGGCAGCTCCCTGGGCCAATACTGACGCTCATGCACGAAAGCGTGGGGATCAAACAGG

>a66eb9cd6a123087ad104a43a2bfa7f9

TTAAACGAAAGCACAACAAAAAGATAGCCAAACTAATACCACCCCAATCACCAGCAATAACACCCACACATACGTTTTACCCAAGAATAGTTAACCTAACCAACGTAACATTCACTAACGAACAGAAACAACTTTTAAACAAAGGGATAAACCACAACCTACACTACACACAGAATAATAACACCATCAAGAACATGGT

>14460154de56fac68aec8c318935c541

TACGGAGGGGGCGAGCGTTGTTCGGAGTGACTGGGCGTAAAGAGCACGTAGGCGGTGTTGTAAGTCATTAGTCAAAGACTAGAGCTCAACTTTAGTAAGGCTAGTGATACTATAATACTAGAGTATCAGAGAGGATTGCAGAATTCCTGGTGTAGCGGTAAAATGCGTAGATATCAGGAGAAATACCGTTAGCGAAGGCGGCAATCTGGCTGGAAACTGACGCTGAGGTGCGAAAGCGTGGGTAGCAAACAGG

>4bd40e2a3663f8ca39dec563dd019522

CACACTTATGTCTTCAGAAAGACAAAATATTTATCCAGGATGCGTTTCAACCATGTCGGTCATTTTCAACTGGCAGTGCCTTGTAGATATTTGTGAAAGATTTTGAATGTGTCTTGTCCATAGAGCCAGTTTGTTCATTAATGATTTTGTTATGTAGTTTTTAACGTAATATATTTAGGCTTTCAAGTAAATTCATTTTGTTTCCTTTTCGCCGGTTGTG

>9889d898689703d136d7ce92fad70d6e

CACCGGCAGCTCGAGTGATGGCCGATTTTATTGGGCCTAAAGCGTCCGTAGCCTGCCGTACGGGTCCGTCGGGAAATCCGCCCGCTCAACGGGCGGACGTCCGGCGGAAACCATACGGCTTGGGACCAGAGGACCCGAGGGGTACGTCGGGGGTAGGAGTGAAATCCCGTAATCCTGGACGGACCGCCGATGGCGAAAGCACGTCGCGAGAACGGATCCGACAGTGAGGGACGAAAGCCAGGGTCTCGAACCGG

>0f0020aa53cc62b10afb5cc8fb5eed2e

TACGGAGGGTGCAAGCGTTAATCGGAATTACTGGGCGTAAAGCGCACGCAGGCGGTTTGTTAAGTCAGATGTGAAATCCCCGAGCTTAACTTGGGAACTGCATTTGAAACTGGCAAGCTAGAGTCTTGTAGAGGGGGGTAGAATTCCAGGTGTAGCGGTGAAATGCGTAGAGATCTGGAGGAATACCGATGGCGAAGGCAGCCATCTGGCCTAACACTGACGCTGAGGTGCGAAAGCATGGGGAGCAAACAGG

>c3362cf17111ca5c614929e0fb80f25d

GACAGAGGATGCAAGCGTTATCCGGAATGATTGGGCGTAAAGCGTCTGTAGGTGGCTTTTCAAGTTCACCGTCAAATCCCCGGGCTCAACCCTGGATAGGCAGTGGAAACTACCAAACTGGAGTACGGTAGGGGCAGAGGGAATTTCCGGTGGAGCGGTGAAATGCGTTGAGATCGGAAAGAACACCAACGGCGAAAGCACTCTGCTGGGCCGACACTAACACTGAGAGACGAAAGCTAGGGGAGAAAATGGG

>87220bfae0f0511ebec7b77c671bd8da

TCGAACCCAAGCCACGTGGTGTAGCGAACCAACACACTACACACCACGAGTACTCCCCCCCCCTTCCAATCAACAAAAAATCACAATACGATTACAGGAAATTGTTCAGAATGTAGCGCCAAATGAAATTACAAAGAGGAGGATTTTGAATATTGCAGAAGGATCGTATTGGTGCAAAACAAACGTTTGCTAGCAGCCTTCCTCACGTGTATACTGCACTGATGCTTCGAGAAATAGTCTGCAGAACTAT

>721f4d8f982c3e8c6f8f4c32360ad5e0

GACAGAGGGTGCAAACGTTGTTCGGAATTACTGGGCGTAAAGCGTGTGTAGGCGGTCTTGTAAGTCGGATGTGAAAGCCCCGGGCTCAACCCGGGAAGTGCACTCGATACTGCGAGACTTGAGTATCGGAGAGGTTGGTGGAATTCTCGGTGTAGAGGTGAAATTCGTAGATATCGAGAGGAACACCGGTGACGAAAGCGGCCAACTGGACGAATACTGACGCTGAGACACGAAAGCGTGGGGAGCAAACAGG

>cc54cbe35b1fdb6fa03828d800f5ad6c

TACCGGCAGCTCAAGTGATGTCCCATATTATTGGGCCTAAAGCGTCCGTAGCTGGCCGACCAAGTCTATCGGGAAATCCACCTGCCCAACAGGTGGGCGTCCGGTAGAAACTGGCCGGCTTGGAACCGGAAGGCTCAGAGAGTACGTCCGGGGTAGGAGTGAAATCCCGTAATCCCGGACGGACTACCGATGGCGAAAGCACTCTGAGAAGACGGCTTCGACAGTGAGGGACGAAAGCTCGGGTCTCGAACCGG

>b7346161b005b9c87f519d24557a7f6d

TACGAAAGGTGCGAGCGTTAATCGGAATTACTGGGCGTAAAGCGCGCGTAGGTGGTGTGTTAAGTCGGATGTGAAAGCCCTGGGCTCAACCTGGGAATGGCATCCGATACTGGCCCGCTAGAGTGCAGTAGAGGGAGGTGGAATTTCCGGTGTAGCGGTGAAATGCGTAGAGATCGGAAGGAACACCAGTGGCGAAGGCGGCCTCCTGGACTGACACTGACACTGAGGTGCGAAAGCGTGGGGAGCGAACAGG

>c8f614dea3859d909c9d623ce57e702f

TAAATTGACAGTGCCTTTGATAACGATGAATATACGACGAAGAAACTGCTACAGCCACGACAACGTCGACGACGAGTAGTCGCGATAAGAATAATTTTCAACATGCACAAAGAGAGACGAGCAGCGAACTTTATCTCATTAGATAAGAAATGCAGCGATAACAAACAGCCGACAGAGGAGTGTACATCCATACAATGTCACAAGCAAGAGAGCTGTACTACAATAAAACATACGCGCATAACA

>cd3db324b2a93dbe71442ba9f1991325

TACGTAGGTCCCGAGCGTTGCCCGGATTTATTGGGCGTAAAGCGAGCGCAGGCGGTTAGATAAGTCTGAAGTTAAAGGCTGTGGCTTAACCATAGTATGCTTTGGAAACTGTTTAACTTGAGTGCAGAAGGGGAGAGTGGAATTCCATGTGTAGCGGTGAAATGCGTAGATATATGGAGGAACACCGGTGGCGAAAGCGGCTCTCTGGTCTGTAACTGACGCTGAGGCTCGAAAGCGTGGGGAGCAAACAGG

>487de539f50da640cd8914cea7821561

TACGAAGGGGGCTAGCGTTGTTCGGATTTACTGGGCGTAAAGCGCACGTAGGCGGATTGTTAAGTCAGAGGTGAAATCCTGGAGCTCAACTCCAGAACTGCCTTTGATACTGGCAATCTAGAGTCCGGAAGAGGTAAGTGGAACTCCTAGTGTAGAGGTGGAATTCGTAGATATTAGGAAGAACACCAGTGGCGAAGGCGGCTTACTGGTCCGGTACTGACGCTGAGGTGCGAAAGCGTGGGGAGCAAACAGG

>38d11e80b48a1a8189599af73f6cac67

TACCGGCAGCCCGAGTGATGGCCGATCTTATTGGGCCTAAAGCGTCCGTAGCTTGCTGTGTAAGTCCATTGGGAAATCCACCTGCTCAACAGGTGGGCGCCCGGTAGAAACTGTGCGGCTTGGGACCGGAAGGCGCGACGGGTACGTCCGGGGTAGGAGTGAAATCCCGTAATCCTGGACGGACCGCCGATGGCGAAAGCACGTCGCGAGAACGGATCCGACAGTGAGGGACGAAAGCCAGGGTCTCGAACCGG

>b4e588bfeae6a42575d6ebf1b46b9b0c

TACGTAGGGTGCAAGCGTTAATCGGAATTACTGGGCGTAAAGCGTGCGCAGGCGGTTATGCAAGACAAAGGTGAAATCCCCGGGCTCAACCTGGGAACTGCCTTTGTGACTGCATGGCTAGAGTACGGTAGAGGGGGATGGAATTCCGCGTGTAGCAGTGAAATGCGTAGATATGCGGAGGAACACCGATGGCGAAGGCAATCCCCTGGACCTGTACTGACGCTCATGCACGAAAGCGTGGGGAGCAAACAGG

>fe7761d647ff884c09ef4772e9224262

TACGTAGGGTGCGAGCGTTAATCGGAATTACTGGGCGTAAAGCGTGCGCAGGCGGTTTGTTAAGACAGATGTGAAATCCCCGGGCTCAACCTGGGAACTGCATTTGTGACTCGCAGGCTAGAGTATGGCAGAGGGGGGTAGAATTCCACGTGTAGCAGTGAAATGCGTAGAGATGTGGAGGAATACCGATGGCGAAGGCAGCCCCCTGGGCCAATACTGACGCTCATGCACGAAAGCGTGGGGAGCAAACAGG

>59c37907de8deef959bd20c402ce1d40

CAACCAAAAGTTCTATAACATTATAAAACGCTGTTTCAAATGTTTTATGCTGAACTAACCTCAAATACAGGTGTCATAATTTAATCTCTAGGATACGGGTGTCAGAATTTTATTATACGTGGAATTAGTTTCTATACTATACGGATTAAAAATGTTTTGATTTATGAAGATACATGATGATGATGATGATGAAGATACATGATGATGTTTTTGATAACTT

>0252e4492d7dc630e74ac9686f285e13

TACGAAGGGGGCTAGCGTTGCTCGGAATCACTGGGCGTAAAGGGTGCGTAGGCGGGTTTTTAAGTCAGGGGTGAAATCCTGGAGCTCAACTCCAGAACTGCCTTTGATACTGAAGATCTTGAGTCCGGGAGAGGTGAGTGGAACTGCGAGTGTAGAGGTGAAATCCGTAGATATTCGCAAGAACACCAGTGGCGAAGGCGGCTCACTGGCCCGGTACTGACGCTGAGGCACGAAAGCGTGGGGAGCAAACAGG

>f81950ea072375bad6e95cc4fa06ef8c

TACGTAGGGTGCGAGCGTTAATCGGAATTACTGGGCGTAAAGCGTGCGCAGGCGGTTTGTTAAGACAGATGTGAAATCCCCGGGCTCAACCTGGAAACTGCATTTGTGACTGGCAGGCTAGAGTATGGCAGAGGGGGGTAGAATTCCACGTGTAGCAGTGAAATGCGTAGAGATGTGGAGGAATACCGATGGCGAAGGCAGCCCCCTGGGCCAATACTGACGCTCATGCACGAAAGCGTGGGGAGCAAACAGG

>979989b424778e22066a6157fccebad9

CAAGGATCAAAAGGCTTTACGAGTTAGGGTGTGGCCTGCTACCGGCGGTTCCACCGCTTTATTGATGTTCTTTCTACGTTGGCACTAGCTCATCTGGTTGCCATTCAAGTTTTTGTTTGAGTCATCTGGTTTTTGTTTCGATCATTCTGCTCACATGTCCATACCATCGTAAT

>2afd0ba10ca0e8e0ebdee6a1c6fc7536

TACGTAGGGTGCGAGCGTTAATCGGAATTACTGGGCGTAAAGCGTGCGCAGGCGGTTTGTTAAGACAGATGTGAAATCCCCGGGCTCAACCTGGGAACTGCATTTGTGACTGGCAGGCTAGAGTATGGCAGAGGGGGGTAGAATTCCACGTGTAGCAGTGAAATGCGTAGAGTTGTGGAGGAATACCGATGGCGAAGGCAGCCCCCTGGGCCAATACTGACGCTCATGCACGAAAGCGTGGGGAGCAAACAGG

>f215817c85b229fb47293b762d77b055

TACGTAGGGTGCGAGCGTTAATCGGAATTACTGGGCGTAAAGCGTGCGCAGGCGGTTTGTTAAGACAGATGTGAAATCCCCGGGCTCAACCTGGGAACTGCATTTGTGACTGGCAGGCTAGAGTATGGCAGAGGGGGGTAGAATTCCACGTGTAGCAGTGAAATGAGTAGAGATGTGGAGGAATACCGATGGCGAAGGCAGCCCCCTGGGCCAATACTGACGCTCATGCACGAAAGCGTGGGGAGCAAACAGG

>ec9d2d24333bab92fe98e3b889899982

TACGTAGGGTGCGAGCGTTAATCGGAATTACTGGGCGTGAAGCGTGCGCATGCGGTTTGTTAAGACAGATGTGAAATCCCCGGGCTCAACCTGGGAACTGCATTTGTGACTGGCAGGCTAGAGTATGGCAGAGGGGGGTAGAATTCCACGTGTAGCAGTGAAATGCGTAGAGATGTGGAGGAATACCGATGGCGAAGGCAGCCCCCTGGGCCAATACTGACGCTCATGCACGAAAGCGTGGGGAGCAAACAGG

>05f631f0fa7fe26664811b71f55dd0c9

TACAGAGGGTGCGAGCGTTAATCGGATTTACTGGGCGTAAAGCGTGCGTAGGCGGCTTATTAAGTCGGATGCGAAATCCCCGAGCTTAACTTGGGAATTGCATTCGATACTGGTGAGCTAGAGTATGGGAGAGGATGGTAGAATTCCAGGTGTAGCGGTGAAATGCGTAGAGATCTGGAGGAATACCGATGGCGAAGGCAGCCATCTGGCCTAATACTGACGCTGAGGTACGAAAGCATGGGGAGCAAACAGG

>23ae48baea02f241cc8cfa5e2a83d86a

TACGAAGGGTGCAAGCGTTAATCGGAATTACTGGGCGTAAAGCGCGCGTAGGTGGTTTAGCAAGTTGGATGTGAAATCCCCGGGCTCAACCTGGGAACTGCATCCAAAACTACTGAGCTAGAGTACGGTAGAGGGTGGTGGAATTTCCTGTGTAGCGGTGAAATGCGTAGATATAGGAAGGAACACCAGTGGCGAAGGCGACCACCTGGACTGATACTGACACTGAGGTGCGAAAGCGTGGGGAGCAAACAGG

>d21c346ee26d1ee9f75a1f212bdf9fbe

CAACCCACTTTTTTCCCGGGCATAATTTTAGTTGTTTCTGCTGAAATATTAGAAACTGTGTTTCTACTTATCTGCAGGGCATCGGCGGCTCGCTGCGTGAAATTTTGAACACGTTGGGGTGGACCGTTGTTAATTTGTTCCTGTTTTAAATAATTATGCAGTCTTTTAACAAATGTTCTCGCTTGATATTTTAACGTCAATCCACAACCAAATTTTCTAACTCTCCCTTGATCCCTCGAC

>9ef8079ac49480a4baf13de90a631ab2

TACGTGAGAGACTAGTGTTATTCATCTTAATTGGGTTTAAAGGGTACCTAGACAGTCAATATAACCTCTAGAATGCTAATACTTGACTAGAGTTTTAAGTAAGAGGGAAGTACTTAAGGAGTAAGAGATGAAATATCTGTGATACCAAAGGGACTCCGTAAAGGCGAAGGCATCCCTTTATCTAAAAACTAACGTTGAAGGACGAAGGCTTAGATAACAAATAGG

>1b42ad06a911d1125ee15689ea3762d6

TACGTAGGGTGCGAGCGTTAATCGGAATTACTGGGCGTAAAGCGTGCTCAGGCGGTTTGTTAAGACAGATGTGAAATCCCCGGGCTCAACCTGGGAACTTCATTTGTGACTGGCAGGCTAGAGTATGGCAGAGGGGGGTAGAATTCCACGTGTAGCAGTGAAATGCGTAGAGATGTGGAGGAATACCGATGGCGAAGGCAGCCCCCTGGGCCAATACTGACGCTCATGCACGAAAGCGTGGGGAGCAAACAGG

>85a8e6d3675daef6bec6b0dc116ef288

TCAAACGTAAGCACAACAAAAAGATAGCCAAACTAATACCACCCCGATCACCAGCAATTACGCCCACACACACTTTTTACCCAAGAATAGTTAACCTCACCGACATAACATTCACTAACGATCAGGAACATCTTTTAAACAAAGGGATAAATCACAACCTACACTACACGCAGAACAATAACACCATAAAGAACTTTAGATGT

>5a2676b27aa16876cffaca5a4dca4669

TACGTAGGGTGCGAGCGTTGTCCGGAATTATTGGGCGTAAAGGGCTTGTAGGCGGTTTGTCGCGTCTGCCGTGAAATCCTCTGGCTTAACTGGGGGCGTGCGGTGGGTACGGGCAGGCTTGAGTGCGGTAGGGGAGACTGGAACTCCTGGTGTAGCGGTGGAATGCGCAGATATCAGGAAGAACACCGGTGGCGAAGGCGGGTCTCTGGGCCGTTACTGACGCTGAGGAGCGAAAGCGTGGGGAGCGAACAGG

>a4e52ef1c9529b4efd9104901523e6b5

TACGGAGGGTGCAAGCGTTAATCGGAATTACTGGGCGTAAAGCGCACGCAGGCGGTCTGTTAAGTCAGATGTGAAATCCCCGGGCTTAACCTGGGAACTGCATTTGAAACTGGCAGGCTTGAGTCTCGTAGAGGGGGGTAGAATTCCAGGTGTAGCGGTGAAATGCGTAGAGATCTGGAGGAATACCGGTGGCGAAGGCGGCCCCCTGGACAAAGACTGACGCTCAGGTGCGAAAGCGTGGGGAGCAAACAGG

>2645e35510e076bf32a4cc6edd7980d6

TACGGAGGGTGCGAGCGTTGTCCGGATTTATTGGGTTTAAAGGGTGCGTAGGCGGCCCGTTAAGTCCGGGGTGAAAGCCCGCTGCTCAACAGCGGAACTGCCCTGGATACTGACGGGCTTGAGTACAGACGAGGTTGGCGGAATGGACTGAGTAGCGGTGAAATGCATAGATACAGTCCAGAACCCCGATTGCGAAGGCAGCTGACTAGGCTGTTACTGACGCTGAGGCACGAAAGCGTGGGGAGCGAACAGG

>e78cbebbb3caf31eae0ee9b06ca90484

TACGGAGGGTGCAAGCGTTAATCGGAATTACTGGGCGTAAAGCACACGCAGGCGGTCTGTCAAGTCGGATGTGAAATCCCCGGGCTCAACCTGGGAACTGCATTCGAAACTGGCAGGCTAGAGTCTTGTAGAGGGGGGTAGAATTCCAGGTGTAGCGGTGAAATGCGTAGAGATCTGGAGGAATACCGGTGGCGAAGGCGGCCCCCTGGACAAAGACTGACGCTCAGGTGCGAAAGCGTGGGGAGCAAACAGG

>aee0dacb66bb3b44e8986f3859fd19d6

TACGTAGGGTCCGAGCGTTGTCCGGAATTATTGGGCGTAAAGAGCTCGTAGGCGGCATGTCGCGTCTGCTGTGAAAGACCGGGGCTTAACTCCGGTTCTGCAGTGGATACGGGCATGCTAGAGGTAGGTAGGGGAAACTGGAATTCCTGGTGTAGCGGTGAAATGCGCAGATATCAGGAGGAACACCGGTGGCGAAGGCGGGTTTCTGGGCCTTACCTGACGCTGAGGAGCGAAAGCATGGGGAGCGAACAGG

>d91b5266ca87c4b9167c8618bac0aab8

TACCGGCAGTCCGAGTGATGGCCGATATTATTGGGCCTAAAGCGTCCGTAGCCGGCCGGCCAAGTCCGTTGGGAAATCGACGCGCTCAACGCGTCGGCGTCCAGCGGAAACTGTCCGGCTTGGGGCCGGAAGACCTGAGGGGTACGTCCGGGGTAGGAGTGAAATCCCGTAATCCTGGACGGACCGCCGATGGCGAAAGCACGTCGCGAGAACGGATCCGACAGTGAGGGACGAAAGCCAGGGTCTCGAACCGG

>eefeefdd587c6a7b4ca133fd10621b28

TACCGGCAGTCCGAGTGATGGCCGATCTTATTGGGCCTAAAGCGTCCGTAGCTGGCCGCACAAGTCCATCGGAAAATCCACCTGCCCAACGGGTGGGCGTCCGGTGGAAACTGTGTGGCTTGGGACCGGAAGGCGCGACGGGTACGTCCGGGGTAGGAGTGAAATCCCGTAATCCCGGACGGACCGCCGGTGGCGAAAGCGCCTCGAGAGGACGGACCCGACGGTGAGGGACGAAAGCTAGGGTCTCGAACCGG

>a27a86fb7a3f23044d1e84f6db778425

CATTTTACCATAGTGTCTGCCTCCACGGGCACGTTTATGGCTTTGCCGGTTATCTTTTAAGACTGTTTCTATTCTGAGACGACGGATCTGCATACGTGGAAATCCGGATGAAATCAGTCTCTCAGTGATACGATCGAGCGGCAGATGGGTCGGATCTTCCGGATACTTATAACTATTACTTTGGCTCAGGGGAGGCACTTTACCTTCATTAAGCAAGCGGCGGTATGTGGAGCACA

>0f037ebbfc9b686b8383bd0c4458a8d4

TACGTAGGTGGCAAGCGTTATCCGGATTTATTGGGCGTAAAGAGAGTGCAGGCGGTTTTCTAAGTCTGATGTGAAAGCCTTCGGCTTAACCGGAGAAGTGCATCGGAAACTGGATAACTTGAGTGCAGAAGAGGGTAGTGGAACTCCATGTGTAGCGGTGGAATGCGTAGATATATGGAAGAACACCAGTGGCGAAGGCGGCTACCTGGTCTGCAACTGACGCTGAGACTCGAAAGCATGGGTAGCGAACAGC

>28e260728976404c3e3e1ef2357c68a6

GACAGAGGATGCAAGCGTTATCCGGAATGATTGGGCGTAAAGCGTCTGTAGGTGGCTTTTCAAGTCCGCCGTCAAATTCCAGGGCTCAACCCTGGACAGGCGGTAGAAACTACCAAGCTGGAGTACGGTAGGGGCAGAGGGAATTTCCGGTGGAGCGGTGAAATGCGTTGAGATCGGAAAGAACACCAACGGCGAAAGCACTCTGCTGGGCCGACACTGACACTGAGAGACGAAAGCTAGGGGAGCAAATGGG

>652d359e5f3dfa01532b61cff3017993

AACGTAGGAGGCGAGCGTTATCCGGATTCATTGGGCGTAAAGGGCGTGTAGGCGGTGACTCAAGTTGGGCGTGAAATCTCCCGGCTCAACCGGGAGGCGCCGTCCAATACTGGGACACTTGAGGGCAGGAGAGGAAAGCGGAATTCCCGGTGTAGTGGTGGAATACTTAGATATCGGGAGGAACACCAGTGGCGAAAGCGGCTTTCTGGTCTGCACCTAACGCTGAGACACGAAAGCTAGGGGAGCAAACTGGG

>53f0dff8885ea42d35a2ffaf0f1a106b

TACGTGAGAGACTAGTGTTATTCATCTTAATTGGGTTTAAAGGGTACCTAGACAGTCAATATAACTTCTATAATGCTAATACTTGACTAGAGTTTTAAGTAAGAGGGAAGTACTTAAAGAGTAAGAGATGAAATATCTGTGATACCAAAGGGACTCCGTAAAGGCGAAGGCATCCCTTTATCTAAAAACTAACGTTGAAGGACGAAGGCTTAGATAACAAATAGG

>fe43f16280c54140360806cf4141d75a

TACATAGGGGGCGAGCGTTATCCTGAATTATTGGGCGTAAAGGGTGCGTAGGCGGTTAAATAAGTTTATGGTCTAAGTGCAATGCTTAACGTTGTGATGCTATAAAAACTGTTTAGCTAGAGTTGGATAGAGGCAAGTGGAATTCCATGTGTAGTGGTAAAATGCGTAAATATATGGAGGAACACCAGAAGCGAAGGCGGCTTGCTGGGTCTTAACTGACGCTGAGGCACGAAAGCGTGGGGAGCAAACAGG

>8bb9848f9774a4e1889905db0478d1e7

TAGGTAGGGTGCGAGCGTTAATCGGAATTACTGGGCGTAAAGCGTGCGCAGGCGGTTTGTTAAGACAAATGTGAAATCCCCGGGCTCAACCTGGGAACTGCATTTGTGACTGGCAGGCTAGAGTATGGCAGAGGGGGGTAGAATTCCACGTGTAGCAGTGAAATGCGTAGAGATGTGGAGGAATACCGATGGCGAAGGCAGCCCCCTGGGCCAATACTGACGCTCATGCACGAAAGCGTGGGGAGCAAACAGG

>d340d6671732b72c3438e68aa88a2a8f

TACCGGCAGCTCGAGTGATGGCCGATGTTATTGGGCCTAAAGCGTCCGTAGCCGGCTGGACAAGTCCGTCGGGAAATCCACGTGCTCAACGCGTGGGCGTCCGGCGGAAACTGTTCGGCTTGGGGCCGGAAGACCCGAGGGGTACGTCTGGGGTAGGAGTGAAATCCCGTAATCCTGGACGGACCGCCGGTGGCGAAAGCGCCTCGGGAAGACGGACCCGACGGTGAGGGACGAAAGCTAGGGTCTCGAACCGG

>1617553164b9b042ac0d432462faf870

GACAGAGGGTGCAAACGTTGTTCGGAATTACTGGGCGTAAAGCGTGTGTAGGCGGTCTTGTAAGTCGGATGTGAAAGCCCCGGGCTCAACCCGGGGAGTGCACTCGATACTGCGAGACTTGAGTATCGGAGAGGTTGGTGGAATTCTCGGTGTAGAGGTGAAATTCGTAGATATCGAGAGGAACACCGGTGGCGAAAGCGGCCAACTGGACGAATACTGACGCTGAGACACGAAAGCGTGGGGAGCAAACAGG

>026b68ad384fbb3f5bb0f6769b1b4fce

TACGTAGGGTGCAAGCGTTAATCGGAATTACTGGGCGTAAAGCGTGCGCAGGCGGTTATATAAGTCAGATGTGAAATCCCCGGGCTCAACCTGGGAACTGCATTTGAGACTGTATGGCTAGAGTGTGTCAGAGGGGGGTAGAATTCCACGTGTAGCAGTGAAATGCGTAGATATGTGGAGGAATACCGATGGCGAAGGCAGCCCCCTGGGATAACACTGACGCTCATGCACGAAAGCGTGGGGAGCAAACAGG

>22c7a39e90ff38a7d03ecb264ca940da

TACGTAGGGGGCGAGCGTTGCTCGGAATTACTGGGCGTAAAGGGTGTGCAGGCGGCCGATTAAGTCAACGATGAAATCCCGAAGCTCAACTTCGGAATGGTCTTTGATACTGATCGGCTTGAGGCTGGTTGAGGAAAGCGGAATTCCCGGTGTAGTGGTGGAATACTTAGATATCGGGAGGAACACCAGCGGCGAAAGCGGCTTTCTGGACTGTGCCTGACGCTGAAGCGCGAAAGCGTGGGTAGCAAACTGGG

>19f8cd5be90d0db4b6553b00f9715c6f

TACGGAGGATGCGAGCGTTATTCGGAATCATTGGGTTTAAAGGGTCTGTAGGCGGGCTATTAAGTCAGGGGTGAAAGGTTTCAGCTTAACTGAGAAATTGCCTTTGATACTGGTAGTCTTGAATATCTGTGAAGTTCTTGGAATGTGTAGTGTAGCGGTGAAATGCTTAGATATTACACAGAACACCGATTGCGGAGGCAGGGGACTAACAGACGATTGACGCTGAGAGACGAGAGCGTGGGGAGCGAACAGG

>eb3495086228fe9c3b5ad2c14d6dbdba

TACGTAGGGTGCGAGCGTTAATCGGAATTACTGGGCGTAAAGCGTGAGCAGGCGGTTTGTTAAGACAGATGTGAAATCCCCGGGCTCAACCTGGGAACTGCATTTGTGACTGGCAGGCTAGAGTATGGCAGAGGGGGGTAGAATTCCACGTGTAGCAATGAAATGCGTAGAGATGTGGAGGAATACCGATGGCGAAGGCAGCCCCCTGGGCCAATACTGACGCTCATGCACGAAAGCGTGGGGAGCAAACAGG

>8ab8ee32fc8fa00043e7cabe68c08f1f

TACAGAGGGTGCAAGAGTTAATCGGATTTACTGGGCGTAAAGCGCGCGTAGGCGGCTAATTAAGTCAAATGTGAAATCCCCGAGCTTAACTTAGGAATTGCATTCGATACTGGTCAGCTAGAGTATGGGAGAGGATGGTAGAATTCCAGGTGTAGCGGTGAAATGCGTAGAGATCTGGAGGAATACCGATGGCGAAGGCAGCCATCTGGCCTAATACTGACGCTGAGGTACGAAAGCATGGGGAGCAAACAGG

>a5cdd4ac6098e0a5b3f5ced2612bd540

TACGTAGGGTGCGAGCGTTAATCGGAATTACTGGGCGTAAAGCGTGCGCAGGAGGTTTGTTAAGACAGATGTGAAATCCCCAGGCTCAACCTGGGAACTGCATTTGTGACTGGCAGGCTAGAGTATGGCAGAGGGGGGTAGAATTCCACGTGTAGCAGTGAAATGCGTAGAGATGTGGAGGAATACCGATGGCGAAGGCAGCCCCCTGGGCCAATACTGACGCTCATGCGCGAAAGCGTGGGGAGCAAACAGG

>3d7b9ab26afbde5f409c939cd48eac65

CACGATTAACCCAAGTCAATAGAAGCCGGCGTAAAGAGTGTTTTAGATCACCCCCTCCCCAATAAAGCTAAAACACACCTGAGTTGTAAAAAACTCCAGTTGACACAAAATAGACTACGAAAGTGGCTTTAACATATCTGAACACACAATAGCTAAGACCCAAACTGGG

>7fb0693199b248ddd10af217027ba947

TACGTAGGGTGCGAGCGTTAATCGGAATTACTGGGCGTAAAGCGTGCGCAGGCGGTTTGTTAAGACAGAGGTGAAATACCCGGGCTCAACCTGGGAACTGCATTTGTGACTGGCAGGCTAGAGTATGGCAGAGGGGGGTAGAATTCCACGTGTAGCAGTGAAATGCGTAGAGATGTGGAGGAATACCGATGGCGAAGGCAGCCCCCTGGGCCAATACTGACGCTCATGCACGAAAGCGTGGGGAGCAAACAGG

>89e6e6dfbfe1d301b2f1d1620b2704f8

TACGTAGGGTGCGAGCGTTAATCGGAATTACTAGGCGTAAAGCGTGCGCAGGCGGTTTGTTAAGACAGATGTGAAATCCCCGGGCTCAACCTGGGAACTGCATTTGTGACTGGCAGGCTAGAGTATGGCAGAGGGGGGTAGAATTCCACGTGTAGCAGTGAAATGCGTAGAGATGTGGAGGAATACCGATGGCGAAGGCAGCCCCCTGGGCCAATACTGACGCTCATGCACGAAAGCGTGGGGAGCAAACAGG

>d55fc7109fb9e529ae36036f8aa0167b

TACCGGCAGCCCGAGTGATGACCGATCTTATTGGGCCTAAAGCGTCCGTAGCCGGCCGAACGGGTTCATCGGGAAATCCACGCGCCCAACGCGTGGGCGTCCGGTGAATACCATTCGGCTTGGGGCCGGAAGACCCAGCGGGTACGTCCGGGGTAGGAGTGAAATCCCGTAATCCTGGACGGACCGCCGATGGCGAAAGCACGCTGGGAAAACGGATCCGACAGTGAGGGACGAAAGCCAGGGTCTCGAACCGG

>ad22787e1bcba17b30befd2d915663c9

TACGTAGGGTGCGAGCGTTAATCGGAATTACTGGGCGTAAAGCGTGCGCAGGCGGTTTGTTAAGACAGATGTGAAATCCCCGGGCTCAACCTGGGAACTGCATTTGTGACTGACAGGCTAGAGTATGGCAGAGGGGGGTAGAATTCCACGTGTAGCAGTGAAATGCGTAGAGATGTGGAGGAATACCGATGGCGAAGGCAGCCCCCTGGGCCAATACTGACGCTCATGCACGAAAGCGTGGGGAGCAAACAGG

>c1520ecfa9c4aa010cea1a8a01f9e6f3

CACGATTAACCCAAGTCAATAGAAGCCGGCGTAAAGAGTGTTTTAGATCACCCCCTCCCCAATAAAGCTAAAACTCACCTGAGTTGTAAAAAACTCCAGTTGACACGAAATAGACTACGAAAGTGGCTTTAACATATCTGAACACACAATAGCTAAGACCCAAACTGGG

>d119d40850715d31821680f45af7de5c

TACGAAGGGGGCTAGCGTTGCTCGGAATCACTGGGCGTAAAGGGTGCGTAGGCGGGTCTTTAAGTCAGGGGTGAAATCCTGGAGCTCAACTCCAGAACTGCCTTTGATACTGAGGATCTTGAGTCCGGAAGAGGTGAGTGGAACTGCGAGTGTAGAGGTGAAATTCGTAGATATTCGTAAGAACACCAGTGGCGAAGGCGGCTCACTGGTCCGGTACTGACGCTGAGGCACGAAAGCGTGGGGAGCAAACAGG

>e8a123a4face7e46bade30ee3777bdc2

TACGTAGGGTGCGAGCGTTAATCGGAATTACTGGGCGTAAAGCGTGCGCAGGCGGTTTGTTAAGACAGATGTGAAATCCCCGGGCTCAACCTGGGAACTGCATTTGTGACTGGCAAGCTAGAGTATGGCAGAGGGGGGTAGAATTCCACGTGTAGCAGTGAAATGCGTAGAGATGTGGAGGAATACCGATGGCGAAGGCAGCCCCCTGGGCCAATACTGACGCTCATGCACGAAAGCGTGGGGAGCAAACAGG

>b7e6c39dc73ed4c3cbc82a0583220153

CACACTAATCTCTTCAGAAAGACAAAATATTTCATCACGATGCATTTCAACCATGACGGTCATTTTTAACTGGCAGTGCCTTATTCGTGAAAGGTTTTGAACGTGTCTTGTCCATAGAGTTCAGTTTGTTCAATAATGATTTTTTTTATGTAATTTGTAACGTAATATATTTAGGCTTTCAAGTAAATTCATTTTGTTTCCTTTTTTGCCGGCTGTG

>8ea5892d164efb254173cdcf378c4908

TACCGGCAGCTCAAGTGATGTCCCATATTATTGGGCCTAAAGCGTCCGTAGCTGGCCGCGCAAGTCCGTCGGGAAATCCACCTGCCCAACAGGTGGGCGTCCGGCGGAAACTGTGTGGCTTGGAACCGGAAGGCTCAGAGAGTACGTCCGGGGTAGGAGTGAAATCCCGTAATCCCGGACGGACTACCGATGGCGAAAGCACTCTGAGAAGACGGCTTCGACAGTGAGGGACGAAAGCTCGGGTCTCAAACCGG

>2171439b2b67e2189fa8009a2e9d6e3f

TACGTAGGGTGCGAGCGTTAATCGGAATTACTGGGCGTAAAGCTTGCGCAGGCGGTTTGTTAAGACAGATGTGAAATCCCCGGGCTCAACCTGGGGACTTCATTTGTGACTGGCAGGCTATAGTATGGCAGAGGGGGGTAGAATTCCACGTGTAGCAGTGAAATGCGTAGAGATGTGGAGGAATACCGATGGCGAAGGCAGCCCCCTGGGCCAATACTGACGCTCATGCACGAAAGCGTGGGGAGCAAACAGG

>bf8e34c229a33d28a85c3625c00e70db

TACGAAGGGGGCTAGCGTTGCTCGGAATCACTGGGCGTAAAGGGTGCGTAGGCGGGTCTTTAAGTCAGGGGTGAAATCCTGGAGCTCAACTCCAGAACTGCCTTTGATACTGAGGATCTTGAGTCCGGAAGAGGTGAGTGGAACTGCGAGTGTAGAGGTGAAATTCGTAGATATTCGCAAGAACACCAGTGGCGAAGTCGGCTCACTGGTCCGGTACTGACGCTGAGGCACGAAAGCGTGGGGAGCAAACAGG

>f330e765325ac47997066c79007f76b7

TACGTAGGGCGCAAGCGTTGTCCGGAATTATTGGGCGTAAAGAGCTTGTAGGTGGCTTGTCGCGTCTGCCGTGAAAACCCGAGGCTCAACCTCGGGCGTGCGGTGGGTACGGGCAGGCTAGAGTGTGGTAGGGGAGACTGGAACTCCTGGTGTAGCGGTGAAATGCGCAGATATCAGGAAGAACACCGATGGCGAAGGCAGGTCTCTGGGCCATTACTGACACTGAGAAGCGAAAGCATGGGTAGCGAACAGG

>90dca90e7e0c14f7161fbb9a0a5d1caa

TACGTAGGGTGCGAGCGTTAATCGGAATTACTGGGCGTAAAGCGTGCGCAGGGGGTTTGTTAAGACAGATGTGAAATCCCCGGGCTCAACCTGGGAACTGCATTTGTGACTGGCAGGCTAGAGTATGGCAGAGGGGGGTAGAATTCCACGTGTAGCAGTGAAATGCGTAGAGATGTGGAGGAATACCGATGGCGAAGGCAGCCCCCTGGGCCAATACTGACGCTCATGCACGAAAGCGTGGGGAGCAAACAGG

>4fabceb15bb71b51400d998ff639ff4c

TACGTAGGGCGCAAGCGTTATCCGGAATTATTGGGCGTAAAGAGCTCGTAGGCGGTTTGTCGCGTCTGCTGTGAAAGTCCGGGGCTCAACTCCGGTTCTGCAGTGGGTACGGGCAGGCTAGAGTGATGTAGGGGAGACTGGAATTCCTGGTGTAGCGGTGAAATGCGCAGATATCAGGAGGAACACCGATGGCGAAGGCAGGTCTCTGGGCATTAACTGACGCCGAGGAGCGAAAGCATGGGGAGCGAACAGG

>aa2b88561f8a32cc39e701c2a67d7e36

TACCGGCAGCTCAAGTGATGTCCAATATTATTGGGCCTAAAGCGTCCGTAGCTGGCCGCGCAAGTCCGTCGGGAAATCCACCCGCTTAACGGGTGGGCGTCCGGCGGAAACTGTGCGGCTTGGGACAGGAAGGCTCGAGGGGTACGTTCGGGGTAGGAGTGAAATCCCGTAATCCTGAACGGACCGCCGATGGCGAAAGCACCTCGAGAGGACTGATCCGACAGTGAGGGACGAAAGCTGGGGTCTCGAACCGG

>5118b52de54fa65ef040b4efb0f51978

ACATTCACAGGCAGGCAGAAGGTATGACGTCATCGGAAATTGTAGTTCCCGACATCAGCAGCCGGCGTAAATTGAATTTGCTGCTCGCTGTGTAAAGGCAACAAGTTTATCCAATACGAGCTGTGTGCATTACAACAAAAAATTTACGCTTCAAGGCAAACGTGATGCATATTTTTTAATTTATAAAATGATGTAATCTATTTTCAAAGCCTAACAT

>2eedc20108688f6ad377cda54f8809b8

AATCTACTATCCAAACCTACAGTGGACACAGTGAAGAAGAAGCAGCTGTCAGATTAAACAGATAACCACCGACAGAACGCTAACAACTACTCTTACCACTTTGTTCACGTTTGACAGCCGTTCCTAGATGCTTGTTTACGTAACTGCTGACAATGAAAATAAACTTGGCATAATCCGTTTA

>2ba93b71cefb6f1f8004f084a1acb9e3

TACGGAGGGGGCTAGCGTTGTTCGGAATTACTGGGCGTAAAGCGCACGTAGGCGGACTGGAAAGTTGGAGGTGAAATCCCAGGGCTCAACCTTGGAACTGCCTTCAAAACTATCAGTCTGGAGTTCGAGAGAGGTGAGTGGAATTCCGAGTGTAGAGGTGAAATTCGTAGATATTCGGAGGAACACCAGTGGCGAAGGCGGCTCACTGGCTCGATACTGACGCTGAGGTGCGAAAGTGTGGGGAGCAAACAGG

>15252a977fb1025f462e10f1e433a2f8

TACGTAGGGTGCGAGCGTTAATCGGAATTACTGGGCGTAAAGCGTGCGCAGGCGGTTTGTTAAGACAGATGTGAAATCCCCGGGCTCAACCTGGGAACTGCATGTGTGACTGGCAGGCTAGAGTATGGCAGAGGGGGGTAGAATTCCACGTGTAGCAGTGAAATGCGTAGAGATGTGGAGGAATACCGATGGCGAAGGCAGCCCCCTGGGCCAATACTGACGCTCATGCACGAAAGCGTGGGGAGCAAACAGG

>6c42b013c96164f1d9af51ac3ee28c5e

TACGTAGGGTGCGAGCGTTAATCGGAATTACTGGGCGTAAAGCGTGCGCAGGCGGTTTGTTAAGACAGATGTGAAATCCCCGGGCTCAACCTGGGAACTGCATTTGTGACTGGCAGGCTAGAGTATGGCAGAGGGGGGTAGAATTCCACGTGTAGCAGTGAAATGCGTAGAGATGTGGAGGAATACCGATGGCGAAGGCAGCCCCCTGGGCCAATACTGACGCTCGTGCACGAAAGCGTGGGGAGCAAACAGG

>a050a90f713b4be99c9ded8902be7514

TACACTTTTGTCTTCAGAAAGACAAAACATTTTTCCAGACTGCGTTTCAACCTTGTCGGTCATTTTCAACTGGCAGTGCCTTGTAGATAGTCGTGGAAGATTTTGGATGTGTCTTGTCCATAGAGTTCAGTTTGTTCATTAATCATTTTGTTATGTAGTTTGTAACGTACTGTATTTAGACTTTCAAGTAAATTCATTTTATTTCCTTTTTTAACGGTTGCG

>08e68b5a3674cbb466394921f59e185e

TACGTAGGGTGCAAGCGTTAATCGGAATTACTGGGCGTAAAGCGTGCGCAGGCGGTTATATAAGACAGATGTGAAATCCCCGGGCTCAACCTGGGAACTGCATTTGTGACTGTATAGCTAGAGTACGGTAGAGGGGGATGGAATTCCGCGTGTAGCAGTGAAATGCTTAGATATGCGGAGGAACACCGATGGCGAAGGTAATCCCCTGGACCTGTACTGACGCTCATGCACGAAAGCGTGGGGAGCAAACAGG

>0aa5d2adc86a2a6aa88112d5ef35015b

CACGTAGGGTGCGAGCGTTGTCCGGAATTATTGGGCGTAAAGAGCTCGTAGGCGGTGTGTCGCGTCGGCCGTGAAAACTTGGGGCTTAACTCTGAGCGTGCGGTCGATACGGGAATCACTTGAGTTCGGCAGGGGAGACTGGAATTCCTGGTGTAGCGGTGAAATGCGCAGATATCTGGAGGAATACCGGTGGCGAAGGCGGCCCCCTGGACGAAGACTGACGCTCAGGTGCGAAAGCGTGGGGAGCAAACAGG

>3cf780f3d3f184641d6f2ce738abbace

CACGATTAACCCAAGTCAATAGAAGCCGGCGTAAAGAGTGTTTTAGGTCACCCCCTCCCCAATAAAGCTAAAACTCACCTGAGTTGTAAAAAACTCCAGTTGACACAAAATAGACTACGAAAGTGGCTTTAACATATCTGAACACACAATAGCTAAGACCCAAACTGGG

>571433790fd93db5c5f3bc2c25a643d5

CACACTTATGTCTTTAGAAAGACAACACATATTTCCAGAATGCATTTCAACCTTGTCGGTGCCTTATACAACTGCCAGTGCCTTGTAGATAGTTGTGGAAGATTTTGAATGTGTCTGGTCCATAGAGTTCAGTTTGTTCGTTAATGATTTTGTTATATAGTTTGTAACGTAATATATTTAAACTTTCAAGTAGATTCATTTTATTTCCTTTTTTGCCGGTTGTG

>dd8229814f2d127e61ac92c5d275db8b

TACGTAGGGTGCAAGCGTTAATCGGAATTACTGGGCGTAAAGCGTGCGCAGGCGGTTATGTAAGACAGATGTGAAATCCCCGGGCTCAACCTGGGAACTGCATTTGTGACTGCATAGCTAGAGTACGGTAGAGGGGGATGGAATTCCGCGTGTAGCAGTGAAATGCGTAGATATGCGGAGGAACACCGATGGCGAAGGCAATCCCCTGGACCTGTACTGACGCTCATGCACGAAAGCGTGGGGAGCAAACAGG

>4a150d824a7756bc2b9541fb5c4774c7

TACCGGCAGCCCGAGTGATGGCCGATATTATTGGGCCTAAAGCGTCCGTAGCTGGCCGCGCAAGTTCGTCGGGAAATCCACTCGCTTAACGAGTGGGCGTCCGGCGAAAACTGTTCGGCTTGGGGCCGGAAGACCCAAGGGGTACGTCCGGGGTAGGAGTGAAATCCCGTAATCCTGAACGGACCGCCGATGGCGAAAGCACCTCGAGAGGACTGATCCGACAGTGAGGGACGAAAGCTGGGGTCTCGAACCGG

>f5a90294901a6e95c91121120baa283d

TACGGAGGGTGCAAGCGTTAATCGGAATTACTGGGCGTAAAGCGCACGCAGGCGGTCTGTTAAGTCAGATGTGAAATCCGCGGGCTTAACCTGGGAACTGCATTTGAAACTGGCAGGCTTGAGTCTCGTAGAGGGGGGTAGAATTCCAGGTGTAGCGGTGAAATGCGTAGAGATCTGGAGGAATACCGGTGGCGAAGGCGGCCCCCTGGACGAAGACTGACGCTCAGGTGCGAAAGCGTGGGGAGCAAACAGG

>a69f247660912d36e29c475d1afc5780

TACGTAGGGTGCGAGCGTTAATCGGAATTACTGGGCGTAAAGCGTGCGCAGGCGGTTTGTTAAGACAGATGTGAAATCCCCGGACTCAACCTGGGAACTGCATTTGTGACTGGCAGGCTAGAGTATGGCAGAGGGGGGTAGAATTCCACGTGTAGCAGTGAAATGCGTAGAGATGTGGAGGAATACCGATGGCGAAGGCAGCCCCCTGGGCCAATACTGACGCTCATGCACGAAAGCGTGGGGAGCAAACAGG

>dbf0f327d48191c177e8508d1804f919

TACGTAGGGTGCGAGCGTTAATCGGAATTACTGGGCGTAAAGCGTGCGCAGGCGGTTTGTTAAGACAGATGTGAAATCCCCGGGCTCAACCTGGGAACTGCATTTGTGACTGGCAGGCTAGAGTATGGCAGAGGGGGGTAGAATTCCACGTGTAGCAGTGAAATGCGTAGAGATGTGGAGGAATACCGATGGCGAAGGCAGCCCCCTGGGCCAATACTGACGCTCATGCATGAAAGCGTGGGGAGCAAACAGG

>08c7f0fd08ae16d41e4d74113d526938

TACGTAGGGTGCGAGCGTTAATCGGAATTACTGGGCGTAAAGCGTGCGCAGGCGGTTTGTTAAGACAGATGTGAAATCCCCGGGCTCAACCTGGGAACTGCATTTGTGACTGGCAGGCTAGAGTATGGCAGAGGGGGGTAGAATTCCACGTGTAGCAGTGAAATGCGTAGAGATGTTGAGGAATACCGATGGCGAAGGCAGCCCCCTGGGCCAATACTGACGCTCATGCACGAAAGCGTGGGGAGCAAACAGG

>379971fc629af960ae9727451c02b56d

TACCGGCAGTCCAAGTGATGGCCGATATTATTGGGCCTAAAGCGTCCGTAGCTTGCTGTGTAAGTCCATTGGGAAATCGACGCGCTCAACGCGTCGGCGTCCGGTAGAAACTGGCCGGCTTGGAACCGGAAGGCTCAGAGAGTACGTCCGGGGTAGGAGTGAAATCCCGTAATCCCGGACGGACTACCGATGGCGAAAGCACTCTGAGAAGACGGCTTCGACAGTGAGGGACGAAAGCTCGGGTCTCAAACCGG

>e551f52ceb35fec63f3945668710910e

TACGTAGGGTGCGAGCGTTAATCGGAATTACTGGGCGTAAAGCGTGCGCAGGCGGTTTGTTAAGACAGATGTGAAATCCCCGGGCTCAACCTGGGAACTGCATTTGTGACTGGCAGGCTAGAGTATGGCAGAGGGGGGTAGAATTCCACGTGTAGCAGTGAAATGCGTAGAGATGTGGAGGAATACCGATGGCGAAGGCAGCCCCCTGGGCCAATACTGACGCTCAGGCACGAAAGCGTGGGGAGCAAACAGG

>d3df483cbb13c7f8c70820fb7e1e6230

TACGTAGGGCGCGAGCGTTGTCCGGAATCATTGGGCGTAAAGCGCGTGTAGGCGGCTGAGTAAGTCTGCTGTGAAAGTCCAGGGCTCAACCCTGGAATGTCGGCGGATACTGCTCAGCTGGAGTACGGGAGGGGCGAGTGGAATTCCTGGTGTAGCGGTGGAATGCGCAGATATCAGGAGGAACACCTATGGCGAAGGCAGCTCGCTGGAACGTTACTGACGCTGAGACGCGAAAGCGTGGGGAGCAAACAGG

>c7e92a6127e8af52665ff93845edd5c8

TACGTGAGAGACTAGTGTTATTCATCTTAATTGGGTTTAAAGGGTACCTAGACAGTCAATATAACTTCTATAATGCTAATACTCGACTAGAGTTTTAAGTAAGAGGGAAGTACTTAAGGAGTAAGAGATGAAATATCTGTGATACCAAAGGGACTCCGTAAAGGCGAAGGCATCCCTTTATCTAAAAACTAACGTTGAAGGACGAAGGCTTAGATAACAAATAGG

>bb4da662834e9ad48c55a73670c6284f

TACGGAGGGTGCGAGCGTTGTCCGGAATCACTGGGCGTAAAGGGCGCGTAGGTGGTGGGGTCAGCGTGTGGTGAAAGCTCGGGGCTCAACCCCGGGTCGGCCATGCGAACTGCCCGACTGGAGCACTGTAGAGGCAGACGGAATTCCGGGTGTAGCGGTGGAATGCGTAGAGATCCGGAAGAACACCGGTGGCGAAGGCGGTCTGCTGGGCAGTTGCTGACACTGAGGCGCGACAGCGTGGGGAGCAAACAGG

>34a790fde4933652ac6e40636cde32c1

TACGTAGGGTGCGAGAGTTAATCGGAATTACTGGGCGTAAAGCGTGCGCAGGCGGTTTGTTAAGACAGATGTGAAATCCCCGGGCTCAACCTGGGAACTGCATTTGTGACTGGCAGGCTAGAGTATGGCAGAGGGGGGTAGAATTCCACGTGTAGCAGTGAAATGCGTAGAGATGTGGAGGAATACCGATGGCGAAGGCAGCCCCCTGGGCCAATACTGACGCTCATGCACAAAAGCGTGGGGAGCAAACAGG

>e26a2732231d74d18bb443320adfaec7

TACGGAGGGTGCAAGCGTTAATCGGAATTACTGGGCGTAAAGCGCACGCAGGCGGTCTGTCAAGTCGGATGTGAAATCCCCGGGCTCAACCTGGGAACTGCATTCGAAACTGGCAGGCTGGAGTCTTGTAGAGGGGGGTAGAATTCCAGGTGTAGCGGTGAAATGCGTAGAGATCTGGAGGAATACCGGTGGCGAAGGCGGTCCCCTGGACAAAGACTGACGCTCAGGTGCGAAAGCGTGGGGAGCAAACAGG

>b0a87493bb42a10b97493d5ffcc414a5

TACGTAGGTGGCAAGCGTTATCCGGAATTATTGGGCGTAAAGCGCGCGTAGGCGGTTTTTTAAGTCTGATGTGAAAGCCCACGGCTCAACCGGGGAGGGTCATTGGAAACTGGGGAACTTGAGTGCAGAAGAGGAGAGTGGAATTCCACGTGTAGCGGTGAAATGCGTAGAGATGTGGAGGAACACCAGTGGCGAAGGCGACTCTCTGGTCTGTAACTGACGCTGAGGGGCGAAAGCGTGGGGAGCAAACAGG

>b565209a8bc025b180bfa172ab53ff63

TACGTAGGGTGCGAGCGTTAATCGGAATTACTGGGCGTAAAGCGTGCGCAGGCGGTTTGTTAAGACAGATGTGAAATCCCCGGGCTCAACCGGGGAACTGCATTTGTGACTGGCAGGCTAGAGTATGGCAGAGGGGGGTAGAATTCCACGTGTAGCAGTGAAATGCGTAGAGATGTGGAGGAATACCGATGGCGAAGGCAGCCCCCTGGGCCAATACTGACGCTCATGCACGAAAGCGTGGGGAGCAAACAGG

>9315fbf6544a47277aab468749e34ced

TACGTAGGGGGCTAGCGTTATCCGGATTTACTGGGCGTAAAGGGTGCGTAGGCGGTCTTTCAAGTCAGGAGTGAAAGGCTACGGCTAAACCGTAGTAAGCTCTTGAAACTGGGAGACTTGAGTGCAGGAGAGGAGAGTGGAATTCCTAGTGTAGCGGTGAAATGCGTAGATATTAGGAGGAACACCAGTTGCGAAGGCGGCTCTCTGGACTGTAACTGACGCTGAGGCACGAAAGCGTGGGGAGCAAACAGG

>e84c0280e5388242196dd7f4e99cc324

TACGTAGGGTGCGAGCGTTAATCGGAATTACTGGGCGTAAAGCGTGCGCAGGCGGTTTGTTAAGACAGATGTGAAATCCCCGGGCTCAACCTGAGAACTGCATTTGTGACTGGCAGGCTAGAGTATGGCAGAGGGGGGTAGAATTCCACGTGTAGCAGTGAAATGCGTAGAGATGTGGAGGAATACCGATGGCGAAGGCAGCCCCCTGGGCCAATACTGACGCTCATGCACGAAAGCGTGGGGAGCAAACAGG

>dfffc1a7a7f71f94f75b529f3fd76812

TTCCTCTTTTTTCAACGAAGTTCCGCAATTCATTTCTATTTGTTGCCATTCATTTGCCTATTTCAATAGATGCCTCTAGTTCGGAACGCTGATACTATACTATCGTTCCATGCGATTCTAGGTCTTCCTCTAAAGTTTTTTTCTTTTTCCTGGTTTCCCATAGCTTTTTGACCAGTCTCTCTGTGCATGCTT

>91d471b974de89131371222d0d05bcc1

TACGAGGGGAGCGAGTGTTGTTCGGTTTTATTGGGCGTAAAGAGTACGTAGGCGGTTTTGTAAGTCAACACTTAAATCTTGAGACTTAATCTCATTACAGGTGTTGATACTGCATAACTATGAACTTAATAGGGGTGAACACAATTCCAAGTGTAGAGGTGAAATTCGTTGATATTTGGAGGAGTACCAAAGGCGAAGGCAGTTCATTGGGTTAAGTTGACGCTGAGGTACGAAAGCGTGGGGAGCAAACAGG

>afcdecd2a479b68a94dbd67c2f8fbe56

TACGTAGGGTGCGAGCGTTAATCGGAATTACTGGGCGTAAAGCGTGCGCAGGCGGTTTGTTAAGACAGATGTGAAATCCCCAGGCTCAACCTGGGAACTGCATTTGTGACTGGCAGGCTAGAGTATGGCAGAGGGGGGTAGAATTCCACGTGTAGCAGTGAAATGCGTAGAGATGTGGAGGAATACCGATAGCGAAGGCAGCCCCCTGGGCCAATACTGACGCTCATGCACGAAAGCGTGGGGAGCAAACAGG

>37bab030949005200b93ef04a280c5e5

CGTTTACTTGATGACGCAATCACTTTCATCAGTTTCGTCATTATCTCACGCCAGCGCGTGCAATTCTCAGCAGCGTGACAATACAATTGAGGCCGACACAGGGAAACAAGTAAATGGATAGACAAATAAACTCGGAAAAAAGATATTGTTTACAGAATTAAGAAAATTTAATTCAAGCCGGCAGGAATTTTGGGTAGGCGAGAAAATAGACGCTTACTAAGTTTTCTAG

>56dab36210be976b791a519d6c2a8be6

TACGTAGGGTGCGAGCGTTAATCGGAATTACTGGGCGTAAAGCGTGCGCAGGCGTTTTGTTAAGACAGATGTGAAATCCCCGGGCTCAACCTGGGAACTGCATTTGTGACTGGCAGGCTAGAGTATGGCAGAGGGGGGTAGAATTCCACGTGTAGCAGTGAAATGCGTAGAGATGTGGAGGAATACCGATGGCGAAGGCAGCCCCCTGGGCCAATACTGACGCTCATGCACGAAAGCGTGGGGAGCAAACAGG

>ecd3cc3f26887d9534d405e21c36e167

TACGTAGGGTGCAAGCGTTAATCGGAATTACTGGGCGTAAAGCGTGCGCAGGCGGTTTGTTAAGACAGATGTGAAATCCCCGGGCTCAACCTGGGAACTGCATTTGTGACTGGCAGGCTAGAGTATGGCAGAGGGAGGTAGAATTCCACGTGTAGCAGTGAAATGCGTAGAGATGTGGAGGAATACCGATGGCGAAGGCAGCCCCCTGGGCCAATACTGACGCTCATGCACGAAAGCGTGGGGAGCAAACAGG

>b23ca268782087d537b1f9d9d12beb93

TACGAGGGGAGCGAGTGTTGTTCGGTTTTATTGGGCGCAAAGGGTGTTCAGGTTGTTCAATAAGTTGATCTCTAAATCTTGGAACTCAACCCCATTCAAGGGGTCAATACTGTTGGACTTGAGCTTACAAGGGACGAATGGAATTCCAAGTGTAGAGGTGAAATTCGTTGATATTTGGGGGAACACCGGAGGCGAAAGCGATTCGTCATACTAAGCTGACACTGAAGACACGAAAGCATGGGGAGCAAACAGG

>acc9971a0ca91051c8e7500e7010408f

TACGTAGGGTGCGAGCGTTAATCTGAATTACTGGGTGTAAATCGTGCGCAGGCGGTTTGTTAAGACAGATGTGAAATCCCCGGGCTCAACCTGGGAACTGCATTTGTGACTGGCAGGCTAGAGTATGGCAGAGGGGGGTAGAATTCCACGTGTAGCAGTGAAATGCGTAGAGATGTGGAGGAATACCGATGGCGAAGGCAGCCCCCTGGGCCAATACTGACGCTCATGCACGAAAGCGTGGGGAGCAAACAGG

>a350b9217996b59c565edf3913dcd999

GCTGGGATTCTGTGTGTTCAATGTATACCGCTGGTTCCTCATTTCGAGTGGAAAGGGAAATTCTTAAGCCCTGTCAGAAAGAGCATCCTACTTGCACAGGGCCTCGAGGCAGTAGCTCGGCGCTCGACTCTGACGCAAAATCCGAACAGCCTAGAAGTGATGAAACATCACACTCCTGAAAAAGACATCAGAGGAGAAAATATTGAAACGTCAAGCAGTAGACAAAAACCTACGCATTCAA

>e260b4de7c71f5a387b24aafce4c1b4f

TACGTAGGGTGCGAGCGTTAATCGGAATTACTGGGCGTAAAGCGTGCGCAGGCGGTTTGTTAAGACAGATGTGAAATCCCCGGGCTCAACCTGGGAACTGCATTTGTGACTGGCAGGCTAGAGTATGGCAGAGGGGGGTAGAATTCCACGTGTAGCAGTGAAATGCGTAGAGATGTGGAGGAATACCGATGGCGAAGGAAGCCCCCTAGGCCAATACTGACGCTCATGCACGAAAGCGTGAGGAGCAAACAGG

>d176697df7e28558a1629b4ea3b6b017

TACGGAGGTGGCAAGCGTTGTTCGGAATTACTGGGCGTAAAGGGTGTGTAGGCGGTTGGCCGTGTTCGTTGTGAAATCTCCCGGGTACTCCGGGAAACTGCATCGAAAGCTGGCCGACTTGAGTGCAGGAGAGGAGAACGGAATTTCCGGTGTAGCGGTGAAATGCGTAGATATCGGAAGGAACGCCGGTGGCGAAAGCGGTTCTCTGGTCTGTTACTGACGCTGAGGCACGAAAGCTAAGGGAGCAAACTGGG

>f51a45db9213288105c40ea145368b94

TACCGGCAGCTCGAGTGATGTCCCATATTATTGGGCCTAAAGCGTCCGTAGCTGGCCAACCAAGTCTATCGGGAAATCCACCTGCCCAACGGGTGGGCGTCCGGTAGAAACTGGCTGGCTTGGAACCGGAAGGCTCAGAGAGTACGTCCGGGGTAGGAGTGAAATCCCGTAATCCCGGACGGACTACCGATGGCGAAAGCACTCTGAGAAGACGGCTTCGACAGTGAGGGACGAAAGCTCGGGTCTCAAACCGG

>a57cc3173b1c7fb8aa68a6a663c3a459

TACGTAGGTGGCGAGCGTTATCCGGATTTACTGGGCGTAAAGGGAGCGTAGGCGGATGATTAAGTGGGATGTGAAATACCCGGGCTCAACTTGGGTGCTGCATTCCAAACTGGTTATCTAGAGTGCAGGAGAGTAGAGTGGAATTCCTAGTGTAGCGGTGAAATGCGTAGAGATTAGGAAGAACACCAGTGGCGAAGGCTACTCTCTGGACTGTAACTGACGCTGAGGCTCGAAAGCGTGGGGAGCAAACAGG

>e55acb4fea59076ba319473e3005097e

CACGTAGGGTGCAAGCGTTATCCGGATTTACTGGGCATAAAGCGCGCGCGGGCGGTGTCGTAAGTTGGATGTGAAATCTTTCGGCTTAACCGGGAGGAGTCATTCAATACTGCGATGCTACGAGGGCAGAAGAGGGGAGCGGAATTCCCGGTGTAGTGGTAAAATGCGTAGAGATCGGGAGGAACACCAGTGGCGAAGGCGGCTCTCTGGTCTGCACCTGACGCTGAGGCGCGAAAGCGTGGGGAGCAAACTGGG

>e26f0ab09c73ab646f179e3acc0d3cca

TACGTAGGGTGCGAGCGTTAATCGGAATTACTGGGCGTAAAGCGTGCGCAGGCGGTTTATTAAGACAGATGTGAAATCCCCGGGCTCAACCTGGGAACTGCATTTGTGACTGGCAGGCTAGAGTATGGCAGAGGGGGGTAGAATTCCACGTGTAGCAGTGAAATGCGTAGAGATGTGGAGGAATACCGATGGCGAAGGCAGCCCCCTGGGCCAATACTGACGCTCATGCACGAAAGCGTGGGGAGCAAACAGG

>85451b5b8c2dd3227af0eb3605eeef79

TACCGGCAGCACGAGTGATGGCCGATATTATTGGGCCTAAAGCGTTCGTAGCCGGCCAGTCAAGTCTGTTGGGAAATCGACGCGCTCAACGCGTCGGCGTCCAGCGGAAACTGATTGGCTTGGGGCCGGAAGATCTGAGGGGTACGTCCGGGGTAGGAGTGAAATCCCGTAATCCTGGACGGACCGCCGGTGGCGAAAGCGCCTCGAGAGGACGGACCCGACGGTGAGGGACGAAAGCTAGGGTCTCGAACCGG

>e23042d877be0096f4a590fda4c898ec

TACGTAGGGTGCGAGCGTTAATCGGAATTACTGGGCGTAAAGCGTGCGCAGGCGGTTTGTTAAGACAGATGTGAAATCCCCGGGCTCAACCTGGGAACTGCATTTGTGACTGGCAGGCTAGAGTATGGCAGAGGGGGGTAGAATTCCACGTGTAGCAGTGAAATGCGTAGAGATGTGGAGGAATACCGATGGCGAAGGCAGCCCCCTAGGCCAATACTGACGCTCATGCACGAAAGCGTGAGGAGCAAACAGG

>1b178f66246e65bc78641e5930da5827

CACGATTAACCCAAGTCAATAGAAGCCGGCGTAAAGAGTGTTTTAGATCACCCCCTCCCCAATAAAGCTAAAACTCACCTGAGTTGTAAAAAACTCCAGTTGACACAAAATAGACTACGAAAGTGGCTTTAACATATCTGAACACACAATATCTAAGACCCAAACTGGG

>a3022e8ed42cb33e0ee95f6c8277c17d

TACGGAGGGTGCAAGCGTTATCCGGATTTATTGGGTTTAAAGGGTCCGTAGGCGGATCCGTAAGTCAGTGGTGAAATCTCATAGCTTAACTATGAAACTGCCATTGATACTGCGGGTCTTGAGTAAGGTAGAAGTAGCTGGAATAAGTAGTGTAGCGGTGAAATGCATAGATATTACTTAGAACACCAATTGCGAAGGCAGGTTACTATGTCTTAACTGACGCTGATGGACGAAAGCGTGGGGAGCGAACAGG

>a60eb4893812f3334f927c532a317de0

TACCGGCAGCTCAAGTGATGTCCCATCTTATTGGGCCTAAAGCGTCCGTAGCTGGCCGCGCAAGTCCATCGGGAAATCCACCTGCTCAACAGGTGGGCGCCCGGTGGAAACTGTGCGGCTTGGGACCGGAAGGCGCGACGGGTACGTCCGGGGTAGGAGTGAAATCCCGTAATCCTGGACGGACCGCCGATGGCGAAAGCACGTCGCGAGAACGGATCCGACAGTGAGGGACGAAAGCCAGGGTCTCGAACCGG

>6c0c202145affc4fdc1026c6a8a69001

TACGGAGGGGGCTAGCGTTGTTCGGAATTACTGGGCGTAAAGCGCGCGTAGGCGGACGGTCAAGTTGGGGGTGAAAGCCCGGGGCTCAACCCCGGAACTGCCTTCAAAACTCCCAGTCTAGAGTTCGAGAGAGGTGAGTGGAACTCCGAGTGTAGAGGTGAAATTCGTAGATATTCGGAAGAACACCAGTGGCGAAGGCGGCTCACTGGCTCGATACTGACGCTGAGGTGCGAAAGTGTGGGGAGCAAACAGG

>a68e6835461d804c7d90d61705b7e7e2

TACGGAGGATGCGAGCGTTATCCGGATTTATTGGGTTTAAAGGGTGCGTAGGTGGTTAATTAAGTCAGCGGTGAAAGTTTGTGGCTCAACCATAAAATTGCCGTCGAAACTGGTTGACTTGAGTATATTTGAGGTAGGCGGAATGCGTGGTGTAGCGGTGAAATGCATAGATATCACGCAGAACTCCGATTGCGAAGGCAGCTTACTAAACTATAACTGACACTGAAGCACGAAAGCGTGGGGATCAAACAGG

>f8b7edb0aa3db4b7f0cdc364efdb36c4

TACGTGAGAGACTAGTGTTATTCATCTTAATTGGGTTTAAAGGGTACCTAGACAGTCAATATAACTTCTAGAATGCTAATACTTGACTAGAGTTTTAAGTAAGAGGGAAGTACTTAAGGAGTAAGAGATGAAATATCTTTGATACCAAAGGGACTCCGTAAAGGCGAAGGCATCCCTTTATCTAAAAACTAACGTTGAAGGACGAAGGCTTAGATAACAAATAGG

>65c4023409626370374c272bdfaa3f98

TACGTGAGAGACTAGTGTTATTCATCTTAACTGGGTTTAAAGGGTACCTAGGCAGTCAATATAACTTCTATAATGCTAATACTTGACTAGAGTTTTAAGTAAGAGGGAAGTACTTAAGGAGTAAGAGATGAAATATCAGTGATACCAAAGGGACTCCGTAAAGGCGAAGGCATCCCTTTATTTAAAAACTAACGTTGAAGGACGAAGGCTTAGATAACAAATAGG

>0e82b35205edd7a80e123a4bbac3bb41

TACCGGCAGCCCGAGTGATGGCCGATCTTATTGGGCCTAAAGCGTCCGTAGCTGGCCGCGCAAGTCCATCGGAAAATCCACCCGCTCAACGGGTGGGCGTCCGGTGGAAACTGTGTGGCTTGGGACAGGAAGGCTCGAGGGGTACGTTCGGGGTAGGAGTGAAATCCCGTAATCCTGGACGGACCGCCGATGGCGAAAGCACGTCGCGAGAACGGATCCGACAGTGAGGGACGAAAGCTGGGGTCTCGAACCGG

>6b8ce5715ace5cd76723e004e9b19026

TACCGGCAGCCCGAGTGATGGCCGATCTTATTGGGCCTAAAGCGTCCGTAGCTGGCCGTACAAGTCCATCGGAAAATCCACCCGCCCAACGGGTGGGCGCCCGGTGGAAACTGCGCGGCTTGGGACCGGAAGGCGCGACGGGTACGCCCGGGGTAGGAGTGAAATCCCGTAATCCTGGACGGACCGCCGATGGCGAAAGCACGTCGCGAGAACGGATCCGACAGTGAGGGACGAAAGCCAGGGTCTCGAACCGG

>8c8fdaf3967c74270d2c3822552a126a

GACGGGGGGGGCAAGTGTTCTTCGGAATGACTGGGCGTCAAGGGCACGTAGGCGGTGAATCGGGTTGAAAGTGAAAGTCGCCAAAAAGTGGCGGAATGCTCTCGAAACCAATTCACTTGAGTGGGACAGAGGAGAGTGGAATTTCGTGTGTAGGGGTGAAATCCGTAGATCTACGAAGGAAGGCCAAAAGCGAAGGCAGCTCTCTGGGTCCCTACCGACGCTGGGGTGCGAAAGCATGGGGAGCGAACAGG

>c3d6d4750b9f36a684f9173716e80af7

CACACTTATGTCTTTAGAAAGACAAAACACTTCTCCAGAATGCATTTCACCCGTGTCGGTCACTTTCAACTGGCAGTAACTTGTTGATATTTGTGTCTTGCCCATAGAGTTCAGTTTGTTCATTAATTATTTTGTTATGTAATTTGTAACGTAATATATTTAGGCTTTCAAGTAAATTCATTTTACTTCCTTTTTGCCGGTTGAG

>86748e6e46eddb955f6918863b3cf191

TACGTAGGTGGCAAGCGTTGTCCGGAATTATTGGGCGTAAAGCGCGCGCAGGCGGCTTCTTAAGTCTGATGTGAAATCTTGCGGCTCAACCGCAAGCGGTCATTGGAAACTGGGAGGCTTGAGTGCAGAAGAGGAGAGTGGAATTCCACGTGTAGCGGTGAAATGCGTAGAGATGTGGAGGAACACCAGTGGCGAAGGCGGCTCTCTGGTCTGTAACTGACGCTGAGGCGCGAAAGCGTGGGGAGCAAACAGG

>19a68fca7301b243e1c490973ad83774

TACCGGCAGTCCAAGTGATGGCCGATATTATTGGGCCTAAAGCGTCCGTAGCTTGCTGTGTAAGTCCATCGGGAAATCCACCCGCCCAACGGGTGGGCGTCCGGTGGAAACTGTGTGGCTTGGGACCGGAAGGCGCGACGGGTACGTCCGGGGTAGGAGTGAAATCCCGTAATCCTGGACGGACCGCCGATGGCGAAAGCACGTCGCGAGAACGGATCCGACAGTGAGGGACGAAAGCTAGGGTCTCGAACCGG

>0e8df85f451dfb77e1f9f45f4feb78f3

TACCGGCAGTCCAAGTGATGGCCGATATTATTGGGCCTAAAGCGTCCGTAGCTTGCTGTGTAAGTCCATTGGGAAATCGACCAGCTCAACTGGTCGGCGTCCGGTGGAAACTACACAGCTTGGGGCCGAGAGACTCAACGGGTACGTCCGGGGTAGGAGTGAAATCCCGTAATCCCGGACGGACTACCGATGGCGAAAGCACTCTGAGAAGACGGCTTCGACAGTGAGGGACGAAAGCTCGGGTCTCAAACCGG

>42556ec29195d58f7a57c69a126b918f

TACGTAGGTGGCAAGCGTTGTCCGGATTTATTGTGCGTAAAGCGAGCGCAGGCGGTTTCTTAAGTCTGATGTGAAAGCCCCCGGCTCAACCGGGGAGGGTCATTGGAAACTGGGAGACTTGAGTGCAGAAGAGGAGAGTGGAATTCCATGTGTAGCGGTGAAATGCGTAGATATATGGAGGAACACCAGTGGCGAAGGCGGCTCTCTGGTCTGTAACTGACGCTGAGGCTCGAAAGCGTGGGGAGCAAACAGG

>8d22bb139a6622ac982417478ebe4106

GACAGAGGGTGCAAACGTTGTTCGGAATTACTGGGCGTAAAGCGTGTGTAGGCGGTCTTGTAAGTCGGATGTGAAAGCCCCGGGCTCAACCCGGGAAGTGCACTCGATACTGCGAGACTTGAGTATCGGAGAGGTTGGTGGAATTCTCGGTGTAGAGGTGAAATTCGTAGATATTGAGAGGAACACCGGTGGCGAAAGCGGCCAACTGGACGAATACTGACGCTGAGACACGAAAGCGTGGGGAGCAAACAGG

>a4f6ac695b7373f08754309aba51c68a

CACACTTACGTCTTCAGAAAGACAAAATATTTCTCCAGGATGCATTTCAACCATGTCGGTCATTTTCAACTGGCAGTGCCTTGTAGATATTTGTGAAAGATTTTGAATGTGTCTTGTCCATAGAGTTCAGTTTGTTCATTAATGATTTTGTTATGTAGTTTGTAACATTTTGTTTCCTTTTTTGCTGGTTGTG

>c926e287f5ed6c240b0098f08a9b31e3

TACCGGCAGCCCGAGTGATGGCCGATCTTATTGGGCCTAAAGCGTCCGTAGCTTGCTGTGTAAGTCCATTGGGAAATCCACGCGCTCAACGCGTGGGCGTCCGGTGGAAACTGCACGGCTTGGGGCCGAGAGACTCGACGGGTACGTCCGGGGTAGGAGTGGAATCCTATAATCCTGGACGGACCACCAATGGGGAAACCACGTTGAGAGACCGGACCCGACAGTGAGGGACGAAAGCCAGGGTCTCGAACCGG

>d0fbc9aff818121519652ff493a83314

TACGTAGGGTGCAAGCGTTAATCGGAATTACTGGGCGTAAAGCGTGCGCAGGCGGTTTCGTAAGTCTGTCGTGAAATCCCCGGGCTTAACCTGGGAATGGCGATGGAGACTGCGAGGCTAGAGTTTGGCAGAGGGGGGTAGAATTCCACGTGTAGCAGTGAAATGCGTAGAGATGTGGAGGAACACCGATGGCGAAGGCAGCCCCCTGGGTCAAAACTGACGCTCATGCACGAAAGCGTGGGGAGCAAACAGG

>f4f5176f82e8c562818211f337295420

TACGTAGGGTGCGAGCGTTAATCGGAATTACTGGGCGTAAAGCGTGCGCAGGCGGTTTGTTAAGACAGATGTGAAATCCCCGGGATCAACCTGGGAACTGCATTTGTGACTGGCAGGCTAGAGTATGGCAGAGGGGGGTAGAATTCCACGTGTAGCAGTGAAATGCATAGAGATGTGGAGGAATACCGATGGCGAAGGCAGCCCCCTGGGCCAATACTGACGCTCATGCACGAAAGCGTGGGGAGCAAACAGG

>101b38b8a865ebd8b70650ebec48379f

TACCGGCAGCCCGAGTGATGGCCGATCTTATTGGGCCTAAAGCGTCCGTAGCCGGCCAGACAAGTCCGTTGGGAAATCGACGTGCCTAACACGTCGGCGTCCAGCGGAAACTGTCTGGCTTGGGGCCGGAAGACTCGAGGGGTACGTCCGGGGTAGGAGTGAAATCCCGTAATCCTGGACGGACCGCCGGTGGCGAAAGCGCCTCGAGAGGACGGACCCGACGGTGAGGGACGAAAGCTAGGGTCTCGAACCGG

>02844c6b58304da5365292f9cfe62179

TACGTAGGGGGCGAGCGTTGCTCGGAATTACTGGGCGTAAAGGGTGTGCAGGCGGCCGATTAGGTCAACGATGAAATCCCGAAGCTCAACTTCGGAATGGTCTTTGATACTGATCGGCTTGAGGCTGGTTGAGGAGAGCGGAATTCCCGGTGGAGCGGTGAAATGCGTAGATATCGGGAGGAACACCAGAGGCGAAGGCGGTTCTCTAAACTTGTCCTGACGCTGAGACACGAAAGCTAGGGGAGCAAACTGGG

>55205e9143090580e7b84eacfe59b263

CACGATTAACCCAAGTCAATAGATGCCGGCGTAAAGAGTGTTTTAGATCACCCCCTCCCCAATAAAGCTAAAACTCACCTGAGTTGTAAAAAACTCCAGTTGACACAAAATAGACTACGAAAGTGGCTTTAACATATCTGAACACACAATAGCTAAGACCCAAACTGGG

>09c0b4f4d8781cdca0a81a60d451dbb9

CACGATTAACCCAAGTCAATAGAAGCCGGCGTAAAGAGTGTTTTAGATAACCCCCTCCCCAATAAAGCTAAAACTCACCTGAGTTGTAAAAAACTCCAGTTGACACAAAATAGACTACGAAAGTGGCTTTAACATATCTGAACACACAATAGCTAAGACCCAAACTGGG

>696f8fc68b014f634145a0c0e24ea07f

TACGTGAGAGACTAGTGTTATTCATCTTAATTGGGTTTAAAGGGTACCTAGGCAGTCAATATAACTTCTATAATGCTAATACTTGACTAGAGTTCTAAGTAAGAGGGAAGTACTTAAGGAGTAAGAGATGAAATATCAGTGATACCAAAGGGACTCCGTAAAGGCGAAGGCATCCCTTTATTTAAAAACTAACGTTGAAGGACGAAGGCTTAGATAACAAATAGG

>73e3875031eba2a6cdc04391f133994c

AAATGGCTACTTTCACGGGTGCAGAACGGGCTAACTGTGTGTTAAGGTTTCATGACACAAACTCTGCAACAACAGTTCAGCATAATTTTTGCACCGAGTGTGGTAAAGATCCTCTTACTAGACCTACAATTTACACTTGACCTCAGAACTTCGTTGAGAGTGGTTGTTCGGTTCAGC

>8b95665b7c7e6cdee461b02916bc04a9

TACGGAGGGTCTAAGCGTTATCCGGATTTATTGGGTTTAAAGGGTGCGTAGGCGGTTTGGTAAGTTAGAGGTGAAATCCCGGGGCTCAACCCTGGAACTGCCTCTGATACTGCCAGGCTAGAGAATGGTTGCTGTGGGCGGAATGTATGGTGTAGCGGTGAAATGCTTAGAGATCATACAGAACACCGATTGCGAAGGCAGCTCACAAAGCCATATCTGACGCTGATGCACGAAAGCGTGGGGAGCAAACAGG

>3bbe766e5c7b65e46522e99d9a6a27a1

CACGTAGGGTGCAAGCGTTATCCGGATTTACTGGGCGTAAAGCGCGCGCGGGCGGTGTCGTAAGTTGGATGTGAAATCTTTCGGCATAACCGGGAGGAGTCATTCAATACTGCGATGCTACGAGGGCAGAAGAGGGGAGCGGAATTCCCGGTGTAGTGGTAAAATGCGTAGAGATCGGGAGGAACACCAGTGGCGAAGGCGGCTCTCTGGTCTGCACCTGACGCTGAGGCGCGAAAGCGTGGGGAGCAAACTGGG

>f826798de79ad8e61d4bd4b7392c4325

TACCGGCAGCCCAAGTGATGGCCGATCTTATTGGGCCTAAAGCGCCCGTAGCTGGCCGCGCAAGTCCATCGGGAAATCCACCTGCTCAACAGGTGGGCGCCCGGTAGAAACTGCGTGGCTTGGGACCGGAAGGCGCGACGGGTACGTCCGGGGTAGGAGTGAAATCCCGTAATCCTGGACGGACCGCCGATGGCGAAAGCACGTCGCGAGAACGGATCCGACAGTGAGGGACGAAAGCCAGGGTCTCGAACCGG

>bafdcc6f7052ec57892ab4de6c794562

TACAGAGGGTGCGAGCGTTAATCGGAATTACTGGGCGTAAAGCGCGCGTAGGCGGTTATTTAAGTCGGATGTGAAATCCCCGAGCTCAACTTGGGAATTGCATTCGATACTGGGTAGCTAGAGTATGGGAGAGGAAGGTAGAATTCCAGGTGTAGCGGTGAAATGCGTAGAGATCTGGAGGAATACCGATGGCGAAGGCAGCCTTCTGGCCTAATACTGACGCTGAGGTGCGAAAGCATGGGGAGCAAACAGG

>efeb045444f102c8c77502e4d9393024

AAAAAGGAAGGAAGACGGAAATAAGAACCACGCAGAAAGCAGACAAAAAAAGACAATGAAATATTAAGATGTTAGAAACGGAAGAACAAGGCGACAGCAAAAAATATGAAGAAATTATAAGTACACAAATAGAGAATTTTCCGAGAAAACGGAAAGTACGATCGTTGAAGAAATACAGAAAAT

>97c5b6c9eb8704f92a063188cae15cf1

TACGGAGGGTGCAAGCGTTAATCGGAATTACTGGGCGTAAAGCGCACGCAGGCGGTCTGTCAAGTCGGATGTGAAATCCCCGGGCTCAACCTGGGAACTGCATCCGAAACTGGCAGGCTAGAGTCCTGTAGAGGGGGGTAGAATTCCAGGTGTAGCGGTGAAATGCGTAGAGATCTGGAGGAATACCGGTGGCGAAGGCGGCCCCCTGGACAAAGACCGACGCTCAGGTGCGAAAGCGTGGGGAGCAAACAGG

>7b673af04ecf62c48a3bd01bcff801c4

TACGGAGGGTGCAAGCGTTATCCGGATTTATTGGGTTTAAAGGGTCCGTAGGCGGACTTATAAGTCAGTGGTGAAATCCTGTCGCTTAACGATAGAACTGCCATTGATACTGTAAGTCTTGAGTATATTTGAGGTAGCTGGAATAAGTAGTGTAGCGGTGAAATGCATAGATATTACTTAGAACACCAATTGCGAAGGCAGGTTACCAAGATATAACTGACGCTGAGGGACGAAAGCGTGGGTAGCGAACAGG

>b0c72e0c96ccb312239ad00cacbbeda6

CACGATTAACCCAAGTCAATAGAAGCCGGCGTAAAGAGTGTTTTAGATCACCCCCTCCCCAATAAAGCTAAAACTCACCTGAGTTGTAAAAAACTCCAGTTGACACAAAATAGACTACGAAAGTGGCTCTAACATATCTGAACACACAATAGCTAAGACCCAAACTGGG

>3816112c3ba5e20fe3c178ea3ed1f6d6

TACGGAGGGGGCTAGCGTTGTTCGGAATTACTGGGCGTAAAGCGCGCGTAGGCGGACGGTCAAGTTGGGGGTGAAAGCCCGGGGCTCAACCCCGGAACTGCCTTCAAAACTGATCGTCTGGAGACCGGGAGAGATGAGTGGAATTCCCAGTGTAGAGGTGAAATTCGTAGATATTGGGAAGAACACCAGTGGCGAAGGCGGCTCACTGGACCGGATCTGACGCTGAGGTGCGAAAGCGTGGGGAGCGAACAGG

>edf11e480afcfbbe9f7be45718c3ff6b

TACCGGCAGCACGAGTGATGGCCGATCTTATTGGGCCTAAAGCGTCCGTAGCCGGCCGAGCAAGTTCGTCGGGAAATCCACCAGCTCAACTGGTGGGCGTCCGGCGAAAACTGTTCGGCTTGGGGCCGGAAGACCCAAGGGGTACGTCCGGGGTAGGAGTGAAATCCCGTAATCCCGGACGGACCGCCAATGGCGAAAGCACCTTGGGAAGACGGACCCGACGGTGAGGGACGAAAGCTGGGGTCTCAAACCGG

>0391ca7b7da7605d116b9e98ea380e46

AGCAACTCTCAGTGCCTTCCTCTTGCTCACGGCCATATGCGTGACAGGGGACCTGCCACCGAACATCCTGGTAATCAGCAAAATATATGACGCGCAGGACGAAGGATCCTGGTGCTCATCGGGCATGACACAGGAGCACAACATTGATATGGGTGCAAGACACCATGTCATAATAAAAAAAAGAACACCCCAGGTGTCCAAATCTCATATTATCATTGTATATTTGATGTCTTAGTTTTATAATTTTTCTTTAC

>ee79f5ad1c65fc5e44586caae7988321

TACGTAGGGTGCGAGCGTTAATCGGAATTACTGGGCGTAAAGCGTGCGCAGGCGGTTTGTTAAGACAGATGTGAAATCCCCGGGCTCAACCTGGGAACTGCATTTGTTACTGGCAGGCTAGAGTATGGCAGAGGGGGGTAGAATTCCACGTGTAGCAGTGAAATGCGTAGAGATGTGGAGGAATACCGATGGCGAAGGCAGCCCCCTGGGCCAATACTGACGCTCATGCACGAAAGCGTGGGGAGCAAACAGG

>6a4a1b2a6ef2a88bcc2a90b0b6393c57

GACGAAGGTGGCGAGCGTTGTTCGGAATCACTGGGCTTAAAGCGCACGTAGGCGGCCTGTCAAGCGCCGTGTGAAATCCCTCGGCTTAACCGAGGAATGGCTTGGCGAACTGGCAGGCTTGAGGCAGGTATGGGCGAGTGGAACTCTTGGTGGAGCGGTGAAATGCGTAGATATCAAGAGGAACGCCGGCGGTGAAGACGACTCGCTGGGCCTGTCCTGACGCTGAGGTGCGAAAGCCAGGGGAGCAAACTGGG

>9f721676c8d8ae1ea5cdc4fd1895572e

TACAGAGGGTGCAAGCGTTAATCGGATTTACTGGGCGTAAAGCGCGCGTAGGCGGCTAATTAAGTCAAATGTGAAATCCCCGAGCTTAACTTGGGAATTGCATTCGATACTGGTTAGCTAGAGTGTGGGAGAGGATGGTAGAATTCCAGGTGTAGCGGTGAAATGCGTAGAGATCTGGAGGAACACCGATGGCGAAGGCAGGTCTCTGGGCATTTACTGACGCTGAGGAGCGAAAGCATGGGGAGCGAACAGG

>1cf4e9fca1cc9ef2a56ea09bf9356b87

TACGGAGGGAGCTAGCGTTATTCGGAATTACTGGGCGTAAAGCGCACGTAGGCGGCTTTGTAAGTTAGAGGTGAAAGCCTGGAGCTCAACTCCAGAATTGCCTTTAAGACTGCATCGCTTGAATCCAGGAGAGGTGAGTGGAATTCCGAATGTAGAGGTGAAATTCGTAGATATTCGGAAGAACACCAGTGGCGAAGGCGGCTCACTGGACTGGTATTGACGCTGAGGTGCGAAAGCGTGGGGAGCAAACAGG

>020e1ba894cf5b5b92e46f82b0c11c34

TACGTAGGTGGCAAGCGTTGTCCGGAATTATTGGGCGTAAAGCGAGCGCAGGCGGTTTCTTAAGTCTGATGTGAAAGCCCCCGGCTCAACCGGGGAGGGTCATTGGAAACTGGGGAACTTGAGTGCAGAAGAGGAGAGTGGAATTCCACGTGTAGCGGTGAAATGCGTAGAGATGTGGAGGAACACCAGTGGCGAAGGCGACTCTCTGGTCTGTAACTGACGCTGAGGAGCGAAAGCGTGGGGAGCAAACAGG

>8b106973970b1f617a9d55e574cdb4cf

TACAGAGGGTGCGAGCGTTAATCGGAATTACTGGGCGTAAAGCGAGTGTAGGTGGCTCATTAAGTCACATGTGAAATCCCCGGGCTTAACCTGGGAACTGCATGTGATACTGGTGGTGCTAGGATATGTGAGAGGGAAGTAGAATTCCAGGTGTAGCGGTGAAATGCGTAGAGATCTGGAGGAATACCGATGGCGAAGGCAGCTTCCTGGCATAATATTGACACTGAGATTCGAAAGCGTGGGTAGCAAACAGG

>7c79f26a299f6a6ed7f719af5a219381

TACGTAGGGTGCGAGCGTTAATCGGAATTACTGGGCGTAAAGCGTGCGCAGGCGGTTTGTTAAGACAGATGTGAAATCCCCGGGCTCAACCTGGGAACTGCATTTGTGACTGGCAGGCTAGAGTATGGCAGAGGGGGGTAGAATTCAACGTGTAGCAGTGAAATGCGTAGAGATGTGGAGGAATACCGATGGCGAAGGCAGCCCCCTGGGCCAATACTGACGCTCATGCACGAAAGCATGGGGAGCAAACAGG

>cd4c8ea560215b8e555439d1471d9f4f

CATTGAACTATCGTGAGAAAGTCAAGCCGCCAAAGGGAATTATATAATAGTAAATATTAGCGTAAATAAACATTTTATTAATAGTTGTAATATATGATAATGTGCAAAAGATAGAGACGATTAAAACGGTGTTTAACAATCGCTTATCGAGACAATATTTAATTA

>eadf7be68efcf12a9345af56620a7a50

ACGATCCACCCATAGATGGATTCGAACTCACAACGCCCTGTTTTGGAAGCGGCAGTGCTGACCACTGCGCACTATGACATGTAATTAGTTATACATTACTTTAGTGAAATTACTTGTAATAGAAATATCGAAACTGCAAAAATCAAAACACGAAAGAATTTTTCTTGCAACGTAAAATCG

>ab6d233f0ef70363ed9c90730cfb1eb9

AACAGAGGATACAAGCGTTATCCGGATTTATTGGGTTTAAAGGGTGCGTAGGTGGTTTTTTAAGTCAGTAGTGAAATCTTAAATCTTAACTTTAAAAGTGCTATTGATACTGATAAACTAGAGTGAGGTTGGAGTAACTGGAATGTGTGGTGGAGCGGTGAAATGCATAGAGATCACACAGAACACCAATCGCGAAGGCATGTTACTAAACATAGACTGACACTGAGGCACGAAAGCATGGGTAGCAAACAGG

>43d3bc56dead272f7ba7a62ca0c1769a

AATCCTTGTGCCGTGAACCGAAATAGTTCCGGAAACGAGGTTCTATTCCAAACTACGAAATGAAGAAGAGGAAGAGGAAGAAGAAGAAATCTGCGAGATCACTATGTGCTTCACACGACGTGATGATCCTATTGCATGCGTTTAGCGAACAACACTATGACCACGCATGCTAAAGCTAAAGGGATAATTATTTT

>f3d6192e11cb491130c8a455eb9fed08

ACTCGATCTGCTGTAATGGGACTAGGTTTGGAATCATTCGTATGCTGTTAATATGGTACTTGTGCTACGATGTTTAGTGGCTACAAGAGACGGATCGTGGTCAGAGTCGCTTTAATGTATTACCGTCGGAGCGGATGTGAGTGTCACTGTAGCGGATGCACTCGGAATAATGTAAAACAGTACAGCAGTACAGTATTAGTCAGGTCGCGTAGCATCTGTTGTGGGTAAAGTAAAAGTCTTATTT

>a3eeb4a759c749b43f253d11a2b01b95

TACCGGCAGCCCGAGTGATGGCCGATCTTATTGGGCCTAAAGCGTCCGTAGCTGGCCGCGCAAGTCCATCGGGAAATCCACCTGCTCAACAGGTGGGCGCCCGGTGGAAACTGTGCGGCTTGGGACCGGAAGGCGCGACGGGTACGCCCGGGGTAGGAGTGAAATCCCGTAATCCTGGACGGACCGCCGATGGCGAAAGCACGTCGCGAGAACGGATCCGACAGTGAGGGACGAAAGCCAGGGTCTCGAACCGG

>53b04ae9dafa9354ad62d01fb8be665c

TACCGGCAGTCCGAGTGATGGCCGATATTATTGGGCCTAAAGCGTCCGTAGCTTGCTGTGTAAGTCCATTGGGAAATCGACCAGCTCAACTGGTCGGCGTCCGGTGGAAACTACACAGCTTGGGGCCGAGAGACTCAACGGGTACGTCCGGGGTAGGAGTGAAATCCCGTAATCCTGGACGGACCACCAATGGGGAAACCACGTTGAGAGACCGGACCCGACAGTGAGGGACGAAAGCCAGGGTCTCGAACCGG

>accccde43fc86696aca738121bbe7dbe

TACGTAGGGTGCGAGCGTTGTCCGGAATTACTGGGCGTAAAGAGCTCGTAGGTGGTTTGTCGCGTCGTCTGTGAAATTCCGGGGCTTAACTCCGGGCGTGCAGGCGATACGGGCATAACTTGAGTACTGTAGGGGAGACTGGAATTCCTGGTGTAGCGGTGAAATGCGCAGATATCAGGAGGAACACCGGTGGCGAAGGCGGGTCTCTGGGCAGTAACTGACGCTGAGGAGCGAAAGCATGGGTAGCGAACAGG

>f5a21cbb2bc8496c2c0a19b97437399e

GTGCTCAGAGAACATCAATCTTTCACTTCGTTCTCACTTTCTCTTTTTACTGCCTCCTCTTCTGACCCTTTTCCACCATACCCCTTCTTCTCCTTTTCGTCATCCCTTTTCGGTCTATTTGTAGCATAATATATTAGATACCCGTGTAGTCCGGCTGACTGAC

>b15ba4b0eba4868c35fbf36382acc0fd

TACCGGCAGCACGAGTGATGGCCGATCTTATTGGGCCTAAAGCGTCCGTAGCCGGCCGAGCAAGTTCGTCGGGAAACCTACCCGCTCAACGGGTAGTCGTCCGGCGAAAACTGTCTGGCTTGGGGCCGGAAGACTCGAGGGGTACGTCCGGGGTAGGAGTGAAATCCCGTAATCCTGGACGGACCGCCGGTGGCGAAAGCGCCTCGAGAGGACGGACCCGACGGTGAGGGACGAAAGCTAGGGTCTCGAACCGG

>a5b57c4923b52d89dc6a48cbaaa24a64

TACGTAGGGTGCGAGCGTTAATCGGAATTACTGGGCGTAAAGCGTGCGCAGGCGGTTTGTTAAGACAGATGTGAAATCCCCGGGCTCAACCTGGGAACTGAATTTGTGACTGGCAGGCTAGAGTATGGCAGAGGGGGGTAGAATTCCACGTGTAGCAGTGAAATGCGTAGAGATGTGGAGGAATACCGATGGCGAAGGCAGCCCCCTGGGCCAATACTGACGATCATGCACGAAAGCGTGGGGAGCAAACAGG

>97ddc265d9ba28add78ecc40069cece7

TACGAAGGGGGCTAGCGTTGCTCGGAATCACTGGGCGTAAAGGGTGCGTAGGCGGGTCTTTAAGTCAGGGGTGAAATCCTGGAGCTCAACTCCAGAACTGCCTTTGATACTGAAGATCTTGAGTTCGGGAGAGGTGAGTGGAACTGCGAGCGTAGAGGTGAAATTCGTAGATATTCGCAAGAACACCAGTGGCGAAGGCGGCTCACTGGCCCGATACTGACGCTGAGGCACGAAAGCGTGGGGAGCAAACAGG

>c23e2f6b26f7a93d8b7daea08bb3fc40

TTGAACCCTAGCCGCATGGTGTAGCGAGCCAATACACCACCGACTATACCGCGGCCTCCAGACTGCAAAGGTACAAATTCATAATCTCAAATAAATTGTGAAAACTACAAAAAAGGATGTAGTTAAAAAAGTGTAAGAAAGTACGTAATACTGCATTAACTGTCAATCCGAGCATTAAATGCAGAAATACGACTGCTGTGCGTAGGGTAGCATGCTCGTAGCGATGTATTTCTCCA

>889ce916ba883c1c3fe92ed4440a34c1

TACGTAGGGTGCGAGCGTTAATCGGAATTACTGGGCGTAAAGCGTGCGCAGGCGGTTTGTTAAGACAGATGTGAAATCCCCGGGCTCAACCTGGGAACTGCATTTGTGACTGGCAGGCTAGAGTATGGCAGAGGGGGGTAGAATTCCACGTGTAGCAGTGAAATGCGTAGAGATGTAGAGGAATACCAATGGCGAAGGCAGCCCCCTGGGCCAATACTGACGCTCATGCACGAAAGCGTGGGGAGCAAACAGG

>79230c5aa75d57413975b01d758c9fe6

TACGTAGGTGGCGAGCGTTGTCCGGATTTATTGGGCGTAAAGGGAGTGTAGGCGGTCTTTTAAGTCTGATGTGAAAGCCCACGGCTCAACCGTGGAGGGTCATTGGAAGCTGGGAGACTTGAGTGCAGAAGAGGAGGGCGGAATTCCATGTGTAGCGGTGAAATGCGTAGATATATGGAGGAACACCAGTGGCGAAGGCGGCTCTCTGGTCTGTAACTGACGCTGAGGCTCGAAAGCGTGGGGAGCAAACAGG

>7116445b94cbb9559e056e564a40b6bb

TACGTAGGGTGCGAGCGTTAATCGGAATTACTGGGCGTAAAGCATGAGCAGGCGGTTTGTTAAGACAGATGTGAAATCCCCGGGCTCAACCTGGGAACTGCATTTGTGACTGGCAGGCTAGAGTATGGCAGAGGGGGGTAGAATTCCACGTGTAGCAGTGAAATGCGTAGAGATGTGGAGGAATACCGATGGCGAAGGCAACCCCCTGGGCCAATACTGACGCTCATGCACGAAAGCGTGGGGAGCAAACAGG

>5bbb66470801c3f985144f933191bf99

CACACTTATGTCTTTAAAAAGACAACACGTTTTTGCAGAATGCATTTCAACCTTCTCGGTCATTTTCAACTGGCAGCGCCTTGTAGATAGTTGCGGAAGATTTTGAATGTTTCTTGTCCATAGAGATCAGTTTGTTCATTAATTATTTTGTTGTGTAGTTTGTAACGTAATATATTTAAACTTTCAAGAAGATTCATTTTATTTCCTTTTTTGCATGTTGTA

>1fbeb65f9fd7704f5592ef4310972e28

TACGTAGGGTGCGAGCGTTAATCGGAATTACTGGGCGTAAAGCGTGCGCAGGCGGTTTGTTAAGATAGATGTGAAATCCCCGGGCTCAACCTGGTAACTGCATTTGTGACTGGCAGGCTAGAGTATGGCAGAGGGGGGTATAATTCCACGTGTAGCAGTGAAATGCGTAGAGATGTGGAGGAATACCGATGGCGAAGGCAGCCCCCTGGGCCAATACTGACGCTCATGCACGAAAGCGTGGGGAGCAAACAGG

>d4acc6cdcb120c1846d9bd2a065ad992

TTAAACGAAAGCACAACAAAAAGATAGCCAAACTAATACCACCCCGATCACCAGCAATAACACCCACACATACGTTTTACCCTAGAATAGTTAACCTAACCAACGTAACATTCACTAACGAACAGAAACAACTTTTAAACAAAGGGATAAACCACAACCTACACTACACACAGAATAATAACACCATCAAGAACATGGT

>6c242c38f20f703694c0501682104d37

CACACTTATGTCTTCAGAAAGACAAAATATTTGTCCAGGATGCATTTCAACCATGTCGGTCATTTTCAACTGGCAGTGCCTTGTAGATTTGTGAAAGATTTTGAATGTGTCTTGTCCATAAAGTTCAGTTTGTTCATTAGTGATTTTGTTATGTAGTTTTTAACGTAATATATTTAGGCTTTCAAGTAAATTCATTTGTTTCCTTTTTTGCCGGTTGTG

>f49a6efe328aaf31d496450138afd401

TACCGGCAGCCCGAGTGATGGCCGATCTTATTGGGCCTAAAGCGTCCGTAGCTGGCCGCACAAGTCCATCGGAAAATCCACCCGCTCAACGGGTGGGCGTCCGGTGGAAACTGTGTGGCTTGGGACCGGAAGGCGCGACGGGTACGTCCGGGGTAGGAGTGAAATCCCGTAATCCTGGACGGACCGCCGATGGCGAAAGCACTCTGAGAAGACGGCTTCGACAGTGAGGGACGAAAGCTCGGGTCTCAAACCGG

>890567a0b6c9d07d7f1fa8c298258575

TACGTAGGGTGCGAGCGTTAATCGGAATTACTGGGCGTAAAGCGTGCGCAGGCGGTTTGTTAAGACAGATGTGAAATCCCCGGGCTCAACCTGGGAACTGCATTTGTGACTGGCAGGCTAGAGTATGGCAGAGGGGGGTAGAATTCCACGTATAGCAGTGAAATACGTAGAGATGTGGAGGAATACCGATGGCGAAGGCAGCCCCCTGGGCCAATACTGACGCTCATGCACGAAAGCGTGGGGAGCAAACAGG

>b9038a6cd6fdd7b0a0c0057517fedbeb

TACGTAGGGTGCAAGCGTTAATCGGAATTACTGGGCGTAAAGCATGCGCAGGCGGTTATGCAAGACAGAGGTGAAATCCCCGGGCTCAACCTGGGAACTGCCTTTGTGACTGCATGGCTAGAGTACGGTAGAGGGGGATGGAATTCCGCGTGTAGCAGTGAAATGCGTAGATATGCGGAGGAACACCGATGGCGAAGGCAATCCCCTGGACCTGTACTGACGCTCATGCACGAAAGCGTGGGGAGCAAACAGG

>580dd49641fc3d7f1bae5e09442726f0

CACGATTAACCTAAGTCAATAGAAGCCGGCGTAAAGAGTGTTTTAGATCACCCCCTCCCCAATAAAGCTAAAACTCACCTGAGTTGTAAAAAACTCCAGTTGACACAAAATAGACTACGAAAGTGGCTTTAACATATCTGAACACACAATAGCTAAGACCCAAACTGGG

>d257ddc5bd93ae1c06012df33d9b1ca7

TACGTAGGGTGCAAGCGTTAATCGGAATTACTGGGCGTAAAGCGTGCGCAGGCGGTTTTGTAAGACAGTGGTGAAATACCCGGGCTCAACCTGGGAACTGCCATTGTGACTGCAAAGCTAGAGTGCGGCAGAGGGGGATGGAATTCCGCGTGTAGCAGTGAAATGCGTAGATATGCGGAGGAACACCGATGGCGAAGGCAATCCCCTGGGCCTGCACTGACGCTAATGCACGAAAGCGTGGGGAGCAAACAGG

>cad2aae68fefcc79e3b15ed9479a8e8a

CAGCATTATAGATACTCAGTTATTAATAGTGAATGGTTATCGATTAATAATATTGAAAAATTCACATTAAATATAAATTATGGCGTGTGAGTGTGTTTGTGGTATGGTTTCATAGATACGTTTTTACTCTGAAATTCCTGGTTAATCATTATGTTCATGGCCGTATTGACGAGACAGTGTTACAGTTTAGCG

>c8e4365cbef170e19d57637594ec072f

TACGTAGGGTGCAAGCGTTAATCGGAATTACTGGGCGTAAAGCGTGCGCAGGCGGTTATGCAAGACAGAGGTGAAATCCCCGGGCTCAACCTGGGAACTGCCTTTGTGACTGTATGGCTAGAGTACGGTAGAGGGGGATGGAATTCCGCGTGTAGCAGTGAAATGCGTAGATATGCGGAGGAACACCGATGGCGAAGGCAATCCCCTGGACCTGTACTGACGCTCATGCACGAAAGCGTGGGGAGCAAACAGG

>db10f599aa14bb2a9825f7f9da018e81

TACGTAGGGTGCGAGCGTTAATCGGAATTACTGGGCGTAAAGCGTGCGCAGGCGGTTTGTTAAGACAGATGTGAAATCCCCGGGCTCAACCTGGGAACTGCATTTGTGACTGGCAGGCTAGAGTATGGCAGAGGGGGGTAGAATTCCACGTGTAGCAGTGAAATGCGTAGAGATGTGGAGGAATACCGATGGCGAAGGCAGCCCCCTGGGCCAATACTGACGCTAATGCACGAAAGCGTGGGGAGCAAACAGG

>431e2fe3198fdaecdaab345c35805143

TACGTAGGGTCCGAGCGTTGTCCGGAATTATTGGGCGTAAAGGGCTCGTAGGCGGTTTGTCACGTCGGGAGTGAAAACTCAGGGCTCAACCCTGAGCGTGCTTCCGATACGGGCAGACTAGAGGGATGCAGGGGAGAACGGAATTCCTGGTGTAGCGGTGGAATGCGCAGATATCAGGAGGAACACCGGTGGCGAAGGCGGTTCTCTGGGCATTACCTGACGCTGAGGAGCGAAAGCATGGGGAGCGAACAGG

>4e125aecd351f585a0ea6087a17f2623

TAGGACAATTACGTGTCTGTTAGGGATAAGGTAGCTTAAGTAAGACAAAGTATCTGGGGAGCTATTATCGAGTAAGTATTATTACGGGAAACCCAATCGTAAGAGGGTCTTTCATCTTTAGTTATACTAATATTAAATTTTTAATTAAAAAGCACGATTAAAGCAGTTTGTTAGGAGAATGTTTACTAATTCCACGG

>5db345a3c750952f1db3597713e5097c

CAAATTTATGTCTTCAGAAAGACAAAATATTTTTCCAGGGTCCATTTTAACCATGTCGGTCATTTTCAACTAGCAGTGCCTTGTAGATATTTGTGAAAGATTTTGAATGTGTCTTGTCCATAGAGTTCAGTTTGTTCATTAATGATTCTTTATGTAGTTTGTAACGTAATATATTTAGGCTTTCAAGTAAATTGATTTTGTTTCCTTTTTCCGGTTGTG

>5765f69ceab0e6c2f9e76f163e881d36

TACGAAGGGTGCAAGCGTTAATCGGAATTACTGGGCGTAAAGCGCGCGTAGGTGGTTTAGCAAGTTGGATGTGAAATCCCCGGGCTCAACCTGGGAACTGCATCCAAAACTACTGAGCTAGAGTACGGTAGAGGGTGGTGGAATTTCCTGTGTAGCGGTGAAATGCGTAGATATAGGAAGGAACACCAGTGGCGAAGGCGACTCTCTGGTCTGTAACTAACGCGGAGGAGCGAAAGCGTGGGGAGCGAACAGG

>db02306d083060e56b139c60ef7149f8

TACGGAGGGTGCGAGCGTTGTCCGGATTTATTGGGTTTAAAGGGTGCGTAGGCGGCTCTTTAAGTCTGGGGTGAAAGCCCGCTGCTCAACAGCGGAACTGCCCTGGATACTGGAGAGCTTGAGTACAGACGAGGTTGGCGGAATGGACAGAGTAGCGGTGAAATGCATAGATACTGTCCAGAACCCCGATTGCGAAGGCAGCTGACTAGGCTGTTACTGACGCTGAGGCACGAAAGCGTGGAGAGCGAACAGG

>b79909bfeb0305a5751370a50117fd45

TACGTAGGGTGCGAGCGTTAATCGGAATTACTAGGCGTAAAGCGTGCGCAGGCGGTTTGTTAAGAAAGATGTGAAATCCCCGGGCTCAACCTGGGAACTGCATTTGTGACTGGCAGGCTAGAGTATGGCAGAGGGGGGTAGAATTCCACGTGTAGCAGTGAAATGCGTAGAGATGTGGAGGAATACCGATGGCGAAGGCAGCCCCCTGGGCCAATACTGACGCTCATGCACGAAAGCGTGGGGAGCAAACAGG

>516a67156dacfa4341327a7e2a22f877

TACGTAGGGGGCGAGCGTTGTCCGGAATTATTGGGCGTAAAGCGCGCGCAGGCGGTCTCTTAAGTCTGATGTGAAAGCCCACGGCTCAACCGTGGAGGGTCATTGGAAACTGGGGGACTTGAGGGCAGGAGAGGAGAGCGGAATTCCACGTGTAGCGGTGAAATGCGTAGAGATGTGGAGGAACACCAGTGGCGAAGGCGGCTCTCTGGCCTGCACCTGACGCTGAGGCGCGAAAGCGTGGGGAGCAAACAGG

>0bb98780c30c32509b60bd6f1ff92603

TACGTAGGGTGCGAGCGTTAATCGGAATTACTGGGCGTAAAGCGTTCGCAGGCGGTTTGTTAAGACAGATGTGAAATCCCCGGGCTCAACCTGGGAACTGCATTTGTGACTGGCAGGCTAGAGTATGGCAGAGGGGGGTAGAATTCCACGTGTAGCAGTGAAATGCGTAGAGATGTGGAGGAATACCGATGGCGAAGGCAGCCCCCTGGGCCAATACTGACGCTCATGCACGAAAGCGTGGGGAGCAAACAGG

>9b25aa5c104e91825446e0d7284af420

CACGTAGGGGGCGAGCGTTGCTCGGAATTACTGGGCGTAAAGGGTGTGCAGGCGGCCGATTAAGTCAACGATGAAATCCCGAAGCTCAACTTCGGAATGGTCTTTGATACTGATCGGCTTGAGGCTGGTTGAGGAGAGCGGAATTCCCGGTGGAGCGGTGAAATGCGTAGATATCGGGAGGAACACCAGAGGCGAATGCGGTTCTCTAAACTTGTCCTGACGCTGAGACACGAAAGCTAGGGGAGCAAACTGGG

>d0471cbf3542d5cc2c200d2d7efc748f

TACGGAGGGGGCTAGCGTTGTTCGGAATCACTGGGCGTAAAGCGCGCGTAGGCGGACTGATTAGTTGGGGGTGAAAGCCCGGAGCTCAACTCCGGAACTGCCTCCAATACTGTCAGTCTTGAGATCGAGAGAGGTAAGCGGAATTCCGAGTGTAGAGGTGAAATTCGTAGATATTCGGAAGAACACCAGTGGCGAAGGCGGCTTACTGGCTCGATACTGACGCTGAGGTGCGAAAGCGTGGGGAGCAAACAGG

>19f1238386b989e133af3bfe029c48fc

TACCGGCAGCCCGAGTGATGGCCGATATTATTGGGCCTAAAGCGTCCGTAGCTTGCTGTGTAAGTCCATTGGAAAATCGACCAGCTCAACTGGTCGGCGTCCGGTGGAAACTACACAGCTTGGGGCCGAGAGACTCAACGGGTACGTCCGGGGTAGGAGTGAAATCCTGTAATCCTGGACGGACCACCAATGGGGAAACCACGTTGAGAGACCGGACCCGACAGTGAGGGACGAAAGCCAGGGTCTCGAACCGG

>92f1720367db58c68a96eceb9feb416a

TACGAAGGGGGCTAGCGTTGCTCGGAATCACTGGGCGTAAAGGGCGCGTAGGCGGCCGATTAAGTCGGGGGTGAAAGCCTGTGGCTCAACCACAGAATTGCCTTCGATACTGGTTGGCTTGAGACCGGAAGAGGACAGCGGAACTGCGAGTGTAGAGGTGAAATTCGTAGATATTCGCAAGAACACCAGTGGCGAAGGCGGCTGTCTGGTCCGGTTCTGACGCTGAGGCGCGAAAGCGTGGGGAGCAAACAGG

>c0181fc48f859922cb74ece335c527c4

TACCGGCAGCACGAGTGATGGCCGATCTTATTGGGCCTAAAGCGTCCGTAGCTGGCCAGACAAGTCCGTTGGGAAATCGGCGTGCCTAACGCGTCGGCGTCCGGCGGAAACTGTTTGGCTTGGGACCGGAAGACCCGAGGGGTACGTCCGGGGTAGGAGTGAAATCCCGTAATCTTGGACGGACCGCCGGTGGCGAAAGCGCCTCGGGAAGACGGATCCGACAGTGAGGGACGAAAGCTGGGGTCTCGAACCGG

>95cc4e0364f4456ca77feec0399d8c56

CAAGGATCAAAAGGCTTTACGAGTTAGGGTGTGGCCTGCTACCGGTGGTTCCACCGCTTTATTGATGTTCTTTCTACGTTGGCACTAGCTCATCTGGTTGCCATTCAAGTTTTTGTTTGAGTCATCTGGTTTTTGTTTCGATCATTCTGCTCACATGTCCATACCATCGTAAT

>1743adb7447efee9854eadaae66e072e

TACCGGCAGTCCGAGTGATGGCCGATATTATTGGGCCTAAAGCGTCCGTAGCTTGCTGTGTAAGTCCGTTGGGAAATCTGCCCGCTTAACGGGCAGGCGTCCAGCGGAAACTGTTCAGCTTGGGACCGGAAGACCTGAGGGGTACGTCTGGGGTAGGAGTGAAATCCCGTAATCCTGGACGGACCGCCGGTGGCGAAAGCGCCTCAGGAGGACGGATCCGACAGTGAGGGACGAAAGCTAGGGTCTCGAACCGG

>8bbca204be401637a61a5074938a09a4

TACGTAGGCGCCAAGCGTTGTTCGGAATTACTGGGCGTAAAGGGCGCCTAGGCGGACGGGTAAGTCGGACGTGAAATCCCTCGGCTCAACCGAGGAATCGCGTCCGATACTGCGCGTCTTGAGTGCAGAAGGGGAGAGTGGAACTCCTGGTGTAGCGGTGAAATGCGTTGATATCAGGATGAACGCCGGTGGCGAAAGCGGCTCTCTAGTCTGTTACTGACGCTGAAGCGCGAAAGCCAGGGGAGCAAACTGGG

>9e0ed8eff698ee874afcb2bf4843e0ba

TACGTAGGGTGCGAGCGTTAATCGGAATTACTGGGCGTAAAGCGTGCGCAGGCGGTTTGTTAAGACAGATGTGAAATCCCCGGGCTCAACCTGGGAACTGCATTTGTGACTGGCAGGCTAGAGTATGGCAGAGGGAGGTAGAATTCCACGTGTAGCAGTGAAATGCGTAGAGATGTGGAGGAATACCGATGGCGAAGGCAGCCCCCTGGGCCAATACTGACGCTCATGCACGAAAGCGTGGGGAGCAAACAGG

>c0aacf9b3175e6bdd2135e4ced8aae38

AAGCAGCATATGGTAATTGTGTGCCAGCAGCCGCGGTAAGTAGGGCTTGAAGTGATGAGGATATCGAAAGGCCTCTTTTAAACCGAAGTTACCAGCTGTTCGGCAATTATCAGCGGCAGTGTTCAAATTAGATACCCCGGTAGTCCGGCTGACTGACTATAGTACCATCTCGTATGCCGTCTTCTGCTTGAAAAAAAAAAAA

>27e8a289c9a8d4275addcc845ed80112

TACAGAGGGTGCAAGCGTTAATCAGAATGACTGGGCGTAAAGGGCGTGTAGGTGGTTAATTAGGTTTGATGTGAAATCCCCGGGCTTAACCTGGGAATTGCGTCGAAAACGGATTAACTCGAGTGAGATAGAGGGTTGTGGAATTTCCGGTGTAGCGGTGAAATGCGTAGATATCGGAAAGAACATCAGTGGCGAAGGCGACAACCTGGATCTTAACTGACACTGAGGCGCGAAGGCGTGGGGAGCAAACAGG

>e9ec150135eba6470a2c4e8e32205930

TACGTAGGTGGCAAGCGTTATCCGGAATTATTGGGCGTAAAGCGCGCGTAGGCGGTTTTTTAAGTCTGATGTGAAAGCCCACGGCTCAACCGTGGAGGGTCATTGGAAACTGGAAAACTTGAGTGCAGAAGAGGAAAGTGGAATTCCATGTGTAGCGGTGAAATGCGCAGAGATATGGAGGAACACCAGTGGCGAAGGCGACTCTCTGGTCTGTAACTGACGCTGATGTGCGAAAGCGTGGGGAGCAAACAGG

>3979ea62484992a05dfbd458e42c1136

TACGTAGGGTGCGAGCGTTAATCGGAATTACTGGGCGTAAAGCGTGCGCAGGCGGTTTGTTAAGACAGATGTGAAATCCCCGGGCTCAACCTGGGAACTGCATTTGTGACTGGCAGGCTAGAGTATGGCAGAGGGGGGTAGAATTCCACGTGTAGCAGTGAAATGCGTAGAGATGTGGAGGAATACCGATGGCGAAGGCAGCCCCCTGGGCCAATACTAACGCTCATGCACGAAAGCGTGGGGAGCAAACAGG

>aa9a359ee5491df7a6fb3c577da72963

TACCGGCAGCCCGAGTGATGGCCGATCTTATTGGGCCTAAAGCGTCCGTAGCTGGCCGCACAAGTCCATCGGAAAATCCACCTGCTCAACAGGTGGGCGCCCGGTGGAAACTGCGCGGCTTGGGACCGGAAGGCGCGACGGGTACGTCCGGGGTAGGAGTGAAATCCCGTAATCCTGGACGGACCGCCGATGGCGAAAGCACGTCGCGAGAACGGATCCGACAGTGAGGGACGAAAGCCAGGGTCTCGAACCGG

>1926880b82ebdbed3f994a5cab4598ef

TACGTAGGTGGCAAGCGTTATCCGGAATTATTGGGCGTAAAGCGCGCGTAGGCGGTTTTTTAAGTCTGATGTGAAAGTCCACGGCTCAACCGTGGAGGGTCATTGGAAACTGGAAAACTTGAGTGCAGAAGAGGAAAGTGGAATTCCATGTGTAGCGGTGAAATGCGCAGAGATATGGAGGAACACCAGTGGCGAAGGCGACTTTCTGGTCTGTAACTGACGCTGATGTGCGAAAGCGTGGGGATCAAACAGG

>099fde62f9593ac11244d4931575a2e6

TACCGGCAGCTCAAGTGATGACCGATATTATTGGGCCTAAAGCGTCCGTAGCCGGCCACGAAGGTTCATCGGGAAATCCGCCAGCTCAACTGGCGGGCGTCCGGTGAAAACCACGTGGCTTGGGACCGGAAGGCTCGAGGGGTACGTCCGGGGTAGGAGTGGAATCCCGTAATCCTGGACGGACCACCGATGGCGAAAGCACCTCGAGAAGACGGATCCGACGGTGAGGGACGAAAGCTAGGGTCTCGAACCGG

>e1d6e1aae36ff549e6e069270500716b

AACAGAGGATACAAGCGTTATCCGGATTTATTGGGTTTAAAGGGTGCGTAGGTGGTTTTTTAAGTCAGTAGTGAAATCTTAAAGCTTAACTTTAAAAGTGCTATTGATACTGATAAACTAGAGTGAGGTTGGAGTAACTGGAATGTGTGGTGGAGCGGTGAAATGCATAAAGATCACACAGAACACCAATCGCGAAGGCATGTTACTAAACATAGACTGACACTGAGGCACGAAAGCATGGGTAGCAAACAGG

>5b76e0cb46574c05f88c216f0cf83bf0

TACCGGCAGCTCGAGTGATGTCCCATATTATTGGGCCTAAAGCGTCCGTAGCTGGCCGACCAAGTCTCCCGGGAAATCCACCCGCCCAACGGGTGGGCGTCCGGTAGAAACTGGCCGGCTTGGAACCGGAAGGCTCAGAGAGTACGTCCGGGGTAGGAGTGAAATCCCGTAATCCCGGACGGACTACCGATGGCGAAAGCACTCTGAGAAGACGGCTTCGACAGTGAGGGACGAAAGCTCGGGTCTCAAACCGG

>052ed87a5bce8789c20b59effda065d6

TACGTAAGGGGCGAGCGTTGTCCGGAATTATTGGGCGTAAAGAGTGCGTAGGCGGCAAATTAAGTCAGATGTGAAAACTAAGGGCTCAACCCATAGATTGCATCTGAAACTGATATGCTTGAGTCAAGGAGAGGAAAGTGGAATTCCTAGTGTAGCGGTGGAATGCGTAGATATTAGGAGGAATACCGGTGGCGAAGGCGACTTTCTGGACTTGAACTGACGCTGAGGCACGAAAGCGTGGGGAGCAAACAGG

>2355994db1cd3586a8b3363a01ff7b3e

TACGTAGGGTGCGAGCGTTAATCGGAATTACTGGGCATAAAGCGTGCGCAGGCGGTTTGTTAAGACAGATGTGAAATCCCCGGGCTCAACCTGGGAACTGCATTTGTGACTGGCAGGCTAGAGTATGGCAGAGGGGGGTAGAATTCCACGTGTAGCAGTGAAATGCGTAGAGATGTGGAGGAATACCGATGGCGAAGGCAGCCCCCTGGGCCAATACTGACGCTCATGCACGAAAGCATGGGGAGCAAACAGG

>633061b5d05001b350eae36d90b16712

AACGTAGGATCCTAGCGTTATCCGAATTTACTGGGCGTAAAGCGCGTGTAGGTGGTTTGGTAAGTTGGATGTGAAAGCTCCTGGCTCAACTGGGAGAGGCCGTTCAAAACTACCAGACTTGAGGGCGGTAGAGGAAGGTGGAATTCCCGGTGTAGTGGTGAAATGCGTAGATATCGGGAGGAACGCCGGTGGCGAAAGCGGCTCTCTGGTCCGCAACTGACGCTGAGGCGCGAAAGCTAGGGGAGCAAACTGGG

>23eb228775bf415a9584cecb3f8f2a2d

TACCGGCAGCCCAAGTGATGGCCGCTGTTATTGGGCCTAAAGCGTCCGTAGCTGGCCGCACAAGTCCGTCGGGAAATCCGCCCGCTCAACGGGCGGCCGTCCGGCGGAACCTGTGTGGCTTGGGACCGGGAGGCCTGAGGGGTACGATCGGGGTAGGAGTGAAATCCTGTAATCCCGCTCGGACCGCCGATGGGGAAACCACCTCAGGAGAACGGATCCGACAGTGAGGGACGAAAGCCAGGGTCTCGAACCGG

>99ce607a758cf7bf528f755ff8711d12

AACAGAGGATACAAGCGTTATCCGGATTTATTGGGTTTAAAGGGTGCGTAGGTGGTTTTTTAAGTCAGTAGTGAAATCTTAAAGCTTAACTTTAAAAGTGCTATTGATACTGATAAACTAGAGTGAGGTTGGAGTAACTGGAATGTGTGGTGGAGCGGTGAAATGCATAGAGATCACACAGAACACCAATCGCGAAGGCATGTTACTAAACATAGACTGACACTGAGGCACGAAAGCATGGATAGCAAACAGG

>644fd07dc284e48b3f9bdbf76078fd2e

TACGTATGGGGCAAGCGTTATCCGGAATTATTGGGCGTAAAGAGTGCGTAGGTGGTGGCTTAAGCGCAGGGTTTAAGGCAATGGCTTAACTATTGTTCGCCTTGCGAACTGGGTCACTTGAGTACAGGAGAGGAAAGCGGAATTCCTAGTGTAGCGGTGAAATGCATAGATATTAGGAGGAACACCGGTGGCGAAGGCGGCTTTCTGGACTGCAACTGACACTGAGGCACGAAAGCGTGGGTAGCAAACAGG

>711c2388901fb381043d22e5636b1ccf

TACGTAGGTGGCAAGCGTTGTCCGGAATTATTGGGCGTAAAGGGCTCGCAGGCGGTTCCTTAAGTCTGATGTGAAAGCCCCCGGCTCAACCGGGGAGGGTCATTGGAAACTGGGGAACTTGAGTGCAGAAGAGGAGAGTGGAATTCCACGTGTAGCGGTGAAATGCGTAGAGATGTGGAGGAACACCAGTGGCGAAGGCGACTCTCTGGTCTGTAACTGACGCTGAGGAGCGAAAGCGTGGGGAGCGAACGGG

>d8d525d4db4d086bfd57ae73c7e74c8d

TACGTAGTGTGCGAGCGTTAATCGGAATTACTGGGCGTAAAGCGTGCGCAGGCGGTTTTTTAAGACAGATGTGAAATCCCCGGGCTCAACCTGGGAACTGCATTTGTGACTGGCAGGCTAGAGTATGGCAGAGGGGGGTAGAATTCCACGTGTAGCAGTGAAATGCGTAGAGATGTGGAGGAATACCGATGGCGAAGGCAGCCCCCTGGGCCAATACTGACGCTCATGCACGAAAGCGTGGGGAGCAAACAGG

>35ffcc3b809d667286737d79670b8de5

TACGTAGGGTGCAAGCGTTATCCGGAATTATTGGGCGTAAAGGGCTCGTAGGCGGTTCGTCGCGTCCGGTGTGAAAGTCCATCGCTTAACGGTGGATCCGCGCCGGGTACGGGCGGGCTTGAGTGCGGTAGGGGAGACTGGAATTCCCGGTGTAACGGTGGAATGTGTAGATATCGGGAAGAACACCAATGGCGAAGGCAGGTCTCTGGGCCGTTACTGACGCTGAGGAGCGAAAGCGTGGGGAGCGAACAGG

>13802cf1e8e92933d02d5c5c095229ff

TACCGGCAGCCCGAGTGATGGCCGATCTTATTGGGCCTAAAGCGTCCGTAGCTGGCCGCGCAAGTCCATCGGAAAATCCACCTGCTCAACAGGTGGGCGCCCGGTGGAAACTGCGCGGCTTGGGACCGGAAGGCGCGACGGGTACGTCCGGGGTAGGAGTGAAATCCCGTAATCCGGGACGGACCGCCGATGGCGAAAGCACGTCGCGAGAACGGATCCGACAGTGAGGGACGAAAGCCAGGGTCTCGAACCGG

>967703ae70e5047665490950858fa3aa

TTTCCAATTATTTATTATAGAGTAACCTTCCTCTTCTCGATTATAGGGAAAGCGGTATGGTATCAGTCGAACCGAACAGAGGATTTGGGATCAACCGCCAACAATCATTCATAATATAAAGCGTGACGTTAACACGAACCGAACGGCATATAGCGAAGACAACGCGAGAAAGTAGACAG

>7cc8be682e7824f5797a47f04e9ff04d

CACGATTAACCCAAGTCAATAGATGCCGGCGTAAAGAGTGTTCTAGATCACCCCCTCCCCAATAAAGCTAAAACTCACCTGAGTTGTAAAAAACTCCAGTTGACACAAAATAGACTACGAAAGTGGCTTTAACATATCTGAACACACAATAGCTAAGACCCAAACTGGG

>b15b147a38d68cd51a0e8cd7295c8783

TACCGGCAGCCCGAGTGATGGCCGATCTTATTGGGCCTAAAGCGTCCGTAGCTGGCCGCACAAGTCCATCGGAAAATCCACCTGCCCAACGGGTGGGCGTCCGGTGGAAACTGTGTGGCTTGGGACCGGAAGGCGCGACGGGTACGTCCGGGGTAGGAGTGAAATCCCGTAATCCTGGACGGACCGCCGATGGCGAAAGCACGTCGCGAGAACGGATCCGACAGTGAGGGACGAAAGCCAGGGTCTCGAACCGG

>c62672c7aad1d894356f587b0255de80

TACGTAGGGTGCGAGCGTTAATCGGAATTACTGGGCGTAAAGCGTGCGCAGGCGGTTTGTTAAGACAGATGTGAAATCCCCGGGCTCAACCTGGGAACTGCATTTGTGACTGGCAGGCTAGAGTATGGCAGAGGGGGGTAGAATTCCACGTGTAGCAGTGAAATGCGTAGAGATGTGGAGGAATACCAATGGCGAAGGCAGCCCCCTGGGCCAATACTGACGCTCATGCACGAAAGCGTGGGGAGCAAACAGG

>83815e2b904838a5960479d6b43702cf

CATTGAACTATCGTGAGAACAGCACGCCGCCAAAGGGAATTATATTATAGTAAATATTAGCGTAAATAAACATTTTATTAATAGTTGTAATATATGAAAATGTGCAGAAGATAGAGACGATGAAAACGGTGTTTAACAATCGCTTATCGAGACAATATTTAATTA

>90542fa2c8a2d1d63cb76d9218d0bbf1

TTCTTACTGCAAACGTATTGTCACACACATATGTTTTGTAGTAATTATTGTGAGCAGCATCCTCTATGTTATAAATGTAATAAGGAAATCTTAAAACAATGATAATAATCTGTTTATAGTAATTAATCTTCATCATCTTCGCCATTCCAGGATTAGAAACCCC

>ec6665f4d7bda38bfb9ebcac845d2067

TACGTAGGGTGCAAGCGTTGTCCGGAATTACTGGGCGTAAAGAGCTCGTAGGTGGTTTGTCACGTCGTCTGTGAAATTCCACAGCTTAACTGTGGGCGTGCAGGCGATACGGGCTGACTTGAGTACTGTAGGGGTAACTGGAATTCCTGGTGTAGCGGTGAAATGCGCAGATATCAGGAGGAACACCGATGGCGAAGGCAGGTTACTGGGCAGTTACTGACGCTGAGGAGCGAAAGCATGGGTAGCAAACAGG

>de56ba5808191bf7ab9551871830fa1f

TACGTGAGAGACTAGTGTTATTCATCTTAATTGGGTTTAAAGGGTACCTAGACAGTCAATATAACTTCTATAATGCTAATACTTGACCAGAGTTTTAAGTAAGAGGGAAGTACTTAAGGAGTAAGAGATGAAATATCTGTGATACCAAAGGGACTCCGTAAAGGCGAAGGCATCCCTTTATCTAAAAACTAACGTTGAAGGACGAAGGCTTAGATAACAAATAGG

>1b4ac868d3fed82f0c73eae3df8fbf06

TACGTGAGAGACTAGTGTTATTCATCTTAATTGGGTTTAAAGGGTACCTAGACAGTCAATATAACTTCTATAATGCTAATACCTGACTAGAGTTTTAAGTAAGAGGGAAGTACTTAAGGAGTAAGAGATGAAATATCTGTGATACCAAAGGGACTCCGTAAAGGCGAAGGCATCCCTTTATCTAAAAACTAACGTTGAAGGACGAAGGCTTAGATAACAAATAGG

>bae232566ba92c6e611c43c446a9b1b0

AGCAACTCTCAGTGCCTTCCTCTTGCTCACGGCCATATGCGTGACAGGGGACCTGCCACCGAACATCCTGGTGATCAGCAAAATATATGACGCGCAGGACGAAGGATCCTGGTGCTCATCGGGCATGACACAGGAGCACAACATTGATATGGGTGCAAGACACCATGTCATAATAAAAAAAAGAACACCCCAGGTGTCCAAATCTCATATTATCATTGTATATTTCATGTCTTAGTTTTATAATTTTTCTTTAC

>a960147baba38b42bf33a918d749da05

TACGTAGGGTGCGAGCGTTAATCGGAATTACTGGGCGTAAAGCGTGCGCAGGCGGTTTGTTAAGACAGATGGGAAATCCCCGGGCTCAACCTGGGAACTGCATTTGTGACTGGCAGGCTAGAGTATGGCAGAGGGGGGTAGAATTCCACGTGTAGCAGTGAAATGCGTAGAGATGTGGAGGAATACCGATGACGAAGGCAGCCCCCTGGGCCAATACTGACGCTCATGCACGAAAGCGTGGGGAGCAAACAGG

>f72aedc63a53047a147ebb0e743b7864

TACGTATGGTGCAAGCGTTATCCGGATTTACTGGGTGTAAAGGGAGCGTAGACGGAGAAGCAAGTCTGGAGTGAAAACCCGGGGCTCAACCCCGGGACTGCTTTGGAAACTGTTTTTCTGGAGTGCCGGAGAGGTAAGCGGAATTCCTAGTGTAGCGGTGAAATGCGTAGATATTAGGAGGAACACCAGTGGCGAAGGCGGCTTACTGGACGGTAACTGACGTTGAGGCTCGAAAGCGTGGGGAGCAAACAGG

>8e9297ea7b861af38de0268d30e6bf38

TACGGAGGATGCGAGCGTTATCCGGATTTATTGGGTTTAAAGGGTGCGTAGGTGGTTAATTAAGTCAGCGGTGAAAGTTTGTGGCTCAACCATAAAATTGCCGTTGAAACTGGTTGACTTGAGTATATTTGAGGTAGACGGAATGCGTGGTGTAGCGGTGAAATGCATAGATATCACGCAGAACTCCGATTGCGAAGGCAGCTTACTAAACTATAACTGACACTGAAGCACGAAAGCGTGGGGATCAAACAGG

>5fb9dc3522602064e3c23ca43cb162e4

TACGTGAGAGACTAGTGTTATTCATCTTAATTGGGTTTAAAGGGTACCTGGACAGTCAATATAACTTCTATAATGCTAATACTTGACTAGAGTTTTAAGTAAGAGGGAAGTACTTAAGGAGTAAGAGATGAAATATCTGTGATACCAAAGGGACTCCGTAAAGGCGAAGGCATCCCTTTATCTAAAAACTAACGTTGAAGGACGAAGGCTTAGATAACAAATAGG

>212ca2de74088a951dad91102d2a9f4c

TACGTAGGGTGCGAGCGTTAATCGGAATTACTGGGCGTAAAGCGTGCGCAGGCGGTTTGTTAAGACAGATGTGAAATCCCCGGGCTCAACCTGGGAACTGCATTTGTGACTGGCAGGCTAGAGTATGGAAGAGGGGGGTAGAATTCCACGTGTAGCAGTGAAATGCGTAGAGATGTGGAGGAATACCGATGGCGAAGGCAGCCCCCTGGGCCAATACTGACGATCATGCACGAAAGCGTGGGGAGCAAACAGG

>b391e6ac0f57761129ebfa7b6c3abede

TACGTAGGGTGCGAGCGTTAATCGGAATTACTGGGCGTAAAGCGTGCGCAGGCGGTTGTGTAAGACAGGCGTGAAATCCCCGGGCTCAACCTGGGAATGGCGCTTGTGACTGCACGGCTGGAGTGCGGCAGAGGGGGATGGAATTCCGCGTGTAGCAGTGAAATGCGTAGATATGCGGAGGAACACCGATGGCGAAGGCAATCCCCTGGGCCTGCACTGACGCTCATGCACGAAAACGTGGGGAGCAAACAGG

>6110f28a1de867a9983a8a28ecf4e533

TACGGAGGGTGCAAGCGTTAATCGGAATTACTGGGCGTAAAGCGCACGCAGGCGGTCTGTCAAGTCAGATGTGAAATCCCCGGGCTTAACCTGGGAACTGCATTTGAAACTGGCAGGCTAGAGTCTTGTAGAGGGGGGTAGAATTCCAGGTGTAGCGGTGAAATGCGTAGAGATCTGGAGGAATACCGGTGGCGAAGGCGGCCCCCTGGACAAAGACTGACGCTCAGGTGCGAAAGCGTGGGGAGCAAACAGG

>ce31d575dac025dd2db4e917c2348c9b

CACGCTTATGTCTTCAGAAATAAACAATTTATTTCCCCATGATGCATTTCAACCATGTCGGCCACTTCCAACTGGCAGTGCCTTCTAGATATTTGTGAAAGATTTTGAATGTGTCTTGTCCATAGAGTTCAGTTTTTTATTAATGATTTTGTTATGTAGTTTGTAACGTAATATATTTTGGCTTTCGAGTAAATTCATTTTGTTTCCATATTTTCCGTTTGTG

>b5e956031fcb670aa5b4e5828f769a92

TACGAAGGGTGCAAGCGTTACTCGGAATTACTGGGCGTAAAGCGTGCGTAGGTGGTTATTTAAGTCCGTTGTGAAAGCCCTGGGCTCGACCTGGGAACTGCAGTGGATACTGGATGACTAGAATGTGGTAGAGGGTAGCGGAATTCCTGGTGTAGCAGTGAAATGCGTAGAGATCAGGAGGAACATCCATGGCGAAGGCAGCTACCTGGACCAACATTGACACTGAGGCACGAAAGCGTGGGGAGCAAACAGG

>bb4e00b81d6ef4649b5857c33e05133d

GACAGAGGGTGCAAACGTTGTTCGGAATTACTGGGCGTAAAGCGTGTGTAGGCGGTCTTGTAAGTCGGATGTGAAAGCCCCGGGCTCAACCCGGGAAGTGCACTCGATACTGCGAGACTTGAGTATCGGAGAGGTTGGTGGAATTCTCGGTGTAGAGGTGAAATTCGTAGATATCGAGAGGAACACCGGTGGCGAAAGCGGCCAACTGGACGAATACTGACGCTGAGACACGAGAGCGTGGGGAGCAAACAGG

>df8f55f5ca981c8cb3dc2db22edc1016

TACCGGCAGCCCGAGTGATGGCCGATCTTATTGGGCCTAAAGCGTCCGTAGCTGGCCGCGCAAGTCCATCGGGAAATCCACCTGCTCAACAGGTGGGCGCCCGGTAGAAACTGTGTGGCTTGGAACCGGAAGGCTCAGAGAGTACGTCCGGGGTAGGAGTGAAATCCCGTAATCCCGGACGGACTACCGATGGCGAAAGCACTCTGAGAAGACGGCTTCGACAGTGAGGGACGAAAGCTCGGGTCTCAAACCGG

>3d35344049d7dee6133e1cffaabca440

AGACGATCTACGATGCAAGGTTCTTCATTGTCTTCCTCAGATATTTAAAGCATAACATCACCATCTTCCGTTACTTATTGAAAACCGTAAGGACTAATATTATTTGTTATTTTAATTTTTTAATCATTTCAGAGACATATTTTTAAAGAAAAATACAATTATAAAGCATAACACTGGCGTTTAAAAAATATATTTCTGTTATTAAGGCTTAATACTAAATAACTATTTATAACAAT

>aa4db0e3321385ed165fcadf4c384f14

TGCGTCACTATGGTAATTGTGTGCCAGCCGCCGCGGTAAGGAAACTGTGAGTGAACAAGTTATGTACAAATACGCGCGGTAGCCGAGTGCGCGCTGGAGAGCTTTACGACCAGAACCCCTGATGGCATTAGAAACCCCGGTAGTCCGGCTGACTGACTTGCTCGTAATCTCGTATGCCGTCTTCTGCTTGAAAAAAAAAAAA

>b90c1454f145171c43fea0054f76d90e

CACGATTAACCCAAGTCAATAGAAGCCGGCGTAAAGAGTGTCTTAGATCACCCCCTCCCCAATAAAGCTAAAACTCACCTGAGTTGTAAAAAACTCCAGTTGACACAAAATAGACTACGAAAGTGGCTTTAACATATCTGAACACACAATAGCTAAGACCCAAACTGGG

>416e8ace40a9987bfc58b796eaf3e4d5

TACGTAGGATCCGAGCGTTGTCCGGAATTACTGGGTGTAAAGGGCGTGTAGGTGGGTCTCTAAGTCGGTGGTGAAATCCTGCGGCTCAACCGCAGAATGGCCTCCGAAACTGGAGATCTTGAGTACGGCAGAGGGAGATGGAATTCATGGTGTAGCGGTGAAATGTGTAGATATCATGAAGAACACCGGTAGCGAAGGCGGTCTCCTGGTCCGTTACTGACACTGAGGCGCGAAAGCGTGGGGAGCAAACTGGG

>0a7e5dcb17555596db48f54e34b3d0bb

TACAGAGGGTGCAAGAGTTAATCGGATTTACTGGGCGTAAAGCGCGCGTAGGCGGCTAATTAAGTCAAATGTGAAATCCCCGAGCTTAACTTAGGAATTGCATTCGATACTGGTCAGCTAGAGTATGGGAGAGGATGGTAGAATTCCAGGTGTAGCGGTGAAATGCGTAGGGATCTGGAGGAATACCGATGGCGAAGGCAGCCATCTGGCCTAATACTGACGCTGAGGTACGAAAGCATGGGGAGCAAACAGG

>d71f1c5102e51f550d027500a0d422cb

TACGTAGGGTGCGAGCGTTAATCGGAATTACTGGGCGTAAAGCGTGCGCAGGCGGTTTGTTAAGACAGATGTGAAATCCCAGGGCTCAACCTGGGAACTGCATTTGTGACTGGCAGGCTAGAGTATGGCAGAGGGGGGTAGAATTCCACGTGTAGCAGTGAAATGCGTAGAGATGTGGAGGAATACCGATGGCGAAGGCAGCCCCCTGGGCCAATACTGACGCTCATGCACGAAAGCGTGGGGAGCAAACAGG

>8fc520f45ff56b3186a52801e14a0f03

TACCGGCAGCCCGAGTGATGGCCGATATTATTGGGCCTAAAGCGTCCGTAGCTGGCCGCACAAGTCCGTCGGGAAATCCACTCGCTTAACGAGTGGGCGTCCGGCGGAAACTGTGCGGCTTGGGACCGGAAGATCCGAGGGGTACGTCCGGGGTAGGAGTGAAATCCTGTAATCCTGGACGGACCACCGATGGCGAAAGCACCTCGGAAGGACGGATCCGACAGTGAGGGACGAAAGCTAGGGTCTCGAACCGG

>3ecb7b0edf106cef7457639cd14f5782

TACGTAGGGTGCGAGCGTTAATCGGAATTACTGGGCGTAAAGCGTGCGCAGGCGGTTTGTTAAGACAGATGTGAAATCCCCGGGCTCAACCTGGGAACTGCATTTGTGACTGGAAGGCTAGAGTATGGCAGAGGGGGGTAGAATTCCACGTGTAGCAGTGAAATGCGTAGAGATGTGGAGGAATACCGATGGCGAAGGCAGCCCCCTGGGCCAATACTGACGCTCATGCACGAAAGCGTGGGGAGCAAACAGG

>7360aa529723d145ab5a84cd7c486ac7

TACCGGCAGCTCGAGTGATGACCGATCTTATTGGGCCTAAAGCGTCCGTAGCTGGCCAAACAAGTCCGTTGGGAAATCGACGTGCCTAACGCGTCGGCGTCCAGCGGAAACTGTTTGGCTTGGGACCGGAAGACCCGAGGGGTACGTCCAGGGTAGGAGTGAAATCCCGTAATCTTGGACGGACCACCGGTGGCGAAAGCGCCTCGGGAAGACGGATCCGACAGTGAGGGACGAAAGCTGGGGTCTCGAACCGG

>fe7f9a4a9ba2f07f51e324d22749a2a8

GACAGAGGGTGCAAACGTTGTTCGGAATTACTGGGCGTAAAGCGTGTGTAGGCGGTCTTGTAAGTCGGATGTGAAAGCCCCGGGCTCAACCCGGGAAGTGCACTCGATACTGCGAGACTTGAGTATCGGAGAGGTTGGTGGAATTCTCGGTGTAGAGGTGAAATTCGTAGATATCGAGAGGAACACTGGTGGCGAAAGCGGCCAACTGGACGAATACTGACGCTGAGACACGAAAGCGTGGGGAGCAAACAGG

>da4d5540430763fed45b4ec4d3c9c301

TACGTAGGGTGCGAGCGTTAATCGGAATTACTGGGCGTAAAGCGTGCGCAGGCGGTTTGTTAAGACAGATGTGAAATCCCCGGGCTCAACCTGGGAACTGCATTTGTGACTGGCAGGCTAGAGTATGGAAGAGGGGGGTAGAATTCCACGTGTAGCAGTGAAATGCGTAGAGATGTGGAGGAATACCGATGGCGAAGGCAGCCCCCTGGGCCAATACTGACGCTCATGCACGAAAGCGTGGGGAGCAAACAGG

>b2840bf0b5aae71899e61a0c6dac0f01

GACGGGGGGGGCAAGTGTTCTTCGGAATGACTGGGCGTAAAGGGCACGTAGGCGGTGAATCGGGTTGAAAGTGAAAGTCGCCAAAAACTGGCGGAATGCTCTCGAAACCAATTCACTTGAGTGAGACAGAGGAGAGTGGAATTTCGTGAGGAGGGGTGAAATCCGAAGATCTACGAAGGAACGCCAAAAGCGAAGGCAGCTCTCTGGGTCCCTACCGACGCTGGGGTGCGAAAGCATGGGGAGCGAACAGG

>e3cfa258f060dba5c2e8ce5bbdfd3118

TACATGAGAGACTAGTGTTATTCATCTTAATTGGGTTTAAAGGGTACCTAGACAGTCAATATAACTTCTATAATGTTATTACTTGACTAGAGTTTTAAGTAAGAGGGAAGTACTTAAGGAGTAAGAGATGAAATATCTGTGATACCAAAGGGACTCCGTAAAGGCGAAGGCATCCCTTTATCTAAAAACTAACGTTGAAGGACGAAGGCTTAGATAACAAATAGG

>d3c722b0117aedab3c00d4424c9a9b8b

TACGTGAGAGACTAGTGTTATTCATCTTAATTGGGTTTAAAGGGTACCTAGACAGTCAATATAACTTCTATAATGCTAATACTTGACTAGAGTTTTAAGTAAGAGGGAAGTGCTTAAGGAGTAAGAGATGAAATATCTGTGATACCAAAGGGACTCCGTAAAGGCGAAGGCATCCCTTTATCTAAAAACTAACGTTGAAGGACGAAGGCTTAGATAACAAATAGG

>ab975b90367587a4709029e1a1dc9021

TACCGGCAGTCCAAGTGATGGCCGCTGTTATTGGGCCTAAAGCGTCCGTAGCCGGCCAGGCAAGTTCGTCGGGAAATCCACCCTCTCAACGGGTGGGCGTCCGGCGAAAACTGTCTGGCTAGGGACCGGAAGACCCGAGGGGTACGTCCAGGGTAGGAGTGAAATCCCGTAATCCTGGACGGACCGCCGGTGGCGAAAGCGCCTCGGGAAGACGGATCCGACGGTGAGGGACGAAAGCTTGGGTCACGAACCGG

>5d52427e8a3e421b6ec8c424892db29d

CCAACACATCAACCATCAGTGACCACGGACCACAGGTCAGACTCTAATACTCGCCCTGAGTTGTCACAAGCAAATAAGGCCACAAAGTCATGGACAAACAAACGCAGCCACCATACGGCAGCAATCGAAAATAAAGAGCACCTGCATATATACATTTAAAACGGACAGTCTTAGTCAATCTGTACTTCCTGCGACTATTTACGG

>f909b6938ad2dff7f0b5aba5076e5f2b

TACGGAGGATCCAAGCGTTATCCGGATTTATTGGGTTTAAAGGGTGCGTAGGCGGCCTGTTAAGTCAGGGGTGAAAGACGGTGGCTCAACCATCGCAGTGCCCTTGATACTGATGGGCTTGAATGAACTAGAGGTAGGCGGAATGTGACAAGTAGCGGTGAAATGCATAGATATGTCACAGAACACCGATTGCGAAGGCAGCTTACTATGGTTTTATTGACGCTGAGGCACGAAAGCGTGGGGATCAAACAGG

>ac1f61421e55c068213c376054061546

GCGGTAATAATGTATCCAAGCATATAGACATCAAATTGATTGACAAATCTATCACATTGTCAAAGTATAGTAGGTATAGAATCATATAACTCTTATCACCTTCATAAAAATAGTCAGTTCCATGTAATTAGATACCCCAGTAGTCCGGCTGACTGACTATAGTACCATCT

>d79a68064e28ab64f10824ab64e32265

TACGTAGGGTGCGAGCGTTAATCGGAATTACTGGGCGTAAAGCGTGCGCAGGCGGTTTGTTAAGACAGATGTGAAATCCCCGGGCTCAACCTGGGAACTGCATTTGTGACTGGCAGGCTAGAGTATTGCAGAGGGGGGTAGAATTCCACGTGTAGCAGTGAAATGCGTAGAGATGTGGAGGAATACCGATGGCGAAGGCAGCCCCCTGGGCCAATACTGACGCTCATGCACGAAAGCGTGGGGAGCAAACAGG

>3738f5445bc173d0ea35ad5794ce39a6

GACCAGATGAATTCCACATTCATATGTTGCAACACGCTCCATCTGATATTCTTGACACGTTATTACAAATTTTTAATAAAATATGGTTCTCTGGCATCTTTCCAACAGCGTGGAAAACAGCAACGATTATTCCCATTCTTAAAGAAAAGAAAACCTTTCATTA

>0ccc7b720225cb8ecbb4b5515cfd611d

GACCGCCAGAAAATTTAGGGGAACCTGGAGGCCGGCTCAGGACAGTAAACACAATGAAAGCAATACTGCGAACAATCTTCGTTTGAAATATTGAATGCCCACTGTATTGAAGTACAGTGTGCTTAAAGTATTTTCCTACGCATTTTTTAAAAGAAATAATTTTTATGATTAGGATAACTTCTTTTCTACAAGTGTTAAAACTCTTACAACCAGCACTAAGAAATTTTTGATAAGCTTTCACTTTAATAACAAATAACCTTGA

>a82ac0a99b1f5f2de7b9ed1f69b8502b

TACGAAGGGGGCTAGCGTTGCTCGGAATCACTGGGCGTAAAGGGTGCGTAGGCGGGTTTTTAAGTCAGGGGTGAAATCCTGGAGCTCAACTCCAGAACTGCCTTTGATACTGAAGATCTTGAGTCCGGGAGAGGTGAGTGGAACTGCGAGTGTAGAGGTGAAATTCGTAGATATTCGCAAGAACACCAGCGGCGAAGGCGGCTCACTGGCCCGGTACTGACGCTGAGGCACGAAAGCGTGGGGAGCAAACAGG

>d25af4bd9195ada4f565db828df2f588

TACGGAGGGTGCAAGCGTTATCCGGATTTATTGGGTTTAAAGGGTTTGTAGGCGGATTTGTAAGTCAGTGGTGAAATCTCACAGCTTAACTGTGAAACTGCCATTGATACTGCAAGTCTTGAGTGTTGTTGAAGTAGCTGGAATAAGTAGTGTAGCGGTGAAATGCATAGATATTACTTAGAACACCAATTGCGAAGGCAGGTTACTAAGCAACAACTGACGCTGATGGACGAAAGCGTGGGGAGCGAACAGG

>bf78dd9424249c6c6ee2438f8c1c8756

TACAGAGGGTGCAAGCGTTAATCGGATTTACTGGGCGTAAAGCGCGCGTAGGCGGCTAATTAAGTCAAATGTGAAATCCCCGAGCTTAACTTGGGAATTGCATTCGATACTGGTTAGCTAGTGTGTGGGAGAGGATGGTAGAATTCCAGGTGTAGCGGTGAAATGCGTAGAGATCTGGAGGAATACCGATGGCGAAGGCAGCCATCTGGCCTAACACTGACGCTGAGGTGCGAAAGCATGGGGAGCAAACAGG

>5acf597617f5eae6daa057fafa310ed9

TCAAACGAAAGCACAACAAAAATATAGCCAAACTAATACCACCCCGATCACCAGCAATAACACCCACACATACGTTTTACCCAAGAATAGTTAACATCACGAACATAACATTCACTAACGATCAGAAACAACTTTTAAACAAAGGGATAAATCACAACCTACACTACACGCAGAACACTATAAGTATACATTGCAGAACACTGTATCAAGAACATTAT

>0485e5cf235bc476d52c58749688439c

TACAGAGGGTGCAAGCGTTAATCGGATTTACTGGGCGTAAAGCGCGCGTAGGCGGCTAATTAAGTCAAATGTGAAATCCCCGAGCTTAACTTGGGAATTGCATTCGTTACTGGTTAGCTAGAGTGTGGGAGAGGATGGTAGAATTCCAGGTGTAGCGGTGAAATGCGTAGAGATCTGGAGGAATACCGATGGCGAAGGCAGCCATCTGGCCTAACACTGACGCTGAGGTGCGAAAGCGTGGGGAGCAAACAGG

>d43c230610130dd2431bc32c3d0c64ca

CATTGAACTATCGTGAGAATGGCACGCCGCCAAAGGGAATTATATTATAGTAAATATTAAAATGGACACAACTCTTACTTGGAAGGTTTGAACATACTGGAATCGAAAGTATTTTTCATCTTCTTCTCCTTCTTCTTCTATACCTCATGGATTAGAAACCCCA

>fccf8e9d807d727697576536b1a42fbb

TACGTAGGGTGCGAGCGTTAATCGGAATTACTGGGCGTAAAGCGTGCGCAGGCGGTTTGTTAAGACAGATGTGAAATCCCCGGGCTCAACCTGGGAACTGCATTTGTGACTGGCAGGCTAGAGTATGGCAGAGGGGGGTAGAATTCCACGTGTAGCAGTGAAATGCGTAGAGATGTGGAGGAATACCGATGGCGAAGACAGTCCCCTGGGCCAATACTGACGCTCATGCACGAAAGCGTGGGGAGCAAACAGG

>507844917604a5cc6e0eb782aafe0618

GCCCCCACGCCCGAGGCGACGACCCCGTGACCACGCCACGAATGATCGAGGCGAGCATAGGGTCGTGCTGACGTGAGCGATATGCCACGGTCGCTGTCGGAGCGTACACCTCGACCGGCGAGCTGCCGACAGCGCTGTAACTGTCGGATGGAGACAAGATGACGTGTCCGAAATGGTCTAGTTTCCTCCTCTTTACTTTTAAATTTCACTGCGTCCTTTCCAGAACTGTTTC

>01ac2a8713daa200e6c0005384f76521

TACGTGAGAGACTAGTGTTATTCATCTTAATTGGGTTTAAAGGGTACCTAGACAGTCAATATAACTTCTATAATGCTAATACTTGACTAGAGTTTTAAGTAAGAGGGAAGTACTTAAGGAGTAAGAGATGAAATATCTGTGATACCAAAGGGACTCCGTAAAGGCGAAGGCATCCCTTTATCTAAAAATTAACGTTGAAGGACGAAGGCTTAGATAACAAATAGG

>8d2094d6b79c6e1e8184e6d7d2f597e2

TACGTAGGGTGCGAGCGTTAATCGGAATTACTGGGCGTAAAGCGTGCGCAGGCGGTTTGTTAAGACAGATGTGAAATCCCCGGGCTCAACCTGGGAACTGCATTTGTGACTGGCAGGCTAGAGTATGGCAGAGGGGGGTAGAATTCCACGTGTAGCAGTGAAATGCGTAGAGATGTGGAGGAATACCGATGGCGAAGGCAGCCCCCTGGGCCAATAGTGACGCTCATGCACGAAAGCGTGGGGAGCAAACAGG

>649d28f50985d48295948e03a6a5c0da

TACCGGCAGTCCGAGTGATGGCCGATATTATTGGGCCTAAAGCGTCCGTAGCCGGCCGGACAAGTCCGTTGGGAAATCGACGTGCCTAACACGTCGGCGTCCAGCGGAAACTGTCCGGCTTGGGGCCGGAAGACCTGAGGGGTACGTCCGGGGTAGGAGTGAAATCCTGTAATCCTGGACGGACCACCAATGGGGAAACCACCTCAGGAAGACGGACCCGACGGTGAGGGACGAAAGCTAGGGTCTCGAACCGG

>d3c2b1c15d26e5fdcab77b372a987628

TACGTAGGGTGCGAGCGTTGTCCGGAATTACTGGGCGTAAAGAGCTCGTAGGTGGTCTGTCGCGTCATTTGTGAAAGCCCGGTGCTTAACTCCGGGTTGGCAGGTGATACGGGCATGACTGGAGTACTGTAGGGGAGACTGGAATTCCTGGTGTAGCGGTGAAATGCGCAGATATCAGGAGGAACACCGGTGGCGAAGGCGGGTCTCTGGGCAGTAACTGACGCTGAGGAGCGAAAGCATGGGTAGCGAACAGG

>061ed8f217547924a73350fc99f34448

TACGTAGGTGGCAAGCGTTGTCCGGATTTATTGGGCGTAAAGCGAGTGCAGGCGGTCCAATAAGTCTGACGTGAAAGCCTTCGGCTCAACCGGAGAATTGCATCAGAAACTGTTGAACTTGAGTGCAGAAGAGGAGAGTGGAACTCCATGTGTAGCGGTGGAATGCGTAGATATATGGAAGAACACCAGTGGCGAAGGCGGCTCTCTGGTCTGCAACTGACGCTGAGGCTCGAAAGCATGGGTAGCGAACAGG

>39e3aa73b81169e5a83b409a8e339a7b

TACGTAGGGTGCGAGCGTTAATCGGAATTACTGGGCGTAAAGCGTGCGCAGGCGGTTTGTTAAGACAGATGTGAAATCCCCGGGCTCAACCTGGGAACTGCATTTGTGACTGGCAGGCTAGAGTATGGCAGAGGGGGGTAGAATTCCACGTGTAGCAGTGAAATGCGTAGAGGTGTGGAGGAATACCGATGGCGAAGGCAGCCCCCTGGGCCAATACTGACGCTCATGCACGAAAGCGTGGGGAGCAAACAGG

>374b724806294b861c4ed5cfb74e2f31

TACGAAGGGGGCTAGCGTTGCTCGGAATCACTGGGCGTAAAGGGCGCGTAGGCGGCCATTCAAGTCGGGGGTGAAAGCCTGTGGCTCAACCACAGAATTGCCTTCGATACTGTTTGGCTTGAGTTTGGTAGAGGTTGGTGGAACTGCGAGTGTAGAGGTGAAATTCGTAGATATTCGCAAGAACACCAGTGGCGAAGGCGGCCAACTGGACCAACACTGACGCTGAGGCGCGAAAGCGTGGGGAGCAAACAGG

>723955eb0d165eb4f7e8ba538e8b0bcf

TACGTAGGGTGCGAGCGTTAATCGGAATTACTGGGCGTAAAGCGTGCGCAGGCGGTTTGTTAAGACAGATGTGAAATCCCCGGGCTCAACCTGGGAACTGCATTTGTGACTGGCAGGCTAGAGTATGGCAGAGGGGGGTAAAATTCCACGTGTAGCAGTGAAATGCGTAGAGATGTGGAGGAATACCGATGGCGAAGGCAGCCCCCTGGGCCAATACTGACGCTCATGCACGAAAGCGTGGGGAGCAAACAGG

>ee0fe9443d9c09304ba1d2d2e317622e

TACGTAGGTGGCAAGCGTTATCCGGATTTATTGGGCGTAAAGAGAGTGCAGGCGGTTTTCTAAGTCTGATGTGAAAGCCTTCGGCTTAACCGGAGAAGTGCATCGGAAGCTGGATAACTTGAGTGCAGAAGAGGGTAGTGGAACTCCATGTGTAGCGGTGGAATGCGTAGATATATGGAAGAACACCAGTGGCGAAGGCGGCTACCTGGTCTGCAACTGACGCTGAGACTCGAAAGCATGGGTAGCGAACAGG

>a54b914857edcc2c5211e94dcba09cfe

TACGTAGGGTGCGAGCGTTAATCGGAATTACTGGGCGTAAAGCGTGCGCAGGCGGTTTGTTAAGACAGATGTGAAATCCCCGGGCTCAACCTGGGAACTGCATTTGTGACTGGCAGGCTAGAGTATGGCAGAGGGGGGTAGAATTCCACGTGTAGCAGTGAAATGCGTAGAGATGTGGAGGAATACCTATGGCGAAGGCAGCCCCCTGGGCCAATACTGACGCTCATGCACGAAAGCGTGGGGAGCAAACAGG

>88b6e5e69b41eb2431fe3afe054cc3b8

ACATTCTTTGTACTTCATTAGTTATTTAATTTACACCACTTGTCACCCCCTTATATTGTACAAATGAGTTTGCTGGCACATTATGTGAAATACACGTTCCTGTTACAACTTATCCCTGACTGCTAAAACATGAACAGCAATATTTCTATGACCACAAAAACTATGGACCAAGAAGCTA

>30cc97f8612e753f1d413b0a8b0dccee

TACGTAGGGGGCAAGCGTTGTCCGGATTTATTGGGCGTAAAGAGCGTGTAGGCGGCTAGGTAGGTGGGTTGTGAAAACTGGAGGCTCAACCTTCAGACGTCGACCCAAACCCCCTGGCTAGAGTCCGGAAGAGGAGAGTGGAATTCCTGGTGTAGCGGTGAAATGCGCAGATATCAGGAAGAACACCCGTGGCGAAGGCGGCTCTCTGGGACGGTACTGACGCTGAGACGCGAAAGCGTGGGGAGCGAACAGG

>42df1d708ce300ca8371c9fdb6342391

TACGGAGGGTGCGAGCGTTAATCGGAATAACTGGGCGTAAAGGGCACGCAGGCGGTGACTTAAGTGAGGTGTGAAAGCCCCGGGCTTAACCTGGGAATTGCATTTCATACTGGGTCGCTAGAGTACTTTAGGGAGGGGTAGAATTCCACGTGTAGCGGTGAAATGCGTAGAGATGTGGAGGAATACCGAAGGCGAAGGCAGCCCCTTTGGAATGTACTGACGCTCATGTGCGAAAGCGTGGGGAGCAAACAGG

>81b98e2eda4509ad90e0d007aa3213a1

CAAATTTACGTCTTCAGAAAGACAAAATATTTTTCCAGGGTCCATTTCAACCATGTCGGTCATTTTCAACTAGCAGTGCCTTGTAGATATTTGTGAAAGATTTTGAATGTGTCTTGTCCATAGAGTTCAGTTGTTCATTAATGATTTTTTTATGTAGTTCGTAACGTAATATATTTAGGCTTTCAAGTAAATTGATTTTGTTTCCTTTTTCCGGTTGCG

>2c0ecb74f8af149bff9f28b29af273c9

TACGTAGGTGGCAAGCGTTATCCGGATTTATTGGGCGTAAAGAGAGTGCAGGCGGTTTTCTAAGTCTGATGTGAAAGCCCCCGGCTCAACCGGGGAGGGTCATTGGAAACTGGGAGACTTGAGTGCAGAAGAGGAGAGTGGAATTCCATGTGTAGCGGTGAAATGCGTAGATATATGGAGGAACACCAGTGGCGAAGGCGGCTCTCTGGTCTGTAACTGACGCTGAGGAGCGAAAGCGTGGGGAGCGAACAGG

>25db98d65284e3e9898bd6f506aeac62

TACCGGCAGCCCGAGTGATGGCCGATCTTATTGGGCCTAAAGCGTCCGTAGCTGGCCGCACAAGTCCATCGGAAAATCCACCCGCTCAACAGGTGGGCGTCCGGTGGAAACTGTGTGGCTTGGGACCGGAAGGCGCGACGGGTACGTCCGGGGTAGGAGTGAAATCCCGTAATCCTGGACGGACCGCCGATGGCGAAAGCACGTCGCGAGAACGGATCCGACAGTGAGGGACGAAAGCCAGGGTCTCGAACCGG

>f9e8e30034eb448c863eeac341ce8544

CACGATTAACCCAAGTCAATAGAAGCCGGCGTAAAGAGTGTTTTAGATCACCCCCTCCCCAATAAAGCTAAAACTCACCTGAGTTGTAAAAAACTCCAGTTGACACAAAATAGATTACGAAAGTGGCTTTAACATATATGAACACACAATAGCTAAGACCCAAACTGGG

>23cdaf486334654440114f5f580e6286

TACGTAGGGTGCGAGCGTTAATCGGAATTACTGGGCGTAAAGCGTGCGCAGGCGGTTTGTTAAGACAGATGTGAAATCCCCGGGCTCAACCTGGGAACTGCATTTGTGACTGGCAGGCTAGAGTATGGCAGAGGGGGGTAGAATTCCACTTGTAGCAGTGAAATGCGTAGAGATGTGGAGGAATACCGATGGCGAAGGCAGCCCCCTGGGCCAATACTGACGCTCATGCACGAAAGCGTGGGGAGCAAACAGG

>91fadb46bbcc997d8f1a419b58efa399

CACACTTATGTCTTCAGAAAGACAAAATATTTCTCCAGGATAAATTTCAACCATGCATGTCGGTCATTTTCAACTGGCAGTGCCTTGTAGATATTTGTGAAAGATTTTGAATGTGTCTTGTCCATAGAGTTGAGTTTGTTCAATAATGATTTTGTTATGTACTTTGTAACGTAATACATTCAGGCTTTCAAGTAAATTCATTTTGTTTACTTCTTTGCCGGTTGTG

>133eb5bfbe5cc0298d6d9fd5fcaed722

TCAAACGTAAGCACAACAGAAAGATAGCCAAACTAATACCACCCCGATCACCAGCAATTACGCCCACACACACTTTTTACCCAAGAATAGTTAACCTCACCAACATAACATTCACTAACGATAAGGAACATCTTTTAAACAAAGGGATAAATCACAACCTACACTACACGCAGAACAATAACACCATAAAGAACATTAT

>f90e8c1337c1f86d68e004fca6d75997

TACGTAGGTGGCAAGCGTTGTCCGGAATTATTGGGCGTAAAGCGCGCGCAGGCGGTCCTTTAAGTCTGATGTGAAATCTTGCGGCTCAACCGCAAGCGGTCATTGGAAACTGGGGGACTTGAGTGCAGAAGAGGAAAGCGGAATTCCACGTGTAGCGGTGAAATGCGTAGAGATGTGGAGGAACACCAGTGGCGAAGGCGGCTTTCTGGTCTGTAACTGACGCTGAGGTGCGAAAGCGTGGGGAGCAAACAGG

>7e6ea8f6a3d82fd1dac6abccaf7c0bd5

TACGTAGGGTGCGAGCGTTAATCGGAATTACTGGGCGTAAAGCGTGCGCAGGCGGTTTGTTAAGACAGATGTGAAATACCCGGGCTCAACCTGGGAACTGCATTTGTGACTGGCAGGCTAGAGTATGACAGAGGGGGGTAGAATTCCACGTGTAGCAGTGAAATGCGTAGAGATGTGGAGGAATACCGATGGCGAAGGCAGCCCCCTGGGCCAATACTGACGCTCATGCACGAAAGCGTGGGGAGCAAACAGG

>a731831ee30c8e955cc738044b1fc536

TACGTAGGGTGCGAGCGTTAATCGGAATTACTGGGCGTAAAGCGTGCGCAGGCGGTTTGTTAAGACAGATGTGAAATCCCCGGGCTCAACCTGGGAACTGCATTTGTGACTGGCAGGCTAGAGTATGGCAGAGGGGGGTAGAATTCCACGTGTAGCAGTGAAATGCGTAGAGATGTGGAGGAATACCGATGGCGAAGGCAACCCCCTGAGCCAATACTGACGCTCATGCACGAAAGCGTGGGGAGCAAACAGG

>f61feda57015e486c7270dcfacdf2dc2

CACGATTAACCCAAGTCAATAGAAGCCGGCGTAAAGAGTGTTTTAGATCACCCCCTCCCCAATAAAGCTAAAACTCACCTGAGTTGTAAAAAACTCCAGTTGATACAAAATAGACTACGAAAGTGGCTTTAACATATCTGAACACACAATAGCTAAGACCCAAACTGGG

>50b75b6c1c9d8cf1c84370f5450e61c4

AACGTAGGAGGCGAGCGTTATCTGGATTCATTGGGCGTAAAGGGCGTGTAGGCGGTGACTCAAGTTGGGCGTGAAATCTCCCGGCTCAACCGGGAGGCGCCGTCCAATACTGGGACACTTGAGGGCAGGAGAGGAAAGCGGAATTCCCGGTGTAGTGGTGGAATACTTAGATATCGGGAGGAACACCAGTGGCGAAAGCGGCTTTCCGGACTGTGCCTGACGCTGAAGCGCGAAAGCGTGGGTAGCAAACTGGG

>45cbbf634df1afd8fa8a4f54417ac627

AATTGTGTGCCAGCCGCCGCGGTAAGGTATTGGGATGAACAATGCAATTTAGTAAAAACTAGATATTTTACATCCTGTTTTTTTTAATAAGGCAGCTGCTGTAGACCTTAGTAGATTTGTTGGTGCATTAGAAACCCGAGTAGTCCGGCTGACTGACTGCGTATACATCTCGTATGCCGTCTTCTGCT

>daef49aa6f6ceb24926a377e794ff433

TACGTAGGGTGCGAGCGTTAATCGGAATTACTGGGCGTAAAGCGTGCGCAGGCGGTTTGTTAAGACAGATGTGAAATCCCCGGGCTCAACCTGGGAACTGCATTCGTGACTGGCAGGCTAGAGTATGGCAGAGGGGGGTAGAATTCCACGTGTAGCAGTGAAATGCGTAGAGATGTGGAGGAATACCGATGGCGAAGGCAGCCCCCTGGGCCAATACTGACGCTCATGCACGAAAGCGTGGGGAGCAAACAGG

>d5e96c404b86dbf304eb1182a089030c

TACGTAGGGTGCGAGCGTTAATCGGAATTACTGGGCGTAAAGCGTGCGCAGGCGGTTTGTTAAGACAGATGTGAAATCCCCGGGCTCAACCTGGGAACTACATTTGTGACTGGCAGGCTAGAGTATGGCAGAGGGGGGTAGAATTCCACGTGTAGCAGTGAAATGCGTAGAGATGTGGAGGAATACCGATGGCGAAGGCAGCCCCCTGGGCCAATACTGACGCTCATGCACGAAAGCGTGGGGAGCAAGCAGG

>20add184d73f0c8333dd1b2b1e26e5e1

TACGTAGGGTGCGAGCGTTAATCGGAATTACTGGGCGTAAAGCGTGCGCAGGCGGTTTGTTAAGACAGATGTGAAATCCCCGGGCTCAACCTGGGAACTGCATTTGTGACTGGCAGGCTAGAGTATGGCAGAGGGGGGTAGAATTCCACGTGTAGCAGTGAAATGCGTAGAGATGTGGAGGAATACCGATGGCGAAGGCAGCCCCCTAGGCCAATACTGACGCTCATACACGAAAGCGTGGGGAGCAAACAGG

>48fc9c01e94335d65ee3c395fdc549a1

TACGTAGGGTGCGAGCGTTAATCGGAATTACTGGGCGTAAAGCGTGCGCAGGCGGTTTGTTAAGACAGATGTGAAATCCCCGGGCTCAACCTGGGAACTGCATTTGTGACTGGCAGGCTAGAGTATGGCAGAGGGGGGTAGAATTACACGTGTAGCAGTGAAATGCGTAGAGATGTGGAGGAATACCGATGGCGAAGGCAGCCCCCTGGGCCAATACTGACGCTCATGCACGAAAGCGTGGGGAGCAAACAGG

>1b3a52c873cfd09044e13dadf8c904fe

TACCGGCAGCTCGAGTGATGTCCAATATTATTGGGCCTAAAGCGTCCGTAGCTGGCCGCGCAAGTCCATCGGGAAATCCACCTGCTCAACAGGTGGGCGCCCGGTAGAAACTGCGTGGCTTGGGACCGGAAGGCGCGACGGGTACGTCCGGGGTAGGAGTGAAATCCCGTAATCCTGGACGGACCGCCGATGGCGAAAGCACGTCGCGAGAACGGATCCGACAGTGAGGGACGAAAGCCAGGGTCTCGAACCGG

>08fc6827e76c18d69b2ac421f0074aee

TACGTAGGTGGCAAGCGTTGTCCGGATTTATTGGGCGTAAAGCGAGCGCAGGCGGTTTCTTAAGTCTGATGTGAAAGCCCCCGGCTCAACCGGGGAGGGTCATTGGAAACTGGGAGACTTGAGTGCAGAAGAGGAAAGTGGAATTCCATGTGTAGCGGTGAAATGCGCAGAGATATGGAGGAACACCAGTGGCGAAGGCGACTTTCTGGTCTGTAACTGACGCTGATGTGCGAAAGCGTGGGGATCAAACAGG

>7d92fcc8e152cfe06616712a69267cc6

CAAGCAGCATATGGTAATTGTGTGCCAGCAGCCGCGGTAACTTGCAGCTTATAAGAACAAACAGAAACTAAAAAACGATCATTATTTTACACAAATACAGGTAACAACTGTAACAGAACTACACAAATTAGAAACCCCTGTAGTCCGGCTGACTGACTCTCGACTTATCTCGTATGCCGTCTTCTGCTTGAAAAAAAAAAAAC

>34b78390543a0b236a54427ff5a3f835

TACGTAGGCAGCAAGCGTTGTTCGGAGTTACTGGGCGTAAAGAGTGCGTAGGCGGTGCCCTAAGTCTGTTGTGAAATCTCCCGGCTTAACTGGGAGGGTGCGGTGGAAACTGGGGTGCTTGAGTGTGGGAGAGGTTGGCGGAATTCCTGGTGTAGCGGTGAAATGCGTAGATATCAGGAGGAACACCGGTGGTGTAGACGGCTAACTGGACCATGACTGACGCTGAGGCACGAAAGCGTGGGTAGCAAACAGG

>16d241861b6dfef2623a8973d654812f

CAAATTTATGTCTTCAGAAAGACAAAATATTTTTCCAGGGTCCATCTCAACCATGTCGGTCATTTTCAACTAGCAGTGCCTTGTAGATATTTGTGAAAGATTTTGAATGTGTCTTGTCCATAGAGTTCAGTTTGTTCATTAATGATTCTTTATGTAGTTTGTAACGTAATATATTTAGGCTTTCAAGTAAATTGATTTTGTTTCCTTTTTCCGGTTGTG

>cae39575f829845e6c1f853055af456b

AACGTAGGATCCTAGCGTTATCCGAATTTACTGGGCGTAAAGCGCGTGTAGGTGGTTTGGTAAGTTGGATGTGAAAGCTCCTGGCTCAACTGGGAGAGGCCGTTCAAAACTACCAGACTTGAGGGCGGTAGAGGAAGGTGGAATTCCCGGTGTAGTGGTGAAATGCGTAGATATCGGGAGGAACACCTGTGGCGAAGGCGGCTTCCTGGGATGCCCCTGACGCTGAGACGCGAAAGCTAGGGGAGCAAACTGGG

>c7e95d23ca9b3fe76ccd5212e8794a2c

CATACTTATGTCGTTAGAAAGACAAAACATTTTTCCAGAATGCATTTCAACCTTCTCGGTCATTTTCAACTGGCAGTGCCTTGTAGATAGTTGTGGAAAATGTTGAATGTGTCTTGTCCATAGAGTTCAGTTTGTTTATTTAAGATTTTGTTATGTAGCTTGTAACGTAGTATATTTAGACTTTCAAGTAAATTCATTTTATTTCTTTTTTTGCCGGTTGTG

>c3e8514ca7153db9c4b552c3efc99d13

TACGTAGGGGGCAAGCGTTGTCCGGAATCATTGGGCGTAAAGCGCGTGTAGGCGGTTCGATAAGTCTGCTGTGAAAGTCCAGGGCTCAACCCTGGGATGCCGGTGGATACTGTCGGGCTAGAGTACGGAAGAGGCGAGTGGAATTCCCGGTGTAGCGGTGAAATGCGCAGATATCGGGAGGAACACCAATTGCGAAGGCAGCTCGCTGGGACGTTACTGACGCTGAGACGCGAAAGCGTGGGGAGCAAACAGG

>f604625bacbecdf591e9690cb4c87751

TACGAAGGTGGCTAGCGTTGTTCGGAATCACTGGGCTTAAAGAGCACGTAGGCGGCCTGTCAAGCGTCTTGTGAAATCCCTCGGCTCAACCGAGGAACGGCTGGGCGAACTGGCAGGCTTGAGGTAGGTAGGGGCGCACGGAACTCTTGGTGGAGCGGTGGAATGCGTAGATATCAAGAGGAACGCCGGTGGCGAAGGCGGTGCGCTGGGCCTATCCTGACGCTCAGGTGCGAAAGCCAGGGGAGCAAACTGGG

>4e5ff310075a50266f9d480989d68398

TACGTAGGGTGCGAGCGTTAATCGGAATTACTGGGCGTAAAGCGTGCGCAGGCGGTTATACAAGACAGGCGTGAAATCCCCGGGCTTAACCTGGGAATGGCGCCTGTGACTGTATAGCTAGAGTGTGTCAGAGGGGGGTAGAATTCCACGTGTAGCAGTGAAATGCGTAGATATGTGGAGGAATACCAATGGCGAAGGCAGCCCCCTGGGATAACACTGACGCTCATGCACGAAAGCGTGGGGAGCAAACAGG

>200c6b428bd98e88f0a2162483e2caf1

GACAGAGGGTGCAAACGTTGTTCGGAATTACTGGGCGTAAAGCGTGTGTAGGCGGCCATGTAAGTTGGATGTGAAAGCCCCGGGCTCAACCCGGGGAGTGCATTCAAAACTGCGTGGCTTGAGTACTGGAGAGGTTGGTAGAATTCTCGGTGTAGAGGTGAAATTCGTAGATATCGAGAGGAATACCGGTGGCGAAGGCGGCCAACTGGACAGATACTGACGCTGAGACACGAAAGCGTGGGGAGCAAACAGG

>b09e79f5e72ceb8d764ee00113f97c79

TACGTGAGAGACTAGTGTTATTCATCTTAATTGGGTTTAAAGGGTACCTAGACAGTCAATATAACTTCTAGAATGCTAATACTTGACTAGAGTTTTAAGTAAGAGGGAAGTACTTAAGGAGTAAGAGATGAAATATCTGTGATACCAAAGGGACTCCGTAAAGGCGAAGGCATCCCTTTATCCAAAAACTAACGTTGAAGGACGAAGGCTTAGATAACAAATAGG

>d75ac4aba564a2f61714d1c078248776

CACACTTATGTCATTACAAAGACAACACGTTTTTGCAGAATGCATTTCAACCTTCTCGGTCATTTTCAACTAGCAGTGCCTTGTAGATAAATGTGGAAGATTTTGAATGTTTCTTTTCCATAGAGTTCAGTTTGTTCATTAATTATTTTGTTGTGTAGTTTGTAACGTAATATATTTAAACTTTCAAGAAGATTCATTTCATTTCCTTTTTTTGCCTGTTGTA

>2e0f9067d6f832f27f8cfca0957f7eed

TACGTGAGAGACTAGTGTTATTCATATTAATTGGGTTTAAAGGGTACCTAGACAGTCAATATAACTTCTAGAATGCTAATACTTGACTAGAGTTTTAAGTAAGAGGGAAGTACTTAAGGAGTAAGAGATGAAATATCTGTGATACCAAAGGGACTCCGTAAAGGCGAAGGCATCCCTTTATCTAAAAACTAACGTTGAAGGACGAAGGCTTAGATAACAAATAGG

>904c75024291129174fa4dc85a2c9a61

TACAGAGGGTGCAAGCGTTAATCGGATTTACTGGGCGTAAAGCGCGCGTAGGCGGCTAATTAAGTCAAATGTGAAATCCCCGAGCTTAACTTGGGAATTGCATTCGTTACTGGTTAGCTAGAGTGTGGGAGAGGATGGTAGAATTCCAGGTGTAGCGGTGAAATGCGTAGAGATCTGGAGGAGTACCGATGGCGAAGGCAGCCATCTGGCCTAACACTGACGCTCAGGTGCGAAAGCGTGGGGAGCAAACAGG

>aca28e4f440b01d56bc951ba98270a87

TACGTAGGGTCCGAGCGTTGTCCGGAATTACTGGGCGTAAAGAGCTCGTAGGTGGTTTGTCGCGTTGTTCGTGAAAACTCACAGCTTAACTGTGGGCGTGCGGGCGATACGGGCAGACTAGAGTACTGCAGGGGAGACTGGAATTCCTGGTGTAGCGGTGGAATGCGCAGATATCAGGAGGAACACCGGTGGCGAAGGCGGGTCTCTGGGCAGTAACTGACGCTGAGGAGCGAAAGCGTGGGTAGCGAACAGG

>85d9cf769aefe7d929ee985c263c826d

TACGAAGGGGGCTAGCGTTGCTCGGAATCACTGGGCGTAAAGGGCGCGTAGGCGGCCTTGTAAGTTGGGGGTGAAAGCCCGTGGCTCAACCACGGAAATGCCTTCGATACTGCTTGGCTTGAGTGTGGTAGAGGTTGGTGGAACTGCGAGTGTAGAGGTGAAATTCGTAGATATTCGCAAGAACACCGGTGGCGAAGGCGGCCAACTGGACCATCACTGACGCTGAGGCGCGAAAGCGTGGGGAGCAAACAGG

>94fa5cda4b45883ec90a83a057e514d5

AGTACGACGACGGTCCATCAATGGCGCCTTCCGAACTGATTAGGACACCTCTGAAGTGATAAACGATAATGTCGATATTTCTGGTAACACAGCTGTGTCATTCTCGAGTAGACCGTCTCAATTTGAGGTGTCTTTTGAGAGGACAGGATATCACTCGTTCTGGAGAGAT

>b6a4048840db3250b2b05456c2b85e93

TACGTAGGGTGCAAGCGTTATCCGGAATTATTGGGCGTAAAGAGCTCGTAGGCGGTTTGTCGCGTCTGCTGTGAAATCCCGAGGCTCAACCTCGGGCCAGCAGTGGGTACGGGCAGACTAGAGTGCGGTAGGGGAGATTGGAATTCCTGGTGTAGCGGTGGAATGCGCAGATATCAGGAGGAACACCGATGGCGAAGGCAGATCTCTGGGCCGTAACTGACGCTGAGGAGCGAAAGGGTGGGGAGCAAACAGG

>7f7173572371dd936b63cc75d96f0f6e

TACGGAGGGTGCAAGCGTTACCCGGAATCACTGGGCGTAAAGGGCGTGTAGGCGGGACCGTAAGTCTGGTTTTAAAGACTGAGGCTCAACCTCAGGAGTGGACTGGATACTGCGATCCTTGACCTCTGGAGAGGCAACCGGAATTCCTGGTGTAGCGGTGGAATGCGTAGATACCAGGAGGAACACCAATGGCGAAGGCAGGTTGCTGGACAGAAGGTGACGCTGAGGCGCGAAAGTGTGGGGAGCAAACCGG

>d4ef667f547f19576a2330ddc3deb3cf

TACGTAGGGTGCGAGCGTTAATCGGAATTACTGGGCGTAAAGCGTGCGCAGGCGGTTTGTTAAGACAGATGTGAAATCCCCGGGCTCAACCTGGGAACTGCATTTGTGACTGGCAGGCTAGAGTATGGCAAAGGGGGGTAGAATTCCACGTGTAGCAGTGAAATGCGTAGAGATGTGGAGGAATACCGATGGCGAAGGCAGCCCCCTGGGCCAATACTGACGCTCATGCACGAAAGCGTGGGGAGCAAACAGG

>1bee305d84ba0b4a1ef599b7f06c9b0c

TACGAAGGGTGCAAGCGTTACTCTGAATTACTGGGCGTAAAGCGTGCGTAGGTGGTTCGTTAAGTCTGATGTGAAAGCCCTGGGCTCAACCTGGGAATTGCATTGGATACTGGCGAGCTAGAGTGCGGTAGAGGGTAGTGGAATTCCCGGTGTAGCAGTGAAATGCGTAGAGATCGGGAGGAACATCCGTGGCGAAGGCGACTGCCTGGACCAGCACTGACACTGAGGCACGAAAGCGTGGGGAGCAAACAGG

>0686bfbbca8578a0c155c5c4992e44f3

TTTCGGGAAGAGATACGGTTACGATTAAGGGAGTTCGGAAGATAATAAGGACTTCAATCCATAAGCTTACCAACGACATAATAACAAAACAGCTGTTCTGGTGTGAACATGTGCAACCGATGAGCGGGGAACGATTATCTGCACTAGGCACCAAGAGGGAGATGGAAACTCCGTGGATGGATAGGTATAGCTGGAG

>ecec99ccb4df23e131708967b378bb7c

TACGGAGGGTGCAAGCGTTAATCGGAATTACTGGGCGTAAAGCGCGCGTAGGTGGCTTGATAAGTCGGATGTGAAATCCCCGGGCTCAACCTGGGAACTGCATCCAAAACTGTCTGGCTAGAGTGTGGTAGAGGGTAGTGGAATTTCCAGTGTAGCGGTGAAATGCGTAGATATTGGAAGGAACACCAGTGGCGAAGGCGACTACCAGGACTAACGCTGACACTGAGGTGCGAAAGCGTGGGGAGCAAACAGG

>96e401bd0d6425acdf9070d1551c949f

TACAAGTAAATCTTTTTTTAAAAAATTAAAAATAATAAAAAAATTTTTGTATTTTAAAAATTTATAAAGGTGAAATTTATAATTTTAAAAAAATTAATTTGATTTTATAAAAATTTAATTTTTAAACTAGGATTAGAAACCCCAGTAGTCCGGCTGACTGACT

>ca67684dff3f1ab49c6392cdc7a0e041

AATCAAGGTGCGTTTTTCAGCCCCCAGATCAATTTAAACTATGAAAATGAGTACCTAGGATCAAGTAGGTGAAAAGCACTACACCTGACACCTGTATGCCTTCCTTGAATGCCGTACGGCTTAAGCCGTAAGTCGTATTGCGGCAGCAATAGCGAGAGAAATTTAAAAAATTGAAAATAGGCTTTTAAATTATTGTGTAATGTCTCCTTCTTGTTCTTCTTCTTCTTTTACTACCCTTATAGG

>3fa218b1b9e45ecad0a054385dd07e16

TACCGGCAGCCCGAGTGATGGCCGATCTTATTGGGCCTAAAGCGTCCGTAGCTGGCCGCACAAGTCCATCGGAAAATCCACCCGCCCAACGGGTGGGCGTCCGGTGGAGACTGTGTGGCTTGGGACCGGAAGGCGCGACGGGTACGTCCGGGGTAGGAGTGAAATCCCGTAATCCTGGACGGACCGCCGATGGCGAAAGCACGTCGCGAGAACGGATCCGACAGTGAGGGACGAAAGCCAGGGTCTCGAACCGG

>9306f1ce6832b623372ef0f72871adc3

GACGAAGGTGGCGAGCGTTGCTCGGAATCACTGGGCTTAAAGCGGGCGTAGGCGGCCCGGCCAGTCGGGTGTGAAATCCCTTGGCTCGACCAAGGAACGGCACCCGATACTACCGGGCTTGAGGCGTCTAGGGGCGACTGGAACTCTCGGTGGAGCGGTGAAATGCGTAGATATCGAGAGGAACGCCAATGGCGAAGACAGGTCGCTGGGGACGTCCTGACGCTGAGGTCCGAAAGCTAGGGGAGCAAACTGGG

>8134e82b700494b3151816343028b77b

TACGGAGGGTGCAAGCGTTAATCGGAATTACTGGGCGTAAGGCGCGCGTAGGTGGTTCAGCAAGTTGGATGTGAAATCCCCGGGCTCAACCTGGGAACTGCATCCAAAACTACTGAGCTAGAGTACGGTAGAGGGTGGTGGAATTTCCTGTGTAGCGGTGAAATGCGTAGATATAGGAAGGAACACCAGTGGCGAAGGCGACCACCTGGACTGATACTGACACTGAGGTGCGAAAGCGTGGGGGGCAAACAGG

>11e7105f3b0034cfd70168da8333e5a1

TACGTAGGGTGCGAGCGTTAATCGGAATTACTGGGCGTAAAGCGTGCGCAGGCGGTTTGTTAAGACAGATGTGAAATCCCCGGGCTCAACCTGGGAACTGCATTTGTGACTGGCAGGCTAGAGTATAGCAGAGGGGGGTAGAATTCCACGTGTAGCAGTGAAATGCGTAGAGATGTGGAGGAATACCGATGGCGAAGGCAGCCCCCTGGGCCAATACTGACGATCATGCACGAAAGCGTGGGGAGCAAACAGG

>155aa3314a0be990fe6841b7e4c3c7e3

AATTACAGTAAGCATGAATAAAGTATAGCATAAATAAAGCTTGAATAAAATTACAGTAAGCATGACTTCGATAAGGAAAATTGAACACAATAATTAAGTTAGTTTAGAAAATATTTCTTTTATTAACATAGCTATAAATAAAACGAATAAAAACAAAATTATTGCAATTGAGATGATGTATATGAGAATGAACTGTGGCGTTTCAAGAAGGGATTAACAGATAAATAATAAGTAGATTAGGG

>ceff1daa0679dde981b7e51cd71a904e

GACAGAGGATGCAAGCGTTATCCGGAATGATTGGGCGTAAAGCGTCTGTAGGTGGCTTTTTAAGTCCGCCGTCAAATCCCAGGGCTCAACCCTGGACAGGCGGTGGAAACTGCCAAGCTGGAGTACGGTAGGGGCAGAGGGAATTTCCGGTGGAGCGGTGAAATGCGCAGAGATCGGAAAGAACACCAACGGCGAAAGCACTCTGCTGGGCCGACACTGACACTGAGAGACGAAAGCTAGGGGAGCAAATGGG

>d925cdfd2bc1f0aaa3fc2d7f7e3e297c

CAGTTTTAAATTCCGATTCAAAGTTTCCAGTATCATGGTTTTCTATTTACCTACGCTGTTGCAGTATAGAAGTACGTATTACGTATGTGATGAACCTATAATCTTTAACGTGTAAGGAAACCCCGGATTAGCGTTGAATATCTTATGATCATGTCATCAGATACAACTCCTGATAGAGCGTCAGAAAAG

>b2fa1507fee16327f5928d4b3b5f2b73

CACATTTATGTCATTAGAAAGACAACACGTTTTTGCAGAATGCATTTCAACCTTCTCGGTCATTTTCAACTGGCAGTGCCTTGTAGATAATTGTGGAAGATTTTGGATGTTTCTTGCTCATAGAGTTCAGTTTGTTTATTAATGATTTTGTTGTGTAGTTTGTAACGTAATATACTTAAACTTTCAAGAAGATTCATTTTATTTCCTTTTTTGCCTGTTGTA

>bef6f5d3a0be8542eecd536522bdddd8

TACGTAGGGTGCGAGCGTTAATCGGAATTACTGGGCGTAAAGCGTGCGCAGGCGGTTTGTTAAGACAGATGTGAAATCCCCGGGCTCAACCTGGGAACTGCATTTGTGACTGGCAGGCTAGAGTATGGCAGAGGGGGGTAGAATTCCACGTGTAGCAGTGAAATGCGTAGAGATGTGGAGGAATACCGATGGCGAAGGCAGACCCCTGGGCCAATACTGACGCTCATGCACGAAAGCGTGGGGAGCAAACAGG

>9cb963a881efbec82e4f6e2bbe1d6dd4

TACGGAGGGTGCAAGCGTTAATCGGAATTACTGGGCGTAAAGCGCACGCAGGCGGTCTGTCAAGTCGGATGTGAAATCCCCGGGCTCAACCTGGGAACTGCATTCGAAACTGGCAGGCTAGAGTCTTGTAGAGGGGGGTAGAATTCCAGGTGTAGCGGCGAAATGCGTAGAGATCTGGAGGAATACCGGTGGCGAAGGCGGCCCCCTGGACAAAGACTGACGCTCAGGTGCGAAAGCGTGGGGAGCAAACAGG

>b74c3132186549748f875ca0e2d307b1

TACGGAGGGGGTTAGCGTTGTTCGGAATTACTGGGCGTAAAGCGCGCGTAGGCGGATTGGAAAGTTGGGGGTGAAATCCCGGGGCTCAACCCCGGAACTGCCTTCAAAACTCCCAGTCTAGAGTTCGAGAGAGGTGAGTGGAACTCCGAGTGTAGAGGTGAAATTCGTAGATATTCGGAAGAACACCAGTGGCGAAGGCGGCTCACTGGCTCGATACTGGCGCTGAGGTGCGAAAGTGTGGGGAGCAAACAGG

>108b41c05c7f58b9e1c82b570c378223

TCAAACGAAAGCACAACAAAAAGATAGCCAAACTAATACCACCCCGATCACCAGCAATAATATCCACACATACGTTTTACTCCAGAATGGTTAACCTCACCAACATAACATTCACTAACGATCAGAAACAACTTTTAAACAAAGAGATAAATCCCAACCTACACCACACGCAGAACAATAACACCATCAAGAAACAGGG

>d0b05768ea6a191b60959ff739e909a7

TACGTAGGTGGCAAGCGTTATCCGGAATTATTGGGCGTAAAGCGCGCGTAGGCGGTTTTTTAAGTCTGATGTGAAAGCCCACGGCTCAACCGTGGAGGGTCATTGGAAACTGGAAAACTTGAGTGCAGAAGAGGAAAGTGGAATTCCACGTGTAGCGGTGAAATGCGCAGAGATATGGAGGAACACCAGTGGCGAAGGCGACTTTCTGGTCTGTAACTGACGCTGATGTGCGAAAGCGTGGGGATCAAACAGG

>bbfc419d8d00188cf5af46498d590177

GTGCTCGAAGAACATCAATCTTTCACTTCGTTTTACTTTCTCTTTTTACTGCCTCCTCTTTTGACCCTTTTCCAACATACCTCTTTCGCTCCTTTTCGTCATCCCTTTTCGGTCTATTTGTAGCATAATATATTAGATACCCCGGTAGTCCGGCTGACTGACT

>142407339819ca5617af6611a25a45dd

CACAAAAAAATTTGTATTAACAAAATTTGTGAAAGACCAGATATTTGTCCAGGATGCATTTCAACCATGTCGGTCATTTTCAACTGGCAGTGCCTTGTAGATATTTGTGAAACATTTTGAATGTGTCTTGTCCATAGAGTTCAGTTTGTGCATTAATGATTTTGTTATGTAGTTTGTAACGTGATATATTTAGGCTTTCAAGTAAATTCATTTTGTTTCATTTTTTGCCGGTTGTG

>46d76db30eb4ca8abe4e71d1c9e8cab6

CACACTTATGTCTTGAGAAAGACAAAATATTTATCCAGGATGCATTTCAACCATGTCGGTCATTTTCAACTGGCAGTGCCTTGTAGATATTTGTGAAATATTTTGAATGTGTCTTGTCCATAGAGTTCAGTTTGTTCATTAATGATTTTGTTATGTAATTTGTAACCTAATATATTTAGCCTATCAAGTAAATTCATTTTGTTTCCTTTTTGACGGTTCTG

>f032fef84e6dc79bccedf321245abafb

TACGTAGGGCGCGAGCGTTATCCGGAATTATTGGGCGTAAAGAGCTCGTAGGCGGTTTGTCGCGTCTGCTGTGAAAGACCGGGGCTCAACTCCGGTTCTGCAGTGGGTACGGGCAGGCTGGAGTGCAGTAGGGGAGACTGGAATTCCTGGTGTAGCGGTGAAATGCGCAGATATCAGGAGGAACACCGATGGCGAAAGCAGGTCTCTGGGCTGTTACTGACGCTGAGGAGCGAAAGCATGGGGAGCGAACAGG

>334facb5ae0c36e05aa73e478c91b376

TACGGAGGGGGCTAGCGTTGTTCGGAATTACTGGACGTAAAGCGCACGTAGGCGGCTTTGTAAGTTAGAGGTGAAAGCCTGGAGCTTAACTCCAGAACTGCCTTTAAGACTGCATCGCTTGAATCCGGGAGAGGTGAGTGGAATTCCGAGTGTAGAGGTGAAATTCGTAGATATTCGGAAGAACACCAGTGGCGAAGGCGGCTCACTGGACCGGTATTGACGCTGAGGTGCGAAAGCGTGGGGAGCAAACAGG

>2c69b472c7f34853923df85468bcfb5e

TACGGAGGGTGCAAGCGTTAATCGGAATTACTGGGCGTAAAGCGCACGCAGGCGGTCTGTCAAGTCGGATGTGAAATCCCCGGGCTCAACCTGGGAACTGCATTCGAAACTGGCAGGCTAGAGTCTTGCAGAGGGGGGTAGAATTCCAGGTGTAGCGGTGAAATGCGTAGAGATCTGGAGGAATACCGGTGGCGAAGGCGGCCCCCTGGACAAAGACTGACGCTCAGGTGCGAAAGCGTGGGGAGCAAACAGG

>8d37e60dc879d49ebd0995f397fb9b85

AAATTCAGCTTTTTGCATAACAAAACTGAACTCGTCAACTCATGTTGATTTTGCAGAGGACGAAAAAGACCGAATCTTACGTTATGACAACAGGTTGCTGTTACTATTTTTGACTTGAGACGATGAGATACTACAACTCGCTACTGCTTTCTTAATTGAATGTAAGCAATAATTCGTAAATTGCTACTGGCTTTGAAATTTACCAGTAGATACAAAATATTTCACTTTC

>af4fa13114bc162bdc45578ada52e27a

CACACTTTGATCCAGGATGCATTTCAACCATGTCGGTCATTTTCAACTGGCAGTGCCTTGTAGATATTTGTGAAAGATTTTGAATGTGTCTTGTCCATAGAGTTCAGTTCGTTCATTAATTATTTTGTTATGCAGTTTGTAACGTAATATATTTAGGCTTTCAAGTAAATTCATTTTGTTTCCTTTTTGCCGGTTGTG

>8979cf65a1e08320922470fe74d7c532

TACCGGCAGCACGAGTGATGGCCGATCTTATTGGGCCTAAAGCGTCCGTAGCCGGCCGAGCAAGTTCGTCGGGAAATCCACCTGCTCAACAGGTGGGCGCCCGGTGGAAACTGTGCGGCTTGGGACCGGAAGGCGCGACGGGTACGTCCGGGGTAGGAGTGAAATCCCGTAATCCTGGACGGACCGCCGATGGCGAAAGCACGTCGCGAGAACGGATCCGACAGTGAGGGACGAAAGCCAGGGTCTCGAACCGG

>86694f7fd91432807f62def7c70b8b08

CACGTATGGTGCGAGCGTTGTCCGGAATTATTGGGCGTAAAGAGCTCGTAGGCGGTGTGTCGCGTCGGCCGTGAAAACTTGGGGCTTAACTCTGAGCGTGCGGTCGATACGGGCATCACTTGAGTTCGGCAGGGGAGACTGGAATTCCTGGTGTAGCGGTGAAATGCGCAGATATCAGGAGGAACACCGGTGGCGAAGGCGGGTCTCTGGGCCGATACTGACGCTGAGGAGCGAAAGCGTGGGGAGCGAACAGG

>a88ec1b3c2891222c1b9b84665460d3f

TACGTAGGGTGCGAGCGTTAATCGGAATTACTGGGCGTAAAGCGTGCGCAGGCGGTTTGTTAAGACAGATGTGAAATCCCCGGGCTCAACCTGGGAACTGCATTTGTGACTGGCAGGCTAGAGTATGGCAGAGGGGGGTAGAATTCCACGTGTAGCAGTGAAATGCGTAGAGATGTGGAGGAATACCGATGGCGAAAGCAGCCCCCTGGGCCAATACTGACGCTCATGCACGAAAGCGTGGGGAGCAAACAGG

>729336664968608d23d3bf8e559c1d55

TCAAACGAAAGCACAACAAAAAGATAGCCAAACTAATACCACCCCGATCACCAGCAATAACACCCACACATACGTTTTACCCAAGAATAGTTAACCTCACCAACATAACATTCACTAACGATCAGAAACACCTTTTAAACAAAGGGATAAATCACAACCTACACTACAAGCAGAACAATAACACCATCAAGAACATTAT

>0e2d370f860f8262fe2d1fd778dd030f

TACGTAGGGTGCGAGCGTTGTCCGGAATTACTGGGCGTAAAGAGCTCGTAGGTGGTTTGTCGCGTCGTCTGTGAAATTCCGGGGCTTAACTCCGGGCGTGCAGGCGATACGGGCATAACTTGAGTACTGTAGGGGAGACTGGAATTCCTGGTGTAGCGGTGAAATGCGCAGATATCAGGAGGAACACCGGTGGCGAAGGCGGGTCTCTGGGCAGTAACTGACGCTGAGGAGCGAAAGCATGGGGAGCGAACAGG

>2d3bfb064457b675a6474c2bf5e25eff

TACGGAGGGGGCTAGCGTTGTTCGGAATTACTGGGCGTAAAGCGCGCGTAGGCGGACGGTCAAGTTGGGGGTGAAGGCCCGGGGCTCAACCCCGGAACTGCCTTCAAAACTGATCGTCTGGAGACCGGGAGAGGTGAGTGGAATTCCCAGTGTAGAGGTGAAATTCGTAGATATTGGGAAGAACACCAGTGGCGAAGGCGGCTCACTGGACCGGATCTGACGCTGAGGTGCGAAAGCGTGGGGAGCGAACAGG

>b31bf1fb109295662c231f3184693565

AGTTTTAATTTTACGGGCACATCCCATGCATAAATAGAAGTAAAGGGTATTGCATAGTGGAATACTATATACCCAAACTTGTAAAAGTAGTTAATAATTCGTGGTTTCGTTCCTCGTCTATCTACCTACCTATCTACCTATCTCTGATGACATTAGAAACCCC

>a7eec406085f3722c7e014629bdf641d

CAGGCTGTGGGTGCTAGACTCCGGTGTCGTCGACTCTCTCGTCAACTTCAGGGTCGCCTGTCCGCCCAAACTGCTCGTCTTCGACATGCAAACCGATCAGCTGGTAAGATACACTACACTGTTGGTAGCTGCTCTCAACATTTACATTAGATACCCGAGTAGT

>2143862f52603160d4415c1d0f2d9ee1

TACGTAGGGGGCGAGCGTTGCTCGGAATTACTGGGCGTAAAGGGTGTGCAGGCGGCCGATTAAGTCAACGATGAAATCCCGAAGCTCAACTTCGGAATGGTCTTTGATACTGATCGGCTTGAGGCTGGTTGAGGAGAGCGGAATTCCCGGTGGAGCGGTGAAATGCGTAGATATCGGGAGGAACACCAGAGGCGAAGGCGGTTCTCTAAACTTGTCCTGACGCTGAGACACGAAGGCTAGGGGAGCAAACTGGG

>487641c0651f4e41b27169b3ceb03146

TACCGGCAGTCCGAGTGATGGCCGATATTATTGGGCCTAAAGCGTCCGTAGCCGGCCGGACAAGTCCGTTGGGAAATCGACGCGCTCAACGCGTCGGCGTCCAGCGGAAACTGTCCGGCTTGGGGCCGGAAGACCTGAGGGGTACGTCCGGGGTAGGAGTGAAATCCTGTAATCCTGGACGGACCACCAATGGCGAAAGCACGTCAAGAGACCGGACCCGACGGTGAGGGACGAAAGCCAGGGTCTCGAACCGG

>c841dc97ae3fa5e916c224d08b960b93

CACACTTATGTCTTCAGAAAGACAAAATATTTATCCACGATGCATTTCAACCATGTCGGTCATTTTCAACTGGCAGTGCCTTGTAGACATTTGTGAAAGATTTTGAATGTGTCTTGTCCATAGAGTTCAGTTTGTTCATTAATGATTTTGTTATGTAGTTTGTAACGTAATATATTTAGGTTATCAAGTAAATTTATTTTGTTTCCTTTTTTCCGGTTGTG

>a14ea7b3d8187093ed43ccb13b783e18

CACGTCTGCCCCCGGTGTGGTTTATGCGATACACGTGTACTGGGGTGGCTGTGAATTAATCAAGACAGGCATAACGCCTTTAGGGAGCAGTTTGCACTTACCCGAATTGAAATTAACAAAACAGTTTGTGGCCGGGTACCGGTGTTGGACGATCAAACTCCAT

>fef465e62a6fc39591d6a83d23b5c9d7

TACGTAGGGTGCGAGCGTTAATCGGAATTACTGGACGTAAAGCGTGCGCAGGCGGTTTGTTAAGACAGATGTGAAATCCCCGGGCTCAACCTGGGAACTGCATTTGTGACTGGCAGGCTAGAGTATGGCAGAGGAGGGTAGAATTCCACGTGTAGCAGTGAAATGCGTAGAGATGTGGAGGAATACCGATGGCGAAGGCAGCCCCCTGGGCCAATACTGACGCTCATGCACGAAAGCGTGGGGAGCAAACAGG

>da9aaf0e8fc1b0721edc595ae76dfa2b

TACGTAGGGTGCAAGCGTTATCCGGAATTATTGGGCGTAAAGAGCTCGTAGGCGGTTTGTCGCGTCTGCTGTGAAATCCCGAGGCTCAACCTCGGGCCTGCAGTGGGTACGGGCAGACTAGAGTGCGGTAGGGGAGATTAGAAACCCCAGTAGTCCGGCTGAC

>0451ef81b64ba7726fbb4e2d1ad6de04

CCAGGGACTTGTGCACTCGTAATAAAAATTTGGTGCTTGTAACAAAATGCTGGAAGTATGATGTTTTGAATAAATACGTTCATGGTAGCTAAATTTACACACTTTAAATTACTGAAATGTGTTCCCTCTTATTCAAAATTTCTTGATTATAACCTCTAGGGACATAAAATCATCAATGAGAGTCTTTAATGGAATGC

>fdd076f7f866641d93bb925218142f06

TACGTAGGTGGCGAGCGTTATCCGGATTTACTGGGCGTAAAGGGAGCGTAGGCGGCTGATTAAGTGGGATGTGAAGTACCCGGGCTCAACTTGGGTGCTGCATTCCAAACTGGTTATCTAGAGTGCAGGAGAGGAGAGTGGAATTCCTAGTGTAGCGGTGAAATGCGTAGAGATTAGGAAGAACACCAGTGGCGAAGGCGACTCTCTGGACTGTAACTGACGCTGAGGCTCGAAAGCGTGGGGAGCAAACAGG

>bc5a96e3e6ee61f53758db65fb27b93d

TACGTAGGGTGCGAGCGTTAATCGGAATTACTGGGCGTAAAGCGTGCGCAGGCGGTGATGTAAGACCGATGTGAAATCCCCTGGCTTAACCTGGGAACTGCATTGGTGACTGCATCGCTGGAGTATGGCAGAGGGGGGTGGAATTCCACGTGTAGCAGTGAAATGCGTAGAGATGTGGAGGAACACCGATGGCGAAGGCAGCCCCCTGGGCCAATACTGACGCTCATGCACGAAAGCGTGGGGAGCAAACAGG

>2f9941fdea46fda8050de5cda1e48c05

AACAGAGGTGGCGAGCGTTGTTCGGAATCACTGGGCATAAAGGGCGCGTAGGTGGCCAGGCAGGTCAGGGGTGAAATCCCCCGGCTCAACCGGGGAATTGCCTTTGAAACCACTTGGCTGGAGCAGAAGAGGGGAGAGTGGAACTCGTGGTGGAGCGGTGAAATGCGTAGATATCACGAGGAACGCCGGCGGCGAAAGCGACTCTCTGGCTTCCTGCTGACACTGAGGCGCGAAAGCTAGGGGAGCAAACTGGG

>0b4432210f154f6ad39b09fcc87c0073

TACCGGCAGCCCAAGTGATGGCCGATCTTATTGGGCCTAAAGCGTCCGTAGCTGGCCACGCAAGTCCATCGGGAAATCCACCCGCTCAACGGGTGGGCGACCGGTAGAAACTGCGTGGCTTGGGACCGGAAGGCGCGACGGGTACGTCCGGGGTAGGAGTGAAATCCCGTAATCCTGGACGGACCGCCGATGGCGAAAGCACGTCGCGAGAACGGATCCGACAGTGAGGGACGAAAGCCAGGGTCTCGAACCGG

>7461ddca005401dddae6b5b865c05119

CACACTTATGTCTTCAGAAAGACAAAATATTTCTCCAGGATAAATTTCAACCATGCATGTCGGTCATTTTCAACTGGCAGTGCCTTGTAGATATTTGTGAAAGATTTTGAATGTGTCTTGTCCATAGAGTTGAGTTTGTTCAATAATGATTTTGCTATGTACTTTGTAACGTAATACATTCAGGCTTTCAAGTAAATTCATTTTGTTTACTTCTTTGCCGGTTGTG

>ad38ae96214151fce59ff29618b6b761

TACGTAGGTGGCAAGCGTTGTCCGGAATTATTGGGCGTAAAGCGCGCGCAGGCGGTCCTTTAAGTCTGATGTGAAAGCCCACGGCTCAACCGTGGAGGGTCATTGGAAACTGGGGGACTTGAGTGCAGAAGAGGAGAGTGGAATTCCACGTGTAGCGGTGAAATGCGTAGAGATGTGGAGGAACACCAGTGGCGAAGGCGACTCTCTGGTCTGTAACTGACGCTGAGGCGCGAAAGCGTGGGGAGCGAACAGG

>a9164d01308501b576afa06256a15b0f

TACGAAGGGGGCTAGCGTTGTTCGGATTTACTGGGCGTAAAGCGCACGTAGGCGGATCGATCAGTCAGGGGTGAAATCCCAGAGCTCAACTCTGGAACTGCCTTTGATACTGTCGATCTGGAGTATGGAAGAGGTGAGTGGAATTCCGAGTGTAGAGGTGAAATTCGTAGATATTCGGAGGAACACCAGTGGCGAAGGCGGCTCACTGGTCCATTACTGACGCTGAGGTGCGAAAGCGTGGGGAGCAAACAGG

>0e29c6a607cd9775f4b3d7c2bace66cb

TACGTAGGGGGCGAGCGTTGTCCGGATTCACTGGGCGTAAAGCGCGCGCAGACGGCTTTCTGCGTCCGGGGTGAAAACCCGGGGCTCAACCCCGGGAGGGCCTTGGATACGGGAGAGCTTGAGGGCCGGAGAGGCAAGGGGAATTCCACGTGTAGCGGTGAAATGCGTAGAGATGTGGAGGAACACCTGTGGCGAAGGCGCCTTGCTGGCCGGTTCCTGACGTTGAGGCGCGAAAGCGTGGGGAGCAAACAGG

>711add8467865c1068bb31d88afa0075

TACGTAGGGGGCGAGCGTTGTCCGGAATTATTGGGCGTAAAGAGCGTGTAGGCGGCCCGGTAAGTCCGCCGTGAAAGCTTGGGGCTCAACCCCAAGTTTGCGGTGGATACTGTCGGGCTAGAGGGTGGTAGAGGCGAGTGGAATTCCCGGTGTAGCGGTGAAATGCGCAGATATCGGGAGGAACACCAGTAGCGAAGGCGGCTCGCTGGGCCATACCTGACGCTGAGACGCGGAAGCGTGGGGAGCGAACAGG

>e63f8196fbfa25f2edec4cc8c8371050

TACGTGAGAGACTAGTGTTATTCATCTTAATTGGGTTTAAAGGGTACCTAGACAGTCAATATAACTTCTATAATGCTAATACTTGACTAGAGTTTTAAGTAAGAGGGAAGTACTTAAGGAGTAAGAGATGAAATATCTGTGATACCAAAGGGACTCCGTAAAGGCGAAGGCATCCCTTTATCTAGAAACTAACGTTGAAGGACGAAGGCTTAGATAACAAATAGG

>15f870b2ae4b655b13a94c2bb1797c7d

TACGTAGGGTGCGAGCGTTAATCGGAATTACTGGGCGTAAAGCGTGCGCAGGCGGTTTGTTAAGACAGATGTGAAATCCCCGGGCTCAACCTGGGAACTGCATTTGTGACTGGCAGGCTAGAGTATGTCAGAGGGGGGTAGAATTCCACGTGTAGCAGTGAAATGCGTAGAGATGTGGAGGAATACCGATGGCGAAGGCAGCCCCCTGGGCCAATACTGACGCTCATGCACGAAAGCGTGGGGAGCAAACAGG

>bd5694354e902555c31436d2f2da343d

CACGATTAACCCAAGTCAATAGAAGCCGGCGTAAAGAGTGTTTTAGATCACTCCCTCCCCAATAAGGCTAAAACTCACCTGAGTTGTAAAAAACTCCAGTTGACACAAAATAGACTACGAAAGTGGCTTTAACATATCTGAACACACAATAGCTGAGACCCAAACTGGG

>2de29ac737c78bb57b8daf156bc03646

GACAGAGGATGCAAGCGTTATCCGGAGTGATTGGGCGTAAAGCGTCTGTAGGTGGCTTTTTAAGTTCGCCGTCAAATCCCAGGGCTCAACCCTGGACAGGTGGTGAAAACTACTAAGCTAGAGTACGGTAGGGGCAGAGGGAATTTCCGGTGGAGCGATAAAATGCGTAGAGATCGGAAGGAACACCAACGGCGAAAGCACTCTGCTGGGCCGACACTGACACTGAGAGACGAAAGCTAGGGGAGCGAATGGG

>e05deff8c0a5a45f5d4cd157e8cf521f

CACACTTATGTCTTTAGAAAGACAAAACATTTCTCCAGACTGCATTTCAACCATGTCGGTCATTTTCAATTTGCAGTGCCTTGTAGATAGTTGTGGAAGATTTTGAACGTGTCTTGTCCATATAGTTCAGATTGTTCATGAATGATTTTGTTATGTAGTTTGTAACGTAGTATACTTAGACATTGAAGTAAATTCATTTTATTTCCTTTTTTGCCGGTTGTG

>86ac46ab62e9fad41c8a3b8d956902db

TACAGTAGCTTCAACACCAGCTAAAATAAAACTCATGTTACATAATTACTGATTGTGTTGTTATTTTGTTAGCCAATTGACGTTTTACGCCTATTATATTTAGATTATAACGTCAATAACAGGTACATAATTTTATTTAAAAAAAAAAGAAATTGAATCCCGTAAAACTCTGTCATCTTATTAACACAGAAACAAACTTTGTCAAAGGAATCGTCACCGTCATGTCTTCTTCGCCATATAAATTCACACAG

>4899704ce6503b650ca0da8b09d21803

TACGTAGGGTGCGAGCGTTAATCGGAATTACTGGGCGTAAAGCGTGCGCAGGCGGTTTGTTAAGACAGATGTGAAATCCCCGGGCTCAACCTGGGAACTGCATTTGTGACTGGCAGGCTAGAGTATGGCAGAGGGGGGTAGAATTCCACGTGTAGCAGTGAAATGCGTAGAGATGTGGAGGAATACCGATGGCGAAGGCAGCCCCCTGGGCAAATACTGACGCTCATGCACGAAAGCGTGGGGAGCAAACAGG

>bb5a81cc6acbb5bcd79dd59ceea648bd

TCAAACGAAAGCACAACAAAAAGATAGCCAAAGTAATACCACCCCGATCACCAGCAATAACACCCACACATATGTTTTACCCAAGAATAGTTAACCTCACCAACATAACATTCACTATCAGAAACACCTTTTAAACAAAGCGATAAATCACAACCTACACTACACGTAGAACAATAACACCATCAAGAACATTGT

>485febe89f9b2eb47e1d18eb2e215c48

TACGTAGGTCCCGAGCGTTGTCCGGATTTATTGGGCGTAAAGCGAGCGCAGGCGGTTAGATAAGTCTGAAGTTAAAGGCTGTGGCTTAACCATAGTACGCTTTGGAAACTGCTTAACTTGAGTGCAGAAGGGGAGAGTGGAATTCCATGTGTAGCGGTGAAATGCGTAGATATATGGAGGAACACCGGTGGCGAAAGCGGCTCTCTGGTCTGTAACTGACGCTGAGGCTCGAAAGCGTGGGGAGCGAACAGG

>1e1e27a25aac5918141ff129ece7fe28

AACAGAGGATACAAGCGTTATACGGATTTATTGGGTTTAAAGGGTGCGTAGGTGGTTTTTTAAGTCAGTAGTGAAATCTTAAAGCTTAACTTTAAAAGTGCTATTGATACTGATAAACTAGAGTGAGGTTGGAGTAACTGGAATGTGTGGTGGAGCGGTGAAATGCATAGAGATCACACAGAACACCAATCGCGAAGGCATGTTACTAAACATAGACTGACACTGAGGCACGAAAGCATGGGTAGCAAACAGG

>12c45c51e1355386db94b23633856d6d

CACGATTAACCCAAGTCAATAGAAGCCGGCGTAAAGAGTGTTTTAGATCACCCCCTCCCCAATAAAGCTAAAACTCATCTGAGTTGTAAAAAACTCCAGTTGACACAAAATAGACTACGAAAGTGGCTTTAACATATCTGAACACACAATAGCTAAGACCCAAACTGGG

>22ae0faba91e24b0fa6ab81536d45ae9

TACGTAGGGTGCGAGCGTTAATCGGAATTACTGGGCGTAAAGCGTGCGCAGGCGGTTTGTTAAGACAGATGTGAAATCCCCGGTCTCAACCTGGGAACTGCATTTGTGACTGGCAGGCTAGAGTATGGCAGAGGGGGGTAGAATTCCACGTGTAGCAGTGAAATGCGTAGAGATGTGGAGGAATACCGATGGCGAAGGCAGCCCCCTGGGCCAATACTGACGCTCATGCACGAAAGCGTGGGGAGCAAACAGG

>c051bf78e1582f167ef9b25ac9c7e4ae

TACGTAGGGTGCGAGCGTTAATCGGAATTACTGGGCGTAAAGCGTGCGCAGGCGGTTTGTTAAGACAGATGTGAAATCCCCGGGCTCAACCTGGGAACTGCATTTGTGACTGGCAGGCTAGAGTATGGCAGAGGGGGGTAGAATTCCACGTGTAGCAGTGAAATGCGTTGAGATGTGGAGGAATACCGATGGCGAAGGCAGCCCCCTGGGCCAATACTGACGCTCATGCACGAAAGCGTGGGGAGCAAACAGG

>f55f5632a5d9312fd5f2448e76cd89e1

TACGTAGGGTGCGAGCGTTAATCGGAATTACTGGACGTAAAGCGTGCGCAGGCGGTTTGTTAAGACAGATGTAAAATCCCCGGGCTCAACCTGGGAACTGCATTTGTGACTGGCAGGCTAGAGTATGGCAGAGGGGGGTAGAATTCCACGTGTAGCAGTGAAATGCGTAGAGATGTGGAGGAATACCGATGGCGAAGGCAGCCCCCTGGACCAATACTGACGCTCATGCACGAAAGCGTGGGGAGCAAACAGG

>338129c875f99b3cc399cfee30d7bbef

TACGATTTCTTTAATTTAAATAGTTAAGTTTCAGTTAATATAACAATAATGTAAAATATCTATAATTTTGGTGAAATATATTTTATTTTTAAAAATTAATTTTATGTCTGAAAAATTTTTGTTTAAACTAGGATTAGATACCCGGGTAGTCCGGCTGACTGAC

>d6cddab96de9008077dd0f815750c7ab

TACGTAGGGCGCGAGCGTTATCCGGAATTATTGGGCGTAAAGAGCTCGTAGGCGGTTTGTCACGTCTGCTGTGAAAGCCCGGGGCTTAACCCCGGGTGTGCAGTGGGTACGGGCAGACTGGAGTGCAGTAGGGGAGACTGGAATTCCTGGTGTAGCGGTGAAATGCGCAGATATCGGGAGGAACACCGATGGCGAAGGCAGGTCTCTGGGCTGTTACTGACGCTGAGGAGCGAAAGCATGGGGAGCGAACAGG

>38c27ceaed634984c1225a82648cf571

TACAGAGGGTGCAAGCGTTAATCGGAATTACTGGGCGTAAAGCGCGCGTAGGTGGTTTGTTAAGTTGGATGTGAAAGCCCCGGGCTCAACCTGGGAACTGCATCCAAAACTGGCAAGCTAGAGTATGGTAGAGGGTGGTGGAATTTCCTGTGTAGCGGTGAAATGCGTAGATATAGGAAGGAACACCAGTGGCGAAGGCGACCACCTGGACTGATACTGACACTGAGGTGCGAAAGCGTGGGGAGCAAACAGG

>5b4549a05fcb9ba6eb28bf2f2df1c359

CAGGGATCGATTGATCTTCGCGACTAGGGTGTACCTGCTACCGTTATGCTGCCATTTACAAAGCATTAATTTGTGTTTTACACAGCCAATGCATGATTCCAACAAATATTCATGACTATGAAATAAATTCGTTGATGACTGGCGGAATTGTCGAAACAGTTACTACTGTGCCCACGAATG

>0a5fa958ab625e8cba8506db0416b1f7

GACGAAGGGGGCTAGCGTTGTTCGGAATTACTGGGCGTAAAGCGCGTGTAGGCGGTTGTTCAAGTCGGGTGTGAAAGCCTTGAGCTCAACTCAAGAAATGCACTCGGTACTGGATGACTAGAGGACCGGAGAGGATAGTGGAATTCCCAGTGTAGTGGTGAAATACGTAGAGATTGGGAAGAACACCAGTGGCGAAGGCGGCTATCTGGACGGTTTCTGACGCTAAGACGCGAAAGCGTGGGGAGCAAACAGG

>1e75957fae35ca6019cae7cbc120ae1f

AGTGTTCATGCAGAAGTAAACGAAGGTGATAGAACTAGAACGTAGTGAAACGTCTGCTGAAATTATCTCGTGCATCGACGCGAATTGTTTTGTTGAGTGGAGTTACAGACAGAGGACTACGAACAACGAGAATTAGAAACCCGAGTAGTCCGGCTGACTGACT

>3851626ca3e1cb1f2d960c2e7fed3290

CACACTTATGTCTTCAGAAAGACAAAATATTTATCCAGGATGCATTTCAACCATGTCGGTCATTTTCAACTGGCAGTGCCTTGTAGACATTTGTGAAAGATTTTGAATGTGTCTTGTCCATAGAGTTCAGTTTGTTCATTAATGATTTTGTTATGTAGTTTGTAACGTAATATATTTAGCCTATCAAGTAAATTCATTTTGTTTCCTTTTTGCCGGTTGTG

>da46929e5c8c87ecc4bd1c5fd5aaea82

TACGTGAGAGACTAGTGTTATTCATCTTAATTGGGTTTAAAGGGTACCTAGACAGTCAATATAACCTCTATAATGCTAATACTTGACTAGAGTTTTAAGTAAGAGGGAAGTACTTAAGGAGTAAGAGATGAAATATCTGTGATACCAAAGGGACTCCGTAAAGGCGAAGGCATCCCTTTATCTAAAAACTAACGTTGAAGGACGAAGGCTTAGATAACAAATAGG

>59b24a2ecdc739b3d30f1bd2871db277

TACGTAGGGTGCGAGCGTTAATCGGAATTACTGGGCGTAAAGCGTGCGCAGGCGGTTTGTTAAGACAGATGTGAAATCCCCGGGCTCAACCTGGGGACTGCATTTGTGACTGGCAGGCTATAGTATGGCAGAGGGGGGTAGAATTCCACGTGTAGCAGTGAAATGCGTAGAGATGTGGAGGAATACCGATGGCGAAGGCAGCCCCCTGGGCCAATACTGACGCTCATGCACGAAAGCGTGGGGAGCAAACAGG

>d2fce88068a0e56c69f57c78b82bb694

CACACTTATGTCTTCAGAAAGACAAAATATTTCCCCATGATGCATTTCAACCATGTCGGTCATTTTCAACTGGCAGTGCCTTGTTGATATTTGTGAAAGATTTTGAATATGTCTTGTCCATAGAGTTGAGTTTGTTCATTAATGATTTTGTTATGTAGTTTGTAACGTAATACATTTAGGCTTTCAAGTAAATTCATTTTGTTTACTTTTTTGCCGGTTGTG

>60babe004399fcdc8660aa0f7e1f60b2

TACGTAGGGTGCGAGCGTTAATCGGAATTACTGGGCTTAAAGCGTGCGCAGGCGGTTTGTTAAGACAGATGTGAAATCCCCGGGCTCAACCTGGGAACTTCATTTGTGACTGGCAGGCTAGAGTATGGCAGAGGGGGGTAGAATTCCACGTGTAGCAGTGAAATGCGTAGAGATGTGGAGGAATACCGATAGCGAAGGCAGCCCCCTGGGCCAATACTGACGCTCATGCACGAAAGCGTGGGGAGCAAACAGG

>dd344587e37f3bed1655a9a191518481

TACCGGCAGCTCAAGTGATGACCGATATTATTGGGCCTAAAGCGTCCGTAGCCTGCCGTGCAAGTCCATCGGGAAATCCACCCGCCCAACGGGTGGCCGTCCGGTGGAAACTGCACGGCTTGGGACCGGAAGGCGCGACGGGTACGTCCAGGGTAGGAGTGAAACCCCGTAATCCTGGACGGACCGCCGATGGCGAAAGCACGTCGCGAGAACGGATCCGACAGTGAGGGACGAAAGCTAGGGTCTCGAACCGG

>aaae1bfb5028c286da14c6bc0ebeb91c

TACGTAGGGTGCAAGCGTTAATCGGAATTACTGGGCGTAAAGCGTGCGCAGGCGGTTATGCAAGACAGAGGTGAAATCCCCGGGCTCAACCTGGGAACTGCCTTTGTGACTGCATGGCTAGAGTACGGTAGAGGGGGATGGAATTCCGCGTGTAGCAGTGAAATGCGTAGATATGCGGAGGAACACCGATGGCGAAGACAATCCCCTGGACCTGTACTGACGCTCATGCACGAAAGCGTGGGGAGCAAACAGG

>e97f6e688b6f7e3cc8d71ce88527aaa7

CACGATTAACCCAAGTCAATAGAAGCCGGCGTAAAGAGTGTTTTAGATCACCCCCTCCCCAATAAAGCTAAAACTCACCTGAGTTGTAAAAAACTCCAGTTGACACAAAATAGACTACGAAAGTGGCGTTAACATATCTGAACACACAATAGCTAAGACCCAAACTGGG

>46ae254487cb88d66bfaf850e08abdb7

TACGATTAACCCAAACTAATTATCTTCGGCGTAAAACGTGTCAACTACAAATAAATAAATAGAATTAAAATCCAACTTATATGTGAAAATTCATTGTTAGGACCTAAACTCAATAACGAAAGTAATTCTAGTCATTTATAATACACGACAGCTAAGACCCAAACTGGG

>81397e00fb09aa52cb0cdbec167941d0

GACCAGATTTCCCAATTCATATGTTTCAACACGCTCCATCTCATATTCTTGCACATTATTAAAATTTTTTATTAAAATATGGTTCTCTAGCATCTTTTCAACAGGTGGAAAACAGCAAAGATTATTCCCATTCACAAAGAAAACAAAAACTCTTCACTATTAG

>18a998ae6ffb28730ab4751dc033753f

TACGTAGGGTGCGAGCGTTAATCGGAATTACTGGGCGTAAAGCGTGCGCAGGCGGTTTGTTAAGACAGATGTGAAATCCCCGGGCTTAACCTGGGAACTGCATTTGTGACTGGCAAGCTAGAGTATGGCAGAGGGGGGTAGAATTCCACGTGTAGCAGTGAAATGCGTAGAGATGTGGAGGAATACCGATGGCGAAGGCAGCTCCCTGGGCCAATACTGACGCTCATGCACGAAAGCGTGGGTAGCAAACAGG

>2c46696a1c67e2aec05d3d2c6c803a2e

TACAGAGGGTGCAAGCGTTAATCGGATTTGCTGGGCGTAAAGCGCGCGTAGGCGGCTAATTAAGTCAAATGTGAAATCCCCGAGCTTAACTTGGGAATTGCATTCGATACTGGTTAGCTAGAGTGTGGGAGAGGATGGTAGAATTCCAGGTGTAGCGGTGAAATGCGTAGAGATCTGGAGGAATACCGATGGCGAAGGCAGCCATCTGGCCTAACACTGACGCTGAGGTGCGAAAGCATGGGGAGCAAACAGG

>3cd123916fa0f9e209c69455de514d82

TACGTAGGGTGCGAGCGTTAATCGGAATTACTGGGCGTAAAGCGTGCGCAGGCGGTTTGTTAAGACAGATGTGAAATCCCCGGGCTCAACCTGTGAACTGCATTTGTGACTGGCAGGCTAGAGTATGGCAGAGGGGGGTAGAATTCCACGTGTAGCAGTGAAATGCGTAGAGATGTGGAGGAATACCGATGGCGAAGGCAGCCCCCTGGGCCAATACTGACGCTCATGCACGAAAGCGTGGGGAGCAAACAGG

>49a3133981d8fbc13153f9c62c849b16

TACGTAGGGTGCGAGCGTTAATCGGAATTACTGGGCGTAAAGCGTGCGCAGGCGGTTTGTTAAGACAGATGTGAAATCCCCGGGCTCAACCTAGGAACTGCATTTGTGACTGGAAGGCTAGAGTATGGCAGAGGGGGGTAGAATTCCACGTGTAGCAGTGAAATGCGTAGAGATGTGGAGGAATACCGATGGCGAAGGCAGCCCCCTGGGCCAATACTGACGCTCATGCACGAAAGCGTGGGGAGCAAACAGG

>5b4798e4090206081de42d76dd9e5363

GACCAGATGATTTCCACTTTCATATGTTGCAACATGCTCCAACTTATGAATTGCTAATACGATCAAAGATGTATTATGGGGCCCCAGCATTTACCTCAGCGATCCCTCACAACCTTAGAAAACTCATCTCTATCCACAAGGTGCTATCCTTCTTACTATAGATGTTACCTC

>5b981762a0bf753cc0bab173e808b30d

ACATTCTTTGTACTTCACTAGTTATTTAATTTACACCACTTGTCACCCCCTTATATTGTACAAACGAGTTTGCTGGCACATTATGTGAAATACACGTTCCTGTTACAGCTTATCCCTGACCTGCTAAAACGTGAAAGCAATATTTCTATGACCACAAAAACTATGGGCCAAGAAGCTA

>f053dc400e83a3abaaa1aeef9dc9fe84

AAATGGCTACTTTCACGGGTGCAGAACGGGCTAACTGTGTGTTAAGGTTTCATGACACAAACTCTGCAACAACAGTTCAGCATAATTTTTGCACCGAGTGTGGTAAAGATCCTCTTACTAGACCTAAAATTTACACTTGACCTCAGAACTTCGTTGAGAGTGGTTGTTCGGTTCAGC

>aafb6ca6537c21f170ece1de474769ab

TACGTAGGTGGCAAGCGTTATCCGGAATTATTGGGCGTAAAGCGCGCGTAGGTGGTTTTTTAAGTCTGATGTGAAAGCCCACGGCTCAACCGTGGAGGGTCATTGGAAACTGGAAAACTTGAGTGCAGAAGAGGAAAGTGGAATTCCATGTGTAGCGGTGAAATGCGCAGAGATATGGAGGAACACCAGTGGCGAAGGCGACTTTCTGGTCTGTAACTGACGCTGATGTGCGAAAGCGTGGGGATCAAACAGG

>ece0c8d175efd7f577ec1416f2bc896d

CACACTTATGTCTTCCGAAAGACAAAATATTTCTCCAGGATGCATTTCAACCATGCCGGTCATTTTCAACTGGCAGTGCCTTGTAGATATTTGTAAAAGACTTTGAATGCCATAGAGTTCAGTTTGTTCATTAATGATTTGAAATGATTTGTTCTTATGTAGTTTGTAACGTAATATATTGAGGCTTTCAAATAAATTCATTTTGTTTCCTTCTTGCCGGTTGTG

>ca8f59b27a5781c2f5cb26bfcc41e0b5

TACCGGCAGCTCAAGTGATGTCCCATATTATTGGGCCTAAAGCGTCCGTAGCTGGCCAACCAAGTCCATCGGGAAATCCACCTGCCCAACGGGTGGGCGTCCGGTGGAAACTGGCTGGCTTGGAACCGGAAGGCTCAGAGAGTACGTCCGGGGTAGGAGTGAAATCCCGTAATCCCGGACGGACTACCGATGGCGAAAGCACTCTGAGAAGACGGCTTCGACAGTGAGGGACGAAAGCTAGGGTCTCAAACCGG

>2cc25e8336079f5b7bc2495ba24a48ad

CACGATTAACCCAAGTCAATAGAAGCCTGCGTAAAGAGTGTTTTAGATCACCCCCTCCCCAATAAAGCTAAAACTCACCTGAGTTGTAAAAAACTCCAGTTGACACAAAATAGACTACGAAAGTGGCTTTAACATATCTGAACACACAATAGCTAAGACCCAAACTGGG

>196664956080ce68c15d05ecfb368124

TACGTAGGGTGCGAGCGTTAATCGGAATTACTGGGCGTAAAGCGTGCGCAGGCGGTTGTGTAAGACAGGCGTGAAATCCCCGGGCTCAACCTGGGAATGGCGCTTGTGACTGCACGGCTGGAGTGCGGCAGAGGGGGATGGAATTACGCGTGTAGCAGTGAAATGCGTAGATATGCGGAGGAACACCGATGGCGAAGGCAATCCCCTGGGCCTGCACTGACGCTCATGCACGAAAGCGTGGGGAGCAAACAGG

>ccf5e9ea952ef8078e2b47e69740a68b

TACCGGCAGCCCAAGTGATGGCCGATCTTATTGGGCCTAAAGCGTCCGTAGCTGGCCGCGCAAGTCCATCGGGAAATCCACCTGCTCAACAGGTGGGCGCCCGGTAGAAACTGTGCGGCTTGGGACCGGAAGGCGCGACGGGTACGTCCGGGGTAGGAGTGAAATCCCGTAATCCTGGACGGACCGCCGATGGCGAAAGCACGTCGCGAGAACGGATCCGACAGTGAGGGACGAAAGCCAGGGTCTCGAACCGG

>023823282901e3e51812a17368847bfc

TCAAACGAAAACACAACAAAAAGCTAGCCAAACTAATACCACTCCGATCCCCAGCAATTACGCCCACACACACTTTTTACCCAAGAATAGTTAACCTCACCGACATAACATTCACTAATGATCAGAAACATCTTTTAAACAAAGGGATAAATCACAACCTACACATTAT

>fc080f8b950228adf64ac0572a926fc2

GACAGAAAGGGCGACCCATTGGAATTATAAATTGGTGTAAAGGATACGTAGCCGGCCGTAAAAATGGCTAGAGTCAAATAGAAGTAATTACAATACCTAGAGTAGGGCTGATATCTTATGATACTAGGGGGAGTGCTAAAGGCGAAGGCGTATTACTAGGTAATGACTGACGGTGAGGTATGAAGGCTAAGGTAGCGATAGGG

>cb70782a34c2abf6652a83d1d094edb5

TACGATTTCTTTAATTTAAATATTTAAGTTTCAGTTAATGTATTTGTAATTTTAAAAAGTTATAATTTTGGTGAAATATATTTTGTCTTAAAAAATTAATTTTATATCTGAAAAATTTTTGTTCAAACTAGGATTAGAAACCCCGGTAGTCCGGCTGACTGAC

>f7ee63db99e4f280ff44d7faebc96de4

TACGGAGGGGGCAAGCGTTGTTCGGATTTACTGGGCGTAAAGGGTTCGTAGGCGGTTGCGTAAGTCTGGTGTGAAATCTTCGCCGCTCAACGGGAAGAGGCCATTGGAAACTGCGCAACTTGAGTGTGGTAGAGGCAAGCGGAATTCCTGGTGTAGCGGTGGAATGTGTAGATATCAGGAAGAACATCTGTGGCGAAGGCGGCTTGCTGGGCCATTACTGACGCTGAGGAACGAAAGCGTGGGGAGCAAACTGGG

>523086cce8ee5004fb2170f0de5cacd0

GTAAAACAGATTTATACATACACAACATTCGTTATAACATACAAATGAAACTACTAAGTTATAAAGAGCATTGCAGGAAACTGAAAGAGAAATGTGAAAAACTAGACAATCAACTGAACAAAGATTATTAGATACCCCGGTAGTCCGGCTGACTGACTATAGTACCA

>577078162157af3815271202add80313

TTAAACGAAAGCACAACAAAAAGATAGCCAAACTAATACCACCCCAATCACCAGCAATAATACCCACACACACGTTTTACCCAAGAATAGTTAACCTAACCAACGTAACATTCACTAACGAACAGAAACAACTTTTAAACAAAGGGATAAACCACAACCTACACTACACACAGAATAATAACACCATCAAGAACATGGT

>a4a184f8ec256cd6c63f32992c5b8d02

TTAAACGAAAGCACAACAAAAAGATAGCCAAACTATACCACCCCAATCACCAGCAATAACACCCGCACATACGTTTTACCCAAGAATAGTTAACCTAACCAACGTAACATTCACTAACGAACAGAAACAACTTTTAAACAAAGGGATAAACCACAACCTACACTACACACAGAATAATAACACCATCAAGAACATGGT

>07536bd2539bbdf3c2d52f7e57b3307c

GACGGGGGGGGCAAGTGTTCTTCGGAATGACTGGGCGTAAAGGGCACGTAGGCGGTGAATCGGGTTGAAAGTGAAAGTCGCCAAAAACAGGCGGAATGCTCTCGAAACCAATTCACTTGAGTGAGACAGAGGAGAGTGGAATTTCGTGAGGAGGGGTGAAATCCGAAGATCTACGAAGGAACGCCAAAAGCGAAGGCAGCTCTCTGGGTCCCTACCGACGCTGGGGTGCGAAAGCATGGGGAGCGAACAGG

>0a04e2b3d70f2374ea081120418f2366

TACGGAGGGCGCGAGCGTTACCCGGATTTACTGGGCGTAAAGGGCGTGTAGGCGGCCTGGGGCGTCCCATGTGAAAGGCCACGGCTCAACCGTGGAGGAGCGTGGGATACGCTCAGGCTAGAGGGTGGGAGAGGGTGGTGGAATTCCCGGAGTAGCGGTGAAATGCGCAGATACCGGGAGGAACGCCGATGGCGAAGGCAGCCACCCGGTCCACTTCTGACGCTGAGGCGCGAAAGCGTGGGGAGCAAACCGG

>0e139f872e149fc9d3e330ddd29f03d3

TACCGGCAGCACGAGTGATGGCCGATCTTATTGGGCCTAAAGCGTCCGTAGCTGGCCAAACAAGTCCGTTGGGAAATCTGCCCGCTTAACGGGCAGGCGTCCAGCGGAAACTGTTCAGCTTGGGACCGGAAGACCTGAGGGGTACGTCTGGGGTAGGAGTGAAATCCCGTAATCCTGGACGGACCGCCGGTGGCGAAAGCGCCTCAGGAGGACGGATCCGACAGTGAGGGACGAAAGCTAGGGTCTCGAACCGG

>80d7864adbb06a30f6497f6f30bb3f7c

AATTGTGTGCCAGCCGCCGCGGTAAGGTATTGGGATGAACAATGCAATTTAGTAAAAACTAGATATTTTACATCCTGTTTTTTTTAATAAGGCAGCTGCTGTAGACCTTAGTAGATTTGTTGGTGCATTAGAAACCCTAGTAGTCCGGCTGACTGACTTGCTCGTAATCTCGTATGCCGTCTTCTGCT

>378dcdcffc34d28e8ddb64546ca36b71

TACCGGCAGCCCGAGTGATGGCCGATCTTATTGGGCCTAAAGCGTCCGTAGCTGGCCGCGCAAGTCCATCGGGAAATCCACCTGCTCAACAGGTGGGCGCCCGGTGGAAACTGTGCGGCTTGGGACCGGAAGGCGCGACGGGTACGTCCGGGGTAGGAGTGAAACCCCGTAATCCTGGACGGACCGCCGATGGCGAAAGCACGTCGCGAGAACGGATCCGACAGTGAGGGACGAAAGCCAGGGTCTCGAACCGG

>82591ead40e0d03a04d932fcdc00eb8e

TACGAAGGGGGCTAGCGTTGTTCGGATTAACTGGGCGTAAAGGGTGCGTAGGCGGGTTTTTAAGTCAGGGGTGAAATCCCGAGGCTCAACCTCGGAACTGCCTTTGATACTGGAAATCTTGAGTCCGGGAGAGGTGAGTGGAACTGCGAGTGTAGAGGTGAAATTCGTAGATATTCGCAAGAACACCAGTGGCGAAGGCGGCTCACTGGCCCGGTACTGACGCTGAGGCACGAAAGCGTGGGGAGCAAACAGG

>509fe3c0453a23b46932364703f38129

TACGTAGGGTGCGAGCGTTAATCGGAATTACTGGGCGTAAAGCGTGCGCAGTCGGTTTGTTAAGACAGATGTGAAATCCCCGGGCTCAACCTGGGAACTGCATTTGTGACTGGCAGGCTAGAGTATGGCAGAGGGGGGTAGAATTCCACGTGTAGCAGTGAAATGCGTAGAGATGTGGAGGAATACCGATGGCGAAGGCAGCCCCCTGGGCCAATACTGACGCTCATGCACGAAAGCGTGGGGAGCAAACAGG

>284db3708e1e4562d4e0c90b7a88afb5

TACGAAGGGTGCAAGCGTTACTCGGAATTACTGGGCGTAAAGCGTGCGTAGGTGGTCGTTTAAGTCCGTTGTGAAAGCCCTGGGCTCAACCTGGGAACTGCAGTGGATACTGGGCGACTAGAGTGTGGTAGAGGGTAGCGGAATTCCTGGTGTAGCAGTGAAATGCGTAGAGATCAGGAGGAACATCCATGGCGAAGGCAGCTACCTGGACCAACACTGACACTGGGGCACGAAAGCGTGGGGAGCAAACAGG

>73e3c2a14e584e848e693c2964e33b1d

GACATAGGTGGCAAGCGTTATTCGGAATTACTAGGCGTAAAGCGAGTGTAGGCGGACTCTTAAGTCCGACGTTAAATCTCCCGGCTTAACTGGGAGCTGTCGTCGGATACTGAGAGCCTCGAATGGGGTAGGGGGCAGCGGAATTCCCGGTGTAGCGGTGAAATGCGTAGATATCGGGAGGAACACCTATGGCGAAAGCAGCTGCCTGGGCCTCTATTGACGCTAAGACTCGAAAGCTGGGGGAGCAAACAGG

>ac007328bce609a715815c6a7fbb9e95

TACGAGGAAGACTAGTGTTATTCATCTTTATTAGGTTTAAAGGGTACCTAGACGGCATATCAAGCCCCAAAAGGGAACAGATATACTAGAGTTTTATGTGAGAGGAATATATTAGTACTATTGGTGTAGAGATGAAATTCTTTGATACTAATAGGACGGATAACAGCAAAAGCAAACCTTTATGTAAAAACTGACGTTGAGGGACGAAGGCTTGGGTCGCGAATAGG

>f586eb9bdbf49f41be28749a48dd34fc

TACGTAGGGTGCGAGCGTTAATCGGAATTACTGGGCGTAAAGCGTGCGCAGGCGATTTGTTAAGACAGATGTGAAATCCCCGGGCTCAACCTGGGAACTGCATTTGTGACTGGCAGGCTAGAGTATGGCAGAGGGGGGTAGAATTCCACGTGTAGCAGTGAAATGCTTAGAGATGTGGAGGAATACCGATGGCGAAGGCAGCCCCCTGGGCCAATACTGACGCTCATGCACGAAAGCGTGGGGAGCAAACAGG

>0f32cf0fe5b3a6f57f8e67e42ed92edf

TACGTAGGGTGCGAGCGTTAATCGGAATTACTGGGCGTAAAGCGTGCGCAGGCGGTTTGTTAAGACAGATGTGAAATCCCCGGGCTCAACCTGGGAACTGCATTTGTGACTGGCAGGCTAGAGTATGGCAGAGGGGGGTAGAATTCCACGTGTAGCAGTGAAATGCGTAGAGATGTGGAGTAATACCGATGGCGAAGGCAGCCCCCTGGGCCAATACTGACGCTCATGCACGAAAGCGTGGGGAGCAAACAGG

>dccf879f8cc28907b79d1cb04fe08337

TTATTTCTATTTCCCAATAATTATCAGGGAAATAGAAATAATTACTAAGGAAATGGAAATAATTACCAAAGAAACAACGAAAATAATCGCAATAACAACAACAATAATTATAACAACCGACAGAACCAGGATCAAAATAATGCTAATAAAAATATAAATTAGA

>d2a81f1b8c355d479ea563ddbb02aab3

TACGTAAAAGACTAGTGTTAGTCATCTTTATTAGGTTTAAAGGGTACCTAGACGGTAAATTAAACTCTAAATGAGTACTTTTTTACTAGAGTTTTATAAGAGAAGGAAGAATTTCTGGAGTAGTGATAAAATACTCTTATACCAGAAGGACTGGTAACAGCGAAGGCATCCTTCTATGTAGAAACTGACGTTGAGGGACGAAGGCTTGGGTAGCAATAAGG

>7efc0b010fa376080171e09e945597ff

GATAGCGTTATGGTAATTGTGTGCCAGCCGCCGCGGTAAGGAAACTGTGAGTGAACAAGTTATGTACAAATACGCGCGGTAGCCGAGTGCGCGCTGGAGAGCTTTACGACCAGAACCCCTGATGGCATTAGATACCCTGGTAGTCCGGCTGACTGACTGATCTACGATCTCGTATGCCGTCTTCTGCTTGAAAAAAAAAAAA

>c6d01fa7ab31c60842697ff48bc7e383

TACGAAGGGTGCAAGCGTTACTCGGAATTACTGGGCGTAAAGCGTGCGTAGGTGGTTCGTTAAGTCTGATGTGAAAGCCCTGGGCTCAACCTGGGAATTGCATTGGATACTGGCGAGCTAGAGTGCGGTAGAGGGTAGTGGAATTCCCGGTGTAGCAGTGAAATGCGTAGAGATCGGGAGGAACATCCGTGGCGAAGGCGACTGCCTGGACCAGCACTGACACTGAGGCACGAAAGCGTGGGGAGCAAACCGG

>b710a48dd60b1266a5f3c537640bf681

CACGATTAACCCAAGTCAATAGAAGCCGGCGTAAAGAGTGTTTTAGATCACCCCCTCCCCAATAAAGCTAAAACTCACCTGAGTTGTAAAAAACTCCAGTTGACACAAAATAGACTACGAAAGTGGCTTTAACATATCTGAACGCACAATAGCTAAGACCCAAACTGGG

>b982fd64e9aec68c30d405e616b64434

AATTGTGTGCCAGCCGCCGCGGTAAGGTATTGGGATGAACAATGCAATTTAGTAAAAACTAGATATTTTACATCCTGTTTTTTTTAATAAGGCAGCTGCTGTAGACCTTAGTAGATTTGTTGGTGCATTAGAAACCCGAGTAGTCCGGCTGACTGACTAACGCTGAATCTCGTATGCCGTCTTCTGCT

>c013e25c0f87d3541576620c9bccc41a

TACGTAGGGTGCAAGCGCTAATCGGAATTACTGGGCGTAAAGCGTGCGCAGGCGGTTTGTTAAGACAGATGTGAAATCCCCGGGCTCAACCTGGGAACTGCATTTGTGACTGGCAGGCTAGAGTATGGCAGAGGGGGGTAGAATTCCACGTGTAGCAGTGAAATGCGTAGAGATGTGGAGGAATACCGATGGCGAAGGCAGCCCCCTGGGCCAATACTGACGCTCATGCACGAAAGCGTGGGGAGCAAACAGG

>bfc0cfe464135b442ee496ed5142bd69

TACGTAGGGTGCGAGCGTTAATCGGAATTACTGGGCGTAAAGCGTGCGCAGGCGGTTTGTTAAGACAGATGTGAAATCCCCGGGCTCAACCTGGGAACTGCATTTGTGACTGGCAGGCTAGAGTATGGCAGAGGGGGGTAGAATTCCACGTGTAGCAGTGAAATGCGTAGAGATGTGGAGGAATACCGATGGCGAAGGCAGCCCCCTGGGCCAATACTGACGCTCATGCACGAAAGCGTGGGAAGCAAACAGG

>d1ff356f2b62162db0887f565a2e96ce

TTAGCTGGGTGATACCCATCACAATTGGAGCATGTGACTGAGTTCTCCGCCACATCGCACACAACGCTGTGTCATATGACAGAAGGGAGAAGAATGTCCATACAGCTGGCAGTTAAAGCTCTTCGGCGGCCAGAGGAGGTTGTTAAGGGGCGTACTTGCACCCTTGCATCCGAAGATACAGGACGATCTGTACTAAATAATTGTCGGCAAAGTTGCCTATCATTCTTAATTTGATAAGAAAGGAGTAT

>5a4fe8c5d058984c6917ebc528576901

TACGGAGGGTGCAAGCGTTATCCGGATTTATTGGGTTTAAAGGGTCCGTAGGCGGATTTGTAAGTCAGTGGTGAAATCTCACAGCTTAACTGTGAAACTGCCATTGATACTGCAAGTCTTGAGTGTTGTTGAAGTAGCTGGAATAAGTAGTGTAGCAATGAAATGCATAGATATTACTTAGAACACCAATTGCGAAGGCAGGTTACTAAGCAACAACTGACGCTGATGGACGAAAGCGTGGGGAGCGAACAGG

>025029fdba038e8e4757db574cde5d78

AGTGTTCATGCAGAAGTAAACGAAGGTGATAGAACTAGAACGTAGTGAAACGTCTGCTGAAATTATCTCGTGCATCGACGCGAATTGTTTTGTTGAGTGGAGTTACAGACAGAGGACTACGAACAACGAGAATTAGAAACCCCTGTAGTCCGGCTGACTGACT

>33fd3dd57121d8d37e03b2746107e110

TACGGAGGGTGCAAGCGTTAATCGGAATTACTGGGCGTAAAGCGCACGCAGGCGGTCTGTCAAGTCGGATGTGAAATCCCCGGGCTCAACCTGGGAACTGCATTCGAAACTGGCAGGCTAGAGTCTTGTAGAGGGGGGTATAATTCCAGGTGTAGCGGTGAAATGCGTAGAGATCTGGAGGAATACCGGTGGCGAAGGCGGCCCCCTGGACAAAGACTGACGCTCAGGTGCGAAAGCGTGGGGAGCAAACAGG

>5b17e9937f9b6df9ef67e54f8c55cc47

CAAACATTACTACGTAAGAGCGGAATCGTGTTTCGTGTAGCGTACAAAGAGATAACAATCTGCTGCTTCAACTATCCAGCAGACTATATCCTCGTTGCTTTAAAGTTGTACCGAAAGACGCGTCGCGGATTCGTCACCCTGTTAGTACGTCACGTCACGTACCACGAGAATGGAGACCAGACGAGCGAAAACAGCTGATTCTTTAGAGATTCTCTAAGTTC

>40a730a53a300658f7ce924b58152f24

CTAGGCTACACAGCACATGCTGAGTAACTTGTAGCAGTGACGGTGGCCAAGTTACACCTTTCTACTTTGTCGTTGGGGTGTGATGTTAAGGACCGTATGAACATCAGAGAATATGTGCGTTCATGTAGATTCCAATATTAGAAACCCCGGTAGTCCGGCTGAC

>4f71ff7539a5a94272a18ad0579c10b4

TACATAGGGGGCAAGCGTTATCCGGAATTATTGGGCGTAAAGGGTGCGTAGGCGGCTAAATAAGTTTATGGTCTAAGTGCAATGCTTAACGTTGTGATGCTATAAAAACTGTTTAGCTAGAGTTGGATAGAGGCAAGTGGAATTCCATGTGTAGTGGTAAAATGCGTAAATATATGGAGGAACACCAGAAGCGAAGGCGGCTTGCTGGGTCTTAACTGACGCTGAGGCACGAAAGCGTGGGGAGCAAACAGG

>2b6761b920ef2a7254876bd5beee9caa

TACGGAGGGTGCAAGCGTTAATCGGAATTACTGGGCGTAAAGCGCACGCGGGCGGTCTGTTAAGTCAGATGTGAAATCCCCGGGCTTAACCTGGGAACTGCATTTGAAACTGGCAGGCTTGAGTCTTGTAGAGGGGGGTAGAATTCCAGGTGTAGCGGTGAAATGCGTAGAGATCTGGAGGAATACCGGTGGCGAAGGCGGCCCCCTGGACAAAGACTGACGCTCAGGTGCGAAAGCGTGGGGAGCAAACAGG

>a3a4f4088deb6f130ded2c0a967a712f

TACGTAGGGTGCAAGCGTTAATCGGAATTACTGGGCGTAAAGCGTGCGCAGGCGGTTATATAAGACAGATGTGAAATCCCCGGGCTCAACCTGGGAACTGCATTTGTGACTGTATGGCTAGAGTACGGTAGAGGGGGATGGAATTCCGCGTGTAGCAGTGAAATGCGTAGATATGCGGAGGAACACCGATGGCGAAGGCAATCCCCTGGACCTGTACTGACGCTCATGCACGAAAGCGTGGGGAGCAAACAGG

>2b3241e445043d858344b89771fad5ae

TACAGAGGGTGCGAGCGTTAATCGGAATTACTGGGCGTAAAGCGCACGTAGATGGCTTTGTAAGTCGGATGTGAAATCCCCGGGCTCAACCTGGGAACTGCATTCGAAACTGCATTGCTCGAATGTGGAAGAGGGAAGCGGAATTCCAGGTGTAGCGGTGAAATGCGTAGATATCTGGAGGAACATCAGTGGCGAAGGCGGCTTCCTGGTCCAACATTGACATTCAGGTGCGAAAGCGTGGGGAGCAAACAGG

>13c27ebd7545d4fc37fe55e8d54226d6

CACACTTATGTCTTCAGAAAGACAAAATATTTATCCAGGATGCATTTCAACCATGTCGGTCATTTTCAACTGGCAGTGCCTTGTAGATATTTGTGAAAGATTTTGAATGTGTCTTGTCCATAGAGTTCAGTTTGTTCATTAATGATTTTGTTATGTAGTTTGTAACGTAATATATTTAGGCTTTCAAGTCAAGTCATTTTGTTTCCTTTTTGCCGGGTGTG

>7c0347061bb315177e7e5606cc40a8bc

CAAGCAGCATATGGTAATTGTGTGCCAGCAGCCGCGGTAACTTGCAGCTTATAAGAACAAACAGAAACTAAAAAACGATCATTATTTTACACAAATACAGGTAACAACTGTAACAGAACTACACAAATTAGATACCCTGGTAGTCCGGCTGACTGACTCTCGACTTATCTCGTATGCCGTCTTCTGCTTGAAAAAAAAAAAAA

>b3686894a510ca8cf3cbe2a8822375ab

TACGTAGGTCCCGAGAGTTGTCCGGATTTATTGGGCGTAAAGCGAGCGCAGGCGGTTTAATAAGTCTGAAGTTAAAGGCAGTGGCTTAACCATTGTTCGCTTTGGAAACTGTTAAACTTGAGTGCAGAAGGGGAGAGTGGAATTCCATGTGTAGCGGTGAAATGCGTAGATATATGGAGGAACACCGGTGGCGAAAGCGGCCCTCTGGGCTGTAACTGACGCTGAGGCTCGAAAGCGTGGGGAGCAAACAGG

>0d07443257a527f2abc8e78ae7a84679

TACGATTTCTTTAATTTAAATATTTAAGTTTCAGTTAATGTAACAATAATATAAAATATCTATAATTTTGGTGAAATATATTTTATCTTTAAAAATTAATTTTATGTCTGAAAAATTTTTGTATAAACTAGGATTAGAAACCCCAGTAGTCCGGCTGACTGAC

>0110bf45e4dc901a5bbbed16e1c2fa1a

TACGTAGGTGGCAAGCGTTGTCCGGAATTATTGGGCGTAAAGGGCTCGCAGGCGGTTCCTTAAGTCTGATGTGAAAGCCCCCGGCTCAACCGGGGAGGGTCATTGGAAACTGGGGAACTTGAGTGCAGAAGAGGAGAGTGGAATTCCACGTGTAGCGGTGAAATGCGTAGAGATGTGGAGGAACACCAGTGGCGAAGGCGACTCTCTGGTCTGTAACTGACGCTGAGGCGCGAAAGCGTGGGGAGCAAACAGG

>18916ff01c5cc30f13e73cab657abe0e

TACGTAGGTGGCAAGCGTTGTCCGGATTTATTGGGCGTAAAGCGAGCGCAGGCGGATTGATAAGTCTGATGTGAAAGCCTTCGGCTCAACCGAAGAACTGCATCAGAAACTGTCAATCTTGAGTGCAGAAGAGGAGAGTGGAACTCCATGTGTAGCGGTGGAATGCGTAGATATATGGAAGAACACCAGTGGCGAAGGCGGCTCTCTGGTCTGTAACTGACGCTGAGGCTCGAAAGCATGGGTAGCGAACAGG

>9105bf1a385ece4450c354324df7b29b

TACGTAGGGTGCGAGCGTTAATCGGAAATACTGGGCGTAAAGCGTGCGCAGGCGGTTTGTTAAGACAGATGTGAAATCCCCGGGCTCAACCTGGGAACTGCATTTGTGACTGGCAGGCTAGAGTATGGCAGAGGGGGGTAGAATTCCACGTGTAGCAGTGAAATGCGTAGAGATGTGGAGGAATACCGATGGCGAAGGCAGCCCCCTGGACCAATACTGACGCTCATGCACGAAAGCGTGGGGAGCAAACAGG

>005b3ee3b6a326b075a3c8caf4fbbfbf

TACGAAGGGGGCTAGCGTTGCTCGGAATCACTGGGCGTAAAGGGTGCGTAGGCGGGTTTTTAAGTCATGGGTGAAATCCTGGAGCTCAACTCCAGAACTGCCTTTGATACTGAAGATCTTGAGTCCGGGAGAGGTGAGTGGAACTGCGAGTGTAGAGGTGAAATTCGTAGATATTCGCAAGAACACCAGTGGCGAAGGCGGCTCACTGGCCCGGTACTGACGCTGAGGCACGAAAGCGTGGGGAGCAAACAGG

>7e1fc8e6ebab8727d30aa8675d17ca9c

TACGAAGGGGGCTAGCGTTGTTCGGATTTACTGGGCGTAAAGCGCACGTAGGCGGATTGTTAAGTGAGAGGTGAAATCCCAGAGCTCAACTCTGGAACTGCCTTTCATACTGGCAATCTAGAGTCCGGAAGAGGTAAGTGGAACTCCTAGTGTAGAGGTGGAATTCGTAGATATTAGGAAGAACACCAGTGGCGAAGGCGGCTTACTGGTCCGGTACTGACGCTGAGGTGCGAAAGCGTGGGGAGCAAACAGG

>a7db6f710c18b0445f5654fce61f0e04

TAGGACAATTACGTGTCTGTTAGGGATAAGGTAGCTTAAGTAAGACAAAGTATCTGGGGAGCTATTATCGAGTAAGTATTATTACGGGAAACCCAATCGTAATAGGGTCTTTCATCTTTAGTTATACTAATATTAAATTTTTAATTAAAAAGCACGATTAAAGCAGTTTGTTAGGAGAATGTTTACTAATTCCACGG

>e9c78d2055cd598e6647f65b1eeb957c

CACACTTATGTCTCCAGAAAGACAAAATATTTATCCAGGATGCATTTCAACCATGTCGGTCATTTTCAACTGGCAGTGCCCTGTAGATATTTGTGAACGATTTTGAATGTGTCTTGTCCATAGAGATCAGTTTGTTCATTAATGATTTTGTTATGTAGTTTGTAACGTAATATATTTAGGCTATCAAGTAAATTCATTTTGTTTCCTTTTTTCCGGTTGTG

>dca2e3e4cab64e09bda16175e3dfa326

TACGTAGGGCGCAAGCGTTATCCGGAATTATTGGGCGTAAAGAGCTTGTAGGCGGTTCGTCGCGTCTGGTGTGAGAGCCCATCGCTTAACGGTGGGTCTGCGCCGGGTACGGGCGGGCTAGAGTGCAGTAGGGGAGACTGGAATTCCCGGTGTAACGGTGGAATGTGTAGATATCGGGAAGAACACCAATGGCGAAGGCAGGTCTCTGGGCTGTTACTGACGCTGAGAAGCGAAAGCGTGGGGAGCGAACAGG

>ae99a1afbbf790b745d03a35b02f26ae

TACGTAGGGTGCGAGCGTTAATCGGAATTACTGGGAGTAAAGCGTGCGCAGGCGGTTTGTTAAGACAGATGTGAAATCCCCGGGCTCAACCTGGGAACTGCATGTGTGACTGGCAGGCTAGAGTATGGCAGAGGGGGGTAGAATTCCACGTGTAGCAGTGAAATGCGTAGAGATGTGGAGGAATACCGATGGCGAAGGCAGCCCCCTGGGCCAATACTGACGCTCATGCACGAAAGCGTGGGGAGCAAACAGG

>f75e3891fed8cff1f0d066b92604fd75

GTGCCAGCCGCCGCGGTAAAACAGTGAGTCATATTTACGGCGCTGATTGAGAAAGGTTAAACATGAAATGTTAAATACTTGGCTTCGAGTTGCAGTTGCCCGGTCAGTTACCTGCCAATCAATTGTATTAGAAACCCTGGTAGTCCGGCTGACTGACTGCGTATACATCTCGTATGCCGTCT

>24730d0b2b0a31b35e360ffa8ec3aae0

TACGTAGGGTGCGAGCGTTAATCGGAATTACTGGGCGTAAAGCGTGCGCAGGCGGTTTGTTAAGACAGATGTGAAATCCCCGGGCTCAACCTGGGAACTGCATTTGTGACTGGCAGGCTAGAGTATGGCAGAGGGGGGTAGAATTCCACGTGTAGCAGTGAAATGCGTAGAGATGTGGAGGAATACCGATGGCGAAGGCAGCCCCCTGGGCCAATACTGACGCTCATGCACGAAAACGTGGGGAGCAAACAGG

>8bc299234bf149709f26d6aa3c6c3007

TACCGGCAGCCCGAGTGATGGCCGATCTTATTGGGCCTAAAGCGTCCGTAGCTGGCCGCGCAAGTCCATCGGGAAATCCACCCGCTCAACAGGTGGGCGCCCGGTGGAAACTGTGTGGCTCGGGATCGGAAGACCCAACGGGTACGTCTTGGGTAGGAGTGAAATCCCGTAATCCTGGACGGACCACCGATAGCGAAAGCACGTTGGGAAGACGAATCCGACGGTGAGGGACGAAAGCCAGGGTCTCGAACCGG

>0d0441f2cd3e2059febc4b33984dfcb5

TACGTAGGGTACGAGCGTTAATCGGAATTACTGGCCGTAAAGCGTGCGCAGGCGGTTTGTTAAGACAGATGTGAAATCCCCGGGCTCAACCTGGGAACTGCATTTGTGACTGGCAGGCTAGAGTATGGCAGAGGGGGGTAGAATTCCACGTGTAGCAGTGAAATGCGTAGAGATGTGGAGGAATACCGATGGCGAAGGCAGCCCCCTGGGCCAATACTGACGCTCATGCACGAAAGCGTGGGGAGCAAACAGG

>345a1546e6ec00fdcadd5bda61e94aba

TACGTAGGGTGCGAGCGTTAATCGGAATTACTGGGCGTAAAGTGTGCGCAGGCGGTTTGTTAAGACAGATGTGAAATCCCCGGGCTCAACCTGGGAACTGCATTTGTGACTGGCAGGCTAGAGTATGGCAGAGGGGGGTAGAATTCCACGTGTAGCAGTGAAATGCGTAGATATGTGGAGGAATACCGATGGCGAAGGCAGCCCCCTGGGCCAATACTGACGCTCATGCACGAAAGCGTGGGGAGCAAACAGG

>7467c965375968d2ff853520d771b434

GACGAAGGTGGCTAGCGTTATTCGGTGTCACTGGGCTTAAAGGGTGTGTAGGCGGAACAGTAAGCGTTTTGTGAAATCCCCCGGCTTAACCGGGGAATTGCTTGATGAACTGCTGTTCTTGAGGCAAGTAGGGGTGCATGGAACTCTTGGTGGAGCGGTGGAATGCGTAGATATCAAGAGGAACGCCGGTGGTGAAGACGCTGCACTATACTTGTCCTGACGCTGAGACACGAAAGCGTGGGGAGCAAACTGGG

>2ed50cc760fc3d7dc5d319262d3dc7c1

CGGTTTACCAAGTATTTGGCAACTTTATGCGTTCATTTTGGGTGTTGAAACTTAAAAATCTCATCTTTTTTTATCGTTAAGGGATTCAGGATCCAGCGAGTAGCTGTCCTGTTCACAAGTAGGATAGCGTGGTTAATGTTACAGCGTTATATCTGGACTACCTGTAATTTCTTCATGATGAGACCCAAGGTTCCAACTGCACGTTATGAAAACCAAACTAGTTTGACACTA

>49f57825e54fad856bfc58eb476c5fec

AACAGAGGATACAAGCGTTATCCGGATTTATTGGGTTTAAAGGGTGCGTAGGTGGTTTTTTAAGTCAGTAGTGAAATCTTAAAGCTTAACTTTAAAAGTGGTATTGATACTGATAAACTAGAGTGAGGTTGGAGTAACTGGAATGTGTGGTGGAGCGGTGAAATGCATAGAGATCACACAGAACACCAATCGCGAAGGCATGTTACTAAACATAGACTGACACTGAGGCACGAAAGCATGGGTAGCAAACAGG

>c5968e79be5a3532b10e3d230953af15

TACGAAGGGGGCTAGCGTTGCTCGGAATCACTGGGCGTAAAGGGTGCGTAGGCGGGTTTTTAAGTCAGGGGTGAAATCCTGGAGCTCAACTCCAGAACTGCCTTTGATACTGAAGATCTTGAGTCCGGGAGAGGTGAGTGGAACTGCGAGTGTAGAGGTGAAATTCGTAGATATTCGCAAGAACACCAGTGGCGAAGGCGGCTCACTGGCCCGGTACTGATGCTGAGGCACGAAAGCGTGGGGAGCAAACAGG

>8174b1daa6fea4a2a99ea226d7675c4a

TACGTAGGGTGCGAGCGTTAATCGGAATTACTGGGCGTAAAGTGTGCGCAGGCGGCTTGTTAAGACAGATGTGAAATCCCCGGGCTCAACCTGGGAACTGCATTTGTGACTGGCAGGCTAGAGTATGGCAGAGGGGGGTAGAATTCCACGTGTAGCAGTGAAATGCGTAGAGATGTGGAGGAATACCGATGGCGAAGGCAGCCCCCTGGGCCAATACTGACGCTCATGCACGAAAGCGTGGGGAGCAAACAGG

>0e131d71ed6fa4ea1c8e9429b9f32ecd

TACGGAGGGTGCAAGCGTTATCCGGAATCATTGGGTTTAAAGGGTCCGCAGGCGGACGTATAAGTCAGTGGTGAAAGCCTACAGCTTAACTGTAGAACTGCCATTGATACTGTATGTCTTGAATTCGGTCGAAGTGGGCGGAATGTGTCATGTAGCGGTGAAATGCATAGATATGACACAGAACACCGATAGCGAAGGCAGCTCACTAGGCCTGAATTGACGCTCAGGGACGAAAGCGTGGGGAGCGAACAGG

>c732b708b951c2ad494a34d0e1eb8b7a

TACCGGCAGCTCAAGTGATGACCGATATTATTGGGCCTAAAGCGTCCGTAGCCGGCCACGAAGGTTCATCGGGAAATCCGCCAGCTCAACTGGCGGGCGTCCGGTGAAAACCACGTGGCTTGGGACCGGAAGGCTCGAGGGGTACGTCCGGTGTAGGAGTGAAATCCCGTAATCCTGGACGGACCACCGATGGCGAAAGCACCTCGAGAAGACGGATCCGACGGTGAGGGACGAAAGCTAGGGTCTCGAACCGG

>1584a8db2c197fdb7c02c8001458ff92

CACGATTAACCCAAGTCAATAGAAGCCGGCGTAAAGAGTGTTTTAGATCACCCCCTCCCCAATAAAGCTAAAACTCACCTGAGTTGTAAAAAACTCCAGTTGACACAGAATAGACTACGAAAGTGGCTTTAACATATCTGAACATACAATAGCTAAGACCCAAACTGGG

>42d75d5345be270c4f59e2fd82fb005d

TACGTAGGGTGCGAGCGTTAATCGGAATTACTGGGCGTAAAGCGTGCGCAGGCGGTTTGTTAAGACAGATGTGAAATCCCCGGGCTCAACCTGGGAACTGCATTTGTGACTGGCAGGCTAGAGTATGGCAGAGGGGGGTAGAATTCCACGTGTAGCAGTTAAATGCGTAGAGATGTGGAGGAATACCGATGGCGAAGGCAGCCCCCTGGGCCAATACTGACGCTCATGCACGAAAGCGTGGGGAGCAAACAGG

>cff60bed359051dd65fe850fd0bd07e1

TACGTAGGGTGCAAGCGTTGTCCGGAATTACTGGGCGTAAAGAGCTCGTAGGCGGTTTGTCGCGTCGTCTGTGAAAACCAGCAGCTCAACTGTTGGCTTGCAGGCGATACGGGCAGACTTGAGTATTTCAGGGGAGACTGGAATTCCTGGTGTAGCGGTGAAATGCGCAGATATCAGGAGGAACACCGGTGGCGAAGGCGGGTCTCTGGGAAATAACTGACGCTGAGGAGCGAAAGCGTGGGTAGCGAACAGG

>8378381d35c85bc0ab8e01a20f551c09

TACGTAGGGTGCGAGCGTTAATCGGAATTACTGGGCGTAAAGCTTGCGCAGGCGGTTTGTTAAGACAGATGTGAAATCCCCGGGCTCAACCTGGGAACTGCATTTGTGACTGGCAGGTTAGAGTATGGCAGAGGGGGGTAGAATTCCACGTGTAGCAGTGAAATGCGTAGAGATGTGGAGGAATACCGATGGCGAAGGCAGCCCCCTGGGCCAATACTGACGCTCATGCACGAAAGCGTGGGGAGCAAACAGG

>66c13e34fddfc3380180f99bea88b8ad

TACGGAGGGGGTTAGCGTTGTTCGGAATTACTGGGCGTAAAGCGCACGTAGGCGGATCAGAAAGTTGGGGGTGAAATCCCGGGGCTCAACCCCGGAACTGCCTTCAAAACTCCCAGTCTAGAGTTCGAGAGAGGTGAGTGGAACTCCGAGTGTAGAGGTGAAATTCGTAGATATTCGGAAGAACACCAGTGGCGAAGGCGGCTCACTGGCTCGATACTGACGCTGAGGTGCGAAAGTGTGGGGAGCAAACAGG

>ed4d1ccc2a05a6413feac3022c5bbb38

TACGTAGGGTGCGAGCGTTAATCGGAATTACTGGGCGTAAAGCGTGCGCAGGCGGTTTGTTAAGACAGATGTGAAATCCCCGGGCTCAACCTGGGAACTGCATTTGTGTCTGGCAGGCTAGAGTATGGCAGAGGGGGGTAGAATTCCACGTGTAGCAGTGAAATGCGTAGAGATGTGGAGGAATACCGATGGCGAAGGCAGCCCCCTGGGCCAATACTGACGCTCATGCACGAAAGCGTGGGGAGCAAACAGG

>51e78791ea4ae715a44161ab3d5f737a

TACGTAGGGTGCGAGCGTTAATCGGAATTACTGGGCGTAAAGCGTGCGCAGGCGGTTTGTCAAGACAGATGTGAAATCCCCGGGCTCAACCTGGGAACTGCATTTGTGACTGGCAGGCTAGAGTATGGCAGAGGGAGGTAGAATTCCACGTGTAGCAGTGAAATGCGTAGAGATGTGGAGGAATACCGATGGCGAAGGCAGCCCCCTGGGCCAATACTGACGCTCATGCACGAAAGCGTGGGGAGCAAACAGG

>1de0492f6809702853d5ca7bea302d5c

TACGTAGGGTGCAAGCGTTGTCCGGAATTACTGGGCATAAAGAGCTCGTAGGTGGTTTGTCGCGTCGTCTGTGAAAGCCCGGGGCTTAACTCCGGGTCTGCAGGCGATACGGGCATAACTAGAGTGCTGTAGGGGAGACTGGAATTCCTGGTGTAGCGGTGAAATGCGCAGATATCAGGAGGAACACCGATGGCGAAGGCAGGTCTCTGGGCAGTAACTGACGCTGAGGAGCGAAAGCATGGGGAGCGAACAGG

>cf3ad529641e8dd4786209006277fe8f

CACACTTATGTCTTCCGAAAGACAAAATATTTCTCCAGGATGTATTTCAACCATGCCGGTCATTTTCAACTGGCAGTGCCTTGTAGATATTTTTAAAAGACTTTGAATGCATCTTGTTCATAGAGTTCAGTTTGTTCATTAATGATTTGTTCTTATGTAGTTTGTAACGTAATATATTGAGGCTTTCAACTAAATTCATTTTATTTCCTTTCTTGCCGGTTGTG

>3f974f982c2e1b436ae56da5b61e64d7

CCGCGGTAATACTGTATCCAAGCATATAGACATCAAATTGATTGCCAAATCTATCATATTGTCAAAGTATAGTAGGTATAAAATCATATAACTCGTATCACCTTCGGAAAAATAGTCAGACATTTAATTAGATACCCTTGTAGTCCGGCTGACTGACTCGAAGTATATCTCG

>9a851d6b2911894bc6fe49053eaad27a

TACCGGCAGCTCAAGTGATGTCCCATATTATTGGGCCTAAAGCGTCCGTAGCTGGCCGACCAAGTCTATCGGGAAATCCACCTGCCCAACAGGTGGGCGTCCGGTAGAAACTGGCCGGCTTGGAACCGGAAGGCTCAGAGAGTACGTCCGGGGTAGGAGTGAAATCCCGTAATCCCGGACGGACTACCGATGGCGAAAGCACTCTGAGAAGACGGCTTCGACAGTGAGGGACGAAAGCTAGGGTCTCGAACCGG

>49db2c2747bd4d0a106385cbbf8ee283

AAACACTGGTGGTCAGTGTAGAGAAATTAAAAAAATAACGGACTCTGTAATATTATAAAACAGAAGAGAATAGTGCTGACCATTCTATTAGAGCAAGTAATATAAGATTAATTAAACTTAACACATATTAGAAACCCCAGTAGTCCGGCTGACTGACTATAGTA

>578c34a49858d16cab7298b69dd606f3

TACGTAGGGTGCGAGCGTTAATCGGAATTACTGGGCGTAAAGCGTGCACAGGCGGTTTGTTAAGACAGATGTGAAATCCCCGGGCTCAACCTGGGAACTGCATTTGTGACTGGCAGGCTAGAGTATGGCAGAGGGGGGTAGAATTCCACGTGTAGCAGTGAAATGCGTAGAGATGTGGAGGAATACCGATGGCGAAGGAAGCCCCCTGGGCCAATACTGACGCTAATGCACGAAAGCGTGGGGAGCAAACAGG

>9d02c46091c1491ba89fd76c728d8538

TACGTAGGGTGCGAGCGTTAATCAGAATTACTGGGCGTAAAGCGTGCGCAGGCGGTTTGTTAAGACAGATGTGAAATCCCCGGGCTCAACCTGGGAACTGCATTTGTGACTGGCAGGCTAGAGTATGGCAGAGGGGGGTAGAATTCCACGTGTAGCAGTGAAATGCGTAGAGATGTGGAGGAATACCGATGGCGAAGGCAGCCCCCTGGGCCAATACTGACGCTCATGCACGAAAGCGTGGGGAGCAAACAGG

>b07e3383335fd65cd3fb0bccb94205a7

GCGGTAATAATGTATCCAAGCATATAGACATCAAATTGATTGACAAATCTATCACATTGTCAAAGTATAGTAGGTATAGAATCATATAACTCTTATCACCTTCATAAAAATAGTCAGTTCCATGTAATTAGATACCCTAGTAGTCCGGCTGACTGACTTCTCTATGATCT

>c5184cbcec2173f862a9d274663764a6

TACGGAGGGTGCAAGCGTTAATCGGAATTACTGGGCGTAAAGCGCACGCAGGCGGTCTGTCAGGTCGGATGTGAAATCCCCGGGCTCAACCTGGGAACTGCATTCGAAACTGGCAGGCTAGAGTCTTGTAGAGGGGGGTAGAATTCCAGGTGTAGCGGTGAAATGCGTAGAGATCTGGAGGAATACCGGTGGCGAAGGCGGCCCCCTGGACAAAGACTGACGCTCAGGTGCGAAAGCGTGGGGAGCAAACAGG

>1a0642cc5962d74de08040fe998e0bcb

TACGTAAGGACCGAGCGCTGTCCGGAATCATTGGGCGTAAAGGGTACGTAGGCGGCTAGAAAAGTTAGAAGTCAAAGGCTATAGCTCAACTATAGTAAGCTTCTAAAACTATTTAGCTTGAGAAATGGAAGGGAAAGTGGAATTCCTAGTGTAGCGGTGGAATGCGCAGATATTAGGAAGAATACCGGTGGCGAAGGCGACTTTCTGGCCATTATCTGACGCTGAGGTACGAAAGCGTGGGTAGCAAACAGG

>72fb8f1f5bf5768a27d40fcec200a688

TACGGAGGGTGCGAGCGTTAATCGGAATTACTGGGCGTAAAGCGCGCGTAGGCGGCGTGATAAGCCGGTTGTGAAAGCCCCGGGCTCAACCTGGGAACGGCATCCGGAACTGTCAGGCTAGAGTGCAGGAGAGGAAGATAGAATTCCCGGTGTAGCGGTGAAATGCGTAGAGATCGGGAGGAATACCAGTGGCGAAGGCGGCCTTCTGGACTGACACTGACGCTGAGGTGCGAAAGCGTGGGTAGCAAACAGG

>e3846fade5112d68ef2b27ab0106477a

TACGAAGGGGGCTAGCGTTGCTCGGAATCACTGGGCGTAAAGGGTGCGTAGGCGGGTCTTTAAGTTAGGGGTGAAATCCTGGAGCTCAACTCCAGAACTGCCTTTGATACTGAAGATCTTGAGTTCGGGAGAGGTGAGTGGAACTGCGAGTGTAGAGGTGAAATTCGTAGATATTCGCAAGAACACCAGTGGCGAAGGCGGCTCACTGGCCCGATACTGACGCTGAGGCACGAAAGCGTGGGGAGCAAACAGG

>ba6caf9f2cb5f41a5ad7580983eccbde

TACGTAGGGTGCGAGCGTTAATCGGAATTACTGGGCGTAAAGCGTGCGCAGGCGGTTTGTTAAGACAGATGTGAAATCCCCGGGTTCAACCTGGGAACTGCATTTGTGACTGGCAGGCTAGAGTATGGCAGAGGGGGGTAGAATTCCACGTGTAGCAGTGAAATGCGTAGAGATGTGGAGGAATACCGATGGCGAAGGCAGCCCCCTGGGCCAATACTGACGCTCATGCACGAAAGCGTGGGGAGCAAACAGG

>e1b2b6ae4af6686429c21926cc0314a0

TACAGAGGATGCAAGCGTTATCCGGAATGATTGGGCGTAAAGCGTCTGTAGGTGGCTTTTTAAGTCCGCCGTCAAATCCCAGGGCTCAACTCTGGACAGGCGGTGGAAACTACCAAGCTGGAGTACGGTAGGGGCAGAGGGAATTTCCGGTGGAGCGGTGAAATGCGTAGAGATCGGAAAGAACACCAACGGCGAAAGCACTCTGCTGGGCCGACACTGACACTGAGAGACGAAAGCTAGGGGAGCGAATGGG

>12f91355e4cab7e650c2e9c99e702748

GCTTCGTATCGCGCTAATTTAATTTTTTTTAATTAGGCTTTTTCACGCTACTCATAAGAGCTAGAAAGTAAAAATGTTAATATTTGATTCTTAATTTTCTGTAAATTTTTTGGATTAATTATATTGGACATAACATCTATGTGAGATATACTTATCTTTTTTTGTCGTGGTAACACACTTATGTCTTTAAAAAGACAACACGTTTTTGCAGAATGCATTTCAACCTTCTCGGTCATTTTCAACTGTTTAACAGAAGCAATAACATCTAAGGTTTAAA

>2aa9ec1b838df930349a77cf47f23b6e

TTTATTTATTTCTTTACTTAGGGATTACACGTCCAGCATCTGGAATTATAGACACGAAAACGGCGAGTAGCTGGCATACTTCGTGCACGGATCGTGTTAAGCTTAAGACTGACAAACAAACTATAGAAATGATCATTAAACATTAGAAACCCCGGTAGTCCGG

>8e8cd1f06be3cb35fc2d83739d19d115

TACGTAGGGTGCGAGCGTTAATCGGAATTACTGGGCGTAAAGCGTGCGCAGGCGGTTTGTTAAGACAGATGTGAAATCCCCGGGCTAAACCTGGGAACTGCATTTGTGACTGGCAGGCTAGAGTATGGCAGAGGGGGGTAGAATTCCACGTGTAGCAGTGAAATGCGTAGAGATGTGGAGAAATACCGATGGCGAAGGCAGCCCCCTGGGCCAATACTGACGCTCATGCACGAAAGCGTGGGGAGCAAACAGG

>9e40142795b7eeeaabec48eb4ceb864f

TACGTAGGGTGCGAGCGTTAATCGGAATTACTGGGCGTAAAGCGTGCGCAGGCGGTTTGTTAAGACAGATGTGAAATCCCCGGGGTCAACCTGGGAACTGCATTTGTGACTGGCAGGCTAGAGTATGGCAGAGGGGGGTAGAATTCCACGTGTAGCAGTGAAATGCGTAGAGATGTGGAGGAATACCGATGGCGAAGGCAGCCCCCTGGGCCAATACTGACGCTCATGCACGAAAGCGTGGGGAGCAAACAGG

>a963ad5e5e15128633542932e52aa93b

TACCGGCAGTCCGAGTGATGGCCGATATTATTGGGCCTAAAGCGTCCGTAGCTTGCTGTGTAAGTCCATTGGGAAATCGACCAGCTCAACTGGTCGGCGTCCGGTGGAAACTACACAGCTTGGGGCCGAGAGACTCAACGGGTACGTCCGGGGTAGGAGTGAAATCCTGTAATCCTGGACGGACCACCAACGGGGAAACCACGTTGAGAGACCGGACCCGACAGTGAGGGACGAAAGCCAGGGTCTCGAACCGG

>c241e7ea7b6a7aa4815c0283f785ec87

TACCGGCAGCTCAAGTGATGTCCCATATTATTGGGCCTAAAGCGTCCGTAGCTGGCCGACCAAGTCTATCGGGAAATCCACCTGCCCAACAGGTGGGCGTCCGGTAGAAACCGGCCGGCTTGGAACCGGAAGGCTCAGAGAGTACGTCCGGGGTAGGAGTGAAACCCCGTAATCCCGGACGGACTACCGATGGCGAAAGCACTCTGAGAAGACGGCTTCGACAGTGAGGGACGAAAGCTAGGGTCTCGAACCGG

>5d288b56e5416cc5081bd3c3140e2e12

TAATTGTGAGCACGCCCGGAATCGTATTTTACGTATTGCTAAAATAATATTGTAGAATATGCGTAACACTGTGCAGAGTAACAAACGTAAGACGTATTCTACGTCAAAAATAAAAAATATTACATTTGAATTTAGCGCACATGCAAAAGTTACTGCACCCTGGGCTTCTTTTTTATCAGATGCAAAAACAAGGTCCACGAAATTATCTTGTTGCTTCTGCGGCGTATTTTGTTTAAT

>3213ec11c0d1e87051303c73dd4209f8

CACGATTAACCCAAGTCAATAGAAGCCGGCGTAAAGAGTGTTTTAGATCACCCCCTCCCCAATAAAGCTAAAACTCACCTGAGTTGTAAAAAACTCCAGTTGACACAAAATAGTCTACGAAAGTGGCTTTAACATATCTGAACACACAATAGCTAAGACCCAAACTGGG

>81c03d7817f8343fca401f5cd1a6ac19

AACAGAGGATACAAGCGTTATCCGGATTTATTGGGTTTAAAGGGTGCGTAGGTGGTTTTTTAAGTCAGTAGTGGAATCTTAAAGCTTAACTTTAAAAGTGCTATTGATACCGATAAACTAGAGTGAGGTTGGAGTAACTGGAATGTGTGGTGGAGCGGTGAAATGCATAGAGATCACACAGAACACCAATCGCGAAGGCATGTTACTAAACATAGACTGACACTGAGGCACGAAAGCATGGGTAGCAAACAGG

>9344bf6b5f504eed8c8d81fc9e475cc1

CACAGTACGTCATCCATCGATGGGCTTCAATCTGTGACCACCTCTCATCTACAGCCTACCTAAGTGTTCTCCCCATCTATAGCCTGTATAATACATTAAATACCTTGCAAATATAACATACATATTCGTACATTGTATTATAATTCATTATAAGGAATAATAATAATGCATATTTA

>1b9b28e04591a9697daee38e053ad023

TAATTGTGTGCCAGCCGCCGCGGTAAGGCAAGAGGCATGGGTCAGCAGCTCTCTCTAGCATGTTAAGGTTCACTGTTGGTGGATTATGGATGTGATGCTCTTGCTGTTGATATTGAGGAAGGTAAGATTAGATACCCTGGTAGTCCGGCTGACTGACTTGCTCGTAATCTCGTATGCCGTCTTCTGCTT

>dc08452d5b96ebc193b0763d5f6ca9a9

TACGTAGGGTGCGAGCGTTAATCGGAATTACTGGGCGTAAAGCGTGCGCAGGCGGTTTTGTAAGACAGGCGTGAAATCCCCGGGCTCAACCTGGGAACTGCGTTTGTGACTGCAAGGCTAGAGTATGGCAGAGGGGGGTGGAATTCCACGTGTAGCAGTGAAATGCGTAGAGATGTGGAGGAACACCGATGGCGAAGGAAGCCCCCTGGGCCAATACTGACGCTCATGCACGAAAGCGTGGGTAGCAAACAGG

>89df6600be0d5acb2d1178069852a1cf

TACGTAGGGTGCGAGCGTTAATCGGAATTACTGGGCGTAAAGCGTGCGCAGGCGGTTTGCTAAGACAGATGTGAAATCCCCAGGCTCAACCTGGGAACTGCATTTGTGACTGGCAGGCTAGAGTATGGCAGAGGGGGGTAGAATTCCACGTGTAGCAGTGAAATGCGTAGAGATGTGGAGGAATACCGATGGCGAAGGCAGCCCCCTGGGCCAATACTGACGCTCATGCACGAAAGCGTGGGGAGCAAACAGG

>4bafd62b363749b94c5f36a1c9af5a9e

TACGTAGGTCCCGAGCGTTGTCCGGATTTATTGGGCGTAAAGCGAGCGCAGGCGGTTTAATAAGTCTGAAGTTAAAGGCAGTGGCTTAACCATTGTTCGCTTTGGAAACTGTTAAACTTGAGTGCAGAAGGGGAGAGTGGAATTCCATGTGTAGCGGTGAAATGCGTAGATATATGGAGGAACACCGGTGGCGAAAGCGGTTCTCTGGTCTGTAACTGACGCTGAGGCTCGAAAGCGTGGTGAGCAAACAGG

>59af98a591877ddce8561fb9d6d7eecb

TACGATTTCTTTAATTTAAATAGTTAAGTTTCAGTTAATGTATATATAATTTTAAATATTTATAATTTTGGTGAAATATATTTTGTTTTAAAAAATTAATTTTATATCTGAAAAATTTTTGTTCAAACTAGGATTAGAAACCCCAGTAGTCCGGCTGACTGAC

>444f3c7c79aac18cf449b3154369550a

TACGATTTCTTTAATTTAAGTGGTTAAGTTTCAGTTAATATAACATTAATATAAAATATCTATAATTTTGGTGAAATATATTTTATCTTTAAAAATTAATTATATGCCTGAAAAATTTTTGTTTAAACTAGGATTAGAAACCCCGGTAGTCCGGCTGACTGAC

>226177f7193540b13571c6729d6680f1

AACGTAGGAGGCGAGCGTTATCCGGATTCATTGGGCGTAAAGGGCGTGTAGGCGGTGACTCAAGTTGGGCGTGAAATCTCCCGGCTCAACCGGGAGGCGCCGTCCAATACTGGGACACTTGAGGGCAGGAGAGGAAAGCGGAATTCCCGGTGTAGTGGTGGAATACTTAGATATCGGGAGGAACACCAGTGGCGAAAGCGGCTTTCTGGACTGTGCCTGACGCTGAAGCGCGAAAGCGTGGGTAGCAAACTGGG

>2f249d386548511c81385c190446f34a

TACGTAGGGTGCGAGCGTTAATCGGAATTACTGGGCGTAAAGCGTGCGCAGGCGGTTTGTTAAGACAGATGTGAAATCCCCGGGCTCAACCTGGGAACTGCATTTGTGACTGGCAGGCTAGAGTATGGCAGAGGGGGGTAGAATTCCACGTGTAGCAGTGAAATGCGTAGAGATGTGGAGGAATACCGATGACGAAGGCAGCCCCATGGGCCAATACTGACGCTCATGCACGAAAGCGTGGGGAGCAAACAGG

>20b93bde81b5c8f5182d71d7ca30c00d

TACGTAGGGTGCGAGCGTTAATCGGAATTACTGGGCGTAAAGCGTGCGCAGGCGGTTTGTTAAGACAGATGTGAAATCCCCGGGCTCAACCTGGGAACTGCATTTGTGACTGGCAGGCTAGAGTATGGCAGAGGGGGGTAGAATTACACGTGTAGCAGTGAAATGCGTAGAGATGTGGAGGAATACCGATGGCGAAGGCAGCCCCCTAGGCCAATACTGACGCTCATGCACGAAAGCGTGAGGAGCAAACAGG

>fa3b4e12a813a9ba7c65069fb96778c2

TACGTAGGGTGCGAGCGTTAATCGGAATTACTGGGCGTAAAGCGTGCGCAGGCGGTTTGTTAAGACAGATGTGAAATCCCCGGGCTCAACCTGGGAACTGCATTTGTGACTGGCAGGCTAGAGTATGGCAGAGGGGGGTAGAATTCCACGTGTAGCAGTGAAATGCGTAGAAATGTGGAGGAATACCGATGGCGAAGGCAGCCCCCTGGGCCAATACTGACGCTCATGCAAGAAAGCGTGGGGAGCAAACAGG

>658aae35fd01535d98b334af1bdbfeaa

TACGTAGGGTGCGAGCGTTAATCGGAATTACTGGGCGTAAAGCGTGCGCAGGCGGTTTGTTAAGACAGATGTGAAATCCCCGGGCTAAACCTGGGAACTGCATTTGTGACTGGCAGGCTAGAGTATGGCAGAGGGGGGTAGAATTCCACGTGTAGAAGTGAAATGCGTAGAGATGTGGAGGAATACCGATGGCGAAGGCAGCCCCCTGGGCCAATACTGACGCTCATGCACGAAAGCGTGGGGAGCAAACAGG

>4c2912bc6b4d834f984dc086baa43a63

TACGTAGGGTGCGAGCGTTAATCGGAATTACTGGGCGTAAAGCGTGCGCAGGCGGTTTGTTAAGACAGATGTGAAATCACCGAGCTCAACCTGGGAACTGCATTTGTGACTGGCAGGCTAGAGTATGGCAGAGGGGGGTAGAATTCCACGTGTAGCAGTGAAATGCGTAGAGATGTGGAGGAATACCGATGGCGAAGGCAGCCCCCTGGGCCAATACTGACGCTCATGCACGAAAGCGTGGGGAGCAAACAGG

>49efa8012e087821c77300111858c6bb

TACCGGCAGCACGAGTGATGGCCGATCTTATTGGGCCTAAAGCGTCCGTAGCCGGCCAGACAAGTCCGTTGGGAAATCGACGCGCTCAACGCGTCGGCGTCCGGCGGAAACTGTTCGGCTTGGGGCCGGAAGACCTGAGGGGTACGTCCGGGGTAGGAGTGAAATCCCGTAATCCCGGACGGACTACCGATGGCGAAAGCACTCTGAGAAGACGGCTTCGACAGTGAGGGACGAAAGCTCGGGTCTCAAACCGG

>a6664287fe5f48c49cd7163f493ce56c

TACGTAGGTGGCAAGCGTTGTCCGGATTTATTGGGCGTAAAGCGAGCGCAGGCGGTTCCTTAAGTCTGCTGTGAAAGCCCCCGGCTCAACCGGGGAGGGTCATTGGAAACTGGGGAACTTGAGTGCAGAAGAGGAGAGTGGAATTCCATGTGTAGCGGTGAAATGCGTAGATATATGGAGGAACACCAGTGGCGAAGGCGACTCTCTGGTCTGTAACTGACGCTGAGGCTCGAAAGCGTGGGTAGCAAACAGG

>224102d609b83a172b11bd9115107f16

TACGGAGGATGCGAGCGTTATCCGGATTTATTGGGTTTAAAGGGTGCGTAGGCGGCCTGTTAAGTCAGCGGTGAAATCTAGGAGCTTAACTCCTAAATTGCCATTGATACTGGCGGGCTTGAGTGTAGATGAGGTAGGCGGAATGCGTGGTGTAGCGGTGGAATGCATAGATATCACGCAGAACTCCGATTGCGAAGGCAGCTTACTAAGGTACAACTGACGCTGAAGCACGAAAGCGTGGGTATCAAACAGG

>2f7617281dce81ff29d5487b61cbda39

AATTGTGTGCCAGCCGCCGCGGTAAGGTATTGGGATGAACAATGCAATTTAGTAAAAACTAGATATTTTACATCCTGTTTTTTTTAATAAGGCAGCTGCTGTAGACCTTAGTAGATTTGTTGGTGCATTAGATACCCTAGTAGTCCGGCTGACTGACTGATCTACGATCTCGTATGCCGTCTTCTGCT

>2740cf2417c92847cc298cbd71dd1fcd

TACGTAGGTGGCAAGCGTTGTCCGGAATTATTGGGCGTAAAGCGCGCGCAGGCGGATAGGTCAGTCTGTCTTAAAAGTTCGGGGCTTAACCCCGTGATGGGATGGAAACTGCCAATCTAGAGTATCGGAGAGGAAAGTGGAATTCCTAGTGTAGCGGTGAAATGCGTAGATATTAGGAAGAACACCAGTGGCGAAGGCGACTTTCTGGACGAAAACTGACGCTGAGGCGCGAAAGCCAGGGGAGCGAACGGG

>ca8c1cfd11ff0a6977f6772630a85de3

TACGTAGGGTGCAAGCGTTAATCGGAATTACTGGGCGTAAAGCGTGCGCAGGCGGTTGTGTAAGTCTGATGTGAAAGCCCCGGGCTTAACCTGGGAACTGCATTGGAGACTGCACAGCTAGAGTGCGTCAGAGGGGGGTAGAATTCCGCGTGTAGCAGTGAAATGCGTAGAGATGCGGAGGAATACCGATGGCGAAGGCAGCCCCCTGGGATGACACTGACGCTCATGCACGAAAGCGTGGGGAGCAAACAGG

>4e563de74c6dcd60c46a1a2f032f416a

TACGTGAGAGACTAGTGTTATTCATCTTAATTGGGTTTAAAGGGTACCTAGACAGTCAATATAACTTCTATAATGCTAACACTTGACTAGAGTTTTAAGTAAGAGGGAAGTACTTAAGGAGTAAGAGATGAAATATCTGTGATACCAAAGGGACTCCGTAAAGGCGAAGGCATCCCTTTATCTAAAAACTAACGTTGAAGGACGAAGGCTTAGATAACAAATAGG

>087cbbb3c836a8bf29742deb5bc0551e

AGTTTTAATTTTACGGGCACATCCCATGCATAAATAGAAGTAAAGGGTATTGCATAGTGGAATACTATATACCCAAACTTGTAAAAGTAGTTAATAATTCGTGGTTTCGTTCCTCGTCTATCTACCTACCTATCTACCTATCTCTGATGACATTAGATACCCC

>46409e3f7cd638211b258418d71f8016

GACCAGATGAATTCCACATTCATATGTTGCAACACGCTCCATCTCATATTCTTGACACATTATTAAAATTTTTATAAAATATGGTTCTCTGGCATCTTTCCAACAGCGTGGAAAACAGCAACGATTATTCCCATTATTAAAGAAAACAAAAACCCTTGATTAT

>e168638098395c855d9b01eab085fc3b

CACGACGTAGTCGCTTCCGTTTTTTTCTGTTGGTGAGCAACATTTGCCAGTCGCTGGTGGACGGTTCATCTTCCTTTATGTTGTCACTGTCTTCATAATACTCAATTGAATCATATACGTCATTACGTTGAGGAGTGTTGGGTATATTATGTTGTTTTTTTATGAAATCGTTTGTACACGCATTGATTGTTGGTCCATATT

>3bdf2c3389e11aa5c8158de29376c784

TACGTAGGGTGCGAGCGTTAATCGGAATTACTGGGCGTAAAGCGTGCGCAGGCGGTTTGTTAAGACAGATGTGAAATCCCCGGGCTCAACCTGGGAACTGCCTTTGTGACTGGCAGGCTAGAGTATGGCAGAGGGGGGTAGAATTCCACGTGTAGCAGTGAAATGCGTAGAGATGTGGAGGAATACCGATGGCGAAGGCAGCCCCCTGGGCCAATACTGACGCTCATGCACGAAAGCGTGGGGAGCAAACAGG

>a4ef8de559b5285400fefd3c251d9a10

CACACTTATGTCTTCAGAAAGACAAAATATTTCTCCAGGATGCATTTCAACCATGTTGGTCATTTTCAACTGGCAGTGCCTTGTAGATGTTTGTAAAAGATTTTGAATGTGTCTTGTCCATAGAGTACAGTTTGTTGAAGCAAACTGAAATATGTTTGTTCAAGCATACATTGGCTTTCAAGTAAATTCATTTTGTGTCCTTTTTTGCCGGTTGTG

>61cd3cfe309b274d16c412db5cbe7bbc

TACGTAGGGTGCGAGCATTAATCGGAATTACTGGGCGTAAAGCGTGCGCAGGCGGTTTGTTAAGACAGATGTGAAATCCCCGGGCTCAACCTGGGAACTGCATTTGTGACTGGCAGGCTAGAGTATGGCAGAGGGGGGTAGAATTCCACGTGTAGCAGTGAAATGCGTAGAGATGTGGAGGAATACCGATGGCGAAGGCAGCCCCCTGGGCCAATACTGACGCTCATGCACGAAAGCGTGGGGAGCAAACAGG

>04473186b449708af6ed8cd47ca78523

GTAATACTGTATCCAAGCATATAGACATCAAATTGATTGACAAATCTATCATATTGTCAAAGTATAGTCGGTATAGAATCATATAACTCTTATCACCTTCGGTAAAATCGTCAGACATTTCATGTAATTAGAAACCCTAGTAGTCCGGCTGACTGACTTGCTCGTAA

>cd78da16a6c901a8e93fa4770af08f92

TACGTAGGGTGCGAGCGTTAATCGGAATTACTTGGCGTAAAGCGTGCGCAGGCGGTTTGTTAAGACAGATGTGAAATCCCCGGGCTCAACCTGGGAACTGCATTTGTGACTGGCAGGCTAGAGTATGGCAGAGGGGGGTAGAATTCCACGTGTAGCAGTGAAATGCGTAGAGATGTGGAGGAATACCGATGGCGAAGGCAGCCCCCTGGGCCAATACTGACGCTCATTCACGAAAGCGTGGGGAGCAAACAGG

>276f9938a9b3d23af07c14e9bf2c7882

TACGATTTCTTTAATTTAAGTGGTTAAGTTTCAGTTAATATAACATTAATATAAAATATCTATAATTTTGGTGAAATATATTTTATCTTTAAAAATTAATTATATGCCTGAAAAATTTTTGTTTAAACTAGGATTAGAAACCCTTGTAGTCCGGCTGACTGAC

>d141bc188777e8e4c082f85605da4b7b

TACGGAGGGTGCAAGCGTTAATCGGGATTACTGGGCGTAAAGCGTGCGCAGGCGGCTGATTAAGTCGGATGTGAGAGCCCCGGGCTCAACCTGGGAATGGCATTCGATACTGGTCAGCTAGAGTCTGGTAGAGGTAAGCGGAATTCCGGGTGTAGCGGTGAAATGCGTAGATATCCGGAGGAACATCAGTGGCGAAGGCGGCTTACTGGACCAAGACTGACGCTCAGGCACGAAAGCGTGGGTAGCAAACAGG

>735bef4cd693442c62f2f6ea1b41a099

TACCGGCAGCCCAAGTGATGGCCGATCTTATTGGGCCTAAAGCGTCCGTAGCTGGCCACGCAAGTCCATCGGGAAATCCACCCGCTCAACGGGTGGGCGACCGGTAGAAACTGCGTGGCTCGGGACCGGAAGGCGCGACGGGTACGTCCGGGGTAGGAGTGAAATCCCGTAATCCTGGACGGACCGCCGATGGCGAAAGCACGTCGCGAGAACGGATCCGACAGTGAGGGACGAAAGCCAGGGTCTCGAACCGG

>75c158c0d6e38a61504484da7825ba26
[truncated: 316,190 more chars]
