# Supplementary material for: Lack of host phylogenetic structure in the gut bacterial communities of New Zealand cicadas and their interspecific hybrids
Source: Sci Rep. 2022 Nov 29;12:20559. doi: 10.1038/s41598-022-24723-3 (PMC9709078; doi:10.1038/s41598-022-24723-3)
Supplement: Supplementary file 3 — Supplementary Information 3. [file 41598_2022_24723_MOESM3_ESM.docx]

>e722caa722d4fa7a2f4cfb5ce80d2c2e

AACAGAGGATACAAGCGTTATCCGGATTTATTGGGTTTAAAGGGTGCGTAGGTGGTTTTTTAAGTCAGTAGTGAAATCTTAAAGCTTAACTTTAAAAGTGCTATTGA

>c478f9383d5ed8f27d69bd5b399584ec

TACGTGAGAGACTAGTGTTATTCATCTTAATTGGGTTTAAAGGGTACCTAGACAGTCAATATAACTTCTATAATGCTAATACTTGACTAGAGTTTTAAGTAAGAGGG

>622f6795f3194365d3964889c859a229

TACAATTTCTTCAATTTAAATTTTAAAAAGTTTCAGTTAATATATTATTTAAATTTTTATAAATAATAATTTTAGTGAAATATATTATTATTATATTTTATTAATTT

>7582d8ad49c53be23195be8f0f03b17f

TACGGAGGATACAAGCGTTATCCGGATTTATTGGGTTTAAAGGGTGCGTAGGTTGTTTTTTAAGTCAGTAGTGAAATCTTAAAGCTTAACTTTAAAAGTGCTATTGA

>ebdf93bd6b5c7c881e7cff194eff9632

TACGTGAGAGACTAGTGTTATTCATCTTAATTGGGTTTAAAGGGTACCTAGGCAGTCAATATAACTTCTATAATGCTAATACTTGACTAGAGTTTTAAGTAAGAGGG

>a4229eed4a0ec67dc4e321ee665a6350

TACGTAGGTGGCAAGCGTTGTCCGGAATTATTGGGCGTAAAGCGCGCGCAGGTGGTTTCTTAAGTCTGATGTGAAAGCCCACGGCTCAACCGTGGAGGGTCATTGGA

>d42da02d1c2665c51ea3b0430c69e656

TACATAGGGGGCAAGCGTTATCCGGATTTATTGGGCGTAAAGGGTGCGTAGGCGGTTAAGTAAGTCTGTGGTTTAAACGCAATGCTCAACATTGTGACGCTATAGAA

>c7b10f43885ae6203a3db7222e15cfe9

TACGTAGGGTGCAAGCGTTAATCGGAATTACTGGGCGTAAAGCGTGCGCAGGCGGTTATGCAAGACAGAGGTGAAATCCCCGGGCTCAACCTGGGAACTGCCTTTGT

>7c642815cca08d0fde9c95039b94adea

TACGTGAGAGACTAGTGTTATTCATCTTAATTGGGTTTAAAGGGTACCTAGACAGTCAATATAACTTCTAGAATGCTAATACTTGACTAGAGTTTTAAGTAAGAGGG

>fb86c0ea8a2676ad5a1b4c1962af8c2e

TACGTAGGGCGCAAGCGTTATCCGGAATTATTGGGCGTAAAGAGCTCGTAGGCGGTTTGTCGCGTCTGCTGTGAAAGTCCGGGGCTCAACTCCGGTTCTGCAGTGGG

>f3d94689b366e3d8e2c1c89be2edcae1

TACAGAGGGTGCAAGCGTTAATCGGAATTACTGGGCGTAAAGCGCGCGTAGGTGGTTTGTTAAGTTGGATGTGAAATCCCCGGGCTCAACCTGGGAACTGCATTCAA

>54071d07dff7c13b6248d3d978c9498a

TACGAAGGGGGCGAGCGTTGTTCGGAATAACTGGGCGTAAAGGGCACGTAGGCGGGTTATTAAGTCAGTGGTGAAATCCCAAGGCTCAACCTTGGAACTGCCTCTGA

>f4cf96807e2e4bc1be64ab97d89ac937

CCCCATGGCGCATTAGAAACCCTAGTAGTCCGGCTGACTGACTATGAGCTCATCTCGTATGCCGTCTTCTGCTTGAAAAAAAAAAACTAGAGTTTTAAGTAAGAGGG

>007b44281789511d4e4e3bea70bb0d7a

CCCCATGGCGCATTAGATACCCGAGTAGTCCGGCTGACTGACTATGAGCTCATCTCGTATGCCGTCTTCTGCTTGAAAAAAAAAAAATAGAGTTTTAAGTAAGAGGG

>30d9030770e03d4c99f83c3bd3157cb1

CCCCATGGCGCATTAGAAACCCTTGTAGTCCGGCTGACTGACTATGAGCTCATCTCGTATGCCGTCTTCTGCTTGAAAAAAAAAATTAACTTTAAAAGTGCTATTGA

>75a44af92bace73219ba8130c5c4881f

TACGATTAACCCAAACTAATTATCTTCGGCGTAAAACGTGTCAACTATAAATAAATAAATAGAATTAAAATCCAACTTATATGTGAAAATTCATTGTTAGGACCTAA

>8a3157775373100bca853e9d05c5f156

CCCCATGGCGCATTAGATACCCCTGTAGTCCGGCTGACTGACTATGAGCTCATCTCGTATGCCGTCTTCTGCTTGAAAAAAAAAAACTAGAGTTTTAAGTAAGAGGG

>a3849caf2c343f007c9d75a3b1437863

TACGTAGGGCGCAAGCGTTATCCGGAATTATTGGGCGTAAAGAGCTCGTAGGCGGTTTGTCGCGTCTGCTGTGAAAGACCGGGGCTCAACTCCGGTTCTGCAGTGGG

>e5546e68ee19f0d3c0b810733c51e72d

TACGTGAGAGACTAGTGTTATTCATCTTAATTGGGTTTAAAGGGTACCTAGACAGTCAATATAACTTCTATAATGCTAATACTTGACTAGAGTTTTAAGGAAGAGGG

>c50094b95aca3490999771b00e78f11e

CCCCATGGCGCATTAGATACCCCAGTAGTCCGGCTGACTGACTATGAGCTCATCTCGTATGCCGTCTTCTGCTTGAAAAAAAAAATTAACTTTAAAAGTGCTATTGA

>81c2a385859fac8fcd4347eaffa8d061

TACGTAGGGCGCGAGCGTTATCCGGAATTATTGGGCGTAAAGAGCTCGTAGGCGGTTTGTTGCGTCTGCTGTGAAAGACCGGGGCTCAACTCCGGTTCTGCAGTGGG

>c76c38915325c0069ab5eeea843feebb

TACGTAGGGTCCAAGCGTTAATCGGAATTACTGGGCGTAAAGCGTGCGCAGGCGGTTGTGCAAGACCGATGTGAAATCCCCGAGCTTAACTTGGGAATTGCATTGGT

>b961b2278240929bac9aa6a799326185

TACGGAGGGTGCAAGCGTTAATCGGAATGACTGGGCGTAAAGCGCACGCAGGCGGTCAATTAAGTTGGATGTGAAATCCCCGGGCTTAACCTGGGAACGGCATCCAA

>bf410fa78cf0487854571aa314d74712

TACGTAGGGTGCAAGCGTTGTCCGGAATTACTGGGCGTAAAGAGCTCGTAGGCGGTTTGTCACGTCGTCTGTGAAAACCCGAGGCTTAACCTCGGGCCTGCAGGCGA

>5444e352c5fba8f465269fc1dc4a7e47

CCCCATGGCGCATTAGAAACCCCTGTAGTCCGGCTGACTGACTATGAGCTCATCTCGTATGCCGTCTTCTGCTTGAAAAAAAAAATCAACCTGGGAACTGCATTCAA

>cfeaa69e96026f2e3cc2742bb79638ee

CCCCATGGCGCATTAGAAACCCGGGTAGTCCGGCTGACTGACTTAACGTCCATCTCGTATGCCGTCTTCTGCTTGAAAAAAAAAATTAACTTTAAAAGTGCTATTGA

>20f4aab62020f60258b53035924ca53a

TACGGAGGGTGCAAGCGTTATCCGGATTTATTGGGTTTAAAGGGTCCGTAGGCGGACTTATAAGTCAGTGGTGAAAGCCTGTCGCTTAACGATAGAACTGCCATTGA

>b5ebc5ebd18a8334c043f81c45bcfc39

CCCCATGGCGCATTAGAAACCCGAGTAGTCCGGCTGACTGACTATGAGCTCATCTCGTATGCCGTCTTCTGCTTGAAAAAAAAAATTAACTTTAAAAGTGCTATTGA

>529078a565c42f6613b0b999caf925fa

TACGAAGGGGGCTAGCGTTGCTCGGAATCACTGGGCGTAAAGGGTGCGTAGGCGGGTCTTTAAGTCAGGGGTGAAATCCTGGAGCTCAACTCCAGAACTGCCTTTGA

>fcb5d3116063a8bfd060616bc3985150

TACAATTTCTTCAATTTAAATTTTTAAAAGTTTCAGTTAATAAATTATTTAAATTTTTATGAATAATAATTTTAGTGAAATATATTATTATTAAATTTTGTTAATTT

>95759bf14ee83a1f3d1ce8a27bbbd5ea

CCCCATGGCGCATTAGATACCCTGGTAGTCCGGCTGACTGACTATGAGCTCATCTCGTATGCCGTCTTCTGCTTGAAAAAAAAAATTAACTTTAAAAGTGCTATTGA

>9ded0b31e09dc75a1cc13f73a35988f9

CCCCATGGCGCATTAGAAACCCGAGTAGTCCGGCTGACTGACTATGAGCTCATCTCGTATGCCGTCTTCTGCTTGAAAAAAAAAATCAACCGTGGAGGGTCATTGGA

>95f767a980404bb339a54735c3ca8ce1

CCCCATGGCGCATTAGAAACCCCAGTAGTCCGGCTGACTGACTTGCGTCAAATCTCGTATGCCGTCTTCTGCTTGAAAAAAAAAATTAACTTTAAAAGTGCTATTGA

>473fa9bd9cbfd84a75cbc46b581b49cc

TACGAGGGGAGCGAGTGTTGTTCGGTTTTATTGGGCGTAAAGGGTATGTAGGCGGTTTTGTAAGTCAACACTTAAATCTTGAGACTTAATCTCATTACAGGTGTTGA

>8ef0bc9d4b2e1199338aa422be50a6d2

TACGTAGGGTGCGAGCGTTGTCCGGAATTATTGGGCGTAAAGAGCTCGTAGGCGGTTTGTCACGTCTGCTGTGAAATCCCGAGGCTCAACCTCGGGTCTGCAGTGGG

>338d535c2a7335ef4ff1ac7252fa4293

CCCCATGGCGCATTAGAAACCCCAGTAGTCCGGCTGACTGACTCGCGATATATCTCGTATGCCGTCTTCTGCTTGAAAAAAAAAATTAACTTTAAAAGTGCTATTGA

>0a99cf347b578d78357da98f724800d1

TACGTAGGGTGCAAGCGTTAATCGGAATTACTGGGCGTAAAGCGTGCGCAGGCGGTTATGTAAGACAGAGGTGAAATCCCCGGGCTCAACCTGGGAACGGCCTTTGT

>1d2180c3b29594855bf0624c7fa54a9a

TACATAGGGGGCGAGCGTTATCCGGAATTATTGGGCGTAAAGGGTGCGTAGGCGGTTAAATAAGTTTATGGTCTAAGTGCAATGCTTAACGTTGTGATGCTATAAAA

>551b1189dd1caa4a9084ce903f777702

CCCCATGGCGCATTAGAAACCCTAGTAGTCCGGCTGACTGACTATGAGCTCATCTCGTATGCCGTCTTCTGCTTGAAAAAAAAAATCAACTCCGGTTCTGCAGTGGG

>a317de863cd094a7a08210b9838ee1e4

TACGAAGGGGGCTAGCGTTGCTCGGAATGACTGGGCGTAAAGGGCGTGTAGGCGGTTTGTACAGTCAGATGTGAAATCCCCGGGCTTAACCTGGGAGCTGCATTTGA

>8ec371c0a06b6b6d3aeac05e01a80af5

CCCCATGGCGCATTAGAAACCCTAGTAGTCCGGCTGACTGACTACTGTGTAATCTCGTATGCCGTCTTCTGCTTGAAAAAAAAAATTAACTTTAAAAGTGCTATTGA

>15a2aa7ab0b365e021624d67096e372f

CCCCATGGCGCATTAGATACCCGGGTAGTCCGGCTGACTGACTCGCGATATATCTCGTATGCCGTCTTCTGCTTGAAAAAAAAAATTAACTTTAAAAGTGCTATTGA

>ce5d2d580d6229327918baa8fa9ada86

CACGATTAACCCAAGTCAATAGAAGCCGGCGTAAAGAGTGTTTTAGATCACCCCCTCCCCAATAAAGCTAAAACTCACCTGAGTTGTAAAAAACTCCAGTTGACACA

>37d0d14a3c772b16f9f54ba2c6cf2579

CCCCATGGCGCATTAGATACCCTTGTAGTCCGGCTGACTGACTCTACGACCATCTCGTATGCCGTCTTCTGCTTGAAAAAAAAAATTAACTTTAAAAGTGCTATTGA

>8c87f6048ecd0ebc46c344bb0b903994

CCCCATGGCGCATTAGAAACCCTGGTAGTCCGGCTGACTGACTGAGACTTAATCTCGTATGCCGTCTTCTGCTTGAAAAAAAAAATTAACTTTAAAAGTGCTATTGA

>959cce8e69971bd15fde7ffa9a85634c

ATATCGTCAGTCCCTGTTCTTAGATGTGATCTTTCCAGAGCCGCACTGAGGCACACACACAACAGGGCACACAGATCAGAAACATCCTAGAATGTCTATTCTTACAT

>af84cdabf7d1edcab5b6c36f773d72ab

TACGGAGGGTGCAAGCGTTATCCGGATTTATTGGGTTTAAAGGGTCCGTAGGCTGATGTGTAAGTCAGTGGTGAAATCTCACAGCTTAACTGTGAAACTGCCATTGA

>831d31b058256b114b3ec42b126b9ce9

CATTGAACTATCGTGAGAAAGTCACGCCGCCAAAGGGAATTATATTATAGTAAATATTGGCGTAAATAAACATTTTATTAATAGTTGTAATATATGAAAATGTGCAG

>0fdfa6b31bf36d17e5c1afc20717d794

TACGGAGGGTGCAAGCGTTATCCGGATTTATTGGGTTTAAAGGGTCCGTAGGCGGATCTGTAAGTCAGTGGTGAAATCTCACAGCTTAACTGTGAAACTGCCATTGA

>0439a94c03ec4ccb21057d8061dd3f96

TTAGAAACCCCAGTAGTCCGGCTGACTGACTTAACGTCCATCTCGTATGCCGTCTTCTGCTTGAAAAAAAAAATGCTAATACTTGACTAGAGTTTTAAGTAAGAGGG

>d7731408036f86ecd8ce5feb427c689e

CCCCATGGCGCATTAGAAACCCTAGTAGTCCGGCTGACTGACTCGCGATATATCTCGTATGCCGTCTTCTGCTTGAAAAAAAAAATTAACTTTAAAAGTGCTATTGA

>cb0ef026c40401552aaf472bdba4b5a7

CCCCATGGCGCATTAGAAACCCTTGTAGTCCGGCTGACTGACTCGAGCTAGATCTCGTATGCCGTCTTCTGCTTGAAAAAAAAAATTAACTTTAAAAGTGCTATTGA

>ee833f326fc082a69433040a260d0226

CCCCATGGCGCATTAGAAACCCTAGTAGTCCGGCTGACTGACTATGAGCTCATCTCGTATGCCGTCTTCTGCTTGAAAAAAAAAATATTATTATATTTTATTAATTT

>e1a57bb2527ea31a3f2a1b168abf322f

CCCCATGGCGCATTAGAAACCCTTGTAGTCCGGCTGACTGACTTGCGTCAAATCTCGTATGCCGTCTTCTGCTTGAAAAAAAAAAACTAGAGTTTTAAGTAAGAGGG

>25bd5b5762c617bada6ac81c2c7a2046

AGGACTTGGAGGAACGACTCTGATAAACTATATGTTGTACACCAGAGGCAGTTCACGGGATTATAACAAATATGCTCAAGACGGAAACTATGGTTGGAGCTTTAAAG

>a2bdbd8a7a27a52bffdbb792b69b067b

TACGAGGGGAGCGAGTGTTGTTCAGTTTTATTGGGCGTAAAGGGTATGTAGGCGGTTTTGTAAGTCAACACTTAAATCTTGAGACTTAATCTCATTATAGCGTTGAT

>c0ca9d845a098b53087b20b5f99d53d8

CATTGAACTATCGTGAGAAAGTCACGCCGCCAAAGGGAATTATATTATAGTAATTATTGGCGTTAATAAACATTTTAGTAATAGTTGTAATATATCATATTGTGTGA

>e8f8e2b87db4f3270b1c415ebabf4a61

TATTCTCGCGCAAACGTAAGCGGCCTCGGCCGCTTAAAGTAGGCCCCGTCGAGGTGCCCTGGACCAACAGCGTCAAGTACCTCGGGCTCCACGTGGATTCCCGACTC

>11bddbe92f40c0c0ef27454ed587fb71

CCAACACATCAACCATCAGTGACCACGGACCACAGGTCAGACTAATACTCGGCCTGAGTTGTCACAAGCAAATAAGGCCACAAAGTCATGGACAAACAAACGCAGCC

>88f032d027978b119f66ef545ebe6e63

CCCCATGGCGCATTAGATACCCCGGTAGTCCGGCTGACTGACTTCCTCATGATCTCGTATGCCGTCTTCTGCTTGAAAAAAAAAATTAACTTTAAAAGTGCTATTGA

>3e318e2b382f870e979cfae29dac2a7c

TGTTGATGTGGCACCTGGAGACGCGTGGTTATTGGCGAAACGTTGCGTTTGTTCTTTACCAACGGTATCAACCGTATAAGAATACGTTTACGCTTAAAATAATTATA

>915a663a931bf1756c47a525ae153572

TACGTAGGGTGCGAGCGTTAATCGGAATTACTGGGCGTAAAGCGTGCGCAGGCGGTCTTGTAAGACAGAGGTGAAATCCCTGGGCTCAACCTAGGAATGGCCTTTGT

>8a8ff2d7090544e92787b8a989497fbe

GACAGAGGATGCAAGCGTTATCCGGAATGATTGGGCGTAAAGCGTCTGTAGGTGGCTTTTCAAGTCCGCCGTCAAATTCCAGGGCTCAACCCTGGACAGGCGGTAGA

>bc1af3c7e26d4403331c7c0e3e5f5f33

TCCATTAGAAACCCGGGTAGTCCGGCTGACTGACTATGAGCTCATCTCGTATGCCGTCTTCTGCTTGAAAAAAAAAACTTAAAGCTTAACTTTAAAAGTGCTATTGA

>0a2a30f1895ddad16718f37697749d81

CCCCATGGCGCATTAGAAACCCTTGTAGTCCGGCTGACTGACTGAGACTTAATCTCGTATGCCGTCTTCTGCTTGAAAAAAAAAATTAACTTTAAAAGTGCTATTGA

>b366b261124ecaf2a4df6e6abb7df0b3

CCCCATGGCGCATTAGAAACCCCTGTAGTCCGGCTGACTGACTGCGATACGATCTCGTATGCCGTCTTCTGCTTGAAAAAAAAAAATAACTTTAAAAGTGCTATTGA

>9c1bd80719510de1e7a5c110aa757165

TACGTAGGTGGCAAGCGTTATCCGGAATTATTGGGCGTAAAGCGCGCGTAGGCGGTTTTTTAAGTCTGATGTGAAAGCCCACGGCTCAACCGTGGAGGGTCATTGGA

>c3104caa1bec19ed00ffa4c6355c0b0f

GACGGGGGGGGCAAGTGTTCTTCGGAATGACTGGGCGTAAAGGGCACGTAGGCGGTGAATCGGGTTGAAAGTGAAAGTCGCCAAAAAGTGGCGGAATGCTCTCGAAA

>743b83f9890e6f77233b0b74f1a88b20

CATTGAACTATCGTGAGAAAGTCACGCCGCCAAAGGGAATTATATTATAGTAATTATTGGCGTTAATAAACATTTTATTAATAGTTGTAATATATGATAATGTGTGA

>0c7fb353b3af9d3320352b0c3a7ec480

CCCCATGGCGCATTAGAAACCCTGGTAGTCCGGCTGACTGACTACGTACGTATCTCGTATGCCGTCTTCTGCTTGAAAAAAAAAATTAACTTTAAAAGTGCTATTGA

>f0b5dd6b84667e01a538d08d9dfea0c6

CCCCATGGCGCATTAGATACCCCTGTAGTCCGGCTGACTGACTCGCGATATATCTCGTATGCCGTCTTCTGCTTGAAAAAAAAAATTAACTTTAAAAGTGCTATTGA

>49368f895b2bf66d4b117f29cec308c8

CCCCATGGCGCATTAGAAACCCTAGTAGTCCGGCTGACTGACTCGCGATATATCTCGTATGCCGTCTTCTGCTTGAAAAAAAAAAACTAGAGTTTTAAGTAAGAGGG

>6085f48d69943fe37ed7d598794a212a

TACGTAGGGTGCGAGCGTTGTCCGGAATTACTGGGCGTAAAGAGCTCGTAGGTGGTTTGTCGCGTCGTCTGTGAAATTCCGGGGCTTAACTTCGGGCGTGCAGGCGA

>4ea8e9bf00f749278ab631c0757c2035

TCCATTAGATACCCGTGTAGTCCGGCTGACTGACTATGAGCTCATCTCGTATGCCGTCTTCTGCTTGAAAAAAAAAAAATACTTGACTAGAGTTTTAAGTAAGAGGG

>2f39f99ef69c90f0aa622aaa2b332a0f

TCCATTAGATACCCCGGTAGTCCGGCTGACTGACTTAACGTCCATCTCGTATGCCGTCTTCTGCTTGAAAAAAAAAACTTAAAGCTTAACTTTAAAAGTGCTATTGA

>14fb596fdd955f131042dadcb9d3a2ad

TCCCGGTGGTCCATTAGAAACCCCAGTAGTCCGGCTGACTGACTGAGACTTAATCTCGTATGCCGTCTTCTGCTTGAAAAAAAAAAAAATTGTGTGCCAGCAGCCGC

>e2cb201b7ee06d27bcc2b93bb6f4f26b

TCCATTAGAAACCCTTGTAGTCCGGCTGACTGACTCTCTAGAGATCTCGTATGCCGTCTTCTGCTTGAAAAAAAAAAAAATATATTATTATTATATTTTATTAATTT

>c1f6cdf6f9d8b29c1a33f32689b5435b

CCCCATGGCGCATTAGAAACCCCTGTAGTCCGGCTGACTGACTGAGACTTAATCTCGTATGCCGTCTTCTGCTTGAAAAAAAAAAATAACTTTAAAAGTGCTATTGA

>5020f1957705b4d630fd6ee5a558d4e8

TACGTAGGGTGCAAGCGTTAATCGGAATTACTGGGCGTAAAGCGTGCGCAGGCGGTTTTGTAAGTCTGTCGTGAAAGCCCCGGGCTCAACCTGGGAATTGCGATGGA

>832e2f08cc56d500023f51a2fb62da29

TACGGAGGGTGCAAGCGTTATCCGGATTTATTGGGTTTAAAGGGTCCGTAGGCGGATTAATAAGTCAGTGGTGAAAGCCCGCAGCTTAACTGTGGAACTGCCATTGA

>3d14e49a63173562474f583036c38bef

TACAATTTCTTCAATTTAAATTTTAAACAGTTTCAGTTAATATATTATTTAAATTTTTATAAATAATAATTTTAGTGAAATATATTATTATTATATTTTATTAATTT

>0d85fd85119111635f8a6240ab8afca5

CCCCATGGCGCATTAGAAACCCTTGTAGTCCGGCTGACTGACTCGCGATATATCTCGTATGCCGTCTTCTGCTTGAAAAAAAAAAAATAGAGTTTTAAGTAAGAGGG

>29aae829cbda7ba44838917cea56c471

CACGGGGGGCGCAAGCGTTATTCGGAATTATTGGGCGTAAAGGGCGCGCAGGCGGTCTTGTCCGTCAGGTGTGAAAGCTCGGGGCTCAACCCCGGAAGTGCACTTGA

>28fff442ae5622e160db454625bfcd28

CCCCATGGCGCATTAGAAACCCCGGTAGTCCGGCTGACTGACTTAACGTCCATCTCGTATGCCGTCTTCTGCTTGAAAAAAAAAAACTAGAGTTTTAAGTAAGAGGG

>c117c4597caa5c670117610d87423102

TACGGAGGGTGCGAGCGTTAATCGGAATAACTGGGCGTAAAGGGCACGCAGGCGGTGACTTAAGTGAGGTGTGAAAGCCCCGGGCTTAACCTGGGAATTGCATTTCA

>5754667a87ac5d4276b29f7077ba2581

AAGACGGCATGAGATCATTAGATACCCTGGTAGTCCGGCTGACTGACTTCCTCATGATCTCGTATGCCGTCTTCTGCTTGAAAAAAAAAATTTAAAAGTGCTATTGA

>539846cc836e808c64760a50a02da96d

TACAGAGGATGCAAGCGTTATCCGGAATGATTGGGCGTAAAGCGTCTGTAGGTGGCTTTTTAAGTCCGCCGTCAAATCCCAGGGCTCAACCCTGGACAGGCGGTGGA

>80c22f0aebcd9f6c6f94ae8ff7299318

CCCCATGGCGCATTAGATACCCCAGTAGTCCGGCTGACTGACTGAGACTTAATCTCGTATGCCGTCTTCTGCTTGAAAAAAAAAATTAACTTTAAAAGTGCTATTGA

>11951bf500bb3c765f7507901059eba0

CATTGAACTATCGTGAGAAAGTCAAGCCGCCAAAGGGAATTATATTATAGTAAATATTGGCGTAAATAAACATTTTATTAATAGTTGTAATATATGAAAATGTGCAG

>69d90088846a8339d349c7337a7a9187

CCCCATGGCGCATTAGATACCCTTGTAGTCCGGCTGACTGACTCGCGATATATCTCGTATGCCGTCTTCTGCTTGAAAAAAAAAATTAACTTTAAAAGTGCTATTGA

>f22f798bbb157a8f9ea175fcfdd055c1

CCCCATGGCGCATTAGAAACCCTTGTAGTCCGGCTGACTGACTACGTACGTATCTCGTATGCCGTCTTCTGCTTGAAAAAAAAAATTAACTTTAAAAGTGCTATTGA

>821292f17a0024b875fb6e1af0bee724

TTGTACAAATCTTTGTAACTGGATCGGTTCCGCACGAGTGTGTGTGCACTTCCTCTTTAGGCACTGCCAGTAAAATCTCATGACTTCTTAGTATCGACGTGTAGGTT

>61ce99d79d9c800da441b7d1cd4d06d6

TACGTAGGGCGCAAGCGTTGTCCGGAATTATTGGGCGTAAAGAGCTCGTAGGCGGTTTGTCGCGTCTGCTGTGAAAACGCGAGGCTTAACCTCGCGCCTGCAGTGGG

>f9b6824ec3092800832831b8a90da226

ACATTCTTTGTACTTCATTAGTTATTTAATTTACACCACTTGTCACCCCCTTATATTGTACAAATGAGTTTGCTGGCACATTATGTGAAATACACGTTCCTGTTACA

>3f3735f7b18698f2aa569c5586ccbed1

TACGTAGGGTGCGAGCGTTATCCGGAATTATTGGGCGTAAAGAGCTCGTAGGCGGTTTGTCGCGTCTGTCGTGAAAGTCCGGGGCTTAACCCCGGATCTGCGGTGGG

>3703b47da4a6361c638cb655d437bc5e

CCCCATGGCGCATTAGAAACCCGTGTAGTCCGGCTGACTGACTACGTACGTATCTCGTATGCCGTCTTCTGCTTGAAAAAAAAAATTAACTTTAAAAGTGCTATTGA

>ca6210b0f8fbafafd0080494472de017

TCCATTAGAAACCCCAGTAGTCCGGCTGACTGACTATGAGCTCATCTCGTATGCCGTCTTCTGCTTGAAAAAAAAAAAAATATATTATTATTATATTTTATTAATTT

>7f8e0bbe49c7c6c948a6aada23cba30d

CATGGTCATCCAGCCAGATTCCGTATTTATGTACGTGACGCTTGACTTTATACACGTGGAAAGCGATGCTTTAACCACCGGGTGAATTGAGTTATAATAGTTAATTT

>3a0243916fd335e64c9dce1b9729c450

TCCATTAGAAACCCCGGTAGTCCGGCTGACTGACTTCCTCATGATCTCGTATGCCGTCTTCTGCTTGAAAAAAAAAACTTAAAGCTTAACTTTAAAAGTGCTATTGA

>30ea10378ea72cbed43dc185657a55f9

GGCATACGAGATCATTAGAAACCCTAGTAGTCCGGCTGACTGACTTCCTCATGATCTCGTATGCCGTCTTCTGCTTGAAAAAAAAAAAACTTTAAAAGTGCTATTGA

>e33a297fa0cb2e6266f12e39d1faba7c

TACAGAGGGTGCAAGCGTTAATCGGAATTACTGGGCGTAAAGCGCGCGTAGGTGGTTTGTTAAGTTGAATGTGAAATCCCCGGGCTCAACCTGGGAACTGCATCCAA

>ba2f768327281682bcc0fe02ff6e6ec5

TACGTAGGGTGCGAGCGTTAATCGGAATTACTGGGCGTAAAGCGTGCGCAGGCGGTTGTGTAAGACAGGCGTGAAATCCCCGGGCTCAACCTGGGAATGGCGCTTGT

>e2632104f2015e93f246b2b575411d38

GACAGAGGGTGCAAACGTTGTTCGGAATTACTGGGCGTAAAGCGTGTGTAGGCGGCCATGTAAGTTGGATGTGAAAGCCCCGGGCTCAACCCGGGAAGTGCATTCAA

>b240ea6e235f98339dd0678f528f5ff5

TACGGAGGGGGCTAGCGTTGTTCGGAATTACTGGGCGTAAAGCGCACGTAGGCGGCTTGGTAAGTTAGAGGTGAAAGCCCAGGGCTCAACCCTGGAATTGCCTTTAA

>63e74c7080c5701f62c85a10e986fa61

TACGAAGGGGGCTAGCGTTGCTCGGAATCACTGGGCGTAAAGGGTGCGTAGGCGGGTTTTTAAGTCAGGGGTGAAATCCTGGAGCTCAACTCCAGAACTGCCTTTGA

>e50ee5a0d02647a8bebd287a7874ef3a

CATTGAACTATCGTGAGAAAGTCAAACCGCCAAAGGGAATTATATTATAGTAAATATTAGCGTAAATAAACATTTTATTAATAGTTGTAATATATGAAAATGTGCAG

>479af1a242209857c2f4ee290130f18f

CCCCATGGCGCATTAGAAACCCGGGTAGTCCGGCTGACTGACTTGCGTCAAATCTCGTATGCCGTCTTCTGCTTGAAAAAAAAAATTAACTTTAAAAGTGCTATTGA

>27464f046b45c46a478dc23233454bf6

CCCCATGGCGCATTAGATACCCGAGTAGTCCGGCTGACTGACTCGAGCTAGATCTCGTATGCCGTCTTCTGCTTGAAAAAAAAAAACTAGAGGTTTAAGTAAGAGGG

>4d03596b63121b8892e8b42e6dc9a9fe

TACGATTTCTTTAATTTAAATAGTTAAGTTTCAGTTAATATAACAATAATATAAAATATCTATAATTTTGGTGAAATATATTTTATCTTGAAAAATTAATTTTATGT

>87b5abd7f7e41d13aa3c3140b1cf5d30

TTAGAAACCCCTGTAGTCCGGCTGACTGACTGTTACAGCATCTCGTATGCCGTCTTCTGCTTGAAAAAAAAAAAAATCTTAAAGCTTAACTTTAAAAGTGCTATTGA

>c68b7df01330e2cfdf0a917de386ae61

TCCATTAGATACCCCTGTAGTCCGGCTGACTGACTGAGACTTAATCTCGTATGCCGTCTTCTGCTTGAAAAAAAAAAATTAAAGCTTAACTTTAAAAGTGCTATTGA

>3655a680863b022fcddb90f59742c875

TACGGAGGGTGCGAGCGTTAATCGGAATTACTGGGCGTAAAGCGCATGCAGGTGGTTCATTAAGTCAGATGTGAAAGCCCGGGGCTCAACCTCGGAACTGCATTTGA

>ec3e76993db28145b0f9271b47a736bf

TACGGAGGGTGCGAGCGTTAATCGGAATGACTGGGCGTAAAGCGCACGCAGGCGGTCAATTAAGTTGGATGTGAAATCCCCGGGCTTAACCTGGGAACGGCATCCAA

>a6693edf30776708316cc3eb62620f1f

CATTGAACTATCGTGAGAGAGTCAAGCCGCCAAAGGGAATTATATTATAGTAAATATTGGCGTAAATAAACATTTTATTAATAGTTGTAATATATGAAAATGTGCAG

>83e4f401ab59fbda29412033b45913e8

TACGAGGGGAGCGAGTGTTGTTCGGTTTTATTGGGCGTAAAGGGTACGTAGGCGGTTTTGTTAGTCAACAATTAAATCTTGGAACTTAATTCCATAGCAGTTGTTGA

>4a78ebd4da8ecb48310fdae8768f6d99

TTAGAAACCCGTGTAGTCCGGCTGACTGACTACTGTGTAATCTCGTATGCCGTCTTCTGCTTGAAAAAAAAAATGCTAATACTTGACTAGAGTTTTAAGTAAGAGGG

>09fd359de8c17c63e194f94ef211d325

CCCCATGGCGCATTAGAAACCCCAGTAGTCCGGCTGACTGACTGTTACAGCATCTCGTATGCCGTCTTCTGCTTGAAAAAAAAAATTAACTTTAAAAGTGCTATTGA

>166c1ea00263a019ee499d2f5d85cbc8

TCCATTAGAAACCCTGGTAGTCCGGCTGACTGACTCTACGACCATCTCGTATGCCGTCTTCTGCTTGAAAAAAAAAACTTAAAGATTAACTTTAAAAGTGCTATTGA

>7a57ca4e2a78e754f17152a186f6d765

CATTGAACTATCGTGAGAAAGTCAAACCGCCAAAGGGAATTATATTATAGTAAATATTGGCGTAAATAAACATTTTATTAATAGTTGTAATATATGAAAATGTGCAG

>0d236be2d7e808c64da544c33eb9129c

GACGGGGGGGGCAAGTGTTCTTCGGAATGACTGGGCGTAAAGGGCACGTAGGCGGTGAATCGGGTTGAAAGTGAAAGTCGCCAAAAACTGGCGGAATGCTCTCGAAA

>f7d2b5b1a960fb31183b66d8a876481f

TACAGAGGGTGCAAGCGTTGTTCGGAATTACTGGGCGTAAAGCGTGCGTAGTCGGTATTGAGAGTCACGGGTGAAATCCCAGGGCTTAACCCTGGAACTGCCTGTGA

>c8435aba9a2c2e408ea4264b81343fa8

TACGAGGGGAGCGAGTGTTGTTCGGTTTTATTGGGCGTAAAGGGTGTTCAGGTTGTTCAATAAGTTGATCTCTAAATCTTGGAACTCAACCCCATTCAAGGGGTCAA

>2b0fe9daf2d063d611a28cc676f5d9d1

AAATTCAGGTAACTGCACATCGTAAGAAATTAGAAACCCTTGTAGTCCGGCTGACTGACTATGAGCTCATCTCGTATGCCGTCTTCTGCTTGAAAAAAAAAAAAAGA

>ae9ebcd8cc2562a16bf6a4c3322b1789

TACGAGGGGAGCGAGTGTTGTTCGGTTTTATTGGGCGTAAAGGGTATGTAGGCGGTTTTGTAAGTCAACAACCAAATCTTGGAACTTAATTCCATAGCGTATGTTGA

>db7fe8e59dad0518333bcaaaec5df36d

TACGGAGGGTGCGAGCGTTAATCGGAATCACTGGGCGTAAAGCGCACGTAGGCTGCTTGGTAAGTCAGGGGTGAAAGCCCGCGGCTCAACCGCGGAATTGCCTTTGA

>6417c062d9eb9d544f49e17358a39883

TACGTAGGGTGCGAGCGTTAATCGGAATTACTGGGCGTAAAGCGTGCGCAGGCGGTTATGTAAGACAGATGTGAAATCCCCGGGCTCAACCTGGGAACTGCATTTGT

>572d135c7702577aee1b0a5666c2e530

TACGTAGGGTGCGAGCGTTGTCCGGAATTACTGGGCGTAAAGGGCTCGTAGGTGGTTTGTCGCGTCGTCTGTGAAATTCCGGGGCTTAACTCCGGGCGTGCAGGCGA

>fce7d43212f63e3f215aa65b2db19b25

TACGTAGGGCGCAAGCGTTATCCGGAATTATTGGGCGTAAAGAGCTCGTAGGCGGTTTGTTGCGTCTGCTGTGAAAGACCGGGGCTCAACTCCGGTTCTGCAGTGGG

>15aee9fc8e5f63ecfb6fe11c65a53878

TCCATTAGAAACCCTTGTAGTCCGGCTGACTGACTCTACGACCATCTCGTATGCCGTCTTCTGCTTGAAAAAAAAAACTTAAAGCTTAACTTTAAAAGTGCTATTGA

>4d0401b47252fba6eaa1ceac148152d8

TACGAAGGGGGCTAGCGTTGCTCGGAATGACTGGGCGTAAAGGGCGTGTAGGCGGTTTGTACAGTTAGATGTGAAATCCCCGGGCTTAACCTGGGAGCTGCATTTAA

>ab2c45cb4d472067e460518c4f6b1e86

CATTGAACTATCGTGAGAAAGTCACGCCGCCAAAGGGAATTATATTATAGTAATTATTGGCGTTAATAAACATTTTATTAATAGTTGTAATATATGATAACGTGTAA

>2f920005d0f15cdd242e60e942460d4f

TACGAAGGGTGCAAGCGTTAATCGGAATTACTGGGCGTAAAGCGCGCGTAGGTGGTTTGATAAGTTGGATGTGAAAGCCCCGGGCTCAACCTGGGAATTGCATCCAA

>c150b70ec7a96a96deb4e1df88d800c2

TCCATTAGATACCCGAGTAGTCCGGCTGACTGACTCGAGCTAGATCTCGTATGCCGTCTTCTGCTTGAAAAAAAAAACTTAAAGCTTAACTTTAAAAGTGCTATTGA

>bb516ed60919c27ab77ee5d052960ef9

CCCCATGGCGCATTAGAAACCCCGGTAGTCCGGCTGACTGACTTACTAGGTATCTCGTATGCCGTCTTCTGCTTGAAAAAAAAAAATAACTTTAAAAGTGCTATTGA

>1ea8a1eed1dd67e4548a0e1de9f01d74

CACATTAGAAACCCCGGTAGTCCGGCTGACTGACTTCTAGACTATCTCGTATGCCGTCTTCTGCTTGAAAAAAAAAACTTAAAGCTTAACTTTAAAAGTGCTATTGA

>6d12e5a56124bef4c1251e2b2b34797d

TACGTAGGGCGCAAGCGTTGTCCGGAATTATTGGGCGTAAAGAGCTCGTAGGCGGTCTGTCGCGTCTGCTGTGAAATCCCGAGGCTCAACCTCGGGCTTGCAGTGGG

>5405d3a5959a9eb18402e41d1aebfde8

TACATAGGGGGCAAGCGTTATCCGGAATTATTGGGCGTAAAGGGTGCGTAGGCGGTTAAATAAGTTTATGGTCTAAGTGCAATGCTCAACATTGTGATGCTATAAAA

>2cd99077c58922830f04d5a01e6d4c64

CACGATTAACCCAAGTCAATAGAAGCCGGCGTAAAGAGTGTTTTAGATCACCCCTCCCCAATAAAGCTAAAACTCACCTGAGTTGTAAAAAACTCCAGTTGACACAA

>f61aad62249765cbadde4b99f3755093

TACAGAGGGTGCAAGCGTTAATCGGAATTACTGGGCGTAAAGCGCGCGTAGGTGGTTTGTTAAGTTGGATGTGAAATCCCCGGGCTCAACCTGGGAACTGCATCCAA

>89918f9f242cb04f080df2e2cac14af6

CTGGCACACAATTACCATATAGTAATTAGATACCCGGGTAGTCCGGCTGACTGACTTCCTCATGATCTCGTATGCCGTCTTCTGCTTGAAAAAAAAAAAAAAAAACA

>0e38276703e868b01a423718bb958d78

TACGGAGGGTGCAAGCGTTAATCGGAATTACTGGGCGTAAAGCGCACGCAGGCGGTCTGTCAAGTCGGATGTGAAATCCCCGGGCTCAACCTGGGAACTGCATTCGA

>8fe4b3db1cb5dd81b54084c497b0f161

TCCATTAGAAACCCGGGTAGTCCGGCTGACTGACTCGAGCTAGATCTCGTATGCCGTCTTCTGCTTGAAAAAAAAAACTTAAAGCTTAACTTTAAAAGTGCTATTGA

>2fb46ef89433436402bc8ff6e24f6a38

TACGAAGGGGGCTAGCGTTGCTCGGAATTACTGGGCGTAAAGGGAGCGTAGGCGGACATTTAAGTCAGGGGTGAAATCCCGGGGCTCAACCTCGGAATTGCCTTTGA

>b18ca441b23ff91c1598a1a733c0fa3e

TTAGAAACCCGAGTAGTCCGGCTGACTGACTTAACGTCCATCTCGTATGCCGTCTTCTGCTTGAAAAAAAAAAAAATCTTAAAGCTTAACTTTAAAAGTGCTATTGA

>19036d25d1f36d78422dba0eeaac113c

TTAGATACCCCTGTAGTCCGGCTGACTGACTTCTAGACTATCTCGTATGCCGTCTTCTGCTTGAAAAAAAAAAAAATCTTAAAGCTTAACTTTAAAAGTGCTATTGA

>bcb8644bd67d653ace4aff1395342bd8

TTCCAGCTCCAATAGCGTATATTAAAGTTGTTGCGGTTAAAAAGCTCGTAGTTGGATCTGTGTGCCACGCTGTCGGTTCACCGCCCGTCGGTGTCAACTGGCATGTC

>83410df232615ff8cc7443522012119a

CCCCATGGCGCATTAGAAACCCTAGTAGTCCGGCTGACTGACTCTCTAGAGATCTCGTATGCCGTCTTCTGCTTGAAAAAAAAAATTAACTTTAAAAGTGCTATTGA

>e1753406085b572782387b71943d3954

TACGAGGGGAGCGAGTGTTGTTCAGTTTTATTGGGCGTAAAGGGTATGTAGGCGGTTTTGTAAGTCAACAATTAAATCTTGGAACTTAATTCCATTACAATTGTTGA

>455e725b3a8fbbf0a5cc1df8c72e91d3

CCCCATGGCGCATTAGATACCCGAGTAGTCCGGCTGACTGACTTAACGTCCATCTCGTATGCCGTCTTCTGCTTGAAAAAAAAAATTAACTTTAAAAGTGCTATTGA

>b132521a1616b789f80df767c96c00c4

CGAACGATCGCATACTTGTCCTCTCATTAGAAACCCTAGTAGTCCGGCTGACTGACTCGCGATATATCTCGTATGCCGTCTTCTGCTTGAAAAAAAAAAAAAAAAAA

>aa86b1ec56338d46eb19f3cc11e7dccc

GTGCTCAAAGAACATCAATCTTTCACTTCGTTCTCACTTTCTCTTTTTACTGCCTCCTCTTCTGACCCTTTTCCACCATACCCCTTCCTCTCCTTTTCGTCATCCCT

>531bc35d211c7cb2bf894ef91a005fab

TCCATTAGAAACCCCGGTAGTCCGGCTGACTGACTGCGATACGATCTCGTATGCCGTCTTCTGCTTGAAAAAAAAAAAAATATATTATTATTATATTTTATTAATTT

>41dd57780b447a70dcf1beea79510f9f

TACGTAGGGTGCAAGCGTTAATCGGAATTACTGGGCGTAAAGCGTGCGCAGGCGGTTTTGTAAGTTTGTCGTGAAATCCCCGGGCTCAACCTGGGAATGGCGATGAA

>3ede73cdc09e2a14e80fcd9628fc6957

TACGAAGGGTGCAAGCGTTACTCGGAATTACTGGGCGTAAAGCGTGCGTAGGTGGTCGTTTAAGTCCGTTGTGAAAGCCCTGGGCTCAACCTGGGAACTGCAGTGGA

>8127f3f22db4fb89fd6e1500e8136738

CATGGTCATCCAGCCAGATTTCGTATTTATGTACGTGACGTTTGACTTTATTCACGTGGAAAGCGATGCTTTAACCACCGGGTGAATTGAGTTATAATAGTTAATTT

>5318afaeec791e784b8106861d23ddbd

TACGAGGGGGGCAAGCGTTGTTCGGAATTATTGGGCGTAAAGGGCGCGTAGGCGGTTTGGCAAGTTTGGTGTGAAATCTTCGGGCTCAACTCGAAGTCTGCACCGAA

>af2942b4ffe57d2be7346230895b4639

CCCCATGGCGCATTAGAAACCCCAGTAGTCCGGCTGACTGACTCTATCGTGATCTCGTATGCCGTCTTCTGCTTGAAAAAAAAAATTAACTTTAAAAGTGCTATTGA

>30846242711111d4fedd325dc5396998

TACGAAGGGGGCTAGCGTTGTTCGGAATCACTGGGCGTAAAGCGCACGTAGGCGGACTTTTAAGTCAGGGGTGAAATCCCAAGGCTCAACCTTGGAACTGCCTTTGA

>44a28c46a3872405f0e2ef031586c803

TACGAGGGGAGCGAGTGTTGTTCAGTTTTATTGGGCGTAAAGGGTGTTCAGGTGGCTAAGCAAGTTAACAACAAAATCTTGAGGCTCAACCTCATAACGTTCGGTTA

>a4cf57b34ed382aaf113e8a900db22db

CCCCATGGCGCATTAGAAACCCTTGTAGTCCGGCTGACTGACTCTACGACCATCTCGTATGCCGTCTTCTGCTTGAAAAAAAAAATTAACTTTAAAAGTGCTATTGA

>6962a281dedc661c23412a22b060e207

TAGTGATGTTTTGGCTGACTTTATCTGCTGCTTTGTTACAGTCAAGCTTAATAAAACCACCATAAACACACACATTAAGTAAGATGCTAACTCAAGTCACACACAAC

>d1c7e31fa94165a22908e39d90dc1970

CAAATTTATGTCTTCAGAAATACAAAATATTTTTCCAGGGTCCATTTGAACCATGTCGGTCATTTTCAACTAGCAGTGCCTTGTAGATATTTGTGAAAGATTTTGAA

>b46e69a2f3d7802337f1b02e76b26e72

TACGTAGGGTGCGAGCGTTAATCGGAATTACTGGGCGTAAAGCGTGCGCAGGCGGTTTTGTAAGACAGGCGTGAAATCCCCGAGCTCAACTTGGGAATGGCGCTTGT

>a79007da1638b2adf9b12d1570117061

ATATCGTCAGTCCCTGTTCTTAGATGTGATCTTTCCAGAGCCGCACTGAGGCACACACACAACAGGGCGCACAGATCAGAAACATCCTAGAATGTCTATTCTTACAT

>2f311c2bf31ec3bff49009b42ab64b4d

CCCCATGGCGCATTAGATACCCTGGTAGTCCGGCTGACTGACTACGTACGTATCTCGTATGCCGTCTTCTGCTTGAAAAAAAAAATTAACTTTAAAAGTGCTATTGA

>d733a05b4615745760e8b49708024979

TACTGTATCCAAGCATATAGACATCAAATTGATTGACAAATCTATCATATTGTCAAAGTATAGTCGGTATAAAATCATATAACTGTTATCACCTTCGGAAAAATCGT

>a5ddd1095793bbd459119b0d5295d80f

CCCCATGGCGCATTAGATACCCCAGTAGTCCGGCTGACTGACTTCCTCATGATCTCGTATGCCGTCTTCTGCTTGAAAAAAAAAAAAAACTTTAAAAGTGCTATTGA

>8302db9771c230804ec90c95e907b984

CAAATTTATGTCTTCAGAAAGACAAAATATTTTTCCAGGGTCCATTTGAACCATGTCGGTCATTTTCAACTAGCAGTGCCTTGTAGATATTTGTGAAAGATTTTGAA

>ee85c7f93730a8e237195a660548234b

TACAGAGGGTGCAAGCGTTAATCGGAATTACTGGGCGTAAAGCGTGCGTAGACGGTTACATAAGTCGGGTGTGAAAGCCCCGGGCTCAACCTGGGAATTGCATTCGA

>0711c5dd4f4264290b852a8d381b1ef0

TACGTAGGGTGCGAGCGTTGTCCGGATTTATTGGGCGTAAAGGGCTCGTAGGTGGTTGATCGCGTCGGAAGTGTAATCTTGGGGCTTAACCCTGAGCGTGCTTTCGA

>305ac3a3ebe700be41388ce18e34ae24

TACGAGGGGAGCGAGTGTTGTTCGGTTTTATTGGGCGTAAAGGGCACTTAGGTTGCTTTATAAGTTAACAACCAAATCTTGGAACTCAATTCCATTCCGGTTGTTAA

>5a3ae5dddebbc8e03909d21f2888884a

TACGTAGGTCCCGAGCGTTGTCCGGATTTATTGGGCGTAAAGCGAGCGCAGGCGGTTAGATAAGTCTGAAGTTAAAGGCTGTGGCTTAACCATAGTACGCTTTGGAA

>6992325e2d343ddfc4d3c503c2a9407d

CATTTTACCATAGTGTCTGCCTCCACGGGCACGTTTATGGCTTTGCCGGTTATCTTTTAAGACTGTTTCCATTCTGAGACGACGGATCTGCATACGTGGAAATCCGG

>16b2c2dd859f4b622a12747f8480a10c

GACAGAGGATGCAAGCGTTATCCGGAATGATTGGGCGTAAAGCGTCTGTAGGTGGCTTTTCAAGTCCGCCGTCAAATCCCAGGGCTCAACCCTGGACAGGCGGTGGA

>6389b25a7a7aaf549eb32e11781ae7de

TACATAGGGGGCAAGCGTTATCCGGAATTATTGGGCGTAAAGGGTGCGTAGGCGGTTAAATAAGTTTATGGTCTAAGTGCAATGCTTAACGTTGTGATGCTATAAAA

>c4235f83fb2febbbacd44d33bb8696fd

TACGTAGGTGGCAAGCGTTATCCGGAATTATTGGGCGTAAAGCGCGCGTAGGTGGTTTTTTAAGTCTGATGTGAAAGCCCACGGCTCAACCGTGGAGGGTCATTGGA

>02a29bb9f1ffb21a0c9947e7d7c5dc5a

TACGGAGGGTGCGAGCGTTGTCCGGAATCACTGGGCGTAAAGGGCGCGTAGGTGGCAGGGGCAAGCGTGCGGTGAAAGCCCGGGGCTCAACTCCGGGTCGGCCGTGC

>74a75731b65980bb93cd933a5c973d96

TACGAGGGGAGCGAGTGTTGTTCGGTTTTATTGGGCGTAAAGGGTATGTAGGCGGTTTTGTAAGTCAATATTTAAATCTTGAGACTTAATCTCATTACAGGTGTTGA

>49323799e1bef25708de08b9663558c0

CTCGTATCATACTACACGCTACACACTGCTTTGCTCTCCTTAACACCAAACATGATATAAAGTTTTACACCAATATAAATCAATATTACACTACAGACCAACGATAC

>518b121a12cbf417ff6a6b4c4980eba1

ACATTCTTTGTACTTCATTAGTTATTTAATTTACACCACTTGTCACCCCCTTATATTGTACAAACGAGTTTGCTGGCACATTTTGTGAAATACACGTTCCTGTTACA

>3d3fe5d2f6fbcd3e3daec1ab997807b8

TACGAGGGGTGCAAGCGTTATTCGGAATTATTGGGCGTAAAGGGTGCGTAGGCGGCATATTAAGTCAACTGTTAAATTCCTCGGCCTAACCGAGGCTCTGCGGTAGA

>d402c4ef43aa19c4646960af9bf1c1bd

TACAGAGGGTGCGAGCGTTAATCGGATTTACTGGGCGTAAAGCGTGCGTAGGCGGCTTTTTAAGTCGGATGTGAAATCCCTGAGCTTAACTTAGGAATTGCATTCGA

>dc46f4801c3a07ad1b60aadcc6a4e90f

TACGTAGGTGGCAAGCGTTGTCCGGATTTATTGGGCGTAAAGCGAGCGCAGGCGGTTTCTTAAGTCTGATGTGAAAGCCCCCGGCTCAACCGGGGAGGGTCATTGGA

>6dfbeb900324194402f8f91cd82fffb6

TACAGAGGGTGCAAGCGTTAATCGGAATTACTGGGCGTAAAGCGCGCGTAGGTGGTTAGTTAAGTTGGATGTGAAATCCCCGGGCTCAACCTGGGAACTGCATTCAA

>ce8fa67177d3850ff97cc22bdcbd28a7

TTCCAGCTCCAATAGCGTATATTAAAGTTGTTGCAGTTAAAACGCCCGTAGTCGAATTTTTGAGGGTAGGAAGGTTAAGAGGGGTGCTGGCTTTTGTTAGTATTCTT

>512b31feef22df0a292b1058ecc54f4a

TACGAGGGGAGCGAGTGTTGTTCGGTTTTATTGGGCGTAAAGGGCACTTAGGCTGTTTTACAAGTTAACAGTTAAATCTTGGAACTCAATTCCATGTCAATTGTTAA

>910310509a4e2e59d2872f753a18e8fd

CATTGAACTACCGTGAGAGAGTCAAGCCGCCAAAGGGAATTATATTATAGTAAATATTGGCGTAAATAAACATTTTATTAATAGTTGTAATATATGAAAATGTGCAG

>c3e223ec5ce9baa46ee2df30666bd7c7

TCCATTAGAAACCCCGGTAGTCCGGCTGACTGACTAGTCGCAGATCTCGTATGCCGTCTTCTGCTTGAAAAAAAAAACTTAAAGCTTAACTTTAAAAGTGCTATTGA

>6961f4967fc0a33bd1a989be6719b11b

TACGGAGGGAGCTAGCGTTGTTCGGAATTACTGGGCGTAAAGCGCACGTAGGCGGCGATTTAAGTCAGAGGTGAAAGCCCGGGGCTCAACCCCGGAACTGCCTTTGA

>05ad55dfd1e6e1b2dcf00e6779816809

CCCCATGGCGCATTAGAAACCCCAGTAGTCCGGCTGACTGACTACGTACGTATCTCGTATGCCGTCTTCTGCTTGAAAAAAAAAATTAACTTTAAAAGTGCTATTGA

>24287e1a8cc5d89eacfe6057315f0fb0

CAAATTTATGTCTTCAGAAAGACAAAATATTTTTCCAGGGTCCATTTCAACCATGTCGGTCATTTTCAACTAGCAGTGCCTTGTAGATATTTGTGAAAGATTTGGAA

>a11f13da9de2f7ac195530c537fba54d

CATTGAACTATCGTGAGAAAGTCAAACCGCCAAAGGGAATTATATTATAGTAAATATTGGCGTAAATAAACATTTTATTAATAGTTGTAATATATGATAATGTGCAG

>ece8c9942ba10428a2be703419033281

CCCCATGGCGCATTAGATACCCGAGTAGTCCGGCTGACTGACTTGCGTCAAATCTCGTATGCCGTCTTCTGCTTGAAAAAAAAAATTAACTTTAAAAGTGCTATTGA

>ea1ff8d1a60726281f3dee01e120ec62

CACGATTAACCCAAGTCAATAGAAGCCGGCGTAAAGAGCGTTTTAGATCACCCCCTCCCCAATAAAGCTAAAACTCACCTGAGTTGTAAAAAACTCCAGTTGACACA

>33fc34ba965c476802f79bcde9830807

CATGGTCATCCAGCCAGATTTCGTATTTATGTACGTGACGCTTGACTTTATTCACGTGGAAAGCGATGCTTTAACCACACGACCGTACCACCGGGTGAATTGAGTTA

>1e669e56f82acd9177cc4f1c01fe42c6

TCATGGACAACGGAGAAGAATATATCCCAACTGAAACAAACCAAGGCATAAGACAAGTTTATTGTCTGTCACCACTACTTTTTAACCTATATCTAGATGATCTCCTC

>0b1942bfde1d546bf08d94c9d0917191

TACAATTTCTTCAATTTAAATTTTTAAAAGTTTCAGTTAATAATTTATTTAAATTTCTATAAATAATAATTTTAGTGAAATATATTATTATTATATTTTATTAATTT

>fdda17bb489459c45b6f37f8a75987a3

TACAATTTCTTCAATTTAAATTTTAAAAAGTTTCAGTTAATAGATTATTTAAATTTTTATAAATAATAATTTTAGTGAAATATATTATTATTATATTTTATTAATTT

>4e71a411b6549830d9ea196eb1286e43

CACGTAGGGTGCGAGCGTTGTCCGGAATTATTGGGCGTAAAGGGCTCGTAGGCGGTTTGTCGCGTCGGGAGTGAAAACTCAGGGCTCAACCCTGTTCGTGCTTCCGA

>50edc2b74dea17d897569bdc4fe7a790

TACGAGGGGAGCGAGTGTTGTTCAGTTTTATTGGGCGTAAAGGGTGTTCAGGTGGCCAAGCAAGTTAACAACAAAATCTTGAGATCCAACCTCATAACGTTCGGTTA

>f349b222f71fdccc2ec4052ede6db715

TACGAGGGGAGCGAGTGTTGTTCGGTTTTATTGGGCGTAAAGGGTATGTAGGCGGTTTTGTAAGTCAACAATTAAATCTTGAAGCTTAACTTCATTAGCGGTTGTTG

>c416ac66d0e2de79799230c4f05e4b1f

TACGGAAGGTCCAGGCGTTATCCGGATTTATTGGGTTTAAAGGGTGCGTAGGCCGTTTGATAAGCGTGCTGTGAAATATAGTGGCTCAACCTCTATCGTGCAGCGCG

>d96d3e77fee6fac0911f3bb567f8c930

CAAATTTATGTCTTCAGAAAGACAAAATATTTTTCCAGGGTCCATTTCAACCATGTCGGTCATTTTCAACTAGCAGTGCCTTGTAGATATTTGTGAAAGATTTTGAA

>e9c8b6cb8bf2cc5b752c4fb0c46325f4

TTAGATACCCCGGTAGTCCGGCTGACTGACTTCTAGACTATCTCGTATGCCGTCTTCTGCTTGAAAAAAAAAAAAATCTTAAAGCTTAACTTTAAAAGTGCTATTGA

>8249fa4e8704ea1313ae2d74c2fe1b5b

TACGTAGGTGGCAAGCGTTGTCCGGAATTATTGGGCGTAAAGCGCGCGCAGGCGGTCCTTTAAGTCTGATGTGAAAGCCCACGGCTCAACCGTGGAGGGTCATTGGA

>1bca6c86eb352ce9621d5f19fc414c89

TACGTAGGGTGCAAGCGTTGTCCGGAATTACTGGGCGTAAAGAGTTCGTAGGCGGTTTGTCGCGTCGTTTGTGAAAACCAGCAGCTCAACTGCTGGCTTGCAGGCGA

>6302d553d353e63904b4cd3675063f8a

TACGAGGGGAGCGAGTGTTGTTCGGTTTTATTGGGCGTAAAGGGCTATTAGGTGGCTCAATAAGTCGATAGTTAAATCTTGGAACTTAATTCCATCTATGTTATCGA

>4f9c0faa2c06ddc8c7c3c8fcaaeb5db9

GGTTATGTTCGTCAACACCGGGCAAACCCGGTCATATAATTTTACCATACACTATAACACGTTGTGGTCGAGATTGCAGTGCGTGGTGAGGTTATGTTCATCATATA

>7460f1c90bd0ce79181d9e43b201a649

CATTGAACTATCGTGAGAAAGTCACGCCGCCAAAGGGAATTGTATTATAGTAAATATTGGCGTAAATAAACATTTTATTAATAGTTGTAATATATGAAAATGTTCAG

>9e704ebb1815e9d9754b7a63c05dfdbc

CCCCATGGCGCATTAGAAACCCCTGTAGTCCGGCTGACTGACTCTATCGTGATCTCGTATGCCGTCTTCTGCTTGAAAAAAAAAATTAACTTTAAAAGTGCTATTGA

>539742e8b29d67a3f470d1a72b350617

TACGATTTTCTAAATTTAATTATGTTAGTTTCAGTTAAAAAATGTGTTGATATTCAATTTTTTTAAATTTTGGTGGAATAAAATATAAATATGTGTTTGATTTTATG

>7480c8664b8750365dabf04a1a66e653

AGGAATGAGATATCCGCATCTTCGATATTCTCATTTGATCGCTGTTCACTCAATTTCTCATTTTTAACGCAACTTAAAAACAATTCTCTAAGATTGCACGTTTCTAA

>b224692f8601f5941e31276ca69dbf99

CCCCATGGCGCATTAGAAACCCCTGTAGTCCGGCTGACTGACTACGTACGTATCTCGTATGCCGTCTTCTGCTTGAAAAAAAAAAACTAGAGTTTTAAGTAAGAGGG

>e1d2b09eac338d0cfd105cab549e0967

TACGGAGGGAGCTAGCGTTGTTCGGAATTACTGGGCGTAAAGCGCGCGTAGGCGGCTACTCAAGTCAGAGGTGAAAGCCCGGGGCTCAACCCCGGAACTGCCTTTGA

>76076d2199562b2ef1aac6c01e02f1c0

TACGTAGGTGGCAAGCGTTGTCCGGAATTATTGGGCGTAAAGCGCGCGCAGGCGGATTGGTCAGTCTGTCTTAAAAGTTCGGGGCTTAACCCCGTGATGGGATGGAA

>0c7fd14dcd26ad935ea932b411ea0340

TACGTATGTCGCAAGCGTTATCCGGAATTATTGGGCTTAAAGGGCATCTAGGCGGTATAACAAGTTGAAGGTGAAAAGCTGTAGCTCAACTATAGTCTTGCCTACAA

>e082d19e463e23c00739626977ce881c

TACGTAGGGTGCAAGCGTTAATCGGAATTACTGGGCGTAAAGCGTGCGCAGGCGGTTTTGTAAGTCTGACGTGAAATCCCCGGGCTCAACCTGGGAATTGCGTTGGA

>27226cce530c5af229486655eb109a85

TCATGGACAACGGAGAAGAATATATCCCAACTGAAACAAACCAAGGCGTAAGACAAGTTTATTGTCTGTCACCACTACTTTTTAACCTATATCTAGATGATCTCCTC

>91357a4d378601f227ab6def4a33f778

TGTTGATGTGGCACCTGGAGACGCCTGGTTATTGGCGAAACGTTGCGTTTGTTCTTTACCAACGGTATCAACCGTATAAGAATACGTTTACGCTTAAAATAATTATA

>211c43a39fafa0b23f4965c041210456

TATTCTCGCGAAAACGTAAGCGGCCTCGGCCGCTTAAAGTAGGCCCCGTCGAGGTGCCCTGGACCAATAGCGTCAAGTACCTCGGGCTCCACGTGGATTCCCGACTC

>3767952a1c612fb2607bd266e3d07b67

TACGAAGGGTGCAAGCGTTACTCGGAATTACTGGGCGTAAAGCGTGCGTAGGTGGTTGTTTAAGTCTGTTGTGAAAGCCCTGGGCTCAACCTGGGAACTGCAGTGGA

>8daaa48f5bd4784e14fa17c24fc9ce37

TACGGAGGGTGCAAGCGTTAATCGGAATTACTGGGCGTAAAGCGCGCGTAGGTGGTTTGATAAGCGAGATGTGAAAGCCCCGGGCTCAACCTGGGAACGGCATTTCG

>54836850bc1e4e64eab05a2115cbdb43

TACGTAGGGTGCGAGCGTTGTCCGGAATTACTGGGCGTAAAGAGCTCGTAGGTGGTTTGTCGCGTCGTTTGTGTAAGCCCGCAGCTTAACTGCGGGACTGCAGGCGA

>00dd827b0da0a8be02bde1ca0b4f52e1

TACGAAGGGGGCTAGCGTTGTTCGGATTTACTGGGCGTAAAGCGCACGTAGGCGGACTTTTAAGTCAGGGGTGAAATCCCGGGGCTCAACCCCGGAACTGCCTTTGA

>92c23189e11e5caaf2353901f0174c73

TCCATTAGAAACCCTTGTAGTCCGGCTGACTGACTCGAGCTAGATCTCGTATGCCGTCTTCTGCTTGAAAAAAAAAAAAATATATTATTATTATATTTTATTAATTT

>e740c56e8123d600df4f5be76c70a662

TACGAAGGGAGCGAGTGTTGTTCGGTTTTATTGGGCGTAAAGAGCACTTAGGCTGTTTTACAAGTTAACAGATAAATCTTGGAACTCAATTCCATGTCAATTGTTAA

>f5bc0189c0e25325e805164466b81d0e

TTAGATACCCCGGTAGTCCGGCTGACTGACTCGAGCTAGATCTCGTATGCCGTCTTCTGCTTGAAAAAAAAAATGCTAATACTTGACTAGAGTTTTAAGTAAGAGGG

>e6e33998e66dba61afeee8a0feb2a0f2

TACGGAGGATCCGAGCGTTATCCGGATTTATTGGGTTTAAAGGGAGCGTAGATGGATGTTTAAGTCAGTTGTGAAAGTTTGCGGCTCAACCGTAAAATTGCAGTTGA

>1daac1863914f16a3bf467bff67b502d

TACGAAGGGAGCTAGCGTTGTTCGGAATCACTGGGCGTAAAGCGCACGTAGGCGGATATGTCAGTCAGGGGTGAAATCCCGGAGCTCAACTTCGGAACTGCCTTTGA

>27af90dca0d43d04d7d01e4ff71b2088

TACGGAGGGAGCTAGCGTTATTCGGAATTACTGGGCGTAAAGCGCACGTAGGCGGCTTTGTAAGTAAGAGGTGAAAGCCCAGAGCTCAACTCTGGAATTGCCTTTTA

>62fd5e751d627576425508028344ef47

TACAGAGGGTGCAAGCGTTAATCGGAATTACTGGGCGTAAAGCGCGCGTAGGCGGCTTGTTAAGTCAAATGTGAAATCCCCGAGCTTAACTTGGGCATTGCATTCGA

>3989ec1ec0a0761dde92e2e7fb6d5d8b

TACGAAAGGTGCAAGCGTTAATCGGAATTACTGGGCGTAAAGCGCGCGTAGGCGGTGTGTTAAGTCGGATGTGAAAGCCCAGGGCTCAACCTTGGAATTGCATCCGA

>a977c0aceecbe162284cb79805cfd0ed

TACGTAGGTCCCGAGCGTTGTCCGGATTTATTGGGCGTAAAGCGAGCGCAGGTGGTTTATTAAGTCTGGTGTAAAAGGCAGTGGCTCAACCATTGTATGCATTGGAA

>281de05f37cf76562bd99bedb91f9fe2

CATTGGACTATCGTGAGAAAGTCAAACCGCCAAAGGGAATTATATTATAGTAAATATTAGCGTAAATAAACATTTTATTAATAGTTGTAATATATGAAAATGTGCAG

>d0d92fdf10aa6645bcaa00d3b6b469ac

AACAGAGGATACAAGCGTTATCCGGATTTATTGGGTTTAAAGGGTGCGTAGGTGGTTTTTTAAGTCAGTAGTTAAATCTTAAAGCTTAACTTTAAAAGTGCTATTGA

>548ba8337453d3f884d2c7def7e61be0

TACGGAGGGTGCAAGCGTTATCCGGATTTACTGGGTTTAAAGGGTGCGTAGGTGGGCAGTTAAGTCAGTGGTGAAATCTCCGAGCTTAACTCGGAAACTGCCATTGA

>d84c4864539748b7688944e85907f6bd

TACGTAGGGTCCAAGCGTTAATCGGAATTACTGGGCGTAAAGCGTGCGCAGGCGGTTGTGCAAGACCGATGTGAAATCCCCGGGCTTAACCTGGGAATTGCATTGGT

>17541b54106e0dc1491c25524aefe0b1

TCCATTAGAAACCCTAGTAGTCCGGCTGACTGACTCGAGCTAGATCTCGTATGCCGTCTTCTGCTTGAAAAAAAAAACTTAAAGCTTAACTTTAAAAGTGCTATTGA

>408fc040f1bff2c286dc8835a6c402ea

TCCGGCTGACTGACTTCCTCATGATCTCGTATTAGAAACCCCAGTAGTCCGGCTGACTGACTTCCTCATGATCTCGTATGCCGTCTTCTGCTTGAAAAAAAAAAAAA

>a0422bb0b17f5bf25bde9a7f7bef6923

TACGTAGGGTGCGAGCGTTAATCGGAATTACTGGGCGTAAAGCGTGCGCAGGCGGTTGTGCAAGACAGATGTGAAATCCCCGGGCTTAACCTGGGAACTGCATTTGT

>b881978490e622ce1da2eabc091f90ee

ACAACAAGGGGAACCTAACAATGAGCCGCCTCGCAATCAATCAATTTTCGCCACGCCACATCAACGCCATTCGTGTAGCACAATTTTTCTTCGCCTATCTACACACA

>79289e19a47b8a6d2e7649416f3bc659

TACAATTTCTTCAATTTTAAATTTATAAAGTTTCAGTAAATATATAATATAATTTTTATAAATAATAATTTTAGTGAAATATGTTATTATTATGTTTTTATGATTTG

>c4ba8ee8853a67f4114e8c4276faf3ad

TACGAAGGGGGCTAGCGTTGCTCGGAATCACTGGGCGTAAAGGGCGCGTAGGCGGCCATTCAAGTCGGGGGTGAAAGCCTGTGGCTCAACCACAGAATTGCCTTCGA

>560ef1fcaa5e5f006b33e57181683323

TACGTAGGGGGCTAGCGTTATCCGGAATTACTGGGCGTAAAGGGTGCGTAGGTGGTTTTTTAAGTCAGAAGTGAAAGGCTACGGCTCAACCGTAGTAAGCTTTTGAA

>539fce76d81cf72a54bda14969ff2bf0

CCCCATGGCGCATTAGAAACCCCAGTAGTCCGGCTGACTGACTTCTAGACTATCTCGTATGCCGTCTTCTGCTTGAAAAAAAAAATTAACTTTAAAAGTGCTATTGA

>95e713bf61412b3e81720679738089d4

TACGTAGGGTGCGAGCGTTGTCCGGATTTACTGGGCGTAAAGAGCTCGTAGGTGGCTTGTCGCGTCGTCTGTGAAAGTCTGGGGCTTAACTCCGGGTGTGCAGGCGA

>35543afe3b1f8293591debf1e5b0ed9e

ACCTTTCAGTTTTATTTTGCGCTTCTTTACATGCGTTTTACTGTTACTTTAACAGAATAGAATTTTCTGTAGATTAGTATCTTACTGTAGATTTGATGTTATTTTTT

>b4b2c59fc400c7a6ace9289a10cf7bd4

CACGATTAACCCAAGTCAATAGAAACCGGCATAAAGGGTGTTTTAGATCAATTCCCCTCAATAAAGCTAAAATTCACGTGAGTTGTAAAAAACTCCAGTTGATACAA

>56e06ca4f4422ace798868a4d4c1bbd1

GGTTATGTTCGTCAACACCGGGCAAACCCGGTCATATAATTTTACCATACACTATAACACGTTGAGGTCGAGATTGCAGTGCAAGGTGAGGTTATGTTATCACATAC

>58bcd959a23b19e4c0dc2e7829866a1c

TACGGAGGGAGCTAGCGTTGTTCGGAATTACTGGGCGTAAAGCGCACGTAGGCGGCTTTGTAAGTCAGAGGTGAAAGCCTGGAGCTCAACTCCAGAACTGCCTTTGA

>35d5ea57c5970d2683e5755b1a76d5ce

CATTGAACTATCGTGAGAAAGTCAAACCGCCAAAGGGAATTATATTATAGTAAATATTGGCGTAAATAAACATTTTAGTAATAGTTGTAATATATGAAAATGTGCAG

>ce2c20574fbe035c5a7b81cd04fb9a39

GCGTTATATACTTTGCAAATACACTATAACATTAGATGCCATTGCCTCCGTTTTGCGGTTATGTTCGTCATATCCTTTTACAATACACTATAACATTAGATACCACA

>68392abbc7f1051185e6b95a0ab32d15

CCCCATGGCGCATTAGAAACCCTAGTAGTCCGGCTGACTGACTCTATCGTGATCTCGTATGCCGTCTTCTGCTTGAAAAAAAAAATTAACTTTAAAAGTGCTATTGA

>dc6b36b8653599189d68615562a90b86

TACGTAGGTGGCAAGCGTTGTCCGGAATTATTGGGCGTAAAGCGCGCGCAGGCGGTCTCTTAAGTCTGATGTGAAAGCCCACGGCTCAACCGTGGAGGGTCATTGGA

>76fdece475c6111718f7ba1a40e5c7e5

TACGATTCATTCAATTTAAATATAAAAGTTTCAGTTATAATTAATTTTGATTACAAATTTTATAAATTTTGGTGAAATATATTTTAATTAATAATTCATAATTTATA

>9008f239fc970c71f68c9ea84ff9661b

TACGTAGGGTGCGAGCGTTGTCCGGAATTACTGGGCGTAAAGAGCTCGTAGGCGGTTTGTCACGTCGTCTGTGAAATCCTAGGGCTTAACCCTGGACGTGCAGGCGA

>e3cac77b6dc93ac704d134356b5d0c35

CATTGAACTATCGTGAGAAAGTCAAACCGCCAAAGGGAATTATATTATAGTAAATATTAGCGTAAATAAACATTTTATTAATAATTGTAATATATGAAAATGTGCAG

>e0ed3af5527c86850fc183771bc140e3

CATTGAACTATCGTGAGAACGGCACGCCGCCAAAGGGAATTATATTATAGTAAATATTAGCGTAAATAAACATTTTATTAATAGTTGTAATATATGAAAATGTGCAG

>1054d03b0fe44b162748079fec718a10

TACGGAGGGTGCGAGCGTTGTCCGGATTTATTGGGTTTAAAGGGTGCGTAGGCGGCCGTTTAAGTCTGGGGTGAAAGCCCGCTGCTCAACAGCGGAACTGCCCTGGA

>d10938af07ea3414a8e8f20944af43da

TACATAGGTGGCAAGCGTTATCCGGATTTATTGGGCGTACAGGGTGCGTAGGTGGTTTGATAAGTTTAGGGTTAAAGGCCGGAGCTCAACTCCGGTTCGCCTTGAAA

>b3081da70a4a0aac0966c39a3015a564

CTGGCACACAATTACCATATAGTAATTAGAAACCCTAGTAGTCCGGCTGACTGACTCTCTAGAGATCTCGTATGCCGTCTTCTGCTTGAAAAAAAAAAAAAAAAAAA

>c2ab158224a93ab3143ef2bb654e622f

TACGTAGGTGGCAAGCGTTGTCCGGAATTATTGGGCGTAAAGCGCGCGCAGGCGGATCAGTCAGTCTGTCTTAAAAGTTCGGGGCTTAACCCCGTGATGGGATGGAA

>32beb655dd7677f4363d605a87267034

TACGGAGGATGCAAGCGTTATCCGGAATTATTGGGCGTAAAGCGTCCGTAGGTGGCTTAGTAAGTCTTTTGTCAAATCGTGCAGCTTAACTGCATACGGGCGGAGGA

>323f31090892d0cc4ba06ec343e82a22

GTGCTCGAAGAACATCAATCTTTCACTTCGTTCTCACTTTCTCTTTTTACTGCCTCCTCTTCTGACCCTTTTCCAACATACCCCTTTCCCTCCTTTTCGTCATCCCT

>d4e91e09f554632039b32584663db34f

CATTGAACTATCGTGAGAAAGTCAAACCGACAAAGGGAATTATATTATAGTAATTATTGGCGTTAATAAACATTTTATTAATAGTTGTAATATATGATAATGTGTGA

>8282974eef52d51c468ed640e906a91c

TCCGGCTGACTGACTTAACGTCCATCTCGTATGCCGTCTTCTGCTTGAAAAAAAAACCCTGTAGTCCGGCTGACTGACTTAACGTCCATCTCGTATGCCGTCTTCTG

>fb8e8b1875cdc4ee30dc6134c625a338

CGTGAAAGCAAAACTTCTATGTAAAGGGGTACCGACACCGACAATATCCACGTTTCTAGGAGACTGTTAAACGTGGGCACAGTTTTTGCGCAGCCGATTTTTCTCAC

>88d6ce791f884c203894ecd22d82d60b

TACAAGTAAATCTTTTTTTAAAAAATTAAAAATAATAAAAAAATTTTTGTATTTTAAAAATTTATAAAGGTGAAATTTATAATTTTAAAAAAATTAATTTGATTTTA

>21e3eb04985602d1515f315d170f4f38

CACACTTATGTCTTCAGAAAGACAAAATATTTCTCCAGGATGCATTTCAACCATGTCGGTCATTTTCAACTGGCAGTGCCTTGTAGTTATTTGTGAAAGATTATGAA

>7fb0122c4c8b7879aee3a36ff3919cac

TACGGAGGATGCGAGCGTTATCCGGGTTTATTGGGTTTAAAGGGTGCGTAGGTGGTTAATTAAGTCAGCGGTGAAAGTTTGTGGCTCAACCATAAAATTGCCGTTGA

>92e6c731bf5ada1afbce63d3dee8b84f

AAGAATACGTTTGAGTTAGTGGGAAGGGAGACCTCAGACAGTACAGACGTATAGTGTTCCACTCAGTAAATAATTATCAGTAAATGTGTTTAAAATAGTTAAAAAAG

>1116b29abcfe8cc0b210f63b326a5cde

CTGGCACACAATTACCATATATAGTAATTAGAAACCCCAGTAGTCCGGCTGACTGACTTCCTCATGATCTCGTATGCCGTCTTCTGCTTGAAAAAAAAAAAAAAAAA

>589bfb362e9a3c6cc4eb501a55f6b34a

TACGGAGGGTGCAAGCGTTAATCGGAATTACTGGGCGTAAAGCGTGCGTAGGCGGTTCGTTAAGTCTGTTGTGAAAGCCCCGGGCTCAACCTGGGAATGGCAATGGA

>57443de71494da1db5a5c6515a2e4033

GAGGTATGGGGAAGGAGATTAGAACCGGCACTTATACGTCTTACAGTGAGGATCGCCGGATACAGAGTGTAATCAATTGTCTTCACTAGCGTAGAGACGGAGCAGTT

>3d758966e02d9144f92494855731cb0c

TACGTAGGGGGCGAGCGTTATCCGGAATCACTGGGCGTAAAGGGTGCGTAGGCGGCCAATAAAGTCTGGGGTGAAAGGCTACGGCTCAACCGTAGTAAGCCTTGGAA

>aeb1e205a0b6e2055c5d1eff873cd830

TACGTAGGGTGCAAGCGTTAATCGGAATTACTGGGCGTAAAGCGTGCGCAGGCAGTTATGCAAGACAGAGGTGAAATCCCCGGGCTCAACCTGGGAACTGCCTTTGT

>0523f3212844b843ea53454a3f7492d9

TACAATTTCTTCAATTTAAATTTTTAAAAGTTTCAGTTAATAAATTATTTAAACTTTTGTGAATAATAATTTTAGTGATATAGATTATTATTTTATTTTGTTAGTTT

>1e4d37258f5d00eedcbfac22fad1411d

TACGTAGGGTGCAAGCGTTGTCCGGAATTATTGGGCGTAAAGAGCTCGTAGGCGGTTTGTCGCGTCTGCTGTGAAAACTGGAGGCTCAACCTCCAGCCTGCAGTGGG

>24a343ce3de8032b819c4e542d4d4a7b

CACGATTAACCCAAGTCAATAGAAGCCGGCGTAAAGAGTGCTTTAGATCACCCCCTCCCCAATAAAGCTAAAACTCACCTGAGTTGTAAAAAACTCCAGTTGACACA

>acb4f52e06b2c103248be5c69c306628

TACGTAGGGTGCGAGCGTTATCCGGAATTATTGGGCGTAAAGAGCTCGTAGGCGGTTTGTCGCGTCTGTCGTGAAAGTCCGGGGCTTAACCCCGGATCTGCGGCGGG

>bdd0149b41989b2661a69b55f6e5aed6

GACAGAGGCCACAAGCGTTAGGCGGAATCACTGGGCTTAAAGCGTGTGTAGGCGGATCGTTAAGTGCCTTGTGAAATCCCCCGGCTCAACCGGGGAACGGCTGGGCA

>ab00840d97a1372a5e98f8099495b7db

CACGGGGGGCGCAAGCGTTATTCGGAATTATTGGGCGTAAAGGGCGCGTAGGCGGCCTGTTAAGTCAGATGTGAAAGCCCGGGGCTCAACCCCGGAAGTGCATTTGA

>d9f2d4bbc6e7a7c17b317a45fc4c3ec7

TACGTAGGGTGCAAGCGTTGTCCGGAATTATTGGGCGTAAAGAGCTCGTAGGCGGTTTGTCGCGTCTGCTGTGAAATCCCGAGGCTCAACCTCGGGCTTGCAGTGGG

>67f8914d8e28d68960c84ce9f2b00078

TACGAAGGGGGCTAGCGTTGTTCGGATTTACTGGGCGTAAAGCGCACGTAGGCGGATTTTTAAGTCAGGGGTGAAATCCCGGGGCTCAACCCCGGAACTGCCTTTGA

>9e9b565db0a37021c1d20ee525e3cb9c

CACACTTATGTCTATAGAAAGACAAAACATTTTTCCAGGATGCATTTCAACCATGTCGGTCATTTTCAACTGGCAGTGCCTTGTAGATATTTGTGAAAGATTTTGAA

>4a50d209046a98b4846783d2d48e3ce3

TACGTAGGGCGCAAGCGTTGTCCGGAATTATTGGGCGTAAAGAGCTCGTAGGCGGTTTGTCGCGTCTGCTGTGAAAACCTAAGGCTCAACCTTGGGCTTGCAGTGGG

>afb1fe47ae6fb3695977137e8662d4e0

GAAAGAGTAGAAGGTGAGACGTTGAAGAAGAAGAAGCAGCAGTCACAAATACACAACACACACGCACAAGGTCGATCCTTTTCAGGGTCGGCTGTAATGCTACCCGC

>02585504331ee403fee074b3bc039e70

TACGGAGGGTGCAAGCGTTATCCGGATTCATTGGGTTTAAAGGGTCCGCAGGCGGGCTTGTAAGTCAGGGGTGAAAGCCGACAGCTTAACTGTCGAACTGCCTTTGA

>95a80282782566b06341c63871cea9da

AACAGAGGATACAAGCGTCATCCGGATTTATTGGGTTTAAAGGGTGCGTAGGTGGTTTTTTAAGTCAGTAGTGAAATCTTAAAGCTTAACTTTAAAAGTGCTATTGA

>031b0bb086e340f74f61acb5afc3d5bc

TACGTAGGGTGCGAGCGTTAATCGGAATTACTGGGCGTAAAGCGTGCGCAGGCGGTTTTTCAAGTCTGATGTGAAAGCCCCGGGCTTAACCTGGGAACTGCGTTGGA

>e96f44422ae65dbea32f43b71b5cb487

TACGGAGGATCCAAGCGTTATCCGGAATCATTGGGTTTAAAGGGTCCGTAGGCGGTCAAGTAAGTCAGTGGTGAAAGCCCATCGCTCAACGGTGGAACGGCCATTGA

>96eb08930fa26a8e3a47aab4a77d4373

TACGTATGTTCCAAGCGTTATCCGGATTTATTGGGCGTAAAGCGAGCGCAGACGGTTATTTAAGTCTGAAGTGAAAGCCCTCAGCTCAACTGAGGAATTGCTTTGGA

>84703df82975bb6b56cbc833d6a5b0a0

TACGAAGGGGGCTAGCGTTGCTCGGAATGACTGGGCGTAAAGGGCGTGTAGGCGGTTTGTACAGTCAGATGTGAAATCCCCGGGCTTAACCCGGGAGCTGCATTTGA

>396dcaa9b6b123e0c60d1ad7f999962d

TACGTAAAATACAAGTGTTATTCATGTTTAATCGGTTTAAAGGGTACCTAGACGGAATATCAAGCCTAAATAAAAAGGGACTAGTATTCTTGAGTTTTATGGAAGAA

>82692c1e9ce56c600bba1b18846b40b6

TACGAAGGGGGCTAGCGTTGCTCGGAATTACTGGGCGTAAAGGGCGCGTAGGCGGACAGTTAAGTTGGGGGTGAAAGCCCGGGGCTCAACCTCGGAAATGCCTTCAA

>725d01ba9b737d771ff765f0e66a8873

TACGTAGGGGGCAAACGTTGTCCGGAATGACTGGGCGTAAAGGGCGAGTAGGTGGTATGCCAAGTCAGAAGTGAAAACTCCGGGCTCAACCTGGAGATTGCTACTGA

>6d4a382538e920b574cd60a28aed3fe9

AACGGAGGATACGAGCGTTATCCGGATTTATTGGGTTTAAAGGGTGCGTAGGTGGTTTTTTAAGTCAGTAGTGAAATCTTAAAGCTTAACTTTAAAAGTGCTATTGA

>628742fadf4e9f56bec7180e1d567f9f

TTTGCCGAGTAAGGTTCGCCGCAATTAATACAAACGCGGCGCCCCGCAGCTGGCACCTTACAATCTTTAGTTTTTGTAAAATCACATTACACAAATATTTGTGTCAG

>4c1e16b7853982553a5b1b09bb1be521

TACAATTTCTTCAATTTAAATTTTTAAAAGTTTCAGTTAATAAATTATTAAAATTTTTGTGAATAATAATTTTAGTGATATAGATTATTATTTTATTTTATTAGTTT

>4a1ec6b27080ca3bb169553daed4711c

GGGGGGGGGGGGGGGGGGGGGGGGGGGGGGGGGGGGGGGGGGGGGGGGGGGGGGGGGGGGGGGGGGGGGGGGGGGGGGGGGGGGGGGGGGGGGGGGGGGGGGGGGGG

>71e225cc636e3e3a808657157b86cf1d

TACAGAGGGTGCAAGCGTTAATCGGATTTACTGGGCGTAAAGCGCGCGTAGGCGGCTAATTAAGTCAAATGTGAAATCCCCGAGCTTAACTTGGGAATTGCATTCGA

>43c32139a164c8f574fef18665ebfc63

ACAAATGGTGAATAGTATAGTGTCAATAATGGCATTAGAAACCCCAGTAGTCCGGCTGACTGACTCTACGACCATCTCGTATGCCGTCTTCTGCTTGAAAAAAAAAA

>05766c2eec3283747721b1a803806476

TACGTAGGGCGCGAGCGTTGTCCGGAATTATTGGGCGTAAAGAGCTCGTAGGCGGCTGGTCGCGTCTGTCGTGAAATCCTCTGGCTTAACTGGGGGCTTGCGGTGGG

>a03bb4211db8e767f7fa0f08c0c12159

TACGAAGGGGGCTAGCGTTGCTCGGAATTACTGGGCGTAAAGGGAGCGTAGGCGGACTGTTTAGTCAGAGGTGAAAGCCCAGGGCTCAACCTTGGAATTGCCTTTGA

>f7d730961bdb9834fb5f5970025d5279

TACGTGAGAGACTAGTGTTATTCATCTTAATTGGGTTTAAAGGGTACCTAGACAGTCAATATAACTTCTATAATGCTAATACTTGACCAGAGTTTTAAGTAAGAGGG

>661d338f5deb9bd8d747c6e5eda307ee

TACGGAGGATCCAAGCGTTATCCGGAATCATTGGGTTTAAAGGGTCCGTAGGCGGTCAGGTAAGTCAGTGGTGAAAGCCCATCGCTCAACGGTGGAACGGCCATTGA

>81379a7f68975f025c0b441b3c03ce1f

CAACACCTTCATATCTTCTTTTTGGGTAGCGTTTTGGTCCTTTGCCTTTCACATTTCCTACGACGTCTTTTCCTGATATTATACCTTCGTCCATTCTATTAGAAACC

>35a5536b40b00f20db12d08540883bb2

GACAGAGGGTGCAAACGTTGTTCGGAATTACTGGGCGTAAAGCGTGTGTAGGCGGTCTTGTAAGTCGGATGTGAAAGCCCCGGGCTCAACCCGGGAAGTGCACTCGA

>47ce608ca97240dd9911b169d9c8df5e

AGGACTTCGAGTACCTATATCGAAACTGACACATACGCCTGAACACTGTTCGAAACAGCCACGTGTACAAGACTGACTAAAACTAAAACAAGCCTTACTGAGGAAAA

>23d1039fdda7ac9e27be6c1f8cb95236

TACAATTTCTTCAATTTAAATTTTTAAAAGTTTCAGTTAATAAATTATTTAAATTTTTATAAATAATAATTTTAGTGAAATATATTATTATTATATTTTATTAATTT

>345f1ba4f01b77d4d343c5b24866baae

TACGTAGGGTGCAAGCGTTGTCCGGAATCACTGGGCGTAAAGAGTTCGTAGGCGGCTTTGTAAGTCAGGTGTGAAAGGCATTGGCTCAACCAATGTAAGCACTTGAA

>b5a26bb734773d05178b23692ad6e37b

TACGTAGGGTGCAAGCGTTAATCGAAATTACTGGGCGTAAAGCGTGCGCAGGCGGTTATGCAAGACAGAGGTGAAATCCCCGGGCTCAACCTGGGAACTGCCTTTGT

>c9053e4d9903d9ad722b9d8f594b0d55

TACCGGCAGTCCGAGTGATGGCCGCTATTATTGGGCCTAAAGCGTCCGTAGCTGGCCGCGCAAGTCCATCGGGAAATCCACCTGCTCAACAGGTGGGCGCCCGGTGG

>88fea80e7f6100b52d98d857d4c56901

TACGTAGGGTGCAAGCGTTGTCCGGAATTATTGGGCGTAAAGAGCTCGTAGGCGGTTTGTCGCGTCTGCTGTGAAAACGCGAGGCTCAACCTCGCGCCTGCAGTGGG

>f741a9c77a3cf328ca21131234db15dd

TACCATACGTCACATTTTATCGCATTATACTAGTAATACTGATATCAAGTACCTTGTCTCGTTTACATTATAACATTTTATGTCGAGCAACGATACCGCATTCCTAA

>cc0316b56fa7c12287fbe066157c71c2

TACGTGAAGGATAAGCGTAATTCATCATCACTAGGTATCAAGCGTCTGTAGGCGGCGAATGTGAGCTAGAGTCTGACTAGGAAATTGGAATTTAATAAGTAAGGATA

>473994145594e385956f5f9bd48370d1

TACGTAGGGGGCAAGCGTTGTCCGGAATCATTGGGCGTAAAGCGCGTGTAGGCGGCCAGATAAGTCCGTTCTGAAAGCCCGGGGCTCAACCCCGGGAGGCGGATGGA

>f50437c271162aaffe9259e1fce390cd

CACGATTCTCTAAATTTAATTATGTTAGTTTCAGTTAAAAATGTAAATGTGTTAATATTAAGTTTTTTAAATTTTGGTGAAATAATGTATAAATATGTGTTTACTTC

>2a8311e2c696eaa7bb5dc9976f5edd6a

CAAATTTATGTCTTCAGAAAGACAAAATATTTTTCCAGGGTCCATTCCAACCGTGTCGGTCATTTTCAACTAGCAGTGCCTTGTAGATATTTGTGAAAGATTTTGAA

>50d03a9ad8897296d3071a13f0682e74

TACGAAGGTGGCTAGCGTTGCTCGGAATTACTGGGCGTAAAGGGAGCGTAGGCGGACATTTAAGTCAGGGGTGAAATCCCGGGGCTCAACCTCGGAATTGCCTTTGA

>4b861ce45092b1cfb7aced02079e804a

TACGTAGGGTGCAAGCGTTAATCGGAATTACTGGGCGTAAAGCGTGCGCAGGCGGTTTTGTAAGTCTGACGTGAAAGCCCCGGGCTCAACCTGGGAATTGCGTTGGA

>b5bdf0d19f2caeea40062018d0de97e7

TACGTAGGTGGCAAGCGTTGTCCGGATTTACTGGGCGTAAAGGGAGCGTAGGTGGATATTTAAGTGGGATGTGAAATACTCGGGCTTAACCTGGGTGCTGCATTCCA

>01378ad0b7e5bae4671c9e6e53c1de36

ACAAATGGTGAATAGTATAGTGTCAATAATGGCATTAGAAACCCTAGTAGTCCGGCTGACTGACTCTACGACCATCTCGTATGCCGTCTTCTGCTTGAAAAAAAAAA

>942a5b017b0ac145eb2f14cab79baaca

TACGGAGGATCCGAGCGTTATCCGGATTTATTGGGTTTAAAGGGTGCGTAGGTGGTTTTATAAGTCAGCGGTGAAAGTTTGCAGCTTAACTGTAAAAATGCCGTTGA

>3bba3031c8bfdd98bb3fc128256b4038

CATGGTCATCCAGCCAGATTTCGTATTTATGTACGTGACGGTTGACTTTATTCACGTGGAAAGCGATGCTTTAACCACACGACCGTACCACCGGGTGAATTGAGTTA

>1513ef2b995dd53cd222475496ef9f35

CCGCTGTGTTCAGTTTGATATACTTTTTGTACAATACACAATAACAGATGCCACAAGCTACACACACACACACACACACACACACACACATAGAAAGTCGTAGGACG

>d7d039b3c9d362704b29117b8f70bb00

GGCAATTAAATGAGATCAAGGGGATACAAATTAGAAACCCTAGTAGTCCGGCTGACTGACTATGAGCTCATCTCGTATGCCGTCTTCTGCTTGAAAAAAAAAAAAAA

>f0e2515e8dd7cebcaa0c7e3d71a92fce

AACAGAGGATACAAGCGCTATCCGGATTTATTGGGTTTAAAGGGTGCGTAGGTGGTTTTTTAAGTCAGTAGTGAAATCTTAAAGCTTAACTTTAAAAGTGCTATTGA

>d52d16872e0fc408eb89072fc22961bb

AACAGAGGATACAAGCGTTATCCGGATTTATTGGGTTTAAAGGGTGCGTAGGTGGTTTTTTAAGTCAGTAGTGAAATCTTAAGGCTTAACTTTAAAAGTGCTATTGA

>6d92c8de1caa0362e1a71fcfa47cc9e0

TACGAGGGGAGCGAGTGTTGTTCGATTTTATTGGGCGTAAAGGGTATGTAGGCGGTTTTGTAAGTCAACGATTAAATCTTGAGGCTTAACTTCATAGTGGTTGTTGA

>f8f5ed3bff0173e5801d73bab3366b98

TCCGGCTGACTGACTGTTACAGCATCTCGTATGCCGTCTTCTGCTTGAATAAAAAACCTTGTAGTCCGGCTGACTGACTGTTACAGCATCTCGTATGCCGTCTTCTG

>7c6850097ed5d1b4c7f5f98ca6ff58e1

CATTGAACTATCGTGAGAAAGTCAAACCGCCAAAGGGAATTATAGAATAGTGAATATTAGCGTAAATAAACATTTTATTAATAGTTGTAATATATGATAATGTGCAG

>3a075f662596f43c57bb98674e95de71

TACAGAGGGTGCGAGCGTTAATCGGAATTACTGGGCGTAAAGCGAGTGTAGGTGGCTCATTAAGTCACATGTGAAATCCCCGGGCTTAACCTGGGAACTGCATGTGA

>7665ba5bfe0327578bcd733885edd928

TAGTGATGTTTTGGCTGACTTTATCTGCTGCTTTGTTACAGTCAAGCTTAATAAAACCACCATATACACACACATCAAGTAAGATGCTAACTCAAGACACACACAAC

>7e209319eef102a150964a5ba9933907

ACAAATGGTGAATAGTATAGTGTCAATAATGGCATTAGAAACCCTTGTAGTCCGGCTGACTGACTCTACGACCATCTCGTATGCCGTCTTCTGCTTGAAAAAAAAAA

>e7132db7d22b8d5e467c4840a648b0d1

TACGTAGGGGGCGAGCGTTATCCGGAATTATTGGGCGTAAAGGGTGCGTAGGCGGCCATTTAAGTCAGGTGTGAAAGGTTACGGCTCAACCGTAATTAGCACTTGAA

>8fcb14fec0e8e64845c252a185b3486e

ACAAATGGTGAATAGTATAGTGTCAATAATGGCATTAGAAACCCGAGTAGTCCGGCTGACTGACTCTACGACCATCTCGTATGCCGTCTTCTGCTTGAAAAAAAAAA

>623567a79acd5d259adc8b645cd5dab7

TACGAAGGGTGCAAGCGTTACTCGGAATTACTGGGCGTAAAGCGTGCGTAGGTGGTTCGTTAAGTCTGATGTGAAAGCCCTGGGCTCAACCTGGGAATTGCATTGGA

>9f796d9fa8e388bd3c7d73904d47542f

TACGTAGGGGGCAAGCGTTATCCGGAATAACTGGGCGTAAAGGGTGCGTAGGCGGCCCTGCAAGTCAGAAGTGAAAGGCTACGGCTCAACCGTAGTAAGCTTTTGAA

>994f4a547b89fb741309f4e34a75efc4

GGTTATGTTCGTCAACACCGGGCAAACCCGGTCATATAATTTTACCATACACTATAACACGTTGAGGTCGAGATTGCAGTGCGTGGTGAGGTTATGTTATCATATAC

>765e5ef2a5dda09d7b2289241d589e0a

TACGGAGGGGGCTAGCGTTGTTCGGAATTACTGGGCGTAAAGCGCACGTAGGCGGCTTTGTAAGTTAGAGGTGAAAGCCTGGGGCTCAACTCCAGAATTGCCTTTAA

>73ead36f3bf192360e6cff0e00949f54

TACCGGCAGCTCAAGTGATGACCGATATTATTGGGCCTAAAGCGTCCGTAGCCGGCCACGAAGGTTCATCGGGAAATCCGCCAGCTCAACTGGCGGGCGTCCGGTGA

>911b9d4415fd95f6b903887c450456d2

TACAGAGGGTGCAAGCGTTAATCGGATTTACTGGGCGTAAAGCGTGCGTAGGTGGCCAATTAAGTCAAATGTGAAATCCCCGAGCTTAACTTGGGAATTGCATTCGA

>a0c1d978c5a1077d488eb140fbab352e

GACAGAGGGTGCAAACGTTGTTCGGAATTACTGGGCGTAAAGCGTGTGTAGGCGGCTATGTAAGTCGGATGTGAAAGCCCTGGGCTCAACCCAGGAAGTGCACTCGA

>09a18f3427b675ce994a22d9b83415ad

TACGTGAGAGACTAGTGTTATTCATCTTAATTGGGTTTAAAGGGTACCTAGACAGTCGATATAACTTCTATAATGCTAATACTTGACTAGAGTTTTAAGTAAGAGGG

>19f9ec584266620a5ea4fc96523ed9f2

TACGGAGGGGGCTAGCGTTGTTCGGAATTACTGGGCGTAAAGCGCACGTAGGCGGACCGGAAAGTCAGAGGTGAAATCCCAGGGCTCAACCTTGGAACTGCCTTTGA

>b229a6abac93a679b48671e149c98789

GTGGTTTTCACCAAATTTTTTTTATCTAAATTAGAAACCCCGGTAGTCCGGCTGACTGACTACTGTGTAATCTCGTATGCCGTCTTCTGCTTGAAAAAAAAAAAAAA

>cdbec509e2044ee1c5aab9fe0177046f

TACGTAGGTGGCAAGCGTTATCCGGAATTATTGGGCGTAAAGCGCGCGCAGGCGGTTTCTTAAGTCTGATGTGAAAGCCCACGGCTCAACCGTGGAGGGTCATTGGA

>6337bc587b54ed7a75360c3da6855063

CATTGCGTAACATCGCGTAATAAATCGCACGTCACAAGTAAAACTATGAGGTAACTAACTGCTTTATGAGATTCGTCATGATCTATTCTTGTCTATTGCGTCATCGC

>3fd496fea294dd4c1c7f91b28abb137d

TACGTAGGGTGCAAGCGTTAATCGGAATTACTGGGCGTAAAGCGTGCGCAGGCGGTTGTGCAAGTCTGATGTGAAAGCCCCGGGCTCAACCTGGGAACGGCATTGGA

>8ad06ddc21a307828500e3a90da420c2

TACAATTTCTTCAATTTTAAATTTATAAAGTTTCAGTAAGTATATAATATAATTTTTATAAATAATAATTTTAGTGAAATATGTTATTATTATATTTTTATGATTTT

>f6a62bd1b9a5d460fd827e0c1d46908e

TACAATTTCTTCAATTTAAATTTTAAAAGGTTTCAGTTAATATATTATTTAAATTTTTATAAATAATAATTTTAGTGAAATATATTATTATTATATTTTATTAATTT

>00237aabaa9e87ea88d7c1883d27910c

ACAAATGGTGAATAGTATAGTGTCAATAATGGCATTAGAAACCCCGGTAGTCCGGCTGACTGACTCTACGACCATCTCGTATGCCGTCTTCTGCTTGAAAAAAAAAA

>1282d6194e73c708a1341e8e7660bf86

TCCGGCTGACTGACTTAACGTCCATCTCGTATGCCGTCTTCTGCTTGAAAAAAAACCCTTGTAGTCCGGCTGACTGACTTAACGTCCATCTCGTATGCCGTCTTCTG

>c1f5f9bcfb339300f4f50aebec65c7db

CATTGAACTATCGTGAGAAAGTCACGCCGCCAAAGAGAATTATATTATAGTAAATATTGGCGTAAATAAACATTTTAGTAATAGTTGTAATATATCATATTGTGTGA

>8fbc90de98f1572f1ae2291aa31a3d3c

TTTCGAGAAGAGATACGGTTACGATTAAGGGAGTTCGGGAGATAATAAGGACTTCAATCCATAAGCTGACCAACGACATAATAACAAAACAGCTGTTCTGGTGTGAA

>5219ccfc5173c90e33a6b53d0f6b1f46

TACGTAGGGTGCAAGCGTTAATCGGAATTACTGGGCGTAAAGCGTGCGCAGGCGGTCCGCTAAGACAGATGTGAAATCCCCGGGCTTAACCTGGGAACTGCATTTGT

>335c596144512a82bae0404748faa042

CTGGCACACAATTACCATATATAGTAATTAGAAACCCCGGTAGTCCGGCTGACTGACTCTCTAGAGATCTCGTATGCCGTCTTCTGCTTGAAAAAAAAAAAAAAACA

>34aae0ee67822cbfc74fb68fd455ea35

TACGAAGGGGGCTAGCGTTGCTCGGAATCACTGGGCGTAAAGGGCGCGTAGGCGGCGTTTTAAGTCGGGGGTGAAAGCCTGTGGCTCAACCACAGAATGGCCTTCGA

>dd52624b69a1485c89cce879755d96fd

CATTGAACTATCGTGAGAAAGTCAAACCGCCAAAGGGAATTATATTATAGTAAATATTGGCGTAAATAAGCATTTTATTAATAATTGTAATATATGAAAATGTGCAG

>a39a64f198edae332ba2d42b2fe968c1

TACGTAGGGTGCGAGCGTTAATCGGAATTACTGGGCGTAAAGCGTGCGCAGGCGGTTTTGTAAGACAGGCGTGAAATCCCCGGGCTCAACCTGGGAACTGCGCTTGT

>416bfe31243c7e256b10e60c8f9d2b15

TACGTAGGTGGCAAGCGTTGTCCGGATTTATTGGGCGTAAAGCGCGCGCAGGCGGTCCTTTAAGTCTGATGTGAAATCTTGCGGCTCAACCGCAAGCGGTCATTGGA

>050df11dc2914cf4b9999d7e753687ef

TACGTAGGGGGCGAGCGTTATCCGGAATTATTGGGCGTAAAGAGTGCGTAGGCGGTTTGTTAAGTCATTTGTAAAAGATCTCAGCCCAACTGAGTAGGGCGAATGAA

>d5cf5577ff77466e97966379a5e2513c

CATTTTACCATAGTGTCTGCCTCCACGGGCACGTTTATGGCTTTGCCGGTTATCTTTTAAGACTATTTCTATTCTGAGACGACGGATCGCATACGTGGAAATCCGGA

>a52ff5b0f3b95c997cde4b68b489acde

GTGCTCGAAGAACATCAATCTTTCACTTTGTTCTCACTTTCTCTTTTTACTGCCTCCTCTTCTGACCCTTTTCCAACATACCCCTTTCGCTCCTTTTCGTCATCCCT

>a43aadbc07d7e5e087e8d36e51e95ef0

CATGGTCATCCAGCCAGATTCCGTATTTATGTACGTGACGCTTGACTTTATACACGTGGAAAGCGATGCTTTAACCACAGGGTGAATTGAGTTATAATAGTTAATTT

>4dbb149ee840b9e4bdafe6d858cb51fb

TACGTAGGGTGCGAGCGTTGTCCGGAATTATTGGGCGTAAAGAGCTTGTAGGCGGTCTGTCGCGTCTGCTGTGAAAGACCGGGGCTTAACTCCGGTTCTGCAGTGGG

>61792144748a9550da8851ca80d92415

AGGATTGGGAGGGACGACTCTGATAAACTACATGATATACACCAGAGGAAGTCCACAGGATTACGACAGATACGCTGCAGATGGTAACTATGGTTGGAGCTATAACG

>04bac551245a4e6ad6a72f77d4a18067

CAAAGATAATGAGGAGAAGAAAACGAAAGGACATAAAGTAGCTGAAGATTTGGTCACAACGAAATGGAAGAGCCGAATGATAGTGAGGTACGCGACCAGGGCAGAAA

>db4dee5a3130aa212ccbf285718288df

CTCATCCTAACTGTCTGTGCAATTAAACATTCAGTTTTCTTATGAGTTAATTGCCTGTGCGCGCGTGTGCATGTGTGTGTGAGGCAACCGCCAGTACGCCATAAGTT

>6698858a6a3eb719ed753a08288f12de

CACACTTATGTCTTCAGAAAGACAAAATATTTCTCCAGGATGCATTTCAACCATGTCGGTCATTTTCAACTGGCAGTGACTTGTAGATATTTGTGAAAGATTTTGAA

>9e33f386b892cb0d73afeba5766b7a01

TACGTAGGGTGCGAGCGTTGTCCGGAATTATTGGGCGTAAAGAGCTCGTAGGCGGTCTGTCACGTCGTTCGTGAAAACTTGGGGCTTAACCCTGAGCTTGCGGTCGA

>73f8612b51b7c4b628eb30af64d0901e

TACGGAGGGTGCGAGCGTTAATCGGAATTACTGGGCGTAAAGCGTACGCAGGCGGTTTGTTAAGCGAGATGTGAAAGCCCCGGGCTCAACCTGGGAACTGCATTTCG

>d6cee8d2d17ea3f91fdfbda7538429ca

CTCGTATCATACTACACGCTACACACTGCTTTGCTCCCGTAACACCAAACAAGATATATAAAGTTTTACACCAATATAAATCAATATTACACTACAGACCAACGATA

>f95f51689fe926d1e278f76ab6a708b3

TACGTAGGTGGCAAGCGTTATCCGGAATTATTGGGCGTAAAGCGCGCGCAGGTGGTTTCTTAAGTCTGATGTGAAAGCCCACGGCTCAACCGTGGAGGGTCATTGGA

>e1046832b33368efecf26d52048929ab

TACGTGAGAGACTAGTGTTATTCATCTTAATTGGGTTTAAAGGGTCCCTAGGCAGTCAATATAACTTCTATAATGCTAATACTTGACTAGAGTTTTAAGTAAGAGGG

>7bf50ecea91c0fcf5e1269aed9ebd664

TCAACAAGATAATCCGTTACAATTCAAGAAAAAATTGACCACACGCAATAATAATGTCAACTATAAAGAATTCCGCACTAATTATAACACAACAACTACAAACACTT

>e1765b48f490d22e4515928a289f6157

CATTGAACTATCGTGAGAAAGGCACGCCGCCAAAGGGAATTATATTATAGTAAATATTGGCGTAAATAAACATTTTATTAATAGTTGTAATATATGCAAATGTGCAA

>acbdb0c4350cc432548aaed17bea052b

TACGGAGGATCCAAGCGTTATCCGGAATCATTGGGTTTAAAGGGTCCGTAGGCGGTCTTATAAGTCAGTGGTGAAATCTCCCCGCTCAACGGGGAAACGGCCATTGA

>b91914768a977da00c42d589e3af0db5

GACAGAGGATGCAAGCGTTATCCGGAATGATTGGGCGTAAAGCGTCTGTAGGTGGCTTTTTAAGTTCGCCGTCAAATCCCAGGGCTCAACCCTGGACAGGCGGTGGA

>9d399dedc1836eeae56d7e9134d6b42a

TACGTAGGGTGCAAGCGTTAATCGGAATTACTGGGCGTAAAGCGTGCGCGGGCGGTTTTGTAAGTCTGTCGTGAAAGCCCCGGGCTCAACCTGGGAATTGCGATGGA

>9f4d2f045b0b6120c86bbcdb97e208e6

TACGTAGGGTGCAAGCGTTAATCGGAATTACTGGGCGTAAAGCGTGCGCAGGCGGTGATATAAGACAGATGTGAAATCCCCGGGCTCAACCTGGGAACTGCATTTGT

>aebd35b3c1a563f8ab5c4b6a1da10cc2

TACGGAGGATCCAAGCGTTATCCGGAATCATTGGGTTTAAAGGGTCCGTAGGCGGACTAATAAGTCAGTGGTGAAATCTCCCGGCTCAACCGGGAAATGGCCATTGA

>6d4e02010e3a9f76512d0237d6ef0a47

CACACTTATGTCTTCAGAAAGACAAAATATTTCTCCAGGATGCATTTCAACCATGTCGGTCATTCTCAACTGGGAGTGCCTTGTAGATATTTGTGAAAGATTTTGAA

>77ef8541a2ed76ac9ba75462d3828d07

GACAGAGGATGCAAGCGTTATCCGGAATGATTGGGCGTAAAGCGTCTGTAGGTGGCTTTTTAAGTCCGCTGTCAAATCCCAGGGCTCAACCCTGGACAGGCGGTGGA

>2cd4d857fa769b1e1ff2b8af98fee8b2

TACGGAGGGAGCTAGCGTTGTTCGGAATTACTGGGCGTAAAGCGCACGTAGGCGGCTTTGTAAGTTAGAGGTGAAAGCCTGGAGCTCAACTCCAGAATTGCCTTTAA

>809a0d719835c8cfa9f7dfb708a8cc63

CATACGTTACAAACTACACAACAAAATCATTAATAAATAAACTGAACTCTATAGACAAGTCACATTCAAAATTTTCCACAACCATCTACAATGCACTGCCAGTTGAA

>d305124abfc1d3933a1878ef3c8c2279

TACGGAGGATCCAAGCGTTATCCGGAATCATTGGGTTTAAAGGGTCCGTAGGCGGTTTAATAAGTCAGTGGTGAAAGCCCATCGCTCAACGGTGGAACGGCCATTGA

>6f52a0977ba41fdf7e22ee474114e77f

TACAATTTCTTCAATTTAAATTTTTAAAAGTTTCAGTTAATAAATCATTTATATTTTTATGAATAATAATTTTAGTGAAATATATTGTTATTTTATTTTATATGTTT

>9476b81db2aa463c4e4e3152a9d045ec

TACGGAGGGTGCAAGCGTTAATCGGAATTACTGGGCGTAAAGCGCACGCAGGCGGTCTGTTAAGTCAGATGTGAAATCCCCGGGCTTAACCTGGGAACTGCATTTGA

>abf2ea08bb6338a3a1fb6a5095db101f

TACGTAGGTGGCAAGCGTTGTCCGGATTTACTGGGCGTAAAGGGAGCGTAGGCGGATTTTTAAGTGGGATGTGAAATACCCGGGCTCAACCTGGGTGCTGCATTCCA

>5f3a0dd2bab11e440fcc57dd7d6aff1f

CCACTGTGTTGAGTGTGATATACTTTTACAATACACTATAACATTGAATACCACAAGCCACACACACACACACACACGCACACAGAAAGTCGTAAGACGCAAACACA

>41372d03d4514d578ad0f8dea6bd4b76

TACGTGAGAGACTAGTGTTATTCGTCTTAATTGGGTTTAAAGGGTACCTAGACAGTCAATATAACTTCTATAATGCTAATACTTGACTAGAGTTTTAAGTAAGAGGG

>55dfbab6e0fc202cfb5f7edff1dfe282

TACAATTTCTTCAATTTAAATTTTAAGAAGTTTCAGTTAATATATTATTTAAATTTTTATAAATAATAATTTTAGTGAAATATATTATTATTATATTTTATTAATTT

>22c761e542411cdedccb6ee3f168fa30

CATGGTCATCCAGCCAGATTCCGTATTTATGTACGTGATGCTTGACTTTATACACGTGGAAAGCGATGCTTTAACCACCGGGTGAATTGAGTTATAATAGTTAATTT

>bb6ec3c9798c9fb3494f568e3deb1c0b

TACGTAGGGGGCAAGCGTTATCCGGAATCACTGGGCGTAAAGGGTGCGTAGGCGGCTAATCAAGCCAGGGGTGAAAGGCTACGGCTTAACCGTAGTAAGCCTTTGGA

>bc99bffab9f4ee3c9cc9c337c2a8f7d0

CATTGAACTATCGTGAGAAAGTCACGCCGCCAAAGGGAATTATATTATAGTAATTATTGGCGTTAACAAACATTTTATTAATAGTTGTAATATATGATAATGTGTGA

>872eebbc6f87c86d0817d5a27ce3dd9c

ACAAATGGTGAATAGTATAGTGTCAATAATGGCATTAGATACCCCAGTAGTCCGGCTGACTGACTCTACGACCATCTCGTATGCCGTCTTCTGCTTGAAAAAAAAAA

>a65971eedc2c1186b10cdeda40193f45

TGCCCGTAGGTTCCTTCGTTTCCGTACCGACGTCGTAACGACACCTACAATTGACAATTTATAAATTAAGTTAATTTATAACAGACAAAATACAAATTTTATACTTG

>690f5efce6003033046f7cfdec9c7ce7

TACGTAGGGTGCAAGCGTTGTCCGGAATTATTGGGCGTAAAGAGCTCGTAGGCGGTCTGTCACGTCGGCTGTGAAAACTTGGGGCTCAACCCCAAGCCTGCAGTCGA

>94a21d3b27e6eb6f7c3ae1ec0b5b3424

CGGTTTACCAAGTATTTGGCAACTTTATGCGTTCATTTTGGGCGTTAAAACTTAAAAATCTCATCTTTTTTTATCGTTAAGGAATTCAGGATCCAGCGAGTAGCTAT

>4fc50a36ef734461dfbe866c93eac160

TACAGAGGGTGCAAGCGTTAATCGGAATTACTGGGCGTAAAGCGCGCGTAGGTGGTTCGTTAAGTTGGATGTGAAATCCCCGGGCTCAACCTGGGAACTGCATTCAA

>7f03487b60779be2f0ccd28ce5a246f9

GTGCTCAAAGAACATCAATCTTTCACTTCCTTCTCACTTTCTCTTTTTACTGCCTCCTCTTCTGACCCTTTCCACCATACGCCTTCCTCTCCTTTTCGTCATCCCTT

>5146645b8259caecb1933da9f33030eb

TGAAAATATCCAACGCACACACGGCGCATGCGCATGTTTACGAACAGCGGGCCTCTAAGCGGTCAGTTGCCTTTCGCGGACTACAGCTGGCGACCCTTGTCGCTCTC

>1a92db186fb4e0ed3c66341e1ead547a

CATTTTACCATAGTGTCTGCCTCCACGGGCACGTTTATGGCTTTGCCGGTTATCTTTTAAGACTGTTTCTATTCTGAGACGACGGATCTGCATACGTGGAAATCCGG

>b942a18bc8523f11614baef286e959c5

CATTTTACCATAGTGTCTGCCTCCACGGGCACGTTTGTGGCTTTGCCGGTTATCTTTTAAGACTGTTTCTATTCTGAGACGACGGATCTGCATACGTGGAAATCCGG

>9f522a28aa76219b608d16bae782d3a2

TACGATTTCTTTAATTTAAATAGTTAAGTTTCAGTTAATATATTAATAATATAAAATAGCTATAATTTTGGTGAAATATATTTTATCTTAAAAAATTATTTTTATGT

>da2879854ea8035ae51750e08aa5d1a5

TACGATTTCTTTAATTTAAATATTTAAGTTTCAGTTAATATAACAATAATATAAAATATCTATAATTTTGGTGAAATATATTTTATCTTTAAGAATTAATTTTATGT

>bfa4e3a9cabcf6335b3c9d70a5e12c1c

CAGCCCAGCGCCCTGGAAACACTACCACGACCGGCTGGAACGATACGAGTGTCATTTTCCTATTCTGTTTTAATATGGCAGGTTATCTATCGATTAGATAAGCTGTG

>4e9e270199b2026abe6ef2313dbb298e

TACGGAGGGTGCAAGCGTTAATCGGAATTACTGGGCGTAAAGCGCACGCAGGCGGTTTGTTAAGTCAGATGTGAAATCCCCGAGCTTAACTTGGGAACTGCATTTGA

>179f8449c5436b6bbb06554831c9ba7b

TACGGAGGATCCGAGCGTTATCCGGATTTATTGGGTTTAAAGGGAGCGTAGATGGGTTGTTAAGTCAGTTGTGAAAGTTTGCGGCTCAACCGTAAAATTGCAATTGA

>a36a7cba60e77dbce9710f734a15c5be

TGTTAAAGCTATGACTCACTATACAGGGCACTAGGAAATACCGTTTATTGCGACAAGGAATGGGGTTTGGTCAGTTTATTTTAGTTTGCTCCTATTACAGTTACAAA

>777543c05cdf136a921d5dd90d6cc448

TACGTAGGTGGCAAGCGTTGTCCGGAATTATTGGGCGTAAAGCGCGCGCAGGTGGTTTAATAAGTCTGATGTGAAAGCCCACGGCTCAACCGTGGAGGGTCATTGGA

>633cc9e347af99511f2521769b1e77e8

TACGGAGGGGGCTAGCGTTGTTCGGAATTACTGGGCGTAAAGCGCACGTAGGCGGCGATTTAAGTCAGGGGTGAAAGCCCGGGGCTCAACCCCGGAATAGCCCTTGA

>1ea328fdd068ade7b9a3a8839173d6d7

TTGGCGTTGGTTCAGCTCTTGTATTAGAAAGCCTCGGTGGCACAGTTCACCACAGTATCTCCATCAGGTATCTCCTTCCTATGGCTACAGTAGGTGGTTTTATCGAT

>39a0560d514b17e42c3e54ee26142562

CAAACGTTTTAGCACGTTTCAATTATAATTCAATATAAAATGTAGTACTTATTCCACGTGTATATCAGTGATATCAAACACGTATGTCGACATGCCAACCATGAATA

>43fc5a79a0498e3ad1a394d50c0a4db9

TACAGAGGGTGCGAGCGTTAATCGGATTTACTGTGCGTAAAGCGTGCGTAGGCGGCTTATTAAGTCGGATGTGAAATCCCCGAGCTTAACTTGGGAATTGCATTCGA

>2cd080a0a692971477a234614276c0d6

TACGAAGGGGGCTAGCGTTGTTCGGAATTACTGGGCGTAAAGCGCACGTAGGCGGACATTTAAGTCAGGGGTGAAATCCCGGGGCTCAACCCCGGAACTGCCTTTGA

>f9daf5b52d8784f0ca6648ef449fd4d0

TACAGAGGGTGCGAGCGTTAATCGGATTTACTGGGCGTAAAGCGTGCGTAGGCGGCTTTTTAAGTCGGATGTGAAATCCCCGAGCTTAACTTGGGAATTGCATTCGA

>b86050351cf965d5a41075e948d50a96

TCCTTTGTCATGATCATCCGCGCACCAAGGAAGTCATCGACTTTATGCCGGCAGCCATTCGGTCGTCAAAAGTTATTCTGCATGGATAGGGTTTGTTACGTTCATTG

>8cf34b78ef14ca7f6d09669f3a299d4c

TTCCTTCAATTTAAATTTATGAAGTTTCAGTTAATATATAATATAATTTTTATAAGTAATAATTTTAGTAAAATATATTATTATTTTATTTTTTTATGGTTTGCCTG

>5fae6c9da4c8a76ebb6ba4111b5f454c

CACACTTATGTCTTCAGAAAGACAAAATATTTCTCCAGGATGCATTTCAACCATGTCGGTCATTTTCAACCGGCAGTGCCTTGTAGATATTTGTGAAAGATTTTGAA

>3dec7b26d8aab3f0fccc1febd32d1fc0

TCAAACGAAAGCACAACAAAAAGATAGCCAAAGTAATACCACCCCGATCACCAGCAATAACACCCACACATATGTTTTACCCAAGAATAGTTAACCTCACCAACATA

>895f0bffe5f7937a57f4055cd64313f0

AACAGAGGATACAAGCGTTATCCTGATTTATTGGGTTTAAAGGGTGCGTAGGTGGTTTTTTAAGTCAGTAGTGAATTCTTAAAGCTTAACTTTAAAAGTGCTATTGA

>6fc0110007778864702b5143016fc497

TACGTAGGTGGCAAGCGTTGTCCGGATTTACTGGGCGTAAAGAGTATGTAGGCGGACATTTAAGTCAGATGTGAAATCCCCGGGCTTAACCTGGGGGCTGCATTTGA

>9339ee45e4f9c555f19d04b760f12ace

TACGATTTCTTTAATTTAAATAGTTAAGTTTCAGTTATTAAAACAATAATATAAAATATCTATAATTTTGGTGAAATATATTTTATCTTTAAAAATTAATTTTATGT

>c4db09cfd36f0ac63d271c0dfd16cb63

CAAAGATAATGAGGAGAAGAAAACGAAAGGACATAAGGTAGCTGAAGATTTGGTCACAACGAAATGGAAGAGCCGAATGATAGTGAGGTACGCGACCAGGGCAGAAA

>b6fea2da58f1e254f5d8d4e09e5cf1a2

CTGGCACACAATTACCATATATAGTAATTAGAAACCCCTGTAGTCCGGCTGACTGACTCGAGCTAGATCTCGTATGCCGTCTTCTGCTTGAAAAAAAAAAAAAAAAA

>c54509de115303a56b11ee277c779598

TACGTAGGGCGCAAGCGTTATCCGGAATTATTGGGCGTAAAGAGCTCGTAGGCGGTTTGTCGCGTCTGCCGTGAAAGTCCGGGGCTCAACTCCGGATCTGCGGTGGG

>db581192f5216da22861d644d8a26820

CATTGAACTATCGTGAGAAAGTCACGCCGCCAAAGGGAATTATATTATAGTAATTATTGGCGTTAATAAACATTTTAGTAATAGTTGTAATATATCATATTGTGCGA

>26bb0bdbea9c59de2c96b47c92991785

CACACTTATGTCTTTAGAAAGACAACACGTTTTTCCAGAATGCATTTCAACCTTCTCGGCCATTTTCAACTGGCAGTGTCTTTTCAATGTGTTTCTGCAAGATAAAC

>84099e839a22a7f65a70b4d9435b0bc2

TACGAGACAGCTAGAAAACTACGTACAAGCTACAGCACCTTGAGACGTCCGACACCTAAGAAAAGTTGTTTTTGACGAGCTTGAATGTAGTCTTACATGCCTGCCAG

>116e0a982b894443513b02040019d572

TATTCTCGCGAAAACGTAATCGGCCTCGGCCGCTTAAATTAGGCTCCGTCGAGGTGCCCTGGACCAATAGCGTCAAGTACCTCGGGCTCCACGTGGATTCCCGACTC

>5c4d7acd918d234b944be18ba925de8a

TACGAAGGGGGCTAGCGTTGCTCGGAATGACTGGGCGTAAAGGGCGCGTAGGCGGACTGGTTAGTCAGACGTGAAATTCCTGGGCTCAACCTGGGGGCTGCGTTTGA

>3bf0127cd8e0da7f320bcb08fc2d348b

TACGTAGGTGGCAAGCGTTGTCCGGATTTATTGGGCGTAAAGCGAGCGCAGGCGGTTCCTTAAGTCTGATGTGAAAGCCCCCGGCTCAACCGGGGAGGGTCATTGGA

>af199630dfb55c65f417db1eb78e0ca6

CACACTTATGTCTTCAGAAAGACAAAATATTTCTCCAGGATGCATTTCAACCATGTCGGTCATTTTCAACTGGCAGTGCCTTGTAGATATTTGTGAAAGATTTTGAA

>622e8cf7a6c8a5b12e521ccec8c2aaad

TGTAATGTCCAAAGAACCGTTAAAACTAATTAGAAACCCTAGTAGTCCGGCTGACTGACTACGTACGTATCTCGTATGCCGTCTTCTGCTTGAAAAAAAAAAAAAAA

>615cef519aad1758bc225f70d5f9283f

CACACACGGTAAACACAGAACGACGCTGAATGAATAAATCGCAAATATATTAATAAACTCACTTTTTATACATACGCCCGATTTACTTGAGAAATCGCGGCACACCA

>7c9135569ca19242ead53377c858b8a9

TACAGTAGCTTCAACACCAGCTAAAATAAAACTCATGTTACATAATTACTGATTGTGTTGTTATTTTGTTAGCCAATTGACGTTTTACGCCTATTATATTTAGATTA

>bd57ab9fa6c31d0c3b53a7cc7fb4071b

TACGTAGGTGGCAAGCGTTGTCCGGATTTATTGGGTTTAAAGGGTGCGTAGGCGGCCCTGTAAGTCAGTGCTGAAATATCCCAGCTTAACTGGGAGGGTGGCATTGA

>03644364405b079b2ff4eff8bf18c470

TCCGGCTGACTGACTTAACGTCCATCTCGTATGCCGTCTTCTGCTTGAAAAAACAAGTAGTCCGGCTGACTGACTTAACGTCCATCTCGTATGCCGTCTTCTGCTTG

>f5989117bdd68fc0c733d9b7e9390623

CATTTTACCATAGTGTCTGCCTCCACGGGCACGTTTATGGCTTTGCCGGTTATCTTTTGAGACTGTTTCTATTCTGAGATGACGGATCTGCATACGTGGAAATCCGG

>42e520ff23e6e8948de9ca6e0cb77725

TACGTAGGGTGCAAGCGTTGTCCGGAATTATTGGGCGTAAAGAGCTCGTAGGCGGCTTGTCGCGTCTGCTGTGAAATCCCGGGGCTCAACCCCGGGCCTGCAGTGGG

>b4b96d37afea19c769d689c7cd1161b4

TACGGAGGATCCAAGCGTTATCCGGATTTATTGGGTTTAAAGGGTGCGTAGGCGGCCTGTTAAGTCAGGGGTGAAATTTTCCAGCTCAACTGGAAGCTTGCCTTTGA

>5fecaf637db2785ed35820ceb314be59

AAACTAAAAAAATATAAACTCCTGTGTAAAGTAGACCCGGTTAATTACGGTGTAAAGAAATTCGTAACTTAATTTTGTTACCAAAAATTAAAAATAATTTCAAAAAT

>adacd30ee0968f5dec16a01a04392cc6

TACGGAGGATCCAAGCGTTATCCGGAATCATTGGGTTTAAAGGGTCCGTAGGCGGTCAGATAAGTCAGTGGTGAAAGCCCATCGCTCAACGGTGGAACGGCCATTGA

>55c58d6eee93457d480967406c132a87

TACGTAGGGTGCGAGCGTTAATCGGAATTACTGGGCGTAAAGCGTGCGCAGGCGGTCTGACAAGTCTGATGTGAAAGCCCCGGGCTTAACCTGGGAACTGCGTTGGA

>3622ae5e18e0d9abf519c34b62ced9a8

TACGGAGGGAGCTAGCGTTGTTCGGAATTACTGGGCGTAAAGCGCACGTAGGCGGCTTTGTAAGTTAGAGGTGAAAGCCTGGAGCTCAACTCCAGAACTGCCTTTAA

>55c974c5fc929137f3e7362382470586

AGGACTTGGAGGAACGACTCTGATAAACTATATGTTGTACACCAGAGGCAGTTCACGGGGTTATAACAAATATGCTCAAGACGGAAACTATGGTTGGAGCTTTAAAG

>fb41ccc4cd0b31288d75f129edff78b5

TACATAGGGGGCAAGCGTTATCCGGATTTATTGGGCGTAAAGGGTGCGTAGGCGGTTAAGTAAGTCTGTGGTCTAAACGCAATGCTCAACATTGTGACGCTATAGAA

>ae38923886aee3cbfcfdce44cb304e65

TACGTAGGGTGCGAGCGTTAATCGGAATTACTGGGCGTAAAGCGTGCGCAGGCGGCTTTGCAAGACAGATGTGAAATCCCCGGGCTCAACCTGGGAACTGCATTTGT

>9a2fb842d0a93686d332d4dff1cbc372

TACGATTAACCCAAACTAATTATCTTCGGCGTAAAACGTGTCAACTATAAATAAATAAATAGAATTAAAATCCAACTTATATGTGGAAATTCATTGTTAGGACCTAA

>21e996af64a146bced53a07a45b11d42

TACAGAGGGTGCAAGCGTTAATCGGATTTACTGGGCGTAAAGCGCGCGTAGGTGGCCAATTAAGTCAAATGTGAAATCCCCGAGCTTAACTTGGGAATTGCATTCGA

>5b3d5a9c178f56156c6d785e2f4e59b8

TCCGGCTGACTGACTTCTAGACTATCTATTAGATACCCTAGTAGTCCGGCTGACTGACTTCTAGACTATCTCGTATGCCGTCTTCTGCTTGAAAAAAAAAAATAATA

>c08faa38b4737d23ef7aa0218a34c8a7

CATTGAACTATCGTGAGAAAGTCAAACCGCCAAAGGGAATTATATTATAGTAAATATTGGCGTAAATAAACATTTTATTAATAGTTGTAATATATGAAAATGTGCAT

>2d3429fb6ab5578b36af3eb51df0658c

TACGTAGGGTCCAAGCGTTAATCGGAATTACTGGGCGTAAAGCGTGCGCAGACGGTTGTGCAAGACCGATGTGAAATCCCCGAGCTTAACTTGGGAATTGCATTGGT

>44966df353bb6afde48dfbc1fa716e00

ACCACAACGCCTTCCCGCCGCGCGCGATGCGCCAAGAAGCTTAAGAAACTTGCATTACAATAAACATAATAACTAACAATTTAATAAGTAAGTAAGTTGAGAGTAAC

>a7bc02471511db10395e961fe673a026

TACGTAGGTGGCAAGCGTTGTCCGGATTTATTGGGCGTAAAGCGAGCGCAGGCGGAAGAATAAGTCTGATGTGAAAGCCCTCGGCTTAACCGAGGAACTGCATCGGA

>10b8054881bff81c24a624bec9b6043e

TACGTGAGAGACTAGTGTTATTCATCTTAATTGGGTTTAAAGGGTACCTAGACAGTCAATATAACTTCTATAATGCTAATACTTGACTAGAGTTCTAAGTAAGAGGG

>fc127f60b5e0fb88d5f1781c6b795b48

CGCACGTCTTATACTGTTTCATCCGGAACTAAGAAACAGTTGAAACCGATCCGATATCTGCCTGCGTCTATCGGCGAGCCCATGTCCAGGATCATGAACCATTGTCG

>142638a074cb9e120fd849306a897921

TACAGAGGGTGCAAGCGTTAATCGGATTTACTGGGCGTAAAGCGTGCGTAGGTGGTCTTTTAAGTCGGATGTGAAATCCCTGAGCTTAACTTAGGAATTGCATTCGA

>dd9ccf13a27595403504f5453a8c5894

TACAATTTTTTCAATTTAAATTTATAAAGTTTCAGTAAATATATAATAGAATGTTTATAAATAATAATTTTAGTGAAATATGTTATTATTATGCTTTCATGATTTGT

>043a982185e371a975971a0be68cbda3

TACGTAGGGTCCGAGCGTTGTCCGGAATTATTGGGCGTAAAGGGCTCGTAGGCGGTTTGTCGCGTCGGGAGTGAAAACTCAGGGCTCAACCCTGAGCGTGCTTTCGA

>4139a0c2b0d54ba4f3f46d270a6329d3

TACAGAGGGTGCAAGCGTTAATCGGAATTACTGGGCGTAAAGCGCACGTAGGCGGTTGTTTAAGTTGGATGTGAAAGCCCCGGGCTCAACCTGGGAATTGCATTCAA

>dff5b274df94597b7056f2b461e070e2

TACGAAGGGGGCTAGCGTTGCTCGGAATGACTGGGCGTAAAGGGCGTGTAGGCGGTTTGTACAGTCAGATGTGGAATCCCCGGGCTTAACCTGGGAGCTGCATTTGA

>fd20257ae8015c90f3e75641bad91504

TTAGAAACATCAGTATAGGCCGCCGAGTTGCTGACTCTCAGACATTGTACCTAGCGTGCGTCATACAGGTTCAATGATGGTTTCCATGAGTCCCCATACGCTCCTGC

>21db271313c73efb1b8c6e518e971426

TATTCTCGCGCAAACGTAAGCGGCCTCGGCCGCTTAAAGTAGGCCCCGTCGAGGTGCCCTGGACCAACAACGTCAAGTACCTCGGGCTCCACGTGGATTCCCGACTC

>e9812af9e30e2469491c0906c173efe4

GTGGTTTTCACCAAATTTTTTTTATCTAAATTAGATACCCGAGTAGTCCGGCTGACTGACTTGCGTCAAATCTCGTATGCCGTCTTCTGCTTGAAAAAAAAAAAAAA

>8a37b6773120b6ce2e358fa42f6f7a01

TACGTAGGGTGCAAGCGTTAATCGGAATTACTGGGCGTAAAGCGTGCGCAGGCGGTTATATAAGACAGATGTGAAATCCCCGGGCTCAACCTGGGAACTGCATTTGT

>94357fb170fa1b1a29ea84da805b9511

TACGTAGGGGGCTAGCGTTATCCGGATTTACTGGGCGTAAAGGGTGCGTAGGCGGTCTTTCAAGTCAGGAGTGAAAGGCTACGGCTCAACCGTAGTAAGCTCTTGAA

>3fe7624811efdf77d93649fbf16dfced

TACGTAGGTGGCAAGCGTTGTCCGGAATTATTGGGCGTAAAGCGCGCGCAGGTGGTTTCTTAAGTCTGATGTGAAAGCCCACGGCTCAATCGTGGAGGGTCATTGGA

>d2e039448ee2aeb678ea15e6ff5661ba

TACGTGAGGGACTAGTGTTATTCATCTTAATTGGGTTTAAAGGGTACCTAGACAGTCAATATAACTTCTATAATGCTAATACTTGACTAGAGTTTTAAGTAAGAGGG

>3d3c1ec0f369fc56942b096f2c65fc50

ATGACAAAATCATTGAACATGTTCAACATATTATTCAAAGTGACACATTATTTCAAAGAAGAAAATCTAACAACCTCTGATGCGTTTCTCTTGTATATGTATATTAG

>1c8ac9a2c31c6c6c9f3bf86c7ef82ad7

GACGGGGGGGGCAAGTGTTCTTCGGAATGACTAGGCGTAAAGGGCACGTAGGCGGTGAATCGGGTTGAAAGTGAAAGTCGCCAAAAACTGGTGGAATGCTCTCGAAA

>9fea791b0cb7ad9ecb5f3e3dc7614b63

GATATATTGAGCTATTTGTAGCTGTGAAGTGTACCAGATATGTTATTAGTATTAATTGAGTTGAGGCGGAGCTCTACAATTACGGACACTTGAAGTGGTGTAGTCTA

>8a5d65b2be64af1fc11cd559e470bd5c

CGAGGACGATTCACGCTGTATTTGGCATTCGGACGGTCTCAGTACTATCGGTACAGCACTTTTGTACAGTGAACGATATGAGGACGCTACAACTATCCAAATTTCTT

>8c0313eb6dd552bca246fe70e9e88139

AACAGAGGATACAAGCGTTATCCGGATTTATTGGGTTTAAAGGGTGCGTAGGTGGTTTTTTAAGTCAGTAGTGAAGTCTTAAAGCTTAACTTTAAAAGTGCTATTGA

>a54e4d5f9a18d20999462689ced36851

GTGCTCAAAGAACATCAATCTTTCACTTCGTTCTCACTTTCTCTTTTTACTGCCTCCTCTTCTGACCCTTTTCCACCATACCCGTTCCTCTCCTTTTCGTCATCCCT

>4290b6e355c7800fc274768538c4f1c4

TGTGTTAGGTCTGAAGCCGGTGACAGACAAAGTAGCGTGTTTTTATCTATTTATACCGTCTGTTGTCCAAGATAACGCCGCCGTTCCCGCAACCCTGCGTCTGTACT

>f3eeb32d109ff354a310581a9b5454b4

CGGTTTACAAAGTATTGGGCAACTTTATGCGTTCATTTTGGGTGTTAAAACTTAAAAATCTCATCTTTTTTTATCGTTAAGGGAATCAAGATCCAGCGAGTAGCTGT

>32baafe2837289d34721a30f6e707c02

CACACTTCCCTACGTCTTTAGAAAGACATAACATTTTTCCAGAATCCTTTTCAATCACCATAGGCCAATATATATTATATAAGTTTATATTTAAATGGCTACATGTA

>f7cf1666475be7658133d99868ce52ed

TCCGGCTGACTGACTAGTCGCAGATCTCGTATGCCGTCTTCTGCTTGAAAAAAAAACCGGGTAGTCCGGCTGACTGACTAGTCGCAGATCTCGTATGCCGTCTTCTG

>98439a3a002bcc93957b3ffdd2cf2941

TACGTAGGTGGCAAGCGTTGTCCGGAATTATTGGGCGTAAAGGGCGTGTAGGTGGATTCCTAAGTCGTGTGTCTAAGTGCGGTGCTCAACACCGTATGGGCGCAGGA

>a42b26462accf466b0edf46b40b1fa7f

CACACGTATGTCTTCCGAAAGACAAAACGTTTCTCCAGGATGCATTTCAACCTTCTCGGTCATTTTCAACTGGCAGTGCCTTGTAGATATTTGAGAAAGATTTTGAA

>336ca8c13df5f96f08765578f40c14b8

CGCTATACATATTCATTTCTTTCAAAGATCGGAAAATAAAGCGTTCTTGTTCAATAAACAAACTTCCCTTAATTATAGTAGTGTTGTTTAAAAGGATTAATTAACCC

>4ca135bf09289c2aaf61833d6a1f39c1

TACGAAGGGGGCTAGCGTTGTTCGGATTTACTGGGCGTAAAGCGCACGTAGGCGGATTGTTAAGTTAGGGGTGAAATCCCAGGGCTCAACCCTGGAACTGCCTTTAA

>217635a19702e65212975019a889f493

TACGGAGGGTGCGAGCGTTAATCGGAATTACTGGGCGTAAAGCGCACGCAGGCGGTTTGTTAAGCTAGATGTGAAAGCCCCGGGCTCAACCTGGGATGGTCATTTAG

>046f54f1c97df6c5de52138084251f1c

TGGCTGCCCTCTATTTCTCTGCCCACTCAACGTAGATTATAATAGCGGCAGTGTCGGAAACGTCTTTCCCCTCTATGCAGTTCCTCGATATAGTCGACGTATTCCGA

>1d43e7acecf94872adced9b6fa9014f1

CACACTTATTTAGAAAGACAAAACATTTTTCCAGAATGGATTTCAACCCTGTCGGTCATTTTCAACTGGCAGTGCCTTGTAGATAGTTGTGGAAAATTTTGAACGTT

>37d5005bf172fc11ace7585084c5e509

CCACCCCCACCGCTGTGTTCATACTTTTTTTTTTTACAATAACAGATGTCACAATTCACACACACACACACACACTTAATGTACATTAGATACCCCTGTAGTCCGGC

>d2634d5e25ebad026785ffbaf44493d4

TACAATTTCATCAATTTAAATTTTTAAAAGTTTCAGTTAATAGATTATTTAAATTTTTGTGAATAATAATTTTAGTGAAATATATTATTATTATTTTATTTTATTAG

>dc0e6f1a956fbb5c46e7006f8057800d

AAGATCTCAAGCCAATCTGGTGCTCTTGCAGCTTGCGAACAGCTACTGGACGGCCTGTAAAAGTGTATGGAAAAGTGGAGATACACCTACGCCTCGGTAGACTTGAG

>6b3afca972bf0ef59b00776dac52ba2e

TATTCTCGCGAAAACGTAAGCGGCCTCGGCCGCTTAAAGTAGGCCCCGTCGAGGTGCCCTGCACCAATAGCGTCAAGTACCTCGGGCTCCACGGGGATTCCCGACTC

>fb444e10e0631b29fad6f120aec77647

ACAACAAGGGGAACCTAACAATGAGCCGCTTCGCAATCAATCAATTTTCGCCACGCCACATCAACGCCATTCGTGTAGCACAATTTTTCTTCGCCTATCTACACACA

>d09b2cf02e271c14e243f9888f5e6497

AGCAGTTTTGTTTAATACATATATATCTTTAACGTACTTGGACACGAACGAACGCTCGAGGTATAAACGTTTTAGGGATTTATTTATAGGGGTGTATATGGAAAGGG

>0c6f4807ac08bdf1b52711dbb9247c7e

CACACTTATGTCTTCAGAAAGACAAAATATTTCTCCAGGATGCATTTCAACCATGTCGCTCATTTTCAACTGGCAGTGCCTTGTAGTTATTTGTGAAAGATTATGAA

>30bb1902fe19246125a2e9bca6f10817

CATTGAACTATCGTGAGAAAGTCGAACCGCCAAAGGGAATTATATTATAGTAATTATTGGCGTTAATAAACATTTTATTAATAGTTGTAATATATGATAATGTGTGA

>3287183b692e17dbc0cc73bdb4382011

CAAAATAATTTAATTTTGTCTTACGCTTGGGTTTTTGGGAACAAGATTATAAAACTGACGCCAAACTTTTTATCAAAAACTATGAAAATACTTTACAATTGATATTA

>d94dad529a0d45fba69e28b43f258c7c

GATTTCGTAATAAATCGGATGTTACAACCAAATTATGACGTCAATAACTGCTTTATGAGATTCGTCATGATCTATTCTTGTCTATTGAGTCATCGCGTATAATTATC

>94d47eb05dad288e2e11f5ac7a91c365

TACGTAGGTGGCAAGCGTTGTCCGGATTTACTGGGCGTAAAGGGTGCGTAGGCGGACAATTAAGTCAGATGTGAAATCCCAGGGCTCAACCTTGGGGCTGCATTTGA

>e3709f992d3273f0fa21084f20dac174

ACAACAAGGGGAACTTAACAATGAGCCGCCTCGCAATCAATCAATTTTCGTCACGCCACATCAACGCCATTCGTGTAGCACAATTTTTCTTCGCCTATCTACACACA

>cb7fe7a47d2fb0691851e284798f011c

CATTTTACCATAGTGTCTGCCTCCACGGGCACGTTTATGGCTTTGCCGGTTATTTTTTAAGACTGTTTCTATTCTGAGATGACGGATCTGCATACGTGGAAATCCGG

>a4ab5a745553cb3573a976c37e951a89

TACGGAGGGGGCTAGCGTTGTTCGGAATTACTGGGCGTAAAGCGCACGTAGGCGGCGATTTAAGTCAGAGGTGAAAGCCTGGAGCTCAACTCCAGAACTGCCTTTGA

>ac00a5e85e6844c53b98bd5cb4e8a840

TACGTGAGAGACTAGTGTTATTCATCTTAATTGGGTTTAAAGGGTACCTAGGCAGTCAATATAACTTCTGTAATGCTAATACTTGACTAGAGTTTTAAGTAAGAGGG

>6c1861b8c18ab41038889a42c6d6e194

CTCGATTAATAACAGACGCGGGAACTGCGTACTGTGCATACGAAATACGTTAATCAAGTCGTTAATCAATCTTGCAGTTCCGCCTACCCGACGAAATCTAACGCAAC

>dc376119ce2b6320d913d46ff8143bb1

AACAGAGGATACAAGCGTTATCCGGATTTATTGGGTTTAAAGGGTGCGTAGGTGGTTTTTTAAGTCAGTAGTGAAATCTTAAAGCCTAACTTTAAAAGTGCTATTGA

>001ed8f062ebe4c5bdd72591415e24f2

ACAAATGGTGAATAGTATAGTGTCAATAATGGCATTAGAAACCCGAGTAGTCCGGCTGACTGACTATGAGCTCATCTCGTATGCCGTCTTCTGCTTGAAAAAAAAAA

>d1cbb5cc9a102bebc88ace177a402df9

AACAGAGGATACAAGCGTTATCCGGATTTATTGGGTTTAAAGGGTGCGTAGGTGGTTTTTTAAGTCAGTAGCGAAATCTTAAAGCTTAACTTTAAAAGTGCTATTGA

>228f423b2dcbd2e9060a368ac49e2440

TAAGGCCTCCTTCTAAGTTGCACTCCACTTGGTATGACCTTGTTCAGCTTTTCCACAATCCTGAGCCATCTAAAGCTTAGTCACCTTCAAGCTAAGATATAAGCTCA

>5d5109fccac9360cf7e97bdc3b470933

AGGAATGAGATATCCGCATCTTCGATATTCTCATTTGATCGCTGTTCACTCTATTTCTCATTTTTAACGCAACTTAAAAACAATTCTCTAAGATTGCACGTTTCTAA

>78c1a0433ffe88e9fb149205ddb641f0

TACGGAGGGGGCTAGCGTTGTTCGGAATTACTGGGCGTAAAGCGCACGTAGGCGGACCAGAAAGTCGGGGGTGAAATCCCGGGGCTCAACCCCGGAACTGCCTTCGA

>c5d0a9c08b4a354fe9187b7876d10074

TTCCAGCTCCAATAGCGTATATTAAAGTTGTTGCGATTAGAAACCCGTGTAGTCCGGCTGACTGACTCGCGATATATCTCGTATGCCGTCTTCTGCTTGAAAAAAAA

>c5124d4dde2f48b221feba338f2749af

TACGGAGGGGGCTAGCGTTGTTCGGAATTACTGGGCGTAAAGCGCACGTAGGCGGCTTGGTAAGTTAGAGGTGAAAGCCTGGAGCTCAACTCCAGAATTGCCTTTAA

>61d8dafece91c14be35a3f34ac4e3c2c

TACGTGAGAGACTAGTGTTACTCATCTTAATTGGGTTTAAAGGGTACCTAGACAGTCAATATAACTTCTATAATGCTAATACTTGACTAGAGTTTTAAGTAAGAGGG

>131de23b96243600a62abb65627ed2d6

CAATATATTAAACATAGAAATTTAATGTAAATATTGAGAGGATTAAATCGATTATTGTAGACCAAGTTCGTGAAGACGAAAACGCAGATTCAAAACAGACAATAAAA

>c57c0e69db1fc793f299cd38e396ba2d

AGATATAATTCGGATTTTATACAGTCAATTTCCCCTTAAAAGACGATGCCAAACCGAACAATGGTATACCTGCCTAGTCGTAATCTCTACTTAGCAATTCGGTGCGC

>298fd559a2671fd2a5d7f8c2fad6f6e3

CATTGGACTATCGTGAGAAAGTCAAACCGCCAAAGGGAATTATATTATAGTAAATATTAGCGTAAATAAACATTTTATTAATAATTGTAATATATGAAAATGTGCAG

>a29c4300497d4e45080cf66f6c1eb84d

TACGTAGGTGGCAAGCGTTGTCCGAAATTATTGGGCGTAAAGCGCGCGCAGGCGGCTATGTAAGTCTGGTGTTAAAGCCCGGGGCTCAACCCCGGTTCGCATCGGAA

>a195d5e010573d597a4f35c7272879e0

GGGTAAGTGCTCTTAACTGCAGGACCTCCAATTTTCTCTTTTAAGAAGCATTTTACAGTTAGCTGGATTTCACATTAGCTTGCCTTCTATTGCTGATACTAGACACT

>81d8b1a1c35c9bffdb8a48b590435898

AACAGAGGATACAAGCGTTATCCGGATTTATTGGGTTTAAAGGGTGCGTAGGTGGTTTCTTAAGTCAGTAGTGAAATCTTAAAGCTTAACTTTAAAAGTGCTATTGA

>9df8a98010078aad1394651b4b115a79

ACAACAAGGGGAACCTAACAATGAGCCGCCTCGCAATCAATCAATTTTCGCCACGCCACATCAACGCCATTCGTGTAGCACAATTTTTCTTCGCCTACCTACACACA

>e858527f60ca27b2c5f84696b2c3a20a

AACAGAGGATACAAGCGTTATCCGGATTTATTGGGTTTAAAGGGTGCGTAGGTGGTTTTTTAAGTCAGTAGTAAAATCTTAAAGCTTAACTTTAAAAGTGCTATTGA

>a6c08766a592b53ac108b4bbb7f5b683

CACACGTGACAAAAAATGGCAACACACGATACAAGCCAAATCTAACAAAGCTCAAACAGTTTCGGTAAAAAAAGGTAGAAATGGTAGAACTGTAGGCGGACTGAGGC

>848fd6d8574db324195d27089a22037d

TACGTAGGGTGCAAGCGTTGTCCGGAATTATTGGGCGTAAAGAGCTCGTAGGCGGTCTGTCGCGTCGGCTGTGAAAACCCGAGGCTCAACCTCGGGCCTGCAGTCGA

>653e343400e6b969e261bc8186776dfa

CCCCATGGCGCATTAGAAACCCTGGTAGTCCGGCTGACTGACTATGAGCTCATCTCGTATGCCGTCTTCTGCTTGAAAAAAAAAATTTTTTTTTTTTTTTTTTTTTT

>7a5540147206a6365128bfc936b014a3

TACGTAGGTGGCAAGCGTTGTCCGGAATTATTGGGCGTAAAGCGCGCGCAGGCGGTTCCTTAAGTCTGATGTGAAAGCCCACGGCTCAACCGTGGAGGGTCATTGGA

>69c735dbe1c11a7dd896f2f418a45fe1

TCTGTTTCGTATGCTCCAATCTCAGTTTTACTCTTTCCTCTGCAGTTATTCCCTCCTCCACTTCTTCTTTAATTACTCTGTGAACCAATCCTGTATGTCTCAAGACA

>67fed31da912b2c4961b78b4506c8baf

AGTCAGCGGTATATAATAAAAAATCCTAAAGTAAGGCAGAGTGCTGAGCATCCGATGATTATGCGTTACCATTCTCGTTATTTATGTAATGTAAGTCAATCACTAAT

>de129cd3aaa6b68e89831fc7f421ce60

TACAATTTCTTCAATTTAAATTTTTAAAAGTTTCAGTTAATAAATTATTTAAATTACTATGAATAATAATTTTAGTGAAATATATTATTATTATATTTTATTAATTT

>45c77fdcedb4b213429adbff235ec20f

CCGCTGTGTTCAGTTTGATATACTTTTTGTACAATACACAATAACAGATGCCACAAGCTACACACACACACACACACACACACACACACACACACACACACACACAC

>a291f63d163b661047e3e9d36686d97d

TACGTAGGGGGCGAGCGTTGTCCGGAATTACTGGGCGTAAAGCGCGCGCAGGTGGCTTGTTAAGTCAGGTGTCAAAATGCGGGGCTCAACCCCGTACCGCACTTGAA

>1826c6364427bbc5334f45a5182c5d05

TGTAGCGACAAGTCCATGTTTTTTATCGGCAAACAATGGGCCGATTGTTTTACTAATTCATTGTTTAATTTCTCCAAAAACTGCAGAATATCGTAACATTCACTGGA

>5641e27ac7854f23b04b9eaac901412a

TACGTAGGGGGCGAGCGTTGTCCGGAATTATTGGGCGTAAAGCGCGCGCAGGCGGCCCTTTAAGTCTGATGTGAAAGCCCGCGGCTCAACCGCGGATGGGCATTGGA

>02b0a37c15af391d9dcfa25dbeea292f

CATGGTCATCCAGCCAGATTTCGTATTTATGTACGTGACGCTTGACTTTATTCACGTGAAAAGCGATGCTTTAACCACACGACCGTACCACCGGGTGAATTGAGTTA

>6f3d0c79f9318686a41a688bf7bfa698

TACGTGAGAGACTAGTGTTATTCATCTTAATTGGGTTTAAAGGGTACCTAGACAGTCAATATAACTTCTATAATGCTAATACTTGGCTAGAGTTTTAAGTAAGAGGG

>12b395473950133daaff28c95f3253f7

GACAAGGGAGACAAGTGTTATTCATCTTTAACAGGTATATAGGGTACCTAGACGGTGTAAAATGGCTTAAATAAGTACCTATTACACTTGAGTTTGATATGGGAGAG

>1de348c777ed3a11794dec5202811d1f

TACGTAGGGTGCAAGCGTTATCCGGAATTATTGGGCGTAAAGAGCTCGTAGGCGGTTTGTCGCGTCTGTCGTGAAAGTCCGGGGCTCAACTCCGGATCTGCGGTGGG

>e4a80978e1066ea017fb3fd4798ea397

CATTGAACTATCGTGAGAAAGTCACGCCGCCAAAGGGAATTATATTATAGTAAATATTGGCGTAAATAAACATTTTATTAATAGTTGTAATATATGAAAATGTTCAG

>38202c7fa941f454cd723e3d99a67513

AACAGAGGATACAAGCGTTATCCGGATTTATTGGGTTTAAAGGGTGCGTAGGTGGTTCTTTAAGTCAGTAGTGAAATCTTAAAGCTTAACTTTAAAAGTGCTATTGA

>cd6fb581c4fe891a979ec93c1168e747

ATCAAATATATCGTGGTCATTCGTGGTTATATATCTGCTTTTGATCTGCCATGTGGTTGTTTAATATTGATGCTTACGAACATGTAATTGTGCATTTAAGTTGTACA

>334607b1d3431b059495bf4b69ef762c

TACCGGCAGCCCGAGTGATGGCCGATCTTATTGGGCCTAAAGCGTCCGTAGCTGGCCGCGCAAGTCCGTTGGGAAATCTGCCCGCTTAACGGGCAGGCGTCCAGCGG

>24ea5f5c3604ec78d562849b3ff9c324

AACAGAGGATACAAGCGTTATCCGGATTTATTGGGTCTAAAGGGTGCGTAGGTGGTTTTTTAAGTCAGTAGTGAAATCTTAAAGCTTAACTTTAAAAGTGCTATTGA

>ae5af40e6eb417b9879bd05a70d055bb

TACGTAGGGTGCGAGCGTTGTCCGGAATTACTGGGCGTAAAGAGCTCGTAGGTGGTTTGTCGCGTCGTCTGTGAAATTCCGGGGCTTAACTCCGGGCGTGCAGGCGA

>f5983ff4b803dd062318f87bd17d1907

ACTGCAGGTCGCGCATACCTGTGAGAGAATCTGTAACACACACACCACGGGTATATGCAGAGAGGTCGCCTACGCTCTACGCTAGTGTGGCACTCAAAGAGAAGACA

>11ae728e83839c25764b3f17d96125ae

TACAATTTCTTCAATTTAAATTTTTAAAAGTTTCAGTTAATAAGTTATTTAAATTTTTATGAATAATAATTTTAGTGAAATATATTATTATTATTATATTTTATTAA

>72be195ddd239716ce58bb99e06f3a40

TACGGAGGGGGCTAGCGTTGTTCGGAATTACTGGGCGTAAAGCGCACGTAGGCGGTTTTGTAAGTCAGAGGTGAAAGCCTGGAGCTCAACTCCAGAACTGCCTTTGA

>922d39eb8c20267a9f42088847e0db7a

CAAATTTATGTCTTCAGAAAGACAAAATATTTTTCTAGGGTCCATTTCAACCATGTCGGTAATTTTCAACTAGCAGTGCCTTGTAGGTATTTGTGAAAGATTTTGAA

>a1912f4bc53ad26dd0ffd16593513011

TACGTAGGGTGCAGGCGTTAATCGGAATTACTGGGCGTAAAGCGCACGTAGGCTGCTTGGTAAGTCAGGGGTGAAAGCCCGCGGCTCAACCGCGGAATTGCCTTTGA

>5884f2dc21e2a2c81f34d5a6da8dce98

ACACAGAACGACGCTGAATGAATAAATCGCAAATATATTAATAAACTCACTTTTTATACATACGCCCGATTTACTTGAGAAATCGCGGCACACCACCACCCCAAAAG

>ba38d0fd7123d5cea8b2e4968eeaaca5

ACAACAAGGGGAACCTAACAATGAGCCGCTTCGCAATCAATCAATTTTCGCCACGCCACATCAACGCTATTCGTGTAGAACAATTTTTCTTCGCCTATCTACACACA

>17ab8d6dddb347e1d226fee1cf956bdc

TACGATTTCTTTAATTTAAATAGTTAGGTTTCAGTTAATATATTAATAATATAAAATAGTTATAATTTTGGTGAAATATATTTTATCTTGAAAAATTATTTTTATGT

>f47ab3d016dba1a6b5f61201d5ada085

CACACACGGTAAACACAGAACGACGCTGAATGAATAAATCGCAATTATATTAATAAACTCACTTTTTATACATACGCCCGATTTCGTTGAGAAATCGCGGAACACCC

>d6c06505ebc8d0d9aa9de96c79538dc9

CAAATTTATGTCTTCAGAAAGACAAAATATTTTTCCAGGGTCCATTTCAACCATGTCGGTCATTTTCAACTAGCAGTGCCTTGTAAATATTTGTGAAAGATTTTGAA

>11adf773c70e0817cc853a5d0be9f279

ACGATCCACCCATAGATGGATTCGAACTCACAACGCCCCGTTTTGGAAGCGGCAGTGCTGACCACTGCGCACTATGACATGTAATTAGTTATACATCACTTTAGTGA

>82402dddbe453eff97f1fa178210ea2b

TTAAAGAAACGCCGTCTACTACGAACGTGTATGAACAGCAAATGTGTGTCACTAACGAAAACAAATTCATTCGTAAAACATACTCGGTACCGCTGCACTATCAAGAG

>e7922c4bf7d590f2b1c3a879a75419c8

CACACTTATGTCTTTAAAAAGACAACACGTTTTGCAGAATGCATTTCAACCTTCTCGGTCATTTTTAACTGGCAGTGCCTTGTAGATAATTGTGGAAGATTTTGAAA

>598f89c39658a7f09e6376d818ac1118

TACGATTTCTTTAATTTAAATAGTTAAGTTTCAGTTAATATAATAATAATATAAAATATCTATAATTTTGGTGAAATATATTTTGTCTTTAAAAATTAATTTTATGT

>5e1bbf34d28e786aceaa37a5db6317cf

TACAGAGGGTGCGAGCGTTAATCGGATTTACTGGGCGTAAAGCGTGCGTAGGTGGCTTTTTAAGTCAAATGTGAAATCCCCGAGCTTAACTTGGGAATTGCATTCGA

>ddb9e7a2410f87686047e72b6acb5a2f

ACGCAAGGAATACACGCTGCATATAACGCGCATGCGCGTACAACGCCAACGTATTATTTTAAATGCATTAAGACCGTTCGCGACGTATTAAAAGCGGGGTCGGTCGG

>63644a1425df21643d5425e216eaa118

CCACCACAACCCCCCTCCGCTATGTTCATACTTTTTTTTTTTTTACAATAACAGATGCCACAATTCACACACACACACACACACACACTTAATGTACATTAGAAACC

>1ddf71219788cd70b74b325296c51705

TATGATCTTCACGTGACATCTCTAGAACAATGCTAGCATTTACACCATGTTTTAAGTCGCACACAAAGAAACCCGCCTACTGTATTTATTGAATTACACCATTATTG

>83cf3986698ad82140b9fcf6d688ff59

TACGTAGGGGGCAAGCGTTATCCGGAATCACTGGGCGTAAAGGGTGCGTAGGCGGCCAATCAAGCCAGGGGTGAAAGGCTACGGCTCAACCGTAGTAAGCCCTTGGA

>db4b6785e2b56098745fda06108b3b02

TACGTAGGGCGCGAGCGTTATCCGGAATTATTGGGCGTAAAGAGCTTGTAGGCGGTTTGTTGCGTCTGCTGTGAAAGACCGGGGCTCAACTCCGGTTCTGCAGTGGG

>465305479651d27c09e190f0ac11bee9

TACGTAGGGCGCAAGCGTTGTCCGGAATTATTGGGCGTAAAGAGCTTGTAGGTGGCTTGTCGCGTCTGCCGTGAAAACCCGAGGCTCAACCTCGGGCGTGCGGTGGG

>fea090ccbcfc22a95dfb7fe49f8c8921

TACGTAGGGTGCGAGCGTTAATCGGAATTACTGGGCGTAAGGCGTGCGCAGGCGGTCTGACAAGTCTGATGTGAAAGCCCCGGGCTTAACCTGGGAACTGCGTTGGA

>cb69d45997f2ec80adbf3c72bada55b5

GACAGAGGATGCAAGCGTTATCCAGAATGATTGGGCGTAAAGTGTCTGTAGGTGGCTTTTCAAGTCCGCCGTCAAATTCCAGGGCTCAACCCTGGACAAGCGGTAGA

>ad9f63b3ecbc782087d047af2bf82a20

TATTCTCGCGAAAACGCAAGCGGCCTCGGCCGCTTAAAGTAGGCTCCGTCGAGGTGCCCTGGACCAATAGCGTCAAGTACCTCGGGCTCCACGTGGATTCCCGACTC

>267e4d90155d404dc1c713bf6f07e767

TACAATTTCTTCATTTAAATTTCAAAAGTTTCAGATAATAAGTTATTTAAATTTTTATGAATAATAATTTTAGTGAAATATATTATTATTTTATTTTATTAATTTGT

>a66a6a662e39e04b807820a3165c760e

TACGTAGAAGACTAGTGTTATTCATCTTTAATAGGTTTAAAGGGTACCTAGACGGTAAAATTAGTCTAAAATATGATACGGTTTTACTAGAGTTATATATAAGGAGG

>720c78e688a06dac412f432f6f3a58d9

TACGTAGGTGGCGAGCGTTGTCCGGATTTACTGGGCGTAAAGGGTGCGTAGGCGGATTCTTAAGTGGGATGTGAAATACCCGGGCTTAACTTGGGTGCTGCATTCCA

>e1cfc520d1163b8de61605b60ec45300

TACGTAGGGTGCAAGCGTTAATCGGAATTACTGGGCGTAAAGCGTGCGCAGGCGGTTTGTTAAGACAGATGTGAAATCCCCGGGCTCAACCTGGGAACTGCATTTGT

>1e1ed43433b1f64d0495e3f7162f1eec

TACAATTTCTTCAATTTTAAATTTATAAAGTTTCAGTAAATATATAATGTAATTTTTATAAATAATAATTTTAGTGAAATATGTTATTATTATGTTTTTATGATTTG

>3fada69d4798a7398f9300735e42ba5a

TACGAAGGGGGCTAGCGTTGTTCGGAATTACTGGGCGTAAAGCGCACGTAGGCGGATCCTTAAGTCAGGGGTGAAATCCCGAGGCTCAACCTCGGAACTGCCTTTGA

>0f36db3e5442fa95142b145c747cfaab

CATTGAACTATCGTGAGAAAGTCAAGCCGCCAAAGGGAATTATAGAATAGTGAATATTAGCGTAAATAAACATTTTATTAATAGTTGTAATATATGATAATGTGCAG

>5d9c701f4b5ef04da00fa6a793954f4a

TACGAAGGGGGCTAGCGTTGCTCGGAATTACTGGGCGTAAAGGGCGCGTAGGCGGACAGTTAAGTTGGGGGTGAAAGCCCGGGGCTCAACCTCGGAATTGCCTTCAA

>94e23940eb814c45af5b444065e6d121

TCCGGCTGACTGACTTCTAGACTATATTAGAAACCCTTGTAGTCCGGCTGACTGACTTCTAGACTATCTCGTATGCCGTCTTCTGCTTGAAAAAAAAAAAAAAAAAA

>08082b78655199540f7e60816122d608

TACGTAGGGGGCAAGCGTTATCCGGATTTACTGGGTGTAAAGGGAGCGTAGGTGGTGTGGTAAGTCAGATGTGAAAGCCCGGGGCTCAACCCCGGGACTGCATTTGA

>dcf652267609108428e5c2d715ab2f25

AACAGAGGATACAAGCGTTATCCGGATTTGTTGGGTTTAAAGGGTGCGTAGGTGGTTTTTTAAGTCAGTAGTGAAATCTTAAAGCTTAACTTTAAAAGTGCTATTGA

>e1589a2515f7f038355e6c08f702e264

TACAATTTCTTGTATTTAAATTTTTAAAAGTTTCAGTCAATAAGATATTTAAATTTGTATAAATAATAATTTTAGTGAAATACATTACTATTTTATTTTATTAATTT

>ef7648fbb745f5c9e4f86ca82e0a79bc

AACGTAGGTCACAAGCGTTGTCCGGAATTACTGGGTGTAAAGGGAGCGCAGGCGGGAAGACAAGTTGGAAGTGAAATCTATGGGCTCAACCCATAAACTGCTTTCAA

>33d3b081e5e72259113f53070ce5f3d4

TACGTAGGTGGCAAGCGTTGTCCGGATTTATTGGGCGTAAAGCGAGCGCAGGCGGTCTTTTAAGTCTGATGTGAAAGCCTTCGGCTTAACCGGAGAAGTGCATCGGA

>24e7836f839c4db614f9dc40b295c28d

GTGGTTTTCACCAAATTTTTTTTATCTAAATTAGAAACCCCAGTAGTCCGGCTGACTGACTTACTAGGTATCTCGTATGCCGTCTTCTGCTTGAAAAAAAAAAAAAA

>24cb92a83a343ddffde24076ecde764e

TACAATTTCTTCAATTTAAATTTTTAAAAGTTTCAGTTAATAAATTATTTAAATTTTTATGAATAATAATTTTAGTGAAATATATTATTATTTTATTTTATTTTATT

>3c9b33f4fd0d8ea72388b23faf60e8cc

TACGTAGGGTGCAAGCGTTGTCCGGAATTATTGGGCGTAAAGAGCTCGTAGGCGGTTCGTCGCGTCGGCTGTGAAAACCCGGAGCTCAACTCCGGGCCTGCAGTCGA

>954f6bd48d1724a887efef94af7acd23

TACGTAGGTGGCAAGCGTTATCCGGAATTATTGGGCGTAAAGCGCGTGTAGGCGGTTTTTTAAGTCTGATGTGAAAGCCCACGGCTCAACCGTGGAGGGTCATTGGA

>d9e15483232f9681ca0aaa98474319f7

CGAGGACGATTCACGCTGTATTTGGCATTCGGACGGTCTCAGTACTATTGGTACAGCACTTTTGTACAGTGAACGATATGAGGACGCTACAACTATCCACATTTCGT

>02bba502b0a98345c0c3810a24e9c7dd

TACGAAGGGGGCTAGCGTTGCCCGGAATCACTGGGCGTAAAGGGTGCGTAGGCGGGTCTTTAAGTCAGGGGTGAAATCCTGGAGCTCAACTCCAGAACTGCCTTTGA

>0693079c126e133d08b25abf5348534a

TACGGAGGGTGCAAGCGTTATCCGGATTCACTGGGTTTAAAGGGTGCGTAGGCGGGTTGGTAAGTCCGTGGTGAAATCCCCAAGCTTAACTTGGGAACTGCCGTGGA

>e039cc5bb4169d98bca7d1ae90900e03

TCCGGCTGACTGACTGTTACAGCATCTCGTATGCCGTCTTCTGCTTGAATAAAAACCCTAGTAGTCCGGCTGACTGACTGTTACAGCATCTCGTATGCCGTCTTCTG

>c25035f51e9708e08aa8cb3c319e408e

CCCCGCTTATTACATTCTTAAACACAACAGTACATTAGGCCTACAATAATAGAACGGAAGGTTCACTTTCTCCCCGAGCGATACACTGGTATTATGTAATGGCGTAC

>456b32499fe4810d6f0572e478db0d67

CAATTACCATAAGTGTAGAGTGTAGATCTCGATTAGAAACCCCAGTAGTCCGGCTGACTGACTCTACGACCATCTCGTATGCCGTCTTCTGCTTGAAAAAAAAAAAA

>f0a8b94a9d4ff91b3dffea82850b1fb3

TACGTAAAAGACTAGTGTTATTCATCTTTAATCGGTATAAAGGGTACCTAGACAGAATGCCAAGCCATAAAAGGAACTAGTATTCTAGAGTTTTATGTGGGAATATA

>44072411a2743f1738ce90f0667d1586

TACGTAGGTGGCAAGCGTTGTCCGGAATTATTGGGCGTAAAGCGCGCGCAGGCGGCTATGTAAGTCTGGTGTTAAAGCCCGGGGCTCAACCCCGGTTCGCATCGGAA

>123721db7b084d021ace44cfe9d3b0b7

GTGCTCGAAGAACATCAATCTTTCACTTCGTTCTCACTTTCTCTTTTTACTGCCTCCTCTTCTGACCCTTTTCCAACATACCCCTTTCGCTCCTTTTCGTCATCCCT

>e753500e91dfcee37ab6d4ec00479e6f

TACGGAGGGTGCGAGCGTTGTCCGGATTTATTGGGTTTAAAGGGTGCGTAGGTGGCTTTTTAAGTCAGTGGTGAAATACAGCCGCTCAACGGTTGAGGTGCCATTGA

>d4aec5b917712ac26e098e2d7a4deb55

TACGTAGGTGGCGAGCGTTGTCCGGATTTACTGGGCGTAAAGGGTGCGTAGGCGGATGTTTAAGTCAGATGTGAAATACCCGAGCTCAACTTGGGTGCTGCATCTGA

>6320d0ab85a438b107ff1dfd43a0ad48

CACACTTATGTCTTTAGAAAAACAAACCATTTCTCCAGGATGCATTTCAACCATGTCGGTCATTTTCAACTGGCAGTGCCTTGTAGATATTTGTGGAAGATTTTGAA

>5a56aa6130717523aacc1d1464d93417

TTCCAGCTCCAATAGCGTATATTAAAGTTGTTGCGATTAGAAACCCGAGTAGTCCGGCTGACTGACTCGCGATATATCTCGTATGCCGTCTTCTGCTTGAAAAAAAA

>d306bd9f8600564f750684ac7401cd4a

TACGTAGGGTGCAAGCGTTAATCGGAATTACTGGGCGTAAAGCGTGCGTAGGCGGTTATGCAAGACAGAGGTGAAATCCCCGGGCTCAACCTGGGAACTGCCTTTGT

>11671b64a595b98e97b220cbd9b87786

GGGAGGGAATTCAGAAGGAAGTATTTAAAGAGCAAAGATGTAAACACAGATTAGAAACCCTAGTAGTCCGGCTGACTGACTACTGTGTAATCTCGTATGCCGTCTTC

>8dad5b403b54b34fb035cdd1d7f077f7

CAAATTTATGTCTTCAGAAAGACAAAATATTTTTCCAGGGTCCATTTCAACCATGTCGGTCATTTTCAACTAGCAGTGTCTTGTAAATATTTGTGAAAGATTTTGAA

>46608c5f4bdbebb3c6e340b4f72720de

TACGAGGGGAGCGAGTGTTGTTCGGTTTTATTGGGCGTAAAGGGCACTTAGGTTGTTCTACAAGTTAACAGTTAAATCTTGGAACTTAATTCCATGGCAACTGTTAA

>a8112e59af2b1eb0eda6763a76c9a561

ATTAACGGATTAGAGAACCGACGTTTCACAGTGATACGGCCGCGATAAATTAACGGATTAGAGAACCGACGTTTCACAGTGATACGGCCGCGATAAATTAACGGATT

>3c807afe4fee0cec4e140ee218ef5c64

TACGGAGGGAGCTAGCGTTATTCGGAATTACTGGGCGTAAAGCGCACGTAGGCGGCTTTGTAAGTTAGAGGTGAAAGCCTGGAGCTCAACTCCAGAATTGCCTTTAA

>2e8e45688162103f036a8fee959e1b84

TACGTAGGGTGCAAGCGTTAATCGGAATTACTGGGCGTAAAGCGTGCGCAGGCGGTTATGCAAGACAGAGGTGAGATCCCCGGGCTCAACCTGGGAACTGCCTTTGT

>68d4f15655b9c8db5ff52db09a48490f

CAAATTTACGTCTTCAGAAAGACAAAATATTTTTCCAGGGTCCATTTCAACCATGTCGGTCATTTTCAACTAGCAGTGCCTTGTAGATATTTGTGAAAGATTTTGAA

>ed937462ff863e7e1cfd1d91bcf238bd

TCCTTTGCCATGATCATCCGCGCACCAAGGAAGTCATCGACTTTATGCCGGCAGCCATTCGGTCGTCAAAAGCTATTCTGCATGGATAGGCTTTGTTACGATCATTG

>ce599941d0b8ba56a388daaaaf88c615

AACAGAGGATACAAGCGTTATCCGGATTTATTGGGTTTAAAGGGTGCGTAGGTGGTTTTTTAAGTCAGTAGTGAAACCTTAAAGCTTAACTTTAAAAGTGCTATTGA

>36dfe69f0075139298a1315804fc4967

ACAACAAGGGGAACCTAACAATGAGCCGCCTCGCAATCAATCAATTTTCGCCACGCCACATCCAACGCCATTCGTGTAGCACAATTTTTCTTCGCCTATCTACACGC

>3e52621c4023febb209577215dbe52ac

TATTCTCGCGCAAACGCAAGCGGCCTCGGCCGCTTAAAGCAGGCCCCGTCGAGGTACTCTGGACCAACAGCGTCAAGTACCTCGGGCTCCACGTGGACTCCCGACTC

>291050ae7abe6c3e5c05562e89c1a35a

CGAGGACGATTCACGCTGTATTTGGCATTCGGACGGTCTCAGTACTATCGGTACAGCACTTTTGTACAGTGAACGATATGAGGACGCTACAACTATCCACATTTCGT

>794bdd5638d02a8f25b0780d6f925bd2

ACTCGTTCAAATTAATTAAACGAACTGTTCGATCACAATGTGTTGTCGTTCCTGCACACAATTAGAAACTAATTAACTGAAAAATGTAACGAACCGAAAACTGCAGA

>58b073cae15d150b523dd97cb29771fb

CAAAAAACTAGAACATGCATCGAAGAGCAACGTCGAAGAAACGCGCGGTGGACGCGGAACAAGAAATCAACATGGACGAAAAGGAGGCGACGCCGGTGGAAGACATC

>367fd0ad5e0d526e2327363283dbec04

TGGAACAGTGTTTGTTGATTGTGCGTAAGCGGTGAAGGTGTCTGCGTCGCAAATATTTAGTGTTTAATCGTTTTTACCCCGTGTTAGAAAGCCTACGACTTCGGTGA

>cc30542beb2bebf93d161707be434a2d

TACAATTTTTTCAACTTAAATTTATAAAGCTTCAGTAAATATATAATGTAATTTTTGTAAATAATAATTTTAGTGAAATATATTATTATTTTGTTTTTATGATCTGT

>00201164204e5023950e5f7887a5959b

TACGGAGGGGGCTAGCGTTGTTCGGAATTACTGGGCGTAAAGCGCACGTAGGCGGCTTTTTAAGTCAGAGGTGAAAGCCCGGAGCTCAACTCCGGAATTGCCTTTGA

>d9214ce7ec793dea90fddd5eff4c8048

TACGTGAGAGACTAGTGTTATTCATCTTAATTGGGTCTAAAGGGTACCTAGACAGTCAATATAACTTCTATAATGCTAATACTTGACTAGAGTTTTAAGTAAGAGGG

>c0d0209c936e7316f8a53b8c9111bad7

TATAATTTCTTCAATTTAAATTTATAAAGTTTCAGTTAATAGAATTATTTAAATTTTTGTGAATAATAATTTTAGTGAAATATATTATTATTATCTTATTTTATTAC

>7f3e4825a624688665d2d490a06ee4f6

CATTTTACCATAGTGTCTGCCTCCACGGGCACGTTTATGGCTTTGCCGGTTATCTTTTAAGTCTGTTTCTATTCTGAGACGACGGATCTGCATACGTGGAAATCCGG

>d3923fabe20cc48732802baf06c10ddc

CATTGCGTAACATCGCGTAATAAATCGCACGTCACAAGTAAAACTATGAGGTAACTAACTGCTTTATCAGATTCGTCATGATCTATTCTTGTCTATTGCGTCATCGC

>14d016d15010edd41bffa3ea371c76a2

TACGGGGGGGGCAAGTGTTATTCGGAATGACTGGGCGTAAAGGGCACGTAGGCGGTGAAAAGGGTGGAAAGTGAAAGTCGCCAAAACACTGGCGGGGTGCTTTCTTG

>7807b5e6da43b435aef4e86a994754a4

TACGTAGGGTCCAAGCGTTAATCGGAATTACTGGGCGTAAAGCGTGCGCAGGCGGTTGTGTAAGACCGATGTGAAATCCCCGAGCTTAACTTGGGAATTGCATTGGT

>65b11d37ab19953d835686e56b168748

TATTCTCGTGCAAACGTAACACTTAAAGTAGGCCCTGACAAGGTGCCCTGGACGCATAGCAGCAAGTACCTCAGGTTCCATGTAGACTCCCGACTCACCTGGCGAAC

>6fb5d739886d0fec51786033c3904ce7

TACGGAGGATCCAAGCGTTATCCGGAATCATTGGGTTTAAAGGGTCCGTAGGCGGTTTAGTAAGTCAGTGGTGAAAGCCCATCGCTCAACGGTGGAACGGCCATTGA

>ae21cce677ee510e2ecfc3c61e731c71

TACGGAGGGTGCAAGCGTTATCCGGATTTATTGGGTTTAAAGGGTCCGTAGGCGGGCTCGTAAGTCAGTGGTGAAAGCCTGCAGCTTAACTGTAGAACTGCCGTTGA

>aeeeb0f5907ab5e4b19d3ef050eb6a27

TACGTAGGGCGCAAGCGTTGTCCGGAATTATTGGGCGTAAAGAGCTCGTAGGCGGTTTGTCGCGTCTGCTGTGAAAACGCGAGGCTCAACCTCGCGCCTGCAGTGGG

>309a18721c7cd4c095440b647e17dee1

TACGTAGGGGGCTAGCGTTATCCGGATTTACTGGGCGTAAAGGGTGCGTAGGTGGTCCTTCAAGTCGGTGGTTAAAGGCTACGGCTCAACCGTAGTAAGCCGCCGAA

>6cf416f7abd9a15f8d574a8b95b33ed6

TACGAAGGGAGCTAGCGTTGCTCGGAATTACTGGGCGTAAAGGGAGCGTAGGCGGACATTTAAGTCAGGGGTGAAATCCCGGGGCTCAACCTCGGAATTGCCTTTGA

>8b3a42ceeb3788bc620d0093e3de0623

ACTTTGCGTCAGACAATGTTATTTTTTATGAACAATGGTTGGTAAACTTTTTTTCTCGCTATAAATTAGAAAACCATATTGTTGTTAAACATATCTCATTAGTTCAA

>97f5053b17c98c00e197e87f9e1cb0a7

CGATTCTGATGTCATAGAAACTCGTCTACAAATGCCCTGAAGACAACAATAAATTTTTTCGAAACGTGTGCGAAAATTCCTTAAATTGTTTCGTTAATTGCCATAAC

>d92d9604b07029b9e2f4d977ce28897a

GGGAGGGAATTCAGAAGGAAGTATTTAAAGAGCAAAGATGTAAACACAGATTAGAAACCCCTGTAGTCCGGCTGACTGACTACTGTGTAATCTCGTATGCCGTCTTC

>fc7114082850be09cc7d78094e0ba1a9

TACAATTTCTTCAATTTAAATTTTTAAAAGTTTCAGTTAATAGATTATTTAAATTTTTATGAATAATAATTTTAGTGAAATATATTATTATTATATTTTATTAATTT

>85734130f68d79672a32e9fdeb9e85c8

TACGAAGGGGGCTAGCGTTGCTCGGAATCACTGGGCGTAAAGGGCGCGTAGGCGGCCTTGTAAGTTGGGGGTGAAAGCCCGTGGCTCAACCACGGAATTGCCTTCGA

>16d1bdbe63d89b3f2ee52d61b206b9cc

TACGTAGGTGGCAAGCGTTGTCCGGATTTATTGGGCGTAAAGCGAGCGCAGGCGGTCTTTTAAGTCTGATGTGAAAGCCCCCGGCTTAACCGGGGAGGGTCATTGGA

>82fe8de7ebea0b99b4a51474c43826f5

CCACCCCCACCGCTGTGTTCATACTTTTTTTTTTTACAATAACAGATGCCACAATTCACACACACTTAATGTACATTAGATACCCTAGTAGTCCGGCTGACTGACTG

>f0614bc6f76566dd6ba9794f29a033d9

TGTTGTGTGACAGTGAAATCTGGATACTAGCAAATTAGGACAGCTGTAGAATTTAGATACTGTACGAGATTTTTACGTCTATTAAAGAATGCAAAAGAAAAGACGGA

>51714dbb7c82151686942c6c017dc6f3

ACAACAAGGGGAACCTAACAATGAGCCGTCTCGCAATCAATCAATTTTCGCCACGCCACATCAACGCCATTCGTGTAGCACAATTTTTCTTCGCCTATCTACACACA

>34f9314b9c650b2b9b01fd20ba7b62b3

CAAATTTACGTCTTCAGAAAGACAAAATATTTTTCCAGGGTACATTTCAACCATGTCGGTCATTTTCAACTAGCAGTGCCTTGTAGATATTTGTGAAAGATTTTGAA

>fdb8d0d3d853f65897aac73381068c79

TACGTAGGGTGCGAGCGTTAATCGGAATTACTGGACGTAAAGCGTGCGCAGGCGGTCTTGTAAGACAGAGGTGAAATCCCTGGGCTCAACCTAGGAATGGCCTTTGT

>950011ed85ff090028e3fd9dd6f3b34d

TTCCAGCTCCAATAGCGTATATTAAAGTTGTTGCGATTAGAAACCCTGGTAGTCCGGCTGACTGACTCGCGATATATCTCGTATGCCGTCTTCTGCTTGAAAAAAAA

>f26d2df26aef848b1bfe7d0750e50c10

CCACCCCCACCGCTGTGTTCATACTTTTTTTTTTTTACAATAACAGATGCCACAATTCACACACACACACTTAATGTACATTAGAAACCCTGGTAGTCCGGCTGACT

>743163dd00cd81187982e1ed9d6f3431

TACGTGAGAGACTAGTGTTATTCATCTTAATTGGGTTTAAAGGGTACCTAGGCAGTCAATATAACTTCTATAATGCTAATGCTTGACTAGAGTTTTAAGTAAGAGGG

>e99314fd8f03ab59eca36c06cb42a811

TATGTGCACACAGATACAGACACAGTCTCTCCCGGTCTCTCACTTAACTAACAGTCTCACTGCTGGGCTGGCTGCATATTAGAAACCCCAGTAGTCCGGCTGACTGA

>eda766a5c87fda5f7d3f1492d3cdd817

AACAGAGGATACAAGCGTTATCCGGATTCATTGGGTTTAAAGGGTGCGTAGGTGGTTTTTTAAGTCAGTAGTGAAATCTTAAAGCTTAACTTTAAAAGTGCTATTGA

>fae781baebff0dc912041c2c066cb3e0

TACGGAGGGTGCAAGCGTTATCCGGATTCACTGGGTTTAAAGGGTGCGTAGGTGGGTTGGTAAGTCAGTGGTGAAATCCCCGAGCTTAACTTGGGAACTGCCATTGA

>5cfaeff9b6bd9ba3de608e8f576c4a60

AACAGAGGATACAAGCGTTATCCGGATTTATTGGGTTTAAAGGGTGCGTAGGTGGTTTTTTAAGTCAGTAGTGAAATCTTAAAGCTTAACTTTAAAAGTGCTACTGA

>9aa9be34aeb66ad176cf2fd44dd1133e

TACGGAGGGTGCAAGCGTTATCCGGATTCACTGGGTTTAAAGGGTGCGTAGGCGGGCATGTAAGTCAGTGGTGAAATCTCCGGGCTTAACTCGGAAACTGCCATTGA

>e4ff217cdff289f3ba4b0a6cc181bbf5

CATTGAACTATCGTGAGAAAATCACGCCGCCAAAGGGAATTATATTATAGTAATTATTGGCGTTAATAAACATTTTATTAATAGTTGTAATATATGATAACGTGTAA

>3ad907d11f05e7364843a24721c06741

TACGGAGGGTGCGAGCGTTAATCGGAATTACTGGGCGTAAAGCGTGCGTAGGCGGATGTTTAAGTCTGTTGTGAAATCCCCGGGCTCAACCTGGGAATGGCATTGGA

>7c514baf951654843eab6f1101754d28

CCCCATGGCGCATTAGATACCCGGGTAGTCCGGCTGACTGACTCAGTAGGTATCTCGTATGCCGTCTTCTGCTTGAAAAAAAAAAAATTATTATATTTTATTAATTT

>77700a3ac171339d775ad74664883a2b

AACAGAGGATATAAGCGTTATCCGGATTTATTGGGTTTAAAGGGTGCGTAGGTGGTTTTTTAAGTCAGTAGTGAAATCTTAAAGCTTAACTTTAAAAGTGCTATTGA

>65ec954d57c890783271a66bc53e4b8c

TACGGAGGATCCGAGCGTTATCCGGATTTATTGGGTTTAAAGGGTGCGTAGGTGGTTTGATAAGTCAGCGGTGAAAGTTTGCAGCTTAACTGTAAAAATGCCGTTGA

>e0c624a91af9ae6ee821e778f1005380

TAAAATTTCTTCAATTTAAATTTTTAAAAGTTTCAGTTAATAAATTATTTACATTTTTATGAATAATAATTTTAGTGAAAATTTGTCTGAGAAACTTTTTTATTAAA

>2b0805ca54eb1c2da89700cd99596e54

TACAATTTCTTCAATTTTAAATTTATAAAGTTTCAGTAATATAACATAATTTTTATAAATAATAATTTTAGTGAAATATGTTATTATTATGTTTTTATGATTTGTCT

>0802ca805baf0226891192e4f8e360a8

TACAGAGGGTGCAAGCGTTAATCGGAATTACTGGGCGTAAAGCGCGCGTAGGTGGTTTGTTAAGTTGGATGTGAAAGCCCCGGGCTCAACCTGGGAACTGCATCCAA

>22f8185fed426d73f3c2a66e1a74803e

TACGATTTCTTTAATTTAAATTGTTAAGTTTCAGTTAAAAAATAATGATAATAATATAAAATAAATAGAATTTTGGTGAAATATATTTTTCTATTGAAAAATTAATT

>29656e18b831da0c1536d62655caa3a5

TACGAAGGGGGCTAGCGTTGCTCGGAATGACTGGGCGTAGAGGGCGTGTAGGCGGTTTGTACAGTCAGATGTGAAATCCCCGGGCTTAACCTGGGAGCTGCATTTGA

>a2f2dcfd58f93cc62ca05be0c3f83462

TACGTGAGAGACTAGTGTTATTCATCTTAATTGGGTTTAAAGGGTACCTAGGCAGTCAATATAACTTCTATAATGCTAATACTTGACTAGAGTTTCAAGTAAGAGGG

>5fa2211fa566759447bd5f8e0c2dfbe2

TACGTGAGAGACTAGTGTTGTTCATCTTAATTGGGTTTAAAGGGTACCTAGACAGTCAATATAACTTCTATAATGCTAATACTTGACTAGAGTTTTAAGTAAGAGGG

>350a3626ddec5fe36c4186aa279d96b8

TATAATTTCTTCAATTTAAATTTTTAAAAGTTTCAGTTAATAAATTATTTAAATTTTTATGAATAATAATTTTAGTGAAATATATTATTATTATATTTTATTAATTT

>45cc18176d964edc0644c99f0d013762

CCAGACACCGTAGTATGCCCCTGTAATCCCAGCAATTGAGAGGAGGCAGAAAGATTAGAAACCCTAGTAGTCCGGCTGACTGACTCGCGATATATCTCGTATGCCGT

>42d97ae1ffd0ef4d0fb90484f8b393eb

TATTCTCGCGAAAACGCAAGCGGCCTGGGCCGCTTAAAGTAGGCCCCGTCGAGGTGCCCTGGACCAACAGCGTCAAGTACCTCGGGCTCCACGTGGATTCCCGACTC

>c737597c60abc222e7a91a91deb751fd

CCCACCGCTGTGTTCACTTTGATATACTTTTTACAATACACAATAACAGATGCCACAAGCCACACACACACACGCACACAGAAAGTCGTAGGACGCAAACACAGTCA

>ca7b4135ad70fcb6d4c8b961a5bdb20f

CGAGACACACTTGGGTCATTGCCATGGCAACGAACATCTGTAGTCTACAGACTGTATCAATCTCCCATGTTGTTTTATACTAGTCGTTATTTTAAAATACTATGAAT

>707dd8f80ef64cd48532518a0befbe9f

GGGAGGGAATTCAGAAGGAAGTATTTAAAGAGCAAAGATGTAAACACAGATTAGAAACCCGAGTAGTCCGGCTGACTGACTACTGTGTAATCTCGTATGCCGTCTTC

>b526292f0d718ae4a27e8547b70b04f0

CACGATTCTCTAAATTTAATTATGTTAGTTTCAGTTAAAATGTAAATGTTAAAATTAAGTTTTTTAAATTTTGGTGGAATAATGTATAAACATGTGTTTACTTCTAT

>c2c18e83c55249bf51aa3df7934b5c9f

TACGTATGGAGCGAGCGTTGTCCGGAATTATTGGGCGTAAAGGGTACGCAGGCGGTTTAATAAGTCGAATGTTAAAGATCGGGGCTCAACCCCGTAAAGCATTGGAA

>1cd9eafee722ee2e3eefefa439877188

TACCGGCAGCCCGAGTGATGGCCGATCTTATTGGGCCTAAAGCGTCCGTAGCTGGCCGCGCAAGTCCATCGGGAAATCCACCTGCTCAACAGGTGGGCGCCCGGTGG

>e3a758236d8dcf54a8a0ad78ed0a33c3

TACGTAGGGTGCAAGCGTTAATCGGAATTACTGGGCGTAAAGCGTGCGCAGGCGGTTATGCAAGACAGAGGTGAAATCCCCGGGCTCAACCTGGGAACTGCTTTTGT

>2aaaee3b3960897e8515eb8742086ce7

CATGGTCATCCAGCCAGATTTCGTATTTATGTACGTGACGTTTGACTTTAGTCACGTGGAAAGCGATGCTTTAACCACCGGGTGAATTGAGTTATAATAGTTAATTT

>0ffcc2f38d8f32689a39ad2356405b55

TACAATTTCTTCAATTTAAATTTTTAAAAGTTTCAGTTAATAAATTATTACAATTTTTGTGAATAATAATTTTAGTGATATAGATTATTATTTTATTTTATTTTATT

>eb771924440abcf463734e865952fdc9

TACGGAGGATCCGAGCGTTATCCGGAATTATTGGGTTTAAAGGGTGCGTAGGCGGCCAAATAAGTCAGGGGTGAAAGTCGGCAGCTCAACTGTCGCAGTGCCTTTGA

>3ed219546461285d97c86252dcfdcadb

CATGGTCATCCAGCCAGATTTCGTATTTATGTACGTGACGTTTGACTTTATTTACGTGGAAAGCGATGCTTTAACCACCGGGTGAATTGAGTTATAATAGTTAATTT

>a97b9d8a1eabb5cbbfed6e814ea7cf51

TACGAAGGGGGCTAGCGTTGCTCGGAATTACTGGGCGTAAAGCGCACGTAGGCGGTTTCTTAAGTCGGGGGTGAAATCCTGGAGCTCAACTCCAGAACTGCCTTCGA

>1a68f5c62df9653361b923ee9dec6c28

TACGGAGGGTGCGAGCGTTATCCGGAATCACTGGGCGTAAAGGGCGTGTAGGCGGGACGTTAAGTCTGGTTTTAAAGACCGCAGCTCAACTGCGGGAGTGGACTGGA

>d9884e8d17f74f14d238323d1c31491f

GTGCTCAAAGAACATCAATCTTTCACTTCGTTCTCACTTTCTCTTTTTACTGCCTCCTCTTCTGACCCTTTTCCACCATACCCCTTCTTCTCCTTTTCGTCATCCCT

>fcb1f993c9b75755525880abac98c324

GTAGCTACTCAGAGCAATCCCACTTTACTACGAACACAATACACTATGGCCATAATGTTAAGGATTACAACATCTTGCTTAACGACTGCAACGGGGCTTAGTGTTTC

>ecdb4312bdd46990712250d10161a3f2

CATTGAACTATCGTGAGAGAGTCAAGCCGCCAAAGGGAATTATATTATAGTAAATATTGGCGTAAATAAACATTTTATTAATAATTGTAATATATGAAAATGTGCAG

>529e4dbe9b21c3a64896a8a9be873f47

AACAGAGGATACAAGCGTTATCCGGATTTATTGGGTTTAGAGGGTGCGTAGGTGGTTTTTTAAGTCAGTAGTGAAATCTTAAAGCTTAACTTTAAAAGTGCTATTGA

>f48927af194dc6c9e6761f32efbb7459

GAGTAGGCCTACTTGCACTTGTTACACTCAACACTGACTGTACGGGGATGTGTCTATTAAAGATTAACATTCTTAAAATGCTGCTGCCTCCTCCGCCGCCCGCTATT

>d543f1ba2a8b3af322fda9645f515a03

TATTCTCGCGCAAACGCAAGCGGCCTCAACCGCTTAAAGTAGGCCCCGTCGAGGTACCCTGGACCAACAGCGTCAAGTACCTCGGGCTCCACGTGGATTCCCGACTC

>d343cca46517b3220378cc053a93ad56

CCAGACACCGTAGTATGCCCCTGTAATCCCAGCAATTGAGAGGAGGCAGAAAGATTAGAAACCCCAGTAGTCCGGCTGACTGACTCGCGATATATCTCGTATGCCGT

>9d49c4162047a8c70a752f5d730b8c4e

GTGGTTTTTACCAAATTTTTTTTATCTAAATTAGAAACCCCTGTAGTCCGGCTGACTGACTGAGACTTAATCTCGTATGCCGTCTTCTGCTTGAAAAAAAAAAAAAA

>7d40d230eacf8dfa7411efff1bbc6ae8

TTCCAGCTCCAATAGCGTATATTAAAGTTGTTGCGATTAGATACCCCTGTAGTCCGGCTGACTGACTCGCGATATATCTCGTATGCCGTCTTCTGCTTGAAAAAAAA

>b8324a10a7c5855f1ff652798104d087

AACAGAGGATACAAGCGTTATCCTGATTTATTGGGTTTAAAGGGTGCGTAGGTGGTTTCTTAAGTCAGTAGTGAATTCTTAAAGCTTAACTTTAAAAGTGCTATTGA

>2a7e9a4cece27cf4f2a27d97837d1d68

CCACCCCCACCGCTGTGTTCATACTTTTTTTACAATAACAGATGTCACAATTCACACACACACACACTTAATGTACATTAGAAACCCCTGTAGTCCGGCTGACTGAC

>5953d348dbfb4b2661f4e84427baf611

TACGTAGGTGGCAAGCGTTGTCCGGAATTATTGGGCGTAAAGGGCTCGCAGGCGGTTCCTTAAGTCTGATGTGAAAGCCCCCGGCTCAACCGGGGAGGGTCATTGGA

>866a4bb509c822ea24452a0248a90856

ACGTACACACCAACGTCCTAGATATCGTAATGAAATATACTTAAAAAAGACCTACGACCCGAGAAAGCCTGGTAGTGTATTGTCGTTGTCTAGGAAGGAGGATACAA

>ab24776a25e4a7efd00807cb574393d3

GACACTTATGTCTTCAGAAAGACAAAATATTTATCCACGATGCATTTCAACCATGTCGGTCATTTTCAACTGGCAGTGCCTTGTAGATATTTGTGAAAGATTTTGAA

>34092362c3ac7372c8127e9f049dc35e

TACGGAGGGTGCAAGCGTTATCCGGATTCACTGGGTTTAAAGGGTGCGTAGGTGGCTTTGTAAGTCAGTGGTGAAAGCCCGGAGCTCAACTCCGGAACTGCCATTGA

>1773692e652d1e2065349539ca133c75

TTCCAGCTCCAATAGCGTATATTAAAGTTGTTGCGATTAGAAACCCCAGTAGTCCGGCTGACTGACTCGCGATATATCTCGTATGCCGTCTTCTGCTTGAAAAAAAA

>d3d26207a6323b5e103bcbf0eb53a550

TGTTAAAGCTATGACTCACTATACAGGGCACTAGGAAATACCGTTTATTGCGACAAGGAATGGTTTGGTCAGTTTATTTTAGTTTGCTCCTATTATAGTTACAAAAC

>2e8b492ef71c86b4e9f9221b4f679b9d

CCACCCCCACCGCTGTGTTCATACTTTTTTTTTTTTACAATAACAGATGCCACAATTCACACACACACACTTAATGTACATTAGATACCCCTGTAGTCCGGCTGACT

>8895edccc6dd10426ae49af34b3cdb9b

TATTCTCGCGAAAACGTAAGCGGCCTTGGCCGCTTAAAGTAGGCCCCGTCGAGGTGCCCTGGACCAATAGCGTCAAGTACCTCGGGCTCCACGTGGATTCCCGACTC

>31c4d4d5d0bbd0b98238d4f64195cd30

TACGAAGGGTGCAAGCGTTAATCGGAATTACTGGGCGTAAAGCGCGCGTAGGTGGTTCGTTAAGTTGGATGTGAAAGCCCCGGGCTCAACCTGGGAACTGCATCCAA

>ef3982965d43e9769fbd2bab0905ccb5

AACAGAGGATACAAGCGTTATCCGGATTTATTGGGTTTAAAGGGTGCGTAGGTGGCTTTTTAAGTCAGTAGTGAAATCTTAAAGCTTAACTTTAAAAGTGCTATTGA

>4d77d95d82ea005574a8a960dcb79400

TACAGAGGTCCCAAGCGTTGTTCGGATTCACTGGGCGTAAAGGGTGCGTAGGCGGTCGGGTAAGTCTGACGTGAAATCTTCAAGCTCAACTTGGAAACTGCGTCGGA

>1c7bff5828621eb8753a724608fe36cd

TACGATTTCTTTAATTTAAATAGTTAAGTTTCAGTTAATATAGCAATAATATAAAATATCTATAATTTTGGTGAAATATATTTTATCTTGAAAAATTAATTTTATGT

>017847afaa498b18ed06a1f198d9b9e3

TACGGAGGATCCGAGCGTTATCCGGATTTATTGGGTTTAAAGGGAGCGTAGGCGGATGCTTAAGTCAGTTGTGAAAGTTTGCGGCTCAACCGTAAAATTGCAGTTGA

>418b995c3a292a5352f54146d365bf5e

TACGTAGGGGGCAAGCGTTATCCGGATTTACTGGGTGTAAAGGGAGCGTAGGTGGTGCGGTAAGTCAGATGTGAAAGCCCGGGGCTCAACCCCGGGACTGCATTTGA

>f1e77692599d886aeb003788da4f5cdc

TACGTAGGGGGCGAGCGTTGTCCGGAATTATTGGGCGTAAAGCGCGCGCAGGCAGTCTCTTAAGTCTGATGTGAAAGCCCACGGCTCAACCGTGGAGGGTCATTGGA

>d43e8a8221700500b0d8c96ed93f17e1

CCACCCCCACCGCTGTGTTCATACTTTTTTTACAATAACAGATGTCACAATTCACACACACACACACTTAATGTACATTAGAAACCCTAGTAGTCCGGCTGACTGAC

>6dba5db64e19a20dd992663ae4ae067f

TACGGAGGGGGCTAGCGTTGTTCGGAATTACTGGGCGTAAAGCGCACGTAGGCGGCTTGGTAAGTTAGAGGTGAAAGCCTGAAGCTCAACTCCAGAATTGCCTTTAA

>0f8f1b85dc7348e9d86fa33c20846ff0

TGTAATGTCCAAAGAACCGTTAAAACTAATTAGAAACCCCTGTAGTCCGGCTGACTGACTACGTACGTATCTCGTATGCCGTCTTCTGCTTGAAAAAAAAAAAAAAA

>35f4a8f478e14dcb95b84b84825b56c0

TCATCTTCAGTAGCCTGCAGATGAGCTGTTCGTGTTCGCGTTCCAGCGAAGATTCTACTGACGTCGGCTTCGACCAATCAGCAGGCAGCAATGAGCAGCAAATACAT

>3444bb83b7ad8d6a47ca479eef6be01e

TACGTAGGGTGCGAGCGTTAATCGGAATTACTGGGCGTAAAGCGTGCGCAGGCGGTTTTGTAAGACAGGCGTGAAATCCCCGGGCTTAACCTGGGAATGGCGCTTGT

>6b593b5efa761a7d87d75d3fc922d731

CATTGAACTATCGTGAGAAAGTCACGCCGCCAAAGGGAATTGTATTATAGTAAATATTGGCGTAAATAAACATTTTATTAATAATTGTAATATGTGAAAATGTGCAG

>a79e7eb7982a20fdaeb0197ac1aaaf9f

AGGAATGAGATATCCGCATCTTCGATATTCTCATTTGATTGCTGTTCACTCTATTTCTCATTTTTAACGCAACTTAAAAACAATTCTCTAAGATTGCACGTTTCTAA

>278efde9868bc9de597b971505bc6fc2

CTATTTCGCTGTGTGATACTTGTAACTATGTGAAATAGCTATTACATTTTAGTTTTTGTTACGAATACTTTTTTCATACTCATTCAAAGTTATTGGTAAGAAACGTG

>ab76376831c72b5b3503c9e6f9993f7c

TACGGAGGGGGTTAGCGTTGTTCGGAATGACTGGGCGTAAAGCGCGCGTAGGCGGGCTGGAAAGTTGGGGGTGAAATCCCGGGGCTCAACCCCGGAATGGCCTTCAA

>70a25eb0c8a6b252d82417d136aacf31

CATTGAACTATCGTGAGAAAGTCACGCCGCCAAAGGGAATTATATTATAGTAATTATTGGCGTTGATAAACATTTTAGTAATAGTTGTAATATATCATATTGTGTGA

>d9b92b364155da3c449c7f70aba17255

CAACTGGTACTGGTAGCTGGCCACTGGTAGTTGCGAAAGCAGGCCAATCTTGTCCAAGTGTGCAAGTGCTTGACCACCACCAGTAAGTCTAGCCAAGCACTAGGTTC

>edc11a44852cc2e60a77de486a735521

AGATGGCAGCTCTACTTGTAACGTGCACCAAAGAGGAAACCGTCCTGTCATTCGTTTTTTGAGTATAATAAGGGTGAACCGAATGAAAGTTTAATATAGTGGTGCAT

>c36df796f954b28e14d5c7b80cd59303

TACGAAGGGGGCTAGCGTTGTTCGGAATTACTGGGCGTAAAGCGCACGTAGGCGGATATTTAAGTCAGGGGTGAAATCCCAGAGCTCAACTCTGGAACTGCCTTTGA

>b06157f696e169e4541b4bb0c000ee66

CCACCACCCCACCGCTGTGTTCATACTTTTTTTTTTTACAATAACAATTCACACACACACACACACACACTTAATGTACATTAGAAACCCCTGTAGTCCGGCTGACT

>7ad72984a5dbf686636a7cb206a3a4ed

CATTGAACTATCGTGAGAGAGTCAAGCCGACAAAGGGAATTATATTATAGTAAATATTGGCGTAAATAAACATTTTATTAATAGTTGTAATATATGAAAATGTGCAG

>beca0abff5a25f58dd3c20082234a46e

TACAGAGGTCTCAAGCGTTGTTCGGAATCACTGGGCGTAAAGCGTGCGTAGGCTGTTTCGTAAGTCGTGTGTGAAAGGCGCGGGCTCAACCCGCGGACGGCACATGA

>987cdc904b9a446a6cd080a8f3570fd2

CCAGACACCGTAGTATGCCCCTGTAATCCCAGCAATTGAGAGGAGGCAGAAAGATTAGAAACCCCTGTAGTCCGGCTGACTGACTCGCGATATATCTCGTATGCCGT

>8fdbaa6875f17a2111b4c7efd52763bd

TGAAAATATCCAACGCACACACGGCGGGCCTCTAACCGGTCAGTTGCCTTTCGCGGACTACAGCTGGCGACCCTTGTCGCTCTCTTTTAATCTTTATTCTCTTTCGC

>435a667d8d3e7e765fea7773c82f230f

AGTTCATATTGTGAAACAAAAAATTTTTTCAAAGTGCACAGATGCAGTTTTTTCAACAAACAACAACAACAACAAATGTGAACGTTAGCTAGCTACTTAGGGTTTGT

>ab8fc169b32c51a96eadbb7f0929f8c8

TACGTGAGAGACTAGTGTTATTCATCTTAATTAGGTTTAAAGGGTACCTAGACAGTCAATATAACTTCTATAATGCTAATACTTGACTAGAGTTTTAAGTAAGAGGG

>bc4b7e31062381cc78f5421839ad4ec8

TACAATTTTTGTCAATTTAAATTTATAAAGTTTCAGTAAATATACAATAGAATGTTTATAAATAATAATTTTAGTGAAATATGTTATTATTATGTTTTCATGATTTG

>6f1711344541ab14d52302c00d85392f

CATTGAACTATCGTGAGAAAATCACGCCGCCAAAGGGAATTATATTATAGTAATTATTGGCGTTAATAAACATTTTATTAATAGTTGTAATATATCATATTGTGTGA

>ef7d1144bf7436f6ccbf94d20f575f7d

GGGAGGGAATTCAGAAGGAAGTATTTAAAGAGCAAAGATGTAAACACAGATTAGAAACCCCGGTAGTCCGGCTGACTGACTACTGTGTAATCTCGTATGCCGTCTTC

>5720387b46c02d5c514b23f1fa45fcc2

TAAAACAACAATGGCCTGGAGGCAATATTTCTGTTCGCCTCGTGGTCCATGCTGGCGGAAATGAAACTAGGTACAGCCCATAATTGCGACGTCAACCAGTAGGCACC

>5642aad55e3638fabde1ec8b9a9cda92

TGTTGATGTGGCACCTGGAGACGCGTGGTTATTGGCGAAACGTTGCGTCTGTTCTTTACCAACGGTATCAACCGTATAAGAATACGTTTACGCTTAAAATAATTATA

>57d3fd03cf06ca1b974dbb96e7f3da29

AACGTAGGGGGCGAGCGTTGTCCGGAATTACTGGGCGTAAAGGGCGTGTAGGCGGCCAATTAAGTCATTCGTGAAACCTGCCGGCTTAACCGGTAGCTTGCGAAAGA

>cb18d479afcf8764593c7eef14e8a58d

TACGTGAGAGACTAGCGTTATTCATCTTAATTGGGTTTAAAGGGTACCTAGACAGTCAATATAACTTCTATAATGCTAATACTTGACTAGAGTTTTAAGTAAGAGGG

>55e6a969d233f9a65058b052239f1f9a

CCACCACCCACCGCTGTGTTCATACTTTTTTCACAATAACAGATGTCACAATTCACACACACACACTTAATGTACATTAGAAACCCCAGTAGTCCGGCTGACTGACT

>b53da8f64abe197cb8dd17881a17a933

TCAAACGAAAGCACAACAAAAAGACAGCCAAACTAATACCACCCCGATCACCAGCAATAACACCCACACATACGTTTTACCCATGAATAGTTAACCTCACCAACATA

>5ca6aed336ec40b8199e8c1f2f68ad97

GACAGAGGATGCAAGCGTTATCCGGAATGATTGGGCGTAAAGCGTCTGTAGGTGGCTTTTCAAGTCCGCCGTCAAATCCCAGGGCTCAACCCTGGACAGGCGGTGGG

>1881843cd0d214508fd51dc62b553ff3

ACAAATGGTGAATAGTATAGTGTCAATAATGGCATTAGAAACCCCTGTAGTCCGGCTGACTGACTATGAGCTCATCTCGTATGCCGTCTTCTGCTTGAAAAAAAAAA

>ee9512fa2111d255acd4121ee6d3e159

TACAGAGGGTGCGAGCGTTAATCGGATTTACTGGGCGTAAAGCGTGCGTAGGCGGCCAATTAAGTCAAATGTGAAATCCCCGAGCTTAACTTGGGAATTGCATTCGA

>4a917a48895eba7fbb16d8d5ba5b3d9f

TACGTGAGAGACTAGTGTTATTCATCTTAATTGGGTTTAAAGGGTACCTAGACAGTCAATATAACTTCTATATTGCTAATACTTGACTAGAGTTTTAAGTAAGAGGG

>e5164120734a8a509a2d67493032670e

TACGTAGGGCGCGAGCGTTATCCGGAATTATTGGGCGTAAAGAGCTCGTAGGCGGTTTGTTGCGTCTGCCGTGAAAGACCGGGGCTCAACTCCGGTTCTGCAGTGGG

>b9e42e2f8e0f030d4c5f96f849af1388

TACGTGAGAGACTAGTGTTATTCATCTTAATTGGGTTTAAAGGGTACCTAGACAGTCTATATAACTTCTATAATGCTAATACTTGACTAGAGTTTTAAGTAAGAGGG

>9400da6a6512574d4d136e4b280fb2a9

TACGGAGGGTGCAAGCGTTATCCGGATTTATTGGGTTTAAAGGGTCCGTAGGCGGATGTGTAAGTCAGTGGTGAAATCTCGCAGCTTAACTGCGAAACTGCCATTGA

>e037b0f2707edc355c2ee5a312c39548

TACGTAGGGGGCAAGCGTTATCCGGATTTACTGGGTGTAAAGGGAGCGTAGACGGAAGAGCAAGTCTGATGTGAAAGGCTGGGGCTTAACCCCAGGACTGCATTGGA

>6ee3efbbdb8f76d600cb5553379b42ac

AAAACCAACAATGTGGTTTTTGACCCTAAATGATTTACACGTTTGAAATCTAACCAAAATAACTGTTTGCTGAAAAACACGATTTGTTTTGCTACAAAATAACACCT

>d82b67e917d0eff577cb6558b023cc3c

ATTCAATAAGTTAAACGGCTTTGAAGTAGTAAAAAAGAGTCATTCGCGGAATAGAGTGCGTCCGCCGTCGAGGAGGGGATCGGAAATCTCTGATGATGAGCTGTGAA

>fac623c58d6bdaf9697d035b426f4fe0

TGCACTGTAGTGCAAACTATTTAAAACGATTAGAAACCCTAGTAGTCCGGCTGACTGACTTAACGTCCATCTCGTATGCCGTCTTCTGCTTGAAAAAAAAAAAAAAA

>ae762b1cdf01b2be3a498423f7e23951

CTATGTCAAGGCCGCAATGAGAATTTCGCTCTAATGTTTTCCTTTATTGTTAACGCGTAGTATGGAATAATCTAGTATTTCGGGGAGGGGTATAAATACCAGTACTC

>a614f884e92d3e01c4e0ebd9b50c3b0c

AGTTTTATCTATTATTAAAAATGGGAAAGTAGTTAAAATCAGTCGACTTGAAACCACTCCCCCCCCCCCGGTGATTGTTAGGCTCTCGAGCCAAAATTTTACTAAAA

>588dc7f34a58a76bcbd035246816450d

CATTGAACTATCGTGAGAGAGTCAAGCCGCCAAAGGGAATTATATTATAGTAAATATTAGCGTAAATAAACATTTTATTAATAGTTGTAATATATGATAATGTGCAG

>753dd064c470c790223b7f3f3560695a

AATTACAGTAAGCATGAATAAAGTATAGCATAAATAAAGCTTGAATAAAATTACAGTAAGCATGACTTCGATAAGAAAAATTGAATACAATAATTAAGTTAGTTTAG

>1889032b07b42544340004583865ba27

CACACTTATGTCTTCAGAAAGACAAAATATTTCTCCAGGATGCATAACCATGTCGGTCATTTTCAACTGGCAGTGTCTTGTATATATTTGTGGAAGATTTTCAATGT

>df148bc18b4fb16430b3d9e9fc95685b

GTGCTCGAAGAACATCAATCTTTCACTTCGTTCTCACTTTCTCTTTTTACTGCCTCCTCTTTTGACCCTTTTCCAACATACCTCTTTCGCTCCTTTTCGTCATCCCT

>b33827fb70c2a45e1850224c8a60f58b

GAAGAGTGATTTTTGACGAGAGTACAGCTCGGAGTCTGATTAGAAACCCTTGTAGTCCGGCTGACTGACTATAGCGCTATCTCGTATGCCGTCTTCTGCTTGAAAAA

>64292828a72e299822a33cd4c2c7ecc3

TACGGAGGGTGCAAGCGTTAATCGGAATTACTGGGCGTAAAGCGCGCGTAGGTGGCTAAGTCAGCCAGGTGTGAAAGCCCCGGGCTCAACCTGGGAACGGCATCTGG

>302377ae2464adf015bc3f1c542db038

TACGAGGGGAGCGAGTGTTGTTCGGTTTTATTGGGCGTAAAGAGCACTTAGGCTGTTTTACAAGTTAACAGTTAAATCTTGGAACTCAATTCCATGCTAATTGTTAA

>b27d01484117e748dbaf877cd6b3a024

TACGAAGGTCCCAAGCGTTGTTCGGAATCACTGGGCGTAAAGGGAGCGTAGGCGGCGTGGTAAGTCAGATGTGAAATCCCGGGGCTCAACCCCGGAACTGCATCCGA

>69f3cc701dd9f44db43dcab8ff66ceb8

CCACCACCCCACCGCTATGTTCATACTTTTTTTTTACAATAACAGATGCCACAATTCACACACACTTAATGTACATTAGAAACCCTTGTAGTCCGGCTGACTGACTG

>43aeba47a2e7acd4c2cd8e382699fd6a

AGGCTCCCGCCCCGCAGACCTCATTGGTCAATTAATTCACTCTTCAGCCTTCTCATTAGAAACCCCAGTAGTCCGGCTGACTGACTCGCGATATATCTCGTATGCCG

>bc6bbc2e89bd89cdb388e5564566eebf

TACAGAGGGTGCAAGCGTTAATCGGAATTACTGGGCGTAAAGCGTGCGTAGACGGTTACATAAGTCGGATGTGAAAGCCCCGGGCTCAACCTGGGAATGGCATTCGA

>b5fc9adb54433e1a84eae1dd15e9df7d

TTAGCTGGGTGAGCTCCCTCGCAGTTAGCGCATTTGGCCGGAGCAGGGCGGGAAACTTCGCATCCCCCGCGATACTCCTGCCCGCAGTGCACACAGCGCGGTAGTAG

>51e4e1b7676eb3cc3af5c0264f5147d5

TATGTGCACACAGATACAGACACAGTCTCTCCCGGTCTCTCACTTAACTAACAGTCTCACTGCTGGGCTGGCTGCATATTAGAAACCCCGGTAGTCCGGCTGACTGA

>d58120bc8a128bba589ab485ffc52ce2

TACGTAGGCGGCAAGCGTTGTCCGGAATTATTGGGCGTAAAGGGAGCGCAGGTGGGACGGTAAGTCTGTCTTAAAAGGCAAGGGCTCAGCCCCTGTAAGGGACAGAA

>35bf68961a222199ec6779e6cb9c7f03

TCCTTTGTCATGATCATCCGCGTACCAAGTAAGTCATCGACTTTATGCCGGCAGCCATTCGGTCGTCAAAAGTTATTCTGCATGGATAGGGTTTGTTACGTTCATTG

>7c4d028463e022b1a621f27cbad1367b

TCAAACGAAAGCACAACAAAAAGATAGCCAAACTAATACCACCCCGATCACCAGCAATAATATCCACACATACGTTTTACCCCAGAATGGTTAACCTCACCAACATA

>8961cafe1293b44ed9bc9b7d134599c4

TATGTGCACACAGATACAGACACAGTCTCTCCCGGTCTCTCACTTAACTAACAGTCTCACTGCTGGGCTGGCTGCATATTAGAAACCCGAGTAGTCCGGCTGACTGA

>1f69499c1755c209e3eac1a4014f5b7c

CCCACGTTTGTTCAAATCTCTTTGTATTATCATTTTATAGTTCATGTCTTACTTTTATGCTATTTTTATAATTTTAATTTTCTTTATAACTTTTTTTGTATTGTTTT

>3457a27ad1e572ca3f4f18669f10aef8

TACGTAGGTGGCAAGCGTTGTCCGGAATTATTGGGCGTAAAGCGCGCGCAGGCGGCCTTTTAAGTCTGATGTGAAAGCCCCCGGCTCAACCGGGGAGGGTCATTGGA

>39816f09a8c6dd3e5706b71c03561835

TATGTGCACACAGATACAGACACAGTCTCTCCCGGTCTCTCACTTAACTAACAGTCTCACTGCTGGGCTGGCTGCATATTAGAAACCCCTGTAGTCCGGCTGACTGA

>0e5523cc113e16378fd5c93abec3c326

TACGTAGGGCGCAAGCGTTATCCGGAATTATCGGGCGTAAAGAGCTCGTAGGCGGTTTGTCGCGTCTGCCGTGAAAGTCCGGGGCTCAACTCCGGATCTGCGGTGGG

>b512b39daf81e1ddfd360a48e7ea9490

TATGTGCACACAGATACAGACACAGTCTCTCCCGGTCTCTCACTTAACTAACAGTCTCACTGCTGGGCTGGCTGCATATTAGAAACCCTAGTAGTCCGGCTGACTGA

>4ac15b7855c08b01ed91c0898dbe582d

TACGTAGGGTGCAAGCGTTGTCCGGAATTATTGGGCGTAAAGAGCTCGTAGGCGGTTTGTCGCGTCTGCTGTGAAATCCCGAGGCTCAACCTCGGGCCTGCAGTGGG

>ce88b07af18c73f10a18480e90f5c94b

TACGTAGGGCGCGAGCGTTATCCGGAATTATTGGGCGTAAAGAGCTCGTAGGCCGTTTGTTGCGTCTGCTGTGAAAGACCGGGGCTCAACTCCGGTTCTGCAGTGGG

>464178b218e65dd5d5031e326382535d

CCAGACACCGTAGTATGCCCCTGTAATCCCAGCAATTGAGAGGAGGCAGAAAGATTAGATACCCCAGTAGTCCGGCTGACTGACTCGCGATATATCTCGTATGCCGT

>05cab82e0e830065d85920d47c0c38e2

CGCATACGGCGGACGCACACAAAAACAAGACTAAAGAGGATAGTCTATCGAGACGTCGCATCAACCCTTTTAAGGGCCACAATACCTTCAACAGCCCTCTTAAGGGC

>1b8090518b736b75c64ce33217d311a2

GGGAGGGAATTCAGAAGGAAGTATTTAAAGAGCAAAGATGTAAACACAGATTAGATACCCTTGTAGTCCGGCTGACTGACTACTGTGTAATCTCGTATGCCGTCTTC

>9be7b3ad8bdee0fdd5de84afaaa77799

TACGAAGGGGGCGAGCGTTATTCGGAATAACTGGGCGTAAAGGGCACGTAGGCGGGTTATTAAGTCAGTGGTGAAATCCCAAGGCTCAACCTTGGAACTGCCTCTGA

>fc2ffb16190e692e347e2b43799f1cfb

CCACCACCCACCGCTGTGTTCATACTTTTTTCACAATAACAGATGTCACAATTCACACACACACACTTAATGTACATTAGAAACCCGAGTAGTCCGGCTGACTGACT

>1569d5cbdf1e833578096c79a3f088c9

TACGGAGGATCCGAGCGTTATCCGGATTTATTGGGTTTAAAGGGAGCGTAGGTGGATTGTTAAGTCAGTTGTGAAAGTTTGCGGCTCAACCGTAAAATTGCAGTTGA

>378554255a41e58ddfc20c9926c2060b

AACAGAGGATACAAGCGTTATCCGGATTTATTGGGTTTAGAGGGTGCGTAGGTGGTTTTTTAAGTCAGTAGTGAAATCTTAAAGCTTAACTTTAAAAGTGCTATCGA

>5c37cf409ef379c909b4cbf3779becba

CATTGAACTATCGTGAGAAAGTCACGCCGCCAAAGGGAATTATATTATAGTAATTATTGGCGTTAATAAACATTTTATTAATAGTTATAATATATGATAATGTGTGA

>ed1dbf1d19a13d7070c00cb8c1c73ed0

AGCGGCGTGCAGTACCAGGAGAGCACTGCTACACACGTATCTATCAGAAACCATGTAGCGCCAACGCGGCAAAGATCGTGATTTGTTCCACGGCAAACAGACTGACC

>65b546f343f4b41dcbe89cc820cb854b

GCCTTGATCCTTTCAGGGACTGTTGAGCGGAGGTTTGAAATGACCTGAAGATAGCTTGAATGCCGAAAGCGCTTGTCAATACCAAAGACAACATAGTGGTTGGTACA

>0480dace57dda0577f87e80173337f68

AGTGGAAGAAGTGTAGGCAGAAACAAATATTGATTGATCAATTGTGGTGAAGAGGAACAAACCCAAAGTGCATTGTAACTTTGGCGGCGATGATGATGATGATGATG

>b277a01206128e3d9f289249006336bb

TACGTAGGTGGCAAGCGTTATCCGGAATTATTGGGCGTAAAGCGCGCGTAGCCGGTTTTTTAAGTCTGATGTGAAAGCCCACGGCTCAACCGTGGAGGGTCATTGGA

>fc338107be050292b06bb4f7e34fe860

CATTGAAACCTCGTGAGAAAGTCACGCCGCCAAAGGGAATTATATTATAGTAATTATTGACGTTAATAAACATTTTAGTAATAGTTGTAATATATCATATTGTGTGA

>75c960e9667278e428fe231256ed8aaa

AACAGAGGATACAAGCGTTATCCGGATTTATTGGGCTTAAAGGGTGCGTAGGTGGTTTTTTAAGTCAGTAGTGAAATCTTAAAGCTTAACTTTAAAAGTGCTATTGA

>ddc1f3c319c3d3ee057c9f4ea5a10f62

CCACCCCCACCGCTGTGTTCATACTTTTATTTTTTTACAATAACAGATGCCACAATTCACACACACACACTTAATGTACATTAGATACCCTAGTAGTCCGGCTGACT

>e1bc611f2f9e83d14df0cf6cbe47fd4f

TACGTAGGTGGCAAGCGTTGTCCGGAATTATTGGGCGTAAAGCGCGCGCAGGTGGTTTCCTAAGTCTGATGTGAAAGCCCACGGCTCAACCGTGGAGGGTCATTGGA

>6e4af874f0fed6360ab331a97299bf96

CCACCACCCCACCGCTGTGTTCATACTTTTTTTTTACAATAACAGATGCCACAATTCACACACACTTAATGTACATTAGAAACCCGAGTAGTCCGGCTGACTGACTT

>a0f1eec951eb8cc51f358c7bb38484d1

TACGGAGGGTGCAAGCGTTATCCGGACTTACTGGGTTTAAAGGGTGCGTAGGTGGGCAGTTAAGTCAGTGGTGAAATCTCCGAGCTTAACTCGGAAACTGCCATTGA

>39bd03001f19732eadf11732dda5ac6a

TTGGAGGCTAGGCGACTATTAGGCAGAGTCTCCGCTGTGACCCGGCGTGTATTAGAAACCGGCGGTAATTGGAGGCTAGGCGACTATTAGGCAGAGTCTTCGCTGTG

>0531970386159fd2c9611bc361bd4b84

CATGGTCATCCAGCCAGATTTCGTATTTATGTACGTGACGCTTGACTTTATTCACGTGGAAAGCGATGCTTCAACCACACGACCGTACCACCGGGTGAATTGAGTTA

>f24f3cbd993d9024ec6e081033a6361c

AGAAAAATACACATAACCTTTCGCTAATGTCTACCCTGGCTGTTGACTTATCGTTGGACTTTTCCTCGTTGCACCGGCTGACGCGTGTATACGCTTACTGTTTTCGG

>0cf2b2109aa57f6874a4ca48de2c3321

ACTAGCGGATTAGAGAACCGACGTTTCACAGTGATACGGCCGCGACTAACTAGCGGATTAGAGAACCGACGTTTCACAGTGATACGGCCGCGATAAATTAACGGATT

>e0afb615b0108d0e5f4996c85fe8c3c3

TACGGAGGGTCCAAGCGTTGTCCGGAATCACTGGGTGTAAAGGGTGTGTAGGCGGGCCTGCAAGTCAGAGGTGAAAGCCCCCGGCCTAACCGGGGAATTGCCTTTGA

>28987bd0461031b7461a4ad41efa52f7

CCCACCGCTGTGTTCAGTTTGATATACTTTTTTTACAATACACAATAACAGATGCCACAAGCCACACACACAGAAAGTCGTAGAACGCAAACGCATTCATATAATTT

>bd767e8dcc44c79d6fb8ddec3afcd74e

GAACAGCTCATGCTTTCCTCCCCAGCCTTTCTCTCCCAGCTTCTATTAGAAACCCTAGTAGTCCGGCTGACTGACTATGAGCTCATCTCGTATGCCGTCTTCTGCTT

>89df5eb3543375ab5e10ed82899a7c4c

CCACCACCCACCGCTGTGTTCATACTTTTTTCACAATAACAGATGTCACAATTCACACACACACACTTAATGTACATTAGAAACCCCTGTAGTCCGGCTGACTGACT

>ad36732625db6d41c5b69a48f403c025

GCAAAGTTACAGAACAGAAGCCTACCCACTGGCAAAATAGAAAGGGTGAGGGTGGCAATTACAGACAATTCCAGAAATTCTGTGACAAGCCGACCGTAAATACTATC

>01f3971c9a321ce65ffe1b98d46424cc

TACGTGAGAGACTAGTGTTATTCATCTTAATTGGGTTTAAGGGGTACCTAGACAGTCAATATAACTTCTATAATGCTAATACTTGACTAGAGTTTTAAGTAAGAGGG

>0bb89e2a7f77c4605a55a338dbf2ba51

CATATTGTAATACACAGAAATTATGAGCGTTTCATAATGCAAATACTATTGAACAAACTGTTGCATCCACAATACGCGTTCAAATTATACTTGGTGCACGGAGAAAT

>db97d739988542505707d475a2320e3a

TACTTTGCCATGATCATCCGCGCACCAAGGAAGTCGTCGATCTTATACCGGCAGCCATTCGGTCGTCAAAAGCTATTCTGCATGGATAGGGTTTGTTACGTTCATTG

>31dd7a77ecea685eb3101b9ece671a71

TACGTAGGTGGCAAGCGTTATCCGGAATTATTGGGCGTAAAGCGCGCGTAGGCGGTTTTTCAAGTCTGATGTGAAAGCCCACGGCTCAACCGTGGAGGGTCATTGGA

>ce06f611b4603cc5971f531b19a1f158

TCATCCTCAATTGCTTCCAGCGACGACACGTTCCAGCAGCTGAAACGTTTAGCACCAACCACGACGCTGGACCACGGCCCTCCAGCTCGGCACGTTTTTATTTCATA

>e7e779e4b8a9b35dc17ca25abd42ad83

AGGCTCCCGCCCCGCAGACCTCATTGGTCAATTAATTCACTCTTCAGCCTTCTCATTAGAAACCCTAGTAGTCCGGCTGACTGACTCGCGATATATCTCGTATGCCG

>f83ef61b77205c6265f1872ea7aaaae3

TACGGGGGGGGCAAGTGTTATTCGGAATGATTGGGCGTAAAGGGCACGTAGGCGGTGAAAAGGGTGGAAAGTGAAAGTCGCCAAAACACTGGCGGGGTGCTTTCTTG

>141135d7e2c1e4afe8c14990a6ff41fa

GAGAAAAACTATCGCCAACAATTAGCGCATTAAGTAGTAGTAACGGCGACGGTAACAGCTGTACGCTACTGATAAGCCAGCAGCCGACGCTACTTCTCTTCTGTCTA

>66f2ec408fb63b5e4adaee6149b43297

TCCTGGACGTTACAGTCCTTGGCGTCTTCGTCAGTAGCCCGGTGTTTCCTCGTCCTTTAGCTTATTGTGTAATGTATTGATATATTGTTATCAGGGCATATATTGTT

>854c435b93f189aa8c81bb60f90aa160

TAAGGTGCGTAAGAGAATGTCTGAGGAAGGCCTTAAGGTGCTGACCAGAAAGAGAGGCGGATTAAAATCCGCGATAGTGCGGATACAATCCGCAGTAAACTCATTTG

>c4d5541ba042f38c102a433282c7c65a

TACGAAGGGAGCTAGCGTCGCTCGGAATTACTGGGCGTAAAGGGAGCGTAGGCGGACATTTAAGTCAGGGGTGAAATCCCGGGGCTCAACCTCGGAATTGCCTTTGA

>f032c92f38606e8a45506b09aa1463ab

TACGGAGGGTGCAAGCGTTAATCAGAATTACTGGGCGTAAAGAGCGCGTAGGTGGTTTTTTAAGTCAGATGTGAAAGCCCTAGGCTTAACCTAGGAACTGCATTTGA

>aec8a3759cd85315a873ccde5e9c0050

TACGTAGGTGGCAAGCGTTATCCGGATTTATTGGGCGTAAAGAGAGTGCAGGCGGTTTTCTAAGTCTGATGTGAAAGCCTTCGGCTTAACCGGAGAAGTGCATCGGA

>6401a5b5f6d87bde734a0064b32e5868

TCCGGCTGACTGACTAGTCGCAGATCTCGTATGCCGTCTTCTGCTTGAAAAAACAAAGTAGTCCGGCTGACTGACTAGTCGCAGATCTCGTATGCCGTCTTCTGCTT

>3532b0df6373498416855f40bc5a53ab

ATTTAAAGTGATATGGCAGTAGCGAAAGTCAATCCAAGTTAAATTCCGTGTGTTGTAGTATTTTGTAACGGGTGATTAATGATATTCAATTGTGAAACCTTAAGTTA

>dac7ba7457caaf12868d412a9d10f6b8

ATCCCGGAAGGCAAGTGACCGACGCCCAGGCGTGGAGGCTCGTAACGGCGCACTTAACAACGTTCCTCGCCTACATGCCGGTTCGTATACAGAGGACGCGAACCATA

>7d742c5a855f091c8e6f9a7b02a937bf

CCACCACCCACCGCTGTGTTCATACTTTTTTCACAATAACAGATGTCACAATTCACACACACACACTTAATGTACATTAGAAACCCTAGTAGTCCGGCTGACTGACT

>b2579047b544e9084e413897af5b6600

TATGTGCACACAGATACAGACACAGTCTCTCCCGGTCTCTCACTTAACTAACAGTCTCACTGCTGGGCTGGCTGCATATTAGATACCCCAGTAGTCCGGCTGACTGA

>f9fcb59cdb5c31b493c968e07600a541

CCACCCCACCGCTGTGTTCGTACTTTTTTTTTTACAATAACAGATGCCACAATTCACACACACACACTTAATGTACATTAGAAACCCCTGTAGTCCGGCTGACTGAC

>f8d84d2186b00566f89e1737717f2f57

ACTGTAGGTCGCGCATACCTGTGAGAGAATCTGTAAAACACACCACGGGTATATGCAGAGAGGTCGCCTACGCTCTACGCGAGTATGGCACTCAAAGAGAAGACAGG

>6e1454b10a88c7214cd142b48c223519

ACAACGAATAAACCAAAAACGCAATACCGAAATAAAATAATATGGAAAGTTTAGGAATTGGAGAAGAAACGAGTAAGTGTGAAAACCTTACTCGAATAAATGAAGTT

>3efb427ddc6fdf29d75cf488b5b051c3

CCACTGTGTTCAGTGTGATATACTTTTACAATACACTATAACATTAGAAACCCTTGTAGTCCGGCTGACTGACTCGCGATATATCTCGTATGCCGTCTTCTGCTTGA

>c4f0ad66302dc05bf4a4f637f864bc7e

CCACCCCCACCGCTGTGTTCATACTTTTTTTTTTTTACAATAACAGATGCCACAATTCACACACACACACTTAATGTACATTAGAAACCCGAGTAGTCCGGCTGACT

>ad5631992e29f38f58f5c725db8192b7

CGGTAGGGCCGCACATTACTGTCGGCCATACTCTGTAATTATTGCAATTACAAGTTGTTGTTAATTTATGTGCGCGTGGCGTTTTAATTACACGTACATAATGTATG

>71c88bb2fbb1a1802a3cd368ef02adb8

TTTTTTTCTCGGAATGAAGGTTCCTGCCTAAAGAACGTGTTGTAGAAACGGAGATGATGTTCACCGTATGTAGGTAATGGCATCGTCAGTGTTACACAGTAACAAAC

>e04768275616a921a90d14f11f5a7043

TGTGTGTTTCCTTCCTCAGTATGTATGTGTTTGTGTTGGATCCGTCACACAATTCTGTTTTTAAAGAAAGGGTGGCAGAGTGCCAGTACAGCGGCATTTTTCTCTAC

>b15605512270123a3e5f1c74271b9994

TACAATTTCTTCAATTTTAATTTATAAAGTTTCAGTTAATATATAATATAATTTTTGTAAATAATAATTTTAGTGAAATATATTATTATTTTGTTTTTATAATTTGC

>879d1913ba4694e0f09c6e4da05e612b

TACGAAGGGGGCTAGCGTTGCTCGGAATGACTGGGCGTGAAGGGCGTGTAGGCGGTTTGTACAGTCAGATGTGAAATCCCCGGGCTTAACCTGGGAGCTGCATTTGA

>2b3a1a141d52930048fdd38c21cd4997

TACAATTTCTTCAATTTAAATTTTTAAAAGTTGCAGTCAATAAATTATTTAAATTGTTATGAACAATAATTTTAGTAAAACATATTTTATTTTATCTTATTAATTTG

>359dac4961a6d687cda575aaa8e9807d

TACATAGGGGGCAAGCGTTATCCGGATTTATTGGGCGTAAAGGGTGCGTAGGCGGTTAAATAAGTCTGTGGTTTAAACGCAATGCTCAACATTGTGACGCTATAGAA

>7b39ce70c85d5e60eecb5d3d5cae7ad9

AATCTACTATCCAAACCTACAGTGGACACAGTGAAGAAGAAGCAGCTGTCAGATTAAACAGATAACAACCACCGACAGAACGCTAACAACTGCTCTTACTACTTTGT

>eca6a9cb3c05a238f72b1d298717c213

CCACCACCCACCGCTGTGTTCATACTTTTTTCACAATAACAGATGTCACAATTCACACACACACACTTAATGTACATTAGAAACCCTGGTAGTCCGGCTGACTGACT

>9b474d317b632e1ba31a8df0655b2959

TACGTAGGGCGCAAGCGTTATCCGGAATTATTGGGCGTAAAGAGCTCGTAGGCGGTTTGTCGCGTCTGCTGTGAAATCCCGAGGCTCAACCTCGGGTCTGCAGTGGG

>b59ecf1091ad6dc8795d18bc033893bf

TACAATTTCTTCAATTTAAATTTTTAAAAGTTTCAGTTAATAAATTATTTAAATTTTTATAAATAAAAATTTTAGTGAAATATATTATTATTATTTTATTAATTTGT

>84decddafd533ecd93cd2a35f0d781c8

CAAATTTATGTCTTCAGAAAGACAAAATATTTTTCCAGGGTCCATTTCAACCATGTCGGTCATTTTCAACTAGCAGTGCCTTGTAGATATTTGTGAAAGATTTTGAG

>fd4ce5f737771c7dab610b7c06403943

CCAGACACCGTAGTATGCCCCTGTAATCCCAGCAATTGAGAGGAGGCAGAAAGATTAGAAACCCCGGTAGTCCGGCTGACTGACTCGCGATATATCTCGTATGCCGT

>76d165b0bc746502f73f09cb5960abad

TATTCTCGCGAAAACGCAAGCGGCCTCGGCCGCTTACAGTAGGCCCCGTCGAGGTGCCCTGGACCAACAGCGTCAAGTACCTCGGGCTCCACGTGGATTCCCGACTC

>5a9944e7b638e2d3fa7a47db883c342e

AGGCATCTATTGCCCATCATTGTGTTGTGCTGCCGCAGCGGACTCATTTCGATGCCACCGCCATTGAGCGAACGGGAAGCGCTTGCGCAGGTTCGCTGACTCGCACC

>c5d0f4fb1e634145bb56eb51c1089fe9

TACAGAGGATGCAAGCGTTATCCGGAATGATTGGGCGTAAAGCGTCTGTAGGTGGCTTTTTAAGTCCGCCGTCAAATCCCAGGGCTCAACTCTGGACAGGCGGTGGA

>3e2aa4c0883fc3f7a1eeb5ce1552b233

AACAGAGGATACAAGCGTTATCCGGATTTATTGGGTTTAAAGGGTGCGTAGGTGGTTTTTTAAGTCAGTAGTGGAATCTTAAAGCTTAACTTTAAAAGTGCTATTGA

>df28b4a5e27562264927c00e52d4218c

CACTTTACCATAGTGTCTGCCTCCACGGGCACGTTTATGGCTTGGCCGGTTATCTTTTAAGACTGTTTCAATTTTGAGACGACGGATCTGCAAACGTGAAAATCCGG

>8f330b662418da5bdcc8e4edd1cae7e4

ATCCGCCAACTGCAGGACAACGTCCAAGAAGACCAACAACTTGCCCTCATCTTCTCCATCCACCACTGGAAATTTCTGGAGAATGCCGGAAATAGAGTTGGGTAGTC

>391648365fbef3015b77d7181aed4ab4

TACGTAGGGTGCAAGCGTTAATCGGAATTACTGGGCGTAAAGCATGCGCAGGCGGTTATGCAAGACAGAGGTGAAATCCCCGGGCTCAACCTGGGAACTGCCTTTGT

>48f0e323e257c2933de46bd99f0525f1

GTGCTCGAAGAACATCAATCTTTCACTTCGTTCTCACTTTCTCTTTTTACTGCCTCCTCTTCCGACCCTTTTCCAACATACCCCTTTCGCTCCTTTTCGTCATCCCT

>c780319b8356ad7b803f13e70591ad03

AGGCTTCTATAGCCCATCACCGTGTCGCGCTGCCGCAGCGGACTCGCCTCGAGGCCACTGCCATTGAGATCCACGGAGCTCTTGGCGGCGTTCTCTTGGTAGCGGCT

>8e1e689be91fbe0bed7055b29d828ea2

CCACCACCCACCGCTGTGTTCATACTTTTTTTACAATAACAGATGTCACAATTCACACACACACACTTAATGTGCATTAGAAACCCTAGTAGTCCGGCTGACTGACT

>d942e38bc68f382b8032a1b52b045bba

TACGGAGGGGACTAGCGTTGTTCGGAATTACTGGGCGTAAAGCGCACGTAGGCGGATTTGTAAGTCAGGGGTGAAATCCCGGGGCTCAACCTCGGAACTGCCTTTGA

>b30d78e223099da0087a339f567b46ba

TACGTAGGATACAAGCGTTATCCGGATTTATTGGGTTTAAAGGGTGCGTAGGTTGTTTTTTAAGTCAGTAGTGAAATCTTAAAGCTTAACTTTAAAAGTGCTATTGA

>7dd46ca929b84362668a9374f7deef79

CCCACCGCTGTGTTCAGTTTGATATACTTTTTTTACAATACACAATAACAGATGCCACAAGCCACACACACACACACCGAGAAAGTCGTAGGACGTAAACGCAGTCA

>c06f87f620d0a10d169e8f6b6217a0e5

TACGGAGGGTGCAAGCGTTATCCGGATTTATTGGGTTTAAAGGGTCCGTAGGCGGATTAGTAAGTCAGTGGTGAAAGCCCGCAGCTCAACTGTGGAACTGCCATTGA

>598bb7682b53186ff039f4604a6db238

TACGAAAGGGGCTAGCGTTGTTCGGATTTACTGGGCGTAAAGGGCACGCAGGCGGTCTTGCCAGTCAGGGGTGAAAGCCCGAGGCTCAACCTCGGAACTGCCTCTGA

>2440251830b1606d1eb0ed71d7f72191

AGGCTCCCGCCCCGCAGACCTCATTGGTCAATTAATTCACTCTTCAGCCTTCTCATTAGAAACCCCTGTAGTCCGGCTGACTGACTCGCGATATATCTCGTATGCCG

>4a16944f8913695ef4a1097e880da39a

TACGTAGGTCCCGAGCGTTATCCGGATTTATTGGGCGTAAAGCGAGCGCAGGCGGTTAGATAAGTCTGAAGTTAAAGGCTGTGGCTTAACCATAGTACGCTTTGGAA

>e0028536d11d45697ef15d608f948777

TACGAAGGGGGCTAGCGTTGCTCGGAATCACTGGGCGTGAAGGGTGCGTAGGCGGGTTTTTAAGTCAGGGGTGAAATCCTGGAGCTCAACTCCAGAACTGCCTTTGA

>0e9ace62f11f355c5f7ff045c37bce9c

TGAAAATATCCAACGCACACATGGCGCATGCGCATGTTTACGAACAGCGGGCCTCTAAGCGGTCAGTTGCCTTTCGCGGACTACAGCTGGCGACCCTTGTCGCTCTC

>eae1d16f2fcca4780cadc2785241168b

TACGTGAGAGACTAGTGTTATTCATCTTAATTGGGTTTAAAGGGTACCTAGGCAGTCAATATAACTTCTATAATGCTGATACTTGACTAGAGTTTTAAGTAAGAGGG

>b2423ee29ba7293f91c4a7b9820f1e52

TACAATTTCTTCAATTTAAATTTTTAAAAGTTTCAGTTAAAAAATTATTTAAATTTTTATAAATAATAATTTTAGTGAAATATATTATTATTATATTTTATTAATTT

>b634f6756c7832ad919447a8692f9c3d

GAAGGGTAGCTGCCTTCTGGATCAAAGGTAGGACGTGTATCGTTGGATAACTCCTGACAGAACAATGTATTGCTGGATCATATTTACGTATGATCGGTTATAGTAAA

>6f8fbe8bb93e6bdd387214c1cf6ddf3d

CATTGAACTATCGTGAGAAAGTCAAACCGCCAAAGGGAATTATATTATAGTAAATATTGGCGTAAATAAACATTTTATTAATAGTTGTAATATATGAAAATGTGCAA

>a7845ccad0cd2a3a994863e8ea37e28f

TTTATTTATTTCTTTACTTAGGGATTACACGTCCAGCATCTGGAATTATAGACACGAAAACGGCGAGTAGCTGGCATACTTCGTGCACGGATCGTGTTAAGCTTAAG

>0d8e0284ecbe6643ea4b350219740b1e

TACAATTTCCTCAATTTAAATTTTTAAAGGTTTCAGTTAATAAGTTATTTAAATTTTTATGAATAATATAATTTGTCTGAGAAACTTTTTTATTAAACTAGGATTAG

>c64d9772eff89869efb5020403957674

TACGAAGGGGGCTAGCGTTGTTCGGAATCACTGGGCGTAAAGGGCGCGTAGGCGGCCGATCAAGTCGGGGGTGAAAGCCTGTGGCTCAACCACAGAATTGCCTTCGA

>b6a3bda25e11f34f365f7c663049132a

TCCGGCTGACTGACTTAACGTCCATCTCGTATGATTAGAAACCCGAGTAGTCCGGCTGACTGACTTAACGTCCATCTCGTATGCCGTCTTCTGCTTGAAAAAAAAAA

>1a4f5a5314fd4d5823aa6c42458a5874

TACGTGAGAGACTAGTGTTATTCATCTTGATTGGGTTTAAAGGGTACCTAGGCAGTCAATATAACTTCTATAATGCTAATACTTGACTAGAGTTTTAAGTAAGAGGG

>e77cce6c4d958f2fdd40d83d605b556b

TCCGGCTGACTGACTTCTAGACTATCTCGTATGCCGTCTTCTGCTTGAAAAAAAAAACCAGTAGTCCGGCTGACTGACTTCTAGACTATCTCGTATGCCGTCTTCTG

>d23545876c29c6f068929f4cf9ce8d25

CGGTTTACCAAGTATTTGGCAACTTTATGCGTTCATTATGGGCGTTAAAACTTAAAAATCTCATCTTTTTTATCGTTAAGGAATTCAGGACCCAGCGAGTAGCTGTC

>518edb941478628e832daabc40850b14

CACACTTATGTCTTTAGAAAGACAAAACGTTTTTCCAGAATGCATTTCACCCTTCTCGGTCATTTTCAACTGACAGTGCCTTGTAGATAGTTGTGGAAGATTTTGAG

>58436fd2e24c1e5a902b8ebecdbdbcce

AGGTGAGAGTTCAAGAAACAAGTCTTGCTACTTCCAGAAGAGACAATATATTAGAAACCCGGGTAGTCCGGCTGACTGACTATAGCGCTATCTCGTATGCCGTCTTC

>2ad3b5c78fe2e057b4b3db21b04d4ab4

TGCCCGTAGGAGTCTTCGTTTCCGTACCAACGCCGTAACGACACCTATAATTGACAATTTATAAATTAAGTTGATTGATAACAGACAAAATACAAATTTTACACTTG

>355c932a16692b98467e701cf4a652cb

CACGACCATGTGCTCGTATAGTTTTCTTGCTTTTAGCCATTTTGAGTTTCACAAATGCGTGGGTCAGCGCCCATGCTTGGTGGACGCATAGGTGCGCACTGCCACAT

>fcb237f9b81615ca86faf61e3dc8ab67

CAAGTTAGGACAATGTTATAATTAAACTGTATCCTAATTAACTGACTTTAATAAAAGCGTTAAACTTGAAAACAATACTCTTTATAAGAGAACGAGAAAGAGAAATG

>6b9433c207e88cb1f84017a80a812843

CCCACCGCTGTGTTCAGTGTGATATACTTTTTACAATACACAATAACAGATGCCACAAGCCACACACACACACACACACACACACACATACATAGAAAGTCATAGGA

>e2c99d6fbdec99fbae2f7ada8d1ed440

ACGTGTTGCAAACGTAATGGACGCCGCTGAAGGCAACGATGCGGTCAATCTTAATCTCTTACACGCTTTTCATGAAACGACTGACAAACGATTCAACAATATGTGGT

>d979df9004b68fa60375d82227b8d163

CCACCACCCACCGCTGTGTTCATACTTTTTTCACAATAACAGATGTCACAATTCACACACACACACTTAATGTACATTAGATACCCCGGTAGTCCGGCTGACTGACT

>89a835883fd231612dda1ab4266fc774

TACGAGGGGAGCGAGTGTTGTTCGGTTTTATTGGGCGTAAAGGGTATGTAGGCGGTTTTGTAAGTCAACGATTAAATCTTGAAGCTTAACTTCATTAGCGGTTGTTG

>9ca9200215e2edf27823608c7cd66697

AACAGGGGATACAAGCGTTATCCGGATTTATTGGGTTTAAAGGGTGCGTAGGTGGTTTTTTATGTCAGTAGTGAAATCTTAAAGCTTAACTTTAAAAGTGCTATTGA

>fe96f148dba3a2de468cad8c0c96c0c4

ATCTCATAACTCCATCATAACTTATAAACGCTAGTCTTCCCCCCCCCATCCAGTCAACTACAGTTTCCATCAATAGACCAATTAGAAACCCCAGTAGTCCGGCTGAC

>b1c41772d23f792a9dae37bc93b4dcd4

TACGGAGGGTGCAAGCGTTATCCGGATTTATTGGGTTTAAAGGGTCCGTAGGCGAATTAATAAGTCAGTGGTGAAAGCCCGCAGCTTAACTGTGGAACTGCCATTGA

>971eebae20c86402e27f9e72159d68da

TACGTGAGAGACTAGTGTTATTCATCTTAATTGGGTTTAAAGGGTACCTAGACAGTCAATATAACTTCTAGAATGCTAATACCTGACTAGAGTTTTAAGTAAGAGGG

>4c525eb574ed454645c7d0925c487bdd

ATTTGATCTGCTGATTTGCAATGGGACTAAGTCTGTTGTGTTAGTATTAGTGTTTTCATTAGGTTTGGCAGGAATTTTTGTTTTGTGAATGTTTTCAGTATTTAGTG

>f4c88a43d4ec04dd2b48e7e6db6a45a6

CCAGACACCGTAGTATGCCCCTGTAATCCCAGCAATTGAGAGGAGGCAGAAAGATTAGAAACCCGAGTAGTCCGGCTGACTGACTCGCGATATATCTCGTATGCCGT

>566877ee1c336fe0ac543e6c20a266b4

TACGTAGGGGGCGAGCGTTGTCCGGAATGATTGGGCGTAAAGCGCGCGCAGGCGGTCCTTTAAGTCTGATGTAAAAGCCCGCGGCTTAACCGCGGAAGGTCATTGGA

>1f8e3f2f746641b0e8ec087193b27eef

GGTGACAACGAAGAAAATGATCTACATGGCAGTGGAATTGAGGAGTGTCATATAATTTTATCATTCACTATATAACATTAGATGCCATTTTGTCTCCGTGACGAGGA

>cc07b55b9599daa99f5403ff729ca0b7

TAGCATAAAAAGAATGTCATATAATTACAGTATACATATTAGAAACCCTGGTAGTCCGGCTGACTGACTATGAGCTCATCTCGTATGCCGTCTTCTGCTTGAAAAAA

>75931abc08fc39e528ce8b308a1905f0

TCCTGCTGATAGCCACACCTGCTGAAATTCACTTTTTTCCAATTTAATAAATGGGACGGGGTAATTTAAAATCAATTGCTCGAATTTTCTGTCATATCATGCTATTC

>0dc419226531f361d7669232162de377

GTAAGGCTTGAAGTGATGAGGATATCGAAAGGCCTCTTTTAAACCGAAGTTACCAGCTGTTCGGCAATTATCAGCGGCAGTGTTCAAATTAGAAACCCCAGTAGTCC

>8866af4184ed0a8b0b198a80c0c8ba72

TACGGGGGGGGCAAGCGTTGTTCGGAATTACTGGGCGTAAAGGGTTCGTAGGTGGTCAACTAAGTCAGACGTGAAATCCCTCAGCTTAACTGGGGAACTGCGTCTGA

>4173fa88e7b651f013a2c021fa114b25

CCACCACAACCCCACCGCGGTGTTCATACTTTTTTTTTTTTTTACAATAACAGATGCCACAATTCACACACACACACACACACACACACTTAATGTACATTAGAAAC

>d6ae8f631868f25bf4a7cd543354c303

TCCTTTGCCATGATCATCCGCGCACCAAGGAAGTCATCGACTTTATGCCGGCAGCCTTTCGGTCGTCGAAAGCTATTCTGCATGGATAGGCTTTGTTACGTTAATTG

>440b2cfd681a07f96fbd2346c46dceb7

CACACTTATGTCTTCAGAAAGACAAAATATTTCTCCAGGATGCATTTTAACCATGTCGGTCATTTTCAACTGGCAGTGCCTTGTAGATATTTGTGAAAGATTTTGAA

>3859cbf749122afc236ea8f660d058f3

TACGTAGGTGGCAAGCGTTGTCCGGAATTATTGGGCGTAAAGCGCGCGCAGGCGGTCTCTTAAGTCTGATGTGAAAGCCCACGGCTCAACCGTGGAGGGCCATTGGA

>c7a56abfd1370aa8df59a225860a3b90

CCCACCGCTGTGTTCAGTGTGATATACTTTTTACAATACACAATAACAGATGCCACAAGCCACACACACACACACACACACACACACACAGAAAGTCGTAGGACGTA

>8492f39ae12c7c55cfe5cc6f9313304c

TCATGGACAACGGAGAAGAATATATCCCAACTGAAACAAACCAAGGCGTAAGACAAGTTTATTGTCTGTCACCCCTACTTTTTAACCTATTTCTAGATGATCTCCTC

>64ba9cbc17368bf4d9caf956dd7a9448

TACGTGAGAGACTAGTGTTATTCATCTTAATTGGGTTTAGAGGGTACCTAGACAGTCAATATAACTTCTATAATGCTAATACTTGACTAGAGTTTTAAGTAAGAGGG

>9195c1bcbd3474d4ad4adde8a85530b9

TACGTAGGGTGCGAGCGTTAATCGGAATTACTGGGCGTAAAGCGTGCGCAGGCGGTCTTGTAATACAGAGGTGAAATCCCTGGGCTCAACCTAGGAATGGCCTTTGT

>dd80405286310a6687bda17cec9bbd29

TACGTGAGAGACTAGTGTTATTCATCCTAATTGGGTTTAAAGGGTACCTAGACAGTCAATATAACTTCTATAATGCTAATACTTGACTAGAGTTTTAAGTAAGAGGG

>5b5c139e26c376191e63f57b010f54ca

TAAAACAACAATGGCCTGGAGGCAATATTTCTGTTCGCCTCGTGGTCCATGCTGGCGGAAATGAAACTAGGTACAGCCCATAATTGTGACGTCAACCAGTAGGCACC

>ea886b35a26262da3195c628bef8709c

TACATAGGGGGCAAGCGTTATCCGGAATTATTGGGCGTAAAGCGTGCGTAGGCGGTTAAATAAGTTTATGGTCTAAGTGCAATGCTCAACATTGTGATGCTATAAAA

>4cb43b505cfc62fe156851558ae142af

TACGTGAGAGACTAGTGTTATTCATCTTAATTGGGTTTAAAGGGTACCTAGGCAGTCAATATAACTTCTAGAATGCTAATACTTGACTAGAGTTTTAAGTAAGAGGG

>d947046c348284dc93004f7f473f5237

CGACTAGGAAACAATAGCCGTGAACCCGTAATATAGGTCGCGTGGAGACCATGACCTTTGCAAAGTTGCGCGCTCAACAAACTTACATACATCTGATACTACTCGTA

>ae77dd5cd71599d1bb15ec1a46786294

TACGAAGGGGGCTAGCGTTGCTCGGAATCACTGGGCGTAAAGGGTGCGTAGGCGGGTCTTTAGGTCAGGGGTGAAATCCTGGAGCTCAACTCCAGAACTGCCTTTGA

>23095121c0027c38f7dd52a1966b7605

TACGAAGGGGGCTAGCGTTGTTTGGAATCACTGGGCGTAAAGCGCACGTAGGCGGACTTTTAAGTCAGGGGTGAAATCCCAAGGCTCAACCTTGGAACTGCCTTTGA

>b405c2fec12b28e2f44288eecd5adc2f

TACGGAGGGGGCTAGCGTTGTTCGGAATTACTGGGCGTAAAGCGCACGTAGGCGGTTATTCAAGTCAGAGGTGAAAGCCCGGGGCTCAACCCCGGAACGGCCTTTGA

>ff18ab5a31292aefdd1ca7065c94c8f4

CACACTTATGTCTTCAGAAAGACAAAATATTTATCCAGGATGCGTTTCAACCATGTCGGTCATTTTTAACTGGCAGTGCCTTGTAGATATTTGTGAAAGATTTTGAA

>1d5bd14f2781ca3ecf14b40aaa1a1ca1

TACGAAGGGTACAAGCGTTACTCGGAATTACTGGGCGTAAAGCGTGCGTAGGTGGTCGTTTAAGTCCGTTGTGAAAGCCCTGGGCTCAACCTGGGAACTGCAGTGGA

>feb383842258fc5f4a8eac511c61a912

TACGATTTCTTTAATTTAAATAGTTAAGTTTCAGTTAGTATAACAATAATATAATATATATATAATTTTGGTGAAATATATTTTATCTTTAAAAATTAATTTTATGC

>36144621a43f300a705ea70ea59d1a39

TAGCATTAACAGTAATCTGATGCACACTTTGACATGTAACATTGTTCACATCATCAGAGGTATATTATGATTGTGTGTGTGTGTGTGTGTGTCTAGTAAGGAATATC

>c2d480e06952986ece23a22631447172

CAAATTTATGTCTTCAGAAAGACAAAATATTTTTCCAGGGTCCATTTGAACCATGTCGGTCATTCTCAACTAGCAGTGCCTTGTAGATATTTGTGAAAGATTTTGAA

>4f68fca323e47c379e62385768b6604c

AGGCTCCCGCCCCGCAGACCTCATTGGTCAATTAATTCACTCTTCAGCCTTCTCATTAGAAACCCGAGTAGTCCGGCTGACTGACTCGCGATATATCTCGTATGCCG

>661983a7f99cb9f8662c338b3e9b742e

CACACTTATGTCTTCAGAAAGACAAGATATTTCCCCATGATGCATTTCAACCATGTCGGTCATTTTCAACTGGCAGTGCCTTGTTGATATTTGTGAAAGATTTTGAA

>4874f4a6440ec6b0770d1f97019fc618

AATCATAAAAACAATAAAAGACTATATGCAATTTATGGAAGACTTAGTATTAGAAACCCTTGTAGTCCGGCTGACTGACTACGTACGTATCTCGTATGCCGTCTTCT

>1dd52fcc4940123110749e2cccb81f69

TACGTAGGTGGCAAGCGTTGTCCGGATTTATTGGGCGTAAAGCGAGCGCAGGCGGAAGAATAGGTCTGATGTGAAAGCCCTCGGCTTAACCGAGGAACTGCATCGGA

>a01301b666fd96922eb6d8e14adda49a

ACTGCTGCTATGCGCTTATGACCCCGGAAACCGATTAGAAACCCCTGTAGTCCGGCTGACTGACTATGAGCTCATCTCGTATGCCGTCTTCTGCTTGAAAAAAAAAA

>cdd66891a8da2ed817e34b52473e0771

TACGGAGGATCCAAGCGTTATCCGGATTTATTGGGTTTAAAGGGTGCGTAGGTGGCCTATTAAGTCAGGGGTGAAAGACGGTAGCTTAACTATCGCAGTGCCTTTGA

>d9cd6a352bacf8076b2dfa325088e275

CCACCCCCACCGCTGTGTTCATACTTTTTTTACAATAACAGATGTCACAATTCACACACACACACACTTAATGTACATTAGATACCCGAGTAGTCCGGCTGACTGAC

>112503287f4bd018b751a8cbc33f25c6

TACGTGAGAGACTGGTGTTATTCATCTTAATTGGGTTTAAAGGGTACCTAGGCAGTCAATATAACTTCTATAATGCTAATACTTGACTAGAGTTTTAAGTAAGAGGG

>241bf83218deab5924d186923f14dc5f

CACGTGGAAAACACCGTGTTCTTTCTTTATCGATTAGTTTCAGTAATTCCCTACAAGATGCAACGAGTGTGCGTGAGCGTATTTGTTCAGTACTGACCACGAGGCAT

>b8f21150c9017bc852d4afa22be52541

GTCGCTACCGATGAAGGTTGTATACCATCCGTAAATAAATGAATTTTACGCTTATTCTAACCCTTACTGTATCGTAATTTTATTCGCTATGCTAAGCGCGGGATTAT

>a56917f7416bee66fb1802672e00fcd1

GTGCTCAAAGAACATCAATCTTTCACTTCGTTCTCACTTTCTCTTTTTACTGCCTCCTCTTCTGAACCTTTCCACCATACCCCTTCCTCTCCTTTTCGTCATCCCTT

>ebc6d7ac0cf56361cfe47400eebcc7d8

CGCCGCTGATGTTGCTTATAGTATACGGCTAAGTAGTGTTTGTAGTTAGTCGAACAATTAATCGATACATTTTCGAGCTACACTACGTTATTAATGGCTTTTCTTTG

>fbb7c88f6eb82cb1a83a8c643f450c1b

TCCGGCTGACTGACTTAACGTCCATCTCGTATGATTAGATACCCCAGTAGTCCGGCTGACTGACTTAACGTCCATCTCGTATGCCGTCTTCTGCTTGAAAAAAAAAA

>5c48801b54db788370be3e294a061bcc

TACAATTTCTTCAATTTAAATTTTTAAACGTTTCAGTTAATAAATTATTTAAATTTTTATGAATAATAATTTTAGTGAAATATATTATTATTTTATTTTATTAGTTT

>80b0fbd432b0d30da1f1b5c6414cfe64

TTCCAGCTCCAATAGCGTATATTAAAGTTGTTGCGATTAGAAACCCCAGTAGTCCGGCTGACTGACTAGTCGCAGATCTCGTATGCCGTCTTCTGCTTGAAAAAAAA

>3471aef3d3fb986ac349776af81a52ab

CCAACAGTACCTTTCCAAGGGACAAATAGGGGATAATTAGAAACCCCTGTAGTCCGGCTGACTGACTATGAGCTCATCTCGTATGCCGTCTTCTGCTTGAAAAAAAA

>81d2defc6248388374676540fdfa50a3

TACAATTTTTTCAATTTAAATGTATAAAGCTTCAGTAAATATATAATGTAATTTTTGTAAATAATAATTTTAGTGAAATATATTATTATTTTGTTTTTATGATCTGT

>2967da20606a80389b70a1324f011c8e

TCCTTTGCCACGATCATCCGCGCACCAAGGAAGTCATCGACTTTATGCCGGCAGCCATTCGGTCGTCAAAAGCTATTCTGCATGGATAGGCTTTGTTACGATCATTG

>fe59991fe78ab3dcf6c805e237475b3f

AATCATAAAAACAATAAAAGACTATATGCAATTTATGGAAGACTTAGTATTAGAAACCCCTGTAGTCCGGCTGACTGACTACGTACGTATCTCGTATGCCGTCTTCT

>57dc52344634e38a2e8a6bce07a95b46

AAGATACAGCTGGTTATGACGACCCGGGGGGGAGGGGATAGATCCTTTTATTTTCGGCGTTGGAAAATGTTGGCTCTAAAAGCATTTTCCACATTACACTCAGATTC

>2b8eb40a48f7030adfc279469047ad01

CCACCCCACCGCTGTGTTCATACTTTTTTTACAATAACAGATGCCACGATTCACACACACACGCTTAATGTACATTAGAAACCCGAGTAGTCCGGCTGACTGACTAT

>e0a5d24e0656061d3fd8da1b6a4fa960

TACGTAGGGGGCGAGCGTTGTCCGGATTTACTGGGCGTAAAGGGTGAGTAGGCGGTAATATGTGTCAGATGTAAAAGGCTAAGGCTTAACCATAGTTAGCATTTGAA

>c4c0cdfd602127a2f702be2055047f43

TACGAAGGGGGCTAGCGTTGCTCGGAATGACTGGGCGTAAAGGGCGTGTAGGCGGTTTGTACGGTTAGATGTGAAATCCCCGGGCTTAACCTGGGAGCTGCATTTAA

>12e40ab8269cddb0bffd3fc8dc9b51a6

CATTGAACTATCGTGAGAACGGCACGCCGCCAAAGGGAATTATATTATAGTAAATATTGGCGTAAATAAACATTTTATTAATAGTTGTAATATATGAAAATGTGCAA

>56e78e2430909425bf970f2a6ad87966

CCACCCCACCGCTGTGTTCATACTTTTTTTACAATAAAAAAGATGCCACAATTCACACACACACACTTAATGTACATTAGAAACCCTGGTAGTCCGGCTGACTGACT

>a4f5d06b8a1ba2e801529fb65cafce1b

CTTGCAGCTTATAAGAACAAACAGAAACTAAAAAACGATCATTATTTTACACAAATACAGGTAACAACTGTAACAGAACTACACAAATTAGAAACCCCAGTAGTCCG

>b0d56f60cd1985d6b78ed9f7f87ae657

TTCCAGCTCCAATAGCGTATATTAAAGTTGTTGCGATTAGAAACCCTAGTAGTCCGGCTGACTGACTAGTCGCAGATCTCGTATGCCGTCTTCTGCTTGAAAAAAAA

>239b65fdcda5f4696fb801166c30f4e0

TCGCCATCACCATCGTCATCATCATCAGCATCATCATCAGTATCATCATCATCATCATCATCATCACACCACCACCACCACCACCACCACCACCATCATCATCATCA

>89b5657ee03259994bb8b978f387cb0b

TACGTGAGAGACTAGTGTTATTCATCATAATTGGGTTTAAAGGGTACCTAGACAGTCAATATAACTTCTATAATGCTAATACTTGACTAGAGTTTTAAGTAAGAGGG

>63466f3f9ef7ff3b027c6c0ae38c3391

AGAAAGAGTAGAAGGTGAGACGTTGAAGAAGAAGAAGCAGCAGTCACAAATACACAACACACACGCACAAGGTCGATCCTTTTCAGGGTCGGCTGTAATGCTACCCG

>3e4df08bebef02cd4489af51ec3298f1

AATCGAGAGGAGTCCGGGGTTAGTGGATGCTTCAACTCAAAAGTTAATCGTCAACATTAAAAGTACTGTAACGTTTGTTCTGGTATAAAATGGACAAGTGAACAATA

>ce0d04b97a2e2bf3cfc1e61e93d57172

CAGTTAAGTACTTTGCTAGTCCGTAACCAGGTCGTTTTTATGATCTGTAACCCACGCAGTGTTACAGTTTACAAATTTATAAAGCAAGATAGTAACCGTGCAACATA

>fa27c0e6b5b7c303b3f8c8f74c9b30c1

TACGTGAGAGACTAGTGTTATTCATCTTAATTGGGTTTAAAGGGTACCTAGGCAGTCAATATAACTTCTACAATGCTAATACTTGACTAGAGTTTTAAGTAAGAGGG

>f04d2a4ca921117d00ef1eb874e0b935

TACGTAGGGTGCGAGCGTTAATCGGAATTACTGGGCGTAAAGCGTGCGCAGGCGGTTTTGTAAGACAGGCGTGAAATCCCCGGGCTTAACCTGGGAATAGCGCTTGT

>9f09c9df1494db766adf80f9fd3c2f4f

AATCATAAAAACAATAAAAGACTATATGCAATTTATGGAAGACTTAGTATTAGAAACCCCAGTAGTCCGGCTGACTGACTACGTACGTATCTCGTATGCCGTCTTCT

>6467d5b79e248bf8305ae0f03ff2da3e

TACGAGGGGAGCGAGTGTTGTTCGGTTTTATTGGACGTAAAGGGTATGTAGGCGGTTTTGTAAGTCAACACTTAAATCTTGAGACTTAATCTCATTACAGGTGTTGA

>067a87f47a7f3735f1e6e733be51b959

TACGTAGGGCGCAAGCGTTGTCCGGAATTATTGGGCGTAAAGAGCTCGTAGGCGGTTTGTCGCGTCTGCTGTGAAAACGCAAGGCTTAACCTTGCGCCTGCAGTGGG

>ac0a8bd4ed4c2c206015eb92f27f6489

CACGTAGGGGGCGAGCGTTGTCCGGAATTACTGGGCGTAAAGCGCGCGTAGGCGGTTGTGTAAGTCAGATGTAAAAGGCATGGGCTCAACCTGTGTGTGCATCTGAA

>e266d1ffaa1a8a2b2890519c771726e2

CCACCACCCCACCGCTGTGTTCATACTTTTTTTTTTTACAATAACAATTCACACACACACACACACACACTTAATGTACATTAGAAACCCTAGTAGTCCGGCTGACT

>527ed279079a3e3d3b5da526f6927082

ATTTAGTTTTGGTAAAGGTCAAGATCCATTTAAATTAGACGAAGGTCGTCTGAGTAATTTAGCAAAAACTGCACATGAAGATAATTGGATAATGATCCAGAAGCCGC

>3843fd38b232c164395a693ba16204d4

AAATGTCCCAACGGTCTTTAAATTAGAGATGACCCTCGTACAATGTACAGTGCAAGTAGAGAATGTTGACCTAACAGAATAACAAACTATCACCCACCGATAATATA

>84175bdfc60295f05c2cffcf5d634738

TACGTAGGGTCCAAGCGTTAATCGGAATTACTGGGCGTAAAGCGTGCGCAGGCGGTTGTGCAAGACCGATGTGAAATCCCAGAGCTTAACTTGGGAATTGCATTGGT

>cf8be1aaa76767e21876f333f389ef54

ACTAAGCTCAACCACTGGCCCTTATGTGCCAATTAGAAACCCTTGTAGTCCGGCTGACTGACTGTTACAGCATCTCGTATGCCGTCTTCTGCTTGAAAAAAAAAAAA

>05486bff2e7fc1ed4fc838b4bb47d34e

CACAAGTAAGACGAGTGTTATTCATCTTTATTAGGTTTAAAGGGTACCTAGACAGTATATTTAGCCAATAAAGGGTACTAATATGCTAGAGTTTTATGGGAGAGTGA

>7678e579328c5e67970fb3ca79d3bc67

CACGTAGGGTGCGAGCGTTGTCCGGAATTATTGGGCGTAAAGGGCTCGTAGGCGGTTCGTCGCGTCGGGAGTGAAAACTCAGGGCTCAACCCTGTTCGTGCTTCCGA

>1782c25de02c5a2a4b564fd0fa8b1146

CATTGAACTATCGTGAGAAAGTCACGCCGCCAAAGGGAATTATATTATAGTAAATATTGGCGTAAATAAGCATTTTATTAATAGTTGTAATATATGATAATGTGTGA

>e1944dfdac1b82d1557784cfbc013084

CAAACTTATGTCTTTAGAAAGACAAAACATTTTTCCAGAATGCATTTCAACCATGTCGGTCATTTTCAATTGGCAGTGCCTTGTAGATAGTTGTGGAAGATTTTGAA

>38fe6c58510446639b3ff35c0448ec4b

CATTGAACTATCGTGAGAAAGTCACGCCGCCAAAGGGAATTATATTATAGTAATTATTGGCGTTAATAAACATTTTATTAATAGTTATAATATATGATAACGTGTGA

>1f950aa99c32beee1702f01907f12841

TACGATTTTCTAAATTTAATTATGTTAGTTTCAGTTAAAAAATGTGTTAATATTAAATTTTTTTAAATTTTGGTGGAATAATATATAAATATGTGTTTAATTTTATG

>29dd3b6a532274654e6023f77a5e9bb5

TACGGAGGGTGCAAGCGTTATCCGGATTTATTGGGTTTAAAGGGTCCGTAGGCGGACTTATAAGTCAGTGGTGAAAGCCTGTCTCTTAACGATAGAACTGCCATTGA

>d540cd21872e79a0ddc90cff03aa5e5c

AGGCTCCCGCCCCGCAGACCTCATTGGTCAATTAATTCACTCTTCAGCCTTCTCATTAGAAACCCTAGTAGTCCGGCTGACTGACTATGAGCTCATCTCGTATGCCG

>8faca86b5aa62fe62a7902461aecc05d

ACAACAAGGGGAACCTAACAATGAGCCGCCTCGCAATCAATCAATTTTCGTCACGCCACATCAACGCCATTTGTGTAGCACAATTTTTCTTCGCCTATCTACACACA

>53513bf63982c6ecdb216f2579daba82

TACGTAGGGTGCGAGCGTTAATCGGAATTACTGGGCGTAAAGCGTGCGCAGGCGGTAATGTAAGACAGATGTGAAATCCCCGGGCTCAACCTGGGAACTGCATTTGT

>473c6bcfae93078326b5f8908985ac66

TACAGTTTCTTCAATTTAAATTTTAAAAAGTTTCAGTTAATAAATTATTTAAATTTTTATGAATAATAATTTTAGTGAAATATGTTATTATTATATTTTATTAATTT

>d471f09754a92cfede60c589eec28919

CGGTGTAGATCTCGTATGCCGTCTTCTGCTTGATTAGATACCCGAGTAGTCCGGCTGACTGACTCGAGCTAGATCTCGTATGCCGTCTTCTGCTTGAAAAAAAAAAA

>c8272953684498402c7580d2e54b8032

TACGAAGGGGGCTAGCGTTGCTCGGAATGACTGGGCGTAAAGGGCGTGTAGGCGGTTTGTACAGTTAGATGTGAAATCCCCGGGCTTAACCTGGGAGCCGCATTTAA

>7497d4665ab77a70bb88ac4aced947da

CCACCACCCCACCGCTGTGTTCATACTTTTTTTTTACAATAACAGATGCCACAATTCACACACACTTAATGTACATTAGAAACCCCAGTAGTCCGGCTGACTGACTT

>7d4738389f3985a41536b5e3713bba8b

TACGAAGGGTGCAAGCGTTACTCGGAATTACTGGGCGTAAAGCGTGCGTAGGTGGTTTGTTAAGTCTGATGTGAAAGCCCTGGGCTCAACCTGGGAATTGCATTGGA

>a0fbb7af0b94be1fb72c73dbc1f1190f

TACAGAGGGTGCAAGCGTTGTTCGGAATTACTGGGCGTAAAGCGCGCGTAGTCGGTATTGAGAGTCACGGGTGAAATCCCAGGGCTTAACCCTGGAACTGCCTGTGA

>48efab1604cdb86f2336e812c71b0437

GACAGAGGGTGCAAACGTTGTTCGGAATTACTGGGCGTAAAGCGTGTGTAGGCGGCCACGTAAGTTGGATGTGAAAGCCCCGGGCTCAACCCGGGAAGTGCATTCAA

>14c7a0b190acc3f20fd0a23025ed0d69

TACGAGGGGGGCGAGCGTTGTTCGGAATAACTGGGCGTAAAGGGCGCGTAGGCGGGCGATTAAGTTAGAGGTGAAATCCCAGGGCTCAACCTTGGAACGGCCTTTAA

>1ff82aaf8b9dd0fc4044fe22459e4cb8

CATTGAACTATCGTGAGAAAGTCACGCCGCCAAAGGGAATTATATTATAGTATTTATTGGCGTTGATAAACATTTTAGTAATAGTTGTAATATATGATATTGTGTGA

>81860be40831177a569a7dd6966d231d

AGGCTCCCGCCCCGCAGACCTCATTGGTCAATTAATTCACTCTTCAGCCTTCTCATTAGAAACCCCGGTAGTCCGGCTGACTGACTCGCGATATATCTCGTATGCCG

>94b19180ba019bd96d3db70d596ca671

TACGATTTCTTTAATTTAAATATTTAAGTTTCAGTTAATATAACAATAATATAAAATATCTATAATTTTGGTGAAATATATTTTATCTTTAAAAATTAATTTTATGT

>d8998b862b664fac65dd0ab4c44fa0b6

AACAGAGGATACAAGCGTTATCCGGATTTATTGGGTTTAAAGGGTGCGTAGGTGGTTTTTTAAGTCGGTAGTGAAATCTTAAAGCTTAACTTTAAAAGTGCTATTGA

>c3e5ef3995c5d37589a673d5a1c65ac3

TACGGGGGGGGCAAGCGTTGTTCGGAATTACTGGGCGTAAAGCGCGTGTAGGCGGCTCTTCAAGTCAGACGTGAAAGCCTCAGGCTCAACCTGAGAACGGCGTTTGA

>017f71616bbbfe6e7be7297a61633fb2

TACAATTTCTTCAATTTAAATTTCTAAAGTTTCAGTAAATATATATAATTTTTATAAATAATAATTTTAGTAAAATATATGTTGTTATTATGTTTTTATGATTTGTC

>238ec2e3b5568910a3ad53ce5db0e553

TTGGCAGTATGGTTGCCTCCGCACAGAGCACACTTAGCAGGCAGGGCTTTGTCTTTTTTACTCACCTTTGTGTCATGTGGTCCTCTACATTTTACGCAAACAAAAGG

>a62dd024dbc10e10a145950004db8c51

ACAAGATGTATAGTACTGACCCAGCCGTTAAGAATCATGTCATCTTACCATAAAAATTAGAAACCCCAGTAGTCCGGCTGACTGACTGTTACAGCATCTCGTATGCC

>fe6d0454f3a4d1b8fa7022cbea392631

CCACAACCCACCGCTGTGTTCATACTTTTTTTTACAATAACAGATGCCACAATTCACACACACACACTTAATGTACATTAGATACCCTGGTAGTCCGGCTGACTGAC

>f78d7a336fc719e83e80c6f9087b190d

GAACAGCTCATGCTTTCCTCCCCAGCCTTTCTCTCCCAGCTTCTATTAGATACCCTAGTAGTCCGGCTGACTGACTATGAGCTCATCTCGTATGCCGTCTTCTGCTT

>a7348ddf1b796934a3cc8255e322eb50

GAACAGCTCATGCTTTCCTCCCCAGCCTTTCTCTCCCAGCTTCTATTAGAAACCCCAGTAGTCCGGCTGACTGACTATGAGCTCATCTCGTATGCCGTCTTCTGCTT

>a38a93ce866cd7e5e1b29dca102ee0fe

AGGCTCCCGCCCCGCAGACCTCATTGGTCAATTAATTCACTCTTCAGCCTTCTCATTAGATACCCCTGTAGTCCGGCTGACTGACTCGCGATATATCTCGTATGCCG

>5ec89275ef18ed104a25ae19c76e75c8

TATGAAGGGGGCTAGCGTTGCTCGGAATGACTGGGCGTAAAGGGCGTGTAGGCGGTTTGTACAGTCAGATGTGAAATCCCCGGGCTTAACCTGGGAGCTGCATTTGA

>4341a62335896b62469202b4aa2af766

TACGTGAGAGACTAGTGTTATTCATCTTAATTGGGTTTAAAGGGTACCTAGACAGTCAATATAACTTCTATAATGCTAATACCTGACTAGAGTTTTAAGTAAGAGGG

>d9784128cca235d5db7ba4c8f870a0e8

TACGTGAGAGACTAGTGTTATTCATCTTAATTGGGTTTAAAGGGTACCTAGGCAGTCAATATAACTTCTATAATGGTAATACTTGACTAGAGTTTTAAGTAAGAGGG

>3da91888a9e161d25c835ad509148ae8

CCGTCTCAAGCATTTTTTTAACAGTGAACAGTTGGTAATTCGTGACGTGGTTAAACACGTGCAAAAGGTATGAAAAAGGAGTACTGAACGTTTGGGGACTCTTCTCC

>ece399be95044b4a04b5c8563b4a6a41

TACGTAGGGCGCGAGCGTTATCCGGAATTATTGGGCGTAAAGAGCTCGTAGGCGGTTTGTTGCGTCTGCTGTGAGAGACCGGGGCTCAACTCCGGTTCTGCAGTGGG

>17e97dfaaa70c521bafb0fcb4734786a

TTCCAGCTCCAATAGCGTATATTAAAGTTGTTGCGATTAGAAACCCGAGTAGTCCGGCTGACTGACTAGTCGCAGATCTCGTATGCCGTCTTCTGCTTGAAAAAAAA

>5d4607778310f43dff27f8d40815bf82

TACGTGAGAGACTAGTGTTATTCATCTTAATTGGGTTTAAAGGGTACCTAGACAGTCAATATAACTTCTATAACGCTAATACTTGACTAGAGTTTTAAGTAAGAGGG

>0b575bad18ae8118cc377caf8d309380

AGGTGAGAGTTCAAGAAACAAGTCTTGCCACTTCCAGAAGAGACGATACATTAGATACCCCAGTAGTCCGGCTGACTGACTATAGCGCTATCTCGTATGCCGTCTTC

>2863b1c5c7ed0295acb899f315fe5f85

TTCCAGCTCCAATAGCGTATATTAAAGTTGTTGCGATTAGAAACCCCGGTAGTCCGGCTGACTGACTAGTCGCAGATCTCGTATGCCGTCTTCTGCTTGAAAAAAAA

>f01113c7678de406c516589db147d20d

CATTGAACTATCGTGAGAACAGCACGCCGCCAAAGGGAATTATATTATAGTAAATATTAGCGTAAATAAACATTTTATTAATAGTTGTAATATATGAAAATGTGCAG

>2dd68f574b54455905b57704fdf2914c

TACGTATGTCGCAAGCGTTATCCGGATTTATTGGGCGTAAAGCGCGTCTAGGTGGTTTGATAAGTCTGATGTGAAAATGCGGGGCTCAACTCCGTATTGCGTTGGAA

>301b51518f053e50d89ef0a7cc049325

CACGATTAACCCAAGTCAATAGAAACCGGCATAAAGGGTGTTTTAGATCAATTCCCCTCAATAAAGCTAAAATTCACGTGAGTCGTAAAAAACTCTAGTTGATACAA

>582b9be2aada582b4c6d6c4a2d2fc9c2

TATTCTCGCGCAAACGTAAGCGTCCTCGGCCGCTTAAAGTAGGCCCCGTCGAGGTGCCCTGGACCAACAGCGTCAAGTACCTCGGGCTCCACGTGGATTCCCAACTC

>c45eb9151fd6bd75cade0c5faebdb31c

TACGTGAGAGACTAGTGTTATTCATCTTAATTGGGTTTAAAGGGTACCTAGACAGTCAATATAACTTCTATAATGCTAATACTTGACTAGTGTTTTAAGTAAGAGGG

>b111b9459c50b0b05e8d25a83820ced4

TAGGCTTACTTATTCCCTGATAAGAAAAGTTTTATGAATGTTGGTCCAAATAGAGCCGTTGAACTATAGAAGAAGATGAATAAAAACCTACAACAATATTATAATAT

>21c7609444d1a1e543cc986db502e348

TACGTAGGGTGCGAGCGTTGTCCGGAATTACTGGGCGTAAAGAGTTCGTAGGCGGTTTGTCGCGTCGTTTGTGAAAACCCGGGGCTCAACTTCGGGCTTGCAGGCGA

>1dc1c9b4f3c3cef88e47162bb29d9c28

TCCGGCTGACTGACTAGTCGCAGATCTCGTATGCATTAGAAACCCCTGTAGTCCGGCTGACTGACTAGTCGCAGATCTCGTATGCCGTCTTCTGCTTGAAAAAAAAA

>136f6bdb2883c4bda8c9f52d7896fac7

TACGTAGGTGGCGAGCGTTGTCCGGAATTACTGGGTGTAAAGGGCGTGTAGGCGGGGATGTAAGTCAGATGTGAAATACCACGGCTTAACCGTGGGGCTGCATCTGA

>50f7a69189a24a731a640b48a6698409

AATTTAAAAGAACGATATGGTATTGTGAAAACCAGTCTATTTAAATTCCGTGTGCTGTAATATTTTGTAATGGGTAATTAATGATATTCAATTGTGAAATCTTAAGT

>3bad231a3966368705dfcb4f323b791b

CGGTTTACCAAGTATTGGGCAACTTTATGCGTTCATTTTGGGTGTTAAAACTTAAAAATCTCATCTTTTTTATCGTTAAGGGATTCAGGATCAAGCGAGTAGCTGTC

>f0bde0fa4b4580b526a1a9e2c0f3c1e8

ACGAAACACTGACCCCGTCAACATTTTGTCATTCGTATAAATGGTGGGTAGCAGGGGAAAGGAACGAGAAAGGGGAAAAATAACGTGCATAAAAAATAAAACATACA

>1f877c78355d2b5c76f4f3c6682ef3d2

CATTGAACTATCGTGAGAGAGTCACGCCGCCAAAGGGAATTATATTATAGTAAATATTGGCGTAAATAAACATTTTATTAATAGTTGTAATATATGAAAATGTGCAG

>5a0e6db9197e65b725fdd5102bf66ad9

TCCTTTGTCATGATCATCCGCGCACCAAGGAAGTCATCGACTTTATGCCGGGCAGCCATTCGGTCGTCAAAAGTTATTCTGCATGGATAGGGTTTGTTACGTTCATT

>0ae1d4dd6ac0031831271fbdfa14c85f

TCCGGCTGACTGACTATAGCGCTATCTCGTATGCCGTCTTCTGCTTGAAAAAAAAAAAGTCCGGCTGACTGACTATAGCGCTATCTCGTATGCCGTCTTCTGCTTGA

>a4161733eb00953b1b6c2d9e12cba783

ACAAGATGTATAGTACTGACCCAGCCGTTAAGAATCATGTCATCTTACCATAAAAATTAGAAACCCTAGTAGTCCGGCTGACTGACTGTTACAGCATCTCGTATGCC

>3556dc267bcc145092f8e214462ea161

ACTAAGCTCAACCACTGGCCCTTATGTGCCAATTAGAAACCCCAGTAGTCCGGCTGACTGACTGTTACAGCATCTCGTATGCCGTCTTCTGCTTGAAAAAAAAAAAA

>60257c52b08ee15071440d24f0a3f2a8

GAACAGCTCATGCTTTCCTCCCCAGCCTTTCTCTCCCAGCTTCTATTAGAAACCCTGGTAGTCCGGCTGACTGACTATGAGCTCATCTCGTATGCCGTCTTCTGCTT

>1e6eb57d2496d0d5e28662b242e4a9ec

CAACACAGCACTACACTTTCCACTCTTGTTTTCAAACAAACTACAATATCGAAACTCAAACACACCGATTGGCAGTACATGATAATAACAAAACACAGAGATTCTGT

>36bb7d82d5a90987f3d78ec0c35dbd0f

CCATTGTGTTAAGTGTGATATACATTTACAATACACTATAACATTAGATGCCACAAGCCACACACACACATACAAACAAACAGAAAGTCGTAGGACGCAAACACAGT

>fc48f95bdc18641b5e846c774c2c99a8

GACGGGGGGGGCAAGTGTTCTTCGGAATGACTGGGCGTAAAGGGCACGTAGGCGGTGAATCGGGTTGAAAGTGAAAGTCGCCAAAAACAGGCGGAATGCTCTCGAAA

>cf9d9ca161b5ca28e949c6d30ac6c07d

TTGGCGGGGTGTTTATCCCCACAGTTCGCGCACTTGGGGTCCTCCGTTGGGGTCTTTTTACAGTCCCCCTTCGGGTGGGACAGCCCGCAGCGCACGCACCGTGGAGT

>f8fff4c6bd788e935490745db4ab1c83

TCCGGCTGACTGACTTAACGTCCATCTCGTATGATTAGAAACCCTGGTAGTCCGGCTGACTGACTTAACGTCCATCTCGTATGCCGTCTTCTGCTTGAAAAAAAAAA

>f6b78d5ab25ac34ae993976a3f8c0964

CCACCCCCACCGCTGTGTTCATACTTTTTTTTTACAATAACAGATGCCACAATTCACACACACACACACTTAATGTACATTAGAAACCCCTGTAGTCCGGCTGACTG

>49be68194d04ac75fa7b2d74e4b827ca

TACAAGTAAGACTAGTGTTATTCATCTTAATTAGGTTTAAAGGGTACCTAGACAGTATTTCTAGCCTCCAAAGGGAACAGATTTACTAGAGTTTTATGTGAGAGGAA

>7525a4b226cebf9e795bbabeef89ebcb

TACGGAGGGTGCAAGCGTTAATCGGAATGACTGGGCGTAAAGCGCACGCAGGGGGTCAATTAAGTTGGATGTGAAATCCCCGGGCTTAACCTGGGAACGGCATCCAA

>29a791284825734f3fbf36b35c5dc1e0

TACGTGAGAGACTAGTGTTATTCATCTTAATTGGGTTTAAAGGGTACCTGGGCAGTCAATATAACTTCTATAATGCTAATACTTGACTAGAGTTTTAAGTAAGAGGG

>341bce752bf8030ecf62e05d5285c114

AATCATAAAAACAATAAAAGACTATATGCAATTTATGGAAGACTTAGTATTAGAAACCCGAGTAGTCCGGCTGACTGACTACGTACGTATCTCGTATGCCGTCTTCT

>fe926ea4eef07f5b9f96150b95705b46

CTACTTCGCTGTGTGATACTTGTAACTATGTGAAATAGCTATTACATTTAATTTTTGTTACGAATACTTTTTTCATACTCATTCAAAGTTATTGGTAAGAAACGTGT

>c4bc3eb1c326ecf133b7b1e4c92832a0

TACGTGAGAGACTAGTGTTATTCATCTTAATTGGGTTTAAAGGGTACCTAGGCAGTCAATATAACTTCTATAAAGCTAATACTTGACTAGAGTTTTAAGTAAGAGGG

>87495343b7ef6b91321f1653bcfc9063

TACAATTTCTTCAATTAAAATTTTTAAAAGTTTCAGTTAATAGATTATTTAAATTTTTATGAATAATAATTTTAGTGAAATATATTATTATTATATTTTATTAATTT

>9855b32aa945a4f413318bd28c238e7e

TACGTAGGGCGCGAGCGTTATCCGGAATTATTGGGCGTAAAGAGCTCGTAGGCGGTTTGCTGCGTCTGCTGTGAAAGACCGGGGCTCAACTCCGGTTCTGCAGTGGG

>9dc51bb8efd87793669a0c437e24d295

CCACCCCCACCGCTGTGTTCATACTTTTTTTACAATAACAGATGTCACAATTCACACACACACACACTTAATGTACATTAGAAACCCGGGTAGTCCGGCTGACTGAC

>4774bd2e9eccd298526aacbfe7448a67

TACGTGAGAGACTAGTGTTATTCATCTTAATCGGGTTTAAAGGGTACCTAGACAGTCAATATAACTTCTATAATGCTAATACTTGACTAGAGTTTTAAGTAAGAGGG

>b4df6b8bb89eaaeef48c98b75688523c

TACGTAGGTGACAAGCGTTGTCCGGATTTATTGGGCGTAAAGCGAGCGCAGGCGGTCCGGTAAGTCTGATGTGAAAGCCCACGGCTCAACCGTGGAACGGCATTGGA

>605341b0e19ac91d82290866e33b3e75

TACGTAGGGCGCAAGCGTTATCCGGAATTATTGGGCGTAAAGAGCTCGTAGGCGGTTTGTCGCGTCTGCTGTGAAAACTGGAGGCTCAACCTCCAGCCTGCAGTGGG

>67ac33470eacd3d4702aaac2daa3f993

AATCAATTATAGACCCTAACGTGTCGATATTGGCGTAACGTTTCGTCTTTCTGACTGTCTCTTACGGCCCACTTTCCCTCTCAGCAGCCCCTTTCGCTGTTTTCCCA

>a8d7f92cc7297bf9559d268842125104

CCCACTGCTGTGTTCATTGTGATATACTTTTTACAATACACAATAACAGATGCCACAAGCCACACACACACACACACACACACACATACATAGAAAGTCGTAGAACG

>00d138fb1cf0aa5385c8fad15a35c7d5

CATTGAACTATCGTGAGAAAGTCAAACCGCCAAAGGGAATTATATTATAGTAAATATTAGCGTTAATAAACATTTTATTAATAGTAGTAATATATGATAATGTGCAG

>d255af868ff33cec1e0a31d2abf857c2

CCATCCAGTCAACTATCGCGCTGTTCCTCGTATTAGAAACCCTAGTAGTCCGGCTGACTGACTTAACGTCCATCTCGTATGCCGTCTTCTGCTTGAAAAAAAAAAAA

>8c034bc9256a0e34dd1d155b36bab674

TACGAAGGGGGCTAGCGTCGCTCGGAATCACTGGGCGTAAAGGGTGCGTAGGCGGGTCTTTAAGTCAGGGGTGAAATCCTGGAGCTCAACTCCAGAACTGCCTTTGA

>4d797bad8c40f786c119b4617fcbe33d

TACGTAGGGTGCGAGCGTTAATCGGAGTTACTGGGCGTAAAGCGTGCGCAGGCGGTTATGTAAGACAGATGTGAAATCCCCGGGCTCAACCTGGGAACTGCATTTGT

>e62a6726ed03f918101ac89d4fa5557c

TCCTTTGCCACGATCATCCGCGCACCAAGGAAGTCATCGACTTCATGCCGGCAGCCATTCGGTCGTCAAAAGTTATTCTGCATGGATAGGCTTTGTTACGTTCATTG

>7e59735f9b632c6a82a624b427025453

CATTGAACCATCGTGAGAAAGTCAAGCCGCCAAAGGGAATTATATTATAGTAAATATTGGCGTAAATAAACATTTTATTAATAGTTGTAATATATGAAAATGTGCAG

>231b6f921d2bd2c556d9bc3e900d381e

TACAGAGGGTGCAAGCGTCAATCGGAATTACTGGGCGTAAAGCGCGCGTAGGTGGTTTGTTAAGTTGGATGTGAAATCCCCGGGCTCAACCTGGGAACTGCATTCAA

>a26ba15f99126dd46e1971da4327e115

TCCGGCTGACTGACTTCCTCATGATCTCGTATGCCGTCTTCTGCTTGATTAAAAACCCCTGTAGTCCGGCTGACTGACTTCCTCATGATCTCGTATGCCGTCTTCTG

>5e27017f7c2562d431f41f7c1ddf4df9

TACGTAGGGCGCGAGCGTTATCCGGAATTATTGGGCGTAAAGAGCTCGTAGGCGGTTTGTTGCGTCTGCTGTGAAAGACCGGGGCTCAACTCCGGTTCTGCGGTGGG

>8ae8feebf2a4519f8e6e00ddc7aea125

CATTGAACTATCGTGAGAATGGCACGCCGCCAAAGGGAATTATATTATAGTAAATATTGGCGTAAATAAACATTTTATTAATAGTTGTAATAGATGAAAATGTGCAG

>c07bcd06a9b19949a52c7d66a7273c7e

TGACTGGTGGCTTCTTTGGAACCTGGCCAGAACGCATTTGAAACTCACTACACTGTTGTACTAACATCGTTAAATTAGAAACCCTAGTAGTCCGGCTGACTGACTAT

>1446df370a430333f72f50c10b4b630e

TATTCTCGCGAAAACGTAAGCGGCCTCGGCCGCTTAAAGTAGGCCCCGTCGAGGTGCCCTGGACCACTAGCGGCAAGTACCTCGGGCTCCACGTGGATTCCCGACTC

>5d1f3ced200bd1410e0323d768922c97

TACGGAGGGTGCAAGCGTTAATCGGAATAACTGGGCGTAAAGCGCACGCAGGCGGTTTGCTAAGCTAGATGTGAAAGCCCCGGGCTCAACCTGGGAACTGCATTTAG

>5cfcad117026db269e6b4949e2dbc3fa

TACGTAGGGCGCAAGCGTTATCCGGAATTATTGGGCGTAAAGAGCTCGTAGGCGGTTTGTCGCGTCTGCTGTGAAATCCGGAGGCTCAACCTCCGGCCTGCAGTGGG

>0f093165eb44b3a6d139584c707329d7

CAAATTTATGTCTTCAGAAAGACAAAATATTTTTCCAGGGTCCATTTCAACCATGTCGGTCATTTTCAACTAGCAGTGCCTTGTAGATATTTGTGAACGATTTTGAA

>eb2d94ecdebcc912a8435c8614ad5ffe

GTGTATTGCCTACTTATGAAACACACACACACACACACACACACACACACACACACACACACACATACACACTGATTAGAAACCCGAGTAGTCCGGCTGACTGACTA

>7c3281a628cd485603e4d3d589750425

GTTGAAGCCTATAAATCCGAATCAGTTAGTTTTGACGTGAAAGAAAGGAATGGATCGGTGAATCGAATGGGCACGTTTCTCACCACCACCACGTTACCGTTATGCAA

>124c74df494bc5853924d167d0d0ae05

TACGAAGGGGGCTAGCGTTGTTCGGAATTACTGGGCGTAAAGCGCATGTAGGCGGATATTTAAGTCAGGGGTGAAATCCCAGAGCTCAACTCTGGAACTGCCTTTGA

>c434ccbcbc565df73ffebba223345145

AATCATAAAAACAATAAAAGACTATATGCAATTTATGGAAGACTTAGTATTAGAAACCCCGGTAGTCCGGCTGACTGACTACGTACGTATCTCGTATGCCGTCTTCT

>272abf0d498a6447797e5cbf2c686353

TACGGAGGATCCGAGCGTTATCCGGATTTATTGGGTTTAAAGGGAGCGTAGATGGATGTTTAAGTCAGTTGTGAAAGTTTGTGGCTCAACCGTAAAATTGCAGTTGA

>1ed32de53a4370f00061fc2c45e259a9

GTAAGGCTTGAAGTGATGAGGATATCGAAAGGCCTCTTTTAAACCGAAGTTACCAGCTGTTCGGCAATTATCAGCGGCAGTGTTCAAATTAGATACCCTAGTAGTCC

>2f05b939d464bc38fea3da4b248fe8d1

TACGATTTCTTTAATTTAAGTGTTTAAGTTTCAGTTAATGAATTAAAAATGTAAAATAGCTATAATTTTGGTGAAATATATTTTATCTTGAAAAATTATTTTTATGT

>dcf451717f5bd01aab5d2307b0f4edaa

TACGTAGGGTGCAAGCGTTAATCGGAATTACTGGGCGTAAAGCGTGCGCAGGCGGTTATGCGAGACAGAGGTGAAATCCCCGGGCTCAACCTGGGAACTGCCTTTGT

>7039fc0f4d76b08974c0ed5ec908c423

AACAGAGGATACAAGCGTTATCCGGACTTATTGGGTTTAAAGGGTGCGTAGGTGGTTTTTTAAGTCAGTAGTGAAATCTTAAAGCTTAACTTTAAAAGTGCTATTGA

>9e39075bc9ae621460c5be1621f9c898

CATTGAGCTATCGTGAGAAAGTCAAGCCGCCAAAGGGAATTATATTATAGTAAATATTGGCGTAAATAAACATTTTATTAATAGTTGTAATATATGAAAATGTGCAG

>4ef747bad406d4fcb257353843e96652

CCACTGTGTTGAGTGTGATATACTTTTACAATACACTATAACATTAGAAACCCGGGTAGTCCGGCTGACTGACTCTACGACCATCTCGTATGCCGTCTTCTGCTTGA

>ffed2799c3e127dfc945aaf0b271e1df

CGAGGACGATTCACGCTGTATTTGGCATTCGGACCGGTCTCAGTACTATCGGTACAGCACTTTTGTACAGTGAACGATATGAGGACGCTACAACTATCCACATTTGC

>58a6352c30cdb52cea339cb176270f56

ACTGCTGTTTGTGTTTGGAAAAACAGACACTAAGGAAATTTATAAGTTTTGTGAAGTTATTGAGGGAACTCTGGAGAGTGACGCAAGTATTAGAAACCCTAGTAGTC

>568bafe8a9224e48fc82121406790303

ACGCAAGGAATACACGCTGCATATAACGCGCATGCGCGTACAACACCAACGTATTATTTTAAATGCATTAAGACCGTTCGGGACGTATTAAAAGCGGGGTCGGTCGG

>cb45887013c517bee8c84a3cdbc430d3

TACGTAGGGGGCAAGCGTTATCCGGATTTACTGGGTGTAAAGGGTGCGTAGGCGGCGAAGTAAGTCAGATGTGAAAGCCCGAGGCTCAACCTCGGGACTGCATTTGA

>3455af2fcaffc5e54545a7d87a18dda3

TACGGAGGGGGCTAGCGTTGTTCGGAATTACTGGGCGTAAAGCGCACGTAGGCGGACTGATCAGTTGGGGGTGAAAGCCCGGAGCTCAACTCCGGAACTGCCTCCAA

>8c983b55298ddd9f52f24cf2dee6a798

AGCACTGAGATACACAGGTAGTAATTATAATATTGTATTGGAAGTTTACTTATAGCGACTTTGCTACTCGAATATTCGTATGAATATATATATGAGAATCACGCCGA

>31fc202b967138f57991eedd6d7c8dab

AACAGAGGATACAAGCGTTATCCAGATTTATTGGGTTTAAAGGGTGCGTAGGTGGTTTTTTAAGTCAGTAGTGAAATCTTAAAGCTTAACTTTAAAAGTGCTATTGA

>26ae6a3a4063326c86469ca163ecad25

TACGTGAGAGACTAGTGTTATTCATCTTAATTGGGTTTAAAGGGTACCTAGACAGTCAATATGACTTCTATAATGCTAATACTTGACTAGAGTTTTAAGTAAGAGGG

>7d55cc71fd9b823fbc4f9b4073af1501

TACGTAGGGTGCGAGCGTTGTCCGGATTTACTGGGTGTAAAGAGCTCGTAGGTGGCTTGTCGCGTCGTCTGTGAAAGTCTGGGGCTTAACTCCGGGTGTGCAGGCGA

>bf13b4b51f9039578f766bc2a215ebbc

CTGGCACACAATTACCATACAGACGTGTTAGATCTATTAGAAACCCCTGTAGTCCGGCTGACTGACTCGAGCTAGATCTCGTATGCCGTCTTCTGCTTGAAAAAAAA

>0ab4e735ce0706fcd5b17e3bd731df42

TACGTAGGGGGCGAGCGTTGTCCGGAATTATTGGGCGTAAAGCGCGCGCAGGCGGTCTCTTAAGTCTGATGTGAAAGCCCACGGCTCAACCGTGGAGGGTCATTGGA

>21dab81db97165797524cae20e9e49f6

CCACCACCCCACCGCTGTGTTCATACTTTTTTTTTACAATAACAGATGCCACAATTCACACACACTTAATGTACATTAGATACCCTAGTAGTCCGGCTGACTGACTT

>f0fb66166377a801020d166276f9dfdd

TACGTAGGGGGCTAGCGTTATCCGGATTTACTGGGCGTAAAGGGTGCGTAGGCGGTCTTTTAAGTCAGGAGTGAAAGGCTACGGCTCAACCGTAGTAAGCTCTTGAA

>5754a26384932c1780103fc7e60ebe57

TTCCAGCTCCAATAGCGTATATTAAAGTTGTTGCGATTAGATACCCCAGTAGTCCGGCTGACTGACTAGTCGCAGATCTCGTATGCCGTCTTCTGCTTGAAAAAAAA

>cd399f370e56cf480554586bc6352b90

CCCACCGCTGTGTTCAGTTTGATATACTTTTTACAATACACAATAACAGATGTCACAAGCCACACACACACACACACACACAGAAAGTCGTAGAACGCAAACACAGT

>44762dd5016508a9049a4f33dbcba339

ACAAGATGTATAGTACTGACCCAGCCGTTAAGAATCATGTCATCTTACCATAAAAATTAGAAACCCGAGTAGTCCGGCTGACTGACTGTTACAGCATCTCGTATGCC

>3c489221577f7095f1d801b36649efdd

TACGTAGGTGGCAAGCGTTGTCCGGAATTATTGGGCGTAAAGCGCGCGCAGGTGGTTTCTTAAGTCTGATGTGGAAGCCCACGGCTCAACCGTGGAGGGTCATTGGA

>d12090ab9028001739c7e5f84a2d58c6

CCACCACCCACCGCTGTGTTCATACTTCTTTTACAATAACAGATGTCACAATTCACACACACACACTTAATGTGCATTAGAAACCCCTGTAGTCCGGCTGACTGACT

>47b051e507c8e3e5239f39a318db846b

TACGTAGGTGGCAAGCGTTGTCCGGAATTATTGGGCGTAAAGCGCGCGCAGGTGGTTTCTTAAGCCTGATGTGAAAGCCCACGGCTCAACCGTGGAGGGTCATTGGA

>0eb169f6cd56802f2bc86e451f03b3b6

GTTTTTACAGAGATAAGGTCTCAAGTCTGGGAATGTGGCTATTATAAATGAGATTTTATGAATTTCATAAAAAGTTCAAAATTAATTTTAGGGAACTCAGTAAACGT

>47573cca1f1c61feefa14fd8e9b2b3e2

CATGGTCATCCAGCCAGATTCCGTATTTATGTACGTGACGCTTGACTTTATACACGTGGAAAGCGATGCTTTAACCACCGGGTGGATTGAGTTATAATAGTTAATTT

>cca44c6a9eb9cf8ef1012ee918bda7c3

TCCATAACACACACACACACCACTTAATTTTAAATCACCACAAATTATACATTCATTAGAAACCCCAGTAGTCCGGCTGACTGACTATAGCGCTATCTCGTATGCCG

>ff8dcc7d46385f7c0186807f0f0e5103

TACGGGGGGGGGCAAGTGTTATTCGGAATAACTGGGCGTAAAGGGCACGTAGGCGGTGAAAAGGGTGGAAATTGAAAGTCGCCAAAACACTGGCGGGGTGCTTTCTT

>b203513b8aa759579351108e71b59c96

TAAACGGCAGCCACAGCCGTCATGATATTTACTGTGGCAATTTGTTCCACATATTACGATGAATGCATTAGATACCCGAGTAGTCCGGCTGACTGACTCTATCGTGA

>f31b26dcfb4e92a69ed3dd630132dcd0

TACGGAGGGTGCAAGCGTTAATCGGAATTACTGGGCGTAAAGCGCGCGTAGGTGGCTTGATAAGCCGGTTGTGAAAGCCCCGGGCTCAACCTGGGAACGGCATCCGG

>78c16a688055a832942e9c8a3894ec3d

TCATGGACAACGGAGAAGAATATATCCCAACTGAAACAAACCAAGGCGTAAGACAAGTTTATCGTCTGTCACCACTACTTTTTAACCTATATCTAGATGATCTCCTC

>13d68d407b45d349fa40fd584a860f95

CCACCACCACCACAGTGCTGAGTGTGATATACTTTTTTTACAATACACAATAACAGATGCCACAAGCCACACACACACACACACACATAGAAAGTCCTAGAACGCAA

>820a3e2743e2aa9f803ec8169f3b47fa

CACTAATTAAAGTGATGTTCTTTTGTAACTAAGTAAAAATAAAACTAATTAAAATGTAAAAATAAATGTAACATTTAGATAACTACAGGACAGCGTGATAACCGTTC

>45d97f29571dd83e0b2332057e677555

CCACCACCCACCGCTGTGTTCACACTTTTTTTTACAATAACAGATGCCACAATTCACACACACACACACACACACACACTTAATGTACATTAGAAACCCTTGTAGTC

>709241f1a71745d0f8868981d0fac47e

TACGTAGGGTCCGAGCGTTGCCCGGAATTATTGGGCGTAAAGGGCTCGTAGGCGGTTTGTCGCGTCGGGAGTGAAAACTCAGGGCTCAACCCTGAGCGTGCTTTCGA

>095b28d7ec61e071f1f4c6d61cb56fdf

TACGTAGGTGGCAAGCGTTGTCCGGATTTACTGGGCGTAAAGGGTGCGTAGGCGGATAATTAAGTCAGATGTGAAATCCCAGGGCTCAACCTTGGGGCTGCATTTGA

>b0f2a2b8ed05d84e0a99645b1c7b01c5

GGCACCAGGAAGATCAGCACAGCCATTCAGGGATTAGAAACCCGAGTAGTCCGGCTGACTGACTTGCGTCAAATCTCGTATGCCGTCTTCTGCTTGAAAAAAAAAAA

>098fa6ebc3e4552e34fceac13db26a56

AATGGTTCGCTGCCGCAGACGTGAGGACTTGCCGCGGTACAGAAGCTTTCAGTGCCTTCTCCGCACTCGCCAATGCGAGATGCGAGGACCAGCAACCTAACTTCCTT

>ae330172c97d312af779fb3ad25b09e2

TACGAAGGGTCCAAGCGTTGTTCGGAATCATTGGGCGTAAAGCGAGCGCAGGCGGATCAGTAAGTCAGGTGTGAAATCTCGAAGCTCAACTTCGAAACTGCGCCTGA

>d401e9eed175b111066f5cf2dd87ef93

CCACCCCACCGCTGTGTTCGTACTTTTTTTTTTACAATAACAGATGCCACAATTCACACACACACACTTAATGTACATTAGATACCCTAGTAGTCCGGCTGACTGAC

>3bdc3ee9934cc5d0c11c5313a18cf14f

TTCCAGCTCCAATAGCGTATATTAAAGTTGTTGCGATTAGATACCCGAGTAGTCCGGCTGACTGACTTGCGTCAAATCTCGTATGCCGTCTTCTGCTTGAAAAAAAA

>fbd80cfb73ac6468ad7e110af21af2d4

TACGTGAGAGACTAGTGTTATTCATCTTAATTGGGTTTATAGGGTACCTAGGCAGTCAATATAACTTCTATAATGCTAATACTTGACTAGAGTTTTAAGTAAGAGGG

>fb788a64c4c0271f785e2ec4f81b4e1b

AACACACGACACACCACCGTCATATAGAAATATGTCTGTCTTTTAACCTTGTGAAGTGACGAGCTGTACGGTATTAGAATTATGTTTTAATAATTATAATTATTTTT

>0adef744fcdddf0c53c6702a7c018142

CATTGAACTATCGTGAGAACGGCACGCCGCCAAAGGGAATTATATTATAGTAAATATTGGCGTAAATAAACATTTTATTAATAGTTGTAATATATGAAAATGTGCAG

>1e4800fdedc9828681e355641f637844

TACGTAGGGTGCAAGCGCTAATCGGAATTACTGGGCGTAAAGCGTGCGCAGGCGGTTATGCAAGACAGAGGTGAAATCCCCGGGCTCAACCTGGGAACTGCCTTTGT

>ea390daf6c4f5a47278ed79d6589a43e

CATACATAACATATACATACTAAGTTGGTCTCTAAATATTAGAAACCCCAGTAGTCCGGCTGACTGACTTGCGTCAAATCTCGTATGCCGTCTTCTGCTTGAAAAAA

>bf2ad5814d25df9faeb2116a4f515c0d

TACGAAGGGGGCGAGCGTTGTTCGGAATAACTGGGCGTAAAGGGCACGTAGGCGGGTTATCAAGTCAGTGGTGAAATCCCAAGGCTCAACCTTGGAACTGCCTCTGA

>e40d07b79a79ce9738443060201930f6

AATCATAAAAACAATAAAAGACTATATGCAATTTATGGAAGACTTAGTATTAGATACCCCTGTAGTCCGGCTGACTGACTACGTACGTATCTCGTATGCCGTCTTCT

>9b3e5fd9d2bfccda8af293f950725ff7

CATGGTCACCCAGCCAGATTTCGTATTTATGTACGTGACGCTTGACTTTATTCACGTGGAAAGCGATGCTTTAACCACACGACCGTACCACCGGGTGAATTGAGTTA

>84a531fc55a3fdff40747997cc6ab652

CATTGAACTATCGTGAGAATGGCACGCCGCCAAAGGGAATTATATTATAGTAAATATTGGCGTAAATAAACATTTTATTAATAGTTGTAATATATGAAAATGTGCAG

>d9c2077e3de7a1eed437b4335137cfdc

CGTATTTCTTGGATGTGCATTGTTTCCAGTTTTGAATTATAAAAAATATCTTAGAAACGTAAGAAAATAATTGAATTTGTATTGACGTTTTCTACTCAATTGGTACG

>7cb7bf4ca4ce8c6d5119a6d309496a89

TGACCGTAGGTTCCTTCGTTTCCGTACCGACGTCGTAACGACACCTACAATTGACAATTTATAAATTAAGTTAATTTATAACAGACAAAATACAAATTTTGTACTTG

>a601a6e07f9646b118ad9e80475c62c2

TACGGAGGGGGCTAGCGTTGTTCGGAATTACTGGGCGTAAAGCGCACGTAGGCGGACCAGAAAGTTGGGGGTGAAATCCCGGGGCTCAACCTCGGAACTGCCCTCAA

>a0b90d3f2f6eb9cd44f3e0683246e482

TACGGAGGGTGCAAGCGTTAATCGGAATCACTGGGCGTAAAGCGCGCGTAGGCCGCTTTCTAAGTCGGACGTGAAAGCCCTCGGCTCAACCGGGGAACTGCGTTCGA

>e615dcc4a4369e68190413b11f7a8e54

CCACCCCCACCGCTGTGTTCATACTTTTTTTACAATAACAGATGCCACAATTCACACACACACACACTTAATGTACATTAGAAACCCCAGTAGTCCGGCTGACTGAC

>d83dd216240225d60104cc981aa20b09

CACACTTATGTCTTCAGAAAGACAAAATATTTCCCCATGATGCATTTCAACCATGTCGGTCATTTTCAACTGGCAGTGCCTTGTTGATATTTGTGAAAGATTTTGAA

>9b72223967e6abc64fc159dcdeabbde0

ACAAGATGTATAGTACTGACCCAGCCGTTAAGAATCATGTCATCTTACCATAAAAATTAGATACCCTAGTAGTCCGGCTGACTGACTGTTACAGCATCTCGTATGCC

>17ddbd46eb422b78d635d8d73bd6fd8d

TACGTGAGAGACTAGTGTTATTCATCTTAATTGGGTTTAAAGGGTACCTAGGCAGTCAATATAACTTCTATAATGCTAATACTTGACTAGGGTTTTAAGTAAGAGGG

>15ae718ed7d78dc036c6b696a5be6449

TACGTAGGTGGCAAGCGTTGTCCGGAATTATTGGGCGTAAAGCGCGCGCAGGTGGTTTCTTAAGTCTGATGTGAAGGCCCACGGCTCAACCGTGGAGGGTCATTGGA

>72d2c2867d6b0d970880cbb22f4b579c

TACGGAGGGTGCGAGCGTTAATCGGAATTACTGGGCGTAAAGCGCGCGTAGGCGGTTAGTTAAGCTGGATGTGAAAGCCCTGGGCTCAACCTGGGAACTGCATTCAG

>e236159edebeb103918da0db9441eebc

TACGTAGGGTGCAAGCGTTAATCGGAATTACTGGGCGTAAAGCGTGCGCAGGCGGTTTTGTAAGACTGTCGTGAAATCCCCGGGCTCAACCTGGGAATGGCGATGGT

>a06d6deec4fe5eb6a213ec38e3857248

GTGATAGAAGTCGTTTTATATAAATGTGTGTGTGTGTGTGTGTGTGTGTGTGTGTGTGTGTGTGTGTGTGTGTGTGTGTGTGTAAGTTACTTACTGAGCAGAAGCTT

>bafd72a05a31b4fed63316c993b561d5

TTCGCTGGATGCGAGCCGCCGCAGTTGCAGCATGTGGGAGCGATCTCCGGTGCAACCGGGCAGTTACCCCGGTGCAACCGGGCAGTTACCCCGGTGATCACCACCAC

>a9ee2adf95b67a9122afa7659c78d181

ACAAGATGTATAGTACTGACCCAGCCGTTAAGAATCATGTCATCTTACCATAAAAATTAGAAACCCCTGTAGTCCGGCTGACTGACTGTTACAGCATCTCGTATGCC

>5d55bc5ed81cb35fdab01f38006dbe7e

TACGGAGGGTGCAAGCGTTAATCGGAATTACTGGGCGTAAAGCGCGCGTAGGCGGTTTGGCAAGCTGGATGTGAAAGCCCCGGGCTTAACCTGGGAACTGCATCCAG

>714aa1615623b66665667fd2eb333a21

TACGTATGGGGCGAGCGTTATCCGGAATTATTGGGCGTAAAGAGTTCGTAGGCGGTCCTTTAAGTCAGAAGTGAAAGGCAGTGGCTCAACCATTGTAAGCTTTTGAA

>3c2bb3d24c6845a47c39ffbb3b35b993

AGAAAGAGTAGAAGGTGAGACGTTGAAGAAGAAGAAGAAGCAGCAGTCACAAATACACAACACACACGCACAAGGTCGATCCTTTTCAGGGTCGGCTGTAATGCTAC

>23a80a63e5c6992016f437e6829c85e0

TACAATTTCATCAATTTAAATTTTTAAAAGTTTCAGTTAATAGATTATTTAAATTTTTGTGAATAATAATTTTCGTGAAATATATTATTATTATTTTATTTTATTAG

>0fb18658111491c9ff4b5b480e8e78ab

TACGTAGGGCGCAAGCGTTATCCGGAATTATTGGGCGTAAAGAGCTCGTAGGCGGTTTGTCGCGTCTGCCGTGAAAGTCCGGGGCTTAACTCCGGATCTGCGGTGGG

>d10233c4a1a9b2351f5db394f60efdf6

TACGGAGGATCCGAGCGTTATCCGGATTTATTGGGTTTAAAGGGAGCGTAGGTGGACAGTTAAGTCAGTTGTGAAAGTTTGCGGCTCAACCGTAAAATTGCAGTTGA

>c854ea80dd391e91eaeea38bcff73711

GACAAGGGAGACGAGTGTTATTCATCTTTAACAGGTATATAGGGTACCTAGACGGTGTACAAAGGCTTAGATAAGTACCTGTTACACTTGAGTTTGATATGTGAGAG

>1e954cb4194847413e67bcd9bd9bd894

TACGTAGGTGGCAAGCGTTGTCCGGATTTATTGGGCGTAAAGCGAGCGCAGGCGGTTTCTTAAGTCTGATGTGAAAGCCCTCGGCTCAACCGGGGAGGGTCATTGGA

>cc231855ec0e093bbc018042d0e8effb

TACGATTCATTCAATTTAAATATTAATGTTTCAGTCTGAGAAATTTAGTGAAATATATTTTAATAAATGATTAATTATTTTTGTCTGCGAAACTTTTATGTTAAAGT

>cba256fdffe299a9bed4885dcb029b53

CCACCCCACCGCTGTGTTCATACTTTTTTTTACAATACACAACAACAGATGCCACAATTCACACACACACACAGACACACACACACACACACAGACACACACACAAG

>7b92a0beaaf0636ef251561ec8823f71

TACAGAGGGTGCAAGCGTTAATTGGAATTACTGGGCGTAAAGCGCGCGTAGGTGGTTTGTTAAGTTGGATGTGAAATCCCCGGGCTCAACCTGGGAACTGCATTCAA

>0eec1383a3762c22222b0c2b8f8246a3

TACGTAGGGTGCGAGCGTTGTCCGGATTTACTGGGCGTAAAGAGCTCGTAGGTGGCTTGTCGCGTCGTCTGTGAAAGTCTGGGGCTTAACTACGGGTGTGCAGGCGA

>a284ae23b3f90fae24f48058c302934f

TACGAAGGGGGCTAGCGTTGCTCGGAATTACTGGGCGTAAAGGGAGCGTAGGCGGACATTTAAGTCGGGGGTGAAATCCCGGGGCTCAACCTCGGAATTGCCTTTGA

>696c93f4ee07ab3386ed2043a112a74c

TACGGGGGGTGCAAGCGTTATTCGGAATTATTGGGCGTAAAGGGCACGCAGGCGGTCCTGTCAGTCAGATGTGAAAGCCCGGGGCTCAACCCCGGACGTGCATTTGA

>1059c5df95e8687228ae4267646b4c59

TACTCATCACCACTAAAGAAGGCTTCCCCAGTCGTAGCCGAATGCCTGCCAGAGCAACCACATTAGATACCCCTGTAGTCCGGCTGACTGACTTAACGTCCATCTCG

>4d9582016a4c7c545b2f42c403e01f24

CCACCACCCACCGCTGTGTTCATACTTTTTTTACAATAACAGATGCCACAATTCACACACACACACACTTATTGTACATTAGAAACCCTAGTAGTCCGGCTGACTGA

>338e534d3e0a72cb0dbdba6aa857599c

ACTAAGCTCAACCACTGGCCCTTATGTGCCAATTAGAAACCCCTGTAGTCCGGCTGACTGACTACGTACGTATCTCGTATGCCGTCTTCTGCTTGAAAAAAAAAAAA

>fb7558e147889508935ae843865de8a9

TACGAGGGGAGCGAGTGTTGTTCAGTTTTATTGGGCGTAAAGGGTATGTAGGCGGTTTTGTAAGTCAACACTTAAATCTTGAGACTTAATCTCATTATAGTGTTGAT

>4fa5c70856acd9186f781b971fd926c9

TACGGAGGATGCAAGCGTTATCCGGAATTATTGGGCGTAAAGCGTCCGTAGGTGGCTTAGTAAGTCTTTTGTCAAATCGCGCAGCTTAACTGCATACGGGCGGAGGA

>c25a44823322d02f7946ff323fb14cac

CCATTACACATGGAGCGGTCACAATATTTTTAAACTTGTGTTCTGTAAATAACTGCCAATGACGTTTGCGTTGCGTACACAGGAAATCTAAGGAACGCGCGGTATTC

>865ac0e3aa4924330945f16ea40ce994

TTAGAAACATCAGTATAGGCCGCCGAGTTGCTGACTCCCAGACACTGTACCTAGCGCGCGTCATACAGGTTCAATGATGGTTTCCATGAGTCCCCATACGCTCCTGC

>99902ac02a583e094ddfd109bb53c9a9

CATTGAACTATCGTGAGAAAGTCAAGCCGCCAAAAGGAATTATATTATAGTAAATATTGGCGTAAATAAACATTTTATTAATGGTTGTAATATATGAAAATGTGCAG

>3707d72abadaf6660eae24f270325296

CCTAATGCAGGCAAAAAGAAGCGGCATCGTAAGCCCCGTGGCGCTCGCTCCCGGGCTCCTGCCAACCCAGCCGCACAGTCCAGACAGCCTGCGCAGCGCGTGAAGAC

>ca39fc6f8d1247fc9f9dcc5bd22a49a8

TACGTAGGTGGCGAGCGTTGTCCGGAATTATTGGGCGTAAAGGGTGCGTAGGCGGTCTATAAAGTCTGATTTGAAAGCCCATGGCTTAACCATGGAGGGAGATTGGA

>856858f2d781665c050fe75c271977e8

TACGTGAGAGACTAGTGTTATTCATCTTAATTGGGTTTAAAGGGTACCTAGACAGTCAATATAACTTCTATAATGCTAATACTCGACTAGAGTTTTAAGTAAGAGGG

>f16121d1e834d3157be35c24289d6680

TGTCCGCACCTAATAGGGCATCAACGGGAGCGGGTTGGTCAAATGTCGGATCGGCAAGAACCAAGTCCTTAGCCGCACTCTTAACTTCTGAAGCTATCGCTGTCAGA

>2d7f72a25b18396d1dedf7a8e216e319

TACGTGAGAGACTAGTGTTATTCATCTTAATTGGGTTTAAAGGGTACCTAGACAGTCAATATAACTTCTATAATGCTAATACTTGACTAGAGTTTAAAGTAAGAGGG

>6c0c43fe76bb936532ebdac2f1ef0aba

TACGAAGGGGGCTAGCGTTGCTCGGAATCACTGGGCGTAAAGGGTGCGTAGGCGGGTTTTTAAGTCGGGGGTGAAATCCTGGAGCTCAACTCCAGAACTGCCTTTGA

>1f74ad5ddda4976bee15b299eebc241c

CATTGAACTATCGTGAGAAAGTCACGCCGCCAAAGGAAATTATATTATAGTAATTATTGGCGTTAATAAACATTTTAGTAATAGTTGTAATATATCATATTGTGTGA

>41ee286c4ae9fa49f4174c40a79d92e9

TACGGAGGGGACTAGCGTTGTTCGGAATTACTGGGCGTAAAGAGTACGTAGGCTGTTTGTCAAGTTGGGTGTGAAAGCCCGGGGCTCAACCCCGGAACTGCACTCAA

>726375d0bc9fcba09c63c4dfbc5a0be2

TACGGAGGGTGCGAGCGTTAATCGGAATTACTGGGCGTAAAGCGCATGCAGGTGGTTTGTTAAGTCAGATGTGAAAGCCCGGGGCTCAACCTCGGAATTGCATTTGA

>54cce1bc65ff65d02e0ce71b26d99248

CATACATAACATATACATACTAAGTTGGTCTCTAAATATTAGAAACCCCGGTAGTCCGGCTGACTGACTTGCGTCAAATCTCGTATGCCGTCTTCTGCTTGAAAAAA

>138eab094e5c05a2777b64361a84331c

CATTGAACTATCGTGAGAAAGTCAAGCCGCCAAAGGGAATTATATTATAGTAAATATTGGCGTAAATAAACATTTTGTTAATAGTTGTAATATATGAAAATGTGCAG

>3c35f4f0990ddb9cca0c6e1d38e0beb2

TTGGCGTTGGTTCAGCTTTTGTATTACAAAGCCTCGGTGGCACAGTTCACCACAGTATCTCCATCAGGTATCTCCTTCCTATGGCTACAGTAGGTGGTTTATCGATC

>412b0b72534d33357e5f30eb45b13dcc

AACAGAGGATACAAGCGTTATCCGGATTTATTGGGTTTAAAGGGTGCGTAGGTGGTTTTTTAAGTCAGTAGTGAAATCTTAAAGCTTAACTTTGAAAGTGCTATTGA

>0e6793ec3573b0c6402c070e3ec7c359

ACAGCCCATTACTAGTCGGATGCGTAAAGGGAAAAATATATGAAGTACACAATTAGTATAATGTTTTTCTACGCGGAATAGAAACGGCGGAATAGGCTTTCATCCAT

>7550d119a79331766a8ab05b0bca2aa9

TACGGAGGATGCGAGCGTTATCCGGATTTATTGGGTTTAAAGGGTGCGTAGGCGGCCTGTTAAGTCAGCGGTGAAATCTAGGAGCTTAACTCCTAAATTGCCATTGA

>a2144c4069b7fc0c7e7892dce14122aa

TACGTAGGGTGCGAGCGTTAATCGGAATTACTGGGCGTAAAGCGTGCGCAGGCGGTCTTGTAAAACAGAGGTGAAATCCCTGGGCTCAACCTAGGAATGGCCTTTGT

>d33f05d3944b55f8f5130bfec2996c75

GGGAGACACATTCAGCAAAGTCGCTTGAAACAATTTCGATGTTACTGTATACATTTATTCACAATATAAAAATATTGTTACACTAAATTTAATTTAATTGGAGTTGA

>92da6ea5a78aa182ac5b0e47320b131c

CATTGAACTATCGTGAGAAAGTCAAGCCGCCAAAGGGAATTATATTATAGTAAATATTAGCGTAAATAAACATTTTATTAATAATTGTAATATATGAAAATGTGCAG

>3d6274e3eb7a94a641d57615ab096439

CACACTTATGTCTTCAGAAAGACAAAATATTTCTCCAGGATGCATTTCAACCATGTCGGTCATTTTCAACTGGCAGTGCCTTGTATTTGTGAAAGATTATGAATGTG

>08f464f3a716bccad6b91c66fc40140f

CCACCACCCCACCGCTGTGTTCATACTTTTTTTTTACAATAACAGATGCCACAATTCACACACACTTAATGTACATTAGATACCCCTGTAGTCCGGCTGACTGACTT

>f34bd13a1e1d72f28f4b7bd3d43a338c

CTTCAGGCCACAGATGAGACCCTGCAGGTTATCTCCGCTGGAGCCCCGGTTCATTAGAAACCCGAGTAGTCCGGCTGACTGACTTGCGTCAAATCTCGTATGCCGTC

>e630bf7fce6497685df18c7733285834

TACGTGAGAGACTAGTGTTATTCATCTTAATTGGGTTCAAAGGGTACCTAGACAGTCAATATAACTTCTATAATGCTAATACTTGACTAGAGTTTTAAGTAAGAGGG

>004012d1a82468188ef0364f0f6c31fe

TGCCCGTAGGTTCCTTCGTTTCCGTACCGACGTCGTAACGACACCTACAATTGACAATTTATAAATTAAGTTAATTTATAACAGACAAAATACAAATTTTATGCTTG

>ebda193f63bceb75e9477f4df5d7c4fa

TACGGAGGGAGCTAGCGTTATTCGGAATTACTGGGCGTAAAGCGCACGTAGGCGGCTTTGTAAGTAAGAGGTGAAAGCCTGGTGCTCAACACCAGAACTGCCTTTTA

>fb68bad4d97b5630adfb2f01b06e14e7

TACAATTTCTTCAATTTAAATTTTTAAAAGTTTCAGTTAATAAATTATTTAAATTTCTATGAATAATAATTTTAGTGAAATATATTATTAATACATTTTATTAATTT

>e4edbeed082b315be6c4430595e2a5df

CATTGAACTATTGTGAGAAAGTCAAACCGCCAAAGGGAATTATATTATAGTAAATATTGGCGTAAATAAACATTTTATTAATAGTTGTAATATATGAAAATGTGCAG

>21bd48f20fc6be36f54b6b04543eea6f

CCACCCCACCGCTGTGTTCATACTTTTTTTACAATAACAGATGCCACGATTCACACACACACGCTTAATGTACATTAGATACCCTGGTAGTCCGGCTGACTGACTAT

>6847eba1ae7f2eb75c8547b7fb61d90d

AACAGAGGATACAAGCGTTATCCGGATTTATTGGGTTTAAGGGGTGCGTAGGTGGTTTTTTAAGTCAGTAGTGAAATCTTAAAGCTTAACTTTAAAAGTGCTATTGA

>d3178b95ecacc1a7783430cf54fe760b

ACTAAGCTCAACCACTGGCCCTTATGTGCCAATTAGAAACCCTAGTAGTCCGGCTGACTGACTACGTACGTATCTCGTATGCCGTCTTCTGCTTGAAAAAAAAAAAA

>d0bd595be7de1c21ab543ea8f4d41c4c

TCAAAGGAAAGCACAACAAAAAGATAGCCAAACTATTACCACCCCGATCCCCAGCAATAACACCCACACATACGTTTTACCCAAGAATAGTTAACCTCACCTACATA

>443c147e58a7f41ffba3b3b27630170f

TACGGAGGGGGTTAGCGCTGTTCGGAATGACTGGGCGTAAAGCGCGCGTAGGCGGGCTGGAAAGTTGGGGGTGAAATCCCGGGGCTCAACCCCGGAATGGCCTTCAA

>ebcf156d3d939c41a136adc9cde37c09

TACGAAGGGGGCTAGCGTTGTTCGGATTTACTGGGCGTAAAGCGCACGTAGGCGGATTGTTAAGTGAGGGGTGAAATCCTGGAGCTCAACTCCAGAACTGCCTTTCA

>13e1ac398bd82190cecf361da0017d70

TACGTGAGAGACTAGTGTTATTCATCTTAATTGGGTTTAAAGGGTACCTAGGCAGTCAATATAACTTCTATAATGCTAATACTTGATTAGAGTTTTAAGTAAGAGGG

>733462a2085a1eaa28f2d4c089cecbd7

TTCCATCTCCGGTAGTATATGTTAAAAGCGTTGCGATTAGAAACCCCAGTAGTCCGGCTGACTGACTCGAGCTAGATCTCGTATGCCGTCTTCTGCTTGAAAAAAAA

>bcb4067eec66f25afab87f4c5402f89b

CATTGAACCATCGTGAGAAAGTCAAGCCGCCAAAGGGAATTATATTATGGTAAATATTGGCGTAAATAAACATTTTATTAATAGTTGTAATATATGAAAATGTGCAG

>f909959126cd8263639fe041b0302a33

CCACCACTGTGTTGAGTGTGATATACTTTTACAATACACTATAACATTAGCTGCCATTTTGTCTCCGTGACGAGGTTAGGTTCGTCATATACTTTTACCATACACTA

>7128eab55e4a8f62c58a128abff76ce3

AGAAAGAGTAGAAGGTGGAGGAGGAGGAGGTGAGACGTTGAAGAAGAAGCAGCAGTCACAAATACACAACACACACGCACAAGTTGGCCCTTTTCGGGGCCAGCTTT

>1ab27d6b4fe62292007e140bd879132f

TACGAAGGGGGCTAGCGTTGTTCGGAATTACTGGGCGTAAAGCGCACGTAGGCGGACATTTAAGTCAGGGGTGAAATCCCTGGGCTCAACCCCGGAACTGCCTTTGA

>36c7172b762635b34f3bee4774ca5b8e

TACGTAGGGGGCAAGCGTTGTCCGGAATTATTGGGCGTAAAGGGTGCGTAGGCGGCCATGTAAGTCAGATGTGAAAGACTACGGCTTAACCGTAGGGTTGCATTTGA

>58747cce050891aa00c05202455769f4

TACAATTTCTTCAATTTAAATTTATAGTTTCAGTTAATAGAATTATTTAATTTTTGTGAATAACAATTTTAGTGAAATATATTATTATTATCTTATTTTATTACTTT

>d056eedbef40b9c8b09cee4bffde1563

TACGTGAGAGACTAGTGTTATTCATCTTAATTGGGTTTAAAGGGCACCTAGGCAGTCAATATAACTTCTATAATGCTAATACTTGACTAGAGTTTTAAGTAAGAGGG

>ffecd7340d8a52a49e76d5a43bc5f745

CACACTTATGTCTTCAGAAAGACAAGATATTTCCCCATGATGCATTTCAACCATGTCGGTCATTTTCAACTGGCGGTGCCTTGTTGATATTTGTGAAAGATTTTGAA

>23c40e8a620ee53a988a93f67b9a70ee

AGTCGTTATTATTACCCCAGGTGTTATGCTAAACCCATTTTCTCACGCTGAGGCTATCTGAATGTATAATGTTATACTTGATACAGGCGAGAGTTGAGAAAAAGTAC

>cf15933723c7e26155985d0e9aef5b2a

AACTGCTGTTTGTGTTTGGAAAGACAGACAATAAGGAAATTCACAAGTTTTGTGAAGTTATTAAGGGAACTATGGAGAATGACGCAAGTATTAGAAACCCCAGTAGT

>118568fa1f0b6b4c8db1c8caddd2e387

TACGTAGGTGGCAAGCGTTGTCCGGAATTATTGGGCGTAAAGCGCGCGCAGGTGGTTTCTTAAGTCTGATGTGAAAGCCCGCGGCTCAACCGTGGAGGGTCATTGGA

>d1f8762be2e5f84143fbe19b2f47417d

TACGTAGGGCGCGAGCGTTATCCGGAATTATTGGGCGTAAAGGGCTCGTAGGCGGTTTGTTGCGTCTGCTGTGAAAGACCGGGGCTCAACTCCGGTTCTGCAGTGGG

>246c646b515c6eb3581c6bf154e36be0

CTGGAGACCGTCTGCAACAGTTACGTGTATTATTGCGTGTATTAGAAACCCCAGTAGTCCGGCTGACTGACTCGCGATATATCTCGTATGCCGTCTTCTGCTTGAAA

>0b0bc3385538b399b8f3397e92ad2a83

TACGTGAGAGACTAGTGTTATTCATCTTAGTTGGGTTTAAAGGGTACCTAGACAGTCAATATAACTTCTATAATGCTAATACTTGACTAGAGTTTTAAGTAAGAGGG

>7f318d86aa73bcb4c3c2ceb47207c941

TACGTAGGGTGCAAGCGTTAATCGGAATTACTGGGCGTAAAGCGTGCGCAGGCGGTTTTGTAAGTCTGTCGTGAAATCCCCGGGCTCAACCTGGGAATGGCGATGGA

>db35c13827086c0a8ea268ddb40430c6

CACACTCATGCCTTTAGAAAGAGGGTTTCAACCATGTCGGTCATTTTCAACTGGCAGTGCCTTGTAGGTATTTGTGGAAGATCTTGTCCATAGAGTTCAGTTTGTTC

>517a6058421c4192c388a94d81abd046

TACAATTTCTTCAATTTAAATTTTTAAAAGTTTCAGTTAATAAATTATTTAAATTTTTATGAATAATAATTTTAGTGAAATACATTATTATTATATTTTATTAATTT

>106e48b1528d448fe49a12674ef4234e

TTGTACAAATCTTTGTAACTGGATCGGTTCCGCACGAGTGTGTGTGCACTTCCTCTTTAGGCACTGCCAGTAAAACCTCATGACTTCTTAGTATCGACGTGTAGGTT

>9bd9b742e4b6dd5c50c7fa04ec266f46

TACGAAGGGGGCTAGCGTTGTTTGGAATCACTGGGCGTAAAGCGCACGTAGGCGGACTTCTAAGTCAGGGGTGAAATCCCAAGGCTCAACCTTGGAACTGCCTTTGA

>41743a989400b6c327059978b5968a56

ACTAGCGGATTAGAGACCGACGTTTCACAGTGATACGGCCGCGATAAACTAGCGGATTAGAGACCGACGTTTCACAGTGATACGGCCGCGATAAACTAGCGGATTAG

>8b6102ddb8c62a6b37e4ddab81f0ad33

CATACATAACATATACATACTAAGTTGGTCTCTAAATATTAGAAACCCGAGTAGTCCGGCTGACTGACTTGCGTCAAATCTCGTATGCCGTCTTCTGCTTGAAAAAA

>70640b58748e7bd228ebd95cab0ebca1

CCACCACCCACCGCTGTGTTCATACTTTTTTTACAATAACAGATGTCACAATTCACACACACACACTTAATGTACATTAGAAACCCCAGTAGTCCGGCTGACTGACT

>819caa1477db99df99a87125b7992e2d

TGACTGGTGGCTTCTTTGGAACCTGGCCAGAACGCATTTGAAACTCACTACACTGTTGTACTAACATCGTTAAATTAGAAACCCTTGTAGTCCGGCTGACTGACTAT

>6c789e76bc0daa0732fbbb6bcfbf4c27

TACGTAGGTGGCGAGCGTTGTCCGGAATTATTGGGCGTAAAGGGTGCGTAGGCGGTCTATTAAGTCTGATTTGAAAGCCCATGGCTTAACCATGGAGGGAGATTGGA

>58ca6f3e16491d14bd11d25d29b09585

TCCGGCTGACTGACTATGAGCTCATCTCGTATGCCGTCTTCTGCTTGAATAAAAACCCTTGTAGTCCGGCTGACTGACTATGAGCTCATCTCGTATGCCGTCTTCTG

>b7779aef89ddf968a3f88870386033dc

ATTTGTTTTCTTAGTACAGACAGACGTACGTGGTGCAGTTGATATTGGAAATGCATTACTATGCATTTTTTATCTTTTCATTGGTACGAATTACACGAGGTCGTTAA

>6e9eced407d6993e312000abd9662ec2

TGGACTCCGAGATGTCTGATAATGAACAAGAAACAGATGGTGAATTAGAAACCCTAGTAGTCCGGCTGACTGACTGCGATACGATCTCGTATGCCGTCTTCTGCTTG

>c3462ff37c890440cc84bc117268d0f0

CAGCTAAACGTAGGATCAGAGAAATACAGCTAGGAGTACAGTGTGCATGTATCTTGTCTGCCCTTGATATGGCACGAGCGGGTTCTCCCTTTTTCCAGCGGATGCTA

>9c705e1ace74401458e45ecb41bed11e

TTTTTTTCGGAATGAAGGTTCCTGCCTAAAGAACGCGTTGTAGAAACGGAGATGATGTTCACCGTATGTAGGTAATGGCATCGTCAGTGTTACACAGTAACAAACGT

>8dcf4d441431604075ada3438181afc9

ACTAAGCTCAACCACTGGCCCTTATGTGCCAATTAGATACCCGAGTAGTCCGGCTGACTGACTGTTACAGCATCTCGTATGCCGTCTTCTGCTTGAAAAAAAAAAAA

>74e80dc65d40077a15d7d9374b14ad7c

TATACCATGTTCGCAGTAGCTACGTAACACAGCCAGCGTATGTGTATGGAGTATTGAGTACTCGCCATATGCGGCAGTGATGTCATTACTGCACCGGGGGAGTGGAC

>cefe79af56c8e43c3c9c434fdf3e431c

AAGTTAAAGAAATGTAAGAAAACTTAAAACACTTATTCAAAAGATTCTATGTTAACTCACTGCGCATTAACTCACATCAAATACATGTAATACACTACTGAACGATT

>40e238232bc994f187d19cfda3d02373

TACGTAGGGGGCAAACGTTGTCCGGAATGACTGGGCGTAAAGGGCGAGTAGGTGGTTTGCCAAGTCAGAAGTGAAAACTCCGGGCTCAACCTGGAGATTGCTACTGA

>8af41196f6eabfdc9bf502e4ca99e1e7

TGGACTCCGAGATGTCTGATAATGAACAAGAAACAGATGGTGAATTAGAAACCCCTGTAGTCCGGCTGACTGACTGCGATACGATCTCGTATGCCGTCTTCTGCTTG

>7232ed1c100285fe7ba3083d3e75fe7b

CATACATAACATATACATACTAAGTTGGTCTCTAAATATTAGAAACCCCTGTAGTCCGGCTGACTGACTTGCGTCAAATCTCGTATGCCGTCTTCTGCTTGAAAAAA

>377ec25a0263c68eb500947d0aa17079

TCCGGCTGACTGACTCGCGATATATCTCGTATGCCGTCTTCTGCTTGAATAAAAACCCCAGTAGTCCGGCTGACTGACTCGCGATATATCTCGTATGCCGTCTTCTG

>b70666ea8fbd724c69f03626dd852823

CTGGAGACCGTCTGCAACATGTTTGTGAAGGTAAACAGTTACGTGTATTATTGCGTGTATTAGAAACCCTAGTAGTCCGGCTGACTGACTGAGACTTAATCTCGTAT

>01e9f36d2171ca8a0682bd27be3d3a9f

TACGTAGGTGGCAAGCGTTATACGGAATTATTGGGCGTAAAGCGCGCGTAGGCGGTTTTTTAAGTCTGATGTGAAAGCCCACGGCTCAACCGTGGAGGGTCATTGGA

>ffff2c00f6fecb8c883b99410b5b9d04

TACGTAGGGAGCGAGCGTTGTCCGGAATTATTGGGCGTAAAGGGTGCGTAGGCGGCCTATTAAGTCTGGTGTGAAAGCCCACAGCTTAACTGTGGAGGGTCATCGGA

>ca70cab275edda81d8a62791bcb0b629

GTGAGCCAACGCACTACCTACTACACAATACCCGTTCCCTATAAGCTTGTATTGTTCCCACATGTCAACAGTTTTAATCTAATTACAAATGAGAAAATTTACAGTAA

>3940425d081d89d3f1bb09c5e9a5f5aa

TACGAAGGGGGCTAGCGTTGCTCGGAATCACTGGGCGTAAAGGGTGCGTAGGCGGGTCTTTAAGTCAGGGGTGGAATCCTGGAGCTCAACTCCAGAACTGCCTTTGA

>b7ff05cf2c0422492be1f84d03d0a2fd

GAGACTCTAAGTTCAAACTAAAGTGGTTCACATTTTGCTCTATGACAACGGCCCTTCTAAGGGCCAACAGAAAGCTCATATGGACCTTGAAGCGGGTTTGTTCAAGC

>be14e466ea57019bc1bd960cf1e3e938

TACGGAGGATCCGAGCGTTATCCGGATTTATTGGGTTTAAAGGGAGCGTAGATGGATATTTAAGTCAGTTGTGAAAGTTTGCGGCTCAACCGTAAAATTGCAGTTGA

>d58cf153deccbd2d64cf75872861daf0

AATCGGTCCGATTTGGCGGGATGACCGTAAAATCGTGTCATACTTGTCTCAAAAAATCCGACAAATTCATCCAGTCTGTTGACTTTTGAACAGTTAATCTTCGTTTC

>ef5a8c8d2a010c6ad0e0cd5529525b1c

TACGTAGGGCGCGAGCGTTATCCGGAATTATTGGGCGTAAAGAGCTCGTAGGCGGTTTGTTGCGTCTGCTGTGAAAGACCGGGGCTCAGCTCCGGTTCTGCAGTGGG

>e5c3dcdf27d82c38ad7419442ac50dab

GACAGGGGATGCAAGCGTTATCCGGAATGATTGGGCGTAAAGCGTCTGTAGGTGGCTCCTTAAGTCCACTGTCAAATCCCAGGGCTCAACCCCGGACAGGCGGTGGA

>8fe28a7f6700a6fd37e4972ce9969104

CATTGAACTATCGTGAGAGAGTCAAGCCGCCAAAGGGAATTATATTATAGTAAATGGTAGCGTAAATAAACATTTTATTAATAGTTGTAATATATGATAATGTGCAG

>546a3140f682b4903b9bb340ce2b15fc

CATACATAACATATACATACTAAGTTGGTCTCTAAATATTAGAAACCCTAGTAGTCCGGCTGACTGACTTGCGTCAAATCTCGTATGCCGTCTTCTGCTTGAAAAAA

>7a5037c631b630afa2b4cdc08c464b4e

AACAGAGGATACAAGCGTTATCCGGATTTATTGGGTTTAAAGGGGGCGTAGGGGGTTTTTTAAGTCAGTAGTGAAATCTTAAAGCTTAACTTTAAAAGTGCTATTGA

>ab7d49323a2ae824a5529174a573184a

AACCAATGATTTAGAGAAAGATGAATGGTAGGACTTGTCCAAATTTTTATGTACATGAAATCACGAAGCTGCTTGGTGATTGTTTTGACTGCTGAAGTACATACAGG

>59d286a6d8bff4c31fd7d4b1a23697eb

ACATTCTTTGTACTTCATTAGTTATTTAATTTACACCACTTGTCACCCCCTTATATTGTACAAATGAGTTTGCTGGCACATTATGCGAAATACACGTTCCTGTTACA

>d3d905b18e85a07c088af9eb6e477a9b

TACGAAGGGGGCTAGCGTTGCTCGGAATCACTGGGCGTAAAGGGTGCGTAGGCGGGTCTTTAAGTCGGGGGTGAAATCCTGGAGCTCAACTCCAGAACTGCCTTTGA

>ca77985424cecf68d558ea0eaed4d494

TACAATTTCTTCAACTTAAATTTAAAAGTTTCAGTTAATAAATTATTTAAATTTTTAAGAATAATAATTTTAGTGAAATATATTATTATTATATTTTATTTATTTGT

>68f9e2bad4356cecccb20d4bbbcbed09

GGTGAAAACACAGTTCGACAACGCGTTTTAATTACGTTCAGCGGTCACGTGAATACAAATTAGAAACCCGAGTAGTCCGGCTGACTGACTATAGCGCTATCTCGTAT

>bcd2d9f532b5ec2a13bf75de50d55b02

TACGTAGGGTGCGAGCGTTGTCCGGAATTATTGGGCGTAAAGAGCTCGTAGGCGGTGTGTCGCGTCGGCCGTGAAAACCTGGGGCTCAACTCTGGGCGTGCGGTCGA

>106fe467ab7e10bcd7aa6f2d337003ec

TACGGGGGGTGCAAGCGTTATTCGGAATTATTGGGCGTAAAGGGCGCGTAGGCGGCCTGTTAAGTCAGATGTGAAAGCCCGGGGCTCAACCCCGGAAGTGCATTTGA

>0c2a2081e58d4d21491a287d55e0ec8b

CTGTGTTGAGTGTGATATACTTTTACAATATATAACACGTGGCGGTAGGGTGGCAACGAAGAAAATGATCTACATGGCAGTGGAGGAATTGAGGAGTGTCATATAAT

>50be508d39cb706510dd881c7a887af5

ACTAGCGGATTAGAGACCGACGTTTCACAGTGAGACGGCCGCGATAAACTAGCGGATTAGAGACCGACGTTTCACAGTGAGACAGCCGCGATAAACTAGCGGATTAG

>87999f361a44432be4d067bc3d64a9ef

TACGTAGGTGGCAAGCGTTGTCCGGATTTATTGGGCGTAAAGCGAGCGCAGGCGGATTGATAAGTCTGATGTGAAAGCCTTCGGCTCAACCGAAGAACTGCATCAGA

>fa18389321f684499e6aa40aa0f8261a

ATCTGCTGTTTGTGTTTGGAAAGACAGACAATAAGGAAATTCACAAGTTTTGTGAAGTTATTAAGGGAACTCTGGAGAATGACGCAAGTATTAGAAGCAGTATACCA

>e9692a6ad3334149da0195ab3dfc7739

TACAATTTCTTCAATTTAAATTTTTAAAAGTTTCAGTTAATAAATTATTAAAATTTTTGTGAATAATAATTTTAGTGATATAAATTATTATTTTATTCTATTAGTTT

>1b22b9dedc8010a23548e532f568a858

ACAAGATGTATAGTACTGACCCAGCCGTTAAGAATCATGTCATCTTACCATAAAAATTAGAAACCCCGGTAGTCCGGCTGACTGACTGTTACAGCATCTCGTATGCC

>97abdf2e27f8605ceace0f4900f55217

TCCGGCTGACTGACTGAGACTTATCTCGTATGCCGTCTTCTGCTTGGAAAAACAAGTAGTCCGGCTGACTGACTGAGACTTAATCTCGTATGCCGTCTTCTGCTTGA

>dd1efff2ed3be89322e55087897c183b

TACGTGAGGGACTAGTGTTATTCATCTTAATTGGGTTTAAAGGGTACCTAGACAGTCAATATAACTTCTATAATGCTAATACTTGGCTAGAGTTTTAAGTAAGAGGG

>ec91f12c9cb67ab0bdf1f4c73e453b9c

CATTGAACTATCGTGAGAGAGTCAAACCGCCAAAGGGAATTATATTATAGTAAATATTAGCGTAAATAAACATTTTATTAATAGTTGTAATATATGATAATGTGCAG

>213ad74d32ad2d1cfdb6441fd7421973

TACAGAGGGTGCGAGCGTTAATCGGATTTACTGGGCGTAAAGCGTGCGTAGGCGGCTTTTTAAGTCGGATGTGAAATCCCTAAGCTTAACTTAGGAATTGCATTCGA

>e9f005ac324a444f2116566646ebf818

TACTGTATCCAAGCATATAGACATCAAATTGATTGACAAATCTATCATATTGTCAAAGTATAGTAGGTATAAAATCATATAACTCGTATCACCTTCGGAAAAATAGT

>42234380893f112090ca300aa2afa371

TACGTGAGAGACTAGTGTTATTCATCTTAATTGGGTTTAAAGGGTACCTAGACAGTCAATATAACTCCTATAATGCTAATACTTGACTAGAGTTTTAAGTAAGAGGG

>d7964476d3e42d82d6c87ec260b6a03d

TGTAATGTCCAAAGAACCGTTAAAACTAATTAGAAACCCTTGTAGTCCGGCTGACTGACTTGCGTCAAATCTCGTATGCCGTCTTCTGCTTGAAAAAAAAAAAAAAA

>a9a1887d1b1f71898d5793a516e71a45

CTAAACATAGCACAGACGTGTTTCCATAAATAAATATTTTCTGTTGTAAAACAAGCCTTATGATGTAATTAGAAACCCCTGTAGTCCGGCTGACTGACTATAGCGCT

>e654ceb1e38fe01974b806e4f868e79a

TACGAAGGGGGCTAGCGTTGCTCGGAATCACTGGGCGTAAAGGGTGCGTAGGCGGGTCTTTAAGTCAGGGGTGAAATCCTGGAGCTCAATTCCAGAACTGCCTTTGA

>5d6246866ab1b74bc017a7ae0512f61b

AAATAATAGAATAGCCTCTCATGCGGACTGTACGAAGCTCCGAGCGCCGAAATTAGTTTGTACGAAACCTTCAATAAATTAGAAACCCTAGTAGTCCGGCTGACTGA

>1138e89b07c33caf875efebb518a6d04

CATTGAACTATCGTGAGAAAGTCAAACCGCCAAAGGGAATTATATTATAGTAAGTATTGGCGTAAATAAACATTTTATTAATAGTTGTAATATATGAAAATGTGCAG

>de8f392aacc880a69e61f864bc4b2c5b

TACGAAGGGGGCTAGCGTTGCTCGGAATGACTGGGCGTAAAGGGCGTGTAGGCGGCTTGTACAGTCAGATGTGAAATCCCCGGGCTTAACCTGGGAGCTGCATTTGA

>8eeeac08ee5cf3b5cd6fdfc301ed9cae

TGCAATTTCTTCAGCTATTATAAGCATGCGCCTATAATAACACAATTATAACGTTTCCGTCTTGTAGGCCTATTGGACCGTTCAATTATTTTCTACTTGCTAATTCT

>9152314a9c7f448c5e4394f37ecfeba3

TACGTAGGAGGCTAGTGTTAGTTATCTTTATTGGGTTTAAAGGGTAAGTAGACGGTAAATTAAACTCTAAACGAGTACTTTTTTACTAGAGTTATATGAGAGAAGGA

>c28fd435a3eba0308aeb9b44287b6c1a

TACGTGAGAGACTAGTGTTATTTATCTTAATTGGGTTTAAAGGGTACCTAGACAGTCAATATAACTTCTATAATGCTAATACTTGACTAGAGTTTTAAGTAAGAGGG

>d9fed7739dcd87a9c6c53b843357f4f3

TACGTAGGGTGCAAGCGTTGTCCGGAATTATTGGGCGTAAAGAGCTCGTAGGCGGTCTGTCACGTCGGGCGTGAAAATCCAGGGCTTAACCCTGGACCTGCGTTCGA

>3d84a420453e95c6d09d6eaa021f8bc0

CATTGAACTATCGTGAGAAAGTCAAGCCGCCAAAGGGAATTATATTATAGTAAATATTGGCGTAAGTAAACATTTTATTAATAGTTGTAATATATGAAAATGTGCAG

>979030f1340ddac0e32fc93a91837a15

AGGCTCCCGCCCCGCAGACCTCATTGGTCAATTAATTCACTCTTCAGCCTTCTCATTAGAAACCCTAGTAGTCCGGCTGACTGACTTGCGTCAAATCTCGTATGCCG

>4ec60b32b3330af9634ff557b7c05638

TACGGAGGGTGCAAGCGTTAATCGGAATTACTGGGCGTAAAGCGCGCGTAGGTGGTTTGGTAAGCGAGATGTGAAAGCCCCGGGCTTAACCTGGGAACGGCATTTCG

>59202fe50af34b698ef04ec46c880376

TACAATTTCTTCAATTTAAATTTATAAAGTTTCAGTTAATAGAATTATTTAAATTTTTGTGAATAATAATTTTAGTGAAATATATTATTATTATCTTATTTTATTAC

>87b622077bd4303c296ba1e2478eeb7f

TTCCATCTCCGGTAGTATATGTTAAAAGCGTTGCGATTAGAAACCCTTGTAGTCCGGCTGACTGACTCGAGCTAGATCTCGTATGCCGTCTTCTGCTTGAAAAAAAA

>a75e8cdf6acf3a8987c2bdd1faa0b5d8

TCCGGCTGACTGACTCGAGCTAGATCTCGTATGCATTAGAAACCCCAGTAGTCCGGCTGACTGACTCGAGCTAGATCTCGTATGCCGTCTTCTGCTTGAAAAAAAAA

>241df05df7c589071dd6e951c551ee3b

TACAGAGGGTGCGAGCGTTAATCGGGATTACTGGGCGTAAAGCGAGTGTAGGTGGCTCATTAAGTCACATGTGAAATCCCCGGGCTTAACCTGGGAACTGCATGTGA

>282f31902fc8d0ddff275ad188bc0833

ACAACGAATAAACGAAAAACGCAAAACCGAAATAAAATAATATGGAAAGTTTAGGAATTGGAGAAGAAACGAGTAAGTGTGAAAACTTACTCGAATAAATGAAGATT

>115463a4360a69983d7867b7d3bb9ad2

TTAGCCGGATGGGCTCCTTTGCAGTTGCAGCATTTAGCTGTGTACTCCGTCGTAGGCTTTTTTTTGGGGACAGATTTGGAGTGTTGCTGAGTAGGTTGGCTGGCGCT

>56b6c3d215e737628d57197eccaffcf5

TACGTAGGTGGCAAGCGTTATCCGGAATTATTGGGCGTAAAGCGCGCGTAGCCGGTTTTTTAAGTCTGATGTGAGAGCCCACGGCTCAACCGTGGAGGGTCATTGGA

>1589680012aa7781838fa23f7daac1db

TACGAGGGGGGCAAGCGTTGTTCGGAATTATTGGGCGTAAAGGGTGCGTAGGCGGTTTGGTAAGTCTCTTGTGAAATCTACAGGCTCAACTTGTAGACTGCAAGGGA

>5740c0109a7eecb47cbfdfd4a8c671ce

TACGTGAGAGACTAGTGTTATTCATCTTAGTTGGGTTTAAAGGGTACCTAGACAGTCAATATAACTTCTATAATGCTAATACTTGACTAGAGTTTTAAGGAAGAGGG

>b88ac6ff136a056b181e151749ff79e6

TACGTAGGGGGCAAGCGTTATCCGGATTTACTGGGTGTAAAGGGAGCGTAGGTGGTTCGGCAAGTCAGATGTGAAAGCCCAGGGCTCAACCCTGGGACTGCATTTGA

>7b81e56cd2751d3e40ba2ffc79c63adf

ACAACAAGGGGAACCTAACAATGAGCCGCCTCGCAATCAATCAATTTTCGCCACGCCACATCAACGCCATTCGTGTAGCACAATTTTCCTTCGCCTATCTACACGCA

>0d2dd2e6e0d6463d6d42fdeee83c23db

CCAACACATCAACCATCAGTGACCACGGACCACAGGTCAGACTAATACTCGGCCTGAGTTGTCACAAGCAAGTAAGGCCACAAAGTCATGGACAAACAAACGCAGCC

>c53c840a07016f2912a1f3da90f6e558

CTTCAGGCCACAGATGAGACCCTGCAGGTTATCTCCGCTGGAGCCCCGGTTCATTAGAAACCCCTGTAGTCCGGCTGACTGACTATGAGCTCATCTCGTATGCCGTC

>bf11a936be988384d09315567120f0b0

CCACCCCACCGCTGTGTTCATACTTTTTTTTTTTTACAATAACAGATGTCACAATTCACACACACTTAATGTACATTAGATACCCCAGTAGTCCGGCTGACTGACTT

>7e1eb7bd4a5faa70322c7c0b9a86809c

CCCACCGCTGTGTTCAGTTTGATATACTTTTTTTACAATACACAATAACAGATGCCACAAGCCACACACACACACACACCGAGAAAGTCGTAGGACGTAAACGCAGT

>febadf1688382b150f9168673eb5ab76

TACGATTTCTTTAATTTAAATAGTTAAGTTTCAGTTAATATAATAGTAATATAAAATATCTATAATTTTGGTGAAATATATTTTATCTTGAAAAATTAATTTTATGT

>1793ebc6c6069c0565684ad0220ab23c

TACAATTTCTTCAATTTAAATTTGTAAAGTTTCAGTTAATAGAATTATTTAAATTTTTGTGAATAATAATTTTAGTGAAATATATTATTATTACCTTATTTTACTGT

>4ea6e69c0e65af49818944787b462f89

CCTGTTTCTACAACGTAAACAGTAGCCGAAAGTCGCTTTAACATGAAAACTAGACTGCGTTGAAAAAGTCAAGGGAACTACGTAATTAGATTAGAAACCCCAGTAGT

>da2c1f145b0b24a23c4893d5dbc54792

TACGTAGGGAGCGAGCGTTGTCCGGAATCATTGGGCGTAAAGGGCGCGTAGGCGGTTTTTTAAGTCAAATGTGAAAATCCAGGGCTTAACTCTGGGACTGCGTTTGA

>782d6157de11472076b24489000d3b20

CCATCCCACCGCTGTGTTCATACTTTTTTTACAATAACAGATGCCACAATTCACACACATACACACACACTTAATGTACATTAGAAACCCCAGTAGTCCGGCTGACT

>86b307c87b340f9bb2ba39e283653839

TTGGCTGGGTGCTTCTCTCCACAGTTTGCGCACTTTGGGTCCTCCTCAGGTGACTTTTTACAGTCACCCTTCGGATTAGAAACCCTAGTAGTCCGGCTGACTGACTA

>d07540f49f4aa81b28e8eb087a717ac9

TGACTGGTGGCTTCTTTGGAACCTGGCCAGAACGCATTTGAAACTCACTACACTGTTGTACTAACATCGTTAAATTAGAAACCCGAGTAGTCCGGCTGACTGACTAT

>24bf1d45a50124546f38ed371a372c04

TACGTAGGTCCCGAGCGTTGTCCGGATTTATTGGGCGTAAAGCGAGCGCAGGCGGTTTGATAAGTCTGAAGTTAAAGGCTGTGGCTCAACCATAGTTCGCTTTGGAA

>44485bc6b6074509bef0ee8512dd5f6b

TAATATCTTTGTACGCCAGGGGCGCAGTAAATTGATTTTGAAAAATACTACATTAGAAACCCTAGTAGTCCGGCTGACTGACTAGTCGCAGATCTCGTATGCCGTCT

>db4ec206f456a97bc7db939b9d32257c

TTCCATCTCCGGTAGTATATGTTAAAAGCGTTGCGATTAGAAACCCTGGTAGTCCGGCTGACTGACTCGAGCTAGATCTCGTATGCCGTCTTCTGCTTGAAAAAAAA

>b209211181602598a932c0cd8185f3eb

TAAAACAACAATGGCCTGGAGGCAATATTTCTGTTCGCCTCGTGGTCCATGCTGGCGGAAATGAAACTGGGTACAGCCCATAATTATGACGTCAACCAGTGCTTACC

>1171a502c7f1a1eee213c72887d3687b

TCCTTTGTCATGATCATCCGCGCACCAAGGAAGTCATCGACTTTATGCCGGCAGCCATTCGGTCGTCAAAAATTATTCTGCATGGATAGGGTTTGTTACGTTCATTG

>65ea358fa192162216f5d190d6fbab85

CCACAACCAACCGCTGTGTTCATACTTTTTTTTTACAATAACAGATGCCACAATTCACACACACACACACACTTAATGTACATTAGAAACCCCGGTAGTCCGGCTGA

>5c6bd4d24cd0b65fa89addd57a634fda

TGTTGATGTGGCACCTGGAGACGCGTGGTTATTGGCGAAACGTTGCGTTTGTTCTTTACCAACGGTATCGACCGTATAAGAATACGTTTACGCTTAAAATAATTATA

>0738bed7f575cc9330f520d366b1b076

GGATACTTCGGGTTAGTTAAATCCGTAATACTTGAAGACGAAAATTCGGTTAGATCGATTACAGAAGATATTGGATATATTAGAAACCCGAGTAGTCCGGCTGACTG

>7eb5b5f702bcd955e2c5849c5fd67f30

TACAGAGGGTGCAAGCGTTAATCGGAATTACTGGGCGTAAAGCGCACGTGGGTGGTTTGTTGAGTTGAATGTGAAATCCCCGGGCTCAACCTGGGAACTGCATCCAA

>30ec5e1c21637118931a83b9abdfb06b

CAACAAAGACAACCCAGACAGTAAAAAGGAGCAAGGAAGGGAACCCTGTTTGCTAAATCTACGAGCATTAGAAACCCCTGTAGTCCGGCTGACTGACTTGCGTCAAA

>aa9c7bbb0602d099a2f7d7a8b23572e1

CCACCACCCACCGCTGTGTTCATACTTTTTTTACAATAACAGATGTCACAATTCACACACACACACTTAATGTACATTAGAAACCCTTGTAGTCCGGCTGACTGACT

>af13cad5126548f346b54b3be0a73b14

TACGAAGGGTGCAAGCGTTACTCGGAATTACTGGGCGTAAAGCGTGCGTAGGTGGTGAGTTAAGTCTGTCGTGAAAGCCCCGGGCTCAACCTGGGAATGGCGATGGA

>cb8d62b005103f2c919f9bdfdf948420

TGAAAATATCCAACGCACACACGGCGCGTGCGCATGTTTACGAACAGCGGGCCTCTAAGCGGTCAGTTGCCTTTCGCGGACTACAGCTGGCGACCCTTGTCGCTCTC

>8464e96aefa9526e37aa2f65eb86acde

AGCTTCCCGTGGCAGCATTAGTGGTGGCAGCTTACAAGCCTCCCCAACGGGGACCGCTTTCGCAAGAATGCAGACACGGCCAGTGTCGTAGTCCTCGGTCCGACTTA

>177fccbef5c7d467aaf55ab3008ff4b1

AACAGAGGATACAAGCGTTATCCGGATTTATTGGGTTTAAAGGATGCGTAGGTGGTTTTTTAAGTCAGTAGTGAAATCTTAAAGCTTAACTTTAAAAGTGCTATTGA

>a83e3744cd61c89346d059752d1ebd36

CCACAACCCACCGCTGTGTTCATACTTTTTTTTTTTTACAATAACAGATGCCACAATTCATAAACACTTAATGTACATTAGATACCCTAGTAGTCCGGCTGACTGAC

>6b2ffe04029060f666db643e8ba8d556

TACTCATCACCACTAAAGAAGGCTTCCCCAGTCGTAGCCGAATGCCTGCCAGAGCAACCACATTAGAAACCCTGGTAGTCCGGCTGACTGACTTAACGTCCATCTCG

>fc1143169ee87012f7eb17722949af4f

TGACTGGTGGCTTCTTTGGAACCTGGCCAGAACGCATTTGAAACTCACTACACTGTTGTACTAACATCGTTAAATTAGATACCCTAGTAGTCCGGCTGACTGACTAT

>0a1b6f8938e756c4e580c10fde1069bf

GAACAGCTCATGCTTTCCTCCCCAGCCTTTCTCTCCCAGCTTCTATTAGATACCCTAGTAGTCCGGCTGACTGACTTGCGTCAAATCTCGTATGCCGTCTTCTGCTT

>1791e06092021726dc5833229218622a

CCACAACCTCCACCGCTATGTTCATACTTTTTTTTTACAATAACAATTCACACACACACACACACACTTAATGTACATTAGAAACCCCTGTAGTCCGGCTGACTGAC

>5f426f21d0a4b78d9176cb09029b302f

CCTAATGCAGTCATAAAGAAGCGGCAACGTAAGCCCCGTGGCGCTAGATCCCGGGCTCCTGCCAACCCAGCCGCACAGTCCAGACAGCCTGCGCAGCGCGTGAAGAC

>e7211ae227182c92039942b24143bb08

GGTGAAAACACAGTTCGACAACGGGTTTTAATTACGTTCAGCGGTCGCTTGAATACAAATTAGAAACCCGAGTAGTCCGGCTGACTGACTATAGCGCTATCTCGTAT

>5893bfa541763e5c6060919de6f2dbe5

GATTGCGTAACATCGCGTAATAAATCGCACGTTACAAGCAAAATTATAAGGTAACTAACTGCTTTATCAGATTCGTCATGATCTATTCTTCTCTATTGCGTCATCGC

>a8dbaec673a33e6bdf203e58188cd7e9

TTCCAGCTCCAATAGCGTATATTAAAGTTGTTGCGATTAGAAACCCCAGTAGTCCGGCTGACTGACTGTTACAGCATCTCGTATGCCGTCTTCTGCTTGAAAAAAAA

>a98b67e3b994f02aca282066debbd027

CACGTAGGGTGCGAGCGTTGTCCGGAATTATTGGGCGTAAAGAGCTCGTAGGCGGTGTGTCGCGTCGGCCGTGAAAACTTGGGGCTTAACTCTGAGCGTGCGGTCGA

>d30495d04b56bee6e67f9c5467532761

CATACATAACATATACATACTAAGTTGGTCTCTAAATATTAGATACCCCGGTAGTCCGGCTGACTGACTTGCGTCAAATCTCGTATGCCGTCTTCTGCTTGAAAAAA

>d5a033aea9afd40b61feb9ceb49183ba

CCACCCCCACCGCTGTGTTCATACTTTTTTTTTTACAATAACAGATGCCACAATTTACACACACACACTTAATGTACATTAGAAACCCGAGTAGTCCGGCTGACTGA

>9b8edb712ba6676ec07954c43872c37c

TGACTGGTGGCTTCTTTGGAACCTGGCCAGAACGCATTTGAAACTCACTACACTGTTGTACTAACATCGTTAAATTAGAAACCCCAGTAGTCCGGCTGACTGACTAT

>e3fb75fa12ca7e5bf54339d97ed40e9c

TACAATTTCTTCAATTGAAATTTGTAGTTTCAGTTAGTATATAATATAATTTTTGTAAATAATAATTTTAGTGAAATATATTATTATTTTGTTTTTATAATTTGTTT

>a70a55cc4da0b25a73bdb27432edfb1b

TATTCTCGCGCAAACGCAAGCGGCCTCGGCCGCTTAAAGTAGGCCCCGTCGAGGTACCCTGGACCAACAGCGTCAAGTACCTCGGGCTCCACGTGGACTCCCGACTC

>35e117957e6303a2e70c98e5d5149eab

AGTTAACAGCGAACTGACCAGCGAGTTTCGTCGCCTCGAAAACCACAAATTAGAAACCCTAGTAGTCCGGCTGACTGACTCTCTAGAGATCTCGTATGCCGTCTTCT

>983a76a1069715276a2c50c93ef94dd0

TCCTTTGCCATGATCATCCGCGCACCAAGAATGGAATGGCGCACCATCGACTTTATGCCGGCAGCCATTCGGTCGTCGAAAGCTATTCTGCATGGATAGGCTTTGTT

>3971be9b9325626ab26d8953526e8551

TTCCAGCTCCAATAGCGTATATTAAAGTTGTTGCGATTAGATACCCCTGTAGTCCGGCTGACTGACTTACTAGGTATCTCGTATGCCGTCTTCTGCTTGAAAAAAAA

>519fba2fc7310e55b422e410ff836c62

TTCTTATTTTTAACTCTGTCAGATCAGAGGTGATTATAGTAATCACTGAACAATCAGAAATCTTTCTTTTTGGCAGTTCTCATTAGAAACCCTTGTAGTCCGGCTGA

>81d9eee5ffab31cd75e558ad6b7ebebd

AAGCGATAGAGAAAAGGCGAGAGCGAAAAAGAAACGGGTGAAGAGAGAAAGTGAGTGAAGTTCAACGTGATGCATATCATTATCACGGAGGAACAGGTTGCTCGTGA

>f4f603f5402bf4d668ec1eecf9293624

TACGGAGGGTGCGAGCGTTAATCGGAATTACTGGGCGTAAAGCGCATGCAGGCGGTCTGTTAAGCAAGATGTGAAAGCCCGGGGCTCAACCTCGGAACTGCATTTGA

>9cf78a28a0ab5245565514a929b5a65b

TACGTGAGAGACTAGTGTTATTCATCTTAATTGGGTTTAAAGGGTACCTAGGCGGTCAATATAACTTCTATAATGCTAATACTTGACTAGAGTTTTAAGTAAGAGGG

>9aaf6f73ff7cc365960924afad9b2294

TACGGGGGGTGCAAGCGTTGTTCGGAATTATTGGGCGTAAAGAGCGTGTAGGCGGTTTGTTAAGTCTGATGTGAAAGCCCTGGGCTCAACCCAGGAAGTGCATTGGA

>821470ed74ac1db6f41394f175c8b2bc

TGGCTGCCCTCTATTTCTCTGCCCACTCAACGTAGATTATAATAGCGTCAGTGTCGGAAACGTCTCCCCTCTATGCAGTTCCTCGATATAGTCGACGTATTCCGATC

>6f2d24da90feee3b02da09ce8f6c56e5

CCACAACCCCAAACCGCTATGTTCATACTTCTTTTTTTACAATAACAGATGCCACAATTCACACACACACACACATTTAATGTACATTCGAAAACACAAGTCACACA

>8668ed82556b5fad8d15a12d65394e33

ACAAATGGTGAACAGTATAGTGTCAATAATGGCATTAGAAACCCCAGTAGTCCGGCTGACTGACTCGCGATATATCTCGTATGCCGTCTTCTGCTTGAAAAAAAAAA

>59f10b821750af0a913ae44f386f77fb

TACGTAGGGCGCGAGCGTTGTCCGGAATTATTGGGCGTAAAGGGCTTGTAGGCGGTTGGTCGCGTCTGCCGTGAAATCCTCTGGCTTAACTGGGGGCGTGCGGTGGG

>f368f9467a277889fa9a258f37267733

TACGGAGGGTGCAAGCGTTATCCGGATTCACTGGGTTTAAAGGGTGCGTAGGTGGGTTTGTAAGTCAGTGGTGAAATCTCCAAGCTTAACTTGGAAACTGCCGTTGA

>d1d81ef0d851895c25c69754733a2885

CCCACCGCTGTGTTCAGTTTGATATACTTTTTTTACAATACACAATAACAGATGCCACAAGCCACACACACACACACACACACACAGAAAGTTGTAGGACGCAAACA

>c9ec89b21ac4b1d819a4cf55d5fcfe63

TTACATAATTACATGGAGCCTCAGACATCTTGGAAGACTCTGAAGAACTATGTTATTGAGATGCAATGCCAAGTCCCGTTAGTTGTTCGTTGTTCCCTCCACGGCTT

>e25e7038b771ea0672cb134b76f225cb

AGTCAGCGGTCTATAATAAAAAATCCTAAAGTAAGGCAGAGTGCTGAGCATCCGGTGATTATGCGTTACCATTCTCGTTATTTATGTAATGTAAGTCAATCACTAAT

>8e0e48b95551e729eebc10e46a01d870

TACGTAGGGTGCAAGCGTTAATCGGAATTACTGGGCGTAAAGCGTGCGCAGGCGGTTTATTAAGACAGATGTGAAATCCCCGGGCTCAACCTGGGAACTGCATTTGT

>1694982c0412bd4bfc52a144fbc1aaff

TACGAAGGGGGCTAGCGTTGTTCGGAATTACTGGGCGTAAAGCGCACGTAGGCGGATATTTAGGTCAGGGGTGAAATCCCAGAGCTCAACTCTGGAACTGCCTTTGA

>4e62681dd00ca6247481f8dca9de21f5

CGGTAGGGCCGCACATTACTGTCGGCCATACTCTGTAATTATTGCAATTACAAGTTGTTGTTAATTTATGTGCGCGTGGCGTTTTAATTACACGTACATACATAATG

>248b2fa93201b059d261364927ad83c2

ACTAAGCTCAACCACTGGCCCTTATGTGCCAATTAGAAACCCGAGTAGTCCGGCTGACTGACTACGTACGTATCTCGTATGCCGTCTTCTGCTTGAAAAAAAAAAAA

>09575ff9dde2525c4af32df0ee566ba2

CCACCCCCACCGCTGTGTTCATACTTTTTTGACAATAACAGATGTCACAATTCACACACACACACACTTAATGTACATTAGAAACCCGAGTAGTCCGGCTGACTGAC

>dc61c46f4922d13151c7807de074492c

CATTGAACTATCGTGAGAAAGTCAAACCGCCATAGGGAATTATATTATAGTAAATATTGGCGTAAATAAACATTTTATTAATAGTTGTAATATATGAAAATGTGCAG

>9249d14882ae9ead55608725389ce140

TACGTAGGTCCCGAGCGTTGTCCGGATTTATTGGGCGTAAAGCGAGCGCAGGTGGTTTATTAAGTCTGGCGTAAAAGGCAGTGGCTCAACCATTGTATGCATTGGAA

>c200712ce4d2abff22c705b69e988e59

CCACCCCCACCGCTGTGTTCATACTTTTATTTTTTTACAATAACAGATGCCACAATTCACACACACACACTTAATGTACATTAGAAACCCGTGTAGTCCGGCTGACT

>d6f69f67f083325a67fec86b79ec05a8

TGGAGTTCCACTTCTCCGCATTCACCACCGCTGTGATCTTCACCCGTCTCCCTGCGCAAGGGCAAAGCGTTATTCTCCACAGTACTGGCTCCTCGGAGTATACATGA

>4c4acc300511687fd7668ff97c4142f6

CCACCACCCACCGCTGTTTTCATACTTTTTTTACAATAACAGATGCCACAATTCACACACACACACACACACTTAATGTACATTAGATACCCTTGTAGTCCGGCTGA

>7dc4080c084f70634f1fe9ba9fd8238d

CCACTGTGTTGAGTGTGATATACTTTTACAATACACTATAACATTAGAAACCCCTGTAGTCCGGCTGACTGACTCTACGACCATCTCGTATGCCGTCTTCTGCTTGA

>2707adc7ad5b62614c48963fb4aaf75e

TTCCATCTCCGGTAGTATATGTTAAAAGCGTTGCGATTAGAAACCCGTGTAGTCCGGCTGACTGACTCGAGCTAGATCTCGTATGCCGTCTTCTGCTTGAAAAAAAA

>007c655eafe7ad28d35385254f9195db

TACGTAGGGCGCAAGCGTTAATCGGAATTACTGGGCGTAAAGCGTGCGCAGGCGGTTATGCAAGACAGAGGTGAAATCCCCGGGCTCAACCTGGGAACTGCCTTTGT

>46fa5952b64acb99761d4692fd5cdf6f

TATCCTGCTGGACGCAGCTAGGATATATGATCAGAAAGGCAAAGTGGAACAAAATTCGTTTTTAATAAACGCCATACAAGATTAGAAACCCTAGTAGTCCGGCTGAC

>86fc9fcb36f0f96635a4a2b7b8de6835

CCACCCCCACAGCTGTGTTCATACTTTTTTTTTTTTACAATAACAGATGCCACAATTTGCACACACACACTTAATGTACATTAGAAACCCTTGTAGTCCGGCTGACT

>8989c786108983eba840fe322c90ff4d

TACGTAGGGGGCGAGCGTTGTCCGGAATTACTGGGCGTAAAGGGTGCGTAGGCGGCCTTGCAAGTCAGATGTGAAAGGCATCGGCTCAACCGATGTAAGCATTTGAA

>effafc7eb1c51a6a8f97720093086e54

TACGTAGGGCGCGAGCGTTATCCGGAATTATTGGGCGTAAAGAGCTCGTAGGCGGCTTGTTGCGTCTGCTGTGAAAGACCGGGGCTCAACTCCGGTTCTGCAGTGGG

>b875fb272f09dea9daff63a09a8fe559

TACGTAGGGTCCAAGCGTTAGTCGGAATTACTGGGCGTAAAGCGTGCGCAGGCGGTTGTGCAAGACCGATGTGAAATCCCCGAGCTTAACTTGGGAATTGCATTGGT

>c044c7bdf833283f59d1b38a15a6c70c

TACGATTTTCTAAATTTAATTATGTTAGGTTCAGTTAAAAAATGTGTTGATATTAAATTTTTTTAAATTTTGGTGGAATAATATATAAATATGTGTTTAATTTTATG

>79d555452e60223fe3a062fe70d7e69f

CACACTTATGTCTCCAGAAAGACAAAATATTTATCCAGGATGCATTTCAACCATGTCGGTCATTTTCAACTGGCAGTGCCTTGTAGATATTTGTGAACGATTTTGAA

>81c88abfc2b50a574f75be49a98941f6

ACTAGAAGAACACATAGAGGCGGACTTTCGGACAACCAATTCGGCTTCCGGAAGGGTCGGTCTACTATTGATGCCGCGGAACGGGCTGTGCAAACTGCCAGAACGGC

>b30991dd9232ba73c59e34b4f9a799ec

TACGTAAGAGACTAGTGTTATTCATCTTAATTAGGTTTAAAGGGTACCTAAACGGTCAGTATAGCTTGTAAAAATGTTAGTATACGACTAGAGTTTTATATGTAAGA

>b409fa43b79e251756bffa153678951a

ACCGCTGTGTTGAGTGTGATATACTTTTTACAATACACAATAACAGATGCCACAAGCCACACACACACACCCACACACATACACACACACACACAGAAAGTCGTAGG

>4c28dadb87a951f92ee210771b61d9c1

TCCGGCTGACTGACTAGTCGCAGATCTCGTATGCCGTCTTCTGCTTGAAAAAAAAACCGAGTAGTCCGGCTGACTGACTAGTCGCAGATCTCGTATGCCGTCTTCTG

>2f1cb68c2e9c5b304cbca6e947e06351

TACAATTTCTTCAATTTAAATTTTTAAAAGTTTCAGTTAATAAATTATTTAAATTTTTGTGAATAATAGTTTTAGTGAAATATATTATTTTATTTTATTAGTTTGTC

>25ff95e98f7184cfc4328eafe3171556

TACGTAGGGGGCAAACGTTGTCCGGAATGACTGGGCGTAAAGGGCGAGTAGGTGGTTTGCCAAGTCAGGAGTGAAAACTCCGGGCTCAACCTGGAGATTGCTACTGA

>9d64784fe8b65a83915c98668f131db5

TAAGGCCTCCTTCTAAGTTGCACTCCACTTGGTATGACCTTGTTCAGCTTTTCCACAATCCTGAGCCATCTAAAGCTTAGTCACCTTCAAGCTAAGATATAAGCTTA

>9c7784512c9b332a85125562a323544d

GACCTAGACTCCGGAGTGCAAAATGTGCACCGATCTTCACCTTTAGATGACGTTTTCATTGTCTCTAAAGGTCATAATTAGAAACCCCGGTAGTCCGGCTGACTGAC

>6e0f7ee48f434104d6be5a7c650c9947

GACAGAGGATGCAAGCGTTATCCGGAATGATTGGGCGTAAAGCGTCTGTAGGTGGCTTTTTAAGTCCGCCGTCAAATCCCAGGGCTCAACCCTGGACAGGCGGTGGA

>fad23a85b2bbb3247653a236e1ba5933

ATTATTCTTCAAATGAGAACATCAGTCACACGAGTGTAAAGTGCTAATTTACTGAGTACATTGCATTGGTTAGTTTCTGCTAATTTTTTGTTGAGTCCTGTGGTTAT

>8ea9ee2050e79bb87798c4bcfb5e0008

CAACAAAGACAACCCAGACAGTAAAAAGGAGCAAGGAAGGGAACCCTGTTTGCTAAATCTACGAGCATTAGATACCCTAGTAGTCCGGCTGACTGACTTGCGTCAAA

>d0e3403b1fbf08c5e08c3b1c71e5fa9d

TACGTAGGGGGCGAGCGTTATCCGGAATTATTGGGCGTAAAGAGTGCGTAGGCGGTTTGTTAAGTCACTTGTAAAAGATCTCAGCCCAACTGAGTAGGGCGAGTGAA

>7915b2c6a4f5f1f4fd039996e068cf43

GTGCTCAAAGAACATCAATCTTTCACTTCGTTCTCACTTTCTCTTTTTACTGCCTCCTCTACTGACCCTTTTCCACCATACCCCTTCCTCTCCTTTTCGTCATCCCT

>9dff8f037f958c5e6b82745c3acfe752

TACAATTTCTTCAATTTAAATTTTTAAAAGTTTCAGTTAATAAACTATTTAAATTTTTATAGATAATAATTTTAGTGAAATATATTATTATTATATTTTATTAATTT

>473ff06d4c3d84b7511eee9f0e55c9f1

TTCCAGCTCCAATAGCGTATATTAAAGTTGTTGCGATTAGAAACCCCTGTAGTCCGGCTGACTGACTTGCGTCAAATCTCGTATGCCGTCTTCTGCTTGAAAAAAAA

>848068cf998c989a9e9e7ba2ffdce58a

TACGAAGGGGGCTAGCGTTGCTCGGAATCACTGGGCGTAAAGGGTGCGTAGGCGGGTCTTTAAGTCAGGGGTGAAATCCTGGAGCTCAACTCCAGAACTGCCTTGAT

>9707085e4eb6d587dd7dcb589d691e42

ATATCGTCAGTCCCTGTTCTTAGATGTGATCTTTCCAGAGCCGCACTGAGGCACACACACAACAGGGCACACAGATCAGAAACATCCTATAATGTCTATTCTTACAT

>9cccfb699237ea934a9f3619b1e091be

GTGTTGAGTGTGATATACTTTTACAATACACTATAACATTAGAAACCCCAGTAGTCCGGCTGACTGACTTAACGTCCATCTCGTATGCCGTCTTCTGCTTGAAAAAA

>653f280cd3baf4eee03ca7858017fce8

TACGTAGGTGGCAAGCGTTGTCCGGAATTATTGGGCGTAAAGCGCGCGCAGGTGGTTTCTTAAGTCTGTTGTGAAAGCCCACGGCTCAACCGTGGAGGGTCATTGGA

>1a2bdf528640463e0b015f0516f22a6e

TGAAATTTGCTTACTGTTTACCGTGCGGAAGAAGAAGGTGAAGAAGAAGAAGAAGAAGAAGAAGAAAAAGAAGAAGAAGAAGGAAAGTTGGAAAAGTCAGTGGCATT

>eaf1a0d291407f0bcc46218261bcb09a

ATTTAAAAGTTGATATGGCATTTGTAAAAGCCAATCTAAGTTAAATTCCGTGGGCTGTAATATTTTGTAACGGGTAATTAATGATATTCAATTGTGAAATCTTAAGT

>0987c6a1e00385744f3ce2142ef58bbb

TTCCATCTCCGGTAGTATATGTTAAAAGCGTTGCGATTAGATACCCTAGTAGTCCGGCTGACTGACTCGAGCTAGATCTCGTATGCCGTCTTCTGCTTGAAAAAAAA

>87036b42497ab91e025d01309b68e137

CACGATTTTCTAAATTTAATTATGTTAGTTTCAGTTAAAAAATGTGTTAATATTAAGTTTTTTAAATTTTGGTGGAATAATATATAAATATGTGTTTAATTTTGTCT

>72520b065e7aa11732b59e05606f859c

TACGAAGGGGGCTAGCGTTGCTCGGAATCACTGGGCGTAAAGGGCGCGTAGGCGGCTGACTTAGTCGAGGGTGAAAGCCCGTGGCTCAACCACGGAATGGCCTTCGA

>886e99ff149e40177cf77f44f46d6429

TACGTAGGTGGCGAGCGTTGTCCGGAATTACTGGGCGTAAAGGGCGCGTAGGCGGCTCTTTAAGTCAGATGTGAAAACCCGAGGCTTAACTTCGGGAGTGCATTTGA

>2d9ae35907da7d09ad7bef3ed00e39bd

TGCCCGTAGGAGTCTTCGTTTCCGTACCAACGCCGTAACGACACCTATAATTGACAATTTATAAATTAAGTCGATTGATAACAGACAAAATACAAATTTTACACTTG

>61e478a8e237fd8afd3a1e58080ecdad

TGTATGAGTGTCTATAGCATTATTGATAGAAGATAGAGGGGTGAGAACAGCCAGAAAGAATGCTACAAGCATGCCTAAACAAGTCTAATCTAGTGTGGGTTTAAGTA

>e10b25bfc1eb1f43cc58b777ad4a450c

CCACCCCACCCCACCGCTGTGTTCATAATTTTTTTACAATAACAGATGCTACAAGCCACACACACACACTTAATGTACATTAGATACCCCAGTAGTCCGGCTGACTG

>f172c17c402ab1bc6e027488e2878079

TACAATTTCTTCAATTTAAATTTTAAAAAGTTTCAGTTAATAGGTTATTTAAATTTTTATAAATAATAATTTTAGTGAAATATATTATTATTATATTTTATTAATTT

>8a29673b29dbf56f46da54d5288a258a

AGATATAATTCGGATTTTATACAGTCAATTTCCCTTTACAAGGTGATGCCAAACCGAACAGTGGTATACCTGCCTAGTCATAATATCTACTTAGCAATTCGGAGCGG

>162ae84a78ccb0db4422371f0282ae2e

TTCCATCTCCGGTAGTATATGTTAAAAGCGTTGCGATTAGAAACCCGTGTAGTCCGGCTGACTGACTTGCGTCAAATCTCGTATGCCGTCTTCTGCTTGAAAAAAAA

>f641ef8847ac6333fba53b80cef77cda

CAAATTTATGTCTTCAGAAAGACAAAGTATTTTTCTAGGGTCCATTTCAACCATGTCGGTAATTTTCAACTAGCAGTGCCTTGTAGATATTTGTGAAAGATTTTGAA

>1832dc74fd7bcfe3d707b7c79fcab5b3

TACGTAGGGTGCAAGCGTTAATCGGAATTACTGGGCGTAAAGCGTGCGCAGGCGGTTATGCAGGACAGAGGTGAAATCCCCGGGCTCAACCTGGGAACTGCCTTTGT

>b1cf751129c38a545f04122ddbe13dcc

CCGTTACCGGTAACAGTGACTGCTGCAGGGACGGTTACAAAGTACGCGTTCCAGGTATCTGTTACCGCGTAACTGCTACATTAGAAACCCGAGTAGTCCGGCTGACT

>b314a62d80f81a1ce79245a7fbf84913

GCGATAACGACTGTAAACAAACGTCAGCACATTCATTCGCGCGCGTGCCGCTGTTGACAAACAAACCTTCTTAAACGCTGCGATTAGAAACCCCAGTAGTCCGGCTG

>2e82facba0553bf4eb87cbee9a2a1e97

TGATAGTACCAATACAAAACCGAACACAAAATGTCCCATATTACTTTCAAGTTCATCAAATAAAATCGAACAAGAAACGTAGAGATATTAGATACCCGGGTAGTCCG

>32b4fc12fb6d297eb232980433629e8d

CCACTGTGTTCAGTGTGATATACTTTTACAATACACTATAACATTAGATACCCTAGTAGTCCGGCTGACTGACTGTTACAGCATCTCGTATGCCGTCTTCTGCTTGA

>4282644e70c81b9d0bea17f9db92cca9

CCACCACCCACCGCTGTGTTCATACTTCTTTTACAATAACAGATGTCACAATTCACACACACACACTTAATGTGCATTAGAAACCCGAGTAGTCCGGCTGACTGACT

>afcf30e409b29af54f9fa0b19331a9ff

TACGTAGGGTGCGAGCGTTGTCCGGAATTACTGGGCATAAAGAGCTCGTAGGTGGTTTGTCGCGTCGTCTGTGAAATTCCGGGGCTTAACTTCGGGCGTGCAGGCGA

>267d4881c87f803856e7b1c4dd88363b

TTCCAGCTCCAATAGCGTATATTAAAGTTGTTGCGATTAGATACCCTTGTAGTCCGGCTGACTGACTGTTACAGCATCTCGTATGCCGTCTTCTGCTTGAAAAAAAA

>5f920abe5c4f3b67e64b0add24fbeb33

TACGGAGGGTGCAAGCGTTAATCGGAATTACTGGGCGTAAAGCGCACGCAGGCGGTTTGTTAAGTCAGATGTGAAATCCCCGGGCTCAACCTGGGAACTGCATCTGA

>67d2286c7a5b6ae4219400c8340e1610

TCCGGCTGACTGACTTGCGTCAAATCTCGTATGATTAGAAACCCTAGTAGTCCGGCTGACTGACTACTGTGTAATCTCGTATGCCGTCTTCTGCTTGAAAAAAAAAA

>fe65ba1cd6911bea76e5f44e051ab99b

CCACCACCCACCGCTGTGTTCATACTTTTTTTACAATAACAGATGTCACAATTCACACACACACACTTAATGTACATTAGAAACCCGAGTAGTCCGGCTGACTGACT

>d3712a00f752aff6d52cd3e6fd560884

CCACCACCCACCGCTGTGTTCATACTTTTTTTACAATAACAGATGTCACAATTCACACACACACACTTAATGTACATTAGATACCCGAGTAGTCCGGCTGACTGACT

>23ea25dcb37149057fddbf8cb84715e2

CAACAAAGACAACCCAGACAGTAAAAAGGAGCAAGGAAGGGAACCCTGTTTGCTAAATCTACGAGCATTAGAAACCCTAGTAGTCCGGCTGACTGACTATGAGCTCA

>1c95cf6a2bbbb97eba5a868634c53a47

TGGACTCCGAGATGTCTGATAATGAACAAGAAACAGATGGTGAATTAGATACCCCAGTAGTCCGGCTGACTGACTGCGATACGATCTCGTATGCCGTCTTCTGCTTG

>130564a87e2739b8c4cf444b80aa9936

TGGACTCCGAGATGTCTGATAATGAACAAGAAACAGATGGTGAATTAGAAACCCGTGTAGTCCGGCTGACTGACTGCGATACGATCTCGTATGCCGTCTTCTGCTTG

>9efa50e7b728764986c92a73490a296b

TGGACTCCGAGATGTCTGATAATGAACAAGAAACAGATGGTGAATTAGATACCCCTGTAGTCCGGCTGACTGACTGCGATACGATCTCGTATGCCGTCTTCTGCTTG

>c0e412a7e9f61c3d5a51479cd1c21604

CCACCCCCACCGCTGTGTTCATACTTTTTTTACAATAACAGATGCCACAATTCACACACACACACACTTAATGTACATTAGAAACCCGTGTAGTCCGGCTGACTGAC

>252a83d9d2e1c1c8eef8b9a9b6faf38d

TCAAGTAGGCTTTGAATAACGCGCATCGATTTGCCGCAATAACGCTCGTTGTCATCGGCCAGTTATTGGGACGCGACAATAACAGCAGGACATTTCACGGAGAGTTA

>af13b3d3b61fe6e57fbb9a6d916d8109

TACGAAGGGGGCTAGCGTTGCTCGGAATTACTGGGCGTAAAGGGCGCGTAGGCGGATCGTTAAGTCAGAGGTGAAATCCCAGGGCTCAACCCTGGAACTGCCTTTGA

>5b6690d2f478e084cfbac56c1b6e7075

TACGTAGGTGGCAAGCGTTGTCCGGATTTATTGGGCGTAAAGCGAGTGCAGGCGGTTCAATAAGTCTGATGTGAAAGCCTTCGGCTCAACCGGAGAATTGCATCAGA

>4dafdfab12dbf4188ad554aab991ebb9

TTAGCTGGGTGAGCTCCCTCGCAGTTTGCGCATTTGGCCGGAGCAGGGCGGGAAACTTCGCAGCCCCCGCGATACTCCTGCCCGCAGTGCACACAGCGCGGTAGTAG

>60dc04dff066a74d9e2ac672891ce43e

ACATAGTTATACACAACTCTCATGTTTTATTATAATTACTTTAGATTAAAGAGTTTGATATGGTTGTAGTACATTCAACTTCAAATTAGATACCCCGGTAGTCCGGC

>a87b9519c5e44db709b968d66774a9ed

ACTAAGCTCAACCACTGGCCCTTATGTGCCAATTAGATACCCTTGTAGTCCGGCTGACTGACTACGTACGTATCTCGTATGCCGTCTTCTGCTTGAAAAAAAAAAAA

>b95183bbc33583dad625f470d65c9fb3

CATTGAACTATCGTGAGAAAGTCACGCCGCCAAAGGGAATTATATTATAGTAATTATTGGCGTTAATAAACATTTTATCAATAGTTGTAATATATGATAACATGTAA

>fdf668181b1e5d48f64876869a13fda1

TGTTAACAAAAACGACGTCATCAGACGTTGCAAGAAAAGTAATAAATCCGTTTTATTTCTGCAGATTTTTGCACTAATAAATACGCAGTCACTAAGTTACACACGAT

>78b7657996e351c334a32ab599b10c77

ACAACATGTATAGTACTGACCCAGCTGTTAAGAATGATGTCATCTTACCATAAAAATTAGAAACCCCAGTAGTCCGGCTGACTGACTGAGACTTAATCTCGTATGCC

>ad5e0863db59fda5e86e22153585958b

ATATCGTCAGTCCCTGTTCTTAGATGTTATCTTTCCAGAGCCGCACTGAGGCACACACACAACAGGGCACACAGATCAGAAACATCCTAGAATGTCTATTCTTACAT

>4e4d25ee4c47ae507df827fc4a2b74dd

CTGGAGACCGTCTGCAACATGTTTGTGAAGGTAAACAGTTACGTGTATTATTGCGTGTATTAGAAACCCGAGTAGTCCGGCTGACTGACTCGCGATATATCTCGTAT

>27983234eda99901cc913422d8eab57b

TTAGCTGGGTGAGCTCCCCCGCAGTTTGCGCATTTGGCCGGAGCAGGGCGGGAAACTTCGCAGCCCCCGCGATACTCCTGCCCGCAGTGCACACAGCGCGGTAGTAG

>5143569d31704882b1bc20917531807b

TACAATTTCTTCAATTTAAATTTTTAAAAGTTTCAGTCAATAAATTATTTAAATATTTATGAATAATAATTTTAGTGAAATATATTATTATTTTATTTTATTAATTT

>624101c7e82a0237f75e8d23a462d244

CCACCACCCCACCGCTGTGTTCATACTTTTTTTTTACAATAACAGATGCCACAATTCACACACACTTAATGTACATTAGAAACCCTGGTAGTCCGGCTGACTGACTT

>e47559fb0879616e25a7d230ab7db77e

CATTGAACTATCGTGAGAAAGTCACGCCGCCAAAGGGTATTATATTATAGTAAATATTGGCGTAAATAAACATTTTATTAATAGTTGTAATATATGATAATGTGTGA

>781e83ddef881eae6a7826677e05eb57

TGTCTGCACCTAATAAGGCATCAACGGGAGCGGGTTGGTCAAAAGTCGGATCGGCAAGAATCAAGTCCTTAGCCGCACTCTCAAACTCTGAAGCTATTGCTGTCAGA

>d33d7daa9a219769f453c05db82c93d5

TACAATAATATAAGACTTACTTTAAAGGAAACACTGTAGTATTAGAAACCCCAGTAGTCCGGCTGACTGACTCTCTAGAGATCTCGTATGCCGTCTTCTGCTTGAAA

>72e2d4156c753128f7e80f6d581cf38c

TTGGCGTTGGTTCAGCTCTTGTATTACAAAGCCTCGGTGGCACAGTTCACCACAGTATCTCCATCAGGTATCTCCTTCCTATGGCTACAGTAGGTGGTTTTATCGAT

>0297c74c79718bd3e2f681b715da48ce

CCCACCGCTGTGTTCAGTGTGATATACTTTTTACAATACACAATAACAGATGCCACAAGCCACACACACACACACACACACACACACACACATACACAGAAAGTCGT

>5d051dc2361de71cb49950af1bbb53c1

CCATCCAGTCAACTATCGCGCTGTTCCTCGTATTAGATACCCCTGTAGTCCGGCTGACTGACTTAACGTCCATCTCGTATGCCGTCTTCTGCTTGAAAAAAAAAAAA

>23f057afc08f14b38e426cbc442462ba

AAGAGACGTTGAACGACCGGTAGTAAGGTGGCGAGAGTCATGAGGCCGGCACAGGAGAATGCTTCTAAGCCTTGAACAGTTCATCATGATGATTATTATTTCGAAAG

>246afc2c71442d606d9a725852f5d4b7

ACCACAGTACAAAACACTATAATATAATGATAAACCTAAATTGTTACCCGAAGTCGTAGGGGGTATTCATCACGGTTGGCATTAGAAACCCCGGTAGTCCGGCTGAC

>22ca7243980b755abbe0d895c2f26352

TACGGAGGGTGCAAGCGTTGTTCGGAATTATTGGGCGTAAAGGGCGCGCAGGCGGTCTTGTCCGTCAGGTGTGAAAGCTCGGGGCTCAACCCCGGAAGTGCACTTGA

>2f9d4ae1b036a998ee2b0dab26bf248c

CACGGGGGGCGCAAGCGTTATTCGGAATTATTGGGCGTAAAGGGCGCGCAGGCGGTCTTGTCGGTCAGGTGTGAAAGCTCGGGGCTCAACCCCGGAAGTGCACTTGA

>6c7f4c59cce866c9779930ad014c051e

TTAGGTTAACTGAAATTGACCCCGGCCGTGAAAGTTTTAAAACTGAATCATAGGACATCATCTAAATGAAGAGTTTAAGTCTTAATTCAGAATGATAAAGAAATAAC

>cca19d069eb0072f7a922291747ce3f0

CATTCCTTGGTGCAAGTATTTCTGCATCTCCCTTCCTCAATCCTATACATTAACCAGAACTGCTTCTCAGAATTAGACAATCTAACTGCTTCTCAGGTATAGACAAT

>99526941c8947fa2229b254ed61c3260

GACAGAGGGTGCAAACGTTGTTCGGAATTACTGGGCGTAAAGCGTGTGTAGGCGGCTAAGTAAGTCGGATGTGAAAGCCCTGGGCTCAACCCAGGAAGTGCACTCGA

>33f463437b12bd14c128564a168889e7

TTGGCGTTGGTTCAGCTCTTGTATTAGAAAGCCTGGGTGGCACAGTTCACCACAGTATCTCCATCAGGTATCTCCTTCCTATGGCTACAGTAGGTGGTTTTATCGAT

>2f33594eb2ce46aee27f80a5e3eb2267

ACTAAGCTCAACCACTGGCCCTTATGTGCCAATTAGAAACCCTGGTAGTCCGGCTGACTGACTACGTACGTATCTCGTATGCCGTCTTCTGCTTGAAAAAAAAAAAA

>5445ff7ea953c29941e3779b353be13b

ACAACGAATAAACGAAAAACGCAAAACCGAAATAAAATAGTATGGAAAGTTTAGAAATTGGAGAAGAAACGAGTAAGTGTGAAAACCTTACTCGAATAAATAAAGTT

>26d89834ba432272d8a63c0cbd9c7cf0

GCGATAACGACTGTAAACAAACGTCAGCACATTCATTCGCGCGCGTGCCGCTGTTGACAAACAAACCTTCTTAAACGCTGCGATTAGATACCCCAGTAGTCCGGCTG

>2d04961f1b75ebf25d5ce4fcd64e193a

GGTGAAAACACAGTTCGACAACGGGTTTTAATTACGTTCAGCGGTCGCTTGAATACAAATTAGAAACCCTAGTAGTCCGGCTGACTGACTATAGCGCTATCTCGTAT

>210e43dcc348490be7f3221a8ef5ebb9

TACGTAGGGCGCAAGCGTTGTCCGGAATTATTGGGCGTAAAGAGCTCGTAGGCGGTCCGTCGCGTCTGCTGTGAAATCCCGAGGCTCAACCTCGGGCTTGCAGTGGG

>819a8566f1d7bfb473156e5d0f0a30ca

CACACTTATGTCTTTCTAAAGATAAGTAGGCCAGCAATTAAAGATAATGTAAAATGATGTTATTTTACTCACCTTTCACTCCAGTGGCGCCCTTCTTCGGCGATAAA

>3c12f0d6c86c4a2d1bb5ef5df35eebdd

TCTGATCAGGTCATAAACTGTGTGAACAAAGACACCGCGAGGCCTTAGACAAAGATGTAGTTTGTCGCCCATATTGTTTAACATATTTTAGATTGACGATCTGGTCA

>002580ec468a10e0e3bda3446f196f91

TACGGAGGGTGCAAGCGTTAATCGGAATTACTGGGCGTAAAGCGCACGCAGGCGGTCTGTCAAGTCGGATGTGAAATCCCCGGGCTTAACCTGGGAACTGCATTCGA

>4df3c7264e017c545dfbcaec0801e234

TATTTAAATGAATGGTATTTAGTTCTACCGTCATCCTTTCCTAAGTAAATTTTAACACTGGTAGTGGGGATCAGAAAAGTGTTTAACAAAATACTGATAAGTGGAAC

>279d594e36729e1a25078f1160cc4912

TTCCAGCTCCAATAGCGTATATTAAAGTTGTTGCGATTAGAAACCCCTGTAGTCCGGCTGACTGACTTACTAGGTATCTCGTATGCCGTCTTCTGCTTGAAAAAAAA

>9bbb018189332396b8a09c49f61d5e90

AACAGAGGATACAAGCGTTATCCGGATTTATTTGGTTTAAAGGGTGCGTAGGTGGTTTTTTAAGTCAGTAGTGAAATCTTAAAGCTTAACTTTAAAAGTGCTATTGA

>01b9731e81142fe0cdf0ddaa6f3ae07e

TACGTAGGTGGCAACCGTTGTCCGGAATTATTGGGCGTAAAGCGCGCGCAGGTGGTTTCTTAAGTCTGATGTGAAAGCCCACGGCTCAACCGTGGAGGGTCATTGGA

>2d6908ef730419ac9e6263b6ac8b7fc7

CATTGAACTATCGTGAGAAAGTCAAACCGCCAAAGGGAATTATATTATAGTAAATAGTAGCGTAAATAAACATTTTATTAATAGTTGTAATATATGATAATGTGCAG

>8cde8b717d43c995c2f920a75391f1d4

TACATAAGGAGCAAGCGTTATCCGGAATTATTGGGCGTAAAGAGCTCGTAGGCGGTCTTAAAAGTCAGGTGTGAAATTATCAGGCTCAACCTGATAAGGTCATCTGA

>eaf82fa3584f5d49582d033c7945f888

TACGGAGGATCCAAGCGTTATCCGGATTTATTGGGTTTAAAGGGAGCGTAGGTGGAAGCCTAAGTCAGTTGTGAAAGTTTGCGGCTCAACCGTAAAATTGCAGTTGA

>b4149799c29b8f9de99ec873d707bf0b

TGGACTCCGAGATGTCTGATAATGAACAAGAAACAGATGGTGAATTAGAAACCCCGGTAGTCCGGCTGACTGACTGCGATACGATCTCGTATGCCGTCTTCTGCTTG

>b5e0894683078ac6c7514831c5b5fe1b

CCAATACTTTGAACCAGTGAATTTACGTTAATATGGTAACGTATTGAAGTTCCGTGTAGCAATCAATATGACATATAACGTCCACGATGTAATCCCAAGGTTACGTA

>92cc2efdbacaedd3e57a870189582216

TGAAAATATCCAACGCACACACGGCGCATGCGCATGTTTACGAACAGCTGGCCTCTAAGCGGTCAGTTGCCTTTCGCGGACTACAGCTGGCGACCCTTGTCGCTCTC

>d546fefae724e4dbb3e898e45aff0155

CCACCACCCACCGCTGTGTTCATACTTTTTTTACAATAACAGATGTCACAATTCACACACACACACTTAATGTGCATTAGATACCCGAGTAGTCCGGCTGACTGACT

>aa42d3b04abc475e22cb0cce3cc1b7ee

TACGGAGGATGCAAGCGTTATCCGGAATTATTGGGCGTAAAGCGTCCGCAGGTGGCACTTCAAGTCTGCTGTCAAAGACCGGGGCTTAACCCTGGAGAGGCAGTGGA

>2df0aabcdce7bc5ad4974e1ec048ed1c

TACGTAGGGTGCAAGCGTTAATCGGAATTACTGGGCGTAAAGCGTGCGCAGGCGGCGATATAAGACAGATGTGAAATCCCCGGGCTCAACCTGGGAACTGCATTTGT

>14674cc6fbbcc67496891ea6667278f0

CCAGACACCGTAGTATGCCCATGTAATCCCAGCAATTGAGAGGAGGCAGAAAGATTAGAAACCCTAGTAGTCCGGCTGACTGACTCGCGATATATCTCGTATGCCGT

>3a35936e5bbef53465326770947a54e1

TTCCAGCTCCAATAGCGTATATTAAAGTTGTTGCGATTAGAAACCCTGGTAGTCCGGCTGACTGACTTGCGTCAAATCTCGTATGCCGTCTTCTGCTTGAAAAAAAA

>b8403b8ae7f15c44dd6893dfc49dc500

TACGTAGGGTCCAAGCGTTAATCGGAATTACTGGGCGTAAAGCGTGCGTAGGCGGTTGTGCAAGACCGATGTGAAATCCCCGAGCTTAACTTGGGAATTGCATTGGT

>4a4c59bba9c1c7cab5a3e40f70a65301

TTCCAGCTCCAATAGCGTATATTAAAGTTGTTGCGATTAGAAACCCCAGTAGTCCGGCTGACTGACTTACTAGGTATCTCGTATGCCGTCTTCTGCTTGAAAAAAAA

>40dfaf73a7eb1d0cee1a99fb6f6e0cb2

TACGGAGGATCCGAGCGTTATCCGGATTTATTGGGTTTAAAGGGGGCGTAGGCGGATGCTTAAGTCAGTTGTGAAAGTTTGCGGCTCAACCGTAAAATTGCAGTTGA

>278b07918c21d821b923ba33776f650d

TTCCAGCTCCAATAGCGTTTATTAAAGTTGTTGCGGTTAAAAAGCTCGTAGTTGGATCTGTGTGCCACGCTGTCGGTTCACCGCCCGTCGGTATCAACTGGCATGTC

>73ac4d5a01191dced55c223eb987c1ff

AGCTCTACATACGACCGTAACGTTGCTTGTTGGCAAAACCGCAAACCGTGCTACAAAGTTTGGACAATAGAGAGCGCTTGTTTACGTACATCGAGCTGTTATTACTT

>21efc09e54917282b816f8948faaf627

TTCTTCTTTCCGAACCGGCGCCGTGAACCACGTTAGCGTGATTATACGGAACTGTTCACTTTACGGGTACTAACAGTGAGGTGGGACGTAATACTGGCCGTGACATG

>a05f6be616e524426bee4a60b14e4048

TACGTAGGTGGCAAGCGTTGTCCGGAATTACTGGGCGTAAAGAGTGCGTAGGCGGATGATTAAGTGAGATGTGAAATACCCGAGCTCAACTTGGGTGCTGCATTTCA

>0b46d9b0e53afb8badc1afc395a84eaf

TACGGAGGGAGCTAGCGTTGTTCGGAATTACTGGGCGTAAAGCGCACGTAGGCGGCTTTTCAAGTCAGGGGTGAAATCCCGGGGCTCAACCCCGGAACTGCCCTTGA

>94eae949ee21cd68489ce5810808ab6f

TACGTAGGGGGCAAACGTTGTCCGGAATGACTGGGCGTAAAGGGCGAGTAGGTGGTTTGCCAAGTCAGAAGTGAAAACTCCGGGCTCAACTTGGAGATTGCTACTGA

>f2851cec2ed26c30b36fed97b086a246

CACTGACAACCTATCGGAGTAAAAACTCGCACCAACTTTAAACTCACAGTAGCATGTGTTTTATAAACAAGCCTCAGGCTCGAAATTCTTTGAAATGATTGTTTTTA

>f8c1cd0ad8078aa4b8143dd0e37eb7f8

TTAAATTTATCATCGCAGTTGACAGTTGGCTAGCATGTGCCCGTTCGCGCTTCCTTGTATATATATGTGTGTGTGTGTGCGTGTGCGTGTGTGTGAAACGATGTACA

>90b412cc95855e25520fdc6f538da5e3

TACAATTTCTTCAATTTTAAATTTATAAAGTTTCAGTAAATATATAACATAATTTTTATAAATAATAATTTTAGTGAAATATGTTGTTATTATGTTTTTATGATTTG

>0855f1eeedcb77e6cf064817c430b105

TGTCCGCACCCAATAGGGCATCAACGGGAGCGGGTCTGTCAAATGTCGGGTCAGCAAGGACCAAGTCCTTGGCTGCAATCTTAACCTCCGAAGCTATTGCTGTCAGA

>e55ae15ce7ab75f757f004fa003e6b9e

GAAAGAGTAGAAGGTGAGACGTTGAAGAAGAAGCAGTCACAAATACACAACACACACGCACAAGGTCGATCCTTTTCAGGGTCGGCTGTAATGCTACCCGCCAACTC

>c0dc6a3a02a80b56791b825a5d72c88b

CGAAAACAATAACAAAAAACGATCAGAGGTCGTAGACTGAAAGCGCCTATTTCCTAATCATTAGAAACCCCAGTAGTCCGGCTGACTGACTACGTACGTATCTCGTA

>9af165ee355a33dce10e3c7f03abfad3

CCACAACCCCCCACCGCTATGTTCATACTTTTTTTTTACAATAACAGATGCCACAATTCACACACACACACACACACACACACACACACACACACACACACTTAATG

>6bb10d0c726e67d00cb7a0c539e9ea18

TACGTAGGTGGCGAGCGTTGTCCGGAATTACTGGGCGTAAAGGGTGCGTAGGCGGATATTTAAGTCAGATGTGAAATCCCCGAGCTTAACTTGGGGGCTGCATTTGA

>55e0bbe9ba02faa33b72404190796036

TACGTAGGGGGCGAGCGTTGTCCGGAATCACTGGGCGTAAAGGGTGCGTAGGCGGTTATATAAGTCAGGTGTAAAAGGCATTGGCTCAACCAATGTAAGCACTTGAA

>7f1db6d9fee3719fe0184cbb6317e259

TACGTAGGGGGCGAGCGTTGTCCGGATTTACTGGGCGTAAAGGGTGAGTAGGCGGTTATATGTGTCAGATGTAAAAGGCTAAGGCTTAACCATAGTTAGCATTTGAA

>ee2c73f019612a95209f8e4cd6318554

TCACCACCCACCGCTGTGTTCATACTTTTTTTACAATAACAGATGCCACAATTCACACACACACACACTTAATGTACATTAGAAACCCTAGTAGTCCGGCTGACTGA

>5fa5e6675bd0b55272ec93e6f1400f88

AAGGAAATTGAAAGTAAACGACTCCTCTGACTGCAATACACCATCATCATCATTAGATACCCTAGTAGTCCGGCTGACTGACTATGAGCTCATCTCGTATGCCGTCT

>703f0674bc5d99d1dc06dee8d379ed0f

ACAACAAGGGGAACCTAACAATGAGCCGCCTCACAATCAATCAATTTTCGCCACGCCACATCAACGCCATTCGTGTAGCACAATTTTTCTTCGCCTATCTACACACA

>01e093f7140a367ad4a751cf7bcbda8f

ATTCTGAAACCTAGAGGAATAACGGTTTATCGCTACACCATTCTATACTCTTCCAGAAGACCCCAGGACTAAAATAATTGTCTCTTAATCTATCTACTAGTTTCCAT

>924ef27258771270525fd89b78f3c649

CTAGGCTACACAGCACATGCTGAGTAACTTGTAGCAGTGACGGTGGCCAAGTTACACCTTTCTACTTTGTCGTTGGGGTGTGATGTTAAGGACCGTATGAACATCAG

>8054e64eb681b38fa1ebc124bcbf9bf5

GAGAAAAACAACCGTATTTGAAGAGGTAAGCAGCTCACAGTATTACATACTGCAGACTATCGTGATAATTAGATACCCTAGTAGTCCGGCTGACTGACTATGAGCTC

>af6adb4716d9b59b5448bed69c21736e

TTGGCTGGGTGCTTCTCTCCACAGTTTGCGCACTTTGGGTCCTCCTCAGGTGACTTTTTACAGTCACCCTTCGGATTAGAAACCCCAGTAGTCCGGCTGACTGACTA

>ebf9aef9cf7b2719d34e8979050cc934

TACAATTTCTTCAATTTAAATTTATAAAGTTTCAGTAAATATATAATATAATTTTTGTAGATAATAATTTTAGTGAAATATATTTTTATTTTGTTTTTATAATCTGT

>3ce8a5a3fdfb044a3232b508302baaf9

TTCTAAAGAGTTCCGTGTGCTGTATGTAATATTTTGTAACGTGTAACGCTTCAAAAATTAATAAAAATAAAATTAATAAATTAATGTCCCTGCTTGGGAGCCAATAA

>777e56e689cd32155ce31ce450870797

GATGCAACAAATTATAAAATAAAAATGTCACGAATGTTTCACAAACCAGCAAGGTTCAGCTTACATTAGATTTGAGAGGGGAAAACAGATACATGTAGAATTACCTC

>b34f1bb19b58e79e79ad809ef002d474

TACGTAGGGTGCGAGCGTTAATCGGAATTACTGGGCGTAAAGCGTGCGCAGGCGGTTTTGTAAGACAGGCGAGAAATCCCCGGGCTCAACCTGGGAACTGCGCTTGT

>2e0afc79f4f9aba8b01fe85fe46ad95d

ACGTTGATCGTTTGACCCCAGAAGAGGTCACAAACACAAAATTTATTCCGCGTCGGTCGGTGTGTACGTTTCGCAACGTTGCGATTAACGGGACACGCGGTATCTGT

>37a8629331722172dd7c71ba9c0da221

AAGATGTCAAGCCAATCTGGTGCTCTTGCAGCTTGCGAACAGCTACTGGACGGCCTGTAAAAGTGTATGGAAAAGTGGAGATACACCTACGCCTCGGTAGACTTGAG

>14fef22c5cbff3571dbc47a7ad7fdbaa

TACAATTTCTAGAAATTAATCCAGCCTTACGCAAAGTGTAATCACAATTAGCGACGTGCGCAAACACTGTTTACCCCACTGGCCCGCTTAAGCCTACTCGACGTTCG

>f9f0fb82fbdaeb8844cd1f17890d5ad8

ACAAATGGTGAATAGTATAGTGTCAATAATGGCATTAGATACCCCTGTAGTCCGGCTGACTGACTACTGTGTAATCTCGTATGCCGTCTTCTGCTTGAAAAAAAAAA

>84e890c7bf41378095203ad9a469f0cd

CCACTGTGTTGAGTGTGATATACTTTTACAATACACTATAACATTAGATACCACAAGCCACACACACACACACATAGAAAGTCGTAGGACGCCAACGCAGTCATATA

>e5534ce2140cab38f20acf3f55b9abb0

TTGGCTGGGTGCTTCTCTCCACAGTTTGCGCACTTTGGGTCCTCCTCAGGTGACTTTTTACAGTCACCCTTCGGATTAGAAACCCGAGTAGTCCGGCTGACTGACTA

>2fc0e9aa0519212cb2a46c444fbb49bd

TACGTGAGAGACTAGTGTTATTCATCTTAATTGGGTTTAAAGGGTACCCAGACAGTCAATATAACTTCTATAATGCTAATACTTGACTAGAGTTTTAAGTAAGAGGG

>1b4211a49494f6176c53e3038c517b94

CACACTTATGTCTTCAGAGAGACAAAATATTTCTCCAGGATGCATTTCAACCATGTCGCTCATTTTCAACTGGCAGTGCCTTGTAGTTATTTGTGAAAGATTATGAA

>be9345619542ce5b840a8689661dc0dc

TACGTAGGGTGCAAGCGTTAATCGGAATTACTGGGCGTAAAGCGTGCGCAGGCGGTTTGTTAAGCCAGATGTGAAATCCCCGGGCTCAACCTGGGAACTGCATTTGT

>7df7be8d9f425e8b71fed12a32ecec88

CGGTTTACCAAGTATTGGGCAACTTTATGCGTTCATTTTGGGTGTTAAAACTTAAAAATCTCATCTTTTTTTATCGTTAAGGGATTCAGGATCCAGCGAGTAGCTGT

>be6217cc4b84b35f67cd82816f4b64ca

ACCACAACGCCTTCCCGCCGCGCGCGATGCGCCAAGAAGCTTAAGAAACTTGCATTACAATAAACATAATAACTAACAATTCAATAAGTAAGTAAGTTGAGAGTAAC

>0a3e7667cc7b3fa12604f5c9b85175a5

CCACCACCCACCGCTGTTTTCATACTTTTTTTACAATAACAGATGCCACAATTCACACACACACACACACACTTAATGTACATTAGATACCCTAGTAGTCCGGCTGA

>f440f72976de956aaada1c3d91449e4d

CCACCCACCGCTGTGTTCATACTTTTTTTACAATACACAATAACAGATGCCACAAGCTACACACTTAATGTACATTAGAAACCCTAGTAGTCCGGCTGACTGACTAG

>9804aeaa8681d25dda17fab39b4dcaa6

TCAAGTAGGCTTTGAATAACGCGCATCAATTTGCCGCAATAACGCTCGTTGTCATTGGCCAGTTATTGGGACGCGACAATAACAGCAGGACATTTCACGGACAGTTA

>d6c5ef1c9b25dd68489e12917111507d

TACGGAGGGGGCTAGCGTTGTTCGGAATTACTGGGCGTAAAGCGCGCGTAGGCGGGCTGGTTAGTTGGGGGTGAAATCCCAGGGCTCAACCCTGGAACTGCCCTCAA

>cbd68550ffc4916700211bd2fd817332

CACTTGAAACATATAGTGCAATCAACACCGCGGGAACATTAGAAACCCTAGTAGTCCGGCTGACTGACTACGTACGTATCTCGTATGCCGTCTTCTGCTTGAAAAAA

>0d5e12af044dde73c1f6fec01d06ab84

TACGTAGGGTGCAAGCGTTAATCGGAATTACTGGGCGTAAAGCGTGCGAAGGCGGTTATGCAAGACAGAGGTGAAATCCCCGGGCTCAACCTGGGAACTGCCTTTGT

>ff1cb0c54d210f6c08ef336b079264eb

CGAGACACACTTGGGTCATTGCCATGGCAACGAACATCTGTAGTCTACAGACTGTATCAATCTCCCATGTTGTTTTATACTAGTCGTTATTTTATTATTATTATTAT

>3e35ee67a3a2b0eebc97146c300e55a1

TACAATTTTTTCAATTTAAATTTATAAAGTTTCAGTAAATATATAGTAGAATGTTTATAAATAATAATTTTAGTGAAATATGTTATTATTATGCTTTCATGATTTGT

>b1e4d01519094ddeb0a8d03dad4fb9ae

CCTAATGCAGGCAAAAAGAAGCGGCAACGTAAGCCCCGTGGCGCTAGATCCCGGGCTCCTGCCAACCCAGCCGCACAGTCCAGACAGCCTGCGCAGCGCGTTAAGAC

>159da48fce0d95863b9c547a15b1ba3f

TGGCTGCCCTCTATTTCTCTGCCCACTCAACGTAGATTATAATAGCGTCAGTGTCGGAAACGTCTTTCCCCTCTGTGCAGTTCCTCGATATAGTCGACGTATTCCGA

>49438c669f47352dc0c43ccc20577e16

TCATGGACAACGGAGAAGAATATATCCCAACTGAAACAAACCAAGGCGTAAGACAGGTTTATTGTCTGTCACCACTACTTTTTAACCTATATCTAGATGATCTCCTC

>1203160fee1201579754e14adfef094e

TTCCAGCTCCAATAGCGTATATTAAAGTTGTTGCGATTAGAAACCCGTGTAGTCCGGCTGACTGACTGTTACAGCATCTCGTATGCCGTCTTCTGCTTGAAAAAAAA

>6ba740cfd6eb36c8209bbd3e7cc3daef

TTCACAACAATATTATTCTTTGGTATAACATATGCCTTACTCTAATTAGATACCCTGGTAGTCCGGCTGACTGACTAGTCGCAGATCTCGTATGCCGTCTTCTGCTT

>1d5cef4947b8c363338c723b954762fe

TGTTAGAGCTATGACTCACTATACACGGCACTAGGAAATACCGTTTATTGCGACAACTCTGAATAACAAGGAGTGGTTTGGCCAGTTTATTTTAGTTTGCTACTAGT

>55b90841fe9dad6ec7797adcd6f030ee

TACGGAGGGAGCTAGCGTTGTTCGGAATTACTGGGCGTAAAGCGCGCGTAGGCGGCTTAGCAAGTTGGGGGTGAAATCCCGGGGCTTAACCTCGGAACTGCCTCCAA

>a73c6ffa1b0fa46f1518212b86d75fc1

GGTGCAACTCGAGTCATTGCATACTCCCCCCGAACCACTTTACAGTTTGTTGATGGATCTGCACACACGACACAGGCATTTTATGGGCAATGTCCGTAAATATAACA

>d0e4d2485205996eada3a47685404fde

TATCCCGCAGCTGAAGGGATACCAATATCCCTTATGACTAATTAGAAACCCTAGTAGTCCGGCTGACTGACTTGCGTCAAATCTCGTATGCCGTCTTCTGCTTGAAA

>845532a0c230bd42b6b06b9dc15a3e27

CCACCACCCACCGATGTGTTCATACTTTTTTTTACAATAACAGATGCCACAATTCACACACACACACTTAATGTACATTAGAAAACACAAGTCACACACACACACAC

>9c94be952b6bd350e485544a1ea0ebfd

AGCTAGCTTCTGGTTCATGGCAACCGCCCGCAATCTCAGAAGCCAACTATTTTCGTGGATATGAGAAATAAAGTTCCGTCTTGTCGTTTATCGTACCCCTCTTAAAC

>9aab90e0ee7afc6d9724db2e65f75c20

TTAGCTGATTGAAATTAGGCTACATGCCTCTCTCTCGATGCCAAATAATAACTTATCGGATTAGAAACCCCAGTAGTCCGGCTGACTGACTATAGCGCTATCTCGTA

>1df052eb0953dcf51a7d2b10b3bd83ef

CGAATTTAGCGGTCATCATCAGCACGTACACATCGCATTTATGCTCATCATGTCCATGATGCCTGTCTCTGTAGAATAATTATAAAATTATAATTACGCGAGTAACG

>abd689a087ec664f4556a85a996acae6

TTCACAACAATATTATTCTTTGGTATAACATATGCCTTACTCTAATTAGAAACCCCAGTAGTCCGGCTGACTGACTAGTCGCAGATCTCGTATGCCGTCTTCTGCTT

>c18b3c11ce56c614ff01676177fec143

CAAGGTTTCCTTTACAACCTTCTTGGTCACCTCCGAGTCTACTATTGTCGTGTCCGTCGGATATATCAACACCACATTTTCCGGGTCTCTTGCAACTACCTTGCGAT

>22e2606faba122fdd832c91ca11c0668

CCAACAGTACCTTTCCAAGGGACAAATAGGGGATAATTAGATACCCGTGTAGTCCGGCTGACTGACTTGCGTCAAATCTCGTATGCCGTCTTCTGCTTGAAAAAAAA

>b20ba3f3f31ad8d5d037c80d63bc9859

CGTTATAGCGACTTTCCCCCTCCATCCAGTCCCTGCAGTTGTGAGGTAACACCGGCAACCCAACAGTCATAGCACGTGCTCCGGCATCCACCTCGAAGCCAGCGCAG

>e51d1f7cb0a519533ac77fb88023a441

CATTGAACTATCGTGAGAAAGTCACGCCGCCAAAGGGAATTATATTGTAGTAAATATTAGCGTAAATAAACATTTTATTAATAGTTGTAATATATGAAAATGTGCAG

>98422ce8befdd02267aee5ce9ea497e4

CACTTGAAACATATAGTGCAATCAACACCGCGGGAACATTAGAAACCCCAGTAGTCCGGCTGACTGACTACGTACGTATCTCGTATGCCGTCTTCTGCTTGAAAAAA

>b3a4d00284a5af73c37779456e48d1fb

TACGTAGGGTGCAAGCGTTAATCGGAATTACTGGGCGTAAAGCGTGCGCAGGCGGTTATGCAAGACAGAGGTGAAATCCCCGGGCCCAACCTGGGAACTGCCTTTGT

>53d842281ca0f5b687f2b82e255bb449

TCACCACCCACCGCTGTGTTCATACTTTTTTTACAATAACAGATGCCACAATTCACACACACACACACTTAATGTACATTAGATACCCCAGTAGTCCGGCTGACTGA

>4f360dc925a31456317f2a8615302793

TACGTAGGGTGCAAGCGTTAATCGGAATTACTGGGCGTTAAGCGTGCGCAGGCGGTTATGCAAGACAGAGGTGAAATCCCCGGGCTCAACCTGGGAACTGCCTTTGT

>c78fae064e1e8cd672584bd9f8c97a9a

CCACCACCCACCGCTGTGTTCATACTTTTTTTACAATAACAGATGTCACAATTCACACACACACACTTAATGTACATTAGAAACCCTGGTAGTCCGGCTGACTGACT

>a0b44abff7ad5af1e88f60dc533cafab

TTCCAGCTCCAATAGCGTATATTAAAGTTGTTGCGGTTAAAAAGCTCGTAGTTGGATCTGTGTGCCACGCTGTCGGTTCACCGCCCGTCGGTGTCAACTGGCGTGTC

>e2861d2b6c1a735e6f58e74e214514c4

TACGAAGGGGGCTAGCGTTGCTCGGAATTACTGGGCGTAAAGGGCGCGTAGGCGGACAGTTAAGTTGGGGGTGAGAGCCCGGGGCTCAACCTCGGAAATGCCTTCAA

>e828c30905fc1841f1ba7feffaf2d1d9

CAAGGTGTTCTGCCTGGGAACAGAAGGACTGACTAGTGGTATTCAAGACAACTAAACGGGCTGTAACAAAACTTATAAAAAGTATTAGATACCCCAGTAGTCCGGCT

>bc8a0921577767036cc38a6a1e5486e2

GGTGAAAACACAGTTCGACAACGGGTTTTAATTACGTTCAGCGGTCGCTTGAATACAAATTAGAAACCCCTGTAGTCCGGCTGACTGACTATAGCGCTATCTCGTAT

>a6a1254e3a8a673324ed081ad76e6075

TACGGAGGGTGCAAGCGTTATCCGGATTTATTGGGTTTAAAGGGTCCGTAGGCGGACTTATAAGTCAGTGGTGGAAGCCCGCAGCTTAACTGTGGAACTGCCATTGA

>40c01aeff196c3161b5981ed93cf1eb5

CATTGAACTATCGTGAGAGAGTCATGCCGCCAAAGGGAATTATATTATAGTAAATATTGGCGTAAATAAACATTTTATTAATAGTTGTAATATATGAAAATGTGCAG

>df8ed3d67ff28d76c959ea77ec2ae7fc

TGGACTCCGAGATGTCTGATAATGAACAAGAAACAGATGGTGAATTAGAAACCCCAGTAGTCCGGCTGACTGACTCGCGATATATCTCGTATGCCGTCTTCTGCTTG

>5392c2f0b58ad29c009adbd32df2e76d

TACGGGGGGGGCAAGCGTTGTTCGGAATTACTGGGCGTAAAGGGTTCGTAGGTGGCCAACTAAGTCAGACGTGAAATCCCTCAGCTTAACTGGGGAACTGCGTCTGA

>eb459a657088ca2f5b67a541a5d2b50d

CCACTGTGTTCAGTGTGATATACTTTCAGAATACACTATAACATTAGAAACCCTAGTAGTCCGGCTGACTGACTGCGATACGATCTCGTATGCCGTCTTCTGCTTGA

>30d5cff22913a9707a4c9e2a9eaf3c46

CCACCACCCACCGCTGTGTTCATACTTTTTTTACAATAACAGATGTCACAATTCACACACACACACTTAATGTGCATTAGAAACCCCGGTAGTCCGGCTGACTGACT

>b50688ff1a896a77ddeb055465b70d9d

AATATAATCCGGGGGTTTTATACGGTCACTGTCTCCTTGCAATATGATAGAAAACCAGCGAGTGATTTCTGCCTAGTCATACTTCTCTATGTAGGCGTTTGGAGCCG

>cfe3f2b1909cc1ebdaa9ff29ecddf459

TACGTAGGGTGCAAGCGTTCATCGGAATTACTGGGCGTAAAGCGTGCGCAGGCGGTTGTGCAAGTCTGATGTGAAAGCCCCGGGCTCAACCTGGGAACGGCATTGGA

>ef90e2bac7d9b4add4db4852ecd530e9

AAGTACTCAAGATGTGCAAAGAACCGTATAATAGAAACAACTGGAATCGCGGATATAATTATGTAAACGCGCTTCTTTGTTTCACTTGTCTTTTATTTGGAGGAGAA

>068f0273cbe0376c2e69999e46716441

TACGATTTCTTTAATTTAAATTATTAAGTTTCAGTTAAAAATAATAATAATATAAAATAACTACAATTTTGGTGAAATATATTTTTTTTCTTGAAAAATTAATTTTA

>8c14d5931e799b7d8e9f9f0668763ed7

TACGAAGGGGGCGAGCGTTGTTCGGAATAACTGGGCGTAAAGGGCGCGTAGGCGGGTAATTAAGTCAGTGGTGAAATCCCAAGGCTCAACCTTGGAACTGCCTCTGA

>0ef6c6a4ef67ecda6d56d5ccff87e212

AGTTAACAGCGAACTGACCAGCGAGTTTCGTCGCCTCGAAAACCACAAATTAGATACCCCTGTAGTCCGGCTGACTGACTCTCTAGAGATCTCGTATGCCGTCTTCT

>60849f75ef78c73c290d1f38eb0f1c31

ACTAAGCTCAACCACTGGCCCTTATGTGCCAATTAGAAACCCTAGTAGTCCGGCTGACTGACTTGCGTCAAATCTCGTATGCCGTCTTCTGCTTGAAAAAAAAAAAA

>bb0307b2fe11e84e37a060f9ca06af71

CCACCACCCACCGCTGTGTTCATATTTTTTTTACAATAACAGATGCCACAATTCACACACTTAATGTACATTAGAAACCCCAGTAGTCCGGCTGACTGACTACGTAC

>72da66c38e28d40afb910d135ae0b4b2

TACGTGAGAGACTAGTGTTATTCATCTTAATTGGGTTTAAAGGGAACCTAGGCAGTCAATATAACTTCTATAATGCTAATACTTGACTAGAGTTTTAAGTAAGAGGG

>272bcf1ac61ddc5721a5906acadc1f02

TACTCATCACCACTAAAGAAGGCTTCCCCAGTCGTAGCCGAATGCCTGCCAGAGCAACCACATTAGAAACCCTTGTAGTCCGGCTGACTGACTCTACGACCATCTCG

>5005aa5e6d7a47b79aa189dddb9db0ee

ATGCAAATTGGGATCACTCGATTGAAATCTTAAACCGCATTAGAAACCCGTGTAGTCCGGCTGACTGACTACGTACGTATCTCGTATGCCGTCTTCTGCTTGAAAAA

>f85c7c3199a1faaf769d77037d339020

TACAGAGGGTGCAAGCGATAATCGGAATTACTGGGCGTAAAGCGTGCGTAGACGGTTACATAAGTCGGGTGTGAAAGCCCCGGGCTCAACCTGGGAATTGCATTCGA

>424d881689d449ec9ae7c33a51f58e71

TGTCCGCACCTAATAGGGCATCAACGGGAGCGGGTTAGTCAAAAGTCGGCGAGATCGGCGAGAACCAAGTCCTTAGCCGCACTCTTAACCTCTGAGGCTATTGCTGT

>26a385ea6a59da9edb99cdb968684e89

TACGAGGGGGGCAAGCGTTGTTCGGAATTATTGGGCGTAAAGGGCGCGTAGGCGGTTTGACAAGTTTGGTGTGAAATCTATGGGCTCAACCCATAGCCTGCATCGAA

>822551675fd1a94b8285e2e5701dbc33

TCCGGCTGACTGACTTACTAGGTAGTATGCCGTCTTCTGCTTGTAATGATACGGCGACCACCGAGATCTACACGTCTAGTGTATGGTAATTGTGTGTCAGCAGCCGC

>91cc374228b15d76400a10c485418f79

TACGTAGGTGGCAAGCGTCGTCCGGAATTATTGGGCGTAAAGCGCGCGCAGGTGGTTTCTTAAGTCTGATGTGAAAGCCCACGGCTCAACCGTGGAGGGTCATTGGA

>38dfe10d1442f8b7eae3ee5f3966e802

ATGATACGGCGACCACCGAGATCTACACCTACTATATATGGTAATTAGAAACCCTAGTAGTCCGGCTGACTGACTATGAGCTCATCTCGTATGCCGTCTTCTGCTTG

>378692910b36fd2e1b07b93595623a97

CCACCACCCACCGCTGTGTTCATACTTTTTTTACAATAACAGATGTCACAATTCACACACACACACTTAATGTGCATTAGATACCCTTGTAGTCCGGCTGACTGACT

>edd8a7e51798bcc9777fdda84b0d200a

TACAATTTCTTCAATTTAAATTTTTAAAAGTTTCAGTTAATAAATTATTTAAATTTTTATGAATAATAATTTTAGTGAAACATATTATTTTATTTTATTAGTTTGCC

>5e7621ca1ca1a946934f1c46f0d85ffb

TAAAATTTCTTCAATTTAAATTTTTAAAAGTTTCAGTTAATAATTTATTTAAATTTTTATGAATAATAATTTTAGTGAAATATATTATTATTTTATTTTATTAGTTT

>e74f3819544348fc15ec5d230fb2bb34

TACGTAGGTGGCAAGCGTTGTCCGGAATTATTGGGCGTAAAGCGCGCACAGGTGGTTTCTTAAGTCTGATGTGAAAGCCCACGGCTCAACCGTGGAGGGTCATTGGA

>dee1692b6b493ee204eaeaa5a309f6c2

ATCTCATAACTCCATCATAACTTATAAACGCTAGTCTTCCCCCCCCCATCCAGTCAACTACAGTTTCCATCAATAGACCAATTAGATACCCGAGTAGTCCGGCTGAC

>280dfbaebbfd766c25a42caeda9ca8a5

TACGTAGGGTGCAAGCGTTAATCGGAATTACTGGGCGTAAAGCGTGCGCAGGCGGTTCGCTAAGACAGATGTGAAATCCCCGGGCTTGACCTGGGAACTGCATTTGT

>506dd227753f755a9ebda04e6b106ca1

ACTTTGCGTCAGACAATGTTATTTTTTATGAACAATGGTTGGTAAACTTTTTTTCTCGCTATAAATTAGAAACCCCTGTAGTCCGGCTGACTGACTTGCGTCAAATC

>86030e278a54929f597f55602d1de819

TACGAAGGGGGCTAGCGTTGCTCGGAATGACTGGGCGTAAAGGGCGTGTAGGCGGTTTGTACAGTCAGATGTAAAATCCCCGGGCTTAACCTGGGAGCTGCATTTGA

>d4494109e2d2a9d35856e2bc54e52a8d

CACACTTATGTCTTTTGATAGACAAAACATTTCAACAGAATGCATTTCAACCTTCTCGATCATTAGAAACCCTAGTAGTCCGGCTGACTGACTGCGATACGATCTCG

>2a14b576f0af3b8b38de8bb866ee3f22

CATTGAACTATCGTGAGAAAGGCACGCCGCCAAAGGGAATTATATTATAGTGAATATTGGCGTAAATAAACATTTTATTAATAGTTGTAATATATGCAAATGTGCAA

>a8a51feb65b59df7f2bb72793cfb607e

TAAACGGCAGCCACAGCCGTCATGATATTTACTGTGGCAATTTGTTCCACATATTACGATGAATGCATTAGAAACCCCTGTAGTCCGGCTGACTGACTCTATCGTGA

>11e71451ba23ed12afe366b8a0383e89

TACGGAGGGTGCGAGCGTTAATCGGAATTACTAGGCGTAAAGCGCATGCAGGTGGTTCATTAAGTCAGATGTGAAAGCCCGGGGCTCAACCTCGGAACTGCATTTGA

>09ee7eca9db2ae4c831ea29f8abde640

CATTGAACTATCGTGAGAAAGTCAAACCGCCAAAGGGAATTATATTATAGTAAATATTGGCGTAAATAAACATTTTAGTAATAGTTGCAATATATGAAAATGTGCAG

>41810553628837ab6f3df940f0987ad4

AATCGTGGTGTAGCTCGCTGCCGCAGACGTGAGGACTTGCCGCGGCGCAGAAACTTTCAGTGCCCTCCCCGCACTCGCCATATGCGAGATGCGAGGACCAGCAACCT

>8d661a54ac0cee3b4fe90af20ff78ea8

TACGAGGGGTGTAAGTGTTAATCGGAATTACTGGGCGTAAAGCGTGCGTTGGTGGTTTGTTAAGCAAGATTAGATACCCTTGTAGTCCGGCTGACTGACTATGAGCT

>38d8c05e93ab4369017bed868655f461

AACAGAGGATACAAGCGTTATCCGGATTTATTGGGTTTAAAGGGTGCGTAGGTGGTTTTTTAAGTCAGTAGTGAAATCTTAAAGCTTAGCTTTAAAAGTGCTATTGA

>992ae5f64cf20249b18e3083f11ef951

CCCACCGCTGTGTTCAGTGTGATATACTTTTTACAATACACAATAACAGATGCCACAAGCCACACACACATACATAGAAAGTCGTAGAACGCAAACACAGACATGAA

>d6bcd35d1d1076b6411ab440271707e1

CCACCACCCACCGCTGTGTTCATACTTTTTTTACAATAACAGATGCCACAATTCACACACACACTTAATGTACATTAGATACCCTAGTAGTCCGGCTGACTGACTCT

>327be5631fb1375b8b3413f8de8ecc05

CAAATTTATGTCTTCAGAAAGACAAAATATTTCTCCAGGGTCCATTTGAACCATGTCGGTCATTTTCAACTAGCAGTGCCTTGTAGATATTTGTGAAAGATTTTGAA

>7bce454c411bab3f86c706a46d893c0a

TACGTAGGGTGCAAGCGTTAATCGGAATTACTGGGCGTAAGGCGTGCGCAGGCGGTTATGCAAGACAGAGGTGAAATCCCCGGGCTCAACCTGGGAACTGCCTTTGT

>45f9a4139f8e232c00e567191cca556f

TCTGTTTCGTATGCTCCAATCTCAGTTTTACTCTTTCCTCTGCAGTTCTTCCCCCTCTCCTCTACTCCTTTAATCACTCTATGAACCAATCCTGAATGTCTCAAGAC

>6e9ed6ce747bc58b5fa661b565d139b3

TACAGAGGGTGCAAGCGTTAATCGGAATTACTGGGCGTAAAGCGCGCGTAGGTGGTTTGTTAAGTTGGATGCGAAATCCCCGGGCTCAACCTGGGAACTGCATTCAA

>5bf82ffd9810ee4a809d275857930007

CCACTGTGTTGAGTGTGATATACTTTTACAATACACTATAACATTAGAAACCCTAGTAGTCCGGCTGACTGACTCTACGACCATCTCGTATGCCGTCTTCTGCTTGA

>ee22d5d4abadb3a39a6c7e82ab029be7

CTCTCTTTGATCTATTAAAGTTTTATTAGCTTATAAGCTACTTGCTATCTGCCATGCATGTCATGTCCTTTATTAGATACCCGTGTAGTCCGGCTGACTGACTCTAC

>953b69f93416c833dbbd750a2d32c628

TACTGTATCCAAGCGTATAGACATCAAATTGATTGACAAATCTATCATATTGTCAAAGTATAGTAGGTATAAAATCATATAACTCGTATCACCTTCGGAAAAATAGT

>a75937ef63adef7202638f94189836f3

TACGTGAGAGACTAGTGTTATTCATCTTAATTGGGTTTAAAGGGTACCGAGACAGTCAATATAACTTCTATAATGCTAATACTTGACTAGAGTTTTAAGGAAGAGGG

>c1b436ddb7a77c8c399edfd846d71046

CTACTGATATACTTTTACAATACACTATAACATTAGAAACCCGGGTAGTCCGGCTGACTGACTAGTCGCAGATCTCGTATGCCGTCTTCTGCTTGAAAAAAAAAAAA

>bc4518d349fa04b068aea91650aa362b

GACGGGGGGGGCAAGTGTTCTTCGGAATGACTGGGCGTAAAGGGCACGTAGGCGGTGAATCGGGTTTAAAGTGAAAGTCGCCAAAAACTGGTGGAATGCTCTCGAAA

>e95fb010e0c8fe50ecd91a8732880eb1

TGTAAATTGTCATATATAAGGTAACCACTATGAGACTTGGTGAATTTCTCATTAGAAACCCTGGTAGTCCGGCTGACTGACTATGAGCTCATCTCGTATGCCGTCTT

>9490139fd4d7e861e6bad6d15c7ca2f9

TACGATTTCTTTAATTTAAATAGTTAAGTTTCAGTTAATATATTAATAATATAAAATAGCTGTAATTTTGGTGAAATATATTTTATCTTAAAAAATTATTTTTATGT

>c80901df1a412c064f0985feadc6a7fe

TACGTAGGTGGCAAGCGTTGTCCGGAATTATTGGGCGTAAAGCGCGCGCAGGTGGTTTCTTAAGTCTGATCTGAAAGCCCACGGCTCAACCGTGGAGGGTCATTGGA

>e87e2324f996263075077433a82e70bd

CACTTTCGTATTATATCGAGACGGTGCCTATTACGCCCTAATTGTGCTCTGTCTTAGCTCATTTTACTTCAGATCACGCCAGATTAGAAACCCCTGTAGTCCGGCTG

>3523474d986d69ab5bfa04c7381cf85a

CAATAGAGCGTCACCGTTTTCGAGGTGCTGTGGCCGAGACCGACAAGGGGCGCAAGTAGATCCCCTCGCGGGAGGCCGGGCCTGGACAACAGCGCATCCGAGGTCAC

>5d8dea53428216bebee5051d1bae699d

ACAGCCTCTCTGCAAATTCCAGTGGTTTGTATATATTACAGATTCTTTCAGGTAATGATGGATCTACAGTTGAACTCCGCCTCCGAACGGCTATGTAAAGAGGTTTA

>4898459f3bae15b3c7b6605de37bb145

CCACTGTGTTCAGTGTGATATACTTTCAGAATACACTATAACATTAGATACTACAAGCCACACACACACACACACACACACACACACACACACACACACACACACAC

>32fcd4fcc5adfd707bf697d20f8e872d

CATGGTCATCCAGCCAGATTCCGTATTTATGTACGTGACGCTTGACTTTATACACGTGGAAAGCGATGCTTTAACCACCGGGTAAATTGAGTTATAATAGTTAATTT

>a6a0169e7f786810b384b6f437a80432

CCATGACGGTATGAACGCCGCCATACCGCTACCGTGTACCGCTGTCTGTGTGTTTGAACCTTAAGACTATCGTCAACACTTACGTGTGCCGGTAATCACCTACGCGG

>911b5748c5c3400550fcfb4f692d13c3

TACGGAGGGGGCTAGCGTTGTTCGGAATTACTGGGCGTAAAGCGCGCGTAGGCGGTGTGTTAAGTCGGATGTGAAAGCCCAGGGCTCAACCCTGGAATTGCATCCGA

>92864cfddc2c3e9f4b9cf821b0867ea2

ACTAAGCTCAACCACTGGCCCTTATGTGCCAATTAGATACCCCTGTAGTCCGGCTGACTGACTGTTACAGCATCTCGTATGCCGTCTTCTGCTTGAAAAAAAAAAAA

>3ae9ad3b5892ad8c7edbbac38914ad4f

TACGAGGGGGGCAAGCGTTGTTCGGAATTATTGGGCGTAAAGGGCGCGTAGGCGGTTTGGCAAGTTTGGTGTGAAATCTTCGGGCTCAACTCGAAGTCTGCATCGAA

>0fa1aa2a3b9f098d0de1716c960f55ad

CCACCACAACCCCCCACCGCTATGTTCATACTTTTTTTTTTTTTTACAATAACAGATGCCACAATTCACACACACACACACACACACTTAATGTACATTAGAAACCC

>efb10276af4dba4efee9d31e7e92a07b

TACGATTTCTTTAATTTAAATAGTTAAGTTTCAGTTAGTATAACAATAATATAAAATATATATAATTTTGGTGAAATATATTTTATCTTTAAAAATTAATTTTATGC

>9d7765abf9d2379458688fe1ce5bcdcb

CATTGAACTATCGTGAGAACGTCAAGCCGCCAAAGGGAATTATATTATAGTAAATATTGGCGTAAATAAACATTTTATTAATAGTTGTAATATATGAAAATGTGCAG

>d720158cd3e1ae8bbfee431d73e281e7

AACGTAGGGGGCAAGCGTTGTCCGGAATTACTGGGCGTAAAGCGCGCGCAGGCGGTTCCTTAAGTCTGATGTGAAAGCCCACGGCTCAACCGTGGAGGGTCATTAGA

>9025d89b456922b57d21591f30ec60e8

GTTTAGAGAGAAATTAAAAGGAGCTTTAAGTTAAATTTTTCATATTAGCAAAAGGTCCAGAGATAGACTACACTTAAATGAAGCCATGCAATGTACAAGAAGTATAA

>983d3ab673fed62de02ef3efe49e548e

AGGATATTTTAACTACGGTATAATGGATGCACCTGAAAGAAAGGAAAAAGTAAGACAAGGAAAAGACAACATAATTTTACACCAGCCACGAGGGTTCCACTGTACGT

>6c88ff23e99963ce83d6d8237a3f0288

TGACTGGTGGCTTCTTTGGAACCTGGCCAGAACGCATTTGAAACTCACTACACTGTTGTACTAACATCGTTAAATTAGAAACCCTGGTAGTCCGGCTGACTGACTAT

>3cabaf37d339dc0305121e77095607de

TGGATCACCTACCGCCTTCTGGATTATGCATCGAGTATAATCGTGTAATTGTGGGTGCTTAGAAAATATTAGATACCCGAGTAGTCCGGCTGACTGACTATAGCGCT

>865c2213b237eafb607b1574c41d4ce4

CATTGAACTATCGTGAGAAAGTCAAGCCGCCAAAGGGAATTATATTATAGTAAATATTGGCGTAAATAGACATTTTATTAATAGTTGTAATATATGAAAATGTGCAG

>b29c0fa6bd7a167bc25a26ee4cfbdb80

TACGTAGGGGGCGAGCGTTGTCCGGAATTATTGGGCGTAAAGGGCGCGTAGGCGGATACTTAAGTCCGGTGTGAAAGATCAGGGCTCAACCCTGAGAGTGCATCGGA

>04f48bd175816e2982a4d347acac7905

TGGACTCCGAGATGTCTGATAATGAACAAGAAACAGATGGTGAATTAGAAACCCGTGTAGTCCGGCTGACTGACTTACTAGGTATCTCGTATGCCGTCTTCTGCTTG

>8d84fc594df8bdb3f282af38c4314a67

CCCACCGCTGTGTTCAGTTTGATATACTTTTTTTACAATACACAATAACAGATGCCACAAGCCACACACACACACACACACACACACAGAAAGTCGTAGGACGTAAA

>f71d2c295dc2cd9bfcf270ae1bd9b5a6

TTGGCTGGGTGCTTCTCTCCACAGTTTGCGCACTTTGGGTCCTCCTCAGGTGACTTTTTACAGTCACCCTTCGGATTAGAAACCCTGGTAGTCCGGCTGACTGACTA

>7bd662e8574307101361ae8a88f54151

CATGGTCATCCAGCCAGATTTCGTATTTATGTACGTGACGCTTGACTTTATTCACGTGGGAAGCGATGCTTTAACCACACGACCGTACCACCGGGTGAATTGAGTTA

>f4c5c497ebbc156edba7114355f87ca2

GGTGAAAACACAGTTCGACAACGCGTTTTAATTACGTTCAGCGGTCACGTGAATACAAATTAGATACCCTGGTAGTCCGGCTGACTGACTATAGCGCTATCTCGTAT

>7cf872b2a3f3405b49181801242ba010

TACGTGAGAGACTAGTGTTATTCATCTTAATTGGGTTTAAAGGGTACCTATACAGTCAATATAATTTCTAGAATGCTAATACTTGACTAGAGTTTTAAGTAAGAGGG

>59ad0e61d4c6f3408466ae500fa9b0e1

TGGACTCCGAGATGTCTGATAATGAACAAGAAACAGATGGTGAATTAGAAACCCCAGTAGTCCGGCTGACTGACTTACTAGGTATCTCGTATGCCGTCTTCTGCTTG

>23be95ee22c784c68ff83163b0ff70fc

TTCCAGCTCCAATAGCGTATATTAAAGTTGTTGCGATTAGAAACCCTAGTAGTCCGGCTGACTGACTTACTAGGTATCTCGTATGCCGTCTTCTGCTTGAAAAAAAA

>d5f077b59731950150064f6e4fcb848a

TACTCATCACCACTAAAGAAGGCTTCCCCAGTCGTAGCCGAATGCCTGCCAGAGCAACCACATTAGAAACCCGAGTAGTCCGGCTGACTGACTTAACGTCCATCTCG

>10139fc3a2cd0e23c04263c44e1ba33f

AAAGGCCAGATTGGCTGTCCGCGGCAAATGAGCGCCGGCCGGAGCAAATGCGATTACCCTGGAATACCGCAACAGTCGCCAATTATCCGGTGCGCGATTTTATAATG

>6eb4dbc91fd43f91d7f950c51054bdd4

CGAGGACGATTCACGCTGTATTTGGCATTCGGACGGTCTCAGTACTATCGGTACAGCACTTTTGTACAGTGAACGATATGAGGACGCTGCAACTATCCAAATTTCTT

>0d7558b2a0a3322969a864284a0f67c5

TACGTAGGGTGCGAGCGTTGTCCGGAATTATTGGGCGTAAAGAGCTCGTAGGCGGTCTGTCGCGTCGTTCGTGAAAACTTGGGGCTTAACCCTGAGCTTGCGGTCGA

>6f7ca18193bd1d9d06e1d3ccca3ee4c5

CACGATTTTCTAAATTTAATTGTGTTAGTTTCAGTTAAAAAAATATGTTAATATAAAATTTTTTAAATTTTGGTGGAATAATATATAAATATGTGTTTAATTTTATG

>4971ecc8fdfedde39d4dbb897df4e57c

TACGTAGGGGGCAAGCGTTATCCGGAATCATTGGGCGTAAAGGGTGCGTAGGCGGTTATGCAAGTTAGAGGTGAAAGGCTACGGCTCAACCGTAGTAAGCCTTTAAA

>59d46125ac3d3fda380edb34341a0b66

TACGGAGGGGGTTAGCGTTGTTCGGAATTACTGGGCGTAAAGCGTACGTAGGCGGATCAGAAAGTTGGGGGTGAAATCCCGGGGCTCAACCCCGGAACTGCCTCCAA

>27075e3ca73ec0122f343bd24e7b5948

TTGTCCACAAGGCAAAAGGAGAGTCGGACGCCGAAGAACAAGATGGAGAAGCTGCATTGGACAATGAAGTTTGGTGCAGACGGGAGAATCTCCTAACTTATGAGTAG

>33e274be0b31c6a5039b217c45b5ff24

CACGGGGGGCGCAAGCGTTATTCGGAATTATTGGGCGTAAAGGGCGCGCAGGCGGTCTTGTCCGTTAGGTGTGAAAGCTCGGGGCTCAACCCCGGAAGTGCACTTGA

>56c36d44a67da0d0bc1c3b7ab4696181

TAAACGGCAGCCGCAGCCGTCATGATATTTACTGTGGCAATTTGTTCCACATATTACGATGAATGCATTAGAAACCCCGGTAGTCCGGCTGACTGACTCTACGACCA

>04bb454c44740ec883531abb171acebb

CGGTTTACCAAGTATACCGTTGGGCAACTTTATGCGTTCATTTTGGGTGTTAAAACTTAAAAATCTCATCTTTTTTTTATCGTTAAGGGATTCAGGATCCAGCGAGT

>70b46fd8a96e291c09013cb4ef0a179d

TACGGAGGGTGCGAGCGTTAATCGGAATCACTGGGCGTAAAGCGCGCGTAGGCGGCGCGATAAGTCAGGCGTGAAAGCCCTCGGCTCAACCGGGGAATTGCGCTTGA

>449a78dc9e2413ff1c78ae064e33dca9

AGAAAGAGTAGAAGGTGGAGGTGAGACGTTGAAGAAGAAGAAGCAGTCAATACAAATACACAACACACACGCACAGTTGGTCCTTTTCGAGGGGCCAGCTTTAATTC

>5e3006f1026da5429ec0af6a5e0d4f5c

CGCAAAGTCAGGTCAAAACGTTTTTTGAAACTGGATCATCACTTACCAATCCAGGTAAGGATTGGGGGTTATACTGAAAAAGCAAAATCGTCAGTAGTAAATGCAGG

>e0fdd1813632a01ee2c3033e61470abe

TGGACTCCGAGATGTCTGATAATGAACAAGAAACAGATGGTGAATTAGAAACCCTAGTAGTCCGGCTGACTGACTACGTACGTATCTCGTATGCCGTCTTCTGCTTG

>06842f513d40c4400c967ebc355edc80

TACGTAGGGCGCAAGCGTTGTCCGGAATTATTGGGCGTAAAGAGCTCGTAGGCGGTTTGTCGCGCCTGCTGTGAAAACGCGAGGCTTAACCTCGCGCCTGCAGTGGG

>628608a5b4f60f78de2183a480c93a2a

CAAAAATTATACAACGCCGCACATGTTCATATGTGTATGCCTAATCCTTTTGGGGGTGAATAAATAATATAACAGATAGGGTTTTGTGGCACAGGAGAGAGTAACCT

>fbc8fd45bdb6320fcb2b775a17018a11

CCACTGTGTTCAGTGTGATATACTTTTACAATACACTATAACATTAGAAACCCCTGTAGTCCGGCTGACTGACTACGTACGTATCTCGTATGCCGTCTTCTGCTTGA

>b588a0ea914875f628e72a4ed202bf8c

TACGTAAGAGACTAGTGTTATTCATCTTAATTAGGTTTAAAGGGTACTCAAACGGTCTGCTTCGCATAAAAAGACTAGAGTTATATAGAAGAAGGTAGTACCTTAAG

>36c270f0cf5ff9bc248a95bef4db235d

TTCCAGCTCCAATAGCGTATATTAAAGTTGTTGCGATTAGAAACCCCTGTAGTCCGGCTGACTGACTCTCTAGAGATCTCGTATGCCGTCTTCTGCTTGAAAAAAAA

>a74d4f36c25c9b58a9e1dcc07ed3f724

AAATAATAGAATAGCCTCTCATGCGGACTGTACGAAGCTCCGAGCGCCGAAATTAGTTTGTACGAAACCTTCAATAAATTAGAAACCCGAGTAGTCCGGCTGACTGA

>ba01645f8c84eb2682ba28d645a5981a

TACGTAGGGGGCAAGCGTTATCCGGAATTATTGGGCGTAAAGAGTACGTAGGTGGTTATGTAAGCGTGGGGTGAAAGGCAATGGCTCAACCATTGTTAGCCTTACGA

>e6bd97b3118eb2bfeda18f5768abb918

TGACCGTAGGTTCCTTCGTTTCCGTACCGACGTCGTAACGACACCTACAATTGACAATTTATAAATTAAGTTAATTTATAACAGACAAAATACAAATTTTATACTTG

>4dbf53ed671461cf9807637d5e056fed

GTGCTCGAAGAACATCAATCTTTCACTTCGTTCTCACTTTCTCTTTTTACTGCCCCCTCTTCTGACCCTTTTCCAACATACCCCTTTCCCTCCTTTTCGTCATCCCT

>1734f9b9c9f4ec49d94c2689216a16a6

TTGGCTGGGTGCTTCTCTCCACAGTTTGCGCACTTTGGGTCCTCCTCAGGTGACTTTTTACAGTCACCCTTCGGATTAGAAACCCTTGTAGTCCGGCTGACTGACTA

>bc80b07acf5725602dec41dd18e8a9f5

TACGGAGGGTGCAAGCGTTACCCGGAATCACTGGGCGTAAAGGGCGTGTAGGCGGCCTGCCAAGTCTGGTTTTAAAGCCTGCGGCTCAACCGCAGATCTGGACTGGA

>1b550f842302e1c3e4a9f7bf840b1697

CGGGTTGAGGGGGACTCGTGTCCATTAGCATGGCATTATGTAGTAAGTGTGGCAGCATGCTCAATGAATTATGCTGCCCGCTTTGGAGTCGCCCCAGTTGTTTGCTT

>bd4832969ee610feda002e8701a2c071

CAGGGTGTTCTGCCTGGGAACAGAAGAACTGACTAGTGGTATTCAAGACAACTAAACGGGCTATGACAAAACTTATAAAAAGTATTAGATACCCTGGTAGTCCGGCT

>10e786057f01234a1783dd4fdff202df

TGTAAATTGTCATATATAAGGTAACCACTATGAGACTTGGTGAATCTCTCATTAGAAACCCGAGTAGTCCGGCTGACTGACTTGCGTCAAATCTCGTATGCCGTCTT

>8309396fa263b06203b8870f22ab4dad

GAACAGCTCATGCTTTCCTCCCCAGCCTTTCTCTCCCAGCTTCTATTAGATACCCGGGTAGTCCGGCTGACTGACTTGCGTCAAATCTCGTATGCCGTCTTCTGCTT

>8898af45c7c5318d82b5b22170720d59

TGAAGTCATTCAGAGTCTTCGCGCGAGCTGCCGGGTAATTAACTTTAATTAAGACAGTTGTGTATACATATACTATACACGACGAAGACGGCATTGTACCGACTGCG

>a5afd0e42d71c638d9b3656a36b7c5e7

TACGTAGGTGGCAAGCGTTGTCCGGAATTATTGGGCGTGAAGCGCGCGCAGGTGGTTTCTTAAGTCTGATGTGAAAGCCCACGGCTCAACCGTGGAGGGTCATTGGA

>ac72b60767371f89918b2d78d25c0682

TACGTAGGTGGCAAGCGTTGTCCGGAATTATTGGGCGTAAAGCGCGCGCAGGTGGTTTCTTGAGTCTGATGTGAAAGCCCACGGCTCAACCGTGGAGGGTCATTGGA

>d921dee0b88f39fb2f5db0bad5827344

CTCAAATCCCTCAATTTAAATCTATAAAGCTTCAGTTACTATATAACATAATTTTTATAAATAATAATTTTGGTGAAATATATTATTATTTTATTTGTATAATTTGT

>b3396ae1536670efc03782b8c0dc4f62

GACAAGATAGGAAAATTGCTATATCTGTGGCTTCGTCAAGTATAGCAGCAACGCTATTAGATACCCTAGTAGTCCGGCTGACTGACTCAGTAGGTATCTCGTATGCC

>1ab058f4c75e61f467208478a0ae670b

AGGACTTGGAGGAACGACTCTGATAAACTATATGTTGTACACCTGAGGCAGTTCACGGGATTATAACAAATATGCCCAAGACGGAAACTATGGTTGGAGCTTTAAAG

>2cf231bcc0b74ccb7e646ba93a4c76f8

CACACACGGTAAACACAGAACGACGCTGAATGAATAAATCGCAAATATATTAATAAATTCACTTTTTATACATACGCCCGATTTCCTTGAGAAATCGCGGCACACCC

>f1f6aa1ac6be6b5a4296f48bee9cae71

TACGTAGGGAGCGAGCGTTGTCCGGAATCATTGGGCGTAAAGGGCGCGTAGGCGGTATTTTAAGTCAAATGTGAAAATCCAGGGCTTAACTCTGGGACTGCGTTTGA

>efd6be6854f01473bbfca3a1f2404fc2

TGGACTCCGAGATGTCTGATAATGAACAAGAAACAGATGGTGAATTAGAAACCCGAGTAGTCCGGCTGACTGACTACGTACGTATCTCGTATGCCGTCTTCTGCTTG

>8685ebe9ad42e1450c8fd7e21aef347c

CGTGCGTCCACTAGCACGCTGACAGCTGTTATATGTTACAGCACCTGTTACTACTCTACATTGACCTCGCTAAAATATTACCGGTAATATTAGAAACCCGTGTAGTC

>523d9e0e3c902a1887ab6dfc8fc1f22f

TCTCAGTTAGACTTGAACTATATTATATATTTAGAAAGGAAACCACCATACCCAGTGACAAACTGCATTAGATACCCTAGTAGTCCGGCTGACTGACTATAGCGCTA

>65197a999c4b0f96f0f72296c21d42e1

AAATGATTATTTATAAGCCCTTAAGCGGTCTAACACCCTTCCATTAGATACCCCTGTAGTCCGGCTGACTGACTATAGCGCTATCTCGTATGCCGTCTTCTGCTTGA

>14658e0e3e2af7a8d2236d01fe878672

CCACCCCCACCGCTGTGTTCATACTTTTTTGACAATAACAGATGTCACAATTCACACACACACACACTTAATGTACATTAGAAACCCGGGTAGTCCGGCTGACTGAC

>00405cbdce42019ea5645ab318fed242

TACGGGGGTGGCAAGCGTTGTCCGGAATTACTAGGCGTAAAGGGCAGGTAGGCGGATATTCAAGTCAGGTGTTAAAGGCATCAGCTCAACTGGTGTACGGCATTTGA

>00c4a010dda81379e49124c3c58e53d5

TACGTAGGGGGCGAGCGTTGTCCGGATTTACTGGGCGTAAAGGGTGCGTAGGCGGTTCTGCAAGTCAGGTGTTAAAGGCATCGGCTTAACCGATGTAAGCATTTGAA

>ec2537a1943510d370bc8dd695d623ab

TAAATGCAATCTTTGATAGTAATCAGCAAAAGTTGTGATTAGAAACCCTTGTAGTCCGGCTGACTGACTATGAGCTCATCTCGTATGCCGTCTTCTGCTTGAAAAAA

>daa29dc9757656e402707765d8da9c1c

CTAAGTTACGTTCAGTTTACCCTAAATTTATTTTTCAACTCGCTTACTTCACGATTCTCATTTGTCCTCATTTTCTACCCACAATTCGTTGCGGCACGCATGCGCAG

>636df8b6877ec5ef8585129cbfac5cef

CGGTAGTGCCGCACATTACTGTCGGCCATACTCTGTAATTATTGCAATTACAAGTTGTTGTTAATTTATGTGCGCGTGGCGTTTTAATTACACGTACATAATGTATG

>8dc1c71b676b09013af4dd6178fd0430

TTCCAGTTCCAATGGCGAATATTAAAGCTGTTGCAATTAGAAACCCCTGTAGTCCGGCTGACTGACTCAGTAGGTATCTCGTATGCCGTCTTCTGCTTGAAAAAAAA

>f41320193cd175810a229968f650d020

GACAAGATAGGAAAATTGCTATATCTGTGGCTTCGTCAAGTATAGCAGCAACGCTATTAGAAACCCGGGTAGTCCGGCTGACTGACTCAGTAGGTATCTCGTATGCC

>cd11aa25a43707aa771b1ca78f1db2d0

GGAGAGTACACCAATATTGTAAAGAGTTGGGATGTCCTAAAACAATTATTACTGACAATGGAACGCAGTTCACCTCTAGAAAATGGTTAAACGGCTTAAACGAATTA

>57601aaa454b900ba8bd82eeb852dd2c

TACGTAGGTGGCAAGCGTTGTCCGGAATTACTGGGTGTAAAGGGCGTGTAGGCGGGGATGTAAGTCAGATGTGAAATTCCGGGGCTCAACCCCGGAGCTGCATCTGA

>6fe4475dc0c595f86105ba8d46b9dde5

TACGTAGGGGGCAAGCGTTGTCCGGAATTATTGGGCGTAAAGCGCGCGCAGGCGGTTTCTTAAGTCTGATGTGAAAGCCCACGGCTCAACCGTGGAGGGTCATTGGA

>a6895a1c4b4a31a489bddae5b0b501c8

CAACTTTTTAATAACAACATACAGCCGTTCATTAACCTCCAAAATGTTAATTACCTCTAGATATATATAGGGTAATTACATCATCTTCCTCCCCCAGTAATGAGCCA

>084fdddb67edc16e38fcc979365fe427

ATAGCCTGAGATATTACATATAGGAAAGATGAAGATATTGGACAAAAGTTAAATAAATTTAGTTATATCTGAGGAAGTTAAAAGAATAAGGCAGGGCGAGGGATAGA

>2af6dad5de32cd0ac68b945c77fd7fe0

CCACCCTCATCGACGCAGCAGATAGTCACCGCTCGCCGTAAACACGTCTTGGAATGAACATCAACCCTCACCCCACCGCGACATATTACGGATTGTAGACAAACCAA

>152ad307cf494c948e5ca4f05474127d

CACGGGGACCACACGAGGCTCCATTGTGTCCGGCTATTTGTGTTTCAATTAAAAGTTTTCATTATTAAACTCGCTACTCACTAAAAGCTGCATATCCCATAATGTGT

>3f76b4b5d0c79681a1a99545fdd7f337

GGAACAAAGACTGTGAATGAACAAGTTATGTACAAATACACGCGGTAGCCGAGTGCGCGCTGGAGAGCTTTACGACCAGAACCCCTGATGGCATTAGAAACCCCAGT

>76534911a07a9e6cb9960810ec09ea89

TACAATTTCTTCAATTTAAATTTTTAAAAGTTTCAGTTAATAAATTATTTAAATTTTTGTGAATAATAATTTTAGTGAAATGTATTATTTTATTTTATTAGTTTGTC

>03cce302bbe3cbcdd3e9565a7ed89d14

CCGTTACCGGTAACAGTGACTGCTGCAGGGACGGTTACAAAGTACGCGTTCCAGGTATCTGTTACCGCGTAACTGCTACATTAGATACCCTAGTAGTCCGGCTGACT

>36552a2e7050d4193d1103f4684e2e7e

TACGATTTCTTTAATTTAAATTATTAAGTTTCAGTTAAAAATAATAATAATATAAAATAACTATAATTTTGGTGAAATATATTTTTTTCTTGAAAAATTAATTTTAT

>2db81dbec4bebde349af5337cf0f6c98

CCACCCCCACCGCTGTGTTCATACTTTTTTGACAATAACAGATGTCACAATTCACACACACACACACTTAATGTACATTAGATACCCCAGTAGTCCGGCTGACTGAC

>877047aad83dc1605e05671104dd7aea

TGTCCGCACCTAATAGGGCATCAACGGGAGCGGGTCGGTCAAATGTCGGATTGGCAAGAACCAGGTCCTTAGCTGCACTCTTAAACCTCTGAAGCTATTGCTGTCAG

>4ed8b9d7a16eb4b7bdde79353e31e591

TCCTTTGTCATGATCATCCGCGCACCAAGGAAGTCATCGACTTTATGCCGGCAGCCATTCGGTCGTCAAAAGTTATTCTGCATGGATAGGCTTTGTTACGTTCATTG

>ef4a364fe7ea7e03f1f6b17acbd050f0

TACGGAGGGTGCAAGCGTTAATCGGAATTACTGGGCGTAAAGCGCGCGTAGGCGGATGATTAAGTCAGATGTGAAAGCCCCGGGCTCAACCTGGGAACTGCATTTGA

>c6d45ffa9597cf8d8cfd38cd8b3b85b9

CACACCCTGAAAGACAAAATATTTCTCCGGGATGCATTTCAACCATGTCGGTCATTTTCAACTGGCAGCGCCTTGTTGTTATTTGTGACATATTTTGAATGTGTCTT

>1573e85355a93ad03d2c64d639a98251

TACGAAAGGTGCAAGCGTTAATCGGAATTGCTGGGCGTAAAGCGCGCGTAGGCGGTGTGTTAAGTCGGATGTGGAAGCCCAGGGCTCAACCTTGGAATTGCATCCGA

>4f71ddbee9fd78fb608e570e537a8328

CACGGGGGGCGCAAGCGTTATTCGGAATTATTGGGCGTAAAGGGCGCGCAGGCGGTCTTGTCCGTCAGGTGTGAAATCTCGGGGCTCAACCCCGGAAGTGCACTTGA

>1df7ade0e264a717f244c02c89600904

CCTCCCCACCGCTATGTTCATACTTTTATTTTTTACAATAACAGATGCTACAATTCACACACACACACACACACTTAATGTACATTAGAAAAACACAAGTCACACAC

>3a0a1df540ca4fe6148e2fd895413de1

TACGTAGGGTGCAAGCGTTAATCGGAATTACTGGGCGTAAAGCGTGCGCAGGCGGTGATATAAGACAGATGCGAAATCCCCGGGCTCAACCTGGGAACTGCATTTGT

>7a7485e4f5794c13973519a138a67d31

ACTTTGCGTCAGACAATGTTATTTTTTATGAACAATGGTTGGTAAACTTTTTTTCTCGCTATAAATTAGAAACCCGAGTAGTCCGGCTGACTGACTTGCGTCAAATC

>8344c91dc88e1e25cb172c7566d3d9ea

TACGTAGGTGGCGAGCGTTGTCCGGAATTACTGGGCGTAAAGGGTGCGTAGGCGGATGCCTAAGTCAGATGTGAAAATCCAGGGCTCAACTCTGGAACTGCATTTGA

>7974060e9e4df1abd79e9324f115a7a7

TACGTAGGGGGCAAGCGTTATCCGGAATCACTGGGCGTAAAGGGTGCGTAGGCGGTCGACAAAGTCTGGGGTGAAAGGCTACGGCTCAACCGTAGTAAGCCTTGGAA

>59d5d19ecc70e8ee760e9258d134e5ad

CCACCACCACCACCACCACTGTGCTGAGTGTGATATACTTTTTTTACAATACACAATAACAGATGCGACAAGCCCCCCCCCCACACACACACACACACACATAGAAA

>82ee4b714cb3488c19ab2314ca23b3ea

TTCCAGCTCCAATAGCGTATATTAAAGTTGTTGCGATTAGAAACCCCGGTAGTCCGGCTGACTGACTTACTAGGTATCTCGTATGCCGTCTTCTGCTTGAAAAAAAA

>6589fede61b98d3748e03bf5e852cb41

CTGGAGACCGTCTGCAACATGTTTGTGAAGGTAAACAGTTACGTGTATTATTGCGTGTATTAGATACCCCAGTAGTCCGGCTGACTGACTCGCGATATATCTCGTAT

>a89026197b832c366b8cc95a63ff0fe5

GACGGGGGGGGCAAGTGTTATTCGGAATGACTGGGCGTAAAGGGCACGTAGGCAGTGAATCAGGTTGAAAGTCAAAGGCGCCAGCTCAACTGGCGGAATGCTTTCAA

>dba082cbe0519b8ca0896f9044cf3dbc

CACAATAGCACACAAAAACAGATTGGGCATCACACTGCGAAGAGAGAAGGAAGGGAATGCGTACTGGCAACCTTGCACTATTATTTGTATATCACTGGCTGTGACTG

>b7abd47b16c9e32d9e6e5594e9b8057e

TACGTGAGAGGCTAGTGTTATTCATCTTAATTGGGTTTAAAGGGTACCTAGGCAGTCAATATAACTTCTGTAATGCTAATACTTGACTAGAGTTTTAAGTAAGAGGG

>32d9b9e42558ab17d2001c5c7a9ea75a

CGGTTTACCAAGTATACCGTTGGGCAACTTTATGCGTTCATTTTGGGTGTTAAAACTTAAAAATCTCATCTCTTTTTATCGTTAAGGGATTCAGGATCAAGCGAGTA

>68af16de16f028c0604b451c8f9c8614

TACGAGGGGAGCGAGTGTTGTTCGGTTTTATTGGGCGTAAAGGGTGTTCAGGTTGTTCAATAAGTTGATCTCCAAATCTTGGAACTCAACCCCATTCAAGGGGTCAA

>5ecfc6b3753338e78b48d88aea7bff2a

GGTGAAAACACAGTTCGACAACGGGTTTTAATTACGTTCAGCGGTCGCTTGAATACAAATTAGATACCCTGGTAGTCCGGCTGACTGACTATAGCGCTATCTCGTAT

>a511c9c62a94c35c39066dd621c0dadd

CACGATTTTCTAAATTTAATTATGTTAGTTTCAGTTAAAAAAAGTGTGTTAATATTAAGTTTTTTAAATTTTGGTGGAATAATATATAAATATGTGTTTAATTTTAT

>735bdbc2a35b92c3c8342b9c7c589dbc

TTCACAACAATATTATTCTTTGGTATAACATATGCCTTACTCTAATTAGATACCCGAGTAGTCCGGCTGACTGACTAGTCGCAGATCTCGTATGCCGTCTTCTGCTT

>5a2b920bd2329a18de5d3192ce4a48eb

TACAAGGAAGACTAGTGTTATTCATCTTAATTAGGTTTAAAGGGTACCTAGACAGTATTTCTAGCCTCCAAAGGGAACAGACTTACTAGAGTTTTATGTGAGAGGAA

>838d90da604ce869dca16e32a812d3c7

GGAGGAGGTTAGGTTCGTCATATACTTTTACAATACACTATAACATTAGATACCCCGGTAGTCCGGCTGACTGACTCTACGACCATCTCGTATGCCGTCTTCTGCTT

>5e0261044b8213387fa483ed37436472

TGTTGATGTGGCACCTGGAGACGCGTGGTTATTGGCGAAACGTTGCGTTTGTTCTTTGCCAACGGTATCAACCGTATAAGAATACGTTTACGCTTAAAATAATTATA

>416171d05e4d9abd4e72aec42e1e1fae

TACGTAGGGTGCGAGCGTTGTCCGGAATTACTGGGCGTAAAGAGCTCGTAGGTGGTTTGTCGCGTCGTTTGTGTGAGCCCGCAGCTTAACTGCGGGACTGCAGGCGA

>a60a222897c51b360f2000c466c40a2d

TACGAAGGGTGCAAGCGTTAATCTGAATTACTGGGCGTAAAGCGCGCGTAGGTGGCTTGATAAGTTGGATGTGAAAGCCCCGGGCTCAACCTGGGAATTGCATCCAA

>60509b25dfdb427e631abbe6adecbe41

CAACAAAGACAACCCAGACAGTAAAAAGGAGCAAGGAAGGGAACCCTGTTTGCTAAATCTACGAGCATTAGATACCCGGGTAGTCCGGCTGACTGACTATGAGCTCA

>27e20bf0af2099027175b7488a67a699

GTGCTCAAAGAACATCAATCTTTCACTTCGTTCTGACTTTCTCTTTTTACTGCCTCCTCTTCTCACCCTTTTCCACCATACCCCTTCCTCTCCTTTTCTTCATCCCT

>e4ac90f800f505a438d52de10c49f261

CATTGAACTATCGTGAGAAAGTCAAACCGCCAAAGGGAATTATATTATAGTAAATATTGGCGTAAATAAACATTTTATTAATAGTTGTAATATATGAAAAAGTGCAG

>e8a511cc948c89a5054300afd730435c

CATGGTCATCCAGCCAGATTCCGTATTTATGTACGTGACGCTTGACTTTATACACGTGGAAAGCGATGCTTTAACCACCGGGTGAATTGAGTTATAATAGGTAATTT

>e04ba759088326d84cf227c28950bdca

TACGTAGGGTGCAAGCGTTAATCGGAATTACTGGGCGTAAAGCGTGCGCAGGCGGTTATGCAAGACAGATGTGAAATCCCCGGGCTCAACCTGGGAACTGCCTTTGT

>61afa12c57832ed408edf5c94b6d9429

TACAATTTCTTCAATTTAAATTTTTAAAAGCTTCAGTTAATAAATAATTTAAATTTTTATAAATAATAATTTTAGTGAAATGTATTATTATTATATTTTATTAATTT

>0b48c3315ce380cce288e81a576c7e0b

ACAGCAACCCCAACTTGGTGTTCTGAGCGGGCTTGCTCAGACTACAGAGATGACAGAACTTCACCCGGGTGGCGATGTCCCGCCCATGCCTTTCCAGATGAAGTTTT

>81c8b9dd78f4c29fc1776ef564634559

TACAGAGGATGCAAGCGTTATCCGGAATGATTGGGCGTAAGGCGTCTGTAGGTGGCTTTTTAAGTCCGCCGTCAAATCCCAGGGCTCAACCCTGGACAGGCGGTGGA

>ec04d8b76202931daf0e333e9883690e

TACGGAGGGGGCTAGCGTTGTTCGGAATTACTGGGCGTAAAGCGCTCGTAGGCGGATTGGTCAGTCAGGGGTGAAAGCCCGGAGCTCAACTCCGGAACTGCCTTTGA

>02855540aea4c74d59e37307fdb6b0da

TACGTAGGGTGCAAGCGTTGTCCGGAATCACTGGGCGTAAAGGGAGCGTAGGTGGTAATTTAAGTCAATTGTAAAAGATCATGGCTCAACCATGGTATGCAGTTGAA

>0a886b1e28d4d91093d00aad5e13905d

ATCTGGTTTGGAGCCTGTACTTGTGCACATTATTAAACTCGAGCTCATGGTAGACACACGTAAGTAAATATTCTGTAGGTTTAAAAGAGACCGTCTGATGTTGTGTG

>1fb778f70a304faa9b4d8180a29efb77

GGTTATATTCGTCATATACTTTTACAATACACTATAACATTAGAAACCCTAGTAGTCCGGCTGACTGACTGCGATACGATCTCGTATGCCGTCTTCTGCTTGAAAAA

>24ebec40ce8f86aeb29dadc125449b4b

CACTTGAAACATATAGTGCAATCAACACCGCGGGAACATTAGAAACCCTGGTAGTCCGGCTGACTGACTACGTACGTATCTCGTATGCCGTCTTCTGCTTGAAAAAA

>f2b995fba1b31e7e1b61cd229db1c0fe

CCCCGGTCGACCCAAAACTACCGACACCTGATTGACTCACTTAGTTCATCCTCTCTGCCCCACGTTGGGCGCCATTTGTCACGTCCCTTCCTGAAGATACTGAGACG

>5a5c6a915edb6a36309730f92c67117b

TACGATTTCTTTAATTTAAGTGTTTAAGTTTCAGTTAATGAATTAAAAATGTAAAATAGCTATAATTTTGGTGAAATATATTTTATCTTAAAAAATTATTTTTATGT

>bd91a264e3e1b4c875485ecfc8fe5a5a

GTCACACCACGGTCTCCGCATACCAACCGTTCTAGAGGTTATCCATGAAAGAAGCAGTAAATTTCATGAACGATTAGAAACCCGAGTAGTCCGGCTGACTGACTGTT

>8a9d1dede7ab23854f0deb6fb99139df

CCACCACCACCACCACCACTGTGCTGAGTGTGATATACTTTTTTTACAATACACAATAACAGATGCCACAAGCTACACACACACACTCACACACACACACACACATA

>2324b316d44286e8400595ed949411f8

TACGTGGGAGACTGGTGTTATTCATCTTAATTGGGTTTAAAGGGTACCTAGACAGTCAATATAACTTCTATAATGCTAATACTTGACTAGAGTTTTAAGTAAGAGGG

>1ee8dde1ef1b131e01faaffde670c70f

CCACTGTGTTGAGTGTGATATACTTTTACAATACACTATAACATTAGAAACCCCGGTAGTCCGGCTGACTGACTCTACGACCATCTCGTATGCCGTCTTCTGCTTGA

>4657929154495df090639cbd984005a1

TACAGGTTGCATTTTAGAGAGGTTATTCATGTAAGAGAAATAAATATACACGTGTTATTTACAAAAATTAGAAACCCTTGTAGTCCGGCTGACTGACTCTCTAGAGA

>b5c46d280df684fda849f2cf9cc7d7fa

TACGTGAGAGACTAGTGTTATTCATCTTAATTGGGTTTAAAGGGTACCTAGACAGTCAATATAACTCCTATAATGCTAATACTTGACCAGAGTTTTAAGTAAGAGGG

>bde9916ec4e3d18d4fbf8743037cd5ec

CCACAGTGTTCAGTGTGTTATACTTTTACAATACACTATAACATTAGAAACCCCGGTAGTCCGGCTGACTGACTACGTACGTATCTCGTATGCCGTCTTCTGCTTGA

>5131b1234c0f41d67a880021a1bd9f5a

CGTTATAGCGACTTTCCCCCTCCACCCAGTCCTTGCAGTTGTGAGGTAACGCCGGCCACCTAAGAGTCATAGCACATGCTCCGGCATCCGCCTCGAATCCAGCGCAG

>b64b72a891b208e25a04be8a1a6f861a

TTGGCTGGGTGCTTCTCTCCACAGTTTGCGCACTTTGGGTCCTCCTCAGGTGACTTTTTACAGTCACCCTTCGGATTAGATACCCTTGTAGTCCGGCTGACTGACTA

>b9342ba99b3f94527da4f3e7dc0a16b6

TCCGGCTGACTGACTTAACGTCCATCTCGTATGCCATTAGAAACCCTAGTAGTCCGGCTGACTGACTTAACGTCCATCTCGTATGCCGTCTTCTGCTTGAAAAAAAA

>703ade695f6e07052be01ea95eec6a5d

CATTGAACTATCGTGAGAGAGTCAAGCCGCCAAAGGGAATTATATTGTAGTAAATATTGGCGTAAATAAACATTTTATTAATAGTTGTAATATATGAAAATGTGCAG

>b1889c8a908053b1ccc71ad302a070c8

TATTCTCGCGAAAACGTAAGCGGCCTCGGCCGCTTAAAGTAGGCCCCGTCGAGGTGCCCTGCACCAATAGCGTCAAGTACCTCGGGCTCCACGTGGATTCCCGGCTC

>e4dd75b628ac8d63b6bb55747bc44fa3

TACGAAGGGGGCTAGCGTTGCTCGGAATCACTGGGCGTAAAGGGCGCGTAGGCGGCTGATTTAGTCGAGGGTGAAAGCCCGTGGCTCAACCACGGAATGGCCTTCGA

>1ba5a39e2e9c87a25c75158dd94b565a

ACAACAAGGGGAACCTAACAATGAGCCGCCTCGCAATCAATCAATTTTCGCCACGCCACATCAACGCAATTCGTGTAGCACAATTTTTCTTCGCCTATCTACACGCA

>3f35239f716460e11e4a757aaabacb02

ACAACAAGGGGAACCTAACAATGAGCCGCCTCGCAATCAATCAATTTTCGTCACGCCATTCGTGTAGCACAATTTTTCTTCGCCTATCTACACACACGCACGCACAC

>a78f435743b00b83c26dfa17b432ec3d

TACAGAGGTCACAAACGTTGTCCGGAATCACTGGGCGTAAGGGGCATGCAGGCGGTTCGGTAAGTCAGGTGTGAAATCTTCTGGCTCAACCCGGAAATTGCATCTGA

>8b6012e0a0efbbdac49e29bdeb54276e

TATGAGTGGTGTAAGTGTTAATCGGAATTACTGGGCGTAAAGCGTGCGTTGGTTGTTTGTTAAGCAAGATTAGAAACCCTGGTAGTCCGGCTGACTGACTATGAGCT

>069e8e218ee26c3fea863cc79d35bb92

TACGAAGGGTGCAAGCGTTACTCGGAATTACTGGGCGTAAAGCGTGCGTAGGTGGTGAGTTAAGTCTGTCGTGAAAGCCCCGGGCTCAACCTGAGAATGGCGATGGA

>290106e6e391a1138ead8bd24aaed4ab

TACGTGAGAGACTAGTGTTATTCACCTTAATTGGGTTTAAAGGGTACCTAGACAGTCAATATAACTTCTATAATGCTAATACTTGACTAGAGTTTTAAGTAAGAGGG

>4fe980e091540baa69128b980c934223

CATGGTCATCCAGCCAGATTTCGTATTTATGTACGTGACGCTTGACTTTATTCACGTGGAAAGCGATGCTTTAACCACACGACCGTACCACCGGGTGAATTGAGTAA

>1bd1625a9122c08759090e55e0d2a461

TACGTAGGGTGCAAGCGTTAACCGGAATTACTGGGCGTAAAGCGTGCGCAGGCAGTTATGCAAGACAGAGGTGAAATCCCCGGGCTCAACCTGGGAACTGCCTTTGT

>715d16d453f1b5d6829053e1f489bd85

TTCCATCTCCGGTAGTATATGTTAAAAGCGTTGCGATTAGAAACCCCAGTAGTCCGGCTGACTGACTCTATCGTGATCTCGTATGCCGTCTTCTGCTTGAAAAAAAA

>1669290e1448ca6af81c6f8bd378a401

CACATGTACAAGACCACGATACAGCGGACGCACGCCAAGCCTGAAGAGGAGACTCATATTTCAACCGCGAAAGCATAAAAAGAAACATATGTGTATATACGTATTTA

>f582acd2802a1729cae7cc2e701f55fb

CCGTTACCGGTAACAGTGACTGCTGCAGGGACGGTTACAAAGTACGCGTTCCAGGTATCTGTTACCGCGTAACTGCTACATTAGAAACCCCTGTAGTCCGGCTGACT

>39f8868d6aca1a6ea958ef8ef9b3eede

TACTGAACGTTATTACCAAAATAATAGGAATTACAATTCCAACAACTACAGTAATAACAACAGGGTTTCCCGACCACGAGTAAATTTCATAAGAGCTGAAGATAATG

>1a610fd245f4af6e7da9f30f185c56fd

TATTCTCGCGCAAACGTAAGCGGCCTCGGCCGCTTAAAGTAGGCCCCGTCGAGATGCCCTGGACCAACAGCGTCAAGTACCTCGGGCTCCACGTGGATTCCCGACTC

>23b7859df3633188f41e4c665e1d70d4

TACAGGTTGCATTTTAGAGAGGTTATTCATGTAAGAGAAATAAATATACACGTGTTATTTACAAAAATTAGATACCCGAGTAGTCCGGCTGACTGACTCTCTAGAGA

>9e634791528b8e08d46aa12cd0a52b0e

TACGTAGGGGGCAAGCGTTATCCGGAATCATTGGGCGTAAAGGGTGCGTAGGCGGCCCTATAGGTCAGAGGTCAAAGGCTACGGCTCAACCGTAGTAAGCCTTTGAA

>eb4b4138ce6d1b5c247a5e28d2b67f18

CTTCAGGCCACAGATGAGACCCTGCAGGTTATCTCCGCTGGAGCCCCGGTTCATTAGATACCCTTGTAGTCCGGCTGACTGACTTGCGTCAAATCTCGTATGCCGTC

>943654eb2f7883d702a25931d6f28ab5

TACGAAGGGTGCAAGCGTTAATCGGAATTACTGGGCGTAAAGCGCGCGTAGGTGGTTTGATAAGTTGGATGTGAAAGCCCCTGGCTCAACCTGGGAATTGCATCCAA

>e10dde8e68c9f6fd43be9b7eac4b882b

AACAGAGGATACAAGCGTTATCCGGATTTATTGGGTTTAAAGGGTGCGTAGGTGGTTTTTTAAGTCAGCAGTGAAATCTTAAAGCTTAACTTTGAAAGTGCTATTGA

>511b3184781b53256275954a5ed6f9d0

TACAATTTCTTCAATTTAAGTTTTTAAAAGTTTCAGTTAATAAATTATTTAAATTTTTATGAATAATAATTTTAGTGAAATATATTATTATTATATTTTATTAATTT

>f845f3f54365984130c3c68161b3f048

TACAATTTCTTCAATTTAAATTTTTAAAAGTTTCAGTTAATAATTTTTTTGAATTTTTATAAATAATAATTTTAGTGAAATATATTATTATTATTATATTTTATTAA

>1de96a0f36d3c00a52eb1b4999100c15

TACGGAGGGTGCGAGCGTTGTCCGGATTTATTGGGTTTAAAGGGTGCGTAGGCGGCCTCATAAGTCCGGGGTGAAAGCCCGTTGCTTAACAACGGAACTGCCCTGGA

>f4b5145abd5f2a7bcc532136f8d2f39c

GTCCAGCGTCTGACAGCCGCTCTGCCCAGCTCAGGTACTTTGTTTTTTGTGTATAACTTACTCAGCCGCCAGGGTTAACTAGTCGTGAGGGGTGAAATAGTTACACC

>e520660bee20d2a0a1c65c2939c9e115

TACGTAGTGGGCAAGCGTTATCCGGATTTACTGGGTGTAAAGGGAGCGTAGGTGGTGCGGTAAGTCAGATGTGAAAGCCCGGGGCTTAACCCTGGGACTGCATTTGA

>15bd29a590d1c475a95a82d5b8bea336

CATTTTACCATAGTGTCTGCCTCCACGGGCACGTTTATGGCTTTGCCGGTTATCTTTTAAGACTGTTTCCATTCTGAGACGACGGATCTGCTTACGTGGAAATCCGG

>9172d65be7a700f9a307461e36cfc891

TACGTAGGGTGCAAGCGTTAATCGGAATTACTGGGCGTAAAGCGTGCGCAGGCGGTTTTGTAAGTCTGTCGTGAAATCCCCGGGCTCAACCTGGGAATTGCGATGGA

>d727ce2a6d50662087f3de432dfd956e

TTCCATCTCCGGTAGTATATGTTAAAAGCGTTGCGATTAGAAACCCTTGTAGTCCGGCTGACTGACTCTATCGTGATCTCGTATGCCGTCTTCTGCTTGAAAAAAAA

>a338049a22b82182c35d8823670e9d2e

TACGTAGGTGGCGAGCGTTGTCCGGATTTACTGGGCGTAAAGGGTGCGTAGGCGGATATTTAAGTCAGATGTGAAATTCCCGGGCTCAACCTGGGAGCTGCATTTGA

>c0703dadb5e19d194a9dbcda3c7f77d9

TACGTAGGTGGCAAGCGTTGTCCGGAATTATTGGGCGTAAAGAGTGCGTAGGCGGTCTATTAAGTCTCATTTGAAAGCCCATGGCTTAACCATGGAGGGTGATGGGA

>2bc2715f4eabdadbc9aa4db4e461cee5

TTCCAGCTCCAATAGCGTATATTAAAGTTGTTGCGATTAGAAACCCGAGTAGTCCGGCTGACTGACTTACTAGGTATCTCGTATGCCGTCTTCTGCTTGAAAAAAAA

>341e71cfc67363ccdb6a618430345d8b

TACGTAGGGGGCGAGCGTTGTCCGGATTTACTGGGCGTAAAGGGTGAGTAGGCGGTTATATGTGTCAGATGTAAAAGGCTATGGCTTAACCATGGTTAGCATTTGAA

>b4e39ee6193eb1e965b3307d5cd09859

TACGTGAGAGACTAGTGTTGTTCATCTTAATTGGGTTTAAAGAGTACCTAGACAGTCAATATAACTTCTATAATGCTAATACTTGACTAGAGTTTTAAGTAAGAGGG

>cef5775ea4977654c12ded12b18ad006

TAGTGATGTTTTGGCTGACTTTATCTGCTGCTTTGTTACAGTCAAGCTTAATAAAACCACCACAAACACACACATTAAGTAAGATGCTAACTCAAGTCACACACAAC

>5ca141f581490c2d40f278b964dd4308

GAGTGACCGTATAAAAGCCAGACTATATTGTGCCCGCGGCAGAATCGGACGCAAGCCACGTGGTGCAGCTAGCCAACATACTACCGACTACACCACGGCCGCCCCTT

>b128af3c890c5400b71d18cfa6be5366

CTGAGTGAGTTAATGAGTGGTAATTAGTTAGAAAATTAAGCTTTGCTGGTCTCTGTCCTCCGCCGTTTCTGTCCTTTTCTTTTCACCCTGACTCTTCTTCAATCTCT

>d3195ca85add50489020318e2f7b9043

TATAATTTCTTCAATTTAAATTTTTAAAAGCTTCAGTTAATAAATTATTTAAATTTTCATGAATAATAATTTTAGTGAAATATGTTATTATTATATTTTATTAATTT

>a77969479846d78bf46049f48aed28ef

TACGGAGGGGGCTAGTGTTGTTCGGAATTACTGGGCGTAAAGCGCACGTAGGCGGACCGGAAAGTCAGAGGTGAAATCCCAGGGCTCAACCTTGGAACTGCCTTTGA

>657e4bf681e1e0604de4df34406fc64f

TACGAAGGGGGCTAGCGTTGTTCGGAATTACTGGGCGTAAAGCGCACGTAGGCGGACATTTAAGTCAGGGGTGAAATCCCGGGGCTCAACCCGGGAACTGCCTTTGA

>cfd3ae02d9811c850083f11d388c92c3

CATTGAACTATCGTGAGAAAGTCACGTCGCCAAAGGGAATTATATTATAGTAATTATTGGCGTTAATAAACATTTTATTAATAGTTGTAATATATGATAACGTGTAA

>265703221123b921f1eabfe3755da1af

CACGGGGGGCGCAAGCGTTATTCGGAATTATTGGGCGTAAAGGGCGCGCAGGCGGTCTTGTCCATCAGGTGTGAAAGCTCGGGGCTCAACCCCGGAAGTGCACTTGA

>cee031c0044f2e3716fbd3dbda02e6f4

TACGTGAGAGACTAGTGTTATTCATCTTAATTGGGTTTAAAGGGTACCTAGACAGTCAATATAACTTCTATAATGCTATTACTTGACTAGAGTTTTAAGTAAGAGGG

>9d06bafc7c1bce4c4d92af5fc41d2f25

TTGGCGTTGGTTCAGCTCTTGTATTAGAAAGCCTCGGTGGCACAGTTTACCACAGTATCTCCATCAGGTATCTCCTTCCTGTGGCTACAGTAGGTGGTTTATCGATC

>18f2e35fef06bbdfe118e04c1a362c18

TGGATCACCTACCGCCTTCTGGATTATGCATCGAGTATAATCGTGTAATTGTGGGTGCTTAGAAAATATTAGAAACCCGTGTAGTCCGGCTGACTGACTATAGCGCT

>983f4a70e41378de5befe04a19c40419

GGAGGAGGAGGTTAGGTTCGTCATATACTTTTACAAATACACTATAACATTAGAAACCCTAGTAGTCCGGCTGACTGACTCTATCGTGATCTCGTATGCCGTCTTCT

>aea89a7782347477369e121e7532dd9c

TACGTAGGGTGCGAGCGTTAATCGGAATTATTGGGCGTAAAGCGAGTGTAGACGGTTATTTAAGCCAGATGTGAAATACCCGAGCCTAACTTGGGAGGTGCATATGG

>55fb34923e16ad2fc73b3a6c2005c137

TTCCATCTCCGGTAGTATATGTTAAAAGCGTTGCGATTAGATACCCCGGTAGTCCGGCTGACTGACTTGCGTCAAATCTCGTATGCCGTCTTCTGCTTGAAAAAAAA

>a3f49815cdd1039f0ca034f592fbe136

AAGGAAATTGAAAGTAAACGACTCCTCTGACTGCAATACACCATCATCATCATTAGAAACCCCAGTAGTCCGGCTGACTGACTATGAGCTCATCTCGTATGCCGTCT

>c2b11dfd18a6519697f483c3bf746809

CAAGGTGTTCTGCCTGGGAACAGAAGGACTGACTAGTGGTATTCAAGACAACTAAACGGGCTGTAACAAAACTTATAAAAAGTATTAGAAACCCTTGTAGTCCGGCT

>332bef79f73f7575ad1f0118639db370

GGAAGCTTATCGTCACCACCTATGTGGTGCCGGTGGTTACCTACGCGATCCCCATATGGGGCTACTTGGCCTTCAGTCTCTCCTGGACCGAGGACTGCGCTGGGCAT

>56888baf7acc75d669c95300b05ca497

AGGAATGAGATATCCGCATCTTCGATATTCTCATTTGATCGCTGTTCACTCAATTTCTCATTTTTAACGCAACTTAAAAACAACTCTCTAAGATTGCACGTTTCTAA

>7ada5320e34bc7e6e34828d36c403e5d

CATTGAACTATCGTGAGAAAGTCACGCCGCCAAAGGGAATTATATTATAGTAATTATTGGCGTTAAAAAACATTTTATTAATAGTTGTAATATATGATAATGTGTGG

>1f6cb5fb0d3dae65756c7571b703b5b6

CATTGAACTATCGTGAGAAAGTCACGCCGCCAAAGGGAATTATATTATAGTAATTATTGGCGTTAACAAACATTTTATTAATAGTTGTAACATATGATAATGTGTGA

>d3e89dbc2e470b36cdcaa8dc2b68f980

TACAGAGGATGCAAGCGTTATCCGGAATGATTGGGCGTAAAGCGTCTGTAGGTGGCTTTTTAAGTCCGCCGTCAAATTCCAGGGCTCAACCCTGGACAGGCGGTGGA

>9b962f3ca011f082847e91e6a902451b

CGTATTTCTTGGATGTGCATTATTTCCAGTTTTCAATTATAAAAAATATCTTAAAAACGTAAGAAAATAATTGAATTTGTATTGACGTTTTCTACTCAATTGGTACG

>0374c54413a496d849a3bf5e3de6361b

TACGTAGGGGGCGAGCGTTATCCGGAATTATTGGGCGTAAAGCGTGCGTAGGCGGCATGTTAAGTCATTTGTAAAAGATCTCAGCCCAACTGAGTAAGGCGAATGAA

>6936392183409430aad8f7fb5f983264

CGAGCGTTCTCCCCTCCCCAACACACACACACTTACCCTACTCACTAGGTTTACCGTTAATTAGAAACCCTAGTAGTCCGGCTGACTGACTCGCGATATATCTCGTA

>b4f1a46e57cbf4e80d5db34c6994581a

GACAGAGGGTGCAAACGTTGTTCGGAATTACTGGGCGTAAAGCGTGTGTAGGCGGCCATGTAAGTTGGATGTGGAAGCCCCGGGCTCAACCCGGGAAGTGCATTCAA

>4b6eed5373d7765258ee3e73bf3dc20a

TACGTAGGTGGCAAGCGTTGTCCGGAATTATTGGGCGTAAAGCGCGCGCAGGTGGTCTCTTAAGTCTGGTGTGAAAGCCCACAGCTTAACTGTGGAGGGTCATCGGA

>3ef19b9b2b89e98e31260f5ac7c30ca3

TCAAGTAGGCTTTGAATAACGCGCATCAATTTGCCGCAATAACGTTCGTTGTCATTGGCCAGTTATTGGGACGCGACAATAACAGCAGGACATTTCACGGAGAGTTA

>4d34207391bb4499a5c2a3b157e0b472

TACGTAGGGTGCGAGCGTTGTCCGGAATTACTGGGCGTAAAGAGCTCGTAGGTGGTCTGTCGCGTCATTTGTGAAAGCCCGGTGCTTAACTCCGGGTTGGCAGGTGA

>18c9cbcd1eb60542c0ea436be24424d2

ATGCAAATTGGGATCACTCGATTGAAATCTTAAACCGCATTAGATACCCTGGTAGTCCGGCTGACTGACTACGTACGTATCTCGTATGCCGTCTTCTGCTTGAAAAA

>9b5a8b46887ddb28bfc91632fece0cf4

CCACAACCCACCGCTGTGTTCATACTTTTTTTTTTACAATAACAGATGCCACAATTCACACACACACAATGTACATTAGAAACCCCTGTAGTCCGGCTGACTGACTT

>cae2b0996d1874340244d00ba8a35d9f

ACAACCAGGGGAACCTAACAATGAGCCGCCTCGCAATCAATCAATTTTCGCCACGCCACATCAACGCCATTCGTGTAGCACAATTTTTCTTCGCCTATCTACACACA

>c0075d468727201ad4cad687d9538257

TAAAACAACAATGGCCTGGAGGCAATATTTCTGTTCACCTCGTGGTCCATGCTGGCGGAGATGAAACTAGGTACAGCCCATAATTATGACGTCAACCAGTGCTTACC

>51beb36ba736cdfe3dc3f37a4ed92062

AGGACTTCGAGTACCTATATCGAAACTGACACATACGCCTGAACACTGTTCGAAACAGCCACGTGTACAAGACTGACTAATACTAAAACAAGCCTTACTGAGGAAAA

>f85581b3052caf206f9626139e0f5a23

TGTCCGCACCTAATAGGGCATCAACGGGAGCGGGTCTGTCAAATGTAGGGTCGGCAAGGACCAAGTCCTTAGCTGCACTCTTAACCTCTGAAGCTATTGCTGTCAGA

>91dbfd8adf81918a08845abec1056e38

TACGTAGGGCGCAAGCGTTGTCCGGAATTATTGGGCGTAAAGAGTTCGTAGGCGGCTTTGTAAGTTAGGTGTGAAAGGCATTGGCTCAACCAATGTAAGCACTTGAA

>8fad4c91336d137ba8b6cdeb35188fae

TACGTAGGGGGCAAGCATTATCCGGAATCACTGGGTGTAAAGGGTGCGTAGGCGGCAAGGCAAGTCAGATGTGAAAGCCCGGAGCTCAACTCCGGGATTGCATTTGA

>957d66973ad38ec9fad58c3dbd36bcc1

ATATCGTCAGTCCCTGTTCTTAGATGTGATCTTTCCAGAGCCGCGCTGAGGCGCACACACAACAGGGCACACAGATCAGAAACATCCTAGAATGTCTATTCTTACAT

>9429e319352eaf9318749c9c26737933

ATGATTCCATAAACACCTGTGAAAACAAATTAGGAATTTTAGAACAAAAAGTAAAACAAATACAACAGACTACTTCACTCGCAAACCAGGTCACTTATATATACAAA

>d598e6c6fb23fe004eccc4e479954ee6

ACTTTGCGTCAGACAATGTTATTTTTTATGAACAATGGTTGGTAAACTTTTTTTCTCGCTATAAATTAGAAACCCTGGTAGTCCGGCTGACTGACTTGCGTCAAATC

>0891753889313cb40b10c3df427f6d0e

CATGCCGAAAACATTGGATGATCGCTATTAGTCGCCCCAAAAATATCTTTCACATAAGGATACTACACATTATAGTTATATTATTGCCAAGTGTAATTTGAAAATTT

>2fe9954f13c4164845106c766eff273e

ACAAATGGTGAACAGTATAGTGTCAATAATGGCATTAGATACCCTGGTAGTCCGGCTGACTGACTCGCGATATATCTCGTATGCCGTCTTCTGCTTGAAAAAAAAAA

>43cd0f7df543960a09bdfb013ff222a9

TACGTAGGGAGCGAGCGTTGTCCGGAATCATTGGGCGTAAAGAGCGCGTAGGTGGATCAGTTAGTCTGCTGTGAAAGTCAAAGGCTCAACCTTTGAAAGCCGGTGGA

>fd7179378d3605a16ab9af39c9c65a83

TACGTAGGGGGCAAGCGTTATCCGGATTTACTGGGTGTAAAGGGAGCGTAGGTGGTGTGGTAAGTCAGATGTGAAAGCCCGGGGCTTAACCCCGGGATTGCATTTGA

>bd9ca60a5802d4e1136a7170a6d05e2e

TACATCTAGACAAGTTGACCAATGATGACGGAATTAGAAACCCGTGTAGTCCGGCTGACTGACTAGTCGCAGATCTCGTATGCCGTCTTCTGCTTGAAAAAAAAAAA

>9947e02ebcacdcb26b0f89e934a1976a

TCTACTGAGATCAGTATGGTAAAATCTACATGAAAAAAACAAGGTTACATTCTGAAAACTAAGTTAGATATTTTCTTTATAATAAAAAGCTAATTTTATAAGGATTC

>25dccb9f2bed64764012017e95d7f331

TACGTAGGGGGCTAGCGTTATCCGGAATTACTGGGCGTAAAGGGTGCGTAGGCGGTCTTTCAAGCCAGAAGTGAAAGGCTACGGCTCAACCGTAGTAAGCTTTTGGA

>ffc065850e6a685a14c8607a9286f1fe

TACGGAGGATGCAAGCGTTATCCGGAATTATTGGGCGTAAAGGGCGCGCAGGCGGTCTTGTCCGTCAGGTGTGAAAGCTCGGGGCTCAACCCCGGAAGTGCACTTGA

>ddbbe06c7563ff11db90519dc9bdde46

TACGGAGGGTGCAAGCGTTAATCGGAATTACTGGGCGTAAAGCGTACGTAGGTGGTTTTGTAAGTTGGATGCGAAAGCCCTGAGCTCAACTTGGGAATGGCATTCAA

>cfc85787b1ad63f305c4b91add500993

AACAGAGGATACAAGCGTTATCCGGATTTATTGGGTTTAAAGGGTGCGCAGGTGGTTTCTTAAGTCAGTAGTGAAATCTTAAAGCTTAACTTTAAAAGTGCTATTGA

>07c3d36f1658d74993680b0196871c99

CCACCACCCACCGCTGTGTTCATACTTCTTTTACAATAACAGATGTCACAATTCACACACACACACTTAATGTGCATTAGATACCCTGGTAGTCCGGCTGACTGACT

>eb2c556b2f2be75e8dd5adb073f43cf6

CTCAAATCCCTCAATTTAAATCTACAAAGCTTCAGTTACTATATAACATAATTTTTATAAATAATAATTTTGGTGAAATATATTATTATTTTATTTGTATAATTTGT

>ec8ed20111f4b70620f608a90a53fe5a

AGGAGAAAAAAGTGTGGCCAAGCCACTTTGTAAGCAACCTAAAGCTTCTTGTCTTGACAAAAAGACCACCGAAATCTTTGAAACTGACTTCACTATCCATGAATTAG

>a4081a4955e87e4e6a1e732eeb9ce4f3

CAAGTTAGGACAATGCTATAATTAAACTGTATCCTAATTAACTGACTTTAATAAAAGCGTTAAACTTGAAAACAATACTCTTTATAAGAGAACGAGAAAGAGAAATG

>4dcd9ffd8a0d09402aa773103899628e

TACGGAGGGGGCTAGCGTTGTTCGGAATTACTGGGCGTAAAGCGCACGTAGGCGGCTAGGTAAGTTAGAGGTGAAAGCCCAGGGCTCAACCCTGGAATTGCCTTTAA

>0ea5db4b4272de1d0ae1348c3d36d322

CAACAAAGACAACCCAGACAGTAAAAAGGAGCAAGGAAGGGAACCCTGTTTGCTAAATCTACGAGCATTAGAAACCCGGGTAGTCCGGCTGACTGACTTGCGTCAAA

>efc1d41f13f9446e726f3ac41d9fd1b1

TTCCATCTCCGGTAGTATATGTTAAAAGCGTTGCGATTAGAAACCCTAGTAGTCCGGCTGACTGACTTGCGTCAAATCTCGTATGCCGTCTTCTGCTTGAAAAAAAA

>36d58011405e3d776c8f1701f933ab01

TCCCCGCAATAGTACCTCCTTCATACAGAAGCATTCCACCATTAGAAACCCCTGTAGTCCGGCTGACTGACTTGCGTCAAATCTCGTATGCCGTCTTCTGCTTGAAA

>c8259adaa08830bbe672ffa81d655a7a

TACGAAGGGGGCTAGCGTTGTTCGGATTTACTGGGCGTAAAGCGCACGTAGGCGGATTGGTCAGTTAGGGGTGAAATCCCAGAGCTCAACTCTGGAACTGCCTTTAA

>d373457c833727d77f113430ad1ae5e7

CCACGTCACTCACGGCCGTAGCATATAATTAAAAAAGCTACACGTGCTTCCTGGGTTGTTAAAAGGAGTGACATCGTAGGCCGTACTTAGGCGAATGGATTCAGTTC

>c8e7ecd62a093653f737a2dbda0b2982

TGGACTCCGAGATGTCTGATAATGAACAAGAAACAGATGGTGAATTAGATACCCCAGTAGTCCGGCTGACTGACTACGTACGTATCTCGTATGCCGTCTTCTGCTTG

>1872f610e810872378d750a1354bb6dc

TACGTAGGGTGCAAGCGTTGTCCGGATTTATTGGGCGTAAAGGGCTCGTAGGCGGTTTGTCGCGTCGGATGTGAAAACTTGGAGCTCAACTCCGAGCCTGCATTCGA

>b816244453ec34c1dfa005c6fea8973a

TACGTAGGGCGCAAGCGTTGTCCGGAATTATTGGGCGTAAAGCGCGCGCAGGCGGTCTGTTAAGTCTGGTGTTTAATCCCGGGGCTCAACCCCGGTACGCATCTGGA

>8303f4b2dbac6c524647f09e7f690801

TACGAAAGGTGCAAGCGTTAATCGGAATTACTGAGCGTAAAGCGCGCGTAGGCGGTGTGTTAAGTCGGATGTGAAAGCCCAGGGCTCAACCTTGGAATTGCATCCGA

>f5dfa79f6454e472bf64df691a27c62b

CACGGGGGGCGCAAGCGTTATTCGGAATTATTGGGCGTAAAGGGCGCGCAGGCGGTCTTGTCCGTCAGGTGTGAAAGTTCGGGGCTCAACCCCGGAAGTGCACTTGA

>370ced9d971d0cb9aefbcd9beb4bd0c0

CAACCACTGTGCTGAGTGTGATATACTTTTTTTACAATACACAATAACAGATGCCACAAGCCACACACACACACAAGCACAACACACACACACACACACACACACAC

>ab03bcbb5571e75e39c646b69209c7af

CTCTCTTTGATCTATTAAAGTTTTATTAGCTTATAAGCTACTTGCTATCTGCCATGCATGTCATGTCCTTTATTAGATACCCCAGTAGTCCGGCTGACTGACTCTAC

>88d78ec2631893bc35267be9fac9280d

TACGTAGGGTGCAAGCGTTGTCCGGAATTACTGGGCGTAAAGAGCTCGTAGGTGGTTTGTCGCGTCGTCTGTGAAATCCCGGGGCTTAACTCCGGGTCTGCAGGCGA

>bb7a6ce8d34047ec01990c160384d594

ATAGATTAAAAGCCACTGTCCCCTGCTGCTGTTTCCTTCAGGGATATTACATTGGGCTCTAGCGGCTGTAGTTAAGCTCGCAATTATTGTGGGTTTCTGGGAAAGCC

>b90be66561a5a7bfb3bf334356c63820

CGTTATCGGCCGCTTACGGACGCTACTAGCACGTATACTTACGCTAACGCGTGTAATGCAGTACAAACAGCGTTTGCTCACCTCGGCAAATCCGTAGTATAAACATT

>2518ebe0bab2dccc54958844156a8546

CCACCACCCCACCGCTGTGTTCATACTTTTTTTTTACAATAACAGATGCCACAATTCACACACACTTAATGTACATTAGATACCCGGGTAGTCCGGCTGACTGACTT

>8446293a86d9cda164ff5dc2abfe2369

CTCGGTTGGTTCTTTGTAGTTAGTTGCGTAAGTCTCGAGTATATATATCATAATTATAATACACAGGTTATGTAACAGTTTCAAGTGATTCACTTATAAAACAAACT

>766c85da073bd6449009cefb53372c29

TTCCAGCTCCAATAGCGTATATTAAAGTTGTTGCGGTTAAAAAGCTCGTAGTTGGATCTGTGCGCCACGCTGTCGGTTCACCGCCCGTCGGTGTCAACTGGCATGTC

>13f13dd6f85e2e294c8fdf959de35b7d

ACAAATGGTGAATAGTATAGTGTCAATAATGGCATTAGAAACCCCAGTAGTCCGGCTGACTGACTACTGTGTAATCTCGTATGCCGTCTTCTGCTTGAAAAAAAAAA

>d87e42009700320db3692ae61403e4f5

TGTCCGCACCTAATAGGGCATCAACGGGAGCGGGTCGGTCAAATGTCGGTTCGGCAAGAACCAGGTCCTTAGCTGCACTCTTAACCTCTGAAGCTATTGCTGTCAGA

>ee858a1b80f9fa005d421366220e6855

TGGACTCCGAGATGTCTGATAATGAACAAGAAACAGATGGTGAATTAGATACCCTTGTAGTCCGGCTGACTGACTCGCGATATATCTCGTATGCCGTCTTCTGCTTG

>2375ac3dd4524c6974f46237de8a3c0c

TACGTAGGGGGCGAGCGTTGTCCGGAATTACTGGGCGTAAAGAGTACGTAGGCGGCTTTATAAGTCAGATGTTAAAGGCTTCGGCTTAACCGAGGTAAGCATTTGAA

>c45b3650304f51aa19aae6fad309d162

GAGAAAAACAACCGTATTTGAAGAGGTAAGCAGCTCACAGTATTACATACTGCAGACGATCGTGATAATTAGAAACCCCTGTAGTCCGGCTGACTGACTATGAGCTC

>eacb21990cdfae18c32fae052d704439

TCCACCGCTGTGTTCAGTTTGATATACTTTGTTTACAATACACAATAACAGATGCCACAAGCCACACACACACACACACACACACACACAGAAAGTCGTAGAACGCA

>52d9d86824cf52ab07e4c0f0954fefd4

CTCTCTTTGATCTATTAAAGTTTTATTAGCTTATAAGCTACTTGCTATCTGCCATGCATGTCATGTCCTTTATTAGAAACCCCAGTAGTCCGGCTGACTGACTCTAC

>2fd50c2724a6a9542061816b4f64db66

TCGAGTCCCTTACGCGCTGTTTAGCGAGCCAACGCACTATCGAATACACAGCGACCACACCCCTATCGTATACGCGTGTAATTAGCAACACAATATTAATTCTTATA

>89936e78ef5930bf7cf5b664d78a4cd2

TACGAAGGGGGCTAGCGTTGCTCGGAATTACTGGGCGTAAAGGGCGCATAGGCGGACAGTTAAGTTGGGGGTGAAAGCCCGGGGCTCAACCTCGGAAATGCCTTCAA

>c8333701ca89a516464ab2872c281ff0

TACAGAGGGTGCAAGCGTTAAACGGAATTACTGGGCGTAAAGCGCGCGTAGGTGGTTAGTTAAGTTGGATGTGAAATCCCCGGGCTCAACCTGGGAACTGCATTCAA

>2587782ca33635d7ceb058ce873e89e0

CTGGATTGCGCTTTGCTAGCGTCCACATTGGAGCAATCTCGTGACCCCTTTTCCGGATTAGCTTTCTGGTTTAGTTTGAAAGCAAAATTAATGAATTTTGTAAGTCT

>60fe68a49096476bea8e1a743e16eb1b

TTCCAGCTCCAATAGCGTATATTAAAGTTGTTGCGATTAGATACCCTAGTAGTCCGGCTGACTGACTCTCTAGAGATCTCGTATGCCGTCTTCTGCTTGAAAAAAAA

>a08e41adad0a118e5759f1f7f108e7a6

TACGAAGGGTGCAAGCGTTACTCGGAATTACTGGGCGTAAAGCGTGCGTAGGTGGTTATTTAAGTCCGTTGTGAAAGCCCTGGGCTCAACCTGGGAACTGCAGTGGA

>fa09cc5311355b16f7d5b331e0adab51

CCGCCGCCGCCGCCACTGTGTTGAGTGTGATATACTTTTACAATATATAACACGTGGCGGTAGGGCGGCAACGAAGAAAATGATCTACACGGCAGTGGAGGAATTGA

>679a46c6e7170c0611b6a1b2fb843d97

TCCTTTGTCATGATCATCCGAGCACCAAGGAAGTCATCGACTTTATGCCGGCAGCCATTCGGTCGTCAAAAGTTATTCTGCATGGATAGGGTTTGTTACGTTCATTG

>7707f81e18b22e391dc32280886ee4dd

CGAGGACGATTCACGCTGTATTTGGCATTCGGACGGTCTCAGTACTATTGGTACAGCACTTCTGTACAGTGAACGATATGAGGACGCTACAACTATCCACATTTCGT

>99d338914266bd42759fd746f0f4fedf

TATGTAGAGTGCGAGCGTTGTCCGGAATTATTGGGCGTAAAGAGCTCGTAGGCGGTTTGTCGCGTCGGCCGTGAAAACCTGCCGCTTAACGGTGGGCGTGCGGTCGA

>698aafea611f80bc643f58a71562fed9

ACAGTATCAAAATAGAGACCTGAATGGAGTTGGGTGTAGCGGTGAGTGAGTGGATGTAGTTCCATGATAGGAGTATGAGACACGTTGTTTTTGTAAGAAAACGTAGT

>8029f46f5f9b397ba8951de634484717

CCACTGTGTTGAGTGTGATATACTTTTACAATACACTATAACATTAGATACCCTTGTAGTCCGGCTGACTGACTCTACGACCATCTCGTATGCCGTCTTCTGCTTGA

>8593124656afb20a301a893d170fbbe8

TAATATCTTTGTACGCCAGGGGCGCAGTAAATTGATTTTGAAAAATACTACATTAGATACCCGGGTAGTCCGGCTGACTGACTAGTCGCAGATCTCGTATGCCGTCT

>94a48aaa7763ea59a9d0db88b0b0aae4

GGTGAAAACACAGTTCGACAACGCGTTTTAATTACGTTCAGCGGTCACGTGAATACAAATTAGATACCCCGGTAGTCCGGCTGACTGACTATAGCGCTATCTCGTAT

>8e55df451ab1699ae4f5cff0d79d7b33

AACAGAGGATACAAGCGTTATCTGGATTTATTAGGTTTAAAGGGTGCGTAGGTGGTTTTTTAAGTCAGTAGTGGAATCTTAAAGCTTAACTTTAAAAGTGCTATTGA

>8d359dce266d7d72709b1593d2aa21ce

CCACAACCCACCGCTGTGTTCATACTTTTTTTTTTTTACAATAACAGATGCCACAATTCACACACACTTAATGTACATTAGATACCCCGGTAGTCCGGCTGACTGAC

>43bc8bbacd1488cf3632c1bbc8831222

CGCAGATGAATGAACGTAGATTTATAAGGTTGCACAAGATAAACAAACAAATCGAAGGACGTTCCATCGCAAAAGACAATTCAGCGTATGCAAATTAGAGTAGAACT

>dd5c6e7526ea3efb156d24da1a375103

AGACAAGCAATTGTTGTTGAGTGAAATACAAGCACTCAGTGACTCGATCAGAAAAAAGCATCACGCGTTGAAGCGCGGCATGGTGGACACGGCGAGTTCTCTCGCCA

>187c9d27f80e30df6da41f4bb34c849d

TAACAATGACGGCAAATGTAATCTATTTTATAATCTTCTCTTAGCCTCAGCGCTAATATCATTTGAATTTGAATAGCATATTATTATTATCATCATCATTTTATTTC

>b04c649ad29861d1f15cf50298bf4dab

GGAAGTGCAGTCTTGTTGGCAGTCTTTATTAGATTCCGTCTTGTTGGCAGTTCTTTCCTCTTATAATATTCATAAATATGGTTCCGTATGGCACTTTCTGAAAAAGA

>48a38afb700a3c7dc07f21266f988f47

TTCCATCTCCGGTAGTATATGTTAAAAGCGTTGCGATTAGATACCCGAGTAGTCCGGCTGACTGACTCTATCGTGATCTCGTATGCCGTCTTCTGCTTGAAAAAAAA

>a718a57ccb8d023bffcf6a8e6466cd67

GATTAAGGTTTGTAGTCGTTACGTATTTCTTGTCTTATTTATGGATTTAGACGTTCATGAAAGCTAACGTAGATAAAAACCTTTCAAAGTTACAAAGTGTAGTGGAA

>089360679f36bc46ae2fbd7497a54d85

TACAATTTCTTCAATTGAAACTTGTAGTTTAAGTTAGTATATAATATAATTTTTGTAAATAATAATTTTAGTGAAATATATTATTATTTTGTTTTTATAATTTGTTT

>3f2ce9e4913af3eb727f909505d19757

TACGGAGGGGGCTAGCGTTATTCGGAATTACTGGGCGTAAAGCGCACGTAGGCGGCTTTGTAAGTTAGAGGTGAAAGCCTGGGGCTCAACTCCAGAATTGCCTTTAA

>dea3ee7e772997e60af45d31f6b73170

GGAGTACAGCAGCAGCTTTCTACTGTCGTCAGCTGAAAGTAATTTATCAGAATCAGTCAAAGCATATTCCCTATAAATTATACATCATGTTTATAAAATGCTTTGAT

>d6028dc79370d1ef2bfcc80161cb07d4

AGTATCCTCTAGAATTTTCAACCGTTTGTTAAGGTCGCATATTATGGAATCCTGCAGACTATAAATAGCCCACACCTGCCGCTTAATTTTTCAGTTTACTTCGGGCG

>e7c0c7a852d4c2d713f6a89e2baaf3d4

CTGGATTGCGCTTTGCTAGCGTCCACATTGGAGCAATCTCGTGACCCCTTTCCCGGATTAGCTTTCTGGTTTAGTTTGAAAGCAAAATTAATGAATTTTGTAAGTCT

>5d8848bbdcb4663a85acf06828c08c4b

CAAACGAATATGTTTTTGTTATTTTTAAGTATAAAAACAATTGTATTACTCGAATTGCTTTTCGGAAGTTCGACACAGCTTACACATTTCTTGAAAAATTTAATGTC

>29693d3ffb1f28c0d33fc5363ad0b276

TACGTAGGGGGCAAGCGTTATCCGGAATCACTGGGCGTAAAGGGTGCGTAGGCGGCCAATAAAGTCAGGGGTGAAAGGCTACGGCTTAACCGTAGTAAGCCTTTGAA

>518c6bf2e75b3bd00656575ccdb908bb

TACGTAGGTGGCAAGCGTTATCCGGAATTATTGGGCGTAAAGCGCGCGTAGGCGGTTTCTTAAGTCTGATGTGAAAGCCCACGGCTCAACCGTGGAGGGTCATTGGA

>50d0f6f6cb2ffbf38eaa21f6c8ea2731

TACGTAGGGTGCAGGCATTGTCTGGAATCACTGGGCGTAAAGAGTTCGTAGGCGGCTTTGTAAGTCAGGTGTGAAAGGCATTGGCTCAACCAATGTAAGCACTTGAA

>7690a4b3e3c9dc2cc9058bbea3f6336b

TACGTAGGGGGCAAGCGTTGTCCGGAATTATTTGGCGTAAAGAGTGCGTAGGCGGTTTGTTAAGTCATTTGTAAAAGATCTCAGCCCAACTGAGTAGGGCGAATGAA

>0d8dc1c423a9e5074b1d3dcfb13fdfa0

CCACCCCACCGCTGTGTTCATACTTTTTTTACAATAACAGATGCCACGATTCACACACACACGCTTAATGTACATTAGAAACCCTTGTAGTCCGGCTGACTGACTAT

>13ac60fd08c7c7e8a39b97d9409a656d

ATGCAAATTGGGATCACTCGATTGAAATCTTAAACCGCATTAGATACCCCGGTAGTCCGGCTGACTGACTACGTACGTATCTCGTATGCCGTCTTCTGCTTGAAAAA

>d116b557a9a8e3ac0c770040a028db5c

TCCGGCTGACTGACTTAACGTCCATCTCGTATATTAGAAACCCGAGTAGTCCGGCTGACTGACTTAACGTCCATCTCGTATGCCGTCTTCTGCTTGAAAAAAAAAAA

>92516b3124ae7f087a8f574ae3728197

CATTGAACTATCGTGAGAAAGTCACGCCGCCAAAGGGAATTATATTATAGTAATTATTGGCGTTAATAAACATTTAGTAATAGTTGTAATATATGATAATGTGTGAA

>886c3d9a282daa6ea81bb06971f9e765

AAATAATAGAATAGCCTCTCATGCGGACTGTACGAAGCTCCGAGCGCCGAAATTAGTTTGTACGAAACCTTCAATAAATTAGATACCCCAGTAGTCCGGCTGACTGA

>b5e9047458c3780379c6590ef2c53080

TTCCAGCTCCAGTAGCGTATATTAAAGTTGTTGCAGTTAAAAAGCTCGTAGTCGAACTTCGGGCCTGGCGGGGCGGTCCGCCTTACGGTGTGCACTGTCCGGCCGGG

>2e74d7d141996c80a3109031abd37098

TGGAACAGTGTTTGTTGATTGTGCGTGAGCGGTGAAGGTGTCTGCGTCGCAAATATTTAGTGTTTAATCGTTTTTACCCCGTGTTAGAAAGCCTACGACTTCGGTGA

>e16a435409b72731c8b8f069b63a16c8

CCCTTTTGTTACTGTGTATTCATTAACTCCCATGTCGGTTTCGTACATCAGAATTCTATGCGTAAAATTGTCCCTGACTATCCTTATTAGAAACCCCAGTAGTCCGG

>9ddbb88335588871f6c773f7f79bab3e

CCACTGTGTTGAGTGTGATATACTTTTACAATACACTATAACATTAGATGCCACAAGCCACACACACACACACACACACATAAAGTCGTAGGACACAAACACAGTCA

>e5c11be097bd999cbac41b99e1519f03

AACAGAGGATACAAGCGTTATCCGGATTTATTGGGTTTAAAGGGTGCGTAGGTGGCTTTTTAAGTCAGTAGTGAAATCTTAAAGCTTAACTTTAAAAGTGCTATCGA

>24e29ea3d4f7b8c6a563eecaac20248f

TACGTAAAAGACTAGTGTTATTCATCTTTAATCGGTATAAAGGGTACCTAGACAGAATGCTAAGCCATAAAAGGAACTAGTATTCTAGAGTTTTATATGGGAATATA

>9e527dd54e122fd9cba640a973e11986

TACAATTCCTTCAATTTAAATTTATAGAGTTTCAGGTATAAATTTTTATAAATAATAATTTTAATTAAATGTATTTTGTTTTATTTTTTTTATTTGGCTGACTAATT

>a24852dbe6ea90b9b27960e83f29ce84

TACGTAGGTGGCAAGCGTTATCCGGAATTATTGGGCGTAAAGCGCGCGTAGGCGGTTTTTTAAGTCTGATGTGTAAGCCCACGGCTCAACCGTGGAGGGTCATTGGA

>65f987731166a982363bf673434a8df3

TGTCCGCACCTAATAAGGCATCAACGGGAGCAGGTTGGTCAAAGGTCGGATCGGCAAGAACCAAGTCCTTAGCCGCACTCTTAACCTCTGAAGCTATTGCTGTCAGA

>52f06ac71cafbd32791bab16bfe4e2fd

AAATAATAGAATAGCCTCTCATGCGGACTGTACGAAGCTCCGAGCGCCGAAATTAGTTTGTACGAAACCTTCAATAAATTAGAAACCCCGGTAGTCCGGCTGACTGA

>de4e1412479e266774a834dd20b5c88b

TACGGAGGGGGCAAGCGTTGTTCGGAATTACTGGGCGTAAAGGGCGCGTAGGCGGTCTGTTGCGTCAGGTGTGAAAGCCCTGGGCTCAACCTAGGAGGTGCACTTGA

>9b8f17386c37e010fbc91f98d641c438

TACAGAGGGTGCGAACGTTGCTCGGATCTACTGGGCGTAAAGCGCGTGTAGGCGGACTCGCAAGTCGGTTGTGAAATCCCTGGGCTTAACCTAGGAACTGCATCCGA

>36d1429eecd0b0587f828c4da7d79c91

ACTATCAATCAGCTGGAAGATGAAATGGGAAAATTCATCAGTGATATCCAACATGCTGCATGGAGTAGTACGCTGGTAATCCGAAGGAAAACTATGGAGTATGGATA

>7c21c2825cec4c017c9f368c4b548cf2

TTCCATCTCCGGTAGTATATGTTAAAAGCGTTGCGATTAGAAACCCTGGTAGTCCGGCTGACTGACTCTATCGTGATCTCGTATGCCGTCTTCTGCTTGAAAAAAAA

>9e68894eecb3f2a622eddb87b2ea0ea6

AACAGAGGATACAAGCGTTATACGGATTTATTGGGTTTAAAGGGTGCGTAGGTGGTTTTTTAAGTCAGTAGTGAAATCTTAGAGCTTAACTTTAAAAGTGCTATTGA

>7e2b820421f40487cd76703798567852

CGTTTATTTTAGTTTGTTGTTATTATAAACATAATTTTAGTTTATATTAATTGTGTGTTCCCAGTATCTAGTGTTTCTAAAATTGTATCATGTTTTGTGTTGATTTG

>411ae9f0dada2a9d8a0e2e6d24a9a91b

TGGATCACCTACCGCCTTCTGGATTATGCATCGAGTATAATCGTGTAATTGTGGGTGCTTAGAAAATATTAGATACCCTTGTAGTCCGGCTGACTGACTATAGCGCT

>591e820a4eea6a545783de66f83e9cd0

GGATACTTCGGGTTAGTTAAATCCGTAATACTTGAAGACGAAAATTCGGTTAGATCGATTACAGAAGATATTGGATATATTAGATACCCTGGTAGTCCGGCTGACTG

>3a4c294317632838a37d031ee98a114b

ACTTTGCGTCAGACAATGTTATTTTTTATGAACAATGGTTGGTAAACTTTTTTTCTCGCTATAAATTAGATACCCGAGTAGTCCGGCTGACTGACTTGCGTCAAATC

>7a947564db89b8b96b52aaff4c724e70

TACGGAGGGTGCAAACGTTGCTCGGAATCATTGGGCGTAAAGCGTGCGTAGGCGGCTTGGCAAGTCGGATGTGAAAGCCCTCGGCTCAACCGAGGAAGTGCATTCGA

>8a95e250f759834e4a7a9eed89e2059c

CCACTCTTATCTATTCGAAGCTTGACATTCTGTGGTTTATGCTATTCAGGAGTTTTAAACTTCGTCTCCCTCTCTTCATCACTCCTCCAACTGTTCCATTAGAAACC

>b5c22d5a625e7ab4dfa0749482ad0354

TGGACTCCGAGATGTCTGATAATGAACAAGAAACAGATGGTGAATTAGATACCCTTGTAGTCCGGCTGACTGACTACGTACGTATCTCGTATGCCGTCTTCTGCTTG

>131f399fa9a865228fe628ff5e3980ce

TACGAAGGGTGCAAGCGTTAATCGGAATTACTGGGCGTAAAGCGCGCGTAGGTTGTTTGATAAGTTGGATGTGAAAGCCCCGGGCTCAACCTGTGAATTGCATCCAA

>fc413b866a6cd22aa388b993501d7828

CACGGGGGGCGCAAGCGTTATTCGGAATTATTTGGCGTAAAGGGCGCGCAGGCTGTCTTGTCCGTCAGGTGTGAAAGCTCGGGGCTCAACCCCGGAAGTGCACTTGA

>138ef010381d8d4baa1b59a24b90cb3c

TACGTAGGGTGCAAGCGTTAATCGGAATTACTGGGCGTAAAGCGTGCGCAGGCGGTCCGCTAAGACAGATGTGAAATCCCCGGGATTAACCTGGGAACTGCATTTGT

>2da5e6812bed98471c6d0b68a3cad0b1

CGTGACCGTATCGCGGTGGGAACACGTCATGTCTGTCCGTATATAGTACACGGAGTTATACGATATCGCCGAGTTCCGTGATCGCCCGCATTAGAAACCCCAGTAGT

>0b56f73b93e4f1bc0f66ddac457a3145

ACAACAAGGGGAACCTAACAATGAGCCGCCTCGCAATCAATCAATTTTCGTCACGCCACATCAACGCCATTCGTGTAGCACAATTTTTCTTCGCCTATCTACACACA

>5bcf9e61116b4b44f0d1ad37c7973ef9

TACGTAAGAGACTAGTGTTATTCATCTTAATTAGGTTTAAAGGGTACCCAGACGGTCAATATAGCTTATAAAATGTTAGTACTTGACTAGAGTTTTATGTAAGAGGG

>199e4c4450ac1298cb94237d3aba6c86

CGAGTGTTCTCCCCCCCTCCCCAACACACACGTACCCTACTCACTACGTTTACCGTTAATTAGATACCCGTGTAGTCCGGCTGACTGACTATGAGCTCATCTCGTAT

>a9e70b5c077ab52af25ab7d4fd4597b6

CCACCGAGATCTACACTCGACGAGTATGGTAATTAGATACCCTAGTAGTCCGGCTGACTGACTCAGTAGGTATCTCGTATGCCGTCTTCTGCTTGAAAAAAAAAAAA

>0cfb75a86bd7397d7574f01684b1dc12

TACAATTCCTTCAATTTAAATTTATACAGTTTCAGGTATACATTTTTCTGTAATTTTTATAAATAATAATTTTAGGTAAATGTGTTTTGTTTTATTTTTATTTATTT

>c4c3c1819b15b42523640e1da6e16666

GGTGACAACGAAGAAAATGATCTACATGGCAGTGGAGGAGGAATTGAGTGTCATATAATTTTATCATTCACTATAACATTAGATGCCATTTTGTCTCCGTGGCGAGG

>b47a8c7e2de2eeac89a18f30e6aa5615

TACGTAGGTGGCGAGCGTTGTCCGGATTTACTGGGCGTAAAGGGAGCGTAGGCGGATTTTTAAGTGAGATGTGAAATACCCGGGCTCAACTTGGGTGCTGCATTTCA

>6560fa0944a4bea92d7c10e1893292fb

TACGTAGGTGGCAAGCGTTGTCCGGAATTATTGGGCGTAAAGAGTACGTAGGCGGTTTGTTAAGTCTGAGGTTAAAGACTGGAGCTCAACTCCAGCACGCCTTGGAA

>c10617b6a5574fba0c177dd47af5b715

TACGGAGGGTGCGAGCGTTAATCGGAATCACTGGGCGTAAAGCGCGCGTAGGCGGCGCGGTAAGTCAGACGTGAAAGCCCTCGGCTCAACCGGGGAATTGCGTTTGA

>dd2a2ce32ef9b8b96ac8b9b7387381f6

TACGGAGGGTGCGAGCGTTAATCGGGATTACTGGGCGTAAAGCGCACGCAGGCGGTTTGTTAAGCTAGATGTGAAAGCCCCGGGCTCAACCTGGGATAGTCATTTAG

>3fa5c779ee69ddc27dd057838ab08e39

TCAAGTAGGCTTTGAATAACGCGCATCAATTTGCCGCAATAACGCTCGTTGTCATTGGCCAGTTATTGGGACGCGACAATAACAGCCGGACATTTCACGGAGAGTTA

>13fcf4bb9a13122c116cdf758d356bfc

CAACTTTTTAATAACAACATACAGCCGTTCATTAGCCTCCAAAATGTTAATTACCTCTAGATAGATACAGGGTAATTACATCATCTTCCTTCCCCAGTAATGAGCCA

>1e1aa7e0f92615301af317ce953bd352

CCACCCCACCGCTGTGTTCATACTTTTTTTACAATAACAGATGCCACGATTCACACACACACGCTTAATGTACATTAGATACCCCAGTAGTCCGGCTGACTGACTAT

>80540ce38adc66cb4f5255c24438060c

TACGAGAGGTCCAAACGTTATTCGGAATCACTGGGCTTACAGAGTTCGTAGGCGGTTCTACAAGTGAGGTGTGAAAGCCCTCGGCTCAACCGAGGAACTGCGCTTCA

>9c7d1723a5516679285a06a7aca76115

TCAGTGTGATATACTTTTTACAATACACTATAACATTAGATACCACAAGCCACACACACACACACAGAAAGTCGTAGGACGTAAACGCAGTCATATAATTTTACTGT

>57161bea8cca61d0d5708d7b12602e0f

TACGTAGGTGGCGAGCGTTGTCCGGATTTACTGGGCGTAAAGGGTGCGTAGGCGGCTGTTTAAGTCAGATGTGAAATACCCGAGCTCAACTTGGGTGCTGCATCTGA

>29487eb1e067bc038123cce6dde92363

TACAATAATATAAGACTTACTTTAAAGGAAACACTGTAGTATTAGATACCCGTGTAGTCCGGCTGACTGACTCTCTAGAGATCTCGTATGCCGTCTTCTGCTTGAAA

>c45104c66f064f0ecbc2f6c48d589561

TACGTAGGGTGCGAGCGTTATCCGGAATTATTGGGCGTAAAGAGCTCGTAGGCGGTTTGTCACGTCTGCTGTGAAAGCCCGGGGCTTAACTCCGGGTGTGCAGTGGG

>ae3988f5964a3ddfc41607a95791541b

TCAGTGTGATATACTTTTTACAATACACTATAACATTAGAAACCCCAGTAGTCCGGCTGACTGACTAGTCGCAGATCTCGTATGCCGTCTTCTGCTTGAAAAAAAAA

>34843fd79b6b4ae31fae46acb1c7ef51

CCATTGTGTTAAGTGTGATATACATTTACAATACACTATAACATTAGATGCCACAAGCCACACACACACACATACAAACAAACAGAAAGTCGTAGGACGCAAACACA

>e75464494e0e6ff1ec08b4e85937169e

TACGTAGGGGGCAAGCGTTATCCGGATTTACTGGGTGTAAAGGGAGCGTAGGTGGTGTGGTAAGTCAGATGTGAAAGCCCAGGGCTCAACCCAGGGACTGCATTTGA

>84f7fdd229e70026c27c5385136e4861

TACATAGGGGGCAAGCGTTATCCGGAATTATTGGGCGTAAAGCGTGCGTAGGCGGTTTATTAAGTCTGATGTGAAATTCCACAGCTTAACTGTGGAGGGTCATTGGA

>ba1986bc8f25592920dfb3e8b825b511

TTGGCGTTGGTTCAGCTCTTGTATTAGAAAGCCTCGGTGGCACAGTTCACCACAGTATCTCCATCAGGTATCTCCTTCCTATGGGTACAGTAGGTGGTTTTATCGAT

>1e25f0a8db20fdcbef25d3bb07629a2c

GGAGCCAGACGTACCACCTGGTGTGTGGAACTCTCTGCGGCAGGCATGAAAACCCACTCTGGAACTACTTCTTGTATTACATTACAGTGGTGCCTCGCATATCGCCC

>ee37fe96353469a705680c7cfd089321

TTGGCGTTGGTTCAGCTTTTGTATTACAAAGCCTCGGTGGCACAGTACACCACAGTATCTCCATCAGGTATCTCCTTCCTATGGCTACAGTAGGTGGTTTATCGATC

>8638f9db4994d1b98ab6bb373b5521fb

TACGTAGGGTCCAAGCGTTAATCGGAATTACTGGGCGTAAAGCGTGCGCAGGCGGTTGTGCAAGACCGATGTGAAATCACCGAGCTTAACTTGGGAATTGCATTGGT

>f5560e5e4db554f7028147ee68d8b239

GGTTTTATATCGCCCGCATATAAAATGTAGATAAAAACAGTGTCTCAGTCGACTAAAAACCACTCCCCGGGCCGGGTGAATACATAGGCTGACGAGCCAAAATTTTA

>fc8041d03e332b88a48bef36df87dff0

ACAACGAATAAACCAAAAACGCAATACCGAAATAAAATAATATGGAAAGTTTAGGAATTGGAGAAGAAAAGAGTAAGTGTGAAAACCTTACTCGAATAAATGAAGTT

>8931bc5b2f9432f271ab333e3a100b64

TACGAAGGGGGCTAGCGTTGCTCGGAATCACTGGGCGTAAAGGGTGCGTAGGTGGATCTTTAAGTCAGGGGTGAAATCCTGGAGCTCAACTCCAGAACTGCCTTTGA

>5993f3ee214f96c550b9f1d38601ae0f

TTCACAACAATATTATTCTTTGGTATAACATATGCCTTACTCTAATTAGAAACCCCTGTAGTCCGGCTGACTGACTAGTCGCAGATCTCGTATGCCGTCTTCTGCTT

>7cfc2526adae585c5299b10004d8c5fc

TGTCTGCACCTAATAGGGCATCAACGGGAGCGGGTCAGTCAAATGTCGGATCGGCAAGATCCAAGTCCTTAGCTGCACTCTTAACCTCTAAAGCTATTGCTGTCAGA

>df5b3feaf6b5052e8cb274052695e86a

CACACTTATATCTTTAGAAAGACAAAACATTTTTCCAGGATGCATTTCAACCATGTCGGTCATTTTCCAACTGGCTATATCATTTTCAATTATAATTACCTATATCA

>2c7b18a40e5f5c8b67efae2b460fb6ed

TACGTAGGGGGCGAGCGTTGTCCGGAATTACTGGGCGTAAAGGGTGCGCAGGCTGTTATGCAAGTCTGCTGTGAAATGCACGGGCTCAACCCGTGAGTTGCGGCGGA

>f6a3a4633f977d17bb8d91fab3c8e34f

TACGTAGGGGGCGAGCGTTATCCGGAATCACTGGGCGTAAAGGGTGCGTAGGCGGCCAAATAAGCCAGAGGTGAAAGGCTACGGCTCAACCGTAGTAAGCCTTTGGA

>12aa39daabbc0fa64936cb6020a1f9c1

GTACTCTGTAGACCATAAACCTTCTACCAGTCTGGAAAAATAAATAAATATTAGAAACCCCAGTAGTCCGGCTGACTGACTTGCGTCAAATCTCGTATGCCGTCTTC

>4972a0003094e1cdacdc442dc58b3e16

CAAATTTATGTCTTCAGAAAGACAAAATATTTTTCCAGGGTCCATTTGAACCATGTCGGTCATTTTCAACTAGCAGTGCCTTATAGATATTTGTGAAAGATTTTGAA

>869947e5586c0164f18915a1be5fcd67

CACGTAAGGGGCGAGCGTTGTTCGGAATTATTGGGCGTAAAGGGCGCGCAGGCGGTCTTGTCCGTCAGGTGTGAAAGCTCGGGGCTCAACCCCGGAAGTGCACTTGA

>2093290fe2d3bc5b286e161d76c884ab

TACGTAGGGGGCAAGCGTTATCAGGATTTACTGGGTGTAAAGAGAGCGTAGGTGGTGTGGTAAGTCAGATGTGAAAGCCCGGGGCTCAACCCCGGGACTGCATTTGA

>e364e848d6ae4bd5a16c38a8237546ba

TACGTAGGGTGCGAGCGTTAATCGGATTTACTGGGCGTAAAGCGTGCGCAGGCGGCCCGTTAAGACAGGTGTGAAATCCCTGGGCTCAACCTAGGAATTGCGCTTGT

>d1d1076049def148d4fad36a0e98921d

TACGTAGGTGGCAAGCGTTGTCCGGAATTATTGGGCGTAAAGCGCGCGCAGGTAGTTTCTTAAGTCTGATGTGAAAGCCCACGGCTCAACCGTGGAGGGTCATTGGA

>0d2dcc81fafcbd65de54ed585f2e87ce

TGCCCGTAGTAGTCTTCGTTTCCGTACCAACGCCGTAACGACACCTATAATTGACAATTTATAAATTAAGTTGATTGATAACAGACAAAATACAAATTTTACACTTG

>bb11ec9753bd0d5895fc4587e66e4077

TACGGAGGTGGCAAATGTTGCTCGGAATCACTGGGCGTAAAGCGCGCGCAGGCGGATTGACAAGTCAAGAGTGAAATCTCTATGCTTAACGTGGAGCTTGCTTTTGA

>87457b421f844a0cab5a17c763b81591

TACGTAGGGAGCAAGCGTTGTCCGGAATCATTGGGCGTAAAGGGCGCGTAGGCGGCCTGCTAAGTCAAGTGTGAAAATCAACGGCTCAACCGTTGAACTGCGCTTGA

>6bdba842ebdc963dac6be57e925ab527

AGGCTCCCGCCCCGCAGACCTCATTGGTCAATTAATTCACTCTTCAGCCTTCTCATTAGATACCCCGGTAGTCCGGCTGACTGACTTGCGTCAAATCTCGTATGCCG

>17e6b8300a0edfc72e25c5ebe08fca0a

CCACCGCTGTGTTCAGTTTGATATACTTTTTTTACAATACACAATAACAGATGCCACAAACCACACACACACACACACACACACAAACAAAAAGTCGTAGGACGTAA

>ccd029aad9a76ad3dab5ddbf050f4e53

TACGTAGGGGGCAAGCGTTATCCGGATTTACTGGGTGTAAAGGGAGCGTAGACGGCTTAGCAAGTCTGAAGTGAAAGCCCGGGGCTCAACCCCGGGACTGCTTTGGA

>2f9a659a7dce6532333a43ddb5d638c7

TACGTAGGGAGCAAGCGTTGTCCGGAATTACTGGGCGTAAAGGGCGCGCAGGCGGTTTGATAAGTCAGATGTGAAAGCCCACGGCTTAACCGTGGAAGTGCATTTGA

>130292139fc6030616dab8c53ab1a437

GACGGAGGGTGCAAGCGTTAATCGGAATTACTGGGCGTAAAGCGCGCGTAGGTGGTTTGGTAAGCGAGATGTGAAAGCCCCGGGCTTAACCTGGGAACGGCATTTCG

>d7885779c399bfd3dd13e17528a0b7d3

GAACTAACATGTTTTAGCGGCTAACGCCACCGTGAAGCGTATTAGAAACCCCGGTAGTCCGGCTGACTGACTATGAGCTCATCTCGTATGCCGTCTTCTGCTTGAAA

>506614d22d8e298e8469b84c9f9e1797

TACGGGGGGGGCAAGTGTTATTCGGAATGACTGGGCGTAAAGGGCACATAGGCGGTGAAAAGGGTGGAAAGTGAAAGTCGCCAAAACACTGGCGGGGTGCTTTCTTG

>0447b1542ef8e6a996b224fe366d5a66

CGCACACACGCATTGTTCCGTTACTCTGTCCTTATGGTCCGAACGAAAAACACCAACTCTTGATTAGATACCCTGGTAGTCCGGCTGACTGACTATAGCGCTATCTC

>828a9b785768fe041719f4c272523c49

CACACTTATGTCTTTAGAAAGACAAAACATTTCCCCAGGATGCATTTCAACCATGTCGGTCATTTTCAACTGGCAGTGCCTTGTAGTTATTTGTGAAAGATTTTGAA

>e3c29583684ad280c12bd0ef748a3d2d

CATTGAACTATCGTGAGAAAGTCACGACGCCAAAGGGAATTATATTATAGTAATTATTGGCGTTAATAAACATTTTATTAATAGTTGTAATATATGAAAATGTGCAG

>f673b055c51be6a37822d6df2c982c7f

TACGTAGGTGGCGAGCGTTATCCGGAATTATTGGGCGTAAAGGGTGCGTAGGCGGCCAATCAAGCCAGGGGTGAAAGGCTACGGCTCAACCGTAGTAAGCCCTTGGA

>0d414db43f0e9f5458f716e78be91d20

CACTTGAAACATATAGTGCAATCAACACCGCGGGAACATTAGAAACCCGAGTAGTCCGGCTGACTGACTACGTACGTATCTCGTATGCCGTCTTCTGCTTGAAAAAA

>346d13453295f725567bc6c870577191

TACGTAGGGTGCGAGCGTTAATCGGAATTATTGGGCGTAAAGCGCGCGCAGGCGGTTTCTTAAGTCTAATGTGAAAGCCCACGGCTCAACCGTGGAGGGTCATTGGA

>fdf88d56ba69e75c2c3fe9bcbc8bae9a

CCACCACCACCACTACTGTGCTGAGTGTGATATACTTTTTTTACAATACACAATAACAGATGCCACAAGCCACACACACACACGCACAACACACACACACACACAGA

>123a59c0830ff2fdd72761c1b8e718ca

ACAGCCTCTCTGCAAATACCAGTGGTGTCGATATATTACAGATTCTTTCAGGTAATGATGGATCTACAGTTGAACACCTGCTCCGAACGGCTAAGTAGAGAGGTTTC

>2eff9731a55d333d382b441f1c0366ea

CATTGAACTATCGTGAGAAAGTCAAACCGCCAAAGGGAATTATATTATAGTAAATATTGGCGTAAGTAAACATTTTATTAATAGTTGTAATATATGAAAATGTGCAG

>a2c6d6b9b30c16043555b272cbc9021e

TTGGCGTTGGTTCAGCTCTTGTATTAGAAAGCCTCGGTGGCACAGTTTACCACAGTATCTCCTACAGGTATCTCCTTCCTGTGGCTACAGTAGGTGGTTTATCGATC

>a406c6e119ecd2c70548740348a9c34c

TACGTAGGGGGCTAGCGTTGTCCGGAATTACTGGGCGTAAAGAGTGCGTAGGCGGATGATTAAGTGAGATGTGAAATACCCGAGCTCAACTTGGGTGCTGCATTTCA

>8d422d4af01b2268cf5805b56d1f12d1

TACAAGGAAGACTAGTGTTATTCATCTTAATTAGGTTTAAAGGGTACCTAGACAGTATTTCTAGCCTCCAAAGGGAACAGACTTACTAGAGTTTTATGTGAGGAAAA

>7de71ae6056bfa558852f2e532cf4f9a

TACGTAGGGGGCGAGCGTTGTCCGGAATCACTGGGCGTAAAGGGTGCGTAGGCGGTCGATAAAGTCTGGGGTGAAAGGCTACGGCTCAACCGTAGTAAGCCTTGGAA

>efb19fd3a1ffaee3491a5638f37859fc

TACGTAGGTGGCAAGCGTTATCCGGAATTATTGGGCGTAAAGCGCGCGCAGGTGGTTTCTTAAGTCTGATGTGAGAGCCCACGGCTCAACCGTGGAGGGTCATTGGA

>47cfa7b2fbf04d19fa125ad807fd3275

CACGATTAACCCAAGTCAATAGAAGCCGGCGTAAAGAGTGTTTTAGATCACCCCCTCCCCGATAAAGCTAAAGCTCACCTGAGTTGTAAAAAACTCCAGTTGACACA

>5957970ad1476d48da7f554b200de251

CTCTCTTTGATCTATTAAAGTTTTATTAGCTTATAAGCTACTTGCTATCTGCCATGCATGTCATGTCCTTTATTAGAAACCCGAGTAGTCCGGCTGACTGACTCTAC

>6479973478688133ff451d59a8091428

TTCCATCTCCGGTAGTATATGTTAAAAGCGTTGCGATTAGAAACCCGGGTAGTCCGGCTGACTGACTCTATCGTGATCTCGTATGCCGTCTTCTGCTTGAAAAAAAA

>2019023e9603ed43deba5c72d1c25f16

TACGTAGGGGGCGAGCGTTGTCCGGAATTATTGGGCGTAAAGCGCGCGCAGGCGGCTGTTTAAGTCTGGTGTTTAATCCTGGGGCTCAACCCCGGGTCGCACTGGAA

>0c9281c4807d06224a3304aac7c8c702

ATTCTGAAACCGGATAACGGATTATCGTCTACACCATTGGATACTCTTCCAGAAGACCCCAGGACTAAAATAATTGTCTCTTAATCTATCTACTAGTTTCCACAACA

>1476f1e7a837c368c63185d8862fa055

TACAGAGGGTGCGAGCGTTAATCGGAATTACTGGGCGTAAAGCGCGCGTAGGCGGCTTGTTAAGTCAAATGTGAAATCCCCGAGCCTAACTTGGGCATTGCATTCGA

>970b52a7872cb4b557046d2040a995aa

TACGTAGGGTGCGAGCGTTGTCCGGAATTATTGGGCGTAAAGAGCTTGTAGGCGGTTTGTTGCGTCGGAAGTGAAAACTCAGGGCTTAACCCTGAGCCTGCTTTCGA

>87ab680cef284e1c37e72f323bf7b769

GAACAGCTCATGCTTTCCTCCCCAGCCTTTCTCTCCCAGCTTCTATTAGAAACCCCTGTAGTCCGGCTGACTGACTTGCGTCAAATCTCGTATGCCGTCTTCTGCTT

>8a283ce62684dac42fe4018a4dd40d07

TACGTAGGTGGCAAGCGTTGTCCGGAACTATTGGGCGTAAAGCGCGCGCAGGTGGTTTCTTAAGTCTGATGTGAAAGCCCACGGCTCAACCGTGGAGGGTCATTGGA

>3db4f3b149ab87f2d0a69e8c0fcc4817

TCCTTTGCAATGCTCATCCGCTCACCAAGAATGGAATGGCGCACCATCGACTTTATGCCGGCAGCCATTCGGTCGTCGAAAGCTATTCTGCATGGATAGGGTTTGTT

>82559090af42a6e5485c5ef55161ecac

TACACTTATCTCTTTAGAAAGACAAAATATTTCTCCAGGATGCAGTTCAACCATGTCGGTCATTTTCAACTGGCAGTGCCTTGTAGATAGTTGTGAAAGAATGTGAA

>9a0c01e610d91886ecf6577ad8acfcc8

GGAGGGTGCACCAGTATTGTGAAGAGTTTGGGAGTCCCAAAACGATTCTCACTGACAATGGAACACAATTTACATCAAAAAAATGGGTGAACGGCTTGAATGAGCTA

>ea4c07068d46ef0d0fcd3e9cbb472991

CACACGTGACAAAAAATGGCAACACACGATACAAGCCAAATCTAACAAAGCTCAAACAGTTTCGGTAAAAAAAGGTAGAAATGGTAGAACCGTAGGCGGACTGAGGC

>4e1919d65244e9ada35a5c30479122d9

CGGCGTGACAATAGTCAAACATATCCCACACAGAGGCAAAATCATGAACGTTTCGTAAAATCTACATATATGCTAACGCAGAACACAAAACCTGTTGATATATGAAC

>86cbc5484dbed4b6721633a461e733ae

CATTGAACTATCGTGAGAAAATCGCGCCGCCAAAGGGAATTATATTATAGTAATTATTGGCGTTAATAAACATTTTATTAATAGTTGTAATATATGATATTGTGTGA

>a89aeb416c77b089ab451d1771b323ed

CATCGATGCCATCAAATTTATTTATAATCTGTCTGATTCATCATCGATGCTCAATTTATCTGACGTCTTCAGCCGGTTGATAATACCAGTTATTATGTTTAACCCCA

>cc5da1fb56e48d651ead7eaab84052ec

TGTAATGTCCAATGAACCGTTAAAACTACTTTGACACCCCAGTGTAGTCTGTATTGTAACAAAAGCATTGTATTAGGAACTAAACAAAAATGTTTCTATTCCTTGTT

>ab5a7ba63acd78833e3407f36edb4af9

TACGGAGGATCCGAGCGTTATCCGGATTTATTGGGTTTAAATGGAGCGTAGATGGGTTGTTAAGTCAGTTGTGAAAGTTTGCGGCTCAACCGTAAAATTGCAATCGA

>7f01ccc832d9b60e67c99c6fe51b2f1d

TACGGAGGGTGCAAGCGTTAATCGGAATTACTGGGCGTAAAGCGCACGCAGGCGGTTCGTTAAGTCAGATGTGAAATCCCCGAGCTTAACTTGGGAACTGCATTTGA

>1dca2fce4f9c032df19bb5cd05878fec

TACGGAGGGTGCAAGCGTTAATCGGAATTACTGAGCGTAAAGCGCACGCAGGCGGTCTGTCAAGTCGGATGTGAAATCCCCGGGCTCAACCTGGGAACTGCATTCGA

>12a74694fbc9eb8ccb15f88867d50bfd

GAACAGCTCATGCTTCCCTCCCCAGCCTTTCTCTCCCAGCTTCCATTAGAAACCCTTGTAGTCCGGCTGACTGACTATGAGCTCATCTCGTATGCCGTCTTCTGCTT

>58cdab7e2eaf4ce4f4165f2429ce3ad0

ATGCAAATTGGGATCACTCGATTGAAATCTTAAACCGCATTAGAAACCCTAGTAGTCCGGCTGACTGACTACGTACGTATCTCGTATGCCGTCTTCTGCTTGAAAAA

>df421fb3e3ba9592e5171a4287cea985

TACAATTTCTTCAATTTAAATTTTTGAAAGTTTCAGTTAATAAATTATTTAAATTTTTATGAATAATAATTTTAGTGAAATATATTATTATTTTATTTTATTAATTT

>b083f31711f5aac4dfa621469026d19d

TACGTAGGGCCCAAGCGTTAATCGGAATTACTGGGCGTAAAGCGTGCGCAGGCGGTTGTGCAAGACCGATGTGAAATCCCCGAGCTTAACTTGGGAATTGCATTGGT

>ac2e8c1040be6959ee8f817e53a7e9ea

GACGCTTATAGCCCACCTTTATATTAGTTACCACAATATTCCACAGCGTTCTTCACTGTTTCGTTGTTATTCTGTATTGGAGGTTGTACAGCGCCACAACCATAACA

>116d7ac56e123b67c486d9bdbd04b8d2

GAACAGCTCATGCTTTCCTCCCCAGCCTTTCTCTCCCAGCTTCTATTAGAAACCCTGGTAGTCCGGCTGACTGACTTGCGTCAAATCTCGTATGCCGTCTTCTGCTT

>aba7939af0054180c19a953e092d4aa3

TACGTAGGGCGCAAGCGTTGTCCGGAATTACTGGGCGTAAAGAGTTCGTAGGCGGATGATTAAGTGAGATGTGAAATACCCGAGCTCAACTTGGGTGCTGCATTTCA

>cc692cbfa66afa113a11a01912415ad6

TACGGAGGGTGCAAGCGTTAATTGGAATTACTGGGCGTAAAGCGCGCGTAGGTGGTTTGATAAGCGAGATGTGAAAGCCCCGGGCTCAACCTGGGAACGGCATTTCG

>2ca8d777a7d8dd4ad2d2cd9a7bd4a60d

TATCCGTGTATCCGTTTTTCAGAGCCTCAAACAGTATTTTTGTTTTAGCACTCATGCTTAAGTTTTTTCCTCAATTACCATCGTTATGTACGTAGTTTTCTCCATAT

>9695cddff4b0a027f42fe557485cc06d

GTTGCACAAAATACAATGCGATACATATTTTATAAAACAGCAAGGTTCACCTTACACTAGTGTTGAGTGACTGGAAAATGTTCGCCTACGGTTACCTCATCTTAACC

>b155402bd6b8bf11de97c989d95e2c45

TACAATTTCTTCAATTTAAATTTTTAAAAGCTTCAGTTAATAAATTATTTAAATTTTTATAAATAATAATTTTAGTGAAATGTATTATTATTATATTTTATTAATTT

>176377bc7420cd93b983ebfae83d0892

TACGGAGGATCCGAGCGTTATCCGGAATTATTGGGTTTAAAGGGTGCGTAGGCGGCCAGATAAGTCAGGGGTGAAAGTCGGCAGCTCAACTGTCGCAGTGCCTTTGA

>959859b448dae319d741dcdb37f80fa9

CTGCGTTCACAGTAGAAATCGTTCCGTAAGATGTCGTCAGACATAAGTGATCGCAGCAATTAATGTCGCTGTGTGCCACGTGACGAATTACACATGAGAGAGAGAGA

>9f20b9ea324a7c9118e1a8369ad75d57

TACGTAGGGCGCAAGCGTTGTCCGGAATTACTGGGCGTAAAGGGCGTGTAGGCGGCCGATCAAGTCAGGTGTGAAATTTCAGGGCTCAACCCTGGACGTGCACTTGA

>1b5c8d5fb2045fecb63a34d10338e40a

TACGAAGGGTGCAAGCGTTAATCGGAATTACTTGGCGTAAAGCGCGCGTAGGTGGTTTGATAAGTTGGATGTGAAAGCCCCAGGCTCAACCTGGGAATTGCATCCAA

>daf56a2fe60f0eeb7adc332e5ff6e3dd

CTGGAGACCGTCTGCAACATGTTTGTGAAGGTAAACAGTTACGTGTATTATTGCGTGTATTAGAAACCCCTGTAGTCCGGCTGACTGACTCGCGATATATCTCGTAT

>38af27982bf302e3b261b5dbc0d5b412

CATGGTCATCCAGCCAGATTTCGTATTTATGTACGTGACGCTTGACTTTATTCACGTGGAAAGCGATGCTTTAACCACACGACCGTACCACCGGGTGAATAGAGTTA

>7601278c24c9389eaa510c745231767c

CCACAACCCACCGCTGTGTTCATACTTTTTTTTTTTACAATAACAGATGCCACAATTCACACACACACAATGTACATTAGATACCCGTGTAGTCCGGCTGACTGACT

>6ab71d03e602f53c605380e0c9e47a27

GACAAGATAGGAAAATTGCTATATCTGTGGCTTCGTCAAGTATAGCAGCAACGCTATTAGAAACCCCTGTAGTCCGGCTGACTGACTCAGTAGGTATCTCGTATGCC

>6c973ea18ccb30dd870339b9898975a7

CATTTTACCATAGTGTCTGCCTCCACGGGCACGTTTATGGCTTTTCCGGTTATCTTTTAAGACTGTTTCTATTCTGAGACGACGGATCTGCATACGTGGAAATCCGG

>1d945ef53578941f271c6bc476a70a23

TACGTGAGAGACTAGTGTTGTTCATCTTAATTGGGTTTAAAGGGTACCTAGACAGTCAATATAACTTCTATAATGCTAATACTCGACTAGAGTTTTAAGTAAGAGGG

>aaae77e7dfd05b64005a64b67c073bde

CACGTAGGGGGCAAGCGTTGTCCGGAATTACTGGGCGTAAAGCGCGCGTAGGCGGTTTTGCAAGTCAGAGGTGAAAACCGGCAGCTCAACTGCAGGCTGGCCTCTGA

>7921d95d70a6d789d6fe767c70e540d7

TACGTAGGGGGCGAGCGTTGTCCGGAATTACTGGGCGTAAAGGGCGTGTAGGCGGCCTTGAAAGTCAGTCGTGAAACCCGACAGCTCAACTGTCGGCTGGCGATTGA

>776a521f344e7eaab40d59fba7e9bf15

TACGTAGGTGGCAAGCGTTGTCCGGATTTACTGGGCGTAAAGGGAGCGTAGGTGGATATTTAAGTGGGATGTGAAATACCCGGGCTTAACCTGGGTGCTGCATTCCA

>43ac3ead7bbd374a7a1cd6f8d1113fec

TTAGCTGGATGTGCCTCAGACCACGGAAGTGATAGCGTTTTTTAGTTATAGTCATAATGTACTGAAGAAACTATCGACAAGAAGATGGGAAGCTAAGAAGATGGCAC

>68de652fac9d390bdffb4c064a130923

TACGATTTCTTTAATTTAAATAGTTAAGTTTCAGTTAATATAACAATAATGTAAAATATCTATAATTTTGGTGAAATATATTTTATCTTTAAAAATTAATTTTATGT

>be811e02eec65d19ff7e5f9146c5bc1d

TGGACTCCGAGATGTCTGATAATGAACAAGAAACAGATGGTGAATTAGAAACCCCTGTAGTCCGGCTGACTGACTACGTACGTATCTCGTATGCCGTCTTCTGCTTG

>b9c4fb1a41758e4c72d66c24d5527731

TACGTAGGGGGCAAGCGTTATCCGGAATTACTGGGTGTAAAGGGTGCGTAGGTGGTATGGCAAGTCAGAAGTGAAAACCCAGGGCTTAACTCTGGGACTGCTTTTGA

>7177e0d3ca714d90fd425492dd647dcb

ACAACAAGGGGAACCTAACAATGAGCCGCCTCGCAATCAATCAATTTTCGCCACGCCACATCAACGCCATTCGTGTAGCAGAATTTTTCTTCGCCTATCTACACACA

>8f27015c047e9c87f62417cda765bf6a

TACGTAGGGCGCAAGCGTTGTCCGGAATTATTGGGCGTAAAGAGCTCGTAGGCGGTTTGTCGCGTCTGGTGTGAAAACTCAAGGCTCAACCTTGAGCTTGCATCGGG

>025178e03a3806a5b8a97ee8ca931ac6

ATGCAAATTGGGATCACTCGATTGAAATCTTAAACCGCATTAGAAACCCCAGTAGTCCGGCTGACTGACTACGTACGTATCTCGTATGCCGTCTTCTGCTTGAAAAA

>24a3ded66c6e29064edda8af25cea5b4

TACGTAGGGTGCGAGCGTTGTCCGGAATTACTGGGCGTAAAGAGCTCGTAGGTGGTTTGTCGCGTCGTCTGTGAAATCCCGGGGCTTAACTTCGGGCGTGCAGGCGA

>93ecdca152ae087d3c0cb8b7aab27776

TACGAAGGTCCCAAGCGTTGTTCGGAATCACTGGGCGTAAAGGGAGCGTAGGCGGCGTGGTAGGTCAGATGTGAAATCCCGGGGCTCAACCCCGGAACTGCATCCGA

>322b039317d4409ab2bee07b71a1d917

TACGTGAGAGACTAGTGTTATTCATCTTAATTGGGTTTAAAGGGTACCTAGACAGTCAATATAACTTCTAGAATCCTAATACTTGACTAGAGTTTTAAGTAAGAGGG

>b9186c2f6474de333b909fce8c9997d1

TGTCCGCACCTAATAGGGCATCAACGGGAGCGGGTCAGTCAAATGTCAGATCGGCAAGAACCAAGTCCTTAGCTGCACTGTTAACCTCTGAAGTTATTGCGGTCATA

>fa8b817b5c973006bcab0f504949b4d6

GTGCAAAAGGTGCCCTATTTGGAAGAGATAAGACATTTTATTCATGAGCATTTTACCTTGAATTGGAATTTATCTTGAAATTAGAAACCCTGGTAGTCCGGCTGACT

>c6a5ef2bd68c1c976b1da2f1cff682fb

TACGTAGGGAGCGAGCGTTGTCCGGAATTACTGGGCGTAAAGGGTGCGTAGGCGGCGATGCAAGTCAGATGTGAAATCCCCGGGCTTAACTCGGGGGGTGCATTTGA

>77608f82196654653cce7f4d3b35c625

GACCAAGACTAAAGACTTGCAGTGCGTGGATCACGACGACGAAGCTCTGTCCATGTATTGTATGGTCTATTAGAAACCCCTGTAGTCCGGCTGACTGACTCTATCGT

>5c81d5e142a36976515ad464ab2eaa5c

TACGTAGGGGGCAAGCGTTATCCGGATTTACTGGGTGTAAAGGGTGCGTAGGCGGCGAAGTAAGTCAGATGTGAAATCCCCGGGCTTAACCTGGGAACTGCATCTGA

>b210c67a87495f458a5bb0f15b256cca

TACGTAGGGGGCGAGCGTTGTCCGGATTTACTGGGCGTAAAGGGTGAGTAGGCGGTTGTATGTGTCAGATGTAAAAGGCTAAGGCTTAACCATAGTTAGCATTTGAA

>8e7760ddb2b9b5427cfc1b7ad2ec6720

TACGTAGGTGGCAAGCGATGTCCGGAATTATTGGGCGTAAAGCGCGCGCAGGCGGTTTCTTAAGTCTGATGTGAAAGCCCACGGCTCAACCGTGGAGGGTCATTGGA

>9ce3739512397750f1b613699679defe

TACGTAGGTGGCAAGCGTTATTCGGAATTATTGGGCGTAAAGCGCGCGCAGGTGGTTTCTTAAGTCTGATGTGAAAGCCCACGGCTCAACCGTGGAGGGTCATTGGA

>b01e26251501dd469083347402e6f1b3

GATATATTGAGCTATTTGTAGCTGTGAAGTGTACCAGATATGTTATTAGTATTAATTGAGTTGAGGCGGAGCTCTACAATTACGGACACTTGAAGTGGTGTAGCCTA

>3d5555018bd22e6702ff18016d5c8041

TTTGTAATCTTCCCGAAAAACAAAAACTAAATAAACAAATTTGTATGTTGATGTTCTGTAGTTATTTCTCCATCACTTCCACTGGTTTAAAAATTTTAATTCACTTT

>27afd56a53240f7fcc2255469ed7b360

TGTCCGCACCTAATAAGGCATCAACGGGAGCGGGTTGGTCAAAAGTCGGATCGGCAAGAACCAAGTCCTTAGCCGCACTCTTAACCTCTGAAGCTATTGCTGTCAAA

>a22e79bffeaf686cec9847102c3c55ed

TACGGAGAGTGCAAGCGTTAATCGGAATTACTGGGCGTAAAGCGTACGCAGGCTGTCAAGTCAGTCAGATGTGAAAGCCCCGGGCTTAACCTGGGAACTGCATTTGA

>7c49ca4823def0ec51283fde6012ee2d

ACAAATGGTGAATAGTATAGTGTCAATAATGGCATTAGAAACCCTTGTAGTCCGGCTGACTGACTACTGTGTAATCTCGTATGCCGTCTTCTGCTTGAAAAAAAAAA

>5956f805fc8dab2508842f0d3e54bc0f

TACGAAGGGGGCTAGCGTTGCTCGGAATCACTGGGCGTAAAGCGCACGTAGGCGGATTGTTAAGTCAGGGGTGAAATCCTGGAGCTCAACTCCAGAACTGCCTTTGA

>4b87dfbe938da7075366c06f9a8d8dd5

TACGGAGGGTGTGAGCGTTATCCGGAATCACTGGGCGTAAAGGGCGTGTAGGCGGGACGTTAAGTCTGGTTTTAAAGACCGCAGCTCAACTGCGGGAGTGGACTGGA

>a0fa4922ee3058d3181fe240a31deb6f

CCACCACAACCCCATCGCTGTGTTCATACTTTTTTTTACAATAACAGATGCCCCAATTCACACACACACACACACACACACACACACTTAATGTACATTAGATACCC

>56835b6a8da8c7728c3eb2726cdf27bc

TACGATTTCTTTAATTTAAATAGTTAAGTTTCAGTTAATGTATTAATAATATAAAATAACTATAATTTTGGTGAAATATATTTTATCTTAAAAAATTAGTTCTATGT

>00c4db2565827d75244d484ca18a2bdf

TACGGAGGATGCGAGCGTTATCCGGATTTATTGGGTTTAAAGGGTGCGTAGGTGGTTAATTAAGTCAGCGGTGAAAGTTTGTGGCTCAACCATAAAATTGCCGTTGA

>e301948a22f54de3e391e91905be1366

GTCGCTACCGATGAAGGTTGTATACCATCCGTAAATAAATGAATTTTACGCTTATTCTAACCCTTACTGTATCGTAGTTTTATTCGCTATGCTAAGCGCGGGATTAT

>1c76d379c4b1b85c751c4d3c50232e5f

CCTAATGCAGGCAAAAAGAAGCGGCAACGTAAGCCCCGTGGCGCTTGATCCCGGGCTCCTGCCAACACAGCCGCACAGTCCAGACAGCCTGCGCAGCGCGTGAAGAC

>66ed8d5e2b90f765b9a1aca972c9a4f7

CATTGAACTATCGTGAGAAAGTCACGCCGCCAAAGGGAATTATATTATAGTAAATATTGGCGTAAATAAACATTGTATTAATAGTTGTAATATATGAAAATGTGCAG

>4fc8826d00450ea18b31a6a41859801f

TACGTGAGAGACTAGTGTTATTCATCTTAATTGGGTTTAAAGGGTACCTAGACAGTCAATATAACTTCTAGAATGCTAATATTTGACTAGAGTTTTAAGTAAGAGGG

>a624f10ded52f594c307b9574ee4d7d4

TACGTGAGAGACTAGTGTTATTCATCTTAATTGGGTTTAAAGGGTACCTAGGCGGTCAGTATAACTTCTATAATGCTAATACTTGACTAGAGTTTTAAGTAAGAGGG

>6f3d29142feb1bbe6fa42127e83af38f

TTCCAGCTCCAATAGCGTATATTAAAGTTGTTGCGATTAGAAACCCTAGTAGTCCGGCTGACTGACTGTTACAGCATCTCGTATGCCGTCTTCTGCTTGAAAAAAAA

>22effd645f1da46ddae51c26b8c9a71a

CCACCCCCACCGCTGTGTTCATACTTTTTTTACAATAACAGATGCCACAATTCACACACACACACACTTAATGTACATTAGAAACCCTGGTAGTCCGGCTGACTGAC

>42ddad4fc426af6c130e684c15894c4e

CCACCCCCACCGCTGTGTTCATACTTTTTTTACAATAACAGATGCCACAATTCACACACACACACACTTAATGTACATTAGATACCCCTGTAGTCCGGCTGACTGAC

>971c1b3fb11a0979ac35c78f20001ae4

TACGTAGGGGGCGAGCGTTGTCCGGAATTACTGGGCGTAAAGGGTGCGTAGGCGGCCAATCAAGTCAGATGTGAAATACCACGGCTCAACTGTGGGGGTGCATTTGA

>50d261baabaa2722376559e592de6090

TACGTAGGGTGCGAGCGTTGTCCGGATTTATTGGGCGTAAAGGGCTCGTAGGTGGTTGATAGCGTCGGAAGTGTAATCTTGGGGCTTAACCCTGAGCGTGCTTTCGA

>af840018873a2cec942468b917c1f40e

ACATTCATTGTACTTCATTAGTTATTTAATTTACACCACTTGTCACCCCCTTATATTGTACAAACGAGTTTGCTGGCACATTATGTGAAATACACGTTCCTGTTACA

>af63c2ded75655e3d5b4d0cf850dca7a

CCACCCCACCGCTGTATTCATACTTTTTTTTTTACAATAACAGATGCTACAATTCACACACACACACACACACTTAATGTACATTAGATACCCTTGTAGTCCGGCTG

>2cedde430f81b59d5aece2b988732324

TACAATTTCTTCAATTTAAATTTTTAAAAGCTTCAGCTAATAAATTATTTAAATTTTTATAAATAATAATTTTAGTGAAATGTATTATTATTATATTTTATTAATTT

>3cc41c19bccb92fe2c9dc3739f4848e7

TACGTAGGGTGCAAGCGTTGTCCGGAATTATGGGGCGTAAAGAGCTCGTAGGCGGTTTGTCGCGTCTGCTGTGAAATCCCGAGGCTCAACCTCGGGCCTGCAGTGGG

>8610df99c4b4546b9daa00d652f50426

AAATCAGACAGTAGTTTCGCACTTGATTTCTCAACCACTGAGCATTCATGTCCATCACCCATCCCTACAGCCAATATTAGAAACCCCAGTAGTCCGGCTGACTGACT

>5f3a62888782edd498ecb3d56b8094de

CACGGGGGGCGCAAGCGTTATTCGGAATTATTGGGCGTAAAGGGCGCGCAGGCGGCCTGGTAAGTCAGATGTGAAAGCCCGGGGCTTAACCCCGGAAGTGCATTTGA

>1ccb0eaae5c8f4c164a6ae7766d3fba2

TACGTAGGTGGCGAGCGTTGTCCGGAATTACTGGGTGTAAAGGGAGCGTAGGCGGGCTTGCAAGTCAGAAGTGAAAGGCTACGGCTCAACCGTAGTAAGCTTTTGAA

>80944bbec78f714c2db31d1f6de5b1e1

TACGTAGGGGGCGAGCGTTATCCGGAATCACTGGGCGTAAAGGGTGCGTAGGCGGCCAATAAAGTCTGGAGTGAAAGGCTACGGCTCAACCGTAGTAAGCCTTGGAA

>5a34fb3318c2dcf940cf6c3429243b37

TTAAAGAAACGCCGTCTACTACGAATGTTTATGGACATCAAATTTGTGTTATTAACGAAAACAAATTCATTCGTAAAACATACCCGGCACCGCTACACTATCAAGAA

>cf3204e72be00f61d237312f8ea13442

CCACCACCCACCGCTGTGTTCATACTTTTTTTACAATAACAGATGTCACAATTCACACACACACACTTAATGTGCATTAGAAACCCGTGTAGTCCGGCTGACTGACT

>80dcebe82845b0613512e1725034fb47

GTCGCTACCGATGAAGGTTGTATACCATCCGTAAATAAATGAATTTTATGCTTATTCTAACCCTTACTGTATCGTAATTTTATTCGCTATGCTAAGCGCGGGATTAT

>61e4746df8ddce97ebe99290b70500d6

ATCACCACACACACACACACACACACACACACACACACACACACACACACACATATTTGTCAAACGCTGAAACTGTTTTCACACCTCGTTACCAATGTACTAAATGA

>8356e2f7317d05f960667983cdb474ca

TACGATTCATTCAATTTAAATATTAATGTTTCAGTCTGAGAAATTTAGTGAAATATATTTTAATAAATGATTAATTATTTTTGTCTGCGTAACTTTTATGTTAAAGT

>0f68e181f500650c4e8712f341785793

GTTAACATCGGACTTTGTTCTTTATACTTGTTCAGACGTGTTACAGTTGCGGTAAATTTTTAGTTCATATTTCCTGTTTATACCCACACACTTGTTCATATTTTTTG

>8dd6bb69fd7b7e626eaf965759063277

CACTTGAAACATATAGTGCAATCAACACCGCGGGAACATTAGATACCCGAGTAGTCCGGCTGACTGACTACGTACGTATCTCGTATGCCGTCTTCTGCTTGAAAAAA

>574017cb3dafff474fea7afbc970c91a

TACGTAGGGGGCAAGCGTTATCCGGATTTACTGGGTGTAAAGGGAGCGTAGACGGTGATGTAAGTCAGATGTGAAAGCCCAGGGCTCAACCCTGGGACTGCATTTGA

>082d688248b0ffb6724dac816e1bb226

TACGTAGGTGGCGAGCGTTGTCCGGAATTATTGGGCGTAAAGAACTCGTAGGCGGTTTGTCGCGTCTGCTGTGAAAACGCGAGGCTTAACCTCGCGCCTGCAGTGGG

>8657938fe647c1722a477c8045fd0913

GAGGATTATTAAGTTAACCCTTTCATAACCTCGGTAAGTCTTCGGTTAATATAGGCCAGGTTGCACTATTTTACCGTAAGGCTGAGAAGTAGGGCAACGCAGTTACC

>1ef542f2696705930f810774dbbec406

CTCTCTTTGATCTATTAAAGTTTTATTAGCTTATAAGCTACTTGCTATCTGCCATGCATGTCATGTCCTTTATTAGAAACCCCGGTAGTCCGGCTGACTGACTCTAC

>f9c9dd61e2b816f0ca3f323cc9c4979b

TACGTAGGGTGCAAGCGTTGTCCGGAATTATTGGGCGTAAAGAGCTCGTAGGCGGTCTGTCGCGTCGGCTGTGAAAACTTGGGGCTCAACCCCAAGCCTGCAGTCGA

>9b093bfb38cf45736235ba0e05b76104

GGAGGGTGCACCAGTATTGTGAAGAGTTTGGGAGTCCCAAAACGATTCTCAGTGACAATGGGACACAGTTCACATCAAAAAAATGGGTGAACGGCTTGAATGAGTTA

>afe671d74f6357a97675d85bae2ddd3f

TACGTAGGTGGCAAGCGTTGTCCGGAATTACTGGGCGTAAAGCGCGCGCAGGTGGTTTCTTAAGTCTGATGTGAAAGCCCACGGCTCAACCGTGGAGGGTCATTGGA

>d4c5137ae6fb8b7d582fd5d445406c7b

TACAATAATATAAGACTTACTTTAAAGGAAACACTGTAGTATTAGAAACCCGAGTAGTCCGGCTGACTGACTCTCTAGAGATCTCGTATGCCGTCTTCTGCTTGAAA

>a7093819c2e897d4019949594d64ead1

TACGTAGGTGGCGAGCGTTGTCCGGAATTACTGGGCGTAAAGGGCGCGTAGGCGGCTCTTTAAGTCAGATGTGAAAACCCAGGGCTTAACTTTGGGAGTGCATTTGA

>2b36e4af6fb925287b1e229ec3c2700f

ACAAATGGTGAATAGTATAGTGTCAATAATGGCATTAGATACCCGAGTAGTCCGGCTGACTGACTACTGTGTAATCTCGTATGCCGTCTTCTGCTTGAAAAAAAAAA

>eb13c60e3e0efeec615f199525307ba1

TACGTAGGGTGCAAGCGTTAATCGGAATTACTGGGCGTAAAGCGTGCGCAGGGGGTTATGCAAGACAGAGGTGAAATCCCCGGGCTCAACCTGGGAACTGCCTTTGT

>ad36ebf5909f35a5770e813e6e87ab54

TACGGAGGGTGCAAGCGTTAATCGGAATTATTGGGCGTAAAGCGCGCGCAGGTGGTTTCTTAAGTCTGATGTGAAAGCCCATGGCTCAACCGTGGAGGGTCATTGGA

>620ff35886105be0958b28ef336043eb

TACGTAGGTGGCAAGCGTTATCCGGAATTACTGGGTGTAAATGGCGTGTAGGCGGGTAAGCAAGTCAGATGTGAAATTCCGAGGCTCAACCTCGGCGCTGCATCTGA

>41865a0fc6e1bdd4a2aacb3646ae4b2e

GAACAGCTCATGCTTTCCTCCTCAGCCTTTCTCTCCCAGCTTCTATTAGAAACCCTAGTAGTCCGGCTGACTGACTATGAGCTCATCTCGTATGCCGTCTTCTGCTT

>0b3eb1b44f9a62a743fca4ed1d8bf0c7

AATCGTGGTGTAGCCAAGGGCTCGCTGCCGCAGACGTGAGGACTTGCCGCAGTGCAGAAACTTTCAGTGCCCTCCCCGCACTCGCCATATGCGAGATGCGAGGACCA

>ee00eedc29602c4b39b7c71b0b9ef293

CATTGAACTATCGTGAGAAAGTCAAACCGCCAAAGGGAATTATATTATAGTAAATATTGGCGTAAATAAACATTTATTTAATAGTTGTAATATATGAAAATGTGCAG

>a16b1b5b6cf28ab76ccb935e479138df

TACAATTTCTTGAATTTAAATTTTTAAAAGTTTCAGTCAATAAGATATTTAAATTTTTATAAATAATAATTTAAGTGAAATACATTACTATTTTATTTTATTAATTT

>1686dde01ba1ef594271c1cd0c26e185

TACGTAGGGGGCGAGCGTTGTCCGGAATTACTGGGCGTAAAGGGTGCGTAGGCGGCTATTTAAGTTGGATGTGAAATACCCGGGCTTAACTTGGGGGGTGCATTCAA

>78bafe4f7413202dda1f6b4a009693fa

TACGTAGGGTGCAAGCGTTGTCCGGAATTATTGGGCGTAAAGAGCTCGTAGGCGGTCTGTCACGTCGGCTGTGAAAACCCGAGGCTCAACCTCGGGCCTGCAGTCGA

>3ff759059c5bb12642929680f0a99720

ACATCAGCTGGAGTGACGCAGTCACACGCGAGTCACGCCGCACAGCCTAAGCCGAAGCGCTTTCGTAAGCCACGCACTACACGTCAGCCTGTAATGGCCAGGCAAAT

>0724b51e0b342d10f9edd2ea714edcf3

TACAGAGGATGCAAGCGTTATCCGGAATGATTGGGCGTAAAGCGCCTGTAGGTGGCTTTTTAAGACCGCCGTCAAATCCCAGGGCTCAACCCTGGACAGGCGGTGGA

>215337a22e87e5cad0a753f201dfec41

TCCGGCTGACTGACTCGCGATATATCTCGTATATTAGAAACCCGTGTAGTCCGGCTGACTGACTCGCGATATATCTCGTATGCCGTCTTCTGCTTGAAAAAAAAAAA

>c4eca3fbbcb4ef6e5ff16a0d1bb71d09

TACGTAGGGTGCGAGCGTTGTCCGGAATTATTGGGCGTAAAGGGCTCGTAGGCGGTTTGTCGCGTCGGGAGTGAAAACCAGGTGCTTAACACCTGGCTTGCTTTCGA

>2362f0f9c8cfe1f0614ce36564fe7612

TACGTAGGGTGCGAGCGTTAATCGGAATTACTGGGCGTAAAGCGTGCGCAGGCGGTAAGTTAAGTCTGATGTGAAAGCCCCGGGCTCAACCTGGGAACTGCATTCGA

>ddacc029e59f9579b28ce70f241be4b1

TACGTAGGTGGCGAGCGTTGTCCGTAATTACTGGGCGTAAAGGATGCGTAGGCGGATTCTTAAGTCAGATGTGAAATACCCGAGCTTAACTTGGGTGCTGCATTCCA

>a84d4abdfe8175649589f98886280f56

TACGAAGGGTGCAAGCGTTAATCGGAATTACTGGGAGTAAAGCGCGCGTAGGTGGTTTGATAAGTTGGATGTGAAAGCCACGGGCTCAACCTGGGAATTGCATCCAA

>d4f55d3cd5b3c3896a203db6847321d7

CTCGAATTAGCCGAATGCACACAACCGGTGTGTTTGTTTTTTTGTCGTCCTTGAGGGAAGGCTCGTCGCTCGGTCGTTGCCCGCAGCCGTTCTTATCGATTGAGTAC

>d1bc58897897a1eeb2b49a90275dd7f5

GGTTATGTTCGTCAACACCGGGCAAACCCGATCATATAATTTTACCATACACTATAACACGTTGAGGTCGAGATTGCAGTGCAAGGTGAGGTTATGTTATCACATAC

>0dc58b805e0b7f6850b35ba4059656dc

TACGATTTCTTTAATTTAAATAGTTAAGTTTCAGCTAATATAATAGTAATATAAAATATCTATAATTTTGGTGAAATATATTTTATCTTGAAAAATTAATTTTATGT

>9b8e421fa37c37acc2f3c12803492c32

ACCACAGTACAAAACACTATAATATAATGATAAACCTAAATTGTTACCCGAAGTCGTAGGGGGTATTCATCACGGTTGGCATTAGATACCCTTGTAGTCCGGCTGAC

>f6b62309f1dfe46a770fd2cd27d96358

GCTTCGTATCACGCTAATTTAATTTTTTTTTAATTAGGCTTTTTCATGCTACTCATAAGAGCTAGAAAGTAAAAATGTTAATATTTGATTCTTAATTTTCTTTCAAT

>332202c3b218898ab33ed67775f90b5f

TCTGCAAACAGCGTACAATAACAATGAGCATTGTCGAAGCTATAACATGGACGATGTGAAACTACGTTATTATTTTGACTGTTCTTGATTCTAAGAGAATAGTAAAG

>32ade0cc9196c96ef09585fb499b6c2a

TAGTTCCTAGTTATAGTTATAAGATTGGGAAGACTATTAGAAACCCGAGTAGTCCGGCTGACTGACTTGCGTCAAATCTCGTATGCCGTCTTCTGCTTGAAAAAAAA

>08cb7b374549ce6dafb1859b12de7463

TACGTAGGGGGTGAGCGTTATCCGGAATCACTGGGCGTAAAGGGTGCGTAGGCGGCCAATAAAGTCTGTGGTGAAAGGCTACGGCTCAACCGTAGTAAGCTTTTGAA

>5b5de9a9df13f1fff6e2db9fa4d44e7d

TACGTAGGTGGCAAGCGTTGTCCGGATTTACTGGGCGTAAAGGGAGCGTAGGCGGATTTTTAAGTGGGATGTGAAATACCCAGGCTCAACCTGGGTGCTGCATTCCA

>ca885ba9a19a7347938e653f2efd062a

TACTCATCACCACTAAAGAAGGCTTCCCCAGTCGTAGCCGAATGCCTGCCAGAGCAACCACATTAGAAACCCCAGTAGTCCGGCTGACTGACTTAACGTCCATCTCG

>bb6a7e8d1bffc1b775e70b63614afbff

CTGGAGACCGTCTGCAACATGTTTGTGAAGGTAAACAGTTACGTGTATTATTGCGTGTATTAGATACCCTAGTAGTCCGGCTGACTGACTCGCGATATATCTCGTAT

>f5f39a0f3d7b6efadf1449edf7cc353f

TACATAGGGGGCGAGCGTTATCCGGAATCATTGGGCGTAAAGGGTGCGTAGGCGGTTAAATAAGTTTATGGTCTAAGTGCAGTGCTTAACGTTGTGATGCTATAAAA

>2a45f5be9d6308e6d9fbd39d863f4197

TAATATCTTTGTACGCCAGGGGCGCAGTAAATTGATTTTGAAAAATACTACATTAGAAACCCCTGTAGTCCGGCTGACTGACTAGTCGCAGATCTCGTATGCCGTCT

>60a812d5b7b1b28e1a0ec0470a0fe3fc

AGGACTTGGAGGAACGACTCTGATAAACTATATGTTGTACACCAGAGGCAACTCACGGGATTATAACAAATATGCTCAAGACGGAAACTATGGTTGGAGCTTTAAAG

>cf1580133270e9961873127f1b7bfa8a

TACGTAGGTGGCAACCGTTGTCCGGAATTGTTGGGCGTAAAGCGCGCGCAGGTGGTTTCTTAAGTCTGATGTGAAAGCCCACGGCTCAACCGTGGAGGGTCATTGGA

>732c7f3f8b2cdf7c758d353a8e54974d

TTCCGAAGCCATTGGGAGTCCCACATCAAATTCTGATGTAAATTTACCCTCTCCTAGTGTGTCCCCGACCTGTTCATCAAAGGGAATTTTAAATTATGTTTTAAAAG

>027e4caaa27533808278f5ad7c621946

TTATTATTTGCACTCAAGCCATTCTTAATTTTAGTCTATTAGACTGACACATCTAAGTTATTAGTATCTTAAAAAAATAATATACATTAGTTAGGCAACATTTTAAC

>66ce27b167a413a9fa659b13e9bf79d1

TGTCCGCACCTAATAGGGCATCATCGGGAGCGGGTCGGTCAAATGTCGGATCGGCAAGAACCAGGTCCTTAGCTGCACTCTTAACCTCTGAGGCTATTGCTGTCGGA

>a64efcc11d6ceb52c592f4224a298fe9

TACGTAGGTGGCGAGCGTTGTCCGGAATTACTGGGCGTAAAGGGCGAGTAGGCGGTACTACAAGTCAGATGTGAAAGGCGAGGGCTCAACCCTCGGATTGCATTTGA

>e9b97269ccbfb76300ff20f904b0141c

TACGTAGGGTGCGAGCGTTGTCCGGAATTATTGGGCGTAAAGAGCTCGTAGGCGGTCTGTCGCGTCTGCTGTGAAAACCCGAGGCTCAACCTCGGGCCTGCAGTGGG

>adfef453e9c77b86331338fc7d141957

TGGACTCCGAGATGTCTGATAATGAACAAGAAACAGATGGTGAATTAGATACCCCTGTAGTCCGGCTGACTGACTCGCGATATATCTCGTATGCCGTCTTCTGCTTG

>a38d13d508666fbbcea6ba964a1de454

CCCACCGCTGTGTTCAGTTTGATATACTTTTTTTACAATACACAATAACAGATGCCACAAGCCACACACACACACACACACACATAGAAAGTCGTAGGACGTAAACG

>1079025f0cc6f11648502511de4e9bd0

AACCAGCACCTCAAGTGGTCAGGAGGATTATTGGGCCTAAAGCATCCGTAGCCTGCTCTGTAAGTTTTCGGTTAAATCTATATGCTCAACGTATAGGCTGCCGAAAA

>4172868dd01077e0ba0dea30e137b432

TACGTAGGGGGCGAGCGTTGTCCGGAATTACTGGGCGTAAAGCGTGCGCAGGTGGCTTGTTAAGTCAGGTGTCAAAATGCGGGGCTCAACCCCGTACCGCACTTGAA

>4e494d7dbf20e848c4107329b7a8a11e

AGAAGGACAAGGGGGGTGAGGTAACTTACTCACCACAGTCACCTACGCAGTGACGACGCCAGTATAAACTGATAACGAATGTTGTACCGTCTGATATCAGAGACGAA

>5f4f75dc5a799637b20b49b150c6da14

CATTGAACTATCGTGAGAAAGTCAAACCGCCAAAGGGAATTATATTATAGTAAATATTGGCGTAAATGAACATTTTATTAATAGTTGTAATATATGATAATGTGCAG

>3e63bb93feedfecf9a33579ab10d9c46

CTGTTGGAGCTTCCACGGCGGCCGGCGAGCGCCCGACACCGGTGACACCTGTACGACCTCTCGGAGGGCCCGAGTCCGGCCGACGTCTACGCCTACGCGGCTTAGCA

>a7f9a0bb9424351b7ac014aac41a6540

TTGGCGTTGTTTCAGCTCTTGTATTAGAAAGCCTTGGTGGCACAGTTCACCACAGTATCTCCATCAGGTATCTTCTTCCTATGGCTACAGCAGATTCGTTTTATCGA

>2be26ac456257313a601f057247770ab

TACGAAGGGGGCTAGCGTTGCTCGGAATCACTGGGCGCAAAGGGTGCGTAGGCGGGTTTTTAAGTCAGGGGTGAAATCCTGGAGCTCAACTCCAGAACTGCCTTTGA

>be671558322b80670d9c9a86b693e5aa

TTCCAGCTCCAATAGCGTATATTAAAGTTGTTGCGGTTAAAAAGCTCGTAGTTGGATCTGTGTGCCACGCTGTCGGTTCACCGCCTGCGGTGTCAACTGGCATGTCT

>568cc0aa2a59d1dea9c1ee0abac245bf

AAATTTTTTTAGAATATTTCAATTATAATCCAGTATAAAATATAGTACTTATCACACGTGTTTATCAATGATGTATAAACATTTATGGCGACATGTGAACCATGAAT

>04e03301bcd02f91ce43a414e29dda93

TCCCCTTTCTTCCCTTTCCCTTTTCCCCCGTTTCCTTTCCTCTCTTCTTTCCCTTTTTCTTTTTTTTTTGTGTAGTGAAATATATTATTATTATATTTTATTAATTT

>63720a58e8d3118fbd98827ef4a90bd1

TATTCTCGCGAAAACGCAAGCGGCCTCGGCCGCTTAAATTAGGCCCCGTCGAGGTGCCCTGGACCAATAGCGTCAAGTACCTCGGGCTCCACGTGGATTCCCGACTC

>d0cfad72d99a39d1cdd7cf233c4b1031

GGAAATGTTTGTTTGTTGTTGTCGCTGCCGGTGTACGACTGTGCTTGTGTGTGTGGTGGGTGTATCTATTTGTCTCTGTAACACTGACAAATCGGTATATACTGTAC

>db0e545713c18f21cbec1d6a6540ca04

TTCCATCTCCGGTAGCATATGTTAAAAGCGTTGCGGTTAAATACTCGTAGTTCGATCTGTGTGCCACGCTGTCGGTTCACCGCCCGTCGGAGCCAACTGTGAGATGT

>c4e3e5a83067fae708e20be5804ec4ea

TACGTAGGGGGCGAGCGTTGTCCGGATTTACTGGGCGTAAAGGGTGAGTAGGCGGTTGTATATGTCAGATGTAAAAGGCACAGGCTTAACTTGTGTAAGCATTTGAA

>caef2acca36c139c5c8d646dffa26832

TACGAAGGGGGCTAGCGTTGCTCGGAATCACTGGGCGTAAAGCGCACGTAGGCGGCTTTTTAAGTCAGGGGTGAAATCCTGGAGCTCAACTCCAGAACTGCCTTTGA

>f932eae69fdcaf9cab4f76ed0c22d5b7

TACGTAGGTGGCAAGCGTTGTCCGGAATTACTGGGCGTAAAGGGCGAGTAGGCGGTAATACAAGTCAGATGTGAAAGGCGAGGGCTCAACCCTCGGATTGCATTTGA

>1932bedce82325fc57d8312f65169bea

TACGTAGGGAGCGAGCGTTGTCCGGAATTACTGGGCGTAAAGGGTGCGTAGGCGGCATGGCAAGTCAGATGTGAAATCCCCGGGCTTAACTCGGGGGGTGCATTTGA

>c89be59b1217ea730b99c73478a967ac

TACGTAGGGGGCAAGCGTTATCCGGAATCATTGGGCGTAAAGGGTGCGTAGGCGGTTATGCAAGTTAGGGGTGAAAGGCTACGGCTCAACCGTAGTAAGCCTTTAAA

>9b16e5d297ff9fbafd554584f7a48e9b

TACGTAGGGTGCAAGCGTTGTCCGGATTTACTGGGCGTAAAGGGTGCGTAGGCGGATTCTTAAGTGGGATGTGAAATACCCGGGCTTAACTTGGGTGCTGCATTCCA

>caeb847ae0796ebba811930c705b693b

TACGTATGGTGCAAGCGTTAATCTGAATTACTGGGCGTAAAGCGTGCGCAGGCGGTCCGCTAAGTCAGATGTGAAATCCCCGGGCTTAACCTGGGAACTGCATTTGT

>1c5299da79efd86b9dee114471f1d37d

TAGACCATTGTAAAACAGTTAGATTTGGACCACGACAAAGTATAAAATTATTATACTTTGGCGGACTTAGCAGAAGCGTACCGACTCTCGACCACGATTAGAAACCC

>ecd7f78313ef5e0057bee4e25f00801e

GTGCTCAGAGAACATCAATCTTTCACTTCGTTCTCACTTTCTCTTTTTACTGCCTCCTCTTCTGACCCTTTTCCACCATACCCCTTCTTCTCCTTTTCGTCATCCCT

>2eb60c5b64b5afa2edc3f453ba689c43

TACAATAATATAAGACTTACTTTAAAGGAAACACTGTAGTATTAGAAACCCTTGTAGTCCGGCTGACTGACTCTCTAGAGATCTCGTATGCCGTCTTCTGCTTGAAA

>e5d910d18d9b60519a06af2bcf55a598

TACGGAGGGTGCAAGCGTTGTTCGGAATCACTGGGCGTAAAGGGCGCGCAGGCGGTTTGATAAGTCAGATGTGAAAGCCCACGGCTTAACCGTGGAAGTGCATTTGA

>2f945df9df6a18c9f417e85b2124cebc

TACGTAGGTGGCGAGCGTTGTCCGGAATTACTGGGTGTAAAGGGAGCGTAGGCGGGCTTGCAAGTTGAATGTTTAATCTATGGGCTCAACCCATAGCTGCGTTCAAA

>4b647ab3bf40bf80f27d1c096f8a2cf1

TACGTAGGTGGCGAGCGTTGTCCGGAATTACTGGGTGTAAAGGGCGTGTAGGCGGGAATGCAAGTCAGATGTGAAATACCATGGCTCAACTGTGGGGCTGCATCTGA

>9f858e22ffcc06db5cef7d1a5ee71eb6

CATGGTCATCCAGCCAGATTTCGTATTTATGAACGTGACGCTTGACTTTATTCACGTGGAAAGCGATGTTTTAACCACACGACCGTACAACCGGGTGAATTGAGTTA

>881c85add2852b9f1e643acdf1b8cff3

GGTTATGTTCGTCACATACTTTTACAATACACTATAACATTAGATACCCCGGTAGTCCGGCTGACTGACTCGCGATATATCTCGTATGCCGTCTTCTGCTTGAAAAA

>b1bd6057fa73c3b90a9b9ed725188e07

AACGTAGGGGGCAAGCGTTGTCCGGAATTACTGGGCGTAAAGAGCGTGTAGGCGGCTGATTAAGTTAGATGTGAAACCTGGAGGCTCAACCACCAGCTTGAATCTAA

>af204b69a114ec4bafde42cd73521e26

TACGTAGGTGGCGAGCGTTGTCCGGAATTACTGGGCGTAAAGGGTGCGTAGGCGGATATTTAAGTCAGATGTGAAATCACCGAGCTTAACTTGGGGCTGCATTTGAA

>8f6fb420296db64132ec7f18d9cefc3f

CACGGGGGGCGCAAGCGTTATTCGGAATTATTGGGCGTAATTGGCGCGCAGGCGGTCTTGTCCGTCAGGTGTGAAAGCTCGTGGCTCAACCCCGGAAGTGCACTTGA

>04adcfedb927ba1678b9861ad03b783e

TACGGAGGGTGCAGGCGTTAATCGGAATTACTGGGCGTAAAGCGCACGCAGGTGGTCTGTCAAGTCGGATGTGAAATCCCCGGGCTCAACCTGGGAACTGCATTCGA

>cf62cec4061a17719152317b8f3b1ed6

TACGTAGGTGGCAAGCGTTATCCGGAATTATTGGGCATAAAGCGCGCGCAGGTGGTTTCTTAAGTCTGATGTGAAAGCCCACGGCTCAACCGTGGAGGGTCATTGGA

>d9a34d1da78deed6be5227d91913471d

GTGTTGAGTGTGATATACTTTTACAATACACTATAACATTAGAAACCCCGGTAGTCCGGCTGACTGACTGAGACTTAATCTCGTATGCCGTCTTCTGCTTGAAAAAA

>009eea4ddee99e9d4b7a6a726bc89023

TACGTGAGGGACTAGTGTTATTCATCTTAATTGGGTTTAAAGGGTACCTAGACAGTCAGTATAACTTCTATAATGCTAATACTTGGCTAGAGTTTTAAGTAAGAGGG

>aa094e41d055404cb035c4749efce350

CACAAGTAAGACGAGTGTTATTCATCTTTATTAGGTTTAAAGGGTACCTAGACAGTATATTTAGCCAATAAAGGGTACTAATATGCTAGAGTGTTATGGGAGAGTGA

>acea7906422db7618be180725eebdd54

GTGCTCAAAGAACATCAATCTTTCACTTCGTTCTAACTTTCTCTTTTTACTGCCTCCTCTTCTGACCCTTTTCCACCATACCCCTTCTTCTCCTTTTCGTCATCCCT

>bdabd2cf364d1dbe1d02297520c1ab5a

CCACCACAACCCACCGCTGTGTTCCACAATAACAACAATAACAGATGCCACAATTCACACACACACACTTAATGTACATTAGAAACCCCAGTAGTCCGGCTGACTGA

>1ddd338de41eced6e3490d3b5b4e2a99

TTACATAATTACATGGAGCCTCAGACATCTTGGAAGACTCTAAAGAACTATGTTATTGAGATGCAATGCCAAGTCCCGTTAGTTGTTCGTTGTTCCCTCCACGGCTT

>984f3c8c7bc4224444245e797a41c407

TAAAGCAACAATGGCCTGGGGCAATATTTCTGTTCGCCTCGTGGTCCATGCTGGCGGAAATGAAACTAGGTACAGCCCATAACTGTGATGTCAACCAGTGCTTACCA

>7748489f554edd23fde6e7ef8564e6f7

ACACGTCCGTCTACAAATGATTCGTCGTGTCCTTTCTAGCACACTATGCTTTGGTTTAACACAATTGTGTAAATAATATTGTAGTGAGTACTGTAAAGCTTTAACCG

>e29bebb274a5e0ccb098b8dc97d008ee

TGTCCGCACCTAATAGGGCATCAACGGGAGCGGGTCGGTCAAATGTCGGATCGGCAAGAACCAAGTGCTTGGCTGCACTCTTAACCGCTGAAGCTATTGCTGTCAGA

>2871a84ecad86d0a1b7f18dcf3526ff0

TACGTAGGGTGCAAGCGTTGTCCGGATTTATTGGGCGTAAAGGGCTCGTAGGCGGTCTGTCGCGTCGGATGTGAAAACTTGGAGCTCAACTCCGAGCCCGCATTCGA

>eed0bb991b2c439c16aa1eacfead666f

TACGGAGGGTGCGAGCGTTAATCGGAATCACTGGGCGTAAAGCGCACGTAGGCTGTTTGGTAAGTCAGGGGTGAAATCCCGCGGCTCAACCGCGGAATTGCCCTTGA

>84e4800a7c4fae121235b59b45264f4a

CCACCACCACCACCACCACTGTGCTGAGTGTGATATACTTTTTTTACAATACACAATAACAGATGCGACAAGCCCCCCCACACACACACACACACACATAGAAAGTC

>1bb166af5ee40c1a3bbcebcd01e9d633

TCTGTCTGCACTAAATTATTATTACTGTGTTCAATTGCTTAGTTACTGCACGAGTGTTATGACGCTTTGAATCAGTTTATTTGTAACGATCAGAGGCGAGGGAAAAT

>5792cdbbf2ed6d7026ba176d3dec6241

TACGTAGGTGGCGAGCGTTGTCCGGAATTACTGGGTGTAAAGGGCGTGAAGGCGGGGATGTAAGTCAGATGTGAAATACCACGGCTTAACCGTGGGGCTGCATCTGA

>f032c70d1eaf54d072b3ff2277f67cd7

TACGGAGGATGCAAGCGTTAATCGGAATTACTGGGCGTAAAGCGCGCGTAGGTGGTGTGTTAAGTCGGATGTGAAAGCCCAGGGCTCAACCTTGGAATTGCATCCGA

>1feebce51b1f076580a83b9ca647a34c

TACGAAAGGTGCAAGCGTTAATCGGAATTACTGGGCGTAAAGCGCGCGTAGGCGGTGTGTTAAGTCGGATGTGAAAGCCCAGTGCTCAACCTTGGAATTGCATCCGA

>2a66e7f63c22aa3b8dcbdb7d713a6d25

TACATAGGGTGCAAGCGTTGTCCGGAATTATTGGGCGTAAAGAGCTCGTAGGTGGTTCGTCACGTCGGATGTGAAAATCTGGGGCTTAACCCCAGACCTGCATTCGA

>6be9bb2e10daeef2ded88c48cc2b96a1

CCTACCGCTGTGTTCAGTGTGATATACTTTTTACAATCCACAATAACAGATGCCACAAGCCCCACACACACACACACACACACACAGAAAGGCGTAGAACGCAAACA

>2e12eb199024d9c683bb7d5cb3b2bb7b

AATAATACGTGCGTTTTATCTGGCATACCCGGACCAGATAAATAACAAGGGCACACATAATACTACATACAAAACACTCTCCCCCCTACCGCACGGCTTGCCCCCGA

>ba0d20f328171d24a134f6fd5f1d234b

CCACCACCACCACCACCACTGTGCTGAGTGTGATATACTTTTTTTACAATACACAATAACAGATGCCACAAGCTACACACACACACACACACACACACACACATAGA

>cd67ceb2b6f2374eb13baa08bed71516

CCACCCCACCGCTGTGTTCATACTTTTTTTACAATAACAGATGCCACAATTCACACACACACACACACACACACACAAGTTCGATGGAGAGGTGGGGGGAGGGCAAG

>7610a1ff13de92266d49b988a9d077fa

TTCCAGCTCCAATAGCGTATATTAAAGTTGTTGCGGTTAAAAAGCTCGTAGTTGGATCTGTGTGCCACGCTGTCGGTTCACCGCCCGTCGATGTCAACTGGCATGTC

>08c24dc814276e50594d28eaf58877da

ACAACGAATAAACGATAAACGCAAAACCGAAATAAAATAATATGGAAAGTTTAGGAATTGGAGAAGAAACTAGTAAGTGTGAAAACCTTACTCGTATAAATGAAGTT

>28c42cca050197f442999da185fcb517

CCACCACTGTGTTCAGTGTGATATACTTTTACGATACACTATAACATTAGAAACCCGAGTAGTCCGGCTGACTGACTGTTACAGCATCTCGTATGCCGTCTTCTGCT

>092d0000babadfd96645a31b2c905d90

TGTCCGCACCTAATAAGGCATCAACGGGAGCGGGTCGATCAAACGTGGGATCGGCAAGAACCAAGTCCCTAGCCGCACTCTTAACTTCTGAAGCTATCGCTGTCAGA

>9daccb758c0ee502c07e76a2f7557ecd

TACGATTCATTCTATTTAAATACAAAAGTTTCAGTTATAATTAATTTTGATTATAAATTTTGGTGAAATATATTTTAATTAATAATTCATAATTTATATCTGAAAAA

>c1c0f8842817290155b113ab298701fc

TACATAGGTCGCAAGCGTTATTCGGATTTATTGGGCGTAAAGCAAGCGCAGGCGGATGAACAAGTTCTGTGTTAAAAGCAGCTGCTCAACAGTTGTTTGCACCGAAT

>78403e5afe7089cc9e8c722c5566e661

TACGATTTCTTTAATTTAAATAGTTAAGTTTCAGTTAGTAAAACAATAATATAAAATATCTATAATTTTGGTGAAATATATTTTATCTTTAAAAATTAATTTTATGT

>386e4876cbb7d0c13af8991f94958bb5

CGTGTTCCTCTCGCTACTTGTATGTTGAATAAAAACGTATATCATATGTTATCTCTGCCTCCTTCCCTCTCATTACACACACACACACACTGCATAGAGACTCTCAC

>6cb4967f6bef9a390753c4e63686a1fc

CTTCAGGCCACAGATGAGACCCTGCAGGTTATCTCTGCTGGAGCCCCGGTTCATTAGAAACCCGAGTAGTCCGGCTGACTGACTATGAGCTCATCTCGTATGCCGTC

>0d4d4f40a71ea1b9e7e01c8e0f5931a9

TACGTGGGAGACTAGTGTTATTCATCTTAATTGGGTTTAAAGGGTACCTAGACAGTCAATATAACTTCTATAATGCTGATACTTGACTAGAGTTTTAAGTAAGAGGG

>45a9aeca820e1351ee58f016f7ecdf0a

CACGATTTTCTAAATTTAATTATGTTAGTTTCAGTTAAAAAAAATGTGTTAATATTAAGTTTTTTTTAATTTTGGTGGAATAATATATAAATATGTGTTTAATTATA

>67122ec7b83f726885bc4c8d220a4f0f

CCACCCCACCGCTGTGTTCATACTTTTTTTACAATACACAATAACAGATGCCACAATTCACACACACACTTAATGTACATTAGAAAACACAAGCCACACACACACAC

>731aacfaadf30b9cac77cf6c776ab2bf

TACGTAGGTGGCGAGCGTTGTCCGGAATTACTGGGCGTAAAGGGCGCGTAGGCGGCCTGATAGGTCAGATGTGAAACCCCCAGGCTCAACTTGGGGCATGCATTTGA

>02d1079827acc68cbe4cef8cb428afd4

TACGTAGGGGGCTAGCGTTATCCGGAATCACTGGGCGTAAAGGGTGCGTAGGCGGCCAATAAAGTCTGGGGTGAAAGGCTACGGCTTAACCGTAGTAAGCCTTGGAA

>48de6e427b54074526c8b79e557ce142

ATATCGTCAGTCCCTGTTCTTAGATGTGATCTTTCCAGAGCCGCACTGAGGCACACACACAACAGGGCGCACAGATCGGAAACATCCTAGAATGTCTATTCTTACAT

>9e12b1810cf2943d74cedd231db0b320

CAACCACTGTGCTGAGTGTGATACTTTTTTACAATACACAATAACAGATGCCACAAGCTACACACACACACACACATACATAGAAAGTCGTAGGACGCAAACACAGT

>255d7a171986d8907caf2f6c0dbdea1f

CACGCTTATGTCTTTAGAAAGACAAAACATTTTTCCAGAATGCATTGTAACCATGTCGGTCATTTTCAACTGCAGTGCCTTGTAGATGTTTGTGGAAGATTTTGAAT

>0edb1953d70fa459c1fc0b2aded0fdac

CCCACCGCTGTGTTTAGTTTGGTATACTTTTTTTACAATACACAATAACAGATGCCACAAGCCACACACACACACACACACACACACACACACAAAGTCGTAGGACG

>af89f2aabcba6f70ce70af233444444b

TACGTAGGTGGCAAACGTTGTCCGGAATGACTGGGCGTAAAGGGCGAGTAGGTGGTATGCCAAGTCAGAAGTGAAAACTCCGGGCTCAACCTGGAGATTGCTACTGA

>d3fc209b612c7834a386c6561eb6b1c4

TACGTAGGGGGCGAGCGTTATCCGGATTTATTGGGCGTAAAGGGTGCGTAGGCGGCCTTGTAAGTCAGGTGTGAAAGGCTACGGCTCAACCGTAGTTAGCACTTGAA

>3f973cd3f16a1066253217366ff93068

AACGTAGGCTCCAAGCGTTGTTCGGAATTACTGGGCGTAAAGCGCGCATAGGTGGTTTGATAAGCGAGATGTGAAGCCCCGGGCTCAACCTGGGAACGGCATTTCGA

>5ae5aadafc4e08bc61f621364ba4c007

TACGTAGGGTGCAAGCGTTAATCGGAATTATTGGGCGTAAAGCGAGTGCAGACGGTTACTTAAGCCAGATGTGAAATCCCCAAGCTTAACTTGGGACGTGCATTTGG

>899b3f9e3fcab762cc9d0a53fd05dcb6

TACGTTTTCCTTTATTTAAATAGTTAAGTTTCAGTTAACATAATAATAATATAAAATAACTATAATTTTGGTGAAATATATTTTATCTTTAAAAATTAATTCTATGT

>666d24c347009cc66e24269441fc9dcb

TCACCACCCCCCACCGCTGTGTTCATACTTTTTTTACAATAACAGATGCCACAATTCACACACACACACACACACACACACACACACACACACACACTTAATGTACA

>6274a478581c4257b91851d255f6fa8f

TCTGTCTGCACTAAATTATTATTACTGCGTTCAATTGCTTAGTTACTGCACGAGTGTTATGACGCGTTGAATCAGTTTATTTGTAACGATCAGAGGCGAGGGAAAAT

>f7586d9922498166c32a9f1f60492832

GAAGAGTGATTTTTAACGAGAGTAACGCTCGGAGTCTAATTAGAAACCCGAGTAGTCCGGCTGACTGACTATAGCGCTATCTCGTATGCCGTCTTCTGCTTGAAAAA

>d2d2d0cf0c8ec37c23de58301af09b7d

TACGTAGGGAGCGAGCGTTGTCCGGAATCATTGGGCGTAAAGGGCGCGTAGGCGGTTTGTTAAGTCAAATGTGAAATACACTGGCTCAACCAGTGGGCTGCGTTTGA

>72772ef56d40b793d9bdd9907000913e

TACGGAGGGTGCAAGCGTTAATCGGAATTACTGGGCGTAAAGCGTACGTAGGTGGTTTTGTAAGTTGGATGTGAAAGCCCTGAGCTCAACTTGGGAATGGCATTCAA

>2492e220218a50bc32b8c4b15e2b85e3

TACGAAGGGGGCTAGCGTTGTTCGGAATCACTGGGCGTAAAGCGCACGTAGGCTGCTTGGTAAGTCAGGGGTGAAAGCCCGCGGCTCAACCGCGGAATTGCCTTTGA

>fda8b9860b2f1bfd1bd63f9158f02790

ACAAATGGTGAATAGTATAGTGTCAATAATGGCATTAGAAACCCTGGTAGTCCGGCTGACTGACTACTGTGTAATCTCGTATGCCGTCTTCTGCTTGAAAAAAAAAA

>ef1571534806544ecced57702823a67d

CGCACTTCCCTCTTTAGAAAGACAAAACATTTCTCCAGAATGCATTTCAACCATGTCGGTCTTTTTCAACTGGCAGTGCCTTGTAGCTAGTTGTAGAAAATGTAGAA

>327c1855992d2976dd147bdc34de578b

TCTGTTTCGTATGCTCCAATCTCAGTTTTACTCTTTCCTCTGCAGTTCTTCCCCCTCTCCCCTACTCCTTTAATCACTCTATGAACCAATCCTGAATGTCTCAAGAC

>da638d492eac0e112f0224b8999b53eb

TACGGGGGGTGCAAGCGTTATTCGGAATTATTGGGCGTAAAGCGCGCGCAGGTGGTCTCTTAAGTCTGATGTGAAAGCCCCCGGCTCAACCGGGGAGGGTCATTGGA

>d2cbc15811097ff9ba3fd136fea924b4

TACGGAGGGGGCTAGCGTTGTTCGGAATTACTGGGCGTAAAGCGCACGTAGGCGGGTATCCAAGTTGGGGGTGAAATCCCGGGGCTCAACCCCGGAACTGCCTCCAA

>c8ede3dc130246a3174655b27a8b1a66

TACGTAGGTGGCGAGCGTTGTCCGGAATTACTGGGCGTAAAGGGTGCGTAGGCGGATATTTAAGTCAGATGTGAAATCCCCGAGCTTAACTTGGGGCTGCATTTGAA

>56f904156c17c41d0443807dd1b3a7f9

TTGGCGTTGGTTCAGCTCTTGTATTACAAAGCCTCGGTGGCACAGTTCACCACAGTATCTCCATCACGTATCTCCTTCCTATGGCTACAGTAGGTGGTTTTATCGAT

>707a5a8af973a0c11c22c61b03072935

CCGATAAGAATACAATTAAGTTATGGGTGTCCAATTTTAGAGCTACCGGTTCAGCTTTGAATTAGAAACCCCTGTAGTCCGGCTGACTGACTCAGTAGGTATCTCGT

>ad1aeadc24de59f4a57fd92b187e844a

AACATTTTATTTTTAATGGTGCTGCAGGTCATTACATAGTTGAAAAAATAGGAAGCAGTTTATTGCTCATTGTAGGTTTTACAAAATCTTATACACATTATTATTTA

>716aa32b9733a0c8fa283a852b09e125

ATTACTGCCAAGCTCTGTCCACACGTAGCCAAGATGACGAAGTTAAGACCTCCCTCTTAAAATTAAAAAAGTCAGTTAACGAATTATTAGAAACCCTTGTAGTCCGG

>b662cf9b37b6a1f73810a6e34647ee83

TACGTAGGGTGCGAGCGTTGTCCGGAATTATTGGGCGTAAAGAGCTCGTAGGCGGCTTGTCGCGTCGGATGTGAAAGCCCGGGGCTTAACCCTGGGTCTGCATTCGA

>63a6ed11fd67be3008eee3635466e1bf

TACGTAGGTGGCGAGCGTTGTCCGGAATTACTGGGCGTAAAGGGTGCGTAGGCGGAATCTTAAGTCAGATGTGAAATTCCTAGGCTCAACCTGGGAACTGCATTTGA

>34d6f9ecb13f9556e287b5f06dca1642

TACGTAGGTGGCAAGCGTTGTCCGGATTTACTGGGCGTAAAGAGTATGTAGGCGGACATTTAAGTCAGATGTAAAATCCCCGGGCTTAACCTGGGGGCTGCATTTGA

>dcbfae2eff504e4424de64dd3fe4db8f

TGTTCCACGCCTCCCAGGCAGCCCGTGTTTAATGTGTATATTACTGTGTCGGATCGTGGAGAGGAAACACGGCGGGCGCGTAGAAGGTTGCGTAACTGCGTCGCAGG

>a81e333c25be787cc19938ff866c46a4

TGGACTCCGAGATGTCTGATAATGAACAAGAAACAGATGGTGAATTAGATACCCCTGTAGTCCGGCTGACTGACTTACTAGGTATCTCGTATGCCGTCTTCTGCTTG

>7ae2b7d1b9b18b099462cea39d180db6

TACGGAGGGGGCTAGCGTTGTTCGGAATTACTGGGCGTAAAGGGCGCGTAGGCGGTATGCCATGTCAGGGGTGAAAGGCCTGGGCTCAACCCAGGAACTGCCTTTGA

>d1572dad6d4259f12f820fc0803eb5da

TACGTAGGGTGCAAGCGTTAATCGGAATTACTGGGCGTAGAGCGCACGCAGGCGGTTTGTTAAGTCAGATGTGAAATCCCCGAGCTTAACTTGGGAACTGCATTTGA

>0d83d2af821b21a30eedea3fc56581f0

ATGCTAACGACGTCATAAATATTTCAGATGAGGTTATTATATTCTGTATACACTTGCCCTCGGTGCTGGCGTGAGCGTCTCGCCCTGTATGCGTGGCGACCGCAAAA

>43dc727f078ac1010cd3bc190403aae0

CTGGAGACCGTCTGCAACATGTTTGTGAAGGTAAACAGTTACGTGTATTATTGCGTGTATTAGAAACCCTTGTAGTCCGGCTGACTGACTCGCGATATATCTCGTAT

>b32de414b4444d8ffe765a51ce6fdfbc

TACGTAGGGTGCGAGCGTTATCCGGAATTATTGGGCGTAAAGAGCTCGTAGGCGGTTTGTCGCGTCTGTCGTGAAAGTCCGGGGCTTAACCCCGGATCAGCGGTGGG

>c43d905b01887554b9ec971c91a31b1f

GACAGAGGATGCAAGCGTTATCCGGAATGATTGGGCGTAAAGCGTCTGTAGGTGGCTTTTTAAGTCTGCCGTCAAATCCCAGGGCTCAACCCTGGACAGGCGGTGGA

>5cad8e8bc72aae6f6cd35b27c286ca9c

AAACCTAGTTCATTATAAAAGGGATAAGGCGCTAGGCAATTAGATACCCTTGTAGTCCGGCTGACTGACTCGCGATATATCTCGTATGCCGTCTTCTGCTTGAAAAA

>48f121e2baf9323f96f06de059abd937

TTGTACAAATCTTTGTAACTGGATCGGTTCCGCACGAGTGTGTGTACACTTCCTCTTTAGGCACTGCCAGTAAAACCTCATGACTTCTTAGTATCGACGTGTAGGTT

>7fdd51a3388785484a89b42917ad6f75

TATTCTCGCGCAAACGTAAGCGGCCTCGGCCGCTTAAAGTAGGCCCCGTCGAGGTGCCCTGGACCAACAGCATCAAGTACCTCGGGCTCCACGTGGATTCCCGACCC

>37fd31fae6ae1ecb8cc62f53ca0f4826

GGGCACCCGTGATAACTCACGCTTCCGCCAACACATGATTTGATACTATCGGTACATACGGAACGCGGCCGTAAATTAGATACCCCAGTAGTCCGGCTGACTGACTT

>41fad35f427c7150ae960acd2ae20da5

TGAAATTTCAGAAAACAATAAAAGCGTTAAACTTTTTTTATAAGGTTTTATGGTGTTTCCATACGATGCAGTGCAGACTTTTCCGCCGTTATTTACTAAAACAGTCG

>cacec7e9066d5ebad72443856029d0ba

AACGTAGGGGGCGAGCGTTGTCCGGAATTACTGGGCGTAAAGGGCGTGTAGGCGGCCCTGCAAGTCAGTCGTGAAACCCGTCGGCTCAACCGGCGGCTGGCGATTGA

>17d1551e84ef4383f6be1cd352317c4e

TACGGAGGATGCAAGCGTTATCCGGATTCATTGGGTTTAAAGGGTGCGCAGGCGGGCTTGTAAGTCAGTGGTGAAATACTGCCGCTTAACGGTAGAATTGCCATTGA

>9c3c94134fea325d0c0bcd5d33217e86

TACGGAGGGGGCTAGCGTTGTTCGGAATTACTGGGCGTAAAGCGCGCGTAGGCGGATCGATCAGTCAGAGGTGAAATCCCGGAGCTCAACTTCGGAACTGCCTTTGA

>1567d2b3e38b2042866d9e68799c18c4

TACGTAGGTGGCAAGCGTTATCCGGAATCACTGGTGTAAAGGGCGTGTAGGCGGGGATGTAAGTCAGATGTGAAATACCACGGCTTAACCGTGGGGCTGCATCTGAA

>3b89a1387eb5c6bd641c8796e5fa363a

TACGTAGAGTGCAAGCGTTGTCCGGAATTATTGGGCGTAAAGAGCTCGTAGGCGGCCTGTCGCGCCGGATGTGAAAATCCAAGGCTCAACCTTGGACCTGCATTCGA

>5ceeeea9d44c1eba5b3d036965b0e071

TTGACTGGGTGAGACCCACGGCAGTTGCAGCATGTTGCAGAGGTCTCCGGTGCGACCCGGATAGTAGCCTCGGTGCTCTCCTAAAGTCTCTGTGGAAAACGGCCAAC

>7dbe29135bc95df78772f95e6f54c9bd

TACGTAGGGGGCAATCGTTGTCCGGAATCACTGGGCGTAAAGCGCGCGTAGGTGGTTTGATAAGTTGGATGTGAAAGACCCGGGCTCAACCTGGGAATTGCATCCAA

>8f1630124018951f0c6150afe7f3e964

TACGAAGGGTGCAAGCGTTAATCGGTATTACTGGGCGTAAAGCGCGCGTAGGTGGTTCGTTAGGTTGGATGTGAAGGCCCCGGGCTCAACCTGGGAACTGCATCCAA

>03d72baa2e780af958d4ef29904fdb39

TACGTAGGGGGCAAGCGTTATCCGGATTTACTGGGTGTAAAGGGAGCGTAGGTGGTGCGGTAAGTCAGATGTGAAAGCCCGGGGCTCAACCCTGGGACTGCATTTGA

>183e0e186e093519902b49a5d4eb62de

TACGGAGGATCCGAGCGTTATCCGGATTTATTGGGTTTAAAGGGAGCGTAGATGGGTTGTTAAGTAAGTTGTGAAAGTTTGCGGCTCAACCGTAAAATTGCAATTGA

>a54a13afd5e6285d9dde5db7e30012ad

TACGGAGGGTGCGAGCGTTAATCGGAATGACTGGGCGTAAAGGGCACGCAGGCGGATAGTTAAGTGAGATGTGAAAGCCCCGGGCTTAACCTGGGAATTGCATTTCA

>c2b0f2b99dc94b681b37e941c6e7accb

TCCTTTGCCATTCGCGGTATCCGCGCACCAAGGAAGTCATCGACTTTATGCCGGCAGCCATTCGGTCGTCAAAAGTTATTCTGCATGGATAGGCTTTGTTACGTTCA

>8d869350e0dd7d09535600524e1e01ba

AAGGAAATTGAAAGTAAACGACTCCTCTGACTGCAATACACCATCATCATCATTAGATACCCGGGTAGTCCGGCTGACTGACTATGAGCTCATCTCGTATGCCGTCT

>4257fb3edd4ae28b94e483b620c84a7e

CTGGAGACCGTCTGCAACATGTTTGTGAAGGTAAACAGTTACGTGTATTATTGCGTGTATTAGATACCCGTGTAGTCCGGCTGACTGACTCGCGATATATCTCGTAT

>71fa48527d6763c8ab69d0598e693387

GAACAGCTCATGCTTTCCTCCCCAGCCTTTCTCTCCCAGCTTCTATTAGAAACCCCAGTAGTCCGGCTGACTGACTTCCTCATGATCTCGTATGCCGTCTTCTGCTT

>887d9771b0c46133a64451813b459659

ACAGCCATTTACTTGCACAAAGTTTTTAACGAATGAGATAATTGAAAACAATAAAAAATGGAAATAGAATTCTTTTAATGAATTTTATTCGTAGTAGTTTACATTTG

>f9e5e08c7d92e9164f83137a95071f85

CCATTACACATGGAGCGGTCACAATATTTATAAACTTGTGTTCTGTAAATAACTGCCAATGACGTTTGCGTTGCGTACACAGGAAATCTAAGGAACGCGCGGTATTC

>a790eb0a034aff0bf9f53d36bc59d030

TGCCCGTAGGTTCCTTCGTTTCCGTACCGACGTCGTAACGACACCTACAATTGACAATTTATAAATTAAGTTTATTTATAACAGACAAAATACAAATTTTATACTTG

>6578b03eedc14d09f34c6e24c78d53b9

TGTCCGCACCTAATAGGGCATCAACGGGAGCGGGTTGGTCAAAAGTTGGGTCGGCGAGAACCAAATCCTTAGCCGCACTCTTAACCTCTGAGGCTATTGCTGTCAGA

>6a67c57d24edd2636b4b709c3516238e

AGTTAACAGCGAACTGACCAGCGAGTTTCGTCGCCTCGAAAACCACAAATTAGAAACCCCGGTAGTCCGGCTGACTGACTCTCTAGAGATCTCGTATGCCGTCTTCT

>ed25cc6d6635881c7f7f40106e8048ee

TTATCAGGGTTATTTTCTTCTCCCTTCTTGAATATAGGGAGCGTCGTGCTAGTTTTCCAGTCTCATGGGACTTCTCCCTTTTCCCGTTAGCTCATCACATAATTTCG

>d22db11d282935964d906b244da6066d

TACGAAGGGGGCTAGCGTTGCTCGGAATCACTGGGCGTAAAGGGCGCGTAGGCGGCCGATTAAGTCGGGGGTGAAAGCCTGTGGCTCAACCACAGAATTGCCTTCGA

>adfbcda2be18f53ef62c1f12859693dc

TACAGAGGGTGCAAGCGTTAATCGGAATTACTGGGCGTAAAGCGTGCGCAGGCGGTGATGTAAGACAGATGTGAAATCCCCGGGCTCAACCTGGGAACTGCATTTGT

>5f3b563f8fc630631e9a7dba070a2a60

TACGAAAGGGGCTAGCGTTGTTCGGAATTACTGGGCGTAAAGCGCACGTAGGCGGCATTGTAAGTTGGGGGTGAAATCCCGGAGCTCAACTCCGGAACTGCCTCCAA

>1ecf9d42f8b6bdec86c8414a53db6bce

CATCACTAGAATAATCACAGTATAGTTTTAAGATATACGATTTCTGTAATTTAGAGAATACCTTTTAACTTCAATACACGATATTAATTTTCTAGGATAAAAGGGCA

>b5afa9b0399803987bdd26c88c45448a

CCACCACCACCACCACCACCACCACCACCACCACCACCACCACCACTACCACCACCACCACCACCACTGTGCTGAGTGGGATATACTTTTTTTACAATACACAATAA

>355d82ac8f911185070901cc41de877a

TACGGAGGGGGCTAGCGTTGTTCGGAATTACTGGGCGTAAAGCGCACGTAGGTGGGTATCCAAGTTGGGGGTGAAATCCCGGGGCTCAACCCCGGAACTGCCTCCAA

>220968520434cfb50fa830fc0f0bb4e7

TACGTATGGAGCAAGCGTTATCCGGATTTACTGGGTGTAAGGGGAGTGTAGGTGGCCAGGCAAGTCAGAAGTGAAAGCCCGGGGCTCAACCCCGGGACTGCTTTTGA

>50b1acab564ecde92911daf1be997f43

TACGTAGGTGGCAAGTGTTGTCCGGAATTATTGGGCGTAAAGCGCGCGCAGGCGGTCTTTTAAGTCTGATGTGAAAACCCACGGCTCAACCGTGGAGGGTCATTGGA

>191b0c042015a816722e9cd964241c01

TACGTAGGGCGCAAGCGTTGTCCAGAATTATTGGGCGTAAAGAGCTCGTAGGCGGTTTGTCTCGTCTGCTGTGAAAACGCGAGGCTTAACCTCGCGCCTGCAGTGGG

>628581ab866a1f599f7846d18dfd8586

TACGAAGGGTGCAAGCGTTAATCGGAATTACTGGGCGTAAAGCGCGTGTAGGTGGTTTGATAAGTTGGATGTGAAAGCCCCTGGCTCAACCTGGGAATTGCATCCAA

>aaf8e6db3ba8982dab94ac32deacbba3

CACGGGGGGCGCAAGCGTTATTCGGAATTATTGGGCGTAAAGGGCGCGCAGGTGGTCTTGTCCGTCAGGTGTGAAAGCTCGTGGCTCAACCCCGGAAGTGCACTTGA

>d3de7dce3358705dd08939ae6eb0b3f1

TACGGAGGATCCGAGCGTTATCCGGATTTATTGGGTTTAAAGGGAGCGTAGATGGATGTTTAAGTCAGTTGTGAAAGTTTGCAGCTCAACCGTAAAATTGCAGTTGG

>65397339086985d0c76f23800929b9f5

GCCTTTATTTGCTTCGCCGCAAAAAAAGCCTCCGAGAAAAACGACGAGAAATTACATTCTTCTTATTGTTGTTGTTGTTGTTATTATTATTTAACTCGAGGTATTTA

>198c52b7341a6c51bee8f220197bd51e

GAACGAAGGCCGGAGATAGACAGCTCACAAGTGGCTGCAAGTCGCGACGGCGCCTCGGAGAAGCGAAGAGTGATTTAGGGGCTCCGGAATGTGAGGCCAAAACCCCA

>fabe7ed61d3c30ae8e7f400c106e4274

CCACTGTGTTGAGTGTGATATACTTTTACAATACACTATAACATTAGATACCACAAGCCACACACACACACACAGAAAGTCGTAGGACTCAAACACAGTCATATAAT

>4ad316db23082fd3d89ffdac322d4bbf

TTGGCGTTGGTTCAGCTCTTGTATTACAAAGCCTCGGTGGCACAGTTCACCACAGTATCTCCATCAGGTATCTCCTCCTTATGGCTACAGTAGGTGGTTTTATCGAT

>dc9c9be2f461883284a99e2e1d90d019

TACAATTTCTCCAATTTAAATGCTTTAAAAGTTTCAGTTAATAAATTATTTAAATTTTTATGAATAATAATTTTAGTGAAATATGTTATTATTATATTTTATTAATT

>9dec3a7e314498b1b11f7ea1e77a2eb4

CTCAAATCCCTCAATTTAAATCTATAAAGCTTCAGTTACTATATAACATAATTTTTATAAATAATAATTTTGGTGAAATATATTATTATTTTATTTTTATAATTTGT

>b219f56aaf9e15bbcdb027d436e271ce

TGATACGGCGACCACCGAGATTTACACCTACTATATATGGTAATTGTGTATTAGATACCCGGGTAGTCCGGCTGACTGACTTCCTCATGATCTCGTATGCCGTCTTC

>cc1d63323e8bf27dca34a538d3ecc9c8

TGCTTCATAACCCACAATGAGTCGGCGTTCGTCGTGTTTTACGACCGTCGAGAGCGGAGAGCAATAAAAGCTAATCGTGAAACCGAATTTACACGATCAAAACAATG

>d6a067c2e729028791ebc2dd4c66276d

GTATCGAACCCATGCCGTCGGCATATCAATCCAACTGACTGGCCCACGGCCATCCTATTCAAAAAACAAAATTATAATTTGTCTAAAAATACTTTTGCAATTAGAAA

>289f29ec06e9c0a72571734760fab2b5

TGGCTACTTTTGTGTTCTGCTTTTCAAGGTACTGACCAAGAGAAAAATACATAAGGATTTCGCACAATAAGTGTTCCAGATTTCACGGAAGTCTGAAGCAGCTATCC

>28702706d24216d99f1c60622d3e1d87

CCACCCCACCGCTGTGTTCATACTTTTTTTACAATACACAATAACAGATGCCACAATTCACACACACACACACACACACACACGCAGAAAGTCGTAGGACGTAAACG

>18caab556aecce953ac95bc99717ad70

ACAAATGGTGAACAGTATAGTGTCAATAATGGCATTAGAAACCCTTGTAGTCCGGCTGACTGACTCGCGATATATCTCGTATGCCGTCTTCTGCTTGAAAAAAAAAA

>859c366a887fda14b94bc66926ed81f4

TACGTAGGGGGCAAGCGTTATCCGGAATTACTGGGTGTAAAGGGTGCGTAGGCGGTAATGCAAGTCAGATGTGAAAGCCCAGGGCTCAACCTTGGGACTGCATTTGA

>7ec689b36b07214354fe4f5be658a52b

TACGTAGGGAGCGAGCGTTGTCCGGAATTACTGGGCGTAAAGGGCACGCAGGCGGTCATATAAGTCAGCTGTTAAATGTATGGGCTTAACCGATGCATGCGGTTGAA

>8bafc6cbd16a3180fa844120735b9a47

AGCACTTAGATAAATAGGCAGTAATTATAACTGTATTGGAAGTTTACTTATAGCGACTTTGCTTATATGAGAATCACGCCGATACTTTTACCGCACCGACTATAAAA

>2fde9d2ccfa7c8f8dbf96bf1fa09edcc

CATTGAACTATCGTGAGAAAGTCACGCCGCCAAAGGGAATTGTATTATAGTAAATATTGGCGTAAATAAACATTTTATTAATAATTGTAATATATGAAAATGTGCAA

>4bf75d63f87406c866fb36e7623a9001

TAAAAGTTGACTTTGTAAAAATCCACTGCCTTCTTATCCTGGCGTGCAAGGCTGTGTACTTTCGAAAGGGTTGGCCGTGATCTTGAAACCGCACACAGAAAGGAAAG

>2cae4f7192ad6383c340e1c471efc322

CCACCCCCACCGCTGTGTTAATACTTTTTGTTTTGTTTACAATAACAGATGCCACAATTCACACACACTTAATGTACATTAGAAACCCTAGTAGTCCGGCTGACTGA

>a6beea9a0c06df2ea53d4a83674d8e73

TACGTAGGTGGCAAGCGTTGTCCGGAGTTATTTGGCGTAAAGCGCGCGCAGGTGGTCTCTTAAGTCTGATGTGAAAGCCCCCGGCTCAACCGGGGAGGGTCATTGGA

>3ef7e5b07c62d052d0f69ba696c0e7c9

CACGGGGGGCGCAAGCGTTATTCGGAATTATTGGGCGTAAAGGGCGCGTAGGCGGTCTTGTCCGTCAGGTGTGAAAGCTCGGGGCTCAACCACAGAAGTGCACTTGA

>66855d9705ee88649f66080232983a8c

CCCACCGCTGTGTTCAGTTTGATATACTTTTTACAATACACAATAACAGATGCCACAAGCCACCTACACAAACACACAGAAAGTCGTAGAACGCAAACGCAGTCATA

>88fd46f86860e35c86fedcf97aea0f14

TACTCATCACCACTAAAGAAGGCTTCCCCAGTCGTAGCCGAATGCCTGCCAGAGCAACTACATTAGAAACCCTTGTAGTCCGGCTGACTGACTACGTACGTATCTCG

>0eba881477c4234317d144c038c22811

ATTCATGCACACTGGTCGCGGTGATTTTCCTGCAATTGCACATATCTTGATTAGAAACCCGAGTAGTCCGGCTGACTGACTTGCGTCAAATCTCGTATGCCGTCTTC

>b5901aa2b6eb7e7b4ad345995bc03c99

AGGTATCGAACACTGGGAGGCTGTTTCGTGCACTTCAGCTGAAACATTATTAGAAACCCTAGTAGTCCGGCTGACTGACTATGAGCTCATCTCGTATGCCGTCTTCT

>57a65955b1a11d81baafa511de37895a

CGTAATGCAGGCAAAAAGAAGCGGCATCGTAAGCCCCGAGGCGCTCGCTCCCGGGCTCCTGCCAACCCAGCCGCACAGTCCAGACAGCCTGCGCAACGCGAGAAGAC

>639b437f1c7ad4c8b8fe2abc02baac83

TACGTAGGTGGCAAGCGTTGTCCGGAATTACTGGGCGTAAAGAGTATGTAGGCGGATGCTTAAGTCAGATGTGAAATACCCGGGCTCAACCCGGGAGCTGCATTTGA

>a951ae59556c550485d65a6ba3ac763f

TACGAGGGGTGCAAGCGTTAATCGGAATTACTGGGCGTAAAGCGCGCGTAGGCGGTTAGTTAAGCTGGATGTGAAAGCCCAGGGCTCAACCTTGGAACTGCATTTGA

>cccec31f414a022b955c1b22a6de2bc6

TACGATTTCTTTAATTTAAATAGTTAAGTTTCAGTTAATATAACAATAATGTAAAATATCTATAATTTTGGTGAAATATATTTTATCTCTAAAAATTAATTTTATGT

>086a5240913f217684fcfd9c6cb5f872

CCCACCACTGTGTTCAGTTTGATATACTTTTTTTTACAATACACAATAACAGATGCCACAAACCACACACACACACACACAGAGAAAGTCGTAGGACGTAAACGCAA

>a53a9635a3c8307c4c453fcb8461884b

TGCCCGTAGGTTCCTTCGTTTCCGTACCGACGTCGTAACGACACCTACAATTGACAATTTATAAATTAAGTTAATTTATAACAGACAAAATACAAATTTCATACTTG

>a9db700d102753facbac52610ab42a84

TACGTAGGGGGCAAGCGTTATCCGGAATTACTGGGCGTAAAGCGCGCGTAGGCGGCTTTGTAAGTCGGATGTGAAAGCCCTGGGCTCAACCTGGGAACTGCATTTCG

>81e0894d4e55b51d6b7b321f6883eef9

TACGTAGGGTGCGAGCGTTGTCCGGAATTACTGGGCGTAAAGAGCTCGTAGGTGGTTTGTCGCGTCGTTTGTGTAATACCGCAGCTTAACTGCGGGGTTGCAGGCGA

>c5c928b95536431439ca656bfe1ac3ca

ATTACAGTAAAGTTTATGCAGAGAAATAAAGTATGAAGTTGGCGCTGTAACACCTAACTCGGTGCAATCAACTACGTTACGGCGCGGCGTGATGAATGTTTTCCTTA

>85e380434f7772011511c8718c6f8c67

TACGTAGGTGGCAAGCGTTGTCCGGATTTATTGGGCGTAAGGCGAGCGCAGGCGGATTGATAAGTCTGATGCGAAAGCCTTCGGCTCAACCGAAGAACTGCATCAGA

>e8768e5cc2cf2f9d004b1f5e0c33cf98

TACGAAGGGGGCTAGCGTTGCTCGGAATGACTGGGCGTAAAGGGCGCGTAGGCGGTTTGCACAGTCAGATGTGAAATTCCTGGGCTTAACCTGTGGGCTGCATTTGA

>05c8cdbaf3c3dd443b9d00d9a552bb5f

TACGAGGGGGGCTAGCGTTGCTCGGAATTACTGGGCGTAAAGGGAGCGTAGGCGGACATTTAAGTCAGGGGTGAAATCCCGGGGCTCAACCTCGGAATTGCCTTTGA

>010b652e9daac6c7f80eaee67b719b6b

CAGTATCAACTCGGAGTTTCACAAGATAGCAAAATAAGCCAAAATATAGGCCTTAAAATTAATACAAAGAAAATGCAGGTGTTGATGAACAATAGAAAAAGGAATGT

>53ab2274ec4b0be1b4aa18c47e1a422e

CCTACATGAGAATTCATTCTTTAATTGCTTTATTCTCTGATTATTTAACTCTTTGTACCTTTACTATTACTTATCACATCCGTTTCTATTGCGCACATTTTTCTCTT

>d729b8a35ae21830201d0db49b085462

TACGGAGGGAGCTAGCGTTGTTCGGAATTACTGGGCATAAAGCGCACGTAGGCGGCTTTGTAAGTTAGAGGTGAAAGCCTGGAGCTCAACTCCAGAATTGCCTTTGA

>455e62ae6ebac218887f88ef3f824a27

AAGGATCGATGCCATGATCTATGCGCGAGAATTCCATATTAAATAGAATCATAACACTTGTCTGTTCAATGTTTCTCAAATTTGACGTGTAGATTCCTGTTACTGCC

>01f6ceb77f298b3a23005755ff733771

AGAGGAGAAACACATCAAACACTAACACAGAACAACAATACACACCACCGAACAACTACCACAACACATAACATCTCAACAAAGACACTTATTAGAAACCCCTGTAG

>a6ef8ad1a677d829fdf957a0d9bbe0ba

TACGTATGGGGCAAGCGTTGTTCGGATTTATTGGGCGTAAAGGGCACGCAGGCTGACTATTAAGTTGGGTGTATAATCTCCAGGCTCAACCTGGATCCTGCACTCAA

>5c3c527ebcb3706ee158d4996ca9ef25

TACGTAGGGAGCAAGCGTTGTCCGGAATTACTGGGCGTAAAGGGTGCGTAGGCGGCATGGTATGTCAGATGTGAAATCCCCGGGCTTAACTCGGGTGGTGCATTTGA

>5d880e0f4956d1619d89a04f424caba6

ACAACAAGGGGAACCTAACAATGAGCCGCCTCGCAATCAATCAATTTTCGCCACGCCACATCAACGCCATTCGTGTAGCACAATTTTTCTTCGGCTATCTACACACA

>f7731162d3e2c1d600d7d486ed0bc10d

CGTAGTGGTGTTAATTTAAAACAATGTGTTTTTGTCATCTCGTCAAACAGGACCGGTGAAAACGCGTACCTAGTGTAAAAAACGGCCCAAGGTAGTTTGGATTTGAA

>fd7d16855426e8f2362b7d5c15c6c764

AACCAGCACCTCAAGTGGTCAGGATGATTATTGGGCCTAAAGCATCCGTAGCCTGCTTTGTAAGTTTTCGGTTAAGTCCATGTGCTCAACGCATGGGCTGCCGAAAA

>c15899d2649e4ed357bb81c67cb6f0df

TACGTAGGTGGCGAGCGTTGTCCGGAATTACTGGGCGTAAAGGGCGCGTAGGCGGCCTAATAAGTCAGATGTGAAAACCCTGGGCTTAACCCTGGGATTGCATTTGA

>fc65c6762311a835b291cbc6e3d0570e

TACGTAGGGGTCGAGCGTTGTCCGGAGTTACTGGGCGTAAAGCGTGTGCAGTCGGCGGCCTGCGCCTGGCGTGAAAGCCCCCGGCTCAACCGGGGAGGGTCGTCGGG

>d451633648be304db3af17f71c584bc7

TACGTAGGGGGCAAGCGTTATCCGGATTTACTGGGTGTAAAGGGAGCGTAGGTGGTGCGGTAAGTCAGATGTGAAAGCCCGGGGCTTAACTCGGGGGGTGCATTTGA

>3c75806a6d77c71a28598af9ec4b2bf2

TACGTAGGTGGCGAGCGTTGTCCGGAATTACTGGGCGTAAAGGATGCGTAGGCGGATTCTTAAGTCAGATGTGAAATACCCGAGCTTAACTTGAGTGCTGCATTAGA

>02638e320334a84dddbf84b2e821d6cc

TACGTAGGGGGCAAACGTTGTCCGGAATGACTGGGCGTAAAGGGCGAGTAGGTGGTATGCCAAGTCAGAAGTAAAAACTCCGGGCTCAACCTGGAGATTGCTACTGA

>36eb0f6cbf1b8c27e6bedd64c30074e3

TTAGAAACATCAGTATAGGCCGCCGAGTTGCTGACTCCCAGACACTGTACCTAGCGCGCGTCATACAGATTCAATGATGGTTTCAATGAGTCCCCATACGCTACTGC

>3ff165f1698ff8e66705f1427e5dc65a

GCCGTACCTTAAAAAGCCTGCCACTACTTGTGCAGGCGGCGCTGTTCCTTCGGCACCGAAGACTGTGCTACCCCCTCGGGGTGACCAGTCAACTTCGGTGCCATCGC

>d28ccf3be6ab3ba5bbc3ec67d5be2703

CTGGAGACCGTCTGCAACATGTTTGTGAAGGTAAACAGTTACGTGTATTATTGCGTGTATTAGAAACCCTGGTAGTCCGGCTGACTGACTCGCGATATATCTCGTAT

>e7b333297f67115ca8fb928354ada9b8

TACGTAGGGTGCAAGCGTTGTCCGGAATTATTGGGCGTAAAGAGCTCGTAGGCGGTTTGTCGCGTCGGCTGTGAAAACTTGGGGCTCAACCCCAAGCCTGCAGTCGA

>73366d454af9e573e6bff07f50e1684a

CAATAATTCAGTCGCAAAGTTTCGTCGTCGTCCTCGTCATTGTTGTTGTTATTATTGTTATTATCATTAGAAACCCCAGTAGTCCGGCTGACTGACTATAGCGCTAT

>36b911a0b432abbcad04eadf636c05eb

TGGCTGCCCTCTATTTCTCTGCCCACTCAACGTAGATTATAATAGCGTCAGTGTCGGAAACGTCTTTCCCCTCTATGCAGTTCCTCGATATAGTCGACGTATTCCGA

>6d0089ed3e7cc32b828854d40809fed3

TATTCTCGCGAAAACGCAAGCGGCCTCGGCCGCTTAAAGTAGGCTCCGTCGAGGTGCCCTGGACCAATAGCGTCAAGTACCTCGGGCTCCACGTGGATTCCCGATTC

>d1714b5d0b4f693058583f87d2d1ae30

ATTTGATCTGCTGATTTGCAATGGGACTAAGTCTGTTGTGTTAGTATCAGTGTTTTCATTAGGTTTGGCAGGAATTTTTGTTTTGTGAATGTTTTCAGTATTTAGTG

>35f9ecb107e885f3e1501f79de645c9b

CTGTTTGACCACGGGCACATTTATTGCTTGGCCGATGATCTTGTAAGTTCCCGTTTCCGTTCTGAGGCGACGGATCTGCATAATTGGTAATGTAGGTGAAATCAGTC

>ea3b204e4a210a792b8127099b646198

CCACTGTGTTGAGTGTGATATACTTTTACAATACACTATAACATTAGATACCACAAGCCACACACACACACACACACAGAAAGTCGTAGGACGCAAACACAGTCATA

>8c886460d37930b66e16de3a75a83f68

CCACGTCACTCACGGCCGTAGCATATCATTAAAAAGCAACACGTGCTTCCTGGGTTGTTAAAATGAGTGACATCGTAGGCCGTACTGGGGCGAATGGATTCAGTTCC

>fa1be45f12fe1c99ac133ff2fbb3728f

ATATCAATAGAATTCTAAGCGTAAATGCACTCCTACTTGCAGACCAACTAGCTATTACGAAAGATACTGAAGATGACGTGCTGAATATCAAAGTGGTAGATTGAGTT

>f7c99d697909ca7c4cb414a3d6c4b084

CCACCACCCACCGCTGTGTTCATACTTTTTTTACAATAACAGATGCCACAATTCACACACACACACTTAATGTACATTAGAAAACACAAGTCACACACACACAAGTT

>b40cd23873af8fb26dc3e63586d91619

AACCAGCACCTCAAGTGGTCAGGATGATTATTGAGCCTAAAGCATCCGTAGCCTGCTTTGTAAGTTTTCGGTTAAATCCATACGCTTAACGTATGGGCTGCCGGGAA

>688409c4a45fd0800e6b53c5c537627c

GACGTAGGGGGCGAGCGTTGTCCGGAATTACTGGGCGTAAAGAGCACGTAGGCGGATGCTTAAGTCAGGTGTGAAAGATCATGGCTCAACCATGGTATGCTGTTGAA

>1e129bf0cc823e51370621d96b9a1869

TACGGAGGGTGCGAGCGTTAATCGGAATTACTGGGCGTAAAGCGTACGCAGGCGGTTTGTTAAGCAAGATGTGAAAGCCCTGGGCTCAACCTAGGAACCGCATTTTG

>7a65a1efb711ee23a47143bd9f6b3cf1

TACGTAGGTGGCAAGCGTTGTCCGGATTTATTGGGCGTAAAGCGCGCGCAGGCGGTCTTTTAAGTCTGATGTGAAAGCCCCCGGCTTAACCGGGGAGGGTCATTGGA

>b3e058c4df1c484b3ce28a2a8fe7adf8

TACGTAGGTGGCAAGCGTTGTCCGGAATTATTGGGCGTAAAGAGCGCGCAGGCGGTTTTTTAAGTCTGATGTGAAAGCCCACGGCTCAACCGTGGAGGGTCATTGGA

>5f9499cc7abe6a6178718931f9ed75d3

TACGTAGGTGGCGAGCGTTGTCCGGAATTACTGGGCGTAAAGGGCGCGTAGGCGGCTTTTTAAGTCAGATGTGAAAACCCGAGGCTTAACTTCGGGACTGCATTTGA

>10f19a68f4d932bf4df8d8710055e78a

TACGTAGGGTCCAAGCGTTAATCGGGATTACTGGGCGTAAAGCGTGCGCAGGCGGTTGTGCAAGACCGATGTGAAATCCCCGGGCTTAACCTGGGAACTGCATTTGT

>2d81d29d27df1d72e2293106c4bab78d

TACGTAGGGTGCAAGCGTTAATCGGAATTACTGGGCGTAAAGCGTACGCAGGCGGTTTGCTAAGCGAGATGTGAAAGCCCCGGGCTCAACCTGGGAACTGCATTTCG

>8ab4ea0e5ee9bef7537c2894aced746c

TACGGAGGGTGCAAGTGTTAATCGGAATTACTGGGCGTAAAGCGCACGCAGGCGGCCTGTCAAGTCGGATGTGAAATCCCCGGGCTCAACCTGGGAACTGCATTCGA

>68305bce56284218ca79e068f241b216

CAACTTTTTAATAACAACATACAGCCGTTCATTAACCTCCAAAATGTTAATTACCTCTAGATAGATATAGGGTAATTACATCATCTTCCTCCCCCAGTAATGAGCCA

>dedc07849b3ada48bf5863bb5e560eb1

TACGGAGGGTGCAAGCGTTAATCGGAATTACTGGGCGTAAAGCGCACGCAGGCTGTTTGTTAAGTCAGATGTGAAATCCCCGGGCTCAACCTGGGAACTGCATTCGA

>c33aac27f578a02eb3f2afa352ef9d10

CCACCCACCGCTGTGTTCATACTTTTTTTTTTACAATAACAGATGTCACAATTCACACACACACACACACTTAATGTACATTAGATACCCCGGTAGTCCGGCTGACT

>40782c13b89c0427e0cff447174616ee

TACGAGGGGGGCTAGCGTTGCTCGGAATTACTGGGCGTAAAGGGAGCGTAGGCGGACATTTAAGTCAGGGGTGAAGTCCCGGGGCTCAACCTCGGAATTGCCTTTGA

>ba5622bd59112a0cc770d08a81933ac3

TATAATTTCTTCAATTTAAATTTTTAAAAGTTTCAGTTAATAAATTATTTAAATTTTTATGAGTAATAATTTTAGTGAAATATATTATTATTATTATATTTTATTAA

>c8b61b01ea175ab1b4f0df5e86bcff3f

TACAATTTCTTCAATTTAAATTTTTAAAAGTTTCAGTTAATAAATTATTTAAATTTTTATGAACAATAATTTTAGTGAAATATATTATTATTATATTTTATTAATTT

>fe1a35c126b963c0ac76b043797549b6

GAACAGCTCATGCTTTCCTCCCCAGCCTTTCTCTCCCAGCTTCTATTAGAAACCCCTGTAGTCCGGCTGACTGACTCGAGCTAGATCTCGTATGCCGTCTTCTGCTT

>493271d733d9225ef6b91a48a41a9826

TACGATTTTCTAAATTTAATTATGTTAGTTTCAGTTAAAAAATGTGTTAATATTAAATTTTTTTAAATTTTGGTGAAATAATATATAAATATGTGTTTAATTTTATG

>6eb2e36586af51df122acad4e3372d2b

CCTAATGCAGGCAAAAAGAAGCGGCAACGTTAGCCCCGTGGCGCTAGATCCCGGGCTCCTGCCAACCCAGCCGCACAGTCCAGACAGCCTGCGCAGCGCGTGAAGAC

>8916fd43a2b348210b2e223e759ee466

TACCGATGTTTTGGTTGACTTGATCTGCTGCTTTGTTGTAGTGGGATCAAATGTGTTGTGTTGGTATTTTAGTGTATTGATTAGAAACCCTAGTAGTCCGGCTGACT

>1256b15327dfc2d9af40001dc51b84ad

AACCGTACAAACCAAAATTTAACATCGTTGAAGATGGGTCTAAGCCCCAATCAAGTGTATAATGCGGACAAGAGCGGTTTCTTTTAGCGGCTTTTAGCAACACACAC

>152fa93c1590055f2fcd8bf2d6044ebb

TGTCCGCACCTAATAAGGCATCAACGGGAGCGGGTTGGTCAAATGTCGGATCGGCAAGAACCAAGTCCTTAGCCGCACTCTTAACCTCTGAAGCTATTGCTGTCAGA

>cbc501d1cbf72c44fbb978ca8349338a

TCCTTCAACGGCTTTTTTGACACATCCGGCGTTAATGACGAATTTGCTGGCTTTAGGAAACCGTTTACAATCATTAAGCCAGCTCACTGTGTTTTTGATTGACAGTT

>93a61ccebb7072d933484eff58efd177

TACGTAGGGGGCAAGCGTTATCCGGAATTACTGGGTGTAAAGGGTGAGTAGGCGGCATGGTAAGTTAGATGTGAAAGCCCGAGGCTTAACCTCGGGATTGCATTTAA

>f2f94f4c5ff4967b6d6138c17b4b3d22

TACGGAGGGTGCGAGCGTTAATCGGAATTACTGGGCGTAAAGCGCATGCAGGTGGTTCATTAAGTCAGATGTGAAAGCCTGGGGCTCAACCTCGGAACCGCATTTTG

>7f7c9551cb452bf9c6fc42d080c0cb41

CCACAACCCACCGCTGTGTTCATACTTTTTTTTTTACAATAACAGATGCCACAATTCACACACACTTAATGTACATTAGAAACCCTTGTAGTCCGGCTGACTGACTC

>850d521d82217a64440ba0051231c4f9

CTCCAGTATATAGTCATGTATTATCTTTATGGTATGTATGGCAAAGACAGAAAAACATCAGTTAAAATTCCACAGAATTTTAATCTAAGAAAATAACCCATTAGATA

>3cfa451ae8db3ee36625bdb994df798f

TGGACTCCGAGATGTCTGATAATGAACAAGAAACAGATGGTGAATTAGAAACCCTAGTAGTCCGGCTGACTGACTTACTAGGTATCTCGTATGCCGTCTTCTGCTTG

>32b0ff9a2669b54f1c7ac391e403e203

TACGTAGGGTGCAAGCGTTGTCCGGAATCACTGGGCGTAAAGAGTTCGTAGGCGGCTTTATAAGTCAGGTGTGAAAGGCATTGGCTCAACCAATGTAAGCACTTGAA

>cd82028035e4db35dbd5e5ef129090a8

AACATCTTTATATTTCTTACTAGTTTTCCAGAGAATATTGTCATTGATTTTCAAGTCATTTTTTTTTACCGGGTAACGTCCCTTAAAACGTTCGAACTATTTTCTTT

>33c230af719baa335a5a0ea0ee547342

ACACTAAACGCGGCGTGAAATCTTAGTCTGATTACGAGTGCAGCGGGGATAAGCAAGCATCGTGCGGGTCTGTGTTTGGATTAAAAGCATTGTGCACGTTACAACTA

>56231d0e6bbcf8596bdc6d3610f3862b

TCAAACGAAAGCACAACAAAAAGATAGCCAAACTAATAATAGCAAAACTAACACGTAGAACAATAACACCATCAAGAACATTATATTAGACACAGAAACAGGGATTC

>1ac5f60c5107876c63a6c0a594fb7c14

ATATTTTCGATTATCATTAAACCTAACCGTCGGAGTAGAATAACGCGTCTCGAGCTCAGTTTACGTACCGTACTTAAGTTTTCAACTGGAATTTTATCTCTGTTTCA

>6f3a29690d0066a499608aa5c89af8e4

AGAAAGAGTAGAAGGTGGAGATGGGGAAGGTGAGACGTTGAAGAAGAAGCAGCAGTCACAAATACACAACACACACGCACAAGGCCGGTCCTTTTCAGGGTCGGCTG

>d3aa2b387da520f207f296a7005bc0a1

TACAATTTCTTCAATTTAAATTTTTAAAAGTTTCAGTTAATAAATTATTTAAATTTTTATGAATAATAGTTTTAGTGAAATATATTATTATTATATTTTATTAATTT

>8e8621002cc73fa94f21acc39529ba2d

TACGAGGGGAGCGAGTGTTGTTCGGTTTTATTGGGCGTGAAGGGTATGTAGGCGGTTTTGTAAGTCAGCAACCAAATCTTGGAACTTAATTCCATAGCGTATGTTGA

>9f69a1c3ad49b3f11c0b52bf6abbb95f

TACGTAGGGTGCGAGCGTTGTCCGGAATTATTGGGCGTAAAGGGCTCGTAGGCGGTTTGTCGCGTCGGGAGTGAAAACACCGGGCTTAACTCGGTGCTTGCTTCCGA

>8fdd3e6e994b0b8712f7a6cfbad43d43

ATACTCCAGGCTATTGAAATAGGATTGCTAAGGTGGTTCAAAGGATGCGTTAACTTTGTGTTATTTTTGACCTATCCTCACAAACCCTTCATCGGGAAAATTCATTG

>6ab06580fbd013ea490cb0ee1f57e11d

TCCCCGTAGTCCATTAGATACCCGAGTAGTCCATTAGAAACCCTGGTAGTCCGGCTGACTGACTCTCTAGAGATCTCGTATGCCGTCTTCTGCTTGAAAAAAAAAAA

>8e77a1f4d96865c1841ac3569570931d

ACAAATGGTGAACAGTATAGTGTCAATAATGGCATTAGATACCCCTGTAGTCCGGCTGACTGACTCGCGATATATCTCGTATGCCGTCTTCTGCTTGAAAAAAAAAA

>046905ab38834098c5e5886a08e0f3e6

CACGTAGGGGGCGAGCGTTGTCCGGAATTACTGGGCGTAAAGCGCGCGTAGGCGGTTGTGTAAGTCAGAGGTGAAATTTTGTGGCTCAACCACAAACGTGCCATTGA

>69afdeefcc80381909163069cbbdb2c2

TACGAAGGGAGCTAGCGTTGTTCGGAATTACTGGGCGTAAAGGGCGCGTAGGCGGCTCTGTAAGTCAGATGTGAAAGCCCCGGGCTTAACCTGGGAACTGCATTTGA

>6ac40e4c767ab04f7c2a3a6df6ce0bc4

TACGTAGGGGGCGAGCGTTATCCGGAATTATTGGGCGTAAAGAGTACGTAGGTGGTTTCGTAAGCGTGGGGTGAAAGACCGAAGCTCAACTTCGGTTAGCCTTGCGA

>c4012675c1ff839970f084ad383e7449

TACGTAGGGGGCAAGCGTTATCCGGAATCACTGGGTGTAAAGGGTGCGTAGGCGGTTTAGCAAGTCAGATGTGAAAGCCCAGGGCTCAACCCAGGGACTGCATTTGA

>d2432d16f5bcd1abbf936a3ce82752c6

TACGTAGGTGGCAAGCGTTGTCCGGATTTACTGGGCGTAAAGGATGCGTAGGTGGATATTTAAGTGGGATGTGAAATACCCGGGCTCAACTTGGGTGCTGCATTCCA

>cc1d6e2ffce9bfe5c3200f498be70e85

TACGTAGGTGGCAAGCGTTGTCCGGATTTACTGGGCGTAAAGGATGCGTAGGCGGATGTTTAAGTGAGATGTGAAATACCCGAGCTCAACTTGGGTGCTGCATTTCA

>2d00c52181193f89dc09d0d574d0a9c8

TACGTAGGTGGCAAGCGTTGTCCGGATTTATTGGGCGTAAAGCGCGCGTAGGCGGTTTTTTAAGTCTGATGTGAAAGCCCCCGGCTCAACCGGGGAGGGTCATTGGA

>ecc7719b63c842bdaafc6a068d19d6c3

AGGCGGTGTTAACCTCAAAACTCAGTCGACCGTTTGGACACGCAAAGCACAATTTTTACACACTTTTTAAAACATAATTATTGTAATGTGTGTGATACGATTCTATA

>b4ad257d2c8057843268f005bc63ddb6

TGATGAGCGAATATCGAATGGAGCTAGAATAAGTACTAGGGTTTTTCTTCAACTTTAATTAGATACCCGAGTAGTCCGGCTGACTGACTCTCTAGAGATCTCGTATG

>7c0e6c6017b9249033503a781f54fb11

CGGTTTACAAAGTATTGGGCAACTTTATGCGTTCATTTTGGGTGTTAAAACTTAAAAATCTCATCTTTTTTTATCGTTAAGGGAATCAAGATCCAGCGAGTAGCTCT

>f33c3591e1839f655269666a5b7ff795

TACGTAGGTGGCAAGCGTTGTCCGGAATTATTGGGCGTAAAGGGTGCGTAGGCGGCTTCTTAAGTCTGATGTGAAAGCCCATGGCTCAACCATGGAGGGTCATTGGA

>3c100dca7ba7993ee09952cab3e90493

TACGTAGGTGGCAAGCGTTGTCCGGAATTATTGGGCGTAAAGCGCGCGCAGGCGGTTTCTTAAGTCTGATGTGAAAGCCCACGGCTCAGCCGTGGAGGGTCATTGGA

>dab26629d1562a07a5d9ad4e7e362d7c

TACGTAGGGGGCAAGCGTTGTCCGGATTTACTGGGCGTAAAGAGTGCGTAGGCGGATGATTAAGTGAGATGTGAAATACCCGAGCTCAACTTGGGTGCTGCATTTCA

>f54b2c4167b515328ab12981b5342f32

CACGTGGGGCGCAAGCGTTATTCGGAATTATTGGGCGTAAAGGGCGCACAGGCGGTCTTGTCCGTCAGGTGTGAAAGCTCGGGGCTCAACCCCGGAAGTGCACTTGA

>5d2a2e0e61ae84cd68e63b3a89c51824

AAGGAAATTGAAAGTAAACGACTCCTCTGACTGCAATACACCATCATCATCATTAGAAACCCGTGTAGTCCGGCTGACTGACTATGAGCTCATCTCGTATGCCGTCT

>71dccdc2a83f9783e775ecb3636aaa38

TACGTGAGAGACTAGTGTTATTCATCTTAATTGGGTTTAAAGGGTGCCTAGACAGTCAATATAACTTCTATAATGCTAATACTTGACTAAAGTTTTAAGTAAGAGGG

>fcba6c59e84e01b122312bcc5cdb4983

ACCGAACTGAGCGTCTCAACCTTGAGGCGAACAACAAACGTACCTTACTGAACTAATACCGTCGAGCTCAACTGACTGAGCTATGCGGCCATTAATTAAATTAATGT

>2cdf432d8aa08ba57fbd52b54b145365

GGAGTACAGCAGCCGCAGCAACAGCTTTCTACTGTCGTCAGCTGAAAGTAATTTATCAGAATCATTCAAAGCATATTCCCTATAAATTATACATCATGTTTATAACA

>50080499540443ffa3ae6bd30b3d0498

CATAATGCAGGCAAAAAGAAGCGGCAACGTAAGCCCCGTGGCGCTAGATCCCGGGCTCCTGCCAACCCAGCCGCACAGTCCGGACAGCCTGCGCAGCGCGTGAAGAC

>41b35dc9be50eff3e3b8018186577d7c

ACTAAGCTCAACCACTGGCCCTTATGTGCCAATTAGAAACCCGGGTAGTCCGGCTGACTGACTGTTACAGCATCTCGTATGCCGTCTTCTGCTTGAAAAAAAAAAAA

>9c896a17065154d844939925ea300afd

TACGTAGGGAGCGAGCGTTGTCCGGAATTACTGGGCGTAAAGGGTGCGTAGGCGGCTTAGCAAGTCAGATGTGAAATCCCCGGGCTTAACTCGGGGGGTGCATTTGA

>9b10bf4daaa68d1af3caf6f3128cad6d

TACGGAGGGTGCAAGCGTTAATCGGAATTACTGGGCGTAAAGCGTACGTAGGCGGTTTGTTCAGTCAGATGTGAAAGCCCCGGGCTTAACCTGGGAACTGCATTTGA

>ed2df51f9434b1c9ca864489245fd404

TACAGAGAGTGCGAGCGTTAATCGGAATTACTGGCGTAAAGCGCACGTAGGTGGATACTTAAGTCGGATGTGAAAGCCCTGGGCTTAACCTAGGAATTGCATTCGAT

>dec3c41bf99f42385f0029bc491120ce

TACGAAAGGGGCTAGCGTCGTTCGGATTTACTGGGCGTAAAGGACACGCAGGCGGTCTTGCCAGTCAGGGGTGAAAGCCCGAGGCTCAACCTCGGAACTGCCTCTGA

>3ffb436f3d54752a83919639045216f2

TAGGTTATTATGATTGCGTAAATCGCGACGGCAAATACAGAGAGTGCGTAGCGGCAGAGTCGTGTGGGTTGCAGCTCTGTAGATGCCGTGGTGTTCGTGCTACGCGA

>ad6007cf6e836f9397165c1a157a3f42

TACGTGAGAGACTAGTGTTATTCATCTTAATTGGGTTTAGAGGGTACCTAGACAGTCGATATAACTTCTATAATGCTAATACTTGACTAGAGTTTTAAGTAAGAGGG

>ec432777f301053b30ed9fb711c33f13

GCTTCGTATCACGCTAATTTAATTTTTTTTTCATTTTTTTTTAATTAGGCTTTTTCATGCTACGCATAAGAGCTAGAAAGTAAAAATGTTGATATTTGATTCTTAAT

>6d018646d1f359fb53afb5d01913bb51

TACGTAGGGTACGAGCGTTGTCCGGAATTATTGGGCGTAAAGAGCTCGTAGGTGGTTGGTCACGTCTGCTGTGGAAACGCAACGCTTAACGTTGCGCGGGCAGTGGG

>d509cd4bd7dafa41b50c65b651bfe6df

TACGTAGGGGGCAAGCGTTGTCCGGAATTATTGGGCGTAAAGCGCACGTAGGCGGCTATTTAAGTCAGATGCGAAAACCTGAGGCTCAACCACAGGCATGCATTTGA

>2e8a80a66128b6a1f79314133759e4d8

TACTGAGGGTGCAAGCGTTAATCGGAATTACTGGGCGTAAAGCGCGCGTAGGTGGCTAAGTCAGCCAGGTGTGGAAGCCCCGGGCTCAACCTGGGAACGGCATCTGG

>51bc166f3d8ac8d88cc86ddf374de788

TACGTAGGGAGCGAGCGTTGTCCGGAATTACTGGGTGTAAAGGGCGTGTAGGCGGGAACGCAAGTCAGATATGAAATACCGAGGCTTAACTTCGGGGCTGCATCTGA

>3aba3565241b93d2eb335122700720e8

TACGGAGGGTGCGAGCGTTAATCGGAATTACTGGGCGTAAAGCGCGCGCAGGCGGTCTGTCAAGTCTGGTGTGAAAGCCCGGGGCTCAACCCCGTAAGTGCACTTGA

>ddca7389cf4c37a50dee95333b8081f1

TACGGAGGGTGCAAGCGTTAATCGGAATCACTGGGCGTAAAGCGCACGTAGGCTGTTTGGTAAGTCAGGGGTGAAATCCCGCGGCTCAACCGCGGAATTGCCTTTGA

>ed07e2e553c704018f12ab37daf07d01

CCACTGTGTTCGGTGTGATATACTTTTACAATACACTATAACATTAGAAACCCGTGTAGTCCGGCTGACTGACTATAGCGCTATCTCGTATGCCGTCTTCTGCTTGA

>771be9be5edc6097e259cbb503af5d35

TACGGAGGATACAAGCGTTATCCGGGTTTATTGGGTTTAAAGGGTGCGTAGGTTGTTTTTTAAGTCAGTGGTGAAGTCTTAAAGCTTAACTTTAAAAGTGCTATTGA

>2263e4b909961506ab9348cb5f2fedea

TACAGAGGGTGCAAGCGTTAATCGGAATTACTGGGCGTAAAGCGCGCGTAGGTGGTTCGTTAAGGTGGATGTGAAATCCCCGGGCTCAACCTGGGAACTGCATTCAA

>7b217216f84f856dbee8ee1a23bf2bf2

CTGGAGACCGTCTGCAACATGTTTGTGAAGGTAAACAGTTACGTGTATTATTGCGTGTATTAGAAACCCCAGTAGTCCGGCTGACTGACTTACTAGGTATCTCGTAT

>d5bbd6d047f8677d159dce1004bf0a92

TACAATTTCTTCAATTTAAATTTTTAAAAGTTTCAGTCAATAAATTATTTAAATATTTATGAATAATAATTTTAGTGAAATATATTTTTATTTTATTTTATTAATTT

>5ed2901be950b9750ece35d8e8ebe7be

CAGCTAGTTAATATACATTCTTTATAACCCCATATTAATTGTTATAGTAGAAACTGTCGATAACGACCTCTGAAAACCGTTGTAACCGGTGCTCGCTAAAAACACCG

>399e8157dd446478b71a9bbc5d0e2202

CCAGCTGAGTTGAAAGTTTGTGTTAATACCTGCGAATTGTATCAGTGGACTTTTTAATGACAACTATTTAATTTTCTGTGTAAAGAGTATTGTAATTTTATTTACTT

>66fb2872c10c7e70050a351fb1df8f08

TGTCCGCACCTAATAGGGCATCAACGGGAACGGGTTGGTCAAATGTCGGATCGGCAAGAACCAAGTCCTTAGCCGCACTCTTAACCTCTGAAGCTACTGCTCTCAGA

>61fd85f7a3e48b9a9431a57f8d00d252

TACAGAGGGTGCGAGCGTTAATCGGAATTACTGGGCGTAAAGCGCGCGTAGGCGGCTTGGTCAGTCGGATGTGAAAGCCCCGGGCTTAACCTGGGAATTGCATTCGA

>80cde438babfa056e2cff76760d766d0

TACGGAGGATCCGAGCGTTATCCGGAATTATTGGGTTTAAAGGGTGCGCAGGCGGGGATCTAAGTCAGGGGTGAAAGACGGTGGCTCAACCATCGCAGTGCCTTTGA

>843484c1aea07054842d6cabe74715a3

AACCAGCACCTCAAGTGGTCAGGAGGATTATTGGGCCTAAAGCATCCGTAGCCTGCTCCGTAAGTTTTCGGTTAAATCTATATGCTCAACGTATAGGCTGCCGAAAA

>d51980387f1f16e0826774f156352fa2

TACGTAGGGAGCAAGCGTTGTCCGGAATTATTGGGCGTAAAGGGTGCGTAGGCGGTTTTGTAAGTCAGATGTGAAATCCCCAGGCTTAACTTGGGAGTTGCATTTGA

>208b58cffe32c9c0065e0eaad2cf7e0e

TACGGAGGGGGTTAGCGTTGTTCGGAATTACTGGGCGTAAAGCGTACGTAGGCGGATTGGAAAGTTGGGGGTGAAATCCCAGGGCTCAACCCTGGAACTGCCTCCAA

>eced58863fd900530ab9939a6e2f702b

TTCGTAGGGGGCGAGCGTTATCCGGATTTATTGGGCGTAAAGGGTGCGTAGGCGGCCTTGTAAGTCAGGTGTGAAAGGCTACGGCTCAACCGTAGTTAGCACTTGAA

>02c75880618597aa5b4c471d8febba47

TACGTAGGGGGCTAGCGTTATCCGGAATTACTGGGCGTAAAGGGTGCGTAGGCGGTCTTTCAAGCCAGAAGTGAAAGGCTACGGCTCAACCGTAGTAAGCTTTTGAA

>d23b93dff7c357b0ee544938ef5646cf

ATATCGTCAGTCCCTGTTCTTAGATGTGATCTTTCCGGAGCCGCACTGGGGCACACACACAACAGGGCACACAGATCAGAAACATCCTAGAATGTCTATTCTTACAT

>79ff53603f4a80a4b16e244f45d52db0

TACGATTCACCCAAACTAATTCTCTTCGGCGTAAAACGTGTCAACTATAAATAAATCAATAGAATTAAAATCCAACTTATATGTGAAAATTCATTGTTAGGACCTAA

>44341a7fb06096d0b29fd9b039f57b07

CACTGACAACCTATCGGAGTAAAAACTCGCACCAACTTTAAACTCACAGTAGCATGTGTTTTAGAAACAAGCCTCAGGCTCGAAATTCTTGAAACGATTGTTTTTAT

>505cfcc2c37bb9fc109400a612d564f5

CACGGGGGGCGCAAGCGTTATTAGGAATTATTGGGCGTAAAGGGCGCGTAGGCGGTCTTGTCCGTCAGGTGTGAAAGCTCGGGGCTCAACCCCAGAAGTGCACTTGA

>b13636d10090204fd08f07423f7d460c

TACGGAGGGTGCAAGCGTTAATCGGAATTACTGAGCGTAAAGCGCACGCAGGCGGTCTGTCAAGTAGGATGTGAAATCCCCGGGCTCAACCTGGGAACTGCATTCGA

>b427fbc3d02fdc86ceadd93040e174cf

TACGTATGGGGCGAGCGTTATCCGGAATTATTGGGCGTAAAGAATTCGTAGGCGGTCCTTTAAGTCAGAAGTGAAAGGCAGTGGCTCGACCATTGTAAGCTTTTGAA

>57da7656b17f1c1d2a5d7750e9d4c51b

TACGTAGGGCGCAAGCGTTAATCGGAATTACTGGGCGTAAAGCGTGCGCAGGCGGTCCACTAAGACAGATGTGAAATCCCCGGGCTTAACCTGGGAACTGCATTTGT

>fed6cc056e4ba6838b5e84b0e222fc42

TACGTAGGTGGCAAGCGTTGTCCGGAATTATTGGGCGTAAAGCGCGCGCAGGCGGTCCTTTAAGTATGATGTGAAAGCCCACGGCTCAACCGTGGAGGGTCATTGGA

>d264b6eb688d71abf7cf827723aaccfa

TACGTAGGGTGCAAGCGTTAATCGGAATTACTGGGCATAAAGCGTGCGCAGGCGGTTATGCAAGACAGAGGTGAAATCCCCGGGCTCAGCCTGGGAACTGCCTTTGT

>dbf808ce50f797f92f7329849cd98bfa

ATAAGTTAAAAGGACTATGCTATAAAAACAAGGCGACTCAGGATAAAAATTAAGTGAATATGACTTACCCAATTAAATTCAATGGAAGTAGGAGGGGATTTGGCAAG

>6ce318baa79293afcadbadc07af506cb

TAAAAGTTGACTTTGTAAAAATCCACTGCCTTCTTATCCTGGCGTGCAAGGCTGTGTACTTTCGATAGGGTTGGCCGTGATCTTGAAACCGCACACAGAAAGGAAAG

>b739b8c1db79137e776f4e9f798e153a

ACAACCAGTAACTTAGATTTGAAAAACTCACAGCGATTTCAGAACGTGATGGTCAGCACCACAACCGTGGCCCCTCGTTCCTATCAAACTAATTACAGTGCACCTAC

>e27532bc6e5652c5dee319d32a904679

TCAAGTAGGCTTTGAATAACGCGCATCGATTTGCCGCAATAACGCTCGTTGTCATCGGCCAGTTATCGGGACGCGACAATAACAGCAGGACATTTCACGGAGAGTTA

>70cb4c8c00c9c17297952caced5e5d5f

TTAGTATGGAAGTGTAGAAAAGTACGAACAGGGAAAATTGAAACCCAGTGTGTAAAAATGTAAGTGGTAACATATAAATCAGAGTATTATGTATACATATATGAATC

>5c898e04daeb26b6b562b22580bdcb94

AACAGAGGATACAAGCGTTATCCGGATTTATTGGGTTTAAAGGGTGCGTAGGTGGTTTTTTAAGTCAGTAGTGAAATCTTCCCGCTTAACTTTCCCCGTGCTCTTGC

>78406140f1c351a515afc5c4a3ec17ac

CAACCCACTTTTTCCCCGGGCGTAATTTCCTTTGTTTCTGCTGAAATATTAGAAACTGTGTTTCTACTTATCTGCAGGGCATCGGCGGCTCGCTGCGTGAAATTTTG

>720406407d33b5624278b515e31991b9

GAAAACTCGATTGGTGCTGCATTCTGGTGCATAGCCGCTGTAGGCAAACGAAGTTGCCGGAAATAATTGTGATTAACACTGTCTGGCTACACCCTTCATCGATCATG

>d019876916a6216397e47b5972fc4c17

ACGATCAACACACGATTCTTCACTCCATCGCCAGCGCTCGCGGACGACGACGACAGCGCAACGTCCAAGGTAAGCGGAATCGAACGTACAAGGGGGAGGGGTACGGG

>6afb0e2015460f14bff38382bb394e6f

GACAGGGGATGCAAGCGTTATCCTGAATGATTGGGCGTAAAGCGTCTGTAGGTGGCTCCTTAAGTCCACTGTCAAATCCCAGGGCTCAACCCCGGACAGGCGGTGGA

>8c0d6e6e7e66bac277a4117a3a540a93

CAACCACTGTGCTGAGTGTGATATACTTTTTTTACAATACACAATAACAGATGCCACAAGCCACACACACACACGCACAACACACACACACACACACACAGAAAGTC

>07247a17b41e0494313215b555a6d173

CATAGGTAACTGCCACTATAAACTAACGAATGGAAAAAACACAATTCAGATTTTTATATTCCTACAAGCGAGACTTATGTCTGTCGACCATTTCTGTATTTGTAGAG

>86565dbca7505ccea9ef51171d25ed26

TATCCGCACCTAATAGTGCACCAACAGGAGCGGGCTGGTCAAAAGTAGGGTCGGCGAGAACCAAGTCCCTAGCCGCACTCTTGACCTCTGAGGCTATTGCGGTCAGA

>7d0af8694d118b537a93da843f8c9151

TGTCCGCACCTAATAGGGCATGAACGGGAGCGGGTTGGTCAAAAGTCGGATCGGCAAGAACCAAGTCCTTAGCCGCACTCTTAACCTCTGAAGCTATTGCTGTCAGA

>522c97cc0508eb6e2c8aebeebb5f9e21

AACGTAGGAGGCGAGCGTTATCCGGATTTACTGGGCGTAAAGCGCGTGTAGGCGGCGAAGAAAGTTGGATGTGAAAGCTCCTGGCTTAACTGGGAGAGGTCGTTCAA

>b0453b912fe43c454c2bc09656da2266

TACGGAGGGTGCAAACGTTGTTCGGAATTACTGGGCGTAAAGCGCGCGTAGGCGGTTTGTTAAGTCAGATGTGAAAGTCCACAGCTCAACTGTGGAAGTGCATTTGA

>091e095eb38d511bf83a49797adcb0f6

TACGTAGGGGGCTAGCGTTGTCCGGATTTACTGGGCGTAAAGGGTGAGTAGGCGGTTGTATATGTCAGATGTAAAAGGCGTGGGCTTAACCTACGTTAGCATTTGAA

>d723f5e2204e4c41763511f6f3e243bc

TACGTAGGTGGCGAGCGTTGTCCGGATTTATTGGGTTTAAAGGGTGCGTAGGTGGTTTTATAAGTCAGCGGTGAAAGTTTGCAGCTTAACTGTAAAAGTGCCGTTGA

>ccea13318cab48e2e85dde738cba4779

TACATAAGGAGCGAGCGCTATCCGGAATTATTGGGCGTAAAGAGCTCGTAGGCGGTCTTAAAAGTCAGGTGTGAAATTATCAGGCTTAACCTGATAAGGTCATCTGA

>658735dc691f29c3369da5ca61c6d18e

TACGTAGGTGGCAAGCGTTGTCCGGATTTACTGGGCGTAAAGGGTGCGTAGGCGGACAATTAAGTCAGATGTGAAATCCCCGGGCTCAACCTGGGGGCTGCATTTGA

>992860a3c9e14f73b40a5358904bef9e

ATTACTGCCAAGCTCTGTCCACACGTAGCCAAGATGACGAAGTTAAGAGCTCCCTTTTAAAATTAAAAAAGTCAGTTAATGAATTATTAGAAACCCGTGTAGTCCGG

>3d7c88d6bd60a3efc2e30fc0d89f4440

AACGGGGGGGACTAGTGTTATTCATCCTGACTGGGCGTAAAGGGCATGTAGGCGGTTTGTTGGGTTTTTTGTAAAATTCTTGGGCTCAACCTGAGGAAGACATTTTA

>4d266c06090442d51cf763818400f1b5

TAAACGGCAGCCACAGCCGTCATGATATTTACTGTGGCAATTTGTTCCACATATTACGATGAATGCATTAGAAACCCCAGTAGTCCGGCTGACTGACTTACTAGGTA

>fa9b7b4a4ed27ce65db9c201b680ebea

TACGACGGGTGCAAGCGTTAATCGGAATTACTGGGCGGAAAGCGCGCGTAGGTGGTTTGATAAGTTGGATGTGAAAGCCCCGGGCTCAACCTGGGAATTGCGTCCAA

>e85faa8eb7f36d2a9014c73d1fa341c0

TACGGAGGGGGCAAGCGTTAATCGGAATTACTGGGCGTAAAGCGCGCGTAGGTGGTTTGATAGGCGAGATGTGAAAGCCCCGGGCTCAACCTGGGAACGGCATTTCG

>666431a738e10f2e719463b8698477ab

CCAAACGTGTTGTCAGTGTTTCAACCACACTTCGTCGGTACTTTACGTCGGATGGCAGAACGTACGTCAGAGATTACAGTGGCAGTCGCTGACTTGATAACACTTTG

>9837833246aa1ec2bffef01c329b736b

ACAATCAAGGGTCGCAAAAGCCCCAACACAGCCAACCAGATTTTTGTATAGCTATAATACAATTAAATACTTTCTCTAAATCATTACAGTTATTCCCAACAACACGT

>1b0747d5a7e31aecf6947f35cabb8bf5

TACGGAGGGGGCTAGCGTTGTTCTGAATTACTGGGCGTAAAGCGCACGTAGGCGGACCAGAAAGTTGGGGGTGAAATCCCGGGGCTCAACCTCGGAACTGCCTTCAA

>51ad66df5595a5e549c84612f975b537

GCTTCCGCAAGAAGACATTTGTACAATTCGCGGGAGACAGGGTGACATGTATGGCCGAAGCGTCTCTGCAGTAACTATCGGGAGTCTGTGAGGAACCGTTATATGTC

>dc17eaaeb3769bbc91a67e928cd0058b

TAGGCTATTAGGGTCAACGTACTCGCCGCCGCTCGCTCACACCTTAACACACGCACCATGGCCAGCGCGCCGTATTGTTGTTAGCCGTTGGAATGTCATTGCAGATG

>caaed9a9c43df885e3c864d22ac61e3c

TTTCAAGATATAGCAGTGTAAAGCTAATCAATGTAAATACCATGTGCATTATGTAACATTTTGTAACGGGTAATACTGATATTCAATTGATTGAAATCTTTAGTTAC

>546e6ae38320cab32fc8107f9110f9bc

TACAATTTCTTCAATTTAAATTTTTAAAAGCTCCAGCTAATAAATTATTTAAATTTTTATAAATAATAATTTTAGTGAAATGTATTATTATTATATTTTATTAATTT

>c1a733272911bcb70d12b7610a42b330

CACAGCGGCTCCCAGCTTTCTCCGGCTTTCTTTTGTTTCCCGGGTTCTTCTGTGGTTTTTTCCTTCCTTCTTTCCTTCTTCCCTCTTTTCTTTAAAAGTGCTATTGA

>26a8f72cc522e74503bd3c0dfeccb58b

ATCTAGTCTCACAAACAAATATGTAACCCGAAAGAGTTAAAAATATAATTCGTTTCCTTTGAGGGTTTTGCATAAATTAAAGTTATGTTTTTAACATTATATTAAAC

>60bc67048211d4f9abb62ab4f080d1f1

TTCCAGCTCCAATAGCGTATATTAAAGTTGTTGCGATTAGATACCCCAGTAGTCCGGCTGACTGACTATAGCGCTATCTCGTATGCCGTCTTCTGCTTGAAAAAAAA

>d46716aea7883eeff345f9cd67741801

GCATCGTGGTTCCGCTTGTTAAGTTGAGCATCACATGAATGAATGACCGAATGGCAGCTCAGAGGCCTGGAATATCTACAGTTATTGAGGTACACCACCCTACCCTA

>9d3ef926dad197c4a80fffbbeafc429b

TACGTAAGGACCGAGCGTTGTCCGGAATCATTGGGCGTAAAGGGTACGTAGGCGGTTAGAAAAGTTAGAAGTGAAAGGCTATAGCTCAACTATAGTAAGCTTTTAAA

>cf5201d217a2b0805fcc7ddc8cded695

TACGTAGGGGGCAAGCGTTATCCGGAATTACTGGGCGTAAAGGGTGCGTAGGCGGCCTTGTAAGTCAGGTGTGAAAGGCATTGGCTCAACCAATGTAAGCACTTGAA

>4604c1d403adaa2165ad2af0691eb915

TTCTTATTTTTAACTCTGTCAGATCAGAGGTGATTATAGTAATCACTGAACAATCAGAAATCTTTCTTTTTGGCAGTTCTCATTAGATACCCGGGTAGTCCGGCTGA

>bfe4854fef65fa8c4048052fdcbc076f

TACGGAGGGTGCAAGCGTTAATCGGAATTACTGGGCGTAAAGCGCGCGTAGGCGGATAATTAAGTCAGATGTGAAAGCCCCGGGCTCAACCTGGGAACTGCATTTGA

>1e0511350f916c44b560f5b0a7d00410

AGACAAGCAATTGTTGAGTGAAATACAAGCACTCAGTGACTCGATCAGAAAAAAGCATCACGCGTTGAAGCGCGGTATGGTGGACACGGCGAGTTCTCTCGCCAAGA

>a95f8dad9d6d520a3ae9e2ebcc436dbd

TACGTAGGGAGCGAGCGTTGTCCGGAATCATTGGGCGTAAAGCGCGCGCAGGCGGTCTTTTAAGTCTGATGTGAAAGTCCACGGCTCAACCGTGGAGGGTCATTGGA

>5df131c66e2630b2c63e2f35d543af15

CATTTTACCATAGTGTCTGCCTCCACGGGCACGTTTATGGCTTTGCCGGTTATCTTTTGAGACTGTTTCTATTCCGAGATGACGGATCCGCATACGTGGAAATCCGG

>7e23c31866cbe480fd67a41cfa3bd497

TACAATTTCTTCAATTTAAATTTTTAAAAGTTTCAGTTAATAAATTATTTAAATTTTTATGAATAATAATTTTAGTGGAATATATTATTATTATATTTTATTAATTT

>e325a270b048949ee0564a9c794c788b

GTAGCAAGTAGTGGTAATTATCTGGTCGATTTCAATTACGTACTGCAATTCATTACCCATTTTAATTGAATTTCTCTTACTTTTACTTCCCACTCCTGATTCAGACT

>1889b82c87add93f83c5012c5cb69c51

GAAGGGCAACGAGTGCAGTAGTGATTGGATTTGTATATTCATACGCTCGTAGTTGCAAGCGTATGTCATGATAACTTATTTTTGTTTCTCACCCTGATGGTTTACGT

>c3d14090f849051fc8d99eaf63ccff26

TACAGAGGGTGCAAGCGTTAATCGGAATTACTGGGCGTAAAGCGCGCGTAGGTGATTTGTTAAGTTGAATGTGAAATCCCCGGGCTCAACCTAGGAACTGCATCCAA

>41fd2e0a073383226db9c3983126893b

ACTGCACCTGGAGATTTAAACATTAGCCATGCAGTGCTAACTCACTTGTGTGACGTAAATCCTATATTTAGTACTCAAATTTACGTCAGCACATAACCTATAAGACG

>7d0246b502fb79b2050bff2fbdf6d21a

TACGTAGGGGGCGAGCGTTGTCCGGAATGACTGGGCGTAAAGGGCGCGTAGGTGGCTGCGCAAGTCTGGAGTGAAAGTCCTGCTTTTAAGGTGGGAATGGCTTTGGA

>ecf6c4890a03cb1fe2e6e5c338a893df

TACGTATGGGGCAAGCGTTGTTCGGAATTATTGGGCGTAAAGGGCGTGTAGGCGGGGAGTTAAGTCAGGTGTGAAATCCATAGGCTCAACCTGTGAACTGCACTTGA

>bccb05adfe582c4a2aa36bb531902a71

AACGTAGGGGGCGAGCGTTGTCCGGAATTACTGGGCGTAAAGGGCGTGTAGGCGGTAAAGTAAGTCCGTAGTGAAACCCGGCAGCTCAACTGCCGGCTTGCTATAGA

>0e279c489a5a3db4c2f4c03bc50e1f51

TACGTAGGGGGCAAGCGTTGTCCGGAATTACTGGGCGTAAAGGGCGTGTAGGCGGTTTTTTAAGTTAGGAGTGAAAACTCGAGGCTTAACCTCGAGACTGCTTCTAA

>27f5df67741ba34ebf224e164f822131

CACGTAGGGGGCGAGCGTTGTCCGGAATTACTGGGCGTAAAGGGTGCGTAGGCGGTCTGTTAAGTCAGATGTGAAAGACCCGGGCTCAACCCGGGATGTGCATCTGA

>10a8fb5b6d349033bd0de82e3bebebd2

TACGTAGGGGGCAAGCGTTGTCCGGAATTACTGGGCGTAAAGGGCGTGTAGGCGGCCATGTATGTCAGATGTGAAATGCCACGGCTCAACCGAGGCACTGCATTTGA

>03f50ba07286e0c0529a49d610252dd4

TACGGAAGGTGCGAGCGTTAATCGGAATTACTGGGCGTAAAGCGCACGTAGGCGGTTTGTTAAGTTGGATGTGAAAGCCCAGGGCTCAACCTTGGAATTGCATCCGA

>ab04b4177ac0eef09078b7f5a1f5ceff

TAGCATTTACAGTAATCTGATGCACACTTTGACATGTCACATTGCTCACATCATCAGAGGTATATGATTGTGTGTGTGTGTGTGTGTGTGTCTAGTAGGGAATATCT

>8ab01497ed693ad7a0ee5f25d60a7965

TGGACTCCGAGATGTCTGATAATGAACAAGAAACAGATGGTGAATTAGAAACCCTGGTAGTCCGGCTGACTGACTTACTAGGTATCTCGTATGCCGTCTTCTGCTTG

>5279305c654b173e8b4cbf52d2ffdefc

TCTGCTTAGTGAATGGCAGAGTATTCACAAACAGTGGTCTGTTGTGTATAACGAATGTAGCGGATAATGCATAACAGTCATACACAAACTCATATAAGCAGGCAGTT

>7e17da8185edc18b42a31ff265c76993

AGTTCATATTGTGAAACAAAAAATTTTTTCAAAGTGCGCAGATGCAGTTTTTTATTTTCAACAAACAACAACAACAAAAATGTGCGTGTCAGCAAGCTACTTAGGTT

>ebf872a556cbcc0982547464f03b0a3a

TCCGGCTGACTGACTCTACGACCATCTCGTATATTAGATACCCGTGTAGTCCGGCTGACTGACTCTACGACCATCTCGTATGCCGTCTTCTGCTTGAAAAAAAAAAA

>8d027fe22a88555526aad3c74388a35d

CTGGTCTACTGGACGTAGGTTTTTCAGCTGTTAAAAATTTTAATCCCTTGTATTTAGATTAATATATATAAATAATACTACATCTGTAGAATTTAATTTTAAACCTT

>e946209c441633e3a13c540c590250cb

CGGTTTACCAAGTATTGGGCAACTTTATGTGTTCATTTTGGGTGTTAAAACTTAAAAATCTCATCTTTTTTATCGTTAAGGGATTCAAGATCCAGCGAGTAGCTGTC

>a659776d83ea437f42fd2c9a7c52fd7e

GTTGCATCTATAGCAGTGGTTCCCTCTTAAAGGCTTAAAGGTATACACAGGCGTTTGCGGTGAGGAGCGGAATGCTCCTTACGCCTGTGGGTATGTGCACGTAAAGA

>968842851bb9e58b41052e3439761d86

GACTAGATGAATAGTACATATGTTGCAACATGCTCCATCTCATATTGTTGATGGACTACTGCAAATTTTTAATGAAATTTGCTCTGTAGTATCTTTCCAGAAGCGCC

>b2cfa527b7fe658db7db0eb60296c5d9

TGTCCGCACTTAATAAGGCATCAACGGGAGCGGGTCGGTCAAATGTAGGATCGGCAAGGACCAAGTCCTTGACTGCACTTTAAACCTGTGAAGCTATTGCTGTCAGA

>b82ce39946dd7eaa704d3bdc166f7f75

TACGGGTGGTGCAAGTGTTATTCCTATTTACTGGGTGTAAAGGGTGTGTAGATGGTATGAAAAGGTTTAAATTAACATGAAATTGTTTTTATACTTTTCTTCTAGAA

>2b956a9e25b945ea4db615b9205e057c

TACGTAGGGGGCGAGCGTTGTCCGGATTTACTGGGCGTAAAGGGTGAGTAGGCGGTAACGTATGTCAGATGTGAAAGGCGAGGGCTAAACCCTCGTAAGCATTTGAA

>1527b0b00df892ee78880e967fbc55a7

TACGAAGGGTGCAAGCGTTAATCGGAATTACTGGGCGTAAAGCGCGCGTAGGTGGTTCAGCAAGTTGGATGTGAAATCCCCGGGCTCAACCTGGGAACTGCATCCAA

>6bd3df270e0532181572b26f678ae308

GTGACAAGGTTACCCGGCGTTGCTCCAGCTGATTGCTTTGTTGGCCTTGAGAAGTGAAACTGTCTGACCCAAGGACTAGCAGACGGTAGTTCTACTAGTTTCAGGAA

>1527f3029d5d4c97caf5d2aa066a396a

TATCCGCACCTAATAGTGCATCAACAGGAGCGGGCTGGTCAAAAGTAGGGTCGGCGAGAACCAAGTCTCTAGCCGCACTCTGAACCTCTGAGGCTATTGCGGTCAGA

>6e67e922870d6d7d814d1eae2ffa63d4

CACACTGTCTTTAGATAGACAAAACATTTCTCCAGGATGCATTTCAACTATGTCCGTCATTTTCAACTGGTAGTGCCTTGTAGATAGTTGTGAAAGATTTTGAATGT

>11f079eb36c67ca3a2ca642f0dd0d75b

AGTAATAGTACACCAACTACACGACATACACATCAACACAGCTCGACTCACGACTAACTAATTTACGGCCGACTTGTCTTGAGAAATCCAATTCGAATTGCATATAC

>49d71fd1b835697595d4521493c0f781

TACGTAGGTGGCAAGCGATGTCCGGATTTACTGGGCGTAAAGAGTATGTAGGCGGACATTTAAGTCAGATGTGAAATCCCCGGGCTTAACCTGGGGGCTGCACTTGA

>fe1055d3806d36d930551ccba0682968

TACGTAGGTGGCGAGCGTTGTCCGGATTTACTGGGCGTAAAGGGTGCGTAGGCGGATTCTTAAGTGGGATGTGAAATACCTGGGCTTAACTTGGGTGCTGCATTCCA

>ead625435766561ffede0961d0306b8d

CAACCACTGTGCTGAGTGTGATATACTTTTTTTACAATACACAATAACAGATGCCACAAGCCACACACACACACACACACACAGAAATTCGTAGGACGTAAACGCAG

>b3bb3bf989da38cba9607e23425cd90f

CAGTAGTTTTTTTACAAAGAGGACAAACATGTAATTAGTATCTCGTCCTTCACACAGTTAATCCGATACACGACAAGTGCCGAACAGAACACAGACTGTGCACGGTT

>c4c4e6afc12bd52738b2fff5139f9abf

TACAGAGGGTGCAAGCGTTAATCGGAATTACTGGGCGTAAAGCGCGCGTAGGTGGTTTGTTAAGTTGGATGTGAGACCCCCGGGCTCAACCTGGGAACTGCATTCAA

>6cc9b652dae754ef97cfcf1130d492f9

CGTTTCTTTCGCGCCGATCTTCGCCTCTCCAGGAATCCCTTCTTCACCGCTTCGCTGTCCGACGCCGAACTACTGTCCGTCTCGGTCGTCGTGTCCTTGGTTTTCCG

>cca1c6a943ffa5d7ef704c5d001f315e

TCCATAAAACACATACATGCACACCACTTAATTTTATCAACACAAATTATACATTCATTAGAAACCCTAGTAGTCCGGCTGACTGACTATGAGCTCATCTCGTATGC

>f4007498c8ad431bacce6ee8f1e1d57f

CACCGTCACCAGGCTCAGTCACCCATATTGCCGTCAACTTGCTCCGCCTCTGCACAGTCACCATCAGATGGTGTCACACTGCAGCCAATAAGATCTTCAGGTATGAT

>8d6536318dc91f8ecdf9fd3f3b6ee957

TACAATTTCTTCAATTTAAATTTTTAAAAGTTTCAGTTAATAAGTTATTTAAATTTTTATGAACAATAATTTTAGTGAAATATATTATTATTATATTTTATTAATTT

>fe6385b934f72710fdaaa75ee407d9f7

GGGTGTTTTAGACAGAAAGACTGAAAGTATAAATACTTCTGTAAAAATAATAATAATAATTGATGTTGATTTCAAAGATAACAAGACTTTTAGCGATTTTGTAAAAT

>71b9f6cd814be1c5b81b513fe9fbecc2

TATGCTTGTGCAGACGCAAGCGGCCGCGGCCGCTTAAAGTAGGCCCCGTCGAGGTGCCCTGGACCAATAACGTCAAGTACCTAGGGCTCCACGTGAACTCCCGACTC

>12825999ab029eed0b085390c9a163c7

TTAGGTAAACTGAAATTGACCCCGGCCGTGAAAGTTTTAAAACTGAATCATAGGACATCATCTAAATGAAAAGTTTAAGTCTTAATTCAGAATGATAAATAAAAAAC

>277a1b7e29109ac331a7157421857913

GGAGGAAATTAGAACAAAAATAATAATTAGTGATCGAATTCTAGAACAGGAGAGCCACTCTAATTATTTAGGAAATGATATTGGCTATGATAGAAATTACGATATTG

>79ad2602ef9847da60271ce43fef3cad

AATTGCTATTTGTGTTTAGAAAAACAGACAATAAGGAAATTCATAAGTTTTATGAAGTTATTAAGGGAACTCTGGAGAATGACGCAAGTATTAGAAACCCTGGTAGT

>80c7d697ffb6c4a552d42497acb08c21

GGTGACAACGAAGAAAATGATCTACATGGCAGTGGAGGAGGAATTGAGTGTCGTATAATTTTACCATTCACTATAACATTAGATGCCATTTTGTCTCCGTGGCGAGG

>ef2affbe987fa763086fbbf8884aeccf

TCCCCGTAGTCCATTAGATACCCGAGTAGTCCATTAGATACCCGGGTAGTCCGGCTGACTGACTCTCTAGAGATCTCGTATGCCGTCTTCTGCTTGAAAAAAAAAAA

>659b43a058009e9d6e760d619509ad8c

TACGTAGGTGGCAAGCGTTGTCCGGAATTATTGGGCGTAAAGAGTGTGTAGGTGGTCTATTAAGTCTGATTTGAAAGCCCATGGCTCAACCATGGAGGGTGATTAGA

>d56718f602d3ca14f7c3ffec5344cc7b

GACGTAGGGGGCGAGCGTTGTCCGGAATTACTGGGCGTAAAGAGCACGTAGGCGGATATTTAAGTCAGGTGTGAAAGACCGCAGCTTAACTGCGGGGTTGCACTTGA

>3721b3546477314a55ea5d32e034d46b

CACGGGGGGCGCAAGCGTTATTCGGAATTATTGGGCGTAAAGGGCGCGCAGGCGGTCTTGTCCGTCAGGTGTGAAAACCCGGGGCTCAACTCCGGGAGCGCACAAGA

>d68425033a8578540794308c2b64ffdc

TACGTATGGTGCAAGCGTTATCCGGATTTACTGGGTGTAAAGGGAGCGCAGGCGGTGCGGCAAGTCTGATGTGAAAGCCCGGGGCTCAACCCCGGTACTGCATTGGA

>38e3a547f8ec2a3cb8e1434baa818be7

TACGTAGGGTGCAAGCGTTAATCGGAATTACTGGGCGTAAAGCGTGCGCAGGCGGTTATATAAGACAGATGTGAAATCCCCGGGCTCAACCTGGGGCCTGCATTTGT

>24c90d5b5cfb9db51fdb9141f89e91cc

TACGGAGGGTGCGAGCGTTAATCGGAATTACTGGGCGTAAAGCGGGCGTAGGCGGCTTGTTAAGTCGGATGTGAAAGCCCCGGGCTTAACCTGGGAATTGCATTCGA

>dcb438836a643298ef7f815ad984d5fa

AACGTAGGGGGCGAGCGTTGTCCGGAATTACTGGGCGTAAAGGGCGTGTAGGCGGCAATGCAAGTCAGGAGTGAAACCCGTCGGCTTAACCGACGGCCTGCTTCTGA

>80371ec8cd150a3937d1fdb0031bcfb3

AGATGGATAACATCCTGTGGTGAATCTCAAACGTGCAGTATTTGTTTGGAATGCAGTATTAGAAGCCATTTTAAAATGTACGTTACTTAGAATTAGAAACCCCTGTA

>4bc227825cb375d9f66ebf7ee9520936

CAATAGAGCGTCACCGTTTTCGAGGTGCTGTGGCCGAGGCCGACAAGGGGCGCAAGTAGATCCCCTCGCGGGAGGCCGGACCTGGACAACAGCGCATCCGAGGTCAC

>c6f20a964c59139f34e8a96504de6919

GCCTCCACGTTTGAGGCCACGGTTCCGTGGCTGTCTCTAGAATTTATCGAAGGGGGCGTAGATTGCGCTGGCGTGGGCGATAATTCTCGGTTAGTTATTAAGTGTTC

>6f623992c36dce10150e5413846cf9a8

TACGTAGGGGGCAAGCGTTATCCGGAATAACTGGGCGTAAAGGGTGCGTAGGCGGCCCTGCAAGTCAGGTGTTAAAGGCATCGGCTTAACCGATGTAAGCATTTGAA

>ac8d15718e8d8d8a2ab337fde946213e

CTTCAGGCCACAGACGAGACCCTGCAGGTTATCTCCGCTGGAGCCCCGGTTCATTAGAAACCCTTGTAGTCCGGCTGACTGACTATGAGCTCATCTCGTATGCCGTC

>0a90be6a0c3813d6051ded498ec1764c

ACAACAAGGGGAACCTAACAATGAGCCGTCTCGCAATCAATCAATTTTCGCCACGCCACATCAACGCCATTCGTGTAGCACAATTTTTTTTCGCCTATCTACACACA

>9ac46f6a9074476384716a410bfa6813

CATTGAACTATCGTGAGAAAGTCAAACCGCCAAAGGGAATTATATTATAGTAAATATTGGCGTAAATAAACATTTTATTTATAGTTGTAATATATGAAAATGTGCAG

>4e028d3d05447a0093ea2aae1e06496d

TACGTAAGAGACTAGTGTTATTCATCTTAATTAGGTTTAAAGGGTACCTAGACGGTCAATATAGCTTCTAGAATGTTAGTACTTGACTAGAGTTTGATATAAGAGGG

>050621ca4c46fb7f1e79057d58c066e9

TACAATTTCTTCAATTTAAATTTTAAAAAGTTTCAGTTAATATATTATTTAACTTTTTATCCCTCCTCTTTTTCGTGCCTTTTATTCTTTTTATATTTTATTAATTT

>3f575e90c7f87bef1d3809b36129b93a

TTTAAGATGTAGCAGTGAAAAGCTAATCTATTTAAATCTCGTGTGCTGTATGTAATATTTTGTAACGGGTAATACTGATATTCAATTGATTGAAATCTTTAGTTACA

>9ccee5b7d960742ea57ecae79242076d

GACGGAGGGGGCTAGCGTTGTTCGGAATGACTGGGCGTAAAGGGCGCGTAGGCGGCAAATCAAGTCAGGTGTGAAAGCCTTGGGCTCAACCCAAGAATTGCGCTTGA

>bb838a317af23446895a473aa48ef46e

TACGTAGGTGGCAAGCGTTGTCCGGAATTATTGGGCGTAAAGCGCGCGCAGGCGGCTTCCCAAGTCCCTCTTAAAAGTGCGGGGCTTAACCCCGTGATGGGAAGGAA

>a2f0272380910573242407afcaa2a739

CACGTAAGTGGCAAGCGTTGTTCGGATTCATTGGGCGTAAAGGGCGCGTAGGCGGCGTTTCAAGCTTGGTGTGAAATATTTCGGCTTAACCGAAAAGACGCGCTGAG

>b852902a2564aa84987fe054cd466a21

TTATGGTGATGAGGTACAGTTGACCTTTATTCAAACTTCACTTATTAGAAACCCGAGTAGTCCGGCTGACTGACTTGCGTCAAATCTCGTATGCCGTCTTCTGCTTG

>b4ca31dc758eeedc5dae8028484e0928

TACGTAGGGGGCAAGCGTTATCCGGATTTACTGGGTGTAAAGGGAGCGTAGACGGTTAAGTAAGTCTGATGTGAAAGCCCAGGGCTCAACCCTGGGACTGCATTGGA

>0f3510609fe58935689c941e186ceaed

TACGTAGGTGGCGAGCGTTGTCCGGAATTACTGGGCGTAAAGGGTGCGTAGGCGGGTATTTAAGTCAGATGTGAAATTCCGGGGCTCAACTTCGGAACTGCATTTGA

>fd026d7d29b5d3e4dc5b32769d31e8ae

TACGGAGGGTGCAAGCGTTGTTCGGAATTATTGGGCGTAAAGCGCGTGTAGGCGGTTTGATAAGTCATTTGTAAAAGATCTCAGCCCAACTGAGTAGGGCGAATGAA

>6d0e4147728e10f958c8efb9b1b438f1

TGTCCGCACCCAATAAGGCATCAACGGGAGCGGGTTGGTCAAAAGTCGGATCGGCAAGAACCAGGTCCCTAGCCACACTCTTAACCTCTGAGGCTATCGCTGTCAGA

>ffbddd4bcdf1cf2b2e98d2446607799b

CAGTTAAACTTACTTTGCTAGTCCGTAACCAGGTCGTTTTTATGATCTGTAACCCACGCAGTGTTACAGTTTACAAATTTATAAAGCAAGATAGTAACCGTGCAACA

>1acdf8fa89ba42c03b6dd221fb0159b6

AGATGGATAACATCCTGTGGTGAATCTCAAACGTGCAGTATTTGTTTGGAATGCAGTATTAGAAACCCTAGTAGTCCGGCTGACTGACTGTTACAGCATCTCGTATG

>420b3b1a6fcc43796e64a895aa7a2703

GAGGATTATTAAGTTAACCCTTTCATAACCTCGGTAAGTCTTCGGTTAATATAGGCCAGGCTGAGAAGTAGGGCAACGTAGTTACCAATACGCCTATTTTTTTTTTC

>d1526177918ed9c382c07c4e6f0d7ae7

CCGTCTTCAGTGGAGCTGCAGACAAGATTACAGTGTCCGCGATCAAGATCTGGGTGGGCCATTACGAGGTGCCGCGCCAAAACGGAGGTTGAAGTTTAAACCTGGTA

>9e10a1f8ac089fea07f58e91b1e93423

TGTAATGTCCAAAGAACCGTTAAAACTAATTTGATACCCCAGTGTAGTCTGTATTGTAACAAAAGCATTGTATTAGGAACTAACCAAAAATGTTTCTATTCCTTCTT

>c836052eafe8291d2b397f2a8e2e120d

TACGTAGGGGGCAAGCGTTATCCGGATTTACTGGGTGTAAAGGGAGCGTAGGTGGTGCGGTAAGTAAGATGTGAAAGACTACGGCTTAACCGTGGGGTTGCATTTGA

>34f345e6de6a277d051dc30439a4c2d3

CCAGGGACTTGTGCACTCGTAATAAAAATTTGGTGCTTGTAACAAAATGCTGGAAGTATGATGTTTTGAATAAATACGTTCATGGTAGCTAAATTTACACACTTTAA

>060b466e236836b8beffec5c8a300f24

ATGCAAATTGGGATCACTCGATTGAAATCTTAAACCGCATTAGATACCCGAGTAGTCCGGCTGACTGACTACGTACGTATCTCGTATGCCGTCTTCTGCTTGAAAAA

>0bc778be50d1ced91604560343422593

GTACGTGAAACAGCTTCTCGGACTCTATTGAAGCTTAAAAAGCAAAGCTATGTTTCGGTTGTTTCTGTAACCGTCTTCAGGCTACGACTAAATTACATAAATTGGGA

>580b2df2cf880dae8d54ad848603e00b

TTGGCGTTGGTTCAGCTCTTGTATTAGAAAGCCTCGGTGGCACAGTTCACCACAGTATCTCCATCAGGTATCTCCTTCCTATGGCTACAGCAGGTTCGTTTTATCGA

>27c3637ea508960c9b9a8dbefde41b3a

TACGTAGGGTGCAAGCGTTAATCGGAATTACTGGGCGTAAAGCGTGCGCGGGCGGTTATGCAAGACAGAGGTGAAATCCCCGGGCTCAACCCGGGAACTGCCTTTGT

>956b7c84df7003b4f900988fe2d238c4

TTCCAGCTCCAATAGCGTATATTAAAGTTGTTGCGATTAGATACCCCAGTAGTCCGGCTGACTGACTGCGATACGATCTCGTATGCCGTCTTCTGCTTGAAAAAAAA

>4a1f56636a752c90a92e9ecdf22042c6

TACAATTTCTTCAACTTAAATTTTTAAAAGTTTCAGTCAATAAGTTATTTAAATTTTTGTGAATATTAATTTTAATAAAACATATTCTTATTTTATTTTACTAATTT

>cc1e46260da6e3e37cae982f80ebddc7

CTGAGTGAGTTAATGAGTGGTAATTAGTTAGAAAATTAAGCTATGCTGGTCTCTGTCCTCCGCCGTTTCTGTCCTTTTCTTTTCACCCTGACTCTTCTTCAATCTCT

>4d946310e1961df9ae12b7b808e69b36

GTCAAAACGTCTGTTACTTTCTGGATAGCCCTCGTATATAAAAAGAAACTAATAAAAACCAGAGAAGAATTAGAAACCCCTGTAGTCCGGCTGACTGACTATAGCGC

>f8cd1542acc1a7599f6fdfd0c8bebbb2

CTGTTTGACCACGGGCACATTTATTGCTTGGCCGATGATCTTGTAAGTTCGCGTCTCCATTCTGAGGCAACGGATCTGCATAATTGGTAATGTAGGTGAAATCAGTC

>d2b48b7fdcc9049c6d30bd7ee77f5966

TTCCAGCTCCAATAGCGTATATTAAAGTTGTTGCGATTAGAAACCCTGGTAGTCCGGCTGACTGACTGTTACAGCATCTCGTATGCCGTCTTCTGCTTGAAAAAAAA

>c00ecf0120c2d10939fa6faa8d9d057a

TACGTAGGGACCAAGCGTTGTTCGGATTTACTGGGCGTAAAGGGCGCGTAGGCGGTTTGTCAAGTCAGTTGTGAAATCTCCGAGCTTAACTCGGAACGGTCAACTGA

>3444c70faaf15d2b5f6c07e706317903

TGTCCGCACCTAATAGGGCATCAACGGGAGCAGGTCGGTCAAATGTCGGATCGGCAAGAACCAAGTCCTTAGCTGCACTCTTAACCTCTGAAGCTATTGCTGTCAGA

>efdec98e5a86bd5fb70fa78737e14ca7

TACGAAGGGGGCTAGCGTTGCTCGGAATCACTGGGCGTAAAGCGCACGTAGGCGGGTCGTTAAGTCAGGGGTGAAATCCTGGAGCTCAACTCCAGAACTGCCCTTGA

>31b3b99e726443f5b163c9672fe0e7ef

CACGGGGGGAGCAAGCGTTGTCCGGAATCACTAGGCGTAAAGGGTAAGTAGGCGGAAGCGAAAGTCAGATGTTAAAGGCACCGGCTCAACCGATGTACGGCATTTGA

>a8a252f95eeceb1979acede0d0b07e5e

TACGTAGGTGGCAAGCGTTGTCCGGAATTACTGGGCGTAAAGGGCGTGTAGGCGGCCTTTTAAGTCAGATGTGAAATCCCAAGGCTTAACCTTGGAACTGCATTTGA

>d54e1b93f66858f3865c20de062c4eb2

TACGGAGGGTGCAAGCGTTAATCGGAATTACTGGGCGTAAAGCGTACGTAGGCGGTGTGTTAAGCTAGATGTGAAAGCCCCGGGCTCAACCTGGGAATAGCATTTAG

>28fd25677aef2a8eedefbeb38f48dc07

TACGGAGGGGGCTAGCGTTGTTCGGAATTACTGGGTGTAAAGCGCACGTAGGCGGGTATCCAAGTTGGGGGTGAAATCCCGGGGCTCAACCCCGGAACTGCCTCCAA

>946a23bb08d929162286f9a967b27803

AAAACCTTCCTCCTTTAAATTGCTTCTTGTCTGGTATTTAGTCATATCAACAAAAAGTAACTAATAGTAATTAGATACCCGGGTAGTCCGGCTGACTGACTTGCGTC

>25ae46b350f88b2ace1edbf01e8258c5

TACGTAGGGGGCGAGCGTTGTCCGGAATTATTGGGCGTAAAGGGTGCGTAGGCGGCCTAGTAAGTCAGATGTGAAAGCCATCGGCTCAACCGATGTAAGCATTTGAA

>c4b3afacc89890dc545b9d352544b5b9

TACGGAGGGTGCGAGCGTTAATCGGAAATACTGGGCGTAAAGCGCACGCAGGCGGTTTGTTAAGCTAGATGTGAAAGCCCGGGGCTCAACCTCGGAACTGCATTTGA

>817e228d0853c94160a934326274b42a

TACGAAGGGGGCTAGCGTTGCTCGGAATTACTGGGCGTAAAGCGCACGTAGGCGGCTTCTTAAGTCGGAGGTGAAATCCTGGAGCTCAACTCCAGAACTGCCTTCGA

>d4c9466223d0c6677a7cd90cc43de07c

TACGTAGGGGGCGAGCGTTGTCCGGAATCATTGGGCGTAAAGGGCGCGTAGGCGGCCTGGTAAGTCAAGTGTGAAAATCAACGGCTCAACCGTTGAACTGCGCTTGA

>dd5850ef533221d34c025be8130418f4

TACGTAGGTGGCGAGCGTTGTCCGGAATTATTGGGCGTAAAGCGTGCGTAGGCGGTCTATTAAGTCTGATTTGAAAGCCCATGGCTTAACCATGGAGGGTGATTGGA

>764f967cb9ecc378a70c3681ec90f21e

TACGTAGGTGGCGAGCGTTGTCCGGAATTATTGGGCGTAAAGGGTGCGTAGGCGGTCTATAAAGTCTGATTTGCAAGCCCATGGCTTAACCATGGAGGGAGATTGGA

>1b1577eb1b05ed851280dc37c0a8d933

TACGTAGGGTGCAAGCGTTAATCGGAATTACTGGGCGTAAGGCGTGCGCAGGCGGTTATGCAAGACAGAGGTGAAATCCCCGGGCTCAACCTGGGAACTGCCCTTGT

>e91bc296ae5165e64cf7fa5c722326dc

TACGGAGGGCGCAAGCGTTGTTCGGAATCACTGGGCATAAAGCGCGCGCAGGCGGGACCGCAAGTCGGAAGTGAAATCCCGGGGCTCAACTCCGGGGCTGCTTACGA

>d521131545c97faa0f57f898c6f4dfef

CACGGGGGGCGCAAGCGTTATTCGGAATTACTAGGCGTAAAGCGCGCGTAGGCGGTTGTGTAAGTCAGATGTAAAAGGCATGGGCTCAACCTGTGTGTGCATCTGAA

>5c0392e08a46ae31261fd27ea95f0828

TACGTAGGGTGCAAGCGTTAATCGGAATCACTGGGCGTAAAGCGTGCGCAGGCGGTCCGCTAAGACAGATGTGAAATCCCCGGGCTTAACCTGGGAACTGCATTGGT

>3baa942fc67754fbb507ac7c2ef8781e

GCAACTACAGCACAATAAACTGTCTACATTTAATAAACAAACTTATTGAATGTTGCAATGAGTACACAAAACTTAGTGTTTAGAAGAAAAAAGGATATTCACAATTT

>fed68b561bb9f3119395e26e4a37ae9b

CAACCACTGTGCTGAGTGTGATATACTTTTTTTACAATACACAATAACAGATGCCACAAGCCACACACACACACAGAAAGTCGTAGGACGTAAACGCAGTCATATAA

>2df0c9a07784080e1b74241a2937cf5a

TACGTAGGGGGCAAGCGTTGTCCGGATTTATTGGGCGTAAAGAGCGTGTAGGCGGCCAGACAGGTCCGTTGTGAAAACTCGAGGCTCAACCTCGAGACGTCGATGGA

>201ae9734911c0d64372d7f171135b0e

TTGTATAAGTTGTGTAAACCAAACATTCAGGTTACTAGTACTGCACTGTATGCATTACGGTTTAGGTTTTCTTGTTTTCCAACCCCCTGATAGCAGGCCGCAGTGTC

>45a064d3387f897b15b262f3e960bb78

TACGGAGGGTGCAAGCGTTATCCGGACTTATTGGGTTTAAAGGGTCCGTAGGCGGGTTATTAAGTCAGTGGTGAAATCTCATAGCTCAACTATGAAACTGCCATTGA

>090a6b0d61e1f3643d106a60d6880a21

TGTTATTTTAATACTACAAGCACGAATTTGTAATACTTTCTAGTGGTGCGAGTGAAATAATCATGGCCACTTGACCAATTTCGTTATAATACCAAGGTGATTCAAAA

>e9cc7ef1af2f2162c22f8d397db8b0b4

GACCTTGAACTCTATCTCTAGCCTGTTTGCAGCATCAAGTAGAAGACTAGATCCTTTTTTCCGTGCACACCACGCCTACAGCCCGCTTGGAAAAGTCAACTGGGTAG

>acd917630bd931ad801eff636986f7d8

TTCTCATGTGTGATGGGCAACGATTTTATTACGTTAAGCGCCTCGCCATGTAATGACGAGTGCAAATAGTGAAGTTTCTGTATATCTGTCAGATCTGCTCTGACATG

>c5b9c00d3ce93a70b82f2e5e9033e0cc

CACGATTTTCTAAATTTAATTATGTTAGTTTCAGTTAAAAAAATGTGTTAATATTAAGTTTTTTAAATTTTGGTGGAATAATATATAAATATATGTTTAATTTTATG

>f3b3a854b466053f92af8871a3c6eaef

TATTCTCGCGCAAACGCAAGCGGCCTCGGCCGCTTAAAGTAGGCCTCGTCGAGGTGCCCTGGACCAACAGCGTCAAGTACCTCGGGCTCCACGTAGATTCCCGACTT

>40600b2864e1da10b4541cdb28aeb147

CACACAACGGAAACGTGTAATCCGCTGAAAACAGTAGTTCAGCATACAGACGTTAACTAGCGTACGTAAAACGGCGCGTGAGACTGGGCGACGCGACGCCCAGTATA

>de17a8d53c922cc5314ed80c85b2d360

CATTGTACTATCGTGAGAAAGTCAAACCACCAAAGGGAATTATATTATAGTAATTATTGGCGTTAATAAACATTTTATTAATAGTTGTAATATATGATAATGTGTGA

>c966c0975d340c1710baf956f196d434

CATTGAACTATCGTGAGAGAGTCAAGCCGCCAAAGGGAATTATATTATAGTAAATATTGGCGTAAATAAACATTTTATTAACAGTTGTAATATGTGAAAATGTGCAG

>4a850df26a51155f1a7600f6b73ad03f

CTACCTCTTTATCTTATAGATAAGAGACATTTAATAAGAGATGTGAATTTTACATTGATAGTAAGTTCAAATTAGAAACCCCGGTAGTCCGGCTGACTGACTTGCGT

>555301123e6c6a7cdcc118daca8cbb03

TACGTAGGTGGCAAGCGTTGTCCGGAATTATTGGGCGTAAAGAGTGCGTAGGTGGTTTCTTAAGTCAGGAGTGAAAGGCTACGGCTCAACCGTAGTAAGCTCTTGAA

>a4be726b91964457753c2602154f88e4

TACGGAGGGTGCAAGCGTTAATCGGAATTACTGGGCGTAAAGCGCGCGTAGGCGGTTTGTTAAGCTAGATGTGAAAGCCCCGGGCTCAACCTGGGAACTGCATTTAG

>03d33ee127f780fb23c67b67a6902e3b

TACGGAGGATGCGAGCGTTATCCGGATTCATTGGGTTTAAAGGGTGCGTAGGCGGACTTATAAGTCAGTGGTGAAAACCTGCAGCTTAACTGTAGAGGTGCCATTGA

>68399d3132d559c5e6e9745e7dd5a917

TATGTAGGTGGCAAGCGTTGTCCGGATTTATTGGGCGTAAAGCGCGCGTAGGCGGTTGTGTAAGTCAGATGTAAAAGGCATGGGCTCAACCTGTGTGTGCATCTGAA

>f55e518125018de8b6f7c2118b46717a

AACGTAGGACGCAAGCGTTATCCGGATTTACTGGGCGTAAAGGGCGCGCAGGCGGTCTTGTCCGTCAGGTGTGAAAGCTCGGGGCTCAACCCCGGAAGTGCACTTGA

>8626f932330128b7f7f638535917f1fc

TACGGAGGGTGCAAGCGTTAATCGGAATTACTGGGCGTAAAGCGCGCGTAGGCGTATGATTAAGTCAGATGTGAAAGCCCAGGGCTTAACCTTGGAACTGCATTTGA

>20445eef2b3d7b41e847d3ec82328d3c

TACGTAGGTGGCGAGCGTTGTCCGGAATTACTGGGCGTAAAGGGTGCGTAGGCGGACAATTAAGTCAGATGTGAAATTCCAGGGCTCAACCTTGGAACTGCATTTGA

>714c2fd3d646a66f09080a92b0c1d5b2

TACGAAAGGTGCAAGCGTTAATCGGAATTACTGGGCGTAAAGCGCACGCAGGCGGTTTGTTAAGCTAGATGTGAAAGCCCCGGGCTCAATCTGGGATGGTCATTTAG

>451f651f57360cfb33efa0a57ad50f97

TACGTAGGTGGCGAGCGTTGTCCGGAATTACTGGGTGTAAAGGGCGTGTAGGCGGGAGTGCAAGTCAGATGTGAAATACCGTGGCTTAACCACGGGGCTGCATCTGA

>c3cc98720b8679796949574ac0c3fb75

TACGGAGGGTGCAAGCGTTAATCGGAATTACTGGGCGTAAAGCGCGCGTTGGTGGTTTGATAAGCGAGATGTGAAAGCCCCGGGCTCAATCTGGGAACGGCATTTCG

>581e21ec2a8c17390fde7246fff475d7

TGTCCGCACCTAATAGGGCATCAAAGGGAGCGGGTCTGTCAAATGTAGGGTCGGCAAGGACCAAGTCCTTGGCTGCACTCTTAACCTCTGAAGCTATTGCTGTCAGA

>4e83312ea35be7dc45b35ed2c7e83f3f

AACTGTCGATTTTACAGGCACTGGCGAGTGTGGTAGTAGATTGGATCACATAACACAGCCAACATCGCTATACCAAACCACAAGTCTCCACTAGTGGCAACACTCAA

>6b30be8fc62f1e4702a679cefd1b52f1

CATTGAACTATCGTGAGAAAGTCACGCCGCCAAAGGGAATTATATTATAGTAATTATTGGCGTTAATAAGCATTTTATTAATAGTTGTAATATATCATATTGAGTGA

>36332b2ef183a2a00dc4d9de53b73f0d

TACGGAGGGTGCAGGCGTTAATCGGAATTACTGGGCGTAAAGCACACGCAGGCGGTCTGTCAAGTCGGATGTGAAATCCCCGGGCTCAACCTGGGAACTGCATTCGA

>ca6d372eeae1b4646bb082c51ea75a79

AGCGAAAGGGGCTAGCGTTGTTCGGATTTACTGGGCGTAAAGGGCACGCAGGCGGTCTTGCCAGTCAGGGGTGAAAGCCCGAGGCTCAACCTCGGAACTGCCTCTGA

>612cfa43592b1714697b5c5ee7ac08f9

TACGGAGGGTGCAAGCGTTAATCGGAATTACTGGGCGTAAAGCGTACGCAGGCTGTCAAGTCAGTCAGATGTGAAAGCCCCGGGCTTAATCTGGGAACTGCATTTGA

>872b857e81b44230ddb826b60149bb62

TACGTAGGGTGCGAGCGTTGTCCGGATTTACTGGGCGTAAAGGGTGCGTAGGCGGCCTTTCAAGTCAGATGTTAAAGGCTTCGGCTTAACCTAGGTAAGCATTTGAA

>724199c598406d6c966c653bb394d43d

TACGAAGGGGGCTAGCGTTGCTCGGAATCACTGGGCGTAAAGCGCACGTAGGCGGCTTCTTAAGTCAGGGGTGAAATCCTGGAGCTCAACTCCAGAACTGCCTTTGA

>f81bac85e92cd1b0bb0ccdd30d051d29

CATGGATGATACAGGAGCTCATTGGGCTGAGACCCAGACAGCGCACACACGATTCGGGTGCTAGACACCCCGCACAGCAACAGTCAGTCAGTAATATTGGTTATCGT

>afeebee1926106e485aeca1ba1a43138

CCACTGTGTTGAGTGTGATATACTTTTACAATACACTATAACATTAGATGCCACAAACCACACACACACACGCACAGAAAGTCGTAGGACGCAAACACAGTCATATA

>7f953d28c973eac4e2e6b82d6ac68775

CTTTAATACCAGCTGAAAAAGTGGACGAAGCGTGGTTAATCATTATGGAGAAGTCAACCCAACATCTAAAATTAGAAACCCGAGTAGTCCGGCTGACTGACTATGAG

>31be1e07c7a08467e1ce550b250b3515

TGACCGTAGGTTCCTTCGTTTCCGTACCGACGTCGTAACGACACCTACAATTGACAATTTATAAATTAAGTTAATTTATAACAGAGAAAATACAAATTTTATACTTG

>2641fb32ba390034ebef068e06cfc956

TTAGCTGGATGCGCTCCCCCGCAGTTTGCGCATTTGGCGGGATCAGAGCGGGAAACTTCGCAGCCCCCGCGATGCTCCTGCCCGCAGCGCACACAGTGCGGGAATAA

>82b1f058c35158df5099f5af896990e1

TACAATTTCTTCAATTTAAATTTTAAAAAGTTTCAGTTAATATATTATTTAAATTTTTATAAATAATAATTTTAGTGAACTCTATTATTATTATCTTTTCTTCCTTT

>ed6803fa8fae1212054da6fe144dcf62

ATGCTCTCGCCCCGCAGACCTCATTGGTCAATTAATTCACTCTTCAGCCTTCTCATTAGAAACCCCAGTAGTCCGGCTGACTGACTCGAGCTAGATCTCGTATGCCG

>eb3150324edbfab5e40e49914e2ab08e

TACGAAGGGGGCTAGCGTTGTTCGGAATTACTGGGCGTAAAGCGCACGTAGGCGGACATTTAAGTCAGGGGTGAAATCCCAGGGCTCAACCCTGGAACTGCCTTTGA

>55cfc5208ce690819a9f53278e7db3b7

GACACAATAAACTACTCCGGAACAAGGATCATCGCCACCTCACAACACCCAACATTAGAAACCCTGGTAGTCCGGCTGACTGACTTGCGTCAAATCTCGTATGCCGT

>53973beab49991c16f8fb2dc4a69ab96

TCAGTTTGATATACTTTTTTTACAATACACAATAACAGATGCCACAAGCCTCACACACACACACAGAAAGTCGTAGAATTTTGCAGTAATACAATTTTACGACAGTC

>e5715886087b8a50d0d241bbf8b477cd

TCCGGCTGACTGACTAGTCGCAGATCTCGTATGCCGTCTTCTGCTTGAAAAAACATGTAGTCCGGCTGACTGACTAGTCGCAGATCTCGTATGCCGTCTTCTGCTTG

>cc663526ab3d226930b767e492259441

GAGGGATAGTGAAGAAACTGGGAATGGGGAAATAAAGGAGTTGCTTCGACAGATGTTTGAAGAAATAAAGGAGATAAGAAAGGAGAATCAAGATTTAAGGGGGGAGA

>e9e43c2030213ec8537baa491ff9627e

TGTCCGCACCCTATAAGGCATCAACGGGAGCGGGTTGGTCAAAAGTCGGATCGGCAAGAACCAGGTCCTTCGCCGCACTCTTAACCTCTGAGACTATCGCTGTCAGA

>951477ccf79a72ec9a6ae13fe9f8b017

TACGGAGGGTGCAAGCGTTATCCGGAATCATTGGGTTTAAAGGGTCCGCAGGCGGATTTATAAGTCAGTGGTGAAAGCCTACAGCTTAACTGTAGAACTGCCATTGA

>b04b881ff3c256e1a0dbf937333b6430

CGGTTTACCAAGTATTTGGCAACTTTATACGTTCAATGTGCGTGTTAAAACTTAAAAATCTCATCTTTTTTATCTTTAAGGGATTCAGGATCCAGCGAGTAGCGTGG

>84575cc9294bc76934d4ca19c6e62d54

TACGGAGGATGCAAGCGTTATCCGGATTCATTGGGTTTAAAGGGTGCGTAGGCGGATTATTAAGTCAGTGGTGAAATCCTGCGGCTCAACCGTAGAACTGCCATTGA

>4e83b2a7b12bcc480eb2dbb2224174cd

TACAGAGGGTGCGAGCGTTAATCGGAATTACTGGGCGTAAAGCGCACGTAGGCGGCTTGGCATGTCGGGTGTGAAATCCCCGGGCTCAACCTGGGAACTGCATCCGA

>f1373d4f008041cb212bcc3ba42d8f84

TACGGAGGGGACTAGCGTTGTTCGGAATTACTGGGCGTAAAGCGCGCGTAGGCGGACTGTTAAGTCAGAGGTGAAATCCCGGGGCTCAACCCCGGAACTGCCTTTGA

>4239e7fd8c9d052ee9a58d10b46474f1

TACGTAGGTGGCGAGCGTTGTCCGGAATTACTGGGCGTAAAGGGTGCGTAGGCGGTCTGTTAAGTCAGATGTGAAAGCCCGGGGCTCAACCCCGGGACTGCATTTGA

>32e299379783d09b3ebee303404d9f49

TACGTAGGGAGCGAGCGTTAATCGGAATTACTGGGCGTAAAGCGCACGTAGGCTGCTTGGTAAGTCAGGGGTGAAAGCCCGCGGCTCAACCGCGGAATTGCCTTTGA

>f84d3a4f071d2c2fa5c12722ec2406c7

CCACCACCACCACCACCACCACTGTGCTGAGTGTGATATACTTTTTTTACAATACACAATAACAGATGCCACAAGCCACACACACACACACACACACACACATAGAA

>b203911cd9c740330b8adab0fa2ed6a8

CAGCAGAAAGCGAAAACGTTACCCTCGTCCATACAGTTCGCGTTAGAGAAATTCGAAAAGAAATCCAACCCTCTGCGGAACGGAGCTTCGACAGCGCCTCGTACAGT

>c7448537329383777f341febecb54133

TACGTAGGTGGCGAGCGTTGTCCGGAATTACTGGGCGTAAAGGGTGCGTAGGCGGTAGGCCAAGTCAGATGTGAAATTCCAGGGCTCAACTCTGGGCGTGCATTTGA

>83cff7813a9cb5043601917151cc3293

TACAGAGGGTGCGAGCGTTAATCGGAATCACTGGGCGTAAAGGGTGCGTAGGCGGCCAATCAAGCCAGGGGTAAAAGGCTACGGCTCAACCGTAGTAAGCCCTTGGA

>c0381dcf8ccdbf9080d15f5ac7af9235

TACGAAGGGGGCTAGCGTTGCTCGGAATGACTGGGCGTAAAGGGCGCGTAGGCGGATTTGTCAGTCAGGCGTGAAATTCCTGGGCTTAACCTGGGGGCTGCGTTTGA

>a12d211a91c9e1ce8b088e5b13dd6665

GACAAGGGAGACGAGTGTTATTCATCTTTAACAGGTATATAGGGTACCTAGACGGTGTACAAAGGCTTTTATAAGTACCTGTTACACTTGAGTTTGATATGTGAGAG

>5357183d1a397dc47dcb3bfd454a8a6e

TGGTCCACATATGTCTAGGAACGTCTTTTCTAATTTATAATTGGAAGTGATGGGTATTAGCTTTCCCTCATGAATCTCGTTTGAAGTTTTAGCCATTTGACACAGAT

>679e95c72f1bd09f5e735a78b9e3e541

CTTCACAAAATAGTGCCGTTCGTCTCATCCCAGTATAAAATTTGTCTGTGAGCCGGCCAGCTGCAAAACAAACAGAGTTCTAGAACGTAAAATCGCGGGCGCCCGCA

>f9f6d1660358aa2ae1e1aa5b4f4df8a3

TTAGCTGATTGAAATTAGGCTACATGCCTCTCTCTCGATGCCAAATAATAACTCATCGGATTAGATACCCTTGTAGTCCGGCTGACTGACTATAGCGCTATCTCGTA

>4e3c19e12742ed3f5bbde50eaa12a891

ATCGTTTTATTACCAATTCTCGCAGCTCCTCTTTTCTTATTCAGCCGCTCCTATTGGCGTAACTCACAATGCTTTATATTTTAGAATTATACTTAACATTGCTTCCA

>ee03992ef9eb1824cc05594e025c796e

TTCCAGCTCCAATAGCGTATATTAAAATTGTTGACGTTAAAAAGCTCGTAGTCGACTTTCGGCCTCAGTCGACCGGTCCGCTCACTGAGTGTGTACTGGACTCGATT

>d5e4e200d37ad86520cc0a2a68d50010

TACGTAGGGGGCAAACGTTGTCCGGATTTATTGGGCGTAAAGCGCGCGCAGGCGGTTTCGTAAGTCTGATGTTAAAGCCCGGGGCTCAACCTCGGTCCGCATTGGAA

>21f139c8045145c94df52371382b9b10

TACGTAGGGGGCGAGCGTTATCCGGAATTATTGGGCGTAAAGCGCACGTAGGCGGACATTTAAGTTGGAGGTGAAAGCCTTGGGCTCAACCCAAGAATTGCCTTTGA

>35b140556a122592545bed7586602d05

TACGGAGGGTGCAAGCGTTGTTCGGAATTACTGGGCGTAAAGGGCGCGTAGGCGGCGTAACAAGTCAGGTGTGAAAGTCCAGGGCTTAACCCTGGAAGTGCATCTGA

>29065934e7e9d422feefe746b8acbf32

TACGTAGGTGGCGAGCGTTGTCCGGAATTACTGGGTGTAAAGAGCGTGTAGGCGGGTGCTTAAGTCAGGTGTGAAAGACCGGGGCTCAACTCCGGGGTTGCACTTGA

>27ad85be3c8374b578e2da8802e1bde6

TACGTAGGTGGCGAGCGTTATCCGGAATTACTGGGCGTAAAGGGTGCGTAGGCGGCCTTGTAAGTCAGAAGTGAAAGGCTACGGCTCAACCGTAGTAAGCTTTTGAA

>cf143e21698c6a47f9e1cfcd1df1fab6

TACGTAGGGGGCGAGCGTTATCCGGAATCATTGGGCGTAAAGGGTGCGTAGGCGGTTATGCAAGTTAGGGGTGAAAGGCTACGGCTCAACCGTAGTAAGCCTTTAAA

>8a883e12c03b89a957278b6952e1218b

TACGTAGGGGGCAAGCGTTGTCCGGAATTACTGGGCGTAAAGGGCGTGTAGGCGGTTTTTTAAGTTAGGAGTGAAAACTCAGGGCTTAACCCTGAGACTGCTTCTAA

>d85409158be7555dd3315c4a61dc55f6

GACCAAGACTAAAGACTTGCAGTGCGTGGATCACGACGACGAAGCTCTGTCCATGTATTGTATGGTCTATTAGAAACCCTAGTAGTCCGGCTGACTGACTCTACGAC

>91e73af15e4b3e11298b53c1f19b0b1a

TTCGCTGGATGCGAGCCGCCGCAGTTGCAGCATGTGGGAGCAGTCTCCGGGGCAACCGGGCAGTTTCCCCGGTGATCGCCACCACAACGCACGCAACGCGGCGCCAT

>febd8b533e9d1321220d72f7b7cf8586

CACACTTATGTCTTTAGAAATACAAACCATTTCTCCAGGATGCATTTCAACCATGTCGGTCATTTTAAACTGGCAGTGCCTTGTAGATATTTGTGAAAGATTTTGAA

>52631ec826053e920c17ea1305b6fd45

TACGTGAGAGACTAGTGTTATTCATCTTAATTGGGGTTAAAGGGTACCTAGACAGTCAATATAACTTCTATAATGCTAATGCTTGACTAGAGTTTTAAGTAAGAGGG

>3b3520ffcd5e97906dfa8ee4c9dad14d

TACGCAGGTGGCGAGCGTTGTCCGGAATTACTGGGTGTAAAGGGAGCGTAGGCGGGCTTGCAAGTTGAATGTTTAATCTATGGGCTCAACCCATAGCTGCGTTCAAA

>d7d26599801458258d5df2eb04bed149

TACGTAGGGTGCAAGCGTTAATCGGAATTACTGGGCGTAAAGCGTGCGCAGGCGGTTCGGAAAGAAAGATGTGAAATCCCAGAGCTTAACTTTGGAACTGCATTTTT

>1b5086cd7f0ca862999e5665663b10c2

TACGTAGGTGGCGAGCGTTGTCCGGAATTACTTGGCGTAAAGGGTGCGTAGGCGGAATCTTAAGTCAGATGTGAAATTCCTAGGCTCAACCTGGGAACTGCATTTGA

>5cd9f3e06ad1844a5555a1b61927db3a

GTCAGTCAAAGGTGGCATCTTCTCCACCGAGCAAACCCGCATCACAGACTTCAATCCACGTCCATCCGCATCTAACACTTTACAGGAATCGTAGAGTACTGCCACAA

>4b0168c17a325ffa243bf5c78c001b94

AATGGTTCGCTGCCGCAGACTTGAGGACTTGCCGCGGTACAGAAGCTTTCAGTGCCTTCTCCGCACTCGCCAATGCGAGATGCGAGGACCAGCAACCTAACTTCCTT

>861d45a3f963ba0795a6b958e2710bc1

TACGTAGGGTGCAAGCGTTAATCGGAATTACTGGGCGTAAAGCGTGCGCAGGCGGTTGTGCAAGACGGATGTGAAATCCCCGGGCTTAACCTGGGAATTGCATTCGT

>29ad8b7d681f47255b01fb8b8ca1e5d5

CACGATTTTCTAAATTTAATTATGTTAGTTTCAGTTAAAAAAAAATGTGTTAATATTAAGTTTTTTAAATTTTGGTGGAATAATATATAAATATGTGTTTAGTTTTA

>656aaa8904f897d66c0286a32800df3f

TACGTAGGTGGCAAGCGTTGTCCGGAATTATTGGGCGTAAAGCGCGCGCGGGCGGTTCCTTAAGTCTGATGTGAAAGCCCACGGCTCAACCGTGGAGGGTCATTGGG

>ebdfc06c951e61c8e97ab91aa770befb

TGTCCGCACCTAATAGGGCATCAACAGGAGCGGGTGGGTCAAATGTCGGATCGGCAAGAACCAGGTCCTTAGCTGCACTCTTAACCTCTGAAGCTATTGCTGTCAGA

>7af5ce56905c7f04fce250f79bf896ad

GACGTAGGGGGCAAGCGTTACCCGGAATCACTGGGCGTAAAGGGAGCGTAGGCGGCCAAGAAGGTTGTATGTTAAAGACGACGGCTTAACCGGAGAGATGCATACAT

>3ce31ff850135bb36e0286af7899d958

TACGTAGGGTGCGAGCGTTGTCCGGAATTATTGGGCGTAAAGGATGCGTAGGCGGCCTTTTAAGTGGGATGTGAAATACCCGGGCTTAACCCGGGAATGGCATTCCA

>402f80793c595b13702481bd27607283

TACGTAGGAGGCTAACGTTATCCGGATTTACTGGGGGTAAAGCGCGTGCAGGCGGTTCGGTAAGTTGGACGTGAAAGCTCCCGGCTCAACTGGGAGAGGTCGTTCAA

>8c15be54e01fc103178c58897bc5df8a

TACAGAGGGTGCGAACGTTGCTCGGATTTACTGGGCGTAAAGCGCGTGTAGGCGGACTCGCAAGTCGGTTGTGAAATCCCTGGGCTTAACCTAGGAACTGCATCCGA

>5e6ad18a42cc16a0d282ff956947e5d5

TACGGAGGATGCGAGCGTTATCCGGATTTATTGGGTTTAAAGGGTACGCAGGCGGTATCGTAAGTTAGTGGTTAAATCTACAGGCTAAACCTGTATACGCCATTAAA

>d43641ccbedaf1c4bb6a9cbdee026d3c

TACGGAGGGTGCGAGCGTTAATCGGAATCACTGGGCGTAAAGCGCACATAGGCTGCTTTGTAAGTCGGATGTGAAAGCCCTCGGCCCAACCGGGGAACTGCATTCGA

>2ebb52ec7ea52b6631a918788f896ed3

TACAGAGGGTGCAAGCGTTGTTCGGAATTATTGGGCGTAAAGGGCGTGTAGGCGGTCTGCTAAGTCATGTGTGAAATCCCTCGGCTTAACCGAGGAACGACGCATGA

>b0367ce7698b1c3516628c5764442c77

TACGGAGGGGGCGAGCGTTGTTCGGAATTACTGGGCGTAAAGGGCGCGTAGGCGGCCATGTCAGTTGGGAGTGAAAGCCCCGGGCTCAACCTGGGAACTGCTCTCAA

>1218d53b009def9695378470b92852bb

TACGTAGGGTGCGAGCGTTAATCGGAATTACTGGGCGTAAAGCGTGCGCAGGTGGTTTTGTAAGTCAGATGTGAAATCCCCGGGCTTAACCTGGGAACTGCGTTTGA

>cbfabc7d35187fbb35d368a92e504596

AACGTTGGACGCAAGCGTTATCCGGATTTACTGGGCGTAAAGAGCGTTGAGGCGGTTCCGTAAGTTGGGCGTGAAAGCCCCGGGCTTAACTCGGGGAGGCCGTTCAA

>2a90e5483542f4126d0c8c706aed0ea9

TACGGTGGGTGCAAGCGTTAATCGGAATTACTGGGCGTAAAGCGCACGCAGGCGGTTTGTTAAGCTAGATATGAAAGCCCCGGGCTCGACCTGGGATGGTCATTTAG

>4dd5efa0ec4cb455b039726b3be7e4cd

TACGGAGGGTGCAAGCGTTAATCGGAATTACTGGGCGTAAAGCGTACGTAGGCGGCGTGTTAAGCAAGATGTGAAAGCCCCGGGCTCAACCTGGGAATTGCATTTTG

>500667123212061304a4a67b76df3b4b

TACGGGGGGTGCAAGCGTTATTCGGTATTATTGGGCGTAAAGAGTGCGTAGGCGGTTTGTTAAGTCATTTGTAAAAGGTCTCAGCCCAACTGAGTAGGGCGAATGAA

>0d6c5e7b970c64bca259733d886aea11

TACGTAGGGTGCAAGCGTTGTCCGGATTTATTGGGCGTAAAGGGCTCGTAGGCGGTCTGTCACGTCGGATGTGAAAACTTGGAGCTCAACTCCGAGCCTGCACTCGA

>11fce62a92c315c054d9d7527da5be56

CACGTAGGGGGCAAGCGTTATCCGGAATAACTGCGCGTAAAGGGTGCGTAGGCGGCCCTGCAAGTCAGAAGTGAAAGGCTACGGCTCAACCGTAGTAAGCTTTTGAA

>b7856359fd39ebd49a806530fc5b08d7

TTATTTATCCTGCAGCCTACGTACAGTACAGTAGTGGTAACTGATAACGTGTAGGTAGGCCTACTGTGTGATGTGCTACGTAAAGTGCTTGATACGGAATAATTTAC

>802e602e4861991b2614b4e655014244

CCACCACCACCACCACCACCACTGTGCTGAGTGTGATATACTTTTTTTACAATACACAATAACAGATGCGACAAGCCCCCCCCCACACACACACACACACACACACA

>644a2afbb93affdb2f86d5e76849333c

GTGCTCGAAGAACATCAATCTTTCACTTCGTTCTCACTTTCTCTTTTTACTGCCCCCTCTTCTGACCCTTTTCCAACATACCCCTTTCGCTCCTTTTCGTCATCCCT

>d611eab89a6486a92d6a9d620ec99752

TACGTAGGGGGCAAGCGTTATCCGGAATAACTGGGCGTAAAGGGTGCGTAGGCGGCCTTGTAAGTCAGAAGTGAAAGGCTACGGCTCAACCGTAGTAAGCTTTTGAA

>2d16d26a0805c8cc3248f21aa1ed15d8

TACGAAAGGTGCGAGCGTTAATCGGAATTACTGGGCGTAAAGAGCGCGTAGGCGGTGTGTTAAGTCGGATGTGAAAGCCCAGGGCTCAACCTTGGAATTGCATCCGA

>b11c2af9055d6b87541451f91034ea1e

TACGAAGGGTGCAAGCGTTAATCGGAATTACTGGGCGTAAAGCGCGCGTAGGTGGTTTGATAAGTTGGATGTGAGAGCCCCGGGCTCAACATGGGAATTGCATCCAA

>d0de813cc10be37b45d4b5b12e1b13f1

TCAGTGTGATATACTTTTTACAATACACAATACCAGATGCCACAAGCCACACACACACAAACAGAAAGTCGTAGAACGGAAACACAGTCATATAATTTTACCGTACA

>c3cc3a105229579848899982e2141146

TGTCCGCACCTAATAGGGCATCAACGGGAGCGGGTCTGTCAAATGTAGGGTCGGCAAGGACCAAGTTCTTGGCTGCACTCTTAACCTCTGAAGCTATTTCTGTCAGA

>70c3987be0f1dab3a11f7dd40d58d48e

AGTAAACACAATATCTGTCAGGACTGCCACTCGTTACTTGATTCAGCACTCTCCTTATATAGTTTAGTTTAGATTCTTCTGTTTTACAGATTGACTCACTTGATTCG

>c3fd5d584e7c4e647d5cb983b3257a5d

TAATATCTTTGTACGCCAGGGGCGCAGTAAATTGATTTTGAAAAATACTACATTAGATACCCCAGTAGTCCGGCTGACTGACTAGTCGCAGATCTCGTATGCCGTCT

>1aa6faa98dcb1cf960d2dfb57ffd4e4b

TACGTAGGTGGCAAGCGTTGTCCGGAATTATTGGGCGTAAAGCGTGCGCAGGTGGTTTCTTAAGTCTGATGTGAAAGCCCACGGCTCAACCGTGGAGGGTCATTGGA

>119ef12d8a5c33e6293c2a2b1ca63b2f

TACGGAGGGCGCGAGCGTTACCCGGATTTACTGGGCGTAAAGGGCGTGTAGGCGGCCTGGGGCGTCCCATGTGAAAGGCCACGGCTCAACCGTGGAGGAGCGTGGGA

>fa942407fa9c805389c3996bf5f2bb8a

TACAATTTCTTCAATTTAAATTTTTAAAAGTTTCAGTTAATAAATTATTTAAATTTTTGTGAATAATAATTTTAGTGAAATAGATTATTATTTTATTTTATTAGTTT

>b9add37728308c50cf89d908dfaddf08

TACAATTTATTCATTTTAAATTTTTAATAGTTTCAGTTAATAAGTTATTTAAATTTTTATGAATAATAATTTTAGTGAAATGTATTATTATTATATTTTATTAATTT

>e3be11478a041ed3fdd0d5642546357f

CAACAACAATTGTGCTGAGTGTGATATACTTTTTTTACAATACACAATAACAGATGCCACACACACACACACACACACAGAAAGTCGTAGGACGTAAACGCAGTCAT

>a941b046e1d0f6145f15d3b8a18662ad

GCCGTAGCAACTTTTTATTACACACATAAATTTCTAATGTTACCTTGCATTGAATTCTCTGAGTAATTTTATTGGAAATGAGTCCAAACACGATGCCGTTACGATTT

>c268b7d20d8fc693098b6e5219ba1511

CCTACCGCTGTGTTCAGCGTGATATACTTTTTACAATACACAATAACAGATGTCACAAGCCACACACACACATACATAGAAAGTCGTAGAACGCAAACACAGTCATA

>e25b2101d1214ecfe91e836823c7fad2

TACGGAGGGTGCAAGCGTTATCCGGATTTATTGGGTTTAAAGGGTCCGTAGGCGGATCCGTAAGTCAGTGGTGAAATCTCACAGCTCAACTGTGAAACTGCCATTGA

>5b0d670443ce4e3a539f3b2055253b6e

TACGTAGGGGGCTAGCGTTATCCGGAATAACTGGGCGTAAAGGGTGCGTAGGCGGCCCTGCAAGTCAGATGTGAAATCCCAGGGCTCAACCTTGGGGCTGCATTTGA

>5abb5aa219b9a69b5a3bb25047ca4f5d

CACGTATGGGGCGAGCGTTATCCGGATTTATTGGGTTTAAAGGGTGCGCAGGCGGGCTTGTAAGTCAGTGGTGAAATCTCAGGGCTTAACTCTGAAACTGCCATTGA

>05ef1ccdcb6db06b94cfce942b07c113

GACGGAGGGAGCAAGCGTTGTCCGGAATTACTGGGCGTAAAGAGCTCGTAGGCGGGGGCCTAAGTCTGGGAGTAAATCCATCGGCTCAACCGGTGAACCATCCTGGA

>2f6b3615ecad431fc3a6353adfa756bd

TACGGAGGATCCGAGCGTTATCCGGATTTATTGGGTTTAAAGGGTGCGTAGGCGGTCTGATAAGTCAGTGGTGAAATACGGCAGCTTAACTGTCGAGGTGCCATTGA

>e5fba1cd8755e115718dc4908dc6ff7c

TACGGAGGGTGCAAGCGTTGTTCGGAATTATTGGGCGTAAAGCGCGTGCAGGCGGTCTGTTAAGTCTGATGTGAAAGCCCCGGGCTCAACCTGGGAAGTGCATTGGA

>a00e34ffa39975b5b6faa16eafe0a70f

TACGTAGGGGGCAAGCGTTATCCGGAATTACTGGGTGTAAAGGGAGAGTAGGCGGCATGATAAGTTAGATGTGAAAGCCCGGAGCTTAACTTCGGGATTGTATTTAA

>6e5c0da2cd0141691336a2f81e8e445b

TACGGGGGGTGCAAGCGTTGTTCGGAATTATTGGGCGTAAAGAGCGTGTAGGCGGCGTGGTAAGTCGGATGTGAAATCCCGGGGCTTAACCCCGGAAGTGCAGTTGA

>4dcaa8921c3adde2947c9760d0a7620b

TACGAAAGGGGCTAGCGTTGTCCGGAATTACTGGGCGTAAAGCGCGCGTAGGTGGTTTGATAAGCGAGATGTGAAAGCCCCGGGCTCAACCTGGGAACGGCATTTCG

>bf8297c109db0243f670c4731584ee17

TACGGAGGGAGCGAGCGTTAATCGGAATTACTGAGCGTAAAGCGCGCGTAGGCGGCTTTGTAAGTCGGATGTGAAAGCCCTGGGCTTAACCTGGGAATGGCACTCGA

>dd66087dd6f43125d27df198af091da6

TACGGAGGGGGCTAGCGTTGTTCGGAATTACTGGGCGTAAAGCGCTCGTAGGCGGATTGGTCAGTCAGGGGTGAAAGCCCGGAGCTCAACTCCGGAACTGCCTTGAT

>32f7c8be7895e062daeee8e64a2e718d

TACGGAGGGTGCAAGCGTTAATCGGAATTACTGGGCGTAAAGCGCATGCAGGCGGTCTGTTAAGCAAGATGTGAAAGCCCCGGGCTCAACCTGGGGACTGCATTTCG

>a06386d79202fdc5f75ccc08ce79fa6b

TACGTAGGTGGCAAGCGTTGTCCGGATTTACTGGGCGTAAAGAGTGTGTAGGCGGATAATTAAGTCAGATGTGAAAGCCCAAGGCTCAACCTTGGAGGTGCATTTGA

>ad1d461cf5227af340e642ddfba58cb1

TACGTAGGTAGGAAGGAACACCAGTGGCGAAGGCGGCACTCTGGACCAATACTGACACTGAGGTGCGAAAGCGTGGGGAGCAAACAGGATTAGAAACCCGGGTAGTC

>9f09ac2533761bc8637f2d5234c58c45

TACGTAGGGCGCAAGCGTTGTCCGGATTTACTGGGCGTAAAGAGTATGTAGGCGGACATTTAAGTCAGATGTGAAATCCCCGGGCTCAACCTGGGGGCTGCATTTGA

>d11e2447b2ed183ee8938be3bfe6de09

TACGTAGGTGGCGAGCGTTGTCCGGAATTACTGGGCGTAAAGGGCGTGTAGGCGGCCTTTTAAGTCAGATGTGAAATCTCAGGGCTTAACCCTGAAACTGCATTTGA

>082b0eb37cb8f9e44ee070a8c06d959c

TACGGAGGGTGCGAGCGTTAATCGGAATTACTAGGCGTAAAGCGTACGCAGGCGGTTTGTTAAGCTAGATGTGAAAGCCCCGGGCTCAACCTGGGATGGTCATTTAG

>e49a4aca1a25d6cb7949707d2cd7974f

TACGGAGGGTGCAAGCGTTGTTCGGAATTATTGGGCGTAAAGCGCGTGTAGGCGGTTTGTTAAGTCTGATGTGAAAGCCCCGGGCTCAACCTGGGAAGTGCATTGGA

>3735a2244d6d2dbe3a3d97524c2c07ac

CAACAAAGACAACCCAGACAGTAAAAAGGAGCAAGGAAGGGAACCCTGTTTGCTAAATCTACGAGCATTAGATACCCGTGTAGTCCGGCTGACTGACTTGCGTCAAA

>a75a7f536a5c52fc48f09e94b1c150e3

AGCCTCAAATCAGTGCCTTCCTGCGAAAGCCAAACGCGTGTAGCGAGGTCCTGATCCCGAACATCTTGGTGAGTAGTGTAAAACGCTATGATCCTGGCGCACATCGG

>240ba38d5441c526427371c924fd69ff

CGCCTCTTAATCTTACACCATCATCGTTAGTCACAGCTGCTAATTACCGCGCGCCTGGGTAACATAACTCACTACCTTGCCGGTACTGTGGGTAACTGTAACTTATT

>ee912be4b3d06ffe138dadd67bceb207

TACTCATCACCACTAAAGAAGGCTTCCCCAGTCGTAGCCGAATGCCTGCCAGAGCAACCACATTAGAAACCCCTGTAGTCCGGCTGACTGACTGCGATACGATCTCG

>713350c6e213d8855d4a42ad6a9fe8f0

CCACCCCCATCGCTGTGTTCATACTTTTTTTACGATAACAGATGCCACAATTCACACACACACACACTTAATGTACATTAGAAAACACAAGTCACACATACACACAC

>ed522084f93b0df5ad508a3e0b27108f

TACGTAGGGGGCGAGCGTTGTCCGGAATCACTGGGCGTAAAGGGTGCGTAGGCGGCCAATCAAGCCAGGGGTGAAAGGCTACGGCTCAACCGTAGTTAGCACTTGAA

>a0e4a248979a119a29827b317f1ddc9f

TACGTAGGTGGCAAGCGTTGTCCGGAGTTATTGGGCGTAAAGCGCTCGCAGGTGGTCTCTTAAGTCTGATGTGAAAGCCCCCGGCTCAACCGGGGAGGGTCATTGGA

>3c8fa0842f6e34b6d269922adc8ab9ba

TACGTAGGTGGCAAGCGTTGTCCGGAATTATTGGGCGTAAAGCGCGCGCAGGCGGTCTCTTAAGTCTGATGTGAAAGCCCCCGGCTCAACCGGGGAAGGTCATTGGA

>c4f2fd60c4b71b2f7162e5519843c9e1

TACGTAGGGGGCGAGCGTTATCCGGAATCACTGGGCGTAAAGAGTGCGTAGGCGGCCAATAAAGTCTGGGGTGAAAGGCTACGGCTCAACCGTAGTAAGACTTGGAA

>747b892ff4723c7258fe0dc4b44fc284

TACGGAGGATCCGAGCGTTATCCGGATTTATTGGGTTTAAAGGGAGCGTAGATGGGTTGTTAAGTCAGTTGTGAAAGTTTGCGGCTCAACAGTAAAATTGCAGTTGA

>385f876e15e9a38171713ac68304865d

TGCCCGTAGGTTCCTTCGTTTCCGTACCGACGTCGTAACGACACCTACAAATGACAATTTATAAATTAAGTTAATTTATAACAGACAAAATACAAATTTTATACTTG

>a8e4856bb8da615eb31d356de5e2ad94

GTTTTACTAGGGTGTGGTACGGAAGTTGTACTATTCTCCATCTATTCTGGACCAATTTCTATAAAATTTGGTTCTCCATTAGAAACCCCAGTAGTCCGGCTGACTGA

>9103f1f4e38c9120126f8b9089707b87

AAGGAAATTGAAAGTAAACGACTCCTCTGACTGCAATACACCATCATCATCATTAGATACCCCTGTAGTCCGGCTGACTGACTATGAGCTCATCTCGTATGCCGTCT

>9ba4d1b47181df1154a4534630d3aae2

TTCGCTGGATGCGAGCCGCCGCAGTTGCAGCATGTGGGTGCAGTCTCCGGAGTAACCGGACAGTTTCCTCGGTGATCGCCCCCACAACGCACGCAACGCGGCGCCAT

>0eedc7c0951b10a9a893d1921c92daae

CCACCACCCACCGCTGTGTTCATACTTTTTTTACAATAACAGATGCCACAATTCACACACACACTTAATGTACATTAGAAAACACACACACAAACAAGTTCGATGGG

>1b226921aabdc423faf068b66cd441fe

AACAGAGGATACAAGCGTTATCCGGATTTATTGGGTTTAAAGGGTGCGTAGGTGGTTTTTTAAGTCAGTAGTGAAATCTTCCAGCTTAACTTTCCCCGTGCTCTTGC

>d33bb35d54df53e4519c8f83a2408859

CATTGAACTCGTGAGAAAGGCACGCCGCCAAAGGGAATTATATTATAGTAAATATTGGCGTAAATAAACATTTTATTAATAGTTGTAATATATGAAAATGTGCAGAA

>c156dd01a62a7d8c69a000ae0ce4d3cd

AAGACGCCTCTTTGAACCTGCGTTGTTCAGTTTTAATTTTCCACTCTAAAATGCTTCCTCATCCCATAAGACCCTGTGCTTCTCACACCACGCATGTTCTGCTAATC

>8336b052ab78d3dc65c1fb0321795f9f

CGCTATACATATTCATTTATTTCAAAGATCGGAAAATAAAGCGTTCTTGTTCAATAAACAAACTTCCCTTAATTATAGTAGTGTTGTTTAAAAGGATTAATTAACCC

>fb25d93e5baf9c9e4fe52598df1d00db

TTCCAGCTCCAATAGCGTATATTAAAGTTGCTGCATTAAGCTCGTATTAGAAACCCTAGTAGTCCGGCTGACTGACTTCCTCATGATCTCGTATGCCGTCTTCTGCT

>f3a1ca0f99462cc995ac34c61b2dd67c

TATCAGGCAAGAGGAAAAGAAAATACAGTTATAAAAAAGCAGATTCTTATCAAGTATTCTAGAGTCTACACTATTTTATTAGAAACCCCGGTAGTCCGGCTGACTGA

>84d207c96a190bc642365f868a28227e

CTAGTATTATTCTACACGCTGCACACCGTTTTACACTCTTTATTCAACACTAAAAAAGATGTAAAGATTCACACGAATATAAATCAATATTACACTATAGACCACCG

>0790a83668112f0e2ec53773bf81f70f

TGTCCGCACCTAATAGGGCATCAACGGGAGCGGGTCGGTCAAATGTCGGATCGGCTGGAACCAGGTCCTTAGCTGCAAACTTAACCTCTGACGCTATTGCTGTCATA

>fcb35815506843cc5110daca08851acb

TACGTAGGGGGCGAGCGTTGTCCGGAATTACTGGGCGTAAAGGGTGCGCAGGCTGTTGTGCGAGTCTGCTGTGAAATGTACCGGCTTAACCGGTAAGTTGCGGCGGA

>9f0b1c6120a6081d631b44fa8198eb70

GACGGGGGGGGCGAGCGTTGTTCGGAGTTACTGGGCGTAAAGGGCGCGCAGGCGGCTTGGGAAGTCTTGGGTGAAAGCCCCCAGCTCAACTGGGGAATGGCCTGAGA

>addb01910a28074cec0d4041b7b0e572

TACGTAGGGGGCAAGCGTTGTCCGGAATCATTGGGCGTAAAGCGCGTGTAGGCGGCCCGGTAAGTCCGCTGTGAAAGTCCAGGGCTCAACCCTGGGATGCCGGTGGA

>25de3849057b5388e3db6e4ce21b8017

TACGGGGGGTGCAAGCGTTGCTCGGAATCACTGGGCGTAAAGCGCGTGTAGGCGGCTTGTCAAGTCAGATGTGAAAGCTCGGGGCTCAACCCCGGAAGTGCACTTGA

>f3d27264c3dc3979d11663d03ff3e186

TACGAGGGGGGCGAGCGTTGTTCGGAATTATTGGGCGTAAAGGGTGCGTAGGCGGTTTGGTAAGTCTTGTGTGAAATCTACAGGCTCAACTTGTAGTCTGCACGAGA

>e6264872b6dcae1acd526ed937754b99

TACGTATGGAGCAAGCGTTATCCGGATTTACTGGGTGTAAAGGGAGTGTAGGTGGCCAGGCAAGTCAGAAGTGAAAGCCCGGGGCTCAACCCCGGGACTGCTTTTGA

>fafc00938b430de038bf707e81a9be5e

TACGAAGGGGGCTAGCGTTGTTCGGAATTACTGGGCGTAAAGCGCACGTAGGCTGATAGTTAAGTGAGGGGTGAAATCCCGAGGCTCAACCTCGGAACTGCCCTTCA

>c645831a64dd15ea780a3e5c080e5f87

TACGTAGGTGGCGAGCGTTGTCCGGAATTACTGGGTGTAAAGGGTGCGTAGGCGGTAATGCAAGTCAGATGTGAAAGCCCGGGGCTCAACCCCGGGGCTGCATTTGA

>332c21ab3f211816c9a46a7b3729a56d

TACGGGGGGTGCAAGCGTTGCTCGGAATCACTGGGCGTAAAGCGCGTGTAGGCGGCTTGTCAAGTCAGATGTGAAAGCCTGCGGCTTAACCGTAGAAGTGCATCTGA

>648d3deaf016461d21d35c15483a18e8

CACGTAGGGTGCGAGCGTTGTCCGGAATTATTGGGCGTAAAGAGCTCGTAGGCGGTGTGTCACGTCGATCGTGAAAACCTGTGGCTTAACTATGGGCGTGCGGTCGA

>406c7af461b6c64a798c383a7283086e

TACGGAGGGTGCAAGCGTTAATCGGAATTACTGGGCGTAAAGCGCGCATAGGCGGCTTCGTCAGTCAGATGTGAAAGCCCCGGGCTTAACCTGGGAACTGCATTTGA

>83e4a42183b89f22054f72339479199a

TACGTAGGTGGCGAGCGTTGTCCGGAATTACTGGGTGTAAAGGGCGTGTAGGCGGGGGTACAAGTCAGATGTGAAATACCGTAGCTTAACTACGGGGCTGCATCTGA

>0986c1a383a748f793a49c9beffd51e8

TACGTAGGGGGCTAGCGTTATCCGGAATTATTGGGCGTAAAGGGTGCGTAGGCGGTCCTACAAGTCAGAGGTGAAAGGCTACGGCTCAACCGTAGTAAGCCTTTGAA

>2c4750ac39e720804071dbcb6a9d160a

TACGGAGGGGGCTAGCGTTGTTCGGAATTACTGGGCGTAAAGCGCGCGTAGGCGGACTATTAAGTCAGGGGTGAAATCCCGGGGCTCAACCCCGGAACTGCCCTTGA

>de825a8ddf3207551d6bc80f97532d23

CACGGAGGGTGCAAGCGTTAATCGGAATCACTGGGCCGGAATTGACGCTGAGGCACGAAAGCGTGGGGAGCAAACAGGATTAGAAACCCCAGTAGTCCGGCTGACTG

>efad76ccdfbbb381a3e055d9fd0687fd

TACGTAGGTGGCAAGCGTTGTCCGGAATTATTGGGCGTAAAACGCGCGCAGGTGGTTCCTTAAGTCTGATGTGAAAGCTCACGGCTCAACCGTGGAGGGTCATTGGA

>dea6373741ae75a3e2414b587b159613

TACGTAGGGGGCAAGCGTTATCCGGAATAATTGGGCGTAAAGGGTGCGTAGGCGGCCCTGCAAGTCAGAAGTGAGAGGCTACGGCTCAACCGTAGTAAGCTTTTGGA

>abefe00c06e4e920807aa7a5f3ea66d2

TACGGAGGGTGCGAGCGTTAATCGGAATTACTGGGCGTAAAGCGCGCGTAGGCGGCTTCGTCAGTCAGATGTGAAAGCCCCGGGCTCAACCTGGGAACTGCATTTGA

>d0bfb90630026320a2b0b33c5996663c

TACGGGGGGTGCAAGCGTTGCTCGGAATCACTGGGCGTAAAGGGCGCGCAGGCGGTCTTGTCCGTCAGGTGTGAAAGCTCGGGGCTCAACCCCGGAAGTGCACTTGA

>e3943f447c53432c5629cb3daa18f810

TACGTAGGTGGCAAGCGTTATCCGGATTTACTGGGCGTAAAGAGTATGTAGGCGGACATTTAAGTCAGATGTGAGATCCCCGGGCTTAACCTGGGGGCTGCATTTGA

>5bffd093346e73936f633e4c0bab03ab

TACGGGGGGTGCAAGCGTTATTCGGAATTATTGGGCGTAAAGGGCGCGTAGGCGGTCTCTTAAGTCAGATGTGAAAGCCCGGGGCTCAACCCCGGAAGTGCATTTGA

>40b460ff3e74bc8b3dfa0276a4c19034

GGTGATTTACGCGTGGCACGCTGGCGGTAATCATGGCGCGCCCACGAATTGCTACAAATCCGTTTCAATTATTGAATCTTACGCTACACTCCGAGTTTCAGTTAACC

>b626f7658168e018fed2464b27fb96a6

TACGTAGGGGGCGAGCGTTATCCGGAATCACTGGGCGTAAAGGGTGCGTAGGCGATTATGCAAGTTAGAGGTGAAAGGCTACGGCTCAACCGTAGTAAGCCTTGGAA

>35b47878b0d27daa2fb75dd0b489e48c

CCCCGTTTAATTACATTCTTAATCACAACAGTGCATTAGGCCTACAACAATAGAACGGAAGGTTCACTTTCTCCCCGAGCGATACATCTGGTATTACGTAATGGCGT

>3344dbb5f1217ea205951bbe8b058f67

CATTGAACTATCGTGAGAATGGCACGCCGCCAAAGGGAATTATATTATAGTAAATATTGGCGTAAATAAACATTTTATTAATAGGTGTAATATATGAAAATGTGCAG

>073a5abfceb2a6b8e812de5be8d20abd

ACGGCCACGTACTTTGTTTTCTGCAGTATTCGGTTACGATGAGTTTTTATATTACGCCTAAAGCGAGCGTTACTGCATAATACGGCCTTGGAGTTTAAAAATAACGG

>a59325947b152193a794569ab2aff79e

TACAATTTCTTCCATTTCCCTTTTCCCCCGTTTCCGTTCCTCTCTTCTTTCCCTTTTTCTCCCTCCTCCTTTTCTTTCTCTCTCTTTTTATTATATTTTATTAATTT

>d2917568b089a10a2f90bcffc5e3c265

TACAATTTCTTCAATTTAAATTTTAAAAAGTTTCAGTTAATATATTATTATTATATTTTATTAATTTGTCTGAGAAACTTTTTTTGCTAGACTAGGATTAGAAACCC

>181b772a57de4cb7b5a2c25332ff45e8

TACAATTTCTTCAATTTAAATTTTTAAAAGTTTCAGTTAATAAATTATTTAAATTTCTATGAATAACAATTTTAGTGAAATATATTATTATTACATTTTATTAATTT

>e5756e47042d66de92f19b58b9bd297c

GAACAGCTCATGCTTTCCTCCCCAGCCTTTCTCTCCCAGCTTCTATTAGATACCCTAGTAGTCCGGCTGACTGACTCTCTAGAGATCTCGTATGCCGTCTTCTGCTT

>eb31929a586b494dbbcec3d679375531

TAAAAGTTGGTTTTGTAAAAATCCACTGCCTTCTTTTCCTGTCGTGCAAGGCTATGTACTTTCGATAGGGTTGGCCGTGATCTTGAAACCGCACACAGGAAGGAAAG

>ab517adb80f89a2cd107784d000ae6c8

TGTCCGCACCTAATAAGGCATCAACGGGAGCGGGTCGGTCAAACATCGGATCGGCAAGAACCAAGTCCTTAGCCGCACTCTTAACCTCTGAAGCTATTGCCGTCAGA

>c309e798c9a94e47b208be8e948f8a8a

TGTCCGCACCTAATAGGGCATCAACGGGAGCGGGTCGGTCAATTGTCGGATCGGCAAAGACCAAGTCCTTGGCTGCATTCTTAACCTCTGAAGCTATTGCTGTCAGA

>0b2f38ada74ec4921d738f7670c706d7

CACGGAGGGTGCAAGCGTTAATCGGAATTATTGGGCGTAAAGGGCGCGCAGGCGGGCTTGTAAGTCAGTGGTGAAATCTCAGGGCTTAACTCTGAAACTGCCATTGA

>ccd2f8698970bcfd968a037f95a350db

CACGGGGGGTGCAAGCGTTATCCGGATTCATTGGGTTTAAAGGGTGCGTAGGCGGGCTATTAAGTCAGTGGTGAAATCCTACAGCTCAACTGTAGAACTGCCGTTGA

>64acce53b7b2d1a0c8aa5d371328b888

TACGTAGGCTCCAAGCGTTGTTCGGAATTACTGGGCGTAAAGCGAGTGTAGGCGGTCCATCAAGTTGGTTGTGAAATCTCCTGGCTCAACTGGGAGGGTGTGACCAA

>f8c63c52b03ae5d35d0a714ff480e94a

TACAGAGGGTGCGATCGTTAATCGGAATTACTGGGCGTAAAGCGCATGTGGGTGGATATTTAAGTCGGATGTGAAAGCCCTGGGCTCAACCTGGGAACTGCATTCGA

>e38dc7df0eebe0829d3f60a02be872c4

TACGTAGGGGGCGAGCGTTGTCCGGATTTACTGGGCGTAAAGGGCGTGTAGGCGGCAATGTAAGTCAGGAGTGAAACCCGTCGGCTTAACCGACGGCCTGCTTCTGA

>412a87e5deedba8800ad33dbb78abe8f

GACGTAGGGCGCAAGCGTTGTCCGGATTTATTGGGCGTAAAGAGCTCGTAGGCGGCTTGTCGCGTCGACTGTGAAAACCCGTGGCTCAACTGCGGGCTTGCAGTCGA

>fd35dfcfab1a19a23da510c9a043f8db

CACGTAGGGGGCGAGCGTTGTCCGGAATTACTGGGCGTAAAGGGCGCGTAGGCGGATGATTTAGTCAGATGTGAAAAATGCAGGCCTAACCTGGATTGTGCATTTGA

>39b2f42768e788e1f8cdca3f8e4cb05a

TACGTATGGGGCGAGCGTTGTTCGGAATTATTGGGTGTAAAGGGCGTCTAGGCGGGAAATTAAGTTAGATGTGAAAGGCGTGGGCTCAACCCGCGAACTGCATCTAA

>227e21f4215b946d5ee3ca690519a266

TACGTAGGTGGCAAGCGTTATCCGGAATTATTGGGCGTAAAGAGGGAGCAGGCGGCTATAAAGGTCTGTGGTGAAAGACTGAAGCTCAACTTCAGTAAGCCATGGAA

>08267ec6efee73d41f129d1034058989

TACGTAGGCGGCGAGCGTTGTCCGGAATTACTGGGTGTAAAGGGTGCGTAGGCGGTTTAGCAAGTCAGATGTGAAAGCCCAGGGCTCAACCCTGGGACTACATTTGA

>cb37d7d6d161c1ccf84f5522d18a111f

TACGGAGGGTGCAAGCGTTGTTCGGAATTATTGGGCGTAAAGAGCATGTAGGCGGGCTATTAAGTCTGGTGTGAAAGCCCGGGGCTCAACCCCGGAAGTGCACTTGA

>28cef3a1a266160a1df7052388ad89c4

TACGAAGGGAGCTAGCGTTGTTCGGAATTACTGGGCGTAAAGCGCACGTAGGCGGATTTGTTAGTCAGGGGTGAAATCCCGGGGCCCAACCTCGGAACTGCCTTTGA

>b9ae562b5e994ab80d7ea9783acdacb0

TACGTAGGGGGCAAGCGTTATCCGGAATCATTGGGCGTAAAGAGTACGTAGGCGGCCATATAAGTCAGAAGTGAAAGGCAGTGGCTCAACCATTGTAAGCTTTTGAA

>ae9266fe4c24180c7a0f7b4f631dddeb

CAATAGAGCGTCACCGTTTTCGAGGTGCTGTGGCCGAGGCCGACAAGGGGCGCAAGTAGATCCCCTCGCGGGAGTCCCGGGGAGCGTACAGCAGCACATCTGTCATC

>b109f26c00e17e9b84e08b43f7f3b683

TGTCCGCACCTAATAGGGCATCAACGGGAGCGGGTTGGTCAAAAGTCGGATCGGCGAGAACCAAGTCCTTAGCCGCACTCTTAACCTCTGAGTCTATTGCTGTCAGA

>5f60fabc0e2b5ef94f48fc8654d9af73

GTAGGGCTTGAAATGATGAGGATACCGAAAGGCATCGACTAAAGCGAAGCAACCAGCTGTTCGGCAATTATCAGCGCCAAACCAGCTGTTCGGCAATTAGATACCCC

>d6a64a9f28e4c2d530f340b50cd8e4fa

TACGAAGGGTGCAAGCGTTACTCGGAATTACTGGGCGTAAAGCGTGCGTAGGCGGTTCGTTAAGTCCGATGTGAAAGCCCTGGGCTCAACCTGGGAACTGCATTGGA

>ff291a1dd8d7bbb3c941ba7be6099210

GGCAAATTGTTTCGTTTACTTGCAATCGCTTGCCAGAATTTTCAATGGCTCCACTAAACGGAAAGTCATATTAAAATTAAACTTTTAATTTCATTCGCAATGCAGCG

>411345d59cd2ef576b4b11b117656030

CCACTGTGTTGAGTGTGATATACTTTTACAATACACTATAACATTAGAAACCCCAGTAGTCCGGCTGACTGACTTAACGTCCATCTCGTATGCCGTCTTCTGCTTGA

>18e0a52ea186489be00a37ddee14c9f8

CCGGACTGTGCCGCCTTGATCCGAACTCCCGACACATAACATACTTAACTGATAGCGTCGTGCTCAACCGACTGAGTTATCCGGCCACTGGTGTTCCTGTAATTTAT

>0147957888ce535022aa8f5302c1fdd3

ACTACATTATCAGGGGCGTCATTTCAGATTTTGGTTGGGGGGGAGGGGGCAAGCACAGAGCACAATAGTGTTTTCCAGGTTTTGTGATTATTTGGTATTATAGAAAA

>6e900e3f85ebcdd0fd5d9ceeaf080fc0

TACAGAGGGTGCAAGCGTTAATCGGAATTACTGGGCGTAAAGCGCACGTAGGCGGTTTTTTAAGTCAGATGTGAAAGCCCCGGGCTCAACCTGGGAATTGCATTTGA

>9c4f57ec9fdca59ea5552749e10c118b

TACGAAGGGGGCTAGCGTTGCTCGGAATCACTGGGAGTAAAGGGCGCGTAGGCGGCCGATTAAGTCGGGGGTGAAAGCCTGTGGCTCAACCACAGAATTGCCTTCGA

>99bd4cb78daf7995b4a7c9ed694f2ba6

GGCCAGATAAACTCCACATTCATATGTTGCAACACGCTCTATCCCATATTCTTGACACATTATTAAAAAGTTTTAATAAAATATGGTTTTCTGGCATCTTTCCAACA

>a5e2426d51749e8a5667e8d9ee42286c

GGAGTACAGCAGCAGCAGTCGTTATCGTCAACGCCCAACTTGATGGCCAGGTCGTTGTCGCAGCCACATCCACATCCGCCACATCCGCATCCGCATCCGCCACATCC

>6eb4d060a33f013e17c50712463405d6

GCGTTATATACTTTGCAAATACACTATAACATTAGATGCCATTGCCTCCGTTTTGCGGTTATGTTCGTCATATCCTTTTACAATACACTATAACATTAGAAACCCGA

>2b0c238c0a90e3d4a91a0079be83b3f5

CGTTAGAAACCGAGGTTGATGGCTGGAACTGAGAGTAATCCCGCTCAGCAGATACAGCATTAGCTATAGGAACTGAAGATGATGATGATGACTGGCAGGGATTCTGC

>7c0dd6b030911ae7b9781d85b51d763d

CTCTTTACGATGTCGCAAACGTTACGACGTTGATGAATTGCCTCTTCTTCTTCTTTTCTTCACCCACAGGTTGACGGACTTCCAATTTCTTCACTTGCAAAGACCGC

>32e42a53aa6b6f2124d0834587f5060c

TACAGAGGGTGCGAGCGTTAATCGGATTTACTGGGCGTAAAGCGTGCGTAGGCGGCTTCTTAAGTCGGATGTGAAATCCCTGAGCTTAACTTAGGAATTGCATTCGA

>28ea4ca65740699de75dd13cbfe1f5a8

GGAGGGTGAGACAGTATAGTGAAGAATTTGGGACACCTAAAGCAATTCTCACTGACAATGGGACACAATTTACGTCAAAGAAATGGGTGAACGGCTTGAATGAGCTA

>d5b80392abdccf306a970aa3450171e2

CCTGTTCATTGATTCGAAATTCGAATCAGTAAGTGAGCGAGATTGAAACGAAAGAAAAAGATTGCCATGAAGAGATCTATTGACCCAGTCAACGAAATTACAGAAAT

>4921b79c8c9358c8cea72559d4955f8d

TATTCTCGCGTAAAGCAAGCGGCCTCGGCCGCTTAAAGTAGGCTCCGTCGACGTACCCTGGACCAACAGCGTAAAGTACCTCGGGCTCCACGTGGACTCCCGACTCA

>d85622ad314fd2d38ce99f1ca2fef15c

CCACAACCCACCGCTATGTTCATACTTTTTTTTTACAATAACAGCTGCCACAATTCACACACACACGCTTAATGTACAGAAGAAAAATGATCTAGACGGCAGTGGTG

>189a6714b361e54a40cd3e8e5144dd6b

CCACTGTGTTGAGTGTGATATACTTTTACAATACACTATAACATTAGATACCACAAGCAAACACACACACACACACACATAGAAAGTAGTAGGACGCAAACGCAGTC

>3278805a40e1799e8236f2cfdadc724b

GTAGCTACTCAGAGCAATCCCACTTTACTACGAACACAATACACTATGGCCATAATTTTAAGGATTACAACATCTTGCTTAACGACTGCAACGAGGTTTAGTGTTTC

>cbd9d803b329fadfb09c64ae80171e36

TGTCCGCACCTAATAGGGCATCAACGGGAGCGGGTCAGTCAAATGTAGGATCGGCAAGAACCAAGTCCTTAGCTGCACTCTTAACCTCTGAAGCTATTGCTGTCAGA

>f2958cc515d56da8a19394d417f5792c

TACGGAGGGTGCAAGCATTGATCGGATGTACTGGGCGTAAAGGGCACGCAGGCGGTCATATAAGTCAGCTGTTAAATGTATGGGCTTAACCGATGCATGCGGTTGAA

>f59c3d96ac1d2afe81253d08ff1cdf11

TACGTAGGGAGCAAGCGTTATCCGGATTTACTGGGTGTAAAGGGCGCGTAGGCGGGACTGCAAGTCAGGTGTGAAATCTGGTGGCTCAACCACCAAACTGCACTTGA

>8970f4edb2eba5757cfae52e514fdd43

TACGGAGGGTGCAAGCGTTGTTCGGAATCATTGGTCGTAAAGAGCGTGTAGGCGGGATGGTATGTCGAATGTGAAAGCTCTAGGCTCAACCTAGGAATTGCATCCGA

>22bd4bb382c85f3581dedc91d4659f58

TACTAGCGGTCCAAGTCGCAGCCATTTTTATTGGGTCTAAAACATCCGTAGCTTGTCAAGTGAGTTTTCTATGAAATCCCGGGTCTTAAGTTCGGGGCGAGTGGAAA

>f0dd5b8e237048cb194537862193c7ec

TACGTAGGGTGCGAGCGTTGTCCGGAATTACTGGGCGTAAAGAGCTCGTAGGCGGTTTGTCGCGTCGTCTGTGAAATTCTGCAGCTTAACTGCAGGCGTGCAGGCGA

>52dbd6bd3bb488d550b3997799c508ef

TACGGAGGGTGCAAGCGTTATCCGGAATCATTGGGCGTAAAGGGTGCGTAGGCGGCCTGACAAGTCAGAGGTGAAAGGCTACGGCTTAACCGTAGTAAGCCTTTGAA

>e38a43dca288f7164fc2c65dedcae2c0

TACGTAGGGGGCAAGCGTTATCCGGATTTACTGGGTGTAAAGAGTGCGTAGGCGGCGGAGTAAGTCAGATGTGAAAGCCCGAAGCTCAACTTCGGGACTGCATTTGA

>c6335060eb054210ce564d3c985f954c

TACGTAGGTGGCAAGCGTTGTCCGGATTTACTGGGCGTAAAGGATGCGTAGGCGGATATTTAAGTCAGATGTGAAAACCCAGGGCTTAACTTTGGGAGTGCATTTGA

>823db9dd9b83aa20a884ac2b76508f94

GACGTAGGGGGCGAGCGTTGTCCGGAATTACTGGGCGTAAAGGGTGCGTAGGCGGCCAATAAAGTCTGAGGTGAAAGGCTACGGCTTAACCGTAGTAAGCCTTGGAA

>2693056ab8633fe945c15e448cdae73f

TACGGAGGGTGCGAGCGTTAATCGGAATTACTGGGCGTAAAGCGCATGCAGGTGGTTCATTAAGTCAGATGTGAAATCCCCGGGCTTAACCTGGGAACTGCATTCAA

>1c59b91c636094d85985b64cf72965c1

TACGGAGGGGGCAAGCGTTGTTCGGAATTACTGGGCGTAAAGGGCGTGTAGGCGGTCTGTTGCGTCAGGTGTGAAAGCCCTGGGCTCAACCTAGGAGTTGCACTTGA

>6f11dc86e475d104883fbbaaa928d9b9

TACGTAGGGGGCGAGCGTTGTCCGGAATTACTGGGCGTAAAGGGTGCGTAGGCGGCTTGTTAAGTTGGATGTGAAATACCCGGGCTCAACCTGGGGGGTGCATTCGA

>56fd86a834c6a2c48ef4158a2bff222e

TACGTAGGGGGCAAGCGTTATCCGGAATAACTGGGCGTAAAGGGTGCGTAGGCAGCCCTGCAAGTCAGAAGTGAAAGGCTACGGGTCAACCGTAGTAAGCTTTTGAA

>3856a7b0dd275e25d3975177f1dc1240

GACGGAGGATCCGAGCGTTATCCAGATTTATTGGGTTTAAAGGGTGCGTAGGTGGTTTTATAAGTCAGCGGTGAAAGTTTGCAGCTTAACTGTAAAAATGCCGTTGA

>5ce9e4e1cd114cacfbc5976c5725fb1d
[truncated: 166,428 more chars]
