# Supplementary material for: Lack of host phylogenetic structure in the gut bacterial communities of New Zealand cicadas and their interspecific hybrids
Source: Sci Rep. 2022 Nov 29;12:20559. doi: 10.1038/s41598-022-24723-3 (PMC9709078; doi:10.1038/s41598-022-24723-3)
Supplement: Supplementary file 4 — Supplementary Information 4. [file 41598_2022_24723_MOESM4_ESM.docx]

>acoustica-rosea_99.NZ.MK.LOH.01

NNNNNNNNNNNNNNNNNNNNNNNNNNNNNNNNNNNNNNNNNNNNNNNNNNNNNNNNNNNNNNNNNNNNNNNNNNNNNNNNNNNNNNNNNNNNNNNNNNNNNNNNNNNNNNNNNNNNNNNNNNNNNNNNNNNNNNNNNNNNNNNNNNNNNNNNNNNNNNNNNNNNNNNNNNNNNNNNNNNNNNNNNNNNNNNNNNNNNNNNNNNNNTATCATTATTTACATTTATTTTATTAAATAATTTATTGGGATTACTCCCCTATATTTTTACAAGTCCAAGTCATTTAGTATTTACAATAAGATTAGCATTACCACTATGGTTATCATTTATACTTTATGGATTTATTAACAATATAAACCATATATTTTGTCATTTAGTTCCGTCAGGAACTCCTAATATTTTAATACCTTTTATAGTTATTATTGAAAGAGTCAGAAATTTAATTCGACCTGGCTCATTGGCTGTCCGGCTAACAGCTAATATAATTGCTGGACATCTTTTAATAACTTTATTGGGTAATTTACCTATAAGTTATGAATTATATTCAGGTATAATCATTATTTTTCAAGTTATATTAATATTATTTGAATTAGCCGTTTGTGTTATTCAATCATATGTATTTATAGTACTTAGAACTTTATATTATAGAGAAGTAAATATTCCCCAGATATCTCCCCTTAATTGATTATTATTNNNNNNNNNNNNNNNNNNNNNNNNNNNNNNNNNNNNNNNNNNNNNNNNNNNNNNNNNNNNNNNNNNNNNNNNNNNNNNNNNNNNNNNNNNNNNNNNNNNNNNNNNNNNNTTTAAACCAACACGACAAAATCATCCTTTAATTAAAATTATTAATAATTCATTAATTGACTTCCCAGCACCATCAAACCTATCTTATTGGTGAAATTTTGGATTTGTATTAGGATTATGTTTAACAATCCAAATTCTAACTGGATTATTTTTATCAATGCATTATAATGCTAATATTATAAATGCTTTTGAAAGATTAAGACATATTTGTCGTGATGTAAATTATGGTTGAATTTTGCGAGTTATTCACGCTAATGGAGCTTCATTATTTTTCATATGTGTTTATCTACATGTAGGACGTGGATTATATTATGGTTCATTCAAATATATTGAAACATGATCTATCGGTGTAATTATATTATTAATACTAATAGCTACCGCTTTCTTAGGATATGTATTACCATGAGGACAAATATCTTTTTGAGGGGCTACAGTTATTACAAACTTATTATCAGCTATTCCTTATTTAGGTGGAATATTAGTTAATTGAATTTGAGGAGGATTTGCAGTTGATAATGCAACACTAACTCGATTTTACTCATTCCACTTTATTTTACCATTCATTGTATTAAGACTAACAATCATTCATTTGTTATATCTACATACAACAGGTTCAAATAATCCACTAGGAATTAATTCCAATAATGACAAAGTTCCTTTTCACCCATACTTCTCCATTAAAGATATTATAAGACTATTTATCTTAATAATTATTTTCTTTATATTAGTCATATTGGAACCCTACATACTAGGGGATCCAGATAATTTTATTCCTGCCAATCCACTTGTAACACCAAAGCACATCCAACCTGAATGATATTTTTTATTTGCCTACGCAATTCTTCGTTCAATTCCTAATAAGTTAGGGGGAGTAATTGCTCTTTTTATATCAATTTTTATTTTAATATTTGTACCATTGTTAAATAATTCCAACTTTATAGGATTAAATAATTACCCAATCAATCAAATCATATTCTGATATATAGTAATCATTCTAATTTTATTAACTTGAATTGGAGCACGACCTGTCGAATTACCTTATATTAACTTAGGAATACTTTTAACACTTATATATTTTTCTTACTTCATTATTGACCCAATAATTAAATCTTTTTGAGACAAATTAATTAGGTAGATGAAAAAATGATTCTTCTCAACTAATCATAAAGATATTGGAACAATATATTTCATTTTTGGTATTTGATCAGGTATAATTGGCACAACTTTAAGAGTTTTAATTCGTGTAGAACTTGGAACTCCAGGTTCCTTTATTGGTGATGATCAAATTTATAATGTAATTGTTACTGCTCATGCTTTCATTATAATTTTTTTTATAGTTATACCAATTATAATTGGTGGATTTGGGAATTGATTAGTCCCTTTAATAATTGGAGCTCCTGATATAGCTTTTCCTCGTATAAATAATATAAGTTTTTGATTATTACCACCCTCTCTAATTCTTATCTTAGTAGGAAGAATAGTTGATAGAGGTGCAGGTACAGGTTGAACAGTTTATCCCCCCTTATCTGCAGGTATTGCGCATTCTGGTTCATGCGTTGACTTAACTATTTTTTCTTTACACCTTGCAGGTGTATCTTCAATTTTAGGTGCTGTAAATTTCATTAGAACAATTTTTAATATACGTTCAATAGGAATTTTATTAGATCGAATACCTTTATTTGTATGATCAGTTTTAATTACTGCATTTTTGTTATTGTTGTCTTTACCTGTTCTAGCAGGTGCTATCACAATATTATTAACAGATCGAAATTTAAATACTTCATTTTTTGACCCTTCCGGGGGGGGAGATCCCATTCTTTATCAACATTTATTTTGATTTTTTGGACATCCTGAAGTTTATATTTTAATTTTACCAGGATTTGGTTTAATTTCTCACATTATTACCCAAGAGAGAGGTAAGATTGAATCTTTTGGTTCATTAGGAATAGTTTATGCTATAATATCAATTGGTATTTTAGGATTTGTTGTATGAGCACATCATATATTTACTGTAGGAATAGATGTTGATACACGTGCATATTTTACATCAGCTACTATAATTATTGCAGTTCCCACTGGAATTAAAGTATTTAGTTGACTCGCAACTTTAAGAGGAATAAAACTTAATATTACATCTTCAGCCTTATGGGCTTTAGGCTTTGTCTTTTTATTTACCATTGGAGGTTTAACCGGCGTGATTCTAGCTAATTCTTCAATTGATATTATATTACATGATACATATTATGTAGTTGCACATTTTCATTATGTTTTATCAATAGGAGCAGTTTTCGCTATTATAGCAAGATTTATTCATTGATTCCCATTATTTACAGGATTAAGATTAAACTCAAATTGATTAAAAATTCATTTTCTATTGATATTTATTGGTGTAAACATAACATTCTTTCCTCAACATTTTTTAGGATTAAGAGGAATACCTCGTCGATATTCAGACTATCCCGATGCTTATATGTCATGAAACATAATTTCATCAATAGGAAGAATTATATCTCTAATTGGAATCTTATTTTTATTATTTATTGTTTGAGAAAGTTTTATTTCAATACGATTAGTATTATATTCTAATAGAATTCAATCCTCTATTGAATGAATACAGAAGTTCCCTCCATCTGAACATTCTTATAATGAAATGCCAATATTAATTCAAATTTCAAATTGATCTTATATTAATATACAGGATGCTGTATCACCATTAATAGAGCAATTAATATTTTTTCATGATCATGTATTAGTCATTTTAATAATAATTACAATTGTTGTTGCTTACATAATAATTATATTANNNNNNNNNNNNNTTATTAATCGATTACTTCTTGAAGGGCAATTAATTGAGTTCATTTGAACTTTATTACCTGCAATAACTTTAATTTTTATTGCATTACCATCATTACGATTATTATACATATTAGACGAAATTAATAATCCATTATTAACATTAAAAATTATTGGTCATCAATGATATTGATCATATGAATACTCAGATTTTTCTGAGGTAGAATTTGATTCATATATAAAATCTATAAACGAAATAAATAAAAATGAATTTCGATTATTAGATGTTGATAATCGAGTAATTCTACCATTTAATATTCAAGTACGACTATTAGTTTCTTCTTTTGATGTTATCCACTCTTGAGCAATACCATCTATAAGACTTAAAGTTGATGCAGTTCCAGGACGACTAAATCAAATAAGAATATTAATTAGTCGTCCTGGTGTATCTTTTGGACAATGTTCTGAAATTTGTGGGGCAAATCATAGATTTATACCTATTGTAATTGAAAGAATTAGAATAAAAATATTTATTAAATGATTAATTAATTATATGAATAATCATCCTTATCATTTAGTTGATTATAGACCCTGACCTTTAACTGGATCAATTGGAGCATTAACTTTTGTTTCCGGTATAGTTATAATATTTCATAAATGTAACTTTATATTATTATATACTGGTATTATATTATTATTAATAACAATAATTCAATGATGACGTGACATTTCCCGAGAAGGAACATTTTTAGGAATACACACAATAATGGTAGTGAATGGTTTGAAAATAGGCATATTATTATTTATTGTATCAGAAGTTCTTTTTTTTGTTTCATTTTTTTGGGGATTTTTTCATAGTAGATTAAGGCCTGTAGTAGAAATTGGTATAATTTGACCTCCTAGAGGTATTTATGTTTTTAATCCAACTCAAGTCCCCTTATTAAATACAATAATTTTATTGTGTTCAGGAATTACAATTACTTGAGCCCATCATTCAATTATAAATGGCAATCATATTAATTCTATCTATAGAATTATGTTAACTGTTATTTTAGGAATATATTTCACTATTTTGCAAGGTTATGAATATTATGAAGCTCCATTTGCAATTAATGATTCCATCTATGGATCTTCTTTTTTTATAGCTACTGGATTTCATGGAATTCACGTAATCATTGGAACAAGATTTATTGTTGTATGTTTATTACGACAAATAAAGTTTCATTTTTCAAAAAATCATCATTTTGGCTTCGAAGCTGCTGCTTGATATTGACATTTTGTTGATGTAGTCTGATTATTTTTATATTTATCAATTTATTGATGAGGAAGATAATAAATTATAGTTTTTAAAAAGAAAAAAAAAATAAATAAATTTAGAGATATTGGCAAATAAGATTTTCAAGCCAAATATATTAACTTATCATAACGATAACGGGGAAGAGTTCCTCGAACTCAAATGAAAAGAAAACACATTATAGAAACTTGAATTGGAAAAACAATTGAATTAATTTTTCCTCCTAAAAACATTAAACAAAATATTATTCTCATAAATAAAATACTAGAATACTCAGCCAAAAAAATAAATGCAAATCTAGAACCTCTATATTCAACATTAAAGCCGGACACTAACTCTGATTCTCCCTCAGAAAAATCAAATGGAGATCGATTAGTTTCAGCTATAGCTGAAGATAATCAACATATTCTTAAGGGAAGAAATAAAAAAATAAATCACACAAACTCCTGAAATATAAACAAATCAATAATGTTATAACTTCTTATTATTATTATAGGACACAATATAATTAGAACCAATCTTACCTCATAAGAAATAGATTGAGCAATTGATCGAATACACCCTAACATTGCATAACTTGAGTTAGAAGATCAACCTGTTAATATTAATGAATAGACTCTTATTCTTGAACAACAAAAAAAAAACAAAATACCAAAATTAAAAGAAACACAATTAATTATATATGGATATAATGATCAAATTAATAATCTATTGAATAATCCTATAATAGGTCTAAAAATATAAATTAAATAATTAGATATTAATGGAATTGTGTTTTCCTTTATAAATAATTTAATAGCATCAGAAATGGGCTGTAAAATACCCAAAAACCCAACTTTATTTGGACCTTTACGAATTTGAATATATCTCAAAACCTTACGCTCCAAAAGAGTAATAAATGCCACTCCAATCAAAATAAATAATACAGTAATTAAAATAGTAATTAAATACAAAAATAATTCTTCTTATATACTTTACATAATTTTCTTATTATTAGGAATTATAATTTCAGTTTCTTCAAACAATTGGCTGGGTTGTTGAATAGGAATTGAAATAAATATAGTTTCATTTTTGCCTATAATGGTAAATAAAATANNNNNNNNNNNNNNNNNNNNNNNNNNNNNNNNNNNNNNNNNNNNNNNNNNNNNNNNNNNNNNNNNNNNNNNNNNNNNNNNNNNNNNNNNNNNNNNNNNNNNNNNNNNNNNNNNNNNNNNNNNNNNNNNNNNNNNNNNNNNNNNNNNNNNNNNNNNNNNNNNNNNNNNNNNNNNNNNNNNNNNNNNNNNNNNNNNNNNNNNNNNNNNNNNNNNNNNNNNNNNNNNNNNNNNNNNNNNNNNNNNNNNNNNNNNNNNNNNNNNNNNNNNNNNNNNNNNNNNNNNNNNNNNNNNNNNNNNNNNNNNNNNNNNNNNNNNNNNNNNNNNNNNNNNNNNNNNNNNNNNNNNNNNNNNNNNNNNNNNNNNNNNNNNNNNNNNNNNNNNNNNNNNNNNNNNNNNNNNNNNNNNNNNNNNNNNNNNNNNNNNNNNNNNNNNNNNNNNNNNNNNNNNNNNNNNNNNNNNNNNNNNNNNNNNNNNNNNNNNNNNNNNNNNNNNNNNNNNNNNNNNNNNNNNNNNNNNNNNNNNNNNNNNNNNNNNNNNNNNNNNNNNNNNNNNNNNNNNNNNNNNNNNNNNNNNNNNNNNNNNNNNNNNNNNNNNNNNNNNNNNNNNNNNNNNNNNNNNNNNNNNNNNNNNNNNNNNNNNNNNNNNNNNNNNNNNNNNNNNNNNNNNNNNNNNNNNNNNNNNNNNNNNNNNNNNNNNNNNNNNNNNNNNNNNNNNNNNNNNNNNNNNNNNNNNNNNNNNNNNNNNNNNNNNNNNNNNNNNNNNNNNNNNNNNNNNNNNNNNNNNNNNNNNNNNNNNNNNNNNNNNNNNATTAATTACATTAAATATGTGTTAGNNNNNNNNNNNNNNNNNNNNNNNNNNNNNNNNNNNNNNNNNNNNNNNNNNNNNNNNNNNNNNNNNNNNNNNNNNNNNNNNNNNNNNNNNNNNNNNNNNNNNNNNNNNNNNNNNNNNNNNNNNNAAATNNNNNNNNNNNNNNNCATCTCGCATTCCATTTTCAAGACACTTTTTTTTAATTGCAGTAATTTTTTTAATTTTTGATGTTGAATTAGTAATTATTATACCTGTAATTTTAGTAATAATTAGATTGAGATCTATTGATATTTATATTATCATATTCATCTTTCTAATTATTTTAACTTTAGGGTTATATCATGAATGATATAATAATATATTAAATTGGNNNNNNNGTATAATTAAAATATTTAATTTCAAAATAATTAAATTTAAGGGAATTCAATGTATCATAATCAAAAAAAACTCCCGTACTCTTCCCGAATTAAATGAAAATAAACTAGAAAAAAATGTACCATGTTGAGTAAATGAAAATATATATAATCTATAACAAGCAGATAAAAATGAAGATAAAATCAATATAATCATTATTAACATATTTCAAGATATTAATCTATTAATAATTATAATTTCACCAGCTAAATTAATTGTAGGTGGACAAGATATATTTCTTGCTGACATTAAAAATCATAATAAAGATATTGAAGGTATAAATGTTAATATACCCTTATTAATTAATAATCTTCGACTATTAGTTCGTTCATACAATATATTTGCTAAACAGAATAATCCAGAAGAACATAAACCATGTCCAATTATTATATAGTATGAACCACATATTCCCCAATTTCTTATAGTTATAATCCCTCTAATCACAAGACCTATATGAGCAACAGATGAATAAGCAATTATTGATTTAATATCAATCTGAATTATACACAGAACTCTAATTATAATACTTCCAATTATTGAAATTGAGATCCATATAAATCCAAATTTATAAAAATAAGAAGGAATAATATACATTACACGAATTAGACCATACCCCCCTAATTTTAATAAAATTCTAGCTAGAATTATTGAACCAGAAACAGGAGCCTCAACATGAGCTTTAGGTAATCAAAAATGAAAAAATAATGGTATTTTAATTAAAAAAGCTATTATTATACCAAAATAAATATAAATATTGACAGAATAATTTATTAATATAANAATACTAGTTATATTATTTTTATAAAGAAAAAAAATTCTTATTAACATTGGTAATGATGCAAATAATGTATAAAACAATAAATAATACCTCGCATCAATACGTTCTGGTTGATATCCCCAACCTATAATAATAACTATAATTGGAATTAATCTAGATTCAAAACAAATATAAAAAACTATAANNNNTGATGTAGAGAAAGAAATAATTAAAAAAATTATTAACATAACACCAAAAATAAAAAAAATAACTTATTATATTTATATATTGGACTAGATACAATTATTAAAAATGAAATTCAAATTCAAAATAATTAAACCATAAGATATTTCATCAATACAAAAATTATAACCTAATATATATAAATTCTTATTTATAAAAACCAAAAATATAATTATCGANACAATAATTATATTCATTAATCATGAATTATAAATTAATAGGGTTAATATAAAACAAAAAAAAACTAATTTTATTGATATAGATATTAATTTATCATTATTATGAGATCGAATTAATATTACTAAGCATGATAAACCCATAACTCCTTCACAGACTCTAAAAACCAAGAAANTTAATAAAATATGTAAATCATATATTTTTATAGAAAAAATAAATGATAAAGAACAAAACATTCTTAAAATAATTAATTCCAATCTTAATGTTGTTATTAAATGTTTTCGATTCATACATAATGAGACTATCCCTGAAAAGAATATAAAGATAAAATATATTAATAATGTTATATTCNNNNNNNNNNNNNNNNNNNNNNNNNNNNNNNNNNNNNNNNNNNNNNNNNNNNNNNNNNNNNNNNNNNNNNNNNNNNNNNNNNNNNNNNNNNNNNNNNNNNNNNNNNNNNNNNNNNNNNNNNNNNNNNNNNNNNNNNNNNNNNNNNNNNNNNNNNNNNNNNNNNNNNNNNNNNNNNNNNNNNNNNNNNNNNNNNNNNNNNNNNNNNNNNNNNNNNNNNNNNNNNNNNNNNNNNNNNNNNNNNNNNNNNNNNNNNNNNNNNNNNNNNNNNNNNNNNNNNNNNNNNNNNNNNNNNNNNNNNNNNNNNNNNNNNNNNNNNNNNNNNNNNNNNNNNNNNNNNNNNNNNNNNNNNNNNNNNNNNNNNNNNNNNNNNNNNNNNNNNNNNNNNNNNNNNNNNNNNNNNNNNNNNNNNNNNNNNNNNNNNNNNNNNNNNNNNNNNNNNNNNNNNNNNNNNNNNNNNNNNNNNNNNNNNNNNNNNNNNNNNNNNNNNNNNNNNNNNNNNNNNNNNNNNNNNNNNNNNNNNNNNNNNNNNNNNNNNNNNNNNNNNNNNNNNNNNNNNNNNNNNTAATTAATAAATTAATATGTCTTGAACATATAGTTTCTATAATTAAATCTTTTGAATAAAATCCTCTCATAAATGGAATTCCACATAATGATATATTAGCAATATTTATTATTGTAATAGTTAATGGTATTTGAAATCTTAAACATCCTATTACACGGATGTCTTGATTATTATTAAAATTATGAATCAAAATTCCTGCGCACAAAAATAAAGTAGCTTTAAATAAAGCATGAACAATTAAATGAAAAAATGATAATATTGGATAACCAAATATAATAATTGTTATTATAATCCCCAATTGTCTTAAAGTTGATAATGCAATAATTTTTTTCAAATCAAACTCAAAATTTGCTCTAATTCCTGATATTAGTATAGTTAAAATTGAAATTAATATAAATACTTGAATATAGTCAAAATTTAAAATAATATTAGAAAATCGAATTATTAAATATACTCCAGCAGTAACAAGTGTAGATGAATGAACTAAAGCAGAAACAGGAGTTGGGGCAGCCATAGCTGCCGGTAATCATGAAGAAAATGGAATTTGAGCTCTCTTTGTAAATCTTGCAATAATAATCATAATTAATATAATTTATATCTTTATTAATAAAAGATAAAAATTTCATGACCCAAAATTTAATATTCATGCAATAACTATTAAAATAGCAACATCTCCCACACGATTCATTAGCGCAGTTAATATTCCTGCATTATTAGAATATAAATTTTGATAATAAATAACTAAACAATAAGAAACTAGACCAAGCCCATCTCAACCAATGATAATTCTAATTATATTAGGTCTAATGATTAATAAAACTATAGATATAACAAACCCTAAAAAAATATAAATAAAACGATTAATGAATTTGTCATTATTTATATATATTCCACTGTATAAAACAACCACTGATGAAATAAATATAACTGTCGAAAGAAATTGTTGATATAAAATCAAAAATTAGAGTCATATAAATATTACATGNNNNNNNNNNNNNNNNNNNNNNNNNNNNNNNNNNNNNNNNNNNNNNNNNNNNNNNNNNNNNNNNNNNNNNNNNNNNNNNNNNNNNNNAAGAATAATAAATATATAAACATTAAATCTTTTATTTATCATGAAAATTGCTATGTACTTAATAATTATTTTATCAACAAATTTCATTTTTATAAAGCACCCCCTATCAATAGGATTAATTTTAATAATACAAACAATATTATCTTGCTTAATTTGTAGTCTTTATTTAAGTTGTTATTTATTTTCTTACATCTTATATCTTATTTTTATTGGTGGTATATTAATTTTATTTATATATATATCAAGAATTGCATCAAATGAAAAATTTATTTATTCAATTAAATTAATAATGTTTAATTTTTCACTTATAACTTTAGTCAATTTAGTCAACATAATTGATTTAAAATCAATAAATATTAGAAGAAATATTATAATATATATAAACCACAACAATTTCATAATAAGAAAAATATATATTATCCCTTCTGGGATAATAACACTAATTTTAACAATTTATTTACTATTTGTTTTAATTATTGTAATTAATATTTTAACAATAAATATACTAACTCTTCGAAGAAGAACTTCTTTCTAACTACACCTTCCGGTACAGTTACTTTGTTACGACTTATCTCATTAATATAATGAGAGTGACGGGCGATATGTACATAAATTAGAGCCAATTTCAATTAATTAAATTTAATTAATTTATTATCAAATCCAATTTCATATTTTTTNNATNATAAAAAATAATTCAATACATAATTAAATGTAACCCATTTTTTCTTTAATATAAACTGCACCTTGACCTGACATTATATNNNNNNNNNNNNNNNNNNNNNNNTTTTCTTATAAAACATTATTCACAGAGATATACAAATTAAATTAAAGTTTTTTCTATCGTGGATTATCAATTATAAAACAGGTTCCTCTGATAAGATAAATTACCGCCAAATTCTTTGAATTTAAAGATCATTTCTAATAATAATCAAGTTATTTTTATCACATTTTTAATAATAGGNNNNNNNNNNNNNNNNNNNNNNNNNNNNNNNNNNNNNNNNNNNNNNNNNNNNNNNNNNNNNNNTATTTCACCAAAATTATAGATATTTTATATTATTGTTATATTAACTGAAACTTAACTATTTAAATTAAAGAAATCGTATAACCGCAACTGCTGGCACGAAATTTGATTCTTTTAAATAAAATTACTAATTCTAATTTTATTAATTAATAATAGTAAATACTGCGCATTTGTTAAAANTACTTCCATTTAGAAATTAAAAACAATCAAAAAATTGCATGTAATATAATTTTAAATTTAAATATTCTTAAACCAGAATTAAACTTCAAATAATAAAATTATTTAATGAGGTCCTTTCGTACTAACATTAAAAATAATTGAGTAGATAGAAACCAACCTGGCTCACGCCGGTTTGAACTCAGATCATGTAAGAATATTAAGGGTCGAACAGACCCAGAAATAATAAATTTTGCTCCAATCTCTATTCTTAATCCAACATCGAGGTCGCAATCATATTTATCGATATGAACTCTTCAAATTAATTACGCTGTTATCCCTAAGGTAATTTATTCTTATAATCAAAAATTTGGATCAATATTTACATAAATTTATGAAATTTATATTAAAAGTTAATTATATTTTAATATCACCCCAACAAAAAAATCAATTAAATAAAAAAAATTAATTAACTGTAAANAAATAAATTTAATNAAGATTTTAAAAATTCTATAGGGTCTTCTCGTCCCACTCACATATTTAAGCCTTTTTACTTAAAAATCAATTTCAATTATTAATATTAATTAAGTTAATTTCTCATTCAATCATTCATTCAAGCCTCCAATTAAAAGACTATTTATTATGCTACCTTTGCACGGTCAATTTACCGCGGCCATTTAATTTTTAATCATTGGGCAGATTAGACTTTTAATTCTTTCTAAAAGACATGTTTTTGATAAACAGGTGAAAATTTTTTTTGCCTAAATTATAAATATTATTTATAANNNNNNNNNNNNNNNNNNNNNNNNNNNNNNNNNNNNNNNNNNNNNNNNNNNNNNNNNNNNNNNNNNNNNNNNNNNNNNNNNNNNNNNNNNNNNNNNNATTATATTTCGGGAAAAATAAATACTTAAAAGTTTTAAATTAACCCTGATACAAAAGGTACAAATTATATTCTACTTAAAATTATTTATAAAGTTNTTTCCTTATCAGTTAATCCAAATTATTTTTTTATATAAAATTACTTTAACACAAAAATTTTTTTTTCAAAATGTNAAAAAACAACTTTTANTAAAATTATATTATGGGATATATTTAATTATAATAATTGAATTGCAGTCAATAGGTGTTATCTATAACTATCCTTAAGTAATGAAGTAAAATAATACATTTAGTTTCGACCTAAAATAAGAATTAGTATTCCTTACTTTTAAATGAAGCCAAAATAGAGGCATTTTATTGTTAATAAAATAATTGAAANTTTATTTCCATTTAAAGAAAAATTAGTTTAATAAAAATATAAATCTGTCAGATTTATGAAACTTTTGAGTATTTTTCTGACTTTTTATTATNTNTTCTACTTTAAATTTGCAATTTAATATTATAAATTAAATATAAGACTTTATGTTAACAGAATCAAACTGTCTATTAATATCAAAAATTAATGTTCTTCATAAACTATAACATTTATAAAGTTTATAAAACATTTCATTTTCATTGAAAAGAGAGACTTAGTCTTGTAAAATTCTATNNNNNNNNNNNNNNNNNNNNNNNNNNNNNNNNNNNNNNNNNNNNNNNNNNNNNNNNNTATCCAATAAATTTTNTTTTATCTTTAAAATCACAATTTAATATTTTTATTAAACTAATTGAATTACTATTTGTAATAAATATTACATTATTAAATTCTAAATTTAAGGCATTAATTTTGCTAAAATAGTTCTAATATGGCAGATTAGTGCAATGAATTTAAGATTCATATATAAAATATNATTTTTTTATTGGAAAATAAGATGCCTGAGTNNGAAAGGGTTATTTTGATAGAATAAATTATGTAATATAATTACTCNNNCATTAAGTGGCTGAAAGTAAGTAATGGTCTCTTAAACCAATTAATAGTAATTAACAAATACTCTTAATGAAAGATAAGCTAAAANTAAAGCTAATGGGTTCATACCTCACTTATGGAAANTTTTCCTCTTTTTATTTAAAAACAAAAGTTACCTTAATATCNNNNNNNNNNNNNNNNNNNNNNNNNNNNNNNNNNTCAGAAAAGACAATACAATCATTTTAAACTCCCAAAGTTTATATTTTACATTTAAACTATTTTCTGAGTTAATTAGCTTAACCAAAAGCATTTATTTTGAAAGTAAAAGAAAAGATATTTAATCTATTAACTTAGGAAACTAGAAATAAAATTAGCTTCTAACTAAATTTTAAAGCGGTTAAATTCCGTTTTTTCCTTGTTTTAATAGTTTAAANNNAAAAATTTAGATCTTGTAAATCTAAGATAACGTAAGTTTTAAAAATAGGTTTTAAGTTATTAANAACTATTATCCTTCAAAGTTAAAAATATANNNNNNNNNATTATTAAGAGAAATAATTTTTCATAAATAAATTTACAGTTTATTGCCTATTTTCGGCCATCCTAATCAAGTTATATTGATTGAACAATATTCTCATTAGTGTAAATAAAGTGCTAATATAGCTTTAACTTG

>angusta_08.NZ.SC.IDN.12

AATTTATTTTCATCATTTGACCCTTCAACTGGATTTCTATCTCTTAATTGACTAAGATCAATAATTCTACTGTTATTTTTACCTTTAACTTACTGATATATTCCTAATCGTTTTATTCTTTTATATAATAAAATTTTAATTTTATTAAATAATGAATTAAATACATTAATAAATTTTAAATCATTGGGAAGATCACTAATATTTTTATCGTTATTTATATTTATTTTATTAAATAACTTATTGGGATTACTTCCATATATTTTTACAAGTCCAAGTCACTTAGTATTCACAATAAGATTAGCGTTACCACTATGATTATCATTTATACTTTATGGATTTATTAATAATATAAATCATATATTTTGTCATTTAGTTCCATCAGGAACTCCTAACATTTTAATACCTTTTATAGTTATTATTGAAAGAATCAGAAATTTAATTCGTCCTGGTTCCTTAGCTGTTCGACTAACAGCCAATATAATTGCTGGACATCTTTTAATAACTTTATTAGGTAATTTACCCATAAATTACGAATTATATTCAGGTATAATTATTATTTTCCAAGTTATATTAATATTATTTGAATTAGCTGTTTGCATCATTCAATCCTACGTATTCATAGTTCTTAGAACTTTATATTATAGAGAAGTAAATATTCCTCAAATATCTCCTCTTAATTGACTATTATTTATTTATTTTNNNNNNNTAATTATTGTTATTATTATATTTATGTATTTTACTTATTTATTAAAATCTAATATAACAGCCAAAAATAAATTAATAGATAGAATAATTTGATTTAAGCCAACACGACAAAATCATCCTTTAATTAAAATTATTAATAATTCATTAATTGACTTCCCAGCACCATCTAATTTATCCTATTGGTGAAATTTTGGATTTATATTAGGATTGTGTTTAATAATCCAAATTATAACTGGATTATTTTTATCAATACATTACAATGCCAACATTATAAACGCTTTTGAAAGATTAAGACACATTTGTCGTGATGTAAACTATGGTTGAATTTTACGAGTCATTCACGCTAATGGAGCTTCATTATTTTTCATTTGTGTTTATCTACATGTAGGACGTGGATTATATTATGGCTCATTCAAATATATTGAAACATGATCTATTGGTGTAATTATACTATTAATATTAATAGCTACCGCTTTCTTAGGGTATGTTTTACCATGAGGACAAATATCTTTTTGAGGAGCTACAGTTATTACAAATTTATTATCAGCTATTCCTTACTTAGGAAGAATATTAGTTAATTGAATTTGAGGAGGATTTGCAGTTGATAACGCAACATTAACTCGATTTTACTCATTCCATTTTATTTTACCATTTATTGTATTAAGACTAACAATTATTCACTTATTATATCTTCATACAACAGGTTCAAATAATCCATTAGGAATTAATTCTAATAATGACAAAGTTCCTTTTCACCCATACTTCTCCATTAAAGATGTTATGAGACTAATTATCTTGATAATTATTTTCTTTATATTAGTTATATTAGAACCCTACATACTAGGGGATCCAGATAATTTTATTCCTGCCAACCCACTTGTTACACCAAAGCATATTCAACCTGAATGATATTTTTTATTTGCCTACGCAATTCTTCGTTCAATTCCTAATAAACTAGGGGGAGTAATTGCTCTTTTTATATCAATTTTTATTTTAATGTTTGTACCTTTATTAAATAATTCTAATTTTATAGGTTTAAATAATTACCCAATTAATCAAATTATATTTTGATATATAGTAATCATTTTAATTTTATTAACCTGAATTGGAGCACGACCAGTTGAATTGCCTTATATTAACTTTGGAATATTTTTAACACTTATATATTTTTCTTACTTTATTATTGACCCAATAATTAAATCTATTTGAGATAAATTAATTAGATAGATGAAAAAATGATTCTTCTCAACTAATCATAAAGATATTGGAACAATATATTTCATTTTTGGTATTTGATCAGGTATAATTGGTACAACTTTAAGAGTTTTAATTCGTGTAGAACTTGGTACTCCAGGTTCATTTATTGGTGATGATCAAATTTATAATGTAATTGTCACTGCTCATGCTTTCATTATAATTTTTTTTATAGTTATACCAATTATAATTGGTGGATTTGGAAATTGGTTAGTTCCTTTAATAATTGGAGCTCCTGATATAGCTTTTCCTCGTATAAATAATATAAGTTTTTGATTATTACCACCCTCTCTAATTCTTATTTTAGTGGGAAGAATAGTTGATAGAGGTGCAGGCACAGGTTGGACAGTTTACCCTCCCCTATCTGCAGGGATTGCACATTCTGGTTCATGTGTTGATTTAACTATTTTTTCTTTACACCTCGCAGGTGTATCTTCAATTTTAGGTGCTGTAAATTTCATTAGAACAATTTTTAATATACGTTCAATAGGAATTTGATTAGATCGAATACCCTTATTTGTATGAGCAGTTTTAATTACTGCATTTTTATTATTGCTGTCTTTACCTGTTTTAGCAGGTGCTATTACAATGTTATTAACAGATCGAAATTTAAATACTTCATTTTTTGATCCTGCAGGAGGGGGTGACCCCATTCTTTATCAACATTTATTTTGATTTTTTGGTCATCCTGAAGTTTATATTTTAATTTTACCAGGATTTGGTTTGATTTCTCACATTATTACCCAGGAAAGAGGTAAAATTGAATCTTTCGGTTCATTAGGAATAATTTATGCTATAATATCAATTGGTATTTTAGGATTTGTTGTATGAGCACATCATATATTCACTGTAGGAATAGATGTTGATACACGTGCATATTTTACATCAGCCACTATAATTATCGCAGTTCCTACTGGAATTAAAGTATTTAGTTGACTTGCAACTTTGAGAGGTATAAAAATCAATATTACATCTTCAGCATTATGGGCTTTAGGATTTGTCTTTTTATTTACTATTGGAGGTTTAACTGGAGTAATTCTAGCTAATTCTTCAATTGATATTATATTACATGATACATATTATGTAGTTGCACATTTTCATTATGTTTTATCTATAGGAGCGGTTTTTGCTATTATAGCAAGATTTATTCATTGATTCCCATTATTTACTGGATTAAGATTAAACTCAAATTGATTAAAAATTCATTTTCTATTGATATTTATTGGCGTAAACATAACATTTTTTCCTCAACATTTTTTAGGATTAAGAGGTATACCTCGTCGGTATTCAGACTATCCTGATGCCTACATATCATGAAACATAATTTCATCAATAGGAAGAATTATATCTTTAGTTGGAATTTTATTTTTATTATTTATTGTTTGAGAAAGTTTTATTTCTATACGGTTAGTATTATATTCTAATAGAATTCAATCTTCTATTGAATGAATACAAAAGTTTCCCCCATCTGAACATTCTTATAATGAAATGCCATTATTAATTCAAATTTCAAATTGATCTTATATTAATATACAGGATGCTGTATCACCATTGATAGAGCAGTTAATATTTTTTCATGATCATGTATTAATCATTTTAATTATAATTACAATTGTTGTTGCTTACATAATAATTATACTAATATTAAATAAAATTATTAATCGTTTACTTCTTGAAGGACAATTTATTGAATTTATTTGAACTTTGTTACCTGCAATAACTTTAATTTTTATTGCACTACCATCATTGCGATTATTATATATATTAGACGAAATTAATAATCCATTATTAACATTAAAAATTATTGGTCATCAATGATACTGATCATATGAATATTCAGATTTTTCTGATGTAGAATTTGATTCATATATAAAATCCATAAATGAAATAAATAAGAATGAATTTCGTTTATTAGATGTAGATAATCGAGTAATTCTACCATTTAATATTCAAGTCCGACTATTAGTTTCTTCTTTCGATGTTATTCACTCTTGAGCAATACCATCTATGAGACTTAAAGTTGATGCAGTACCAGGCCGACTAAATCAAATAAGAATATTAATTAGTCGGCCTGGTGTATCATATGGACAATGTTCCGAAATCTGTGGTGCAAACCACAGATTTATGCCTATTGTAATTGAGAGAATTAGAATAAAAATATTTATTAAATGATTAATTAATTATATGAATAATCATCCTTATCATATAGTTGATTATAGACCCTGACCTTTAACTGGATCAATTGGAGCATTAACTTTTGTTTCTGGTATAGTTATAATATTTCATAAATGTAACTTTGTATTATTATATATTGGTATTTTACTGTTACTAATAACAATAATTCAATGATGACGTGATATTTCTCGAGAAGGAACATTTTTAGGAATACACACAATAATGGTAGTGAACGGTTTAAAAATAGGTATATTATTATTTATTGTATCAGAAATTCTTTTTTTTGTTTCATTTTTTTGGGGATTTTTCCATAGTAGATTAAGACCTGTAGTAGAAATTGGCATAATTTGACCCCCTAGAGGTATTTATGTCTTTAATCCAACTCAAGTCCCTTTATTAAATACAATAATTTTACTATGTTCAGGTATTACAATTACTTGGGCCCATCATTCAATTATATGTGGCAATCATATTAATTCTATTTATAGAATTATATTAACTGTTATTTTAGGTATATATTTCACTATTCTACAAGGTTATGAATATTATGAAGCTCCATTTGCAATTAATGATTCCATTTATGGATCCTCTTTTTTTATAGCTACTGGATTTCATGGAATCCATGTAATTATTGGAACAAGATTTATTACTGTATGTTTATTACGACAAATAAAGTTTCATTTCTCAAAAAATCATCATTTTGGTTTTGAAGCTGCTGCTTGATATTGACATTTTGTTGATGTAGTTTGACTATTTTTATATTTATCAATTTATTGATGAGGTAGCTAGTAAATTATAGTTTTTAAAAAGAAAAAAAAAATAAATAAATTTAAAGATACTGGTAAATAAGATTTTCAAGCTAAATATATTAATTTATCATACCGATAACGAGGAAGAGTTCCTCGGACTCAAATAAAAAGAAAACACATTATAGAAACTTGAATTGGAAAAACAATTGAACTAATTTTTCCTCCTAAAAATATTAAACAAAATATTATTCTCATAAATAAAATACTTGAATACTCAGCCAAGAAAATAAATGCAAATCTAGAACCTCCATATTCAACATTAAAGCCAGAAACTAATTCTGATTCTCCCTCAGAAAAATCAAATGGAGATCGATTAGTTTCAGCTAAAGCTGAAGATAATCAACATATTCTTAATGGAAGAAATAAAAAAATAAATCACATATACTCCTGAAATATAAACAAATCAATAATATTATAACTTCTTATTATTATTATAGGACATAACATAATTAAAACCAATCTTACTTCATAAGAAATAGATTGAGCAATTGATCGAATACATCCTAGCATTGCATAACTTGAATTAGAAGATCAACCTGTTAATATTAATGAATAAACTCTCATTCTTGAACAACAAAAAAAAAATAAAATACCAAAATTAAAAGAAACACAATTAATTATATATGGATATAATGATCAAATCAACAATCTATTAAATAATCCTATTACAGGCCTAAAAATATAAATTAAATAATTAGATATTAATGGAATTGTACTTTCCTTTATAAATAATTTAATAGCATCAGAAATAGGCTGCAAGATACCCAAAAATCCAACTTTATTTGGACCTTTACGAATTTGAATATATCTCAAAACCTTACGCTCCAAAAGAGTAATAAATGCCACTCCAATCAAAATAAATAATAAAGTAATTAAAATAGTAATTAAATACNNNNNNNNNNNNNNNNNNNNNNNNNNNNNNNNNNNNNNNNNNNNNNNNNNNNNNNNNNNNNNNNNNNNNNNNNNNNNNNNNNNNNNNNNNNNNNNNNNNNNNNNNNNNNNNNNNNNNNNNNNNNNNNNNNNNNNNNNNNNNNNNNNNNNNNNNNNNNNNNNNNNNNNNNNNNNNNNNNNNNNNNNNNNNNNNNNNNNNNNNNNNNNNNNNNNNNNNNNNNNNNNNNNNNNNNNNNNNNNNNNTGATTATACTTATATAATTGTAATTAGATTAATAATTAAAATTGGGTGTCCTCCTTTCCATTTTTGATATGTATCTGTTATTGAGGGTTTAACTTAAATAGTATGTTTTATTTTAATAACTATTCAAAAAATTATTCCTTTAATTATATTGTCATATTTAAATATTAATATAAGATTATTTATTGTAATAGCATGTATTTGAGGATGCATTAGAGGGCTGGGTTATTCTTCAATGCGTAAAATCATTGCTTACTCCTCAATTTATAATTTAAGATGAATTTTTAGAGGTATTGTTATTATTAATTATTCATGGCTGATTTACTATTTTATTTATTCATTTACATTATTAGCTGTATGTTATATATTTAAATTATTTAATATTAATTATATTAATCAATTTATTATAGTATCTTTTAATTTTATAAAGTCAATTATAATAATATGTATTTTTATGTCTATGGGTGGATTACCACCCTTCTTGGGGTTTTTCCCTAAGTTAATCATAATTTACTGTTTACTGTTAAATAACATAATATTTATTTGTATTATGTTGTTAATAACAGCTTTGATTATTTTGTTTTTTTATTTACGAATTTTAATTACAACACTGATAATAAATACAATTTCAATAAAAAGAATTATCATGAGAGTTTCATATACCTATTACATTGCTGGAATATTTTCATTATTTGGAATAATTTTTTTTTCATTAATTACATTAAATATGTGTTAGATCTATAATATTTTGATCTATTCNNTTATTTTAATATTTTTATTAATATTTCTAATATTATTATTGTTATATATTTCTTATAAATCGTTAAAAGATCGAGAAAAATCATCTCCATTTGAGTGTGGTTTTAGGCCATTTGAATCATCTCGTATTCCATTTTCAAGACACTTTTTTTTAATTGCAGTAATTTTTTTAATTTTTGATGTTGAATTGGTAATTATTATACCCGTAATTTTAGTAATAATTAGATTAAAAGTTATTGATATTTATATTATTATATTAATCTTCTTAATTATTTTAACTTTAGGATTATATCATGAATGATATAATAATATATTAAATTGANNNNNNNGTTTAATTAAAATATTTAATTTCAAAATAATTAAATTTAAGGGAATTCAATGTATTATAATCAAGAAAAACTCCCGAACTCTTCCAGAATTAAATGAGAATAAACTAGAAAAAAATATACCATGTTGAGTAAATGAAAATATATATAATCTATAACAAGCAGATAGAAATGATGATAAAATCAATAAAATTATTATTAATATATTTCAAGATATTAATCTATTAATAATTATAATTTCACCAGCTAAATTAATTGTAGGTGGACAGGACATATTTCTTGCCGACATTAAAAATCATAATAAAGATATTGAAGGCATAAATGTCAATATACCCTTATTAATTAATAATCTTCGACTATTAGTTCGCTCATATAATATATTTGCTAAACAAAATAATCCAGAAGAACATAAACCATGTCCAATTATTATATAATATGACCCACATAATCCCCAATTTCTTATAGTTATAATTCCTCTAATTACAAGACCTATGTGGGCCACAGATGAATAAGCAATTATTGATTTAATATCAATCTGAATTATGCATAGAATTCTAATTAAAATACTTCCAATCATTGAAATTGAGATCCATAAAAATCCAAATTTATAAAAATAAGAAGGAATAATATATATTATACGAATTAAACCATATCCTCCTAATTTTAGTAAAATTCTAGCTAAAATTATTGAACCAGAAACAGGAGCTTCAACATGAGCTTTAGGTAATCAAAAATGAAAAAATAATGGTATTTTAATTAAAAAAGCCATTATTATACCAAAATAAATATAAATATTGACATAATAATTTATTAACATAAAAATACTGGTTATATTATTTTTATAAAGAAAAAAAATTCTCATTAACATTGGTAGTGACGCAAATAATGTATAGAATAATAAATAATATCTTGCATCAATACGTTCTGGTTGATAGCCCCAACCTATAATAATAATTATAATTGGAATTAATCTAGACTCAAAACAAATATAAAAAATTATAATTCTTGATGTAGAAAAAGAAAAAATCAAAAAAATTATTAACATAAAACCAAAAATAAAAAAAATATTTTATTACGTTTATACATTGGGCTAGAAACAATTATTAAAAATGAAATCCAAATACAAAATAATTAGACCATAAGATATTTCATCAATATAAAAATTATAGCCTAACATATATAAATTCTTATTTATAAAAACCAAAAATATAATTACCAAAACAATAATTATATTTATTAATCATGAATTATAAATTAATAGGGTTAGTATAAAGCAAAAAAAAACTAATTTTATTGACATAGATATTAATTTATCATTATTATGAGATCGAATTAATATTACTAAACATGATAAACCCATCACTCCTTCACAAACTCTAAAAACCAAGAAAATTAATAAAATATGCAAATCATATATTTTTATAGAAAAAATAAATGATAAAGAACAAAATATTCTTAAAATAATTAATTCCAATCTTAACGTTGTTATTAAATGTTTTCGATTCATACATAATGAAACTATCCCTGAAAAGAACATAGAGATAAAATATATTAATAATGTTATATTCTATAAGTATAAAACAATAATTCAAACAATAAATGAAATTATTTGAATCTTAAAATTATTATTTATCAATTTATAATTTAATGATATTATTATTTTTAAAGTTTTTAAAATTCCTTGGGGTCCAAGGTTTTCACCTCATCCCATATCAATTGATTTTTGAAATATAAGTGATTTACTTATTAATAACATTTGATTATGAAACGTTGACAATTGTTTTATAAATCACATTGATCCAAAAAATTCATAAACAATTTTTATTTTAGTTTTAGAATAAATTGACATTTCATAACCCAATCAAATTCCTAATATTGAAAATATTAATGCCAATAATTTACCCTCAATAGGTATAAAAATTATAATTGGATCATTAAATATTAATCATCTTAATATAGATCCCGAAAAAATTGAGTAAATAGATAAAATAATAATTCTCTTAATTATATTATTTATATTTTCACTTAATAATCTTAATTTATAGAAATTGTAATTTATATTTATTGAAAAAAATGTCAAACGGGCAGAATAAAAAGAAGTTAAACCAATACCAATATATATAAATATTATAGTTAATAAATTAATATGTCTAGAACATATAGTTTCTATAATTAAATCTTTTGAATAAAATCCTCTCATAAATGGAATTCCACATAATGACATATTAGCAATATTTATTATCGTAATAGTTAATGGCATTTGAAATCTTAAACATCCTATTACACGAATATCTTGATTATTATTAAAATTGTGAATCAAAATGCCTGCACACAAAAATAAAGTAGCTTTAAATAAAGCATGAACAATTAAATGAAAAAACGATAATATTGGATAACCAAATATAATAATTGTTATTATAATCCCTAACTGTCTCAAAGTTGATAATGCAATAATTTTTTTTAAATCAAACTCAAAATTTGCTCTAATTCCTGATATTAATATAGTTAAAATTGAAATTAACATAAATACTTGAATATAATCAAAATTCAAGATAATATTAGAAAACCGAATTATTAAGTATACTCCAGCAGTGACAAGTGTAGATGAATGAACCAAAGCAGATACAGGTGTTGGAGCAGCCATAGCTGCTGGTAATCATGAAGAAAATGGAATTTGAGCTCTCTTTGTAAATCTTGCAATAATAATCATAATCAATATAATTTATATCTTTATTAATAAAAGATAAAAATTTCATGACCCAAAATTTAATATTCATGCAATAGCTATTAAAATAGCAACATCTCCCACACGATTCATTAATGCAGTTAATATTCCTGCATTATTAGAATATAAATTTTGATAATAAATAACTAAACAATAAGAAACTAATCCAAGTCCATCTCAACCAATAATAATTCTCATTATATTAGGTCTAATAATTAATAAGACCATAGATATAACAAATCCTAAAACAATATAAATAAAACGATTAATAAATTTGTCATTATTTATATATATTCCACTATATAAAACAACCACTGATGAAATAAATATAACTGTTGAAAGAAATTGTTGATATAAAATCAAAAATTATAGTCATATAAATATTACATGAATTAATTGAAAATTATTCATTCCAATATAAATATATAATTATTTTTGAAATTCATTGAATATTAATATTAATAGACTTAGGAATATAAGAATAATAAATATATAAACATTAAACTTTTTATTTATCATGAAAATTACTATGTATTTAATAGTTATTTTATCAATAAATTTTATTTTTATAAAGCACCCTTTATCAATAGGATTAATTTTAATAATACAAACAGTATTGTCTTGCTTAATTTGTAGACTTTATTTAAGTTGTTATTTATTTTCTTATATTTTATATCTTATTTTTATTGGTGGTATATTAATTTTATTTATATATATATCAAGAATTGCATCAAATGAAAAATTTATTTACTCCATTAAACTAATAATGTTTAATTTTTTATTTTTAACTTTAATTAATTTAATCAATATAATTGATTTAAAATCACTAAATATTAGAAGAAATATAATAATATATATAAACCACAACAACTTTATAATAAGAAAAATATATATTATTCCTTCTGGAATAATAACACTAATTTTAACAATTTATTTACTATTTGTTTTAATTATTGTAATTAATATTTTAACAATAAATATATTAACTCTTCGAAGAAGAGTCTCTTTCTAACTACACCTTCCGGTACAGTTACTTTGTTACGACTTATCTCATTAATATTATGAGAGTGACGGGCGATATGTACATAAATTAGAGCCAATTTCAATTAATTAAATTTAATTAATTTATTATCAAATCCAATTTCATATTTCTTNNAACTTAAAAAATAATTCAATAAATAGCTAATTGTAACCCATTTCTTCTTTAATATAAACTGCACCTTGACCTGACATTATATATAANGTAATATAGTATGAAAATATTTCTTATAAAACATTCTTGACAGAGATATACAAATTAAATTAAAGTTTTTTCTATCGTGGACTATCAATTATAAAACAGGTTCCTCTGATAAGATAAATTACCGCCAAATTCTTTGAATTTAAAGATCATTTCTAATAATAATCAAGTTATTTTTATCACATTTTTAATAATAGGGTATCTAATCCTAGTTTAAACAAAAATTTTTCAGACATAAAATTAATTTTTCAAGATAAAATATATTTCACCAAAATTATAGATATTTTATATTATTGTTATATTAACTGAAACTTAACTATTTAAATTAAAGAAATCGTATAACCGCAACTGCTGGCACGAAATTTGATTCTTTTAAATAAAATTACTAATTCTAATATTATTAATTAATAATAATAAATACTGCGCATTTATTCAAANTATTTCCATTTAGAAATTAAAAACAATTAAAAAATTGCATGTAATATAATTTTAAATTTAAATTATANTAAACCAGAATTTAACTTCAAATAATAAAATTATTTAATGAGGTCCTTTCGTACTAACATTAAAAATAATTGAATAGATAGAAACCAACCTGGCTCACGCCGGTCTGAACTCAGATCATGTAAGAATATTAAGGGTCGAACAGACCCAGAAATAATAAATTTTGCTCCAATCCCTATTCTTAATCCAACATCGAGGTCGCAATCATATTTATCGATATGAACTCTTCAAATTAATTACGCTGTTATCCCTAAGGTAATTTATTCTTATAATCAAAAATTTGGATCAATATTCACATAAATTTATGAAATTTTTATTAAAAGTTAATTATATTTTAATATCACCCCAACAAAAAAATTAATTAAATAAAAAAAATTAATTAACTATAAANAAATAAATTTAATNAAAATTTTAAAAATTCTATAGGGTCTTCTCGTCCCACTCACATATTTAAGCTTTTTTACTTAAAAATCAATTTCAATTATTAATATTAATTAAGTTAATTTCTCATTCAATCATTCATTCAAGCCTCCAATTAAAAGACTATTTATTATGCTACCTTTGCACGGTCAATTTACCGCGGCCATTTAATTTATAATCATTGGGCAGATTAGACTTTTAATTCATTCTAAAAGACATGTTTTTGATAAACAGGTGAAAATTTTTTTTGCCTAAATTATAAATATTATTTACAACCAAAAAACCAGATATCATATAATTTGATAAAATATCATTTCCAGAATAAATTTATTAATATATATACAATAATAACTAACAAATNTAAACTAAATCATTATATTTCGGGAAAAATAAATACTTAAAAGTCTTCAATTAACCCTGATACAAAAGGTACAAATTATATTCTACTTAAAACTATTTATAAAGCTNTTNCCCTATCGGTCAATCCAAATTATTTTTTTATATAAAATTACTTTAACACAAAAATTTTTTTTTTGAAATGTNAAAAAACAAATTTTANTGAAATTATAATATGGGATATATTTAATTATAATAATTGAATTGCAATCAATAGGTGTTATCTATAACTATCCTTAAGTAATGAAGTAAAATAATACATTTAGTTTCGACCTAAAATAAGAATTANAATTCCTTGCTTTTGAATGAAGCCAAACTAGAGGCATTTTATTGTTAATAAAATAATTGAAANTTTATTTCCATTTAAAGAAAAATTAGTTTAACTAAAATATAAATCTGTCAGATTTATGAAACTTTTGAGTATTTTTCTGACTTTTTGTAGNNTNTTCTACTTTAAATTTGCAATTTAATATTATAAATTAAATATAAGACTNNNNNNNNNNNNNNNNNNNNNNNNNNNNNNNNNNNNNNNNNNNNNNNNNNNNNNNNNNNNNNNNTTTATAAAGTTTATAAAACATTTCATTTTCATTGAAAAGAGAGACTTAGTCTTATAAAATTCTATTAGTATATGAGTATATTTAACTTCCAATTAAAAGGATTAATTTTATTAAATAGAGTATATCCAATAAATCTNNTTTTATCTTTAAAATCACAATTTAATATTTTTATTAAACTAATTGAATTACTATTTGTAATAAATATTACATTATTAAATTCTAAATTTAAGGCATTAATTTTGCTAAAATAGTTCTAATATGGCAGATTAGTGCAATGAATTTAAGATTCATATATAAAATATNATTTTTTTATTGGAANNNNNNNNNNNNNNNNNNNNNNNNNNNNNNNNNNNNNNNNNNNNNNNNNNNNNNNNNNNNNNNNNCATTAAGTGGCTGAAAGTAAGTAATGGTCTCTTAAACCAATTAATAGTAATTAACAAATACTCTTAATGNNNNNNNNNNNNNNNNNNNNNNNNNNNNNNNNNNNNNNNNNNNNNNNNNNNNNNNNNNNNNNNNNTTTAAAAACAAAAGTTACCTTAATATCTTCAATATTATGCTCTTAATTAAGCTATTTAAATTCAGAAAAGATAAAACAATCATTTTAAACTTCCAAAGTTTATATTTTATATTTAAACTATTTTCTGAGTTAATTAGCTTAACCNAAAGCATTTATTTTGAAAGTAAAAGAAAAGATANTTAATCTATTAACTTAGGAAACCAGAAATAAAATTAGCTTCTAACTAACTTTTAAAGCGGTTAAATTCCGTTTTTTCCTTGTTTTAATAGTTTAATNNNAAAAATTTAGATCTTGTAAATCTAAGATAACGTAAGTTTTAAAAATAGGTTTTAAGTTATTAANAACTATTATCCTTCAAAGTTAAAAATATAATTANATTTATTATTAAGAGAAATAATTTTTCATAAATAAATTTACAGTTTATTGCCTATTTTCAGCCATCCTAATCAAGTTATATTGATTGAACAATATTCTCATTAGTGTAAATAAAGTGCTAATATAGCTTTAACTCG

>murihikua_08.NZ.OL.RSC.01

AATTTATTTTCATCATTTGACCCTTCAACTGGATTTATATCTCTTAATTGACTAAGATCAATAATTCTACTGTTATTTTTACCTCTAACTTACTGATATATTCCTAATCGTTTTATTCTTTTATATAATAAAATTTTAATTTTATTAAACAATGAATTAAATACATTAATAAATTTTAAATCATTAGGAAGATCATTAATATTTTTATCATTATTTATATTTATTTTATTAAATAACTTATTGGGATTACTTCCATATATTTTTACAAGTCCAAGTCACTTAGTATTCACAATAAGATTAGCATTACCACTATGATTATCATTTATACTTTATGGATTTATTAATAATATAAATCATATATTTTGTCATTTAGTTCCATCAGGAACTCCTAATATTTTAATACCTTTTATAGTTATTATTGAAAGAATCAGAAATTTAATTCGTCCTGGTTCCTTAGCTGTTCGACTAACAGCTAATATAATTGCTGGACATCTTTTAATAACTTTATTAGGTAATTTACCCATAAATTATGAATTATATTCAGGCATAATTATTATTTTCCAAGTTATATTAATATTATTTGAATTAGCTGTTTGCATCATTCAATCCTATGTATTCATAGTTCTTAGAACTTTATATTATAGAGAAGTAAATATTCCTCAAATATCTCCTCTTAATTGACTATTATTTATTTATTTTNNNNNNNCAATAATTGTTATTATTATATTTATGTATTTTACTTGTTTATTAAAATCTGATATAACAGTCAATAAAAAATTAATAGATAGAGTAATTTGATTTAAGCCAACACGACAAAATCATCCTTTAATTAAAATTATTAATAATTCATTAATTGACTTCCCAGCACCATCTAATTTATCCTATTGGTGAAATTTTGGATTTGTATTAGGATTGTGTTTAATAATCCAAATTATAACTGGATTATTTTTATCAATACATTATAGTGCCAACATTATAAACGCTTTTGAAAGATTAAGACACATTTGTCGTGATGTAAACTATGGTTGAATTTTACGAGTCATTCACGCTAATGGAGCTTCATTATTTTTCATTTGTGTTTATCTACATGTAGGACGTGGATTATATTATGGCTCATTCAAATATATTGAAACATGATCTATTGGTGTAATTATTCTATTAATATTAATAGCTACCGCTTTCTTAGGATATGTTTTACCATGAGGACAAATATCTTTTTGAGGAGCTACAGTTATTACAAATTTATTATCAGCTATTCCTTACTTAGGAAGAATATTAGTTAATTGAATTTGAGGAGGATTTGCAGTTGATAACGCAACACTAACTCGATTTTACTCATTCCACTTTATTTTACCATTTATTGTATTAAGACTAACAATTATTCACTTATTATATCTTCATACAACAGGTTCAAATAATCCATTAGGAATTAATTCTAATAATGACAAAGTTCCTTTTCACCCATACTTTTCCATTAAAGATGTTATAAGACTAATTATCTTAATAATTATTTTCTTTATATTAATTATATTAGAACCCTACATACTAGGGGATCCAGATAATTTTATTCCTGCCAACCCACTTGTTACACCAAAGCATATTCAACCTGAATGATATTTTTTATTTGCCTACGCAATTCTTCGTTCAATTCCTAATAAACTAGGGGGAGTAATTGCTCTTTTTATATCAATTTTTATTTTAATGTTTGTACCTTTATTAAATAATTCTAATTTTATAGGTTTAAATAATTACCCAATTAATCAAATTATATTTTGATATATAGTAATCATTCTAATTTTATTAACCTGAATTGGAGCACGACCAGTTGAATTGCCTTATATTAACTTTGGAATATTTTTAACACTTATATATTTTTCTTACTTTATTATTGACCCAATAATTAAATCTATTTGAGATAAATTAATTAGATAGATGAAAAAATGATTCTTCTCAACTAATCATAAAGATATTGGAACAATATATTTCATTTTTGGTATTTGATCAGGTATAATTGGTACAACTTTAAGAGTTTTAATTCGTGTAGAACTTGGAACTCCAGGTTCATTTATTGGTGATGATCAAATTTATAATGTAATTGTCACTGCTCATGCTTTCATTATAATTTTTTTTATAGTTATACCAATTATAATTGGTGGATTTGGAAATTGGTTAGTTCCTTTAATAATTGGAGCTCCTGATATAGCTTTTCCTCGTATAAATAATATAAGTTTTTGATTATTACCACCCTCTCTAATTCTTATTTTAGTGGGAAGAATGGTTGATAGAGGTGCAGGCACAGGTTGAACAGTTTACCCCCCCCTATCTGCAGGAATTGCACATTCTGGTTCATGTGTTGATTTAACTATTTTTTCTTTACACCTCGCAGGTGTATCTTCAATTTTAGGTGCTGTAAATTTCATTAGAACAATTTTTAATATACGTTCAATAGGAATTTGATTAGATCGAATACCCTTATTTGTATGAGCAGTTTTAATTACTGCATTTTTATTATTGCTGTCTTTACCTGTTTTAGCAGGTGCTATTACAATGTTATTAACAGATCGAAATTTAAATACTTCATTTTTTGACCCTGCAGGAGGGGGTGACCCAATTCTTTATCAACATTTATTTTGATTTTTTGGTCATCCTGAAGTTTATATTTTAATTTTACCAGGATTTGGTTTAATTTCTCATATTATTACCCAGGAAAGAGGTAAAATTGAATCTTTCGGTTCATTAGGAATAATTTATGCTATAATATCAATTGGTATTTTAGGATTTGTTGTATGAGCACACCATATATTCACTGTAGGAATAGATGTTGATACACGTGCATATTTTACATCAGCCACTATAATTATCGCAGTTCCTACTGGAATTAAAGTATTTAGTTGACTTGCAACTTTGAGAGGTATAAAAATCAATATTACATCTTCAGCATTATGGGCTTTAGGATTTGTCTTTTTATTTACTATTGGAGGTTTAACTGGAGTAATTCTAGCTAATTCTTCAATTGATATTATATTACATGATACATATTATGTAGTTGCACATTTTCATTATGTTTTATCTATAGGAGCGGTTTTTGCTATTATAGCAAGATTTATTCATTGATTCCCATTATTTACTGGATTAAGATTAAACTCAAATTGATTAAAAATTCATTTTCTATTGATATTTATTGGTGTAAACATAACATTTTTTCCTCAACATTTTTTAGGATTAAGAGGTATACCTCGTCGGTATTCAGATTATCCTGATGCCTATATATCATGAAACATAATTTCATCAATAGGAAGAATTATATCTTTAGTTGGAATTTTATTTTTATTATTTATTGTTTGAGAAAGTTTTATTTCTATACGGTTAGTATTATATTCTAATAGAATTCAATCTTCTATTGAATGAATACAAAAGTTTCCCCCATCTGAACATTCTTATAATGAAATGCCATTATTAATTCAAATTTCAAATTGATCTTATATTAATATACAGGATGCTGTATCACCATTGATAGAGCAGTTAATATTTTTTCATGATCATGTATTAATCATTTTAATTATAATTACAATTGTTGTTGCTTACATAATAATTATACTAATATTAAATAAAATTATTAATCGTTTACTTCTTGAAGGACAATTCATTGAATTTATTTGAACTTTGTTACCTGCAATAACTTTAATTTTTATTGCACTACCATCATTGCGATTATTATATATATTAGACGAAATTAATAATCCATTATTAACATTAAAAATTATTGGTCATCAATGATACTGATCATATGAATATTCAGATTTTTCTGATGTAGAATTTGATTCATATATAAAATCCATAAATGAAATAAATAAGAATGAATTTCGTTTATTAGATGTAGATAATCGAGTAATTCTACCATTTAATATTCAAGTCCGACTATTAGTTTCTTCTTTCGATGTTATTCACTCTTGAGCAATACCATCCATGAGACTTAAAGTTGATGCAGTACCAGGCCGACTAAATCAAATAAGAATATTAATTAGTCGGCCTGGTGTATCATATGGACAATGTTCCGAAATCTGTGGTGCAAACCACAGATTTATGCCTATTGTAATTGAGAGAATTAGAATAAAAATATTTATTAAATGATTAATTAATTATATGAATAATCATCCTTATCATATAGTTGATTATAGACCCTGACCTTTAACTGGATCAATTGGAGCATTAACTTTTGTTTCTGGTATAGTTATAATATTTCATAAATGTAACTTTATATTATTATATATTGGTATTTTACTATTACTAATAACAATAATTCAATGATGACGTGATATTTCTCGAGAAGGAACATTTTTAGGAATACACACAATAATGGTAGTGAACGGTTTAAAAATAGGTATATTATTATTTATTGTATCAGAAGTTCTTTTTTTTGTTTCATTTTTTTGGGGATTTTTCCATAGTAGATTAAGACCTGTAGTAGAAATTGGCATAATTTGACCCCCTAGAGGTATCTATGTCTTTAATCCAACTCAAGTCCCTTTATTAAATACAATAATTTTATTATGTTCAGGTATTACAATTACTTGGGCTCATCATTCAATTATATGTGGCAATCATATTAATTCTATTTATAGAATTATATTAACTGTTATTTTAGGTATATATTTCACCATTCTACAAGGTTATGAATATTATGAAGCTCCATTTGCAATTAATGATTCCATCTATGGATCCTCTTTTTTTATAGCTACTGGATTTCATGGAATCCATGTAATTATTGGAACAAGATTTATTACCGTATGTTTATTACGACAAATAAAGTTTCATTTCTCAAAAAATCATCATTTTGGTTTTGAAGCTGCTGCTTGATATTGACATTTTGTTGATGTAGTTTGACTATTTTTATATTTATCAATTTATTGATGAGGTAGCTAGTAAATTATAGTTTTTAAAAAGAAAAAAAAAATAAATAAATTTAAAGATACTGGTAAATAAGATTTTCAAGCTAAATATATTAATTTATCATACCGATAACGAGGAAGAGTTCCTCGGACTCAAATAAAAAGAAAACACATTATAGAAACTTGAATTGGAAAAATAATTGAACTAATTTTTCCTCCTAGAAATATTAAACAAAATATTATTCTCATAAATAAAATACTTGAATACTCAGCCAAGAAAATAAATGCAAATCTAGAACCTCCATATTCAACATTAAAGCCAGAAACTAATTCTGATTCTCCCTCAGAAAAATCAAATGGAGATCGATTAGTTTCAGCTAAAGCTGAAGATAATCAACATATTCTTAATGGAAGAAATAAAAAAATAAATCATATAAACTCCTGAAATATAAACAAATCAATAATATTATAACTTCTTATTATTATTATAGGACATAATATAATTAAAACCAATCTTACTTCATAAGAAATAGATTGAGCAATTGATCGAATACATCCTAGCATTGCATAACTTGAATTAGAAGATCAACCTGTTAATATTAATGAATAAACTCTCATTCTTGAACAACAAAAAAAAAATAAAATACCAAAATTAAAAGAAACACAATTAATTATATATGGATATAATGATCAAATCAACAATCTATTGAATAATCCTATTACAGGCCTAAAAATATAAATTAAATAATTAGATATTAATGGAATTGTATTTTCCTTTATAAATAATTTAATAGCATCAGAAATAGGCTGCAAGATACCCAAAAATCCAACTTTATTTGGACCTTTACGAATTTGAATATATCTCAAAACCTTACGCTCCAAAAGAGTAATAAATGCCACTCCAATCAAAATAAATAATAAAGTAATTAAAATAGTAATTAAATACAAAAATAATTCTTCTTATTTACTTTATATAATTTTCTTACTATTAGGAATTATAATTTCAGTTTCTTCAAACAATTGGCTGGGTTGTTGAATAGGAATTGAAATAAATATAGTTTCATTTTTGCCCATAATGGCAAATAAAATAAGAATTTATGCTTCAGAATCAATAATCAAATACTTTATTATCCAAAGAATGGGATCGAGTCTATTATTAATAACTATTATTATNNNNNNNNNNNNNNNTGATTTAAATTATATAATTATAATTAGATTAATAATTAAAATTGGCTGTCCTCCTTTCCATTTTTGATATGTATCTGTTATTGAAGGTTTAGCTTGAATAGTATGTTTTATTTTAATAACTATTCAAAAGATTATTCCTTTAATTATGTTATCATATTTAAATGTTAATATAAGATTATTTATTGTTATATCATGTATATGAGGGTGCATTGGAGGGCTGGGCTATTCTTCAATGCGTAAAATCATTGCTTACTCCTCAATTTATAATTTAAGATGAATTTTTAGAGGTATTGTTATTATTAATTATTCATGGCTGATTTACTATTTTATTTATTCATTTACATTATTAGCTGTATGTTATATATTTAAATTATNNNNNNTTAATTATATTAATCAATTTATTATAGTATCTTTTAATTTTATAAAGTCAATTATAATAATATGTATTTTTATGTCTATGGGTGGTTTACCACCCTTCTTGGGGTTTTTCCCTAAATTAATCATAATTTACTGTTTACTGCTAAATAACATAATATTTATTTGTATTATACTATTAATAACAGCTTTGATTATTTTGTTTTTTTATTTACGAATTTTAATTACAACACTGATAATAAATACAATTTCAATAAAAAGAATTATCATGAGAGTTTCATATACCTATTACATTGCTGGAATATTTTCATTATTTGGAATAATTTTTTTTTCATTAATTACATTAAATATGTGTTAGATCTATAATATTTTGATCTATTCNNTTATTTTAATATTTTTATTAATATTTCTAATATTNNNNNNNNNNNNTATTTCTTATAAATCTATAAAAGATCGAGAAAAATCATCCCCATTTGAGTGTGGTTTTAGGCCATTTGAATCATCTCGTATTCCATTTTCAAGACACTTTTTTTTAATTGCAGTAATTTTTTTAATTTTTGATGTTGAATTGGTAATTATTATACCCGTAATTTTAGTAATAATTAGATTAAAAGTTATTGATATTTATATTATTATATTAATCTTCTTAATTATTTTAACTTTAGGATTATATCATGAATGATATAATAATATATTAAATTGANNNNNNNGTTTAATTAAAATATTTAATTTCAAAATAATTAAATTTAAGGGAATTCAATGTATTATAATCAAGAAAAACTCCCGAACTCTTCCAGAATTAAATGAGAATAAACTAGAAAAAAATATACCATGTTGAGTAAATGAAAATATATATAATCTATAACAAGCAGATAGAAATGATGATAAAATCAATAAAATTATTATTAATATATTTCAAGATATTAATCTATTAATAATTATAATTTCACCAGCTAGATTAATTGTAGGTGGACAAGACATATTTCTTGCCGACATTAAAAATCATAATAAAGATATTGAAGGCATAAATGTCAATATACCCTTATTAATTAATAATCTTCGACTATTAGTTCGCTCATATAATATATTTGCTAAACAAAATAATCCAGAAGAACATAAACCATGTCCAATTATTATATAATATGACCCACATAATCCCCAATTTCTTATAGTTATAATTCCTCTAATTACAAGACCTATGTGGGCCACAGATGAATAAGCAATCATTGATTTAATATCAATCTGAATTATACATAAAATTCTAATTAAAATACTTCCAATCATTGAAATTGAGATCCATAAAAATCCAAATTTATAAAAATAAGAAGGAATAATATATATTATACGAATTAAACCATAACCTCCTAATTTTAGTAAAATTCTAGCTAAAATTATTGAACCAGAAACAGGAGCTTCAACATGAGCTTTAGGTAATCAAAAATGAAAAAATAGTGGTATTTTAATTAAAAAAGCCATTATTATACCAAAATAAATATAAATATTGACAGAATAATTTATTAACATAAAAACACTAGTTATATTATTTTTATAAAGAAAAAAAATTCTCATTAACATTGGTAGTGACGCAAATAATGTATAGAATAATAAATAATATCTTGCATCAATACGTTCTGGTTGATAACCCCAACCTATAATAATAATTATAATTGGAATTAATCTAGACTCAAAACAAATATAAAAAATTATAATTCTTGATGTAGAAAAAGAAAAAATCAAAAAAATTATCAACATAACACCAAAAATAAAAAAAATATTTTATTACGTTTATATATTGGGCTAGAAACAATTATTAAAAATGAAATCCAAATTCAAAATAATTAGACCATAAGATATTTCATCAATATAAAAATTATAGCCTAACATATATAAATTCTTATTTATAAAAACCAAAAATATAATTATCAAAATAATAAGTATATTTATTAATCATGAATTATAAATTAATAGGGTTAATATAAAGCAAAAAAAAACTAATTTTATTGACATAGATATTAATTTATCATTATTATGAGATCGAATTAATATTACTAAACATGATAAACCCATCACCCCTTCACAAACTCTAAAAACCAAGAAAATTAATAAAATATGCAAATCATATATTTTTATAGAAAAAATAAATGATAAAGAACAAAATATTCTTAAAATAATTAATTCCAATCTTAACGTTGTTATTAAATGTTTTCGATTCATACATAATGAAACTATCCCTGAAAAGAACATAGAGATAAAATATATTAATAATGTTATATTCTATAAATATAAAACAATAATTCAAACAATAAATGAAATTATTTGAATCTTAAAATTATTATTTATCAATTTATAATTTAATGATATTATTATTTTTAAAGTTTTTAAAATTCCTTGGGGTCCAAGGTTTTCACCTCATCCCATATCAATTGATTTTTGAAGTATAAGTGATTTACTTATTAATAACATTTGATTATGAAAAGTTGACAATTGTTTTATAAATCACATTGATCCAAAAAATTCATAAACAATTTTTATTTTAGTTTTAGAATAAATTGACATTTCATAACCCAATCAAATTCCTAATATTGAAAATATTAATGCCAATAATTTACCCTCAATAGGTATGAAAATTATAATTGGATCATTAAATATTAATCATCTTAATATAGATCCCGAAAAAATTGAGTAAATAGATAAAATAATAATTCTCTTAATTATATTGTTTATATTTTCACTTAATAATCTTAATTTATAAAAATTGTAATTTATATTTATTGAAAAAAATGTCAAACGGGCAGAATAAAAAGAAGTTAAACCAATACCAATATATATAAATATTATAATTAATAAATTAATATGTCTAGAACATATAGTTTCTATAATTAAATCTTTTGAATAAAATCCTCTCATAAATGGAATTCCACATAATGACATGTTAGCAATATTTATTATCGTAATAGTTAATGGTATTTGAAATCTTAAACATCCTATTACACGAATATCTTGATTATTATTAAAATTGTGAATCAAAATGCCTGCACACAAAAATAAAGTAGCTTTAAATAAAGCATGAACAATTAAATGAAAAAACGATAATATTGGATAACCAAATATGATAATTGTTATTATAATCCCTAACTGTCTCAAAGTTGATAATGCAATAATTTTTTTTAAATCAAACTCAAAATTTGCTCTAATTCCTGATATTAATATAGTTAAAATTGAAATTAATATAAATACTTGAATATAATCAAAATTCAAGATAACATTAGAAAACCGAATTATTAAGTATACTCCAGCAGTGACAAGTGTAGATGAATGAACCAAAGCAGATACAGGTGTTGGAGCAGCCATAGCTGCTGGTAATCATGAAGAAAATGGAATTTGAGCTCTTTTTGTAAATCTTGCAATAATAATCATAATCAATATAATTTATATCTTTATTAATAAAAGATAAAAATTTCATGACCCAAAATTTAATATTCATGCAATAGCTATTAAAATAGCAACATCTCCCACACGATTCATTAATGCAGTTAATATTCCTGCATTATTAGAATATAAATTTTGATAATAAATAACTAAGCAATAAGAAACTAATCCAAGTCCATCTCAACCAATAATAATTCTCATTATATTAGGTCTAATAATTAATAAGATCATAGATATAACAAATCCTAAAACAATATAAATAAAACGATTAATAAATTTGTCATTATTTATATATATTCCACTATATAAAACAACCACTGATGAAATAAATATAACTGTTGAAAGAAATTGTTGATATAAAATCAAAAATTATAGTCATATAAATATTACATGAATTAATTGAAAATTATTCATTCCAATATAACTATGTAATTATTTTTGAAATTCATTGAATATTAATATTAATAGACTTAAGAATATAAGAATAATAAATATATAAACATTAAACTTTTTATTTATCATGAAAATTGCTATGTATTTAATAGTTATTTTATCAATAAATTTTATTTTTATAAAGCACCCTTTATCAATAGGATTGATTTTAATAATACAAACATTATTGTCTTGCTTAATTTGTAGACTTTATTTAAGTTGTTATTTATTTTCTTATATCTTATATCTTATTTTTATTGGTGGTATATTAATTTTATTTATATATATATCAAGAATTGCATCAAATGAAAAATTTATTTACTCCATTAAACTAATAATGTTTAATTTTTTATTTTTAACTTTAATTAATTTAATCAATATAATTGATTTAAAATCACTAAATATTAGAAGAAATATAATAATATACATAAACCACAACAACTTTATAATAAGAAAAATATATATTATTCCTTCCGGAATAATAACACTAATTTTAACAATTTATTTGCTATTTGTTTTAATTATTGTAATTAATATTTTAACAATAAATATACTAACTCTTCGAAGAAGAGCCTCTTTCTAACTACACCTTCCGGTACAGTTACTTTGTTACGACTTATCTCATTAATATTATGAGAGTGACGGGCGATATGTACATAAATTAGAGCCAATTTCAATTAATTAAATTTAATTAATTTATTATCAAATCCAATTTCATATTTCTTNNAACTTAAAAAATAATTCAATAAATAACTAATTGTAACCCATTTCTTCTTTAATATAAACTGCACCTTGACCTGACATTATATATAANGTAATATAGTACGAAAATATTTCTTATAAAACATTCTTGACAGAGATATACAAATTAAATTAAAGTTTTTTCTATCGTGGACTATCAATTATAAAACAGGTTCCTCTGATAAGATAAATTACCGCCAAATTCTTTGAATTTAAAGATCATTTCTAATAATAATCAAGTTATTTTTATCACATTTTTAATAATAGGGTATCTAATCCTAGTTTAAACAAAAATTTTTCAGACATAAAATTAATTTTTCAAGATAAAATATATTTCACCAAAATTATAGATATTTTATATTATTGTTATATTAACTGAAACTTAACTATTTAAATTAAAGAAATCGTATAACCGCAACTGCTGGCACGAAATTTGATTCTTTTAAATAAAATTACTAATTCTAATATTATTAATTAATAATAATAAATACTGCGCATTTATTCAAANTATTTCCATTTAGAAATTAAAAACAATTAAAAAATTGCATGTAATATAATTTTAAATTTAAATTATANTAAACCAGAATTAAACTTCAAATAATAAAATTATTTAATGAGGTCCTTTCGTACTAACATTAAAAATAATTGAGTAGATAGAAACCAACCTGGCTCACGCCGGTTTGAACTCAGATCATGTAAGAATATTAAGGGTCGAACAGACCCAGAAATAATAAATTTTGCTCCAATCCCTATTCTTAATCCAACATCGAGGTCGCAATCATATTTATCGATATGAACTCTCCAAATTAATTACGCTGTTATCCCTAAGGTAATTTATTCTTATAATCAAAAATTTGGATCAATATTCACATAAATTTATGAAATTTTTATTAAAAGTTAATTATATTTTAATATCACCCCAACAAAAAAATTAATTAAATAAAAAAAATTAATTAACTATAAANAAATAAATTTAATNAAAATTTTAAAAATTCTATAGGGTCTTCTCGTCCCACTCACATATTTAAGCTTTTTTACTTAAAAATCAATTTCAATTATTAATATTAATTAAGTTAATTTCTCATTCAATCATTCATTCAAGCCTCCAATTAAAAGACTATTTATTATGCTACCTTTGCACGGTCAATTTACCGCGGCCATTTAATTTATAATCATTGGGCAGATTAGACTTTTAATTCATTCTAAAAGACATGTTTTTGATAAACAGGTGAAAATTTTTTTTGCCTAAATTATAAATATTATTTACAACCAAAAAACCAGATATCATATAATTTGATAAAATATCATTTCCAGAATAAATTTATTAATATATATACAATAATAACTAACAAATNTATACTAAATCATTATATTTCGGGAAAAATAAATACTTAAAAGTCTTCAATTAACCCTGATACAAAAGGTACAAATTATATTCTACTTAAAACTATTTATAAAGCTNTTNCCCTATCGGTCAATCCAAATTATTTTTTTATATAAAATTACTTTAACACAAAAATTTTTTTTTTGAAATGTAAAAAAACAAATTTTANTGAAATTATAATATGGGATATATTTAATTATAATAATTGAATTGCAATCAATAGGTGTTATCTATAACTATTCTTAAGTAATGAAGTAAAATAATACATTTAGTTTCGACCTAAAATAAGAATTANAATTCCTTGCTTTTGAATGAAGCCAAACTAGAGGCATTTTATTGTTAATAAAATAATTGAAANTTTATTTCCATTTAAAGAAAAATTAGTTTAACTAAAATATAAATCTGTCAGATTTATGAAACTTTTGAGTATTTTTCTGACTTTTTGTAGNNTNTTCTACTTTAAATTTGCAATTTAATATTATAAATTAAATATAAGACTTTATGTTAGCAGAATTAAACTGTCTATTAATATCAAAAATTAATGTTCTTCATAAACTATAACATTTATAAAGTTTATAAAACATTTCATTTTCATTGAAAAGAGAGACTTAGTCTTATAAAATTCTATTAGTATATAAGTATATTTAACTTCCAATTAAAAGGATTAATTTTATTAAATAGATTATATCCAATAAATTTNNTTTTATCTTTAAAATCACAATTTAATATTTTTATTAAACTAATTGAATTACTATTTGTAATAAATATTACATTATTAAATTCTAAATTTAAGGCATTAATTTTGCTAAAATAGTTCTAATATGGCAGATTAGTGCAATGAATTTAAGATTCATATATAAAATATNATTTTTTTATTGGAAAATAAGGTGCCTGAATNAAAAAGGATTATTTTGATAGAATAAATTATGTAATAAAATTACTCTTACATTAAGTGGCTGAAAGTAAGTAATGGTCTCTTAAACCAATTAATAGTAATTAACAAATACTCTTAATGAAAGATAAGCTNAAANTAAAGCTAATGGGTTCATACCTCATTTATGGAAANTCTTCCTCTTTTTATTTAAAAACAAAAGTTACCTTAATATCTTCAATATTACGCTCTTAATTAAGCTATTTAAATTCAGAAAAGATAAAACAATCATTTTAAACTTCCAAAGTTTATATTTTATATTTAAACTATTTTCTGAGTTAATTAGCTTAACCNAAAGCATTTATTTTGAAAGTAAAAGAAAAGATATTTAATCTATTAACTTAGGAAACCAGGAATAAAATTAGCTTCTAACTAACTTTTAAAGCGGTTAAATTCCGTTTTTTCCTTGTTTTAATAGTTTAATNNNAAAAATTTAGATCTTGTAAATCTAAGATAACGTAAGTTTTAAAAATAGGTTTTAAGTTATTAANAACTATTATCCTTCAAAGTTAAAAATATAATTANATTTATTATTAAGAGAAATAATTTTTCATAAATAAATTTACAGTTTATTGCCTATTTTCAGCCATCCTAATCAAGTTACATTGATTGAACAATATTCTCATTAGTGTAAATAAAGTGCTAATATAGCTTTAACTTG

>westlandica-north-inland_12.NZ.BR.MMK.17

NNNNNNNNNNNNNNNNNNNNNNNNNNNNNNNNNNNNNNNNNNNNNNNNNNNNNNNNNNNNNNNNNNNNNNNNNNNNNNNNNNNNNNNNNNNNNNNNNNNNNNNNNNNNNNNNNNNNNNNNNNNNNNNNNNNNNNNNNNNNNNNNNNNNNNNNNNNNNNNNNNNNNNNNNNNNNNNNTAAGTCATTGGGAAGATCATTAGTATTTTTATCGTTATTTATATTTATTTTATTAAATAACTTATTGGGTTTACTTCCATATATTTTTACGAGTCCAAGTCATTTAGTATTCACAATAAGATTAGCGTTACCACTATGATTATCATTTATACTTTATGGATTTATTAATAATATAAATCACATATTTTGTCATTTAGTTCCATCAGGAACTCCTAATATTTTAATACCTTTTATAGTTATAATTGAAAGAGTCAGAAATTTAATTCGTCCTGGTTCCTTGGCTGTTCGACTAACAGCTAATATAATTGCTGGACATCTATTAATAACTTTATTAGGTAATTTACCCATAAGTTACGAATTATATTCAGGCCTAATTATTATTTTTCAGGTTATATTAATATTATTTGAATTAGCTGTTTGTATTATTCAATCCTACGTATTTATAGTTCTTAGAACTTTATATTATAGAGAAGTAAATATCCCTCAAATATCTCCTCTTAATTGACTATTANNNNNNNNNNNNNNNNNNNNNNNNNNNNNNNNNNNNNNNNNNNNNNNNNNNNNNNNNNNNNNNNNNNNNNNNNNNNNNNNNNNNNNNNNNNNNNNNNNNNNNNNNNNNNNNTTTAAGCCAACACGACAAAATCATCCTTTAATTAAAATTATTAACAATTCATTAATTGACTTTCCAGCACCATCTAATTTATCTTATTGGTGAAATTTTGGATTTGTATTAGGATTATGCTTAATAATCCAAATTATAACTGGATTATTTTTATCAATGCATTATAATGCCAACATTATAAACGCTTTCGAAAGATTAAGACATATTTGTCGTGATGTAAACTATGGTTGAATTTTACGAGTCATTCACGCTAATGGAGCTTCATTATTTTTCATTTGTGTATATATACACGTAGGACGTGGATTATATTATGGATCATTCAAATATATTGAAACATGATCTATTGGTGTAATCATATTATTAATATTAATAGCTACCGCTTTCTTAGGATATGTTTTACCATGAGGACAAATATCTTTTTGAGGAGCTACAGTTATTACAAATTTATTATCAGCTATTCCTTACTTAGGAAGAATACTAGTTAATTGAATTTGAGGAGGATTTGCAGTTGATAATGCAACACTAACTCGATTTTATTCATTCCACTTTATTTTACCATTTATTGTATTAAGATTAACAATTATTCACTTACTATATCTTCATACAACAGGTTCTAATAATCCATTAGGAATTAATTCTAATAATGACAAAGTCCCTTTTCACCCATACTTCTCCATTAAGGATATTATGAGACTAATTATCTTAATAATTATTTTCTTTATATTAGTTATATTGGAACCATACATGCTAGGAGATCCAGATAATTTTATTCCTGCTAATCCACTAGTAACACCAAAACATATTCAACCTGAATGATATTTTTTATTTGCCTATGCAATTCTTCGATCAATTCCTAATAAATTAGGAGGAGTGATTGCTCTCTTCATGTCAATTTTTATTTTAATGTTTGTACCTTTATTAAATAATTCTAATTTTATAGGTTTAAATAACTACCCAATTAATCAAATTATATTTTGATATATAGTAATCATTCTAATTTTATTAACCTGAATTGGAGCACGACCTGTTGAATTACCTTATATTAACTTTGGAATATTTTTAACACTTATATATTTTTCTTACTTTATTATTGACCCAATAATTAAATCTATTTGAGACAAATTAATTAGATAGATGAAAAAATGATTCTTCTCAACTAATCATAAAGATATTGGAACAATATATTTTATTTTTGGTATTTGATCAGGTATAATTGGTACAACTTTAAGAGTTTTAATTCGTGTAGAACTTGGAACTCCAGGTTCATTTATTGGTGATGATCAAATTTATAATGTAATTGTCACTGCTCATGCTTTCATTATAATTTTTTTTATAGTTATACCAATTATAATTGGTGGATTTGGAAATTGATTAGTTCCTTTAATAATTGGAGCTCCTGATATAGCTTTTCCTCGTATAAATAATATAAGTTTTTGATTATTACCACCCTCTCTAATTCTTATTTTGGTGGGAAGAATAGTTGATAGAGGTGCAGGAACAGGTTGAACAGTTTACCCTCCTTTATCTGCAGGGATTGCACATTCTGGTTCATGTGTTGATTTAACTATTTTCTCTTTACACCTCGCAGGTGTATCTTCAATTTTAGGTGCTGTAAATTTCATTAGAACAATTTTTAATATACGTTCAATAGGAATTTGATTAGATCGAATACCCTTATTTGTATGAGCAGTTTTAATTACTGCATTCTTGTTATTATTATCTTTACCTGTTTTAGCAGGTGCTATTACAATATTATTAACAGATCGAAATTTAAATACTTCATTTTTTGACCCTGCAGGGGGGGGAGACCCAATTCTTTATCAGCATTTATTTTGATTTTTTGGTCATCCTGAAGTTTATATTTTAATTTTACCAGGATTTGGTTTAATTTCTCATATTATTACCCAGGAAAGAGGTAAAATTGAATCTTTTGGTTCATTAGGAATAATTTATGCTATGATATCAATTGGTATTTTAGGATTTGTTGTATGAGCACATCATATATTTACTGTAGGAATAGACGTTGATACACGTGCATATTTCACATCAGCTACTATAATTATTGCAGTTCCTACTGGAATTAAAGTATTTAGTTGACTCGCAACTTTGAGAGGTATAAAAATCAATATTACATCTTCAGCTTTATGGGCTTTAGGATTTGTATTTTTATTTACTATTGGAGGTTTAACCGGAGTAATTTTAGCTAATTCTTCAATTGATATTATATTACATGATACATACTATGTAGTTGCACATTTCCATTATGTTTTATCTATAGGAGCAGTTTTTGCCATTATAGCAAGATTTATTCATTGGTTTCCATTATTCACAGGAATAAGATTAAACTCAAATTGATTAAAAATTCATTTTCTATTGATATTTATTGGTGTAAATATAACATTTTTTCCCCAACATTTTTTGGGACTAAGAGGAATACCTCGTCGGTATTCAGACTATCCTGACGCCTATATGTCATGAAACATAATTTCATCAATAGGAAGAATTATATCTTTAGTCGGAATTTTATTTTTGTTATTCATTGTTTGAGAAAGTTTTATTTCCATACGATTAGTATTATATTCTAATAGAATTCAATCTTCTATTGAATGAATACAGAAGTTTCCCCCATCTGAACATTCTTATAATGAAATGCCATTATTAATTCAAATTTCAAATTGATCTTATATTAATATACAGGATGCTGTATCACCATTGATAGAGCAGTTAATATTTTTTCATGATCATGTATTAATTATTTTAATTATAATTACAATTGTTGTTGCTTATATAATAATAATACTAATATTAAATAAAATTATTAATCGTTTACTTCTCGAAGGACAATTAATTGAATTTATTTGAACTTTGTTACCTGCAATAACTTTAATTTTTATTGCATTACCATCATTGCGATTATTATATATATTAGACGAAATTAATAATCCATTATTAACATTGAAAATTATTGGTCATCAATGATACTGATCATATGAATATTCAGATTTTTCTGATGTAGAATTTGATTCATATATAAAATCCATAAATGAAATAAATAAAAATGAATTTCGTTTATTAGATGTAGATAATCGAGTAATTCTACCATTTAATATTCAAGTCCGACTATTAGTTTCTTCTTTTGATGTTATTCACTCTTGAGCAATACCATCTATAAGACTTAAAGTTGATGCAGTACCAGGACGATTAAATCAAATAAGAATATTAATTAGTCGTCCTGGTATATCATATGGACAATGTTCTGAAATTTGTGGAGCAAATCATAGATTTATGCCTATTGTAATTGAAAGAATTAGAATAAAAATATTTATTAAATGATTAATTAATTATATGAATAATCATCCTTATCATATAGTTGATTATAGACCCTGACCTTTAACTGGATCAATTGGAGCATTAACTTTTGTTTCTGGTATAGTTATAATGTTTCATAAATGTAACTTTATATTATTATATATAGGTATTTTATTATTACTAATAACAATAATTCAATGATGACGTGATATTTCCCGAGAGGGAACATTTTTAGGAATACATACAATAATGGTAGTGAACGGTTTGAAAATAGGTATATTATTATTTATTGTATCAGAAGTTCTTTTTTTTGTTTCATTTTTTTGGGGATTTTTTCATAGTAGATTAAGACCTGTAGTTGAAATTGGCATAATTTGACCCCCTAGAGGTATTTATGTTTTTAATCCAACTCAAGTCCCTTTATTAAATACAATAATCTTATTATGTTCAGGAATTACAATTACTTGGGCTCATCATTCAATTATGTGTGGAAATCATATTAATTCTATTAATAGAATTATATTAACTGTTATTTTAGGTATATATTTCACCATTCTGCAAGGTTACGAATATTACGAAGCTCCATTTGCAATTAATGATTCTATCTATGGGTCATCTTTTTTTATAGCTACTGGATTTCATGGAATCCATGTAATTATTGGAACAAGATTTATTACCGTATGTTTATTACGACAAATAAAGTTTCATTTCTCAAAAAATCATCACTTTGGTTTTGAAGCTGCTGCTTGATATTGACATTTTGTTGATGTAGTTTGATTATTTTTATATTTATCAATTTATTGATGAGGAAGATAGTAAATTATAGTTTTTAAAAAGAAAAAAAAAATAAATAGATTTAAAGATACTGGTAAATAAGATTTTCAAGCTAAATATATTAATTTATCATACCGATAACGAGGAAGAGTTCCTCGGACTCAAATGAAAAGAAAACATATCATAGAAACTTGAATTGGAAAAACAATTGAACTAATTTTTCCCCCTAGAAATATTAAACAAAATATTATTCTCATAAACAAAATACTTGAATATTCAGCCAGGAAAATAAATGCAAAGCTAGAACCTCCATATTCAACATTAAAACCAGAAACTAACTCTGATTCTCCCTCAGAAAAATCAAATGGAGATCGATTAGTTTCAGCTAAAGCTGAAGATAATCAACATATTCTTAATGGAAGAAATAAAAAAATAAATCAAACAAACTCCTGAAATATAAACAAATCAATAATATTATAGCTTCTGATTATTATTATAGGACACAATATAATCAAAACCAATCTTACTTCATAAGAAATAGATTGAGCAATTGATCGAATACATCCCAACATTGCATAACTTGAATTAGAAGATCAACCTGTTAATATTAATGAATAAACTCTCATTCTTGAACAACAAAAAAAAAATAAAATACCAAAATTAAAAGAAACACAATTAATTATATATGGATATAATGATCAAATCAACAATCTATTAAATAATCCTATTACAGGCCTAAAAATATAAATTAAATAATTAGATATTAATGGAATTGTGTTTTCCTTTATAAATAATTTAATAGCATCAGAAATAGGCTGCAAGATACCCAAAAATCCAACTTTATTTGGACCTTTACGAATTTGAATATATCTCAAAACCTTTCGCTCCAAAAGAGTAATAAATGCCACTCCAATCAAAATAAATAATACAGTAATTAAAATAGTAATTAAATACNNNNNNNNNNNNNNNNNNNNNNNNNNNNNNNNNNNNNNNNNNNNNNNNNNNNNNNNNNNNNNNNNNNNNNNNNNNNNNNNNNNNNNNNNNNNNNNNNNNNNNNNNNNNNNNNNNNNNNNNNNNNNNNNNNNNNNNNNNNNNNNNNNNNNNNNNNNNNNNNNNNNNNNNNNNNNNNNNNNNNNNNNNNNNNNNNNNNNNNNNNNNNNNNNNNNNNNNNNNNNNNNNNNNNNNNNNNNNNNNNNNNNNNNNNNNNATATAATTATAATTAGATTAATAATTAAAATTGGCTGTCCTCCTTTCCATTTTTGATATGTATCTGTTATTGAAGGTTTAACTTGAATAGTATGTTTTATTTTAATAACTATTCAAAAGATTATTCCTTTAATTATATTATCATATTTAAATGTTAATATAAGATTATTTATTGTTATAGCATGTATATGAGGATGCATTGGAGGGCTGGGCTATTCTTCAATGCGTAAAATCATTGCTTATTCTTCAATCTATAATTTAAGATGAATTTTTAGAGGTATTGTTATTATTAACTATTCATGGCTGATTTATTATTTTATTTATTCATTTACATTATTAGCTGTATGCTATATATTTAAATTATTTAATATTAATTATATTAATCAATTTATTATAGTATCTTTTAATTTTATAAAGTCAATTATAATAATATGTATTTTTATATCTATGGGGGGACTTCCCCCCTTCTTAGGATTTTTCCCTAAATTAATTATAATTTACTGTTTACTGATAAATAACATAATATTCATTTGCATTATGTTATTAATAACAGCTTTGATTATTTTGTTTTTTTATTTACGAATTTTAATTACAACATTAATAATAAATACAATCTCAATAAAAAGAATTATCATAAGAGTTTCATATACCTATTATATTGCTGGAATATTTTCATTATTTGGAATAATTTTTTTTTCATTAATTACATTAAATATGTGTTAGATCTATAATATTTTGATTTATTCNNTTANNNNNNNNNNNNNNNNNNNNNTTNNNNNNNNNNNNNNNNNNNNNNNNNCTTATAAATCTATAAAAGATCGAGAAAAATCGTCCCCATTTGAGTGTGGTTTTAGACCGTTTGAATCATCTCGCATTCCATTTTCAAGACACTTTTTTTTAATTGCAGTAATTTTTTTAATTTTTGATGTTGAATTGGTAATTATTATACCTGTAATTTTAGTAATAATTAGACTAAAAGTTATTGATATTTATATTATTATATTAATCTTCTTAATTATTTTAACTTTAGGATTATATCATGAATGATATAATAATATATTAAATTGANNNNNNNGTTTAATTAAAATAGTTAATTTCAAAATAATTAAATTTAGGGGAATTCAATGCATCATAATAAGGAAAAACTCCCGAACTCTTCCAGAATTAAATGAAAATAAACTAGAAAAAAATATACCATGTTGAGTAAATGAAAATATATATAATCTATAACAAGCAGATAAAAATGATGATAAAATTAATAAAATCATTATTAATATATTTCAAGATATTAATCTATTAATAATCATAATTTCACCAGCTAGATTAATAGTAGGTGGACAAGACATATTTCTTGCCGACATTAAAAATCATAATAAAGATATTGAAGGTATAAATGTCAATATACCCTTATTAATTAATAATCTTCGACTATTAGTTCGCTCATACAATATATTTGCTAAACAAAATAATCCAGAAGAACATAAACCATGTCCAATTATTATATAATATGAACCACATAATCCTCAATTTCTTATAGTTATAATTCCTCTAATCACAAGACCTATATGAGCCACAGATGAATAAGCAATTATTGATTTAATATCAATCTGAATTATACATAGAATTCTAATCAAAATACTTCCAATTATTGAAATTGAGATTCATAAAAATCCAAATTTATAAAAATAAGAAGGAATAATATATATTATTCGAATTAAACCATATCCTCCTAGTTTTAGTAAAATTCTAGCTAAAATTATTGAACCAGAAACAGGAGCTTCAACATGAGCTTTAGGTAATCAAAAATGAAAAAATAGTGGTATTTTAATTAAAAAAGCCATTATTATACCAAAATAAATATAAATATTGACAGAATAATTCATTAACATAAAAATACTAGTTATATTATTTTTATAAAGGAAAAAAATTCTCATTAACATTGGTAATGATGCAAATAATGTATAAAATAATAAATAATACCTTGCATCAATACGTTCTGGTTGATAACCTCAACCTATAATAATAATTATAATTGGAATTAATCTAGATTCAAAACAAATATAAAAAATTATAATTCTTGATGTAGAAAAAGAAATAATCAAAAAAATTATTAACATAACACCAAAAAGAGAAAAAATATTTTATTACGTTTATATATTGGGCTAGAAACAATTATTAAAAATGAAATCCAAATTTAAAATAATTAGACCATAAGATATTTCATCAATATAAAAATTATAACCTAACATATATAAACTCTTATTTATAAAAACCAAAAATATAATTATCAAAACAATAATTATATTTATTAATCATGAATTATAAGTTAATAGGGTTAATATGAAACAAAAAAAAACTAACTTTATTGACATAGATATTAATTTATCATTATTATGAGATCGAATTAATATTACTAAGCATGATAAGCCTATTACTCCTTCACAAACTCTAAAAACCAAGAAAATTAGTAAAATATGCAAATCATATATTTTTATAGAAAAAATAAATGATAAAGAACAAAACATTCTTAAAATAATTAATTCCAATCTTAGTGCTGTTATTAAATGTTTTCGATTCATGCATAATGAAATTATCCCTGAAAAGAACATGGAGATAAAATATATTAATAATGTTATATTCTAAAAATATAAAACAATAATTCAAACAATAAATGAAATTATTTGAATCTTAAAATTATTATTTATCAATTTATAATTTAATGATGTTATTATTTTTAAAGTTTTTAAAATTCCTTGAGGGCCAAGGTCTTCACCTCATCCCATATCAATTGATTTTTGAAATATAAATGATTTATTTATCAATAACATTTGATTATGAAAAGTTGACAATTGTTTTATAAATCACATTGATCCAAAAAACTCGTAAACAATTTTTATTTTAGTTTTAGAATAAATTGACATTTCATAACCCAATCAAATTCCTAATATTGAAAATATTAATGCTAATAATTTACCCTCAATAGGTATAAAAATCATAATCGGATCATTAAATATTAATCATCTTAATATAGATCCTGAGAAAATTGAGTAAATAGATAAAATAATAATTCTTTTAATTATATTATTTATATTTTCACTTAATGATCTTAATTTATAAAAATTATAATTTATATTTATTGAAAAGAATGTCAAACGGGCAGAATAAAAGGAAGTTAAACCAATACCAATATACATAAATATCATAATTAATAAATTAATATTATTAGAACATATAGTTTCTATAATTAAATCTTTTGAGTAAAATCCTCTTATAAATGGAATTCCACATAATGACATATTAGCAATATTTATTATTGTAATAGTTAATGGTATTTGAAATCTTAAACATCCTATTATACGAATATCTTGATTGTTGTTAAAATTGTGAATCAAAATCCCTGCACACAAAAATAAAGTAGCTTTAAATAAAGCATGAACAATTAAATGAAAAAATGATAATATTGGATAACCAAATATAATAATTGTTATTATAATCCCTAACTGTCTCAAAGTTGATAGTGCAATAATTTTTTTTAAATCAAACTCAAAATTTGCTCTAATTCCTGATATTAATATAGTTAAAATTGAAATTAATATAAATACTTGAATATAATCAAAATTCAAGATAATGTTAGAAAATCGAATTATTAAATATACACCAGCAGTGACAAGTGTAGATGAATGAACCAAAGCAGATACAGGAGTTGGAGCAGCTATAGCTGCTGGTAATCATGAAGAAAATGGAATTTGAGCTCTCTTTGTAAATCTTGCAATAATAATTATAATCAATATAATTTATATCTTTATTAATAAAAGATAAAAATTTCATGACCCAAAATTTAATATTCATGCAATAGCTATTAAAATAGCAACATCTCCCACACGATTCATTAATGCAGTTAATATTCCTGCATTATTAGAGTATAAATTTTGATAATAAATAACTAAGCAATAAGAAACTAATCCAAGTCCATCTCAACCAATAATAATTCTAATTATATTAGGTCTAATGATTAATAATACTATGGATATAACAAATCCTAAAACAATATAGATAAAACGATTAATAAATTTGTCATTATTTATATATATTCCACTATATAAAACAACCACTGATGAAATAAATATAACTGTTGAAAGAAATTGTTGATATAAAATCAAAAATTATAGTCATATAAATATTACATGAATTAATTGAAAATTATTCATTCCAATATAACTATATAATTATTTTTGAAATTCATTGAATATTAATATTAATAGACTTAGGAATATAAGAATAATAAATATATAAACATTAAACTTTTTATTTATCATGAAAATTGCCATGTATTTGATAATTATATTATCAATAAATTTTATTTTTATAAAGCACCCTTTATCAATAGGATTAATTTTAATAATACAAACAGTTTTGTCTTGCTNNNNNNNNNNNNNNNNNNNNNNNNNNNNNNNNNNNNNNNNNNNNNNNNNNNNNNNNNNNNNNNNNNNNNNNNNNNNNNNNNNNNNNNNNNNNNNNNNNNNNNNNNNNNNNNNNNNNNNNNNNNNNNNNNNNNNNNNNNNNNNNNNNNNNNNNNNNTTTTTAACTTTAATTAATTTAATAAATATAATTGATTTAAAATCACTAAATATTATAAGAAACATAGTAATATATATAAACCACAACAACTTTATAATAAGAAAAATATATATTATTCCTTCTGGAATAATAACACTAATTTTAACAATTTATTTACTATTTGTTTTAATTATTGTAATTAATATTTTAACAATAAATATATTAACCCTTCGAAGAAGAACTTCTTTCTAACTACACCTTCCGGTACAGTTACTTTGTTACGACTTATCTCATTAATATAATGAGAGTGACGGGCGATATGTACATAAATTAGAGCCAATTTCAATTAATTAAATTTAATTAATTTATTATCAAATCCAATTTCATATTTCTTNNAATTTAAAAAATAATTCAATAAATAATTAATTGTAACCCATTTCTTCTTTAATATAAACTGCACCTTGACCTGACATTAAATATAANGTAATATATTATGAAAATTTTTCTTATAAAACATTCTTGACAGAGATATACAAATTAAATTAAAGTTTTTTCTATCGTGGACTATCAATTATAAAACAGGTTCCTCTGATAAGATAAATTACCGCCAAATTCTTTGAATTTAAAGATCATTTCTAATAATAATCAAGCTATTTTTATCACATTTTTAATAATAGGGTATCTAATCCTAGTTTAAACAAAAATTTTTCAGACATAAAATTAATTTTTCAAGATAAAATATATTTCACCAAAATTATAGATATTTTATATTATTGTTATATTAACTGAAACTTAACTATTTAAATTAAAGAAATCGTATAACCGCAACTGCTGGCACGAAATTTGATTCTTTTAAATAAAATTACTAATTCTAATTTTATTAATTAATAATAATAAATACTGCGCATTTATTCAAANTATTTCCATTTAGAAATTAAAAACAATTAAAAAATTGCATGTAATATAATTTTAAATTTAAATTATCNTAAACTAGAATTAAACTTCAAATAATAAAATTATTTAATGAGGTCCTTTCGTACTAACATTAAAAATAATTGAGTAGATAGAAACCAACCTGGCTCACGCCGGTTTGAACTCAGATCATGTAAGAATATTAAGGGTCGAACAGACCCAGAAATAATAAATTTTGCTCCAATTCCTATTCTTAATCCAACATCGAGGTCGCAATCATATTCATCGATATGAACTCTTCAAATTAATTACGCTGTTATCCCTAAGGTAATTTATTCTTATAATCAAAAATTTGGATCAATATTCACATAAATTTATGAAATTTTTATTAAAAGTTAATTATATTTTAATATCACCCCAACAAAAAAATCAATTAAATAAAAAAAATTAATTAACTATAAANAAATAAACTTAATNAAGATTTTTAAAATTCTATAGGGTCTTCTCGTCCCACTCATATATTTAAGCTTTTTTACTTAAAAATCAATTTCAATTGTTAATACTAATTAAGTTAATTTTTCATTCAATCATTCATTCAAGCCTCCAATTAAAAGACTATTTATTATGCTACCTTTGCACGGTCAATTTACCGCGGCCATTTAATTTATAATCATTGGGCAGATTAGACTTTTAATTCATTCTAAAAGACATGTTTTTGATAAACAGGTGAAAATTTTTTTTGCCTAAATTATAAATATTATTTACAACCAAAAAACCAGATATCATATAATTTGATAAAATATCATTTCCAGAATAATTTCATTAATATATATACAATAATAACTAACAAATNTATACTAAATCATTATATTTCGGGAAAAATAAATACTTAAAAATCTTTAATTAACCCTGATACAAAAGGTACAAATTATATTCTACTTAAAACTATTTATAAAGTTNTTNCCCTATCGGTTAATCCAAATTATTTTTTTATATAAAATTACTTTAACACAAAAATTTTTTTTTCAAAATGTAAAAAAACAAATTTAANTGAAATTATAATATGGGATATATTTAATTATAATAATTGAATTGCAATCAATAGGTGTTATCTATAACTATCCTTAAGTAATGAAGTAAAATAATACATTTAGTTTCGACCTAAAATAAGAATCANAATTCCTTGCTTTTAAATGAAGCCAAACTAGAGGCATTTTATTGTTAATAAAATAATTGAAANTTTATTTCCATTTAAAGAAAAATTAGTTTAATTAAAATATAAACCTGTCAGATTTATGAAGCTCTTGAGTATTTTTCTGACTTTTTGTGATNTNTTCTACTTTAAATTTGCAATTTAATATTATAAATTAAATATAAGACTNNNNNNNNNNNNNNNNNNNNNNNNNNNNNNNNNNNNNNNNNNNNNNNNNNNNNNNNNNNNNNNNTTTATAAAGTTTATAAAACATTTCATTTTCATTGAAAAGAGAGACTTGGTCTTATAAAATTCTATTAGTATATAAGTATATTTAACTTCCAATTAAAAGGATTAATTTTATTAAATAGAATATATCCAATAAATTTNNTTTTATCTTTAAAATCACAATTTAATATTTTTATTAAACTAATTGAATTACTATTTGTAATAAATATTACATTATTAAATTCTAAATTTAAGGCATTAATTTTGCTAAAATAGTTCTAATATGGCAGATTAGTGCAATGAATTTAAGATTCATATATAAAATATNATTTTTTTATTGGAANNNNNNNNNNNNNNNNNNNNNNNNNNNNNNNNNNNNNNNNNNNNNNNNNNNNNNNNNNNNNNNNNCATTAAGTGGCTGAAAGTAAGTAATGGTCTCTTAAACCAATTAATAGTAATTAACAAATACTCTTAATGNNNNNNNNNNNNNNNNNNNNNNNNNNNNNNNNNNNNNNNNNNNNNNNNNNNNNNNNNNNNNNNNNTTTAAAAACAAAAGTTACCTTAATATCTTCAATATTATGCTCTTAATTAAGCTATTTAAATTCAGAAAAGATAAAACAATCATTTTAAACTTCCAAAGTTTATATTTTATATTTAAACTATTTTCTGAGTTAATTAGCTTAACCTAAAGCATTTATTTTGAAAGTAAAAGAAAAGATAATTAATCTATTAACTTAGGAAACCAGAAATAAAATTAGCTTCTAACTAACTTTTAAAGCGGTTAAATTCCGTTTTTTCCTTGTTTTAATAGTTTAATNNNAAAAATTTAGATCTTGTAAATCTAAGATAACATAAGTTTTAAAAATAGGTTTTAAGTTATTAANAACTATTATCCTTCAAAGTTAAAAATATAATTANATTTATTATTAGGAGAAATAATTTTTCATAAATAAATTTACAGTTTATTGCCTATTTTCGGCCATCCCAATCAAGTTATATTGATTGAACAATACTCTCATTAGTGTAAATAAAGTGCTAATATAGCTTTAACTTG

>flemingi_11.NZ.NN.LGU.02

AATTTATTTTCATCATTTGATCCTTCAACTGGGTTTTTATCTCTTAATTGATTAAGATCAATAATTCTACTATCATTTTTACCTCTAACTTACTGGTATATTCCTAATCGTTTTATTATTTTATACAATAAGGTTTTAATTTCATTAAATAATGAATTAAATATATTAATAAATTATAAATCATTGGGAAGATCATTAATACTTTTGTCCTTATTTATATTTATTCTATTAAATAATTTATTAGGCTTACTTCCATATATTTTTACAAGTTCAAGTCATTTAGTATTTACAATAAGATTAGCATTACCATTATGATTATCATTCATACTTTATGGATTTATTAATAATATAAATCATATATTTTGTCACTTAGTTCCTTCAGGAACTCCTAATATTTTAATGCCTTTTATAGTTATTATTGAAAGAGTTAGAAACTTAATTCGTCCCGGTTCCTTAGCTGTTCGACTAACAGCTAATATAATTGCTGGACATCTTTTGATAACTTTATTAGGCAACTTACCTATAAGTTATGAATTATATTCAGGCTTAATTATTATTTTTCAAGTTACATTAATATTATTTGAATTAGCTGTTTGTGTTATTCAATCTTATGTATTTATAGTACTTAGAACTTTATATTATAGAGAAGTAAATATTCCACAAATATCTCCTCTTAATTGATTATTATTTATTTATTTTNNNNNNNTAATTATTGTTATTATTATATTTATGTATTTTACTTATCTATTAAAATCTAATATAACAGCCAAAAACAAATTAATAGATAGAATAATTTGATTTAAGCCAACACGACAAAATCATCCTTTAATTAAAATTATTAATAATTCATTAATTGACTTTCCAGCACCATCTAATTTATCTTATTGGTGAAATTTTGGATTCGTATTAGGATTGTGCTTAATAATCCAGATTATAACTGGATTATTTTTATCAATACATTATAATGCCAACATTATAAATGCTTTCGAAAGATTAAGACATATTTGTCGTGATGTAAATTATGGTTGAATTTTACGAGTCATCCACGCTAATGGAGCTTCATTATTTTTTATTTGCGTCTATCTACATGTAGGACGTGGATTATATTATGGATCATATAAATACATTGAAACATGATCTATTGGCGTAATTATATTATTAATATTAATAGCTACAGCTTTCTTGGGATATGTTCTACCGTGAGGACAAATATCTTTTTGGGGAGCTACAGTTATTACAAATTTATTATCAGCTATTCCTTATCTAGGAAGAATATTAGTTAACTGAATTTGAGGTGGGTTTGCAGTTGATAACGCAACACTAACTCGATTCTATTCATTCCACTTTATTTTACCATTCATTGTATTAAGATTAACAATTATTCACTTATTATATCTTCATACAACAGGTTCAAATAATCCACTAGGAATTAATTCTAATAATGACAAAGTTCCTTTTCACCCATACTTTTCCATTAAAGATATTATAAGACTAATTATATTAATAATTATTTTCTTTATACTAATTATATTGGAGCCTTACATTCTAGGGGATCCAGACAATTTTATTCCTGCCAACCCACTTGTAACACCAAAGCATATTCAACCTGAATGATATTTTTTATTTGCCTATGCAATTCTTCGTTCAATTCCTAATAAATTAGGAGGAGTAATTGCTCTTTTCATATCAATTTTTATTTTAATGTTTGTACCTTTATTAAATAATTCTAACTTTATAGGATTAAATAATTACCCAATTAATCAAATTATATTTTGATATATAGTAATTATTTTAATCTTATTAACTTGAATTGGAGCACGACCTGTTGAATTGCCTTACATTAACTTTGGAATATTTTTAACACTTATATATTTTTCTTACTTCATTATTGACCCAATAATTAAATCTACTTGAGACAAGTTAATTAGGTAGATGAAAAAATGATTCTTCTCAACTAATCATAAAGATATTGGAACAATATATTTCATTTTTGGTATTTGATCAGGTATAATTGGTACAACTTTAAGAGTTTTAATTCGTGTAGAGCTTGGAACTCCAGGTTCATTTATTGGTGATGATCAAATTTATAACGTAATTGTTACTGCTCATGCTTTCATTATAATTTTTTTCATGGTTATGCCAATTATAATTGGTGGATTCGGAAATTGACTAGTTCCTTTAATGATTGGAGCTCCTGATATAGCTTTTCCTCGTATAAATAATATAAGTTTTTGATTATTACCCCCTTCTCTAATTCTTATTTTAGTGGGAAGACTAGTTGATAGAGGTGCAGGCACCGGCTGAACAGTTTATCCTCCCCTATCTGCCGGGATTGCTCATTCTGGTTCATGTGTTGATTTAACTATTTTTTCCTTACATCTTGCAGGTGTATCTTCAATTCTAGGTGCTGTTAATTTTATTAGAACAATCTTTAATATACGTTCAATAGGAATTTGATTGGATCGAATGCCCTTATTTGTGTGATCAGTTTTAATTACTGCATTTTTGTTATTGTTGTCTTTACCTGTTCTAGCAGGAGCTATTACAATGTTACTAACAGATCGAAATTTAAATACTTCATTTTTTGATCCTGCAGGAGGGGGAGATCCAATTCTTTATCAACATTTGTTTTGATTTTTTGGTCATCCTGAAGTTTATATTTTAATTTTACCAGGATTTGGTTTAATTTCTCATATTATTACCCAAGAGAGAGGTAAAATTGAATCTTTTGGTTCATTAGGAATAGTCTATGCTATAATATCAATTGGTATTTTAGGATTTGTTGTATGGGCACATCACATATTTACTGTAGGAATAGATGTTGATACACGTGCATATTTCACATCAGCTACTATAATTATTGCAGTTCCTACTGGAATTAAAGTATTTAGCTGACTTGCAACTTTAAGAGGTATAAAAATCAATATTACATCTTCAGCTTTATGAGCTTTAGGATTTGTTTTTTTATTTACTATTGGAGGATTAACTGGAGTAATTTTAGCTAATTCTTCAATTGATATTATATTACATGATACATACTATGTGGTTGCACATTTTCATTATGTTTTATCAATAGGAGCAGTTTTTGCTATTATAGCAAGATTTATTCATTGATTCCCATTATTTACAGGATTAAGATTAAACTCAAATTGATTGAAAATTCATTTTCTATTAATATTTATCGGTGTAAATATAACATTCTTTCCTCAACATTTCTTAGGATTAAGAGGAATACCTCGTCGGTACTCAGACTATCCTGATGCTTACATGTCATGAAACATAATTTCATCAATAGGAAGAATTATATCTTTAGTTGGAATTTTATTTTTATTATTTATTGTTTGAGAAAGTTTTATTTCAATACGGTTAGTATTATATTCTAATAGAATTCAATCTTCTATTGAATGAATACAAAAGTTTCCTCCATCTGAACATTCTTACAACGAAATGCCATTATTAATTCAAATTTCAAATTGATCTTATATTAATATACAGGATGCTGTATCACCATTAATAGAGCAGTTAATATTTTTTCATGATCATGTATTAATTATTCTAATTATAATTACAATTGTTGTTGCTTACATAATAATTATATTAATATTAAATAAAATTATTAATCGTTTACTTCTTGAAGGACAATTAATTGAGTTTATTTGAACTTTGTTACCTGCAATGACTTTAATTTTTATTGCATTACCATCATTGCGATTATTATATATATTAGACGAAATTAATAATCCATTACTAACATTAAAAATTATTGGTCATCAATGATACTGATCATATGAATATTCAGATTTTTCTGATGTGGAATTTGATTCATATATAAAATCTATTAACGAAATAAATAAAAATGAATTTCGTTTATTAGATGTAGATAATCGAGTAATCTTACCATTTAATATTCAAGTCCGACTACTAGTATCTTCTTTCGATGTTATTCACTCTTGAGCAATACCATCAATAAGTCTTAAAGTTGATGCAGTACCAGGACGACTAAATCAAATAAGAATATTAATTAGTCGTCCTGGTGTGTCATATGGGCAATGCTCTGAAATTTGTGGGGCAAATCATAGATTTATGCCTATTGTAATTGAAAGAATTAGAATAAAAATATTTATTAAATGATTAATTAGTTATATGAATAATCATCCTTATCATATAGTTGATTATAGACCCTGACCTCTAACTGGATCAATTGGAGCATTAACTTTTGTTTCTGGTATAATTATAATATTTCATAAATGTAACTTTATATTATTATATATTGGTATTTTACTGTTATTAATAACAATAATTCAATGATGACGTGACATTTCCCGAGAGGGGACATTTTTAGGAATACACACAATAATGGTAGTGAACGGTTTAAAAATAGGTATATTATTATTTATTGTATCAGAAGTTCTTTTTTTTGTTTCGTTTTTTTGAGGATTTTTTCATAGTAGATTAAGACCTGTGGTAGAAATTGGAATAATTTGACCACCCATGGGTATTTATGTCTTTAACCCAACTCAAGTCCCTTTATTAAATACAATAATCCTATTATGTTCAGGAATCACAATTACTTGAGCTCATCATTCAATTATATGTGGCAACCATATTAATTCTATTTATAGAATTATGTTAACTGTTATTTTAGGGATATATTTTACCTTTCTACAAGGTTATGAATATTATGAAGCTCCATTTGCAATTAATGATTCCATTTATGGATCTTCTTTTTTTATAGCTACTGGATTCCATGGAATCCATGTAATTATTGGAACAAGATTTATTATTGTATGTTTATTACGACAAATAAAGTTTCATTTCTCAAGAAATCATCATTTTGGTTTTGAAGCTGCCGCTTGATATTGACATTTTGTTGATGTAGTATGATTATTCTTATATTTATCAATTTATTGATGAGGAAGATAATAAATTATAGTTTTTAAAAAAAAATAAAAAACAAATAAATTTAGAGATACTGGTAAATAAGATTTTCAAGCTAAATATATTAATTTATCATAACGATAACGAGGAAGAGTCCCTCGAACTCAAATAAAAAGAAAACATATTATAGAAACTTGAATTGGAAAAACAATTGAACTAATTATTCCTCCTAAGAATATCAAACAAAATATTATTCTCATAAATAAAATACTTGAATACTCAGCCAAGAAAATAAATGCAAATCTAGAACCTCCATATTCAACATTAAAACCAGATACTAACTCTGATTCTCCCTCAGAAAAATCAAATGGAGATCGATTAGTTTCAGCTAAAGCTGATGATAATCAACATATTCTTAATGGAAGGAATAAAAAAATAAATCACACAAACTCCTGAAATATAAATAAATCAATAATATTATATCTTCTTATTATTATTATAGGACATAATATAATCAAAACCAATCTTACTTCATAAGAAATAGATTGAGCAATTGATCGAATACATCCTAATATTGCATAACTTGAATTAGAAGATCAACCTGTTAATATTAATGAATAGACTCTTATTCTTGAACAGCAAAAAAAAAATAAAATACCAAAATTAAAAGAAACACAATTAATTATATATGGATATAATGATCAAATCAATAATCTATTGAATAATCCTATTACAGGTCTAAAAATATAAATTAAATAATTAGATATTAATGGAATTGTATTTTCCTTTATAAATAATTTAATAGCATCAGAAATAGGCTGCAAGATACCCAAAAATCCAACTTTATTTGGACCTTTACGAATTTGAATATATCTCAAAATCTTTCGTTCCAAAAGAGTAATAAATGCTACTCCAATTAAAATAAATAATACAGTAATTAAAATAGTGATTAAATACAAAAATAATTCTTCTTATTTACTTTATATAATTTTCTTATTATTAGGAATTATAATTTCAGTTTCTTCAAACAATTGGCTGGGTTGTTGAATAGGAATTGAAATAAATATAGTTTCGTTTTTGCCTATAATATCAAATAAAATAAGAATTTACGCTTCAGAATCAATAATTAAATATTTTATTATTCAAAGAATGGGATCGAGTTTATTATTAATATCTATTATTATTNCGTGCGTTGCATTTGATTTAAATTATATAATTATAATTAGATTAATAATTAAAATTGGCTGTCCTCCTTTCCATTTTTGATATGTCTCTGTTATTGAAGGTTTAACTTGAATAGTGTGCTTTATTTTAATAACTATTCAAAAGATTATTCCTTTAATTATATTATCATATTTAAATGTTAATTTAAGATTATTTATTGTTATAGCATGTATTTGAGGATGCATTGGAGGGCTGGGTTATTCTTCAATGCGTAAAATTATTGCTTATTCCTCAATTTATAATTTAAGATGAATTTTTAGAGGTATTGTTATTATTAACTATTCATGGCTGATTTATTATTTTATTTATTCATTTACATTACTAGCTGTATGTTATATATTTAAAATATTTAATATTAATTACATTAATCAATTTATTATAGTATCTTTTAATTTTATAAAGTCAATTATAATAATGTGTATTTTTATGTCTATAGGAGGATTACCTCCTTTCTTGGGATTTTTTCCTAAATTAATTATAATCTACTGTTTAGTACTAAATAATATAATATTTATTTGTATTATATTATTAATAACAGCTCTGATTATTTTGTTTTTTTATTTACGAATTTTAATTACAACACTAATAATAAATACAATTTCAATGAAAAGAATTATTATGAGAGTTTCATATACTTATTATATTGCTGGAATATTTTCATTATTTGGAATAATTTTTTTTTCATTAATTACATTAAATATGTGTTAGATCTATAATATTTTGATTTATTCNNTAATTTTAATAGTTTTACTAATATTTTTATTTTTATTATTGATATTTATTTCCTTTAAATCTATAAAAGATCGAGAAAAATCATCCCCATTTGAATGTGGTTTTAGACCATTTGAATCATCTCGTATTCCATTCTCAAGACACTTTTTTTTAATTGCAGTAATTTTTTTAATTTTTGATGTTGAATTAGTGATTATTATACCTGTAATTTTAGTAATGATCAGGTTAAAAGTTATTGATATTTATATTATTATATTAATCTTCTTAATTATTTTAACTTTAGGATTATATCATGAATGACATAATAATATATTAAATTGANNNNNNNTTTTAATCAAAATATTTAATTTCAAAATAATTAAATTTAAAGGAATTCAATGTATTATAATCAAAAAAAACTCCCGAACTCTTCCAGAATTAAATGAAAATAAACTGGAAAAAAATATACCATGTTGAGTAAATGAAAATATATATAATCTATAGCAAGCAGATAAAAATGAAGACAAAATTAATAAAATCATTATTAATATATTTCAAGATATTAATCTATTAATAATTATAATTTCACCAGCTAAATTAATTGTAGGTGGACAAGATATATTTCTTGCTGACATTAAGAACCATAATAAAGATATTGAAGGTATAAATGTCAACATACCCTTATTAATTAATAATCTTCGACTATTAGTTCGTTCATACAATATATTTGCTAAACAAAATAATCCAGAAGAACATAAACCATGTCCAATTATTATATAATATGACCCACATAATCCCCAATTTCTTATAGTCATAATACCTCTAATCACAAGACCTATGTGAGCCACAGATGAATAGGCAATTATTGATTTAATATCAATCTGAATTATACATAAAATTCTAATTAAAATACTTCCAATTATTGAAACTGAAATCCATAAAAATCCAAATTTATAAAAATAGGAAGGAATAATATACATCACACGAATTAAACCATACCCTCCTAATTTTAGTAAAATTCTAGCTAAAATTATTGAACCAGAAACAGGAGCTTCAACATGAGCTTTAGGCAACCAAAAATGAAAAAATAATGGTATTTTAATTAGGAAAGCCATTATTATACCAAAATAAATATAAATATTAACAGAATAATTTATTAACATAAAAATACTAGTTATATTATTTTTATAAAGAAAAAAAATTCTTATTAATATTGGTAATGATGCAAATAATGTATAAAATAATAAATAATATCTTGCATCAATACGTTCTGGTTGATAACCCCAACCTATAATAATAATTATAATTGGAATTAATCTAGATTCAAAACAAATATAAAAAATTATAATTCTTGATGTAGAAAAAGAAATAATCAAAAAAATTATTAACACAAAACCAAAAATAAAAAAAATAATTTATTACGTTTATATATTGGACTAGAAATAATTATTACAAATGAAATTCAAATTCAAAATAATTAAACCATAAGATATTTCATCAATATAAAAATTATAACCTAATATATATAAACTCTTATTTATAAAAACCAAAAATATAATTATCAGAACAATAATTATATTTATTAATCATGAATTATAAATTAATAGGGTTAGTATAAAGCAAAAAAAAACTAACTTTATTGATATAGATATTAATTTATCATTATGATGAGATCGAATTAATATTACTAAGCATGATAAACCTATTACTCCTTCACAAACTCTAAAAACCAAAAAGATTAATAAGATATGCAAATCATATATTTTTATGGAAAAAATAAATGATAAAGAGCAAAATATTCTTAAAATAATTAATTCTAATCTTAATGTTGTTATTAAGTGTTTTCGATTTATACATAATGAAACTATACCTGAAAAGAATATAGAGATAAAATATATTAATAATGTTATATTCTATAAATATATAACAACAATTCAAACAATAAATGAAATTATTTGAATCTTAAAATTATTATTTATCAATTTATAATTTAATGATATCACTATTTTTAAAGTTTTTAAAATTCCTTGGGGCCCAAGGTTCTCACCTCACCCCATATCAATTGATTTTTGAAATATAAGTGATTGATTTATTAAAAACATTTGATTATGAAAAGTTGACAATTGTTTTATAAACCACATTGATCCAAAGAATTCGTAAACAATTTTTATTTTAGTTTTAGAAAAAATTGATATTTCATAACCCAATCAAATTCCTAATATTGAAAATATTAATGCTAATAATTTACCCTCAATAGGTATAAAAATTATAATTGGATCATTAAATATTAATCATCTTAATATAGACCCCGAAAAAATTGAGTAAATAGACAAAATAATAATTCTTTTAATTATATTATTTATACTTTCATTTAATGATCTTAATTTATAAAAATTGTAATTTATATTTATTGAAAAAAATGTCAATCGGGCAGAATAAAAGGAAGTTAAACCAATACCAATATATATAAATATTATAATTAGTAAATTAATATGCCCAGAACATATAGTTTCTATAATTAAATCTTTTGAGTAAAATCCTCTCATAAATGGAATTCCACATAATGACATATTAGCAATATTTATTATTGTAATAGTTAAAGGTATTTGAAATCTTAAACATCCTATTAAACGAATGTCTTGATTGTTATTGAAATTGTGAATTAAAATTCCTGCACACAAAAATAAAGTAGCTTTAAATAAAGCATGAACAATCAAATGAAAAAATGATAATATTGGATAGCCAAATATAATAATTGTTATCATAATTCCCAGCTGTCTTAAAGTTGATAATGCAATAATTTTTTTCAAATCAAACTCAAAATTAGCTCTAATTCCTGATATTAATATAGTTAAAATTGAAATTAATATAAATAATTGAATATAATCAAAATTCAAGATAATATTAGAAAATCGAATTATTAAATATACTCCAGCAGTGACAAGTGTGGATGAATGAACCAAAGCAGATACAGGTGTTGGAGCAGCTATAGCTGCTGGCAACCATGAAGAAAATGGAATTTGAGCTCTCTTTGTAAATCTTGCGATAATAATCATAATCAATATAATTTATATTCTTATTAATANAAGATAAAAGTTTCATGATCCAAAGTTTAATATTCATGCAATAGCTATTAAAATAGCAACATCCCCTACACGATTCATTAATGCAGTTAATATTCCTGCATTATTAGAATATAAATTTTGATAATAAATAACTAAGCAATAAGAAACTAAACCAAGTCCATCTCAACCAATAATAATTCTTATTATATTAGGTCTAATAATTAATAAAACTATAGACATTACAAACCCTAAAACAATATAAATAAAACGATTAATGAATTTGTCATTATTTATATATATACCACTATATAAAACAACCACTGATGAAATAAATATAACTGTCGAAAGAAATTGTTGATATAAAATCAAAAATTATAGTCATGTAAATATTGCATGAATTAATTGAAAATTATTCATTCTAATANNNNNNNNNNNNNNNNNNNNNNNNNNNNNNNNNNNNNNNNNNNNNNNNNNNNNNNNNNNNNNNNNNNNNNNNNNNNNNNNNNNNNNNNNNNNNNNTATCATGAAAATTGCTATGTATTTAATAATTATTTTATCAATAAATTTTATTTTCATAAAGCACCCTTTATCAATAGGATTAATTTTAATAATACAAACAGTATTATCTTGCCTAATTTGTAGACTTTACTTAAGTTGTTATTTATTTTCTTATATCTTATATCTTATTTTTATTGGTGGTATATTAATTTTATTGTCATTTGCATNNNNNNNNNNNNNNNNNNNNNNNNNNNNNNNNNNNNNNNNNNNNNNNNNNNNNNNNNNNNNNNNNNNNNNNNNNNNNNNNNNNNNNNNNNNNNNNNNNNNNNNATCACTNNNNNNNNNNNNNNNNNNNNNNNNNNNNNNNNNNNNNNNNNNNNNNNNNNNNNNNNNNNNNNNNNNNNNNNNNNNNNNNNNNNNNNNNNNNNNNNNNTTAATTTATTTACTATTTGTTTTAATTATTGTAATTAATATTTTAACAATAAATATGTTAACTCTTCGAAGAAGAACTTCTTTCTAACTACACCTTCCGGTACAGTTACTTTGTTACGACTTATCTCATTAATATTATGAGAGTGACGGGCGATATGTACATAAATTAGAGCTAATTTCAATTAATTAAATTTAATTAATTTATTATCAAATCCAATTTCATATTTTTTNNAATTTAAAAAATAATTCAATATATAATTAATTGTAACCCATTTTTTCTTTAATATAAACTGCACCTTGACCTGACATTATATATAANATAATATAGTATGAAAATATTTCTTATAAAACATTCTTGACAGAGATATACAAATTAAATTAAAGTTTTTTCTATCGTGGAATATCAATTATAAAACAGGTTCCTCTGATAAGATAAATTACCGCCAAATTCTTTGAATTTAAAGATCATTTCTAATAATAATCAAGTTATTTTTATCACATTTTTAATAATAGGGTATCTAATCCTAGTTTAAACAAAAATTTTTCAGACATAAAATTAATTTTTCAAGATAAAATATATTTCACCAAAATTATAGATATTTTATATTATTGCTATATTAACTGAAACTTAACTATTTAAATTAAAGAAATCGTATAACCGCAACTGCTGGCACGAAATTTGATTCTTTTAAATAAAATTACTAATTCTAATCTTATTAATTAATAATAATAAATACTGCGAATTTATTAAAANTATTTTCATTTAGAAATCAAAAACAATCAAAAAATTGCATGTAATATAATTTTAAATTTAAATTGTCTTAAACCAGAATTAAACTTCAAATAATAAAATTATTTAATGAGGTCCTTTCGTACTAACATTAAAAATAATTGAGTAGATAGAAACCAACCTGGCTCACGCCGGTCTGAACTCAGATCATGTAAGAATATTAAGGGTCGAACAGACCCAGAAATAATAAATTTTGCTCCAATCCCTATTCTTAATCCAACATCGAGGTCGCAATCATATTTATCGATATGAACTCTTCAAATTAATTACGCTGTTATCCCTAAGGTAATTTATTCTTATAATCAAAAATTTGGATCAATATTTACATAAATTTATGAAATTTTTATTAAAAGTTAGTTATATTTTAATATCACCCCAACAAAAAAATAAATTAAATAAAAAAAATTAATTAACTATAAANAAATAAACTCAATNAAAATTTTAAAAATTCTATAGGGTCTTCTCGTCCCACTCATATATTTAAGCTTTTTTACTTAAAAATCAATTTCAATTGTTAATATTAATTAAGTTAATTTCTCATTCAATCATTCATTCAAGCCTCCAATTAAAAGACTATTTATTATGCTACCTTTGCACGGTCAATTTACCGCGGCCATTCAATTTTTAATCATTGGGCAGATTAGACTTTTAATTCATTCTAAAAGACATGTTTTTGATAAACAGGTGAAAATTTTTTTTGCCTAAATTATAAACATTATTCATAACTAAAAAACCAGATATCATATAATTTGATAAAATGTCATTTCCAGAATAAATTTATTAATATATATACAATAATAACTAACAAATNTATACTAAATCATTATATTTCGGGAAAAATAAATACTTAAAAGTCTTTAATTAACCCTGATACAAAAGGTACAAATTACATTCTACTTATAACTATTTATAAAGTTNTCACCCTCTCAGTCAATCCAAATTATTTTTTTATATAAAATTACTTTAACACAAAAATTTTTTTTTCAAAATATNAAAAAACAACTTTTANTAAAATTATATTATGGGATATATTTAATTATAATAATTGAATTGCAGTCAATAGGTGTTAACTATAACTATCCTTAAGTAATGAAGTAAAATATTACATTTAGTTTCGACCTAAAATAAGAATTANAATTCCTTACTTTTAAATGAAGCCAAAATAGAGGCATTTTATTGTTAATAAAATAATTGAAANTTTATTTCCATTTAAAGAAAAATTAGTTTAATTAAAATATAAATCTGTCAGATTTATGAAACTTCTGAGTATTTTTCTGACTTTTTGTGATNTNTTCTACTTTAAATTTGCAATTTAATATTATAAATTAAATATAAGACTTTATGTTAGCAGAATTAAACTGTCTATTAATATCAAAAATTAATGTTCTTCATAAACTATAACATTTATAAAGTTTACAAAACATTTCATTTTCATTGAAAAGAGAGACTTAGTCTTATAAGATTCTATTAGTATATAAGTATATTTAACTTCCAATTAAAAGGATTAATTTTATTAAATAGAATATATCCAATAAATTTNNTTTTATCTTTAAAATCACAATTTAATATTTTTATTAAACTAATTGAATTACTATTTGTAATAAATATTACATTATTAAATTCTAAATTTAAGGCATTAATTTTGCTAAAATAGTTCTAATATGGCAGATTAGTGCAATGAATTTAAGATTCATATATAAAATATNATTTTTTTATTAGAAAATAAGATGCCTGAATNNAAAAGGATTATTTTGATAGAATAAATTATGTAATAGAATTACTCNNNCATTAAGTGGCTGAAAGTAAGTAATGGTCTCTTAAACCAATTAATAGTAATTAACAAATACTCTTAATGAAAGATAAGCTAAAATTAAAGCTAATGGGTTCATACCTCACTTATGGAAANTTTTCCTCTTTTTATTTAAAAACAAAAGTTACCTTAATATCTTCAATATTATGCTCTTAATTAAGCTATTTAAACTCAGAAAAGATAAAACAATCATTTTAAACTCCCAAAGTTTATATTTTATATTTAAACTATTTTCTGAGTTAATTAGCTTAACCNAAAGCATTTATTTTGAAAGTAAAAGAAAAGATATTAAATCTATTAACTTAGGAAACTAGAAATAAAATTAGCTTCTAACTAACTTTTAAAGCGGTTAAATTCCGTTTTTTCCTTGTTTTAATAGTTTAATNNNAAAAATTTAGATCTTGTAAATCTAAGATAACATAAGTTTTAAAAATAGGTTTTAAGTTATTAANAACTATTATCCTTCAAAGTTAAAAATATAATTANATTTATTATTAGAAGAAACATTTTTTCATAAATAAATTTACAGTTTATTACCTATTTTCGGCCATCCTAATCAAGTTATATTGATTAAACAATATTCTCATTAGTGTAAATAAAGTGCTTATATAGCTTTAACTTG

>subalpina_01.NZ.WN.RIM.01

AATTTATTTTCATCATTTGATCCTTCAACTGGATTTTTATCTCTTAATTGATTAAGATCAATTATTCTACTATCATTTCTACCTCTAACTTACTGATATATTCCTAATCGTTTTATTCTTTTATACAATAAAATTTTAATTTCATTAAATAATGAATTAAATATATTAATAAATTATAAATCATTGGGAAGATCATTAATACTTTTATCGTTATTTATATTTATTCTATTAAATAATTTATTAGGCTTACTTCCATATATTTTTACAAGTTCAAGTCATTTAGTATTTACAATAAGATTAGCATTACCTTTATGATTATCATTCATACTTTATGGATTTATTAATAATATAAATCATATATTTTGCCACTTAGTTCCGTCAGGAACTCCCAATATTTTAATGCCTTTTATAGTTATTATTGAAAGAGTTAGAAACTTAATTCGTCCCGGTTCTTTAGCTGTTCGATTAACAGCTAATATAATTGCTGGGCATCTTTTAATAACCTTATTAGGAAATTTACCTATAAGTTATGAATTATATTCAGGCTTTATTATTATTTTTCAAGTTACATTAATATTATTTGAATTAGCTGTTTGTGTTATTCAATCTTATGTATTTATAGTACTTAGAACTTTATATTATAGAGAAGTAAATATTCCCCAAATATCTCCTCTTAATTGATTATTATTTATTTATTTTATTATTGTAATTATTGTTATTATTATATTTATGTATTTTACTTATCTATTAAAATCTAATATAACAGCAAAAAATAAATTAATAGATAGAATAATTTGATTTAAACCTACACGACAAAATCATCCTTTAATTAAAATTATTAACAATTCATTAATTGACTTTCCAGCACCATCTAATTTATCTTATTGGTGAAATTTTGGATTTGTATTAGGATTGTGTTTAATAATCCAAATTATAACTGGATTATTTTTATCAATACATTATAATGCCAGCATTATAAATGCTTTCGAAAGATTAAGACATATTTGTCGTGATGTAAATTATGGTTGAATTTTACGAGTCATCCACGCTAATGGAGCTTCATTATTTTTTATTTGTGTTTATCTACATGTAGGACGTGGATTATACTATGGATCATACAAATACATTGAAACATGATCTATTGGTGTAATTATGTTATTAATATTAATAGCTACAGCTTTCTTGGGATATGTTCTACCGTGAGGACAAATATCTTTTTGGGGGGCTACAGTTATTACAAATTTATTATCAGCTATTCCTTATTTAGGAAGAATATTAGTTAACTGAATTTGAGGTGGGTTTGCAGTTGATAACGCAACACTAACTCGATTCTACTCATTCCACTTTATTTTACCATTCATTGTATTAAGATTAACAATTATTCACTTATTGTATCTTCATACAACAGGTTCAAATAATCCACTAGGAATTAATTCTAATAATGACAAAGTTCCTTTTCACCCATATTTTTCCATTAAAGATATTATGAGACTAATTATATTAATAATTATTTTCTTTATATTAGTTATATTGGAGCCTTATATTCTAGGGGATCCAGATAATTTCATTCCTGCCAATCCACTTGTAACACCAAAGCATATTCAACCTGAATGATATTTTTTATTTGCCTATGCAATTCTTCGTTCAATTCCTAACAAATTAGGAGGAGTAATTGCTCTTTTCATATCAATTTTTATTTTAATGTTTGTACCTTTATTAAATAATTCTAACTTTATAGGATTAAATAATTATCCAATTAATCAAATTATATTTTGATATATAGTAATCATCTTAATTTTATTAACTTGAATTGGAGCACGACCTGTTGAATTGCCTTACATTAACTTTGGAATATTTTTAACACTTATATATTTTTCTTACTTCATTATTGACCCAATAATTAAATATACTTGAGACAAGTTAGTTAGGTAGATGAAAAAATGATTCTTCTCAACTAATCATAAAGATATTGGAACAATATATTTTATTTTTGGTATTTGATCAGGTATAATTGGTACAACTTTAAGAGTTTTAATTCGTGTAGAACTTGGAACTCCAGGTTCATTTATTGGTGATGATCAAATTTATAATGTAATTGTTACTGCTCATGCTTTCATTATAATTTTTTTCATGGTTATGCCAATTATAATTGGTGGATTCGGAAATTGACTAGTTCCTTTAATAATTGGAGCTCCTGATATAGCTTTTCCTCGTATAAATAATATAAGTTTTTGATTATTACCCCCTTCCCTAATTCTTATTTTAGTGGGAAGAATAGTTGATAGAGGTGCGGGTACCGGCTGAACAGTTTATCCTCCCCTATCTGCCGGGATTGCACATTCTGGTTCATGTGTTGATTTGACTATTTTTTCCTTACATCTCGCAGGTGTATCTTCAATTTTAGGTGCTGTTAATTTCATTAGAACAATCTTTAATATACGTTCAATAGGAATTTGATTAGATCGAATGCCCTTATTTGTATGAGCAGTTTTAATTACTGCATTTTTGTTATTGTTATCTTTGCCTGTTTTAGCAGGAGCTATTACAATGTTATTAACAGATCGAAATTTAAATACTTCATTTTTTGACCCTGCAGGGGGGGGAGATCCAATTCTTTACCAACATTTGTTTTGATTTTTTGGTCATCCTGAAGTTTATATTTTAATTTTACCAGGATTTGGTTTAATTTCTCATATTATTACCCAAGAGAGAGGTAAAATTGAATCTTTTGGTTCATTAGGAATAATCTATGCTATAATATCAATTGGTATTTTAGGATTTGTTGTATGGGCACATCACATATTTACTGTAGGAATAGATGTTGATACACGTGCATATTTTACATCAGCTACTATAATTATTGCAGTTCCTACTGGAATTAAAGTATTTAGTTGACTTGCAACTTTAAGAGGTATAAAAATCAATATTACATCTTCAGCTTTATGGGCTTTAGGATTTGTTTTTCTATTTACTATTGGAGGCTTGACTGGAGTAATTCTAGCTAATTCTTCAATTGATATTATATTACATGATACATACTATGTGGTTGCACATTTTCATTATGTATTATCAATAGGAGCAGTTTTTGCTATTATAGCAAGATTTATTCATTGGTTTCCATTATTTACAGGATTAAGATTAAACTCAAATTGATTGAAAATTCATTTTCTATTAATATTTATTGGTGTAAATATAACATTCTTTCCTCAACATTTCTTAGGATTAAGAGGAATACCTCGTCGGTACTCAGATTATCCTGATGCTTATATATCATGAAACATAATTTCATCAATAGGAAGAATTATATCTTTAGTTGGAATCTTATTTTTATTATTTATTGTTTGAGAAAGTTTTATTTCAATACGGTTAGTATTATATTCTAATAGAATTCAATCTTCTATTGAATGAATACAAAAGTTTCCTCCATCTGAACATTCTTACAACGAAATGCCATTATTAATTCAAATTTCAAATTGATCTTATATTAATATACAGGATGCTGTATCACCATTAATAGAGCAGTTAATATTTTTTCATGATCATGTATTAATTATTTTAATTACAATCACAATTGTTGTTGCTTACATAATAATAATATTAATATTAAATAAAATTATTAATCGTTTACTTCTTGAAGGACAATTAATTGAATTTATTTGAACTTTATTACCTGCAATAACTTTAATTTTTATTGCATTACCATCATTGCGATTATTATATATATTAGACGAAATTAGTAATCCATTACTGACATTAAAAATTATTGGTCATCAATGATACTGATCATATGAATATTCAGATTTTTCTGATGTAGAATTTGATTCATATATAAAATCTATTAACGAAATAAATAAAAATGAATTTCGTTTATTAGATGTAGATAATCGAGTAATTCTACCATTTAATATTCAGGTCCGACTATTAGTATCTTCTTTCGATGTTATTCACTCTTGAGCAATACCATCAATAAGACTTAAAGTTGATGCAGTACCAGGACGACTAAATCAAATAAGAATATTAATTAGTCGTCCTGGTATATCATATGGACAATGCTCTGAAATTTGTGGGGCAAATCATAGATTTATGCCCATTGTAATTGAAAGAATTAGAATAAAAATATTTATTAAATGATTAATTAGTTATATGAATAATCATCCTTATCATATAGTTGATTATAGACCCTGACCTCTAACTGGATCAATTGGAGCATTAACTTTTGTTTCTGGCATAATTATAATGTTTCATAAATGTAACTTTATATTATTATATATCGGTATTTTACTATTATTAATAACAATAATTCAATGATGACGTGACATTTCTCGAGAGGGAACATTTTTAGGAATACACACTATAATGGTAGTGAATGGTTTAAAAATAGGCATATTATTATTTATTGTATCGGAAGTTCTTTTTTTTGTTTCATTTTTTTGGGGATTTTTTCATAGTAGATTAAGACCTGTGGTAGAAATTGGAATAATTTGACCACCTATGGGCATTTATGTCTTTAATCCAACTCAAGTCCCTTTATTAAATACAATAATTCTATTATGCTCAGGAATCACAATTACTTGAGCTCATCATTCAATTATATGTGGCAACCACATTAATTCTATTTATAGAATTATGCTAACTGTTATTCTAGGGATATATTTCACCTTTCTGCAAGGTTATGAGTATTATGAAGCTCCATTTGCAATTAATGATTCCATTTATGGATCTTCTTTTTTTATAGCTACTGGATTCCATGGAATCCATGTAATTATTGGAACAAGATTTATTATTGTATGCTTATTACGACAAATAAAGTTCCATTTTTCAAGAAATCATCATTTTGGTTTTGAAGCTGCCGCTTGATATTGACATTTTGTTGATGTAGTATGATTATTTCTATATTTATCAATTTATTGATGAGGAAGATAATAAATTATAGATTTTAAAAAAAAATAAAAAATAAATAAATTTAGAGATACCGGTAAATAAGATTTTCAAGCTAAATACATTAATTTATCATAACGATAACGAGGAAGAGTACCTCGAACTCAAATGAAAAGAAAACATATTATAGAAACTTGAATTGGAAAGACAATTGAACTAATTTTTCCTCCTAAAAATATTAAACAAAATAATATTCTCATAAATAAAATACTTGAATACTCAGCCAAAAAAATAAATGCAAATCTAGAACCTCCATATTCAACATTAAAGCCAGATACTAACTCTGATTCTCCCTCAGAGAAATCAAATGGAGATCGATTAGTTTCAGCTAAAGCTGATGATAATCAACATATTCTTAATGGAAGAAATAAAAAAATAAATCACACAAACTCCTGAAATATAAACAAATCAATAATATTATAACTTCTTATTATTATTATAGGACACAACATAATCAAAACCAATCTTACTTCATAAGAAATAGATTGAGCAATTGATCGAATACATCCTAATATTGCATAACTTGAATTAGAAGATCAACCTGTTAATATTAATGAATAGACTCTTATTCTTGAACAACAAAAAAAAAATAAAATACCAAAATTAAAAGAAACACAATTAATTACATATGGATATAATGATCAAATCAATAGTCTATTGAACAATCCTATTACAGGTCTAAAAATATAAATTAAATAATTAGATATTAATGGAATTGTATTTTCCTTTATAAATAATTTAATAGCATCAGAAATAGGCTGTAAGATACCTAAAAATCCAACTTTATTTGGACCTTTACGAATTTGAATATATCTCAAAACCTTTCGCTCCAAAAGAGTAATAAATGCCACTCCAATTAAAATAAACAATACAGTAATTAAAATAGTAATTAAATACAAAAATAATTCTTCTTATTTACTTTATATAATTTTTCTATTGTTAGGGATTATAATTTCAGTTTCTTCAAACAATTGGCTGGGTTGTTGAATAGGAATTGAAATAAATATAGTTTCATTTTTGCCTATAATATCAAATAAAATAAGAATTTACGCTTCAGAATCAATAATTAAATATTTTATTATTCAAAGAATGGGATCGAGTTTATTATTAATATCNNNNNNNNNNNNNNNNNNNNNNTTTGATTTAAATTATATAATTATAATTAGATTAATAATTAAAATTGGCTGTCCTCCTTTCCATTTTTGATATGTCTCTGTTATTGAAGGTTTAACTTGGATAGTATGTTTTATTTTAATAACTATTCAAAAGATTATTCCTTTAATTATATTATCATATTTAAATGTTAATTTAAGATTATTTATTGTTATAGCATGTATTTGAGGATGCATTGGAGGGCTGGGTTATTCTTCAATGCGTAAAATTATTGCTTATTCCTCAATCTATAATTTAAGATGAATTTTTAGAGGTATTGTTATTATTAACTATTCATGGCTGATTTATTATTTTGTTTATTCATTTACATTATTAGCTGTATGCTATATATTTAAAATATTTAATATTAATTACATTAATCAATTTATTATAGTATCTTTTAATTTTATAAAGTCAATTATAATAATGTGTATTTTTATGTCTATAGGAGGCCTACCTCCTTTCTTGGGATTTTTTCCTAAATTAATTATAATCTACTGTTTACTACTAAATAATATAATATTTATTTGTATTATATTATTAATAACAGCTCTGATTATTTTGTTTTTTTATTTACGAATTTTAATTACAACACTAATAATAAATACAATTTCAATGAAAATAATTATAATGAGAGTTTCATGTACTTATTATATTGCTGGAATATTTTCATTATTTGGAATAATTTTTTTTTCATTAATTACATTAAATATATGTTAGNNNNNNNNNNNNNNNNNNNNNNNNNNNNNNNNNNNNNTTATANNAATANNNNNNNNNWTAWTATTAATATTTATTTCCTTTAAATCTATAAAAGATCGAGAAAAATCATCCCCATTTGAATGTGGTTTTAGACCATTTGAATCATCTCGTATTCCATTCTCAAGACACTTTTTTTTAATTGCAGTAATTTTTTTAATTTTTGATGTTGAATTGGTAATTATTATACCTATGATTTTAGTAATAATTAGATTAAAAATTATTGATATTTATATTATTATATTAATCTTCTTAATTATTTTAACTTTAGGATTATATCATGAATGACATAATAATATATTAAATTGANNNNNNNGTTTAATCAAAATATTTAATTTCAAAATAATTAAATTTAACGGAATTCAATGTATTATAATCAAAAAAAACTCCCGGACTCTTCCAGAATTAAATGAAAATAAACTGGAAAAATATATACCATGTTGAGTAAATGAAAATATATATAATCTATAGCAAGCAGATAAAAATGAAGATAAAATCAATAAAATCATTATTAATATATTTCAAGATATTAATCTATTAATAATTATAATTTCACCAGCTAAATTAATTGTCGGTGGACAAGACATATTTCTTGCTGACATTAAAAATCATAATAAAGATATTGAAGGTATAAATGTCAACATACCCTTATTAATTAATAATCTTCGACTATTAGTTCGCTCATACAATATATTTGCTAAACAAAATAATCCAGAAGAACATAAACCATGTCCAATTATTATATAATATGATCCACACAATCCCCAATTTCTTATAGTTATAATACCTCTAATCACAAGACCTATGTGAGCCACAGATGAGTAAGCAATTATTGATTTAATATCAATCTGAATTATGCATAGAATTCTAATTAAAATACTTCCAATTATTGAAACTGAAATCCATAAAAATCCAAATTTATAAAAATAAGAAGGAATAATATATATCACACGAATTAAACCATACCCTCCTAATTTTAATAAAATTCTAGCTAAAATTATTGAACCAGAAACAGGAGCCTCAACATGAGCTTTAGGTAACCAAAAATGAAAAAATAATGGTATTTTAATTAAGAAAGCCATTATTATACCAAAATAAATATAAATATTAACAGAATAATTTATTAACATAAAAATACTAGTTATATTATTTTTATAAAGAAAAAAAATTCTTATTAATATTGGTAATGATGCAAATAATGTATAAAATAATAAATAATATCTTGCATCAATACGTTCTGGTTGATAACCCCAACCTATAATAATAATCATAATTGGAATTAATCTAGATTCAAAACAAATATAAAAAATTATAATTCTTGATGTAGAAAAAGAAATAATCAAAAAAATTATTAACATAACACCAAAAATAAAAAAAATAATTTATTACGTTTATATATTGGACTAGAGACAATTATTAGAAATGAAATTCAAATTCAAAATAATTAAACCATAAGAAATTTCATCAATATAAAAATTATAGCCTAATATATATAAACTCTTATTTATAAAAATCAAAAATATAATTATCAAAACAATAATTATATTTATTAATCATGAATTATAAATTAATAGGGTTAATATAAAGCAAAAAAAAACTAACTTTATTGACATAGATATTAATTTATCATTATTATGAGATCGAATTAATATTACTAAGCATGATAAACCTATTACTCCCTCACAAACTCTAAAAACCAAAAAGATTAATAAAATATGCAAATCATATATTTTTATAGAAAAAATAAATGATAAAGAACAAAATATTCTTAAAATAATTAATTCTAATCTTAATGTTGTTATTAAATGTTTTCGATTCATACATAATGAAACTATACCTGAAAAGAATATAGAGATAAAATATATTAATAATGTTATATTCTATAAATATATAACAACAATTCAAACAATAAATGAAATTATTTGAATCTTAAAATTATTATTTATTAATTTATAATTTAGTGATATCACTATTTTTAAAGTTTTTAAAATTCCTTGGGGACCAAGGTTCTCACCTCACCCCATATCAATTGATTTTTGAAATATAAGTGATTGATTTATTAAAAACATTTGATTATGAAACGTTGACAATTGTTTTATAAACCACATTGATCCAAAGAATTCGTAAACAATTTTTATTTTAGTCTTAGAAAAAATTGATATTTCATAACCCAATCAAATTCCTAATATTGAAAATATTAATGCTAACAATTTACCCTCAATAGGTATAAAAATTATAATTGGATCATTAAATATTAATCATCTTAATATAGACCCCGAAAAAATTGAGTAAATAGACAAAATAATAATTCTTTTAATTATATTATTTATACTTTCATTTAATGATCTTAATTTATAAAAATTGTAATTTATATTTATTGAAAAAAATGTCAAGCGGGCAGAATAAAAGGAAGTTAAACCAATACCAATATATATAAATATTATAATTAATAAATTAATATGCCCAGAACATATAGTTTCTATAATTAAATCTTTTGAGTAAAATCCTCTCATAAAGGGAATTCCACATAATGATATATTAGCAATATTTATTATTGTAATAGTTAATGGTATTTGAAATCTTAAACATCCTATTAAACGAATGTCTTGATTATTATTGAAATTGTGAATCAAAATTCCTGCGCACAAAAATAAAGTAGCTTTAAATAAAGCATGAACAATCAAATGAAAAAATGATAATATTGGATAACCAAATATAATAATTGTTATTATAATTCCCAGCTGTCTTAAAGTTGATAATGCAATAATTTTTTTCAAATCAAACTCAAAATTTGCTCTAATTCCTGATATTAATATAGTTAAAATTGAAATTAGTATAAATACTTGAATATAATCAAAATTCAAGATAATATTAGAAAATCGAATTATTAAATACACTCCAGCAGTAACAAGTGTGGATGAATGTACCAAAGCAGATACAGGTGTTGGAGCAGCTATAGCTGCTGGCAACCATGAAGAAAATGGAATTTGAGCTCTCTTTGTAAATCTTGCAATAATAATCATAATCAATATAATTTATATTCTTATTAATANAAGATAAAAGTTTCATGACCCAAAATTTAATATTCATGCAATAGCTATTAAAATAGCAACATCCCCTACACGGTTCATCAATGCAGTTAATATTCCTGCATTATTAGAATATAAATTTTGATAATAAATAACTAAACAATAAGAAACTAAACCAAGTCCATCTCAACCAATAATAATTCTTATCATATTAGGCCTAATAATTAATAAAACTATAGATATAACAAACCCTAAAACAATATAAATAAAACGATTAATGAATTTGTCATTATTTATATATATTCCACTATACAAAACAACCACTGATGAAATAAATATAACTGTCGAGAGAAATTGTTGATATAAAATCAAAAATTATAGTCATATAAATATTGCATGAATTAATTGAAAATTATTCATTCTAATATAATTATATAATTATTTTTGAAACTCATTGAATATTAATATTAACAAACTCATAAATATAAGAATAATGAATATATAAACATTAAATTTTTTATTTATCATGAAAATTGCTATGTATTTAATAATTATTTTATCAATAAATTTTATTTTCATAAAGCACCCTTTATCAATAGGATTAATTTTAATAATACAAACAGTATTATCTTGCCTAATTTGTAGACTTTACTTAAGTTGTTATTTATTTTCTTATATCTTATATCTTATTTTTATTGGTGGTATATTAATTTTATTTATATATATATCAAGAATTGCATCAAATGAAAAATTTATCTATTCAATTAAATTAATAATATTTAATTTTTTCTCTTTAACTTTAATTAATTTAATCAAAATAATTGATTTAAAATCACTAAATATTAGAAGAAATATTATAATATATATAAATCACAACAACTTTATAATAAGAAAAATATATATTATTCCTTCTGGAATAATAACACTAATTCTAACAATTTATTTATTATTTGTTTTAATTATTGTAATTAATATTTTAACAATAAATATACTAACTCTTCGAAGAAGAACTTCTTTCTAACTACACCTTCCGGTACAGTTACTTTGTTACGACTTATCTCATTAATATTATGAGAGTGACGGGCGATATGTACATAAATTAGAGCTAATTTCAATTAATTAAATTTAATTAATTTATTATCAAATCCAATTTCATATTTTTTNNAGTTTAAAAAATAATTCAATACATAATTAATTGTAACCCATTTTTTCTTTAATATAAACTGCACCTTGACCTGACATTATATATAANATAATATAGTATGAAAATATTTCTTATAAAACATTCTTGACAGAGATATACAAATTAAATTAAAGTTTTTTCTATCGTGGATTATCAATTATAAAACAGGTTCCTCTGATAAGATAAATTACCGCCAAATTCTTTGAATTTAAAGATCATTTCTAATAATAATCAAGTTATTTTTATCACATTTTTAATAATAGGGTATCTAATCCTAGTTTAAACAAAAATTTTTCAGACATAAAATTAATTTTTCAAGATAAAATATATTTCACCAAAATTATAGATATTTTATATTATTATTATATTAACTGAAACTTAACTANNNNNNNNNNNNNNNNNNNNNNNNNNNNNNNNNNNNNNNNNNNNNNNNNNNNNNNNNNNNNNNNNNNNNNNNNNNNNNNNNNNNNNNNNNNNNNNNNNNNNNNNNNNNNNNNNNNNNNNNNNNNNNNNNNNNNNNNNNNNNNNNNNNNNNNNNNNNNNNNNNNNNNNNNNNNNNNNNNNNNNNNNNNNNNNNNNNNNNNNNNNNNNNNATAATAAAATTATTTAATGAGGTCCTTTCGTACTAACATTAAAAATAGTTGAGTAGATAGAAACCAACCTGGCTCACGCCGGTCTGAACTCAGATCATGTAAGAATATTAAGGGTCGAACAGACCCAGAAATAATAAATTTTGCTCCAATCCCTATTCTTAATCCAACATCGAGGTCGCAATCATATTTATCGATATGAACTCTTCAAATTAATTACGCTGTTATCCCTAAGGTAATTTGTTCTTATAATCAAAAATTTGGATCAATATTTACATAAATTTATGAAATTTTTATTAAAAGTTAATTATATTTTAATATCACCCCAACAAAAAAATCAATTAAATAAAAAAAATTAATTAACTATAAANAAATAGACTCAATNAAAATTTTAAAAATTCTATAGGGTCTTCTCGTCCCACTCACATATTTAAGCTTTTTTACTTAAAAATCAATTTCAATTGTTAATATTAATTAAGTTAATTTCTCATTCAATCATTCATTCAAGCCTCCAATTAAAAGACTATTTATTATGCTACCTTTGCACGGTCAATTTACCGCGGCCATTCAATTTTTAATCATTGGGCAGATTAGACTTTTAATTCATTCTAAAAGACATGTTTTTGATAAACAGGTGAAAATTTTTTTTGCCTAAATTATAAACATTATTCATAACCAAAAAACCAGATATCATATAATTTGATAAAATGTCATTTCCAGAATAAATTTATTAATATATATACAATAATAACTAACAAATNTATACTAAATCATTATATTTCGGGAAAAATAAATACTTAAAAGTCTTTAATTAACCCTGATACAAAAGGTACAAATTACATTCTACTTATAAATATTTATAAAGTTNTCACCCTCTCAGTTAATCCAAATTATTTTTTTATATAAAATTACTTTAACACAAAAATTTTTTTTTCAAAATGTAAAAAAACAACTTTTANTAAAATTATATTATGGGATATATTTAATTATAATAATTGAATTGCAGTCAATAGGTGTTAACTATAACTATCCTTAAGTAATGAAGTAAAATATTACATTTAGTTTCGACCTAAAATAAGAATTANAATTCCTTACTTTTAAATGAAGCCAAAATAGAGGCATTTTATTGTTAATAAAATAATTGAAANTTTATTTCCATTTAAAGAAAAATTAGTTTAATTAAAATATAAACCTGTCAGATTTATGAAACTTTTAAGTATTTTTCTGACTTTTTGTGATNTNTTCTACTTTAAATTTGCAATTTAATATTATAAATTAAATATAAGACTTTATGTTAACAGAATTAAACTGTCTATTAATATCAAAAATTAATGTTCTTCATAAACTATAACATTTATAAAGTTTATAAAACATTTCATTTTCATTGAAAAGAGAGACTTAGTCTTATAAGATTCTATTAGTATATAAGTATATTTAACTTCCAATTAAAAGGATTAATTTNNNNNNNNNNNNTNTATCCAATAAATTTNNTTTTATCTTTAAAATCACAATTTAATATTTTTATTAAACTAATTGAATTACTACTTGTAATAAATATTACATTATTAAATTCTAAATTTAAGGCATTAATTTTGCTAAAATAGTTCTAATATGGCAGATTAGTGCAATGAATTTAAGATTCATATATAAAATATNATTTTTTTATTAGAAAATAAGATGCCTGAATNNAAAAGGATTATTTTGATAGAATAAATTATGTAATAAAATTACTCNNNCATTAAGTGGCTGAAAGTAAGTAATGGTCTCTTAAACCAATTAATAGTAATTAACAAATACTCTTAATGAAAGATAAGCTAAAANTAAAGCTAATGGGTTCATACCTCACTTATGGAAANTTTTCCTCTTTTTATTTAAAAACAAAAGTTACCTTAATATCTTCAATATTATGCTCTTAATTAAGCTATTTAAATTCAGAAAAGATAAAACAATCATTTTAAACTCCCAAAGTTTATATTTTATATTTAAACTATTTTCTGAGTTAATTAGCTTAACCNAAAGCATTTATTTTGAAAGTAAAAGAAAAGATATTAAATCTATTAACTTAGGAAACTAGAAATAAAATTAGCTTCTAACTAACTTTTAAAGCGGTTAAATTCCGTTTTTTCCTTGTTTTAATAGTTTAATNNNAAAAATTTAGATCTTGTAAATCTAAGATAACATAAGTTTTAAAAATAGGTTTTAAGTTATTAANAACTATTATCCTTCAAAGTTAAAAATATAATTANATTTATTATTAGAAGAAACATTTTTTCATAAATAAATTTACAGTTTATTACCTATTTTCGGCCATCCTAATCAAGTTATATTGATTAAACAATATTCTCATTAGTGTAAATAAAGTGCTTATATAGCTTTAACTTG

>horologium_14.NZ.NC.NIG.05

NNNNNNNNNNNNNNNNNNNNNNNNNNNNNNNNNNNNNNNNNNNNNNNNNNNNNNNNNNNNNNNNNNNNNNNNNNNNNNNNNNNNNNNNNNNNNNNNNNNNNNNNNNNNNNNNNNNNNNNNNNNNNNNNNNNNNNNNNNNNNNNNNNNNNNNNNNNNNNNNNNNNNNNNNNNNNNNNNNNNNNNNNNNNNNNNNNNNNNNNNNNNNNNNNNNNNNNNNNNNNNATTCTATTAAATAATTTATTAGGTTTACTTCCATATATTTTCACAAGTCCAAGTCATTTAGTATTTACAATAAGATTAGCACTACCACTATGATTATCATTCATACTTTATGGGTTTATTAATAATATAAATCATATATTTTGTCATTTAGTTCCGTCAGGAACTCCTAATATTTTAATACCTTTTATAGTTCTTATTGAAAGAGTTAGAAATTTAATTCGTCCTGGTTCCTTGGCTGTACGGCTAACAGCTAATATAATTGCTGGGCATCTGTTAATAACCTTATTAGGTAATTTACCTATAAGTTATGAACTATATTCAGGTATAATCATTATTTTTCAAGTTATACTAATATTATTTGAATTAGCTGTTTGTGTAATTCAATCATATGTATTTATAGTACTTAGAACTTTATATTATAGAGAAGTAAATATTCCTCAAATATCTCCTCTAAATTGATTATTATTTATNNNNNNNNNNNNNNNNNNNNNNNNNNNNNNNNNNNNNNNNNNNNNNNNNNNNNNNNNNNNNNNNNNNNNNNNNNNNNNNNNNNNNNNNNNNNNNNNNNNNNNNNNNTTTAAGCCAACACGACAAAATCATCCTTTAATTAAAATTATTAATAATTCATTAATTGACTTTCCGGCACCATCTAATTTATCTTATTGATGAAATTTTGGATTTGTATTAGGATTATGCTTAATAATCCAAATTATAACTGGATTATTTTTATCAATACACTATAATGCCAACATTATGAATGCTTTTGAAAGATTAAGACATATCTGTCGTGATGTAAATTATGGTTGAATTTTACGAGTTATTCACGCTAATGGGGCTTCATTATTTTTCATCTGTGTTTATTTACATGTAGGGCGTGGATTATATTATGGTTCATTTAAATATATTGAAACATGATCTATTGGTGTAATTATATTATTAATATTAATAGCTACCGCTTTCTTAGGATATGTTTTACCGTGAGGACAAATATCTTTCTGAGGGGCTACAGTTATTACAAATTTATTATCAGCTATCCCTTATTTAGGTGGAATATTAGTTAATTGAATTTGAGGAGGATTCGCAGTTGACAATGCAACATTAACTCGATTTTACTCATTCCACTTTATTTTACCATTCATTGTACTAAGACTAACAATTATTCACTTATTATATCTTCATACAACAGGTTCAAATAATCCTCTAGGAATTAATTCTAATAATGACAAAGTCCCATTTCACCCATTCTTCTCCATTAAGGATATTATGAGACTATTTATCTTAATAATTGTTTTCTTTATATTAGTCATATTGGAACCATATATACTAGGGGACCCAGATAATTTCACTCCTGCCAATCCACTTGTAACACCAAAGCATATTCAACCTGAATGATATTTTTTATTTGCCTACGCAATTCTTCGTTCAATTCCTAATAAGTTAGGAGGAGTAATTGCTCTTTTTATATCAATCTTTATTTTAATGTTTGTACCTTTGTTAAATAATTCTAATTTTATAGGATTAAATAATTACCCAATTAACCAAATTATATTTTGATATATAGTAATTATTCTAATTTTATTAACTTGAATTGGAGCACGACCTGTCGAATTACCTTATATTAACTTTGGAATATTTTTAACACTTTTATATTTTTCTTACTTCATTATTGACCCAATAATTAAATCTATTTGAGACAAATTAATTAGGTAGATGAAAAAATGATTCTTCTCAACTAATCATAAAGATATTGGAACAATATATTTCATTTTCGGTATTTGATCAGGTATAATTGGTACAACTTTAAGAGTTTTAATTCGTGTAGAACTTGGAACCCCAGGTTCATTTATTGGTGATGATCAAATTTATAATGTAATTGTTACTGCTCATGCTTTCATTATAATTTTTTTTATAGTTATGCCAATTATAATCGGTGGATTTGGAAATTGATTAGTCCCTTTAATAATTGGAGCTCCTGATATAGCTTTTCCTCGTATAAATAATATAAGTTTTTGATTGTTACCACCTTCCCTAATTCTTATATTAGTGGGAAGAATAGTTGATAGAGGTGCAGGTACAGGTTGAACAGTTTATCCACCTTTATCTGCAGGTATTGCACATTCTGGTTCATGTGTTGATTTAACTATTTTTTCTTTACATCTTGCAGGTGTATCATCAATTTTAGGTGCTGTAAACTTCATTAGAACAATTTTCAATATACGTTCAATAGGAATTTGATTAGATCGAATACCACTATTTGTATGAGCAGTTTTAATTACTGCATTTTTGTTATTACTATCTTTACCTGTTTTAGCAGGCGCTATTACAATGTTATTAACAGATCGAAATTTAAATACTTCATTTTTTGACCCTGCAGGAGGGGGAGATCCAATTCTTTATCAGCATTTATTTTGATTTTTTGGTCACCCTGAAGTTTATATTTTAATTTTACCAGGATTTGGTTTAATTTCTCACATTATCACTCAAGAAAGAGGTAAAATTGAATCTTTCGGCTCATTAGGAATAATTTATGCTATAATATCAATTGGTATTTTAGGATTTGTTGTGTGAGCACATCATATATTCACTGTTGGAATAGATGTTGATACACGTGCATATTTTACATCAGCCACTATAATTATTGCAGTTCCTACTGGAATCAAAGTATTTAGTTGACTCGCAACTTTGAGAGGTATAAAAATTAATATTACATCTTCAGCCTTATGGGCTTTAGGATTTGTCTTTTTATTTACTATTGGAGGCTTGACCGGAGTGATTCTAGCTAATTCTTCAATTGATATTATATTACATGATACATATTATGTAGTTGCACATTTTCACTATGTTTTATCAATAGGAGCAGTTTTTGCTATTATAGCAAGATTCATTCATTGATTTCCATTATTTACAGGATTAAGATTAAACTCAAATTGATTAAAAATTCATTTTATATTGATATTTATCGGTGTAAATATAACATTCTTTCCTCAACATTTTTTAGGATTAAGAGGAATACCTCGTCGATATTCAGATTATCCTGATGCTTACATGTCATGAAACATAATTTCATCAATAGGGAGAGTCATGTCTTTAATTGGAATTTTATTTTTATTATTTATTGTTTGAGAAAGTTTTATTTCAATACGATTAGTATTATATTCTAATAGAATTCAATCTTCTATTGAATGAATACAAAAATTCCCTCCATCTGAACATTCTTATAATGAAATGCCATTATTAATTCAAATTTCAAATTGATCTTATATTAATATACAGGATGCTATATCACCATTAATAGAGCAATTAATATTTTTTCATGANNNNNNNNNNNNNNNTTTAATTATAATTACAATTATTGTTGCTTATATAATAATTATATTAATATTGAATAAAATTATTAATCGTTTACTTCTCGAAGGGCAATTAATTGAATTTATTTGAACTTTATTACCTGCAATAACTTTAATTTTTATTGCATTACCATCATTACGATTATTATATATATTAGACGAAATTAATAATCCATTATTAACATTAAAAATTATTGGTCACCAATGATATTGATCATATGAATATTCAGATTTTTCTGATGTAGAATTTGATTCATATATAAAATCCATAAATGAAATAAGTAAAAATGAATTTCGCTTATTAGATGTAGATAATCGAGTAATTTTACCATTTAATATTCAAGTCCGGCTATTAGTTTCTTCTTTTGATGTTATTCACTCCTGAGCAATGCCATCTATAAGACTCAAAGTTGATGCAGTGCCAGGACGATTAAATCAAATAAGAATATTAATTAGTCGTCCTGGTATATCTTATGGACAATGTTCTGAAATTTGTGGGGCAAATCACAGATTTATACCCATTGTAATTGAAAGAATTAGAATAAAAATATTTATTAAATGATTAATTAATTATATGAATAATCATCCTTACCATATAGTTGATTACAGACCCTGACCTTTAACTGGATCAATTGGAGCATTAACTTTTGTTTCCGGTATAGTTATAATATTTCATAAATGTAACTTTATATTACTATATACTGGTATTTTATTATTATTAATAACAATAATTCAATGATGACGTGATATTTCCCGAGAGGGAACATTCTTAGGAATACACACAATAATGGTAGTAAACGGTTTAAAAATAGGAATATTACTATTTATTGTATCAGAAGTTCTTTTTTTTGTTTCATTTTTTTGGGGATTTTTTCATAGTAGATTAAGACCTGTAGTAGAAATTGGTATAATTTGACCTCCAAGGGGTATTTATGTTTTTAATCCGACTCAAGTCCCTTTATTAAATACAATAATTTTATTATGTTCAGGGATCACAATTACTTGAGCCCATCATTCAATTATAAGTGGTAATCATATTAATTCTATCTATAGAATTATGTTAACTGTTATTTTAGGCATATACTTCACTATTCTGCAAGGTTATGAATATTATGAAGCTCCATTTGCAATTAATGATTCCATCTATGGATCTTCTTTTTTTATAGCTACTGGGTTCCATGGAATTCACGTAATCATCGGAACAAGATTTATTATTGTATGCTTATTACGACAAATAAAGTTTCATTTTTCAATAAATCATCATTTTGGCTTCGAAGCTGCTGCTTGATATTGACATTTTGTTGATGTGGTCTGATTATTTTTATATTTATCAATTTATTGATGAGGAAGATAATAAATCATAGTTTTTAAAAAAAGAAAAAAAATAAATAAATTTAGAGATACCGGTAAATAAGATTTTCAAGCTAAATATATTAATTTATCATAACGATAACGAGGAAGAGTTCCTCGAACTCAAATAAAAAGAAAACATATTATAGAAACTTGAATTGGAAAAACAATTGAATTAATTTTTCCTCCTAAAAACATCAAACAAAATAATATTCTCATGAATAAAATACTTGAATACTCAGCCAAAAAAATAAATGCAAATCTAGAACCTCTATATTCAACATTAAAACCAGATACTAACTCTGATTCTCCCTCAGAAAAATCAAATGGAGATCGATTAGTTTCAGCTAAAGCTGAAGATAATCAACACATTCTTAAGGGAAGAAATAAAAAAATAAATCACACAAACTCCTGAAATATAAACAAATCAATAATATTGTAACTTCTTATTATTATCATAGGACACAATATAATTAAAACTAATCTTACCTCATAAGAAATTGATTGAGCAATTGATCGAATACATCCTAATATTGCATAACTTGAGTTAGAAGATCAACCTGTCAATATTAATGAATAAACTCTCATTCTTGAACAACAAAAAAAAAATAAAACACCAAAATTAAAAGAAACACAATTAATTATATATGGATATAATGATCAAATCAATAGTCTATTGAATAATCCTATAATAGGTCTAAAAATATAAATTAAATAATTAGATATTAATGGAATTGTATTTTCCTTTATAAATAATTTAATAGCATCAGAAATAGGCTGTAAAATGCCCAAAAATCCAACTTTATTTGGACCTTTACGAATTTGAATATATCTTAAAACCTTACGCTCCAAAAGAGTAATAAATGCCACTCCAATTAAAATAAATAATACAGTAATTAAAATAGTAATTAAGTACAAAAATAATTCTTCTTATATACTTTATTTAATTTTCTTACTATTAGGAATTATAATTTCAGTTTCTTCAAACAATTGGCTGGGTTGTTGAATAGGAATTGAAATAAATATAGTTTCATTTTTGCCCATAATGGCAAACAAGACAAGAATTTATGCTTCAGAATCAATAATTAAATATTTTATTATNNNNNNNNNNNNNNNNNNNNNNNNNNNNNNNNNNNNNNNNNNNNNNNNNNNNNNNNNNNNNNNNNNNNNNNNNNNNNNNNNNNNNNNNNNNNNNNNNNNNNNNNNNNNNNNNNNNNNNNNNNNNNNNNNNNNNNNNNNNNNNNNNNNNNNNNNNNNNNNNNNNNNNNNNNNNNNNNNNNNNNNNNNNNNNNNNNNNNNNNNNNNNNNNNNNNNNNNNNNNNNNNNNNNNNNNNNNNNNNNNNNNNNNNNNNNNNNNNNNNNNNNNNNNNNNNNNNNNNNNNNNNNNNNNNNNNNNNNNNNNNNNNNNNNNNNNNNNNNNNNNNNNNNNNNNNNNNNNNNNNNNNNNNNNNNNNNNNNNNNNNNNNNNNNNNNNNNNNNNNNNNNNNNNNNNNNNNNNNNNNNNNNNNNNNNNNNNNNNNNNNNNNNNNNNNNNNNNNNNNNNNNNNNNNNNNNNNNNNNNNNNNNNNNNNNNNNNNNNNNNNNNNNNNNNNNNNNNNNNNNNNNNNNNNNNNNNNNNNNNNNNNNNNNNNNNNNNNNNNNNNNNNNNNNNNNNNNNNNNNNNNNNNNNNNNNNNNNNNNNNNNNNNNNNNNNNNNNNNNNNNNNNNNNNNNNNNNNNNNNNNNNNNNNNNNNNNNNNNNNNNNNNNNNNNNNNNNNNNNNNNNNNNNNNNNNNNNNNNNNNNNNNNNNNNNNNNNNNNNNNNNNNNNNNNNNNNNNNNNNNNNNNNNNNNNNNNNNNNNNNNNNNNNTGGAATAATTTTTTTTTCATTAATTACATTAAATATGTGTTAGATCTATAATATTTTGATTTATTCNNTTATTNNNNNNNNNNNNNNNNNNNNNNNNNNNNNNNNNNNNNNNNNNNNNNNNNNNNNNNNNNNNNNNNNNNNNNNNNNNNNNNNNNNNNNNNNNNNNNNNNNNNNNNNNNNNNNNNNNNNNNNNNNNNNNNNNNNNNNNNNNNNNNNNNNNNNNNNNNNNNNNNNNNNNNNNNNNNNNNNNNNNNNNNNNNNNNNNNNNNNNNNNNNNNNNNNNNNAATTAGATTAAAAGTTATTGATATTTATATTATTATATTAATTTTCTTAATTATCTTAACTTTAGGGCTATATCATGAATGATATAATAATATATTAAGTTGANNNNNNNGTGTANNNNNNNNNNNNNNNNNNNNNNNNNNNNNNNNNNNNNNNNNNNNNNNNNNNNNNNNNNNNNNNNNNNNNNNNNNNNNNNNNNNNNNNNNNNNNNNNNNNNNNNNNNNNNNNNNNNNNNNNNNNNNNNNNNNNNNNNNNNNNNNNNNNNNNNNNNNNNNNNNNNNNNNNNNNNNNNNNNNNNNNNNNNNNNNNNNNNNNNNNNNNNNNNNNNNNNNNNNNNNNNNNNNNNNNNNNNNNNNNNNNNNNNNNNNNNNNNNNNNNNNNNNNNNNNNNNNNNNNNNNNNNNNNNNNNNNNNNNNNNNNNNNNNNNNNNNNNNNNNNNNNNNNNNNNNNNNNNNNNNNNNNNNNNNNNNNNNNNNNNNNNNNNNNNNNNNNNNNNNNNNNNNNNNNNNNNNNNNNNNNNNNNNNNNNNNNNNNNNNNNNNNNNNNNNNNNNNNNNNNNNNNNNNNNNNNNNNNNNNNNNNNNNNNNNNNNNNNNNNNNNNNNNNNNNNNNNNNNNNNNNNNNNNNNNNNNNNNNNNNNNNNNNNNNNNNNNNNNNNNNNNNNNNNNNNNNNNNNNNNNNNNNNNNNNNNNNNNNNNNNNNNNNNNNNNNNNNNNNNNNNNNNNNNNNNNNNNNNNNNNNNNNNNNNNNNNNNNNNNNNNNNNNNNNNNNNNNNNNNNNNNNNNNNNNNNNNNNNNNNNNNNNNNNNNNNNNNNNNNNNNNNNNNNNNNNNNNNNNNNNNNNNNNNNNNNNNNNNNNNNNNNNNNNNNNNNNAGCTATTATTATACCAAAATAAATATAAATATTGACAGAATAATTTATTAACATAAAAATACTAGTTATATTATTTTTATAAAGAAAAAAAATTCTTATTAACATTGGTAATGATGCAAATAATGTATAAAACAATAAATAATACCTCGCATCAATACGTTCTGGCTGATAACCCCAACCTATAATAATAATTATAATTGGAATTAATCTAGATTCAAAACAAATATAAAAAATTATAATTCTTGATGTAGAAAAAGAAATAATCAAAAAAATCATTAACATAACACTAAAAATAAAAAAAACAACTTATTACGTTTATATATTGGACTAGAAACAATTATTAGGAATGAAATTCAAATTTAAAATAATTAAACCATAAGACATTTCATCAATATAAAAATTATAGCCTAATATATATAAATTCTTATTTATAAAAACCAAAAATATAATTATCAAGACAATAATTATATTTATTAATCACGAATTATAAATTAGTAGGGTTAATATAAAGCAAAAAAAAACTAATTTTATTGATATGGATATTAATTTATCATTATTATGAGATCGAATTAATATTACTAAGCAGGATAAACCTATAACTCCCTCACAGACTCTAAAGACCAAGAAAATTAATAAAATATGCAAATCATATATTTTTATGGAAAAAATAAATGATAAAGAACAAAATATTCTTAAAATAATTAACTCCAATCTTAATGTTGTTATTAAATGTTTTCGATTCATGCATAATGAAACTATCCCAGAGAAGAATATAAAGGTAAAATATATTAATAATGTTATATTCTATAAATATAAAACAATAATTCAAACAATAAATGAAATTATCTGAATCTTAAAATTATTATTTATCAATTTGTAATTTAATGATATTATTATTTTTAAAGTTTTCAAAATCCCTTGGGGCCCAAAGTTTTCTCCCCATCCCATGTCAACTGATTTTTGAAATATAAGTGACTTATTTATCAATAACATTTGATTATGAAACGTTGACAATTGTTTTATAAATCACATTGATCCAAAAAATTCATTAAAAATTTTTATTTTAGTTTTAGAAAAAATTGATATTTCATAACCCAATCAAATTCCTAATATTGAAAATAATAATGCTAATAATTTACCCTCAATAGGTATTAAAATCATAATTGGATCATTAAATATTAATCATCTTAATATAGATCCCGAAAAAATTGAGTAAATAGATAAAATAATAATTCTTTTANNNNNNTTATTTATACTTTCATTTAATGATCTTAATTTATAAAAATTATAATTTATATTTATTGAAAAAAATGTCAAACGGGCAGAATAAAAAGAAGTTAAACCAATACCAATATATATAAATATTATAATTAATAAATTAACGTGTCTAGAACATATAGTTTCTATAATTAAATCTTTTGAATAAAATCCTCTCATGAAGGGAATTCCACATAAAGACATATTAGCAATATTTATTATTGTAATAGTTAATGGTATTTGAAATCTTAAACATCCTATTATACGAATATCTTGATTATTATTAAAATCGTGAATCAAAATTCCTGCACACAAAAATAAAGTAGCTTTAAATAAAGCATGAACAATTAAATGAAAAAATGATAATATTGGATAACCAAATATAATGATCGTTATTATAATCCCCAACTGTCTTAAAGTTGATAATGCAATAATTTTTTTTAAATCAAACTCAAAATTTGCTCTAATTCCTGATATTAGCATAGTTAAAATTGAAATTAATATAAATAATTGAATATAATCAAAATTCAAAATAATATTAGAAAATCGAATTATTAGATATACCCCAGCAGTAACAAGTGTAGATGAATGAACCAAAGCTGAGACAGGGGTTGGGGCAGCCATAGCTGCCGGTAATCATGAAGAAAATGGAATTTGAGCTCTCTTTGTAAATCTTGCAATAATAATCATAATTATTATAATTNNNNNNNNNNNNNNNNNAAGATAAAAATTTCATGACCCAAAATTTAATATTCATGCAATAGCTATTAAAATTGCAACATCTCCCACACGATTCATTAATGCAGTTAATATTCCTGCATTATTAGAGTATAAATTTTGATAATAAATAACTAGGCAATAAGAAACTAGACCAAGTCCATCTCAACCAACAATAATTCTCATTATATTAGGTCTAATGATTAATAAAACTATTGATATAACAAACCCTAAAACAATATAAATAAAACGATTAATGAATTTATCATTATTTATATATATTCCACTGTATAAAACAACCACTGAGGAAATAAATATAACTGTTGAAAGAAATTGTTGATATAAAATCAAAAATTATAGTCATATAAATATTACATGAATTAATTGAAAATTATTCATTCTANNNNNNNNNNNNNNNNNNNNNNNNNNNNNNNNNNNNNNNNNNNNNNNNNNNNNNNNNNNNNNNNNNNNNNNNNNNNNNNNNNNNNNNNNNNNNNNNNNNNNNATGAAAATTGCTATGTACTTAATAATTATTTTATCAATAAATTTCATTTTCATAAAACACCCTTTATCAATAGGATTGATTTTAATAATACAAACAGTATTATCTTGCTTAATTTGTAGACTTTATTTAAGTTGTTATTTATTTTCTTATATCTTATATCTTATTTTTATTGGTGGTATATTAATTTTATTTATATATATATCAAGAATTGCATCAAATGAAAAATTTATTTACTCAATTAAATTAATAATGTTTAATTTTTTAATTTTAACTTTAATTTTAAAAATAAACATAATTGATTTAAAATCACCAAATATTATAAGAAACATCATAATATATATAAACCACAACAACTTTATAATAAGAAAAATATATATTATTCCTTCCGGAATAATAAGACTAATTTTAACAATTTATTTACTATTTGTTTTAATTATTGTAATTAATATTTTAACAATAAGTATGTCAACTCTTCGAAGAAGAATCTCTTTCTAACTACACCTTCCGGTACAGTTACTTTGTTACGACTTATCTCATTAATGTTATGAGAGTGACGGGCGATATGTACATAAATTAGAGCCAATTTCAATTAATTAAATTTAATTAATTTATTGTCAAATCCAATTTCATATTTTTTTTAATTTAAAAAATAATTCAATACATAATTAATTGTAACCCATTTTTTCTTTAATATAAACTGCACCTTGACCTGACATTAAATATACNATAATATATTATGAAAATATTTCTTATAAAACATTCTTGACAGAGATATACAAATTAAATTAAAGTTTTTTCTATCGTGGATTATCAATTATAAAACAGGTTCCTCTGATAAGATAAATTACCGCCAAATTCTTTGAATTTAAAGATCATTTCTAATAATAATCAAGTTATTTTTATTACATTTTTAATAATAGGGTATCTAATCCTAGTTTAAACAAAAATTTTTCAGACATAAAATTAATTTTTAAAGACAAAATATATTTCACCAAAATTATAGATATTTTATATTATTATTATATTAACTGAAACTTAACTATTTAAATTAAAGAAATCGTATAACCGCAACTGCTGGCACGAAATTTGATTCNNNNNNNNNNNNNNNNNNNNNNNNNNNNNNNNNNNNNNNNNNNNNNNNNNNNNNNNNNNNNNNNNNNNNNNNNNNNNNNNNNNNNNNNNNNNNNNNNNNNNNNNNNNNNNNNNNNNNNNNNNNNNNNNNNNNNNNNNNNNNNNNNNNNNNNNNNNNNATAATAAAATTATTTAATGAGGTCCTTTCGTACTAACATTAAAAATAATTGAGTAGATAGAAACCAACCTGGCTCACGCCGGTCTGAACTCAGATCATGTAAGAATATTAAGGGTCGAACAGACCCAGAAATAATAAATTTTGCTCCAATCCCTATTCTTAATCCAACATCGAGGTCGCAATCATATTTATCGATATGAACTCTTCAAATTAATTACGCTGTTATCCCTAAGGTAATTTATTCTTATAATCAAAAATTTGGATCAATATTTACATAAATTTATGAAATTTATATTAAAAGTTAGTTTTATTTTAATATCACCCCAACAAAAAAATTAATTAAATAAAAAAAATTAATTAACTATAAANAAATAAACTTAATNAAAATTTTAAAAATTCTATAGGGTCTTCTCGTCCCACTCACATATTTAAGCTTTTTTACTTAAAAATCAATTTCAATTATTAACATTAATTAAGTTAATTTCTCATTCAATCATTCATTCAAGCCTCCAATTAAAAGACTATTTATTATGCTACCTTTGCACGGTCAATTTACCGCGGCCATTTAATTTTTAATCATTGGGCAGATTAGACTTTTAATTCTTTCTAAAAGACATGTTTTTGATAAACAGGTGAAAATTTTTTTTGCNNNNNNNNNNNNNNNNNNNNNNNNNNNNNNNNNNNNNNNNNNNNNNNNNNNNNNNNNNNNNNNNNNNNNNNNNNNNNNNNNNNNATATATACAATAATAACTAACAAATNTAAACTAAATCATTATATTTCGGGAAAAATAAATATTTAAAAGTCTTCAATTAACCCTGATACAAAAGGTACAAATTATATTCTACTTAAAACTANTTATAAAGTCNTCTCCTTATCAGTTAATCCAAATTATTTTTTTATATAAAATTACTTTAACACAAAAATTTTTTTTTTAAAAATGAAAAAAACAAATTTTANTAAAATTATATTATGGGATATATTTAATTATAATAATTGAATTGCAGTCAATAGGTGTTAACTATAACTATCCTTAAGTAATGAAGTAAAATAATACATTTAGTTTCGACCTAAAATAAGAATTAGAATTCCTTGCTTTTAAATGAAGCCAAAATAGAGGCATTTTAATGTTAATAAAATAATTGAAANTTTAATTCCATTTAAAGAAAAATTAGTTTAATTAAAATGTAAATCTGTCAGATTTATGAAACTTTTGAGTATTTTTCTGAATTTTTGTGATNTNTTCTACTTTAAATTTGCAATTTAATATTATGAATTAAATATAAGACTTTATGTTAACAGAATTAAACTGTCTATTAATATCAAAAATTAATGTTCTTCATAAACTATAACATTTATAAAGTTTATAAAACATTTCATTTTCATTGAAAAGAGAGACTTAGTCTTATAAAATTCTATTAGTATATAAGTATATTTAACTTCCAATTAAAAGGATTAATTTTATTAAATAGAATANNNNNNNNNNNNNNNNNNNNNNNNNNNNNNNNNNNNNNNNNNNNNNNNNNNNNNNNNNNNNNNNTACTATTTGTAATAAATATTACATTATTAAATTCTAAATTTAAGGCATTAATTTTGCTAAAATAGTTCTAATGTGGCAGATTAGTGCAATGAATTTAAGATTCATATATAAAATANNTATTTTTTATTAGAAAATAAGATGCCTGAATNNAAAAGGATTATTTTGATAGAATAAATTATGTAATATAATTACTCNNNCATTAAGTGGCTGAAAGTAAGTAATGGTCTCTTAAACCAATTAATAGTAATTAACAAATACTCTTAATGAAAGATAAGCTAAAANTAAAGCTAATGGGTTCATACCTCACTTATGGAAANTTTTCCTCTTTTTATTTAAAAACAAAAGTTACCTTAATATCTTCAATATTATGCTCTTAATTAAGCTATTTAAATTCAGAAAAGATAAAAAAATCATTTTAAACTCCCAAAGTTTATATTTTATATTTAAATTATTTTCTGAGTTAATTAGCTTAACCAAAAGCATTTATTTTGAAAGTAAAAGAAAAGATATTTAATCTATTAACTTAGGAAACTAGAAATAAAATTAGCTTCTAACTAACTTTTAAAGCGGTTAAATTCCGTTTTTTCCTTGTTTTAATAGTTTAATNNNAAAAATTTAGATCTTGTAAATCTAAGATAACGTAAGTTTTAAAAATAGGTTTTAAGTTATTAANAACTATTATCCTTCAAAGTTAAAAATATAATTANATTTATTATCAGGAGAAATAATTTTTCATAAATAAATTTACAGTTTATTGCCTATTTTCGGCCATCCTAATCAAGTTATATTGATTAAACAATATTCTCATTAGTGTAAATAAAGTGCTAATATAGCTTTAACTTG

>aotea-east_02.NZ.HB.POR.08

NNNNNNNNNNNNNNNNNNNNNNNNNNNNNNNNNNNNNNNNNNNNNNNNNNNNNNNNNNNNNNNNNNNNNNNNNNNNNNNNNNNNNNNNNNNNNNNNNNNNNNNNNNNNNNNNNNNNNNNNNNNNNNNNNNNNNNNNNNNNNNNNNNNNNNNNNNNNNNNNNNNNNNNNNNNNNNNNNNNNNNNNNNNNNNNNNNNNNNNNNNNNNNNNNNNNNNNNNNNNNNNNNNNNNNNNNNNNNNNNNNNGGTTTACTTCCGTATATTTTCACTAGCTCAAGTCATCTAGTATTTACAATAAGATTAGCATTACCATTATGATTATCATTCATACTTTATGGATTTATTAATAATATAAATCATATATTTTGTCATTTAGTTCCATCTGGAACTCCTAATATTTTAATGCCTTTTATAGTTCTTATTGAAAGAGTCAGAAATTTAATTCGACCTGGCTCCTTAGCTGTCCGACTAACAGCTAACATAATTGCTGGACATCTTTTAATAACCTTATTAGGTAATTTACCTATAAGGTATGAATTATATTCAGGTATTATTATTGTTTTTCAAGTTATATTAATATTATTTGAATTAGCTGTGTGTATTATTCAATCATATGTATTTATAGTTCTTAGAACTTTATATTATAGAGAAGTAAATNNNNNNNNNNNNNNNNNNNNNNNNNNNNNNNNNNNNNNNNNNNNNNNNNNNNNNNNNNNNNNNNNNNNNNNNNNNNNNNNNNNNNNNNNNNNNNNNNNNNNNNNNNNNNNNNNNNNNNNNNNNNNNNNNNNNNNNNNNNNNNNNTTTAAGCCAACACGACAAAATCATCCTTTAATTAAAATTATTAATAATTCATTAATTGACTTCCCAGCACCATCTAATTTATCTTATTGATGAAATTTTGGATTTATATTAGGATTATGCTTAACAATTCAAATTTTAACTGGATTATTTTTATCAATACATTATAATGCTAATATCATAAATGCTTTTGAAAGATTAAGACATATTTGCCGTGATGTAAACTATGGTTGAATCTTACGTGTTATTCACGCTAATGGAGCTTCATTGTTTTTCATTTGTGTATATTTACATGTAGGACGTGGATTATATTATGGATCATTCAAATATATTGAAACATGATCTATTGGTGTAATTATATTATTAATATTAATAGCTACTGCTTTCTTAGGATATGTTTTACCGTGAGGGCAAATATCTTTTTGAGGAGCCACAGTCATTACAAATCTATTATCAGCAATTCCTTATTTAGGTGGAATGTTAGTTAATTGAATTTGAGGAGGATTTGCAGTTGACAATGCAACACTAACTCGATTTTACTCTTTTCATTTTATTTTACCATTCATTGTATTAAGACTAACAATTATTCATCTATTATATTTACATACAACAGGTTCAAATAATCCATTAGGAATTAATTCCAATAATGACAAAGTCCCATTTCACCCATACTTCTCCATTAAGGATATTATAAGCTTATTTTTATTAATAATTATTTTCTTTATATTAGTCATGTTAGAACCCTATATACTAGGAGATCCAGATAATTTTATCCCTGCCAATCCACTTGTAACACCAAAACATATTCAACCTGAATGATACTTTTTATTTGCCTACGCAATCCTCCGTTCAATTCCTAATAAACTAGGAGGAGTAATTGCTCTTTTTATATCAATTTTTATTTTAATGTTTGTACCTTTATTAAATAATTCTAATTTTATAGGATTAAATAATTACCCAATTAATCAAATTATATTTTGATATATAGTAATAATTTTAATTTNNNNNNNNNNNNNNNNNNNNNNNNNNNNNNNNNNNNNNNNNNNNNNNNNNNNNNNNNNNNNNNNNNNNNNNNNNNNNNNNNNNNNNNNNNNNNNNNNNNNNNNNNNNNNNNNNNNNNNNNNNNNNNNNNNNNNNATGAAAAAATGATTCTTCTCAACTAATCATAAAGATATTGGAACAATATATTTCATTTTCGGTATTTGATCAGGTATAATTGGTACAACTTTAAGAGTTTTAATTCGTGTAGAACTTGGAACTCCAGGTTCATTTATTGGTGATGATCAAATTTATAATGTAATTGTCACTGCTCATGCTTTCATTATAATTTTTTTTATGGTAATACCAATTATAATTGGTGGATTTGGAAATTGATTAGTCCCCTTAATAATTGGTGCTCCTGATATAGCTTTTCCTCGTATAAATAATATGAGTTTTTGATTATTACCACCTTCTCTAATTCTTATTTTAGTGGGAAGAATAGTTGATAGAGGTGCAGGCACAGGTTGAACAGTTTACCCTCCATTATCTGCAGGTATTGCACATTCTGGTTCATGTGTTGATCTAACTATTTTTTCTCTACACCTTGCAGGTGTATCCTCAATTTTAGGTGCTGTAAATTTCATTAGAACAATTTTTAACATACGTTCAATGGGAATTTGATTAGATCGTATACCTTTATTTGTATGAGCAGTTTTAATTACTGCATTTTTATTATTACTGTCACTACCCGTTTTAGCAGGCGCTATTACAATATTGTTAACAGATCGAAATTTAAATACTTCATTTTTTGATCCTGCAGGAGGAGGGGATCCTATTCTTTATCAGCATTTATTTTGATTTTTTGGTCATCCTGAAGTCTATATTTTAATTTTACCAGGATTTGGTTTAATTTCTCATATTATTACTCAAGAGAGAGGTAAAATTGAATCTTTTGGCTCATTAGGAATAATTTATGCCATAATATCAATTGGTATTTTAGGATTTGTTGTATGAGCACATCATATATTCACTGTAGGAATAGATGTTGATACACGTGCATATTTTACATCAGCTACTATAATTATTGCAGTTCCCACTGGAATTAAAGTATTTAGCTGACTTGCAACTTTGAGAGGTATAAAAATTAATATTACATCTTCAGCCCTATGAGCATTGGGGTTTGTCTTTTTATTTACTATTGGGGGTTTAACTGGAGTTATTCTAGCCAATTCTTCAATTGATATTATATTACATGATACATATTATGTAGTTGCACATTTCCATTATGTTTTATCAATAGGAGCAGTATTTGCTATTATAGCAAGATTTATCCATTGGTTTCCATTATTTACAGGATTAAGATTAAACTCAAATTGATTAAAAATTCATTTTCTATTAATATTTATTGGTGTAAATATAACATTCTTTCCTCAACATTTTTTAGGATTAAGAGGAATACCTCGTCGTTATTCAGATTATCCTGATGCTTATATATCATGAAACATAATTTCATCAATAGGAAGAATTATATCTTTAATTGGAATTTTATTTTTATTATTTATTGTCTGGGAAAGCTTTATTTCAATACGATTAGTATTATATTCTAATAGAATTCAATCTTCTATTGAATGAATACAAAAATTTCCCCCATCTGAACATTCATATAATGAAATGCCATTGTTAATTCAAATTTCAAATTGATCTTATATTAATATACAGGATGCTGTATCACCATTAATAGAACAATTAATATTTTTTCATGATCATGTGTTGGTAATTTTAATTATAATTACAATTATTGTTGCTTACATAATAGTTATGTTAATATTAAATAAAATTATTAATCGCTTACTTCTTGAAGGACAGTTAATTGAATTTATTTGAACTTTATTGCCTGCAATAACTTTAATTTTTATTGCATTACCATCATTACGATTATTATATATATTAGACGAAATTAATAATCCATTATTAACATTAAAAATTATTGGTCACCAATGATATTGATCATATGAATACTCAGATTTTTCTGATGTAGAATTTGATTCATATATAAAATCTATAAACGAGATAAATAAAAACGAATTTCGTTTATTAGATGTAGATAATCGAGTAATTCTACCATTTAATATTCAAGTCCGACTATTAGTTTCTTCTTTTGATGTTATTCATTCCTGAGCAATACCATCAATAAGACTTAAAGTTGATGCAGTGCCAGGACGATTAAATCAAATAAGAATATTAATTAATCGTCCCGGTGTATCTTATGGACAATGCTCTGAAATTTGTGGAGCAAATCATAGATTTATGCCTATCGTGATTGAAAGAATTAGAATAAAAATATTTATTAAATGATTAATTAATTATATGAATAATCACCCTTACCATATAGTTGACTATAGACCCTGACCTTTAACTGGATCAATTGGAGCATTAACTTTTGTTTCCGGTATAGTTATAATATTTCATAAATGTAATTTTATATTATTATATATTGGTATTTTATTATTATTAATAACAATAATTCAATGATGACGTGACATTTCCCGAGAAGGAACATTTTTAGGAATGCACACAATAATAGTAGTGAATGGTTTAAAAATAGGCATATTATTATTTATTGTATCAGAAATTCTTTTTTTTGTATCATTTTTTTGAGCATTTTTTCATAGTAGATTAAGACCTGTAGTAGAAATTGGTATAATTTGACCTCCTAGAGGTATTTATGTTTTTAATCCGACTCAAGTTCCATTATTAAATACAATAATTTTATTATGTTCAGGAATTACAATTACTTGAGCACATCATTCAATCATAAATGGTAATCACATTAATTCTATCTATAGAATTATATTAACTATTATTTTAGGTATATATTTCACTATTCTGCAAGGTTATGAATATTATGAAGCCCCGTTTGCAATTAATGATTCTATCTATGGATCTTCTTTTTTTATAGCTACTGGATTTCATGGAATTCACGTAATTATTGGAACAAGATTTATTACTGTATGCTTATTACGACAAATAAAGTTTCATTTCTCAATAAATCATCATTTTGGCTTCGAAGCTGCTGCTTGATATTGACATTTTGTTGAAGTAGTCTGATTATTCTTATATTTATCAATTTATTGATGAGGAGGATAATAAATTATAGTTTTTAAAAAGAAAAAAAAAACAAATAAATTCAGAGATACCGGTAAATAAGATTTTCAAGCTAAATATATTAATTTGTCATAACGATAACGAGGAAGAGTACCTCGAATCCAAATAAAAAGAAAACAAATTATAGAAACTTGAATTGGAAAAACAATTGAATTAATTTTTCCTCCTAAAAACATTAAACAAAATAATATTCTTATAAATAAAATACTTGAATACTCAGCCAAAAAAATAAATGCAAATCCAGAACCTCTATATTCAACATTAAAGCCAGAAACTAACTCTGATTCTCCCTCAGAAAAATCAAATGGAGATCGATTAGTCTCAGCTAAAGCTGAAGATAACCAACATATTCTTAAAGGAAGAAATAAAAAAATAAATCACACAAATTCCTGAAATATAAACAAATCAATAATATTATAACTTCTTATTATTATTATAGGACACAATATAATTAAAACCAATCTTACCTCATAAGAAATTGATTGAGCAATTGATCGAATGCATCCCAACATTGCATAACTTGAATTAGAAGATCAACCTGTTAATATTAATGAATAAACTCTTATTCTTGAGCAACAAAAAAAAAATAAAATACCAAAATTAAAAGAAACACAATTAATAAAATATGGATACAATGATCAAATCAACAAACTATTGAATAATCCTATAATAGGCCTAAAAATATAAATTAAATAATTAGATATTAATGGAATTGTATTTTCCTTTATAAATAATTTAATAGCATCAGAAATAGGCTGCAAAATACCCAAAAATCCAACTTTATTTGGGCCTTTACGGATTTGAATATATCTTAAAACTTTACGTTCCAAAAGAGTAATAAATGCCACTCCAATCAAAATAAATAATACAGTAACTAAAATCGTAATTAAATACAAAAATAATTCTTCTTATATACTTTATATAATTTTCTTAATTTTAGGAATTATAATTTCAATTTCTTCAAACAATTGGCTGGGTTGTTGAATGGGGATTGAAATAAATATAGTTTCATTTTTGCCTATAATGGCAAACAAAATAAGAATTTATACTTCGGAATCAATAATTAAATATTTTATCATTCAAAGAATAGGATCGAGTTTATNNNNNNNNNNNNNNNNNNNNNNNNNNNNNNNNNNNNNNNNNNNNNNNNNNNNNNNNNNNNNNNNNNNNNNNNNNNNNNNNNNNNNNNNNNNNNNNNNNNNNNNNNNNNNNNNNNNNNNNNNNNNNNNNNNNNNNNNNNNNNNNNNNNNNNNNNNNNNNNNNNNNNNNNNNNNNNNNNNNNNNNNNNNNNNNNNNNNNNNNNNNNNNNNNNNNNNNNNNNNNNNNNNNNNNNNNNNNNNNNNNNNNNNNNNNNNNNNNNNNNNNNNNNNNNNNNNNNNNNNNNNNNNNNNNNNNNNNNNNNNNNNNNNNNNNNNNNNNNNNNNNNNNNNNNNNNNNNNNNNNNNNNNNNNNNNNNNNNNNNNNNNNNNNNNNNNNNNNNNNNNNNNNNNNNNNNNNNNNNNNNNNNNNNNNNNNNNNNNNNNNNNNNNNNNNNNNNNNNNNNNNNNNNNNNNNNNNNNNNNNNNNNNNNNNNNNNNNNNNNNNNNNNNNNNNNNNNNNNNNNNNNNNNNNNNNNNNNNNNNNNNNNNNNNNNNNNNNNNNNNNNNNNNNNNNNNNNNNNNNNNNNNNNNNNNNNNNNNNNNNNNNNNNNNNNNNNNNNNNNNNNNNNNNNNNNNNNNNNNNNNNNNNNNNNNNNNNNNNNNNNNNNNNNNNNNNNNNNNNNNNNNNNNNNNNNNNNNNNTAAGAGTTTCATATACTTATTATATTGCTGGAGTATTTTCATTATTTGGAATAATTTTTTTATCATTAATTACATTAAATATTTGTTAGNNNNNNNNNNNNNNNNNNNNNNNNNNNNNNNNNNNNNNNNNNNNNNNNNNNNNNNNNNNNNNNNNNNNNNNNNNNNNNNNATANNNNNNNNNNNNNNNNNNNNNNNNNNNNNNNNNNNNNNNNNNNNNNNNNNNNNNNNNNNNNNNNNNNNNNNNNNNNNNNNNNNNNNNNNNNNNNNNNNNNNNNNNNNNNNNNNNNNNNNNNNNNNNNNNNNNNNNNNNNNNNNNNNNNNNNNNNNNNNNNNNNNNNNNNNNNNNNNNNNNNNNNNNNNNNNNNNNNNNNNNNNNNNNNNNNNNNNNNNNNNNNNNNNNNNNNNNNNNNNNNNNNNNNNNNNNNNNNNNNNNNNNNNNNNNNNNNATTAAAATATTTAATTTCAAAATAATTAAATTTAACNNNNNNNNNNNNNNNNNNNNNNNNNNNNNNNNNNNNNNNNNNNNNNNNNNNNNNNNNNNNNNNNNNNNNNNNNNNNNNNNNNNNNNNNNNNNNNNNNNNNNNNNNNNNNNNNNNNNNNNNNNNNNNNNNNNNNNNNNNNNNNNNNNNNNNNNNNNNNNNNNNNNNNNNNNNNNNNNNNTATTAATAATTATAATCTCACCAGCTAAATTAATTGTAGGAGGACAAGATATATTTCTTGCCGACATTAAAAATCATAATAAAGATATTGAAGGCATAAATGTCAATATACCCTTATTAATTAATAATCTTCGACTATTAGTTCGCTCATACAATATGTTTGCTAAACAAAATAATCCAGAAGAACATAAACCATGGCCAATTATCATATAATATGACCCACATATTCCCCAATTTCTTATAGTTATAATTCCTCTAATCACAAGACCTATATGAGCAACAGAAGAATAAGCAATTATTGATTTAATGTCAATTTGAATTATACATAGAATTCTAATCAAAATACTTCCAATTATTGAAATTGAAATCCATATAAACCCAAATTTGTAAAAATAAGAAGGAATAATAAACATTATACGAATTAAACCATATCCACCTAATTTTAACAAAATTCTAGCTAAAATTATTGAACCAGAAACAGGAGCTTCAACATGAGCTTTTGGTAATCAAAAATGAAAAAATAATGGTATTTTAATTAAAAAAGCTATCATTATACCAAAATAAATATAAATATTAACAGAACAATTTATTAACATAAAAATTCTAGTTATATTATTTTTATAAAGAAAAAAAATTCTTATCAATATTGGTATTGATGCAAATAATGTATAAAATAATAAATAATATCTTGCATCAATACGTTCTGGTTGATAGCCCCAACCTATAATAATAATTATAATTGGAATTAATCTTGATTCAAAACAAATGTAAAAAACTATAATTCTTGATGTAGAAAAAGAAATAATCAAAAAAATCATTAACATAACACCAAAAATAAAAAAAATAATTTATTATGCTTATATATTGGACTAGAAACAATTATTAAAAATGAAATTCAAATTTAAAATGATTAAACCATAAGATATTTCATCAATATAAAAATTATAGCCTAATATATATAAATTTTTATTTATAAAAACCAAAAATATAATTATCAAAACAATAATTATATTTATTAATCATGAATTGTAAATTAATAGGGTTAATATAAAGCAAAAAAAAATTAGTTTTATTGATATAGATATTAATTTATCATTATTATGAGATCGAATTAATATAACTAAACATGATAAACCTATTACTCCCTCACAAACTCTAAAAACCAAGAAAATTAATAAAATATGTAAATCATATATTTTTATGGAAAAAATAAATGATAAAGAACAAAATATCCTTAAAATAATTAATTCCAATCTTAATGCCGTTATTAAATGTTTTCGATTTATACATAATGAAATTATTCCTGAAAAGAATATAGAGGTAAAATATATTAATAATGTTATATTCNNNNNNNNNNNNNNNNNNNNNNNNNNNNNNNNNNNNNNNNNNNNNNNNNNNNNNNNNTTATTCATCAATTTATAATTTAATGACATTATTATTTTTAAAGTTTTCAAAATCCCTTGGGGCCCAATGTTTTCACCTCATCCCATATCAATTGATTTTTGAAATATGAATGATTTACTTATCAATAACATTTGATTATGAAACGTTGACAATTGCTTTATAAACCATATTGAACCAAAAAACTCATAAACAATTTTTATTTTAGTTTTAGAAAAAATTGATATTTCGTAACCTAACCAGATTCCTGATATTGAAAAAATCAATGCCAATAATTTGCCCTCTAAAGGCATTAAAATTATAATTGGATCATTAAATATTAATCATCTTAATATAGATCCCGAAAAAATTGAATAAATAGATAAAATAATAATTCTTTTAATTATGTTATTTATACTTTCATTCAATGATCTTAACTTGTAGAAATTATAATTTATATTTATTGAAAAAAATGTCAAACGGGCAGAATAGAAGGAAGTTAAACCAATACCAATATATATAAATATTATAATTAATAAATTAATGTGTCTAGAACACATAGTTTCTATAATTAAATCTTTTGAATAAAATCCACTCAAAAATGGAATTCCACATAATGATATATTAGCAATATTCATTATTGTAATAGTTAATGGTATTTGAAATCTTAAACATCCTATTACACGAATATCTTGATTATTATTAAAATTGTGAATCAAAATTCCTGCACACAAAAATAAAGTAGCTTTAAATAAAGCATGAACAATTAAGTGAAAAAATGATAACATTGGATAACCAAATATAATAATTGTTATTATAATTCCCAGCTGTCTTAAAGTTGATAATGCAATAATTTTTTTTAAATCAAATTCAAAATTTGCTCTAATTCCCGATATTAATATAGTTAAAATTGAAATTAATATAAATAATTGAATATAATCGAAATTTAAAATAATATTAGAAAATCGAATTATTAAATATACTCCAGCAGTAACAAGTGTAGATGAATGAACCAAAGCAGAAACAGGAGTTGGAGCAGCCATAGCTGCTGGTAATCATGAAGAAAATGGAATTTGAGCACTCTTTGTAAATCTCGCAATAATAATCATAATTAATATAATTTATATCTTTATTAATAAAAGATAAAAATTTCATGACCCAAAATTTAATATTCATGCAATAGCTATTAAAATAGCAACATCTCCCACACGATTCATTAATGCAGTTAATATACCCGCATTATTAGAATATAAATTTTGATAATAAATAACTAAGCAATAAGAAACTAAACCAAGTCCATCTCAACCAATAATAATTCTCATTATATTAGGTCTAATAATTAATAAAACTATAGATATAACAAACCCCATAACAATATAAATAAAACGATTAATGAATTTATCATTATTTATATATATTCCACTATATAAAACAACCACTGATGAAATAAATATAACTGTTGAAAGAAATTGTTGATATAAAATCAAAAATTATAGTCATATAAATATTACATGAATTAATTGAAAATTATTCACTCCAATATAATTATATAATTATTTTTAAAATTCATTGAATATTAATATTAGTAAACTCATAAATATAAGAATAATAAATATATAAACATTAAATTTTTTATTTATCATGAAAATTATTATATATTTAATAATTATTTTATCAATAAATTTTNNNNNNNNNNNNNNNNNNNNNNNNNNNNNNNNNNNNNNNNNNNNNNNNNNNNNNNNNNNNNNNNNNNNNNNNNNNNNNNNNNNNNNNNNNNNNNNNNNNNNNNNNNNNNNNNNNNNNNNNNNNNNNNNNNNNNNNNNNNNNNNNNNNNNNNNNNNNNNNNNNNNNNNNNNNNNNNNNNNNNNNNNNNNNNNNNNNNNNNNNNNNNNNNNNNNNNNNNNNNNNNNNNNNNNNNNNNNNNNNNNNNNNNNNNNNNNNNNNNNNNNNNNNNNNNNNNNNNNNNNNNNNNNNNNNNNNNNNNNNNNNNNNNNNNNNNNNNNNNNNNNNNNNNNNNNNNNNNNNNNNNNNNNNNNNNNNNNNNNNNNNNNNNNTTATTTACTATTTGTTTTAATTATTGTAATTAATATTTTAACAACAAATATGTTGACTCTTCGAAGAAGAACTNNNNNNNNNNNNNNNNNNNNNNNNNNNNNNNNNNNNNNNNNNNNNNNNNNNNNNNNNNNNNNNNNNNNNNNNNNNNNNNNNNNNNNNNNNNNNNNNNNNNNNNNNNNNNNNNNNNNNNNNNNNNNNNNNNNNNNNNNNNNNNNNNNNNNNNNNNNNNNNNNNNNNNNNNNNNNNNNNNNNNNNNNNNNNNNNNNNNNNNNNNNNNNNNNNNNNNNNNNNNNNNNNNNNNNNNNNNNNNNNNNNNNNNNNNNNNNNNNNNNNNNNNNNNNNNNNNNNNNNNNNNNNNNNNNNNNNNNNNNNNNNNNNNNNNNNNNNNNNNNNNNNNNNNNNNNNNNNNNNNNNNNNNNNNNNNNNNNNNNNNNNNNNNNNNNNNNNNNNNNNNNNNNNNNNNNNNNNNNNNNNNNNNNNNNNNNNNNNNNNNNNNNNNNNNNNNNNNNNNNNNNNNNNNNNNNNNNNNNNNNNNNNNNNNNNNNNNNNNNNNNNNNNNNNNNNNNNNNNNNNNNNNNNNNNNNNNNNNNNNNNNNNNNNNNNNNNNNNNNNNNNNNNNNNNNNNNNNNNNNNNNNNNNNNNNNNNNNNNNNNNNNNNNNNNNNNNNNNNNNNNNNNNNNNNNNNNNNNNNNNNNNNNNNNNNNNNNNNNNNNNNNNNNNNNNNNNNNNNNNNNNNNNNNNNNNNNNNNNNNNNNNNNNNNNNNNNNNNNNNNNNNNNNNNNNNNNNNNNNNNNNNNNNNNNNNNNNNNNNNNNNNNNNNNNNNNNNNNNNNNNNNATAATAAAATTATTTAATGAGGTCCTTTCGTACTAACATTAAAAATAATTAAGTAGATAGAAACCAACCTGGCTCACGCCGGTTTGAACTCAGATCATGTAAGAATATTAAGGGTCGAACAGACCCAGAAATAATAAATTTTGCTCCAATCCCTATTCTTAATCCAACATAGAGGTCGCAATCATATTTATCGATATGAACTCTTCAAATTAATTACGCTGTTATCCGTAAGGTAAGTTATTCTTATAATCAAAAATTTGGATCAATATTAACATAAATTTATGAAATTTTTATTAAAAGTTAATTATATTTTAATATCACCCCNNNNNNNNNNNNNNNNNNNNNNNNNNNNNNNNNNNNNNNNNNNNNNNNNNNNNNNNNNNNNNNNNNNNNNNNNNNNNNNNNNNNNNNNNNNNNNNNNNNNNNNNNNNNNNNNNNNNNNNNNNNNNNNNNNNNNNNNNNNNNNNNNNNNNNNNNNNNNNNNNNNNNNNNNNNNNNNNNNNNNNNNNNNNNNNNNNNNNNNNNNNNNNNNNNNNNNNNNNNNNNNNNNNNNNNNNNNNNNNNNNNNNNNNNNNNNNNNNNNNNNNNNNNNNNNNNNNNNNNNNNNNNNNNNNNNNNNNNNNNNNNNNNNNNNNNNNNNNNNNNNNNNNNNNNNNNNNNNNNNNNNNNNNNNNNNNNNNNNNNNNNNNNNNNNNNNNNNNNNNNNNNNNNNNNNNNNNNNNNNNNNNNNNNNNNNNNNNNNNNNNNNNNNNNNNNNNNNNNNNNNNNNNNNNNNNNNNNNNNNNNNNNNNNNNNNNNNNNNNNNNNNNNNNNNNNNNNNNNNNNNNNNNNNNNNNNNNNNNNNNNNNNNNNNNNNNNNNNNNNNNNNNNNNNNNNNNNNNNNNNNNNNNNNNNNNNNNNNNNNNNNNNNNNNNNNNNNNNNNNNNNNNNNNNNNNNNNNNNNNNNNNNNNNNNNNNNNNNNNNNNNNNNNNNNNNNNNNNNNNNNNNNNNNNNNNNNNNNNNNNNNNNNNNNNNNNNNNNNNNNNNNNNNNNNNNNNNNNNNNNNNNNNNNNNNNNNNNNNNNNNNNNNNNNNNNNNNNNNNNNNNNNNNNNNNNNNNNNNNNNNNNNNNNNNNNNNNNNNNNNNNNNNNNNNNNNNNNNNNNNNNNNNNNNNNNNNNNNNNNNNNNNNNNNNNNNNNNNNNNNNNNNNNNNNNNNNNNNNNNNNNNNNNNNNNNNNNNNNNGACTTTTTATGATNTNTTCTACTTTAAATTTGCAATTTAATATTATAAATTAAATATAAGACTTTATGTTAACAGAATTAAACTGTCTATTAATATCAAAAATTAATGTTCTTCATAAACTATAACANNNNNNNNNNNNNNNNNNNNNNNNNNNNNNNNNNNNNNNNNNNNNNNNNNNNNNNNNNATTCTATTAGTATATAAGTATATTTAACTTCCAATTAAAAGGATTAATTTTNNNNNNNNNNNNNTATCCAATAAATTTNNTTTTATCTTTAAAATCACAATTTAATATTTTTGTTAAACTAATTGAATTACTATTTGTAATAAATATTACATTATTAAATTCTAAATTTAAGGCATTAATTTTGCTAAAATAGTTCTAATATGGCAGATTAGTGCAATGAATTTAANNNNNNNNNATAAAATANNTTTTTTTTATTGGAAAGTAAGATGCATGAAACGAAAAGGATTATTTTGATAGAATAAATCATGTAATAAAATTACTCNNNCATTAAGTGGCTGAAAGTAAGTAATGGTCTCTTAAACCAATTANNNNNNNNNNNNNNNNNNNNNNNNNNAAAGATAAGCTAAAGTTAAAGCTAATGGGTTCATACCTCACTTATGGAAATTTTTCCTCTTTTTANNNNNNNNNNNNNNNNNNNNNNNNNNNNNNNNNNNNNNNNNNNNNNNNNNNNNNNNNNNNNTCAGAAAAGATANNATGATCATTTTAAACTTCCAAAGTTTATATTTTATATTTAAATTATTTTCTGNNNNNNTTAGCTTAAACAAAAGCATTTATTTTGAAAGTAAAAGAAAAGATATTTAATCTATTAACTTNNNNNNNNNNNNNNNNNNNNNNNNNNNNNNNNNNNNNNNNNNNNNNNNNNNNNNNNNNNNNNNNNGTTTTAATAGTTTAATNNNAAAAACTTAGATCTTGTAAATCTAAATTAACGTAAGTTTTAAAAATAGGTTTTAAGTTATTGANAACTATTATCCTTCAAAGTTAAAAATATAATTANATTTATTATTAGGAGAAATAATTTTTCATAAATAAATTTACAGTTTATTGCCTATTATCGGCCATCCTAANNNNNNNNNNNNNNNNNNNNNNNNNNNNNNNNNNNNNNNNNNNNNNNNNNNNNNNNNNNNNNNNNN

>muta-NI-east_02.NZ.HB.OCB.01

AACTTATTTTCATCATTTGACCCTTCAACTGGATTTTTATCATTAAATTGATTAAGATCAATAATTCTACTATTATTTATACCTTTAACTTATTGATATATTCCTAATCGTTTTATTCTTCTATATAACAAAATTTTAATTTTATTAAATAATGAATTAAATATATTAATAAACTATAAATCATTAGGAAGATCATTAATATTTTTATCATTATTTATATTTATTCTACTAAATAATTTATTGGGCTTACTTCCGTATATTTTCACTAGTCCAAGTCATCTAGTATTTACAATAAGATTAGCATTACCACTATGATTATCATTCATACTTTATGGATTTATTAATAACATAAATCATATATTTTGTCATTTAGTTCCATCCGGAACTCCTAATATTTTAATGTCTTTTATAGTTCTCATTGAAAGAGTCAGAAATTTAATTCGTCCTGGTTCCTTAGCTGTTCGATTGACAGCTAATATAATTGCTGGACATCTTTTGATAACTTTATTAGGTAACTTACCTATAAGGTATGAATTATATTCAGGTATTATTATTATTTTTCAAGTTATATTAATATTATTTGAATTAGCTGTTTGTATTATTCAATCATATGTATTTATAGTTCTTAGAACTTTATATTATAGAGAAGTAAATATTCCTCAAATATCTCCTCTTAATTGATTATTATTTATTTATTNNNNNNNNNNNNNNNNNNNNNNNNNTATATTTATATATTTTACTTATCTATTAAAATCTAACATAACAGCCAAAAATAAATTAATAGATAGAATAATTTGATTTAAGCCAACACGACAAAATCATCCTTTAATTAAAATTATTAATAATTCACTAATTGACTTCCCAGCACCATCTAATTTATCTTATTGATGAAATTTTGGATTTGTATTAGGATTATGCTTAACGATTCAAATTTTAACTGGATTATTTTTGTCAATACATTATAATGCCAATATCATAAATGCTTTTGAAAGATTAAGACATATTTGCCGTGATGTAAACTATGGTTGAATTTTACGTGTTATTCACGCTAATGGGGCTTCATTGTTTTTCATTTGTGTATATTTACATGTAGGACGTGGATTATATTATGGATCATTCAAATATATTGAAACATGATCTATTGGTGTAATTATATTATTAATATTAATAGCTACTGCTTTCTTAGGATATGTTTTACCGTGAGGACAAATATCTTTTTGAGGAGCCACAGTCATTACAAATTTATTATCAGCCATTCCTTATTTAGGTGGAATATTAGTTAATTGAATTTGAGGAGGATTTGCAGTTGACAATGCAACATTAACTCGATTTTACTCCTTTCATTTTATTTTACCATTCATTGTATTAAGAATAACAATTATTCACCTATTATATTTACATACAACAGGTTCAAATAATCCATTAGGAATTAATTCCAACAATGACAAAGTCCCATTTCACCCATACTTCTCCATTAAGGATATTATAAGCCTATTTTTATTAATAATTATTTTCTTTATATTAGTCATGTTAGAACCCTATATACTAGGGGATCCAGATAATTTTATTCCTGCCAATCCACTTGTAACACCAAAACATATTCAACCTGAATGATACTTTTTATTTGCCTACGCAATCCTCCGTTCAATTCCTAATAAACTAGGAGGAGTAATTGCTCTTTTTATATCAATTTTCATTTTAATGTTTGTACCTTTATTAAATAATTCTAATTTTATAGGATTAAATAATTACCCAATTAATCAAATTATATTTTGATATATAGTAATAATTTTAATTTTATTAACTTGAATTGGAGCACGACCTGTCGAATTACCTTATGTTAACTTTGGAATATTTTTAACAATTATATATTTCTCTTACTTCATTATTGACCCAATAATTAAATCTATTTGAGATAAATTAATTAGATAGATGAAAAAATGATTCTTCTCAACTAATCATAAAGATATTGGAACAATATATTTCATTTTTGGTATTTGATCAGGTATAATTGGTACAACTTTAAGAGTTTTAATTCGCGTAGAACTTGGAACTCCTGGTTCATTTATTGGTGATGATCAAATTTATAATGTAATTGTCACTGCTCATGCTTTCATTATAATTTTTTTTATGGTAATACCAATTATAATTGGTGGATTTGGAAATTGATTAGTCCCCCTAATAATTGGAGCTCCTGATATAGCTTTTCCTCGTATAAATAATATGAGTTTTTGATTATTACCACCTTCCCTAATTCTTATTTTAGTGGGAAGAATAGTTGATAGAGGTGCAGGTACAGGCTGAACAGTTTACCCTCCTTTATCCGCAGGTATTGCACATTCTGGTTCATGTGTTGATTTAACTATTTTTTCTCTACACCTTGCAGGTGTATCTTCAATTTTAGGTGCTGTAAATTTCATTAGAACAATTTTTAATATACGTTCAATAGGAATTTGATTGGATCGCATACCTTTATTTGTATGAGCAGTCTTAATTACTGCATTTTTATTATTATTGTCATTACCCGTTTTAGCAGGAGCTATTACAATATTGTTAACAGATCGAAATTTAAATACTTCGTTTTTCGATCCTGCAGGAGGGGGAGATCCTATTCTTTATCAACATTTATTTTGGTTTTTTGGTCATCCTGAAGTCTATATTTTAATTTTACCAGGATTTGGTTTGATTTCTCACATTATTACTCAAGAAAGAGGAAAAGTTGAATCTTTTGGATCATTAGGAATAATTTATGCCATAATATCAATTGGTATTTTAGGATTTGTTGTATGAGCACATCATATATTTACTGTAGGAATAGATGTTGATACACGTGCATATTTTACATCAGCTACTATAATTATTGCAGTTCCCACTGGAATTAAAGTATTCAGTTGACTTGCAACTTTGAGAGGTATAAAAATTAATATTACGTCTTCAGCCCTATGAGCGTTAGGATTTGTCTTTTTATTTACTATTGGGGGTTTAACTGGAGTTATTCTAGCCAATTCTTCAATTGATATTATATTACATGATACATATTATGTAGTTGCACATTTCCATTATGTTTTATCAATAGGAGCAGTATTTGCTATTATAGCAAGATTTATTCATTGGTTTCCATTATTTACAGGATTGAGATTAAACTCAAATTGATTAAAAATTCATTTTCTATTAATATTTATTGGTGTAAATATAACATTCTTTCCTCAACATTTTTTAGGATTAAGAGGAATACCTCGTCGTTATTCAGACTATCCTGATGCTTATATATCATGAAACATAATTTCATCAATAGGAAGAATTATATCTTTAATTGGAATTTTATTTTTATTATTTATTGTTTGGGAAAGCTTTATTTCAATACGATTAGTATTATATTCTAATAGAATTCAATCTTCTATTGAATGAATACAAAAATTCCCCCCATCTGAACATTCATATAATGAAATGCCATTGTTAATTCAAATTTCAAATTGATCTTATATTAATATACAGGATGCTGTATCACCATTAATAGAACAATTAATATTTTTTCATGATCATGTGTTAGTAATTTTAATTATAATTACAATTGTTGTTGCTTACATAATAATTATATTAATATTAAATAAAATTATTAATCGTTTACTTCTTGAAGGACAGTTAATTGAATTTATTTGAACTTTATTGCCTGCAATGACTTTAATTTTTATTGCATTACCATCATTACGATTATTATATATATTAGACGAAATTAATAATCCACTACTAACATTAAAAATTATTGGTCACCAGTGATATTGATCATATGAATATTCAGATTTTTCTGATGTAGAATTTGATTCATATATAAAATCTATAAACGAAATAAATAAAAACGAATTTCGTTTATTAGATGTAGATAATCGAGTGATTCTGCCATTTAATATTCAAGTCCGACTATTAATTTCTTCTTTTGATGTTATTCATTCTTGAGCAATACCATCAATAAGACTTAAAGTTGATGCAGTGCCAGGACGACTAAATCAAATAAGAATATTAATTAGTCGTCCTGGTGTATCTTACGGACAATGTTCTGAAATTTGTGGAGCAAATCATAGATTTATGCCTATTGTAATTGAAAGAATTAGAATAAAAATATTTATTAAATGATTAATTAATTATATGAATAATCACCCTTATCATATAGTTGACTATAGACCCTGACCTTTAACTGGATCAATTGGAGCATTAACTTTTGTTTCCGGTATAGTTATAATATTTCATAAATGTAATTTTGTATTATTATACATTGGTATTTTATTATTATTAATAACAATAATTCAATGATGACGTGATATTTCTCGAGAAGGGACATTTTTAGGAATACACACAATAATAGTAGTAAATGGTTTGAAAATAGGCATATTATTATTTATTGTATCAGAAATTCTTTTTTTTGTATCATTTTTTTGAGCATTTTTTCATAGTAGATTAAGACCTGTAGTAGAAATTGGTATAATTTGACCTCCTAGAGGTATTTATGCTTTTAATCCGACTCAAGTTCCATTATTAAATACAATAATTTTATTATGCTCAGGAATTACAATTACTTGAGCACATCATTCAATCATAAATGGTGATCACCTTAATTCTATCTATAGAATTATATTAACTGTTATTTTAGGTATATATTTCACTATTCTGCAAGGTTATGAATATTATGAAGCCCCATTTGCAATTAATGATTCTATCTATGGATCTTCTTTTTTTATAGCTACTGGATTTCATGGAATTCACGTAATTATTGGAACAAGATTTATTACTGTATGCTTATTACGACAAATAAAGTTTCATTTCTCAATAAATCATCATTTTGGCTTTGAAGCTGCTGCTTGATATTGACATTTTGTTGATGTAGTATGATTATTTTTATATTTATCAATTTATTGATGAGGAAGATAATAAATTATAGTTTTTAAAAAGAAAAAAAAAACAAATAAATTCAGAGATACCGGTAAATAAGATTTTCAAGCCAAATATATTAATTTGTCATAACGATAACGAGGAAGGGTACCCCGAATCCAAATGAAAAGAAAACAAATTATAGAAACTTGAATTGGAAAAACAATTGAATTAATTTTTCCTCCTAAAAACATTAAACAAAATAATATTCTCATAAATAAAATACTTGAATACTCAGCCAAAAAAATAAATGCAAATCCAGAACCTCTATATTCAACATTAAAGCCAGAAACTAACTCAGATTCTCCCTCAGAAAAATCAAATGGAGATCGATTAGTCTCAGCTAAAGCTGAAGATAATCAACACATTCTTAAAGGAAGAAACAAAAAAATAAATCACACAAATTCCTGAAATACAAACAAATCAATAATATTATAACTTCTTATTATTATTATAGGACACAATATAATTAAAACTAATCTTACCTCATAAGAAATAGATTGAGCAATTGATCGAATGCACCCTAATATTGCATAACTTGAATTAGAAGATCAACCTGTTAATATTAATGAATAAACTCTTATTCTTGAACAACAAAAAAAAAATAAAATACCAAAATTAAAAGAAACACAATTAATGAAATATGGATACAATGATCAAATCAACAGACTATTAAATAATCCTATAATAGGCCTAAAAATATAAATTAAATAATTAGATATTAATGGAATTGTATTTTCCTTTATAAATAATTTAATAGCATCAGAAATAGGCTGCAAAATACCTAAAAATCCAACTTTATTTGGGCCCTTACGAATTTGAATATATCTTAGAACCTTACGTTCCAAAAGAGTAATAAATGCCACTCCAATCAAAATAAATAATACAGTAACTAAAATCGTAATTAAATACAAAAATAATTCTTCTTATATACTTTATATAATTTTCTTAATTTTAGGAATTATAATTTCAATTTCTTCAAACAATTGGCTGGGTTGTTGAATAGGAATTGAAATAAATATAGTTTCATTTTTGCCTATAATGGCAAACAAAATAAGAATTTATGCTTCGGAATCAATAATTAAATATTTTATCATTCAAAGAATAGGATCGAGTTTGTTATTAATAACTATTATTATTNNNAATATAATAATTGATTTAAATTATATAATTATAGTTAGATTAATAATTAAAATTGGCTGTCCTCCTTTTCATTCTTGATATGTTTCTGTTATTGAAGGTTTAACTTGAATAGTATGTTTTATTTTAATAACTATTCAAAAAATTATTCCTTTAATTATACTATCATATTTAAATATTAATATAAGATTATTTATTATTATATCATGCATTTGAGGATGTATTGGAGGGCTGGGCTATTCTTCAATACGTAAAATCATTGCTTATTCATCAATTTATAATTTAAGATGAATTTTTAGAGGTATTATTATTATTAATTACTCATGGCTGATTTATTATTTTATTTATTCATTTACATTATTAGCTGTATGTTATATATTTAAAATGTTTAATATTAATTATATTAATCAATTTATTATAGTATCTTTTAATTTTATAAAATCAATCATAATAATGTGTATTTTTATATCTATAGGGGGATTACCCCCCTTTTTAGGGTTTTTTCCTAAATTAATTATAATTTACTGCTTATTATTAAATAATATAATACTCATTTGTATTATATTATTAATAACAGCTCTGATTATTTTATTTTTTTATTTACGAATCTTAATTACAACACTAATAATAAATACAATTTCAATAAAAAGAATCGTCATAAGAGTTTCATATACTTATTATATTGCTGGAGTATTTTCATTATTTGGAATAATTTTTTTATCATTAATTACATTAAATATTTGTTAGATTTATAATATTTTGATTTATTCNNTTACCTTAATATTTTTACTAATATTTTTATTATCATTATTAATATATATTTCCTATAAATCTATAAAAGATCGAGAAAAATCATCACCATTTGAGTGTGGATTTAGACCATTTGAATCATCTCGTATTCCATTTTCTAGACACTTTTTCTTAATTGCAGTAATTTTTTTAATTTTTGATGTTGAATTGGTAATTATTATACCTGTAATTTTAGTAATAATTAGATTAAAAACTATTGATATTTATGTTATTATATTAATCTTCTTAATTATTTTGACTTTAGGATTATATCATGAATGATATAATAATATATTAAATTGANNNNNNNGTCTAATTAAAATATTTAATTTCAAAATAATTAAATTTAAGGGAATCCAATGTATCATAATCAAAAAAAACTCTCGAACTCTTCCAGAATTAAATGAAAACAAACTGGAAAAAAATATACCATGTTGAGTAAATGAAAATATATATAATCTATAACAAGCAGATAAAAATGAAGATAAAATCAATAAAACCATTATTAACATGTTTCAAGATATTAATCTATTAATAATTATAATTTCACCAGCTAAATTAATTGTAGGAGGACAAGATATATTTCTTGCCGACATTAAAAATCATAATAAAGATATTGAAGGCATAAATGTCAATATACCCTTATTAATTAATAATCTTCGACTATTAGTTCGCTCATACAATATGTTTGCTAAACAAAATAATCCAGAAGAACATAAACCATGTCCAATCATTATATAATATGACCCACATATTCCCCAATTTCTTATAGTTATAATTCCTCTAATCACAAGACCTATATGAGCAACAGATGAATAAGCAATTATTGATTTAATGTCAATTTGAATTATACATAAAATTCTAATTAAAATACTTCCAATTATTGAAATTGAAATCCATATAAACCCAAATTTGTAAAAATAAGAAGGAATAATGTACATTATACGAATTAAACCATATCCACCTAATTTTAACAAAATTCTAGCTAAAATTATTGAACCAGAAACAGGAGCTTCAACATGAGCTTTTGGTAATCAAAAATGAAAAAATAATGGTATTTTAATTAAAAAAGCTATTATTATACCAAAATAAATATAAATATTAATAGAACAACTTATTAATATAAAAATTATAGTTATATTATTTTTATAAAGAAAAAAAATTCTTATCAATATTGGTAATGATGCAAATAATGTATAAAATAATAAATAATATCTTGCATCAATACGTTCTGGTTGATAGCCCCAACCTATGATAATAATTATAATTGGAATTAATCTTGATTCAAAACAAATGTAAAAAACTATAATTCTTGATGTAGAAAAAGAAATAATCAAAAAAATCATTAACATAACACCAAAAATAAAAAAAATAATCTATTATGTTTATATATTGGACTAGAAACAATTATTAAAAATGAAATTCAAATTTAAAATGATTAAACCATAAGATATTTCATCAATATAAAAATTATAGCCTAATATATATAAATTTTTATTTATAAAAACCAAAAATATAACTATCAAAACAATAATTATATTTATTAATCATGAATTATAAATTAATAGGGTTAATATAAAGCAAAAAAAAACTAGTTTTATTGATATAGATATTAATTTATCATTATTATGAGATCGAATTAATATAACTAAACACGATAAACCTATTACTCCTTCACAAACTCTAAAAACCAAGAAAATTAATAAAATATGTAAATCATATATTTTTATGGAAAAAATAAATGATAAAGAACAAAATATTCTTAAAATAATTAATTCCAATCTTAATGCTGTTATTAAATGTTTTCGATTTATACATAATGAAATTATCCCTGAAAAAAATATAGAGGTAAAATATATTAATAATGTTATATTCTATAAATATAAAACAATAATTCAAATAATGAATGTAATTATTTGAATCTTAAAATTATTATTCATCAATTTATAATTTAATGATATTATTATTTTTAAAGTTTTCAAAATCCCTTGGGGCCCAAAGTTTTCACCTCATCCCATATCAATTGATTTTTGAAATATGAATGATTTACTTATCAATAATATTTGATTATGAAACGTTGACAATTGCTTTATAAACCACATTGAACCAAAAAACTCATAAACAATTTTTATTTTAGTTTTAGAAAAAATTGATATCTCGTAACCCAACCAGATTCCTGATATTGAAAAAATCAATGCCAATAATTTGCCCTCTATAGGCATTAAAATTATAATTGGATCATTAAATATTAATCATCTTAATATAGATCCCGAAAAAATTGAATAAATAGATAAGATAATAATTCTTTTAATTATGTTATTTATTCTTTCATTCAATGATCTTAACTTATAAAAATTATAATTTATATTTATTGAAAAAAATGTCAAACGGGCAGAATAGAAGGAAGTTAAGCCAATACCAATATATATAAATATTATAATTAATAAATTAATGTGTCTAGAACACATAGTTTCTATAATTAAATCTTTTGAATAAAATCCACTCAAAAATGGAATTCCACATAATGATATATTAGCAATATTCATTATTGTAATAGTTAGTGGTATTTGAAACCTTAAACATCCTATTACACGAATATCTTGATTATTATTAAAATTGTGAATCAAAATTCCTGCACACAAAAATAAAGTAGCTTTAAATAAAGCATGAACAATTAAGTGAAAAAATGATAACATTGGATAACCAAATATAATAATTGTTATTATAATTCCCAGCTGTCTTAAAGTTGATAATGCAATAATTTTTTTTAAATCAAATTCAAAATTTGCTCTAATTCCCGATATTAATATAGTTAAAATTGAAATTAATATAAATAATCTAATATAATCAAAATTCAAAATAATATTAGAAAATCGAATTATTAAATATACTCCAGCAGTAACAAGTGTGGATGAATGAACCAAAGCAGAAACAGGAGTTGGAGCAGCCATAGCTGCTGGTAATCATGAAGAAAATGGAATTTGAGCACTCTTTGTAAATCTCGCAATAATAATTATAATTAATATAATTTATATCTTTATTAATAAAAGATAAAAATTTCATGACCCAAAATTTAATATTCATGCAATAGCTATTAAAATAGCAACATCCCCCACACGATTCATTAATGCAGTTAATATGCCTGCATTATTAGAATATAAATTTTGATAATAAATAACTAAGCAATAAGAAACTAAACCAAGTCCATCTCAACCAATAATAATTCTCATTATATTAGGTCTAATGATTAATAAAACTATAGATATAACAAACCCCATAACAATATAAATAAAACGATTAATGAATTTATCATTATTTATATATATTCCACTATATAAAACAACTACTGATGAAATAAATATAACTGTTGAAAGAAATTGTTGATATAAAATCAAAAATTATAGTCATATAAATATTACATGAATTAATTGAAAATTATTCACTCCAATATAATTATATAATTATTTTTGAAATTCATTGAATATTAATATTAGTAAACTTATAAATATAAGAATAATAAATATATAAACATTAAATTTTTTATTTATCATGAAAATTGTTATGTATTTAATAATTATTTTATCAATAAATTTTATTTTCATAAAACATCCCTTATCAATAGGGTTAATTTTAATAATACAAACAATTCTATCTTGCTTAATCTGTAGACTTTATTTAAGTTGTTATTTATTTTCTTATATCTTATATCTTATTTTTATTGGTGGTATATTAATTCTATTTATATATATATCAAGAATTGCATCAAATGAAAAATTTATTTATTCTATTAAATTAATAATGCTTAATTTTTTAATTTTAATTTTAACTAATTTTATCAATATAATTGATTTAAAAACACTAAATATTAAAAGAAATATTATAACATATATAAACCACAACAATTTAATAATAAGAAAACTATATATTATTCCTTCTGGAATAATAACACTAATATTAACAATTTATTTATTATTTGTTTTAATTATTGTAATTAATATTTTAACAACAAATATATTGACTCTTCGAAGAAGAACTTCTTTCTAACTACACCTTCCGGTACAGTTACTTTGTTACGACTTATCTCATTAATATCATGAGAGTGACGGGCGATATGTACATAAATTAGAGCTAATTTCAATTAATTAAATTTAATTAATTTATTATCAAATCCAATTTCATATTTTCTNNNNNNNNNNAAATAATCCAATAAATAATTAATTGTAACCCATTTTTTCTTCAATATAAACTGCACCTTGACCTGACATTCTAAATATNATAATATAATATGAAAATTTTTCTTATAAAACATTCTTGACAGAGATATACAAATTAAATTAAAGTTTTTTCTATCGTGGACTATCAATTATAAAACAAGTTCCTCTGATAAGATAAATTACCGCCAAATTCTTTGAATTTAAAGATCATTTCTAATAATAATCAAGTTATTTTAATCACATTTTTAATAATAGGGTATCTAATCCTAGTTTATACAAAAATTTTTCAGACATAAAATTAATTTTTAAAGATAAAATATATTTCACCAAAATTATAGATATTTTATATTATTGTTATATTAACTGAAACTTAAATATTTAAATTAAAGAAATCGTATAACCGCAACTGCTGGCACGAAATTTGATTCTTTTAAATAAAATTACTAATTCTAATTTTATTAATTAATAATGATAAATACTGCGTATTTATTAAAAATATTTCCATTTAGAAATTAAAAACAATTAAAAAAGTGCATGTAATATAATTTTAAATTTAAATTTTCNTAAACTAGAATTAAACTTCAAATAATAAAATTATTTAATGAGGTCCTTTCGTACTAACATTAAAAATAATTAAGTAGATAGAAACCAACCTGGCTCACGCCGGTTTGAACTCAGATCATGTAAGAATATTAAGGGTCGAACAGACCCAGAAATAATAAATTTTGCTCCAATCCCTATTCTTAATCCAACATCGAGGTCGCAATCATATTTATCGATATGAACTCTTCAAATTAATTACGCTGTTATCCCTAAGGTAATTTATTCTTATAATCAAAAATCTGGATCAATATTAACATAAATTTATGAAATTTTTATTAAAAGTTAATTATATTTTAATATCACCCCAACAAAAAAATCAATTAAATAAAATAAATTAATTAACTATAAANAAATAAACCTAATNAAGATTTTAAAAATTCTATAGGGTCTTCTCGTCCCACTCATTCATTTAAGCTTTTTTACTTAAAAATCAATTTCAATTATTAATATTAATTAAGTTAATTTCTCATTCAATCATTCATTCAAGCCTCCAATTAAAAGACTATTTATTATGCTACCTTTGCACGGTCAATTTACCGCGGCCATTTAATTTTTAATCATTGGGCAGATTAGACTTTTAATTATTTCTAAAAGACACGTTTTTGATAAACAGGTGAAAATTATTTTTGCCTAAATAATAAATATTATTTATAACTAAAAAACCAGATATCATATAATTTGATAAAATATCATTTCCAGAATAAATTTATTAATATATATACAATAATAACTAACAAATNTAAACTAAATCATTATATTTCGGGAAAAATAAATATTTAAAAGTATTCAATTAACCCTGATACAAAAGGTACAAATTATATTTTACTTAAAATTATTTATAAAGTNNTTTCCCTCTCAGTTAATCAAAATTATTTTTTTATATAAAATTACTTTAACACAAAAATTTTTTTTTCAAAATGTAAAAAACAACTATATANTAAAATTATATTATGGGATATATTTAATTATAATAATTGAATTGCAGTCAATAGGTGTTAACTATAACTATCCTTAAGTAATGAAGTAAAATAATACATTTAGTTTCGACCTAAAATAAGAATTAGAATTCCTTGCTTTTAAATGAAGCCAAAATAGAGGCATTTTATTGTTAATAAAATAATTGAAANTTTATTTCCATTTAAAGAAAGATTAGTTTAATTAAAATATAAATTTGTCAGGTTTATGAAACTTTTAAGTATTTTTCTGACTTTTTATGATNTNTTCTACTTTAAATTTGCAATTTAATATTATAAATTAAATATAAGACTTTATGTTAACAGAATTAAACTGTCTATTAATATCAAAAATTAATGTTCTTCATAAACTATAACATTTATAAAGTTTATAAAACATTTCATTTTCATTGAAAAGAGAGACTTGGTCTTATAAAATTCTATTAGTATATAAGTATATTTAACTTCCAATTAAAAGGATTAATTTTATTAAATAGAATATATCCAATAAATTTNNTTTTATCTTTAAAATCACAATTTAATATTTTTGTTAAACTAATTGAATTACTATTTGTAATAAATATTACATTATTAAATTCTAAATTTAAGGCATTAATTTTGCTAAAACAGCTCTAATATGGCAGATTAGTGCAATGAATTTAAGATTCATATATAAAATANNTTTTTTTTATTGGAAAATAAGATGCCTGAAATGAAAAGGATTATTTTGATAGAATAAATCATGTAATGAAATTACTCNNNCATTAAGTGGCTGAAAGTAAGTAATGGTCTCTTAAACCAATTAATAGTAATTAACATATACTCTTAATGAAAGATAAGCTAAAGTTAAAGCTAATGGGTTCATACCTCACTTATGGAAANTTTTCCTCTTTTTATTTAAAAACAAAAGTTACTTTAATATCTTCAATATTATGCTCTTTATTAAGCTATTTAAATTCAGAAAAGATANNATGATCATTTTAAACTCCCAAAGTTTATATTTTAAATTTAAATTATTTTCTGAGTTAATTAGCTTAAATAAAAGCATTTATTTTGAAAGTAAAAGAAAAGATAGTTAATCTATTAACTTAGGAAACAAGAAATAAAATTAGCTTCTAACTAACTTTTAAAGCGGTTAAATTCCGTTTTTTCCTTGTTTTAATAGTTTAATNNNAAAAACTTAGATCTTGTAAATCTAGATTAACGTAAGTTTTAAAAATAGGTTTTAAGTTATTGANAACTATTATCCTTCAAAGTTAAAAATATAATTANATTTATTATTAGGAGAAATAATTTTTCATAAATAAATTTACAGTTTATTGCCTATTATCGGCCATCCTAATCAAGTTATATTGATTGAACAATATTCTCATTAGTGTAAATAAAGTGCTAATTTAGCTTTAACTTG

>nelsonensis_05.NZ.MB.TFL.01

AACTTATTTTCATCATTTGACCCTTCAACTGGATTTCTATCTCTTAATTGATTAAGATCAATAATTCTACTGTTATTTATACCTTTAACTTATTGATATATTCCTAATCGTTTTATTCTTTTATATAACAAAATTTTAATTTTATTAAATAATGAATTAAATATATTAATAAACTATAAATCATTAGGAAGATCATTAATATTTTTATCGTTATTTATATTTATTCTATTAAATAATTTATTAGGTTTACTTCCGTATATTTTCACTAGTCCAAGTCATCTAGTATTTACAATAAGATTAGCATTACCACTATGGTTATCATTTATACTTTATGGATTTATTAATAATATAAATCATATATTTTGTCATTTAGTTCCATCTGGAACTCCCAATATTTTAATGCCTTTTATAGTTCTTATTGAAAGAGTTAGAAATTTAATTCGACCTGGCTCCTTAGCTGTCCGACTAACAGCTAACATAATTGCTGGACATCTTTTGATAACCTTATTAGGTAACTTACCTATAAGATATGAATTATATTCAGGTATTATTATTATTTTTCAAGTTATATTAATATTATTTGAATTGGCTGTTTGTATTATTCAATCATACGTATTTATAGTTCTTAGAACTTTATATTATAGAGAAGTAAGTATTCCTCAAATATCTCCTCTTAATTGATTATTATTTGTTTATTTTNNNNNNNTAATTATTGTTATTANTATATTTATATATTTTACCTATCTATTAAAATCTGATACAACAGCTAAAAATAAATTAATAGATAGAATAATTTGATTTAAGCCAACACGACAAAATCATCCTTTAATTAAAATTATTAATAATTCATTAATTGACTTCCCAGCACCATCTAATTTATCTTATTGATGAAATTTTGGATTTGTATTAGGATTATGCTTAACAATTCAAATTTTAACTGGATTATTTTTATCAATACATTATAATGCCAATATCATAAATGCTTTTGAAAGATTAAGACATATTTGCCGTGATGTAAACTATGGTTGAATTTTACGTGTTATTCACGCTAATGGAGCTTCATTGTTTTTTATTTGTGTATATTTACATGTAGGACGTGGATTATATTATGGATCATTCAAATATATTGAAACATGATCTATTGGTGTAATTATATTATTAATATTAATAGCTACTGCTTTCTTAGGATATGTTTTACCGTGAGGGCAAATATCTTTTTGAGGAGCCACAGTCATTACAAATTTATTATCAGCCATTCCTTATTTAGGTGGAATATTAGTTAATTGAATTTGAGGAGGATTTGCAGTTGACAATGCAACACTAACTCGATTTTACTCTTTTCATTTTATTTTACCATTCATTGTATTAAGACTAACAATTATTCACTTATTATATTTGCATACAACAGGTTCAAATAATCCATTAGGAATTAACTCCAACAATGACAAAGTTCCATTTCACCCATACTTCTCCATTAAGGATATTATAAGCCTATTTTTATTAATAATTATTTTCTTTATATTAATCATGTTAGAACCCTATATACTAGGGGATCCAGATAATTTTATTCCTGCTAATCCACTTGTAACACCAAAACATATTCAACCTGAATGATACTTTTTATTTGCCTACGCAATCCTCCGTTCAATTCCTAATAAACTAGGAGGAGTAATTGCTCTTTTTATATCAATTTTCATTTTAATGTTTGTACCTTTATTAAATAATTCTAATTTTATAGGATTAAATAATTATCCAATTAATCAAATTATATTTTGATATATAGTAATAATTTTAATTTTATTAACTTGAATTGGAGCACGACCTGTCGAATTACCTTATATTAACTTTGGAATATTTTTAACACTTATATATTTCTCTTACTTTATTATTGACCCAATAATTAAATCTATTTGAGATAAATTAATTAGATAGATGAAAAAATGATTCTTCTCAACTAATCATAAAGATATTGGAACAATATATTTCATTTTTGGTATTTGATCAGGTATAATTGGTACAACTTTAAGAGTTTTAATTCGTGTAGAACTTGGAACCCCAGGTTCATTTATTGGTGATGATCAAATTTATAATGTAATTGTCACTGCTCATGCTTTCATTATAATTTTTTTTATGGTAATACCAATTATAATTGGTGGATTTGGAAATTGATTAGTCCCTTTAATAATTGGAGCTCCTGATATAGCTTTTCCTCGTATAAATAATATGAGTTTTTGATTATTACCACCTTCCCTAATTCTTATTTTAGTGGGAAGAATAGTTGATAGAGGTGCAGGCACAGGTTGAACAGTTTACCCTCCTTTATCCGCAGGTATTGCACATTCTGGTTCATGTGTTGATTTAACTATTTTTTCTCTACACCTTGCAGGTGTATCTTCAATTTTAGGTGCTGTAAATTTCATTAGAACAATTTTTAATATACGTTCAATAGGAATTTGATTGGATCGCATACCTTTATTTGTATGAGCAGTTTTAATTACTGCATTCTTATTATTATTGTCACTACCCGTTTTAGCAGGCGCTATCACAATATTGTTAACAGATCGAAATTTAAATACTTCGTTTTTTGATCCTGCAGGAGGGGGAGATCCAATTCTTTATCAACATTTATTTTGATTTTTTGGTCACCCTGAAGTCTATATTTTAATTTTACCAGGATTTGGTTTAATTTCTCACATTATTACTCAAGAGAGAGGTAAAATTGAATCTTTTGGCTCATTAGGAATAATTTATGCCATAATATCAATTGGTATTTTAGGATTTGTTGTGTGAGCACATCATATATTTACTGTAGGAATAGATGTTGATACACGTGCATATTTTACATCAGCTACTATAATTATTGCAGTTCCCACTGGAATTAAAGTATTTAGTTGACTTGCAACTTTGAGAGGTATAAAAATTAATATTACATCTTCAGCCCTATGAGCATTAGGGTTTGTCTTTTTATTTACTATTGGGGGTTTAACTGGGGTTATTCTAGCCAATTCTTCAATTGATATTATGTTACATGATACATATTATGTAGTTGCACATTTCCATTATGTTTTATCAATAGGAGCAGTGTTTGCTATTATAGCAAGATTTATTCATTGGTTTCCATTATTTACAGGACTAAGATTAAACTCAAATTGATTAAAAATTCATTTTCTATTAATATTTATTGGTGTAAATATAACATTCTTTCCTCAACATTTTTTAGGATTAAGAGGAATACCTCGTCGTTATTCAGACTATCCTGATGCTTATATATCATGAAACATAATTTCATCAATAGGAAGAATTATATCTTTAATTGGAATTTTATTTTTATTATTTATTGTTTGAGAAAGCTTTATTTCAATACGATTAGTATTATATTCTAATAGAATTCAATCTTCTATTGAATGAATACAAAAATTCCCCCCATCTGAACATTCATATAATGAAATGCCATTGTTAATTCAAATTTCAAATTGATCTTATATTAATATACAGGATGCTGTATCACCATTAATAGAACAATTAATATTTTTTCATGATCATGTGTTAGTAATTTTAATTATAATTACAATTGTTGTTGCTTACATAATAGTTATATTAATATTAAATAAAATTATTAATCGCTTACTTCTTGAAGGACAGTTAATTGAATTTATTTGAACTTTATTGCCTGCAATAACTTTAATTTTTATTGCATTACCATCATTACGATTATTATATATATTAGACGAAATTAATAATCCATTACTAACATTAAAAATTATTGGTCACCAGTGATATTGATCATATGAATATTCAGATTTTTCTGATGTAGAATTTGATTCATATATAAAATCTATAAACGAAATAAATAAAAACGAATTTCGTTTATTAGATGTAGATAATCGAGTAATTTTACCATTTAATATTCAAGTCCGACTATTAGTTTCTTCTTTTGATGTTATTCATTCTTGAGCAATGCCATCAATAAGACTTAAAGTTGATGCAGTGCCAGGACGATTAAATCAAATAAGAATATTAATTAGTCGTCCCGGTATTTCTTATGGACAATGTTCTGAAATTTGTGGAGCAAATCATAGATTTATGCCTATCGTAATTGAAAGAATTAGAATAAAAATATTTATTAAATGATTAATTAATTATATGAATAATCACCCTTATCATTTAGTTAATTATAGACCCTGACCTTTAACTGGATCAATTGGAGCATTAACTTTTGTTTCCGGTATAGTTATAATATTTCATAAATGTAATTTTATATTATTATATATTGGTATCTTATTATTATTAATAACAATAATTCAATGATGACGTGACATTTCCCGAGAAGGAACATTTTTAGGAATACACACAATAATAGTAGTGAATGGTTTGAAAATAGGCATATTATTATTTATTGTATCAGAAATTCTTTTTTTTGTATCATTTTTTTGAGGATTTTTTCATAGTAGATTAAGACCTGTAGTAGAAATTGGTATAATTTGACCTCCTAGAGGTATTTATGTTTTTAATCCGACTCAAGTTCCATTATTAAATACAATAATTTTATTATGTTCAGGAATTACAATTACTTGAGCACATCATTCAATCATAAATGGTAATCACATTAATTCTATCTATAGAATTATGTTAACTGTTATTTTAGGTATATATTTCACTATTCTGCAAGGTTATGAATATTATGAAGCCCCATTTGCAATTAATGATTCCATCTATGGATCTTCTTTTTTTATAGCTACTGGATTTCATGGAATCCACGTAATTATTGGAACAAGATTTATTACTGTATGCTTATTACGACAAATAAAGTTTCATTTCTCAATAAATCATCATTTTGGCTTCGAAGCTGCTGCTTGATACTGACATTTTGTTGATGTAGTCTGATTATTTCTATATCTATCAATTTATTGATGAGGAAGATAATAAATTATAGTTTTTAAAAAGAAAAAAAAAACAAATAAATTAAGAGATACCGGTAAATAAGATTTTCAAGCCAAATATATTAATTTGTCATAACGATAACGAGGAAGAGTACCCCGAATCCAAATGAAAAGAAAACAAATTATAGAAACTTGAATTGGAAAAACAATTGAATTAATTTTCCCTCCTAAAAACATTAAACAAAATAATATTCTCATAAATAAAATACTTGAATACTCAGCCAAAAAAATAAATGCAAACCCAGAACCTCTGTATTCAACATTAAAGCCAGAAACTAACTCTGATTCTCCCTCAGAAAAATCAAATGGAGATCGATTAGTCTCAGCTAAAGCTGAAGATAATCAACATATTCTTAAAGGAAAAAATAAAAAAATAAATCACACAAATTCCTGAAATATAAACAAATCAATAATATTATAACTTCTCATTATTATTATAGGACACAATATAATTAAAACCAATCTTACCTCATAAGAAATAGATTGAGCAATTGATCGAATGCATCCTAATATTGCATAACTTGAATTAGAAGATCAACCTGTCAATATTAATGAATAAACTCTTATTCTTGAGCAACAAAAAAAAAATAAAATACCAAAATTAAAAGAAACACAATTAATGAAATATGGATACAATGATCAAATCAACAAACTATTGAATAATCCTATAATAGGCCTAAAAATATAAATTAAATAATTAGATACTAATGGAATTGTATTCTCCTTTATAAATAATTTAATAGCATCAGAAATAGGCTGCAAAATACCCAAAAATCCAACTTTATTTGGGCCCTTACGGATTTGAATATATCTTAAAACCTTACGTTCCAAAAGAGTAATAAATGCCACTCCAATCAAAATAAATAATACAGTAACTAAAATCGTAATTAAATACAAAAATAATTCTTCTTATATACTTTATATAATTTTCTTAATTTTAGGAATTATAATTTCAATTTCTTCAAACAATTGGCTGGGTTGTTGAATAGGAATTGAAATAAATATAGTTTCATTTTTGCCTATAATGGCAAACAAAATAAGAATTTATGCTTCGGAATCAATAATTAAATATTTTATTATTCAAAGAATAGGATCGAGTTTATTATTAATAACTATTATTATTNNNAATATNNNNATTGATTTAAATTATATAATTATAGTTAGATTAATAATTAAAATTGGCTGTCCTCCTTTCCATTCTTGATATGTTTCTGTTATTGAGGGTTTGACTTGAATAGTATGTTTTATTTTAATAACTATTCAAAAAATTATTCCTTTGATTATATTATCATATTTAAATGTTAATATAAGATTATTTATTATTATGTCATGCATTTGAGGATGTATTGGAGGGCTGGGCTATTCTTCAATACGTAAAATCGTTGCTTATTCATCAATTTATAATTTAAGATGAATTTTTAGAGGTATTATTATTATTAATTACTCATGGCTGATTTATTATTTTATTTATTCATTTACATTATTAGCTGTATGTTATATANNNNNNNNNNNNNNNATTAATTATATTAATCAATTCATTATAGTATCTTTTAATTATATAAAATCAATCATAATAATGTGTATTTTTATATCAATGGGGGGATTGCCCCCCTTTTTAGGGTTTTTCCCTAAATTAATTATAATTTACTGCTTATTATTAAATAACATAATATTTATTTGTATTATGTTATTAATAACAGCTCTGATTATTTTATTTTTTTATTTACGAATCTTAATTACAACATTAATAATAAATACAATTTCAATAAAAATAATTGTTATAAGAATTTCATATACTTACTATATTGCTGGAGTATTTTCATTATTTGGAATAATTTTTTTATCATTAATTACATTAAATATTTGTTAGATTTATAATATTTTGATTTATTCNNTTATTTTAATNNNNNNNNNNNNNNNNNNNNNNNNNNNNNNNNNNNNNNNNNNNNNNNNNNCTATAAAAGATCGAGAAAAATCATCACCATTTGAGTGTGGATTTAGCCCATTTGAGTCATCTCGTATTCCATTTTCGAGACACTTTTTCTTAATTGCAGTAATTTTTTTAATTTTTGATGTTGAATTGGTAATTATTATACCTGTAATTTTAGTAATAATTAGATTAAAAACTATTGATATTTATATTATTATATTAATCTTCTTAATTATTTTAACTTTAGGATTATATCATGAATGATATAATAATATATTAAATTGANNNNNNNGTCTAATTAAAATATTTAATTTCAAAATAATTAAATTTAAGGGAATCCAATGTATCATAATCAAGAAAAACTCCCGAACTCTTCCAGAATTAAATGAAAATAAACTAGAAAAAAATATACCATGTTGAGTAAATGAAAATATATATAATCTATAACAAGCAGATAAAAATGAAGATAAAATTAATAAAACCATTATTAGCATATTTCAAGATATTAATCTATTAATAATTATAATCTCACCAGCTAAATTAATTGTAGGAGGACAAGATATATTTCTTGCCGACATTAAAAATCATAATAAAGATATTGAAGGCATGAATGTCAATATACCCTTATTAATTAATAATCTTCGACTATTAGTTCGCTCATATAATATGTTTGCTAAACAAAATAATCCAGAAGAACATAAACCATGACCAATCATTATATAATATGATCCACATATTCCCCAATTTCTTATAGTTATAATTCCTCTAATCACAAGACCTATATGAGCAACAGATGAATAAGCAATTATTGATTTAATGTCAATTTGAATTATACATAGAATTCTAATTAAAATACTTCCAATTATTGAAATTGAGATCCATATAAACCCAAATTTGTAAAAATAAGAAGGAATAATGTACATTATACGAATTAAACCATATCCACCTAATTTTAACAAAATTCTAGCTAAAATTATTGAACCAGAAACAGGAGCTTCAACATGAGCTTTTGGTAACCAAAAATGAAAAAATAATGGTATTTTAATTAAAAAAGCTATTATTATACCAAAATAAATATAAATATTAACAGAGCAACTTATTAACATAAAAATTCTAGTTATATTATTTTTATAAAGAAAAAAAATTCTTATCAATATTGGTAATGATGCAAATAATGTATAAAATAATAAATAATATCTTGCATCAATACGTTCTGGTTGATAACCCCAACCTATAATAATAATTATAATTGGAATTAATCTTGATTCAAAACAAATGTAAAAAACTATAATTCTTGATGTAGAAAAAGAAATAATCAAAAAAATCATTAACATAACACCAAAAATAAAAAAAATAATTTATTATGTTTATATATTGGACTAGAAACAATTATTAAAAATGAAATTCAAATCTAAAATAATTAAACCATAAGATATTTCATCAATATAAAAATTATAACCTAATATATATAAATTTTTACTTGTAAAAACCAAAAATATAATTATCAAAACAATAATTATATTTATTAATCATGAATTATAAATTAATAGGGTTAATATAAAGCAAAAAAAAACTAGTTTTATTGATATAGATATTAATTTATCATTATTATGAGATCGAATTAATATAACTAAACATGACAAACCTATTACTCCTTCACAAACTCTAAAAACCAGGAAAATTAATAAAATATGTAAATCATATATTTTTATGGAAAAAATAAATGATAAAGAACAAAAAATTCTTAAAATAATTAATTCCAATCTTAATGCTGTTATTAAATGTTTTCGATTTATACATAATGAAATTATCCCTGAAAAGAATATAAAGGTAAAATATATTAATAATGTTATATTCTATAAATATAAAACAATAATTCAAATAATGAATGTAATTATTTGAATCTTAAAATTATTATTCATCAATTTATAATTTAATGACATTATTATTTTTAAAGTTTTCAAAATACCTTGGGGCCCAAGGTTTTCACCTCATCCCATATCAATTGATTTTTGAAATATGAATGATTTACTTATCAATAACATTTGATTATGAAACGTTGACAATTGCTTTATAAACCACATTGAACCAAAAAACTCATAAACAATTTTTATTTTAGTTTTAGAAAAAATTGATATCTCATAACCCAATCAGATTCCTGATATTGAAAAAATCAATGCCAATAATTTGCCCTCTATAGGCATTAAAATTATAATTGGATCATTAAATATTAATCATCTTAATATAGATCCCGAAAAAATTGAATAAATAGATAAAATAATAATTCTTTTAATTATGTTATTTATACTTTCATTTAATGATCTTAACTTATAGAAATTATAATTTATATTTATTGAAAAAAATGTCAAACGGGCAGAATAAAAGGANNTTAAACCAATACCAATATATATAAATATTATAATTAATAAATTAATGTACCTAGAACATATAGTTTCTATAATTAAATCTTTTGAATAAAATCCACTCAAAAATGGAATTCCACATAATGATATATTAGCAATATTCATTATTGTAATAGTTAATGGTATTTGGAATCTTAAACATCCTATTACACGAATATCTTGATTATTATTAAAATTATGAATCAAAATTCCTGCACACAAAAATAAAGTAGCTTTAAATAAAGCATGAACAATTAAGTGAAAAAATGATAACATTGGATAACCAAATATAATAATTGTTATTATAATTCCCAGCTGTCTTAAAGTTGATAATGCAATAATTTTTTTTAAATCAAATTCAAAATTTGCTCTAATTCCTGATATTAATATAGTTAAAATTGAAATTAATATAAATAATTGAATATAATCGAAATTCAAAATAATATTAGAAAATCGAATTATTAAATATACTCCAGCAGTAACAAGTGTAGATGAATGAACCAAAGCAGAAACAGGAGTTGGAGCGGCCATAGCTGCTGGTAATCATGAAGAAAATGGAATTTGAGCACTCTTTGTAAATCTCGCAATAATAATTATAATTAATNNNNNNNNNNNNNNNNNNNNTAAAAGATAAAAATTTCATGACCCAAAATTTAATATTCATGCAATAGCTATTAAAATAGCAACATCTCCCACACGATTCATTAATGCAGTTAATATACCTGCATTATTAGAATATAAATTTTGATAATAAATAACTAAACAATAAGAAACTAAACCAAGTCCATCTCAACCAATAATAATTCTCATTATATTAGGTCTAATGATTAATAAAACTATAGATATAACAAACCCCATAACAATATAAATAAAACGATTAATGAATTTATCATTATTTATATATATTCCACTATATAAAACAACCACTGATGAAATAAATATAACTGTTGAAAGAAATTGTTGATATAAAATCAAAAATTATAGTCATATAAATATTACATGAATTAATTGAAAATTATTCACTCCAATATAATTATATAATTATTTTTGAAATTCATTGAATATTAATATTAGTAATCTCATAAATNNNNNNNNNNNNAATATATAAACATTAAATTTTTTATTTATCATGAAAATTGCTATGTATTTAATAATTATTTTATCAATAAATTTTATTTTCATAAAACATCCCTTATCAATAGGATTAATTTTAATAATACAAACAGTTGTATCTTGCTTAATTTGTAGACTTTACTTAAGTTGTTATTTATTTTCTTATATCTTATATCTTATTTTTATTGGTGGTATATTAATTCTATTTATATATATATCAAGAATTGCATCAAATGAAAAATTTATTTATTCTATTAAATTAATAATGCTTAATTTTTTAATTTTAACTTTAACTAATTTTATCAATATAATTGATTTAAAATCACTAAATATTAAAAGAAATATTATAATATATATAAACCACAGCAATTCTATAATAAGAAAACTATATATCATCCCTTCTGGAATAATAACATTAATATTAACAATTTATTTATTATTTGTTTTAATTATTGTAATTAATATTTTAACAACAAATATATTGACTCTTCGAAGAAGAACTTCTTTCTAACTACACCTTCCGGTACAGTTACTTTGTTACGACTTATCTCATTAATATCATGAGAGTGACGGGCGATATGTACATAAATTAGAGCCAATTTCAATTAATTAAATTTAATTAATTTATTATCAAATCCAATTTCTTATTTTCTNNNNNNNNNNAAATAATCCAATAAATAATTAATTGTAACCCATTTTTTCTTTAATATAAACTGCACCTTGACCTGACATTATAAATATNATGATATAATACGAAAATTTTTCTTATAAAACATTCTTGACAGAGATATACAAATTAAATTAAAGTTTTTTCTATCGTGGATTATCAATTATAAAACAGGTTCCTCTGATAAGATAAATTACCGCCAAATTCTTTGAATTTAAAGATCATTTCTAATAATAATCAAGTTATTTTTATCACATTTTTAATAATAGGGTATCTAATCCTAGTTTATACAAAAATTTTTCAGACATAAAATTAATTTTTAAAGATAAAATATATTTCACCAAAATTATAGATATTTTATATTATTGTTACATTAACTGAAACTTAAATATTTAAATTAAAGAAATCGTATAACCGCAACTGCTGGCACGAAATTTGATTCTTTTAAATAAAATTACTAATTCTAATTTTATTAATTAATAATAATAAATACTGCGCATTTATTCAAAATATTTCCATTTAGAAATTAAAAACAATTAAAAAAGTGCATGTAATATAATTTTAAATTTAAATCTTCTTAAACTAGAATTAAACTTCAAATAATAAAATTATTTAATGAGGTCCTTTCGTACTAACATTAAAAATAATTAAGTAGATAGAAACCAACCTGGCTCACGCCGGTTTGAACTCAGATCATGTAAGAATATTAAGGGTCGAACAGACCCAGGAATAATAAATTTTGCTCCAATCCCTATTCTTAATCCAACATCGAGGTCGCAATCATATTTATCGATATGAACTCTTCAAATTAATTACGCTGTTATCCCTAAGGTAATTTATTCTTATAATCAAAAATTTGGATCAATATTAACATAAATTTATGAAATTTTTATTAAAAGTTAATTATATTTTAATATCACCCCAACAAAAAAATCAATTAAATAAAATAAATTAATTAACTATAAANAAACAAACTTAATNAAAATTTTAAAAATTCTATAGGGTCTTCTCGTCCCACTTATTCATTTAAGCTTTTTTACTTAAAAATCAATTTCAATTATTAATATTAATTAAGTTAATTTCTCATTCAATCATTCATTCAAGCCTCCAATTAAAAGACTATTTATTATGCTACCTTTGCACGGTCAATTTACCGCGGCCATTTAATTTTTAATCNNNNNNNNNNNNNNNNNNNNNNNNNNNNNNNNNNNNNNNNNNNNNNNNNNNNNNNNNNNNNNNNNNNNNNNNNNNNNNNNNNNNNNNNNNNNNNNCTAAGAAACCAGATATCATATAATTTGATAAAATGTCATTTCCAGAATAAATTTATTAATATATATACAATAATAANNNNNNNNNNNNNNNNNNNNNNNTATATTTCGGGAAAAATAAATTTTTAAAAGTATTCAATTAACCCTGATACAAAAGGTACAAATTATATTTTACTTAAAATTATTTATAAAGTNNTTTCCCTCTCAGTTAATCAAAATTATTTTTTTATATAAAATTACTTTAACACAAAAATTTTTTTTTCAAAATGTAAAAAACAACTATATANTAAAATTATATTATGGGATATATTTAATTATAATAATTGAATTGCAGTCAATAGGTGTTAACTATAACTATCCTTAAGTAATGAAGTAAAATAATACATTTAGTTTCGACCTAAAATAAGAATTAGAATTCCTTGCTTTTAAATGAAGCCAAAGTAGAGGCATTTTATTGTTAATAAAATAATTGAAANTTTATTTCCATTTAAAGAAAGATTAGTTTAATTAAAATATAAATCTGTCAGGTTTATGAAACTTTTGAGTATTTTTCTGACNTTTTATGATNTNTTCTACTTTAAATTTGCAATTTAATATTATAAATTAAATATAAGACTTTATGTTAACAGAATTAAACTGTCTATTAATATCAAAAATTAATGTTCTTCATAAACTATAACATTTATAAAGTTTATAAAACATTTCATTTTCATTGAAAAGAGAGACTTGGTCTTATAAAATTCTATTAGTATATGAGTATGTTTAACTTCCAATTAAAAGGATTAATTTTATTAAATAGAATATATCCAATAAATTTNNTTTTATCTTTAAAATCACAATTTAATATTTTTGTTAAACTAATTGAATTACTATTTGTAATAAATATTACATTATTAAATTCTAAATTTAAGGCATTAATTTTGCTAAAATAGTTCTAATATGGCAGATTAGTGCAATGAATTTAAGATTCATATATAAAATATNTTTTTTTTATTGGAAAATAAGATGCCTGAAATGAAAAGGATTATTTTGATAGAATAAATCATGTAATAAAATTACTCNNNCATTAAGTGGCTGAAAGTAAGTAATGGTCTCTTAAACCAATTAATAGTAATTAACATATACTCTTGATGAAAGATAAGCTAAAGTTAAAGCTAATGGGTTCATACCTCACTTATGGAAANTTTTCCTCTTTTTATTTAAAAACAAAAGTTACTTTAATATCTTCAATATTATGCTCTTTATTAAGCTATTTAAATTCAGAAAAGATANNATGATCATTTTAAACTCCCAAAGTTTATATTTTATATTTAAATTATTTTCTGAGTTAATTAGCTTAAACAAAAGCATTTATTTTGAAAGTAAAAGAAAAGATATTTAATCTATTAACTTAGGAAACTAGAAATAAAATTAGCTTCTAACTAACTTTTAAAGCGGTTAAATTCCGTTTTTTCCTTGTTTTAATAGTTTAATNNNAAAAACTTAGATCTTGTAAATCTAGATTAACGTAAGTTTTAAAAATAGGTTTTAAGTTATTGANAACTATTATCCTTCAAAGTTAAAAATATAATTANATTTATTATTAGGAGAAATAATTTTTCATAAATAAATTTACAGTTTATTGCCTATTATCGGCCATCCTAATCAAGTTATATTGATTGAACAATATTCTCATTAGTGTAAATAAAGTGCTAAATTAGCTTTAACTTG

>muta-SI_12.NZ.NC.BAL.01

NNNNNNNNNNNNNNNNNNNNNNNNNNNNNNNNNNNNNNNNNNNNNNNNNNNNNNNNNNNNNNNNNNNNNNNNNNNNNNNNNNNNNNNNNNNNNNNNNNNNNNNNNNNNNNNNNNNNNNNNNNNNNNNNNNNNNNNNNNNNNNNNNNNNNNNNNNNNNNNNNNNNNNNNNNNNNNNNNNNNNNNNNNNNNNNNNNNNNNNNNNNNNNNNNNNNNNNNNNNNNNNNNNNNNNNNNNNNNNNNNNNNNNNNNNNNNNNNNNNNNNNNNNNNNNNNNNNNNNNNNNNNNNNNNNAAGAAGACTGGNNNNNNNNNNNNNNNNNNNNNNNNNNNNNNNNNNNNNNNNNNNNNNNNNNNNNNNNNNNNNNNNNNNNNNNNNNNNNNNNNNNNNNNNNNNNNNNNNNNNNNNNNNNNNNNNNNNNNNNNNNNNNNNNNNNNNNNNNNNNNNNNNNNNNNNNNNNNNNNNNNNNNNNNNNNNNNNNNNNNNNNNNNNNNNNNNNNNNNNNNNNNNNNNNNNNNNNNNNNNNNNNNNNNNNNNNNNNNNNNNNNNNNNNNNNNNNNNNNNNNNNNNNNNNNNNNNNNNNNNNNNNNNNNNNNNNNNNNNNNNNNNNNNNNNNNNNNNNNNNNNNNNNNNNNNNNNNNNNNNNNNNNNNNNNNNNNNNNNNNNNNNNNNNNNNNNNNNNNNNNNNNNNNNNNNNNNNNNNNNNNNNNNNNNNNNNNNNNNNNNNNNNNNNNNNNNNNNNNNNNNNNNNNNNNNNNNNNNNNNNNNNNNNNNNNNNNNNNNNNNNNNNNNNNNNNNNNNNTTTAAACCAACACGACAAAATCATCCTTTAATTAAAATTATTAATAATTCATTAATTGACTTCCCAGCACCATCTAATTTATCTTATTGATGAAATTTTGGATTTGTATTAGGATTATGCTTAATGATTCAAATTTTAACTGGATTATTTTTATCAATACATTATAATGCCAATATCATAAATGCTTTTGAAAGATTAAGACATATTTGCCGTGATGTAAACTATGGTTGAATTTTACGTGTTATTCACGCTAATGGAGCTTCATTGTTTTTCATTTGTGTATATTTACATGTAGGACGTGGATTATATTATGGATCATTCAAATATATTGAAACATGATCTATTGGTGTAATTATATTATTAATATTAATAGCTACTGCTTTCTTAGGATATGTTCTACCGTGAGGACAGATATCTTTTTGAGGAGCCACAGTCATTACAAATCTATTATCAGCCATTCCTTATTTAGGTGGAATGTTAGTTAATTGAATTTGAGGAGGATTTGCAGTTGACAATGCAACACTAACTCGATTTTACTCTTTTCATTTTATTTTACCATTCATTGTATTGAGAATAACAATTATTCACTTATTATATTTACATACAACAGGTTCAAATAACCCATTAGGGGTTAACTCAAACAATGACAAAGTCCCATTTCACCCATACTTCTCCATTAAGGATATTATAAGCCTATTTTTATTAATAATTATTTTCTTTATATTAATCATGTTAGAACCATATATACTAGGGGACCCAGATAATTTTATTCCTGCCAATCCACTTGTAACACCAAAACATATTCAACCTGAATGATACTTTTTATTTGCCTACGCAATCTTACGTTCAATTCCTAATAAACTAGGAGGAGTAATTGCTCTTTTTATATCAATTTTCATTTTAATGTTTGTACCTTTATTAAATAATTCTAATTTTATAGGATTAAATAATTACCCAATTAATCAAATTATATTTTGATATATANNNNNNATTTTAATTTTATTAACTTGAATTGGAGCACGACCTGTCGAATTACCTTATATTAACTTTGGAATATTATTAACACTTATATACTTCTCTTACTTCATTATTGACCCAATAATTAAATCTATTTGAGATAAATTAATTAGATAGATGAAAAAATGATTCTTCTCAACTAATCATAAAGATATTGGAACGATATATTTCATTTTTGGTATTTGATCAGGTATAATTGGTACAACTTTAAGAGTTTTAATTCGTGTAGAACTTGGAACTCCAGGTTCATTTATTGGTGATGATCAAATTTATAATGTAATTGTCACTGCTCATGCTTTCATTATAATTTTTTTTATAGTAATACCAATTATAATTGGTGGATTTGGAAATTGATTAGTCCCCTTAATAATTGGAGCTCCTGATATAGCTTTTCCTCGTATAAATAATATGAGTTTTTGATTATTACCACCTTCCCTAATTCTTATTTTAGTGGGAAGAATGGTTGATAGAGGTGCAGGCACAGGTTGAACAGTTTACCCCCCTTTATCCGCAGGTATTGCACATTCTGGTTCATGTGTTGATTTAACTATTTTCTCTCTACACCTTGCAGGTGTATCTTCAATTTTAGGTGCTGTTAATTTCATTAGAACAATTTTTAATATACGTTCAATAGGAATTTGATTGGATCGTATACCTTTATTTGTATGAGCAGTTTTAATTACTGCATTTTTATTATTATTGTCATTACCCGTTTTAGCAGGAGCTATCACAATATTGTTAACAGATCGAAATTTAAATACTTCGTTTTTTGATCCTGCAGGAGGGGGAGATCCTATTCTTTATCAGCATTTATTTTGATTTTTTGGTCATCCTGAAGTCTATATTTTAATTTTACCAGGGTTTGGTTTGATTTCTCACATTATTACTCAAGAGAGAGGTAAAATTGAGTCTTTTGGTTCATTAGGAATAATTTATGCCATAATATCAATTGGTATTTTAGGATTTGTTGTATGAGCACATCATATATTTACTGTAGGAATAGATGTTGATACACGTGCATATTTTACATCAGCTACTATAATTATTGCAGTTCCCACTGGAATTAAAGTATTCAGTTGACTTGCAACTTTGAGAGGTATAAAAATTAATATTACATCATCAGCACTATGAGCGTTAGGGTTTGTCTTTTTATTTACTATTGGGGGTTTAACTGGAGTTATTCTAGCCAATTCTTCAATTGATATTATATTACATGATACATATTATGTAGTTGCTCATTTCCATTATGTTTTATCAATAGGAGCAGTATTTGCTATTATAGCAAGATTTATTCACTGGTTTCCATTATTTACAGGATTAAGATTAAACTCAAATTGATTAAAAATTCATTTTTTATTAATATTTATTGGTGTAAATATAACATTCTTTCCTCAACATTTTTTAGGATTAAGAGGAATACCTCGTCGCTATTCAGACTATCCTGATGCTTATATATCATGAAACATAATTTCATCAATAGGAAGAATTATATCTTTAGTTGGAATTTTATTTTTATTATTTATTGTTTGGGAAAGCTTTATTTCAATACGATTAGTATTATATTCTAATAGAATTCAATCTTCTATTGAATGAATACAAAAATTCCCCCCATCTGAACATTCATATAATGAAATGCCATTGTTAATTCAANNNNNNNNNNNNNNNNNNNNNNNNNNNNNNNNNNNNNNNNNNNNNNNNNNNNNNNNNNNNNNNNNNNNNNNNNNNNNNNNNNNNNNNNNNNNNNNNNNNNNNNNNNNNNNNNNNNNNNNNNNNNNNNNNNNNNNNNTATTAAATAAAATTATTAATCGCTTACTTCTTGAAGGACAGTTAATTGAATTTATTTGAACTTTATTGCCTGCAATAACTTTAATTTTTATTGCATTACCATCATTACGATTATTATATATATTAGACGAAATTAATAATCCATTACTAACATTAAAAATTATTGGTCACCAATGATATTGATCATATGAATATTCAGATTTTTCTGATGTAGAATTTGATTCATATATAAAATCTATAAATGAAATAAATAAAAACGAATTTCGTTTATTAGATGTAGATAATCGAGTAATTCTACCATTTAATATTCAAGTCCGACTATTAATTTCTTCTTTTGATGTTATTCATTCTTGAGCAATACCATCAATAAGACTTAAAGTTGATGCAGTGCCGGGACGACTAAATCAAATAAGAATATTAATTAGTCGTCCCGGTGTATCTTATGGACAATGTTCTGAAATTTGTGGAGCAAATCATAGATTTATGCCTATTGTAATTGAAAGAATTAGAATAAAAATATTTATTAACTGATTAATTAANNNNNNNNNNNNNNNNNNNNNNNNNNNNNNNNNNNNNNNNNNNNNNNNNNNNNNNNNNNNNNNNNNNNNNNNNNNNNNNNNNNNNNNNNNNNNNNNNNNNNNNNNNNNNNNNNNNNNNNNNNNNNNNNNNNNNNNNNNNNNNNNNNNNNNNNNNATAGTACTTCAATGATGACGTGACATTTCCCGAGAGGGAACATTTTTAGGAATACACACAATAATAGTAGTGAATGGTTTGAAAATAGGCATATTATTATTTATTGTATCAGAAATTCTTTTTTTTATATCATTTTTTTGAGCATTTTTTCATAGTAGATTAAGACCTGTAGTAGAAATTGGTATAATTTGACCTCCTAGAGGTATTTATGCTTTTAATCCAACTCAAGTTCCATTATTAAATACAATAATTTTATTATGTTCAGGAATTACAATTACTTGAGCTCATCACTCAATCATAAATGGTAATCACATTAATTCTATCTATAGAATTATATTAACTGTTATTTTAGGTATATATTTCACTATTCTTCAAGGTTATGAATATTATGAAGCCCCATTTGCAATTAATGATTCTATCTATGGATCTTCTTTTTTTATAGCTACTGGATTTCATGGAATTCATGTAATTATTGGAACAAGATTTATTACTGTGTGCTTATTACGACAAATAAAGTTTCATTTTTCAATAAATCATCATTTTGGCTTTGAAGCTGCTGCTTGATATTGACATTTTGTTGATGTAGTATGATTATTTTTATATTTATCAATTTATTGATGAGGAAGATAATAAATTATAGTTTTTAAAAAGAAAAAAAAAACAAATAAATTCAGAGATACCGGTAAATAAGATTTTCAAGCTAAATATATTAATTTGTCATAACGATAACGAGGAAGAGTACCTCGAATCCAAATGAAAAGAAAACAAATTATAGAAACTTGAATTGGAAAAACAATTGAATTAATTTTCCCTCCTAAAAACATTAAACAAAATAATATTCTCATAAATAAAATACTTGAATACTCAGCCAAAAAAATAAATGCAAATCCAGAACCTCTATATTCAATATTAAAGCCAGAAACTAACTCTGATTCTCCCTCAGAAAAATCAAAAGGAGATCGATTAGTTTCAGCTAAAGCTGAAGATAATCAGCACATTCTTAAAGGAAGAAACAAAAAAATAAATCACACAAATTCCTGAAATATAAACAAATCAATAATATTATAACTTCTTATTATTATTATAGGACACAATATAATTAAAACCAATCTTACCTCATAAGAAATAGATTGAGCAATTGATCGAATGCATCCTAATATTGAATAACTTGAATTAGAAGATCAACCTGTTAATATTAATGAATAAACTCTTATTCTTGAGCAACAAAAAAAAAATAAAATACCAAAATTAAAAGAAACACAATTAATAAGATATGGATATAATGATCAAATCAACAGACTATTAAATAATCCTATAATAGGCCTAAAAATATAAATTAAATAATTAGATATTAATGGAATTGTATTTTCCTTTATAAATAATTTAATAGCATCAGAAATAGGCTGCAAAATACCCAAAAATCCAACTTTATTTGGGCCCTTACGAATTTGAATATATCTTAAAACCTTACGCTCCAAAAGCGTAATAAATGCCACTCCAATCAAAATAAATAATANNNNNNNNNNNNNNNNNNNNNNNNNNNNNNNNNNNNNNNNNNNNNNNNNNNNNNNNNNNNNNNNNNNNNNNNNNNNNNNNNNNNNNNNNNNNNNNNNNNNNNNNNNNNNNNNNNNNNNNNNNNNNNNNNNNNNNNNNNNNNNNNNNNNNNNNNNNNNNNNNNNNNNNNNNNNNNNNNNNNNNNNNNNNNNNNNNNNNNNNNNNNNNNNNNNNNNNNNNNNNNNNNNNNNNNNNNNNNNNNNNNNNNNNNNNNNNNNNNNNNNNNNNNNNNNNNNNNNNNNNNNNNNNNNNNNNNNNNNNNNNNNNNNNNNNNNNNNNNNNNNNNNNNNNNNNNNNNNNNNNNNNNNNNNNNNNNNNNNNNNNNNNNNNNNNNNNNNNNNNNNNNNNNNNNNNNNNNNNNNNNNNNNNNNNNNNNNNNNNNNNNNNNNNNNNNNNNNNNNNNNNNNNNNNNNNNNNNNNNNNNNNNNNNNNNNNNNNNNNNNNNNNNNNNNNNNNNNNNNNNNNNNNNNNNNNNNNNNNNNNNNNNNNNNNNNNNNNNNNNNNNNNNNNNNNNNNNNNNNNNNNNNNNNNNNNNNNNNNNNNNNNNNNNNNNNNNNNNNNNNNNNNNNNNNNNNNNNNNNNNNNNNNNNNNNNNNNNNNNNNNNNNNNNNNNNNNNNNNNNNNNNNNNNNNNNNNNNNNNNNNNNNNNNNNNNNNNNNNNNNNNNNNNNNNNNNNNNNNNNNNNNNNNNNNNNNNNNNNNNNNNNNNNNNNNNNNNNNNNNNNNNNNNNNNNNNNNNNNNNNNNNNNNNNNNNNNNNNNNNNNNNNNNNNNNNNNNNNNNNNNNNNNNNNNNNNNNNNNNNNNNNNNNNNNNNNNNNNNNNNNNNNNNNNNNNNNNNNNNNNNNNNNNNNNNNNNNAAGAAAAAGGANNNNNNNNNNNNNNNNNNNNNNNNNNNNNNNNNNNNNNNNNNNNNNNNNNNNNNNNNNNNNNNNNNNNNNNNNNNNNNNNNNNNNNNNNNNNNAGNNNNNNNNNNNNNNNNNNNNNNNNNNNNNNNNNNNNNNNNNNNNNNNNNNNNNNNNNNNNNNNNNNNNNNNNNNNNNNNNNNNNNNNNNNNNNNNNNNNNNNNNNNNNNNNNNNNNNNNNNNNNNNNNNNNNNNNNNNNNNNNNNNNNNNNNNNNNNNNNNNNNNNNNNNNNNNNNNNNNNNNNNNNNNNNNNNNNNNNNNNNNNNNNNNNNNNNNNNNNNNNNNNNNNNNNNNNNNNNNNNNNNNNNNNNNNNNNNNNNNNNNNNNNNNNNNNNNNNNNNNNNNNNNNNNNNNNNNNNNNNNNNNNNNNNNNNNNNNNNNNNNNNNNNNNNNNNNNNNNNNNNNNNNNNNNNNNNNNNNNNNNNNNNNNNNNNNNNNNNNNNNNNNNNNNNNNNNNNNNNNNNNNNNNNNNNNNNNNNNNNNNNNNNNNNNNNNNNNGAGTAAAATGAAAATAAACTGGAAAAAAATATACCATGTTGAGTAAATGAAAATATATATAATCTATAACAAGCAGATAAAAATGAAGATAAAATCAATAAAATCATTATTAACATGTTTCAAGATATTAATCTATTAATAATTATAATCTCACCAGCTAAATTAATTGTAGGAGGACAAGATATATTTCTTGCCGACATTAAAAATCATAATAAAGATATTGAAGGCATAAATGTCAATATACCCTTATTAATTAATAATCTTCGACTATTAGTTCGTTCATACAATATGTTTGCTAAACAAAATAATCCAGAAGAACATAAACCATGACCAATCATTATATAATATGACCCACATATTCCCCAATTTCTTATAGTTATAATTCCTCTAATCACAAGACCTATATGAGCAACAGATGAATAAGCAATTATTGATTTAATGTCAATTTGAATTATACATAGAATTCTAATTAAAATACTTCCAATTATTGAAATTGAAATCCATATAAACCCAAATTTGTAAAAATAAGAAGGAATAATGTACATTATACGAATTAAACCATATCCACCTAATTTTAACAAAATTCTAGCTAAAATTATTGAACCAGAAACAGGAGCTTCAACATGAGCTTTTGGTAATCAAAAATGAAAAAATAATNNNNNNNNNNNNNNNNNNNNNNNNNNNNNNNNNNNNNNNNNNNNNNNNNNNNNNNNNNNNNNNNNNNNNNNNNNNNNNNNNNNNNNNNNNNNNNNNNNNNNNNNNNNNNNNNNNNNNNNNNNNNNNNNNNNNNNNNNNNNNNNNNNNNNNNNNNNNNNNNNNNNNNNNNNNNNNNNNNNNNNNNNNNNNNNNNNNNNNNNNNNNNNNNNNNNNNNNNNNNNNNNNNNNNNNNNNNNNNNNNNNNNNNNNNNNNNNNNNNNNNNNNNNNNNNNNNNNNNNNNNNNNNNNNNNNNNNNNNNNNNNNNNNNNNNNNNNNNNNNNNNNNNNNNNNNNNNNNNNNNNNNNNNNNNNNNNNNNNNNNNNNNNNNNNNNNNNNNNNNNNNNNNNNNNNNNNNNNNNNNNNNNNNNNNNNNNNNNNNNNNNNNNNNNNNNNNNNNNNNNNNNNNNNNNNNNNNNNNNNNNNNNNNNNNNNNNNNNNNNNNNNNNNNNNNNNNNNNNNNNNNNNNNNNNNNNNNNNNNNNNNNNNNNNNNNNNNNNNNNNNNNNNNNNNNNNNNNNNNNNNNNNNNNNNNNNNNNNNNNNNNNNNNNNNNNNNNNNNNNNNNNNNNNNNNNNNNNNNNNNNNNNNNNNNNNNNNNNNNNNNNNNNNNNNNNNNNNNNNNNNNNNNNNNNNNNNNNNNNNNNNNNNNNNNNNNNNNNNNNNNNNNNNNNNNNNNNNNNNNNNNNNNNNNNNNNNNNNNNNNNNNNNNNNNNNNNNNNNNNNNNNNNNNNNNNNNNNNNNNNNNNNNNNNNNNNNNNNNNNNNNNNNNNNNNNNNNNNNNNNNNNNNNNNNNNNNNNNNNNNNNNNNNNNNNNNNNNNNNNNNNNNNNNNNNNNNNNNNNNNNNNNNNNNNNNNNNNNNNNNNNNNNNNNNNNNNNNNNNNNNNNNNNNNNNNNNNNNNNNNNNNNNNNNNNNNNNNNNNNNNNNNNNNNNNNNNNNNNNNNNNNNNNNNNNNNNNNNNNNNNNNNNNNNNNNNNNNNNNNNNNNNNNNNNNNNNNNNNNNNNNNNNNNNNNNNNNNNNNNNNNNNNNNNNNNNNNNNNNNNNNNNNNNNNNNNNNNNNNNNNNNNNNNNNNNNNNNNNNNNNNNNNNNNNNNNNNNNNNNNNNNNNNNNNNNNNNNNNNNNNNNNNNNNNNNNNNNNNNNNNNNNNNNNNNNNNNNNNNNNNNNNNNNNNNNNNNNNNNNNNNNNNNNNNNNNNNNNNNNNNNNNNNNNNNNNNNNNNNNNNNNNNNNNNNNNNNNNNNNNNNNNNNNNNNNNNNNNNNNNNNNNNNNNNNNNNNNNNNNNNNNNNNNNNNNNNNNNNNNNNNNNNNNNNNNNNNNNNNNNNNNNNNNNNNNNNNNNNNNNNNNNAATTAATAAATTAATGTGTCTAGAACACATAGTTTCTATAATTAAATCTTTTGAATAAAATCCACTCAAAAATGGAATTCCACATAATGATATATTAGCAATATTCATTATTGTAATAGTTAGTGGTATTTGAAATCTTAAACATCCTATTACACGAATATCTTGATTATTATTAAAATTGTGAATCAAAATTCCTGCACACAAAAATAAAGTAGCTTTAAATAAAGCATGAACAATTAAGTGAAAAAATGATAACATTGGATAACCAATTATAATAATTGTTATTATAATTCCCAGTTGACTTAAAGTTGACAATGCAATAATTTTTTTTAAATCAAATTCAAAATTTGCTCTAATTCCCGATATTAATATAGTTAAAATTGAAATTAATATAAATAATTGAATATAATCAAAATTCAAAATAATATTAGAAAATCGAATTATTAAATATACTCCAGCAGTAACAAGTGTAGATGAATGAACCAAAGCAGAAACAGGAGTTGGAGCAGCCATAGCTGCTGGTAACCATGAAGAAAATGGAATTTGAGCACTCTTTGTAAATCTCGCAATAATAACTATAATTAAAANNNNNNNNNNNNNNNNNNNNNNAAGATAAAAATTTCATGACCCAAAATTTAATATTCATGCAATAGCTATTAAAATAGCAACATCTCCCACACGATTCATTAATGCAGTTAATATGCCTGCATTATTAGAATATAAATTTTGATAATAAATAATTAAGCAATAAGAAACTAAACCAAGTCCATCTCAACCAATAATAATTCTCATTATATTAGGTCTAATGATTAATAAGACTATAGATATAACAAACCCCATAACAATATAAATAAAACGATTAATGAATTTATCATTATTTATATATATTCCACTATATAAAACAACCACTGATGAAATAAATATAACTGTTGAAAGAAATTGTTGATATAAAATCAAAAATTATAGTCATATAAATATTACATGAATTAATTGNNNNNNNNNNNNNNNNNNNNNNNNNNNNNNNNNNNNNNNNNNNNNNNNNNNNNNNNNNNNNNNNNNNNNNNNNNNNNNNNNNNNNNNNNNNNNNNNNNNNNNNNNNNNNNNNNNNNNNNNNNNNNNNNNNNNNNNNNNNNNNNNNNNNNNNNNNNNNNNNNNNNNNNNNNNNNNNNNNNNNNNNNNNNNNNNNNNNNNNNNNNNNNNNNNNNNNNNNNNNNNNNNNNNNNNNNNNNNNNNNNNNNNNNNNNNNNNNNNNNNNNNNNNNNNNNNNNNNNNNNNNNNNNNNNNNNNNNNNNNNNNNNNNNNNNNNNNNNNNNNNNNNNNNNNNNNNNNNNNNNNNNNNNNNNNNNNNNNNNNNNNNNNNNNNNNNNNNNNNNNNNNNNNNNNNNNNNNNNNNNNNNNNNNNNNNNNNNNNNNNNNNNNNNNNNNNNNNNNNNNNNNNNNNNNNNNNNNNNNNNNNNNNNNNNNNNNNNNNNNNNNNNNNNNNNNNNNNNNNNNNNNNNNNNNNNNNNNNNNNNNNNNNNNNNNNNNNNNNNNNNNNNNNNNNNNNNNNNNNNNNNNNNNNNNNNNNNNNNNNNNNTTTACTCTTCGAAGAAGAACTNNNNNNNNNNNNNNNNNNNNNNNNNNNNNNNNNNNNNNNNNNNNNNNNNNNNNNNNNNNNNNNNNNNNNNNNNNNNNNNNNNNNNNNNNNNNNNNNNNNNNNNNNNNNNNNNNNNNNNNNNNNNNNNNNNNNNNNNNNNNNNNNNNNNNNNNNNNNNNNNNNNNNNNNNNNNNNNNNNNNNNNNNNNNNNTTATANNNNNNNNNNNNNNNNNNNNNNNNNNNNNNNNNNNNNNNNNNNNNNNNNNNNNNNNNNNNNNNNNNNNNNNNNNNNNNNNNNNNNNNNNNNNNNNNNNNNNNNNNNNNNNNNNNNNNNNNNNNNNNNNNNNNNNNNNNNNNNNNNNNNNNNNNNNNNNNNNNNNNNNNNNNNNNNNNNNNNNNNNNNNNNNNNNNNNNNNNNNNNNNNNNNNNNNNNNNNNNNNNNNNNNNNNNNNNNNNNNNNNNNNNNNNNNNNNNNNNNNNNNNNNNNNNNNNNNNNNNNNNNNNNNNNNNNNNNNNNNNNNNNNNNNNNNNNNNNNNNNNNNNNNNNNNNNNNNNNNNNNNNNNNNNNNNNNNNNNNNNNNNNNNNNNNNNNNNNNNNNNNNNNNNNNNNNNNNNNNNNNNNNNNNNNNNNNNNNNNNNNNNNNNNNNNNNNNNNNNNNNNNNNNNNNNNNNNNNNNNNNNNNNNNNNNNNNNNNNNNNNNNNNNNNNNNNNNNNNNNNNNNNNNNNNNNNNNNNNNNNNNNNNNNNNNNNNNNNNNNNNNNNNNNNNNNNNNNNNNNNNNNNNNNNNNNNNNNNNNNNNNNNNNNNNNNNNNNNNNNNNNNNNNNNNNNNNNNNNNNNNNNNNNNNNNNNNNNNNNNNNNNNNNNNNNNNNNNNNNNNNNNNNNNNNNNNNNNNNNNNNNNNNNNNNNNNNNNNNNNNNNNNNNNNNNNNNNNNNNNNNNNNNNNNNNNNNNNNNNNNNNNNNNNNNNNNNNNNNNNNNNNNNNNNNNNNNNNNNNNNNNNNNNNNNNNNNNNNNNNNNNNNNNNNNNNNNNNNNNNNNNNNNNNNNNNNNNNNNNNNNNNNNNNNNNNNNNNNNNNNNNNNNNNNNNNNNNNNNNNNNNNNNNNNNNNNNNNNNNNNNNNNNNNNNNNNNNNNNNNNNNNNNNNNNNNNNNNNNNNNNNNNNNNNNNNNNNNNNNNNNNNNNNNNNNNNNNNNNNNNNNNNNNNNNNNNNNNNNNNNNNNNNNNNNNNNNNNNNNNNNNNNNNNNNNNNNNNNNNNNNNNNNNNNNNNNNNNNNNNNNNNNNNNNNNNNNNNNNNNNNNNNNNNNNNNNNNNNNNNNNNNNNNNNNNNNNNNNNNNNNNNNNNNNNNNNNNNNNNNNNNNNNNNNNNNNNNNNNNNNNNNNNNNNNNNNNNNNNNNNNNNNNNNNNNNNNNNNNNNNNNNNNNNNNNNNNNNNNNNNNNNNNNNNNNNNNNNNNNNNNNNNNNNNNNNNNNNNNNNNNNNNNNNNNNNNNNNNNNNNNNNNNNNNNNNNNNNNNNNNNNNNNNNNNNNNNNNNNNNNNNNNNNNNNNNNNNNNNNNNNNNNNNNNNNNNNNNNNNNNNNNNNNNNNNNNNNNNNNNNNNNNNNNNNNNNNNNNNNNNNNNNNNNNNNNNNNNNNNNNNNNNNNNNNNNNNNNNNNNNNNNNNNNNNNNNNNNNNNNNNNNNNNNNNNNNNNNNNNNNNNNNNNNNNNNNNNNNNNNNNNNNNNNNNNNNNNNNNNNNNNNNNNNNNNNNNNNNNNNNNNNNNNNNNNNNNNNNNNNNNNNNNNNNNNNNNNNNNNNNNNNNNNNNNNNNNNNNNNNNNNNNNNNNNNNNNNNNNNNNNNNNNNNNNNNNNNNNNNNNNNNNNNNNNNNNNNNNNNNNNNNNNNNNNNNNNNNNNNNNNNNNNNNNNNNNNNNNNNNNNNNNNNNNNNNNNNNNNNNNNNNNNNNNNNNNNNNNNNNNNNNNNNNNNNNNNNNNNNNNNNNNNNNNNNNNNNNNNNNNNNNNNNNNNNNNNNNNNNNNNNNTNNNNNNNNNNNNNNNNNNNNNNNNNNNNNNNNNNNNNNNNNNNNNNNNNNNNNNNNNNNNNNNNNNNNNNNNNNNNNNNNNNNNNNNNNNNNNNNNNNNNNNNNNNNNNNNNNNNNNNNNNNNNNNNNNNNNNNNNNNNNNNNNNNNNNNNNNNNNNNNNNNNNNNNNNNATTCTATTAGTATATAAGTATATTTAACTTNNNNNNNNNNNNNNNNNNNNNNNNNNNNNNNNNNNNNNNNNNNNNNNNNNNNNNNNNNNNNNNNNNNNNNNNNNNNNNNNNNNNNNNNNNNNNNNNNNNNNNNNNNNNNNNNNNNNNNNNNNNNNNNNNNNNNNNNNNNNNNNNNNNNNNNNNNNNNNNNNNNNTCTAATATGGCAGATTAGTGCAATGAATTTAAGATTCATATATAAAATANNNNNNNNNNNNNNNNNNNNNNNNNNNNNNNNNNNNNNNNNNNNNNNNNNNNNNNNNNNNNNNNNNNNNNNNNNNNNNNNNNNNNNNNNNNNNNNNNNNNNNNNNNNNNNNNNNNNNNNNNNNNNNNNNNNNNNNNNNNNNNNNNNNNNNNNNNNNNNNNNNNNNNNNNNNNNNNNNNNNNNNNNNNNNNNNNNNNNNNNNNNNNNNNNNNNNNNNNNNNNNNNNNNNNNNNNNNNNNNNNNNNNNNNNNNNNNNNNNNNNNNNNNNNNNNNNNNNNNNNNNNNNNNNNNNNNNNNNNNNNNNNNNNNNNNNNNNNNNNNNNNNNNNNNNNNNNNNNNNNNNNAGTTAATTAGCTTAAACAAAAGCATTTATTTTGAAAGTAAAAGAAAAGATAATTAATCTATTAACTTNNNNNNNNNNNNNNNNNNNNNNNNNNNNNNNNNNNNNNNNNNNNNNNNNNNNNNNNNNNNNNNNNNNNNNNNNNNNNNNNNNNNNNNNNNNNNNNNNNNNNNNNNNNNNNNNNNNNNNNNNNNNNNNNNNNNNNNNNNNNNNNNNNNNNNNNNNNNNNNNNNNNNNNNNNNNNNNNNNNNNNNNNNNNNNNNNNNNNNNNNNNNNNNNCATAAATAAATTTACAGTTTATTGCCTATTATCGGCCATCCTAANNNNNNNNNNNNNNNNNNNNNNNNNNNNNNNNNNNNNNNNNNNNNNNNNNNNNNNNNNNNNNNNNN

>aotea-west_02.NZ.TO.RCG.14

NNNNNNNNNNNNNNNNNNNNNNNNNNNNNNNNNNNNNNNNNNNNNNNNNNNNNNNNNNNNNNNNNNNNNNNNNNNNNNNNNNNNNNNNNNNNNNNNNNNNNNNNNNNNNNNNNNNNNNNNNNNNNNNNNNNNNNNNNNNNNNNNNNNNNNNNNNNNNNNNNNNNNNNNNNNNNNNNNNNNNNNNNNNNNNNNNNNNNNNNNNNNNNNNNNNNNNNNNNNNNNNNNNNNATAAATAATTTATTGGGTTTACTTCCGTATATTTTCACTAGCCCAAGTCATCTAGTATTTACAATAAGATTAGCATTACCATTATGATTATCATTCATACTTTATGGATTTATTAATAATATAAATCATATATTTTGTCATTTAGTTCCATCCGGAACTCCTAATATTTTAATGCCTTTTATAGTTCTTATTGAAAGAGTAAGAAATTTAATTCGACCTGGCTCTTTAGCTGTCCGACTGACAGCTAACATAATTGCTGGTCATCTTTTGATAACCTTATTAGGTAACTTACCCATAAGGTATGAATTATATTCAGGTATTATTATTATTTTTCAAGTTATATTAATATTATTTGAATTAGCTGTTTGTATTATTCAATCATATGTATTTATAGTTCTTAGAACTTTATATTATAGAGAAGTAAATATTCCTCAAATATCNNNNNNNNNNNNNNNNNNNNNNNNNNNNNNNNNNNNNNNNNNNNNNNNNNNNNNNNNNNNNNNNNNNNNNNNNNNNNNNNNNNNNNNNNNNNNNNNNNNNNNNNNNNNNNNNNNNNNNNNNNNNNNNNNNTTTAAGCCAACACGACAAAATCATCCTTTAATTAAAATTATTAATAATTCATTAATTGACTTCCCAGCACCATCTAATTTATCTTATTGATGAAATTTTGGATTTGTATTAGGATTATGCTTAACAATTCAAATTTTAACTGGATTATTCTTATCAATACATTATAATGCCAATATCATAAATGCTTTTGAAAGATTAAGACATATTTGCCGTGATGTAAACTATGGTTGAATCTTACGTGTTATTCACGCCAATGGAGCTTCATTGTTTTTCATTTGTGTATATTTACATGTAGGACGTGGATTATATTATGGATCATTCAAATATATTGAAACATGATCTATTGGTGTAATTATATTATTAATATTAATAGCTACTGCTTTCTTAGGATATGTTTTACCGTGAGGGCAAATATCTTTTTGAGGAGCCACAGTCATTACAAATTTATTATCAGCCATTCCTTATTTAGGTGGAATATTAGTTAATTGAATTTGAGGAGGATTTGCAGTTGACAATGCAACACTAACTCGATTTTACTCTTTTCATTTTATTTTACCATTCATTGTATTAAGACTAACAATTATTCATCTATTATATTTACATACAACAGGTTCAAATAATCCATTAGGAATTAACTCCAATAATGACAAAGTCCCATTTCACCCATACTTCTCCATTAAGGATATTATAAGCCTATTTTTATTAATAATTATTTTCTTTATATTAGTCATATTAGAACCCTATATACTAGGGGATCCAGATAATTTTATTCCTGCCAATCCACTTGTAACACCAAAACATATTCAACCTGAATGATACTTTTTATTTGCCTACGCAATCCTCCGTTCAATTCCTAATAAACTAGGAGGAGTAATTGCTCTTTTTATATCAATTTTCATTTTAATGTTTGTACCTTTATTGAATAATTCTAATTTTATAGGATTAAATAATTACCCAATTAATCAAATTATATTTTGATATATAGTAATAATTTTAATTTTATTAACTTGAATTGGAGCACGACCTGTCGAATTACCTTATATTAACTTTGGAATATTTTTAACACTTATATATTTCTCTTACTTCATTATTGACCCAATAATTAAATCTATTTGAGATAAATTAATTAGATAGATGAAAAAATGATTCTTCTCAACTAATCATAAAGATATTGGAACAATATATTTCATTTTTGGTATTTGATCAGGTATAATTGGCACAACTTTAAGAGTTTTAATTCGTGTAGAACTTGGAACTCCAGGTTCATTTATTGGTGATGATCAAATTTATAATGTAATTGTTACTGCTCATGCTTTCATTATAATTTTTTTTATGGTAATACCCATTATAATTGGTGGATTTGGAAATTGATTAGTCCCCCTAATAATTGGAGCTCCTGATATAGCTTTTCCTCGTATAAATAATATGAGATTTTGATTATTACCACCTTCCTTAATTCTTATTTTAGTGGGAAGAATAGTTGATAGAGGTGCAGGCACAGGTTGAACAGTTTATCCTCCCTTATCTGCAGGTATTGCACATTCTGGTTCATGTGTTGATTTAACTATTTTTTCTCTACACCTTGCAGGTGTATCTTCAATTTTAGGTGCTGTAAATTTCATTAGAACAATTTTTAATATACGTTCAATGGGAATTTGATTGGATCGCATACCTTTATTTGTATGAGCAGTTTTAATTACTGCATTTTTATTATTATTATCACTACCCGTTTTAGCAGGCGCTATCACAATATTATTAACAGATCGAAATTTAAATACTTCATTTTTTGATCCTGCAGGAGGGGGGGATCCTATTCTTTATCAACATTTATTTTGATTTTTTGGTCATCCTGAAGTCTATATTTTAATTTTACCAGGATTTGGTTTAATTTCTCATATTATTACTCAAGAGAGAGGTAAAATTGAATCTTTTGGCTCATTAGGAATAATTTATGCAATAATATCAATTGGTATTTTAGGATTTGTTGTATGAGCACACCATATGTTTACTGTAGGAATAGATGTTGATACACGTGCATATTTTACATCAGCTACTATAATTATTGCAGTTCCTACTGGAATTAAAGTATTTAGTTGACTTGCAACTTTGAGAGGTATAAAAATTAATATTACATCTTCAGCCCTATGAGCGTTAGGATTTGTTTTTTTATTTACTATTGGGGGTTTAACTGGAGTTATTCTAGCCAATTCTTCAATTGATATTATATTACATGATACATATTATGTAGTTGCACATTTCCATTATGTTTTATCAATGGGAGCAGTATTTGCTATTATAGCAAGATTTATTCATTGGTTTCCATTATTTACAGGATTAAGATTAAACTCAAATTGATTAAAAATTCATTTTCTATTAATATTTATTGGTGTAAATATAACATTCTTTCCTCAACATTTTTTAGGATTAAGAGGGATACCTCGTCGCTATTCAGACTATCCTGATGCTTACATATCATGAAACATAATTTCATCAATAGGAAGAATTATATCTTTAATTGGAATTTTATTTTTATTATTTATTGTTTGGGAAAGCTTTATTTCAATACGATTAGTATTATATTCTAATAGAATTCAATCTTCTATTGAATGAATACAAAAATTCCCCCCATCTGAACATTCATATAATGAAATGCCATTGTTAATTCAANNNNNNNNNNNNNNNNNNNNNNNNNNNNNNNNNNNNNNNNNNNNNNNNNNNNNNNNNNNNNNNNNNNNNNNNNNNNNNNNNNNNNNNNNNNNNNNNNNNNNNNNNNNNNNNNNNNNNNNNNNNNNNNNNNNNNNNNNNNNNNNNNNNNNNNNNNNNNNNNNNNNNNNNNNNNNNNNNNNNNNNNNNNNNNNNNNNNNNNNNNNNNNNNNNNNNNNNNNNNNNNNNNNNNNNNNNNNNNNNNNNNNNNNNNTATATATATTAGACGAAATTAATAATCCGTTACTAACATTAAAAATTATTGGTCACCAATGATATTGATCATATGAATATTCAGATTTTTCTGATATAGAATTTGATTCATATATAAAATCTATAAATGAGATAAATAAAAACGAATTTCGTTTATTAGATGTAGATAATCGAGTAATTCTACCATTTAATATCCAAGTCCGACTATTAGTTTCTTCTTTTGATGTAATTCATTCTTGAGCAATACCATCAATAAGACTTAAAGTTGATGCAGTGCCAGGACGATTAAATCAAATAAGAATATTAATTAGTCGTCCAGGTGTATCTTATGGACAATGCTCTGAAATTTGTGGAGCAAATCATAGATTTATGCCTATCGTAATTGAAAGAATTAGAATAAAAATATTTACTAGATGATTAATTAATTATATGAATAATCACCCTTATCATATAGTTGACTATAGACCCTGACCTTTAACTGGATCAATTGGAGCATTAACTTTTGTTTCCGGTATAGTTATAATATTCCATAAATGTAATTTTATATTATTATATATTGGTATTTTATTATTATTAATAACAATAATTCAATGATGACGTGACATTTCCCGGGAAGGGACATTTTTAGGAATACACACAATAATAGTAGTGAATGGTTTGAAAATAGGCATATTATTATTTATTGTATCAGAAATTCTTTTTTTTGTATCATTTTTTTGAGCATTTTTTCATAGTAGATTAAGACCTGTAGTAGAAATTGGTATAATTTGACCTCCTAGAGGTATTTATGTTTTTAATCCGACTCAAGTTCCATTATTAAATACAATAATTTTATTATGTTCAGGAATTACAATTACTTGAGCACATCATTCAATCATAAATGGTAATCACATTAATTCTATCTATAGAATTATATTAACTGTTATTTTAGGTATATATTTCACTGTTCTACAAGGTTATGAATATTATGAAGCTCCATTTGCAATTAATGATTCTATCTATGGATCTTCTTTTTTTATAGCTACTGGATTCCATGGAATTCACGTAGTTATTGGAACAAGATTTATTACTGTATGCTTATTACGAATAATAAAGTTTCATTTCTCAATAAATCATCATTTTGGCTTCGAAGCTGCTGCTTGATATTGACATTTTGTTGATGTAGTCTGATTATTTTTATATTTATCAATTTATTGATGAGGAAGATAATAAATTATAGTTTTTAAAAAGAAAAAAAAAACAAATAAATTCAGAGATACCGGTAAATAAGATTTTCAAGCCAAATATATCAATTTATCATAACGATAACGAGGAAGGGTGCCCCGAATTCAAATGAAAAGAAAACAAATTATAGAAACTTGAATTGGAAAAACAATTGAATTAATTTTTCCCCCTAAAAACATTAAACAAAATAATATTCTCATAAATAAAATACTTGAATACTCAGCCAAAAAAATAAATGCAAACCCAGAACCTCTATATTCAACATTAAAGCCAGAAACTAACTCTGATTCTCCCTCAGAAAAATCAAATGGAGATCGATTAGTCTCAGCTAAAGCTGAAGATAATCAACATATTCTTAAAGGAAGAAACAAAAAAATAAATCACACAAATTCCTGAAATATAAACAAATCAATAATATTATACCTTCTTATTATTATTATAGGACACAATATAATTAAAACCAATCTTACCTCATAAGAAATTGATTGAGCAATTGATCGAATGCATCCTAGCATTGAATAACTTGAATTAGAAGATCAACCTGTCAATATTAATGAATAAACTCTTATTCTTGAACAACAAAAAAAAAATAAAATACCAAAATTAAAAGAAACACAATTAATAAAATAAGGATACAATGATCAAATCAACAGACTATTGAATAATCCTATAATAGGTCTAAAGATATAAATTAAATAATTAGATATTAATGGAATTGTATTTTCCTTTATAAATAATTTAATAGCATCAGAAATAGGCTGCAAAATACCCAAAAATCCAACTTTATTTGGGCCCTTACGGATTTGAATATATCTTAAAACTTTACGTTCCAAAAGAGTAATAAATGCCACTCCAATCAAAATAAATAATACAGTAACTAAAATCGTAATTAAATACNNNNNNNNNNNNNNNNNNNNNNNNNNNNNNNNNNNNNNNNNNNNNNNNNNNNNNNNNNNNNNNNNNNNNNNNNNNNNNNNNNNNNNNNNNNNNNNNNNNNNNNNNNNNNNNNNNNNNNNNNNNNNNNNNNNNNNNNNNNNNNNNNNNNNNNNNNNNNNNNNNNNNNNNNNNNNNNNNNNNNNNNNNNNNNNNNNNNNNNNNNNNNNNNNNNNNNNNNNNNNNNNNNNNNNNNNNNNNNNNNNNNNNNNNNNNNNNNNNNNNNNNNNNNNNNNNNNNNNNNNNNNNNNNNNNNNNNNNNNNNNNNNNNNNNNNNNNNNNNNNNNNNNNNNNNNNNNNNNNNNNNNNNNNNNNNNNNNNNNNNNNNNNNNNNNNNNNNNNNNNNNNNNNNNNNNNNNNNNNNNNNNNNNNNNNNNNNNNNNNNNNNNNNNNNNNNNNNNNNNNNNNNNNNNNNNNNNNNNNNNNNNNNNNNNNNNNNNNNNNNNNNNNNNNNNNNNNNNNNNNNNNNNNNNNNNNNNNNNNNNNNNNNNNNNNNNNNNNNNNNNNNNNNNNNNNNNNNNNNNNNNNNNNNNNNNNNNNNNNNNNNNNNNNNNNNNNNNNNNNNNNNNNNNNNNNNNNNNNNNNNNNNNNNNNNNNNNNNNNNNNNNNNNNNNNNNNNNNNNNNNNNNNNNNNNNNNNNNNNNNNNNNNNNNNNNNNNNNNNNNNNNNNNNNNNNNNNNNNNNNNNNNNNNNNNNNNNNNNNNNNNNNNNNNNNNNNNNNNNNNNNNNNNNNNNNNNNNNNNNNNNNNNNNNNNNNNNNNNNNNNNNNNNNNNNNNNNNNNNNNNNNNNNNNNNNNNNNNNNNNNNNNNNNNNNNNNNNNNNNNNNNNNNNNNNNNNNNNNNNNNNNNNNNNNNNNNNNNNNNNNNNNNNNNNNNNNNNNNNNNNNNNNNNNNNNNNNNNNNNNNNNNNNNNNNNNNNNNNNNNNNNNNNNNNNNNNNATAATTAAAATTNNNNNNNNNNNNNNNNNNNNNNNNNNNNNNNNNNNNNNNNNNNNNNNNNNNNNNNNNNNNNNNNNNNNNNNNNNNNNNNNNNNNNNNNNNNNNNNNNNNNNNNNNNNNNNNNNNNNNNNNNNNNNNNNNNNNNNNNNNNNNNNNNNNNNNNNNNNNNNNNNNNNNNNNNNNNNNNNNNNNNNNNNNNNNNNNNNNNNNNNNNNNNNNNNNNNNNNNNNNNNNNNNNNNNNNNNNNNNNNNNNNNNNNNNNNNNNNNNNNNNNNNNNNNNNNNNNNNNNNNNNNNNNNNNNNNNNNNNNNNNNNNNNNNNNNNNNNNNNNNNNNNNNNNNNNNNNNNNNNNNNNNNNNNNNNNNNNNNNNNNNNNNNNNNNNNNNNNNNNNNNNNNNNNNNNNNNNNNNNNNNNNNNNNNNNNNNNNNNNNNTTCCAGAATTAAATGAAAATAAACTGGAAAAAAATATACCATGTTGAGTAAATGAAAATATATATAATCTATAACAAGCAGACAAAAATGAAGATAAAATCAATAAAACCATTATTAATATATTTCAAGATATTAATCTATTAATAATTATAATCTCACCAGCTAAATTAATTGTAGGAGGACAAGATATATTTCTTGCCGATATTAAAAATCATAATAAAGATATTGAAGGTATAAATGTCAATATACCCTTATTAATTAATAATCTTCGACTATTAGTTCGCTCATACAATATGTTTGCTAAACAAAATAATCCAGAAGAACATAAACCATGACCAATCATTATATAATATGACCCACATATTCCCCAATTTCTTATAGTTATAATTCCTCTAATCACAAGACCTATGTGAGCAACAGATGAATAAGCAATTATTGATTTAATGTCAATTTGAATTATACATAAAATTCTAATTAAAATACTTCCAATTATTGAAATTGAGATCCATAAAAACCCAAATTTGTAAAAATAAGAAGGAATAATATACATTATACGAATTAAACCATATCCACCTAATTTTAACAAAATTCTAGCTAAAATTATTGAACCAGAAACAGGAGCTTCAACATGAGCTTTTNNNNNNNNNNNNNNNNNNNNNNNNNNNNNNNNNNNNNNNNNNNNNNNNNNNNNNNNNNNNNNNNNNNNNNNNNNNNNNNNNNNNNNNNNNNNNNNNNNNNNNNNNNNNNNNNNNNNNNNNNNNNNNNNNNNNNNNNNNNNNNNNNNNNNNNNNNNNNNNNNNNNNNNNNNNNNNNNNNNNNNNNNNNNNNNNNNNNNNNNNNNNNNNNNNNNNNNNNNNNNNNNNNNNNNNNNNNNNNNNNNNNNNNNNNNNNNNNNNNNNNNNNNNNNNNNNNNNNNNNNNNNNNNNNNNNNNNNNNNNNNNNNNNNNNNNNNNNNNNNNNNNNNNNNNNNNNNNNNNNNNNNNNNNNNNNNNNNNNNNNNNNNNNNNNNNNNNNNNNNNNNNNNNNNNNNNNNNNNNNNNNNNNNNNNNNNNNNNNNNNNNNNNNNNNNNNNNNNNNNNNNNNNNNNNNNNNNNNNNNNNNNNNNNNNNNNNNNNNNNNNNNNNNNNNNNNNNNNNNNNNNNNNNNNNNNNNNNNNNNNNNNNNNNNNNNNNNNNNNNNNNNNNNNNNNNNNNNNNNNNNNNNNNNNNNNNNNNNNNNNNNNNNNNNNNNNNNNNNNNNNNNNNNNNNNNNNNNNNNNNNNNNNNNNNNNNNNNNNNNNNNNNNNNNNNNNNNNNNNNNNNNNNNNNNNNNNNNNNNNNNNNNNNNNNNNNNNNNNNNNNNNNNNNNNNNNNNNNNNNNNNNNNNNNNNNNNNNNNNNNNNNNNNNNNNNNNNNNNNNNNNNNNNNNNNNNNNNNNNNNNNNNNNNNNNNNNNNNNNNNNNNNNNNNNNNNNNNNNNNNNNNNNNNNNNNNNNNNNNNNNNNNNNNNNNNNNNNNNNNNNNNNNNNNNNNNNNNNNNNNNNNNNNNNNNNNNNNNNNNNNNTTATTCATCAATTTATAATTTAATGACATTATTATTTTTAAAGTTTTCAAAATCCCTTGGGGCCCAAGGTTTTCACCTCATCCCATATCAATTGATTTTTGAAATATGAATGATTTACTTATCAATAATATTTGATTATGAAACGTTGATAATTGCTTTATAAACCACATTGAACCAAAAAACTCATAAACAATTTTTATTTTAGTTTTAGAAAAAATTGATATTTCGTAACCCAACCAGATTCCTAATATTGAAAAAATCAATGCCAATAATTTACCCTCTAAAGGCATTAAAATTATAATTGGATCATTAAATATTAATCATCTTAATATAGATCCCGAAAAAATTGAATAAATAGANNNNNNNNNNNNNNNNNNNNNNNNNNNNNNNNNNNNNNNNNNNNNNNNNNNNNNNNNNNNNNNNNNNNNNNNNNNNNNNNNNNNNNNNNNNNNNNNNNNNNNNNNNNNNNNNNNNNNNNNNNNNNNNNNNNNNNNNNNNNNNNNNNNNNNNNNNNNNNNNNNNNNNNNNNNNNNNNNNNNNNNNNNNNNNNNNNNNNNNGAATAAAATCCACTCAAAAATGGAATTCCACATAATGATATATTAGCAATATTCATTATTGTAATAGTTAATGGTATTTGAAATCTTAAACATCCTATTACACGAATATCTTGATTATTATTAAAATTGTGAATCAAAATTCCTGCACACAAAAATAAAGTAGCTTTAAATAAAGCATGAACAATTAAGTGAAAAAATGATAACATTGGATAACCAAATATAATAATTGTTATTATAATTCCCAGCTGTCTTAAAGTTGATAGTGCAATAATTTTTTTTAAATCAAATTCAAAATTTGCTCTAATTCCCGATATTAATATAGTTAAAATTGAAATTAATATAAATAATTGAATATAATCGAAATTTAAAATAATATTAGAAAATCGAATTATTAAATATACTCCAGCAGTAACAAGTGTAGATGAGTGAACCAGAGCTGATACAGTAGTTGGAGCAGCTATAGCTGCTGGTAATCATGAAGCAAATGGAATTTGAGCACTCTTTGTAAATCTCGCAATAATAATTATANNNNNNNNNNNNNNNNNNNNNNNNNNNNNNNNNNNNNNNNNNNNNNNNNNNNNNNNNNNNNNNNNNNNNNNNNNNNNNNNNNNNNNNNNNNNNNNNNNNNNNNNNNNNNNNNNNNNTAATANNNNNNNNNNNNNNNNNNNNATATTTTGATAATAAATAACTAAGCAATAAGAAACTAAACCAAGTCCATCTCAACCAATAATAATTCTCATTATATTAGGTCTAATGATTAATAAAACTATAGATATAACAAACCCCATAACAATATAAATAAAACGATTAATGAATTTGTCATTATTTATATATATTCCACTATATAAAACAACCACTGATGAAATAAATATAACTGTTGAAAGAAATTGTTGATATAAAATCAAAAATTATAGTCATATAAATATTACATGAATTAATTGAAAATTATTCACTCCAATATNNNNNNNNNNNNNNNNNNNNNNNNNNNNNNNNNNNNNNNNNNNNNNNNNNNNNNNNNNNNNNNNNNNNNNNNNNNNNNNNNNNNNNNNNNNNNNNNNNNNNNNNNNNNNNNNNNNNNNNNNNNNNNNNNNNNNNNNNNNNNNNNNNNNNNNNNNNNNNNNNNNNNNNNNNNNNNNNNNNNNNNNNNNNNNNNNNNNNNNNNNNNNNNNNNNNNNNNNNNNNNNNNNNNNNNNNNNNNNNNNNNNNNNNNNNNNNNNNNNNNNNNNNNNNNNNNNNNNNNNNNNNNNNNNNNNNNNNNNNNNNNNNNNNNNNNNNNNNNNNNNNNNNNNNNNNNNNNNNNNNNNNNNNNNNNNNNNNNNNNNNNNNNNNNNNNNNNNNNNNNNNNNNNNNNNNNNNNNNNNNNNNNNNNNNNNNNNNNNNNNNNNNNNNNNNNNNNNNNNNNNNNNNNNNNNNNNNNNNNNNNNNNNNNNNNNNNNNNNNNNNNNNNNNNNNNNNNNNNNNNNNNNNNNNNNNNNNNNNNNNNNNNNNNNNNNNNNNNNNNNNNNNNNNNNNNNNNNNNNNNNNNNNNNNNNNNGAAGAAGAACTNNNNNNNNNNNNNNNNNNNNNNNNNNNNNNNNNNNNNNNNNNNNNNNNNNNNNNNNNNNNNNNNNNNNNNNNNNNNNNNNNNNNNNNNNNNNNNNNNNNNNNNNNNNNNNNNNNNNNNNNNNNNNNNNNNNNNNNNNNNNNNNNNNNNNNNNNNNNNNNNNNNNNNNNNNNNNNNNNNNNNNNNNNNNNNNNNNNNNNNNNNNNNNNNNNNNNNNNNNNNNNNNNNNNNNNNNNNNNNNNNNNNNNNNNNNNNNNNNNNNNNNNNNNNNNNNNNNNNNNNNNNNNNNNNNNNNNNNNNNNNNNNNNNNNNNNNNNNNNNNNNNNNNNNNNNNNNNNNNNNNNNNNNNNNNNNNNNNNNNNNNNNNNNNNNNNNNNNNNNNNNNNNNNNNNNNNNNNNNNNNNNNNNNNNNNNNNNNNNNNNNNNNNNNNNNNNNNNNNNNNNNNNNNNNNNNNNNNNNNNNNNNNNNNNNNNNNNNNNNNNNNNNNNNNNNNNNNNNNNNNNNNNNNNNNNNNNNNNNNNNNNNNNNNNNNNNNNNNNNNNNNNNNNNNNNNNNNNNNNNNNNNNNNNNNNNNNNNNNNNNNNNNNNNNNNNNNNNNNNNNNNNNNNNNNNNNNNNNNNNNNNNNNNNNNNNNNNNNNNNNNNNNNNNNNNNNNNNNNNNNNNNNNNNNNNNNNNNNNNNNNNNNNNNNNNNNNNNNNNNNNNNNNNNNNNNNNNNNNNNNNNNNNNNNNNNNNNNNNNNNNNNNNNNNNNNNNNNNNNNNNNNATAATAAAATTATTTAATGAGGTCCTTTCGTACTAACATTAAAAATAATTAAGTAGATAGAAACCAACCTGGCTCACGCCGGTTTGAACTCAGATCATGTAAGAATATTAAGGGTCGAACAGACCCAGAAATAATAAATTTTGCTCCAATCCCTATTCTTAATCCAACATAGAGGTCGCAATCATATTTATCGATATGAACTCTTCAAATTAATTACGCTGTTATCCGTAAGGTAATTTATTCTTATAATCAAAAATTTGGATCAATATTAACATAAATTTATGAAATTTTTATTNNNNNNNNNNNNNNNNNNNNNNNNNNNNNNNNNNNNNNNNNNNNNNNNNNNNNNNNNNNNNNNNNNNNNNNNNNNNNNNNNNNNNNNNNNNNNNNNNNNNNNNNNNNNNNNNNNNNNNNNNNNNNNNNNNNNNNNNNNNNNNNNNNNNNNNNNNNNNNNNNNNNNNNNNNNNNNNNNNNNNNNNNNNNNNNNNNNNNNNNNNNNNNNNNNNNNNNNNNNNNNNNNNNNNNNNNNNNNNNNNNNNNNNNNNNNNNNNNNNNNNNNNNNNNNNNNNNNNNNNNNNNNNNNNNNNNNNNNNNNNNNNNNNNNNNNNNNNNNNNNNNNNNNNNNNNNNNNNNNNNNNNNNNNNNNNNNNNNNNNNNNNNNNNNNNNNNNNNNNNNNNNNNNNNNNNNNNNNNNNNNNNNNNNNNNNNNNNNNNNNNNNNNNNNNNNNNNNNNNNNNNNNNNNNNNNNNNNNNNNNNNNNNNNNNNNNNNNNNNNNNNNNNNNNNNNNNNNNNNNNNNNNNNNNNNNNNNNNNNNNNNNNNNNNNNNNNNNNNNNNNNNNNNNNNNNNNNNNNNNNNNNNNNNNNNNNNNNNNNNNNNNNNNNNNNNNNNNNNNNNNNNNNNNNNNNNNNNNNNNNNNNNNNNNNNNNNNNNNNNNNNNNNNNNNNNNNNNNNNNNNNNNNNNNNNNNNNNNNNNNNNNNNNNNNNNNNNNNNNNNNNNNNNNNNNNNNNNNNNNNNNNNNNNNNNNNNNNNNNNNNNNNNNNNNNNNNNNNNNNNNNNNNNNNNNNNNNNNNNNNNNNNNNNNNNNNNNNNNNNNNNNNNNNNNNNNNNNNNNNNNNNNNNNNNNNNNNNNNNNNNNNNNNNNNNNNNNNNNNNNNNNNNGAAAGATTAGTTTAATTAAAATATAAATCTGTCAGGTTTATGAAACTTTTGAGTATTTTTCTNNNNNNNNNNNNNNNNNNNNNNNNNNNNNNNNNNNNNNNNNNNNNNNNNNNNNNNNNNNNNNNNNNNNNNNNNNNNNNNNNNNNNNNNNNNNNNNNNNNNNNNNNNNNNNNNNNNNNNNNNNNNNNNNNNNNNNNNNNNNNNNNNNNNNNNNNNNNNNNNNNNNNNNNNNNNNNNNNNNNNNNNNATTCTATTAGTATATAAGTATATTTAACTTCCAATTAAAAGGANNNNNNNNNNTAGTTAGATTANNNNNNNNNNNNNNNNNNNNNNNNNNNNNNNNNNNNNNNNNNNNNNNNNNNNNNNNNNNNNNNNTACTACTTGTAATAAATATTACATTATTAAATTCTAAATTTAAGGCATTAATTTTGCTAAAATAGTTCTAATATGGCAGNNNNNNNNNNNNNNNNNNNNNNNNNNNNNNNNNNNNNNNNNNNNNNNNNNNNNNNNNNNNNNNNNNNNNNNNNNNNNNNNNNNNNNNNNNNNNNNNNNNNNNNNNNNNNNNNNNNNNNCATTAAGTGGCTGAAAGTAAGTAATGGTCTCTTAAACCAATTAATAGTAATTAACATATACTCTTAATGNNNNNNNNNNNNNNNNNNNNNNNNNNNNNNNNNNNNNNNNNNNNNNNNNNNNNNNNNNNNNNNNNNNNNNNNNNNNNNNNNNNNNNNNNNNNNNNNNNNNNNNNNNNNNNNNNNNNNNNNNNNNNNNNNNNNNNNNNNNNNNNNNNNNNNNNNNNNNNNNNNNNNNNNNNNNNNNNNNNNNNNNNNNNNNNNAGTTAATTAGCTTAAACAAAAGCATTTATTTTGAAAGTAAAAGAAAAGATATTTAATCTATTAACTTNNNNNNNNNNNNNNNNNNNNNNNNNNNNNNNNNNNNNNNNNNNNNNNNNNNNNNNNNNNNNNNNNNNNNNNNNNNNNNNNNNNNNNNNNNNNNNNNNNNNNNNNNNNNNNNNNNNNNNNNNNNNNNNNNNNNNNNNNNNNNNNNNNNNNNNNNNNNNNNNNNNNNNNNNNNNNNNNNNNNNNNNNNNNTATTAGGAGAAATCATTTTTCATAAATAAATTTACAGTTTATTGCCTATTATCGGCCATCCTAANNNNNNNNNNNNNNNNNNNNNNNNNNNNNNNNNNNNNNNNNNNNNNNNNNNNNNNNNNNNNNNNNN

>muta-tuta-hybrid_12.NZ.NC.WAI.14

NNNNNNNNNNNNNNNNNNNNNNNNNNNNNNNNNNNNNNNNNNNNNNNNNNNNNNNNNNNNNNNNNNNNNNNNNNNNNNNNNNNNNNNNNNNNNNNNNNNNNNNNNNNNNNNNNNNNNNNNNNNNNNNNNNNNNNNNNNNNNNNNNNNNNNNNNNNNNNNNNNNNNNNNNNNNNNNNNNNNNNNNNNNNNNNNNNNNNNNNNNATTTATTTTTAATTTTANTTATTCTACTAAATAATTTATTGGGTTTACTTCCGTATATTTTCACTAGCCCAAGTCATCTAGTGTTCACAATAAGATTAGCATTACCACTATGATTATCATTCATACTTTATGGATTTATTAACAATATAAATCATATATTTTGTCATTTAGTTCCATCCGGAACTCCTAATATTTTAATACCTTTTATAGTTCTTATTGAAAGAGTCAGAAATCTAATTCGTCCTGGCTCCTTAGCTGTCCGACTAACAGCTAATATAATTGCTGGACATCTTTTGATAACCTTATTAGGTAACCTACCTATAAGGTATGAATTATACTCAGGTATTATTATTATTTTTCAAGTTATATTAATATTATTTGAATTAGCTGTTTGTGTTATTCAATCATATGTATTTATAGTTCTTAGAACTTTATATTATAGAGAAGTAAATATTCCTCAAATATCTCCTCTTAATTGATTATTATTTNNNNNNNNNNNNNNNNNNNNNNNNNNNNNNNNNNNNNNNNNNNNNNNNNNNNNNNNNNNNNNNNNNNNNNNNNNNNNNNNNNNNNNNNNNNNNNNNNNNNNNNNNNNNTTTAAGCCAACACGACAAAATCATCCTTTAATTAAAATTATTAATAATTCATTAATTGACTTCCCAGCACCATCTAATTTATCTTATTGATGAAATTTTGGATTTGTATTAGGATTATGCTTAACAATTCAAATTTTAACTGGATTATTTTTATCAATACATTATAATGCCAATATCATAAATGCTTTTGAAAGATTAAGACATATTTGCCGTGATGTAAACTATGGTTGAATTTTACGTGTTATTCACGCTAATGGGGCTTCATTATTTTTCATTTGTGTATATTTACATGTAGGACGTGGATTATATTATGGATCATTCAAATATATTGAAACATGATCTATTGGTGTAATTATATTATTAATATTAATAGCTACTGCTTTCTTAGGATATGTTTTACCGTGAGGTCAGATATCTTTTTGAGGAGCCACAGTCATTACAAATTTATTATCAGCAATTCCTTATTTAGGTGGAATATTAGTTAATTGAATTTGAGGAGGATTTGCAGTTGACAATGCAACACTAACTCGATTTTACTCTTTTCATTTTATTTTACCATTTATTGTATTAAGATTAACAATTATTCACCTATTATATTTACATACAACAGGTTCAAATAATCCATTAGGAATTAACTCCAACAATGACAAAGTCCCATTTCACCCATACTTCTCCGTTAAGGATATTATAAGCCTATTTTTATTAATAATTATTTTCTTTATATTAGTTATGTTAGAACCCTATATACTAGGGGATCCAGATAATTTTATTCCTGCCAATCCACTTGTGACACCAAAACATATTCAACCTGAATGATACTTTTTATTTGCCTACGCAATCCTCCGTTCAATTCCTAATAAACTAGGAGGAGTAATTGCTCTTTTTATATCAATTTTCATTTTAATGTTTGTACCTTTATTAAATAATTCTAATTTTATAGGATTAAATAATTACCCAATTAATCAAATTATATTTTGATATATAGTAATAATTTTAATTTTACTAACTTGAATTGGAGCACGACCTGTCGAATTACCTTATATCAACTTTGGAATATTTTTAACAATTATATATTTTTCTTACTTCATTATTGATCCAATAATTAAATCTACTTGAGATAAATTAATTAGATAGATGAAAAAATGATTCTTCTCAACTAATCATAAAGATATTGGAACAATATATTTCATTTTTGGTATTTGATCAGGTATAATTGGTACAACTTTAAGAGTTTTAATTCGTGTAGAACTTGGAACTCCTGGTTCATTTATTGGTGATGATCAGATTTATAATGTAATTGTCACTGCTCATGCTTTCATTATAATTTTTTTTATGGTAATACCAATTATAATTGGTGGATTTGGAAATTGATTAGTCCCTTTAATAATTGGAGCTCCTGATATAGCTTTTCCTCGTATAAATAATATGAGTTTTTGACTATTACCACCTTCCCTAATTCTTATTTTAGTTGGAAGAATAGTTGATAGAGGTGCAGGCACAGGTTGAACAGTTTACCCTCCTTTATCCGCAGGTATTGCACATTCTGGTTCATGTGTTGATTTAACTATTTTTTCTCTACACCTTGCAGGTGTATCTTCAATTTTAGGTGCTGTGAATTTCATTAGAACAATTTTTAATATACGTTCAATAGGAATTTGATTGGATCGCATACCTTTATTTGTATGGGCAGTTTTAATTACTGCATTTTTATTATTATTATCGTTACCCGTTTTAGCAGGCGCTATTACAATATTGTTAACAGATCGAAATTTAAATACTTCATTTTTTGATCCTGCAGGAGGAGGAGATCCTATTCTTTATCAGCATTTATTTTGATTTTTTGGTCATCCTGAAGTCTATATTTTAATTTTACCAGGATTTGGTTTAATTTCTCACATTATTACTCAAGAGAGAGGTAAAATTGAATCTTTTGGCTCATTAGGAATAATTTATGCCATAATATCAATTGGTATTTTAGGATTTGTTGTATGAGCACATCATATATTTACTGTAGGAATAGATGTTGATACACGTGCATATTTTACATCAGCTACCATAATTATTGCAGTTCCCACTGGAATTAAAGTATTTAGTTGACTTGCAACTTTGAGAGGTATAAAAATTAATATTTCATCTTCAGCCCTATGAGCGTTAGGGTTTGTCTTTTTATTTACTATTGGAGGTTTAACTGGAGTTATTCTAGCCAATTCTTCAATTGATATTATATTACATGATACATATTATGTAGTTGCACATTTCCATTATGTTTTATCAATAGGAGCAGTATTTGCTATTATAGCAAGATTTATTCATTGATTTCCATTATTTACAGGATTAAGATTAAACTCAAATTGATTAAAAATTCATTTTCTATTAATATTTATTGGTGTAAATATAACATTCTTTCCTCAACATTTTTTAGGATTAAGAGGAATACCTCGTCGTTATTCAGACTATCCTGATGCTTATATGTCATGAAATATAGTTTCATCAATAGGAAGAATTATATCTTTAATTGGAATTTTATTTTTATTATTTATTGTTTGGGAAAGCTTTATTTCAATACGATTAGTATTATATTCTAATAGAATTCAATCTTCTATTGAATGAATACAAAAGTTTCCCCCGTCTGAACATTCATATAATGAAATGCCATTGTTAATTCAAATTTCAAATTGATCTTATATTAATATACAGGATGCTGTATCACCATTAATAGAACAATTAATATTTTTTCATGATCATGTATTAGTAATTTTAATTATAATTACAATTGTTGTTGCTTATATAATAGTTATATTAATATTAAATAAAATCATTAATCGCTTACTTCTTGAAGGACAATTAATTGAATTTATTTGAACTCTATTACCTGCAATAACTTTAATTTTTATTGCGTTACCATCATTACGATTATTATATATATTAGACGAAATTAATAATCCATTATTGACATTAAAAATTATTGGTCACCAATGATATTGATCATATGAATATTCAGATTTTTCTGATGTAGAATTTGATTCGTATATAAAATCTATAAACGAAATAAATAAAAACGAATTTCGTTTACTAGATGTAGATAATCGAGTAATTCTACCATTTAATATTCAAGTCCGACTATTAGTTTCTTCTTTTGATGTTATTCATTCTTGAGCAATACCATCAATAAGACTTAAAGTTGATGCAGTACCAGGACGATTAAATCAAATAAGAATATTAATTAGTCGTCCCGGCGTATCTTATGGACAATGTTCTGAAATTTGTGGAGCAAATCATAGATTCATGCCTATCGTAATTGAAAGAATTAGAATAAAAATATTTATTAAATGATTAATTAATTATATGAATAATCACCCTTATCATATAGTTGACTATAGACCCTGACCTTTAACTGGATCAATTGGAGCATTAACCTTTGTTTCCGGTATAGTTATAATATTCCATAAATGTAATTTTATATTATTATATATTGGTATTTTATTATTATTAATAACAATAATTCAATGATGACGTGACATTTCCCGAGAAAGAACGTTTTTAGGAATACACACAATAATAGTAGTGAGTGGTTTGAAAATAGGCATATTATTATTTATTGTATCAGAAATTCTTTTTTTTGTATCATTTTTTTGAGGATTTTTTCATAGTAGATTAAGACCTGTAGTAGAAATTGGTATAATTTGACCTCCTAGAGGTATTTATGTTTTTAATCCAACTCAAGTTCCATTATTAAATACAATAATTTTATTATGTTCAGGAATTACAATTACTTGAGCACATCATTCAATTATAAATGGTAATCACATTAATTCTGTTTATAGAATTATGTTAACTGTTATTTTAGGTATATATTTCACTATTCTGCAAGGCTATGAATATTATGAAGCCCCATTTGCAATTAATGATTCTATCTATGGATCTTCTTTTTTTATAGCTACTGGATTTCATGGAATTCACGTAATTATTGGAACAAGATTTATTACTGTATGCTTATTACGACAAATAAAGTTTCATTTCTCAATAAATCATCATTTTGGCTTCGAAGCTGCTGCTTGATATTGACATTTTGTTGATGTAGTCTGATTATTTTTATATTTATCAATTTATTGATGAGGAAGATAATAAATTATAGTTTTTAAAAAGAAAAAAAAAATAAATAAATTCAGAGATACCGGTAAATAAGATTTTCAAGCCAAATATATTAATTTGTCATAACGATAACGAGGAAGGGTGCCCCGAATCCAAATAAAAAGAAAACAAATTATAGAAACTTGAATTGGAAAAATAATTGAATTAATTTTACCTCCTAAAAACATTAAACAAAATAATATTCTCATAAATAAAATACTTGAATACTCAGCCAAAAAAATAAATGCAAACCCAGAACCTCTATATTCAACATTAAAGCCAGAAACTAACTCTGATTCTCCTTCAGAAAAATCAAATGGAGATCGATTAGTCTCAGCTAAAGCCGAAGATAATCAACATATTCTCAAAGGAAGAAACAAAAAAATAAATCACACAAATTCCTGAAATATAAACAAATCAATAATATTATAACTTCTTATTATTATTATAGGACATAATATAATTAAAACCAATCTTACCTCATAAGAAATAGATTGAGCAATTGATCGAATGCATCCTAATATTGCATAACTTGAATTAGAAGATCAACCTGTCAATATTAATGAATAAACTCTTATTCTTGAACAACAAAAAAAAAATAAAATACCAAAATTAAAAGAAACACAATTAATGAAATATGGATACAATGATCAAATCAACAAACTATTGAATAATCCTATAATAGGTCTAAAAATATAAATTAAATAATTAGATATTAATGGAATTGTATTTTCCTTTATAAATAATTTAATAGCATCAGAAATAGGCTGCAAAATACCTAAAAATCCAACTTTATTTGGGCCTTTACGAATTTGAATATATCTTAAAACCTTACGCTCCAAAAGAGTAATAAATGCCACTCCAATTAAAATAAATAATACAGTAATTAAAATTGTAATTAAATACAAAAACAATTCTCCTTATATACTTTATATAATTTTCTTAATTTTAGGAATTATAATTTCAATTTCTTCAAACAATTGGCTGGGTTGTTGAATGGGAATTGAAATAAATATAGTTTCATTTTTGCCTATAATGGCAAACAAAATAAGAATTTATGCTTCGGAATCAATAATTAAATATTTTATCATTCAAAGAATAGGATCGAGTTTATTATTAATAACTATTATTATTNNNAATATAATAATTGATTTAAATTATATAATTATAGTTAGATTAATAATTAAAATTGGCTGTCCTCCTTTCCATTCTTGATATGTTTCTGTTATTGAAGGTTTGACTTGAATAGTATGTTTTATTTTAATAACTATTCAAAAAATTATTCCTTTGATTATATTATCATATTTAAATRTWWATATAWKRYWAYKYAYYAYYMTMTYATKYMWTTKWKKRTRTWTTRKMRGGYWKRRYTRKTMYWMMWTRMGTMWWAWYWYKKSTWRWWCWTSRAWYTMYARKTTMAGWNNAATTTTTAGAGGTATTRTTATTATTAWTNNNNNNNGKYWWWWWTMWTRKYTKATTTATTCAWTTACATTATTAGCTGTATGYTATATATTTAATTTATTTAATATTAACTATTTTAATCAATTTATTATAGTATCTTATAATTTTATAAAATCAATCATAATAATGTGCATTTTTATATCTATGGGGGGATTACCCCCCTTTTTAGGGTTTTTCCCTAAATTAATTATAATTTACTGCTTATTGTTAAATAACATAATACTTATTTGTATTATGTTATTAATAACAGCTCTGATTATTTTATTTTTTTATTTACGAATCTTAATTACAACATTAATAATAAATACAATTTCAATAAAAAGAATTGTCATAAGAGTTTCATATACTTATTATATTGTTGGAGTATTTTCATTATTTGGAATAATCTTTTTATCATTAATTACATTAAATATTTGTTAGATTTATAATATTTTGATTTATTCNNTTATTTTAATATTTTTACTAATATTTTTATTATCATTATTAATATATGTTTCCCATAAATCTATAAAAGATCGAGAAAAATCATCACCATTTGAGTGTGGATTTAGACCATTTGAATCATCTCGTATTCCATTTTCGAGACACTTTTTCTTAATTGCAGTAATTTTTTTAATTTTTGATGTTGAATTGGTAATTATTATACCTGTAATTTTAGTAATAATTAGATTAAAAACTATTGATATTTATATTATTATATTAATCTTCTTAATTATTTTGACCTTAGGATTATATCATGAATGATATAATAATATATTAAATTGANNNNNNNGTGTAATTAAAATATTTAATTTCAAAATAATTAAATTTAAGGGAATCCAATGTATCATAATCAAAAAAAACTCCCGAACTCTTCCAGAATTAAATGAAAATAAACTGGAAAAAAATATACCATGTTGAGTAAATGAAAATATATATAATCTATAACAAGCAGATAGAAATGAAGATAAAATCAATAAAATCATTATTAACATATTTCAAGATATTAATCTATTAATAATTATAATTTCACCAGCTAAATTAATTGTTGGAGGACATGATATATTTCTTGCCGATATTAAAAATCATAATAAAGATATTGAAGGCATAAATGTCAATATACCCTTATTAATTAATAATCTTCGACTATTAGTTCGCTCATACAATATGTTTGCTAAACAAAATAATCCAGAAGAACATAAACCATGACCAATCATTATATAATATGACCCACATATTCCCCAATTTCTTATAGTTATAATTCCTCTAATCACTAGACCTATATGAGCAACAGATGAATAAGCAATTATTGATTTGATGTCAATTTGAATTATACATAAAATTCTAATTAAAATTCTTCCAATTATTGAAATTGAGATCCATATAAACCCAAATTTGTAAAAATAAGAAGGAATAATGTACATCATACGAATTAAACCGTATCCACCTAATTTTAACAAAATTCTAGCCAAAATTATTGAACCAGAAACAGGAGCTTCAACATGAGCTTTTGGTAACCAAAAATGAAAAAATAATGGTATTTTAATTAAAAAAGCTATTATTATACCAAAATAAATATAAATATTAACAGAACAACTTATTAACATAAAAATTATGGTTATGTTATTTTTATAAAGAAAAAAAATTCTTATTAATATTGGTAATGATGCAAATAATGTATAAAATAATAAATAATATCTTGCATCAATACGTTCTGGTTGATAACCCCAACCTATAATAATAATTATAATTGGAATTAATCTTGATTCAAAACAAATATAAAAAACTATAATTCTTGATGTGGAAAAAGAAATAATCAAAAAAATCATTAACATAACACCAAAAATAAAAAAAATAACTTATTATGTTTATATATTGGACTAGAAACAATTATTAAAAATGAAATTCAAATTNNNNNNNNNNNNNNNNNNNNNNNNNNNNNNNNNNNNNNNNNNNNNNNNNNNNNNNNNNNNNNNNNNNNNNNNNNNNNNNNNNNNNNNNNNNNNNNNNNNNNNNNNNNNNNNNNNNNNNNNNNNNNNNNNNNNNNNNNNNNNNNNNNNNNNNNAAAAAAAAACTAATTTTATTGATATAGATATTAATTTATCATTATTATGAGATCGAATTAATATAACTAAACATGATAAACCTATTACTCCTTCACAAACTCTAAAAACCAAGAAAATTAATAAAATATGTAAATCATATATTTTTATAGAAAAAATAAATGATAAAGAACAAAATATTCTTAAAATAATTAATTCCAATCTTAATGCTGTTATTAAATGTTTTCGATTTATACATAATGAAATTATCCCTGAAAAGAATATAGAGGTAAAATATATTAATAATGTTATATTCTATAAATATAAAATAATAATTCAGATAATGAATGTAATTATTTGAATCTTAAAATTATTATTCATCAATATATAATTTAATGACATTATTATTTTTAAAGTTTTCAAAATCCCTTGGGGCCCAAGGTTTTCACCTCATCCCATATCAATTGATTTTTGAAATATGAATGATTTACTTATTAATAACATTTGATTATGAAACGTTGACAATTGCTTTATAAACCACATTGAACCAAAAAACTCATAAACAATTTTTATTTTAGTTTTAGAAAAAATTGATATCTCATATCCCAACCAGATTCCTGATATTGAAAAAATCAATGCCAATAATTTGCCCTCTATAGGCATTAAAATTATAATTGGATCATTAAATATTAACCATCTTAATATAGATCCCGAAAAAATTGAATAAATAGATAAAATAATAATTCTTTTAATTATGTTATTTATACTTTCATTCAATGATCTTAACTTATAAAAATTATAATTTACATTTATTGAAAAAAATGTCAACCGGGCAGAATAAAATGAAGTTAAACCAATACCAATATATATAAATATTATAATTAATAAATTAATGTGTCTAGAACACATAGTTTCTATAATTAAATCTTTTGAATAAAATCCACTCAAAAATGGAATTCCACATAATGATATATTAGCAATGTTCATTATTGTAATAGTTAGTGGTATTTGAAATCTTAAACATCCTATTACACGAATATCTTGATTATTATTAAAATTGTGAATCAAAATTCCTGCACACAAAAATAAAGTAGCTTTAAATAAAGCATGAACAATTAAGTGAAAAAATGATAACATTGGATAGCCAAATATAATAATTGTTATTATAATTCCCAGTTGTCTTAGAGTTGATAATGCAATGATTTTTTTTAAATCAAATTCAAAATTTGCTCTAATTCCTGATATTAATATAGTTAAAATTGAAATTAATATAAATAATTGAATATAATCGAAGTTCAAAATAATATTAGAAAATCGAATTATTAAATATACTCCAGCAGTAACAAGTGTAGATGAATGAACCAAAGCAGAAACAGGAGTTGGAGCAGCCATAGCTGCTGGTAATCATGAAGAAAATGGAATTTGAGCACTCTTTGTAAACCTCGCAATAATAATTATAATTAATATAATTTATNNNNNNNNNNNNNNNNNNNNNNNNNNNNNNNNNNNNNNNNNNNNNNNNNNNNNNNNNNNNNNNNNNNNNNNNNNNNNNNNNNNNNNNNNNNNNNNNNNNNNNNNNNNNNNNNNNNNNNNNNNNNNNNNNNNNNNNNNNNNNNNNNNNNNNNNNNNNNNNNNNNNNNNNNNNNNNNNNNNNNNNNNNNNNNNNNNNNNNNNNNNNNNNNNNNNNNNNNNNNNNNNNNNNNNNNNNNNNNNNNNNNNNNNNNNNNNNNNNNNNNNNNNNNNNNNNNNNNNNNNNNNNNNNNNNNNNNNNNNNNNNNNNNNNNNNNNNNNNNNNNNNNNNNNNNNNNNNNNNNNNNNNNNNNNNNNNNNNNNNNNNNNNNNNNNNNNNNNNNNNNNNNNNNNNNNNNNNNNNNNNNNNNNNNNNNNNNNNNNNNNNNNNNNNNNNNNNNNNNNNNNNNNNNNNNNNNNNNNNNNNNNNNNNNNNNNNNNNNNNNNNNNNNNNNNNNNNNNATTAAATTTTTTATTTATCATGAAAATTGCTATGTATTTAATAATTATTTTATCAATAAATTTTATTTTCATGAAACATCCCTTATCAATAGGATTAATTTTAATAATACAAACAGTTCTATCTTGCTTAATTTGTAGACTTTACTTAAGTTGTTATTTATTTTCTTATATCTTATATCTTATTTTTATTGGTGGTATATTAATTCTATTTATATATATATCAAGAATTGCATCAAATGAAAAATTTATTTATTCTATTAAATTAATAATGCTTAATTTTTTAATTTTAACTTTAACCAATTTTATCAATATAATTGATTTAAAATCACTAAATATTAANNNNNNNNNNNNNNNNNNNNNNNNNNNNNNNNNNNTTATAATAAGAAAACTATATATCATCCCTTCTGGAATAATAACACTAATATTAACAATTTATTTATTATTTGTTTTAATTATTGTAATTAATATTTTAACAACAAATATATTGACTCTTCGAAGAAGAATTTCTTTCTAACTACACCTTCCGGTACAGTTACTTTGTTACGACTTATCTCATTAATATTATGAGAGTGACGGGCGATATGTACATAAATTAGAGCCAATTTCAATTAATTAAATTTAATTAATTTATTATCAAATCCAATTTCATATTTTCTNNNNNNNNNNAAATAATCCAATAAATAATTAATTGTAACCCATTTTTTCTTTAATATAAACTGCACCTTGACCTGACATTCTAAATATNATAATATAATATGAAAATTTTTCTTATAAAACATTCTTGACAGAGATATACAAATTAAATTAAAGTTTTTTCTATCGTGGATTATCAATTATAAAACAGGTTCCTCTGATAAGATAAATTACCGCCAAATTCTTTGAATTTAAAGATCATTTCTAATAATAATCAAGTTATTTTTATCACATTTTTAATAATAGGGTATCTAATCCTAGTTTATACAAAAATTTTTCAGACATAAAATTAATTTTTAAAGATAAAATATATTTCACCAAAATTTTAGATATTTTATATTATTGTTATATTAACTGAAACTTAACTATTTAAATTAAAGAAATCGTATAACCGCAACTGCTGGCACAAAATTTGATTCTTTTAAATAAAATTACTAATTCTAATTTTATTAATTTATAATAATAAATACTGCGCATTTATTCAAAATATTTCCATTTAGAAATTAAAAACAATTAAAAAAGTGCATGTAATATAATTTTAAATTTAAATTTTNNTAAACTAGAATTAAACTTCAAATAATAAAATTATTTAATGAGGTCCTTTCGTACTAACATTAAAAATAATTAAGTAGATAGAAACCAACCTGGCTCACGCCGGTTTGAACTCAGATCATGTAAGAATGTTAAGGGTCGAACAGACCCAGGAATAATAAATTTTGCTCCAATCCCTATTCTTAATCCAACATCGAGGTCGCAATCATATTTATCGATATGAACTCTTCAAATTAATTACGCTGTTATCCCTAAGGTAATTTATTCTTATAATCAAAAATTTGGATCAATATTAACATAAATTTATGAAATTTTTATTAAAAGTTAATTATATTTTAATATCACCCCAACAAAAAAATCAATTAAATAAAATAAATTAATTAACAATTAGNAAATNNNNNNNNNNNNNNNNNNNNNNNNNNNNNNNNNNNNNNNNNNNNNNNNNNNNNNNNNNNNNNNNNNNNNNNNNNNNNNNNNNNNNNNNNNNNATATTAATNNNNNNNNNNNNNNNNNNNNNNNNNNNNNNNNNNNNNNNNNNNNNNNNNNNNNNNNNNNNNNNNNNNNNNNNNNNNNNNNNNNNNNNNNNNNNNNNNNNNNNNNNNNNNNNNNNNNNNNNNNNNNNNNNNNNNNNNNNNNNNNNNNNNNNNNNNNNNNNNNNNNNNNNNNNNNNNNNNNNNNNNNNNNNNNNNNNNNNNNCTAAAAAACCAGATATCATATAATTTGATAAAATGTCATTTCCAGAATAAATTTATTAATATATATACAATAATAACTAACAAATNTACACTAAATCATTATATTTCGGGAAAAATAAATATTTAAAAGTATTCAATTAATCCTGATACAAAAGGTACAAATTATATTTTACTTAAAATTATTTATAAAATNNTTTCCCTCTCAGTTAATCAAAATTATTTTTTTATATAAAATTACTTCAACACAAAAATTTTTTTTTCAAAATGTAAAAAACAACTATATANTAAAATTATATTATGGGATATATTTAATTATAATAATTGAATTGCAGTCAATAGGTGTTAACTATAACTATCCTTAAGTAATGAAGTAAAATAATACATTTAGTTTCGACCTAAAATAAGAATTAGAGTTCCTTGCTTTTAAATGAAGCCAAAATGGAGGCATTTTATTGTTAATAAAATAATTGAAANTTTATTTCCATTTAAAGAAAAATTAGTTTAATTAAAATATAAATCTGTCAGGTTTATGAAACTTTTGAGTATTTTTCTGACTTTTTATCATNTNTTCTACTTTAAATTTGCAATTTAATATTATAAATTAAATATAAGACTTTATGTTAACAGAATTAAACTGTCTATTAATATCAAAAATTAATGTTCTTCATAAACTATAACATTTATAAAGTTTATAAAACATTTCATTTTCATTGAAAAGAGAGACTTGGTCTTATAAAACTCTATTAGTATATAAGTATATTTAACTTCCAATTAAAAGGATTAATTTTATTAAATAGAATATATCCAATAAATTTNNATTTTATCTTTAAATCACAATATAATATTTGTATTAAACTAATTGAATTACTATTTGTAATAAATATTACATTATTAAATTCTAAATTTAAGGCATTAATTTTGCTAAAATAGTTCTAATATGGCAGATTAGTGCAATGAATTTAAGATTCATATATAAAATANNTTTTTTTTATTGGAAAATAAGATGCCTGAGATGAAAAGGATTATTTTGATAGAATAAATCATGTAATAATATTACTCNNNCATTAAGTGGCTGAAAGTAAGTAATGGTCTCTTAAACCAATTAATAGTAATTAACATATACTCTTAATGAAAGATAAGCTAAAGTTAAAGCTAATGGGTTCATACCTCACTTATGGAAANTTTTCCTCTTTTTATTTAAAAACAAAAGTTACTTTAATATCTTCAATATTATGCTCTTTATTAAGCTATTTAAATTCAGAAAAGATANNATGATCATTTTAAACTCCCAAAGTTTATATTTTATATTTAAATTATTTTCTGAGTTAATTAGCTTATATNAAAGCATTTATTTTGAAAGTAAAAGAAAAGATATTTAATCTATTAACTTAGGAAACTAGAAATAAAATTAGCTTCTAACTAACTTTTAAAGCGGTTAAATTCCGTTTTTTCCTTGTTTTAATAGTTTAATNNNAAAAACTTAGATCTTGTAAATCTAGATTAACGTAAGTTTTAAAAATAGGTTTTAAGTTATTGANAACTATTATCCTTCAAAGTTAAAAATATAATTANATTTATTATTAGGAGAAATAATTTTTCATAAATAAATTTACAGTTTATTGCCTATTATCGGCCATCCTAATCAAGTTATATTGATTAAACAATATTCTCATTAGTGTAAATAAAGTGCTAATTTAGCTTTAACTTG

>tuta-clade2-northeastSI_02.NZ.NN.TTA.35

NNNNNNNNNNNNNNNNNNNNNNNNNNNNNNNNNNNNNNNNNNNNNNNNNNNNNNNNNNNNNNNNNNNNNNNNNNNNNNNNNNNNNNNNNNNNNNNNNNNNNNNNNNNNNNNNNNNNNNNNNNNNNNNNNNNNNNNNNNNNNNNNNNNNNNNNNNNNNNNNNNNNNNNNNNNNNNNNNNNNNNNNNNNNNNNNNNNNNNNNNNNTTTGTCATTATTTATATTTATTCTACTAAATAATTTATTGGGTTTACTTCCGTATATTTTCACTAGCCCAAGTCATCTAGTGTTCACAATAAGATTAGCATTACCACTATGATTATCATTCATAATTTATGGATTTATTAACAATATAAATCATATATTTTGTCATTTAGTTCCATCCGGAACTCCTAATATTTTAATACCTTTTATAGTTCTTATTGAAAGAGTCAGAAATCTAATTCGACCTGGCTCTTTAGCTGTCCGACTAACAGCTAATATAATTGCTGGACATCTTTTGATAACCTTATTAGGTAACCTACCTATAAGGTATGAATTATACTCAAGTATTATTATTATTTTTCAAGTTATATTAATATTATTTGAATTAGCTGTTTGTGTTATTCAATCATATGTATTTATAGTTCTTAGAACTTTATATTATAGAGAAGTAAATNNNNNNNNNNNNNNNNNNNNNNNNNGATCAATATTNNNNNNNNNNNNNNNNNNNNNNNNNNNNNNNNNNNNNNNNNNNNNNNNNNNNNNNNNNNNNNNNNNNNNNNNNNNNNNNNNNNNNNNNNNNNNNNNNNNNNNNNNNNNNTTTAAGCCAACACGACAAAATCATCCTTTAATTAAAATTATTAATAATTCATTAATTGACTTCCCAGCACCATCTAATTTATCTTATTGATGAAATTTTGGATTTGTATTAGGATTATGCTTAACAATTCAAATTTTAACTGGATTATTTTTATCAATACATTATAATGCCAATATCATAAATGCTTTTGAAAGATTAAGACATATTTGCCGTGATGTAAACTATGGTTGAATTTTACGTGTTATTCACGCTAATGGGGCTTCATTATTTTTCATTTGTGTATATTTACATGTAGGACGTGGATTATATTATGGATCATTTAAATATATTGAAACATGATCTATTGGTGTAATTATATTATTAATATTAATAGCTACTGCTTTCTTAGGATATGTTTTACCGTGAGGTCAGATATCTTTTTGAGGAGCCACAGTCATTACAAATTTATTATCAGCAATTCCTTATTTAGGTGGAATATTAGTTAATTGAATTTGAGGAGGATTTGCAGTTGACAATGCAACACTAACTCGATTTTACTCTTTTCATTTTATTTTACCATTTATTGTATTAAGATTAACAATTATTCACCTATTATATTTACATACAACAGGTTCAAATAATCCATTAGGAATTAACTCCAACAATGACAAAGTCCCATTTCACCCATACTTCTCCGTTAAGGATATTATAAGCCTATTTTTATTAATAATTATTTTCTTTATATTAGTTATGTTAGAACCCTATATATTAGGGGATCCAGATAATTTCATTCCTGCCAATCCACTTGTGACACCAAAACATATTCAACCTGAATGATACTTTTTATTTGCCTACGCAATCCTCCGTTCAATTCCTAATAAACTAGGAGGAGTAATTGCTCTTTTTATATCAATTTTCATTTTAATGTTTGTANNNNNNNNNNNNNNNNNNNNNNNNNNNNNNTTAAATAATTACCCAATTAATCAAATTATATTTTGATATATAGTAATAATTTTAATTTTACTAACTTGAATTGGAGCACGACCTGTCGAATTACCTTATATCAACTTTGGAATATTTTTAACAATTATATATTTTTCTTACTTCATTATTGATACAATAATTAAATCTATTTGAGATAAATTAATTAGATAGATGAAAAAATGATTCTTCTCAACTAATCATAAAGATATTGGAACAATATATTTCATTTTTGGTATTTGATCAGGTATAATTGGTACAACTTTAAGAGTTTTAATTCGTGTAGAACTTGGAACTCCTGGTTCATTTATTGGTGATGATCAGATTTATAATGTAATTGTCACTGCTCATGCTTTCATTATAATTTTTTTTATGGTAATACCAATTATAATTGGTGGATTTGGAAATTGATTAGTCCCTTTAATAATTGGAGCTCCTGATATAGCTTTTCCTCGTATAAATAATATGAGTTTTTGACTATTACCACCTTCCCTAATTCTTATTTTAGTGGGAAGAATAGTTGATAGAGGTGCAGGCACGGGTTGAACAGTTTACCCTCCTTTATCCGCAGGTATTGCACATTCTGGTTCATGTGTTGATTTAACTATTTTTTCTCTACACCTTGCAGGTGTATCTTCAATTTTAGGTGCTGTGAATTTTATTAGAACAATTTTTAATATACGTTCAATAGGAATTTGATTGGATCGCATACCTTTATTTGTATGGGCAGTTTTAATTACTGCATTTTTATTATTATTATCATTACCCGTTTTAGCAGGCGCTATTACAATATTGTTAACAGATCGAAATTTAAATACTTCATTTTTTGATCCTGCAGGAGGAGGAGATCCTATTCTTTATCAGCATTTATTTTGATTTTTTGGTCATCCTGAAGTCTATATTTTAATTTTACCAGGATTTGGTTTAATTTCTCACATTATTACTCAAGAGAGAGGTAAAATTGAATCTTTTGGCTCATTAGGAATAATTTATGCCATAATATCAATTGGTATTTTAGGATTTGTTGTATGAGCACATCATATATTTACTGTAGGAATAGATGTCGATACACGTGCATATTTTACATCAGCTACCATAATTATTGCAGTTCCCACTGGAATTAAAGTATTTAGTTGACTTGCAACTTTGAGAGGTATAAAAATTAATATTTCATCTTCAGCCCTATGAGCGTTAGGGTTTGTCTTTTTATTTACTATTGGAGGTTTAACTGGAGTTATTCTAGCCAATTCTTCAATTGATATTATATTACATGATACATATTATGTAGTTGCACATTTCCATTATGTTTTATCAATAGGAGCAGTATTTGCTATTATAGCAAGATTTATTCATTGATTTCCATTATTTACAGGATTAAGATTAAACTCAAATTGATTAAAAATTCATTTTCTATTAATATTTATTGGTGTAAATATAACATTCTTTCCTCAACATTTTTTAGGATTAAGAGGAATACCTCGTCGTTATTCAGACTATCCTGATGCTTATATGTCATGAAATATAGTTTCATCAATAGGAAGAATTATATCTTTAGTTGGAATTTTATTTTTATTATTTATTGTTTGGGAAAGCTTTATTTCAATACGATTAGTATTATATTCTAATAGAATTCAATCTTCTATTGAATGAATACAAAAGTTTCCCCCATCTGAACATTCATATAATGAAATGCCATTGTTAATTCAAATTTCAAATTGATCTTATATTAATATACAGGATGCTGTATCACCATTAATAGAACAATTAATTTTTTTTCATGATCATGTATTAGTTATTTTAATTATAATTACAATTGTTGTTGCTTATATAATATTTATATTAATATNNNNTAAAATCATTAATCGCTTACTTCTTGAAGGACAATTAATTGAATTTATTTGAACTCTATTACCTGCAATAACTTTAATTTTTATTGCGTTACCATCATTACGATTATTATATATATTAGACGAAATTAATAATCCATTATTAACATTAAAAATTATTGGTCACCAATGATATTGATCATATGAATATTCAGATTTTTCTGATGTAGAATTTGATTCATATATAAAATCTATAAACGAAATAAATAAAAACGAATTTCGTTTACTAGATGTAGATAATCGAGTAATTCTACCATTTAATATTCAAGTCCGACTATTAGTTTCTTCTTTTGATGTTATTCATTCTTGAGCAATACCATCAATAAGACTTAAAGTTGATGCAGTACCAGGACGATTAAATCAAATAAGAATATTAATTAGTCGTCCCGGCGTATCTTATGGACAATGTTCTGAAATTTGTGGAGCAAATCATAGATTTATGCCTATCGTAATTGAAAGAATTAGAATAAAAATATTTATTAAATGGTTAATTAATTATATGAATAATCACCCTTATCATATAGTTGACTATAGACCCTGACCTTTAACTGGATCAATTGGAGCATTAACTTTTGTTTCCGGTATAGTTATAATATTCCATAAATGNNNNNNNNNNNNNNNNNNNNNNNNNNNNNNNNNNNNNNTAATAACAATAATTCAATGATGACGTGACATTTCCCGAGAAGGAACTTTTTTAGGAATACACACAATAATAGTAGTGAGTGGTTTGAAAATAGGCATATTATTATTTATTGTATCAGAAATTCTTTTTTTTGTATCATTTTTTTGAGGATTTTTTCATAGTAGATTAAGACCTGTAGTAGAAATTGGTATAATTTGACCTCCTAGAGGTATTTATGTTTTTAATCCAACTCAAGTTCCATTATTAAATACAATAATTTTATTATGTTCAGGAATTACAATTACTTGAGCACATCATTCAATCATAAATGGTAATCACATTAATTCTATTTATAGAATTATGTTAACTGTTATTTTAGGTATATATTTCACTATTCTGCAAGGCTATGAATATTATGAAGCCCCATTTGCAATTAATGATTCTATCTATGGATCTTCTTTTTTTATAGCTACTGGATTTCATGGAATTCACGTAATTATTGGAACAAGATTTATTACTGTATGCTTATTACGACAAATAAAGTTTCATTTCTCAATAAATCACCATTTTGGCTTCGAAGCTGCTGCTTGATATTGACATTTTGTTGATGTAGTCTGATTATTTTTATATTTATCAATTTATTGATGAGGAAGATAATAAATTATAGTTTTTAAAAAGAAAAAAAAAATAAATAAATTCAGAGATACCGGTAAATAAGATTTTCAAGCCAAATATATTAATTTGTCATAACGATAACGAGGAAGGGTGCCCCGAATCCAAATAAAAAGAAAACAAATTATAGAAACTTGAATTGGAAAAACAATTGAATTAATTTTACCTCCTAAAAACATTAAACAAAATAATATTCTCATAAATAAAATACTTGAATACTCAGCCAAAAAAATAAATGCAAACCCAGAACCTCTATATTCAACATTAAAGCCAGAAACTAACTCTGATTCTCCCTCAGAAAAATCAAATGGAGATCGATTAGTCTCAGCTAAAGCTGAAGATAATCAACATATTCTCAAAGGAAGAAACAAAAAAATAAATCACACAAATTCCTGAAATATAAACAAATCAATAATATTATAACTTCTTATTATTATTATAGGACATAATATAATTAAAACCAATCTTACCTCATAAGAAATAGATTGAGCAATTGATCGAATGCATCCTAATATTGCATAACTTGAATTAGAAGATCAACCTGTCAATATTAATGAGTAAACTCTTATTCTTGAGCAACAAAAAAAAAATAAAATACCAAAATTAAAAGAAACACAATTAATGAAATATGGATACAATGATCAAATCAACAAACTATTGAATAATCCTATAATAGGTCTAAAAATATAAATTAAATAATTAGATATTAATGGAATTGTATTTTCCTTTATAAATAATTTAATAGCATCAGAAATAGGCTGCAAAATACCTAAAAATCCAACTTTATTTGGACCTTTACGAATTTGAATATATCTTAAAACCTTACGTTCCAAAAGAGTAATAAATGCCACTCCAATTAAAATAAATAATACAGTAATTAAAATTGTAATTAAATACAAAAACAATTCTTCTTATATACTTTATATAATTTTCTTAATTTTAGGAATTATAATTTCAATTTCTTCAAACAATTGGCTGGGTTGTTGAATAGGAATTGAAATAAATATAGTTTCATTTTTGCCTATAATGGCAAACAAAATAAGAATTTATGCTTCGGAATCAATAATTAAATATTTTATCATTCAAAGAATAGGATCGAGTTTATTATTAATAACTNNNNNNNNNNNNNNNNNNNNNNNNNNNNNNNNNNNNNNNNNNNNNNNNNNNNNNNNNNNNNNNNNNNNNNNNNNNNNNNNNNNNNNNNNNNNNNNNNNNNNNNNNNNNNNNNNNNNNNNNNNNNNNNNNNNNNNNNNNNNNNNNNNNNNNNNNNNNNNNNNNNNNNNNNNNNNNNNNNNNNNNNNNNNNNNNNNNNNNNNNNNNNNNNNNNNNNNNNNNNNNNNNNNNNNNNNNNNNNNNNNNNNNNNNNNNNNNNNNNNNNNNNNNNNNNNNNNNNNNNNNNNNNNNNNNNNNNNNNNNNNNNNNNNNNNNNNNNNNNNNNNNNNNNNNNNNNNNNNNNNNNNNNNNNNNNNNNNNNNNNNNNNNNNNNNNNNNNNNNNNNNNNNNNNNNNNNNNNNNNNNNNNNNNNNNNNNNNNNNNNNNNNNNNNNNNNNNNNNNNNNNNNNNNNNNNNNNNNNNNNNNNNNNNATTTTTATATCTATGGGTGGATTACCCCCCTTTTTAGGGTTTTTCCCTAAATTAATTATAATTTACTGCTTATTGTTAAATAACATAATACTTATTTGTATTATGTTATTAATAACAGCTCTGATTATTTTATTTTTTTATTTACGAATCTTAATTACAACATTAATAATAAATACAATTTCAATAAAAAGAATTGTCATAAGAGTTTCATATACTTATTATATTGTTGGAGTATTTTCATTATTTGGAATAATCTTTTTATCATTAATTACATTAAATATTTGTTAGNNNNNNNNNNNNNNNNNNNNNNNNNNNNNNNNNNNNNNNNNNNNNNNNNNNNNNNNNNNNNNNNNNNNNNNNNNNNNNNNNNNNNNNNNNNNNNNNNNNNNNNNNNNNNNNNNNNNNNNNNNNNNNNNNNNNNNNNNNNNNNNNNNNNNNNNNNNNNNNNNNNNNNNNNNNNNNNNNNNNNNNNNNNNNNNNNNNNNNNNNNNNNNNNNNNNNNNNNNNNNNNNNNNNNNNNNNNNNNNNNNNNNNNNNNNNNNNNNNNNNNNNATTTATATTATTATATCAATCTTCTTAATTATTTTGACCTTAGGATTATATCATGAATGATATAATAATATATTAAATTGANNNNNNNGTGTANNNNNNNNNNNNNNNNNNNNNNNNNNNNNNNNNNNNNNNNNNNNNNNNNNNNNNNNNNNNNNNNNNTCCCGAACTCTTCCAGAATTAAATGAAAATAAACTGGAAAAAAATATACCATGTTGAGTAAATGAAAATATATATAATCTATAACAAGCAGATAGAAATGAAGATAAAATCAATATAATCATTATTAACATATTTCAAGATATTAATCTATTAATAATTATAATTTCACCAGCTAAATTAATTGTAGGAGGACATGATATATTTCTTGCCGATATTAAAAATCATAATAAAGATATTGAAGGCATAAATGTCAATATACCCTTATTAATTAATAATCTTCGACTATTAGTTCGCTCATACAATATGTTTGCTAAACAAAATAATCCAGAAGAACATAAACCATGACCAATCATTATATAATATGACCCACATATTCCCCAATTTCTTATAGTTATAATTCCTCTAATCACTAGACCTATATGAGCAACAGATGAATAAGCAATTATTGATTTGATGTCAATTTGAATTATACATAAAATTCTAATTAAAATTCTTCCAATTATTGAAATTGAGATCCATATAAACCCAAATTTGTAAAAATAAGAAGGAATAATGTACATCATACGAATTAAACCGTATCCACCTAATTTTAACAAAATTCTAGCCAAAATTATTGAACCAGAAACAGGAGCTTCAACATGAGCTTTTGGTAACCAAAAATGAAAAAATAATGGTATTTTAATTAAAAAAGCTATTATTATACCAAAATAAATATAAATATTAACAGAACAACTTATTAACATAAAAATTATGGTTATGTTATTTTTATAAAGAAAAAAAATTCTTATTAATATTGGTAATGATGCAAATAATGTATAAAATAATAAATAATATCTTGCATCAATACGTTCTGGTTGATAACCCCAACCTATAATAATAATTATAATTGGAATTAATCTTGATTCAAAACAAATATAAAAAACTATAATTCTTGATGTGGAAAAAGAAATAATCAAAAAAATCATTAACATAACACCAAAAATAAAAAAAATAACTTATTATGTTTATATATTGGACTAGAAACAATTATTAAAAATGAAATTCAAATTNNNNNNNNNNNNNNNNNNNNNNNNNNNNNNNNNNNNNNNNNNNNNNNNNNNNNNNNNNNNNNNNNNNNNNNNNNNNNNNNNNNNNNNNNNNNNNNNNNNNNNNNNNNNNNNNNNNNNNNNNNNNNNNNNNNNNNNNNNNNNNNNNNNNNNNNNNNNNNNNNNNNNNNNNNNNNNNNNNNNNNNNNNNNNNNNNNNNNNNNNNNNNNNNNNNNNNNNNNNNNNNNNNNNNNNNNNNNNNNNNNNNNNNNNNNNNNNNNNNNNNNNNNNNNNNNNNNNNNNNNNNNNNNNNNNNNNNNNNNNNNNNNNNNNNNNNNNNNNNNNNNNNNNNNNNNNNNNNNNNNNNNNNNNNNNNNNNNNNNNNNNNNNNNNNNNNNNNNNNNNNNNNNNNNNNNNNNNNNNNNNNNNNNNNNNNNNNNNNNNNNNNNNNNNNNNNNNNNNNNNNNNNTATAAATATAAAACAATAATTCAGATAATGAATGTAATTATTTGAATCTTAAAATTATTATTCATCAATATATAATTTAATGACATTATTATTTTTAAAGTTTTCAAAATCCCTTGGGGCCCAAGGTTTTCACCTCATCCCATATCAATTGATTTTTGAAATATGAATGATTTACTTATTAATAACATTTGATTATGAAACGTTGACAATTGCTTTATAAACCACATTGAACCAAAAAACTCATAAACAATTTTTATTTTAGTTTTAGAAAAAATTGATATCTCGTATCCCAACCAGATTCCTGATATTGAAAAAATCAATGCCAATAATTTGCCCTCTATAGGCATTAAAATTATAATTGGATCATTAAATATTNNNNNNNNNNNNNNNNNNNNNNNNNNNNNNNNNNNNATAGATAAAATAATAATTCTTTTAATTATGTTATTTATACTTTCATTCAATGATCTTAACTTATAAAAATTATAATTTACATTTATTGAAAAAAATGTCAACCGGGCAGAATAAAATGAAGTTAAACCAATACCAATATATATAAATATTNNNNNNAATAAATTAATGTGTCTGGAACACATAGTTTCTATAATTAAATCTTTTGAATAAAATCCACTCAAAAATGGAATTCCACATAATGATATATTAGCAATATTCATTATTGTAATAGTAAGTGGTATTTGAAATCTTAAACATCCTATTATACGAATATCTTGATTATTATTAAAATTGTGAATCAAAATTCCTGCACACAAAAATAAAGTAGCTTTAAATAAAGCATGAACAATTAAGTGAAAAAATGATAACATTGGATAGCCAAATATAATAATTGTTATTATAATTCCCAGTTGTCTTAGAGTTGATAATGCAATGATTTTTTTTAAATCAAATTCAAAATTTGCTCTAATTCCTGATATTAATATAGTTAAAATTGAAATTAATATAAATAATTGAATATAATCGAAGTTCAAAATAATATTAGAAAATCGAATTATTAAATATACTCCAGCAGTAACAAGTGTAGATGAATGAACCAAAGCAGAAACAGGAGTTGGAGCAGCCATAGCTGCTGGTAATCATGAAGAAAATGGAATTTGAGCACTCTTTGTAAACCTCGCAATAATAATTATAATTAATNNNNNNNNNNNNNNNNNNNNNNNNNNNNNNNNNNNNNNNNNNNNNNNNNNNNNNNNNNNNNNNNNNNNNNNNNNNNNNNNNNNNNNNNNNNNNNNNNNNNNNNNNNNNNNNNNNNNNNNNNNNNNNNNNNNNNNNNNNNNNNNNNNNNNNNNNNNNNNNNNNNNNNNNNNNNNNNNNNNNNNNNNNNNNNNNNNNNNNNNNNNNNNNNNNNNNNNNNNNNNNNNNNNNNNNNNNNNNNNNNNNNNNNNNNNNNNNNNNNNNNNNNNNNNNNNNNNNNNNNNNNNNNNNNNNNNNNNNNNNNNNNNNNNNNNNNNNNNNNNNNNNNNNNNNNNNNNNNNNNNNNNNNNNNNNNNNNNNNNNNNNNNNNNNNNNNNNNNNNNNNNNNNNNNNNNNNNNNNNNNNNNNNNNNNNNNNNNNNNNNNNNNNNNNNNNNNNNNNNNNNNNNNNNNNNNNNNNNNNNNNNNNNNNNNNNNNNNNNNNNNNNNNNNNNNNNNNNNNNNNNNNNNNNNNNNNNNNNNNNNNNNNATGAAAATTGCTATGTATTTAATAATTATTTTATCAATAAATTTTATTTTCATGAAACATCCCTTATCAATAGGATTAATTTTAATAATACAAACAGTTCTGTCTTGCTTAATTTGTAGACTTTACTTAAGTTGTTATTTATTTTCTTATATCTTANNNNNNNNNNNNNNNNNNNNNNNNNNNNNNNNNNNNNNNNNNNNNNNNNNNNNNNNNNNNNNNNNNNNNNNNNNNNNNNNNNNNNNNNNNNNNNNNNNNNNNNNNNNNNNNNNNNNNNNNNNNNNNNNNNNNNNNNNNNNNNNNNNNNNNNNNNNNNNNNNNNNNNNNNNNNNNNNNNNNNNNNNNNNNNNNNNNNNNNNNNNNNNNNNNNNNNNNNNNNNNNNNNNNNNNNNNNNNNNNNNNNNNNNNNNNNNNNNNNNNNNNNNNNNTGTTTTAATTATTGTAATTAATATTTTAACAACAAATATATTGACTCTTCGAAGAAGAATTTCTTTCTAACTACACCTTCCGGTACAGTTACTTTGTTACGNNNNNNNNNNNNNNNNNNNNNNNNNNNNNNNNNNNNNNNNNNNNNNNNNNNNNNNAATTTCAATTAATTAAATTTAATTAATTTATTATCAAATCCAATTTCATATTTTCTNNAANCTAGAAAATAATCCAATAAATAATTAATTGTAACCCATTTTTTCTTTAATATAAACTGCACCTTGACCTGACATTCTAAATATNATAATATAATATGAAAATTTTTCTTATAAAACATTCTTGACAGAGATATACAAATTAAATTAAAGTTTTTTCTATCGTGGATTATCAATTATAAAACAGGTTCCTCTGATAAGATAAATTACCGCCAAATTCTTTGAATTTAAAGATCATTTCTAATAATAATCAAGTTATTTTTATCACATTTTTAATAATAGGGTATCTAATCCTAGTTTATACAAAAATTTTTCAGACATAAAATTAATTTTTAAAGATAAAATATATTTCATTAAAATTATAGATATTTTNNNNNNNNNNNNNNNNNNNNNNNNNNNNNNNNNNNNNNNNNNNNNNNNNNNNNNNNNNNNNNNNNNNNNNNNNNNNNNNNNNNNNNNNNNNNNNNNNNNNNNNNNNNNNNNNNNNNNNNNNNNNNNNNNNNNNNNNNNNNNNNNNNNNNNNNNNNNNNNNNNNNNNNNNNNNNNNNNNNNNNNNNNNNNNNNNNNNNNNNNNNNNNNNNNNNNNNNNNNNNNNNNNNNNNNNNNNNNNNNATAATAAAATTATTTAATGAGGTCCTTTCGTACTAACATTAAAAATAATTAAGTAGATAGAAACCAACCTGGCTCACGCCGGTTTGAACTCAGATCATGTAAGAATGTTAAGGGTCGAACAGACCCAGGAATAATAAATTTTGCTCCAATCCCTATTCTTAATCCAACATCGAGGTCGCAATCATATTTATCGATATGAACTCTTCAAATTAATTACGCTGTTATCCGTAAGGTAATTTATTCTTATAATCAAAAATTTGGATCAATATTAACATAAATTTATGAAATTTTTATTAAAAGTTAANNNNNNNNNNNNNNNNNNNNNNNNNNNNNNNNNNNNNNNNNNNNNNNNNNNNNNNNNNNNNNNNNNNNNNNNNNNNNNNNNNNNNNNNNNNNNNNNNNNNNNNNNNNNNNNNNNNNNNNNNNNNNNNNNNNNNNNNNNNNNNNNNNNNNNNNNNNNNNNNNNNNNNNNNNNNNNNNNNNNNNNNNNNNNNNNNNNNNNNNNNNNNNNNNNNNNNNNNNNNNNNNNNNNNNNNNNNNNNNNNNNNNNNNNNNNNNNNNNNNNNNNNNNNNNNNNNNNNNNNNNNNNNNNNNNNNNNNNNNNNNNNNNNNNNNNNNNNNNNNNNNNNNNNNNNNNNNNNNNNNNNNNNNNNNNNNNNNNNNNNNNNNNNNNNNNNNNNNNNNNNNNNNNNNNNNNNNNNNNNNNNNNNNNNNNNNNNNNNNNNNNNNNNNNNNNNNNNNNNNNNNNNNNNNNNNNNNNNNNNNNNNNNNNNNNNNNNNNNNNNNNNNNNNNNNNNNNNNNNNNNNNNNNNNNNNNNNNNNNNNNNNNNNNNNNNNNNNNNNNNNNNNNNNNNNNNNNNNNNNNNNNNNNNNNNNNNNNNNNNNNNNNNNNNNNNNNNNNNNNNNNNNNNNNNNNNNNNNNNNNNNNNNNNNNNNNNNNNNNNNNNNNNNNNNNNNNNNNNNNNNNNNNGGGATATATTTAATTATAATAATTGAATTGCAGTCAATAGGTGTTAACTATAACTATCCTTAAGTAATGAAGTAAAATAATACATTTAGTTTCGACCTAAAATAAGAATTAGAGTTCCTTGCTTTTAAATGAAGCCAAAATGGAGGCATTTTATTGTTAATAAAATAATTGAAANTTTATTTCCATTTAAAGAAAAATTNNNNNNNNNNNNNNNNNNNNNNNNNNNNNTTAGGAANNNNNNNNNNNNNNNNNNGACTTTTTATCATNTNTTCTACTTTAAATTTGCAATTTAATATTATAAATTAAATATAAGACTTTATGTTAACAGAATTAAACTGTCTATTAATATCAAAAATTAATGTTCTTCATAAACTATAACATTTATAAAGTTTATAAAACATTTCATTTTCATTGAAAAGAGAGACTTGGTCTTATAAAATTCTATTAGTATATAAGTATATTTAACTTCCAATTAAAAGGATTAATTTTATTAAATAGNNNNNNNNNNNNNNNNNNNNNNNNNNNNNNNNNNNNNNNNNNNNNNNNNNNNNNNNNNNNNNNNNNNNTACTATTTGTAATAAATATTACATTATTAAATTCTAAATTTAAGGCATTAATTTTGCTAAAATAGTTCTAATATGGCAGATTAGTGCAATGAATTTAAGATTCATATATAAAATATNTTTTTTTTATTGGAAAATAAGATGCCTGAGATGAAAAGGATTATTTTGATAGAATAAATCATGTAATAATATTACTCNNNCATTAAGTGGCTGAAAGTAAGTAATGGTCTCTTAAACCAATTAATAGTAATTAACATATACTCTTAATGAAAGATAAGCTAAAGTTAAAGCTAATGGGTTCATACCTCACTTATGGAAANTTTTCCTCTTTTTATTTAAAAACAAAAGTTACTTTAATATCTTCAATATTATGCTCTTTATTAAGCTATTTAAATTCAGAAAAGATANNATGATCATTTTAAACTCCCAAAGTTTATATTTTATATTTAAATTATTTTCTGAGTTAATTAGCTTATATNAAAGCATTTATTTTGAAAGTAAAAGAAAAGATATTTAATCTATTAACTTAGGAAACTAGAAATAAAATTAGCTTCTAACTAACTTTTAAAGCGGTTAAATTCCGTTTTTTCCTTGTTTTAATAGTTTAATNNNAAAAACTTAGATCTTGTAAATCTAGATTAACGTAAGTTTTAAAAATAGGTTTTAAGTTATTGANAACTATTATCCTTCAAAGTTAAAAATATAATTANATTTATTATTAGGAGAAATAATTTTTCATAAATAAATTTACAGTTTATTGCCTATTATCGGCCATCCTAANNNNNNNNNNNNNNNNNNNNNNNNNNCTCATTAGTGTAAATAAAGTGCTAATTTAGCTTTAACTTG

>paxillulae_18.NZ.KA.KAI.3

NNNNNNNNNNNNNNNNNNNNNNNNNNNNNNNNNNNNNNNNNNNNNNNNNNNNNNNNNNNNNNNNNNNNNNNNNNNNNNNNNNNNNNNNNNNNNNNNNNNNNNNNNNNNNNNNNNNNNNNNNNNNNNNNNNNNNNNNNNNNNNNNNNNNNNNNNNNNNNNNNNNNNNNNNNNNNNNNNNNNNNNNNNNNNNNNNNNNNNNNNNNNNNNNCATTATTTATATTTATTCTAATAAATAATTTATTGGGTTTACTTCCATATATTTTCACTAGTTCAAGTCATCTAGTATTTACAATAAGATTAGCATTACCACTATGATTATCATTCATACTTTATGGATTTATTAATAATATAAATCATATATTTTGTCATTTAGTTCCGTCAGGAACTCCTAATATTTTAATGCCTTTTATAGTTATTATTGAAAGAGTCAGAAATTTAATTCGTCCCGGTTCCTTAGCCGTTCGACTAACAGCTAACATAATTGCTGGACATCTTCTAATAACCTTACTAGGCAATTTACCTATAAGGTATGAGTTATATTCAGGTATAATTATTATTTTTCAAGTTATATTAATATTATTTGAACTAGCTGTTTGCATCATTCAATCATATGTATTTATAGTTCTTAGAACTTTATATTATAGAGAAGTTAATATTCCTCAAATATCTCCTCTTAATTGATTATTATTTATTTATTTTNNNNNNNNNNNNNNNNNNNNNNNNNNNNNNNNNNNNNNNNNNNNNNNNNNNNNNNNNNNNNNNNNNNNNNNNNNNNNNNNNNNNNNNNNNNNNNNNNNNTTTAAGCCAACACGACAAAATCATCCCTTAATTAAAATTATTAATAATTCATTAATTGACTTCCCAGCACCATCTAATTTATCTTATTGATGAAATTTTGGATTTGTATTAGGATTATGCTTAACAATTCAAATTTTAACTGGATTATTTTTATCAATGCACTATAATGCCAATATTATAAATTCTTTTGAAAGATTAAGACATATTTGCCGTGATGTAAACTATGGTTGAATTTTACGTGTTATTCACGCTAATGGAGCTTCATTATTTTTCATTTGTGTATATTTACATGTAGGACGTGGATTATATTATGGATCATTCAAATATATTGAGACATGATCAATTGGTGTAATTATATTATTAATATTAATAGCTACTGCCTTCTTAGGATATGTTTTACCGTGAGGGCAAATATCTTTTTGAGGAGCCACAGTAATTACAAATTTATTATCAGCTATTCCTTACTTAGGTGGAATATTAGTTAATTGAATTTGAGGAGGATTTGCAGTTGACAATGCAACATTAACTCGATTTTATTCTTTTCATTTTATTTTACCATTTATTGTATTAAGACTAACAATTATTCACCTATTGTATTTACATACAACAGGTTCAAACAATCCACTGGGAATTAACTCTAACAATGATAAAGTCCCATTTCACCCATATTTCTCCATTAAAGATATTATAAGCCTATTTTTCTTAATAATTATTTTCTTTATATTAGTCATATTAGAACCCTATATACTAGGGGATCCAGACAATTTTATTCCTGCTAATCCACTTGTAACACCAAAACATATTCAACCTGAATGATATTTTTTATTTGCATACGCAATTCTCCGTTCAATTCCCAATAAACTAGGAGGAGTAATTGCTCTTTTTATATCAATTTTCATTTTAATGTTTGTACCATTATTAAATAATTCAAATTTTATAGGATTAAATAATTACCCAATTAATCAAATTATATTTTGATATATAGTAATAATTTTAATTTTATTAACTTGAATTGGAGCACGACCTGTTGAATTACCTTATATTAATTTTGGAATATTTTTAACACTTATATATTTCTCTTACTTCATTATTGACCCAATAATTAAATCTATTTGAGACAAATTAATTAGATAGATGAAAAAATGATTCTTCTCAACTAATCATAAAGACATTGGAACAATGTATTTCATTTTTGGCATTTGATCAGGTATAATTGGTACAACTTTAAGAGTTTTGATTCGTGTAGAACTTGGAACTCCAGGCTCATTTATTGGTGATGATCAAATTTATAATGTAATTGTCACTGCCCATGCTTTCATTATAATTTTTTTTATAGTCATGCCAATTATGATTGGCGGATTTGGAAATTGATTAGTCCCCTTAATAATTGGAGCTCCTGATATAGCTTTTCCTCGTATAAATAATATAAGTTTTTGATTATTACCACCTTCCCTAATTCTTATTTTAGTGGGAAGAATAGTTGACAGAGGTGCAGGTACAGGTTGAACAGTTTACCCCCCTTTATCTGCAAGAATTGCACACTCTGGTTCATGTGTTGATTTGACTATTTTTTCTTTACATCTTGCAGGTGTATCTTCAATTTTAGGTGCTGTAAATTTCATTAGAACAATTTTTAATATGCGTTCAATAGGAATTTGATTGGATCGTATACCTTTATTTGTATGAGCAGTTTTAATTACTGCATTTTTATTATTATTGTCACTACCCGTTTTAGCAGGCGCTATCACAATATTGTTAACAGATCGAAATTTAAATACTTCATTTTTTGATCCTGCAGGAGGAGGAGATCCTATTCTTTATCAACATTTATTTTGATTTTTTGGTCATCCTGAAGTCTATATTTTAATTCTACCAGGATTTGGTTTAATTTCTCACATTATTACTCAAGAAAGAGGTAAGATTGAGTCTTTTGGCTCGTTAGGAATAATTTATGCTATAATATCAATTGGCATTTTAGGATTTGTTGTATGAGCACATCATATATTTACTGTAGGAATAGATGTTGATACGCGCGCATATTTTACATCAGCTACTATAATTATTGCAGTTCCCACTGGAATTAAAGTATTTAGTTGACTCGCAACTTTGAGAGGTATAAAAATTAATATTTCATCTTCAGCTCTATGAGCTTTAGGATTTGTCTTTTTATTTACTATTGGAGGTTTAACTGGAGTAATTCTAGCCAATTCTTCAATTGATATTATATTACATGATACATATTATGTAGTTGCACATTTTCATTATGTTTTATCAATAGGAGCAGTCTTTGCTATTATAGCAAGCTTTATTCACTGATTTCCATTATTTACAGGATTAAGATTAAATTCAAATTGATTAAAAATTCATTTTCTATTAATATTTATTGGTGTAAATATAACATTCTTTCCTCAACATTTTTTAGGATTAAGAGGAATACCTCGTCGGTATTCAGACTATCCTGATGCTTATATGTCATGAAATATAATTTCATCAATAGGAAGAATCATATCTTTAGTTGGAATTTTATTTTTATTATTTATTGTTTGGGAAAGCTTTATTTCAATACGATTAGTATTATATTCTAATAGAATTCAATCCTCTATTGAATGAATACAAAAATTTCCTCCATCTGAACATTCATATAATGAAATGCCATTGTTAATTCAAATTTCAAATTGATCTTATATTAATATACAGGATGCTGTATCACCATTAATAGAGCAGTTAATATTTTTTCATGATCATGTATTAGTAATTTTAATTATAATTACAATTGTTGTTGCTTACATAATAATTATATTAATATTAAATAAAATTATTAATCGCTTACTTCTTGAAGGACAGTTAATTGAATTTATTTGAACTTTATTGCCTGCAATAACTTTAATTTTTATTGCATTACCATCATTACGATTATTATATATATTAGACGAAATTAATAATCCATTATTAACATTAAAAATTATTGGCCACCAATGATATTGATCATATGAATATTCAGATTTTTCTGATGTAGAATTTGATTCATATATAAAATCTATAAACGAAATAAATAAAAACGAATTTCGTTTATTAGATGTAGATAATCGAGTAATTCTACCATTTAATATTCAAGTCCGACTATTAGTTTCTTCTTTTGATGTTATTCACTCTTGAGCAATACCATCAATAAGACTAAAAGTTGATGCAGTGCCAGGACGATTAAATCAAATAAGAATATTAATTAGTCGTCCCGGTGTCTCTTACGGACAATGTTCTGAAATTTGTGGAGCAAATCATAGATTTATGCCTATCGTAGTTGAAAGAATTGGAATAAAAATATTTATTAAATGATTAATTAATTATATGAACAATCACCCTTATCATATAGTTGATTATAGGCCCTGACCTTTAACTGGATCAATTGGAGCACTAACTTTTGTTTCCGGTATAGTTATAATATTTCATNNNNNNNNNNNNNNNNNNNNNNNNNNNGGTATTTTATTATTATTAATAACAATAATTCAATGATGACGTGACATTTCCCGAGAAGGAACATTTTTAGGAATACATACAATAATAGTAGTAAATGGTTTGAAGATAGGCATATTATTATTTATTGTATCAGAAATTCTTTTTTTTGTCTCATTTTTTTGAGGATTTTTTCATAGTAGATTAAGGCCTGTAGTAGAAATTGGTATAATTTGACCTCCCAGAGGTATTTATGTTTTTAATCCGACTCAAGTTCCATTGTTAAATACAATAATTTTATTATGTTCAGGAATTACAATTACTTGAGCACATCATTCAATCATAAGTGGTAATCATATTAATTCTATCTATAGAATTATGTTAACTGTTATTTTAGGTATATATTTTACTATTCTGCAAGGTTATGAGTATTATGAAGCCCCATTTGCAATCAATGATTCTATCTATGGATCTTCTTTTTTTATAGCTACCGGATTTCATGGAATTCACGTAATTATTGGAACAAGATTTATTACTGTATGCTTAGTACGACAAATAAAGTTTCATTTCTCAAGAAATCATCATTTTGGTTTCGAAGCTGCTGCTTGATATTGACATTTTGTTGATGTAGTCTGATTATTTTTATATTTATCAATTTATTGATGAGGAAGATAATAAATTATAGTTTTTAAAAATAAAAAAAAAACAAATAAATTCAGAGATACTGGTAAATAAGATTTTCAAGCCAAATATATTAATTTGTCATAACGATAACGAGGAAGGGTTCCTCGGACCCAAATGAAAAGAAAACAAATCATAGAAACTTGAATTGGAAAAACAATTGAATTAATTTTTCCTCCTAAAAACATTAAACAAAATAGTATTCTCATAAATAGAATACTTGAATATTCAGCTAAAAAAATAAATGCAAATCCAGAACCTCTATATTCAACATTAAAGCCAGAAACTAACTCTGACTCTCCCTCAGAAAAATCAAATGGAGATCGATTAGTTTCAGCTAAAGCTGAGGACAATCAACATATTCTTAAAGGAAGAAACAAAAAAATAAATCAAATAAATTCCTGAAATATAAATAAATCAATAATATTATAACTTCTTATTATTATTATAGGACACAATATAATTAAAACCAATCTTACTTCATAAGAAATAGACTGAGCAATTGACCGAATACATCCTAATATTGCATAACTTGAATTAGAAGATCAACCTGTTAATATTAATGAATAAACTCTTATTCTTGAACAACAAAAAAAAAATAAAATACCAAAATTAAAAGAAACACAATTAATAAAATATGGATATAATGATCAAATCAACAGACTATTAAATAATCCTATAATAGGACTAAAAATATAAATTAAATAATTAGATATTAATGGGATTGTATTTTCCTTTATAAACAATTTAATAGCATCAGAAATAGGCTGCAAAATACCCAAAAATCCAACTTTATTTGGGCCTTTACGAATTTGAATATATCTTAAAACCTTACGCTCCAAAAGAGTAATAAATGCCACTCCAATCAAAATAAATAATACAGTAATTAAAATCGTAATTAAATACAAAAATAATTCTTCTTATATACTTTATATAATTTTCTTAATTTTAGGAATTATAATTTCAGTTTCTTCAAACAATTGGCTGGGTTGTTGAATAGGAATTGAAATAAATATAGTTTCATTTTTGCCTATAATGGTAAACAAAATAAGAATTTATGCTTCGGAATCAATAATTAAATATTTTATTATTCAAAGAATGGGATCGAGTTTATTATTAATATCTATTATNNNNNNNNNNNNNNNNNNNNNNNNNNATTATATAATTATAGTTAGATTAATAATTAAAATTGGCTGTCCTCCTTTTCATTTTTGATATGTCTCTGTTATTGAAGGTTTAACTTGAATAGTATGTTTTATTTTAATAACTATTCAAAAAATTATTCCTTTAATTTTATTATCATATTTAAATGTTAATTTAAGATTATTTATTATTATATCATGTATTTGGGGATGTATTGGAGGGCTGGGCTATTCTTCAATACGTAAAATCATTGCTTATTCATCAATTTATAATTTAAGATGAATTTTTAGAGGTATTATTATTATTAACTATTCATGGCTGATTTATTATTTTATTTATTCATTCACATTATTAGCTGTATGCTATATATTNAAAATATTTAACATTAATTATATTAATCAATTTATTATAGTATCTTTCAATTTTATGAAATCAATCATAATAATGTGTATTTTTATATCTATGGGGGGATTACCCCCCTTTTTAGGGTTTTTCCCTAAATTGATTATAATCTACTGTTTATTATTAAATAACATAATACTTATTTGTGTCATATTATTGATAACAGCTCTAATTATTTTATTTTTTTATTTACGAATTTTAATTACAACATTAATAATAAATACAATTTCAATAAAAAGAATTGTTATAAGAGTTTCATATACTTATTATGTTGCTGGAGTATTTTCATTATTTGGAATAATTTTTTTATCATTAATTACATTAAACATTTGTTAGATTTATAATATCTTGATTTATTCNNTTATTTTAATATTTTTACTAATATTTTTATTATCACTATTAATATATATTTCCTATAAATCTATAAAAGATCGAGAAAAATCATCACCATTTGAGTGTGGATTTAGCCCATTTGAATCATCTCGTATTCCATTTTCGAGACACTTTTTCTTAATTGCAGTAATTTTTTTAATTTTTGATGTTGAATTAGTAATTATTATACCTGTAATTTTAGTAATAATTAGATTAAAAACTATTGATATTTATGTTATTATATTAATCTTCTTAATTATTTTGACTTTAGGATTATATCATGAATGATATAATAATATATTAAATTGANNNNNNNATCTAATTAAAATATTTAATTTCAAAATAATTAAATTTAATGGAATTCAATGTATCATAATCAAAAAAAATTCCCGAACTCTTCCAGAATTAAATGAAAATAAACTGGAAAAAAATATACCATGTTGGGTAAATGAAAATATATATAATCTATAACAAGCAGATAAAAATGAAGATAAAATTAATAAAACCATTATTAATACATTTCAAGATATTAATCTATTAATAATTATAATTTCACCAGCCAAATTAATTGTAGGTGGACAAGATATATTTCTTGCCGATATTAAAAATCATAATAAAGATATTGAGGGCATAAATGTTAATATACCCTTATTAATTAATAATCTTCGACTATTAGTTCGCTCATACAATATGTTTGCTAAACAAAATAATCCAGAAGAACATAAACCATGCCCAATCATTATATAATATGACCCACATATTCCTCAATTTCTTATAGTTATAATTCCTCTAATCACAAGACCTATGTGAGCAACAGATGAATAAGCAATTATTGATTTAATGTCAATCTGAATTATACATAGAATTCTAATTAAAATACTTCCAATTATTGAAATTGAAATTCATATAAATCCAAATTTGTAAAAATAAGAAGGAATAATATAAATTATACGAATTAAACCATATCCACCTAATTTTAACAAAATTCTAGCTAAAATTATTGAACCAGAAACAGGAGCTTCAACATGAGCTTTTGGTAACCAAAAATGAAAAAATAATGGTATTTTAATTAAAAAAGCTATTATTATACCAAAATAAATATAAATATTAACAGAACAACTTATTAACATAAAAATTCTAGTTATATTATTTTTATAAAGAAAAAAAATTCTTATCAATATTGGTAATGATGCAAATAATGTATAAAATAATAAATAATATCTTGCATCAATACGTTCTGGCTGATAGCCCCAACCTATAATAATAATTATAATTGGAATTAATCTAGATTCAAAACAAATGTAAAAAATTATAATTCTTGATGTAGAAAAAGAAATAATCAAAAAAATCATTAACATAACACCAAAAATAAAAAAAATAATTTATTACGTTTATATGTTGGACTAGAAACAATTATTAAAAATGAAATTCAAATTTAAAATAATTAAACCATAAGACATTTCATCAATATAAAAATTATAGCCTAATATATATAAATTCTTATTTATAAAAACCAAAAATATAATTATCAAAACAATAATTATATTTATTAATCATGAATTATAAATTAATAGGGTTAATATAAAGCAAAAAAAAACTAGTTTTATTGATATAGATATTAATTTATCATTATTATGGGATCGAATTAATATAACTAAACATGATAAACCTATTACTCCTTCACAAACTCTAAAAACCAAGAAAATTAATAAAATATGTAAATCATATATTTTTATGGAAAAAATAAATGATAAAGAACAAAATATTCTTAAAATGATTAATTCCAATCTTAATGATGTCATTAAATGTTTTCGATTTATACATAATGAAATTATTCCTGAAAAGAATATAGAAATAAAATATATTAATAATGTTATATTCTATAAATATAAAACAATAATTCAAATAATGAATGTAATTATTTGAATCTTAAAATTATTATTCATCAATTTATAATTTAATGATATTATTATTTTTAAAGTTTTTAAAATCCCCTGGGGACCAAGGTTTTCACCTCATCCCATATCAATAGATTTTTGAAATATGAATGATTTACTTATTAATAATATTTGATTATGAAAAGTTGACAATTGCTTTATAAATCACATTGAACCAAAAAACTCATAAAAAATTTTTATTTTAGTTTTAGAAAAAATCGATATTTCGTAACCCAATCAGATTCCTAATATTGAAAAGATTAATGCTAATAATTTACCCTCTATAGGCATCAAAATTATGATTGGATCATTAAATATTAATCATCTTAACATAGATCCCGAAAAAATTGAATAAATAGATAAGATAATAATTCTTTTAATTATGTTATTTATTCTTTCATTCAATGATCTTAACTTATAGAAATTATAATTTATATTTATTGAAAAAAATGTCAAACGAGCAGAATAAAAGGAAGTTAGACCAATACCAATATATATAAATATTATAATTAATAAATTAATGTGCCTAGAACATATAGTTTCTATAATTAGATCTTTTGAATAAAATCCACTCAAAAATGGAATTCCACATAATGATATGTTAGCAATATTCATTATTGTAATAGTTAATGGTATTTGAAACCTTAAACATCCTATTACACGAATATCTTGATTATTATTAAAATTATGAATTAAAATTCCCGCACATAAAAATAAAGTAGCTTTAAATAAAGCATGAACAATTAAATGAAAAAATGATAACATTGGATAACCAAATATAATAATTGTTATTATAATTCCCAGCTGTCTTAAAGTTGATAGTGCAATGATTTTTTTTAAATCAAATTCAAAATTTGCTCTAATTCCTGATATTAATATAGTTAAAATTGAAATTAATATAAATAATTGAATATAATCAAAATTCAAAATAATATTAGAAAATCGAATTATTAAATATACTCCAGCAGTGACAAGTGTAGATGAATGAACCAAAGCAGAAACAGGAGTTGGAGCAGCCATGGCTGCTGGTAATCATGAAGAAAATGGAATTTGAGCACTCTTTGTAAATCTTGCAATAATAATTATAATCAATATAATTTATATCTTTATTAATAAAAGATAAAAATTTCATGACCCAAAATTTAATATTCATGCAATAGATATTAAAATAGCAACATCTCCCACACGATTCATTAATGCAGTTAATATTCCTGCATTATTAGAATATAAATTTTGATAATAAATAACTAAACAATAAGAGACTAAACCAAGTCCATCTCAACCAATAATAATTCTCATTATATTAGGACTAATAATTAATAAAACTATAGATATAACAAACCCTATAACAATATAAATAAAACGATTAATGAATTTGTCATTATTTATATACATTCCACTATATAAAACAACCACTGACGAAATAAATATAACTGTTGAAAGAAATTGTTGATATAAAATCAAAAATTATAGTCATATAAATATTACATGAATTAATTGAAAATTATTCACTCCANNNNNNNNNNNNNNNNNNNNNNNNNNNNNNNNNNNNNNNNNNNNNNNNNNNNNNNNNNNNNNNNNNNNNNNNNNNNNNNNNNNNNNNNNNNNNNNNTTTATCATGAAAATTGTTATGTATCTAATAATTATTTTATCAATAAATTTTATTTTTATAAAACATCCTCTATCAATAGGATTAATTTTAATAATACAAACAATACTATCTTGCTTAATTTGTAGATTTTATTTAAGTTGTTACTTATTTTCTTATATCTTATATCTTATTTTTATTGGTGGTATGTTAATTTTATTTATATATATATCAAGAATTGCATCAAATGAAAAATTTATTTATTCTATTAAANNNNNNNNNNNNNNNNNNNNNNNNNNNNNNNNNNNNNNNNNNNNNNNNNNNNNNNNTTTAAAAGCATTAAATATTATAATAAATATTATAACATATACAAACCACAACAATTTTATGATAAGAAAAATGTATATTATTCCTTCTGGAATAATAACACTAATGTTAACAATTTACTTATTATTTGTTTTAATTATTGTAATTAATATTTTAACAACAAATATGTTAACTCTTCGAAGAAGAACTTCTTTCTAACTACACCTTCCGGTACAGTTACTTTGTTACGACTTATCTCATTAATATCATGAGAGTGACGGGCGATATGTACATAAATTAGAGCCAATTTCAATTAATTAAATTTAATTAATTTATTATCAAATCCAATTTCATATTTTCTNNAATTTAGAAAATAATCCAATACATAATTAATTGTAACCCATTTTTTCTTTAATATAAACTGCACCTTGACCTGACATTCTAAATACNATAATATAATATGAAAATATTTCTTATAAAACATTCTTGACAGAGATATACAAATTAAACTAAAGTTTTTTCTATCGTGGATTATCAATTATAAAACAGGTTCCTCTGATAAGATAAATTACCGCCAAATTCTTTGAATTTAAAGATCATTTCTAATAATAATCAAGTTATTTTTATCACATTTTTAATAATAGGGTATCTAATCCTAGTTTATACAAAAATTTTTCAGACATAAAATTAATTTTTAAAGATAAAATATATTTCACCAAAATTATAGATATTTTATATTATTGTTATATTAACTGAAACTTAAATATTTAAATTAAAGAAATCGTATAACCGCAACTGCTGGCACGAAATTTGATTCTTCTAAATAAAATTACTAATTCTAATTTTATTAATTAATAATAGTAAATACTGCGCATTTATTCAAANNNNNNNNNNNNNNNNNNNNNNNNNNNNNNNNNNNNNNNNNNNNNNNNNNNNNNNNNNNNNNNNNNNNNNNNNNNNNNNNNNNNNNNNNNATAATAAAATTATTTAATGAGGTCCTTTCGTACTAACATTAAAAATAACTAAGTAGATAGAAACCAACCTGGCTCACGCCGGTCTGAACTCAGATCATGTAAGAATATTAAGGGTCGAACAGACCCAGGAATAATAAATTTTGCTCCAATCCCTATTCTTAATCCAACATCGAGGTCGCAATCATATTTATCGATATGAACTCTCCAAATTAATTACGCTGTTATCCCTAAGGTAATTTATTCTTATAATCAAAAATTTGGATCAATATTAACATAAATTTATGAAATTTTTATTAAAAGTTAATTATATTTTAATATCACCCCAACAAAAAAATCAATTAAATAAAAATAATTAATTAACTATAAANAAATAAACTTAATNAAGATTTTAAAAATTCTATAGGGTCTTCTCGTCCCACTCATTCATTTAAGCTTTTTTACTTAAAAATCAATTTCAATTATTAATATTAATTAAGTTAATTTCTCATTCAATCATTCATTCAAGCCTCCAATTAAAAGACTATTTATTATGCTACCTTTGCACGGTCAATTTACCGCGGCCATTTAATTTTTAATCATTGGGCAGATTAGACTTTTAATTCCTTCTAAAAGACATGTTTTTGATAAACANNNNNNNNNNNNNNNNNNNNNNNNNNNNNNNNNNNNNNNNNNCCAAAAAACCAGATATCATATAATTTGGTAAAATGTCATTTCCAGAATAAATTTATTAATATATATACAATAATAACTAACAAATNTATACTAAATCATTATATTTCGGGAAAAATAAATATTTAAAAGTATTCAATTAACCCTGATACAAAAGGTACAAATTATATTTTACTTAAAATTATTTATAAAGCNNTTTTCCTCTCAGTTAATCAAAATTATTTTTTTATATAAAATTACTTTAACACAAAAATTTTTTTTTCAAAATATAAAAAACAACTATATANTAAAATTATATTATGGGATATATTTAATTATAATAATTGAATTGCAATCAATAGGTGTTAACTATAACTATCCTTAAGTAATGAAGTAAAATAATACATTTAGTTTCGACCTAAAATAAGAATTAGAATTCCTTGCTTTTAAATGAAGCCAAAATAGAGGCACTTTATTGTTAATAAAATAATTGAAANTTTATTTCCATTTAAAGAAAAATTAGTTTAATTAAAATATAAATCTGTCAGGTTTATGAAACTTTTGAGTATTTTTCTGACTTTTCATGATNTATTCTACTTTAAATTTGCAATTTAATATTATAAATTAAATATAAGACTTTATGTTAACAGAATTAAACTGTCTATTAATATCAAAAATTAATGTTCTTCATAAACTATAACATTTATAAAGTTTATAAAACATTTCATTTTCATTGAAAAAAGAGACTTAGTCTTATAAAATTCTATTAGTATATAGGTATATTTAACTTCCAATTAAAAGGATTAATTTTATTAAATAGAATATATCCAATAAATTTNNTTTTATCTTTAAAATCACAATTTAATGTTTTTATTAAACTAATTGAATTACTATTTGTAATAAATATTACATTATTAAATTCTAAATTTAAAGCATTAATTTTGCTAAAATAGTTCTAATATGGCAGATTAGTGCAATGAATTTAAGATTCATATATAAAATANNTTTTTTTTATTGGAAAATAAGATGCCTGAGGCAAAAAGGATTATTTTGATAGAATAAATTATGTAATAAAATTACTCNNNCATTAAGTGGCTGAAAGTAAGTAATGGTCTCTTAAACCAATTAATAGTAATTAACAAATACTCTTAATGAAAGATAAGCTAAAATTAAAGCTAATGGGTTCATACCTCATTTATGGAAANTTTTCCTCTTTTTATTTAAAAACAAAAGTTACTTTAATATCTTCAATATTATGCTCTTAATTAAGCTATTTAAATTCAGAAAAGATGNNATGATCATTTTAAACTTCCAAAGTTTATATTTTATATTTAAATTATTTTCTGAGTTAATTAGCTTAGACAAAAGCATTTATTTTGAAAGTAAAAGAAAAGATATTTAATCTATTAACTTAGGAAACTAGAAATAAAATTAGCTTCTAACTAACTTTTAAAGCGGTTAAATTCCGTTTTTTCCTTGTTTTAATAGTTTAATNNNAAAAACTTAGATCTTGTAAATCTAGATTAACGTAAGTTTTAAAAATAGGTTTTAAGTTATTAANAACTATTATCCTTCAAAGTTAAAAATATAATTANATTTATTATTAGGAGAAATAATTTTTCATAAATAAATTTACAGTTTATTGCCTATTCTCGGCCATCCCAATCAAGTTATATTGATTGAACAATACTCTCATTAGTGTAAATAAAGTGCTATCTTAGCTTTAACTTG

>longula_K.longula1997

NNNNNNNNNNNNNNNNNNNNNNNNNNNNNNNNNNNNNNNNNNNNNNNNNNNNNNNNNNNNNNNNNNNNNNNNNNNNNNNNNNNNNNNNNNNNNNNNNNNNNNNNNNNNNNNNNNNNNNNNNNNNNNNNNNNNNNNNNNNNNNNNNNNNNNNNNNNNNNNNNNNNNNNNNNNNNNNNNNNNNNNNNNNNNNNNNNNNNNNNNNNNNNNNNNNNNNNNNNNNNNNNNNNNNNNNNNNNNNNNTTGGGATTACTCCCGTATATTTTTACTAGTCCAAGTCATTTAGTATTTACAATAAGATTAGCATTACCATTATGATTATCATTCATACTTTATGGATTTATTAATAATATAAATCATATATTTTGTCATTTAGTTCCATCTGGAACTCCTAATATTTTAATGCCTTTTATAGTTCTTATTGAAAGAGTCAGAAATTTAATTCGTCCTGGCTCCTTGGCTGTCCGACTGACAGCTAACATAATTGCTGGACATCTTTTAATAACCTTATTAGGTAATTTACCTATAAGGTATGAATTATATTCAGGTATTATTATTGTTTTTCAAGTTATATTAATATTATTTGAATTAGCTGTGTGTATTATTCAATCATATGTATTTATAGTTCTTAGAACTTTATATTATAGAGAAGTAAATNNNNNNNNNNNNNNNNNNNNNNNNNNNNNNNNNNNNNNNNNNNNNNNNNNNNNNNNNNNNNNNNNNNNNNNNNNNNNNNNNNNNNNNNNNNNNNNNNNNNNNNNNNNNNNNNNNNNNNNNNNNNNNNNNNNNNNNNNNNNNNNNTTTAAGCCAACACGACAAAATAATCCTTTAATTAAAATTATTAATAATTCATTAATTGACTTCCCAGCACCATCTAATTTATCTTATTGATGAAATTTTGGATTTGTATTAGGATTATGCTTAACAATTCAAATTTTAACTGGATTATTTTTATCAATACATTACAATGCCAATATCGTAAATGCTTTTGAAAGATTAAGACATATTTGCCGTGATGTAAACTATGGTTGAATCTTACGTGTTATTCACGCTAATGGAGCTTCATTGTTTTTCATTTGTGTATATTTACATGTAGGACGTGGATTATATTATGGATCATTCAAATATATTGAAACATGATCTATTGGTGTAATTATATTATTAATATTAATAGCTACTGCTTTCTTAGGATATGTTTTACCGTGAGGGCAAATATCTTTTTGAGGAGCCACAGTCATTACAAATCTATTATCAGCAATTCCTTATTTAGGTGGAATGTTAGTTAATTGAATTTGAGGGGGATTTGCAGTTGACAATGCAACACTAACTCGATTTTACTCTTTTCATTTTATTTTACCATTCATTGTATTAAGACTAACAATTATTCACCTATTATATTTACATACAACAGGTTCGAATAATCCATTAGGAATTAACTCCAATAATGACAAAGTCCCATTTCACCCATACTTCTCCATTAAGGATATTATAAGCCTATTTTTATTAATAATTATTTTCTTTATATTAGTCATGTTAGAACCCTATATACTAGGGGATCCAGATAATTTTATCCCTGCCAATCCACTTGTAACACCAAAACATATTCAACCTGAATGATACTTTTTATTTGCCTACGCAATCCTCCGTTCAATTCCTAATAAACTAGGAGGAGTGATTGCTCTTTTTATATCAATTTTTATTTTAATGTTTGTACCTTTATTAAATAATTCTAATTTTATAGGATTAAATAATTACCCAATTAATCAAATTATATTTTGATATATAGTAATAATTTTAATTTTATTAACTTGAATTGGAGCACGACCTGTCGAATTACCTTATATTAACTTTGGAATATTTTTAACACTTATATATTTCTCTTACTTCATTATTGACCCAATAATTAAATCTATTTGAGATAAATTAATTAGATAGATGAAAAAATGATTCTTCTCAACTAATCATAAAGATATTGGAACAATATATTTCATTTTTGGTATTTGATCAGGTATAATTGGTACAACTTTAAGAGTTTTAATTCGTGTAGAACTTGGAACTCCAGGTTCATTTATTGGTGATGATCAAATTTATAATGTAATTGTCACTGCTCATGCTTTCATTATAATTTTTTTTATGGTAATACCAATTATAATTGGTGGATTTGGAAATTGATTAGTCCCCTTAATAATTGGAGCCCCTGATATAGCTTTTCCTCGTATAAATAATATGAGTTTTTGATTATTACCACCTTCTCTAATTCTTATTTTAGTGGGAAGAATAGTTGATAGAGGTGCAGGTACAGGTTGAACAGTTTACCCTCCCTTATCTGCAGGTATTGCACATTCTGGTTCATGTGTTGATCTAACTATTTTTTCTCTACACCTTGCAGGTGTATCTTCAATTTTAGGTGCTGTAAATTTCATTAGAACAATTTTTAATATACGTTCAATGGGAATTTGATTGGATCGTATACCTTTATTTGTATGAGCAGTTTTAATTACTGCATTTTTATTATTATTGTCACTACCCGTTTTAGCAGGCGCTATCACAATATTGTTAACAGATCGAAATTTAAATACTTCATTTTTTGATCCTGCAGGAGGGGGGGATCCTATTCTTTATCAACATTTATTTTGATTTTTTGGTCATCCTGAAGTCTACATTTTAATTTTACCAGGATTTGGCTTAATTTCTCATATTATTACTCAAGAGAGAGGTAAAATTGAATCTTTTGGCTCATTAGGAATAATTTATGCCATAATATCAATTGGTATTTTAGGATTTGTTGTATGAGCACATCATATATTCACTGTAGGAATAGATGTTGATACACGTGCATATTTTACATCAGCTACTATAATTATTGCAGTTCCCACTGGAATTAAAGTATTTAGTTGACTTGCAACTTTGAGAGGTATAAAAATTAATATTACATCTTCAGCCCTATGAGCATTGGGGTTTGTCTTTTTATTTACTATTGGGGGTTTAACTGGAGTTATTTTAGCCAATTCTTCAATTGATATTATATTACATGATACATATTATGTAGTTGCACATTTCCATTATGTTTTATCAATAGGAGCAGTATTTGCTATTATAGCAAGATTTATCCATTGGTTTCCATTATTTACAGGATTAAGATTAAACTCAAATTGATTAAAAATTCATTTTCTATTAATATTTATTGGTGTAAATATAACATTCTTTCCTCAACATTTTTTAGGATTAAGAGGAATACCTCGTCGTTATTCAGATTATCCTGATGCTTATATATCATGAAACATAATTTCATCAATAGGAAGAATTATATCTTTAATTGGAATTTTATTTTTATTATTTATTGTCTGGGAAAGCTTTATTTCAATACGATTAGTATTATATTCTAATAGAATTCAATCTTCTATTGAATGAATACAAAAATTCCCCCCGTCTGAACATTCATATAATGAAATGCCATTGTTAATTCAAATTTCAAATTGATCTTATATTAATATACAGGATGCTGTATCACCATTAATAGAACAATTAATATTTTTTCATGATCATGTGTTGGTAATTTTAATTATAATTACAATTGTTGTTGCTTACATAATAGTAATGTTAATATTAAATAAAATTATTAATCGCTTACTTCTTGAAGGACAGTTAATTGAATTTATTTGAACTTTATTGCCTGCAATAACTTTAATTTTTATTGCATTACCATCATTACGATTATTATATATATTAGACGAAATTAATAATCCATTATTAACATTAAAAATTATTGGTCACCAATGATATTGATCATATGAATATTCAGATTTTTCTGATGTAGAATTTGATTCATATATAAAATCTATAAACGAGATAAATAAAAACGAATTTCGTTTATTAGATGTAGATAATCGAGTAATTTTACCATTTAATATTCAAGTCCGACTATTAGTTTCTTCTTTTGATGTTATTCATTCCTGAGCAATACCATCAATAAGACTTAAAGTTGATGCAGTGCCAGGACGATTGAATCAAATAAGAATATTAATTAGTCGTCCAGGTGTATCTTATGGACAATGCTCTGAAATTTGTGGAGCAAATCATAGATTTATGCCTATCGTAATTGAAAGAATTAGAATAAAAATATTTATTAGATGATTAATTAATTATATGAATAATCACCCTTATCATATAGTTGACTATAGACCCTGACCTTTAACTGGATCAATTGGAGCATTAACTTTTGTTTCCGGTATAGTTATAATATTTCATAAATGTAATTTTATATTATTATATGTTGGTATTTTATTATTATTAATAACAATAATTCAATGATGACGTGACATTTCCCGAGAAGGAACATTTTTAGGAATACACACAATAATAGTAGTGAATGGTTTAAAAATAGGCATATTATTATTTATTGTATCAGAAATTCTTTTTTTTGTATCATTTTTTTGAGCATTTTTTCATAGTAGATTAAGACCTGTAGTAGAAATTGGTATAATTTGACCTCCTAGAGGTATTTATGTTTTTAATCCGACTCAAGTTCCATTATTAAATACAATAATTTTATTATGTTCAGGAATTACAATTACTTGAGCACATCATTCAATCATAAATGGTAATCACATTACTTCTATCTATAGAATTATATTAACTGTTATTTTAGGTGTATACTTCACTATTCTGCAAGGTTATGAATATTATGAAGCTCCATTTGCAATTAATGATTCTATCTATGGATCTTCTTTTTTTATAGCTACTGGATTCCATGGTATTCACGTAATTATTGGAACAATATTTATTACTGTATGCTTATTACGACAAATAAAGTTTCATTTTTCAAAAAATCATCATTTTGGTTTCGAAGCTGCTGCTTGATATTGACATTTTGTTGATGTAGTCTGATTATTTTTATATTTATCAATTTATTGATGAGGAGGATAATAAATTATAGTTTTTAAAAAAAAAAAAAAAACAAATAAATTCAGAGATACCGGTAAATAAGATTTTCAAGCTAAATATATTAATTTGTCATAACGATAACGAGGAAGAGTACCTCGAATCCAAATGAAAAGAAAACAAATTATAGAAACTTGAATTGGAAAAACAATTGAATTAATTTTTCCTCCTAAAAACATTAAACAAAATAATATTCTTATAAATAAAATACTTGAATACTCAGCCAAAAAAATAAATGCAAATCCAGAACCTCTATATTCAACATTAAAACCAGAAACTAACTCTGATTCTCCCTCAGAAAAATCAAATGGAGATCGATTAGTCTCAGCTAAAGCTGAAGATAATCAACATATTCTTAAAGGAAGAAATAAAAAAATAAATCACACAAATTCCTGAAATATAAACAAATCAATAATATTATAACTTCTTATTATTATTATAGGACACAATATAATTAAAACCAATCTTACCTCATAAGAAATTGATTGAGCAATTGATCGAATGCATCCCAACATTGCATAACTTGAATTAGAAGATCAACCTGTTAATATTAAGGAATAAACTCTTATTCTTGAGCAACAAAAAAAAAATAAAATACCAAAATTAAAAGAAACACAATTAATAAGATATGGATACAATGATCAAATCAACAGACTATTGAACAATCCTATAATAGGCCTAAAAACATAAATTAAATAATTAGATATTAATGGAATTGTATTTTCCTTTATAAATAATTTAATAGCGTCAGAAATAGGCTGCAAAATACCCAAAAATCCAACTTTATTTGGGCCCTTACGGATTTGAATGTATCTTAAAACTTTACGTTCCAAAAGAGTAATAAATGCCACCCCAATCAAAATAAATAATACAGTAACTAAAATCGTAATTAAATACNNNNNNNNNNNNNNNNNNNNNNNNNNNNNNNNNNNNNNNNNNNNNNNNNNNNNNNNNNNNNNNNNNNNNNNNNNNNNNNNNNNNNNNNNNNNNNNNNNNNNNNNNNNNNNNNNNNNNNNNNNNNNNNNNNNNNNNNNNNNNNNNNNNNNNNNNNNNNNNNNNNNNNNNNNNNNNNNNNNNNNNNNNNNNNNNNNNNNNNNNNNNNNNNNNNNNNNNNNNNNNNNNNNNNNNNNNNNNNNNNNNNNNNNNNNNNNNNNNNNNNNNNNNNNNNNNNNNNNNNNNNNNNNNNNNNNNNNNNNNNNNNNNNNNNNNNNNNNNNNNNNNNNNNNNNNNNNNNNNNNNNNNNNNNNNNNNNNNNNNNNNNNNNNNNNNNNNNNNNNNNNNNNNNNNNNNNNNNNNNNNNNNNNNNNNNNNNNNNNNNNNNNNNNNNNNNNNNNNNNNNNNNNNNNNNNNNNNNNNNNNNNNNNNNNNNNNNNNNNNNNNNNNNNNNNNNNNNNNNNNNNNNNNNNNNNNNNNNNNNNNNNNNNNNNNNNNNNNNNNNNNNNNNNNNNNNNNNNNNNNNNNNNNNNNNNNNNNNNNNNNNNNNNNNNNNNNNNNNNNNNNNNNNNNNNNNNNNNNNNNNNNNNNNNNNNNNNNNNNNNNNNNNNNNNNNNNNNNNNNNNNNNNNNNNNNNNNNNNNNNNNNNNNNNNNNNNNNNNNNNNNNNNNNNNNNNNNNNNNNNNNNNNNNNNNNNNNNNNNNNNNNNNNNNNNNNNNNNNNNNNNNNNNNNNNNNNNNNNNNNNNNNNNNNNNNNNNNNNNNNNNNNNNNNNNNNNNNNNNNNNNNNNNNNNNNNNNNNNNNNNNNNNNNNNNNNNNNNNNNNNNNNNNNNNNNNNNNNNNNNNNTCATAAGAGTTTCGTATACTTATTATATTGCTGGATTATTTTCATTATTTGGAATAATTTTTTTATCATTAATTACATTAAATATTTGTTAGNNNNNNNNNNNNNNNNNNNNNNNNNNNNNNNNNNNNNNNNNNNNNNNNNNNNNNNNNNNNNNNNNNNNNNNNNNNNNNNNNNNNNNNNNNNNNNNNNNNNNNNNNTCATCACCATTTGAGTGTGGATTTAGGCCATTTGAATCATCTCGTATTCCATTTTCGAGACACTTTTTCTTAATTGCAGTAATCTTTTTAATTTTTGATGTTGAATTGGTAATTATTATACCTGTAATTTTAGTAATAATTAGATTAAAAACTATTGATATTTATGTTATTATATTAATCTTCTTAATTATTNTGACTTTAGGATTATATCATGAATGATATAATAATATATTAAATTGANNNNNNNGTCTAATTAAAATATTTAATTTCAAAATAATTAAATTTAATGGAATTCAATGTATTATAATCAAAAAAANNNNNNNNNNNNNNNNNNNNNNNNNNNANAATAAACTGGAAAAAAATATACCATGTTGAGTAAATGAAAATATATATAATCTATAACAAGCAGATAAAAATGAAGATAAAATCAATAAAACCATTATTAACATATTTCAAGATATTAATCTATTAATAATTATAATCTCACCAGCTAAATTAATTGTAGGAGGACAAGATATATTTCTTGCTGACATTAAAAATCATAATAAAGATATTGAAGGCATAAATGTCAATATACCCTTATTAATTAATAATCTTCGACTATTAGTTCGCTCATACAATATGTTTGCTAAACAAAATAATCCAGAAGAACATAAACCATGACCAATCATTATATAATATGACCCACATATTCCCCAATTTCTTATAGTTATAATTCCTCTAATCACAAGACCTATATGAGCAACAGATGAATAAGCAATTATTGATTTAATGTCAATTTGAATTATACATAGAATTCTAATTAAAATACTTCCAATTATTGAAATTGAGATCCATATAAACCCAAATTTGTAAAAAAAAGAAGGAATAATGAACATTATACGAATTAAACCATATCCACCTAATTTTAACAAAATTCTAGCTAAAATTATTGAACCGGAAACAGGAGCTTCAACATGAGCTTTTGGTAATCAAAAATGAAAAAATAATGGTATTTTAATTAAAAAAGCTATTATTATACCAAAATAAATATAAATATTAACAGAACAACTTATTAACATAAAAATTCTAGTTATATTATTTTTATAAAGAAAAAAAATTCTTATCAATATTGGTAATGATGCAAATAATGTATAAAATAATAAATAATATCTTGCATCAATANNNNNNNNNNNNNNNNNNNNNNNNNNNNNNNNNNNNNNNNNNNNNNNNNNNNNNNNNNNNNNNNNNNNNNNNNNNNNNNNNNNNNNNNNNNNNNNNNNNNNNNNNNNNNNNNNNNNNNNNNNNNNNNNNNNNNNNNNNNNNNNNNNNNNNNNNNNNNNNNNNNNNNNNNNNNNNNNNNNNNNNNNNNNNNNNNNNNNNNNNNNNNNNNNNNNNNNNNNNNNNNNNNNNNNNNNNNNNNNNNNNNNNNNNNNNNNNNNNNNNNNNNNNNNNNNNNNNNNNNNNNNNNNNNNNNNNNNNNNNNNNNNNNNNNNNNNNNNNNNNNNNNNNNNNNNNNNTAATAGGGTTAATATAAAGCAAAAAAAAATTAGTTTTATTGATATAGATATTAATTTATCATTATTATGAGATCGAATTAATATAACTAAACATGATAAACCTATTACTCCCTCACAAACTCTAAAAACCAAGAAAATTAATAAAATATGTAAATCATATATTTTTATGGAAAAAATAAATGATAAAGAACAAAATATTCTTAAAATAATTAATTCCAATCTTAATGCTGTTATTAAATGTTTTCGATTTATACATAATGAAATTATACCTGAAAAGAATATAGAGGTAAAATATATTAATAATGTTACATTCTATAAATATAAAACAATAATTCAAATAATGAATGTAATTATTTGAATCTTAAAATTATTATTCATCAATTTATAATTTAATGACATTATTATTTTTAAAGTTTTCAAAATCCCTTGGGGCCCAAGGTTTTCACCTCATCCCATATCAATTGATTTTTGAAATATGAATGATTTACTTATCAATAACATTTGATTATGAAACGTTGACAATTGCTTTATAAACCACATTGAACCAAAAAACTCATAAACAATTTTTATTTTAGTTTTAGAAAAAATTGATATTTCATAACCCAACCAGATTCCTAATATTGAAAAAATCAATGCCAATAATTTGCCCTCTAAAGGCATTAAAATTATAATTGGATCATTAAATATTAATCATCTTAATATAGATCCCGAAAAAATTGAATAAATAGATAAGATAATAATTCTTTTAATCATGTTATTTATACTTTCATTCAATGATCTTAACTTATAGAAATTATAATTTATATTTATTGAAAAAAATGTCAAACGGGCAGAATAAAAGGAAGTTAAACCAATACCAATATATATAAATATTATAATTAATAAATTAATGTGCCTAGAACACATAGTTTCTATAATTAAATCTTTTGAATAAAATCCACTCAAAAATNNNNNNNNNNNNNNNNNTATATTAGCAATATTCATTATTGTAATAGTTAATGGTATTTGAAATCTTAAACATCCTATTAAACGAATATCTTGATTATTATTAAAATTGTGAATCAAAATTCCTGCACACAAAAATAAAGTAGCTTTAAATAAAGCATGAACAACTAAGTGAAAAAATGATAACATTGNNNNNNNNNNNNNNNNNNNNNNNNNNNNNNNNNNNNNNNNNNNNNNNNNNNNNNNNNNNNNNNNNNNNNNNNNNNNNNNNNNNNNNNNNNNNNNNNNNNNNNNNNNNNNNNNNNNNNNNNNNNNNNNNNNNNNNNNNNNNNNNNNNNNNNNNNNNNNNNNNNNNNNNNNNNNNNNNNNNNNNNNNNNNNNNNNNNNNNNNNNNNNNNNNNNNNNNNNNNNNNNNNNNNNNNNNNNNNNNNNNNNNNNNNNNNNNNNNNNNNNNNNNNNNNNNNNNNNNNNNNNNNNNNNNNNNNNNNNNNNNNNNNNNNNNNNNNNNNNNNNNNNNATAATTTATATCTTTATTAATAAAAGATAAAAATTTCATGACCCAAAATTTAATATTCATGCAATAGCTATTAAAATAGCAACATCTCCCACACGATTCATTAATGCAGTTAATATACCCGCATTATTAGAATATAAATTTTGATAATAAATAACTAAGCAATAAGAAACTAATCCAAGTCCATCTCAACCAATAATAATTCTCATTATATTAGGTCTAATAATTAATAAAACTATAGATATAACAAACCCCATAACAATATAAATAAAACGATTAATGAATTTATCATTATTTATATATATTCCACTATATAAAACAACCACTGATGAAATAAATATAACTGTTGAAAGAAATTGTTGATATAAAATCAAAAATTATNNNNNNNNNNNNNNNNNNNNNNNNNNNNNNNNNNNNNNNNNNNNNNNNNNNNNNNNNNNNNNNNNNNNNNNNNNNNNNNNNNNNNNNNNNNNNNNNNNNNNNNNNNNNNNNNNNNNNNNNNNNNNNNNNNNNNNNNNNNNNNNNNNNTTGAAAATTGTTATATATTTAATAATNNNNNNNNCAATAAATTTTATTTTCATAAAACATCCCTTATCAATAGGATTAATTTTAATAATACAAACAATTCTATCTTGCTTAATTTGTAGACTTTACTTAAGTTGTTATTTATTTTCTTATATCTTATATCTTATTTTTATTGGTGGTATATTAATTCTATTTATATATATATCAAGAATTGCATCAAATGAAAAATTTATCTATTCTATTAAATTAATAATGCTTAATTTTTTAATTTTAACTTTAACTAATTTTATCAATATAATTGATTTAAAATCACTAAATATTAAAATGAATATTATAACATATATAAACCACAACAATTTTATAATAAGAAAGCTATATATTATCCCTTCTGGGATGATAACACTAATATTAACAATTTATTTACTATTTGTTTTAATTATTGTAATTAATATTTTAACAACAAATATATTAACTCTTCGAAGAAGAACTTCTTTCTAACTACACCTTCCGGTACAGTTACTTTGTTACGACTTATCTCATTAATATCATGAGAGTGACGGGCGATATGTACATAAATTAGAGCCAATTTCAATTAATTAAATTTAATTAATTTATTATCAAATCCAATTTCATATTTTCTNNAANCTAGAAAGTAATCCAATAAATAATTAATTGTAACCCATTTTTTCTTTAATATAAACTGCACCTTGACCTGACATTATAAATATNATAATATAATATGAAAATCTTTCTTATAAAACATTCTTGACAGAGATATACAAATTAAATTAAAGTTTTTTCTATCGTGGATTATCAATTATAAAACAGGTTCCTCTGATAAGATAAATTACCGCCAAATTCTTTGAATTTAAAGATCATTTCTAATAATAATCAAGTTATTTTTATCACATTTTTAATAATAGGGTATCTAATCCTAGTTTATACAAAAATTTTCCAGACATAAAATTAATTTTTAAATATAAAATATATTTCACCAAAATTATAGATATTTTATATTATTGTTATATTAACTGAAACTTAAATATTTAAATTAAAGAAATCNNNNNNNNNNNNNNNNNNNNNNNNNNNNNNNNNNNNNNNNNNNNNNNNNNNNNNNNNNNNNNNNNNNNNNNNNNNNNNNNNNNNNNNNNNNNNNNNNNNNNNNNNNNNNNNNNNNNNNNNNNNNNNNNNNNNNNNNNNNNNNNNNNNNNNNNNNNNNNNNNNNNNNNNNNNNNNNNNNNNNNNNNNNNNNNATAATAAAATTATTTAATGAGGTCCTTTCGTACTAACATTAAAAATAATTAAGTAGATAGAAACCAACCTGGCTCACGCCGGTTTGAACTCAGATCATGTAAGAATATTAAGGGTCGAACAGACCCAGGAATAATAAATTTTGCTCCAATCCCTATCCTTAATCCAACATCGAGGTCGCAATCATATTTATCGATATGAACTCTTCAAATTAATTACGCTGTTATCCCTAAGGTAATTTATTCTTATAATCAAAAATTTGGATCAATATTAACATAAATTTATGAAATTTTTATTAAAAGTTAATTATATTTTAATATCACCCCAACAAAAAAATCAATTAAATAAAATAAATTAATTANNNNNNNNNNNNNNNNNNNNNNNNNNNNNNNNNNNNNNNNNNNNNNNNNNNNNNNNNNNNNNNNNNNNNNNNNNNNNNNNNNNNNNNNNNNNNNNNNNNNNNNNNNNNNNNNNNNNNNNNNNNNNNNNNNNNNNNNNNNNNNNNNNNNNNNNNNNNNNNNNNNNNNNNNNNNNNNNNNNNNNNNNNNNNNNNNNNNNNNNNNCATTTAATTTTTAATCATTGGGCAGATTAGACTTTTAATTCTTTCTAAAAGACATGTTTTTGATAAACAGGTGAAAATTATTTTTGCCTAAATANNNNNNNNNNNNNNNNNNNNNNNNNNNNNNNNNNNNNNNNNNNNNNNNNNNNNNNNNNNNNNNNNNNNNNNNNNNNNNNNNNNNNNNNNNNNNNNNNNNNNNNNNNNNNNNNNNNNNNNNNNNNNNNNNNNNNNNNNNNNNNNNNNNNNNNNNNNNNNNNNNNNNNNNNNNNNNNNNNNNNNNNNNNNNNNNNNNNNNNNNNNNNNNNNNNNNNNNNNNNNNNNNNNNNNNNNNNNNNNNNNNNNNNNNNNNNNNNNNNNNNNNNNNNNNNNNNNNNNNNNNNNNNNNNNNNNNNNNNNNNNNNNNNNNNNNNNNGGGATATATTTAATTATAATAATTGAATTGCAGTCAATAGGTGTTAACTATAACTATCCTTAAGTAATGAAGTAAAATAATACATTTAGTTTCGACCTAAAATAAGAATTAGAATTCCTTGCTTTTAAATGAAGCCAAAATAGAGGCATTTTATTGTTAATAAAATAATTGAAANTTTATTTCCATTTAAANNNNNNNNNNNNNNNNNNNNNNNNNNNNNNNNNNNNNNNNNNNNNNNNNNNNNNNNNNNNNNGACTTTTTATGATNTNTTCTACTTTAAATTTGCAATTTAATATTATAAATTAAATATAAGACTNNNNNNNNNNNNNNNNNNNNNNNNNNNNNNNNNNNNNNNNNNNNNNNNNNNNNNNNNNNNNNNNTTTATAAAGTTTATAAAACATTTCATTTTCATTGAAAAGAGAGACTTGGTCTTATAAAATTCTATTAGTATATAAGTATATTTAACTTCCAATTAAAANNNNNNNNNNNNNNNNNNNNNNNNNNNNNNNNNNNNNNNNNNNNNNNNNNNNNNNNNNNNNNNNNNNNNNNNNNNNNNNNNNNNNAATTACTATTTGTAATAAATATTACATTATTAAATTCTAAATTTAAGGCATTATTTTTGCTAAAACAGTTCTAATATGGCAGATTAGTGCAATGAATTTAAGATTCATATATAAAATATNTTTTTTTTATTGGAANNNNNNNNNNNNNNNNNNNNNNNNNNNNNNNNNNNNNNNNNNNNNNNNNNNNNNNNNNNNNNNNNCATTAAGTGGCTGAAAGTAAGTAATGGTCTCTTAAACCAATTAANNNNNNNNNNNNNNNNNNNNNNNNNNNNNNNNNNNNNNNNNNNNNNNNNNNNNNNNNNNNNNNNNNNNNNNNNNNNNNNNNNNNNNNNNNTTTAAAAACAAAAGTTACTTTAATATCTTCAATATTATGCTCTTTATTAAGCTATTTAAATTCAGAAAAGATANNATGATCATTTTAAACTTCCAAAGTTTATATTTTATATTTAAATTATTTTCTGAGTTAATTAGCTTAAACAAAAGCATTTATTTTGAAAGTAAAAGAAAAGATATTTAATCTATTAACTTAGGAAACTAGAAATAAAATTAGCTTCTAACTAACTTTTAAAGCGGTTAAATTCCGTTTTTTCCTTGTTTTAATAGTTTAATNNNAAAAACTTAGATCTTGTAAGTCTAGATTAACGTAAGTTTTAAAAATAGGTTTTAAGTTATTGANAACTATTATCCTTCAAAGTTAAAAATATAATTANATTTATTATTAGGAGAAATAATTTTTCATAAATAAATTTACAATTTATTGCCTATTATCGGCCATCCTAANNNNGTTATATTGATTGAACAATATTCTCATTAGTGTAAATAAAGTGCTAATTTAGCTTTAACTTG

>tuta-clade1-northwestSI_05.NZ.MB.TFL.03

NNNNNNNNNNNNNNNNNNNNNNNNNNNNNNNNNNNNNNNNNNNNNNNNNNNNNNNNNNNNNNNNNNNNNNNNNNNNNNNNNNNNNNNNNNNNNNNNNNNNNNNNNNNNNNNNNNNNNNNNNNNNNNNNNNNNNNNNNNNNNNNNNNNNNNNNNNNNNNNNNNNNATTAATAAATTATAAATCATTAGGAAGATCATTAATATTTTTGTCATTATTTATATTTATTCTACTAAATAATTTATTGGGTTTACTTCCGTATATTTTCACTAGCTCAAGTCATCTAGTGTTCACAATAAGATTAGCATTACCACTATGATTATCATTCATACTTTATGGATTTATTAACAATATAAATCATATATTTTGTCATTTAGTTCCATCCGGAACTCCTAATATTTTAATACCTTTTATAGTTCTTATTGAAAGAGTCAGAAATCTAATTCGTCCTGGCTCCTTAGCTGTCCGACTAACAGCTAATATAATTGCTGGACATCTTTTGATAACCTTATTAGGTAACCTACCTATAAGGTATGAATTATACTCAGGTATTATTATTATTTTTCAAGTTATATTAATATTATTTGAATTAGCTGTTTGTGTTATTCAATCATATGTATTTATAGTTCTTAGAACTTTATATTATAGAGAAGTAAATATTCCTCAAATATCTCCTCTTAATTGATTATTATTTATTTATNNNNNNNNNNNNNNNNNNNNNNNNNNNNNNNNNNNNNNNNNNNNNNNNNNNNNNNNNNNNNNNNNNNNNNNNNNNNNNNNNNNNNNNNNNNNNNNNNNNNNNTTTAAGCCAACACGACAAAATCATCCTTTAATTAAAATTATTAATAATTCATTAATTGACTTCCCAGCACCATCTAATTTATCTTATTGATGAAATTTTGGATTTGTATTAGGATTATGCTTAACAATTCAAATTTTAACTGGATTATTTTTATCAATACATTATAATGCCAATATCATAAATGCTTTTGAAAGATTAAGACATATTTGCCGTGATGTAAACTATGGTTGAATTTTACGTGTTATTCACGCTAATGGGGCTTCATTATTTTTCATTTGTGTATATTTACATGTAGGACGTGGATTATATTATGGATCATTCAAATATATTGAAACATGATCTATTGGTGTAATTATATTATTAATATTAATAGCTACTGCTTTCTTAGGATATGTTTTACCGTGAGGTCAGATATCTTTTTGAGGAGCCACAGTCATTACAAATTTATTATCAGCAATTCCTTATTTAGGTGGAATATTAGTTAATTGAATTTGAGGAGGATTTGCAGTTGACAATGCAACACTAACTCGATTTTACTCTTTTCATTTTATTTTACCATTTATTGTATTAAGATTAACAATTATTCACCTATTATATTTACATACAACAGGTTCAAATAATCCATTAGGAATTAACTCCAACAATGACAAAGTCCCATTTCACCCATACTTCTCCGTTAAGGATATTATAAGCCTATTTTTATTAATAATTATTTTCTTTATATTAGTTATGTTAGAACCCTATATACTAGGGGATCCAGATAATTTTATTCCTGCCAATCCACTTGTGACACCAAAACATATTCAACCTGAATGATACTTTTTATTTGCCTACGCAATCCTCCGTTCAATTCCTAATAAACTAGGAGGAGTAATTGCTCTTTTTATATCAATTTTCATTTTAATGTTTGTACCTTTATTAAATAATTCTAATTTTATAGGATTAAATAATTACCCAATTAATCAAATTATATTTTGATATATAGTAATAATTTTAATTTTACTAACTTGAATTGGAGCACGACCTGTCGAATTACCTTATATCAACTTTGGAATATTTTTAACAATTATATATTTTTCCTACTTCATTATTGATCCAATAATTAAATCTACTTGAGATAAATTAATTAGATAGATGAAAAAATGATTCTTCTCAACTAATCATAAAGATATTGGAACAATATATTTCATTTTTGGTATTTGATCAGGTATAATTGGTACAACTTTAAGAGTTTTAATTCGTGTAGAACTTGGAACTCCTGGTTCATTTATTGGTGATGATCAGATTTATAATGTAATTGTCACTGCTCATGCTTTCATTATAATTTTTTTTATGGTAATACCAATTATAATTGGTGGATTTGGAAATTGATTAGTCCCTTTAATAATTGGAGCTCCTGATATAGCTTTTCCTCGTATAAATAATATGAGTTTTTGACTATTACCACCTTCCCTAATTCTTATTTTAGTGGGAAGAATAGTTGATAGAGGTGCAGGCACAGGTTGAACAGTTTACCCTCCTTTATCCGCAGGTATTGCACATTCTGGTTCATGTGTTGATTTAACTATTTTTTCTCTACACCTTGCAGGTGTATCTTCAATTTTAGGTGCTGTGAATCTCATTAGAACAATTTTTAATATACGTTCAATAGGAATTTGATTGGATCGCATACCTTTATTTGTATGGGCAGTTTTAATTACTGCATTTTTATTATTATTATCGTTACCCGTTTTAGCAGGCGCTATTACAATATTGTTAACAGATCGAAATTTAAATACTTCATTTTTTGATCCTGCAGGAGGAGGAGATCCTATTCTTTATCAGCATTTATTTTGATTTTTTGGTCATCCTGAAGTCTATATTTTAATTTTACCAGGATTTGGTTTAATTTCTCACATTATTACTCAAGAGAGAGGTAAAATTGAATCTTTTGGCTCATTAGGAATAATTTATGCCATAATATCAATTGGTATTTTAGGATTTGTTGTATGAGCACATCATATATTTACTGTAGGAATAGATGTTGATACACGTGCATATTTTACATCAGCTACCATAATTATTGCAGTTCCCACTGGAATTAAAGTATTTAGTTGACTTGCAACTTTGAGAGGTATAAAAATTAATATTTCATCTTCAGCCCTATGAGCGTTAGGGTTTGTCTTTTTATTTACTATTGGAGGTTTAACTGGAGTTATTCTAGCCAATTCTTCAATTGATATTATATTACATGATACATATTATGTAGTTGCACATTTCCATTATGTTTTATCAATAGGAGCAGTATTTGCTATTATAGCAAGATTTATTCATTGATTTCCATTATTTACAGGATTAAGATTAAACTCAAATTGATTAAAAATTCATTTTCTATTAATATTTATTGGTGTAAATATAACATTCTTTCCTCAACATTTTTTAGGATTAAGAGGAATACCTCGTCGTTATTCAGACTATCCTGATGCTTATATGTCATGAAATATAGTTTCATCAATAGGAAGAATTATATCTTTAATTGGAATTTTATTTTTATTATTTATTGTTTGGGAAAGCTTTATTTCAATACGATTAGTATTATATTCTAATAGAATTCAATCTTCTATTGAATGAATACAAAAGTTTCCCCCATCTGAACATTCATATAATGAAATGCCATTGTTAATTCAAATTTCAAATTGATCTTATATTAATATACAGGATGCTGTATCACCATTAATAGAACAATTAATATTTTTTCATGATCATGTATTAGTAATTTTAATTATAATTACAATTGTTGTTGCTTATATAATAGTTATATTAATATTAAATAAAATCATTAATCGCTTACTTCTTGAAGGACAATTAATTGAATTTATTTGAACTCTATTACCTGCAATAACTTTAATTTTTATTGCGTTACCATCATTACGATTATTATATATATTAGACGAAATTAATAATCCATTATTAACATTAAAAATTATTGGTCACCAATGATATTGATCATATGAATATTCAGATTTTTCTGATGTAGAATTTGATTCGTATATAAAATCTATAAACGAAATAAATAAAAACGAATTTCGTTTACTAGATGTAGATAATCGAGTAATTCTACCATTTAATATTCAAGTCCGACTATTAGTTTCTTCTTTTGATGTTATTCATTCTTGAGCAATACCATCAATAAGACTTAAAGTTGATGCAGTACCAGGACGATTAAATCAAATAAGAATATTAATTAGTCGTCCCGGCGTATCTTATGGACAATGTTCTGAAATTTGTGGAGCAAATCATAGATTTATGCCTATCGTAATTGAAAGAATTAGAATAAAAATATTTATTAAATGATTAATTAATTATATGAATAATCACCCTTATCATATAGTTGACTATAGACCCTGACCTTTAACTGGATCAATTGGAGCATTAACCTTTGTTTCCGGTATAGTTATAATATTCCATAAATGTAATTTTATATTATTATATATTGGTATTTTATTATTATTAATAACAATAATTCAATGATGACGTGACATTTCCCGAGAAGGAACGTTTTTAGGAATACACACAATAATAGTAGTGAGTGGTTTGAAAATAGGCATATTATTATTTATTGTATCAGAAATTCTTTTTTTTGTATCATTTTTTTGAGGATTTTTTCATAGTAGATTAAGACCTGTAGTAGAAATTGGTATAATTTGACCTCCTAGAGGTATTTATGTTTTTAATCCAACTCAAGTTCCATTATTAAATACAATAATTTTATTATGTTCAGGAATTACAATTACTTGAGCACATCATTCAATTATAAATGGTAACCACATTAATTCTGTTTATAGAATTATGTTAACTGTTATTTTAGGTATATATTTCACTATTCTGCAAGGCTATGAATATTATGAAGCCCCATTTGCAATTAATGATTCTATCTATGGATCTTCTTTTTTTATAGCTACTGGATTTCATGGAATTCACGTAATTATTGGAACAAGATTTATTACTGTATGCTTATTACGACAAATAAAGTTTCATTTCTCAATAAATCATCATTTTGGCTTCGAAGCTGCTGCTTGATATTGACATTTTGTTGATGTAGTCTGATTATTTTTATATTTATCAATTTATTGATGAGGAAGATAATAAATTATAGTTTTTAAAAAGAAAAAAAAAATAAATAAATTCAGAGATACCGGTAAATAAGATTTTCAAGCCAAATATATTAATTTGTCATAACGATAACGAGGAAGGGTGCCCCGAATCCAAATAAAAAGAAAACAAATTATAGAAACTTGAATTGGAAAAATAATTGAATTAATTTTACCTCCTAAAAACATTAAACAAAATAATATTCTCATAAATAAAATACTTGAATACTCAGCCAAAAAAATAAATGCAAACCCAGAACCTCTATATTCAACATTAAAGCCAGAAACTAACTCTGATTCTCCTTCAGAAAAATCAAATGGAGATCGATTAGTCTCAGCTAAAGCCGAAGATAATCAACATATTCTCAAAGGAAGAAACAAAAAAATAAATCACACAAATTCCTGAAATATAAACAAATCAATAATATTATAACTTCTTATTATTATTATAGGACATAATATAATTAAAACCAATCTTACCTCATAAGAAATAGATTGAGCAATTGATCGAATGCATCCTAATATTGCATAACTTGAATTAGAAGATCAACCTGTCAATATTAATGAATAAACTCTTATTCTTGAACAACAAAAAAAAAATAAAATACCAAAATTAAAAGAAACACAATTAATGAAATATGGATACAATGATCAAATCAACAAACTATTGAATAATCCTATAATAGGTCTAAAAATATAAATTAAATAATTAGATATTAATGGAATTGTATTTTCCTTTATAAATAATTTAATAGCATCAGAAATAGGCTGCAAAATACCTAAAAATCCAACTTTATTTGGGCCTTTACGAATTTGAATATATCTTAAAACCTTACGCTCCAAAAGAGTAATAAATGCCACTCCAATTAAAATAAATAATACAGTAATTAAAATTGTAATTAAATACAAAAACAATTCTTCTTATATACTTTATATAATTTTCTTAATTTTAGGAATTATAATTTCAATTTCTTCAAACAATTGGCTGGGTTGTTGAATGGGAATTGAAATAAATATAGTTTCATTTTTGCCTATAATGGCAAACAAAATAAGAATTTATGCTTCGGAATCAATAATTAAATATTTTATCATTCAAAGAATAGGATCGAGTTTATTATTAATAACTATTATTATTNNNAATATAATANNNNNNNNNNNNNNNNNNNNNNNNNNNNNNNNAATAATTAAAATTGGCTGTCCTCCTTTCCATTCTTGATATGTTTCTGTTATTGAAGGTTTGACTTGAATAGTATGTTTTATTTTAATAACTATTCAAAAAATTATTCCTTTGATTATATTATCATATTTAAATGTTAATATAAGACTATTTATTATTATATCATGCATTTGAGGATGTATTGGAGGGCTGGGCTATTCTTCAATACGTAAAATCATTGCTTATTCATCAATTTATAATTTAAGATGAATTTTTAGAGGTATTATTATTATTAATTACTCATGGCTAATTTATTATTTTATTTATTCATTTACATTATTAGCTGTATGTTATATATTTAAAATATTTAATATTNNNNNNNNNNNNNNNNNNNNNNTAGTATCTTATAATTTTATAAAATCAATCATAATAATGTGCATTTTTATATCTATGGGGGGATTACCCCCCTTTTTAGGGTTTTTCCCTAAATTAATTATAATTTACTGCTTATTGTTAAATAACATAATACTTATTTGTATTATGTTATTAATAACAGCTCTGATTATTTTATTTTTTTATTTACGAATTTTAATTACAACATTAATAATAAATACAATTTCAATAAAAAGAATTGTCATAAGAGTTTCATATACTTATTATATTGTTGGAGTATTTTCATTATTTGGAATAATCTTTTTATCATTAATTACATTAAATATTTGTTAGATTTATAATATTTTGATTTATTCNNTTATTTTAATATTTTTACTAATATTTTTATTATCATTATTAATATATGTTTCCCATAAATCTATAAAAGATCGAGAAAAATCATCACCATTTGAGTGTGGATTTAGACCATTTGAATCATCTCGTATTCCATTTTCGAGACACTTTTTCTTAATTGCAGTAATTTTTTTAATTTTTGATGTTGAATTGGTAATTATTATACCTGTAATTTTAGTAATAATTAGATTAAAAACTATTGATATTTATATTATTATATTAATCTTCTTAATTATTTTGACCTTAGGATTATATCATGAATGATATAATAATATATTAAATTGANNNNNNNGTGTANNNNNNNNNNNNNNNNNNNNNNNNNNNNNNNNNNNNNNNNNNNNNNNNNNNNNNNNNNNNNNNNNNNNNNNNNNNNNNNNNNNNNNNNNNNNNNNNNNNNNNNNNNNNNNNNNNNNNNNNNNNNNNNNNNNNNNNNNNNNNNNNNNNNNNNNNNNNNNNNNNNNNNNNNNNNNNNNNNNNNNNNNNNNNNNNNNNNNNNNNNNNNNNNNNNNNNNNNNNATAATTATAANNNNNNNNNNNNNNNNNNNNNNNNNNNNNNNNNNNNNNNNNNNNNNNNNNNNNNNNNNNNNNNNNNNNNATTTTGAANNNNNNNNNNNNNNNNNNNNNNNATTCATTGATAATATTNNAGTATTAGTAAACTCATAAANNNNNNNNNNNNNNNNNNNNNNNNNNNNNNNNNNNNNNNNNNNNNNNNNNNNNNNNNNNNNNNNNNNNNNNNNNNNNNNNNNNNNNNNNNNNNNNNNNNNNNNNNNNNNNNNNNNNNNNNNNNNNNNNNNNNNNNNNNNNNNNNNNNNNNNNNNNNNNNNNNNNNNNNNNNNNNNNNNNNNNNNNNNNNNNNNNNNNNNNNNNNNNNNNNNNNNNNNNNNNNNNNNNNNNNTATAAGAATAATAANNNNNNNNNNNNNNNNNNNNNNNNNNNNNNNNNNNNNNNNNNNNNNNNNNNNNNNNNNNNNNNNNNNNNNNNNNNNNNNNNNNNNNNNNNNNNNNNNNNNNNNNNNNNNNNNNNNNNNNNNNNNNNNNNNNNNNNNNNNNNNNNNNNNNNNNNNNNNNNNNNNNNNNNNNNNNNNNNNNNNNNNNNNNNNNNNNNNNNNNNNNNNNNNNNNNNNNNNNNNNNNNNNNNNNNNNNNNNNNNNNNNNNNNNNNNNNNNNNNNNNNNNNNNNNNNNNNNNNNNNNNNNNNNNNNNNNNNNNNNNNNNNNNNNNNNNNNNNNNNNNNNNNNNNNNNNNNNNNNNNNNNNNNNNNNNNNNNNNNNNNNNNNNNNNNNNNNNNNNNNNNNNNNNNNNNNNNNNNNNNNNNNNNNNNNNNNNNNNNNNNNNNNNNNNNNNNNNNNNNNNNNNNNNNNNNNNNNNNNNNNNNNNNNNNNNNNNNNNNNNNNNNNNNNNNNNNNNNNNNNNNNNNNNNNNNNNNNNNNNNNNNNNNNNNNNNNNNNNNNNNNNNNNNNNNNNNNNNNNNNNNNNNNNNNNNNNNNNNNNNNNNNNNNNNNNNNNNNNNNNNNNNNNNNNNNNNNNNNNNNNNNNNNNNNNNNNNNNNNNNNNNNNNNNNNNNNNNNNNAAAAACTAATTTTATTGATATAGATATTAATTTATCATTATTATGAGATCGAATTAATATAACTAAACATGATAAACCTATTACTCCTTCACAAACTCTAAAAACCAAGAAAATTAATAAAATATGTAAATCATATATTTTTATAGAAAAAATAAATGATAAAGAACAAAATATTCTTAAAATAATTAATTCCAATCTTAATGCTGTTATTAAATGTTTTCGATTTATACATAATGAAATTATCCCTGAAAAGAATATAAAGGTAAAATATATTAATAATGTTATATTCTATAAATATAAAATAATAATTCAGATAATGAATGTAATTATTTGAATCTTAAAATTATTATTCATCAATATATAATTTAATGACATTATTATTTTTAAAGTTTTCAAAATCCCTTGGGGCCCAAGGTTTTCACCTCATCCCATATCAATTGATTTTTGAAATATGAATGATTTACTTATTAATAACATTTGATTATGAAACGTTGACAATTGCTTTATAAACCACATTGAACCAAAAAACTCATAAACAATTTTTATTTTAGTTTTAGAAAAAATTGATATCTCATATCCCAACCAGATTCCTGATATTGAAAAAATCAATGCCAATAATTTGCCCTCTATAGGCATTAAAATTATAATTGGATCATTAAATATTAACCATCTTAATATAGATCCCGAAAAAATTGAATAAATAGATAAAATAATAATTCTTTTAATTATGTTATTTATACTTTCATTCAATGATCTTAACTTATAAAAATTATAATTTACATTTATTGAAAAAAATGTCAACCGGGCAGAATAAAATGAAGTTAAACCAATACCAATATATATAAATATTATAATTAATAAATTAATGTGTCTAGAACACATAGTTTCTATAATTAAATCTTTTGAATAAAATCCACTCAAAAATGGAATTCCACATAATGATATATTAGCAATGTTCATTATTGTAATAGTTAGTGGTATTTGAAATCTTAAACATCCTATTACACGAATATCTTGATTATTATTAAAATTGTGAATCAAAATTCCTGCACACAAAAATAAAGTAGCTTTAAATAAAGCATGAACAATTAAGTGAAAAAATGATAACATTGGATAGCCAAATATAATAATTGTTATTATAATTCCCAGTTGTCTTAGAGTTGATAATGCAATGATTTTTTTTAAATCAAATTCAAAATTTGCTCTAATTCCTGATATTAATATAGTTAAAATTGAAATTAATATAAATAATTGAATATAATCGAAGTTCAAAATAATATTAGAAAATCGAATTATTAAATATACTCCAGCAGTAACAAGTGTAGATGAATGAACCAAAGCAGAAACAGGAGTTGGAGCAGCCATAGCTGCTGGTAATCATGAAGAAAATGGAATTTGAGCACTCTTTGTAAACCTCGCAATAATAATTATAATTAATATAATTTATATCNNNATTAATANAAGATAAAAATTTCATGACCCAAAATTTAATATTCATGCAATAGCTATTAAAATAGCAACATCTCCCACACGGTTCATTAATGCAGTTAATATACCTGCATTATTAGAATATAAATTTTGATAATAAATAACTAAGCAATAAGAAACTAAACCAAGTCCATCTCAACCAATAATAATTCTAATTATATTAGGTCTAATGATTAATATAACTATAGATATAACAAAACCCATAACAATATAAATAAAACGATTAATAAATTTATCATTATTTATATATATTCCACTATATAAAACAACCACTGATGAAATAAATATAACTGTTGAAAGAAATCGTTGATATAAAATCAAAAATTATAGTCATATAAATATTACATGAATTAATTGAAAATTATTCATTCCAATATAATTATNNNNNNNNNNNNNNNNNNNNNNNNNNNNNNNNNNNNNNNNNNNNNNNNNNNNNNNNNNNNNNNNNNNNNNNNNNNNNNNNNNNNNNNNNNNNATGAAAATTGCTATGTATTTAATAATTATTTTATCAATAAATTTTATTTTCATGAAACATCCCTTATCAATAGGATTAATTTTAATTATACAAACAGTTCTGTCTTGCTTAATTTGTAGACTTTACTTAAGTTGTTATTTATTTTCTTATATCTTATATCTTATTTTTATTGGTGGTATATTAATTCTATTTATATATATATCAAGAATTGCATCAAATGAAAAATTTATTTATTCTATTAAATTAATAATGCTTAATTTTTTAATTTTAACTTTAACCAATTTTATCAATATAATTGATTTAAAATCACTAAATATTAAAAGAAATATTATAATATATATAAACCACAACAATTTTATAATAAGAAAACTATATATCATCCCTTCTGGAATAATAACACTAATATTAACAATTTATTTATTATTTGTTTTAATTATTGTAATTAATATTTTAACAACAAATATATTGACTCTTCGAAGAAGAATTTCTTTCTAACTACACCTTCCGGTACAGTTACTTTGTTACGACTTATCTCATTAATATTATGAGAGTGACGGGCGATATGTACATAAATTAGAGCCAATTTCAATTAATTAAATTTAACTAATTTATTATCAAATCCAATTTCATATTTTCTNNAANATAGAAAATAATCCAATAAATAATTAATTGTAACCCATTTTTTCTTTAATATAAACTGCACCTTGACCTGACATTCTAAATATNATAATATAATATGAAAATTTTTCTTATAAAACATTCTTGACAGAGATATACAAATTAAATTAAAGTTTTTTCTATCGTGGATTATCAATTATAAAACAGGTTCCTCTGATAAGATAAATTACCGCCAAATTCTTTGAATTTAAAGATCATTTCTAATAATAATCAAGTTATTTTTATCACATTTTTAATAATAGGGTATCTAATCCTAGTTTATACAAAAATTTTTCAGACATAAAATTAATTTTTAAAGATAAAATATATTTCACCAAAATTTTAGATATTTTATATTATTGTTATATTAACTGAAACTTAAATATTTAAATTAAAGAAATCGTATAACCGCAACTGCTGGCACGAAATTTGATTCTTTTAAATAAAATTACTAATTCTAATTTTATTAATTTATAATAATAAATACTGCGCATTTATTCAAAATATTTCCATTTAGAAATTAAAAACAATTAAAAAAGTGCATGTAATATAATTTTAAATTTAAATTTNNTTAAACTAGAATTAAACTTCAAATAATAAAATTATTTAATGAGGTCCTTTCGTACTAACATTAAAAATAATTAAGTAGATAGAAACCAACCTGGCTCACGCCGGTTTGAACTCAGATCATGTAAGAATGTTAAGGGTCGAACAGACCCAGGAATAATAAATTTTGCTCCAATCCCTATTCTTAATCCAACATCGAGGTCGCAATCATATTTATCGATATGAACTCTTCAAATTAATTACGCTGTTATCCCTAAGGTAATTTATTCTTATAATCAAAAATTTGGATCAATATTAACATAAATTTATGAAATTTTTATTAAAAGTTAATTATATTTTAATATCACCCCAACAAAAAAATCAATTAAATAAAATAAATTAATTAACTATAAANAAACAAATTTAATNAAAATTTTAAAAATTCTATAGGGTCTTCTCGTCCCACTCATTCATTTAAGCTTTTTTACTTAAAAATCAATTTCAATTATTAATATTAATTAAGTTAATTTCTCATTCAATCATTCATTCAAGCCTCCAATTAAAAGACTATTTATTATGCTACCTTTGCACGGTCAATTTACCGCGGCCATTTAATTTTTAATCATTGGGCAGATTAGACTTTTAATTCTTTCTAAAAGACATGTTTTTGATAAACAGGTGAAAATTATTTTTGCCTAAATAATAAATATTATTTATAACTAAAAAACCAGATATCATATAATTTGATAAAATGTCATTTCCAGAATAAATTTATTAATATATATACAATAATAACTAACAAATNTACACTAAATCATTATATTTCGGGAAAAATAAATATTTAAAAGTATTCAATTAATCCTGATACAAAAGGTACAAATTATATTTTACTTAAAATTATTTATAAAATNNTTTCCCTCTCAGTTAATCAAAATTATTTTTTTATATAAAATTACTTCAACACAAAAATTTTTTTTTCAAAATGTAAAAAACAACTATATANTAAAATTATATTATGGGATATATTTAATTATAATAATTGAATTGCAGTCAATAGGTGTTAACTATAACTATCCTTAAGTAATGAAGTAAAATAATACATTTAGTTTCGACCTAAAATAAGAATTAGAGTTCCTTGCTTTTAAATGAAGCCAAAATGGAGGCATTTTATTGTTAATAAAATAATTGAAANTTTATTTCCATTTAAAGAAAAATTAGTTTAATTAAAATATAAATCTGTCAGGTTTATGAAACTTTTGAGTATTTTTCTGACTTTTTATCATNTNTTCTACTTTAAATTTGCAATTTAATATTATAAATTAAATATAAGACTTTATGTTAACAGAATTAAACTGTCTATTAATATCAAAAATTAATGTTCTTCATAAACTATAACATTTATAAAGTTTATAAAACATTTCATTTTCATTGAAAAGAGAGACTTGGTCTTATAAAATTCTATTAGTATATAAGTATATTTAACTTCCAATTAAAAGGATTAATTTTATTAAATAGAATANNNNNNNNNNNNNNNNNNNNNNNNNNNNNNNNNNNNNNNNNNNNNNNNNNNNNNNNNNNNNNNNTACTATTTGTAATAAATATTACATTATTAAATTCTAAATTTAAGGCATTAATTTTGCTAAAATAGTTCTAATATGGCAGATTAGTGCAATGAATTTAAGATTCATATATAAAATANNTTTTTTTTATTGGAAAATAAGATGCCTGAGATGAAAAGGATTATTTTGATAGAATAAATCATGTAATAATATTACTCNNNCATTAAGTGGCTGAAAGTAAGTAATGGTCTCTTAAACCAATTAATAGTAATTAACATATACTCTTAATGAAAGATAAGCTAAAGTTAAAGCTAATGGGTTCATACCTCACTTATGGAAANTTTTCCTCTTTTTATTTAAAAACAAAAGTTACTTTAATATCTTCAATATTATGCTCTTTATTAAGCTATTTAAATTCAGAAAAGATANNATGATCATTTTAAACTCCCAAAGTTTATATTTTATATTTAAATTATTTTCTGAGTTAATTAGCTTATATNAAAGCATTTATTTTGAAAGTAAAAGAAAAGATATTTAATCTATTAACTTAGGAAACTAGAAATAAAATTAGCTTCTAACTAACTTTTAAAGCGGTTAAATTCCGTTTTTTCCTTGTTTTAATAGTTTAATNNNAAAAACTTAGATCTTGTAAATCTAGATTAACGTAAGTTTTAAAAATAGGTTTTAAGTTATTGANAACTATTATCCTTCAAAGTTAAAAATATAATTANATTTATTATTAGGAGAAATAATTTTTCATAAATAAATTTACAGTTTATTGCCTATTATCGGCCATCCTAATCAAGTTATATTGATTAAACAATATTCTCATTAGTGTAAATAAAGTGCTAATTTAGCTTTAACTTG

>cutora-cumberi-midNI_02.NZ.TO.RCG.03

NNNNNNNNNNNNNNNNNNNNNNNNNNNNNNNNNNNNNNNNNNNNNNNNNNNNNNNNNNNNNNNNNNNNNNNNNNNNNNNNNNNNNNNNNNNNNNNNNNNNNNNNNNNNNNNNNNNNNNNNNNNNNNNNNNNNNNNNNNNNNNNNNNNNNNNNNNNNNNNNNNNNNNNNNNNNNNNNNNNNNNNNNNNNNNNNNNNNNNNNNNNTTTATCATTATTTATATTTATTCTACTAAATAATTTATTAGGTTTACTTCCATATATTTTCACAAGTCCAAGTCACTTAGTATTTACAATAAGATTAGCATTACCATTATGATTATCATTCATACTTTATGGATTTATTAATAATATAAATCATATATTTTGCCATCTAGTTCCATCAGGAACTCCTAATATTTTAATACCTTTCATAGTTATTATTGAAAGAATCAGAAATTTAATTCGTCCTGGTTCATTGGCTGTCCGTCTTACAGCTAATATAATTGCTGGACATCTTTTAATAACTTTATTAGGTAACTTACCTATAAGTTATGAAATATACTCAGGTATAATCATTATTTTTCAAGTTATATTAATATTATTTGAATTAGCTGTTTGTGTAATTCAATCATATGTATTTATAGTACTTAGAACTTTATATTATAGAGAAGTAAATATTCCTCAAATATCTCCTNNNNNNNNNNNNNNNNNNNNNNNNNNNNNNNNNNNNNNNNNNNNNNNNNNNNNNNNNNNNNNNNNNNNNNNNNNNNNNNNNNNNNNNNNNNNNNNNNNNNNNNNNNNNNNNNNNNNNNNNNNNNNNTTTAAACCAACACGACAAAATCATCCTTTGATCAAAATTATTAATAATTCATTAATTGACTTTCCAGCACCATCTAATTTATCTTATTGGTGAAATTTTGGATTTGTTTTAGGATTATGTTTAATAATCCAAATTTTAACTGGATTATTTTTATCAATACATTATAATGCCAATATTATAAATGCTTTTGAAAGATTAAGTCATATCTGTCGTGATGTAAATTATGGTTGAATTTTACGAGTTATTCACGCTAATGGAGCTTCATTATTTTTCATCTGTGTCTATTTACATGTAGGACGTGGATTATACTATGGCTCATTTAAATATGTTGAAACATGATCTATTGGTGTAATTATGTTATTAATATTAATAGCTACCGCTTTCTTAGGATATGTTTTGCCGTGAGGTCAAATATCTTTTTGAGGGGCTACAGTTATTACAAATTTATTATCAGCCATTCCTTATTTAGGTAGAACATTAGTTAATTGAATTTGAGGAGGATTTGCAGTTGATAATGCAACACTAACTCGATTTTACTCATTCCACTTTATCTTACCATTCATTGTATTAAGACTAACAATTATTCACTTGTTGTATCTTCATACAACAGGTTCAAATAATCCTCTAGGGATTAATTCTAATAATGATAAAGTCCCCTTTCACCCATATTTCTCAATTAAGGATATTATGAGACTATTTATCTTAATAATTATTTTTTTTATATTAGTCATATTAGAACCCTACATACTAGGAGATCCAGATAATTTTATTCCCGCCAATCCACTTGTAACACCAAAGCATATTCAACCTGAATGATATTTTTTATTTGCCTACGCAATTCTTCGCTCAATTCCTAACAAGTTAGGAGGAGTAATTGCTCTTTTTATATCAATTNNNNNNNNNNNNNNNNNNNNNNNNNNNNNNNNNNNNNNNNNNNNNNNNNNNNNNNNNNNNNNNNNNNNNNNNNNNNNNNNNNNNNNNNNNNNNNNNNNNNNNNNNNNNNNNNNNNNNNNNNNNNNNNNNNNNNNNNNNNNNNNNNNNNNNNNNNNNNNNNNNNNNNNNNNNNNNNNNNNNNNNNNNNNNNNNNNNNNNNNNNNNNNNNNNNNNNNNNNNNNNNNNNNNNNNNNNNNNNNNATGAAAAAATGATTTTTCTCAACTAATCATAAAGATATTGGAACAATATATTTCATTTTTGGTATTTGATCAGGTATAATTGGTACAACTTTAAGAGTTTTAATTCGTGTAGAACTTGGAACCCCAGGTTCATTTATTGGTGATGATCAAATTTATAATGTAATTGTCACTGCTCACGCTTTCATTATAATTTTTTTTATAGTTATGCCAATTATAATTGGAGGATTTGGAAATTGATTAGTCCCTTTAATAATTGGAGCCCCTGATATAGCCTTTCCTCGTATAAATAATATAAGTTTTTGGTTATTACCACCCTCTTTAACTCTTATTTTAGTGGGAAGAATAGTTGATAGAGGTGCAGGTACAGGTTGAACAGTTTACCCCCCTTTATCTGCAGGTATTGCTCATTCTGGCTCATGTGTTGATTTAACTATTTTTTCTCTACATCTTGCAGGTGTATCCTCAATTTTAGGTGCTGTAAACTTCATTAGAACAATTTTTAATATACGTTCAATAGGAATTTGATTAGATCGTATACCTTTATTTGTATGAGCAGTTTTAATTACTGCATTTTTGTTATTATTGTCTTTACCTGTTTTAGCAGGCGCTATCACAATGTTGCTAACAGATCGAAATTTAAATACTTCATTTTTTGACCCTGCAGGGGGGGGTGATCCTATTTTATATCAACATTTATTTTGATTTTTTGGTCACCCTGAAGTTTATATTTTAATTTTACCAGGATTTGGTTTAATTTCTCACATTATTACTCAAGAGAGAGGTAAAATTGAATCTTTTGGATCATTAGGAATAATTTATGCTATAATATCAATTGGCATTTTGGGATTTGTTGTGTGAGCACATCATATATTTACTGTAGGAATAGATGTTGATACACGTGCATATTTTACATCAGCCACTATAATTATTGCAGTTCCTACTGGAATTAAAGTATTTAGTTGACTAGCAACTTTGAGAGGTATAAAAATTANNNNNNNNNNNNNNNNNNNNNNNNNNNNNNNNNNNNTCTGTTTGTCTGNNNNNNNNNNNNNNNNNNNNNNNNNNNNNNNNNNNNNNNNNNNNNNNNNNNNNNNNNNNNNNNNNNNNNNNNNNNNNNNNNNNNNNNNNNNNNNNNNNNNNNNNNNNNNNNNNNNNNNNNNNNNNNNNNNNNNNNNNNNNNNNNNNNNNNNNNNNNNNNNNNNNNNNNNNNNNNNNNNNNNNNNNNNNNNNNNNNNNNNNNNNNNNNNNNNNNNNNNNNNNNNNNNNNNNNNNNNNNNNNNNNNNNNNNNNNNNNNNNNNNNNNNNNNNNNNNNNNNNNNNNNNNNNNNNNNNNNNNNNNNNNNNNNNNNNNNNNNNNNNNNNNNNNNNNNNNNNNNNNNNNNNNNNNNNNNNNNNNNNNNNNNNNNNNNNNNNNNNNNNNNNNNNNNNNNNNNNNNNNNNNNNNNNNNNNNNNNNNNNNNNNNNNNNNNNNNNNNNNNNNNNNNNNNNNNNNNNNNNNNNNNNNNNNNNNNNNNNNNNNNNNNNNNNNNNNNNATTTCNNNNNNNNNNNNNNNNNNNNNNNNNNNNNNNNNNNNNNNNNNNNNNNNNNNNNNNNNNNNNNNNNNNNNNNNNNNNNNNNNNNNNNNNNNNNNNNNNNNNNNNNNNNNNNNNNNNNNNNNNNNNNNNNNNNNNNNNNNNNNNNNNNNNNNNNNNNNNNNNNNNNNNNNNNNNNNNNNNNNNNNNNNNNNNNNNNNNNNNNNNNNNNNNNNNNNNNNNNNNNNNNNNNNNNNNNNNNNNNNNNNNNNNNNNNNNNNNNNNNNNNNNNNNNNNNCATTATTAACATTAAAAATTATTGGTCATCAATGATATTGATCATATGAATATTCAGATTTTTCTGATGTAGAATTTGATTCATATATAAAATCTATAAGCGAAATAAATAAAAATGAATTTCGCTTATTAGATGTAGATAATCGAGTAATTCTACCATTTAATAGTCAAGTCCGACTATTAATTTCTTCTTTTGATGTTATTCATTCCTGAGCAATGCCATCTATAAGTCTAAAAGTTGACGCAGTGCCAGGACGACTAAATCAAATAAGAATATTAATTAGTCGTCCTGGTGTGTCATATGGACAATGTTCCGAGATTTGTGGGGCAAATCACAGATTCATGCCCATTGTAATTGAAAGAATTAGAATAAAAATATTTATTAAATGATTAATTAATTACATGAATAATCATCCTTATCATATAGTTGATTATAGACCCTGACCTTTAACTGGATCAATTGGAGCATTAACTTTTGTTTCCGGTATAGTTATAATATTTCATAAATGTAACTTTATATTACTATATACTGGTATTTTATTATTATTAATAACAATAATTCAATGATGACGTGACATTTCTCGAGAAGGAACATTTTTAGGAATACACACAATAATAGTAGTAAACGGTTTAAAAATAGGAATACTATTATTTATTGTATCAGAAATTCTTTTTTTTGTTTCATTTTTTTGGGGATTTTTCCATAGTAGATTAAGACCTGTGGTAGAAATTGGCATAATTTGACCCCCTAGAGGTATCTATGTGTTTAATCCAACACAAGTACCTTTATTGAATACAATAATTTTATTATGTTCAGGAATCACAATTACTTGAGCACATCATTCAATTATAAATGGTAATCATATCAATTCTATCTATAGAATTATGTTAACTGTTATTTTAGGTATATACTTCACTATTCTGCAAGGATATGAATATTACGAAGCTCCATTTGCAATTAATGATTCCATCTATGGATCTTCTTTTTTTATAGCTACTGGATTTCATGGAATTCACGTAATCATTGGAACAAGATTTATTATTGTATGCTTAATACGACAAATAAAATTTCATTTCTCAAAAAATCATCATTTTGGCTTTGAAGCTGCTGCTTGATATTGGCATNNNNNNNNNNNNNNNNNNNNNNNNNNNNNNNNNNNNNNNNNNNNNNNNNNNNNNNNNTAAATTATAGTTTTTAAAAAAAAGAAAAAAATAAATAAATTTAGAGATACCGGTAAATAAGATTTTCAAGCTAAATATATTAATTTATCATAACGATAACGGGGAAGAGTTCCTCGAACTCAAATGAAAAAAAAACACATTATAGAAACTTGAATTGGAAAGACAACTGAATTAATTTTTCCTCCTAAAAACATTAAACAAAATATTATTCTCATAAATAAAATACTTGAATACTCAGCCAAAAAAATAAATGCAAATCTAGAACCTCTATATTCAACATTAAAGCCAGATACTAACTCTGATTCTCCCTCAGAAAAATCAAATGGAGACCGATTAGTTTCAGCTAAAGCTGAAGATAATCAACACATTCTTAAGGGAAGAAACAAAAAAATAAATCATATAAACTCCTGAAATATAAACAAATCAATAATATTATAACTTCTTATTATTATTATAGGACATAATATAATTAAAACCAATCTTACCTCATAAGAAATAGATTGAGCAATCGATCGAATACATCCCAATATTGCATAACTTGAATTAGAAGATCAGCCTGTTAATATTAATGAATAAACTCTTATTCTTGAACAACAAAAAAAAAATAAAATGCCAAAATTAAAAGAAACACAATTAATTATATATGGATATAATGATCAAATCAATAATCTATTGAATAATCCTATAATAGGTCTAAAAATATAAATTAAATAATTAGATATTAATGGAATTGTATTTTCCTTTATAAATAATTTAATAGCATCAGAAATAGGCTGTAAAATACCCAAAAATCCAACTTTATTTGGACCTTTACGAATTTGAATATATCTCAAAACCTTGCGTTCCAAAAGAGTAATAAATGCCACTCCAATCAAAATAAATAACACAGTAATTAAAATAGTAATTAAATACAAAAATAATTCTTCTTATATACTTTACATAATTTTCTTACTATTAGGAATTATAATTTCAGTTTCTTCAAACAATTGGCTGGGTTGTTGAATAGGAATTGAAATAAATATAGTTTCATTTTTGCCCATAATGGCAAACAAAATAAGAATTTATGCTTCGGAATCAATAATTAAATATTTTATTATTCAAAGAATGGGATCGAGTTTNNNNNNNNNNNNNNNNNNNNNNNNNNNNNNNNNNNNNNNNNNNNNNNNNNNNNNNNNNNNNNNNNNNNNNNNNNNNNNNNNNNNNNNNNNNNNNNNNNNNNNNNNNNNNNNNNNNNNNNNNNNNNNNNNNNNNNNNNNNNNNNNNNNNNNNNNNNNNNNNNNNNNNNNNNNNNNNNNNNNNNNNNNNNNNNNNNNNNNNNNNNNNNNNNNNNNNNNNNNNNNNNNNNNNNNNNNNNNNNNNNNNNNNNNNNNNNNNNNNNNNNNNNNNNNNNNNNNNNNNNNNNNNNNNNNNNNNNNNNNNNNNNNNNNNNNNNNNNNNNNNNNNNNNNNNNNNNNNNNNNNNNNNNNNNNNNNNNNNNNNNNNNNNNNNNNNNNNNNNNNNNNNNNNNNNNNNNNNNNNNNNNNNNNNNNNNNNNNNNNNNNNNNNNNNNNNNNNNNNNNNNNNNNNNNNNNNNNNNNNNNNNNNNNNNNNNNNNNNNNNNNNNNNNNNNNNNNNNNNNNNNNNNNNNNNNNNNNNNNNNNNNNNNNNNNNNNNNNNNNNNNNNNNNNNNNNNNNNNNNNNNNNNNNNNNNNNNNNNNNNNNNNNNNNNNNNNNNNNNNNNNNNNNNNNNNNNNNNNNNNNNNNNNNNNNNNNNNNNNNNNNNNNNNNNNNNNNNNNNNNNNNNNNNNNNNNNNNNNNNNNNNNNNNNNNNNNNNNNNNNNNNNNNNNNNNNNNNNNNNNNNNNNNNNNNNNNNNNNNNNNNNNNNNNNNNNNNNNNNNNNNNNNNNNNNNNNNNNNNNNNNNNNNNNNNNNNNNNNNNNNNNNNNNNNNNNNNNNNNNNNNNNNNNNNNNNNNNNNNNNNNNNNNNNNNNNNNNNNNNNNNNNNNNNNNNNNNNNNNNNNNNNNNNNNNNNNNNNNNNNNNNNNNNNNNNNNNNNNNNNNNNNNNNNNNNNNNNNNNNNNNNNNNNNNNNNNNNNNNNNNNNNNNNNNNNNNNNNNNNNNNNNNNNNNNNNNNNNNNNNNNNNNNNNNNNNNNNNNNNNNNNNNNNNNNNNNNNNNNNNNNNNNNNNNNNNNNNNNNNNNNNNNNNNNNNNNNNNNNNNNNNNNNNNNNNNNNNNNNNNNNNNNNNNNNNNNNNNNNNNNNNNNNNNNNNNNNNNNNNNNNNNNNNNNNNNNNNNNNNNNNNNNNNNNNNNNNNNNNNNNNNNNNNNNNNNNNNNNNNNNNNNNNNNNNNNNNNNNNNNNNNNNNNNNNNNNNNNNNNNNNNNNNNNNNNNNNNNNNNNNNNNNNNNNNNNNNNNNNNNNNNNNNNNNNNNNNNNNNNNNNNNNNNNNNNNNNNNNNNNNNNNNNNNNNNNNTTAATCTATTAATAATTATAATTTCACCAGCTAAATTAATTGTGGGGGGACAAGACATATTTCTTGCTGACATTAAAAATCATAATAAAGATATTGAAGGTATGAATGTCAATATACCCTTATTAATTAATAATCTTCGACTATTAGTTCGTTCATACAACATATTTGCTAAACAAAATAATCCAGAAGAACATAAACCATGCCCAATTATTATATAATATGAACCACATATTCCCCAATTTCTTATGGTTATAATTCCTCTAATCACAAGACCTATATGAGCAACAGATGAATAAGCAATTATTGATTTAATATCAATCTGAATTATACACAGAATTCTAATTAAAATACTTCCAATTATTGAAATTGAGATCCATATAAATCCAAATTTATAAAAATAAGAAGGAATAATAAACATTACACGAATCAAACCATATCCACCTAATTTTAGTAAAATTCTAGCTAAAATTATTGAACCAGAAACAGGGGCTTCAACATGAGCTTTAGGCAGTCAAAAATGAAAAAATAATGGTATTTTAATTAAAAAAGCTATTATTATACCAAAGTAAATATAAATATTGACAGAATAATTTATTAACATAAAAACACTAGTTATATTATTTTTATAAAGAAAAAAAATTCTTATTAATATTGGTAATGATGCAAATAATGTATAGAATAGTAAATAATAACTCGCATCAATACGTTCTGGCTGATATCCCCAACCTATAATAATAATTATAATTGGAATTAATCTAGATTCAAAACAAATATAAAAAATTATAATTCTTGATGTAGAAAAAGAAATAATCAAAAAAATTATTAGCATAATACCAAAAATAAAAAAAATAACTTATTATGTTTATATATTGGACTAGAAACAATTATTAAAAATGAAATTCAAATTCAAAATAATTAAACCATAAGACATTTCATCAATATAAAAATNNNNNNNNNNNNNNNNNAAATTCTTATTTATAAAAACCNNNNNNNNNNNNNNNNNNNNNNNNNNNNNNNNNNNNNNNNNTTGATTCTGAATTAATANNNNNNNNNNNNNNNNNNNNNNNNNNNNNNNNNNNNNNNNNNNNNNNNNNNNNNNNNNNNNNNNNNNNNNNNNNNNNNNNNNNNNNNNNNNNNNNNNNNNNNNNNNNNNNNNNNNNNNNNNNNNNNNNNNNNNNNNNNNNNNNNNNNNNNNNNNNNNNNNNNNNNNNNNNNNNNNNNNNNNNNNNNNNNNNNNNNNNNNNNNNNNNNNNNNNNNNNNNNNNNNNNNNNNNNNNNNNNNNNNNNNNNNNNNNNNNNNNNNNNNNNNNNNNNNNNNNNNNNNNNNNNNNNNNNNNNNNNNNNNNNNNNNNNNNNNNNNNNNNNNNNNNNNNNNNNNNNNNNNNNNNNNNNNNNNNNNNNNNNNNNNNNNNNNNNNNNNNNNNNNNNNNNNNNNNNNNNNNNNNNNNNNNNNNNNNNNNNNNNNNNNNNNNNNNNNNNNNNNNNNNNNNNNNNNNNNNNNNNNNNNNNNNNNNNNNNNNNNNNNNNNNNNNNNNNNNNNNNNNNNNNNNNNNNNNNNNNNNNNNNNNNNNNNNNNNNNNNNNNNNNNNNNNNNNNNNNNNNNNNNNNNNNNNNNNNNNNNNNNNNNNNNNNNNNNNNNNNNNNNNNNNNNNNNNNNNNNNNNNNNNNNNNNNNNNNNNNNNNNNNNNNNNNNNNNNNNNNNNNNNNNNNNNNNNNNNNNAAATATCNNNNNNNNNNNNNNNNNNNNNNNNNNNNNNNNNNNNNNNNNNNNNNNNNNNNNNNNNNNNNNNNNNNNNNNNNNNNNNNNNNNNNNNNNNNNNNNNNNNNNNNNNNNNNNNNNNNNNNNNNNNNNNNNNNNNNNNNNNNNNNNNNNNNNNNNNNNNNNNNNNNNNNNNNNNNNNNNNNNNNNNNNNNNNNNNNNNNNNNNNNNNNNNNNNNNNNNNNNNNNNNNNNNNNNNNNNNNNTTTGAATAAAATCCTCTCATAAATGGAATTCCACATAATGACATATTAGCAATATTTATTATTGTAATAGTTAATGGTATTTGAAATCTTAAACATCCTATCACACGGATATCTTGATTATTATTAAAATTGTGAATCAAAATTCCTGCACACAAAAATAAAGTAGCTTTAAATAGAGCATGAACAATTAAATGAAAAAATGATAATATTGGATAACCAAATATAATAATTGTTATTATAATCCCCAATTGTCTTAAAGTTGATAATGCAATAATTTTTTTCAAATCAAACTCAAAATTTGCTCTAATTCCTGATATTAATATAGTTAAAATTGAAATTAATATAAATAATTGAATATAATCAAAATTCAAAATAATATTAGAAAATCGAATTATTAAATATACTCCAGCAGTAACAAGTGTAGATGAATGAACCAAGGCAGAAACAGGAGTTGGAGCAGCTATAGCTGCTGGTAATCATGAAGAAAATGGAATTTGAGCTCTTTTTGTAAATCTTGCAATAATAATTATAATTANNNNNNNNNNNNNNNNNNNNNNNNNNNNNNNNNNNNNNNNNNNNNNNNNNNNTAATATTCATGCAATAGCCATTAAAATAGCAACATCTCCAACACGATTCATTAGTGCAGTTAATATTCCTGCATTATTAGAATATAAATTTTGATAGTAAATAACTAGGCAATAAGAAACTAGACCAAGTCCATCTCAACCAATAATAATTCTCATTATATTAGGTCTAATGATTAATAAAATTATAGATATAACAAACCCTAAAACAATATAAATAAAACGATTAATAAATTTATCATTATTTATATATATTCCACTATATAAAACAACCACTGATGAAATAAATATAACTGTTGAAAGAAATTGTTGATATAAAATCAAAAATTATAGTCATATAAATATTACATGAATTANNNNNNNNNNNNNNNNNNNNNNNNNNNNNNNNNNNNNNNNNNNNNNNNNNNNNNNNNNNNNNNNNNNNNNNNNNNNNNNNNNNNNNNNNNNNNNNNNNNNNNNNNNNNNNNNNNNNNNNNNNNNNNNNNNNNNNNNNNNNNNNNNNNNNNNNNNNNNNNNNNNNNNNNNNNNNNNNNNNNNNNNNNNNNNNNNNNNNNNNNNNNNNNNNNNNNNNNNNNNNNNNNNNNNNNNNNNNNNNNNNNNNNNNNNNNNNNNNNNNNNNNNNNNNNNNNNNNNNNNNNNNNNNNNNNNNNNNNNNNNNNNNNNNNNNNNNNNNNNNNNNNNNNNNNNNNNNNNNNNNNNNNNNNNNNNNNNNNNNNNNNNNNNNNNNNNNNNNNNNNNNNNNNNNNNNNNNNNNNNNNNNNNNNNNNNNNNNNNNNNNNNNNNNNNNNNNNNNNNNNNNNNNNNNNNNNNNNNNNNNNNNNNNNNNNNNNNNNNNNNNNNNNNNNNNNNNNNNNNNNNNNNNNNNNNNNNNNNNNNNNNNNNNNNNNNNNNNNNNNNNNNNNNNNNNNNNNNTATTGTAATCAATATTTTAACAATAAATATATTAACTCTTCGAAGAAGAACCNNNNNNNNNNNNNNNNNNNNNNNNNNNNNNNNNNNNNNNNNNNNNNNNNNNNNNNNNNNNNNNNNNNNNNNNNNNNNNNNNNNNNNNNNNNNNNNNNNNNNNNNNNNNNNNNNNNNNNNNNNNNNNNNNNNNNNNNNNNNNNNNNNNNNNNNNNNNNNNNNNNNNNNNNNNNNNNNNNNNNNNNNNNNNNNNNNNNNNNNNNNNNNNNNNNNNNNNNNNNNNNNNNNNNNNNNNNNNNNNNNNNNNNNNNNNNNNNNNNNNNNNNNNNNNNNNNNNNNNNNNNNNNNNNNNNNNNNNNNNNNNNNNNNNNNNNNNNNNNNNNNNNNNNNNNNNNNNNNNNNNNNNNNNNNNNNNNNNNNNNNNNNNNNNNNNNNNNNNNNNNNNNNNNNNNNNNNNNNNNNNNNNNNNNNNNNNNNNNNNNNNNNNNNNNNNNNNNNNNNNNNNNNNNNNNNNNNNNNNNNNNNNNNNNNNNNNNNNNNNNNNNNNNNNNNNNNNNNNNNNNNNNNNNNNNNNNNNNNNNNNNNNNNNNNNNNNNNNNNNNNNNNNNNNNNNNNNNNNNNNNNNNNNNNNNNNNNNNNNNNNNNNNNNNNNNNNNNNNNNNNNNNNNNNNNNNNNNNNNNNNNNNNNNNNNNNNNNNNNNNNNNNNNNNNNNNNNNNNNNNNNNNNNNNNNNNNNNNNNNNNNNNNNNNNNNNNNNNNNNNNNNNNNNNNNNNNNNNNNNNNNNNNNNNNNNNNNNNNNNNNNNNNNNNNNNNNNNNNNNNNNNATAATTAAATTATTTAATGAGGTCCTTTCGTACTAACATTAAAAATAATTGAGTAGATAGAAACCAACCTGGCTCACGCCGGTTTGAACTCAGATCATGTAAGAATATTAAGGGTCGAACAGACCCAGAAATAATAAATTTTGCTCCAATCCCTATTCTTAATCCAACATCGAGGTCGCAATCATATTTATCGATATGAACTCTTCAAATTAATTACGCTGTTATCCCTAAGGTAATTTATTCTTATAATCAAAAATTTGGANNNNNAGCTATTTAAATTTANNNNNNNNNNNNNNNNNNNNNNNNNNNNNNNNNNNNNNNNNNNNNNNNNNNNNNNNNNNNNNNNNNNNNNNNNNNNNNNNNNNNNNNNNNNNNNNNNNNNNNNNNNNNNNNNNNNNNNNNNNNNNNNNNNNNNNNNNNNNNNNNNNNNNNNNNNNNNNNNNNNNNNNNNNNNNNNNNNNNNNNNNNNNNNNNNNNNNNNNNNNNNNNNNNNNNNNNNNNNNNNNNNNNNNNNNNNNNNNNNNNNNNNNNNNNNNNNNNNNNNNNNNNNNNNNNNNNNNNNNNNNNNNNNNNNNNNNNNNNNNNNNNNNNNNNNNNNNNNNNNNNNNNNNNNNNNNNNNNNNNNNNNNNNNNNNNNNNNNNNNNNNNNNNNNNNNNNNNNNNNNNNNNNNNNNNNNNNNNNNNNNNNNNNNNNNNNNNNNNNNNNNNNNNNNNNNNNNNNNNNNNNNNNNNNNNNNNNNNNNNNNNNNNNNNNNNNNNNNNNNNNNNNNNNNNNNNNNNNNNNNNNNNNNNNNNNNNNNNNNNNNNNNNNNNNNNNNNNNNNNNNNNNNNNNNNNNNNNNNNNNNNNNNNNNNNNNNNNNNNNNNNNNNNNNNNNNNNNNNNNNNNNNNNNNNNNNNNNNNNNNNNNNNNNNNNNNNNNNNNNNNNNNNNNNNNNNNNNNNNNNNNNNNNNNNNNNNNNNNNNNNNNNNNNNNNNNNNNNNNNNNNNNNNNNNNNNNNNNNNNNNNNNNNNNNNNNNNNNNNNNNNNNNNNNNNNNNNNNNNNNNNNNNNNNNNNNNNNNNNNNNNNNNNNNNNNNNNNNNNNNNNNNNNNNNNNNNNNNNNNNNNNNNNNNNNNNNNNNNNNNNNNNNNNNNNNNNNNNNNNNNNNNNNNNNNNNNNNNGAAAAATTAGTTTAATNAAAATATAAATCTGTCAGATTTATGAAACTTTTGAGTATTTTTCTGACTTCTTATGATNTNTTCTACTTTAAATTTGCAATTTAATATTATAAATTAAATATAAGGCTTTATGTTAACAGAATTAAACTGTCTATTAATATCAAAAATTAATGTTCTTCATAAACTATAACANNNNNNNNNNNNNNNNNNNNNNNNNNNNNNNNNNNNNNNNNNNNNNNNNNNNNNNNNNNNNNNNNNNNNNNNNNNNNNNNNNNNNNNNNNNNNNNNNNNNNNNNNNNNNNNNNNNNNNNNNNNNNNNNNNNNNNNNNNNNNNNNNNNNNNAGACACAATTGGANNNNNNNNNNNNNNNNNNNNNNNTACTATTTGTAATAAATATTACATTATTAAATTCTAAATTTAAAGCATTAATTTTGCTAAAATAGTNNNNNNNNNNNNNNNNNNNNNNNNNNNNNNNNNNNNNNNNNNNNNNNNNNNNNNNTTTTCTTGTAGNNNNNNNNNNNNNNNNNNNNNNNNNNNNNNNNNNNNGAATAAATTATGTAATATAATTACTCNNNCATTAAGTGGCTGAAAGTAAGTAATGGTCTCTTAAACCAATTAATAGTAATTAACAAATACTCTTAATGAAAGATAAGCTAAAANTAAAGCTAATGGGTTCATACCTCACTTATGGAAATTTTTCCTCTTTTTANNNNNNNNNNNNNNNNNNNNNNNNNNNNNNNNNNNNNNNNNNNNNNNNNNNNNNNNNNNNNNNNNNNNNNNNNNNNNNNNNNNNNNNNNNNNNNNNNNNNNNNNNNNNNNNNNNNNNNNNNNNNNNNNNNNNNNNNNNNNNNNNNNNNNNNNNNNNNNNNNNNNNNNNNNNNNNNNNNNNNNNNNNNNNNNNNNNNNNNNNNNNNNNNNNNNNNNNNNNNNNNNNNNNNNNNNNNNNNNNNNNNNNNNNNNNNNNNNNNNNNNNNNNNNNNNNNNNNNNNNNNNNNNNNNNNNNNNNNNNNNNNNNNNNNNNNNNNNNNNNNNNNNNNNNNNNGTTATTAATAACTATTANNNNNNNNNNNNNNNNNNNNNNNNNNNNNNNNTATTAGGAGAAATAATTTTTCATAAATAAATTTACAGTTTATTGCCTATTATCGGCCATCCTAANNNNNNNNNNNNNNNNNNNNNNNNNNNNNNNNNNNNNNNNNNNNNNNNNNNNNNNNNNNNNNNNNN

>cutora-northauckNI_02.NZ.ND.MAU.09

NNNNNNNNNNNNNNNNNNNNNNNNNNNNNNNNNNNNNNNNNNNNNNNNNNNNNNNNNNNNNNNNNNNNNNNNNNNNNNNNNNNNNNNNNNNNNNNNNNNNNNNNNNNNNNNNNNNNNNNNNNNNNNNNNNNNNNNNNNNNNNNNNNNNNNNNNNNNNNNNNNNNNNNNNNNNNNNNNNNNNNNNNNNNNNNNNNNNNNNNNNNNNNNNNNNNNNNNNNNNNNNNNNNNNNNNNNNNNNNNNNNNNNNNNNNNNNNNNNNNNNNNNNNNNTCCAAGTCACTTAGTATTTACAATAAGATTAGCATTACCATTATGATTATCATTCATACTTTATGGATTTGTTAATAATATNNNNNNNNNNNNNNNNCATCTAGTTCCATCAGGAACTCCTAATATTTTAATACCTTTCATAGTTATTATTGAAAGAATCAGAAATTTAATTCGTCCTGGTTCATTGGCTGTCCGTCTTACAGCTAATATAATTGCTGGACATCTTTTAATAACTTTATTAGGTAACTTACCTATAAATTATGAAATATACTCAGGTATAATCATTATTTTTCAAGTTATATTAATATTATTTGAATTAGCTGTTTGTGTAATTCAATCATATGTATTTATAGTACTTAGAACTTTATATTATAGAGAAGTAAATNNNNNNNNNNNNNNNNNNNNNNNNNNNNNNNNNNNNNNNNNNNNNNNNNNNNNNNNNNNNNNNNNNNNNNNNNNNNNNNNNNNNNNNNNNNNNNNNNNNNNNNNNNNNNNNNNNNNNNNNNNNNNNNNNNNNNNNNNNNNNNNNTTTAAACCAACACGACAAAATCATCCTTTGATTAAAATTATTAATAATTCATTAATTGACTTTCCAGCACCATCTAATTTATCTTATTGGTGAAATTTTGGATTTGTTTTAGGATTGTGTTTAATAATCCAAATTTTAACTGGATTATTTTTATCAATACATTATAATGCCAATATTATAAATGCTTTTGAAAGATTAAGTCATATCTGTCGTGATGTAAATTATGGTTGAATTTTACGAGTTATTCACGCTAATGGAGCTTCATTATTTTTCATCTGTGTCTATTTACATGTAGGACGTGGATTGTACTATGGCTCATTTAAATATGTTGAAACATGATCTATTGGTGTAATTATGTTATTAATATTAATAGCTACCGCTTTCTTAGGATATGTTTTGCCGTGAGGTCAAATATCTTTTTGAGGGGCTACAGTTATTACGAATTTATTATCAGCCATTCCTTATTTAGGTAGAACATTAGTTAATTGAATTTGAGGAGGATTTGCAGTTGATAATGCAACACTAACTCGATTTTACTCATTCCACTTTATCTTACCATTCATTGTATTAAGACTAACAATTATTCACTTGTTGTATCTTCATACAACAGGTTCAAATAATCCTCTAGGGATTAATTCTAATAATGATAAAGTCCCCTTTCACCCATATTTCTCAATTAAGGATATTATGAGACTATTTATCTTAATAATTATTTTTTTTATATTAGTCATATTAGAACCCTACATACTAGGGGATCCAGATAATTTTATTCCCGCCAATCCACTTGTAACACCAAAGCACATCCAACCTGAATGATATTTTTTATTTGCCTACGCAATTCTTCGCTCAATTCCTAATAAGTTAGGAGGAGTAATTGCTCTTTTTATATCAATCTTTATTTTAATGTTTGTACCTTTATTAAATAATTCCAATTTCATAGGATTAAATAGTTACCCAATTAATCAAATTATATTTTGATATATAGTAATCATTTTAATTTTATTAACTTGAATTGGAGCACGACCTGTCGAAATACCTTACATTAACTTTGGAATATTTTTAACACTTATATATTTTTCTTACTTCATTATTGACCCAATAATTAAATCTATTTGAGACAAATTAATTAGGTAGATGAAAAAATGATTTTTCTCAACTAATCATAAAGATATTGGAACAATATATTTCATTTTTGGTATTTGATCAGGTATAATTGGTACAACTTTAAGAGTTTTAATTCGTGTAGAACTTGGAACCCCAGGTTCCTTTATTGGTGATGATCAAATTTATAATGTAATTGTCACTGCTCACGCTTTCATTATAATTTTTTTTATAGTTATGCCAATTATAATTGGAGGATTTGGAAATTGATTAGTCCCTTTAATAATTGGAGCCCCTGATATAGCCTTTCCTCGTATAAATAATATAAGTTTTTGATTATTACCACCCTCTTTAACTCTTATTTTAGTGGGAAGAATAGTTGATAGAGGTGCAGGTACAGGTTGAACAGTTTACCCCCCTTTATCTGCAGGTATTGCTCATTCTGGCTCATGTGTTGATTTAACTATTTTTTCTCTACATCTTGCAGGTGTATCCTCAATTCTAGGTGCTGTAAACTTCATTAGAACAATTTTTAATATACGTTCAATAGGAATTTGATTAGATCGTATACCTTTATTTGTATGAGCAGTTTTAATTACTGCATTTTTGTTATTATTGTCTTTACCTGTTTTAGCAGGCGCTATCACAATGTTGTTAACAGATCGAAATTTAAATACTTCATTTTTTGACCCTGCAGGAGGGGGTGATCCTATTTTATATCAACATTTATTTTGATTTTTTGGTCACCCTGAAGTTTATATTTTAATTTTACCAGGATTTGGTTTAATTTCTCACATTATTACTCAAGAGAGAGGTAAAATTGAATCTTTTGGATCATTAGGAATAATTTATGCTATAATATCAATTGGCATTTTGGGATTTGTTGTGTGAGCACATCATATATTTACTGTAGGAATAGATGTTGATACACGTGCATATTTTACATCAGCCACTATAATTATTGCAGTTCCTACTGGAATTAAAGTATTTAGTTGACTAGCAACTTTGAGAGGTATAAAAATTAATATTACATCTTCAGCTTTATGGGCTCTAGGGTTTGTGTTTTTATTTACTATTGGAGGTTTGACTGGTGTGATTTTAGCTAATTCTTCAATTGATATTATATTACATGATACATACTATGTAGTTGCGCATTTTCATTATGTTTTATCAATAGGAGCAGTTTTTGCTATCATGGCAAGATTTATTCATTGATTTCCATTATTTACAGGATTAAGATTAAACTCAAATTGATTAAAAATTCATTTTTTATTAATATTTATAGGTGTAAATATAACATTCTTTCCTCAACATTTTTTAGGATTAAGAGGAATACCCCGTCGATATTCAGATTATCCTGATGCTTATATATCATGAAATATAATTTCATCAATAGGAAGAATTATATCTTTAATTGGAATTTTATTATTATTATTTATTGTTTGAGAAAGTTTTATTTCAATACGTTTAGTATTATATTCTAATAGAATTCAATCTTCTATTGNNNNNNNNNNNNNNNNNNNNNNNNNNNNNNNNNNNNNNNNNNNNNNNNNNNNNNNNNNNNNNNNNNNNNNNNNNNNNNNNNNNNNNNNNNNNNNNNNNNNNNNNNNNNNNNNNNNNNNNNNNNNNNNNNNNNNNNNNNNNNNNNNNNNNNNNNNNNNNNNNNNNNNNNNNNNNNNNNNNNNNNNNNNNNNNNNNNNNNNNNNNNNNNNNNNNNNNNNNNNNNNNNNNNNNNNNNGTCAATTAATTGAATTTATTTGAACTTTATTACCTGCAATAACTTTAATTTTTATTGCATTACCATCATTACGATTATTATATATATTGGACGAAATTAATAACCCATTATTAACATTAAAAATTATTGGTCATCAATGATATTGATCATATGAATATTCAGATTTTTCTGATGTAGAATTTGATTCATATATAAAATCTATAAGCGAAATAAATAAAAATGAATTTCGCTTATTAGATGTAGATAATCGAGTAATTCTACCATTTAATAGTCAAGTCCGACTATTAATTTCTTCTTTTGATGTTATTCATTCCTGAGCAATGCCATCTATAAGTCTAAAAGTTGACGCAGTGCCAGGACGACTAAATCAAATAAGAATATTAATTAGTCGTCCTGGTGTATCATATGGACAATGTTCCGAGATTTGTGGGGCAAATCACAGATTCATGCCCATTGTAATTGAAAGAATTAGAATNNNNNNNNNNNNNNNNNNNNNNNNNNNNNNNATGAATAATCATCCTTATCATATAGTTGATTATAGACCCTGACCTTTAACTGGATCAATTGGAGCATTAACTTTTGTTTCCGGTATAGTTANNNNNNNNNNNNNNNNNNNNNNNNNNNNNNNNNNNNNNNNNNNNNNNNNNNNNNNNNNNNNNNNNNNNNNNNNNNNNNNNNNNNNNNNNNNNNNNNNNNNNNNNNNNNNNNNNNNNNNNNNNNNAGTAGTAAACGGTTTAAAAATAGGAATACTATTATTTATTGTATCAGAAATTCTTTTTTTTGTTTCATTTTTTTGAGGATTTTTCCATAGTAGATTAAGACCTGTGGTAGAAATTGGCATAATTTGACCCCCTAGAGGTATCTATGTATTTAATCCAACACAAGTACCTTTATTGAATACAATAATTTTATTATGTTCAGGAATCACAATTACTTGAGCCCATCATTCAATTATAAATGGTAATCATATCAATTCTATCTATAGAATTATGTTAACTGTTATTTTAGGTATATACTTCACTATTCTGCAAAGATATGAATATTATGAAGCTCCATTTGCAATTAATGATTCCATTTATGGATCTTCTTTTTTTATAGCTACTGGATTTCATGGAATTCACGTAATCATTGGAACAAGATTTATTATTGTATGCTTAATACGACAAATAAAATTTCATTTCTCAAAAAATCATCATTTTGGCTTTGAAGCTGCTGCTTGATATTGACATTTTGTTGATGTAGTTTGATTATTTCTATATTTATCAATTTATTGATGAGGAAGATAANNNNNNNNNNNNNNNNNNNNNNNNAAAAAAATAAATAAATTTAGAGATACCGGTAAATAAGATTTTCAAGCTAAATATATTAATTTATCATAACGATAACGGGGAAGAGTTCCTCGAACTCAAATGAAAAAAAAACACATCATAGAAACTTGAATTGGAAAGACAACTGAATTAATTTTTCCTCCTAAAAACATTAAACAAAATATTATTCTCATAAATAAAATACTTGAATACTCAGCCAAAAAAATAAATGCAAATCTAGAACCTCTATATTCAACATTAAAGCCAGATACTAACTCTGATTCTCCCTCAGAAAAATCAAATGGAGATCGATTAGTTTCAGCTAAAGCTGAAGATAATCAACACATTCTTAAGGGAAGAAACAAAAAAATAAATCATATAAACTCCTGAAATATAAACAAATCAATAATATTATAACTTCTTATTATTATTATAGGACATAATATAATTAAAACCAATCTTACCTCATAAGAAATAGATTGAGCAATCGATCGAATACATCCCAATATTGCATAACTTGAATTAGAAGATCAGCCTGTTAATATTAATGAATAAACTCTTATTCTTGAACAACAAAAAAAAAATAAAATGCCAAAATTAAAAGAAACACAATTAAATATATATGGATACAATGATCAAATCAATAATCTGTTGATTAATACTATTNNNGGTCTAAAAATATAAATTAAATAGTTAGATATTAATGGAATAGTATGTTCCATTATGAATAATTTAATANNNNNNNNNNNNNNNNNNNNNNNNNNNNNNNNNNNNNNNNNNNNNNNNNNNNNNNNNNNNNNNNNNNNNNNNNNNNNNNNNNNNNNNNNNNNNNNNNNNNNNNNNNNNNNNNNNNNNNNNNNNNNNNNNNNNNNNNNNNNNNNNNNNNNNNNNAAAAATAATTCTTCTTATATACTTTACATAATTTTCTTACTATTAGGAATTATAANNNNNNNNNNTTCAAACAATTGGCTGGGTTGTTGAATAGGAATTGAAATAAATATAGTTTCATTTTTGCCCATAATGGCAAACAAAATAAGAATTTATGCTTCGGAATCAATAATTAAATATTTTATTATTCAAAGAATGGGATCGAGTTTGTTATTAATNNNNNNNNNNNNNNNNNNNNNNNNNNNNNNNNNNNNNNNNNNNNNNNNNNNNNNNNNNNNNNNNNNNNNNNNNNNNNNNNNNNNNNNNNNNNNNNNNNNNNNNNNNNNNNNNNNNNNNNNNNNNNNNNNNNNNNNNNNNNNNNNNNNNNNNNNNNNNNNNNNNNNNNNNNNNNNNNNNNNNNNNNNNNNNNNNNNNNNNNNNNNNNNNNNNNNNNNNNNNNNNNNNNNNNNNNNNNNNNNNNNNNNNNNNNNNNNNNNNNNNNNNNNNNNNNNNNNNNNNNNNNNNNNNNNNNNNNNNNNNNNNNNNNNNNNNNNNNNNNNNNNNNNNNNNNNNNNNNNNNNNNNNNNNNNNNNNNNNNNNNNNNNNNNNNNNNNNNNNNNNNNNNNNNNNNNNNNNNNNNNNNNNNNNNNNNNNNNNNNNNNNNNNNNNNNNNNNNNNNNNNNNNNNNNNNNNNNNNNNNNNNNNNNNNNNNNNNNNNNNNNNNNNNNNNNNNNNNNNNNNNNNNNNNNNNNNNNNNNNNNNNNNNNNNNNNNNNNNNNNNNNNNNNNNNNNNNNNNNNNNNNNNNNNNNNNNNNNNNNNNNNNNNNNNNNNNNNNNNTTTTATTTTTTTATTTACGAATTTTAATTACAACGTTAATAATAAGAACAATTTCAATAAAAAGAATTGTCATTAGAGTTTCGTGAACTTATTATATTGCCGGGATATTTTCATTATTTGGAATAATTTTTTTTTCATTAATTACATTAAATATGTGTTAGNNNNNNNNNNNNNNNNNNNNNNNNNNNNNNNNNNNNNNNNNNNNNNNNNNNNNNNNNNNNNNNNNNNNNNNNNNNNNNNNNNNNNNNNNNNNNNNNNNNNNNNNNNNNNNNNNNNNNNNNNNNNNNNNNNNNNNNNNNNNNNNNNNNNNNNNNNNNNNNNNNNNNNNNNNNNNNNNNNNNNNNNNNNNNNNNNNNNNNNNNNNNNNNNNNNNNNNNNNNNNNNNNNNNNNNNNNNNNNNNNNNNNNNNNNNNNNNNNNNNNNNNNNNNNNNNNNNNNNNNNNNNNNNNNNNNNNNNNNNNNNNNNNNNNNNNNNNNNNNNNNNNNNNNNNNNNNNNNNNNNNNNNNNNNNNNNNNNNNNNNNNNNNNNNNNNNNNNNNNNNNNNNNNNNNNNNNNNNNNNNNNNNNNNNNNNNNNNNNNNNNNNNNNNNNNNNNNNNNNNNNNNNNNNNNNNNNNNNNNNNNNNNNNNNNNNNNNNNNNNNNNNNNNNNNNNNNNNNNNNNNNNNNNNNNNNNNNNNNNNNNNNNNNNNNNNNNNNNNNNNNNNNNNNNNNNNNNTTTCAAGATATTAATCTATTAATAATTATAATTTCACCAGCTAAATTAATTGTGGGGGGACAAGACATATTTCTTGCTGACATTAAAAATCATAATAAAGATATTGAAGGTATGAATGTCAATATACCCTTATTAATTAATAATCTTCGACTATTAGTTCGCTCATACAATATATTTGCTAAACAAAATAATCCAGAAGAACATAAACCATGCCCAATTATTATATAATATGAACCGCATATTCCCCAATTTCTTATGGTCATAATACCTCTAATCACAAGACCTATGTGAGCAACAGATGAATAAGCAATTATTGATTTAATATCAATTTGAATTATACACAGAATTCTAATTAAAATACTTCCAATTATTGAAATTGAAATCCATATAAATCCAAATTTATAAAAATAAGAAGGAATAATAAATATTACACGAATCAAACCATATCCACCCAATTTTAATAAAATTCTAGCTAAAATTATTGAACCAGAAACAGGGGCTTCAACATGAGCTTTAGGCAATCAAAAATGAAANNNNNNNNNNNNNNNNNNNNNNNNNNNNNNNNNNNNNNNNNNNNNNNNNNNNNNNNNNNNNNNNNNNNNNNNNNNNNNNNNNNNNNNNNNNNNNNNNNNNNNNNNNNNNNNNNNNNNNNNNNNNNNNNNNNNNNNNNNNNNNNNNNNNNNNNNNNNNNNNNNNNNNNNNNNNNNNNNNNNNNNNNNNNNNNNNNNNNNNNNNNNNNNNNNNNNNNNNNNNNNNNNNNNNNNNNNNNNNNNNNNNNNNNNNNNNNNNNNNNNNNNNNNNNNNNNNNNNNNNNNNNNNNNNNNNNNNNNNNNNNNNNNNNNNNNNNNNNNNNNNNNNNNNNNNNNNNNNNNNNNNNNNNNNNNNNNNNNNNNNNNNNNNNNNNNNNNNNNNNNNNNNNNNNNNNNNNNNNNNNNNNNNNNNNNNNNNNNNNNNNNNNNNNNNNNNNNNNNNNNNNNNNNNNNNNNNNNNNNNNNNNNNNNNNNNNNNNNNNNNNNNNNNNNNNNNNNNNNNNNNNNNNNNNNNNNNNNNNNNNNNNNNNNNNNNNNNNNNNNNNNNNNNNNNNNNNNNNNNNNNNNNNNNNNNNNNNNNNNNNNNNNNNNNNNNNNNNNNNNNNNNNNNNNNNNNNNNNNNNNNNNNNNNNNNNNNNNNNNNNNNNNNNNNNNNNNNNNNNNNNNNNNNNNNNNNNNNNNNNNNNNNNNNNNNNNNNNNNNNNNNNNNNNNNNNNNNNNNNNNNNNNNNNNNNNNNNNNNNNNNNNNNNNNNNNNNNNNNNNNNNNNNNNNNNNNNNNNNNNNNNNNNNNNNNNNNNNNNNNNNNNNNNNNNNNNNNNNNNNNNNNNNNNNTATAAGTATAAAACAATAATTCAAACAATAAATGAAATTATCTGAATCTTAAAATTATTATTTATCAACTTATAATTTAATGATATTACCATTTTTAAAGTATTTAAAATCCCTTGGGGTCCAAGGTTTTCGCCTCATCCTATATCAACTGATTTTTGAAATATAAGTGATTTACTTATTAATAATATTTGATTGTGAAACGTTGACAATTGTTTTATAAATCACATTGATCCAAAAAATTCATAAACAATTTTTATTTTAGTTTTAGAAAAAATTGATATTTCATAACCTAATCAAATTCCTAATATTGAAAAAAATAATGCCAATAATTTACCTTCAACAGGTAATAAAATTATAATCGGATCATTAAATATTAATCATCTTAGTATAGAACCCGAAAAAATTGAGTAAATAGACAAAATAATAATTCNNNNNNNNNNNNNNNNNNNNNNNNNNNNNNNNNNNNNNNNNNNNNNNNNNNNNNNNNNNNNNNNNNNNNNNNNNNNNNNNNNNNNNNNNNNNNNNNNNNNNNNNNNNNNNNNNNNNNNNNNNNNNNNNNNNNNNNNNNNNNNNNNNNNNNNNNNNNNNNNNNNNNNNNNNNNNNNNNNNNNNNNNNNNNNNNNNNNNNNNNNNNNNNNNNNNNNNNNNNNNNNNNNNNNNNNNNNNNNNNNNNNNNNNNNNNNNNNNNNNNNNNNNNNNNNNNNNNNNNNNNNNNNNNNNNNNNNNNNNNNNNNNNNNNNNNNNNNNNNNNNNNNNNNNNNNNNNNNNNNNNNNNNNNNNNNNNNNNNNNNNNNNNNNNNNNNNNNNNNNNNNNNNNNNNNNNNNNNNNNNNNNNNNNNNNNNNNNNNNNNNNNNNNNNNNNNNNNNNNNNNNNNNNNNNNNNNNNNNNNNNNNNNNNNNNNNNNNNNNNNNNNNNNNNNNNNNNNNNNNNNNNNNNNNNNNNNNNNNNNNNNNNNNNNNNNNNNNNNNNNNNNNNNNNNNNNNNNNNNNNNNNNNNNNNNNNNNNNNNNNNNNNNNNNNNNNNNNNNNNNNNNNNNNNNNNNNNNNNNNNNNNNNNNNNNNNNNNNNNNNNNNNNNNNNNNNNNNNNNNNNNNNNNNNNNNNNNNNNNNNNNNNNNNNNNNNNNNNNNNNNNNNNNNNNNNNNNNNNNNNNNNNNNNNNNNNNNNNNNNNNNNNNNNNNNNNNNNNNNNNNNNNNNNNNNNNNNNNNNNNNNNNNNNNNNNNNNNNNNNNNNNNNNNNNNNNNNNNNNNNNNNNNNNNNNNNNNNNNNNNNNNNNNNNNNNNNNNNNNNNNNNNNNNNNNNNNNNNNNNNNNNNNNNNNNNNNNNNNNNNNNNNNNNNNNNNNNNNNNNNNNNNNNNNNNNNNNNNNNNNNNNNNNNNNNNNNNNNNNNNNNNNNNNNNNNNNNNNNNNNNNNNNNNNNNNNNNNNNNNNNNNNNNNNNNNNNNNNNNNNNNNNNNNNNNNNNNNNNNNNNNNNNNNNNNNNNNNNNNNNNNNNNNNNNNNNNNNNNNNNNNNNNNNNNNNNNNNNNNNNNNNNNNNNNNNNNNNNNNNNNNNNNNNNNNNNNNNNNNNNNNNNNNNNNNNNNNNNNNNNNNNNNNNNNNNNNNNNNNNNNNNNNNNNNNNNNNNNNNNNNNNNNNNNNNNNNNNNNNNNNNNNNNNNNNNNNNNNNNNNNNNNNNNNNNNNNNNNNNNNNNNNNNNNNNNNNNNNNNNNNNNNNNNNNNNNNNNNNNNNNNNNNNNNNNNNNNNNNNNNNNNNNNNNNNNNNNNNNNNNNNNNNNNNNNNNNNNNNNNNNNNNNNNNNNNNNNNNNNNNNNNNNNNNNNNNNNNNNNNNNNNNNNNNNNNNNNNNNNNNNNNNNNNNNNNNNNNNNNNNNNNNNNNNNNNNNNNNNNNNNNNNNNNNNNNNNNNNNNNNNNNNNNNNNNNNNNNNNNNNNNNNNNNNNNNNNNNNNNNNNNNNNNNNNNNNNNNNNNNNNNNNNNNNNNNNNNNNNNNNNNNNNNNNNNNNNNNNNNNNNNNNNNNNNNNNNNNNNNNNNNNNNNNNNNNNNNNNNNNNNNNNNNNNNNNNNNNNNNNNNNNNNNNNNNNNNNNNNNNNTTACTATTTGTTTTAATTATTGTAATCAATATTTTAACAATAAATATATTAACTCTTCGAAGAAGAACCNNNNNNNNNNNNNNNNNNNNNNNNNNNNNNNNNNNNNNNNNNNNNNNNNNNNNNNNNNNNNNNNNNNNNNNNNNNNNNNNNNNNNNNNNNNNNNNNNNNNNNNNNNNNNNNNNNNNNNNNNNNNNNNNNNNNNNNNNNNNNNNNNNNNNNNNNNNNNNNNNNNNNNNNNNNNNNNNNNNNNNNNNNNNNNNNNNNNNNNNNNNNNNNNNNNNNNNNNNNNNNNNNNNNNNNNNNNNNNNNNNNNNNNNNNNNNNNNNNNNNNNNNNNNNNNNNNNNNNNNNNNNNNNNNNNNNNNNNNNNNNNNNNNNNNNNNNNNNNNNNNNNNNNNNNNNNNNNNNNNNNNNNNNNNNNNNNNNNNNNNNNNNNNNNNNNNNNNNNNNNNNNNNNNNNNNNNNNNNNNNNNNNNNNNNNNNNNNNNNNNNNNNNNNNNNNNNNNNNNNNNNNNNNNNNNNNNNNNNNNNNNNNNNNNNNNNNNNNNNNNNNNNNNNNNNNNNNNNNNNNNNNNNNNNNNNNNNNNNNNNNNNNNNNNNNNNNNNNNNNNNNNNNNNNNNNNNNNNNNNNNNNNNNNNNNNNNNNNNNNNNNNNNNNNNNNNNNNNNNNNNNNNNNNNNNNNNNNNNNNNNNNNNNNNNNNNNNNNNNNNNNNNNNNNNNNNNNNNNNNNNNNNNNNNNNNNNNNNNNNNNNNNNNNNNNNNNNNNNNNNNNNNNNNNNNNNNNNNNNNNNNNNNNNNNNNNNNNNNNNNNNNNNNNNNNNNNNNNNNNNNNNNNNNNNNNNNNNNNNNNNNNNNNNNNNNNNNNNNNNNNNNNNNNNNNNNNNNNNNNNNNNNNNNNNNNNNNNNNCACGCCGGTCTGAACTCAGATCATGTAAGAATATTAAGGGTCGAACAGACCCAGAAATAATAAATTTTGCTCCAATCCCTATTCTTAATCCAACATCGAGGTCGCAATCATATTTATCGATATGAACTCTTCAAATTAATTACGCTGTTATCCCTAAGGTAATTTATTCTTATAATCAAAAATTTGGATCAACATTTACATAAATNNNNNNNNNNNNNNNNNNNNNNNNNNNNNNNNNNNNNNNNNNNNNNNNNNNNNNNNNNNNNNNNNNNNNNAAANNNNNNNNNNNNNNNNNNNNNNNNNNNNNNNNNNNNNNNNNNNNNNNNNNNNNNNNNNNNNNNNNNNNNNNNNNNNNNNNNNNNNNNNNNNNNNNNNCAATTCAAACAATAAATGANNNNNNNNNNNNNNNNNNNNNNNNNNNNNNNNNNNNNNNNNNNNNNNNNNNNNNNNNNNNNNNNNNNNNNNNNNNNNNNNNNNNNNNNNNNNNNNNNNNNNNNNNNNNNNNNNNNNNNNNNNNNNNNNNNNNNNNNNNNNNNNNNNNNNNNNNNNNNNNNNNNNNNNNNNNNNNNNNNNNNNNAANNNNNNNNNNNNNNNNNNNNNNNNNNNNNNNNNNNNNNNNNNNNNNNNNNNNNNNNNNNNNNNNNNNNNNNNNNNNNNNNNNNNNNNNNNNNNNNNNNNNNNNNNNNNNNNNNNNNNNNNNNNNNNNNNNNNNNNNNNNNNNNNNNNNNNNNNNNNNNNNNNNNNNNNNNNNNNNNNNNNNNNNNNNNNNNNNNNNNNNNNNNNNNNNNNNNNNNNNNNNNNNNNNNNNNNNNNNNNNNNNNNNNNNNNNNNNNNNNNNNNNNNNNNNNNNNNNNNNNNNNNNNNNNNNNNNNNNNNNNNNNNNNNNNNNNNNNNNNNNNNNNNNNNNNATTATAATAATTGAATTGCAGTCAATAGGTGTTATTTATAACTATCCTTAAGTAATGAAGTAAAATAATACATTTAGTTTCGACCTAAAATAAGAATTAGAATTCCTTACTTTTAAATGAAGCCAAAATAGAGGCATTTTATTGTTAATAAAATAATTGAAANTTTATTTCCATTTAAANNNNNNNNNNNNNNNNNNNNNNNNNNNNNNNNNNNNNNNNNNNNNNNNNNNNNNNNNNNNNNNNNTTCTTATGATNTNTTCTACTTTAAATTTGCAATTTAATATTATAAATTAAATATAAGGCTNNNNNNNNNNNNNNNNNNNNNNNNNNNNNNNNNNNNNNATTAATGTTCTTCATAAACTATAACATTTATAAAGTTTATAAAACATTTCATTTTCATTGAAAAGAGAGACTTAGTCTTATAAAATTCTATTAGTATATAAGTATATTTAACTTCCAATTAAAAGGATTNNNNNNNNNNNNNNNNNNNNNNNNNNNNNNNNNNNNNNNNNNNNNNNNNNNNNNNNNNNNNNNNNNNNNNNNNNNNNNNNNNNNNNNNNNNNNNNNNNNNNNNNNNNNNNNNNNNNNNNNNNNNNNNNNNNNNNNNNNNNNNNNNNNNNNNNNNNNNNNNNNNNNNNNNNNNNNNNNNNNNNNNNNNNNNNNNNNNNNNNNNNNNNNNNNNNNNNNNNNNNNNNNNNNNNNNNNNNNNNNNNNNNNNNNNNNNNNNNNNNNNNNNNNNNNNNNNNNNNNNNNNNNNNNNNNNNNNNNNNNNNNNNNNNNNNNNNNNNNNNNNNNNNNNNNNNNNNNNNNNNNNNNNNNNAAAGATAAGCTAAAANTAAAGCTAATGGGTTCATACCTCACTTATGGAAATTTTTCCTCTTTTTATTTAAAAACAAAAGTTACCTTAATATCTTCAATATTATGCTCTTAATTAAGCTATTTAAATNNNNNNNNNNNNNNNNNNNNNNNNNNNNNNNNNNNNNNNNNNNNNNNNNNNNNNNNNNNNNNNNNNAGTTAATTAGCTTAACCAAAAGCATTTATTTTGAAAGTAAAANNNNNNNNNNNNNNNNNNNNNNNNNAGGAAACTAGAAATAAAATTAGCTTCTAACTAACTTTTAAAGCGGTTAAATTCCGTTTTTTCCTTNNNNNNNNNNNNNNNNNNNNNNNNNNNNNNNNNNNNNNNNNNNNNNNNNNNNNNNNNNNNNNNNNAGGTTTTAAGTTATTAANAACTATTATCCTTCAAAGTTAAAAATATAATTANATTTATTATTAGGAGAAATAATTTTTCATAAATAAATTTACAGTTTATTGCCTATTATCGGCCATCCTAANNNNNNNNNNNNNNNNNNNNNNNNNNNNNNNNNNNNNNNNNNNNNNNNNNNNNNNNNNNNNNNNNN

>peninsularis_03.NZ.MC.BPN.02

NNNNNNNNNNNNNNNNNNNNNNNNNNNNNNNNNNNNNNNNNNNNNNNNNNNNNNNNNNNNNNNNNNNNNNNNNNNNNNNNNNNNNNNNNNNNNNNNNNNNNNNNNNNNNNNNNNNNNNNNNNNNNNNNNNNNNNNNNNNNNNNNNNNNNNNNNNNNNNNNNNNNNNNNNNNNNNNNNNNNNNNNNNNGAAGATCATTAATATTTTTATCGTTATTTATATTTATTCTATTAAATAATTTACTAGGGTTACTTCCATATATTTTCACAAGTCCAAGTCACTTGGTATTTACAATAAGATTAGCATTACCACTATGATTATCATTCATACTTTATGGATTTATTAATAATATAAATCATATATTTTGTCATTTAGTTCCTTCAGGAACTCCTAATATTTTAATACCTTTTATAGTTCTTATTGAAAGAGTTAGAAATTTAATTCGACCTGGTTCCTTAGCTGTTCGACTAACAGCTAATATAATTGCTGGTCATCTTTTAATAACTTTATTAGGTAATTTACCTATAAATTATGAATTATATTCAGGTATAATCATTATTTTTCAAGTTATATTAATATTATTTGAATTAGCTGTTTGTATTATTCAATCATATGTGTTTATAGTACTTAGAACTTTATATTATAGAGAAGTAAATNNNNNNNNNNNNNNNNNNNNNNNNNNNNNNNNNNNNNNNNNNNNNNNNNNNNNNNNNNNNNNNNNNNNNNNNNNNNNNNNNNNNNNNNNNNNNNNNNNNNNNNNNNNNNNNNNNNNNNNNNNNNNNNNNNNNNNNNNNNNNNNNTTTAAGCCAACACGACAAAATCATCCTTTGATTAAAATTATTAATAATTCATTAATTGATTTCCCAGCACCATCTAACTTATCATATTGGTGAAATTTTGGATTTGTTTTAGGATTATGTTTAACAATTCAAATCCTAACTGGATTATTTTTATCAATACATTATAATGCCAATATTATAAATGCTTTTGAAAGATTAAGACATATCTGTCGTGATGTAAATTATGGTTGAATTTTACGAGTTATTCACGCTAATGGAGCTTCATTATTTTTCATCTGTGTTTACCTACATGTAGGGCGTGGATTATATTATGGTTCATTCAAATATATTGAAACATGATCTATTGGTGTAATTATATTATTAATATTAATAGCTACCGCTTTTTTAGGATATGTTTTACCTTGAGGACAAATATCTTTTTGAGGGGCTACAGTTATTACAAATTTATTATCAGCTATTCCTTATTTGGGTGGAATATTAGTTAATTGAATTTGAGGAGGATTTGCAGTTGACAATGCAACACTAACTCGATTTTACTCATTCCACTTTATTTTACCATTCATTGTATTAAGATTAACAATTATTCACTTACTCTATCTTCATACAACAGGTTCAAATAATCCACTAGGAATTAATTCTAATAATGACAAAGTTCCTTTTCACCCATATTTTTCTATTAAGGATATTATGAGACTATTTATCTTAATAATTATTTTCTTTATATTAGTCATATTGGAACCCTACATACTAGGGGATCCAGATAATTTTATTCCTGCCAATCCACTTGTAACACCAAAACATATTCAACCTGAATGATATTTTTTATTTGCCTACGCAATTCTTCGTTCAATTCCTAATAAGTTAGGAGGAGTAATTGCTCTTTTTATATCAATTTTTATTTTAATATTTGTACCCTTGTTAAATAATTCAAATTTTATAGGATTAAATAATTACCCAATTAATCAAATCATATTTTGATATATAGTAATCATTCTAATTTTATTAACTTGAATTGGGGCACGACCTGTTGAATTACCTTATATTAATTTTGGAATATTTTTAACACTTATATATTTTTCTTACTTCATTACTGACCCAATAATTAAATCTATTTGAGACAAATTAATTAGGTAGATGAAAAAATGATTCTTCTCAACTAATCATAAAGATATTGGAACAATATATTTCATTTTTGGTATTTGATCAGGCATAATTGGTACAACTTTAAGAGTTTTAATTCGTGTAGAACTTGGAACTCCAGGTTCATTTATTGGTGATGATCAAATTTATAATGTAATTGTCACTGCTCATGCTTTCATCATAATTTTTTTTATAGTTATGCCAATTATAATTGGTGGATTTGGAAATTGATTAGTCCCTTTGATAATTGGGGCTCCTGATATAGCTTTTCCTCGTATAAATAATATAAGTTTTTGATTATTACCACCCTCTCTAATTCTTATTTTAGTGGGAAGAATAGTTGATAGAGGTGCAGGTACAGGTTGAACAGTATATCCCCCTCTATCTGCAGGTATTGCACATTCTGGTTCATGTGTTGATTTAACTATTTTTTCTTTACACCTTGCAGGTGTGTCTTCAATTCTAGGTGCTGTGAATTTCATTAGAACAATTTTTAATATGCGTTCAATAGGAATTTGATTAGATCGAATACCCTTGTTTGTATGAGCAGTTTTAATTACTGCATTTTTGTTATTGTTGTCCTTACCTGTTTTAGCAGGTGCTATCACAATATTATTAACAGATCGAAATTTAAATACTTCATTTTTTGACCCTGCTGGAGGGGGTGATCCCATTCTTTATCAACATTTATTTTGATTTTTTGGTCACCCTGAAGTTTATATTTTAATCCTACCAGGATTTGGTTTAATTTCTCACATTATTACTCAAGAGAGAGGTAAAATTGAATCTTTTGGTTCATTAGGAATAATTTATGCTATAATATCAATTGGTATCTTGGGATTCGTTGTGTGAGCACATCATATATTTACTGTAGGAATAGATGTTGATACACGTGCATATTTCACATCAGCCACTATAATTATTGCAGTCCCTACTGGAATTAAAGTATTTAGTTGACTCGCAACTTTGAGAGGTATAAAAATTAATATTACATCTTCAGCCTTATGGGCTTTAGGATTTGTCTTTTTATTTACCATTGGAGGTTTAACTGGAGTAATTTTAGCTAATTCTTCAATTGATATTATATTACATGATACATATTATGTAGTTGCACATTTTCATTATGTTTTATCAATAGGAGCAGTTTTTGCTATTATAGCAAGATTTATCCATTGATTTCCATTATTTACAGGATTAAGATTAAACTCAAATTGATTAAAAATTCATTTTCTATTAATATTTATTGGTGTAAATATAACATTCTTTCCTCAACATTTTTTAGGATTGAGAGGAATGCCTCGTCGATATTCAGACTATCCTGATGCTTATATGTCATGAAATATAATTTCATCAATAGGAAGAATTATATCTCTAGTTGGAATTTTATTTTTATTATTTATTGTTTGAGAAAGTTTTATTTCAATACGATTAGTATTATATTCTAATAGAATTCAATCTTCGATTGAATGAATACAGAAGTTCCCTCCGTCTGAACATTCTTATAATGAAATGCCATTATTAATTCAAATTTCAAATTGATCTTATATTAATATACAGGATGCTGTATCACCATTAATAGAGCAATTAATATTTTTTCATGATCATGTATTAGNNNNNNNNNNNNNNNNNNNNNNNNNNNNNNNNNNNNNNNNNNNNNNNNNNNNNNNNNNNNNNNTCATTAATCGTTTACTTCTTGAAGGACAATTAATTGAATTTATTTGAACTTTATTACCTGCAATAACTTTAATTTTTATTGCATTACCATCATTACGATTATTATATATATTAGACGAAATTAATAATCCACTATTAACACTAAAAATTATTGGTCATCAATGATATTGATCATATGAATATTCAGATTTTTCTGATGTAGAATTTGATTCATATATAAAATCTATAAACGAAATAAATAAAAATGAATTTCGCTTACTAGATGTAGATAATCGAGTGATTCTTCCATTTAATATTCAAGTCCGATTACTAATTTCTTCTTTTGATGTTATTCACTCTTGAGCAATACCATCTATAAGACTTAAAGTTGATGCAGTTCCAGGACGACTAAATCAAATAAGAATATTAATTAGTCGTCCTGGTGTATCTTATGGACAATGTTCTGAAATTTGTGGGGCAAATCACAGATTTATACCTATTGTAATCGAAAGAATTAGAATAAAGNNNNNNNNNNNNNNNNNNNNNNNNNNNATGAATAACCATCCTTATCATATAGTTGATTATAGACCTTGACCTTTAACTGGATCAATTGGAGCATTAACTTTTGTTTCTGGTATAGTTATAATATTTCATAAATATAACTTTATATTACTATATACTGGTATTTTATTATTATTAATAACAATAATTCAATGATGACGTGACATTTCCCGAGAGGGAACATTTTTAGGAATACACACAATAATGGTAGTTAGTGGTTTAAAAATAGGAATATTATTATTTATTGTATCAGAAGTTCTTTTTTTTGTTTCATTTTTTTGGGGATTTTTTCATAGTAGATTAAGACCTGTGGTAGAAATTGGTATAATTTGACCTCCTAGAGGTATTTATGTTTTTAACCCAACTCAAGTCCCTTTATTGAATACAATAATTTTATTATGTTCAGGAATTACAATTACTTGAGCTCATCATTCAATTATAAATGGTAATCATATTAATTCTATCTATAGAATTATGCTAACTGTTATTTTAGGTATATATTTCACTATTCTACAAGGATATGAATATTATGAAGCTCCATTTGCAATTAACGATTGCATTTATGGATCTTCGTTTTTTATAGCTACTGGATTTCATGGAATTCACGTAATTATTGGAACAAGATTTATTATTGTATGTTTATTACGACAAGTAATATTTCATTTCTCAAGAAAACATCATTTTGGCTTCGAAGCTGCTGCTTGATATTGACATTTTGTTGATGTAGTCTGATTATTTTTATATTTATCAATTTATTGATGAGGAAGATAGNNNNNNNNNNNNNNNNNNNNNNNNNNNNNNNNNNNNNAATTAAGAGATACCGGTAAATAAGATTTTCAAGCCAAATATATTAATTTATCATAACGATAACGAGGAAGAGTTCCTCGAACTCAAATGAAAAGAAAACACATTATAGAAACTTGAATTGGAAAAACAATTGAATCAATTTTTCCTCCTAAAAACATTAAACAAAATATTATTCTCATAAATAAAATACTTGAATACTCAGCTAAAAAAATAAATGCAAATCTAGAACCTCTATATTCAACATTAAAGCCTGATACTAACTCTGATTCTCCCTCAGAAAAATCAAATGGAGATCGATTAGTTTCAGCTAAAGCTGAAGATAATCAACATATTCTTAATGGAAGAAATAAAAAAATAAATCACATAAACTCCTGAAATATAAACAAATCAATAATATTATAACTTCTTATTATTATTATAGGACACAATATAATTAAAACCAATCTTACTTCATAAGAAATAGATTGAGCAATTGATCGAATACATCCTAACATTGCATAACTTGAGTTAGAAGATCAACCTGTTAATATTAGTGAATAAACTCTTATTCTTGAACAACAAAAAAAAAATAAAATACCAAAATTAAAAGAAACACAATTAATTATATATGGATATAATGATCAAATCAATAATCTATTGAATAATCCTATAATAGGTCTAAAAATATAAATTAAATAATTAGATATTAATGGAATTGTATTTTCCTTCATAAATAATTTAATAGCATCAGAAATAGGCTGTAAAATACCCAAAAATCCAACTTTATTTGGACCTTTACGAATTTGAATATATCTCAAAACCTTACGTTCCAAGAGAGTAATAAATGCCACTCCAATCAAAATAAATAATACAGTAATTAAAATAGTAATTAAATACAAAAATAATTCTTCTTATATACTTTATATAATTTTCTTATTATTAGGAATTATAATTTCAGTTTCTTCAAACAATTGGCTGGGTTGTTGAATGGGAATTGAAATAAATATAGTTTCATTTTTGCCCATAATGGCAAACAAGATAAGAATTTATGCTTCGGAATCAATAATTAAATATTTTATTATTCAAAGAATGNNNNNNNNNNNNNNNNNNNNNNNNNNNNNNNNNNNNNNNNNNNNNNNNNNNNNNNNNNNNNNNNNNNNNNNNNNNNNNNNNNNNNNNNNNNNNNNNNNNNNNNNNNNNNNNNNNNNNNNNNNNNNNNNNNNNNNNNNNNNNNNNNNNNNNNNNNNNNNNNNNNNNNNNNNNNNNNNNNNNNNNNNNNNNNNNNNNNNNNNNNNNNNNNNNNNNNNNNNNNNNNNNNNNNNNNNNNNNNNNNNNNNNNNNNNNNNNNNNNNNNNNNNNNNNNNNNNNNNNNNNNNNNNNNNNNNNNNNNNNNNNNNNNNNNNNNNNNNNNNNNNNNNNNNNNNNNNNNNNNNNNNNNNNNNNNNNNNNNNNNNNNNNNNNNNNNNNNNNNNNNNNNNNNNNNNNNNNNNNNNNNNNNNNNNNNNNNNNNNNNNNNNNNNNNNNNNNNNNNNNNNNNNNNNNNNNNNNNNNNNNNNNNNNNNNNNNNNNNNNNNNNNNNNNNNNNNNNNNNNNNNNNNNNNNNNNNNNNNNNNNNNNNNNNNNNNNNNNNNNNNNNNNNNNNNNNNNNNNNNNNNNNNNNNNNNNNNNNNNNNNNNNNNNNNNNNNNNNNNNNNNNNNNNNNNNNNNNTTATTTTGTTTTTTTATTTACGAATTTTAATTACAACACTAATAATAAACACAATTTCAATAAAAAGAATTGTAATGAGAGTTAAATATACTTATTATATTGCTGGGATATTTTCATTATTTGGAATAATTTTTTTTTCACTAATTACATTAAATATGTGTTAGNNNNNNNNNNNNNNNNNNNNNNNNNNNNNNNNNNNNNNNNNNNNNNNNNNNNNNNNNNNNNNNNNNNNNNNNNNNNNNNNNNNNNNNNNNNNNNNNNNNNNNNNNNNNNNNNNNNNNNNNNNTGGTTTCAGGCCATTTGAATCATCTCGTATTCCATTTTCAAGACACTTTTTTTTAATTGCAGTAATTTTTTTAATTTTTGATGTTGAATTAGTAATTATTATACCTGTAATTTTAGTAATAATTAGACTGAAAGCTATTGATATTTATATTATTATATTAATTTTCCTAATTATTTTAACTTTAGGGCTATATCATGAATGATATAATAATATATTAAATTGANNNNNNNGTATAATTAAAATATTTAATTTCAAAATAATTAAATTTAATGGAATTCAATGTATTATAATCAAGAAAAACTCCCGCACTCTTCCTGAATTAAATGAAAATAGACTGGAAAAAAATATACCATGTTGAGTAAATGAAAATATATATAATCTATAACAAGCAGATAAAAATGAGGATAAAATCAATATAATCATTATTAACATGTTTCAAGACATTAATCTATTAATAATTATAATTTCACCGGCTAAATTAATTGTAGGTGGACAAGACATATTTCTTGCTGACATTAAAAATCATAGTAAAGATATTGAAGGTATAAATGTCAATATACCCTTATTAATTAATAATCTTCGACTATTAGTTCGTTCATACAATATATTTGCTAAACAAAATAATCCAGAAGAACATAAACCATGACCAATTATTATATAATATGATCCACATATTCCCCAATTTCTTATGGTTATAATCCCTCTAATCACAAGACCTATATGAGCAACAGATGAATAAGCAATTATTGATTTAATATCAATCTGAATTATACACAGAATTCTAATTAAAATACTTCCAAGTATTGAAATTGAAATCCATATAAATCCAAATTTATAAAAATAAGAAGGAATAATGTACATCATACGAATTAAACCATAACCCCCTAATTTTAGTAAAATACTAGCTAAAATTATTGAACCAGAAACAGGAGCTTCAACATGAGCTTTAGGCAATCAAAAATGAAAAAATAACGGTATTTTAATTAAAAAAGCTATTATTATACCAAAATAAATATAAATATTGACAGAATAATTTATTAATATGAAAATACTAGTTATATTATTTTTATAAAGAAAAAAAATTCTTATTAACATTGGTAATGATGCAAATAATGTATAAAATAATAAATAATAACTCGCATCAATACGTTCTGGTTGATAACCCCAACCTATAATAATAATCATAATTGGAATTAATCTAGATTCAAAAAAAATATAAAAAATTATAATTCTTGATGTAGAAAAAGAAATAATTAAAAAAATTNNNNNNNNNNNNNNNNNNNNNNNNNNNNNNNNNNNNNNNNNNNNNNNNNNNNNNNNNNNNNNNNNNNNNNNNNNNNNNNNNNNNNNNNNNNNNNNNNNNNNNNNNNNNNNNNNNNNNNNNNNNNNNNNNNNNNNNNNNNNNNNNNNNNNNNNNNNNNNNNNNNNNNNNNNNNNNNNNNNNNNNNNNNNNNNNNNNNNNNNNNNNNNNNNNNNNNNNNNNNNNNNNNNNNNNNNNNNNNNNNNNNNNNNNNNNNNNNNNNNNNNNNNNNNNNNNNNNNNNNNNNNNNNNNNNNNNNNNNNNNNNNNNNNNNNNNNNNNNNNNNNNNNNNNNNNNNNNNNNNNNNNNNNNNNNNNNNNNNNNNNNNNNNNNNNNNNNNNNNNNNNNNNNNNNNNNNNNNNNNNNNNNNNNNNNNNNNNNNNNNNNNNNNNNNNNNNNNNNNNNNNKNNNNNNNNNNNNNNNNNNNNNNNNNNNNNNNNNNNNNNNNNNNNNNNNNNNNNNNNNNNNNNNNNNNNNNNNNNNNNNNNNNNNNNNTATAAATATATAACAATAATTCAAATAATAAATGAAATTATCTGAATCTTAAAATTATTATTTATCAATTTATAATTTAATGACATCATTATTTTTAAAGTTTTTAAAATCCCTTGGGGCCCAAGGCTTTCACCTCATCCCATATCAACTGATTTTTGAAATATAAATGATTTATTTATTAATAACATTTGATTATGAAATGTTGACAATTGTTTTATAAATCACATCGATCCAAAAAATTCATAAACAATTTTTATTTTAGTTTTAGAAAAAATTGATATTTCATAACCCAATCAAATTCCTAATATTGAAAAAAATAATGCCAACAATTTACCCTCAGTAGGCATTAAAATTATAATTGGATCATTAAACATTAATCATCTTAATATAGATCCAGAAAAAATTGAATAAATAGATAAAATAACAATTCTTTTAATCATATTATTTATACTTTCATTTAATGATCTTAATTTATAAAAATTNNNNNNNNNNNNNNNNNNNNNNNNNNNNNNNNNNNNNNNNNNNNNNNNNNNNNNNNNNNNNNNNNNNNNNNNNNNNNNNNNNNNNNNNNNNNNNATATATCTAGAACATATAGTTTCTATAATTAAATCTTTTGAATAAAATCCTCTTATAAATGGAATTCCACATAATGATATATTAGCAATATTTATTATTGTAATAGTTAATGGTATTTGAAATCTTAAACATCCTATTACACGGATGTCTTGATTGTTATTAAAATTGTGAATCAAAATTCCTGCACACAGGAATAAAGTAGCTTTAAATAAAGCATGAACAATTAAATGAAAAAATGATAATATGGGATAACCAAATATAGTAATTGTTATTATAATCCCCAACTGTCTTAAAGTTGATAATGCAATAATTTTTTTTAAATCAAACTCAAAATTTGCTCTAATTCCAGATATTAGCATAGTTAAAATTGAAATTAATATAAATAATTGAATATAATCAAAGTTCAAAATAATATTAGAAAATCGAATTATTAAATATACTCCAGCAGTAACAAGTGTAGATGAATGAACTAAAGCAGAAACAGGAGTTGGAGCAGCCATGGCTGCTGGTAATCATGAAGAAAATGGAATTTGAGCTCTCTTTGTGAATCTTGCAATAATAACCATAATTAATATAATTTATATCTTTATTAATAAAAGATAAAAATTTCATGACCCAAAATTTAATATTCATGCAATAGCTATTAAAATAGCAACATCTCCCACTCGATTTATTAGTGCAGTTAATATTCCTGCATTATTAGAATATAAATTTTGGTAATAAATAACTAGACAGTAAGAAACTAGACCAAGACCATCTCAACCAATAATAATTCTCATTATATTAGGTCTAATAATTAATAAAACTATGGATATAACAAACCCCAAAACAATATAAATAAAACGATTAATGAATTTATCATTATTTATATATACTCCACTGTATAAAACAACCACTGATGAAATAAATATAACTGTCGAAAGAAATTGTTGATATAAAATCAAAAATTATAGTCATATAAATATTACATGAATTAATTGAAGATTATTCATTCTANNNNNNNNATATAATTATTTTTGAAATTCATTGAATATTAATATTAGCAGACTTATAAATATAAGAATAATGAATATGTAAACATTAAATTTTTTATTTATCNNNNNNNNNNNNNNNNNNNNNNNNNNNNNNNNNNNNNNNNNNNNNNNNNNNNNNNNNNNNNNNNNNNNNNNNNNNNNNNNNNNNNNNNNNNNNNNNNNNNNNNNNNNNNNNNNNNNNNNNNNNNNNNNNNNNNNNNNNNNNNNNNNNNNNNNNNNNNNNNNNNNNNNNNNNNNNNNNNNNNNNNNNNNNNNNNNNNNNNNNNNNNNNNNNNNNNNNNNNNNNNNNNNNNNNNNNNNNNNNNNNNNNNNNNNNNNNNNNNNNNNNNNNNNNNNNNNNNNNNATTCAATCAACATAATTGATTTAAAATCACTAAATATTAAAATAAACATTATAATATATATAAACCACAACAATTTTATAATAAGAAAAATATACATTATACCTTCTGGGATAATAACACTGATTTTAACAATTTATTTACTATTTGTTTTAATTATTGTAATTAATATTTTAACAATAAATATATCAACTCTTCGAAGAAGAATTNNNNNNNNNNNNNNNNNNNNNNNNNNNNNNNNNNNNNNNNNNNNNNNNNNNNNNNNNNNNNNNNNNNNNNNNNNNNNNNNNNNNNNNNNNNNNNNNNNNNNNNNNNNNNNNNNNNNNNNNNNNNNNNNNNNNNNNNNNNNNNNNNNNNNNNNNNNNNNNNNNNNNNNNNNNNNNNNNNNNNNNNNNNNNNNNNNNNNNNNNNNNNNNNNNNNNNNNNNNNNNNNNNNNNNNNNNNNNNNNNNNNNNNNNNNNNNNNNNNNNNNNNNNNNCANNNNNNNNNNNNNNNNNNNNNNNNNNNNNNNNNNNNNNNNNNNNNNNNNNNNNNNNNNNNNNNNNNNNNNNNNNNNNNNNNNNNNNNNNNNNNNNNNNNNNNNNNNNNNNNNNNNNNNNNNNNNNNNNNNNNNNNNNNNNNNNNNNNNNNNNNNNNNNNNNNNNNNNNNNNNNNNNNNNNNNNNNNNNNNNNNNNNNNNNNNNNNNNNNNNNNNNNNNNNNNNNNNNNNNNNNNNNNNNNNNNNNNNNNNNNNNNNNNNNNNNNNNNNNNNNNNNNNNNNNNNNNNNNNNNNNNNNNNNNNNNNNNNNNNNNNNNNNNNNNNNNNNNNNNNNNNNNNNNNNNNNNNNNNNNNNNNNNNNNNNNNNNNNNNNNNNNNNNNNNNNNNNNNNNNNNNNNNNNNNNNNNNNNNNNNNNNNNNNNNNNNNNNNNNNNNNNNNNNNNNNNNNNNNNNNNNNNNNNNNNNNNNNNNNNNNNNNNNNNNNNNNNNNNNNNNATAATAAAATTATTTAATGAGGTCCTTTCGTACTAACATTAAAAACAATTGAGTAGATAGAAACCAACCTGGCTCACGCCGGTTTGAACTCAGATCATGTAAGAATATTAAGGGTCGAACAGACCCAGAAATAATAAATTTTGCTCCAATCTCTATTCTTAATCCAACATCGAGGTCGCAATCATATTTATCGATATGAACTCTTCAAATTAATTACGCTGTTATCCCTAAGGTAATTTATTCTTATAATCAAAAATTTGGATCAATATTTACATAAATTTATGAAATTTCTATTAAAAGTTAATTATATTTTAATATCACCCCAACAAAAAAATCAATTAAGTAAATAAAATTAATTAACTGTAAANAAATAAACTTAATNAAGATTTTAAAAATTCTATAGGGTCTTCTCGTCCCACTCACATATTTAAGCTTTTTTACTTAAAAATCAATTTCAATTATTAATATTAATTAAGTTAATTTCTCATTCAACCATTCATTCAAGCCTCCAATTAAAAGACTATTTATTATGCTACCTTTGCACGGTCAATTTACCGCGGCCATTTAATTTNTAATCATTGGGCAGATTAGACTTTTAATTCTTGCTAAAAGACATGTTTTTGATAAACAGGTGAAAATTGTTTTTGCNNNNNNNNNNNNNNNNNNNNNNNNNNNNNNNNNNNNNNNNNNNNNNNNNNNNNNNNNNNNNNNNNNNNNNNNNNNNNNNNNNNNNNNNNNNNNNNNNNNNNNNNNNNNNNNNNNNNNNNNNNNNNNNNNNNNNNNNNNNNNNNNNNNNNNNNNNNNNNNNNNNNNNNNNNNNNNNNNNNNNNNNNNNNNNNNNNNNNNNNNNNNNNNNNNNNNNNNNNNNNNNNNNNNNNNNNNNNNNNNNNNNNNNNNNNNNNNNNNNNNNNNNNNNNNNNNNNNNNNNNNNNNNNNNNNNNNNNNNNNNNNNNNNNNNNNNNNNNNNNNNGGGATATATTTAATTATAATAATTGAATTGCAGTCAATAGGTGTTTTTTATAACTATCCTTAAGTAATGAAGTAAAACAATACATTTAGTTTCGACCTAAAATAAGAATTAGAATTCCTTACTTTTAAATGAAGCCAAAATAGAGGCATTTTATTGTTAATAAAATAATTGAAANTTTATTTCCATTTAAANNNNNNNNNNNNNNNNNNNNNNNNNNNNNNNNNNNNNNNNNNNNNNNNNNNNNNNNNNNNNNGAATTTTTATGATTTNTTCTACTTTAAATTTGCAATTTAATATTATAAATTAAATATAAGACTTTATGTTAACAGAATTAAACTGTCTATTAATATCAAAAATTAATGTTCTTCATAAACTATAACATTTATAAAGTTTATAAAACATTTCATTTTCATTGAAAAGAGAGACTTAGTCTTATAAAATTCTATTAGTATATAAGTATATTTAACTTCCAATTAAAAGGATTAATTTGNNNNNNNNNNNNNTATCCAATAAATTTTNTTTTATCTTTAAAATCACAATTTAATATTTTTATTAAACTAATTGAATTACTATTTGTAATAAACATTACATTTTTAAATTCTAAATTTAAAGCATTAATTTTGCTAAAATAGTTCTAATATGGCAGATTAGTGCAATGAATTTAAGATTCATATATAAAATATNATTTTTTTATTGGAAGATAAGATGCCTGAATNGAAAAGGGTTATTTTGATAGAATAAATTATGTAATATAGTTACTCNNNNNNNNNNNNNNNNNNNNNNNNNNNNNNNNNNNNNNNNNNNNNNNNNNNNNNNNNNNNNNNNNNNNNNNNAAAGATAAGCTAAAATTAAAGCTAATGGGTTCATACCTCACTTATGGAAANTTTTCCTCTTTTTATTTAAAAACAAAAGTTACCTTAATATCTTCAATATTATGCTCTTAATTAAGCTATTTAAATNNNNNNNNNNNNNNNNNNNNNNNNNNNNNNNNNNNNNNNNNNNNNNNNNNNNNNNNNNNNNNNNNNAGTTAATTAGCTTAACCAAAAGCATTTATTTTGAAAGTAAAAGAAAAGATATTTAATNNNNNNNNNNAGGAAACTAGAAATAAAATTAGCTTCTAACTAACTTTTAAAGCGGTTAAATTCCGTTTTTTCCTTNNNNNNNNNNNNNNNNNNNNNNNNNNNNNNNNNNNNNNNNNNNNNNNNNNNNNNNNNNNNNNNNNAGGTTTTAAGTTATTAANAACTATTATCCTTCAAAGTTAAAAATATAATTATATTTATTATTAGGAGAAATAATTTTTCATGAATAAATTTACAGTTTATTACCTATTTTCGGTCATCCTAANNNNNNNNNNNNNNNNNNNNNNNNNNNNNNNNNNNNNNNNNNNNNNNNNNNNNNNNNNNNNNNNNN

>ochrina_14.NZ.AK.PNL.04

NNNNNNNNNNNNNNNNNNNNNNNNNNNNNNNNNNNNNNNNNNNNNNNNNNNNNNNNNNNNNNNNNNNNNNNNNNNNNNNNNNNNNNNNNNNNNNNNNNNNNNNNNNNNNNNNNNNNNNNNNNNNNNNNNNNNNNNNNNNNNNNNNNNNNNNNNNNNNNNNNNNNNTTAATAAATTATAAATCACTAGGAAGATCATTAATATTTTTATCGTTATTTATATTTATTATACTAAATAACTTGTTAGGTTTACTTCCATATATTTTCACAAGTTCAAGTCACTTAGTATTTACAATAAGATTAGCATTACCATTATGATTGTCATTTATGCTTTATGGATTTATTAATAATATAAATCATATATTTTGCCATCTAGTTCCTTCAGGAACTCCTAATATTTTAATACCTTTTATAGTTATTATTGAAAGAGTCAGTAACTTAATTCGTCCTGGTTCACTAGCTGTCCGGCTAACAGCTAATATAATTGCTGGGCATCTTTTAATAACTTTATTAGGTAATTTGCCTATAAATTATGAATTATATTCAGGTATAATCATTATTTTTCAAATTATATTAATATTATTTGAATTAGCTGTTTGTGTAATTCAATCATATGTATTTATAGTGCTTAGAACTTTATATTATAGAGAAGTAAATNNNNNNNNNNNNNNNNNNNNNNNNNNNNNNNNNNNNNNNNNNNNNNNNNNNNNNNNNNNNNNNNNNNNNNNNNNNNNNNNNNNNNNNNNNNNNNNNNNNNNNNNNNNNNNNNNNNNNNNNNNNNNNNNNNNNNNNNNNNNNNNNTTTAAGCCAACACGACAAAATCATCCTTTGATTAAAATTATTAACAATTCATTAATTGACTTTCCAGCACCATCTAACTTATCTTATTGGTGAAATTTTGGATTTGTATTAGGATTATGTTTAACAATCCAAATTCTAACTGGATTATTTTTATCAATACATTATAATGCCAATATTATAAATGCTTTTGAAAGATTAAGCCACATTTGTCGTGATGTAAATTATGGTTGAATCTTACGAGTTATTCACGCTAATGGAGCTTCATTATTTTTCATCTGTGTTTATCTGCATGTAGGACGTGGATTATATTATGGTTCATTTAAATATATTGAAACATGATCTATTGGTGTAATTATACTATTAATATTAATAGCTACCGCTTTTTTAGGATATGTTTTGCCATGAGGACAAATATCTTTCTGGGGGGCCACAGTTATTACAAATTTATTATCAGCCATTCCTTATTTAGGTAGAATATTAGTTAATTGAATTTGAGGAGGATTTGCAGTTGACAATGCAACACTAACTCGATTTTACTCATTCCACTTTATTTTACCATTTATTGTATTAAGACTAACAATTATTCACTTGCTGTATCTTCATACAACAGGTTCAAATAATCCTCTAGGAATTAATTCAAATAATGACAAAGTCCCCTTTCACCCATATTTTTCCATTAAGGATATTATAAGACTATTTATCTTAATAATTATTTTTTTTATATTAGTCATATTGGAACCCTATATACTAGGGGATCCAGATAATTTTATTCCTGCTAATCCACTTGTAACACCAAAGCATATTCAACCTGAATGATACTTTTTATTTGCATACGCAATTCTTCGTTCAATTCCTAACAAATTAGGAGGAGTAATTGCTCTTTTTATATCAATTTTTATTTTAATGTTCGTACCTTTACTAAATAATTCTAATTTCATAGGATTAAATAATTACCCGATTAATCAAATTATTTTTTGATATATAGTAATCATCTTAATTTTATTAACTTGAATTGGAGCACGACCTGTCGAATTACCTTATATTAACTTTGGAATATTTTTAACACTTATATATTTTTCTTACTTTATTATTGACCCAATAATTAAATCTATTTGAGACAAATTAATTAGATAGATGAAAAAATGATTCTTCTCAACTAATCATAAAGATATTGGAACAATATATTTTATTTTTGGTATTTGATCGGGTATAATTGGTACAACTTTAAGAGTTTTAATTCGTGTAGAACTCGGAACTCCAGGTTCTTTCATTGGTGATGATCAAATTTATAATGTAATTGTCACTGCTCATGCTTTCATTATAATTTTTTTTATAGTTATGCCAATTATAATTGGCGGATTTGGAAATTGATTAGTCCCTCTAATAATTGGAGCCCCTGATATAGCTTTTCCTCGTATAAATAACATAAGTTTTTGATTATTACCACCATCTTTAACTCTCATTTTAGTGGGAAGAATAGTTGATAGAGGTGCAGGTACAGGTTGAACAGTTTACCCCCCTCTGTCTGCGGGTATTGCACATTCTGGTTCATGTGTTGATTTAACTATTTTTTCCCTACACCTTGCAGGTGTATCTTCAATTTTAGGTGCTGTGAATTTCATTAGAACAATTTTTAATATACGTTCAATAGGAATTTGACTAGATCGTATACCATTATTTGTCTGAGCAGTTTTAATTACTGCATTTTTATTATTATTGTCTTTACCTGTTTTAGCAGGCGCTATTACAATATTATTAACAGATCGGAATTTAAATACTTCATTTTTTGATCCTGCAGGAGGAGGAGATCCTATTCTTTATCAACATTTATTTTGATTTTTTGGTCACCCTGAAGTTTATATTTTAATCTTGCCAGGATTTGGTTTAATTTCTCATATTATTACTCAAGAGAGAGGCAAAATTGAATCTTTTGGATCATTAGGAATAATTTATGCTATAATATCAATTGGTATTTTAGGATTTGTTGTGTGAGCACATCATATATTTACTGTAGGAATAGATGTTGACACACGTGCATATTTTACATCAGCCACTATAATTATTGCAGTTCCTACTGGAATTAAAGTATTTAGTTGACTTGCAACTTTGAGAGGTATAAAAATTAATATTACATCTTCAGCTTTATGAGCTTTAGGATTCGTATTTTTATTTACTATTGGGGGTTTAACCGGAGTAATTTTAGCTAATTCTTCAATTGATATTATACTACATGATACATATTATGTAGTTGCACATTTTCATTATGTTTTATCAATAGGAGCAGTTTTTGCTATCATAGCAAGATTTATTCATTGATTTCCATTATTTACAGGATTGAGATTAAACTCAAATTGATTAAAAATTCATTTTCTATTGATATTTATAGGTGTAAATATAACATTCTTTCCACAACATTTCTTGGGATTAAGAGGAATACCACGTCGATATTCAGATTATCCTGATGCTTATATATCATGAAACATGATTTCATCAATAGGAAGAATTATATCTTTAATTGGAATTCTACTTTTATTATTTATTGTTTGGGAAAGTTTTATTTCAATACGTTTAGTATTATATTCTAATAGAATTCAATCTTCTATTGAATGAATACAAAAATTTCCTCCATCTGAACATTCTTATAATGAAATGCCATTATTAATTCAAATTTCAAATTGATCTTATATTAATATACAGGATGCTGTATCACCATTAATAGAGCCGTTAATATTTTTTCNNNNNNNNNNNNNNNNNNNNNNNNNNNNNNNNNNNNNNNNNNNNNNNNNNNNNNNNNNNNNNNNNNNNNNNNNNNNNNNNNNNNNNNNNNNNNNNNNNNNNNNNNNNNNNNNNNNNNNNNNNNNNNNNNNNNNNNNNNNNNNNNNNNNNNNNNNNNNNNNNNNNNNNNNNNNNNNNNNNNNNNNNNNNNNNNNNNNNNNNNNNNNNNNNNNNNNNNNNNNNNNNNNNNNNNNNNNNNNNNNNNNNNNNNNNNNNNNNNNNNNNNNNNNNNNNNNNNNNNNNNNNNNNNNNNNNNNNNNNNNNNNNNNNNNNNNNNNNNNNNNNNNNNNNNNNNNNNNNNNNNNNNNNNNNNNNNNNNNNNNNNNNNNNNNNNNNNNNNNNNNNNNNNNNNNNNNNNNNNNNNNNNNNNNNNNNNNNNNNNNNNNNNNNNNNNNNNNNNNNNNNNNNNNNNNNNNNNNNNNNNNNNNNNNNNNNNNNNNNNNNNNNNNNNNNNNNNNNNNNNNNNNNNNNNNNNNNNNNNNNNNNNNNNNNNNNNNNNNNNNNNNNNNNNNNNNNNNNNNNNNNNNNNNNNNNNNNNNNNNNNNNNNNNNNNNNNNNNNNNNNNNNNNNATGAATAATCATCCTTATCATATAGTTGATTATAGACCCTGACCTTTAACTGGATCAATTGGAGCATTAACTTTTGTTTCCGGTATAGTAATAATATTTCATAAATGTAACTTTATATTACTATATACTGGTATTTTATTATTACTAATAACAATAATTCAATGATGACGTGATATTTCTCGAGAAGGAACATTTTTAGGAATACATACATTAATGGTAGTTAATGGTTTGAAAATAGGAATATTATTATTTATTGTATCGGAAGTTCTTTTCTTTGTTTCATTTTTTTGGGGATTTTTCCATAGTAGATTAAGACCTGTAGTAGAAATTGGCATAATATGACCTCCTAGAGGTATTTATGTTTTTAATCCAACCCAAGTACCTCTATTAAATACAATAATTTTATTATGCTCAGGAATTACAATTACTTGAGCTCACCATTCAATTATAAGTGGTAATCATATTAATTCTGTTTATAGAATTATCTTAACTGTTATTTTAGGTATATATTTCACTATTTTACAAGGTTATGAATATTATGAAGCTCCATTTGCAATTAATGATTCCATTTATGGGTCATCTTTTTTTATAGCTACTGGATTTCATGGAATTCACGTAATCATTGGAACAAGATTTATTATTGTATGCTTAATACGACAAATAAGATTTCATTTTTCAAGAAATCATCACTTTGGCTTCGAAGCTGCTGCTTGATATTGACATTTTGTTGACGTAGTTTGATTATTTTTATATTTATCAATTTATTGATGAGGAAGATAATAAATTATAGTCTTTAATAGGAAGAAAAAAATAAATAAATTTAGAGATACCGGTAAATAAGATTTTCAAGCCAAATATATTAATTTATCATAACGATAACGGGGAAGAGTTCCCCGAACTCAAATGAAAAAAAAACACATTATAGAAACTTGAATTGGAAAAACAATTGAATTAATTTTTCCTCCTAAAAACATTAAACAAAATATTATTCTCATAAATAAAATACTTGAATACTCAGCCAAAAAAATAAATGCAAACCTAGAACCTCTATATTCAACATTATAACCAGAAACTAACTCTGATTCTCCCTCAGAGAAATCAAATGGAGATCGATTAGTTTCAGCTAAAGCTGAAGATAATCAACACATTCTTAAAGGAAGAAATAAAAAAATAAATCAAATATACTCCTGATATATAAACAAATCAATAATATTATAACTTCTTATTATCATTATAGGACATAACATAATTAAAACCAATCTTACTTCATAAGAAATAGATTGAGCAATTGATCGAATACATCCCAATATTGCATAACTTGAATTTGAAGATCAACCTGTTAATATTAATGAATAAACTCTTATTCTTGAACAACAAAAAAAAAATAAAACACCAAAATTAAAAGAAACACAATTAATCATGTATGGATATAATGATCAAATCAACAATCTATTGAATAATCCTATAACAGGTCTAAAAATATAAATTAAATAATTAGATATTAATGGAATTGTATTTTCCTTTATAAATAATTTAATGGCATCAGAAATAGGCTGTAAAATACCCAAAAATCCAACTTTATTTGGACCTTTACGAATTTGAATATATCTCAAAACCTTTCGTTCCAAAAGAGTAATAAATGCCACTCCAATCAATATAAATAACACAGTAATTAAAATAGTAATTAAATACNNNNNNNNNNNNNNNNNNNNNNNNNNNNNNNNNNNNNNNNNNNNNNNNNNNNNNNNNNNNNNNNNNNNNNNNNNNNNNNNNNNNNNNNNNNNNNNNNNNNNNNNNNNNNNNNNNNNNNNNNNNNNNNNNNNNNNNNNNNNNNNNNNNNNNNNNNNNNNNNNNNNNNNNNNNNNNNNNNNNNNNNNNNNNNNNNNNNNNNNNNNNNNNNNNNNNNNNNNNNNNNNNNNNNNNNNNNNNNNNNNNNNNNNNNNNNNNNNNNNNNNNNNNNNNNNNNNNNNNNNNNNNNNNNNNNNNNNNNNNNNNNNNNNNNNNNNNNNNNNNNNNNNNNNNNNNNNNNNNNNNNNNNNNNNNNNNNNNNNNNNNNNNNNNNNNNNNNNNNNNNNNNNNNNNNNNNNNNNNNNNNNNNNNNNNNNNNNNNNNNNNNNNNNNNNNNNNNNNNNNNNNNNNNNNNNNNNNNNNNNNNNNNNNNNNNNNNNNNNNNNNNNNNNNNNNNNNNNNNNNNNNNNNNNNNNNNNNNNNNNNNNNNNNNNNNNNNNNNNNNNNNNNNNNNNNNNNNNNNNNNNNNNNNNNNNNNNNNNNNNNNNNNNNNNNNNNNNNNNNNNNNNNNNNNNNNNNNNNNNNNNNNNNNNNNNNNNNNNNNNNNNNNNNNNNNNNNNNNNNNNNNNNNNNNNNNNNNNNNNNNNNNNNNNNNNNNNNNNNNNNNNNNNNNNNNNNNNNNNNNNNNNNNNNNNNNNNNNNNNNNNNNNNNNNNNNNNNNNNNNNNNNNNNNNNNNNNNNNNNNNNNNNNNNNNNNNNNNNNNNNNNNNNNNNNNNATTTTATTTTTTTATTTACGAATTTTAATTACAACGTTAATAATGAGAACAATTTCAATAAAAAGAATTGTTATAAGAGTTTCACATACTTACTATATTGCTGGAATATTTTCATTATTTGGAATAATCTTTTTTTCATTAATTACATTAAATATGTGTTAGATTTATAATNNNNNNNNNNNNNNNNNNNNNNNNNNNNNNNNNNNNNNNNNNNNNNNNNNNNNNNNNNNNNNNNNNNNNNNNNNNNNNNNNNNNNNNNNNNNNNNNNNNNNNNNNNNNNNNNNNNNNNNNNNNNNNNNNNNNNNNNNNNNNNNNNNNNNNNNNNNNNNNNNNNNNNNNNNNNNNNNNNNNNNNNNNNNNNNNNNNNNNNNNNNNNNNNNNNNNNNNNNNNNNNNNNNNNNNNNNNNNNNNNNNNNNNNNNNNNNNNNNNNNNNNNNNNNNNNNNNNNNNNNNNNNNNNNNNNNNNNNNNNNNNNNNNNNNNNNNNNNNNNNNNNNNNNNNNNNNNNNNNNNNNNNNNNNNNNNNNNNNTTAATTTCAAAATAATTAAATTTAATGGAATTCAATGTATTATAATCAAGAAAAACTCCCGGACTCTTCCTGAATTAAATGAAAATAAACTGGAAAAAAATACACCATGTTGAGTATATGAAAATATATATAATCTATAACAAGCAGATAAAAATGAGGATAAAATCAATAAAATCATTATTAATCTATTTCAAGATATTAATCTATTGATAATTATAATTTCACCAGCTAAATTAATTGTAGGGGGACAAGATATATTTCTTGCTGACATTAAAAATCATAATAAAGATATTGAGGGTATAAATGTCAACATACCCTTATTGATTAATAATCTTCGACTATTAGTTCGTTCATATAATATATTTGCTAAGCAAAATAATCCAGAAGAACATAAACCATGTCCAATTATTATATAATATGACCCAAATATTCCCCAATTTCTTATAGTCATAATACCTCTAATTACAAGACCTATGTGAGCAACAGATGAATAAGCAATTATTGATTTAATATCAATCTGAATTATACACAGAATTCTAATCAAAATACTCCCAATTATTGAAATTGAAATCCATATAAATCCAAATTTATAAAAATAAGAAGGAAAAATAAACATTACACGAATCAAACCATATCCACCTAATTTTAATAAAATTCTAGCTAAAATTATTGAACCAGAAACAGGAGCTTCAACATGAGCTTTAGGTAATCAAAAGTGAAAAAATAATGGTATTTTAATTAAAAAAGCCATTATTATACCAAAATAAATATAAATATTGACAGAATAATTTATTAACATAAAAATNNNNNNNNNNNNNNNNNNNNNNNNNNNNNNNNNNNNNNNNNNNNNNNNNNNNNNNNNNNNNNNNNNNNNNNNNNNNNNNNNNNNNNNNNNNNNNNNNNNNNNNNNNNNNNNNNNNNNNNNNNNNNNNNNNNNNNNNNNNNNNNNNNNNNNNNNNNNNNNNNNNNNNNNNNNNNNNNNNNNNNNNNNNNNNNNNNNNNNNNNNNNNNNNNNNNNNNNNNNNNNNNNNNNNNNNNNNNNNNNNNNNNNNNNNNNCGTTTATATATTGGACTGGATACAATTATTAAAAATGAAATTCAAATACAAAATAATTAAACCATATGATATTTCATCAATATAAAAATTATAACCTAATATATATAAATTCTTATTTATAAAAACCAAAAATATAATTATCAAAACGATAATCATATTTATTAATCATGAATCATAAATTAATAGGGTTAATATAAAACAAAAAAAAACTAATTTTATTGATATAGATATTAATTTATCATTATTATGAGACCGAATTAATATAACTAAACATGATAAACCCATTACTCCTTCACAGACTCTAAAGACCAAAAAAATCAATAAAATATGCAAATCATATATTTTTATAGAGAAAAAAAATGACAAAGAACAAAATATTCTTAAGATAATTAATTCCAATCTTAGCGTTGTTATTAAATGTTTTCGATTTATACATAATGAAACTNNNNNNNNNNNNNNNNNNNNNNNNNNNNNNNNNNNNNNNNNNNNNNNNNNNNNNNNNNNNNNNNNNNNNNNNNNNNNNNNNNNNNNNNNNNNNNNNNNNNNNNNNNNNNNNNNNNNNNNNNNNNNNNNNNNNNNNNNNNNNNNNNNNNNNNNNNNNNNNNNNNNNNNNNNNNNNNNNNNNNNNNNNNNNNNNNNNNNNNNNNNNNNNNNNNNNNNNNNNNNNNNNNNNNNNNNNNNNNNNNNNNNNNNNNNNNNNNNNNNNNNNNNNNNNNNNNNNNNNNNNNNNNNNNNNNNNNNNNNNNNNNNNNNNNNNNNNNNNNNNNNNNNNNNNNNNNNNNNNNNNNNNNNNNNNNNNNNNNNNNNNNNNNNNNNNNNNNNNNNNNNNNNNNNNNNNNNNNNNNNNNNNNNNNNNNNNNNNNNNNNNNNNNNNNNNNNNNNNNNNNNNNNNNNNNNNNNNNNNNNNNNNNNNNNNNNNNNNNNNNNNNNNNNNNNNNNNNNNNNNNNNNNNNNNNNNNNNNNNNNNNNNNNNNNNNNNNNNNNNNNNNNNNNNNNNNNNNNNNNNNNNNNNNNNNNNNNNNNNNNNNNNNNNNNNNNNNNNNNNNNNNNNNNNNNAATTAGTAAATTAATATGTCTAGAACATATAGTTTCTATAATTAAATCTTTTGAATAAAATCCTCTTATAAATGGACTTCCACATAATGACATATTAGCAATATTTATTATTGTAATAGTTAATGGTATCTGAAATCTTAAACATCCTATTACACGAATATCTTGATTATTATTAAAATTGTGAATCAAGATTCCCGCACATAAAAATAAAGTAGCCTTAAATAAAGCATGAACAATTAAGTGAAAAAATGATAATATTGGATAGCCAAATATAATAATTGTTATTATAATCCCCAACTGTCTTAAAGTTGATAATGCAATAATTTTTTTTAAATCAAACTCGAAATTGGCTCTAATTCCTGATATTAATATAGTTAAAATTGAAATTAACATAAATACTTGAATGTAATCAAAATTCAAAATAATATTAGAAAATCGAATTATTAAATATACTCCAGCAGTAACAAGAGTAGATGAATGAACCAAGGCAGAAACAGGAGTTGGAGCGGCCATAGCCGCTGGTAATCATGAGGAAAATGGAATCTGAGCTCTCTTTGTAAATCTTGCAATAATAATCATGACTAATATAATTTATATCTTTATTAATAAAGGATAAAAATTTCATGACCCAAAATTTAATATTCATGCAATAGCTATTAAAATAGCAACATCTCCCACACGATTCATTAATGCAGTTAATATTCCTGCATTATTAGAATATAAATTTTGATAGTAAATAACTAAACAATAAGAAACTAAACCAAGTCCATCTCAACCAATAATAATTCTCATTATATTAGGTCTAATAATTAATAAAATTATAGATATAACAAATCCCAAAACAATATAAATAAAACGATTAATAAATTTATCATTATTTATATATATTCCACTATATAAAACAACTACTGATGAAATAAATATAACTGTCGAAAGAAATTGCTGATATAAAATCAAAAATTATAGTCATATAAATATTACATGAATTAATTGAAGATTATTCNNNNNNNNNNNNNNNNNNNNNNNNNNNNNNNNNNNNNNNNNNNNNNNNNNNNNNNNNNNNNNNNNNNNNNNNNNNNNNNNNNNNNNNNNNNNNNNNNNNNNNNNNNNNNNNNNNNNNNNNNNNNNNNNNNNNNNNNNNNNNNNNNNNNNNNNNNNNNNNNNNNNNNNNNNNNNNNNNNNNNNNNNNNNNNNNNNNNNNNNNNNNNNNNNNNNNNNNNNNNNNNNNNNNNNNNNNNNNNNNNNNNNNNNNNNNNNNNNNNNNNNNNNNNNNNNNNNNNNNNNNNNNNNNNNNNNNNNNNNNNNNNNNNNNNNNNNNNNNNNNNNNNNNNNNNNNNNNNNNNNNNNNNNNNNNNNNNNNNNNNNNNNNNNNNNNNNNNNNNNNNNNNNNNNNNNNNNNNNNNNNNNNNNNNNNNNNNNNNNNNNNNNNNNNNNNNNNNNNNNNNNNNNNNNNNNNNNNNNNNNNNNNNNNNNNNNNNNNNNNNNNNNNNNNNNNNNNNNNNNNNNNNNNNNNNNNNNNNNNNNNNNNNNNNNNNNNNNNNNNNNNNNNNNNNNNNNNNNNNNNNNNNNNNNNNNNNNNNNNNNNNNNNNNNNNNNNNNNNNNNNNNNNNNNNNNNNNNNNNNNNNNNNNNNNNNNNNNNNNNNNNNNNNNNNNNNNNNNNNNNNNNNNNNNNNNNNNNNNNNNNNNNNNNNNNNNNNNNNNNNNNNNNNNNNNNNNNNNNNNNNNNNNNNNNNNNNNNNNNNNNNNNNNNNNNNNNNNNNCAATTCAAACAATAAATGANNNNNNNNNNNNNNNNNNNNNNNNNNNNNNNNNNNNNNNNNNNNNNNNNNNNNNNNNNNNNNNNNNNNNNNNNNNNNNNNNNNNNNNNNNNNNNNNNNNNNNNNNNNNNNNNNNNNNNNNNNNNNNNNNNNNNNNNNNNNNNNNNNNNNNNNNNNNNNNNNNNNNNNNNNNNNNNNNNNNNNNNAATCATTTGAATNNNNNNNNNNNNNNNNNNNNNNNNNNNNNNNNNNNNNNNNNNNNNNNNNNNNNNNNNNNNNNNNNNNNNNNNNNNNNNNNNNNNNNNNNNNNNNNNNNNNNNNNNNNNNNNNNNNNNNNNNNNNNNNNNNNNNNNNNNNNNNNNNNNNNNNNNNNNNNNNNNNNNNNNNNNNNNNNNNNNNNNNNNNNNNNNNNNNNNNNNNNNNNNNNNNNNNNNNNNNNNNNNNNNNNNNNNNNNNNNNNNNNNNNNNNNNNNNNNNNNNNNNNNNNNNNNNNNNNNNNNNNNNNNNNNNNNNNNNNNNNNNNNNNNNNNNNNNNNNNNNNNNNNNNNNNNNNNNNNNNNNNNNNNNNNNNNNNNNNNNNNNNNNNNNNNNNNNNNNNNNNNNATAATAAAATTATTTAATGAGGTCNTTTCGTACTAACATTAAAAATAGTTGAGTAGATAGAAACCAACCTGGCTCACGCCGGTCTGAACTCAGATCATGTAAGAATATTAAGGGTCGAACAGACCCAGAAATAATAAATTTTGCTCCAATCCCTATTCTTAATCCAACATCGAGGTCGCAATCATATTTATCGATATGAACTCTTCAAATTAATTACGCTGTTATCCGTAAGGTAATTTATTCTTATAATCAAAAATTTGGANNNNNAGCTATTTAAATTTATAAANNNNNNNNNNNNNNNNNNNNNNNNNNNNNNNNNNNNNNNNNNNNNNNNNNNNNNNNNNNNNNNNNNNNNNNNNNNNNTATANAAACAANNNNNNNNNNNNNNNNNNNNNNNNNNNNNNNNNNNNNNNNNNNNNNNNNNNNNNNNNNNNNNNNNNNNNNNNNNNNNNNNNNNNNNNNNNNNNNNNNNNNNNNNNNNNNNNNNNNNNNNNNNNNNNNNNNNNNNNNNNNNNNNNNNNNNNNNNNNNNNNNNNNNNNNNNNNNNNNNNNNNNNNNNNNNNNNNNNNNNNNNNNNNNNNNNNNNNNNNNNNNNNNNNNNNNNNNNNNNNNNNNNNNNNNNNNNNNNNNNNNNNNNNNNNNNNNNNNNNNNNNNNNNNNNNNNNNNNNNNNNNNNNNNNNNNNNNNNNNNNNNNNNNNNNNNNNNNNNNNNNNNNNNNNNNNNNNNNNNNNNNNNNNNNNNNNNNNNNNNNNNNNNNNNNNNNNNNNNNNNNNNNNNNNNNNNNNNNNNNNNNNNNNNNNNNNNNNNNNNNNNNNNNNNNNNNNNNNNNNNNNNNNNNNNNNNNNNNNNNNNNNNNNNNNNNNNNNNNNNNNNNNNNNNNNNNNNNNNNNNNNNNNNNNNNNNNNNNNNNNNNNNNNNNNNNNNNNNNNNNNNNNNNNNNNNNNNNNNNNNNNNNNNNNNNNNNNNNNNNNNNNNNNNNNNNNNNNNNNNNNNNNNNNNNNNNNNNNNNNNNNNNNNNNNNNNNNNNNNNNNNNNNNNNNNNNNNNNNNNNNNNNNNNNNNNNNNNNNNNNNNNNNNNNNNNNNNNNNNNNNNNNNNNNNNNNNNNNNNNNNNNNNNNNNNNNNNNNNNNNNNNNNNNNNNNNNNNNNNNNNNNNNNNNNNNNNNNNNNNNNNNNNNNNNNNNNNNNNNNNNNNNNNNNNNNNNNNNNNNNNNNNNNNNNNNGACTTTTCATGATNTNTTCTACTTTAAATTTGCAATTTAATATTATAAATTAAATATAAGACTNNNNNNNNNNNNNNNNNNNNNNNNNNNNNNNNNNNNNNNNNNNNNNNNNNNNNNNNNNNNNNNNNNNNNNNNNNNNNNNNNNNNNNNNNNNNNNNNNNNNNNNNNNNNNNNNNNNNNNNNNNATTTTATTAGTATATAAGTATATTTAACTTCCAATTAAAAGGATTAATTTTATTAAATAAAATANNNNNNNNNNNNNNNNNNNNNNNNNNNNNNNNNNNNNNNNNNNNNNNNNNNNNNNNNNNNNNNNTACTATTTGTAATAAATATTACATTATTAAATTCTAAATTTAAGGCATTNTTTTTGCTAAAATAGTTCTAATATGGCAGATTAGTGCAATGAATTTAAGATTCATATATAAAATATATTTTTTTTATTGGAANNNNNNNNNNNNNNNNNNNNNNNNNNNNNNNNNNNNNNNNNNNNNNNNNNNNNNNNNNNNNNNNNNNNNNNNNNNNNNNNNNNNNNNNNNNNNNNNNNNNNNNNNNNNNNNNNNNNNNNNNNNNNNNNNNNNNNNNNNNNNNNNNNNNNNNNNNNNNNNNNNNNNNNNNNNNNNNNNNNNNNNNNNNNNNNNNNNNNNNNNNNNNNNNNNNNNNNNNNNNNNNNNNNNNNNNNNNNNNNNNNNNNNNNNNNNNNNNNNNNNNNNNNNNNNNNNNNNNNNNNNNNNNNNNNNNNNNNNNNNNNNNNNNNNNNNNNNNNNNNNNNNNNAGTTAATTAGCTTAACCAAAAGCATTTATTTTGAAAGTAAAAGAAAAGATATTTAATCTATTAACTTNNNNNNNNNNNNNNNNNNNNNNNNNNNNNNNNNNNNNNNNNNNNNNNNNNNNNNNNNNNNNNNNNNNNNNNNNNNNNNNNNNNNNNNNNNNNNNNNNNNNNNNNNNNNNNNNNNNNNNNNNNNNNNNNNNAGGTTTTAAGTTATTAANAACTATTATCCTTCAAAGTTAAAAATATAATTANATTTATTATTAGGAGAAATATTTTTTCATAAATAAATTTACAGTTTATTGCCTATTATCGGCCATCCTAANNNNNNNNNNNNNNNNNNNNNNNNNNNNNNNNNNNNNNNNNNNNNNNNNNNNNNNNNNNNNNNNNN

>westlandica-south_11.NZ.WD.OKT.33

NNNNNNNNNNNNNNNNNNNNNNNNNNNNNNNNNNNNNNNNNNNNNNNNNNNNNNNNNNNNNNNNNNNNNNNNNNNNNNNNNNNNNNNNNNNNNNNNNNNNNNNNNNNNNNNNNNNNNNNNNNNNNNNNNNNNNNNNNNNNNNNNNNNNNNNNNNNNNNNNNNNNNNNNNNNNNNNNNNNNNNNNNNNNNNNNNNNNNNNNNNNNNNNNNNNNNNNNNNNNNNNNNNNNNNNNNNNNNNNNNNNNNNNNNNNNNNNNNNNNNNNNNNNNNNNNNNNNNNNNNNNNNNNNNNNNNNNNNNNNNNNNNNNNNNNNNNNNNNNNNNNNNNNNNNNNNNNNNNNNNNNNNNNNNNNNNNNNNNNNNNNNNNNNNNNNNNNNNNNNNNNNNNNNNNNNNNNNNNNNNNNNNNNNNNNNNNNNNNNNNNNNNNNNNNNNNNNNNNNNNNNNNNNNNNNNNNNNNNNNNNNNNNNNNNNNNNNNNNNNNNNNNNNNNNNNNNNNNNNNNNNNNNNNNNNNNNNNNNNNNNNNNNNNNNNNNNNNNNNNNNNNNNNNNNNNNNNNNNNNNNNNNNNNNNNNNNNNNNNNNNNNNNNNNNNNNNNNNNNNNNNNNNNNNNNNNNNNNNNNNNNNNNNNNNNNNNNNNNNNNNNNNNNNNNNNNNNNNNNNNNNNNNNNNNNNNNNNNNNNNNNNNNNNNNNNNNNNNNNNNNNNNNNNNNNNNNNNNNNNNNNNNNNNNNNNNNNNNNNNNNNNNNNNNNNNNNNNNNNNNNNNNNNNNNNNNNNNNNNNNNNNNNNNNNNNNNNNNNNNNNNNNNNNNNNNNNNNNNNNNNNNNNNNNNNNNNNNNNTTAATAATTCATTAATTGACTTTCCAGCACCATCCAATTTATCTTATTGGTGAAATTTCGGATTTGTATTAGGATTATGCTTAATAATCCAAATTATAACTGGATTATTTTTATCAATACATTATAATGCCAACATTATAAACGCTTTCGAAAGATTAAGACATATTTGTCGTGATGTAAACTATGGTTGAATTTTACGAGTCATTCACGCTAATGGAGCTTCATTATTTTTCATTTGTGTATATTTACACGTAGGACGTGGATTATATTATGGATCATTCAAATATATTGAAACATGATCTATTGGTGTAATTATACTATTAATATTAATAGCTACTGCTTTCTTAGGATATGTTTTACCATGAGGACAAATATCTTTTTGAGGAGCTACAGTTATTACAAACTTATTATCAGCTATTCCTTACTTAGGAAGAATATTAGTTAATTGAATTTGAGGAGGATTTGCAGTTGATAATGCAACACTAACTCGATTTTATTCATTCCATTTTATTTTACCATTTATTGTATTAAGACTAACAATTATTCACTTACTATATCTCCATACAACAGGTTCAAACAATCCATTAGGAATTAATTCTAATAATGACAAAGTTCCATTTCACCCATTCTTTTCCATTAAGGATATTATAAGATTAATTATCTTAATAATTATTTTCTTTATATTAGTTATATTGGAACCCTATATACTAGGGGATCCAGATAATTTTATTCCTGCTAATCCACTGGTAACACCAAAGCATATTCAACCTGAATGATATTTTTTATTTGCCTACGCAATTCTTCGCTCAATTCCTAATAAACTAGGAGGAGTAATTGCTCTTTTCATGTCAATTTTTATTTTAATGTTTGTACCNNNNNNNNNNNNNNNNNNNNNNNNNNNNNNNNNNNNNNNNNNNNNNNNNNNNNNNNNNNNNNNNNNNNNNNNNNNNNNNNNNNNNNNNNNNNNNNNNNNNNNNNNNNNNNNNNNNNNNNNNNNNNNNNNNNNNNNNNNNNNNNNNNNNNNNNNNNNNNNNNNNNNNNNNNNNNNNNNNNNNNNNNNNNNNNNNNNNNNNNNNNNNNNNNNNNNNNNNNNNATGAAAAAATGATTCTTCTCAACTAATCATAAAGATATTGGAACAATATATTTCATTTTTGGTATTTGATCAGGTATAATTGGTACAACTTTAAGAGTTTTAATTCGTGTCGAACTTGGAACTCCAGGTTCATTTATTGGTGATGATCAAATTTATAATGTAATTGTCACTGCTCACGCTTTCATTATAATTTTTTTTATAGTTATACCAATTATAATTGGCGGATTTGGAAATTGACTAGTTCCTTTAATAATTGGAGCTCCTGATATAGCCTTTCCTCGTATAAATAATATAAGTTTTTGATTATTACCTCCTTCTCTAATTCTTATTTTGGTGGGGAGAATAGTTGATAGAGGTGCAGGCACAGGTTGAACAGTTTATCCCCCTCTATCTGCAGGAATTGCACATTCTGGTTCATGTGTTGATTTAACTATTTTCTCTTTACACCTCGCGGGTGTATCTTCAATTTTAGGTGCTGTAAATTTCATTAGAACAATTTTTAATATACGTTCAATAGGAATTTGATTAGATCGAATACCTTTATTTGTATGAGCAGTTTTAATTACTGCATTCTTGTTATTACTATCTTTACCTGTTTTAGCAGGTGCTATTACAATGTTATTAACAGATCGAAATTTAAATACTTCATTTTTTGACCCTGCAGGAGGGGGGGACCCAATTCTTTATCAACATTTATTTTGATTTTTTGGTCATCCTGAAGTCTATATTTTAATTTTACCAGGATTTGGTTTAATTTCTCATATTATTACCCAAGAGAGAGGCAAAATCGAATCTTTTGGTTCATTAGGAATAATTTATGCTATAATATCAATTGGTATTCTAGGATTTGTTGTATGAGCACATCATATATTCACTGTAGGAATAGACGTTGATACACGTGCATATTTTACATCAGCTACTATAATTATTGCAGTTCCTACTGGAATTAAAGTATTTAGTTGACTCGCAACTTTGAGAGGTATAAAAATCAATATTACATCTTCAGCCTTATGGGCTTTAGGATTTGTTTTTTTATTTACTATTGGAGGTTTAACTGGAGTAATTTTAGCTAATTCTTCAATTGATATTATATTGCATGATACATATTATGTAGTTGCACATTTCCATTATGTTTTATCTATAGGAGCAGTTTTTGCTATTATAGCAAGATTTATTCATTGGTTTCCATTATTTACAGGACTGAGATTAAACTCAAATTGATTAAAAATTCATTTCCTATTGATATTTATTGGTGTAAATATAACATTTTTTCCTCAACATTTTTTGGGATTAAGAGGAATACCTCGTCGGTATTCAGACTATCCTGATGCTTATATGTCATGAAACATAATTTCATCAATAGGAAGAATTATATCTTTAGTTGGAATTTTATTTTTATTATTTATTGTCTGAGAAAGTTTTATTTCTATACGATTAGTATTATATTCCAATAGAATTCAATCTTCTATTGAATGAATACAAAAGTTTCCCCCATCTGAACATTCTTATAATGAAATGCCATTATTAATTCAANNNNNNNNNNNNNNNNNNNNNNNNNNNNNNNNNNNNNNNNNNNNNNNNNNNNNNNNNNNNNNNNNNNNNNNNNNNNNNNNNNNNNNNNNNNNNNNNNNNNNNNNNNNNNNNNNNNNNNNNNNNNNNNNNNNNNNNNNNNNNNNNNNNNNNNNNNNNNNNNNNNNNNNNNNNNNNNNNNNNNNNNNNNNNNNNNNNNNNNNNNNNNNNNNNNNAACTTTAATTTTTATTGCACTACCATCATTGCGATTATTATATATATTAGACGAAATTAATAATCCATTATTAACATTGAAAATTATTGGTCATCAGTGATACTGATCATATGAATACTCAGATTTTTCTGATGTAGAATTTGATTCATATATAAAATCCATAAACGAAATAAATAAGAATGAATTTCGTTTATTAGATGTAGATAACCGAGTAATTTTACCATTTAATATTCAAGTCCGACTAATAGTTTCTTCTTTTGATGTAATTCACTCTTGAGCAATACCATCTATGAGACTTAAAGTTGATGCAGTACCAGGACGATTAAATCAAATAAGAATATTAATTAGTCGTCCTGGCGTATCATATGGACAATGTTCTGAAATTTGTGGAGCAAATCATAGATTTATACCTATTGTAATTGAAAGAATTAGAATGAAAATATTTATTAAATGATTAATTAATTATNNNNNNNNNNNNNNNNNNNNNNNNNNNNNNNNNNNNNNNNNNNNNNNNNNNNNNNNNNNNNNNNNNNNNNNNNNNNNNNNNNNNNNNNNNNNNNNNNNNNNNNNNNNNNNNNNNNNNNNNNNNNNNNNNNNNNNNNNNNNNNNNNNNNNNNNNNNNNNNNNNNNNNNNNNNNNNNNNNNNNNNNNNNNNNNNNNNNNNNNNNNNNNNNNNNNNNNNNNNNNNNNNNNNNNNNNNNNNNNNNNNNNNNNNNNNNNNNNNNNNNNNNNNNNNNNNNNNNNNNNNNNNNNNNNNNNNNNNNNNNNNNNNNNNNNNNNNNNNNNNNNNNNNNNNNNNNNNNNNNNNNNNNNNNNNNNNNNNNNNNNNNNNNNNNNNNNNNNNNNCCCTTTATTAAATACAATAATTTTATTATGTTCAGGAATTACAATTACTTGAGCTCATCATTCAATTATGTGTGGCAATCATATTAATTCTATTTATAGAATTATATTAACTGTTATTTTAGGTATATATTTTACCATTCTACAAGGTTATGAATATTACGAAGCTCCATTTGCAATTAATGATTCCATCTATGGGTCCTCTTTTTTTATAGCTACTGGATTTCATGGGATTCATGTAATTATTGGAACAAGATTTATTACTGTATGTTTATTACGACAAATAAAGTTCCATTTCTCAAGAAATCATCACTTTGGTTTTGAAGCTGCTGCTTGATATTGACATTTTGTTGATGTAGTCTGATTATTTTTATATTTATCAATTTATTGATGAGGTAGATAGNNNNNNNNNNNNNNNNNNNNNNNNNNNNNNNNNNNNAAATTTAGAGATACTGGTAAATAAGATTTTCAAGCCAAATATATTAATTTATCATACCGATAACGAGGAAGAGTTCCTCGGACTCAAATGAAAAGAAAACAAATTATAGAAACTTGAATTGGAAAAACAATTGAACTAATTTTTCCTCCTAGAAATATTAAACAAAATATTATTCTCATAAATAAAATACTTGAATACTCAGCCAAGAAAATAAATGCAAATCTAGAACCCCCATATTCAACATTAAAACCAGAAACTAACTCTGATTCTCCCTCAGAAAAATCAAATGGAGATCGATTAGTTTCAGCTAAAGCTGAAGATAATCAACACATTCTTAATGGAAGAAATAAAAAAATAAATCACACAAACTCCTGAAATATAAATAAATCAATAATATTATAACTTCTAATTATTATTATAGGACACAATATAATCAAAACCAATCTTACTTCATAAGAAATAGATTGAGCAATCGATCGAATACATCCTAACATTGCATAACTTGAATTAGAAGATCAACCTGTTAATATTAATGAATAAACTCTTATTCTTGAACAACAAAAAAAAAATAAAATACCAAAATTAAAAGAAACACAATTAATTATATATGGATATAATGATCAAATCAACAATCTATTGAATAATCCTATTACAGGCCTAAAAATATAAATTAAATAATTAGATATTAATGGAATTGTATTTTCCTTCATAAATAATTTAATAGCATCAGAAATAGGCTGCAAGATACCCAAAAATCCAACTTTATTTGGACCTTTACGAATTTGAATATATCTCAAAACCTTACGCTCCAAAAGAGTAATAAATGCCACTCCAATCAAAATAAATAATACAGTAATTAAAATAGTAATTAAATACAAAAATAATCCTTCTTATTTACTTTATATAACTTTCTTACTATTAGGAATTATAATTTCAGTATCTTCAAACAATTGGCTGGGTTGTTGAATAGGAATTGAAATAAATATAGTTTCATTTTTGCCCATAATGGCAAATAAAATAAGAATTTATGCTTCAGAATCAATAATCAAATATTTTATTATCCAAAGAATGGGNNNNNNNNNNNNNNNNNNNNNNNNNNNNNNNNNNNNNNNNNNNNNNNNNNNNNNNNNNNNNNNNNNNNNNNNNNNNNNNNNNNNNNNNNNNNNNNNNNNNNNNNNNNNNNNNNNNNNNNNNNNNNNNNNNNNNNNNNNNNNNNNNNNNNNNNNNNNNNNNNNNNNNNNNNNNNNNNNNNNNNNNNNNNNNNNNNNNNNNNNNNNNNNNNNNNNNNNNNNNNNNNNNNNNNNNNNNNNNNNNNNNNNNNNNNNNNNNNNNNNNNNNNNNNNNNNNNNNNNNNNNNNNNNNNNNNNNNNNNNNNNNNNNNNNNNNNNNNNNNNNNNNNNNNNNNNNNNNNNNNNNNNNNNNNNNNNNNNNNNNNNNNNNNNNNNNNNNNNNNNNNNNNNNNNNNNNNNNNNNNNNNNNNNNNNNNNNNNNNNNNNNNNNNNNNNNNNNNNNNNNNNNNNNNNNNNNNNNNNNNNNNNNNNNNNNNNNNNNNNNNNNNNNNNNNNNNNNNNNNNNNNNNNNNNNNNNNNNNNNNNNNNNNNNNNNNNNNNNNNNNNNNNNNNNNNNNNNNNNNNNNNNNNNNNNNNNNNNNNNNNNNNNNNNNNNNNNNNNNNNNNNNNNNNNNNNNNNNNNNNNNNNNNNNNNNNNNNNNNNNNNNNNNNNNNNNNNNNNNNNNNNNNNNNNNNNNNNNNNNNNNNNNNNNNNNNNNNNNNNNNNNNNNNNNNNNNNNNNNNNNNNNNNNNNNNNNNNNNNNNNNNNNNNNNNNNNNNNNNNNNNNNNNNNNNNNNNNNNNNNNNNNNNNNNNNNNNNNNNNNNNNNNNNNNNNNNNNNNNNNNNNNNNNNNNNNNNNNNNNNNNNNNNNNNNNNNNNNNNNNNNNNNNNNNNNNNNNNNNNNNNNNNNNNNNNNNNNNNNNNNNNNNNNNNNNNNNNNNNNNNNNNNNNNNNNNNNNNNNNNNNNNNNNNNNNNNNNNNNNNNNNNNNNNNNNNNNNNNNNNNNNNNNNNNNNNNNNNNNNNNNNNNNNNNNNNNNNNNNNNNNNNNNNNNNNNNNNNNNNNNNNNNNNNNNNNNNNNNNNNNNNNNNNNNNNNNNNNNNNNNNNNNNNNNNNNNNNNNNNNNNNNNNNNNNNNNNNNNNNNNNNNNNNNNNNNNNNNNNNNNNNNNNNNNNNNNNNNNNNNNNNNNNNNNNNNNNNNNNNNNNNNNNNNNNNNNNNNNNNNNNNNNNNNNNNNNNNNNNNNNNNNNNNNNNNNNNNNNNNNNNNNNNNNNNNNNNNNNNNNNNNNNNNNNNNNNNNNNNNNNNNNNNNNNNNNNNNNNNNNNNNNNNNNNNNNNNNNNNNNNNNNNNNNNNNNNNNNNNNNNNNNNNNNNNNNNGTAATTATAAATTNNNNNNNNNNNNNNNNNNNNNNNNNNNNNNNNNNNNNNNNNNNNNNNNNNNNNNNNNNNNNNNNNNNNNNNNNNNNNNNNNNNNNNNNNNNNNNNNNNNNNNNNNNNNNNNNNNNNNNNNNNNNNNNNNNNNNNNNNNNNNNNNNNNNNNNNNNNNNNNNNNNNNNNNNNNNNNNNNNNNNNNNNNNNNNNNNNNNNNNNNNNNNNNNNNNNNNNNNNNNNNNNNNNNNNNNNNNNNNNNNNNNNNNNNNNNNNNNNNNNNNNNNNNNNNNNNNNNNNNNNNNNNNNNNNNNNNNNNNNNNNNNNNNNNNNNNNNNNNNNNNNNNNNNNNNNNNNNTTGAAATNNNNNNNNNNNNNNNNNNNNNNNNNNNNNNNNNNNNNNNNNNNNNNNNNNNNNNNNNNNNNNNNNNNNNNNNNNNNNNNNNNNNNNNNNNNNNNNNNNNNNNNNNNNNNNNNNNNNNNNNNNNNNNNNNNNNNNNNNNNNNNNNNNNNNNNNNNNNNNNNNNNNNNNNNNNNNNNNNNNNNNNNNNNNNNNNNNNNNNNNNNNNNNNNNNNNNNNNNNNNNNNNNNNNNNNNNNNNNNNNNNNNNNNNNNNNNNNNNNNNNNNNNNNNNNNNNNNNNNNNNNNNNNNNNNNNNNNNNNNNNNNNNNNNNNNNNNNNNNNNNNNNNNNNNNNNNNNNNNNNNNNNNNNNNNNNNNNNNNNNNNNNNNNNNNNNNNNNNNNNNNNNNNNNNNNNNNNNNNNNNNNNNNNNNNNNNNNNNNNNNNNNNNNNNNNNNNNNNNNNNNNNNNNNNNNNNNNNNNNNNNNNNNNNNNNNNNNNNNNNNNNNNNNNNNNNNNNNNNNNNNNNNNNNNNNNNNNNNNNNNNNNNNNNNNNNNNNNNNNNNNNNNNNNNNNNNNNNNNNNNNNNNNNNNNNNNNNNNNNNNNNNNNNNNNNNNNNNNNNNNNNNNNNNNNNNNNNNNNNNNNNNNNNNNNNNNNNNNNNNNNNNNNNNNNNNNNNNNNNNNNNNNNNNNNNNNNNNNNNNNNNNNNNNNNNNNNNNNNNNNNNNNNNNNNNNNNNNNNNNNNNNNNNNNNNNNNNNNNNNNNNNNNNNNNNNNNNNNNNNNNNNNNNNNNNNNNNNNNNNNNNNNNNNNNNNNNNNNNNNNNNNNNNNNNNNNNNNNNNNNNNNNNNNNNNNNNNNNNNNNNNNNNNNNNNNNNNNNNNNNNNNNNNNNNNNNNNNNNNNNNNNNNNNNNNNNNNNNNNNNNNNNNNNNNNNNNNNNNNNNNNNNNNNNNNNNNNNNNNNNNNNNNNNNNNNNNNNNNNNNNNNNNNNNNNNNNNNNNNNNNNNNNNNNNNNNNNNNNNNNNNNNNNNNNNNNNNNNNNNNNNNNNNNNNNNNNNNNNNNNNNNNNNNNNNNNNNNNNNNNNNNNNNNNNNNNNNNNNNNNNNAGTCTCTAAAATTCCTTGGGGACCAAAATTTTCACCTCATCCCATATCAATTGATTTTTGAAATATAAGTGATTTACTTATTAATAACATTTGATTATGAAACGTTGACAATTGTTTTATAAATCACATTGATCCAAAAAATTCGTAAACAATTTTTATTCTAGTTTTAGAATAAATTGACATTTCATAGCCCAATCAAATTCCTAATATTGAAAATATTAATGCCAATAATTTGCCCTCAATAGGTATAAAAATTATAATCGGATCATTAAATATTAATCATCTTAATATAGATCCCGAAAAAATTGAGTAAATAGATAAAATAATAATTCTTTTAACTATATTATTTATATTTTCATTTAATGATCTTAATTTATAAAAATTATAATTTATATTTATTGAAAAAAACGTCAAACGGGCAGAATAAAAAGAAGTCAAACCAATACCAATATATATAAATATTATAATTAATAAATTAATATGTCCAGAACATATAGTTTCTATAATTAAATCTTTTGAGTAAAATCCTCTTATAAATGGAATCCCACATAATGACATATTAGCAATATTTATTATTGTAATAGTTAATGGTATTTGAAATCTTAAACATCCTATTATACGAATATCTTGATTGTTATTAAAATTGTGAATCAAAATTCCCGCACACAAAAATAAAGTAGCTTTAAATAAAGCATGAACAATTAAATGAAAGAATGATAATATTGGATAACCGAATATAATGATTGTTATTATAATCCCTAACTGTCTCAAAGTTGATAATGCAATAATTTTTTTTAAATCAAACTCAAAATTTGCTCTAATTCCTGATATTAATATAGTTAAAATTGAAATTAATATAAATACTTGAATATAATCAAAATTCAAGATAATATTAGAAAATCGAATTATTAAATATACACCAGCAGTGACAAGTGTAGATGAATGAACCAAAGCAGATACAGGAGTTGGAGCAGCTATAGCTGCTGGTAATCATGAAGAAAATGGAATTTGAGCTCTCTTTGTAAATCTTGCAATAATAATTATAATCAATATAATTNNNNNNNNNNNNNNNNNNNNNNNNNAATTTCATGACCCAAAATTTAATATTCATGCAATAGCTATTAAAATAGCAACATCTCCCACACGGTTTATTAGTGCAGTTAATATTCCTGCATTATTAGAATATAAATTTTGATAATAAATAACTAAACAATAAGAAACTAACCCAAGACCATCTCAACCAATAATAATTCTCATTATATTAGGTCTAATAATCAATAAGACTATAGATATAACAAATCCTAAAACAATATAAATAAAACGATTAATAAATTTGTCATTATTTATATATATTCCACTATACAAAACAACCACTGATGAAATAAATATAACTGTTGAAAGAAATTGTTGATATAAAATCAAAAATTATAGTCATATAAATATTACATGAATTAATTGAAGATTATTCATTCCAATATTACTATNNNNNNNNNNNNNNNNNNNNNNNNNNNNNNNNNNNNNNNNNNNNNNNNNNNNNNNNNNNNNNNNNNNNNNNNNNNNNNNNNNNNNNNNNNNNNNNNNNNNNNNNNNNNNNNNNNNNNNNNNNNNNNNNNNNNNNNNNNNNNNNNNNNNNNNNNNNNNNNNNNNNNNNNNNNNNNNNNNNNNNNNNNNNNNNNNNNNNNNNNNNNNNNNNNNNNNNNNNNNNNNNNNNNNNNNNNNNNNNNNNNNNNNNNNNNNNNNNNNNNNNNNNNNNNNNNNNNNNNNNNNNNNNNNNNNNNNNNNNNNNNNNNNNNNNNNNNNNNNNNNNNNNNNNNNNNNNNNNNNNNNNNNNNNNNNNNNNNNNNNNNNNNNNNNNNNNNNNNNNNNNNNNNNNNNNNNNNNNNNNNNNNNNNNNNNNNNNNNNNNNNNNNNNNNNNNNNNNNNNNNNNNNNNNNNNNNNNNNNNNNNNNNNNNNNNNNNNNNNNNNNNNNNNNNNNNNNNNNNNNNNNNNNNNNNNNNNNNNNNNNNNNNNNNNNNNNNNNNNNNNNNNNNNNNNNNNNNNNNNNNNNNNNNNNNNNNNNNNNNNNNNNNNNNNNNNNNNNNNNNNNNNNNNNNNNNNNNNNNNNNNNNNNNNNNNNNNNNNNNNNNNNNNNNNNNNNNNNNNNNNNNNNNNNNNNNNNNNNNNNNNNNNNNNNNNNNNNNNNNNNNNNNNNNNNNNNNNNNNNNNNNNNNNNNNNNNNNNNNNNNNNNNNNNNNNNNNNNNNNNNNNNNNNNNNNNNNNNNNNNNNNNNNNNNNNNNNNNNNNNNNNNNNNNNNNNNNNNNNNNNNNNNNNNNNNNNNNNNNNNNNNNNNNNNNNNNNNNNNNNNNNNNNNNNNNNNNNNNNNNNNNNNNNNNNNNNNNNNNNNNNNNNNNNNNNNNNNNNNNNNNNNNNNNNNNNNNNNNNNNNNNNNNNNNNNNNNNNNNNNNNNNNNNNNNNNNNNNNNNNNNNNNNNNNNNNNNNNNNNNNNNNNNNNNNNNNNNNNNNNNNNNNNNNNNNNNNNNNNNNNNNNNNNNNNNNNNNNNNNNNNNNNNNNNNNNNNNNNNNNNNNNNNNNNNNNNNNNNNNNNNNNNNNNNNNNNNNNNNNNNNNNNNNNNNNNNNNNNNNNNNNNNNNNNNNNNNNNNNNNNNNNNNNNNNNNNNNNNNNNNNNNNNNNNNNNNNNNNNNNNNNNNNNNNNNNNNNNNNNNNNNNNNNNNNNNNNNNNNNNNNNNNNNNNNNNNNNNNNNNNNNNNNNNNNNNNNNNNNNNNNNNNNNNNNNNNNNNNNNNNNNNNNNNNNNNNNNNNNNNNNNNNNNNNNNNNNNCGATNNATAATAAAATTATTTAATGAGGTCCTTTCGTACTAACATTAAAAATAATTGAGTAGATAGGAATCAACCTGGCTCACGCCGGTCTGAACTCAGATCATGTAANNNNNNNNNNNNNNNNNNNNNNNNNNNNNNNNNNNNNNNNNNNNNNNNNNNNNNNNNNNNNNNNNNNNNNNNNNNNNNNNNNNNNNNNNNNNNNNNNNNNNNNNNNNNNNNNNNNNNNNNNNNNNNTAAGGTAATNNNNNNNNNNNNNNNNNNNNNNNNNNNNNNNNNNNNNNNNNNNNNNNNNNNNNNNNNNNNNNNNNNNNNNNNNNNNNNNNNNNNNNNNNNNNNNNNNNNNNNNNNNNNNNNNNNNNNNNNNNNNNNNNNNNNNNNNNNNNNNNNNNNNNNNNNNNNNNNNNNNNNNGATCTNNNNNNNNNNNNNNNNNNNNNNNNNNNNNNNNNNNNNNNNNNNNNNNNNNNNNNNNNNNNNNNNNNNNNNNNNNNNNNNNNNNNNNNNNNNNNNNNNNNNNNNNNNNNNNNNNNNNNNNNNNNNNNNNNNNNNNNNNNNNNNNNNNNNNNNNNNNNNNNNNNNNNNNNNNNNNNNNNNNNNNNNNNNNNNNNNNNNNNNNNNNNNNNNNNNNNNNNNNNNNNNNNNNNNNNNNNNNNNNNNNNNNNNNNNNNNNNNNNNNNNNNNNNNNNNNNNNNNNNNNNNNNNNNNNNNNNNNNNNNNNNNNNNNNNNNNNNNNNNNNNNNNNNNNNNNNNNNNNNNNNNNNNNNNNNNNNNNNNNNNNNNNNNNNNNNNNNNNNNNNNNNNNNNNNNNNNNNNNNNNNNNNNNNNNNNNNNNNNNNNNNNNNNNNNNNNNNNNNNNNNNNNNNNNNNNNNNNNNNNNNNNNNNNNNNNNNNNNNNNNNNNNNNNNNNNNNNNNNNNNNNNNNNNNNNNNNNNNNNNNNNNNNNNNNNNNNNNNNNNNNNNNNNNNNNNNNNNNNNNNNNNNNNNNNNNNNNNNNNNNNNNNNNNNNNNNNNNNNNNNNNNNNNNNNNNNNNNNNNNNNNNNNNNNNNNNNNNNNNNNNNNNNNNNNNNNNNNNNNNNNNNNNNNNNNNNNNNNNNNNNNNNNNNNNNNNNNNNNNNNNNNNNNNNNNNNNNNNNNNNNNNNNNNNNNNNNNNNNNNNNNNNNNNNNNNNNNNNNNNNNNNNNNNNNNNNNNNNNNNNNNNNNNNNNNNNNNNNNNNNNNNNNNNNNNNNNNNNNNNNNNNNNNNNNNNNNNNNNNNNNNNNNNNNNNNNNNNNNNNNNNNNNNNNNNNNNNNNNTTATGTTAGCAGAATCAAACTGTCTATTAATAACAAAAATTAATGTTCTTCATAAACTATAACANNNNNNNNNNNNNNNNNNNNNNNNNNNNNNNNNNNNNNNNNNNNNNNNNNNNNNNNNNATTCTATTAGTACATAAGTATNNNNNNNNNNNNNNNNNNNNNNNNNNNNNNNNNNNNNNNNNNNNNNNNNNNNNNNNNNNNNNNNNNNNNNNNNNNNNNNNNNNNNNNNNNNNNNNNNNNNNNNNNNNTGCTATTTGTAATAAATATTACATTATTAAATTCTAAATTTAAGGCATTAATTTTGCTAAAATAGTTCTAATATGGCAGATTAGTGCAATGAATTTAAGATTCNNNNNNNNNNNNNNNNNNNNNNNNNNNNNAATAAGGTGCCTGAATNAAAAAGGGTTATTTTGATAGAATAAATTATGTAATGAAATTACTCNNNCATTAAGTGGCTGAAAGTAAGTAATGGNNNNNNNNNNNNNNNNNNNNNNNNNNNNNNNNNNNNNNNNNNAAAGATAAGCTAAAANTAAAGCTAATGGGTTCATACCTCACTTATGGAAANTCTTCCTCTTTTTANNNNNNNNNNNNNNNNNNNNNNNNNNNNNNNNNNNNNNNNNNNNNNNNNNNNNNNNNNNNNNNNNNNNNNNNNNNNNNNNNNNNNNNNNNNNNNNNNNNNNNNNNNNNNNNNNNNNNNNNNNNNNNNNNNNNNNNNNNNNNNNNNNNNNNNNNNNNNNNNNNNNNNNNNNNNNNNNNNNNNNNNNNNNNNNNNNNNNNNNNNNNNNNNNNNNNNNNNNNNNNNNNNNNNNNNNNNNNNNNNNNNNNNNNNNNNNNNNNNNNNNNNNNNNNNNNNNNNNNNNNNNNNNNNNNNNNNNNNNNNNNNNNNNNNNNNNNNNNNNNNNNNNNNNNNNNNNNNNNNNNNNNNNNNNNNNNNNNNNNNNNNNNNNNNNNNNNNNNNNNNTATTAAGAGAAATAATTTTTCATAAATAAATTTACAGTTTATTGCCTATTTTCGGCCATCCTAANNNNNNNNNNNNNNNNNNNNNNNNNNNNNNNNNNNNNNNNNNNNNNNNNNNNNNNNNNNNNNNNNN

>cauta_99.NZ.99N14

NNNNNNNNNNNNNNNNNNNNNNNNNNNNNNNNNNNNNNNNNNNNNNNNNNNNNNNNNNNNNNNNNNNNNNNNNNNNNNNNNNNNNNNNNNNNNNNNNNNNNNNNNNNNNNNNNNNNNNNNNNNNNNNNNNNNNNNNNNNNNNNNNNNNNNNNNNNNNNNNNNNNNNNNNNNNNNNNNNNNNNNNNNNNNNNNNNATTAATATTTCTATCATTATTTATATTTATTCTATTAAATAATTTATTAGGATTACTTCCTTATATTTTTACAAGTCCAAGTCATTTGGTATTTACAATAAGATTAGCATTACCCTTATGACTATCATTTATACTTTATGGATTTATTAATAATGTAAATCATATATTTTGCCATTTAGTTCCATCAGGAACTCCCAATATTTTAATACCTTTTATAGTTCTTATTGAAAGAACTAGAAATTTAATTCGCCCTGGTTCACTAGCTGTTCGATTAACAGCTAATATAATTGCTGGACATCTTTTAATAACTTTATTAGGTAATTTACCTATAAATTATGAATTTTACTCAGGTTTAATTATTATTTTTCAAATCATACTAATATTATTTGAATTAGCTGTTTGCATTATTCAATCCTATGTATTTATAGTACTTAGAACTTTATATTATAGAGAAGTAAATATTCCTCAAATGTCTCCTCTAAATTGATTATTATTAATTTATTTTNNNNNNNNNNNNNNNNNNNNNNNNNNNNNNNNNNNNNNNNNNNNNNNNNNNNNNNNNNNNNNNNNNNNNNNNNNNNNNNNNNNNNNNNNNNNNNNNNNNTTTAAGCCAACACGACAAAATCACCCTTTAATTAAAATTATTAATAATTCATTAATTGACTTTCCTGCACCATCTAATTTATCTTATTGATGAAATTTTGGATTTGTATTAGGATTATGTTTAACAATCCAAATTTTAACTGGATTATTTTTATCAATACATTATAATGCTAATATTATAAATGCTTTTGAAAGATTAAGACACATCTGTCGTGACGTGAATTATGGTTGAATCTTACGAATTATTCACGCTAATGGAGCATCATTATTTTTCATTTGTGTTTACCTACATGTAGGACGAGGATTATATTATGGCTCATTCAAATACATTGAAACATGATCTATTGGTGTAATTATGCTGTTAATATTAATAGCTACCGCTTTCTTAGGATATGTCCTACCATGAGGACAAATATCTTTTTGAGGGGCCACAGTAATCACAAATTTACTATCAGCTATTCCATATTTAGGAGGAATATTAGTCAATTGAATTTGAGGAGGATTTGCAGTTGATAATGCAACATTGACTCGATTTTATTCATTTCACTTTATCTTACCATTTATTGTATTAAGATTAACAATTATTCACTTATTATACCTCCATACAACAGGATCAAATAACCCATTAGGAATTAATTCTAACAATGATAAAGTTCCATTTCATCCTTATTTCTCTATTAAAGATATTATAAGACTATTTATCTTAATAATTATTTTCTTCACAATAGTTATATTAGACCCCTATTTATTAGGGGATCCAGATAATTTTATTCCCGCCAATCCACTCGTAACACCAAAGCACATTCAACCTGAATGATATTTTTTATTTGCCTATGCAATTTTACGATCAATTCCTAATAAATTAGGGGGAGTAATTGCTCTTTTTATATCAATTTTCATTTTAATGTTTGTACCCTTATTAAATAATTCAAATTTCATAAGATTAAATAATTACCCAATTAATCAAATTATATTTTGATATTTAGTAACCATTTTAATTTTATTAACTTGAATTGGAGCACGGCCTGTTGAACTACCTTATATTAATTTTGGAATATTTCTAACAATTATATATTTTTCTTACTTTATTGTTGACCCAATAATTAAATCTATTTGAGATAAATTAATTAGATAGATGAAAAAATGATTCTTCTCAACTAATCATAAAGATATTGGAACTATATATTTCATTTTTGGTATTTGATCAGGTATAATTGGTACAACCTTAAGAGTTTTAATTCGTATTGAACTTGGGACTCCAGGTTCATTTATTGGCGATGATCAAATTTATAATGTAATTGTCACTGCTCATGCTCTCATCATAATCTTTTTTATAGTTATACCAATTATAATTGGTGGATTTGGAAATTGATTAGTTCCTTTGATAATTGGAGCTCCCGATATGGCTTTTCCTCGTATAAATAACATAAGTTTTTGATTACTCCCCCCATCTTTAACTCTTCTTTTGGTTGGAAGAATAGTTGATAGAGGTGCAGGTACAGGTTGAACGGTATATCCTCCTTTATCTGCAGGAGTTGCACATTCTGGTTCATGTGTTGATTTAACTATTTTTTCTTTACACCTAGCGGGTGTGTCATCAATTTTGGGAGCTGTAAATTTCATTAGAACAATTTTTAATATACGTTCAGTAGGAATTTGATTAGATCGAATACCTTTATTTGTATGAGCAGTTTTAATCACTGCATTTTTATTACTATTATCTTTACCTGTTTTAGCAGGTGCTATTACAATACTATTAACAGATCGAAATTTAAATACTTCATTTTTTGACCCTGCAGGGGGAGGAGACCCAATTCTTTATCAACATTTATTTTGATTTTTTGGGCATCCTGAAGTTTATATTTTAATTTTACCAGGATTTGGTTTAATTTCTCATATTATTACCCAAGAGAGAGGTAAGATTGAATCTTTTGGTTCATTAGGAATAATTTATGCTATAATATCAATTGGTATTTTAGGATTTGTTGTATGAGCACATCATATATTCACTGTAGGAATAGATGTTGATACACGTGCATATTTCACATCAGCTACCATAATTATTGCAGTTCCAACTGGAATTAAAGTATTCAGTTGACTTGCAACTTTAAGAGGTATAAAAATCAACATTACATCTTCAGCCTTATGAGCTTTAGGATTTGTATTCTTATTTACTATTGGAGGTTTAACTGGAGTAATTCTAGCTAATTCTTCAATTGATATTATATTGCACGATACATACTATGTAGTTGCACATTTTCATTATGTTTTATCAATAGGAGCAGTTTTTGCTATCATGGCAAGTTTTATTCACTGATTTCCATTATTTACAGGATTAACATTAAATATAAACTGATTAAAAATTCATTTTATATTAATATTTGTTGGTGTAAATATGACATTTTTCCCTCAACATTTTTTGGGATTAAGAGGTATACCTCGTCGATATTCAGATTATCCTGATGCTTATATATCATGAAATATAATTTCATCAATAGGAAGAATTATATCTTTAATTGGAATTATATTTTTGTTATTTATTGTTTGAGAAAGTTTTATTTCAACACGATTAGTTTTATATTCTAATAGAATTCAATCTTCTATTGAGTGAATACAAAAGTTTCCCCCATCTGAACATTCTTATAATGAAATGCCATTATTAATTCAAATTTCAAATTGATCTTATATTAACATACAAGATGCTGTATCACCATTAATAGAACAGTTAATATTTTTTCATGATCATGTATTAGTTATTCTAATNNNNNNNNNNNNNNNNNNNNNNNNNNNNNNNNNNNNNNNNNNNNNNNNNNNNNNNNNNNNCCGCCTTCTTTTAGAAGGTCAGTTAATTGAATTTATTTGAACTTTATTACCTGCAATAACTTTAATTTTTATTGCATTACCATCATTACGATTATTATATATATTAGACGAAATTAATAATCCATTAGTAACATTAAAAATTATTGGTCATCAATGATATTGATCATATGAATATTCAGATTTCTCTGATATAGAATTTGATTCCTATATAAAGTCTAAAAATGAAATAGATAAAAATGAATTTCGTTTGTTGGATGTAGATAATCGAGTAATTCTTCCATTTAACATTCAAATTCGATTATTGATTTCTTCTTTTGATGTTATTCATTCTTGAGCAATACCATCAATGAGACTTAAAGTTGATGCAGTACCAGGACGATTAAATCAAATAAGAATACTGATTAGTCGTCCAGGTATCTCTTATGGACAATGTTCTGAAATTTGTGGAGCAAATCATAGATTTATGCCTATTGTAATTGAAAGAATTAGAATGAAAATATTTATTAAATGGTTAATTAATTATATGAATAATCATCCTTATCATATAGTTGACTATAGACCCTGACCTTTAACTGGATCAATTGGGGCAATAACTTTTGTTTCTGGTATAATTATAATATTTCATAAATGAAACTTTATATTATTATATATTGGCATTTTACTGTTATTAATAACAATAATTCAATGATGGCGTGACATCTCCCGAGAAGGGACATTTTTAGGAATACATACAATAATGGTAGCGAATGGTTTAAAAATAGGAATACTATTATTTATTGTATCAGAGATTCTTTTTTTTGTTTCATTTTTTTGAGGATTTTTTCATAGCAGATTAAGACCTGTAGTAGAAATTGGTATGATTTGACCACCATTAGGAATTTATGTTTTCAATCCAACTCAAGTTCCATTATTAAATACAATAATTTTATTATGTTCAGGAATTACAATTACTTGAGCACATCACTCAATTATAAATGGCAATCATATTAATTCTATTTATAGAATTATATTAACTATTATTTTAGGAATATATTTTACCATTTTGCAAGGCTATGAATATTATGAAGCACCATTTGCAATTAATGATTCTATTTATGGATCTTCTTTTTTTATGGCTACTGGATTTCATGGAATTCACGTAATTATTGGAACAATATTCATTATTGTATGTTTATTACGACAAATAAATTTTCATTTTTCAAGAAATCATCACTTTGGTTTTGAAGCTGCTGCTTGATATTGGCATTTTGTTGATGTAGTTTGACTATTTTTATATTTATCAATTTATTGNNNNNNNNNNNNNTAAATTAAAGTTTTTAAAAACAAAAAAAAAATAAATAAATTAAGAGATACTGGCAAATAACATTTTCAAGCTAAATATATTAATTTATCATAACGATAACGAGGAAGAGTTCCTCGAACTCAAATGAAAAGAAAACATATTATAGAAACTTGAATTGGAAAAACAACTGAATTAATTTTTCCTCCTAAAAATATTAAACAAAATATTATTCTCATAAATAAAATACTAGAATATTCAGCCAAAAAAATAAATGCAAATCTGGAACCTCTATACTCAACATTAAATCCAGATACTAATTCTGATTCTCCCTCAGAAAAATCAAATGGTGACCGGTTAGTTTCAGCTAAAGCTGATGACAATCAGCATATTCTTAAGGGAAGAAATAAGAAAATAAATCACATTACTTCCTGAAATATAAATAAATCAATAATATTATATCTTATTATTATTATCATAGGACATAGTATAATCAAAACTAATCTTACTTCATAAGAAATTGACTGAGCAATAGATCGAATACATCCTAATATTGCATAGCTTGAATTAGAAGATCAACCTGTTAATATCAATGAATAAACTCTTATTCTTGAACAACAAAAAAAAAATATAACACCAAAATTAAAAGAAACACAATTAATTATATAAGGATATAATGATCAAATTAATAATCTATTAAACAAACCTATAATAGGTCTAAAAATATAAATTAAATAATTAGATATTAATGGAATTGTATTTTCCTTTATAAACAACTTAATAGCATCAGAAATAGGCTGCAAAATACCTAAAAATCCAACTTTATTTGGACCTTTACGAATTTGAATATATCTCAAAACTTTTCGCTCCAAAAGAGTAATAAATGCCACCCCAATCAAAATAAATAAAACAGTAATTAAAATAGTAATTAAATACNNNNNNNNNNNNNNNNNNNNNNNNNNNNNNNNNNNNNNNNNNNNNNNNNNNNNNNNNNNNNNNNNNNNNNNNNNNNNNNNNNNNNNNNNNNNNNNNNNNNNNNNNNNNNNNNNNNNNNNNNNNNNNNNNNNNNNNNNNNNNNNNNNNNNNNNNNNNNNNNNNNNNNNNNNNNNNNNNNNNNNNNNNNNNNNNNNNNNNNNNNNNNNNNNNNNNNNNNNNNNNNNNNNNNNNNNNNNNNNNNNNNNNNNNNNNNNNNNNNNNNNNNNNNNNNNNNNNNNNNNNNNNNNNNNNNNNNNNNNNNNNNNNNNNNNNNNNNNNNNNNNTGAGGGTTTAACTTGAATGGTATGTTTTATCTTAATAACTATTCAAAAAATCATTCCATTAATTATATTATCATATTTAAATGTTAATATAAGATTATTTATTGTGATATCATGTATTTGAGGATGTATTGGAGGGCTGGGATATTCTTCAATGCGTAAAATTATTGCTTATTCTTCAATTTATAATTTAAGATGGATTTTTAGAGGTATTATTATTATGAACTACTCATGGCTACTTTATTACTTAATTTATTCATTTACATTATTAGCTGTGTGTTATATATTTTATACATTAAATATTAACTATATTAATCAATTTATTATGGTATCTTTAAATTTTATGAAATCTATTATAATAATGTGTATTTTTATATCTATAGGGGGACTACCCCCTTTCTTAGGGTTTTTTCCTAAATTGATTATAATTTACTGTTTATTATTAAATAATATGATATTTGTATGTATTTTATTATTAATAACAGCACTAATTGTTTTATTTTTCTATTTACGAATCATAATTACAACATTAATAATAAATACAATTTCAATAAAAAGAATTCTCATGAAAGTTTCATATTCTTATTATATTGCTGGAACATTTTCATTGTTTGGAACAATTTTCTTTTCACTAATTACATTAAATATATGTTAGNNNNNNNNNNNNNNNNNNNNNNNNNNNNNNNNNNNNNNNNNNNNNNNNNNNNNNNNNNNNNNNNNNNNNNNNNNNNNNNNNNNNNNNNNNNNNNNNNNNNNNNNNNNNNNNNNNNNNNNNNNNNNNNNNNNNNNNNNNNNNNNNNNNNNNNNNNNNNNNNNNNNNNNNNNNNNNNNNNNNNNNNNNNNNNNNNNNNNNNNNNNNNNNNNNNNNNNNNNNNNNNNNNNNNNNNNNNNNNNNNNNNNNNNNNNNNNNNNNNNNNNNNNNNNNNNNNNNNNNNNNNNNNNNNNNNNNNNNNNNNNNNNNNNNNNNNNNNNNNNNNNNNNNNNNNNNNNNNNNNNNNNNNNNNNNNNNNNNNNNNNNNNNNNNNNNNNNNNNNNNNNNNNNNNNNNNNNNNNNNNNNNNNNNNNNNNNNNNNNNNNNNNNNNNNNNNNNNNNNNNNNNNNNNNNNNNNNNNNNNNNNNNNNNNNNNNNNNNNNNNNNNNNNNNNNNNNNNNNNNNNNNNNNNNNNNNNNNNNNNNNNNNNNNNNNNNNNNNNNNNNNNNNNNNNNNNNNNNNNNNNNNNNNNNNNNNNNNNNNNNNNNNNNNNNNNNNNNNNNNNNNNNNNNNNNNNNNNNNNNNNNNNNNNNNNNNNNNNNNNNNNNNNNNNNNNNNNNNNNNNNNNNNNNNNNNNNNNNNNNNNNNNNNNNNNNNNNNNNNNNNNNNNNNNNNNNNNNNNNNNNNNNNNNNNNNNNNNNNNNNNNNNNNNNNNNNNNNNNNNNNNNNNNNNNNATAATTATATTATATNNNNNNNNNNNNNNNNNNNNNNNNNNNNNNNNNNNNNNNNNNNNNNNNNNNNNNNNNNNNNNNNNNNNNNNNNNNNNNNNNNNNNNNNNNNNNNNNNNNNNNNNNNNNNNNNNNNNNNNNNNNNNNNNNNNNNNNNNNNNNNNNNNNNNNNNNNNNNNNNNNNNNNNNNNNNNNNNNNNNNNNNNNNNNNNNNNNNNNNNNNNNNNNNNNNNNNNNNNNNNNNNNNNNNNNNNNNNNNNNNNNNNNNNNNNNNNNNNNNNNNNNNNNNNNNNNNNNNNNNNNNNNNNNNNNNNNNNNNNNNNNNNNNNNNNNNNNNNNNNNNNNNNNNNNNNNNNNNNNNNNNNNNNNNNNNNNNNNNNNNNNNNNNNNNNNNNNNNNNNNNNNNNNNNNNNNNNNNNNNNNNNNNNNNNNNNNNNNNNNNNNNNNNNNNNNNNNNNNNNNNNNNNNNNNNNNNNNNNNNNNNNNNNNNNNNNNNNNNNNNNNNNNNNNNNNNNNNNNNNNNNNNNNNNNNNNNNNNNNNNNNNNNNNNNNNNNNNNNNNNNNNNNNNNNNNNNNNNNNNNNNNNNNNNNNNNNNNNNNNNNNNNNNNNNNNNNNNNNNNNNNNNNNNNNNNNNNNNNNNNNNNNNNNNNNNNNNNNNNNNNNNNNNNNNNNNNNNNNNNNNNNNNNNNNNNNNNNNNNNNNNNNNNNNNNNNNNNNNNNNNNNNNNNNNNNNNNNNNNNNNNNNNNNNNNNNNNNNNNNNNNNNNNNNNNNNNNNNNNNNNNNNNNNNNNNNNNNNNNNNNNNNNNNNNNNNNNNNNNNNNNNNNNNNNNNNNNNNNNNNNNNNNNNNNNNNNNNNNNNNNNNNNNNNNNNNNNNNNNNNNNNNNNNNNNNNNNNNNNNNNNNNNNNNNNNNNNNNNNNNNNNNNNNNNNNNNNNNNNNNNNNNNNNNNNNNNNNNNNNNNNNNNNNNNNNNNNNNNNNNNNNNNNNNNNNNNNNNNNNNNNNNNNNNNNNNNNNNNNNNNNNNNNNNNNNNNNNNNNNNNNNNNNNNNNNNNNNNNNNNNNNNNNNNNNNNNNNNNNNNNNNNNNNNNNNNNNNNNNNNNNNNNNNNNNNNNNNNNNNNNNNNNNNNNNNNNNNNNNNNNNNNNNNNNNNNNNNNNNNNNNNNNNNNNNNNNNNNNNNNNNNNNNNNNNNNNNNNNNNNNNNNNNNNNNNNNNNNNNNNNNNNNNNNNNNNNNNNNNNNNNNNNNNNNNNNNNNNNNNNNNNNNNNNNNNNNNNNNNNNNNNNNNNNNNNNNNNNNNNNNNNNNNNNNNNNNNNNNNNNNNNNNNNNNNNNNNNNNNNNNNNNNNNNNNNNNNNNNNNNNNNNNNNNNNNNNNNNNNNNNNNNNNNNNNNNNNNNNNNNNNNNNNNNNNNNNNNNNNNNNNNNNNNNNNNNNNNNNNNNNNNNNNNNNNNNNNNNNNNNNNNNNNNNNNNNNNNNNNNNNNNNNNNNNNNNNNNNNNNNNNNNNNNNNNNNNNNNNNNNNNNNNNNNNNNNNNNNNNNNNNNNNNNNNNNNNNNNNNNNNNNNNNNNNNNNNNNNNNNNNNNNNNNNNNNNNNNNNNNNNNNNNNNNNNNNNNNNNNNNNNNNNNNNNNNNNNNNNNNNNNNNNNNNNNNNNNNNNNNNAGACGGGCAGAATAAAAGGAAGTTAAACCAATACCAATATATATAAATATCATAATTAAAAAATTAATATTATTAGAACACATAGTTTCTATAATTAAATCTTTTGAGTAAAATCCTCTCATAAATGGAATTCCACATAATGATATATTAGCAATATTTATTATTGTAATAGTTAGTGGCATCTGAAATCTTAAGCATCCTATTATACGAATATCTTGATTACTATTAAAATTGTGAATCAAAACTCCCGCACATAGAAATAAAGTAGCCTTAAATAAAGCATGAACAATTAAGTGAAAAAATGACAATATTGGATAACCAAACATAATAATTGTTATTATAATTCCTAATTGTCTCAAAGTTGATAATGCAATAATTTTTTTTAAATCAAATTCAAAATTTGCTCTAATTCCGGATATCAATATAGTTAAAATTGAAATTAATATAAATATTTGAATATAATCAAAATTCATAATAATATAAGAAAATCGAATTATTAAATAAACTCCAGCAGTAACAAGTGTTGATGAATGGACCAGAGCTGATACAGGAGTTGGAGCAGCCATAGCTGCCGGCAATCATGAAGAAAATGGAATTTGAGCTCTTTTTGTAAATCCTGCAATAATAACTATAATTAATATAATTCATATCTTTATTAATAAAAGATAAAAATTTCATGAACCAAAATTCAATATTCATGAAATAACTATTAAAATAGCAACATCCCCTACACGATTCATTAATGCAGTTAATATTCCTGCATTATTAGAATATAAATTTTGATAATAAATAACCAAACAATATGAAACTAATCCAAGTCCATCTCACCCAATAATAATTCTAATTATATTAGGTCTAATAATTAATAAAATTATAGACATCACAAATCCTATAACAATATAAATAAAACGATTAATAAATTTATCATTAATTATATATACTCCACTATATAAAATAACTACTGATGAAATAAATATAACTGTCGAGAGAAATTGTTGATATAAAATCAAAAATTATAGTCATATAAATATTACATGAATTAATTGAAAATTATTCATTCCANNNNNNNNNNNNNNNNNNNNNNNNNNNNNNNNNNNNNNNNNNNNNNNNNNNNNNNNNNNNNNNNNNNNNNNNNNNNNNNNNNNNNNNNNNNNNNNNNNNNNNNNNNNNNNNNNNNNNNNNNNNNNNNNNNNNNNNNNNNNNNNNNNNNNNNNNNNNNNNNNNNNNNNNNNNNNNNNNNNNNNNNNNNNNNNNNNNNNNNNNNNNNNNNNNNNNNNNNNNNNNNNNNNNNNNNNNNNNNNNNNNNNNNNNNNNNNNNNNNNNNNNNNNNNNNNNNNNNNNNNNNNNNNNNNNNNNNNNNNNNNNNNNNNNNNNNNNNNNNNNNNNNNNNNNNNNNNNNNNNNNNNNNNNNNNNNNNNNNNNNNNNNNNCTTTAACTTTAATTTACTTTATCAACATAATTGATTTAAAATCATTAAACATCACAAATAATATAATAATATACAAAAATCACAGTAACTTTATAATAAGAAAAATATATATTATCCCTTCTGGGATAATAACACTAATTTTAACAATTTATTTACTATTTGTTTTAATTATTGTAATTAACATATTAACAATAAATATAATAACTCTTCGAAGAAGAACTNNNNNNNNNNNNNNNNNNNNNNNNNNNNNNNNNNNNNNNNNNNNNNNNNNNNNNNNNNNNNNNNNNNNNNNNNNNNNNNNNNNNNNNNNNNNNNNNNNNNNNNNNNNNNNNNNNNNNNNNNNNNNNNNNNNNNNNNNNNNNNNNNNNNNNNNNNNNNNNNNNNNNNNNNNNNNNNNNNNNNNNNNNNNNNNNNNNNNNNNNNNNNNNNNNNNNNNNNNNNNNNNNNNNNNNNNNNNNNNNNNNNNNNNNNNNNNNNNNNNNNNNNNNNNNNNNNNNNNNNNNNNNNNNNNNNNNNNNNNNNNNNNNNNNNNNNNNNNNNNNNNNNNNNNNNNNNNNNNNNNNNNNNNNNNNNNNNNNNNNNNNNNNNAATCATTTTAATTTAAAAAAATTTATTAANNNNNNNNNNNNNNNNNNNNNNNNNNNNNNNNNNNNNNNNNNNNNNNNNNNNNNNNNNNNNNNNNNNNNNNNNNNNNNNNNNNNNNNNNNNNNNNNNNNNNNNNNNNNNNNNNNNNNNNNNNNNNNNNNNNNNNNNNNNNNNNNNNNNNNNNNNNNNNNNNNNNNNNNNNNNNNNNNNNNNNNNNNNNNNNNNNNNNNNNNNNNNNNNNNNNNNNNNNNNNNNNNNNNNNNNNNNNNNNNNNNNNNNNNNNNNNNNNNNNNNNNNNNNNNNNNNNNNNNNNNNNNNNNNNNNNNNNNNNNNNNNNNNNNNNNNNNNNNNNNNNNNNNNNNNNNNNNNNNNNNNNNNNNNNNNNNNNNNNNNNNNNNNNATAATAAAATTATTTAATGAGGTCCTTTCGTACTAACATTAAAAATTATTAAGTAGATAGAAACCAACCTGGCTCACGCCGGTTTGAACTCAGATCATGTAAGAATATTAAGGGTCGAACAGACCCAGAAATAATAAATTTTGCTCCAATCCCTATTCTTAATCCAACATCGAGGTCGCAATCATATTTATCGATATGAACTCTTCAAATTAATTACGCTGTTATCCCTAAGGTAATTTGTTCTTATAATCAAAAATTTGGATCAAAAGTTACATAAATTCATGAAATTTTTATTAAAAGTTAATTATATTTTAATATCACCCCAACAAAAAAACCATTTAAATAAAAAAAATTAATTAACTATAAANAAATAAACTTAATNAAGATTTTAAAAATTCTATAGGGTCTTCTCGTCCCACTTACATATTTAAGCTTTTTTACTTAAAAATCAATTTCAATTATTAATATTAATTAAGTCAATTTCTCATTCAATCATTCATTCAAGCCTCCAATTAAAAGACTATTTATTATGCTACCTTTGCACGGTCAATTTACCGCGGCCATTTAATTTTTAATCATTGGGCAGATTAGACTTTTAATTCTTTCTAAAAGACATGTTTTTGATAAACAGGTGAAAATTTTTTTTGCCTAAATTATAAATATTATTCATAANNNNNNNNNNNNNNNNNNNNNNNNNNNNNNNNNNNNNNNNNNNNNNNNNNNNNNNNNNNNNNNNNNNNNNNNNNNNNNNNNNNNNNNNNNNNNNNNNNNNNNNNNNNNNNNNNNNNNNNNNNNNNNNNNNNNNNNNNNNNNNNNNNNNNNNNNNNNNNNNNNNNNNNNNNNNNNNNNNNNNNNNNNNNNNNNNNNNNNNNNNNNNNNNNNNNNNNNNNNNNNNNNNNNNNNNNNNNNNNNNNNNNNNNNNNNNNNNNNNNNNNNNNNNNNNNNNNNNNNNNNNNNNNNNNNNNNNNNNNNNNNNNNNNNNNNNNNNNNNNNNNNNNNNNNNNNNNNNNNNNNNNNNNNNNNNNNNNNNNNNNNNNNNNNNNNNNNNNNNNNNNNNNNNNNNNNNNNNNNNNNNNNNNNNNNNNNNNNNNNNNNNNNNNNNNNNNNNNNNNNNNNNNNNNNNNNNNNNNNNNNNNNNNNNNNNNNNNNNNNNNNNNNNNNGAAAAATTAGTTTAATTAAAATATAAATCTGTCAGATTTATGACACTTTTAGGTATTTTTCTGAATTTTTATGATNTNTTCTACTTTAAATTTGCAATTTAATATTATAAATTAAATACAAGACTNNNNNNNNNNNNNNNNNNNNNNNNNNNNNNNNNNNNNNNNNNNNNNNNNNNNNNNNNNNNNNNNNNNNNNNNNNNNNNNNNNNNNNNNNNNNNNNNNNNNNNNNNNNNNNNNNNNNNNNNNNNNNNNNNNNNNNNNNNNNNNNNNNNNNNNNNNNNNNNNNNNNNNNNNNNNNNNNNNNNNNNNNNNNNNNNNNNNNNNNNNNNNNNNNNNNNNNNNNNNNNNNNNNNNNNNNNNNNNNNNNNNNNNNNNTACTATTTGTAATAAATATTACATTATTAAATCCTAAATTTAAGGCATCAATTTTGCTAAAACAGTTCTAATATGGCAGATTAGTGCAATGAATTTAAGATTCATATATAAAATATATTTTTTTTATTAGAANNNNNNNNNNNNNNNNNNNNNNNNNNNNNNNNNNNNNNNNNNNNNNNNNNNNNNNNNNNNNNNNNCATTAAGTGGCTGAAAGTAAGTAATGGTCTCTTAAACCAATTAATAGTAATTAACAAATACTCTTAATGNNNNNNNNNNNNNNNNNNNNNNNNNNNNNNNNNNNNNNNNNNNNNNNNNNNNNNNNNNNNNNNNNNNNNNNNNNNNNNNNNNNNNNNNNNNNNNNNNNNNNNNNNNNNNNNNNNNNNNNNNNNNNNNNNNNNNNNNNNNNNNNNNNNNNNNNNNNNNNNNNNNNNNNNNNNNNNNNNNNNNNNNNNNNNNNNAGTTAATTAGCTTAACAAAAAGCATTTACTTTGAAAGTAAAAGAAAAGATATTTAATCTATTAACTNNNNNNNNNNNNNNNNNNNNNNNNNNNNNNNNNNNNNNNNNNNNNNNNNNNNNNNNNNNNNNNNNNNNNNNNNNNNNNNNNNNNNNNNNNNNNNNNNNNNNNNNNNNNNNNNNNNNNNNNNNNNNNNNNNNAGGTTTTAAGTTATTAANAACTATTATCCTTCAAAGTTAAAAATATAATTATATTTATTATTAGGAGAAATAGCTTTTCATAAATAAATTTACAATTTATTGCCTATTATCGGCCATCCTAANNNNNNNNNNNNNNNNNNNNNNNNNNNNNNNNNNNNNNNNNNNNNNNNNNNNNNNNNNNNNNNNNN

>westlandica-north-coast_11.NZ.NN.KOB.10

NNNNNNNNNNNNNNNNNNNNNNNNNNNNNNNNNNNNNNNNNNNNNNNNNNNNNNNNNNNNNNNNNNNNNNNNNNNNNNNNNNNNNNNNNNNNNNNNNNNNNNNNNNNNNNNNNNNNNNNNNNNNNNNNNNNNNNNNNNNNNNNNNNNNNNNNNNNNNNNNNNNNNNNNNNNNNNNNNNNNNNNNNNNNNNNNNNNNNNNNNNNNNNNNNNNNNNNNNNNNNNNNNNNNNNNNNNNNNNNNNNNNNNNNNNNNNNNNNNNNNNNNNNNNNNNNNNNNNNNNNNNNNNTCACAATAAGATTAGCGTTACCACTATGATTATCATTTATACTTTATGGATTTATTAACAATATAAATCACATATTTTGTCATTTAGTTCCATCAGGAACTCCTAGCATTTTAATACCTTTTATAGTTATAATTGAAAGAGTCAGAAATTTAATTCGTCCTGGTTCCTTGGCTGTTCGACTAACAGCTAATATAATTGCTGGACATCTATTAATAACTTTATTAGGTAACTTACCTATAAGTTATGAATTATATTCAGGTCTAATCATTATTTTTCAGGTTATATTAATATTATTTGAATTAGCTGTTTGTATTATTCAATCCTACGTATTTATAGTTCTTAGAACTTTATATTATAGAGAAGTAAATNNNNNNNNNNNNNNNNNNNNNNNNNNNNNNNNNNNNNNNNNNNNNNNNNNNNNNNNNNNNNNNNNNNNNNNNNNNNNNNNNNNNNNNNNNNNNNNNNNNNNNNNNNNNNNNNNNNNNNNNNNNNNNNNNNNNNNNNNNNNNNNNTTTAAGCCAACACGACAAAATCATCCTTTAATTAAAATTATTAATAATTCATTAATTGACTTTCCAGCACCATCTAATTTATCTTATTGGTGAAATTTTGGATTTGTATTAGGATTATGCTTAATAATCCAAATTATAACTGGATTATTTTTATCAATGCATTATAATGCCAACATTATAAACGCTTTCGAAAGATTAAGACATATTTGTCGTGATGTAAACTATGGTNNNNNNNNNNNNNNNNNNNNNNNNNNNNNNNNNNNNNNNNNNNNNNNNNNNNNNNNNNNNNNNNNNNNNNNNNNNNNNNNNNNNNNNNNNNNNNNNNNNNNNNNNNNNNNNNNNNNNNNNNNNNNNNNNNNNNNNNNNNNNNNNNNNNNNNNNNNNNNNNNNNNNNNNNNNNNNNNNNNNNNNNNNNNNNNNNNNNNNNNNNNNNNNNNNNNNNNNNNNNNNNNNNNNNNNNNNNNNNNNNNNNNNNNNNNNNNNNNNNNNNNNNNNNNNNNNNNNNNNNNNNNNNNNNNNNNNNNNNNNNNNNNNNNNNNNNNNNNNNNNNNNNNNNNNNNNNNNNNNNNNNNNNNNNNNNNNNNNNNNNNNNNNNNNNNNNNNNNNNNNNNNNNNNNNNNNNNNNNNNNNNNNNNNNNNNNNNNNNNNNNNNNNNNNNNNNNNNNNNNNNNNNNNNNNNNNNNNNNNNNNNNNNNNNNNNNNNNNNNNNNNNNNNNNNNNNNNNNNNNNNNNNNNNNNNNNNNNNNNNNNNNNNNNNNNNNNNNNNNNNNNNNNNNNNNNNNNNNNNNNNNNNNNNNNNNNNNNNNNNNNNNNNNNNNNNNNNNNNNNNNNNNNNNNNNNNNNNNNNNNNNNNNNNNNNNNNNNNNNNNNNNNNNNNNNNNNNNNNNNNNNNNNNNNNNNNNNNNNNNNNNNNNNNNNNNNNNNNNNNNNNNNNNNNNNNNNNNNNNNNNNNNNNNNNNNNNNNNNNNNNNNNNNNNNNNNNNNNNNNNNNNNNNNNNNNNNNNNNNNNNNNNNNNNNNNNNNNNNNNNNNNNNNNNNNNNNNNNNNNNNNNNNNNNNNNNNNNNNNNNNNNNNNNNNNNNNNNNNNNNNNNNNNNNNNNNNNNNNNNNNNNNNNNNNNNNNNNNNNNNNNNNNATGAAAAAATGATTCTTCTCAACTAATCATAAAGATATTGGAACAATATATTTCATTTTTGGTATTTGATCAGGTATAATTGGTACAACTTTAAGAGTTTTAATTCGTGTAGAACTTGGAACTCCAGGTTCATTTATTGGTGATGATCAAATTTATAATGTAATTGTCACTGCTCATGCTTTCATTATAATTTTTTTTATAGTTATACCAATTATAATTGGTGGATTTGGAAATTGATTAGTTCCTTTAATAATTGGAGCTCCTGATATAGCTTTTCCTCGTATAAATAATATAAGTTTTTGATTATTACCACCCTCTCTAATTCTTATTTTGGTGGGAAGAATAGTTGATAGAGGTGCAGGAACAGGTTGAACAGTTTACCCTCCTTTATCTGCAGGAATTGCACATTCTGGTTCATGTGTTGATTTAACTATTTTCTCTTTACACCTCGCAGGTGTATCTTCAATTTTAGGTGCTGTAAATTTCATTAGAACAATTTTTAATATACGTTCAATAGGAATTTGATTAGATCGAATACCCTTATTTGTATGAGCAGTTTTAATTACTGCATTCTTGTTATTATTATCTTTACCTGTTTTAGCAGGTGCTATTACAATATTATTAACAGATCGAAATTTAAATACTTCATTTTTTGACCCTGCAGGGGGGGGAGACCCAATTCTTTATCAGCATTTATTTTGATTTTTTGGTCATCCTGAAGTTTATATTTTAATTTTACCAGGATTTGGTTTAATTTCTCATATTATTACCCAGGAAAGAGGTAAAATTGAATCTTTTGGTTCATTAGGAATAATTTATGCTATAATATCAATTGGTATTTTAGGATTTGTTGTATGAGCACATCATATATTCACTGTAGGAATAGACGTTGACACACGTGCATATTTTACATCAGCTACTATAATTATTGCAGTTCCTACTGGAATTAAAGTATTTAGTTGACTCGCAACTTTGAGAGGTATGAAAATCAATATTACATCTTCAGCCTTATGGGCTCTAGGATTTGTATTTTTATTTACTATTGGAGGTTTAACCGGGGTAATTTTAGCTAATTCTTCAATTGATATTATATTACATGATACATACTATGTAGTTGCACATTTCCATTATGTTTTATCTATAGGAGCAGTTTTTGCTATTATAGCAAGATTTATTCATTGGTTTCCATTATTCACAGGAATAAGATTAAACTCAAATTGATTAAAAATTCATTTTCTATTGATATTTATTGGTGTAAATATAACATTTTTTCCTCAACATTTTTTGGGACTAAGAGGAATACCTCGTCGGTATTCAGACTATCCTGACGCCTATATGTCATGAAACATAATTTCATCAATAGGAAGAATTATATCTTTAGTCGNNNNNNNNNNNNNNNNNNNNNNNNNNNNNNNNNNNNNNNNNNNNNNNNNNNNNNNNNNNNNNNNNNNTAGAATTCAATCTTCTATTGAATGAATACAGAAATTTCCCCCATCTGAACATTCTTATAATGAAATGCCATTATTAATTCAAATTTCAAATTGATCTTATATTAATATACAGGATGCTGTATCGCCATTGATAGAGCAGTTAATATTTTTTCATGATCATGTATTAATTATTTTAATTATAATTACAATTGTTGTTGCTTACATAATAATAATACTAATATTAAATAAAATTATTAATCGTTTACTTCTTGAAGGAAAATTTATTGAATTTATTTGAACTTTATTACGCGCAATAACTTTAATTTTTATTGCACTACCATCATTGCGATTATTATATATATTAGACGAAATTAATAATCCATTATTAACATTGAAAATTATTGGTCATCAATGATACTGATCATATGAATATTCAGATTTTTCTGATGTAGAATTTGATTCATATATAAAATCCATAAATGAAATAAATAAAAATGAATTTCGTTTATTAGATGTAGATAATCGAGTAATTCTACCATTTAATATTCAAGTACGACTATTAGTTTCTTCTTTTGATGTTATTCACTCTTGAGCTATACCATCTATGAGACTTAAAGTTGATGCAGTACCAGGACGATTAAATCAAATAAGAATATTAATTAGTCGTCCTGGTATATCATATGGACAATGTTCTGAAATTTGTGGAGCAAATCATAGATTTATGCCTATTGTAATTGAAAGAATTAGAATGAAAATATTTATTAAATGATTAATTAATTATATGAATAATCATCCTTATCATATAGTCGATTATAGACCCTGACCTTTAACTGGATCAATTGGAGCATTAACTTTTGTTTCTGGTATAGTTATAATGTTTCATAAATGTAACTTTATATTATTGTATATAGGTATTTTATTATTACTAATAACAATAATTCAATGATGACGTGATATTTCCCGAGAGGGAACATTTTTAGGAATACATACAATAATGGTAGTGAACGGTTTGAAAATAGGCATATTATTATTTATTGTATCAGAAGTTCTTTTTTTTGTTTCATTTTTTTGGGGATTTTTCCATAGTAGATTAAGACCTGTAGTTGAAATTGGCATAATTTGACCCCCTAGAGGTATTTATGTTTTTAATCCAACTCAAGTCCCTTTATTGAATACAATAATCTTATTATGTTCAGGAATTACAATTACTTGGGCTCACCATTCAATTATATGTGGCAATCATATTAATTCTATTTATAGAATTATATTAACTGTTATTTTAGGTATATATTTCACCATTCTACAAGGTTACGAATATTACGAAGCTCCATTTGCAATTAATGATTCTATCTATGGGTCCTCTTTTTTTATAGCTACTGGATTTCATGGAATTCATGTAATTATTGGAACAAGATTTATTACCGTATGTTTATTACGGCAAATAAAGTTTCATTTCTCTAAAAATCATCACTTTGGTTTTGAAGCTGCTGCTTGATATTGACATTTTGTTGATGTAGTTTGATTATTTTTATATTTATCNNNNNNNNNNNNNNNNNNNNNNNNNNNNNNNNNNNNNNNNNNNNNNNNNNNNNNNNNNNNNNNNNNNNNNNNNNNNNNNNNNNNNNNNNNNNNNNNNNNNNNNNNNNNNNNNNNNNNNNNNNNNNNNNNNNNNNNNNNNNNNNNNNNNNNNNNNNNNNNNNNNNNNNNNNNNNNNNNNNNNNNNNNNNNNNNNNNNNNNNNNNNNNNNNNNNNNNNNNNNNNNNNNNNNNNNNNNNNNNNNNNNNNNNNNNNNNNNNNNNNNNNNNNNNNNNNNNNNNNNNNNNNNNNNNNNNNNNNNNNNNNNNNNNNNNNNNNNNNNNNNNNNNNNNNNNNNNNNNNNNNNNNNNNNNNNNNNNNNNNNNNNNNNNNNNNNNNNNNNNNNNNNNNNNNNNNNNNNNNNNNNNNNNNNNNNNNNNNNNNNNNNNNNNNNNNNNNNNNNNNNNNNNNNNNNNNNNNNNNNNNNNNNNNNNNNNNNNNNNNNNNNNNNNNNNNNNNNNNNNNNNNNNNNNNNNNNNNNNNNNNNNNNNNNNNNNNNNNNNNNNNNNNNNNNNNNNNNNNNNNNNNNNNNNNNNNNNNNNNNNNNNNNNNNNNNNNNNNNNNNNNNNNNNNNNNNNNNNNNNNNNNNNNNNNNNNNNNNNNNNNNNNNNNNNNNNNNNNNNNNNNNNNNNNNNNNNNNNNNNNNNNNNNNNNNNNNNNNNNNNNNNNNNNNNNNNNNNNNNNNNNNNNNNNNNNNNNNNNNNNNNNNNNNNNNNNNNNNNNNNNNNNNNNNNNNNNNNNNNNNNNNNNNNNNNNNNNNNNNNNNNNNNNNNNNNNNNNNNNNNNNNNNNNNNNNNNNNNNNNNNNNNNNNNNNNNNNNNNNNNNNNNNNNNNNNNNNNNNNNNNNNNNNNNNNNNNNNNNNNNNNNNNNNNNNNNNNNNNNNNNNNNNNNNNNNNNAAAAATAATTCTTCTTATTTACTTTATATAATTTTCTTACTATTGGGAATTATAATTTCAGTTTCTTCAAACAATTGGCTGGGTTGTTGAATAGGGATTGAAATAAATATAGTTTCATTTTTGCCCATAATGGCAAATAAAATAAGAATTTACGCTTCAGAATCAATAATCAAATATTTTATTATCCAAAGAATGGGATCGAGTCTATTATTAATNNNNNNNNNNNNNNNNNNNNNNNNNNNNNNNNNNNNNNNNNNNNNNNNNNNNNNNNNNNNNNNNNNNNNNNNNNNNNNNNNNNNNNNNNNNNNNNNNNNNNNNNNNNNNNNNNNNNNNNNNNNNNNNNNNNNNNNNNNNNNNNNNNNNNNNNNNNNNNNNNNNNNNNNNNNNNNNNNNNNNNNNNNNNNNNNNNNNNNNNNNNNNNNNNNNNNNNNNNNNNNNNNNNNNNNNNNNNNNNNNNNNNNNNNNNNNNNNNNNNNNNNNNNNNNNNNNNNNNNNNNNNNNNNNNNNNNNNNNNNNNNNNNNNNNNNNNNNNNNNNNNNNNNNNNNNNNNNNNNNNNNNNNNNNNNNNNNNNNNNNNNNNNNNNNNNNNNNNNNNNNNNNNNNNNNNNNNNNNNNNNNNNNNNNNNNNNNNNNNNNNNNNNNNNNNNNNNNNNNNNNNNNNNNNNNNNNNNNNNNNNNNNNNNNNNNNNNNNNNNNNNNNNNNNNNNNNNNNNNNNNNNNNNNNNNNNNNNNNNNNNNNNNNNNNNNNNNNNNNNNNNNNNNNNNNNNNNNNNNNNNNNNNNNNNNNNNNNNNNNNNNNNNNNNNNNNNNNNNNNNNNNNNNNNNNNNNNNNNNNNNNNNNNNNNNNNNNNNNNNNNNNNNNNNNNNNNNNNNNNNNNNNNNNNNNNNNNNNNNNNNNNNNNNNNNNNNNNNNNNNNNNNNNNNNNNNNNNNNNNNNNNNNNNNNNNNNNNNNNNNNNNNNNNNNNNNNNNNNNNNNNNNNNNNNNNNNNNNNNNNNNNNNNNNNNNNNNNNNNNNNNNNNNNNNNNNNNNNNNNNNNNNNNNNNNNNNNNNNNNNNNNNNNNNNNNNNNNNNNNNNNNNNNNNNNNNNNNNNNNNNNNNNNNNNNNNNNNNNNNNNNNNNNNNNNNNNNNNNNNNNNNNNNNNNNNNNNNNNNNNNNNNNNNNNNNNNNNNNNNNNNNNNNNNNNNNNNNNNNNNNNNNNNNNNNNNNNNNNNNNNNNNNNNNNNNNNNNNNNNNNNNNNNNNNNNNNNNNNNNNNNNNNNNNNNNNNNNNNNNNNNNNNNNNNNNNNNNNNNNNNNNNNNNNNNNNNNNNNNNNNNNNNNNNNNNNNNNNNNNNNNNNNNNNNNNNNNNNNNNNNNNNNNNNNNNNNNNNNNNNNNNNNNNNNNNNNNNNNNNNNNNCCCGAACTCTTCCAGAATTAAATGAAAATAAACTGGAAAAAAATATACCATGTTGAGTAAATGAAAATATATATAATCTATAACAAGCAGATAAAAATGATGATAAAATTAATAAAATCATTATTAACATGTTTCAAGATATTAATCTATTAATAATCATAATTTCACCAGCTAGATTAATAGTAGGTGGACAAGACATATTTCTTGCTGACATTAAAAATCATAATAAAGATATTGAAGGCATAAAGGTCAATATACCCTTATTAATTAATAATCTTCGACTATTAGTTCGCTCATATAATATATTTGCTAAACAAAATAATCCAGAAGAACATAAACCATGTCCAATTATTATATAATATGAACCACACAATCCTCAATTTCTCATAGTTATAATTCCTCTAATTACAAGACCTATATGAGCCACAGATGAATAAGCAATTATTGATTTAATATCAATCTGAATTATACATAGAATTCTAATTAAAATACTTCCAATTATTGAAATTGAGATTCATAAAAATCCAAATTTATAAAAATAAAAAGGAATAATATATATCATTCGAATTAAACCATATCCTCCTAGTTTTNNNNNNNNNNNNNNNNNNNNNNNNNNNNNNNNNNNNNNNNNNNNNNNNNNNNNNNNNNNNNNNNNNNNNNNNNNNNNNNNNNNNNNNNNNNNNNNNNNNNNNNNNNNNNNNNNNNNNNNNNNNNNNNNNNNNNNNNNNNNNNNNNNNNNNNNNNNNNNNNNNNNNNNNNNNNNNNNNNNNNNNNNNNNNNNNNNNNNNNNNNNNNNNNNNNNNNNNNNNNNNNNNNNNNNNNNNNNNNNNNNNNNNNNNNNNNNNNNNNNNNNNNNNNNNNNNNNNNNNNNNNNNNNNNNNNNNNNNNNNNNNNNNNNNNNNNNNNNNNNNNNNNNNNNNNNNNNNNNNNNNNNNNNNNNNNNNNNNNNNNNNNNNNNNNNNNNNNNNNNNNNNNNNNNNNNNNNNNNNNNNNNNNNNNNNNNNNNNNNNNNNNNNNNNNNNNNNNNNNNNNNNNNNNNNNNNNNNNNNNNNNNNNNNNNNNNNNNNNNNNNNNNNNNNNNNNNNNNNNNNNNNNNNNNNNNNNNNNNNNNNNNNNNNNNNNNNNNNNNNNNNNNNNNNNNNNNNNNNNNNNNNNNNNNNNNNNNNNNNNNNNNNNNNNNNNNNNNNNNNNNNNNNNNNNNNNNNNNNNNNNNNNNNNNNNNNNNNNNNNNNNNNNNNNNNNNNNNNNNNNNNNNNNNNNNNNNNNNNNNNNNNNNNNNNNNNNNNNNNNNNNNNNNNNNNNNNNNNNNNNNNNNNNNNNNNNNNNNNNNNNNNNNNNNNNNNNNNNNNNNNNNNNNNNNNNNNNNNNNNNNNNNNNNNNNNNNNNNNNNNNNNNNNNNNNNNNNNNNNNNNNNNNNNNNNNNNNNNNNNNNNNNNNNNNNNNNNNNNNNNNNNNNNNNNNNNNNNNNNNNNNNNNNNNNNNNNNNNNNNNNNNNNNNNNNNNNNNNNNNNNNNNNNNNNNNNNNNNNNNNNNNNNNNNNNNNNNNNNNNNNNNNNNNNNNNNNNNNNNNNNNNNNNNNNNNNNNNNNNNNNNNNNNNNNNNNNNNNNNNNNNNNNNNNNNNNNNNNNNNNNNNNNNNNNNNNNNNNNNNNNNNNNNNNNNNNNNNNNNNNNNNNNNNNNNNNNNNNNNNNNNNNNNNNNNNNNNNNNNNNNNNNNNNNNNNNNNNNNNNNNNNNNNNNNNNNNNNNNNNNNNNNNNNNNNNNNNNNNNNNNNNNNNNNNNNNNNNNNNNNNNNNNNNNNNNNNNNNNNNNNNNNNNNNNNNNNNNNNNNNNNNNNNNNNNNNNNNNNNNNNNNNNNNNNNNNNNNNNNNNNNNNNNNNNNNNNNNNNNNNNNNNNNNNNNNNNNNNNNNNNNNNNNNNNNNNNNNNNNNNNNNNNNNNNNNNNNNNNNNNNNNNNNNNNNNNNNNNNNNNNNNNNNNNNNNNNNNNNNNNNNNNNNNNNNNNNNNNNNNNNNNNNNNNNNNNNNNNNNNNNNNNNNNNNNNNNNNNNNNNNNNNNNNNNNNNNNNNNNNNNNNNNNNNNNNNNNNNNNNNNNNNNNNNNNNNNNNNNNNNNNNNNNNNNCTTATAAATGGAATTCCACATAATGACATATTAGCAATATTTATTATTGTAATAGTTAATGGTATTTGAAATCTTAAACATCCTATTACACGAATATCTTGATTGTTATTAAAATTGTGAATCAAAATCCCTGCACACAAAAATAAAGTAGCTTTAAATAAAGCATGAACAATTAAATGAAAAAATGATAATATTGGATAACCAAATATAATAATTGTTATTATAATCCCTAACTGTCTCAAAGTTGATAATGCAATAATTTTTTTTAAATCAAACTCAAAATTTGCTCTAATTCCTGATATTAATATAGTTAAAATTGAAATTAATATAAATAATTGAATATAATCAAAATTCAAGATAATGTTAGAAAATCGAATTATTAAATATACACCAGCAGTGACAAGTGTAGATGAATGAACCAAAGCAGATACAGGAGTTGGAGCAGCTATAGCTGCTGGTAATCATGAAGAAAATGGAATTTGAGCTCTCTTTGTAAATCTTGCAATAATAANNNNNNNNNNNNNNNNNNNNNNNNNNNNNNNNNNNNNNNNNNNNNNNNNNNNNNNNNNNNNNNNNNNNNNNNNNNNNNNNNNNNNNNNNNNNNNNNNNNNNNNNNNNNNNNNNNNNNNNNNNNNNNNNNNNNNNNNNNNNNNNNNNNNNNNNNNNNNNNNNNNNNNNNNNNNNNNNNNNNNNNNNNNNNNNNNNNNNNNNNNNNNNNNNNNNNNNNNNNNNNNNNNNNNNNNNNNNNNNNNNNNNNNNNNNNNNNNNNNNNNNNNNNNNNNNNNNNNNNNNNNNNNNNNNNNNNNNNNNNNNNNNNNNNNNNNNNNNNNNNNNNNNNNNNNNNNNNNNNNNNNNNNNNNNNNNNNNNNNNNNNNNNNNNNNNNNNNNNNNNNNNNNNNNNNNNNNNNNNNNNNNNNNNNNNNNNNNNNNNNNNNNNNNNNNNNNNNNNNNNNNNNNNNNNNNNNNNNNNNNNNNNNNNNNNNNNNNNNNNNNNNNNNNNNNNNNNNNNNNNNNNNNNNNNNNNNNNNNNNNNNNNNNNNNNNNNNNNNNNNNNNNNNNNNNNNNNNNNNNNNNNNNNNNNNNNNNNNNNNNNNNNNNNNNNNNNNNNNNNNNNNNNNNNNNNNNNNNNNNNNNNNNNNNNNNNNNNNNNNNNNNNNNNNNNNNNNNNNNNNNNNNNNNNNNNNNNNNNNNNNNNNNNNNNNNNNNNNNNNNNNNNNNNNNNNNNNNNNNNNNNNNNNNNNNNNNNNNNNNNNNNNNNNNNNNNNNNNNNNNNNNNNNNNNNNNNNNNNNNNNNNNNNNNNNNNNNNNNNNNNNNNNNNNNNNNNNNNNNNNNNNNNNNNNNNNNNNNNNNNNNNNNNNNNNNNNNNNNNNNNNNNNNNNNNNNNNNNNNNNNNNNNNNNNNNNNNNNNNNNNNNNNNNNNNNNNNNNNNNNNNNNNNNNNNNNNNNNNNNNNNNNNNNNTTATTTACTATTTGTTTTAATTATTGTAATTAATATTTTAACAATAAATATATCAACCCTTCGAAGAAGGACTNNNNNNNNNNNNNNNNNNNNNNNNNNNNNNNNNNNNNNNNNNNNNNNNNNNNNNNNNNNNNNNNNNNNNNNNNNNNNNNNNNNNNNNNNNNNNNNNNNNNNNNNNNNNNNNNNNNNNNNNNNNNNNNNNNNNNNNNNNNNNNNNNNNNNNNNNNNNNNNNNNNNNNNNNNNNNNNNNNNNNNNNNNNNNNNNNNNNNNNNNNNNNNNNNNNNNNNNNNNNNNNNNNNNNNNNNNNNNNNNNNNNNNNNNNNNNNNNNNNNNNNNNNNNNNNNNNNNNNNNNNNNNNNNNNNNNNNNNNNNNNNNNNNNNNNNNNNNNNNNNNNNNNNNNNNNNNNNNNNNNNNNNNNNNNNNNNNNNNNNNNNNNNNNNNNNNNNNNNNNNNNNNNNNNNNNNNNNNNNNNNNNNNNNNNNNNNNNNNNNNNNNNNNNNNNNNNNNNNNNNNNNNNNNNNNNNNNNNNNNNNNNNNNNNNNNNNNNNNNNNNNNNNNNNNNNNNNNNNNNNNNNNNNNNNNNNNNNNNNNNNNNNNNNNNNNNNNNNNNNNNNNNNNNNNNNNNNNNNNNNNNNNNNNNNNNNNNNNNNNNNNNNNNNNNNNNNNNNNNNNNNNNNNNNNNNNNNNNNNNNNNNNNNNNNNNNNNNNNNNNNNNNNNNNNNNNNNNNNNNNNNNNNNNNNNNNNNNNNNNNNNNNNNNNNNNNNNNNNNNNNNNNNNNNNNNNNNNNNNNNNNNNNNNNNNNNNNNNNNNNNNNNNNNNNNNNNNNNNNNNNNNNNNNNNNNNNNNNNNNNNNNNNNNNNNNNNNNNNNNNNNNNNNNNNNNNNNNNNNNNNNNNNNNNNNNNNNNNNNNNNNNNNNNNNNNNNNNNNNNNNNNNNNNNNNNNNNNNNNNNNNNNNNNNNNNNNNNNNNNNNNNNNNNNNNNNNNNNNNNNNNNNNNNNNNNNNNNNNNNNNNNNNNNNNNNNNNNNNNNNNNNNNNNNNNNNNNNNNNNNNNNNNNNNNNNNNNNNNNNNNNNNNNNNNNNNNNNNNNNNNNNNNNNNNNNNNNNNNNNNNNNNNNNNNNNNNNNNNNNNNNNNNNNNNNNNNNNNNNNNNNNNNNNNNNNNNNNNNNNNNNNNNNNNNNNNNNNNNNNNNNNNNNNNNNNNNNNNNNNNNNNNNNNNNNNNNNNNNNNNNNNNNNNNNNNNNNNNNNNNNNNNNNNNNNNNNNNNNNNNNNNNNNNNNNNNNNNNNNNNNNNNNNNNNNNNNNNNNNNNNNNNNNNNNNNNNNNNNNNNNNNNNNNNNNNNNNNNNNNNNNNNNNNNNNNNNNNNNNNNNNNNNNNNNNNNNNNNNNNNNNNNNNNNNNNNNNNNNNNNNNNNNNNNNNNNNNNNNNNNNNNNNNNNNNNNNNNNNNNNNNNNNNNNNNNNNNNNNNNNNNNNNNNNNNNNNNNNNNNNNNNNNNNNNNNNNNNNNNNNNNNNNNNNNNNNNNNNNNNNNNNNNNNNNNNNNNNNNNNNNNNNNNNNNNNNNNNNNNNNNNNNNNNNNNNNNNNNNNNNNNNNNNNNNNNNNNNNNNNNNNNNNNNNNNNNNNNNNNNNNNNNNNNNNNNNNNNNNNNNNNNNNNNNNNNNNNNNNNNNNNNNNNNNNNNNNNNNNNNNNNNNNNNNNNNNNNNNNNNNNNNNNNNNNNNNNNNNNNNNNNNNNNNNNNNNNNNNNNNNNNNNNNNNNNNNNNNNNNNNNNNNNNNNNNNNNNNNNNNNNNNNNNNNNNNNNNNNNNNNNNNNNNNNNNNNNNNNNNNNNNNNNNNNNNNNNNNNNNNNNNNNNNNNNNNNNNNNNNNNNNNNNNNNNNNNNNNNNNNNNNNNNNNNNNNNNNNNNNNNNNNNNNNNNNNNNNNNNNNNNNNNNNNNNNNNNNNNNNNNNNNNNNNNNNNNNNNNNNNNNNNNNNNNNNNNNNNNNNNNNNNNNNNNNNNNNNNNNNNNNNNNNNNNNNNNNNNNNNNNNNNNNNNNNNNNNNNNNNNNNNNNNNNNNNNNNNNNNNNNTNNNNNNNNNNNNNNNNNNNNNNNNNNNNNNNNNNNNNNNNNNNNNNNNTTATGTTAGCAGAATTAAACTGTCTATTAATATCAAAAATTAATGTTCTTCATAAACTATAACANNNNNNNNNNNNNNNNNNNNNNNNNNNNNNNNNNNNNNNNNNNNNNNNNNNNNNNNNNNNNNNNNNNNNNNNNNNNNNNNNNNNNNNNNNNNNNNNNNNNNNNNNNNNNNNNNNNNNNNNNNNNNNNNNNNNNNNNNNNNNNNNNNNNNNNNNNNNNNNNNNNNNNNNNNNNNNNNNNNNNNNNNNNNNNNNNNNNNNNNNNNNNNNNNNNNNNNNNNNNNNNNNNNNNNNNNNNNNNNNNNNNNNNNNNNNTCTAATATGGCAGATTAGTGCAATGAACTTAAGATTCATATATAAAATATNATTTTTTTATTGGAAAATAAGATGCCTGAATNAAAAAGGGTTATTTTGATAGAATAAATTATGTAATAAAATTACTCNNNCATTAAGTGGCTGAAAGTAAGTAATGGTCTCTTAAACCAATTAATAGTAATTAACAAATACTCTTAANNAAAGATAAGCTAAAANTAAAGCTAATGGGTTCATACCTCACTTATGGAAANTCTTCCTCTTTTTANNNNNNNNNNNNNNNNNNNNNNNNNNNNNNNNNNNNNNNNNNNNNNNNNNNNNNNNNNNNNNNNNNNNNNNNNNNNNNNNNNNNNNNNNNNNNNNNNNNNNNNNNNNNNNNNNNNNNNNNNNNNNNNNNNNNNNNNNNNNNNNNNNNNNNNNNNNNNNNNNNNNNNNNNNNNNNNNNNNNNNNNNNNNNNNNNNNNNNNNNNNNNNNNNNNNNNNNNNNNNNNNNNNNNNNNNNNNNNNNNNNNNNNNNNNNNNNNNNNNNNNNNNNNNNNNNNNNNNNNNNNNNNNNNNNNNNNNNNNNNNNNNNNNNNNNNNNNNNNNNNNNNNNNNNNNNNNNNNNNNNAACTATTATNNNNNNNNNNNNNNNNNNNNNNNNNNNNNNNNNNNNNGAGAAATAGCTTTTCATAAATAAATTTACAGTTTATTGCCTATTTTCGGCCATCCCAANNNNNNNNNNNNNNNNNNNNNNNNNNNNNNNNNNNNNNNNNNNNNNNNNNNNNNNNNNNNNNNNNN

>campbelli-northSI_04.NZ.MB.ISF.03

AATTTATTTTCATCATTTGATCCTTGTACTGGATTTCTTTCTCTTAATTGATTAAGATCAATAATTTTGTTATTATTTTTGCCTCTTACTTATTGATATATACCAAACCGTTTTATTATTTTTTATAATAAAATTTTATTTTTATTAAATAATGAATTAAATATATTAATAAATTATAAATCATTAGGGAGATCATTAATATTTTTATCACTATTTACATTTATCCTATTAAACAATTTATTAGGATTACTTCCTTATATTTTCACTAGCTCTAGTCATCTAGTATTTACAATAAGATTAGCGTTACCATTATGATTATCATTTATGCTTTATGGATTTATTAATAATGTTAATCATATATTTTGTCATATAGTTCCATCAGGAACTCCTAATATTTTAATACCTTTTATAGTTATTATTGAAAGAATTAGAAATTTAATTCGACCGGGATCATTAGCTGTTCGTTTAACAGCTAACATAATTGCTGGTCATCTTTTAATAACTCTGCTTGGTAATTTACCTATAAACTATGAATATTATTCAGGTATGATTATTATTTTTCAAATCTCGTTAATATTATTTGAATTAGCCGTTTGTATAATTCAGTCTTATGTTTTTATAGTACTTAGAACTTTATATTATAGAGAAGTGAATATTCCTCAAATGTCTCCATTAAATTGATTATTATTTATTTATTTTATTATAATAATNNNNNNNNNNNNNNNNNNNNNNNNNNNNNNNNNNNNNNNNAAATCCAATCTAATAACTGATAAAAAGTTAATAGATAGAATAATTTGATTTAAACCAACACGACAAAATCATCCCTTAATTAAAATTATTAATAATTCATTAATTGATTTCCCAGCTCCATCTAATTTATCTTATTGATGAAACTTCGGATTTGTATTAGGATTGTGCTTAATAATTCAAATCTTTACTGGATTATTTTTATCAATACATTATAATGCTAACATTGTTAGAGCTTTTGAAAGATTAAGTCATATTTGTCGAGATGTAAATTATGGGTGATTCCTACGAATCATTCACGCTAATGGAGCTTCATTATTTTTTATTTGTGTCTACTTACATGTAGGACGTGGATTATATTATGGTTCATATAAATATATGGAAACATGGTCTATTGGTGTAATCATATTATTAATATTAATAGCTACTGCTTTCTTAGGATATGTATTACCATGAGGACAAATATCCTTCTGAGGAGCAACAGTTATTACAAATTTACTTTCAGCTATTCCTTATTTAGGAATAACACTGGTTAATTGAATTTGAGGTGGATTTGCAGTTGATAATGCTACACTAACTCGATTTTACTCATTCCATTTTATTTTACCATTTATTGTTTTGAGATTAACAATTATTCATTTATTATACTTACACACAACAGGTTCAAATAATCCCTTAGGGATTAACTCAAATAATGATAAAGTTCCATTTCATCCATATTTTTCTATTAAAGATATTATAAGGCTCTTTATCTTAATGATTATATTTTTAATATTTGTTATATTAGAACCATATATATTAGGTGATCCTGATAATTTTACTCCTGCTAACCCTTTAGTTACACCAAAACATATTCAACCTGAATGATATTTTTTATTTGCTTATGCTATTCTTCGATCAATTCCTAATAAATTAGGAGGAGTGATTGCTCTTTTTATATCAATTTTTATTTTAATATTTGTACCTTTATTAAACAATTCTAATTTCATGGGATTAAATAATTATCCAATAAATCAAATTTTATTTTGATATTTATTAACTATCTTAATTTTGTTAACTTGAATTGGGGCCCGCCCTGTAGAATTACCTTATATTAACTTTGGCATGTTATTAACATTAATATATTTCTTTTACTTTATTATTGATCCTTTAATTAAATCTTTTTGAGATAAATTAATTAGATAGATGAAAAAATGATTCTTCTCAACTAATCATAAAGACATTGGAACAATGTATTTCATTTTTGGTATTTGATCTGGTATAATTGGTACAACTTTGAGAGTTCTAATTCGTGTAGAACTTGGAACTCCGGGTTCATTTATTGGTGATGATCAGATTTATAATGTAATTGTTACTGCTCATGCTTTTATTATAATTTTTTTTATAGTTATGCCTATTATAATTGGGGGATTTGGGAATTGATTAGTCCCATTGATAATTGGAGCTCCTGATATAGCTTTCCCTCGTATAAATAATATAAGTTTTTGATTATTGCCTCCATCATTAATTCTTATTTTAGTGGGAAGAATAGTTGATAGAGGTGCAGGTACTGGTTGAACAGTCTATCCTCCATTATCTAGTGGGGTAGCACATTCCGGTTCATGTGTAGATTTAACTATTTTCTCTCTTCATCTTGCAGGTGTATCTTCAATTTTAGGTGCTGTAAATTTTATTAGAACAATTTTTAATATACGATCAGTGGGTATTTGATTAGATCGTATACCTTTATTTGTGTGGTCAGTTTTAATTACTGCATTTTTGTTATTATTATCTTTACCTGTTCTAGCAGGTGCTATTACTATGTTATTAACAGATCGAAATTTAAATACTTCATTTTTTGACCCTGCGGGTGGGGGTGATCCAATTTTATATCAACACTTATTTTGATTTTTTGGTCATCCCGAAGTTTATATTTTAATCTTACCAGGGTTTGGTTTAATTTCTCATATTATTACACAAGAAAGAGGTAAAATTGAATCCTTTGGTTCATTAGGGATAATCTATGCTATAATATCAATTGGTATTTTAGGATTTGTTGTATGGGCACATCATATATTTACTGTAGGTATAGATGTTGATACACGTGCATATTTTACATCAGCTACTATAATTATTGCAGTTCCAACTGGAATTAAAGTGTTCAGTTGACTTGCAACTTTAAGAGGAATAAAAATTAATATCACATCTTCAGCTTTATGAGCCCTAGGATTTGTTTTTTTATTTACAATTGGTGGCTTAACTGGAGTAATTTTAGCTAATTCTTCAATTGATATTATATTACATGATACATATTATGTAGTTGCTCATTTTCACTATGTTCTGTCAATAGGAGCAGTTTTTGCTATTATGGCAAGATTTATTCACTGATTTCCTCTTTTTACAGGAATAACATTAAATTCAAATTGATTAAAAATTCATTTTTTATTAATATTTATTGGTGTAAATATAACATTTTTTCCTCAACATTTCTTAGGACTAAGAGGGATGCCTCGCCGATATTCAGATTATCCTGATGCTTATATATCATGAAATATAATTTCGTCAATAGGAAGAATTATATCCTTAATCGGAATTATATTTTTATTATTTATTGTGTGGGAAAGATTTATTTCAATGCGGTTAGTATTATATACTAATAGAATTCAGTCTTCTATTGAATGAATACAGAAATTTCCTCCATCTGAACATTCTTATAATGAAATGCCATTATTAATTCAAATTTCAAATTGATCTTATATAAATATACAGGACGCTGTATCACCATTAATAGAACAATTAATATTTTTTCATGATCATGTATTAATTATTTTAATTATAATTACAATTATTGTTGCTTATATAATATTAATATTGGTATTAAATAAAATTATTAATCGTTTACTCCTTGAGGGACAATTAATTGAATTTATTTGAACTTTATTACCTGCAATAACTTTAATTTTTATTGCATTACCATCATTACGACTATTATATATATTAGATGAAATTAATAATCCGTTATTAACATTAAAAGTTATTGGTCATCAATGATATTGATCATATGAATATTCAGATTTTTTTGATGTAGAATTTGATTCTTATATAAAATCTACAGTTGATATAAAAAAAAATGAATTTCGTTTATTAGATGTTGATAATCGAGTAATTTTACCATTCAATATTCAAATTCGATTGTTGATTTCCTCTTTTGATGTTATTCACTCTTGAGCAATACCATCTATAAGATTGAAAGTTGATGCAGTTCCAGGACGGTTGAATCAGATAAGTATACTAATTAGTCGTCCAGGTATTTCTTATGGTCAATGTTCTGAAATCTGTGGTGCAAATCACAGATTTATACCTATTGTTATTGAAAGAATTAGAATAAAAATATTTATTGACTGATTAGTTAATTATATGACTAATCATCCTTATCATCTAGTTGATTATAGCCCATGACCTTTAACTGGATCAATTGGAGCTCTAAGTTTTGTATCTGGTACAATTATAATATTTCATAAATATAATTTTATTCTATTATATATTGGTATATCCTTATTATTATTAACAATAGTTCAATGATGACGTGACATTTCTCGAGAAGGAACATTCTTGGGAATACATACAATAGTAGTAGTAAATGGTTTAAAAATAGGTATATTATTATTTATTGTATCAGAAATTCTTTTTTTTGTTTCTTTTTTTTGAGGATTTTTTCATAGAAGGTTAAGACCTGTGGTAGAAATTGGAATAATTTGACCTCCCATGGGTATTAATGTTTTTAACCCAACCCAAGTTCCTCTATTGAATACAATAATTTTATTATGTTCAGGAATTACAATCACTTGAGCTCATCATTCAATTATAAATGGTAATCACATTGATTCTATCTATAGAATTACTTTAACTATTATTTTAGGATTATACTTTACTGCTTTACAAGGTTATGAATACTATGAAGCTCCCTTCGCAATCAATGATTCCATTTATGGTTCTTCTTTTTTTATAGCTACTGGATTTCATGGCATTCATGTAATTATTGGAACAACATTTATTATTGTGTGTTTAGTACGACAAATAAATTTTCACTTTTCAATTAATCACCATTTTGGTTTTGAAGCTGCTGCTTGATATTGACATTTTGTTKMTGKARWWWKWTTWTTWWWWYAYTTRTYWWTYTMCTKMTKMRKASKWKAMTAAATTATAGTTTTTAAGAAAAAAAAATAAATAAATAAATTAAGAGAAACTGGCAAATAAGATTTCCAAGCTAAATATATTAATTTGTCGTATCGATAACGAGGAAGAGTCCCTCGTACTCAAATAAAGAAAAAACATAATAGAGATACTTGAATAGGGAAAATAATTGAGTCAATTTTTCCTCCTAAAAATATTAAACAAAATATTATTCTTATAAACAAAATACTAGAATATTCGGCTAGAAAAATAAATGCGAAACCAGCACCTCTATACTCAACATTAAATCCAGAAACCAATTCTGATTCTCCCTCAGAAAAATCAAATGGAGAACGATTTGTTTCAGCTAAAGCCGAAGAAATTCAACACATTCTTAATGGAAGAAATAAAAAAATAAACCATACAGATTTTTGAAATAAAAATAAATCAAGAATATTATAACTTTTAATTATTATTATAGGACATAATATAATTAAAACTAATCTTACCTCATAGGAAATAGATTGAGCAATTGAACGAATACACCCTAATATTGCATAACTTGAATTAGAAGATCATCCTGTCAATATTAAAGAATAAACACTTATTCTTGAACAACAAAAAAAAAATAAAATACCAAAATTAAAAGAAACACAATTAATTATATAAGGGTATAGTGACCATAATAATAATCTATTAAATAATCCTATAACAGGACTAAAAATATAAATTAAATAATTAGACATTAATGGAATAGTATTTTCTTTTATAAATAATTTAATAGCATCTGAAATAGGTTGTAAAATACCCAAAAATCCAACTTTATTTGGTCCCTTACGAATTTGAATATATCTTAAAACTTTGCGTTCTAAGAGAGTAATAAATGCCACTCCAATTAAAATAAATAAAATAGTAATAAAAATTGTAATTAAATACAAAAATAATTCTTCTTATTTATTATATTTGGTTTTCTTATTTTTTGGAATTATAATTTCAATTTCTTCCAACAATTGGCTGGGTTGTTGAATGGGAATTGAAATAAATATAGTTTCATTTTTGCCTATTATGGCAAATAAAATAAGAATTTATGCTTCGGAATCAATAATTAAATATTTTATCATTCAAAGAATGGGATCTAGTTTATTATTAATATCTATTATCATTNNNAGTATAATAATTGACTTAAATTATATAATTATAATTAGATTAATAATTAAAATTGGGTGTCCTCCATTTCATTTTTGATATGTTTCTGTTATTGAGGGTTTATCTTGAATGGTATGTTTTATTTTAATAACCATTCAAAAAATTATTCCTATAGTTATATTATCATATTTGAATGTAAATTTAAGGTTATTTATTATTATAGCATGTATTTGAGGATGTATTGGAGGGCTGGGTTATTCTTCAATACGTAAAATTATTGCTTATTCTTCAATTTATAATTTAAGATGAATTTTTAGAGGTATTATAATTATTAATTATTCATGATTAATTTATTACTTTATTTATTCATTTACATTAATAGCTGTATGTTATATGTTTAATTTATTTAATATTAATTATATTAATCAATTTGTTATATCTTCTTTTAATTTTTCAAAATCAGTTATAATAATAATTATTTTTATATCTATAGGTGGATTACCACCTTTCTTAGGGTTTTTCCCTAAATTAATTATAATTTACTGTTTATTGTTAAATAAAATATTATTTATTTGTGTTTTACTGTTAATAACAGCTTTAATTGTATTATTTTTTTATTTACGAATTGTAATTACAACATTAATAATAAATACTATTTCAATAAAAAGAATTGTTATTGAATCTTCATATTTTTATTATGTTGTTAGAATATTTTCATTATTTGGAATAATTTTTTTGTCTTTGATTACATTAAATTTATGTTAGNNNNNNNNNNNNNNNNNNNNNNNNNNNNNNTTAATGTTTTTATTAACATTTTTATTGTCACTATTAATATTTATTTCTTACAAATCTATAAAAGATCGAGAGAAATCTTCTCCATTTGAATGTGGATTCAATCCATTTGAGTCTTCTCGTATTCCATTCTCAAGACATTTTTTTTTAATTGCAGTAATTTTCTTAATTTTTGATGTAGAATTAGTGATTATTATACCTGTTATTTTAGTAATAAATGGATTAAATATAATTGATATTTATATTATTATATTCATTTTTTTAATTATTTTAACTATAGGTCTATATCATGAATGATATAATAATATATTAAGTTGANNNNNNNGTTTAATTAAAATATTTAATTTTAAAATAATTAAATTCAATGGAATTCAGTGTATTATAATTAAAAAGAATTCCCGGACTCTTCCAGAATTAAATGAAAATAAACTAGAAAAAAATATGCCATGCTGAGTATATGAAAATATATATAATCTATAACAAGCAGATAAAAATGAAGATAAAATCAACAAAACCATTATTAATATATTTCAAGATATTAATCTATTAATAATTATAATTTCACCTGCCAAATTAAGTGTAGGAGGACACGATATATTTCTAGCTGACATTAAAAATCATATTAAAGATATTGAAGGTATAAATGTCAATATTCCCTTATTAATTAATAATCTCCGTCTATTAGTTCGTTCATATAATATATTTGCTAAACAAAATAATCCAGAAGAACAAAGACCATGACCAATTATTATATAATAAGAACCGTAAATTCCTCAATTTGTTATAGTTATAATCCCTCTAATCACAAGACCTATGTGAGCTACAGATGAATAAGCAATTATTGATTTGATATCAATTTGAATTATACAAAGAATTCTAATTAAAATTCTCCCAATTATTGAAATGGAAATTCACACAAATCCAAATTTATAAAAATATAAAGGAATAATATTTATTATTCGAATAATACCATAACCTCCTAACTTTAATAGAATTCTAGCTAGAATTATTGATCCAGAAACAGGTGCTTCAACATGAGCCTTTGGTAATCAAAAATGAAAAAATAGTGGTATTTTAATTAAAAAAGCTATTATCATGCCAACATAAATATAAATATTAATAGGATAAACTATTAATATAAAAATATTAGTTATATTTATTTTATAAAGAAAAAAAATTCTTATTAATATTGGTAATGATGCAAATAATGTATAAAATAATAAATAATATCTTGCATCAATACGTTCTGGCTGATATCCCCAGCCCATAATAATAATTATAATTGGAATTAATCTAGATTCAAAAAAGATATAAAAAATTATAATTCTTGATGTAGAAAAAGAAATAATTAAAAAAATTATTAACACAACACTAAAAATAAAAAAAATAATTTATTATNNNNNNNNNNNNNNNNNNNNNNNNNNNNNNNNNNNNNNNNNNNNNNNNNNNNNNNNNNNNNNNNNNNNNNNNNNNNNNNNNNNNNNNNNNNNNNNNNNNNNNNNNNNNNNNNNNNNNNNNNNNNNNNNNNNNNNNATTATTATAATAATAATTATATTTATCAATCATGAATTATAAATAAGTAGGGTTAATATCAAGCAAGAAAAAATCAATTTTATAGATATTGATATTAATTTATCATTACTATGAGAACGAATTAATGTAACTAAACATGATAATCCCATTACTCCTTCACAAACTCTAAAAACCAAGAAAATTAACAAGATATATATATCATATATTTTTATAGAAAAAATAAATGACAAAGAACAAAATATTCTTAAGATAATTAATTCTAATCTCAATGTTGTTATCAAATGTTTTCGATTTATGCATAACGAAATTATTCCAGAAAGGAATATAGAAAAAAAATATATTAATAGTGTTATATTCTATAAATATATAATAATAATTCAGACAATAAATGAAATTATTTGANNNNNNNNNNNNNNNNNNNNNNNNNNNNNNNNNNNNNNNNNNNNNNNNNNNNNNNNNNNNNNNNNNNNNNNNNNNNNNNNNNNNNNNCCTCATCCCATATCAATAGATTTTTGAAATATTAATGAATTAGTTATTAAAAATATTTGATTATGGAATGTTGATATTTGTTTTATAAATCATATTGAACCAAAAAACTCATAAATAATTTTTATTTTAGTTTTAGAATAAATTGATATCTCATAACCTAATCAAATTCCTAATGCTGAAAATATTAATGCTATTAATTTACCTTCAATAGGTAGTAAAATTATAATTGGATCATTAAATATTAATCATCTTAATATAGATCCAGAAATAATTGAATAAATAGATAAAATAATAATTCTTTTAATTATATCATTTAAGTTTTCACTTAATGATCTTATTTTATAAAAGTTAAAATTTATATTTATTGAAAAATATGTTAAACGAGCAGAATAAAATGAAGTTAATCCAATCCCAATATATATGAATATTATAATTAACATATTAATATTATTAGAACATATGGATTCTATAATTAAATCTTTTGAATAAAATCCTCTTATGAATGGAATTCCACATAATGATATATTAGCAATATTTATTATTGTAATAGTTAAAGGTATTTGAAATCTTAAACATCCTATTACACGAATATCTTGATTATTATTAAAATTATGAATTAAAATACCTGCACATAAAAATAATGTAGCTTTAAATAAAGCATGAACAATTAAATGAAAAAAAGACAATATAGGATAACCAAATATAATAATTGTTATTATGATTCCTAATTGTCTTAAAGTTGATAGAGCAATAATTTTTTTTAAATCAAATTCAAAATTTGCTCTAATTCCTGATATTAACATAGTTAAAATTGAAATTAACATGAATACTTGAATGTAATCAAAGTTTAAAAAAATGTTGGAAAATCGAATTATTAAATATACTCCAGCAGTAACAAGAGTAGACGAGTGAACTAAAGCTGATACAGGGGTTGGAGCAGCTATTGCTGCTGGTAATCAGGAAGAAAAAGGAATTTGAGCTCTCTTAGTAAATCTTGCAATAATAATTAGTATTATTATAATTTATATCTTTATTAATAAAAGATAAAAATTTCATGATCCAAAGTTTAATATTCATGAAATAGTTATTAAAATAGCAACATCTCCAATACGATTTATTAATGCAGTTAATATTCCTGCATTATTAGAGTATAAATTTTGATAGTAAATAACTAAACAATAAGAGACTAAACCAAGACCATCCCAACCAATAATAATTCTTATTATATTAGGTCTAATAATTAACAAAATTATAGATATTACAAATCCTAATACAATATAAATAAAACGACTAATAAACTTATCATTACTCATATATATTCCACTATATAAAATAACTACTGATGAAATAAACATAACTGTTGAAAGAAAATGTTGATATAAAATCAAAAATTATAGTTATATAAATATTGCATGAATTAATTGAAAATTATTCATTCTAATATAATTATATAATTATTTTTGAAATTTATTGAACAATAATATTAATATTCTCATAATTATAAGAATACTAAACATATAAATATTAAATTTTTTGTTTATCATGAAAATTATTATGTTCTTAATAATTATTTTTTCAATTAATTTTATTTTCATGAAACATCCTTTATCAATAGGAATAATTTTATTAACACAAACAATATTTTCTTGTTTAATTTGTAGATTATATTTAAATTGTTATTTATTTTCTTATGTTTTATTCCTCATTTTTATTGGTGGAATATTAATTTTATTTATATATATATCAAGAATTGCATCAAATGAAAAATTTTTTTTTTCTACAAAATTAATAATATTAAATTTTTTATCTTTAAGTTTAATTATTTTATTCGACATAGTTGAACTAAAAACATTAAGTATTAGTAAAAATATTGTTATATATATAACTTACAATGATTATATAATAATAAAAATATATACTATTCCTTCTGGCATAATAACTCTAATTCTCACAATTTATTTATTATTTGTTTTAATTATTGTAATTAATATTTTAACAGTCAATATATTAACTCTTCGGAGAAGAACTTCTTTCTAACTACACCTTCCGGTACAGTTACTTTGTTACGACTTATCTCAATAAAATTATGAGAGTGACGGGCGATATGTACATAATTTAGAGCTAATTTCAATTAATTAAATTTAATTAATTTATTGTCAAATCCAATTTCATATTTCTATATNNNAAAAAAATAATCCATTAAATAATTAATTGTAACCCATTTTTTCTTTAATATAAACTGCACCTTGACCTGACATTAAATATATAATTATATAATATGAAAATTTTTCTTATAAAACATTCTTGACAGAGATATACAAGTTAAACTAAAGTTTATTCTATCGTGGATTATCAATTATAAAACAGGTTCCTCTGATAAGATAAATTACCGCCAAATTCTTTGAATTTAAAGATCATTTCTAATAATAATCAAGTTAATTTTATCACATTTTTAATAATAGGGTATCTAATCCTAGTTTATAAAAAAATTTTTCAGACATAAAAATAATTTTTCAAGATAAAATATATTTCACCAAAATTATAACTATTTTATATTATTAATATATTAACTGAAACCTAACTATTTAAATTAAAGAAATCGTATAACCGCAACTGCTGGCACGAAATTTGATTCTTTTAAATAAAATTACTAACTCTAATATTATTAATTAATAATAATAAATACTGCGCTTTATTNNNAATATATTCCATTTAGAAACTGATAACAATTAAAAAATTGCATGTAATATAATTTTAAATTTAAATNNTATTAAACTAGAATTTAACTTCTAATAATAAAATTATTTAATGAGGTCCTTTCGTACTAACATTAAAAATATTTGAGTAGATAGAAACCAACCTGGCTCACGCCGGTCTGAACTCAGATCATGTAAGAATATTAAGGGTCGAACAGACCCAGAAATAATAAATTTTGCTCCAATTCCTATCCTTAATCCAACATCGAGGTCGCAATCATATTTATCGATATGAACTCTCCAAATTAATTACGCTGTTATCCCTAAGGTAATTTATTCTTATAATCAAAATTTTGGATCAATAATTACATGTATTTATGAAACTTTTATTAAAAGTTAATTATATTTTAATATCACCCCAACAAAAAAATTAATTAAATAAAAAAAATTAATTAACTATTTANAAATAAATTCAATNAAAATTTTAAAAATTCTATAGGGTCTTCTCGTCCCACTCATATATTTAAGCTTTTTTACTTAAAAATCAATTTCAATTGTTAATATTAATTAAGTCAATTTTTCATCCAATCATTCATTCAAGCCTCCAATTAAAAGACTATTTATTATGCTACCTTTGCACGGTCAATTTACCGCGGCCATTTAATTTTNAATCATTGGGCAGATTAGACTTTTAATTCTTACTAAAAGACATGTTTTTGATAAACAGGTGAAAATTTTTTTTGCCTAAATTATAAATATTAATTATAACCAAAAAACCAGATATCATATAATTTGATAAAATATCATTTCCAAAATAAATTTATTAATATATATACAATAACAACTAACAAATNTATCTTAAATCATTATATTTCGGGAAAAATAAATTTTTAAAATTTTTTAATTAACCCTGATACAAAAGGTACAAATTATATTTTACTTATAATTATTTATAANATTATAACCTTGTCAGTAAATCTGAATTATTTTTTAATATAAAATTACTTTAACATAAAAATTTTTTTTTTAAAATGTAAAAACCAATTAATATCCAAGATTAAATTATGGAATGTATTTAATTATAATAATTGAATTGCAATCAATAGGTGTTGNNTTTAACCATTCTTAAGTAATGAAGTAAAGTATTACGTTTAGTTTCGACCTAAGATAAGAATAATTAGTCCATTACTTTAAGTGAAGCCAAGTAAGAGGCGTTTTATTGTTAATAAAATAATTGAAATTTTATTTCCATTTAAAGAAAAATTAGTTTAATAAAAATATAAATCTGTCAGATTTTTGATACTTTTAAGTATTTTTCTGACTTTTANNNTTTTTGACTACTTTAAATTTGCAATTTAATATTATTAATTAAATATAAGACTTTATGTTAGCAGAATTAAACTGTCTATTAATATCAAAAATTAATGTTCTTCATAAACTATAACATTTATAAAGTTTATAAAACATTTCATTTTCATTGAAAAGAGGGACTTAGTCTTATAAAWTWYTAWWWKTWYRTGMRYWTWTYYNNNNNNNNNNNNNNNNNNNNNNNNNNNNNNNNNNNNNNNTATCCAATAAATTTATTTTTATCTTTAAAATCACAATTTAATATTTTTATTAAACTAATTGAATTACTATTTGTAATATATATTACAATATTAAATTCTAAATTTAATGCATTAATTTTGCTAAAACAGTTCTAATATGGCAGATTAGTGCAATGAATTTAAGATTCATATATAAAATATTATTTTTATTNNNAGAAATAAGATGCCTGAANAGTAAAGGATTATTTTGATAGAATAAATTATGTAATTTAATTACTCTTACATTAAGTGGCTGAAAGTAAGTAATGGTCTCTTAAACCAATTAATAGTAATTAACAAATACTCTTAATGAAAGATAAGCTAAGATNAAAGCTAATGGGTTCATACCTCACTTATGGAAATNATTCCTCTTTTTATTTAAAAACAAAAGTTATCTTAATATCTTCAATATTATGCTTTTAATTAAGCTATTTAAATTCAGAAAAGATAATATAATCATTTTAAACTCCCAAAGTTTATATTTTTAAAATAAATTATTTTCTGAGTTAATTAGCTTAATTAAAAGCATTTACTTTGAAAGTAAAAGAAAAGATTAGTAATCTATTAACTTAGGAAACTAGAAATAAAATTAGCTTCTAACTAACTTTTAAAGCGGTTAAATTCCGTTTTTTCCTTGTTTTAATAGTTTAAGTAAAAAAATTTAGATCTTGTAAATCTAAGATAACATAAGTTTTAAAAATAGGTTTTAAGTTANTTAAAACTGTTATCCTTCAAAGTTAAAAATATAATTNTATTTATTATTAGAAGAAATAATTTTTCATAAATAAATTTACAGTTTATTACCTATTTTCGGTCATTCTAATTAAGTTATATTGATTAAACAACATTCTCATTAGCGTAAATAAAGTGCTAAAATAGCTTTAACTTA

>campbelli-southSI_02.NZ.OL.FRL.04

AGTTTATTTTCATCATTTGATCCTTGTACTGGATTTCTTTCTCTTAATTGATTAAGATCAATAATTTTGTTATTATTTTTGCCTTTAACTTATTGATATATACCAAATCGTTTTGTTATTTTATATAATAAAATTTTACTTTCATTAAATAATGAATTAAATATATTGATAAATTATAAATCATTAGGAAGATCATTAATATTTTTATCACTATTTACATTTATTTTATTAAACAATTTATTAGGATTACTTCCTTATATTTTTACTAGTTCTAGTCATCTTGTGTTTACAATAAGATTGGCGTTGCCATTATGACTATCATTTATACTTTATGGATTTATTAATAATGTTAATCATATATTTTGTCATATAGTTCCATCAGGAACTCCTAATATTTTAATACCTTTTATAGTTATTATTGAAAGAGTTAGAAATTTAATTCGACCAGGATCATTAGCTGTTCGTTTAACAGCTAATATAATTGCTGGTCATCTTTTAATAACTCTACTTGGTAATTTGCCTATAAATTATGAATATTATTCAGGTGTGATTATTATTTTTCAAATCTTGTTAATATTATTTGAATTAGCCGTTTGTATAATTCAGTCCTATGTTTTTATAGTGCTTAGAACTTTATACTATAGAGAAGTAAATATTCCTCAAATGTCTCCATTAAATTGATTATTATTTATATATTTTATTNNNNNNNNNNNNNNNNNNNNNNNNNNNNNNNNNNNNNNNNNNNNNNNNNAATCCAATCTAATAACTAATAAAAAGTTAATAGATAGAATAATTTGATTTAAACCAACACGACAAAATCATCCCTTAATTAAAATTATTAATAATTCATTAATTGATTTTCCAGCCCCATCTAATTTATCTTATTGATGAAACTTTGGATTTGTATTAGGATTGTGTTTAATAATTCAAATCTTTACTGGATTATTTTTATCAATACATTATAATGCAAGTATTGTAAGAGCTTTTGAAAGACTAAGTCACATTTGTCGAGATGTAAATTATGGATGATTCTTACGAATCATTCACGCTAATGGAGCCTCATTATTTTTTATTTGTGTATATTTACATGTAGGACGTGGATTATATTATGGTTCATACAAATATATTGAAACATGATCTATTGGTGTAGTTATATTATTAATATTAATAGCTACTGCTTTTTTAGGTTATGTATTACCATGAGGACAAATATCCTTCTGAGGAGCTACAGTTATTACAAATTTACTTTCAGCTATTCCTTATTTAGGAATAACACTGGTTAATTGAATTTGAGGTGGATTTGCAGTTGATAATGCTACATTAACTCGATTTTATTCCTTCCATTTTATTTTACCATTTATTGTATTAAGATTAACAATTATTCATTTACTATACTTACACACAACAGGTTCAAATAATCCTTTAGGGATTAATTCGAATAATGATAAAGTTCCATTTCATCCATATTTTTCTATTAAAGATATTATAAGACTCTTTATGTTAATAATTATATTTTTAATATTTGTTATATTAGAACCATATATATTAGGTGATCCTGACAATTTCACTCCTGCTAACCCTTTAGTTACACCAAAACATATTCAACCTGAATGATATTTTTTATTTGCTTATGCTATTCTTCGATCAATTCCTAATAAATTAGGAGGAGTAATTGCTCTTTTTATATCAATTTTTATTTTAATATTTGTACCTTTGTTAAATAATTCTAATTTCATAGGATTAAATAATTATCCAATAAATCAAATTCTATTTTGATATTTATTAACTATCTTAATTTTGTTAACTTGAATTGGGGCCCGTCCTGTTGAATTACCTTATATTAATTTCGGAATATTATTAACATTAATATATTTCTTTTACTTTATTATTGATCCTTTAATTAAATCTTTTTGAGATAAATTAATTAGATAGATGAAAAAATGATTTTTCTCAACTAATCATAAAGACATTGGAACAATATATTTCATTTTTGGTATTTGATCTGGTATAATTGGTACAACTTTGAGAGTTTTAATTCGTGTAGAACTTGGAACTCCAGGTTCATTTATTGGTGATGATCAGATTTATAATGTAATCGTTACTGCTCATGCTTTTATTATAATTTTTTTTATAGTCATGCCTATTATAATTGGGGGATTTGGAAATTGGTTAATTCCATTAATAATTGGAGCTCCTGATATAGCTTTCCCTCGTATAAATAATATAAGTTTTTGATTGCTACCTCCATCATTAATTCTTATTTTAGTGGGAAGAATAGTTGATAGAGGTGCAGGTACTGGTTGAACAGTTTATCCTCCACTATCTAGTGGAGTAGCACATTCTGGCTCATGTGTCGATTTAACTATTTTCTCCTTACATCTTGCAGGTGTATCCTCAATTTTAGGTGCTGTAAATTTTATTAGAACAATTTTTAATATACGATCAGTGGGTATTTGATTAGATCGTATACCTTTATTTGTGTGGTCAGTCTTGATTACTGCATTCTTGTTATTGTTATCTTTACCTGTTCTAGCAGGTGCTATTACGATATTATTAACAGATCGAAATTTAAATACTTCATTTTTTGACCCTGCAGGGGGAGGTGACCCAATTTTATATCAGCATTTATTTTGATTTTTTGGTCATCCTGAAGTTTATATTTTAATTTTGCCAGGGTTTGGTTTAATTTCTCATATTATTACACAAGAAAGAGGTAAAATTGAATCCTTTGGTTCATTAGGAATAATTTATGCTATAATATCAATTGGTATTTTAGGATTTGTTGTGTGGGCACATCATATGTTTACTGTAGGTATGGACGTTGATACGCGTGCATATTTTACATCAGCTACTATAATTATTGCAGTTCCAACTGGAATTAAAGTGTTTAGTTGACTTGCAACTTTAAGAGGAATAAAAATTAATATTACATCTTCAGCTTTATGAGCCTTAGGATTTGTTTTTTTATTTACAATTGGTGGTTTAACTGGAGTAATTTTAGCTAATTCTTCAATTGATATTATATTACATGATACATATTACGTAGTTGCTCATTTTCATTATGTCCTATCAATAGGAGCAGTTTTTGCTATTATGGCAAGATTTATTCATTGATTTCCTCTATTTACAGGAATGACATTAAATTCAAATTGATTAAAAATTCATTTTTTATTAATATTTATTGGTGTAAATATAACATTTTTTCCTCAACATTTCTTAGGACTAAGAGGTATACCTCGCCGATATTCAGATTATCCTGATGCTTATATATCATGAAATATAATTTCATCAATAGGAAGAATTATATCCTTAATCGGAATTATATTTTTATTATTTATTGTATGGGAAAGATTTATTTCAATGCGATTAGTGTTATATTCTAATAGAATTCAGTCTTCTATTGAATGAATGCAGAAATTTCCTCCATCTGAACATTCTTATAATGAAATGCCATTATTAATTCAAATTTCAAATTGATCTTATATAAATATACAGGACGCTGTATCACCATTAATAGAACAATTAATATTTTTTCATGATCATGTATTAATTATTTTAATTATAATTACAATTATTGTTGCTTATATAATATTAATATTGGTATTAAATAAAATTATTAATCGTTTACTTCTTGAGGGACAATTAATTGAATTTATTTGAACTTTATTACCTGCAATAACTTTAATTTTTATTGCATTACCATCTTTACGACTATTATATATATTGGATGAAATTAATAATCCGTTATTGACATTAAAAGTTATTGGTCATCAATGATATTGATCATATGAATATTCAGATTTTTTTGATGTAGAATTTGATTCTTATATAAAATCTACAATTGATATAAGGAAAAATGAATTTCGTTTATTAGATGTTGATAATCGAGTAATTTTACCATTCAATATTCAAATTCGATTGTTGATTTCTTCTTTTGATGTTATTCATTCTTGAGCAATACCTTCTATAAGATTAAAAGTTGATGCAGTTCCGGGACGGTTGAATCAGATAAGAATATTAATTAGTCGTCCAGGTATTTCTTATGGTCAATGTTCTGAAATCTGTGGTGCAAATCACAGATTTATACCTATTGTTATTGAAAGAATTAGAATGAAAATATTTATTAACTGATTAGTTAATTATATGACTAATCATCCTTATCATCTAGTTGATTATAGCCCATGACCTTTAACTGGATCAATTGGAGCTTTAAGTTTTGTATCTGGTATAATTATAATGTTTCATAAATATAATTTTATTCTATTATATATTGGTATATTTTTGTTGTTATTAACAATAATTCAATGATGACGTGACATTTCTCGAGAAAGAACATTTTTGGGAATACATACAATAGTAGTAGTAAATGGCTTAAAAATAGGTATATTACTATTTATTGTATCAGAAATTTTTTTTTTCGTTTCTTTTTTTTGAGGATTTTTTCATAGAAGATTAAGACCTGTAGTAGAAATTGGAATAATTTGACCTCCTATGGGTATCAATGTTTTTAATCCAACTCAAGTTCCTTTATTGAACACAATAATTTTATTATGTTCAGGAATTACAATCACTTGAGCTCATCATTCAATTATAAATGGTAATCACATTGATTCTATTTATAGAATTACTTTAACTATTATTTTAGGACTCTACTTCACTGCTTTACAAGGTTATGAATACTATGAAGCTCCTTTCGCAATTAATGATTCCATTTATGGTTCTTCTTTTTTTATGGCTACTGGATTTCATGGTATTCACGTAATTATTGGGACAACATTTATTATTGTGTGTTTAATACGACAGATAAATTTTCACTTTTCAATTAATCACCATTTTGGTTTTGAGGCCGCTGCTTGATATTGACATTTTGTTGATGTAGTTTGATTATTTTTGTATTTATCAATTTACTGATGAGGAGGATAATAAATTATAGTTTTTAGGAAAAAAAAATAAATAAACAAATTAAGAGATACTGGCAAATAAGATTTTCAAGCTAAATATATTAACTTGTCATATCGATAACGAGGAAGAGTTCCTCGTACTCAAATAAAGAAAAAACATAGTAAAGATACTTGAATAGGAAAAATAATTGAATCAATTTTTCCTCCTAAAAATATTAAACAAAATATTATTCTTATAAATAAAATACTAGAATATTCAGCTAGAAAAATGAATGCGAAACCTGCACCTCTATATTCAACATTAAATCCAGAAACCAATTCTGATTCTCCCTCAGAAAAATCAAATGGAGAACGATTTGTTTCAGCTAAAGCCGAGGAAATTCAACATATTCTTAATGGAAGAAATAAAAAAATAAATCATACAGATTTTTGAAATAAAAATAAATCAAGAATATTATAACTTTTAATTATTATTATAGGACATAATATAATTAAAACCAATCTTACCTCATAGGAAATAGATTGAGCAATTGAGCGAATACATCCTAATATTGCATAGCTTGAATTAGAAGATCATCCTGTTAATATTAAAGAATAAACACTTATTCTTGAACAACAAAAAAAAAATAAAATACCAAAATTAAAAGAAACACAATTAATTATATAAGGATATAGTGACCATAATAATAATCTATTAAATAATCCTATAACAGGACTAAAAATATAGATTAAATAATTAGACATTAATGGAATAGTATTTTCCTTTATAAATAATTTAATAGCATCAGAAATGGGTTGTAAAATACCCAAAAATCCAACTTTATTAGGTCCCTTACGAATCTGAATATATCTTAAAACTTTACGTTCCAAGAGAGTGATAAATGCCACTCCAATTAAAATGAACAAAATAGTAATAAAAATTGTAATTAAATACAAAAATAATTCTTCTTATTTACTATATTTAGTTTTCTTATTTTTTGGAATTATAATTTCAATTTCTTCTAACAATTGGCTGGGTTGTTGGATGGGAATTGAAATAAATATAGTTTCATTTTTGCCTATTATGGCAAATAAAATAAGAATTTATGCTTCAGAATCAATAATCAAATATTTTATCATTCAAAGAATGGGATCTAGTTTATTATTAATATCTATTATCATTNNNAGAATAATAATTGATTTAAATTATATAATTATAATTAGATTAATAATTAAAATTGGGTGTCCCCCATTTCATTTTTGATATGTTTCTGTTATTGAGGGGCTATCTTGAATGGTATGTTTTATTTTAATAACCATTCAAAAAATTATTCCTATAGTTATATTATCATATTTGAATGTAAATATAAGGTTATTTATTATTATAGCATGTATTTGAGGATGTATTGGAGGGCTGGGATATTCTTCAATACGTAAAATTATTGCTTATTCTTCAATTTATAATTTAAGATGAATTTTTAGAGGTATTATAATTATTAATTATTCATGAATAATTTATTACTTTGTTTATTCATTTATATTAATGGCTGTATGTTATATATTTAATTTATTTAATATTAATTATATTAATCAATTTATTATATCTTCTTTTAATTTTTCAAAATCAGTCATAATGATGATTATTTTTATGTCTATAGGTGGATTACCTCCTTTCTTGGGGTTTTTTCCTAAATTAATTATAATTTACTGTTTATTATTAAATAAAATATTATTTATTTGTATTATACTGTTAATAACAGCTCTAATTGTTTTATTTTTCTATTTACGAATTGTGATTACAACATTAATAATAAATACTATTTCAATAAAAATAATTGTTATTGAAGCTTCATATTTTTATTATATTGTTAGAATATTTTCATTATTTGGAATAATTTTTTTGTCATTAATTACATTAAATTTATGTTAGATTNNNNNNNNNNNNNNNNNNNNNNNNNNNNNNNNNNNNNNNNNNNNNNNNNNNNNNNNNNNNNNNNNNNNNNNNNNNNNNNNNNNNNNNNNNNNNNNNNNNNNNNNNNNNNNNNNNNNNNNNNNNNNNNNNNNNNNNNNNNNNNNNNNNNNNNNNNNNNNNNNNNNNNNNNNNNNNNNNNNNNNNNNNNNNNNNNNNNNNNNNNNNNNNNNNNNNNNNNNNNNNNNNNNNNNNNNNNNNNNNNNNNNNNNNNNNNNNNNNNNNNNNNNNNNNNNTATAATTATTTTTTTAATTATTTTGACTATAGGTTTATACCATGAATGATATAATAATATATTAAGTTGANNNNNNNGTTTAATTAAGATATTTAATTTTAAAATAATTAAATTTAATGGAATTCAGTGTATTATAATTAAAAAGAATTCCCGGACTCTTCCAGAATTAAAAGAAAATAAACTAGAAAAAAATATACCATGTTGAGTATATGAAAATATATATAATCTATAACAAGCAGATAGAAATGAAGATAAAATTAATAAAACTATTATTAATATATTTCAAGATATTAATCTATTAATAATTATAATTTCACCTGCTAAATTAAGTGTAGGGGGGCAAGATATATTTCTCGCTGACATTAAAAATCATATTAAAGATATTGAAGGTATGAATGTTAATATTCCTTTATTAATTAATAATCTCCGTCTATTAGTTCGTTCATATAATATATTTGCTAAGCAAAATAATCCAGAAGAACAAAGACCATGACCAATTATTATATAATAAGAACCATAAATTCCTCAATTTGTTATAGTTATAATTCCTCTAACTACAAGACCTATGTGAGCTACAGATGAATAAGCAATTATTGATTTAATATCAATTTGAATTATACAAAGAATTCTAATTAAGATTCTTCCAATTATTGAAATGGAAATTCACACAAACCCAAATTTATAAAAATATAAAGGAATAATATTCATTATTCGAATAATACCATATCCTCCTAACTTTAATAGAATTCTGGCTAGAATTATTGATCCAGAAACAGGTGCTTCAACATGAGCCTTTGGTAGTCAAAAATGAAAAAATAGTGGTATTTTAATTAAAAAAGCTATTATCATACCAATATAAATGTAAATATTAATGGGATAAACTATTAATATGAAAATATTAGTTATATTTATTTTATAAAGAAACAAAATTCTTATTAACATTGGTAATGATGCAAATAATGTATAAAATAATAAATAATATCTTGCATCAATACGTTCTGGCTGGTAGCCCCAGCCTATAATAATAATCATAATTGGAATTAATCTAGATTCAAAAAAGATATAAAAAATTATAATTCTTGATGTAGAAAAAGAAATAATTAAAAAAATTATTAACATAACACCAAAAGTAAAAAAAACAATTTATTATATTTATATATTGGACTAGAAATAATTATTAAAAATGAGATTCAAATTTAAAATAATTAAACCATAAGATATTTCATCAATATAAAAATTATAGCCTAATATAAATAAATTTTTATTTACAAAAATTAAAAATAAAATTATTACAATAATAATTATATTTATTAATCATGAATTATAAATAAGTAGGGTTAATATCAAACAAGAAAAAATTAATTTTATAGATATTGATATTAATTTATCATTACTATGAGAACGAATTAATGTAACTAAACATGATAATCCCATTACTCCTTCACAAACTCTAAAAACTAAGAAAATTAACAAGATATATATATCATACATTTTTATAGAAAAGATAAATGACAAAGAGCAAAATATTCTTAAGATAATTAATTCTAATCTTAATGTTGTTATCAAATGTTTTCGATTTATGCATAACGAAATTATTCCAGAAATGAATATAGAAAAAAAATATGTTAATAGTGTTATATTCTATAAATATATAATAATAATTCAGATAATAAATGAAATTATTTGAATCTTAAAATTATTATTTGTTAATTTATAATTTAATAATGTTATTATTTTTAAAGTTTTCAAAATTCCTTGAGGTCCAAGGGTTTCACCTCATCCTATATCAATAGATTTTTGAAATATTAATGAATTAGTTATTAAAAATATTTGATTATGGAATGTTGATATTTGTTTTATAAATCATATCGAACCAAAAAACTCATAAATAATTTTTATTTTAGTCTTAGAAAAAATTGATATTTCATAACCTAATCAAATCCCTAATGCTGAAAATATTAATGCTATTAATTTACCTTCAATAGGTATTAAAATTATAATTGGATCATTAAATATTAATCATCTTAATATAGATCCAGAAATAATTGAATAAATAGATAAAATAATGATTCTTTTAATTATATTATTTAAGTTTTCACTTAATGATCTCATTTTATAAAAGTTAAAATTTATATTTATTGAAAAATATGTTAAACGAGCAGAATAAAATGAAGTTAATCCAATCCCAATATATATGAGTATTATAATCAATATATTAATATTGTTAGAACATATGGATTCTATAATTAAATCTTTTGAATAAAATCCTCTTATGAATGGAATTCCACATAATGATATATTAGCAATATTTATTATTGTAATAGTTAAAGGTATTTGAAATCTTAAACATCCTATTACACGAATATCTTGATTATTATTAAAATTATGAATTAAAATACCTGCACACAAAAACAATGTAGCTTTAAATAAAGCATGAACAATTAAATGGAAAAAAGATAATATAGGATAACCAAATATAATAATTGTTATTATGATTCCTAATTGTCTTAAAGTTGATAGAGCAATAATTTTTTTTAAATCAAATTCAAAATTTGCTCTAATTCCTGATATTAACATAGTTAAAATTGAAATTAACATAAATACTTGAATATAATCAAAGTTAACAAAAATGTTAGAAAATCGAATTATTAAGTATACTCCAGCAGTAACAAGAGTAGATGAGTGAACTAAAGCTGATACAGGGGTTGGAGCGGCTATTGCCGCTGGCAATCAAGAAGAAAAAGGGATTTGAGCTCTCTTAGTAAATCTTGCAATAATAATTAGTATTATTATAATTTATATCTTTATTAATAAAAGATGAAAATTTCATGATCCAAAATTTAATATTCATGAAATAACTATTAAAATAGCAACATCCCCAATCCGATTCATTAATGCAGTTAATATTCCTGCATTATTAGAATATAAATTTTGATAATAAATAACTAAACAGTAAGAGACTAAACCGAGACCGTCTCAACCAATAATAATTCTTATTATATTAGGTCTAATAATTAATAAAATTATAGATATTACAAATCCTAATACAATATAAATAAAACGACTAATAAATTTATCATTATTTATATATATTCCACTATATAAAATAACTACTGATGAAATAAATATAACTGTTGAAAGAAAATGTTGATATAAAATCAAAAATTATAGTTATATAAATATTGCATGAATTAATTGAAAATTATTCATTCTAATATAATTATATAATTATTTTTAAAATTTATTGAACAATAATATTAATACTCTCATAATTATAAGAATACTAAACATATAAATATTAAATTTTTTGTTTATCATGAAAATTATTATATTCTTAATAATTATTTTTTCAATTAATTTTATTTTCATAAAACATCCTTTATCAATAGGAATGATTTTATTAACACAAACAATATTTTCTTGTTTGATTTGTAGATTATATTTAAATTGTTATTTATTTTCTTATATTTTATTCCTTATTTTTATTGGTGGAATATTAATTTTATTCATATATATATCAAGAATTGCATCAAATGAAAAATTTTTTTTTTCTACAAAATTAATAATATTAAATTTTTTATTTTTAAGTTTAATCAGTTTATTTAATATAACTGAACTTAAAATATTAAGTACTAGAAAAAATATTGCAATATATGTAACTTACAATGATTATATAATAATAAAAATATATACTATTCCTTCTGGCATAATAACTCTAATTCTCACAATTTATTTATTATTTGTTTTAATTATTGTGATTAATATTTTAACAGTCAATATATTAACTCTTCGGAGAAGAATTTCTTTCTAACTACACCTTCCGGTACAGTTACTTTGTTACGACTTATCTCAATAAAATTATGAGAGTGACGGGCGATATGTACATAATTTAGAGCTAATTTCAATTAATTAAATTTAATTAATTTATTGTCAAATCCAATTTCATATTTTTATGCNNNAAAAAAATAATCCATTAAATAACTAATTGTAACCCATTTTTTCTTTAATATAAACTGCACCTTGACCTGACATTATATATATAANTATATAATATGAAAATTTTTCTTATAAAACATTCTTGACAGAGATATACAAGTTAAATTGAAGTTTTATCTATCGTGGATTATCAATTATAAAACAGGTTCCTCTGATAAGATAAATTACCGCCAAATTCTTTGAATTTAAAGATCATTTCTAATAATAATCAAGTTAATTTTATCACATTTTTAATAATAGGGTATCTAATCCTAGTTTATAAAAAAATTTTTCAGACATAAAAATAATTTTTTAAGATAAAATATATTTCACCAAAATTATAGCTATTTTATATTATTAATATATTAACTGAAACTTAACTATTTAAATTAAAGAAATCGTATAACCGCAACTGCTGGCACGAAATTTGATTCTTTTAAATAAAATTACTAATTCTAATATTATTAATTACTAATAATAAATACTGCGCTTTATTNNNAATATATTCCATTTAGAAACTAAAAACAATTAAAAAATTGCATGTAATATAATTTTAAATTTAAATNNTATTAAACTAGAATTTAACTTCTAATAATAAAATTATTTAATGAGGTCCTTTCGTACTAGCATTAAAAATATCTGAGTAGATAGAAACCGACCTGGCTCACGCCGGTCTGAACTCAGATCATGTAAGAATATTAAGGGTCGAACAGACCCAGAAATAATAAATTTTGCTCCAATTCCTATTCTTAATCCAACATCGAGGTCGCAATCATATTTATCGATATGAACTCTCCAAATCAATTACGCTGTTATCCCTAAGGTAATTTATTCTTATAATCAAAATTTTGGATCAATAATTACATATATTTATGAAACTTTTATTAAAAGTTAGTTATATTTTAATATCACCCCAACAAAAAAATTACTTAAATAAAAAAAATTAATTAACTATTTANAAATAAACTCAAGNAAAATTTTAAAAATTCTATAGGGTCTTCTCGTCCCACTCATATATTTAAGCTTTTTTACTTAAAAATCAATTTCAATTGTTAATATTAATTAAGTCAATTTTTCATCCAATCATTCATTCAAGCCTCCAATTAAAAGACTATTTATTATGCTACCTTTGCACGGTCAATTTACCGCGGCCATTTAATTTTNAATCATTGGGCAGATTAGACTTTTAATTCTTACTAAAAGACATGTTTTTGATAAACAGGTGAAAATTNNNNNNNNNNNNNNNNNNNNNNATATTAATTACTAAAAAACCAGATATCATATAATTTGATAAAATATCATTTCCAAAATAAATTTATTAATATATATACAATAACAACTAACAAATNTATTTTAAATCATTATATTTCGGGAAAAATAAATTTTTAAAATTTTTTAATTAACCCTGATACAAAAGGTACAAATTATATTTTACTTATAATTATTTATAANATTTTTACCTTGCCAGTAAATCTGAATTATTTTTTAATATAAAATTACTTTAACACAAAAATTTTTTTTTTAAAATGTAAAAACCAATTATTTTCTGAGGTTAAATTATGGAATGTATTTAATTATAATAATTGAATTGCAATCAATAGGTGTTGNNTTTAACCATTCTTAAGTAGTGAAGTAAAATATTACGTTTAGTTTCGACCTAAAATAAGAATAATTAGTTCCTTACTTTAAGTGAAGCCAAGTAAGAGGCGTTTTATTGTTAATAAAATAATTGAAATATTATTTCCATTTAAAGAAAAATTAGTTTAATAAAAATATAAATCTGTCAGATTTTTGATACTTTCAAGTATTTTTCTGATTTTTANNNNTTTTATCTACTTTAAATTTGCAATTTAATATTATTAATTAAATATAAGACTTTATGTTAGCAGAATTAAACTGTCTATTAATATCAAAAATTAATGTTCTTCATAAACTATAACATTTATAAAGTTTATAAAACATTTCATTTTCATTGAAAAGAGGGACTTAGTCTTGTAAAATTCTATTAGTATATAAGTATGTTTAATTTCCAATTAAAAGGATTAATTTTATTAAATAGAATATATCCAATAAATTTATTTTTATCTTTAAAATCACAATTTAATATTTTTATTAAACTAATTGAATTACTATTTGTAATATATATTACAATATTAAATTCTAAATTTAATGCATTAATTTTGCTAAAACAGTTCTAATATGGCAGATTAGTGCGATGAATTTAAGATTCATATATAAAATATTATTTTTATTNNNAGAAATAAGATGCCTGAANAGTAAAGGATTATTTTGATAGAATAAATTATGTAATTTAATTACTCTTACATTAAGTGGCTGAAAGTAAGTAATGGTCTCTTAAACCAATTAATAGTAATTAACAAATACTCTTAATGAAAGATAAGCTAAGATNAAAGCTAATGGGTTCATACCTCATTTATGGAAATNATTCCTCTTTTTATTTAAAAACGAAAGTTACCTTAATATCTTCAATATTATGCTCTCAATTAAGCTATTTAAATTCAGAAAAGATAATATAATCATTTTAAACTTCCAAAGTTTATATTTTTAAAATAAATTATTTTCTGAGTTAATTAGCTTAATTAAAAGCATTTATTTTGAAAGTAAAAGAAAAGATTAATAATCTATTAACTTAGGAAACTAGAAATAAAATTAGCTTCTAACTAACTTTTAAAGCGGTTAAATTCCGTTTTTTCCTTGTTTTAATAGTTTAAGCAAAAAAATTTAGATCTTGTAAATCTAAGATAACATAAGTTTTAAAAATAGGTTTTAAGTTANTTAAAACTATTATCCTTCAAAGTTAAAAATATAATTNTATTTATTATTAGGAGAAATAATTTTTCATAAATAAATTTACAGTTTATTACCTATTTTCGGTCATCCTAATTAAGTTATGTTGATTAAACAACATTCTCATTAGTGTAAATAAAGTGCTAAAATAGCTTTAACTTA

>oromelaena_02.NC.TBS.01

AATTTATTTTCATCATTTGATCCTTGTACTGGATTTCTCTCTCTTAATTGGTTAAGATCAATAATTTTATTATTATTTTTGCCTTTGACTTATTGATATATACCAAATCGTTTTATTATTTTATATAATAAAATTTTATTTTCATTAAATAATGAATTAAACATGTTAATAAATTATAAATCATTAGGTAGATCATTAATATTTTTATCACTATTTACATTTATTTTATTAAACAATTTATTAGGATTACTTCCTTATATTTTCACTAGTTCTAGTCATTTAGTGTTTACAATAAGATTAGCATTACCATTATGATTATCATTTATACTTTATGGATTTGTTAACAATATTAATCATATATTTTGCCATATAGTTCCATCTGGAACTCCTAATATTTTAATACCTTTTATAGTTATTATTGAAAGAATTAGAAATTTAATTCGACCAGGGTCATTAGCTGTTCGTTTAACAGCTAATATAATTGCTGGCCATCTTTTAATAACTCTACTCGGTAATTTGCCTATAAATTATGAATATTATTCAGGTATAATTATTATTTTTCAAATCTTGTTAATATTATTTGAATTAGCTGTTTGTATAATTCAGTCTTATGTTTTCATAGTACTTAGAACTTTATATTACAGAGAAGTGAATATCCCTCAAATGTCTCCATTAAATTGATTATTATTTATTTATTTTATTNNNNNNNNNNNNNNNNNNNNNNNNNNNNNNNNNNNNNNNNNNNTATTAAAATCTAATCTAATAACTAATAAAAAGTTAATAGATAGAATAATTTGATTTAAACCAACACGACAAAATCATCCCTTAATTAAAATTATTAATAATTCATTAATTGATTTCCCAGCCCCATCTAATTTATCTTATTGATGAAACTTTGGATTTGTATTAGGATTATGTTTAATAATTCAAATCTTTACCGGATTATTTTTATCAATACATTATAATGCTAATATTGTAAGAGCTTTTGAAAGATTGAGCCACATTTGTCGAGATGTAAATTATGGATGATTCCTACGAATCATTCACGCTAATGGAGCTTCATTATTTTTTATTTGTGTTTATTTACATGTAGGACGTGGATTATATTATGGTTCATATAAATATATTGAAACATGATCCATTGGTGTAATCATATTATTGATATTAATAGCTACTGCTTTTTTAGGATATGTATTACCATGAGGACAAATATCCTTCTGAGGAGCCACAGTTATTACAAATTTACTTTCAGCTATTCCTTATTTAGGAATAACACTGGTTAATTGAATCTGAGGTGGATTTGCAGTTGATAATGCTACATTAACTCGATTTTATTCATTCCATTTTATTTTACCATTTATTGTATTGAGATTAACAATTATTCATTTATTATACTTACACACAACAGGTTCAAATAATCCTTTAGGAATTAATTCTAATAATGATAAAGTACCATTTCATCCATATTTTTCTATTAAAGATATTATAAGACTCTCTATTTTAATAGTTATATTTTTAATATTTGTTATATTAGAGCCATACATATTAGGAGATCCTGACAATTTTACTCCCGCTAACCCTTTAGTTACACCAAAACATATTCAACCTGAATGATATTTTTTATTTGCTTATGCTATTCTTCGATCAATTCCTAATAAATTAGGAGGAGTAATTGCTCTTTTTATATCAATTTTTATTTTAATATTTGTACCTTTGTTAAACAATTCTAATTTTATGGGATTAAATAATTACCCAATAAATCAAATTTTATTCTGATATTTATTAACCATTTTAATTTTGTTAACTTGAATTGGGGCCCGTCCTGTTGAATTACCTTATATTAACTTCGGAATATTATTAACATTAATATATTTCTTTTACTTTATTATTGATCCATTAACTAAATCTTTTTGAGATAAATTAATTAGATAGATGAAAAAATGATTCTTCTCAACTAATCATAAAGACATTGGAACAATGTATTTCATTTTTGGTATTTGATCTGGTATAATTGGTACAACTTTAAGAGTTTTAATTCGTGTAGAACTTGGAACTCCAGGTTCATTTATTGGTGATGATCAAATTTATAACGTAATCGTCACTGCTCATGCTTTTATTATAATTTTTTTTATAGTTATGCCTATTATAATTGGGGGATTTGGAAATTGATTAGTCCCACTAATAATTGGAGCTCCTGATATAGCTTTCCCCCGTATAAACAATATGAGTTTTTGGTTGCTTCCCCCATCATTAATTCTTATTTTAGTAGGAAGAATAGTTGATAGAGGTGCAGGTACTGGTTGAACAGTTTATCCTCCACTATCTAGTGGAGTAGCACATTCTGGTTCATGTGTCGATTTGACTATTTTTTCATTACATCTTGCAGGTGTATCCTCAATTTTAGGTGCTGTAAATTTTATTAGAACAATCTTTAATATACGATCAGTGGGCATTTGATTAGATCGAATACCTTTATTTGTGTGATCAGTTTTAATTACTGCATTTTTGTTATTATTATCTTTACCTGTTCTAGCAGGTGCTATTACGATATTATTAACAGATCGAAATTTAAATACTTCATTTTTTGATCCTGCAGGTGGAGGTGATCCAATTTTATATCAGCATTTATTTTGATTTTTTGGTCATCCCGAAGTTTATATTTTAATTTTACCAGGTTTTGGTTTAATTTCTCACATTATTACACAAGAAAGAGGTAAAATTGAATCCTTTGGTTCATTAGGAATAATTTATGCTATAATATCAATTGGTATTTTAGGATTTGTCGTGTGGGCACATCATATATTTACTGTAGGAATAGACGTTGATACACGTGCATATTTTACATCAGCTACTATAATCATTGCAGTTCCAACTGGAATTAAAGTATTTAGTTGACTTGCAACTTTAAGAGGAATAAAAATCAATATCACATCTTCAGCTTTATGAGCCTTAGGATTTGTTTTTTTATTTACAATTGGTGGTTTAACTGGAGTGATTCTAGCTAATTCTTCAATTGATATTATGTTACATGATACATATTATGTAGTTGCTCATTTTCACTATGTTCTATCAATAGGAGCAGTTTTTGCTATTATAGCAAGATTTATTCACTGATTTCCCCTATTCACAGGAATAACATTGAATTCAAATTGATTAAAAATTCATTTTTTATTAATATTTATTGGTGTAAACATAACATTTTTTCCTCAACATTTCTTAGGACTAAGAGGTATACCTCGCCGATATTCAGATTATCCTGATGCTTATATATCATGAAATATAATTTCATCAATAGGAAGAATTATATCTTTAATCGGGATTATACTTTTATTATTTATTGTGTGAGAAAGATTTATTTCAATACGATTAGTATTATATTCTAATAGTATTCAGTCTTCTATTGAATGAATACAGAAATTCCCCCCATCAGAACATTCTTATAATGAAATGCCATTATTAATTCGAATTTCAAATTGATCTTATATAAATATACAGGACGCTATATCACCATTAATAGAACAATTAATATTTTTTCATGATCATGTATTAGTTATTTTAATTATAATTACAATTATTGTCGCTTATATAATATTAATATTGGTATTAAATAAAATTATTAATCGTTTACTTCTTGAAGGACAATTAATTGAATTTATTTGAACTTTATTACCTGCAATAACTTTAATTTTTATTGCATTACCATCATTACGATTATTATATATGTTGGATGAAATTAATAATCCACTATTAACATTAAAAGTTATTGGTCATCAATGATATTGATCATATGAATATTCAGATTTTTTTGATGTAGAATTTGATTCATATATAAAATCTACAATTGATATAAGAAAGAATGAATTTCGTTTATTAGATGTTGACAATCGAGTAATTTTACCATTCAATATTCAAATTCGATTGTTGATTTCCTCTTTTGATGTTATTCATTCTTGAGCAATACCATCTATAAGATTAAAAGTTGATGCAGTTCCAGGACGATTAAATCAAATAGGAATATTAATTAGTCGTCCAGGTATTTCTTATGGTCAATGTTCTGAAATCTGTGGTGCAAATCACAGATTTATGCCTATTGTTATTGAAAGAATTAGAATGAAAATATTTATTGACTGATTAATTAATTATATGACTAATCATCCTTATCATTTAGTTGATTATAGCCCATGACCTTTGACTGGATCAATTGGAGCTCTAAGTTTTGTCTCCGGTATAATTATAATATTTCATAAATATAATTTTATTCTATTATATATTGGTATATTTTTATTGTTATTAACAATAATTCAATGATGACGTGATATTTCTCGAGAGAGAACATTCTTGGGAATGCATACAATAGTAGTAGTAAATGGTTTAAAAATAGGTATATTACTATTTATTGTATCGGAAATTCTTTTTTTCGTTTCTTTTTTTTGAGGATTTTTTCATAGAAGATTAAGACCTGTGGTAGAAATTGGAATAATTTGACCCCCCATGGGTATTAATGTGTTTAATCCAACTCAAGTTCCTTTATTGAACACAATAATTTTATTATGTTCAGGAATTACAATTACTTGAGCTCATCATTCAATTATAAATGGTAATCACATTGATTCTATTTATAGAATTACTTTAACTATTATTTTAGGATTATATTTCACTGCTTTACAAGGTTATGAATATTATGAAGCTCCTTTCGCAATTAATGATTCCATTTATGGTTCTTCTTTTTTTATGGCTACTGGATTTCATGGTATTCACGTAATTATTGGAACAACATTTATTATTGTGTGTTTAATACGACAAATAAATTTTCATTTTTCAATTAATCATCATTTTGGTTTTGAAGCTGCAGCTTGATATTGACATTTTGTTGATGTAGTTTGATTATTTTTATATTTATCAATTTACTGGTGAGGAGGATAATAAATTATAGTTTTTAAAAAAAAAAAATAAATAAATAAATTAAGAGACACTGGCAAATAAGATTTTCAAGCTAAATATATTAATTTGTCATATCGATATCGAGGAAGTGTACCTCGTACTCAAATAAAGAAAAAACATAATATTGATACTTGAATAGGAAAAATAATTGAATCAATTTTTCCTCCTAAGAATATTAAACAAAATATTATTCTTATAAACAAAATACTAGAATATTCAGCTAAAAAAATAAATGCGAAACCAGCACCTCTATATTCAACATTAAATCCAGAAACCAATTCTGACTCTCCCTCAGAAAAATCAAATGGAGAACGATTTGTTTCGGCTAAAGCCGAAGAAATTCAACATATTCTTAATGGAAGAAATAAAAAAATAAATCATACAGATTTCTGAAATAAAAATAAATCAAGAATATTATAACTTTTAATCATTATTATAGGACATAATATAATTAAAACTAATCTTACTTCATAAGAAATAGATTGAGCAATTGAACGAATACATCCTAATATTGCATAACTTGAATTAGAAGATCATCCTGTTAATATTAAAGAATAAACACTTATTCTTGAACAGCAAAAAAAAAATAAAACACCAAAATTAAAAGAAACACAATTAATTATATAAGGATATAGTGACCATAATAATAATCTATTAAATAGTCCTATAATAGGACTAAAAATATAAATTAAATAATTAGACATTAATGGAATAGTATTTTCCTTTATAAATAATTTAATAGCATCGGAAATAGGTTGTAAAATACCCAAAAATCCAACTTTATTCGGTCCTTTACGAATTTGAACATATCTTAAAACTTTACGTTCCAAAAGAGTAATAAATGCTACTCCAATTAAAATAAATAAAACAGTAATAAAAATTGTAATTAAATACAAAAATAATTCTTCCTATTTACTATATTTAGTTTTCTTATTTTTTGGAATTATAATTTCAATTTCTTCTAACAATTGGCTGGGTTGTTGAATAGGAATTGAAATAAATATAGTTTCATTTTTGCCTATTATGGCAAATAAAATAAGAATTTATGCTTCGGAATCAATAATTAAATATTTTATCATTCAAAGAATGGGATCTAGTTTATTATTAATATCTATTATCATTNNNAGTATAATAATTGATTTAAATTATATAATTATAATTAGATTAATAATTAAAATTGGGTGTCCTCCATTTCATTTTTGATATGTATCTGTTATTGAGGGATTATCTTGAATGGTATGTTTTATTTTAATAACTATTCAAAAAATTATTCCTATAGTTATATTATCATATTTAAATGTAAATATAAGATTATTTATTATTATGGCATGTATTTGAGGATGTATTGGAGGGCTGGGATATTCTTCAATACGTAAAATTATTGCTTACTCTTCAATTTATAATTTAAGATGAATTTTTAGAGGCATTATAATTATTAATTATTCATGAATAATTTATTATTTTGTTTATTCGTTTACATTAATGGCTGTATGCTATATGTTTAATTTATTTAATANNNNNNNNNNNNNNNNNNNNNNNNNNNNNNNNNNNNNNNNNNNNNNNNNNNNNNNNNNNNNNNNNNNNNNNNNNNNNNNNNNNNNNNNNNNNNNNNNNNNNNNNNNNNNNNNNNNNNNNATTATAATTTATTGTCTATTATTAAATAAAATATTATTTATTTGTATTTTACTATTGATGACAGCTTTAATTGTTTTATTTTTTTATTTACGAATTGTGATTACAACATTGATAATAAATACTATTTCAATAAAAAGAATTATTATTGAAGCTTCATATTTTTATTATGTTGTTAGAATATTTTCATTATTTGGAATAATTTTTTTGTCATTAATTACATTAAATTTATGTTAGATTTATAATANNNNNNNNNNNNNNNNNNNNNNNNNNNNNNNNNNNNNNNNNNNNNNNNNNNNNNNNNNNNNNNNNNNNNNNNNNNNNNNNNNNNNNNNNNNNNNNNNNNNNNNNNNNNNNNNNNNNNNNNNNNNNNNNNNNNNNNNNNNNNNNNNNNNNNNNNNNNNNNNNNNNNNNNNNNNNNNNNNNNNNNNNNNNNNNNNNNNNNNNNNNNNNNNNNNNNNNNNNNNNNNNNNNNNNNNNNNNNNNNNNNNNNNNNNNNNNNNNNNNNNNNNNNNNNNNATTTTTTTAATTATTTTAACTATAGGCCTATACCATGAATGATATAATAATATATTAAGTTGANNNNNNNGTTTANNNNNAATATTTAATTTTAAAATAATTAAGTTCAATGGAATTCAGTGTATTATAATCAAAAAAAATTCCCGGACTCTTCCAGAATTAAATGAAAATAAACTAGAAAAAAATATACCATGTTGAGTATATGAAAATATATATAATCTATAACAAGCAGATAAAAATGAAGATAAAATCAATAAAACTATTATTAATATATTTCAAGATATTAATCTATTAATAATTATAATTTCACCTGCTAAATTAAGTGTAGGGGGACAAGATATATTTCCTGCTGACATTAAGAATCATATTAAAGATATTGAAGGTATAAATGTCAATATCCCCTTATTGATTAATAATCTTCGTCTATTAGTTCGTTCGTATAATATATTTGCTAAACAAAATAATCCAGAAGAACAAAGACCATGACCAATTATTATATAATACGAACCATAAATTCCTCAATTTGTTATAGTTATAATTCCTCTAACTACAAGACCTATATGAGCTACAGATGAATAAGCAATTATTGATTTAATATCAATTTGAATTATACAAAGAATTCTAATTAAAATTCTCCCAATTATTGAAATAGAAATTCATACAAACCCAAATTTATAAAAATATATAGGAATAATATTCATTATGCGAATAATACCATAACCTCCTAACTTCAATAAAATTCTAGCTAGAATTATTGATCCAGAAACAGGTGCTTCAACATGAGCTTTTGGTAATCAAAAATGAAAAAATAATGGTATTTTAATTAAAAAAGCTATTATCATGCCAATATAAATATAAATATTAATAGGATANNNNNNNNNNNNNNNNNNNNNNNNNNNNNNNNNNNNNNNNNNNNNNNNNNNNNNNNNNNNNNNNNNNNNNNNNNNNNNNNNNNNNNNNNNNNNNNNNNNNNNNNNNNNNNNNNNNNNNNNNNNNNNNNNNNNNNNNNNNNNNNNNNNNNNNNNNNNNNNNNNNNNNNNNNNNNNNNNNNNNNNNNNNNNNNNNNNNNNNNNNNNNNNNNNNNNNNNNNNNNNNNNNNNNNNNNNNNNNNNNNNNNNNNNNNNNNNNNNNNNNNNNNNNNNNNNNNNNNNNNNNNNNNNNNNNNNNNNNNNNNNNNNNNNNNNNNNNNNNNNNNNNNNNNNNNNNNNNNNNNNNNNNNNNNNNNNNNNNNNNNNNNNNNNNNNNNNNNNNNNNNNNNNNNNNNNNNNNNNNNNNNNNNNNNNNNNNNNNATAATTATATTTATTAATCATGAATTATAAATAAGTAGGGTTAATATAAAACAAGAAAAAATCAATTTTATAGATATTGATATTAATTTATCATTTCTATGAGAACGAATTAATGTAACTAGGCATGATAATCCTATTACTCCTTCACAAACTCTAAAAACCAAGAAAATTAATAAAATATGTATATCATACATTTTTATAGAAAAAATAAATGACAAAGAACAAAATATTCTTAAGATAATTAATTCTAATCTTAATGTTGTTATTAAATGTTTTCGATTTATGCATAACGAAATTATTCCAGAAAGGAATATAGAAGAAAAATATATTAATAGTGTTATATTCTATAAATATATANNAATAATTCAGATAATAAATGAAATTATTTGAATTTTAAAATTATTATTTGTTAATTTGTAATTTAATGACATTATTATTTTCAAAGTTTTTAAAATTCCTTGAGGTCCAAGTTTTTCACCTCATCCCATATCAATGGATTTTTGAAATATTAATGAATTAGTTATTAAAAATATTTGATTATGGAATGTTGATATTTGTTTTATAAATCATATTGAACCAAAAAACTCATAAATAATTTTTATTTTAGTCTTAGAAAAAACTGATATTTCATAACCTAATCAAATTCCTAATGTTGAAAATATTAATGCTATCAATTTGCCTTCAATAGGTATTAAAATTCTAATTGGATCATTAAATATTAATCATCTTAATATAGATCCAGAAATAATTGAATATATAGATAAAATAATGATTCTTTTAATTATATCATTTATATTTTCACTTAATGATCTCATTTTATAAAAGTTAAAGTTTATGCTTATTGAAAAATATGTTAAACGAGCAGAATAAAATGAAGTTAATCCAATCCCAATATATATGAGTGTTATAATTAATATGTTAATACTATTAGAACACATAGACTCTATAATTAAATCTTTTGAATAAAATCCTCTTATGAATGGAATTCCACATAATGATATATTGGCAATATTTATTATTGTAATAGTTAAAGGTATTTGAAATCTTAAACATCCTATTACACGAATATCTTGATTGTTATTAAAATTATGAATTAAAATACCTGCACACAAAAACAATGTAGCTTTAAATAAAGCATGAACAATTAAATGAAAAAAAGATAATATAGGATAACCAAATATAATAATTGTTATCATAATTCCTAATTGTCTTAAAGTTGACAGAGCAATAATTTTTTTTAAATCAAATTCAAAATTTGCTCTAATTCCCGATATTAATATAGTTAAAATTGAAATTAACATGAATACTTGAATATAATCAAAGTTCATAAAAATATTAGAAAATCGAATTATTAAATATACTCCGGCAGTAACAAGAGTGGATGAGTGAACTAAAGCTGATACGGGGGTTGGAGCAGCTATTGCTGCTGGTAATCAAGAGGAAAAAGGAATCTGAGCTCTCTTAGTAAATCTTGCAATAATAATTAGTATTATTATAATTTATANNNNNNNNNNNNNNNNNNNNNNNNNNNNNNNNNNNNNNNNNNNNNNNNNNNNNNNNNNNNNNNNNNNNNNNNNNNNNNNNNNNNNNNNNNNNNNNNNNNNNNNNNNNNNNNNNNNNNNNNNNNNNNNNNNNNNNNNNNNNNNNNNNNNNNNNNNNNNNNNNNNNNNNNNNNNNNNNNNNNNNNNNNNNNNNNNNNNNNNNNNNNNNNNNNNNNNNNNNNNNNNNNNNNNNNNNNNNNNNNNNNNNNNNNNNNNNNNNNNNNNNNNNNNNNNNNNNNNNNNNNNNNNNNNNNNNNNNNNNNNNNNNNNNNNNNNNNNNNNNNNNNNNNNNNNNNNNNNNNNNNNNNNNNNNNNNNNNNNNNNNNNNNNNNNNNNNNNNNNNNNNNNNNNNNNNNNNNNNNNNNNNNNNNNNNNNNNNNNNNNNNNNNNNNNNNNNNNNNNNNNNNNNNNNNNNNNNNNNNNNNNNNNNNNNNNNNNNNNNNNNNNNNNNNNNNNNNNNNNNNNNNNNNNNNNNNNNATGAAAATTATTATGCTCTTAATAATCATTTTTTCAATTAATTTTATTTTCATGAAGCATCCTTTATCAATAGGGATAATTTTATTAATACAAACAATATTTTCTTGCTTAATTTGTAGATTTTATTTAAGTTGTTATTTATTTTCTTACATTTTATTTCTTATTTTTATTGGTGGAATATTAATTTTATTTATATATATATCAAGAATTGCATCAAACGAAAAATTTTTTTTTTCTACAAAATTAATAATATTGAACTTTTTATTTTTAGGTTTAATCAGTTTATTTAATATAGTTGACTTAAAAACATTAAATACTAGGAAAAATATTGTTATATATGTAACTTACAATGATTATATGATAATAAAAATATATATTATTCCTTCTGGTATAATAACTCTAATTCTCACAATTTATTTACTATTTGTTTTAATTATTGTAATTAATATTTTAACAGTCAATATATTAACTCTTCGGAGAAGAATTTCTTTCTAACTACACCTTCCGGTACAGTTACTTTGTTACGACTTATCTCAATAAAATTATGAGAGTGACGGGCGATATGTACATAATTTAGAGCTAATTTCAATTAATTAAATTTAATTAATTTATTATCAAATCCAATTTCATATTCTTTTATNNAAAAAAAATAATCCACTAAATAATTAATTGTAACCCATTTTTTCTTCAATATAAACTGCACCTTGACCTGACATTAAATATATAATTATATAATATGAAAATTTTTCTTATAAAACATTCTTGACAGAGATATACAAGTTAAATTGAAGTTTTTTCTATCGTGGATTATCAATCATAAAACAGATTCCTCTGATAAGATAAATTACCGCCAAATTCTTTGAATTTAAAGATCATTTCTAATAATAATCAAGTTAATTTTATCACATTTTTAATAATAGGGTATCTAATCCTAGTTTATAAAAAAATTTTTCAGACATAAAAATAATTTTTAAAGATAAAATATATTTCACCAAAATTATAACTATTTTATATTATTAATATANNNNNNNNNNNNNNNNNNNNNNNNNNNNNNNNNNNNNNNNNNNNNNNNNNNNNNNNNNNNNNNNNNNNNNNNNNNNNNNNNNNNNNNNNNNNNNNNNNNNNNNNNNNNNNNNNNNNNNNNNNNNNNNNNNNNNNNNNNNNNNNNNNNNNNNNNNNNNNNNNNNNNNNNNNNNNNNNNNNNNNNNNNNNNNNNNNNNNNNNNNNNNNNNNNNNNNNNNNNNNNNNNNATAATAAAATTATTTAATGAGGTCCTTTCGTACTAACATTAAAAATAACTGAGTAGATAGAAACCAACCTGGCTCACGCCGGTTTGAACTCAGATCATGTAAGAATATTAAGGGTCGAACAGACCCAGAAATAATAAATTTTGCTCCAATTCCTATTCTTAATCCAACATCGAGGTCGCAATCCTATTTATCGATATGAACTCTCCAAATTAATTACGCTGTTATCCCTAAGGTAATTTATTCTTATAATCAAAATTTTGGATCAATAATTACATATATTTATGAAACTTTTATTAAAAGTTAATTATATTTTAATATCACCCCAACAAAAAAATTACTTAAATAAAAAAAATTAATTAACTAAATANAAATAAATTCAAANAAAATTTTAAAAATTCTATAGGGTCTTCTCGTCCCACTCATATATTTAAGCTTTTTTACTTAAAAATCAATTTCAATTATTAATATTAATTAAGTCAATTTCTCATTCAATCATTCATACAAGCCTCCAATTAAAAGACTATTTATTATGCTACCTTTGCACGGTCAATTTACCGCGGCCATTCAATTTTNAATCATTGGGCAGATTAGACTTTTAATTCTTACTAAAAGACATGTTTTTGATAAACAGGTGAAAATTNNNNNNNNNNNNNNNNNNNNNNNNNNNNNNNNNNNNNNNNNNNNNNNNNNNNNNNNNNNNNTAAATATTATTTATAAAANNNNNNNNNNNNNNNNNNNNNNNNNNNNNNNNNNNNNNNNNNNNNNNNNNNNNNNNNNNNNNNNNNNNNNNNNNNTTATACTAATTTAATCANNNNNNNNNNNNNNNNNNNNNNNNNNNNNNNNNNNNNNNNNNNNNNNNNNNNNNNNNNNNNNNNNNNNNNNNNNNNNNNNNTTGTAATATAANNNNNNNNNNNNNNAAAAATTTTTTTTTATAAATATAAAAACCAATTATTTTCTAAGGTCAAATTATGGAATGTATTTAATTATAATAATTGAATTGCAGTCAATAGGTGTTGNNTATAACCATTCTTAAGTAATGAAGTAATATATTACATTTAGTTTCGACCTAAAATAAGAATAATCAATTCCTTGCTTTAAATGAAGCCAAGTAAGAGGCATTTTATTGTTAATAAAATAATTGAAATTTTGTTTCCATTTAAAGAAAAATTAGTTTAATAAAAATATAAATCTGTCAGATTTTTGATACTTTCAAGTATTTTTCTGACTTTTANNNTTTTTATCTACTTTAAATTTGCAATTTAATATTATGAATTAAATATAAGACTTTATGTTAGCAGAATTAAACTGTCTATTAATATCAAAAATTAATGTTCTTCATAAACTATAACATTTATAAAGTTTATAAAACATTTCATTTTCATTGAAAAGAGGGACTTAGTCTTATAAAATTCTATTAGTATATAAGTATATTTAACTTCCAATTAAAAGGATTAATTTTATTAAATAGAATANNNNNNNNNNNNNNNNNNNNNNNNNNNNNNNNNNNNNNNNNNNNNNNNNNNNNNNNNNNNNNNNTACTATTTGTAATAAATATTACAATATTAAATTCTAAATTTAATGCATTAATTTTGCTAAAACAGTTCTAATATGGCAGATTAGTGCAATGAATTTAAGATTCATATATAAAATATTATTTTTATTNNNAGAAATAAGATGCCTGAANGGCAAAGGATTATTTTGATAGAATAAATTATGTAATTTAATTACTCTTACATTAAGTGGCTGAAAGTAAGTAATGGTCTCTTAAACCAATTAATAGTAATTAACAAATACTCTTAATGAAAGATAAGCTAAGATNAAAGCTAATGGGTTCATACCTCACTTATGGAAATNATTCCTCTTTTTATTTAAAAACAAGAGTTACCTTAATATCTTCAATATTATGCTCTTAATTAAGCTATTTAAATTCAGAAAAGATAATATAATCATTTTAAACTCCCAAAGTTTATATTTTTTAAATAAATTATTTTCTGAGTTAATTAGCTTAATTAAAAGCATTTATTTTGAAAGTAAAAGAAAAGATTGATAATCTATTAACTTAGGAAACTAGAAATAAAATTAGCTTCTAACTAACTTTTAAAGCGGTTAAATTCCGTTTTTTCCTTGTTTTAATAGTTTANNNAAAAAAATTTAGATCTTGTAAATCTAAGATAACATAAGTTTTAAAAATAGGTTTTAAGTTANTTAAAACTGTTATCCTTCAAAGTTAAAAATATAATTNTATTTATTATTAGGAGAAATAATTTTTCATAAATAAATTTACAGTTTATTACCTATTTTCGGTCATCCTAATTAAGTTATGTTGATTAAACAACATTCTCATTAGTGTAAATAAAGTGCTAAAATAGCTTTAACTTA

>clamitans_03.NZ.SC.HPS.03

NNNNNNNNNNNNNNNNNNNNNNNNNNNNNNNNNNNNNNNNNNNNNNNNNNNNNNNNNNNNNNNNNNNNNNNNNNNNNNNNNNNNNNNNNNNNNNNNNNNNNNNNNNNNNNNNNNNNNNNNNNNNNNNNTAAAATTTTATTTTCATTAAATAACGAATTAAACATATTAATAAATTACAAATCATTAGGTAGATCATTAATATTTTTATCACTATTTACATTTATTTTATTAAACAATTTATTGGGATTACTTCCTTATATTTTCACTAGTTCTAGTCATTTAGTGTTTACAATAAGATTAGCATTACCATTATGATTATCATTTATGATTTATGGATTTATTAATAATGTTAATCATATATTTTGCCATATAGTTCCATCAGGAACTCCTAATATTTTAATGCCTTTTATAGTTATTATTGAAAGAATTAGAAATTTAATTCGACCTGGGTCATTAGCTGTTCGTTTAACAGCTAATATAATTGCTGGTCATCTTTTAATAACTCTACTTGGTAATTTGCCTATAAATTATGAATATTATTCAAGTATAATTATTATTTTTCAAATCTTGTTGATATTATTTGAATTAGCCGTTTGTATAATTCAGTCTTATGTTTTTATAGTACTTAGAACTTTATATTACAGAGAAGTGAATATTCCTCAAATATCCNNNNNNNNNNNNNNNNNNNNNNNNNNNNNNNNNNNNNNNNNNNNNNNNNNNNNNNNNNNNNNNNNNNNNNNNNNNNNNNNNNNNNNNNNNNNNNNNNNNNNNNNNNNNNNNNNNNNNNNNNNNNNNNNNTTTAAACCAACACGACAAAATCATCCCTTAATTAAAATTATTAATAATTCATTAATTGATTTTCCAGCCCCATCTAATTTATCTTATTGATGAAACTTTGGATTTGTATTAGGATTATGCTTAATAATTCAAATCTTTACTGGATTATTTTTATCAATACATTATAATGCTAGTATTGTAAGAGCTTTTGAAAGATTAAGGCACATTTGTCGAGATGTAAATTATGGATGATTCCTGCGAATCATTCACGCTAATGGAGCTTCATTATTTTTTATTTGTGTTTATTTACATGTAGGACGTGGATTATATTATGGTTCATATAAATATATTGAAACATGATCTATTGGTGTAATCATATTACTAATATTAATAGCTACTGCTTTCTTAGGATATGTATTACCATGAGGACAAATATCTTTCTGAGGAGCTACAGTTATTACAAATTTACTTTCAGCTATTCCTTATTTAGGAATAACACTGGTTAATTGAATTTGAGGTGGATTTGCAGTTGATAATGCTACATTAACTCGATTTTACTCATTCCATTTTATTTTGCCATTTATTGTATTGAGATTAACAATTATTCATTTATTATACTTACACACAACAGGTTCAAACAATCCTTTAGGAATTAATTCTAATAATGATAAAGTACCATTTCATCCATATTTTTCTATTAAAGATATTATAAGACTATCTATTCTGATAGTTATATTTTTAATATTTGTTATATTAGAGCCATATATATTGGGAGATCCTGACAATTTTACTCCCGCTAATCCTTTAGTTACACCAAAACATATTCAACCTGAATGATATTTTTTATTTGCTTATGCTATTCTTCGATCAATTCCTAATAAATTAGGAGGAGTAATTGCTCTTTTTATATCAATTTTTATTTTAATATTTGTACCTTTGTTAAACAATTCTAATTTTATGGGATTAAATAATTATCCAATAAATCAAATTTTATTCTGATATTTATTAACCATTTTAATTTTGTTGACTTGAATTGGGGCCCGTCCTGTTGAATTACCTTATATTAACTTCGGAATATTATTAACATTAATATATTTCTTTTACTTTATTATTGACCCATTAACTAAATCTTTTTGAGATAAATTAATTAGATAGATGAAAAAATGATTCTTCTCAACTAATCATAAGGACATTGGAACAATATATTTCATTTTTGGTATTTGATCTGGTATAATTGGTACAACTTTAAGAGTTCTAATTCGTGTAGAACTTGGAACTCCAGGTTCATTTATTGGTGATGATCAAATTTATAACGTAATCGTTACTGCTCATGCTTTTATTATAATTTTTTTTATGGTTATGCCTATTATAATTGGGGGATTTGGAAATTGATTAGTCCCACTAATAATTGGAGCTCCTGATATAGCTTTCCCCCGTATAAACAATATGAGTTTTTGGTTGTTACCCCCCTCATTAATTCTTATTTTAGTAGGAAGAATAGTTGATAGAGGTGCAGGTACTGGTTGAACAGTCTATCCTCCACTATCTAGTGGAGTAGCACATTCTGGTTCATGTGTCGATTTAACTATTTTTTCATTACATCTTGCAGGTGTATCCTCAATTTTAGGTGCTGTAAATTTTATTAGAACAATCTTTAATATACGATCAGTGGGTATTTGATTAGATCGAATACCTTTATTTGTGTGATCTGTATTAATTACTGCATTTTTGTTATTATTATCTTTACCTGTTCTAGCAGGTGCTATTACGATATTATTAACAGATCGAAATTTAAATACTTCATTTTTTGATCCTGCAGGTGGAGGTGATCCAATTTTATATCAGCATTTATTTTGATTTTTTGGTCATCCCGAAGTTTATATTTTAATTTTACCAGGTTTTGGTTTAATTTCTCACATTATTACACAAGAAAGAGGTAAAATTGAATCCTTTGGTTCATTGGGAATAATTTATGCTATAATATCAATTGGTATTTTAGGATTTGTCGTGTGGGCACATCATATATTTACTGTAGGAATAGACGTTGACACACGTGCATATTTTACATCAGCTACTATAATTATTGCAGTTCCAACTGGAATTAAGGTGTTTAGTTGACTTGCAACTTTAAGAGGAATAAAAATTAATATTACATCTTCGGCTTTATGAGCCTTAGGATTTGTTTTTTTATTTACAATTGGTGGTTTAACTGGAGTAATTTTAGCTAATTCTTCAATTGATATTATGTTACATGATACATATTATGTAGTTGCTCATTTTCACTATGTTCTGTCAATAGGAGCAGTTTTTGCTATTATAGCAAGATTTATTCACTGATTTCCCCTATTTACAGGAATAACATTGAATTCAAATTGATTAAAAATTCATTTTTTATTAATATTTATTGGTGTAAACATAACATTTTTTCCTCAACATTTCTTAGGATTAAGAGGTATACCTCGCCGATATTCAGATTATCCTGATGCTTATATATCATGAAATATAATTTCATCAATAGGAAGAATTATATCTTTAATCGGAATTATACTTTTATTATTTATTGTGTGGGAAAGATTTATTTCAATACGATTAGTATTATATTCTAATAGTATTCAATCTTCTATTGAATGAATACAGAAATTTCCCCCATCTGAACATTCTTATAATGAAATGCCATTATTAATTCGAATTTCAAATTGATCTTATATAAATATACAGGACGCTATATCACCATTAATAGAACAATTAATATTTTTTCATGATCATGTATTAGTTATTTTAATTATAATTACAATTATTGTCGCTTATATAATATTAATATTGGTAATAAATAAAATTATTAATCGTTTACTTCTTGAGGGACAATTAATTGAATTTATTTGAACTTTATTACCTGCAATAACTTTAATTTTTATTGCATTACCATCATTACGACTATTATATATGTTGGATGAAATCAATAATCCACTATTAACATTAAAAGTTATTGGTCATCAATGATATTGATCATATGAATATTCAGATTTTTTTGATGTAGAATTTGATTCATATATAAAATCTACAATTGATATAAGAAAAAATGAATTTCGTTTATTAGATGTTGATAATCGAGTAATTTTACCATTTAATATTCAAATTCGGTTGTTAATTTCCTCTTTTGATGTTATTCATTCTTGAGCAATACCATCTATAAGATTAAAAGTTGATGCAATTCCAGGACGATTAAATCAAATAGGAATATTAATTAGTCGTCCAGGTATTTCTTATGGTCAATGTTCTGAAATCTGTGGTGCAAATCACAGATTTATGCCCATTGTTATTGAAAGAATTAGAATGAAAATGTTCATTGACTGATTAATTAATTATATGACTAATCATCCTTATCATTTAGTTGATTATAGCCCATGACCTTTGACTGGATCAATTGGAGCTCTAAGTTTTGTTTCCGGTATAATTATAATATTNNNNAAATATAATTTTATTCTATTATATATTGGTATATTTTTATTGTTATTAACAATAATTCAATGATGACGTGATATTTCTCGAGAGAGAACATTCTTGGGAATGCATACAGTAGTAGTAGTAAATGGTTTAAAAATAGGTATATTACTATTTATTGTATCAGAAATTCTTTTTTTCGTTTCTTTTTTTTGAGAATTTTTTCATAGAAGATTAAGACCTGTGGTAGAAATTGGAATAATTTGGCCTCCCATGGGTATTAATGTGTTTAATCCAACACAAGTTCCTCTATTGAACACAATAATTTTATTATGTTCAGGAATTACAATTACTTGAGCTCATCATTCAATCATAAATGGTAATCACATTGATTCTATTTATAGAATTACTTTAACTATTATTTTAGGATTATACTTCACTGCTTTACAAGGTTATGAATATTATGAAGCTCCTTTTGCAATTAATGATTCCATTTATGGTTCTTCTTTTTTTATGGCTACTGGATTTCATGGTATTCACGTAATTATTGGAACAACATTTATTATTGTGTGTTTAATACGACAAATAAATTTTCATTTTTCAATTAATCATCATTTTGGTTTTGAAGCTGCTGCTTGATATTGACATTTTGTTGATGTAGTTTGATTATTTTTATATTTATCAATTTACTGATGAGGGGGATAATAAATTATAGTTTTTAAAAAAAAAAAATAAATAAATAAATTAAGAGATACTGGCAAATAAGATTTTCAAGCTAAATATATTAATTTATCATATCGATAGCGAGGAAGTGTCCCTCGTACTCAAATAAAGAAAAAACATAATATTGATACTTGAATAGGAAAAATAATTGAATCAATTTTCCCTCCTAAAAATATTAAACAAAATATTATTCTTATAAACAAAATACTAGAATATTCAGCTAGAAAAATAAACGCAAAACCAGCACCTCTATATTCAACATTAAATCCAGAAACCAATTCTGACTCTCCCTCAGAAAAATCAAATGGAGAACGATTTGTTTCAGCTAAAGCCGAAGAAATCCAACATATTCTTAATGGAAGAAATAAAAAAATAAATCATACAGATTTCTGAAATAAAAATAAATCAAGAATATTATAACTTTTAATTATTATTATAGGACATAATATAATTAAAACTAATCTTACCTCATAAGAAATAGATTGAGCAATTGAACGAATACATCCTAATATTGCATAACTTGAATTAGAAGATCATCCTGTTAATATTAAAGAGTAAACACTTATTCTTGAACAGCAAAAAAAAAATAAAATACCAAAATTAAAAGAAACACAATTAATTATATAAGGATATAGTGACCATAATAATAATCTATTAAATAGTCCTATAATAGGACTAAAAATATAAATTAAATAATTAGACATTAATGGAATAGTATTTTCCTTTATAAATAATTTAATAGCATCGGAAATAGGTTGTAAAATACCCAAAAATCCAACTTTATTTGGTCCTTTACGAATTTGAACATATCTTAAAACTTTACGTTCCAAAAGAGTAATAAATGCTACTCCAATTAAAATGAATAAAACAGTAATAAAAATTGTAATTAAATACNNNNNNNNNNNNNNNNNNNNNNNNNNNNNNNNNNNNNNNNNNNNNNNNNNNNNNNNNNNNNNNNNNNNNNNNNNNNNNNNNNNNNNNNNNNNNNNNNNNNNNNNNNNNNNNNNNNNNNNNNNNNNNNNNNNNNNNNNNNNNNNNNNNNNNNNNNNNNNNNNNNNNNNNNNNNNNNNNNNNNNNNNNNNNNNNNNNNNNNNNNNNNNNNNNNNNNNNNNNNNNNNNNNNNNNNNNNNNNNNNNNNNNNNNNNNNNNNNNNNNNNNNNNNNNNNNNNNNNNNNNNNNNNNNNNNNNNNNNNNNNNNNNNNNNNNNNNNNNNNNNNNNNNNNNNNNNNNNNNNNNNNNNNNNNNNNNNNNNNNNNNNNNNNNNNNNNNNNNNNNNNNNNNNNNNNNNNNNNNNNNNNNNNNNNNNNNNNNNNNNNNNNNNNNNNNNNNNNNNNNNNNNNNNNNNNNNNNNNNNNNNNNNNNNNNNNNNNNNNNNNNNNNNNNNNNNNNNNNNNNNNNNNNNNNNNNNNNNNNNNNNNNNNNNNNNNNNNNNNNNNNNNNNNNNNNNNNNNNNNNNNNNNNNNNNNNNNNNNNNNNNNNNNNNNNNNNNNNNNNNNNNNNNNNNNNNNNNNNNNNNNNNNNNNNNNNNNNNNNNNNNNNNNNNNNNNNNNNNNNNNNNNNNNNNNNNNNNNNNNNNNNNNNNNNNNNNNNNNNNNNNNNNNNNNNNNNNNNNNNNNNNNNNNNNNNNNNNNNNNNNNNNNAATTTACTGTTTATTATTAAATAAAATATTACTTATTTGTATTTTATTATTAATGACAGCTTTAATTGTTTTATTTTTCTATTTACGAATTGTGATTACAACATTGATAATAAATACTATTTCAATAAAAATAATTATTATTGAAGTTTCATATTTTTATTATATTGTTAGAATATTTTCATTATTTGGAATAATTTTTTTGTCATTAATTACATTAAATTTATGTTAGNNNNNNNNNNNNNNNNNNNNNNNNNNNNNNNNNNNNNNNNNNNNNNNNNNNNNNNNNNNNNNNNNNNNNNNNNNNNNNNNNNNNNNNNNNNNNNNNNNNNNNNNNNNNNNNNNNNNNNNNNNNNNNNNNNNNNNNNNNNNNNNNNNNNNNNNNNNNNNNNNNNNNNNNNNNNNNNNNNNNNNNNNNNNNNNNNNNNNNNNNNNNNNNNNNNNNNNNNNNNNNNNNNNNNNNNNNNNNNNNNNNNNNNNNNNNNNNNNNNNNNNNNNNNNNNNNNNNNNNNNNATTTTTTTAATTATTTTAACTATGGGCCTATATCATGAATGATATAATAATATATTAAGTTGANNNNNNNGTTTANNNNNNNNNNNNNNNNNNNNNNNNNNNNNNNNNNNNNNNNNNNNNNNNNNNNNNNNNNNNNNNNNNNNNNNTACTCTTCCAGAATTAAATGAAAATAAACTAGAAAAAAATATACCATGTTGAGTATATGAAAATATATATAATCTATAACAAGCAGATAAAAATGAAGATAAAATTAATAAAACTATTATTAATATATTTCAAGATATTAATCCATTAATAATTATAATTTCACCTGCTAAATTAAGTGTAGGGGGGCAAGATATATTTCTTGATGACATTAAGAATCATATTAAAGATATTGAAGGTATAAATGTCAATATCCCCTTATTGATTAATAATCTCCGTCTATTAGTTCGTTCATATAATATATTTGCTAAACAAAATAATCCAGAAGAACAAAGACCATGACCAATTATTATATAATATGAACCATAAATTCCTCAATTTGTTATAGTTATAATTCCTCTAACTACAAGACCTATATGGGCTACAGATGAATAAGCAATTATTGATTTAATATCAATTTGAATTATACAAAGAATTCTAATTATAATTCTCCCAATTATTGAAATAGAAATTCACACAAACCCAAATTTATAAAAATATATAGGAATAATATTCATTATACGAATAATACCATAACCTCCCAACTTCAATAAAATTCTAGCTAGAATCATTGATCCAGAAACAGGTGCTTCAACATGAGCTTTTGGTAATCAAAAATGAAAAAATAATGGCATTTTAATTAAAAAAGCTATTATTATGCCAATATAAATATAAATATTAATAGGATAACCTATTAACATAAAAATATTAGTTATATTTTTTTTATAAAGAAAAAAAATTCTTATTAACATTGGTAATGATGCAAATAATGTATAAAATAATAAATAATATCTTGCATCAATACGTTCAGGTTGATATCCCCAACCCATAATAATAATCATAATTGGAATTAATCTAGATTCAAAAAAAATATAAAAAATTATAATTCTTGATGTAGAGAAAGAAATGATTAGAAAAATTATTAACACAACNNNNNNNNNNNNNNNNNNNNNNNNNNNNNNNNNNNNNNNNNNNNNNNNNNNNNNNNNNNNNNNNNNNNNNNNNNNNNNNNNNNNNNNNNNNNNNNNNNNNNNNNNNNNNNNNNNNNNNNNNNNNNNNNNNNNNNNNNNNNNNNNNNNNNNNNNNNNNNNNNNNNNNNNNNNNNNNNNAATTATATTTATTAATCATGAATTATAAATAAGTAGGGTTAATATCAAGCAAGAAAAAATCAATTTTATAGATATCGATATTAATTTATCATTTCTATGAGAACGAATTAATGTAACTAGGCATGATAATCCCATTACTCCTTCACAAACTCTAAAAACCAAGAAAATTAGCAAAATATGTATATCATACATTTTTATAGAAAAAATAAATGACAAAGAACAAAATATTCTTAAGATAATTAATTCTAATCTTAATGTTGTTATTAAATGTTTTCGATTTATACATAACGAAATTATTCCAGAAAGGAATATAGAAGAAAAATATATTAATAATGTTATATTCTATAAATATATANNNNNNNNNNNAACAATAAATGAAATTATCTGAATCTTAAAGTTATTATTTATTAATTTATAATTTAATGACATTATTATTTTTAAAGTTTTCAAAATTCCTTGAGGTCCAAGATTTTCACCCCATCCCATATCAATGGATTTTTGAAATATTAATGAATTAGTTATTAAAAATATTTGATTATGGAATGTTGATATTTGTTTTATAAATCATATTGAACCAAAAAACTCATAAATAATTTTTATTTTAGTCTTAGAAAAAACTGATATTTCATAACCTAATCAAATTCCTAATGTTGAAAATATTAATGCCATCAATTTGCCTTCAATAGGCATTAAAATTCTAATTGGATCATTAAATATTAATCATCTTAATATAGATCCAGAAATAATTGAATAAATAGATAAAATAATGATTCTTTTAATTATATCATTTATATTTTCACTTAATGATCTCATTTTATAAAAGTTAAAGTTTATACTTATCGAAAAATATGTTAAACGAGCAGAGTAAAATGAAGTTAATCCAATCCCAATATATATGAATACTATAATTAATATATTGATATTATTAGAACATATAGACTCTATAATTAAATCTTTTGAATAAAATCCTCTTATGAATGGAATTCCACATAATGATATATTAGCAATATTTATTATTGTAATAGTTAAAGGCATTTGAAATCTTAAACATCCTATTATACGAATATCTTGATTGTTATTAAAATTATGAATTAAAATACCTGCACACAAAAACAATGTAGCTTTAAATAAAGCATGAACAATTAAATGAAAAAAAGATAACATAGGATAACCAAATATAATAATTGTTATCATAATTCCTAATTGTCTTAAAGTTGATAGAGCAATAATTTTTTTTAAATCAAATTCAAAATTTGCTCTAATTCCCGATATTAATATAGTTAAAATTGAAATTAACATAAATACTTGAATATAATCAAAGTTTATAAAAATATTAGAAAATCGAATTATTAAATATACTCCGGCAGTAACAAGAGTGGATGAGTGAACTAAAGCTGATACAGGGGTTGGAGCAGCTATTGCTGCTGGTAATCAAGAAGAAAAAGGAATCTGAGCTCTCTTAGTAAATCTTGCAATAATAATTAGTATTATTATAATTTATNNNNNNNNNNNNNNNNNNNNNNNNNNNNNNNNNNNNNNNNNNNNNNNNNNNNNNNNNNNNNNNNNNNNNNNNNNNNNNNNNNNNNNNNNNNNNNNNNNNNNNNNNNNNNNNNNNNNNNNNNNNNNNNNNNNNNNNNNNNNNNNNNNNNNNNNNNNNNNNNNNNNNNNNNNNNNNNNNNNNNNNNNNNNNNNNNNNNNNNNNNNNNNNNNNNNNNNNNNNNNNNNNNNNNNNNNNNNNNNNNNNNNNNNNNNNNNNNNNNNNNNNNNNNNNNNNNNNNNNNNNNNNNNNNNNNNNNNNNNNNNNNNNNNNNNNNNNNNNNNNNNNNNNNNNNNNNNNNNNNNNNNNNNNNNNNNNNNNNNNNNNNNNNNNNNNNNNNNNNNNNNNNNNNNNNNNNNNNNNNNNNNNNNNNNNNNNNNNNNNNNNNNNNNNNNNNNNNNNNNNNNNNNNNNNNNNNNNNNNNNNNNNNNNNNNNNNNNNNNNNNNNNNNNNNNNNNNNNNNNNNNNNNNNNNNNNNNNATGAAAATTATTATGCTATTAATAATTATTTTTTCAATTAATTTTATTTTTATGAAACATCCTTTATCAATAGGGATAATTTTATTAATACAAACAATATTTTCTTGTTTAATTTGTAGATTTTATTTAAGTTGTTATTTATTTTCTTATATTTTATTTCTTATTTTTATTGGTGGAATATTAATTTTATTTATATACATATCAAGAATTGCATCAAATGAAAAATTTTTTTTTTCTACAAAATTAATAATATTAAACTTTTTATTTTTAGGTTTAATCAGTTTATTTAATATAATTGACTTAAAAACATTAAATACTAGTAAAAATATTGTAACATATGTAACTTACAATGATTATATGATAATAAAAATATATATTATTCCTTCTGGTATAATAACTCTAATTCTCACAATTTATTTACTATTTGTTTTAATTATTGTAATTAATATTTTAACAGTAAATATATTAACTCTTCGGAGAAGAATTTCTTTCTAACTACACCTTCCGGTACAGTTACTTTGTTACGACTTATCTCAATAAAATTATGAGAGTGACGGGCGATATGTACATAATTTAGAGCTAATTTCAATTAATTAAATTTAATTAATTTATTATCAAATCCAATTTCATATTATTTTATNNNAAAATAATAATCCATTAAATAATTAATTGTAACCCATTTTTTCTTCAATATAAACTGCACCTTGACCTGACATTAAATATATAATTATATAATATGAAAATTTTTCTTATAAAACATTCTTGACAGAGATATACAAGTTAAATTGAAGTTTTTTCTATCGTGGATTATCAATCATAAAACAGGTTCCTCTGATAAGATAAATTACCGCCAAATTCTTTGAATTTAAAGATCATTTCTAATAATAATCAAGTTAATTTTATCACATTTTTAATAATAGGGTATCTAATCCTAGTTTATAAAAAAATTTTTCAGACATAAAAATAATTTTTCAAGATAAAATATATTTCACCAAAATTATAGTTATTTTNNNNNNNNNNNNNNNNNNNNNNNNNNNNNNNNNNNNNNNNNNNNNNNNNNNNNNNNNNNNNNNNNNNNNNNNNNNNNNNNNNNNNNNNNNNNNNNNNNNNNNNNNNNNNNNNNNNNNNNNNNNNNNNNNNNNNNNNNNNNNNNNNNNNNNNNNNNNNNNNNNNNNNNNNNNNNNNNNNNNNNNNNNNNNNNNNNNNNNNNNNNNNNNNNNNNNNNNNNNNNNNNNNNNNNNNNNNNNNNNATAATAAAATTATTTAATGAGGTCCTTTCGTACTAACATTAAAAATAGCTGAGTAGATAGAAACCAACCTGGCTCACGCCGGTTTGAACTCAGATCATGTAAGAATATTAAGGGTCGAACAGACCCAGAAATAATAAATTTTGCTCCAATTTCTATTCTTAATCCAACATCGAGGTCGCAATCTTATTTATCGATATGAACTCTCCAAATTAATTACGCTGTTATCCCTAAGGTAATTTATTCTTATAATCAAAATTTTGGATCAATAATTACATATATTTATGAGACTTTTATTAAAAGTTAATTATATTTTAATGTCACCCCAACAAAAAAATTACTTAAATAAAAAAAATTAATTATCTACATANAAATAAATTCAAGNAAAATTTTAAAAATTCTATAGGGTCTTCTCGTCCCACTCATATATTTAAGCTTTTTTACTTAAAAATCAATTTCAATTATTAATATTAATTAAGTCAATTTCTCATTCAATCATTCATACAAGCCTCCAATTAAAAGACTATTTATTATGCTACCTTTGCACGGTCAATTTACCGCGGCCATTTAATTTTNAATCATTGGGCAGATTAGACTTTTAATTCTTACTAAAAGACATGTTTTTGATAAACAGGTGAAAATTTTTTTTGCCTAAATTATAAATATTATTAATAACCAAAAAACCAGATATCATATAATTTGATAAAATGTCATTTCCAGAATAAATTTATTAATATATATACAATAACAACTAACAAATNTATTCTAAATCATTATATTTCGGGAAAAATAAATTTTTAAAATTTTTCAATTAACCCTGATACAAAAGGTACAAATTATATTTTACTTATAATTATTTATAANATTTTTTCCTTATCAGTAAATCCGAATTATTTTTTTATATAAAATTACTTTAACATAAAAATTTTTTTTTATAAATGTAAAAACCAATTATTTTCTAAGGCCAAATTATGGAATGTATTTAATTATAATAATTGAATTGCAATCAATAGGTGTTGNNTTTAACCATTCTTAAGTAATGAAGTAAAATATTACATTTAGTTTCGACCTAAAATAAGAATAATCAATTCCTTACTTTAAATGAAGCCAAGTAAGAGGCATTTTATTGTTAATAAAATAATTGAAATTTTGTTTCCATTTAAAGAAAAATTAGTTTAATAAAAATATAAATCTGTCAGATTTTTGATACTTTTAAGTATTTTTCTGACTTTTANNNTTTTTATCTACTTTAAATTTGCAATTTAATATTATGAATTAAATATAAGACTNNNNNNNNNNNNNNNNNNNNNNNNNNNNNNNNNNNNNNNNNNNNNNNNNNNNNNNNNNNNNNNNTTTATAAAGTTTATAAAACATTTCATTTTCATTGAAAAGAGGGACTTAGTCTTATAAAGTTCTATTAGTATATAAGTATATTTAACTTCCAATTAAAAGGATTAATTTAATTAAATAGAATANNNNNNNNNNNNNNNNNNNNNNNNNNNNNNNNNNNNNNNNNNNNNNNNNNNNNNNNNNNNNNNNTACTATTTGTAATAAATATTACAGTATTAAATTCTAAATTTAATGCATTAATTTTGCTAAAACAGTTCTAATATGGCAGATTAGTGCAATGAATTTAAGATTCATATATAAAATATTATTTTTATTNNNAGANNNNNNNNNNNNNNNNNNNNNNNNNNNNNNNNNNNNNNNNNNNNNNNNNNNNNNNNNNNNNNNNNCATTAAGTGGCTGAAAGTAAGTAATGGTCTCTTAAACCAAATAATAGTAATTAACAAATACTCTTAATGNNNNNNNNNNNNNNNNNNNNNNNNNNNNNNNNNNNNNNNNNNNNNNNNNNNNNNNNNNNNNNNNNTTTAAAAACAAAAGTTACTTTAATATCTTCAATATTACGCTCTTAATTAAGCTATTTAAATTCAGAAAAGATAATATAATCATTTTAAACTCCCAAAGTTTATATTTTTTAAATAAATTATTTTCTGAGTTAATTAGCTTAATTAAAAGCATTTATTTTGAAAGTAAAAGAAAAGATTTATAATCTATTAACTTAGGAAACTAGAAATAAAATTAGCTTCTAACTAACTTTTAAAGCGGTTAAATTCCGTTTTTTCCTTGTTTTAATAGTTTAAGAAAAAAAATTTAGATCTTGTAAATCTAAGATAACATAAGTTTTAAAAATAGGTTTTAAGTTANTTAAAACTGTTATCCTTCAAAGTTAAAAATATAATTNTATTTATTATTAGGAGAAATAATTTTTCATAAATAAATTTACAGTTTATTACCTATTTTCGGTCATCCTAATTAAGTTATGTTGATCAAACAACATTCTCCTTAGTGTAAATAAAACGCTAAAATAGCTTTAACTTA

>mangu_10.NZ.MC.HUT.06
[truncated: 196,806 more chars]
