# Supplementary material for: Lack of host phylogenetic structure in the gut bacterial communities of New Zealand cicadas and their interspecific hybrids
Source: Sci Rep. 2022 Nov 29;12:20559. doi: 10.1038/s41598-022-24723-3 (PMC9709078; doi:10.1038/s41598-022-24723-3)
Supplement: Supplementary file 5 — Supplementary Information 5. [file 41598_2022_24723_MOESM5_ESM.docx]

#### Tables

##### Table S1: Sample metadata

| Sample Metadata | | | | | | | | | | | | | | |
| --- | --- | --- | --- | --- | --- | --- | --- | --- | --- | --- | --- | --- | --- | --- |
| Cicada tissue samples dissected and sequenced (16s V4 rRNA amplicons) | | | | | | | | | | | | | | |
|  |  |  |  |  |  |  |  |  |  |  |  |  |  |  |
| ID | Country | State | Locality | Dataset | Genus | Species | Tissue Type | Sex | Stage | Elevation (m) | Habitat | Latitude | Longitude | Collection Date |
|  |  |  |  |  |  |  |  |  |  |  |  |  |  |  |
| JK-02 | NZ | HB | ESK | B1 | Kikihia | muta-NI | gut | male | adult | 24 | grass | -39.3879 | 176.8219 | 2017 |
| JK-26 | NZ | WN | HVY | B1 | Kikihia | muta-NI | gut | male | adult | 46 | grass | -41.1342 | 175.0151 | 2010 |
| JK-88 | NZ | MC | BPT | B1 | Kikihia | muta-SI | gut | male | adult | 4 | grass | -43.78 | 172.7882 | 2008 |
| JK-91 | NZ | MC | BPT | B1 | Kikihia | muta-SI | gut | male | adult | 4 | grass | -43.78 | 172.7882 | 2008 |
| JK-112 | NZ | DN | SGB | B1 | Kikihia | muta-SI | gut | male | adult | 6 | grass | -44.9475 | 171.1405 | 2008 |
| JK-115 | NZ | DN | SGB | B1 | Kikihia | muta-SI | gut | male | adult | 6 | grass | -44.9475 | 171.1405 | 2008 |
| JK-29 | NZ | KA | WRD | B1 | Kikihia | muta-SI | gut | male | adult | 33 | grass | -41.8266 | 174.1374 | 2012 |
| JK-46 | NZ | KA | CLV | B1 | Kikihia | muta-SI | gut | male | adult | 37 | grass | -42.1532 | 173.9033 | 2012 |
| JK-52 | NZ | KA | CLV | B1 | Kikihia | muta-SI | gut | male | adult | 37 | grass | -42.1532 | 173.9033 | 2012 |
| JK-151 | NZ | NC | BAL | B1 | Kikihia | muta-SI | gut | male | adult | 193 | grass | -42.8676 | 172.7716 | 2012 |
| JK-67 | NZ | KA | WKK | B1 | Kikihia | muta-tuta-hybrid | gut | male | adult | 129 | grass | -42.3797 | 173.5219 | 2012 |
| JK-70 | NZ | KA | WKK | B1 | Kikihia | muta-tuta-hybrid | gut | male | adult | 129 | grass | -42.3797 | 173.5219 | 2012 |
| JK-73 | NZ | KA | WKK | B1 | Kikihia | muta-tuta-hybrid | gut | male | adult | 129 | grass | -42.3797 | 173.5219 | 2012 |
| JK-76 | NZ | KA | WKK | B1 | Kikihia | muta-tuta-hybrid | gut | male | adult | 129 | grass | -42.3797 | 173.5219 | 2012 |
| JK-133 | NZ | NC | WAI | B1 | Kikihia | muta-tuta-hybrid | gut | male | adult | 224 | grass | -42.6804 | 172.9553 | 2012 |
| JK-136 | NZ | NC | WAI | B1 | Kikihia | muta-tuta-hybrid | gut | male | adult | 224 | grass | -42.6804 | 172.9553 | 2012 |
| JK-139 | NZ | NC | WAI | B1 | Kikihia | muta-tuta-hybrid | gut | male | adult | 224 | grass | -42.6804 | 172.9553 | 2012 |
| JK-142 | NZ | NC | WAI | B1 | Kikihia | muta-tuta-hybrid | gut | male | adult | 224 | grass | -42.6804 | 172.9553 | 2012 |
| JK-145 | NZ | NC | WAI | B1 | Kikihia | muta-tuta-hybrid | gut | male | adult | 224 | grass | -42.6804 | 172.9553 | 2012 |
| JK-58 | NZ | NC | WAI | B1 | Kikihia | muta-tuta-hybrid | gut | male | adult | 224 | grass | -42.6804 | 172.9553 | 2012 |
| JK-61 | NZ | NC | WAI | B1 | Kikihia | muta-tuta-hybrid | gut | male | adult | 224 | grass | -42.6804 | 172.9553 | 2012 |
| JK-100 | NZ | MB | HNC | B1 | Kikihia | muta-tuta-hybrid | gut | male | adult | 340 | grass | -42.5305 | 172.7956 | 2012 |
| JK-94 | NZ | MB | HNC | B1 | Kikihia | muta-tuta-hybrid | gut | male | adult | 340 | grass | -42.5305 | 172.7956 | 2012 |
| JK-109 | NZ | MB | HNB | B1 | Kikihia | muta-tuta-hybrid | gut | male | adult | 346 | grass | -42.5329 | 172.8131 | 2011 |
| JK-118 | NZ | MB | HNB | B1 | Kikihia | muta-tuta-hybrid | gut | male | adult | 346 | grass | -42.5329 | 172.8131 | 2011 |
| JK-121 | NZ | NC | NOW | B1 | Kikihia | muta-tuta-hybrid | gut | male | adult | 475 | grass | -42.5781 | 172.6487 | 2011 |
| JK-124 | NZ | NC | NOW | B1 | Kikihia | muta-tuta-hybrid | gut | male | adult | 475 | grass | -42.5781 | 172.6487 | 2011 |
| JK-127 | NZ | NC | NOW | B1 | Kikihia | muta-tuta-hybrid | gut | male | adult | 475 | grass | -42.5781 | 172.6487 | 2011 |
| JK-08 | NZ | KA | WBS | B1 | Kikihia | muta-tuta-hybrid | gut | male | adult | 495 | grass | -42.4869 | 173.2018 | 2017 |
| JK-44 | NZ | KA | WBS | B1 | Kikihia | muta-tuta-hybrid | gut | male | adult | 495 | grass | -42.4869 | 173.2018 | 2017 |
| JK-11 | NZ | NN | COL | B1 | Kikihia | tuta-clade1-northwestSI | gut | male | adult | 7 | grass | -40.681 | 172.6707 | 2001 |
| JK-14 | NZ | NN | COL | B1 | Kikihia | tuta-clade1-northwestSI | gut | male | adult | 7 | grass | -40.681 | 172.6707 | 2001 |
| JK-32 | NZ | NN | PAJ | B1 | Kikihia | tuta-clade1-northwestSI | gut | male | adult | 31 | grass | -40.799 | 172.7459 | 2013 |
| JK-17 | NZ | NN | WCR | B1 | Kikihia | tuta-clade1-northwestSI | gut | male | adult | 34 | grass | -40.5799 | 172.6274 | 2001 |
| JK-41 | NZ | NN | TTA | B1 | Kikihia | tuta-clade2-northeastSI | gut | male | adult | 0 | grass | -40.5495 | 172.7216 | 2002 |
| JK-43 | NZ | NN | RAB | B1 | Kikihia | tuta-clade2-northeastSI | gut | male | adult | 0 | grass | -41.2822 | 173.1294 | 2017 |
| JK-20 | NZ | MB | PRI | B1 | Kikihia | tuta-clade2-northeastSI | gut | male | adult | 19 | grass | -41.3367 | 173.7644 | 2001 |
| JK-23 | NZ | MB | PRI | B1 | Kikihia | tuta-clade2-northeastSI | gut | male | adult | 19 | grass | -41.3367 | 173.7644 | 2001 |
| JK-103 | NZ | MB | TWI | B1 | Kikihia | tuta-clade2-northeastSI | gut | male | adult | 20 | grass | -41.3378 | 173.7606 | 2008 |
| JK-85 | NZ | NN | ABC | B1 | Kikihia | tuta-clade2-northeastSI | gut | male | adult | 387 | grass | -41.5696 | 172.6882 | 2012 |
| K58b | NZ | KA | CLW | B2 | Kikihia | muta-NI | bacteriome | male | adult | 25 | grass | -42.1561 | 173.9111 | 2018 |
| K60b | NZ | KA | BDB | B2 | Kikihia | paxillulae | bacteriome | male | adult | 158 | shrub | -42.2617 | 173.77 | 2018 |
| K56g | NZ | WN | ASB | B2 | Amphipsalta | cingulata | gut | female | adult |  |  |  |  | 2017 |
| K21g | NZ | AK | TPE | B2 | Amphipsalta | cingulata | gut | male | adult | 4 |  | -36.84 | 174.6617 | 2018 |
| 17NzWnNev21g | NZ | WN | NEV | B2 | Amphipsalta | cingulata | gut | male | adult | 70 |  | -41.302 | 174.8292 | 2017 |
| K18g | NZ | DN | HAM | B2 | Kikihia | acoustica-rosea | gut | male | adult | 32 | shrub | -45.3254 | 170.8176 | 2018 |
| K54g | NZ | KA | WBS | B2 | Kikihia | acoustica-rosea | gut | male | adult | 495 | shrub | -42.4869 | 173.2018 | 2017 |
| K35g | NZ | MK | MCR | B2 | Kikihia | acoustica-rosea | gut | male | adult | 639 | shrub | -43.8113 | 170.1119 | 2002 |
| K13g | NZ | DN | WAI | B2 | Kikihia | angusta | gut | male | adult | 0 | grass | -45.2321 | 170.8693 | 2018 |
| K36g | NZ | WA | FLP | B2 | Kikihia | aotea-east | gut | male | adult | 5 | grass | -41.2533 | 175.9192 | 2017 |
| K41g | NZ | WA | FLP | B2 | Kikihia | aotea-east | gut | male | adult | 5 | grass | -41.2533 | 175.9192 | 2017 |
| K22g | NZ | AK | TEA | B2 | Kikihia | aotea-west | gut | male | adult | 22 | grass | -36.8423 | 174.6551 | 2018 |
| K28g | NZ | AK | TEA | B2 | Kikihia | aotea-west | gut | male | adult | 22 | grass | -36.8423 | 174.6551 | 2018 |
| K66g | NZ | AK | TEA | B2 | Kikihia | aotea-west | gut | male | adult | 22 | grass | -36.8423 | 174.6551 | 2018 |
| 17NzAkPkr01g | NZ | AK | PKR | B2 | Kikihia | aotea-west | gut | male | adult | 153 | grass | -36.8706 | 174.555 | 2017 |
| K40g | NZ | AK | PKR | B2 | Kikihia | cauta | gut | male | adult | 153 | forest | -36.8706 | 174.555 | 2017 |
| K47g | NZ | AK | OLK | B2 | Kikihia | cauta | gut | male | adult | 332 | forest | -36.8834 | 174.5384 | 2017 |
| K20g | NZ | AK | TPE | B2 | Kikihia | cutora-northauckNI | gut | male | adult | 4 | shrub | -36.84 | 174.6617 | 2018 |
| K64g | NZ | AK | TEA | B2 | Kikihia | cutora-northauckNI | gut | male | adult | 22 | shrub | -36.8423 | 174.6551 | 2018 |
| K17g | NZ | KA | BDB | B2 | Kikihia | flemingi | gut | male | adult | 158 | forest | -42.2617 | 173.77 | 2018 |
| K59g | NZ | KA | BDB | B2 | Kikihia | flemingi | gut | male | adult | 158 | forest | -42.2617 | 173.77 | 2018 |
| K32g | NZ | MB | RWR | B2 | Kikihia | horologium | gut | male | adult | 934 | shrub | -42.3302 | 172.7758 | 2017 |
| K33g | NZ | MB | RWR | B2 | Kikihia | horologium | gut | male | adult | 934 | shrub | -42.3302 | 172.7758 | 2017 |
| K45g | NZ | CO | OMS | B2 | Kikihia | murihikua | gut | male | adult | 846 | shrub | -45.3338 | 169.2623 | 2017 |
| K26g | NZ | AK | HEL | B2 | Kikihia | muta-NI | gut | male | adult | 6 | grass | -36.6669 | 174.4505 | 2018 |
| K27g | NZ | AK | HEL | B2 | Kikihia | muta-NI | gut | male | adult | 6 | grass | -36.6669 | 174.4505 | 2018 |
| K65g | NZ | AK | HEL | B2 | Kikihia | muta-NI | gut | male | adult | 6 | grass | -36.6669 | 174.4505 | 2018 |
| K58g | NZ | KA | CLW | B2 | Kikihia | muta-SI | gut | male | adult | 25 | grass | -42.1561 | 173.9111 | 2018 |
| K50g | NZ | NN | ATA | B2 | Kikihia | nelsonensis | gut | male | adult | 11 | shrub | -41.2557 | 173.3004 | 2017 |
| K8g | NZ | KA | KAI | B2 | Kikihia | paxillulae | gut | male | adult | 67 | shrub | -42.4196 | 173.6876 | 2018 |
| K10g | NZ | KA | BDB | B2 | Kikihia | paxillulae | gut | male | adult | 158 | shrub | -42.2617 | 173.77 | 2018 |
| K60g | NZ | KA | BDB | B2 | Kikihia | paxillulae | gut | male | adult | 158 | shrub | -42.2617 | 173.77 | 2018 |
| K57g | NZ | MC | RVS | B2 | Kikihia | peninsularis | gut | male | adult | 420 | shrub | -43.8028 | 172.8417 | 2018 |
| K7g | NZ | MC | RVR | B2 | Kikihia | peninsularis | gut | male | adult | 420 | shrub | -43.8028 | 172.8417 | 2018 |
| K16g | NZ | SD | MAH | B2 | Kikihia | tuta-clade2-northeastSI | gut | male | adult | 14 | grass | -41.2906 | 173.845 | 2018 |
| K29g | NZ | SD | MAH | B2 | Kikihia | tuta-clade2-northeastSI | gut | male | adult | 14 | grass | -41.2906 | 173.845 | 2018 |
| K19g | NZ | BR | FWD | B2 | Kikihia | westlandica-north-inland | gut | male | adult | 29 | grass | -41.7492 | 171.4828 | 2018 |
| K52g | NZ | BR | MMK | B2 | Kikihia | westlandica-north-inland | gut | male | adult | 170 | grass | -41.7995 | 172.314 | 2017 |
| K11g | NZ | KA | WMA | B2 | Maoricicada | campbelli-northSI | gut | male | adult | 77 |  | -41.8825 | 174.0453 | 2018 |
| K34g | NZ | MB | ISL | B2 | Maoricicada | campbelli-northSI | gut | male | adult | 1397 |  | -42.1766 | 172.7946 | 2017 |
| K12g | NZ | SC | HPU | B2 | Maoricicada | campbelli-southSI | gut | male | adult | 735 |  | -44.3444 | 170.5906 | 2018 |
| 17NzBrMrv01g | NZ | BR | MRV | B2 | Maoricicada | hamiltoni-northSI | gut | male | adult | 560 |  | -42.3803 | 172.3146 | 2017 |
| K43g | NZ | BR | MRV | B2 | Maoricicada | hamiltoni-northSI | gut | male | adult | 560 |  | -42.3803 | 172.3146 | 2017 |
| K39g | NZ | TO | TAS | B2 | Maoricicada | iolanthe | gut | male | adult | 991 |  | -38.6959 | 176.1631 | 2017 |
| K48g | NZ | NN | RAB | B2 | Rhodopsalta | cruentata | gut | male | adult | 0 |  | -41.2822 | 173.1294 | 2017 |
| K38g | NZ | WA | FLP | B2 | Rhodopsalta | cruentata | gut | male | adult | 5 |  | -41.2533 | 175.9192 | 2017 |
| K15g | NZ | SD | MAH | B2 | Rhodopsalta | cruentata | gut | male | adult | 14 |  | -41.2906 | 173.845 | 2018 |
| K30g | NZ | SD | MAH | B2 | Rhodopsalta | cruentata | gut | male | adult | 14 |  | -41.2906 | 173.845 | 2018 |
| K24g | NZ | BR | FWD | B2 | Rhodopsalta | cruentata | gut | male | adult | 29 |  | -41.7492 | 171.4828 | 2018 |
| 17NzBrMat01g | NZ | BR | MAT | B2 | Rhodopsalta | cruentata | gut | male | adult | 173 |  | -41.8058 | 172.3247 | 2017 |
| K42g | NZ | WA | FLP | B2 | Rhodopsalta | leptomera | gut | male | adult | 5 |  | -41.2533 | 175.9192 | 2017 |
| K55g | NZ | AK | BTC | B2 | Rhodopsalta | leptomera | gut | male | adult | 11 |  | -36.8863 | 174.4522 | 2017 |
| K23g | NZ | AK | BTB | B2 | Rhodopsalta | leptomera | gut | male | adult | 12 |  | -36.8928 | 174.4488 | 2018 |
| K61g | NZ | AK | BTB | B2 | Rhodopsalta | leptomera | gut | male | adult | 12 |  | -36.8928 | 174.4488 | 2018 |
| K31g | NZ | SC | HPT | B2 | Rhodopsalta | microdora | gut | male | adult | 735 |  | -44.3444 | 170.5905 | 2017 |
| K56r | NZ | WN | ASB | B2 | Amphipsalta | cingulata | reproductive | female | adult |  |  |  |  | 2017 |
| K66r | NZ | AK | TEA | B2 | Kikihia | aotea-west | reproductive | male | adult | 22 | grass | -36.8423 | 174.6551 | 2018 |
| K40r | NC | AK | PKR | B2 | Kikihia | cauta | reproductive | male | adult | 153 | forest | -36.8706 | 174.555 | 2017 |
| K47r | NZ | AK | OLK | B2 | Kikihia | cauta | reproductive | male | adult | 332 | forest | -36.8834 | 174.5384 | 2017 |
| K17r | NZ | KA | BDB | B2 | Kikihia | flemingi | reproductive | male | adult | 158 | forest | -42.2617 | 173.77 | 2018 |
| K58r | NZ | KA | CLW | B2 | Kikihia | muta-SI | reproductive | male | adult | 25 | grass | -42.1561 | 173.9111 | 2018 |
| K50r | NZ | NN | ATA | B2 | Kikihia | nelsonensis | reproductive | male | adult | 11 | shrub | -41.2557 | 173.3004 | 2017 |
| K60r | NZ | KA | BDB | B2 | Kikihia | paxillulae | reproductive | male | adult | 158 | shrub | -42.2617 | 173.77 | 2018 |
| K57r | NZ | MC | RVS | B2 | Kikihia | peninsularis | reproductive | male | adult | 420 | shrub | -43.8028 | 172.8417 | 2018 |
| K53r | NZ | BR | MMK | B2 | Kikihia | westlandica-north-inland | reproductive | male | adult | 170 | grass | -41.7995 | 172.314 | 2017 |
| K43r | NZ | BR | MRV | B2 | Maoricicada | hamiltoni-northSI | reproductive | male | adult | 560 |  | -42.3803 | 172.3146 | 2017 |
| K44r | NZ | BR | MRV | B2 | Maoricicada | hamiltoni-northSI | reproductive | male | adult | 560 |  | -42.3803 | 172.3146 | 2017 |
| K48r | NZ | NN | RAB | B2 | Rhodopsalta | cruentata | reproductive | male | adult | 0 |  | -41.2822 | 173.1294 | 2017 |
| K55r | NZ | AK | BTC | B2 | Rhodopsalta | leptomera | reproductive | male | adult | 11 |  | -36.8863 | 174.4522 | 2017 |
| E70 | NZ | CH | CAR | B3 | Kikihia | longula | egg |  |  | 100 |  | -44.3283 | 176.2183 | 2006 |
| E69 | NZ | CH | TUK | B3 | Kikihia | longula | egg |  |  | 240 |  | -44.0748 | 176.5966 | 2006 |
| E92 |  |  |  | B3 | Maoricicada | otagoensis | egg |  |  | 960 |  | -45.6203 | 167.9521 | 1995 |
| E93 |  |  |  | B3 | Maoricicada | otagoensis | egg |  |  | 960 |  | -45.6203 | 167.9521 | 1995 |
| E89 |  |  |  | B3 | Maoricicada | tenuis | egg |  |  | 1231 |  | -41.5759 | 173.3278 | 1995 |
| E90 |  |  |  | B3 | Maoricicada | tenuis | egg |  |  | 1231 |  | -41.5759 | 173.3278 | 1995 |
| E3 |  |  |  | B3 | Platypedia | putnami | egg |  |  | 2306 |  | 37.25 | -108.4167 |  |
| P1Egg |  |  |  | B3 | Platypedia | putnami | egg |  |  | 2306 |  | 37.25 | -108.4167 |  |
| P2Egg |  |  |  | B3 | Platypedia | putnami | egg |  |  | 2306 |  | 37.25 | -108.4167 |  |
| P15 | NZ | AK | PNL | B3 | Amphipsalta | cingulata | gut | male | adult | 19 |  | -36.7861 | 175.0209 | 2014 |
| P11 | NZ | NC | NOX | B3 | Amphipsalta | strepitans | gut | male | adult | 349 |  | -42.5707 | 172.6781 | 2012 |
| P12 | NZ | NC | NOX | B3 | Amphipsalta | strepitans | gut | male | adult | 349 |  | -42.5707 | 172.6781 | 2012 |
| P13 | NZ | DN | PUK | B3 | Amphipsalta | strepitans | gut | male | adult | 1263 |  | -45.38 | 170.11 | 2009 |
| P14 | NZ | DN | PUK | B3 | Amphipsalta | strepitans | gut | male | adult | 1263 |  | -45.38 | 170.11 | 2010 |
| P55 | NZ | SC | LDN | B3 | Kikihia | angusta | gut | male | adult | 149 | grass | -44.7817 | 170.87 | 2008 |
| P56 | NZ | SC | LDN | B3 | Kikihia | angusta | gut | male | adult | 149 | grass | -44.7817 | 170.87 | 2008 |
| P57 | NC | SC | LDN | B3 | Kikihia | angusta | gut | male | adult | 149 | grass | -44.7817 | 170.87 | 2008 |
| P58 | NZ | SC | LDN | B3 | Kikihia | angusta | gut | male | adult | 149 | grass | -44.7817 | 170.87 | 2008 |
| P59 | NZ | SC | LDN | B3 | Kikihia | angusta | gut | male | adult | 149 | grass | -44.7817 | 170.87 | 2008 |
| P48 | NZ | OL | NLW | B3 | Kikihia | angusta | gut | male | adult | 373 | grass | -44.4833 | 169.2475 | 2008 |
| P60 | NZ | MB | BAV | B3 | Kikihia | angusta | gut | male | adult | 1164 | grass | -42.1615 | 172.821 | 2003 |
| P61 | NZ | MB | BAV | B3 | Kikihia | angusta | gut | male | adult | 1164 | grass | -42.1615 | 172.821 | 2003 |
| P62 | NZ | MB | BAV | B3 | Kikihia | angusta | gut | male | adult | 1164 | grass | -42.1615 | 172.821 | 2003 |
| P63 | NZ | MB | BAV | B3 | Kikihia | angusta | gut | male | adult | 1164 | grass | -42.1615 | 172.821 | 2003 |
| P64 | NZ | MB | BAV | B3 | Kikihia | angusta | gut | male | adult | 1164 | grass | -42.1615 | 172.821 | 2003 |
| P82 | NZ | GB | NUH | B3 | Kikihia | aotea-east | gut | male | adult | 10 | grass | -39.0442 | 177.7377 | 2002 |
| P29 | NZ | ND | TAN | B3 | Kikihia | cauta | gut | male | adult | 5 | forest | -34.6293 | 172.9673 | 2003 |
| P28 | NZ | CL | WAD | B3 | Kikihia | cauta | gut | male | adult | 6 | forest | -36.8431 | 175.6641 | 2002 |
| P32 | NZ | BP | HAW | B3 | Kikihia | cutora-cumberi-midNI | gut | male | adult | 200 | shrub | -37.8901 | 177.5543 | 2003 |
| P30 | NZ | BP | PYE | B3 | Kikihia | cutora-cumberi-midNI | gut | male | adult | 354 | shrub | -37.8631 | 176.1251 | 2002 |
| P31 | NZ | WO | MKE | B3 | Kikihia | cutora-cumberi-midNI | gut | male | adult | 369 | shrub | -38.4628 | 175.3952 | 2003 |
| P78 | NZ | WD | FOX | B3 | Kikihia | horologium | gut | male | adult | 239 | shrub | -43.4972 | 170.0427 | 2003 |
| P22 | NZ | MK | TGP | B3 | Kikihia | horologium | gut | male | adult | 686 | shrub | -43.74 | 170.13 | 2002 |
| P21 | NZ | NC | TEM | B3 | Kikihia | horologium | gut | male | adult | 910 | shrub | -42.9139 | 171.5588 | 2002 |
| P70 | NZ | CH | CAR | B3 | Kikihia | longula | gut | female | adult | 100 |  | -44.3283 | 176.2183 | 2006 |
| P69 | NZ | CH | TUK | B3 | Kikihia | longula | gut | female | adult | 240 |  | -44.0748 | 176.5966 | 2006 |
| P72 | NZ | CH | CAR | B3 | Kikihia | longula | gut | male | adult | 100 |  | -44.3283 | 176.2183 | 2006 |
| P71 | NZ | CH | TUK | B3 | Kikihia | longula | gut | male | adult | 240 |  | -44.0748 | 176.5966 | 2006 |
| P73 | NZ | CH | TUK | B3 | Kikihia | longula | gut | male | adult | 240 |  | -44.0748 | 176.5966 | 2006 |
| P46 | NZ | OL | NLW | B3 | Kikihia | murihikua | gut | male | adult | 373 | shrub | -44.4833 | 169.2475 | 2008 |
| P47 | NZ | OL | NLW | B3 | Kikihia | murihikua | gut | male | adult | 373 | shrub | -44.4833 | 169.2475 | 2008 |
| P49 | NZ | OL | NLW | B3 | Kikihia | murihikua | gut | male | adult | 373 | shrub | -44.4833 | 169.2475 | 2008 |
| P50 | NZ | OL | RSC | B3 | Kikihia | murihikua | gut | male | adult | 391 | shrub | -44.502 | 168.7825 | 2008 |
| P51 | NZ | OL | RSC | B3 | Kikihia | murihikua | gut | male | adult | 391 | shrub | -44.502 | 168.7825 | 2008 |
| P52 | NZ | OL | RSC | B3 | Kikihia | murihikua | gut | male | adult | 391 | shrub | -44.502 | 168.7825 | 2008 |
| P53 | NZ | OL | RSC | B3 | Kikihia | murihikua | gut | male | adult | 391 | shrub | -44.502 | 168.7825 | 2008 |
| P54 | NZ | OL | RSC | B3 | Kikihia | murihikua | gut | male | adult | 391 | shrub | -44.502 | 168.7825 | 2008 |
| P67 | NZ | WN | OTK | B3 | Kikihia | muta | gut | male | adult | 113 | grass | -40.8615 | 175.2346 | 2002 |
| P66 | NZ | SC | PFR | B3 | Kikihia | muta-SI | gut | male | adult | 302 | grass | -43.9027 | 171.253 | 2003 |
| P77 | NZ | NN | WAK | B3 | Kikihia | nelsonensis | gut | male | adult | 46 | shrub | -41.2563 | 173.303 | 2003 |
| P74 | NZ | NN | ABC | B3 | Kikihia | nelsonensis | gut | male | adult | 387 | shrub | -41.5696 | 172.6882 | 2002 |
| P75 | NZ | NN | HHR | B3 | Kikihia | nelsonensis | gut | male | adult | 675 | shrub | -41.0217 | 172.895 | 2003 |
| P68 | NZ | ND | MGP | B3 | Kikihia | ochrina | gut | male | adult | 98 | shrub | -35.1957 | 173.4827 | 2003 |
| P33 | NZ | WN | WNU | B3 | Kikihia | ochrina | gut | male | adult | 211 | shrub | -41.2493 | 174.9212 | 2002 |
| P34 | NZ | WN | WNU | B3 | Kikihia | ochrina | gut | male | adult | 211 | shrub | -41.2493 | 174.9212 | 2002 |
| P35 | NZ | WN | WNU | B3 | Kikihia | ochrina | gut | male | adult | 211 | shrub | -41.2493 | 174.9212 | 2002 |
| P36 | NZ | WN | WNU | B3 | Kikihia | ochrina | gut | male | adult | 211 | shrub | -41.2493 | 174.9212 | 2002 |
| P25 | NZ | MB | PIN | B3 | Kikihia | scutellaris | gut | male | adult | 65 | forest | -41.3483 | 173.6376 | 2002 |
| P26 | NZ | MB | PIN | B3 | Kikihia | scutellaris | gut | male | adult | 65 | forest | -41.3483 | 173.6376 | 2002 |
| P20 | NZ | NN | CBR | B3 | Kikihia | subalpina | gut | male | adult | 186 | forest | -41.0319 | 172.7976 | 2002 |
| P17 | NZ | FD | NTE | B3 | Kikihia | subalpina | gut | male | adult | 290 | forest | -45.142 | 167.9098 | 2002 |
| P19 | NZ | NN | KHA | B3 | Kikihia | subalpina | gut | male | adult | 339 | forest | -40.6366 | 172.558 | 2002 |
| P16 | NZ | BR | HOP | B3 | Kikihia | subalpina | gut | male | adult | 438 | forest | -42.5911 | 172.4472 | 2002 |
| P18 | NZ | MK | MCV | B3 | Kikihia | subalpina | gut | male | adult | 750 | forest | -43.7352 | 170.0956 | 2002 |
| P65 | NZ | NN | TTA | B3 | Kikihia | tuta-clade1-northwestSI | gut | male | adult | 0 | grass | -40.5495 | 172.7216 | 2002 |
| P81 | NZ | SD | TEN | B3 | Kikihia | tuta-clade2-northeastSI | gut | male | adult | 8 | grass | -41.1208 | 173.76 | 2013 |
| P79 | NZ | BR | IRO | B3 | Kikihia | westlandica-north-inland | gut | male | adult | 133 | grass | -41.7867 | 172.031 | 2002 |
| P80 | NZ | WD | RBF | B3 | Kikihia | westlandica-south | gut | male | adult | 77 | grass | -43.941 | 169.2921 | 2006 |
| P76 | NZ | WD | KAN | B3 | Kikihia | westlandica-south | gut | male | adult | 143 | grass | -42.81 | 171.1551 | 2004 |
| P23 | NZ | NN | KAI | B3 | Kikihia |  | gut | male | adult | 35 |  | -40.5761 | 172.6338 | 2002 |
| P24 | NZ | NN | KAI | B3 | Kikihia |  | gut | male | adult | 35 |  | -40.5761 | 172.6338 | 2002 |
| P27 | NZ | NN | KAI | B3 | Kikihia |  | gut | male | adult | 35 |  | -40.5761 | 172.6338 | 2002 |
| P101 | US | NC | Brood VII | B3 | Magicicada | septendecim | gut | female | adult | 377 |  | 42.9598 | -76.193 | 2018 |
| P102 | US | NC | Brood VII | B3 | Magicicada | septendecim | gut | female | adult | 377 |  | 42.9598 | -76.193 | 2018 |
| P100 | US | NC | Brood VII | B3 | Magicicada | septendecim | gut | male | adult | 377 |  | 42.9598 | -76.193 | 2018 |
| P98 | US | NC | Brood VII | B3 | Magicicada | septendecim | gut | male | adult | 377 |  | 42.9598 | -76.193 | 2018 |
| P99 | US | NC | Brood VII | B3 | Magicicada | septendecim | gut | male | adult | 377 |  | 42.9598 | -76.193 | 2018 |
| P43 | NZ | MC | QUR | B3 | Maoricicada | campbelli-northSI | gut | male | adult | 447 |  | -43.6747 | 171.332 | 2011 |
| P8 | NZ | MC | PUD | B3 | Maoricicada | campbelli-northSI | gut | male | adult | 492 |  | -43.5776 | 171.5283 | 2011 |
| P10 | NZ | OL | FRL | B3 | Maoricicada | campbelli-southSI | gut | male | adult | 369 |  | -45.3975 | 168.5936 | 2002 |
| P4 | NZ | OL | NLW | B3 | Maoricicada | campbelli-southSI | gut | male | adult | 373 |  | -44.4833 | 169.2475 | 2008 |
| P5 | NZ | OL | NLW | B3 | Maoricicada | campbelli-southSI | gut | male | adult | 373 |  | -44.4833 | 169.2475 | 2008 |
| P7 | NZ | OL | NLW | B3 | Maoricicada | campbelli-southSI | gut | male | adult | 373 |  | -44.4833 | 169.2475 | 2008 |
| P83 | NZ | OL | RSC | B3 | Maoricicada | campbelli-southSI | gut | male | adult | 391 |  | -44.502 | 168.7825 | 2008 |
| P83x2 | NZ | OL | RSC | B3 | Maoricicada | campbelli-southSI | gut | male | adult | 391 |  | -44.502 | 168.7825 | 2008 |
| P3 | NZ | NC | NIG | B3 | Maoricicada | cassiope | gut | female | adult | 1444 |  | -42.91 | 171.5798 | 2014 |
| P38 | NZ | KA | FYF | B3 | Maoricicada | cassiope-northSI | gut | male | adult | 1066 |  | -42.3242 | 173.5976 | 2003 |
| P2 | NZ | NN | STE | B3 | Maoricicada | cassiope-northSI | gut | male | adult | 1164 |  | -40.794 | 172.4601 | 2014 |
| P39 | NZ | NC | NIG | B3 | Maoricicada | cassiope-northSI | gut | male | adult | 1444 |  | -42.91 | 171.5798 | 2014 |
| P41 | NZ | MC | HTK | B3 | Maoricicada | clamitans | gut | male | adult | 1068 |  | -43.5324 | 171.5394 | 2012 |
| P91 |  |  |  | B3 | Maoricicada | clamitans | gut | male | adult | 1526 |  | -44.2271 | 169.7799 | 1995 |
| P6 | NZ | KA | KAI | B3 | Maoricicada | lindsayi | gut | male | adult | 67 |  | -42.4196 | 173.6876 | 2002 |
| P9 | NZ | NC | NCH | B3 | Maoricicada | lindsayi | gut | male | adult | 80 |  | -42.8064 | 173.2743 | 2002 |
| P40 | NZ | NC | WTF | B3 | Maoricicada | lindsayi | gut | male | adult | 419 |  | -42.5752 | 172.654 | 2012 |
| P45 | NZ | MC | HUT | B3 | Maoricicada | mangu | gut | male | adult | 1581 |  | -43.4957 | 171.5363 | 2014 |
| P1 | NZ | MK | LTM | B3 | Maoricicada | oromelaena | gut | male | adult | 916 |  | -43.6601 | 170.1779 | 2002 |
| P42 | NZ | NC | NIG | B3 | Maoricicada | oromelaena | gut | male | adult | 1444 |  | -42.91 | 171.5798 | 2014 |
| P92 |  |  |  | B3 | Maoricicada | otagoensis | gut | female | adult | 960 |  | -45.6203 | 167.9521 | 1995 |
| P93 |  |  |  | B3 | Maoricicada | otagoensis | gut | female | adult | 960 |  | -45.6203 | 167.9521 | 1995 |
| P37 | NZ | CO | RSR | B3 | Maoricicada | otagoensis | gut | male | adult | 1489 |  | -45.0401 | 168.8015 | 2001 |
| P44 | NZ | MK | OHA | B3 | Maoricicada | phaeoptera | gut | male | adult | 1526 |  | -44.2271 | 169.7799 | 2002 |
| P89 |  |  |  | B3 | Maoricicada | tenuis | gut | female | adult | 1231 |  | -41.5759 | 173.3278 | 1995 |
| P90 |  |  |  | B3 | Maoricicada | tenuis | gut | female | adult | 1231 |  | -41.5759 | 173.3278 | 1995 |
| P87 |  |  |  | B3 | Maoricicada | tenuis | gut | male | adult | 1231 |  | -41.5759 | 173.3278 | 1995 |
| P88 |  |  |  | B3 | Maoricicada | tenuis | gut | male | adult | 1231 |  | -41.5759 | 173.3278 | 1995 |
| P88x2 |  |  |  | B3 | Maoricicada | tenuis | gut | male | adult | 1231 |  | -41.5759 | 173.3278 | 1995 |
| P96 | US | FL | TRA | B3 | Neotibicen | apalachicola | gut | male | adult | 35 |  | 30.4854 | -84.3856 | 2018 |
| P97 | US | FL | TRA | B3 | Neotibicen | davisi | gut | male | adult | 35 |  | 30.4854 | -84.3856 | 2018 |
| P85 | NZ | BR | SEW | B3 | nymph | nymph | gut |  | nymph | 864 |  | -42.4034 | 171.3426 | 2014 |
| P86 |  |  |  | B3 | nymph | nymph | gut |  | nymph | 1587 |  | -44.631 | 168.8876 |  |
| P84 |  |  |  | B3 | nymph | nymph | gut |  | nymph | 2017 |  | -45.0501 | 168.8311 |  |
| P84x2 |  |  |  | B3 | nymph | nymph | gut |  | nymph | 2017 |  | -45.0501 | 168.8311 |  |
| P94 |  |  |  | B3 | Caledopsalta | Caledopsalta sp. | gut | male | adult | 657 |  | -21.6167 | 165.8 | 1995 |
| P95 |  |  |  | B3 | Caledopsalta | Caledopsalta sp. | gut | male | adult | 657 |  | -21.6167 | 165.8 | 1995 |
| P1FG |  |  |  | B3 | Platypedia | putnami | gut | female | adult | 2306 |  | 37.25 | -108.4167 |  |
| P2FG |  |  |  | B3 | Platypedia | putnami | gut | female | adult | 2306 |  | 37.25 | -108.4167 |  |
| P3FG |  |  |  | B3 | Platypedia | putnami | gut | male | adult | 2306 |  | 37.25 | -108.4167 |  |
| P4FG |  |  |  | B3 | Platypedia | putnami | gut | male | adult | 2306 |  | 37.25 | -108.4167 |  |
| P5FG |  |  |  | B3 | Platypedia | putnami | gut | male | adult | 2306 |  | 37.25 | -108.4167 |  |

#####

##### Table S2: Results of Beta regressions using either unweighted or weighted pairwise UniFrac distances as response variables and different combinations of explanatory variables (elevation, phylogeny, and habitat) across seven independently run models. Values represent effect sizes with standard errors in parentheses. Asterisks are included when effect sizes are significant (p << 0.01).

|  | Unweighted UniFrac | | | Weighted UniFrac | | |
| --- | --- | --- | --- | --- | --- | --- |
| Dataset/Model | Different Habitats (UniFrac) | Elevation Difference (UniFrac) | Phylogenetic (Cophenetic) Distance (UniFrac) | Different Habitats (Weighted UniFrac) | Elevation Difference (Weighted UniFrac) | Phylogenetic (Cophenetic) Distance (Weighted UniFrac) |
| Model 1 (Elevation) | | | | | | |
| B2 |  | 0 (0) |  |  | 0.001 (0)* |  |
| B3 |  | 0 (0) |  |  | 0 (0) |  |
| Model 2 (Elevation + Phylogeny) | | | | | | |
| B2 |  | 0 (0) | 0.199 (0.166) |  | 0.001 (0)* | -0.037 (0.266) |
| B3 |  | 0 (0) | 0.12 (0.262) |  | 0 (0) | 0.349 (0.216) |
| Model 3 (Elevation + Habitat) | | | | | | |
| B2 | 0.009 (0.042) | 0 (0) |  | -0.046 (0.066) | 0.001 (0)* |  |
| B3 | 0.022 (0.05) | 0 (0) |  | 0.019 (0.041) | 0 (0) |  |
| Model 4 (Elevation + Phylogeny + Habitat) | | | | | | |
| B2 | -0.008 (0.044) | 0 (0) | 0.209 (0.177) | -0.048 (0.07) | 0.001 (0)* | 0.025 (0.281) |
| B3 | 0.016 (0.055) | 0 (0) | 0.085 (0.289) | -0.012 (0.045) | 0 (0) | 0.375 (0.238) |
| Model 5 (Phylogeny) | | | | | | |
| B2 |  |  | 0.201 (0.159) |  |  | 0.525 (0.266) |
| B3 |  |  | -0.07 (0.244) |  |  | 0.131 (0.203) |
| Model 6 (Phylogeny + Habitat) | | | | | | |
| B2 | -0.008 (0.042) |  | 0.211 (0.169) | -0.057 (0.069) |  | 0.601 (0.281) |
| B3 | 0.026 (0.055) |  | -0.122 (0.268) | 0.001 (0.045) |  | 0.128 (0.223) |
| Model 7 (Habitat) | | | | | | |
| B2 | 0.009 (0.04) |  |  | -0.006 (0.066) |  |  |
| B3 | 0.016 (0.05) |  |  | 0.012 (0.041) |  |  |

##### Table S3: Results of PERMANOVA analysis of the effect of various explanatory variables (elevation, species, and habitat) on weighted UniFrac distances among gut microbial communities across datasets. NA represents values that could not be computed.

|  | B1 | | | B2 | | | B3 | | |
| --- | --- | --- | --- | --- | --- | --- | --- | --- | --- |
|  | R2 | p | betadisp | R2 | p | betadisp | R2 | p | betadisp |
| elevation | 0.04 | 0.03 | NA | 0.03 | 0.03 | NA | 0.03 | 0.05 | NA |
| species | 0.06 | 0.19 | 0.74 | 0.04 | 0.30 | 0.02 | 0.04 | 0.17 | 0.00 |
| habitat | NA | NA | NA | 0.25 | 0.36 | 0.61 | 0.24 | 0.08 | 0.24 |

##### Table S4: General 16S V4 rRNA qPCR data and associated data from 16S V4 rRNA amplicon sequencing.

| Sample name | Species | Extract quantification Qubit (ng/ul) | PCR amplicon QIAxcel quantifications (ng/ul) | Predicted quantity of 16S based on amplicon dataset | Initial copy number estimate (average) | Description of amplicon taxon makeup | Number of total reads? | % Ophio/host reads | Number of bacterial reads (post-filtering) | Number of filtered reads | Percent filtered reads |
| --- | --- | --- | --- | --- | --- | --- | --- | --- | --- | --- | --- |
|  |  |  |  |  |  |  |  |  |  |  |  |
| Emeraldamp control | Pcr control | 2.38 (problem) | 0 | very low | 0.1 |  | Not sequenced | Not sequenced | Not sequenced | Not sequenced | Not sequenced |
| water control | Pcr control | too low (less than 1 ng/ul) | 0 | very low | 0.2 |  | Not sequenced | Not sequenced | Not sequenced | Not sequenced | Not sequenced |
| Powersoil control | powersoil? | too low (less than 1 ng/ul) | 0 | very low | 0.3 |  | 24366 | 3.2 | 19046 | 5320 | 21.8 |
| Plate control | plate control (dneasy) | too low (less than 1 ng/ul) | 0 | very low | 0.4 |  | 4755 | 0 | 472 | 4283 | 90.1 |
| Dneasy control 1 | dneasy | too low (less than 1 ng/ul) | 0 | very low | 0.3 |  | 30856 | 0 | 5183 | 25673 | 83.2 |
| Dneasy control 2 | dneasy | too low (less than 1 ng/ul) | 0 | very low | 0.5 |  | 1871 | 0 | 1677 | 194 | 10.4 |
| JK-02 | muta-NI 17.NZ.HB.BSK.01 | 6.58 | 0.69 | low | 36.2 | mix of many contributing only ~50% | 22128 | 30.8 | 10241 | 11887 | 53.7 |
| JK-112 | muta-SI 08.NZ.DN.SGB.07 | 8.37 | 0.46 | low | 46.8 | mix of many contributing only ~60% | 19478 | 35.9 | 10114 | 9364 | 48.1 |
| D-K40-FG | cauta 17.NZ.AK.PKR.3 | too low (less than 1 ng/ul) | 0.3 | low | 0.2 | three middle abundance taxa in graph making about 15% | 88557 | 18.8 | 19179 | 69378 | 78.3 |
| D-K30-FG | cruentata 18.NZ.SD.MAH.2 | too low (less than 1 ng/ul) | 0 | low | 23.3 | mix of a bunch of middling abundance | 256915 | 15.8 | 4281 | 252634 | 98.3 |
| P63-gut | angusta 03.NZ.MB.BAV.11 | 1.49 | 0 | low | 6.1 | no high abundance taxa in graph | 24692 | 8.2 | 11943 | 12749 | 51.6 |
| P78-gut | horologium 03.NZ.WD.FOX.07 | 2.67 | 0 | low | 20.1 | no high abundance taxa in graph | 20103 | 57.4 | 3474 | 16629 | 82.7 |
| JK-58 | muta-tuta hybrid 12.NZ.NC.WAI.13 | 9.92 | 2.31 | mid | 66.7 | ~60% Pseudomonas | 43291 | 46 | 4425 | 38866 | 89.8 |
| JK-145 | muta-tuta-hybrid 12.NZ.NC.WAI.24 | 9 | 1.77 | mid | 37.3 | possible contaminant signal | 29163 | 31 | 15398 | 13765 | 47.2 |
| D-K33-FG | horologium 17.NZ.MB.RWR.2 | 1.48 | 3.4 | mid | 25.3 | mix of four middling abundance taxa | 143165 | 92.9 | 5770 | 137395 | 96 |
| D-K23-FG | leptomera 18.NZ.AK.BTB.2 | too low (less than 1 ng/ul) | 1.1 | mid | 93.1 | mix of six middling abundance taxa | 82808 | 61.8 | 15675 | 67133 | 81.1 |
| P10-gut | campbelli-southSI 02.NZ.OL.FRL.4 | 1.38 | 0 | mid | 0.4 | ~50% Acetobacter | 80474 | 1.1 | 10167 | 70307 | 87.4 |
| P91-gut | clamitans unknown6 (02.NZ.MK.OHA) | 1.66 | 0 | mid | 22.4 | 50% Cupriavidus | 24636 | 32.1 | 6114 | 18522 | 75.2 |
| JK-52 | muta 12.NZ.KA.CLV.13 | 6.39 | 12.64 | high | 352.2 | 100% Enterobacteriaceae | 47778 | 0.8 | 47360 | 418 | 0.9 |
| JK-29 | muta-SI 12.NZ.KA.WRD.01 | 7.3 | 13.18 | high | 50.3 | 100% Chryseobacterium | 23557 | 0.7 | 23313 | 244 | 1 |
| D-K45-FG | murihikua 17.NZ.CO.OMS.2 | too low (less than 1 ng/ul) | 3 | high | 5.6 | 85% Phytoplasma | 140897 | 43.4 | 56361 | 84536 | 60 |
| D-K60-FG | paxillulae 18.NZ.KA.BDB.2 | too low (less than 1 ng/ul) | 2.4 | high | 1.4 | 100% Phytoplasma | 156454 | 5.2 | 133495 | 22959 | 14.7 |
| P74-gut | nelsonensis 02.NZ.NN.ABC.02 | 3.1 | 0.35 | high | 12.9 | 100% Phytoplasma | 121718 | 1.8 | 102270 | 19448 | 16 |
| P54-gut | muruhikua 08.NZ.OL.RSC.13 | 8.8 | 6.28 | high | 179.8 | 50% Micrococcaceae | 33674 | 0.1 | 33622 | 52 | 0.2 |

#####

#### Supplementary Figures

#####

#####

#####
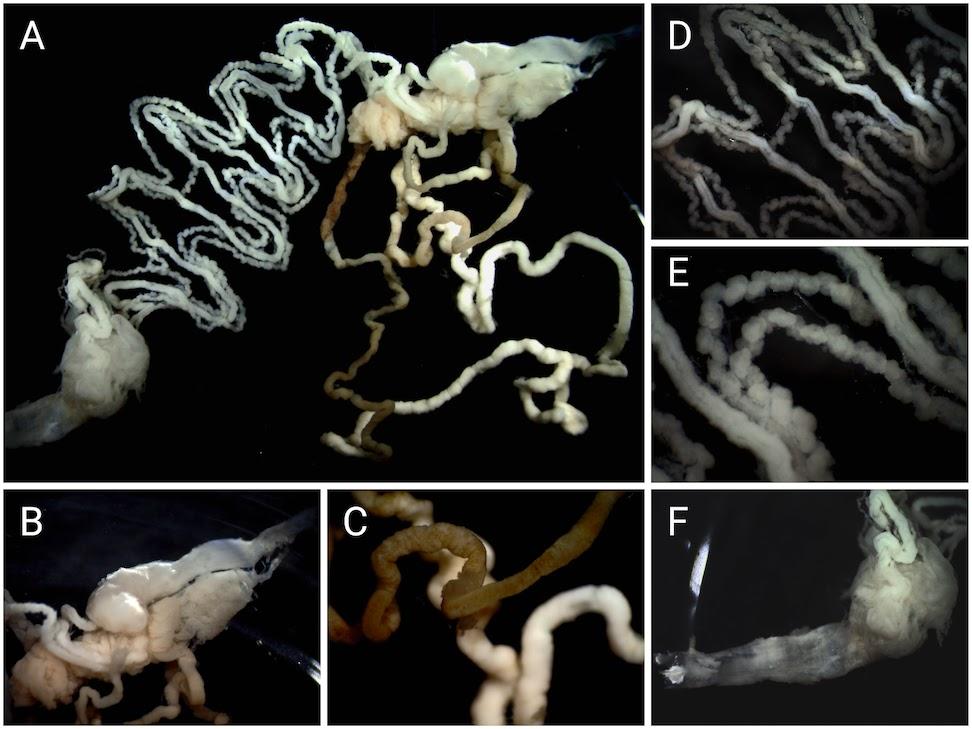


#####

##### Fig. S1: Image of gut tissue dissected from nymphal *Magicicada septendecim*. (A) Complete gut. (B) Filter chamber. (C) Midgut. (D-E) Malphigian tubules and hindgut. (F) Rectum. Adult gut anatomy is identical to that of the nymph in these hemimetabolous insects and varies little across the family.

#####

#####
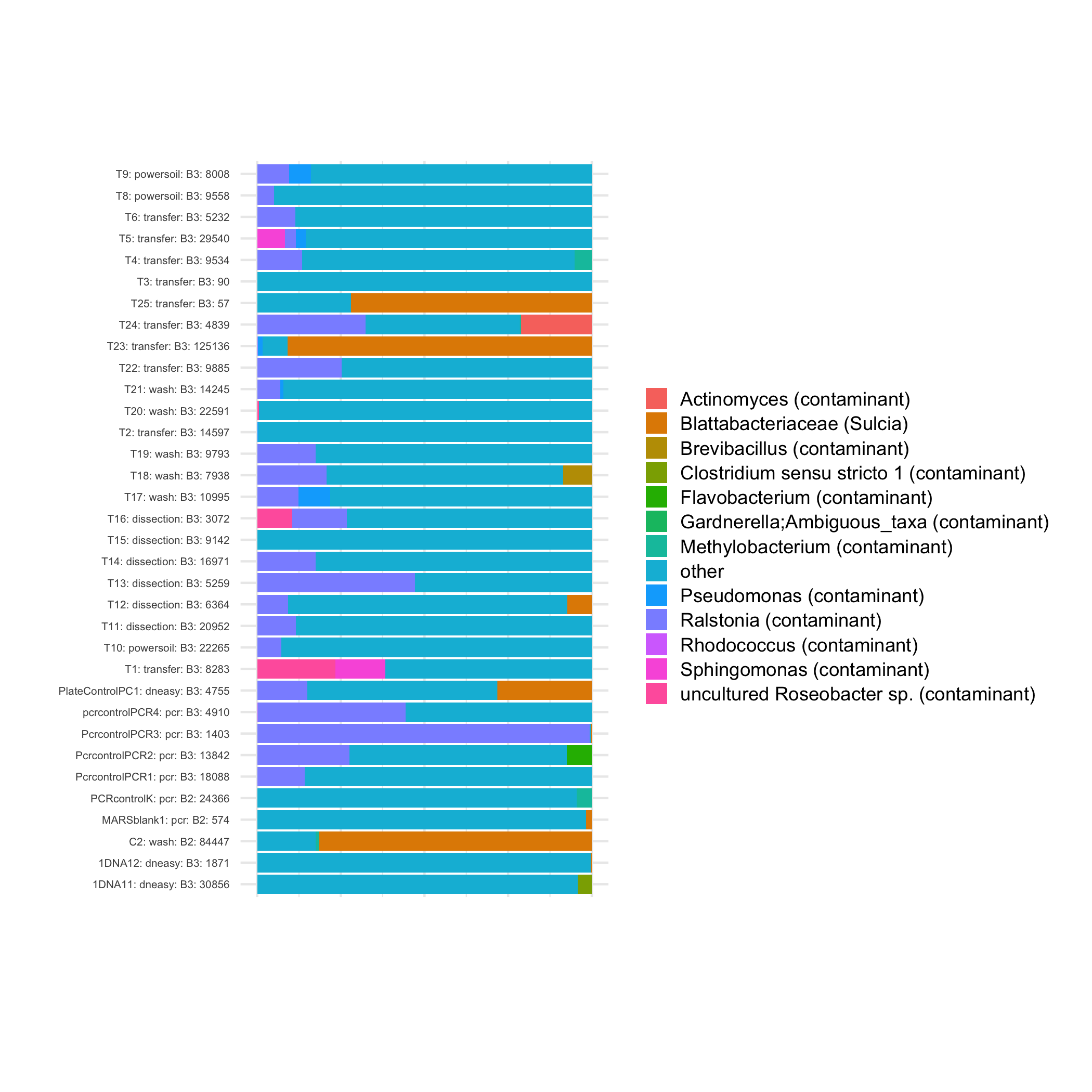


#####

##### Fig. S2: Relative abundance of a subset of putative contaminants identified with Decontam. Sample labels include control type (powersoil, transfer, wash, dissection, dneasy, and pcr), the dataset in which the control was produced (B2 or B3), and the total abundance of all ASVs before dataset filtering.

#####

#####
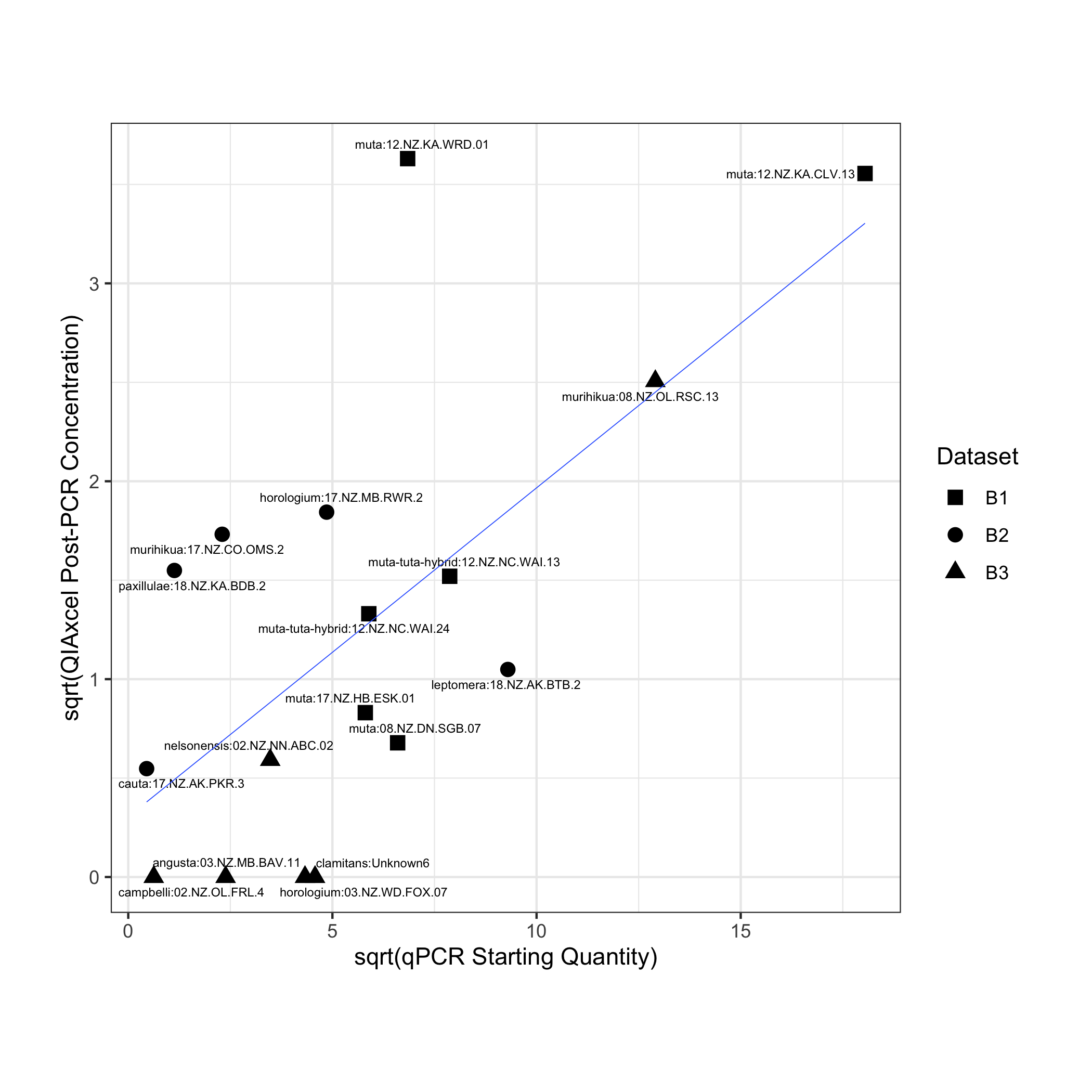


#####

##### Fig. S3: The relationship between total DNA post-PCR DNA concentration and qPCR absolute abundance of 16S rRNA in a subset of samples spanning different datasets. Data based on amplification with the same 16S V4 rRNA primers used to sequence microbial communities.

#####
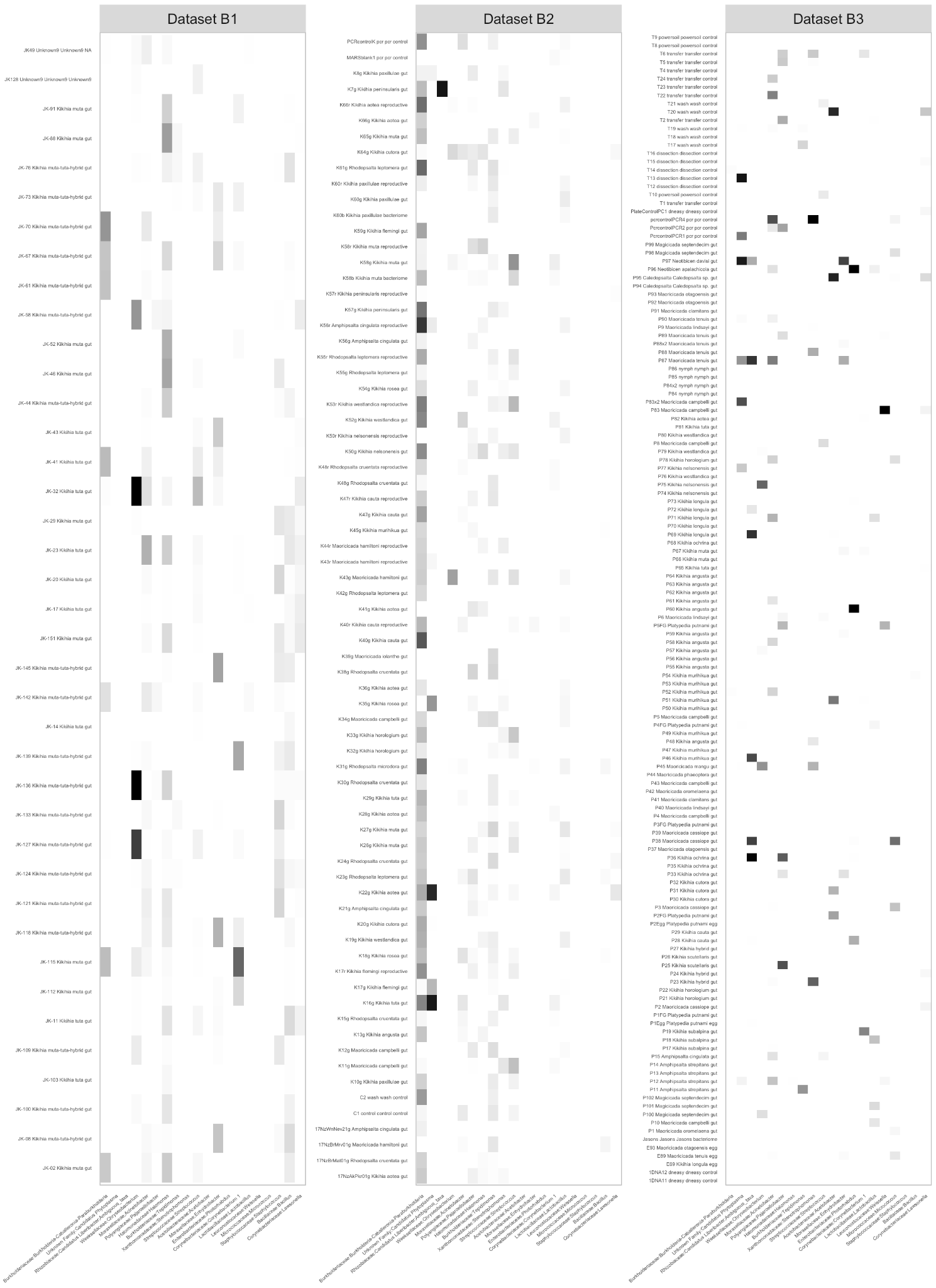
Fig. S4: Relative abundance of major bacterial genera across specimens after dataset filtering. Solid black cells correspond to a relative abundance of 100% and white cells indicate absence of any particular bacterial group.


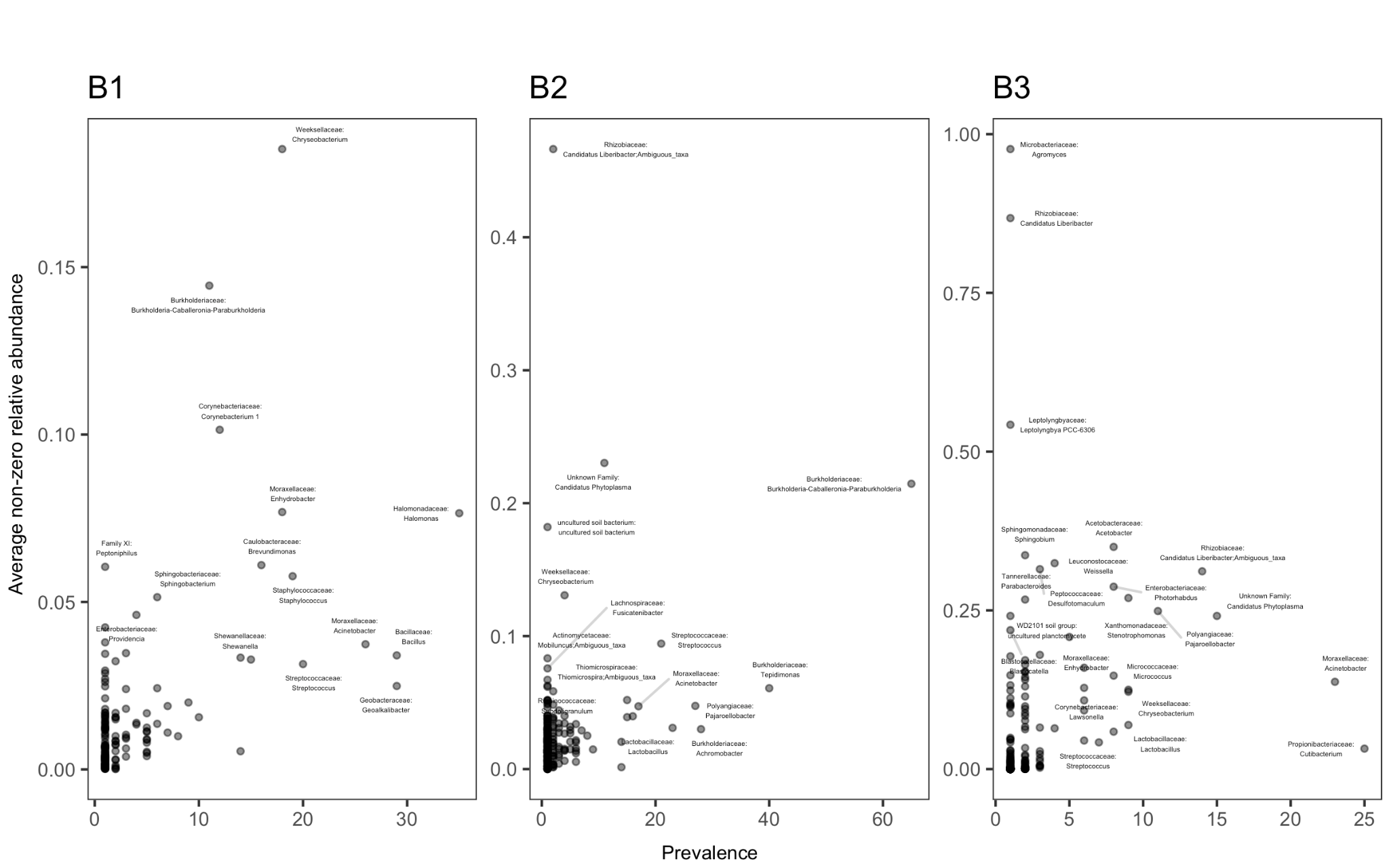


#####

##### Fig. S5: Relationship between average relative abundance across all samples and prevalence across all samples per Genus-level bacterial ASV in each post-filtered dataset. Labeled taxa were included in relative abundance and ordination analyses in Fig. 2B-C. Note that relative abundances were calculated as the average of all non-zero values across samples.

#####

#####

#####
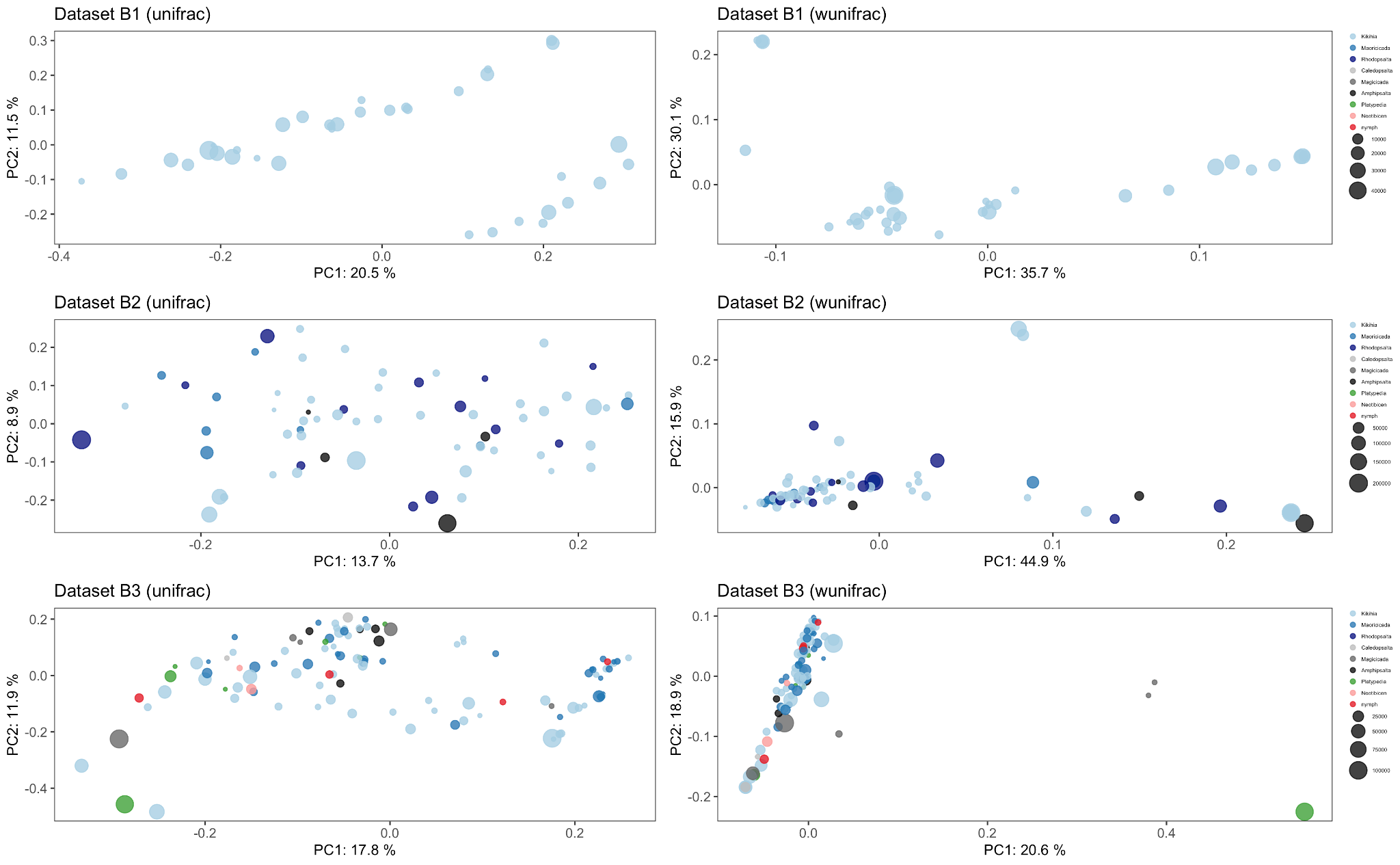


#####

##### Fig. S6: PCoA ordinations of post-filtered datasets of individual samples using either unweighted (left column) or weighted (right column) UniFrac distances. Colors correspond to host genera and point sizes correspond to the total abundance of ASVs after filtering. Axis labels contain the percent variation explained by either the first or second principle components.


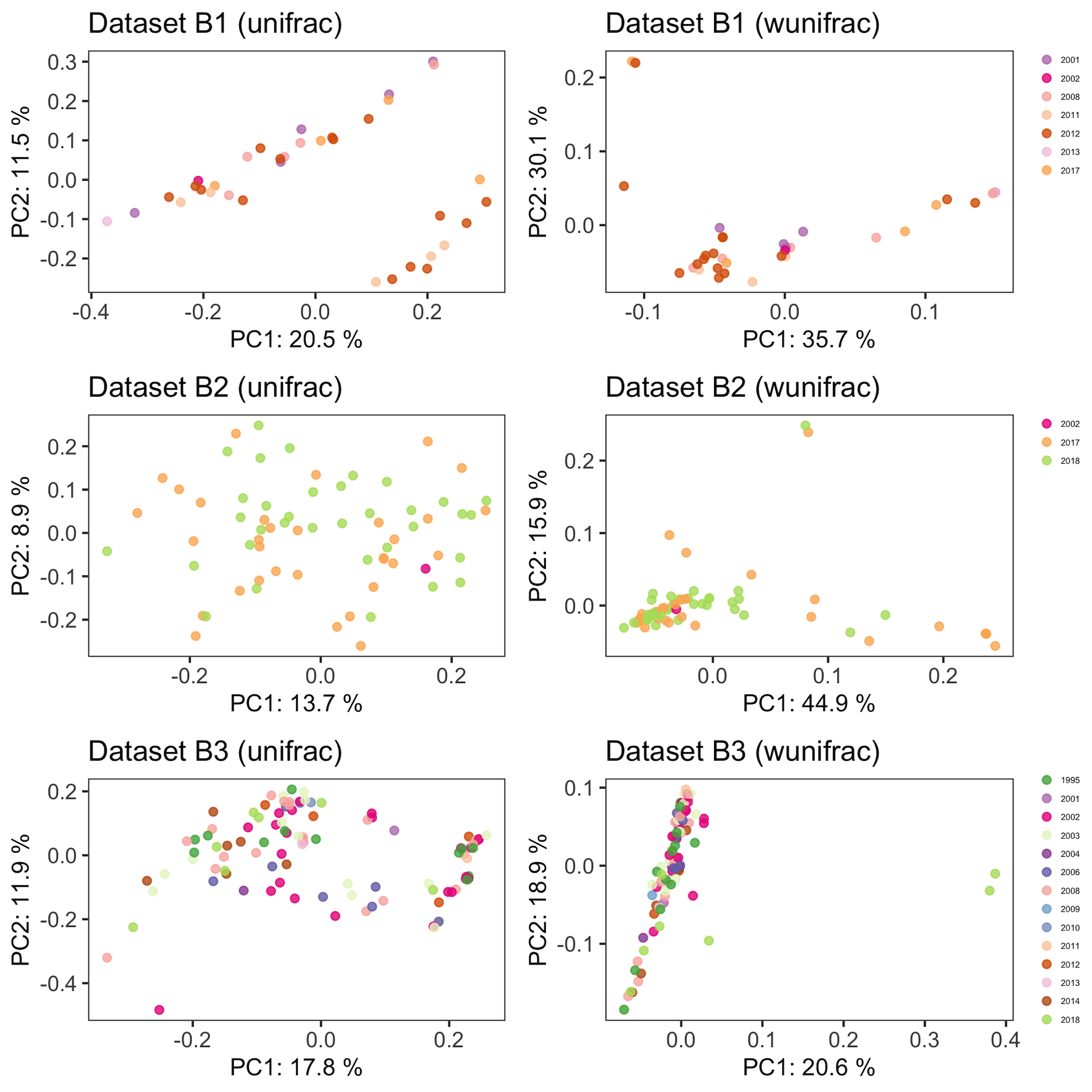


##### Fig. S7: PCoA ordinations of post-filtered datasets of individual samples using either unweighted (left column) or weighted (right column) UniFrac distances. Colors correspond to the year in which the sample was collected. Axis labels contain the percent variation explained by either the first or second principle components.

#####
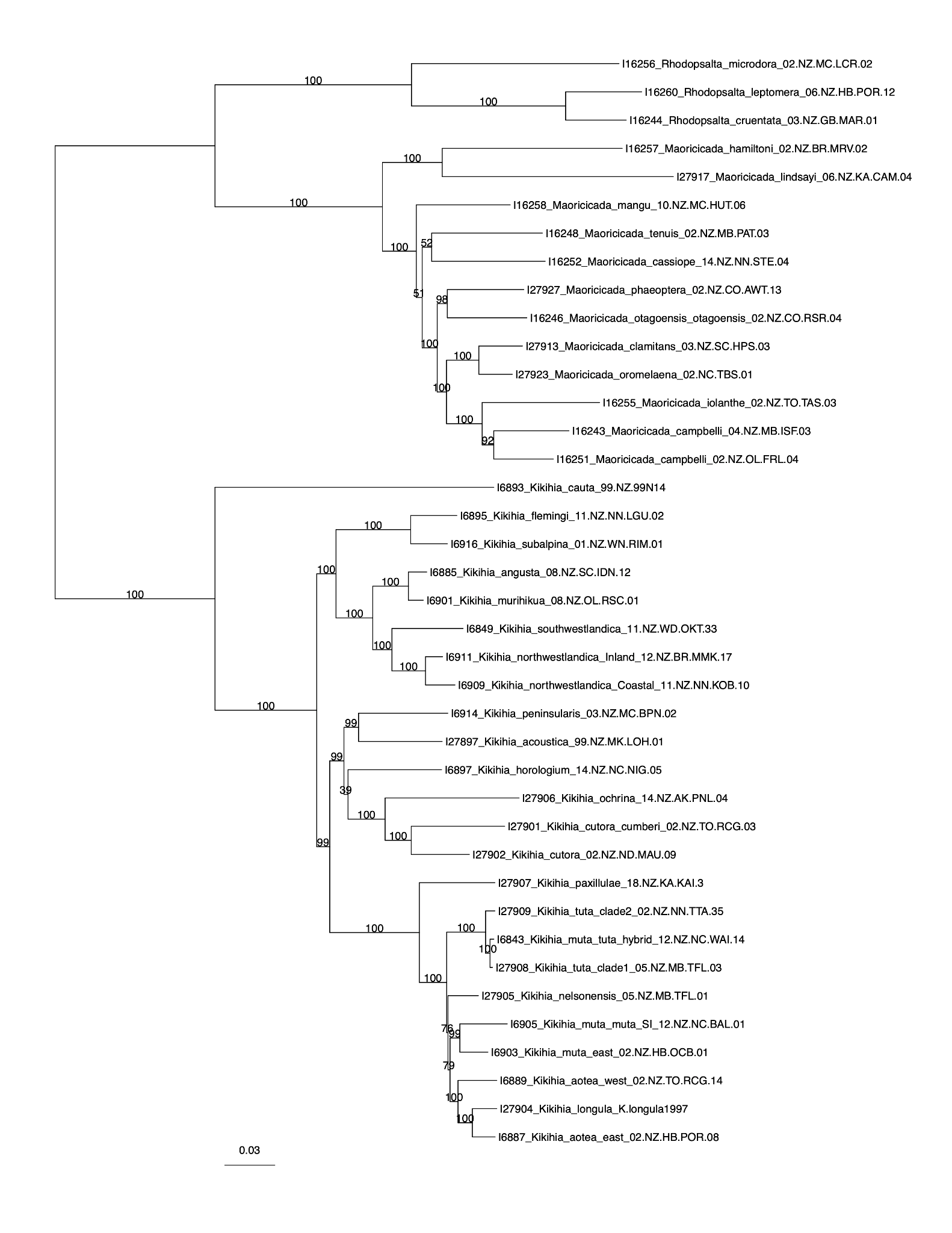


##### Fig. S8: Maximum likelihood phylogeny of mitochondrial genomes from New Zealand cicadas with bootstrap support values.

#####


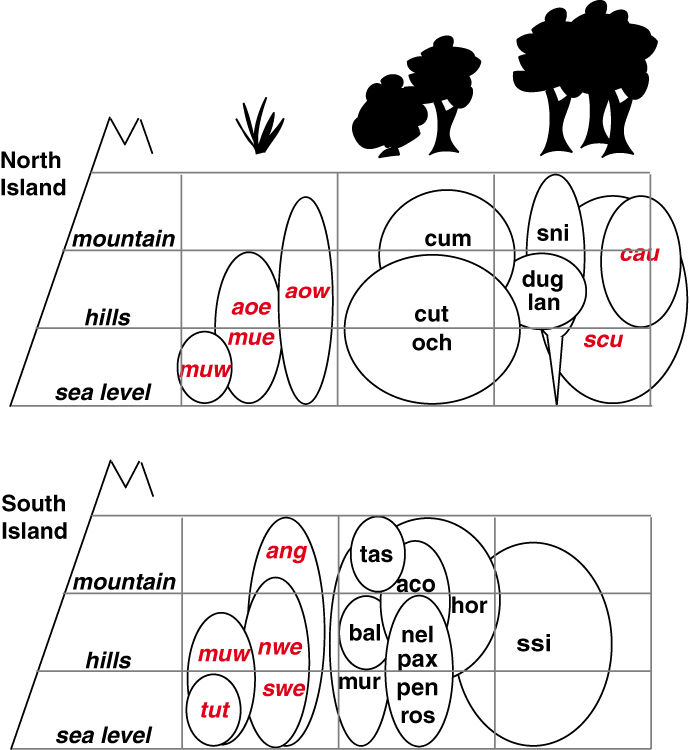


#####

##### Fig. S9: Drawing of hypothetical niche space by Kathy Hill. Distributions are based on elevation and vegetation of various Kikihia species: muw (western muta), mue (eastern muta), aoe (eastern aotea), aow (western aotea), och (ochrina), cut (cutora), cum (cumberi), lan (laneorum), dug (dugdalei), sni (subalpina north island), scu (scutellaris), cau (cauta), tut (tuta), swe (southern westlandica), nwe (northern westlandica), ang (angusta), mur (murihikua), bal (balaena), tas (tasmani), ros (rosea), pen (peninsularis), pax (paxillulae), nel (nelsonensis), aco (acoustica), hor (horologium), ssi (subalpina south island).

##### 
